# Fli1 Downregulation in Scleroderma Myeloid Cells Has Profibrotic and Proinflammatory Effects

**DOI:** 10.3389/fimmu.2020.00800

**Published:** 2020-05-19

**Authors:** Andreea M. Bujor, Fatima El Adili, Arshi Parvez, Grace Marden, Maria Trojanowska

**Affiliations:** ^1^Division of Rheumatology, Department of Medicine, Arthritis and Autoimmune Diseases Research Center, Boston University School of Medicine, Boston, MA, United States; ^2^Division of Rheumatology, Department of Medicine, Whitaker Cardiovascular Institute, Boston University School of Medicine, Boston, MA, United States

**Keywords:** scleroderma, Fli1, monocytes, macrophages, fibroblasts, fibrosis

## Abstract

Scleroderma (SSc) is an autoimmune connective tissue disease characterized by immune dysregulation, vasculopathy, and fibrosis. We have previously demonstrated that low Fli1 expression in SSc fibroblasts and endothelial cells plays an important role in SSc pathogenesis. Cells of myeloid and lymphoid origin also express Fli1 and are dysregulated in patients with SSc, playing key roles in disease pathogenesis. However, the role for immune Fli1 in SSc is not yet clear. Our aim was to elucidate whether Fli1 contributes to the immune dysregulation seen in SSc. Comparison of the expression of Fli1 in monocytes, B- and T-cell fractions of PBMCs isolated from SSc patients and healthy controls (HC), showed an increase in Fli1 levels in monocytes. We used siRNA transfected human myeloid cells and mouse peritoneal macrophages obtained from *Fli1^*flox/flox*^LysMCre^+/+^* mice, and found that markers of alternative macrophage activation were increased with Fli1 deletion. Coculture of Fli1-deficient myeloid cells and primary human or mouse fibroblasts resulted in a potent induction of collagen type I, independent of TGFβ upregulation. We next analyzed global gene expression profile in response to Fli1 downregulation, to gain further insight into the molecular mechanisms of this process and to identify differentially expressed genes in myeloid cells. Of relevance to SSc, the top most upregulated pathways were hallmark IFN-γ and IFN-α response. Additionally, several genes previously linked to SSc pathogenesis and fibrosis in general were also induced, including CCL2, CCL7, MMP12, and CXCL10. ANKRD1, a profibrotic transcription co-regulator was the top upregulated gene in our array. Our results show that Fli1-deficient myeloid cells share key features with cells from SSc patients, with higher expression of profibrotic markers and activation of interferon responsive genes, thus suggesting that dysregulation of Fli1 in myeloid cells may contribute to SSc pathogenesis.

## Introduction

Scleroderma (SSc) is an autoimmune connective tissue disease characterized by immune dysregulation, vasculopathy, and fibrosis. There is no cure for SSc and therapies are at best modestly effective. Immune cell dysregulation occurs early in the course of the disease and it involves both the innate and adaptive systems (1, 2). Despite significant advances in the field, the exact mechanism by which immune dysregulation contributes to vasculopathy and fibrosis is currently unclear.

Depending on the environment, macrophages (Mø) can acquire distinct functional phenotypes: classical, proinflammatory Mø (C-Mø), and alternative, pro-fibrotic Mø (A-Mø), which are just extremes on a continuum of activation states (3). In response to Th_1_ cytokines, including IL-1 and IFNγ, Mø secrete proinflammatory cytokines IL-12, IL-23, IL-1, and TNFα. In contrast, Th_2_ type cytokine (IL-4/13) stimulation leads to differentiation into the profibrotic Mø phenotype, with expression of the CD163 and CD204 markers and secretion of the IL-10, TGFβ, and CCL18, followed by tissue fibrosis (4). Flow cytometry analysis of SSc-PBMCs (peripheral blood mononuclear cells) revealed a higher proportion of monocytes (Mo), which showed expression of CD163 and CD204, while these markers were not present in PBMCs from healthy controls (HC) (5). CD163+/CD204 + cells were also identified in SSc skin biopsies, not only in the perivascular regions, but also between thickened collagen bundles (5). When SSc Mo from ILD patients were stimulated *in vitro* with LPS (lipopolysaccharide), which normally induces differentiation into C-Mø, there was increased expression of CD163 compared to control Mo (6). A comprehensive meta-analysis of transcriptomic data sets from skin biopsies of three large independent SSc patient populations identified a conserved set of genes across the SSc patients, with one subset containing genes characteristic of alternative Mø activation (7).

Monocytes derived from SSc patients may contribute to fibrogenesis via secretion of profibrotic factors, elevated in the skin and serum of SSc patients, is expressed by fibroblasts in SSc and plays an active role early in the disease pathogenesis by recruiting Mo and fibrocytes into tissues. Blockade of CCL2 prevented fibrosis in several animal models of SSc, including sclerodermatous graft-versus-host disease and bleomycin induced skin fibrosis (8, 9). Monocytes also secrete CCL2, which in turn may act as a profibrotic stimulus on fibroblasts, leading to secretion of TGFβ and extracellular matrix production (10). TGFβ further enhances CCL2 production, leading to a complex cascade of feedback regulation. CCL7/MCP-3 (monocyte chemoattractant protein-3), a chemotactic protein closely related to CCL2, is overexpressed by mononuclear cells and fibroblasts in SSc. Apart from promoting the recruitment of immune cells, CCL7 also has direct profibrotic effects on fibroblasts, and its expression is stimulated by TGFβ (11). Another characteristic of SSc Mo is enhanced migration. SSc-interstitial lung disease Mo express higher levels of CCR2 (receptor for CCL2) and lower levels of caveolin-1, both proven to increase the Mo migratory capacity (12, 13). Up-regulation of CCL2 and CCR2 was also reported on macrophages in the skin of early diffuse SSc (14). While all these studies strongly support a role for the mononuclear phagocytic system in SSc, their pathogenetic mechanism is far from clear.

Fli1, a member of the Ets family of transcription factors, is expressed in endothelial cells, fibroblasts and immune cells. Fli1 knockout mice die during embryogenesis due to a defect in vessel maturation (15). Abnormal expression of Fli1 is seen in autoimmune diseases, including systemic lupus erythematosus and SSc, where it plays important roles in pathogenesis (16, 17). Fli1 plays a key role in repressing collagen genes in healthy tissues and its deficiency likely contributes to the upregulated matrix production in SSc (18). Recent studies also suggest that Fli1 is critical for vessel maturation and stabilization. Mice with a conditional knockout of Fli1 in endothelial cells displayed abnormal skin vasculature, with greatly compromised vessel integrity and markedly increased vessel permeability, similar to SSc vasculopathy (19). Fli1 plays an important role in regulating mononuclear phagocyte cell development. Monocytes, Mø and dendritic cells populations were increased in the Fli1^ΔCTA/ΔCTA^ mice (lacking the C-terminal regulatory domain) compared with wild-type littermates, via de-repression of the Flt3L promoter (20). Additionally, Fli1 deficiency induced CXCL13 expression in murine peritoneal macrophages (21). Given the important role that it plays in SSc pathogenesis, our aim was to elucidate whether Fli1 contributes to the immune dysregulation seen in this disease.

## Materials and Methods

### Cell Isolation and Culture

Informed consent was obtained from all subjects, and the study was conducted in compliance with Institutional Review Board guidelines. PBMCs from SSc and HC were isolated by Ficoll Paque gradient centrifugation. CD14+ monocytes were isolated via positive selection from fresh PBMCs using EasyStep human monocyte isolation kit (Stem Cell Technologies, Cambridge, MA catalog # 18058) according to manufacturer’s instructions. B cells were then isolated from remaining PBMCs using EasySep Human CD19 positive selection kit II (Stem Cell Technologies, Cambridge, MA catalog # 17754), and T cells through negative selection (CD14-/CD19- cells). Human dermal fibroblasts were isolated from the forearm of HC and cultured as previously described (22). Cells in passages 2–5 were used for experiments. For experiments using mouse cells, dermal fibroblasts were isolated from the back of the mice after shaving and overnight collagenase digestion. Mature quiescent resident mouse peritoneal macrophages were isolated according to a previously published protocol (23). For coculture experiements, THP1 cells, or mouse peritoneal macrophages were either directly seeded on top of fibroblasts or inside cell inserts with 0.4 μm membrane pore size (Corning, NY, product number 353493).

### Generation of Myeloid-Cell Specific Fli1-Knockout Mice

**FIGURE 1:**
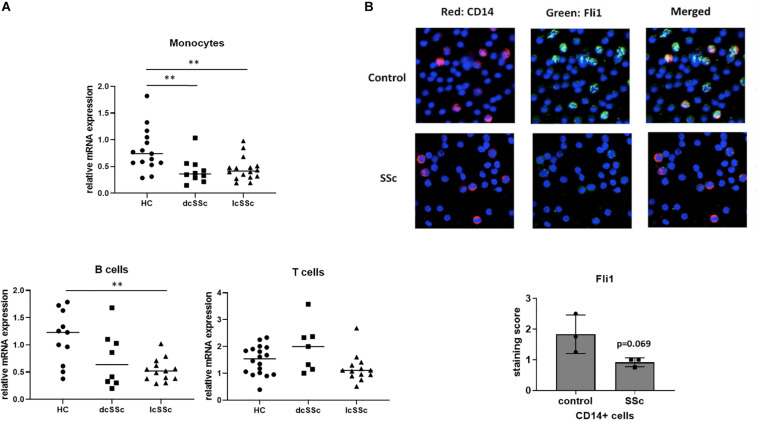
Monocytes isolated from SSc patients have low levels of Fli1. **(A)** Monocytes, T and B lymphocytes were isolated from healthy controls (HC) and SSc patients (diffuse-dcSSc or limited-lcSSc) and mRNA levels of Fli1 were analyzed using quantitative RT-PCR. One way ANOVA (GraphPad Prims 7) was used to compare SSc to control. **(B)** Isolated peripheral blood mononuclear cells (PBMCs) from SSc and healthy controls (HC) were stained with CD14 for monocytes (red) and Fli1 (green) antibodies, and DAPI was used to counterstain the nuclei. Representative images and quantification are shown (*n* = 3 each). **p < 0.01.

All experimental procedures were approved by the Boston University Animal Care and Use Committee and conducted in accordance with the guidelines of the National Institutes of Health. The floxed Fli1 mice was generated using a Fli1 targeting vector purchased from the KOMP repository (UCDavis, Sacramento, CA, Clone name: HTGR06010_A_1_C02, Figure 1). 129 agouti embryonic stem cell lines harboring an insertion with the KOMP vector were generated at the Transgenic Mouse Core at Harvard Medical School (Boston, MA, United States) and used for blastocyst injection to generate chimeric mice, which were then selected for germline transmission. Heterozygous mice with the targeted gene mutation were then crossed with transgenic C57BL/6J mice expressing *FLP1* recombinase (FlpE) in all tissues, under the human β-actin promoter (transgenic B6.Cg-Tg (ACT*FlpE*)9205Dym/J, available from The Jackson Laboratory, United States, Stock number 005703). The resultant Fli1^*flox/flox*^ mice were further crossed to C57BL/6 mice for 10 generations.

For the generation of Fli1 conditional knockout mice, mice expressing the Cre recombinase under the control of the myeloid-specific Lyz2 promoter were purchased from the Jackson Laboratory (B6.129P2-*Lyz2^TM 1(cre)Ifo^*/J, Bar Harbor, ME, United States) and crossed with Fli1^*flox/flox*^ mice.

Six to eight-week-old homozygous mice were used for all experiments. The genotyping primers suggested by Jackson Laboratory were used for the Cre mice and for the Fli1^*flox/flox*^ mice the following primers were used: gactcaaaccagggaaagttgc (3′ loxP site, forward), ttgggaaggtggaatctagcag (3′ loxP site, reverse), acctttgctccacacatctga (5′ loxP site, forward), accttggttacaggactgagtg (5′ loxP, reverse).

### Isolation of Peritoneal Macrophages From Mice

Mice were euthanized following an IACUC approved animal protocol, then 5 cc germ-free PBS was flushed into the peritoneal cavity and the peritoneal lavage fluid was collected. Cells were centrifuged (350 *g* for 5 min at 4°), and plated in RPMI media for 2 h, then unattached cells were washed twice with sterile PBS. Attached macrophages were then harvested using trypsin.

### Immunofluorescence

Cells were washed twice with 1 × PBS and fixed in acetone:methanol 1:1 for 15 min at room temperature, followed by three washes in PBS. Cells were permeabilized with 0.25% Triton X-100 for 15 min followed by three washes in PBS and 1 h blocking in 3% bovine serum albumin (BSA) in PBS at room temperature. Cells were incubated with anti- Fli1 (mouse anti-human, BD Bioscience #554266), CD14 (rabbit monoclonal, Abcam, Cambridge, MA, United States) and Collagen type I (Southern Biotech, #1310-01), and CD163 (mouse antihuman #MCA1853, BioRad, hercules, CA, United States) primary antibodies (1:100 in 1% BSA in PBS) at 4°C overnight followed by three washes in PBS. The bound antibody was detected using anti-rabbit (Alexa Fluor 594, Invitrogen) and anti-mouse (Alexa Fluor 488, Invitrogen) secondary antibodies (1:500 each) for 1 h. Coverslips were washed three times in PBS in the dark and then mounted on glass slides using Vectashield mounting medium with DAPI (Vector Laboratories Inc, Burlingame, CA, United States). Slides were blinded, and ten random fields were examined using an Olympus microscope attached to a digital camera. Semi-quantitative evaluation of staining results for Fli1 expression in CD14 positive cells from SSc patients and controls PBMCs was independently assessed in a blind manner by two experienced investigators. Staining was scored using 7 scores: 0, 0.5, 1, 1.5, 2, 2.5, and 3, according to the intensity, with results expressed as the mean ± standard deviation.

### Inhibition of Protein Expression by Small Interfering RNA (siRNA)

For the inhibition of Fli1 expression using siRNA, THP1 cells or dermal fibroblasts were grown to 80% confluence and transiently transfected with 50 nm Fli1 siRNA (Dharmacon, Fli1 ON-TARGETplus SMARTpool – a mixture of 4 siRNAs provided as a single reagent for enhanced potency and specificity), or the corresponding concentration of scrambled non-silencing siRNA (Scr, Dharmacon, On-TARGETplus Non-targeting Control Pool) for 48 h. Cells were then serum starved overnight and treated as indicated.

### Quantitative RT-PCR

Total RNA was extracted using TRI Reagent and 1 μg was converted to cDNA as previously described (22). Quantitative real time RT–PCR was performed using SYBR Green mixture (Applied Biosystems, Carlsbad, CA, United States) on a StepOnePlus Real-Time PCR system using 1 μl of cDNA in triplicate with beta actin as internal control. The sequences of the primers used are provided in Supplementary Table S1.

### Global Gene Expression Profile

THP1 cells were grown in RPMI media and Fli1 expression was inhibited using siRNA as described above. Cells were then converted to M_0_ macrophages 24 h later using 10 ng/ml PMA (Phorbol 12-myristate 13-acetate) for 4 h, then media changed and cells treated with 100 ng/ml M-CSF (macrophage colony stimulating factor) for 48 h. Cells were lysed using TRIzol and RNA was then extracted using Zymo Research kit and integrity analyzed using Agilent Bioanalyzer. Purity of the RNA samples was confirmed using a NanoDrop spectrophotometer.100 ng of high quality RNA (RIN > 9.0) with Biotin labeling was performed using the WT Plus reagent kit (Affymetrix, Santa Clara, CA, United States) according to the manufacturer’s protocol. The labeled, fragmented DNA was hybridized to the Affymetrix Human Gene 2.0 ST Array for 18 h in a GeneChip Hybridization oven 640 at 45°C with rotation (60 rpm). The hybridized samples were washed and stained using an Affymetrix fluidics station 450. Raw Affymetrix CEL files were normalized to produce Entrez Gene-identifier-specific expression values using the implementation of the Robust Multiarray Average (RMA) in the affy Bioconductor package (version 1.36.1), using R version 2.15.1 and the Brainarray hugene20sthsentrezgcdf R package (version 23.0.0). Raw and processed microarray data have been deposited in the Gene Expression Omnibus (GEO), Series GSE144625.

### Statistical Analysis

One-way analysis of variance (ANOVA) followed by Tukey’s multiple comparisons test was used for comparisons of differences between three or more groups. Unpaired *t*-test was used when only two groups were compared. All statistical analyses were performed using *GraphPad Prism 8* software (La Jolla, CA, United States). A *p* value of <0.05 was considered significant.

## Results

### Monocytes Isolated From Scleroderma Patients Have Low Levels of Fli1

To investigate whether Fli1 contributes to immune abnormalities in SSc, we first evaluated the expression levels of Fli1 in T cell, B cells and monocytes isolated from SSc patients and healthy controls (HC). The mRNA levels of Fli1 were decreased in monocytes isolated from both limited and diffuse SSc patients, compared to HC (Figure 1A). The levels of Fli1 were not significantly different in T or B cells, except for B cells from limited SSc patients, which had lower Fli1 compared to controls. The protein levels of Fli1 were then investigated in patient’s monocytes by immunofluorescence staining using CD14 and Fli1 specific antibodies and PBMCs, confirming the downregulation in SSc patients (Figure 1B).

### Conditioned Media From Fli1 Deficient Mø Has Profibrotic Effects on Fibroblasts

**FIGURE 2:**
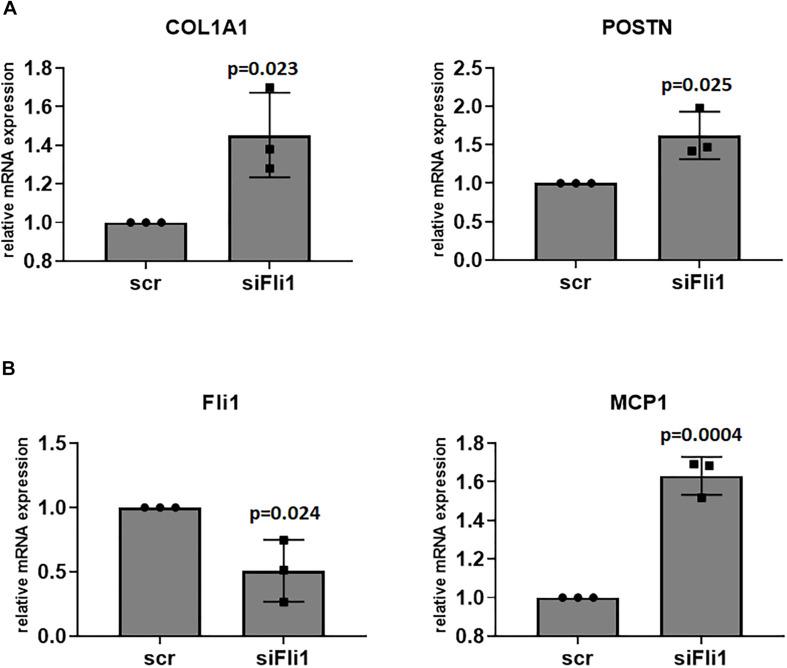
**(A)** Conditioned media from Fli1 deficient Mø has profibrotic effects on fibroblasts. Confluent, serum starved human dermal fibroblast were treated with conditioned media from scr and siFli1 treated THP1 cells, and after 6 h cell pellets were collected and mRNA levels of Collagen (COL1A1) and POSTN (periostin) were analyzed via RT-PCR. **(B)** Downregulation of Fli1 in fibroblasts induces CCL2/MCP1 mRNA levels. Confluent, serum starved human dermal fibroblast were treated with scr and siFli1 for 48 h then cell pellets were collected and mRNA levels of CCL2 were analyzed via RT-PCR. Mean ± SD.

Scleroderma monocytes can contribute to fibrosis and fibroblast activation via several mechanisms, including enhanced differentiation into alternatively activated macrophages and secretion of profibrotic molecules. We next treated human dermal fibroblasts with conditioned media from Fli1 depleted THP1 cells to assess whether low levels of Fli1 contributes to their ability to enhance fibrosis. As seen in Figure 2A, conditioned media from Mø with low Fli1 induced periostin and type I collagen gene expression in human dermal fibroblasts, supporting a profibrotic role for Fli1 deletion in Mø. Interestingly, CCL2 was upregulated in fibroblasts treated with Fli1 siRNA, suggesting that Fli1-deficient fibroblasts could recruit monocytes in early disease stages (Figure 2B). In turn, a soluble factor secreted by Fli1-deficient Mø may induce expression of profibrotic genes in fibroblasts, thus potentially contributing to SSc fibrosis.

### Expression of the Alternative Macrophage Activation Markers Is High in Fli1 Deficient Cells

**FIGURE 3:**
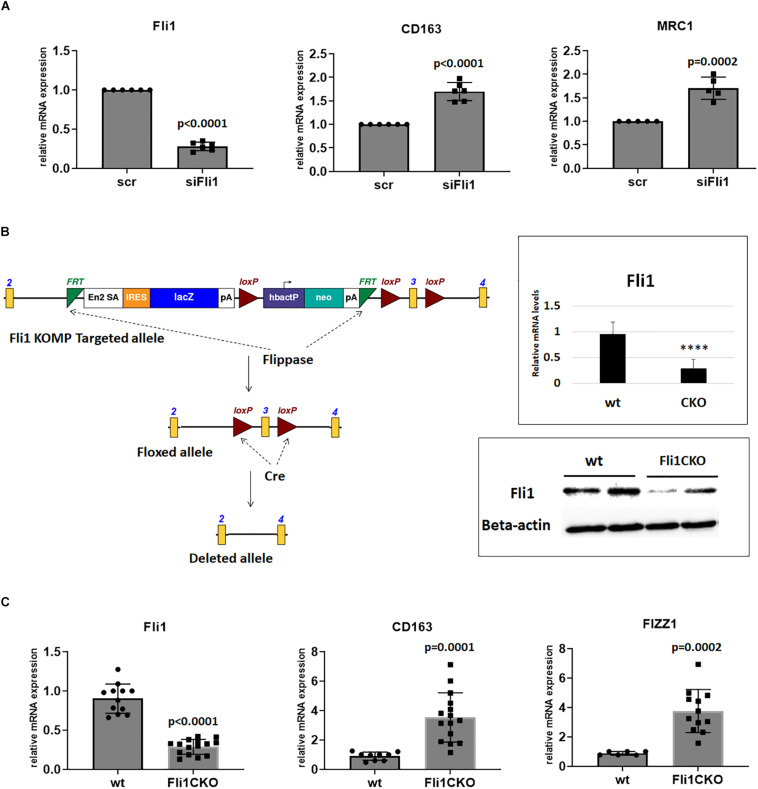
Expression of the A-Mø markers is high in Fli1 deficient cells. **(A)** Human THP1 cells were treated with Fli1 and scr siRNA and then 24 h later with 10 ng PMA for 4 h, then mRNA extracted after total 48 h, and relative mRNA levels of CD163 and MRC1 were quantified by RT-PCR (*n* = 4). Mean ± SD. **(B)** Generation of myeloid specific Fli1 knockout mice (*Fli1^*flox/flox*^LysMCre^+/+^*, Fli1 CKO). Schematic outline of the Fli1 KOMP targeting construct and Fli1 floxed allele. Neo: neomycin resistance gene. Flippase: FLP recombinase to remove the Neo cassette. The structures of the mutant allele after *in vivo* Cre-mediated recombination in myeloid cells lacks exon 3. Upper right panel shows relative mRNA levels of Fli1 in peritoneal macrophages isolated from wt and Fli1CKO mice, and bottom right panel shows protein levels of Fli1 in wt and Fli1CKO. Two mice were pooled for protein levels in each group. Beta actin was used as control for loading. **(C)** Peritoneal macrophages were collected from wt and Fli1 CKO mice and mRNA extracted and RT-PCR used to quantify levels of CD163, and FIZZ1. Individual mice used for each sample. ****p < 0.0001.

To assess whether low Fli1 seen in SSc monocytes skews them toward differentiation into A-Mø, we next used Fli1-depleted THP1 cells, and treated them with 10 ng/ml phorbol-12-myristate-13-acetate (PMA) for 4 h to induce transdifferentiation into Mø (24). Downregulation of Fli1 in these cells resulted in an induction of the mRNA levels of CD163, MRC1, both linked to A-Mø (Figure 3A).

While wildly used for the study of the myeloid cell functions *in vitro*, THP1 cells are secondary, immortalized cells, phenotypically, and functionally different from primary Mo. To validate these findings, we asked whether deletion of Fli1 in primary mouse Mø would skew them toward an alternative activation phenotype and influence development of fibrosis.

### Targeted Ablation of the Fli1 Gene in Mouse Myeloid Cells

Fli1 knockout mice die during embryogenesis due to a defect in vessel maturation (15), precluding assessment of the function of Fli1 in myeloid cells in these mice. To examine the function of Fli1 in mouse myeloid cells, we generated mice with *Fli1* gene selectively ablated in cells of myeloid origin. Fli1^*flox/flox*^ mice were generated as described in section “Materials and Methods,” and crossed with LysMCre transgenic mice that express the Cre-recombinase under the transcriptional control of the myeloid-specific Lyz2 promoter. Cre-mediated recombination in the resultant *Fli1^*flox/flox*^LysMCre^+/+^* mice results in excision of exon 3 from the Fli1 gene (Figure 3B). To confirm that excision of the exon 3 of Fli1 results in a corresponding loss of Fli1 protein, we performed western blot analysis on protein extracts from peritoneal Mø harvested from the *Fli1^*flox/flox*^LysMCr*e^+/+^. Figure 3B (right, bottom) shows that Fli1 was significantly downregulated in the transgenic mice compared to wild types.

Next, we compared the expression of A-Mø markers in peritoneal Mø isolated from wild type and *Fli1^*flox/flox*^LysMCre^+/+^*mice. Similar to results in THP1 cells, the mRNA levels of CD163 and the mouse specific A-Mø marker FIZZ1 were significantly induced in mice with conditional Fli1 deletion (Figure 3C).

Collectively, these results suggest that decreased expression of Fli1 in SSc monocytes may directly contribute to fibrogenesis via imparting cells a selective bias toward alternative macrophage activation and secretion of soluble profibrotic mediators.

### Depletion of Fli1 in Myeloid Cells Does Not Induce TGFβ Gene Expression

**FIGURE 4:**
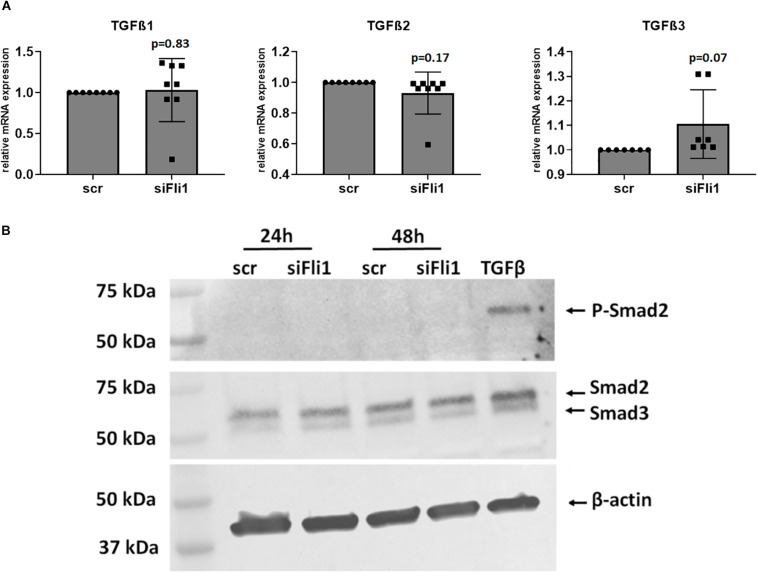
Fli1 downregulation in myeloid cells does not induce TGFβ pathway. **(A)** THP1 cells were transfected as described with scr and Fli1 siRNA and levels of TGFβ isoforms 1, 2, and 3 were measured at the mRNA levels 48 h later using RT-PCR. *n* > 3 each, not statistically significant. **(B)** THP1 monocytes were transfected with siRNA and 48 h later they were plated directly over confluent, serum starved human dermal fibroblasts. After 24 and 48 h, cells were washed and cell layers collected and protein extracted. Levels of P-Smad2 (Ser465/467) and total Smad2/3 were assessed by Western Blot, with TGFβ as positive control.

TGFβ is a central mediator of fibrosis and a key molecule involved in SSc pathogenesis (25, 26). Secretion of TGFβ by myeloid cells has been implicated in the induction of fibrogenesis (27). As an initial step to test whether these molecules mediate the profibrotic phenotype induced by Fli1 downregulation in myeloid cells, we measured the mRNA levels after siRNA mediated depletion of Fli1 in THP1 cells and in peritoneal *Fli1^*flox/flox*^LysMCre^+/+^* macrophages compared to wild type mice. No significant changes in the levels of TGFβ were found (Figure 4A). TGFβ patway activation can be a result of either increased TGFβ protein levels, enhanced activation of latent TGFβ, or enhanced receptor expression. To further explore a potential contribution of the TGFβ pathway in this process, we next assessed phosphorylation levels of its main downstream target, Smad2. There was no induction in P-Smad2 in fibroblasts cocultured with Fli1-depleted THP1 cells (Figure 4B). Collectively, these results suggest that a TGFβ-independent mechanism may be responsible for the fibrogenic effects of low Fli1 in myeloid cells.

### Coculture of Myeloid Cells and Fibroblasts Enhances the Profibrotic Effects Seen With Low Fli1

**FIGURE 5:**
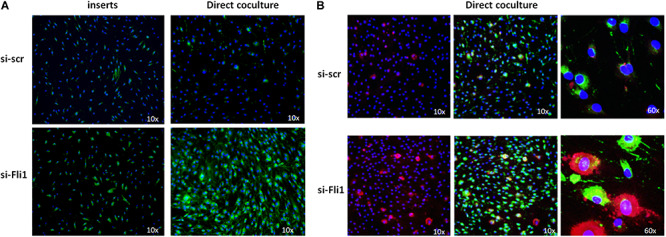
**(A)** Effects on collagen deposition in indirect (inserts) and direct coculture of human dermal fibroblasts and siRNA-treated THP1 cells. THP1 monocytes were transfected with siRNA and 48 h later they were plated either inside inserts or directly over confluent, serum starved fibroblasts. Cells were washed and immunofluorescent staining using specific anti-Collagen type I antibodies was done on day 5. DAPI was used to counterstain the nuclei. Representative images sown, *n* = 3 each. **(B)** Chamber slides were used for direct coculture experiments as described above and cells were stained with CD163 for A-Mø (red) and Collagen type I (green) antibodies, and DAPI was used to counterstain the nuclei. Representative images are shown (*n* = 3 each).

Isolated cell cultures do not reflect the complexity of cell-cell interaction *in vivo*, and direct contact of myeloid cells and fibroblasts may be required to fully achieve the profibrotic phenotype. To better assess paracrine and juxtacrine effects of myeloid-fibroblast cell-cell signaling, we used an experimental model of both non-contacting and direct contact co-culturing. To study the paracrine effects of myeloid-fibroblast co-cultures, we used transwell inserts that provided physical separation of the two cell types while allowing free cytokine transport between them. When cells were co-cultured in direct, physical contact with each other, there was a potent induction of collagen (Figure 5A, right panel). Similar, but less pronounced upregulation of collagen was seen when the two cells types were not in direct contact with each other (Figure 5A, left panel). In a separate experiment, we assessed the expression of CD163 on THP1 cells with low Fli1 in coculture with fibroblasts, and confirmed at the protein levels by immunofluorescent staining that this marker of alternative macrophage activation is induced under these experimental conditions as well (Figure 5B). Altogether, these results indicate that direct cell-cell contact between activated macrophages and fibroblasts may be required for the full fibrogenic effect of Fli1 depletion in myeloid cells.

### Global Gene Expression Profile in Fli1-siRNA Treated THP1 Cells Reveals Upregulation of Pro-Inflammatory and Migration-Related Genes

**FIGURE 6:**
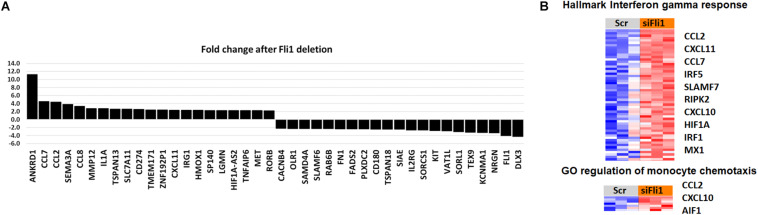
Global gene expression profile in Fli1-siRNA treated myeloid cells. **(A)** The top twenty most up and down-regulated genes after Fli1 deletion in myeloid cells (*p* > 0.001). **(B)** Heatmap of gene-expression of IFN-γ and monocyte migration pathways. Blue indicates repressed mRNA levels and red elevated levels.

Identification of molecular pathways and genes that are significantly associated with Fli1 downregulation might help unravel the mechanisms of myeloid-induced fibrosis. We next downregulated Fli1 expression via siRNA in PMA-treated THP1 monocytes (24), then performed microarray analysis using the Affimetrix GeneChip human 2.0 ST gene array. The expression of Fli1 was reduced approximately 3.7-fold in the samples treated with siFli1 compared to the scrambled controls. A total of 778 genes were identified as significantly differentially expressed in cells with low Fli1 (*p* < 0.005). The top twenty most up and down-regulated genes (*p* > 0.001) are shown in Figure 6A. Gene ontology analysis revealed multiple biological functions that were significantly enriched with siRNA treatment. Notably, several pathways related to activation of inflammatory programs (hallmark IFN-γ & IFN-α response, and hallmark TNFα signaling via NFKB), as well as pathways related to immune cell migration, were among the top upregulated biological pathways (Figure 6B). Amongst the alternative activation markers only CD163 was significantly upregulated in the microarray analysis (1.5 fold, *p* = 0.00073). Detailed heatmaps of the leading edge genes of all gene sets with FDR *q* < 0.25 are available in Supplementary Data Sheet S1.

### Validation of the Differentially Expressed Genes by Real-Time PCR

**FIGURE 7:**
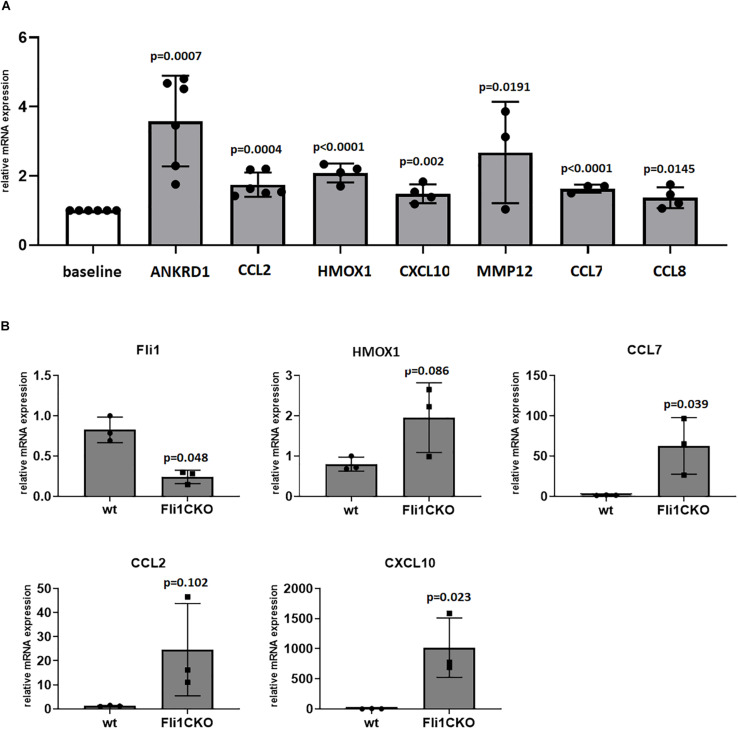
Validation of differentially expressed genes via quatitative RT-PCR. Results in THP1 cells treated with siRNA in **(A)** or peritoneal macrophages isolated from Fli1CKO mice and wt mice in **(B)**. mRNA levels of selected genes of interest were analyzed by RT-PCR in ≥3 separate experiments and the results are presented as bar graphs with controls arbitrarily set at 1. scr, scrambled.

In order to assess the validity of the microarray results, we used QRT-PCR to compare the expression of top genes of interest that had significant expression changes in the microarray analyses. The following genes were selected: ANKRD1, CCL2, CCL7, CCL8, CXCL10, HMOX1, and MMP12. Results are presented in Figure 7A. The top differentially expressed gene in the microarray was ANKRD1, which was induced by 11-fold in cells with low Fli1. This upregulation was confirmed by quantitative RT-PCR analysis, and analysis of CT values revealed that ANKRD1 is expressed at very low levels in primary Mo, and not expressed in quiescent human dermal fibroblasts (data not shown). Next, we used peritoneal macrophages from *Fli1^*flox/flox*^LysMCre^+/+^* mice and validated our microarray results for the genes presented in Figure 7B. Of note, several interferon response genes, including CCL7, CCL2, and CXCL10 were upregulated in these mice, similar to published results in SSc patients. Our microarray data identified multiple Fli1 targets in myeloid cells that had differential expression compared to controls and that are relevant to SSc, thus potentially mediating the altered phenotype of myeloid cells with low Fli1 expression seen in our study.

## Discussion

We show here that monocytes from patients with systemic sclerosis have decreased levels of the transcription factor Fli1, and provide new evidence for an antifibrotic role for Fli1 in these cells. SiRNA mediated downregulation of Fli1 in human myeloid cells, and via Cre mediated targeted disruption in mouse myeloid cells resulted in key changes in their phenotype, with acquisition of alternative, profibrotic features and activation of key interferon regulated genes, similar to what has been described in SSc patients. This suggested that decreased levels of Fli1 in Mo/Mø in SSc patients may contribute to fibrosis via alternative Mø activation and secretion of pro-fibrotic and pro-inflammatory cytokines, with paracrine activation of fibroblasts.

The mononuclear phagocytic system in SSc is central to fibrogenesis, and may contribute to fibrosis via enhanced Mo migration into injured tissues, differentiation of Mo into fibrocytes or A-Mø, and secretion of various pro-fibrotic mediators (28).

It is challenging to elucidate the diverse role of myeloid cells in driving fibrosis, due to their vast plasticity and functional diversity, all depending on a multitude of variables, including tissue environment, injury specific co-signals, and particular disease etiology. Alterations in Mø polarization may contribute to SSc pathogenesis, with a higher proportion of CD163 and CD204 positive Mo reported in SSc blood and skin biopsies (5, 7, 29). However, the mechanism that drives this phenotypic change is still elusive. We found low levels of Fli1 in SSc monocytes and increased expression of A-Mø markers in cells with low Fli1, both in human and mice. Our observations thus identify Fli1 as one of the factors that possibly orchestrates the changes described in SSc myeloid cells.

While helpful to appreciate the heterogeneity and functions of Mø, characterizing them as classical vs. alternatively activated based on the initial stimulus or a mere handful of markers is not ideal and does not reflect the complex *in vivo* microenvironment. There are likely differences in Mø phenotype that span beyond this classification. While this study shows that the expression of alternative activation markers is elevated in cells with low Fli1, the observation that cells have a profibrotic phenotype is of particular relevance, as it implies that decreased Fli1 in myeloid cells has distinctive consequences on cell function.

Myeloid cells derived from SSc patients may contribute to fibrosis via secretion of profibrotic factors, including TGFβ (25). Although we found that conditioned media from Mo with low Fli1 induced fibrosis via direct stimulation of fibroblasts, we failed to find an increase in mRNA levels of TGFβ in Fli1-depleted myeloid cells, and there was no activation of the TGFβ/Smad2 pathway, suggesting that other factors might mediate their profibrotic effects on fibroblasts. Based on the microarray analysis, we identified further potential candidates. To that extent, CCL2 and CCL7 were among the top upregulated genes after Fli1 downregulation in Mø in our study. Earlier reports showed that both these chemokines are increased in SSc serum and can enhance collagen synthesis, but a direct role for CCL2 on fibroblasts is controversial (9–11). Using quantitative RT-PCR analysis, we were unable to find expression of the CCL2 receptor CCR2 in human dermal fibroblasts (data not shown), suggesting that secretion of CCL2 by myeloid cells with low Fli1 does not directly contribute to collagen upregulation. This is in agreement with previously published studies that found no expression of CCL2 receptors and no direct effect of CCL2 stimulation or CCL2 blocking on collagen production by fibroblasts (30, 31). However, indirect fibrogenic effects of CCL2 may still contribute to SSc tissue fibrosis, and there is abundant published evidence to support this notion (32). Fibroblasts serve as a source of cytokines and chemokines that influence the microenvironment, and earlier studies have shown that coculture of myeloid cells and fibroblasts enhances fibrosis (33). SSc fibroblasts are known to secrete CCL2, which attracts monocytes to the site of injury. In our study deletion of Fli1 in both myeloid cells and fibroblasts enhanced CCL2 mRNA levels, thus potentially creating an unbalanced cytokine response that could lead to tissue damage and fibrosis. While the profibrotic phenotype of myeloid cells in SSc may be due to a combination of factors, our finding suggest that the increased CCL2 and CCL7 levels in SSc patients (31, 34–36) may be in part due to Fli1 downregulation in Mo and could contribute to fibrosis.

Of relevance to SSc, CXCL10 and CXCL11 are IFN-γ inducible chemokines that are upregulated in SSc (37, 38) and that showed a twofold induction in response to Fli1 deletion in our study. Interestingly, it was previously found that CXCL10 is expressed early in the disease and associated with a worse outcome, including more severe pulmonary fibrosis (39). Whether overexpression of or CXCL11 contributes to fibrosis remains to be determined.

MMP12 is a matrix metalloproteinase produced at high levels by IL-4- and IL-13- alternative-activated macrophages, and has been implicated in development of fibrosis. MMP-12 deficiency reduced myocardial fibrosis following myocardial infarction and angiotensin II infusion, liver, and lung fibrosis after Schistosoma mansoni infection and lung fibrosis after bleomycin infusion in mice. Proposed mechanisms are via suppression of specific ECM-degrading MMPs and decreased matrix degradation, induction of alternative-macrophage infiltration and PDGF production, and activation of TGF-β signaling pathway (40–43). Importantly, MMP-12 was increased in the serum, alveolar macrophages and dermal inflammatory infiltrates in SSc patients, and its expression correlated with the severity of skin and lung fibrosis (44). MMP12 was induced in response to Fli1 downregulation in myeloid cells, suggesting that MMP12 may contribute to the profibrotic effects we observed in our system. However, MMP12 expression was not enhanced in mice with deletion of Fli1, suggesting there are differences the response to Fli1 downregulation between mice and human cells.

We also found elevated expression of HMOX1, a stress-inducible protein upregulated by various oxidative and inflammatory signals, with immunomodulatory and anti-inflammatory properties. It has been reported that HMOX-1 induction drives the phenotypic shift to M2 macrophages, however, a previous study found that HMOX-1 was expressed at lower levels in SSc compared to controls.

Interestingly, the top, most highly expressed gene in our microarray was ANKRD1 (ankryn repeat domain 1), a transcription co-regulator expressed predominantly in cardiac muscle, with roles in heart and skin fibrosis (45, 46). Very little is known about the role of ANKRD1 in immune cells. We found that ANKRD1 is expressed in cells with low Fli1, but is found at very low levels or not expressed in primary monocytes. This is consistent with previous studies showing that ANKRD1 was sharply and dramatically induced in immune cells during wound healing, but mostly absent in intact skin (47). Interestingly, the profibrotic molecule TGFβ was also shown to induce ANKRD1 expression in vascular smooth muscle cells (48). Given the importance of ANKRD1 in regulating fibrosis and cardiac hypertrophy, further studies are required to assess if its induction in response to low Fli1 in Mo/Mø contributes to their fibrogenic effects.

A number of studies looking at transcriptional profiling of peripheral blood cells from SSc patients revealed significantly increased expression of both type I and type II IFN-inducible genes (49, 50). High levels of both IFN-regulated genes and alternative Mo/Mø activation markers were shown in SSc PBMCs and fibrotic lung tissue (51, 52). It has been suggested that IFN-γ may play a role in the early stages of SSc, in which inflammation and vasculopathy are predominant features (53, 54). Our microarray data shows that downregulation of Fli1 in myeloid cells resulted in induction of numerous interferon-regulated genes, with both type I and type II interferon responses being recorded. Significantly, mice with targeted deletion of Fli1 in myeloid cells showed similar response in the interferon related genes CCL2, CCL7, and CXCL10, confirming microarray data.

Beyond regulating the expression of alternative activation markers and other profibrotic genes, we recorded Fli1 regulation of migration-associated genes, which may control the migratory properties of monocytes *in vivo*, allowing infiltration of tissues and thus potentially contributing to the inflammatory infiltrates seen in the lesional skin in SSc and to exaggerated tissue fibrosis.

Our study has several limitations. Firstly, THP1 cells were used for the experiments involving the effects of Fli1 downregulation in human myeloid cells. While widely used in published studies, these are immortalized cells and may not accurately reflect what occurs in primary cell lines. Nevertheless, we were able to reproduce our results in macrophages from mice with conditional deletion of Fli1, suggesting that findings in human cells could be accurate. Moving forward, it will be of interest to characterize the *in vivo* consequences in loss of Fli1 in myeloid cells, however, this was beyond the scope of this paper. Secondly, while we provide solid evidence that the loss of Fli1 in myeloid cells is profibrotic, we failed to unravel the exact mechanism that leads to this outcome. Our results however, suggest that a number of genes could contribute to this effect, including CCL2, CCL7, MMP12, CXCL10, and ANKRD1.

In summary, myeloid cells with low Fli1 reproduce key features with myeloid cells from SSc patients, with higher expression of profibrotic markers and activation of interferon responsive genes, thus suggesting that Fli1-deficient Mø may contribute to SSc fibrosis. Further work will be required to establish the exact mechanistic details of these findings, but microarray analyses suggests that a combination of factors could be responsible for the modulation of the observed profibrotic effects of myeloid cells with low Fli1 on fibroblasts.

## Data Availability Statement

The datasets generated for this study can be found in the NCBI Gene Expression Omnibus (GEO: Series GSE144625) database.

## Ethics Statement

The studies involving human participants were reviewed and approved by the Boston University, IRB. The patients/participants provided their written informed consent to participate in this study. The animal study was reviewed and approved by Boston University Institutional Animal Care & Use (IACUC).

## Author Contributions

AB contributed to the study design, data curation, statistical analysis and interpretation of the data, and drafted and revised the manuscript. FE performed the staining, coculture, Q-PCR experiments, and participated in the analysis interpretation and revised the manuscript. AP performed Q-PCR experiments in THP1 cells and revised the manuscript. GM participated breeding and genotyping of the mice, and Q-PCR analyses, and in the analysis interpretation, and critically revised the manuscript. MT contributed to the study conceptions and design and revised the manuscript. All authors read and approved the final manuscript.

## Conflict of Interest

The authors declare that the research was conducted in the absence of any commercial or financial relationships that could be construed as a potential conflict of interest.
