# Supplementary material for: Fli1 Downregulation in Scleroderma Myeloid Cells Has Profibrotic and Proinflammatory Effects
Source: Front Immunol. 2020 May 19;11:800. doi: 10.3389/fimmu.2020.00800 (PMC7248379; doi:10.3389/fimmu.2020.00800)
Supplement: Supplementary file 2 [file Table_1.DOCX]

| **Gene** | **Species** | Forward | Reverse |
| --- | --- | --- | --- |
| Beta actin | human | aggatggcaagggacttcctgtaa | aatgtcgcggaggacctttgattgc |
| Fli1 | human | aaccgggtcaatgtgtggaa | caccgacagagcctccttaat |
| ANKRD1 | human | gcgcccgagataagttgct | cctcacaggcgataagatgct |
| MSR1 | human | tgcaaacctgtgcattgatgag | aggaaatgacacattcctgcg |
| Hmox1 | human | aaacttcagagggggcgaag | gctgccacattagggtgtct |
| CXCL10 | human | gaaagcagttagcaaggaaagg | gacatataactccatgtagggaagtg |
| MMP12 | human | ccactgcttctggagctctt | gcgtagtcaacatcctcacg |
| CCL7 | human | gaaagcctctgcagcacttc | aatctgtagcagcaggtagttgaa |
| CCL8 | human | gaagtttttgaagagggtgaga | tgcttgaagtttcactggcatc |
| COL1A1 | human | ccagaagaactggtacatcagca | cgccatactcgaactggaat |
| MRC1 | human | atgagaaccgggattgcagg | cccagtgcttgcacacaaat |
| Periostin | human | aagactgcttcagggagacac | cactgagaacgaccttccctta |
| CCL2 | human | aggagagcagagagtggaaatg | ccctgtacatctgctcctgttt |
| CD163 | human | gggttgttctgttggccattt | tcctcttgaggaaactgcaagc |
|  |  |  |  |
|  |  |  |  |
| Beta actin | mouse | aaggccaaccgtgaaaagat | gtggtacgaccagaggcatac |
| MMP12 | mouse | ttgtggataaacactactggaggt | aaatcagcttggggtaagca |
| Fli1 | mouse | ctctatagtcgcagtaggcg | ccagtggtcaacacttaccca |
| CD163 | mouse | tctcagtgcctctgctgtca | cgccagtctcagttccttct |
| CCL7 | mouse | ttctgtgcctgctgctcata | ttgacatagcagcatgtggat |
| CCL2 | mouse | catccacgtgttggctca | gatcatcttgctggtgaatgagt |
| FIZZ1 | mouse | ccctccactgtaacgaagactc | cacacccagtagcagtcatcc |
| Hmox1 | mouse | aggctaagaccgccttcct | tgtgttcctctgtcagcatca |
| CXCL10 | mouse | gctgccgtcattttctgc | tctcactggcccgtcatc |
|  |  |  |  |

Table 1. Human and mouse primer sequences used in Q-RT-PCR.
