# Supplementary material for: Fli1 Downregulation in Scleroderma Myeloid Cells Has Profibrotic and Proinflammatory Effects
Source: Front Immunol. 2020 May 19;11:800. doi: 10.3389/fimmu.2020.00800 (PMC7248379; doi:10.3389/fimmu.2020.00800)
Supplement: Supplementary file 1 [file Data_Sheet_1.PDF]

HALLMARK_INTERFERON_GAMMA_RESPONSE
NES = 3.12, p = 0.0000, FDR q = 0.0000

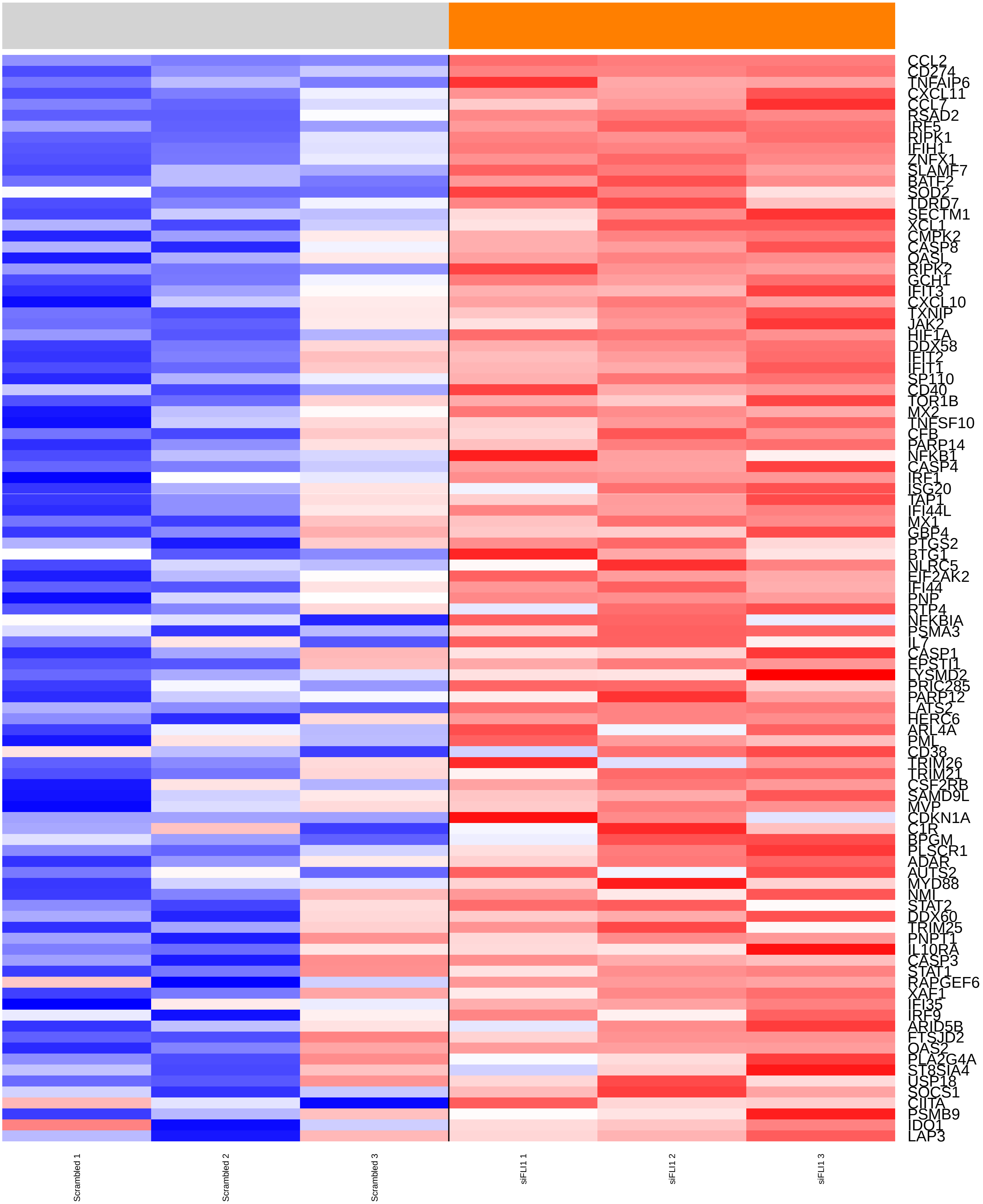

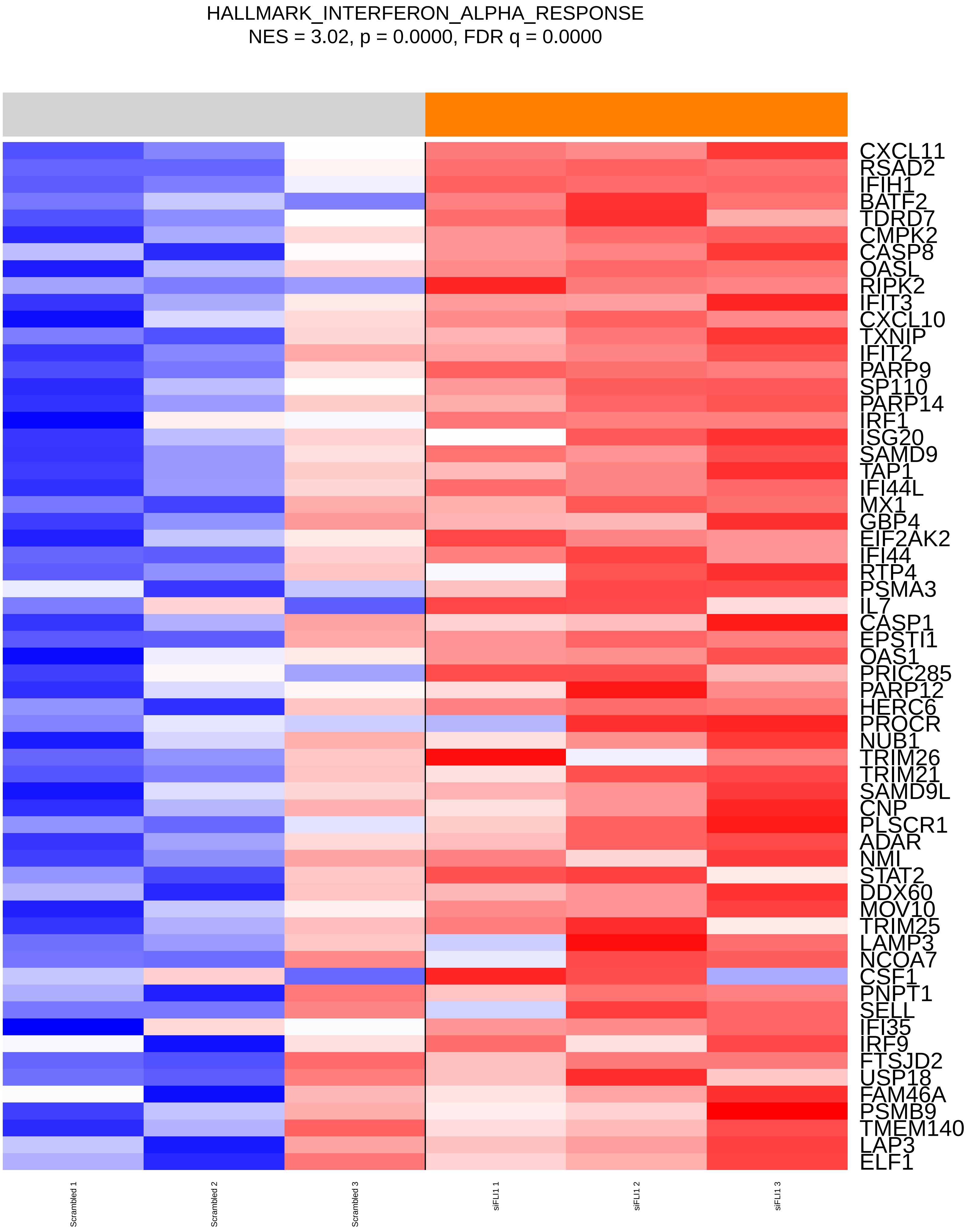
HALLMARK_INTERFERON_ALPHA_RESPONSE
NES = 3.02, p = 0.0000, FDR q = 0.0000
CXCL11
RSAD2
IFIH1
BATF2
TDRD7
CMPK2
CASP8
OASL
RIPK2
IFIT3
CXCL10
TXNIP
IFIT2
PARP9
SP110
PARP14
IRF1
ISG20
SAMD9
TAP1
IFI44L
MX1
GBP4
EIF2AK2
IFI44
RTP4
PSMA3
IL7
CASP1
EPSTI1
OAS1
PRIC285
PARP12
HERC6
PROCR
NUB1
TRIM26
TRIM21
SAMD9L
CNP
PLSCR1
ADAR
NMI
STAT2
DDX60
MOV10
TRIM25
LAMP3
NCOA7
CSF1
PNPT1
SELL
IFI35
IRF9
FTSJD2
USP18
FAM46A
PSMB9
TMEM140
LAP3
ELF1
Scrambled 1
Scrambled 2
Scrambled 3
siFLI1 1
siFLI1 2
siFLI1 3

HALLMARK_TNFA_SIGNALING_VIA_NFKB
NES = 2.69, p = 0.0000, FDR q = 0.0000

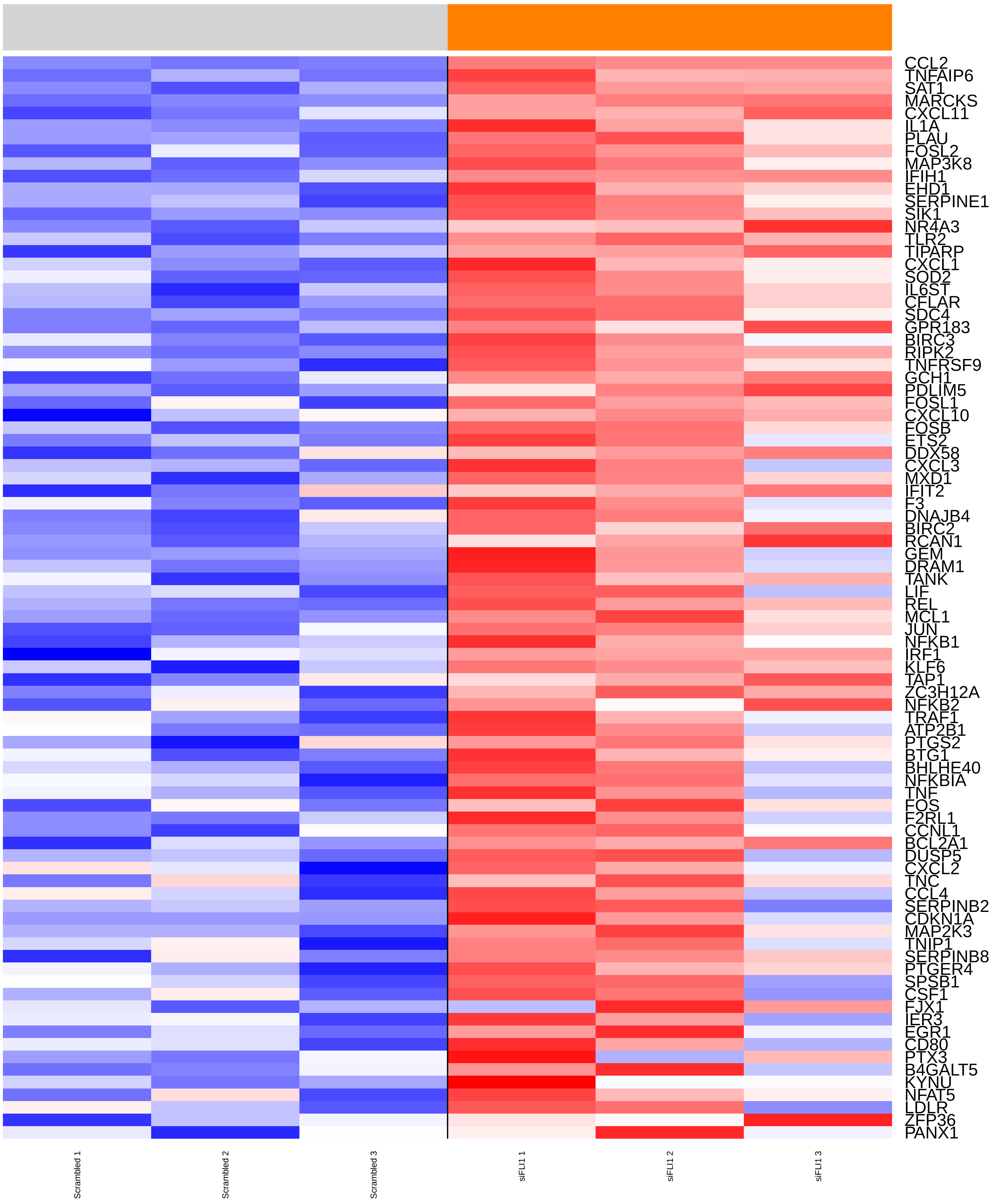

GO_POSITIVE_REGULATION_OF_LEUKOCYTE_MIGRATION
NES = 2.46, p = 0.0000, FDR q = 0.0003

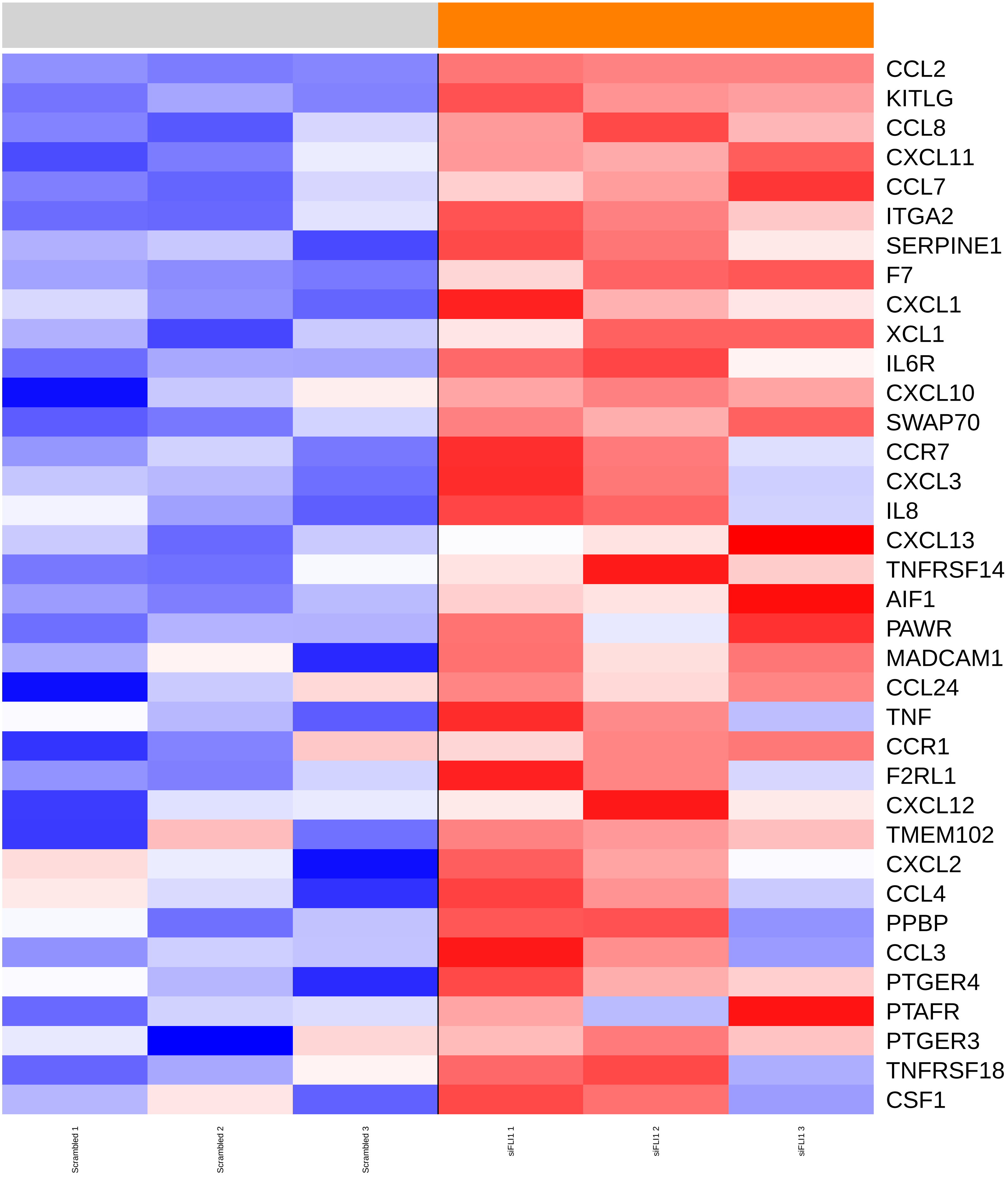

GO_REGULATION_OF_LEUKOCYTE_MIGRATION
NES = 2.40, p = 0.0000, FDR q = 0.0002

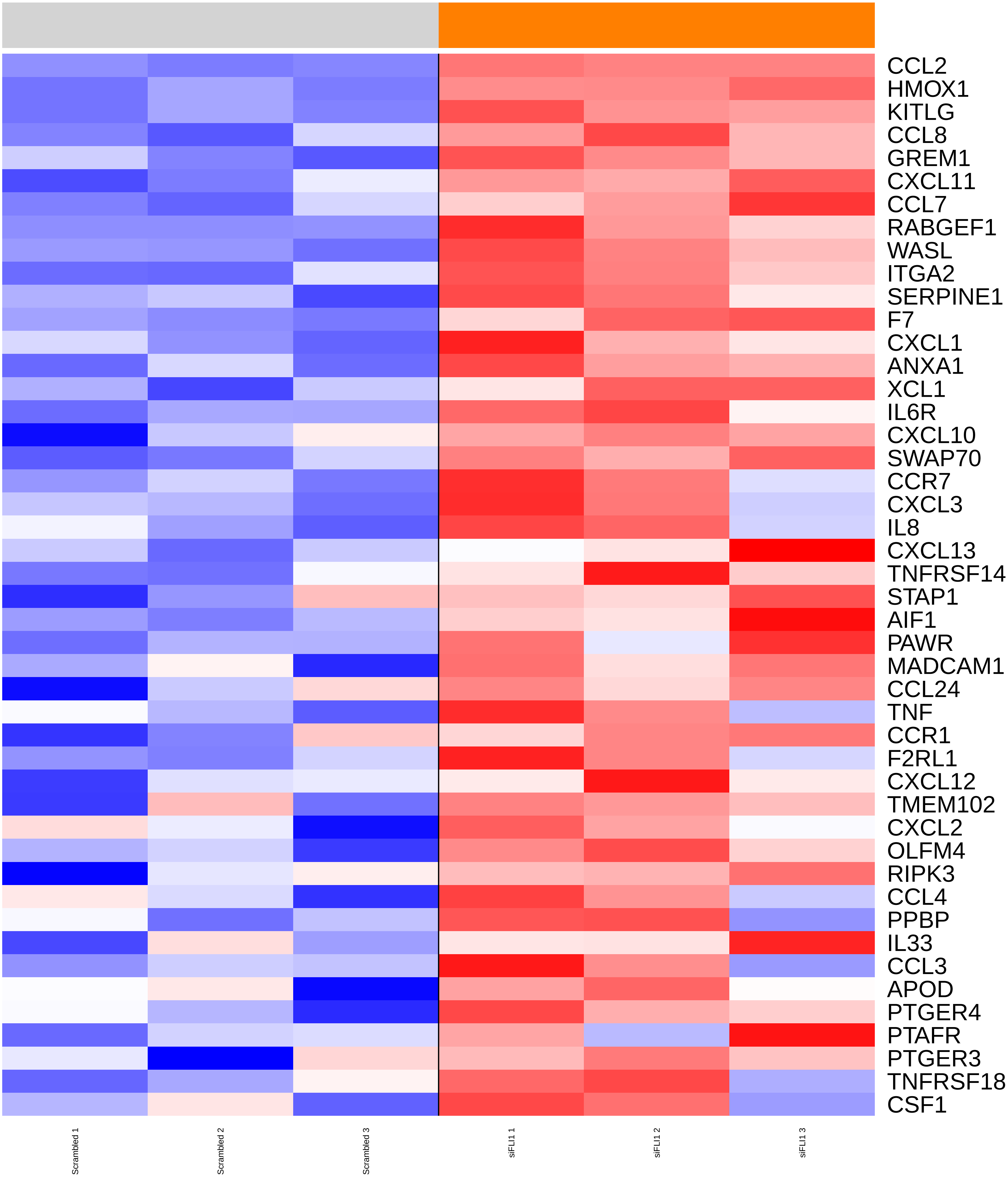

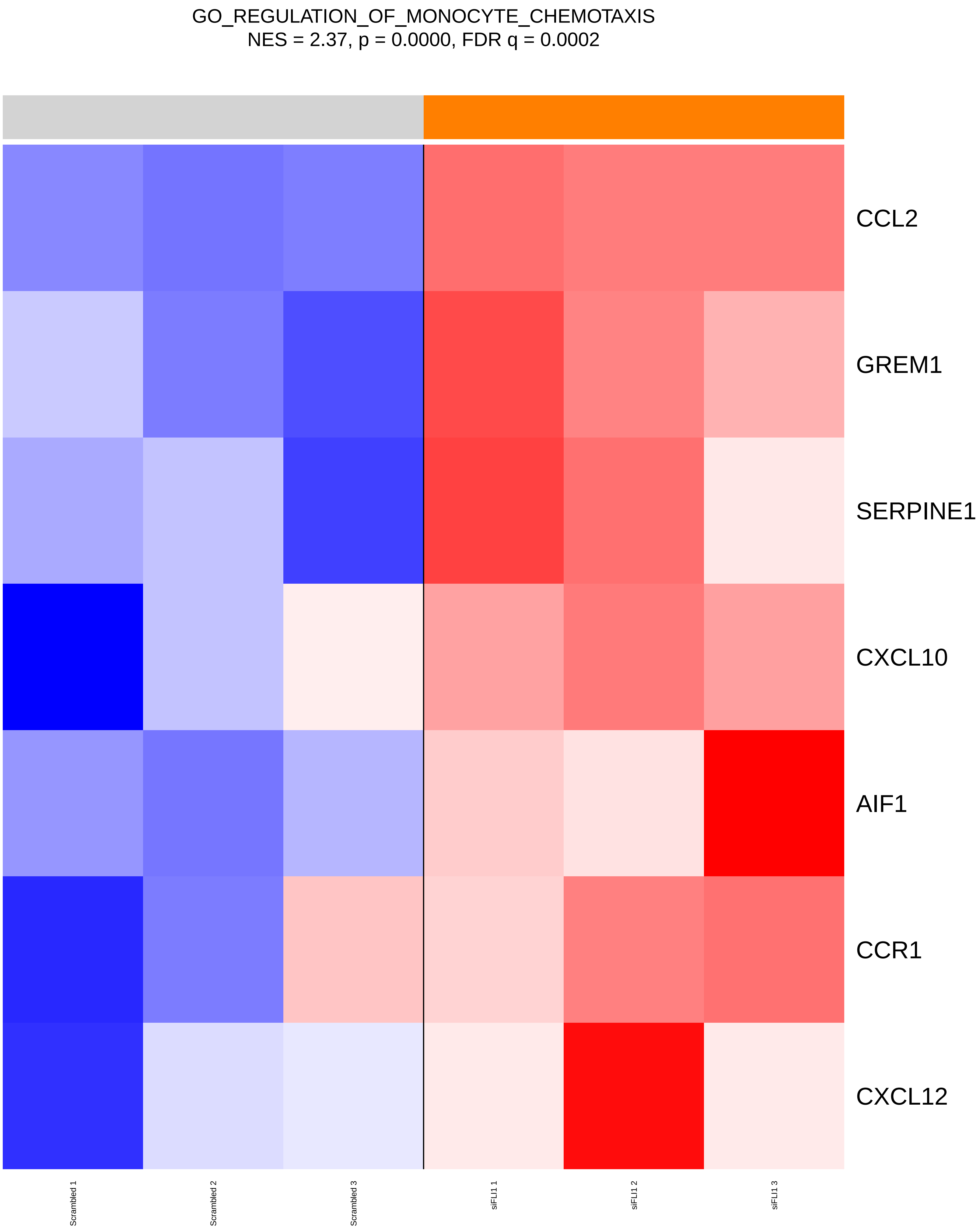
GO_REGULATION_OF_MONOCYTE_CHEMOTAXIS
NES = 2.37, p = 0.0000, FDR q = 0.0002
CCL2
GREM1
SERPINE1
CXCL10
AIF1
CCR1
CXCL12
Scrambled 1
Scrambled 2
Scrambled 3
siFLI1 1
siFLI1 2
siFLI1 3

KEGG_NOD_LIKE_RECEPTOR_SIGNALING_PATHWAY
NES = 2.34, p = 0.0000, FDR q = 0.0002

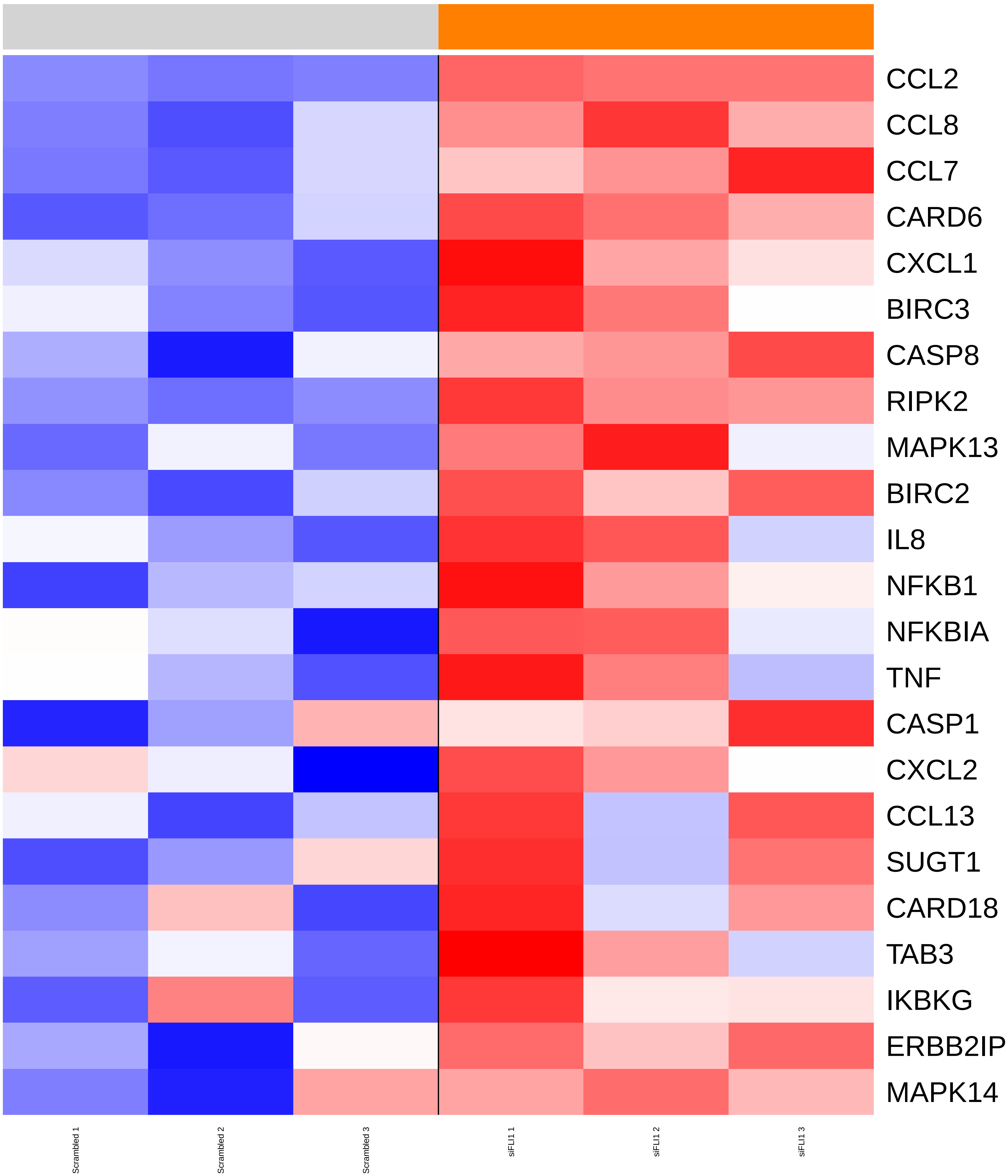

GO_CELLULAR_RESPONSE_TO_INTERLEUKIN_6
NES = 2.31, p = 0.0000, FDR q = 0.0009

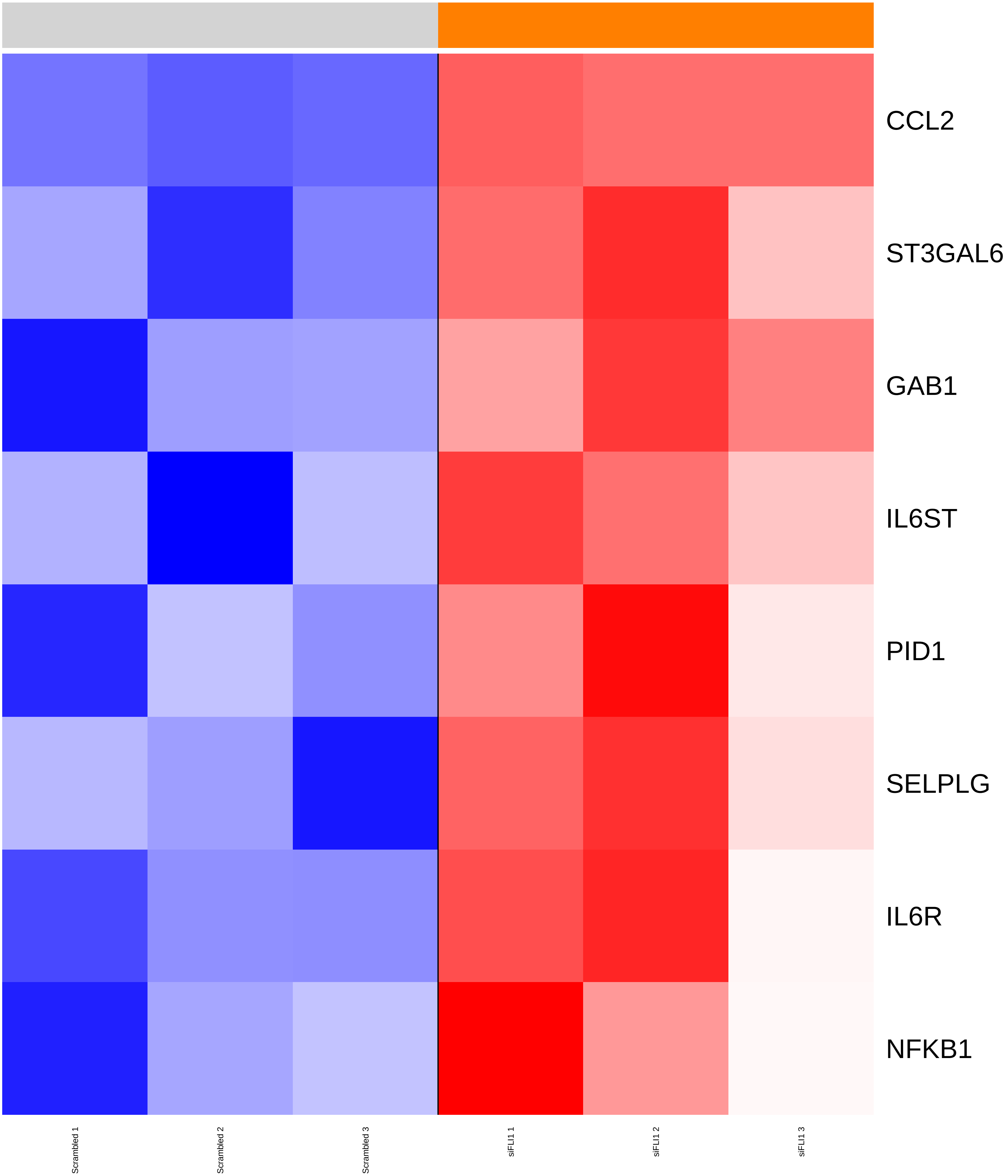

GO_RESPONSE_TO_INTERLEUKIN_6
NES = 2.25, p = 0.0000, FDR q = 0.0032

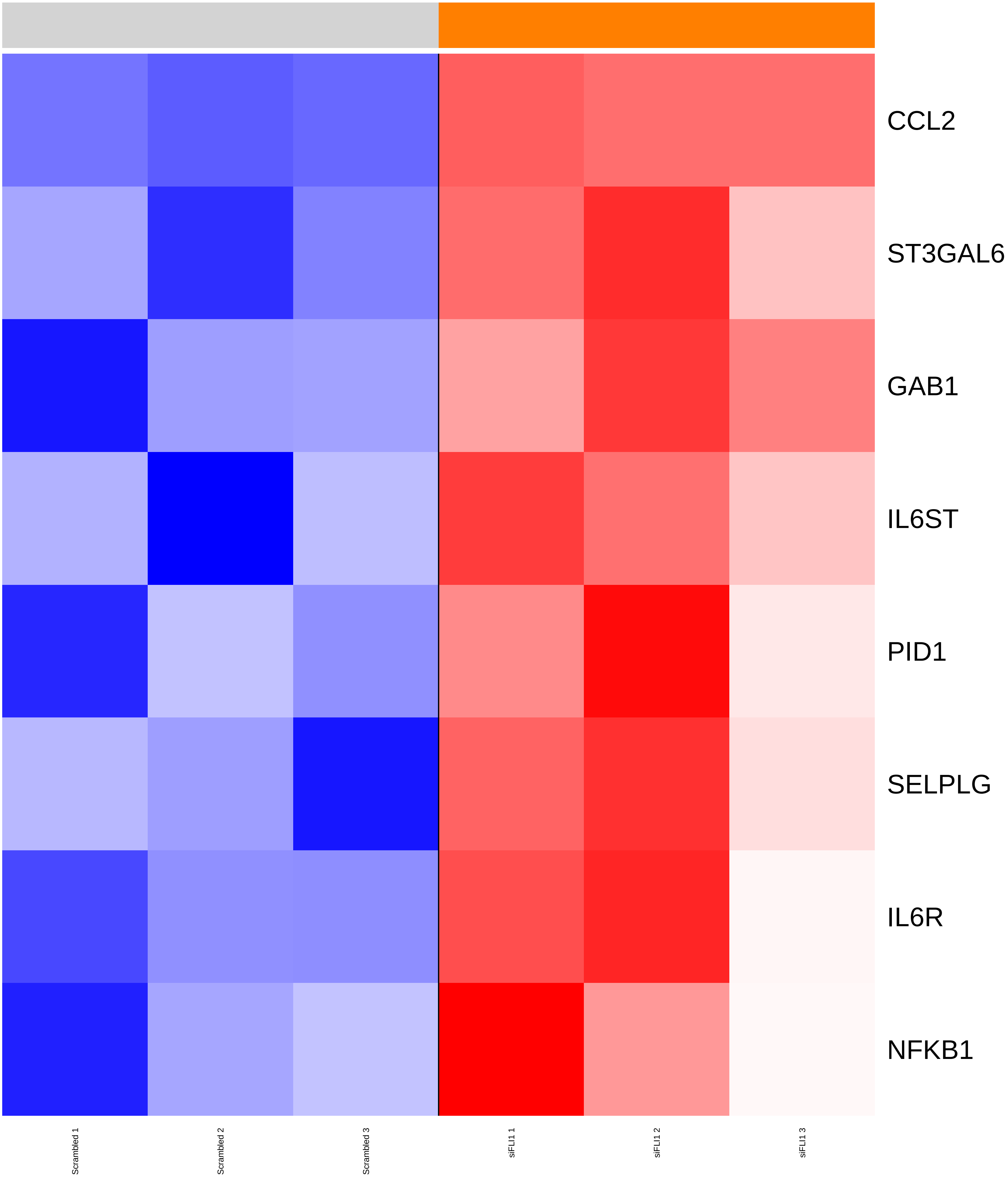

GO_POSITIVE_REGULATION_OF_LEUKOCYTE_CHEMOTAXIS
NES = 2.23, p = 0.0000, FDR q = 0.0042

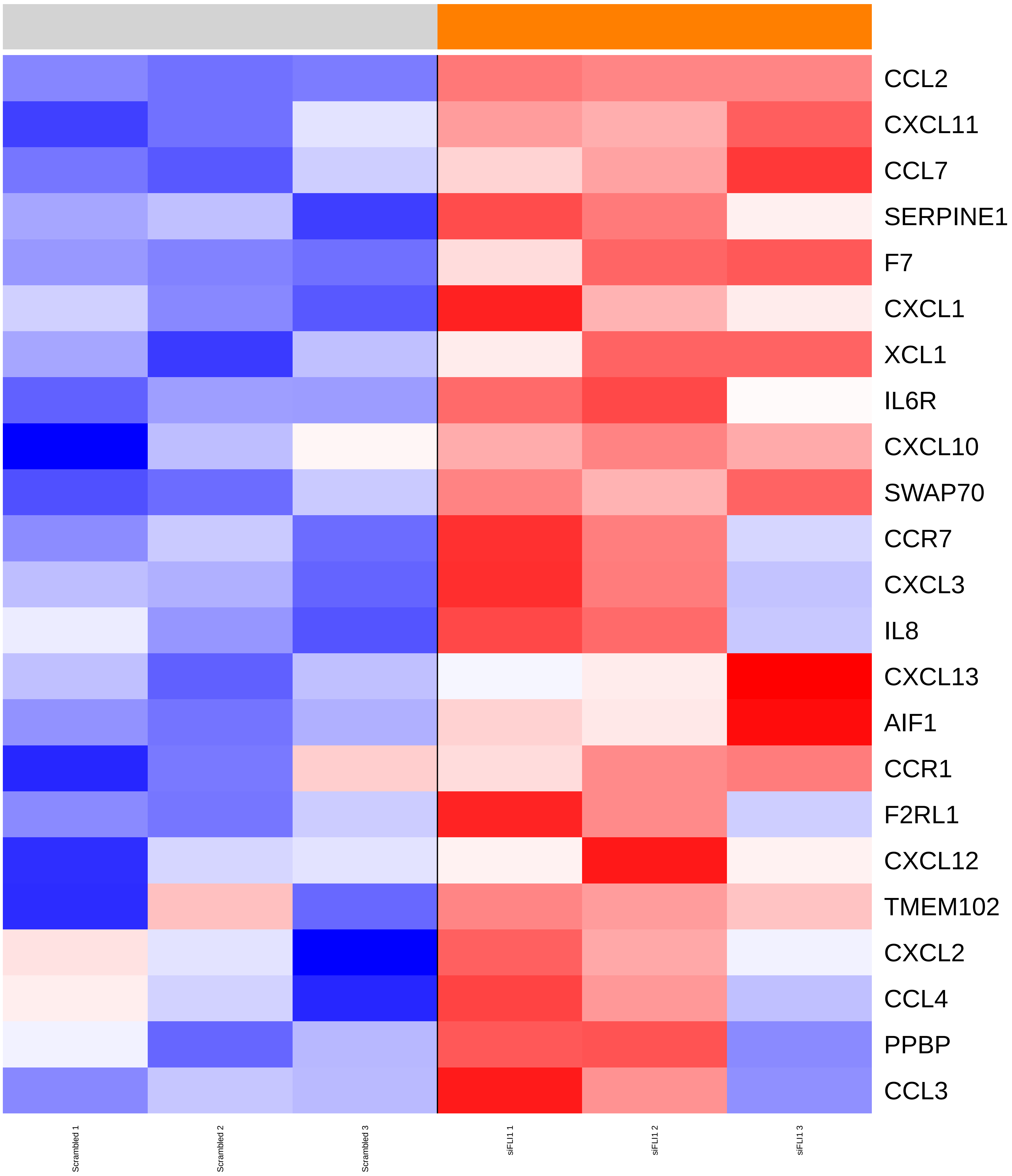

GO_STAT_CASCADE
NES = 2.21, p = 0.0000, FDR q = 0.0046

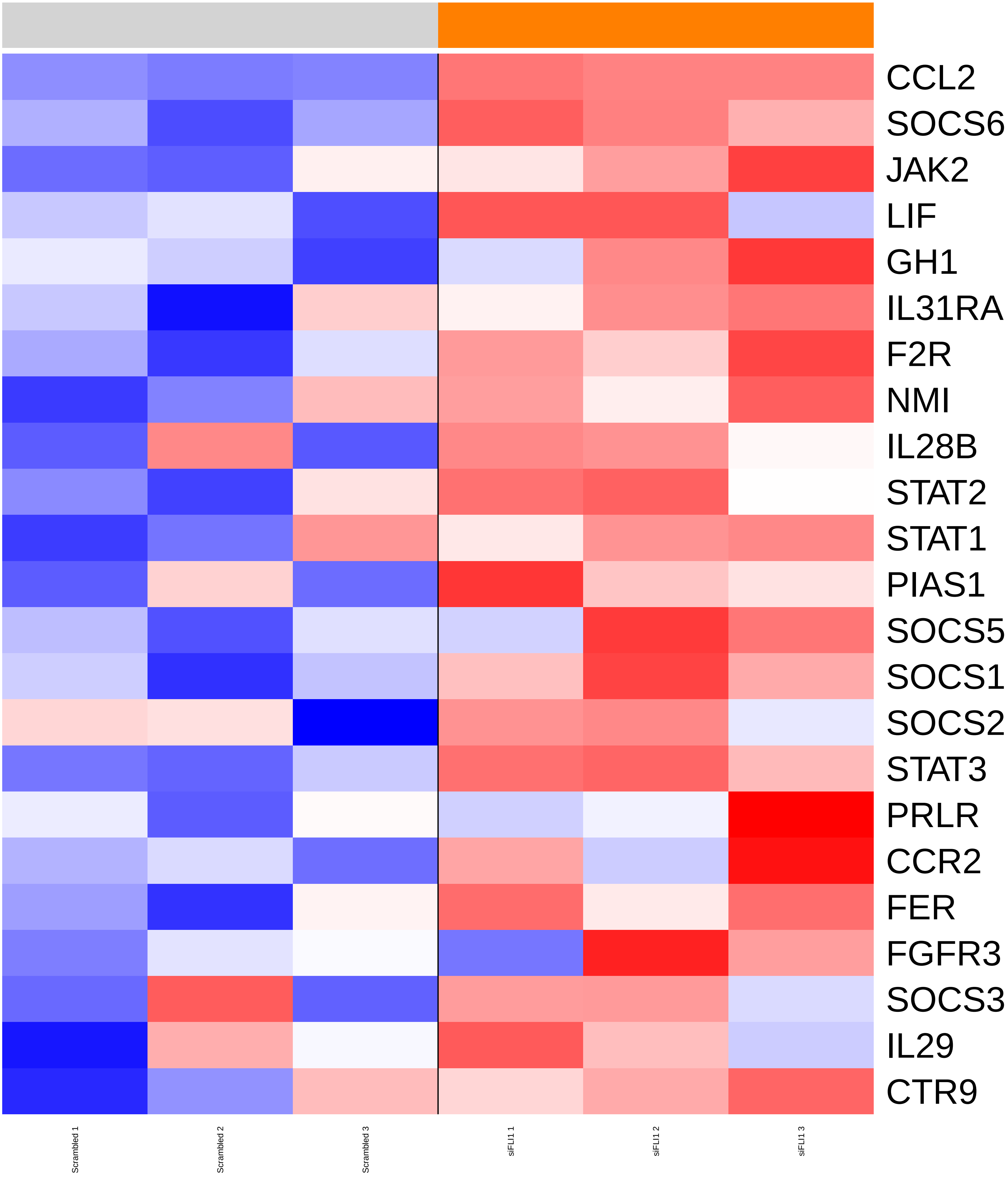

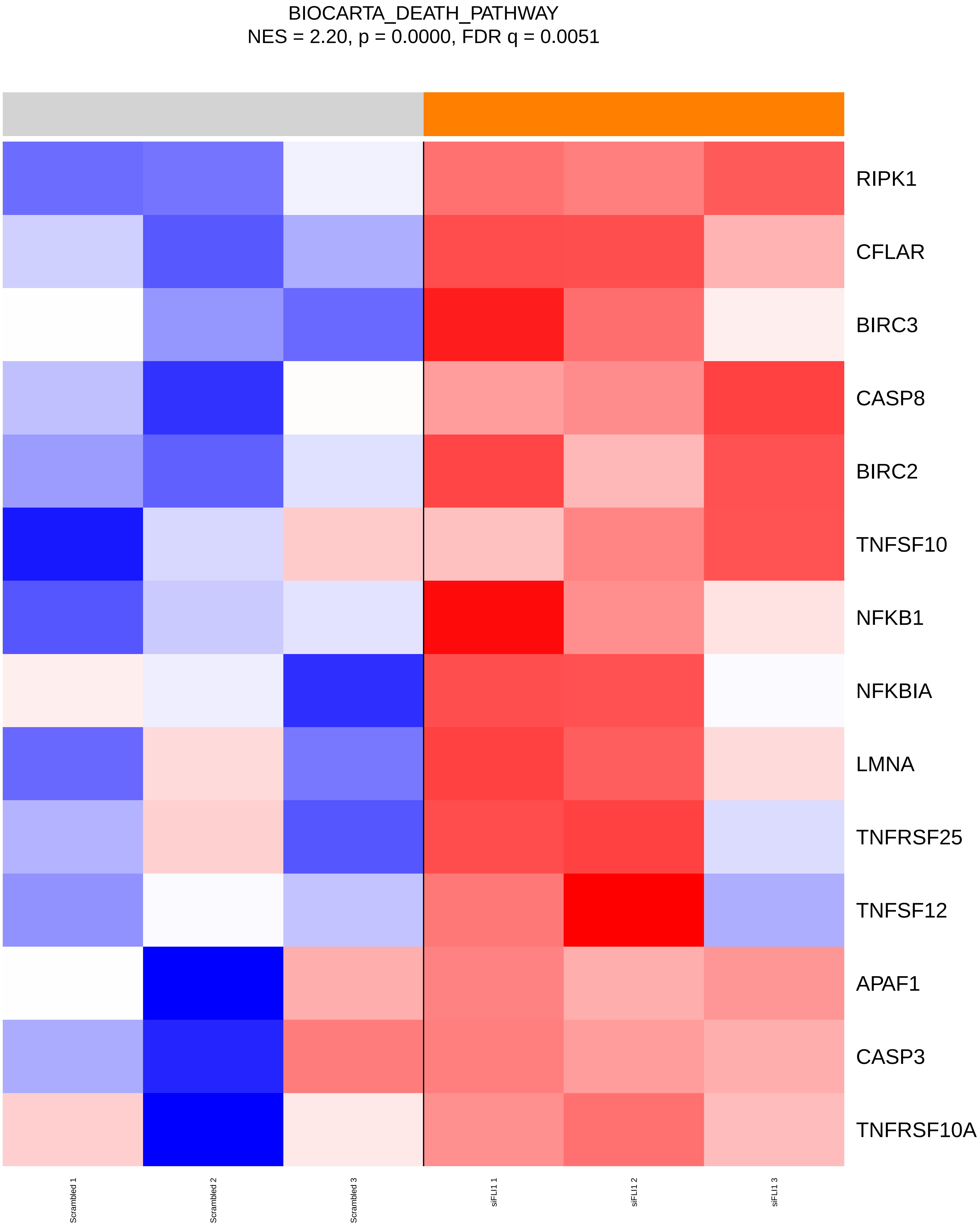

BIOCARTA_DEATH_PATHWAY
NES = 2.20, p = 0.0000, FDR q = 0.0051
RIPK1
CFLAR
BIRC3
CASP8
BIRC2
TNFSF10
NFKB1
NFKBIA
LMNA
TNFRSF25
TNFSF12
APAF1
CASP3
TNFRSF10A
Scrambled 1
Scrambled 2
Scrambled 3
siFLI1 1
siFLI1 2
siFLI1 3

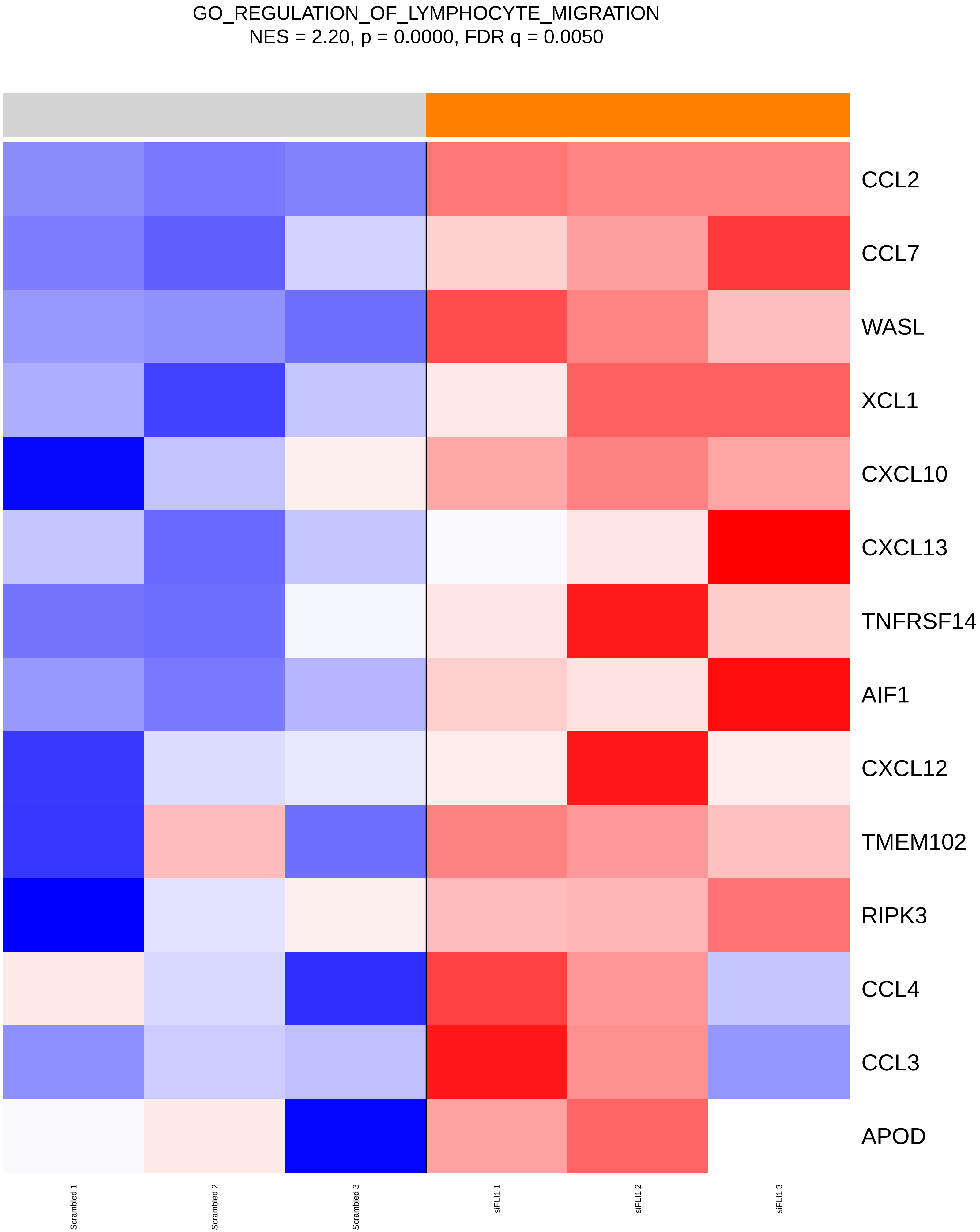
GO_REGULATION_OF_LYMPHOCYTE_MIGRATION
NES = 2.20, p = 0.0000, FDR q = 0.0050
CCL2
CCL7
WASL
XCL1
CXCL10
CXCL13
TNFRSF14
AIF1
CXCL12
TMEM102
RIPK3
CCL4
CCL3
APOD
Scrambled 1
Scrambled 2
Scrambled 3
siFLI1 1
siFLI1 2
siFLI1 3

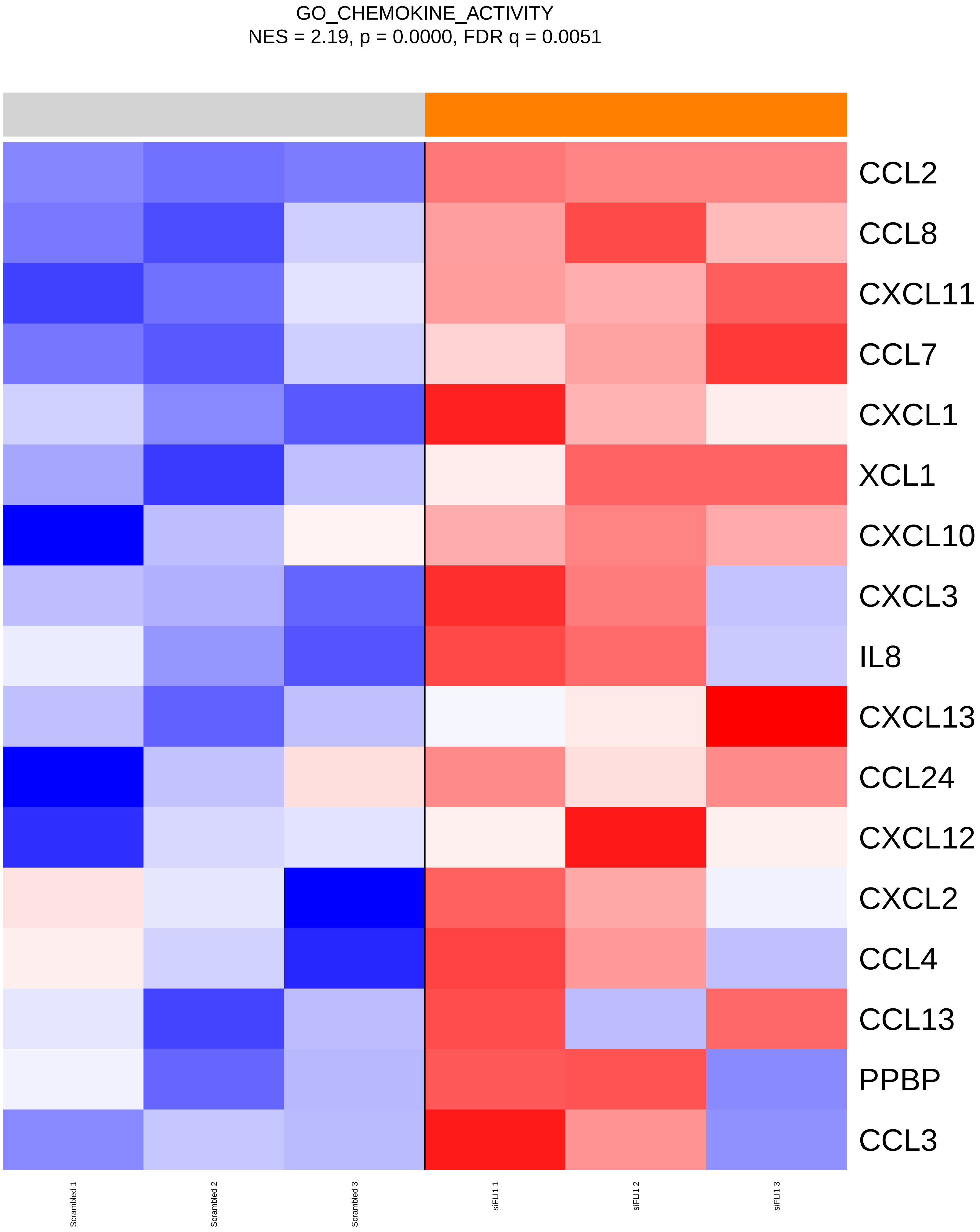
GO_CHEMOKINE_ACTIVITY
NES = 2.19, p = 0.0000, FDR q = 0.0051
CCL2
CCL8
CXCL11
CCL7
CXCL1
XCL1
CXCL10
CXCL3
IL8
CXCL13
CCL24
CXCL12
CXCL2
CCL4
CCL13
PPBP
CCL3
Scrambled 1
Scrambled 2
Scrambled 3
siFLI1 1
siFLI1 2
siFLI1 3

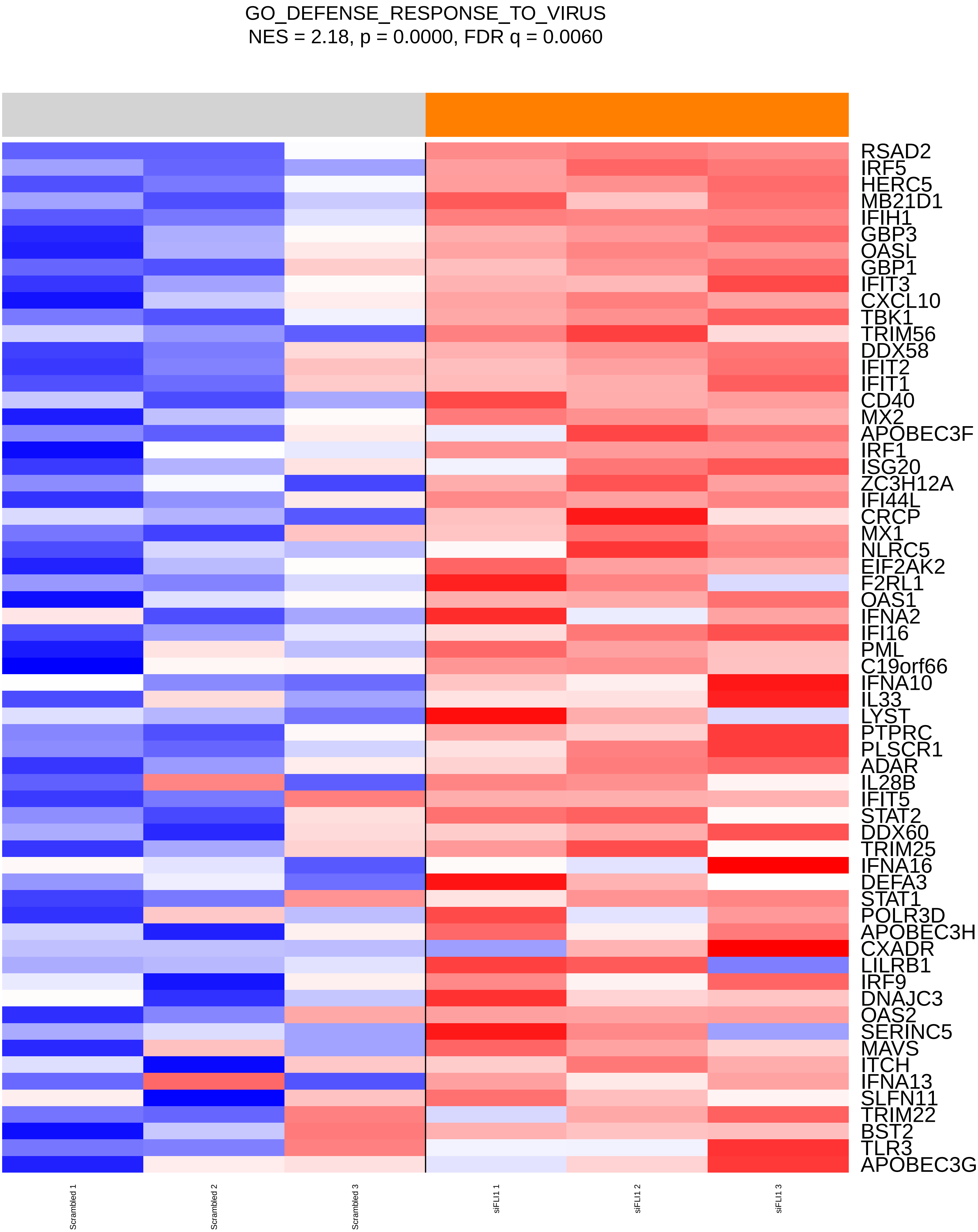
GO_DEFENSE_RESPONSE_TO_VIRUS
NES = 2.18, p = 0.0000, FDR q = 0.0060
RSAD2
IRF5
HERC5
MB21D1
IFIH1
GBP3
OASL
GBP1
IFIT3
CXCL10
TBK1
TRIM56
DDX58
IFIT2
IFIT1
CD40
MX2
APOBEC3F
IRF1
ISG20
ZC3H12A
IFI44L
CRCP
MX1
NLRC5
EIF2AK2
F2RL1
OAS1
IFNA2
IFI16
PML
C19orf66
IFNA10
IL33
LYST
PTPRC
PLSCR1
ADAR
IL28B
IFIT5
STAT2
DDX60
TRIM25
IFNA16
DEFA3
STAT1
POLR3D
APOBEC3H
CXADR
LILRB1
IRF9
DNAJC3
OAS2
SERINC5
MAVS
ITCH
IFNA13
SLFN11
TRIM22
BST2
TLR3
APOBEC3G
Scrambled 1
Scrambled 2
Scrambled 3
siFLI1 1
siFLI1 2
siFLI1 3

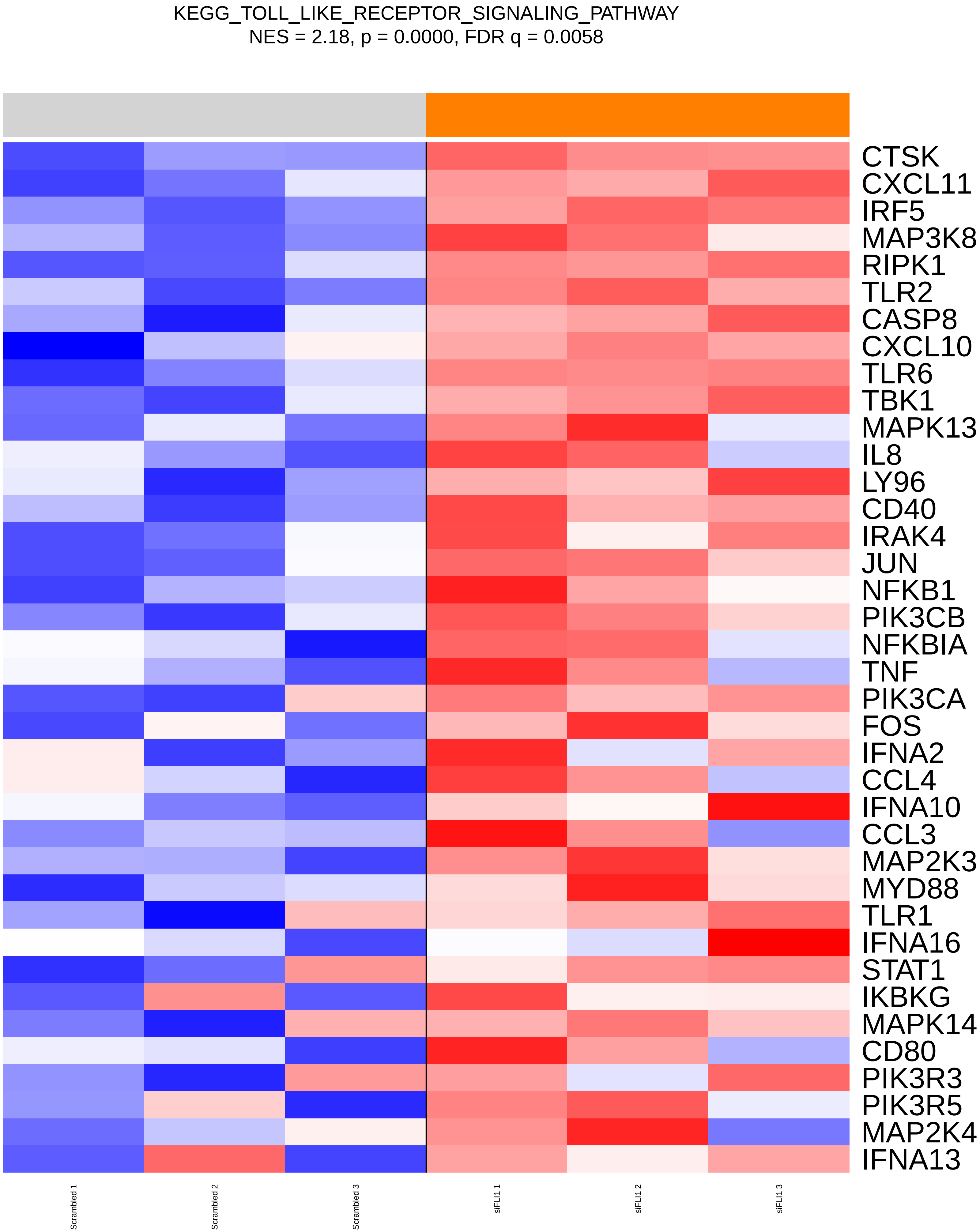
KEGG_TOLL_LIKE_RECEPTOR_SIGNALING_PATHWAY
NES = 2.18, p = 0.0000, FDR q = 0.0058
CTSK
CXCL11
IRF5
MAP3K8
RIPK1
TLR2
CASP8
CXCL10
TLR6
TBK1
MAPK13
IL8
LY96
CD40
IRAK4
JUN
NFKB1
PIK3CB
NFKBIA
TNF
PIK3CA
FOS
IFNA2
CCL4
IFNA10
CCL3
MAP2K3
MYD88
TLR1
IFNA16
STAT1
IKBKG
MAPK14
CD80
PIK3R3
PIK3R5
MAP2K4
IFNA13
Scrambled 1
Scrambled 2
Scrambled 3
siFLI1 1
siFLI1 2
siFLI1 3

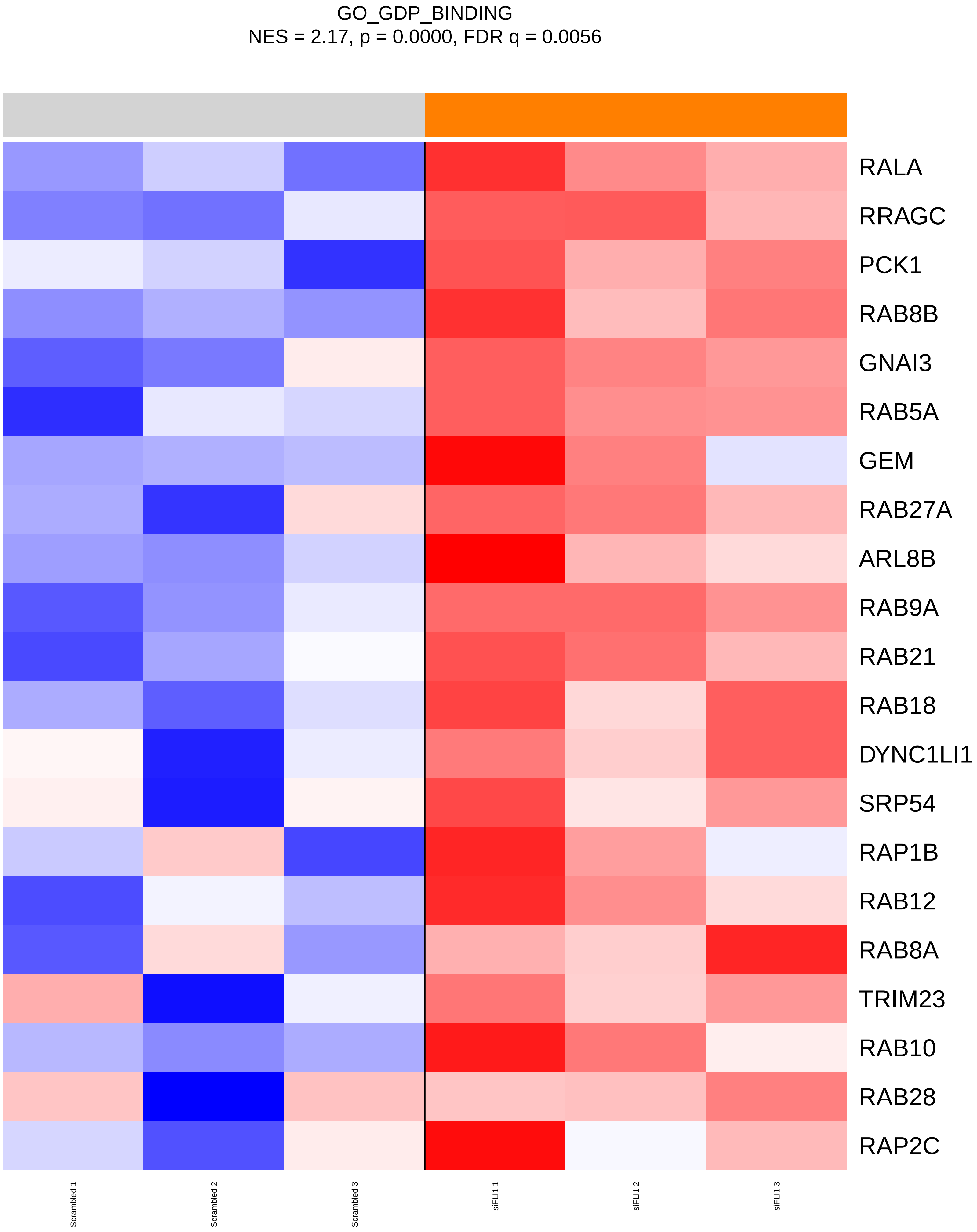
GO_GDP_BINDING
NES = 2.17, p = 0.0000, FDR q = 0.0056
RALA
RRAGC
PCK1
RAB8B
GNAI3
RAB5A
GEM
RAB27A
ARL8B
RAB9A
RAB21
RAB18
DYNC1LI1
SRP54
RAP1B
RAB12
RAB8A
TRIM23
RAB10
RAB28
RAP2C
Scrambled 1
Scrambled 2
Scrambled 3
siFLI1 1
siFLI1 2
siFLI1 3

REACTOME_RIG_I_MDA5_MEDIATED_INDUCTION_OF_IFN_ALPHA_BETA_PATHWAYS
NES = 2.16, p = 0.0000, FDR q = 0.0064

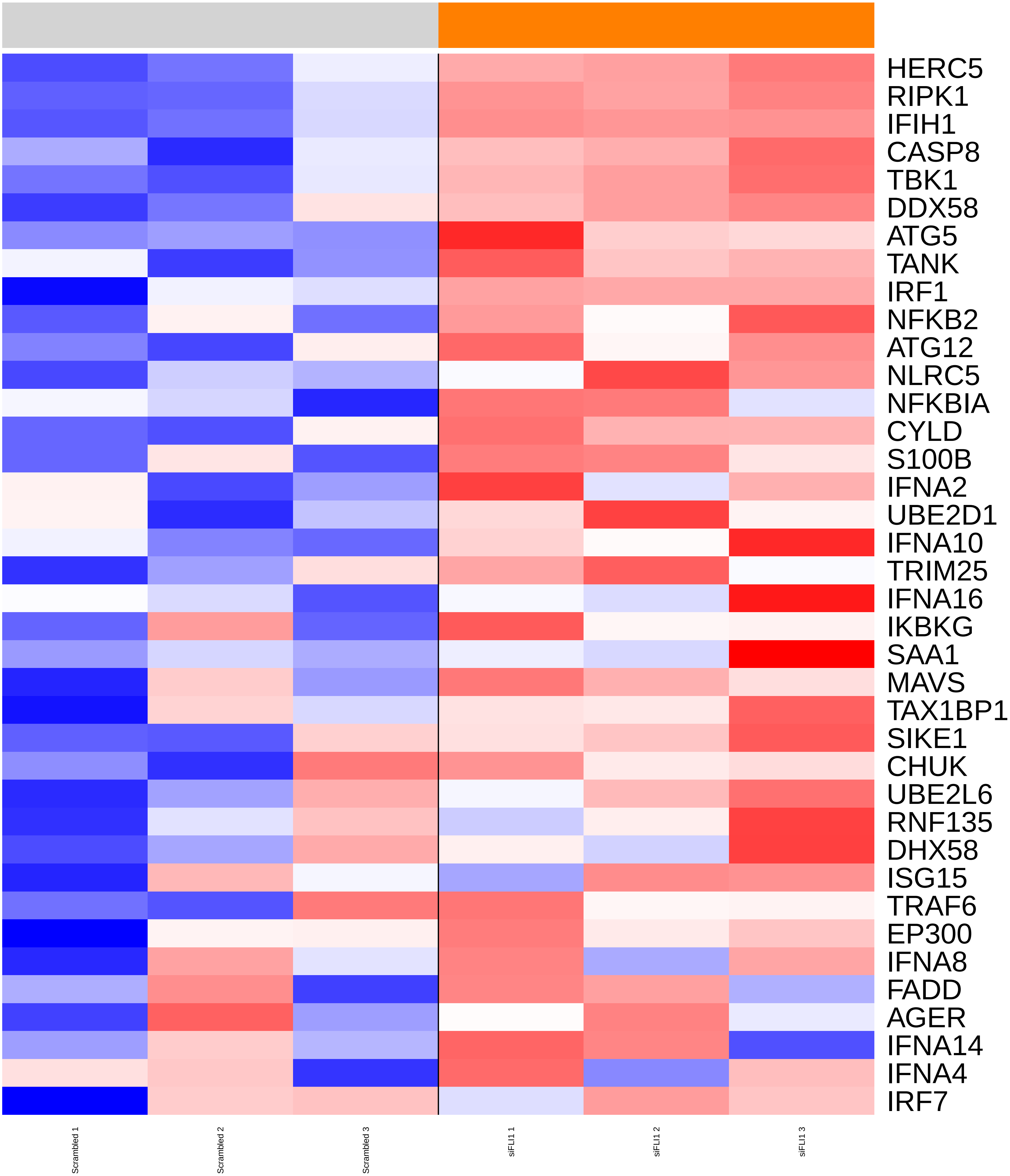

REACTOME_CHEMOKINE_RECEPTORS_BIND_CHEMOKINES
NES = 2.16, p = 0.0000, FDR q = 0.0062

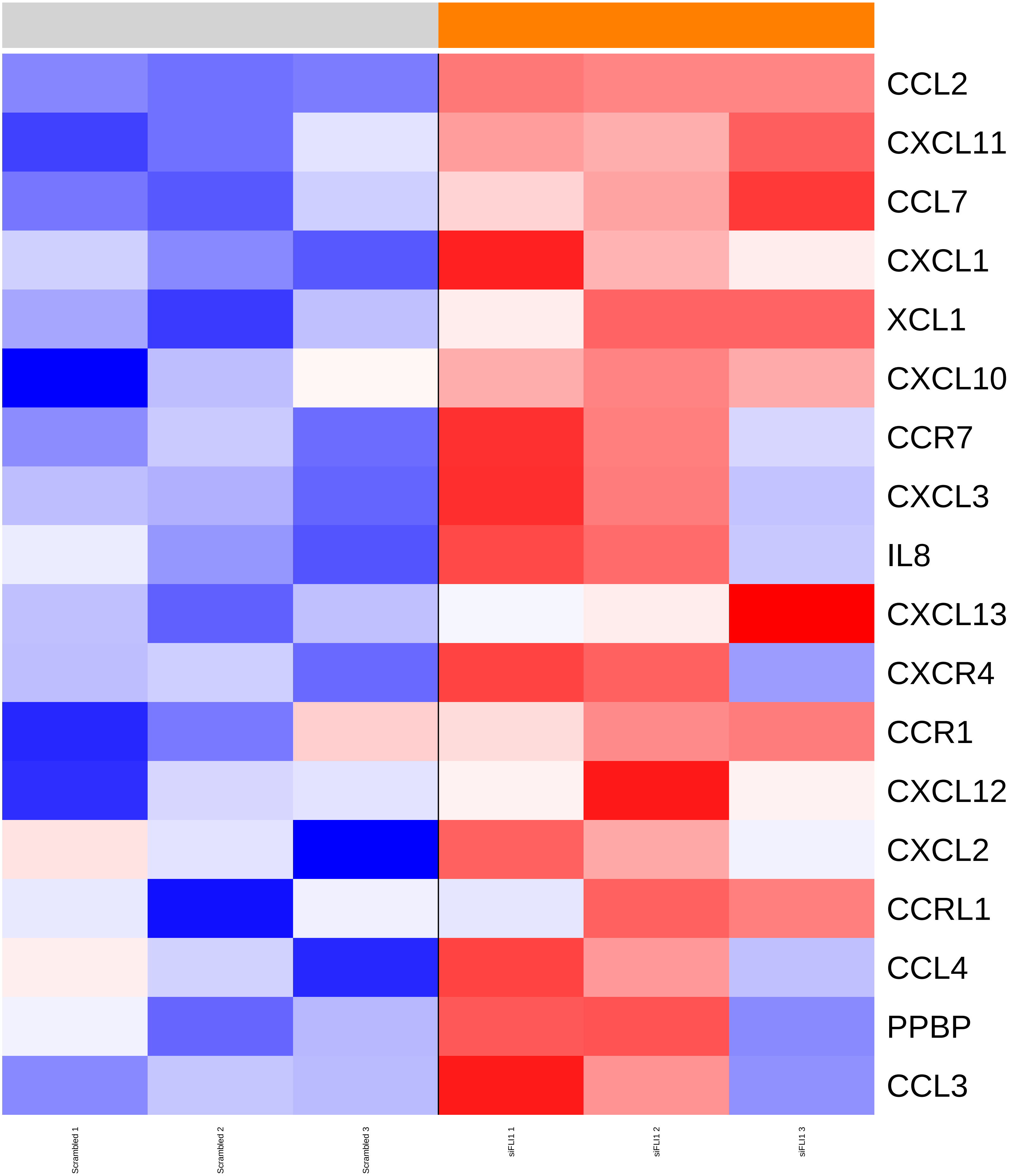

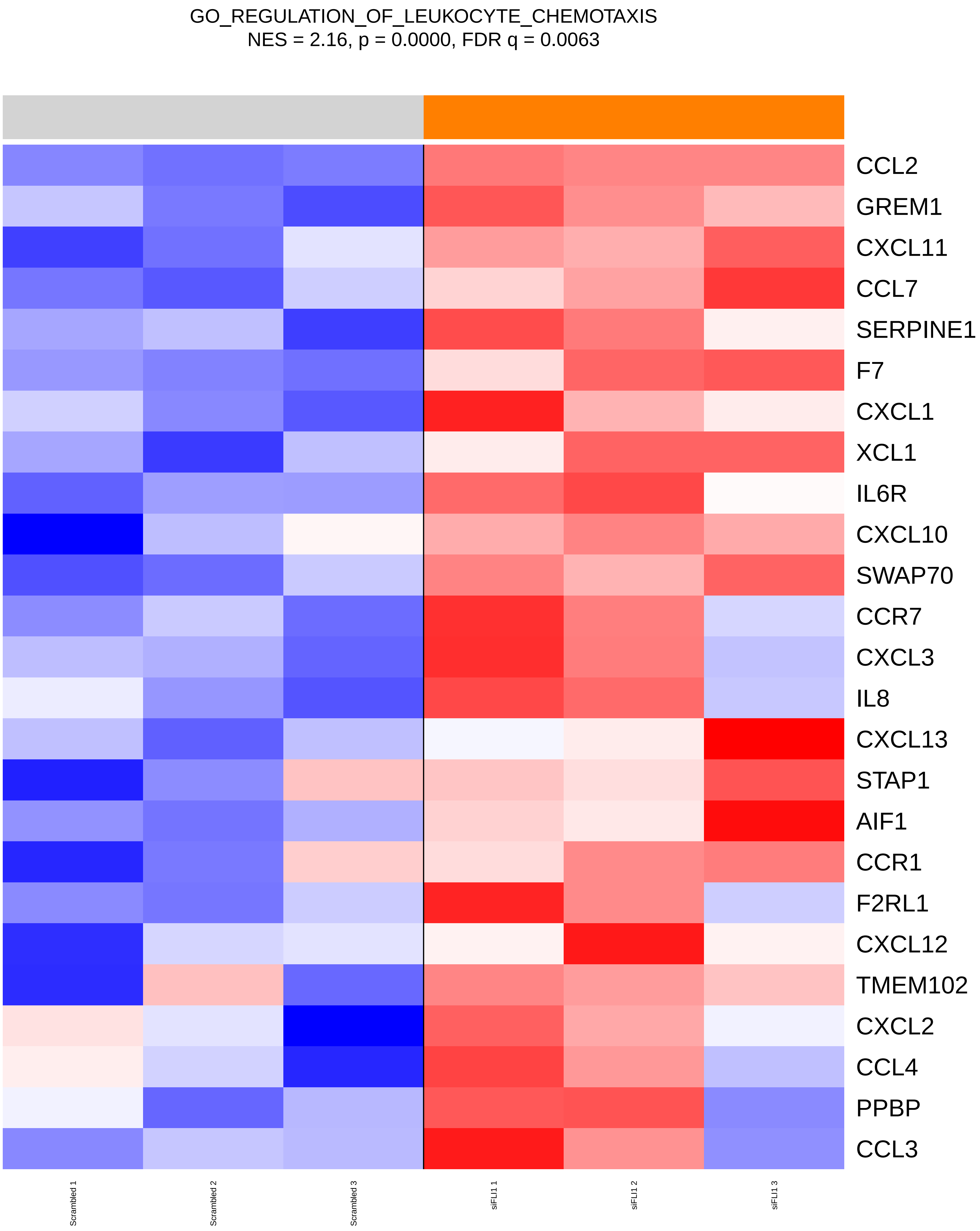
GO_REGULATION_OF_LEUKOCYTE_CHEMOTAXIS
NES = 2.16, p = 0.0000, FDR q = 0.0063
CCL2
GREM1
CXCL11
CCL7
SERPINE1
F7
CXCL1
XCL1
IL6R
CXCL10
SWAP70
CCR7
CXCL3
IL8
CXCL13
STAP1
AIF1
CCR1
F2RL1
CXCL12
TMEM102
CXCL2
CCL4
PPBP
CCL3
Scrambled 1
Scrambled 2
Scrambled 3
siFLI1 1
siFLI1 2
siFLI1 3

GO_POSITIVE_REGULATION_OF_SMOOTH_MUSCLE_CELL_MIGRATION
NES = 2.14, p = 0.0000, FDR q = 0.0065

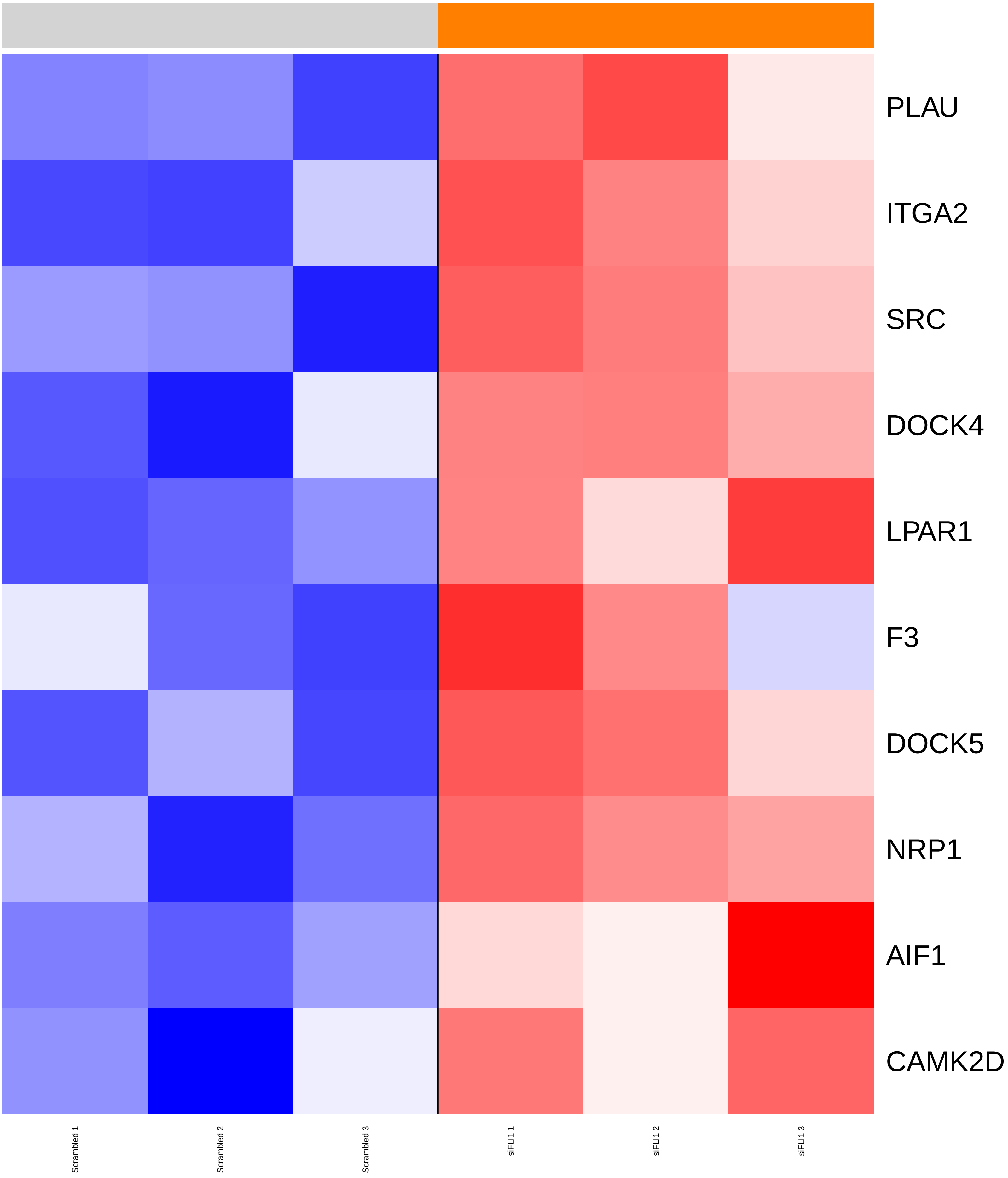

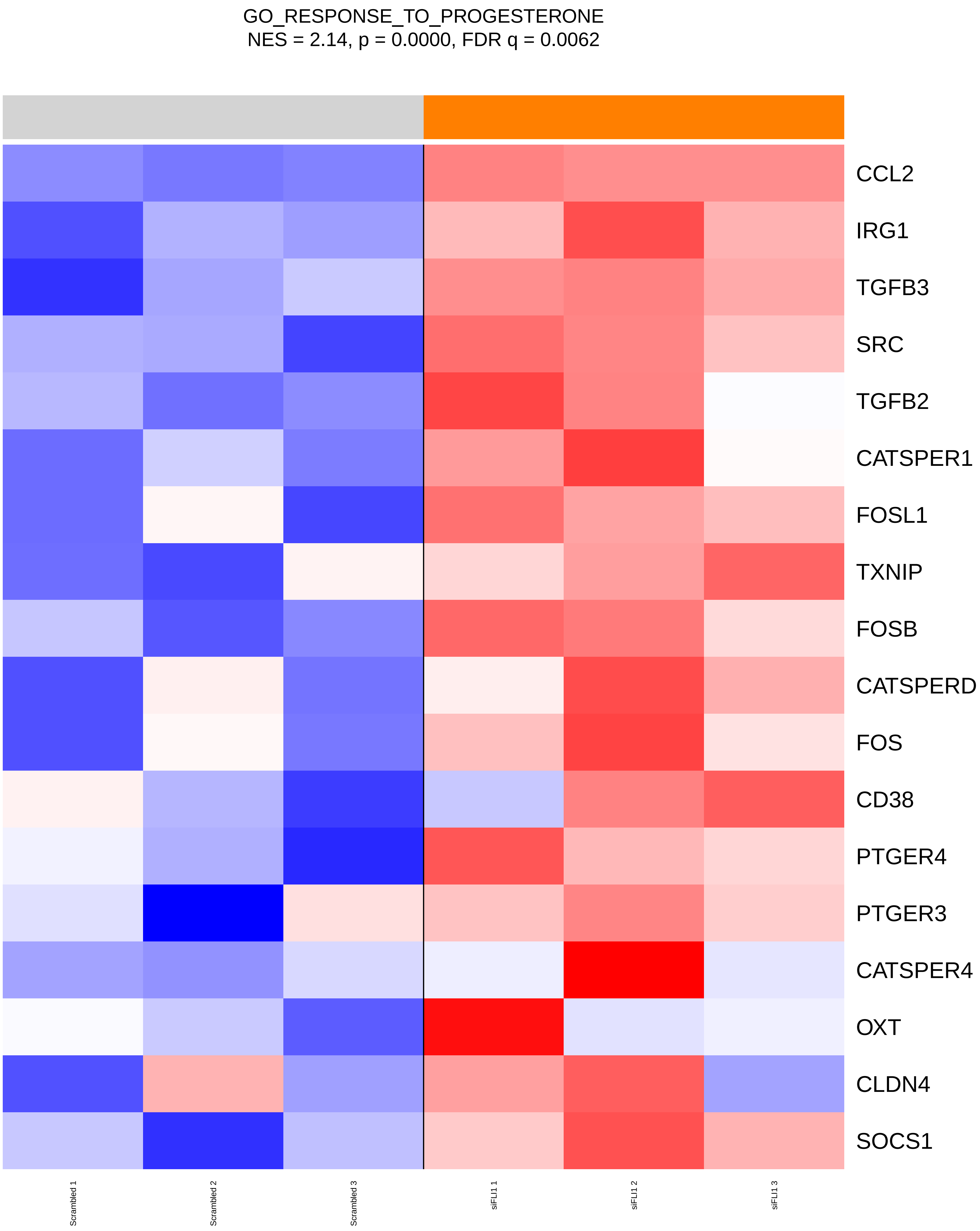
GO_RESPONSE_TO_PROGESTERONE
NES = 2.14, p = 0.0000, FDR q = 0.0062
CCL2
IRG1
TGFB3
SRC
TGFB2
CATSPER1
FOSL1
TXNIP
FOSB
CATSPERD
FOS
CD38
PTGER4
PTGER3
CATSPER4
OXT
CLDN4
SOCS1
Scrambled 1
Scrambled 2
Scrambled 3
siFLI1 1
siFLI1 2
siFLI1 3

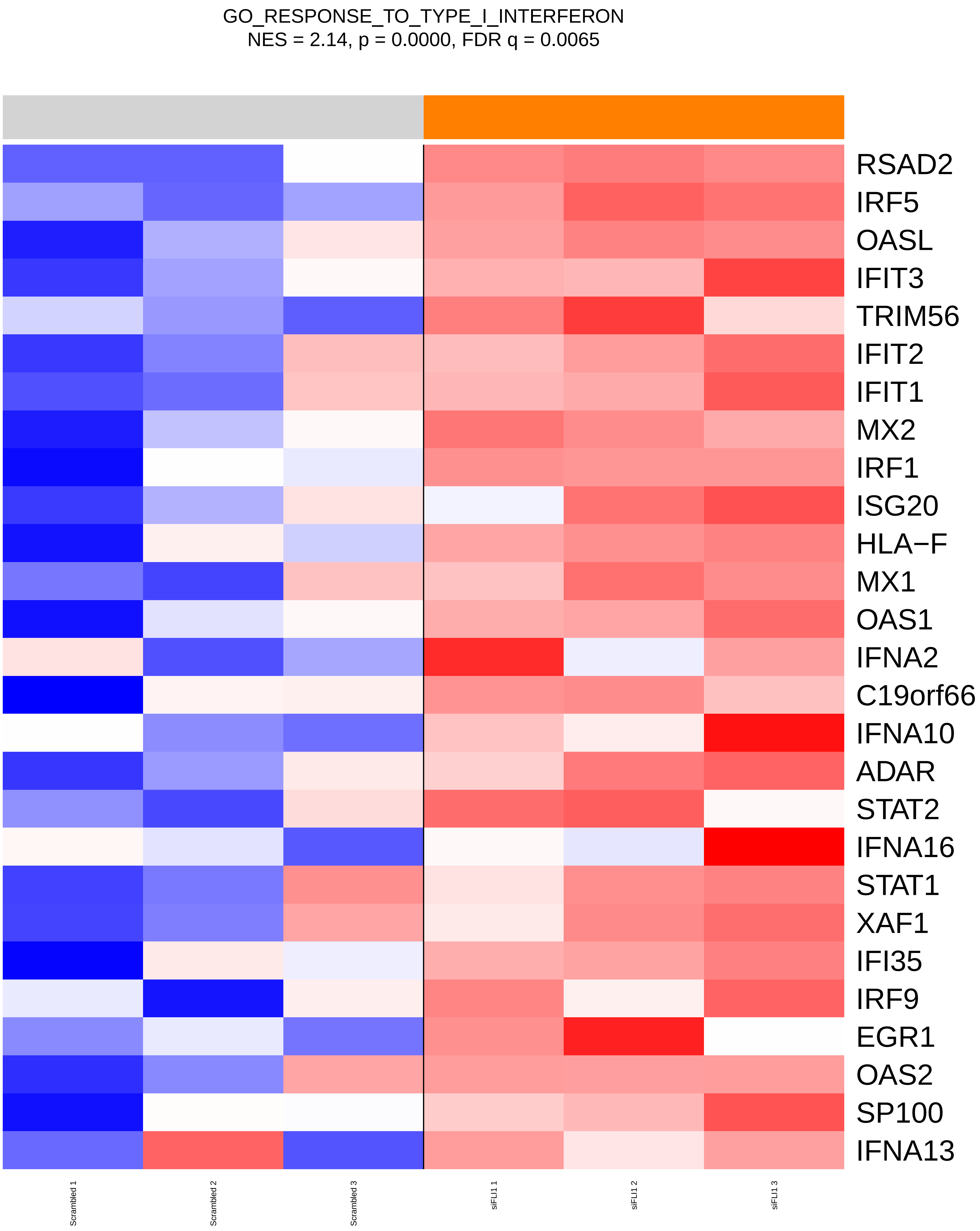
GO_RESPONSE_TO_TYPE_I_INTERFERON
NES = 2.14, p = 0.0000, FDR q = 0.0065
RSAD2
IRF5
OASL
IFIT3
TRIM56
IFIT2
IFIT1
MX2
IRF1
ISG20
HLA−F
MX1
OAS1
IFNA2
C19orf66
IFNA10
ADAR
STAT2
IFNA16
STAT1
XAF1
IFI35
IRF9
EGR1
OAS2
SP100
IFNA13
Scrambled 1
Scrambled 2
Scrambled 3
siFLI1 1
siFLI1 2
siFLI1 3

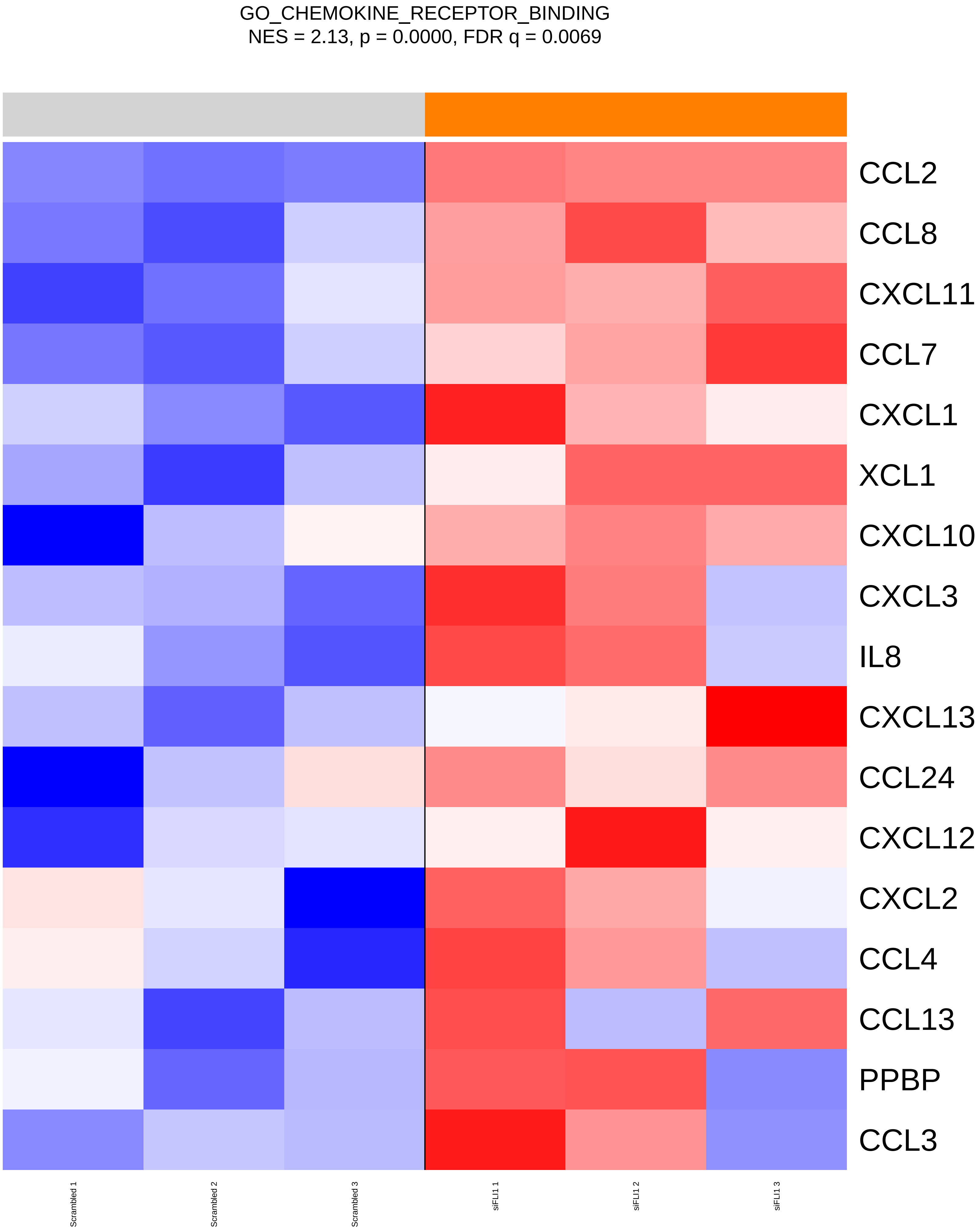
GO_CHEMOKINE_RECEPTOR_BINDING
NES = 2.13, p = 0.0000, FDR q = 0.0069
CCL2
CCL8
CXCL11
CCL7
CXCL1
XCL1
CXCL10
CXCL3
IL8
CXCL13
CCL24
CXCL12
CXCL2
CCL4
CCL13
PPBP
CCL3
Scrambled 1
Scrambled 2
Scrambled 3
siFLI1 1
siFLI1 2
siFLI1 3

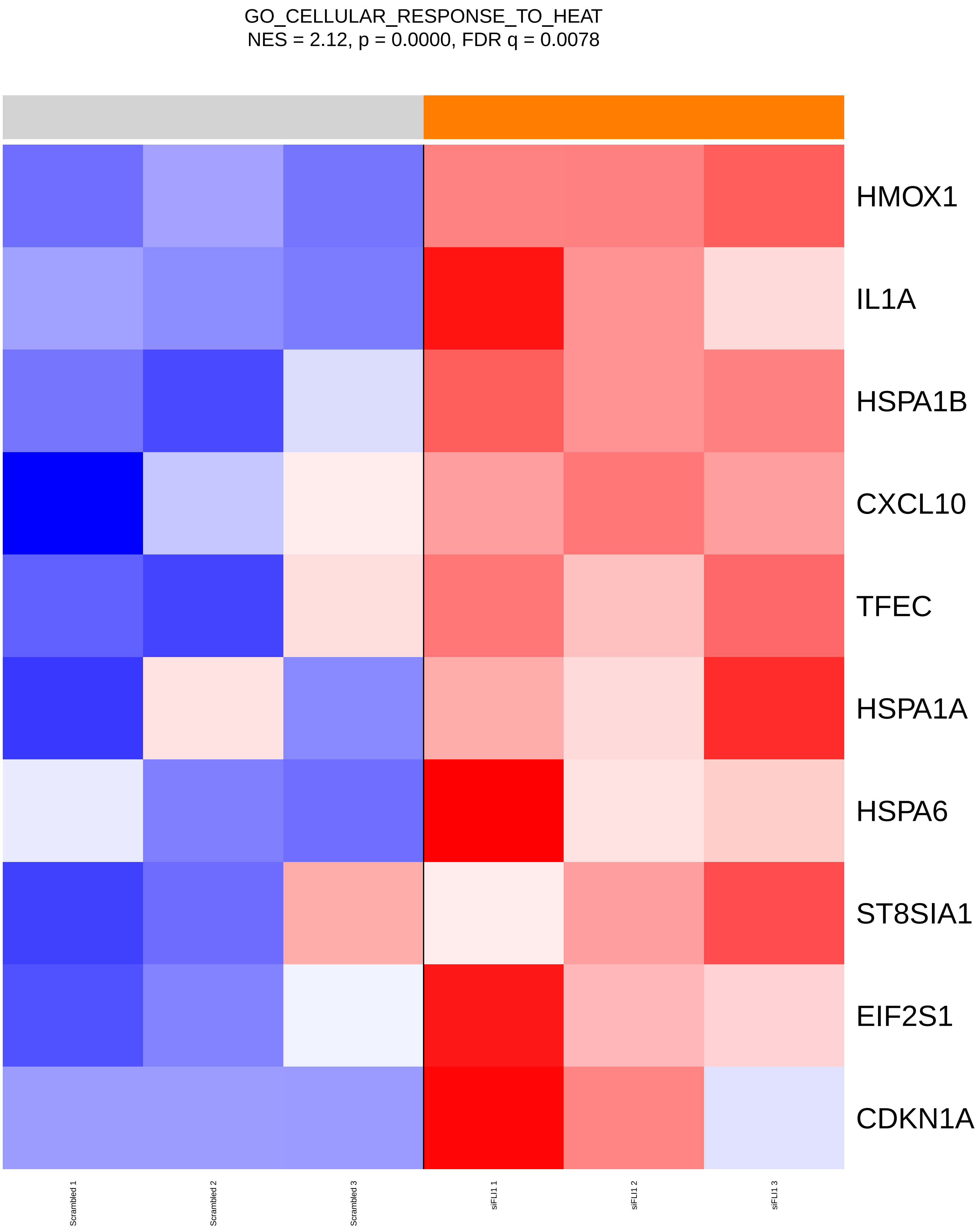
GO_CELLULAR_RESPONSE_TO_HEAT
NES = 2.12, p = 0.0000, FDR q = 0.0078
HMOX1
IL1A
HSPA1B
CXCL10
TFEC
HSPA1A
HSPA6
ST8SIA1
EIF2S1
CDKN1A
Scrambled 1
Scrambled 2
Scrambled 3
siFLI1 1
siFLI1 2
siFLI1 3

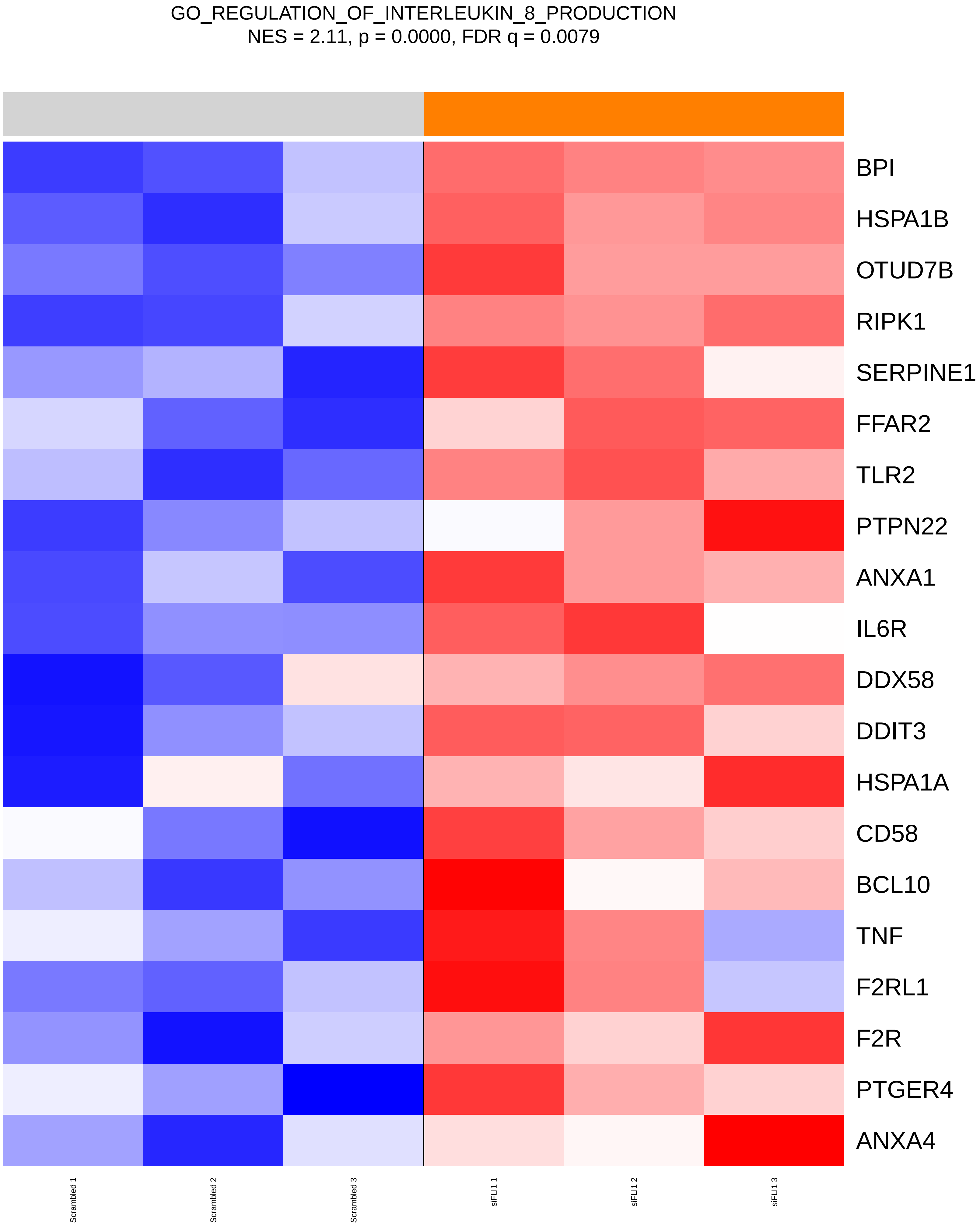
GO_REGULATION_OF_INTERLEUKIN_8_PRODUCTION
NES = 2.11, p = 0.0000, FDR q = 0.0079
BPI
HSPA1B
OTUD7B
RIPK1
SERPINE1
FFAR2
TLR2
PTPN22
ANXA1
IL6R
DDX58
DDIT3
HSPA1A
CD58
BCL10
TNF
F2RL1
F2R
PTGER4
ANXA4
Scrambled 1
Scrambled 2
Scrambled 3
siFLI1 1
siFLI1 2
siFLI1 3

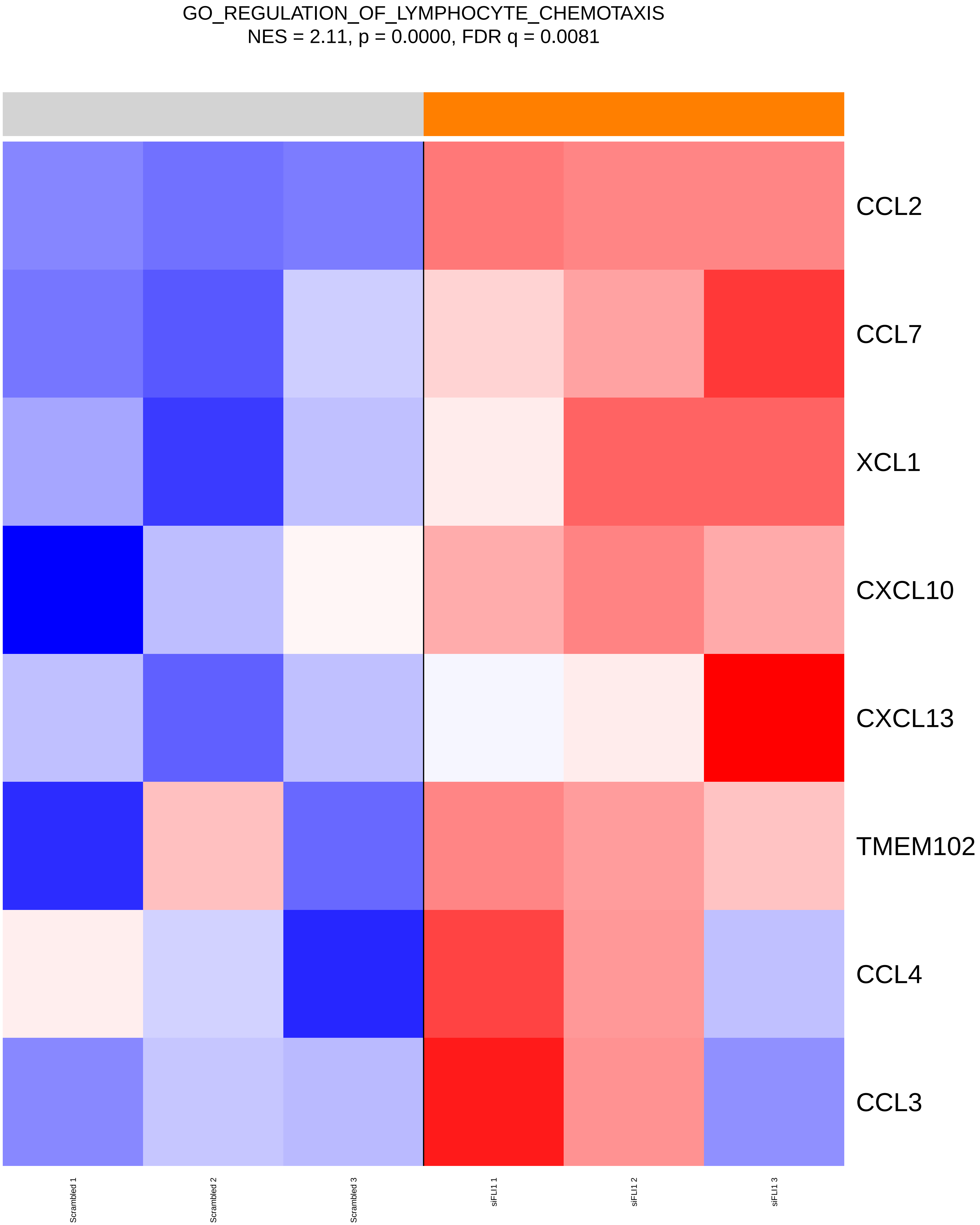
GO_REGULATION_OF_LYMPHOCYTE_CHEMOTAXIS
NES = 2.11, p = 0.0000, FDR q = 0.0081
CCL2
CCL7
XCL1
CXCL10
CXCL13
TMEM102
CCL4
CCL3
Scrambled 1
Scrambled 2
Scrambled 3
siFLI1 1
siFLI1 2
siFLI1 3

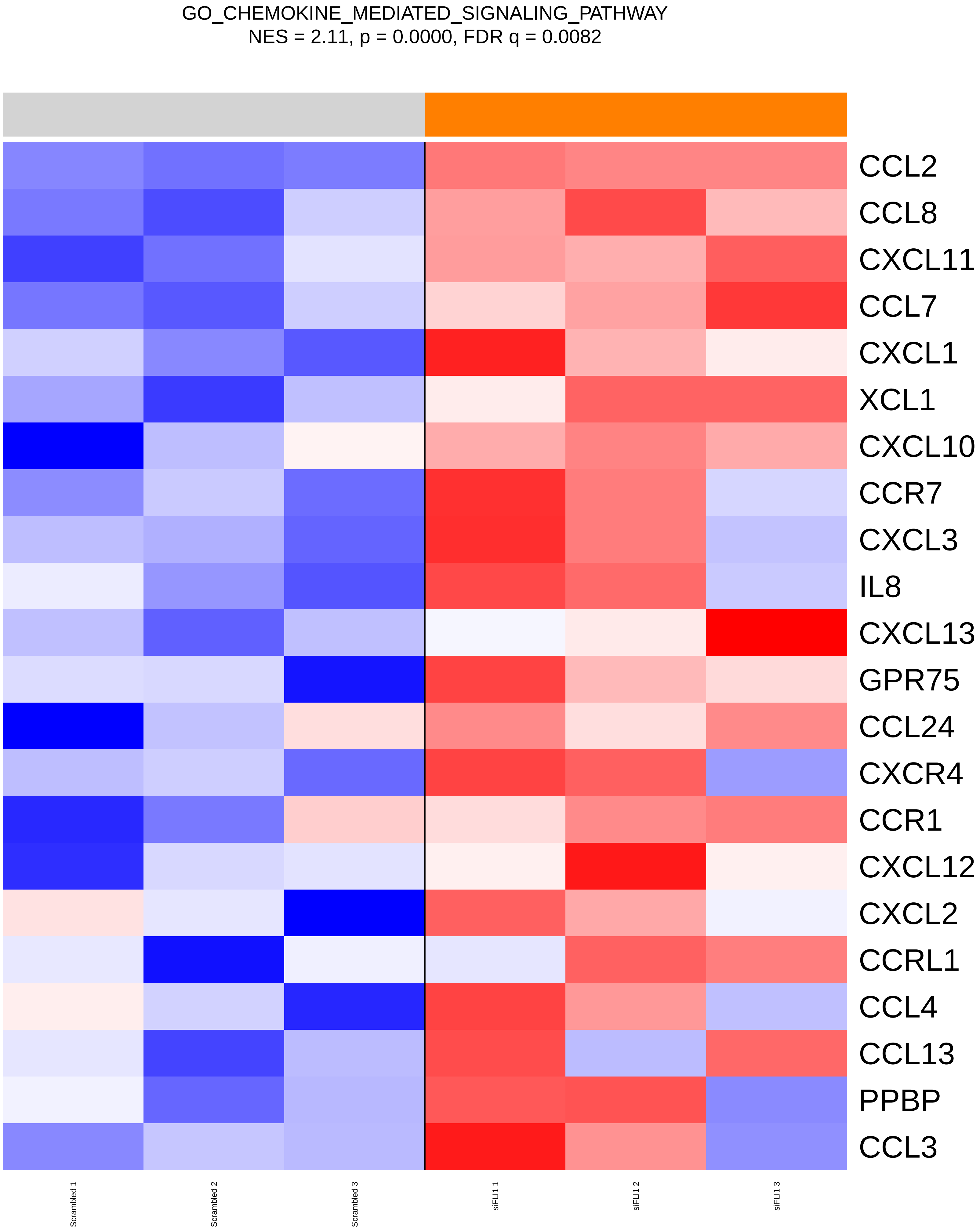
GO_CHEMOKINE_MEDIATED_SIGNALING_PATHWAY
NES = 2.11, p = 0.0000, FDR q = 0.0082
CCL2
CCL8
CXCL11
CCL7
CXCL1
XCL1
CXCL10
CCR7
CXCL3
IL8
CXCL13
GPR75
CCL24
CXCR4
CCR1
CXCL12
CXCL2
CCRL1
CCL4
CCL13
PPBP
CCL3
Scrambled 1
Scrambled 2
Scrambled 3
siFLI1 1
siFLI1 2
siFLI1 3

GO_CXCR_CHEMOKINE_RECEPTOR_BINDING
NES = 2.09, p = 0.0000, FDR q = 0.0099

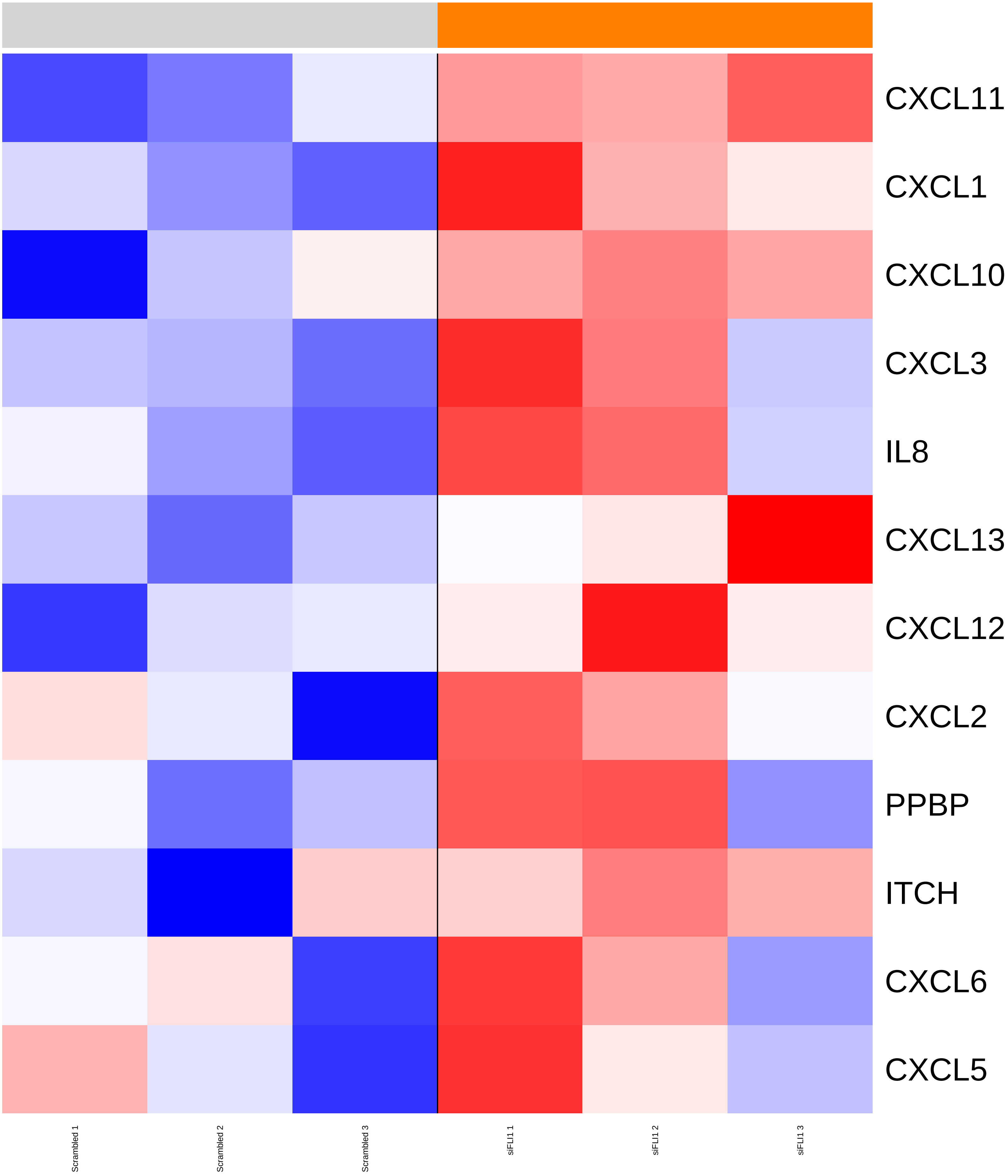

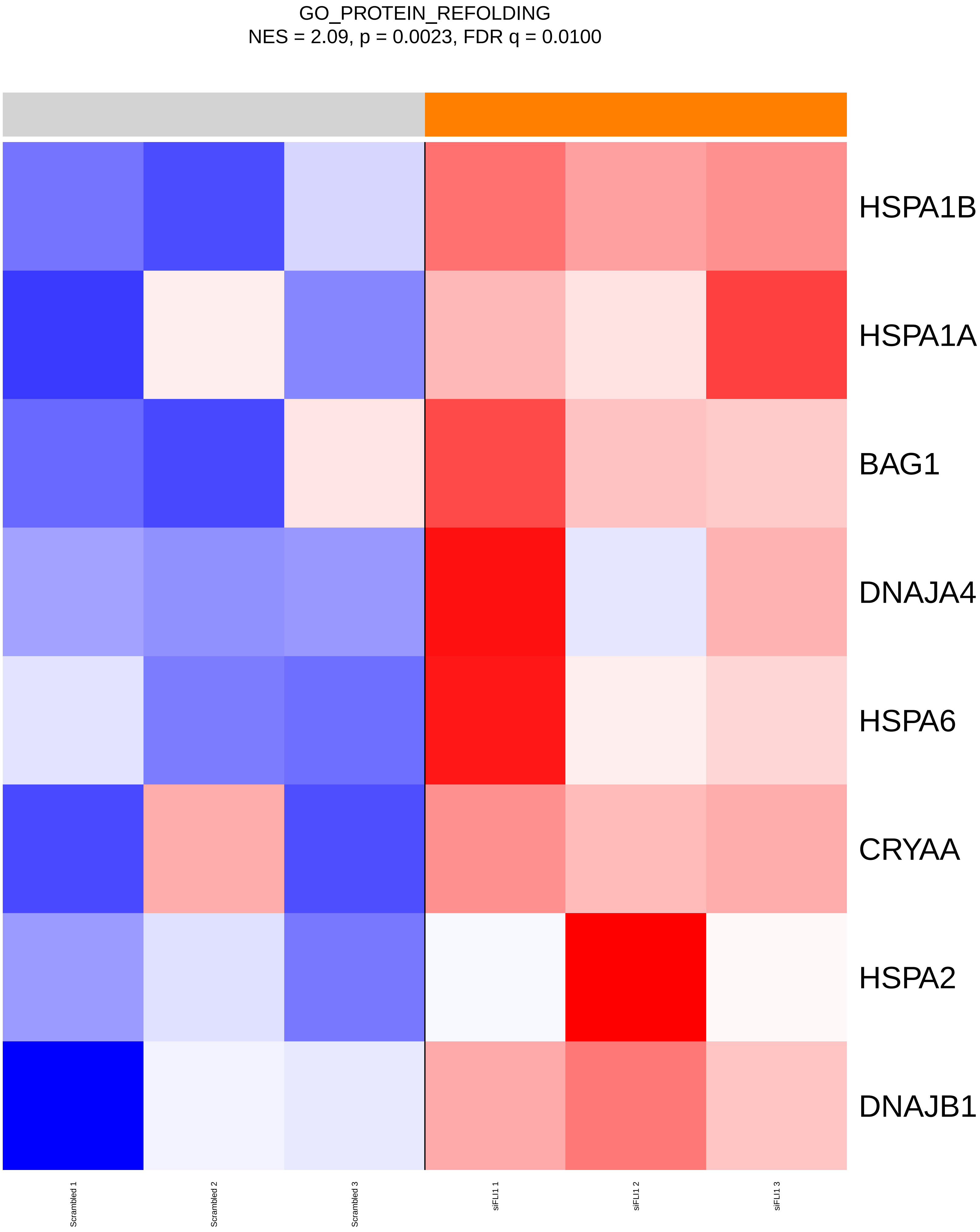
GO_PROTEIN_REFOLDING
NES = 2.09, p = 0.0023, FDR q = 0.0100
HSPA1B
HSPA1A
BAG1
DNAJA4
HSPA6
CRYAA
HSPA2
DNAJB1
Scrambled 1
Scrambled 2
Scrambled 3
siFLI1 1
siFLI1 2
siFLI1 3

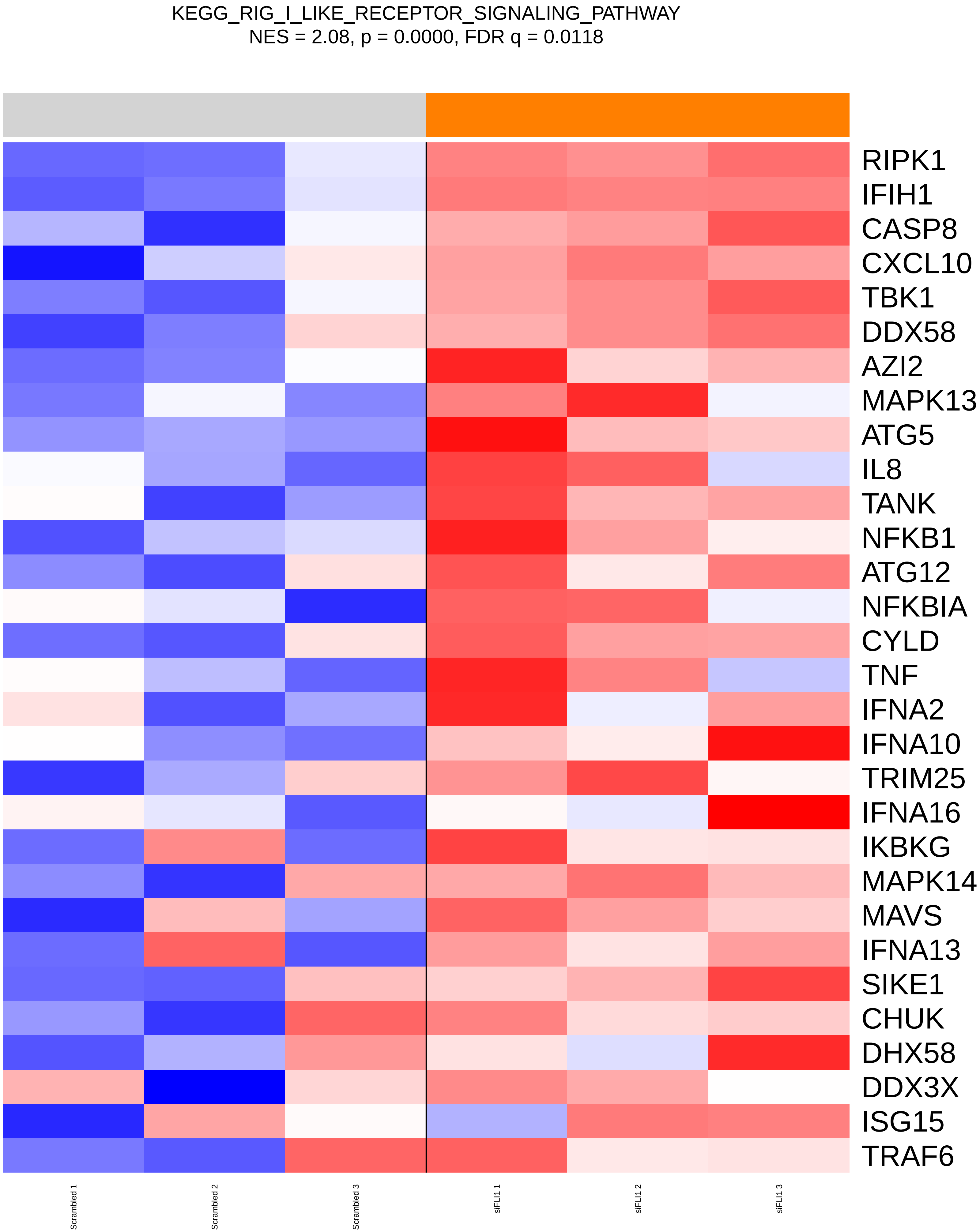
KEGG_RIG_I_LIKE_RECEPTOR_SIGNALING_PATHWAY
NES = 2.08, p = 0.0000, FDR q = 0.0118
RIPK1
IFIH1
CASP8
CXCL10
TBK1
DDX58
AZI2
MAPK13
ATG5
IL8
TANK
NFKB1
ATG12
NFKBIA
CYLD
TNF
IFNA2
IFNA10
TRIM25
IFNA16
IKBKG
MAPK14
MAVS
IFNA13
SIKE1
CHUK
DHX58
DDX3X
ISG15
TRAF6
Scrambled 1
Scrambled 2
Scrambled 3
siFLI1 1
siFLI1 2
siFLI1 3

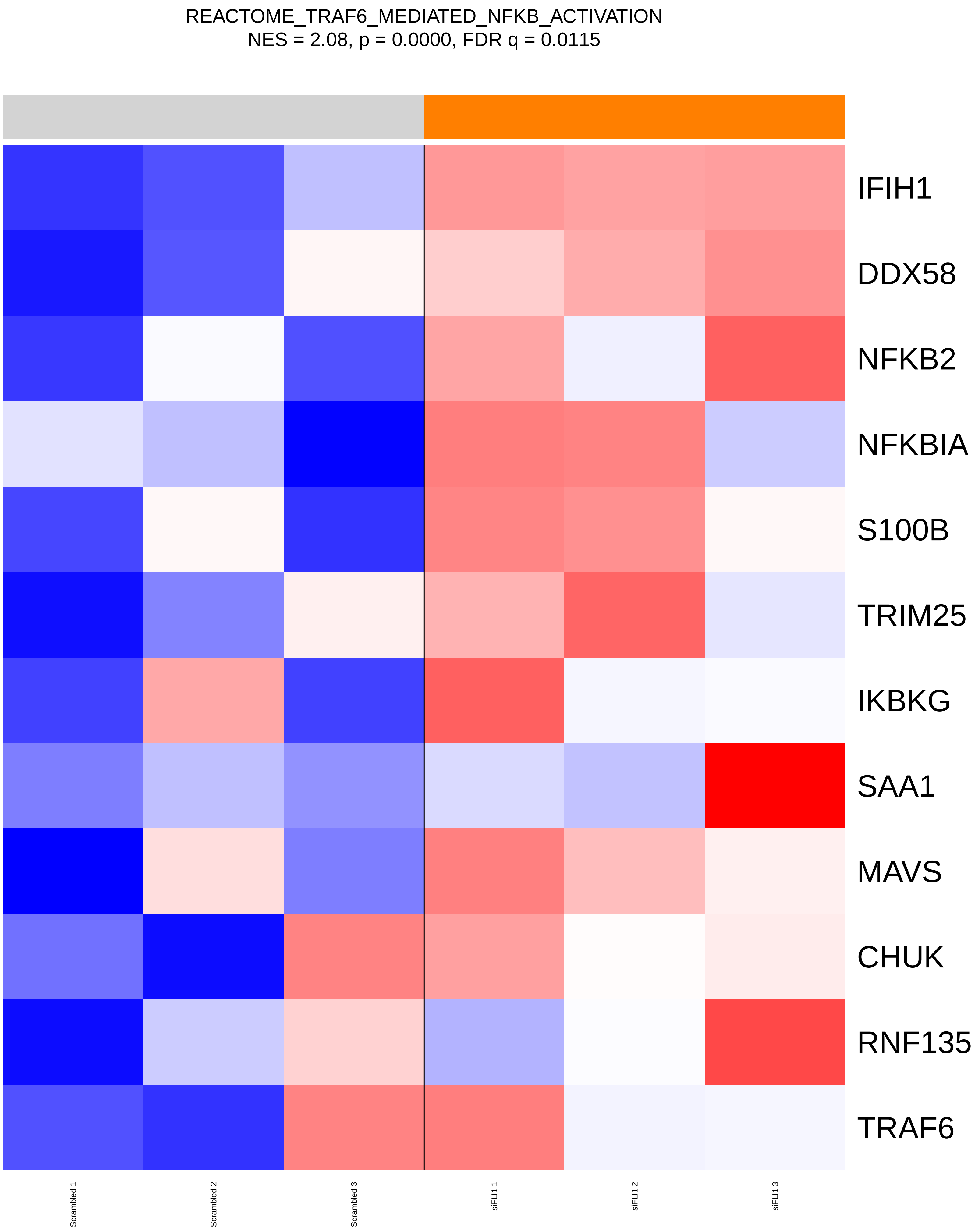
REACTOME_TRAF6_MEDIATED_NFKB_ACTIVATION
NES = 2.08, p = 0.0000, FDR q = 0.0115
IFIH1
DDX58
NFKB2
NFKBIA
S100B
TRIM25
IKBKG
SAA1
MAVS
CHUK
RNF135
TRAF6
Scrambled 1
Scrambled 2
Scrambled 3
siFLI1 1
siFLI1 2
siFLI1 3

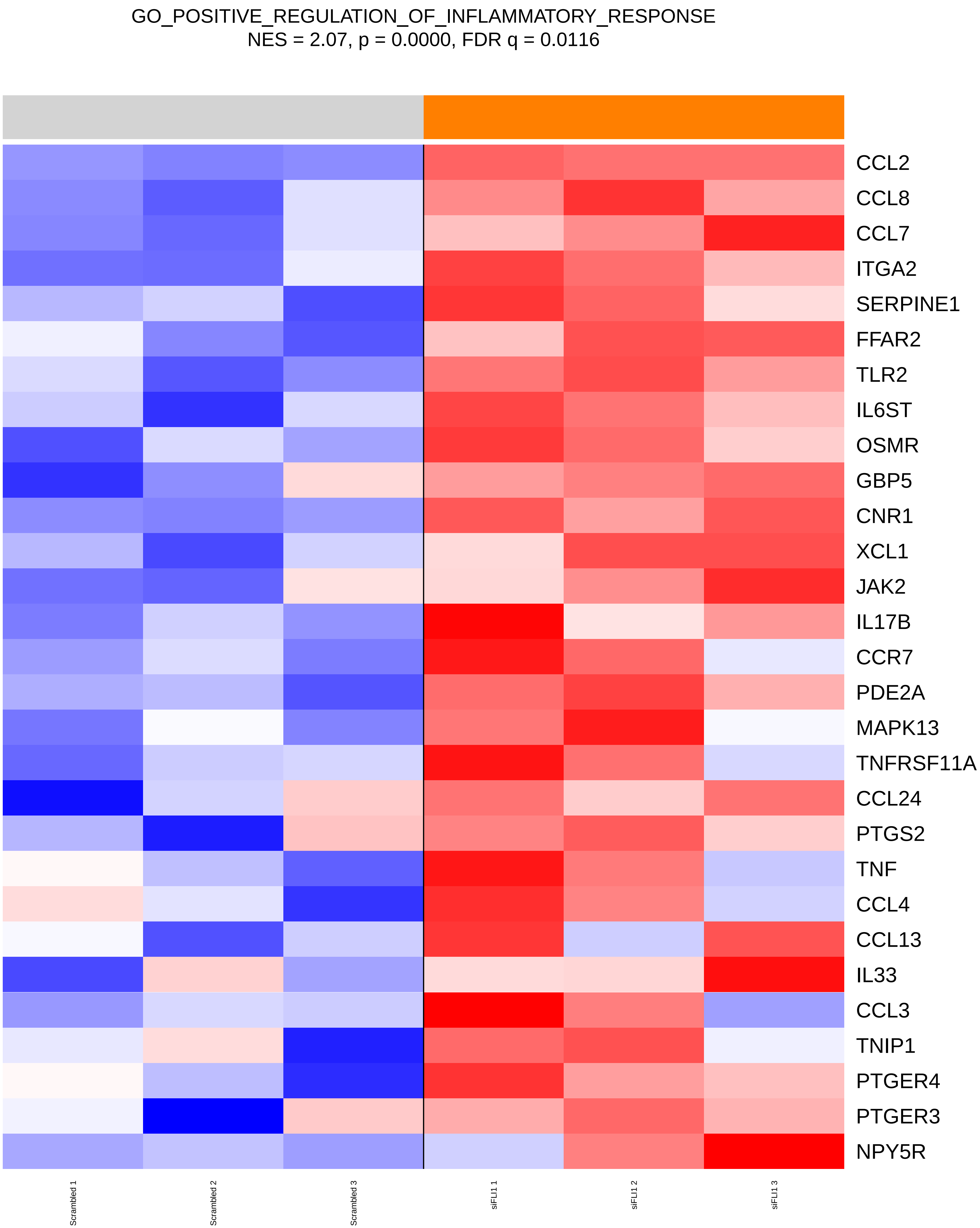
GO_POSITIVE_REGULATION_OF_INFLAMMATORY_RESPONSE
NES = 2.07, p = 0.0000, FDR q = 0.0116
CCL2
CCL8
CCL7
ITGA2
SERPINE1
FFAR2
TLR2
IL6ST
OSMR
GBP5
CNR1
XCL1
JAK2
IL17B
CCR7
PDE2A
MAPK13
TNFRSF11A
CCL24
PTGS2
TNF
CCL4
CCL13
IL33
CCL3
TNIP1
PTGER4
PTGER3
NPY5R
Scrambled 1
Scrambled 2
Scrambled 3
siFLI1 1
siFLI1 2
siFLI1 3

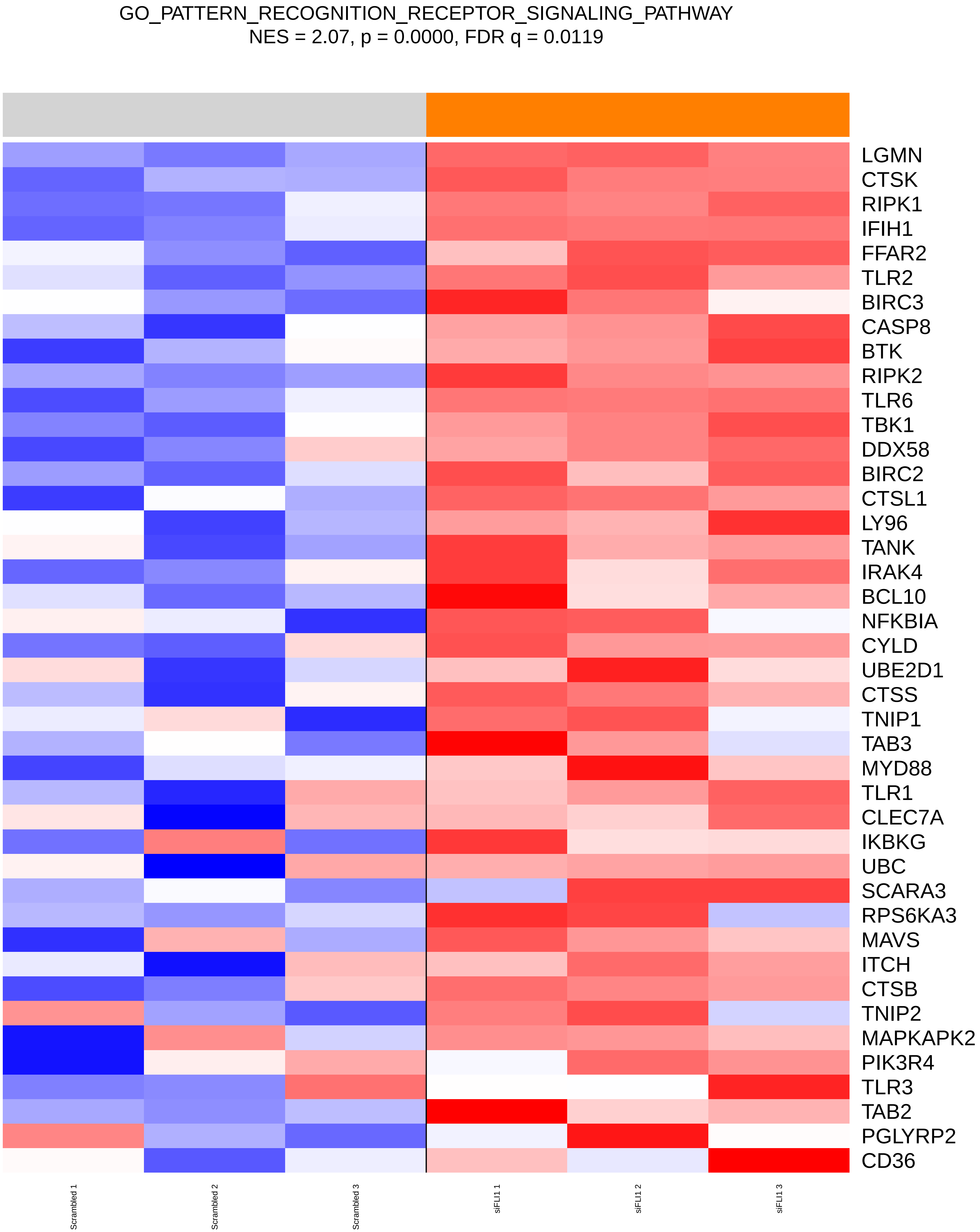
GO_PATTERN_RECOGNITION_RECEPTOR_SIGNALING_PATHWAY
NES = 2.07, p = 0.0000, FDR q = 0.0119
LGMN
CTSK
RIPK1
IFIH1
FFAR2
TLR2
BIRC3
CASP8
BTK
RIPK2
TLR6
TBK1
DDX58
BIRC2
CTSL1
LY96
TANK
IRAK4
BCL10
NFKBIA
CYLD
UBE2D1
CTSS
TNIP1
TAB3
MYD88
TLR1
CLEC7A
IKBKG
UBC
SCARA3
RPS6KA3
MAVS
ITCH
CTSB
TNIP2
MAPKAPK2
PIK3R4
TLR3
TAB2
PGLYRP2
CD36
Scrambled 1
Scrambled 2
Scrambled 3
siFLI1 1
siFLI1 2
siFLI1 3

GO_RESPONSE_TO_INTERLEUKIN_1
NES = 2.07, p = 0.0000, FDR q = 0.0118

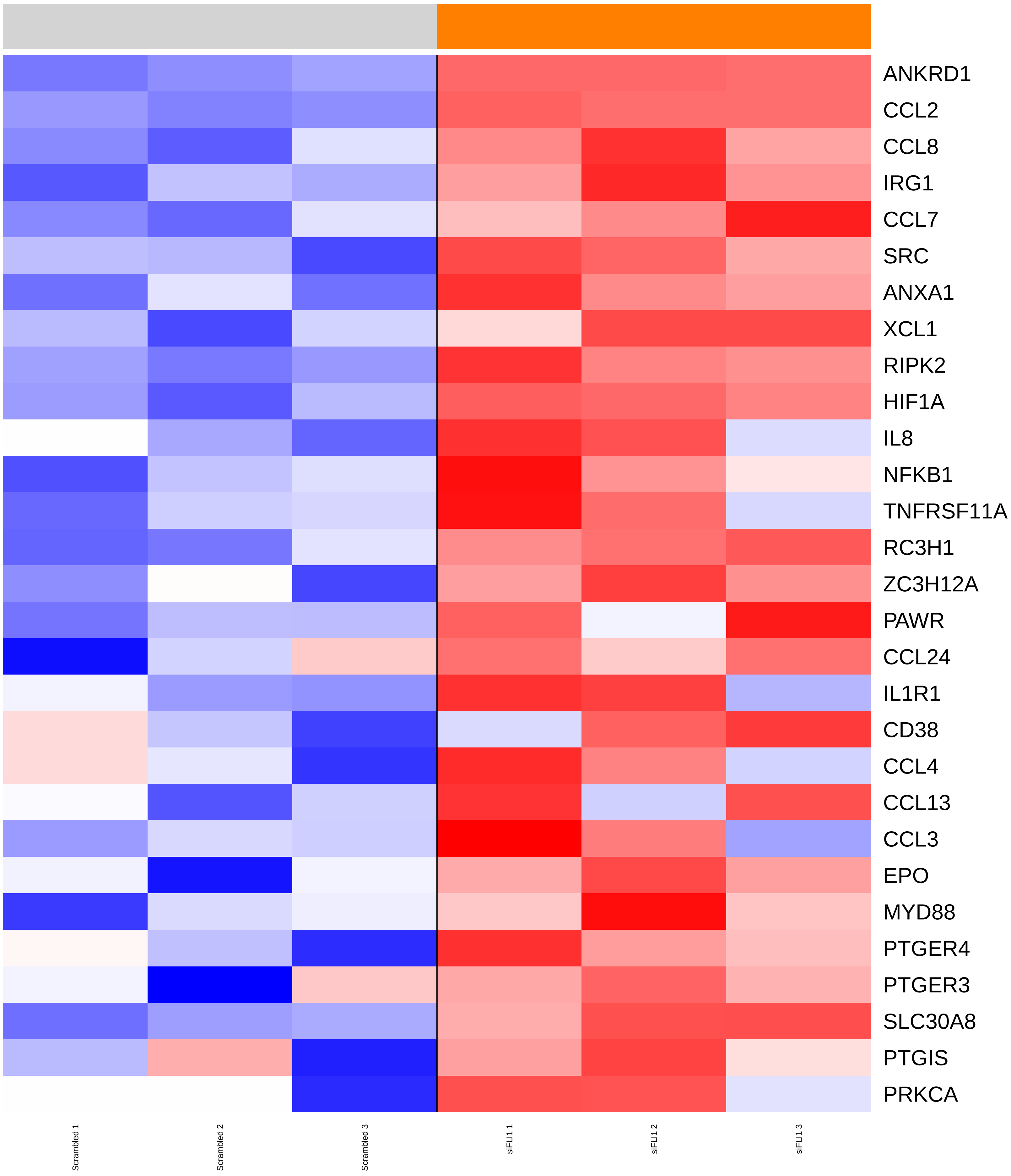

GO_REGULATION_OF_NECROTIC_CELL_DEATH
NES = 2.07, p = 0.0000, FDR q = 0.0115

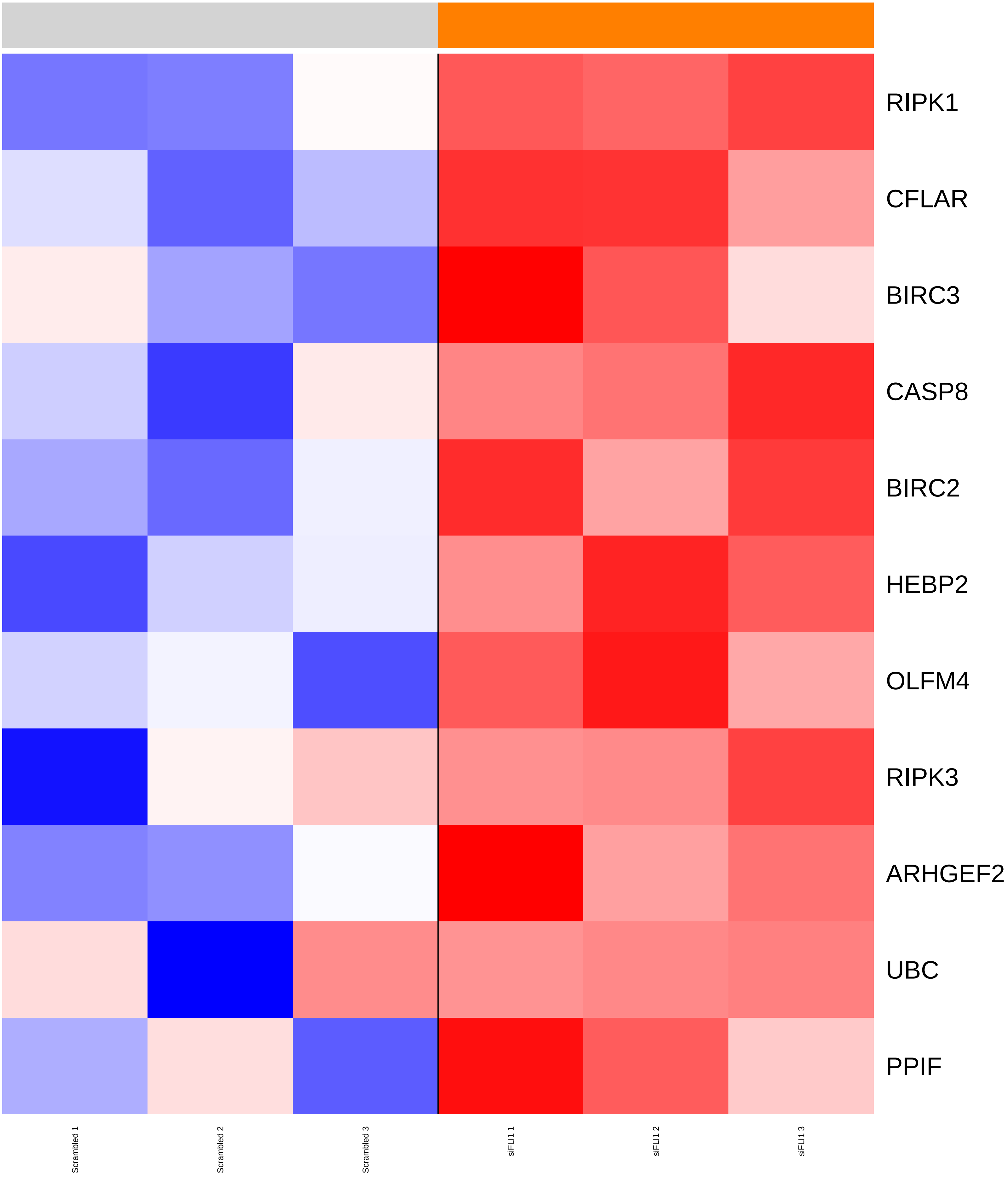

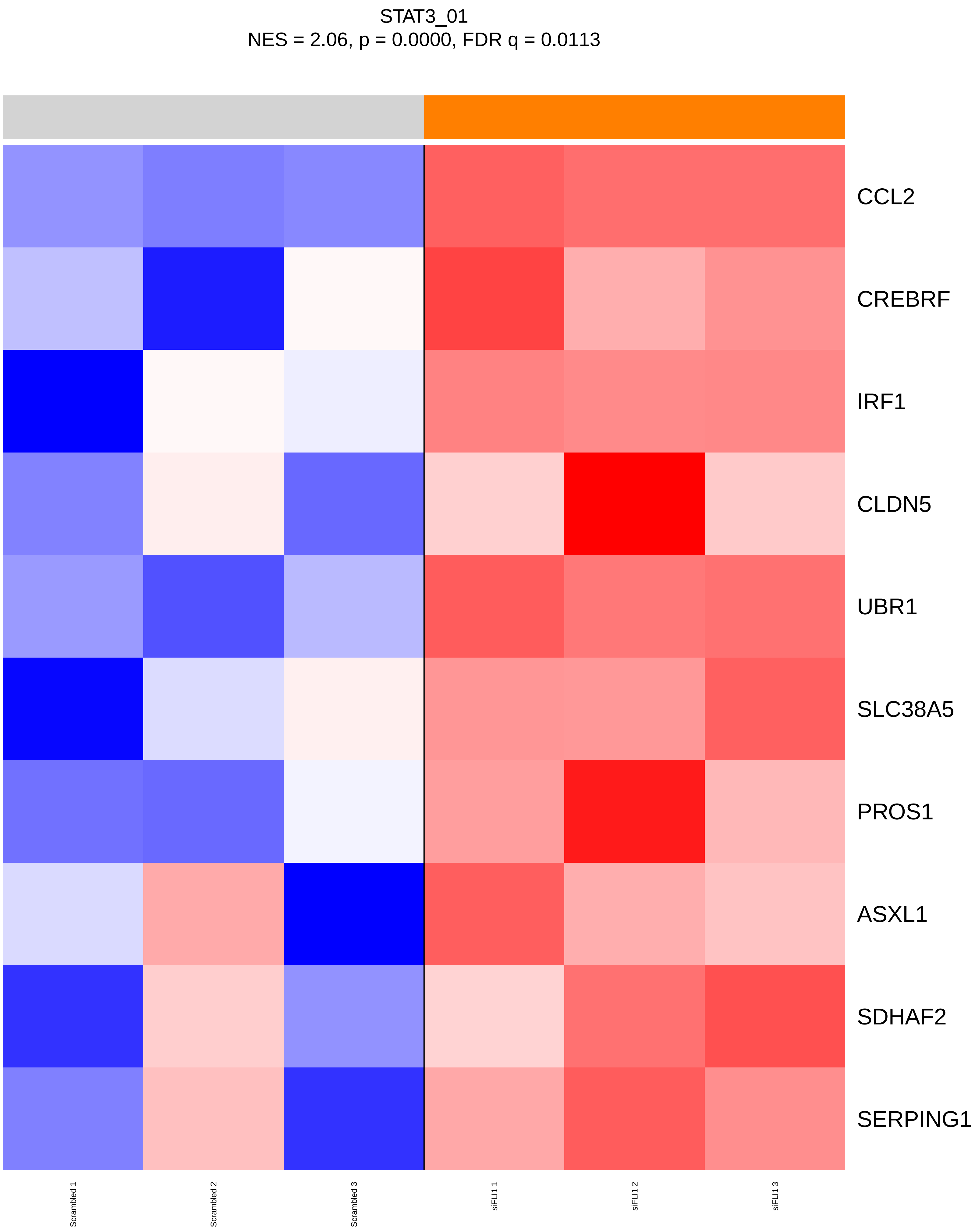
STAT3_01
NES = 2.06, p = 0.0000, FDR q = 0.0113
CCL2
CREBRF
IRF1
CLDN5
UBR1
SLC38A5
PROS1
ASXL1
SDHAF2
SERPING1
Scrambled 1
Scrambled 2
Scrambled 3
siFLI1 1
siFLI1 2
siFLI1 3

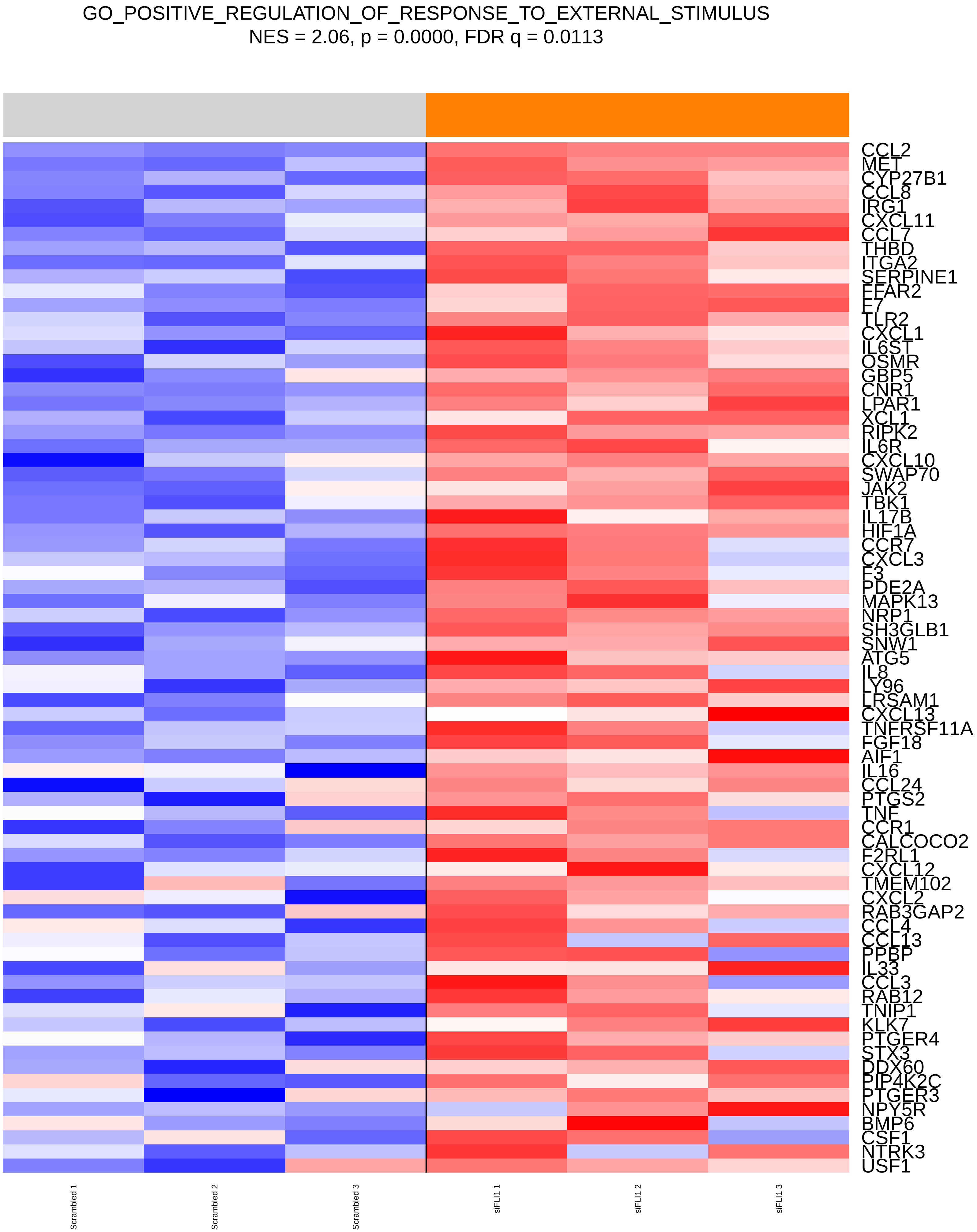
GO_POSITIVE_REGULATION_OF_RESPONSE_TO_EXTERNAL_STIMULUS
NES = 2.06, p = 0.0000, FDR q = 0.0113
CCL2
MET
CYP27B1
CCL8
IRG1
CXCL11
CCL7
THBD
ITGA2
SERPINE1
FFAR2
F7
TLR2
CXCL1
IL6ST
OSMR
GBP5
CNR1
LPAR1
XCL1
RIPK2
IL6R
CXCL10
SWAP70
JAK2
TBK1
IL17B
HIF1A
CCR7
CXCL3
F3
PDE2A
MAPK13
NRP1
SH3GLB1
SNW1
ATG5
IL8
LY96
LRSAM1
CXCL13
TNFRSF11A
FGF18
AIF1
IL16
CCL24
PTGS2
TNF
CCR1
CALCOCO2
F2RL1
CXCL12
TMEM102
CXCL2
RAB3GAP2
CCL4
CCL13
PPBP
IL33
CCL3
RAB12
TNIP1
KLK7
PTGER4
STX3
DDX60
PIP4K2C
PTGER3
NPY5R
BMP6
CSF1
NTRK3
USF1
Scrambled 1
Scrambled 2
Scrambled 3
siFLI1 1
siFLI1 2
siFLI1 3

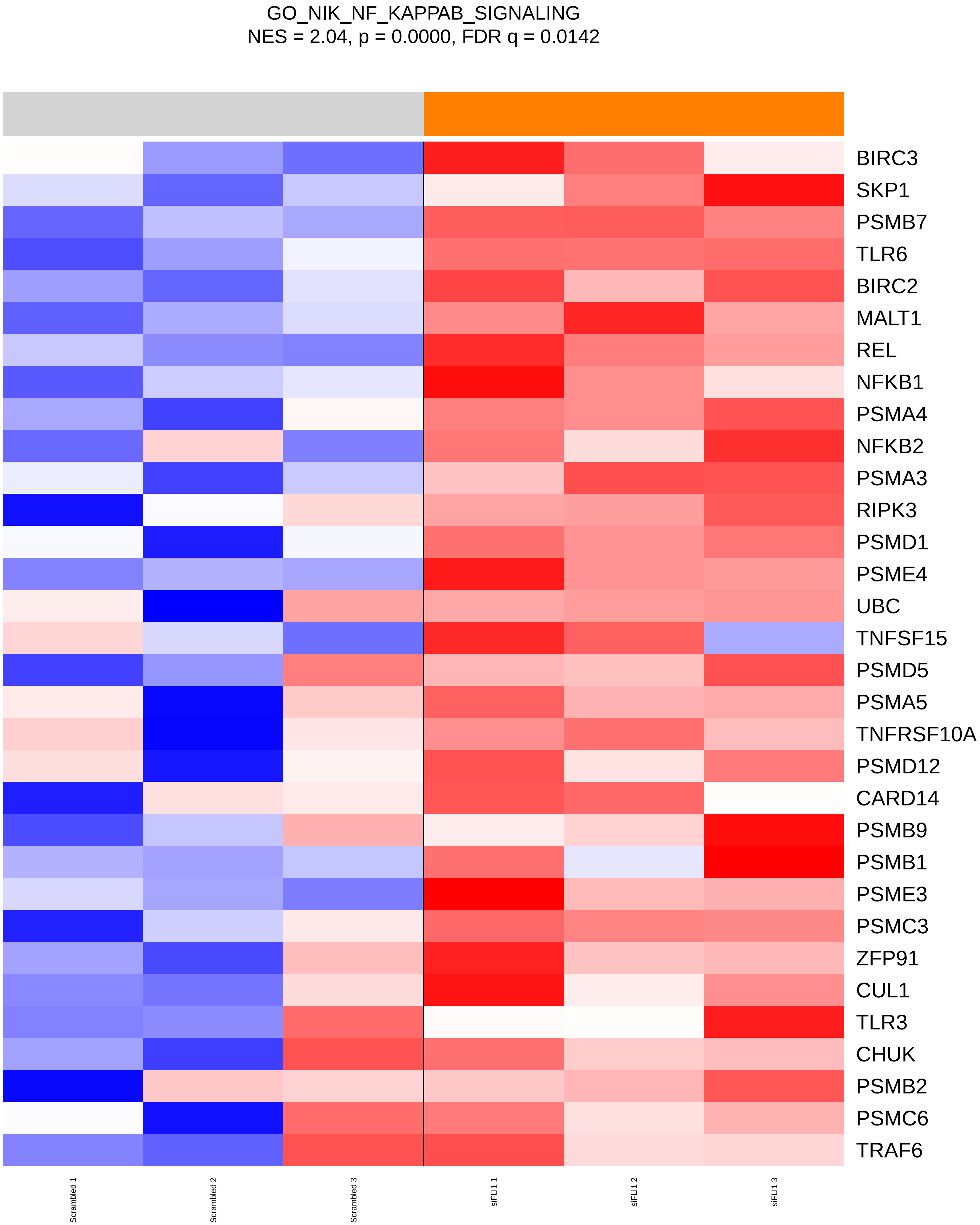
GO_NIK_NF_KAPPAB_SIGNALING
NES = 2.04, p = 0.0000, FDR q = 0.0142
BIRC3
SKP1
PSMB7
TLR6
BIRC2
MALT1
REL
NFKB1
PSMA4
NFKB2
PSMA3
RIPK3
PSMD1
PSME4
UBC
TNFSF15
PSMD5
PSMA5
TNFRSF10A
PSMD12
CARD14
PSMB9
PSMB1
PSME3
PSMC3
ZFP91
CUL1
TLR3
CHUK
PSMB2
PSMC6
TRAF6
Scrambled 1
Scrambled 2
Scrambled 3
siFLI1 1
siFLI1 2
siFLI1 3

REACTOME_INTERFERON_ALPHA_BETA_SIGNALING
NES = 2.04, p = 0.0000, FDR q = 0.0139

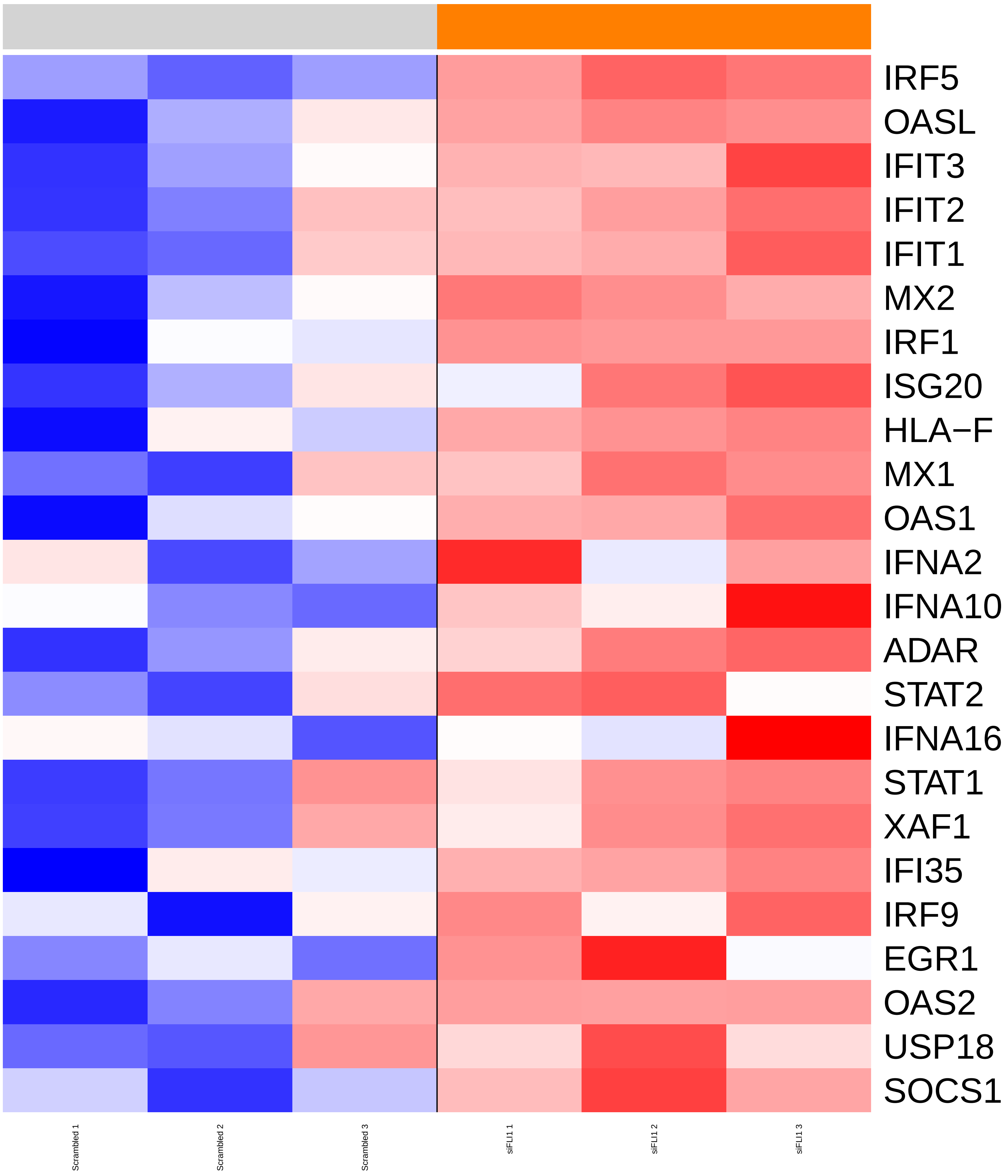

GO_POSITIVE_REGULATION_OF_CHEMOTAXIS
NES = 2.04, p = 0.0000, FDR q = 0.0142

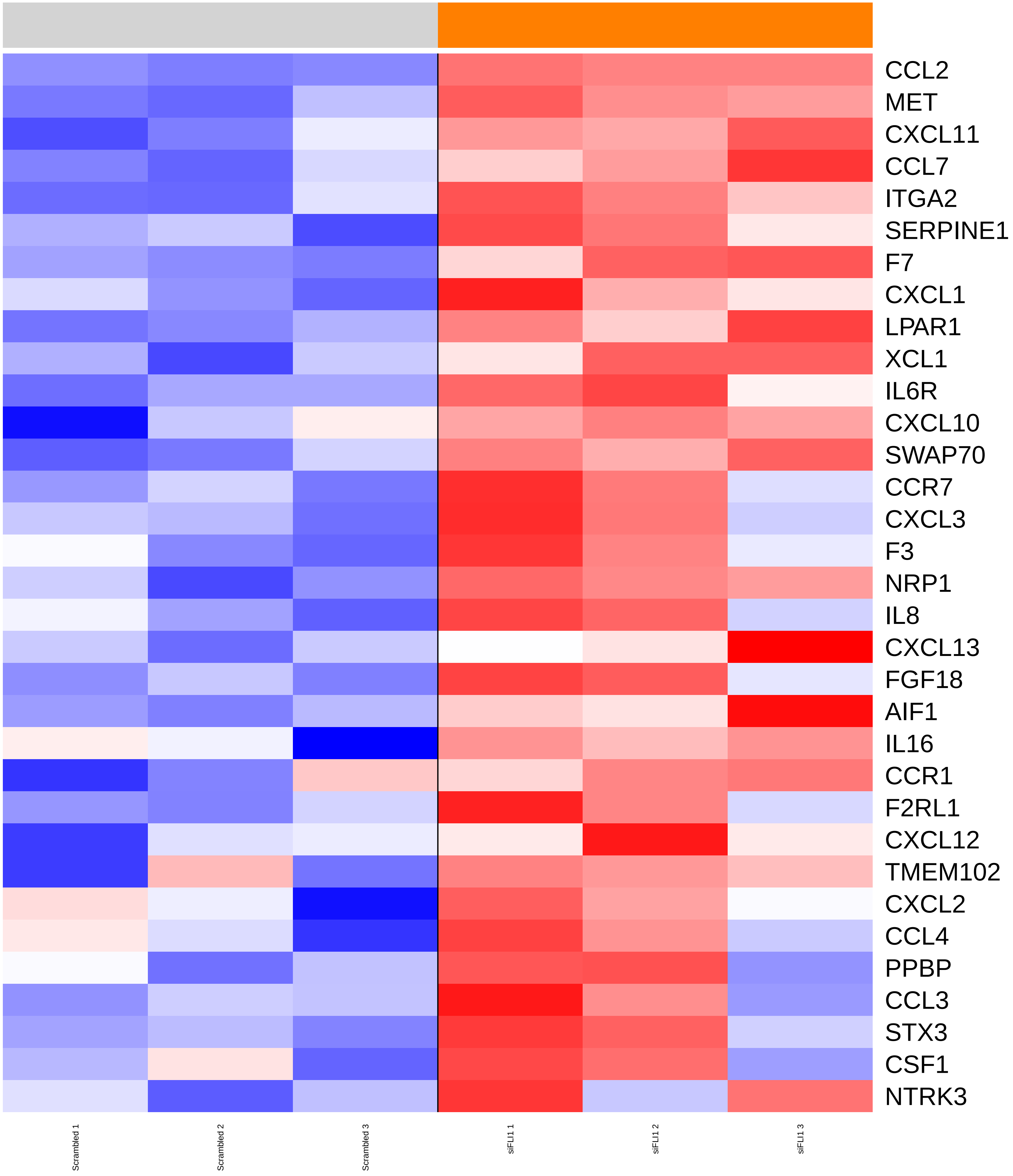

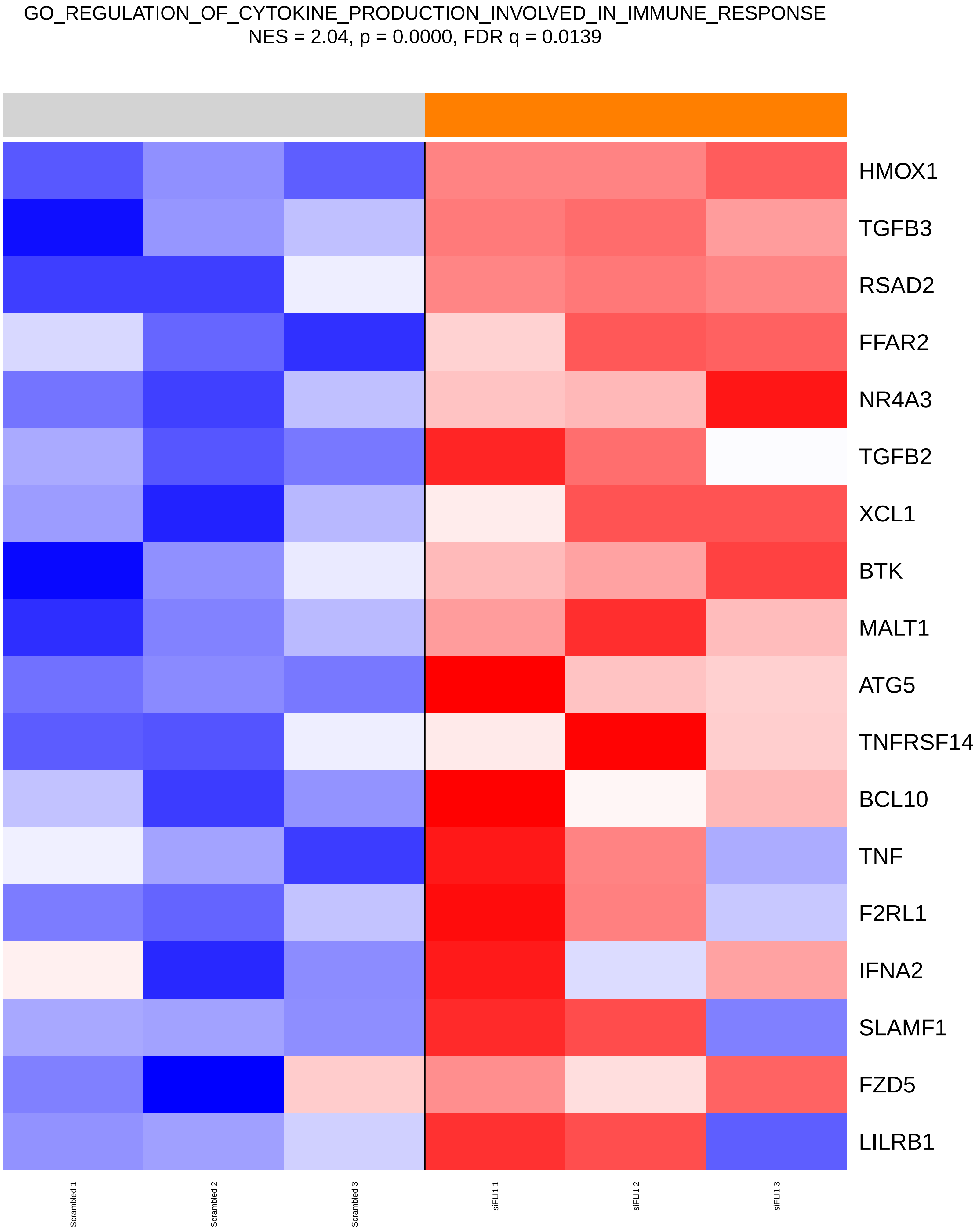
GO_REGULATION_OF_CYTOKINE_PRODUCTION_INVOLVED_IN_IMMUNE_RESPONSE
NES = 2.04, p = 0.0000, FDR q = 0.0139
HMOX1
TGFB3
RSAD2
FFAR2
NR4A3
TGFB2
XCL1
BTK
MALT1
ATG5
TNFRSF14
BCL10
TNF
F2RL1
IFNA2
SLAMF1
FZD5
LILRB1
Scrambled 1
Scrambled 2
Scrambled 3
siFLI1 1
siFLI1 2
siFLI1 3

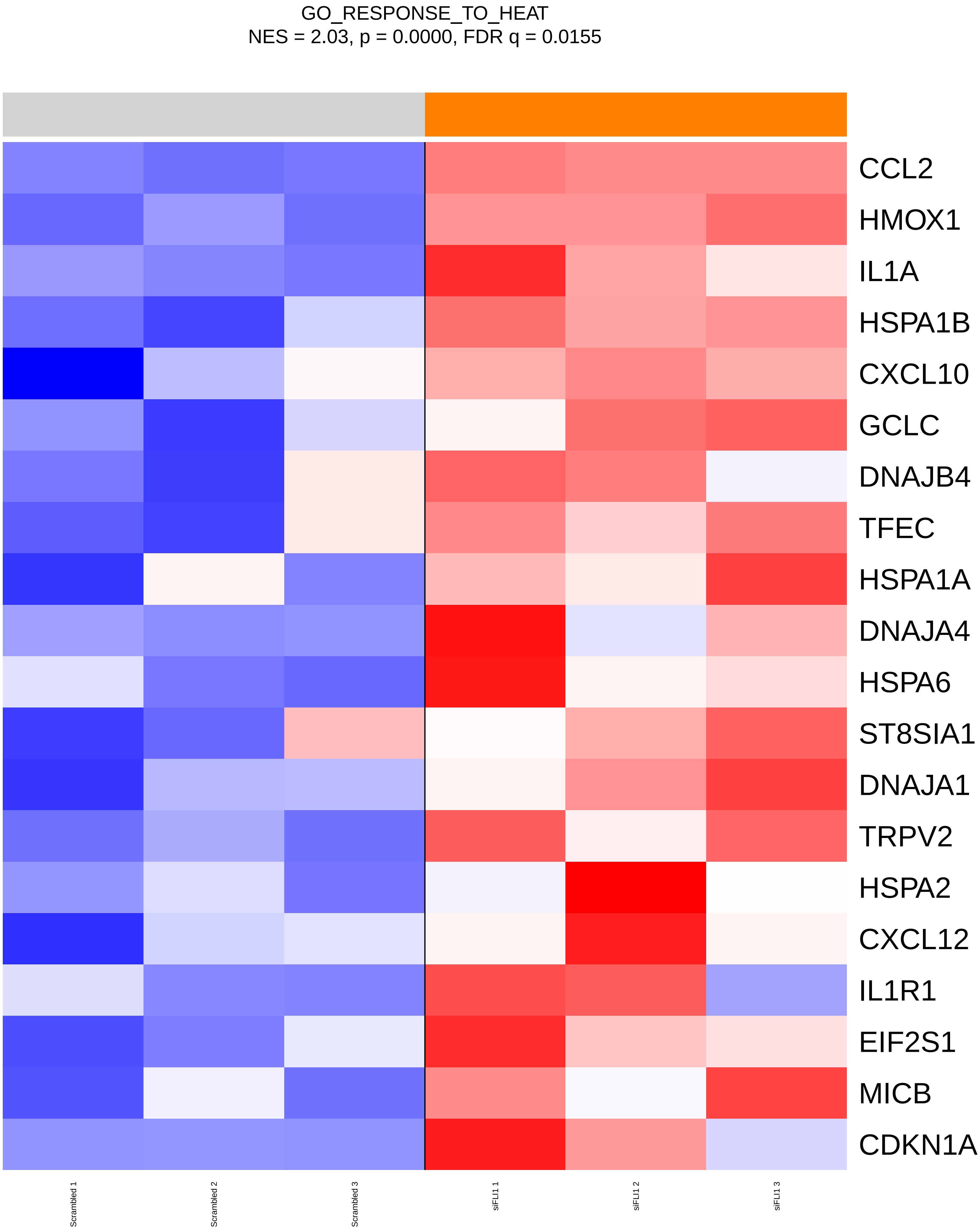
GO_RESPONSE_TO_HEAT
NES = 2.03, p = 0.0000, FDR q = 0.0155
CCL2
HMOX1
IL1A
HSPA1B
CXCL10
GCLC
DNAJB4
TFEC
HSPA1A
DNAJA4
HSPA6
ST8SIA1
DNAJA1
TRPV2
HSPA2
CXCL12
IL1R1
EIF2S1
MICB
CDKN1A
Scrambled 1
Scrambled 2
Scrambled 3
siFLI1 1
siFLI1 2
siFLI1 3

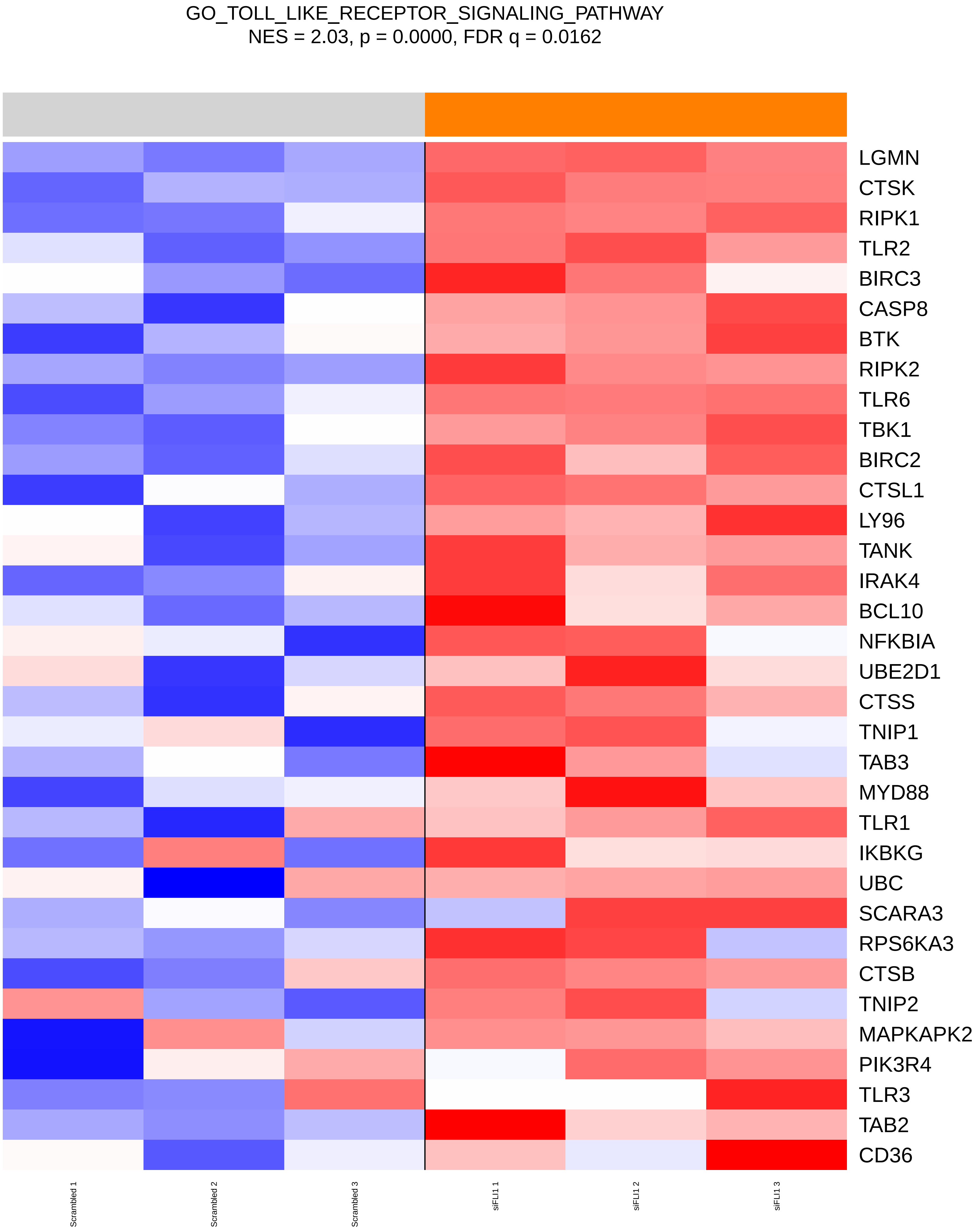
GO_TOLL_LIKE_RECEPTOR_SIGNALING_PATHWAY
NES = 2.03, p = 0.0000, FDR q = 0.0162
LGMN
CTSK
RIPK1
TLR2
BIRC3
CASP8
BTK
RIPK2
TLR6
TBK1
BIRC2
CTSL1
LY96
TANK
IRAK4
BCL10
NFKBIA
UBE2D1
CTSS
TNIP1
TAB3
MYD88
TLR1
IKBKG
UBC
SCARA3
RPS6KA3
CTSB
TNIP2
MAPKAPK2
PIK3R4
TLR3
TAB2
CD36
Scrambled 1
Scrambled 2
Scrambled 3
siFLI1 1
siFLI1 2
siFLI1 3

GO_NEGATIVE_REGULATION_OF_VIRAL_GENOME_REPLICATION
NES = 2.00, p = 0.0000, FDR q = 0.0209

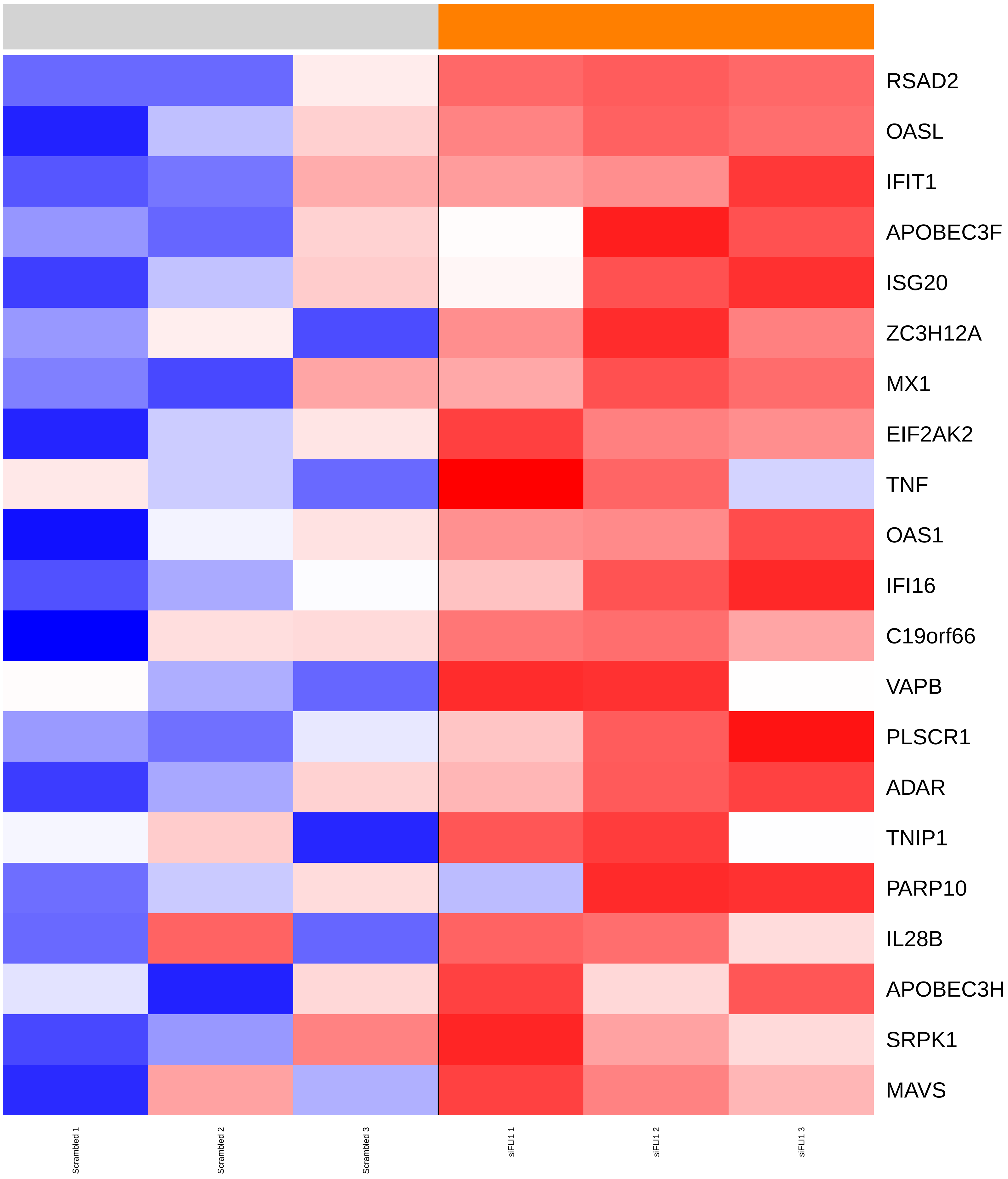

GO_REGULATION_OF_VASCULAR_ENDOTHELIAL_GROWTH_FACTOR_PRODUCTION
NES = 1.99, p = 0.0000, FDR q = 0.0226

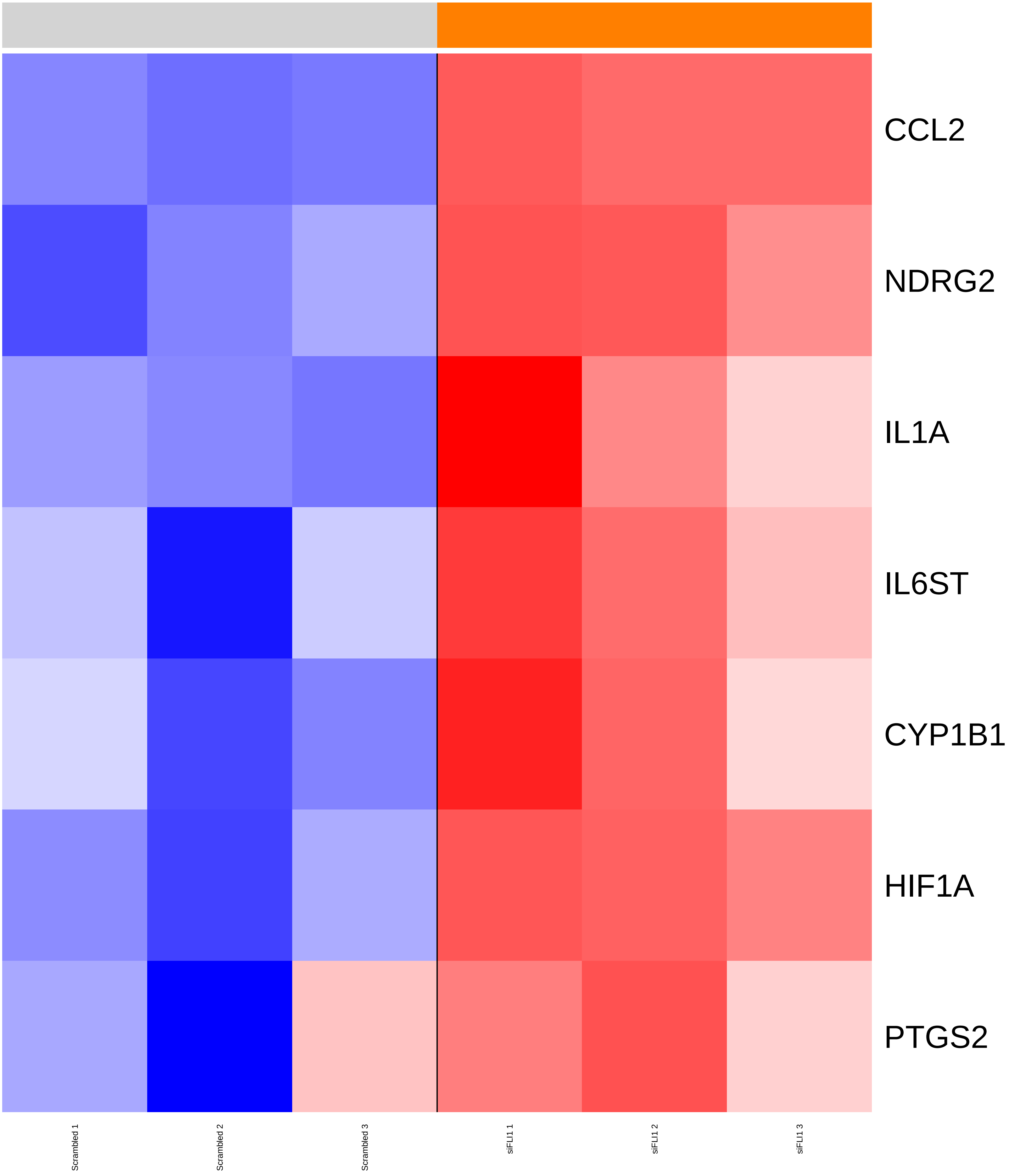

GO_ACTIVATION_OF_INNATE_IMMUNE_RESPONSE
NES = 1.99, p = 0.0000, FDR q = 0.0224

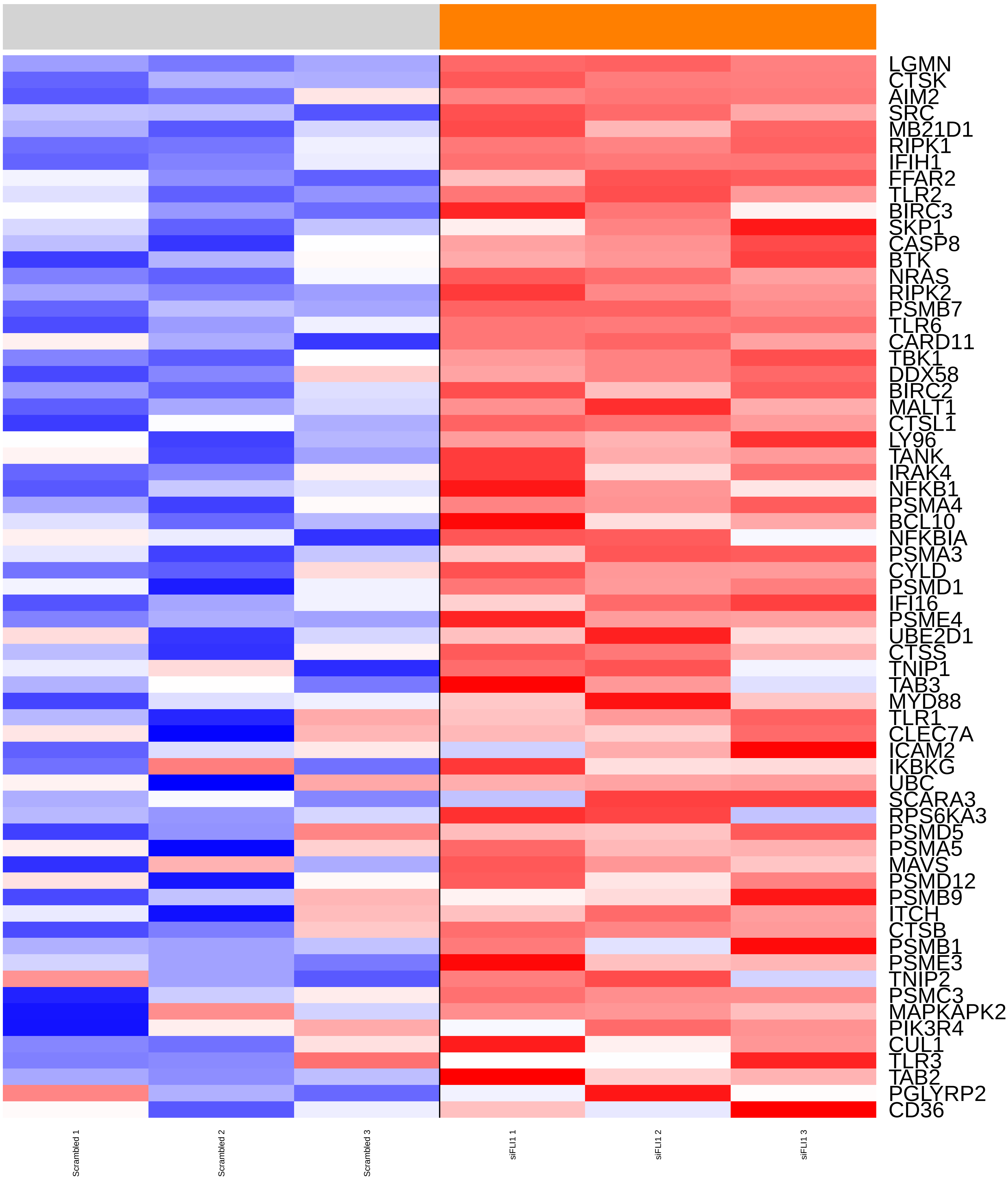

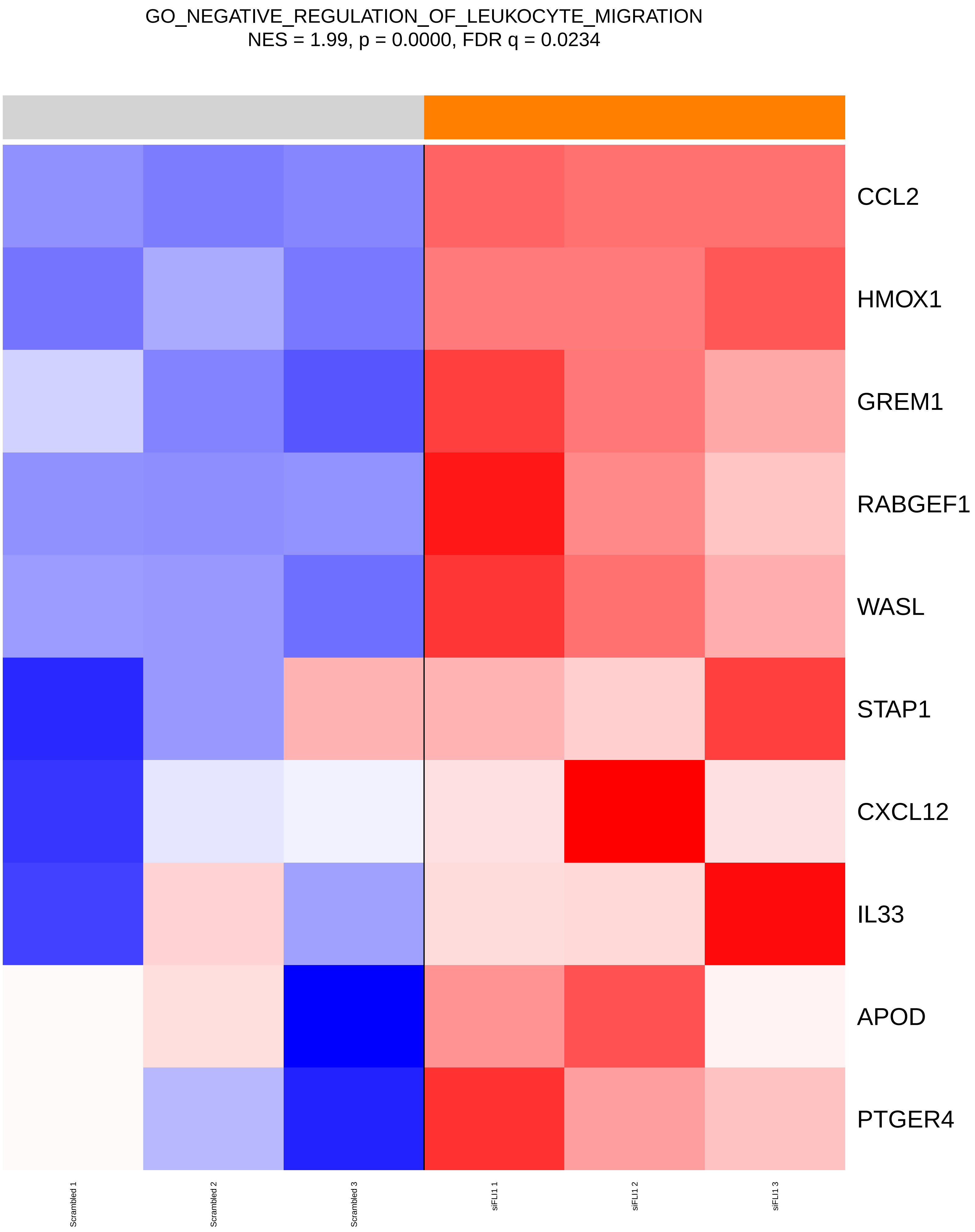
GO_NEGATIVE_REGULATION_OF_LEUKOCYTE_MIGRATION
NES = 1.99, p = 0.0000, FDR q = 0.0234
CCL2
HMOX1
GREM1
RABGEF1
WASL
STAP1
CXCL12
IL33
APOD
PTGER4
Scrambled 1
Scrambled 2
Scrambled 3
siFLI1 1
siFLI1 2
siFLI1 3

KEGG_CYTOSOLIC_DNA_SENSING_PATHWAY
NES = 1.98, p = 0.0000, FDR q = 0.0242

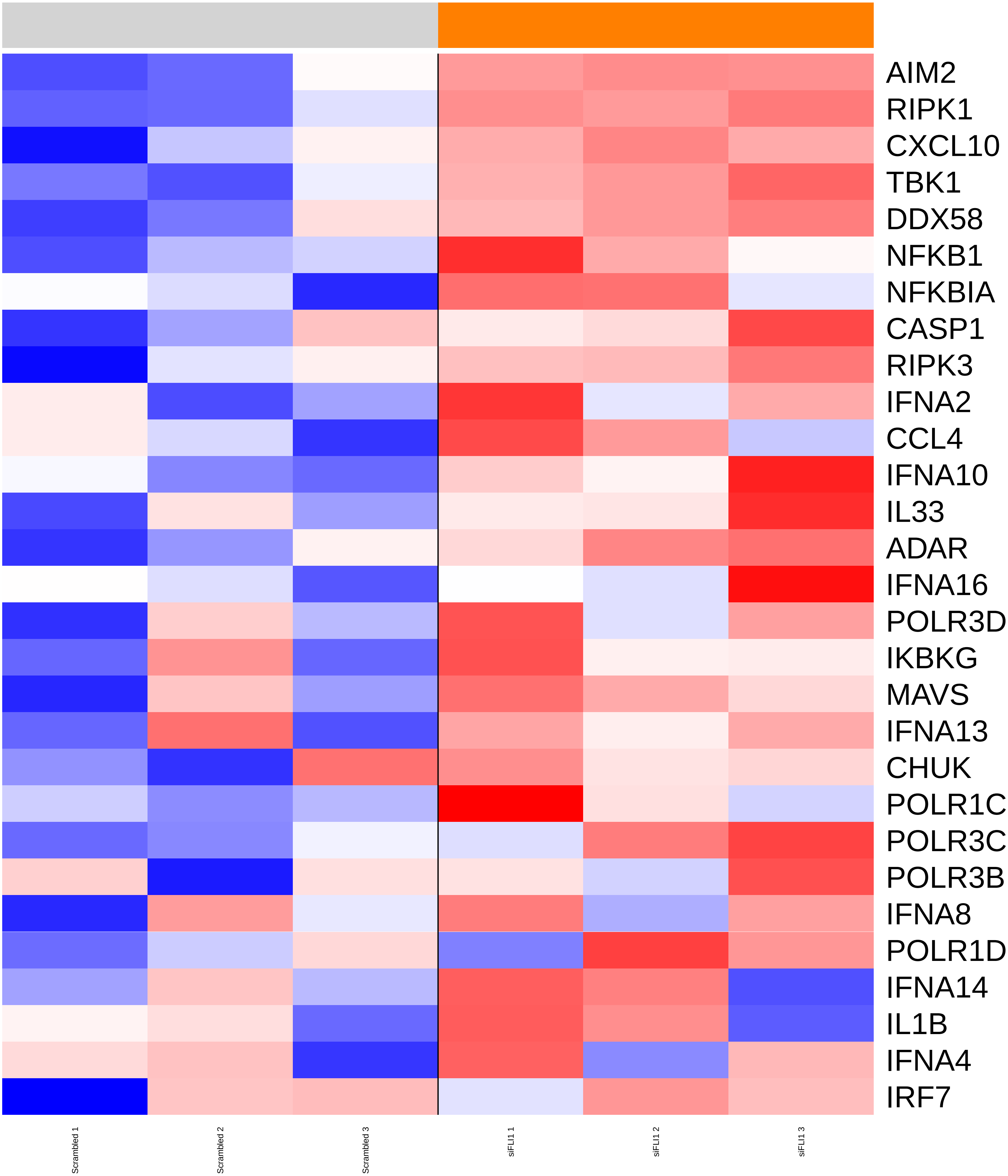

REACTOME_RIP_MEDIATED_NFKB_ACTIVATION_VIA_DAI
NES = 1.98, p = 0.0025, FDR q = 0.0240

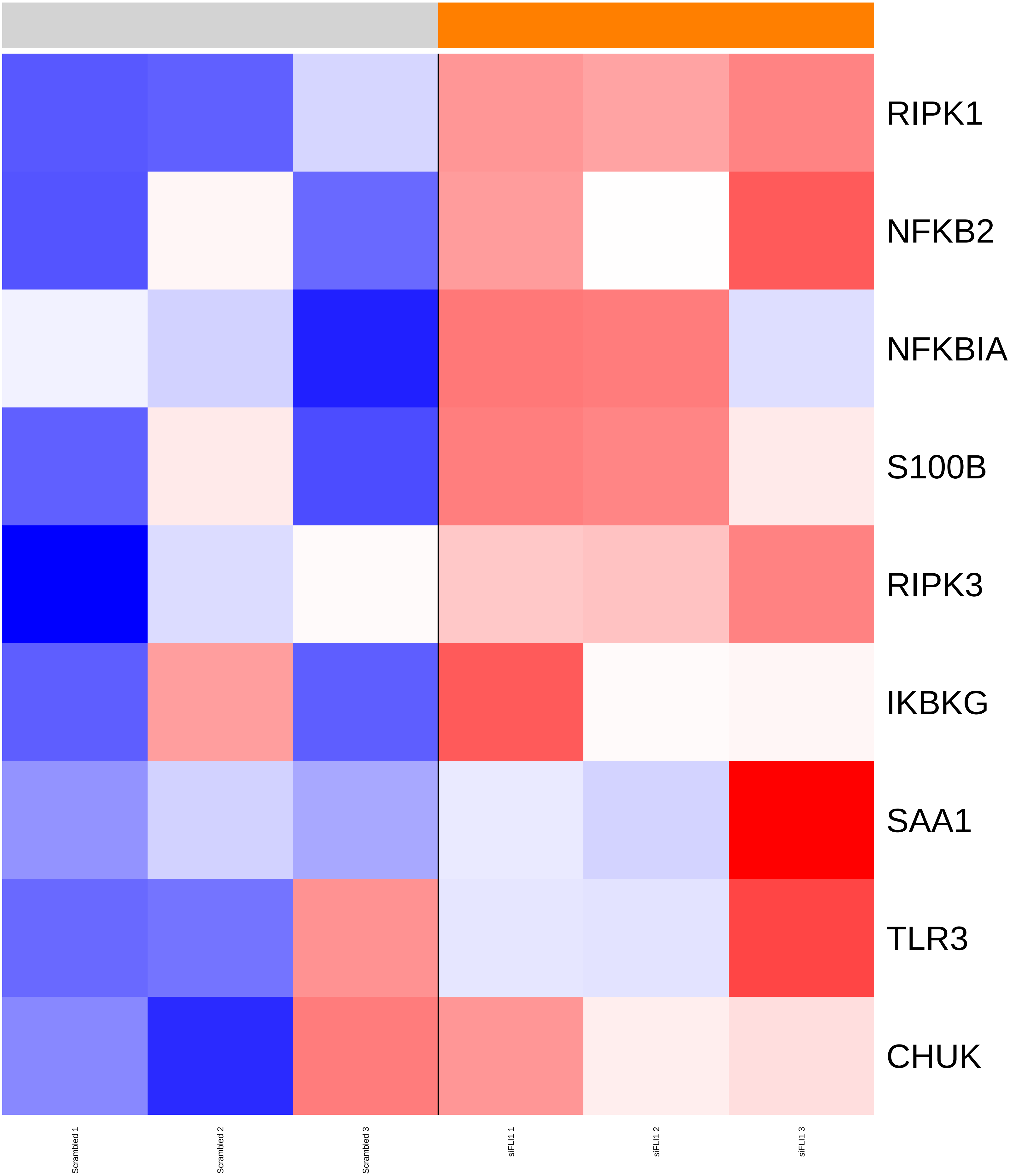

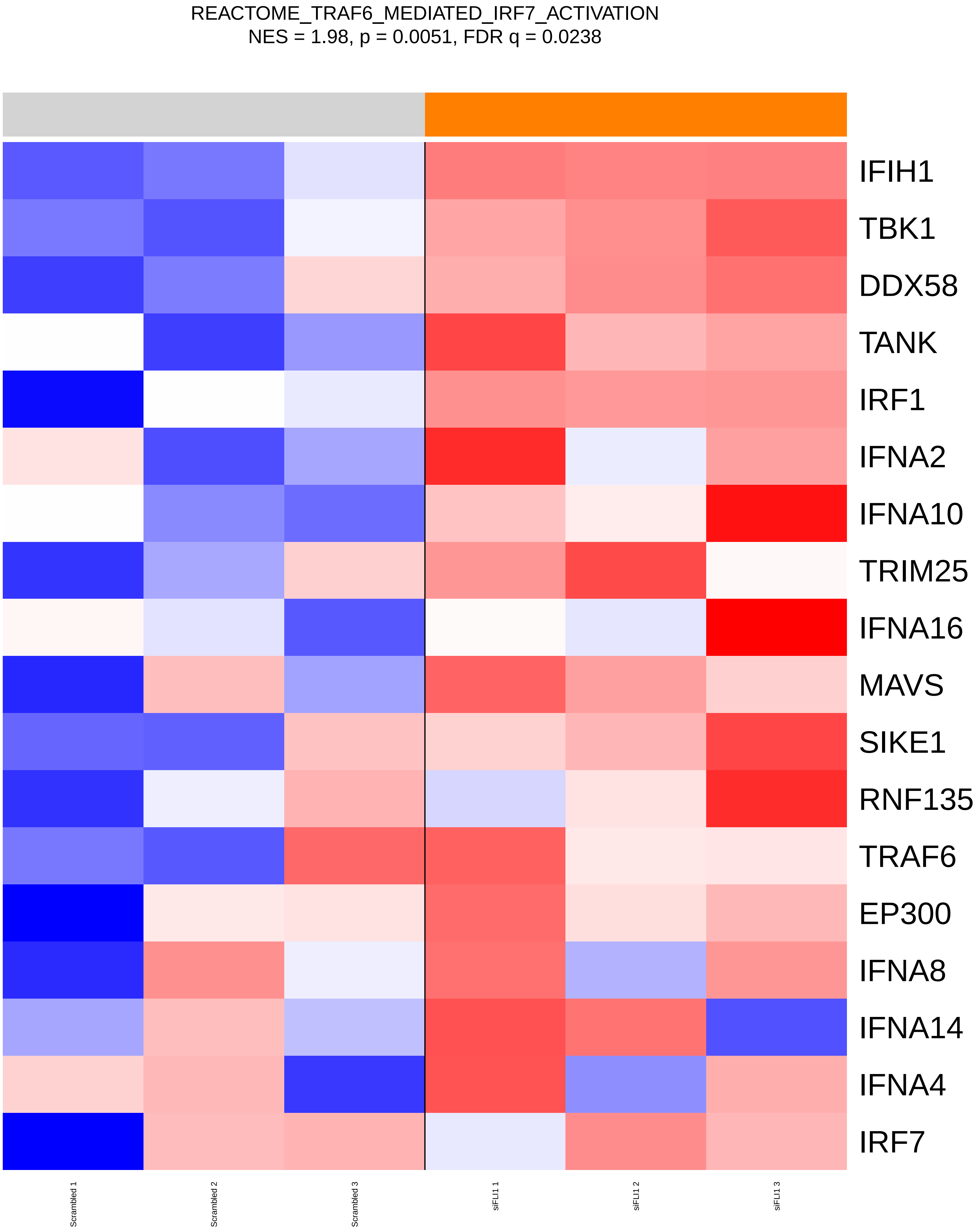
REACTOME_TRAF6_MEDIATED_IRF7_ACTIVATION
NES = 1.98, p = 0.0051, FDR q = 0.0238
IFIH1
TBK1
DDX58
TANK
IRF1
IFNA2
IFNA10
TRIM25
IFNA16
MAVS
SIKE1
RNF135
TRAF6
EP300
IFNA8
IFNA14
IFNA4
IRF7
Scrambled 1
Scrambled 2
Scrambled 3
siFLI1 1
siFLI1 2
siFLI1 3

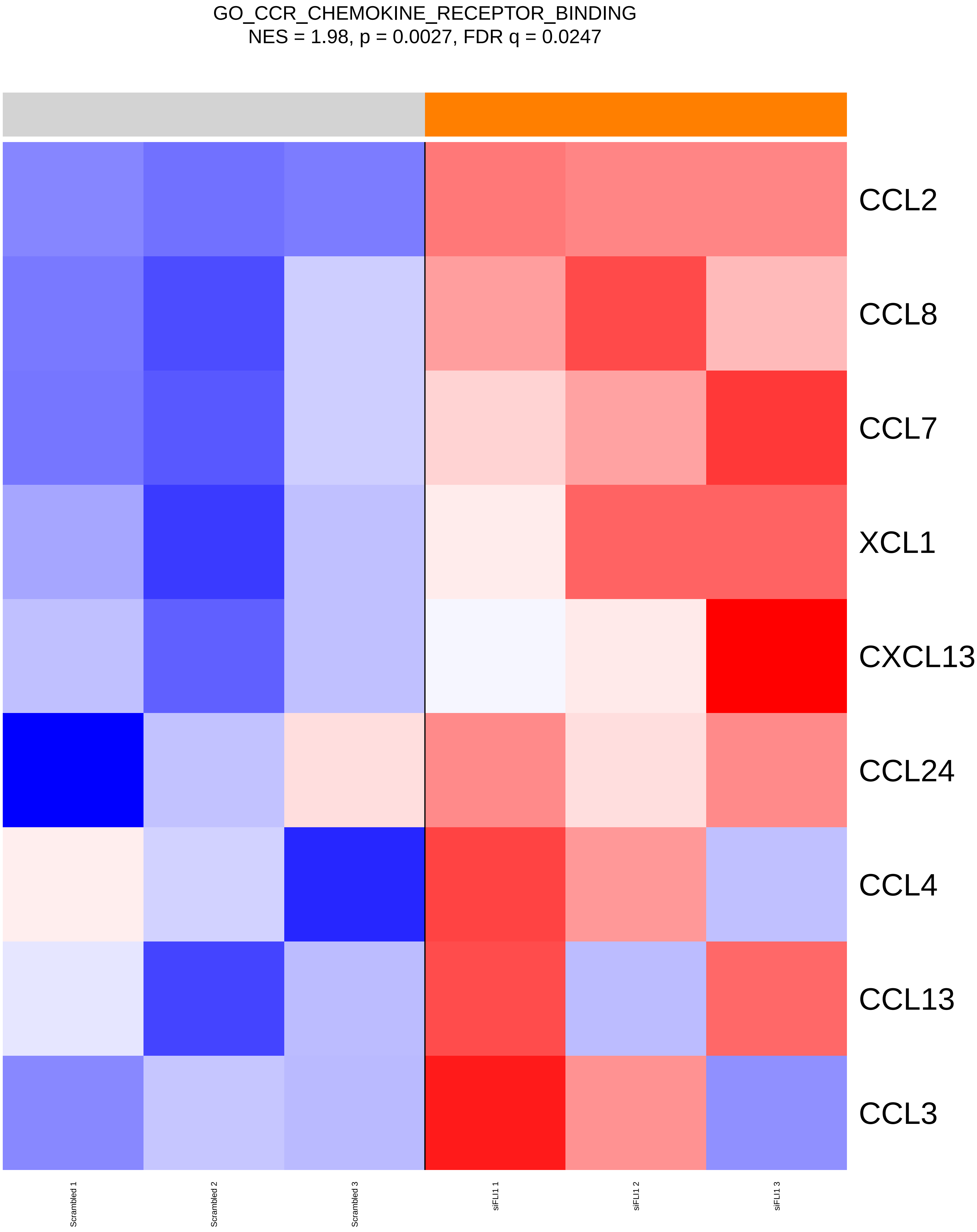
GO_CCR_CHEMOKINE_RECEPTOR_BINDING
NES = 1.98, p = 0.0027, FDR q = 0.0247
CCL2
CCL8
CCL7
XCL1
CXCL13
CCL24
CCL4
CCL13
CCL3
Scrambled 1
Scrambled 2
Scrambled 3
siFLI1 1
siFLI1 2
siFLI1 3

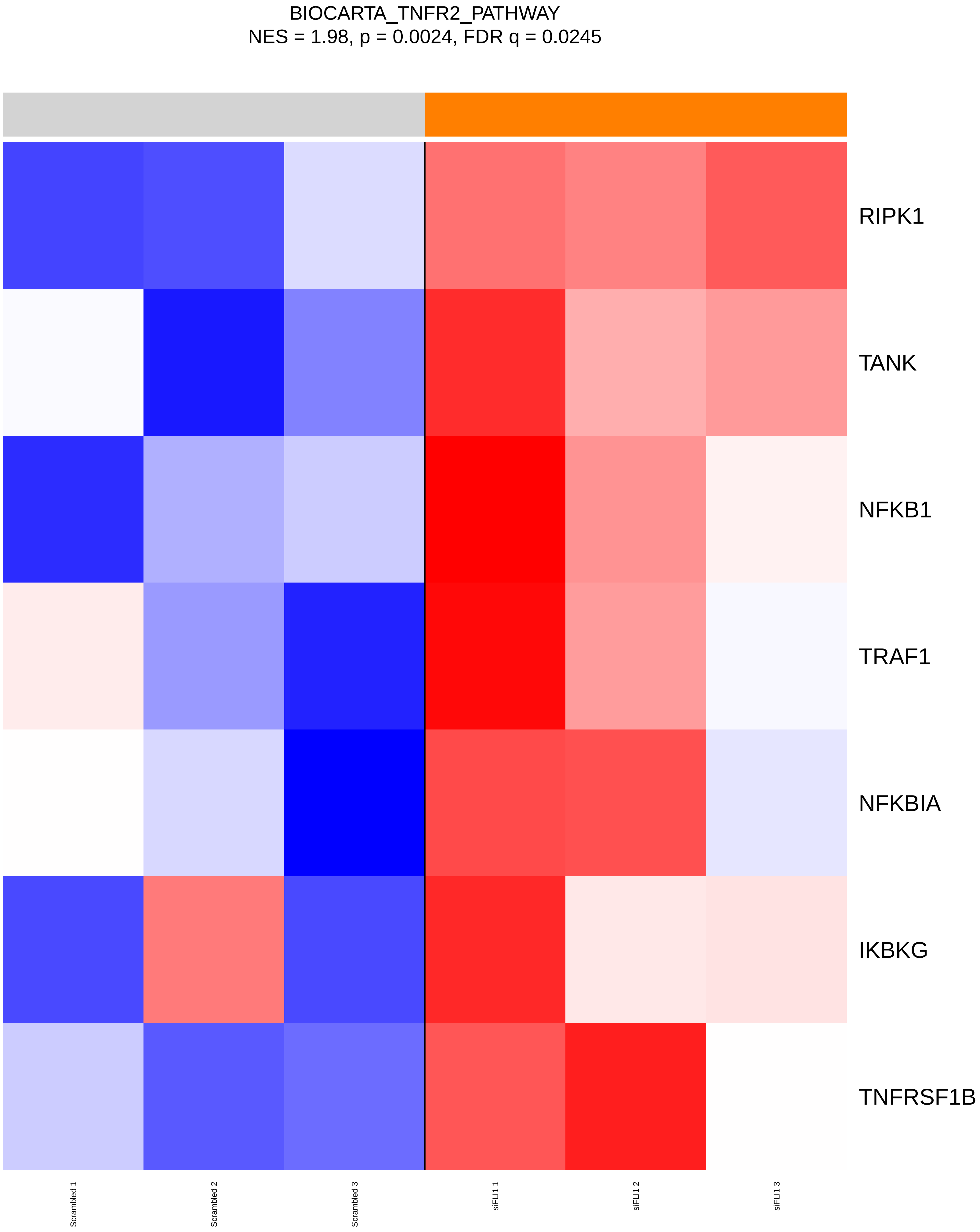

BIOCARTA_TNFR2_PATHWAY
NES = 1.98, p = 0.0024, FDR q = 0.0245
RIPK1
TANK
NFKB1
TRAF1
NFKBIA
IKBKG
TNFRSF1B
Scrambled 1
Scrambled 2
Scrambled 3
siFLI1 1
siFLI1 2
siFLI1 3

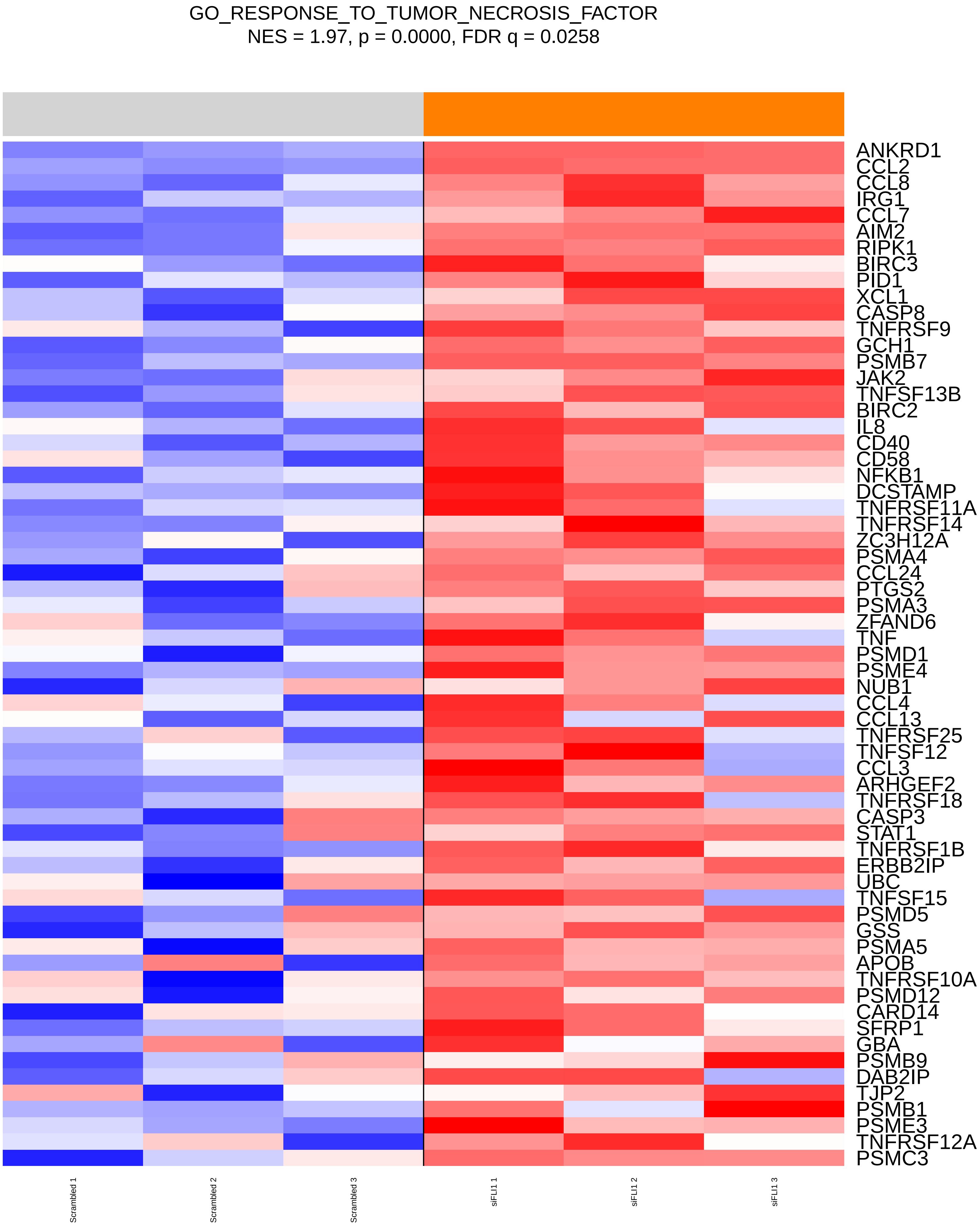
GO_RESPONSE_TO_TUMOR_NECROSIS_FACTOR
NES = 1.97, p = 0.0000, FDR q = 0.0258
ANKRD1
CCL2
CCL8
IRG1
CCL7
AIM2
RIPK1
BIRC3
PID1
XCL1
CASP8
TNFRSF9
GCH1
PSMB7
JAK2
TNFSF13B
BIRC2
IL8
CD40
CD58
NFKB1
DCSTAMP
TNFRSF11A
TNFRSF14
ZC3H12A
PSMA4
CCL24
PTGS2
PSMA3
ZFAND6
TNF
PSMD1
PSME4
NUB1
CCL4
CCL13
TNFRSF25
TNFSF12
CCL3
ARHGEF2
TNFRSF18
CASP3
STAT1
TNFRSF1B
ERBB2IP
UBC
TNFSF15
PSMD5
GSS
PSMA5
APOB
TNFRSF10A
PSMD12
CARD14
SFRP1
GBA
PSMB9
DAB2IP
TJP2
PSMB1
PSME3
TNFRSF12A
PSMC3
Scrambled 1
Scrambled 2
Scrambled 3
siFLI1 1
siFLI1 2
siFLI1 3

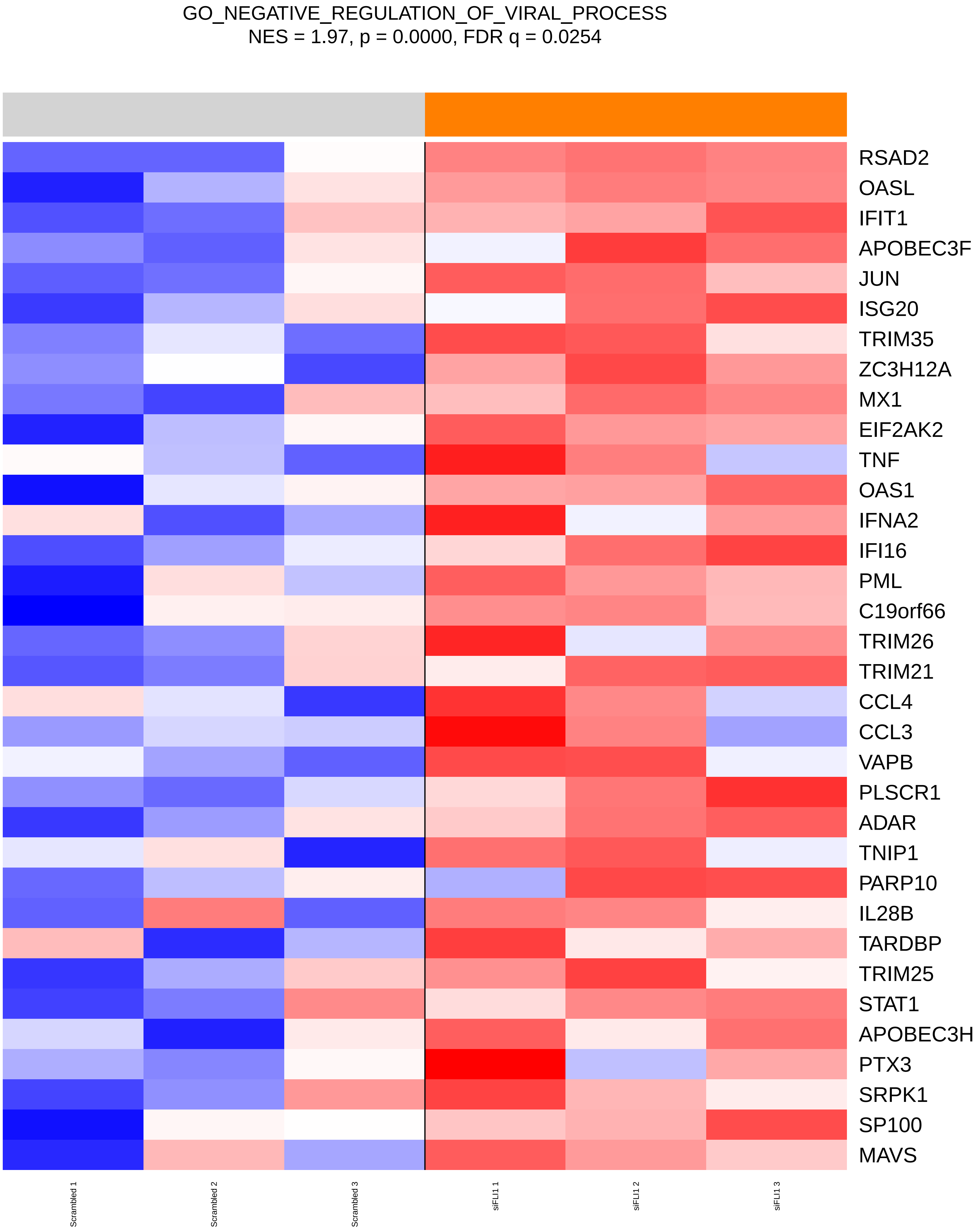
GO_NEGATIVE_REGULATION_OF_VIRAL_PROCESS
NES = 1.97, p = 0.0000, FDR q = 0.0254
RSAD2
OASL
IFIT1
APOBEC3F
JUN
ISG20
TRIM35
ZC3H12A
MX1
EIF2AK2
TNF
OAS1
IFNA2
IFI16
PML
C19orf66
TRIM26
TRIM21
CCL4
CCL3
VAPB
PLSCR1
ADAR
TNIP1
PARP10
IL28B
TARDBP
TRIM25
STAT1
APOBEC3H
PTX3
SRPK1
SP100
MAVS
Scrambled 1
Scrambled 2
Scrambled 3
siFLI1 1
siFLI1 2
siFLI1 3

BIOCARTA_IL10_PATHWAY
NES = 1.97, p = 0.0000, FDR q = 0.0253

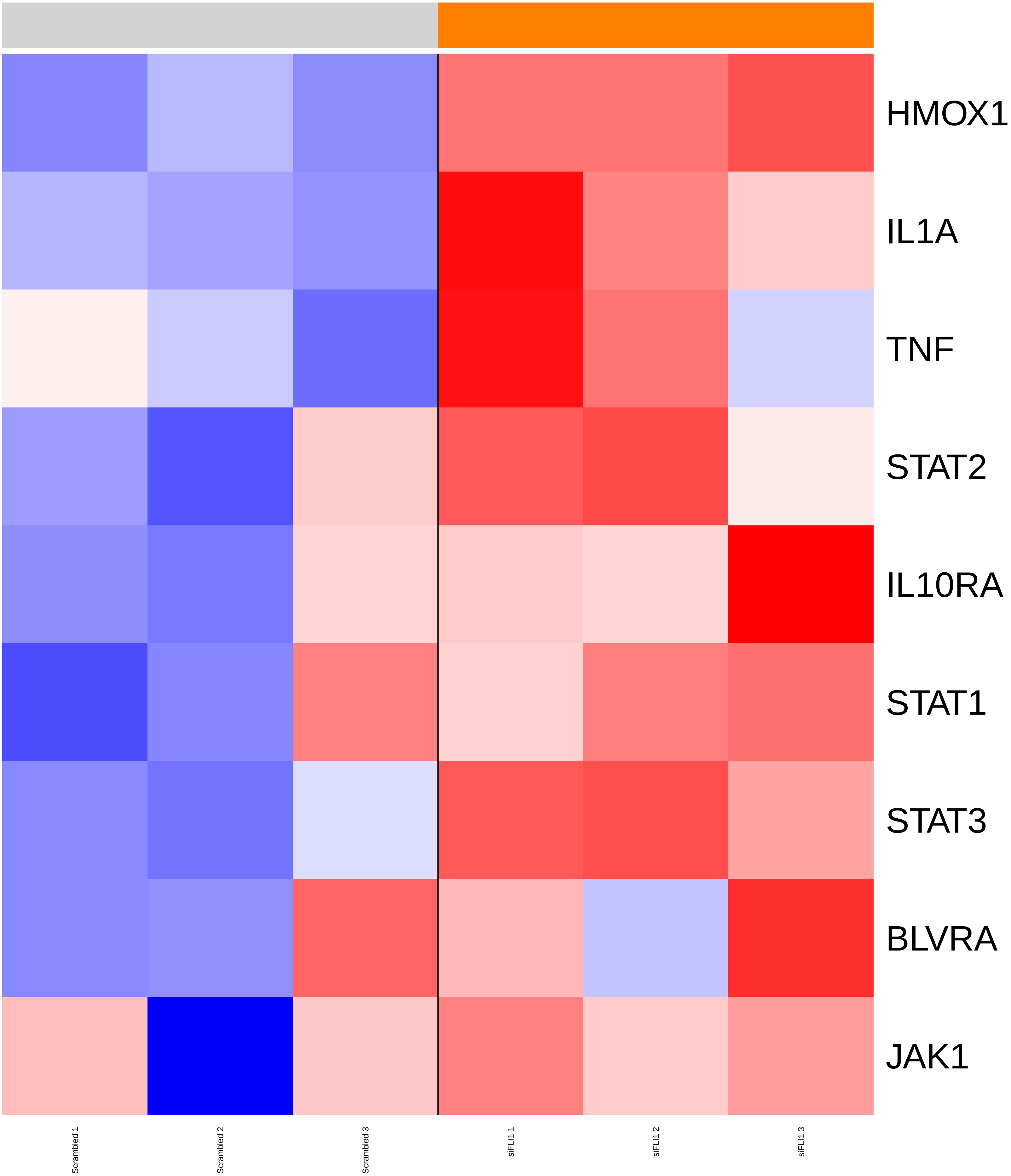

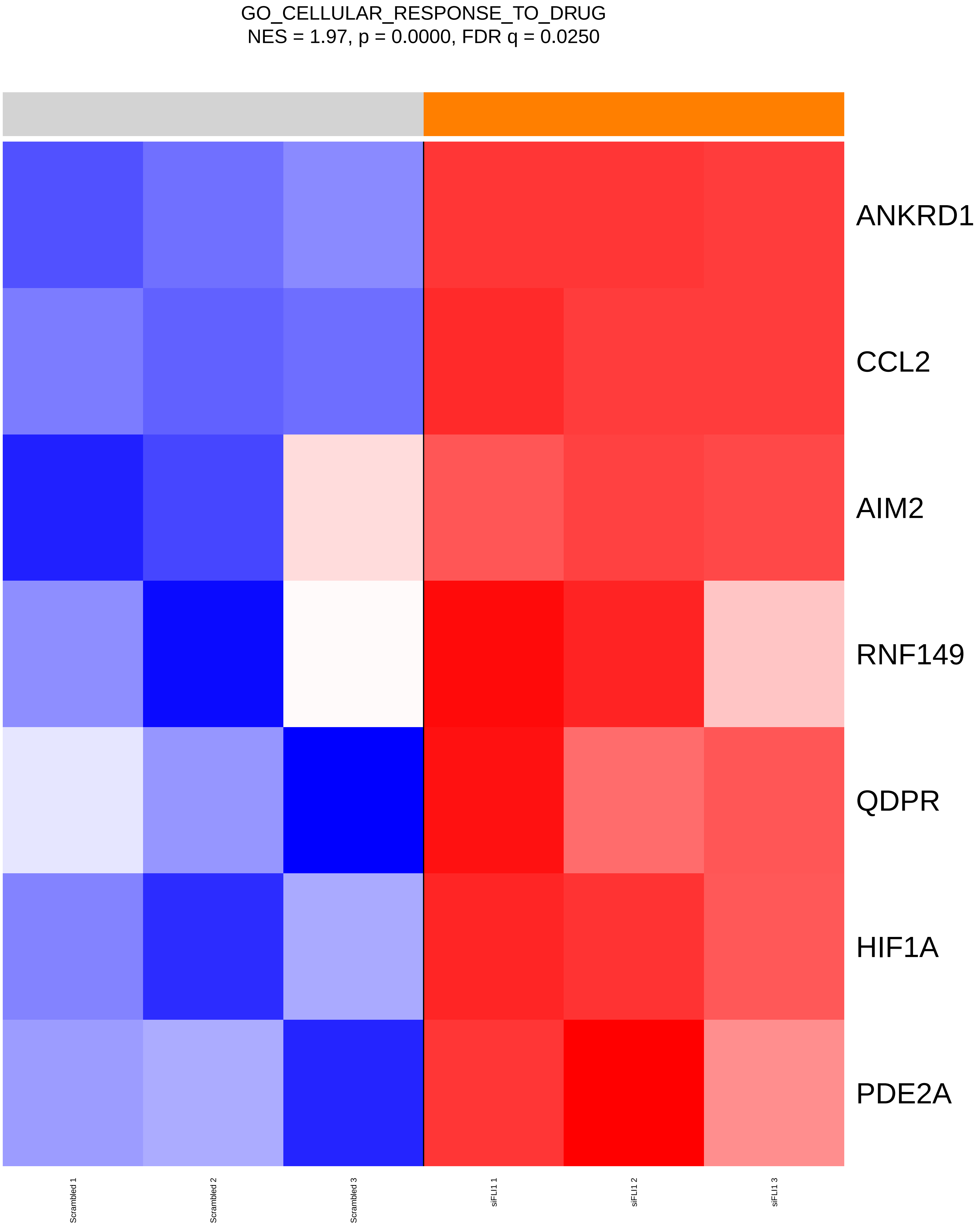
GO_CELLULAR_RESPONSE_TO_DRUG
NES = 1.97, p = 0.0000, FDR q = 0.0250
ANKRD1
CCL2
AIM2
RNF149
QDPR
HIF1A
PDE2A
Scrambled 1
Scrambled 2
Scrambled 3
siFLI1 1
siFLI1 2
siFLI1 3

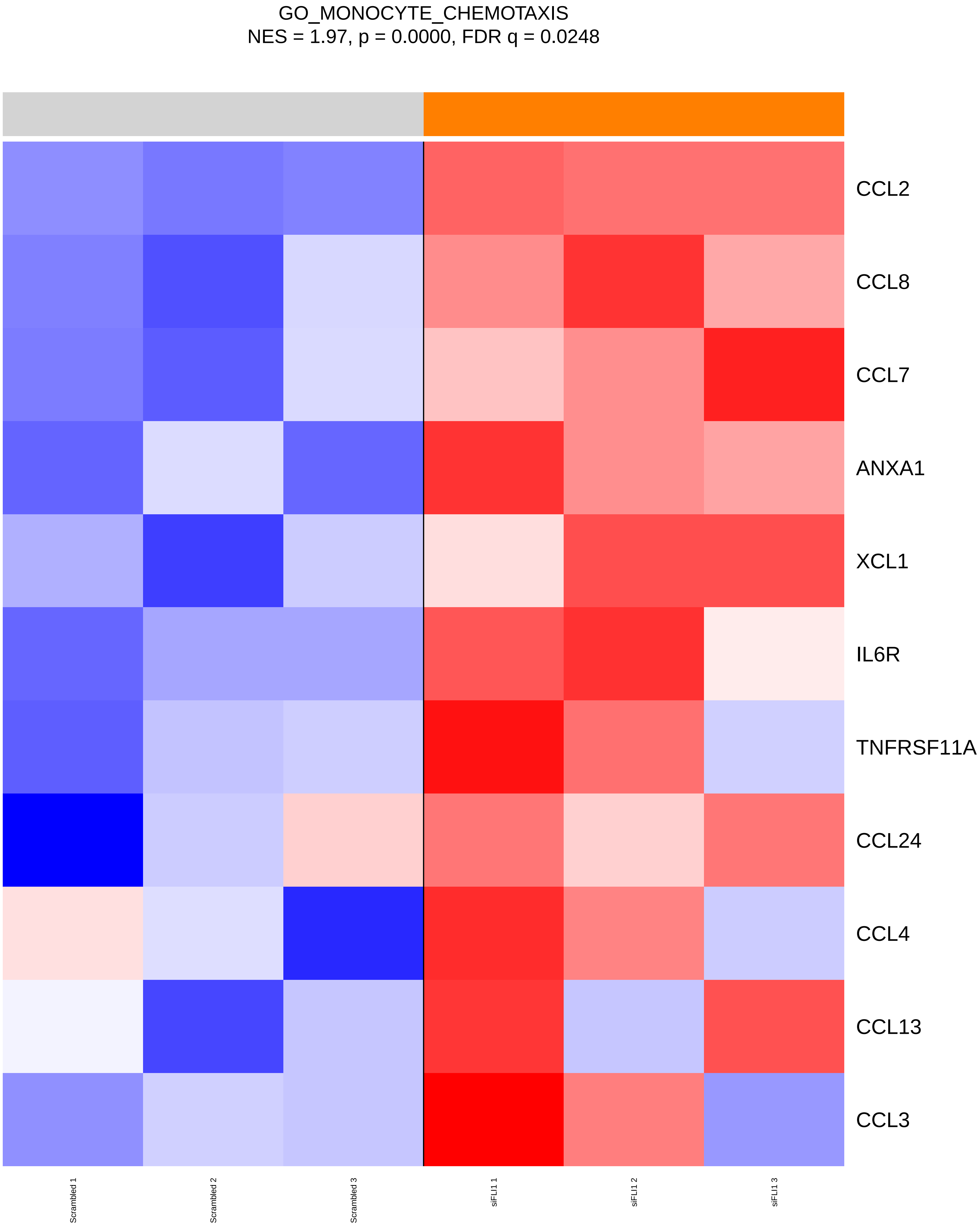
GO_MONOCYTE_CHEMOTAXIS
NES = 1.97, p = 0.0000, FDR q = 0.0248
CCL2
CCL8
CCL7
ANXA1
XCL1
IL6R
TNFRSF11A
CCL24
CCL4
CCL13
CCL3
Scrambled 1
Scrambled 2
Scrambled 3
siFLI1 1
siFLI1 2
siFLI1 3

GO_CYTOKINE_RECEPTOR_BINDING
NES = 1.97, p = 0.0000, FDR q = 0.0244

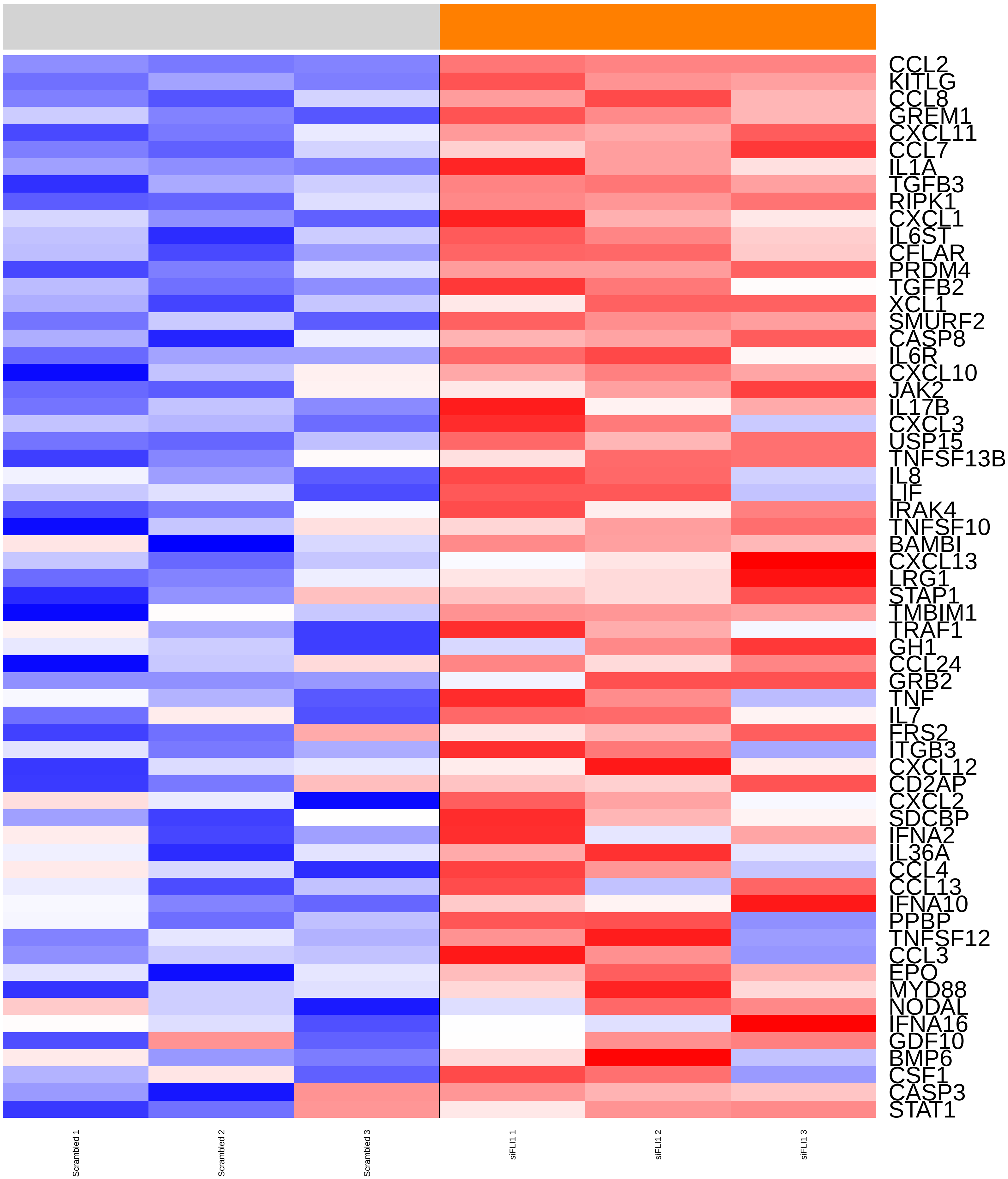

GO_POSITIVE_REGULATION_OF_DEFENSE_RESPONSE
NES = 1.96, p = 0.0000, FDR q = 0.0248

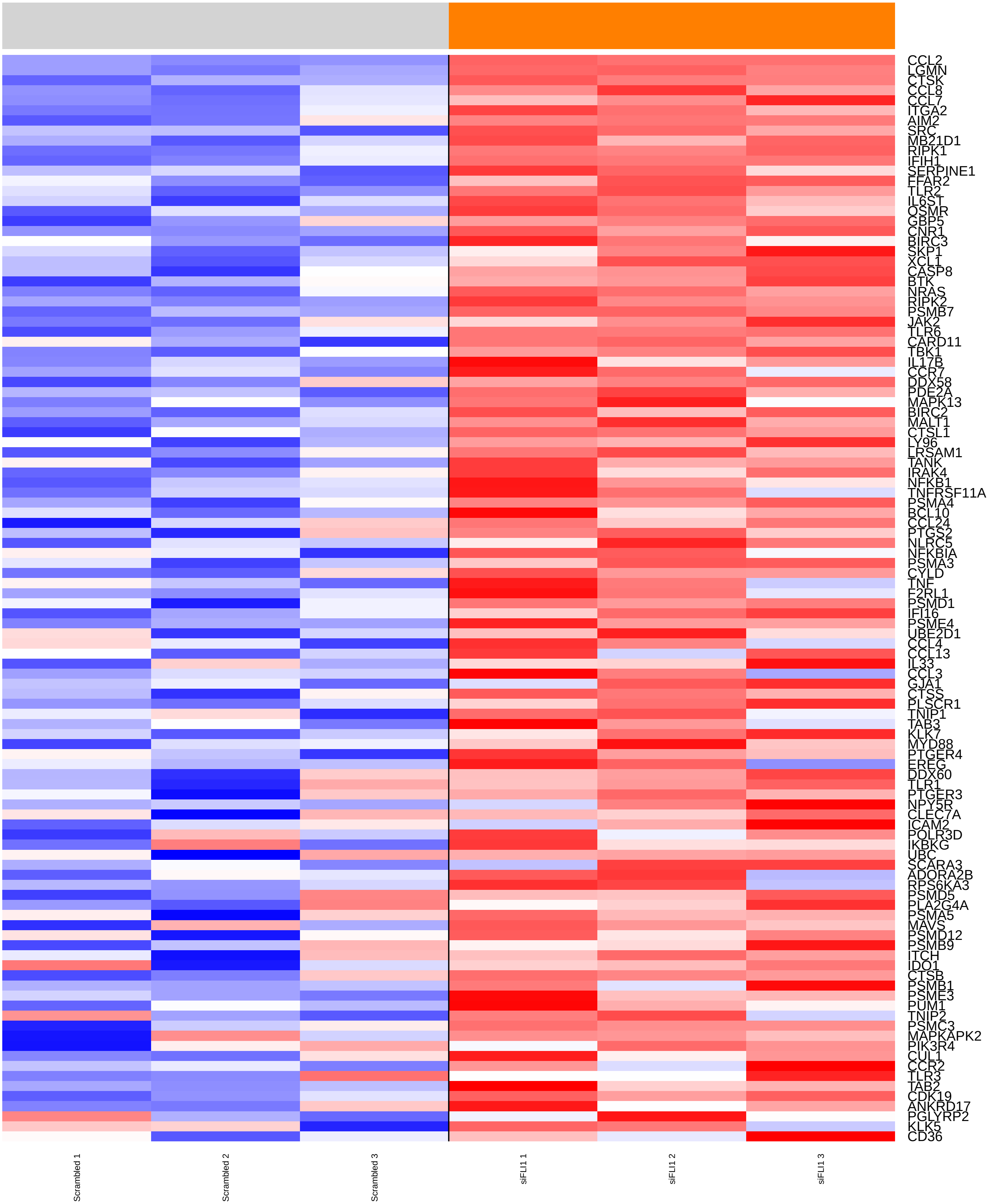

GO_REGULATION_OF_HYDROGEN_PEROXIDE_INDUCED_CELL_DEATH
NES = 1.96, p = 0.0025, FDR q = 0.0252

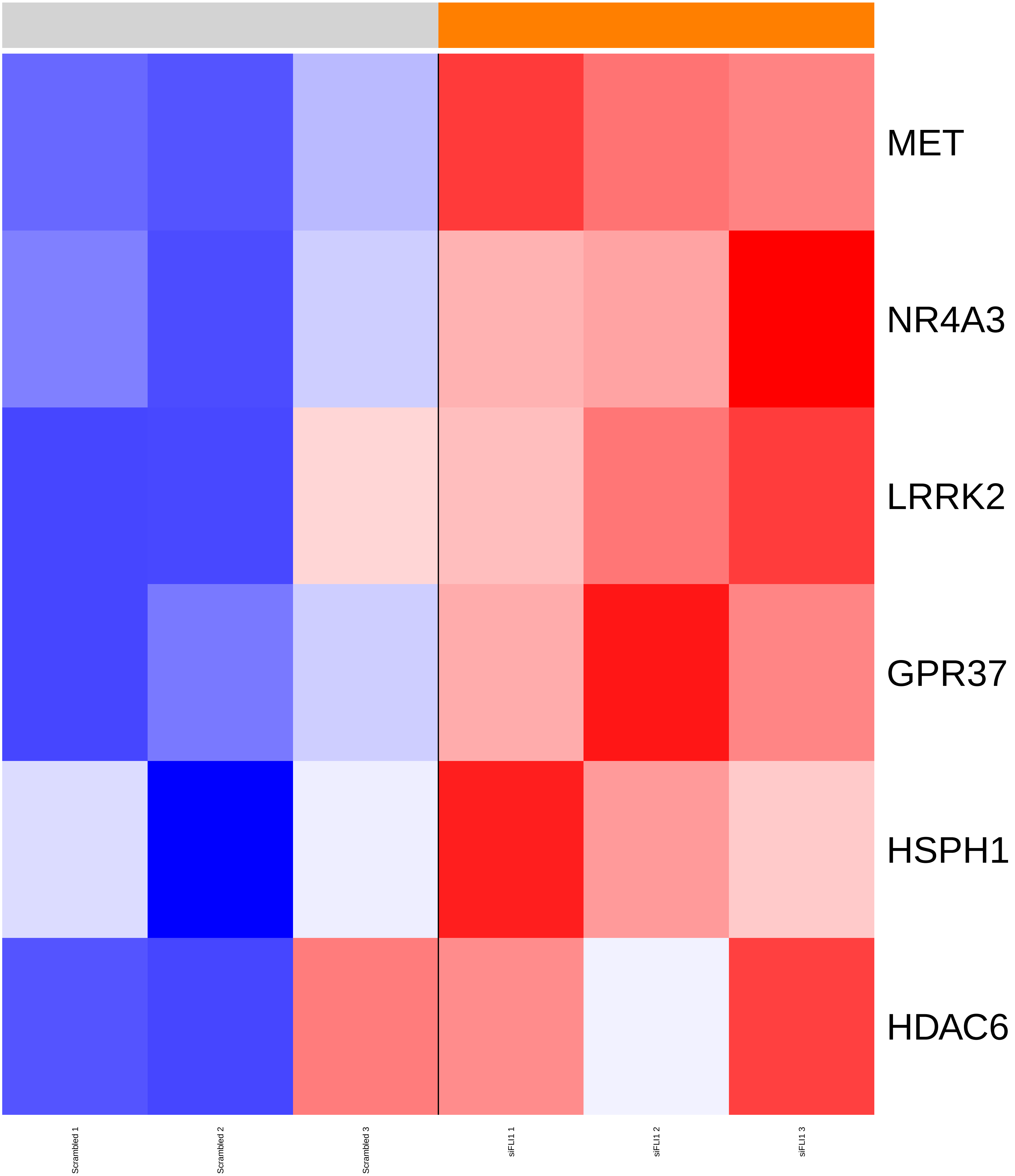

KEGG_LEISHMANIA_INFECTION
NES = 1.96, p = 0.0000, FDR q = 0.0255

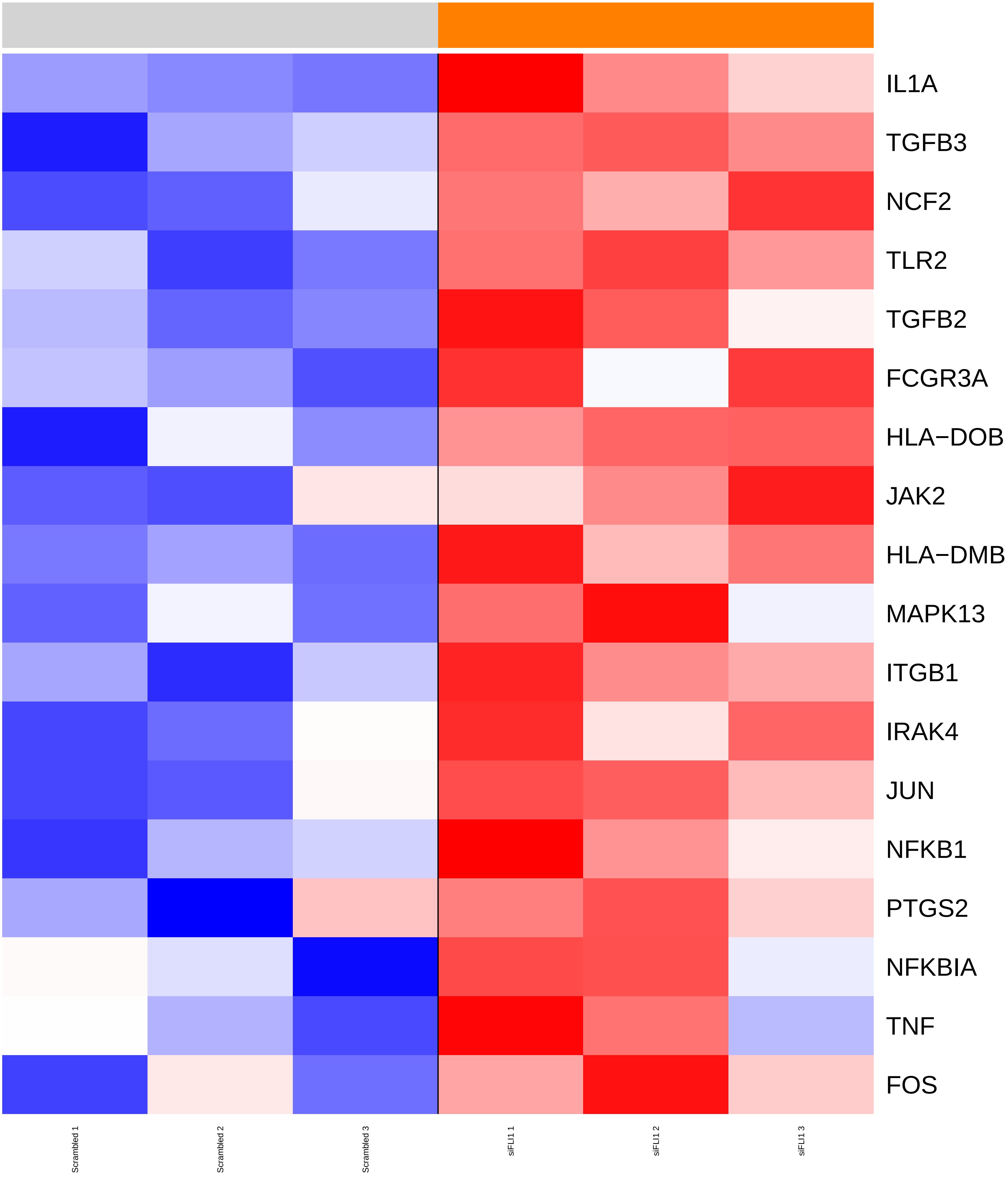

HALLMARK_IL6_JAK_STAT3_SIGNALING
NES = 1.95, p = 0.0000, FDR q = 0.0260

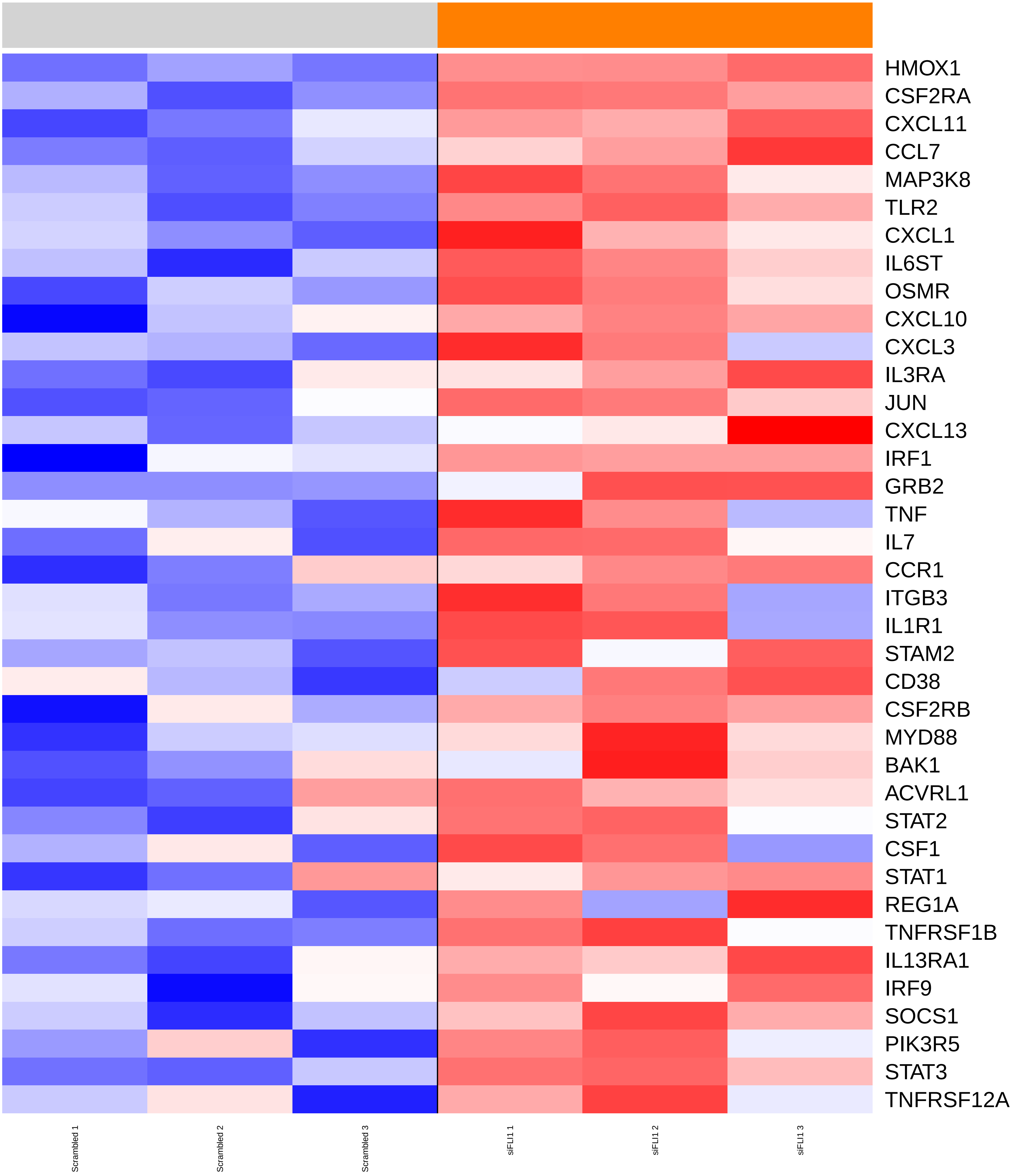

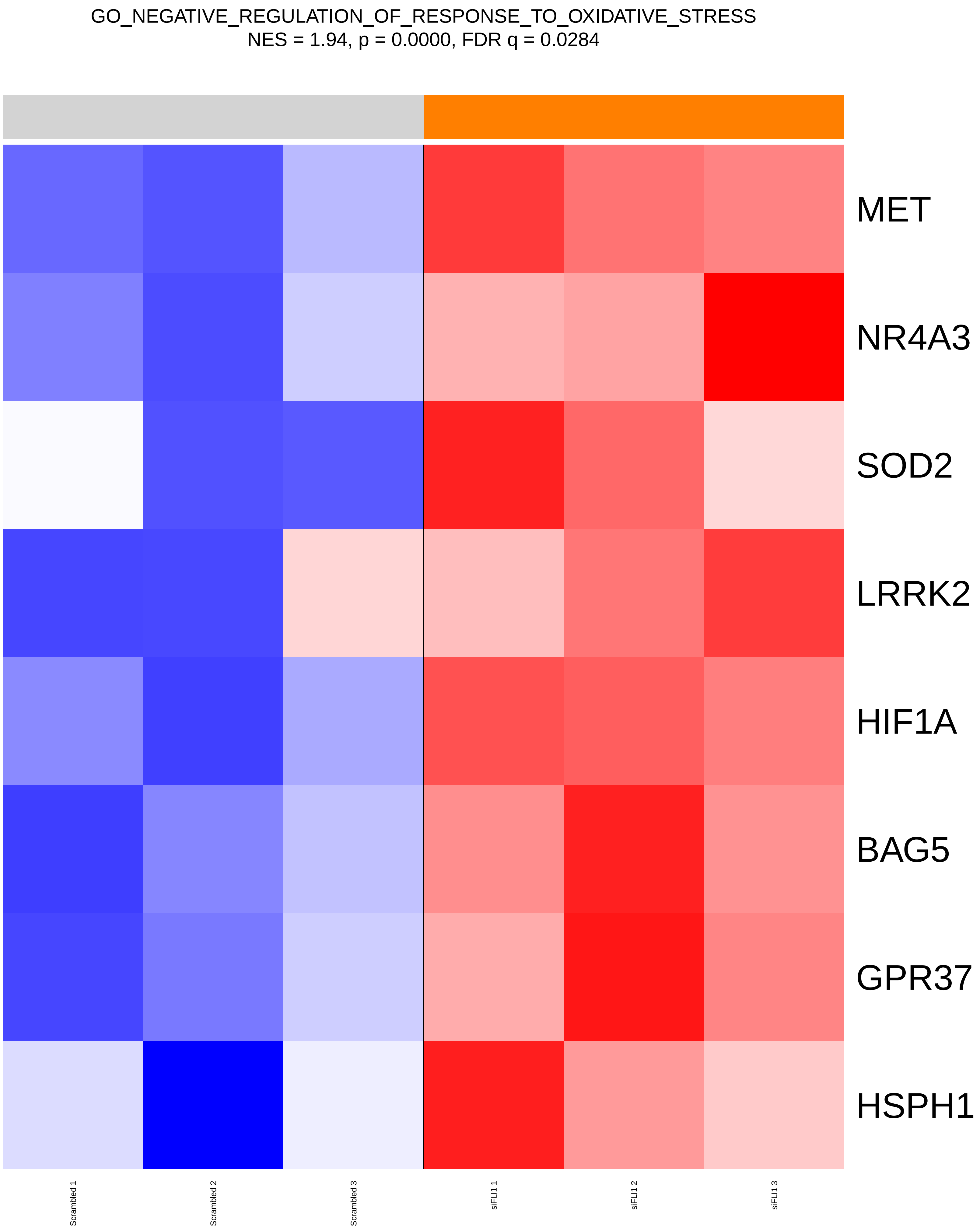
GO_NEGATIVE_REGULATION_OF_RESPONSE_TO_OXIDATIVE_STRESS
NES = 1.94, p = 0.0000, FDR q = 0.0284
MET
NR4A3
SOD2
LRRK2
HIF1A
BAG5
GPR37
HSPH1
Scrambled 1
Scrambled 2
Scrambled 3
siFLI1 1
siFLI1 2
siFLI1 3

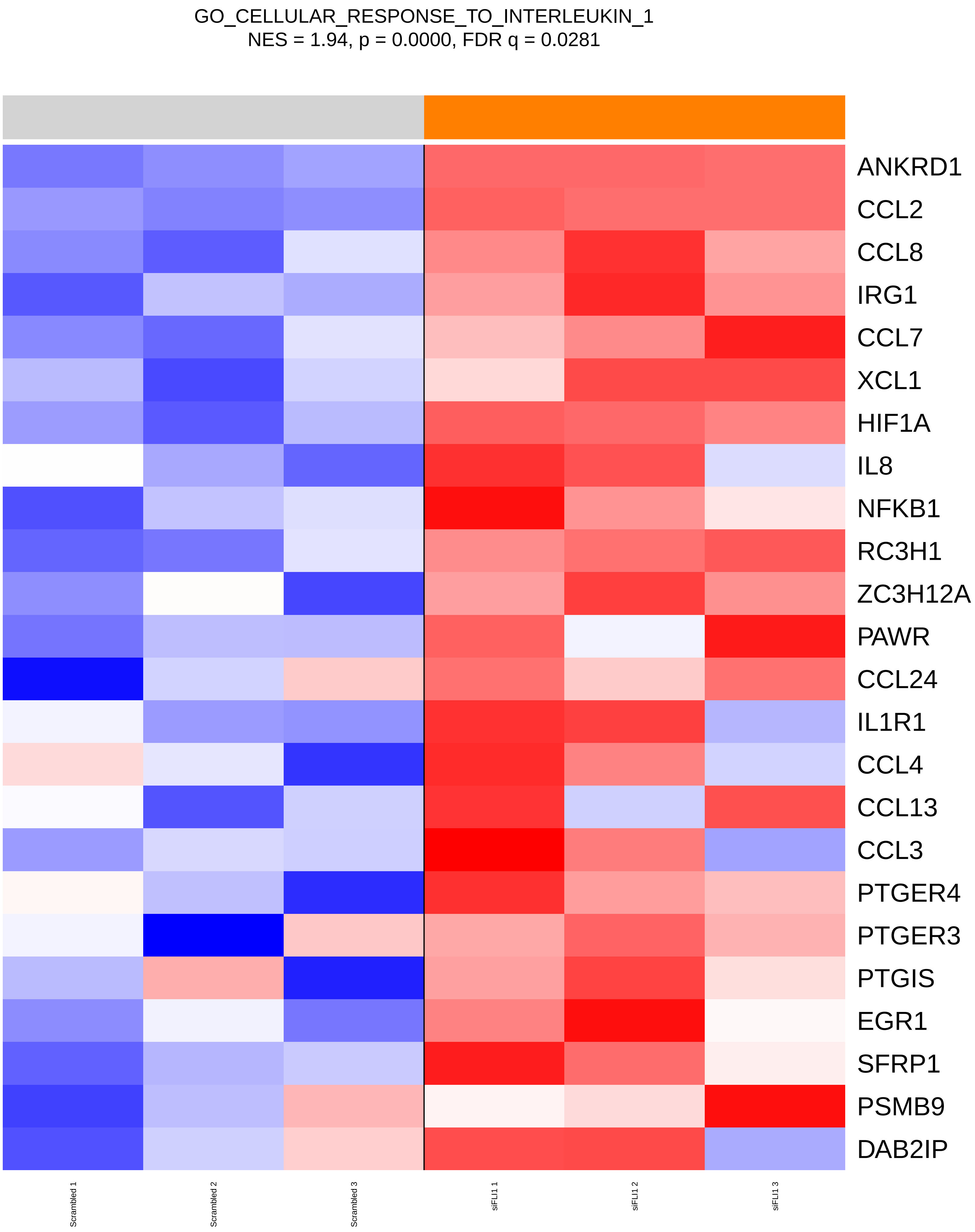
GO_CELLULAR_RESPONSE_TO_INTERLEUKIN_1
NES = 1.94, p = 0.0000, FDR q = 0.0281
ANKRD1
CCL2
CCL8
IRG1
CCL7
XCL1
HIF1A
IL8
NFKB1
RC3H1
ZC3H12A
PAWR
CCL24
IL1R1
CCL4
CCL13
CCL3
PTGER4
PTGER3
PTGIS
EGR1
SFRP1
PSMB9
DAB2IP
Scrambled 1
Scrambled 2
Scrambled 3
siFLI1 1
siFLI1 2
siFLI1 3

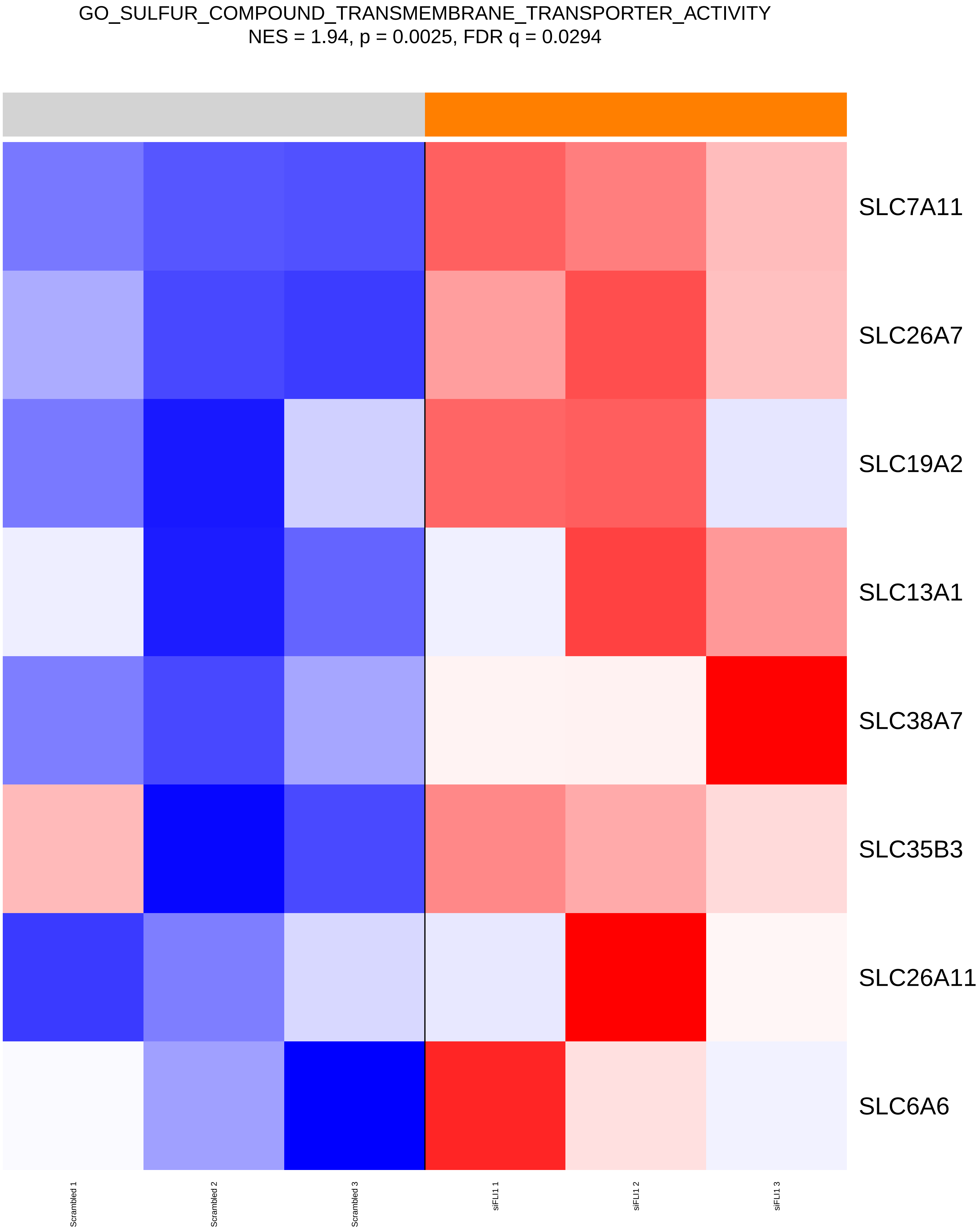
GO_SULFUR_COMPOUND_TRANSMEMBRANE_TRANSPORTER_ACTIVITY
NES = 1.94, p = 0.0025, FDR q = 0.0294
SLC7A11
SLC26A7
SLC19A2
SLC13A1
SLC38A7
SLC35B3
SLC26A11
SLC6A6
Scrambled 1
Scrambled 2
Scrambled 3
siFLI1 1
siFLI1 2
siFLI1 3

BIOCARTA_NFKB_PATHWAY
NES = 1.94, p = 0.0025, FDR q = 0.0297

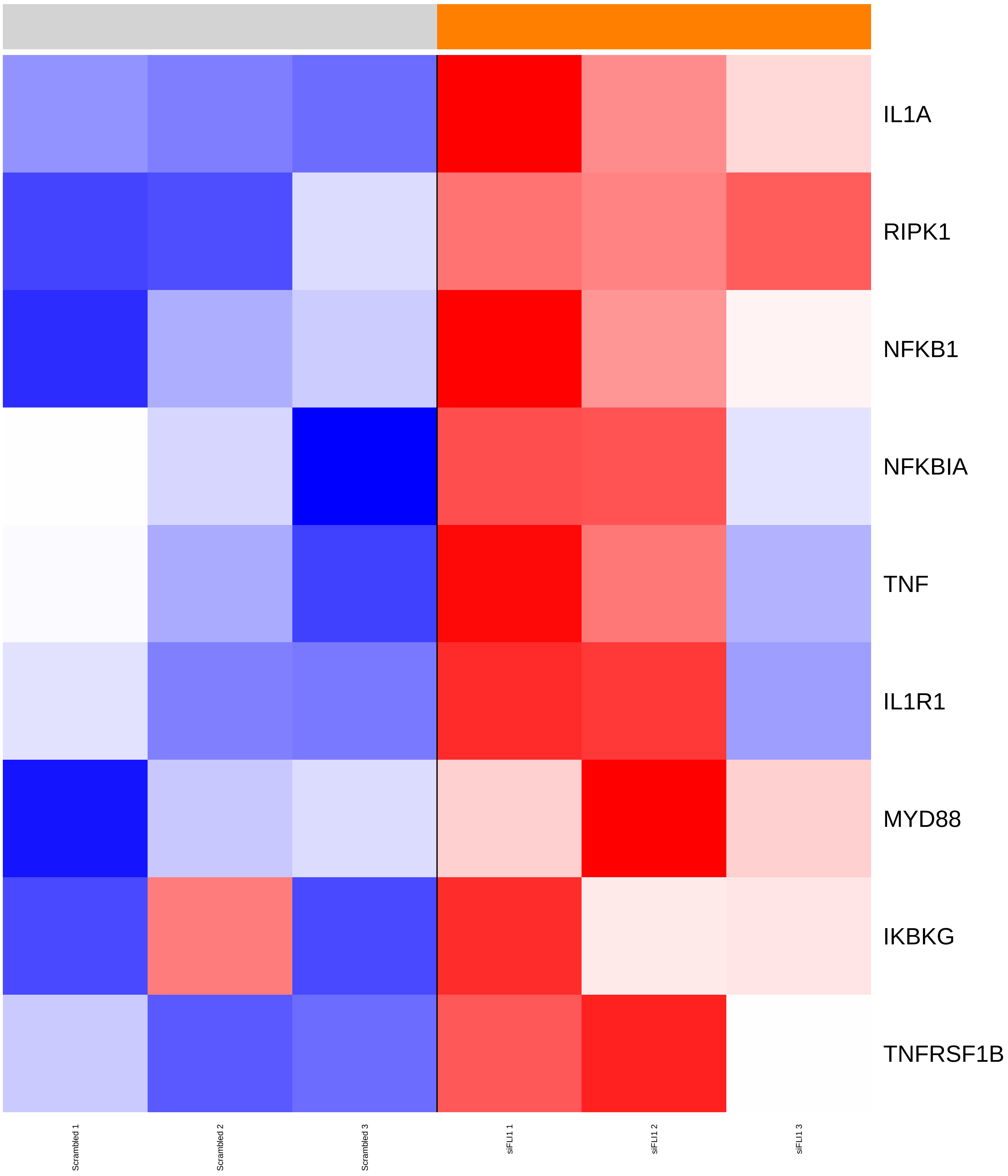

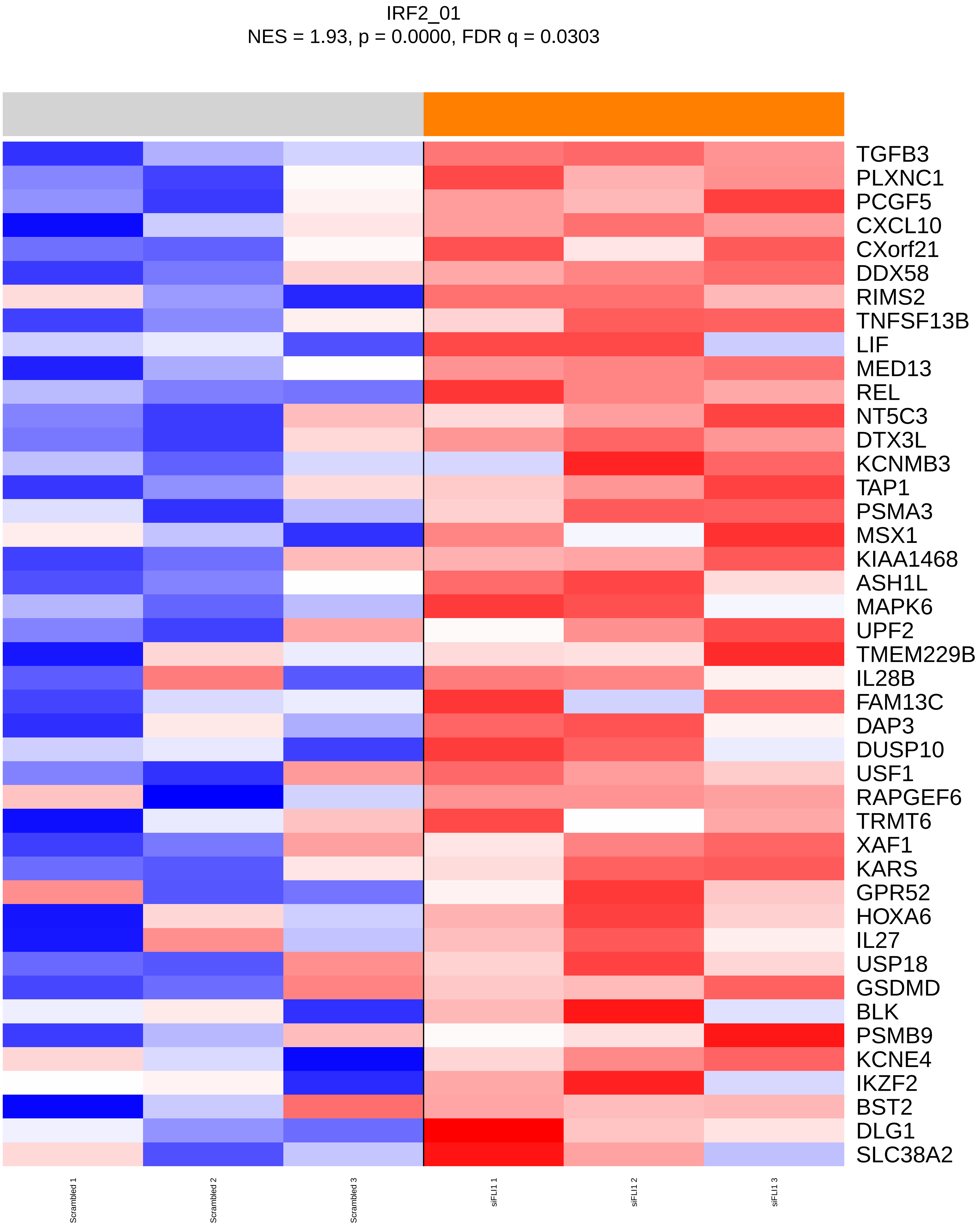
IRF2_01
NES = 1.93, p = 0.0000, FDR q = 0.0303
TGFB3
PLXNC1
PCGF5
CXCL10
CXorf21
DDX58
RIMS2
TNFSF13B
LIF
MED13
REL
NT5C3
DTX3L
KCNMB3
TAP1
PSMA3
MSX1
KIAA1468
ASH1L
MAPK6
UPF2
TMEM229B
IL28B
FAM13C
DAP3
DUSP10
USF1
RAPGEF6
TRMT6
XAF1
KARS
GPR52
HOXA6
IL27
USP18
GSDMD
BLK
PSMB9
KCNE4
IKZF2
BST2
DLG1
SLC38A2
Scrambled 1
Scrambled 2
Scrambled 3
siFLI1 1
siFLI1 2
siFLI1 3

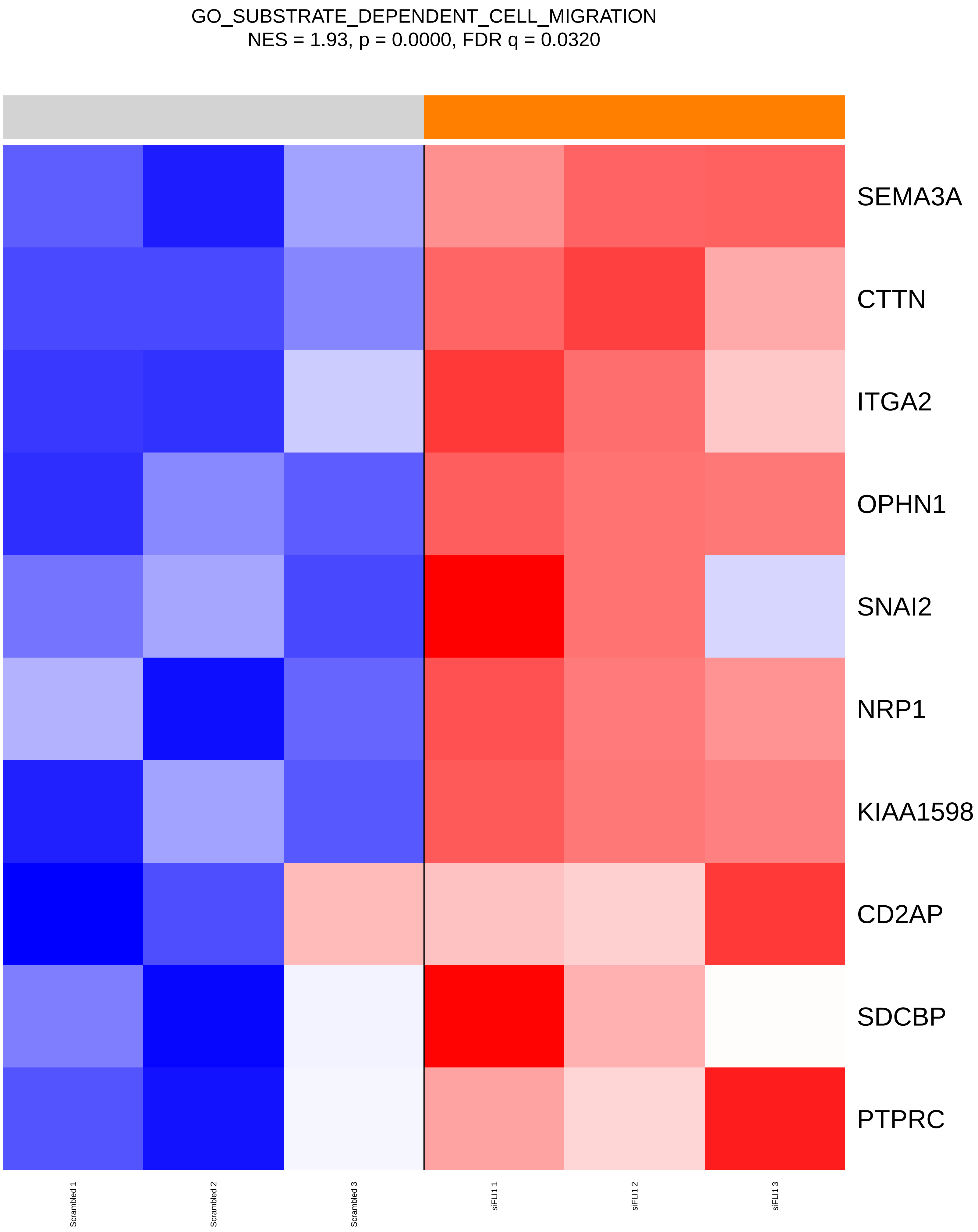
GO_SUBSTRATE_DEPENDENT_CELL_MIGRATION
NES = 1.93, p = 0.0000, FDR q = 0.0320
SEMA3A
CTTN
ITGA2
OPHN1
SNAI2
NRP1
KIAA1598
CD2AP
SDCBP
PTPRC
Scrambled 1
Scrambled 2
Scrambled 3
siFLI1 1
siFLI1 2
siFLI1 3

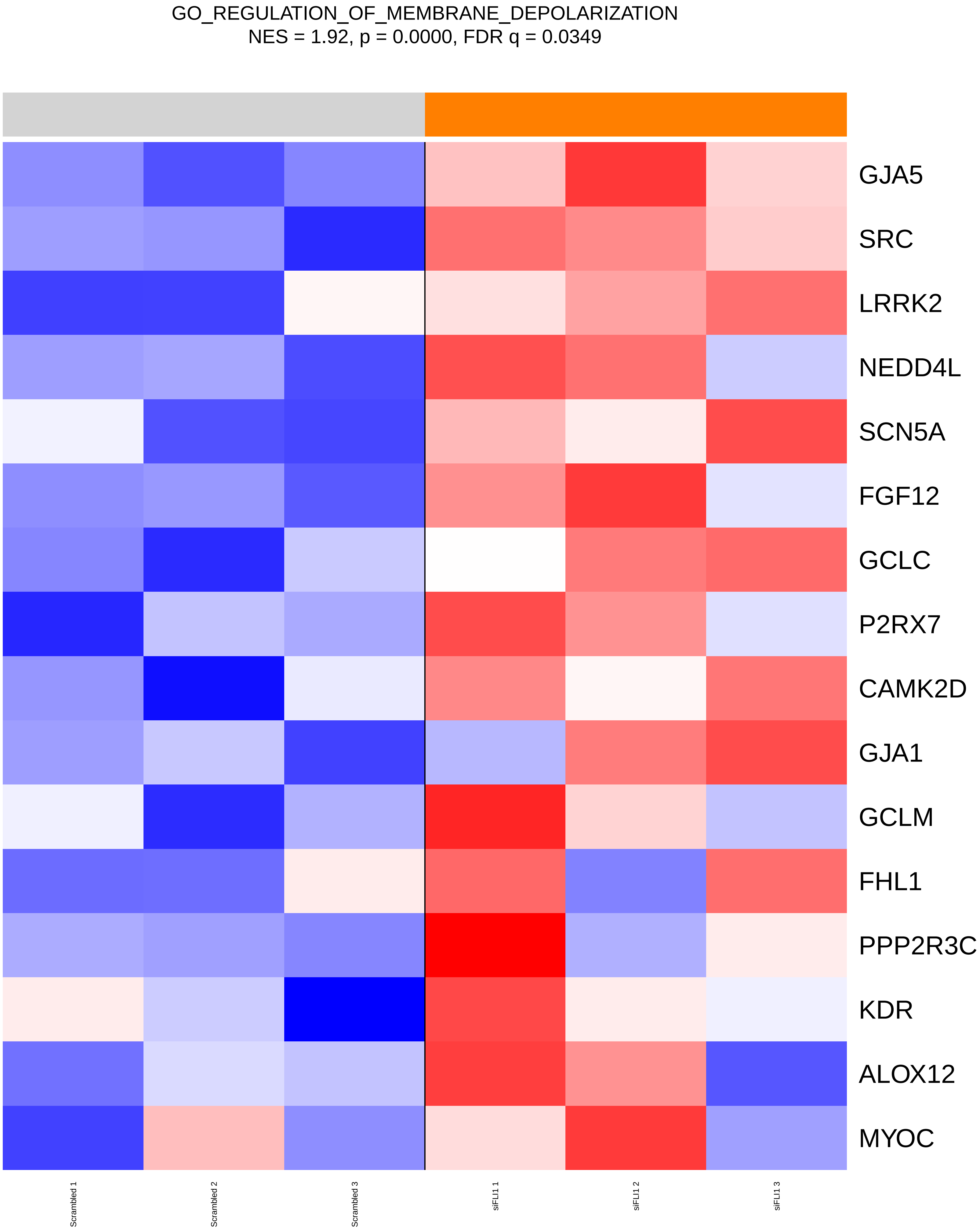

GO_REGULATION_OF_MEMBRANE_DEPOLARIZATION
NES = 1.92, p = 0.0000, FDR q = 0.0349
GJA5
SRC
LRRK2
NEDD4L
SCN5A
FGF12
GCLC
P2RX7
CAMK2D
GJA1
GCLM
FHL1
PPP2R3C
KDR
ALOX12
MYOC
Scrambled 1
Scrambled 2
Scrambled 3
siFLI1 1
siFLI1 2
siFLI1 3

GO_NEGATIVE_REGULATION_OF_RESPONSE_TO_REACTIVE_OXYGEN_SPECIES
NES = 1.92, p = 0.0025, FDR q = 0.0345

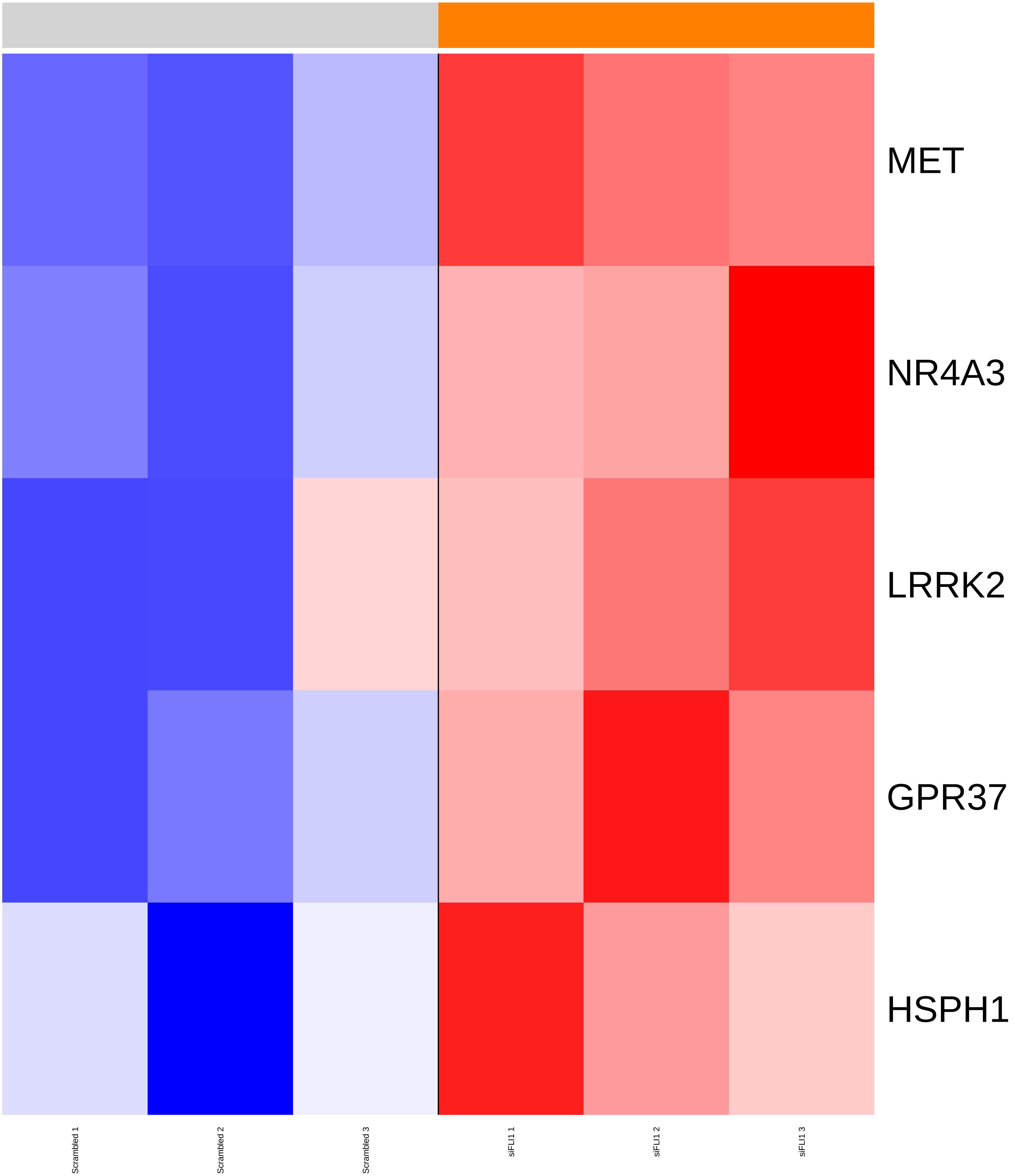

KEGG_ANTIGEN_PROCESSING_AND_PRESENTATION
NES = 1.91, p = 0.0000, FDR q = 0.0374

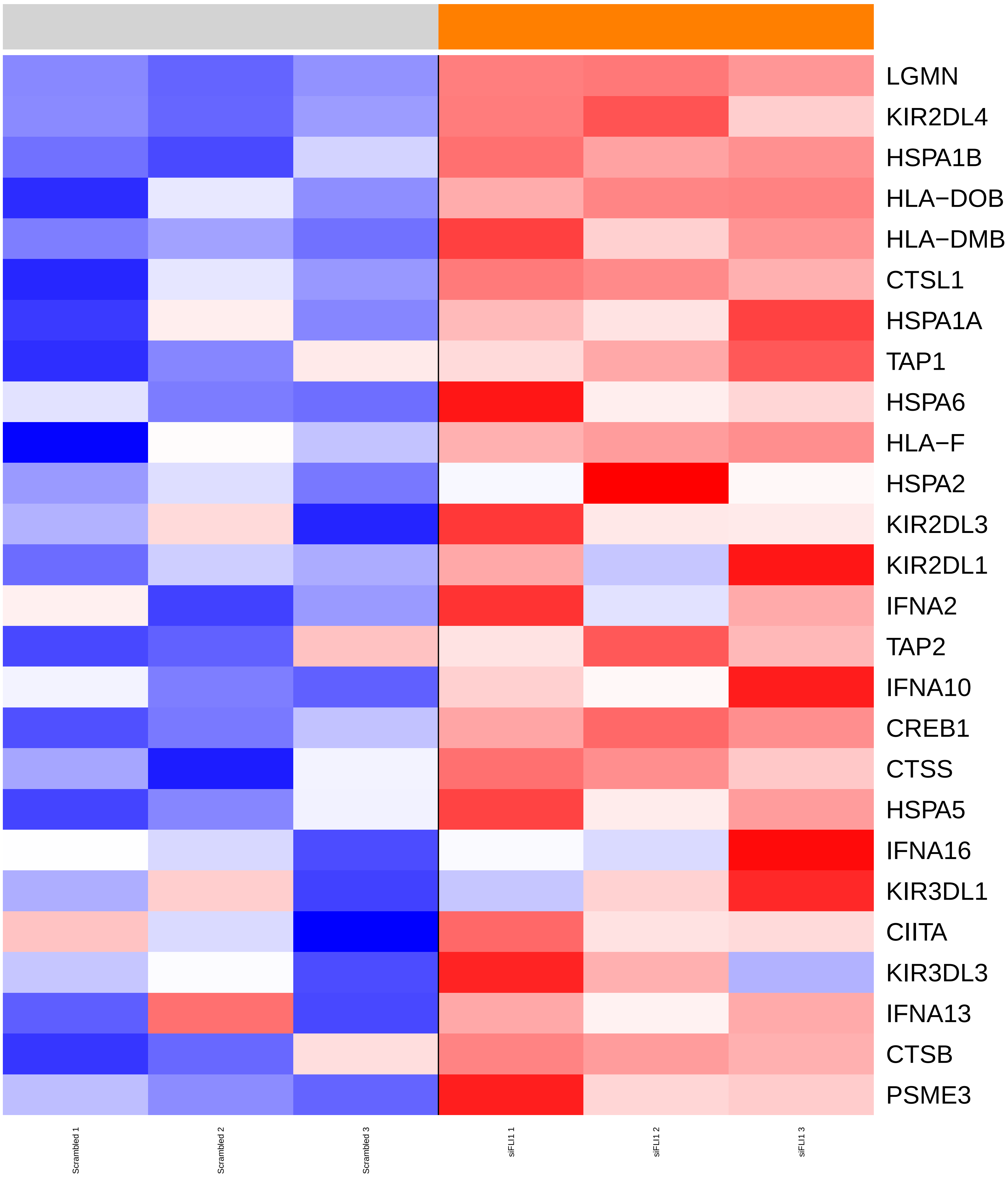

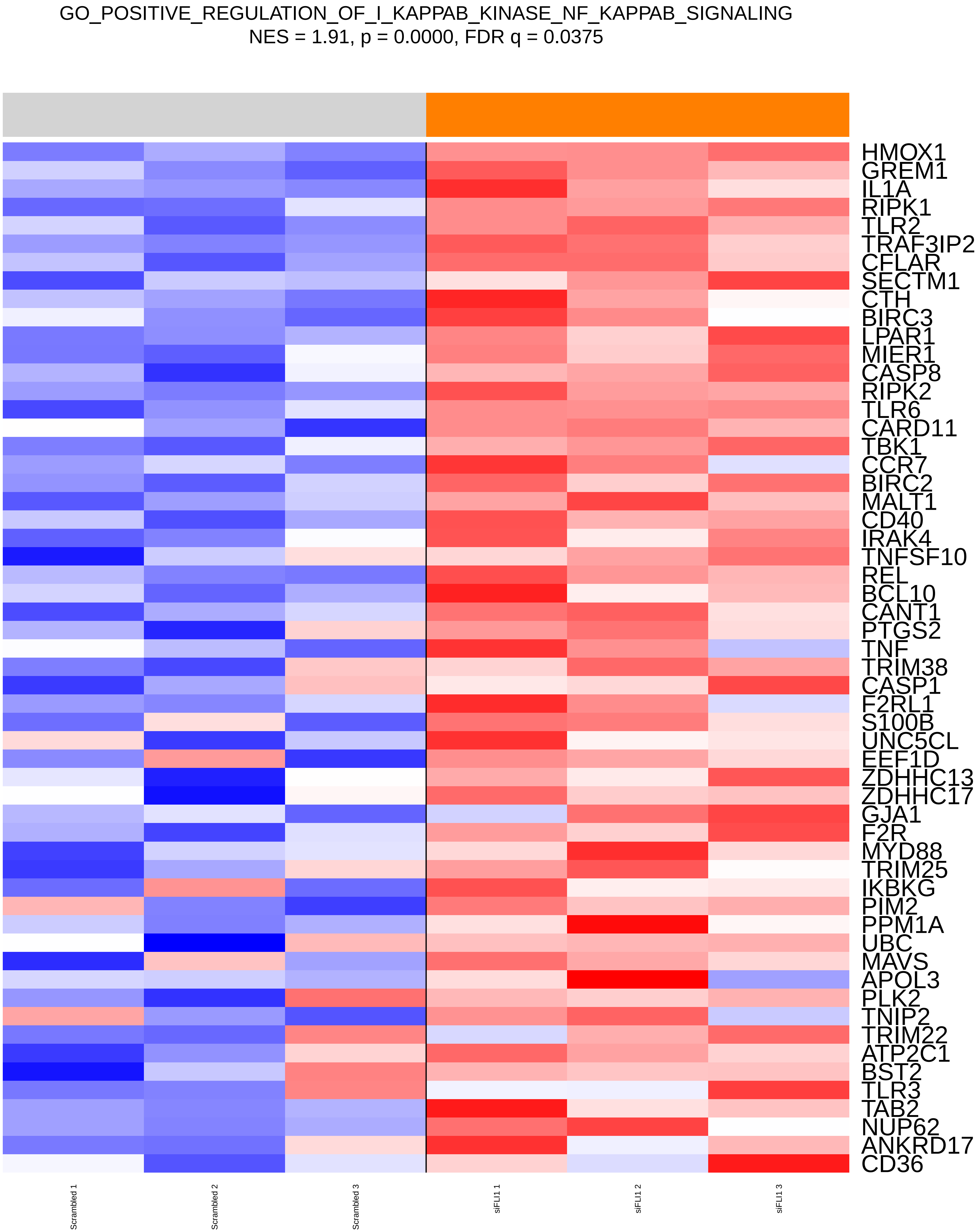
GO_POSITIVE_REGULATION_OF_I_KAPPAB_KINASE_NF_KAPPAB_SIGNALING
NES = 1.91, p = 0.0000, FDR q = 0.0375
HMOX1
GREM1
IL1A
RIPK1
TLR2
TRAF3IP2
CFLAR
SECTM1
CTH
BIRC3
LPAR1
MIER1
CASP8
RIPK2
TLR6
CARD11
TBK1
CCR7
BIRC2
MALT1
CD40
IRAK4
TNFSF10
REL
BCL10
CANT1
PTGS2
TNF
TRIM38
CASP1
F2RL1
S100B
UNC5CL
EEF1D
ZDHHC13
ZDHHC17
GJA1
F2R
MYD88
TRIM25
IKBKG
PIM2
PPM1A
UBC
MAVS
APOL3
PLK2
TNIP2
TRIM22
ATP2C1
BST2
TLR3
TAB2
NUP62
ANKRD17
CD36
Scrambled 1
Scrambled 2
Scrambled 3
siFLI1 1
siFLI1 2
siFLI1 3

REACTOME_ACTIVATION_OF_NF_KAPPAB_IN_B_CELLS
NES = 1.91, p = 0.0000, FDR q = 0.0370

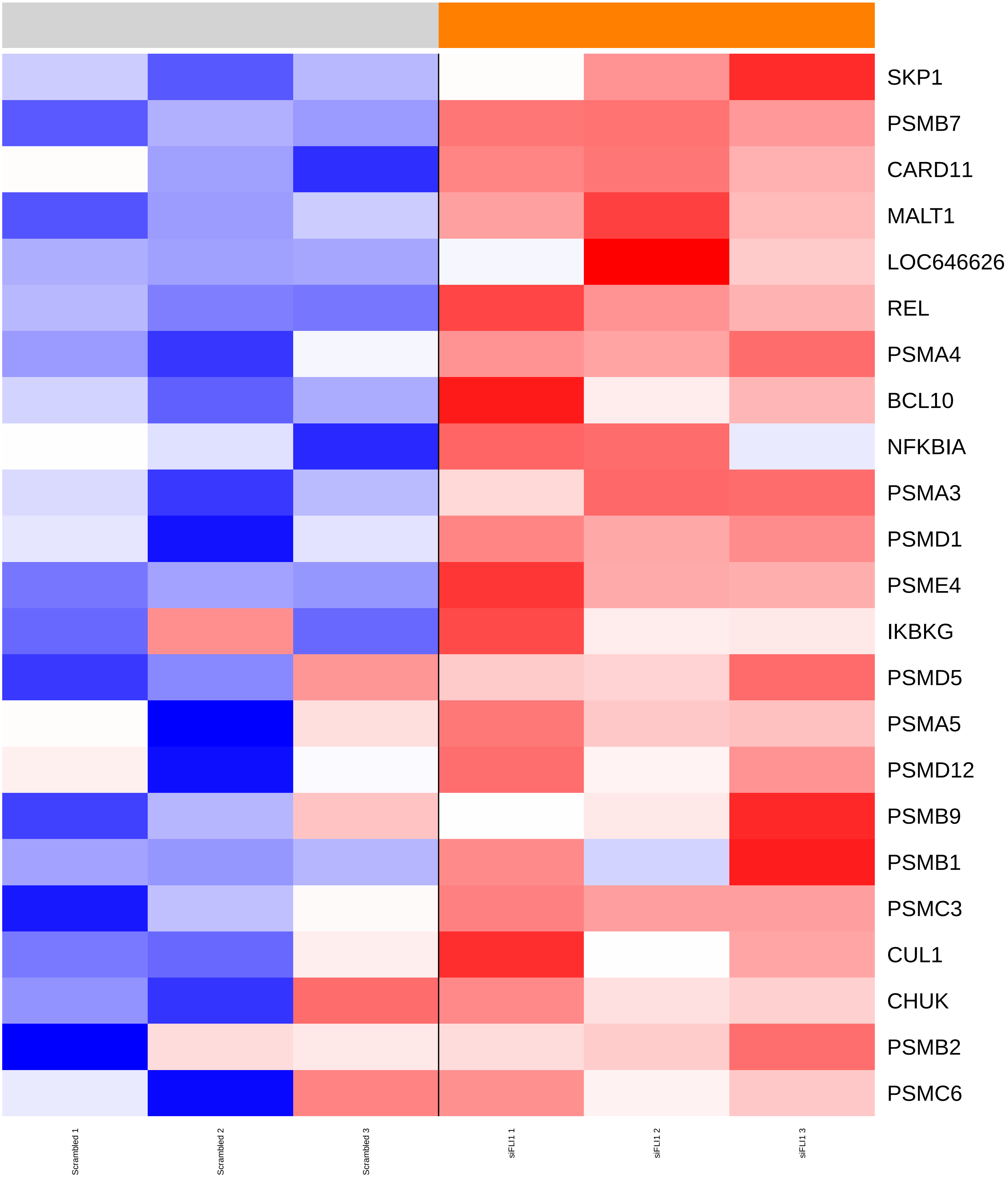

GO_POSITIVE_REGULATION_OF_RESPONSE_TO_WOUNDING
NES = 1.90, p = 0.0000, FDR q = 0.0366

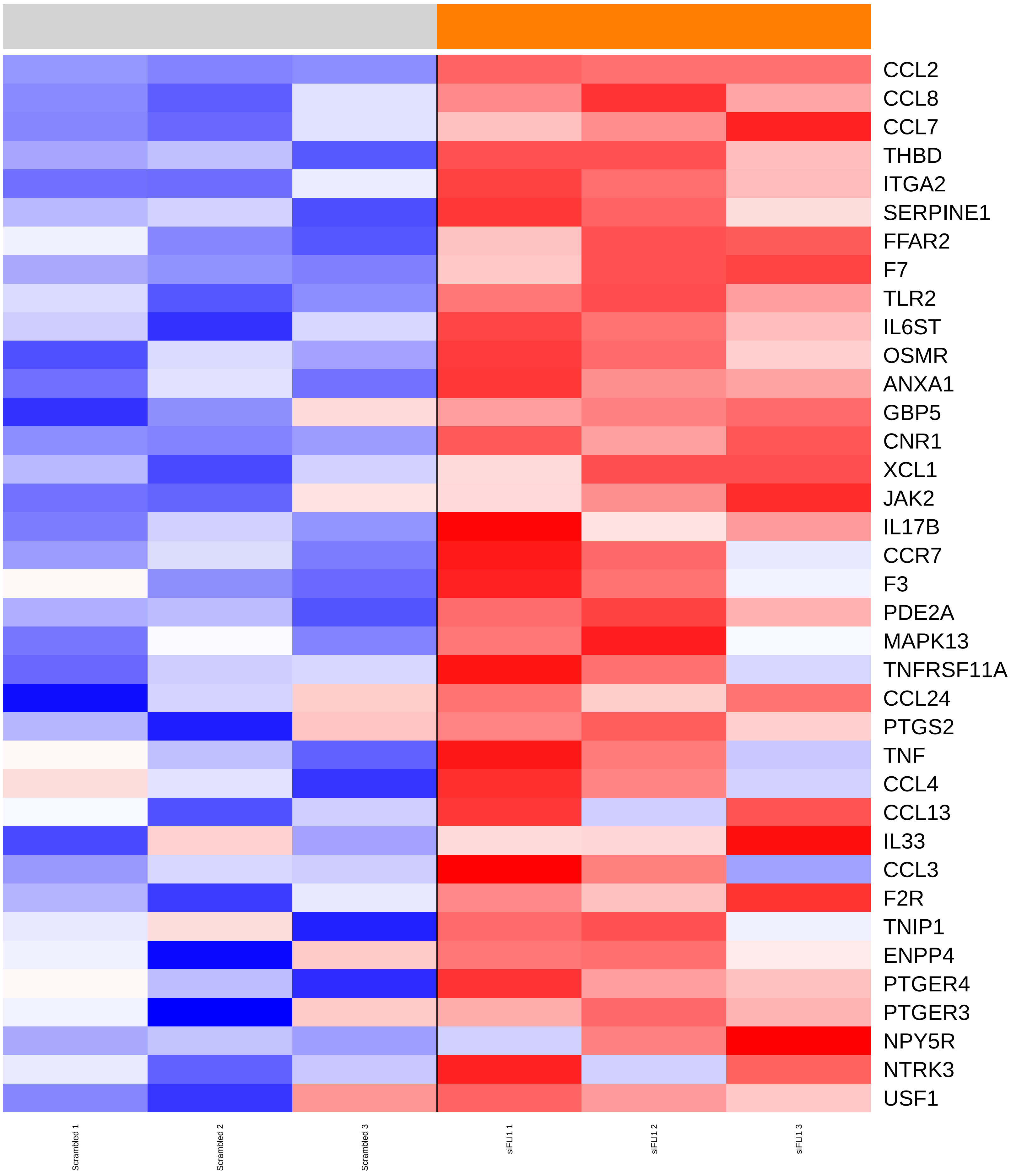

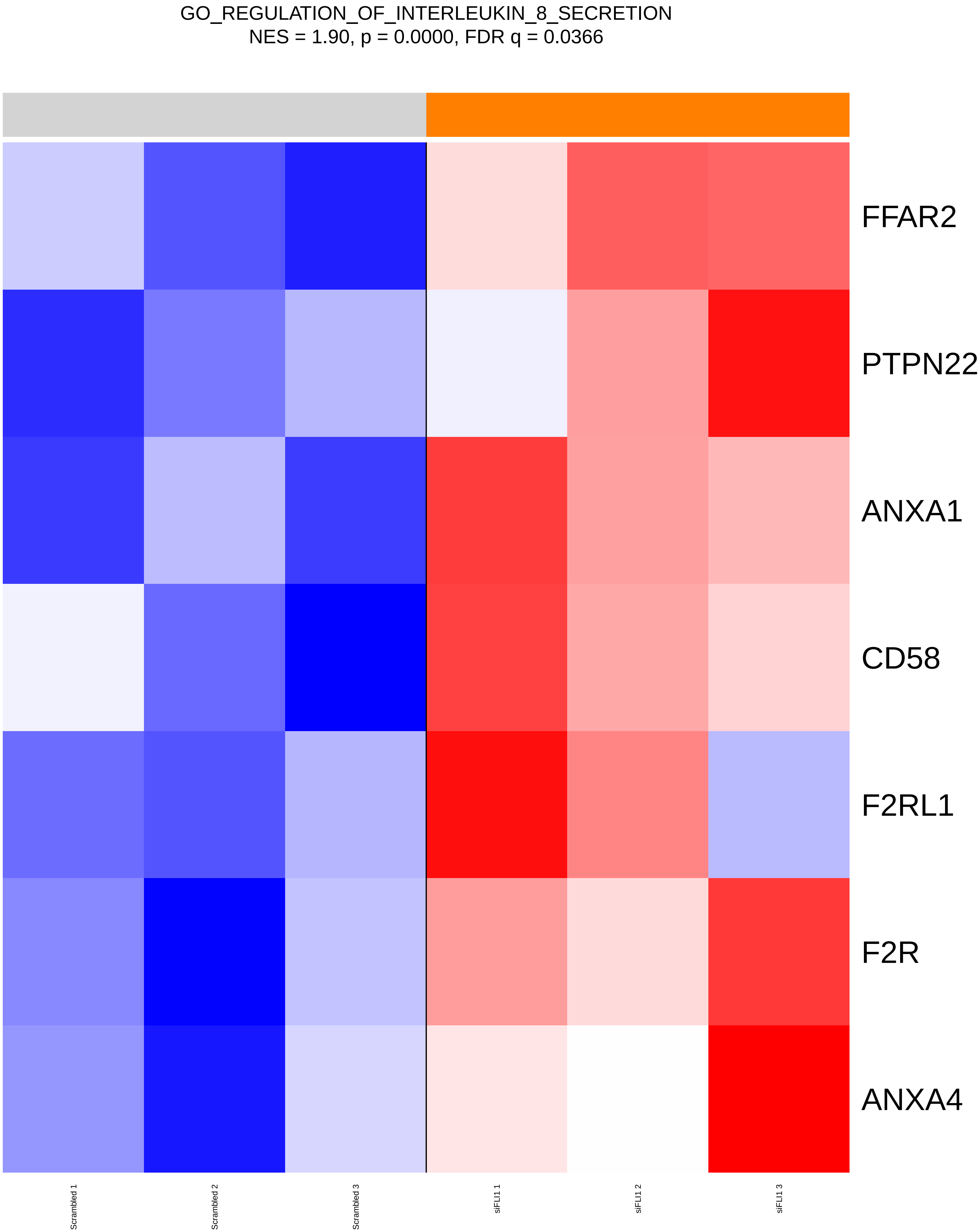
GO_REGULATION_OF_INTERLEUKIN_8_SECRETION
NES = 1.90, p = 0.0000, FDR q = 0.0366
FFAR2
PTPN22
ANXA1
CD58
F2RL1
F2R
ANXA4
Scrambled 1
Scrambled 2
Scrambled 3
siFLI1 1
siFLI1 2
siFLI1 3

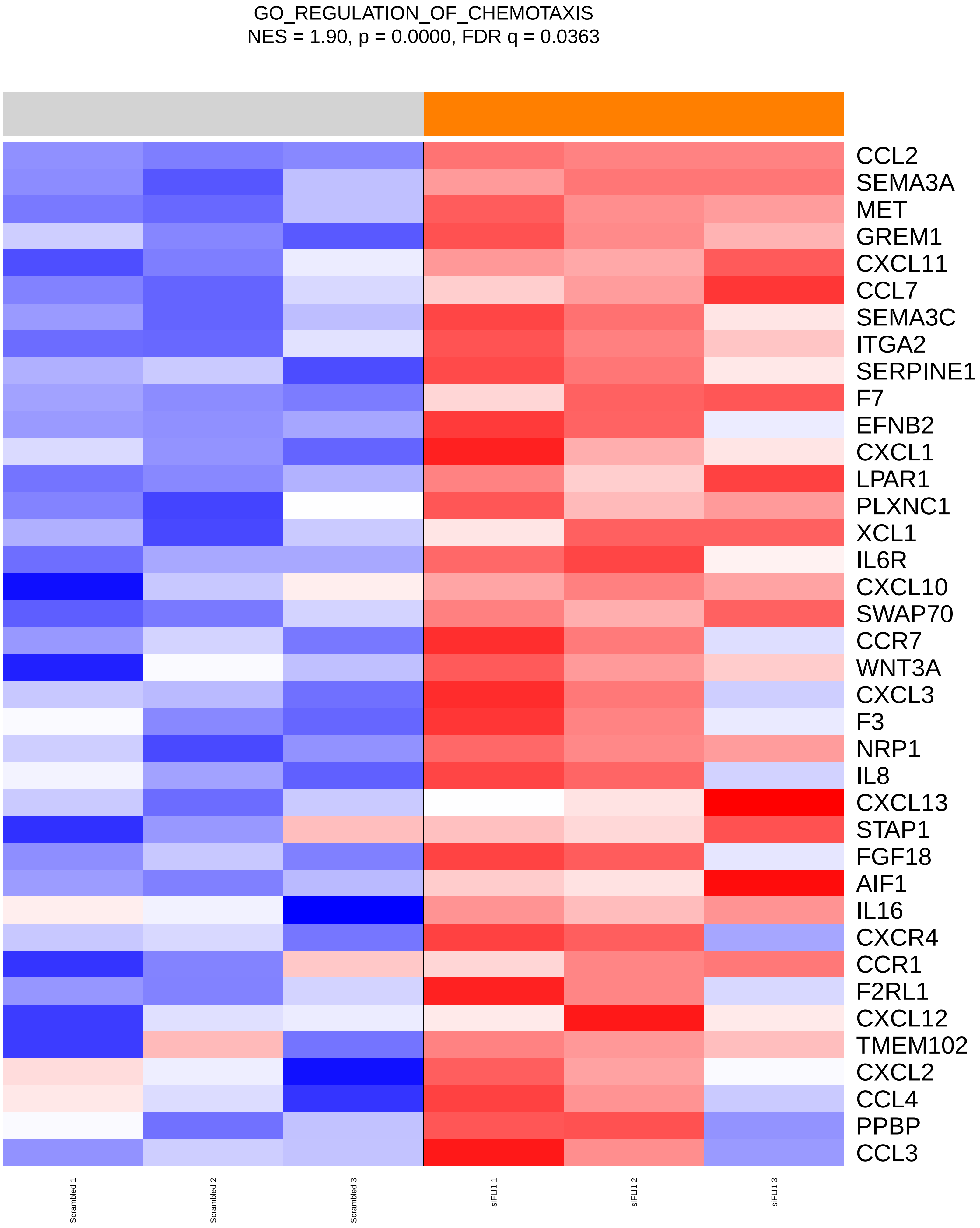
GO_REGULATION_OF_CHEMOTAXIS
NES = 1.90, p = 0.0000, FDR q = 0.0363
CCL2
SEMA3A
MET
GREM1
CXCL11
CCL7
SEMA3C
ITGA2
SERPINE1
F7
EFNB2
CXCL1
LPAR1
PLXNC1
XCL1
IL6R
CXCL10
SWAP70
CCR7
WNT3A
CXCL3
F3
NRP1
IL8
CXCL13
STAP1
FGF18
AIF1
IL16
CXCR4
CCR1
F2RL1
CXCL12
TMEM102
CXCL2
CCL4
PPBP
CCL3
Scrambled 1
Scrambled 2
Scrambled 3
siFLI1 1
siFLI1 2
siFLI1 3

GO_RESPONSE_TO_VIRUS
NES = 1.90, p = 0.0000, FDR q = 0.0359

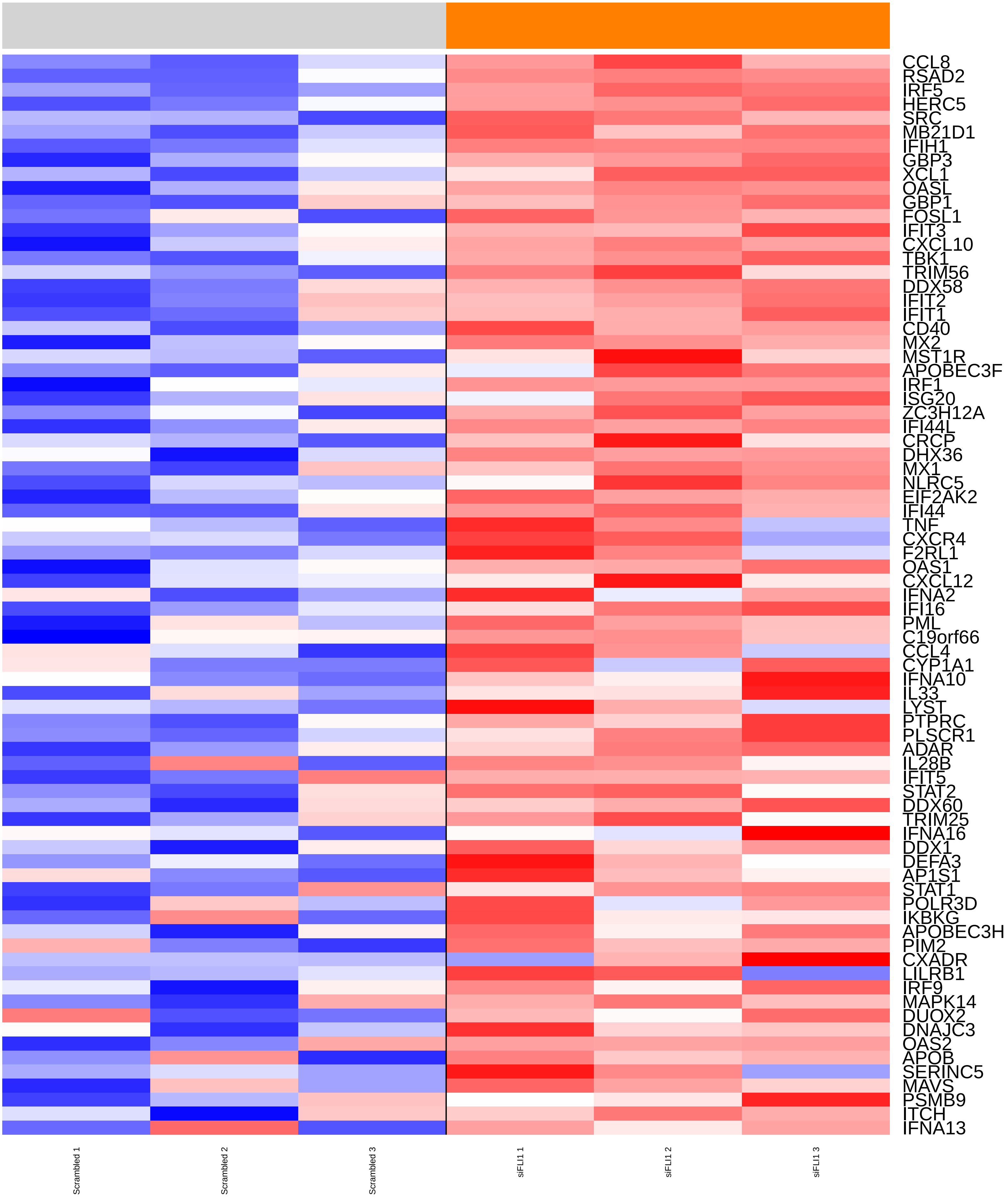

GO_NEGATIVE_REGULATION_OF_TYPE_I_INTERFERON_PRODUCTION
NES = 1.90, p = 0.0000, FDR q = 0.0366

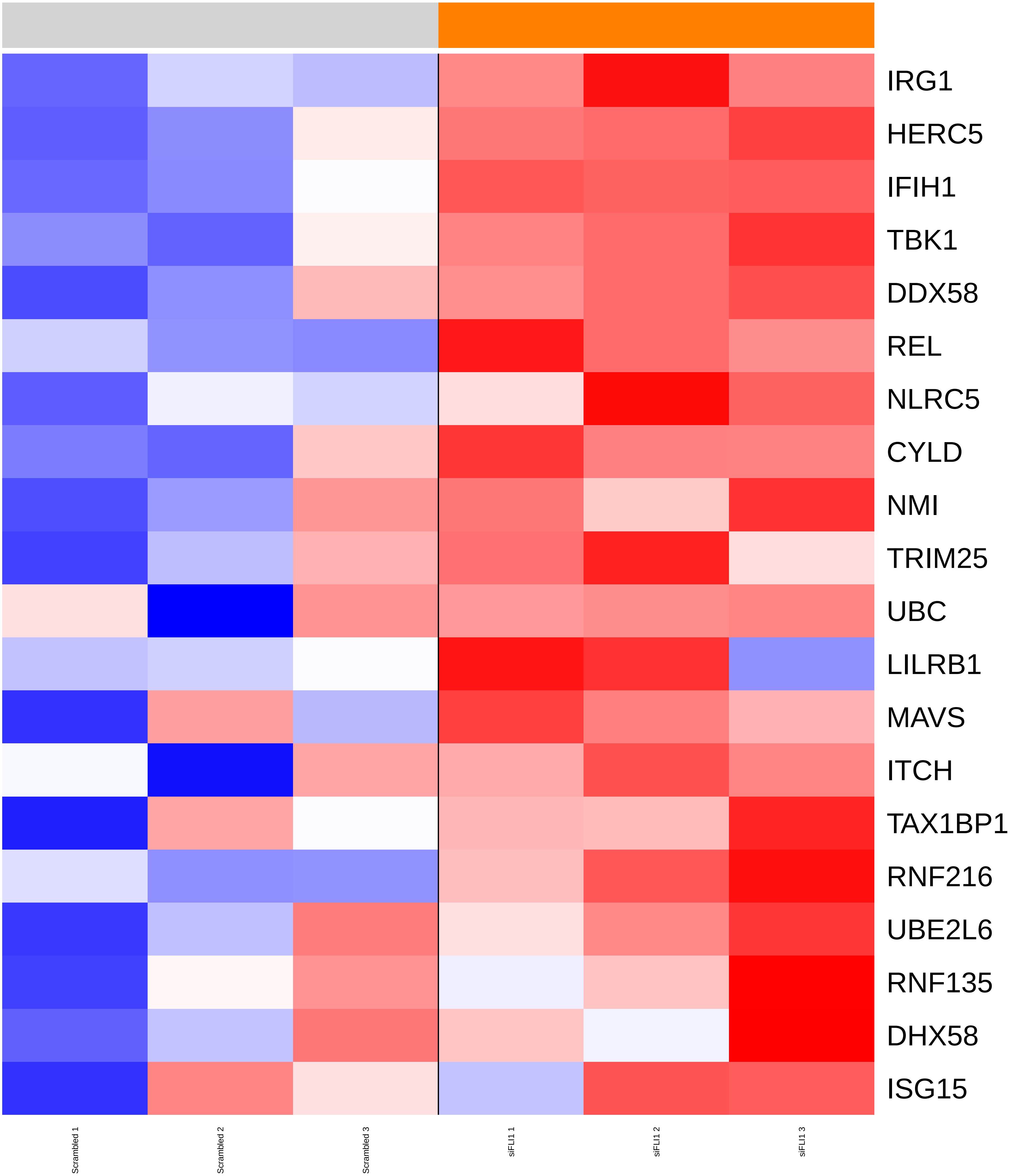

GO_REGULATION_OF_TOLL_LIKE_RECEPTOR_4_SIGNALING_PATHWAY
NES = 1.89, p = 0.0046, FDR q = 0.0382

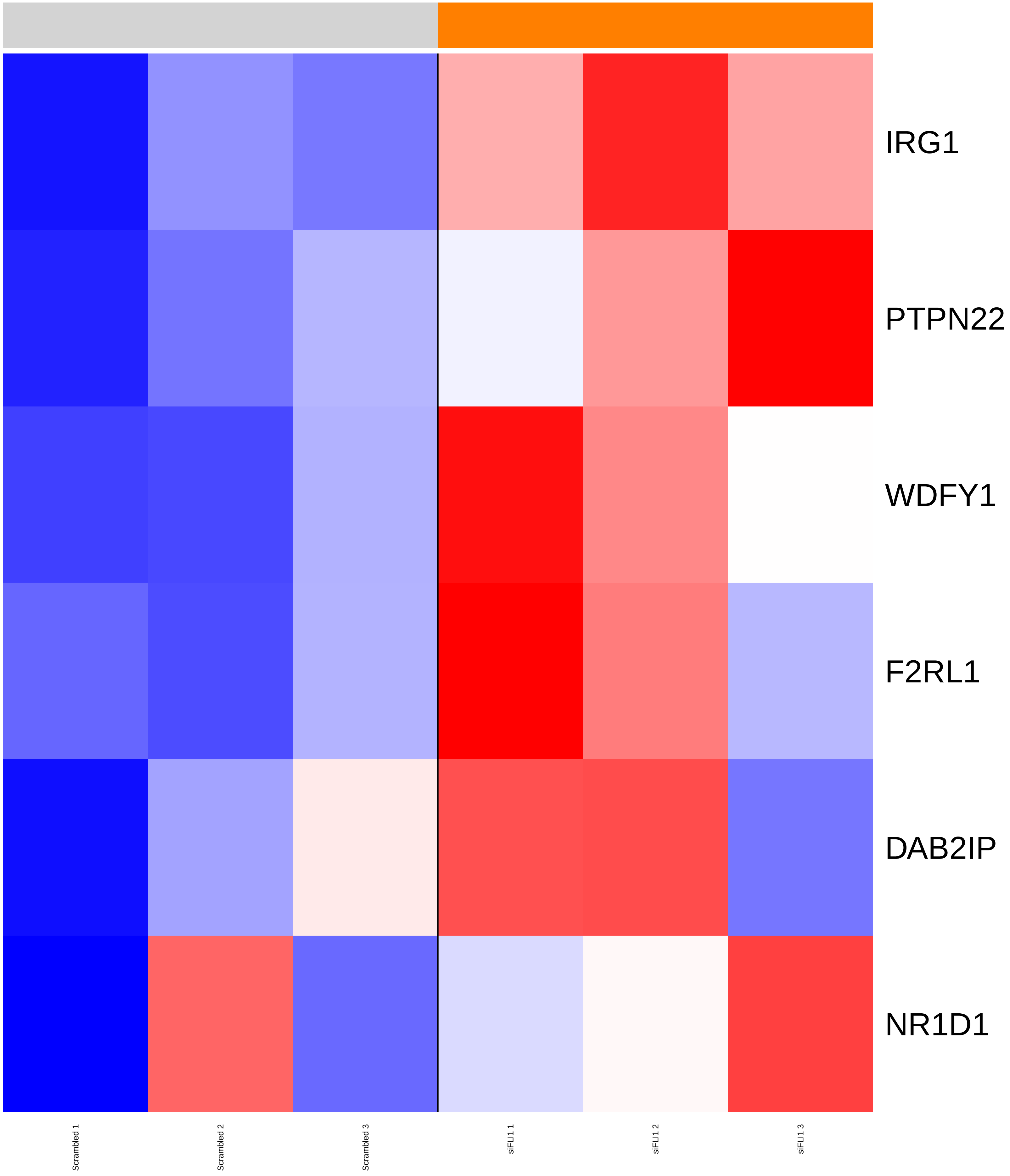

BIOCARTA_CCR5_PATHWAY
NES = 1.89, p = 0.0075, FDR q = 0.0389

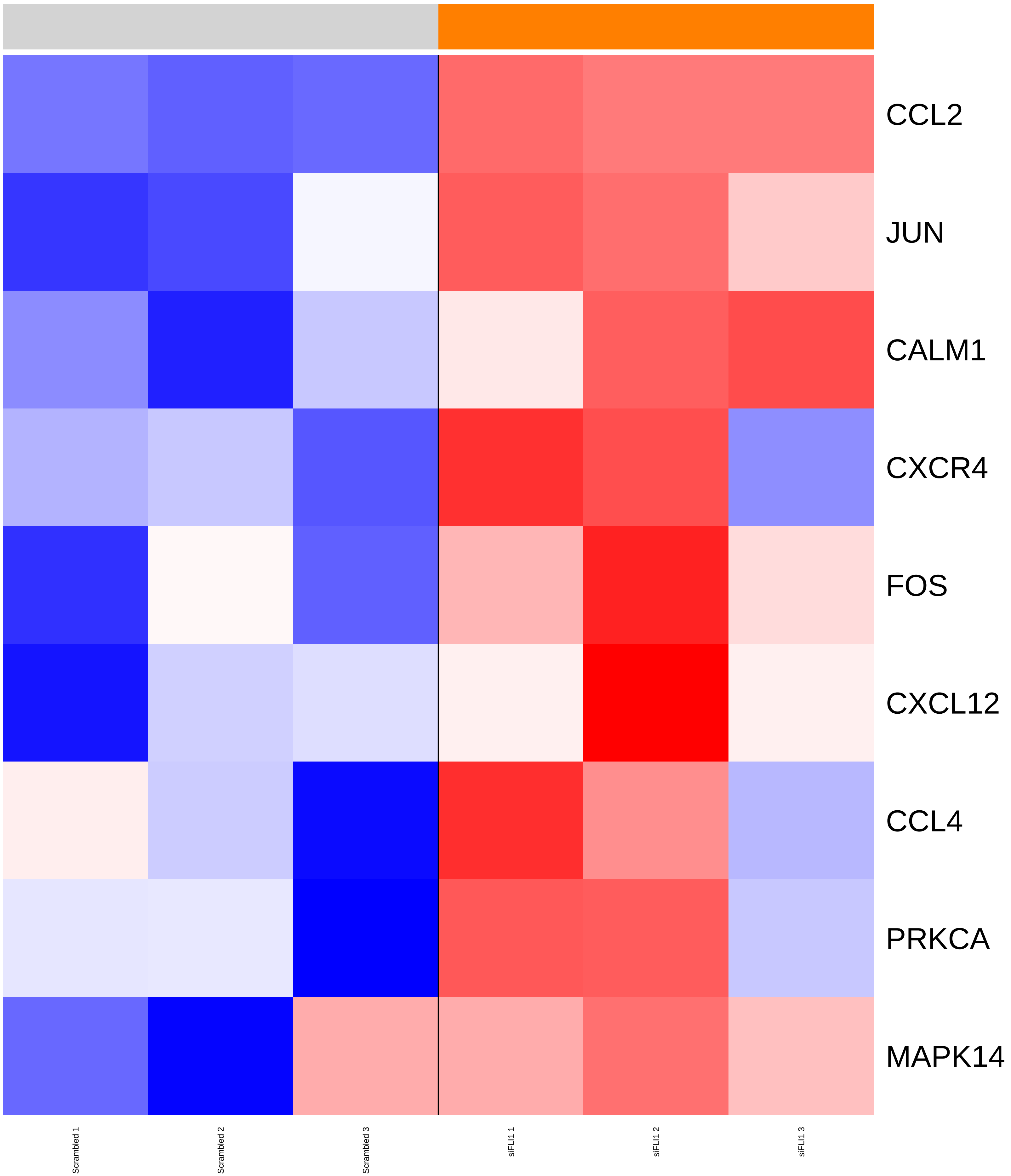

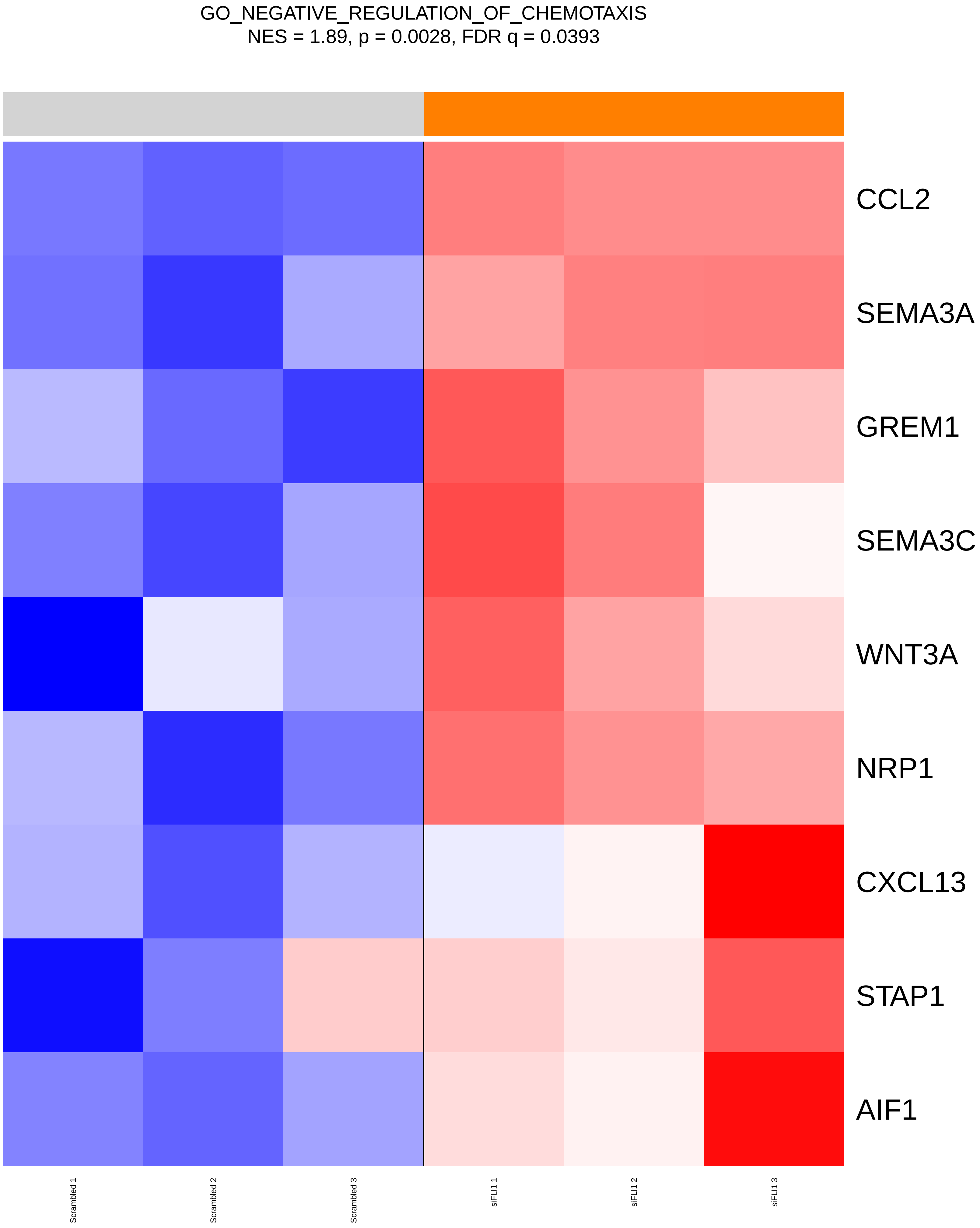
GO_NEGATIVE_REGULATION_OF_CHEMOTAXIS
NES = 1.89, p = 0.0028, FDR q = 0.0393
CCL2
SEMA3A
GREM1
SEMA3C
WNT3A
NRP1
CXCL13
STAP1
AIF1
Scrambled 1
Scrambled 2
Scrambled 3
siFLI1 1
siFLI1 2
siFLI1 3

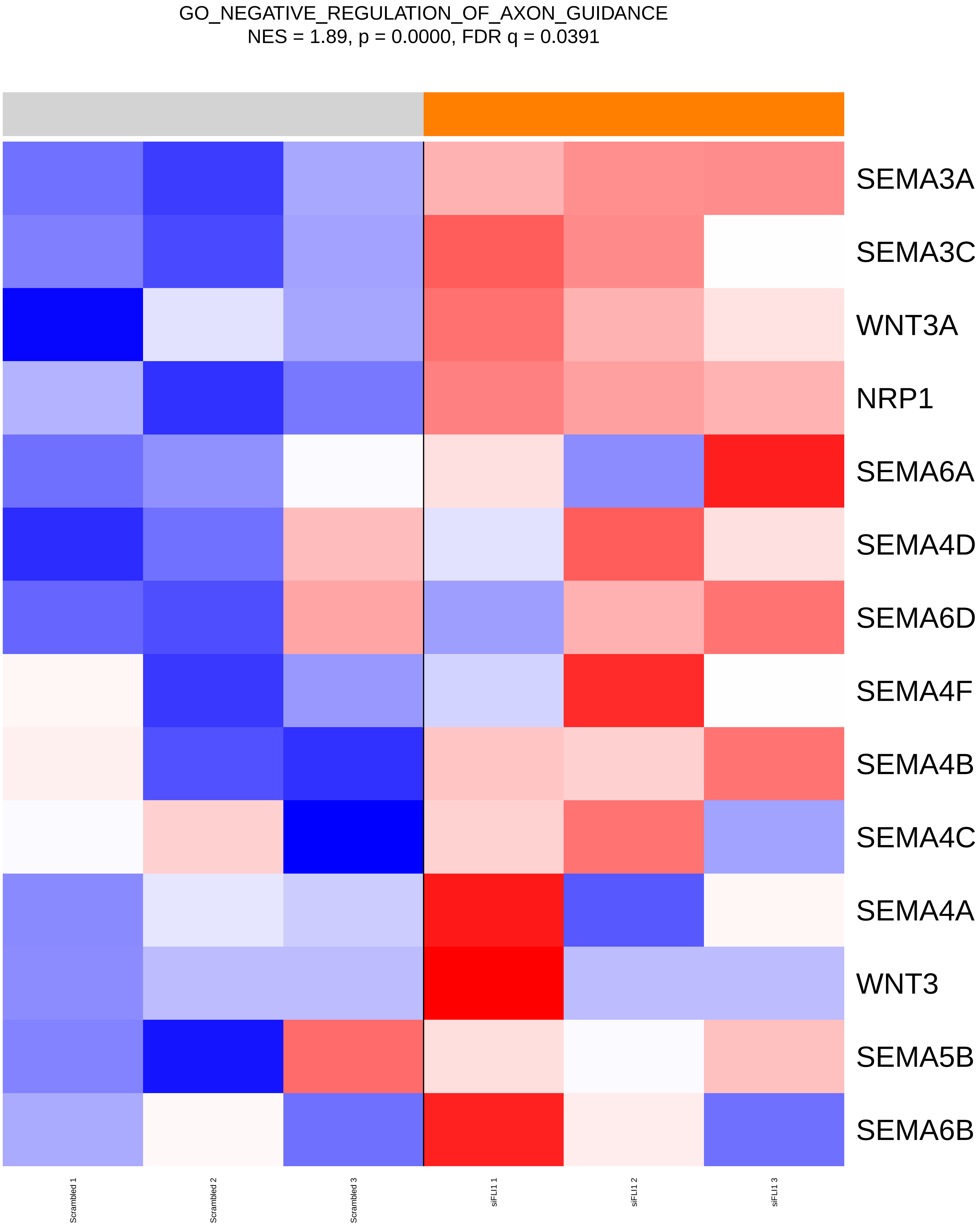
GO_NEGATIVE_REGULATION_OF_AXON_GUIDANCE
NES = 1.89, p = 0.0000, FDR q = 0.0391
SEMA3A
SEMA3C
WNT3A
NRP1
SEMA6A
SEMA4D
SEMA6D
SEMA4F
SEMA4B
SEMA4C
SEMA4A
WNT3
SEMA5B
SEMA6B
Scrambled 1
Scrambled 2
Scrambled 3
siFLI1 1
siFLI1 2
siFLI1 3

HALLMARK_INFLAMMATORY_RESPONSE
NES = 1.88, p = 0.0000, FDR q = 0.0396

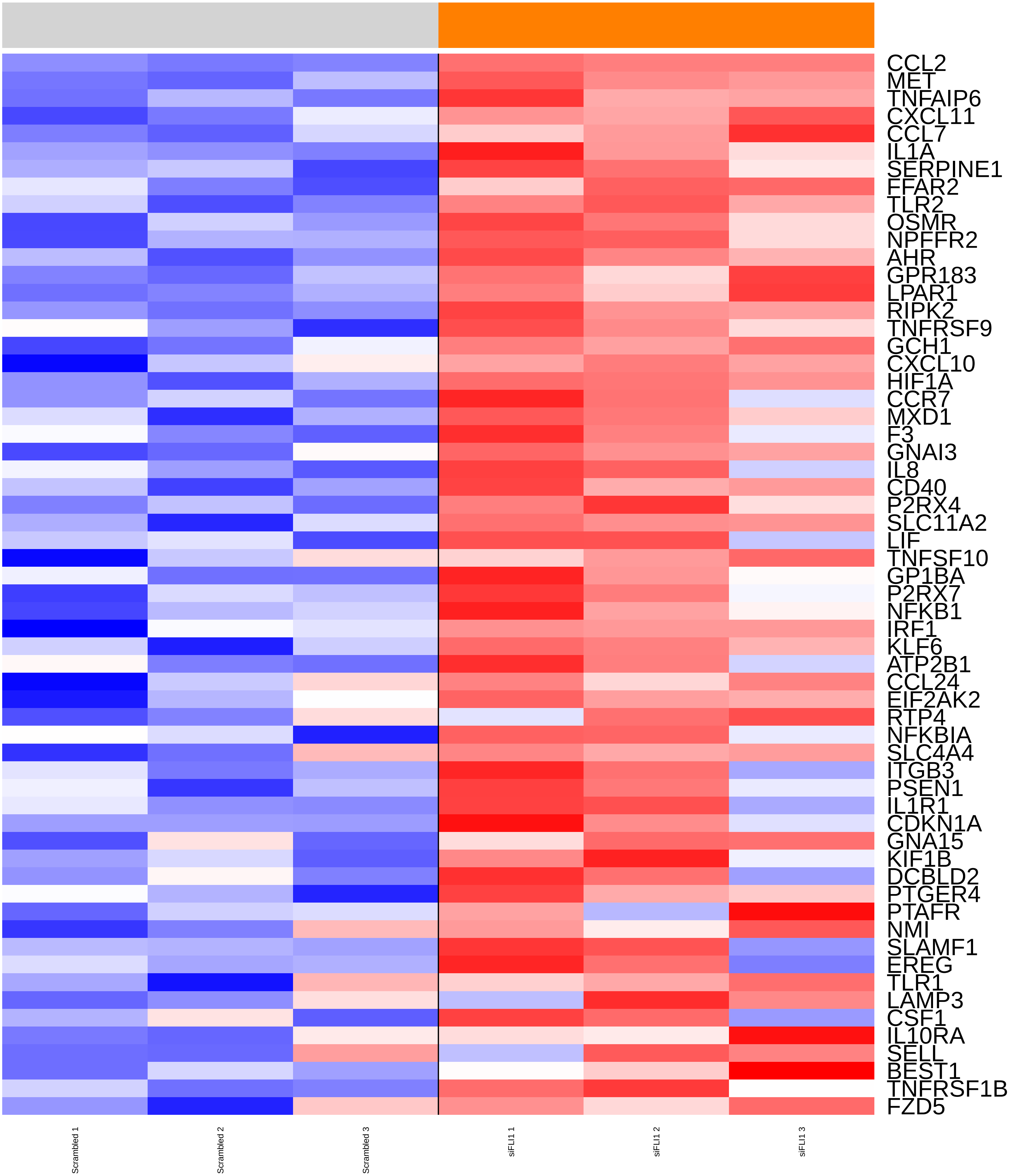

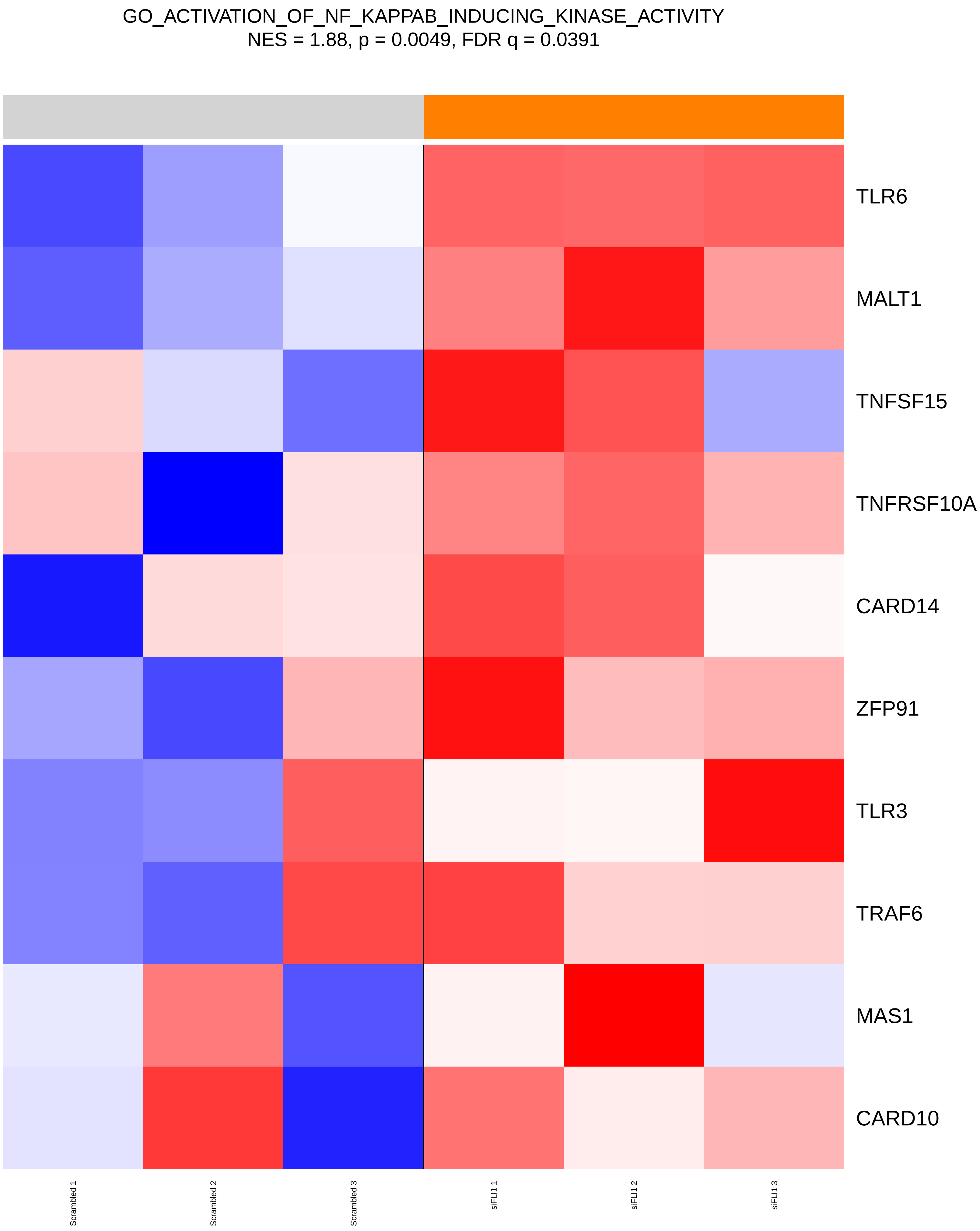
GO_ACTIVATION_OF_NF_KAPPAB_INDUCING_KINASE_ACTIVITY
NES = 1.88, p = 0.0049, FDR q = 0.0391
TLR6
MALT1
TNFSF15
TNFRSF10A
CARD14
ZFP91
TLR3
TRAF6
MAS1
CARD10
Scrambled 1
Scrambled 2
Scrambled 3
siFLI1 1
siFLI1 2
siFLI1 3

GO_POSITIVE_REGULATION_OF_SMALL_GTPASE_MEDIATED_SIGNAL_TRANSDUCTION
NES = 1.88, p = 0.0000, FDR q = 0.0407

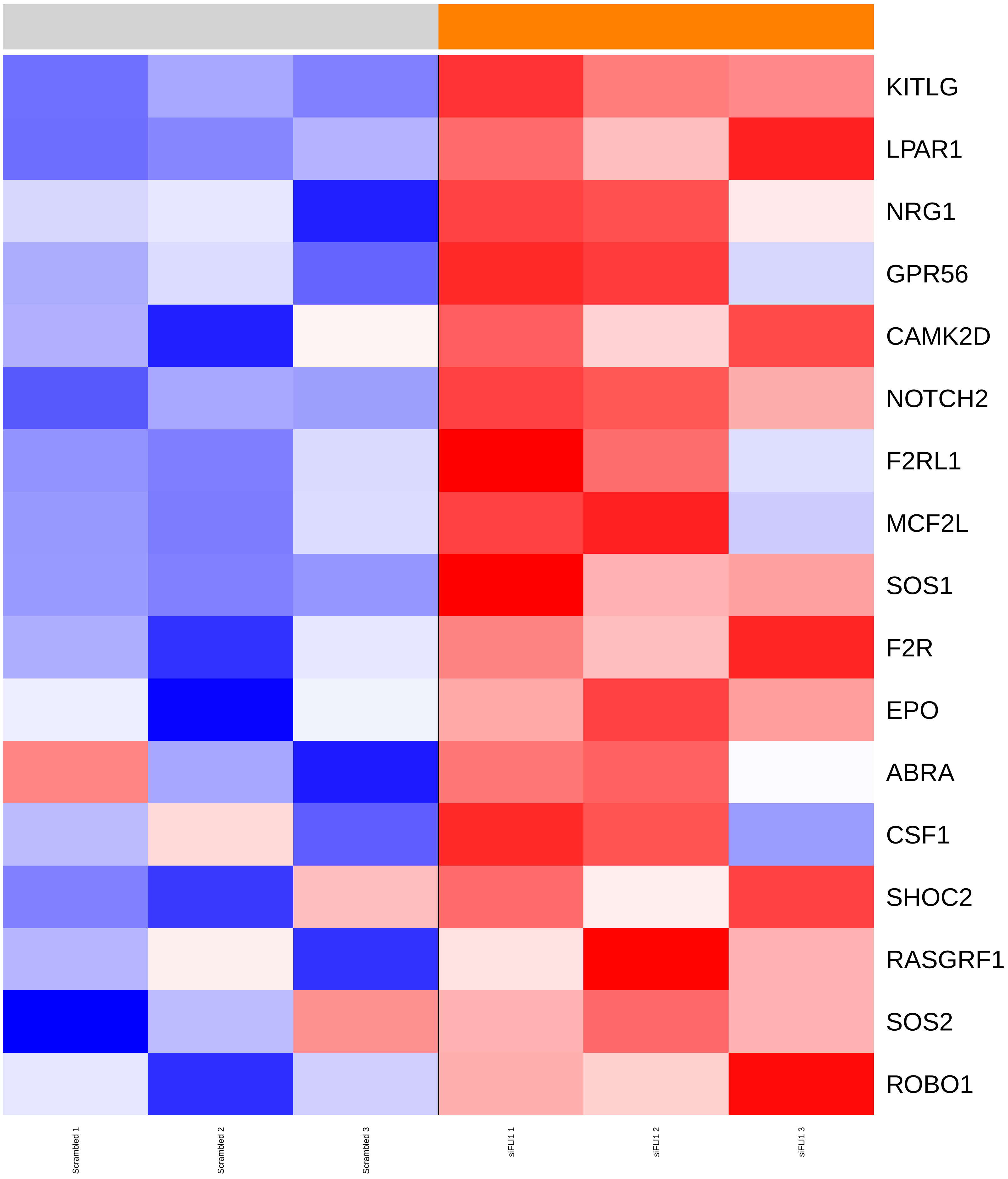

GO_POSITIVE_REGULATION_OF_ACUTE_INFLAMMATORY_RESPONSE
NES = 1.88, p = 0.0026, FDR q = 0.0404

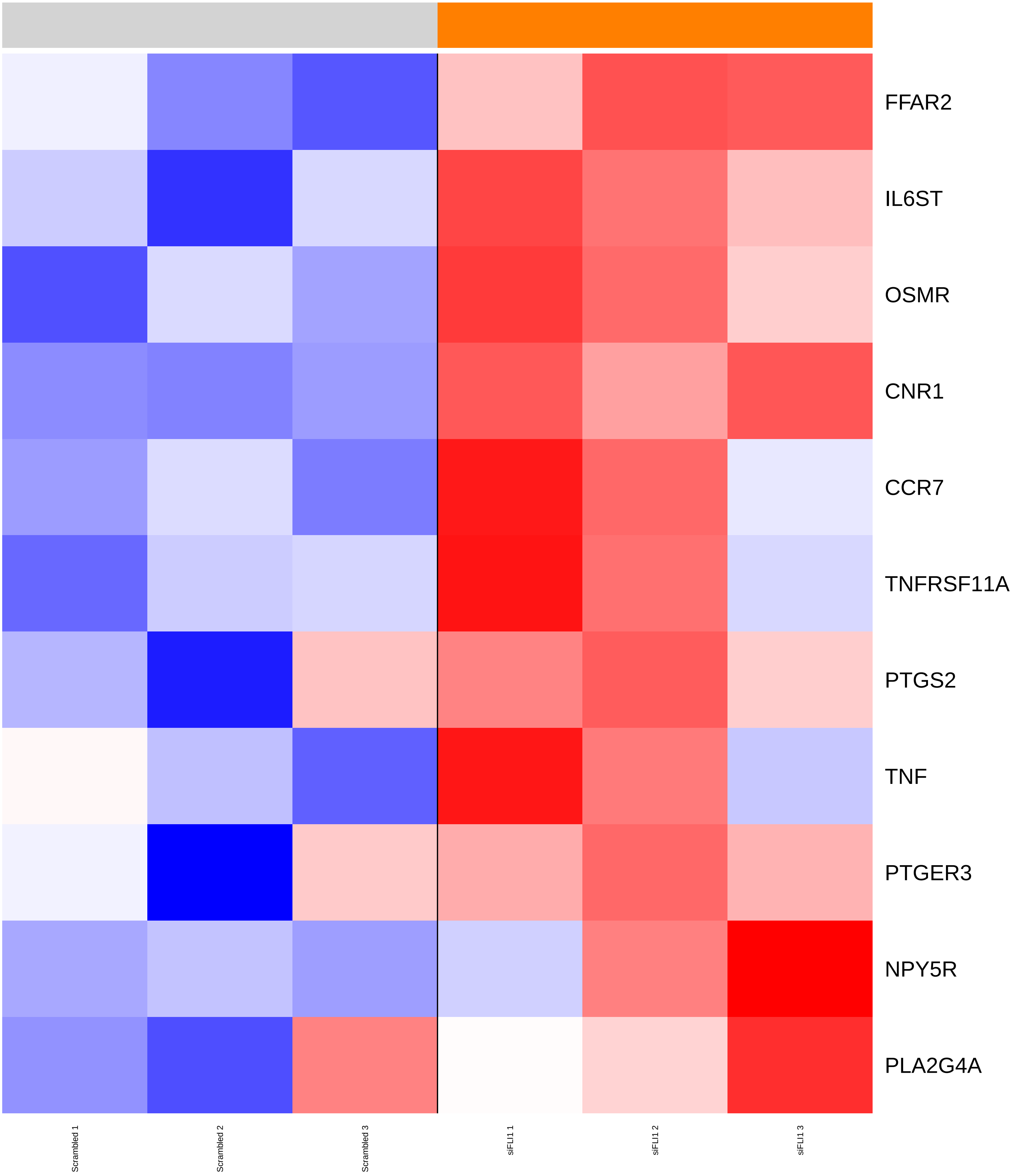

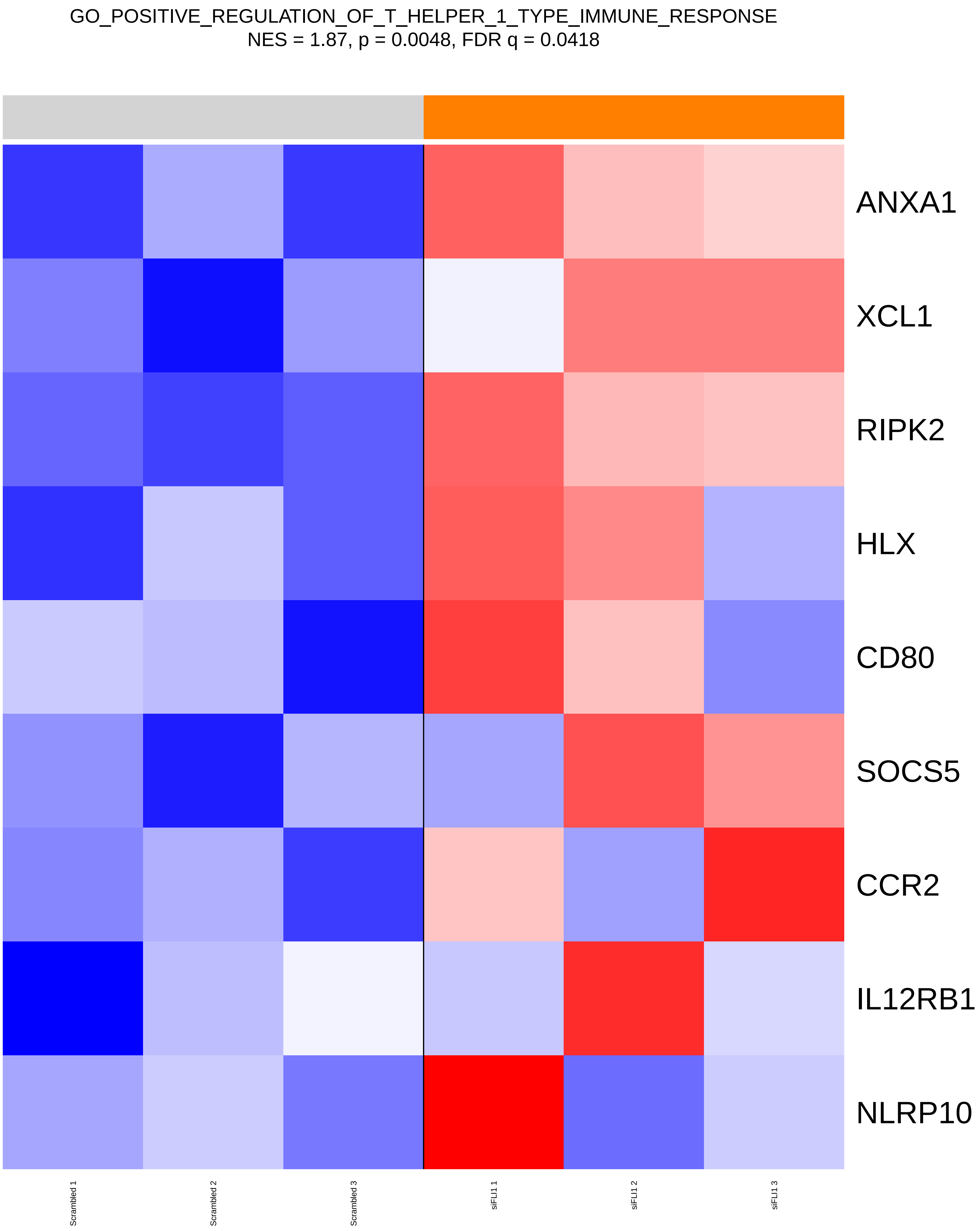
GO_POSITIVE_REGULATION_OF_T_HELPER_1_TYPE_IMMUNE_RESPONSE
NES = 1.87, p = 0.0048, FDR q = 0.0418
ANXA1
XCL1
RIPK2
HLX
CD80
SOCS5
CCR2
IL12RB1
NLRP10
Scrambled 1
Scrambled 2
Scrambled 3
siFLI1 1
siFLI1 2
siFLI1 3

GO_POSITIVE_REGULATION_OF_INTERLEUKIN_8_PRODUCTION
NES = 1.87, p = 0.0000, FDR q = 0.0427

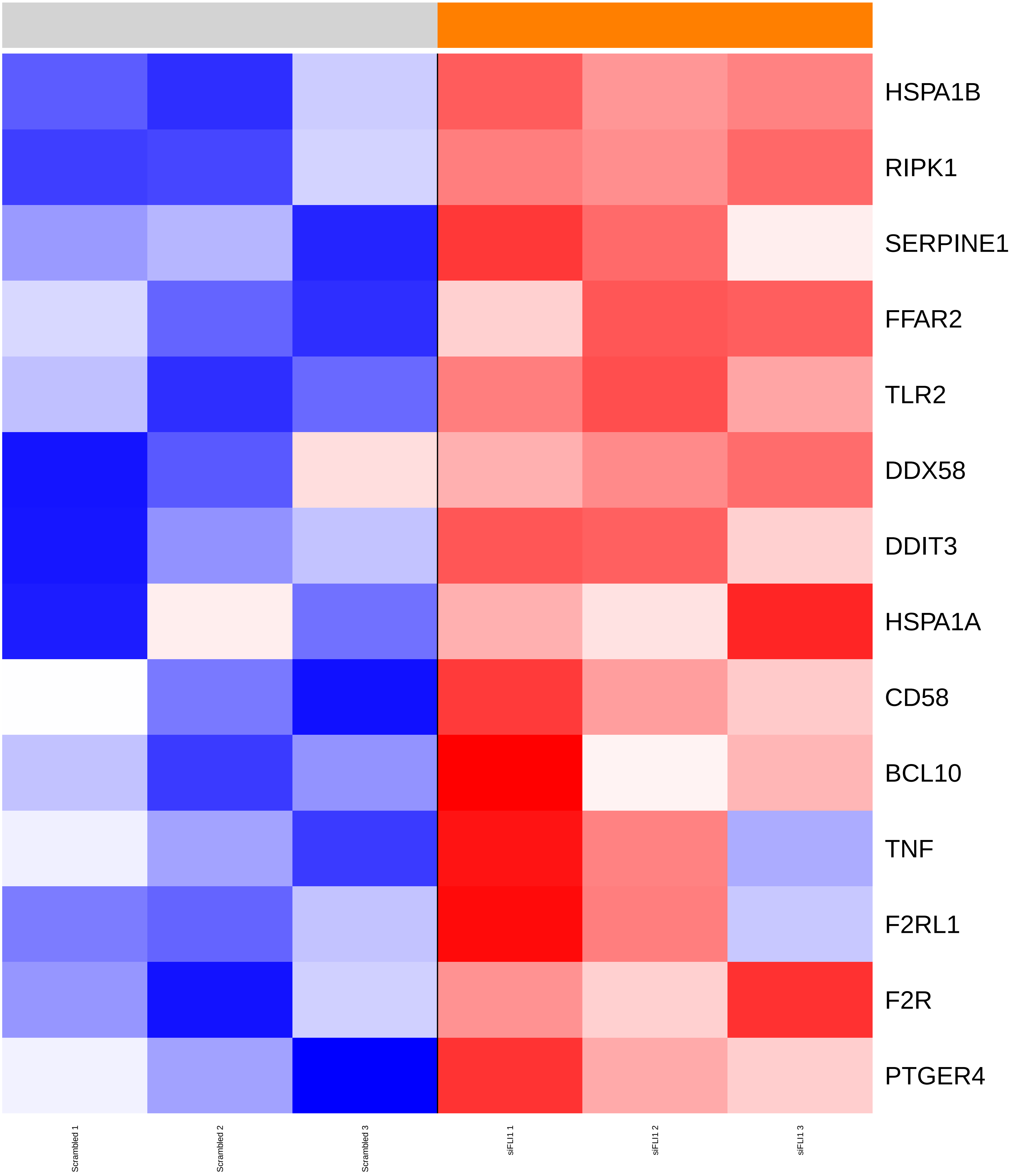

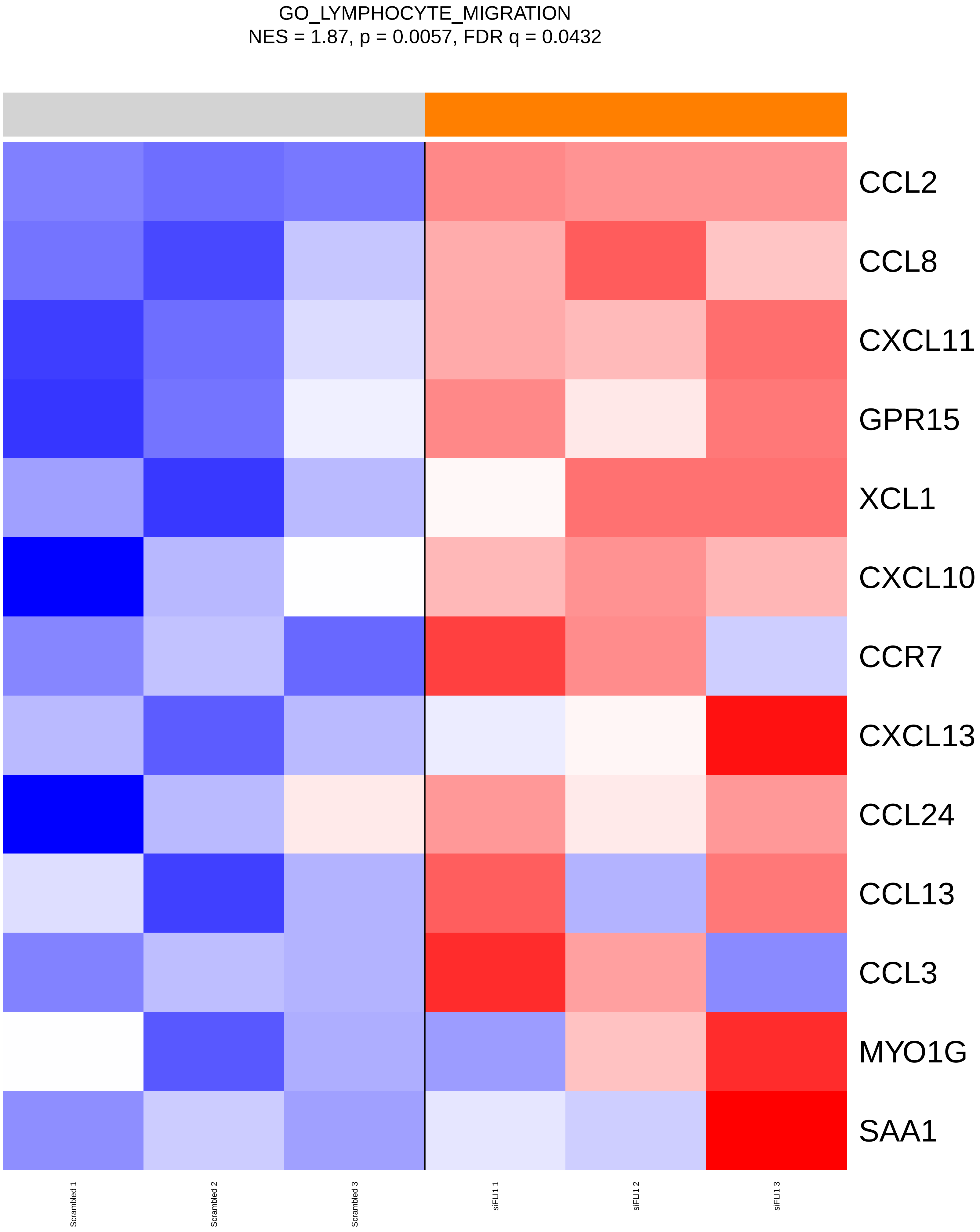
GO_LYMPHOCYTE_MIGRATION
NES = 1.87, p = 0.0057, FDR q = 0.0432
CCL2
CCL8
CXCL11
GPR15
XCL1
CXCL10
CCR7
CXCL13
CCL24
CCL13
CCL3
MYO1G
SAA1
Scrambled 1
Scrambled 2
Scrambled 3
siFLI1 1
siFLI1 2
siFLI1 3

GO_NEGATIVE_REGULATION_OF_PROTEIN_TYROSINE_KINASE_ACTIVITY
NES = 1.87, p = 0.0025, FDR q = 0.0428

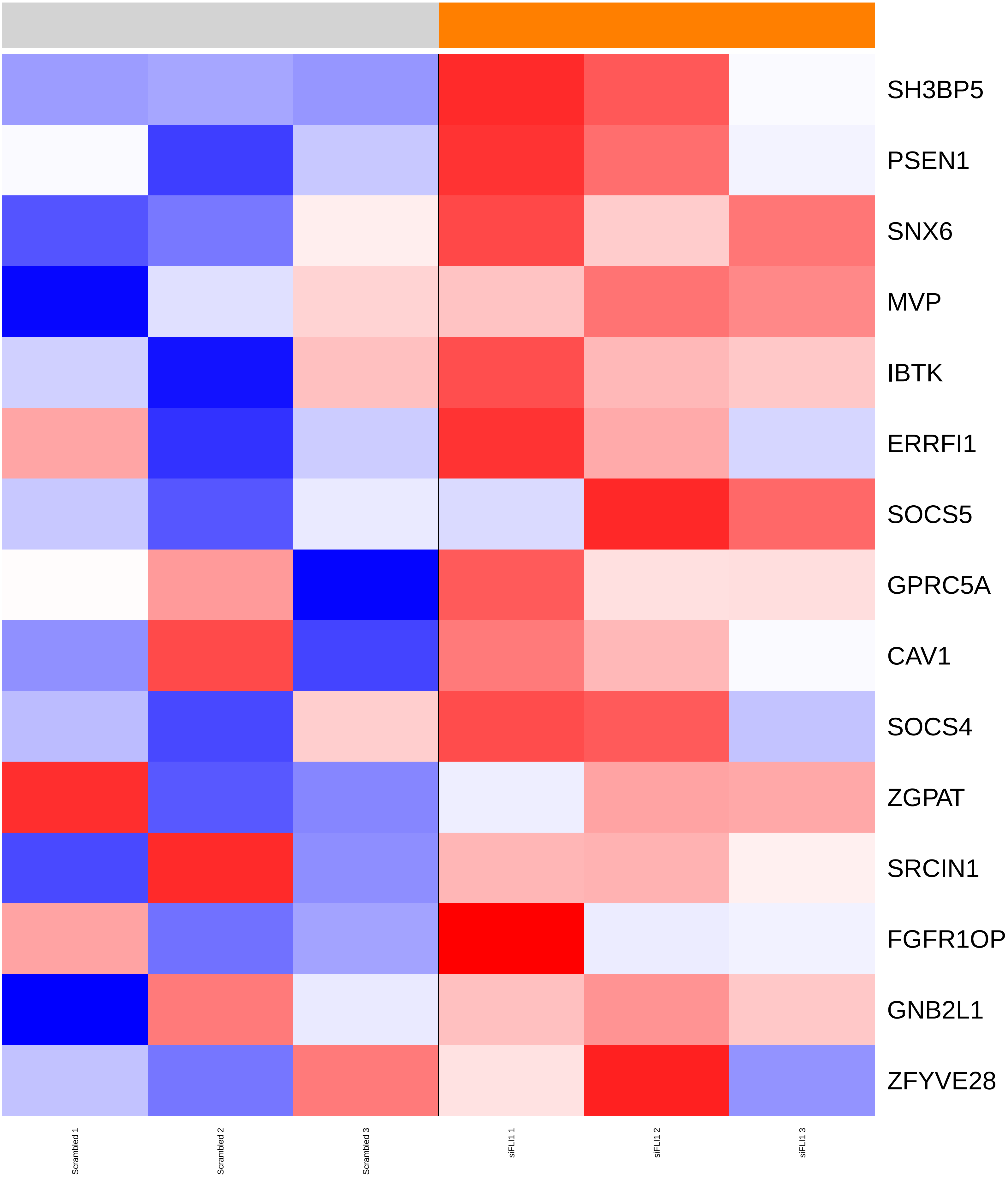

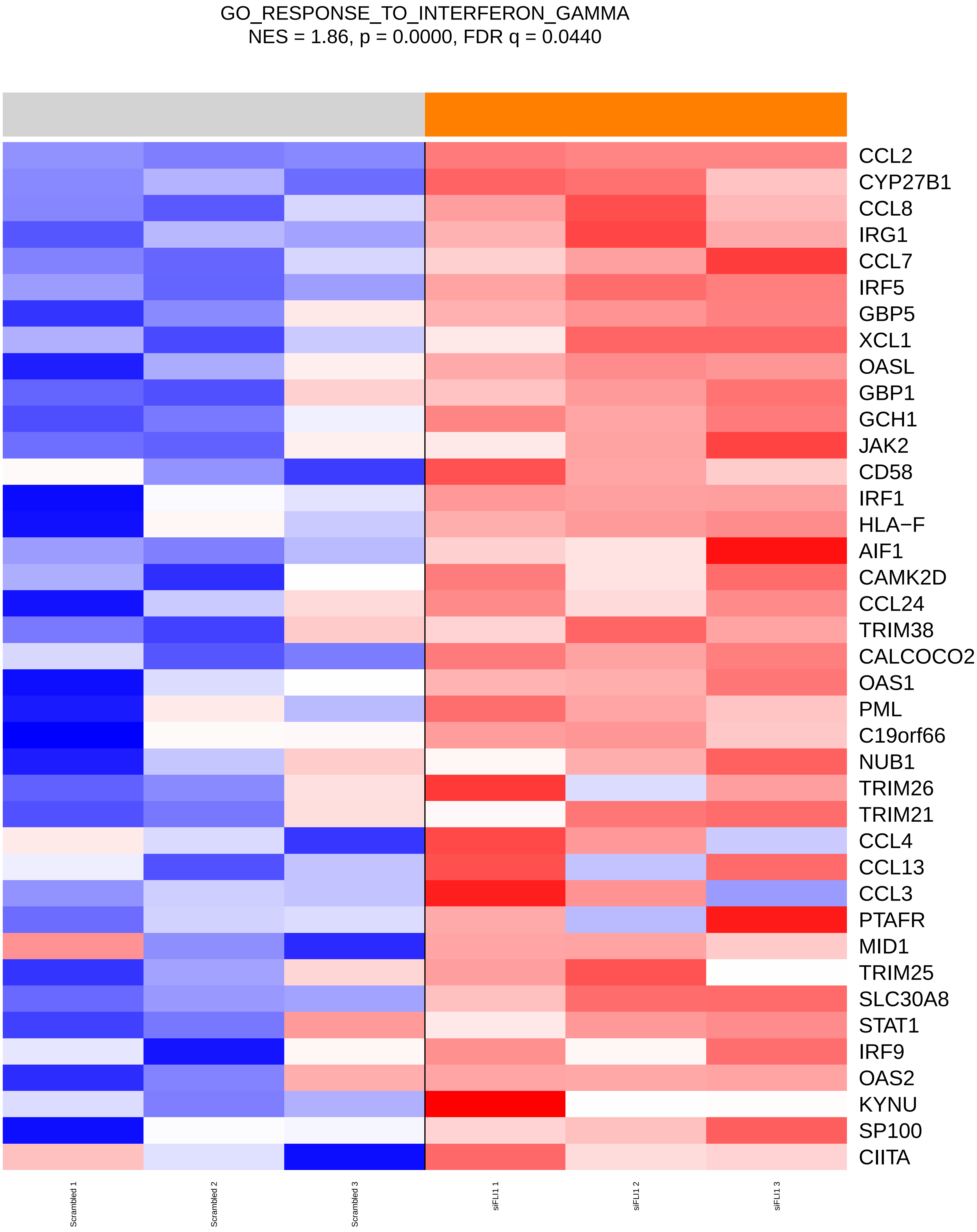

GO_RESPONSE_TO_INTERFERON_GAMMA
NES = 1.86, p = 0.0000, FDR q = 0.0440
CCL2
CYP27B1
CCL8
IRG1
CCL7
IRF5
GBP5
XCL1
OASL
GBP1
GCH1
JAK2
CD58
IRF1
HLA−F
AIF1
CAMK2D
CCL24
TRIM38
CALCOCO2
OAS1
PML
C19orf66
NUB1
TRIM26
TRIM21
CCL4
CCL13
CCL3
PTAFR
MID1
TRIM25
SLC30A8
STAT1
IRF9
OAS2
KYNU
SP100
CIITA
Scrambled 1
Scrambled 2
Scrambled 3
siFLI1 1
siFLI1 2
siFLI1 3

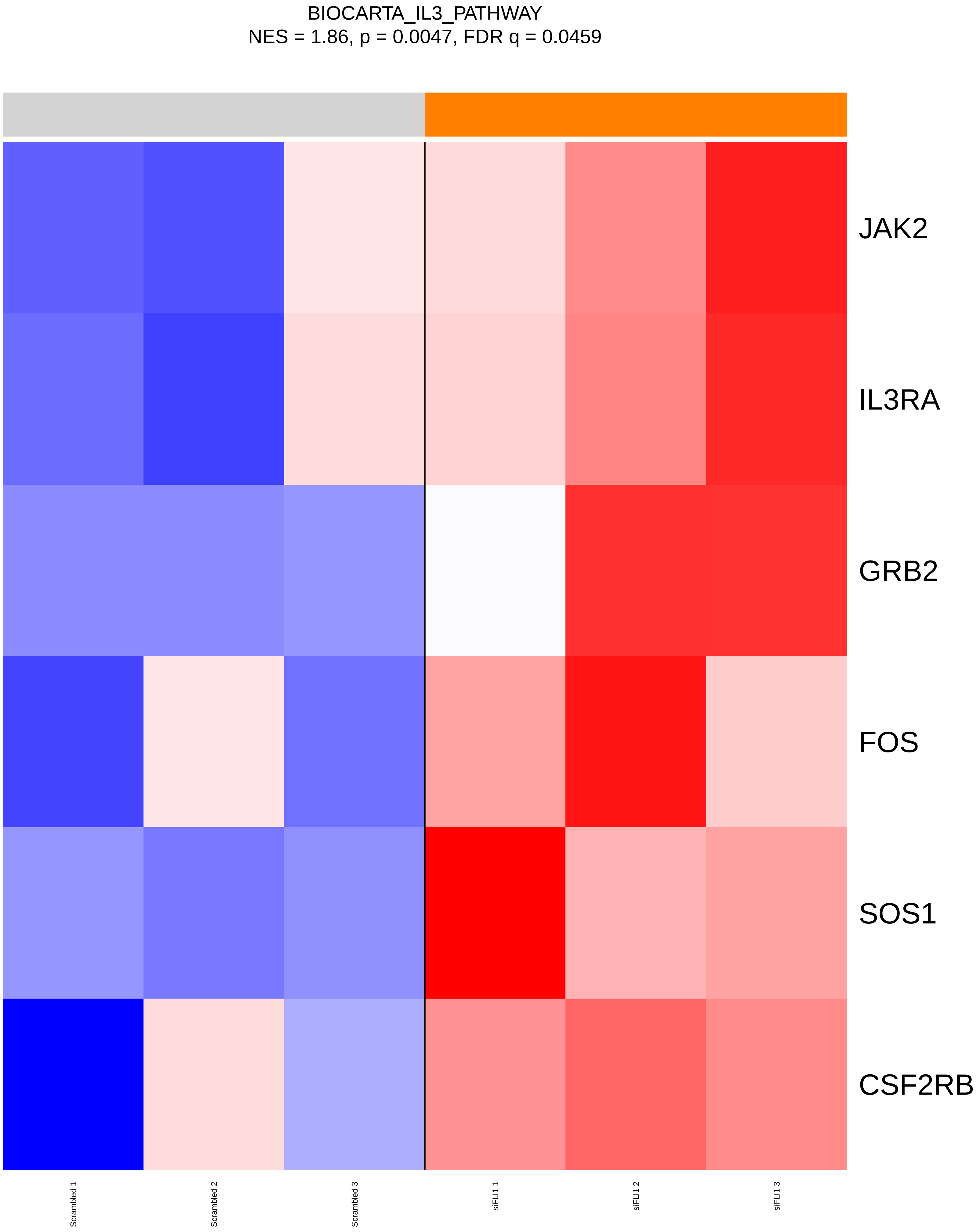
BIOCARTA_IL3_PATHWAY
NES = 1.86, p = 0.0047, FDR q = 0.0459
JAK2
IL3RA
GRB2
FOS
SOS1
CSF2RB
Scrambled 1
Scrambled 2
Scrambled 3
siFLI1 1
siFLI1 2
siFLI1 3

BIOCARTA_MET_PATHWAY
NES = 1.86, p = 0.0000, FDR q = 0.0455

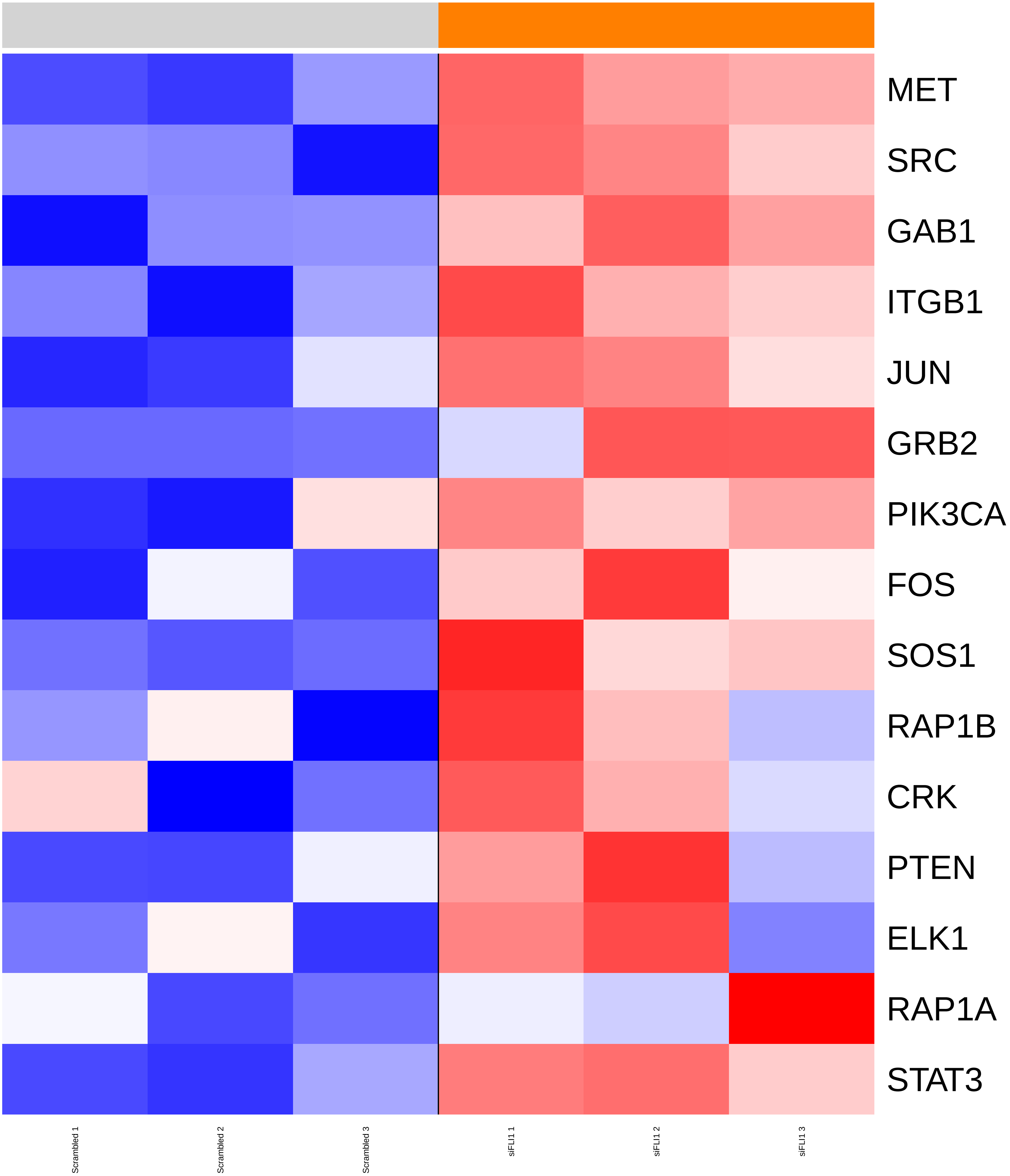

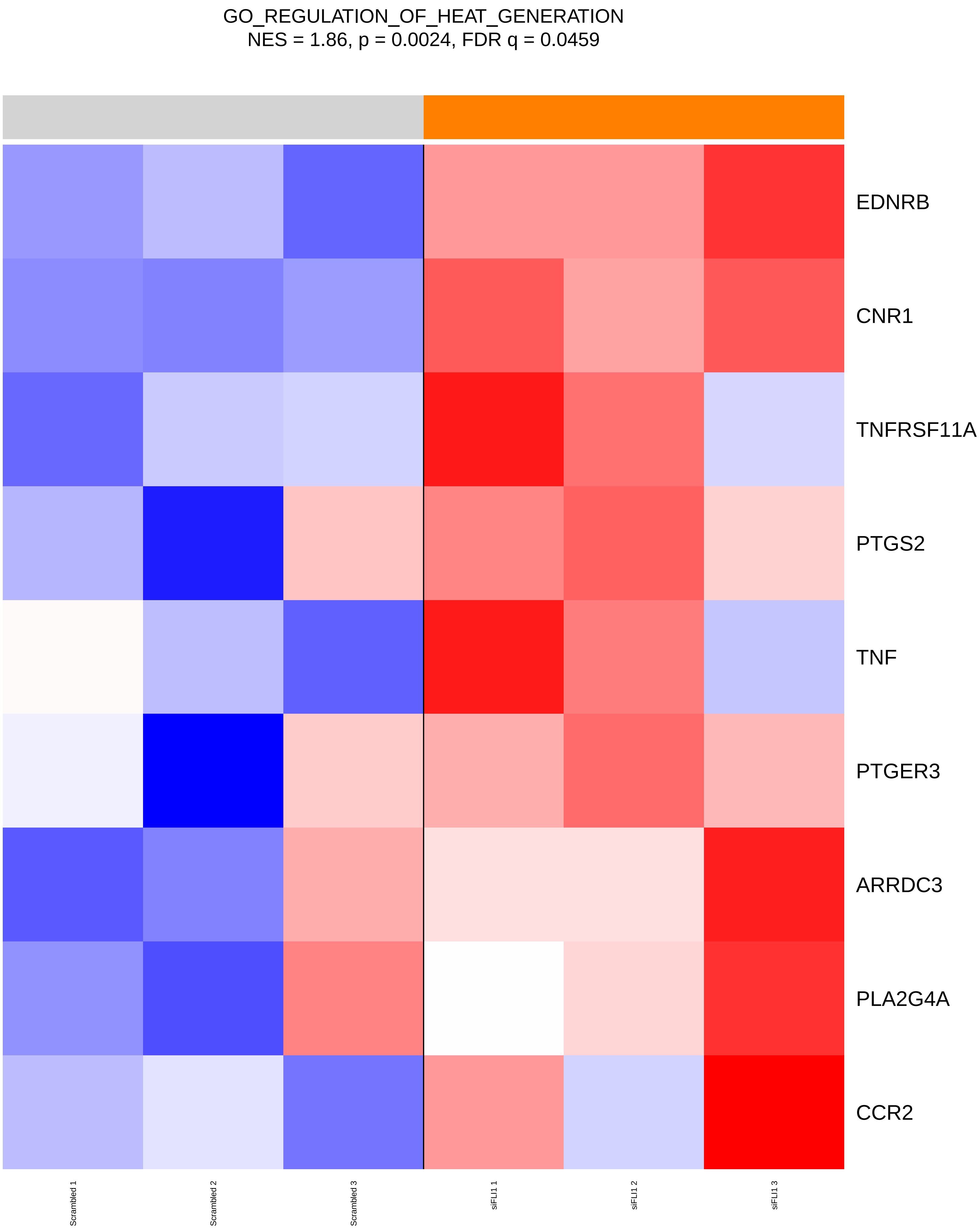

GO_REGULATION_OF_HEAT_GENERATION
NES = 1.86, p = 0.0024, FDR q = 0.0459
EDNRB
CNR1
TNFRSF11A
PTGS2
TNF
PTGER3
ARRDC3
PLA2G4A
CCR2
Scrambled 1
Scrambled 2
Scrambled 3
siFLI1 1
siFLI1 2
siFLI1 3

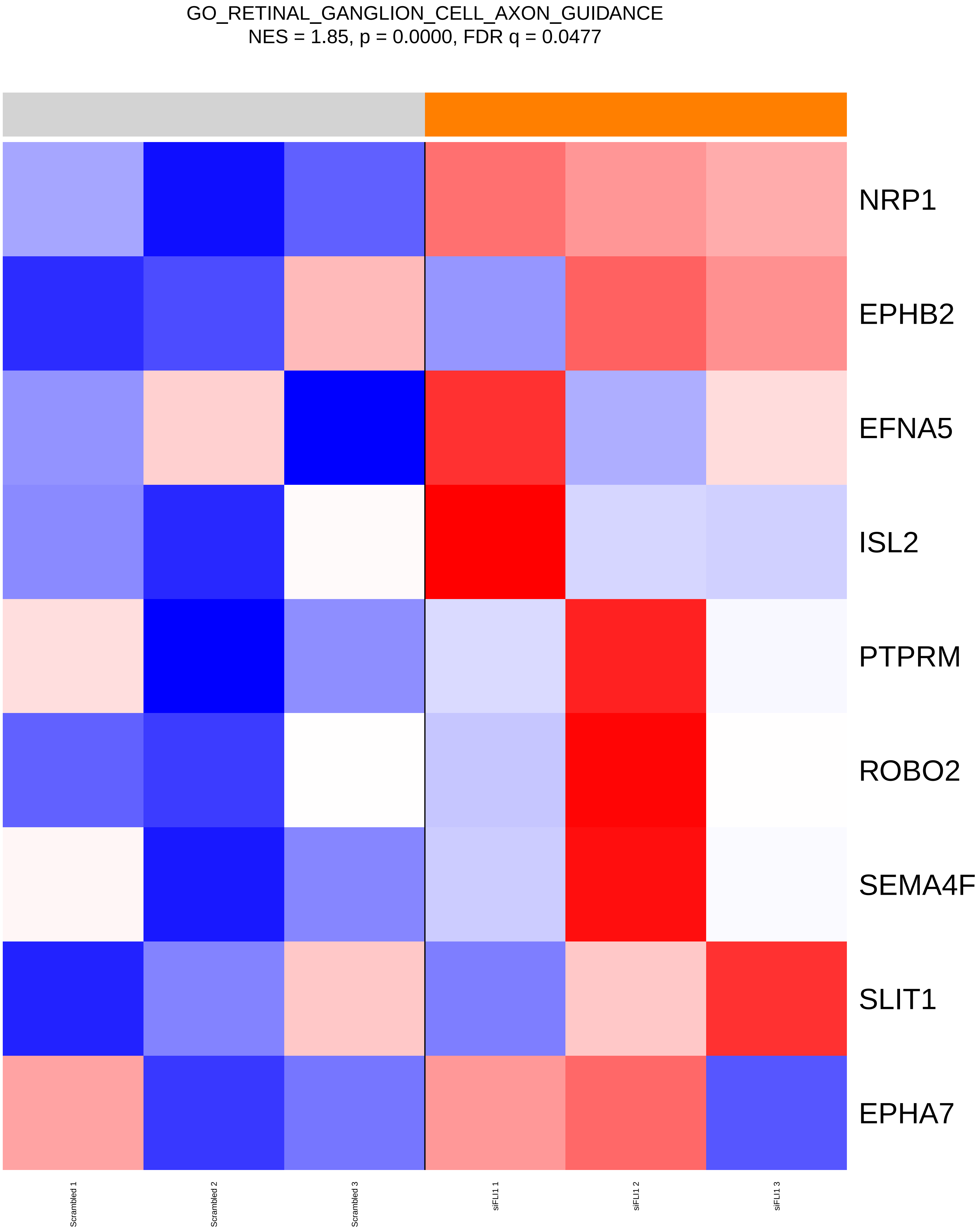
GO_RETINAL_GANGLION_CELL_AXON_GUIDANCE
NES = 1.85, p = 0.0000, FDR q = 0.0477
NRP1
EPHB2
EFNA5
ISL2
PTPRM
ROBO2
SEMA4F
SLIT1
EPHA7
Scrambled 1
Scrambled 2
Scrambled 3
siFLI1 1
siFLI1 2
siFLI1 3

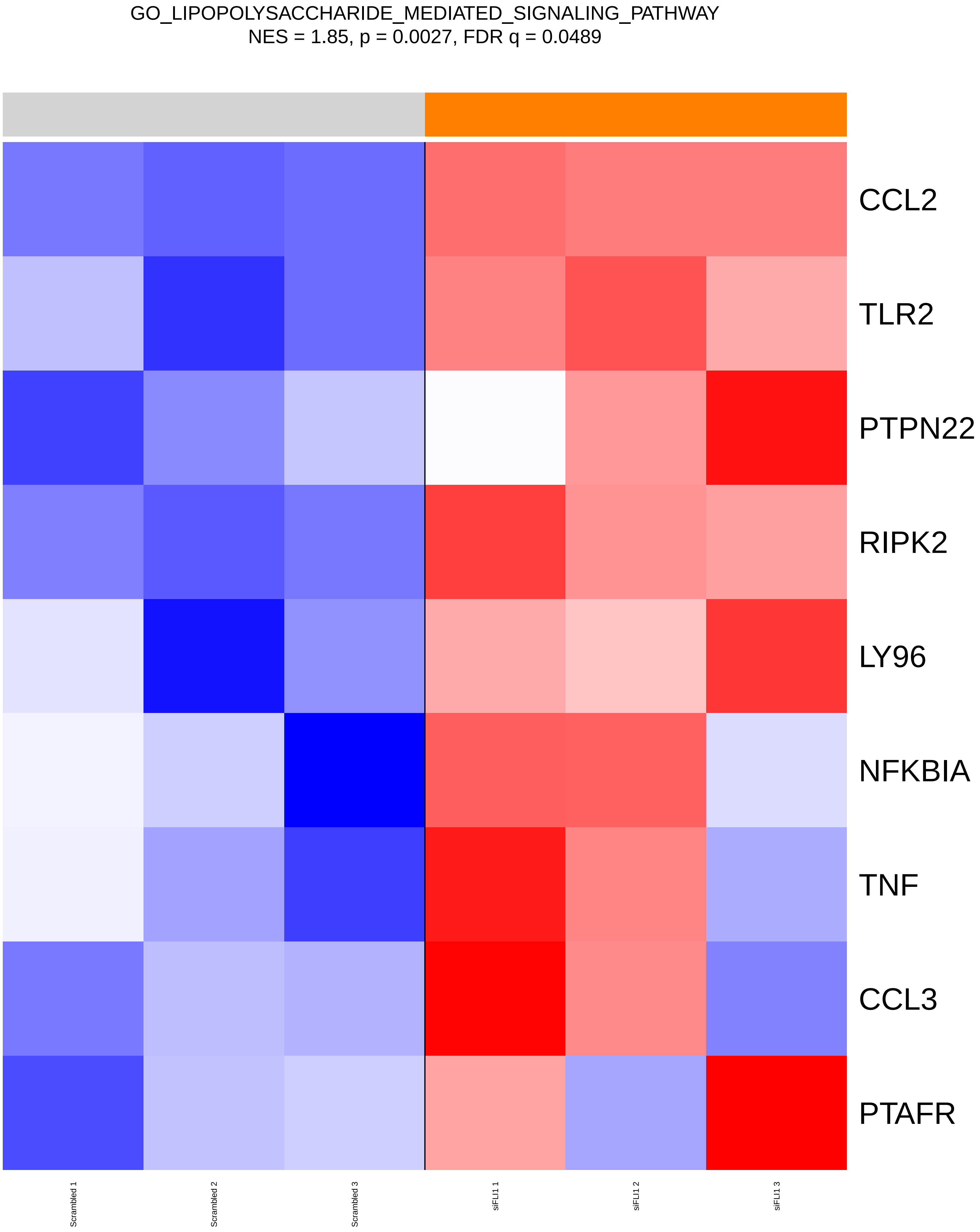
GO_LIPOPOLYSACCHARIDE_MEDIATED_SIGNALING_PATHWAY
NES = 1.85, p = 0.0027, FDR q = 0.0489
CCL2
TLR2
PTPN22
RIPK2
LY96
NFKBIA
TNF
CCL3
PTAFR
Scrambled 1
Scrambled 2
Scrambled 3
siFLI1 1
siFLI1 2
siFLI1 3

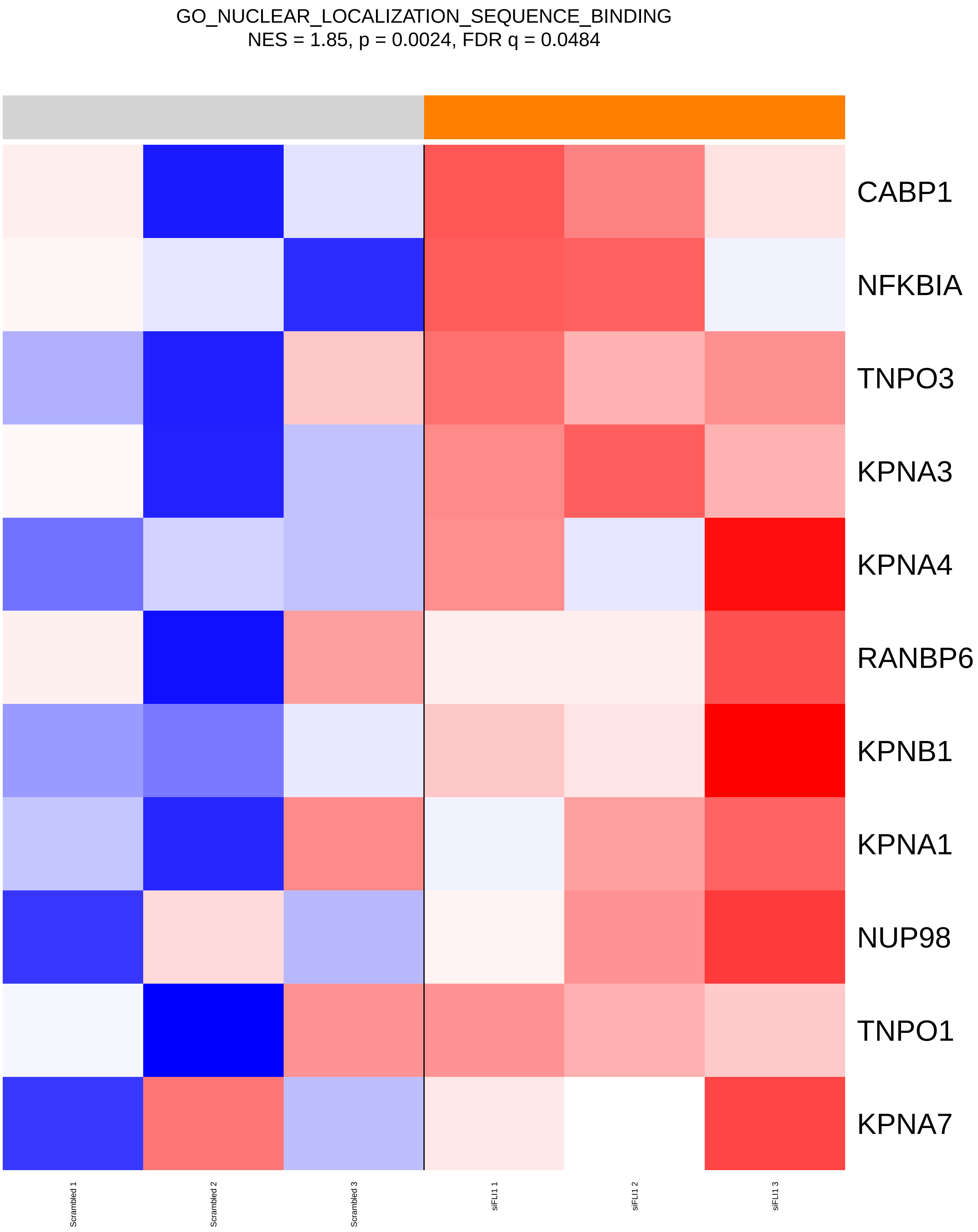
GO_NUCLEAR_LOCALIZATION_SEQUENCE_BINDING
NES = 1.85, p = 0.0024, FDR q = 0.0484
CABP1
NFKBIA
TNPO3
KPNA3
KPNA4
RANBP6
KPNB1
KPNA1
NUP98
TNPO1
KPNA7
Scrambled 1
Scrambled 2
Scrambled 3
siFLI1 1
siFLI1 2
siFLI1 3

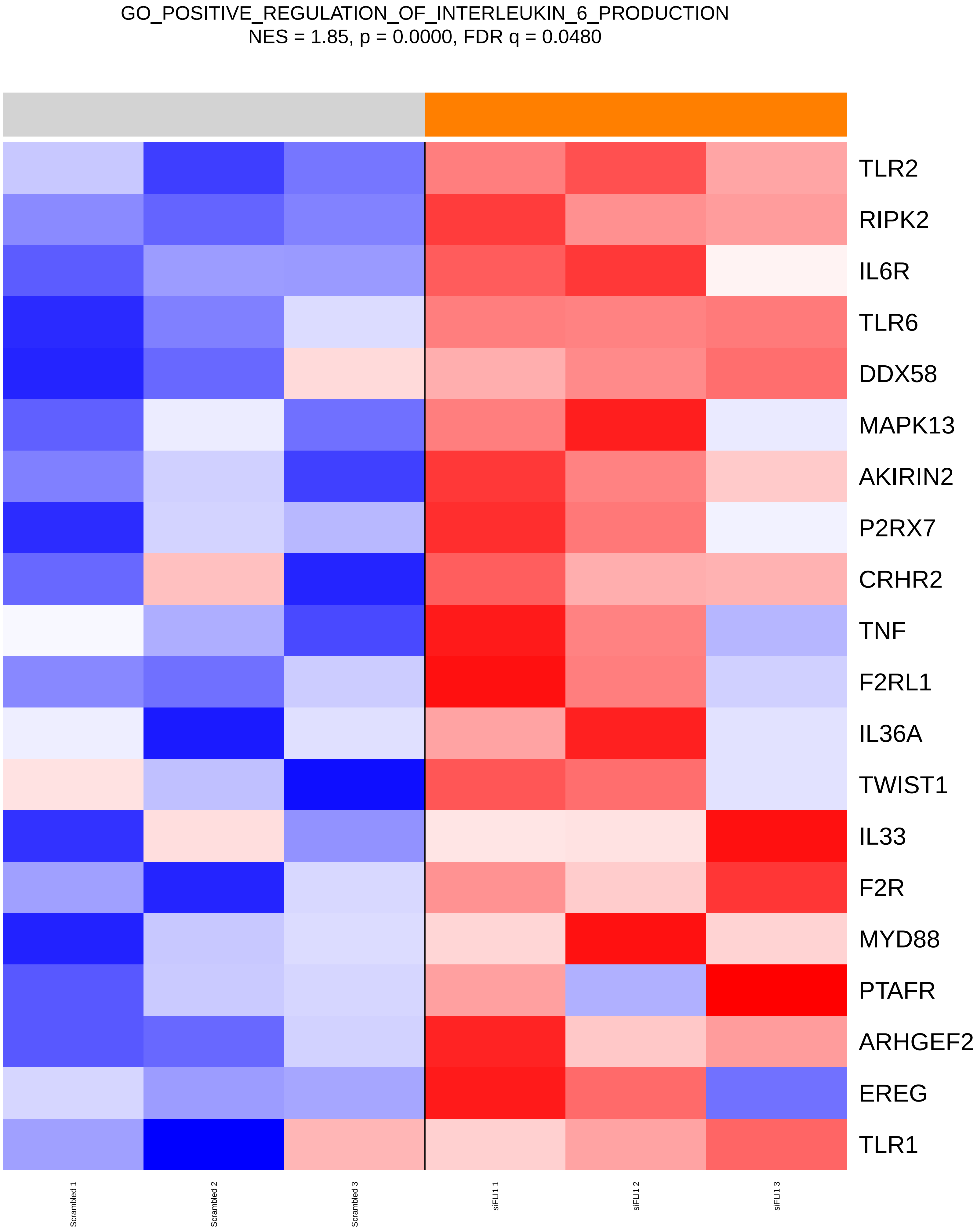
GO_POSITIVE_REGULATION_OF_INTERLEUKIN_6_PRODUCTION
NES = 1.85, p = 0.0000, FDR q = 0.0480
TLR2
RIPK2
IL6R
TLR6
DDX58
MAPK13
AKIRIN2
P2RX7
CRHR2
TNF
F2RL1
IL36A
TWIST1
IL33
F2R
MYD88
PTAFR
ARHGEF2
EREG
TLR1
Scrambled 1
Scrambled 2
Scrambled 3
siFLI1 1
siFLI1 2
siFLI1 3

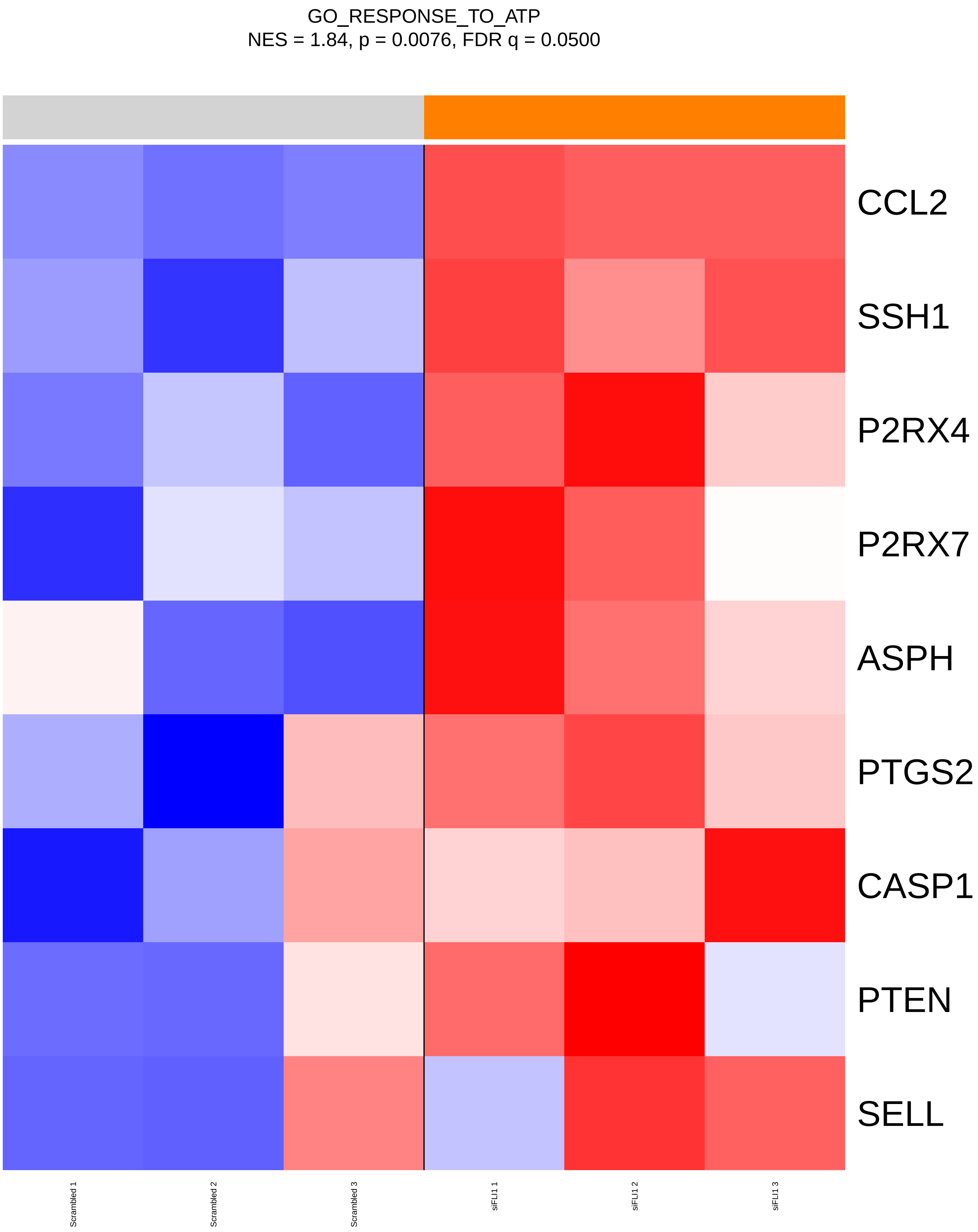

GO_RESPONSE_TO_ATP
NES = 1.84, p = 0.0076, FDR q = 0.0500
CCL2
SSH1
P2RX4
P2RX7
ASPH
PTGS2
CASP1
PTEN
SELL
Scrambled 1
Scrambled 2
Scrambled 3
siFLI1 1
siFLI1 2
siFLI1 3

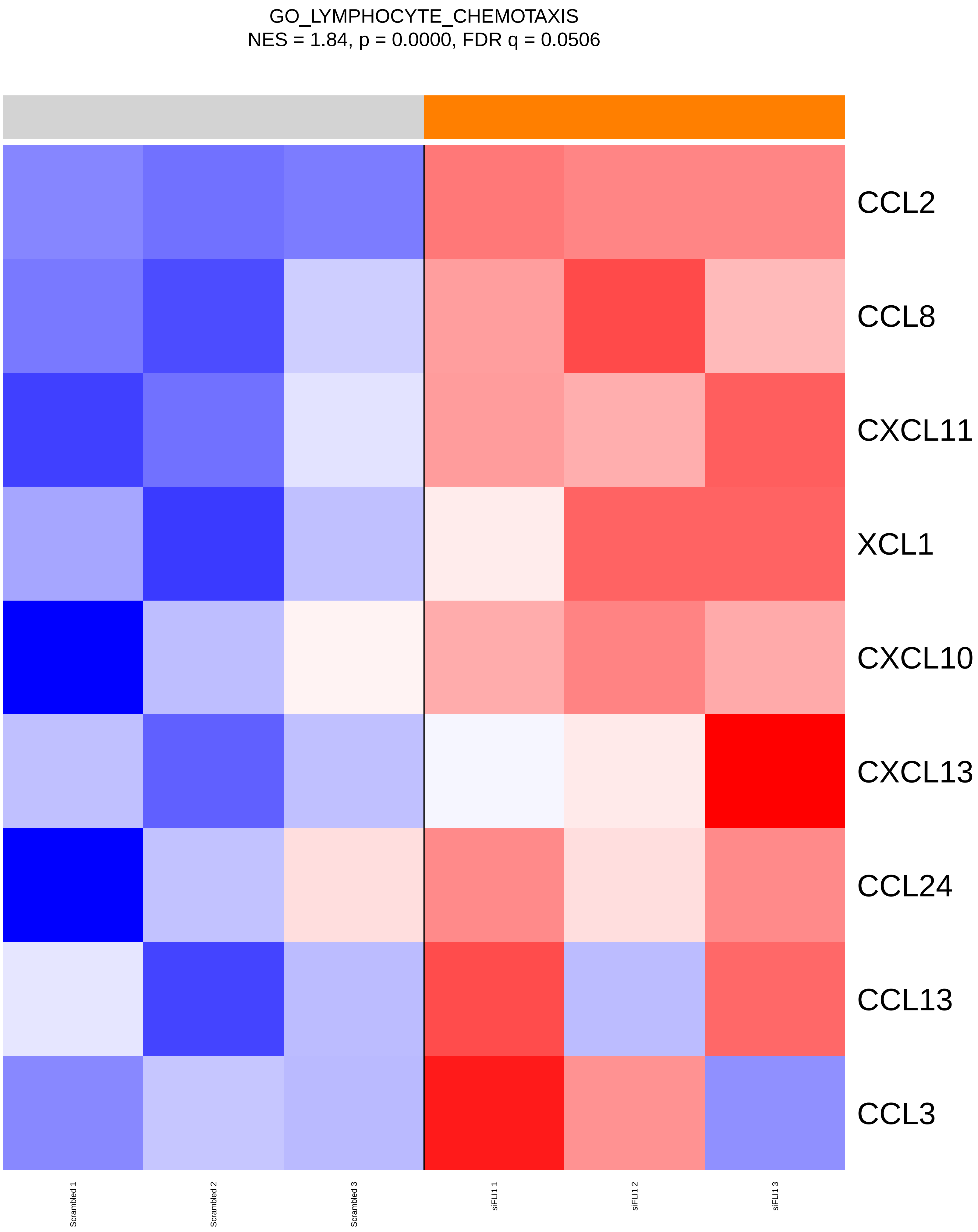
GO_LYMPHOCYTE_CHEMOTAXIS
NES = 1.84, p = 0.0000, FDR q = 0.0506
CCL2
CCL8
CXCL11
XCL1
CXCL10
CXCL13
CCL24
CCL13
CCL3
Scrambled 1
Scrambled 2
Scrambled 3
siFLI1 1
siFLI1 2
siFLI1 3

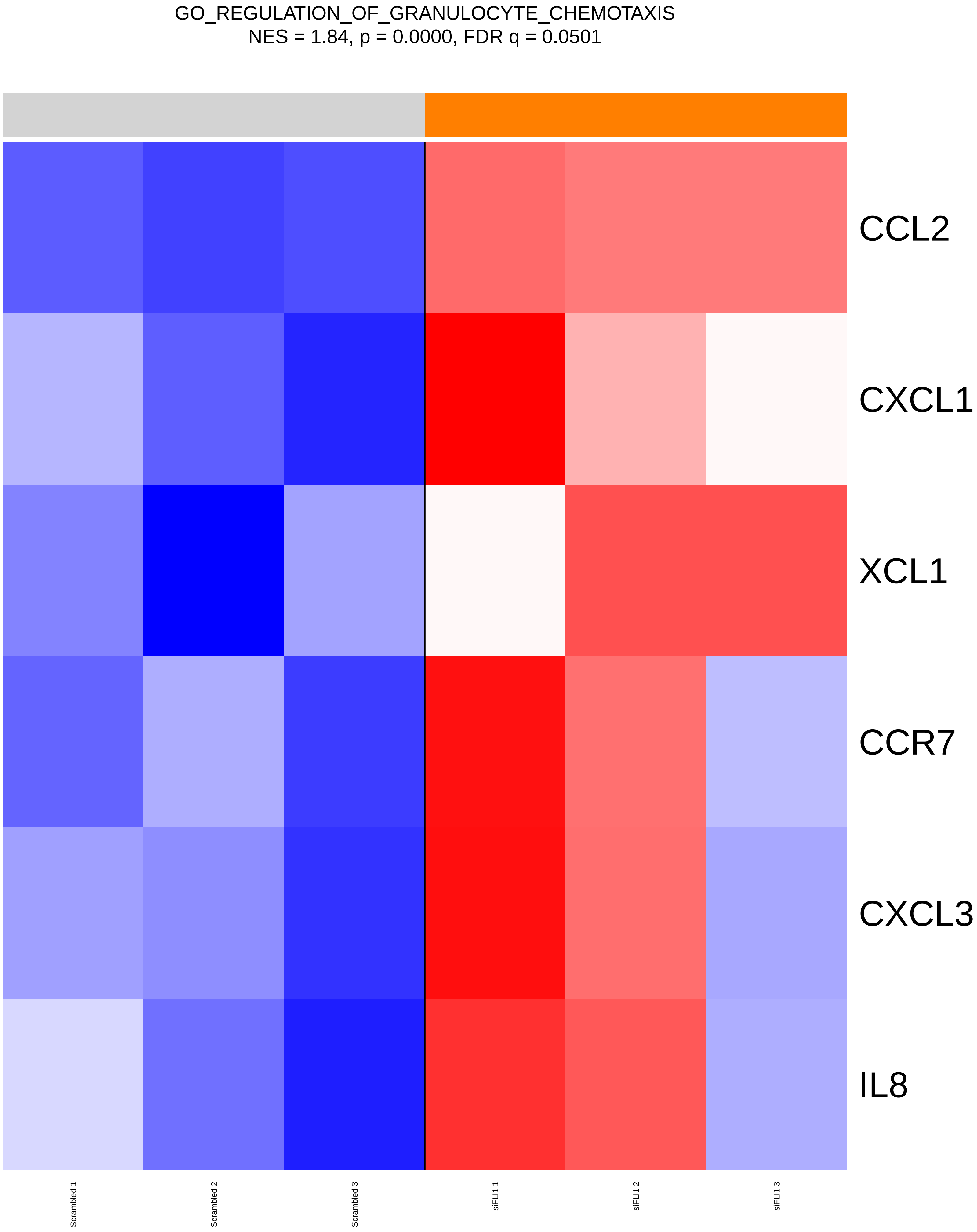
GO_REGULATION_OF_GRANULOCYTE_CHEMOTAXIS
NES = 1.84, p = 0.0000, FDR q = 0.0501
CCL2
CXCL1
XCL1
CCR7
CXCL3
IL8
Scrambled 1
Scrambled 2
Scrambled 3
siFLI1 1
siFLI1 2
siFLI1 3

GO_POSITIVE_REGULATION_OF_TOLL_LIKE_RECEPTOR_SIGNALING_PATHWAY
NES = 1.84, p = 0.0050, FDR q = 0.0500

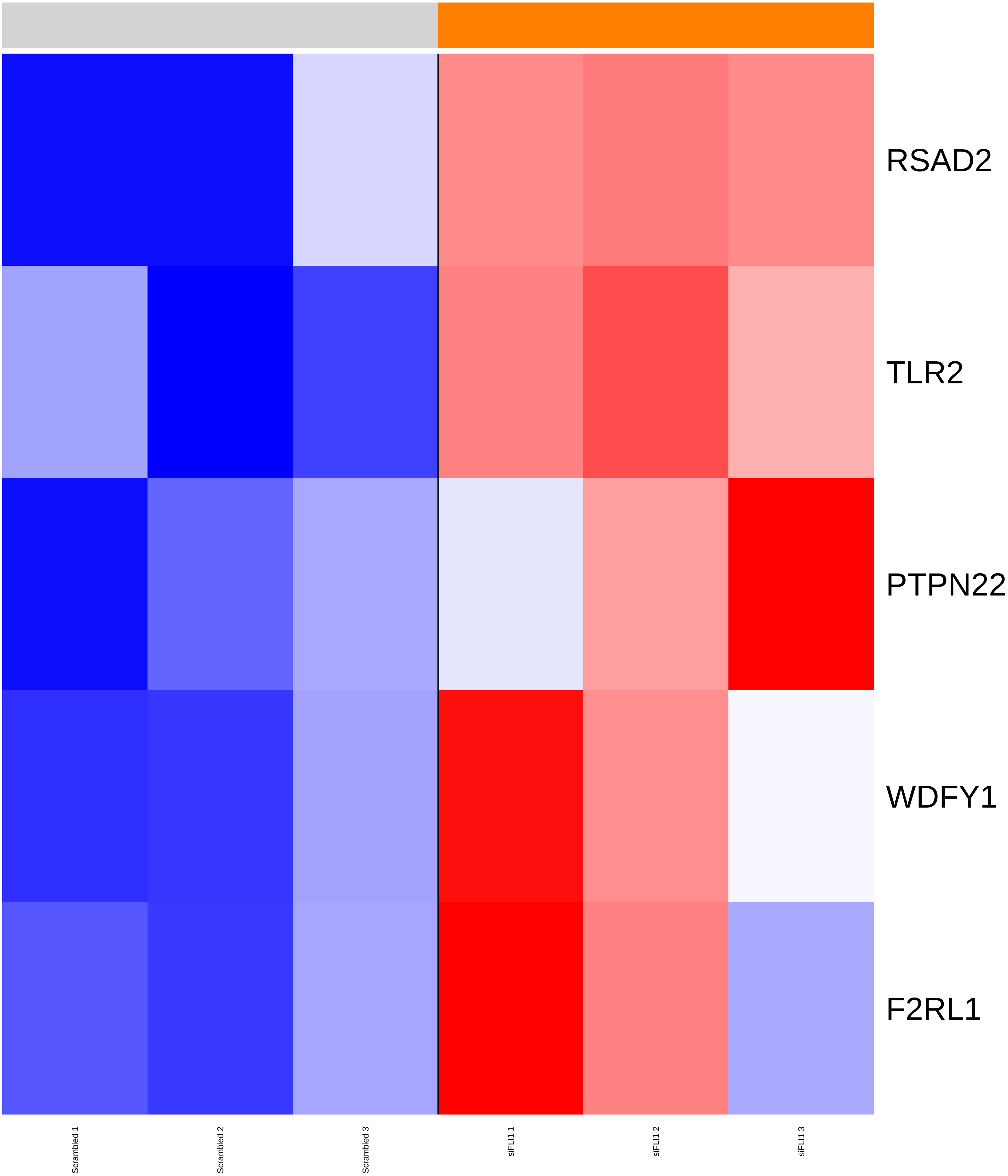

GO_REGULATION_OF_OXIDATIVE_STRESS_INDUCED_CELL_DEATH
NES = 1.84, p = 0.0000, FDR q = 0.0507

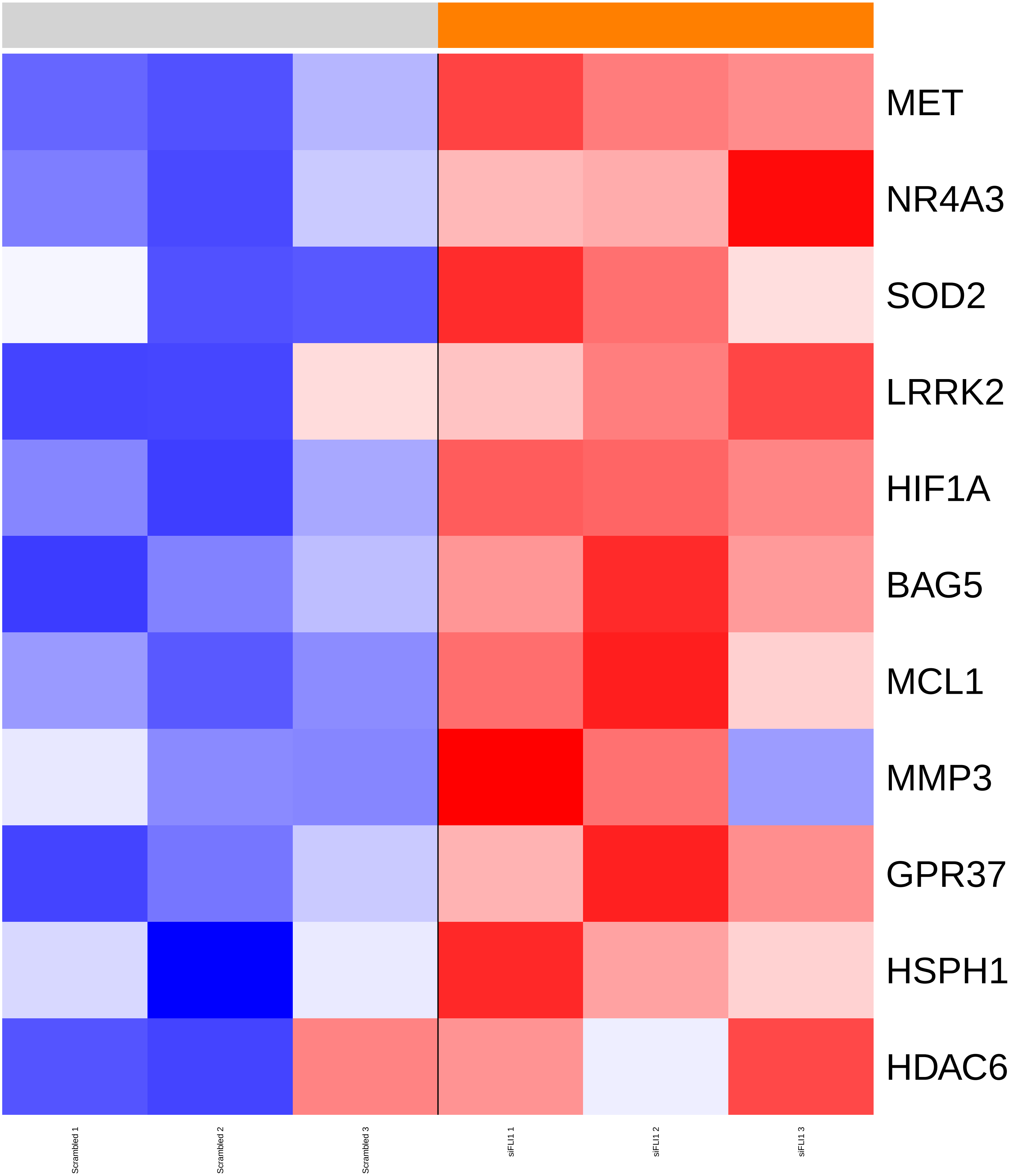

REACTOME_NEGATIVE_REGULATORS_OF_RIG_I_MDA5_SIGNALING
NES = 1.83, p = 0.0026, FDR q = 0.0518

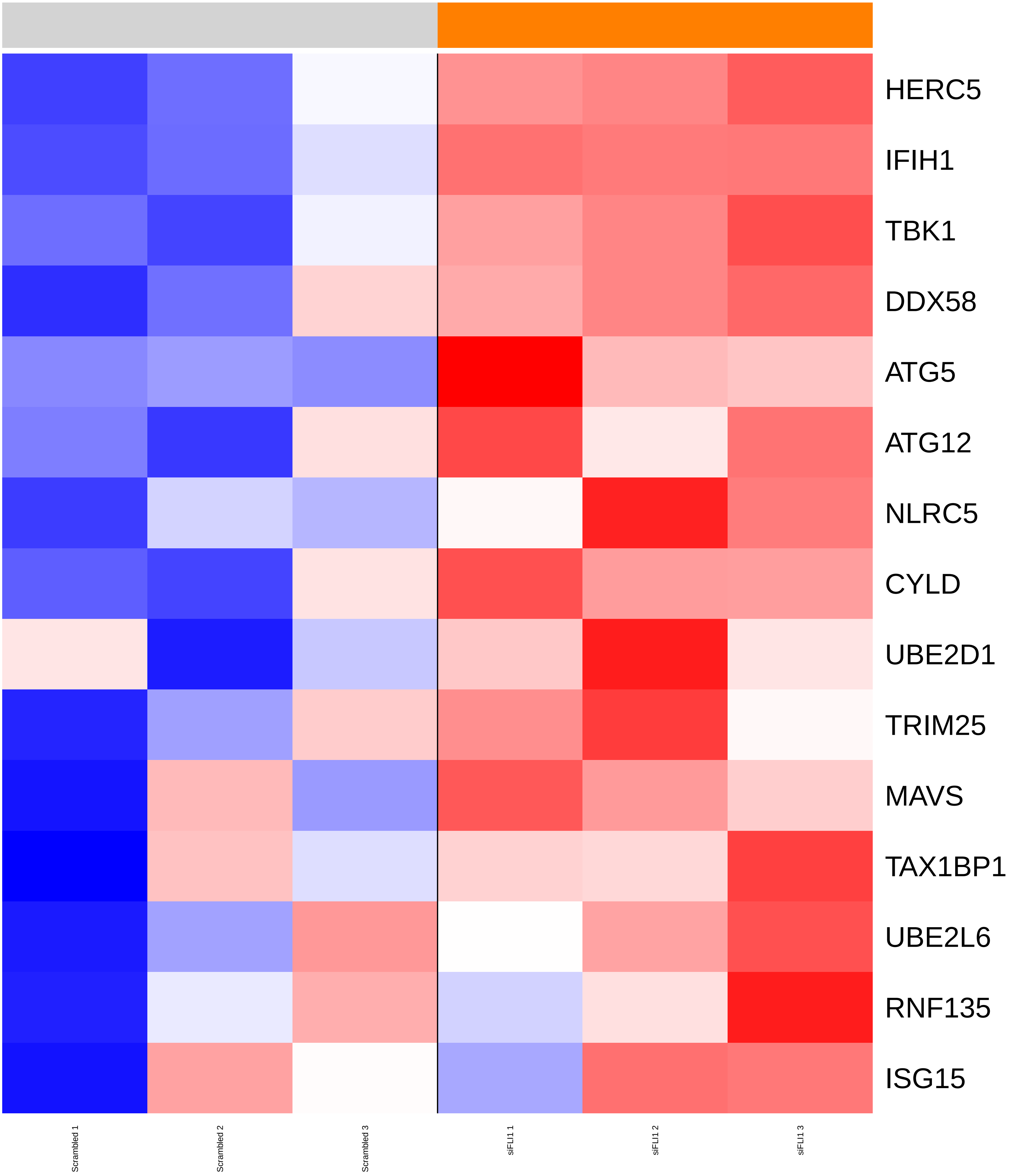

GO_POSITIVE_REGULATION_OF_MULTICELLULAR_ORGANISMAL_METABOLIC_PROCESS
NES = 1.83, p = 0.0047, FDR q = 0.0540

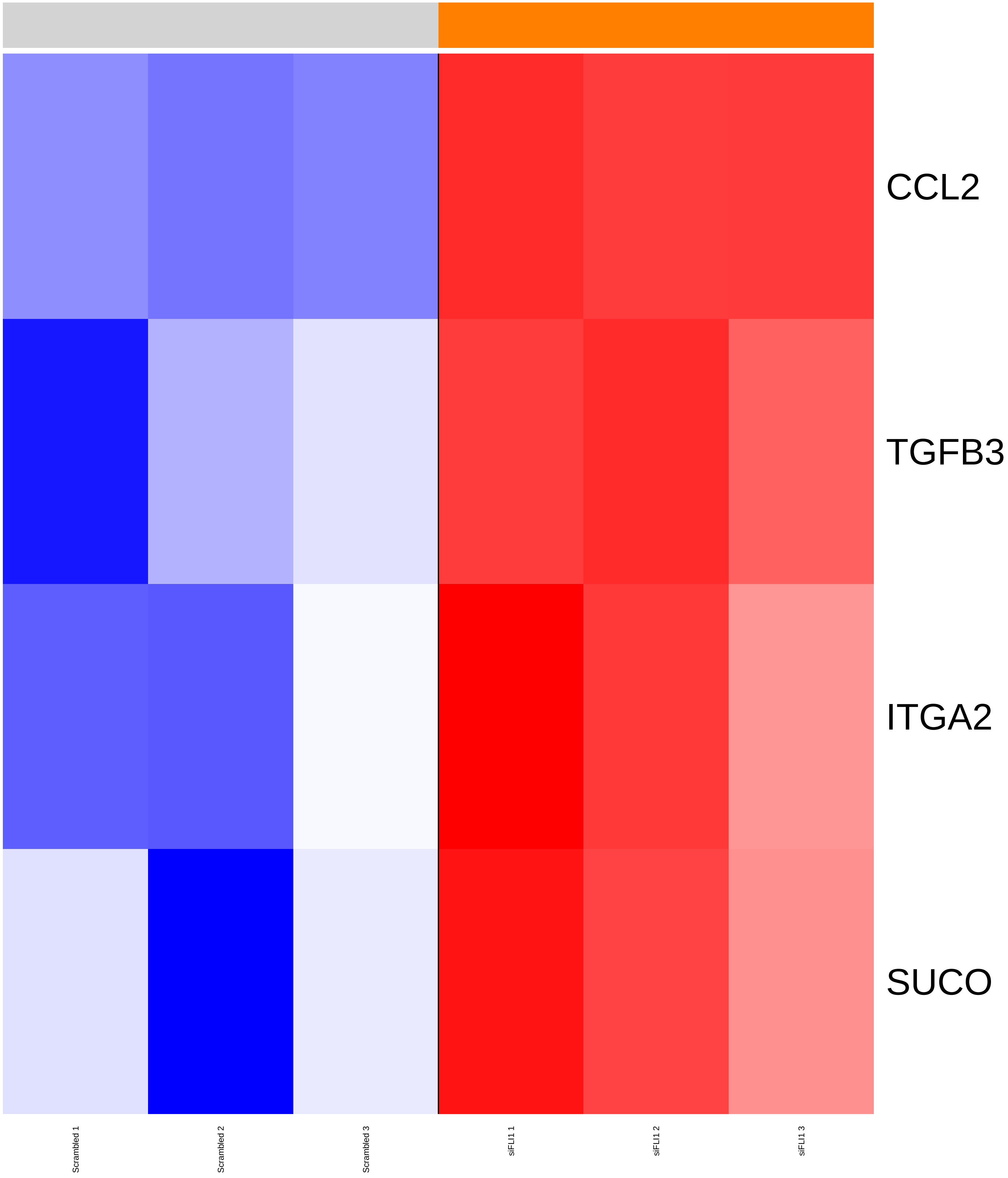

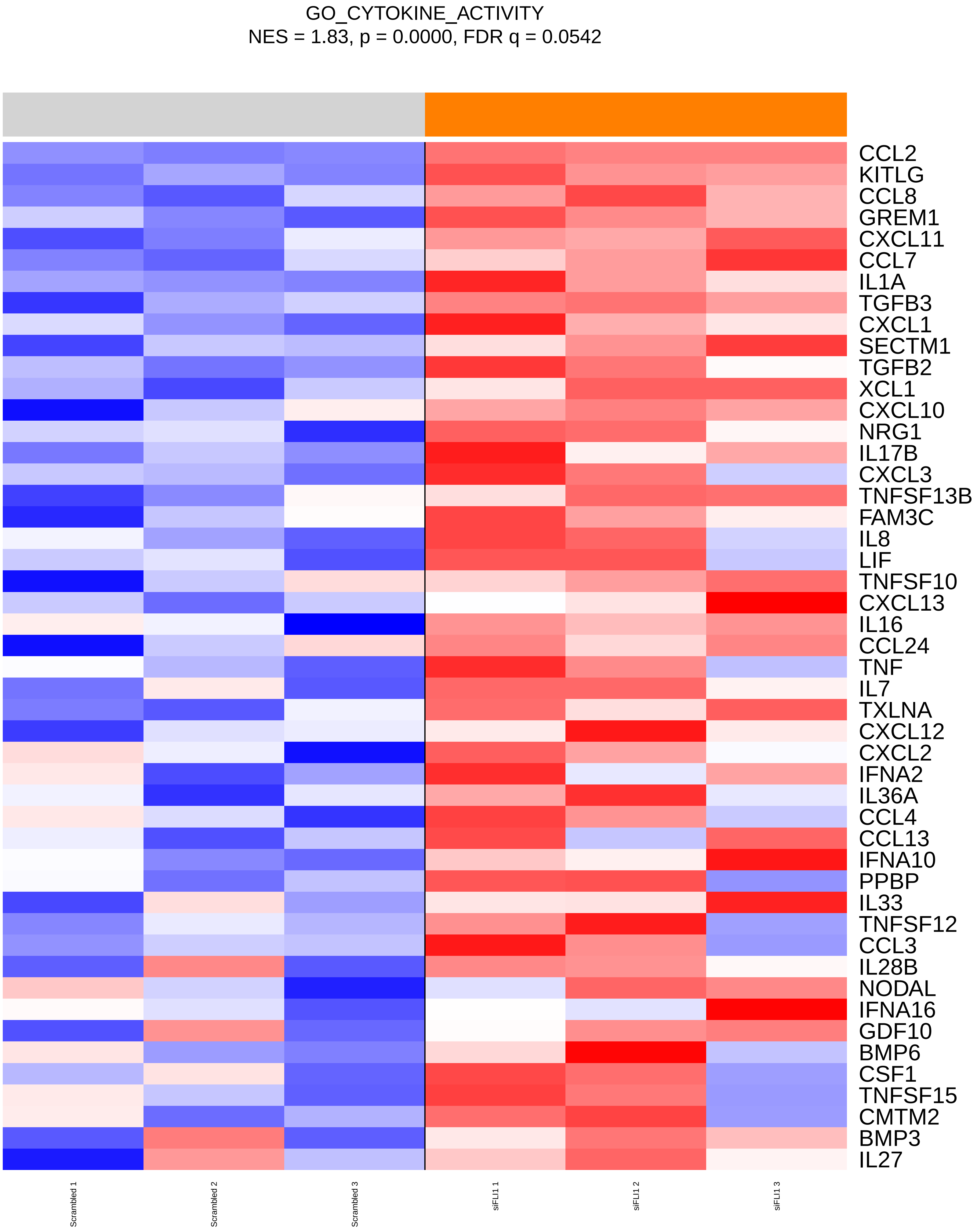
GO_CYTOKINE_ACTIVITY
NES = 1.83, p = 0.0000, FDR q = 0.0542
CCL2
KITLG
CCL8
GREM1
CXCL11
CCL7
IL1A
TGFB3
CXCL1
SECTM1
TGFB2
XCL1
CXCL10
NRG1
IL17B
CXCL3
TNFSF13B
FAM3C
IL8
LIF
TNFSF10
CXCL13
IL16
CCL24
TNF
IL7
TXLNA
CXCL12
CXCL2
IFNA2
IL36A
CCL4
CCL13
IFNA10
PPBP
IL33
TNFSF12
CCL3
IL28B
NODAL
IFNA16
GDF10
BMP6
CSF1
TNFSF15
CMTM2
BMP3
IL27
Scrambled 1
Scrambled 2
Scrambled 3
siFLI1 1
siFLI1 2
siFLI1 3

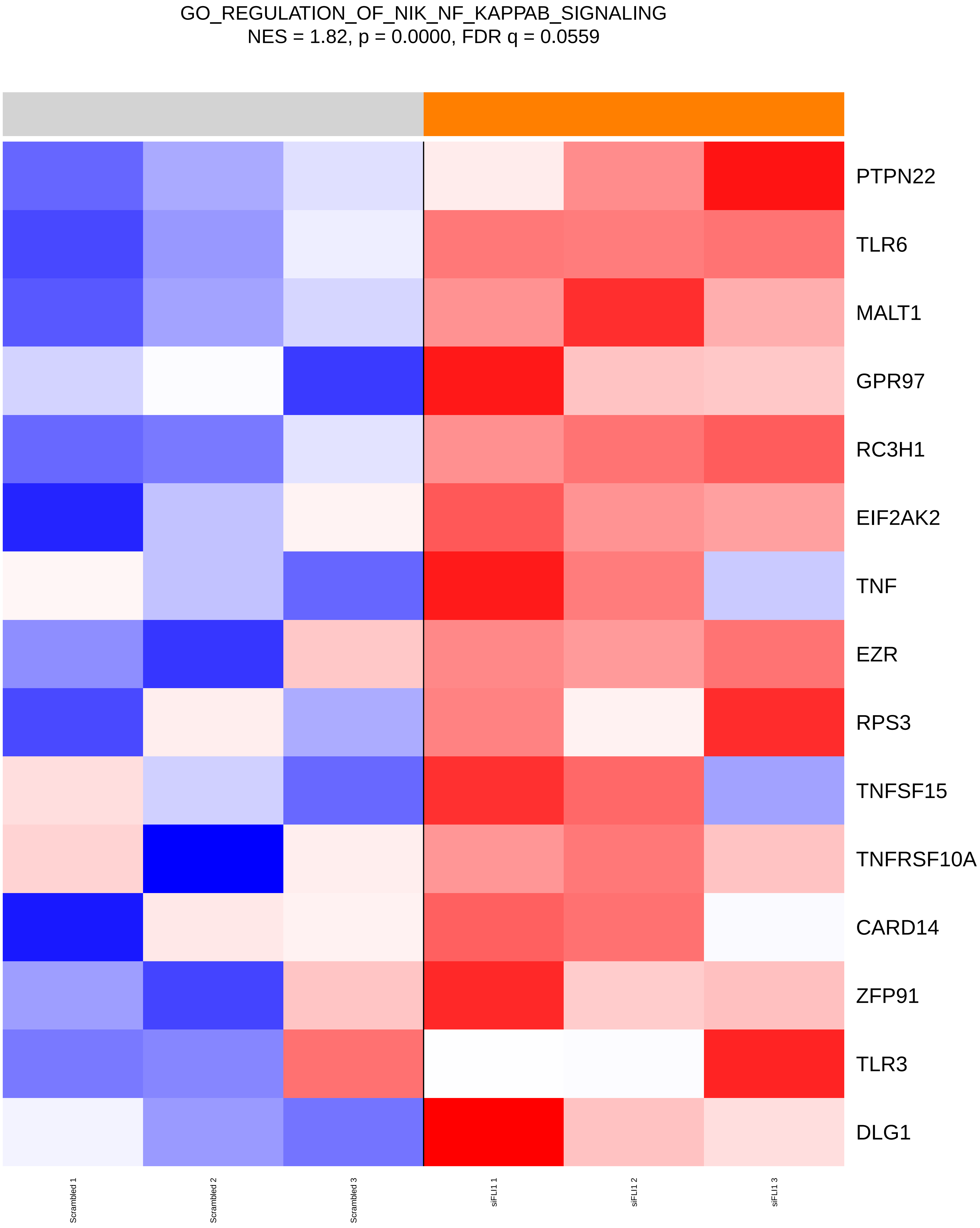
GO_REGULATION_OF_NIK_NF_KAPPAB_SIGNALING
NES = 1.82, p = 0.0000, FDR q = 0.0559
PTPN22
TLR6
MALT1
GPR97
RC3H1
EIF2AK2
TNF
EZR
RPS3
TNFSF15
TNFRSF10A
CARD14
ZFP91
TLR3
DLG1
Scrambled 1
Scrambled 2
Scrambled 3
siFLI1 1
siFLI1 2
siFLI1 3

REACTOME_NUCLEOTIDE_BINDING_DOMAIN_LEUCINE_RICH_REPEAT_CONTAINING_RECEPTOR_NLR_SIGNALING_PATHWAYS
NES = 1.82, p = 0.0000, FDR q = 0.0559

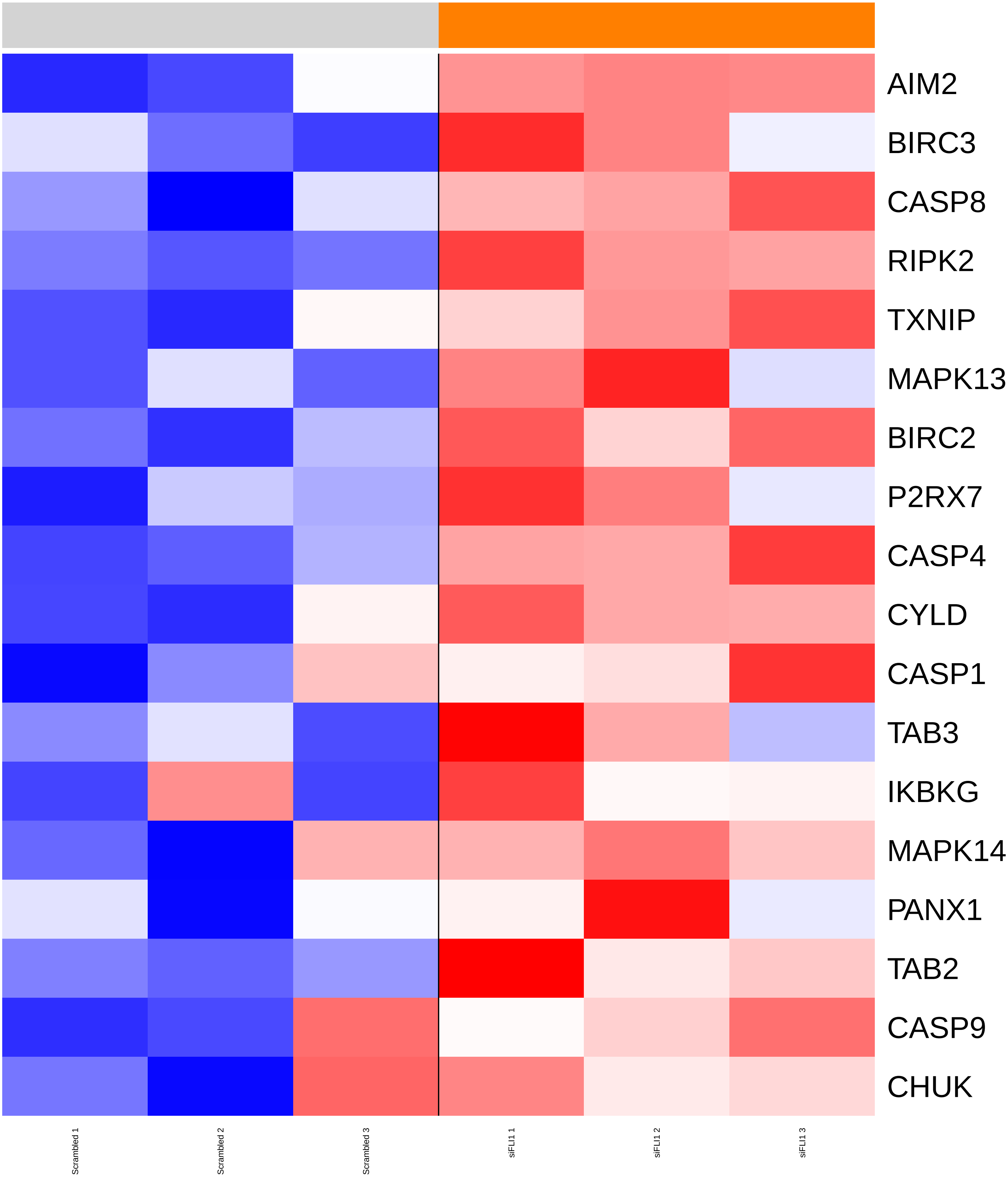

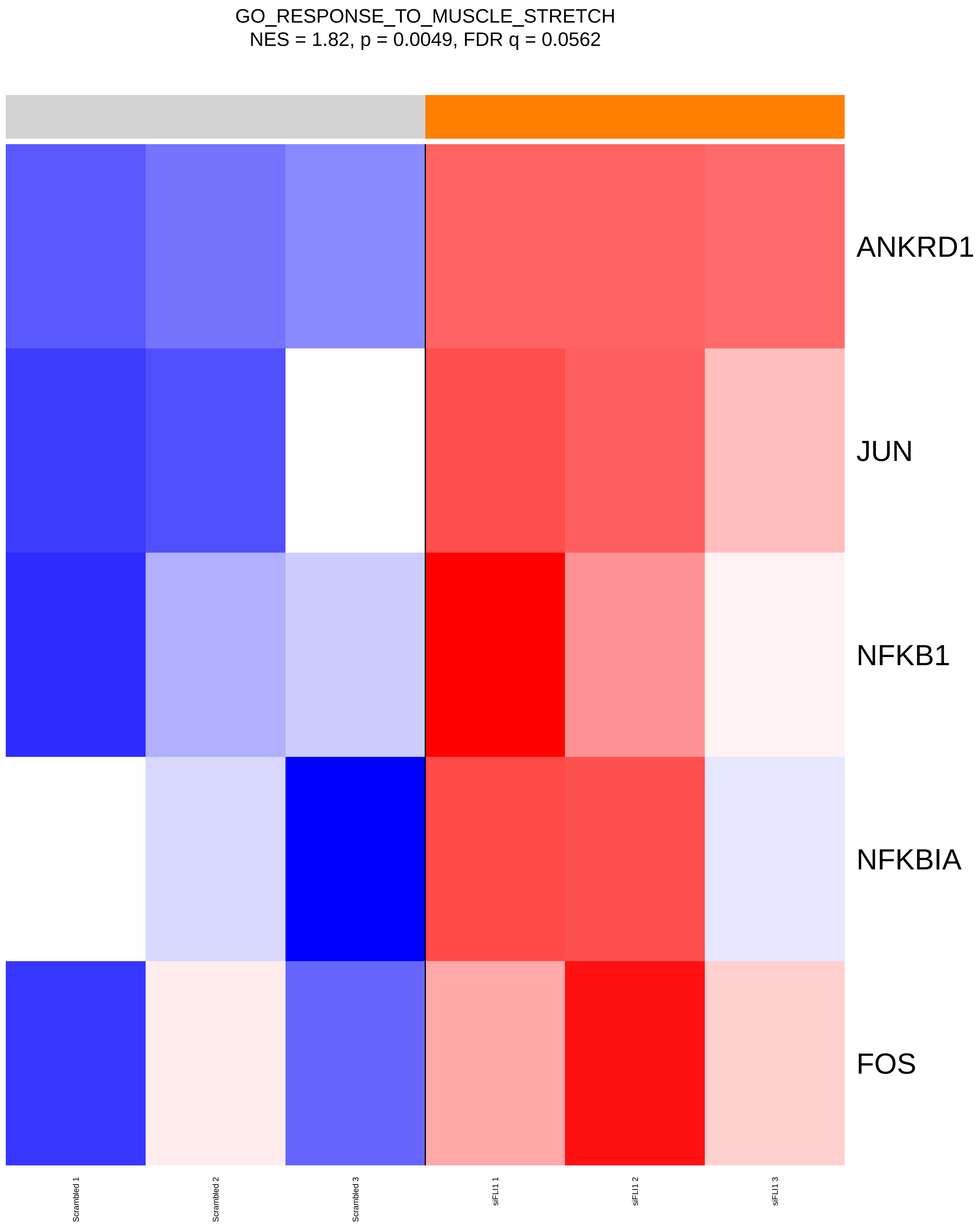
GO_RESPONSE_TO_MUSCLE_STRETCH
NES = 1.82, p = 0.0049, FDR q = 0.0562
ANKRD1
JUN
NFKB1
NFKBIA
FOS
Scrambled 1
Scrambled 2
Scrambled 3
siFLI1 1
siFLI1 2
siFLI1 3

GO_CYTOKINE_MEDIATED_SIGNALING_PATHWAY
NES = 1.82, p = 0.0000, FDR q = 0.0565

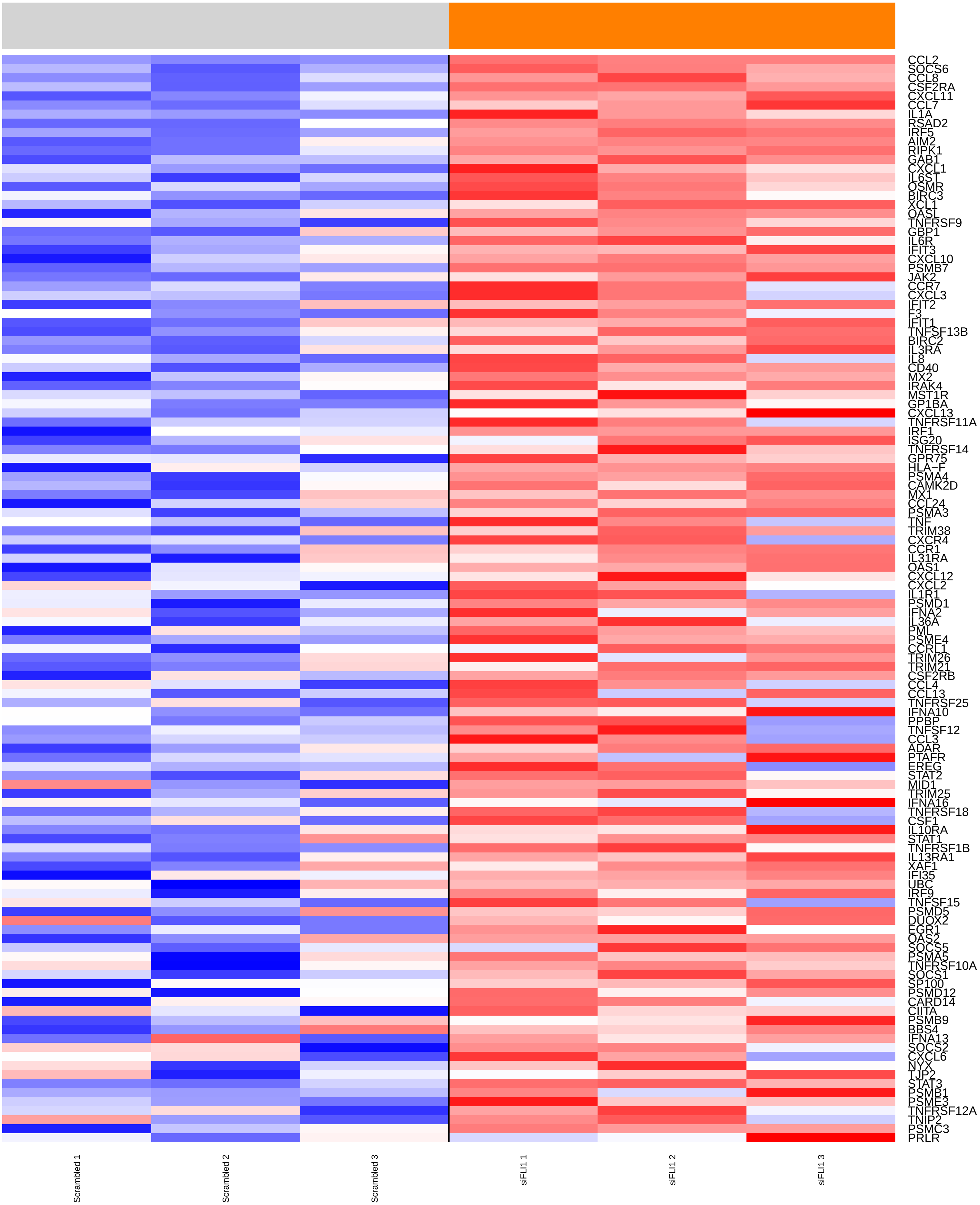

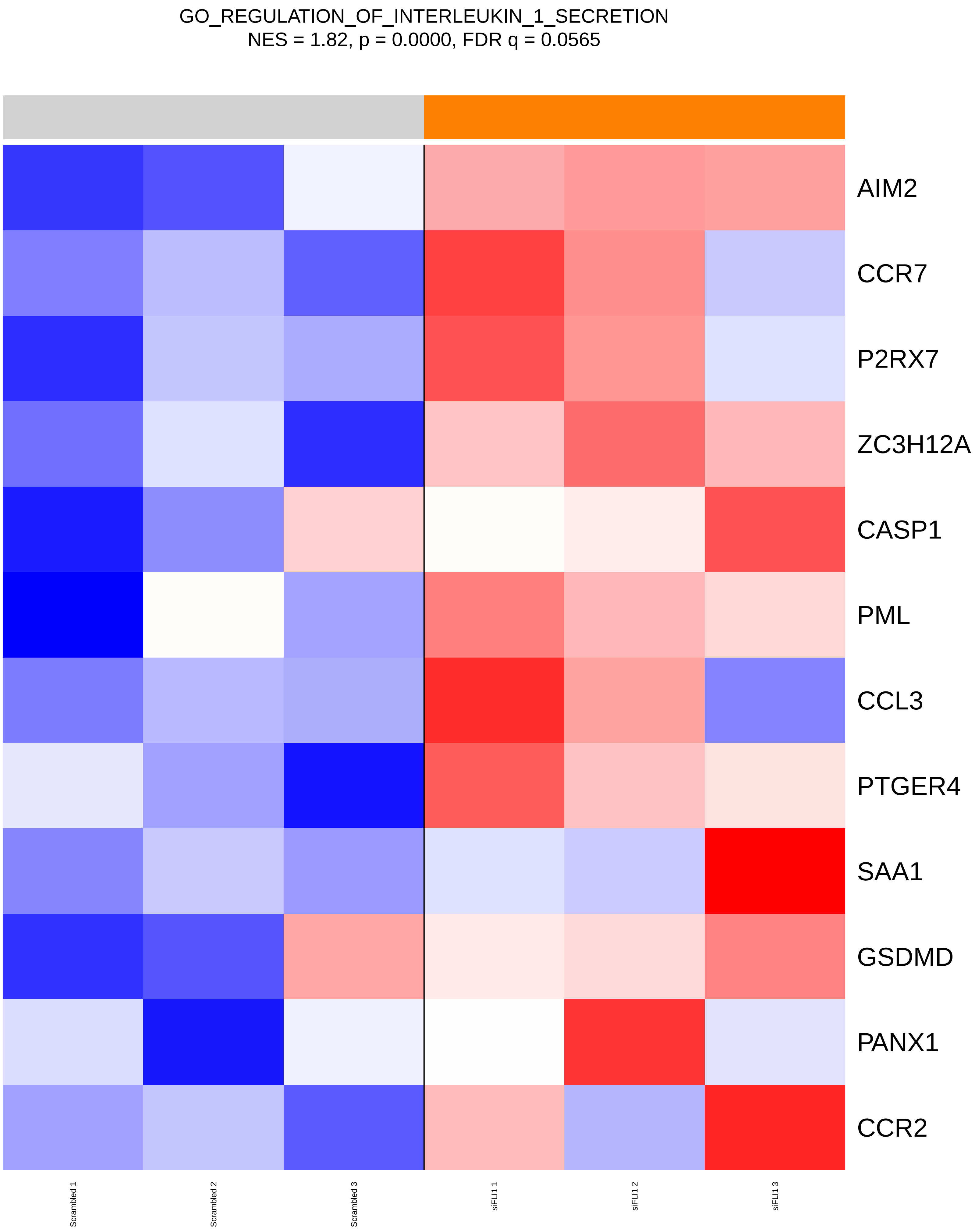
GO_REGULATION_OF_INTERLEUKIN_1_SECRETION
NES = 1.82, p = 0.0000, FDR q = 0.0565
AIM2
CCR7
P2RX7
ZC3H12A
CASP1
PML
CCL3
PTGER4
SAA1
GSDMD
PANX1
CCR2
Scrambled 1
Scrambled 2
Scrambled 3
siFLI1 1
siFLI1 2
siFLI1 3

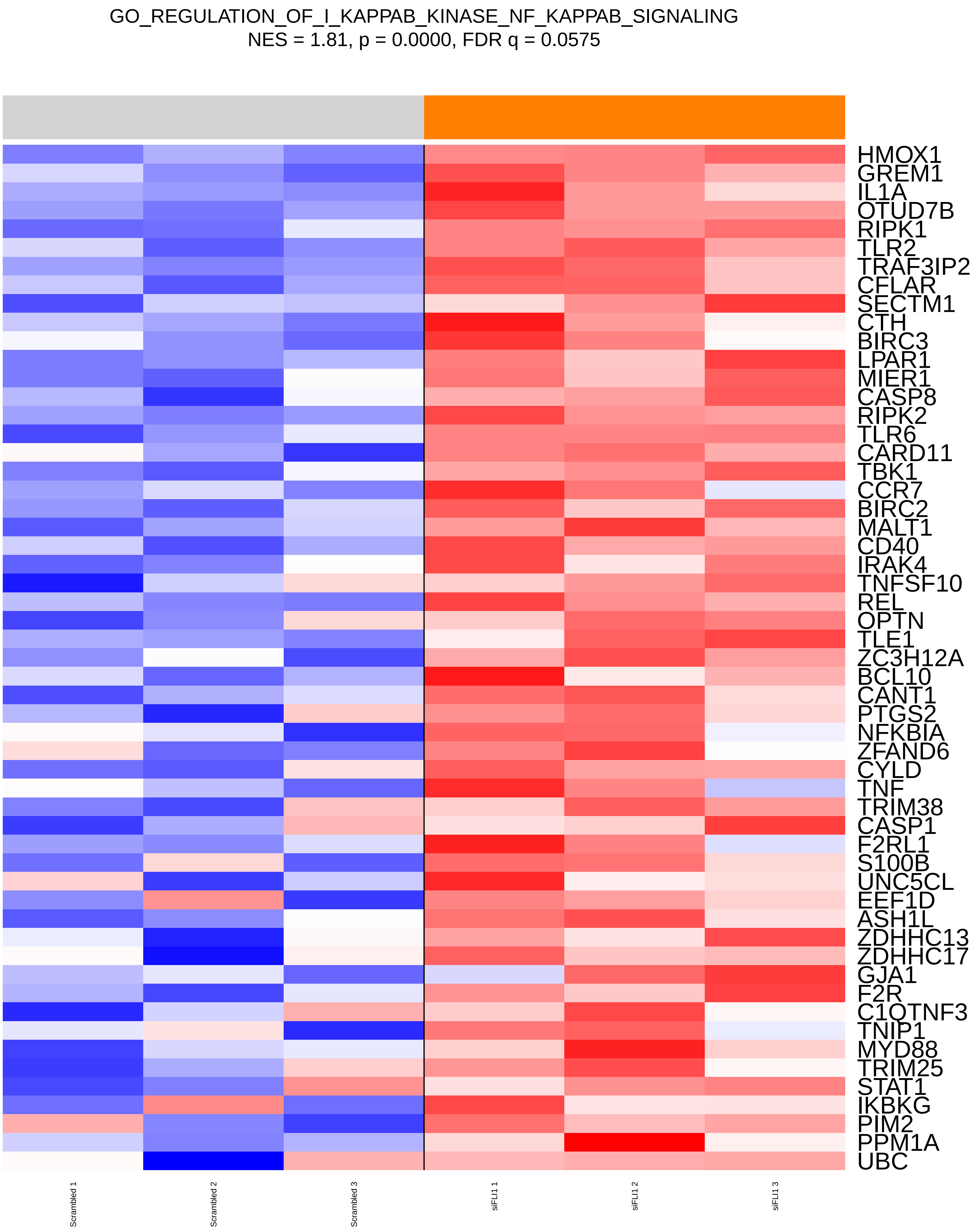

GO_REGULATION_OF_I_KAPPAB_KINASE_NF_KAPPAB_SIGNALING
NES = 1.81, p = 0.0000, FDR q = 0.0575
HMOX1
GREM1
IL1A
OTUD7B
RIPK1
TLR2
TRAF3IP2
CFLAR
SECTM1
CTH
BIRC3
LPAR1
MIER1
CASP8
RIPK2
TLR6
CARD11
TBK1
CCR7
BIRC2
MALT1
CD40
IRAK4
TNFSF10
REL
OPTN
TLE1
ZC3H12A
BCL10
CANT1
PTGS2
NFKBIA
ZFAND6
CYLD
TNF
TRIM38
CASP1
F2RL1
S100B
UNC5CL
EEF1D
ASH1L
ZDHHC13
ZDHHC17
GJA1
F2R
C1QTNF3
TNIP1
MYD88
TRIM25
STAT1
IKBKG
PIM2
PPM1A
UBC
Scrambled 1
Scrambled 2
Scrambled 3
siFLI1 1
siFLI1 2
siFLI1 3

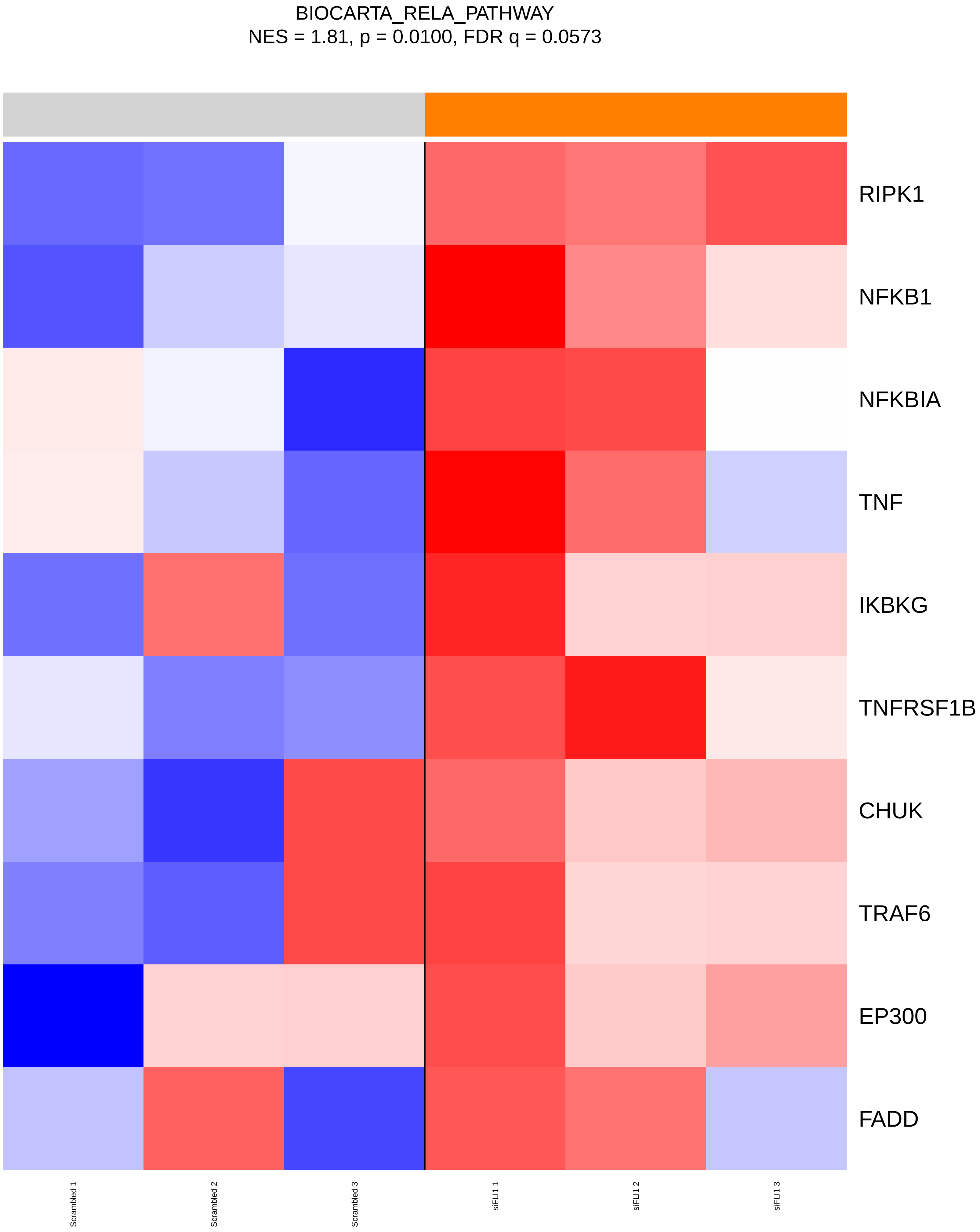
BIOCARTA_RELA_PATHWAY
NES = 1.81, p = 0.0100, FDR q = 0.0573
RIPK1
NFKB1
NFKBIA
TNF
IKBKG
TNFRSF1B
CHUK
TRAF6
EP300
FADD
Scrambled 1
Scrambled 2
Scrambled 3
siFLI1 1
siFLI1 2
siFLI1 3

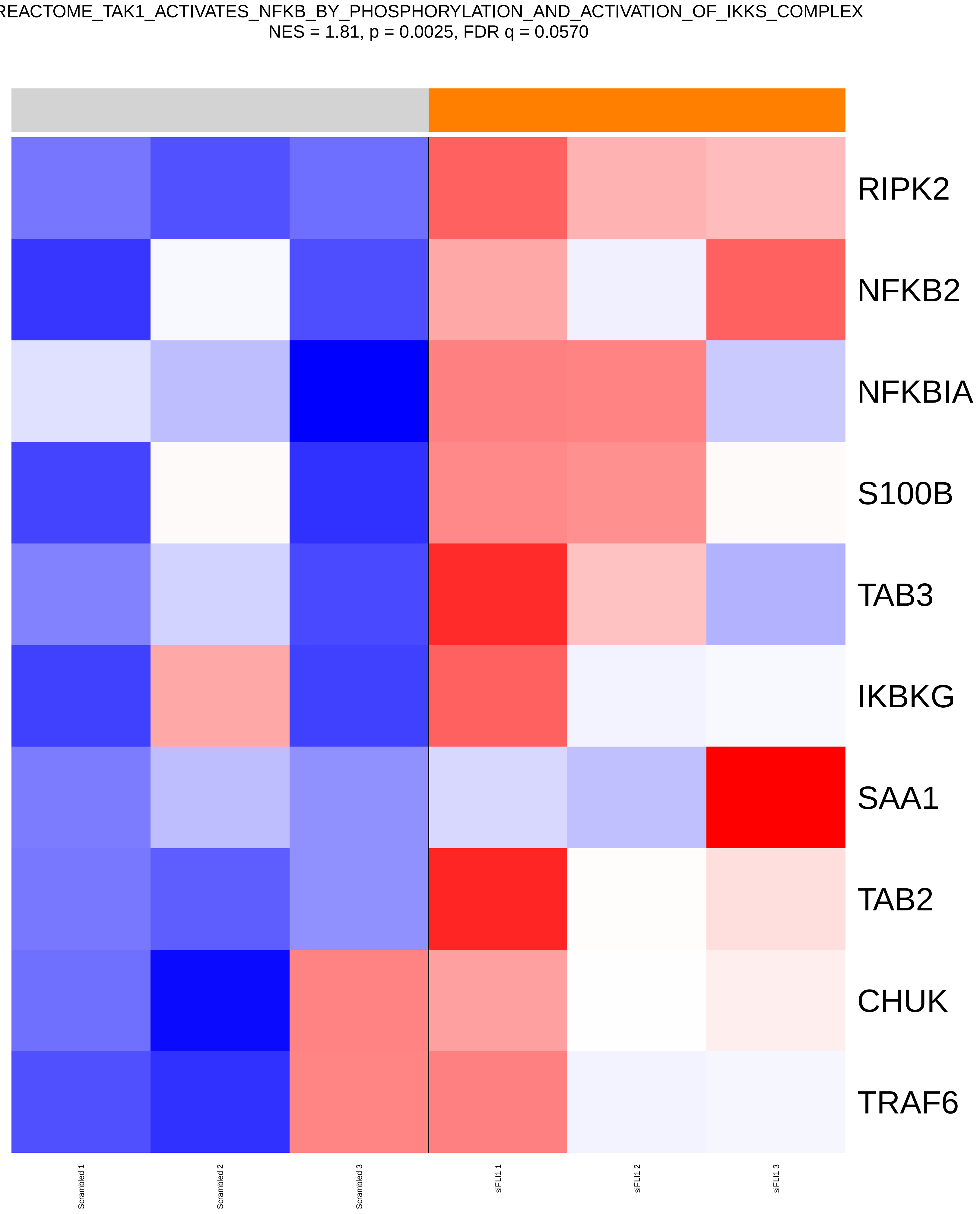
REACTOME_TAK1_ACTIVATES_NFKB_BY_PHOSPHORYLATION_AND_ACTIVATION_OF_IKKS_COMPLEX
NES = 1.81, p = 0.0025, FDR q = 0.0570
RIPK2
NFKB2
NFKBIA
S100B
TAB3
IKBKG
SAA1
TAB2
CHUK
TRAF6
Scrambled 1
Scrambled 2
Scrambled 3
siFLI1 1
siFLI1 2
siFLI1 3

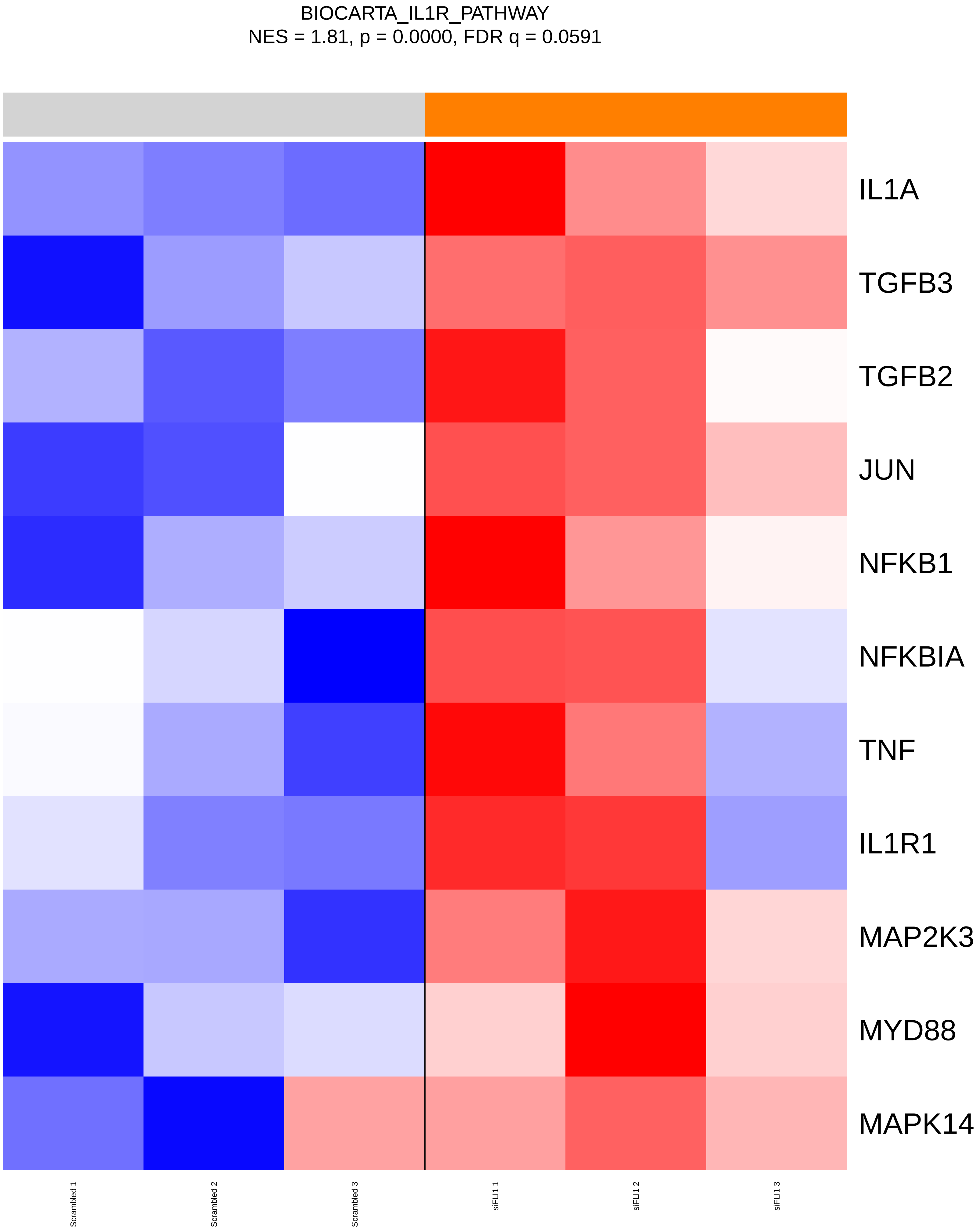
BIOCARTA_IL1R_PATHWAY
NES = 1.81, p = 0.0000, FDR q = 0.0591
IL1A
TGFB3
TGFB2
JUN
NFKB1
NFKBIA
TNF
IL1R1
MAP2K3
MYD88
MAPK14
Scrambled 1
Scrambled 2
Scrambled 3
siFLI1 1
siFLI1 2
siFLI1 3

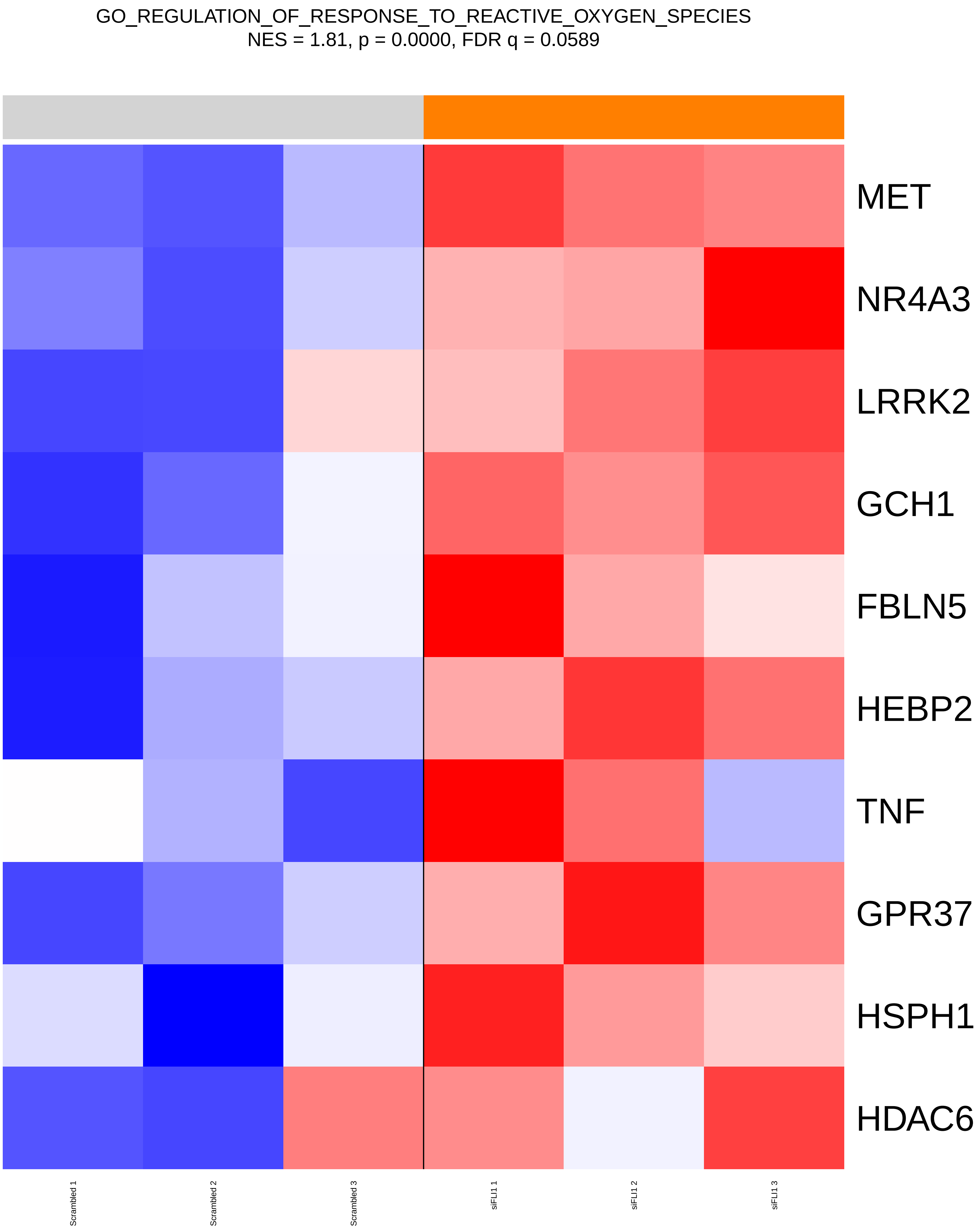
GO_REGULATION_OF_RESPONSE_TO_REACTIVE_OXYGEN_SPECIES
NES = 1.81, p = 0.0000, FDR q = 0.0589
MET
NR4A3
LRRK2
GCH1
FBLN5
HEBP2
TNF
GPR37
HSPH1
HDAC6
Scrambled 1
Scrambled 2
Scrambled 3
siFLI1 1
siFLI1 2
siFLI1 3

GO_POSITIVE_REGULATION_OF_LYMPHOCYTE_MIGRATION
NES = 1.80, p = 0.0069, FDR q = 0.0600

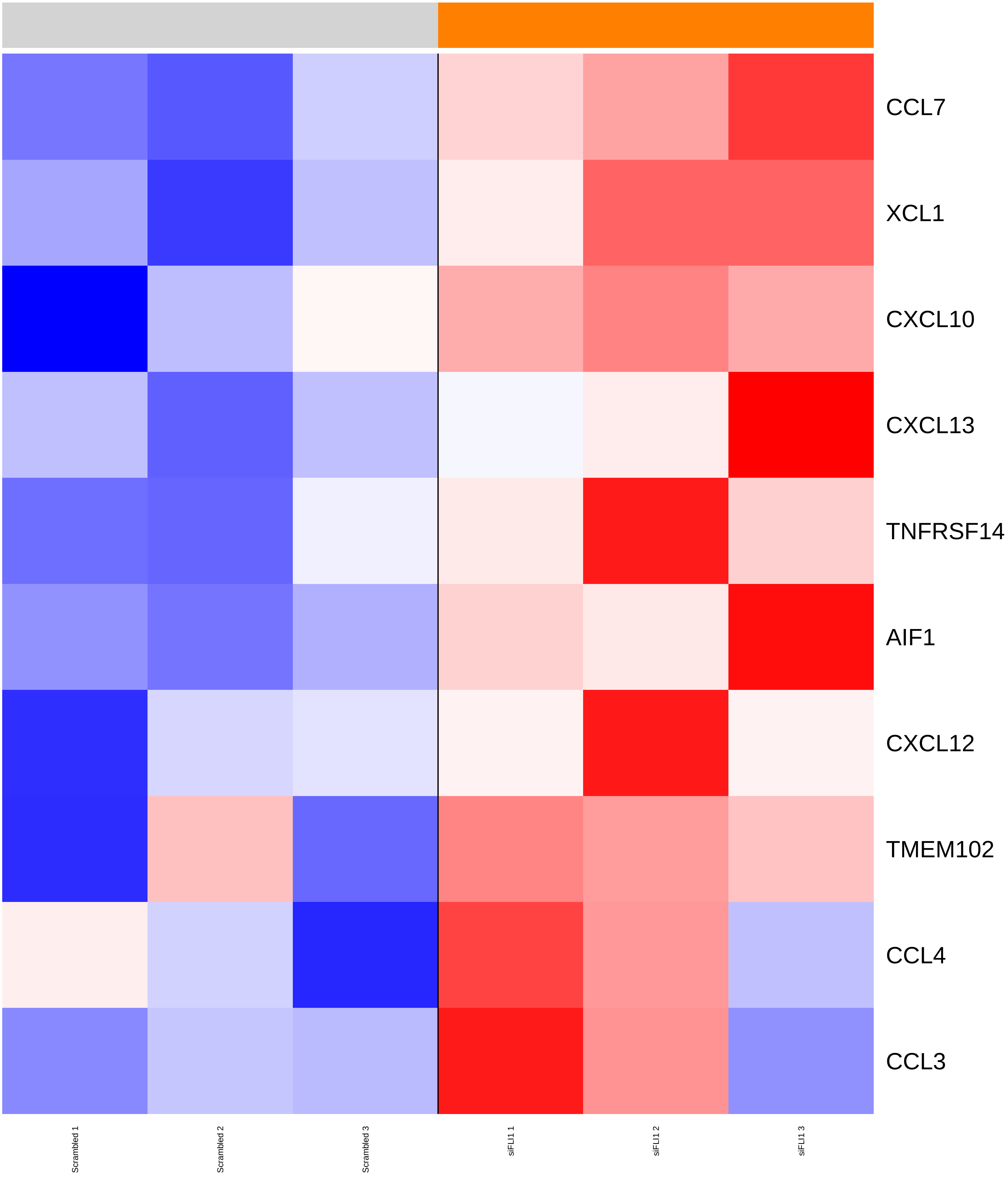

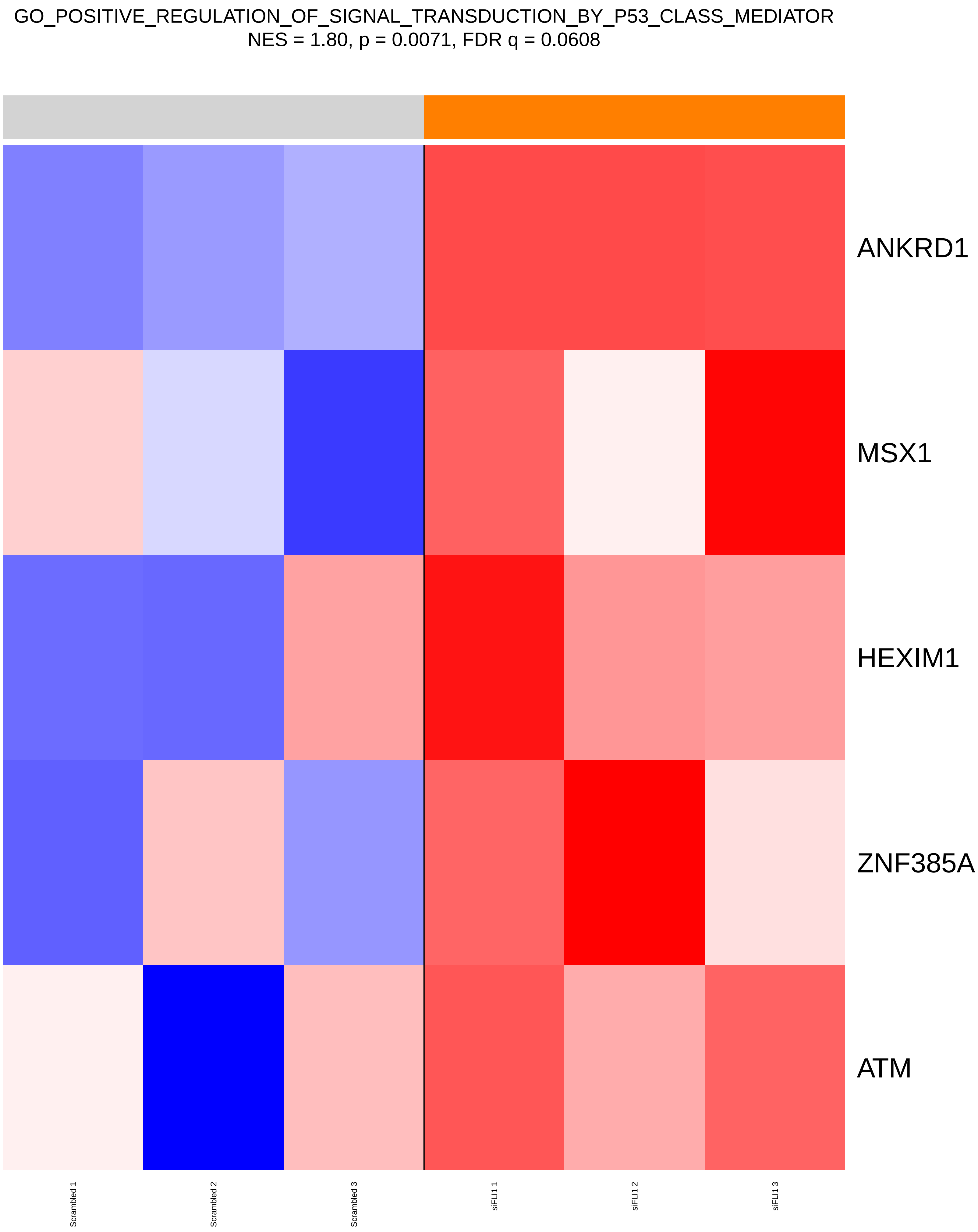
GO_POSITIVE_REGULATION_OF_SIGNAL_TRANSDUCTION_BY_P53_CLASS_MEDIATOR
NES = 1.80, p = 0.0071, FDR q = 0.0608
ANKRD1
MSX1
HEXIM1
ZNF385A
ATM
Scrambled 1
Scrambled 2
Scrambled 3
siFLI1 1
siFLI1 2
siFLI1 3

IRF_Q6
NES = 1.80, p = 0.0000, FDR q = 0.0605

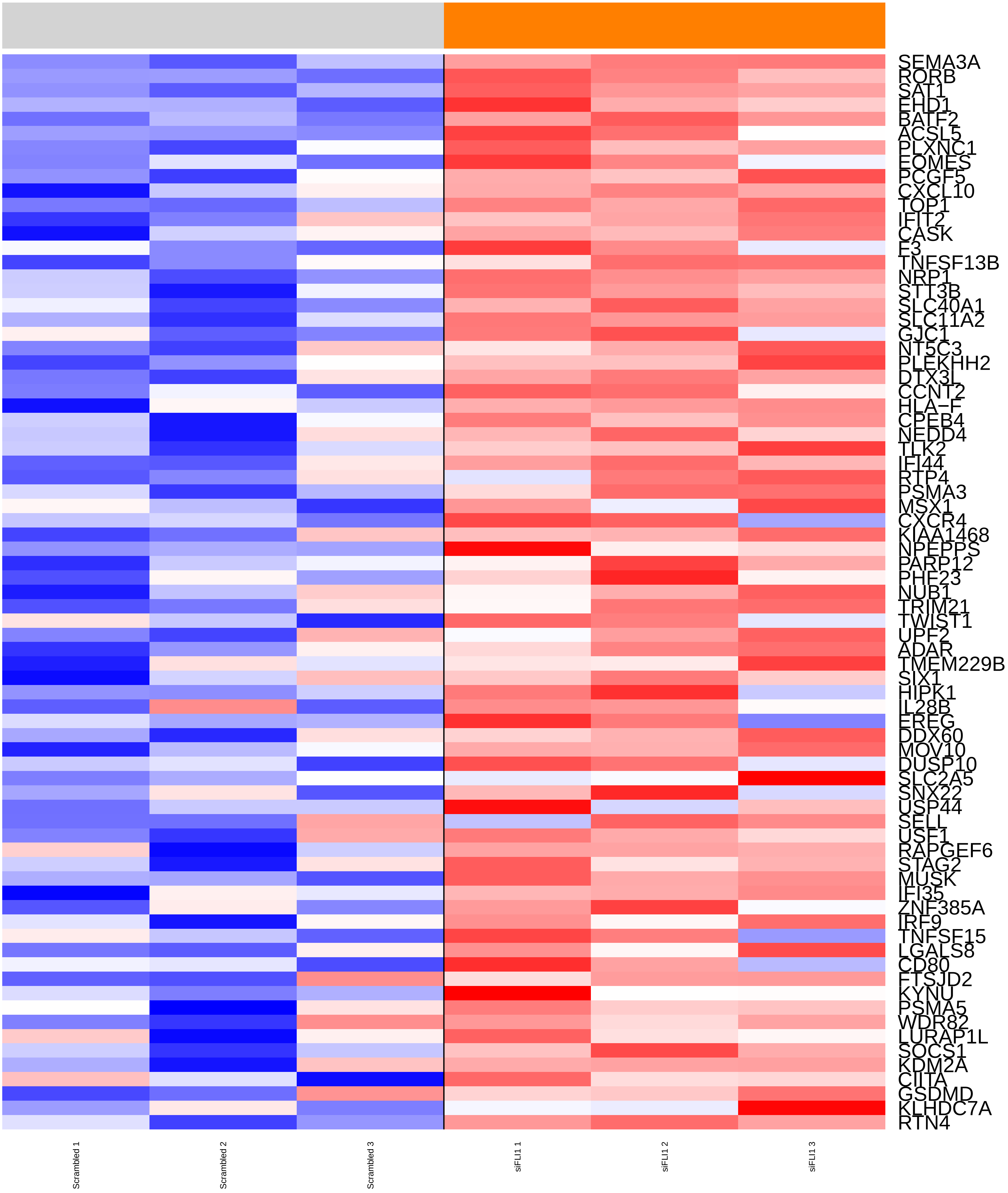

GO_NEGATIVE_REGULATION_OF_EXTRINSIC_APOPTOTIC_SIGNALING_PATHWAY_VIA_DEATH_DOMAIN_RECEPTORS
NES = 1.80, p = 0.0000, FDR q = 0.0609

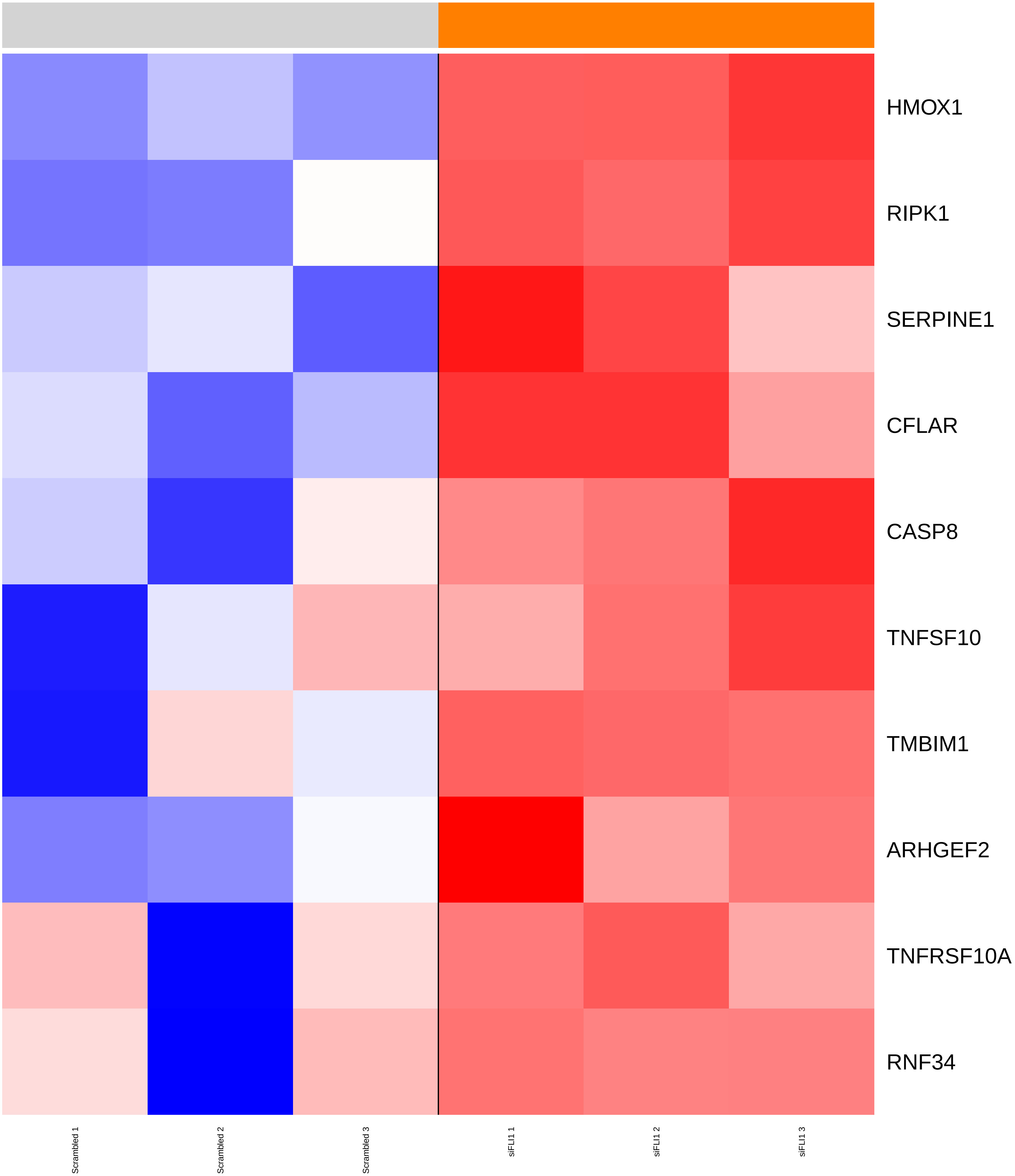

GO_RESPONSE_TO_MOLECULE_OF_BACTERIAL_ORIGIN
NES = 1.80, p = 0.0000, FDR q = 0.0623

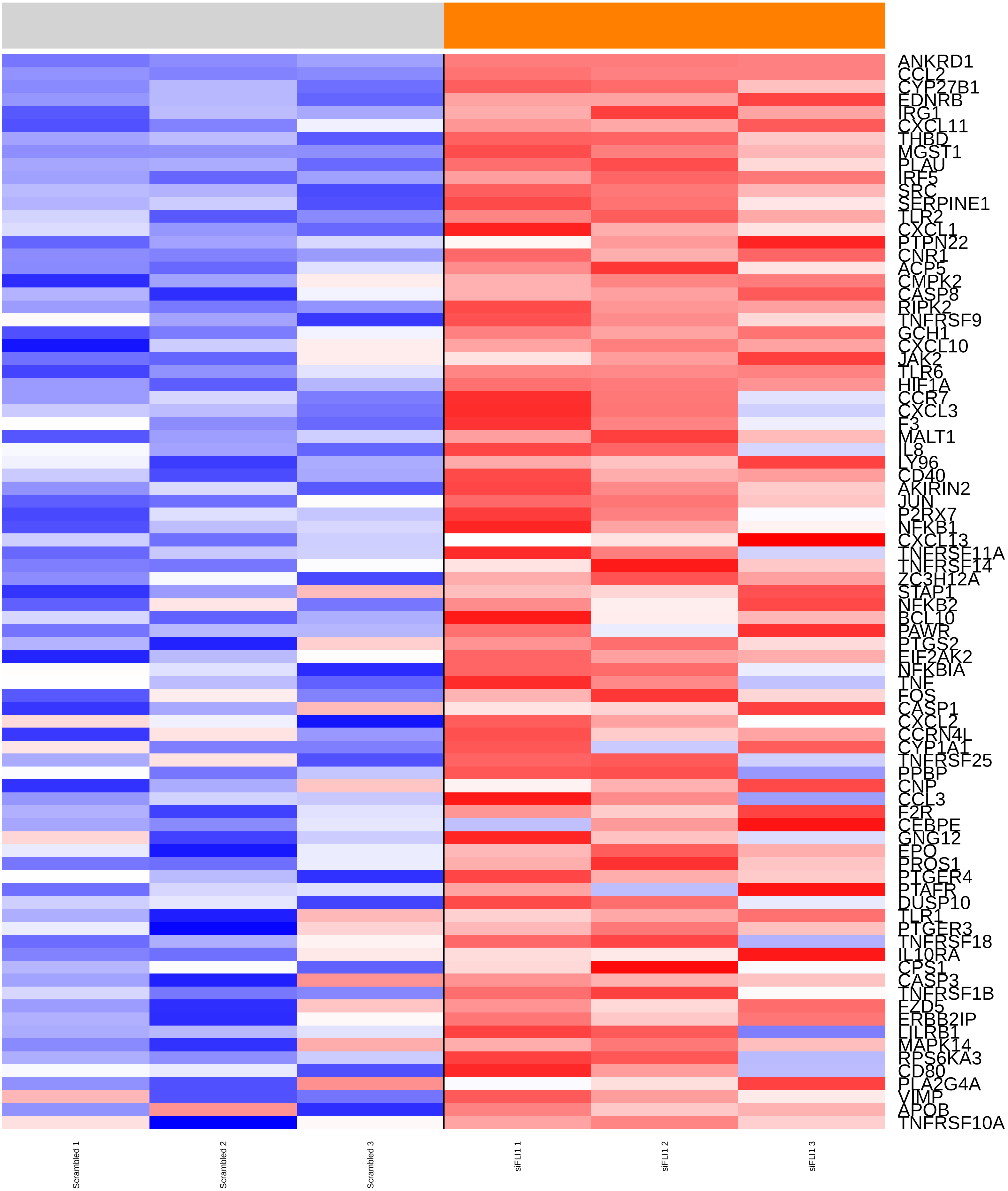

GO_REGULATION_OF_T_CELL_RECEPTOR_SIGNALING_PATHWAY
NES = 1.80, p = 0.0026, FDR q = 0.0619

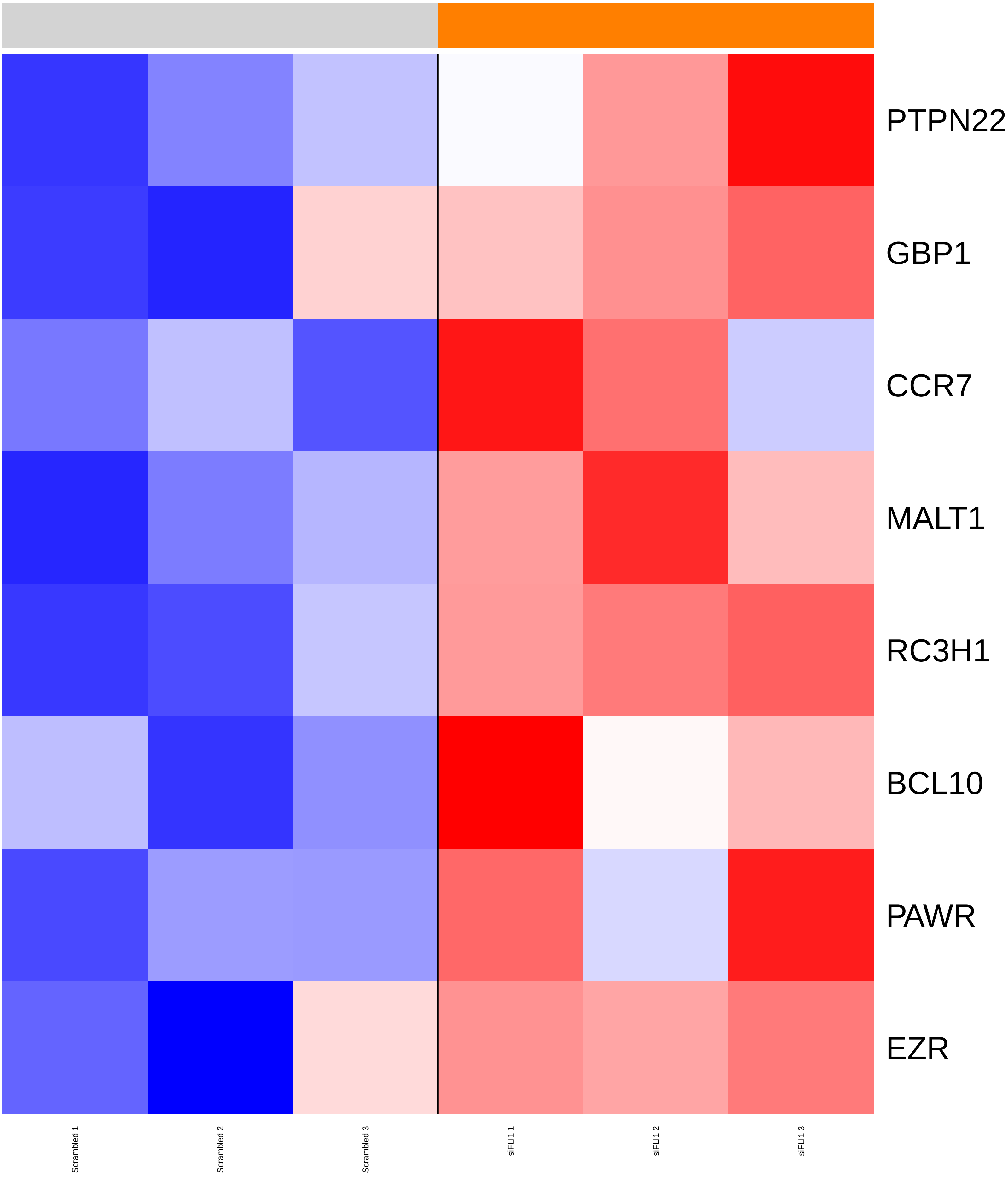

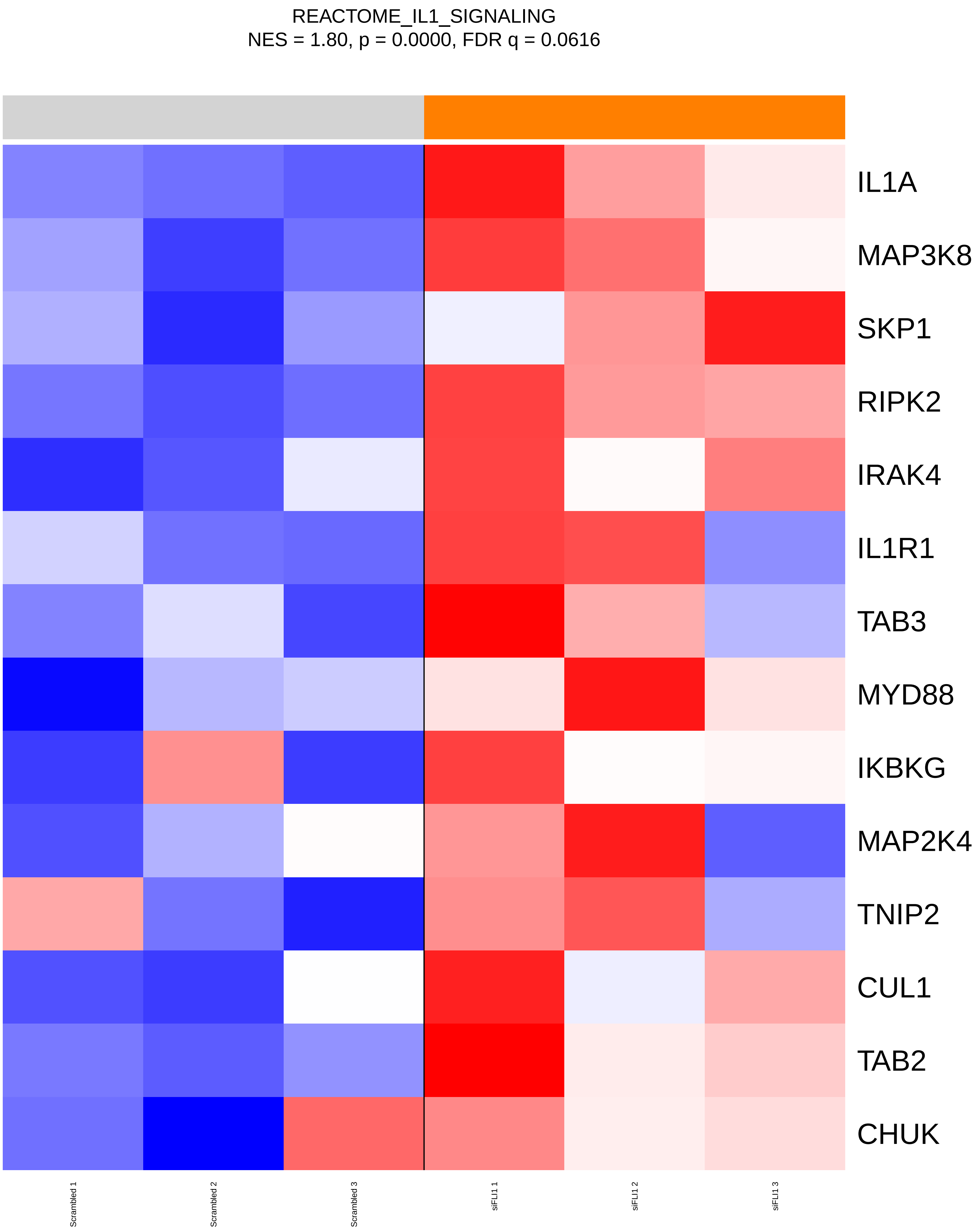

REACTOME_IL1_SIGNALING
NES = 1.80, p = 0.0000, FDR q = 0.0616
IL1A
MAP3K8
SKP1
RIPK2
IRAK4
IL1R1
TAB3
MYD88
IKBKG
MAP2K4
TNIP2
CUL1
TAB2
CHUK
Scrambled 1
Scrambled 2
Scrambled 3
siFLI1 1
siFLI1 2
siFLI1 3

GO_POSITIVE_REGULATION_OF_CYTOKINE_PRODUCTION
NES = 1.79, p = 0.0000, FDR q = 0.0624

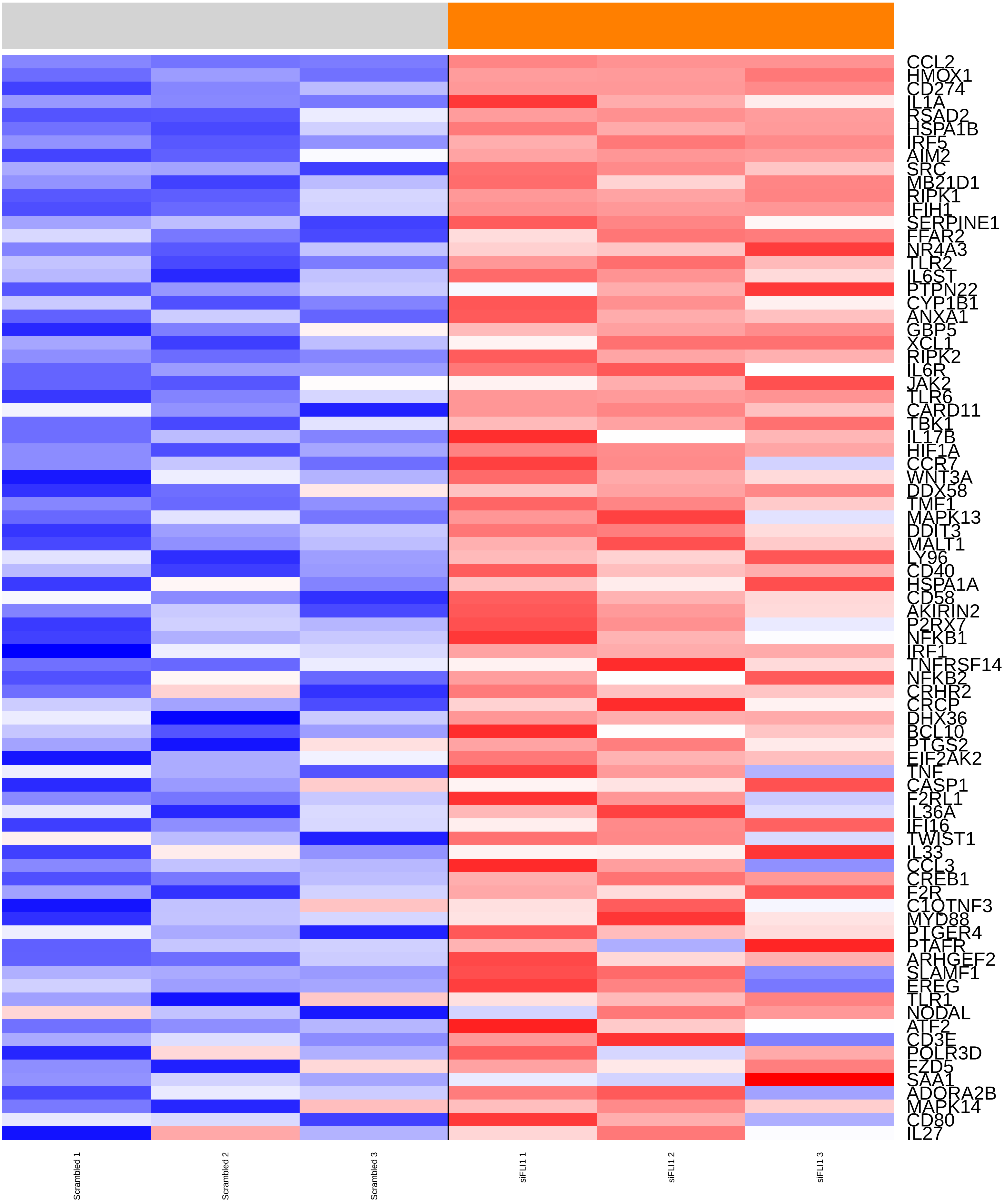

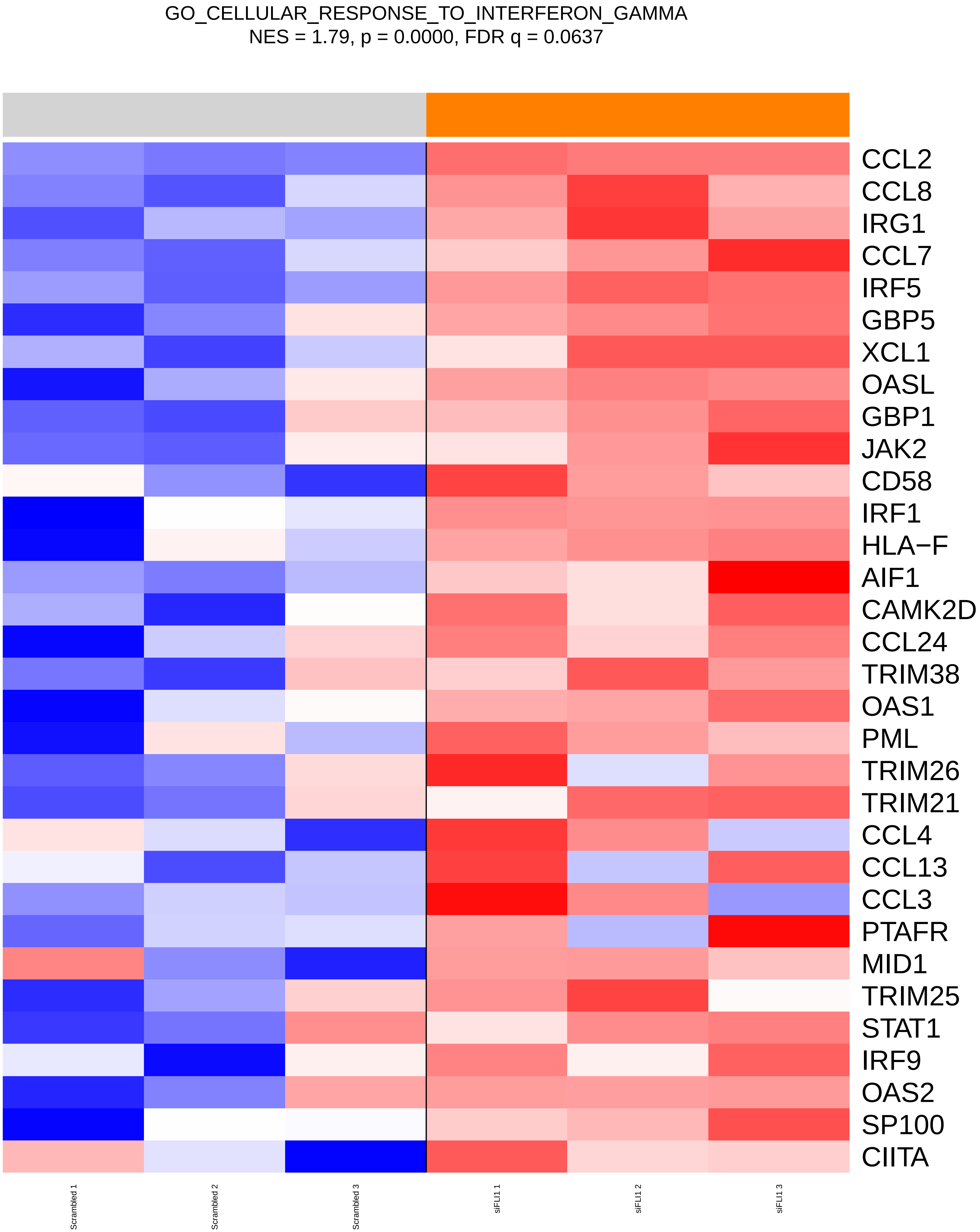
GO_CELLULAR_RESPONSE_TO_INTERFERON_GAMMA
NES = 1.79, p = 0.0000, FDR q = 0.0637
CCL2
CCL8
IRG1
CCL7
IRF5
GBP5
XCL1
OASL
GBP1
JAK2
CD58
IRF1
HLA−F
AIF1
CAMK2D
CCL24
TRIM38
OAS1
PML
TRIM26
TRIM21
CCL4
CCL13
CCL3
PTAFR
MID1
TRIM25
STAT1
IRF9
OAS2
SP100
CIITA
Scrambled 1
Scrambled 2
Scrambled 3
siFLI1 1
siFLI1 2
siFLI1 3

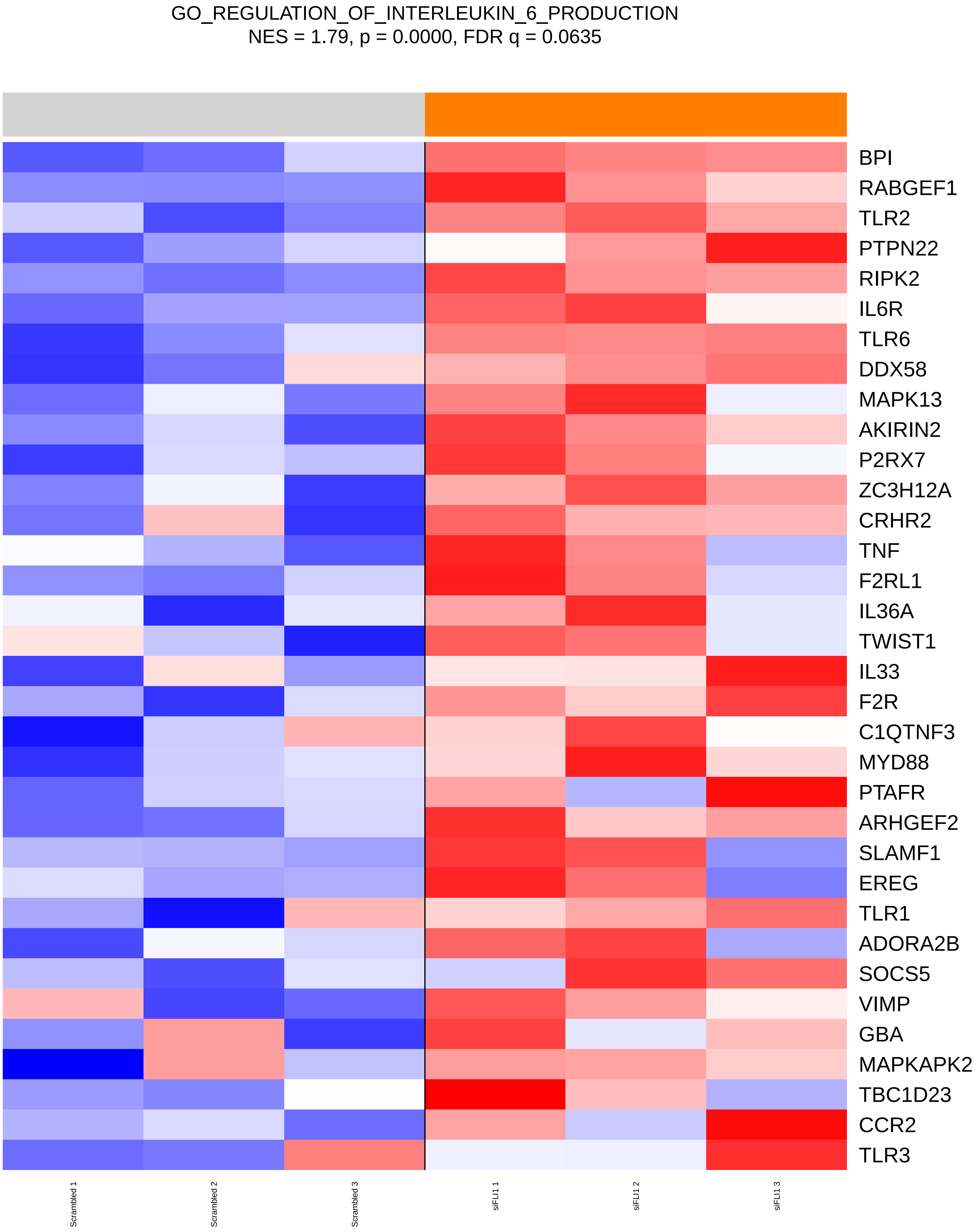
GO_REGULATION_OF_INTERLEUKIN_6_PRODUCTION
NES = 1.79, p = 0.0000, FDR q = 0.0635
BPI
RABGEF1
TLR2
PTPN22
RIPK2
IL6R
TLR6
DDX58
MAPK13
AKIRIN2
P2RX7
ZC3H12A
CRHR2
TNF
F2RL1
IL36A
TWIST1
IL33
F2R
C1QTNF3
MYD88
PTAFR
ARHGEF2
SLAMF1
EREG
TLR1
ADORA2B
SOCS5
VIMP
GBA
MAPKAPK2
TBC1D23
CCR2
TLR3
Scrambled 1
Scrambled 2
Scrambled 3
siFLI1 1
siFLI1 2
siFLI1 3

GO_NEGATIVE_REGULATION_OF_CYTOKINE_PRODUCTION
NES = 1.79, p = 0.0000, FDR q = 0.0641

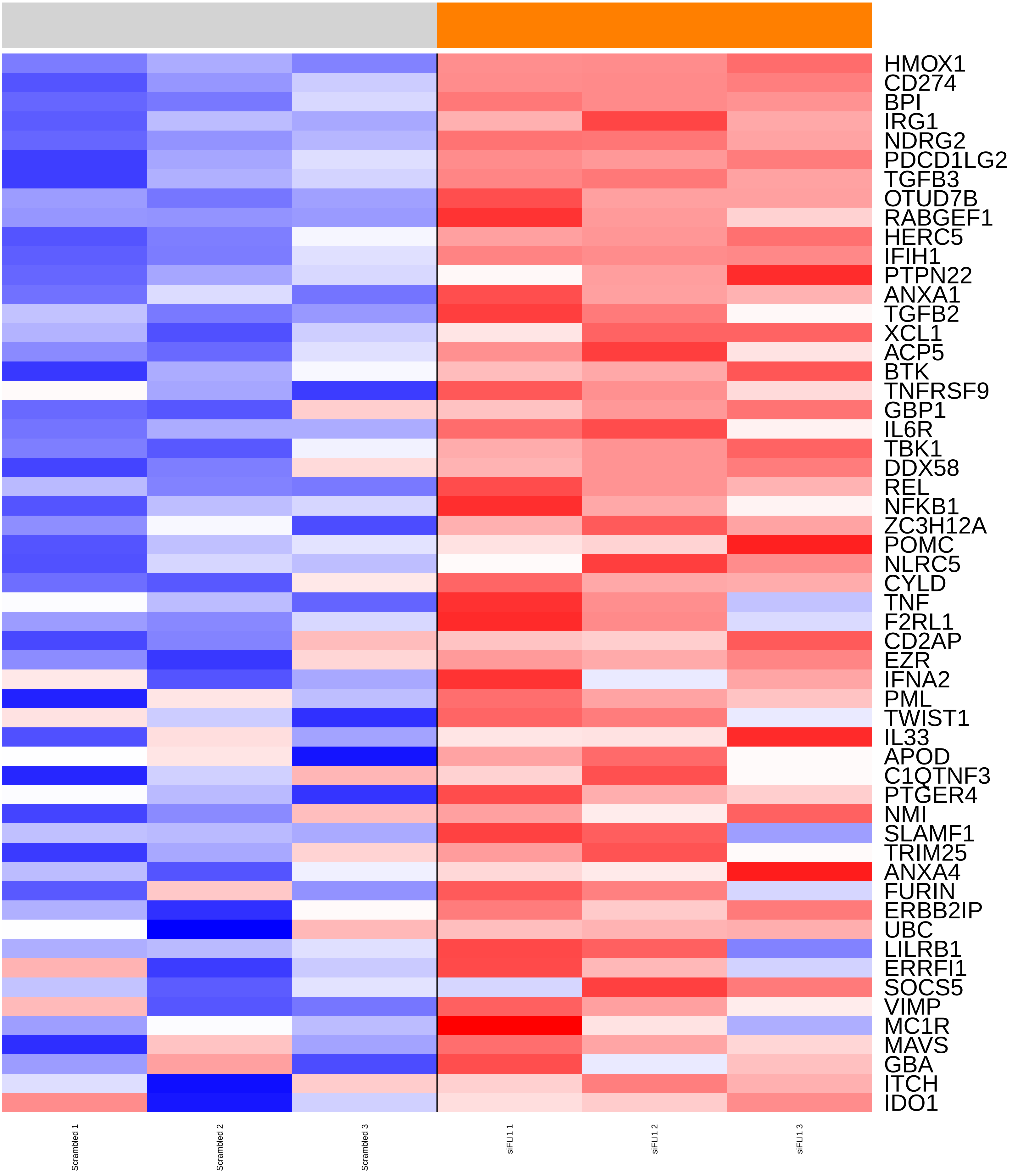

GO_REGULATION_OF_EARLY_ENDOSOME_TO_LATE_ENDOSOME_TRANSPORT
NES = 1.77, p = 0.0067, FDR q = 0.0721

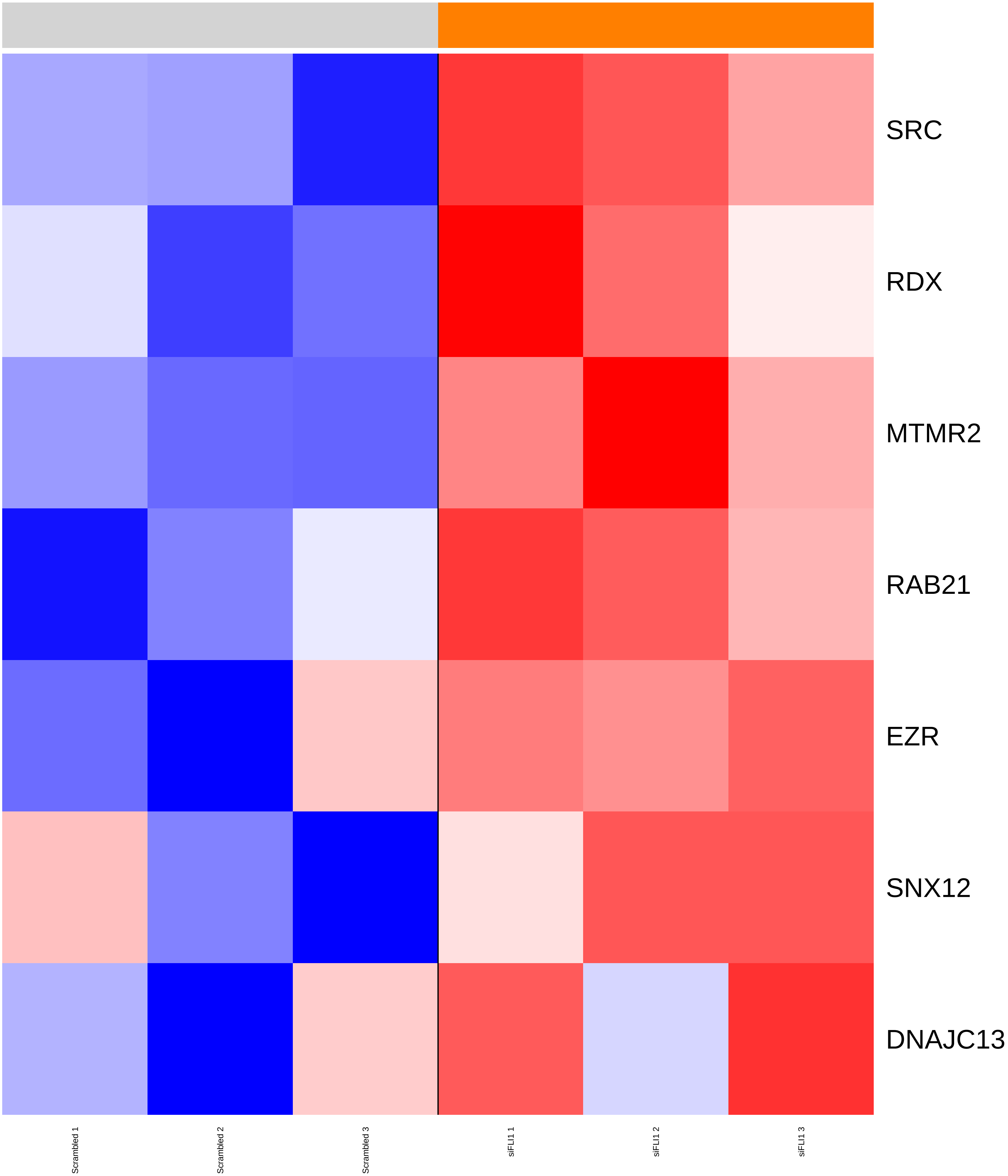

GO_NEGATIVE_REGULATION_OF_EXTRINSIC_APOPTOTIC_SIGNALING_PATHWAY
NES = 1.77, p = 0.0000, FDR q = 0.0717

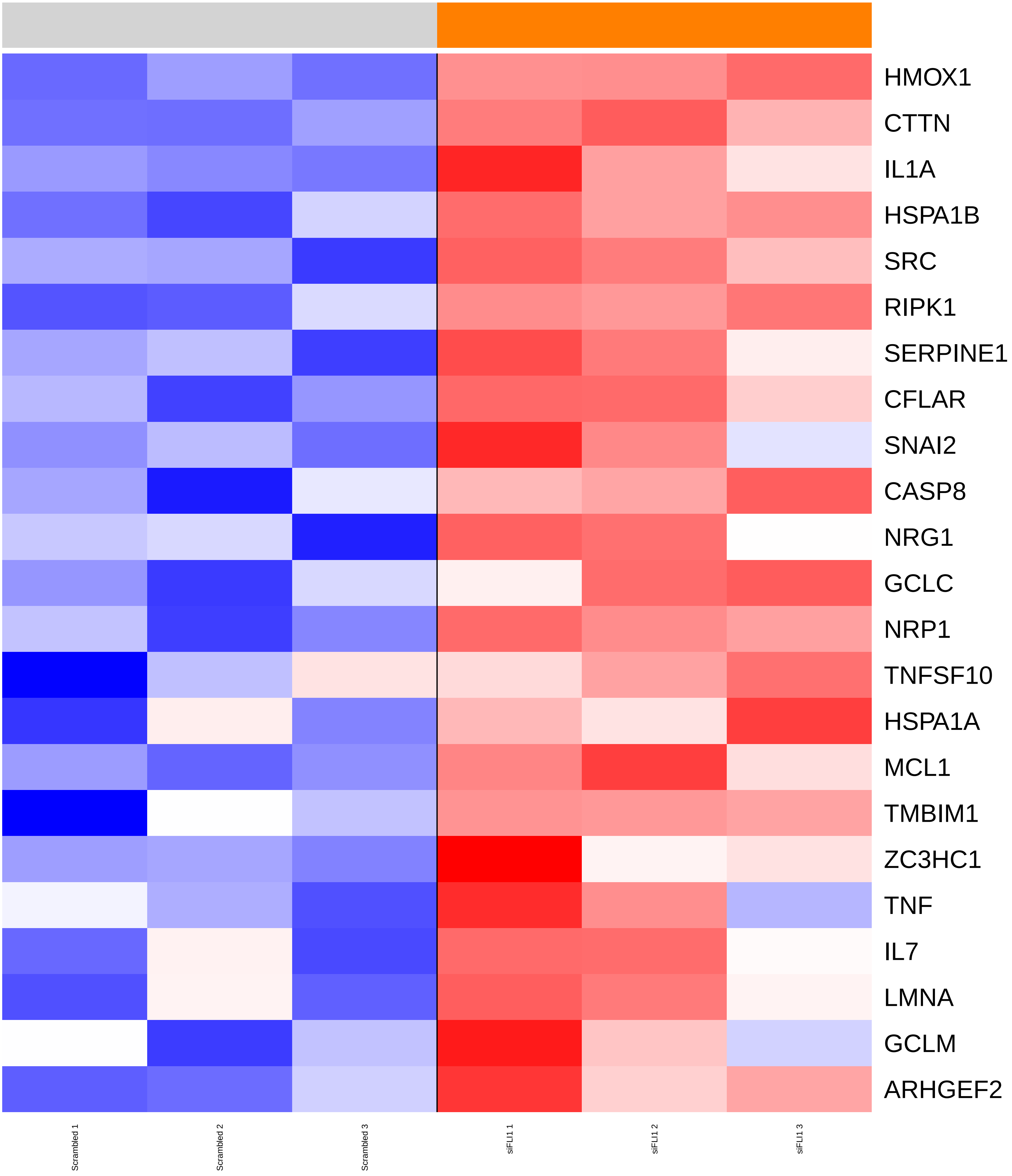

GO_NEGATIVE_REGULATION_OF_LYMPHOCYTE_DIFFERENTIATION
NES = 1.77, p = 0.0078, FDR q = 0.0713

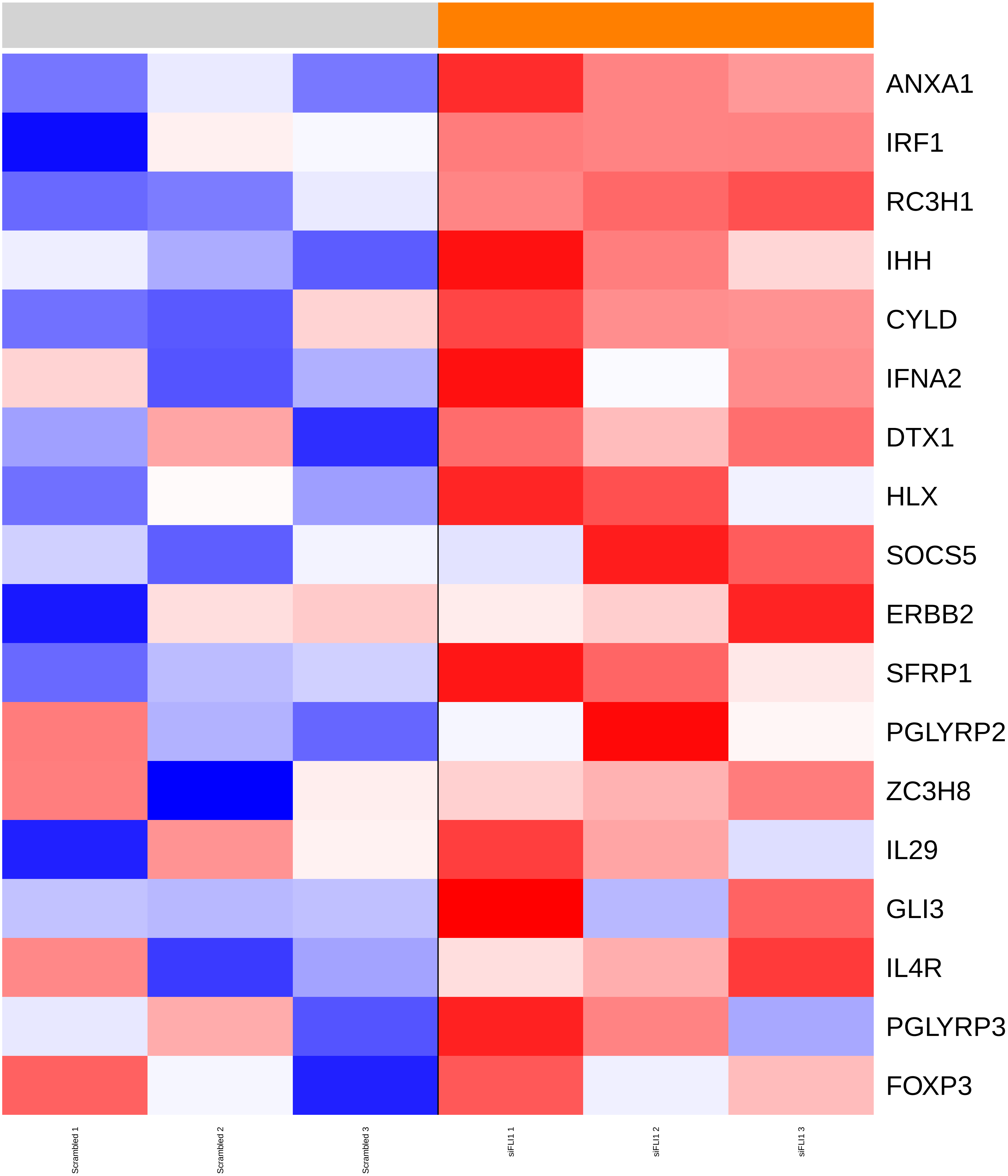

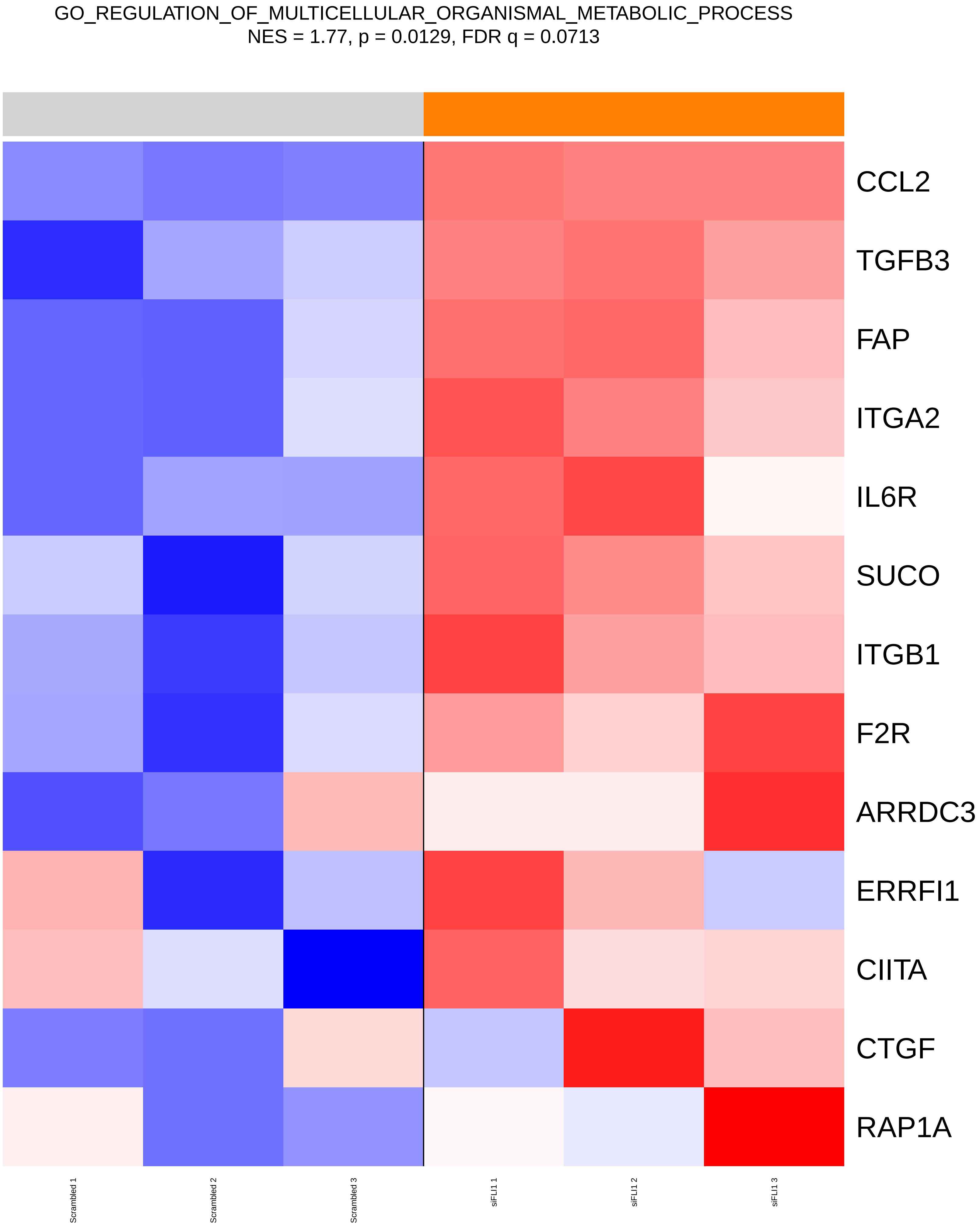
GO_REGULATION_OF_MULTICELLULAR_ORGANISMAL_METABOLIC_PROCESS
NES = 1.77, p = 0.0129, FDR q = 0.0713
CCL2
TGFB3
FAP
ITGA2
IL6R
SUCO
ITGB1
F2R
ARRDC3
ERRFI1
CIITA
CTGF
RAP1A
Scrambled 1
Scrambled 2
Scrambled 3
siFLI1 1
siFLI1 2
siFLI1 3

GO_RESPONSE_TO_TEMPERATURE_STIMULUS
NES = 1.77, p = 0.0000, FDR q = 0.0711

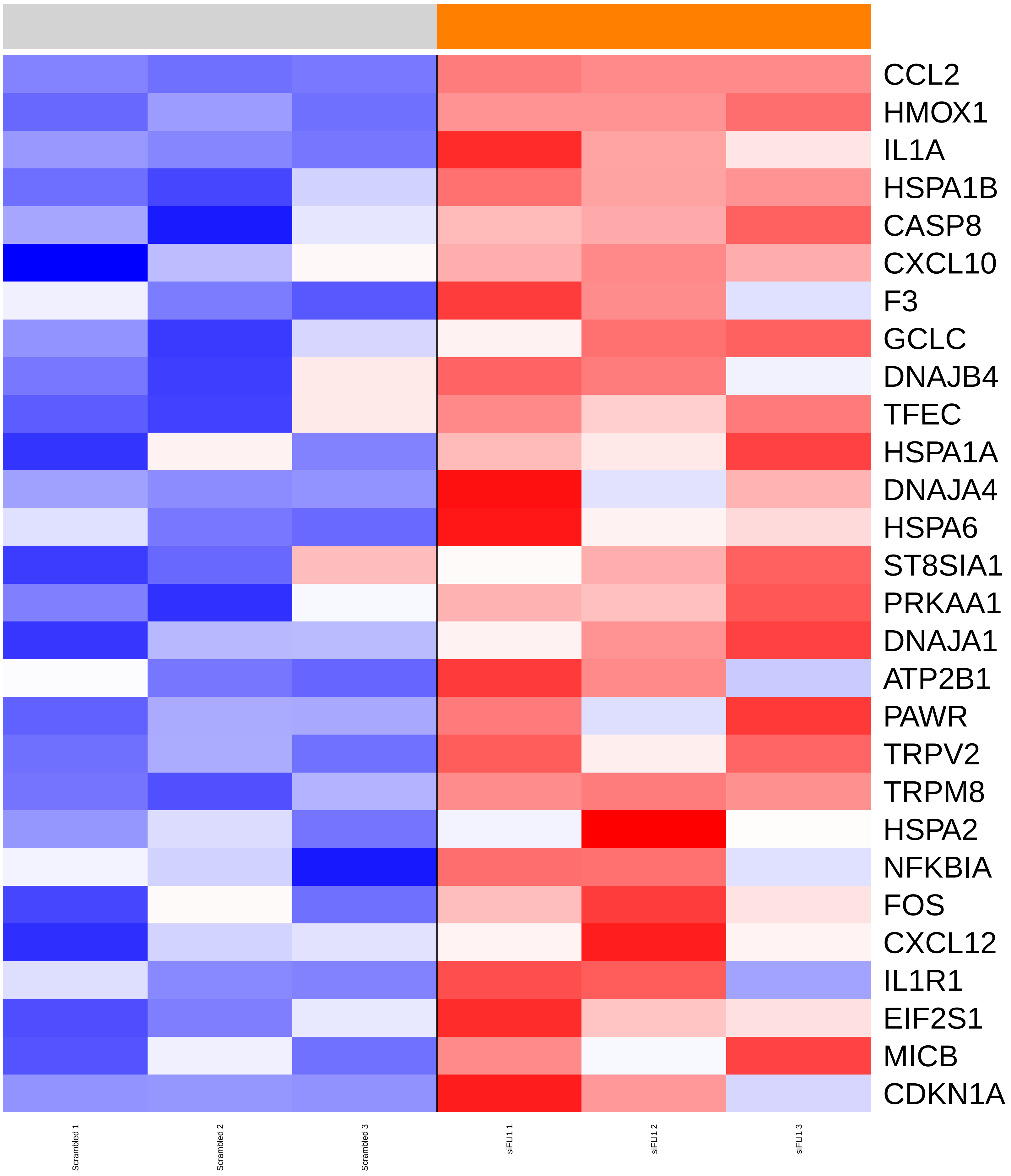

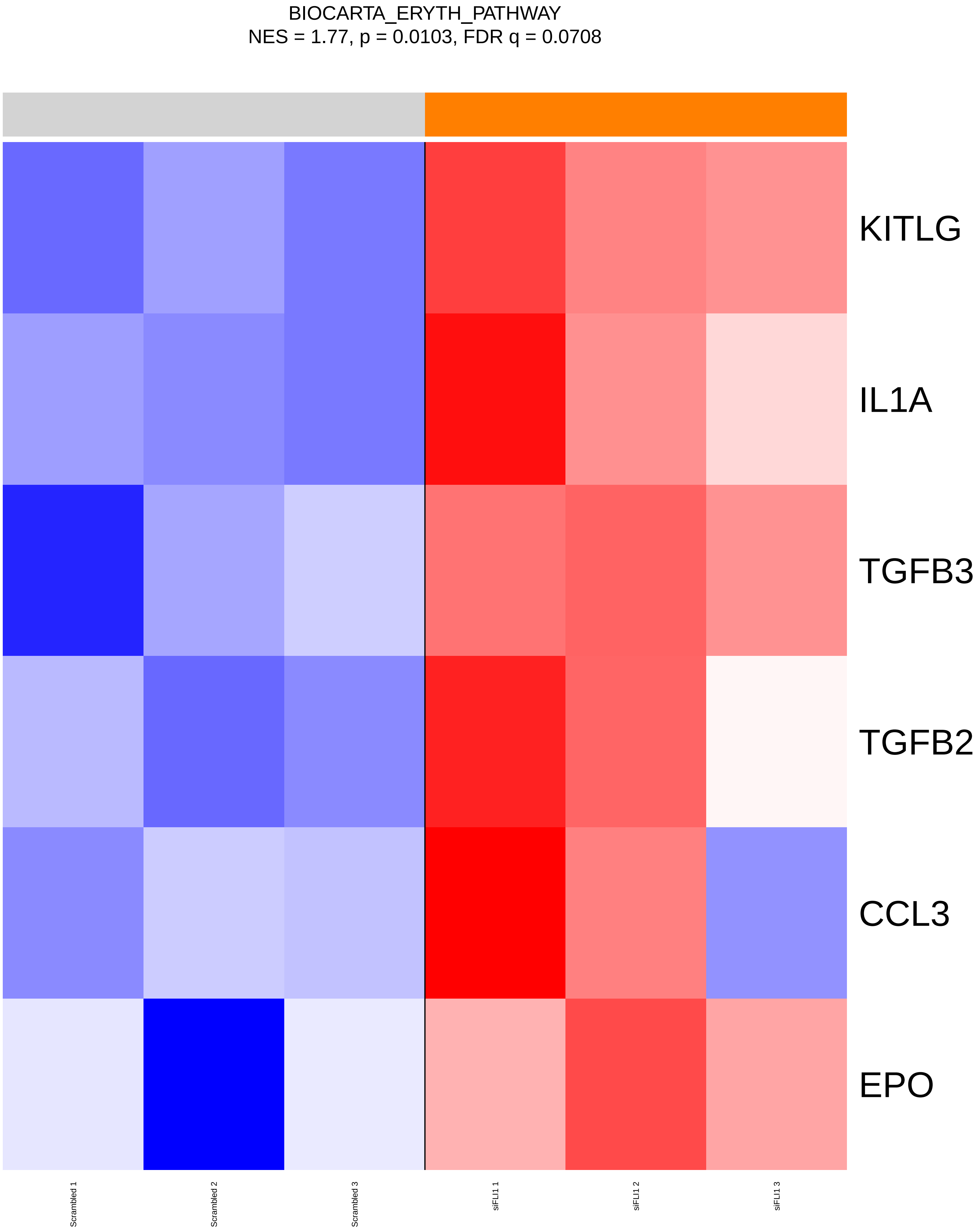
BIOCARTA_ERYTH_PATHWAY
NES = 1.77, p = 0.0103, FDR q = 0.0708
KITLG
IL1A
TGFB3
TGFB2
CCL3
EPO
Scrambled 1
Scrambled 2
Scrambled 3
siFLI1 1
siFLI1 2
siFLI1 3

BIOCARTA_STRESS_PATHWAY
NES = 1.77, p = 0.0105, FDR q = 0.0708

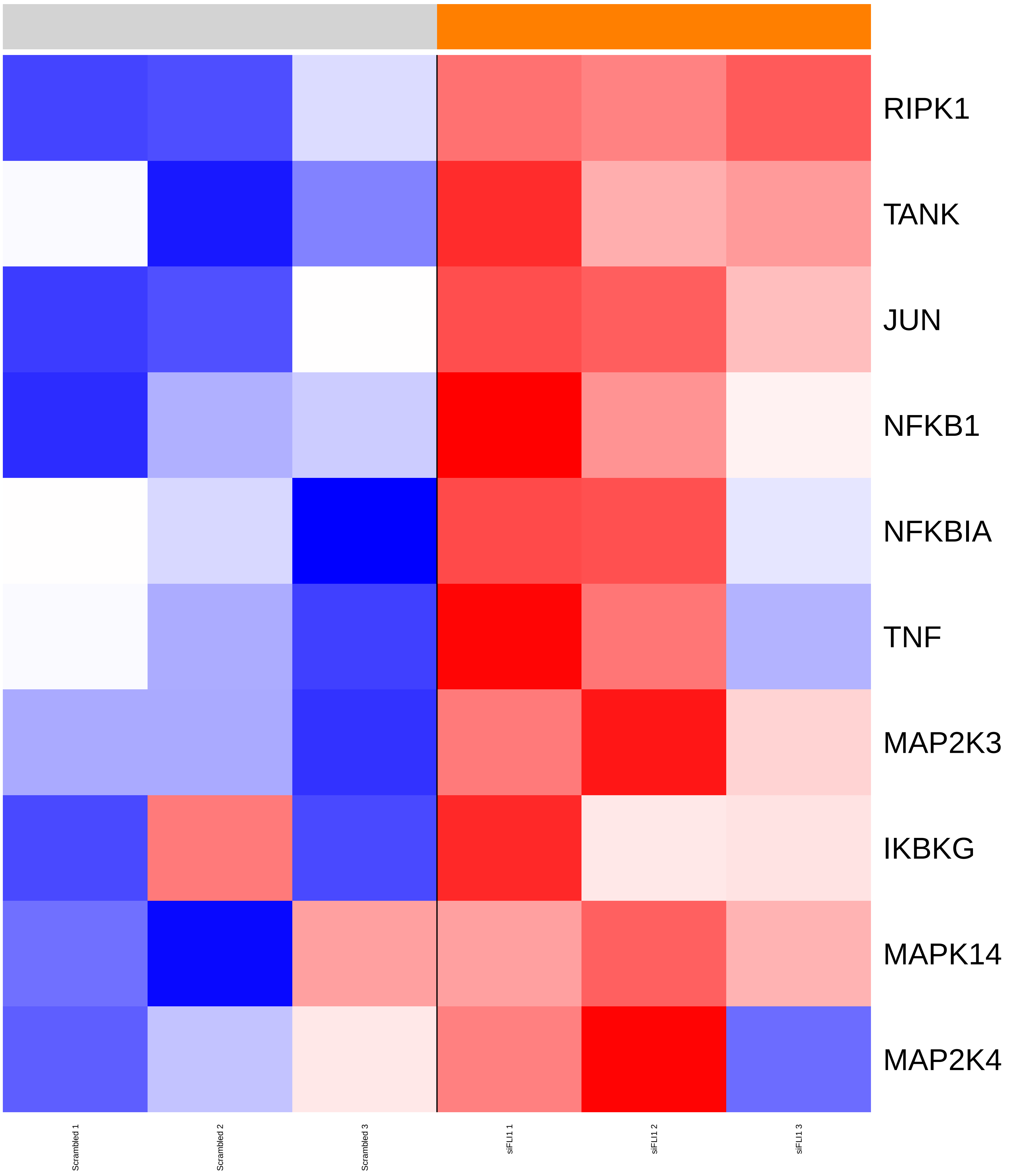

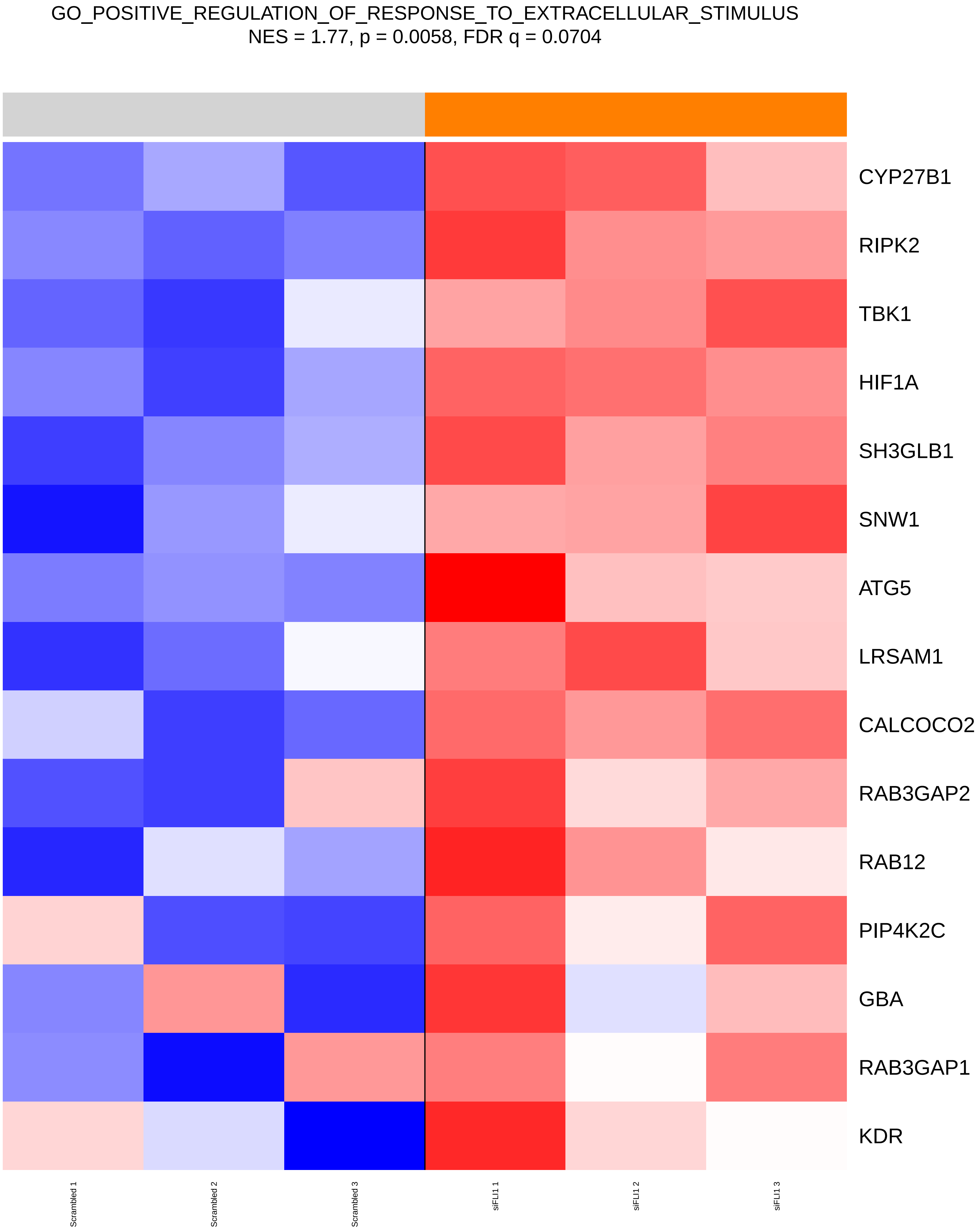

GO_POSITIVE_REGULATION_OF_RESPONSE_TO_EXTRACELLULAR_STIMULUS
NES = 1.77, p = 0.0058, FDR q = 0.0704
CYP27B1
RIPK2
TBK1
HIF1A
SH3GLB1
SNW1
ATG5
LRSAM1
CALCOCO2
RAB3GAP2
RAB12
PIP4K2C
GBA
RAB3GAP1
KDR
Scrambled 1
Scrambled 2
Scrambled 3
siFLI1 1
siFLI1 2
siFLI1 3

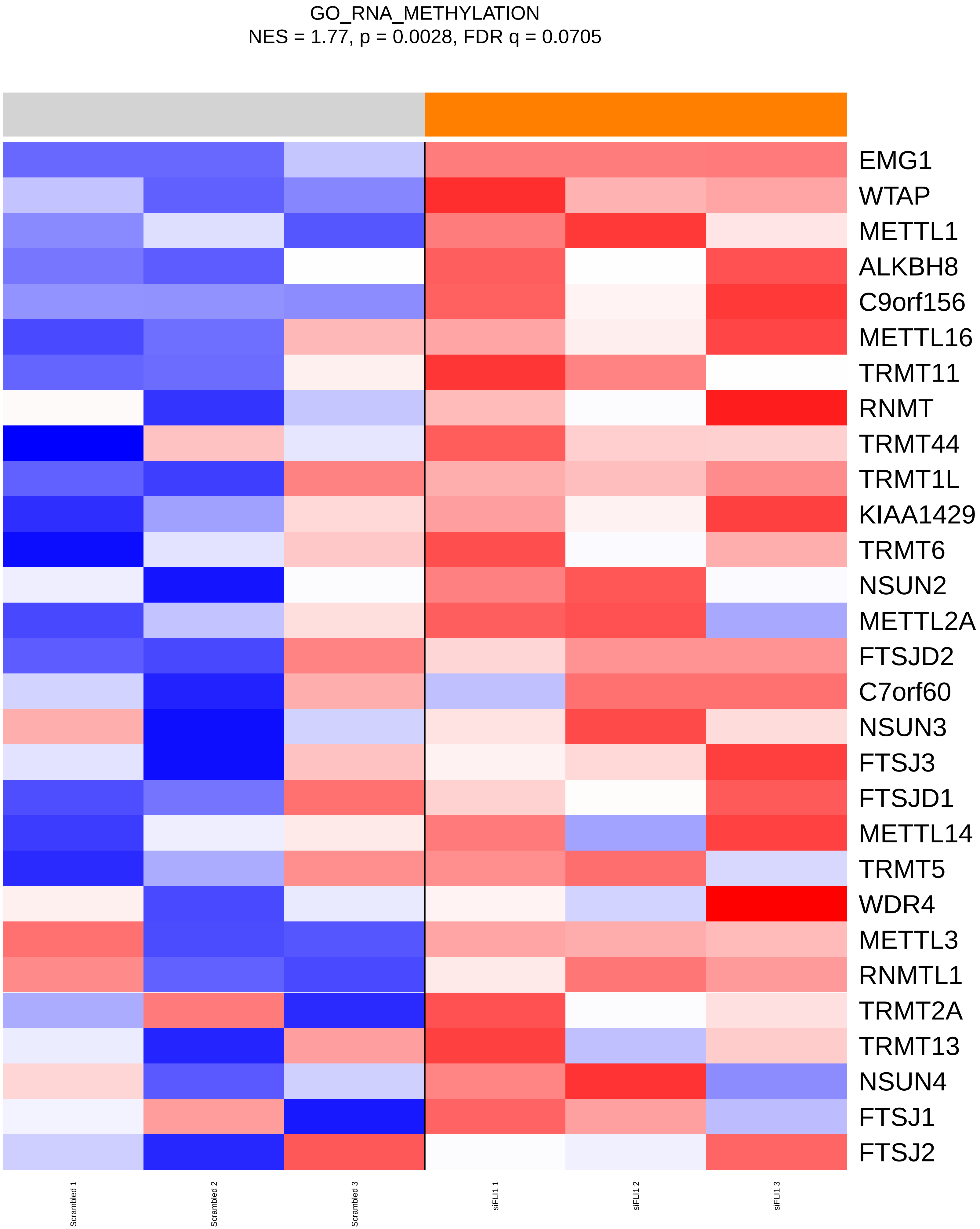
GO_RNA_METHYLATION
NES = 1.77, p = 0.0028, FDR q = 0.0705
EMG1
WTAP
METTL1
ALKBH8
C9orf156
METTL16
TRMT11
RNMT
TRMT44
TRMT1L
KIAA1429
TRMT6
NSUN2
METTL2A
FTSJD2
C7orf60
NSUN3
FTSJ3
FTSJD1
METTL14
TRMT5
WDR4
METTL3
RNMTL1
TRMT2A
TRMT13
NSUN4
FTSJ1
FTSJ2
Scrambled 1
Scrambled 2
Scrambled 3
siFLI1 1
siFLI1 2
siFLI1 3

GO_REGULATION_OF_DNA_DAMAGE_RESPONSE_SIGNAL_TRANSDUCTION_BY_P53_CLASS_MEDIATOR
NES = 1.77, p = 0.0025, FDR q = 0.0700

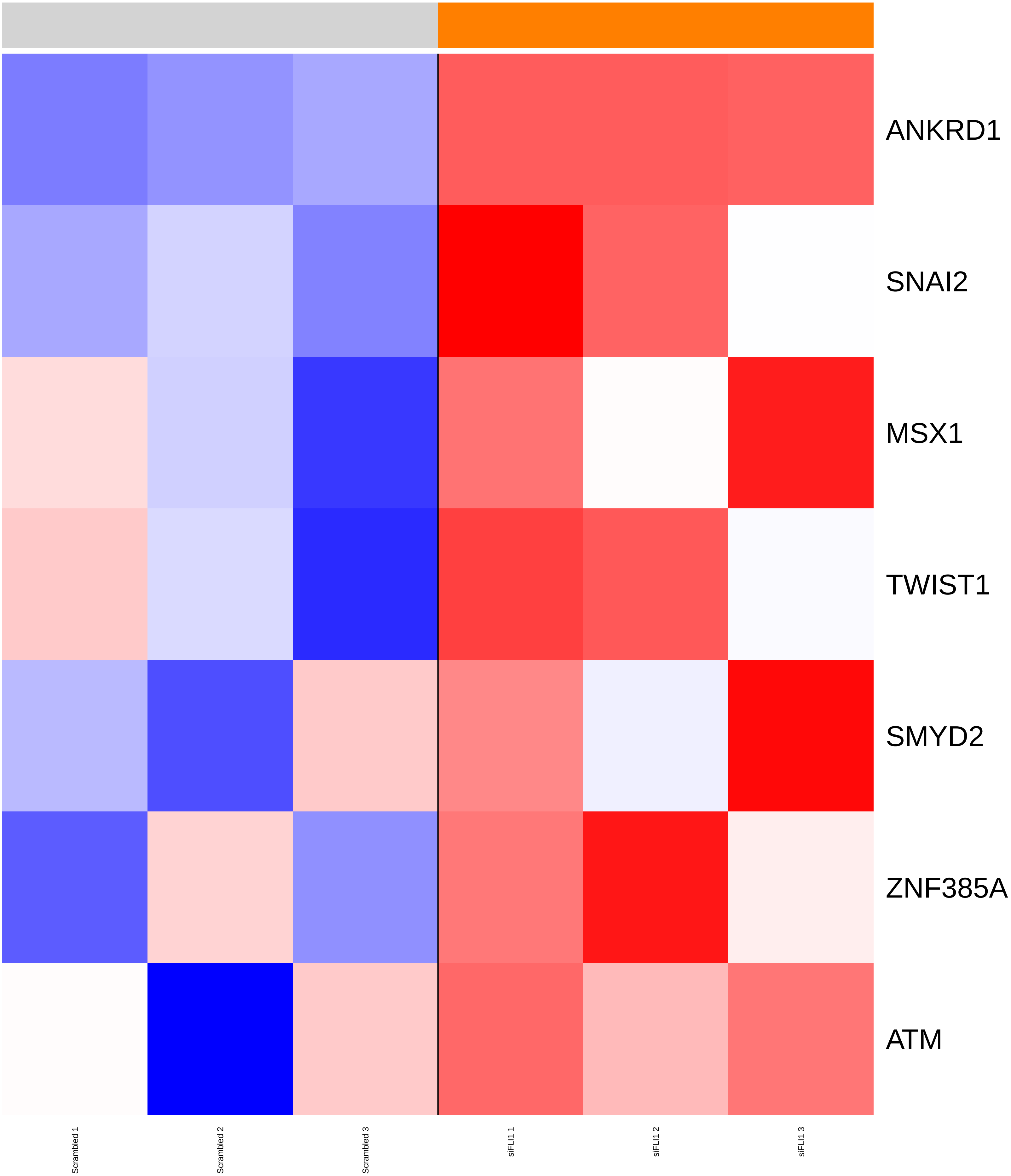

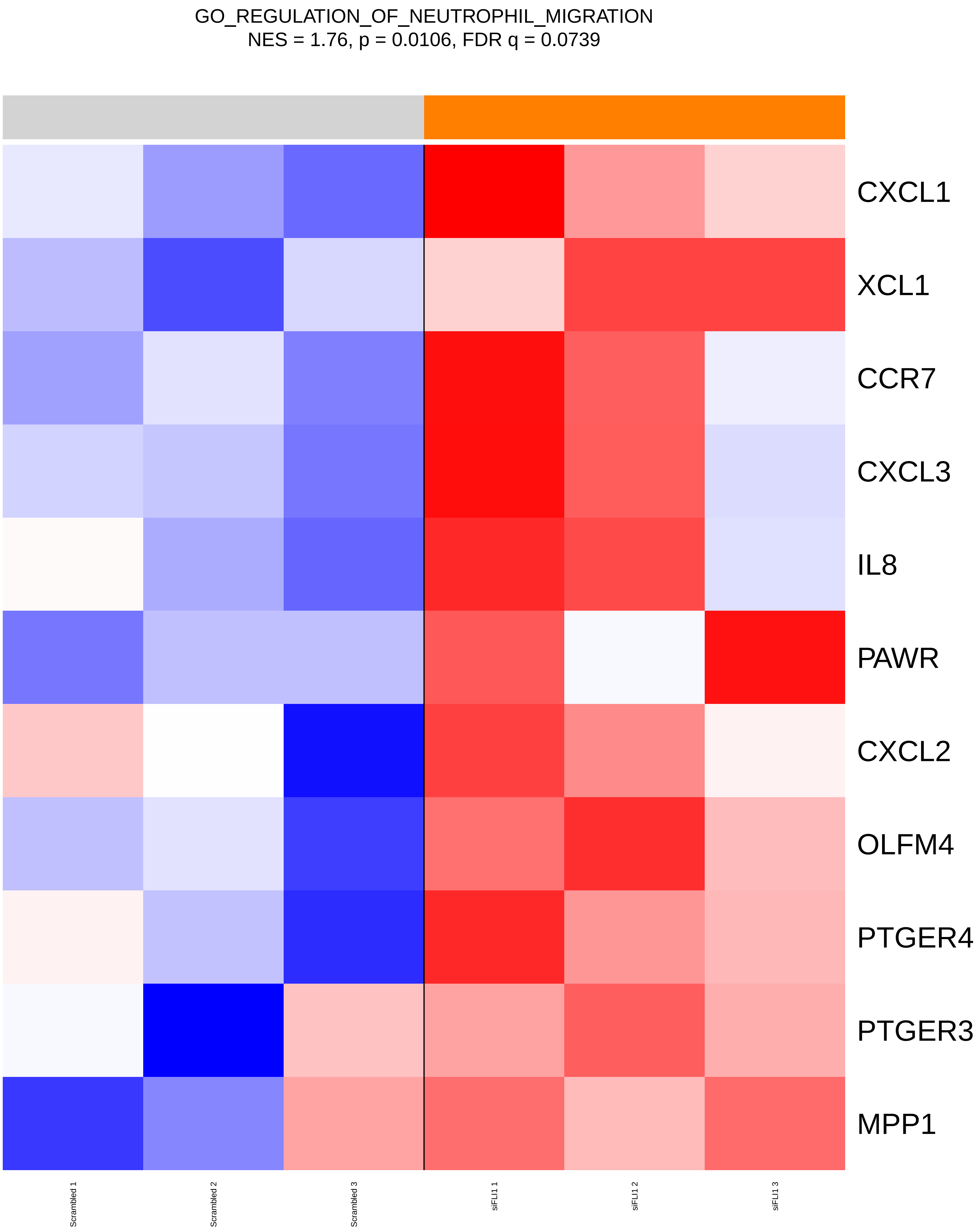
GO_REGULATION_OF_NEUTROPHIL_MIGRATION
NES = 1.76, p = 0.0106, FDR q = 0.0739
CXCL1
XCL1
CCR7
CXCL3
IL8
PAWR
CXCL2
OLFM4
PTGER4
PTGER3
MPP1
Scrambled 1
Scrambled 2
Scrambled 3
siFLI1 1
siFLI1 2
siFLI1 3

GO_NEGATIVE_REGULATION_OF_ADHERENS_JUNCTION_ORGANIZATION
NES = 1.76, p = 0.0091, FDR q = 0.0738

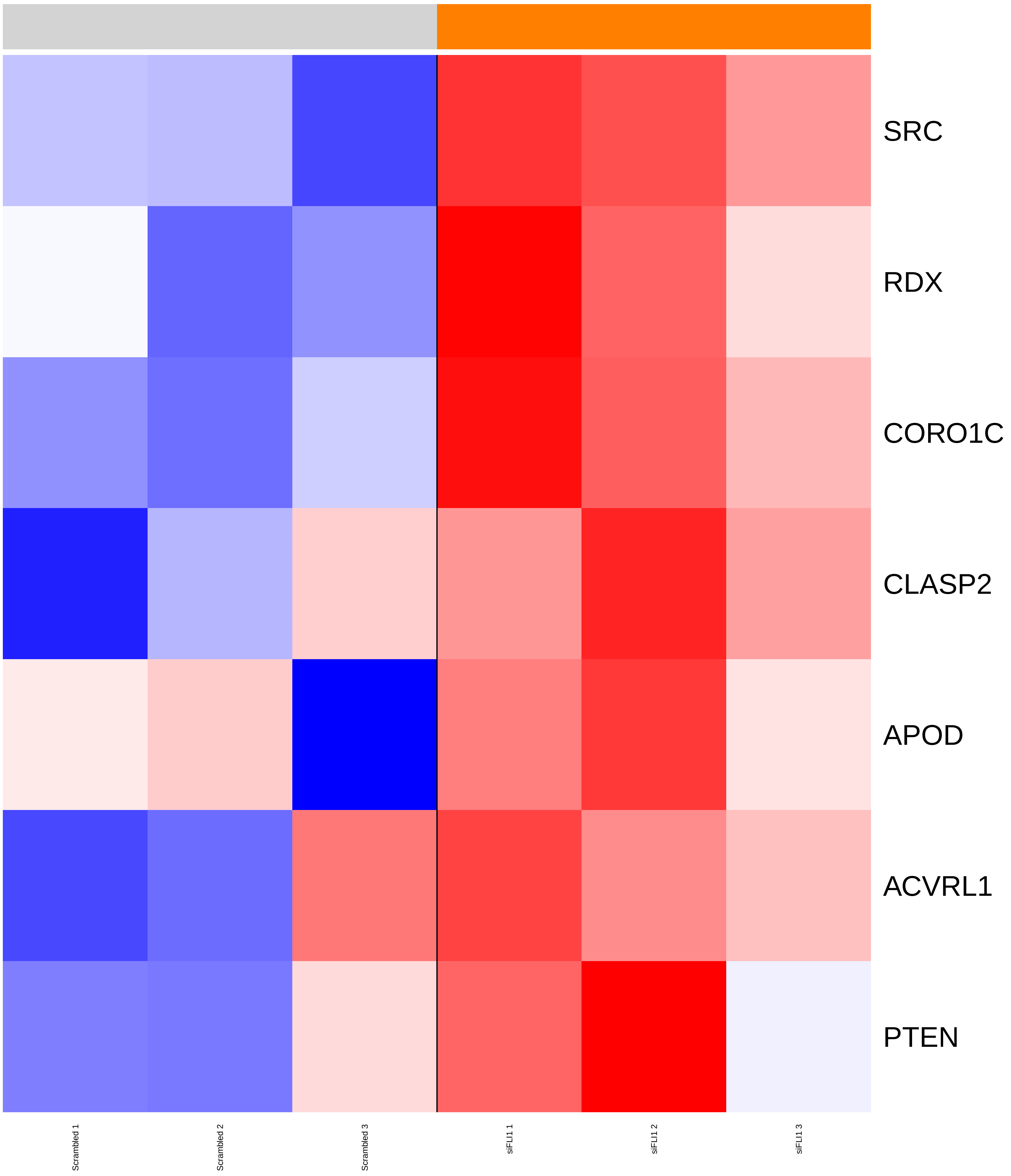

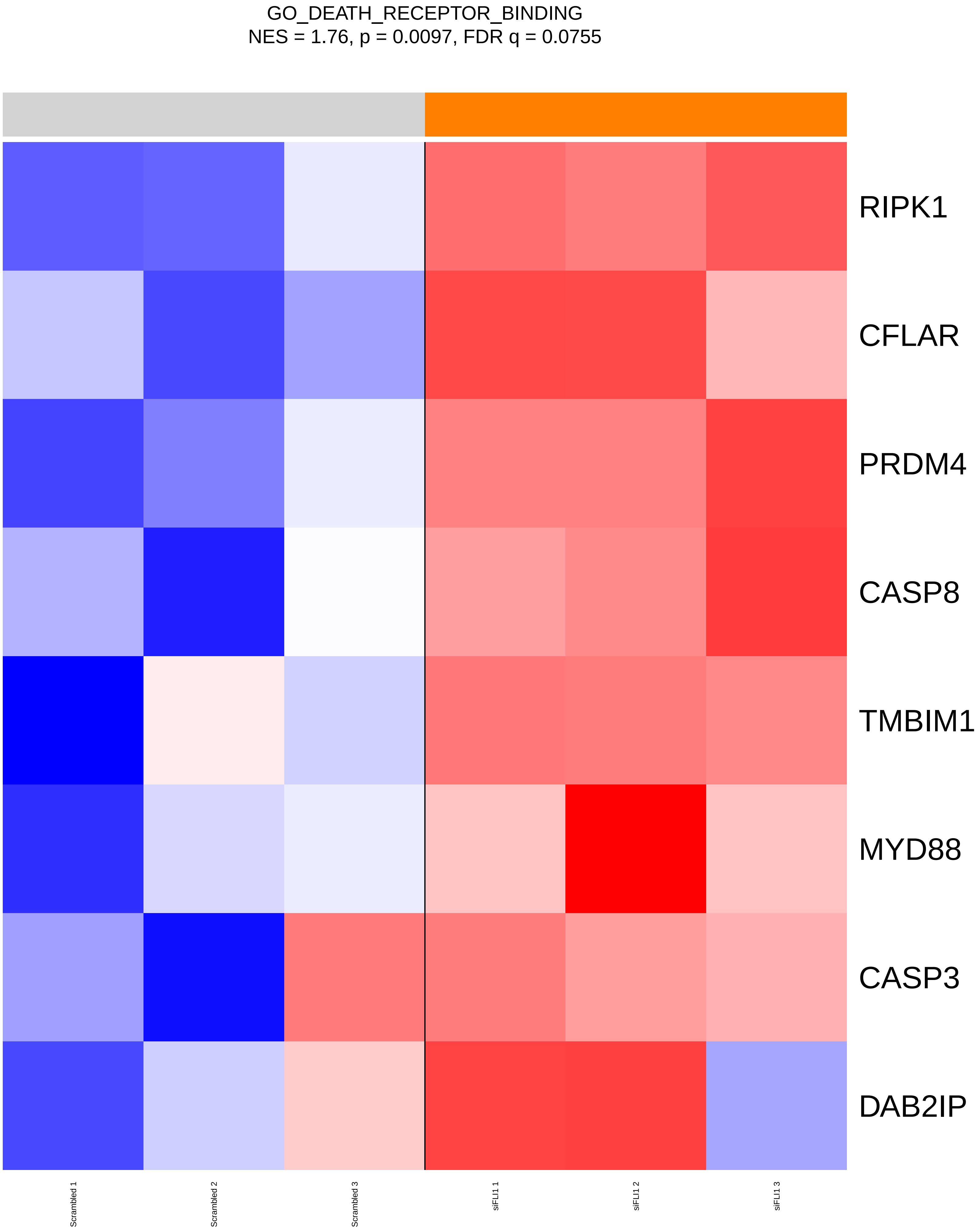

GO_DEATH_RECEPTOR_BINDING
NES = 1.76, p = 0.0097, FDR q = 0.0755
RIPK1
CFLAR
PRDM4
CASP8
TMBIM1
MYD88
CASP3
DAB2IP
Scrambled 1
Scrambled 2
Scrambled 3
siFLI1 1
siFLI1 2
siFLI1 3

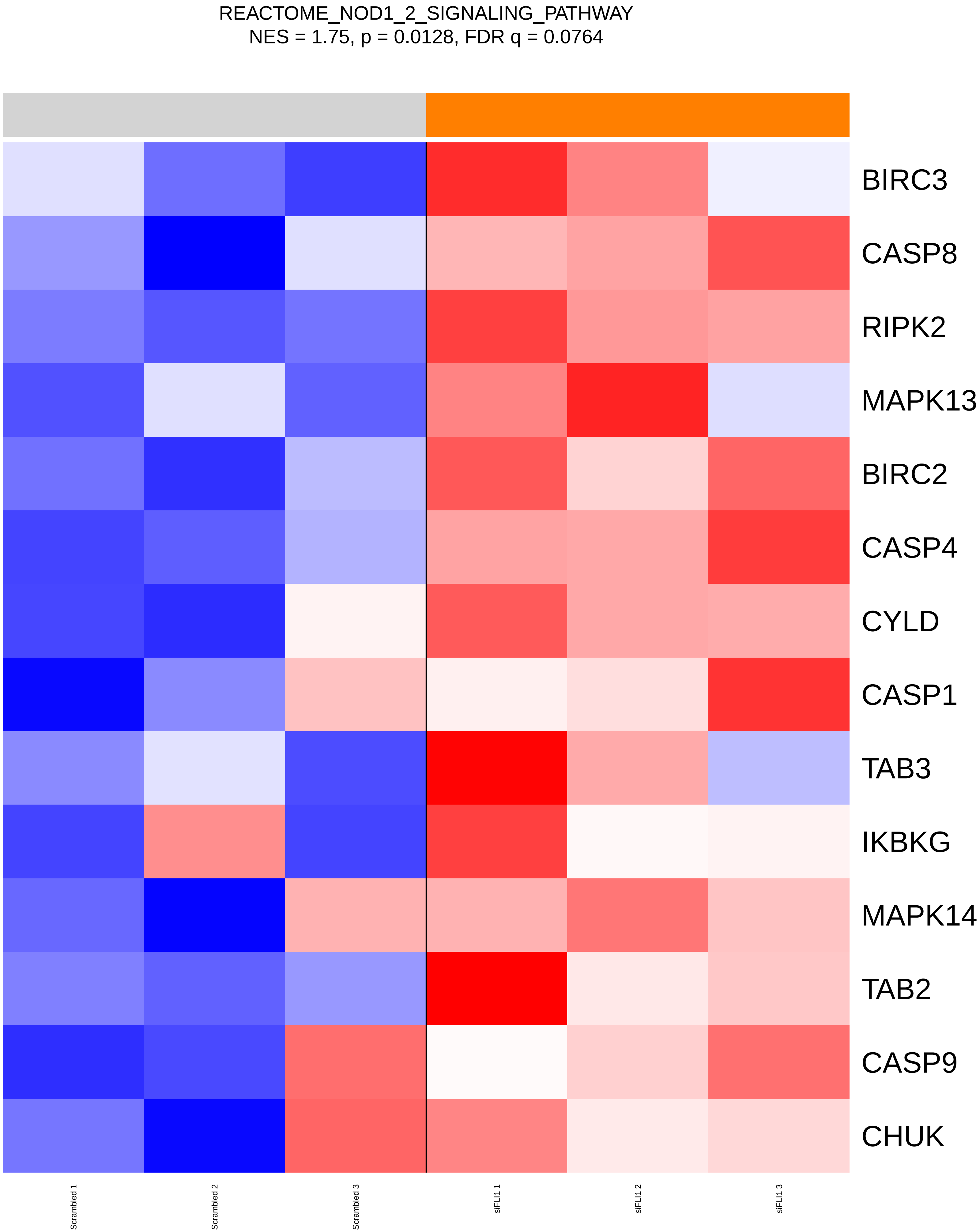
REACTOME_NOD1_2_SIGNALING_PATHWAY
NES = 1.75, p = 0.0128, FDR q = 0.0764
BIRC3
CASP8
RIPK2
MAPK13
BIRC2
CASP4
CYLD
CASP1
TAB3
IKBKG
MAPK14
TAB2
CASP9
CHUK
Scrambled 1
Scrambled 2
Scrambled 3
siFLI1 1
siFLI1 2
siFLI1 3

GO_REGULATION_OF_INNATE_IMMUNE_RESPONSE
NES = 1.75, p = 0.0000, FDR q = 0.0759

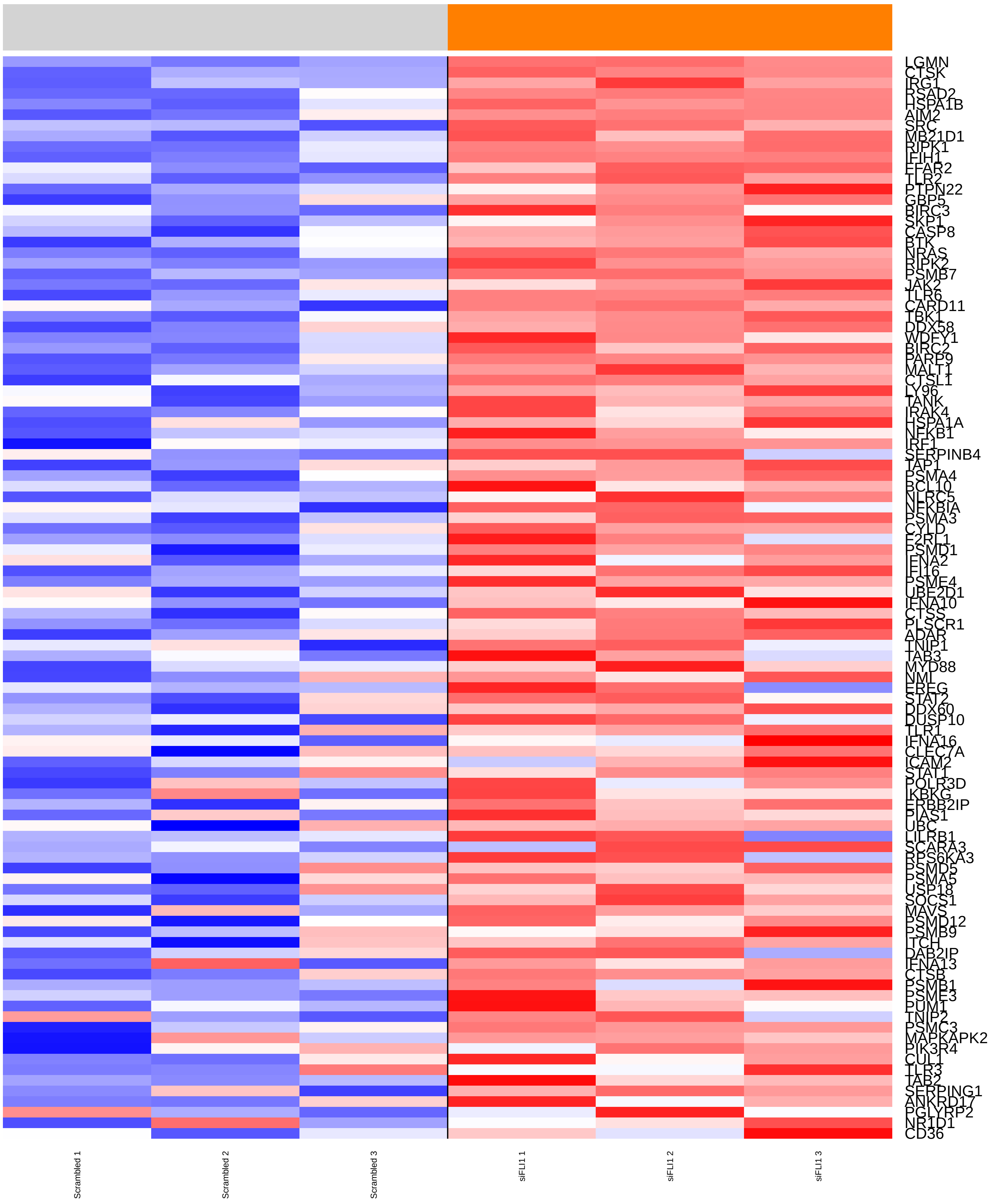

GO_REGULATION_OF_TYPE_I_INTERFERON_PRODUCTION
NES = 1.75, p = 0.0000, FDR q = 0.0756

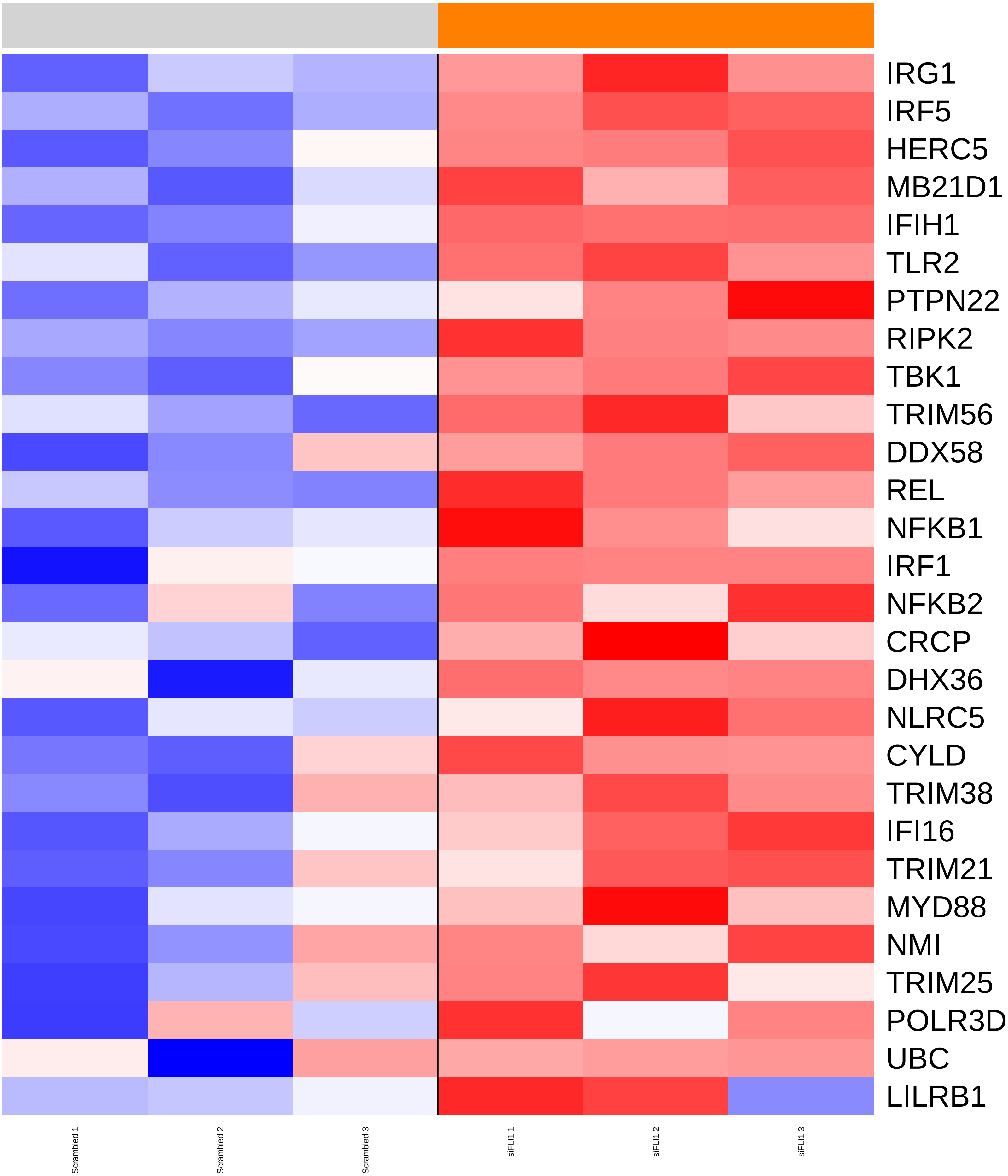

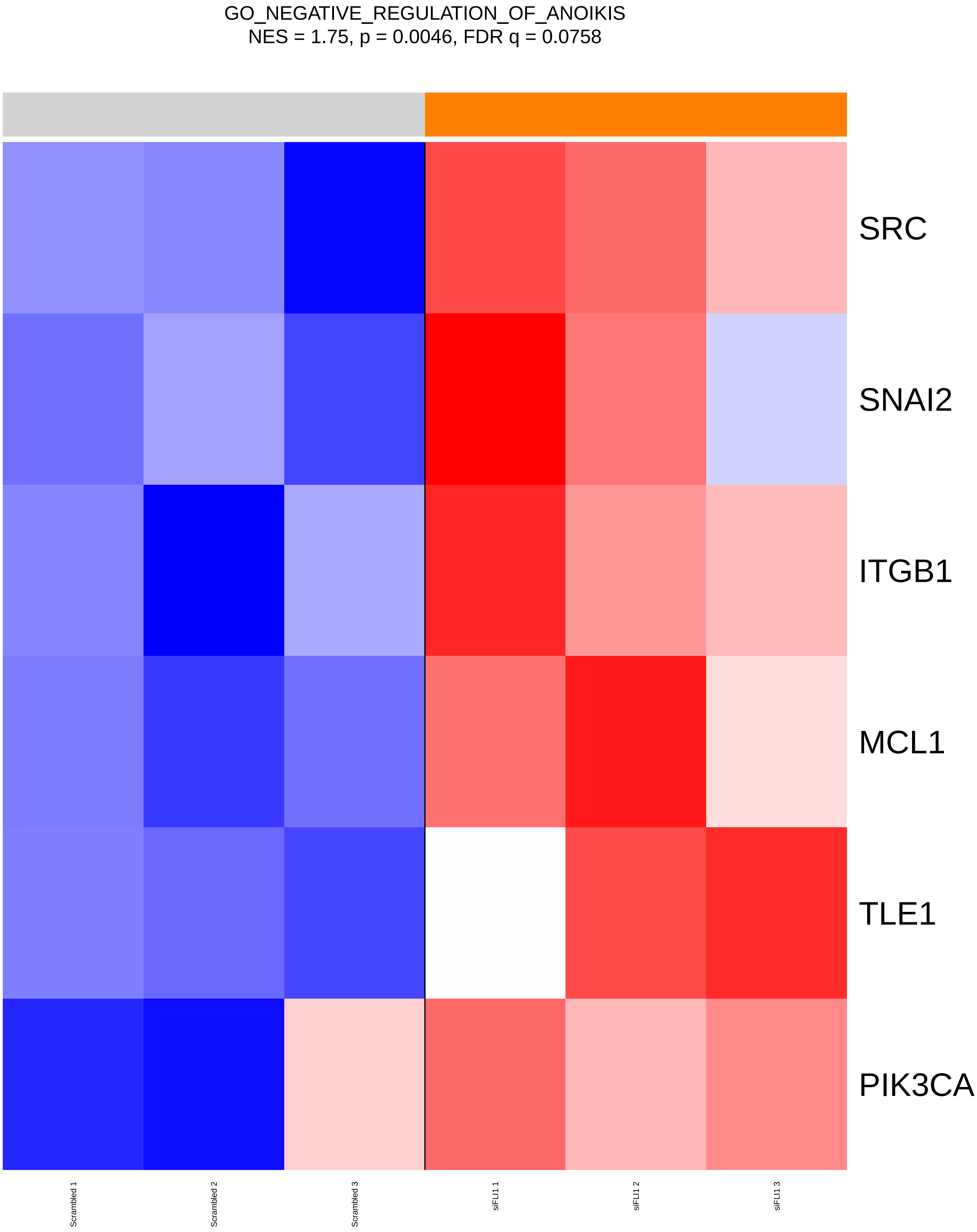
GO_NEGATIVE_REGULATION_OF_ANOIKIS
NES = 1.75, p = 0.0046, FDR q = 0.0758
SRC
SNAI2
ITGB1
MCL1
TLE1
PIK3CA
Scrambled 1
Scrambled 2
Scrambled 3
siFLI1 1
siFLI1 2
siFLI1 3

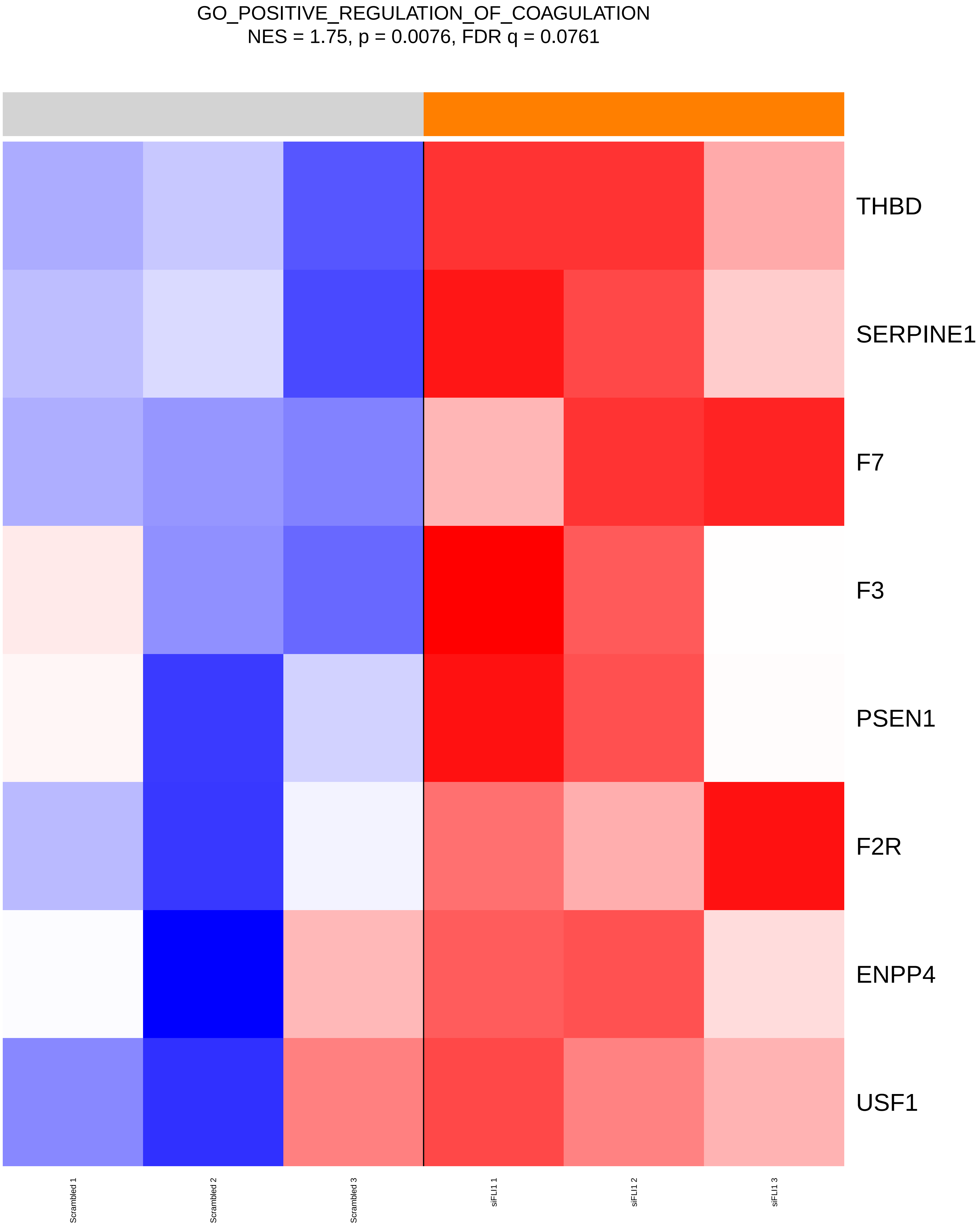
GO_POSITIVE_REGULATION_OF_COAGULATION
NES = 1.75, p = 0.0076, FDR q = 0.0761
THBD
SERPINE1
F7
F3
PSEN1
F2R
ENPP4
USF1
Scrambled 1
Scrambled 2
Scrambled 3
siFLI1 1
siFLI1 2
siFLI1 3

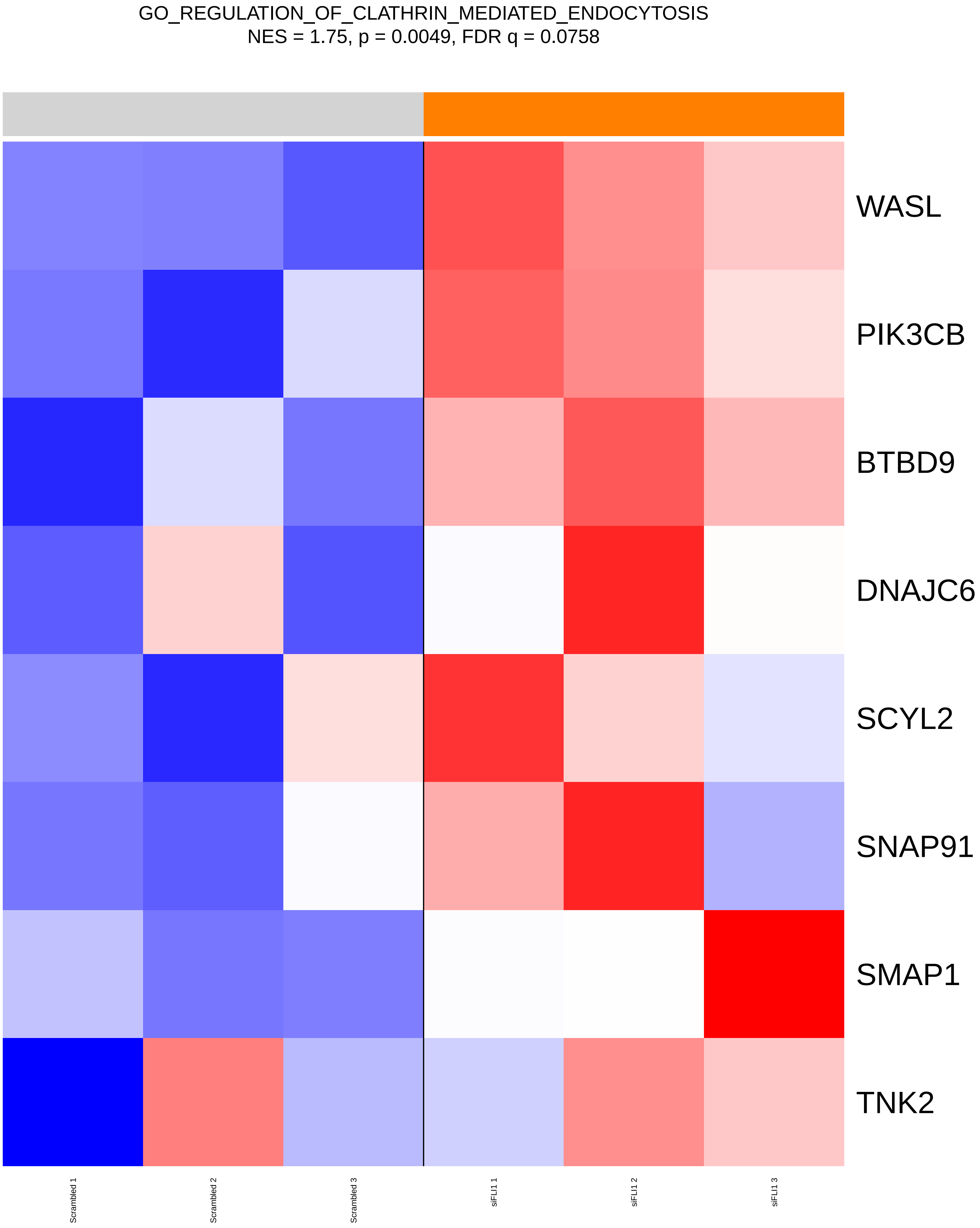
GO_REGULATION_OF_CLATHRIN_MEDIATED_ENDOCYTOSIS
NES = 1.75, p = 0.0049, FDR q = 0.0758
WASL
PIK3CB
BTBD9
DNAJC6
SCYL2
SNAP91
SMAP1
TNK2
Scrambled 1
Scrambled 2
Scrambled 3
siFLI1 1
siFLI1 2
siFLI1 3

KEGG_CHEMOKINE_SIGNALING_PATHWAY
NES = 1.75, p = 0.0000, FDR q = 0.0767

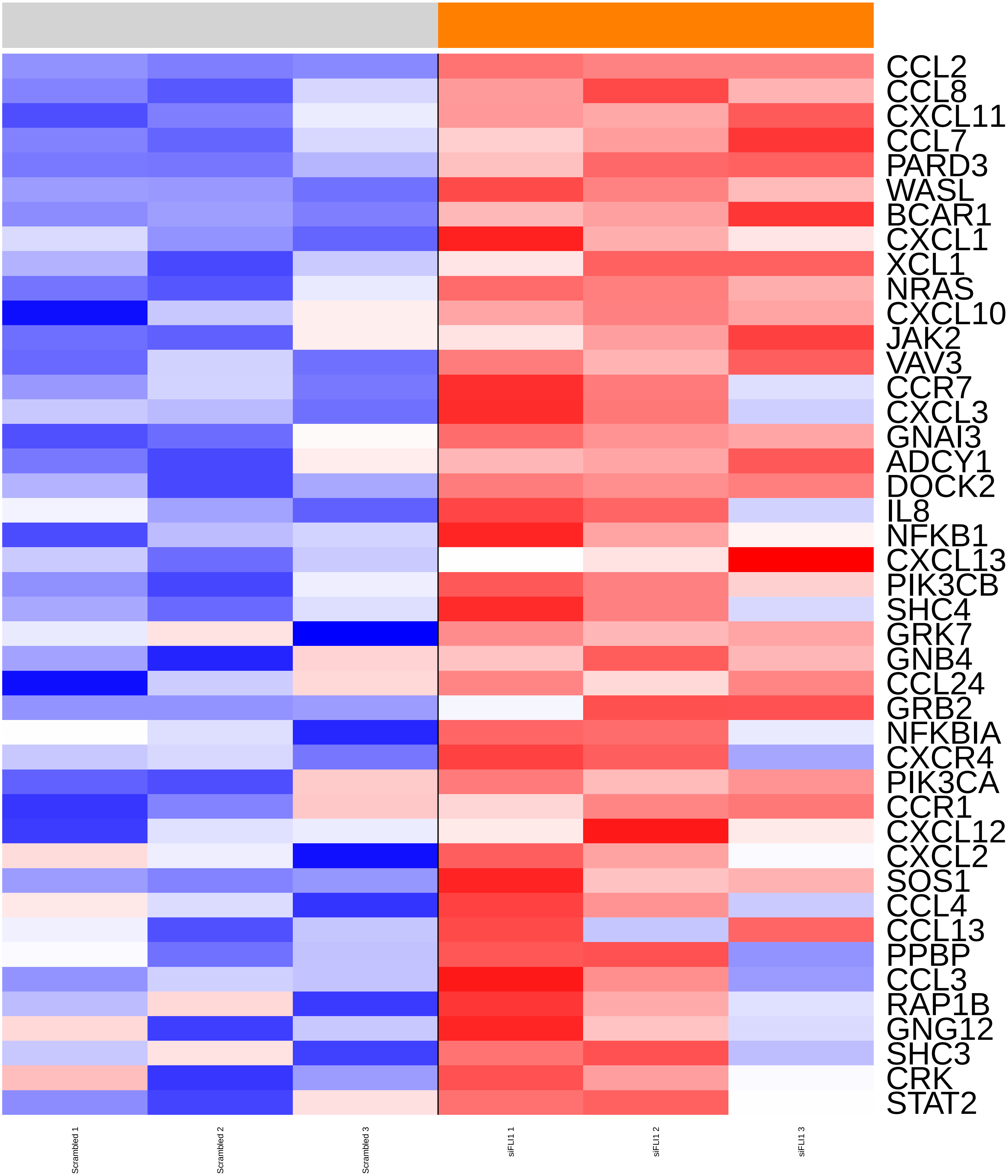

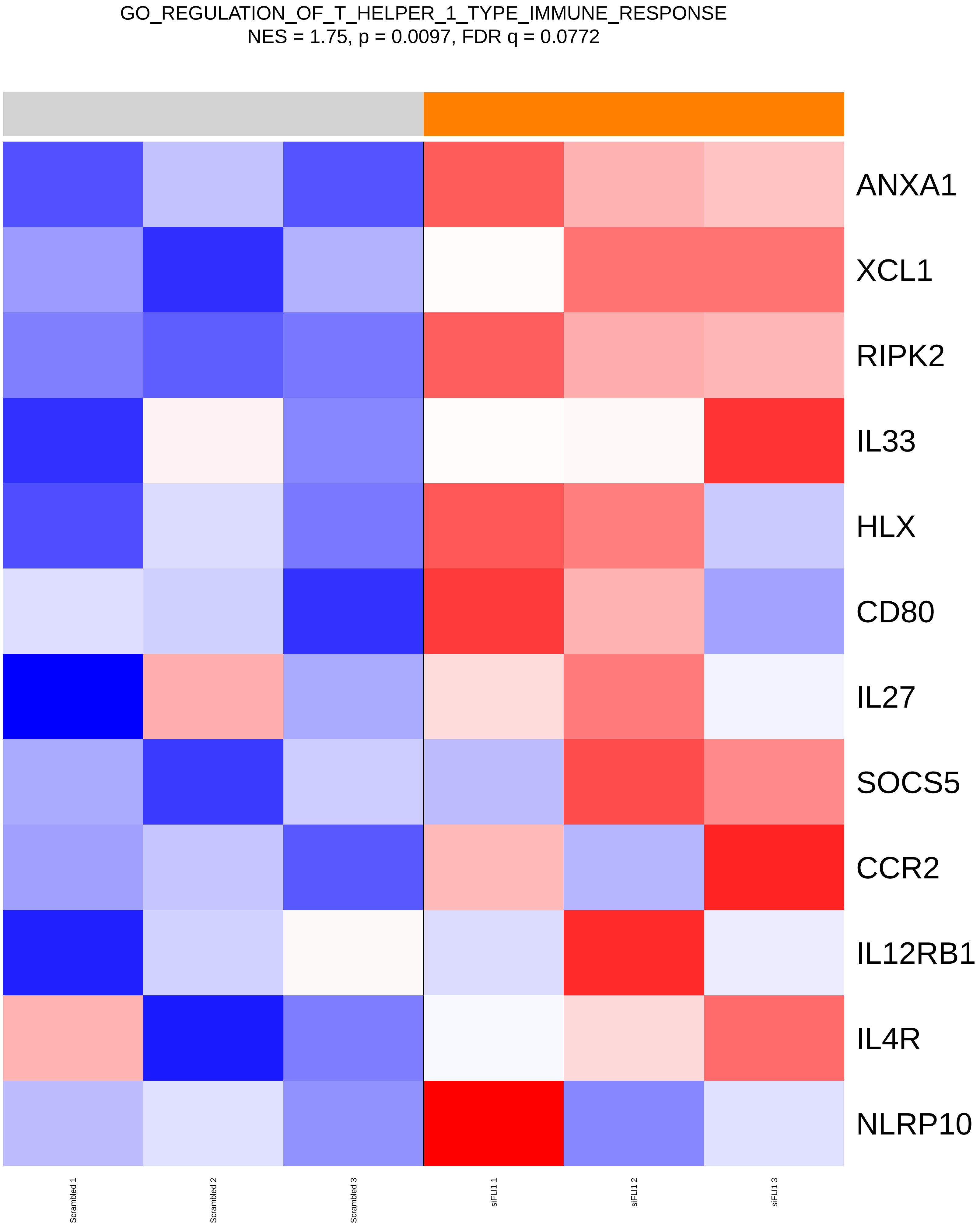
GO_REGULATION_OF_T_HELPER_1_TYPE_IMMUNE_RESPONSE
NES = 1.75, p = 0.0097, FDR q = 0.0772
ANXA1
XCL1
RIPK2
IL33
HLX
CD80
IL27
SOCS5
CCR2
IL12RB1
IL4R
NLRP10
Scrambled 1
Scrambled 2
Scrambled 3
siFLI1 1
siFLI1 2
siFLI1 3

REACTOME_CYTOKINE_SIGNALING_IN_IMMUNE_SYSTEM
NES = 1.75, p = 0.0000, FDR q = 0.0769

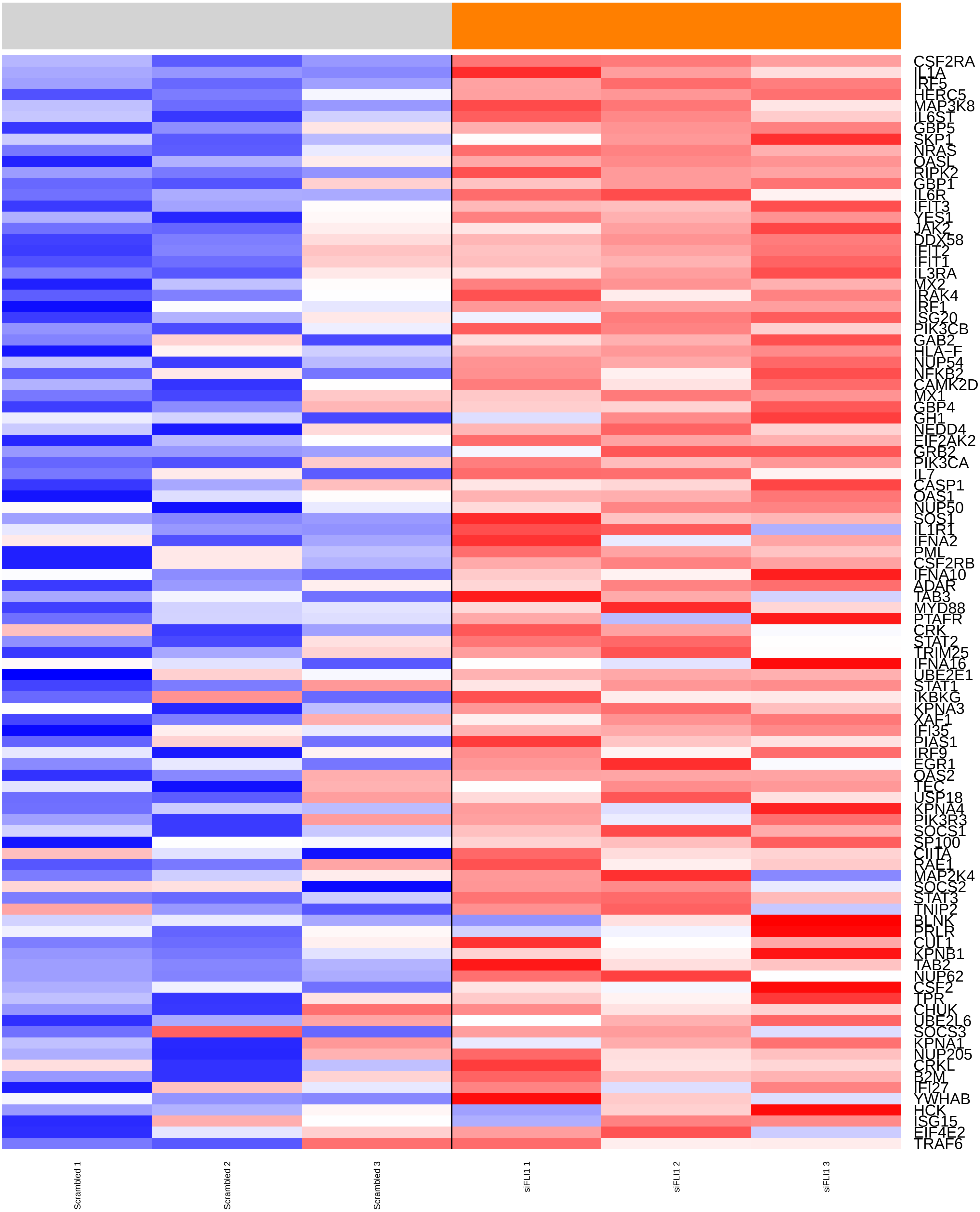

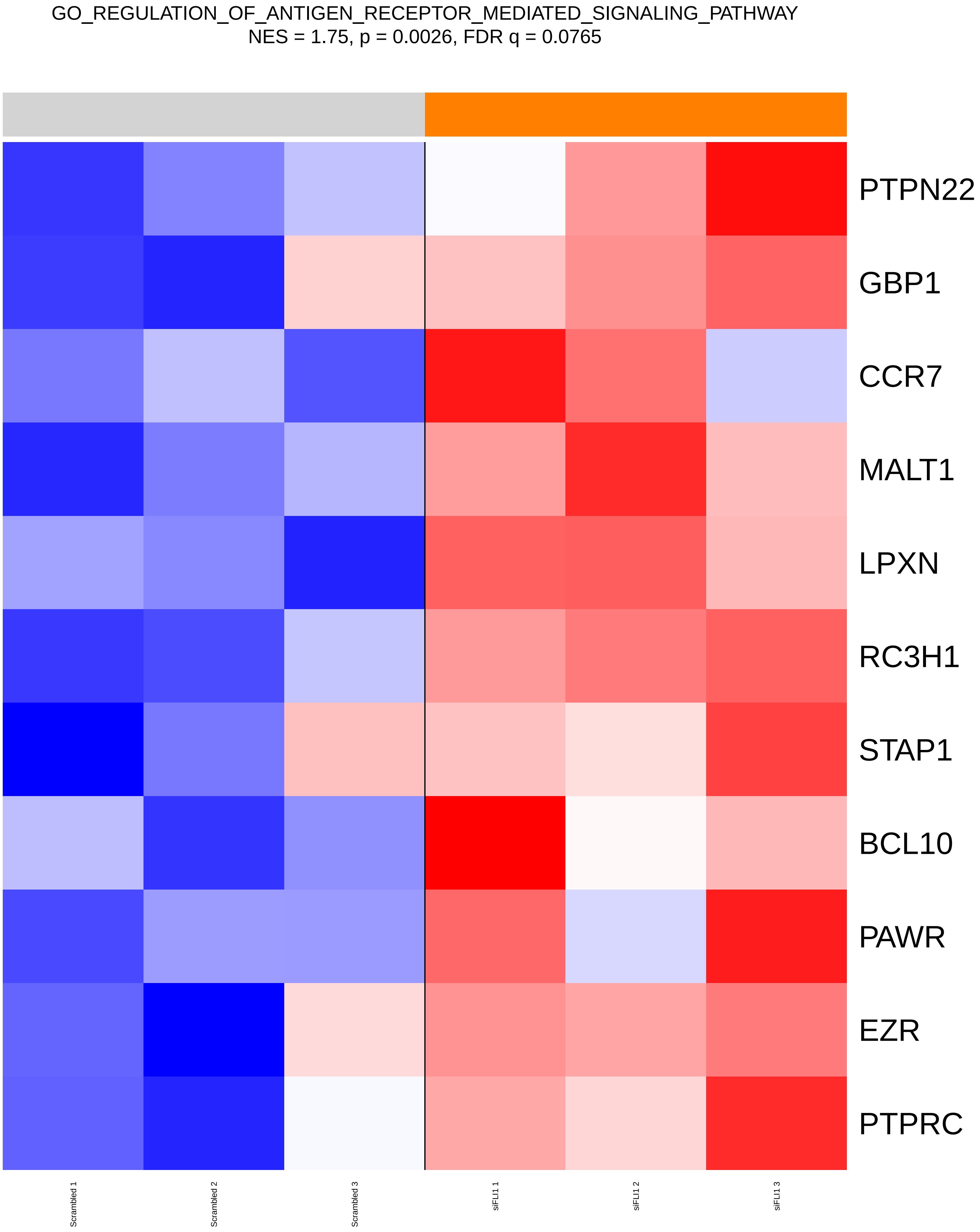

GO_REGULATION_OF_ANTIGEN_RECEPTOR_MEDIATED_SIGNALING_PATHWAY
NES = 1.75, p = 0.0026, FDR q = 0.0765
PTPN22
GBP1
CCR7
MALT1
LPXN
RC3H1
STAP1
BCL10
PAWR
EZR
PTPRC
Scrambled 1
Scrambled 2
Scrambled 3
siFLI1 1
siFLI1 2
siFLI1 3

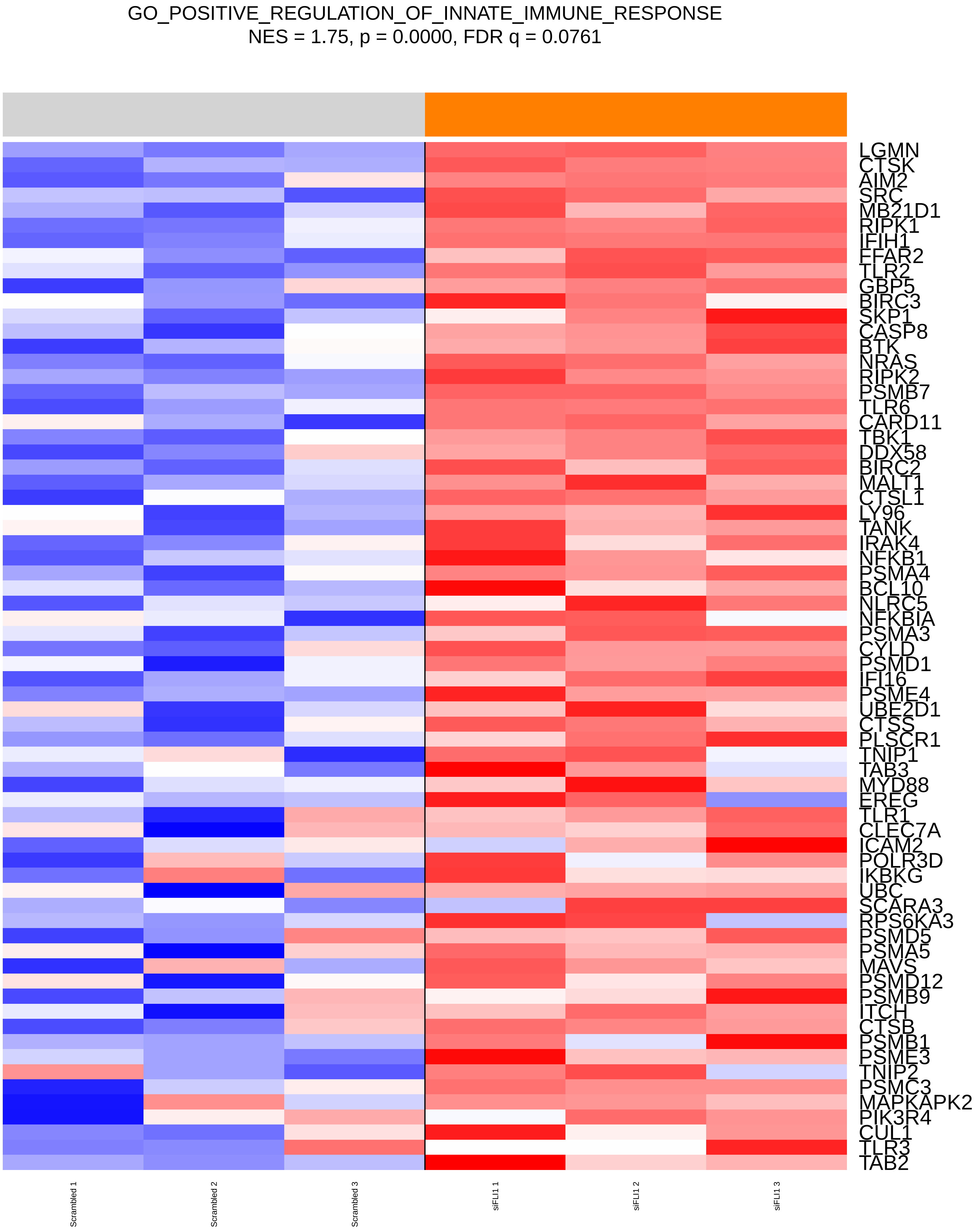
GO_POSITIVE_REGULATION_OF_INNATE_IMMUNE_RESPONSE
NES = 1.75, p = 0.0000, FDR q = 0.0761
LGMN
CTSK
AIM2
SRC
MB21D1
RIPK1
IFIH1
FFAR2
TLR2
GBP5
BIRC3
SKP1
CASP8
BTK
NRAS
RIPK2
PSMB7
TLR6
CARD11
TBK1
DDX58
BIRC2
MALT1
CTSL1
LY96
TANK
IRAK4
NFKB1
PSMA4
BCL10
NLRC5
NFKBIA
PSMA3
CYLD
PSMD1
IFI16
PSME4
UBE2D1
CTSS
PLSCR1
TNIP1
TAB3
MYD88
EREG
TLR1
CLEC7A
ICAM2
POLR3D
IKBKG
UBC
SCARA3
RPS6KA3
PSMD5
PSMA5
MAVS
PSMD12
PSMB9
ITCH
CTSB
PSMB1
PSME3
TNIP2
PSMC3
MAPKAPK2
PIK3R4
CUL1
TLR3
TAB2
Scrambled 1
Scrambled 2
Scrambled 3
siFLI1 1
siFLI1 2
siFLI1 3

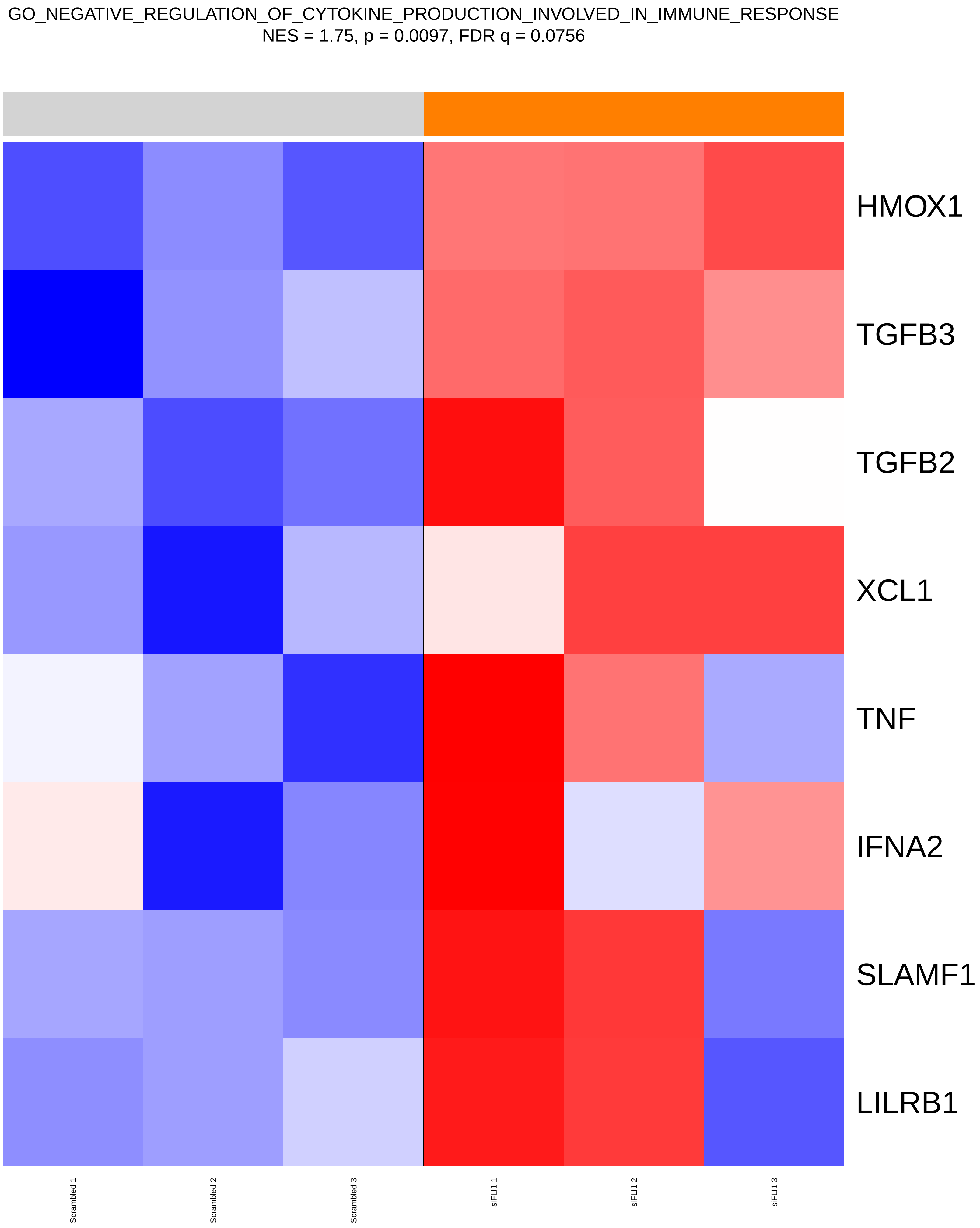
GO_NEGATIVE_REGULATION_OF_CYTOKINE_PRODUCTION_INVOLVED_IN_IMMUNE_RESPONSE
NES = 1.75, p = 0.0097, FDR q = 0.0756
HMOX1
TGFB3
TGFB2
XCL1
TNF
IFNA2
SLAMF1
LILRB1
Scrambled 1
Scrambled 2
Scrambled 3
siFLI1 1
siFLI1 2
siFLI1 3

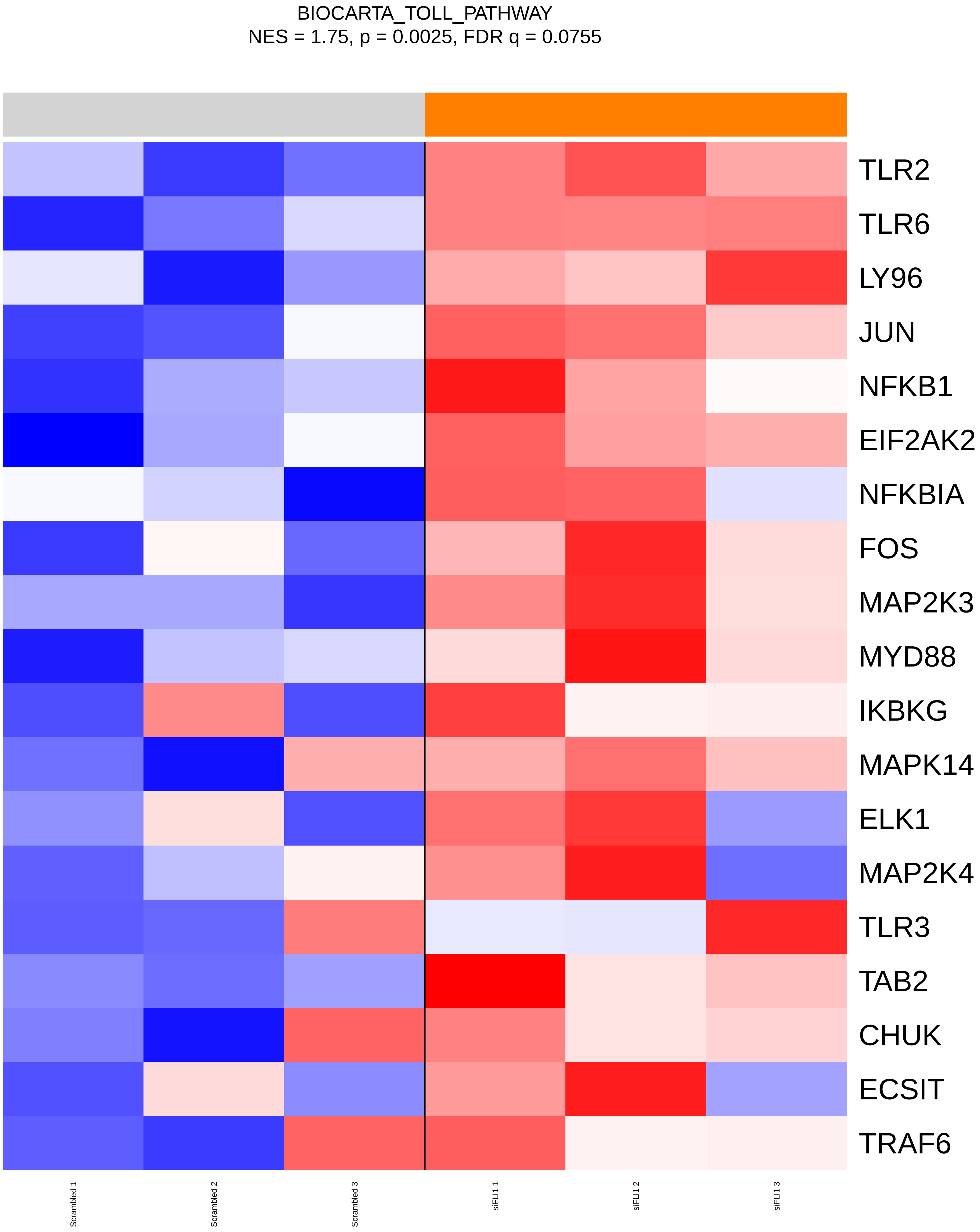
BIOCARTA_TOLL_PATHWAY
NES = 1.75, p = 0.0025, FDR q = 0.0755
TLR2
TLR6
LY96
JUN
NFKB1
EIF2AK2
NFKBIA
FOS
MAP2K3
MYD88
IKBKG
MAPK14
ELK1
MAP2K4
TLR3
TAB2
CHUK
ECSIT
TRAF6
Scrambled 1
Scrambled 2
Scrambled 3
siFLI1 1
siFLI1 2
siFLI1 3

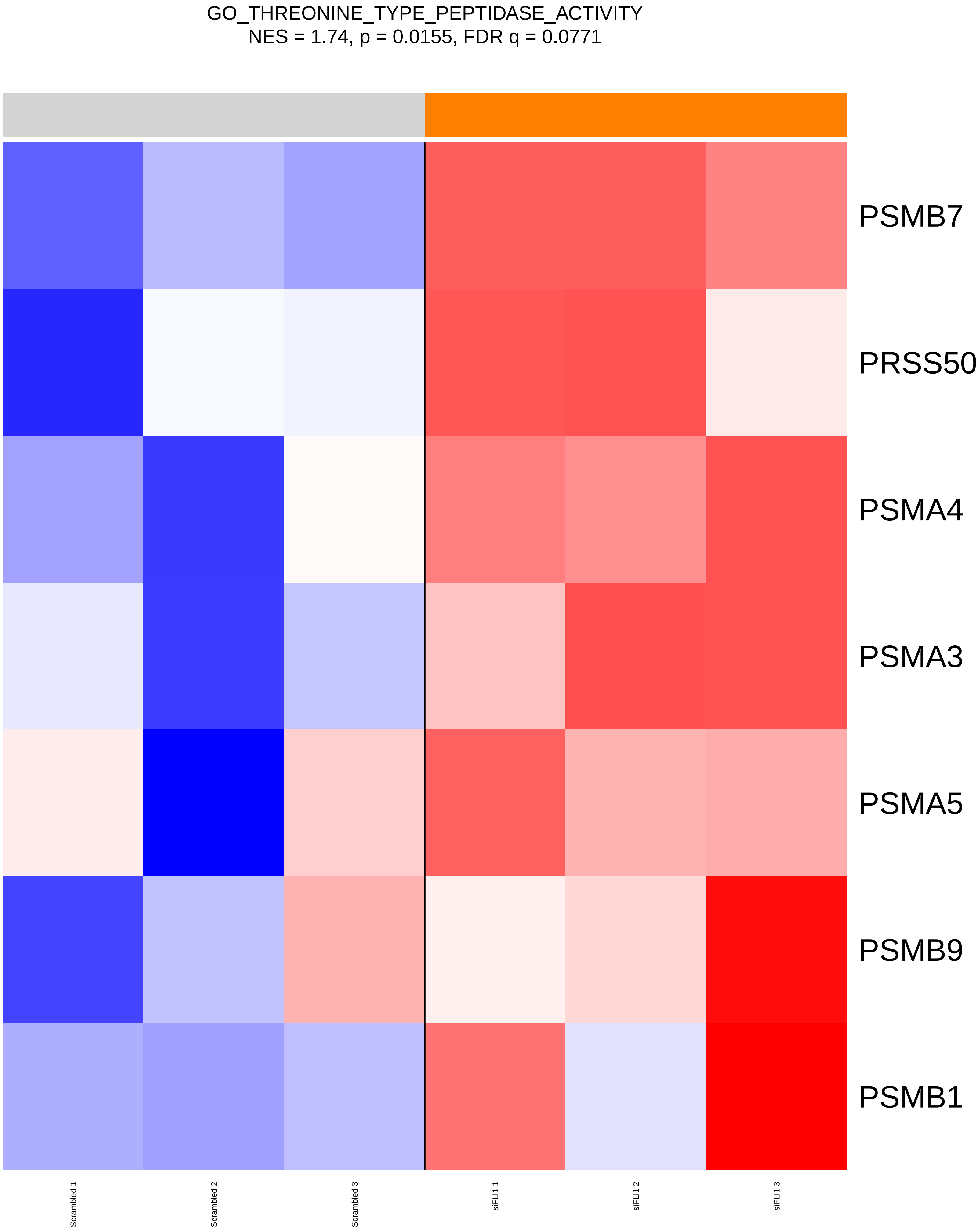
GO_THREONINE_TYPE_PEPTIDASE_ACTIVITY
NES = 1.74, p = 0.0155, FDR q = 0.0771
PSMB7
PRSS50
PSMA4
PSMA3
PSMA5
PSMB9
PSMB1
Scrambled 1
Scrambled 2
Scrambled 3
siFLI1 1
siFLI1 2
siFLI1 3

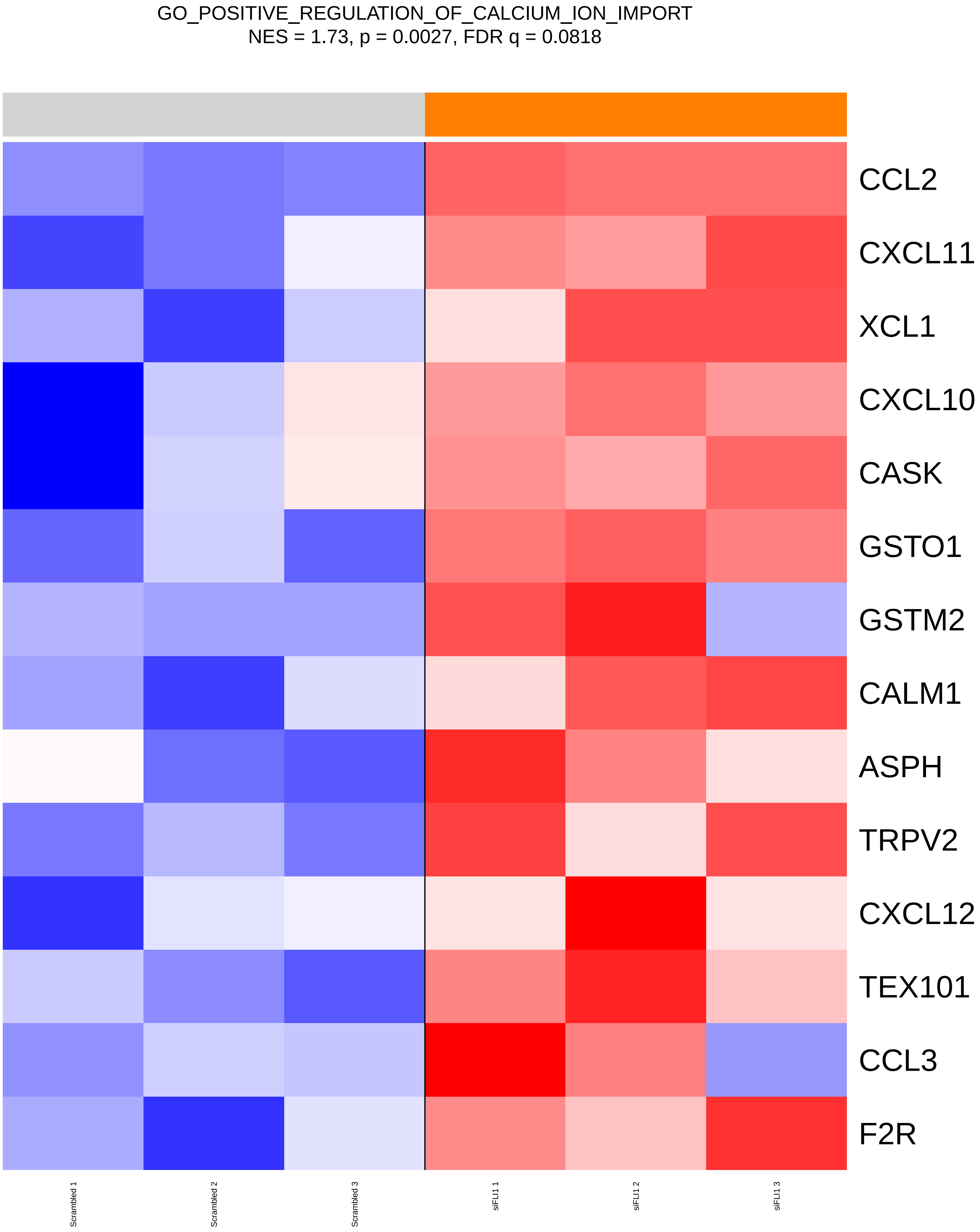
GO_POSITIVE_REGULATION_OF_CALCIUM_ION_IMPORT
NES = 1.73, p = 0.0027, FDR q = 0.0818
CCL2
CXCL11
XCL1
CXCL10
CASK
GSTO1
GSTM2
CALM1
ASPH
TRPV2
CXCL12
TEX101
CCL3
F2R
Scrambled 1
Scrambled 2
Scrambled 3
siFLI1 1
siFLI1 2
siFLI1 3

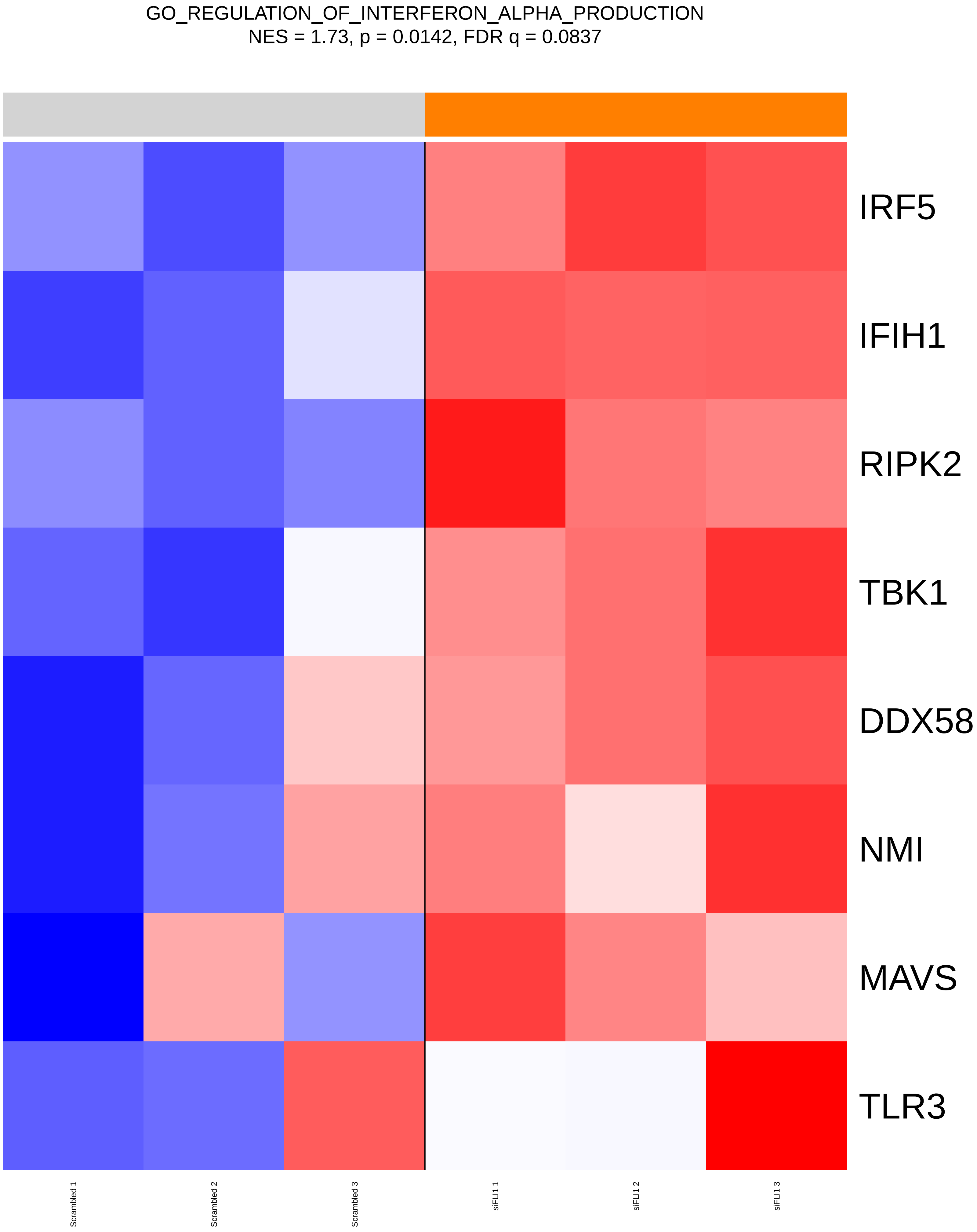
GO_REGULATION_OF_INTERFERON_ALPHA_PRODUCTION
NES = 1.73, p = 0.0142, FDR q = 0.0837
IRF5
IFIH1
RIPK2
TBK1
DDX58
NMI
MAVS
TLR3
Scrambled 1
Scrambled 2
Scrambled 3
siFLI1 1
siFLI1 2
siFLI1 3

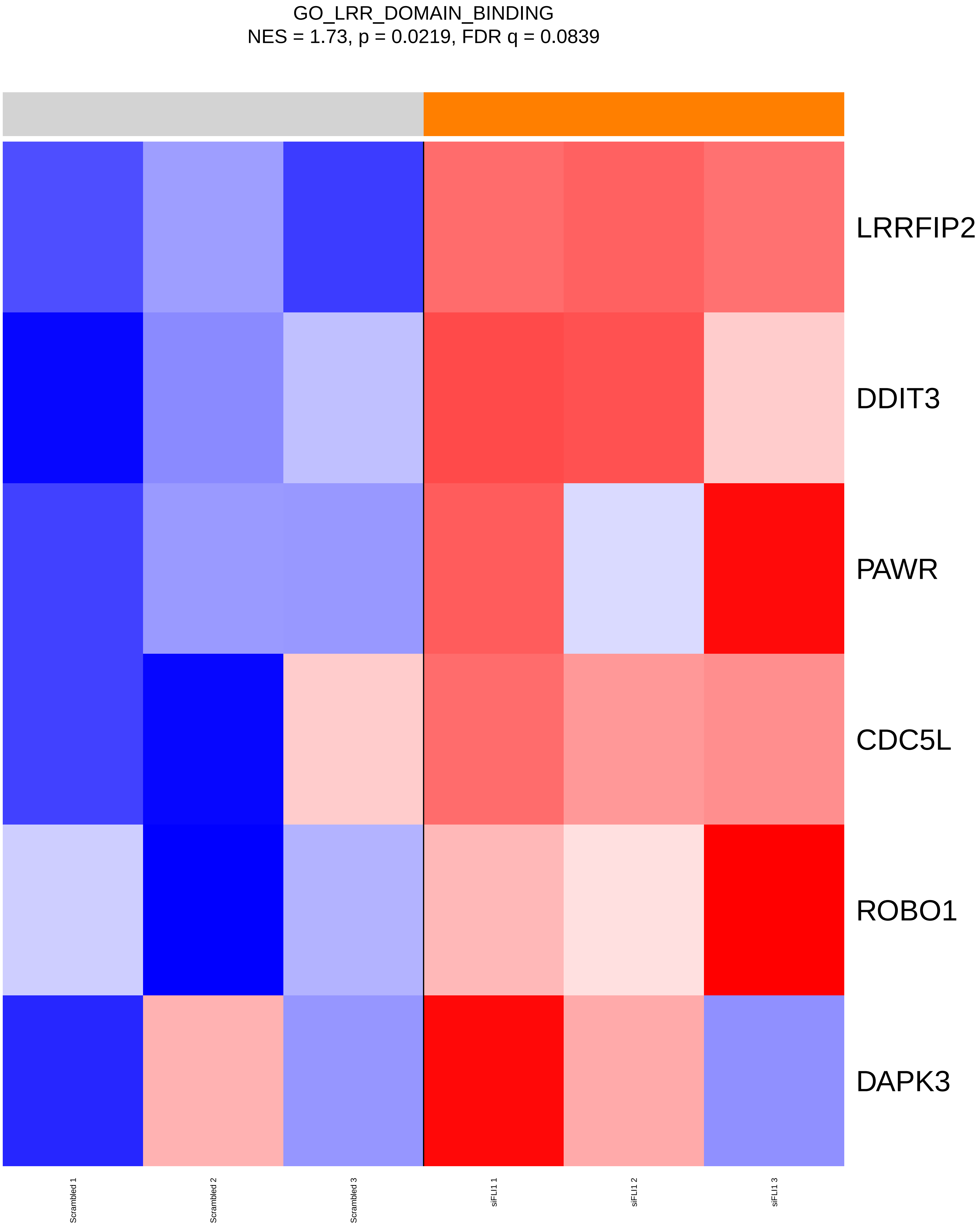
GO_LRR_DOMAIN_BINDING
NES = 1.73, p = 0.0219, FDR q = 0.0839
LRRFIP2
DDIT3
PAWR
CDC5L
ROBO1
DAPK3
Scrambled 1
Scrambled 2
Scrambled 3
siFLI1 1
siFLI1 2
siFLI1 3

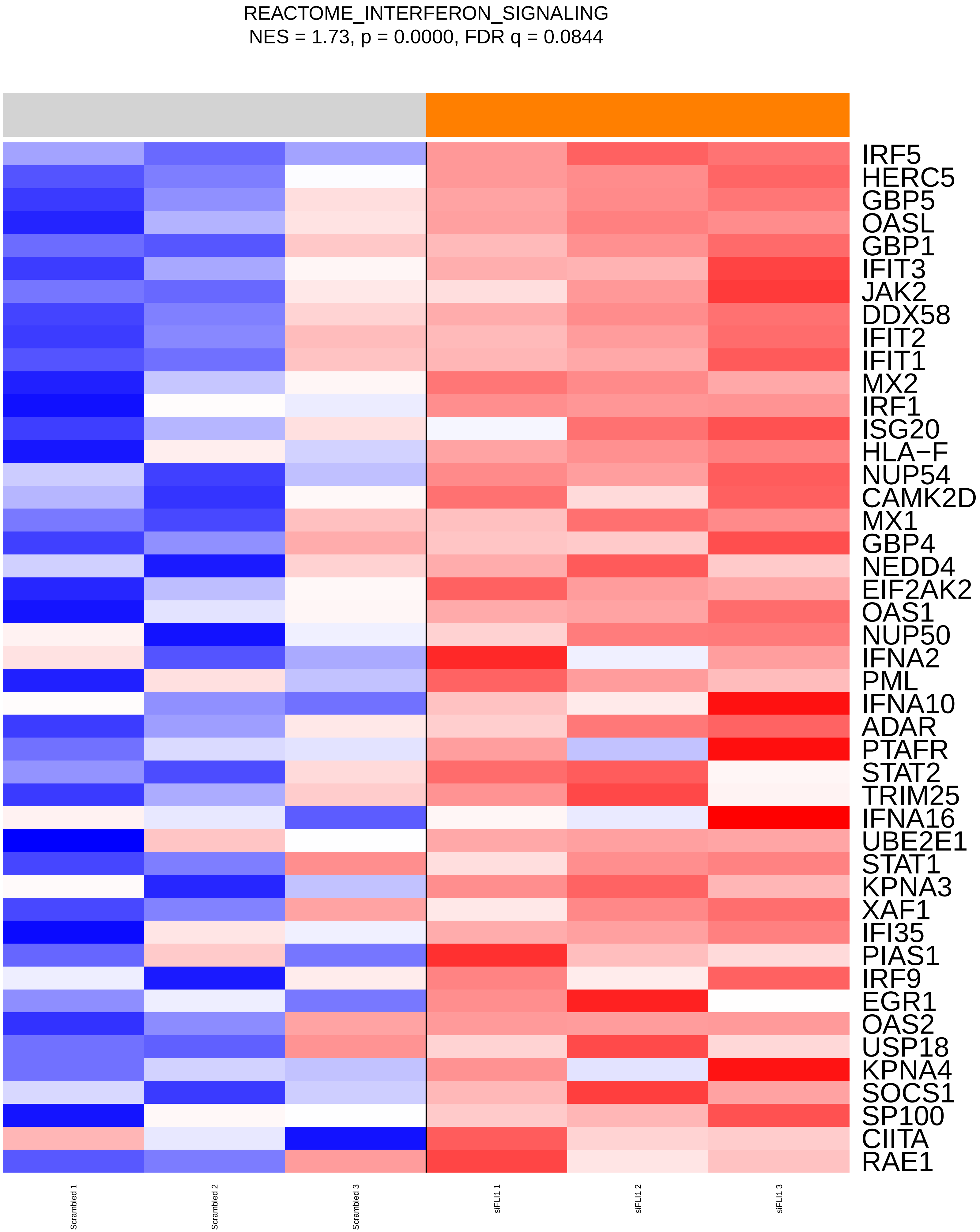
REACTOME_INTERFERON_SIGNALING
NES = 1.73, p = 0.0000, FDR q = 0.0844
IRF5
HERC5
GBP5
OASL
GBP1
IFIT3
JAK2
DDX58
IFIT2
IFIT1
MX2
IRF1
ISG20
HLA−F
NUP54
CAMK2D
MX1
GBP4
NEDD4
EIF2AK2
OAS1
NUP50
IFNA2
PML
IFNA10
ADAR
PTAFR
STAT2
TRIM25
IFNA16
UBE2E1
STAT1
KPNA3
XAF1
IFI35
PIAS1
IRF9
EGR1
OAS2
USP18
KPNA4
SOCS1
SP100
CIITA
RAE1
Scrambled 1
Scrambled 2
Scrambled 3
siFLI1 1
siFLI1 2
siFLI1 3

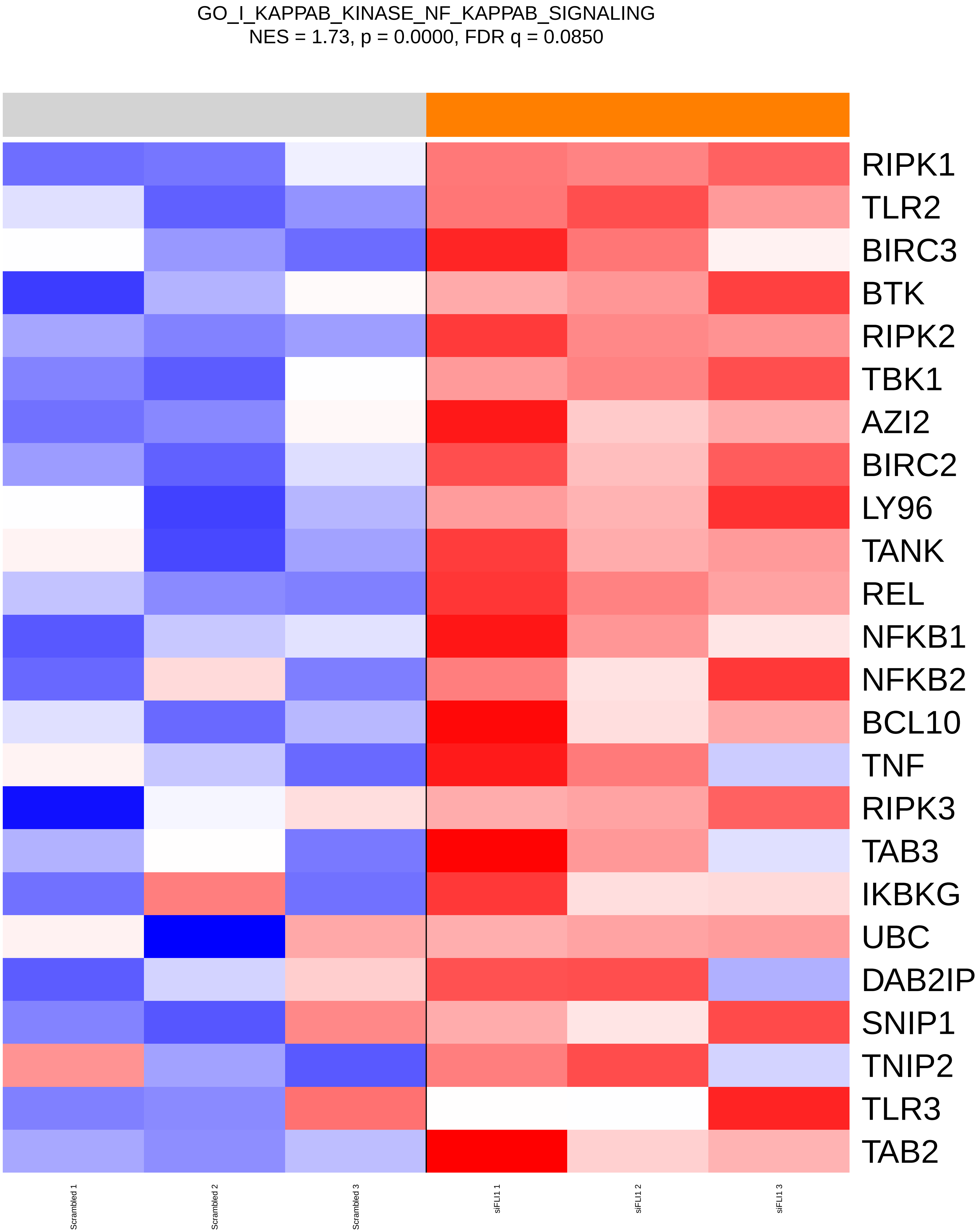
GO_I_KAPPAB_KINASE_NF_KAPPAB_SIGNALING
NES = 1.73, p = 0.0000, FDR q = 0.0850
RIPK1
TLR2
BIRC3
BTK
RIPK2
TBK1
AZI2
BIRC2
LY96
TANK
REL
NFKB1
NFKB2
BCL10
TNF
RIPK3
TAB3
IKBKG
UBC
DAB2IP
SNIP1
TNIP2
TLR3
TAB2
Scrambled 1
Scrambled 2
Scrambled 3
siFLI1 1
siFLI1 2
siFLI1 3

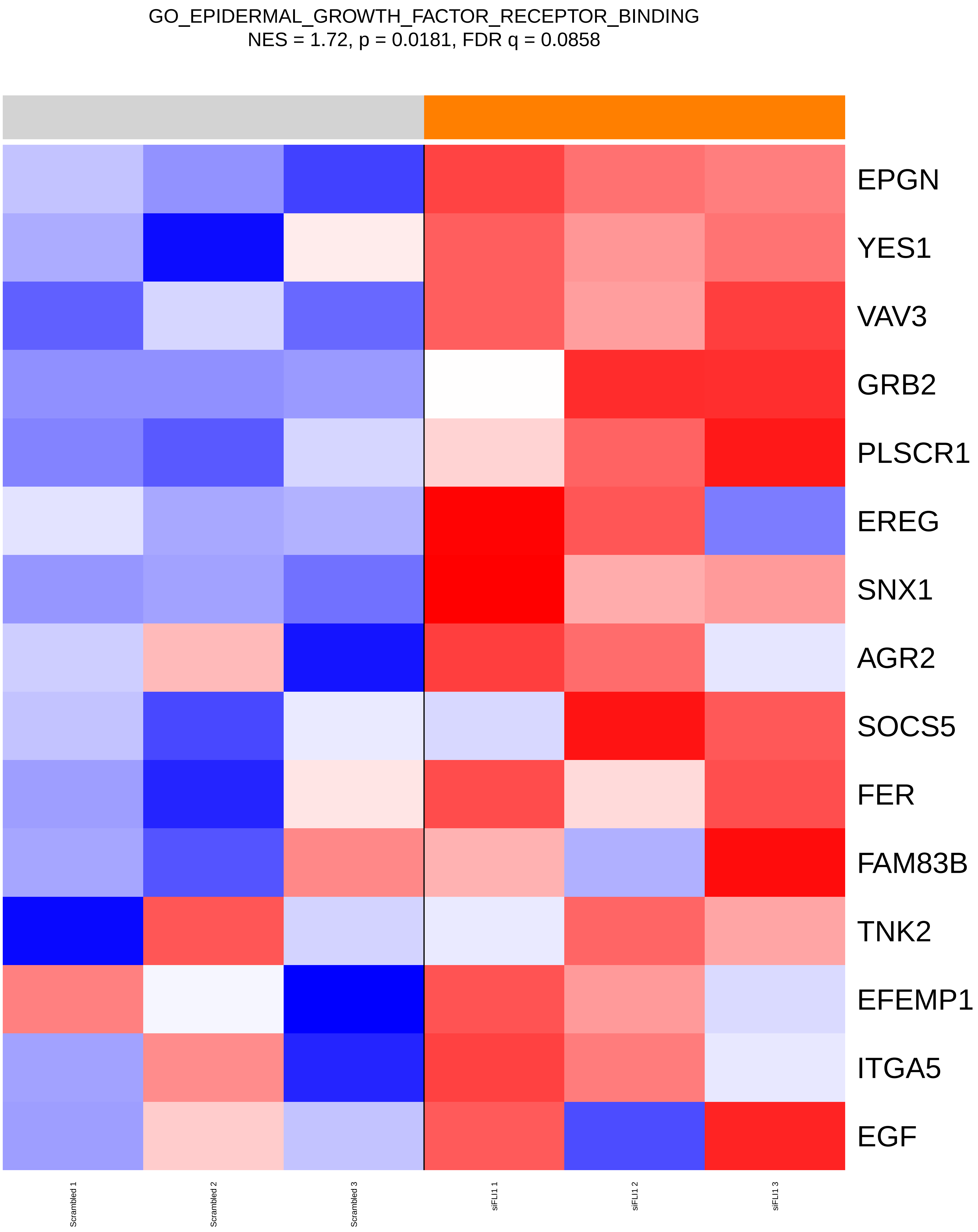

GO_EPIDERMAL_GROWTH_FACTOR_RECEPTOR_BINDING
NES = 1.72, p = 0.0181, FDR q = 0.0858
EPGN
YES1
VAV3
GRB2
PLSCR1
EREG
SNX1
AGR2
SOCS5
FER
FAM83B
TNK2
EFEMP1
ITGA5
EGF
Scrambled 1
Scrambled 2
Scrambled 3
siFLI1 1
siFLI1 2
siFLI1 3

GO_POSITIVE_REGULATION_OF_INTERFERON_ALPHA_PRODUCTION
NES = 1.72, p = 0.0099, FDR q = 0.0867

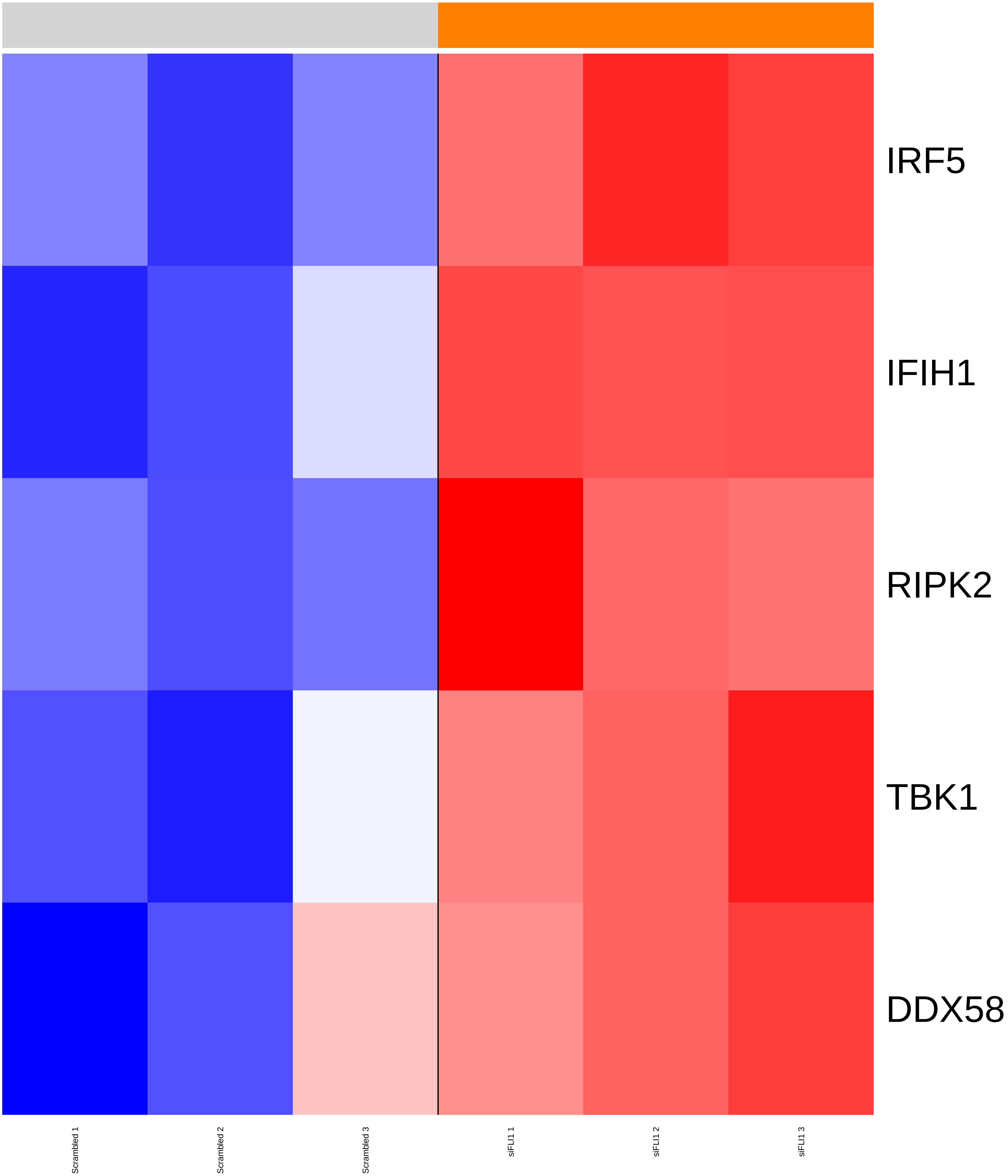

GO_DICARBOXYLIC_ACID_TRANSMEMBRANE_TRANSPORTER_ACTIVITY
NES = 1.72, p = 0.0100, FDR q = 0.0866

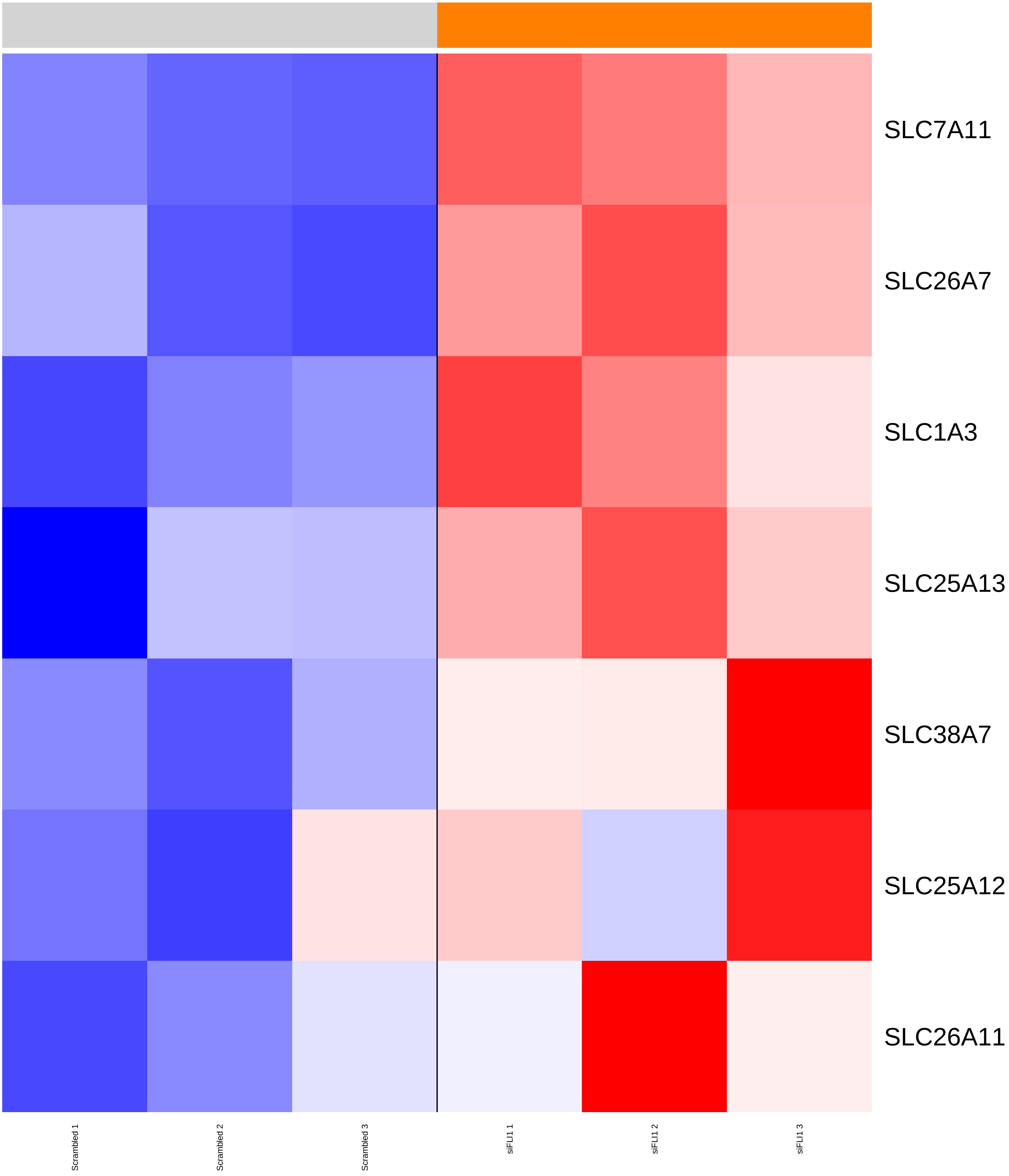

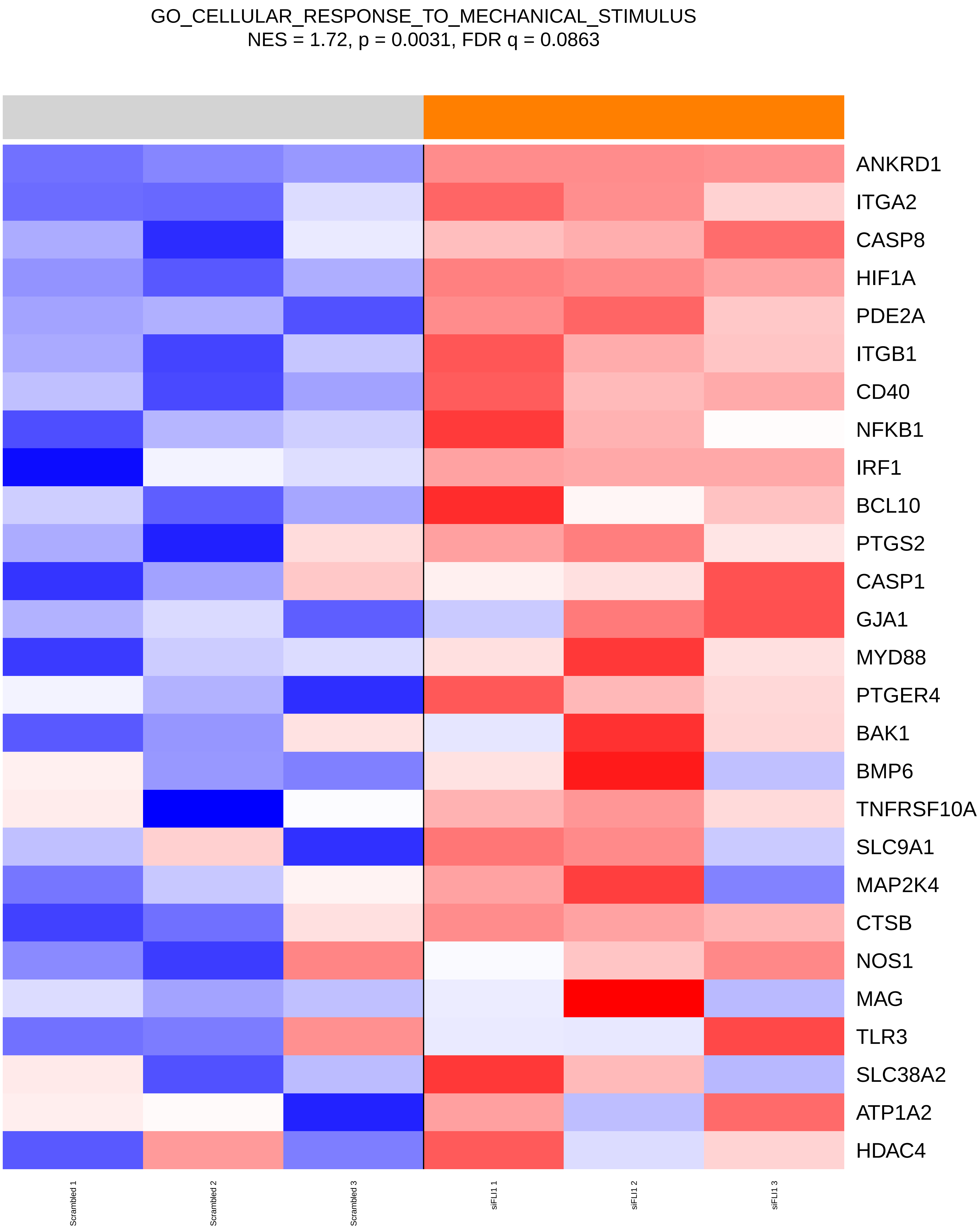
GO_CELLULAR_RESPONSE_TO_MECHANICAL_STIMULUS
NES = 1.72, p = 0.0031, FDR q = 0.0863
ANKRD1
ITGA2
CASP8
HIF1A
PDE2A
ITGB1
CD40
NFKB1
IRF1
BCL10
PTGS2
CASP1
GJA1
MYD88
PTGER4
BAK1
BMP6
TNFRSF10A
SLC9A1
MAP2K4
CTSB
NOS1
MAG
TLR3
SLC38A2
ATP1A2
HDAC4
Scrambled 1
Scrambled 2
Scrambled 3
siFLI1 1
siFLI1 2
siFLI1 3

GO_LEUKOCYTE_CHEMOTAXIS
NES = 1.72, p = 0.0033, FDR q = 0.0863

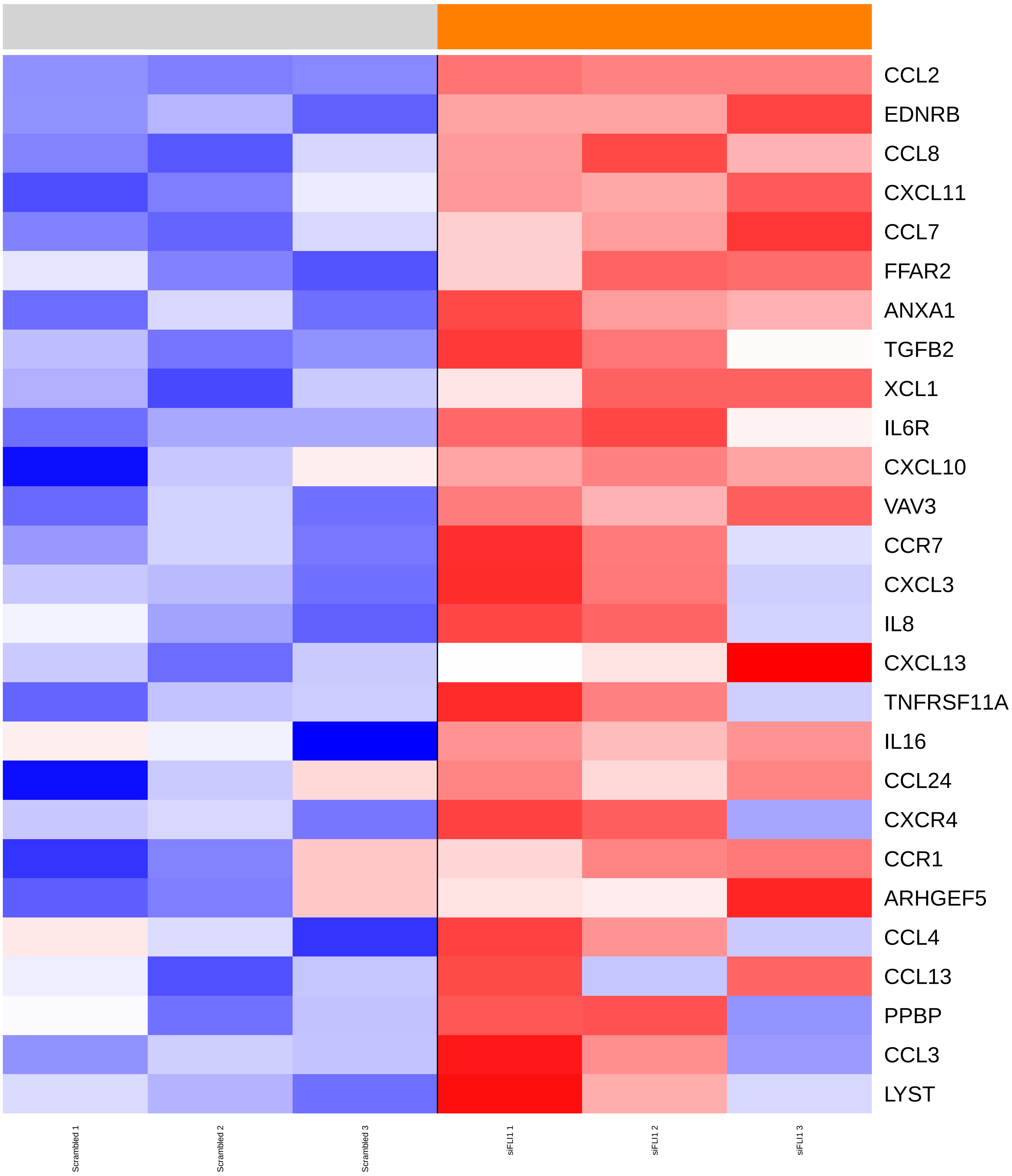

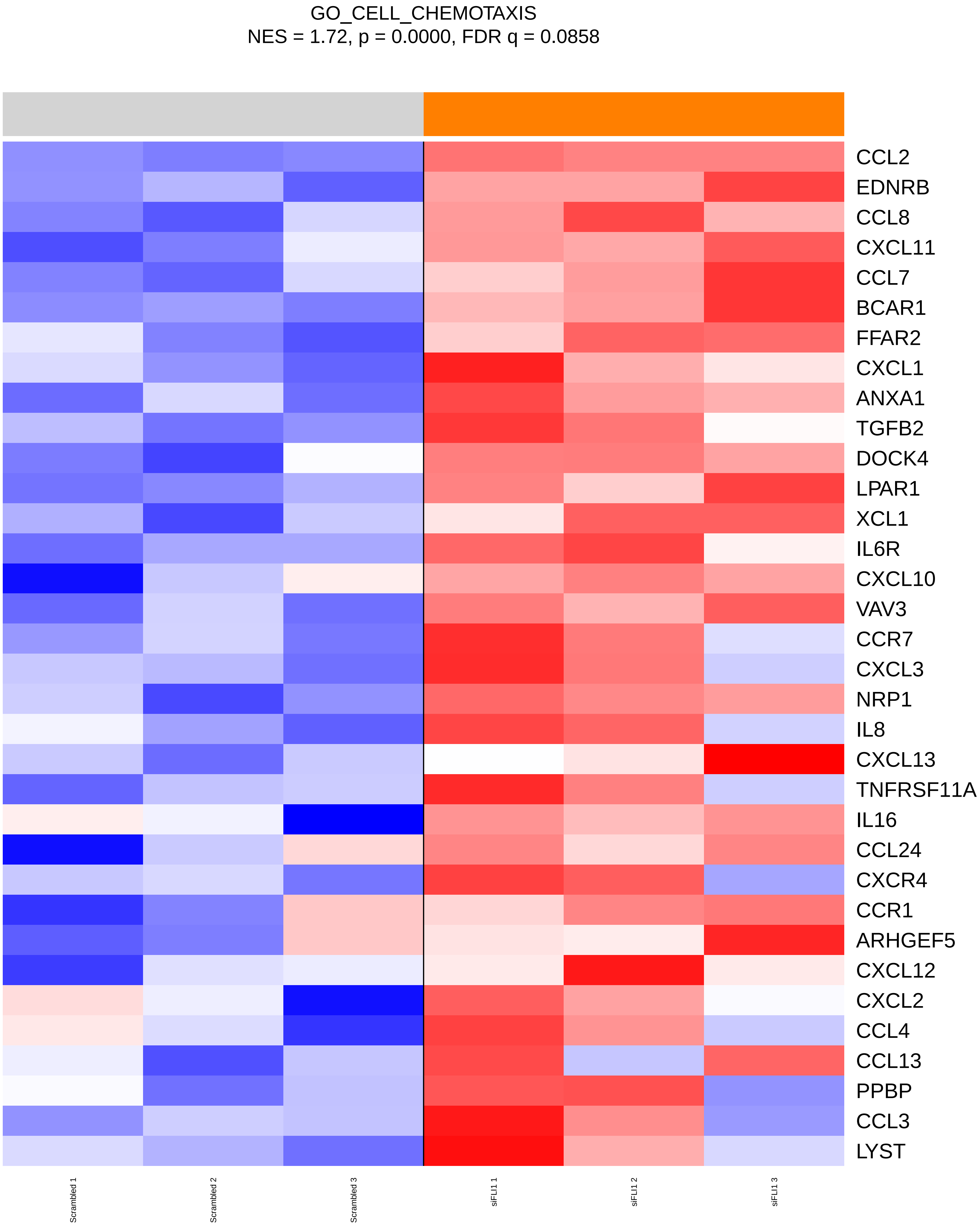
GO_CELL_CHEMOTAXIS
NES = 1.72, p = 0.0000, FDR q = 0.0858
CCL2
EDNRB
CCL8
CXCL11
CCL7
BCAR1
FFAR2
CXCL1
ANXA1
TGFB2
DOCK4
LPAR1
XCL1
IL6R
CXCL10
VAV3
CCR7
CXCL3
NRP1
IL8
CXCL13
TNFRSF11A
IL16
CCL24
CXCR4
CCR1
ARHGEF5
CXCL12
CXCL2
CCL4
CCL13
PPBP
CCL3
LYST
Scrambled 1
Scrambled 2
Scrambled 3
siFLI1 1
siFLI1 2
siFLI1 3

GO_SALIVARY_GLAND_DEVELOPMENT
NES = 1.72, p = 0.0101, FDR q = 0.0865

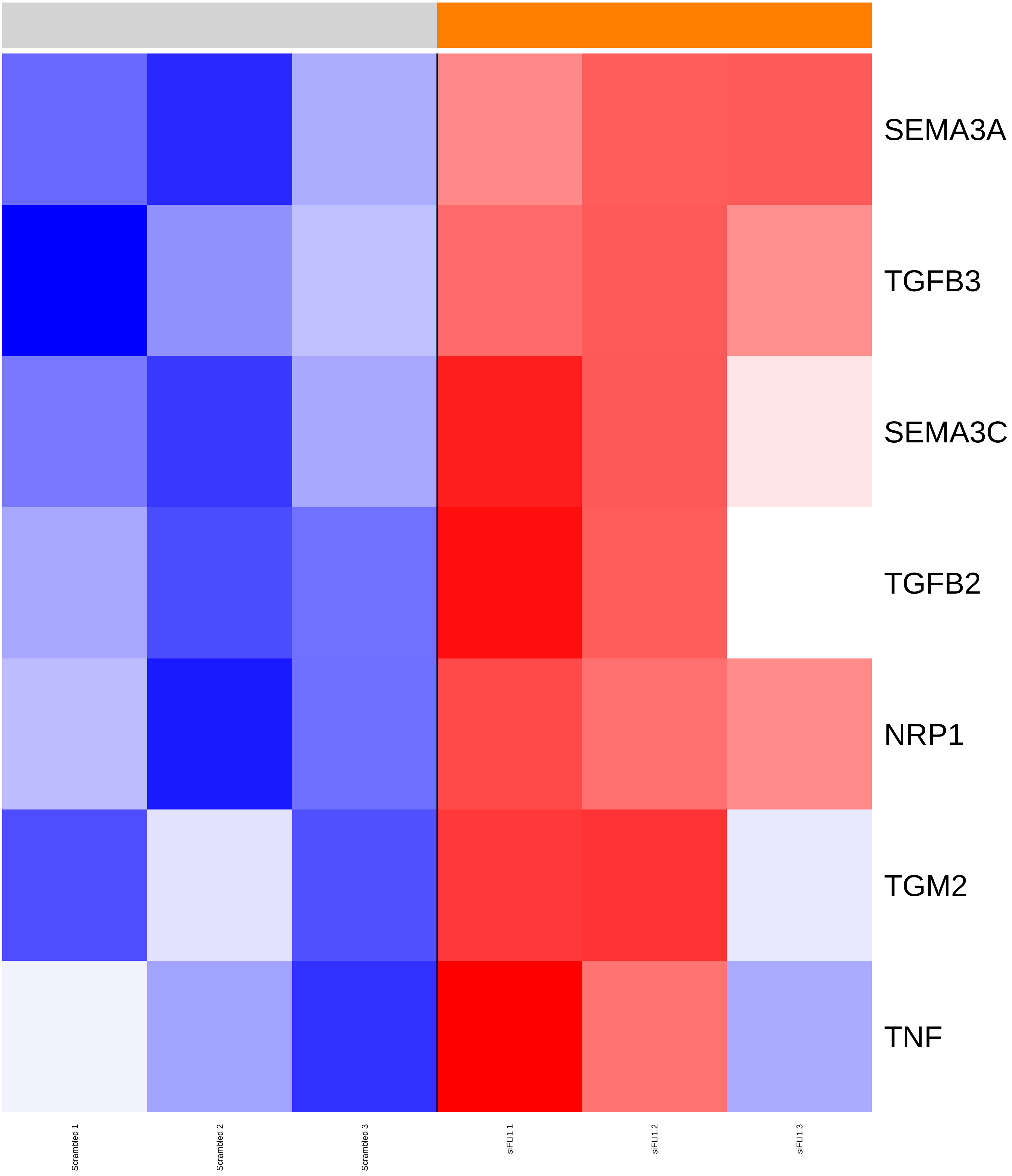

GO_POSITIVE_REGULATION_OF_CYTOKINE_PRODUCTION_INVOLVED_IN_IMMUNE_RESPONSE
NES = 1.72, p = 0.0096, FDR q = 0.0860

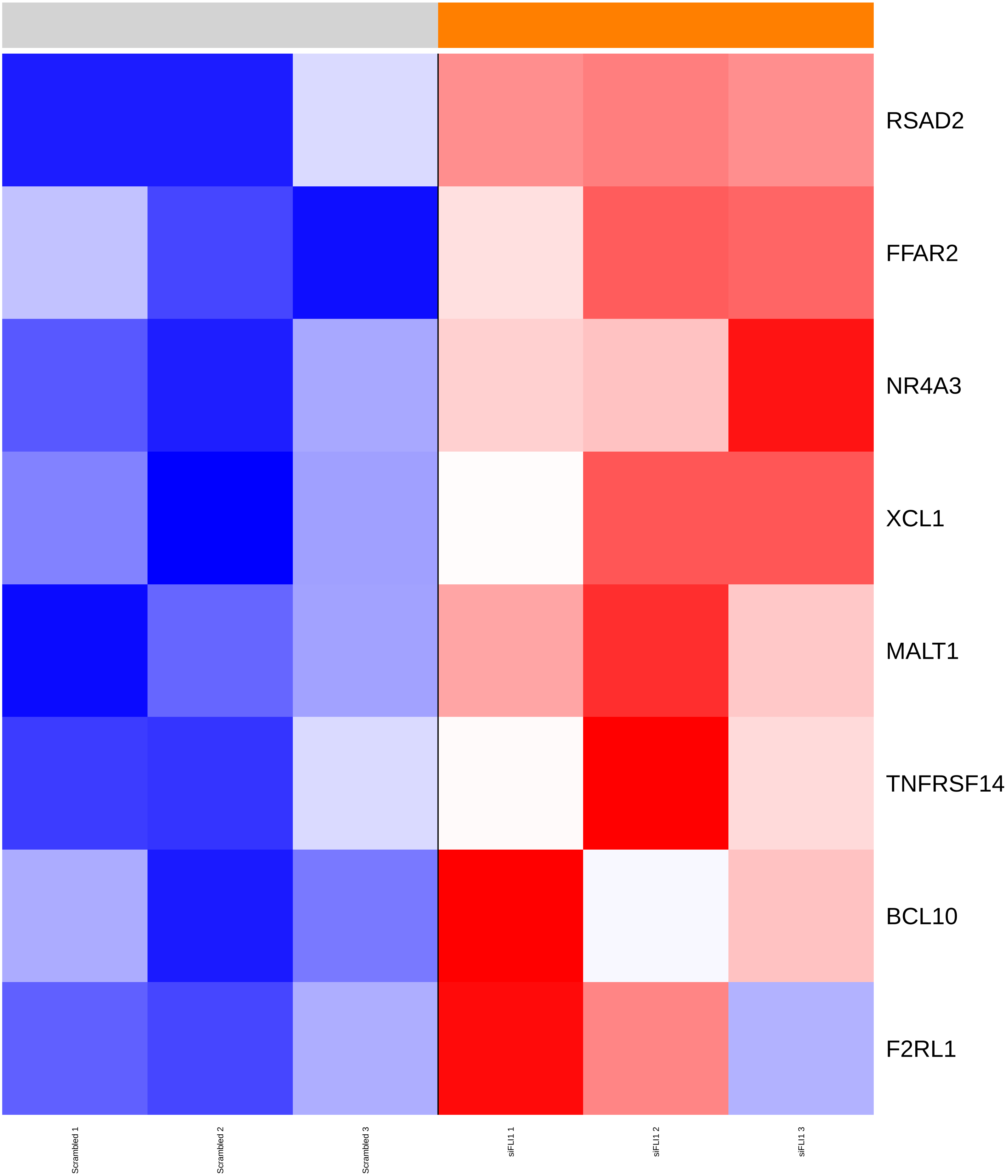

GO_HEAT_SHOCK_PROTEIN_BINDING
NES = 1.72, p = 0.0000, FDR q = 0.0861

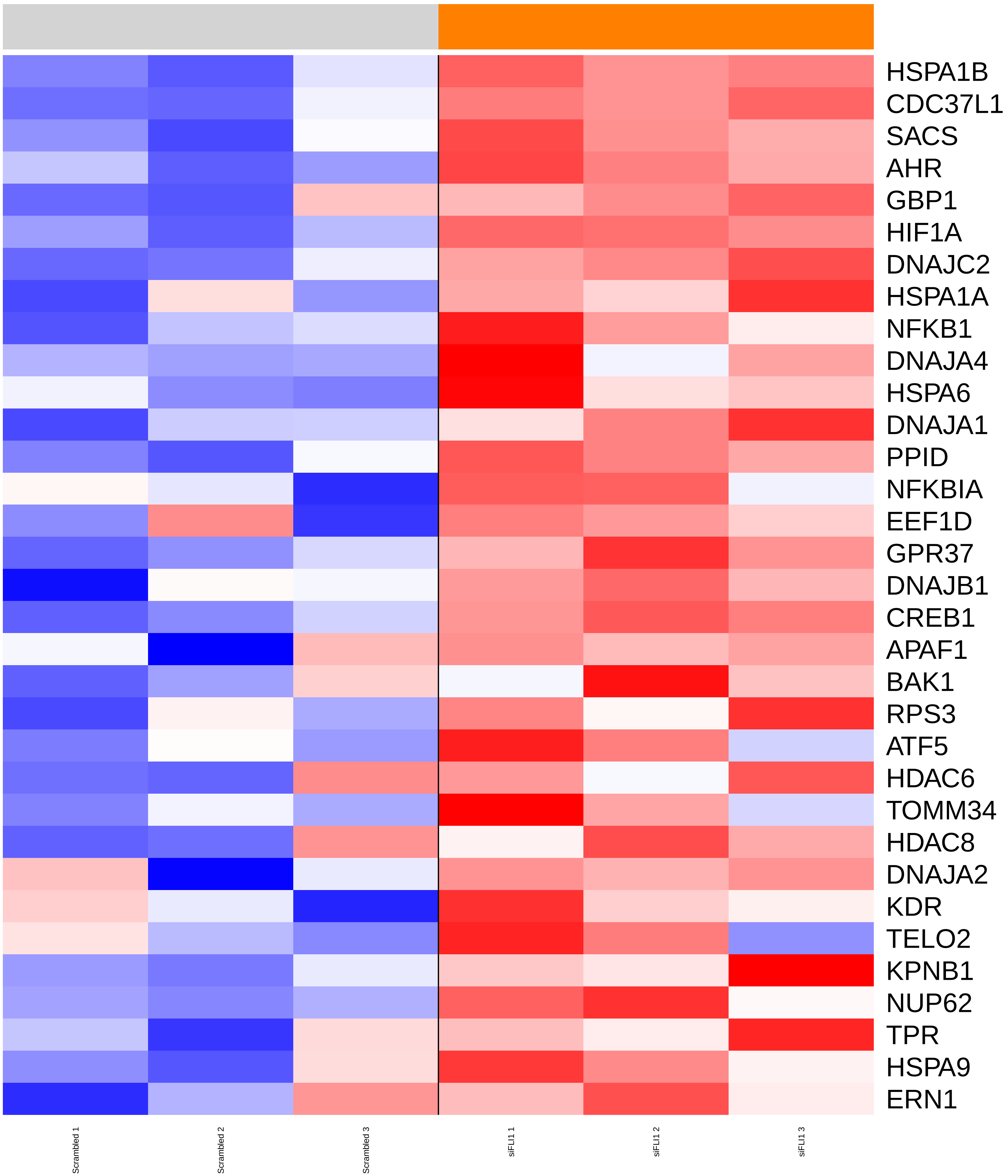

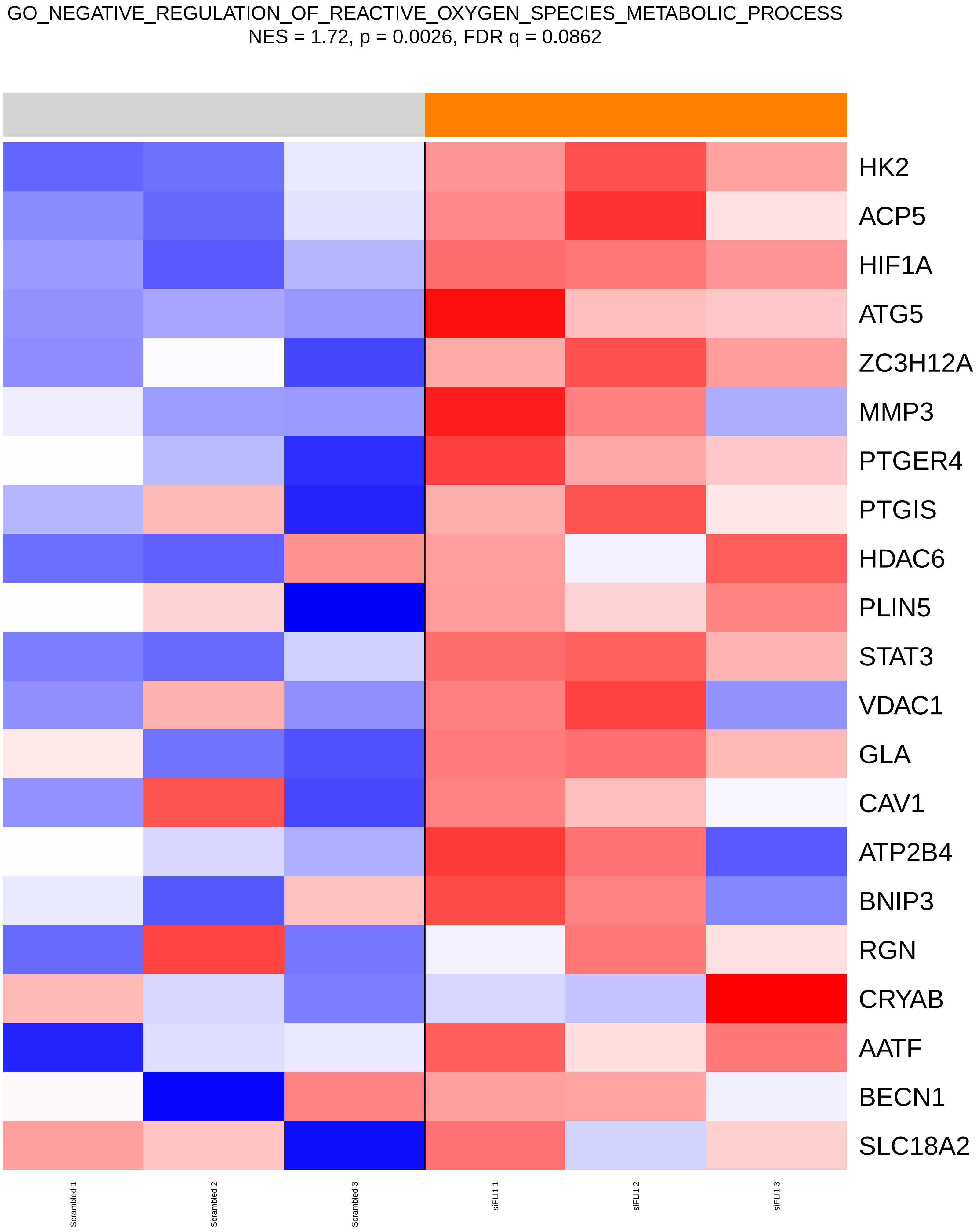
GO_NEGATIVE_REGULATION_OF_REACTIVE_OXYGEN_SPECIES_METABOLIC_PROCESS
NES = 1.72, p = 0.0026, FDR q = 0.0862
HK2
ACP5
HIF1A
ATG5
ZC3H12A
MMP3
PTGER4
PTGIS
HDAC6
PLIN5
STAT3
VDAC1
GLA
CAV1
ATP2B4
BNIP3
RGN
CRYAB
AATF
BECN1
SLC18A2
Scrambled 1
Scrambled 2
Scrambled 3
siFLI1 1
siFLI1 2
siFLI1 3

GO_CYTOPLASMIC_PATTERN_RECOGNITION_RECEPTOR_SIGNALING_PATHWAY
NES = 1.71, p = 0.0026, FDR q = 0.0878

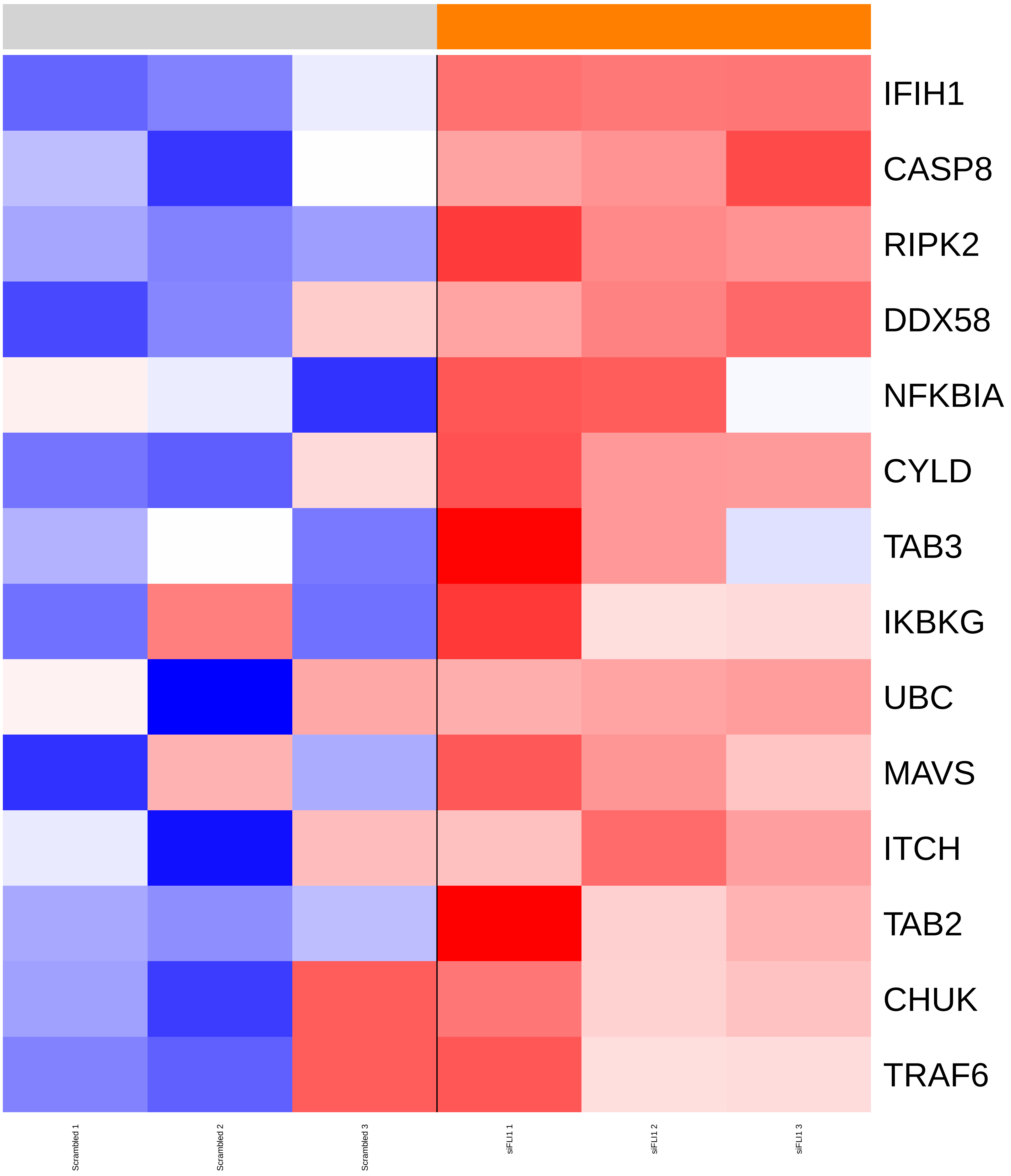

GO_ER_NUCLEUS_SIGNALING_PATHWAY
NES = 1.71, p = 0.0027, FDR q = 0.0919

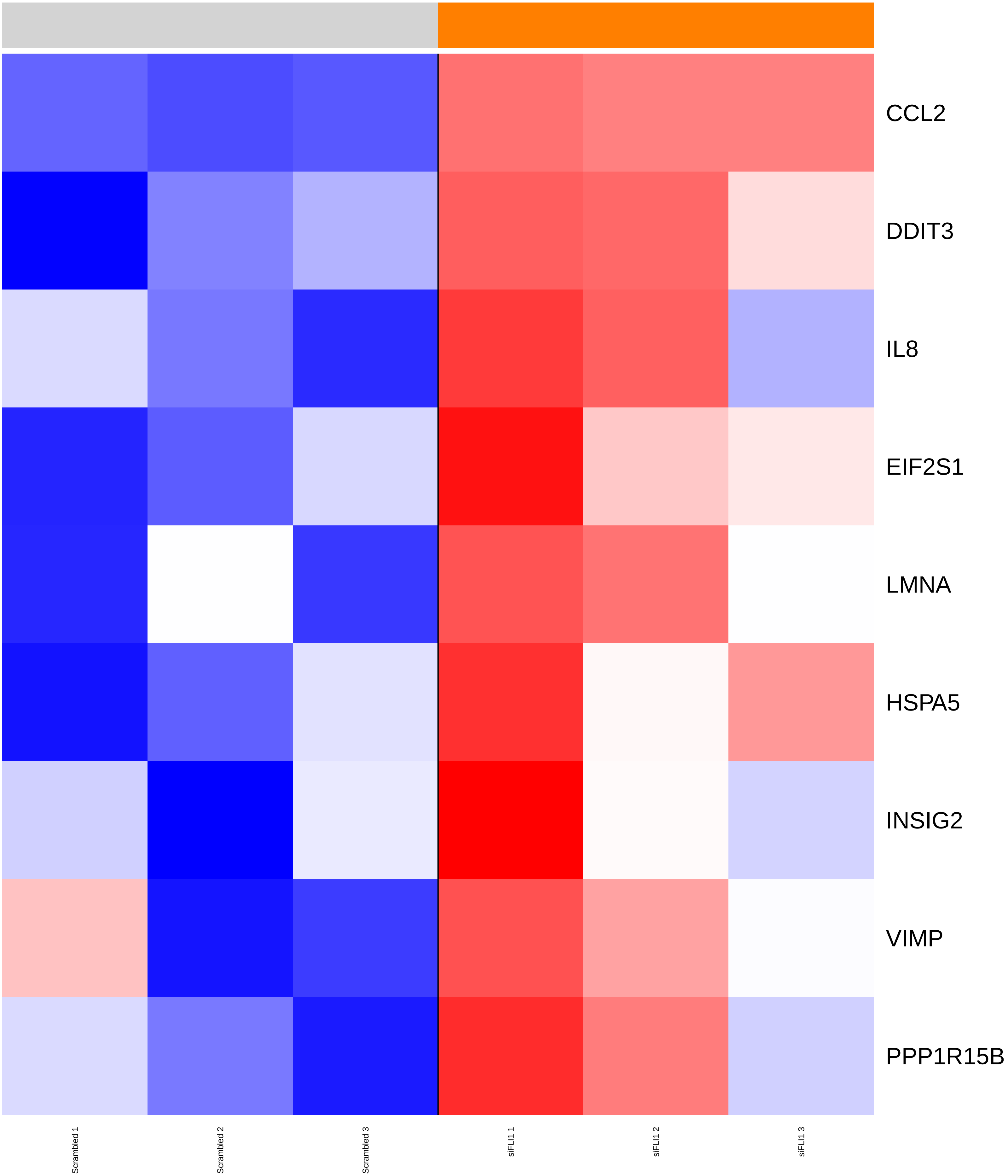

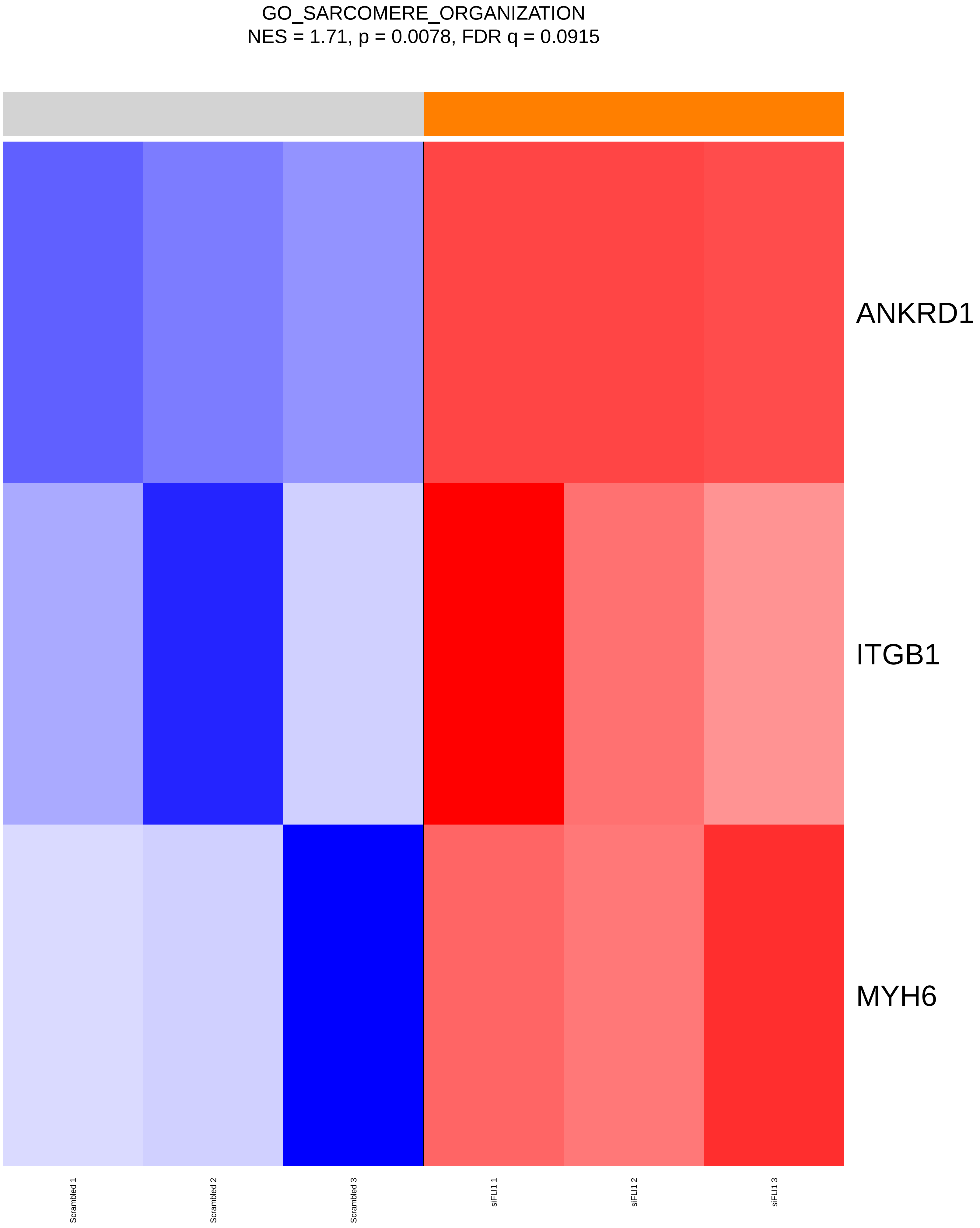
GO_SARCOMERE_ORGANIZATION
NES = 1.71, p = 0.0078, FDR q = 0.0915
ANKRD1
ITGB1
MYH6
Scrambled 1
Scrambled 2
Scrambled 3
siFLI1 1
siFLI1 2
siFLI1 3

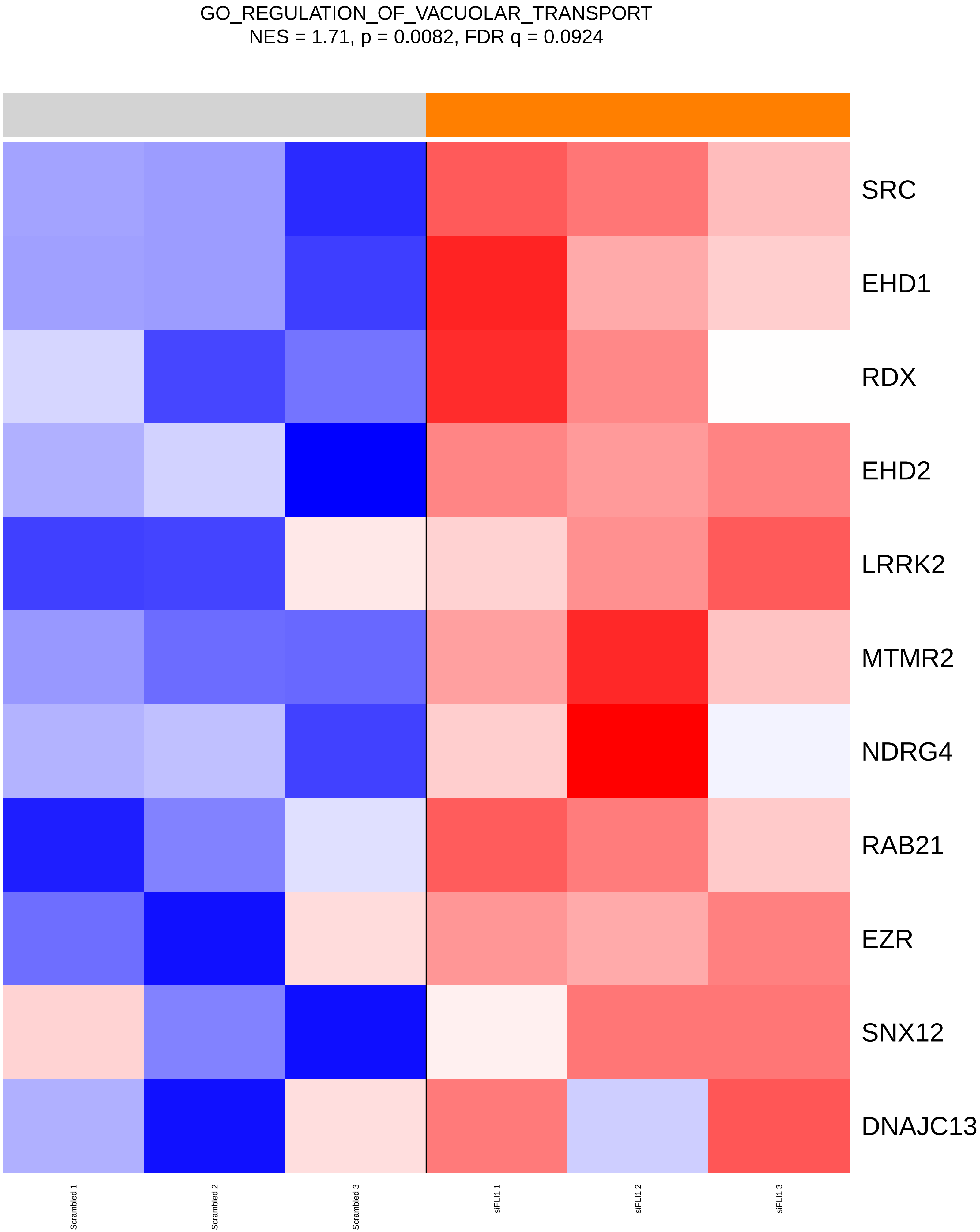
GO_REGULATION_OF_VACUOLAR_TRANSPORT
NES = 1.71, p = 0.0082, FDR q = 0.0924
SRC
EHD1
RDX
EHD2
LRRK2
MTMR2
NDRG4
RAB21
EZR
SNX12
DNAJC13
Scrambled 1
Scrambled 2
Scrambled 3
siFLI1 1
siFLI1 2
siFLI1 3

GO_RESPONSE_TO_INTERFERON_BETA
NES = 1.70, p = 0.0120, FDR q = 0.0962

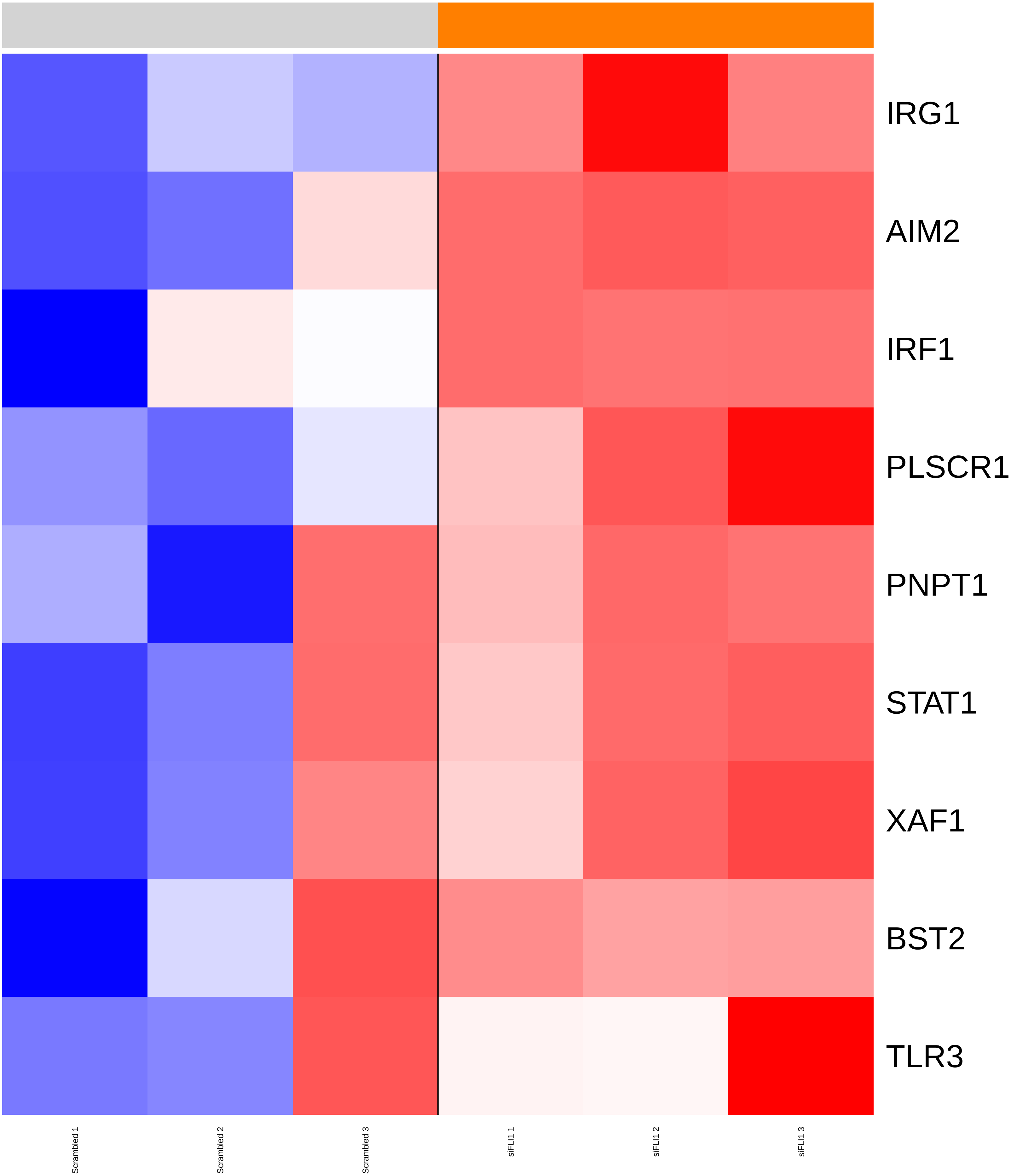

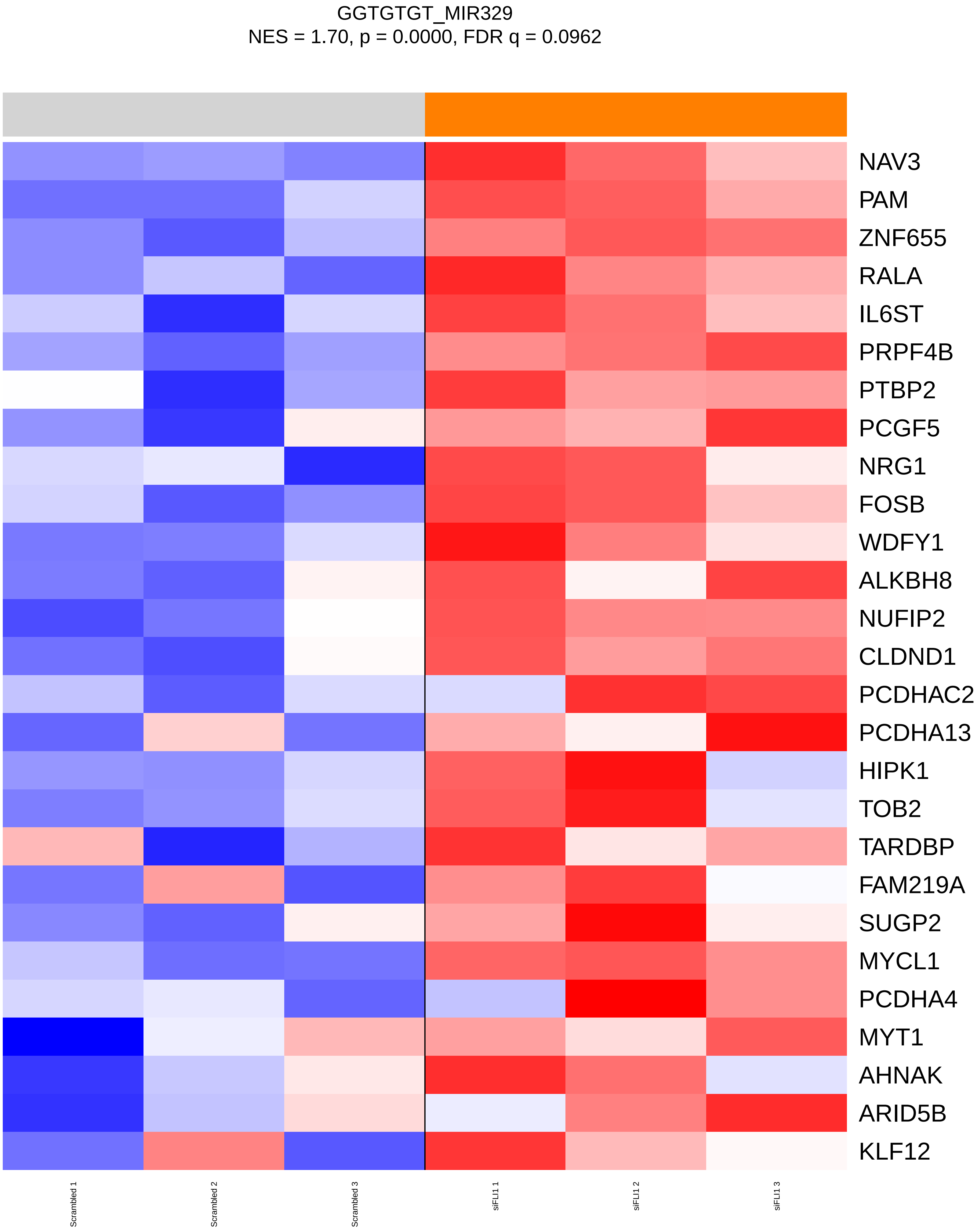
GGTGTGT_MIR329
NES = 1.70, p = 0.0000, FDR q = 0.0962
NAV3
PAM
ZNF655
RALA
IL6ST
PRPF4B
PTBP2
PCGF5
NRG1
FOSB
WDFY1
ALKBH8
NUFIP2
CLDND1
PCDHAC2
PCDHA13
HIPK1
TOB2
TARDBP
FAM219A
SUGP2
MYCL1
PCDHA4
MYT1
AHNAK
ARID5B
KLF12
Scrambled 1
Scrambled 2
Scrambled 3
siFLI1 1
siFLI1 2
siFLI1 3

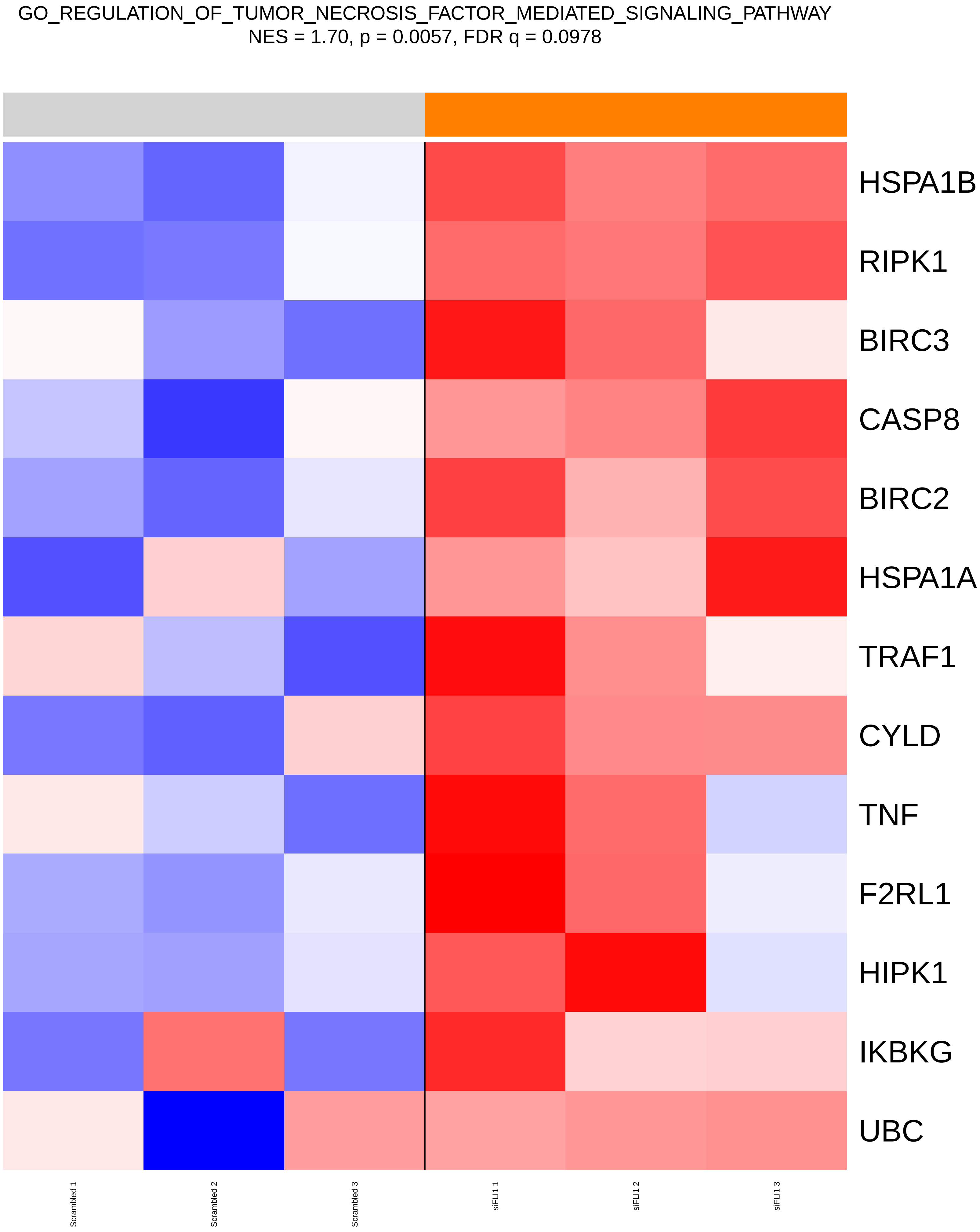
GO_REGULATION_OF_TUMOR_NECROSIS_FACTOR_MEDIATED_SIGNALING_PATHWAY
NES = 1.70, p = 0.0057, FDR q = 0.0978
HSPA1B
RIPK1
BIRC3
CASP8
BIRC2
HSPA1A
TRAF1
CYLD
TNF
F2RL1
HIPK1
IKBKG
UBC
Scrambled 1
Scrambled 2
Scrambled 3
siFLI1 1
siFLI1 2
siFLI1 3

GO_MYD88_INDEPENDENT_TOLL_LIKE_RECEPTOR_SIGNALING_PATHWAY
NES = 1.70, p = 0.0054, FDR q = 0.0983

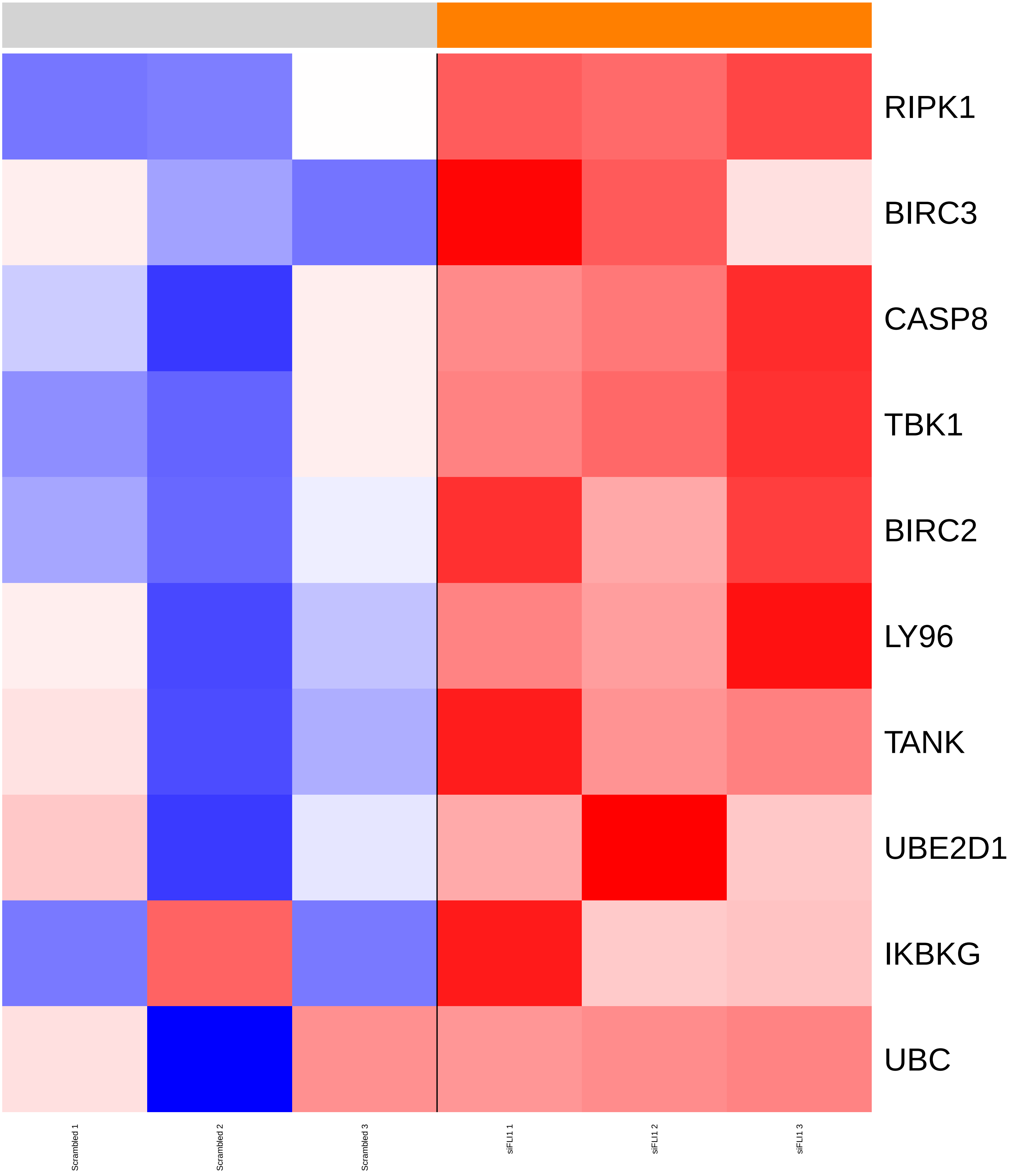

GO_POSITIVE_REGULATION_OF_LOCOMOTION
NES = 1.69, p = 0.0000, FDR q = 0.1044

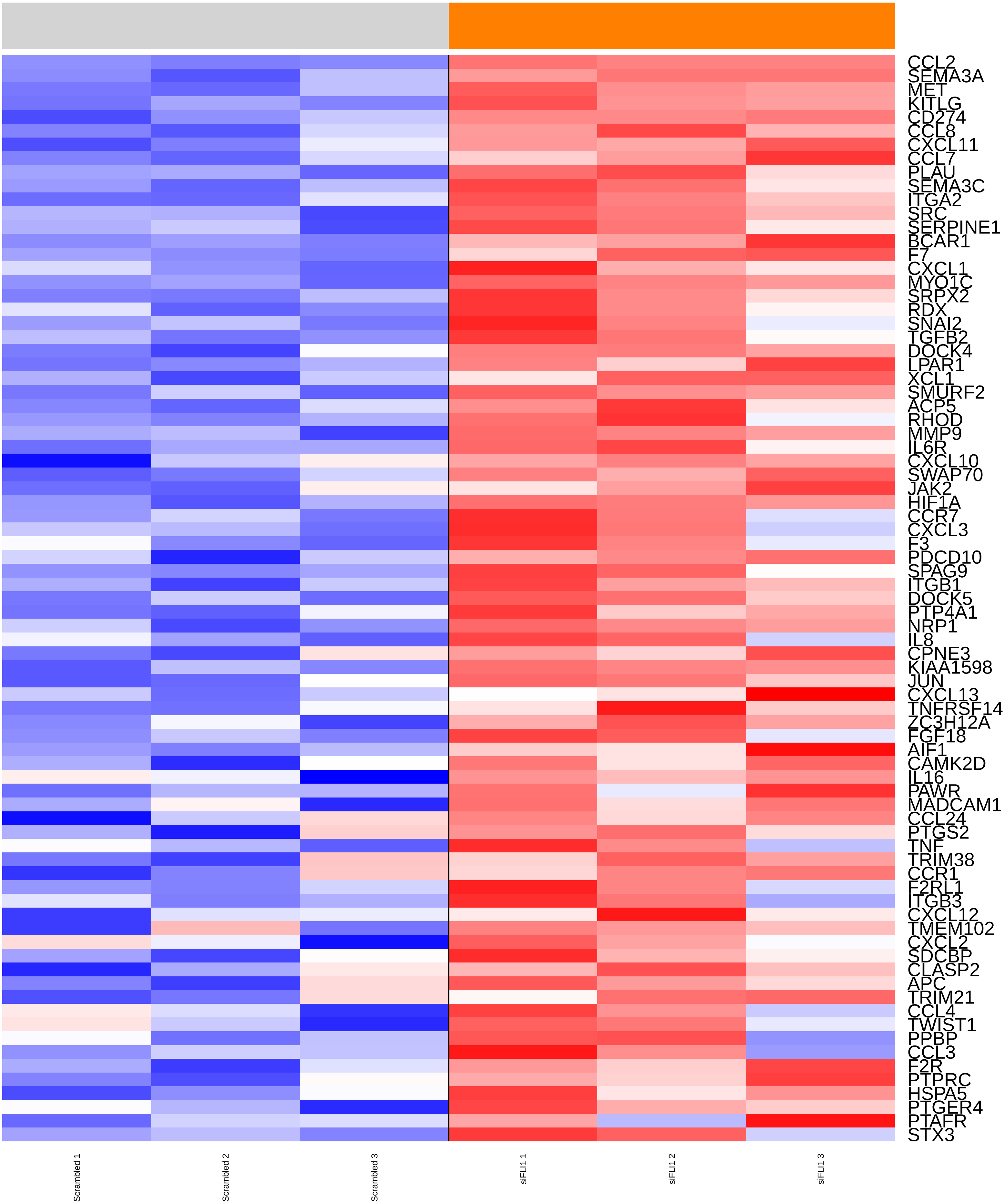

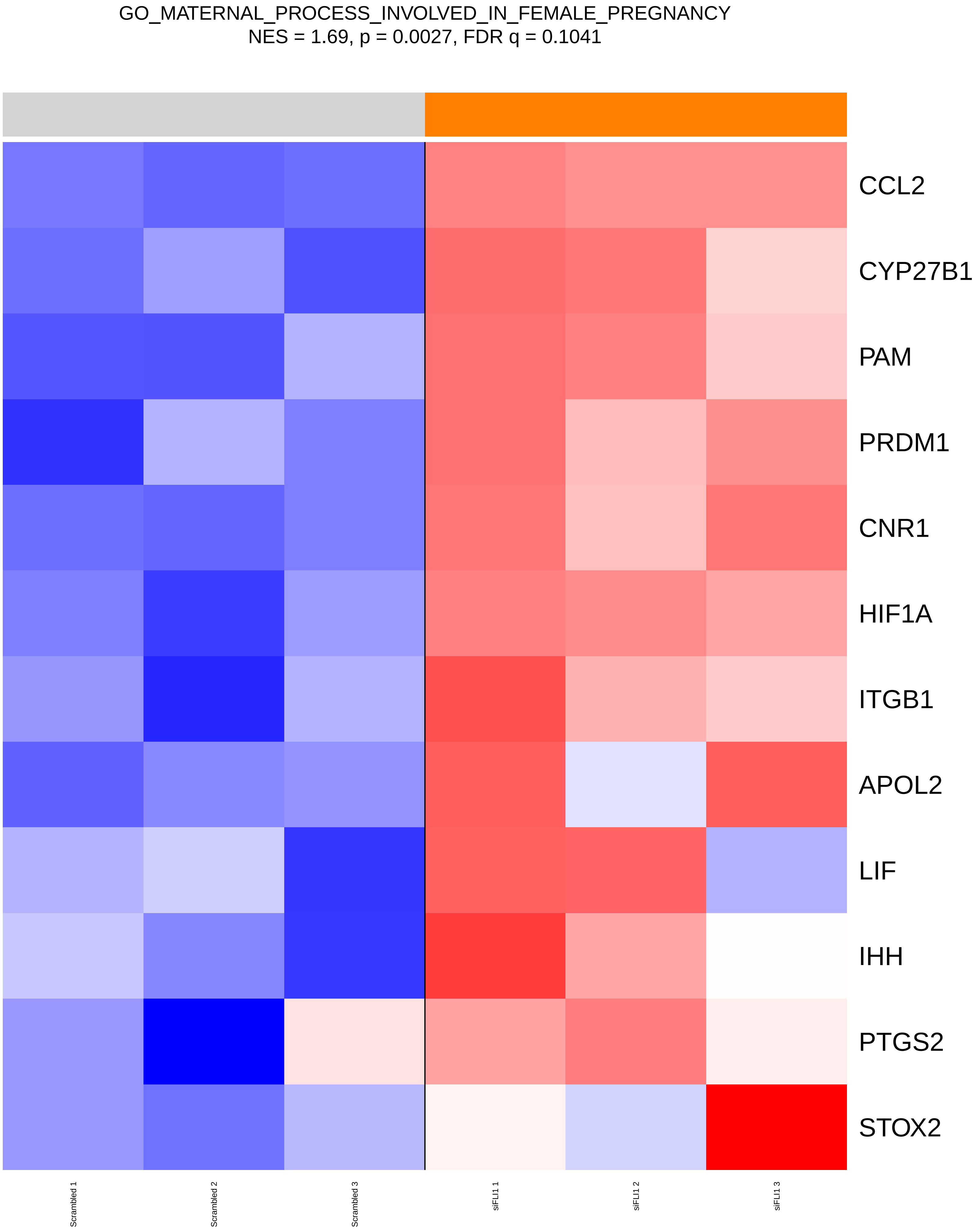
GO_MATERNAL_PROCESS_INVOLVED_IN_FEMALE_PREGNANCY
NES = 1.69, p = 0.0027, FDR q = 0.1041
CCL2
CYP27B1
PAM
PRDM1
CNR1
HIF1A
ITGB1
APOL2
LIF
IHH
PTGS2
STOX2
Scrambled 1
Scrambled 2
Scrambled 3
siFLI1 1
siFLI1 2
siFLI1 3

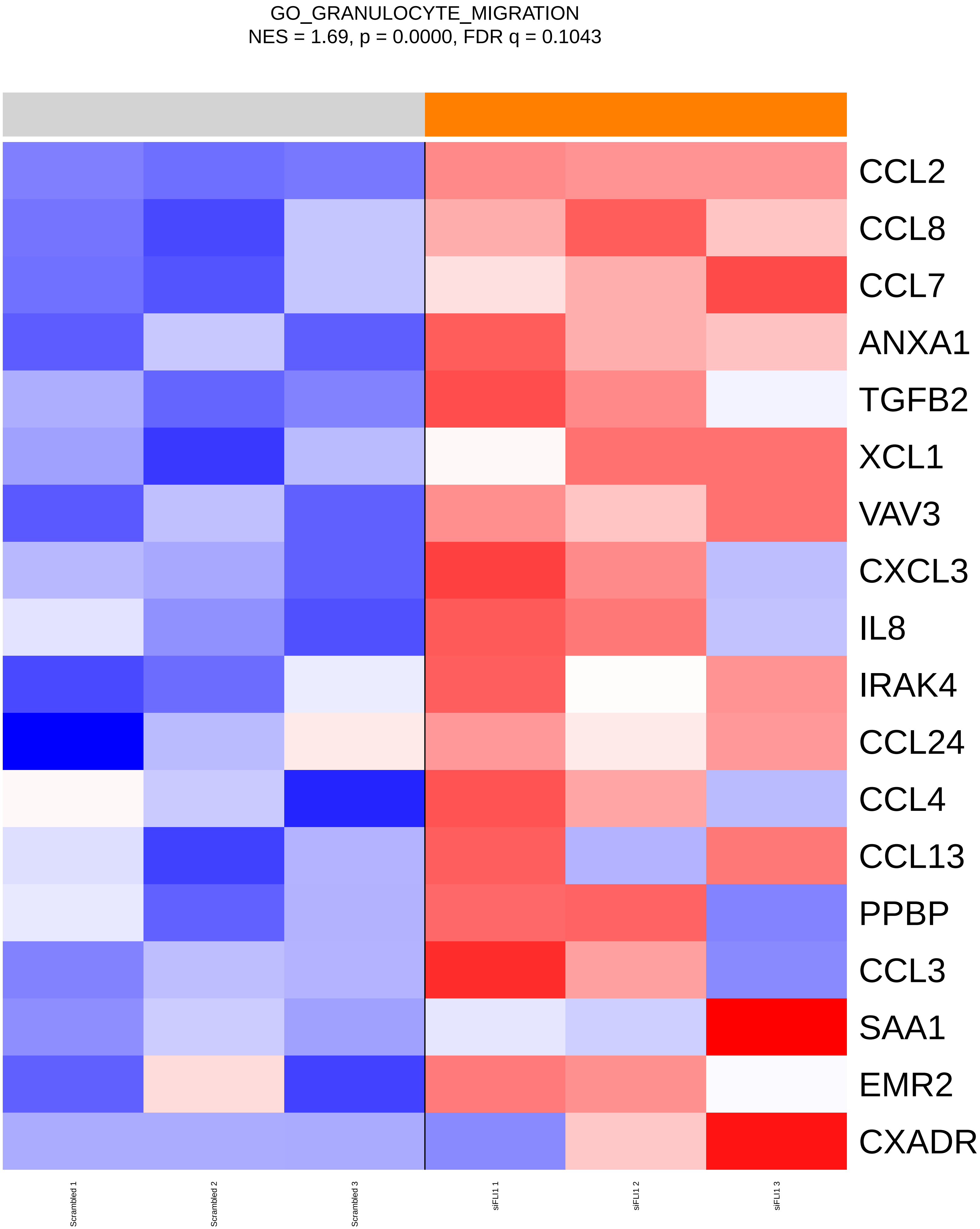
GO_GRANULOCYTE_MIGRATION
NES = 1.69, p = 0.0000, FDR q = 0.1043
CCL2
CCL8
CCL7
ANXA1
TGFB2
XCL1
VAV3
CXCL3
IL8
IRAK4
CCL24
CCL4
CCL13
PPBP
CCL3
SAA1
EMR2
CXADR
Scrambled 1
Scrambled 2
Scrambled 3
siFLI1 1
siFLI1 2
siFLI1 3

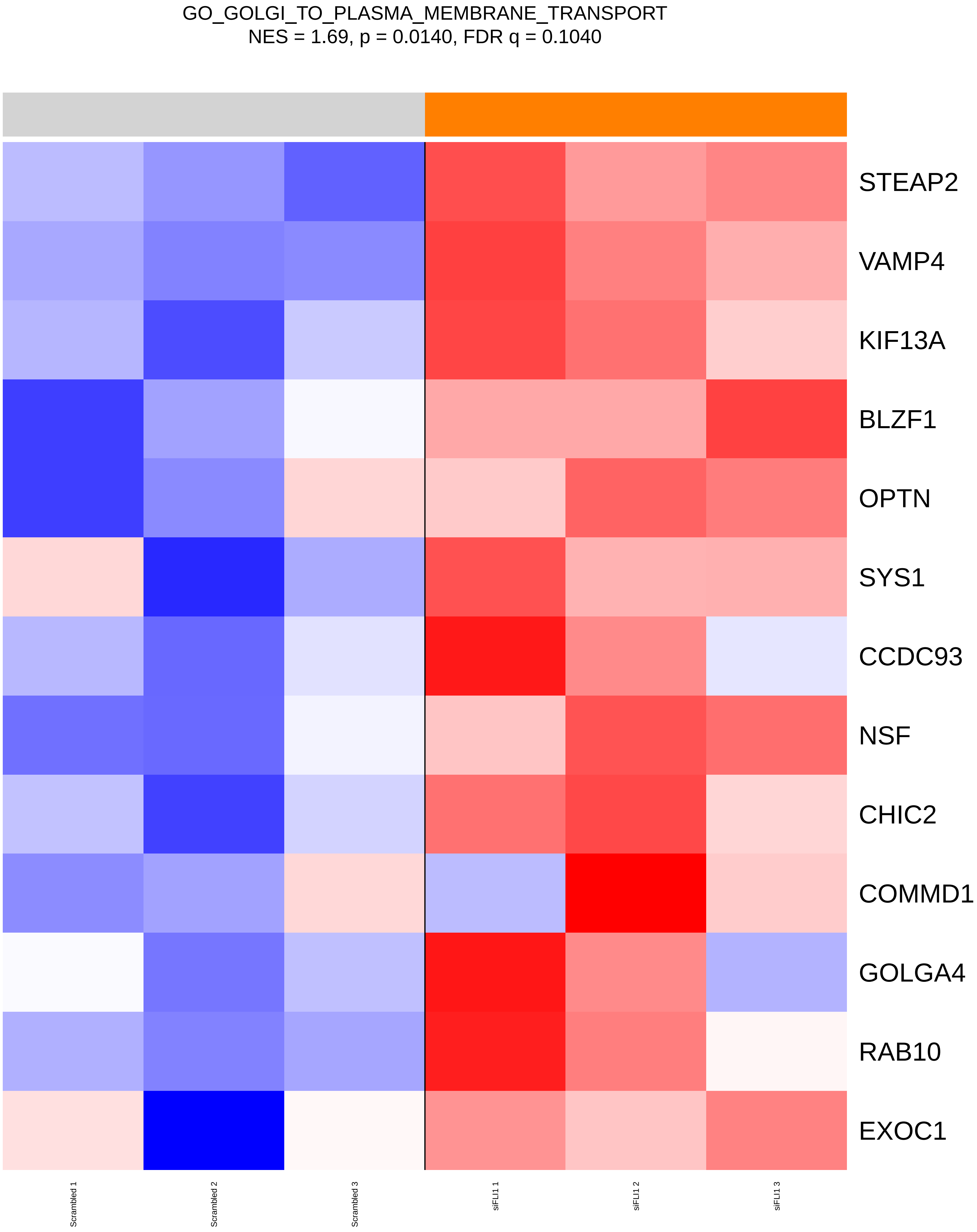
GO_GOLGI_TO_PLASMA_MEMBRANE_TRANSPORT
NES = 1.69, p = 0.0140, FDR q = 0.1040
STEAP2
VAMP4
KIF13A
BLZF1
OPTN
SYS1
CCDC93
NSF
CHIC2
COMMD1
GOLGA4
RAB10
EXOC1
Scrambled 1
Scrambled 2
Scrambled 3
siFLI1 1
siFLI1 2
siFLI1 3

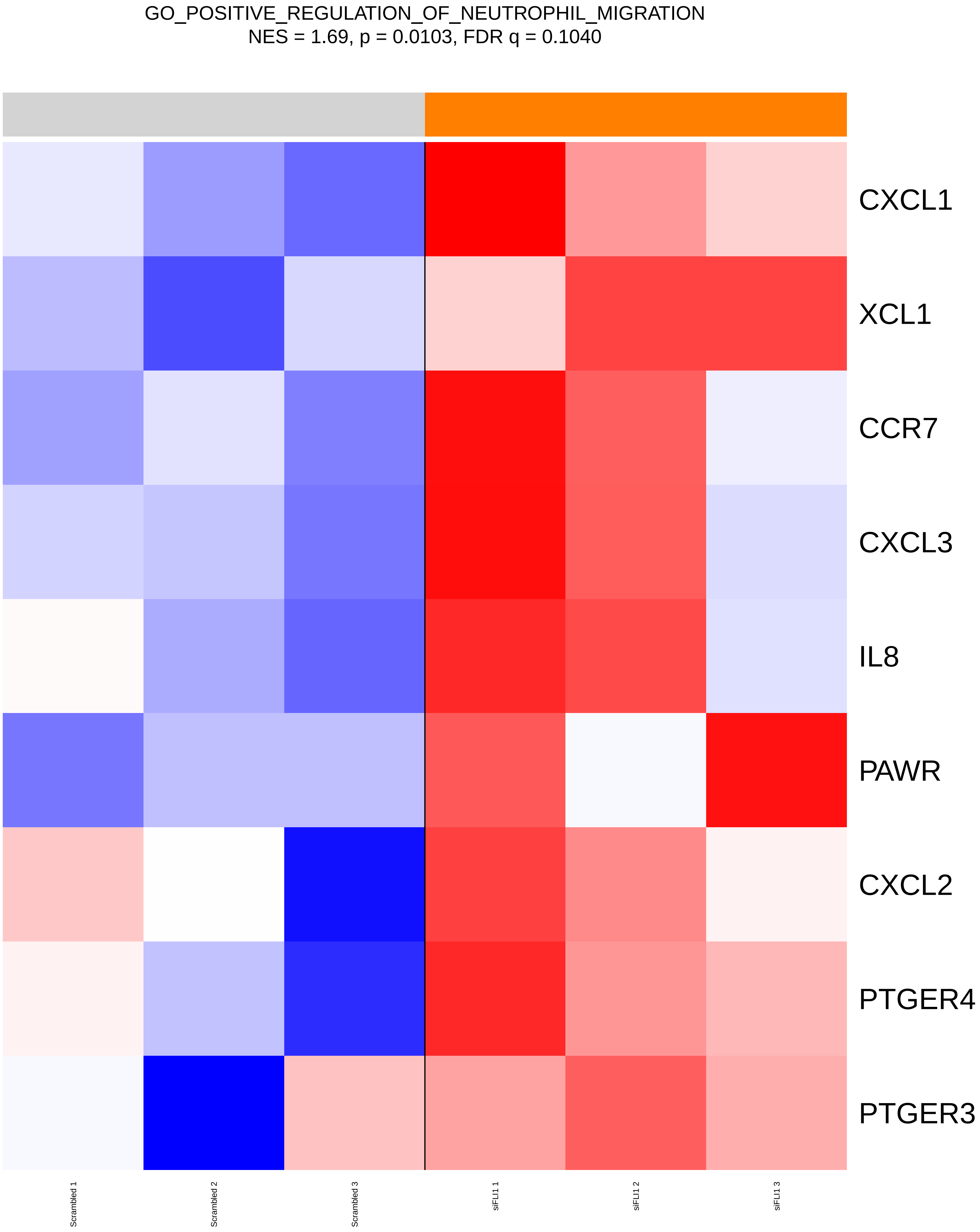
GO_POSITIVE_REGULATION_OF_NEUTROPHIL_MIGRATION
NES = 1.69, p = 0.0103, FDR q = 0.1040
CXCL1
XCL1
CCR7
CXCL3
IL8
PAWR
CXCL2
PTGER4
PTGER3
Scrambled 1
Scrambled 2
Scrambled 3
siFLI1 1
siFLI1 2
siFLI1 3

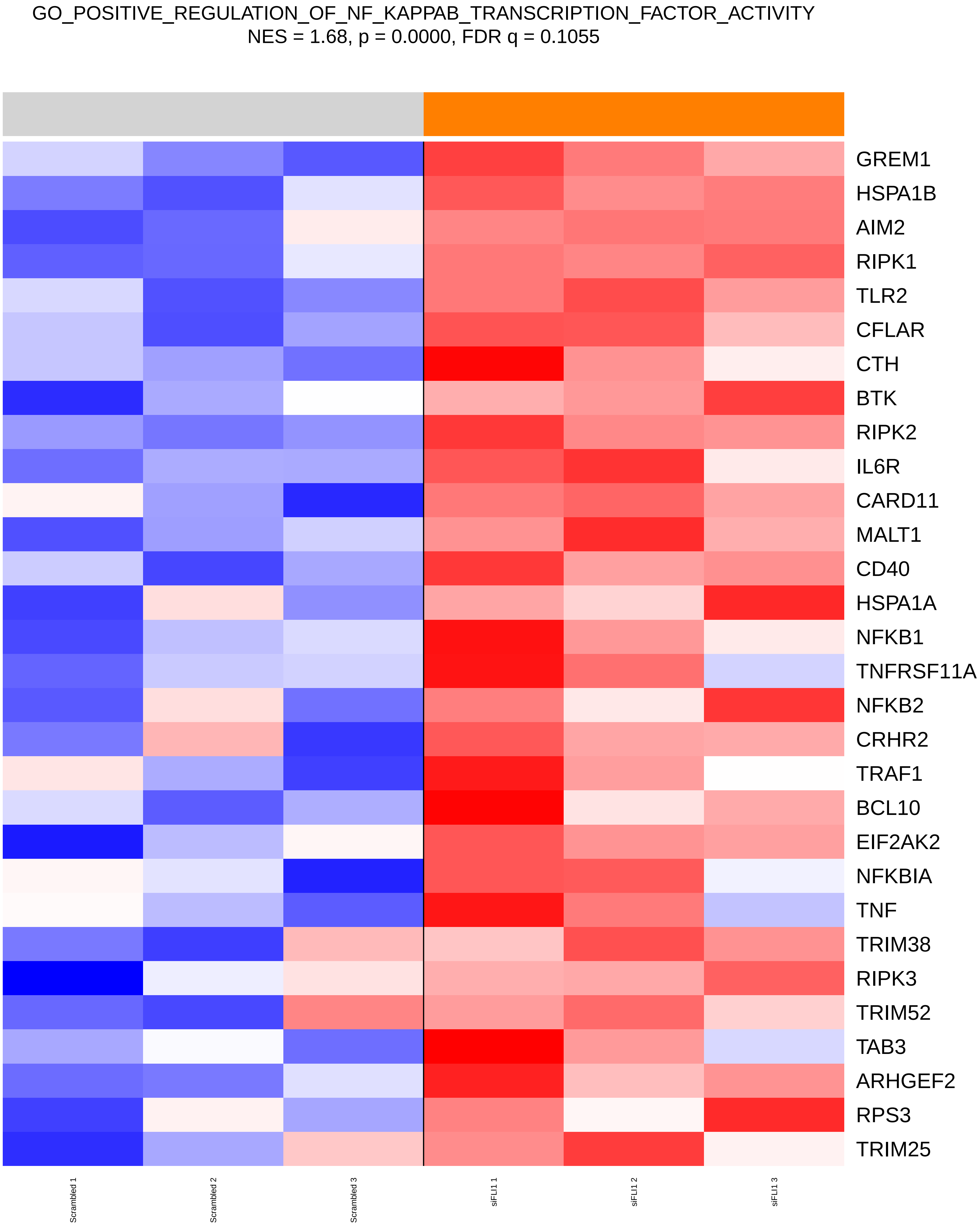
GO_POSITIVE_REGULATION_OF_NF_KAPPAB_TRANSCRIPTION_FACTOR_ACTIVITY
NES = 1.68, p = 0.0000, FDR q = 0.1055
GREM1
HSPA1B
AIM2
RIPK1
TLR2
CFLAR
CTH
BTK
RIPK2
IL6R
CARD11
MALT1
CD40
HSPA1A
NFKB1
TNFRSF11A
NFKB2
CRHR2
TRAF1
BCL10
EIF2AK2
NFKBIA
TNF
TRIM38
RIPK3
TRIM52
TAB3
ARHGEF2
RPS3
TRIM25
Scrambled 1
Scrambled 2
Scrambled 3
siFLI1 1
siFLI1 2
siFLI1 3

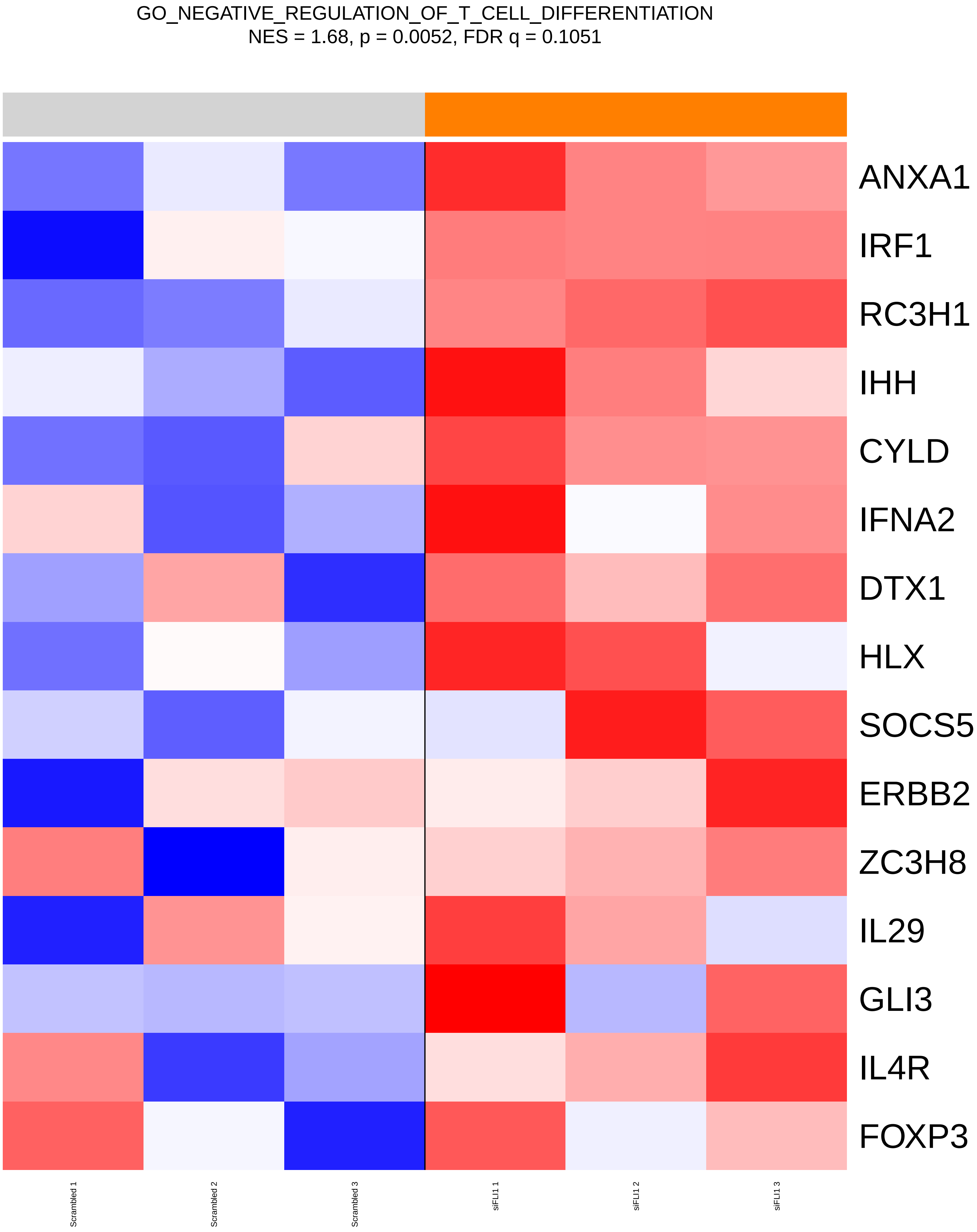
GO_NEGATIVE_REGULATION_OF_T_CELL_DIFFERENTIATION
NES = 1.68, p = 0.0052, FDR q = 0.1051
ANXA1
IRF1
RC3H1
IHH
CYLD
IFNA2
DTX1
HLX
SOCS5
ERBB2
ZC3H8
IL29
GLI3
IL4R
FOXP3
Scrambled 1
Scrambled 2
Scrambled 3
siFLI1 1
siFLI1 2
siFLI1 3

GO_REGULATION_OF_BONE_REMODELING
NES = 1.68, p = 0.0081, FDR q = 0.1058

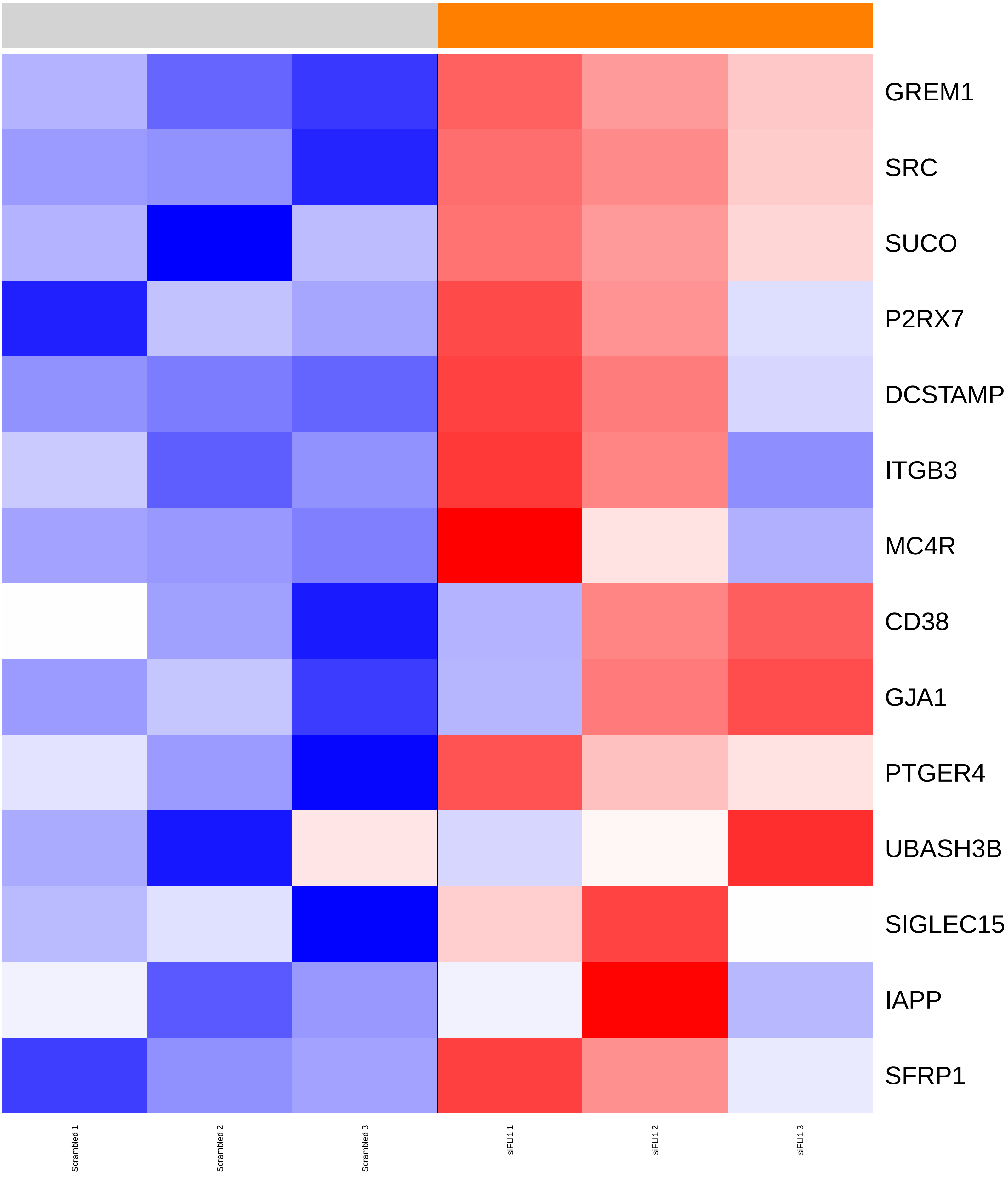

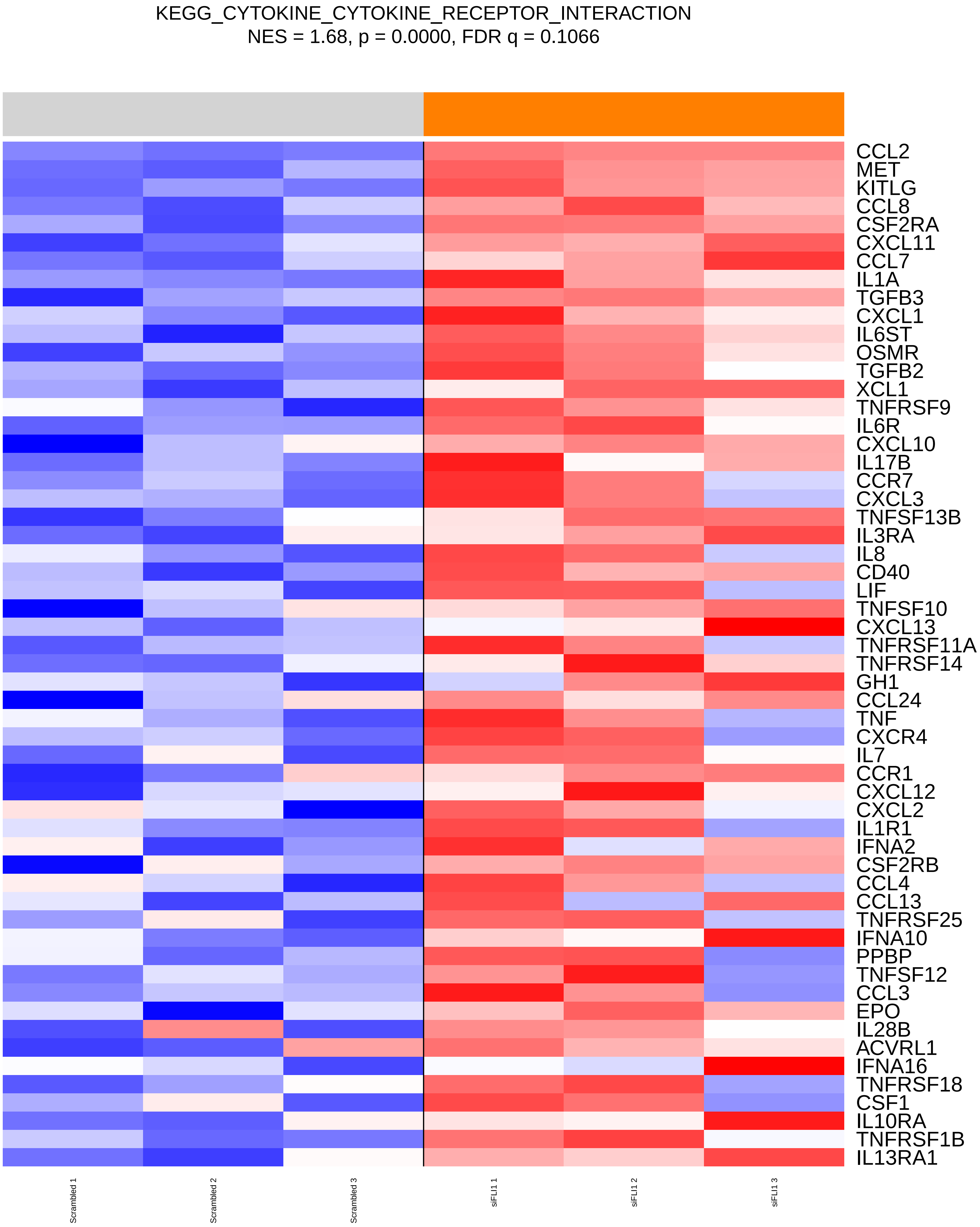
KEGG_CYTOKINE_CYTOKINE_RECEPTOR_INTERACTION
NES = 1.68, p = 0.0000, FDR q = 0.1066
CCL2
MET
KITLG
CCL8
CSF2RA
CXCL11
CCL7
IL1A
TGFB3
CXCL1
IL6ST
OSMR
TGFB2
XCL1
TNFRSF9
IL6R
CXCL10
IL17B
CCR7
CXCL3
TNFSF13B
IL3RA
IL8
CD40
LIF
TNFSF10
CXCL13
TNFRSF11A
TNFRSF14
GH1
CCL24
TNF
CXCR4
IL7
CCR1
CXCL12
CXCL2
IL1R1
IFNA2
CSF2RB
CCL4
CCL13
TNFRSF25
IFNA10
PPBP
TNFSF12
CCL3
EPO
IL28B
ACVRL1
IFNA16
TNFRSF18
CSF1
IL10RA
TNFRSF1B
IL13RA1
Scrambled 1
Scrambled 2
Scrambled 3
siFLI1 1
siFLI1 2
siFLI1 3

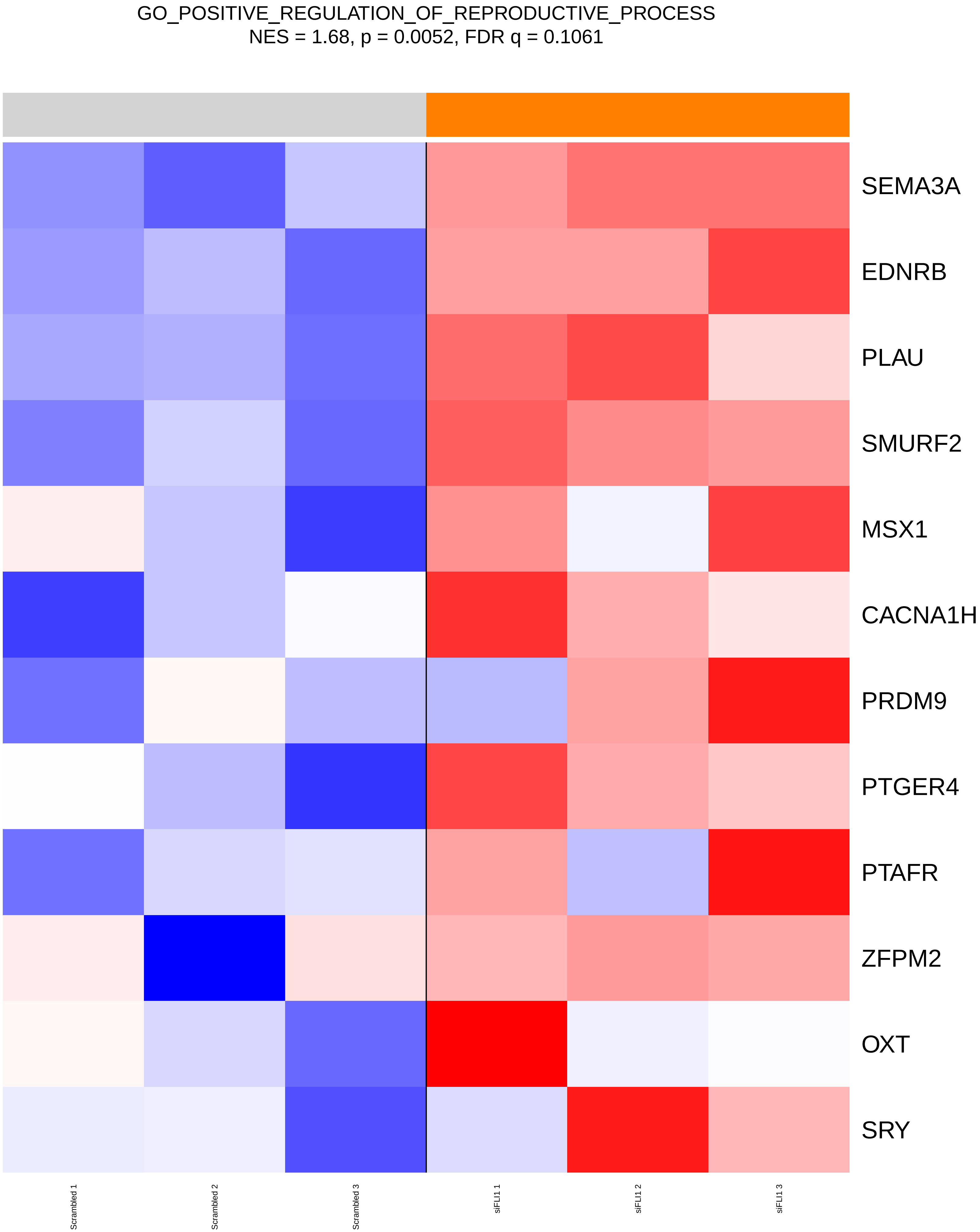

GO_POSITIVE_REGULATION_OF_REPRODUCTIVE_PROCESS
NES = 1.68, p = 0.0052, FDR q = 0.1061
SEMA3A
EDNRB
PLAU
SMURF2
MSX1
CACNA1H
PRDM9
PTGER4
PTAFR
ZFPM2
OXT
SRY
Scrambled 1
Scrambled 2
Scrambled 3
siFLI1 1
siFLI1 2
siFLI1 3

GO_VESICLE_DOCKING
NES = 1.68, p = 0.0028, FDR q = 0.1055

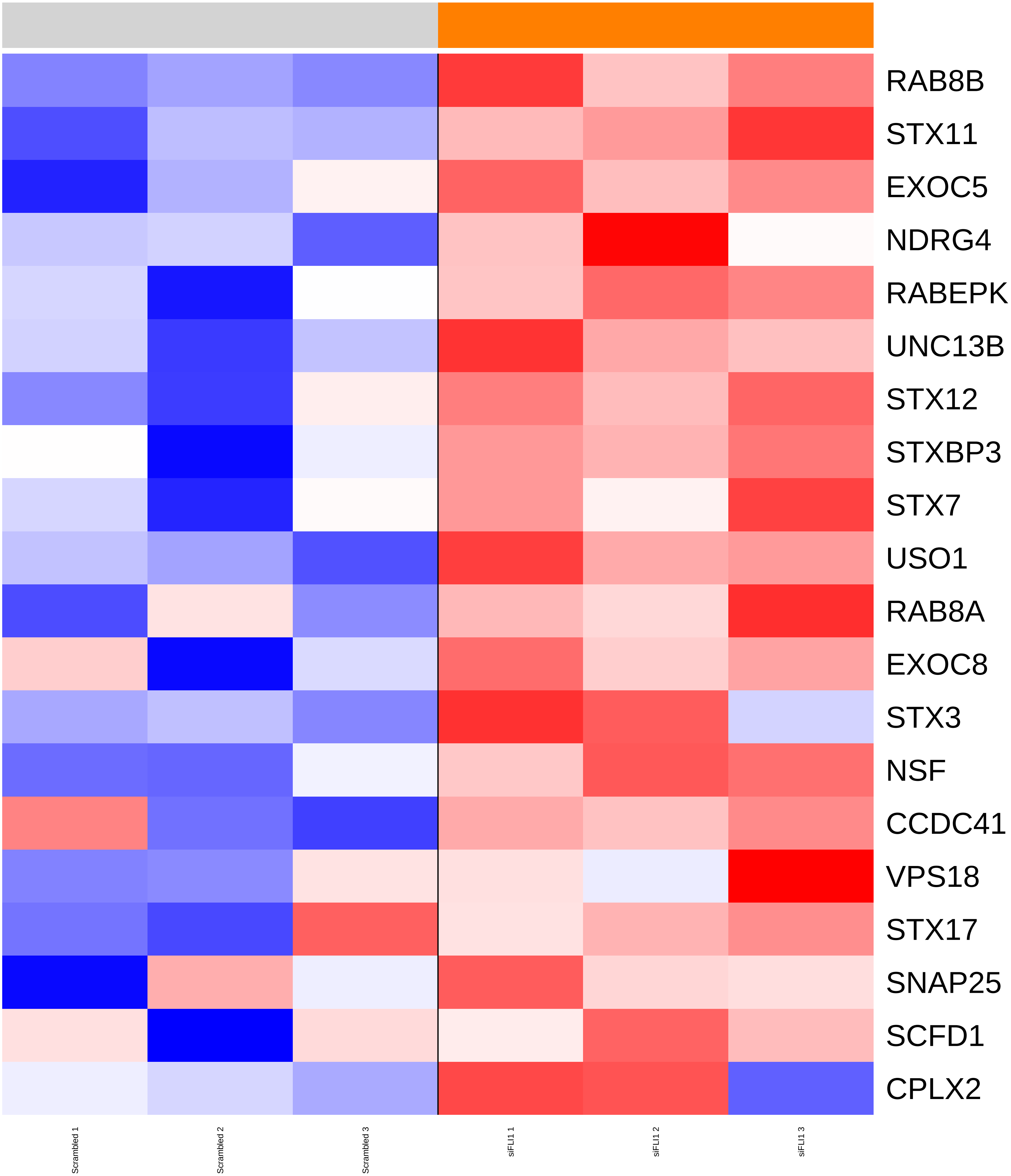

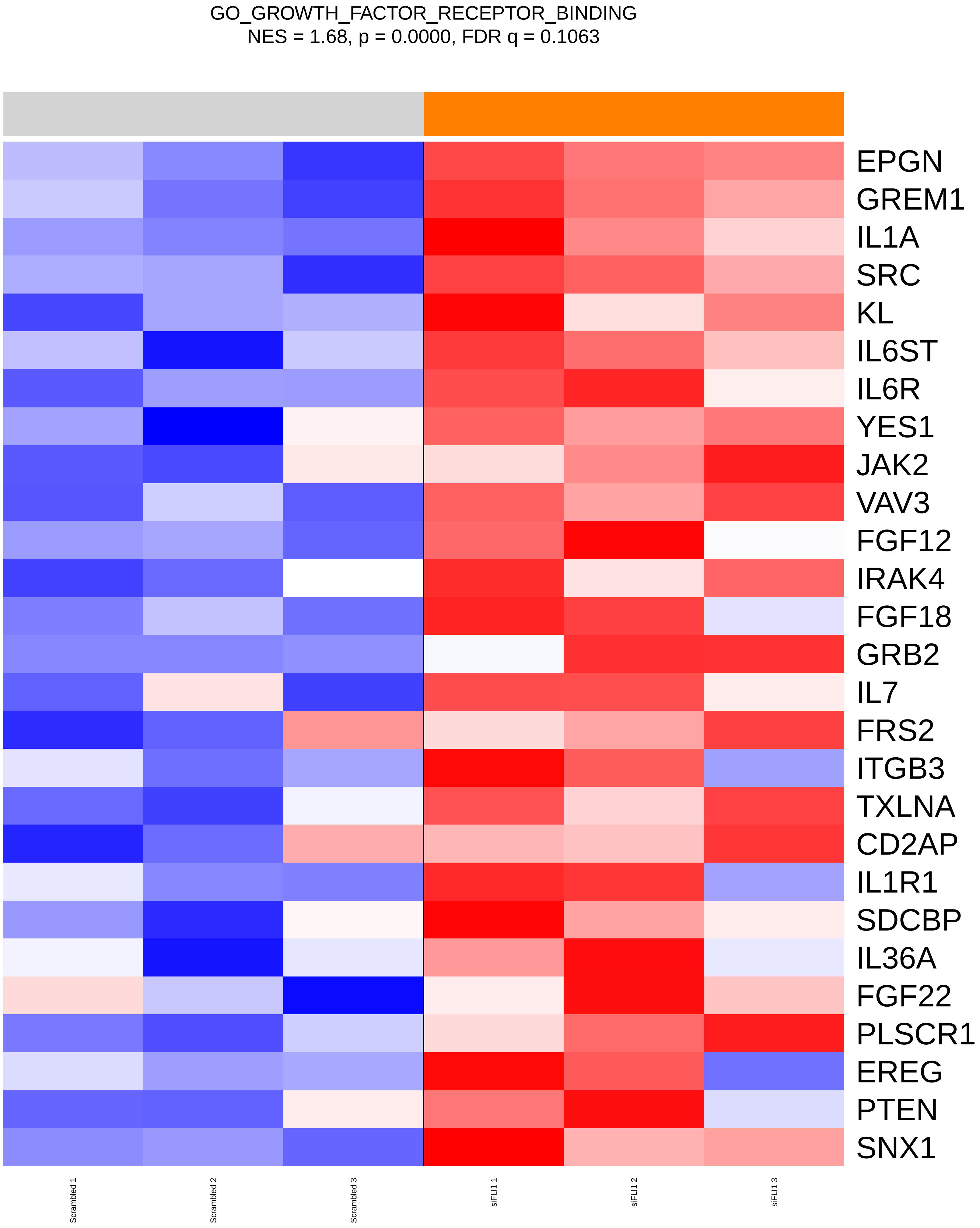
GO_GROWTH_FACTOR_RECEPTOR_BINDING
NES = 1.68, p = 0.0000, FDR q = 0.1063
EPGN
GREM1
IL1A
SRC
KL
IL6ST
IL6R
YES1
JAK2
VAV3
FGF12
IRAK4
FGF18
GRB2
IL7
FRS2
ITGB3
TXLNA
CD2AP
IL1R1
SDCBP
IL36A
FGF22
PLSCR1
EREG
PTEN
SNX1
Scrambled 1
Scrambled 2
Scrambled 3
siFLI1 1
siFLI1 2
siFLI1 3

GO_TUMOR_NECROSIS_FACTOR_MEDIATED_SIGNALING_PATHWAY
NES = 1.68, p = 0.0000, FDR q = 0.1079

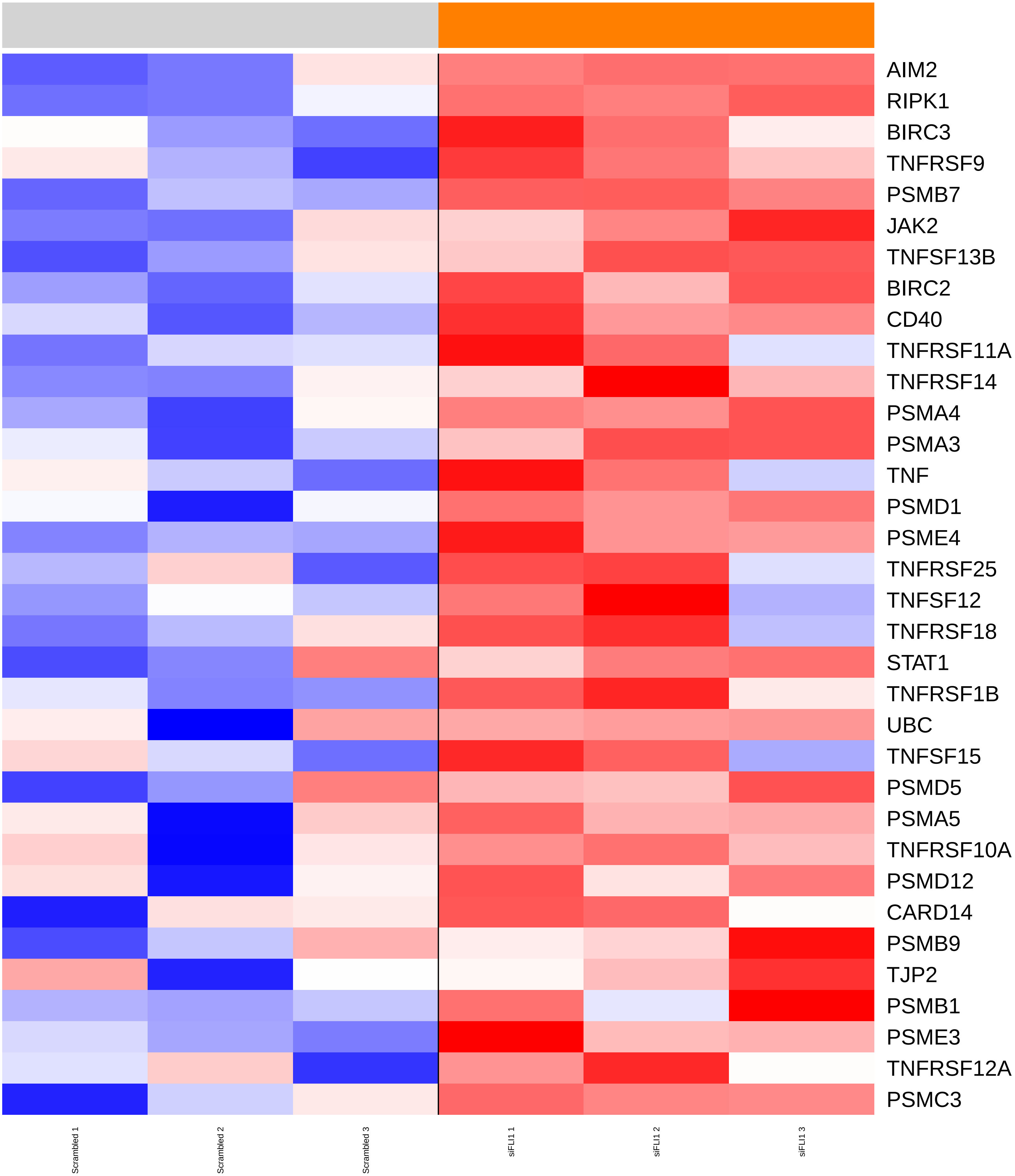

REACTOME_ACTIVATED_TAK1_MEDIATES_P38_MAPK_ACTIVATION
NES = 1.68, p = 0.0274, FDR q = 0.1076

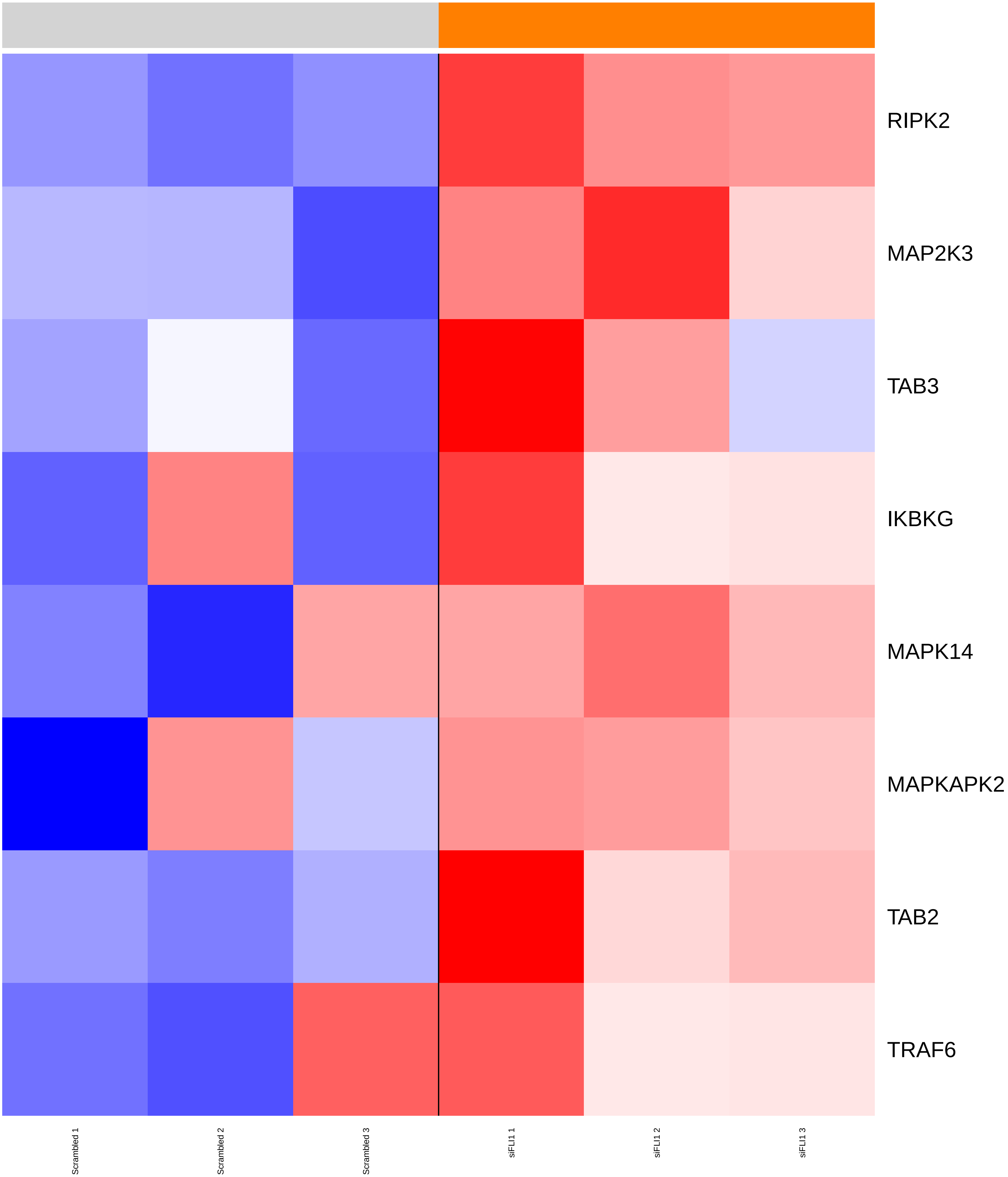

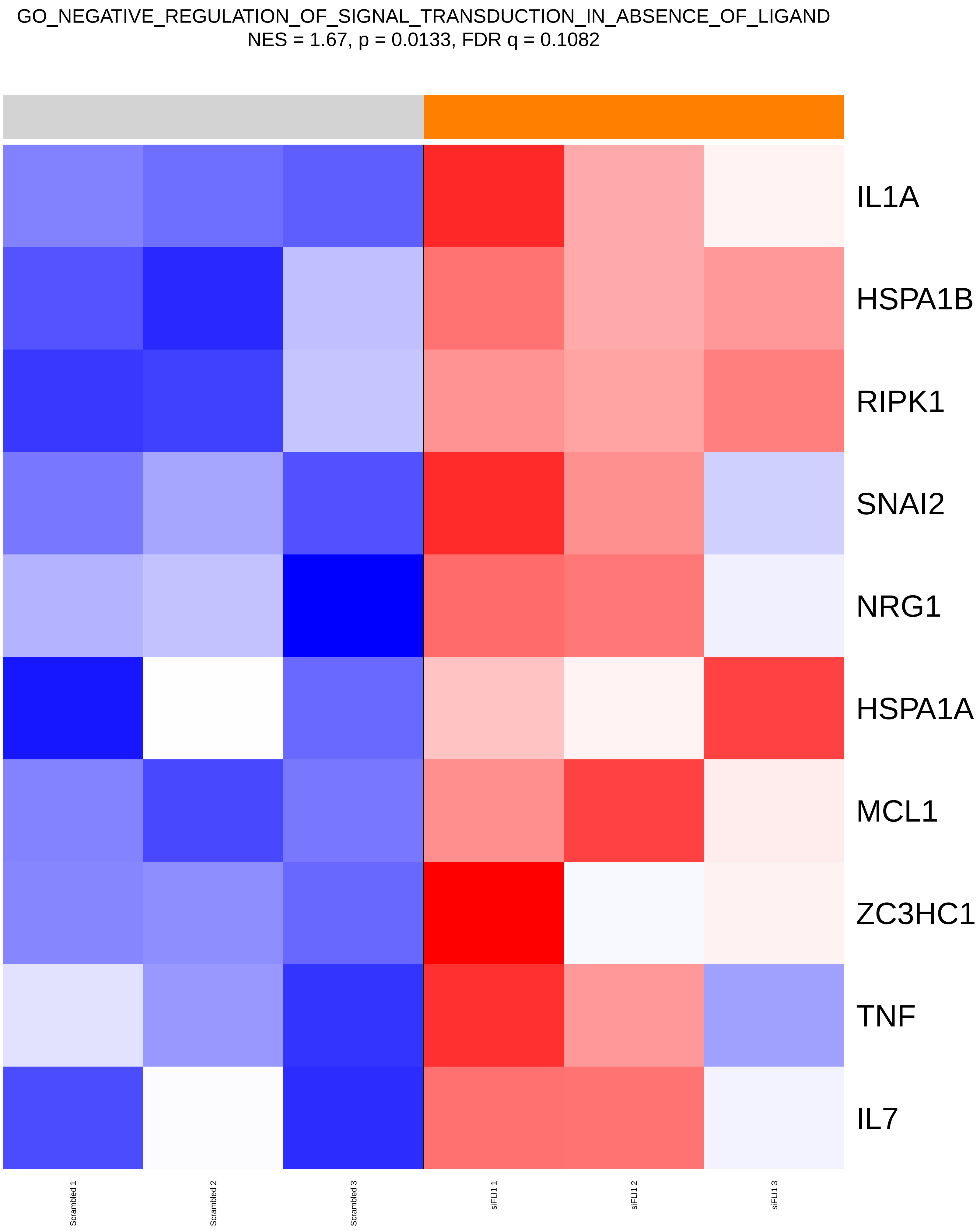
GO_NEGATIVE_REGULATION_OF_SIGNAL_TRANSDUCTION_IN_ABSENCE_OF_LIGAND
NES = 1.67, p = 0.0133, FDR q = 0.1082
IL1A
HSPA1B
RIPK1
SNAI2
NRG1
HSPA1A
MCL1
ZC3HC1
TNF
IL7
Scrambled 1
Scrambled 2
Scrambled 3
siFLI1 1
siFLI1 2
siFLI1 3

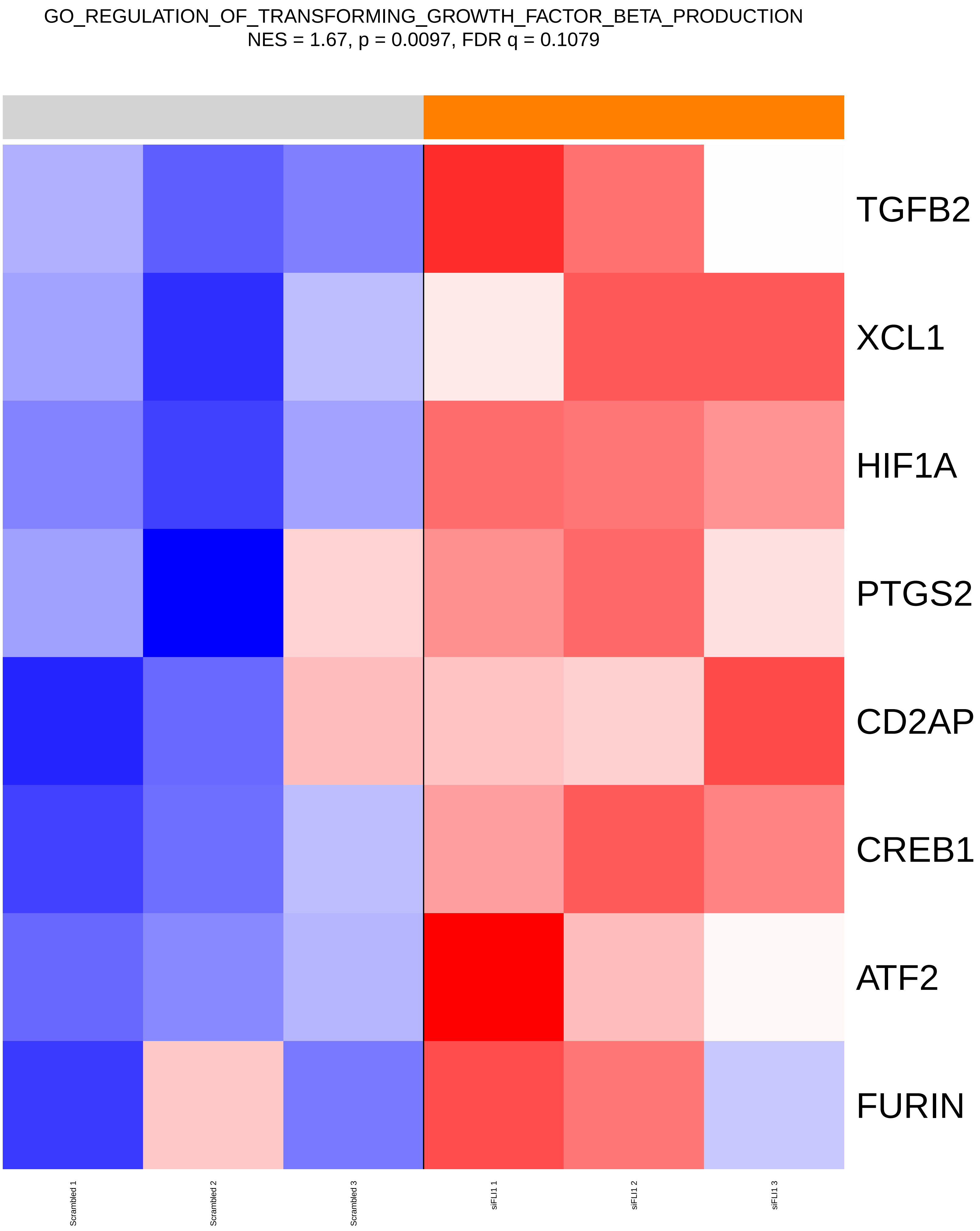
GO_REGULATION_OF_TRANSFORMING_GROWTH_FACTOR_BETA_PRODUCTION
NES = 1.67, p = 0.0097, FDR q = 0.1079
TGFB2
XCL1
HIF1A
PTGS2
CD2AP
CREB1
ATF2
FURIN
Scrambled 1
Scrambled 2
Scrambled 3
siFLI1 1
siFLI1 2
siFLI1 3

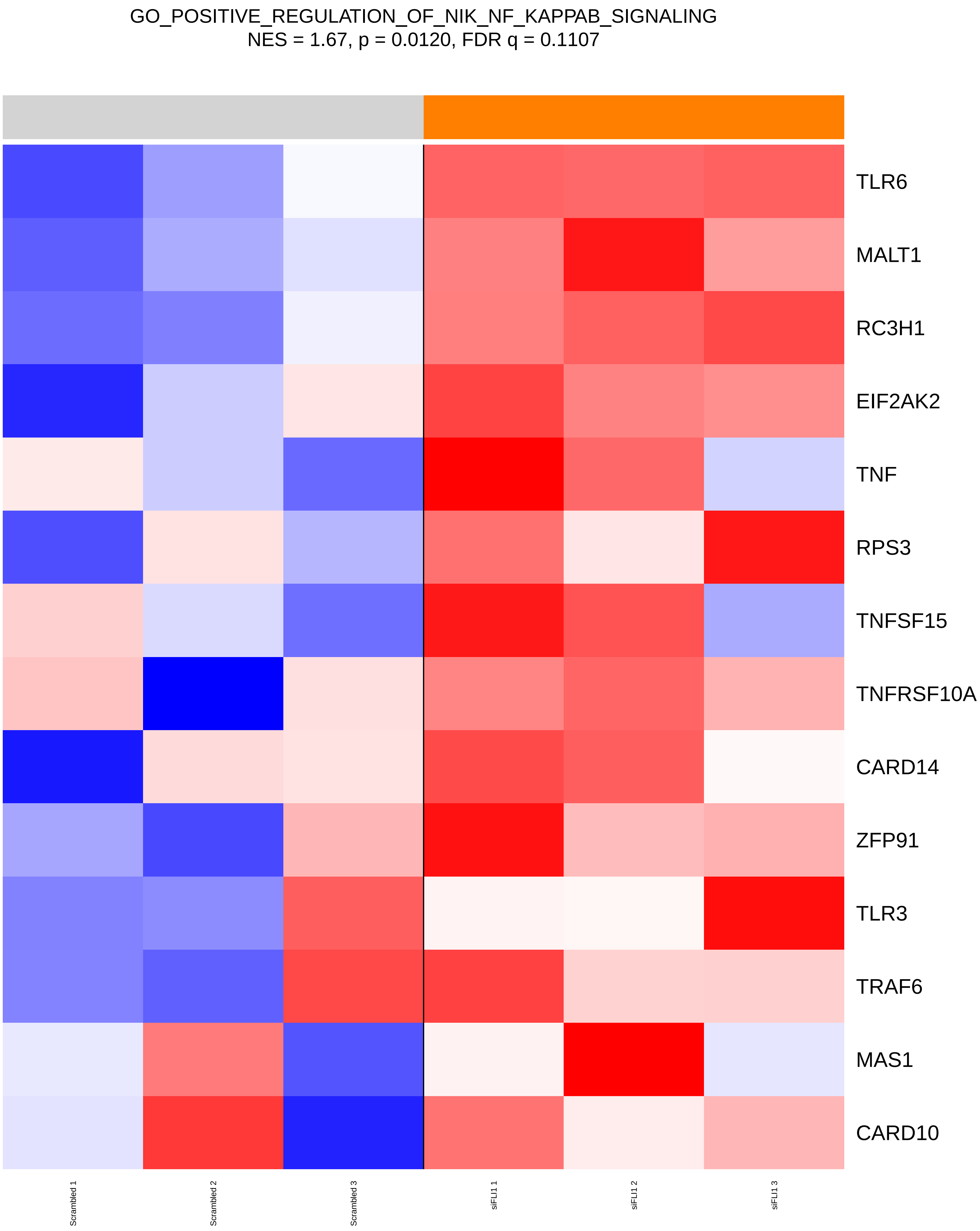
GO_POSITIVE_REGULATION_OF_NIK_NF_KAPPAB_SIGNALING
NES = 1.67, p = 0.0120, FDR q = 0.1107
TLR6
MALT1
RC3H1
EIF2AK2
TNF
RPS3
TNFSF15
TNFRSF10A
CARD14
ZFP91
TLR3
TRAF6
MAS1
CARD10
Scrambled 1
Scrambled 2
Scrambled 3
siFLI1 1
siFLI1 2
siFLI1 3

GO_NEGATIVE_REGULATION_OF_PRODUCTION_OF_MOLECULAR_MEDIATOR_OF_IMMUNE_RESPONSE
NES = 1.67, p = 0.0125, FDR q = 0.1121

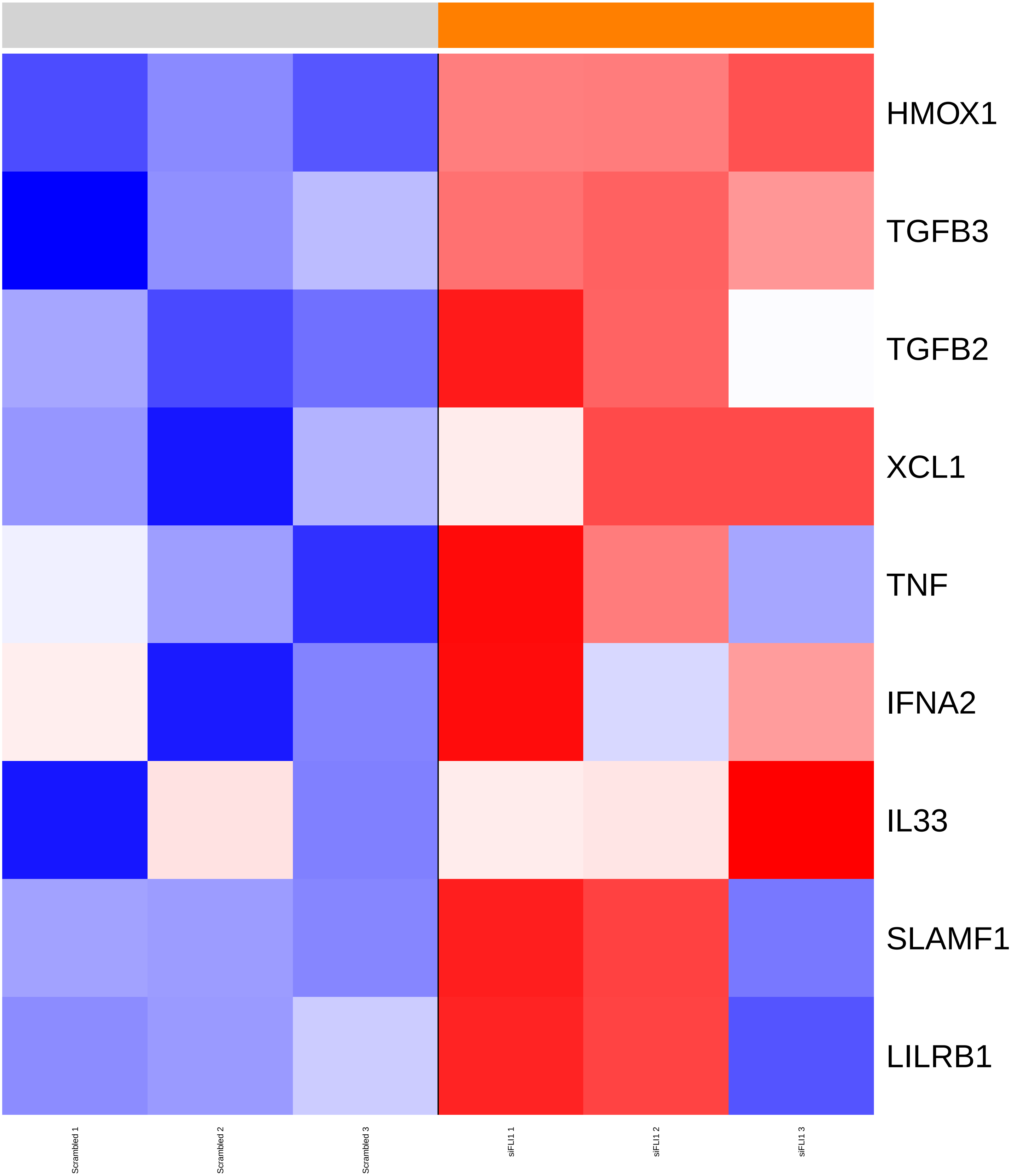

HALLMARK_PROTEIN_SECRETION
NES = 1.66, p = 0.0031, FDR q = 0.1140

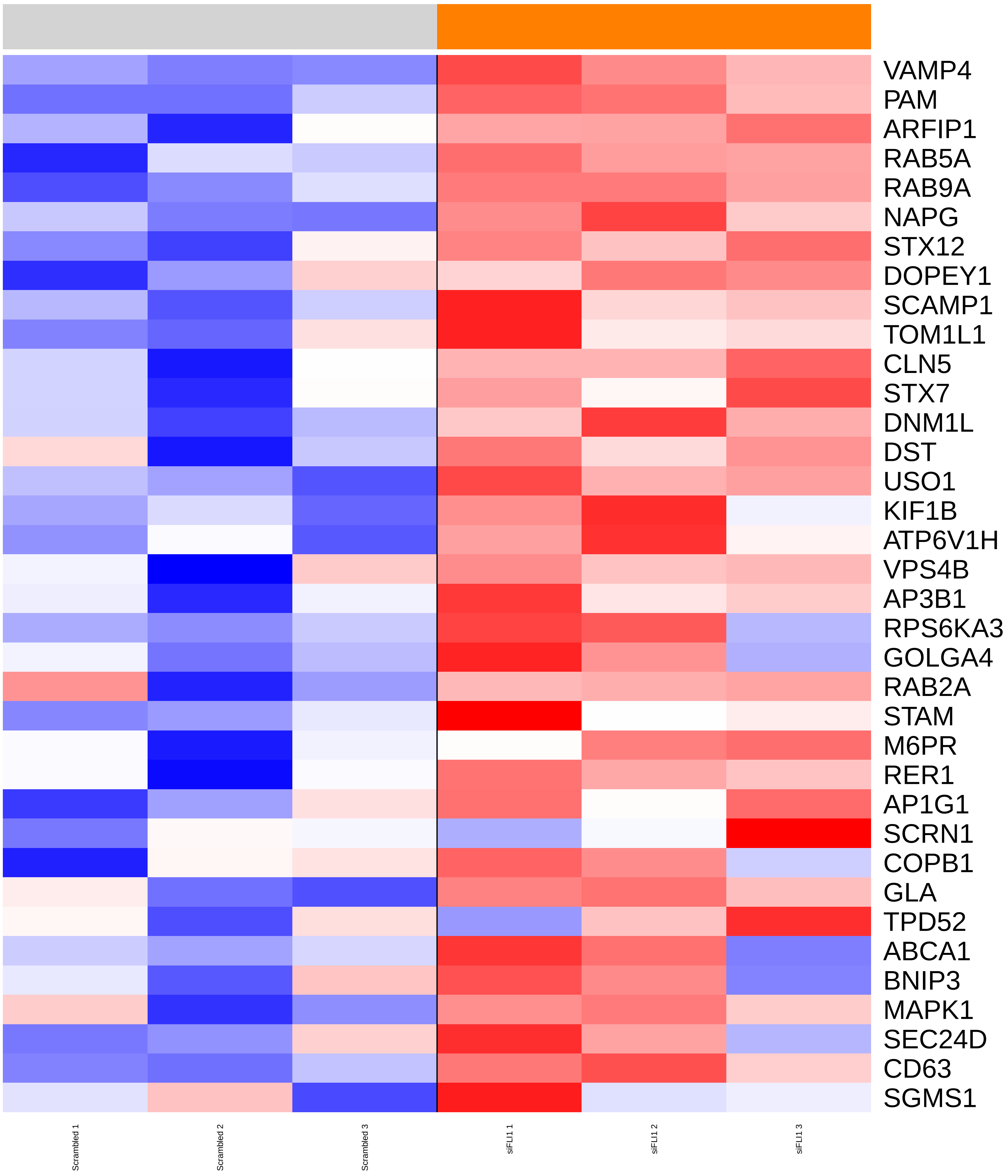

GO_K63_LINKED_POLYUBIQUITIN_BINDING
NES = 1.66, p = 0.0192, FDR q = 0.1148

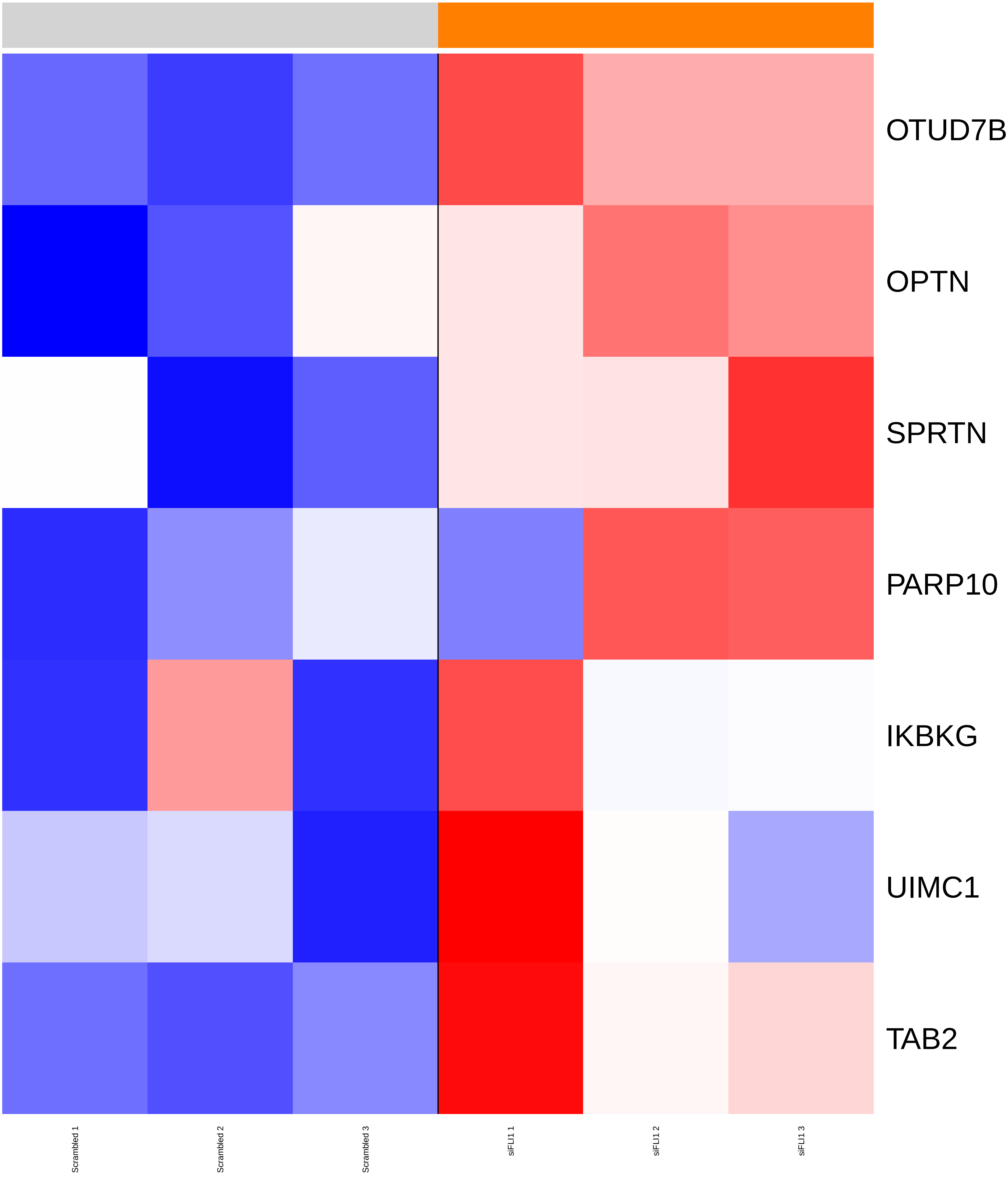

GO_REGULATION_OF_CELLULAR_EXTRAVASATION
NES = 1.66, p = 0.0222, FDR q = 0.1154

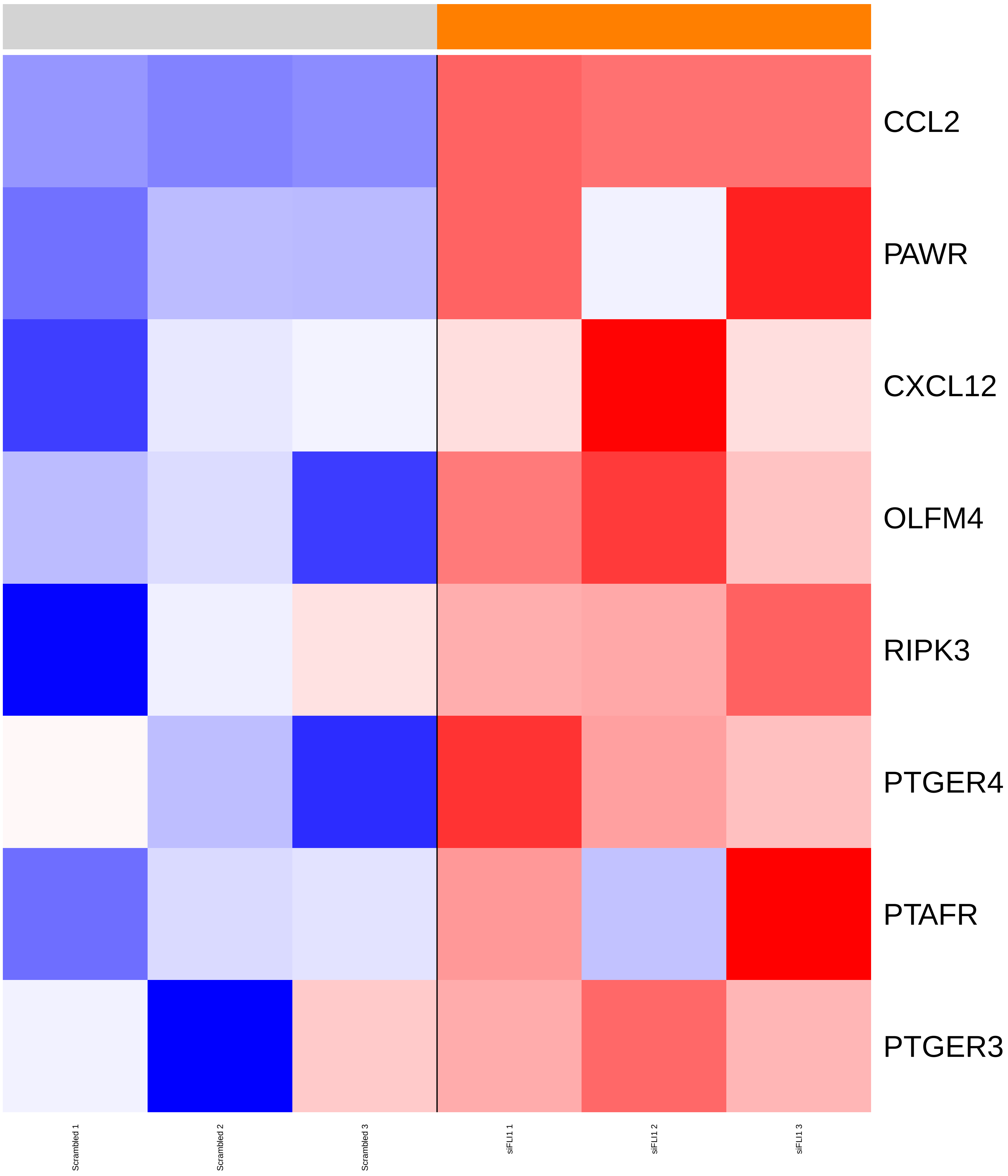

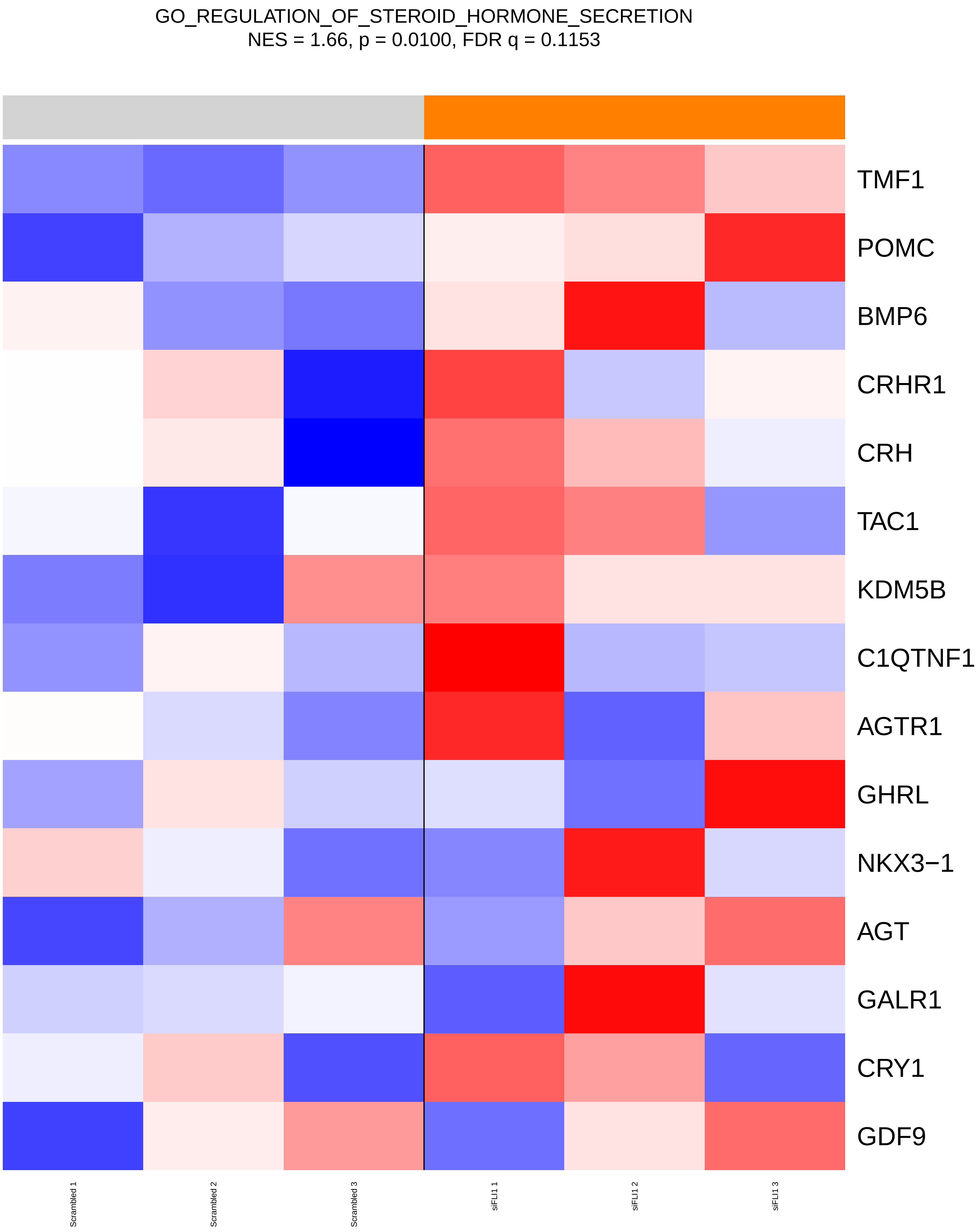
GO_REGULATION_OF_STEROID_HORMONE_SECRETION
NES = 1.66, p = 0.0100, FDR q = 0.1153
TMF1
POMC
BMP6
CRHR1
CRH
TAC1
KDM5B
C1QTNF1
AGTR1
GHRL
NKX3−1
AGT
GALR1
CRY1
GDF9
Scrambled 1
Scrambled 2
Scrambled 3
siFLI1 1
siFLI1 2
siFLI1 3

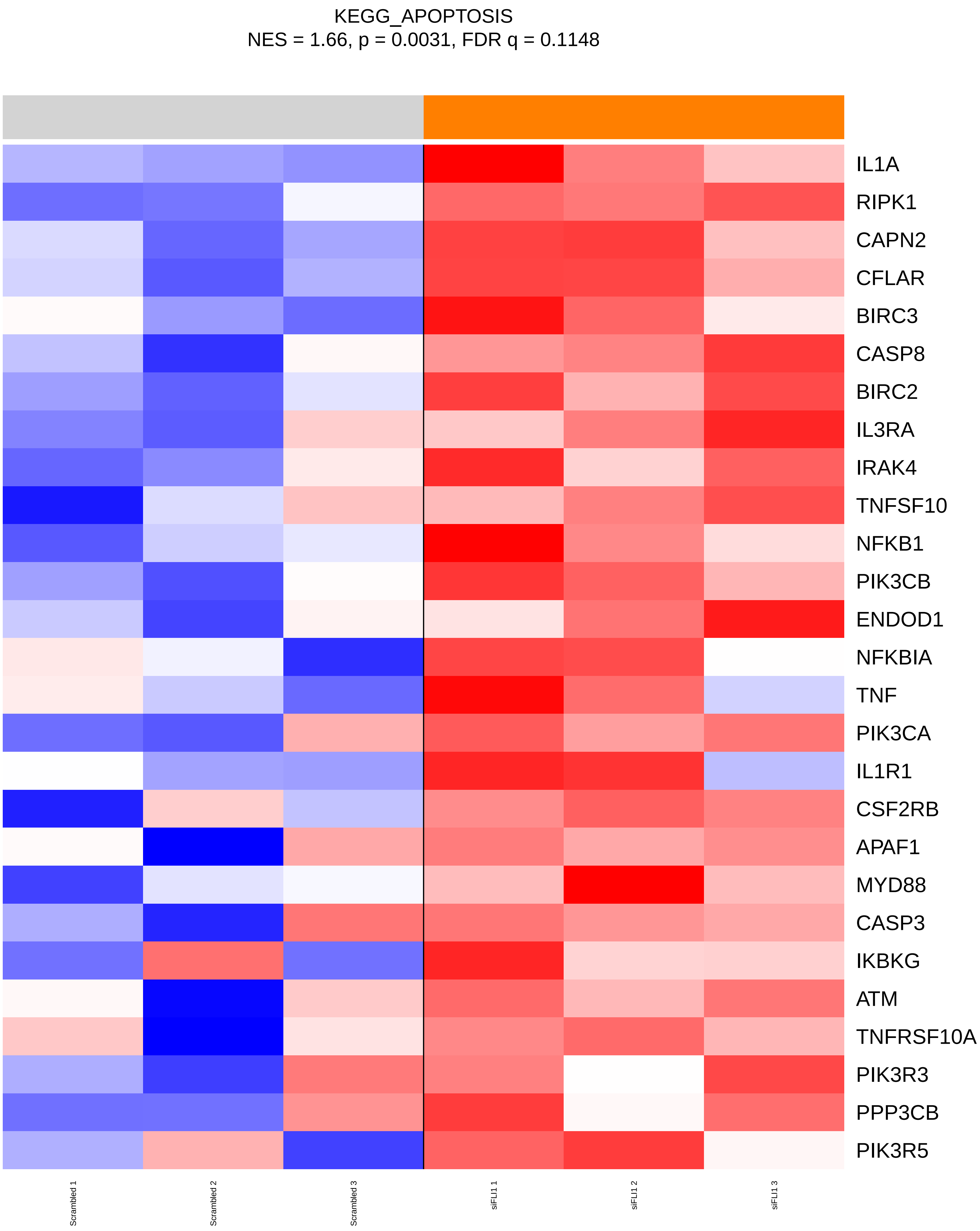

KEGG_APOPTOSIS
NES = 1.66, p = 0.0031, FDR q = 0.1148
IL1A
RIPK1
CAPN2
CFLAR
BIRC3
CASP8
BIRC2
IL3RA
IRAK4
TNFSF10
NFKB1
PIK3CB
ENDOD1
NFKBIA
TNF
PIK3CA
IL1R1
CSF2RB
APAF1
MYD88
CASP3
IKBKG
ATM
TNFRSF10A
PIK3R3
PPP3CB
PIK3R5
Scrambled 1
Scrambled 2
Scrambled 3
siFLI1 1
siFLI1 2
siFLI1 3

GO_NEGATIVE_REGULATION_OF_DNA_BIOSYNTHETIC_PROCESS
NES = 1.65, p = 0.0196, FDR q = 0.1207

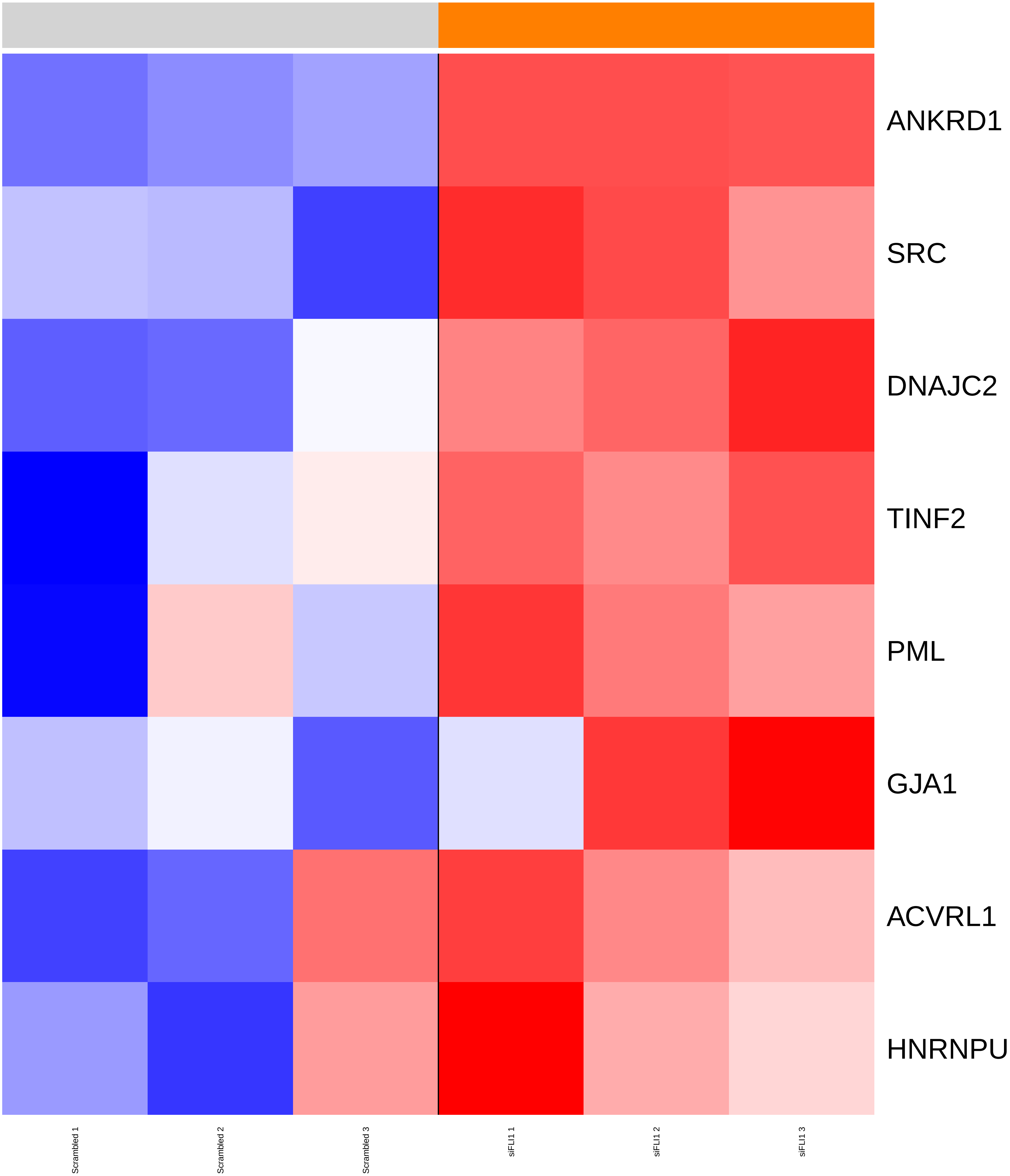

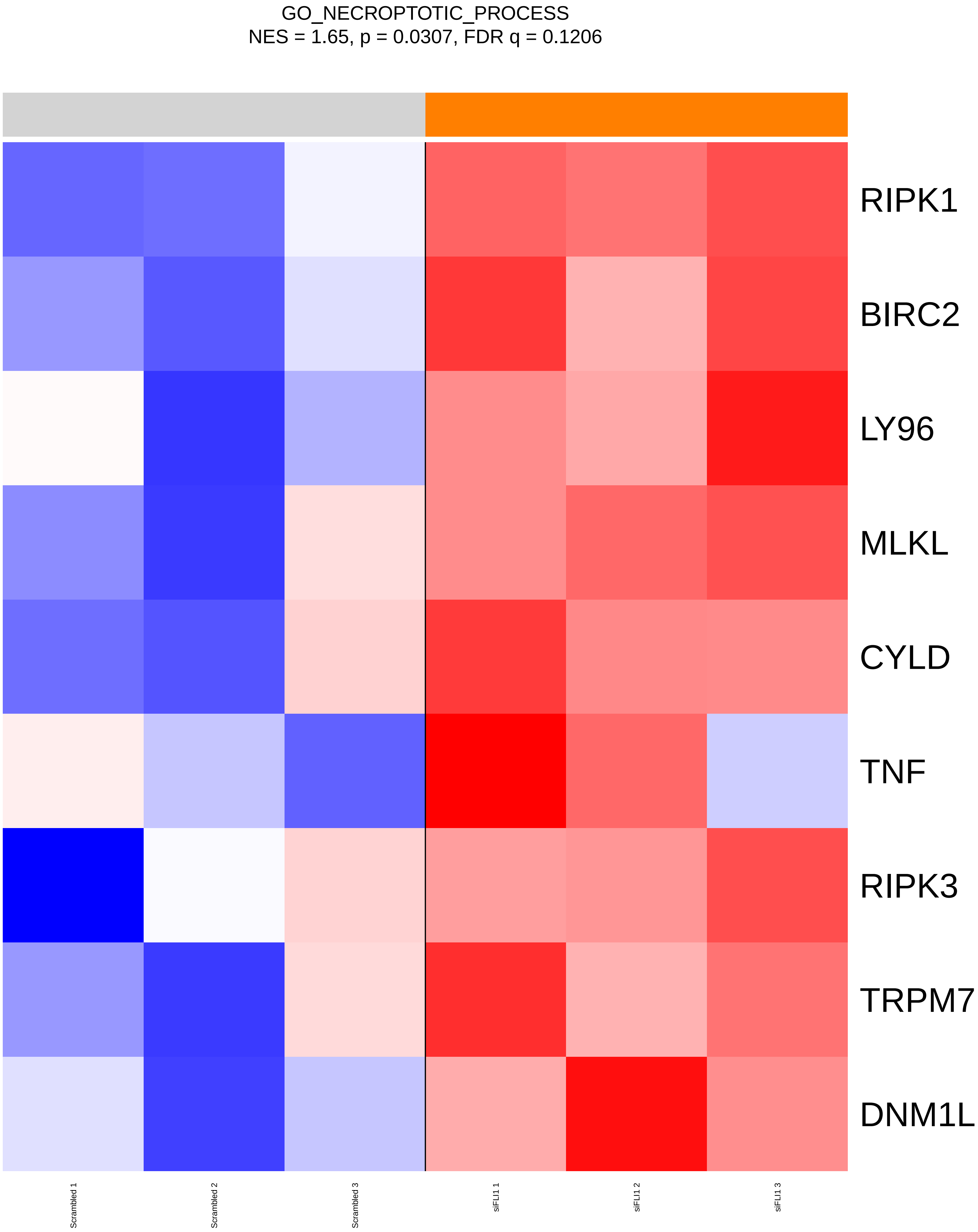
GO_NECROPTOTIC_PROCESS
NES = 1.65, p = 0.0307, FDR q = 0.1206
RIPK1
BIRC2
LY96
MLKL
CYLD
TNF
RIPK3
TRPM7
DNM1L
Scrambled 1
Scrambled 2
Scrambled 3
siFLI1 1
siFLI1 2
siFLI1 3

GO_REGULATION_OF_AUTOPHAGY
NES = 1.65, p = 0.0000, FDR q = 0.1210

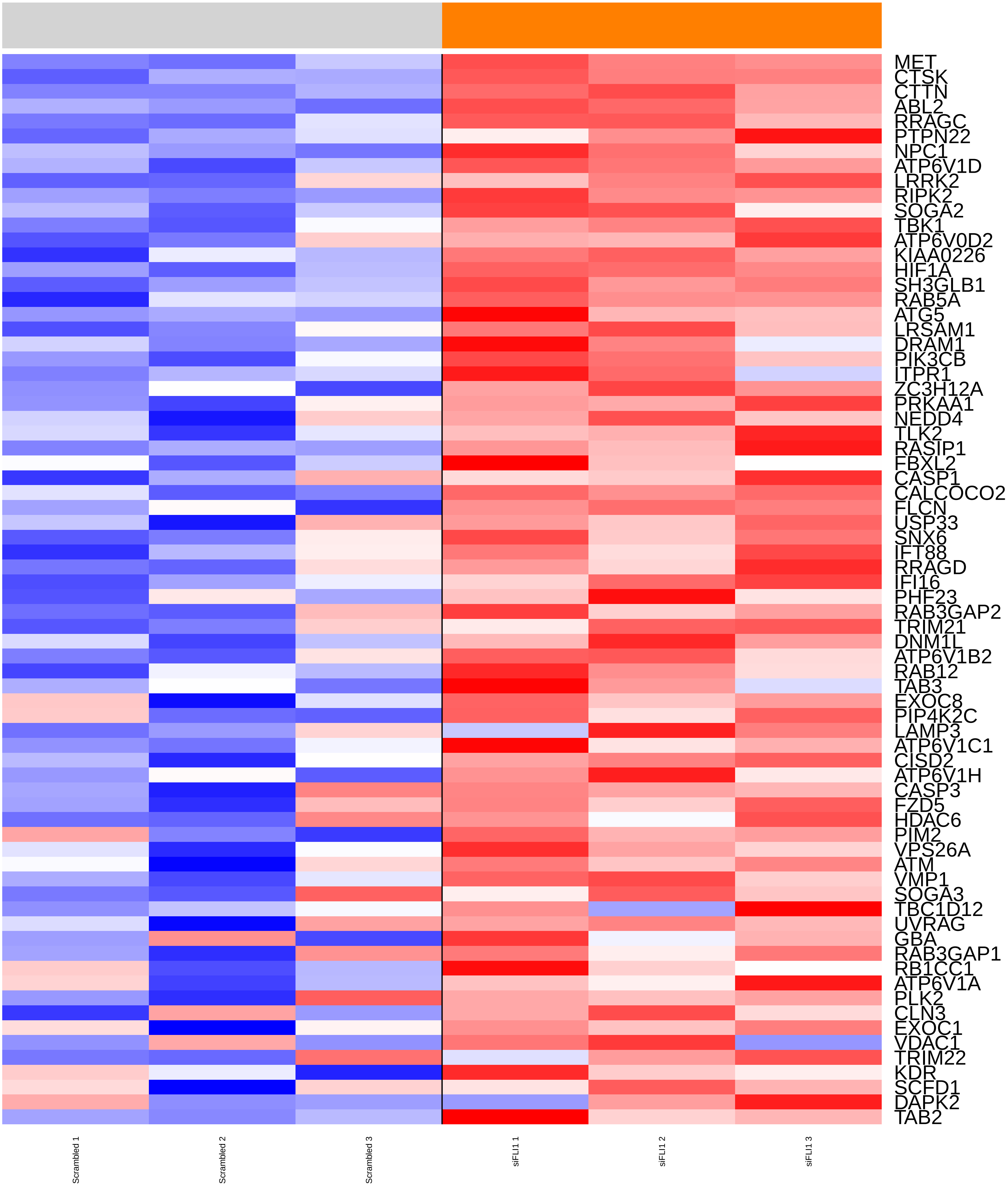

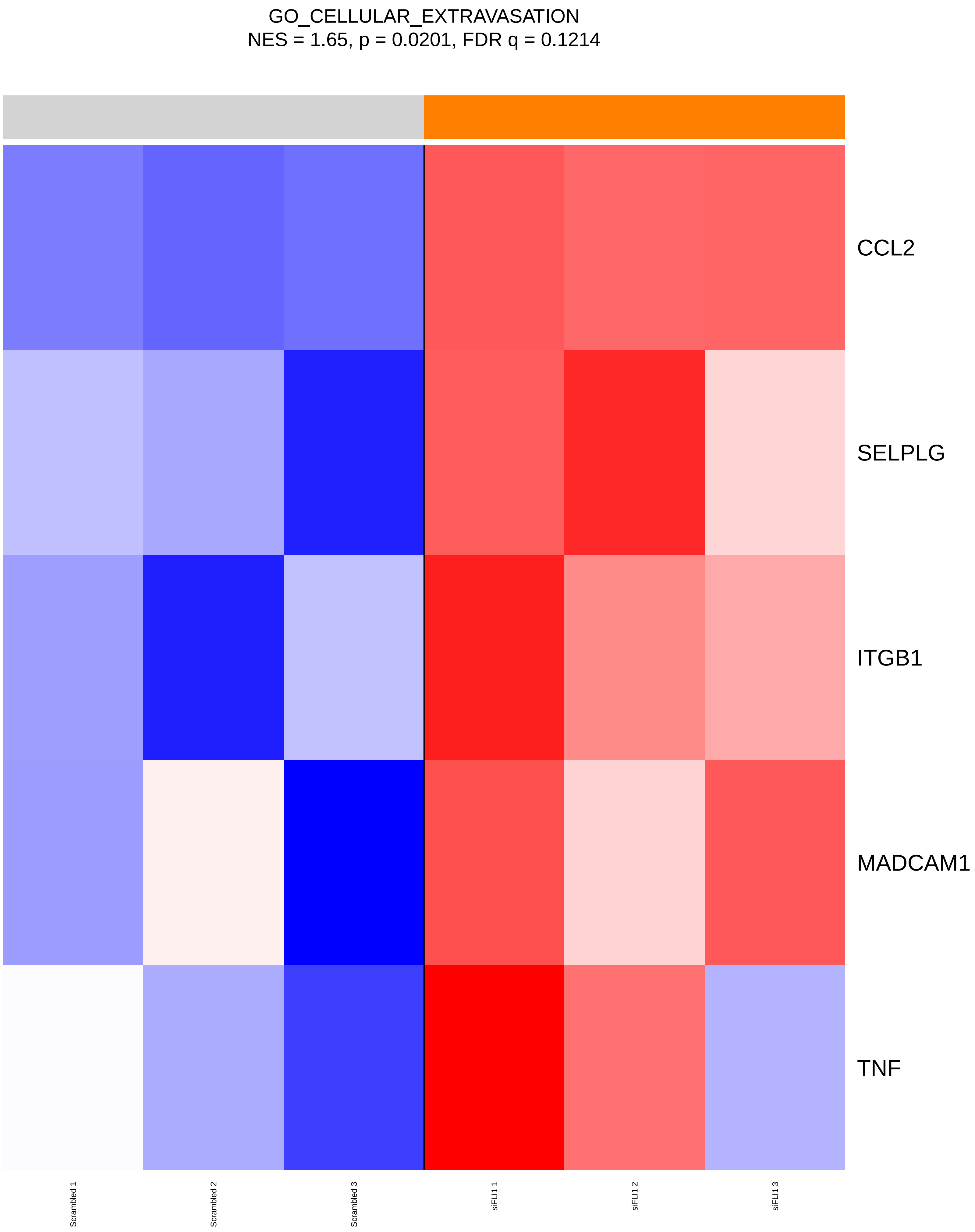
GO_CELLULAR_EXTRAVASATION
NES = 1.65, p = 0.0201, FDR q = 0.1214
CCL2
SELPLG
ITGB1
MADCAM1
TNF
Scrambled 1
Scrambled 2
Scrambled 3
siFLI1 1
siFLI1 2
siFLI1 3

GO_REGULATION_OF_EXTRINSIC_APOPTOTIC_SIGNALING_PATHWAY
NES = 1.65, p = 0.0000, FDR q = 0.1242

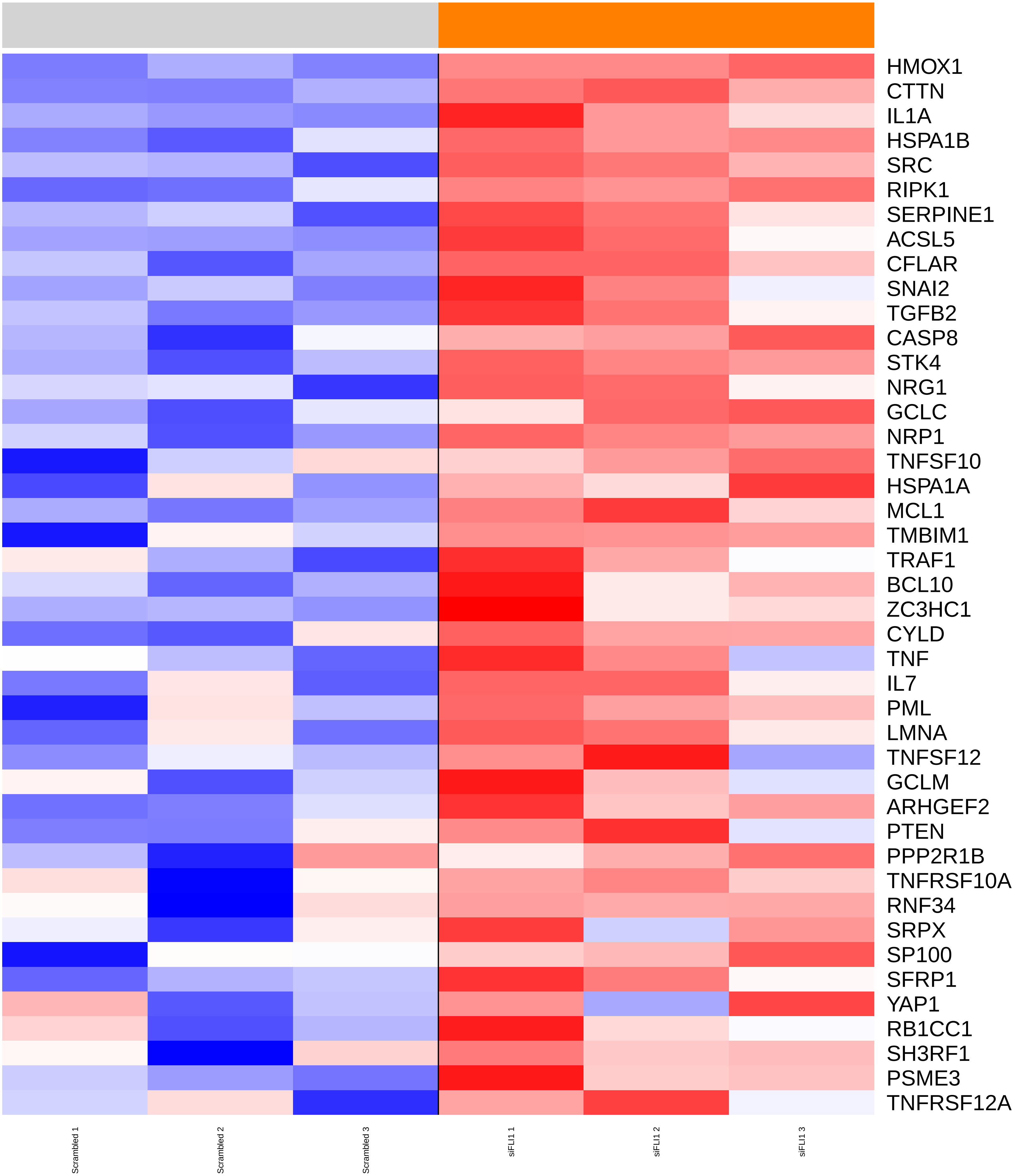

BIOCARTA_CDMAC_PATHWAY
NES = 1.64, p = 0.0325, FDR q = 0.1256

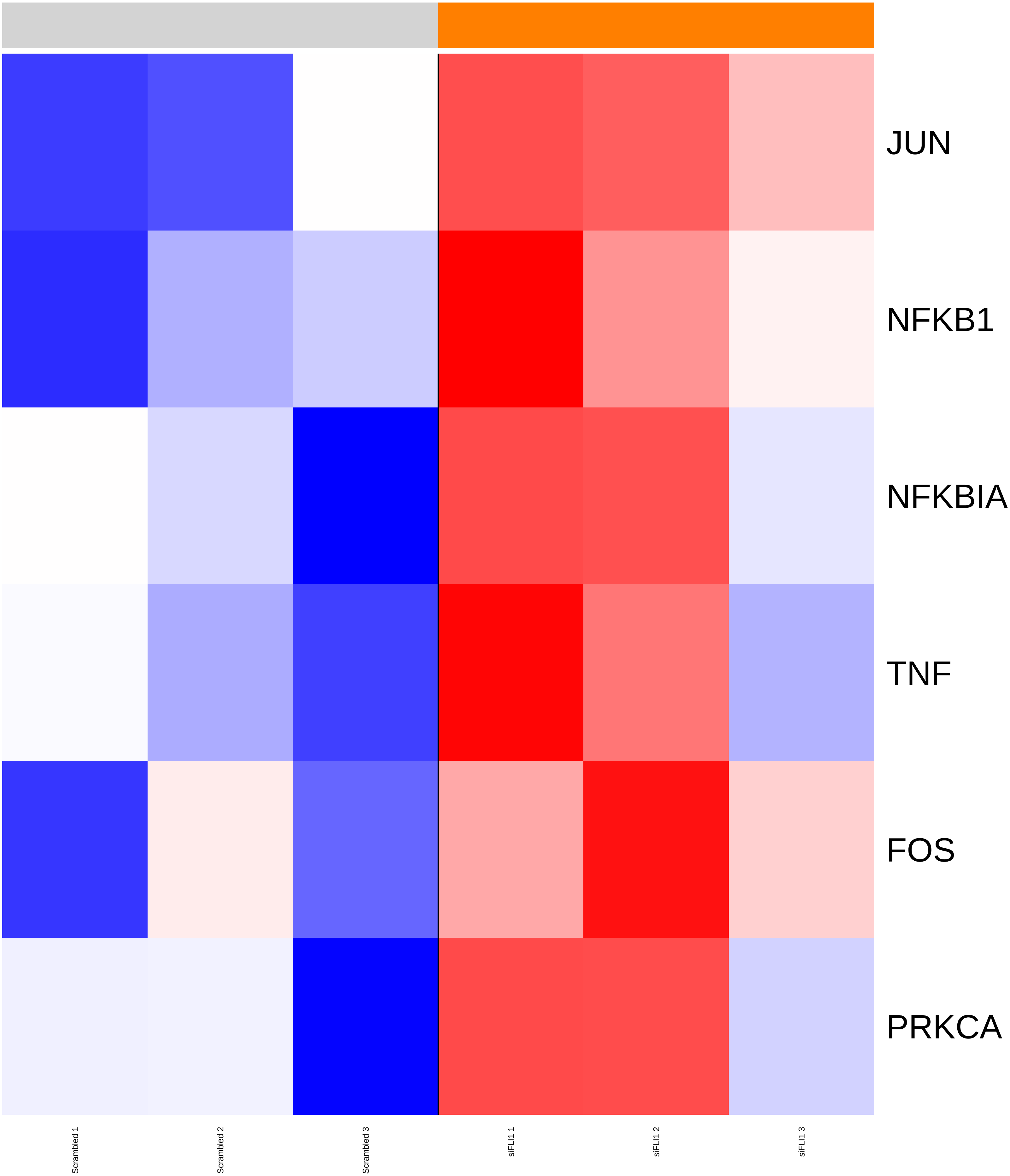

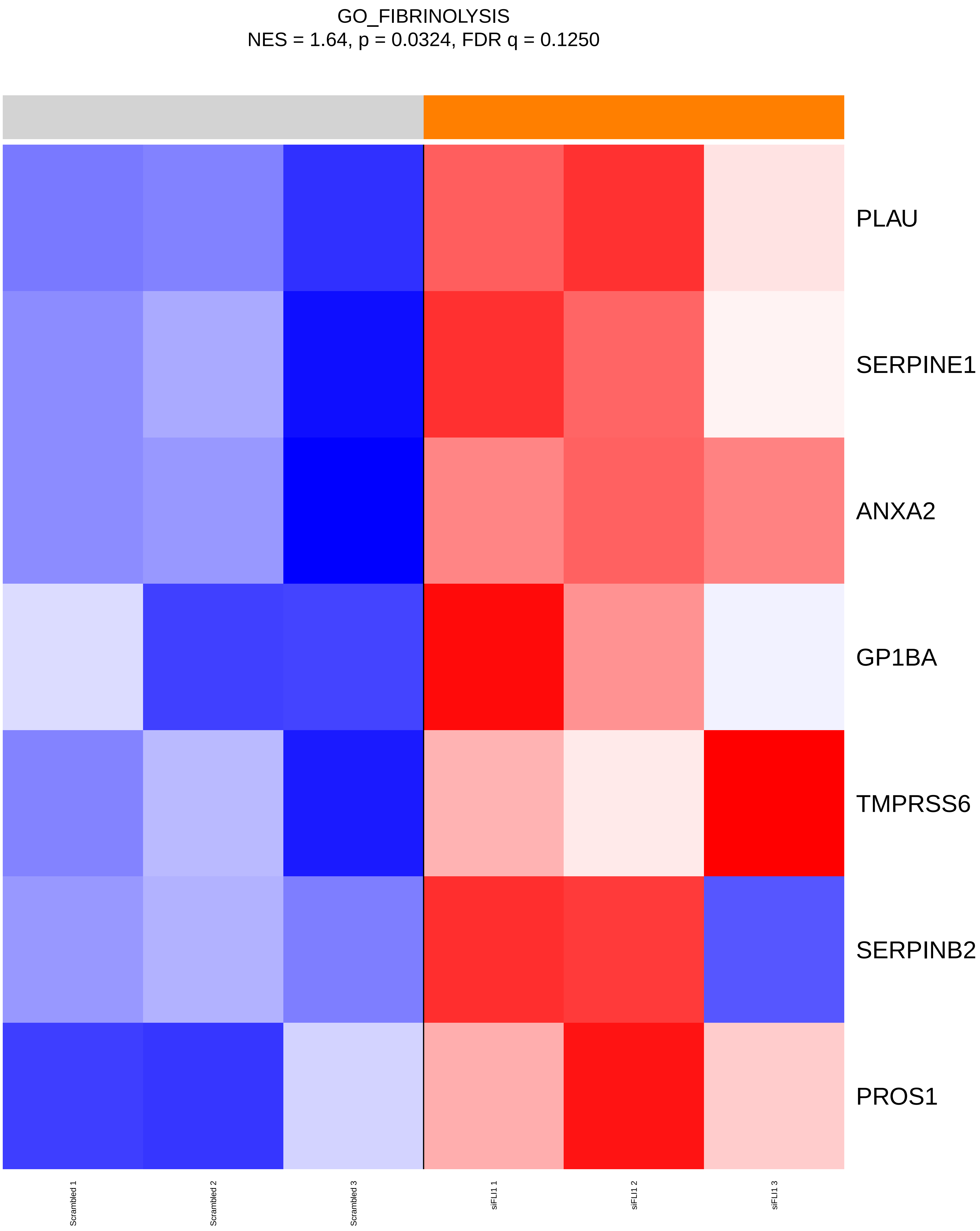
GO_FIBRINOLYSIS
NES = 1.64, p = 0.0324, FDR q = 0.1250
PLAU
SERPINE1
ANXA2
GP1BA
TMPRSS6
SERPINB2
PROS1
Scrambled 1
Scrambled 2
Scrambled 3
siFLI1 1
siFLI1 2
siFLI1 3

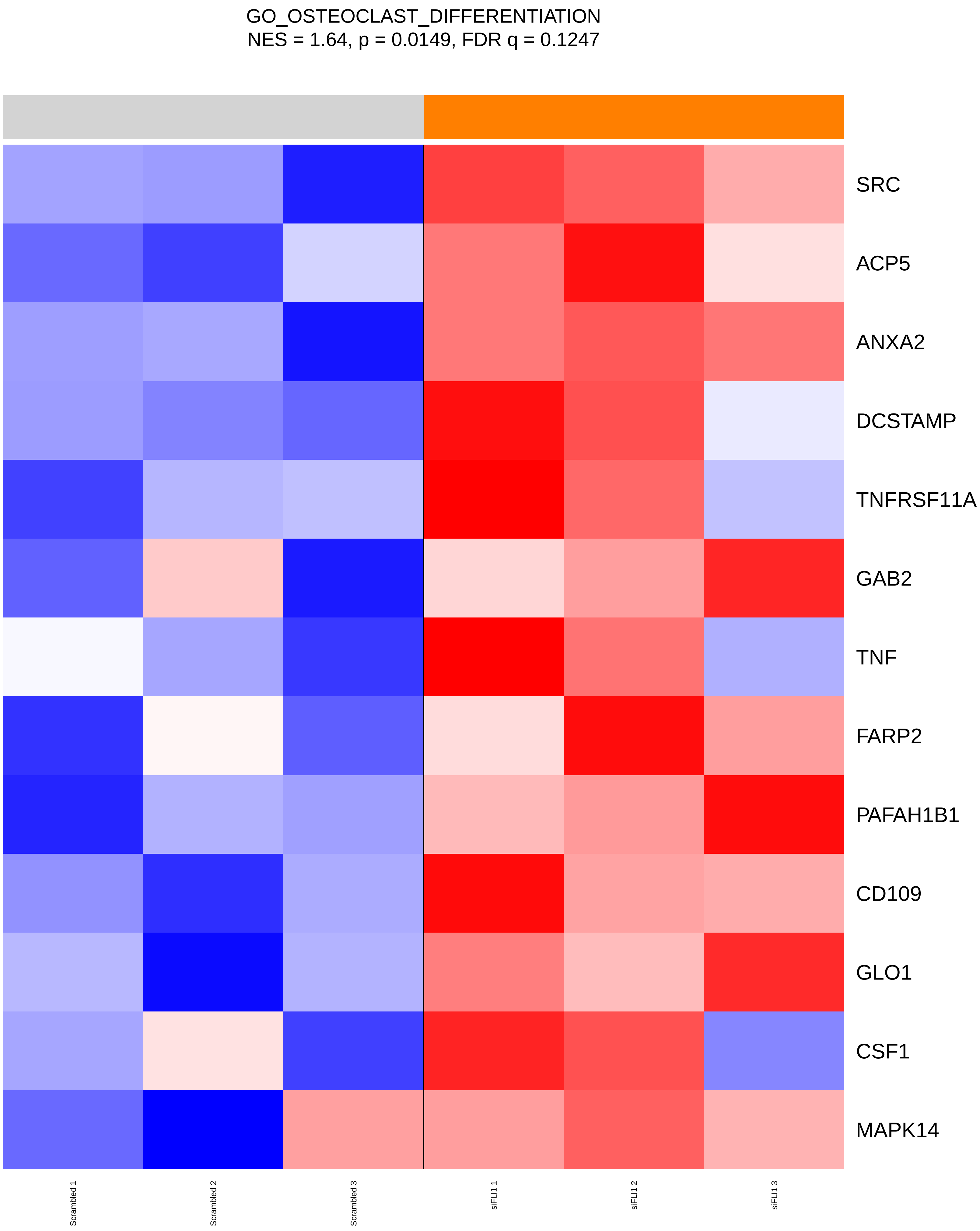

GO_OSTEOCLAST_DIFFERENTIATION
NES = 1.64, p = 0.0149, FDR q = 0.1247
SRC
ACP5
ANXA2
DCSTAMP
TNFRSF11A
GAB2
TNF
FARP2
PAFAH1B1
CD109
GLO1
CSF1
MAPK14
Scrambled 1
Scrambled 2
Scrambled 3
siFLI1 1
siFLI1 2
siFLI1 3

GO_REGULATION_OF_MONONUCLEAR_CELL_MIGRATION
NES = 1.64, p = 0.0242, FDR q = 0.1261

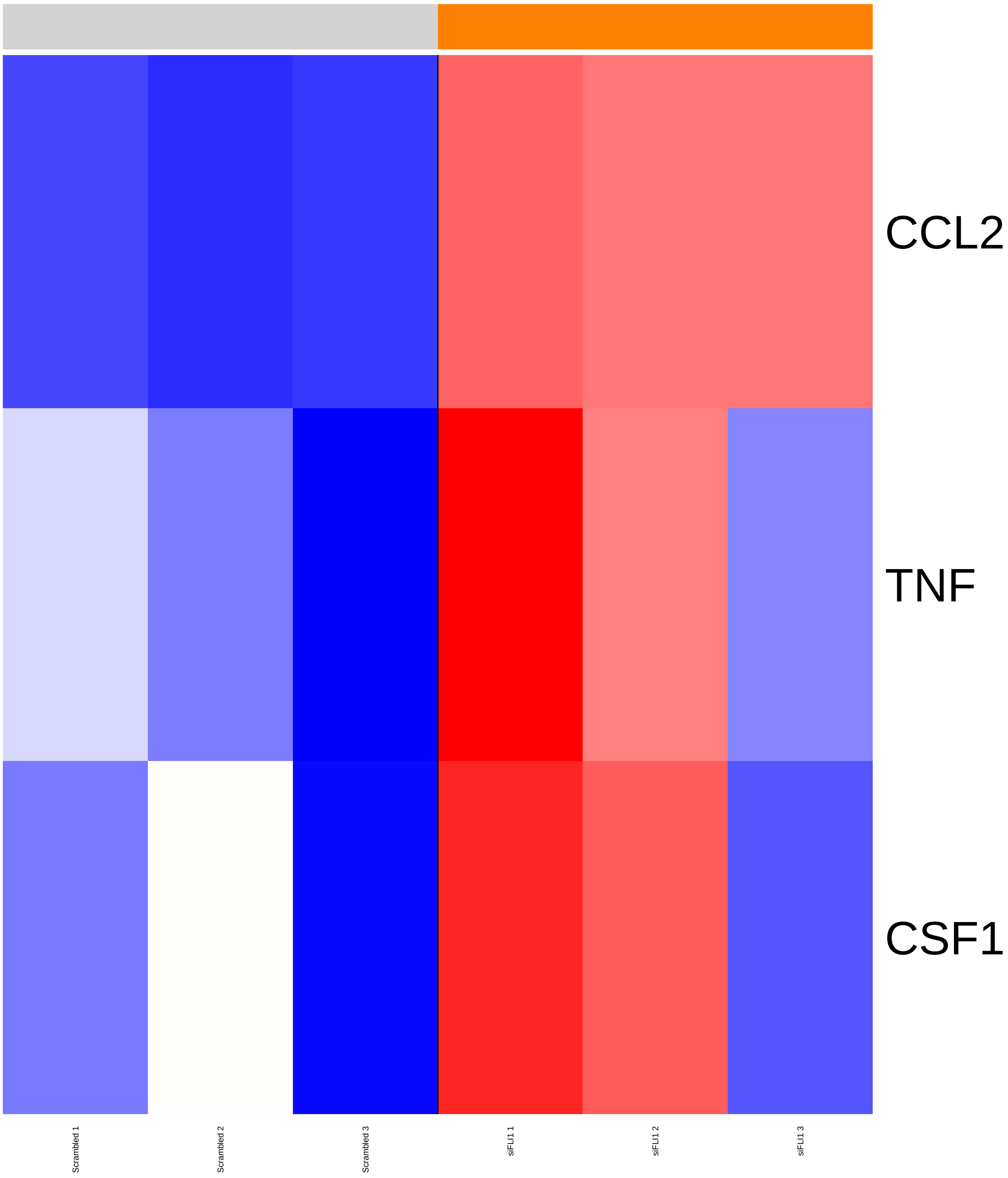

GO_REGULATION_OF_TOLL_LIKE_RECEPTOR_SIGNALING_PATHWAY
NES = 1.64, p = 0.0157, FDR q = 0.1262

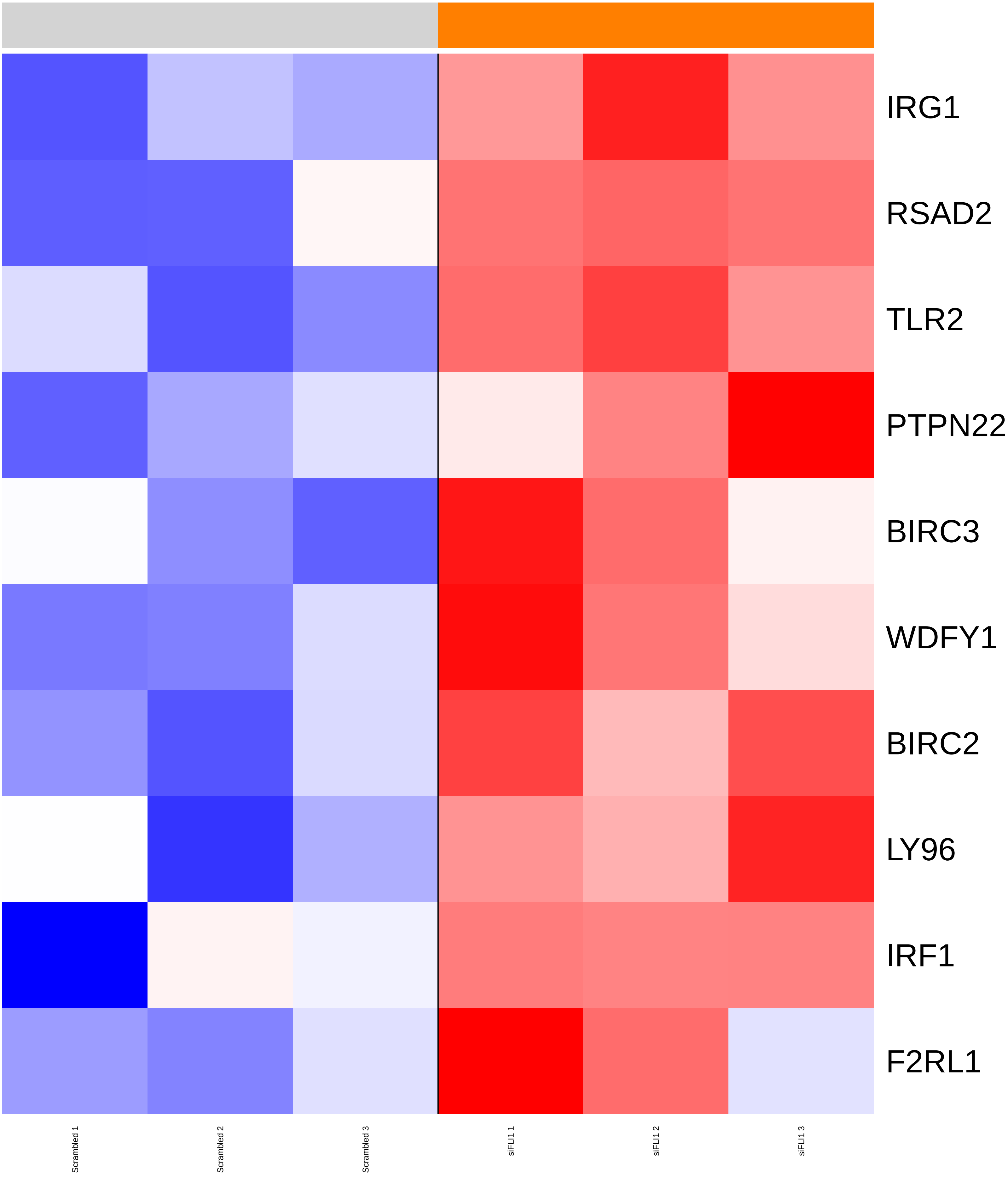

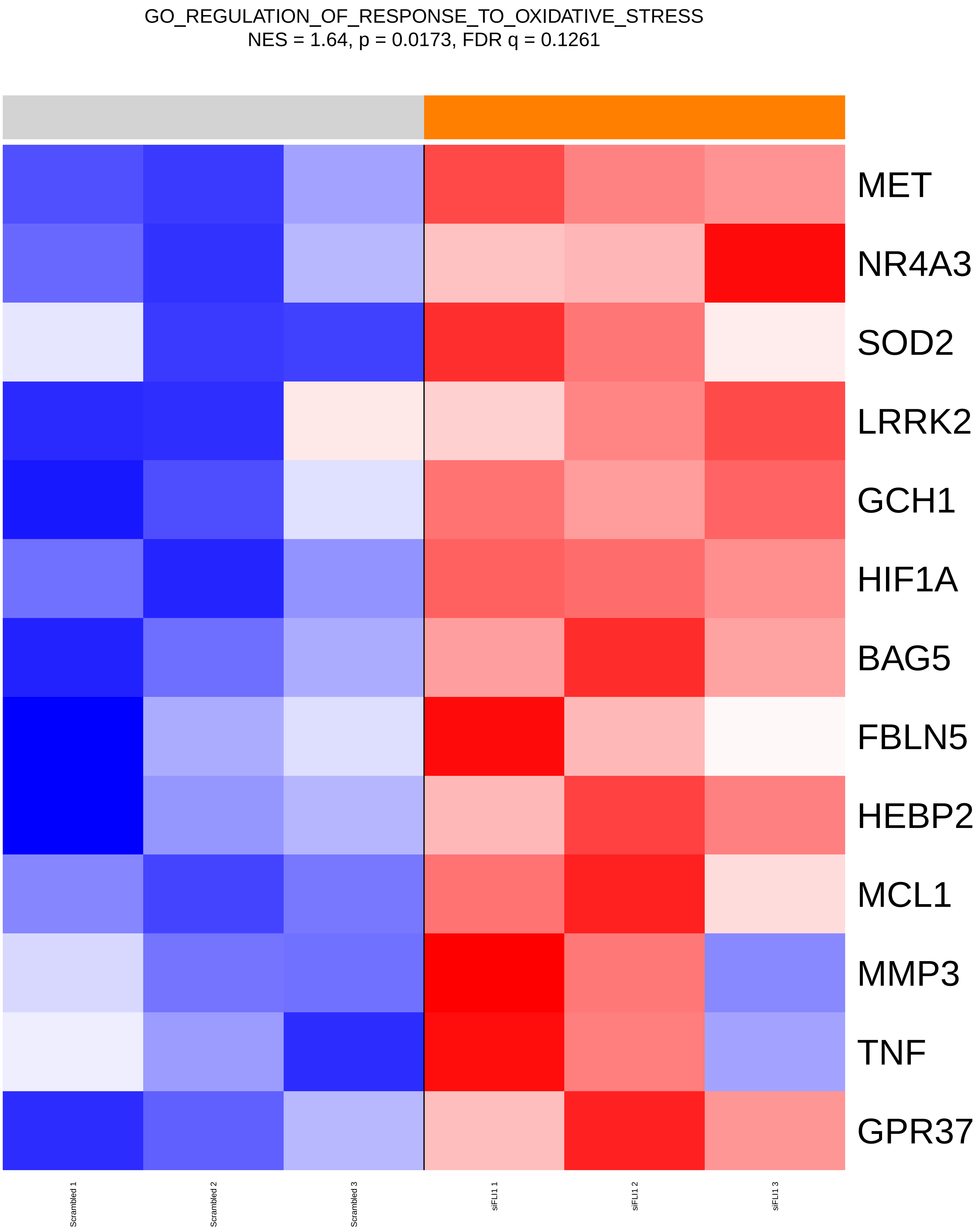
GO_REGULATION_OF_RESPONSE_TO_OXIDATIVE_STRESS
NES = 1.64, p = 0.0173, FDR q = 0.1261
MET
NR4A3
SOD2
LRRK2
GCH1
HIF1A
BAG5
FBLN5
HEBP2
MCL1
MMP3
TNF
GPR37
Scrambled 1
Scrambled 2
Scrambled 3
siFLI1 1
siFLI1 2
siFLI1 3

ATTACAT_MIR3803P
NES = 1.64, p = 0.0000, FDR q = 0.1260

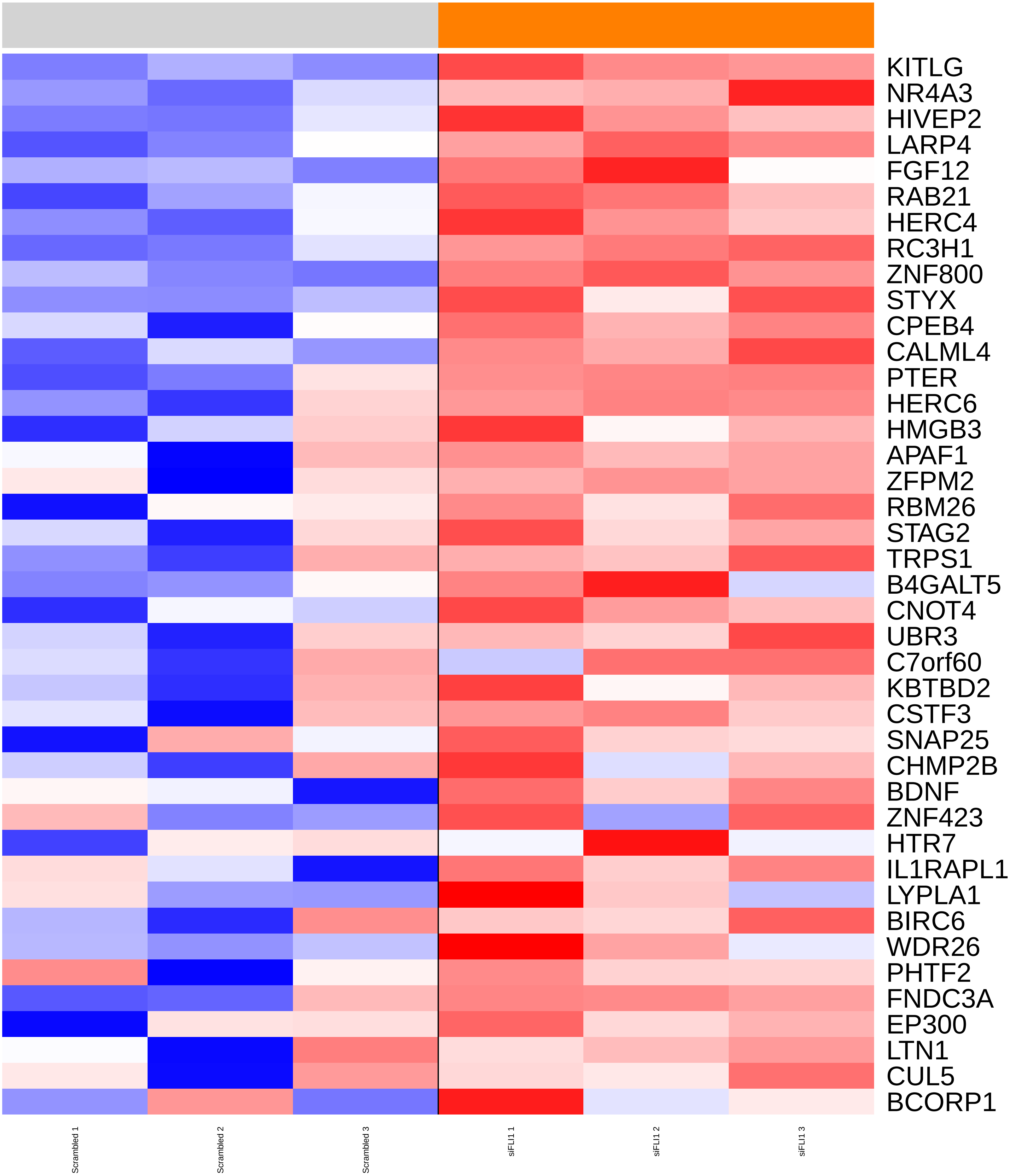

GO_BICARBONATE_TRANSMEMBRANE_TRANSPORTER_ACTIVITY
NES = 1.64, p = 0.0178, FDR q = 0.1258

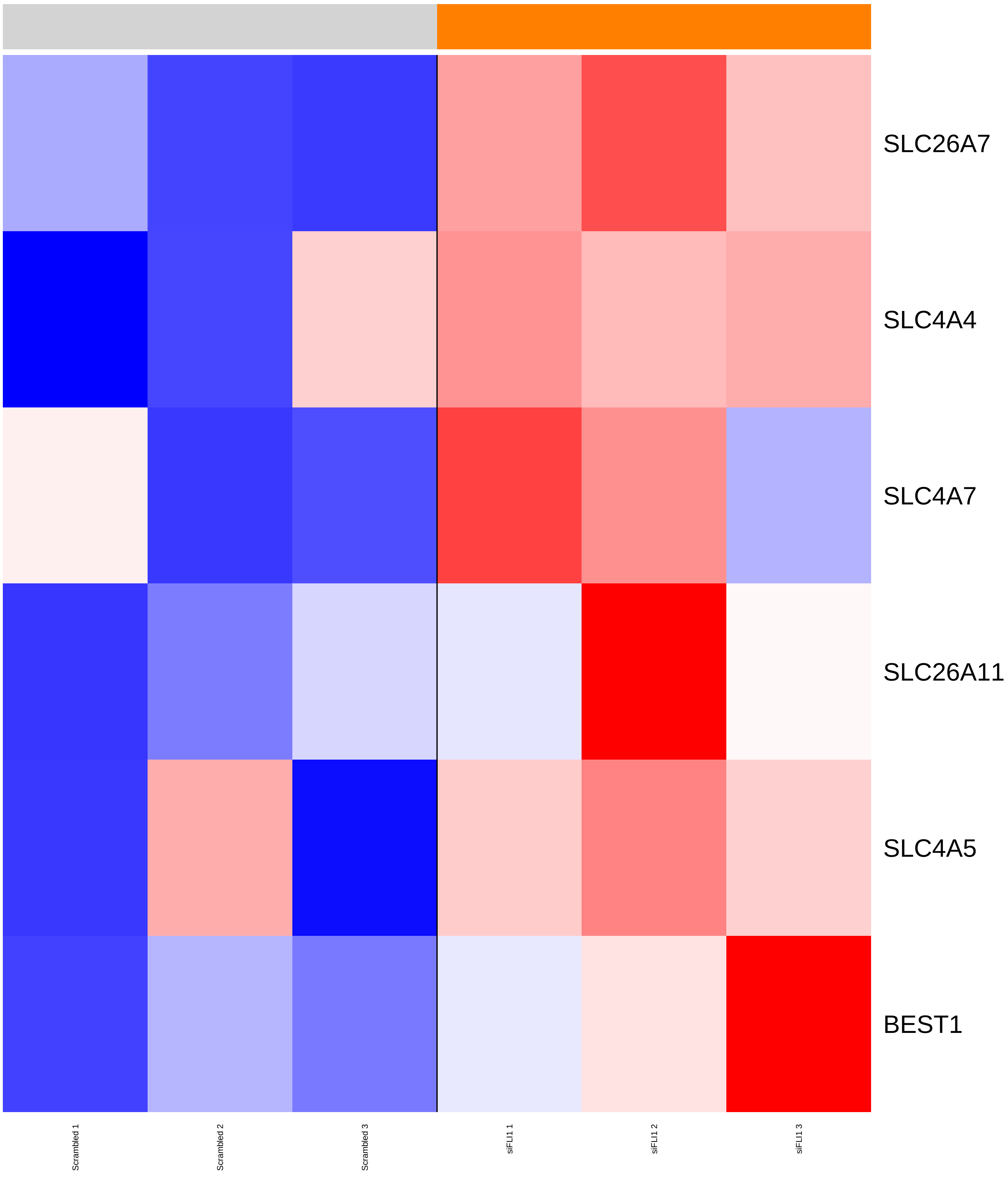

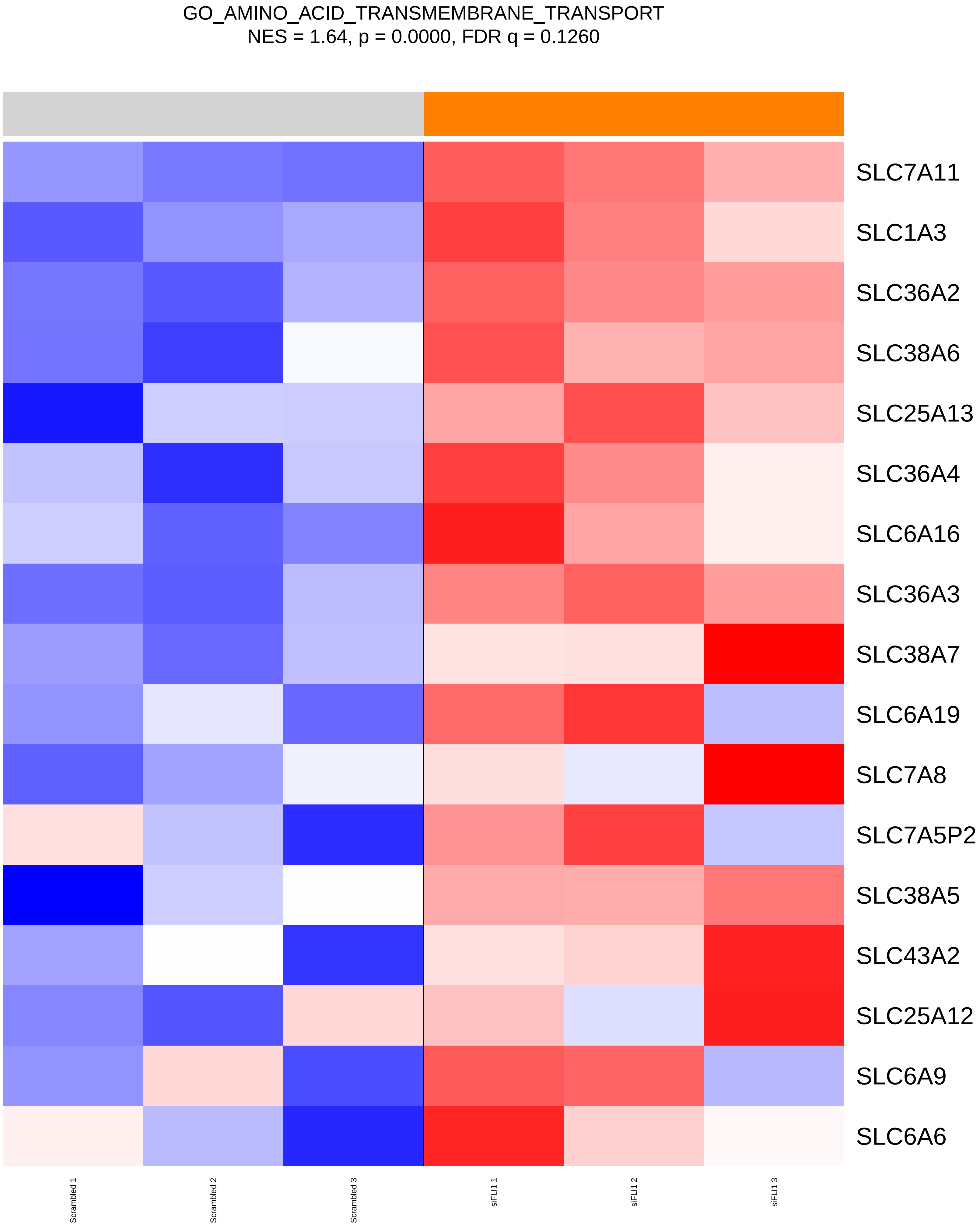
GO_AMINO_ACID_TRANSMEMBRANE_TRANSPORT
NES = 1.64, p = 0.0000, FDR q = 0.1260
SLC7A11
SLC1A3
SLC36A2
SLC38A6
SLC25A13
SLC36A4
SLC6A16
SLC36A3
SLC38A7
SLC6A19
SLC7A8
SLC7A5P2
SLC38A5
SLC43A2
SLC25A12
SLC6A9
SLC6A6
Scrambled 1
Scrambled 2
Scrambled 3
siFLI1 1
siFLI1 2
siFLI1 3

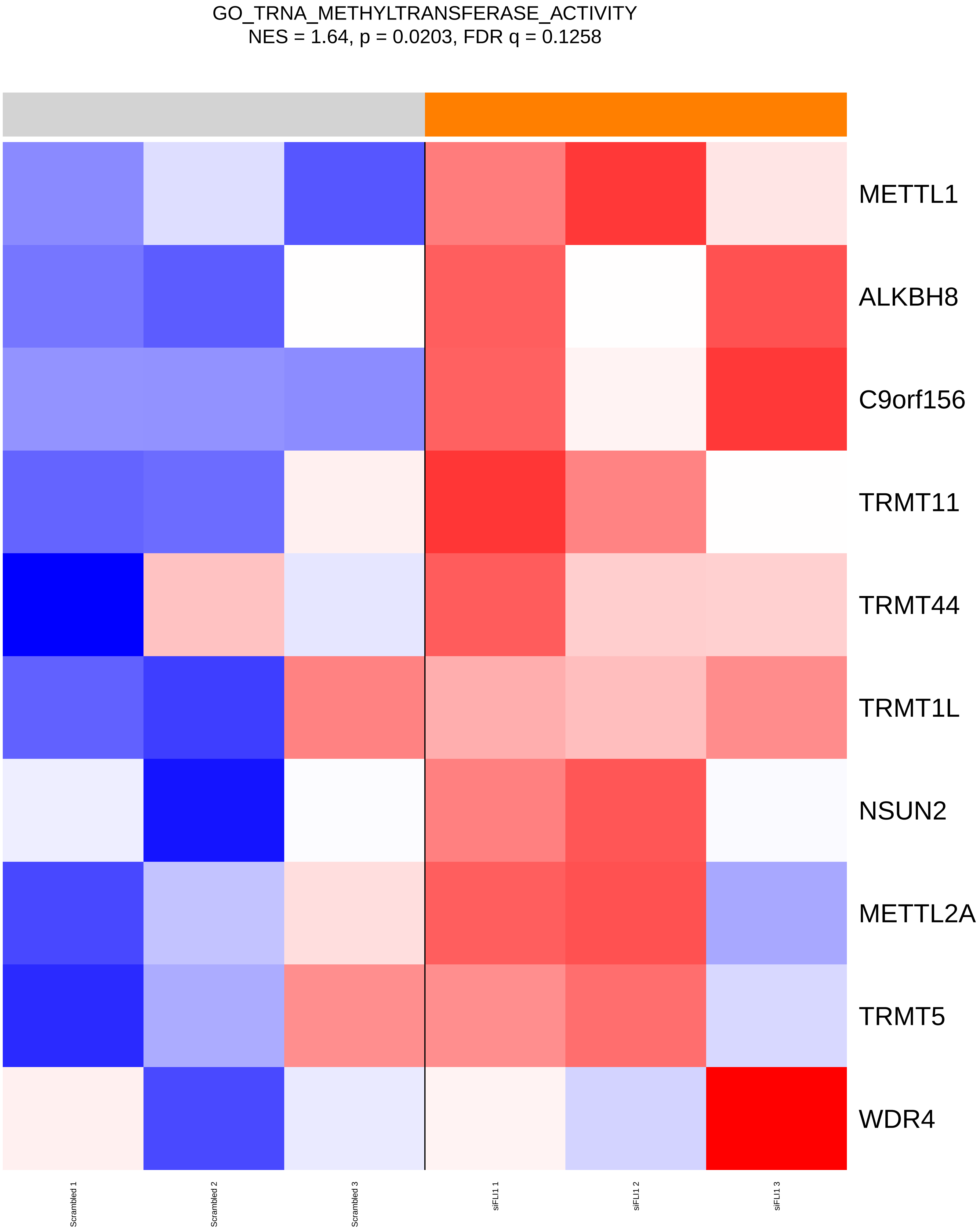
GO_TRNA_METHYLTRANSFERASE_ACTIVITY
NES = 1.64, p = 0.0203, FDR q = 0.1258
METTL1
ALKBH8
C9orf156
TRMT11
TRMT44
TRMT1L
NSUN2
METTL2A
TRMT5
WDR4
Scrambled 1
Scrambled 2
Scrambled 3
siFLI1 1
siFLI1 2
siFLI1 3

GO_PARTURITION
NES = 1.64, p = 0.0174, FDR q = 0.1255

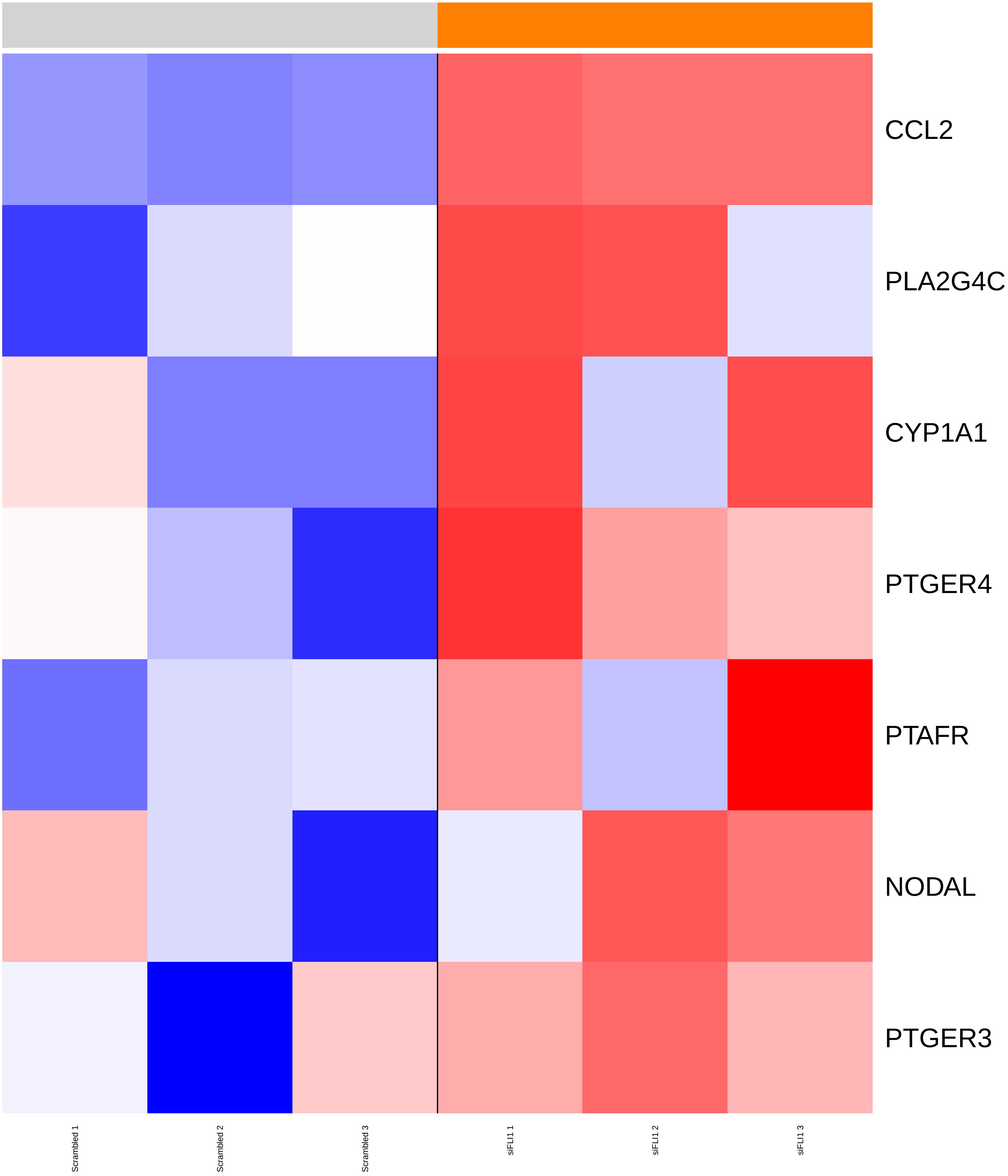

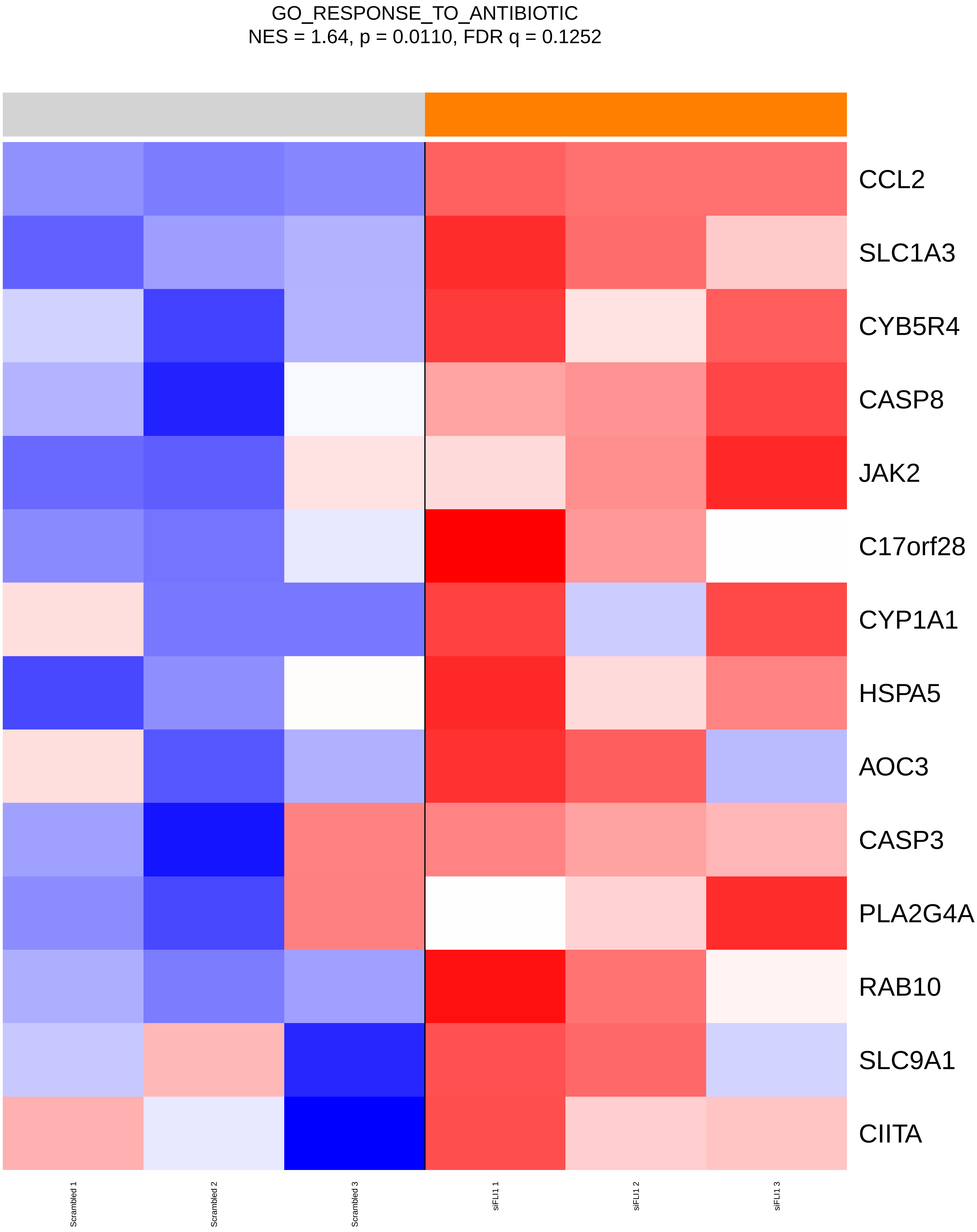
GO_RESPONSE_TO_ANTIBIOTIC
NES = 1.64, p = 0.0110, FDR q = 0.1252
CCL2
SLC1A3
CYB5R4
CASP8
JAK2
C17orf28
CYP1A1
HSPA5
AOC3
CASP3
PLA2G4A
RAB10
SLC9A1
CIITA
Scrambled 1
Scrambled 2
Scrambled 3
siFLI1 1
siFLI1 2
siFLI1 3

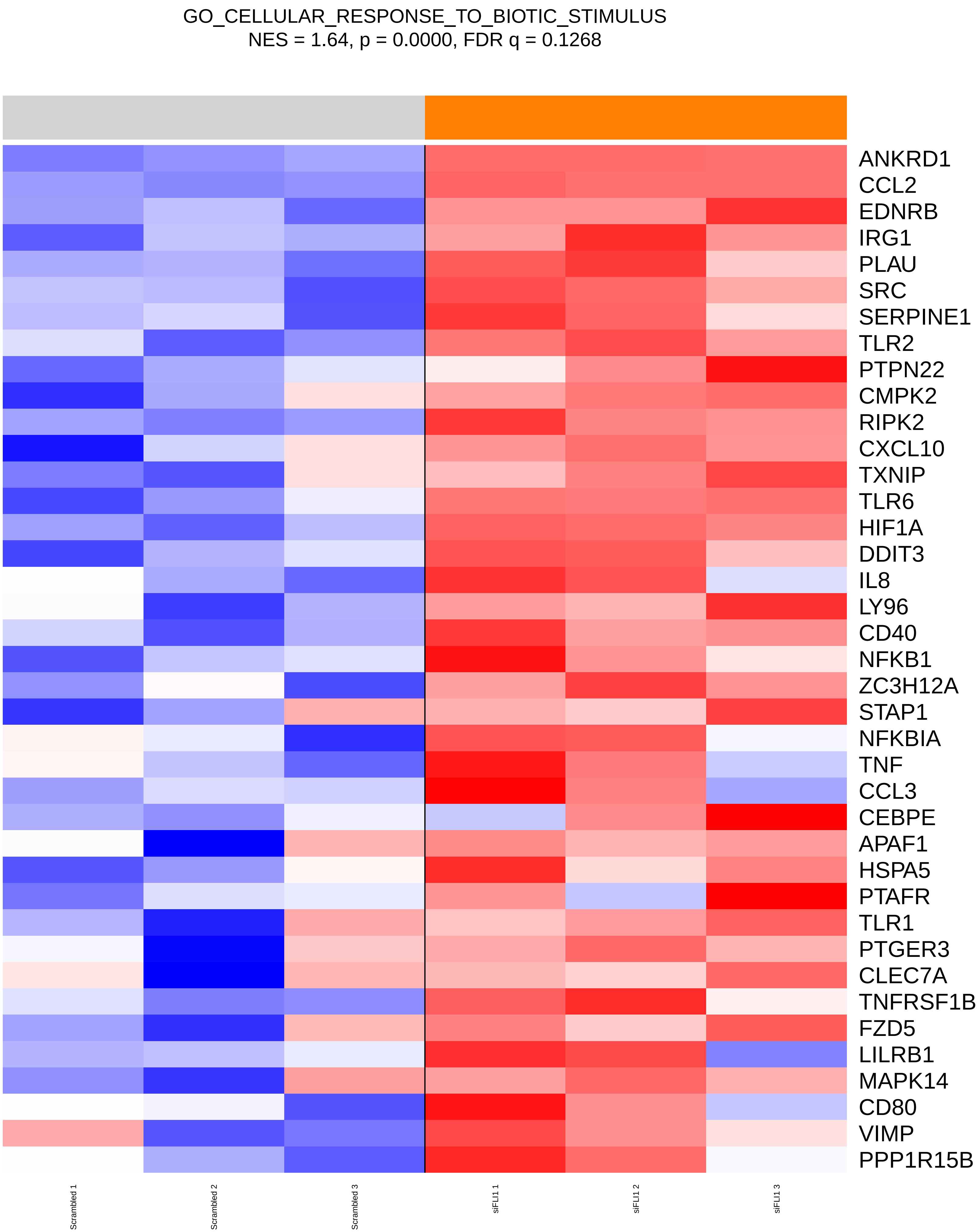
GO_CELLULAR_RESPONSE_TO_BIOTIC_STIMULUS
NES = 1.64, p = 0.0000, FDR q = 0.1268
ANKRD1
CCL2
EDNRB
IRG1
PLAU
SRC
SERPINE1
TLR2
PTPN22
CMPK2
RIPK2
CXCL10
TXNIP
TLR6
HIF1A
DDIT3
IL8
LY96
CD40
NFKB1
ZC3H12A
STAP1
NFKBIA
TNF
CCL3
CEBPE
APAF1
HSPA5
PTAFR
TLR1
PTGER3
CLEC7A
TNFRSF1B
FZD5
LILRB1
MAPK14
CD80
VIMP
PPP1R15B
Scrambled 1
Scrambled 2
Scrambled 3
siFLI1 1
siFLI1 2
siFLI1 3

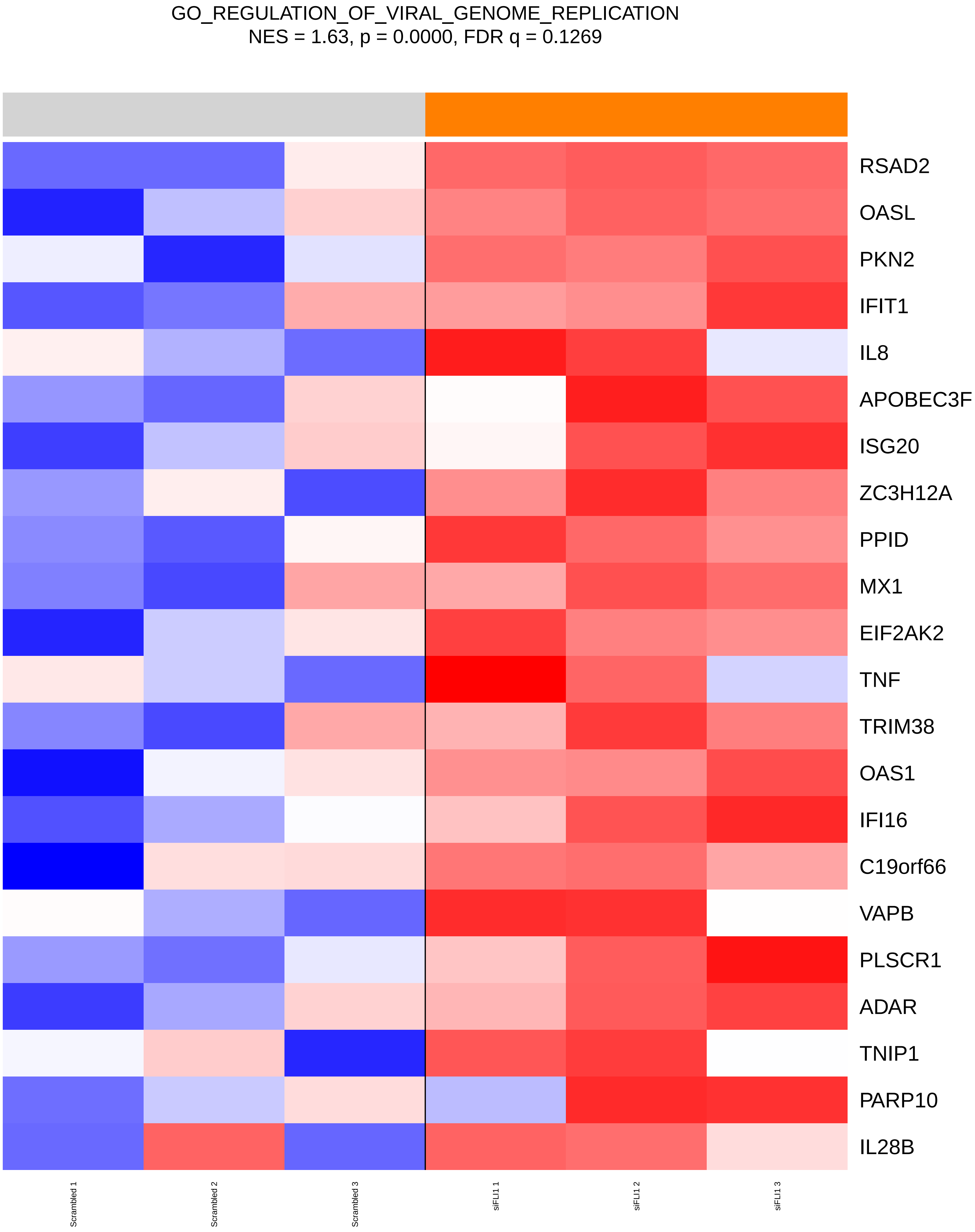

GO_REGULATION_OF_VIRAL_GENOME_REPLICATION
NES = 1.63, p = 0.0000, FDR q = 0.1269
RSAD2
OASL
PKN2
IFIT1
IL8
APOBEC3F
ISG20
ZC3H12A
PPID
MX1
EIF2AK2
TNF
TRIM38
OAS1
IFI16
C19orf66
VAPB
PLSCR1
ADAR
TNIP1
PARP10
IL28B
Scrambled 1
Scrambled 2
Scrambled 3
siFLI1 1
siFLI1 2
siFLI1 3

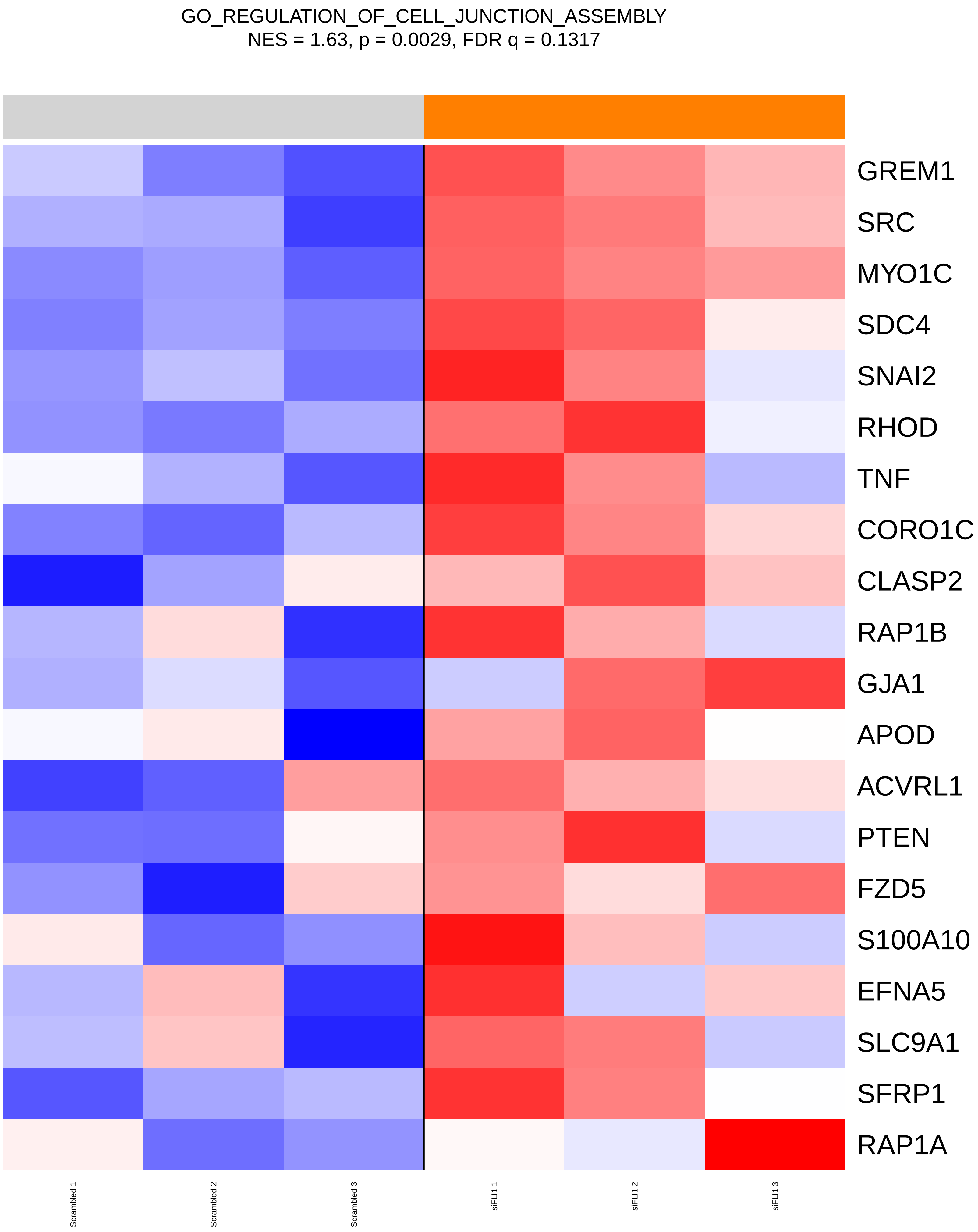
GO_REGULATION_OF_CELL_JUNCTION_ASSEMBLY
NES = 1.63, p = 0.0029, FDR q = 0.1317
GREM1
SRC
MYO1C
SDC4
SNAI2
RHOD
TNF
CORO1C
CLASP2
RAP1B
GJA1
APOD
ACVRL1
PTEN
FZD5
S100A10
EFNA5
SLC9A1
SFRP1
RAP1A
Scrambled 1
Scrambled 2
Scrambled 3
siFLI1 1
siFLI1 2
siFLI1 3

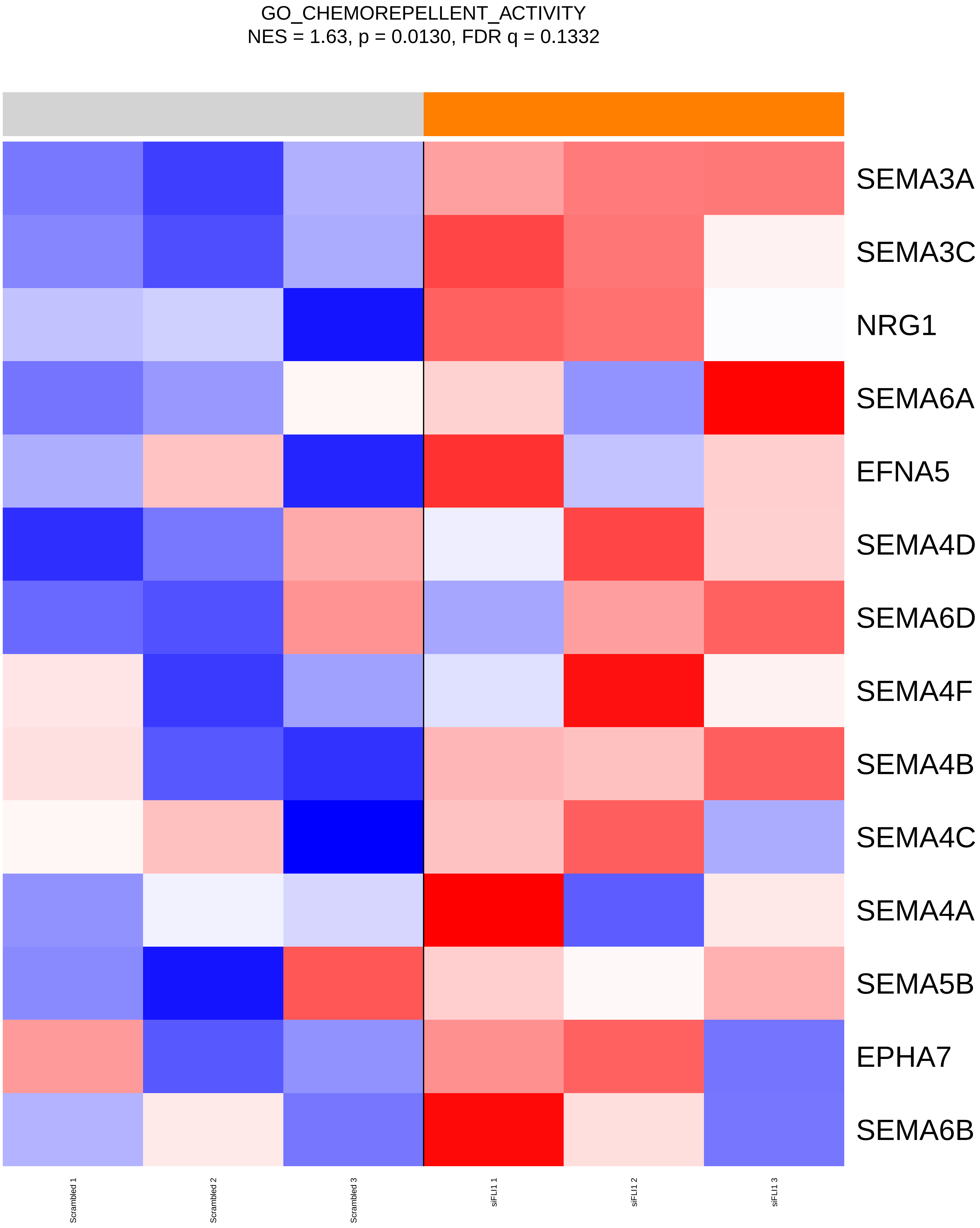
GO_CHEMOREPELLENT_ACTIVITY
NES = 1.63, p = 0.0130, FDR q = 0.1332
SEMA3A
SEMA3C
NRG1
SEMA6A
EFNA5
SEMA4D
SEMA6D
SEMA4F
SEMA4B
SEMA4C
SEMA4A
SEMA5B
EPHA7
SEMA6B
Scrambled 1
Scrambled 2
Scrambled 3
siFLI1 1
siFLI1 2
siFLI1 3

TTTTGAG_MIR373

NES = 1.63, p = 0.0000, FDR q = 0.1335

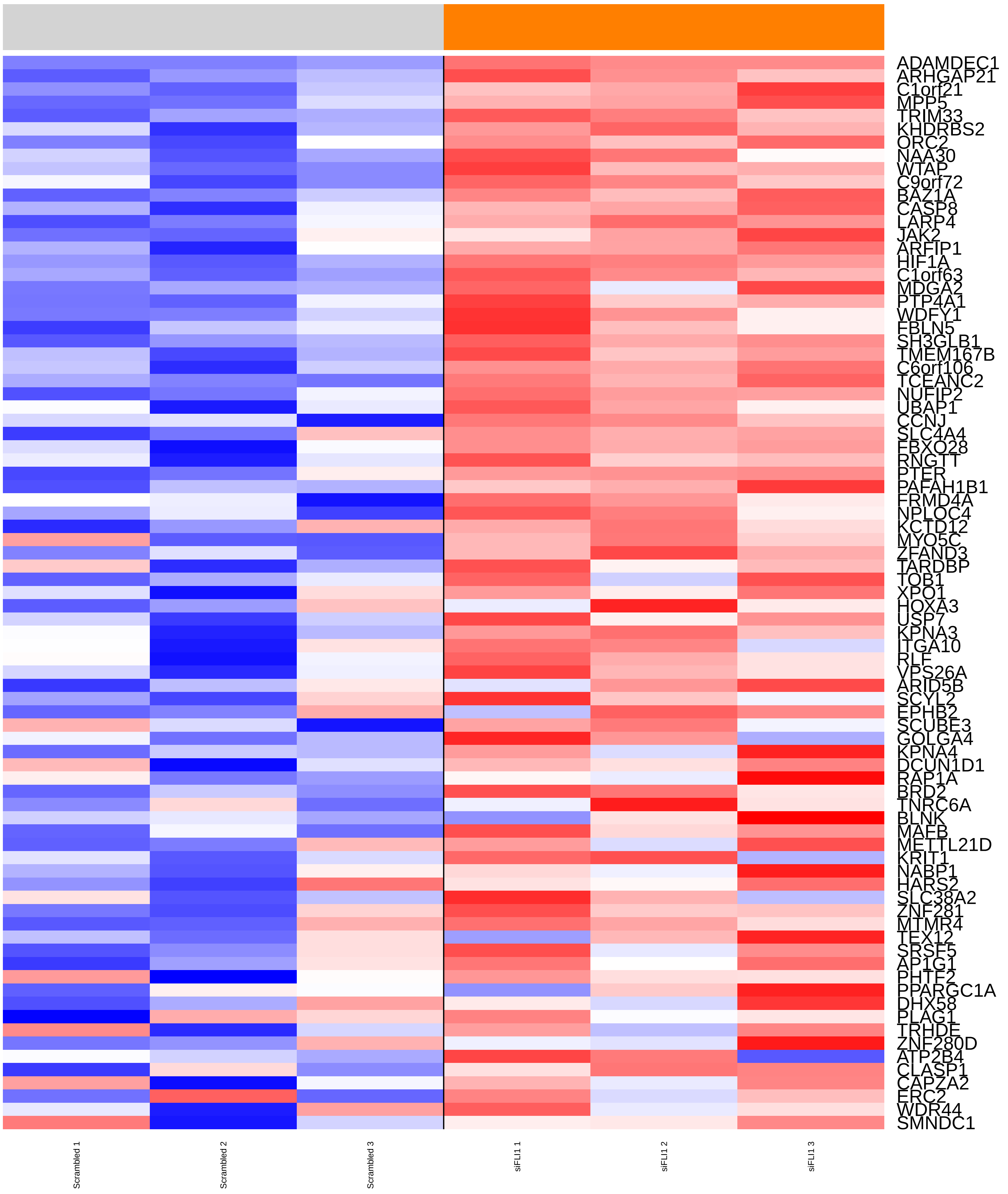

GO_RNA_METHYLTRANSFERASE_ACTIVITY
NES = 1.63, p = 0.0198, FDR q = 0.1335

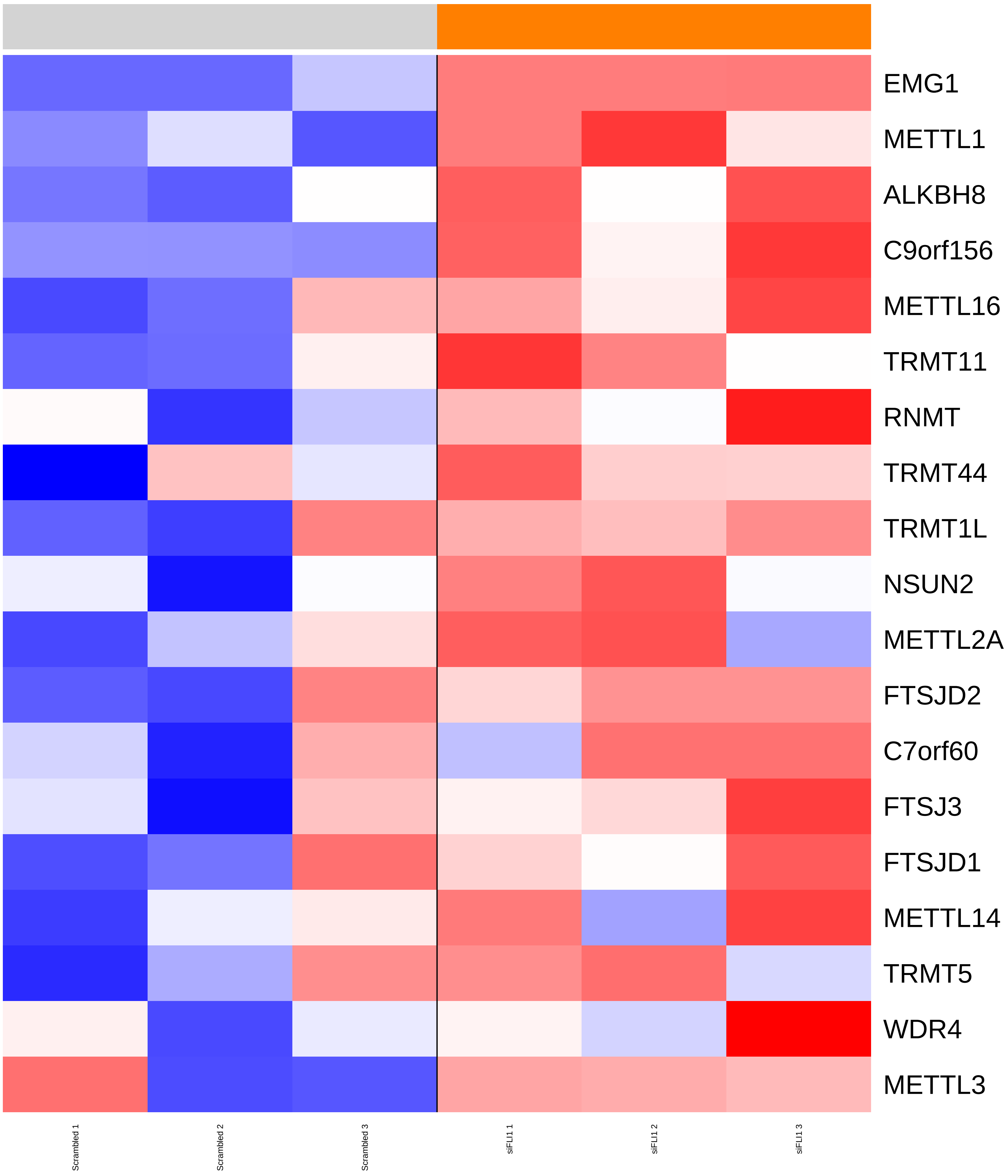

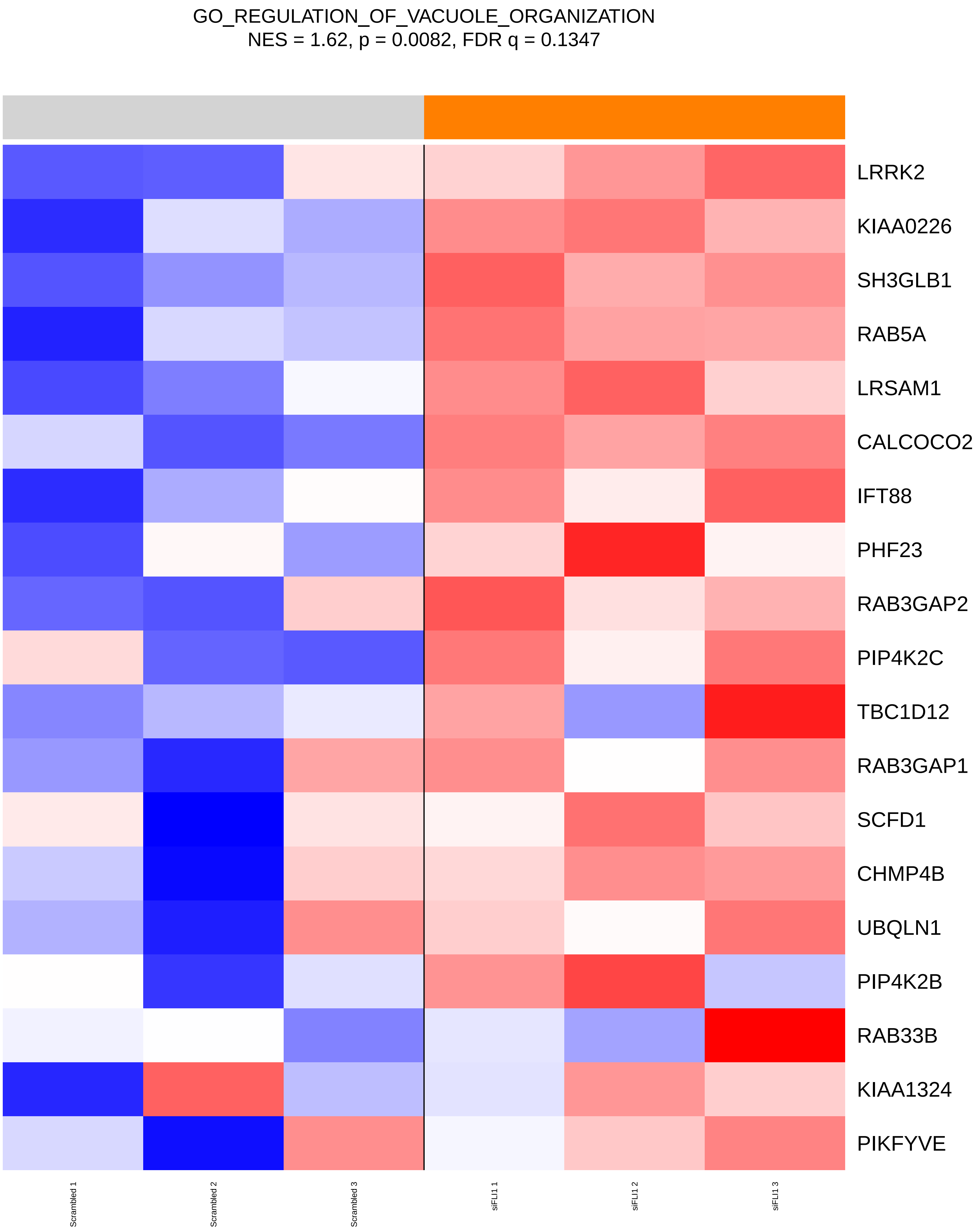
GO_REGULATION_OF_VACUOLE_ORGANIZATION
NES = 1.62, p = 0.0082, FDR q = 0.1347
LRRK2
KIAA0226
SH3GLB1
RAB5A
LRSAM1
CALCOCO2
IFT88
PHF23
RAB3GAP2
PIP4K2C
TBC1D12
RAB3GAP1
SCFD1
CHMP4B
UBQLN1
PIP4K2B
RAB33B
KIAA1324
PIKFYVE
Scrambled 1
Scrambled 2
Scrambled 3
siFLI1 1
siFLI1 2
siFLI1 3

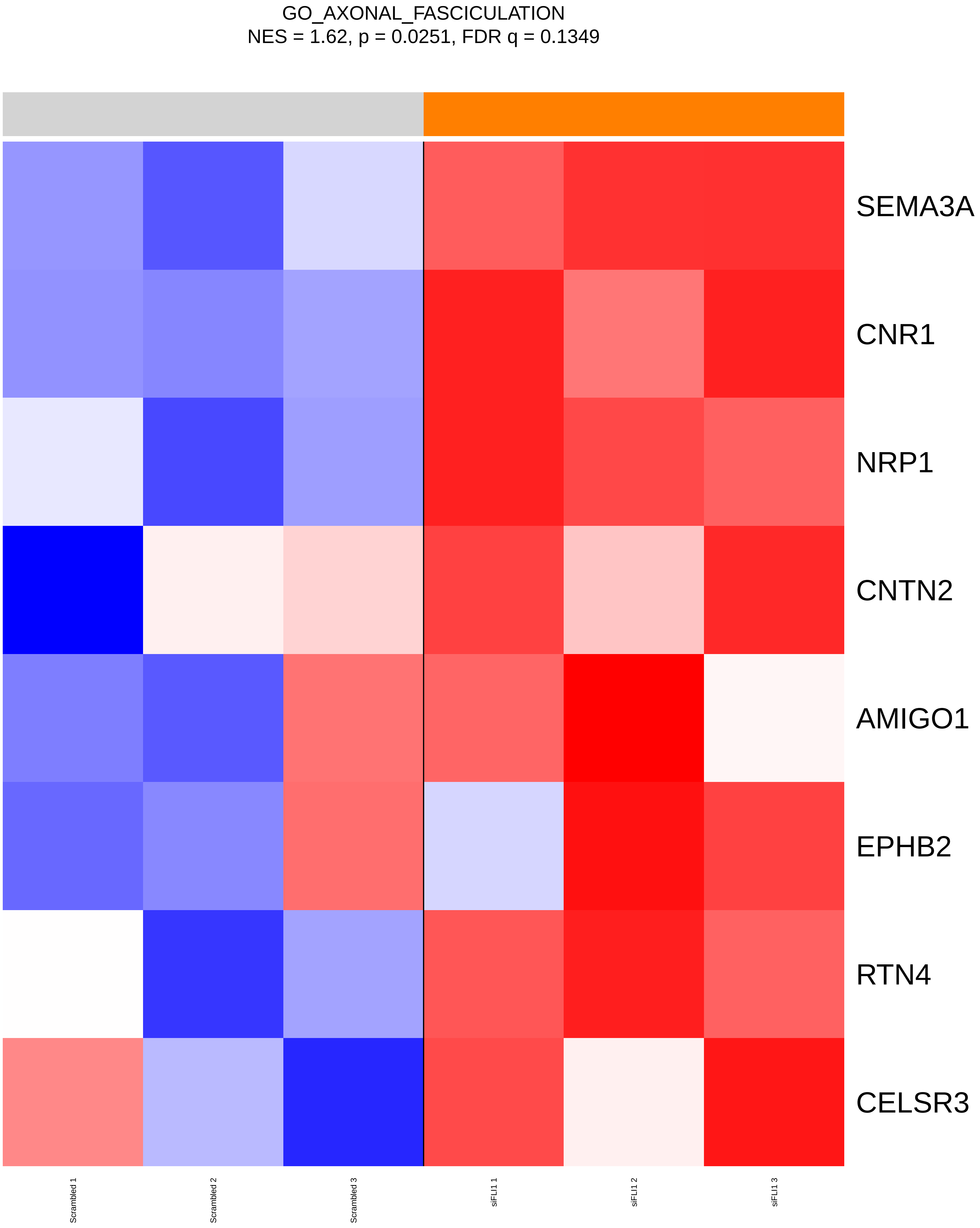
GO_AXONAL_FASCICULATION
NES = 1.62, p = 0.0251, FDR q = 0.1349
SEMA3A
CNR1
NRP1
CNTN2
AMIGO1
EPHB2
RTN4
CELSR3
Scrambled 1
Scrambled 2
Scrambled 3
siFLI1 1
siFLI1 2
siFLI1 3

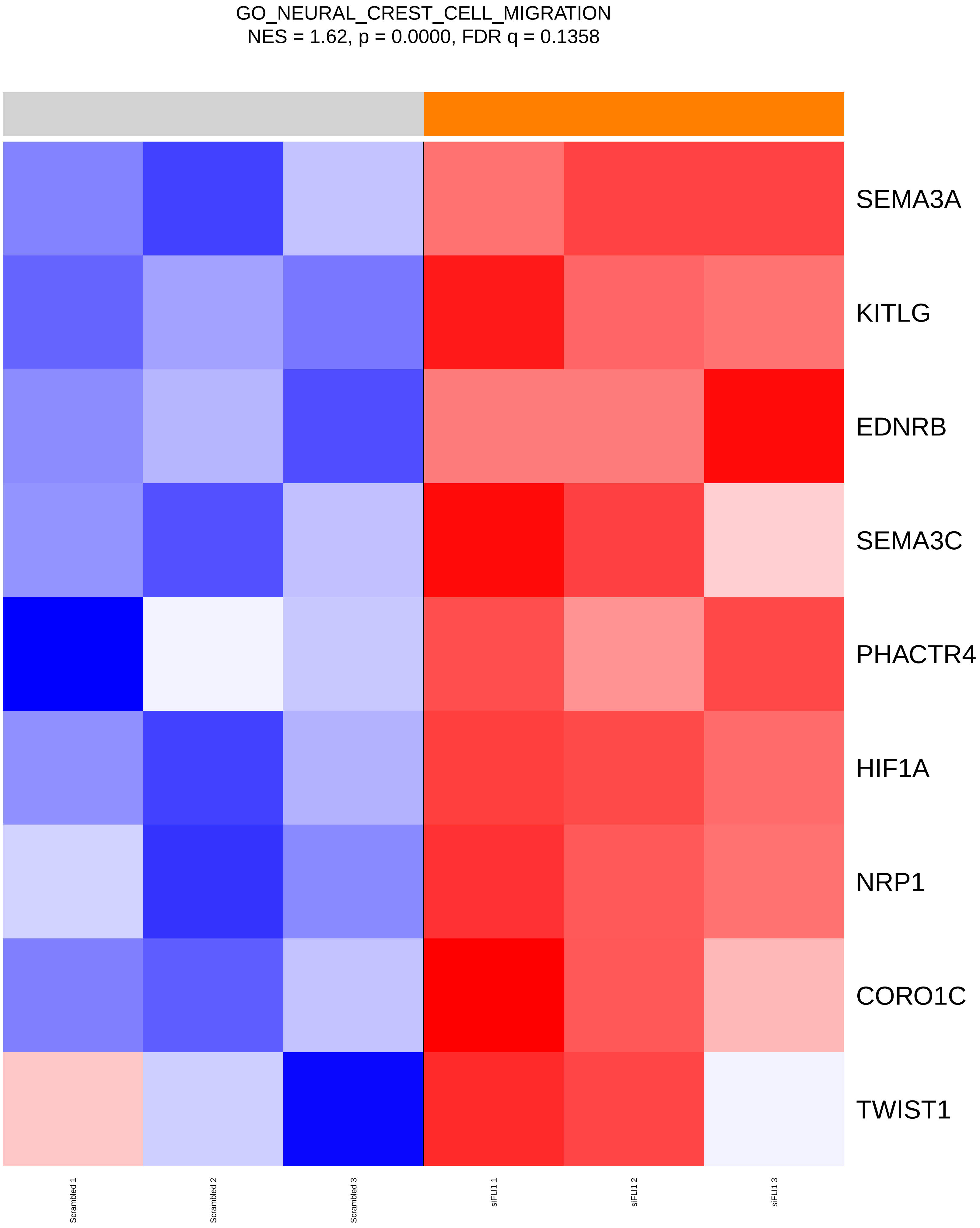
GO_NEURAL_CREST_CELL_MIGRATION
NES = 1.62, p = 0.0000, FDR q = 0.1358
SEMA3A
KITLG
EDNRB
SEMA3C
PHACTR4
HIF1A
NRP1
CORO1C
TWIST1
Scrambled 1
Scrambled 2
Scrambled 3
siFLI1 1
siFLI1 2
siFLI1 3

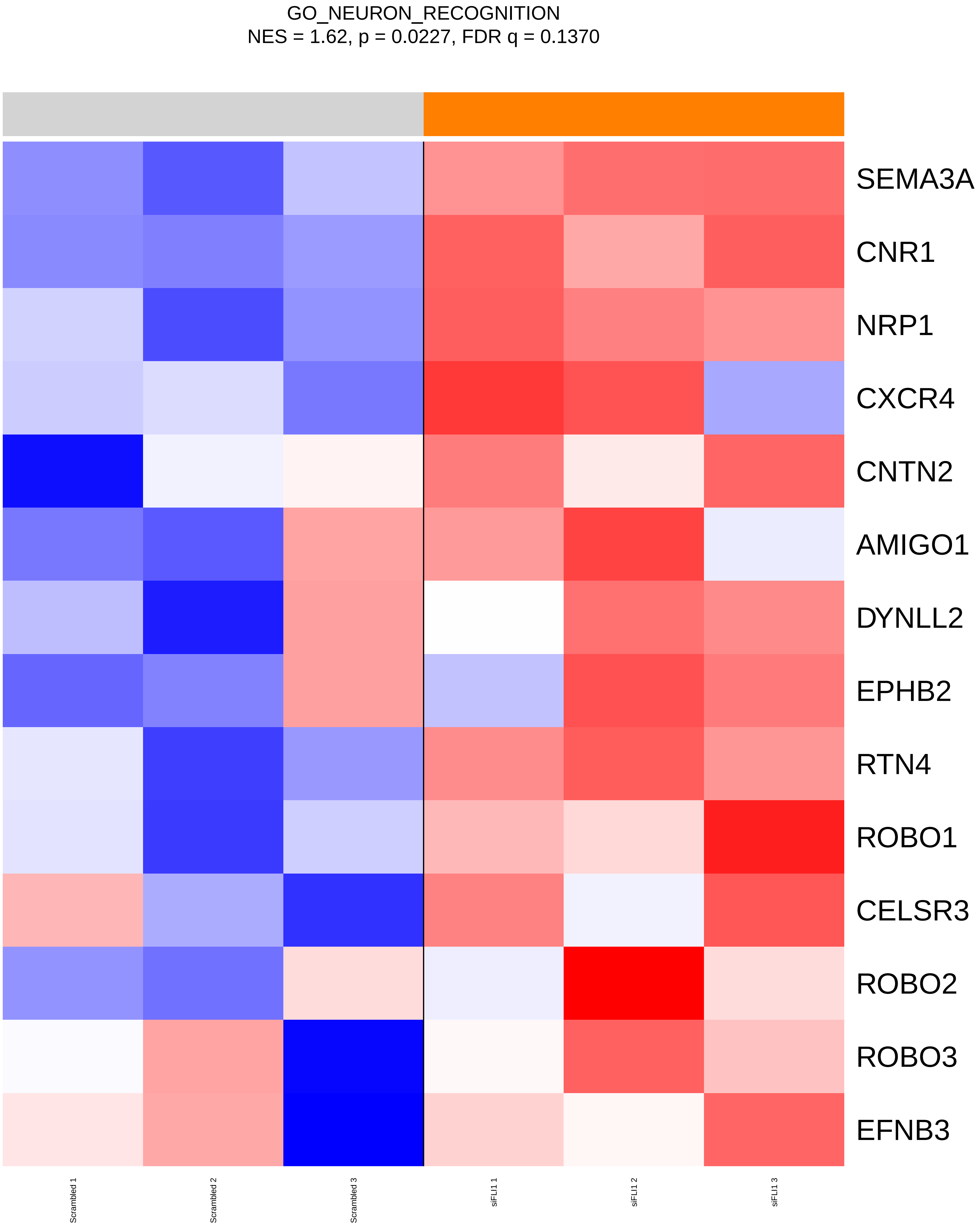
GO_NEURON_RECOGNITION
NES = 1.62, p = 0.0227, FDR q = 0.1370
SEMA3A
CNR1
NRP1
CXCR4
CNTN2
AMIGO1
DYNLL2
EPHB2
RTN4
ROBO1
CELSR3
ROBO2
ROBO3
EFNB3
Scrambled 1
Scrambled 2
Scrambled 3
siFLI1 1
siFLI1 2
siFLI1 3

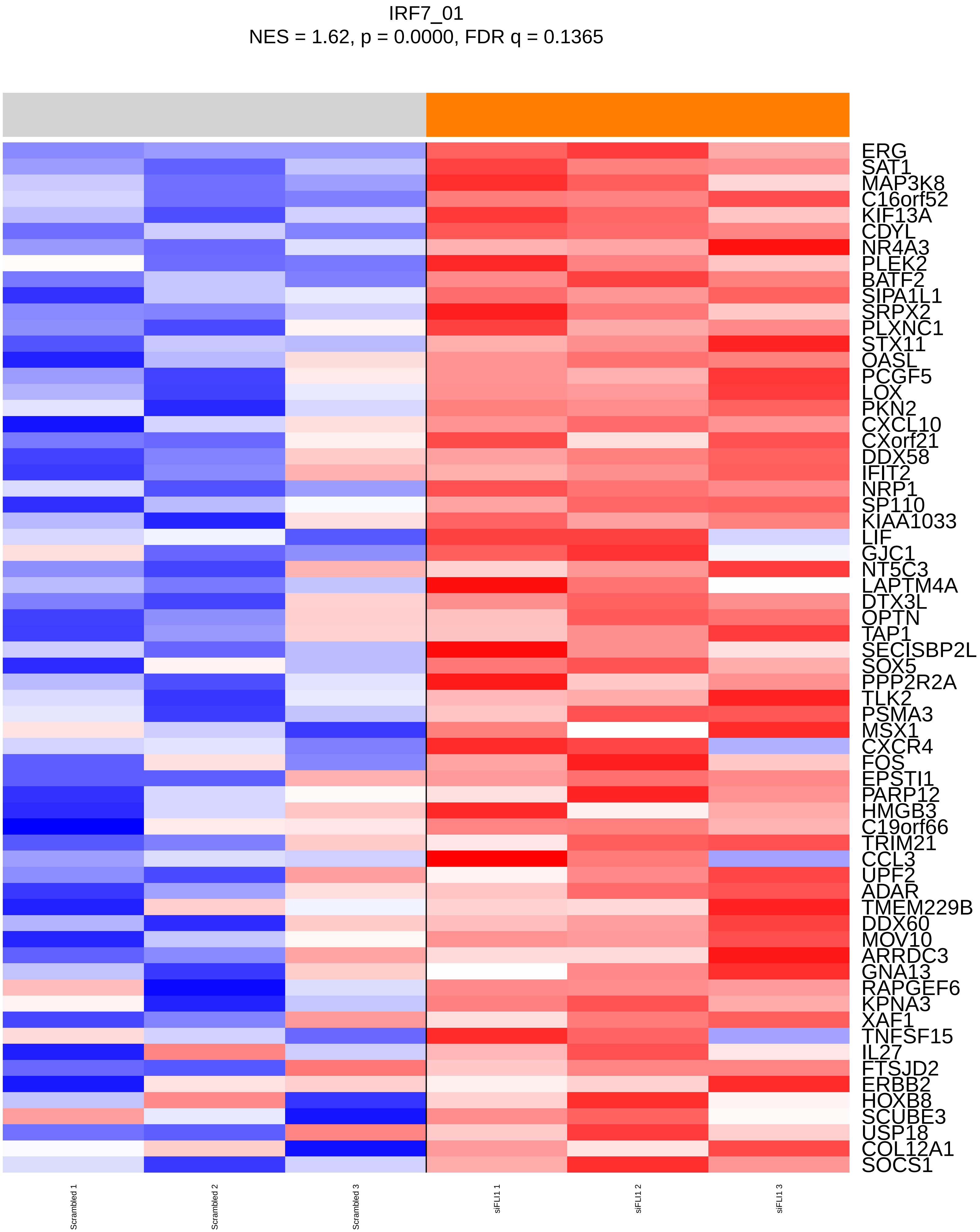
IRF7_01
NES = 1.62, p = 0.0000, FDR q = 0.1365
ERG
SAT1
MAP3K8
C16orf52
KIF13A
CDYL
NR4A3
PLEK2
BATF2
SIPA1L1
SRPX2
PLXNC1
STX11
OASL
PCGF5
LOX
PKN2
CXCL10
CXorf21
DDX58
IFIT2
NRP1
SP110
KIAA1033
LIF
GJC1
NT5C3
LAPTM4A
DTX3L
OPTN
TAP1
SECISBP2L
SOX5
PPP2R2A
TLK2
PSMA3
MSX1
CXCR4
FOS
EPSTI1
PARP12
HMGB3
C19orf66
TRIM21
CCL3
UPF2
ADAR
TMEM229B
DDX60
MOV10
ARRDC3
GNA13
RAPGEF6
KPNA3
XAF1
TNFSF15
IL27
FTSJD2
ERBB2
HOXB8
SCUBE3
USP18
COL12A1
SOCS1
Scrambled 1
Scrambled 2
Scrambled 3
siFLI1 1
siFLI1 2
siFLI1 3

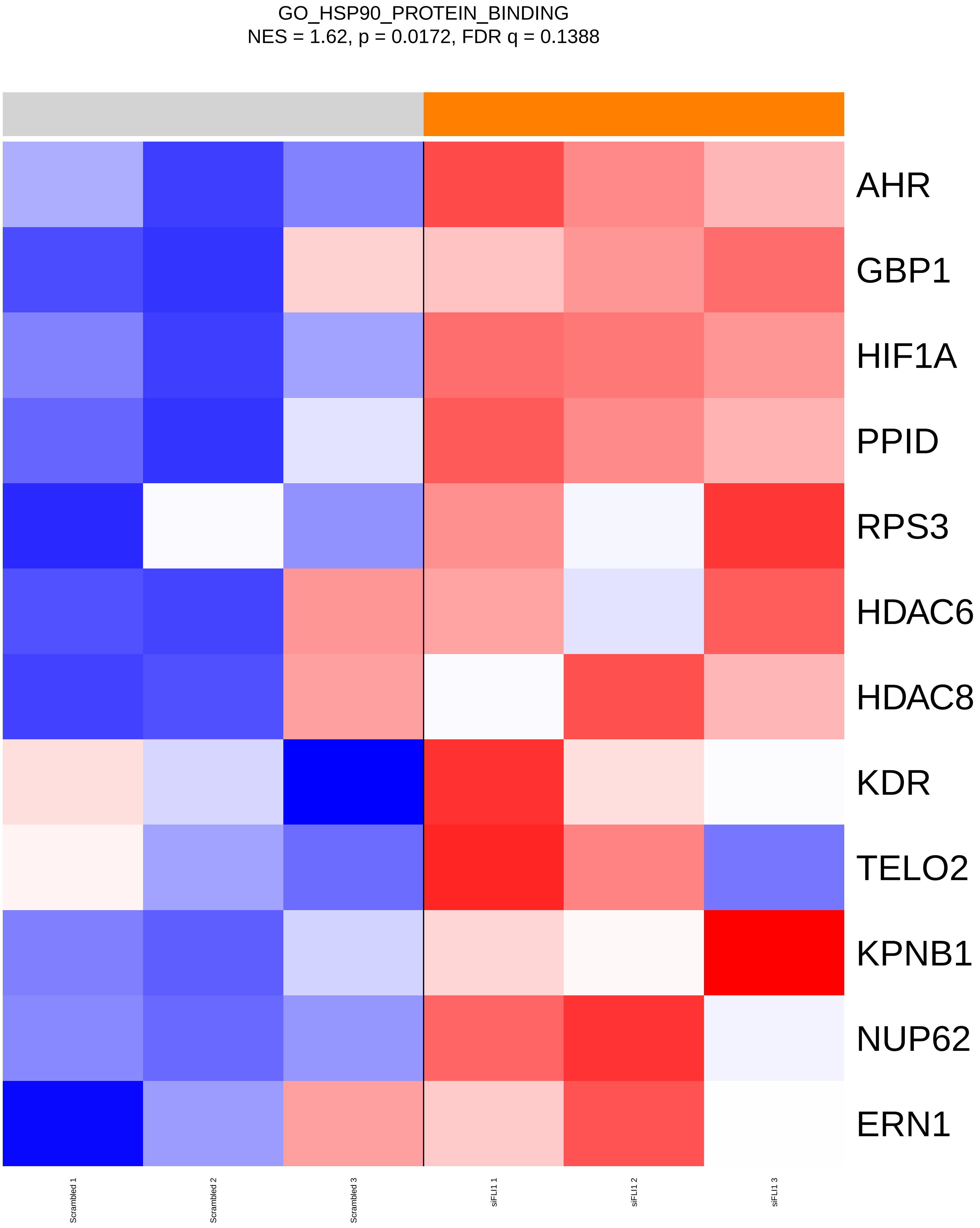
GO_HSP90_PROTEIN_BINDING
NES = 1.62, p = 0.0172, FDR q = 0.1388
AHR
GBP1
HIF1A
PPID
RPS3
HDAC6
HDAC8
KDR
TELO2
KPNB1
NUP62
ERN1
Scrambled 1
Scrambled 2
Scrambled 3
siFLI1 1
siFLI1 2
siFLI1 3

GO_NEGATIVE_REGULATION_OF_CELL_JUNCTION_ASSEMBLY
NES = 1.62, p = 0.0316, FDR q = 0.1384

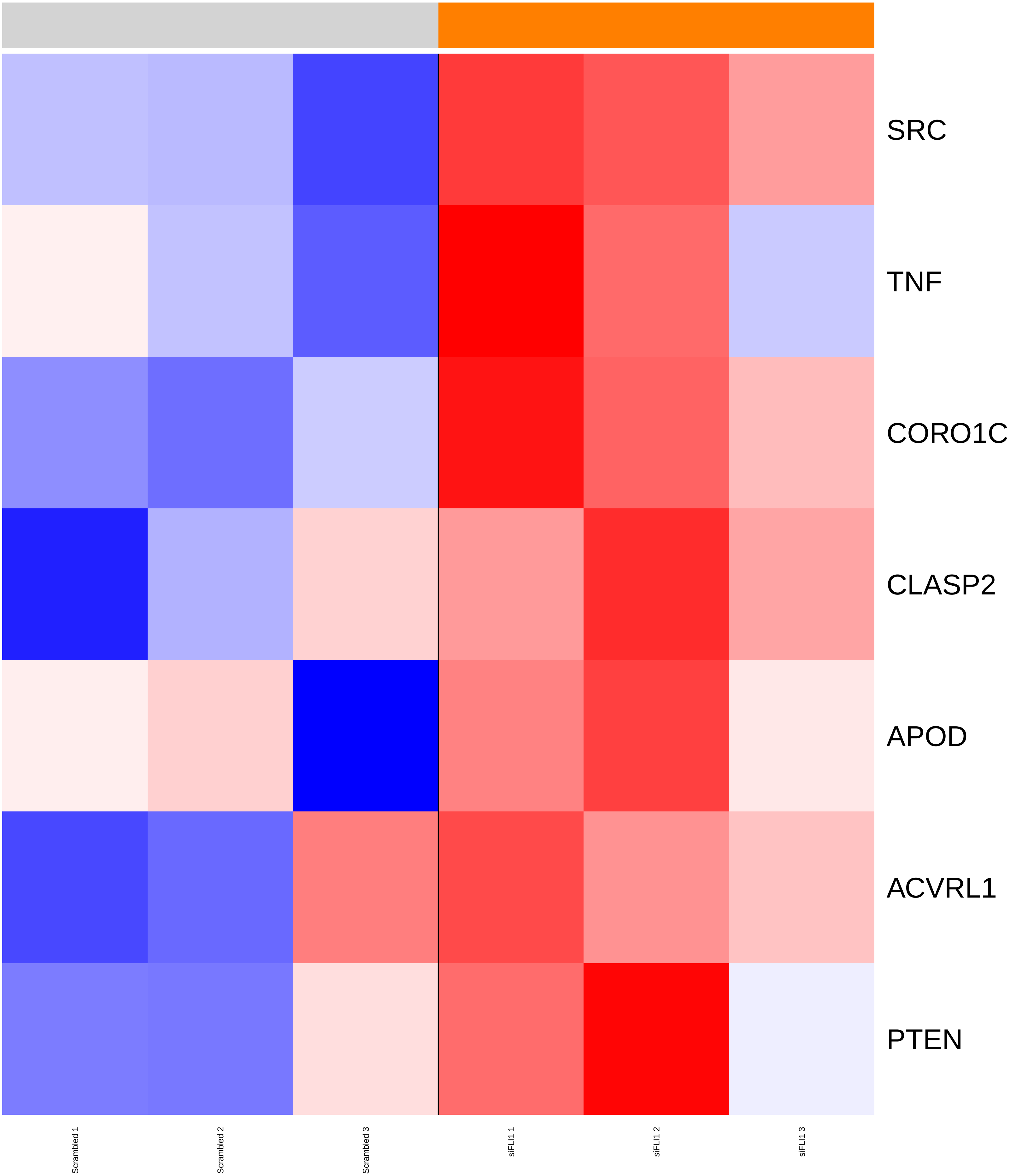

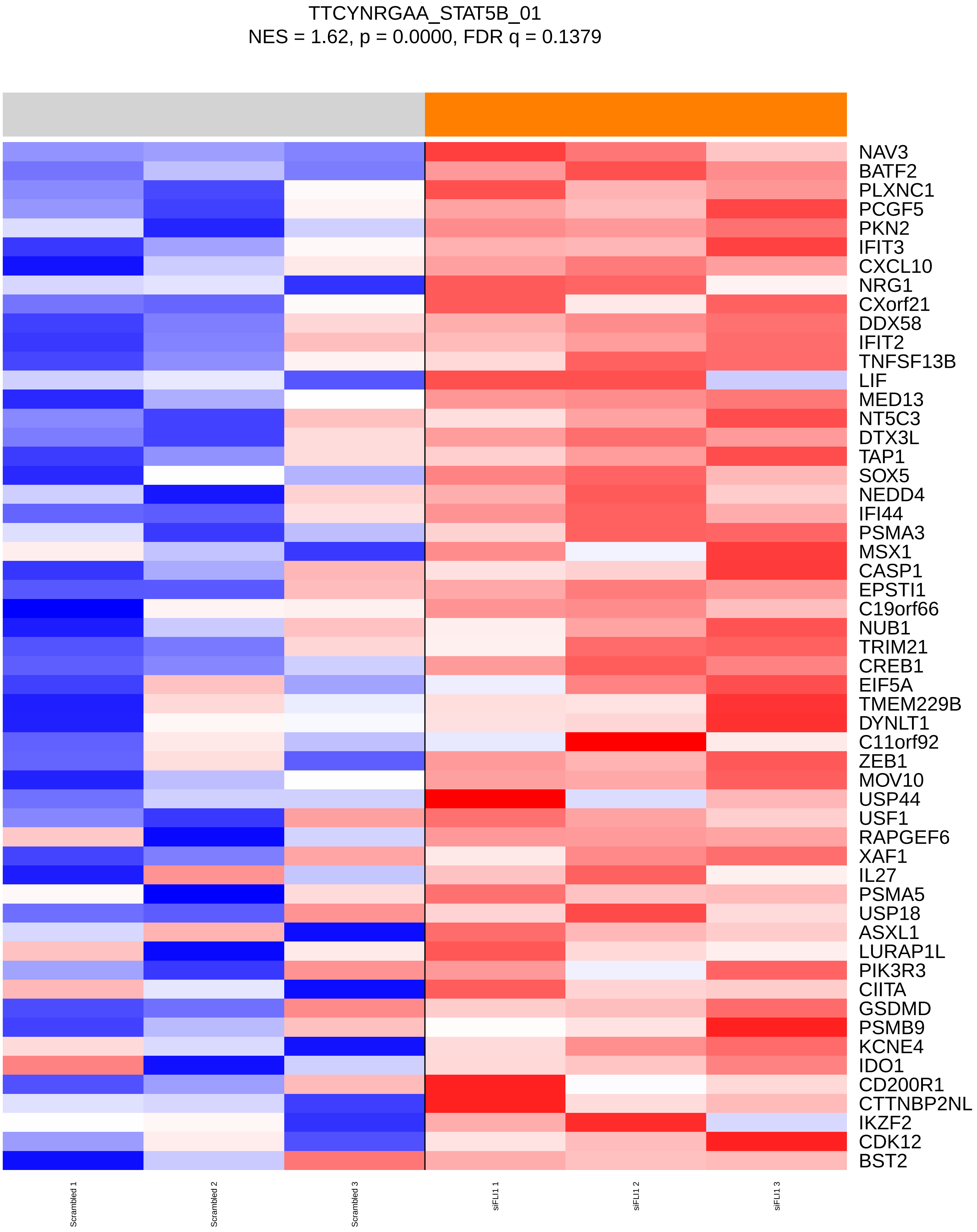
TTCYNRGAA_STAT5B_01
NES = 1.62, p = 0.0000, FDR q = 0.1379
NAV3
BATF2
PLXNC1
PCGF5
PKN2
IFIT3
CXCL10
NRG1
CXorf21
DDX58
IFIT2
TNFSF13B
LIF
MED13
NT5C3
DTX3L
TAP1
SOX5
NEDD4
IFI44
PSMA3
MSX1
CASP1
EPSTI1
C19orf66
NUB1
TRIM21
CREB1
EIF5A
TMEM229B
DYNLT1
C11orf92
ZEB1
MOV10
USP44
USF1
RAPGEF6
XAF1
IL27
PSMA5
USP18
ASXL1
LURAP1L
PIK3R3
CIITA
GSDMD
PSMB9
KCNE4
IDO1
CD200R1
CTTNBP2NL
IKZF2
CDK12
BST2
Scrambled 1
Scrambled 2
Scrambled 3
siFLI1 1
siFLI1 2
siFLI1 3

GO_REGULATION_OF_ANOIKIS
NES = 1.61, p = 0.0287, FDR q = 0.1428

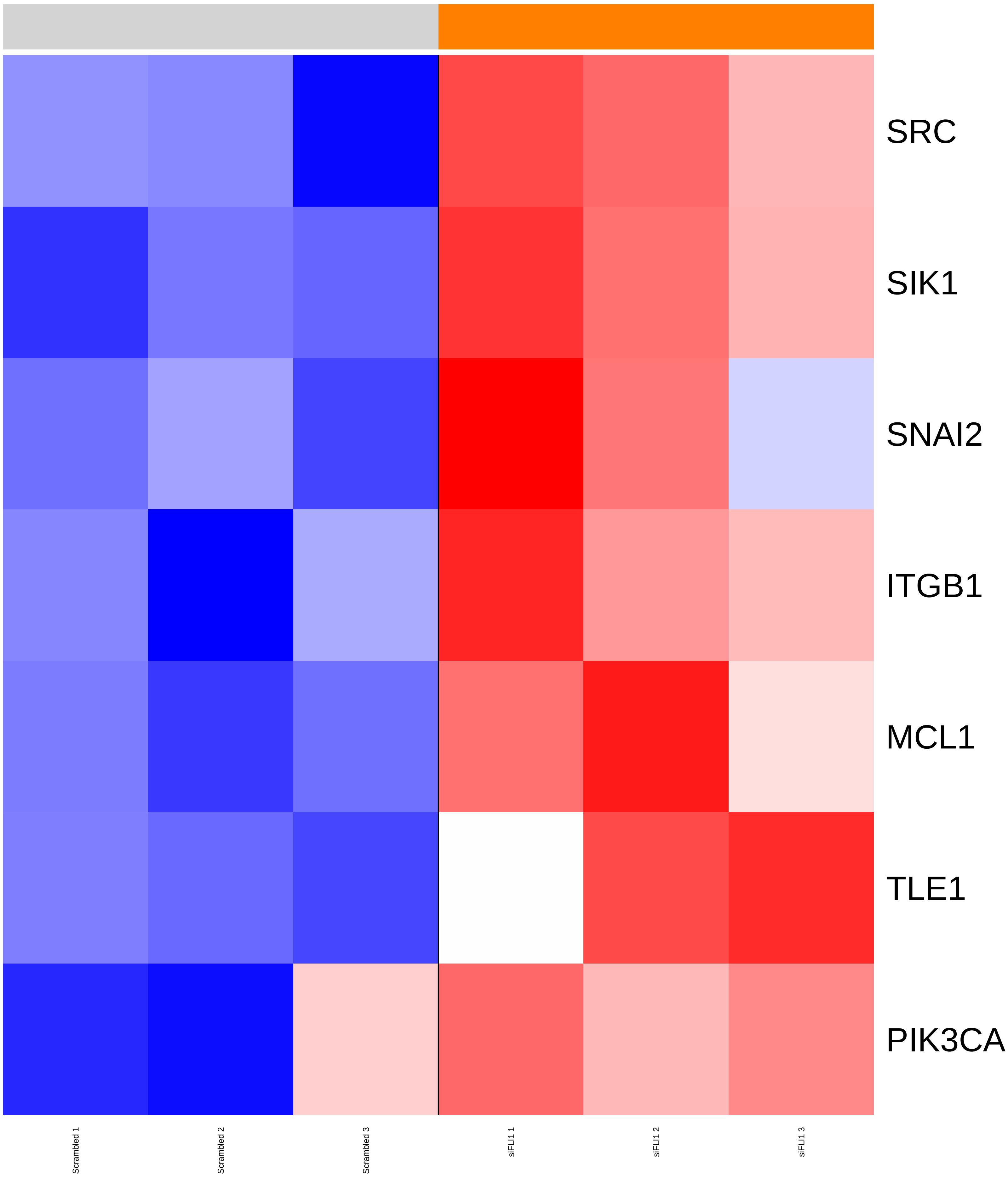

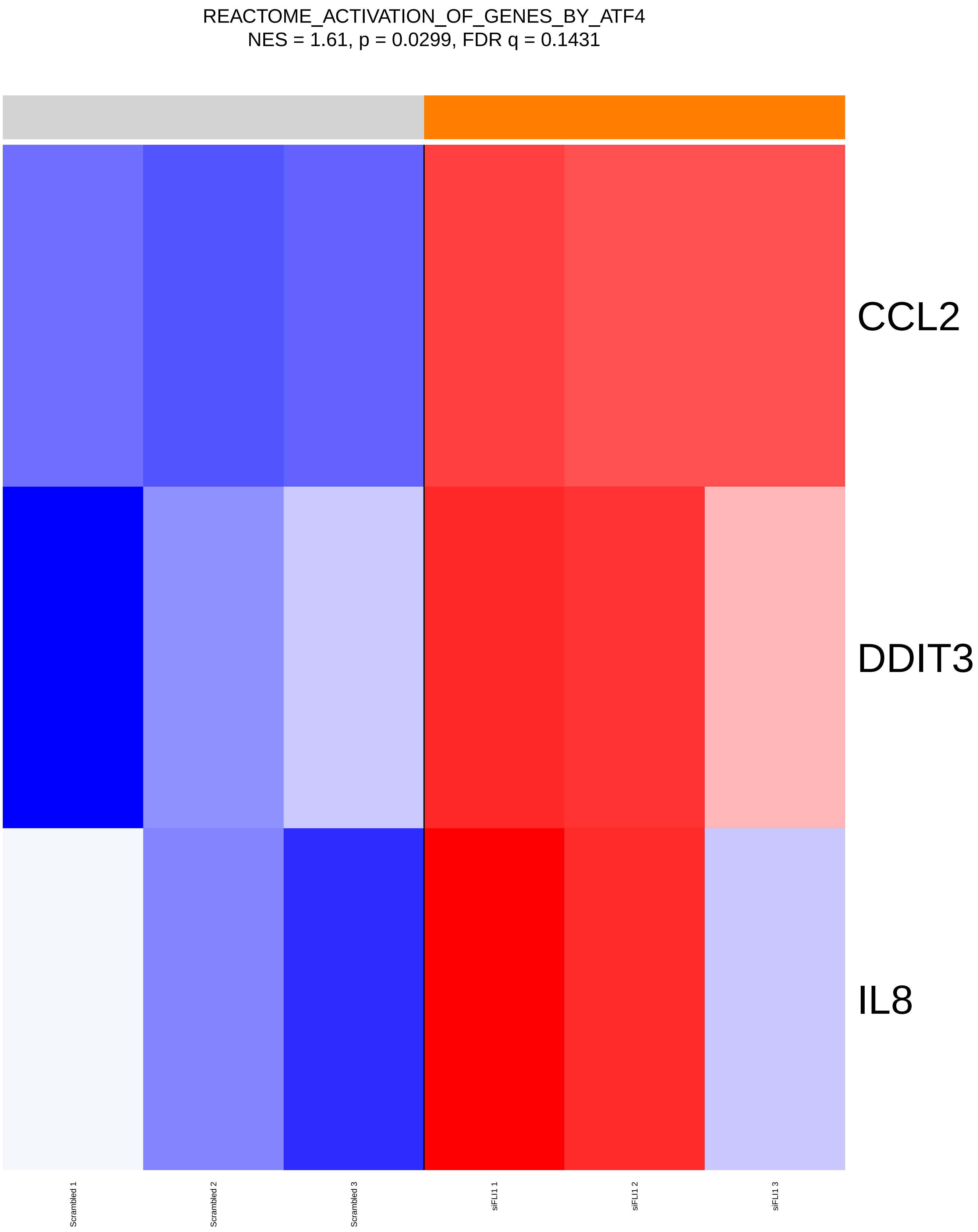
REACTOME_ACTIVATION_OF_GENES_BY_ATF4
NES = 1.61, p = 0.0299, FDR q = 0.1431
CCL2
DDIT3
IL8
Scrambled 1
Scrambled 2
Scrambled 3
siFLI1 1
siFLI1 2
siFLI1 3

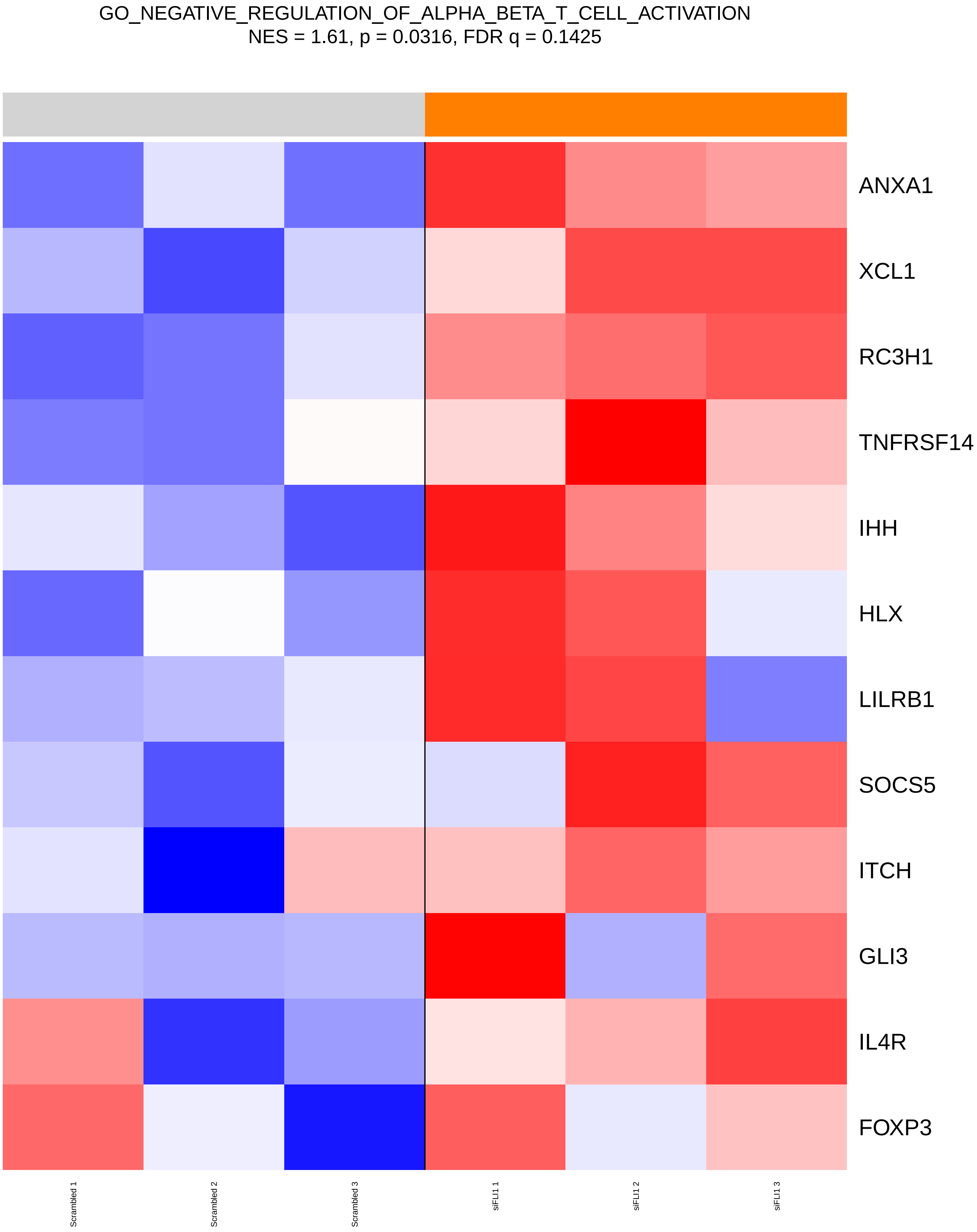
GO_NEGATIVE_REGULATION_OF_ALPHA_BETA_T_CELL_ACTIVATION
NES = 1.61, p = 0.0316, FDR q = 0.1425
ANXA1
XCL1
RC3H1
TNFRSF14
IHH
HLX
LILRB1
SOCS5
ITCH
GLI3
IL4R
FOXP3
Scrambled 1
Scrambled 2
Scrambled 3
siFLI1 1
siFLI1 2
siFLI1 3

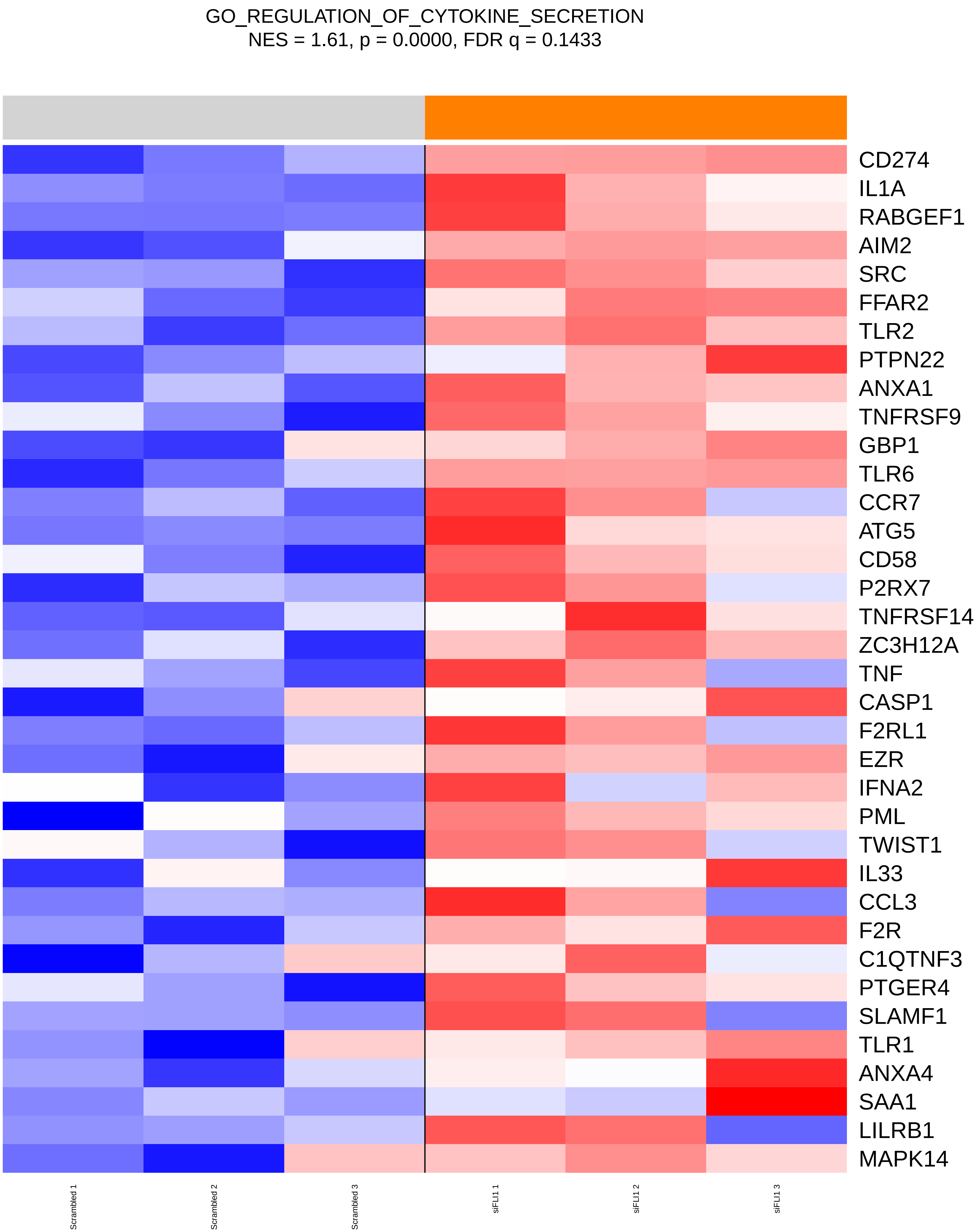
GO_REGULATION_OF_CYTOKINE_SECRETION
NES = 1.61, p = 0.0000, FDR q = 0.1433
CD274
IL1A
RABGEF1
AIM2
SRC
FFAR2
TLR2
PTPN22
ANXA1
TNFRSF9
GBP1
TLR6
CCR7
ATG5
CD58
P2RX7
TNFRSF14
ZC3H12A
TNF
CASP1
F2RL1
EZR
IFNA2
PML
TWIST1
IL33
CCL3
F2R
C1QTNF3
PTGER4
SLAMF1
TLR1
ANXA4
SAA1
LILRB1
MAPK14
Scrambled 1
Scrambled 2
Scrambled 3
siFLI1 1
siFLI1 2
siFLI1 3

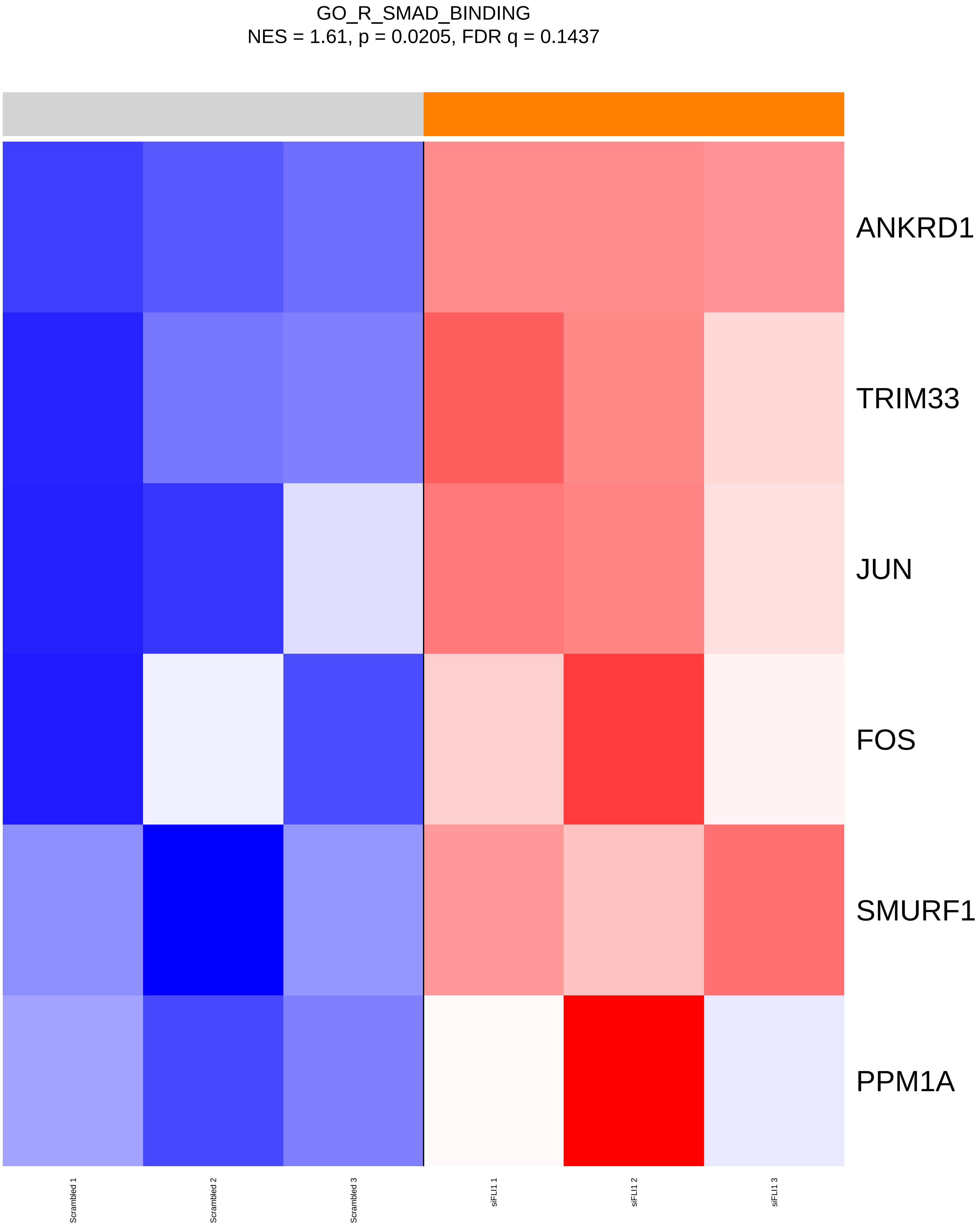
GO_R_SMAD_BINDING
NES = 1.61, p = 0.0205, FDR q = 0.1437
ANKRD1
TRIM33
JUN
FOS
SMURF1
PPM1A
Scrambled 1
Scrambled 2
Scrambled 3
siFLI1 1
siFLI1 2
siFLI1 3

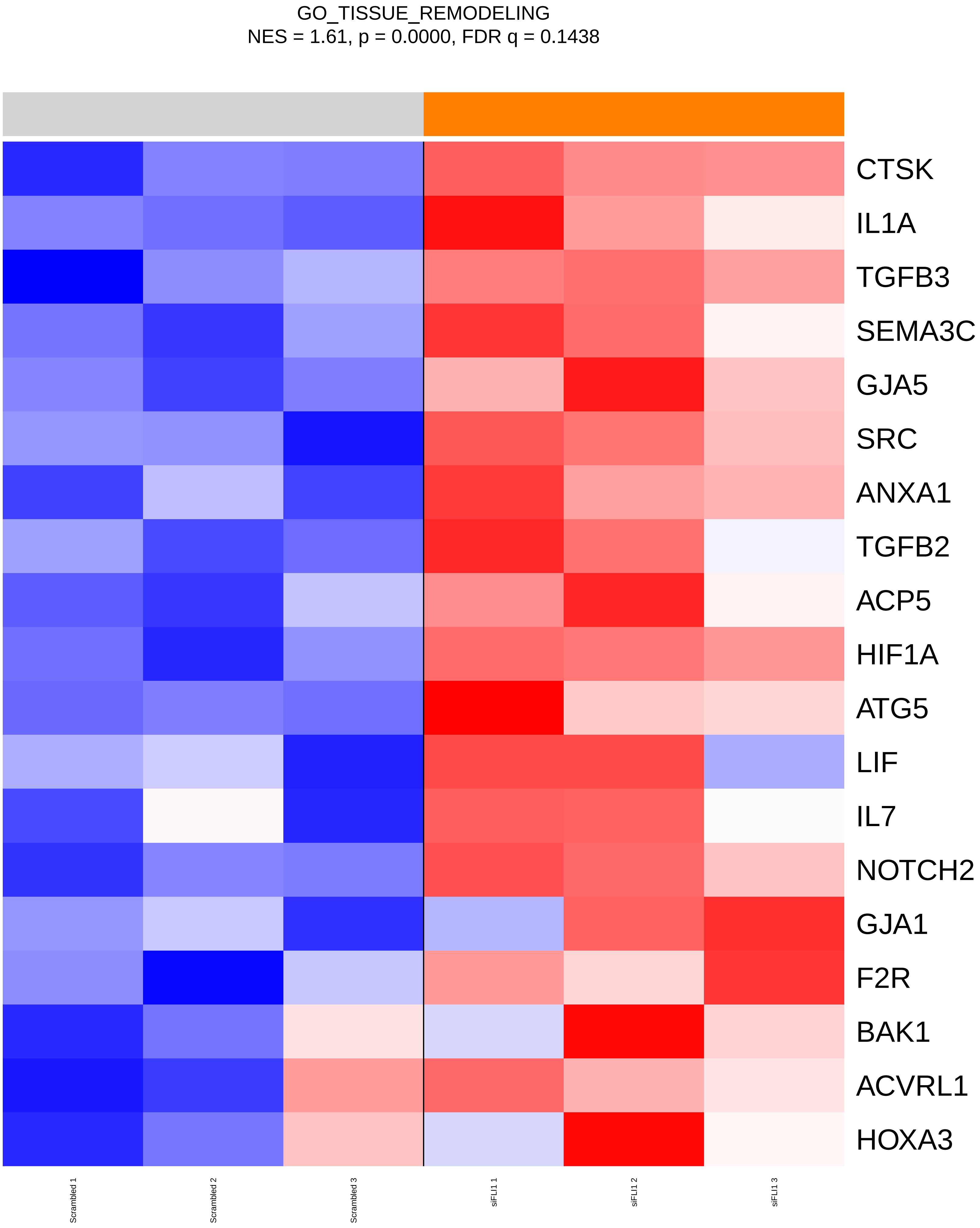
GO_TISSUE_REMODELING
NES = 1.61, p = 0.0000, FDR q = 0.1438
CTSK
IL1A
TGFB3
SEMA3C
GJA5
SRC
ANXA1
TGFB2
ACP5
HIF1A
ATG5
LIF
IL7
NOTCH2
GJA1
F2R
BAK1
ACVRL1
HOXA3
Scrambled 1
Scrambled 2
Scrambled 3
siFLI1 1
siFLI1 2
siFLI1 3

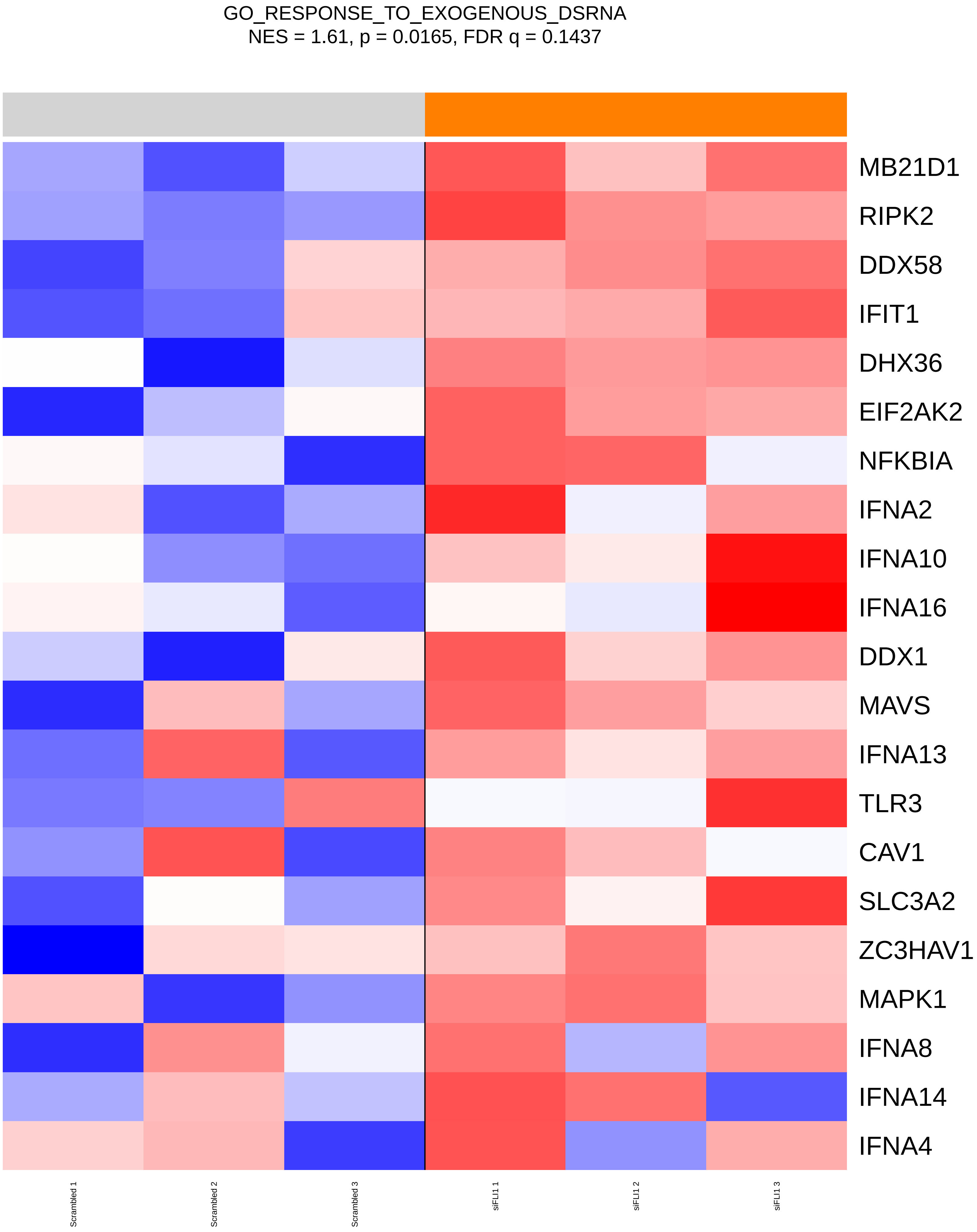

GO_RESPONSE_TO_EXOGENOUS_DSRNA
NES = 1.61, p = 0.0165, FDR q = 0.1437
MB21D1
RIPK2
DDX58
IFIT1
DHX36
EIF2AK2
NFKBIA
IFNA2
IFNA10
IFNA16
DDX1
MAVS
IFNA13
TLR3
CAV1
SLC3A2
ZC3HAV1
MAPK1
IFNA8
IFNA14
IFNA4
Scrambled 1
Scrambled 2
Scrambled 3
siFLI1 1
siFLI1 2
siFLI1 3

GO_REGULATION_OF_PRODUCTION_OF_MOLECULAR_MEDIATOR_OF_IMMUNE_RESPONSE
NES = 1.60, p = 0.0034, FDR q = 0.1444

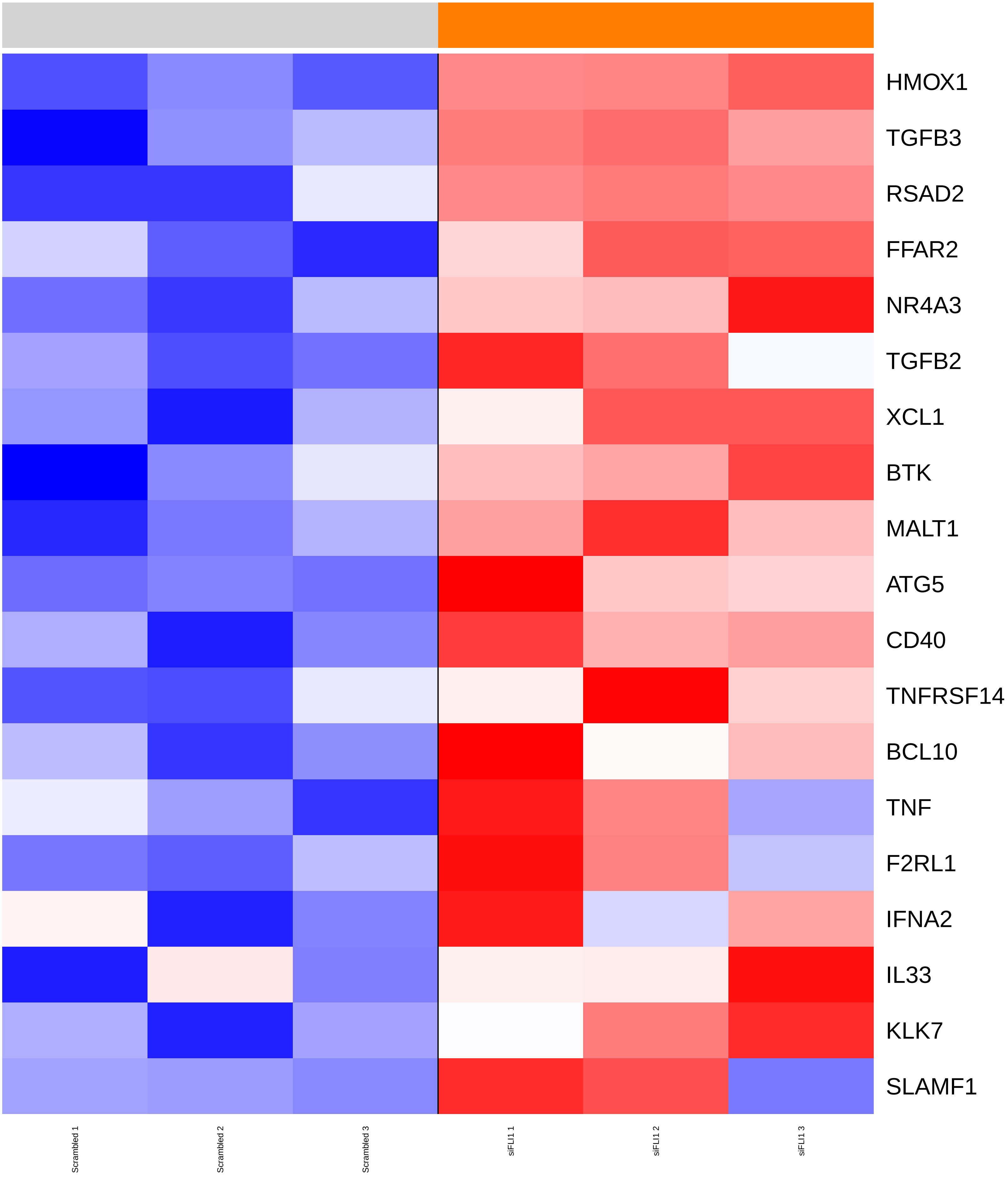

BIOCARTA_HSP27_PATHWAY
NES = 1.60, p = 0.0254, FDR q = 0.1454

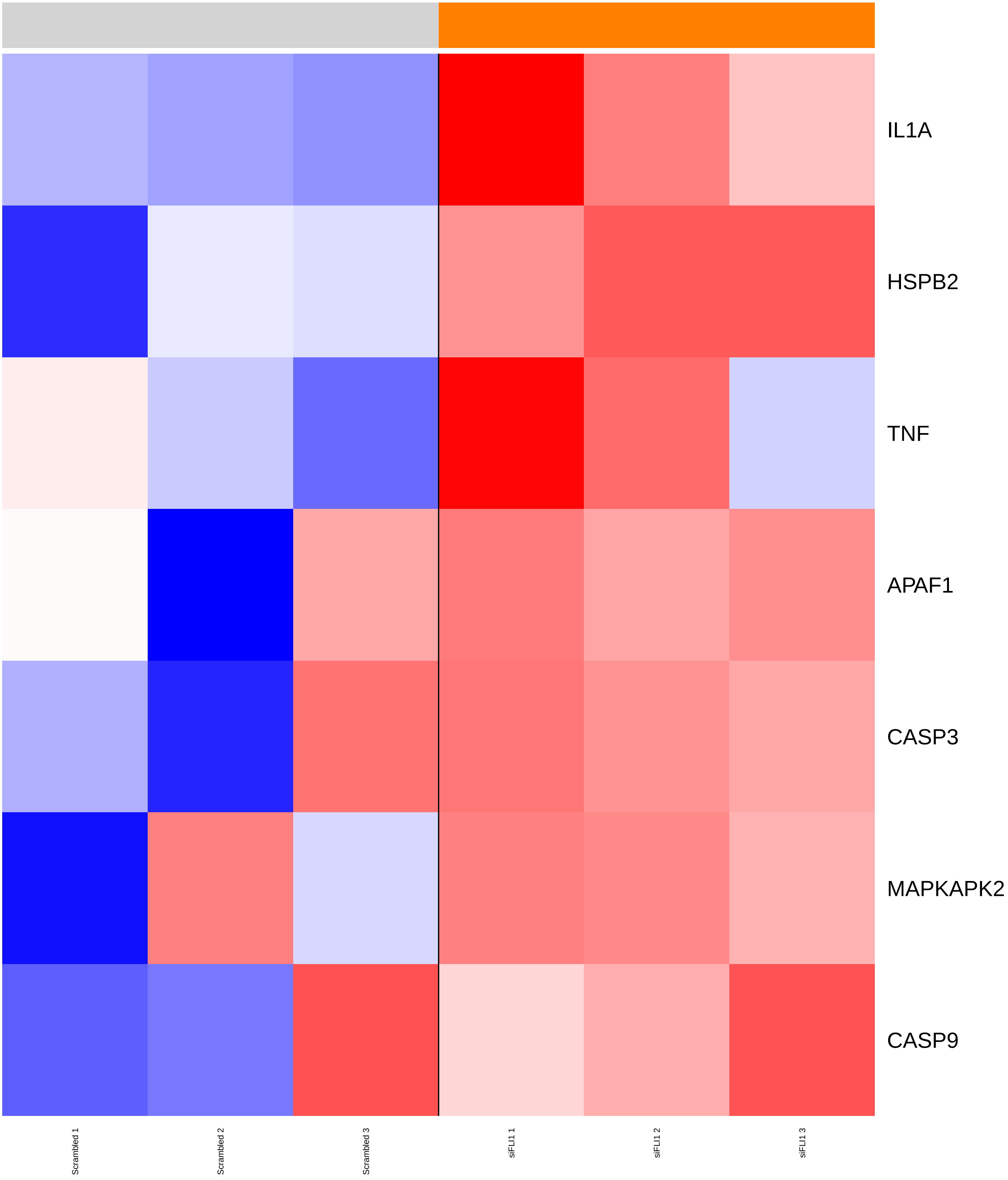

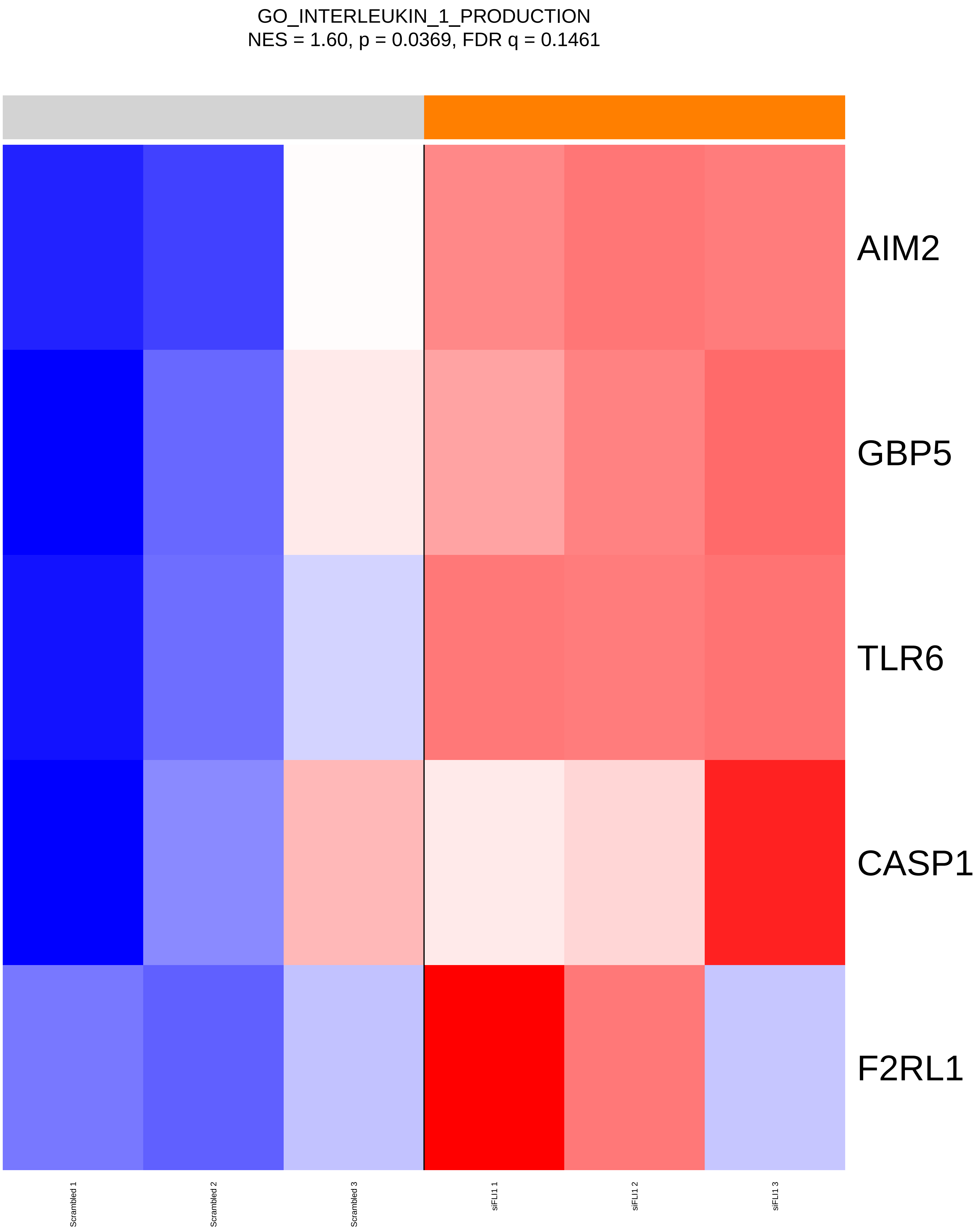
GO_INTERLEUKIN_1_PRODUCTION
NES = 1.60, p = 0.0369, FDR q = 0.1461
AIM2
GBP5
TLR6
CASP1
F2RL1
Scrambled 1
Scrambled 2
Scrambled 3
siFLI1 1
siFLI1 2
siFLI1 3

GO_RESPONSE_TO_ORGANOPHOSPHORUS
NES = 1.60, p = 0.0000, FDR q = 0.1455

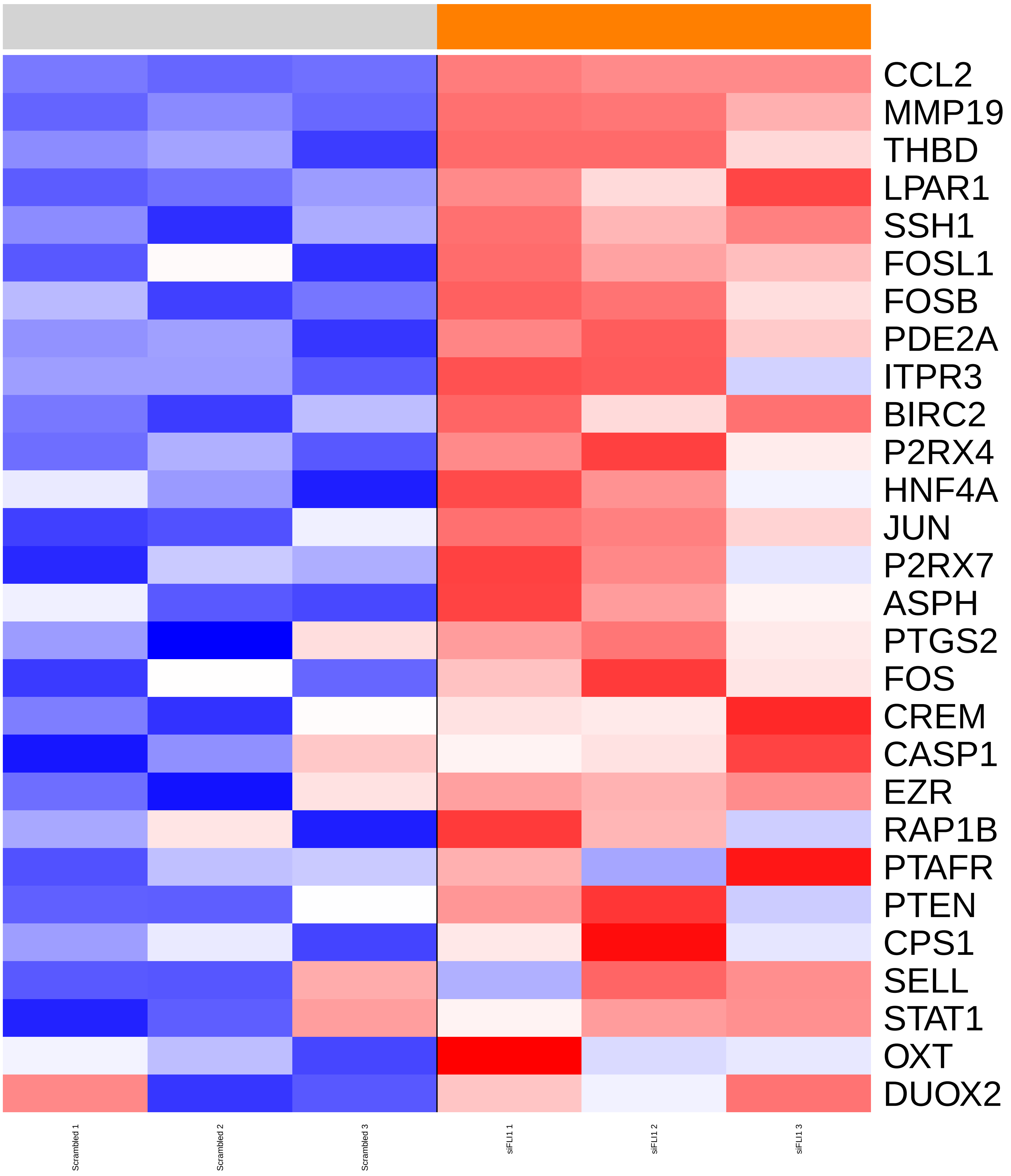

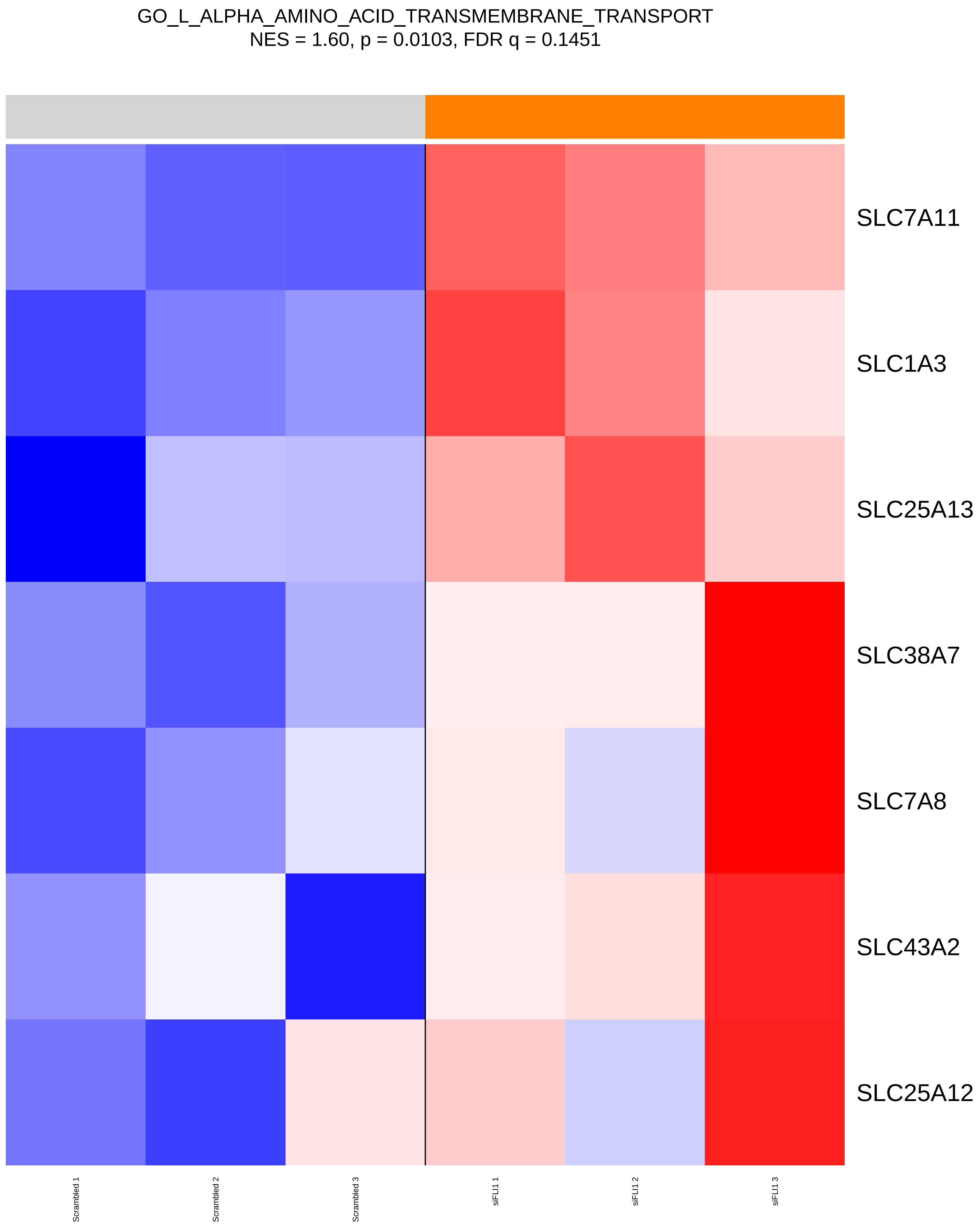
GO_L_ALPHA_AMINO_ACID_TRANSMEMBRANE_TRANSPORT
NES = 1.60, p = 0.0103, FDR q = 0.1451
SLC7A11
SLC1A3
SLC25A13
SLC38A7
SLC7A8
SLC43A2
SLC25A12
Scrambled 1
Scrambled 2
Scrambled 3
siFLI1 1
siFLI1 2
siFLI1 3

GO_MEMBRANE_DOCKING
NES = 1.60, p = 0.0060, FDR q = 0.1455

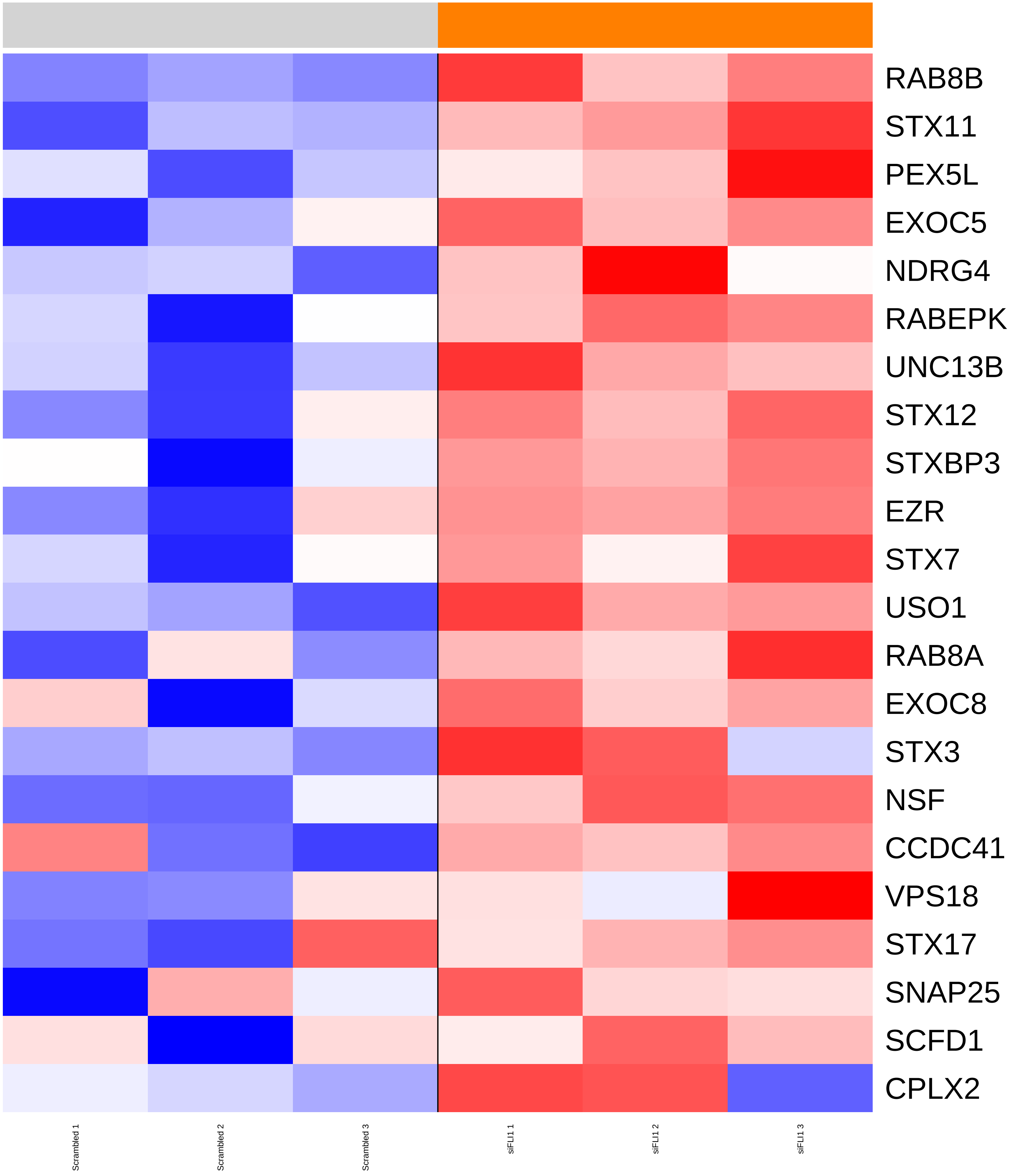

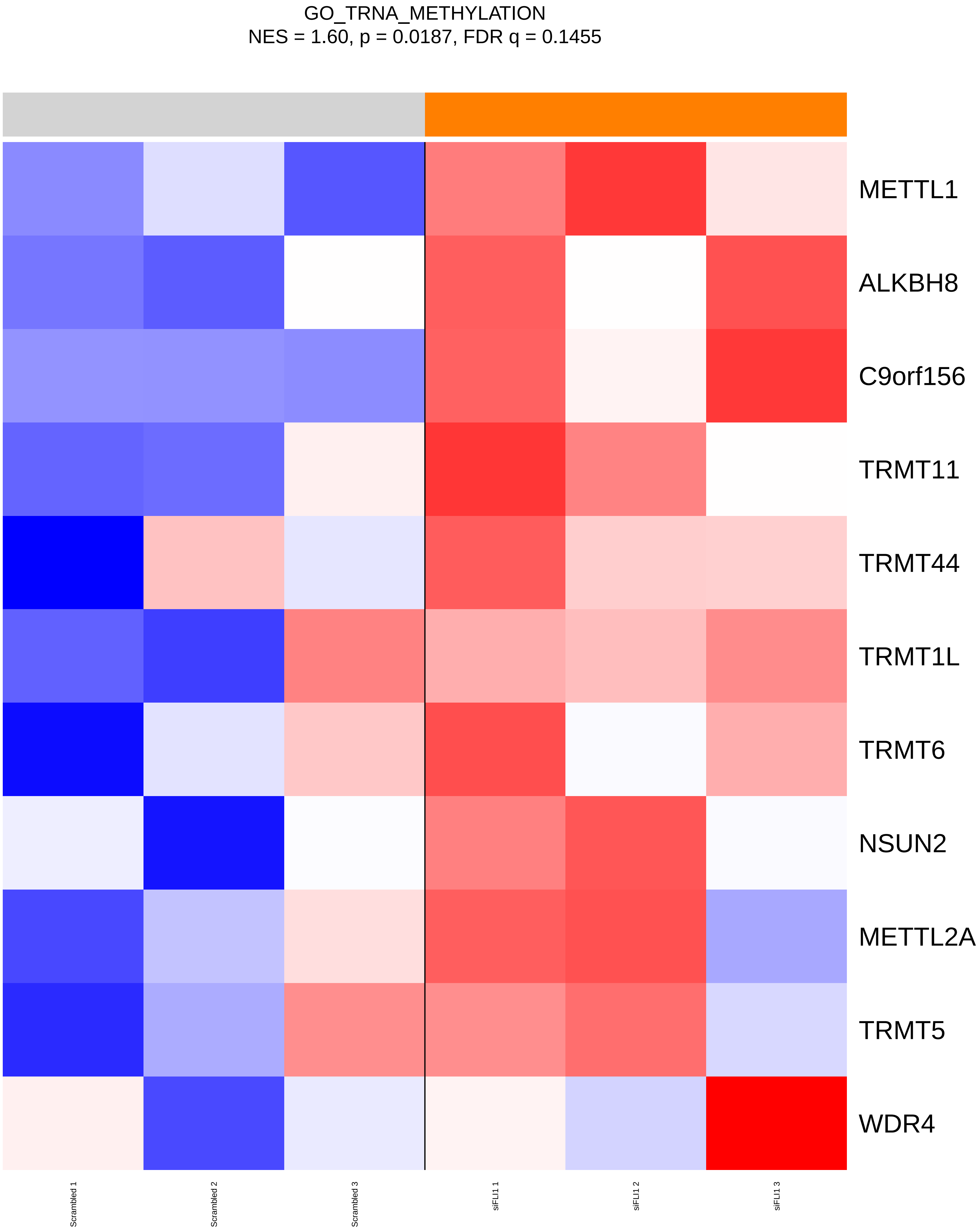
GO_TRNA_METHYLATION
NES = 1.60, p = 0.0187, FDR q = 0.1455
METTL1
ALKBH8
C9orf156
TRMT11
TRMT44
TRMT1L
TRMT6
NSUN2
METTL2A
TRMT5
WDR4
Scrambled 1
Scrambled 2
Scrambled 3
siFLI1 1
siFLI1 2
siFLI1 3

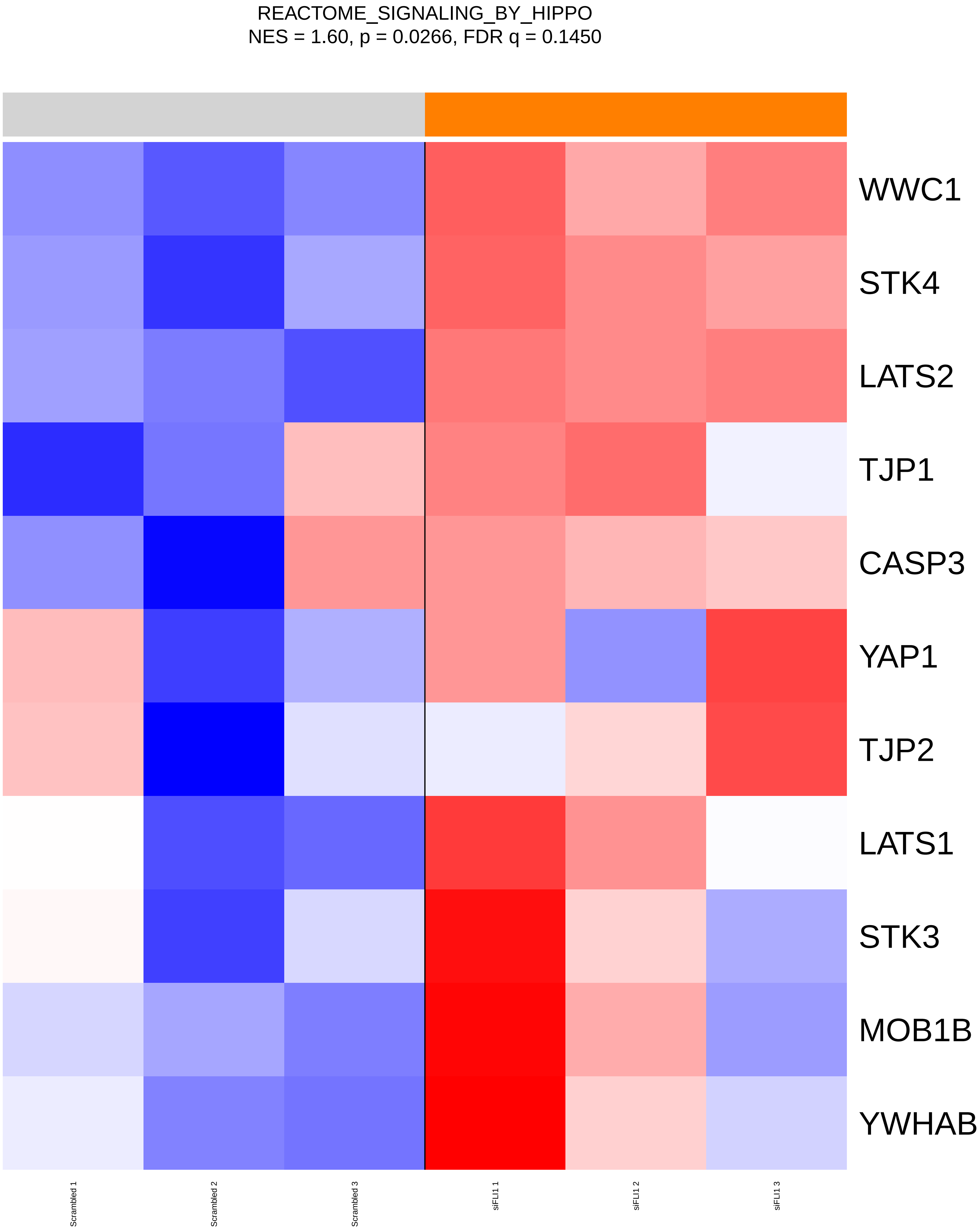
REACTOME_SIGNALING_BY_HIPPO
NES = 1.60, p = 0.0266, FDR q = 0.1450
WWC1
STK4
LATS2
TJP1
CASP3
YAP1
TJP2
LATS1
STK3
MOB1B
YWHAB
Scrambled 1
Scrambled 2
Scrambled 3
siFLI1 1
siFLI1 2
siFLI1 3

GO_ENDOCYTIC_RECYCLING
NES = 1.60, p = 0.0313, FDR q = 0.1453

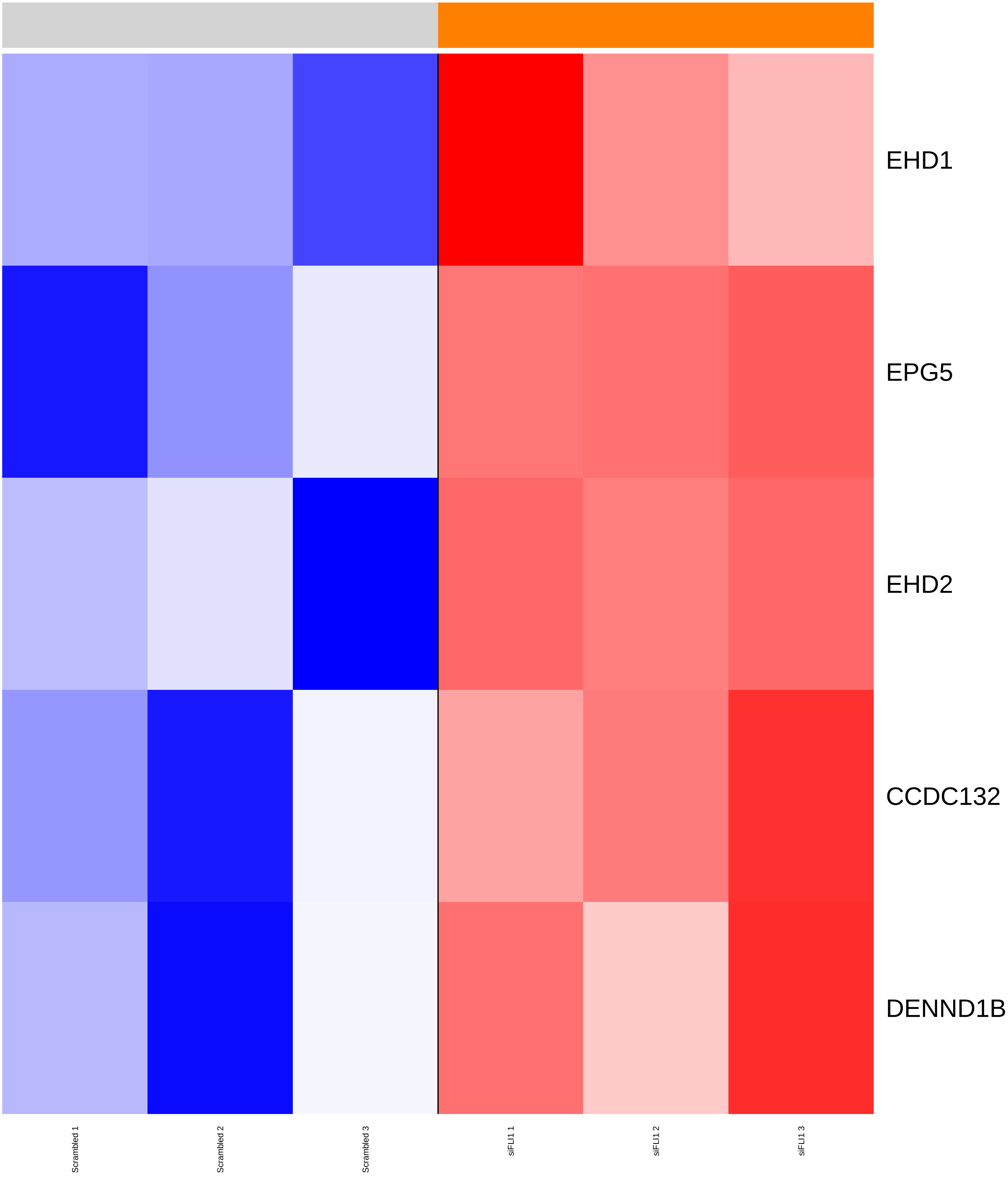

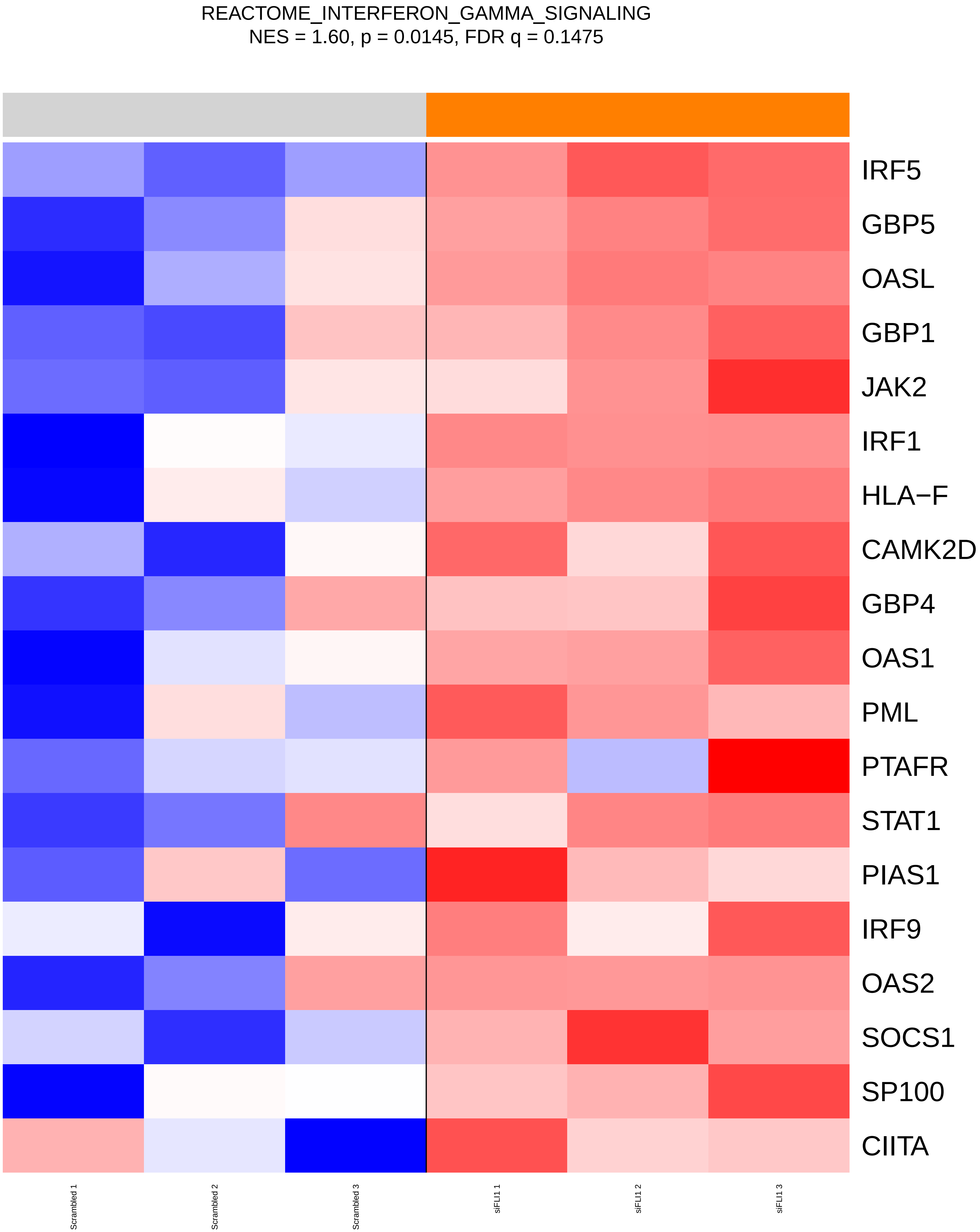
REACTOME_INTERFERON_GAMMA_SIGNALING
NES = 1.60, p = 0.0145, FDR q = 0.1475
IRF5
GBP5
OASL
GBP1
JAK2
IRF1
HLA−F
CAMK2D
GBP4
OAS1
PML
PTAFR
STAT1
PIAS1
IRF9
OAS2
SOCS1
SP100
CIITA
Scrambled 1
Scrambled 2
Scrambled 3
siFLI1 1
siFLI1 2
siFLI1 3

GO_REGULATION_OF_MITOCHONDRIAL_DEPOLARIZATION
NES = 1.60, p = 0.0204, FDR q = 0.1478

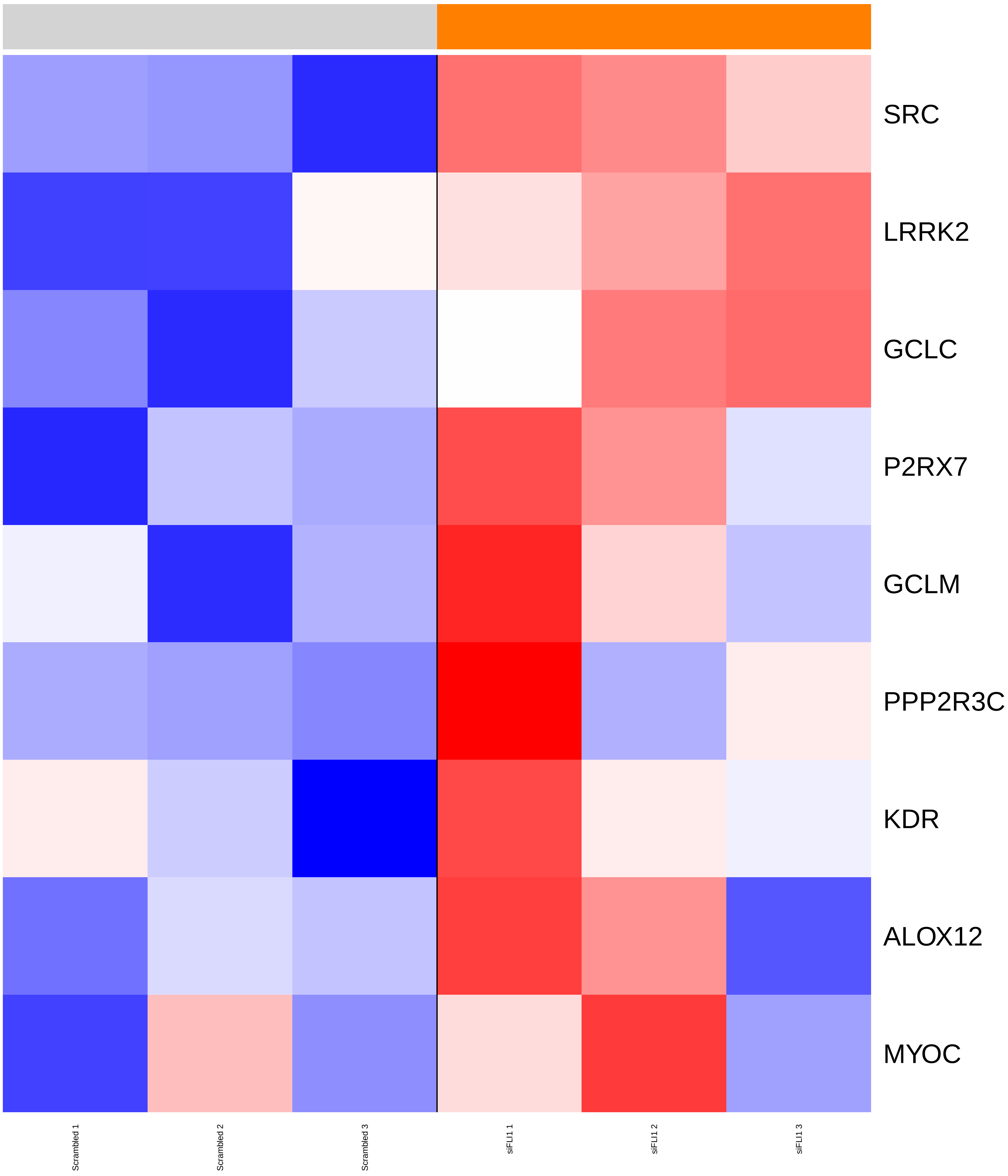

NFKAPPAB65_01
NES = 1.59, p = 0.0000, FDR q = 0.1483

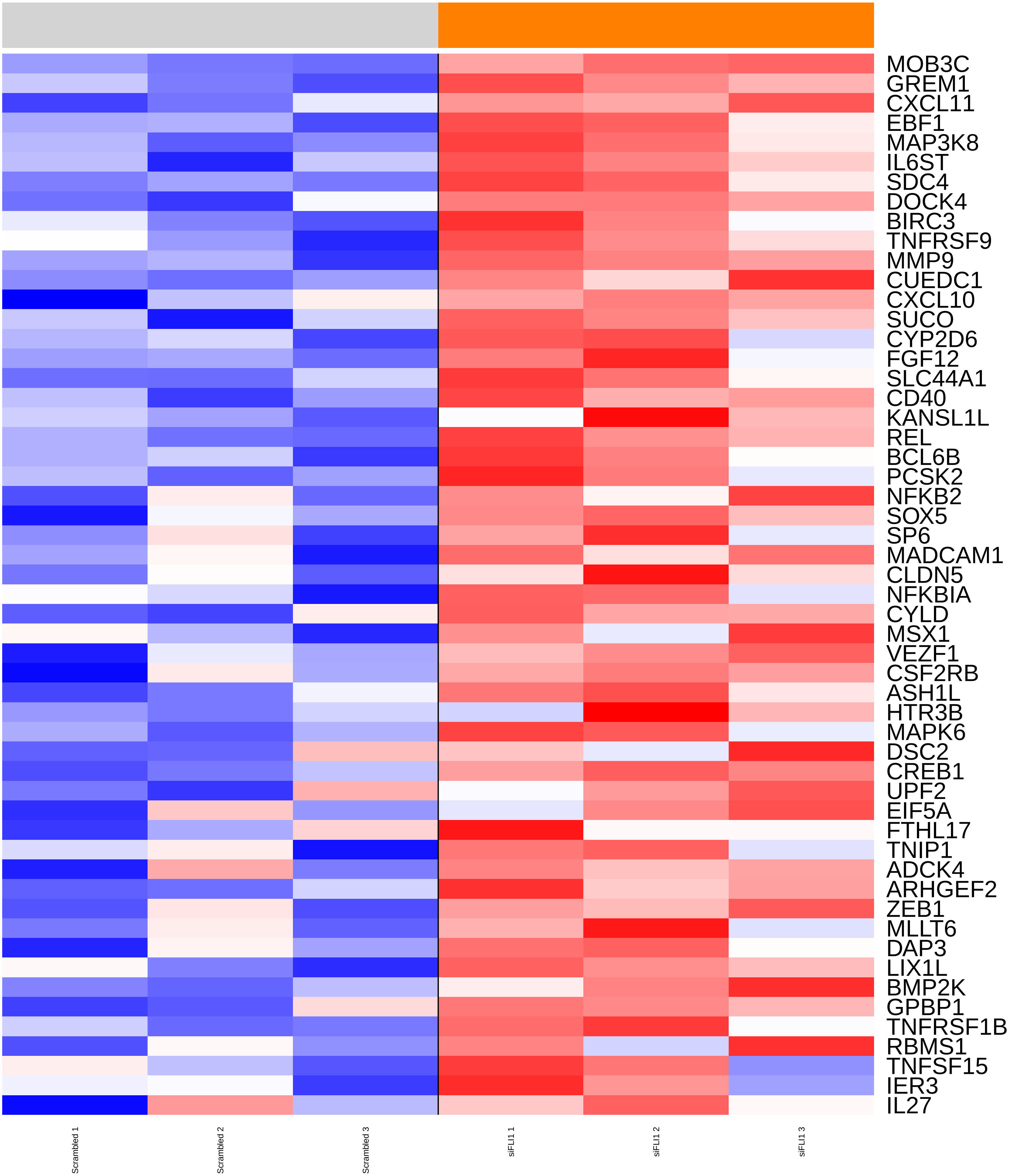

GO_MAGNESIUM_ION_TRANSMEMBRANE_TRANSPORT
NES = 1.59, p = 0.0368, FDR q = 0.1481

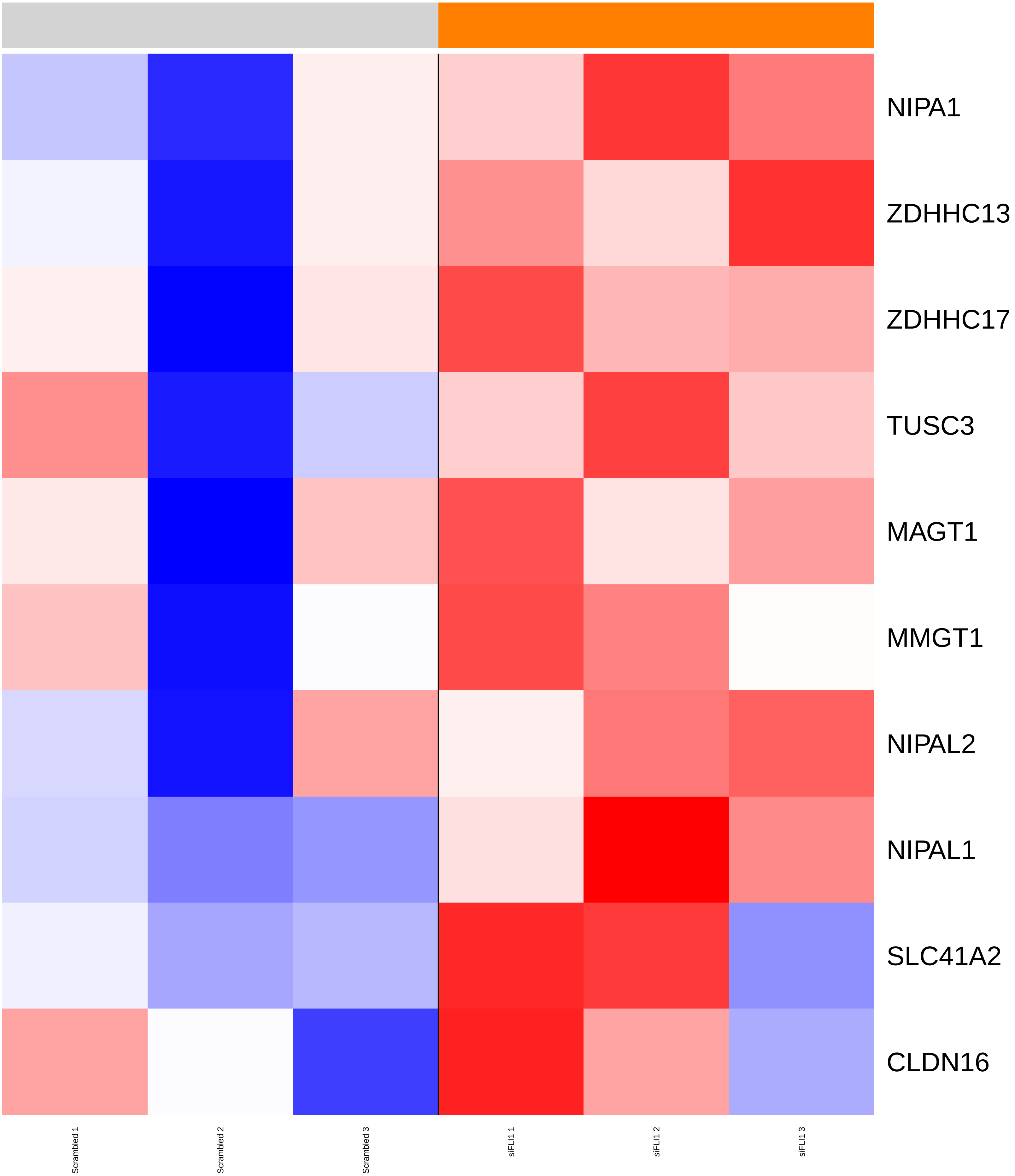

GO_REGULATION_OF_INTERFERON_BETA_PRODUCTION
NES = 1.59, p = 0.0080, FDR q = 0.1493

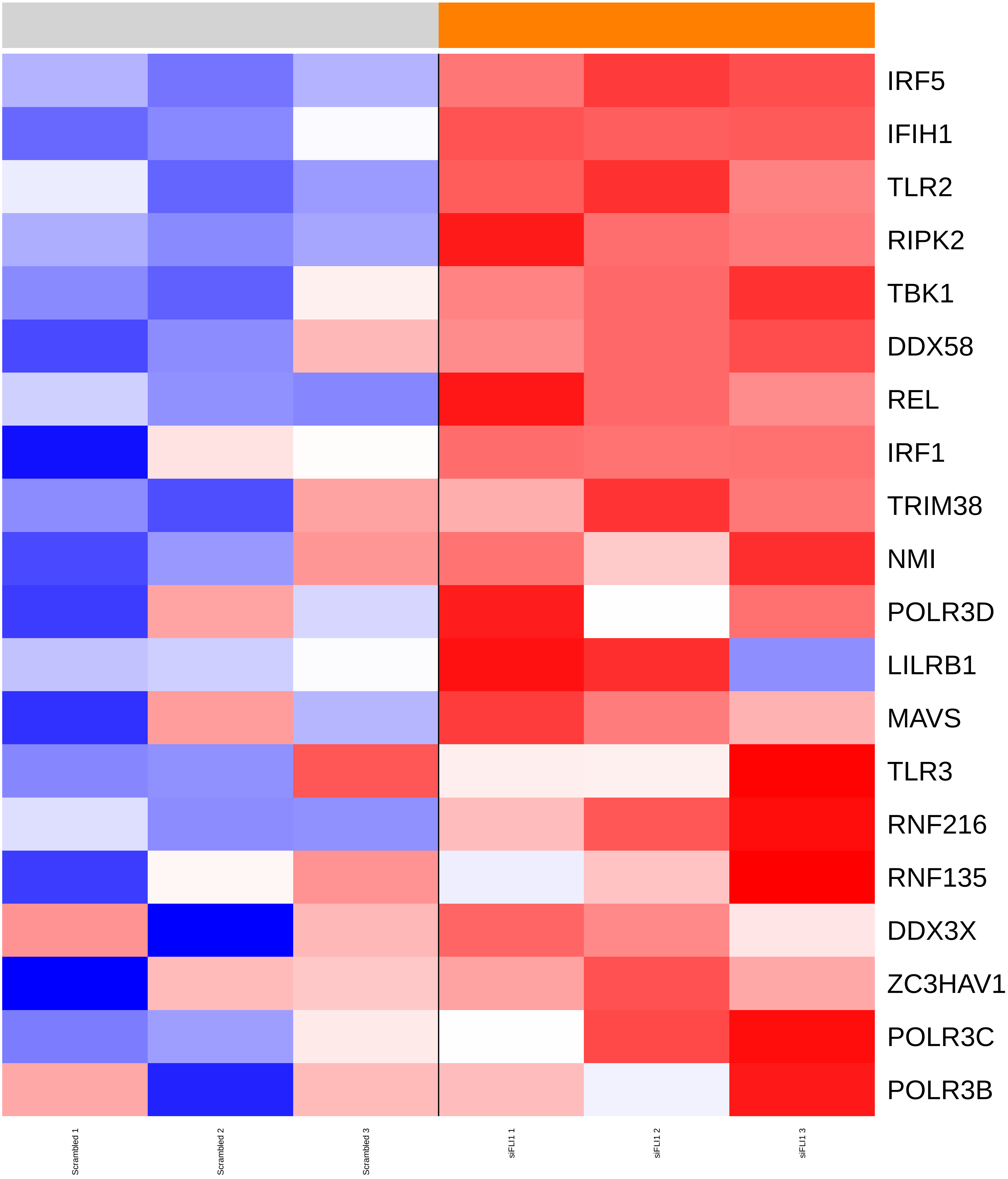

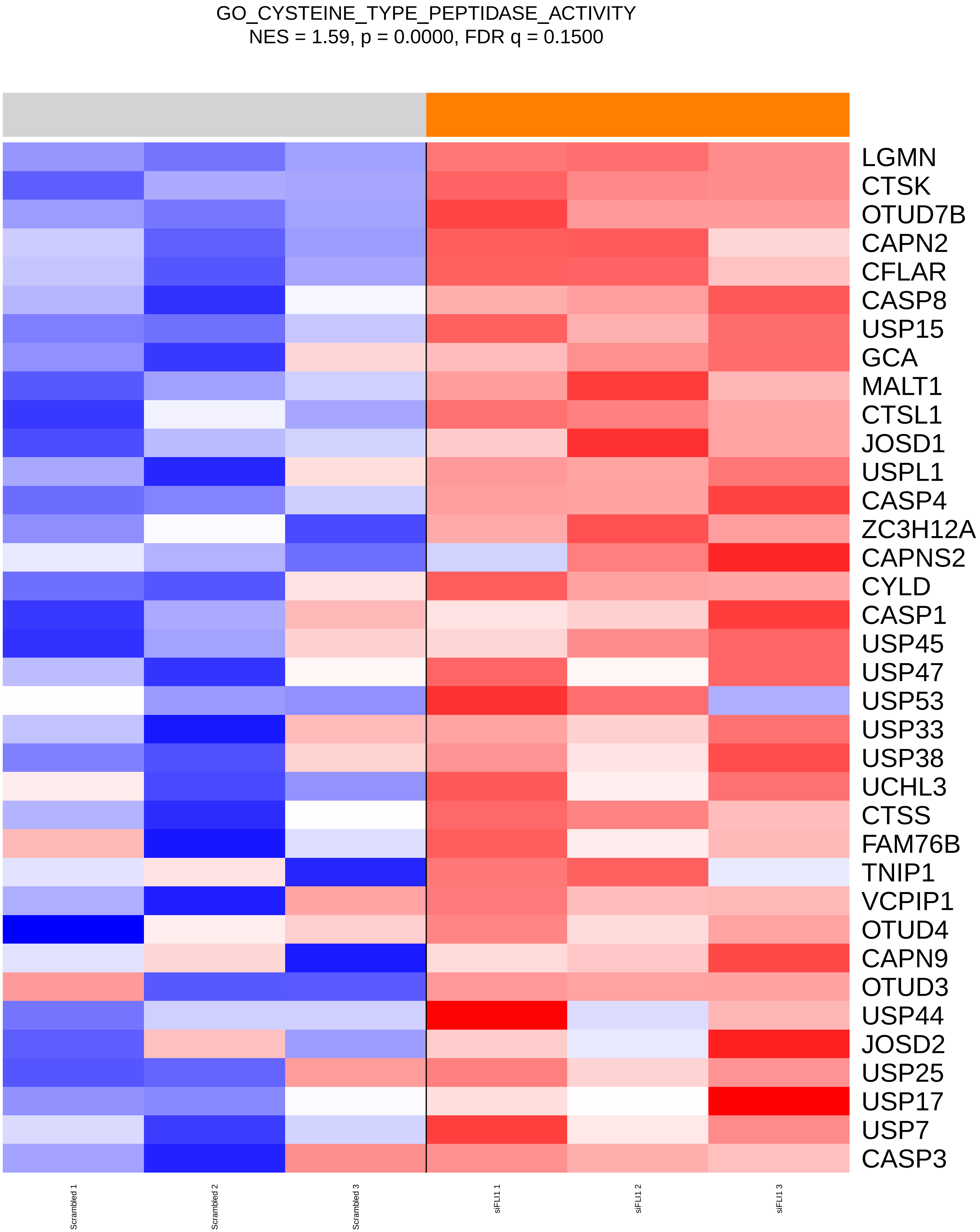
GO_CYSTEINE_TYPE_PEPTIDASE_ACTIVITY
NES = 1.59, p = 0.0000, FDR q = 0.1500
LGMN
CTSK
OTUD7B
CAPN2
CFLAR
CASP8
USP15
GCA
MALT1
CTSL1
JOSD1
USPL1
CASP4
ZC3H12A
CAPNS2
CYLD
CASP1
USP45
USP47
USP53
USP33
USP38
UCHL3
CTSS
FAM76B
TNIP1
VCPIP1
OTUD4
CAPN9
OTUD3
USP44
JOSD2
USP25
USP17
USP7
CASP3
Scrambled 1
Scrambled 2
Scrambled 3
siFLI1 1
siFLI1 2
siFLI1 3

GO_REGULATION_OF_ENDOCRINE_PROCESS
NES = 1.59, p = 0.0140, FDR q = 0.1512

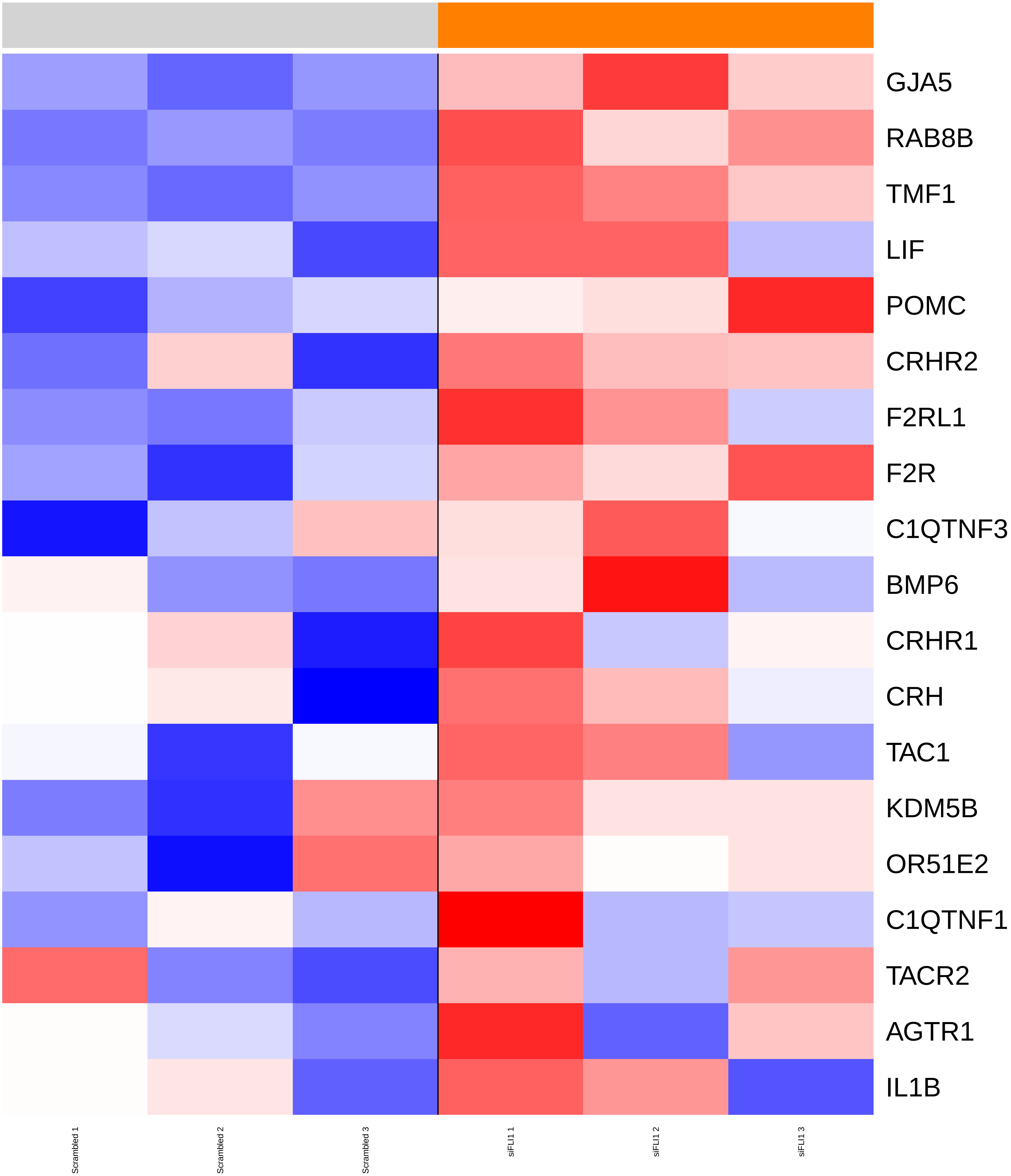

CREL_01
NES = 1.59, p = 0.0000, FDR q = 0.1507

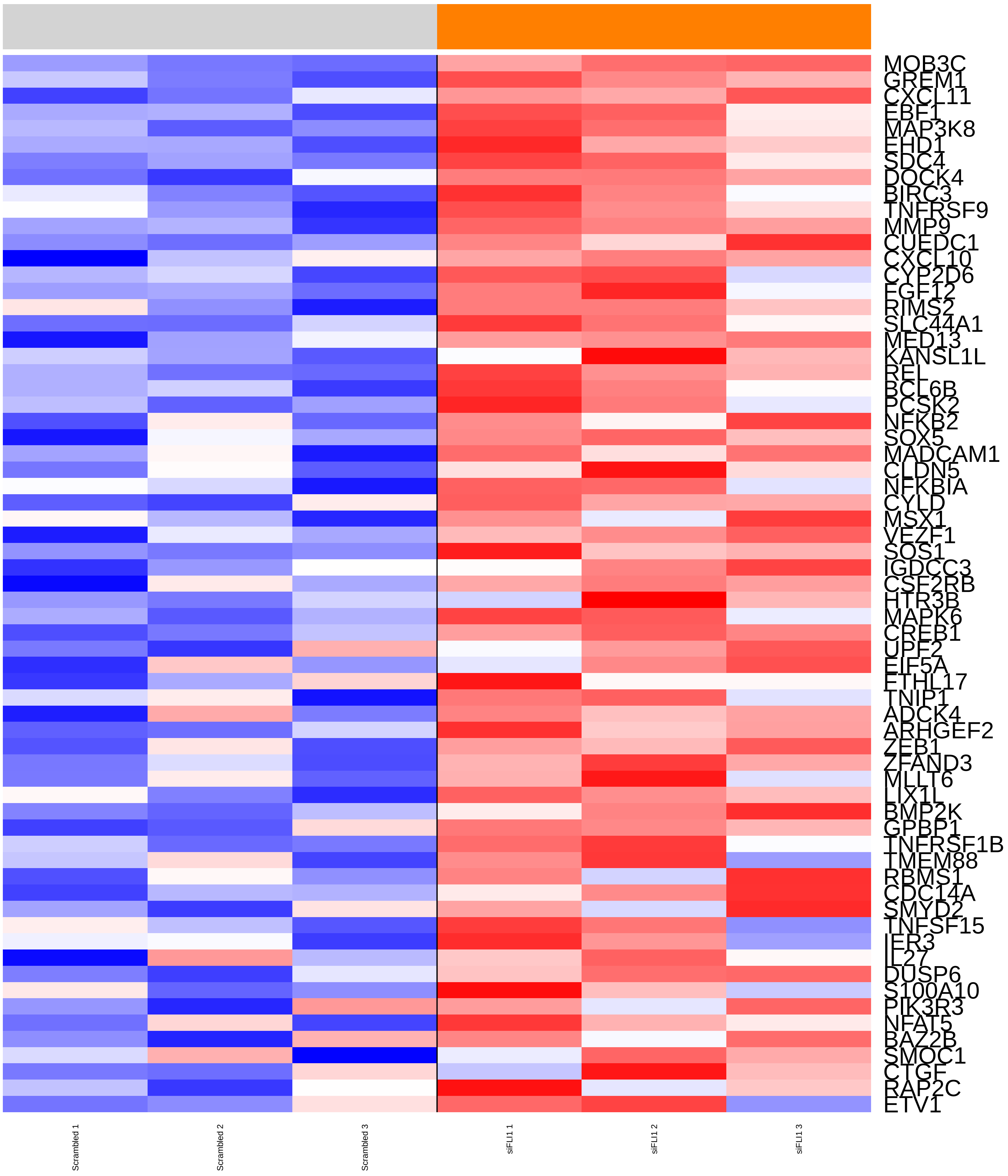

NFKB_Q6_01
NES = 1.59, p = 0.0000, FDR q = 0.1504

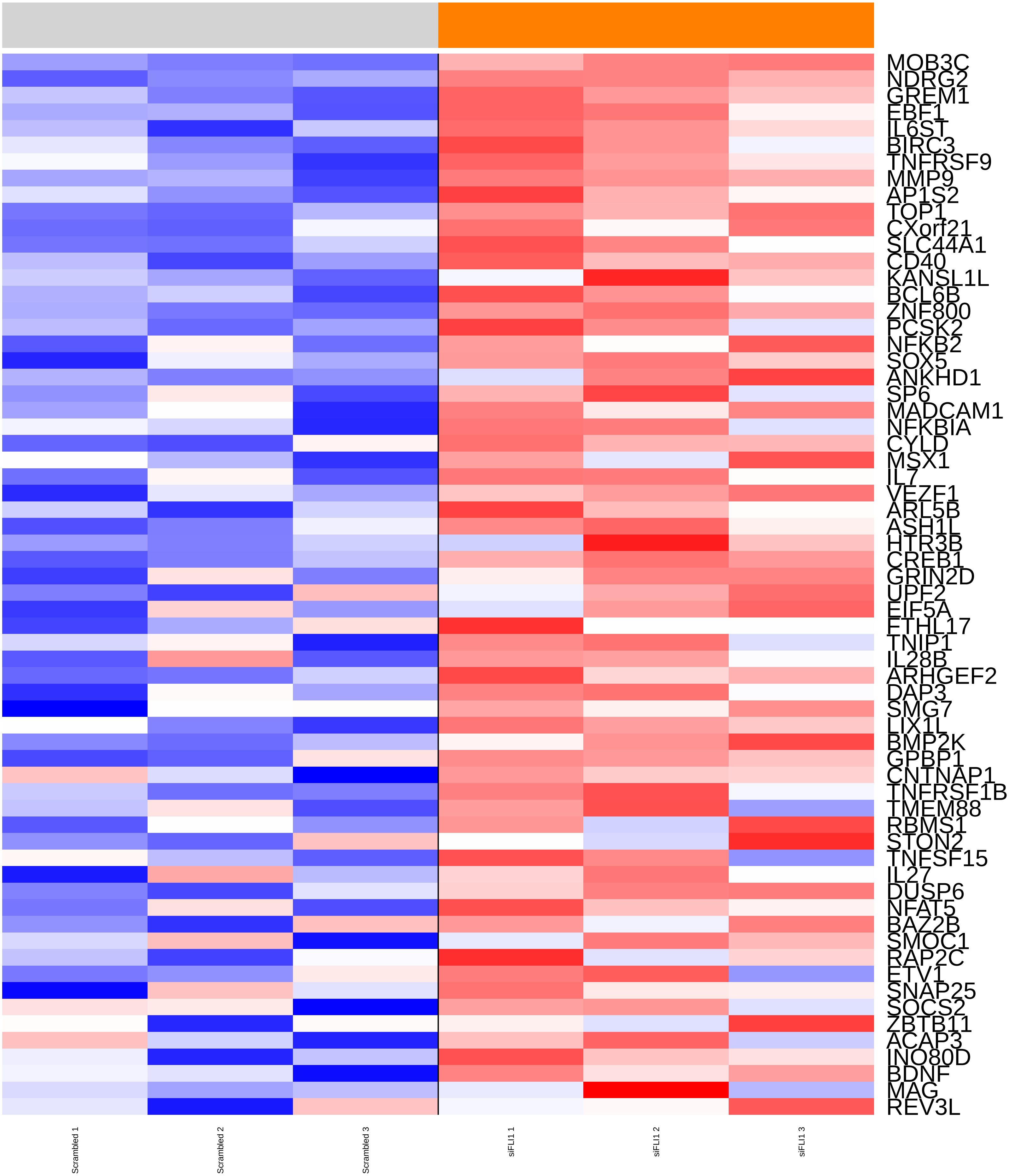

ACTGCAG_MIR173P
NES = 1.59, p = 0.0000, FDR q = 0.1533

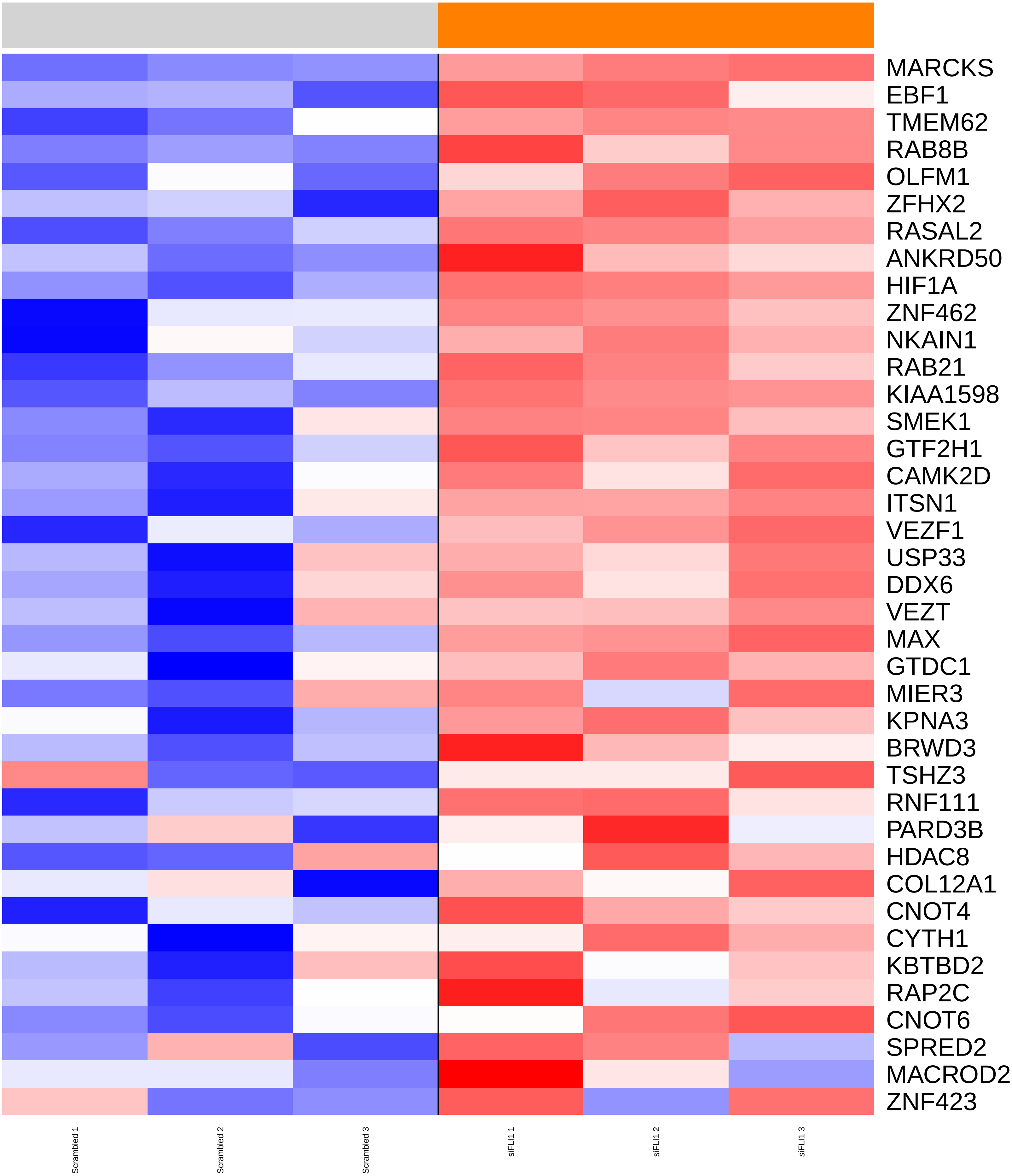

GO_POSITIVE_REGULATION_OF_STRESS_ACTIVATED_PROTEIN_KINASE_SIGNALING_CASCADE
NES = 1.59, p = 0.0000, FDR q = 0.1537

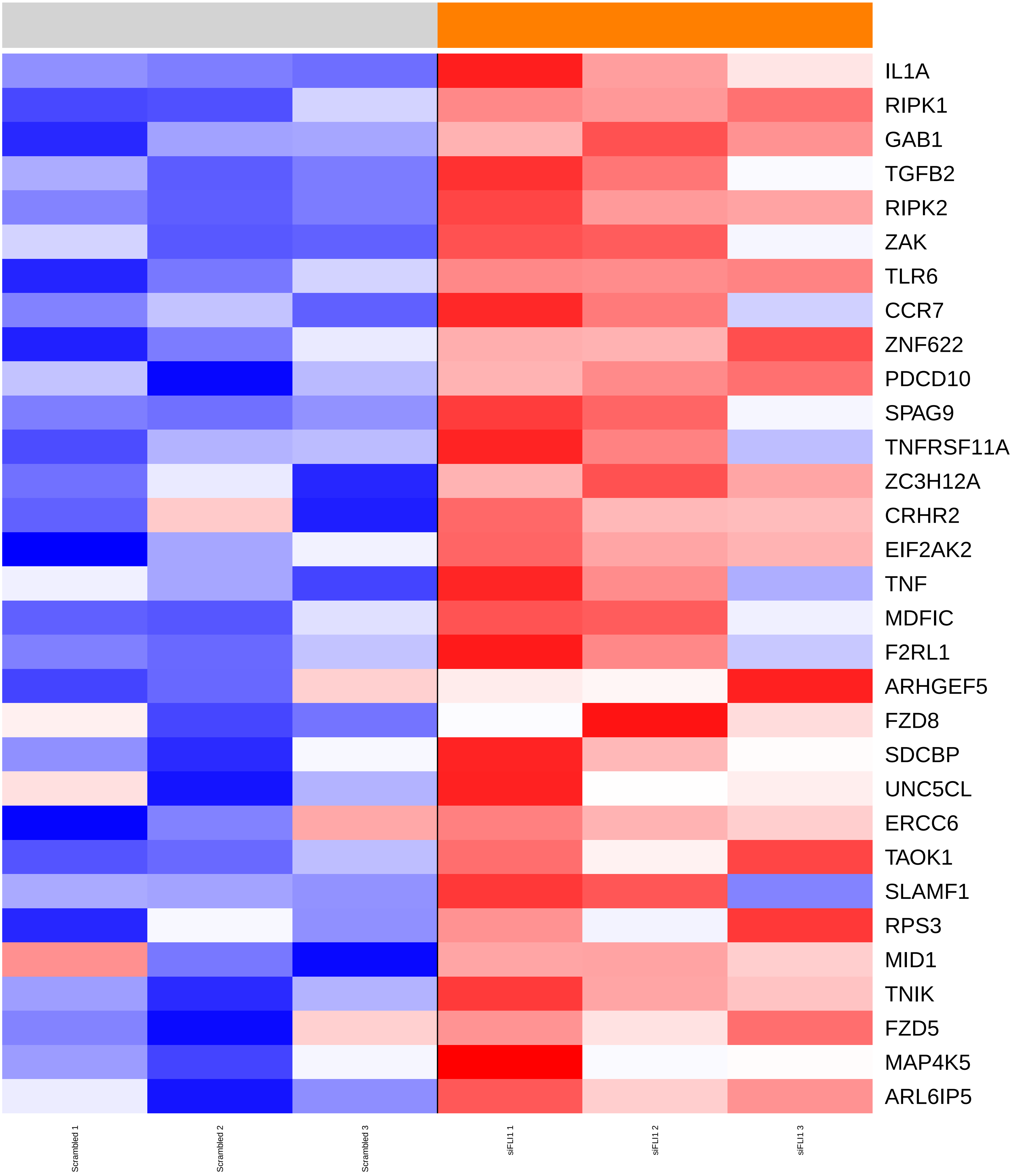

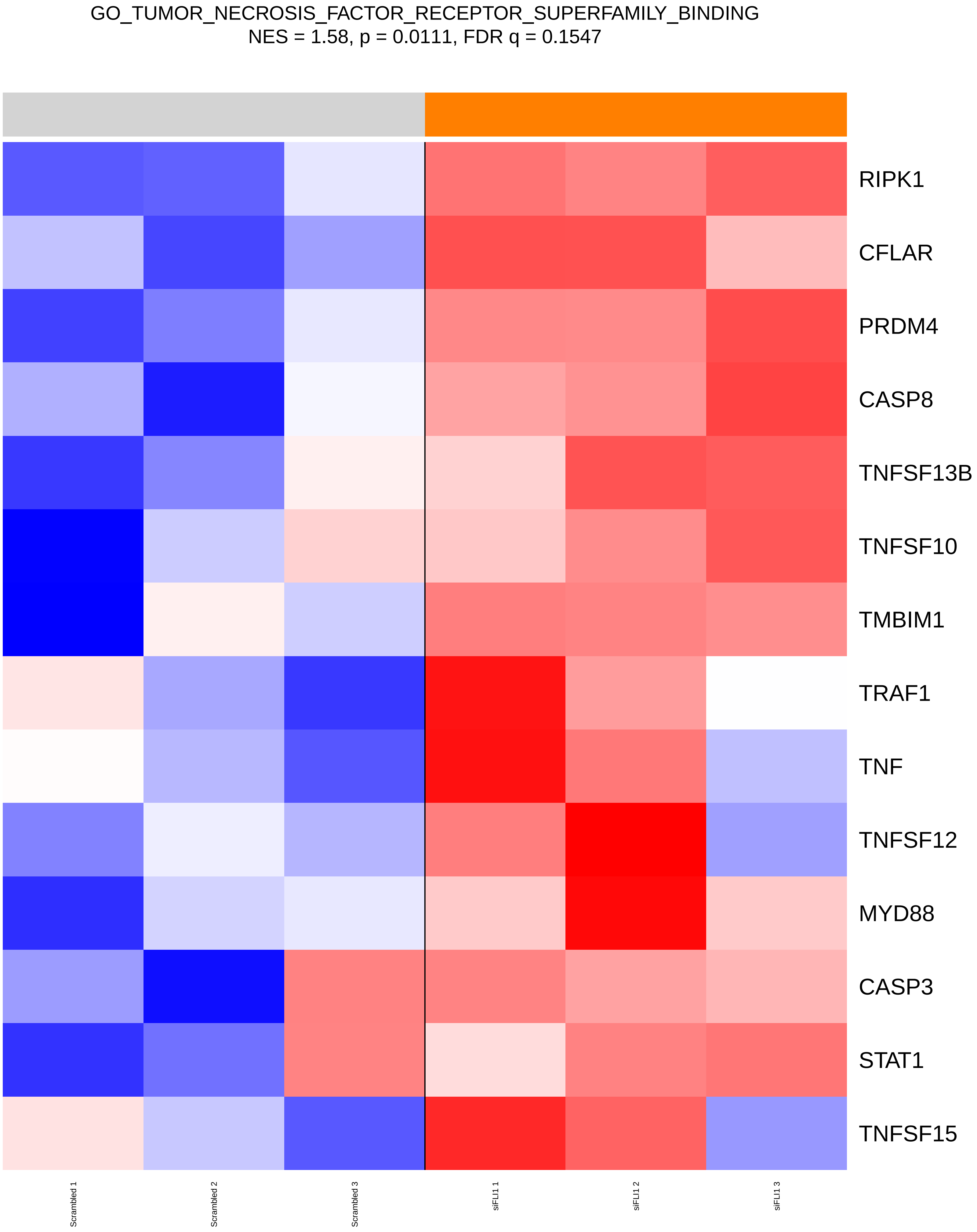
GO_TUMOR_NECROSIS_FACTOR_RECEPTOR_SUPERFAMILY_BINDING
NES = 1.58, p = 0.0111, FDR q = 0.1547
RIPK1
CFLAR
PRDM4
CASP8
TNFSF13B
TNFSF10
TMBIM1
TRAF1
TNF
TNFSF12
MYD88
CASP3
STAT1
TNFSF15
Scrambled 1
Scrambled 2
Scrambled 3
siFLI1 1
siFLI1 2
siFLI1 3

GO_REGULATION_OF_AUTOPHAGOSOME_ASSEMBLY
NES = 1.58, p = 0.0108, FDR q = 0.1556

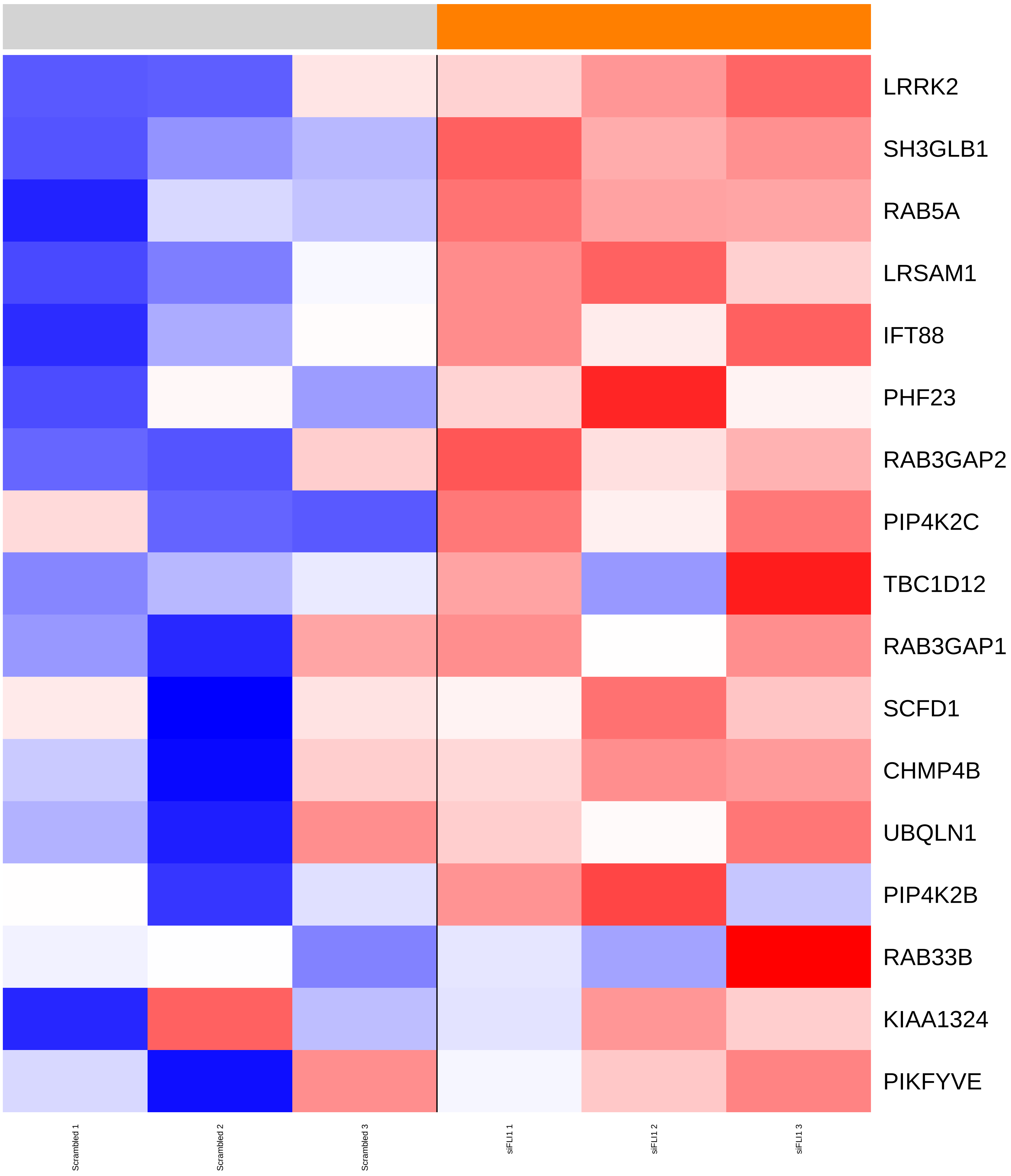

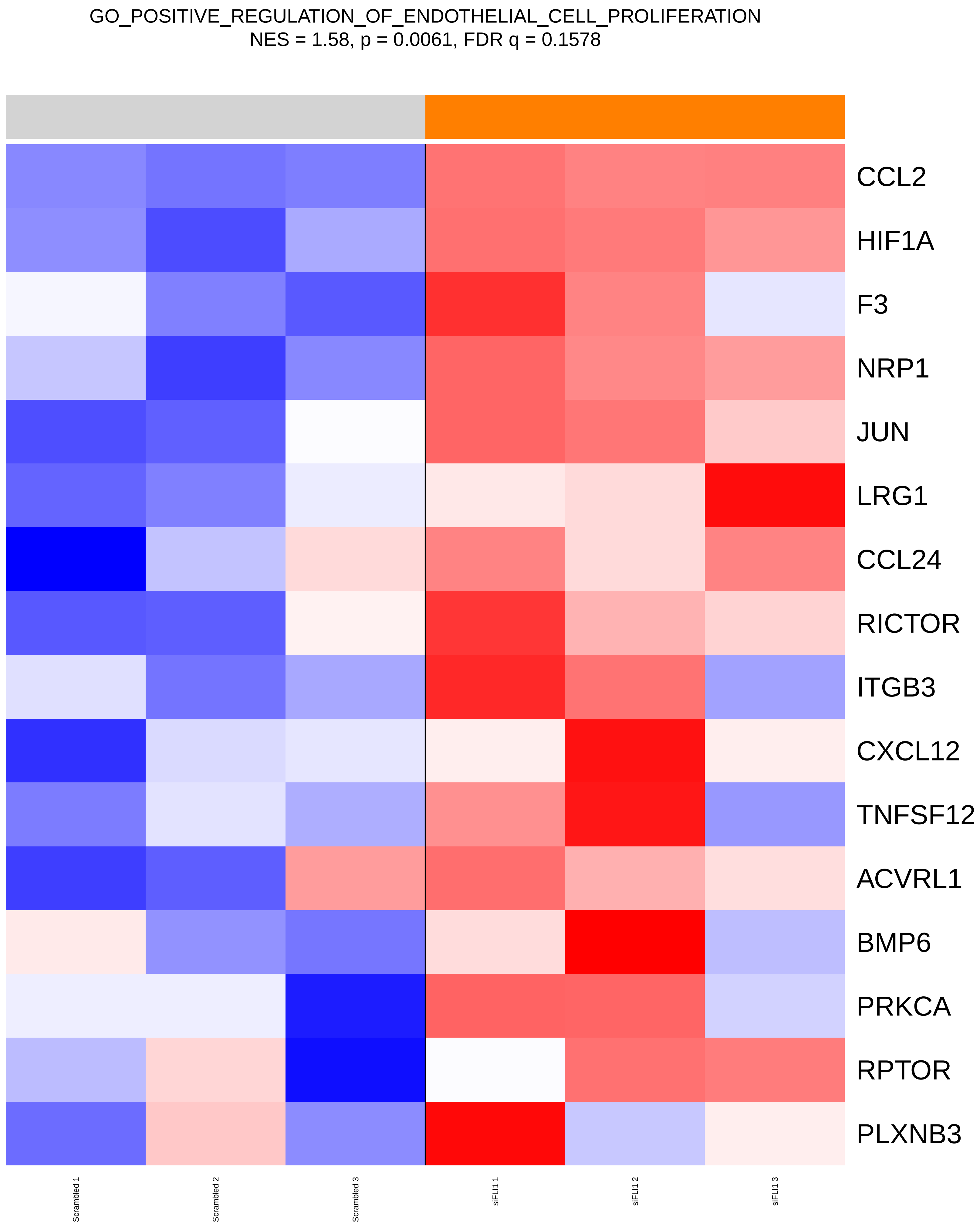
GO_POSITIVE_REGULATION_OF_ENDOTHELIAL_CELL_PROLIFERATION
NES = 1.58, p = 0.0061, FDR q = 0.1578
CCL2
HIF1A
F3
NRP1
JUN
LRG1
CCL24
RICTOR
ITGB3
CXCL12
TNFSF12
ACVRL1
BMP6
PRKCA
RPTOR
PLXNB3
Scrambled 1
Scrambled 2
Scrambled 3
siFLI1 1
siFLI1 2
siFLI1 3

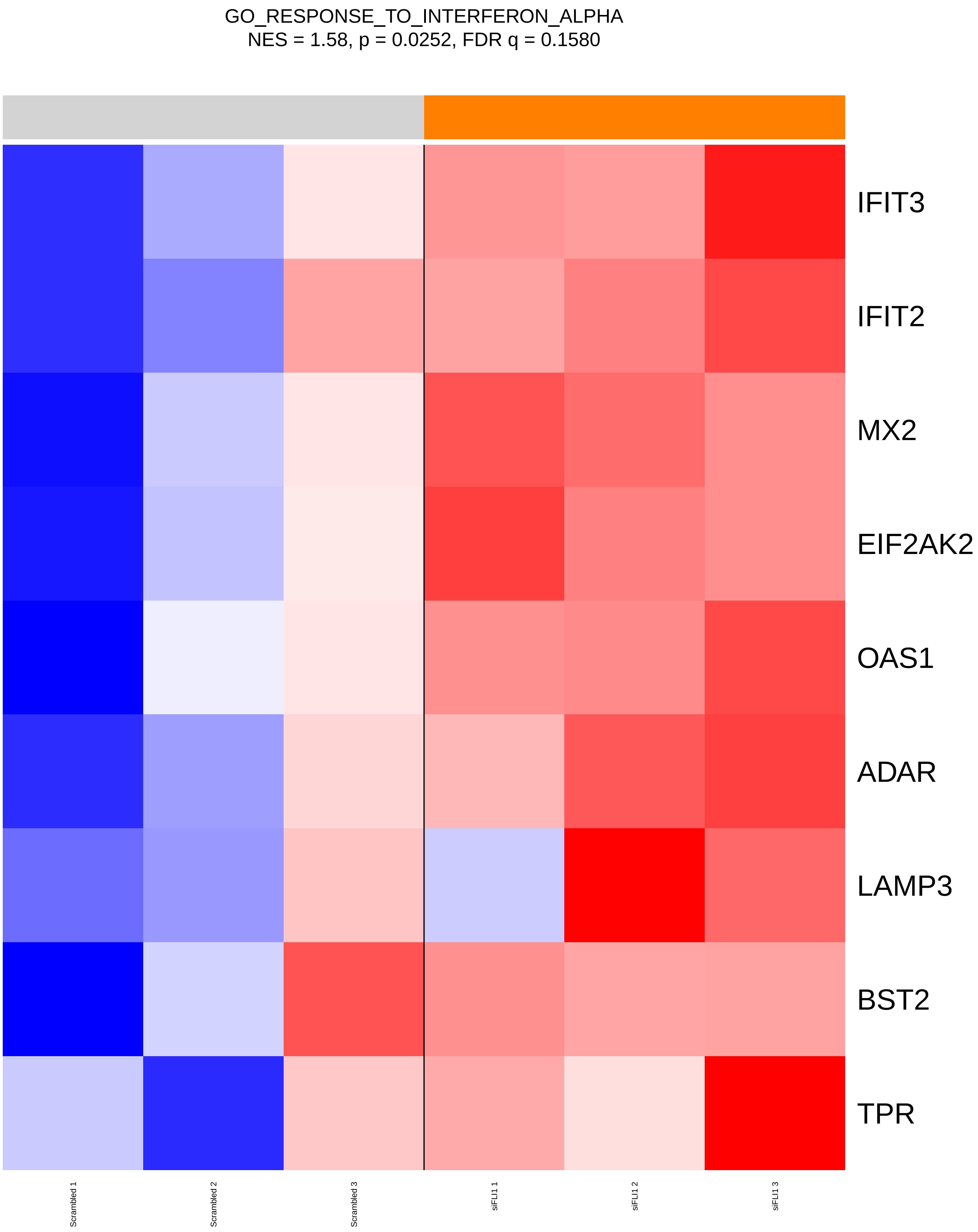
GO_RESPONSE_TO_INTERFERON_ALPHA
NES = 1.58, p = 0.0252, FDR q = 0.1580
IFIT3
IFIT2
MX2
EIF2AK2
OAS1
ADAR
LAMP3
BST2
TPR
Scrambled 1
Scrambled 2
Scrambled 3
siFLI1 1
siFLI1 2
siFLI1 3

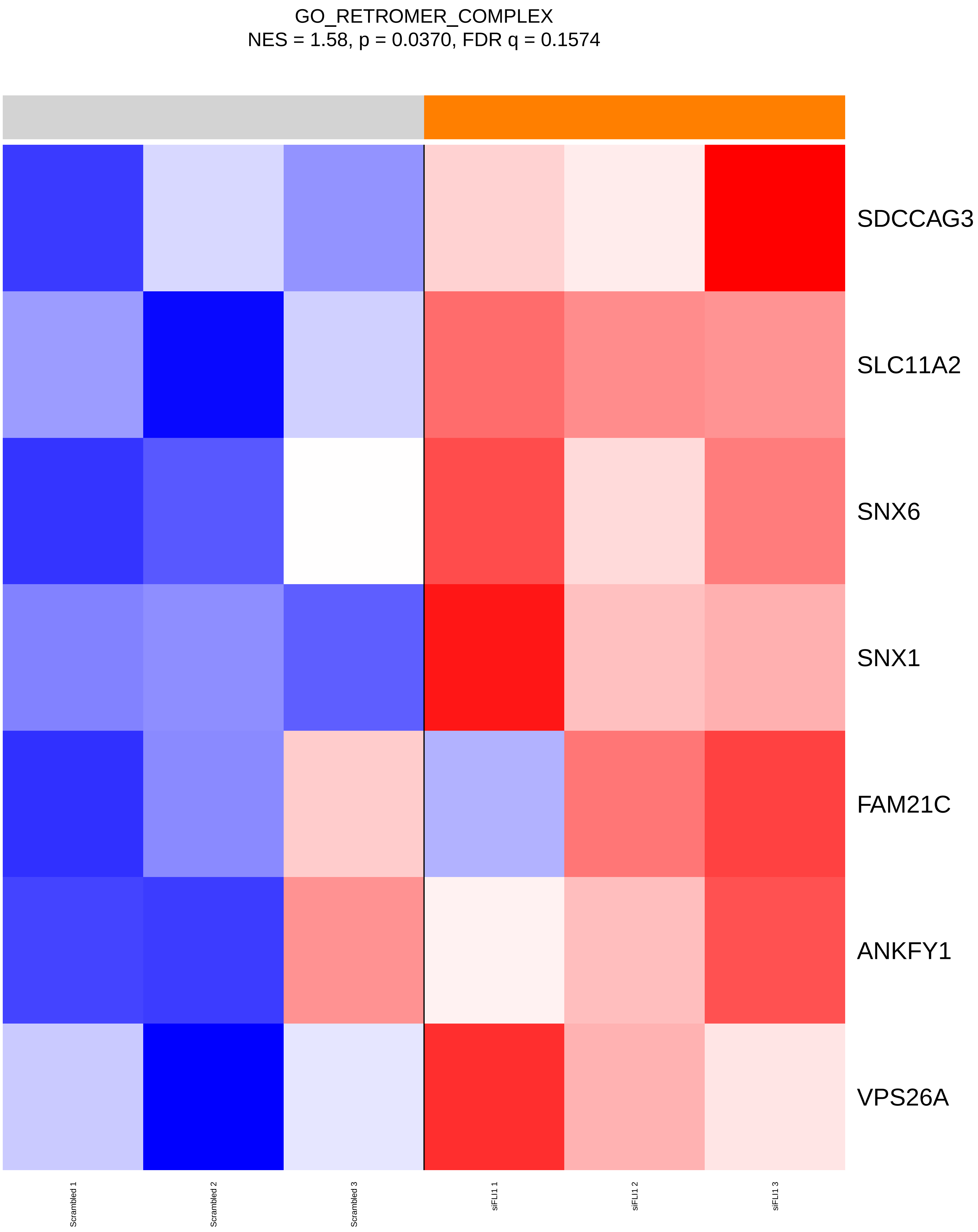
GO_RETROMER_COMPLEX
NES = 1.58, p = 0.0370, FDR q = 0.1574
SDCCAG3
SLC11A2
SNX6
SNX1
FAM21C
ANKFY1
VPS26A
Scrambled 1
Scrambled 2
Scrambled 3
siFLI1 1
siFLI1 2
siFLI1 3

GO_AMINE_CATABOLIC_PROCESS
NES = 1.58, p = 0.0279, FDR q = 0.1573

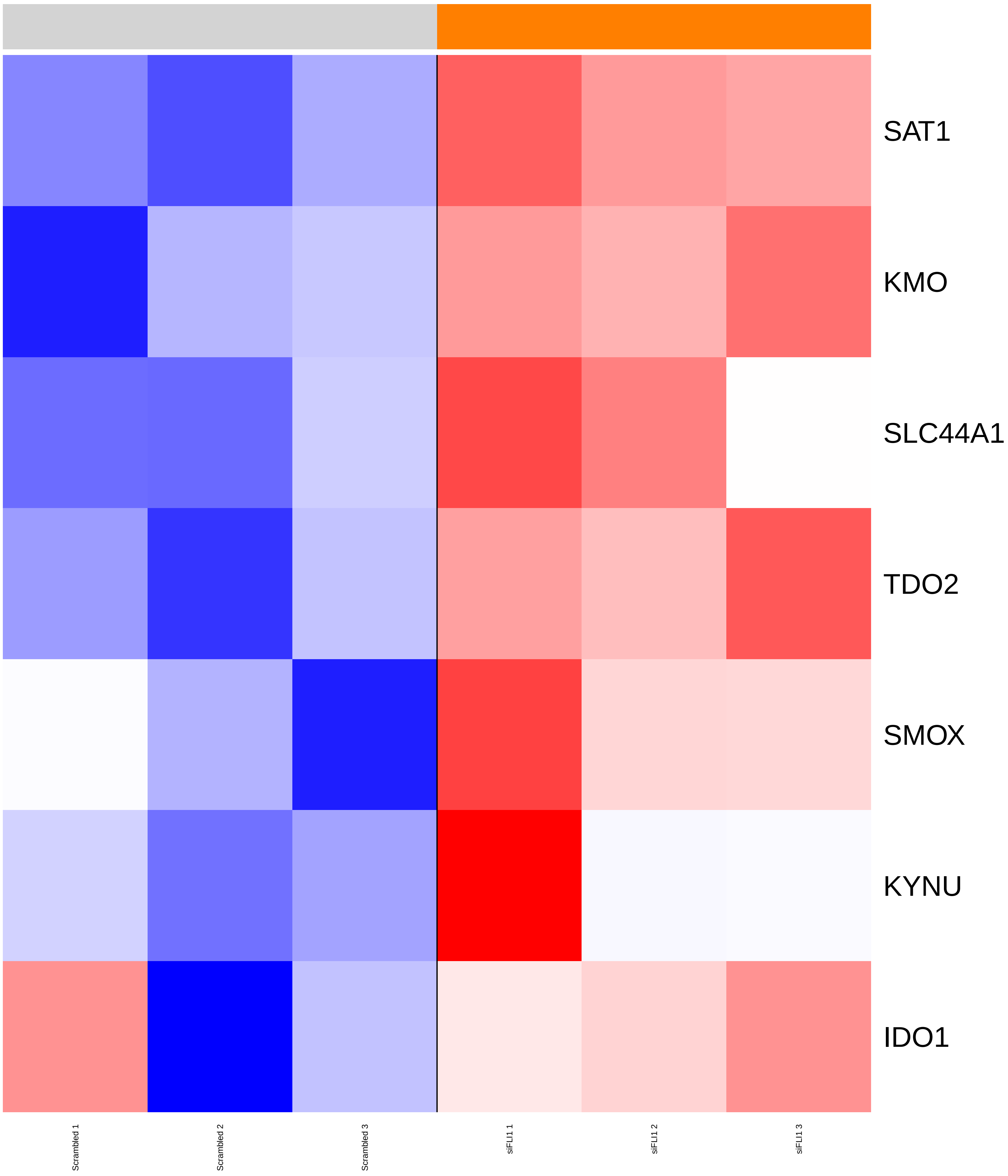

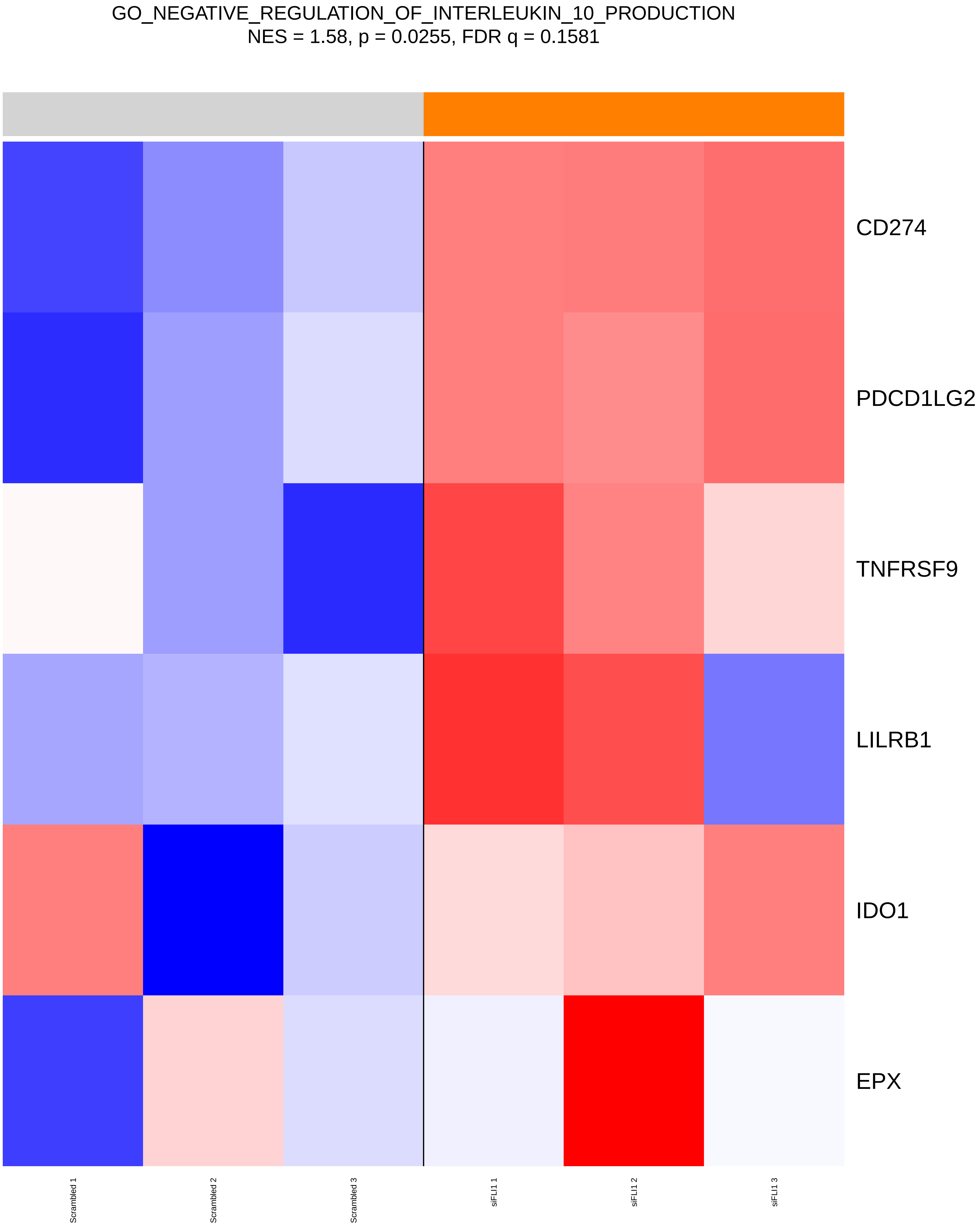

GO_NEGATIVE_REGULATION_OF_INTERLEUKIN_10_PRODUCTION
NES = 1.58, p = 0.0255, FDR q = 0.1581
CD274
PDCD1LG2
TNFRSF9
LILRB1
IDO1
EPX
Scrambled 1
Scrambled 2
Scrambled 3
siFLI1 1
siFLI1 2
siFLI1 3

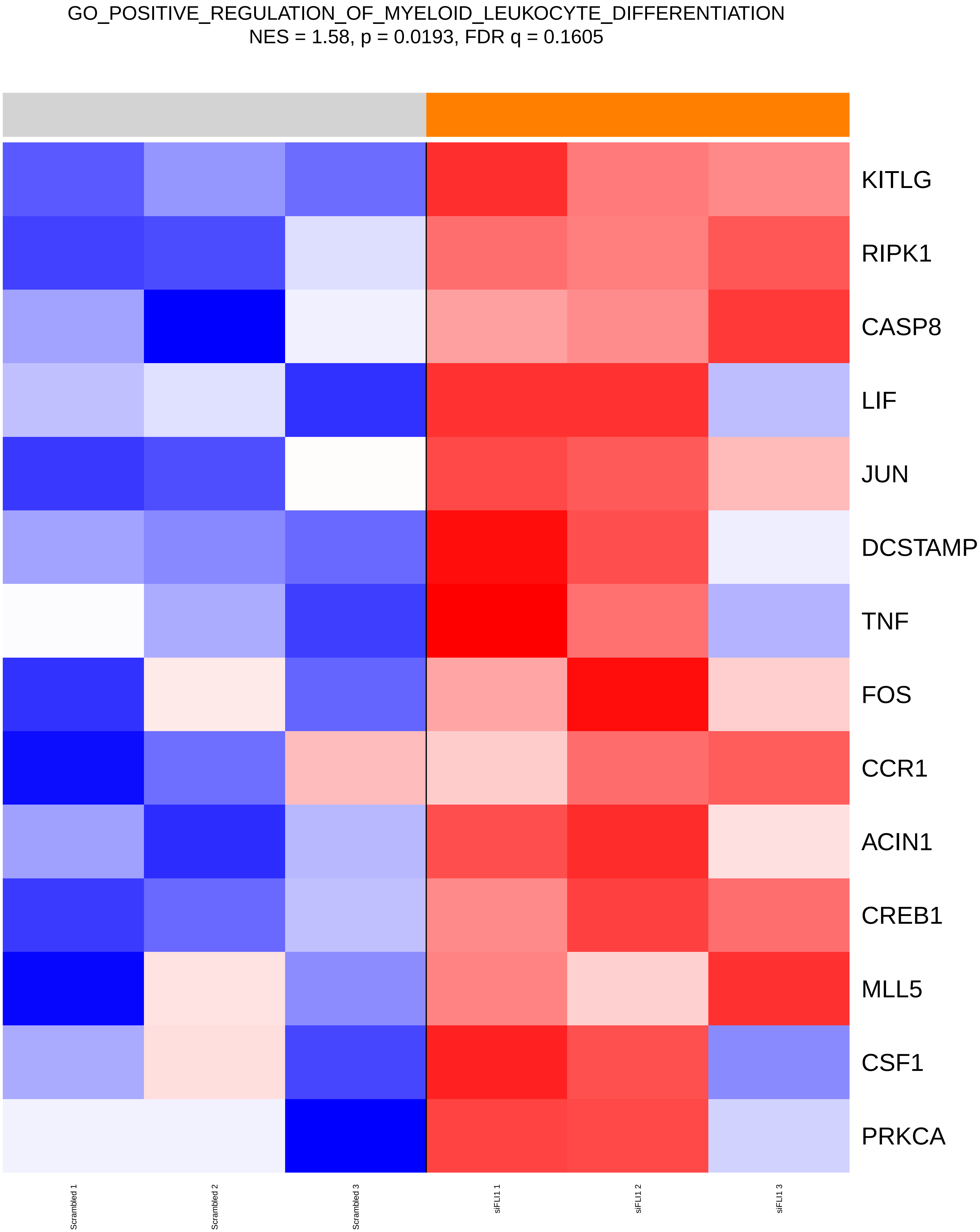
GO_POSITIVE_REGULATION_OF_MYELOID_LEUKOCYTE_DIFFERENTIATION
NES = 1.58, p = 0.0193, FDR q = 0.1605
KITLG
RIPK1
CASP8
LIF
JUN
DCSTAMP
TNF
FOS
CCR1
ACIN1
CREB1
MLL5
CSF1
PRKCA
Scrambled 1
Scrambled 2
Scrambled 3
siFLI1 1
siFLI1 2
siFLI1 3

GO_REGULATION_OF_TUMOR_NECROSIS_FACTOR_SUPERFAMILY_CYTOKINE_PRODUCTION
NES = 1.57, p = 0.0031, FDR q = 0.1609

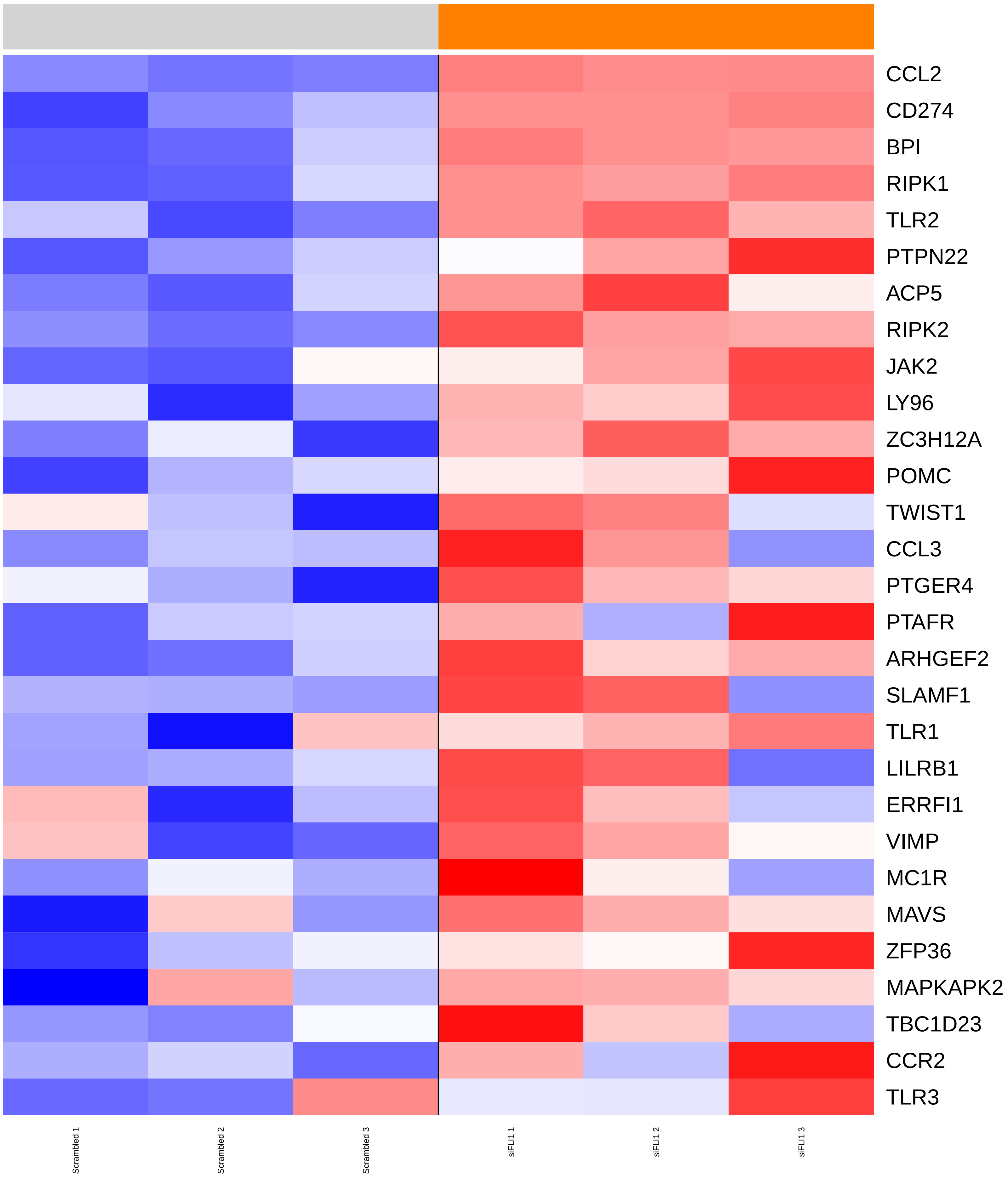

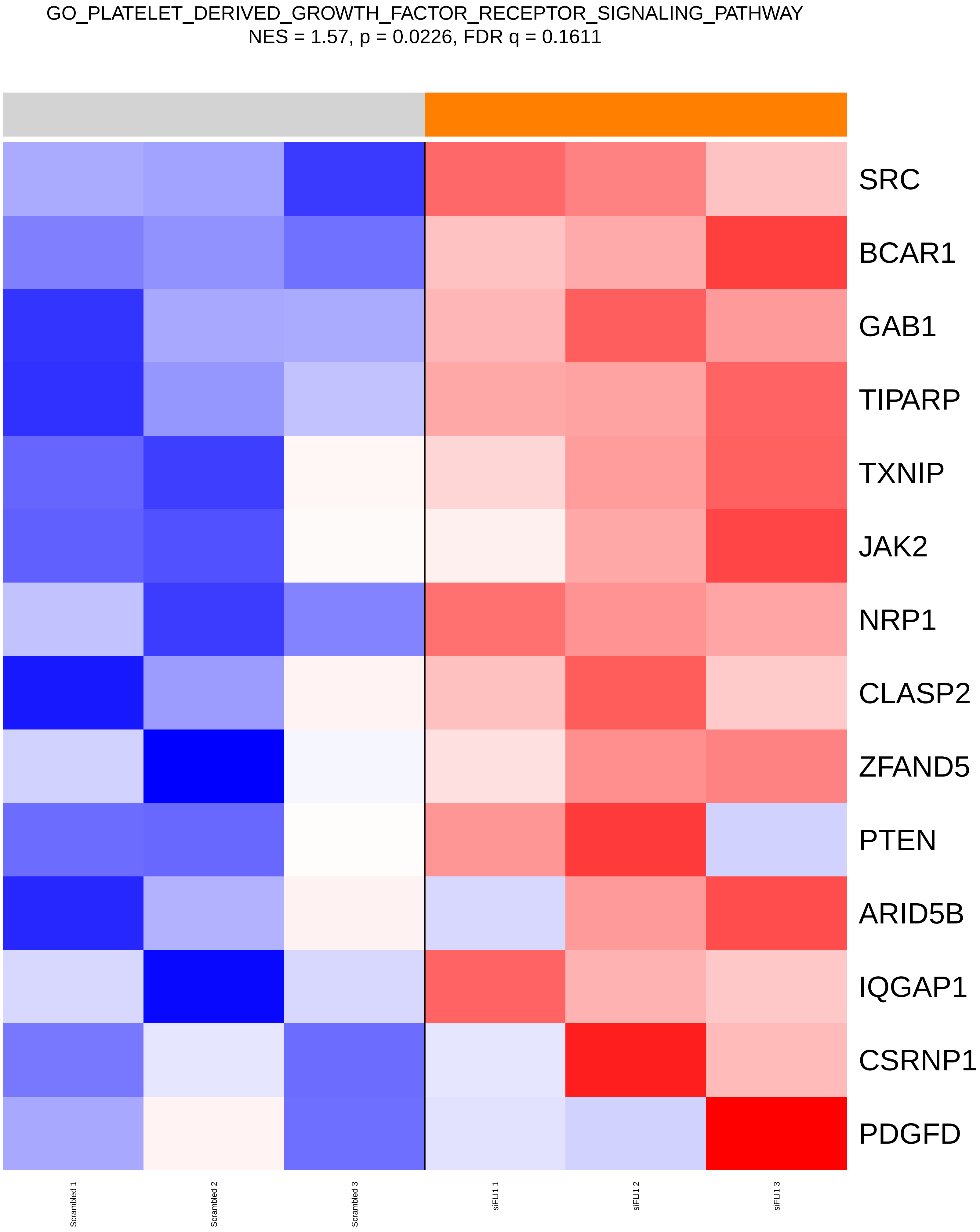
GO_PLATELET_DERIVED_GROWTH_FACTOR_RECEPTOR_SIGNALING_PATHWAY
NES = 1.57, p = 0.0226, FDR q = 0.1611
SRC
BCAR1
GAB1
TIPARP
TXNIP
JAK2
NRP1
CLASP2
ZFAND5
PTEN
ARID5B
IQGAP1
CSRNP1
PDGFD
Scrambled 1
Scrambled 2
Scrambled 3
siFLI1 1
siFLI1 2
siFLI1 3

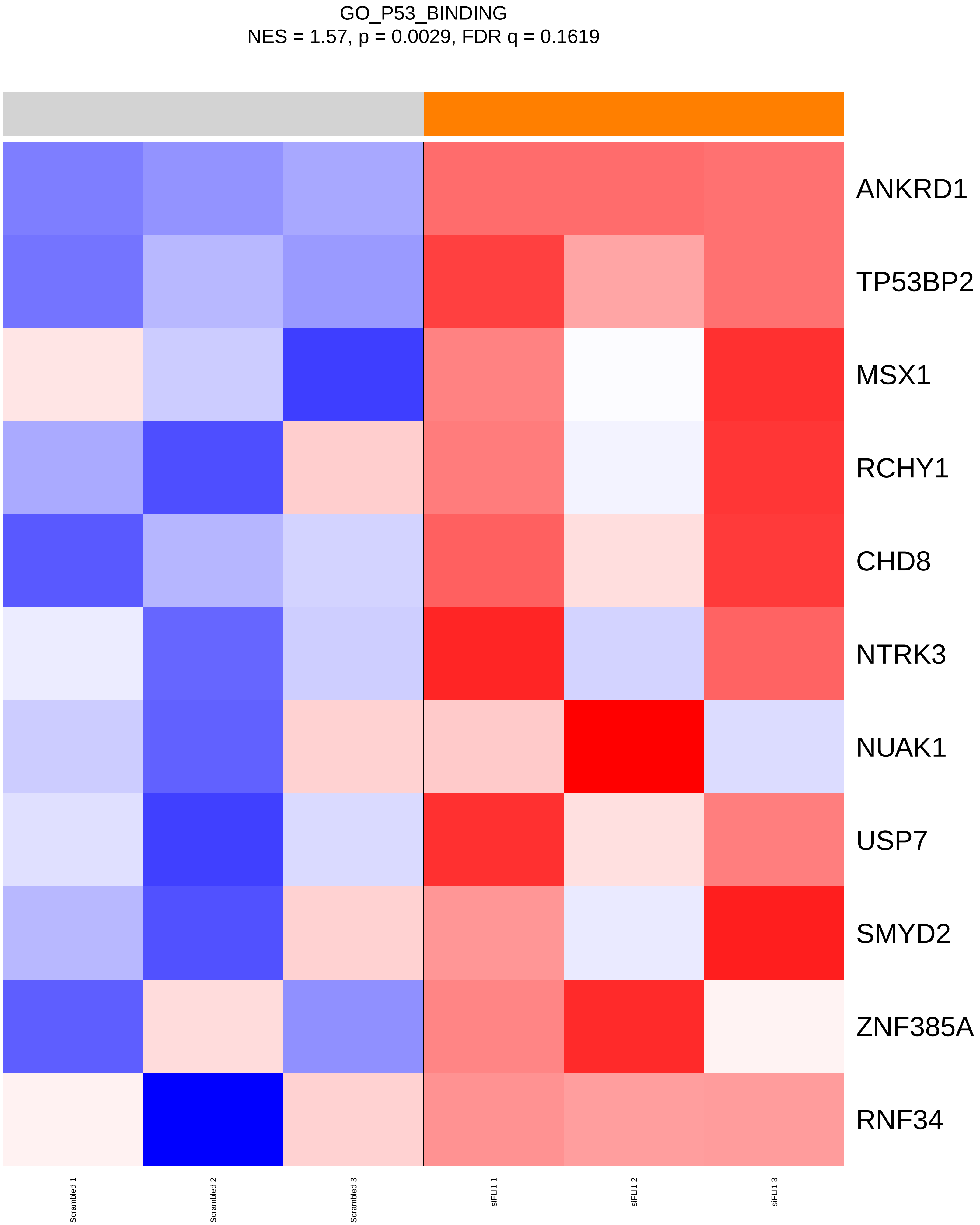
GO_P53_BINDING
NES = 1.57, p = 0.0029, FDR q = 0.1619
ANKRD1
TP53BP2
MSX1
RCHY1
CHD8
NTRK3
NUAK1
USP7
SMYD2
ZNF385A
RNF34
Scrambled 1
Scrambled 2
Scrambled 3
siFLI1 1
siFLI1 2
siFLI1 3

GO_REGULATION_OF_LEUKOCYTE_APOPTOTIC_PROCESS
NES = 1.57, p = 0.0032, FDR q = 0.1618

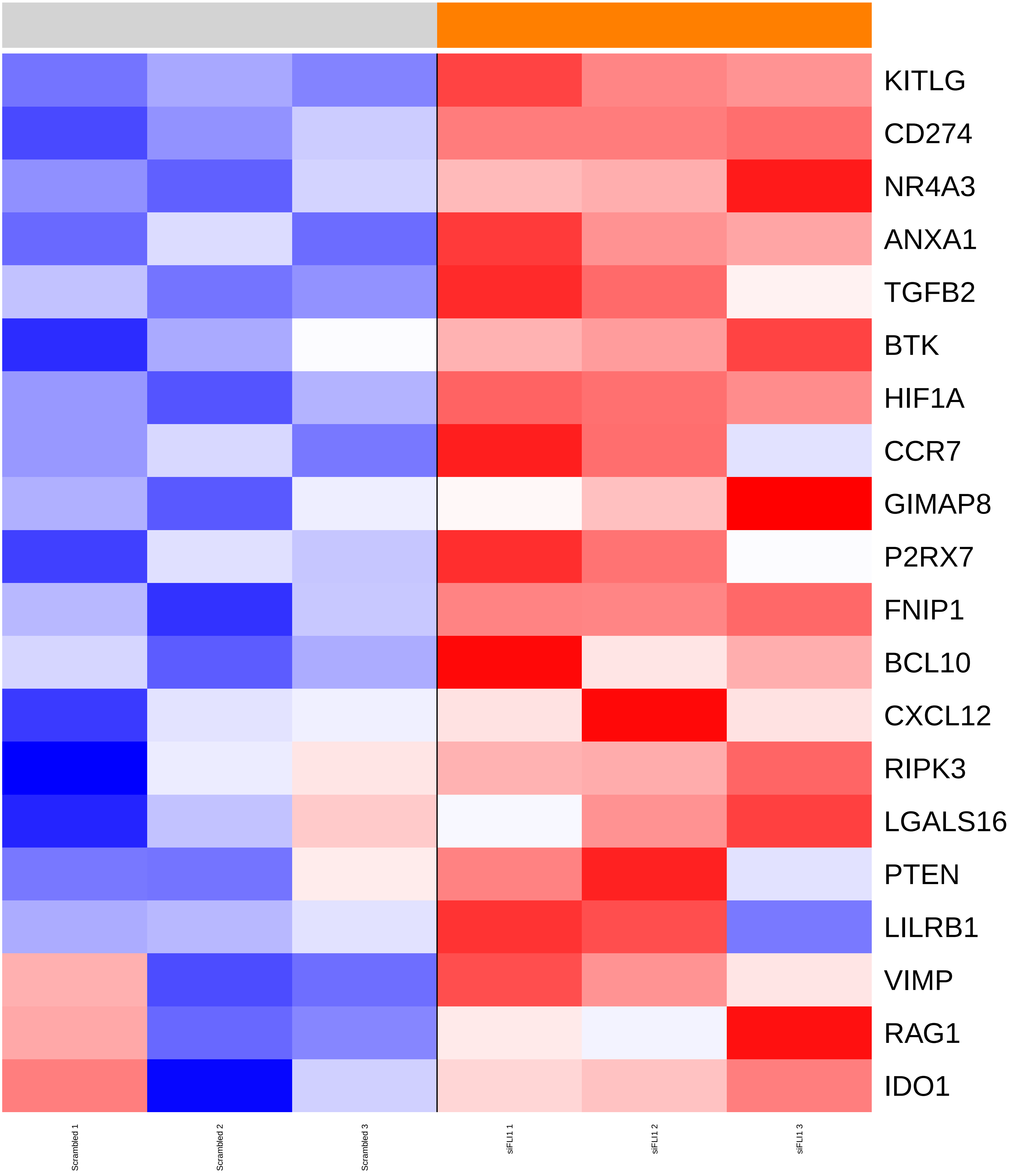

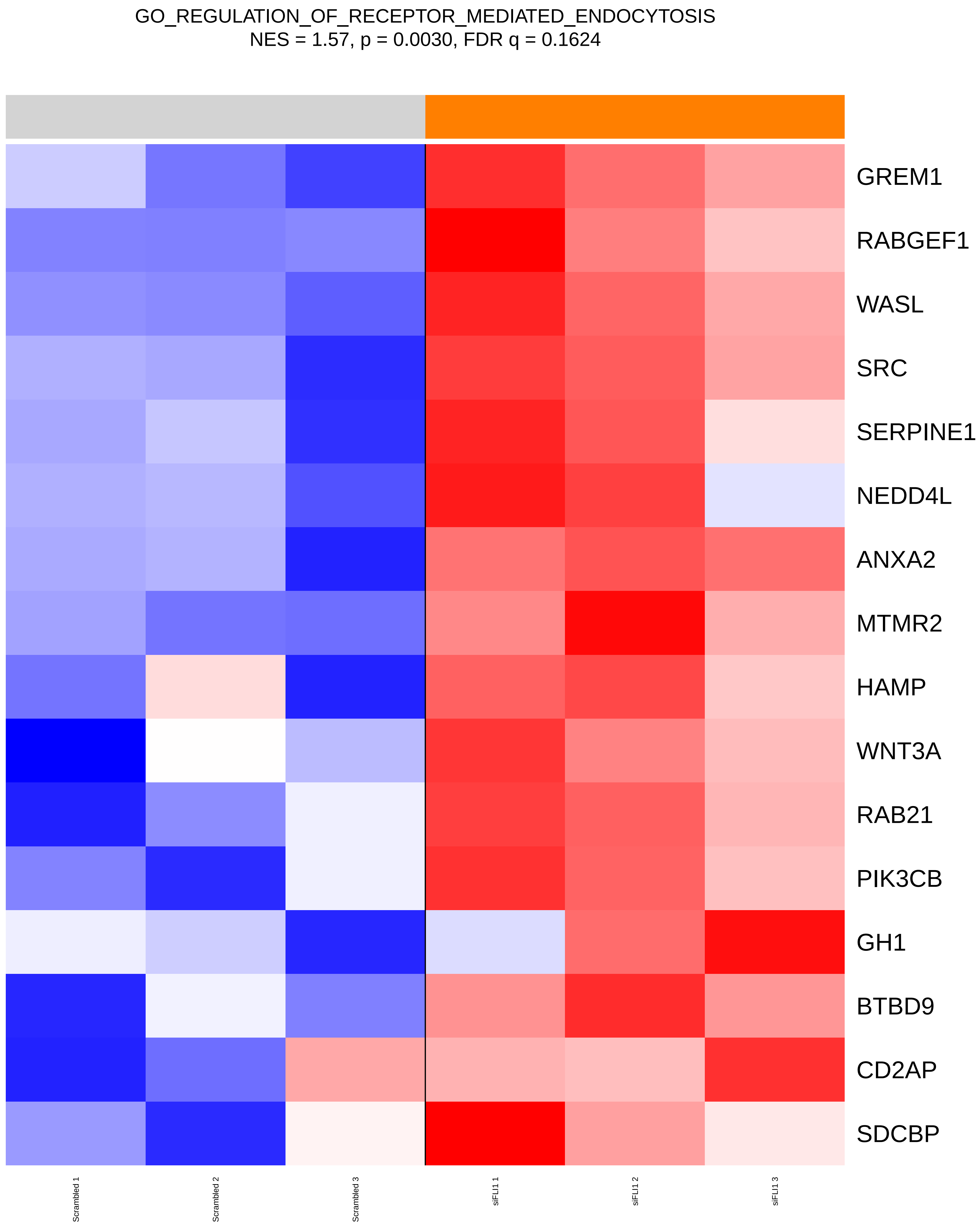
GO_REGULATION_OF_RECEPTOR_MEDIATED_ENDOCYTOSIS
NES = 1.57, p = 0.0030, FDR q = 0.1624
GREM1
RABGEF1
WASL
SRC
SERPINE1
NEDD4L
ANXA2
MTMR2
HAMP
WNT3A
RAB21
PIK3CB
GH1
BTBD9
CD2AP
SDCBP
Scrambled 1
Scrambled 2
Scrambled 3
siFLI1 1
siFLI1 2
siFLI1 3

GO_NUCLEOTIDE_BINDING_DOMAIN_LEUCINE_RICH_REPEAT_CONTAINING_RECEPTOR_SIGNALING_PATHWAY
NES = 1.57, p = 0.0266, FDR q = 0.1624

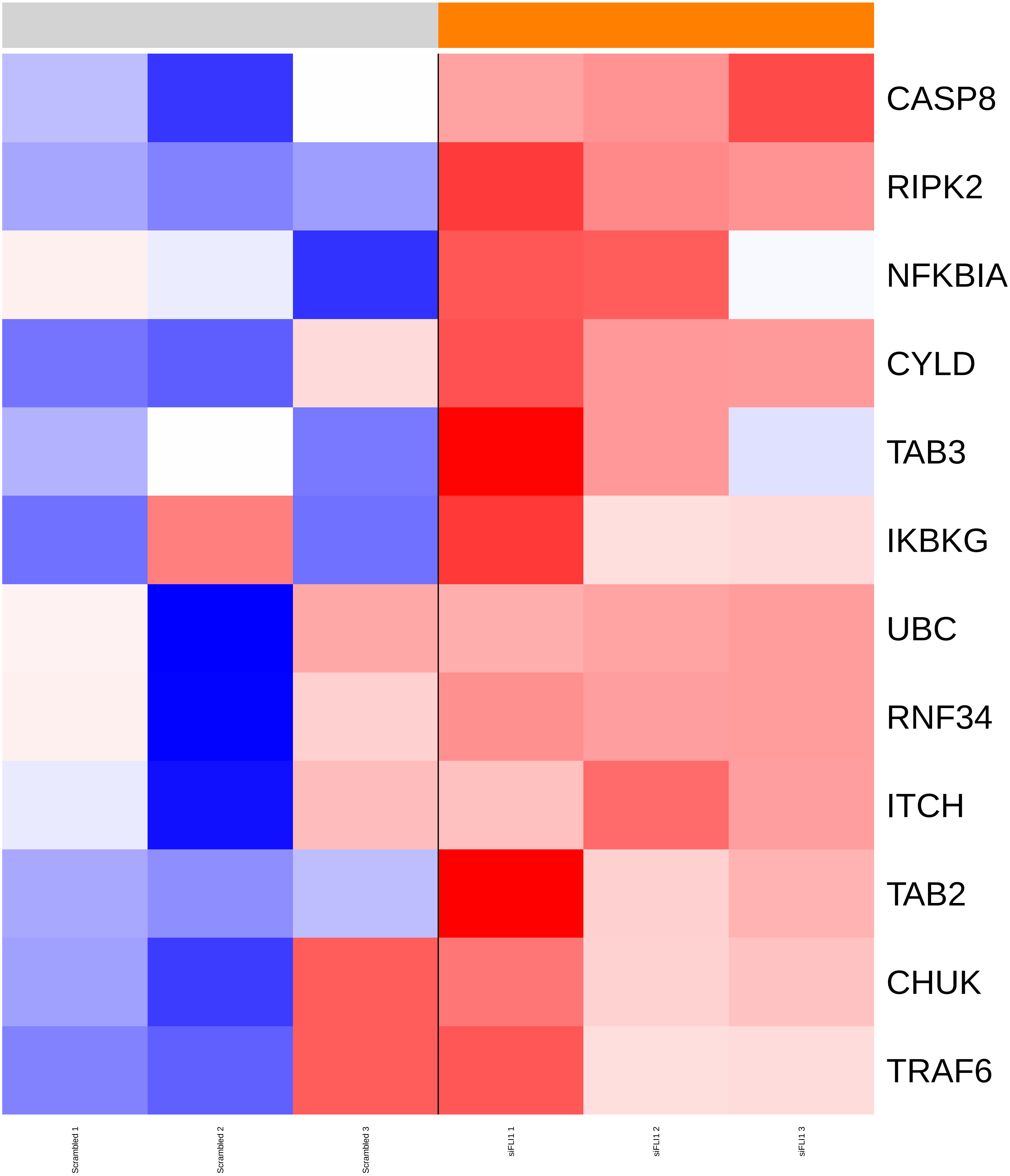

REACTOME_SIGNALLING_TO_RAS
NES = 1.57, p = 0.0165, FDR q = 0.1624

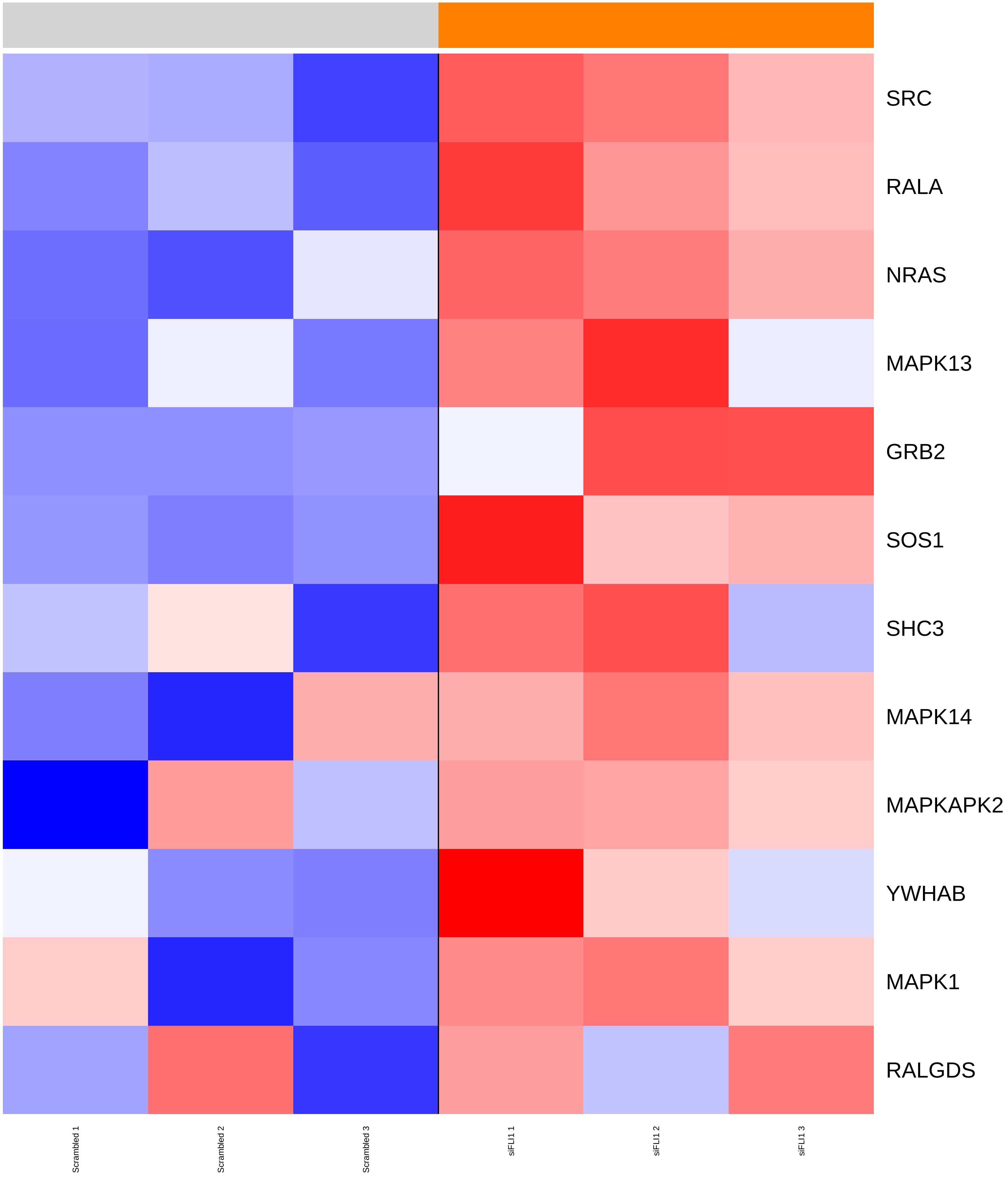

GO_NEGATIVE_CHEMOTAXIS
NES = 1.57, p = 0.0230, FDR q = 0.1644

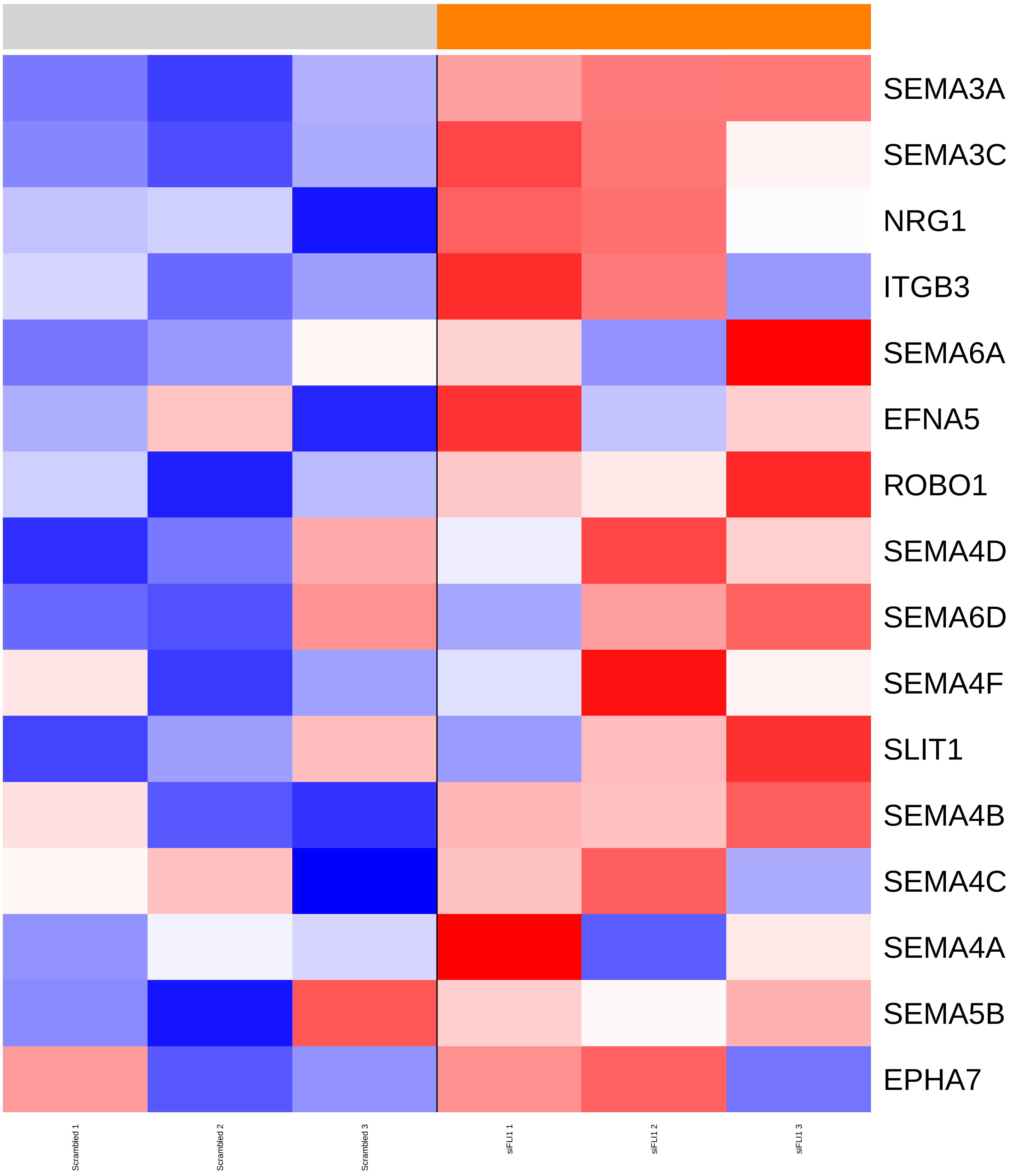

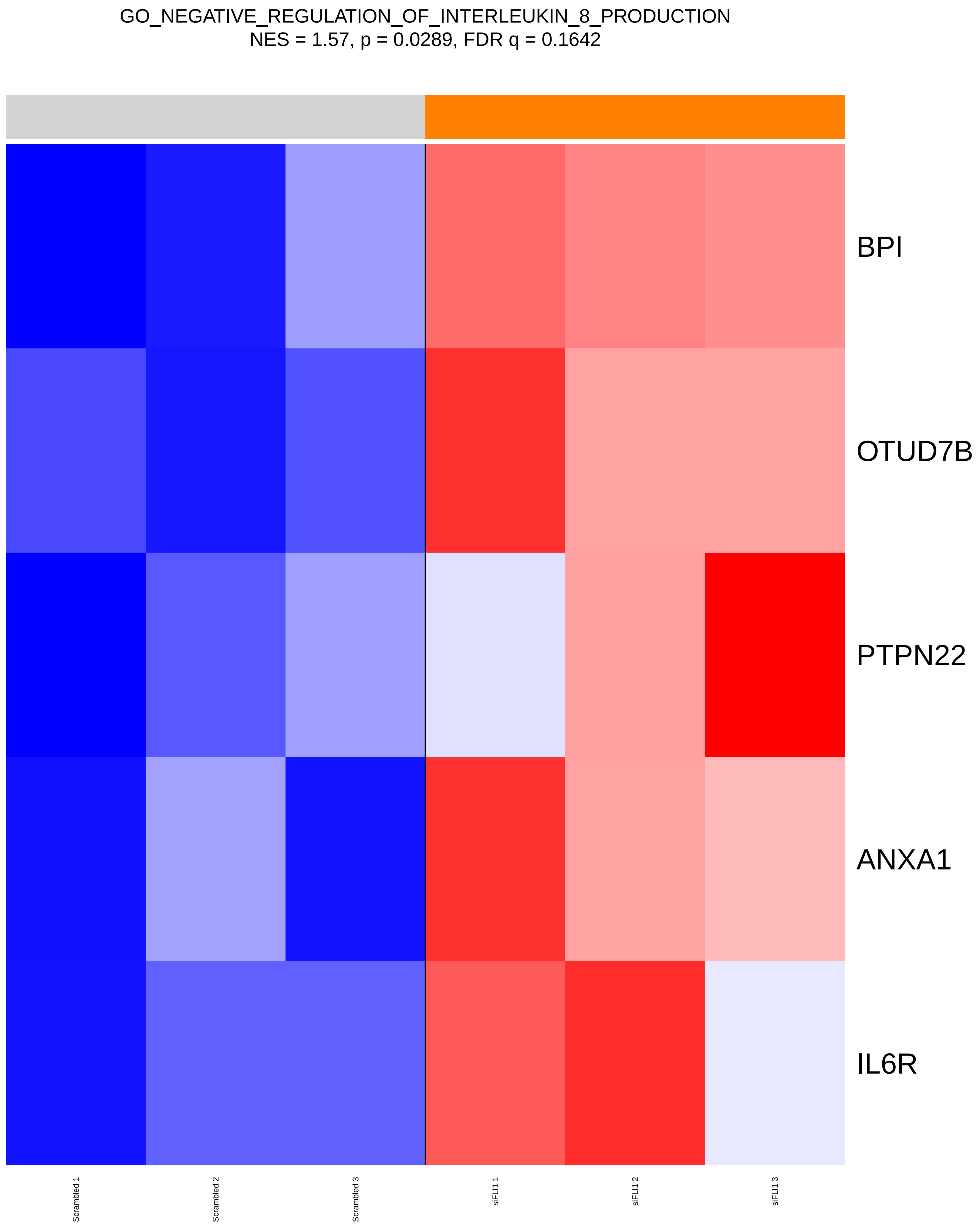
GO_NEGATIVE_REGULATION_OF_INTERLEUKIN_8_PRODUCTION
NES = 1.57, p = 0.0289, FDR q = 0.1642
BPI
OTUD7B
PTPN22
ANXA1
IL6R
Scrambled 1
Scrambled 2
Scrambled 3
siFLI1 1
siFLI1 2
siFLI1 3

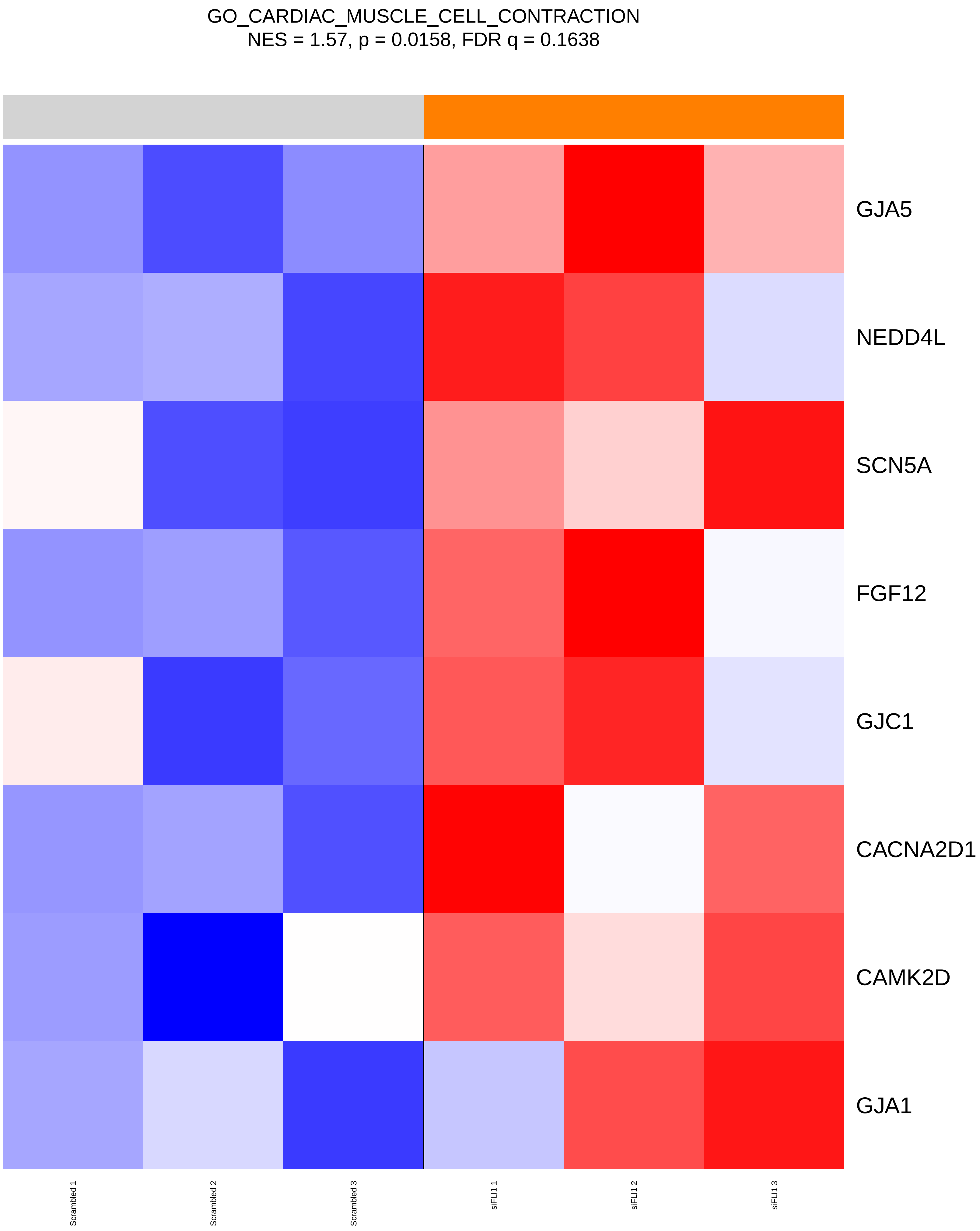
GO_CARDIAC_MUSCLE_CELL_CONTRACTION
NES = 1.57, p = 0.0158, FDR q = 0.1638
GJA5
NEDD4L
SCN5A
FGF12
GJC1
CACNA2D1
CAMK2D
GJA1
Scrambled 1
Scrambled 2
Scrambled 3
siFLI1 1
siFLI1 2
siFLI1 3

HALLMARK_COMPLEMENT
NES = 1.57, p = 0.0000, FDR q = 0.1639

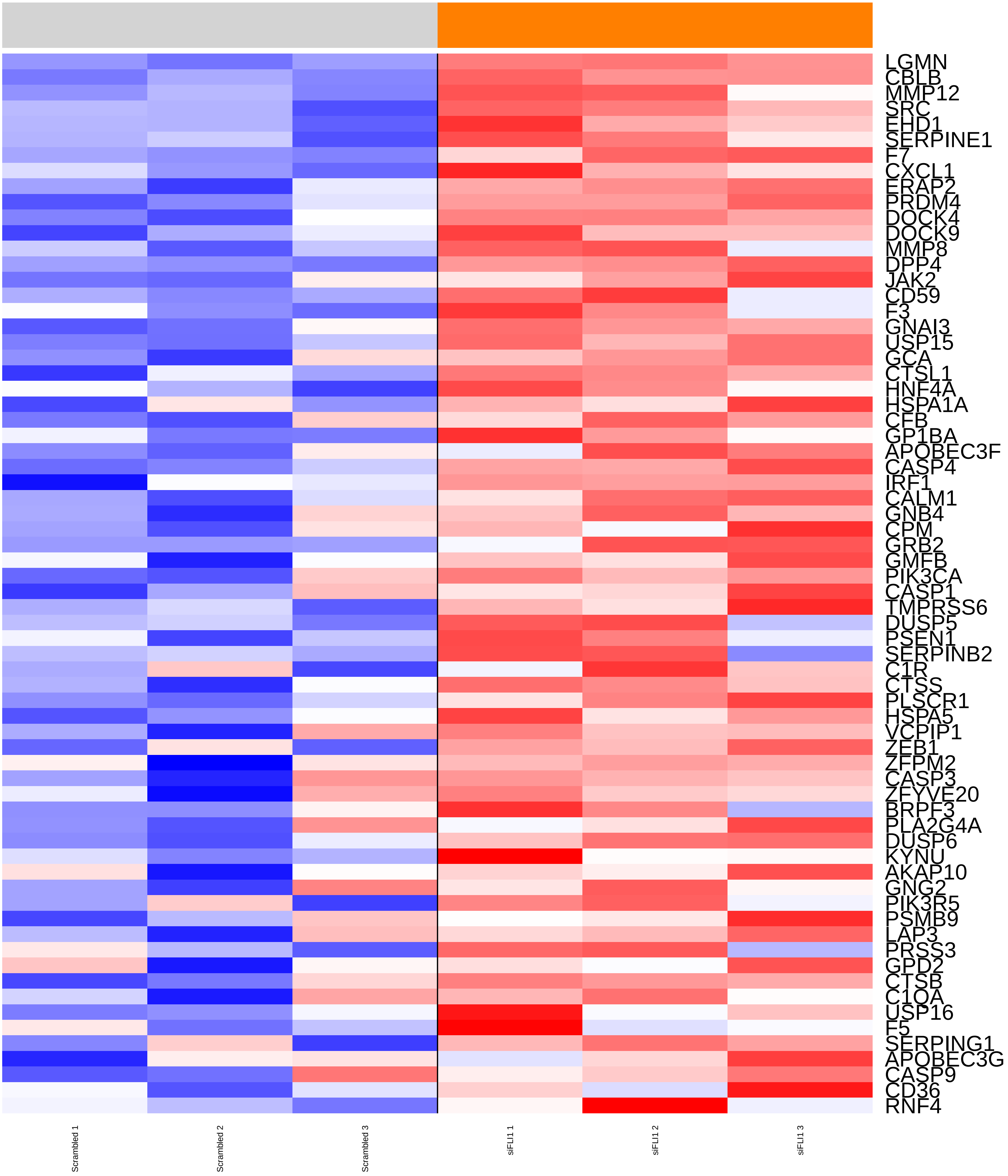

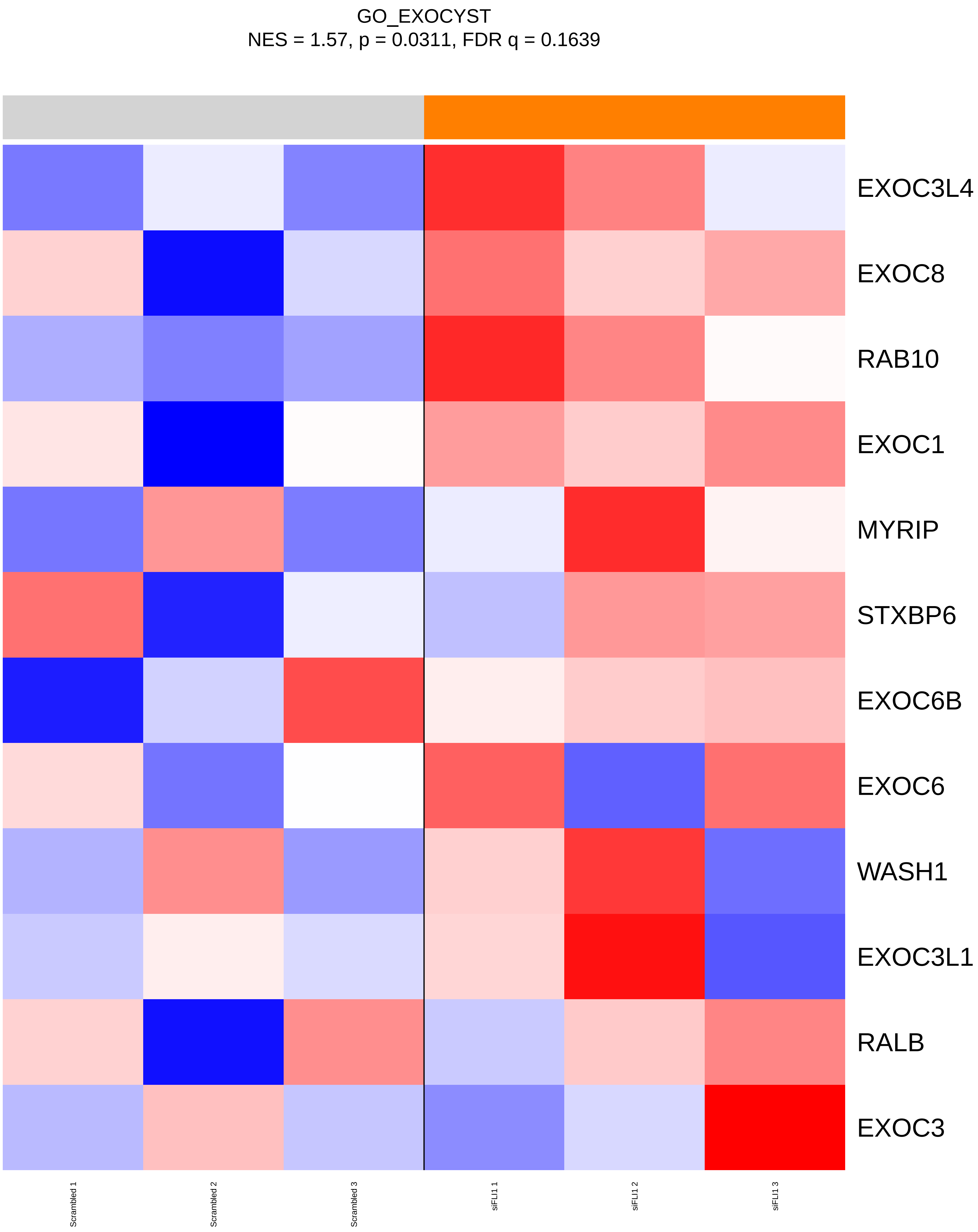
GO_EXOCYST
NES = 1.57, p = 0.0311, FDR q = 0.1639
EXOC3L4
EXOC8
RAB10
EXOC1
MYRIP
STXBP6
EXOC6B
EXOC6
WASH1
EXOC3L1
RALB
EXOC3
Scrambled 1
Scrambled 2
Scrambled 3
siFLI1 1
siFLI1 2
siFLI1 3

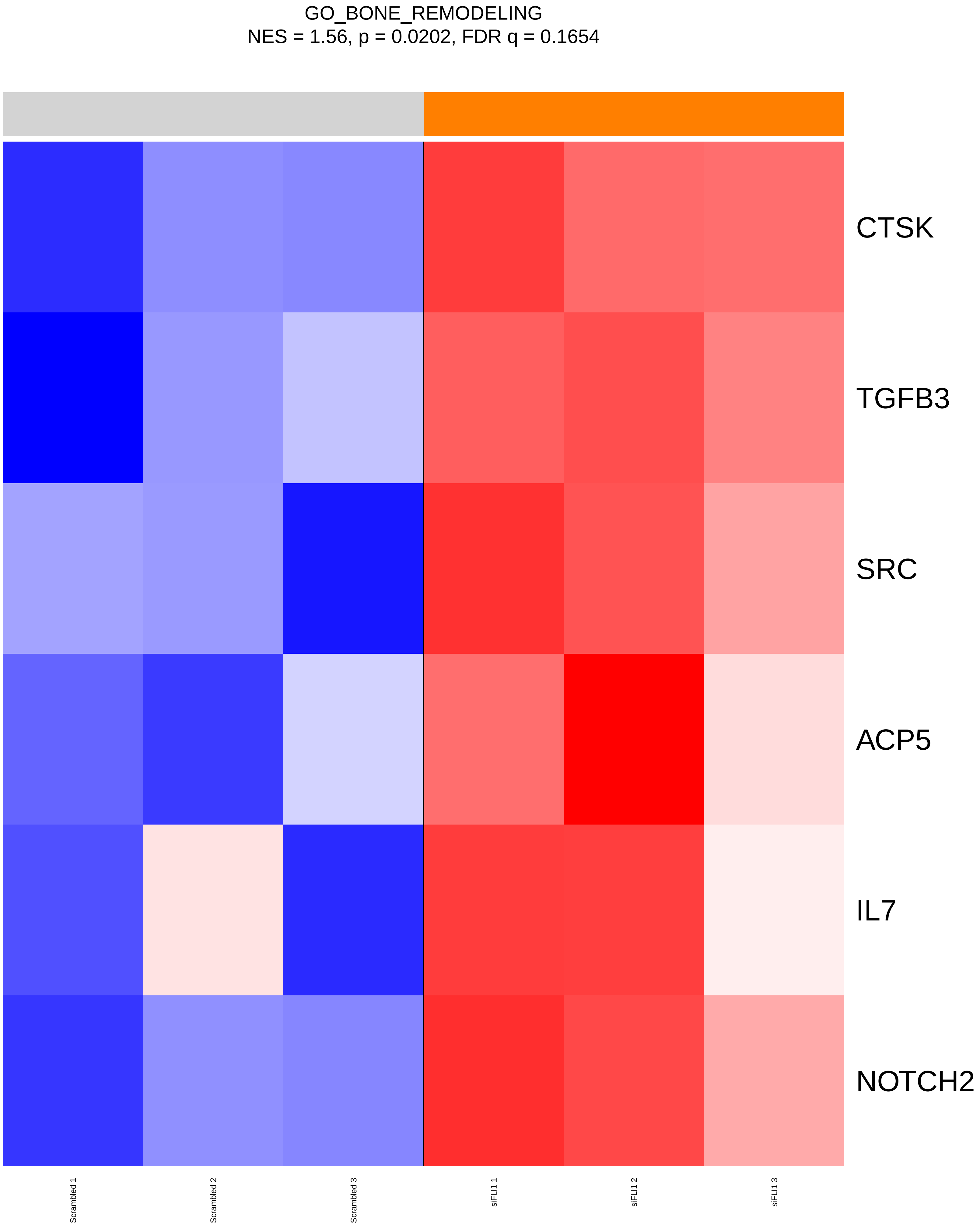
GO_BONE_REMODELING
NES = 1.56, p = 0.0202, FDR q = 0.1654
CTSK
TGFB3
SRC
ACP5
IL7
NOTCH2
Scrambled 1
Scrambled 2
Scrambled 3
siFLI1 1
siFLI1 2
siFLI1 3

REACTOME_TOLL_RECEPTOR_CASCADES
NES = 1.56, p = 0.0101, FDR q = 0.1656

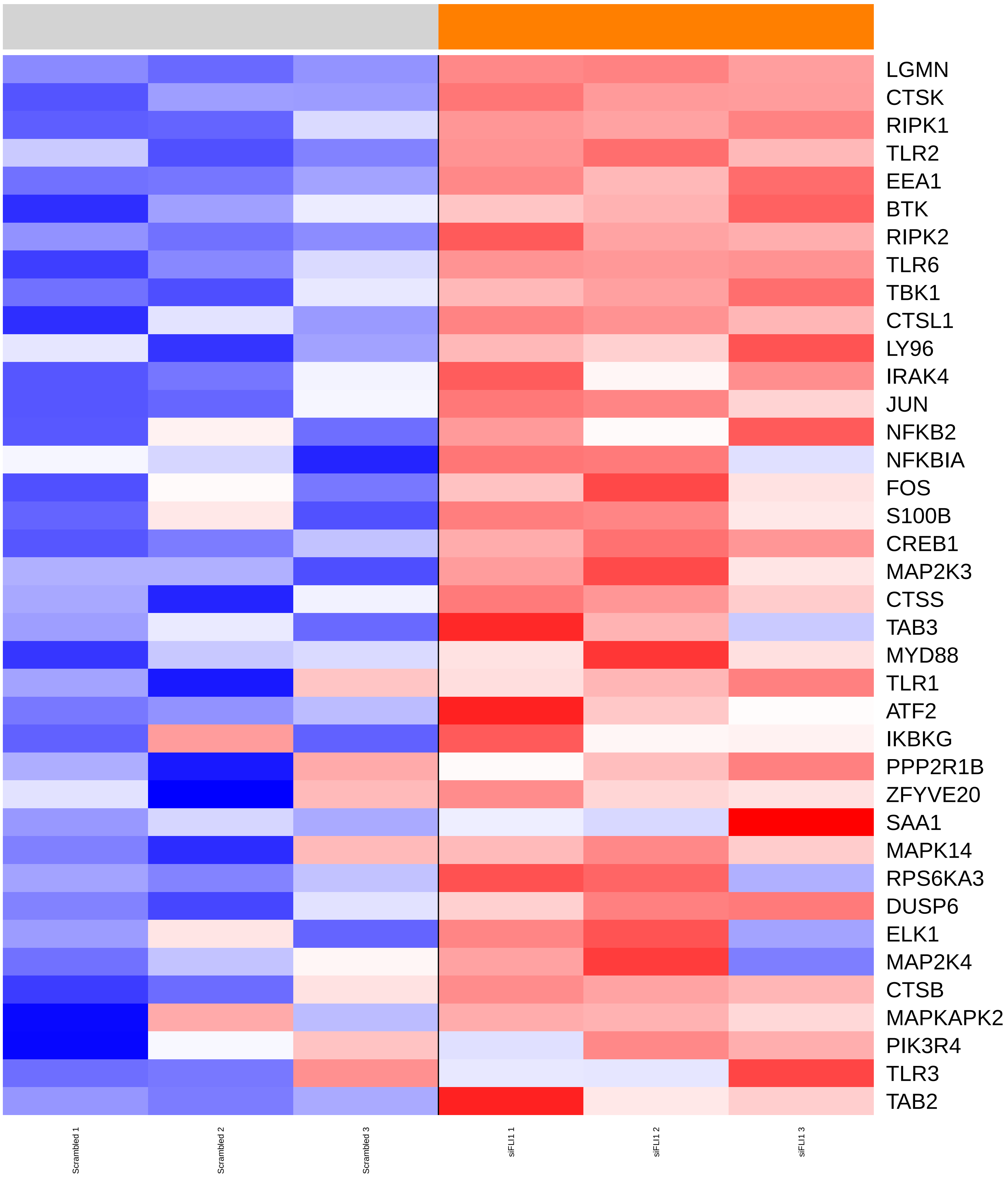

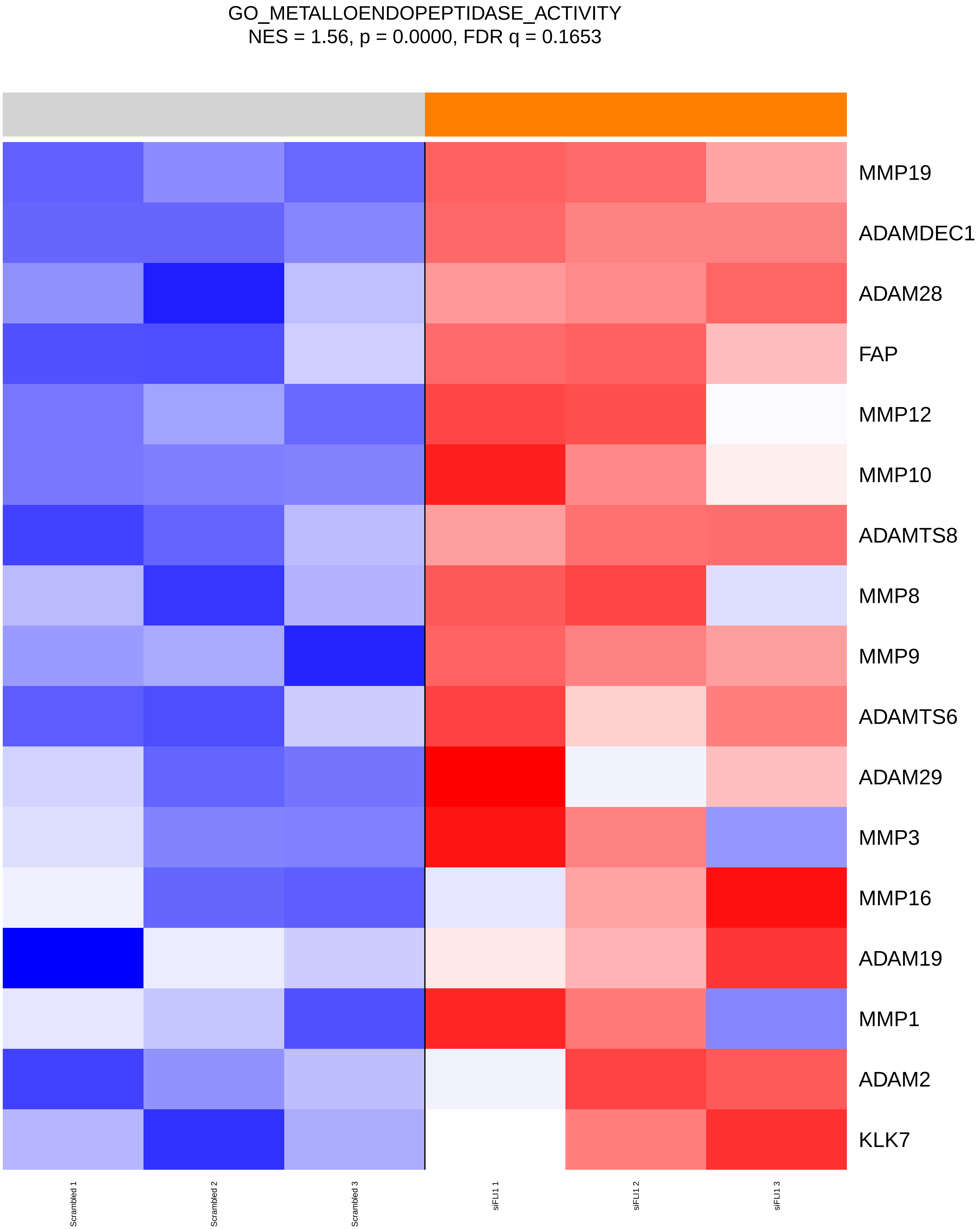

GO_METALLOENDOPEPTIDASE_ACTIVITY
NES = 1.56, p = 0.0000, FDR q = 0.1653
MMP19
ADAMDEC1
ADAM28
FAP
MMP12
MMP10
ADAMTS8
MMP8
MMP9
ADAMTS6
ADAM29
MMP3
MMP16
ADAM19
MMP1
ADAM2
KLK7
Scrambled 1
Scrambled 2
Scrambled 3
siFLI1 1
siFLI1 2
siFLI1 3

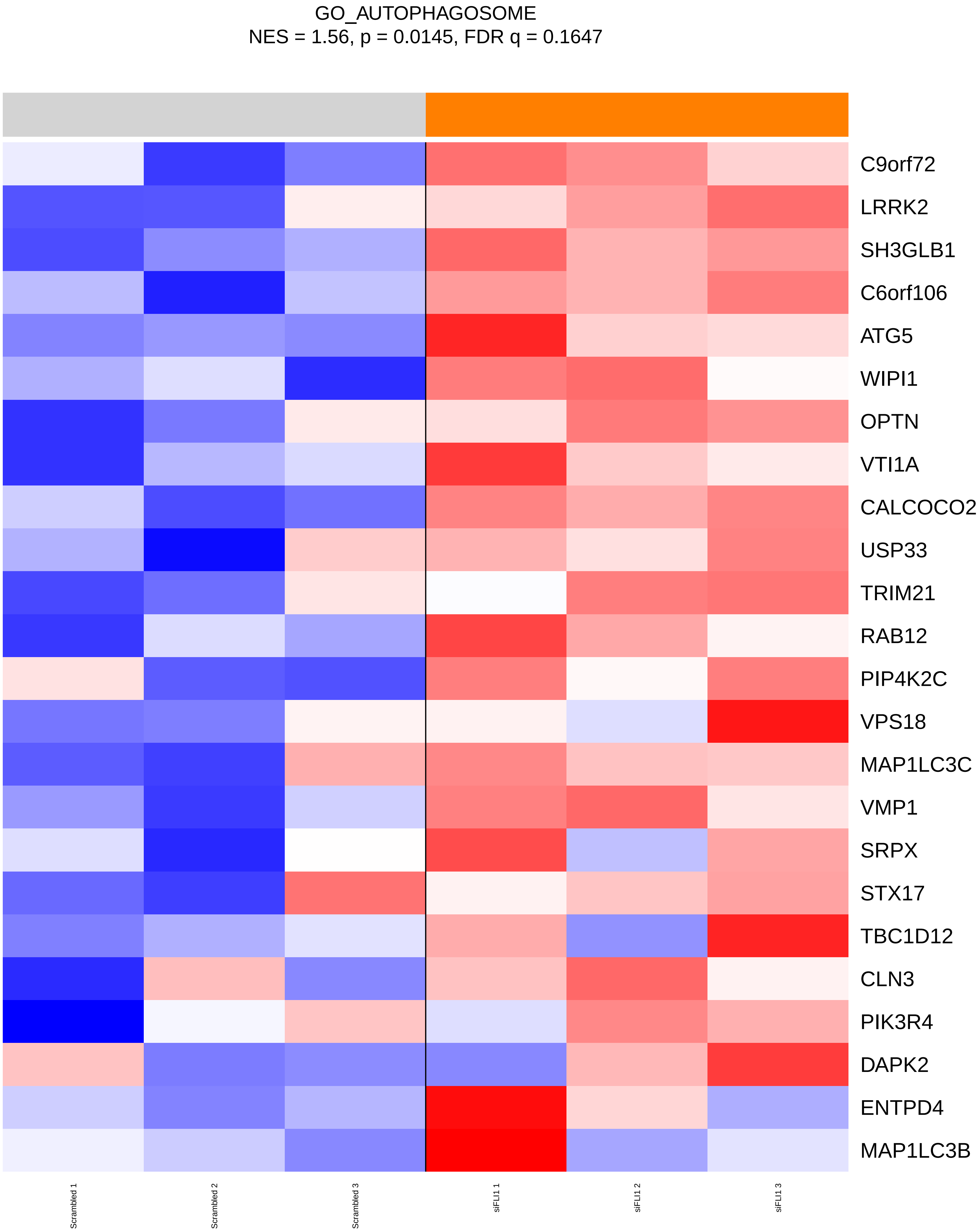
GO_AUTOPHAGOSOME
NES = 1.56, p = 0.0145, FDR q = 0.1647
C9orf72
LRRK2
SH3GLB1
C6orf106
ATG5
WIPI1
OPTN
VTI1A
CALCOCO2
USP33
TRIM21
RAB12
PIP4K2C
VPS18
MAP1LC3C
VMP1
SRPX
STX17
TBC1D12
CLN3
PIK3R4
DAPK2
ENTPD4
MAP1LC3B
Scrambled 1
Scrambled 2
Scrambled 3
siFLI1 1
siFLI1 2
siFLI1 3

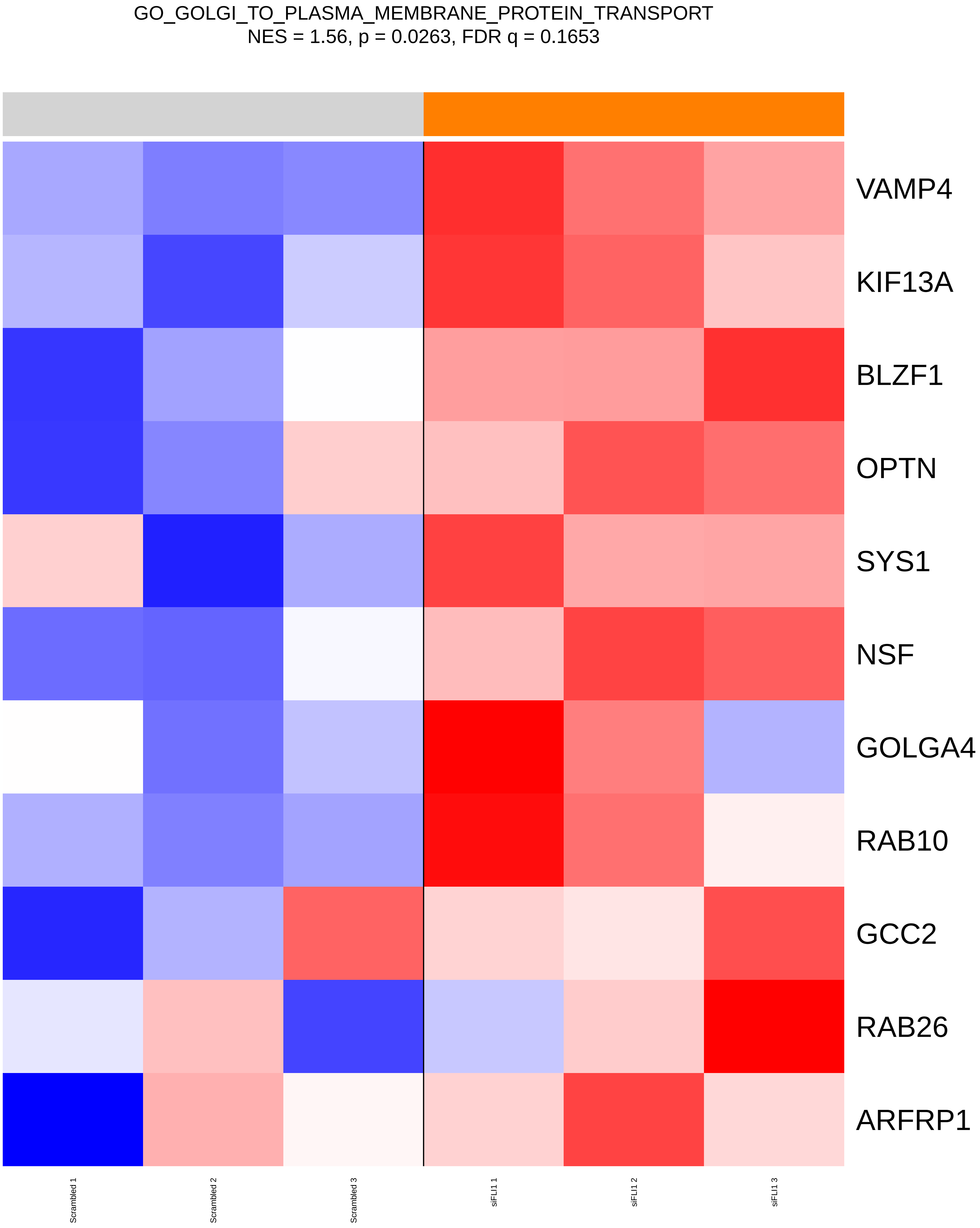
GO_GOLGI_TO_PLASMA_MEMBRANE_PROTEIN_TRANSPORT
NES = 1.56, p = 0.0263, FDR q = 0.1653
VAMP4
KIF13A
BLZF1
OPTN
SYS1
NSF
GOLGA4
RAB10
GCC2
RAB26
ARFRP1
Scrambled 1
Scrambled 2
Scrambled 3
siFLI1 1
siFLI1 2
siFLI1 3

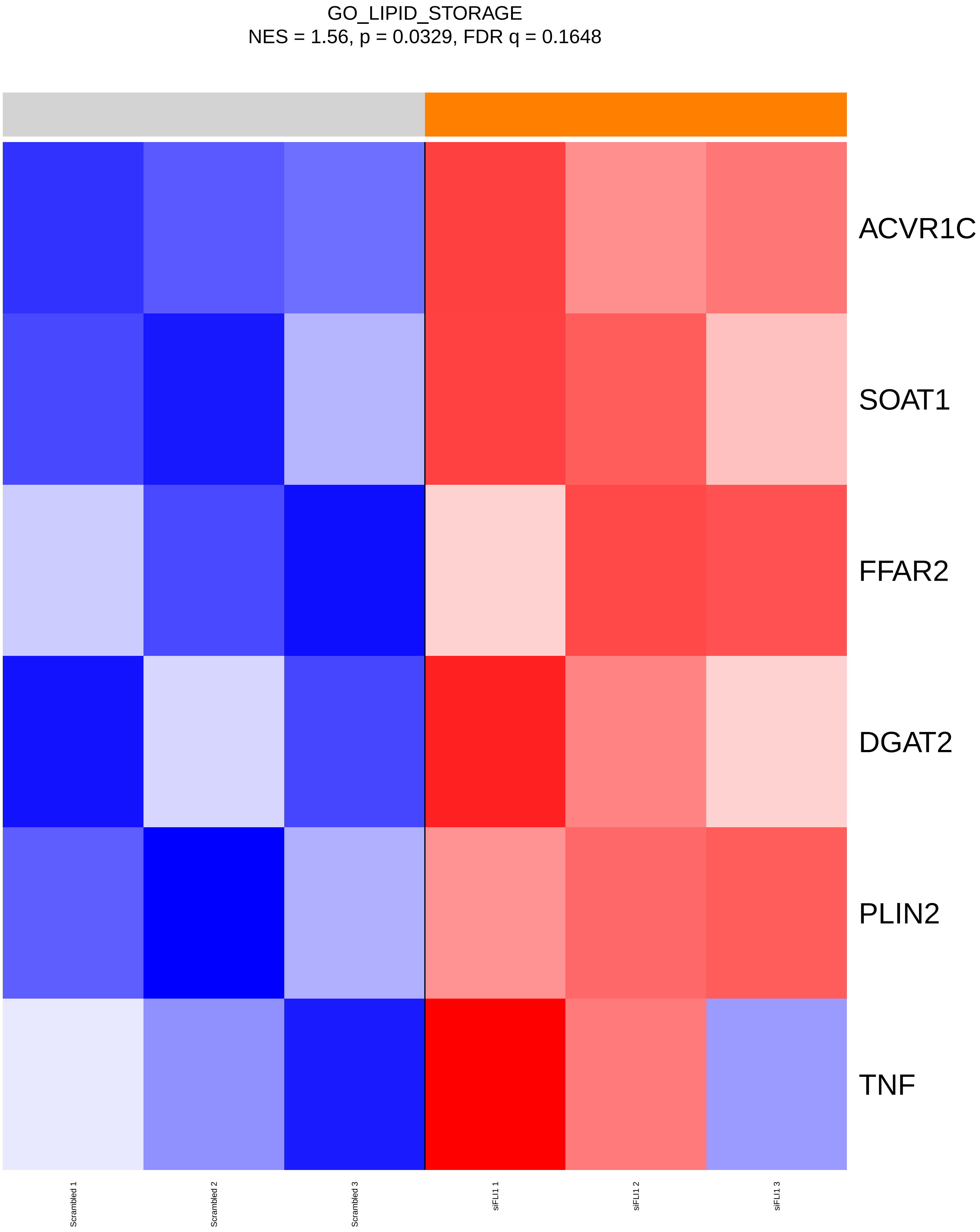
GO_LIPID_STORAGE
NES = 1.56, p = 0.0329, FDR q = 0.1648
ACVR1C
SOAT1
FFAR2
DGAT2
PLIN2
TNF
Scrambled 1
Scrambled 2
Scrambled 3
siFLI1 1
siFLI1 2
siFLI1 3

GO_REGULATION_OF_INFLAMMATORY_RESPONSE
NES = 1.56, p = 0.0000, FDR q = 0.1649

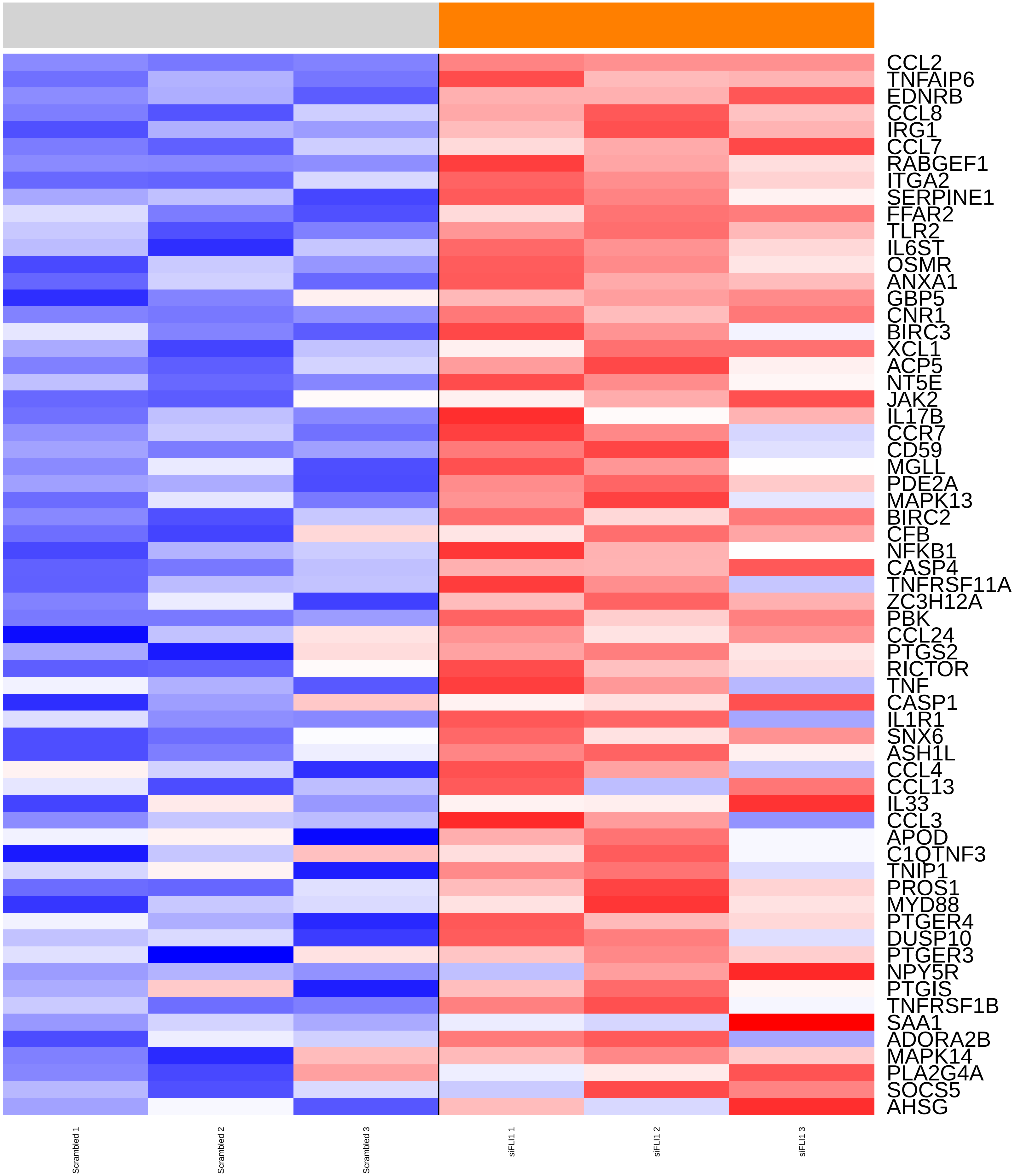

GO_BONE_RESORPTION
NES = 1.56, p = 0.0466, FDR q = 0.1662

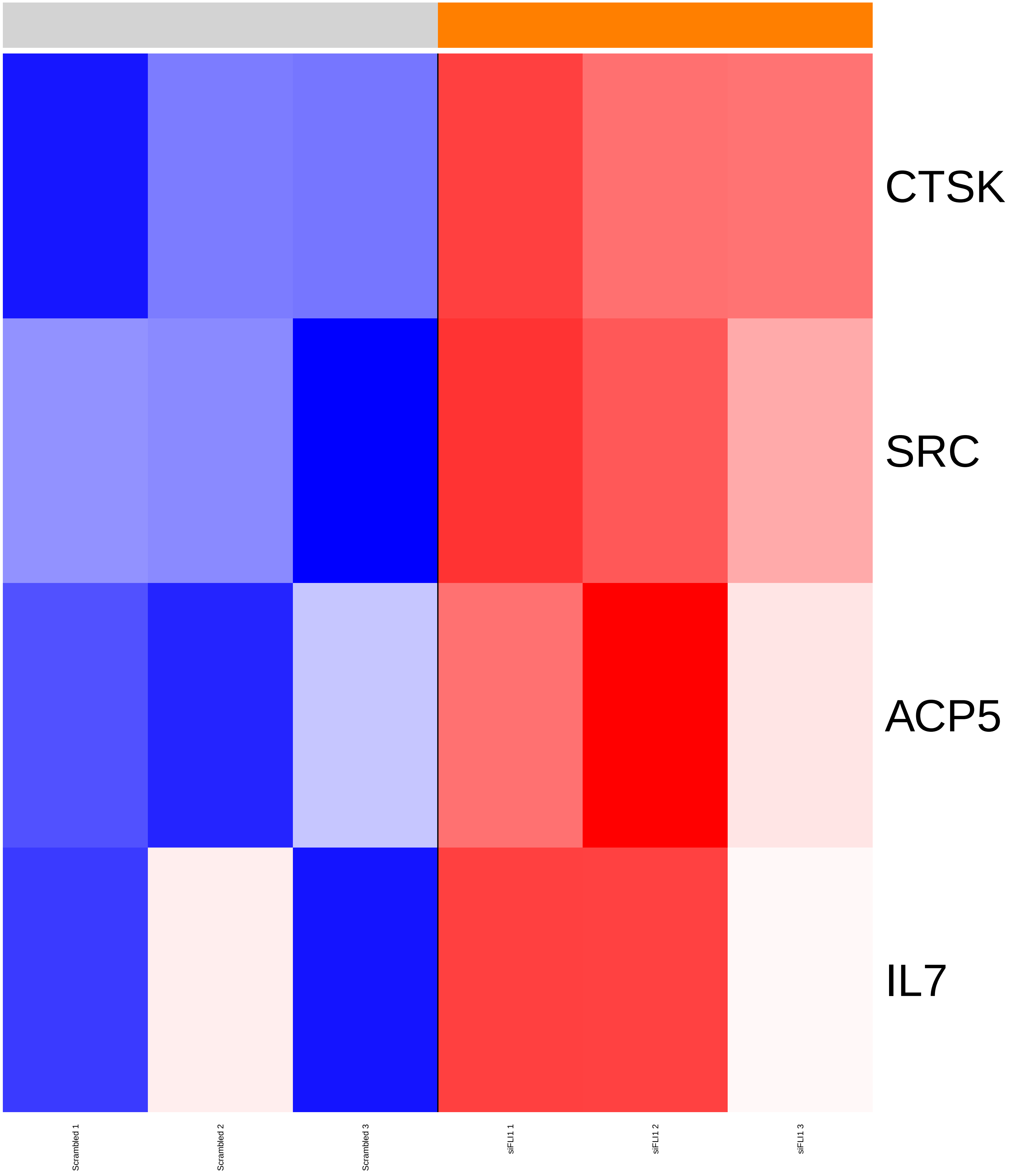

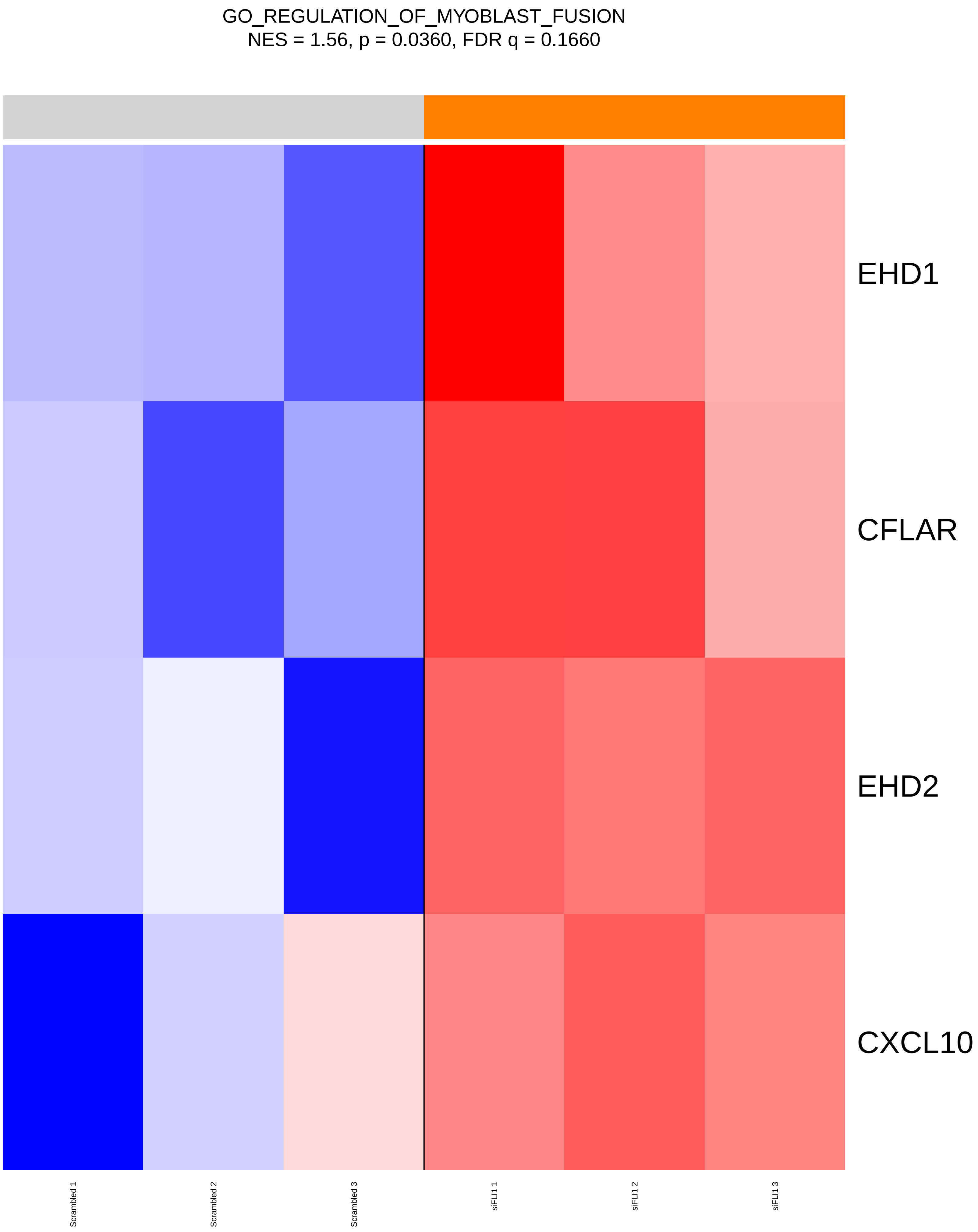
GO_REGULATION_OF_MYOBLAST_FUSION
NES = 1.56, p = 0.0360, FDR q = 0.1660
EHD1
CFLAR
EHD2
CXCL10
Scrambled 1
Scrambled 2
Scrambled 3
siFLI1 1
siFLI1 2
siFLI1 3

GO_POSITIVE_REGULATION_OF_CHONDROCYTE_DIFFERENTIATION
NES = 1.56, p = 0.0417, FDR q = 0.1672

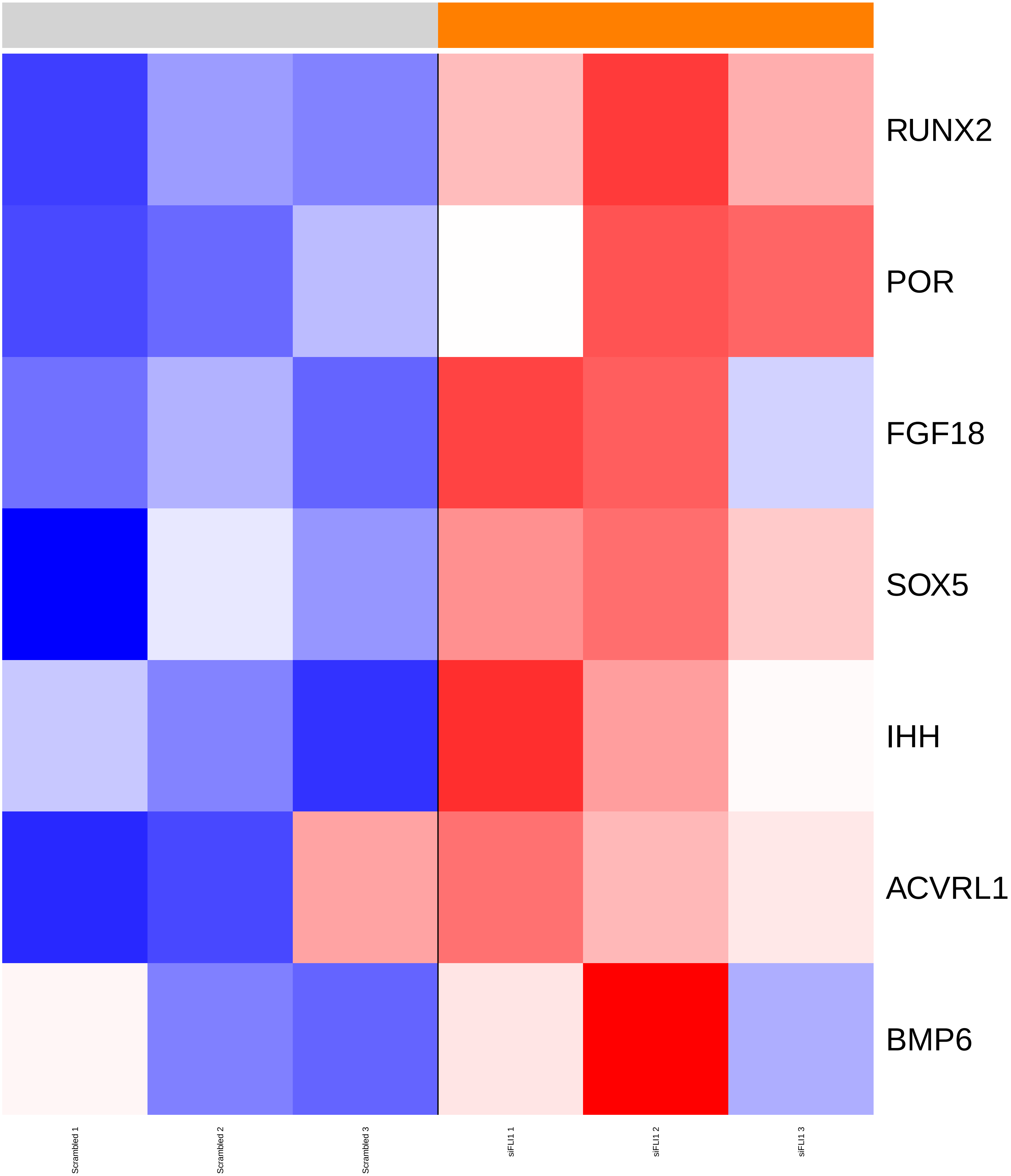

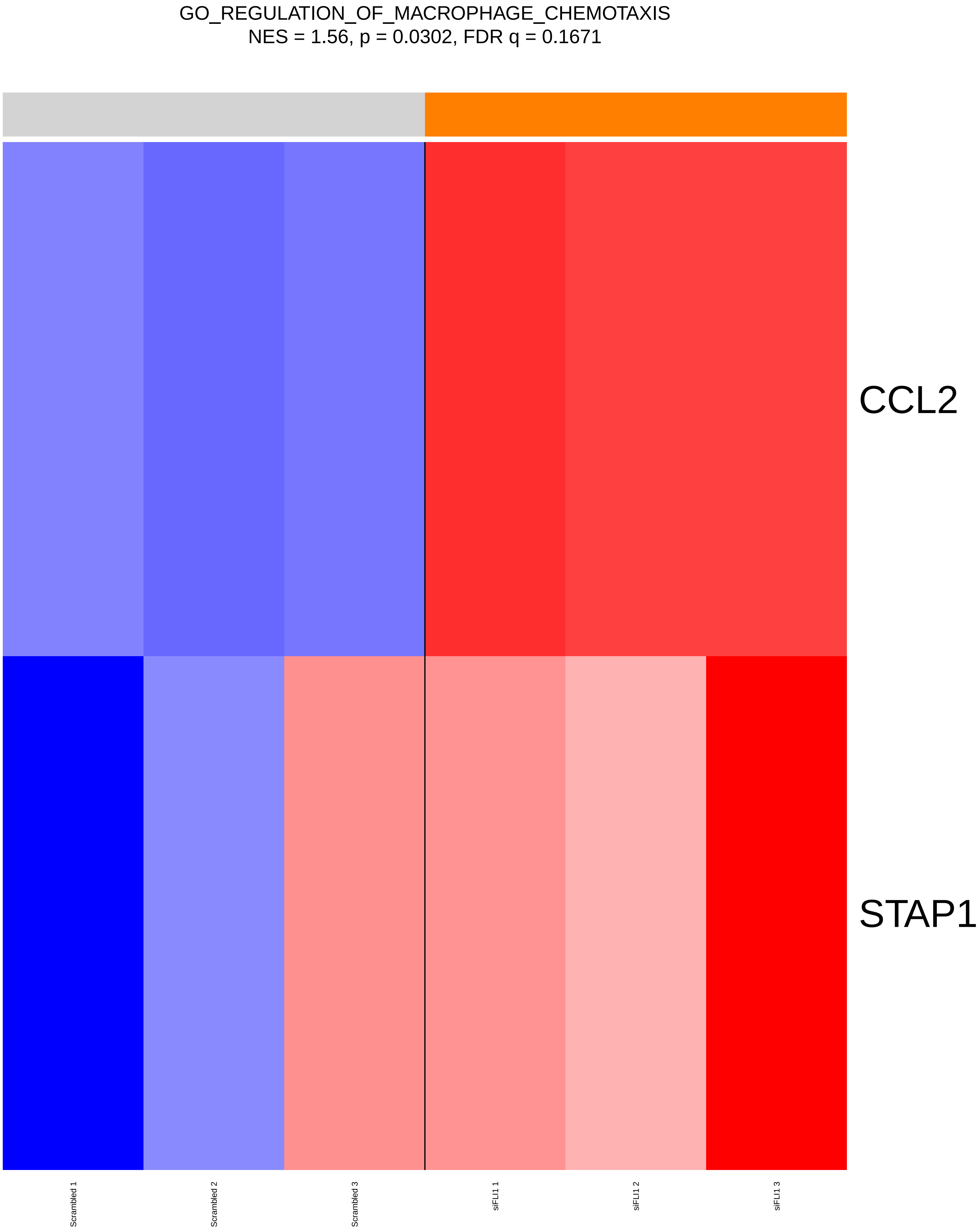
GO_REGULATION_OF_MACROPHAGE_CHEMOTAXIS
NES = 1.56, p = 0.0302, FDR q = 0.1671
CCL2
STAP1
Scrambled 1
Scrambled 2
Scrambled 3
siFLI1 1
siFLI1 2
siFLI1 3

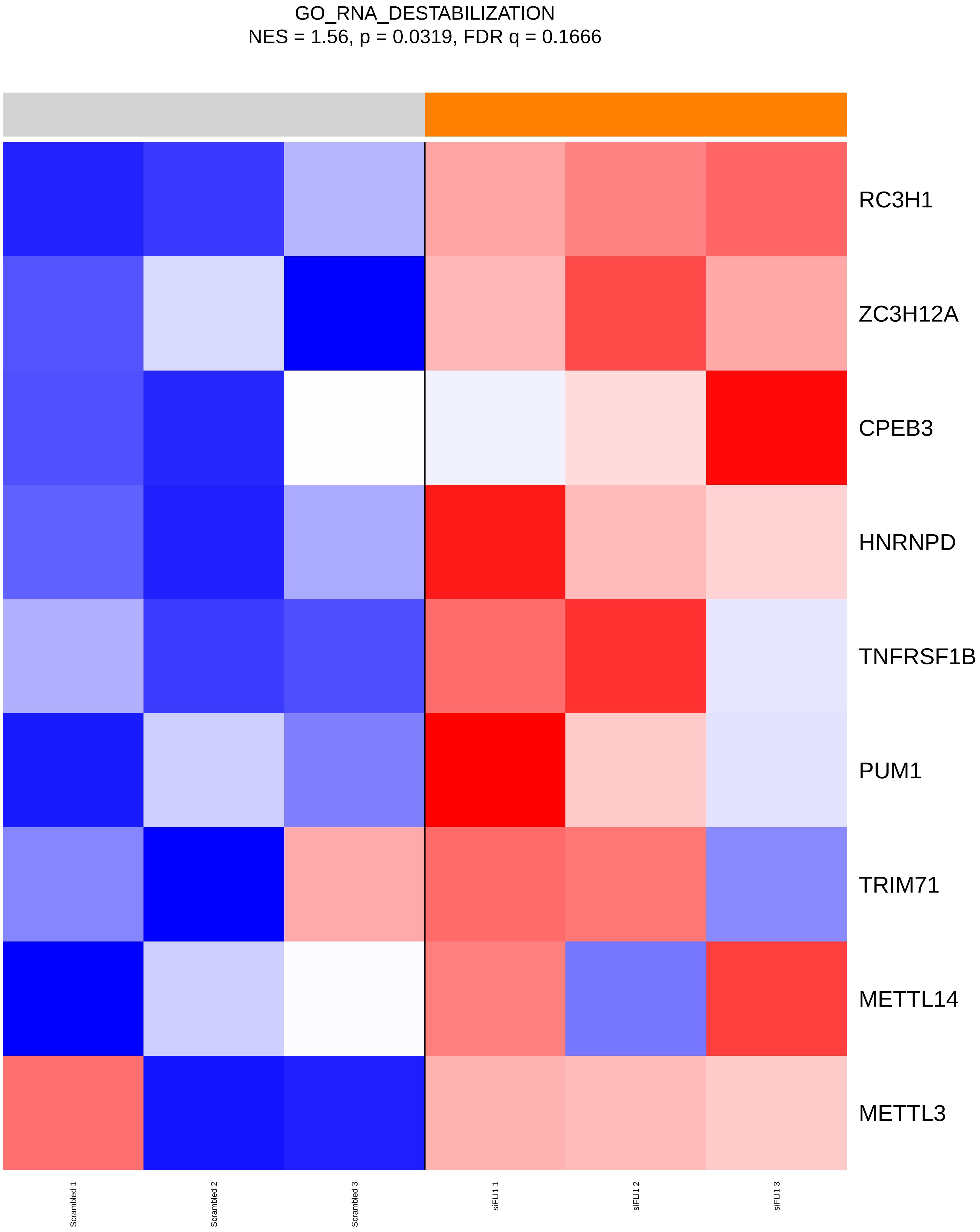
GO_RNA_DESTABILIZATION
NES = 1.56, p = 0.0319, FDR q = 0.1666
RC3H1
ZC3H12A
CPEB3
HNRNPD
TNFRSF1B
PUM1
TRIM71
METTL14
METTL3
Scrambled 1
Scrambled 2
Scrambled 3
siFLI1 1
siFLI1 2
siFLI1 3

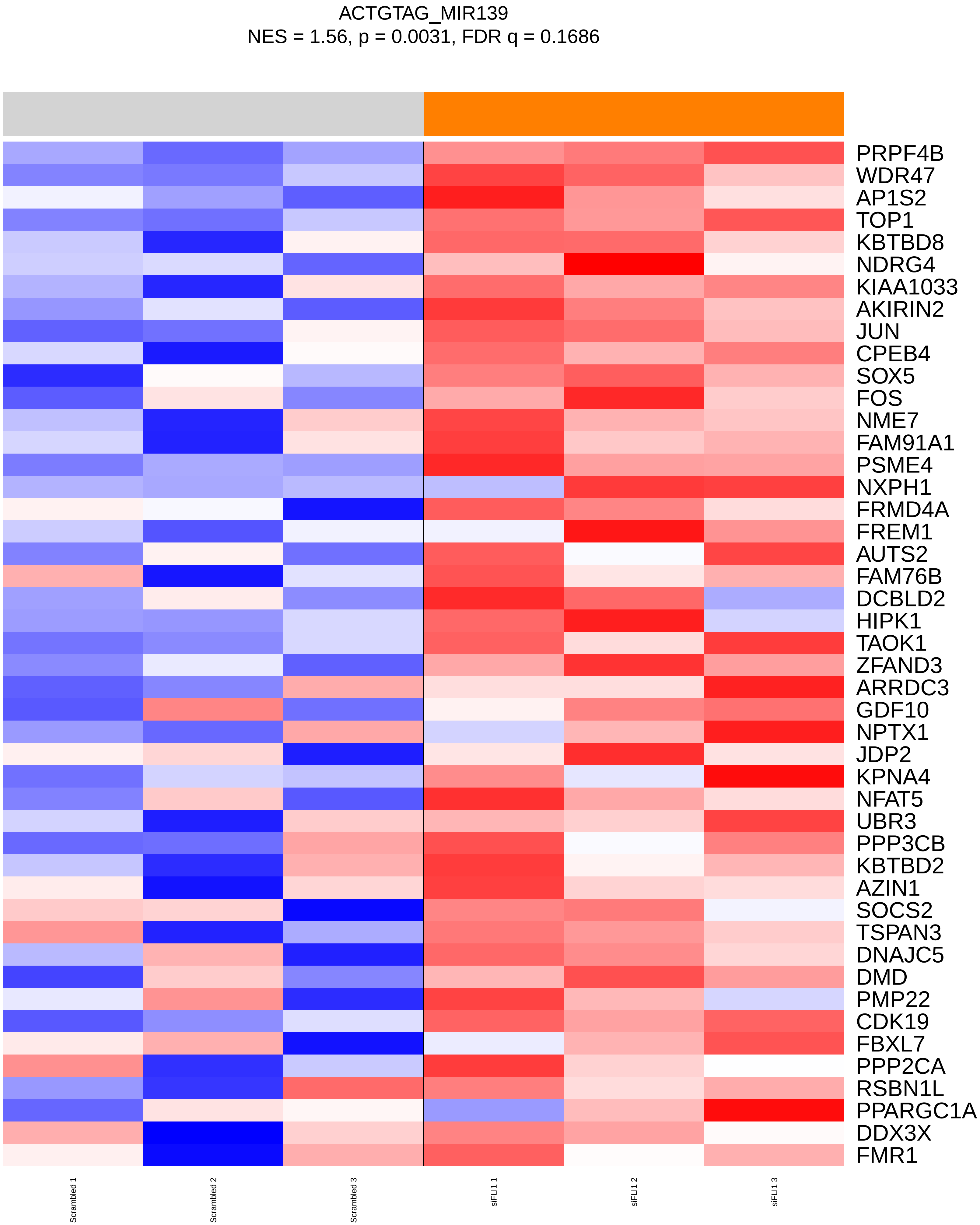
ACTGTAG_MIR139
NES = 1.56, p = 0.0031, FDR q = 0.1686
PRPF4B
WDR47
AP1S2
TOP1
KBTBD8
NDRG4
KIAA1033
AKIRIN2
JUN
CPEB4
SOX5
FOS
NME7
FAM91A1
PSME4
NXPH1
FRMD4A
FREM1
AUTS2
FAM76B
DCBLD2
HIPK1
TAOK1
ZFAND3
ARRDC3
GDF10
NPTX1
JDP2
KPNA4
NFAT5
UBR3
PPP3CB
KBTBD2
AZIN1
SOCS2
TSPAN3
DNAJC5
DMD
PMP22
CDK19
FBXL7
PPP2CA
RSBN1L
PPARGC1A
DDX3X
FMR1
Scrambled 1
Scrambled 2
Scrambled 3
siFLI1 1
siFLI1 2
siFLI1 3

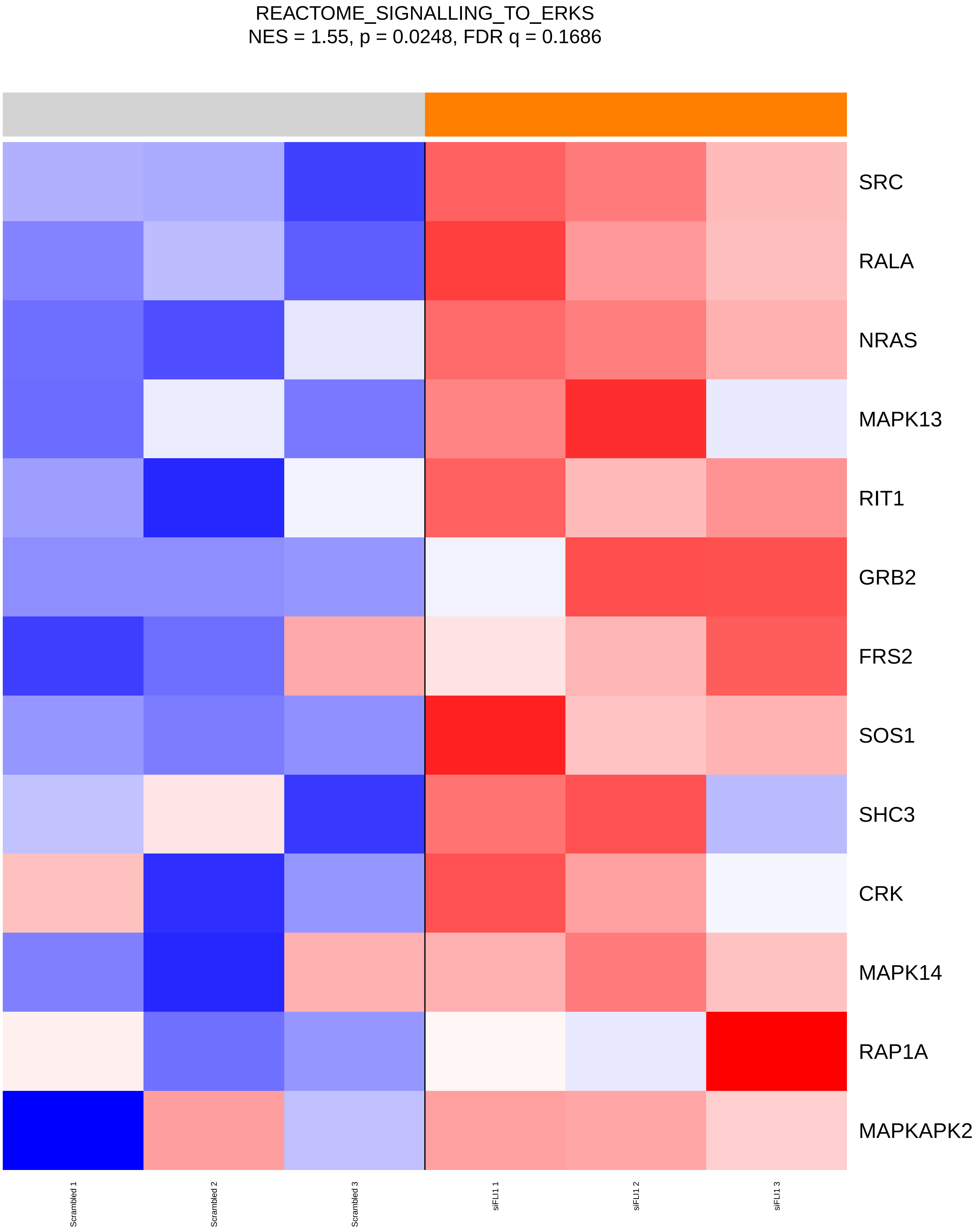
REACTOME_SIGNALLING_TO_ERKS
NES = 1.55, p = 0.0248, FDR q = 0.1686
SRC
RALA
NRAS
MAPK13
RIT1
GRB2
FRS2
SOS1
SHC3
CRK
MAPK14
RAP1A
MAPKAPK2
Scrambled 1
Scrambled 2
Scrambled 3
siFLI1 1
siFLI1 2
siFLI1 3

GO_REGULATION_OF_T_CELL_MIGRATION
NES = 1.55, p = 0.0306, FDR q = 0.1694

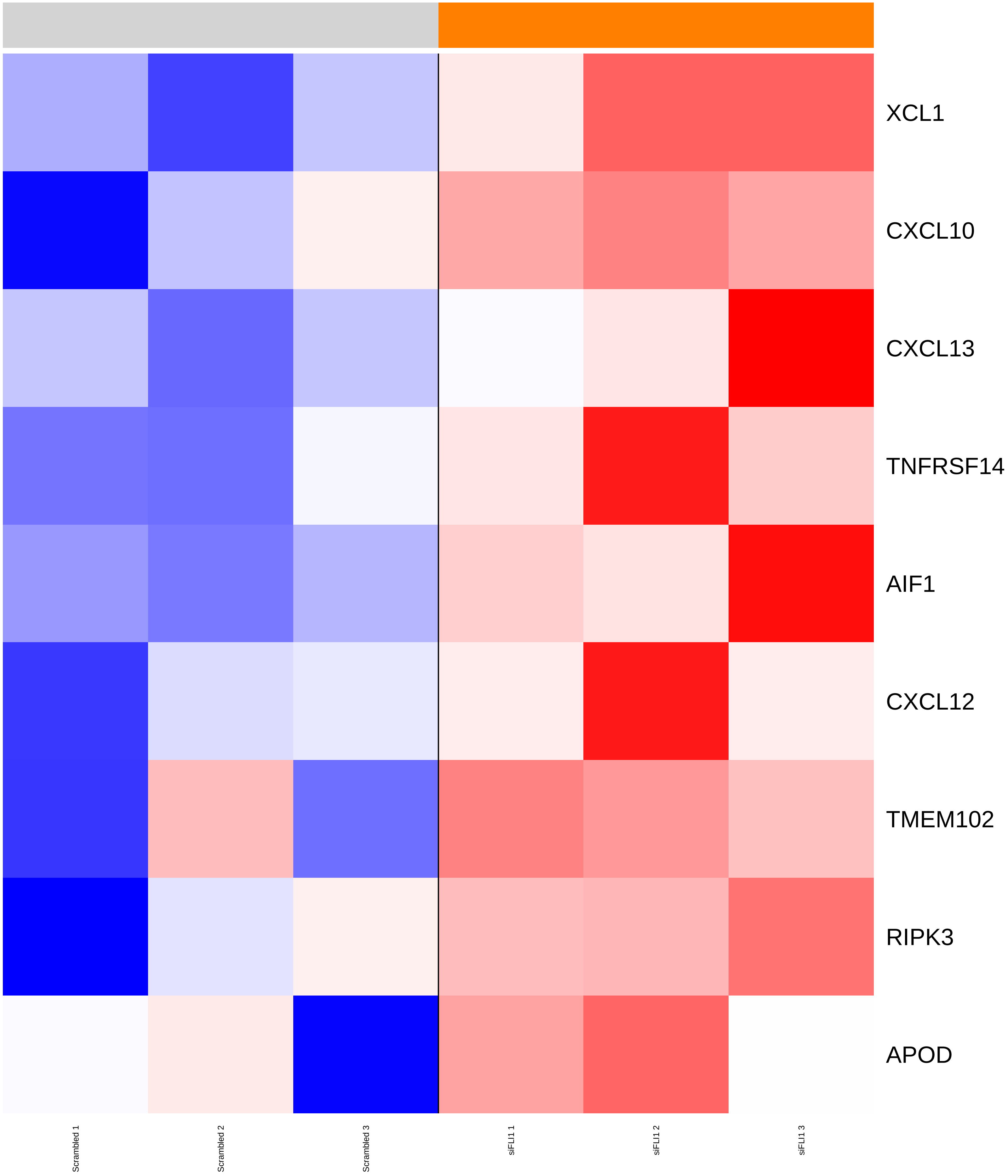

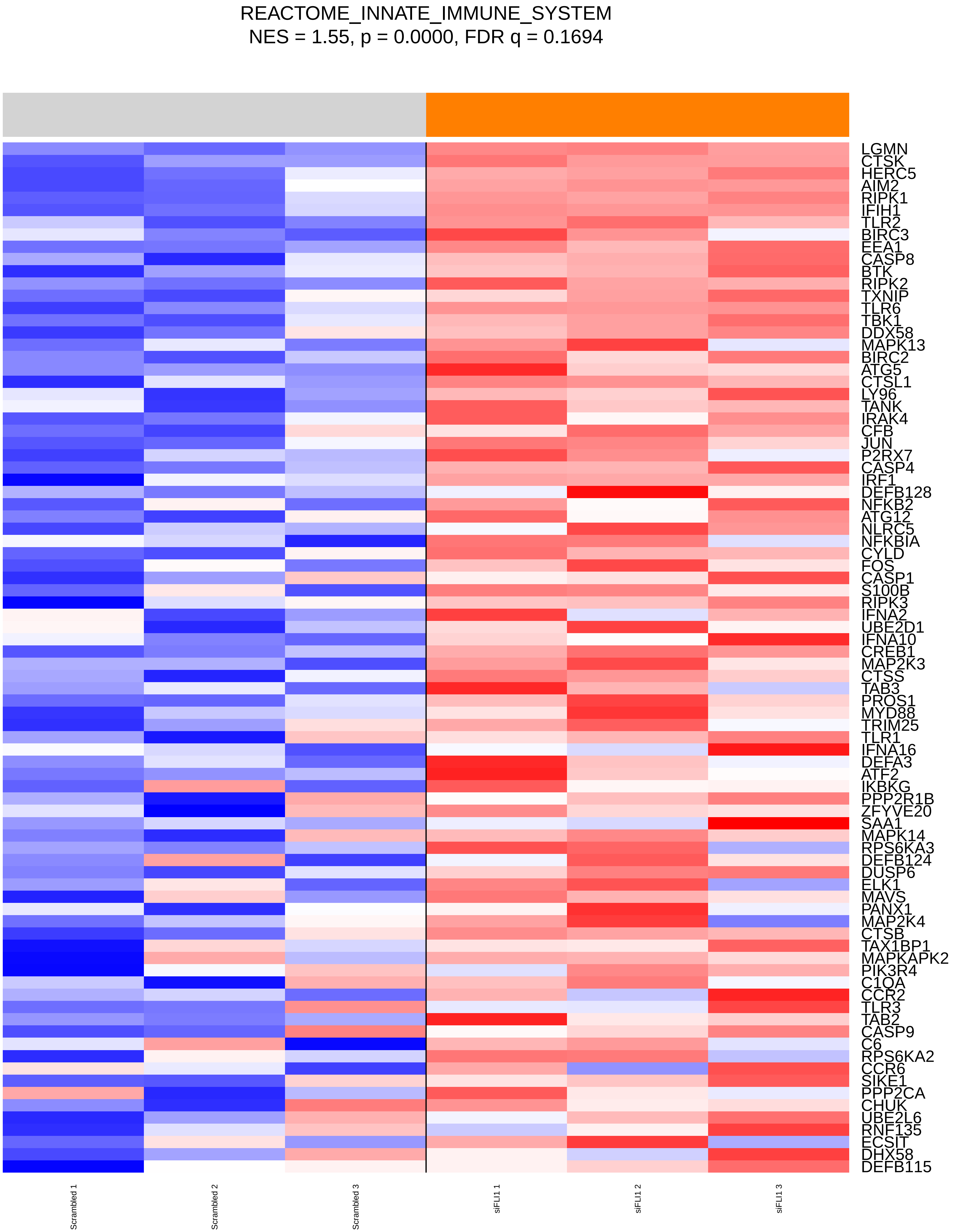
REACTOME_INNATE_IMMUNE_SYSTEM
NES = 1.55, p = 0.0000, FDR q = 0.1694
LGMN
CTSK
HERC5
AIM2
RIPK1
IFIH1
TLR2
BIRC3
EEA1
CASP8
BTK
RIPK2
TXNIP
TLR6
TBK1
DDX58
MAPK13
BIRC2
ATG5
CTSL1
LY96
TANK
IRAK4
CFB
JUN
P2RX7
CASP4
IRF1
DEFB128
NFKB2
ATG12
NLRC5
NFKBIA
CYLD
FOS
CASP1
S100B
RIPK3
IFNA2
UBE2D1
IFNA10
CREB1
MAP2K3
CTSS
TAB3
PROS1
MYD88
TRIM25
TLR1
IFNA16
DEFA3
ATF2
IKBKG
PPP2R1B
ZFYVE20
SAA1
MAPK14
RPS6KA3
DEFB124
DUSP6
ELK1
MAVS
PANX1
MAP2K4
CTSB
TAX1BP1
MAPKAPK2
PIK3R4
C1QA
CCR2
TLR3
TAB2
CASP9
C6
RPS6KA2
CCR6
SIKE1
PPP2CA
CHUK
UBE2L6
RNF135
ECSIT
DHX58
DEFB115
Scrambled 1
Scrambled 2
Scrambled 3
siFLI1 1
siFLI1 2
siFLI1 3

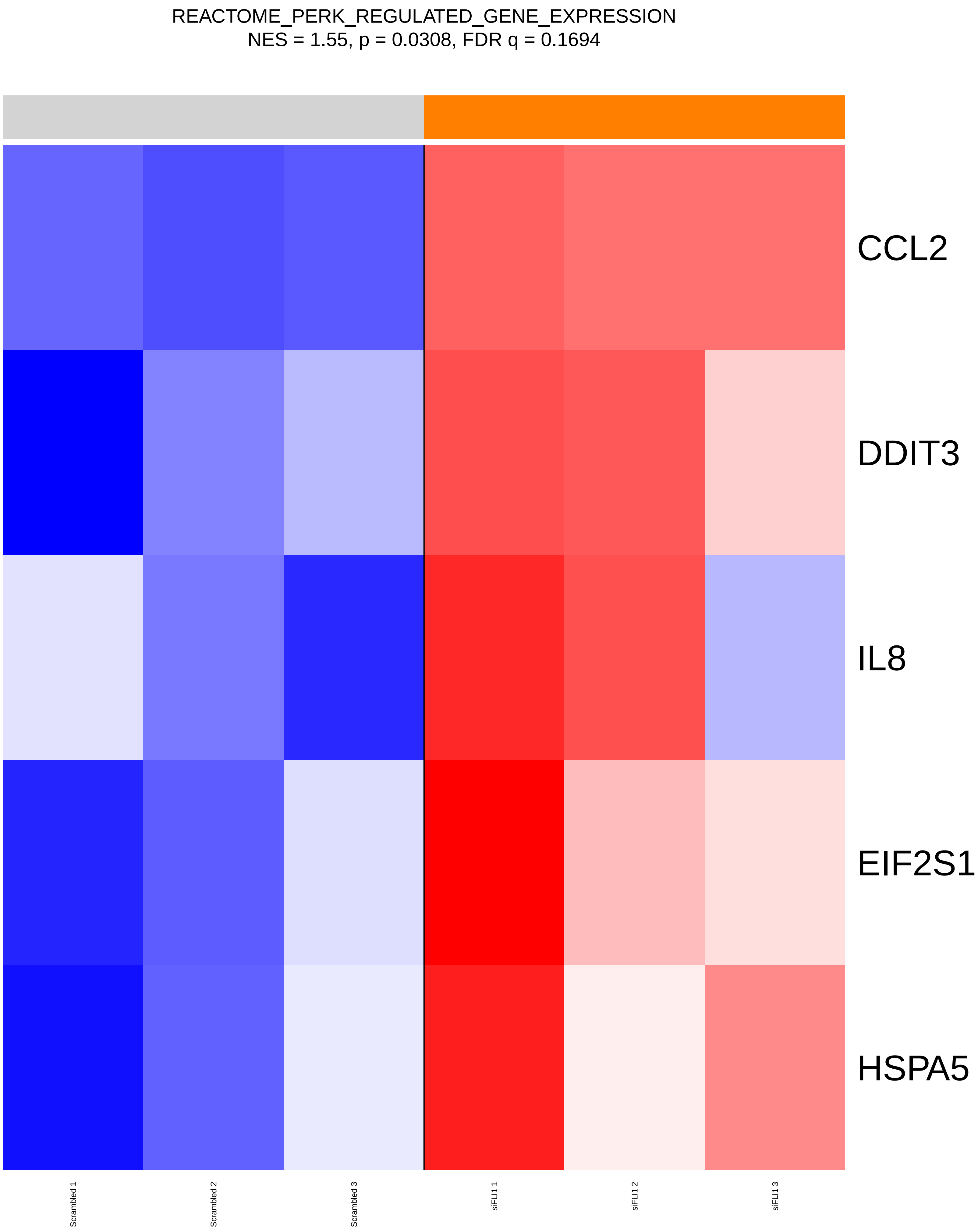
REACTOME_PERK_REGULATED_GENE_EXPRESSION
NES = 1.55, p = 0.0308, FDR q = 0.1694
CCL2
DDIT3
IL8
EIF2S1
HSPA5
Scrambled 1
Scrambled 2
Scrambled 3
siFLI1 1
siFLI1 2
siFLI1 3

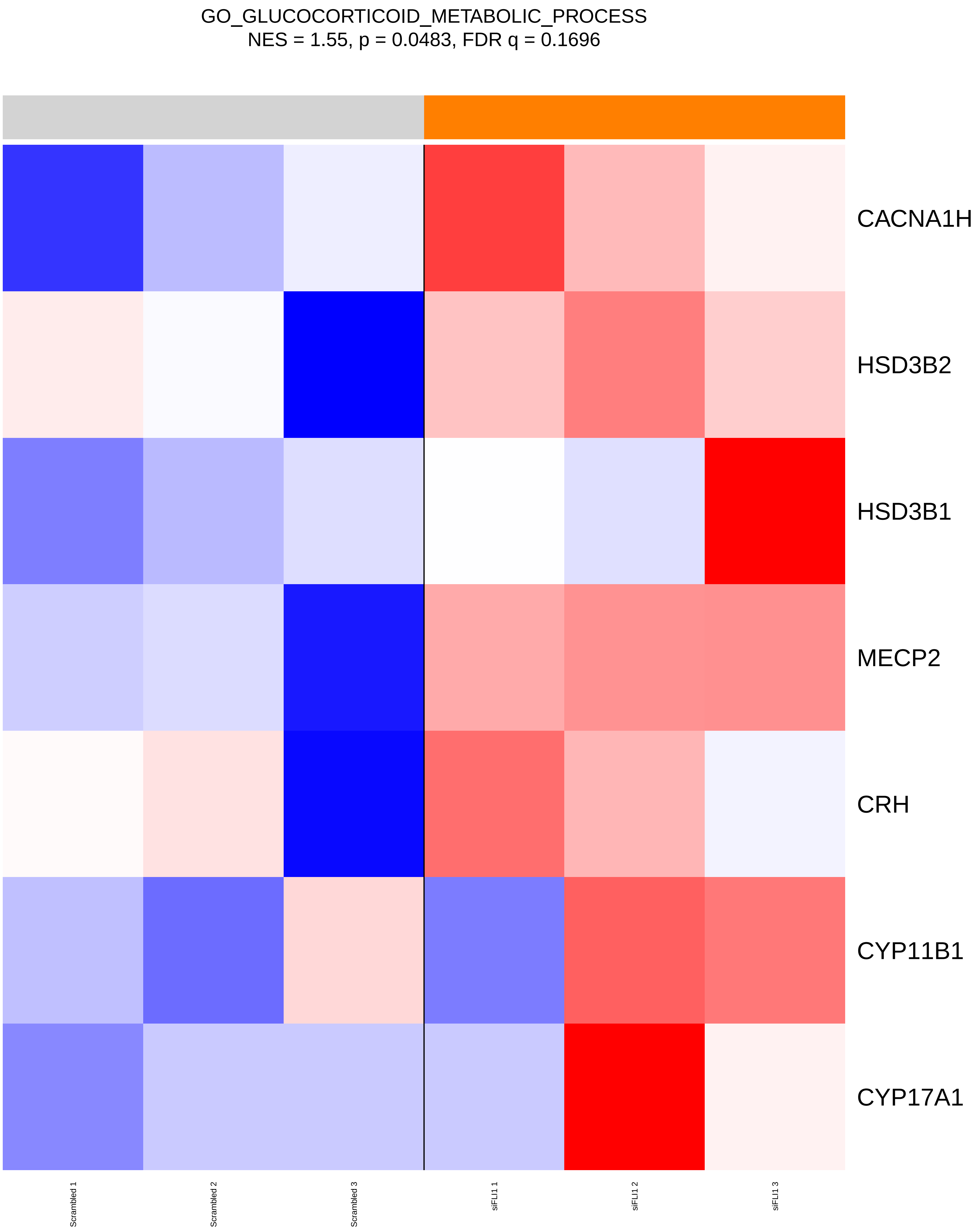
GO_GLUCOCORTICOID_METABOLIC_PROCESS
NES = 1.55, p = 0.0483, FDR q = 0.1696
CACNA1H
HSD3B2
HSD3B1
MECP2
CRH
CYP11B1
CYP17A1
Scrambled 1
Scrambled 2
Scrambled 3
siFLI1 1
siFLI1 2
siFLI1 3

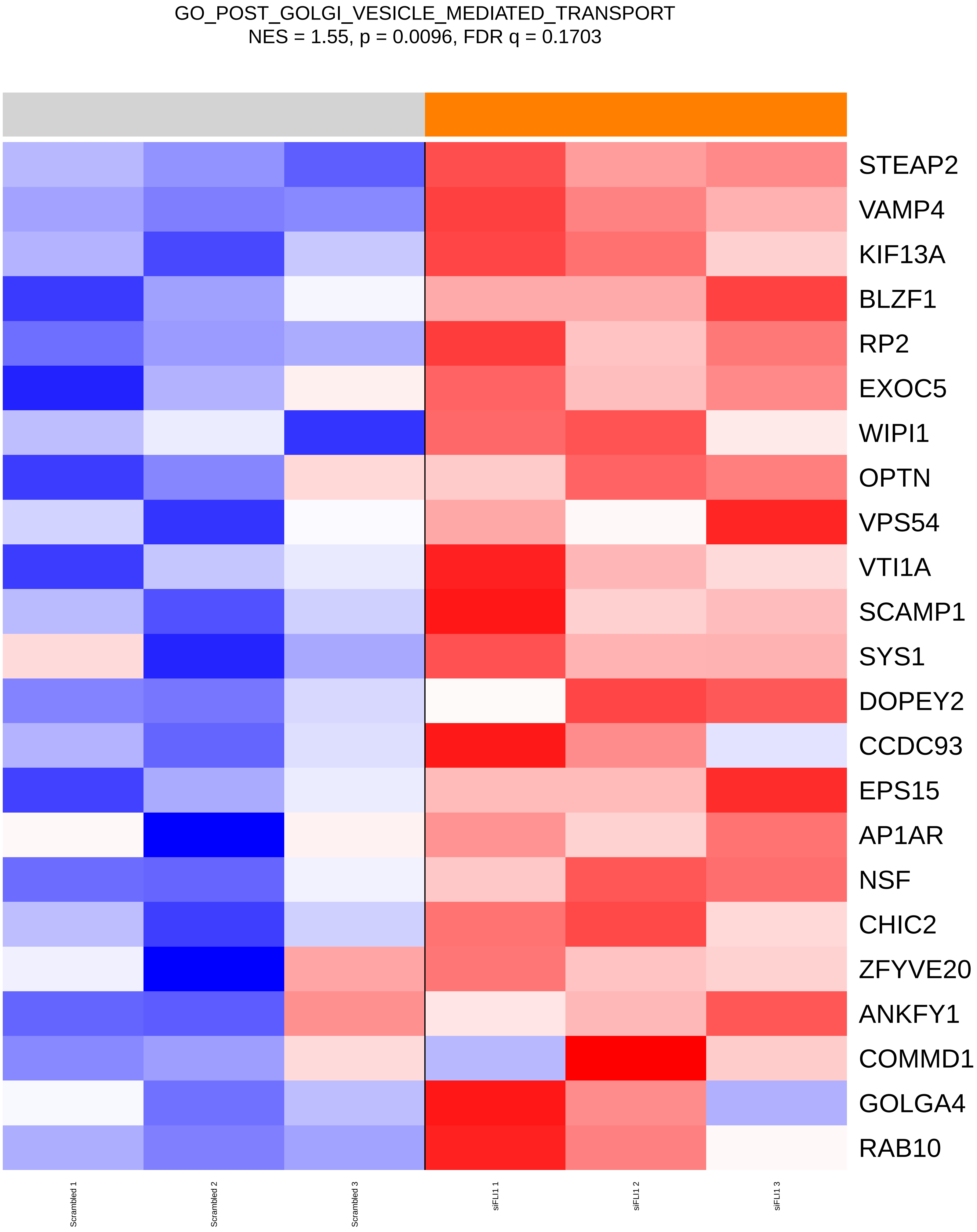
GO_POST_GOLGI_VESICLE_MEDIATED_TRANSPORT
NES = 1.55, p = 0.0096, FDR q = 0.1703
STEAP2
VAMP4
KIF13A
BLZF1
RP2
EXOC5
WIPI1
OPTN
VPS54
VTI1A
SCAMP1
SYS1
DOPEY2
CCDC93
EPS15
AP1AR
NSF
CHIC2
ZFYVE20
ANKFY1
COMMD1
GOLGA4
RAB10
Scrambled 1
Scrambled 2
Scrambled 3
siFLI1 1
siFLI1 2
siFLI1 3

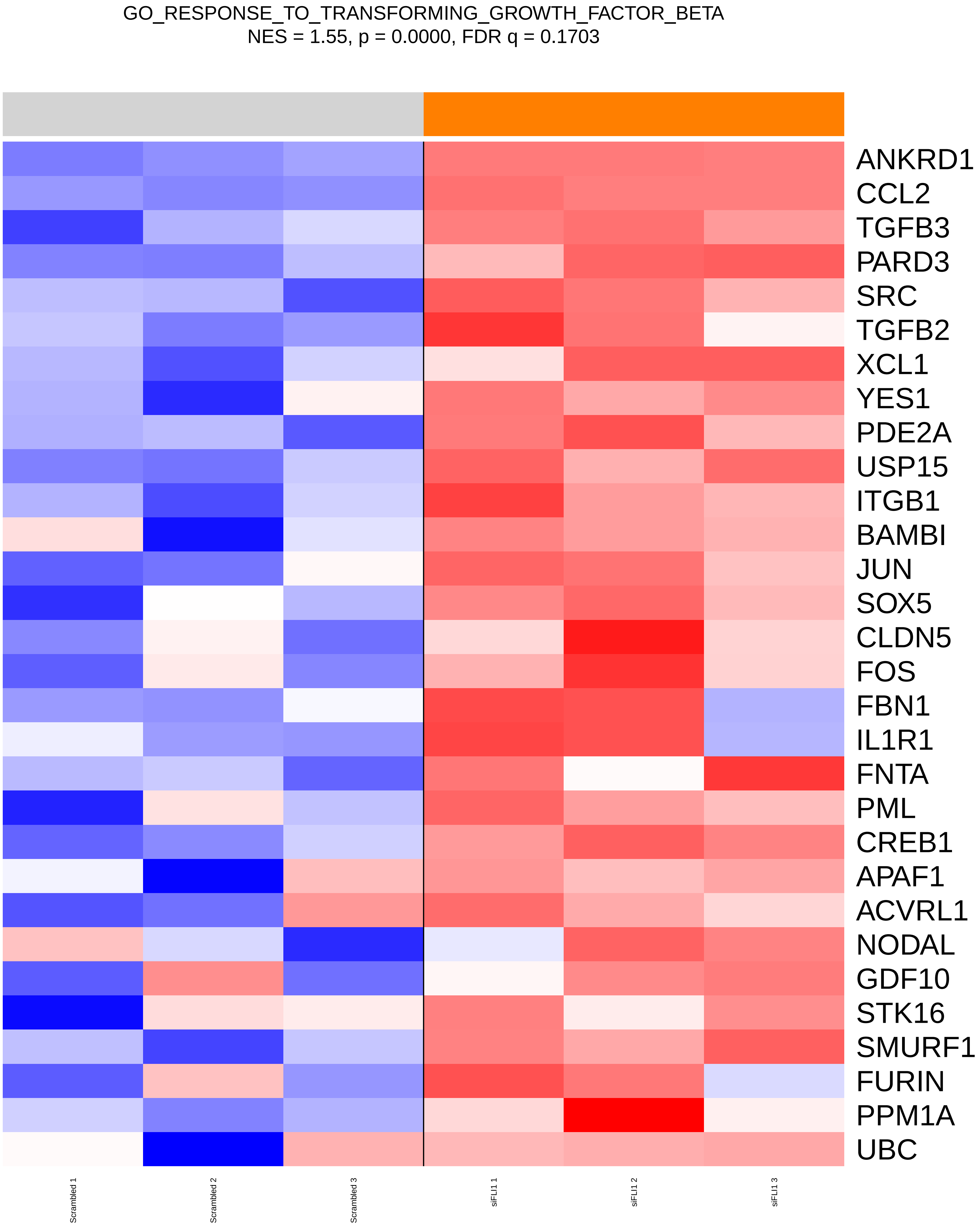
GO_RESPONSE_TO_TRANSFORMING_GROWTH_FACTOR_BETA
NES = 1.55, p = 0.0000, FDR q = 0.1703
ANKRD1
CCL2
TGFB3
PARD3
SRC
TGFB2
XCL1
YES1
PDE2A
USP15
ITGB1
BAMBI
JUN
SOX5
CLDN5
FOS
FBN1
IL1R1
FNTA
PML
CREB1
APAF1
ACVRL1
NODAL
GDF10
STK16
SMURF1
FURIN
PPM1A
UBC
Scrambled 1
Scrambled 2
Scrambled 3
siFLI1 1
siFLI1 2
siFLI1 3

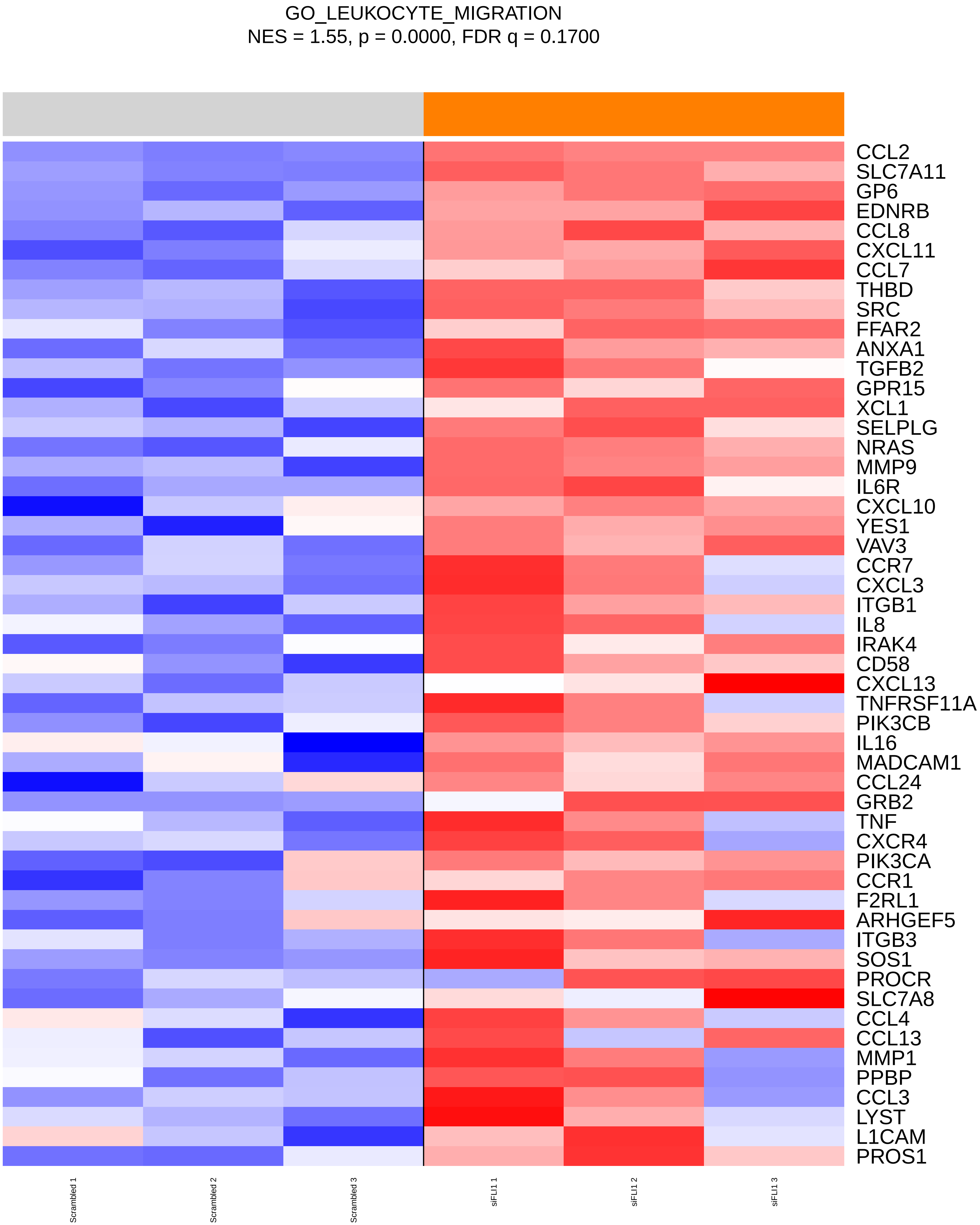
GO_LEUKOCYTE_MIGRATION
NES = 1.55, p = 0.0000, FDR q = 0.1700
CCL2
SLC7A11
GP6
EDNRB
CCL8
CXCL11
CCL7
THBD
SRC
FFAR2
ANXA1
TGFB2
GPR15
XCL1
SELPLG
NRAS
MMP9
IL6R
CXCL10
YES1
VAV3
CCR7
CXCL3
ITGB1
IL8
IRAK4
CD58
CXCL13
TNFRSF11A
PIK3CB
IL16
MADCAM1
CCL24
GRB2
TNF
CXCR4
PIK3CA
CCR1
F2RL1
ARHGEF5
ITGB3
SOS1
PROCR
SLC7A8
CCL4
CCL13
MMP1
PPBP
CCL3
LYST
L1CAM
PROS1
Scrambled 1
Scrambled 2
Scrambled 3
siFLI1 1
siFLI1 2
siFLI1 3

TGCACTG_MIR148A_MIR152_MIR148B
NES = 1.55, p = 0.0000, FDR q = 0.1718

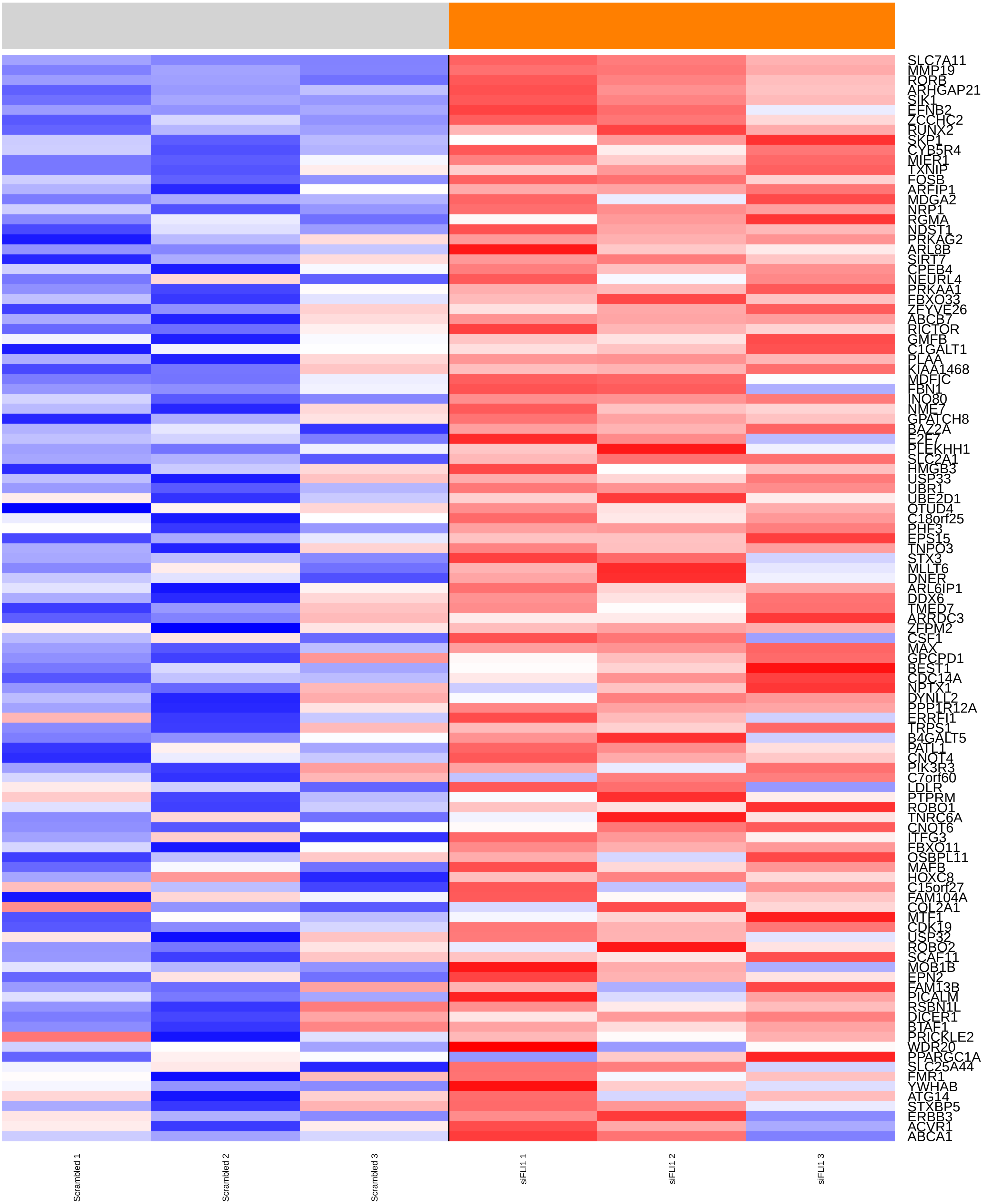

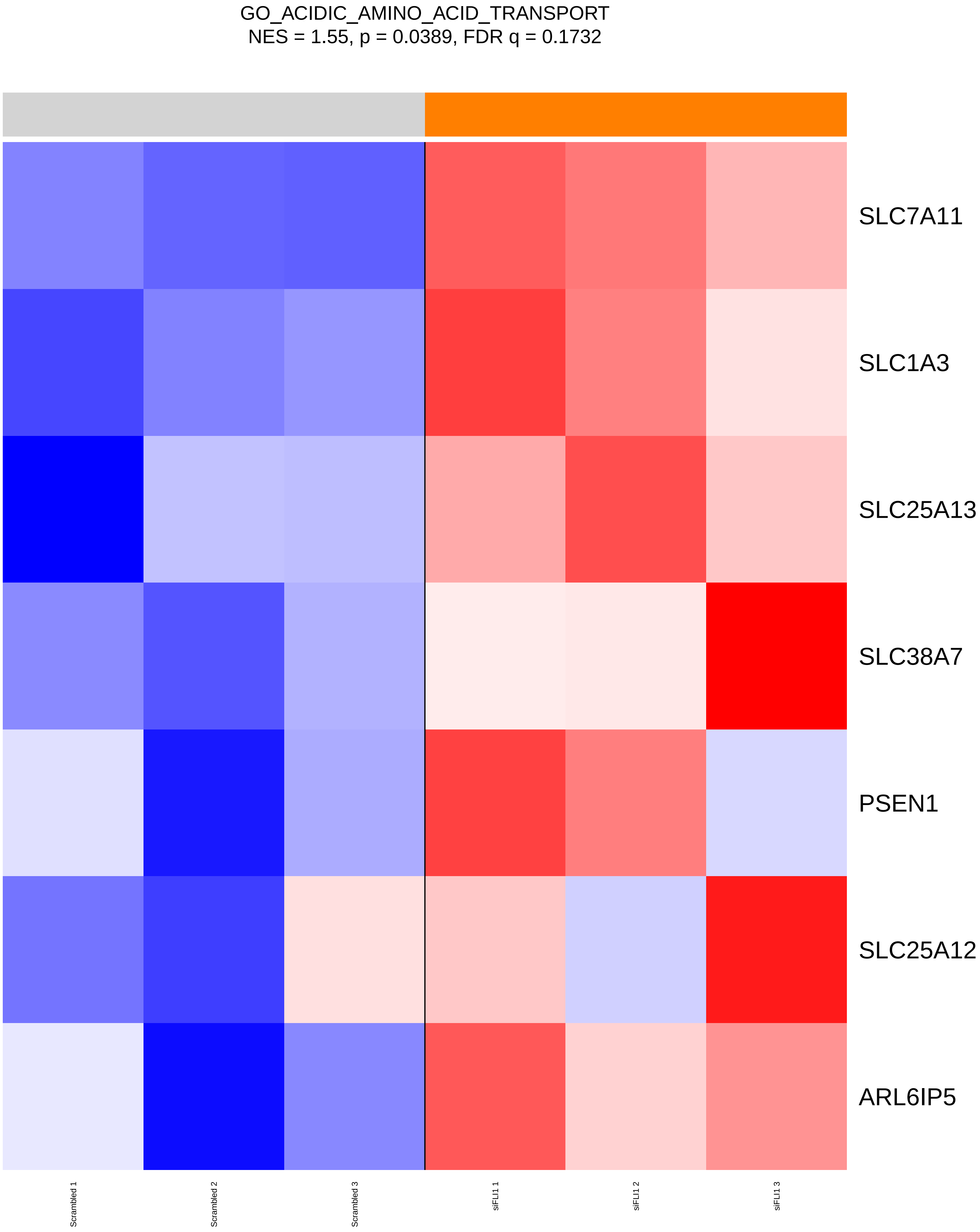
GO_ACIDIC_AMINO_ACID_TRANSPORT
NES = 1.55, p = 0.0389, FDR q = 0.1732
SLC7A11
SLC1A3
SLC25A13
SLC38A7
PSEN1
SLC25A12
ARL6IP5
Scrambled 1
Scrambled 2
Scrambled 3
siFLI1 1
siFLI1 2
siFLI1 3

GO_NEGATIVE_REGULATION_OF_IMMUNE_SYSTEM_PROCESS
NES = 1.55, p = 0.0000, FDR q = 0.1735

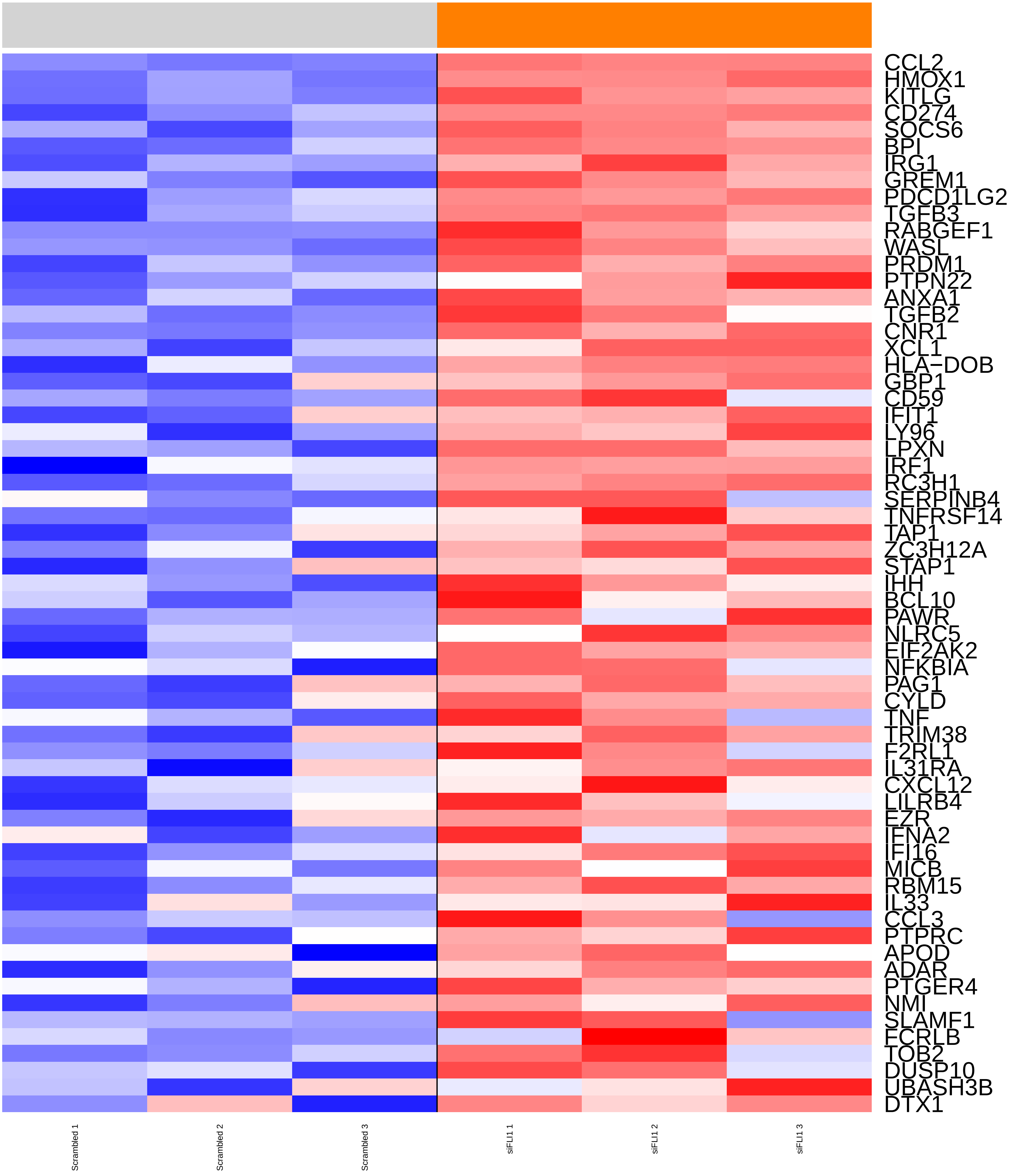

GO_ERBB2_SIGNALING_PATHWAY
NES = 1.54, p = 0.0305, FDR q = 0.1746

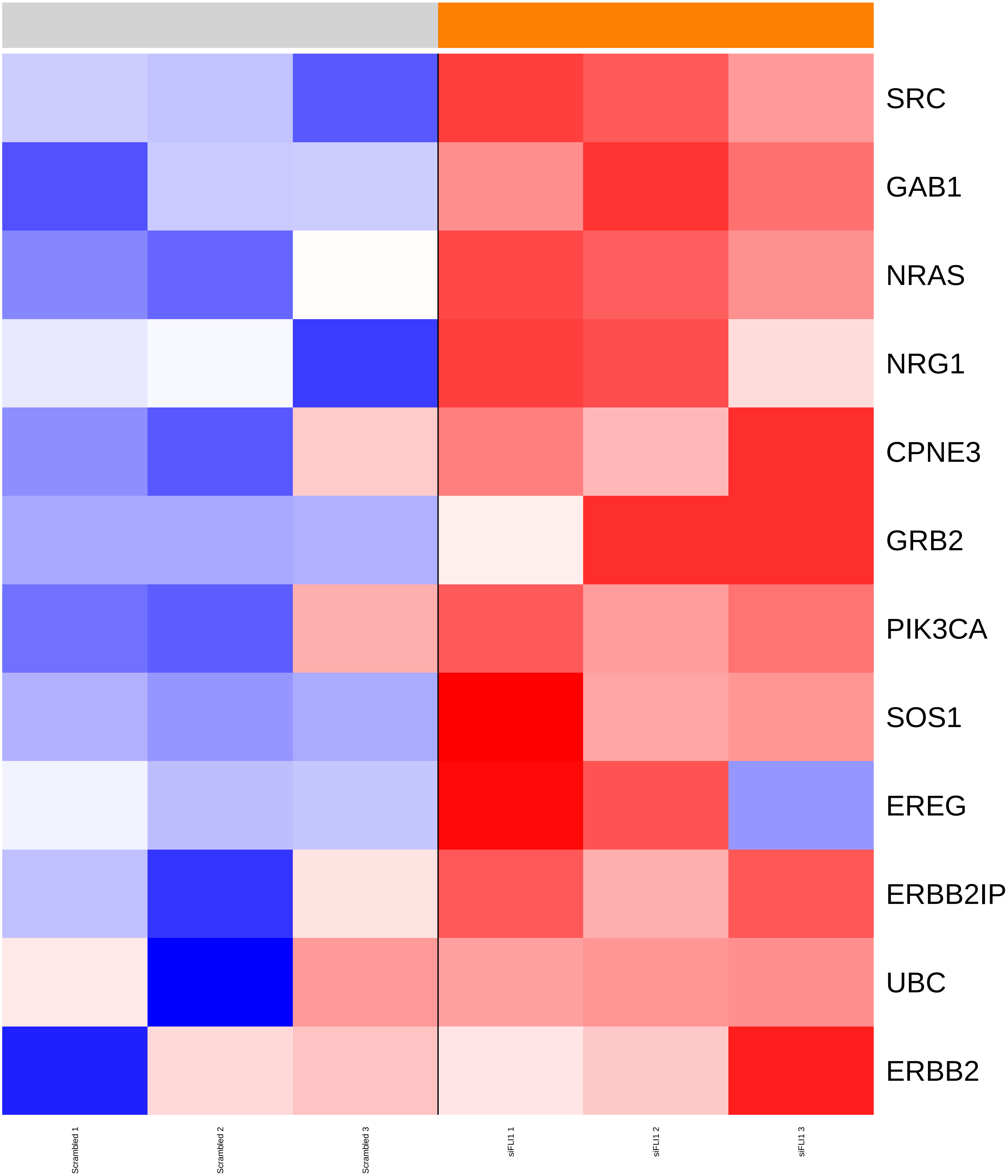

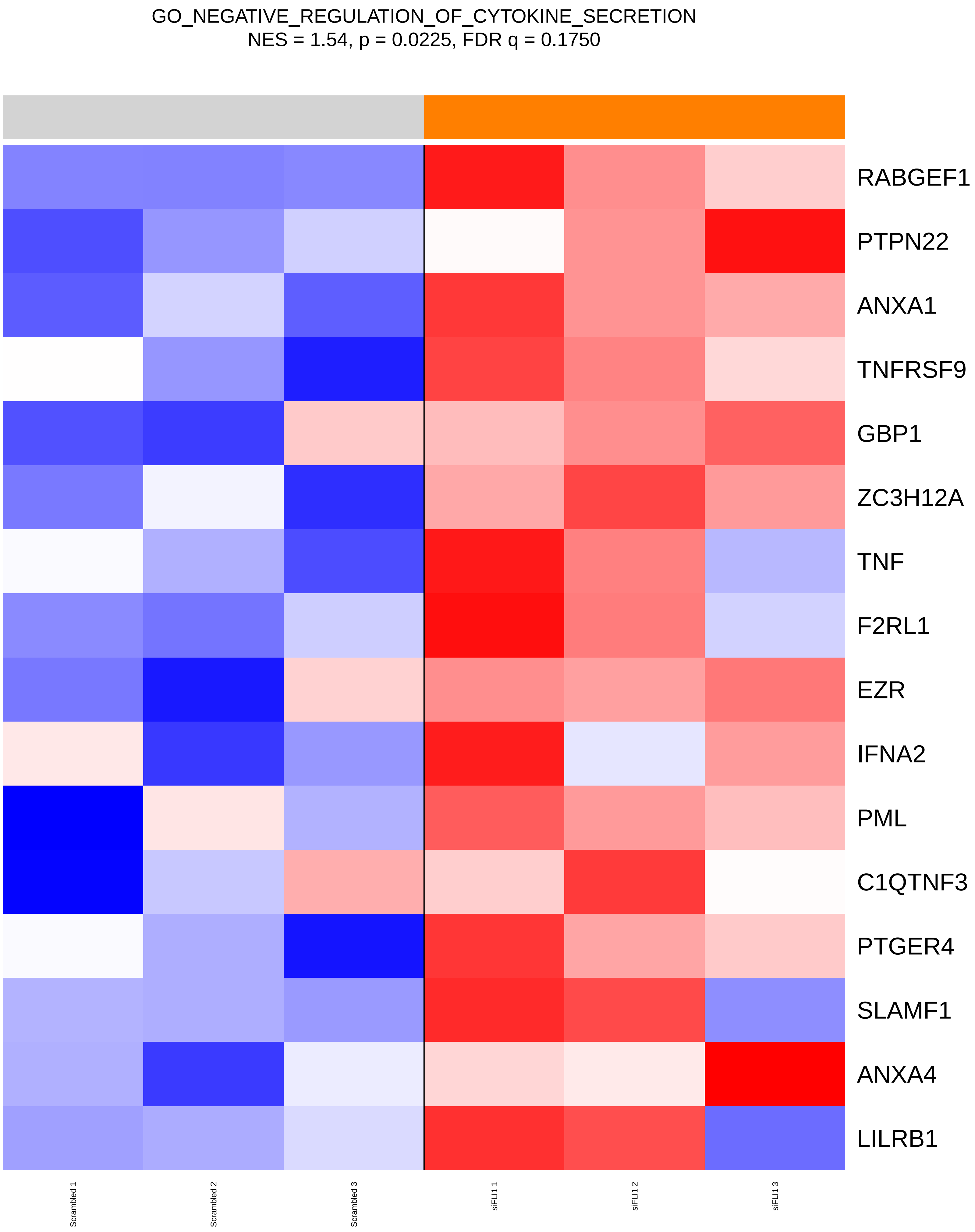
GO_NEGATIVE_REGULATION_OF_CYTOKINE_SECRETION
NES = 1.54, p = 0.0225, FDR q = 0.1750
RABGEF1
PTPN22
ANXA1
TNFRSF9
GBP1
ZC3H12A
TNF
F2RL1
EZR
IFNA2
PML
C1QTNF3
PTGER4
SLAMF1
ANXA4
LILRB1
Scrambled 1
Scrambled 2
Scrambled 3
siFLI1 1
siFLI1 2
siFLI1 3

GO_FC_EPSILON_RECEPTOR_SIGNALING_PATHWAY
NES = 1.54, p = 0.0035, FDR q = 0.1753

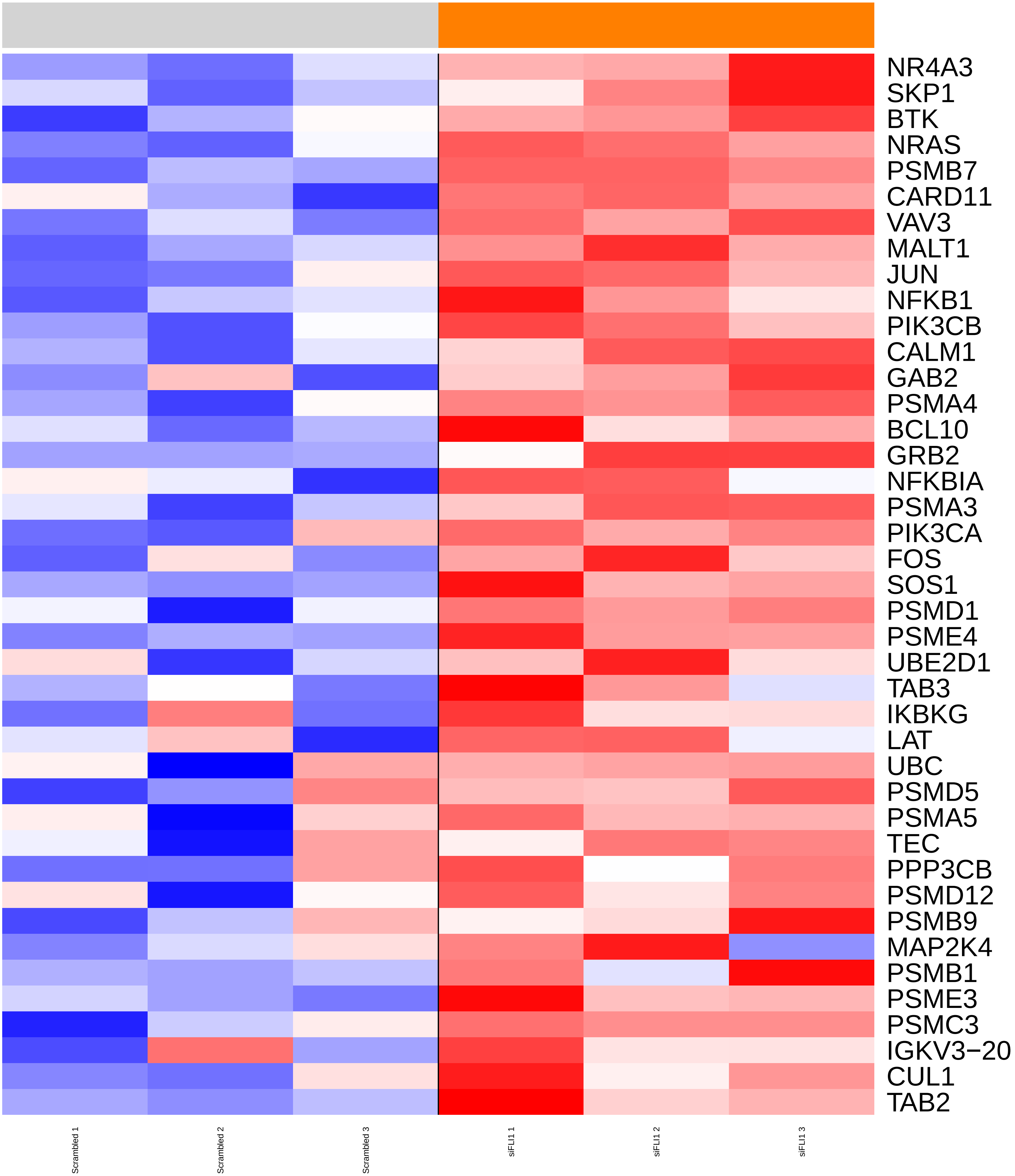

GO_POSITIVE_REGULATION_OF_INTERLEUKIN_1_SECRETION
NES = 1.54, p = 0.0537, FDR q = 0.1751

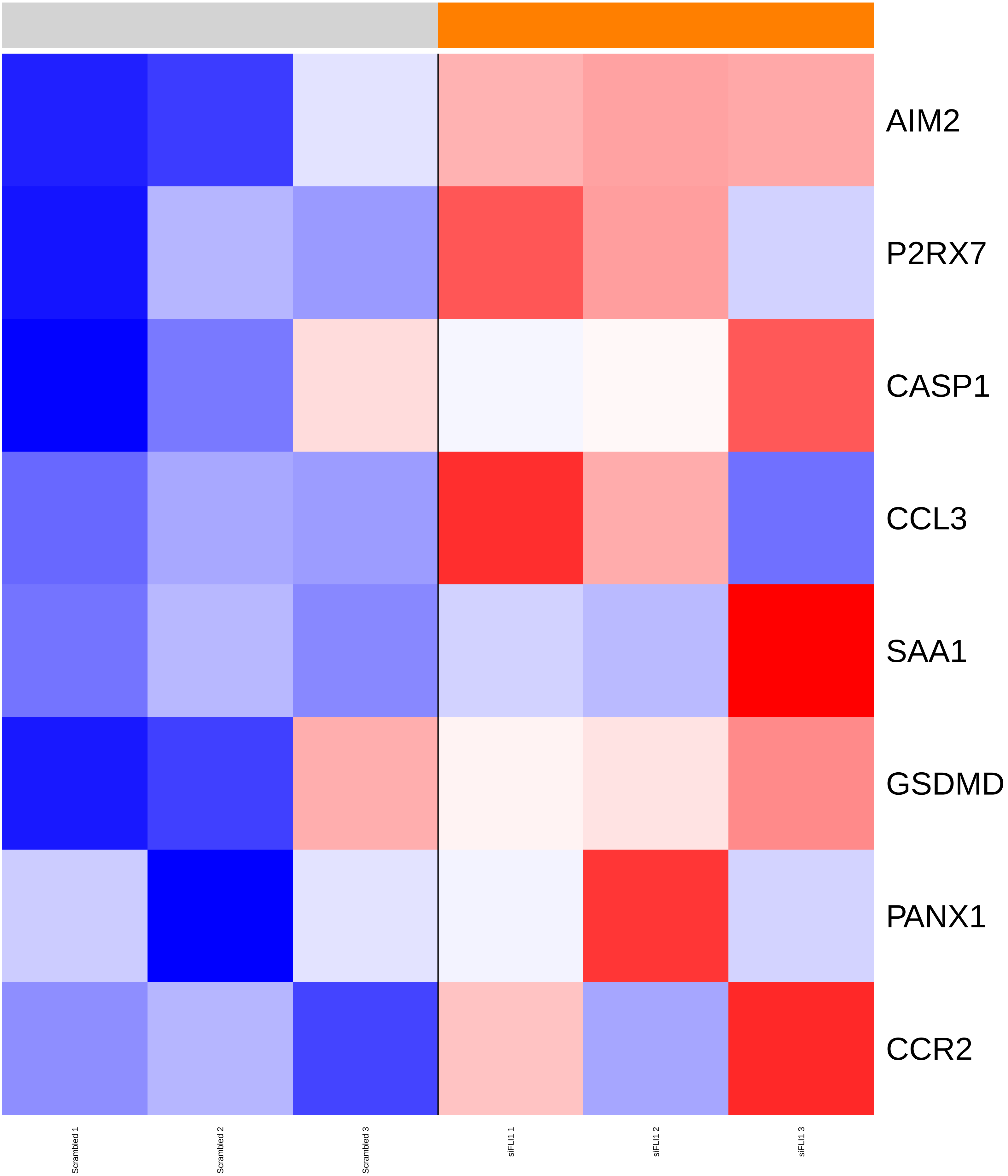

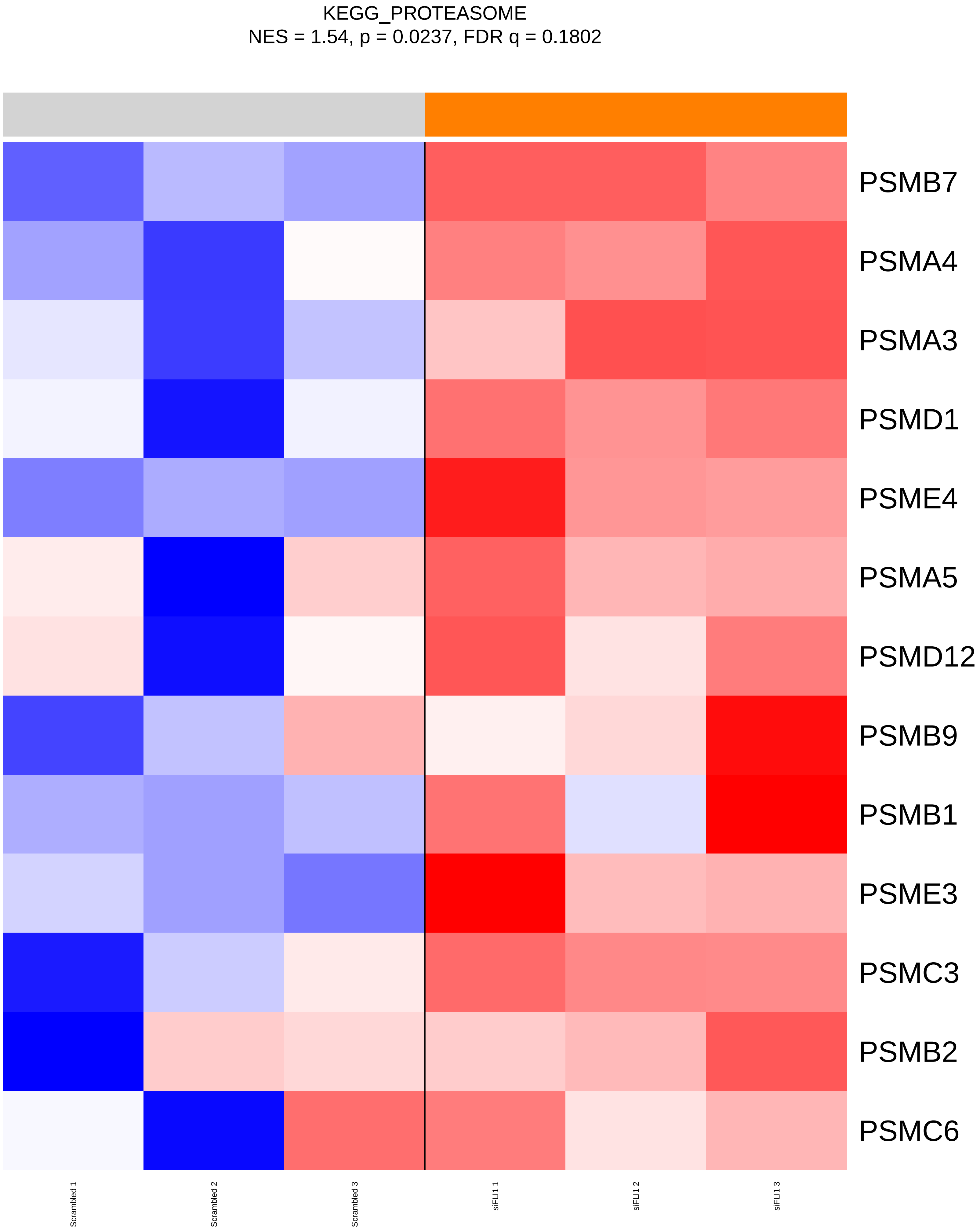
KEGG_PROTEASOME
NES = 1.54, p = 0.0237, FDR q = 0.1802
PSMB7
PSMA4
PSMA3
PSMD1
PSME4
PSMA5
PSMD12
PSMB9
PSMB1
PSME3
PSMC3
PSMB2
PSMC6
Scrambled 1
Scrambled 2
Scrambled 3
siFLI1 1
siFLI1 2
siFLI1 3

GO_UBIQUITIN_LIKE_PROTEIN_SPECIFIC_PROTEASE_ACTIVITY
NES = 1.54, p = 0.0089, FDR q = 0.1803

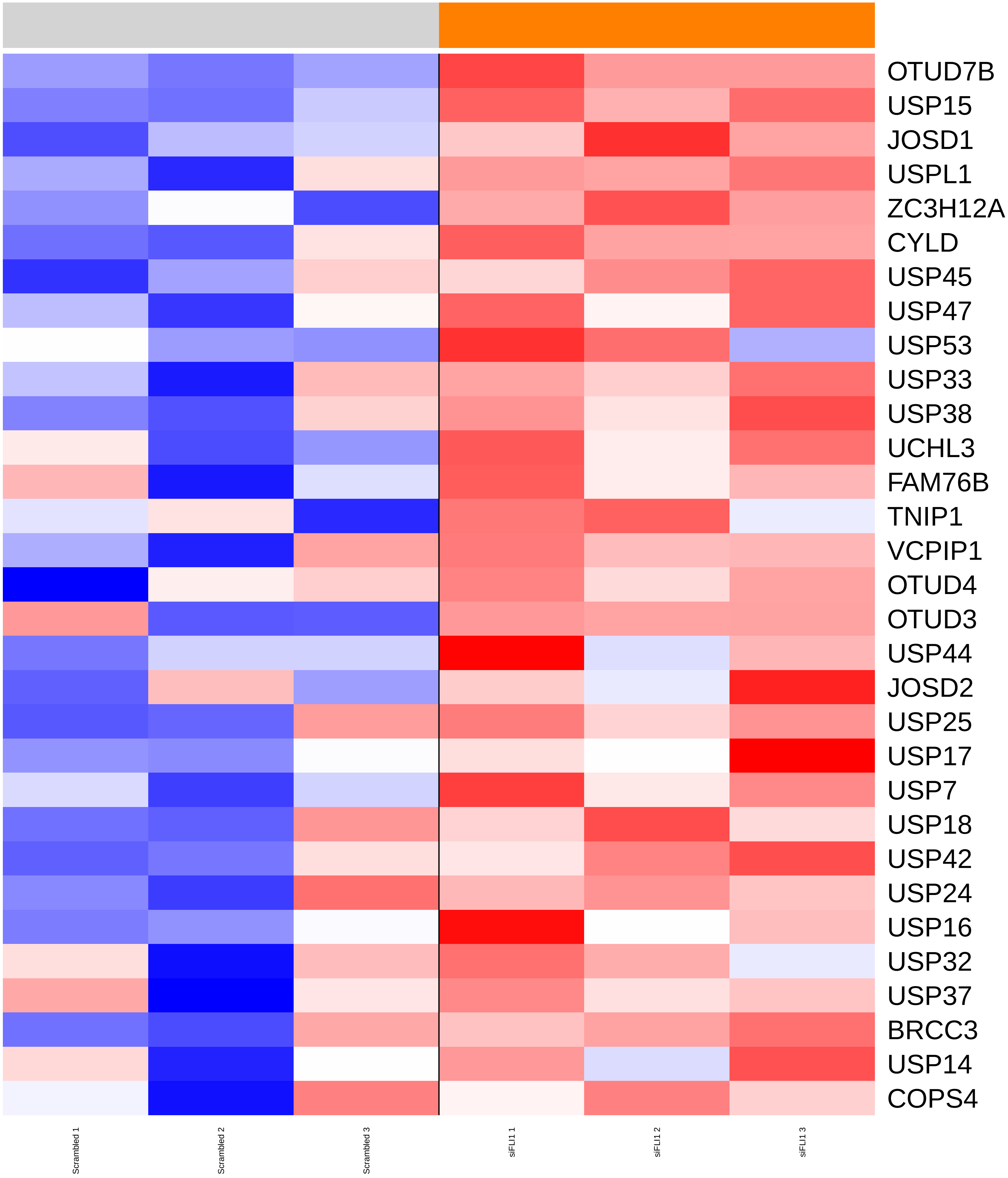

GO_PROTEIN_KINASE_B_SIGNALING
NES = 1.54, p = 0.0239, FDR q = 0.1804

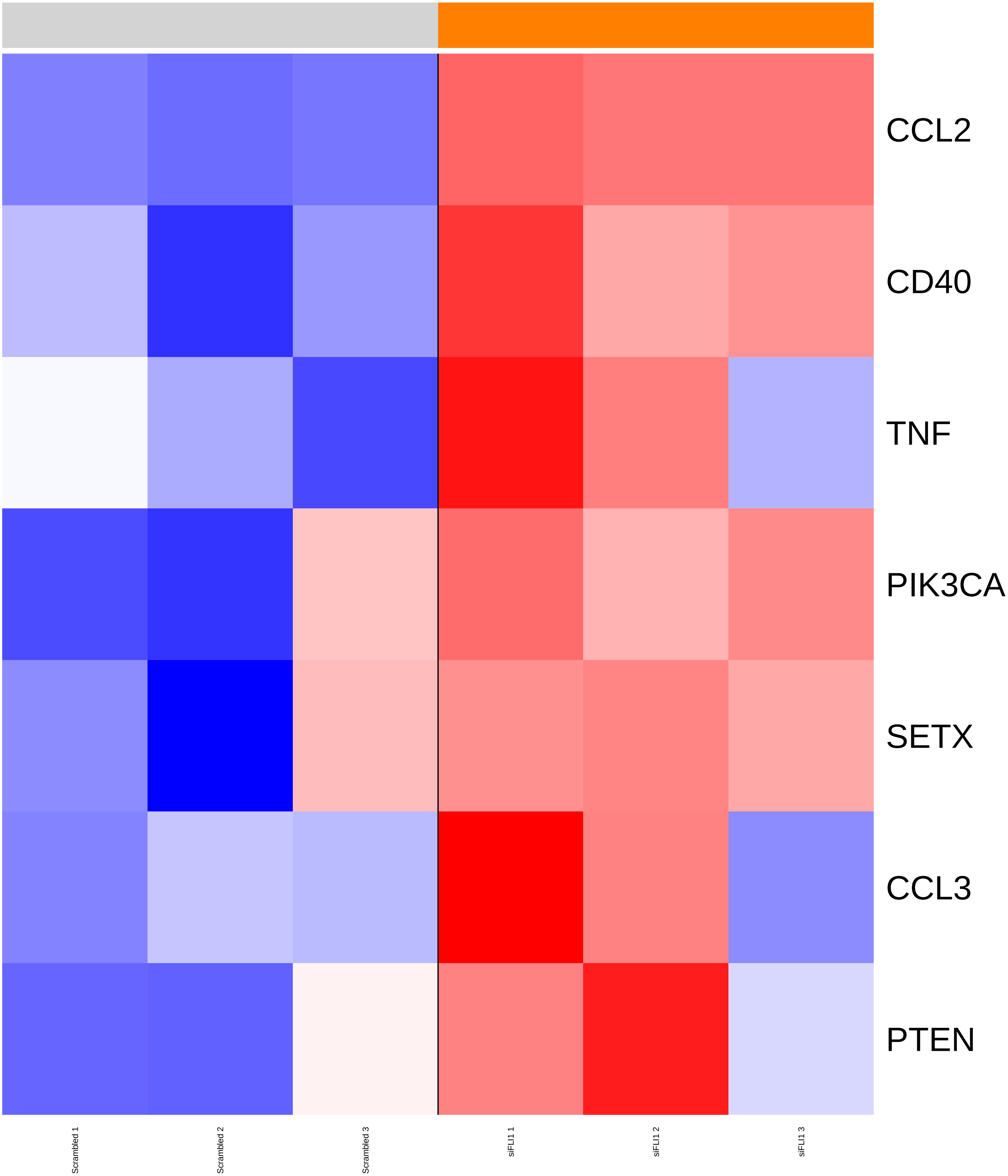

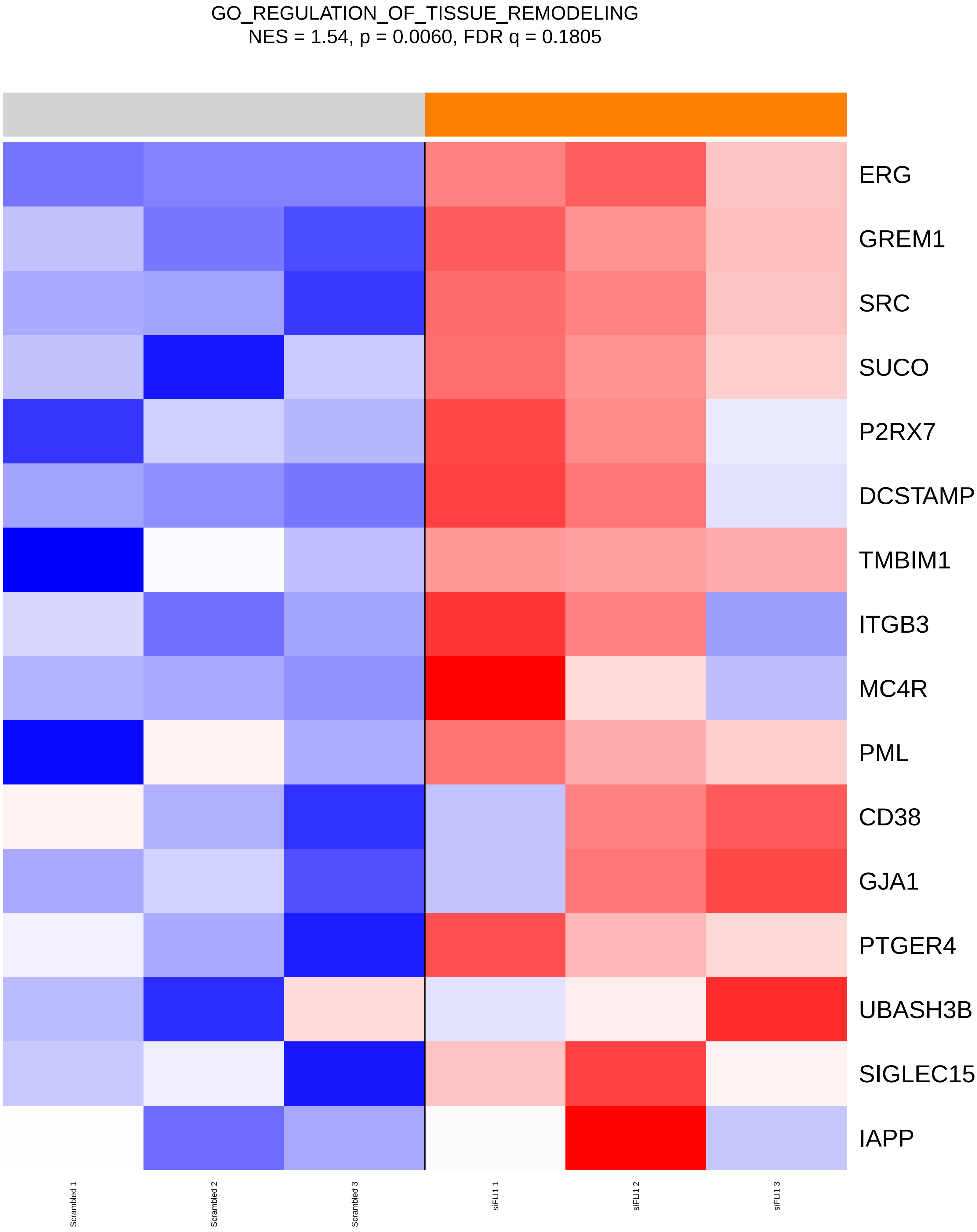

GO_REGULATION_OF_TISSUE_REMODELING
NES = 1.54, p = 0.0060, FDR q = 0.1805
ERG
GREM1
SRC
SUCO
P2RX7
DCSTAMP
TMBIM1
ITGB3
MC4R
PML
CD38
GJA1
PTGER4
UBASH3B
SIGLEC15
IAPP
Scrambled 1
Scrambled 2
Scrambled 3
siFLI1 1
siFLI1 2
siFLI1 3

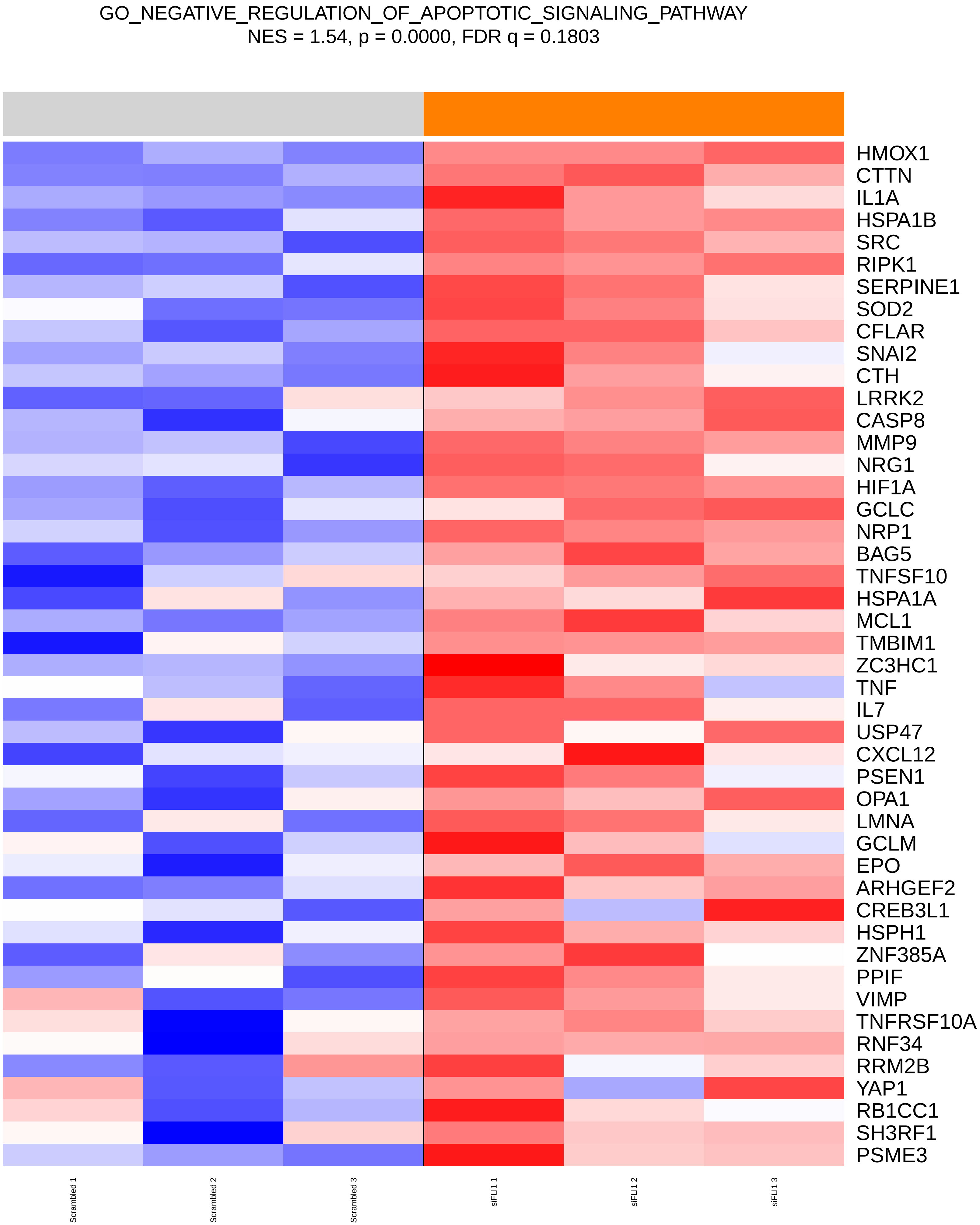
GO_NEGATIVE_REGULATION_OF_APOPTOTIC_SIGNALING_PATHWAY
NES = 1.54, p = 0.0000, FDR q = 0.1803
HMOX1
CTTN
IL1A
HSPA1B
SRC
RIPK1
SERPINE1
SOD2
CFLAR
SNAI2
CTH
LRRK2
CASP8
MMP9
NRG1
HIF1A
GCLC
NRP1
BAG5
TNFSF10
HSPA1A
MCL1
TMBIM1
ZC3HC1
TNF
IL7
USP47
CXCL12
PSEN1
OPA1
LMNA
GCLM
EPO
ARHGEF2
CREB3L1
HSPH1
ZNF385A
PPIF
VIMP
TNFRSF10A
RNF34
RRM2B
YAP1
RB1CC1
SH3RF1
PSME3
Scrambled 1
Scrambled 2
Scrambled 3
siFLI1 1
siFLI1 2
siFLI1 3

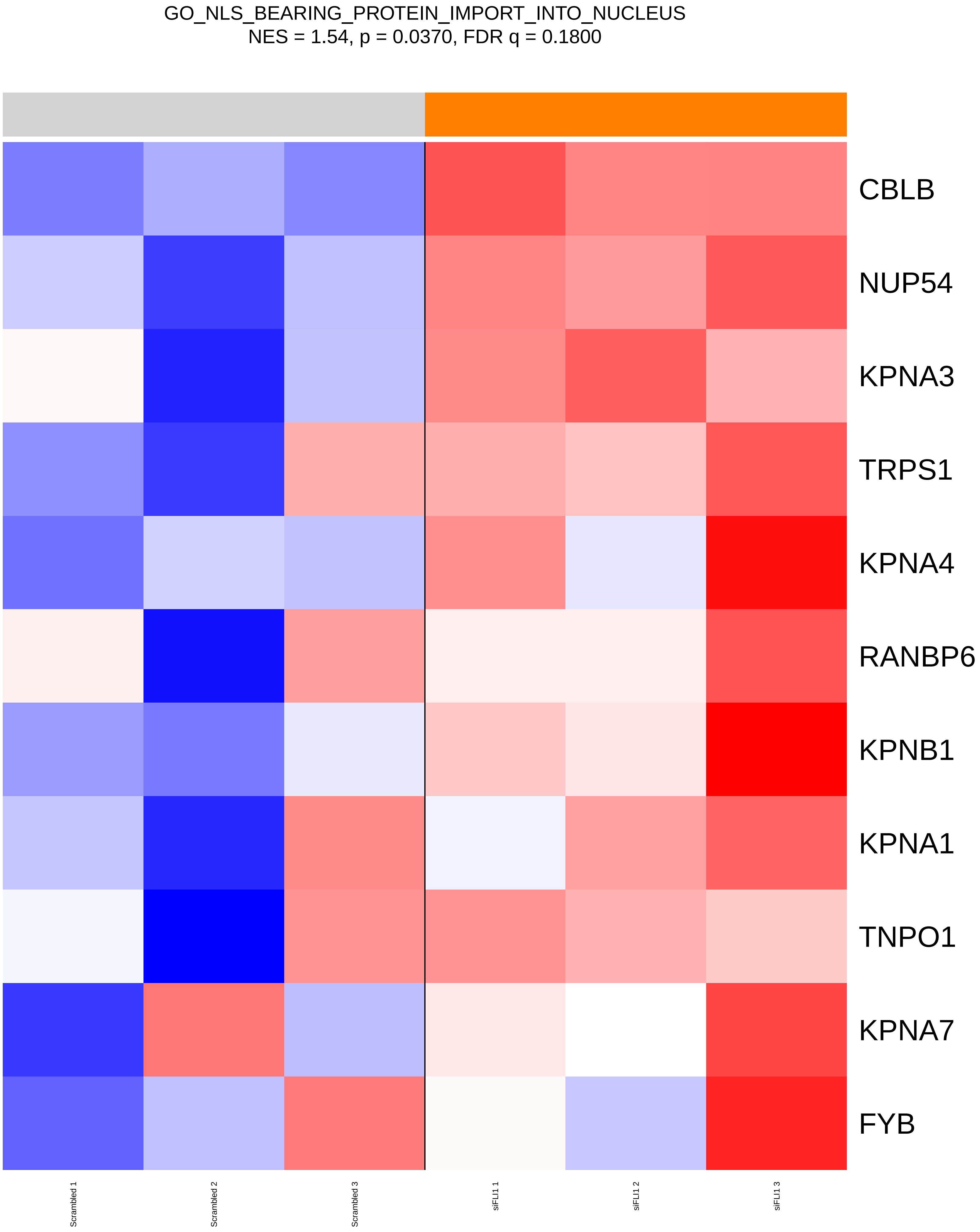
GO_NLS_BEARING_PROTEIN_IMPORT_INTO_NUCLEUS
NES = 1.54, p = 0.0370, FDR q = 0.1800
CBLB
NUP54
KPNA3
TRPS1
KPNA4
RANBP6
KPNB1
KPNA1
TNPO1
KPNA7
FYB
Scrambled 1
Scrambled 2
Scrambled 3
siFLI1 1
siFLI1 2
siFLI1 3

GO_REGULATION_OF_NUCLEASE_ACTIVITY
NES = 1.53, p = 0.0366, FDR q = 0.1795

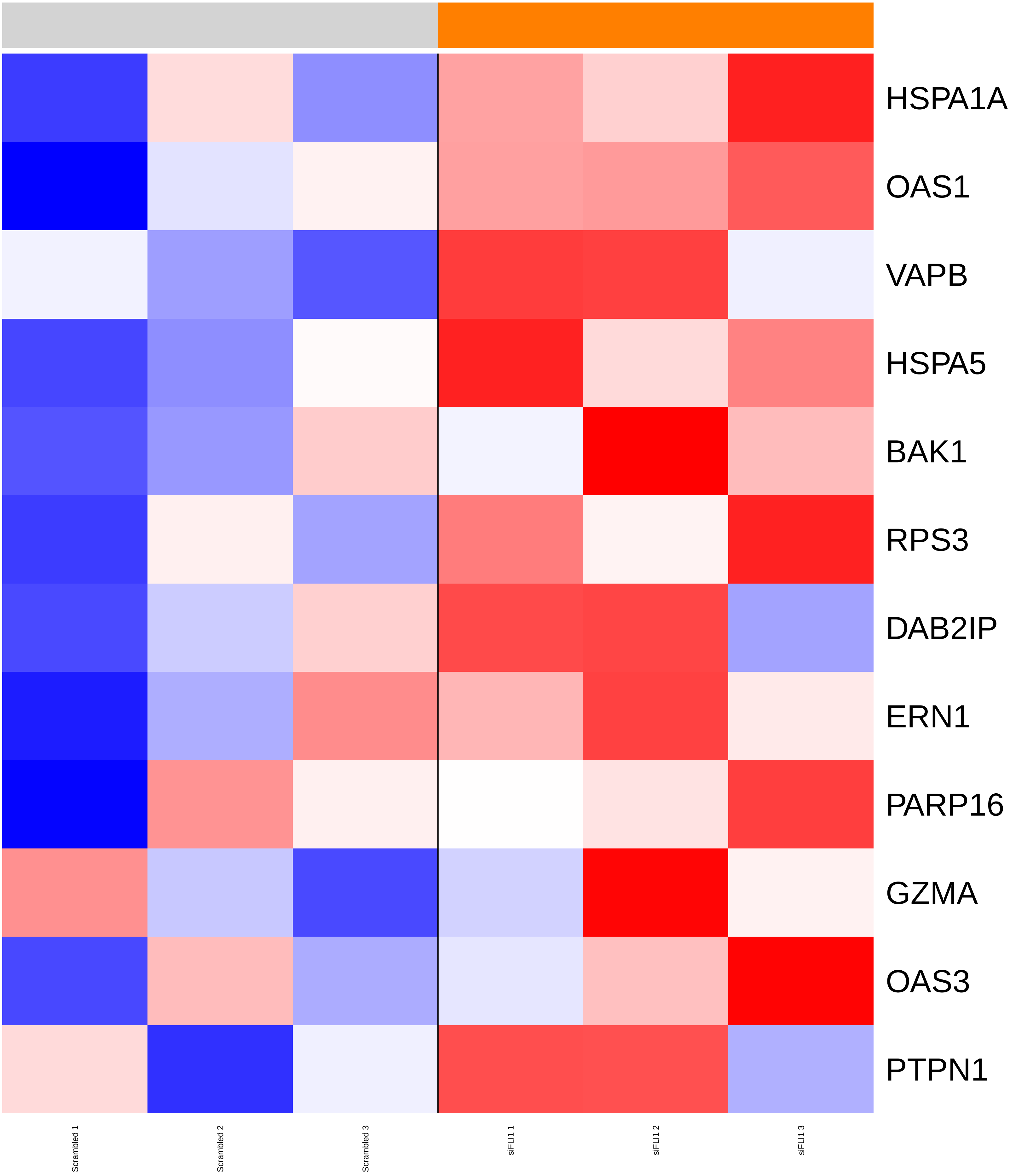

GO_BRANCHING_INVOLVED_IN_SALIVARY_GLAND_MORPHOGENESIS
NES = 1.53, p = 0.0433, FDR q = 0.1800

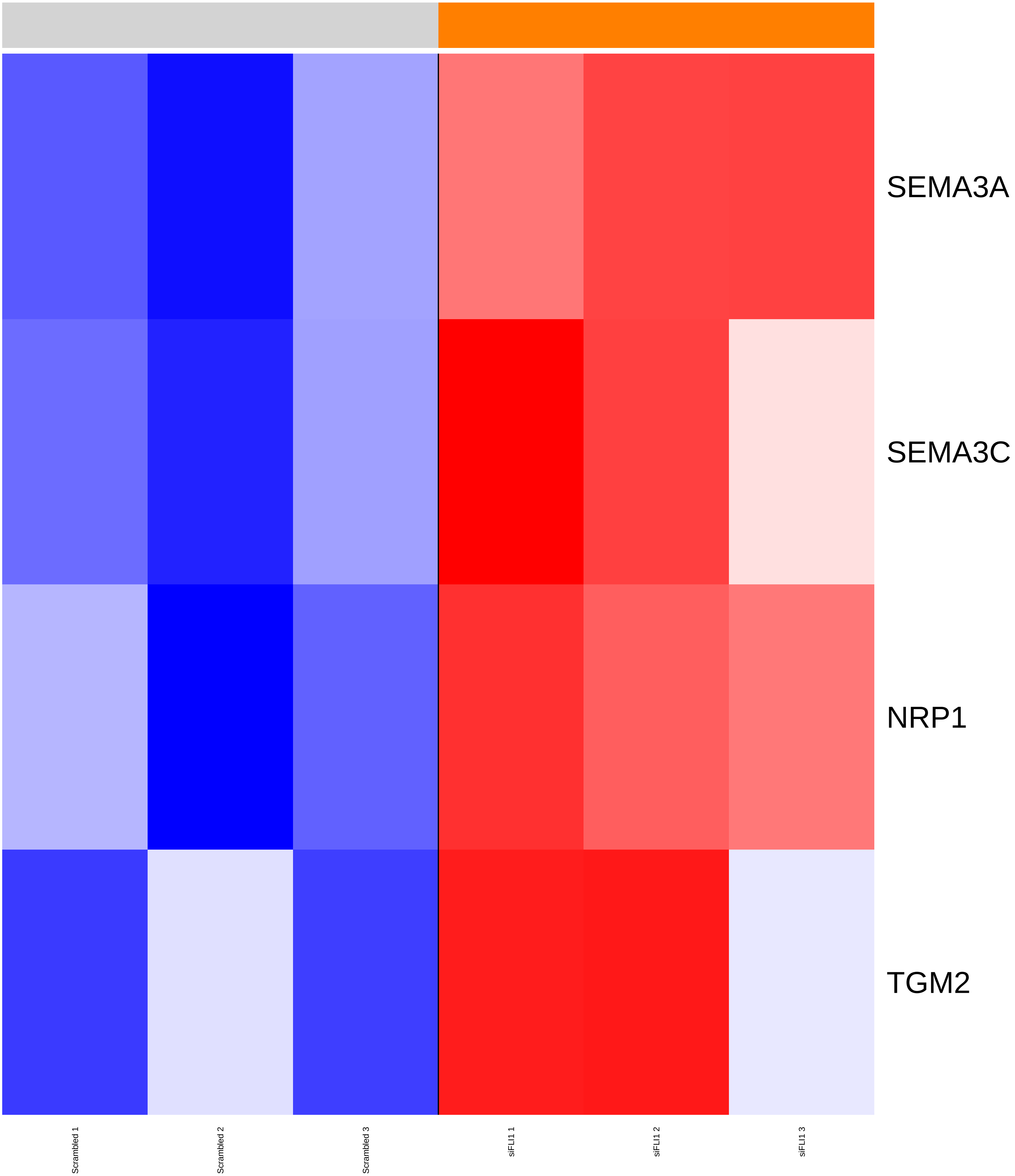

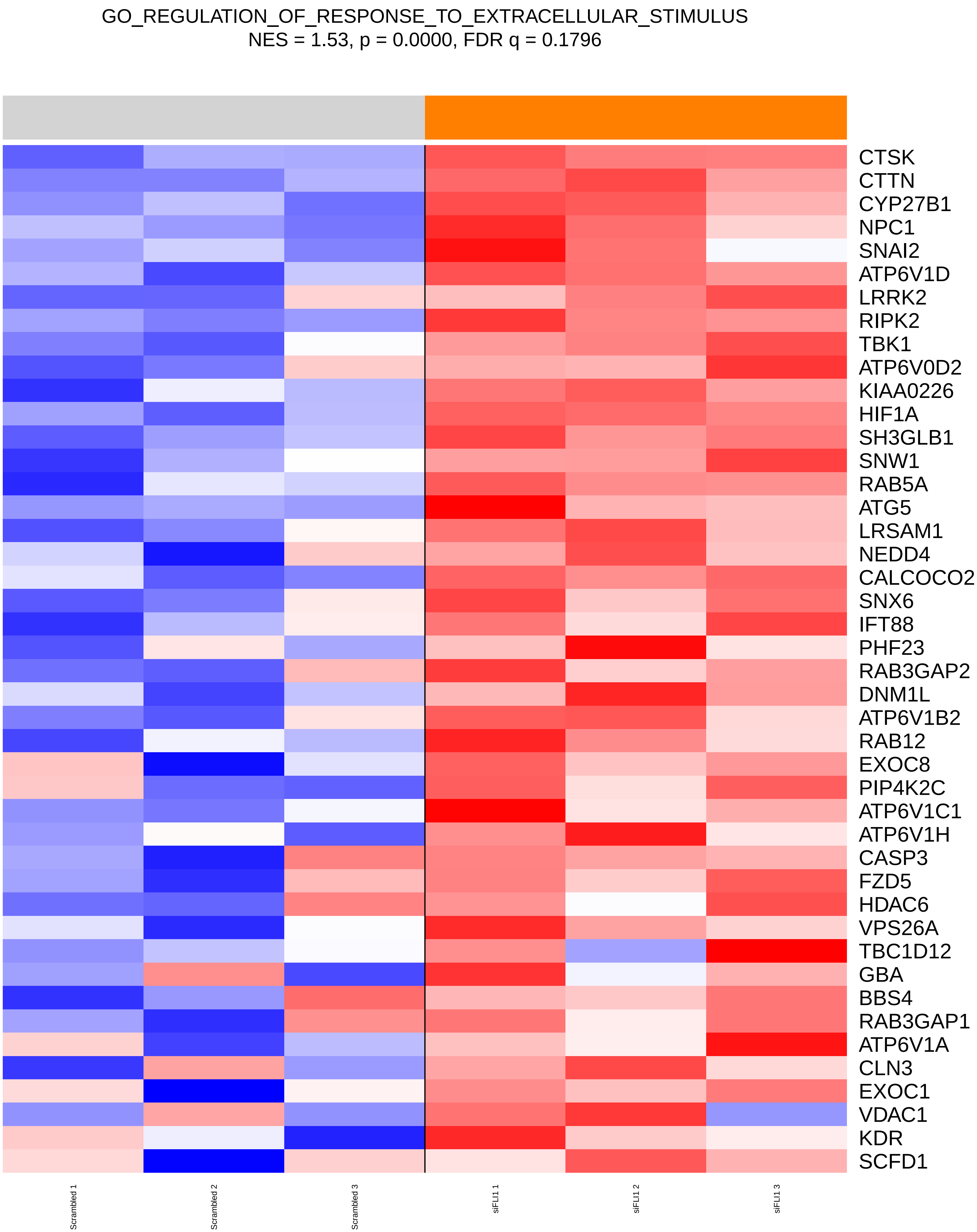
GO_REGULATION_OF_RESPONSE_TO_EXTRACELLULAR_STIMULUS
NES = 1.53, p = 0.0000, FDR q = 0.1796
CTSK
CTTN
CYP27B1
NPC1
SNAI2
ATP6V1D
LRRK2
RIPK2
TBK1
ATP6V0D2
KIAA0226
HIF1A
SH3GLB1
SNW1
RAB5A
ATG5
LRSAM1
NEDD4
CALCOCO2
SNX6
IFT88
PHF23
RAB3GAP2
DNM1L
ATP6V1B2
RAB12
EXOC8
PIP4K2C
ATP6V1C1
ATP6V1H
CASP3
FZD5
HDAC6
VPS26A
TBC1D12
GBA
BBS4
RAB3GAP1
ATP6V1A
CLN3
EXOC1
VDAC1
KDR
SCFD1
Scrambled 1
Scrambled 2
Scrambled 3
siFLI1 1
siFLI1 2
siFLI1 3

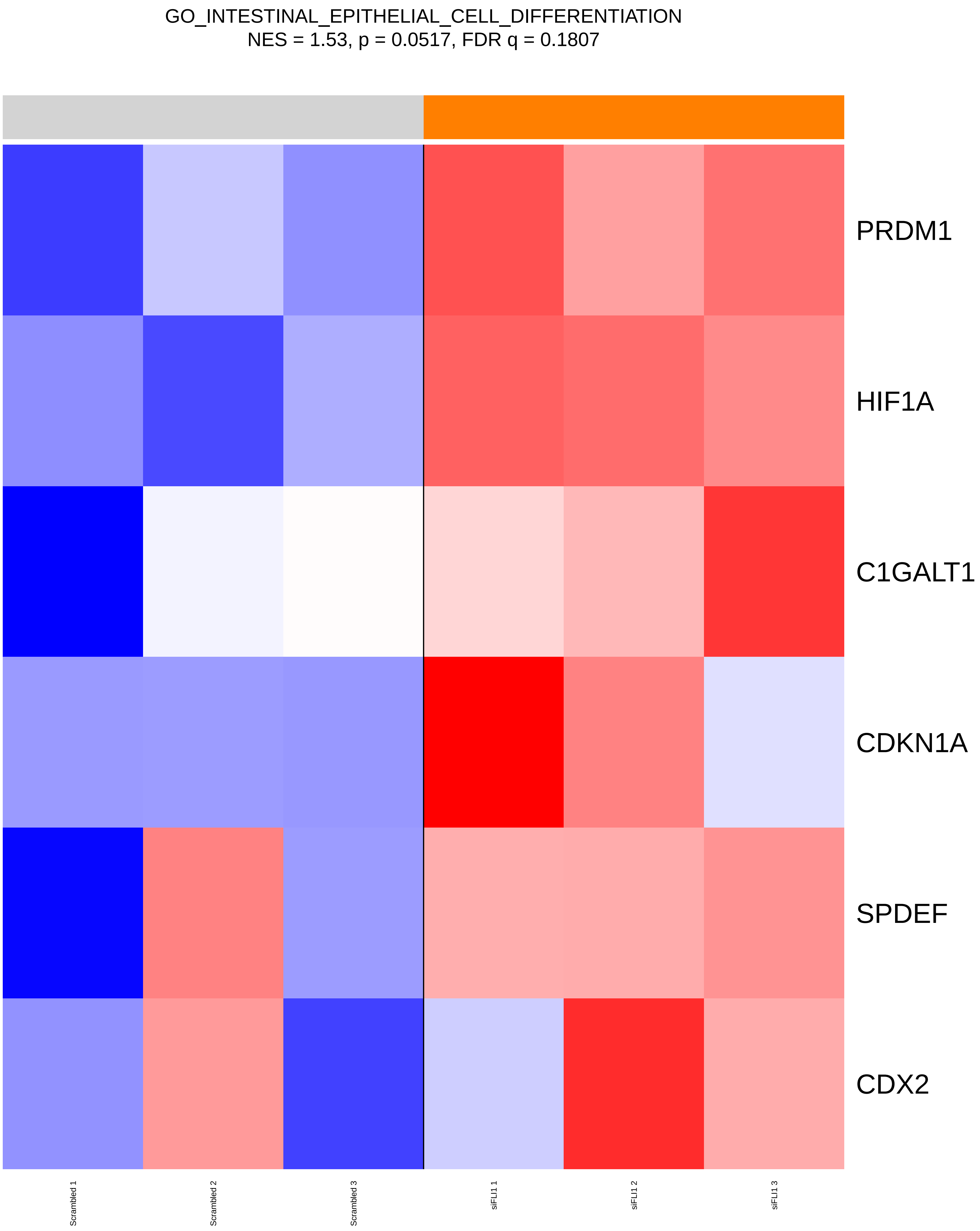
GO_INTESTINAL_EPITHELIAL_CELL_DIFFERENTIATION
NES = 1.53, p = 0.0517, FDR q = 0.1807
PRDM1
HIF1A
C1GALT1
CDKN1A
SPDEF
CDX2
Scrambled 1
Scrambled 2
Scrambled 3
siFLI1 1
siFLI1 2
siFLI1 3

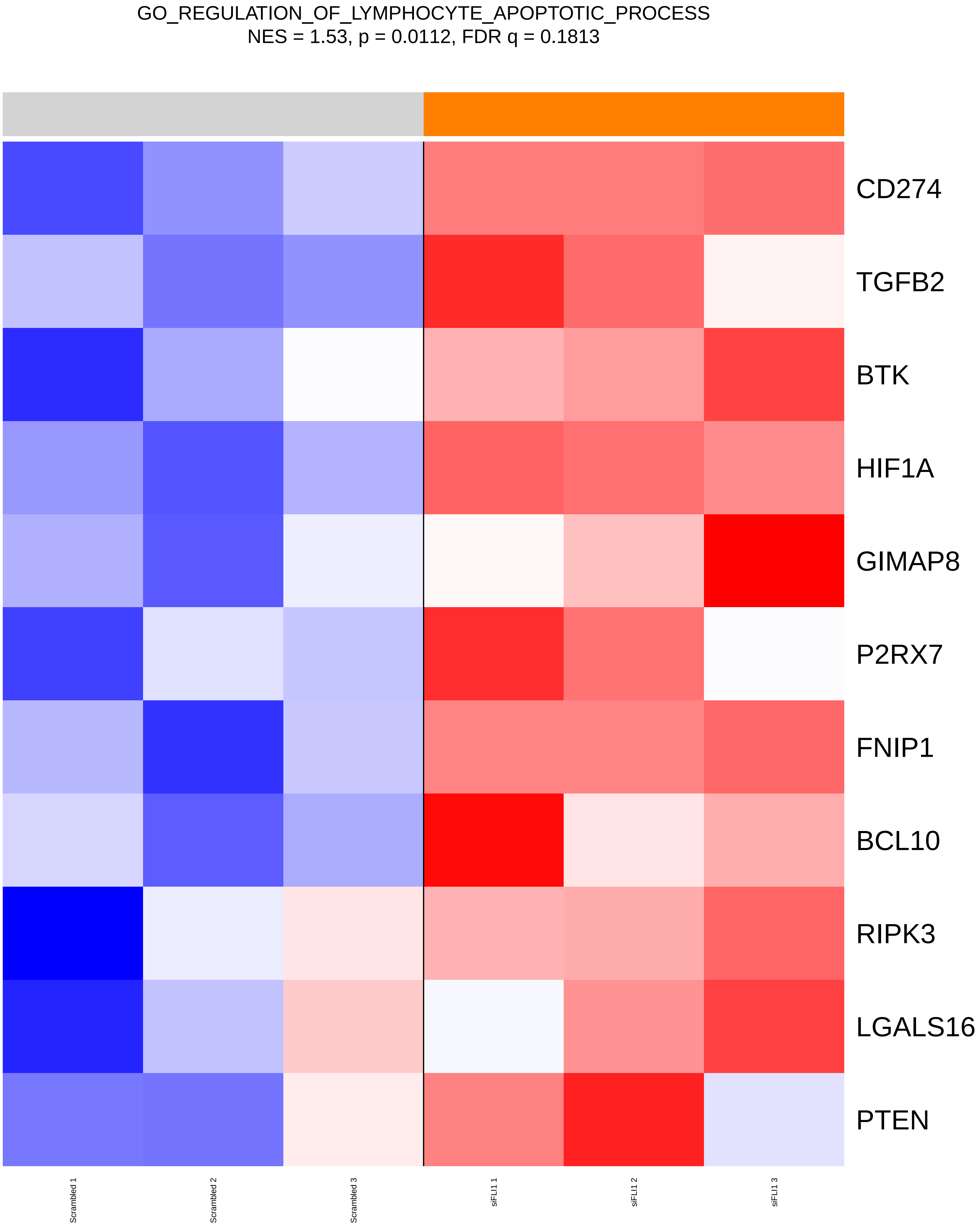

GO_REGULATION_OF_LYMPHOCYTE_APOPTOTIC_PROCESS
NES = 1.53, p = 0.0112, FDR q = 0.1813
CD274
TGFB2
BTK
HIF1A
GIMAP8
P2RX7
FNIP1
BCL10
RIPK3
LGALS16
PTEN
Scrambled 1
Scrambled 2
Scrambled 3
siFLI1 1
siFLI1 2
siFLI1 3

GO_ORGANELLE_FUSION
NES = 1.53, p = 0.0036, FDR q = 0.1832

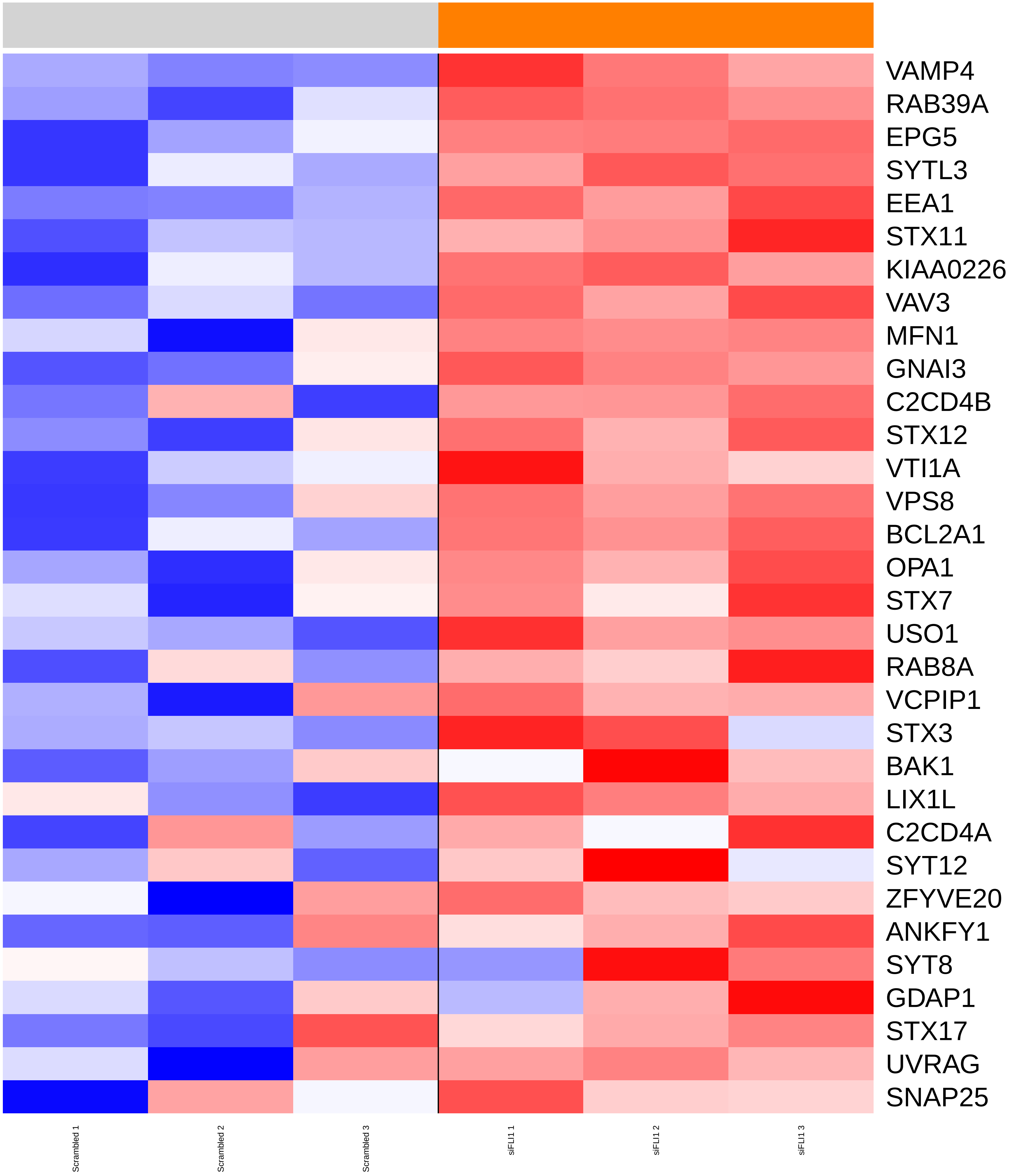

GO_REGULATION_OF_RESPONSE_TO_WOUNDING
NES = 1.53, p = 0.0000, FDR q = 0.1830

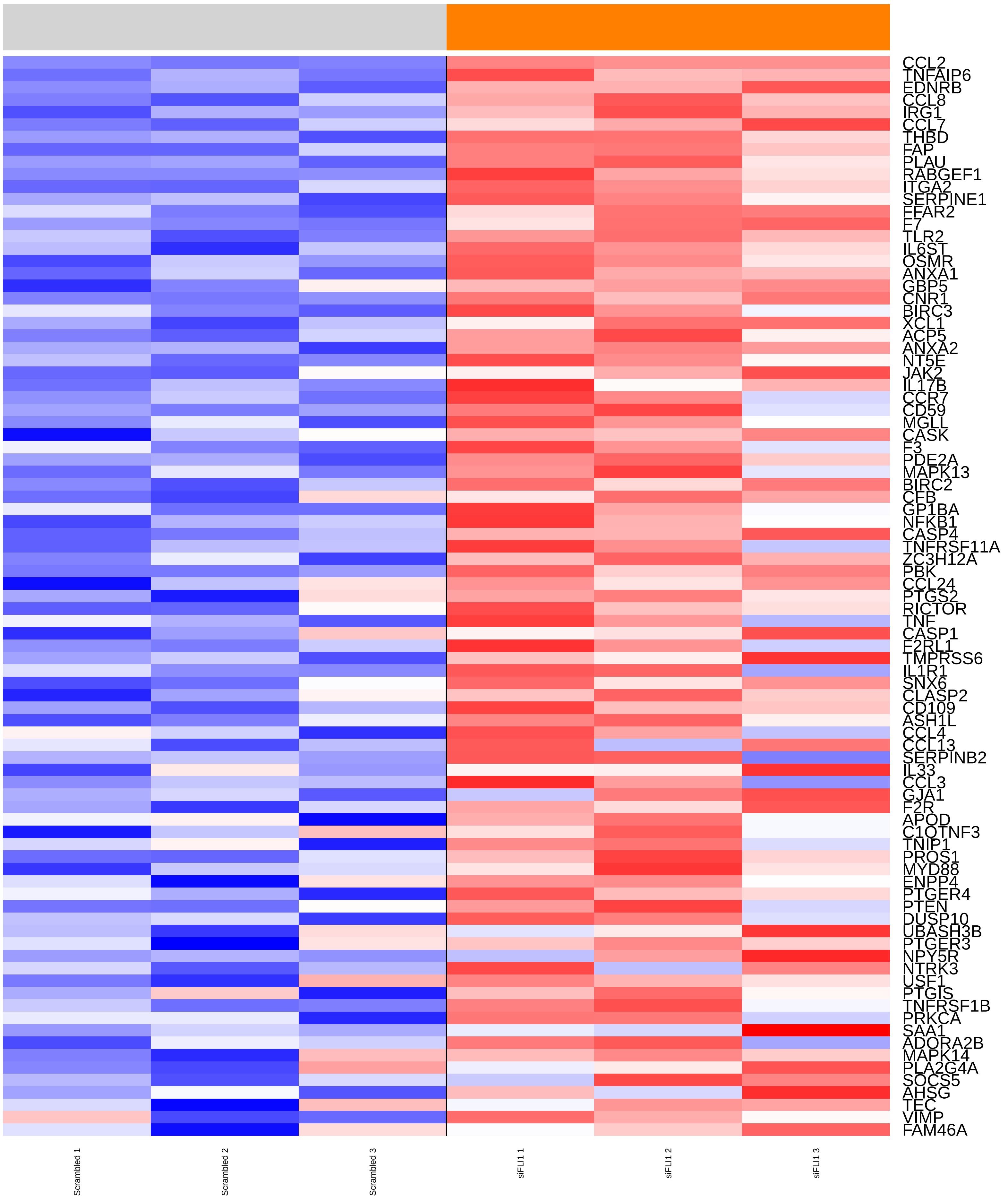

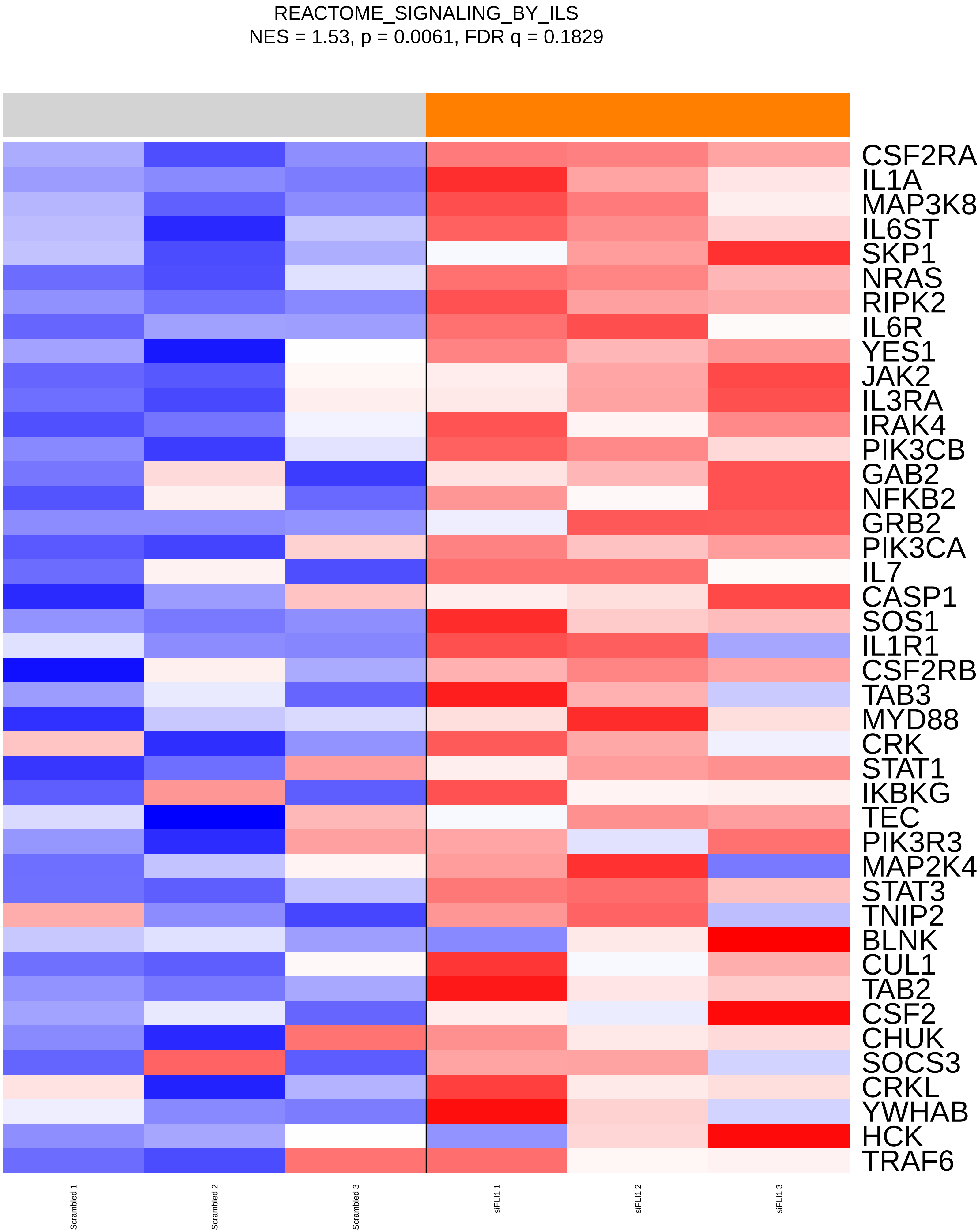
REACTOME_SIGNALING_BY_ILS
NES = 1.53, p = 0.0061, FDR q = 0.1829
CSF2RA
IL1A
MAP3K8
IL6ST
SKP1
NRAS
RIPK2
IL6R
YES1
JAK2
IL3RA
IRAK4
PIK3CB
GAB2
NFKB2
GRB2
PIK3CA
IL7
CASP1
SOS1
IL1R1
CSF2RB
TAB3
MYD88
CRK
STAT1
IKBKG
TEC
PIK3R3
MAP2K4
STAT3
TNIP2
BLNK
CUL1
TAB2
CSF2
CHUK
SOCS3
CRKL
YWHAB
HCK
TRAF6
Scrambled 1
Scrambled 2
Scrambled 3
siFLI1 1
siFLI1 2
siFLI1 3

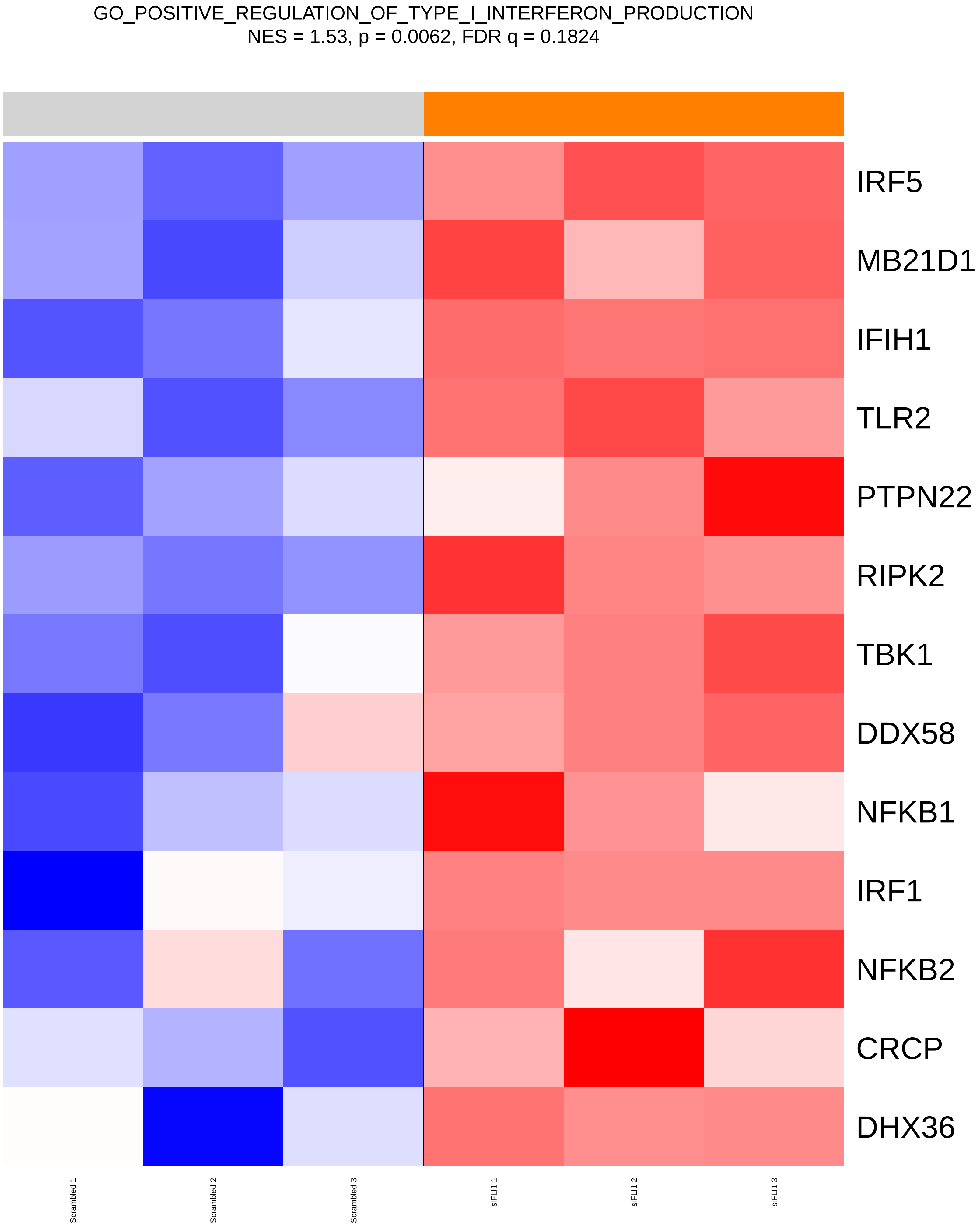
GO_POSITIVE_REGULATION_OF_TYPE_I_INTERFERON_PRODUCTION
NES = 1.53, p = 0.0062, FDR q = 0.1824
IRF5
MB21D1
IFIH1
TLR2
PTPN22
RIPK2
TBK1
DDX58
NFKB1
IRF1
NFKB2
CRCP
DHX36
Scrambled 1
Scrambled 2
Scrambled 3
siFLI1 1
siFLI1 2
siFLI1 3

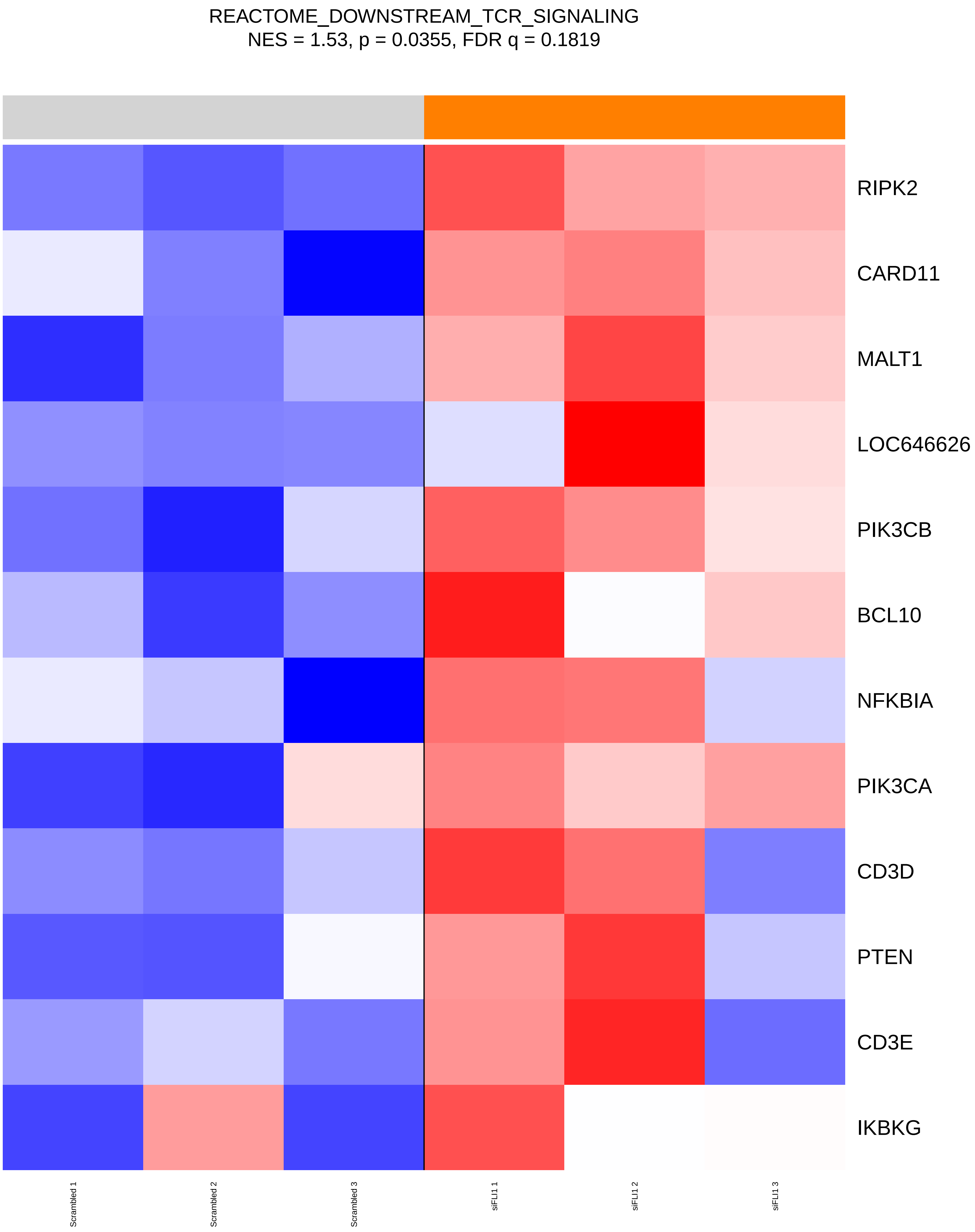
REACTOME_DOWNSTREAM_TCR_SIGNALING
NES = 1.53, p = 0.0355, FDR q = 0.1819
RIPK2
CARD11
MALT1
LOC646626
PIK3CB
BCL10
NFKBIA
PIK3CA
CD3D
PTEN
CD3E
IKBKG
Scrambled 1
Scrambled 2
Scrambled 3
siFLI1 1
siFLI1 2
siFLI1 3

TTGCACT_MIR130A_MIR301_MIR130B
NES = 1.53, p = 0.0000, FDR q = 0.1826

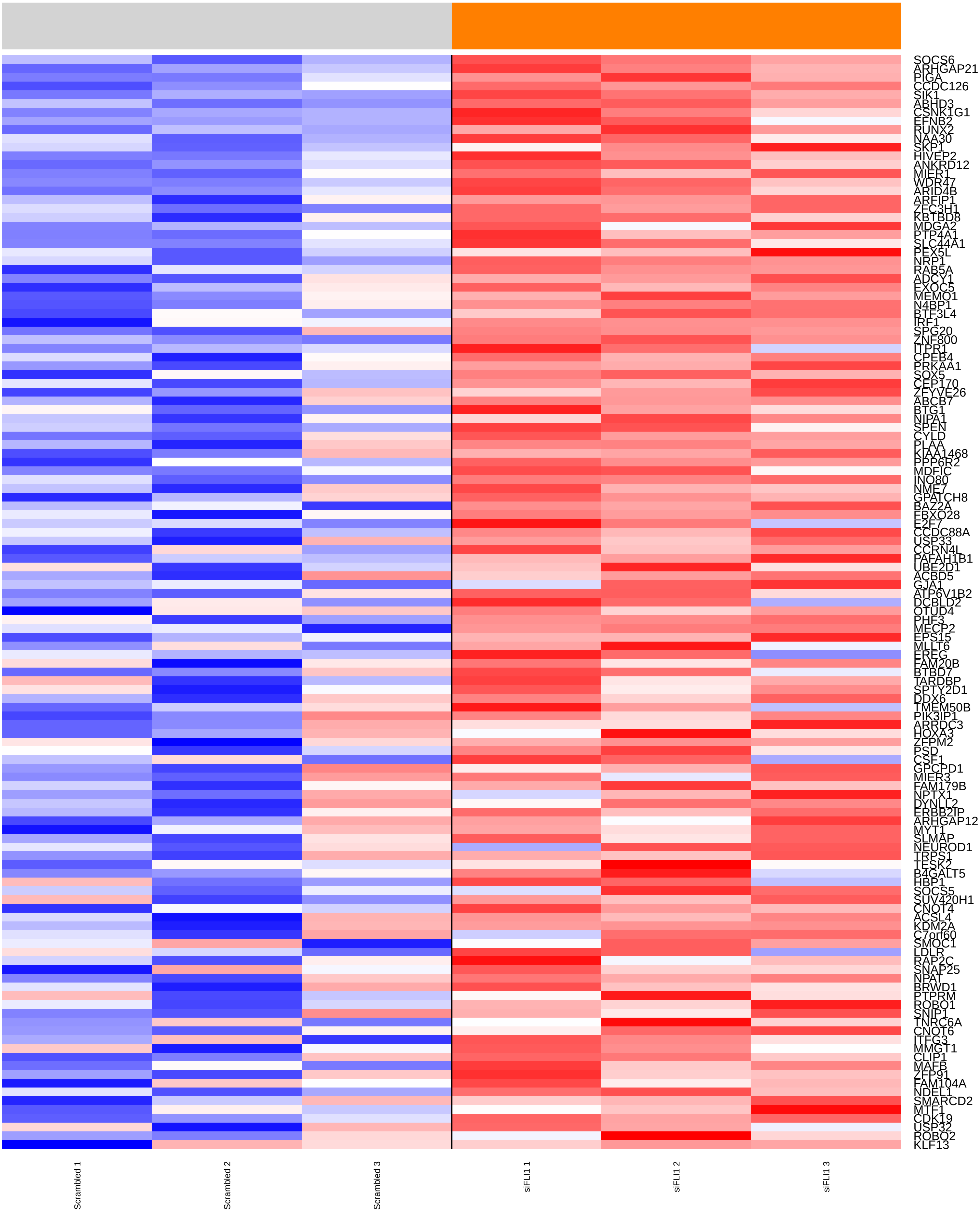

IRF1_01
NES = 1.53, p = 0.0000, FDR q = 0.1834

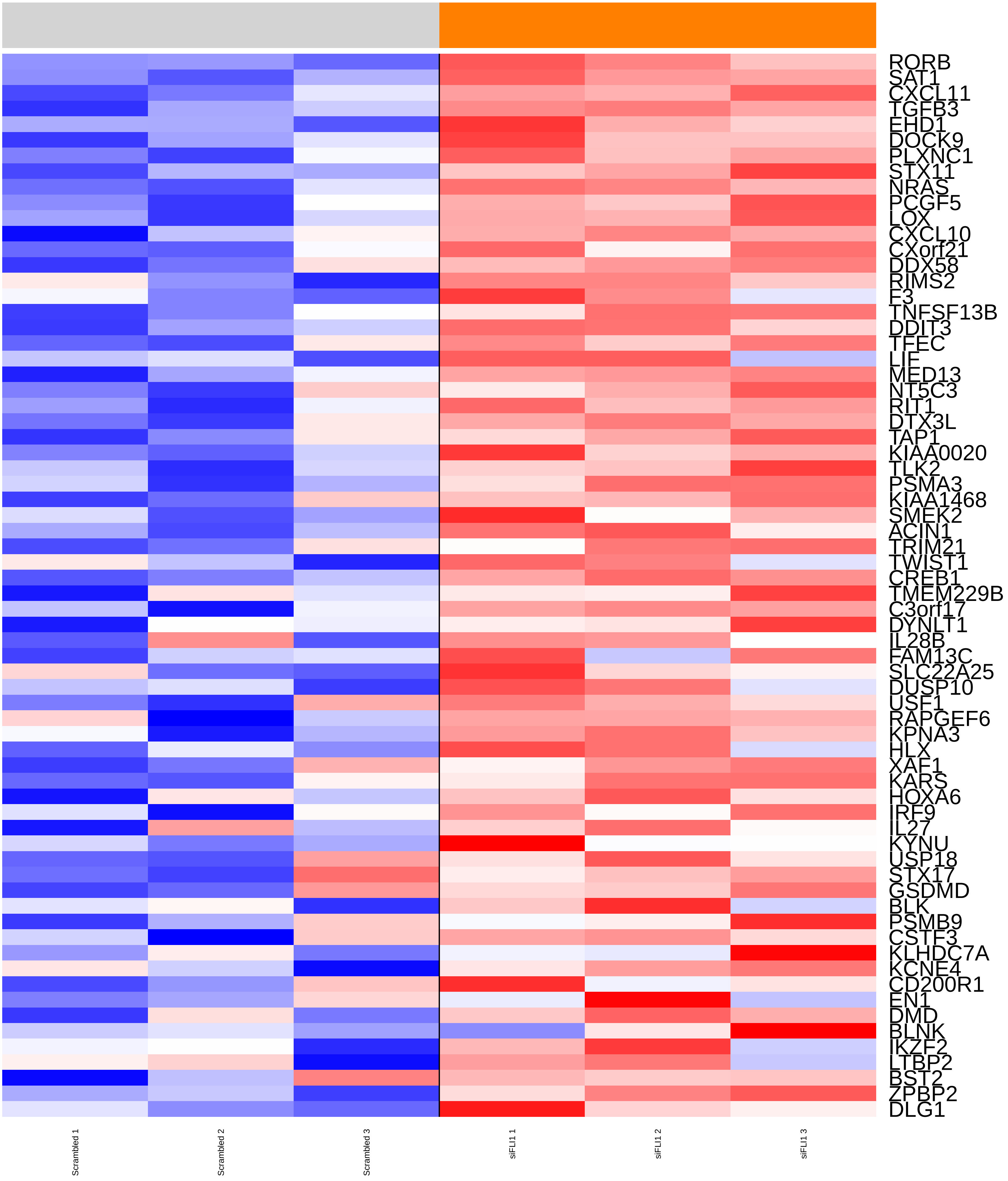

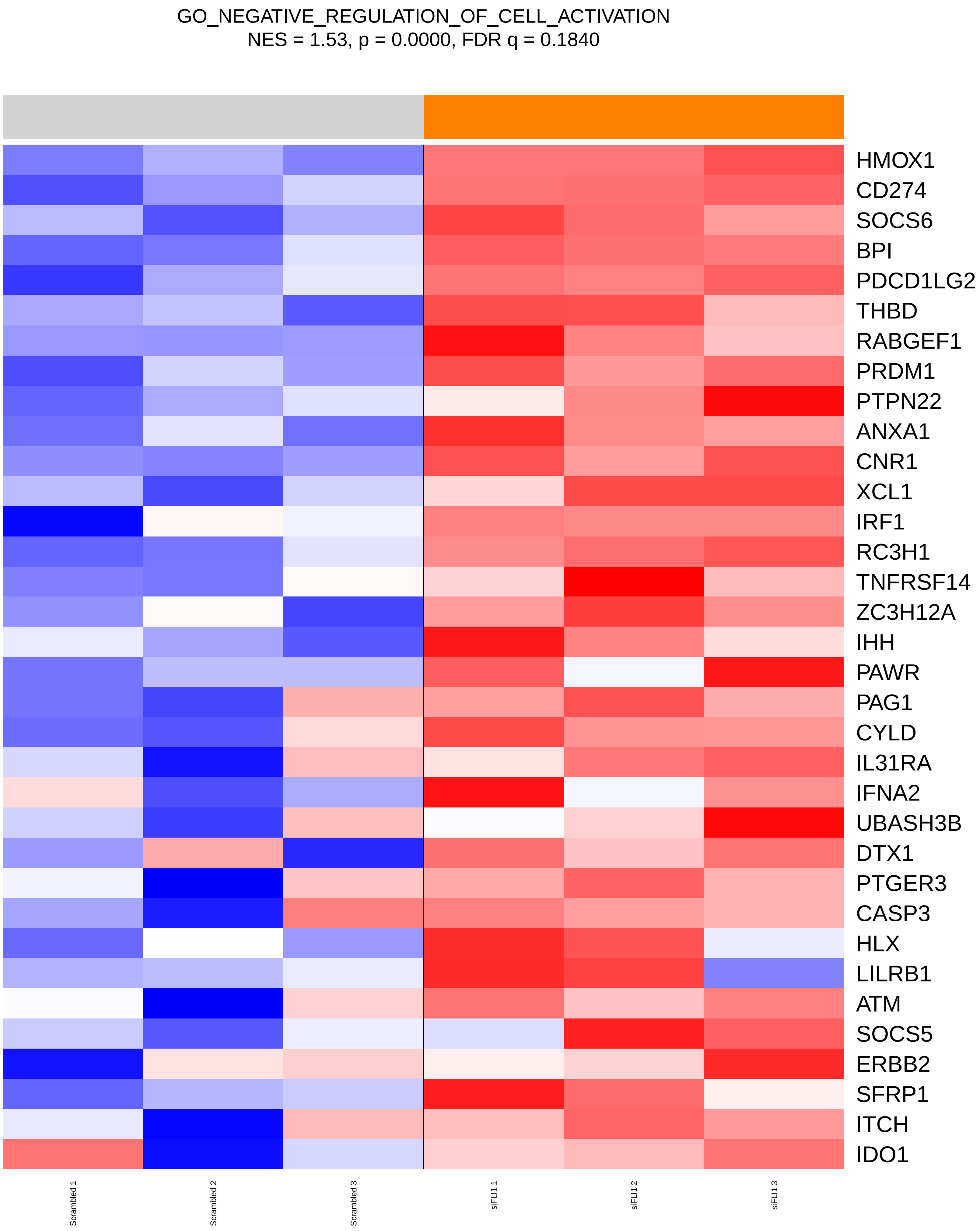
GO_NEGATIVE_REGULATION_OF_CELL_ACTIVATION
NES = 1.53, p = 0.0000, FDR q = 0.1840
HMOX1
CD274
SOCS6
BPI
PDCD1LG2
THBD
RABGEF1
PRDM1
PTPN22
ANXA1
CNR1
XCL1
IRF1
RC3H1
TNFRSF14
ZC3H12A
IHH
PAWR
PAG1
CYLD
IL31RA
IFNA2
UBASH3B
DTX1
PTGER3
CASP3
HLX
LILRB1
ATM
SOCS5
ERBB2
SFRP1
ITCH
IDO1
Scrambled 1
Scrambled 2
Scrambled 3
siFLI1 1
siFLI1 2
siFLI1 3

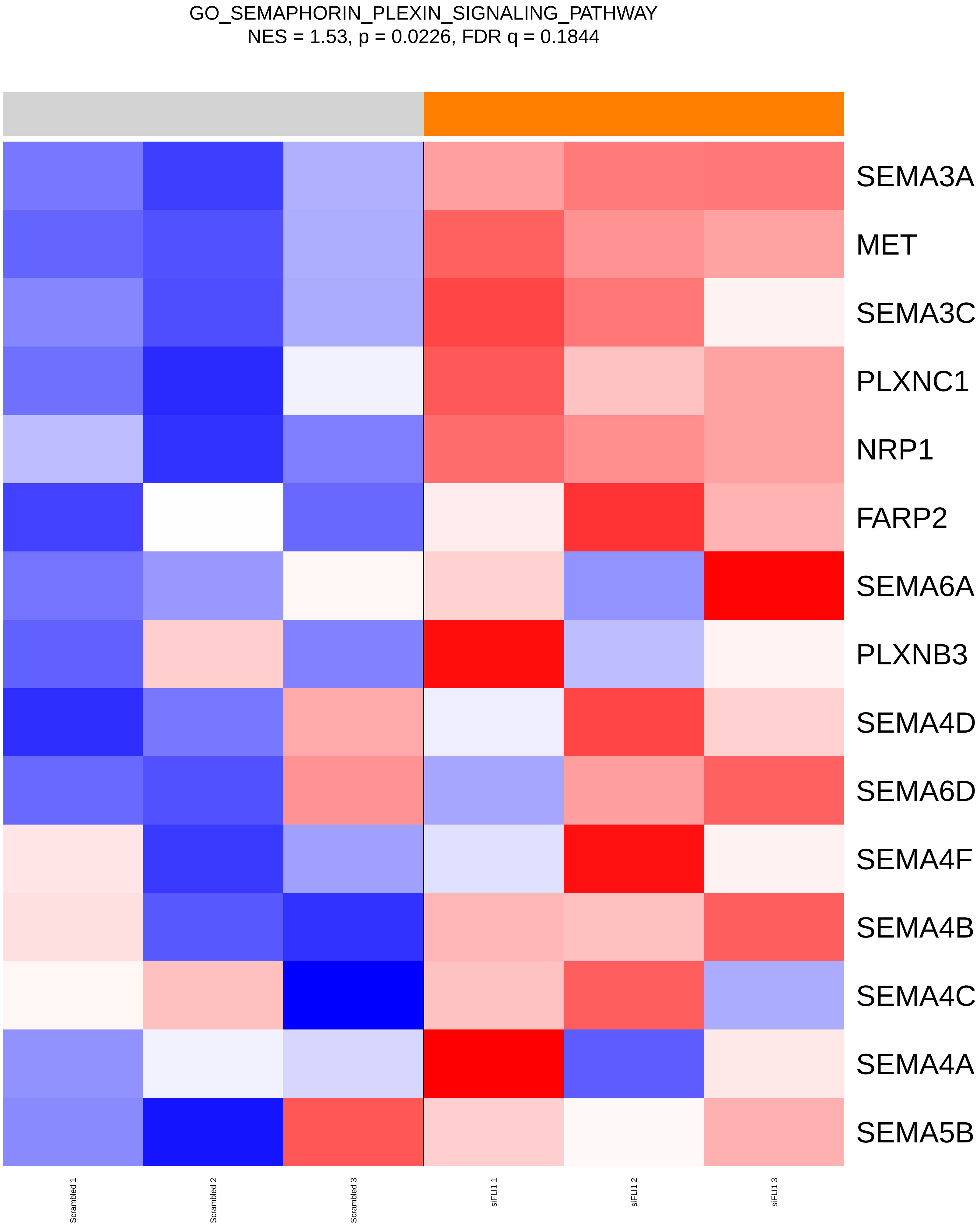
GO_SEMAPHORIN_PLEXIN_SIGNALING_PATHWAY
NES = 1.53, p = 0.0226, FDR q = 0.1844
SEMA3A
MET
SEMA3C
PLXNC1
NRP1
FARP2
SEMA6A
PLXNB3
SEMA4D
SEMA6D
SEMA4F
SEMA4B
SEMA4C
SEMA4A
SEMA5B
Scrambled 1
Scrambled 2
Scrambled 3
siFLI1 1
siFLI1 2
siFLI1 3

GO_NEGATIVE_REGULATION_OF_HOMOTYPIC_CELL_CELL_ADHESION
NES = 1.53, p = 0.0031, FDR q = 0.1839

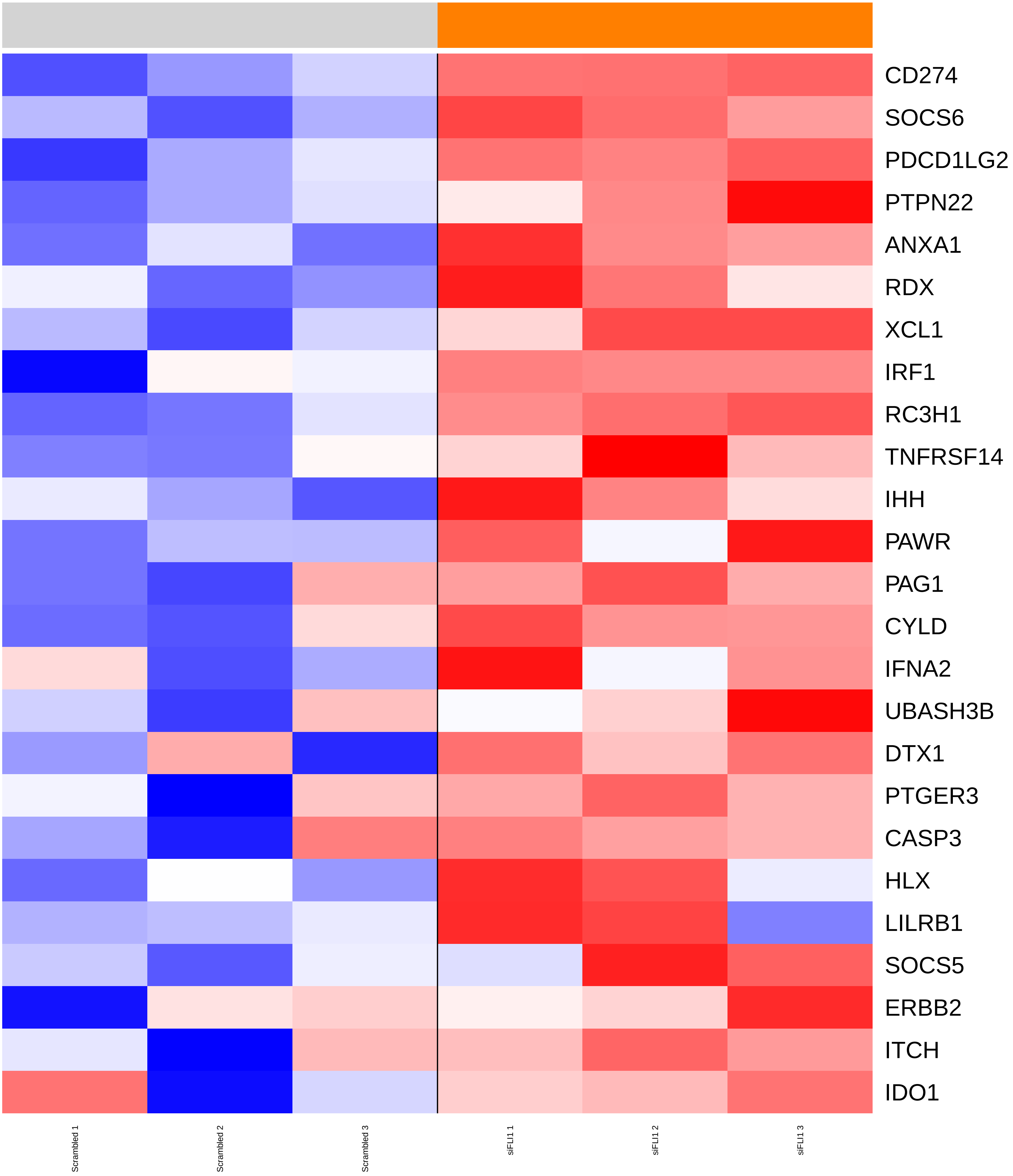

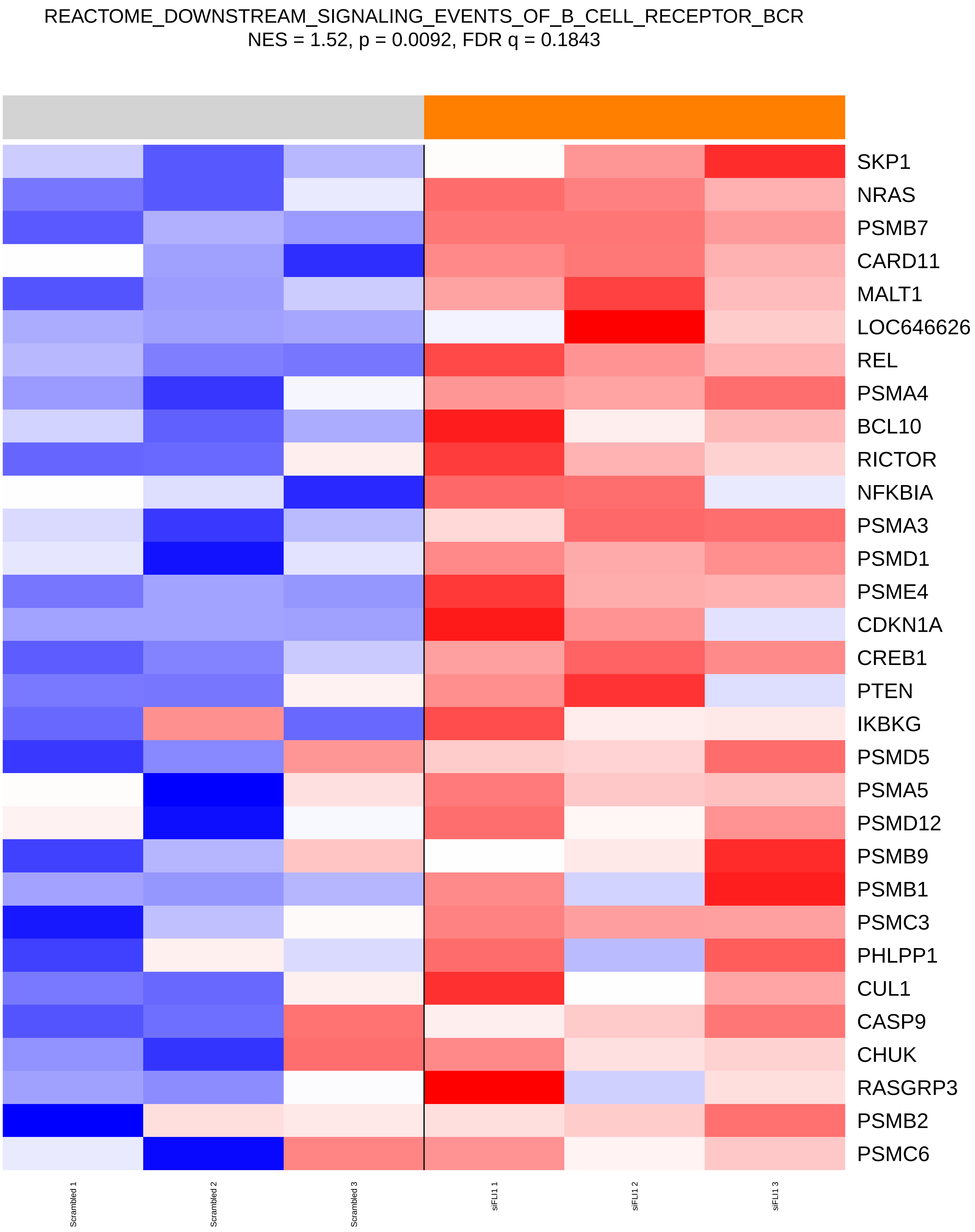
REACTOME_DOWNSTREAM_SIGNALING_EVENTS_OF_B_CELL_RECEPTOR_BCR
NES = 1.52, p = 0.0092, FDR q = 0.1843
SKP1
NRAS
PSMB7
CARD11
MALT1
LOC646626
REL
PSMA4
BCL10
RICTOR
NFKBIA
PSMA3
PSMD1
PSME4
CDKN1A
CREB1
PTEN
IKBKG
PSMD5
PSMA5
PSMD12
PSMB9
PSMB1
PSMC3
PHLPP1
CUL1
CASP9
CHUK
RASGRP3
PSMB2
PSMC6
Scrambled 1
Scrambled 2
Scrambled 3
siFLI1 1
siFLI1 2
siFLI1 3

GO_POSITIVE_REGULATION_OF_INTERFERON_BETA_PRODUCTION
NES = 1.52, p = 0.0405, FDR q = 0.1857

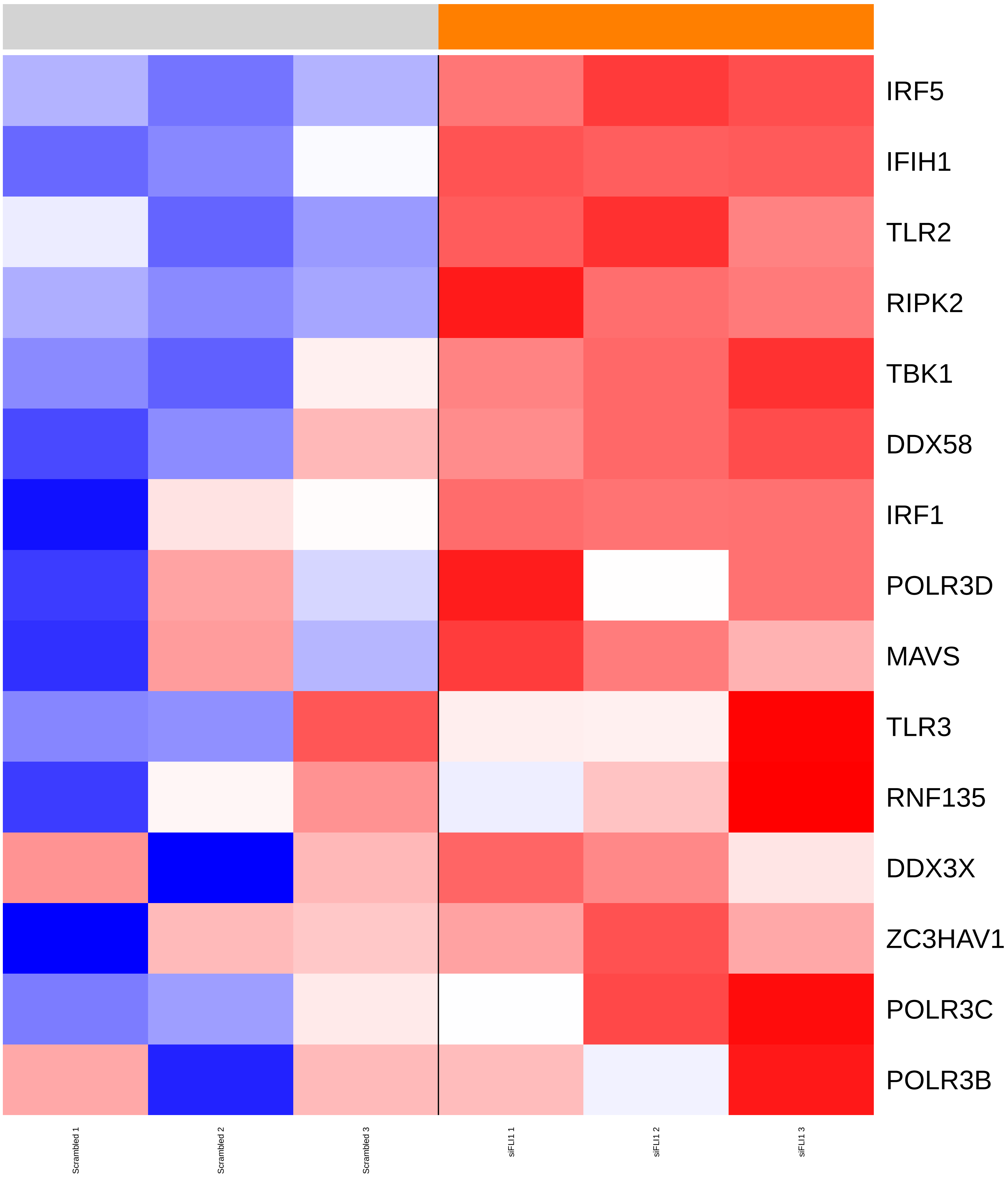

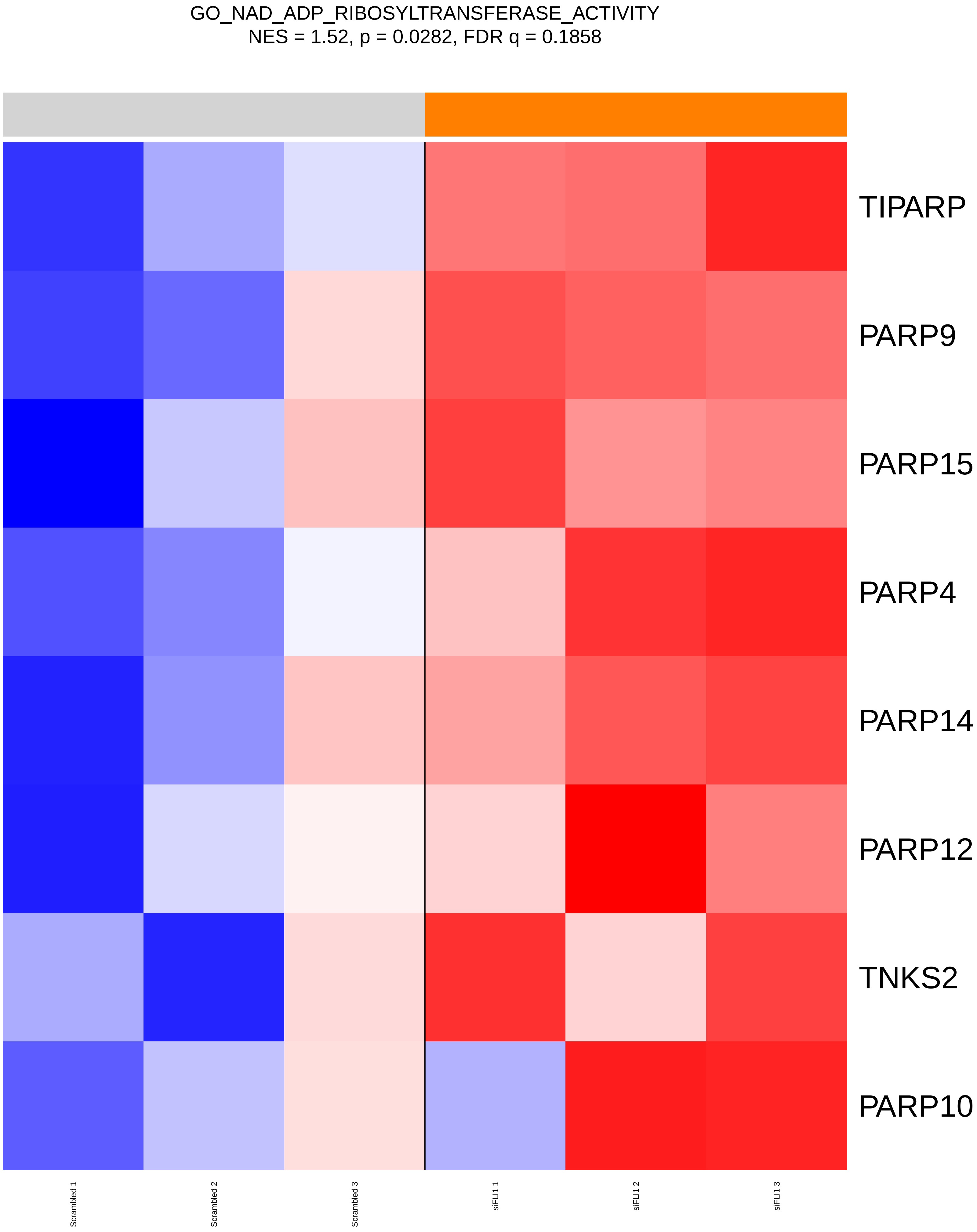
GO_NAD_ADP_RIBOSYLTRANSFERASE_ACTIVITY
NES = 1.52, p = 0.0282, FDR q = 0.1858
TIPARP
PARP9
PARP15
PARP4
PARP14
PARP12
TNKS2
PARP10
Scrambled 1
Scrambled 2
Scrambled 3
siFLI1 1
siFLI1 2
siFLI1 3

GO_SEMAPHORIN_RECEPTOR_BINDING
NES = 1.52, p = 0.0427, FDR q = 0.1865

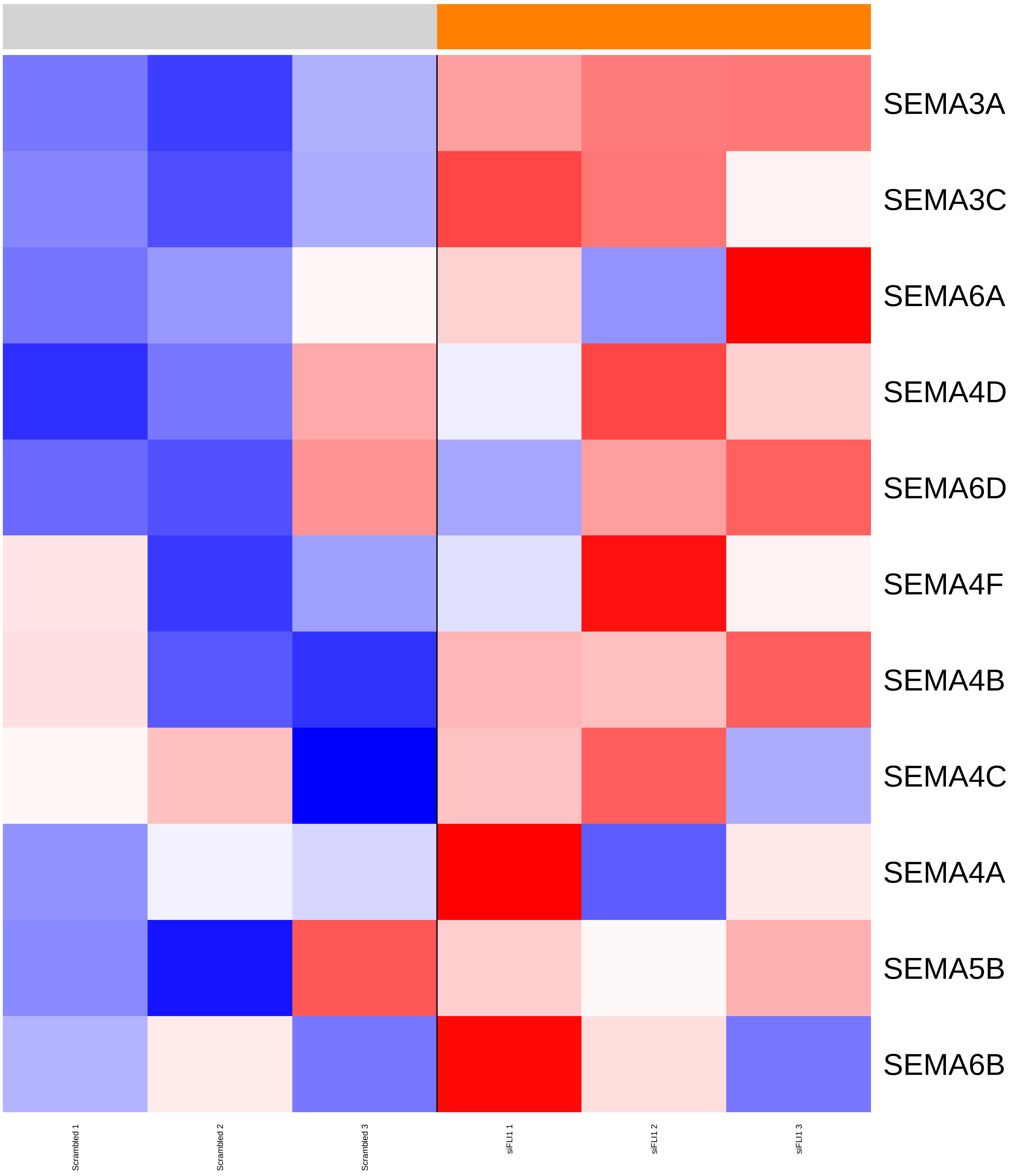

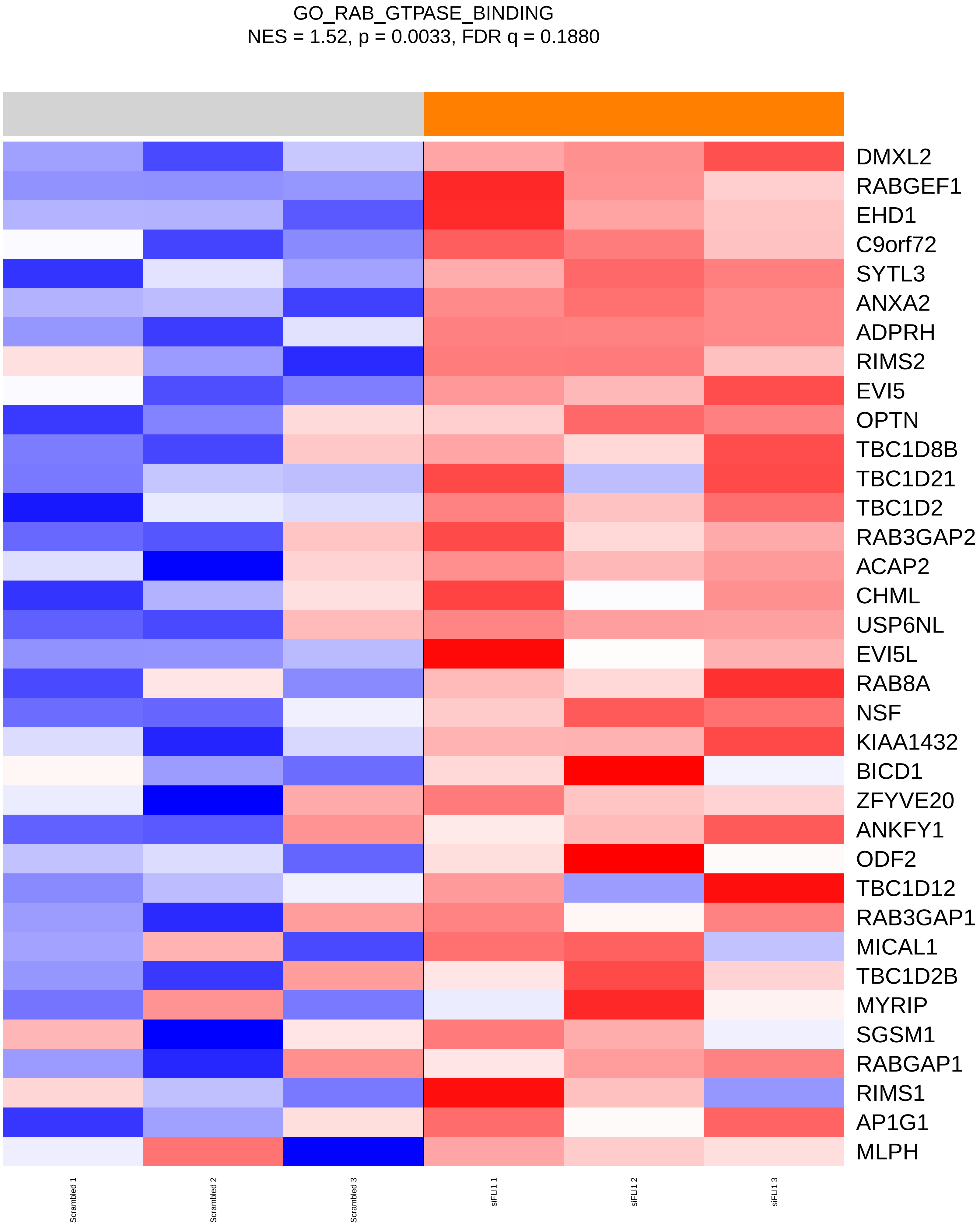
GO_RAB_GTPASE_BINDING
NES = 1.52, p = 0.0033, FDR q = 0.1880
DMXL2
RABGEF1
EHD1
C9orf72
SYTL3
ANXA2
ADPRH
RIMS2
EVI5
OPTN
TBC1D8B
TBC1D21
TBC1D2
RAB3GAP2
ACAP2
CHML
USP6NL
EVI5L
RAB8A
NSF
KIAA1432
BICD1
ZFYVE20
ANKFY1
ODF2
TBC1D12
RAB3GAP1
MICAL1
TBC1D2B
MYRIP
SGSM1
RABGAP1
RIMS1
AP1G1
MLPH
Scrambled 1
Scrambled 2
Scrambled 3
siFLI1 1
siFLI1 2
siFLI1 3

REACTOME_REGULATION_OF_KIT_SIGNALING
NES = 1.52, p = 0.0450, FDR q = 0.1875

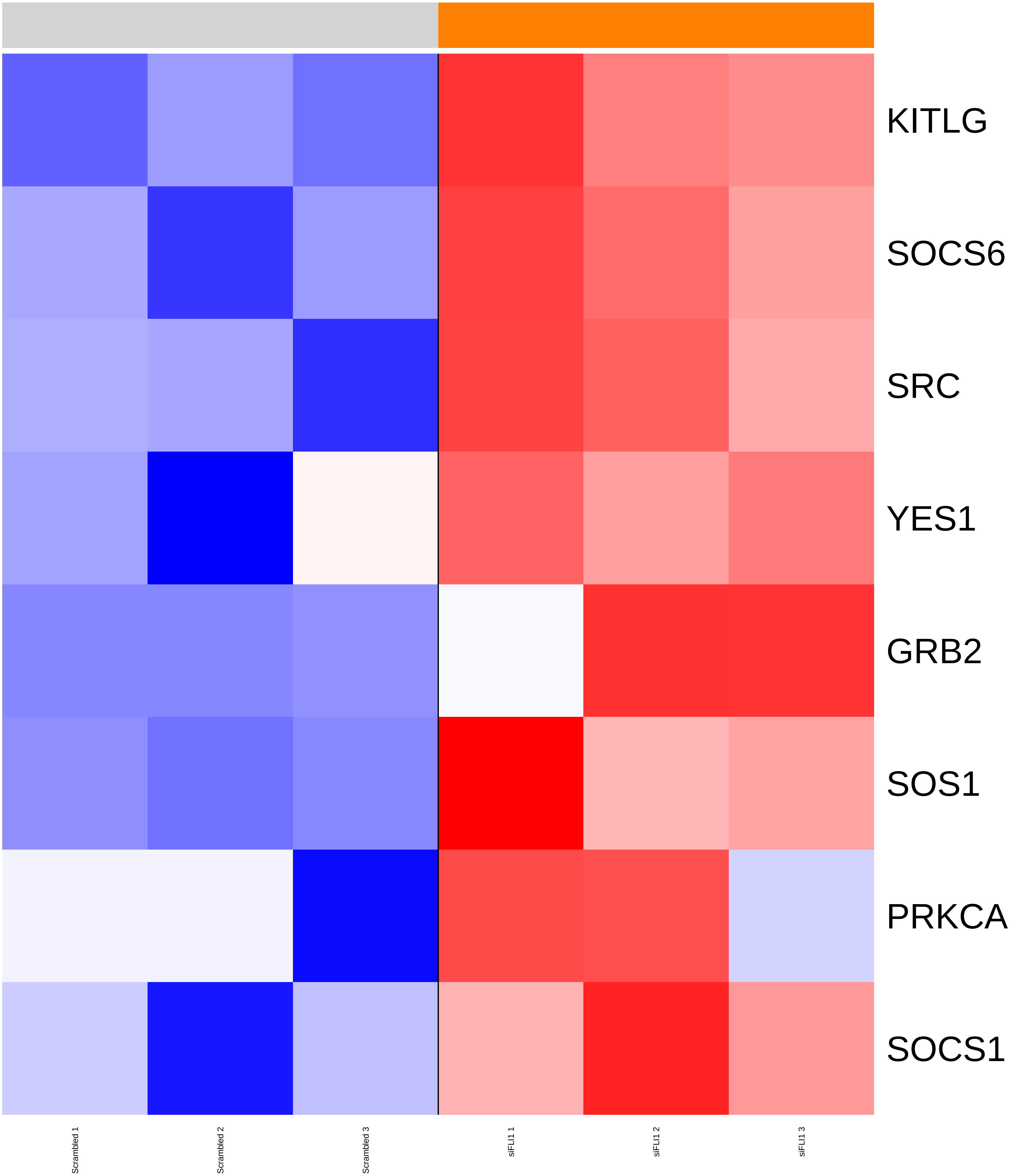

REACTOME_REGULATION_OF_ORNITHINE_DECARBOXYLASE_ODC
NES = 1.52, p = 0.0184, FDR q = 0.1872

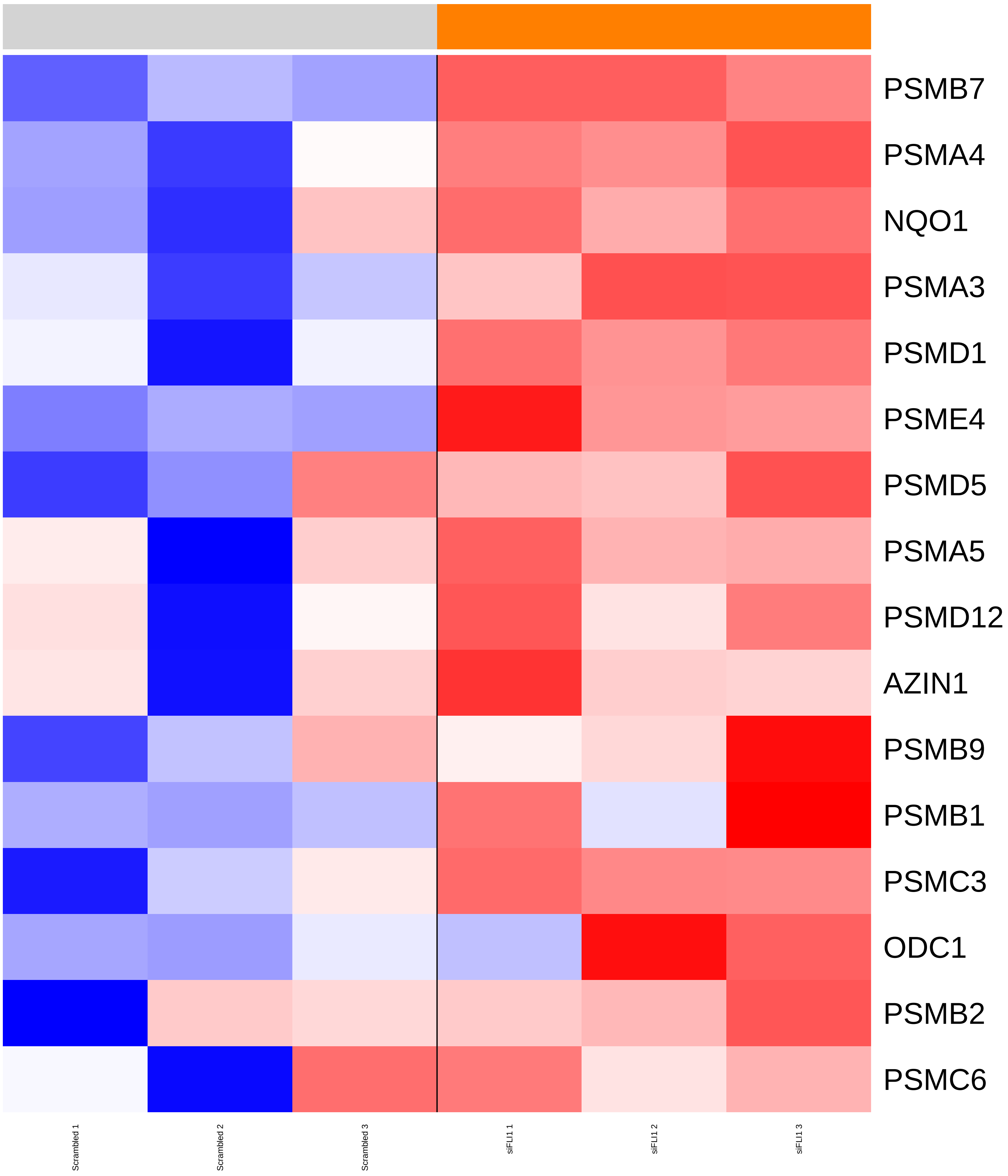

GO_MYELOID_LEUKOCYTE_MIGRATION
NES = 1.52, p = 0.0065, FDR q = 0.1871

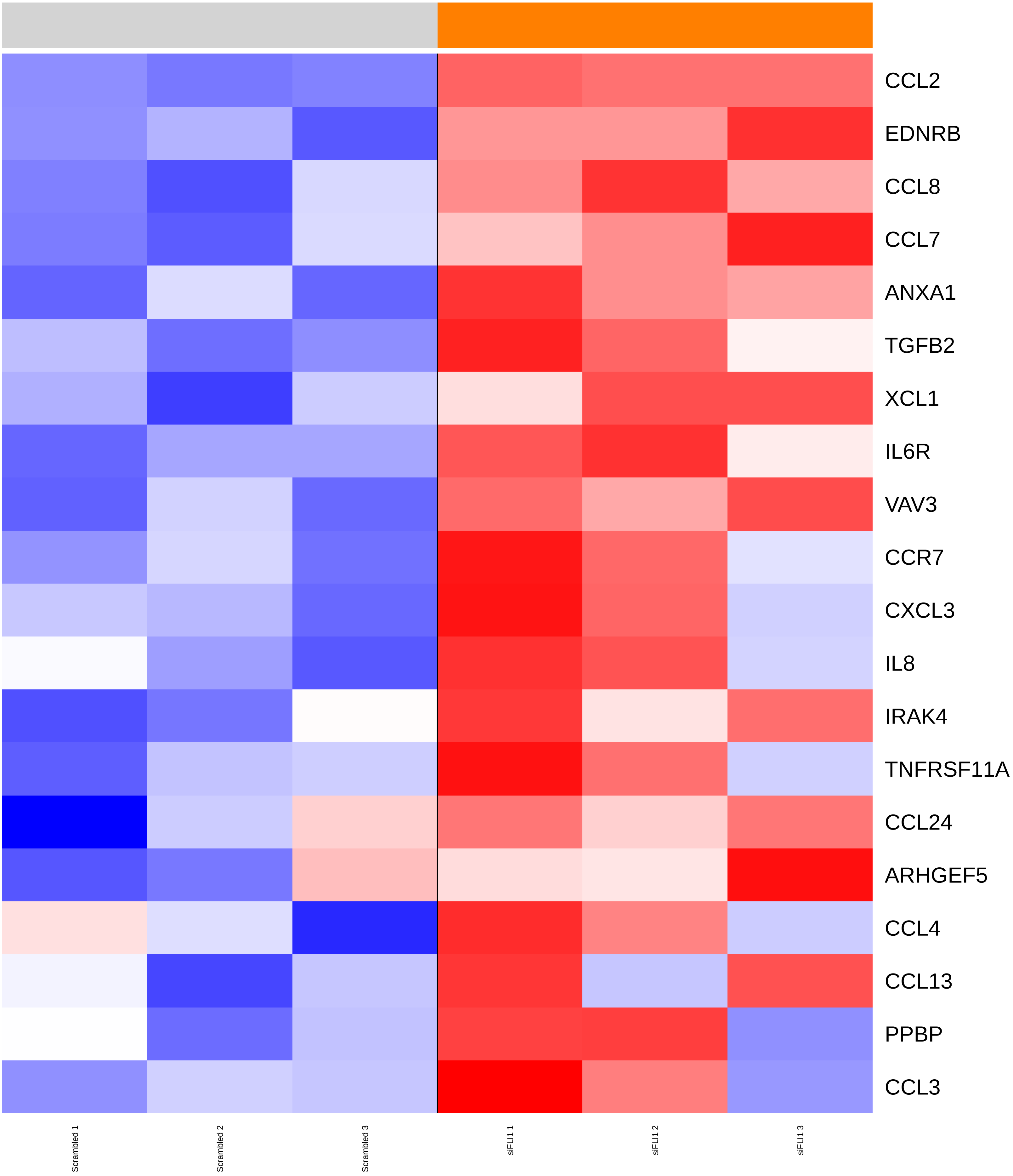

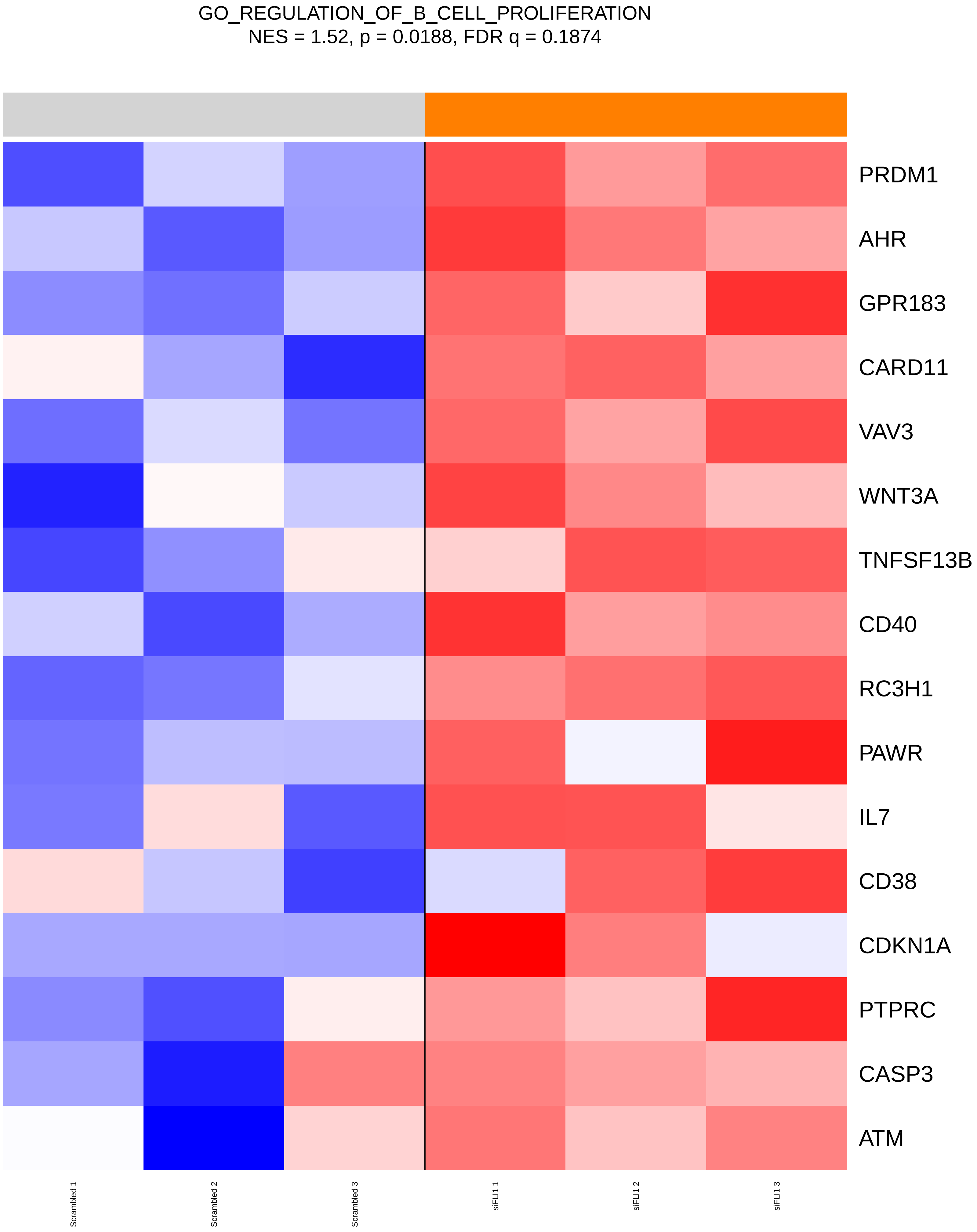
GO_REGULATION_OF_B_CELL_PROLIFERATION
NES = 1.52, p = 0.0188, FDR q = 0.1874
PRDM1
AHR
GPR183
CARD11
VAV3
WNT3A
TNFSF13B
CD40
RC3H1
PAWR
IL7
CD38
CDKN1A
PTPRC
CASP3
ATM
Scrambled 1
Scrambled 2
Scrambled 3
siFLI1 1
siFLI1 2
siFLI1 3

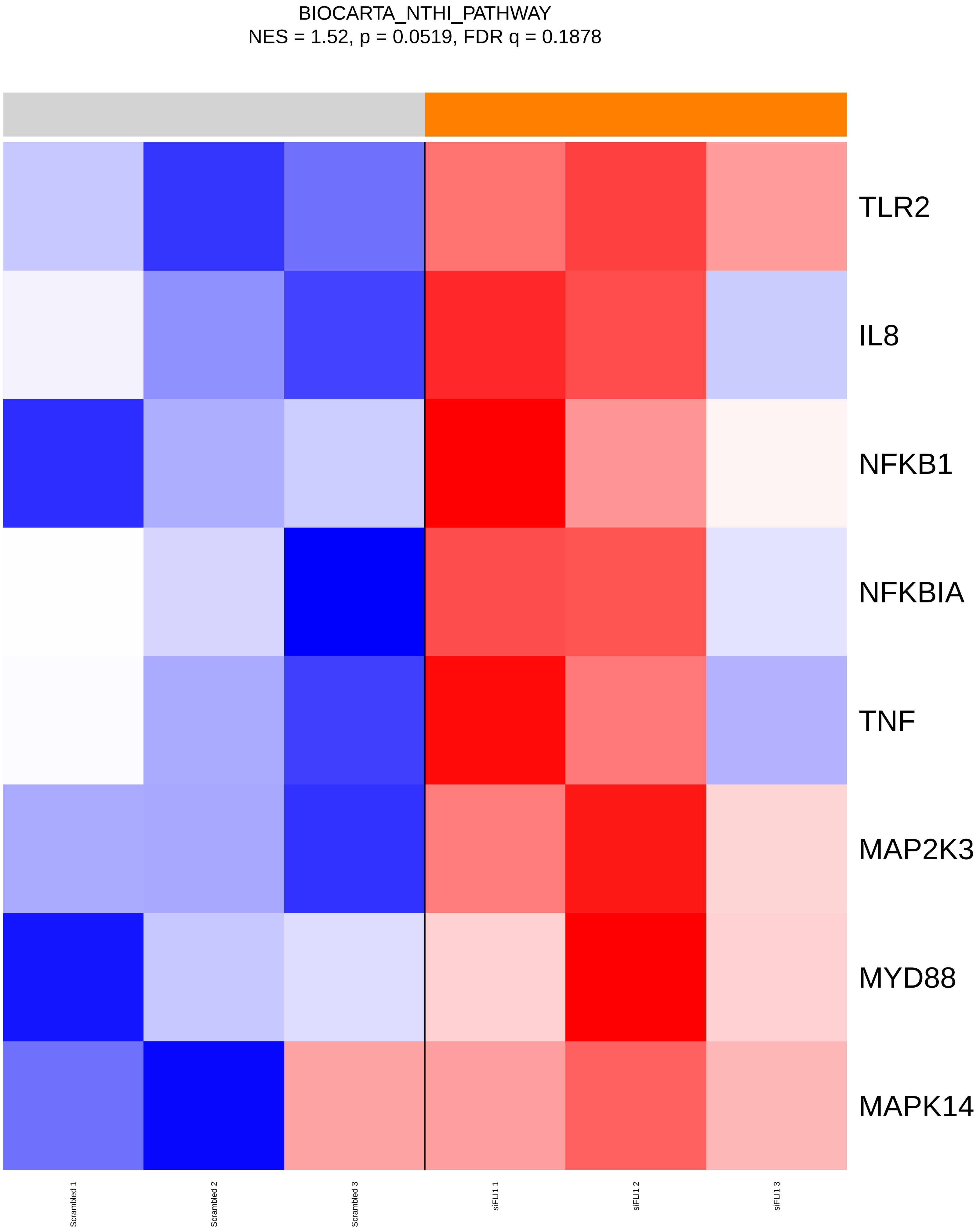
BIOCARTA_NTHI_PATHWAY
NES = 1.52, p = 0.0519, FDR q = 0.1878
TLR2
IL8
NFKB1
NFKBIA
TNF
MAP2K3
MYD88
MAPK14
Scrambled 1
Scrambled 2
Scrambled 3
siFLI1 1
siFLI1 2
siFLI1 3

GO_REGULATION_OF_SYNCYTIUM_FORMATION_BY_PLASMA_MEMBRANE_FUSION
NES = 1.52, p = 0.0318, FDR q = 0.1873

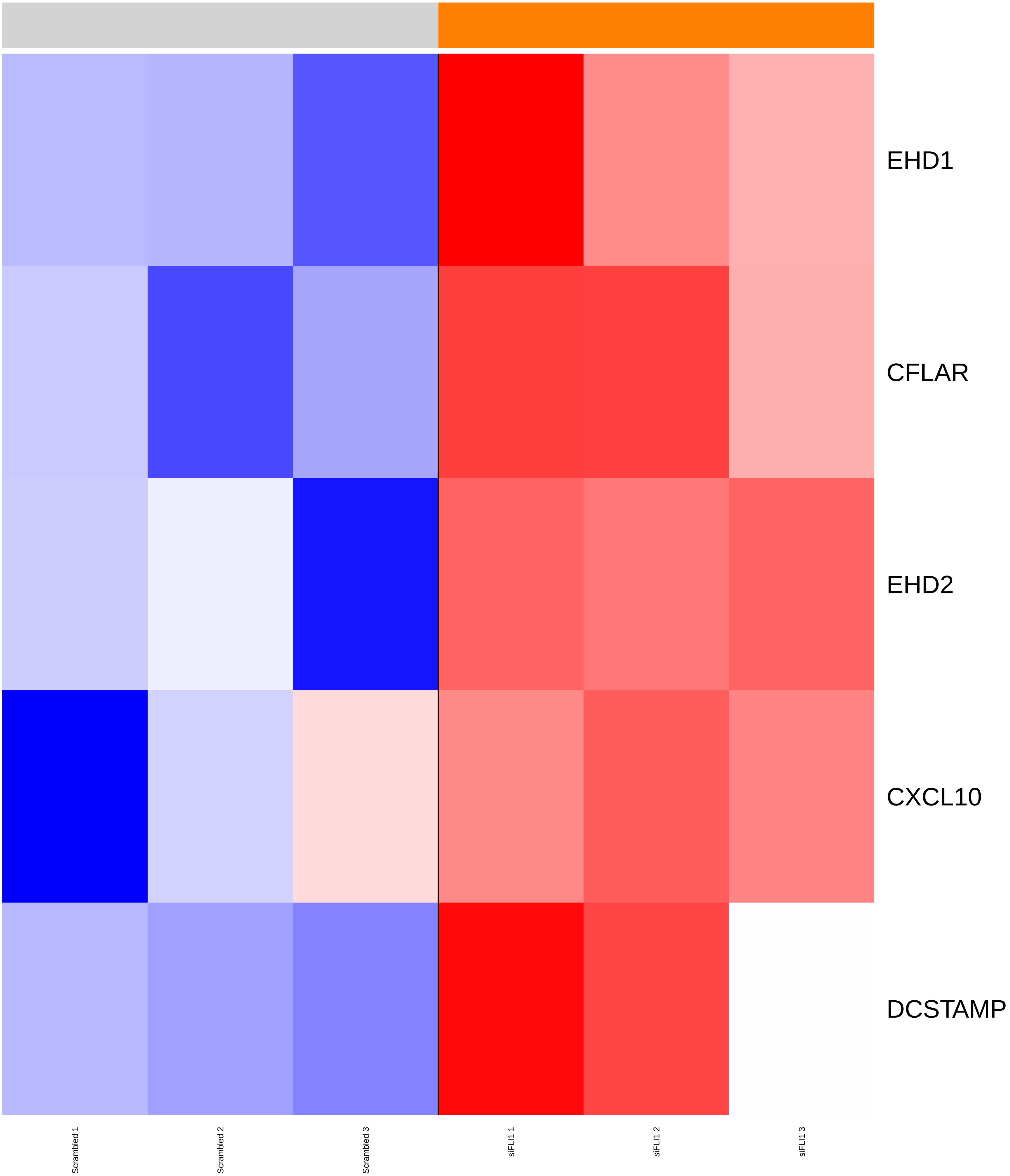

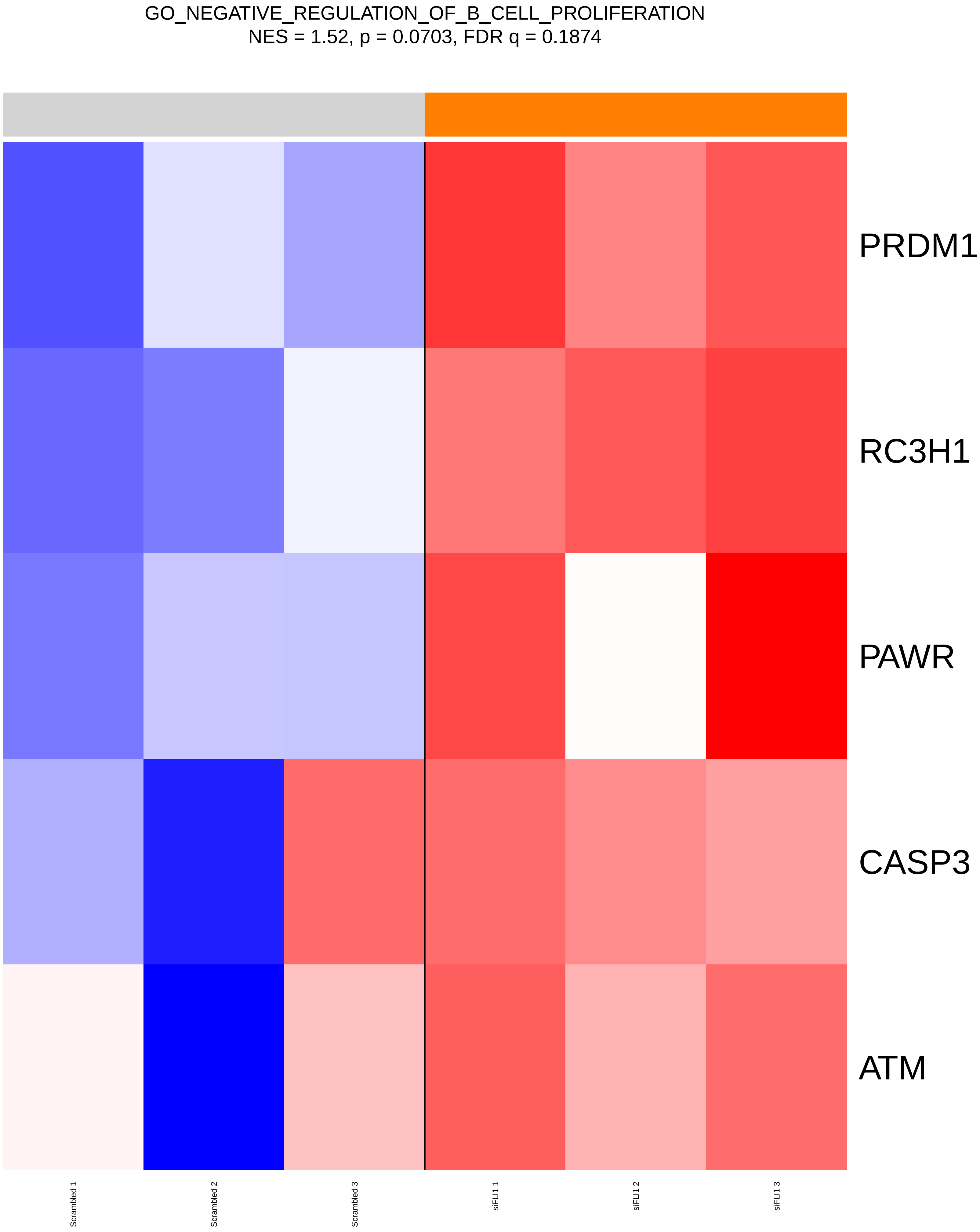
GO_NEGATIVE_REGULATION_OF_B_CELL_PROLIFERATION
NES = 1.52, p = 0.0703, FDR q = 0.1874
PRDM1
RC3H1
PAWR
CASP3
ATM
Scrambled 1
Scrambled 2
Scrambled 3
siFLI1 1
siFLI1 2
siFLI1 3

GO_INFLAMMATORY_RESPONSE
NES = 1.52, p = 0.0000, FDR q = 0.1877

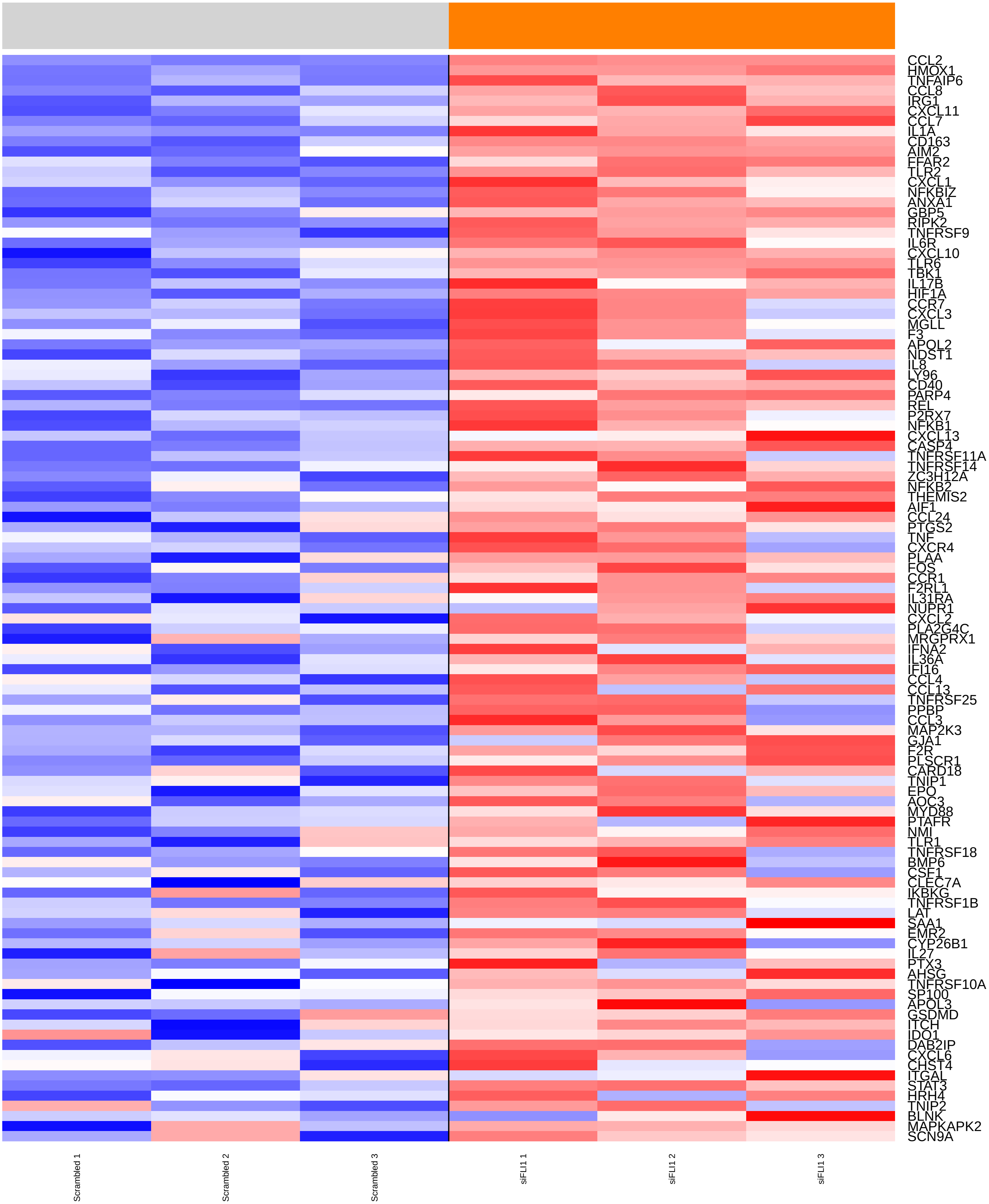

GO_REGULATION_OF_CORTICOSTEROID_HORMONE_SECRETION
NES = 1.52, p = 0.0360, FDR q = 0.1881

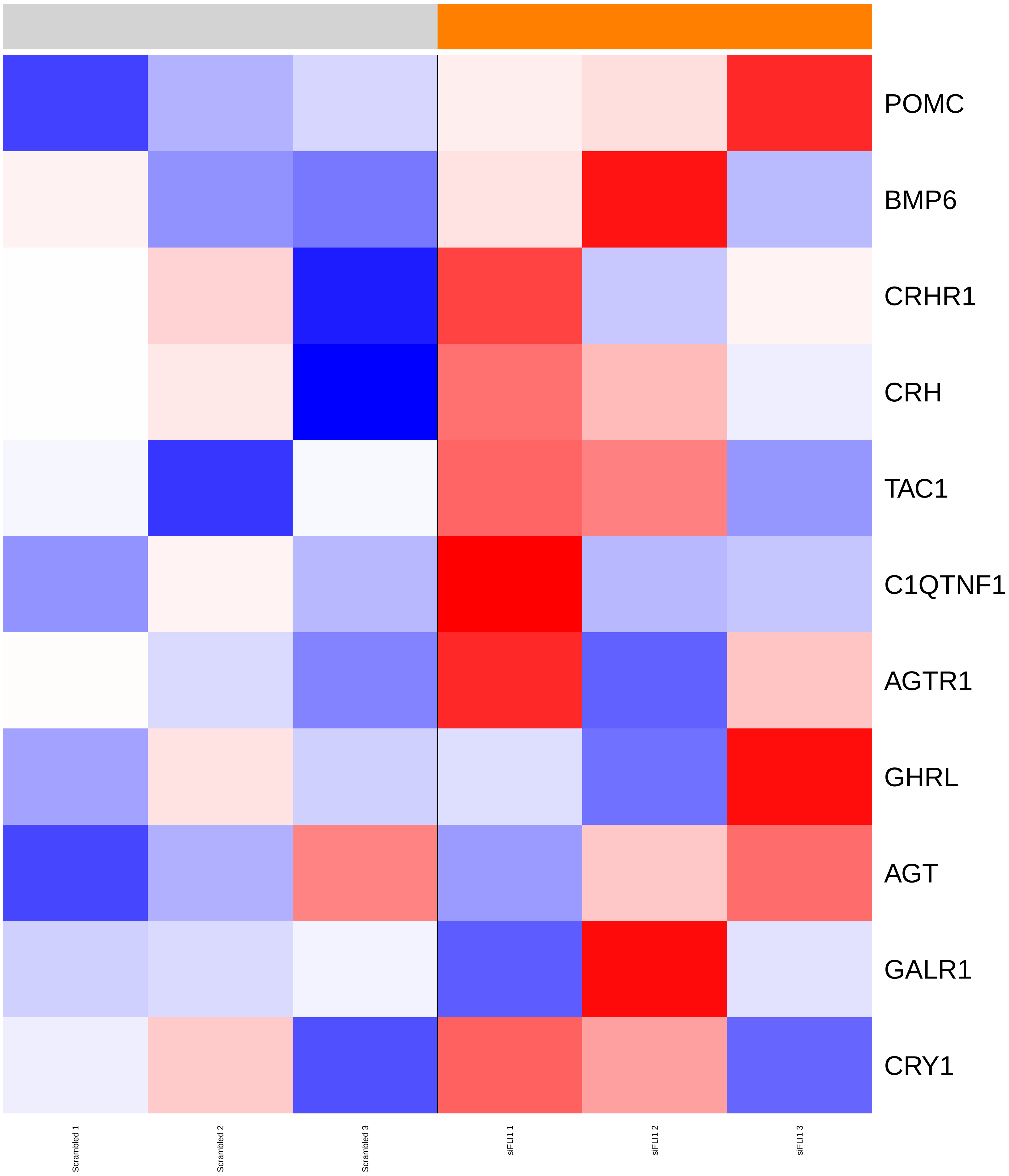

GO_L_AMINO_ACID_TRANSMEMBRANE_TRANSPORTER_ACTIVITY
NES = 1.51, p = 0.0197, FDR q = 0.1890

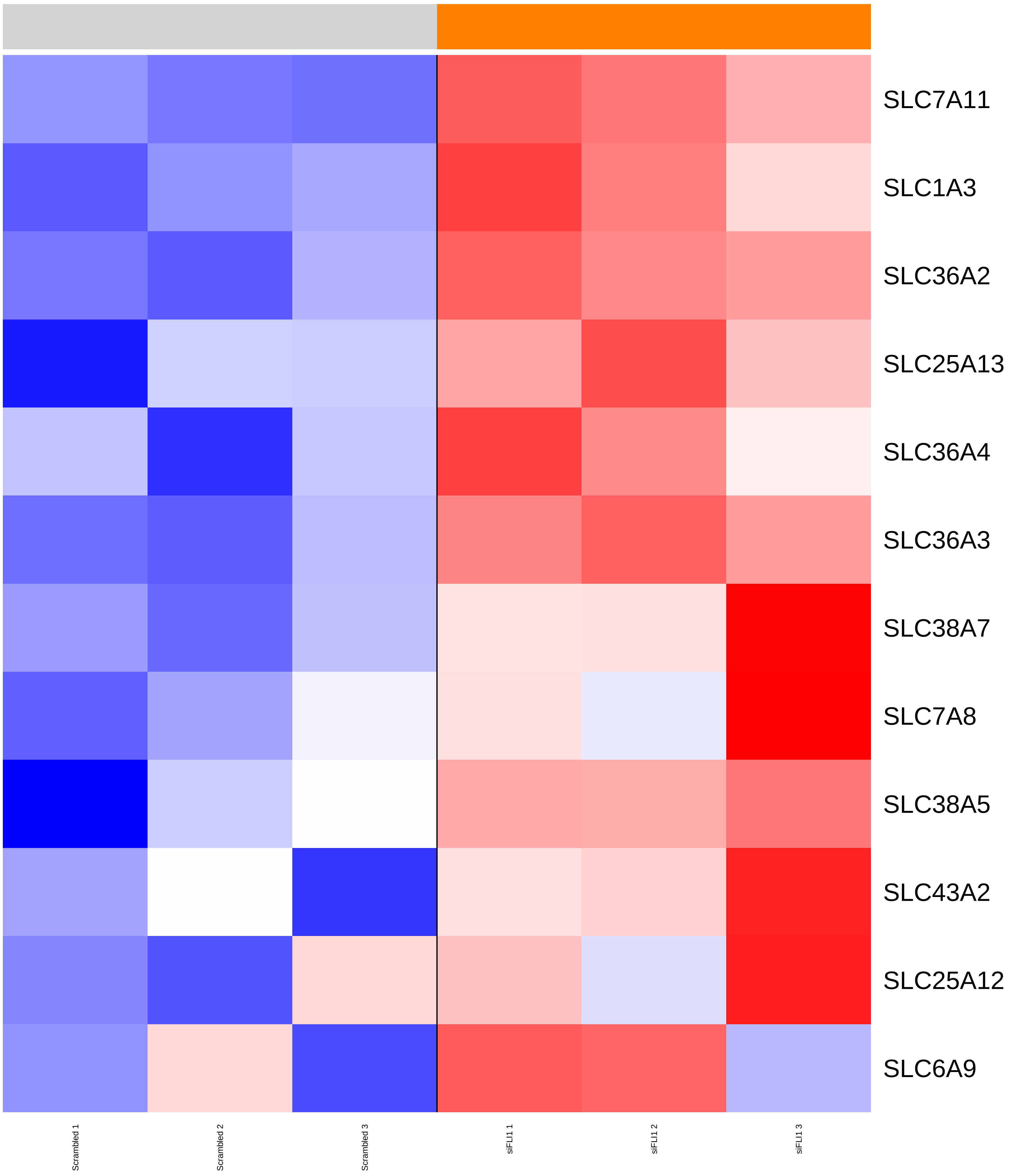

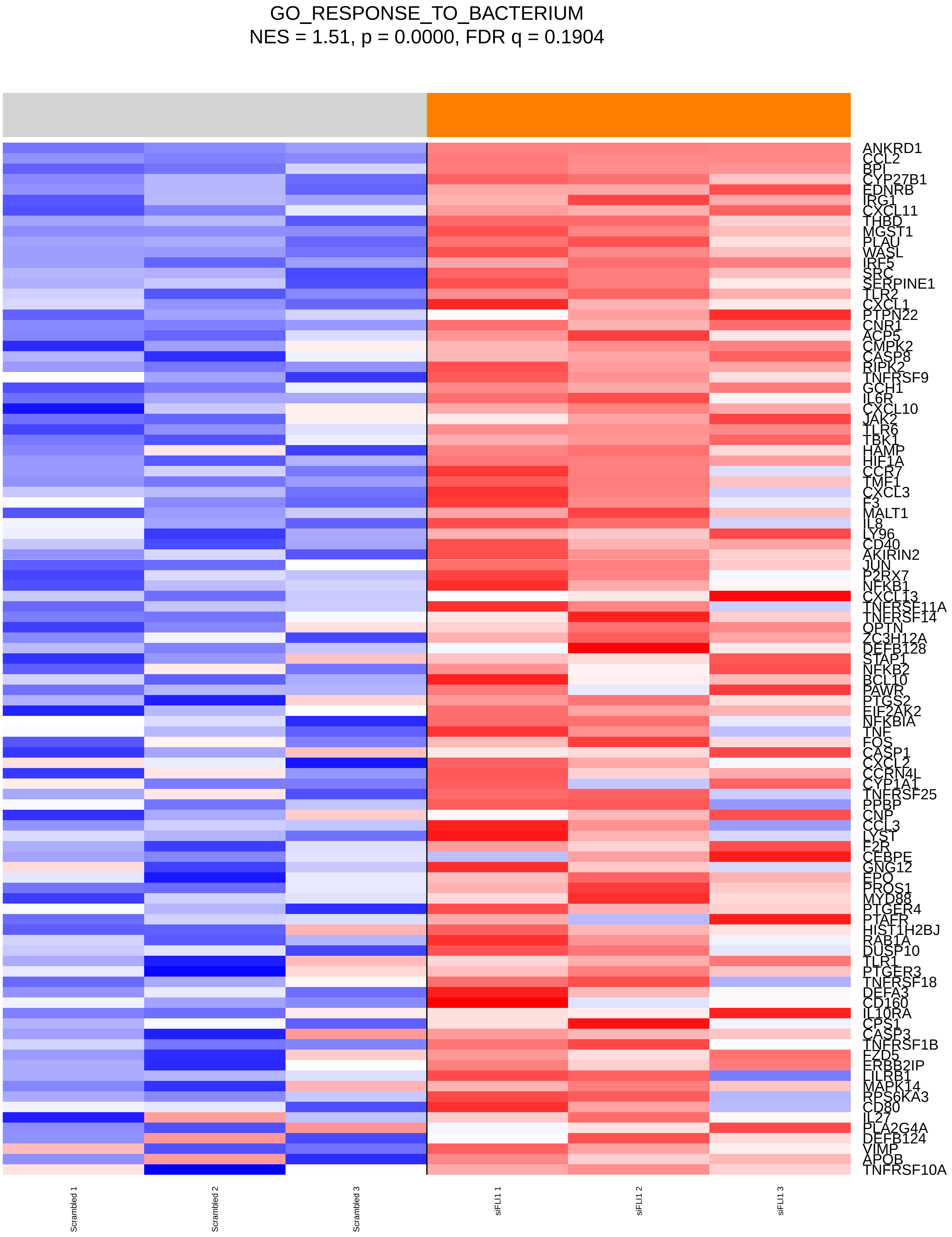
GO_RESPONSE_TO_BACTERIUM
NES = 1.51, p = 0.0000, FDR q = 0.1904
ANKRD1
CCL2
BPI
CYP27B1
EDNRB
IRG1
CXCL11
THBD
MGST1
PLAU
WASL
IRF5
SRC
SERPINE1
TLR2
CXCL1
PTPN22
CNR1
ACP5
CMPK2
CASP8
RIPK2
TNFRSF9
GCH1
IL6R
CXCL10
JAK2
TLR6
TBK1
HAMP
HIF1A
CCR7
TMF1
CXCL3
F3
MALT1
IL8
LY96
CD40
AKIRIN2
JUN
P2RX7
NFKB1
CXCL13
TNFRSF11A
TNFRSF14
OPTN
ZC3H12A
DEFB128
STAP1
NFKB2
BCL10
PAWR
PTGS2
EIF2AK2
NFKBIA
TNF
FOS
CASP1
CXCL2
CCRN4L
CYP1A1
TNFRSF25
PPBP
CNP
CCL3
LYST
F2R
CEBPE
GNG12
EPO
PROS1
MYD88
PTGER4
PTAFR
HIST1H2BJ
RAB1A
DUSP10
TLR1
PTGER3
TNFRSF18
DEFA3
CD160
IL10RA
CPS1
CASP3
TNFRSF1B
FZD5
ERBB2IP
LILRB1
MAPK14
RPS6KA3
CD80
IL27
PLA2G4A
DEFB124
VIMP
APOB
TNFRSF10A
Scrambled 1
Scrambled 2
Scrambled 3
siFLI1 1
siFLI1 2
siFLI1 3

GO_THIOL_DEPENDENT_UBIQUITIN_SPECIFIC_PROTEASE_ACTIVITY
NES = 1.51, p = 0.0202, FDR q = 0.1907

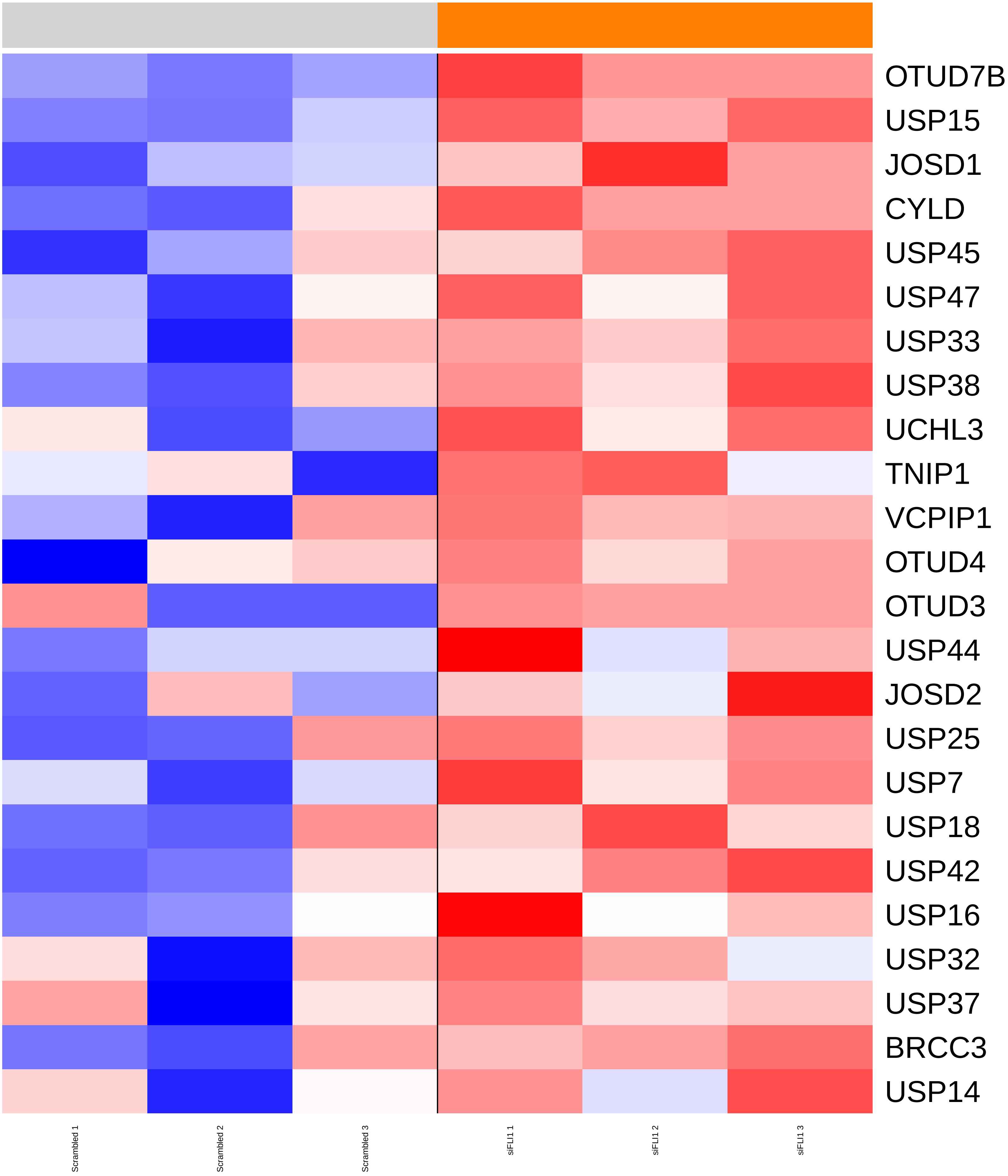

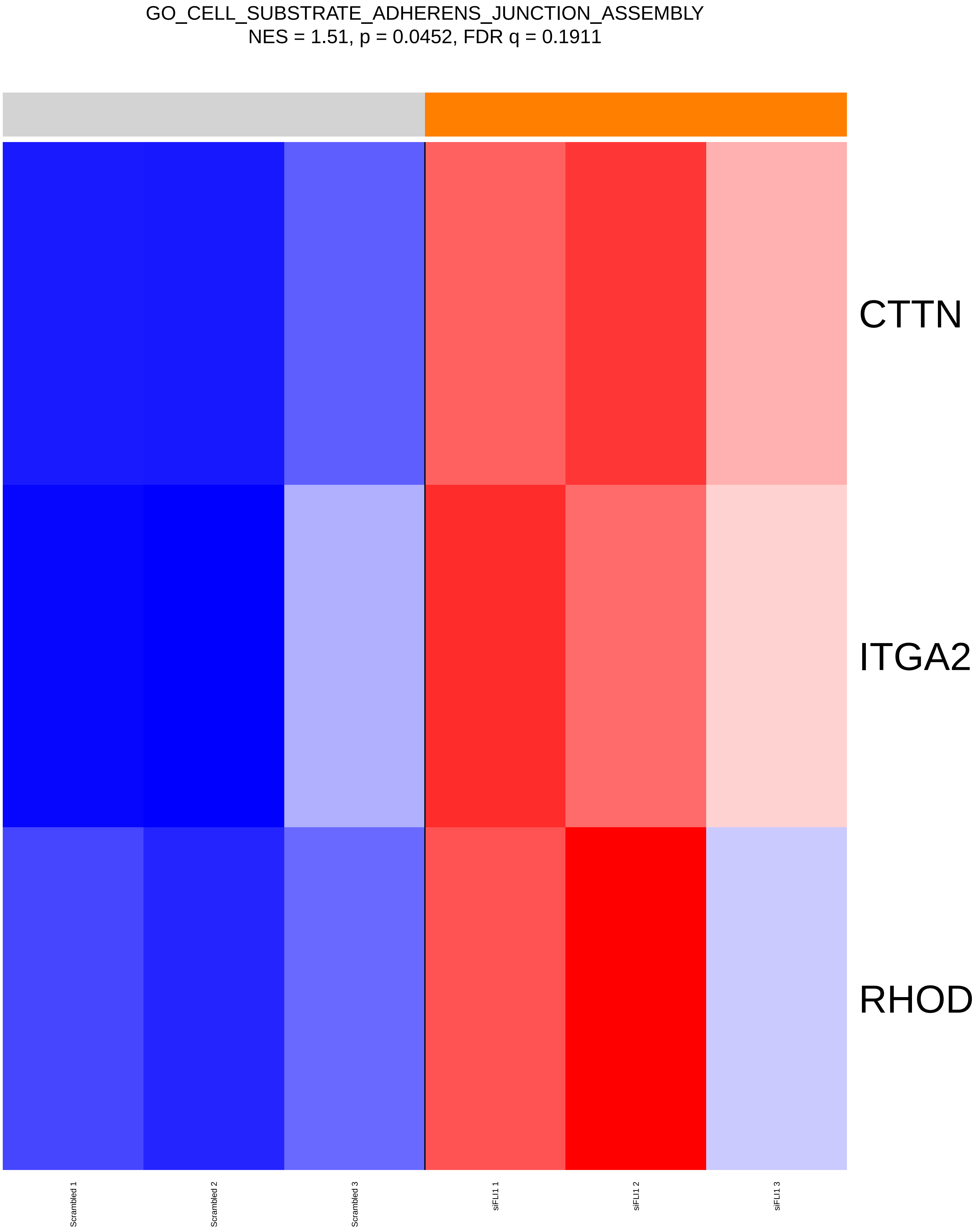
GO_CELL_SUBSTRATE_ADHERENS_JUNCTION_ASSEMBLY
NES = 1.51, p = 0.0452, FDR q = 0.1911
CTTN
ITGA2
RHOD
Scrambled 1
Scrambled 2
Scrambled 3
siFLI1 1
siFLI1 2
siFLI1 3

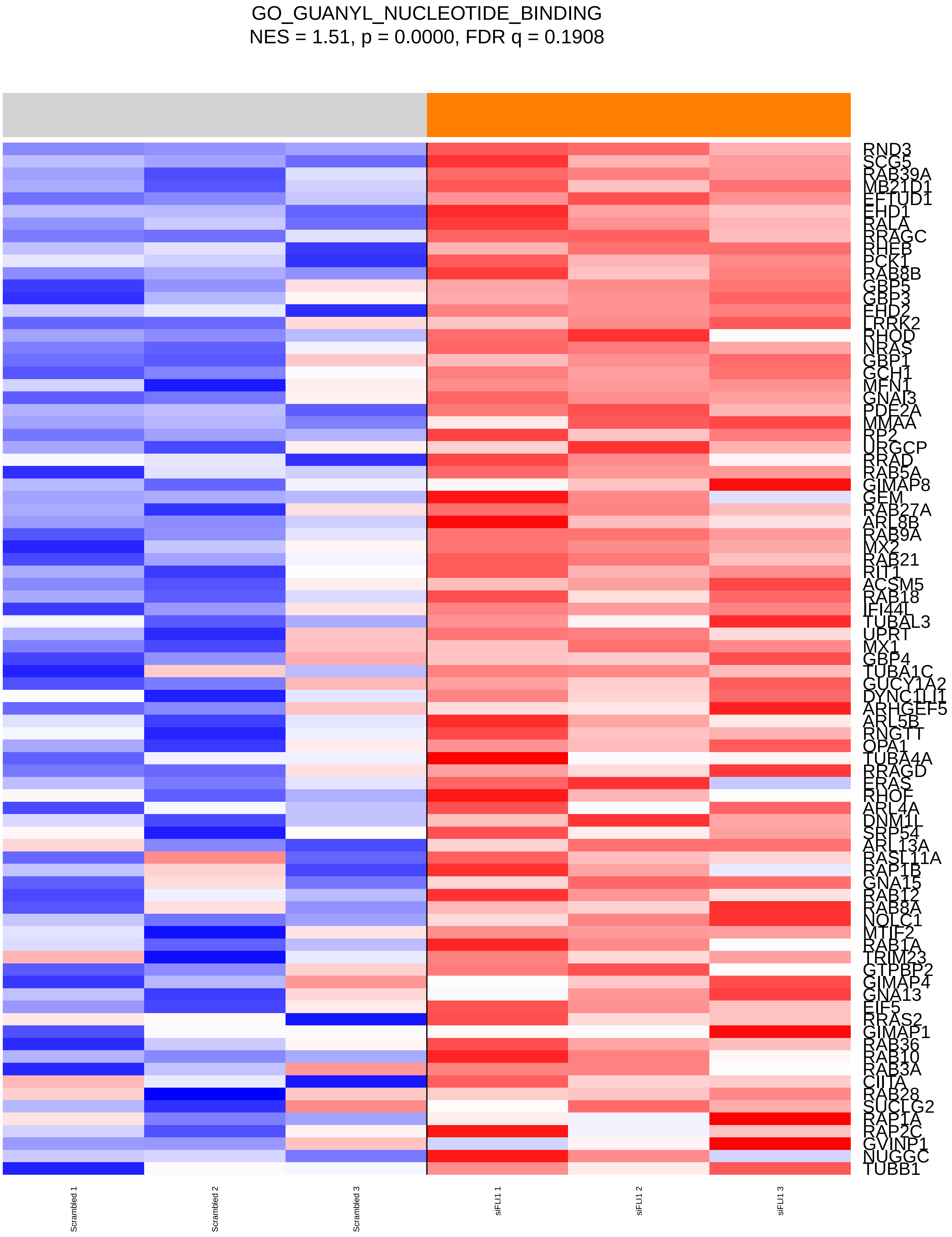
GO_GUANYL_NUCLEOTIDE_BINDING
NES = 1.51, p = 0.0000, FDR q = 0.1908
RND3
SCG5
RAB39A
MB21D1
EFTUD1
EHD1
RALA
RRAGC
RHEB
PCK1
RAB8B
GBP5
GBP3
EHD2
LRRK2
RHOD
NRAS
GBP1
GCH1
MFN1
GNAI3
PDE2A
MMAA
RP2
URGCP
RRAD
RAB5A
GIMAP8
GEM
RAB27A
ARL8B
RAB9A
MX2
RAB21
RIT1
ACSM5
RAB18
IFI44L
TUBAL3
UPRT
MX1
GBP4
TUBA1C
GUCY1A2
DYNC1LI1
ARHGEF5
ARL5B
RNGTT
OPA1
TUBA4A
RRAGD
ERAS
RHOF
ARL4A
DNM1L
SRP54
ARL13A
RASL11A
RAP1B
GNA15
RAB12
RAB8A
NOLC1
MTIF2
RAB1A
TRIM23
GTPBP2
GIMAP4
GNA13
EIF5
RRAS2
GIMAP1
RAB36
RAB10
RAB3A
CIITA
RAB28
SUCLG2
RAP1A
RAP2C
GVINP1
NUGGC
TUBB1
Scrambled 1
Scrambled 2
Scrambled 3
siFLI1 1
siFLI1 2
siFLI1 3

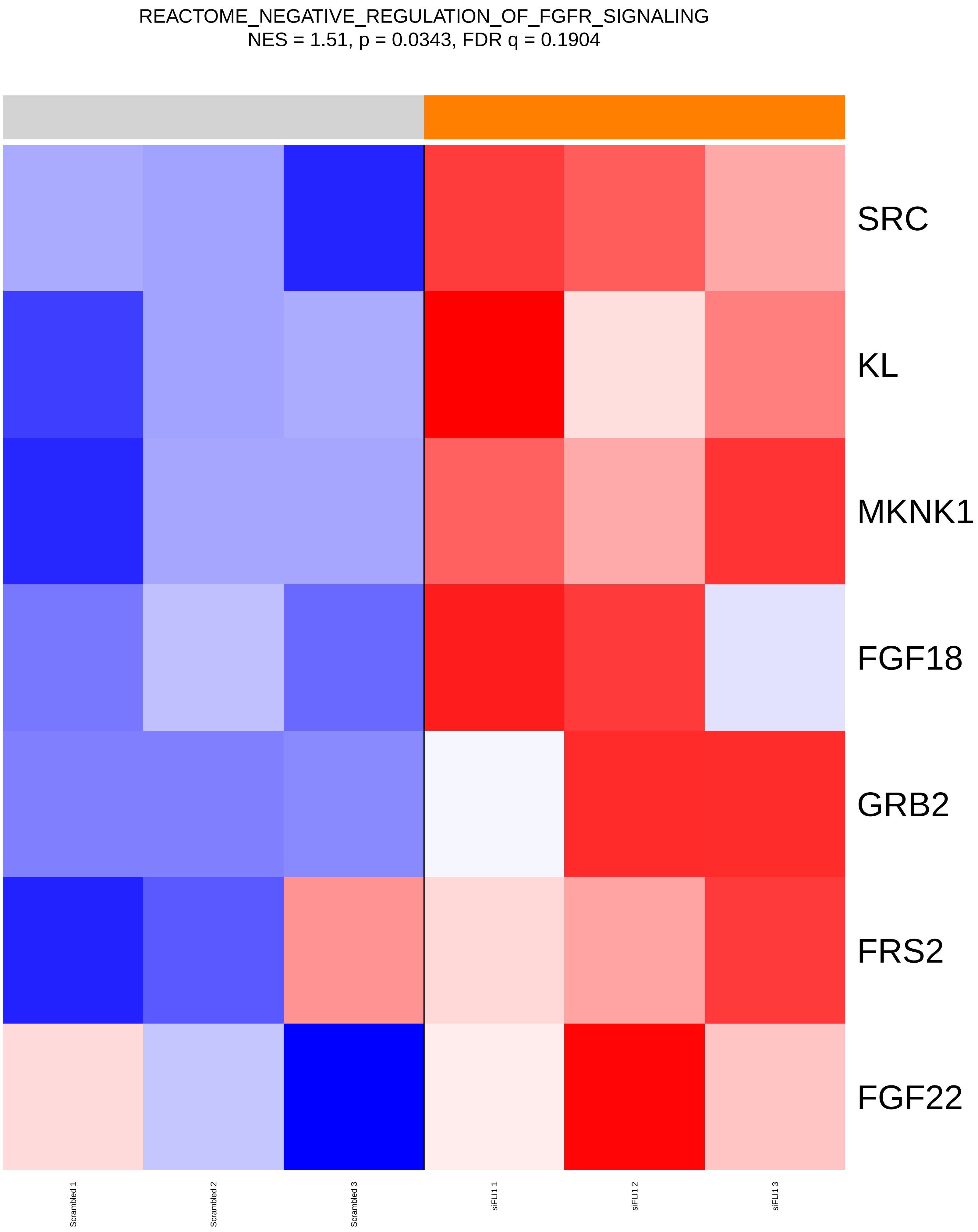

REACTOME_NEGATIVE_REGULATION_OF_FGFR_SIGNALING
NES = 1.51, p = 0.0343, FDR q = 0.1904
SRC
KL
MKNK1
FGF18
GRB2
FRS2
FGF22
Scrambled 1
Scrambled 2
Scrambled 3
siFLI1 1
siFLI1 2
siFLI1 3

ATTCTTT_MIR186
NES = 1.51, p = 0.0000, FDR q = 0.1899

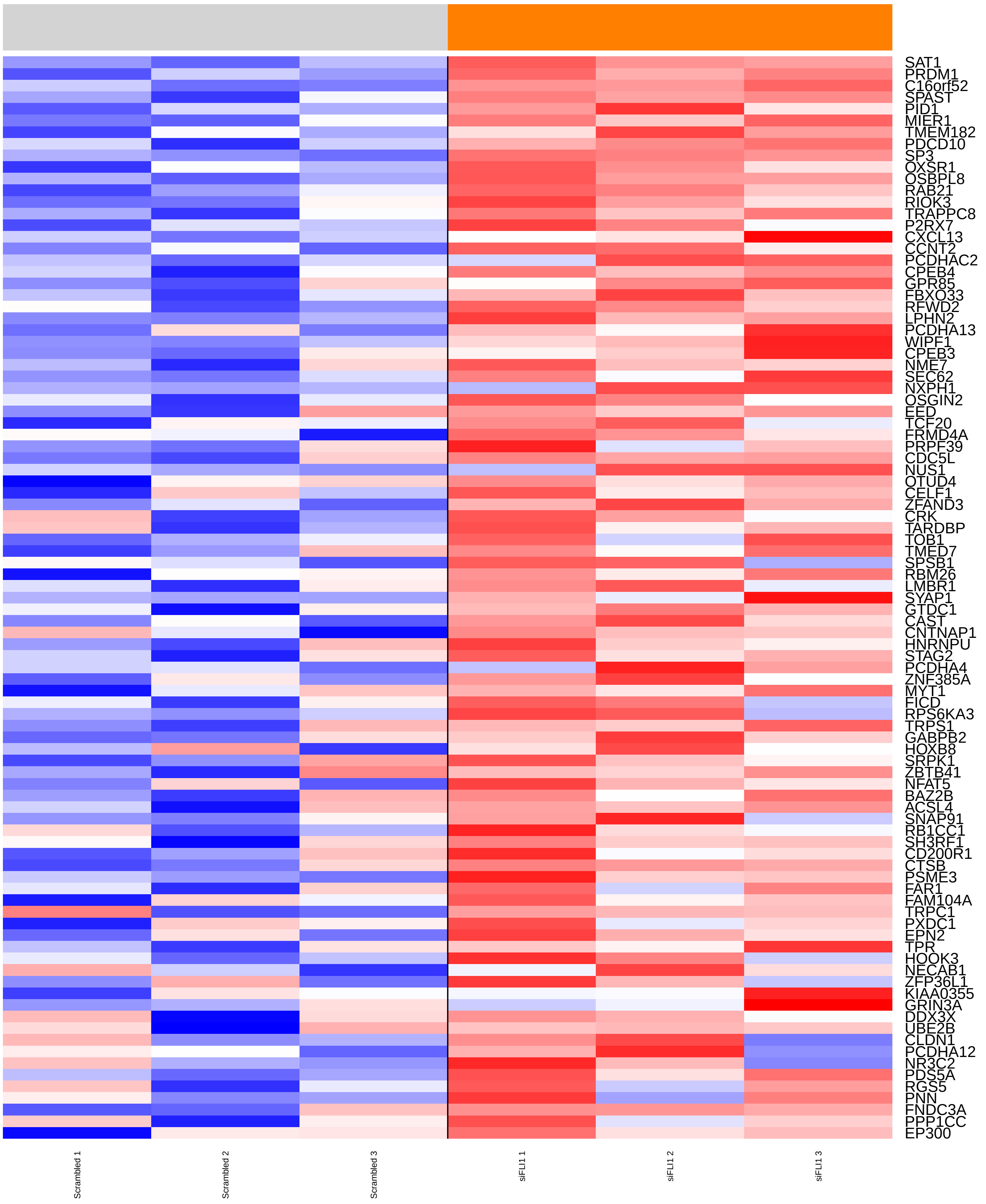

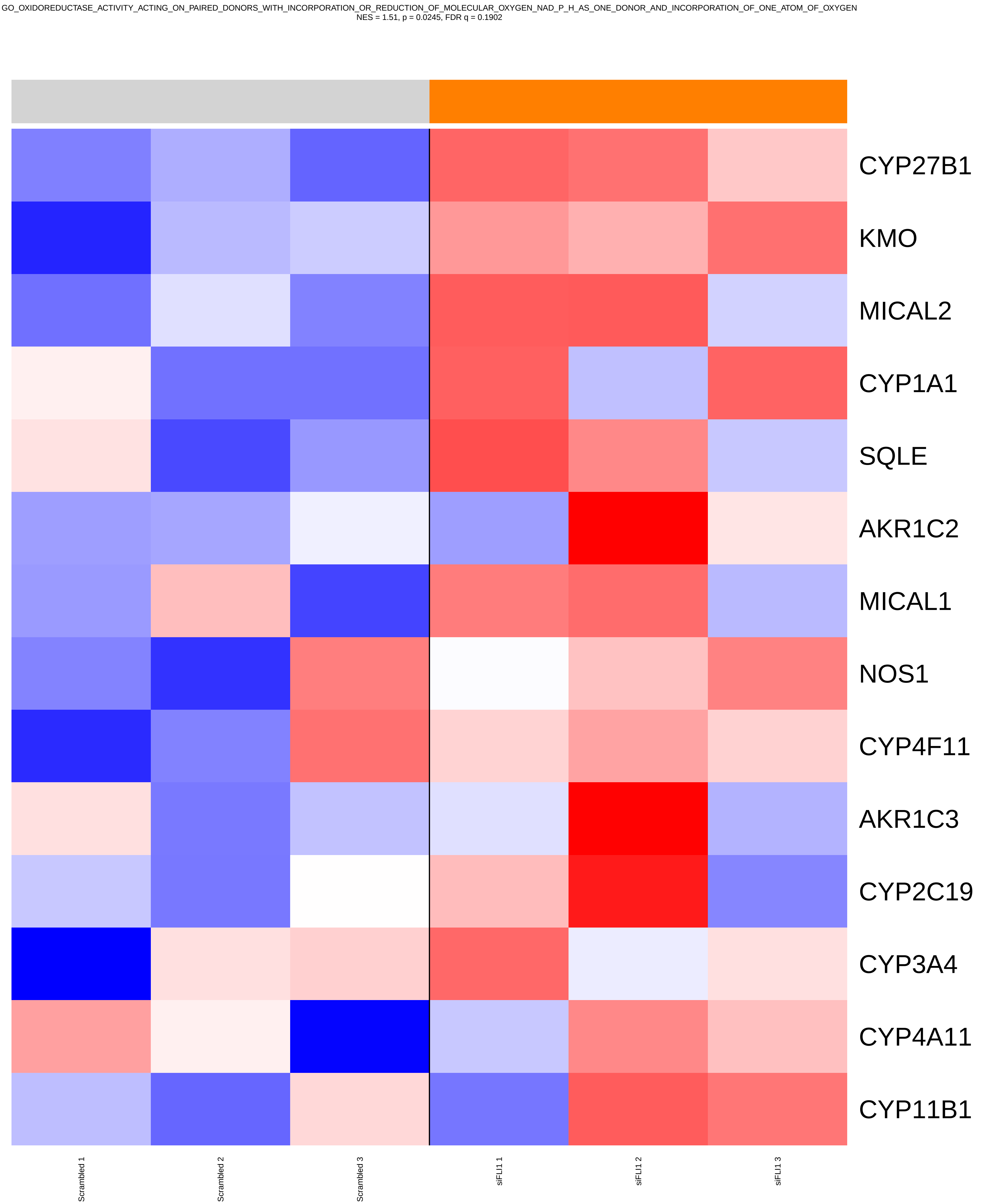
GO_OXIDOREDUCTASE_ACTIVITY_ACTING_ON_PAIRED_DONORS_WITH_INCORPORATION_OR_REDUCTION_OF_MOLECULAR_OXYGEN_NAD_P_H_AS_ONE_DONOR_AND_INCORPORATION_OF_ONE_ATOM_OF_OXYGEN
NES = 1.51, p = 0.0245, FDR q = 0.1902
CYP27B1
KMO
MICAL2
CYP1A1
SQLE
AKR1C2
MICAL1
NOS1
CYP4F11
AKR1C3
CYP2C19
CYP3A4
CYP4A11
CYP11B1
Scrambled 1
Scrambled 2
Scrambled 3
siFLI1 1
siFLI1 2
siFLI1 3

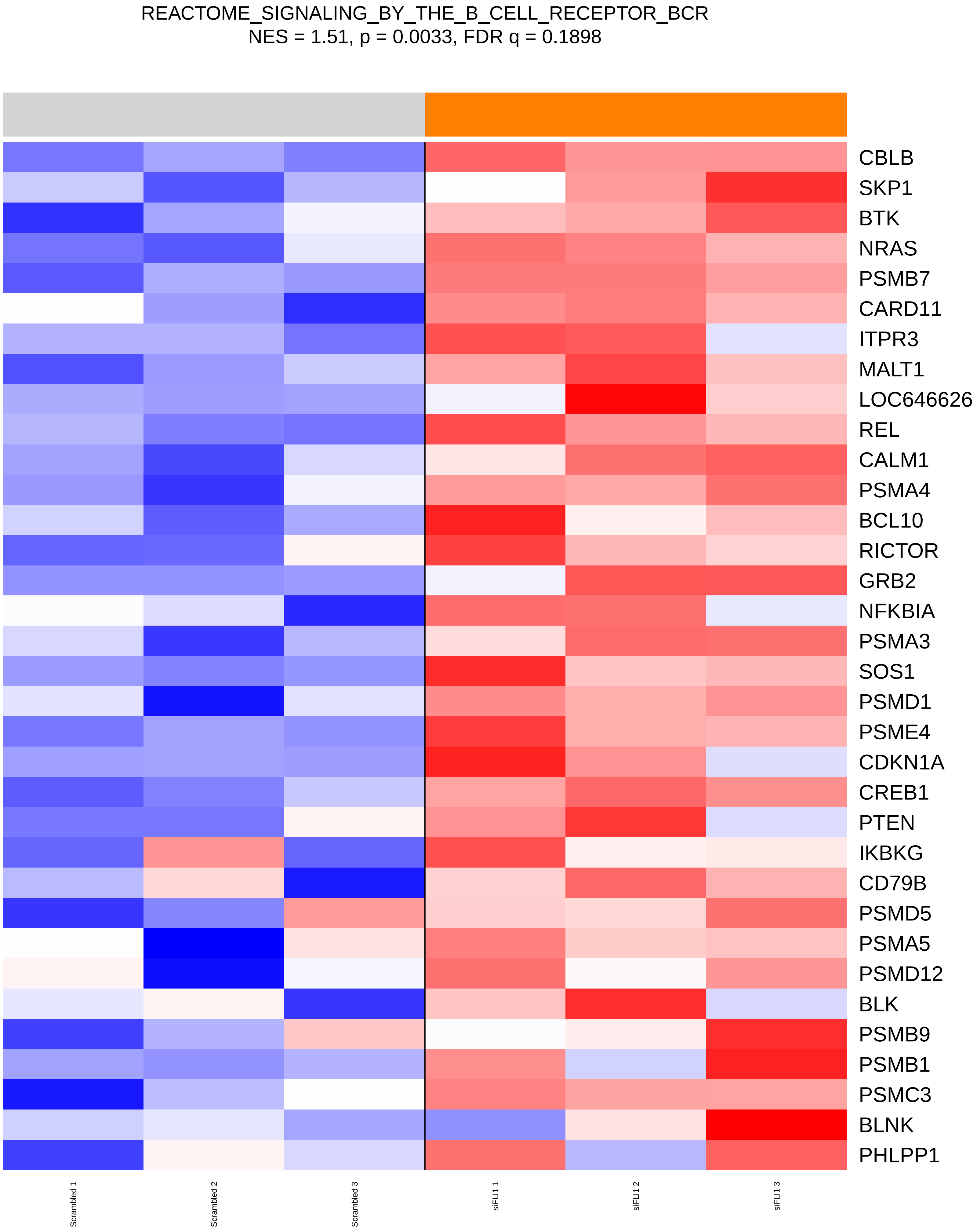
REACTOME_SIGNALING_BY_THE_B_CELL_RECEPTOR_BCR
NES = 1.51, p = 0.0033, FDR q = 0.1898
CBLB
SKP1
BTK
NRAS
PSMB7
CARD11
ITPR3
MALT1
LOC646626
REL
CALM1
PSMA4
BCL10
RICTOR
GRB2
NFKBIA
PSMA3
SOS1
PSMD1
PSME4
CDKN1A
CREB1
PTEN
IKBKG
CD79B
PSMD5
PSMA5
PSMD12
BLK
PSMB9
PSMB1
PSMC3
BLNK
PHLPP1
Scrambled 1
Scrambled 2
Scrambled 3
siFLI1 1
siFLI1 2
siFLI1 3

GO_ENDOSOMAL_PART
NES = 1.51, p = 0.0000, FDR q = 0.1893

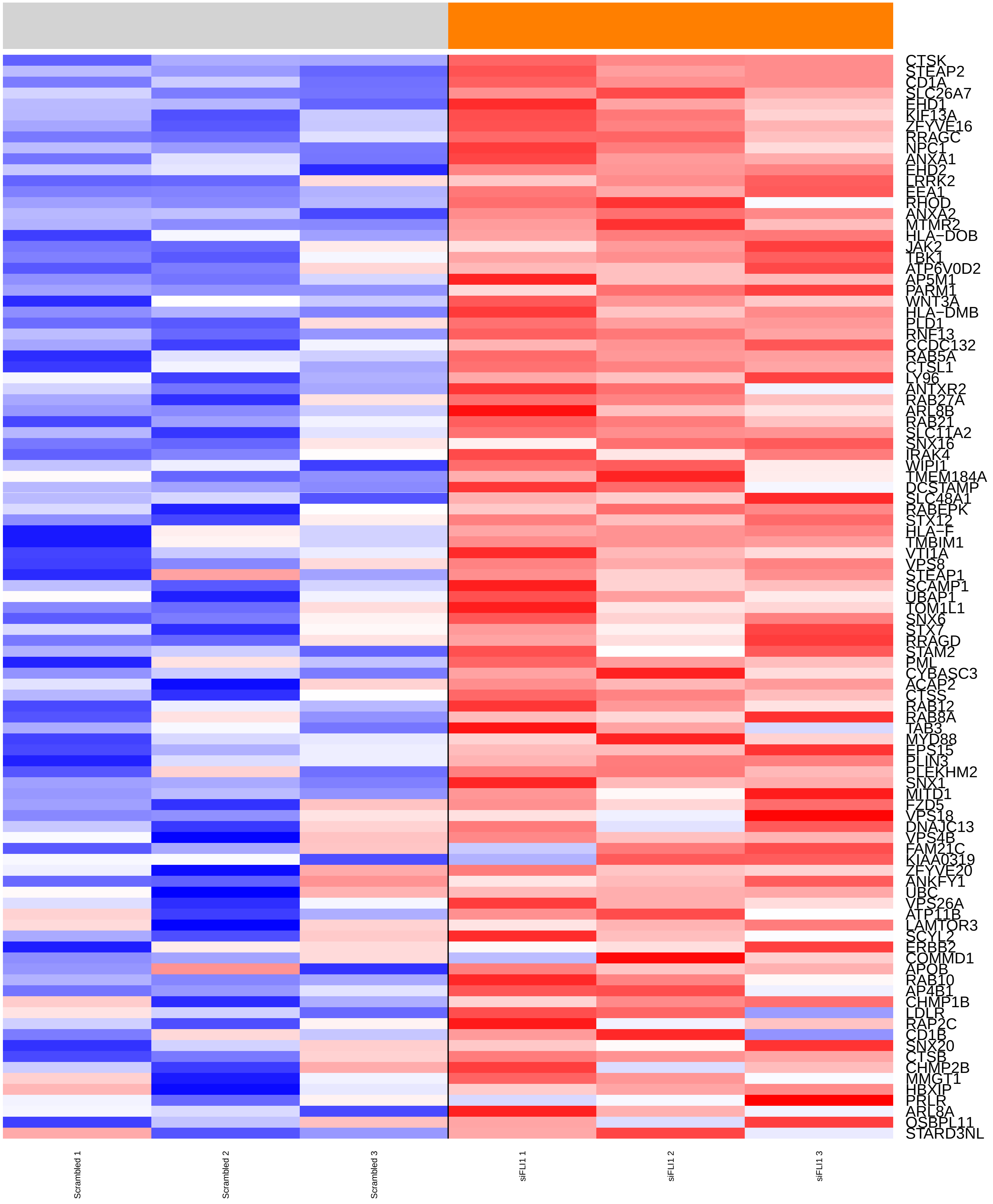

KEGG_RENAL_CELL_CARCINOMA
NES = 1.51, p = 0.0143, FDR q = 0.1890

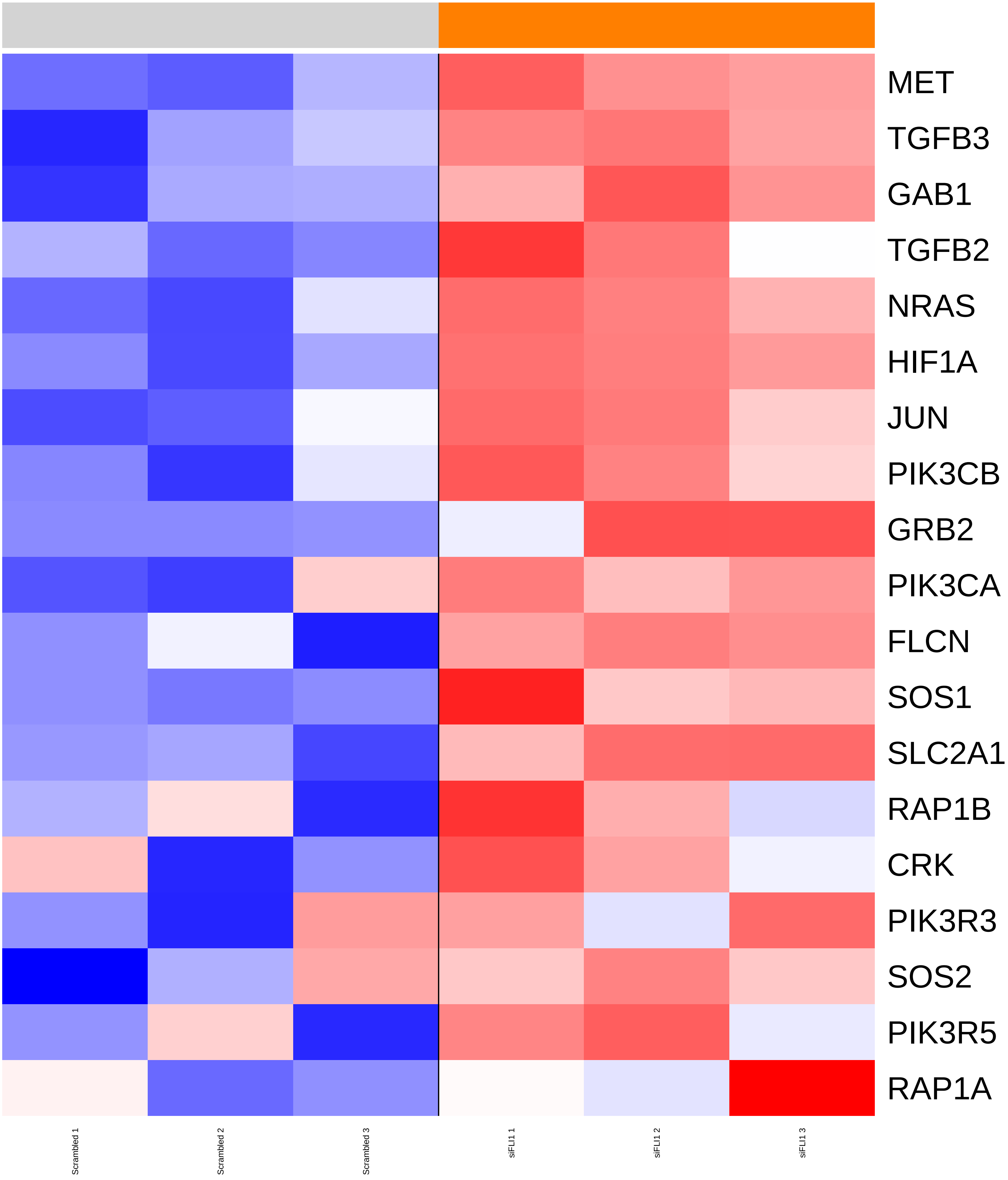

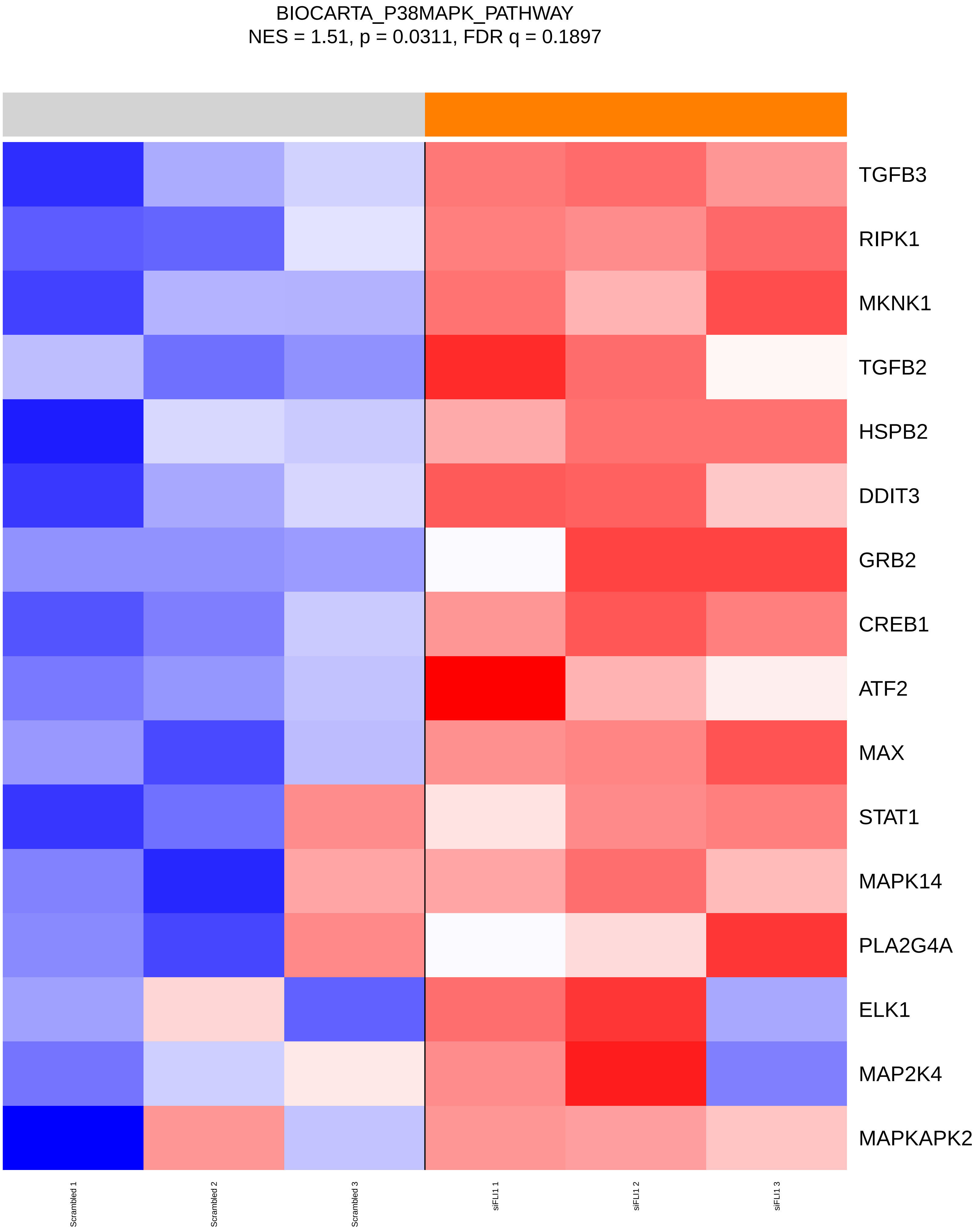

BIOCARTA_P38MAPK_PATHWAY
NES = 1.51, p = 0.0311, FDR q = 0.1897
TGFB3
RIPK1
MKNK1
TGFB2
HSPB2
DDIT3
GRB2
CREB1
ATF2
MAX
STAT1
MAPK14
PLA2G4A
ELK1
MAP2K4
MAPKAPK2
Scrambled 1
Scrambled 2
Scrambled 3
siFLI1 1
siFLI1 2
siFLI1 3

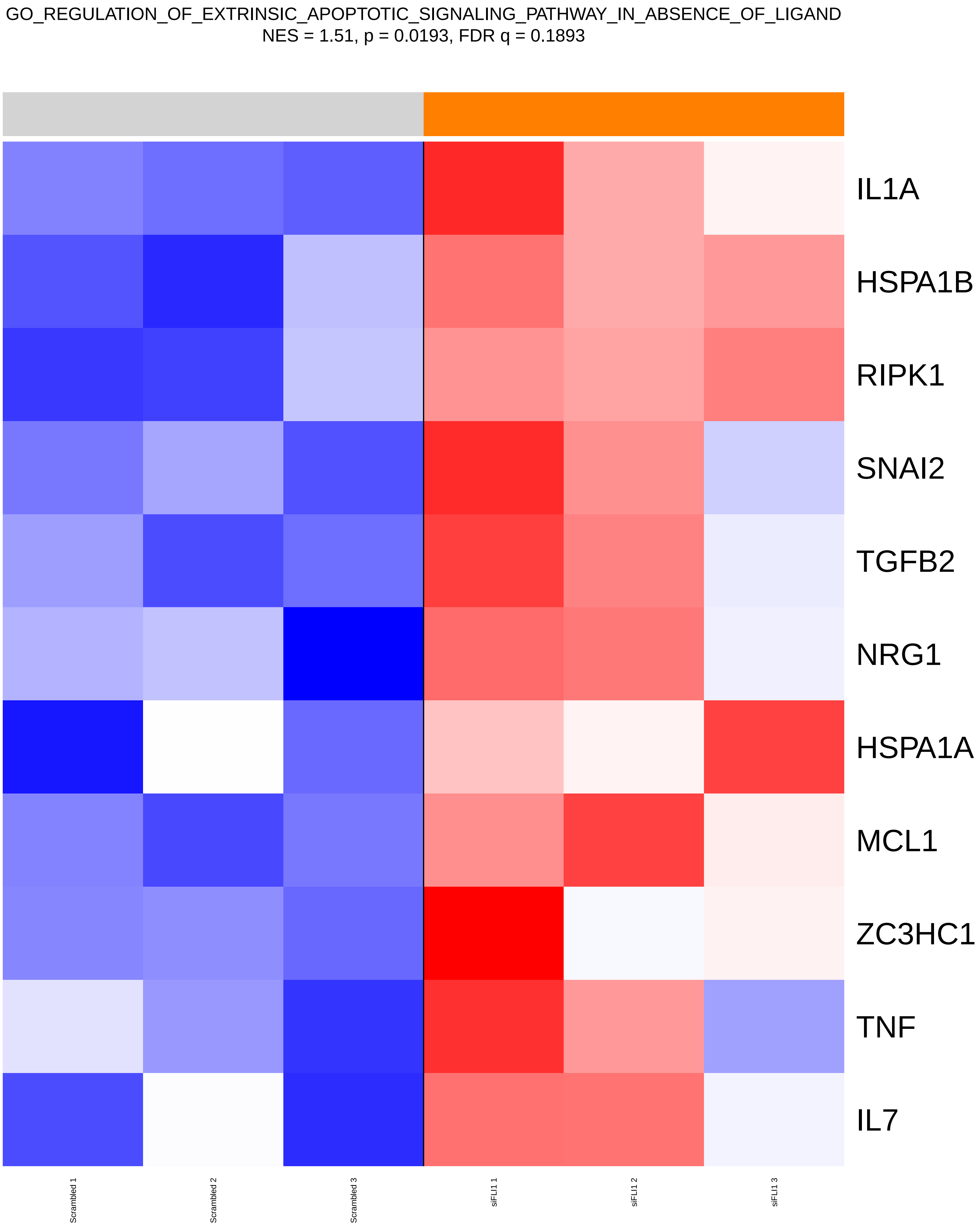
GO_REGULATION_OF_EXTRINSIC_APOPTOTIC_SIGNALING_PATHWAY_IN_ABSENCE_OF_LIGAND
NES = 1.51, p = 0.0193, FDR q = 0.1893
IL1A
HSPA1B
RIPK1
SNAI2
TGFB2
NRG1
HSPA1A
MCL1
ZC3HC1
TNF
IL7
Scrambled 1
Scrambled 2
Scrambled 3
siFLI1 1
siFLI1 2
siFLI1 3

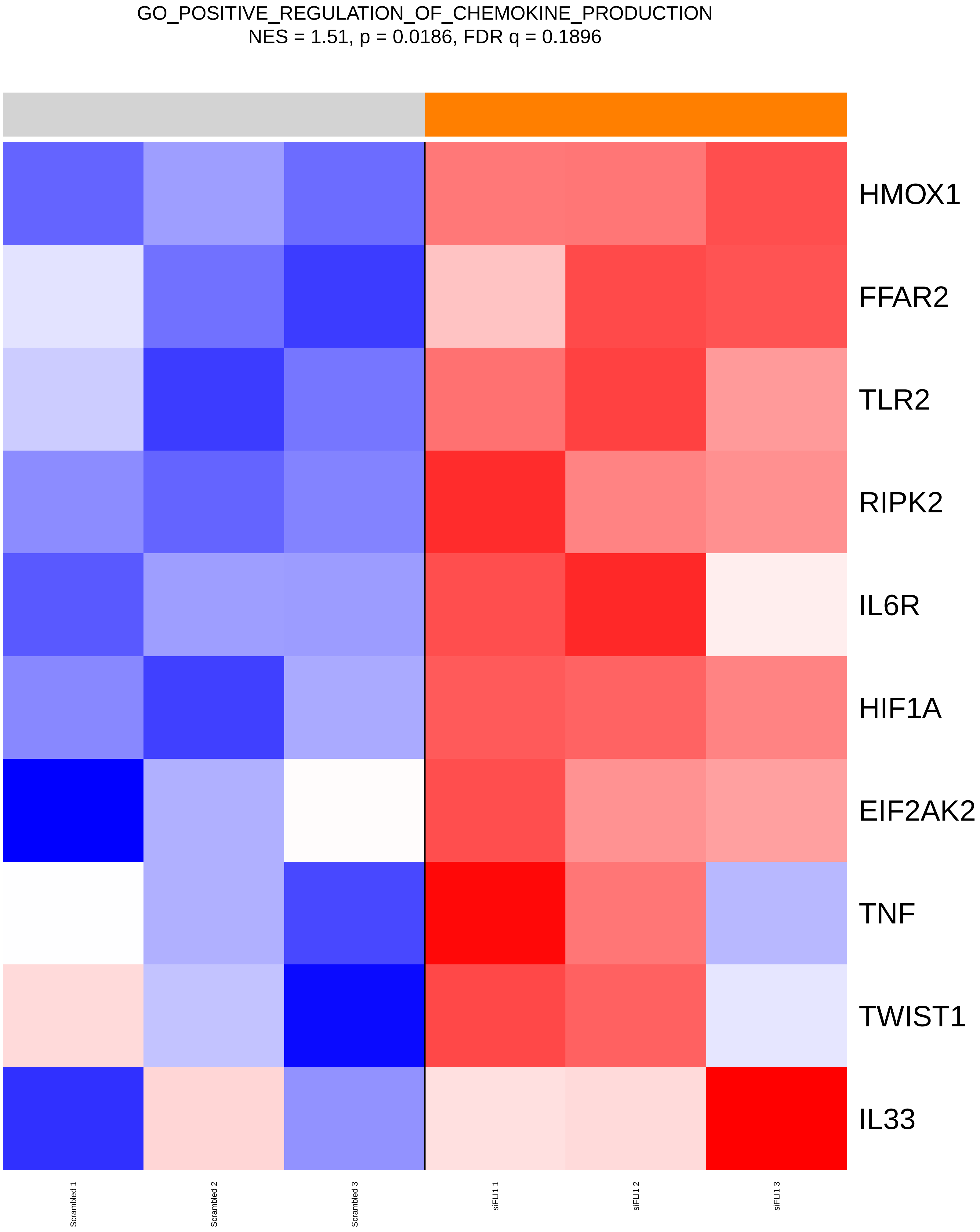
GO_POSITIVE_REGULATION_OF_CHEMOKINE_PRODUCTION
NES = 1.51, p = 0.0186, FDR q = 0.1896
HMOX1
FFAR2
TLR2
RIPK2
IL6R
HIF1A
EIF2AK2
TNF
TWIST1
IL33
Scrambled 1
Scrambled 2
Scrambled 3
siFLI1 1
siFLI1 2
siFLI1 3

GO_INNATE_IMMUNE_RESPONSE_ACTIVATING_CELL_SURFACE_RECEPTOR_SIGNALING_PATHWAY
NES = 1.51, p = 0.0000, FDR q = 0.1903

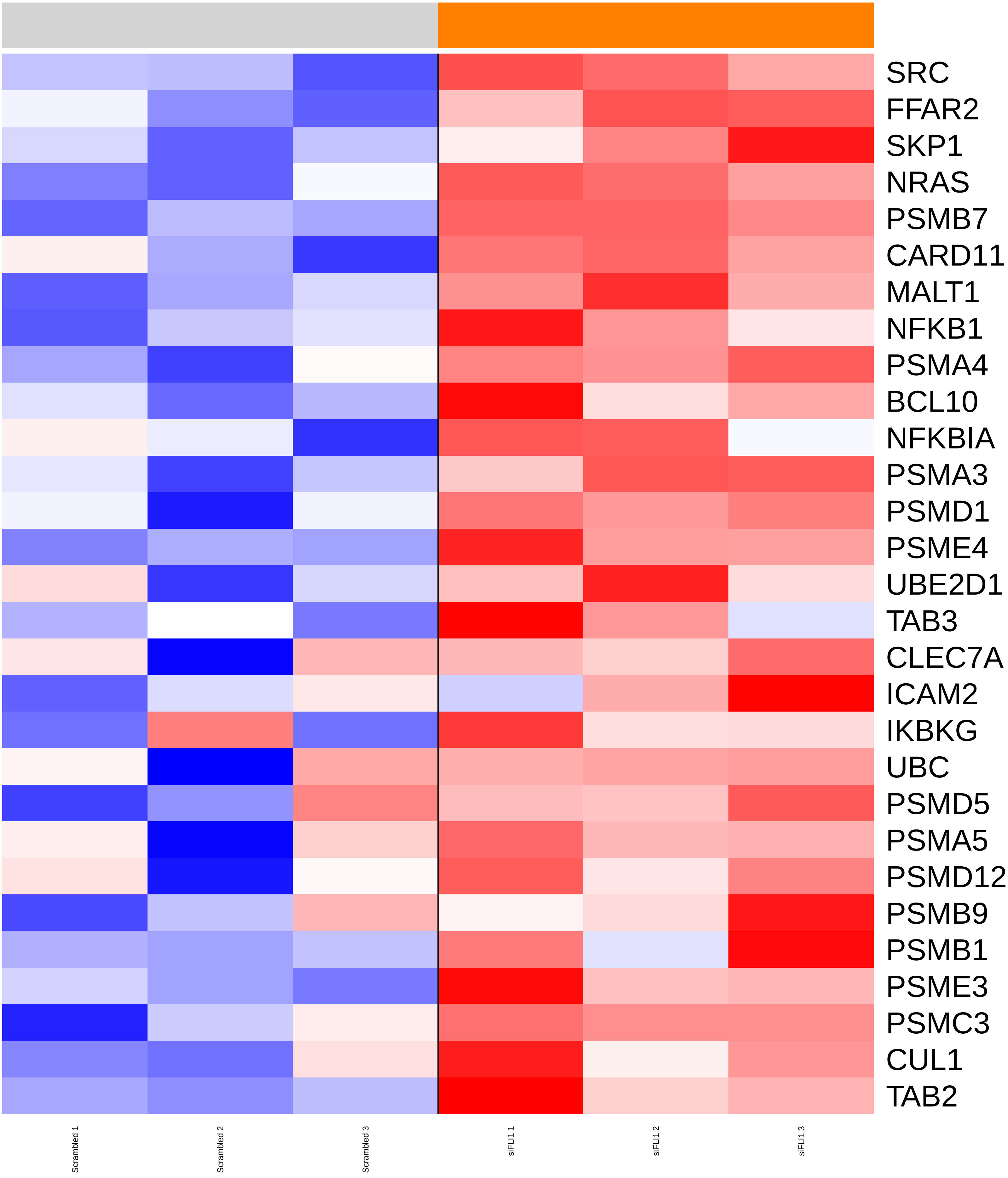

REACTOME_INFLAMMASOMES
NES = 1.51, p = 0.0475, FDR q = 0.1899

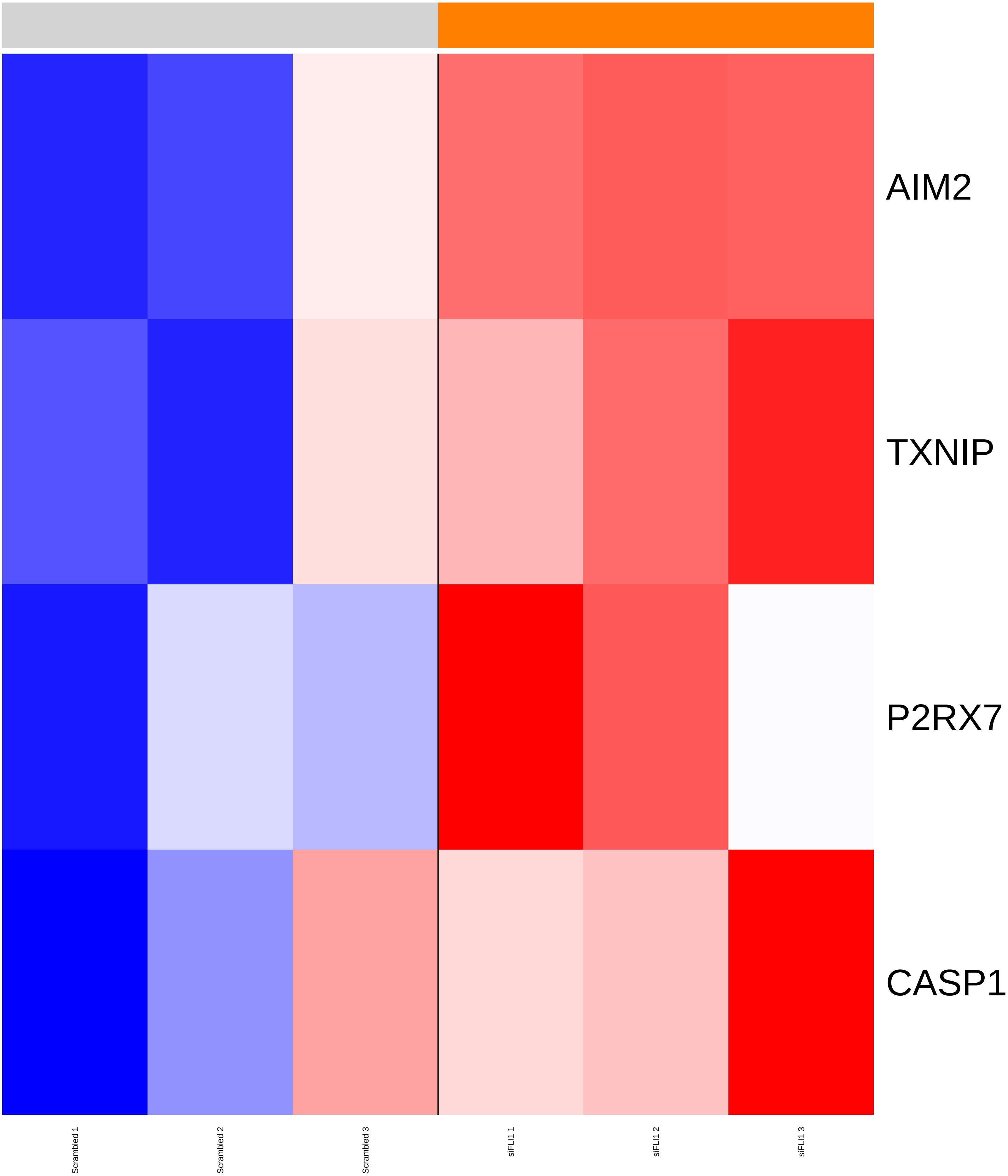

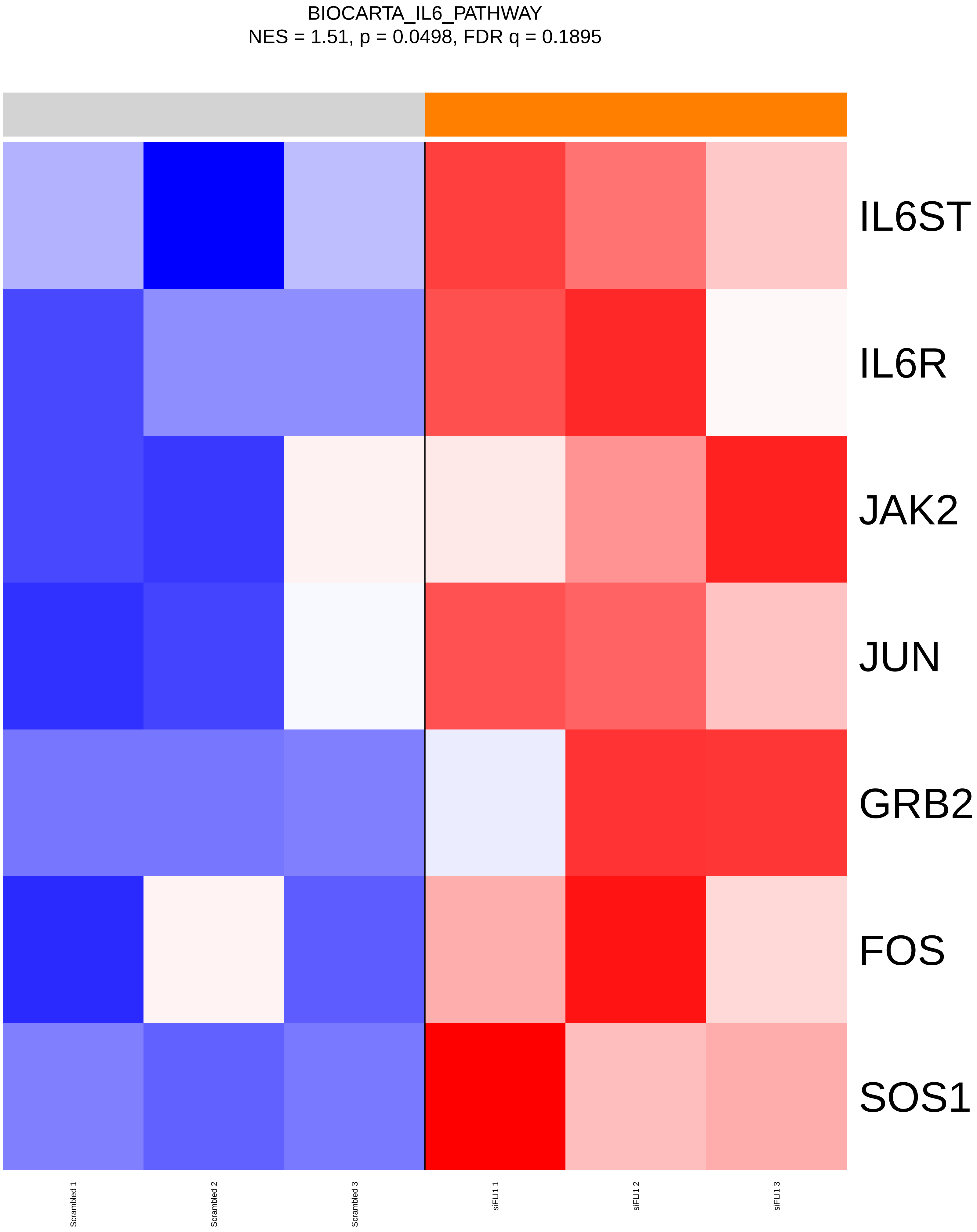
BIOCARTA_IL6_PATHWAY
NES = 1.51, p = 0.0498, FDR q = 0.1895
IL6ST
IL6R
JAK2
JUN
GRB2
FOS
SOS1
Scrambled 1
Scrambled 2
Scrambled 3
siFLI1 1
siFLI1 2
siFLI1 3

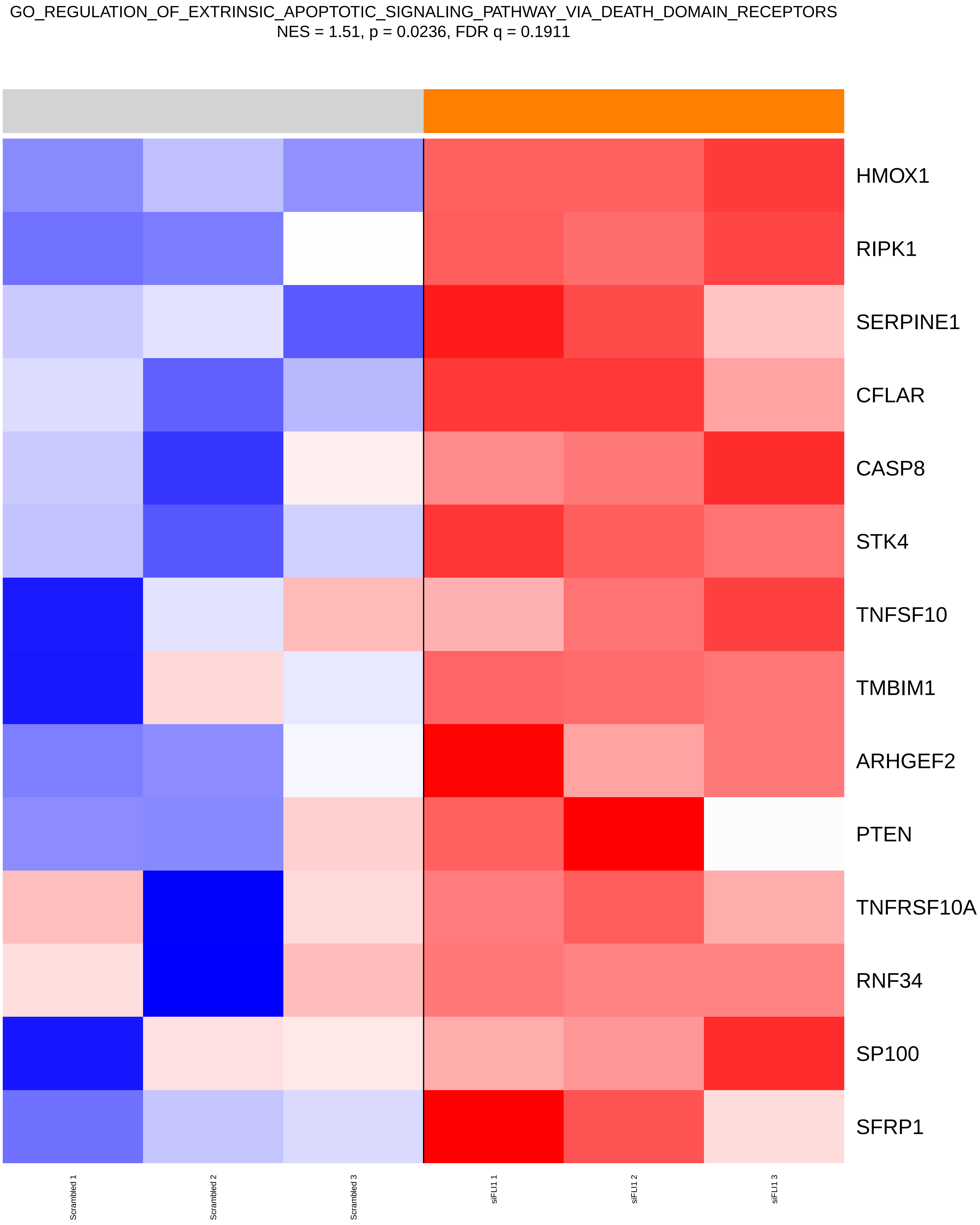
GO_REGULATION_OF_EXTRINSIC_APOPTOTIC_SIGNALING_PATHWAY_VIA_DEATH_DOMAIN_RECEPTORS
NES = 1.51, p = 0.0236, FDR q = 0.1911
HMOX1
RIPK1
SERPINE1
CFLAR
CASP8
STK4
TNFSF10
TMBIM1
ARHGEF2
PTEN
TNFRSF10A
RNF34
SP100
SFRP1
Scrambled 1
Scrambled 2
Scrambled 3
siFLI1 1
siFLI1 2
siFLI1 3

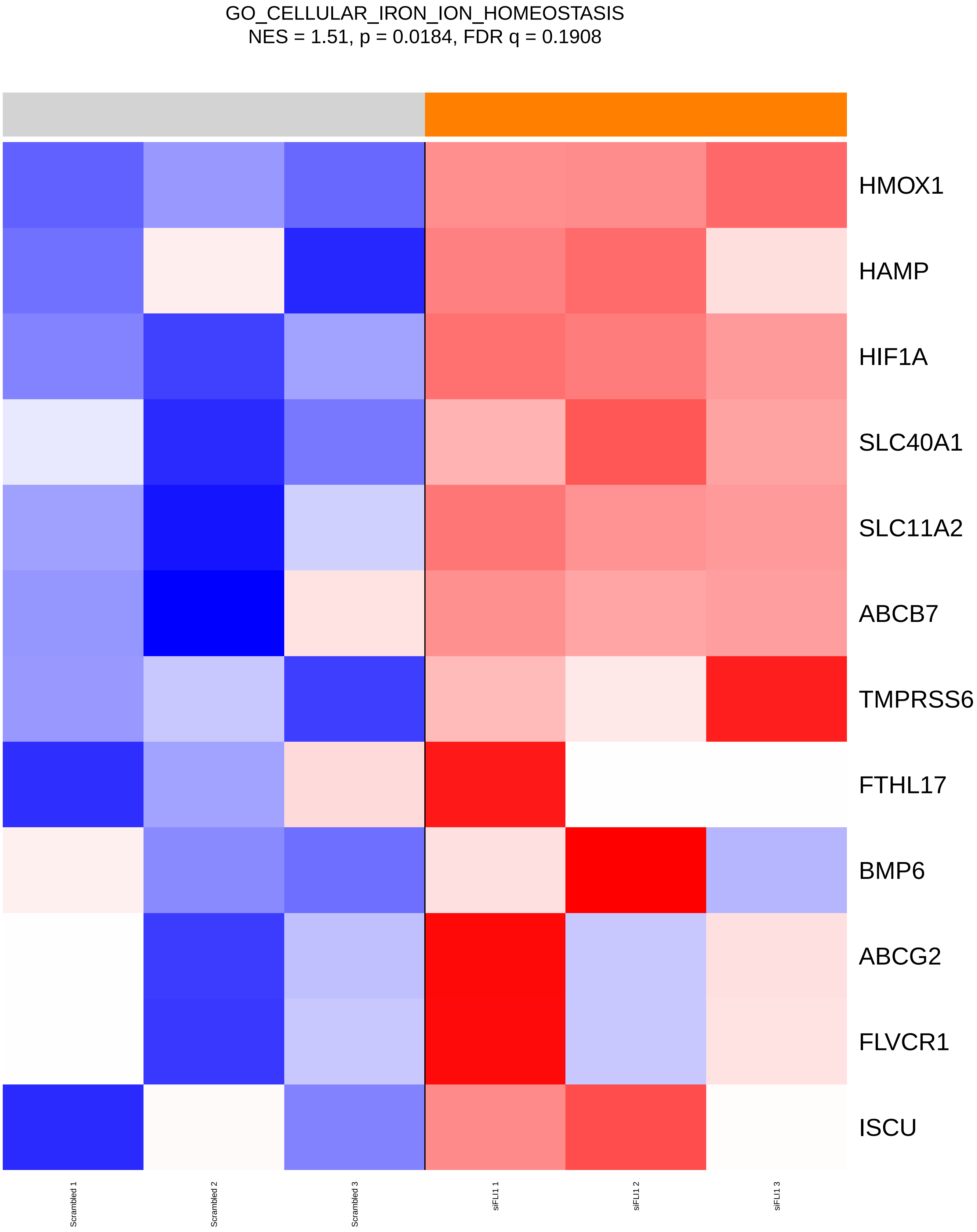
GO_CELLULAR_IRON_ION_HOMEOSTASIS
NES = 1.51, p = 0.0184, FDR q = 0.1908
HMOX1
HAMP
HIF1A
SLC40A1
SLC11A2
ABCB7
TMPRSS6
FTHL17
BMP6
ABCG2
FLVCR1
ISCU
Scrambled 1
Scrambled 2
Scrambled 3
siFLI1 1
siFLI1 2
siFLI1 3

GO_POSITIVE_REGULATION_OF_EPITHELIAL_CELL_PROLIFERATION
NES = 1.50, p = 0.0037, FDR q = 0.1920

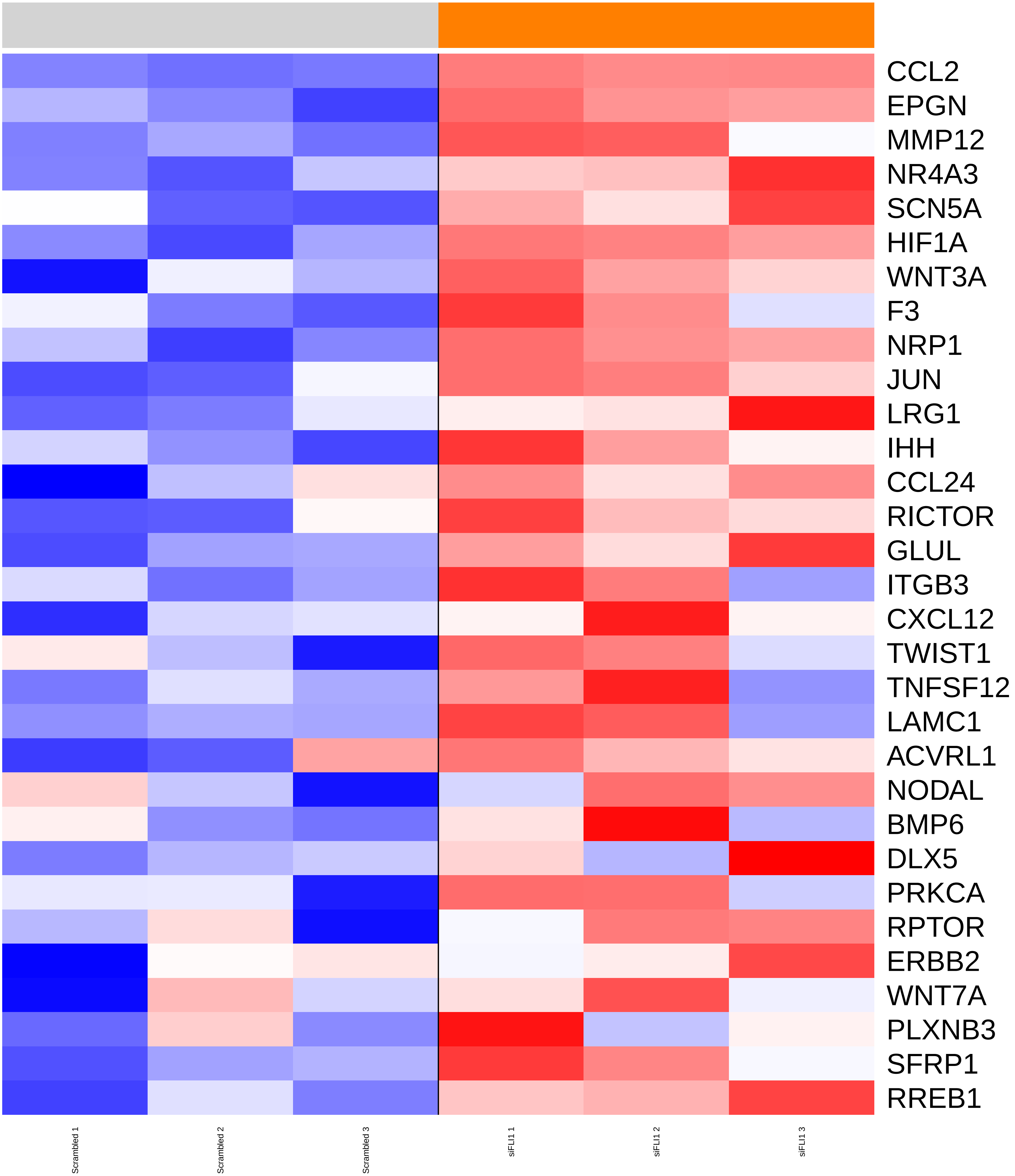

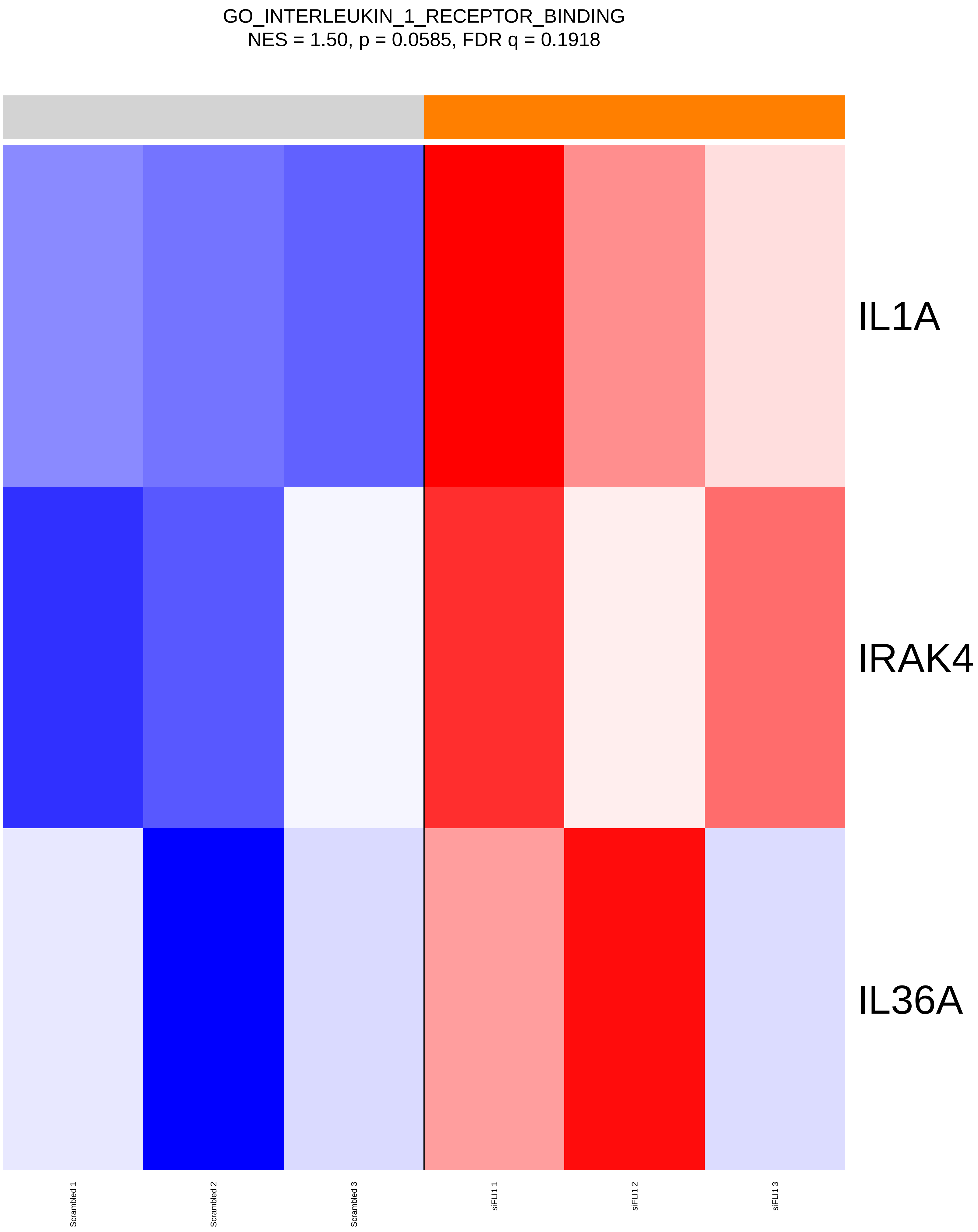
GO_INTERLEUKIN_1_RECEPTOR_BINDING
NES = 1.50, p = 0.0585, FDR q = 0.1918
IL1A
IRAK4
IL36A
Scrambled 1
Scrambled 2
Scrambled 3
siFLI1 1
siFLI1 2
siFLI1 3

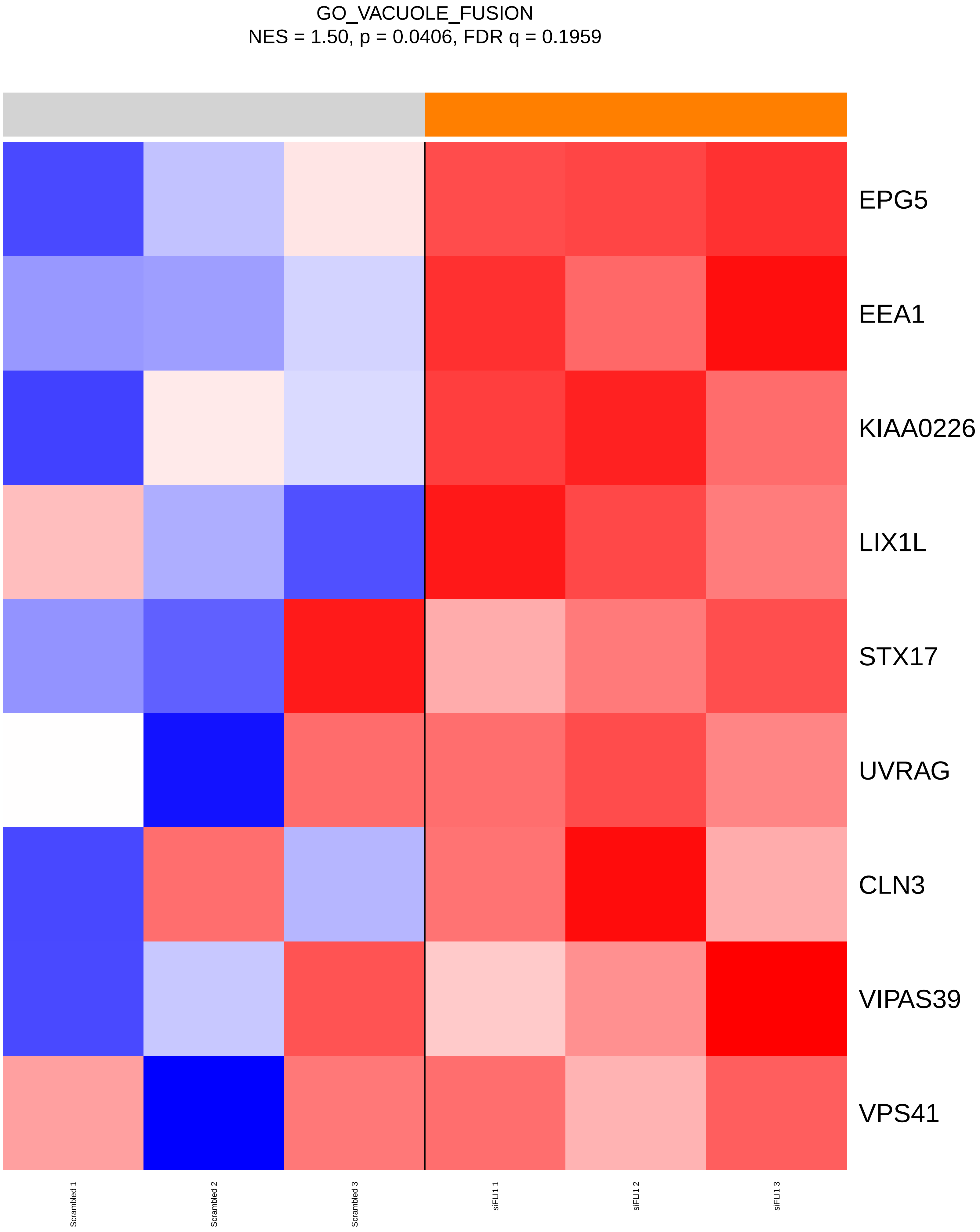

GO_VACUOLE_FUSION
NES = 1.50, p = 0.0406, FDR q = 0.1959
EPG5
EEA1
KIAA0226
LIX1L
STX17
UVRAG
CLN3
VIPAS39
VPS41
Scrambled 1
Scrambled 2
Scrambled 3
siFLI1 1
siFLI1 2
siFLI1 3

GO_REGULATION_OF_PROTEIN_SECRETION
NES = 1.50, p = 0.0000, FDR q = 0.1959

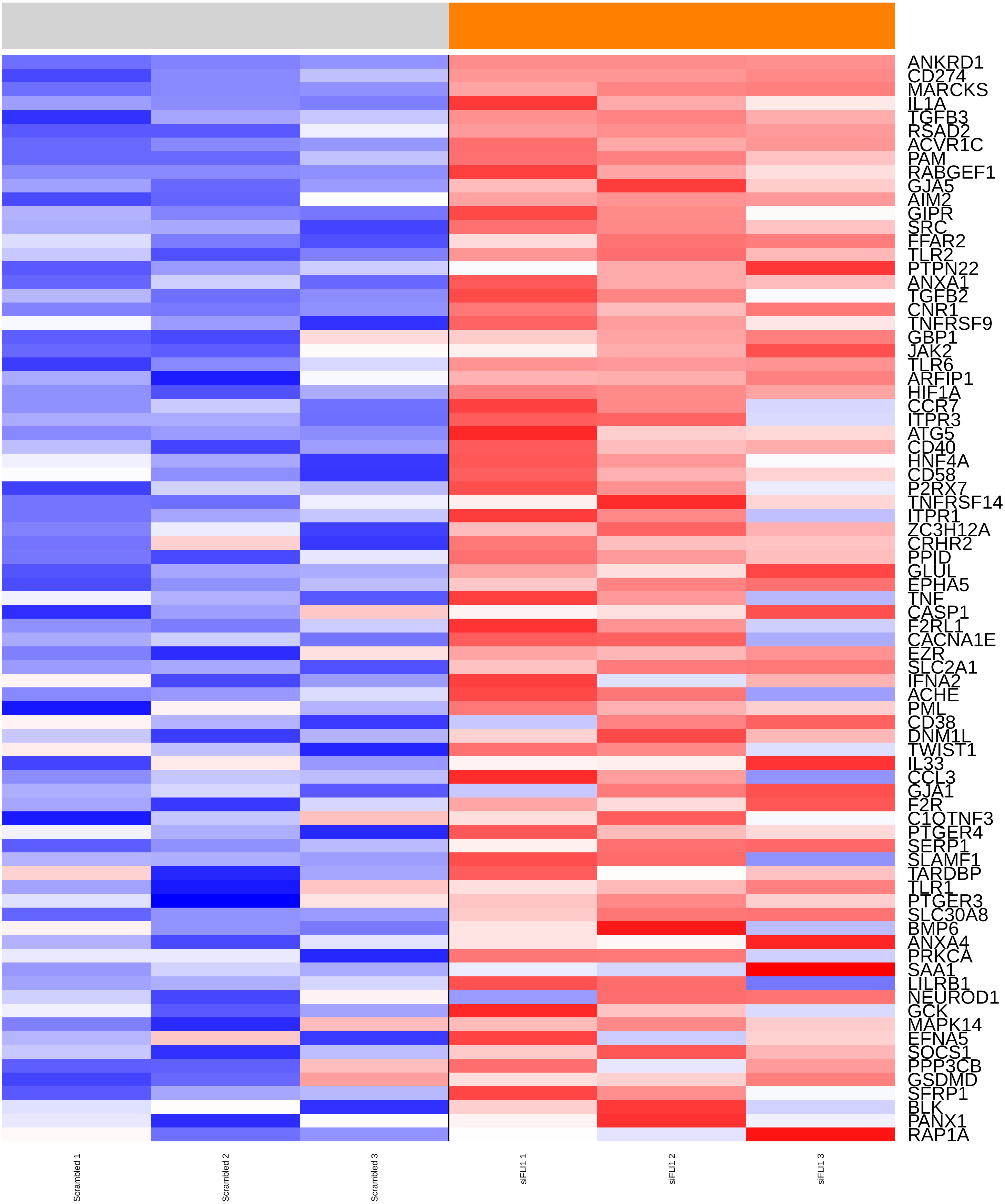

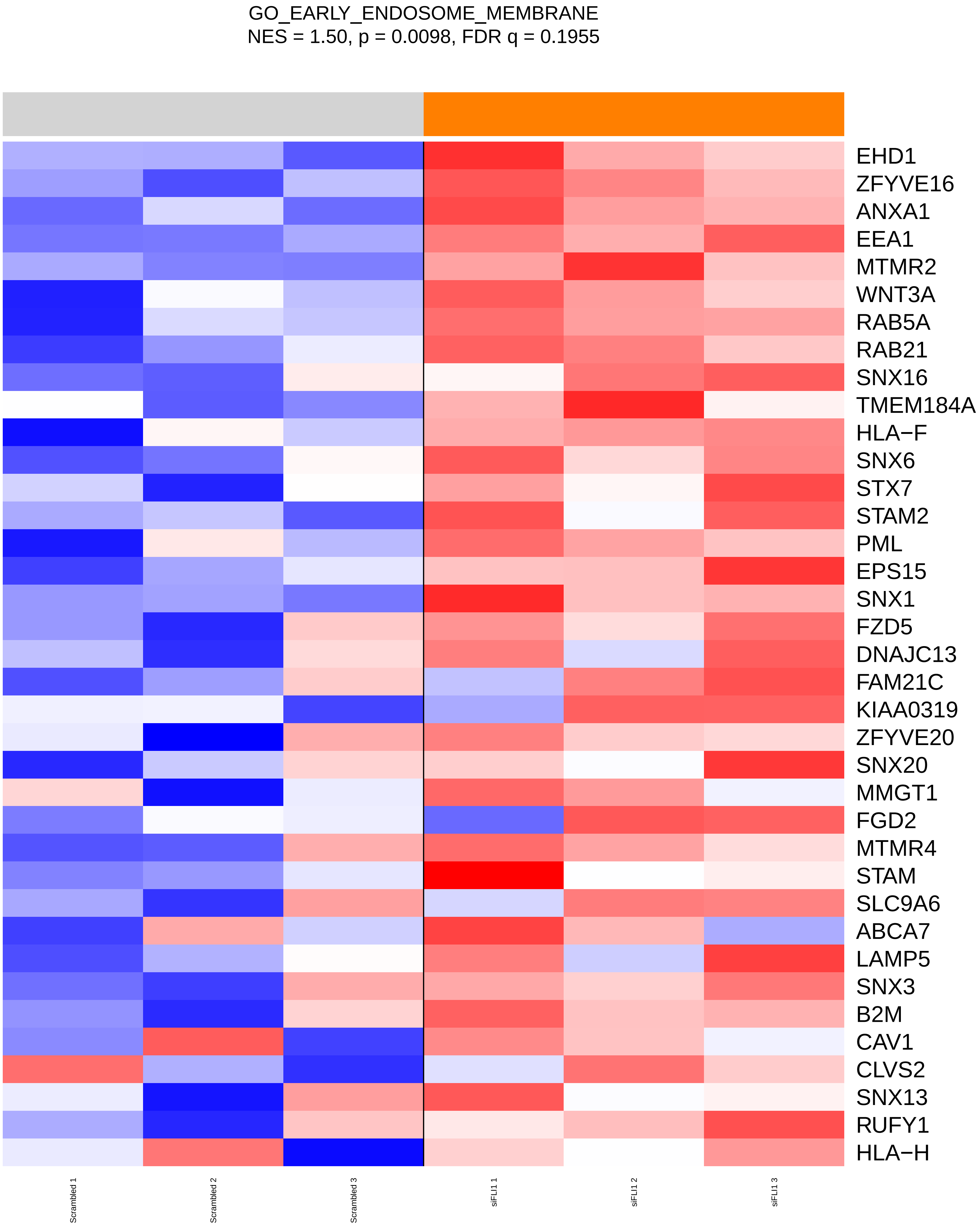
GO_EARLY_ENDOSOME_MEMBRANE
NES = 1.50, p = 0.0098, FDR q = 0.1955
EHD1
ZFYVE16
ANXA1
EEA1
MTMR2
WNT3A
RAB5A
RAB21
SNX16
TMEM184A
HLA−F
SNX6
STX7
STAM2
PML
EPS15
SNX1
FZD5
DNAJC13
FAM21C
KIAA0319
ZFYVE20
SNX20
MMGT1
FGD2
MTMR4
STAM
SLC9A6
ABCA7
LAMP5
SNX3
B2M
CAV1
CLVS2
SNX13
RUFY1
HLA−H
Scrambled 1
Scrambled 2
Scrambled 3
siFLI1 1
siFLI1 2
siFLI1 3

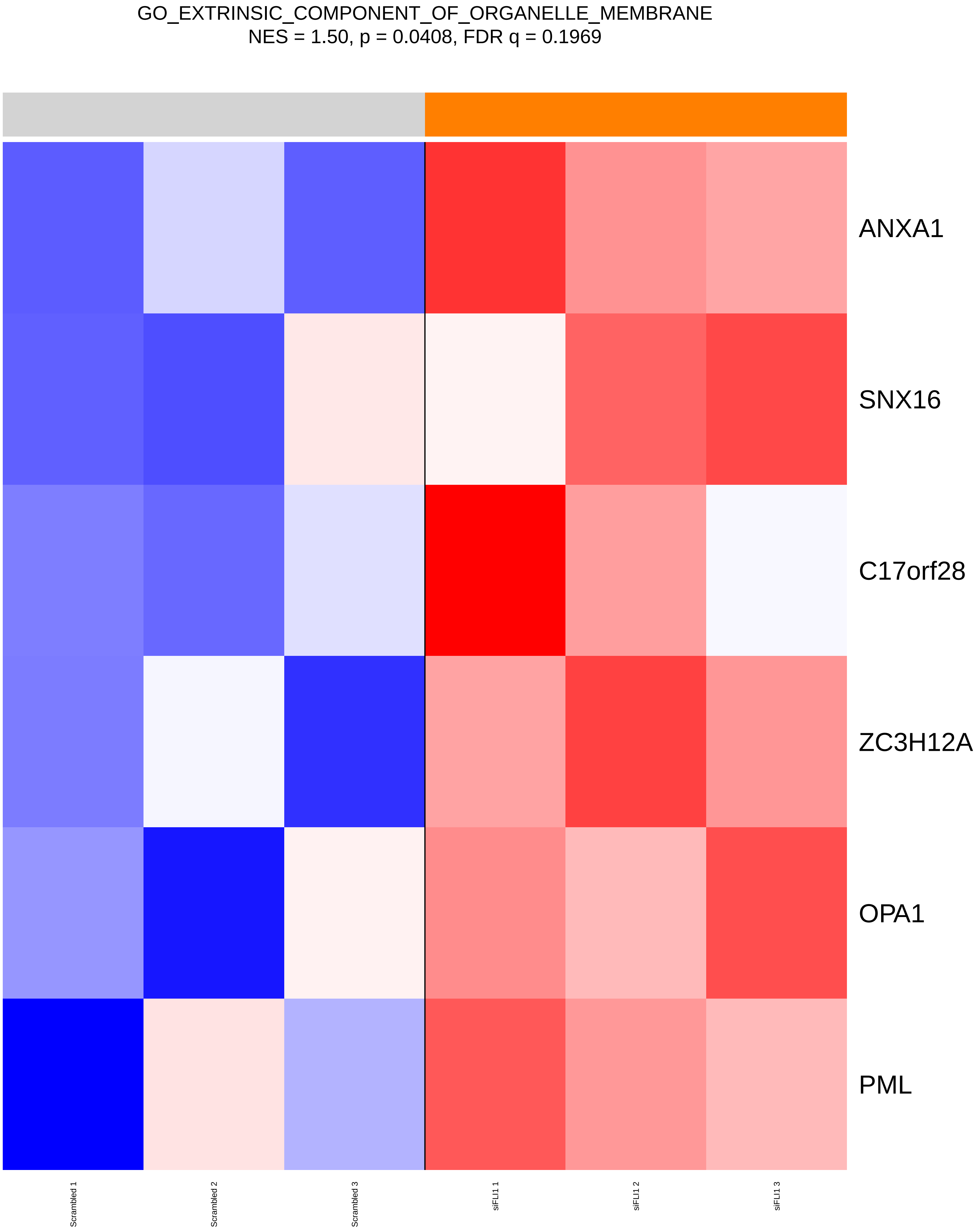
GO_EXTRINSIC_COMPONENT_OF_ORGANELLE_MEMBRANE
NES = 1.50, p = 0.0408, FDR q = 0.1969
ANXA1
SNX16
C17orf28
ZC3H12A
OPA1
PML
Scrambled 1
Scrambled 2
Scrambled 3
siFLI1 1
siFLI1 2
siFLI1 3

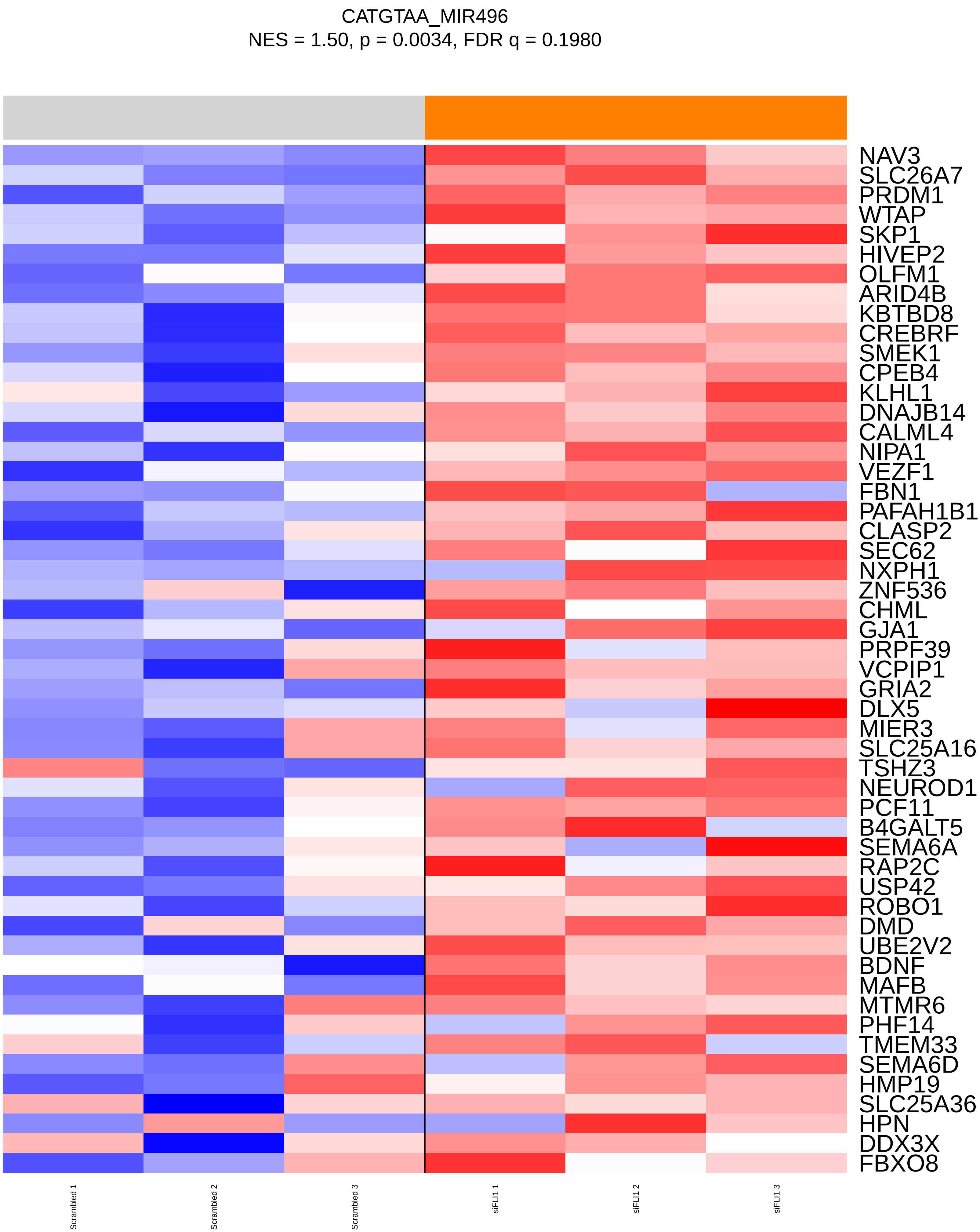
CATGTAA_MIR496
NES = 1.50, p = 0.0034, FDR q = 0.1980
NAV3
SLC26A7
PRDM1
WTAP
SKP1
HIVEP2
OLFM1
ARID4B
KBTBD8
CREBRF
SMEK1
CPEB4
KLHL1
DNAJB14
CALML4
NIPA1
VEZF1
FBN1
PAFAH1B1
CLASP2
SEC62
NXPH1
ZNF536
CHML
GJA1
PRPF39
VCPIP1
GRIA2
DLX5
MIER3
SLC25A16
TSHZ3
NEUROD1
PCF11
B4GALT5
SEMA6A
RAP2C
USP42
ROBO1
DMD
UBE2V2
BDNF
MAFB
MTMR6
PHF14
TMEM33
SEMA6D
HMP19
SLC25A36
HPN
DDX3X
FBXO8
Scrambled 1
Scrambled 2
Scrambled 3
siFLI1 1
siFLI1 2
siFLI1 3

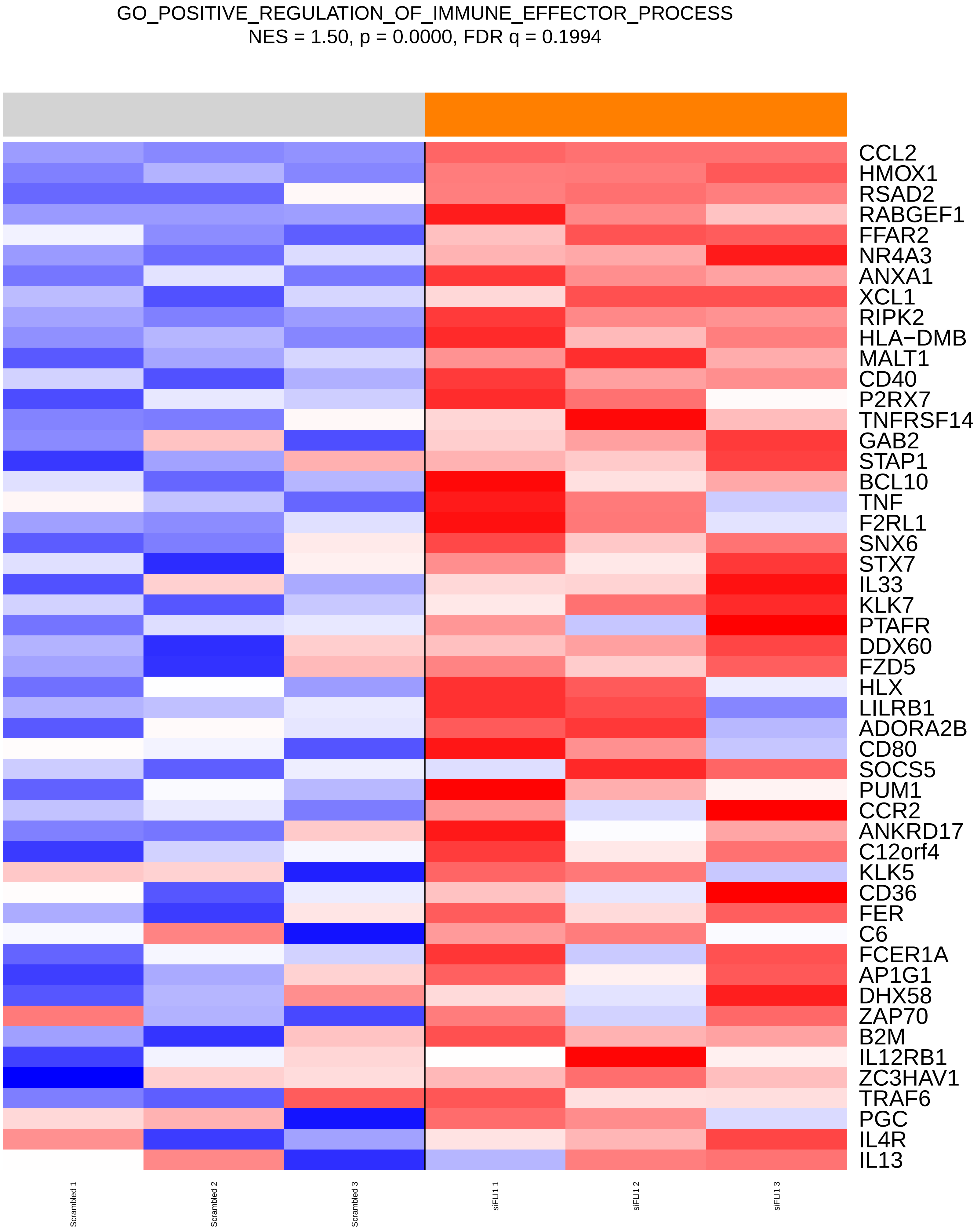
GO_POSITIVE_REGULATION_OF_IMMUNE_EFFECTOR_PROCESS
NES = 1.50, p = 0.0000, FDR q = 0.1994
CCL2
HMOX1
RSAD2
RABGEF1
FFAR2
NR4A3
ANXA1
XCL1
RIPK2
HLA−DMB
MALT1
CD40
P2RX7
TNFRSF14
GAB2
STAP1
BCL10
TNF
F2RL1
SNX6
STX7
IL33
KLK7
PTAFR
DDX60
FZD5
HLX
LILRB1
ADORA2B
CD80
SOCS5
PUM1
CCR2
ANKRD17
C12orf4
KLK5
CD36
FER
C6
FCER1A
AP1G1
DHX58
ZAP70
B2M
IL12RB1
ZC3HAV1
TRAF6
PGC
IL4R
IL13
Scrambled 1
Scrambled 2
Scrambled 3
siFLI1 1
siFLI1 2
siFLI1 3

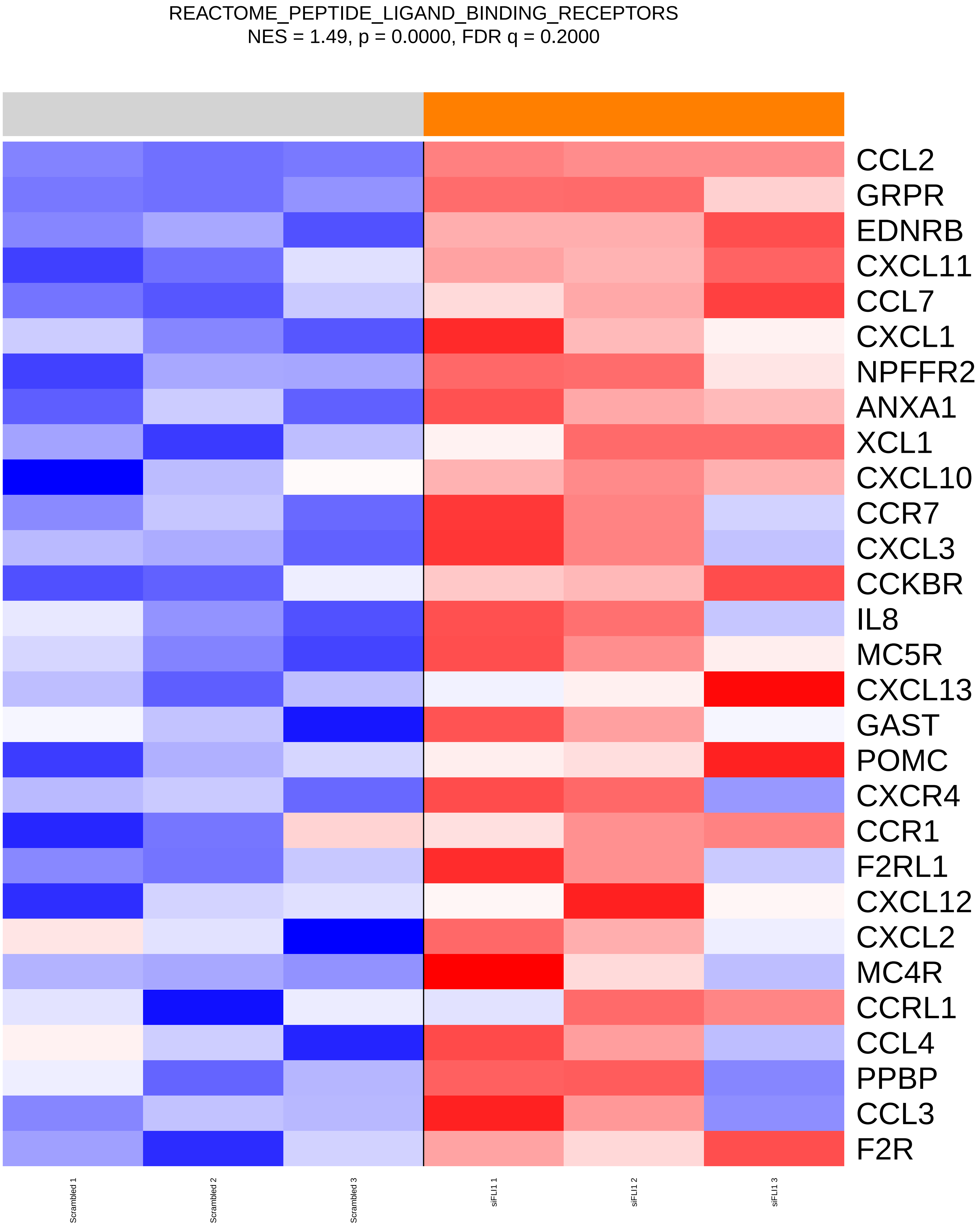
REACTOME_PEPTIDE_LIGAND_BINDING_RECEPTORS
NES = 1.49, p = 0.0000, FDR q = 0.2000
CCL2
GRPR
EDNRB
CXCL11
CCL7
CXCL1
NPFFR2
ANXA1
XCL1
CXCL10
CCR7
CXCL3
CCKBR
IL8
MC5R
CXCL13
GAST
POMC
CXCR4
CCR1
F2RL1
CXCL12
CXCL2
MC4R
CCRL1
CCL4
PPBP
CCL3
F2R
Scrambled 1
Scrambled 2
Scrambled 3
siFLI1 1
siFLI1 2
siFLI1 3

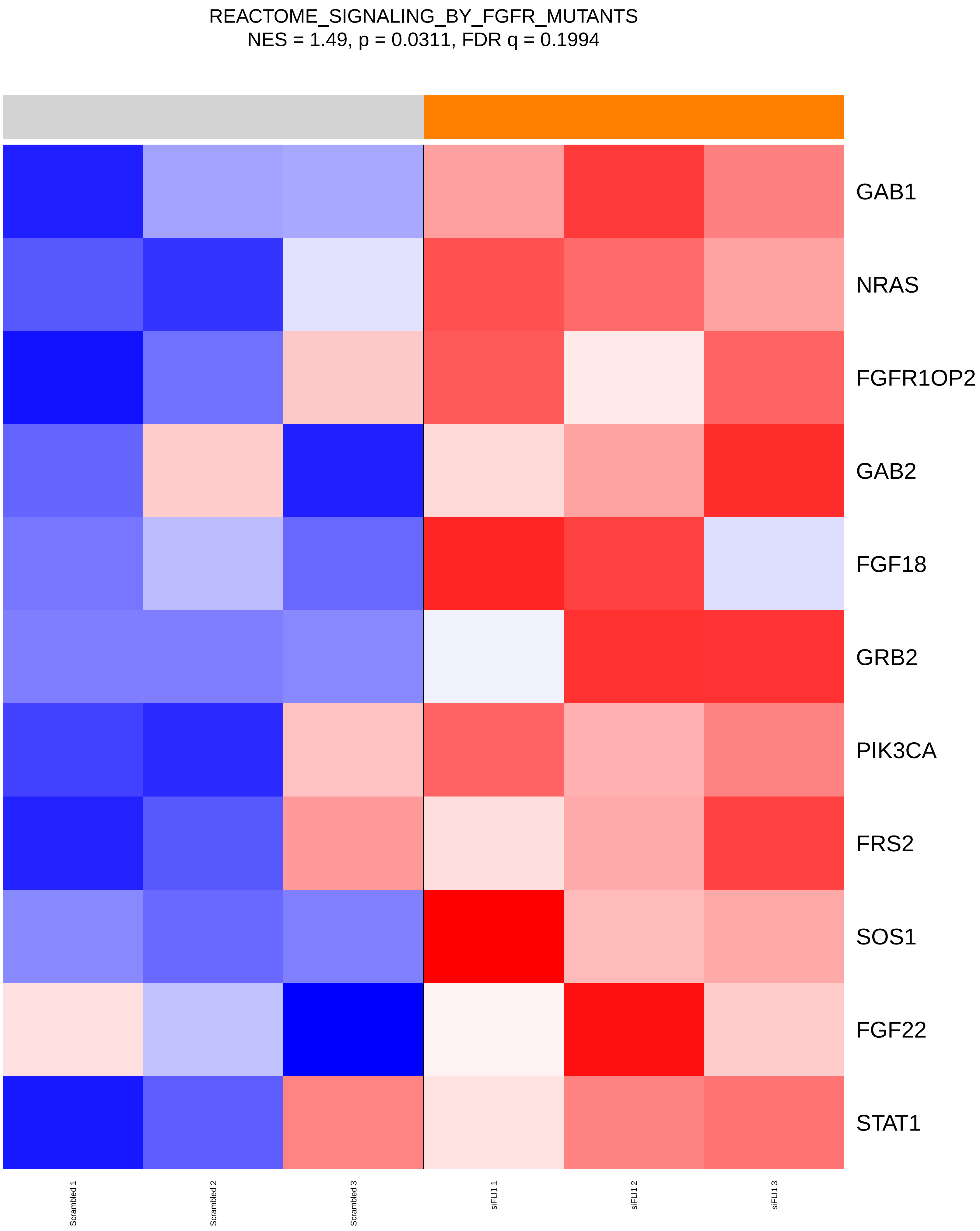
REACTOME_SIGNALING_BY_FGFR_MUTANTS
NES = 1.49, p = 0.0311, FDR q = 0.1994
GAB1
NRAS
FGFR1OP2
GAB2
FGF18
GRB2
PIK3CA
FRS2
SOS1
FGF22
STAT1
Scrambled 1
Scrambled 2
Scrambled 3
siFLI1 1
siFLI1 2
siFLI1 3

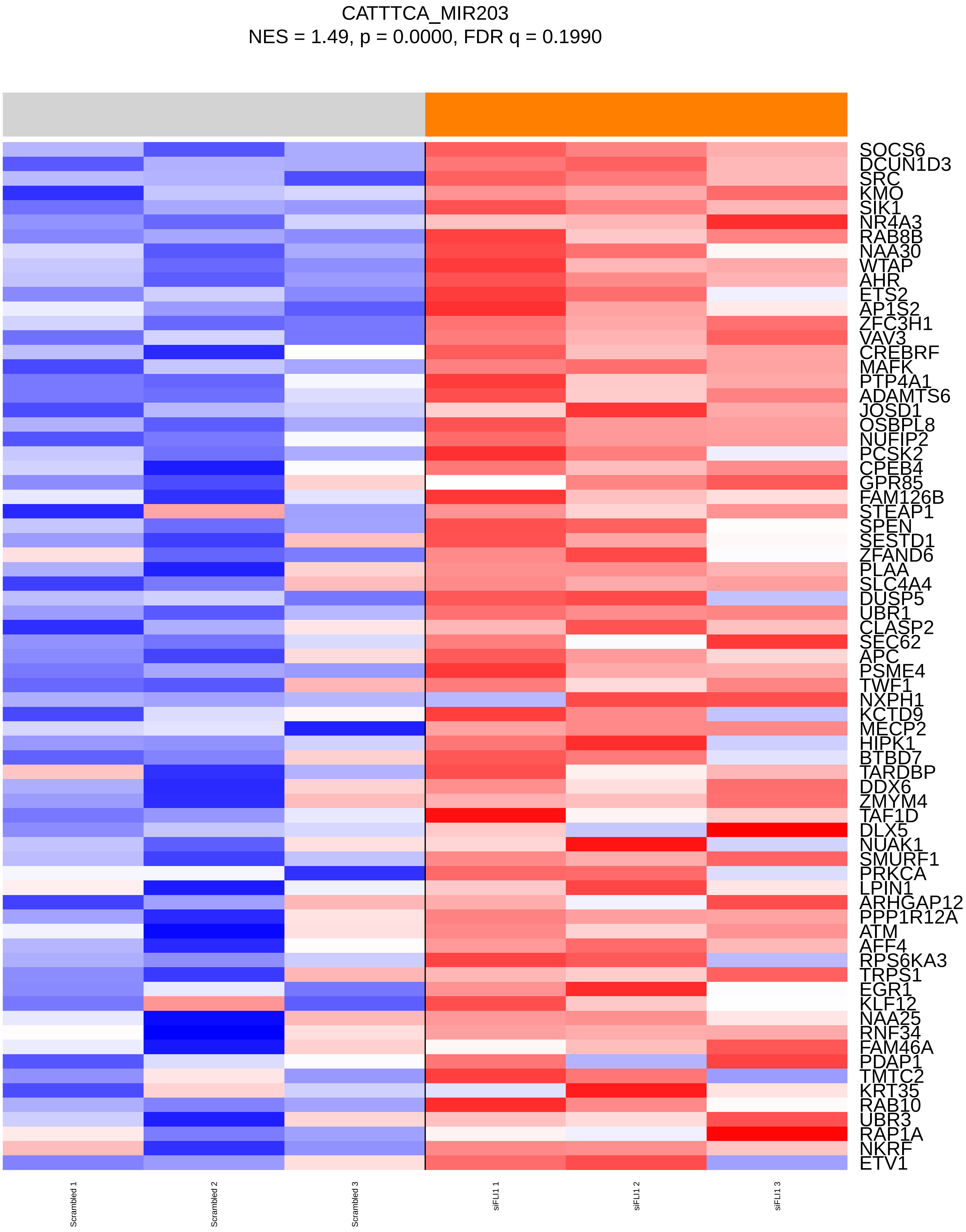
CATTTCA_MIR203
NES = 1.49, p = 0.0000, FDR q = 0.1990
SOCS6
DCUN1D3
SRC
KMO
SIK1
NR4A3
RAB8B
NAA30
WTAP
AHR
ETS2
AP1S2
ZFC3H1
VAV3
CREBRF
MAFK
PTP4A1
ADAMTS6
JOSD1
OSBPL8
NUFIP2
PCSK2
CPEB4
GPR85
FAM126B
STEAP1
SPEN
SESTD1
ZFAND6
PLAA
SLC4A4
DUSP5
UBR1
CLASP2
SEC62
APC
PSME4
TWF1
NXPH1
KCTD9
MECP2
HIPK1
BTBD7
TARDBP
DDX6
ZMYM4
TAF1D
DLX5
NUAK1
SMURF1
PRKCA
LPIN1
ARHGAP12
PPP1R12A
ATM
AFF4
RPS6KA3
TRPS1
EGR1
KLF12
NAA25
RNF34
FAM46A
PDAP1
TMTC2
KRT35
RAB10
UBR3
RAP1A
NKRF
ETV1
Scrambled 1
Scrambled 2
Scrambled 3
siFLI1 1
siFLI1 2
siFLI1 3

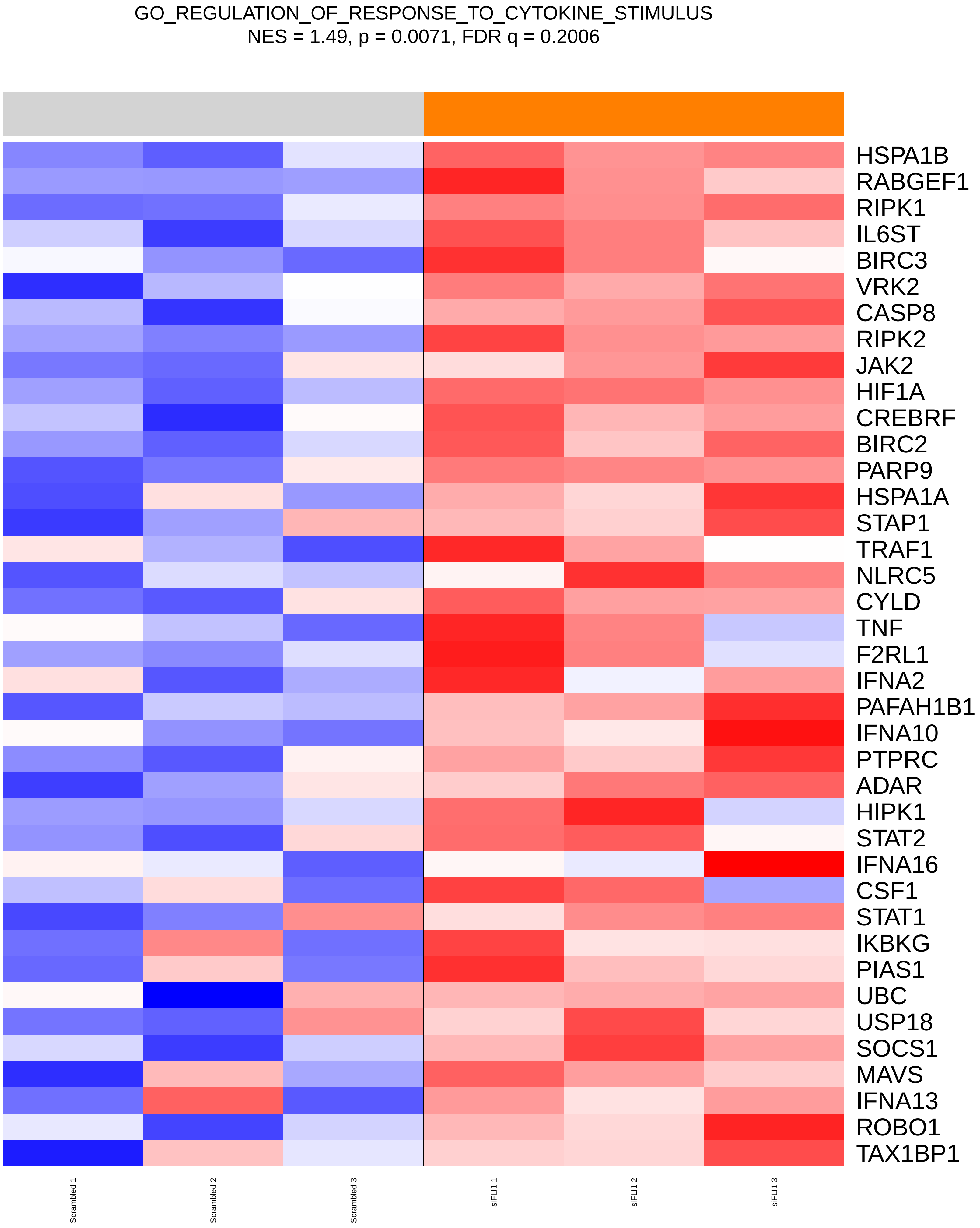
GO_REGULATION_OF_RESPONSE_TO_CYTOKINE_STIMULUS
NES = 1.49, p = 0.0071, FDR q = 0.2006
HSPA1B
RABGEF1
RIPK1
IL6ST
BIRC3
VRK2
CASP8
RIPK2
JAK2
HIF1A
CREBRF
BIRC2
PARP9
HSPA1A
STAP1
TRAF1
NLRC5
CYLD
TNF
F2RL1
IFNA2
PAFAH1B1
IFNA10
PTPRC
ADAR
HIPK1
STAT2
IFNA16
CSF1
STAT1
IKBKG
PIAS1
UBC
USP18
SOCS1
MAVS
IFNA13
ROBO1
TAX1BP1
Scrambled 1
Scrambled 2
Scrambled 3
siFLI1 1
siFLI1 2
siFLI1 3

GO_RIBOSOMAL_LARGE_SUBUNIT_BIOGENESIS
NES = 1.49, p = 0.0224, FDR q = 0.2004

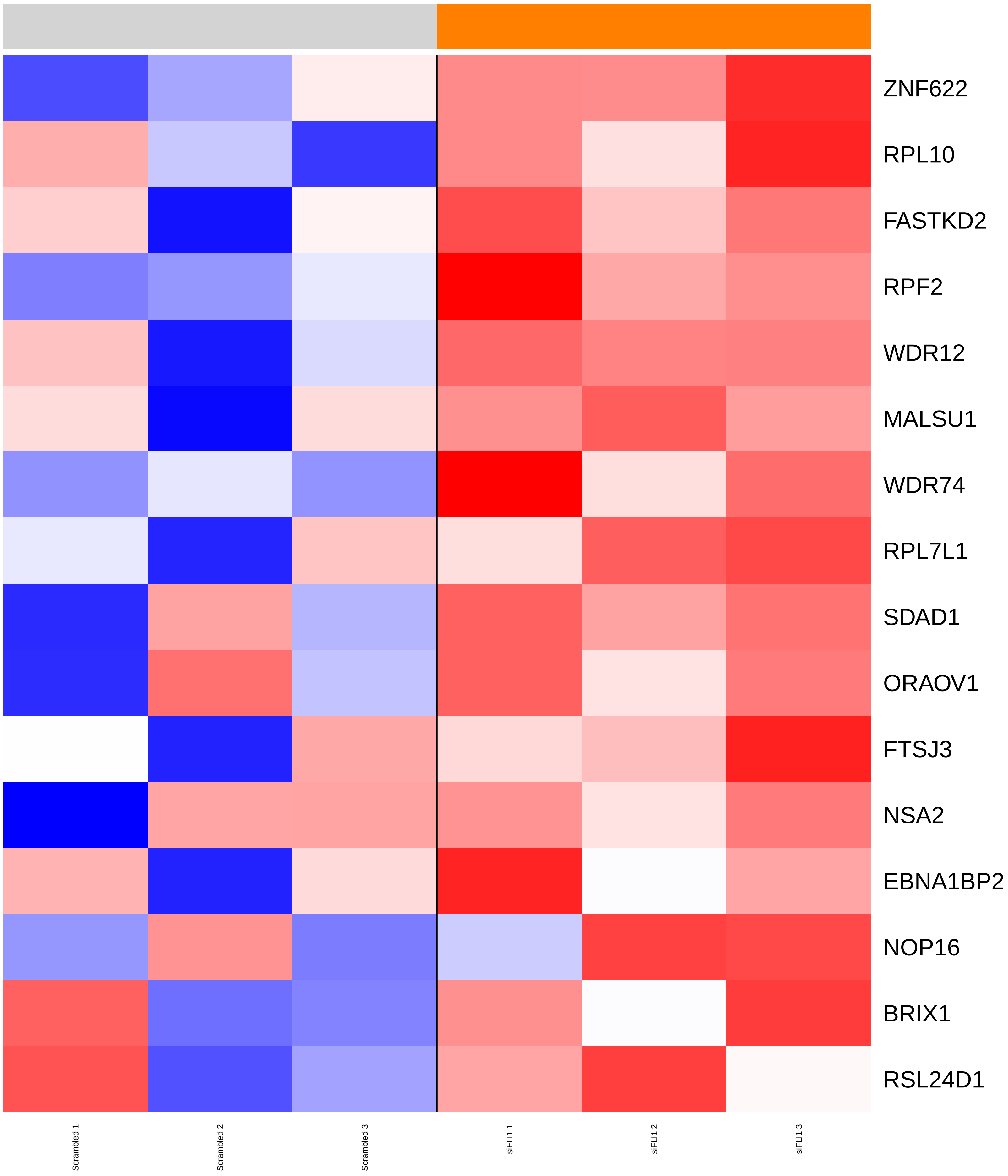

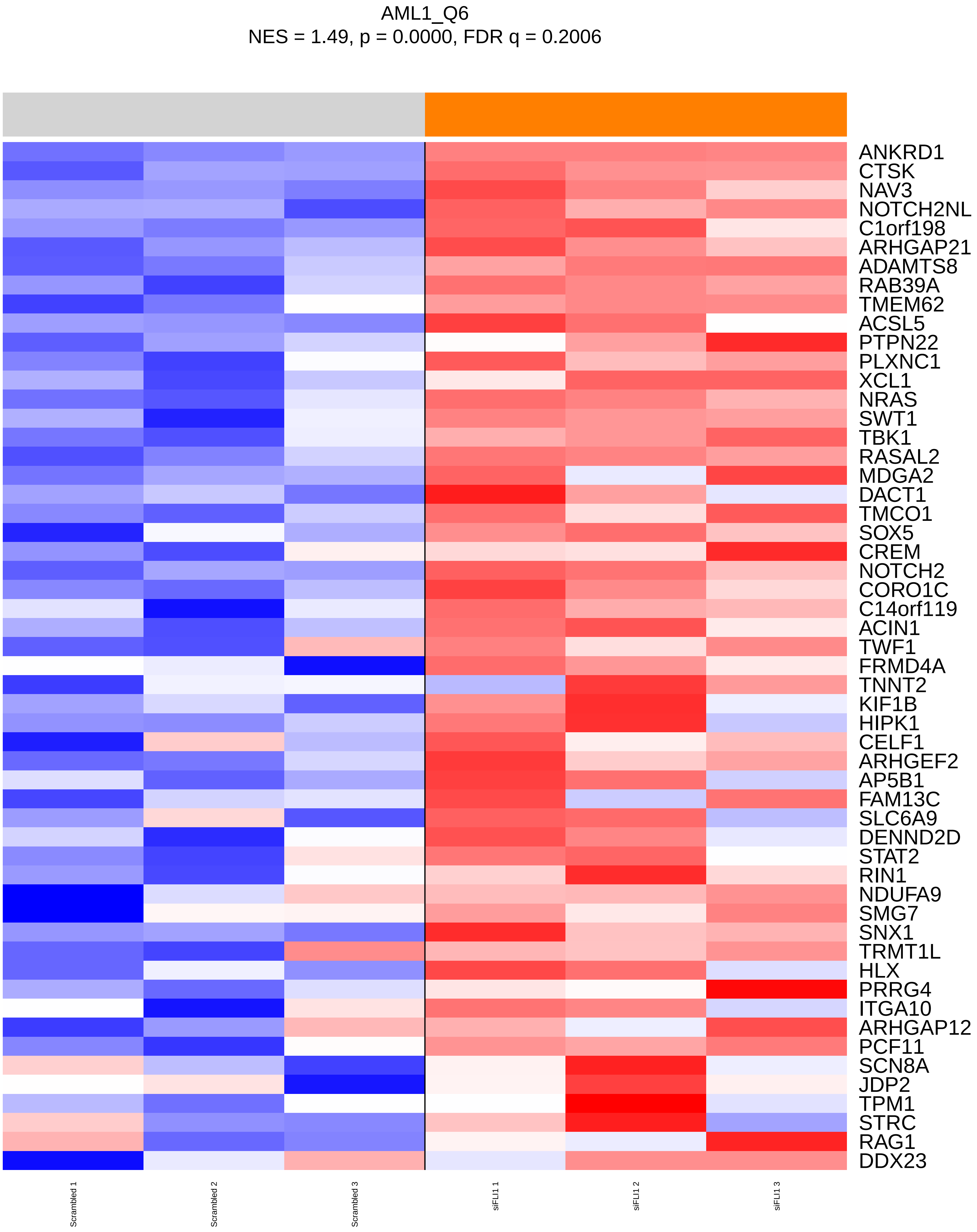
AML1_Q6
NES = 1.49, p = 0.0000, FDR q = 0.2006
ANKRD1
CTSK
NAV3
NOTCH2NL
C1orf198
ARHGAP21
ADAMTS8
RAB39A
TMEM62
ACSL5
PTPN22
PLXNC1
XCL1
NRAS
SWT1
TBK1
RASAL2
MDGA2
DACT1
TMCO1
SOX5
CREM
NOTCH2
CORO1C
C14orf119
ACIN1
TWF1
FRMD4A
TNNT2
KIF1B
HIPK1
CELF1
ARHGEF2
AP5B1
FAM13C
SLC6A9
DENND2D
STAT2
RIN1
NDUFA9
SMG7
SNX1
TRMT1L
HLX
PRRG4
ITGA10
ARHGAP12
PCF11
SCN8A
JDP2
TPM1
STRC
RAG1
DDX23
Scrambled 1
Scrambled 2
Scrambled 3
siFLI1 1
siFLI1 2
siFLI1 3

GO_POSITIVE_REGULATION_OF_MAPK_CASCADE
NES = 1.49, p = 0.0000, FDR q = 0.2001

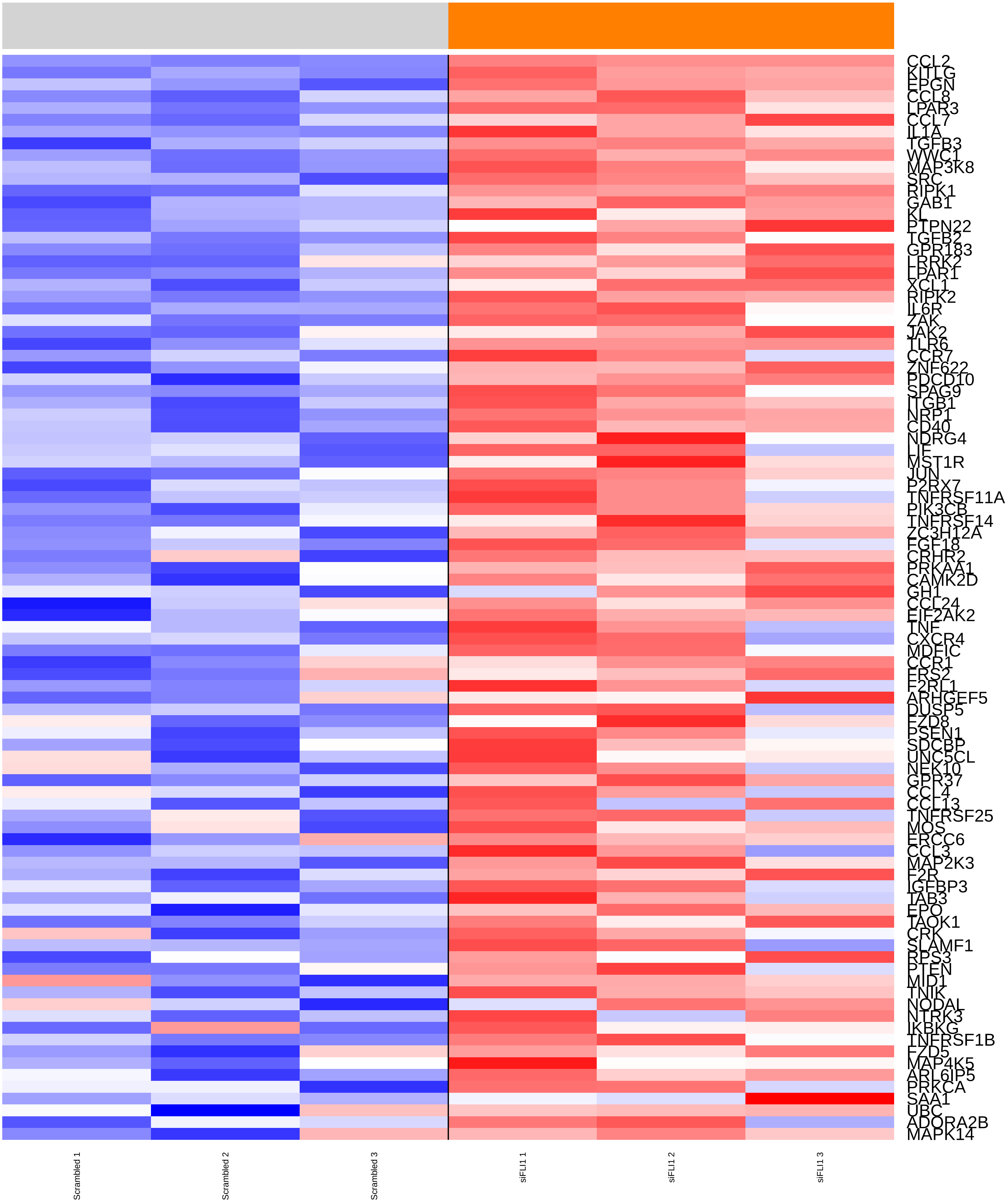

REACTOME_DESTABILIZATION_OF_MRNA_BY_AUF1_HNRNP_D0
NES = 1.49, p = 0.0260, FDR q = 0.2010

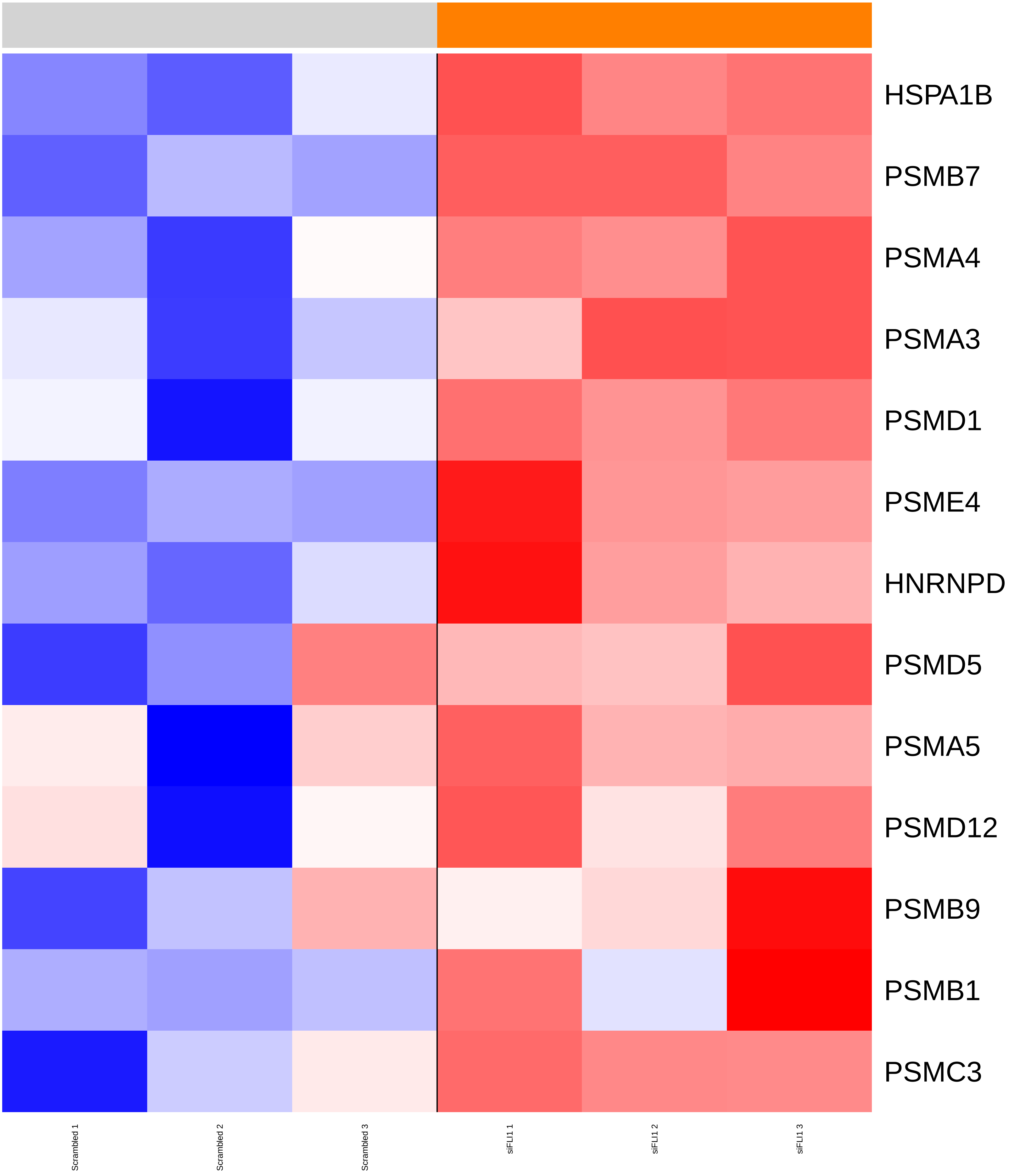

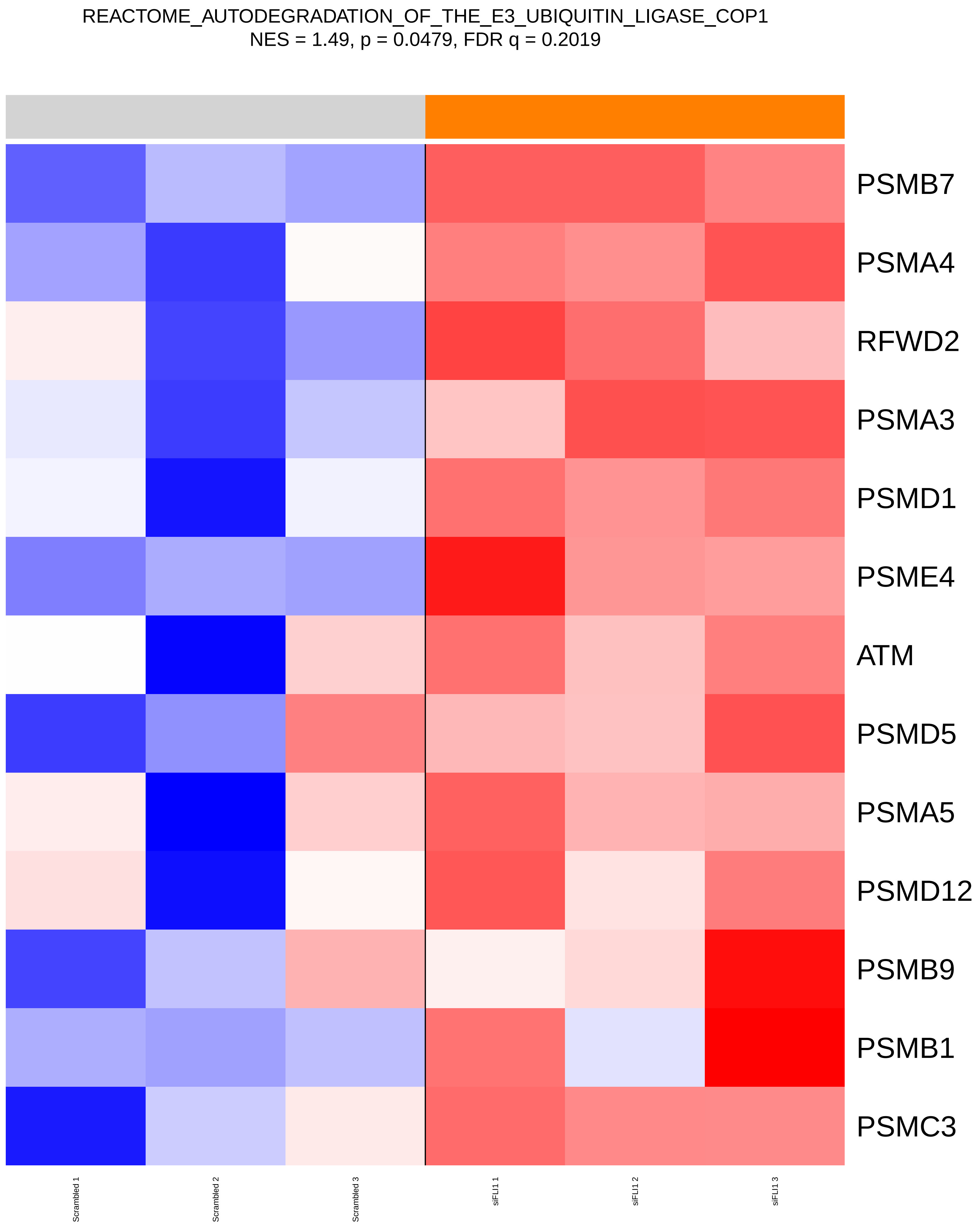
REACTOME_AUTODEGRADATION_OF_THE_E3_UBIQUITIN_LIGASE_COP1
NES = 1.49, p = 0.0479, FDR q = 0.2019
PSMB7
PSMA4
RFWD2
PSMA3
PSMD1
PSME4
ATM
PSMD5
PSMA5
PSMD12
PSMB9
PSMB1
PSMC3
Scrambled 1
Scrambled 2
Scrambled 3
siFLI1 1
siFLI1 2
siFLI1 3

REACTOME_REGULATION_OF_IFNA_SIGNALING
NES = 1.49, p = 0.0369, FDR q = 0.2022

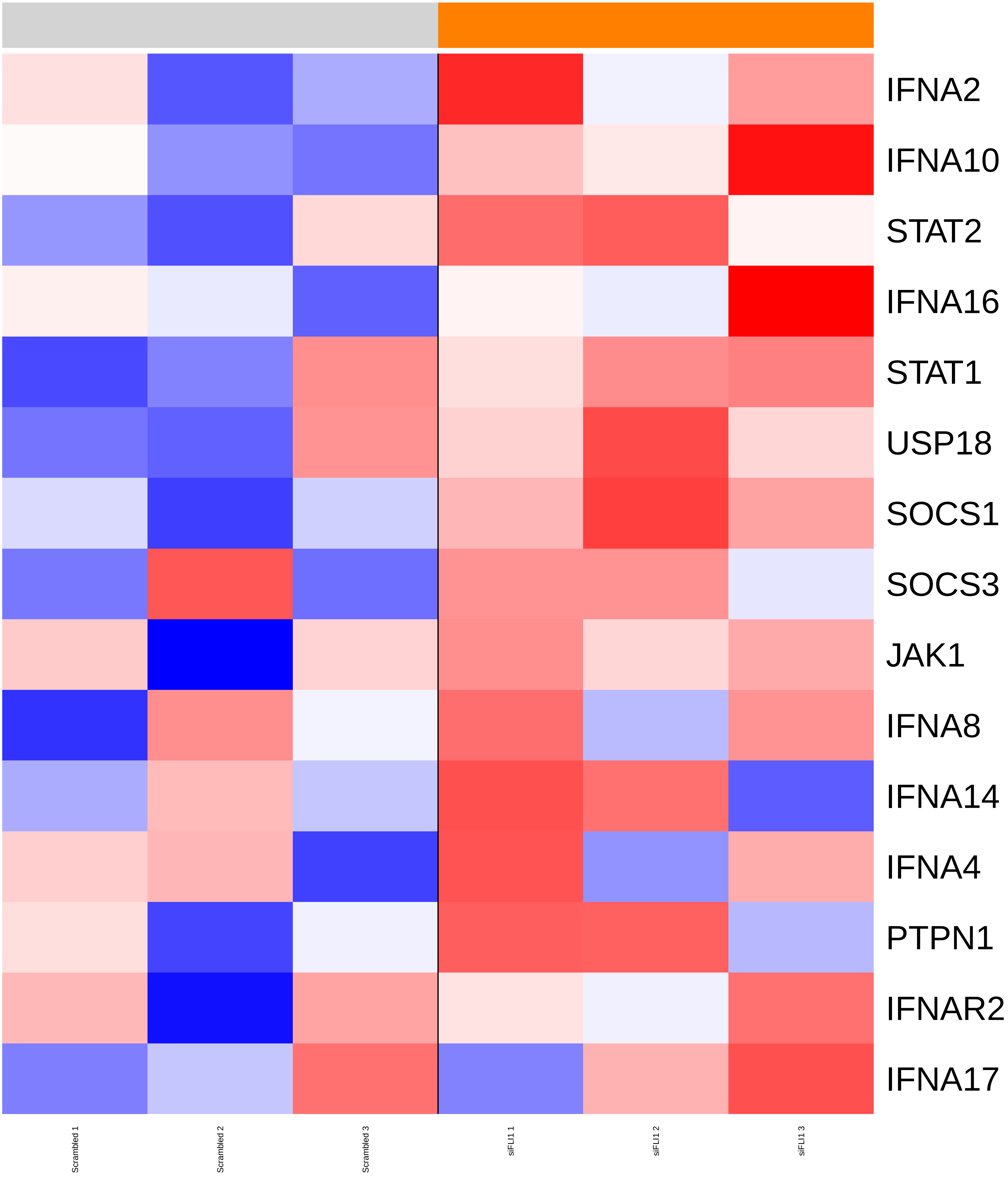

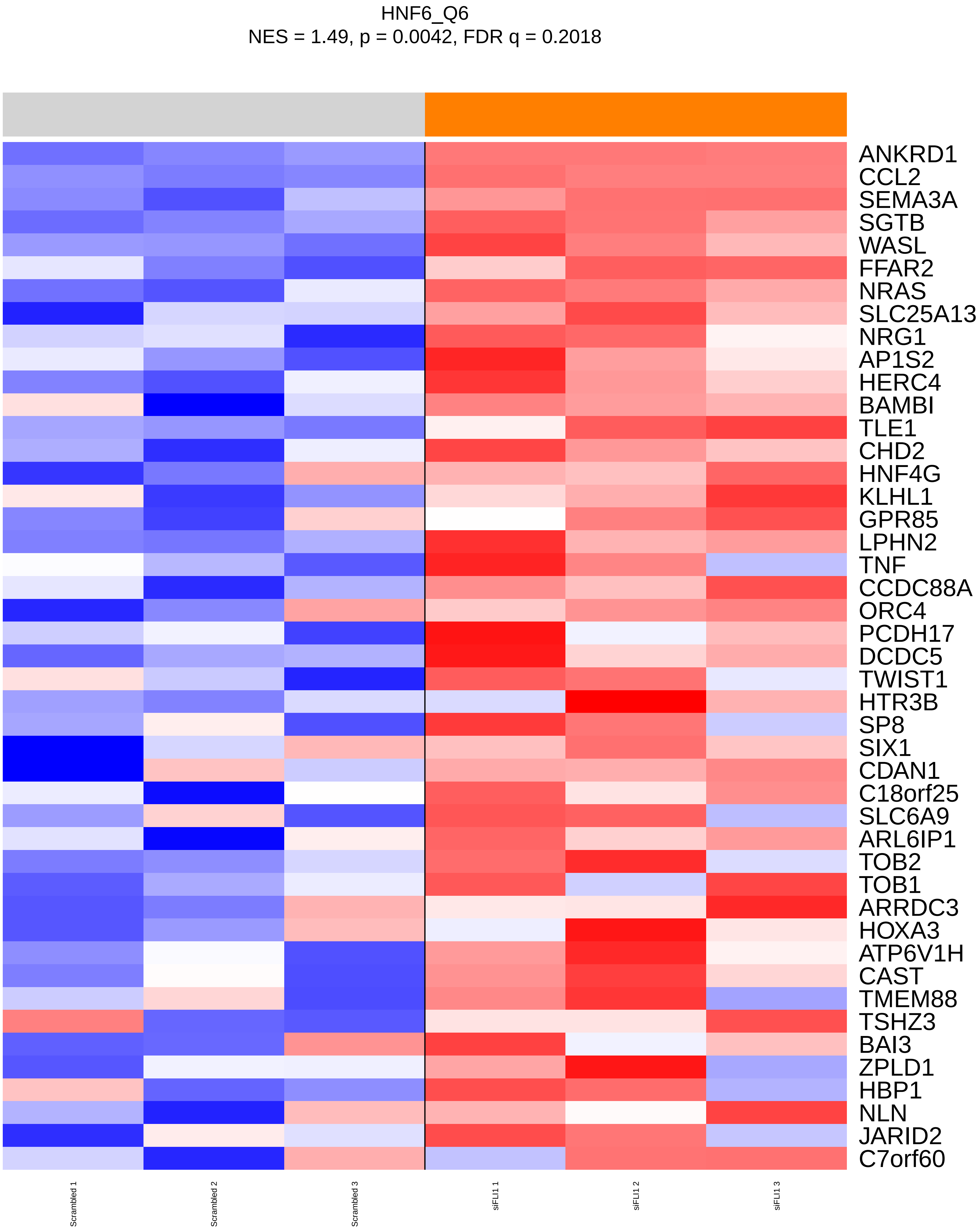
HNF6_Q6
NES = 1.49, p = 0.0042, FDR q = 0.2018
ANKRD1
CCL2
SEMA3A
SGTB
WASL
FFAR2
NRAS
SLC25A13
NRG1
AP1S2
HERC4
BAMBI
TLE1
CHD2
HNF4G
KLHL1
GPR85
LPHN2
TNF
CCDC88A
ORC4
PCDH17
DCDC5
TWIST1
HTR3B
SP8
SIX1
CDAN1
C18orf25
SLC6A9
ARL6IP1
TOB2
TOB1
ARRDC3
HOXA3
ATP6V1H
CAST
TMEM88
TSHZ3
BAI3
ZPLD1
HBP1
NLN
JARID2
C7orf60
Scrambled 1
Scrambled 2
Scrambled 3
siFLI1 1
siFLI1 2
siFLI1 3

GO_GRANULOCYTE_ACTIVATION
NES = 1.49, p = 0.0663, FDR q = 0.2013

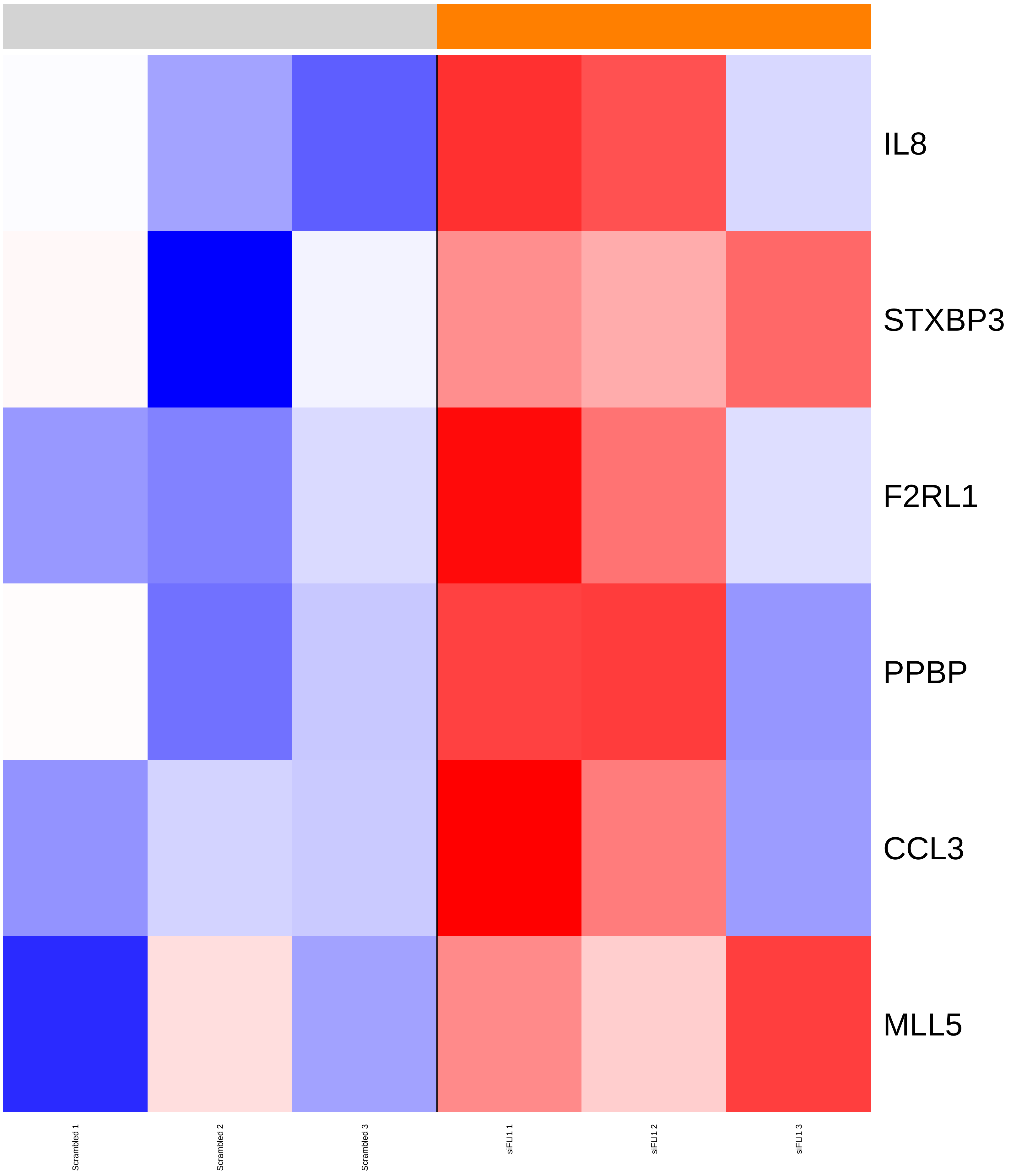

GO_CELLULAR_RESPONSE_TO_INTERFERON_BETA
NES = 1.49, p = 0.0505, FDR q = 0.2008

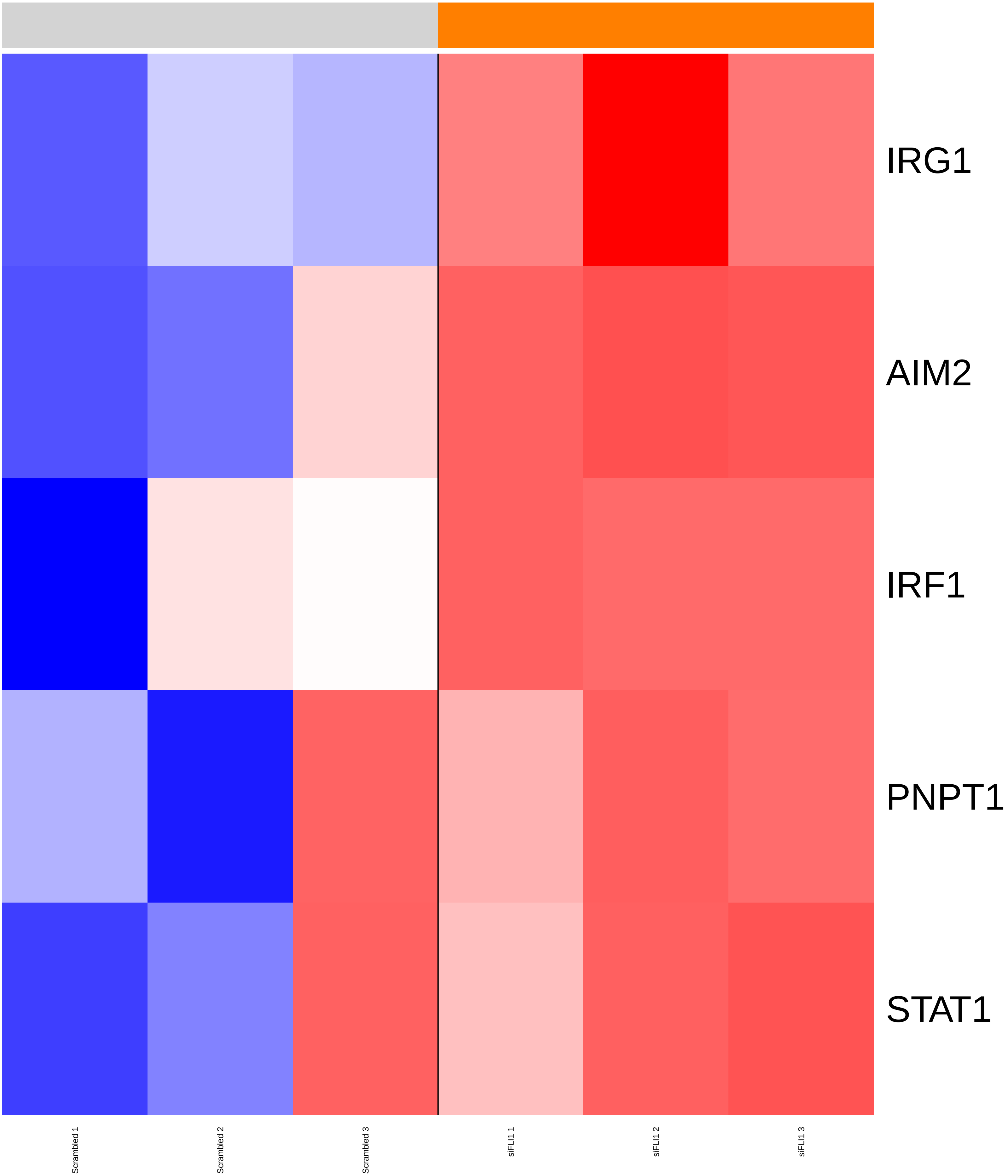

GO_VIRAL_GENOME_REPLICATION
NES = 1.49, p = 0.0588, FDR q = 0.2007

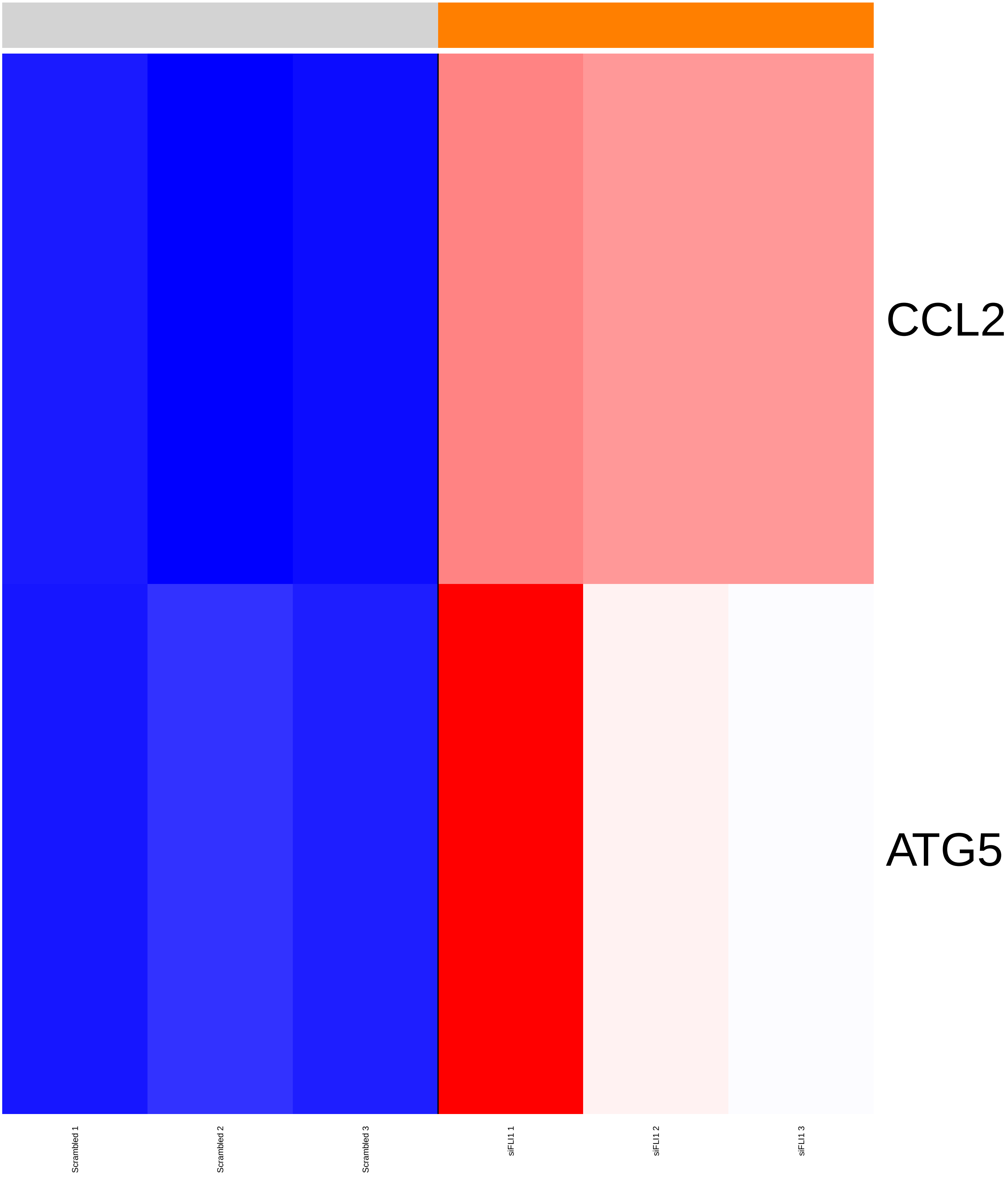

GO_REGULATION_OF_NITRIC_OXIDE_SYNTHASE_BIOSYNTHETIC_PROCESS
NES = 1.49, p = 0.0590, FDR q = 0.2009

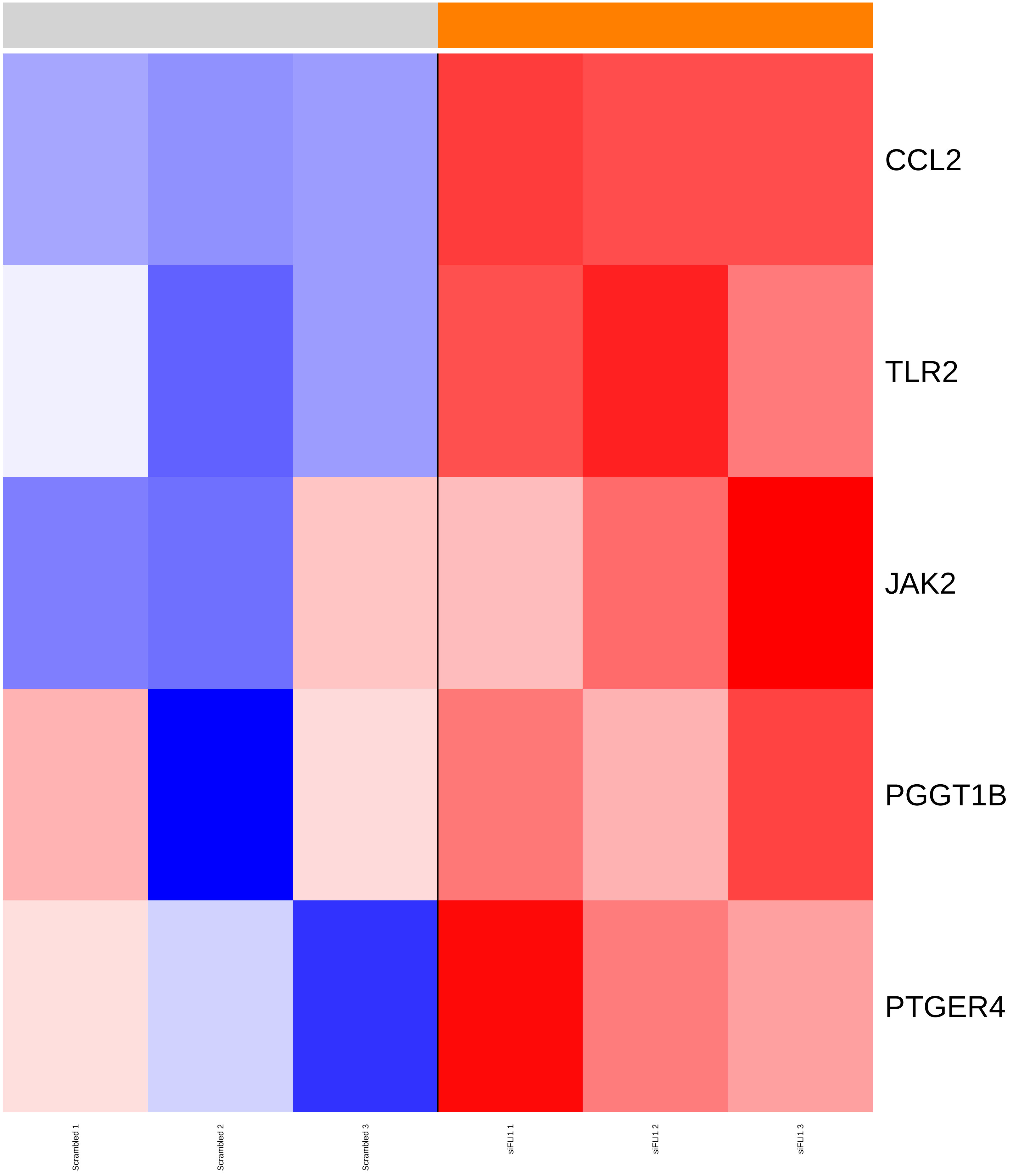

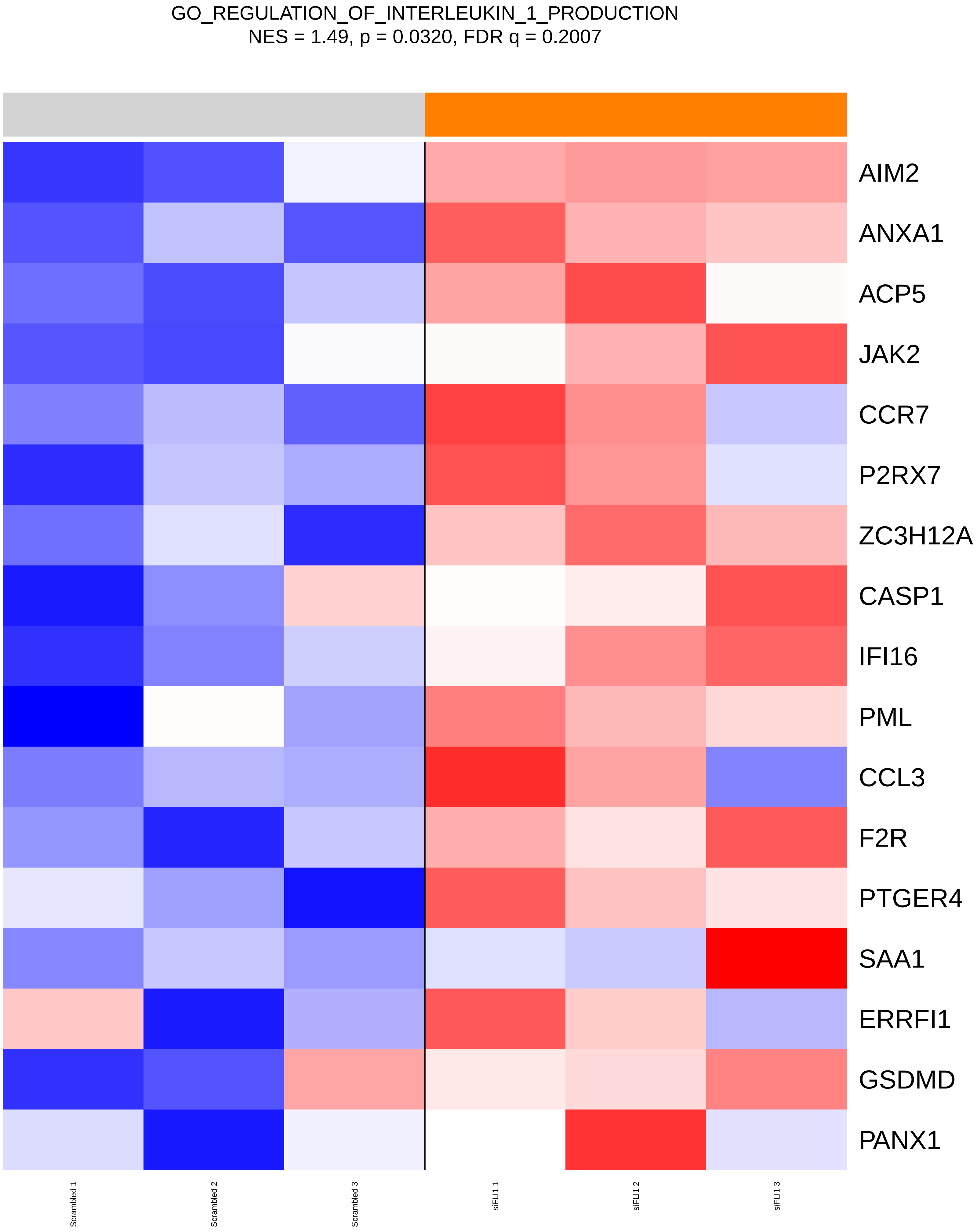
GO_REGULATION_OF_INTERLEUKIN_1_PRODUCTION
NES = 1.49, p = 0.0320, FDR q = 0.2007
AIM2
ANXA1
ACP5
JAK2
CCR7
P2RX7
ZC3H12A
CASP1
IFI16
PML
CCL3
F2R
PTGER4
SAA1
ERRFI1
GSDMD
PANX1
Scrambled 1
Scrambled 2
Scrambled 3
siFLI1 1
siFLI1 2
siFLI1 3

AML1_01
NES = 1.49, p = 0.0000, FDR q = 0.2005

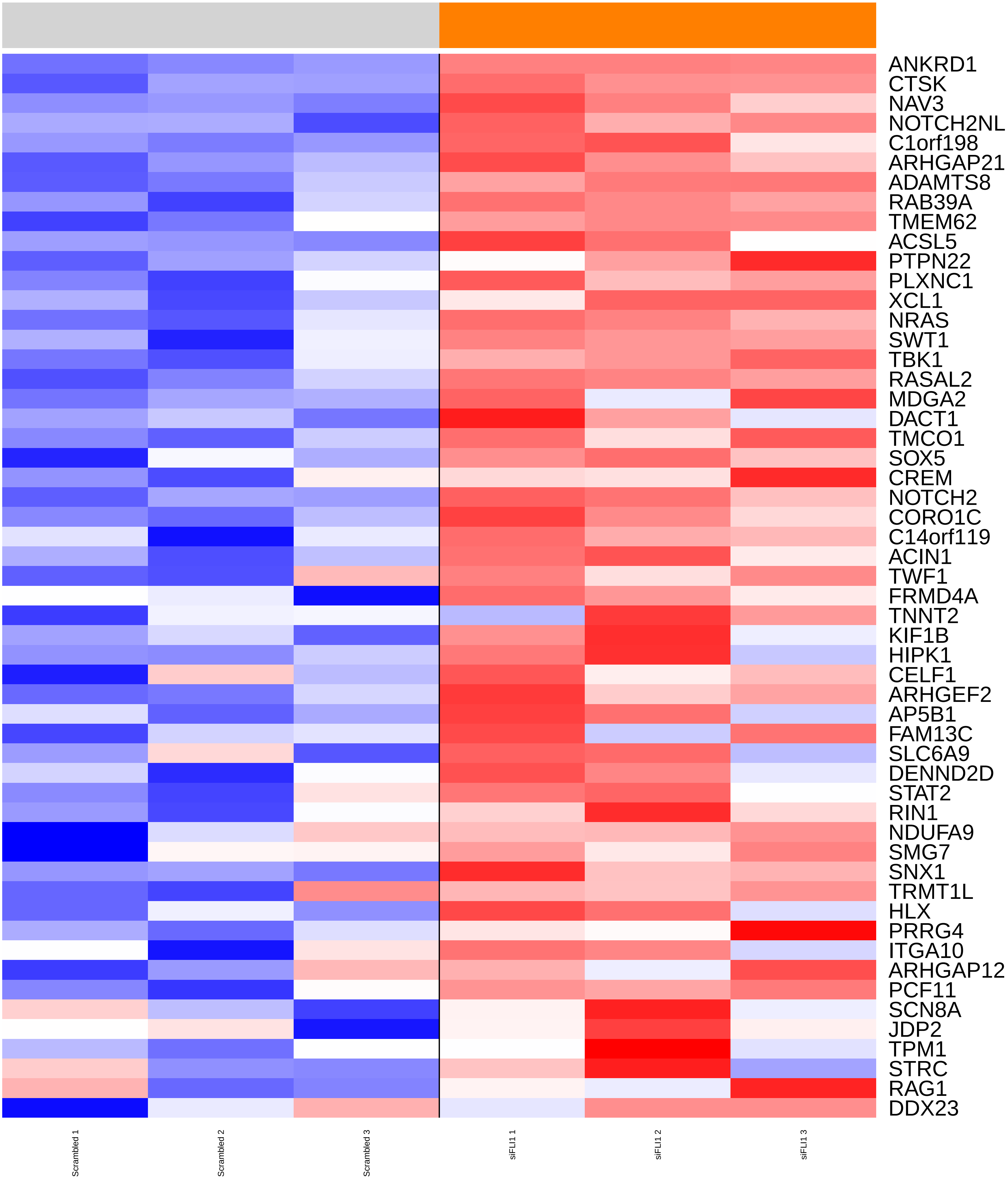

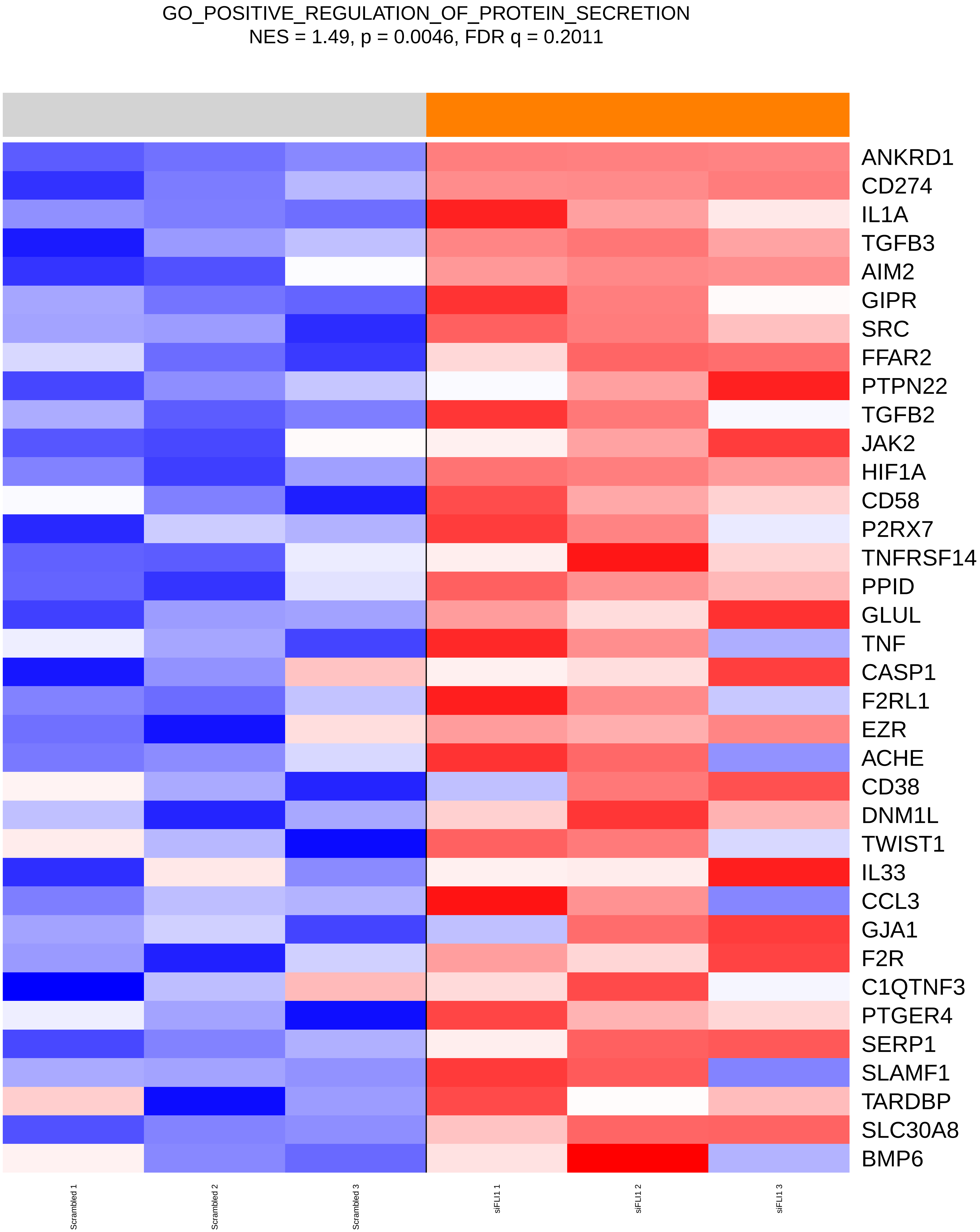
GO_POSITIVE_REGULATION_OF_PROTEIN_SECRETION
NES = 1.49, p = 0.0046, FDR q = 0.2011
ANKRD1
CD274
IL1A
TGFB3
AIM2
GIPR
SRC
FFAR2
PTPN22
TGFB2
JAK2
HIF1A
CD58
P2RX7
TNFRSF14
PPID
GLUL
TNF
CASP1
F2RL1
EZR
ACHE
CD38
DNM1L
TWIST1
IL33
CCL3
GJA1
F2R
C1QTNF3
PTGER4
SERP1
SLAMF1
TARDBP
SLC30A8
BMP6
Scrambled 1
Scrambled 2
Scrambled 3
siFLI1 1
siFLI1 2
siFLI1 3

BIOCARTA_MITOCHONDRIA_PATHWAY
NES = 1.49, p = 0.0484, FDR q = 0.2017

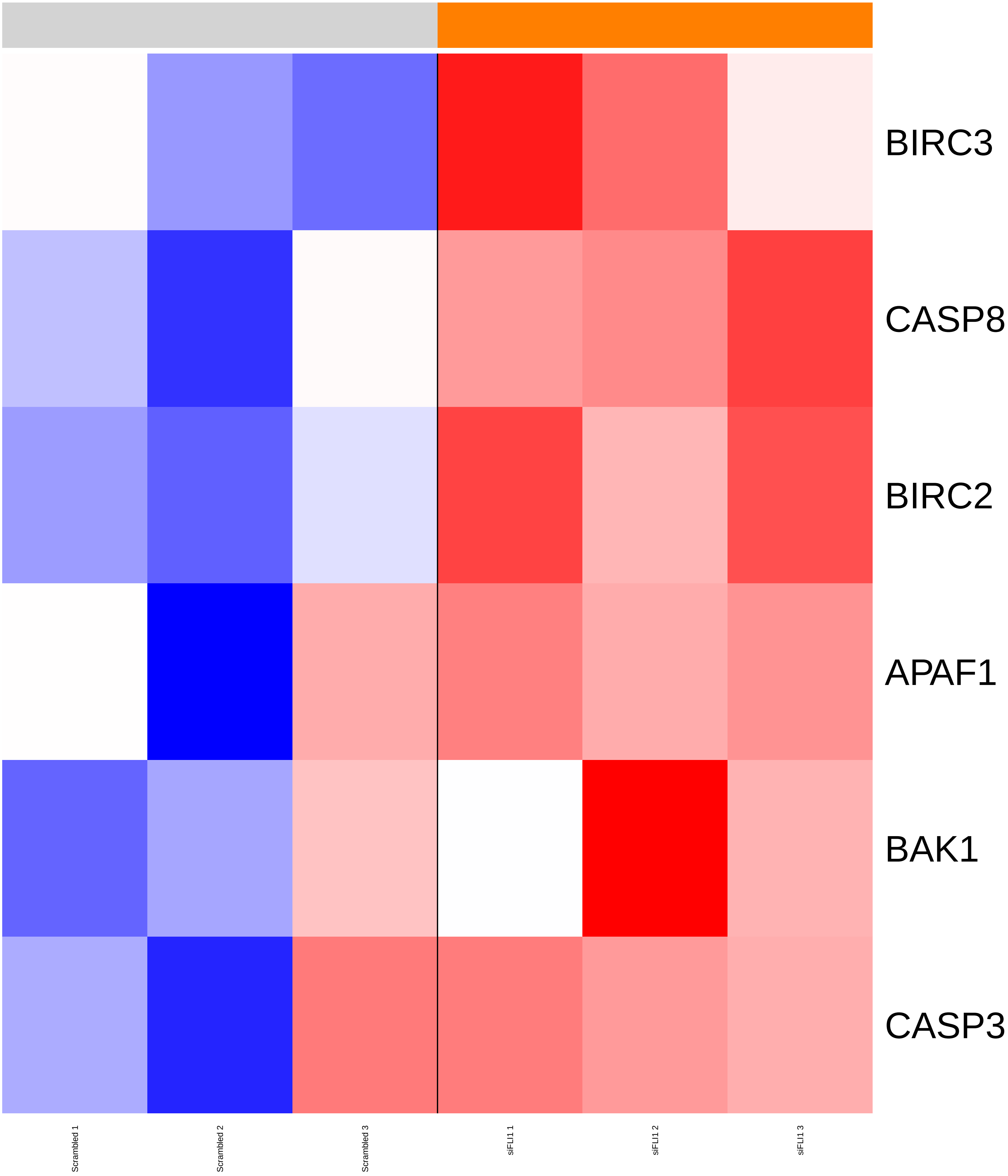

GO_REGULATION_OF_ENDOCYTOSIS
NES = 1.48, p = 0.0000, FDR q = 0.2019

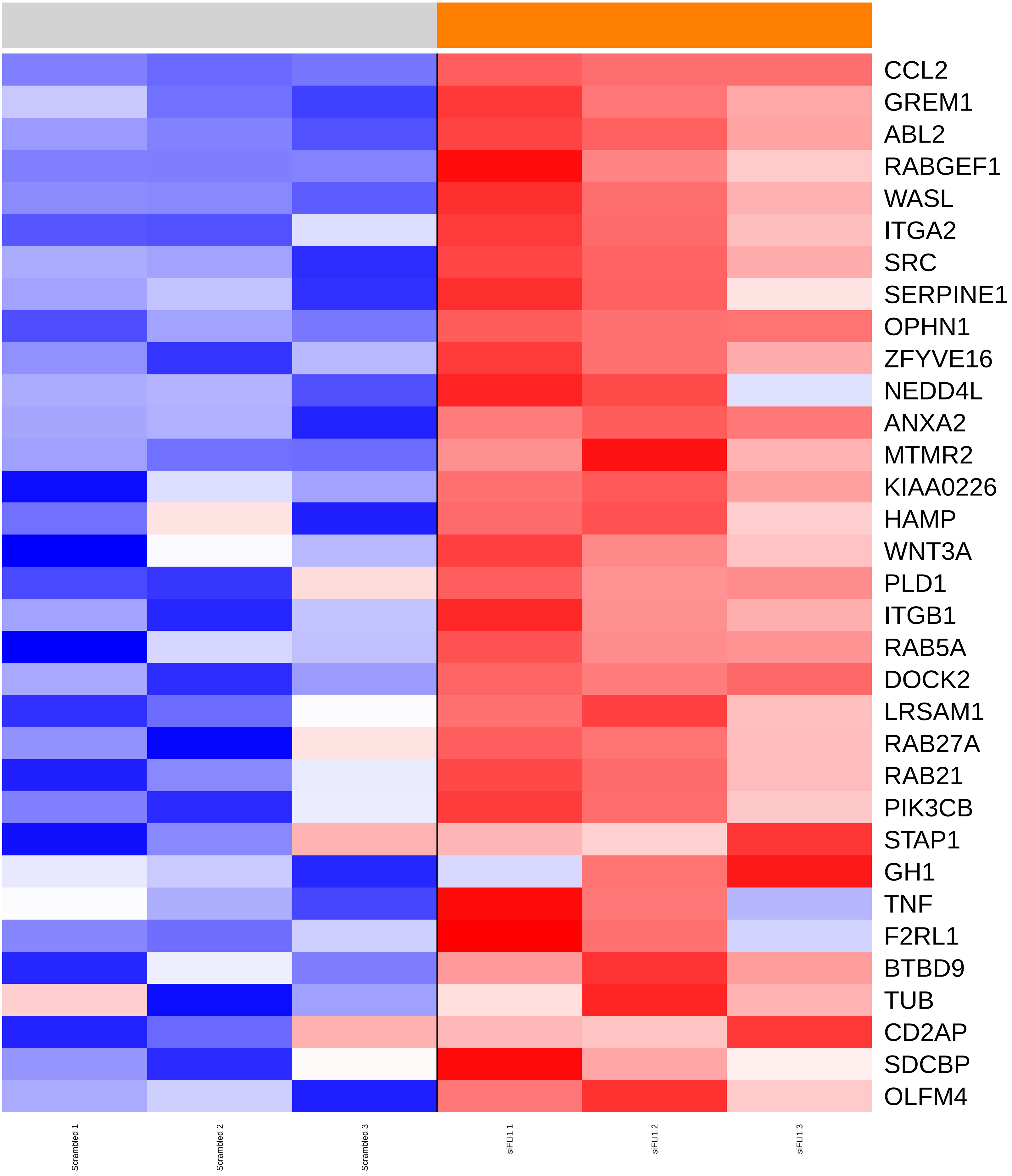

ATCATGA_MIR433
NES = 1.48, p = 0.0100, FDR q = 0.2020

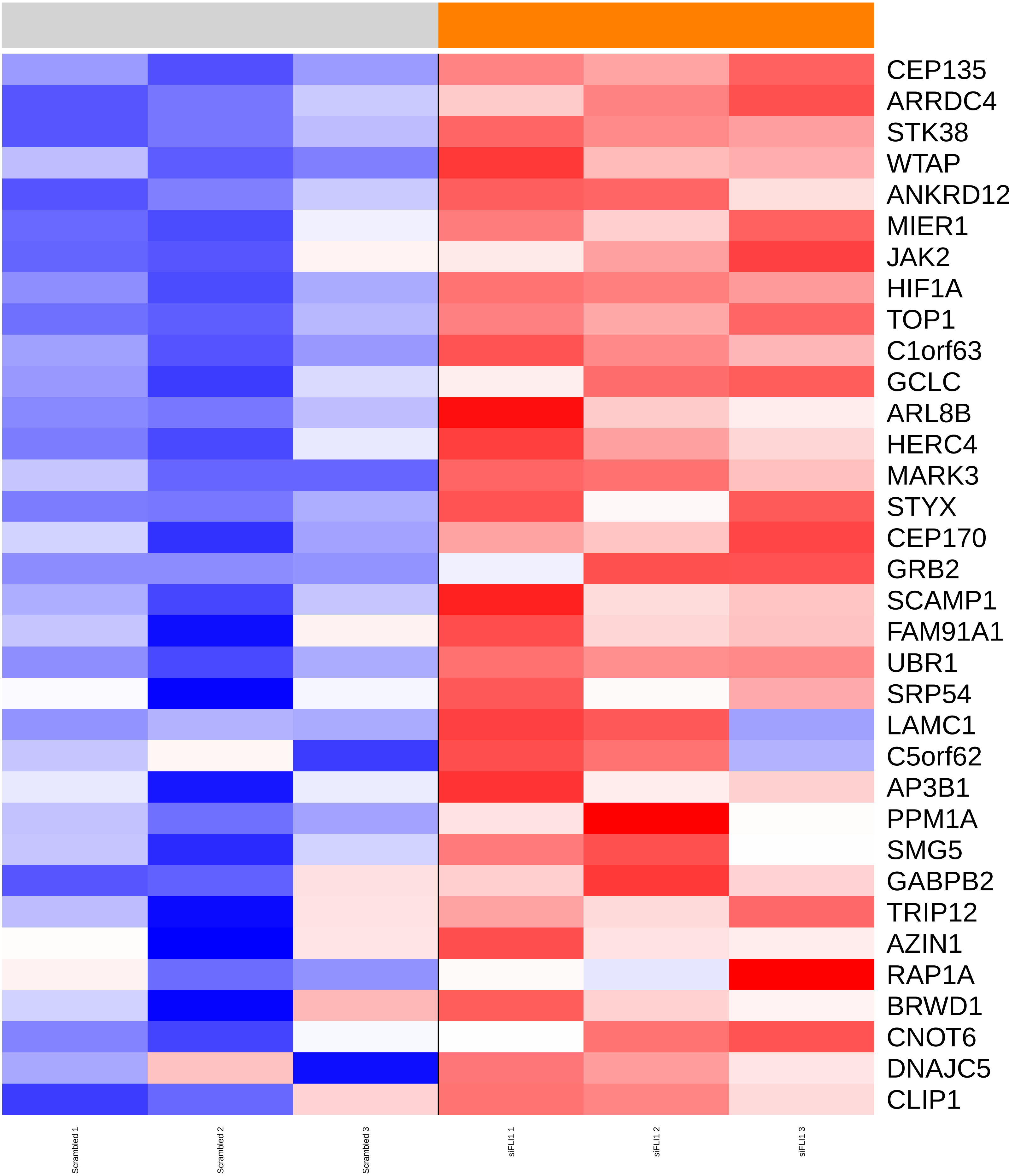

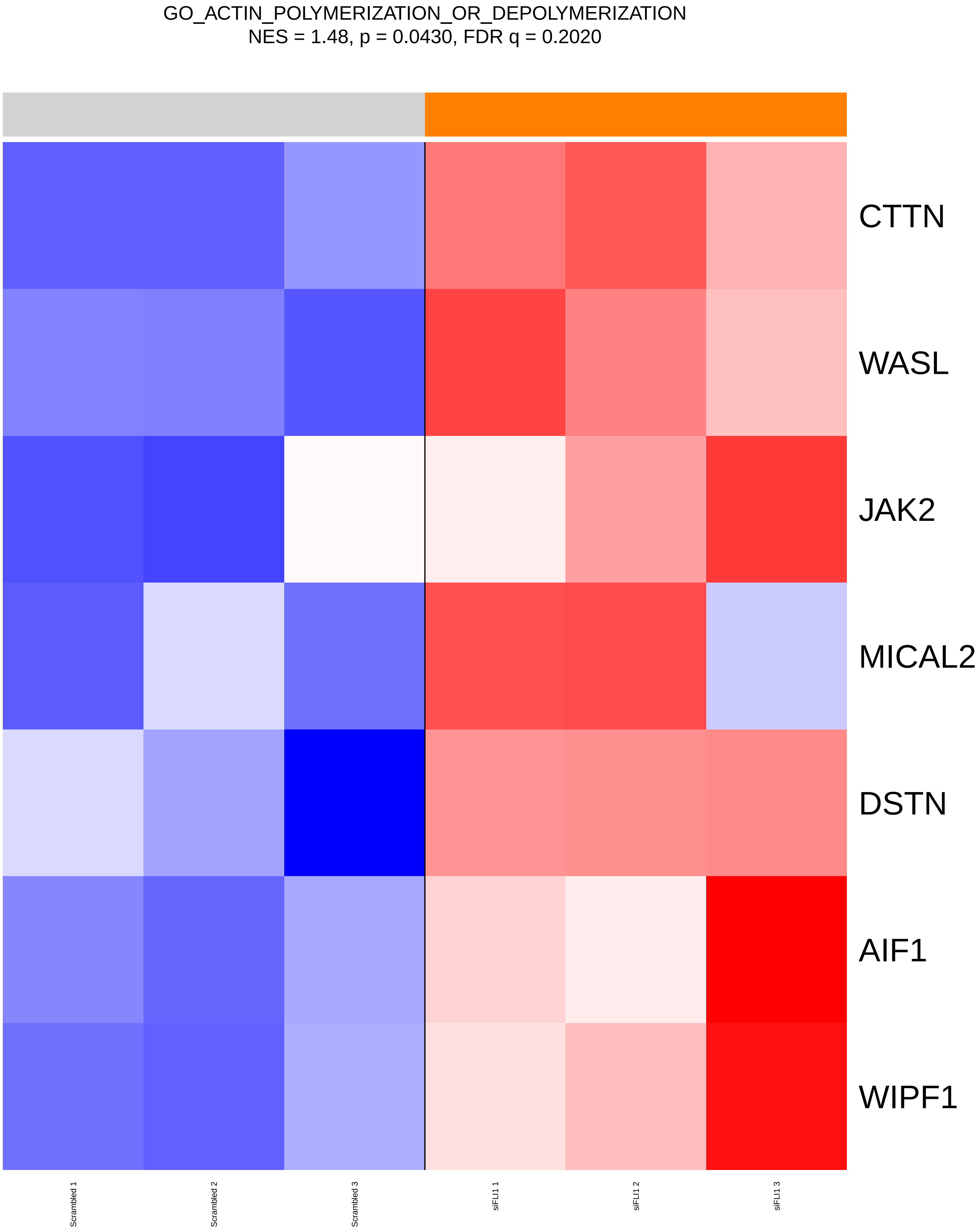

GO_ACTIN_POLYMERIZATION_OR_DEPOLYMERIZATION
NES = 1.48, p = 0.0430, FDR q = 0.2020
CTTN
WASL
JAK2
MICAL2
DSTN
AIF1
WIPF1
Scrambled 1
Scrambled 2
Scrambled 3
siFLI1 1
siFLI1 2
siFLI1 3

GO_MYOFILAMENT
NES = 1.48, p = 0.0444, FDR q = 0.2031

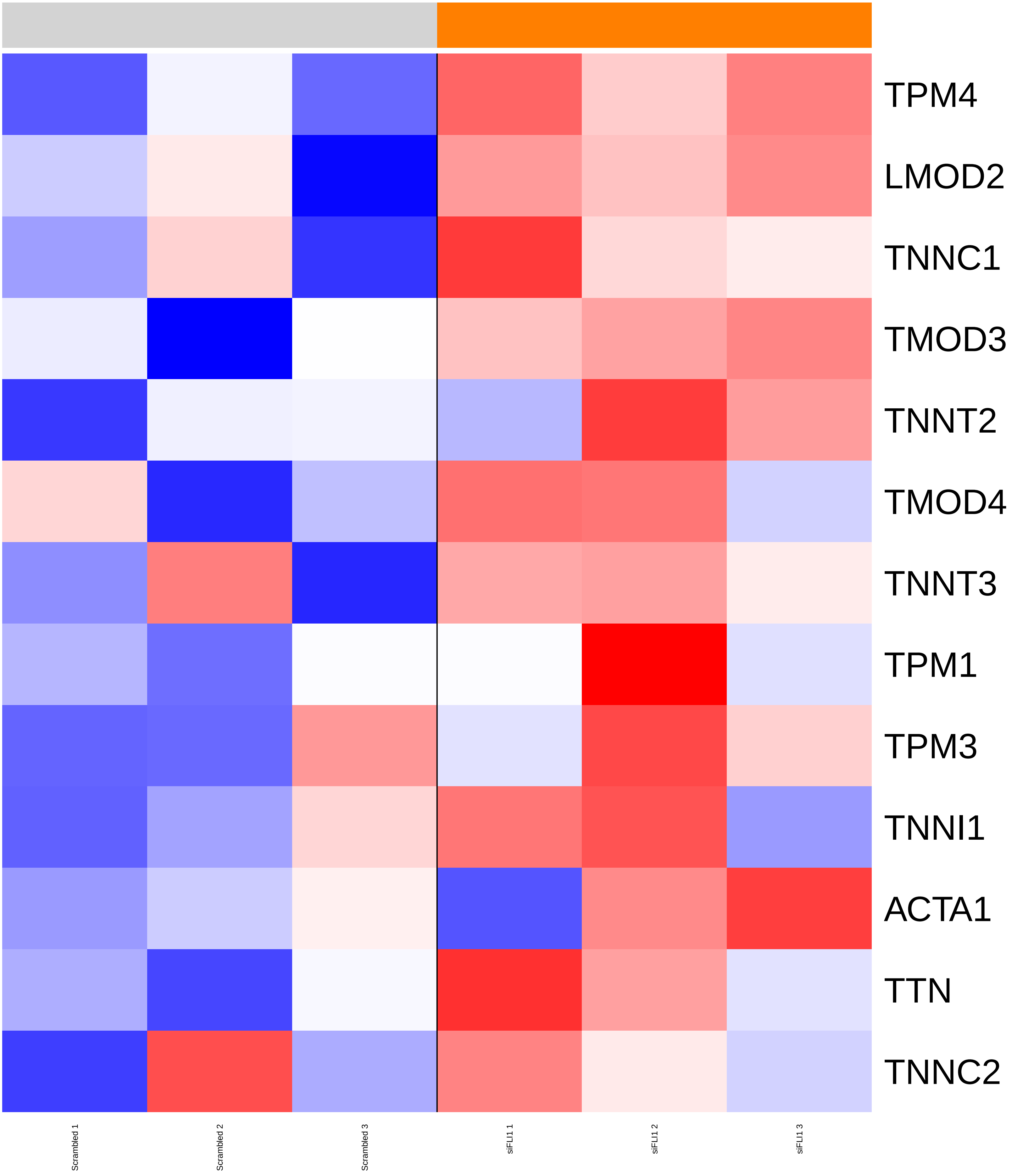

GO_NEGATIVE_REGULATION_OF_LEUKOCYTE_PROLIFERATION
NES = 1.48, p = 0.0355, FDR q = 0.2038

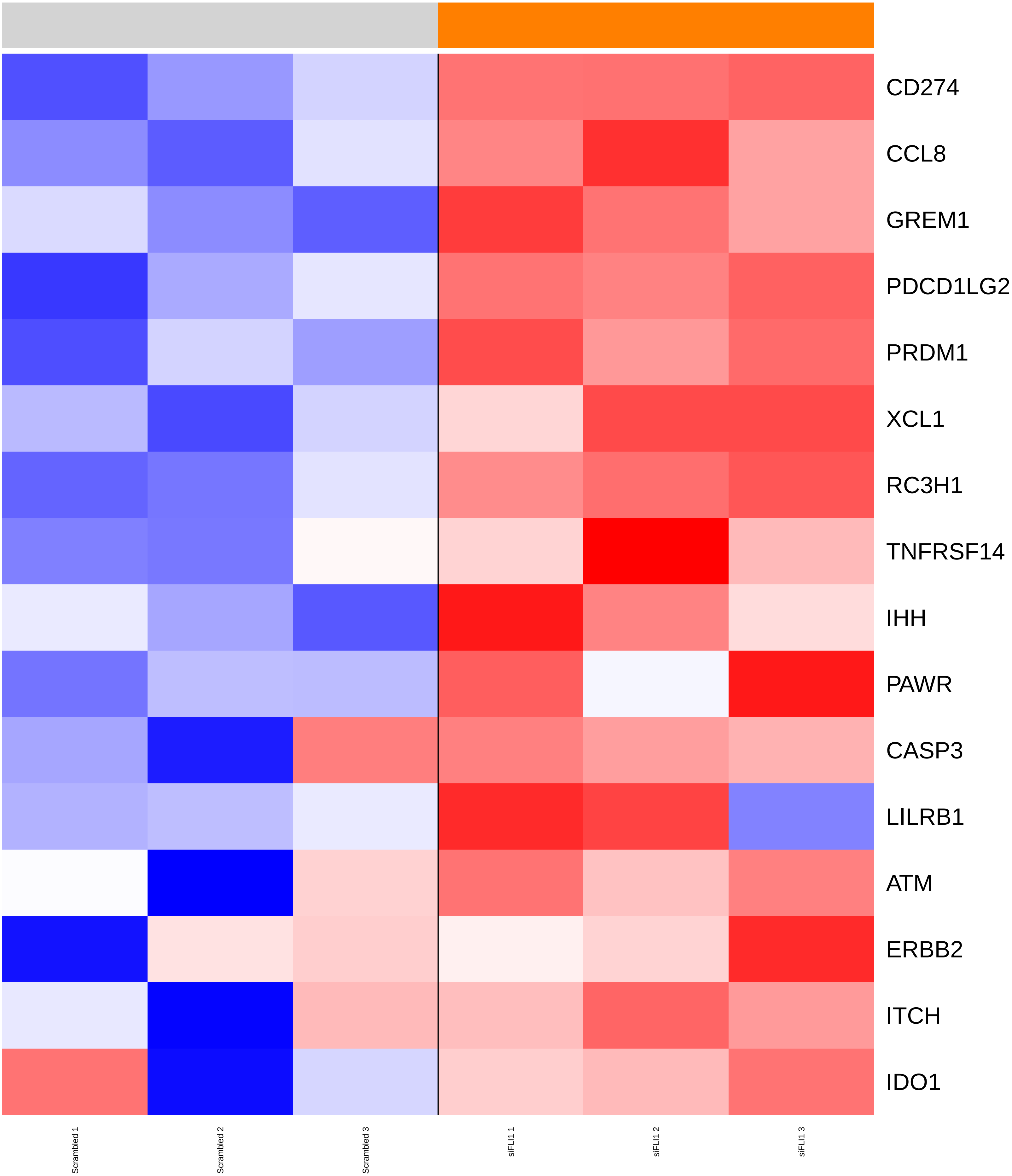

TAATGTG_MIR323
NES = 1.48, p = 0.0000, FDR q = 0.2073

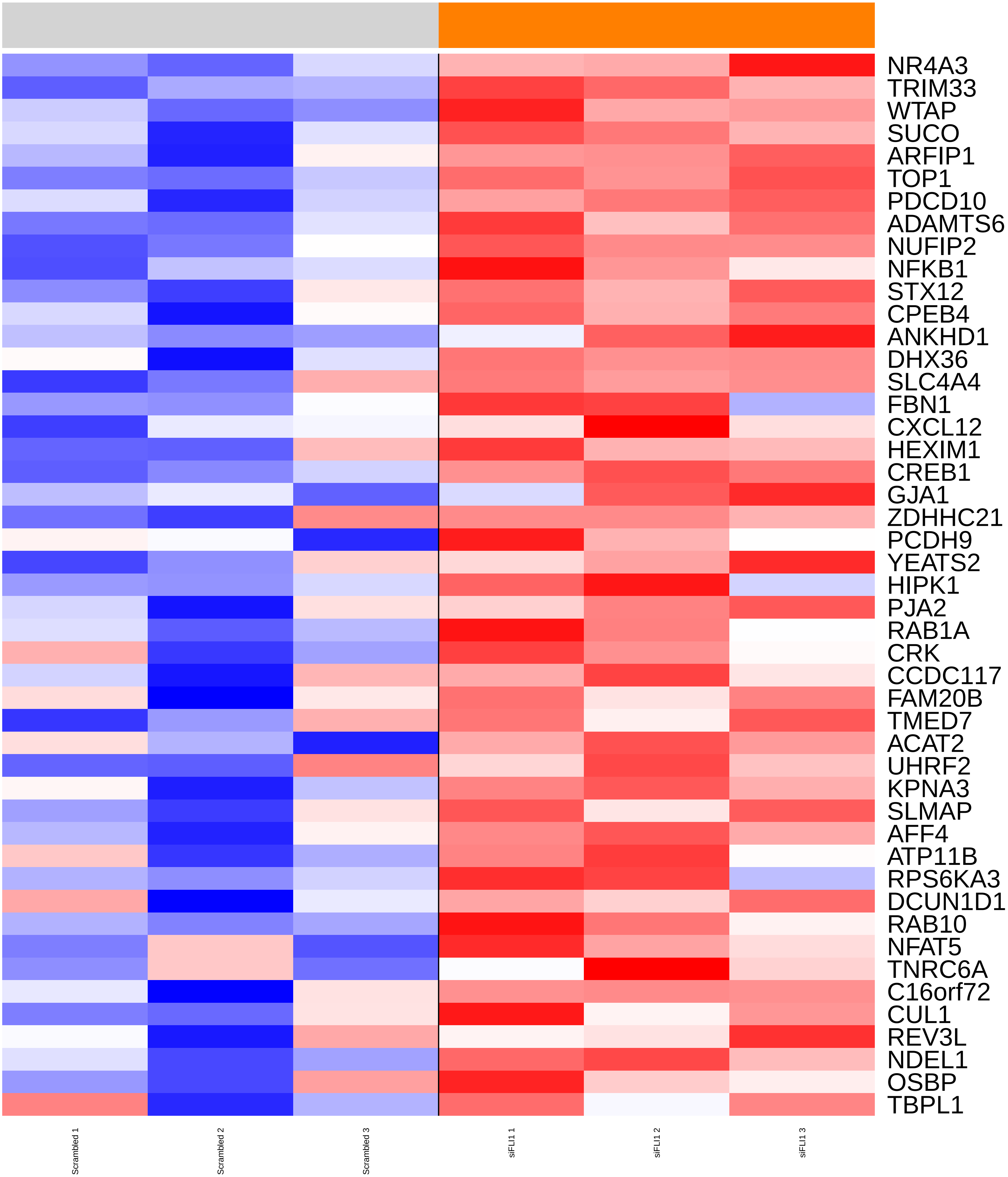

GO_POSITIVE_REGULATION_OF_CYSTEINE_TYPE_ENDOPEPTIDASE_ACTIVITY_INVOLVED_IN_APOPTOTIC_SIGNALING_PATHWAY
NES = 1.48, p = 0.0699, FDR q = 0.2071

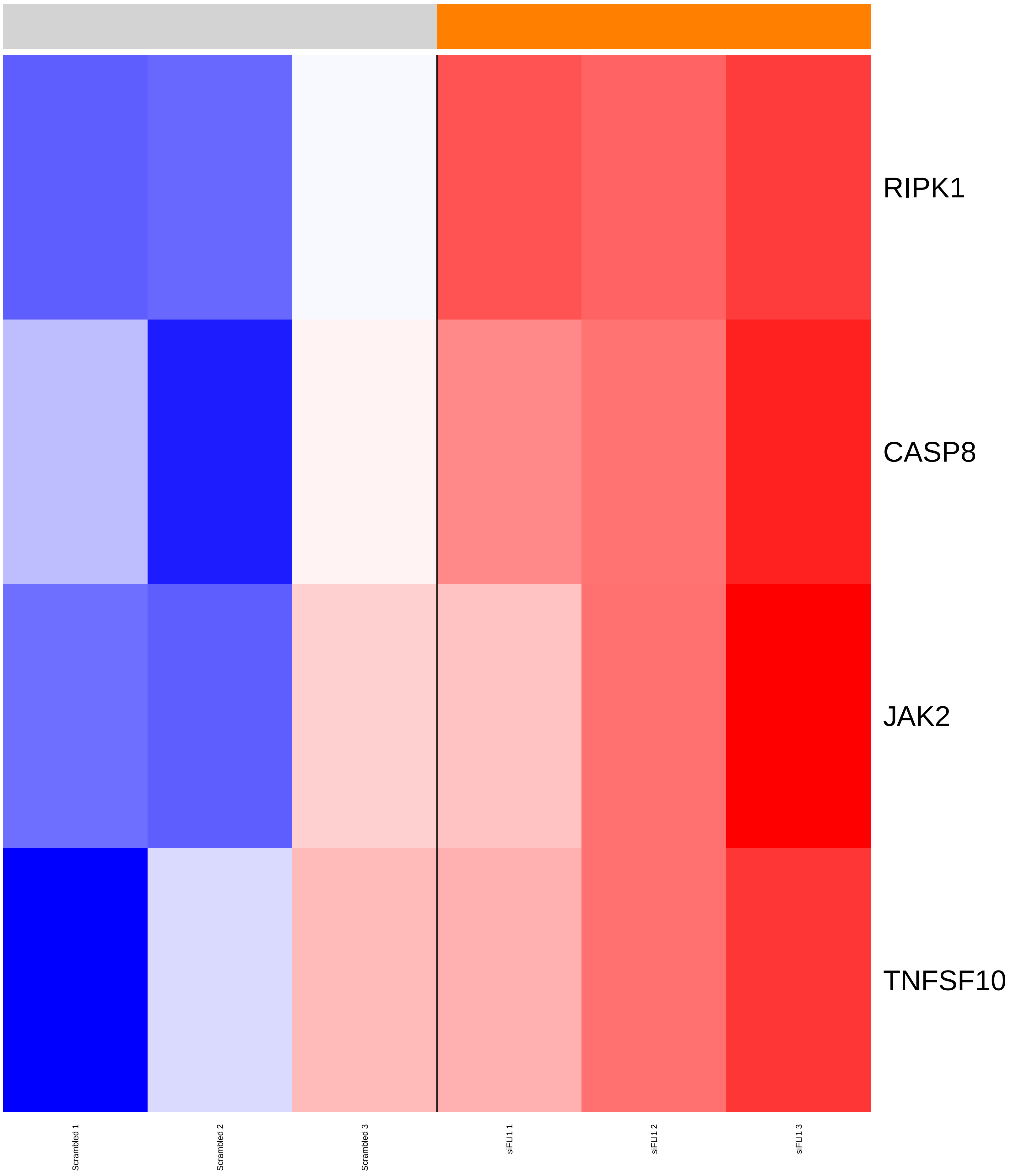

GO_RESPONSE_TO_GAMMA_RADIATION
NES = 1.48, p = 0.0366, FDR q = 0.2067

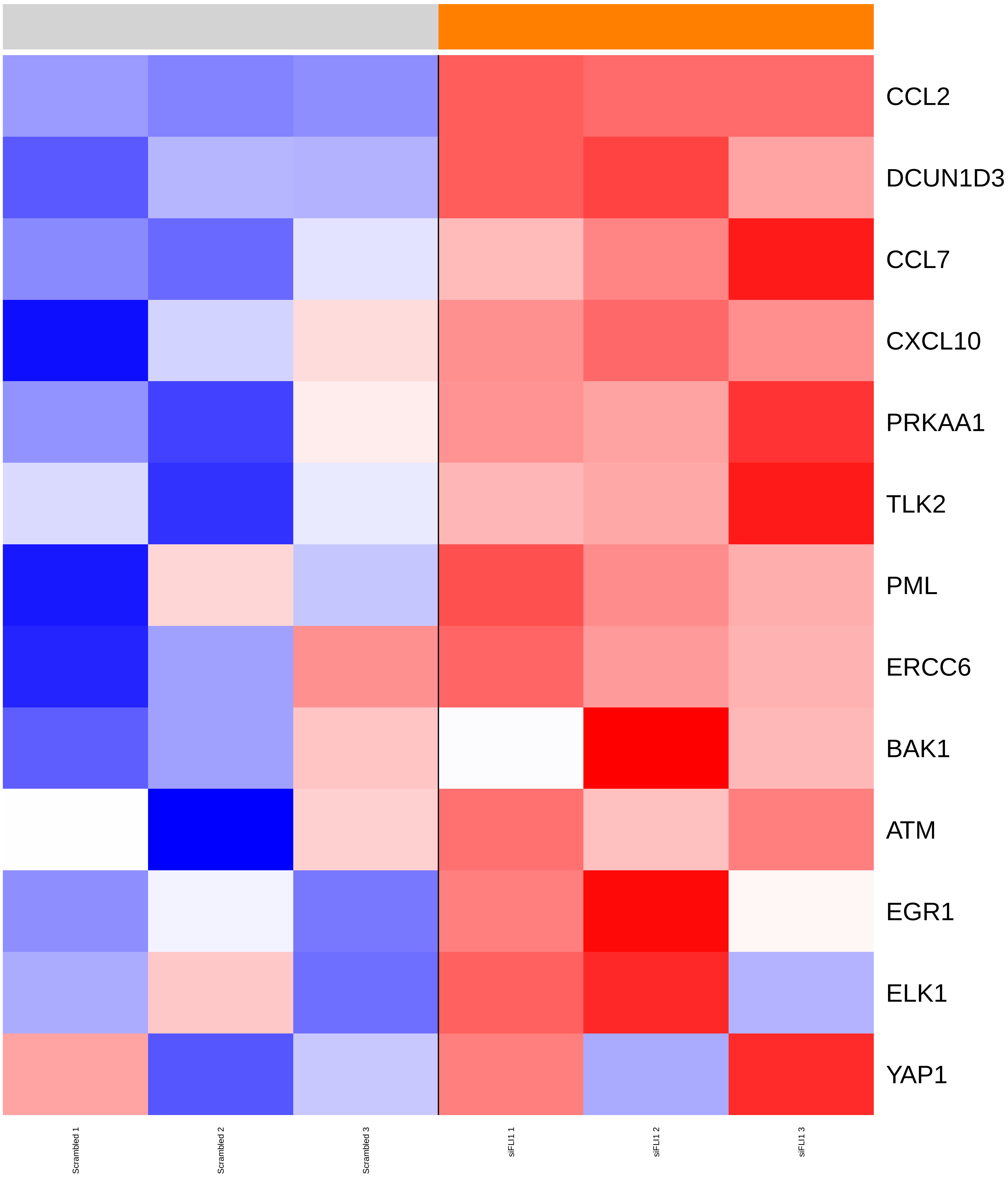

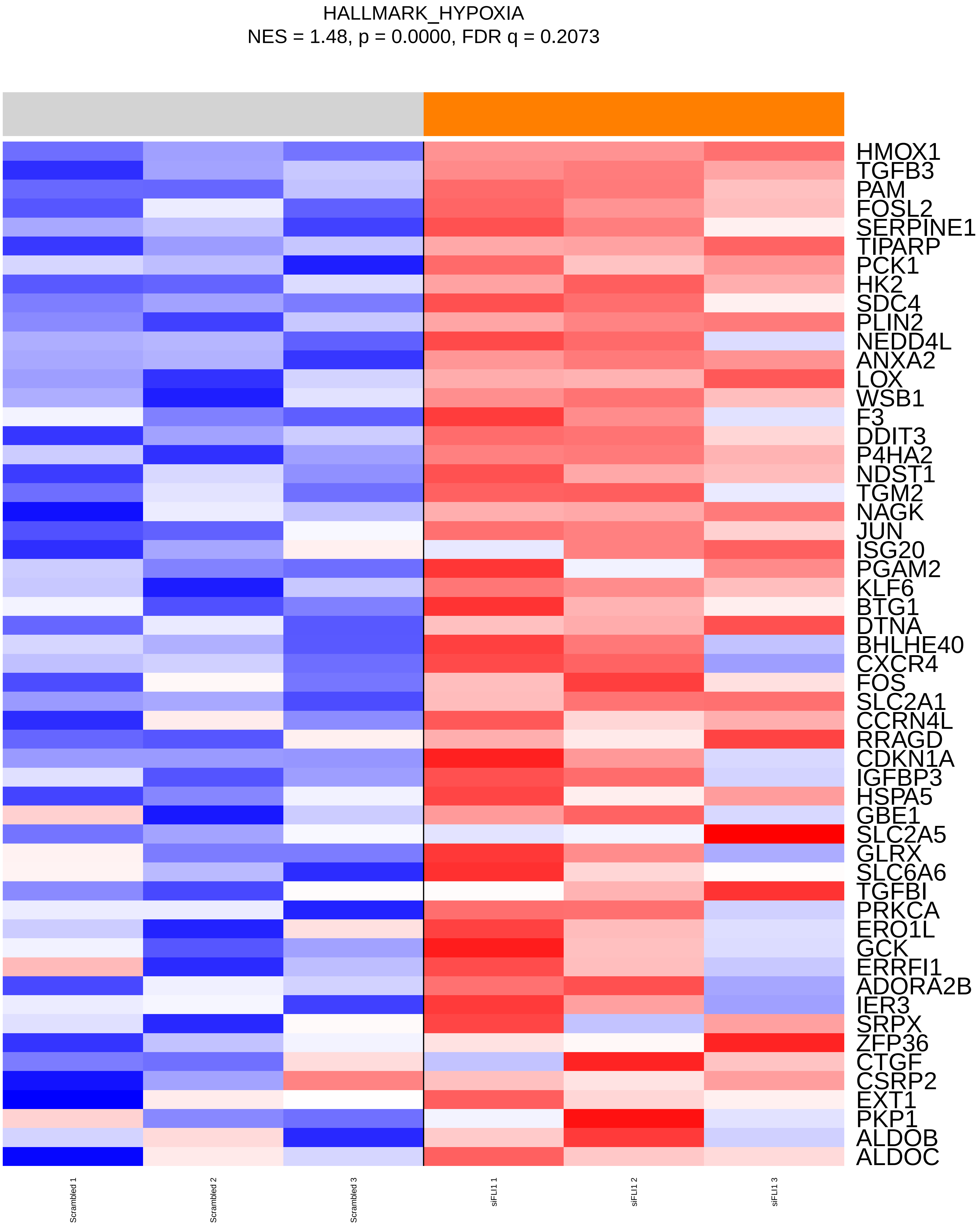
HALLMARK_HYPOXIA
NES = 1.48, p = 0.0000, FDR q = 0.2073
HMOX1
TGFB3
PAM
FOSL2
SERPINE1
TIPARP
PCK1
HK2
SDC4
PLIN2
NEDD4L
ANXA2
LOX
WSB1
F3
DDIT3
P4HA2
NDST1
TGM2
NAGK
JUN
ISG20
PGAM2
KLF6
BTG1
DTNA
BHLHE40
CXCR4
FOS
SLC2A1
CCRN4L
RRAGD
CDKN1A
IGFBP3
HSPA5
GBE1
SLC2A5
GLRX
SLC6A6
TGFBI
PRKCA
ERO1L
GCK
ERRFI1
ADORA2B
IER3
SRPX
ZFP36
CTGF
CSRP2
EXT1
PKP1
ALDOB
ALDOC
Scrambled 1
Scrambled 2
Scrambled 3
siFLI1 1
siFLI1 2
siFLI1 3

GO_RESPONSE_TO_MURAMYL_DIPEPTIDE
NES = 1.48, p = 0.0515, FDR q = 0.2073

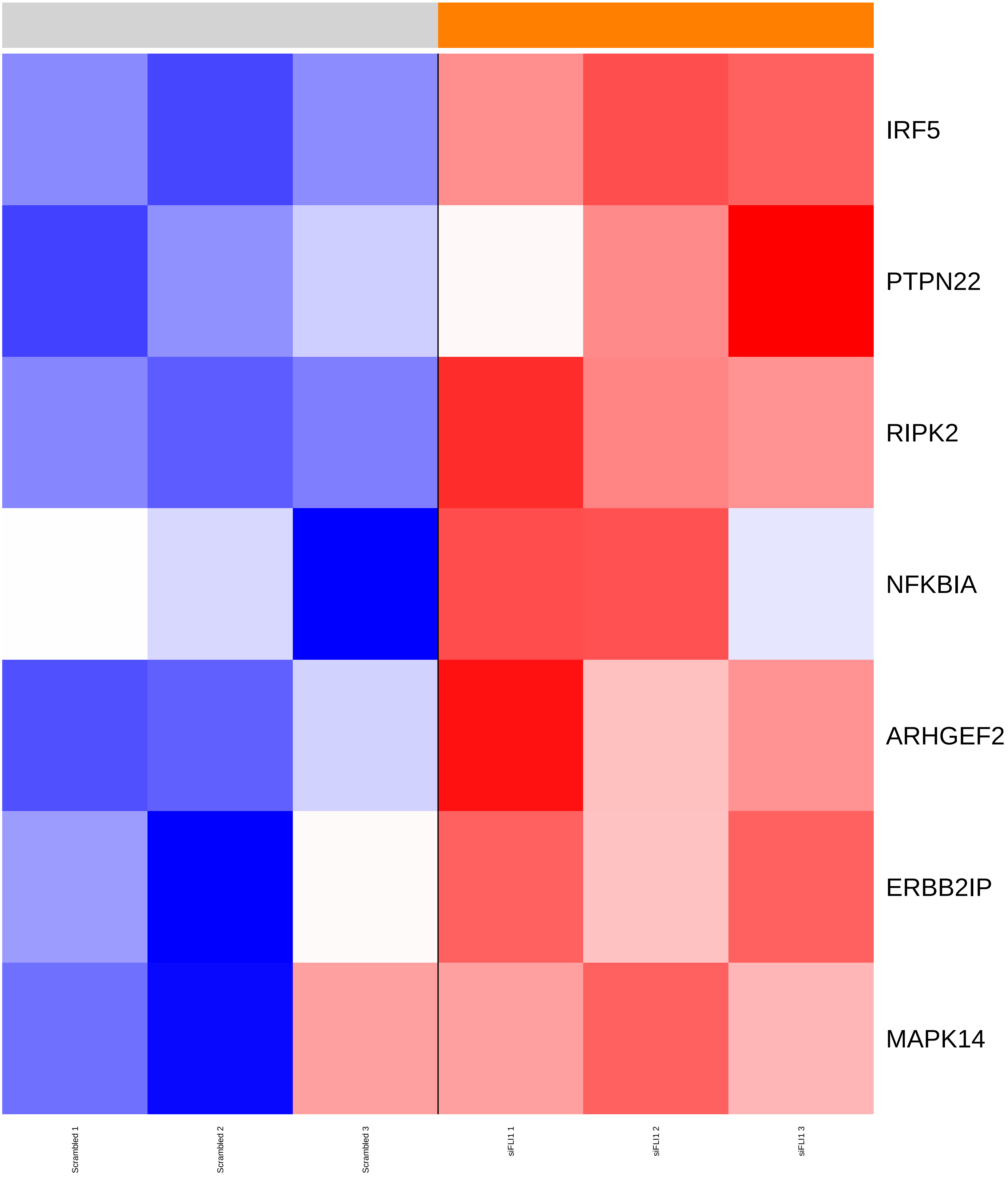

GO_NEGATIVE_REGULATION_OF_RESPONSE_TO_EXTERNAL_STIMULUS
NES = 1.48, p = 0.0000, FDR q = 0.2075

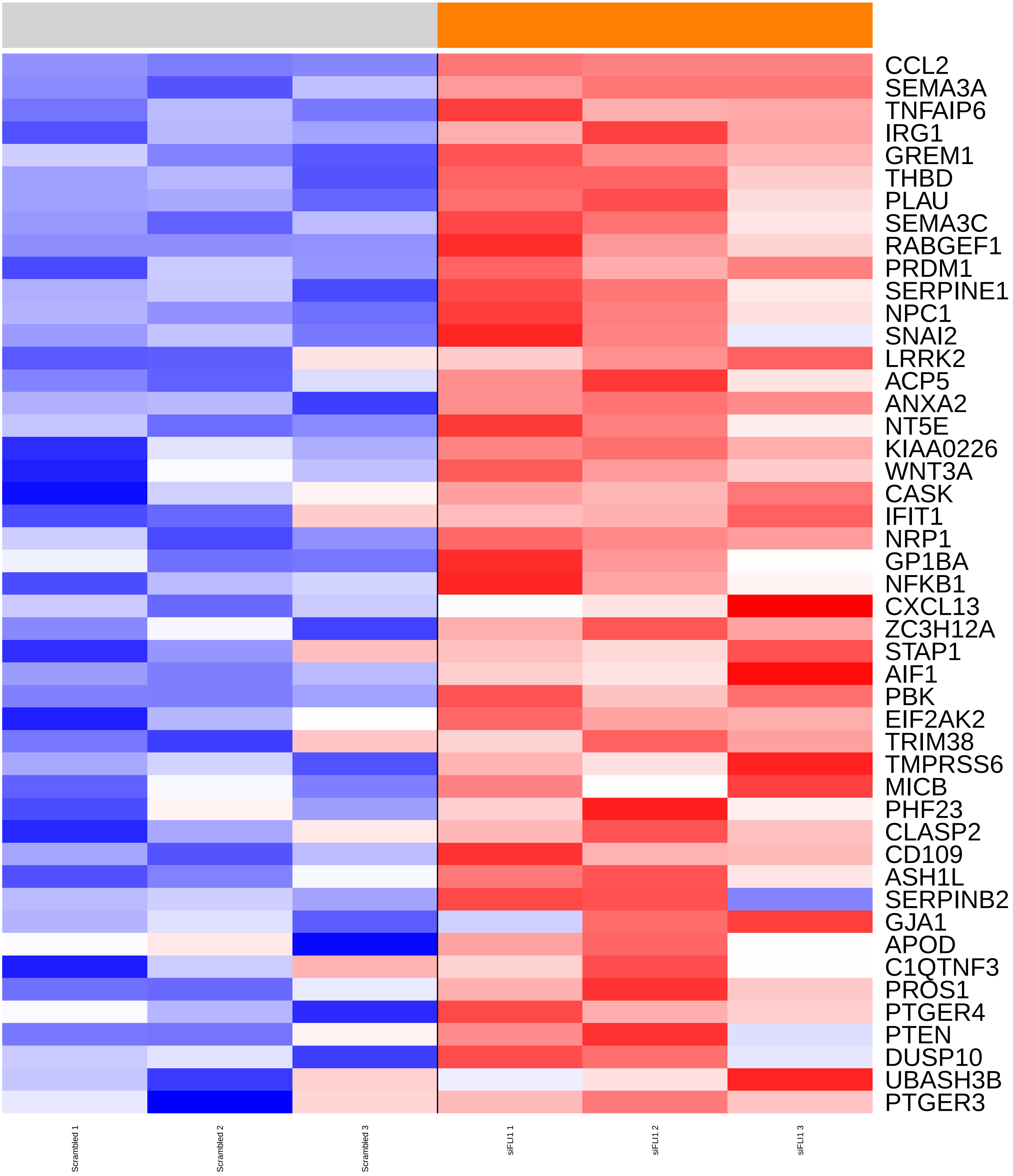

TCCATTKW_UNKNOWN
NES = 1.48, p = 0.0000, FDR q = 0.2075

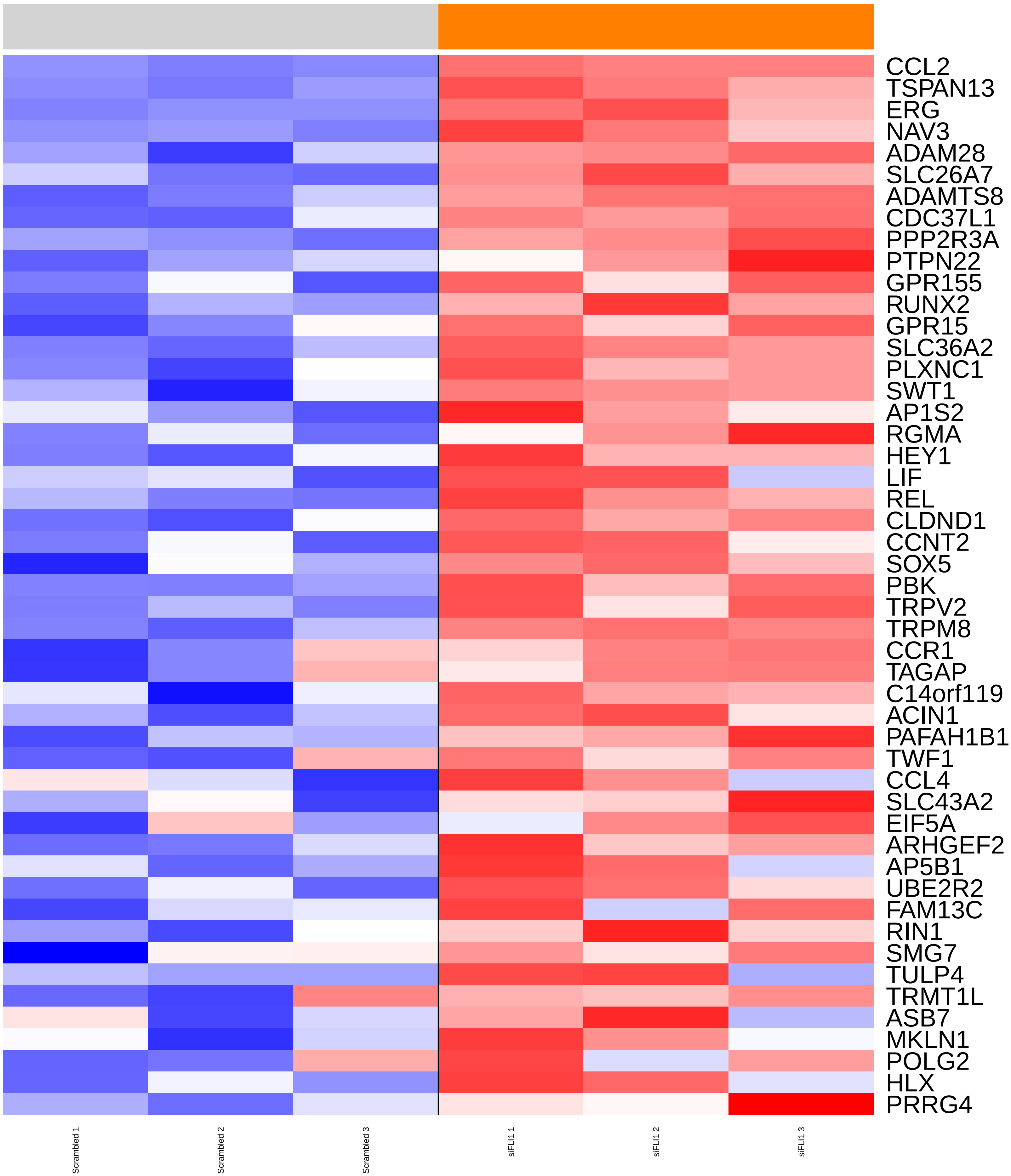

GO_EYE_PHOTORECEPTOR_CELL_DEVELOPMENT
NES = 1.47, p = 0.0360, FDR q = 0.2099

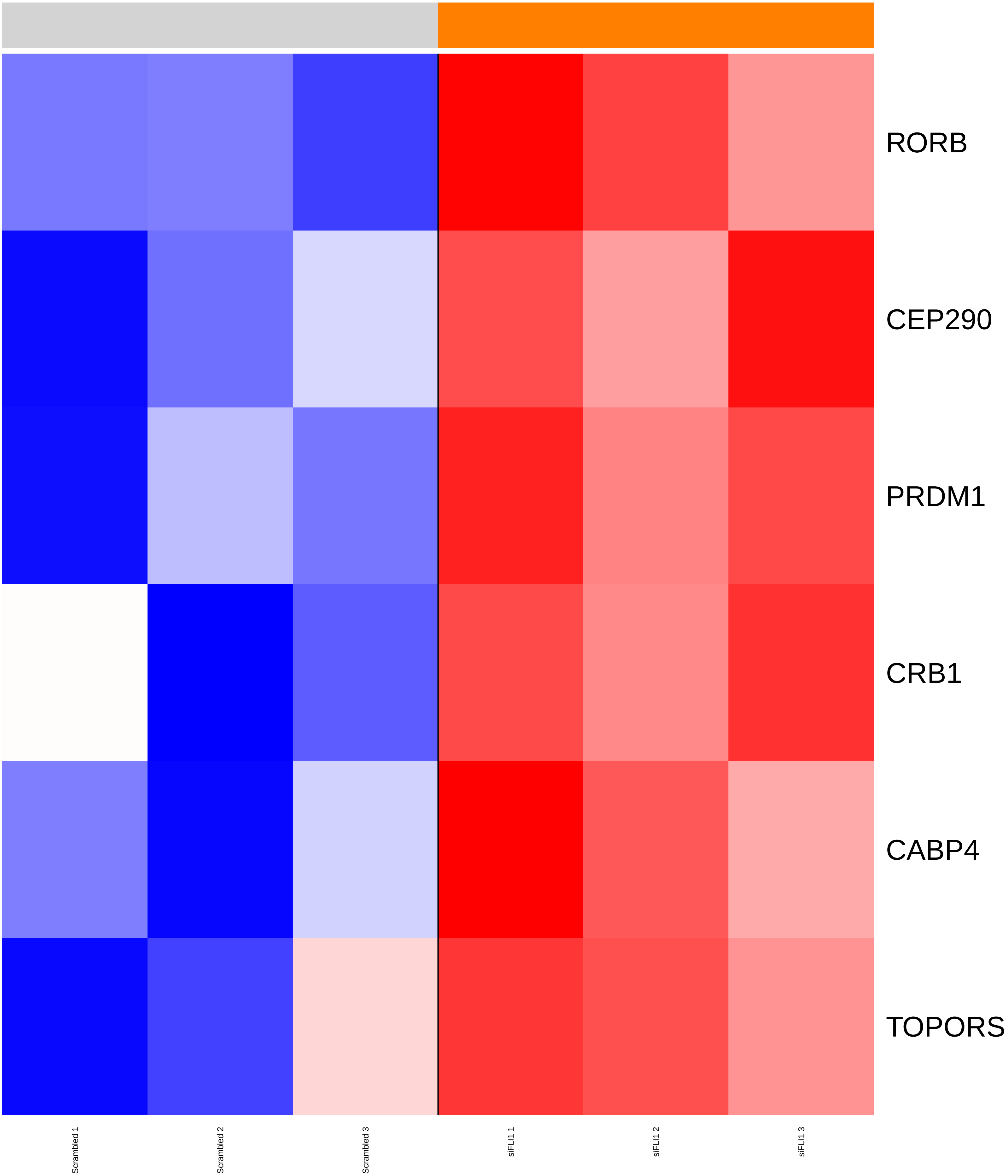

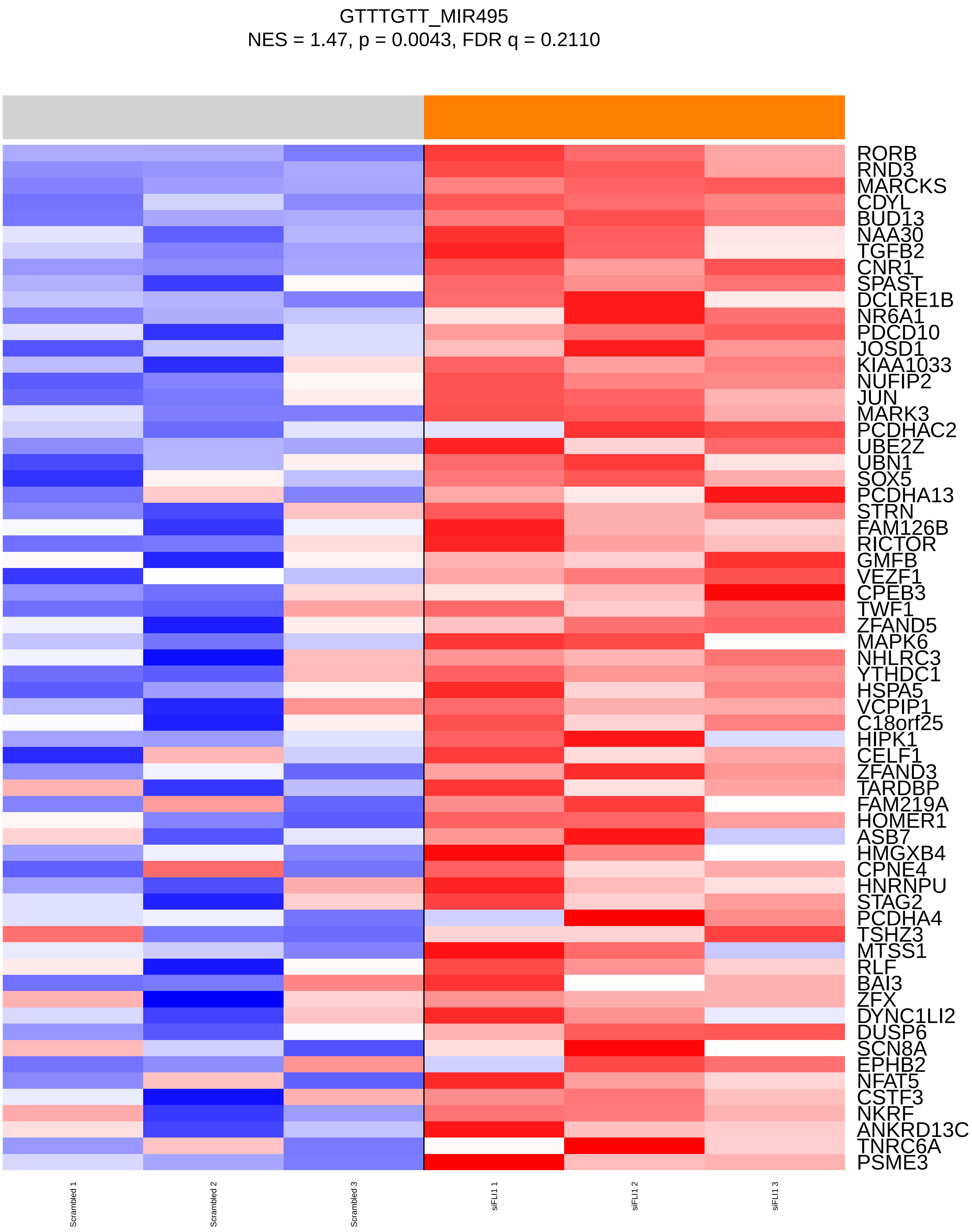
GTTTGTT_MIR495
NES = 1.47, p = 0.0043, FDR q = 0.2110
RORB
RND3
MARCKS
CDYL
BUD13
NAA30
TGFB2
CNR1
SPAST
DCLRE1B
NR6A1
PDCD10
JOSD1
KIAA1033
NUFIP2
JUN
MARK3
PCDHAC2
UBE2Z
UBN1
SOX5
PCDHA13
STRN
FAM126B
RICTOR
GMFB
VEZF1
CPEB3
TWF1
ZFAND5
MAPK6
NHLRC3
YTHDC1
HSPA5
VCPIP1
C18orf25
HIPK1
CELF1
ZFAND3
TARDBP
FAM219A
HOMER1
ASB7
HMGXB4
CPNE4
HNRNPU
STAG2
PCDHA4
TSHZ3
MTSS1
RLF
BAI3
ZFX
DYNC1LI2
DUSP6
SCN8A
EPHB2
NFAT5
CSTF3
NKRF
ANKRD13C
TNRC6A
PSME3
Scrambled 1
Scrambled 2
Scrambled 3
siFLI1 1
siFLI1 2
siFLI1 3

GO_CELLULAR_RESPONSE_TO_EXTERNAL_STIMULUS
NES = 1.47, p = 0.0000, FDR q = 0.2109

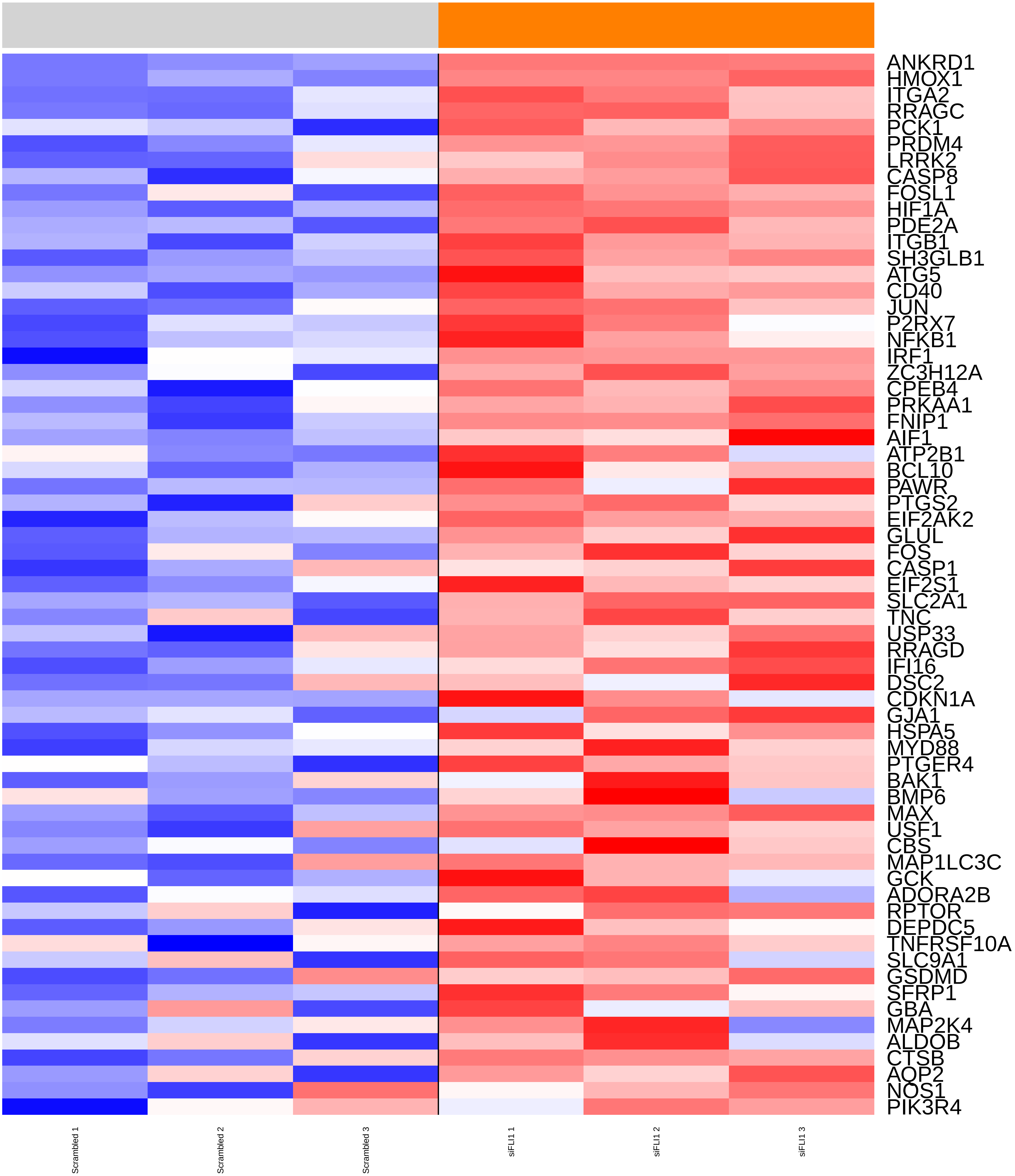

GO_HISTONE_DEMETHYLASE_ACTIVITY
NES = 1.47, p = 0.0697, FDR q = 0.2119

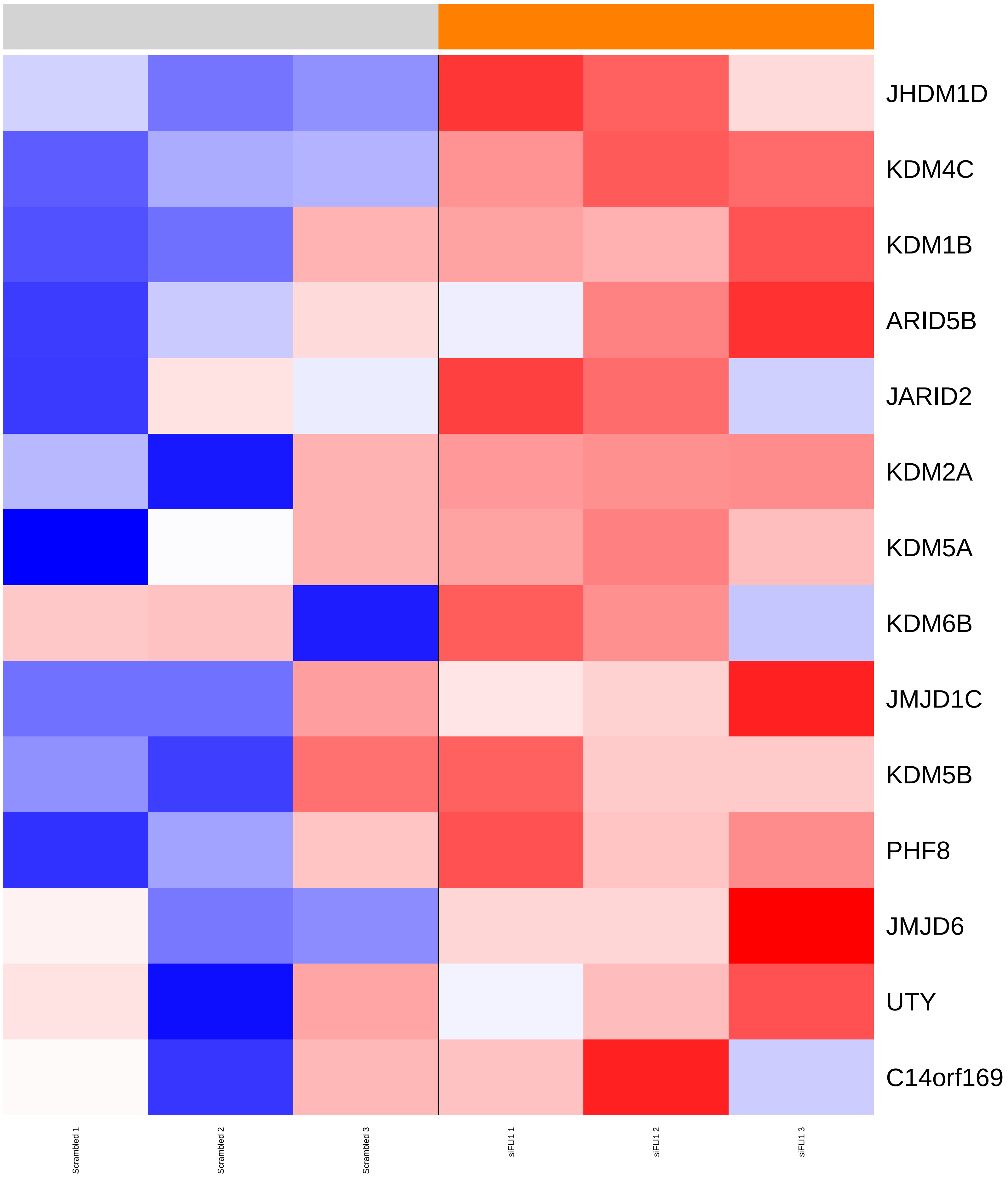

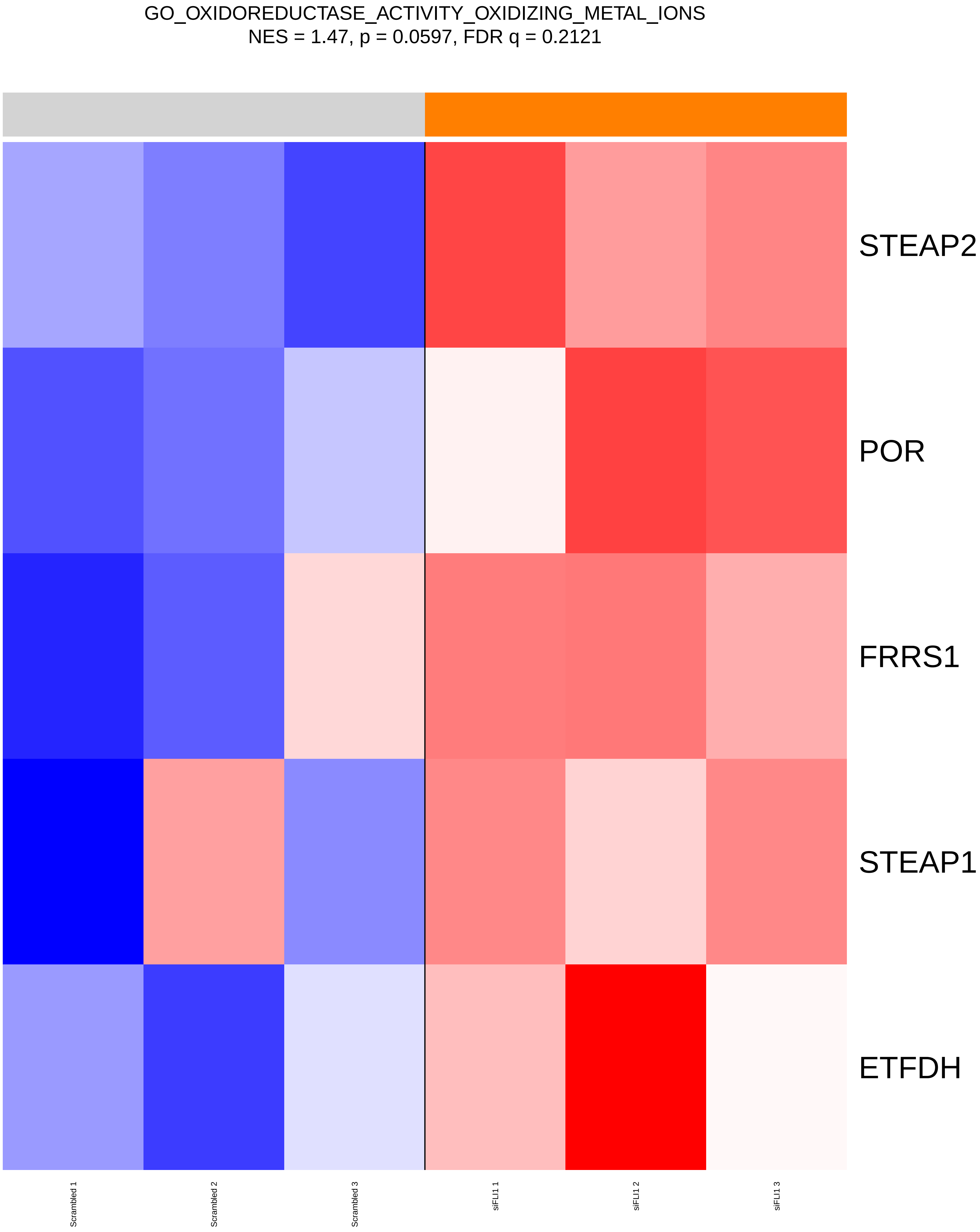
GO_OXIDOREDUCTASE_ACTIVITY_OXIDIZING_METAL_IONS
NES = 1.47, p = 0.0597, FDR q = 0.2121
STEAP2
POR
FRRS1
STEAP1
ETFDH
Scrambled 1
Scrambled 2
Scrambled 3
siFLI1 1
siFLI1 2
siFLI1 3

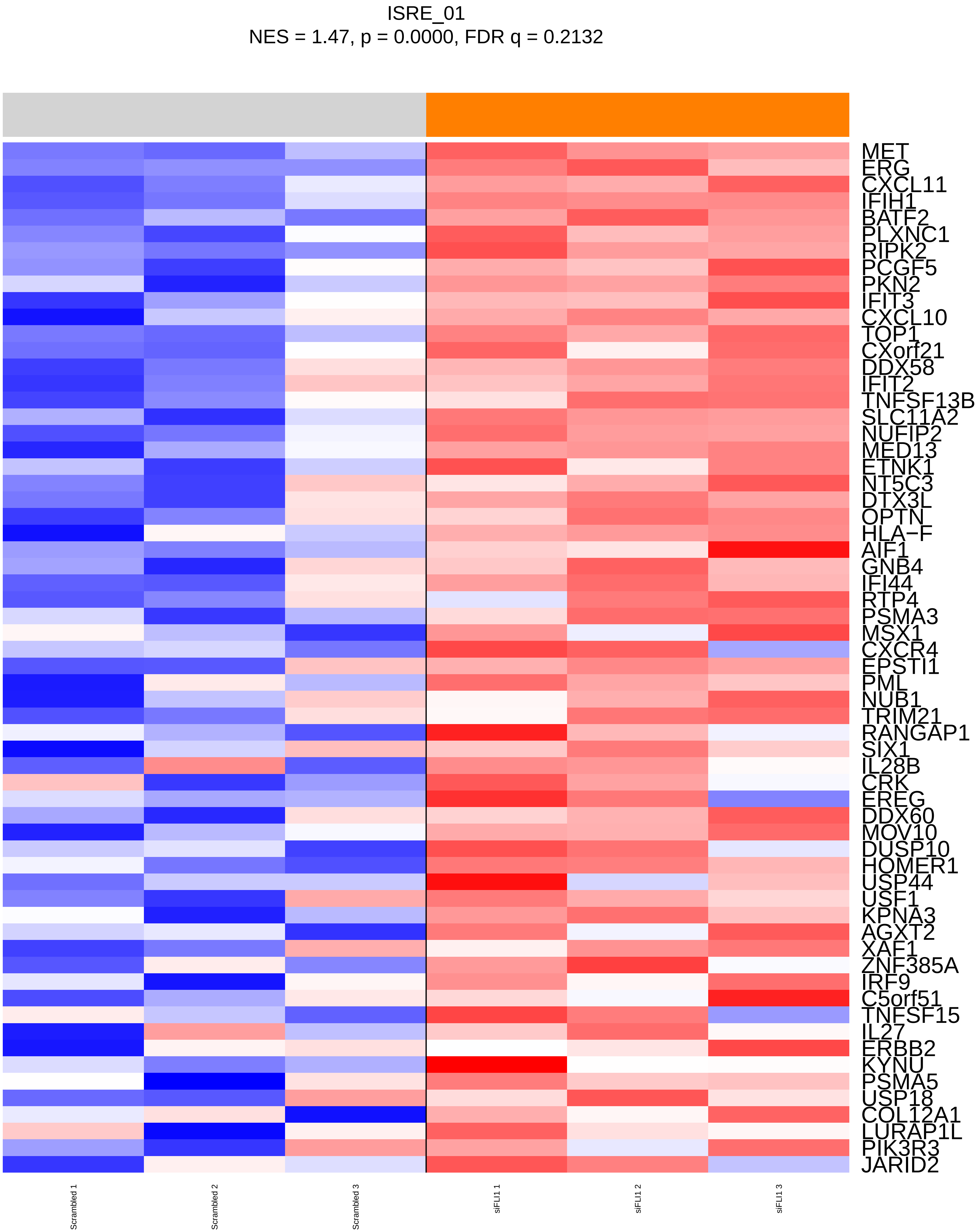
ISRE_01
NES = 1.47, p = 0.0000, FDR q = 0.2132
MET
ERG
CXCL11
IFIH1
BATF2
PLXNC1
RIPK2
PCGF5
PKN2
IFIT3
CXCL10
TOP1
CXorf21
DDX58
IFIT2
TNFSF13B
SLC11A2
NUFIP2
MED13
ETNK1
NT5C3
DTX3L
OPTN
HLA−F
AIF1
GNB4
IFI44
RTP4
PSMA3
MSX1
CXCR4
EPSTI1
PML
NUB1
TRIM21
RANGAP1
SIX1
IL28B
CRK
EREG
DDX60
MOV10
DUSP10
HOMER1
USP44
USF1
KPNA3
AGXT2
XAF1
ZNF385A
IRF9
C5orf51
TNFSF15
IL27
ERBB2
KYNU
PSMA5
USP18
COL12A1
LURAP1L
PIK3R3
JARID2
Scrambled 1
Scrambled 2
Scrambled 3
siFLI1 1
siFLI1 2
siFLI1 3

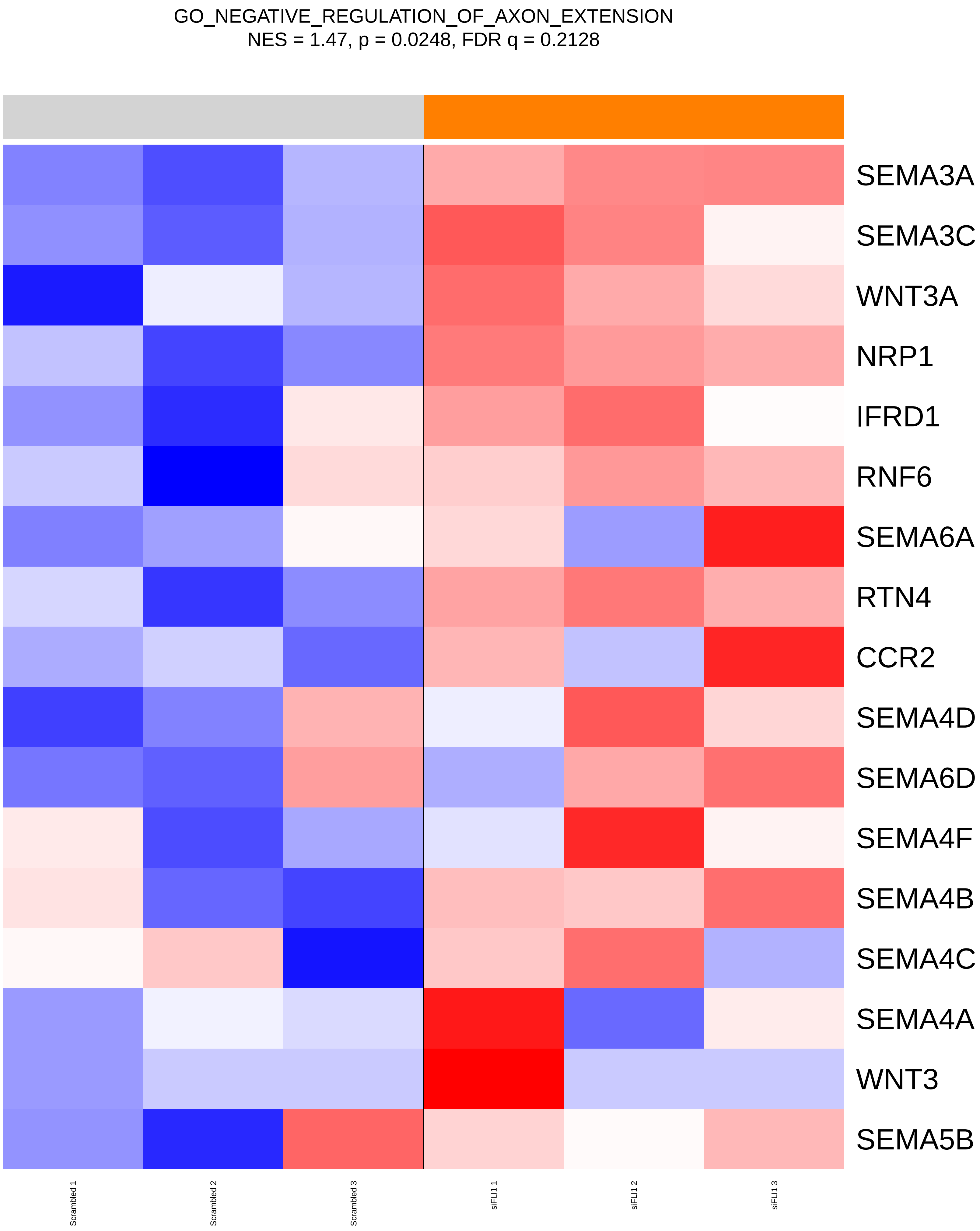
GO_NEGATIVE_REGULATION_OF_AXON_EXTENSION
NES = 1.47, p = 0.0248, FDR q = 0.2128
SEMA3A
SEMA3C
WNT3A
NRP1
IFRD1
RNF6
SEMA6A
RTN4
CCR2
SEMA4D
SEMA6D
SEMA4F
SEMA4B
SEMA4C
SEMA4A
WNT3
SEMA5B
Scrambled 1
Scrambled 2
Scrambled 3
siFLI1 1
siFLI1 2
siFLI1 3

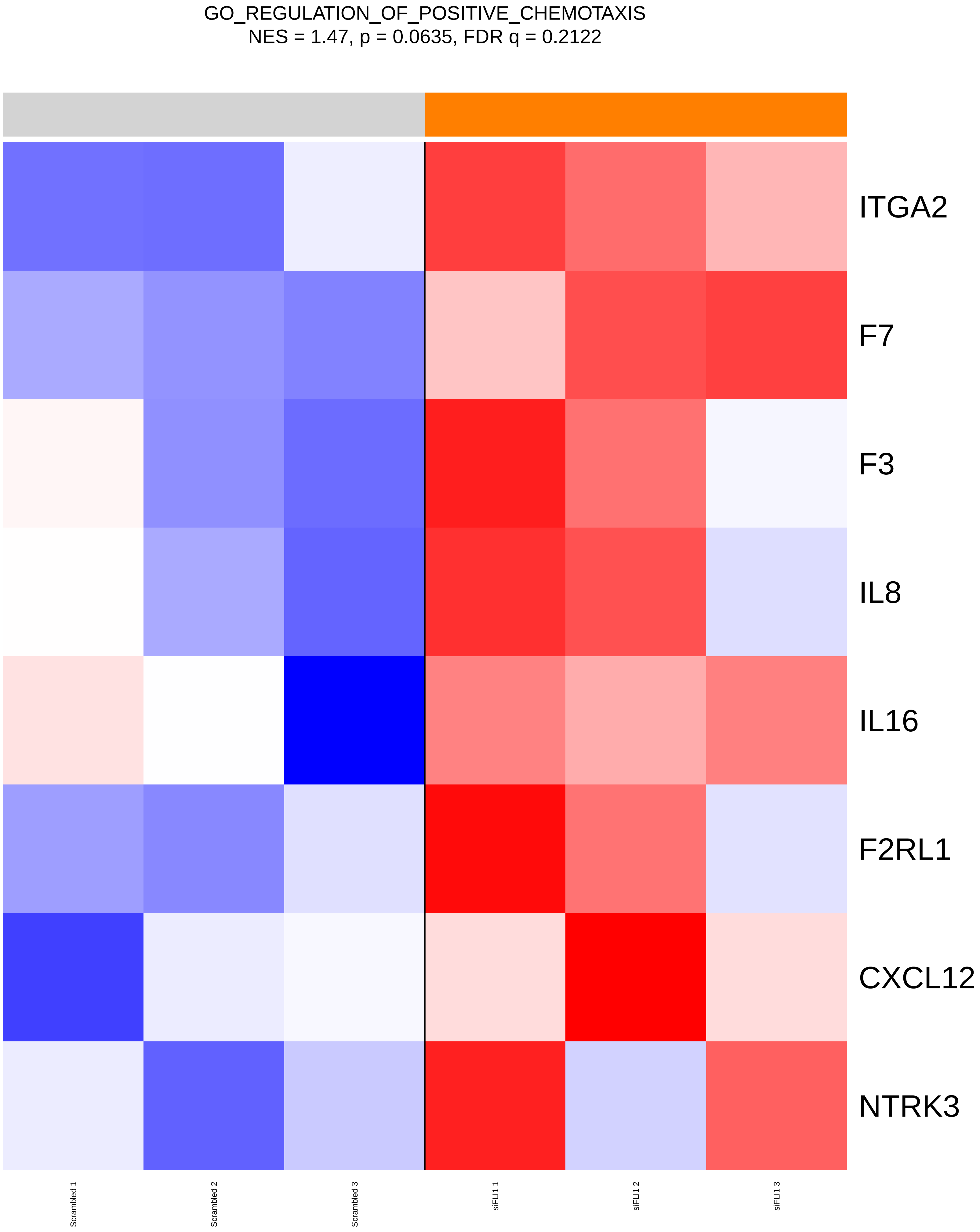
GO_REGULATION_OF_POSITIVE_CHEMOTAXIS
NES = 1.47, p = 0.0635, FDR q = 0.2122
ITGA2
F7
F3
IL8
IL16
F2RL1
CXCL12
NTRK3
Scrambled 1
Scrambled 2
Scrambled 3
siFLI1 1
siFLI1 2
siFLI1 3

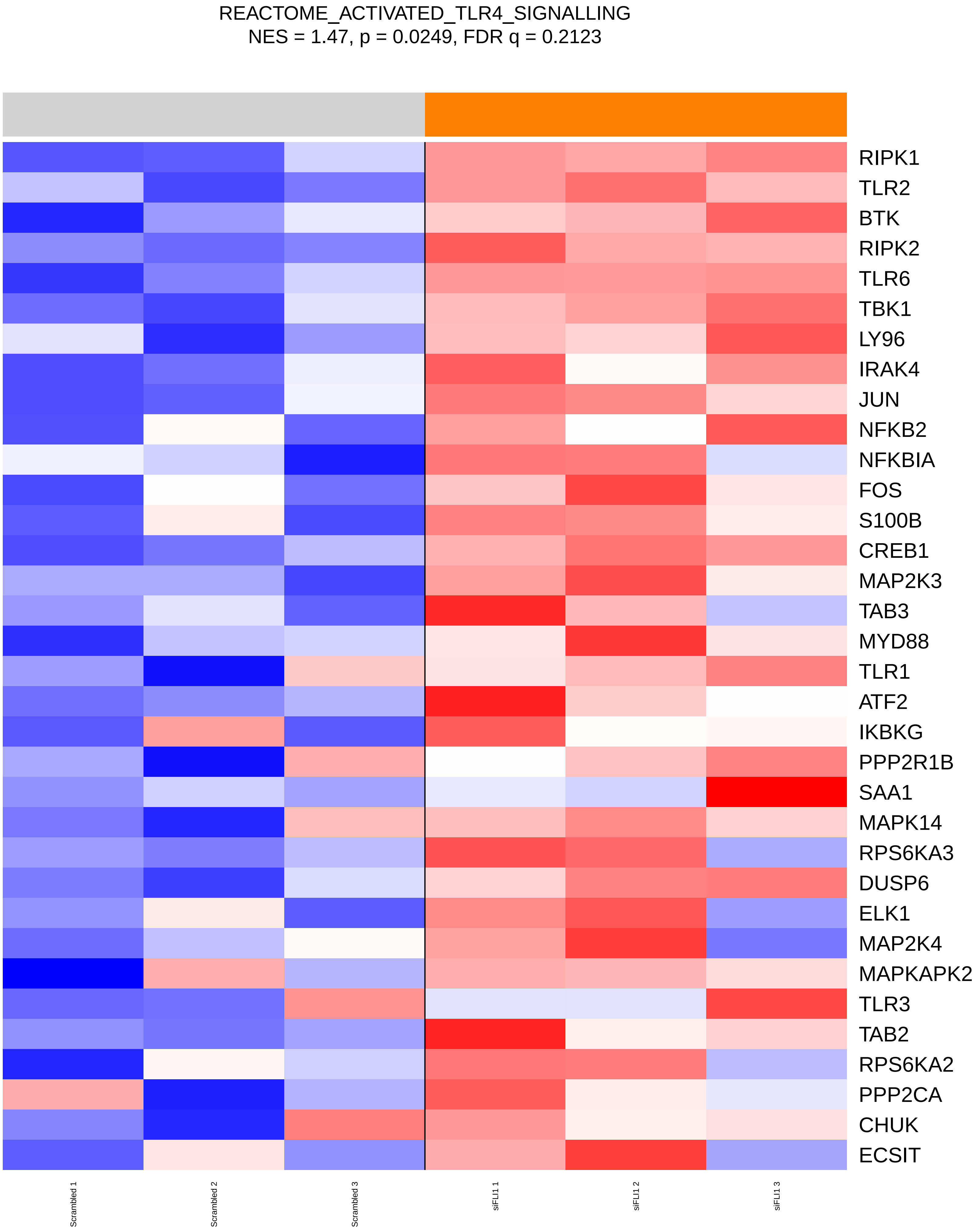
REACTOME_ACTIVATED_TLR4_SIGNALLING
NES = 1.47, p = 0.0249, FDR q = 0.2123
RIPK1
TLR2
BTK
RIPK2
TLR6
TBK1
LY96
IRAK4
JUN
NFKB2
NFKBIA
FOS
S100B
CREB1
MAP2K3
TAB3
MYD88
TLR1
ATF2
IKBKG
PPP2R1B
SAA1
MAPK14
RPS6KA3
DUSP6
ELK1
MAP2K4
MAPKAPK2
TLR3
TAB2
RPS6KA2
PPP2CA
CHUK
ECSIT
Scrambled 1
Scrambled 2
Scrambled 3
siFLI1 1
siFLI1 2
siFLI1 3

GO_N_TERMINAL_PROTEIN_AMINO_ACID_ACETYLATION
NES = 1.47, p = 0.0786, FDR q = 0.2120

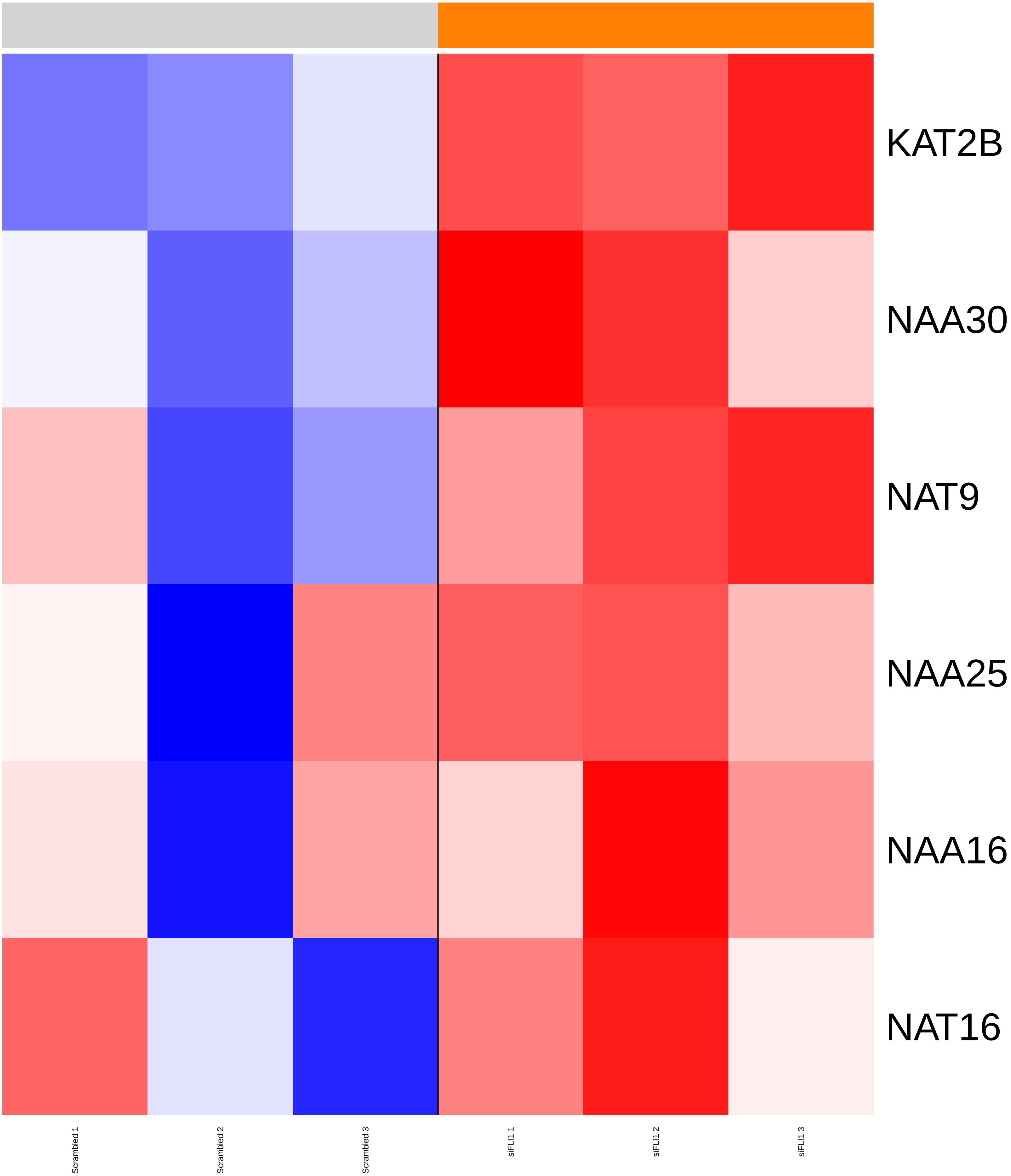

GO_REGULATION_OF_ADHERENS_JUNCTION_ORGANIZATION
NES = 1.47, p = 0.0338, FDR q = 0.2122

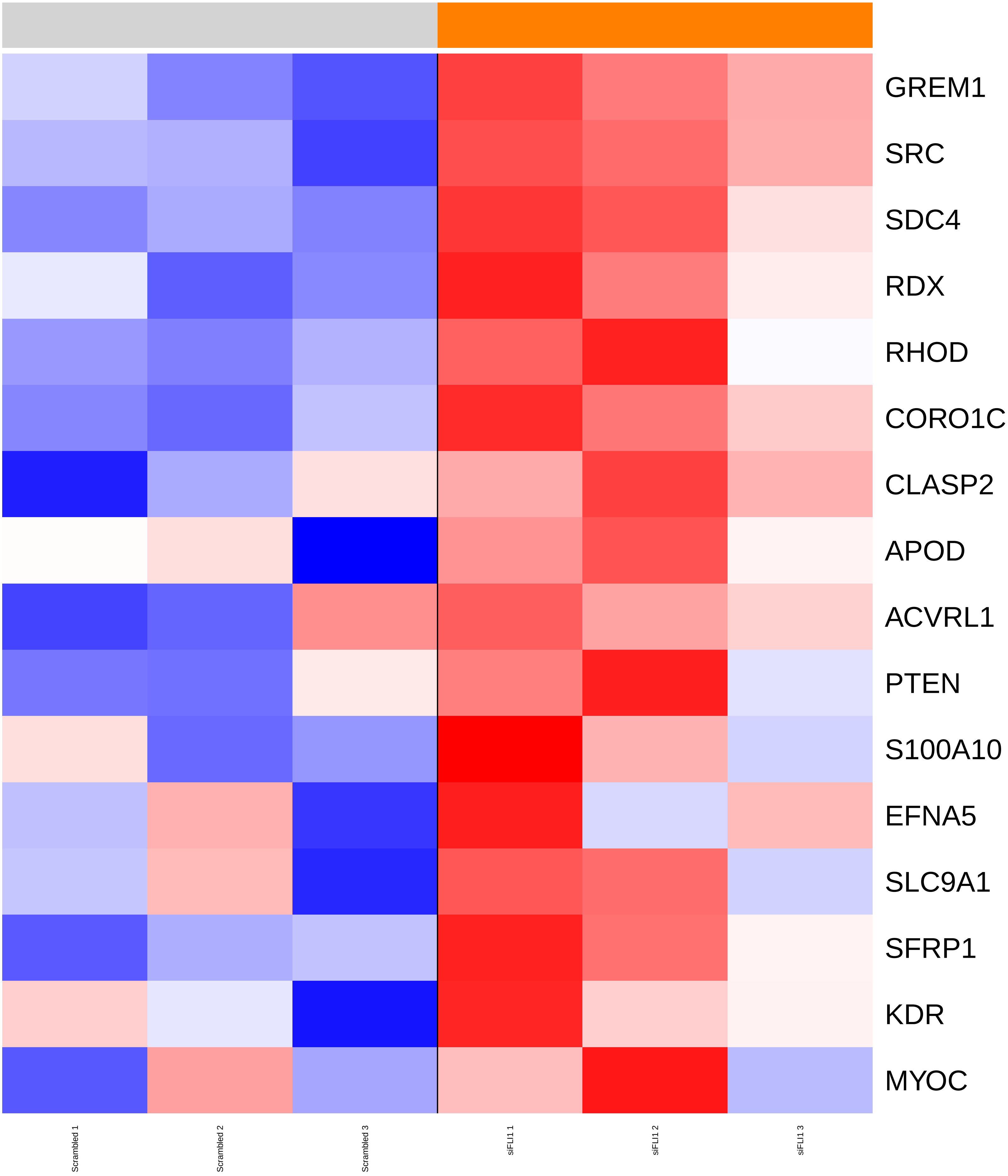

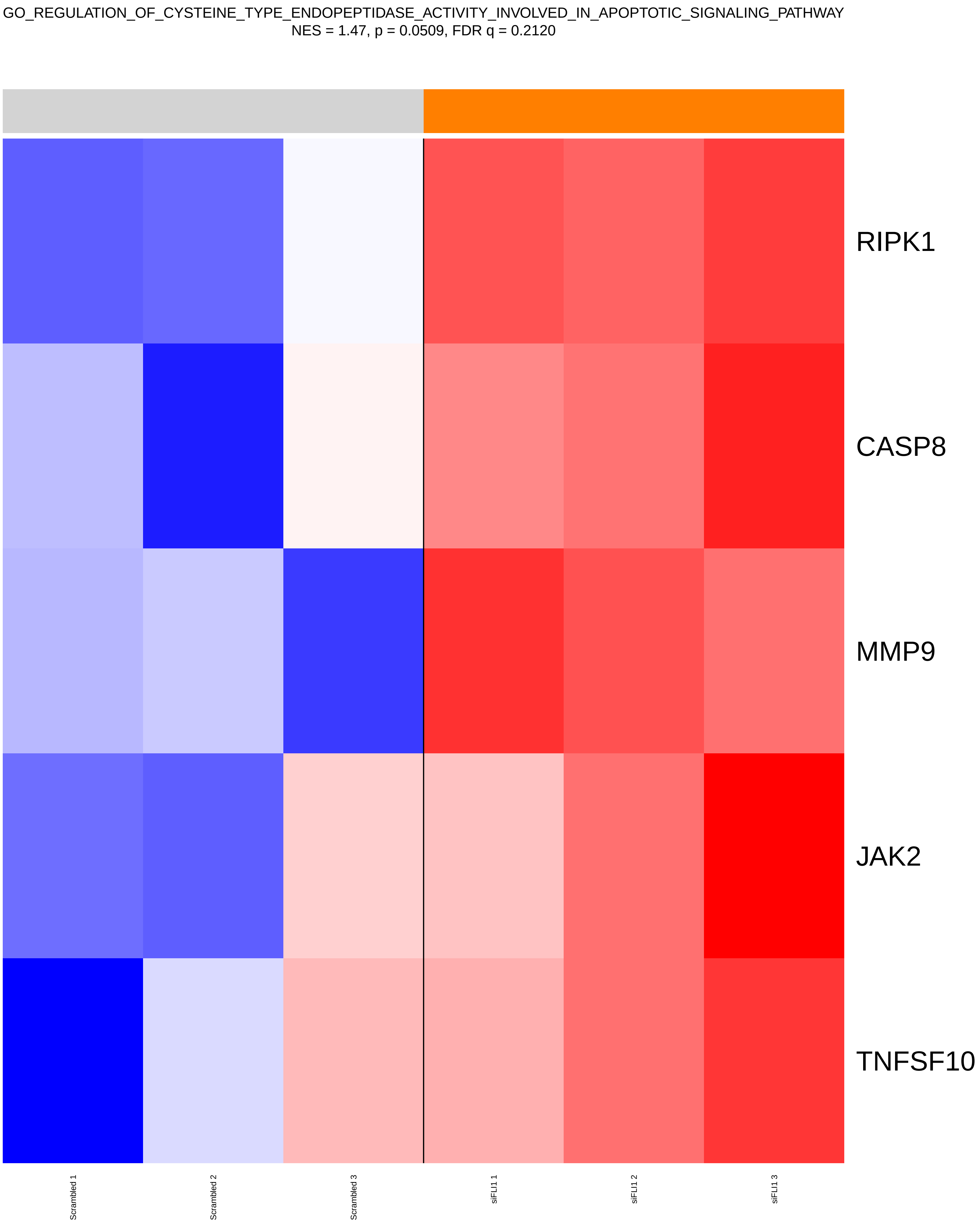
GO_REGULATION_OF_CYSTEINE_TYPE_ENDOPEPTIDASE_ACTIVITY_INVOLVED_IN_APOPTOTIC_SIGNALING_PATHWAY
NES = 1.47, p = 0.0509, FDR q = 0.2120
RIPK1
CASP8
MMP9
JAK2
TNFSF10
Scrambled 1
Scrambled 2
Scrambled 3
siFLI1 1
siFLI1 2
siFLI1 3

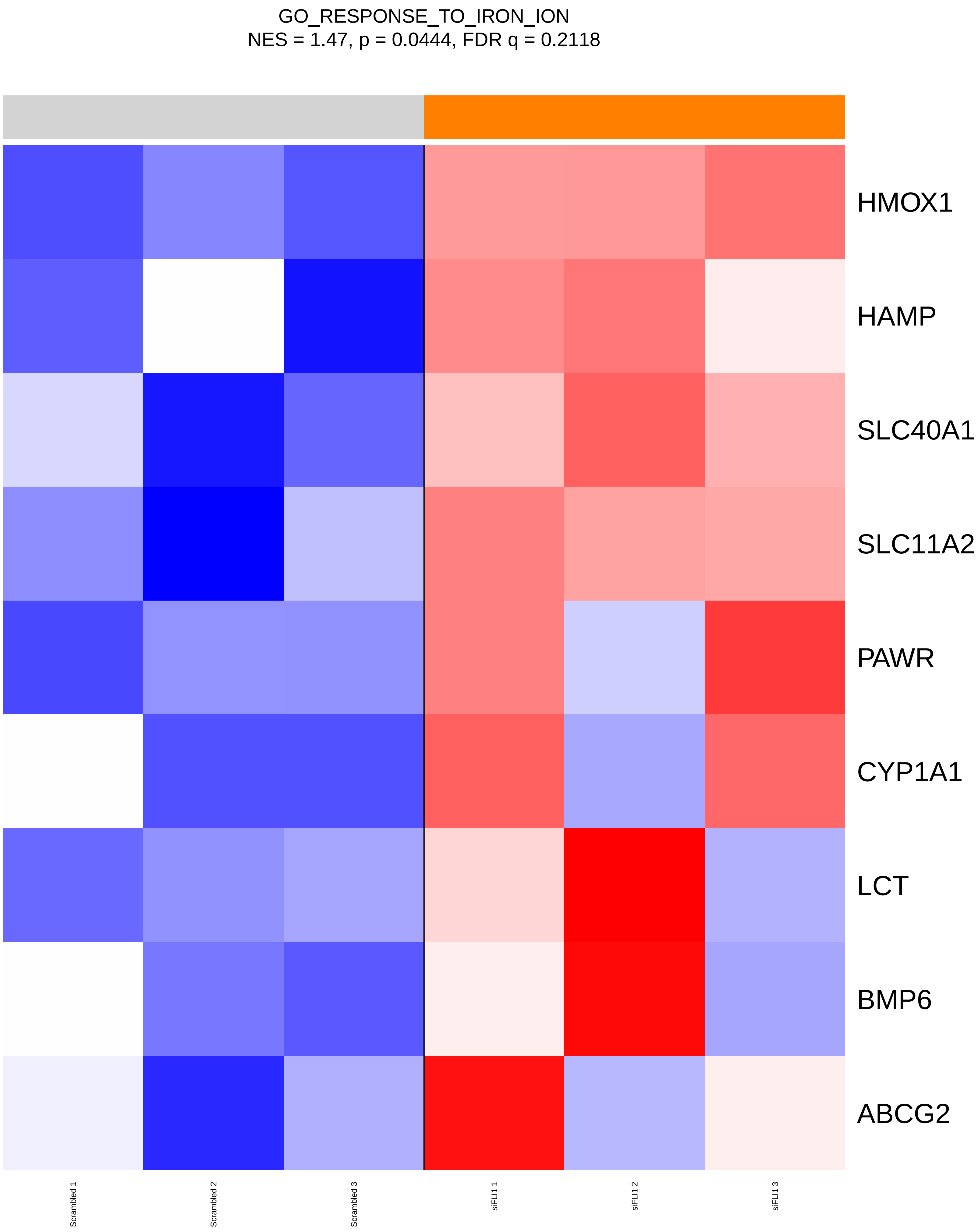
GO_RESPONSE_TO_IRON_ION
NES = 1.47, p = 0.0444, FDR q = 0.2118
HMOX1
HAMP
SLC40A1
SLC11A2
PAWR
CYP1A1
LCT
BMP6
ABCG2
Scrambled 1
Scrambled 2
Scrambled 3
siFLI1 1
siFLI1 2
siFLI1 3

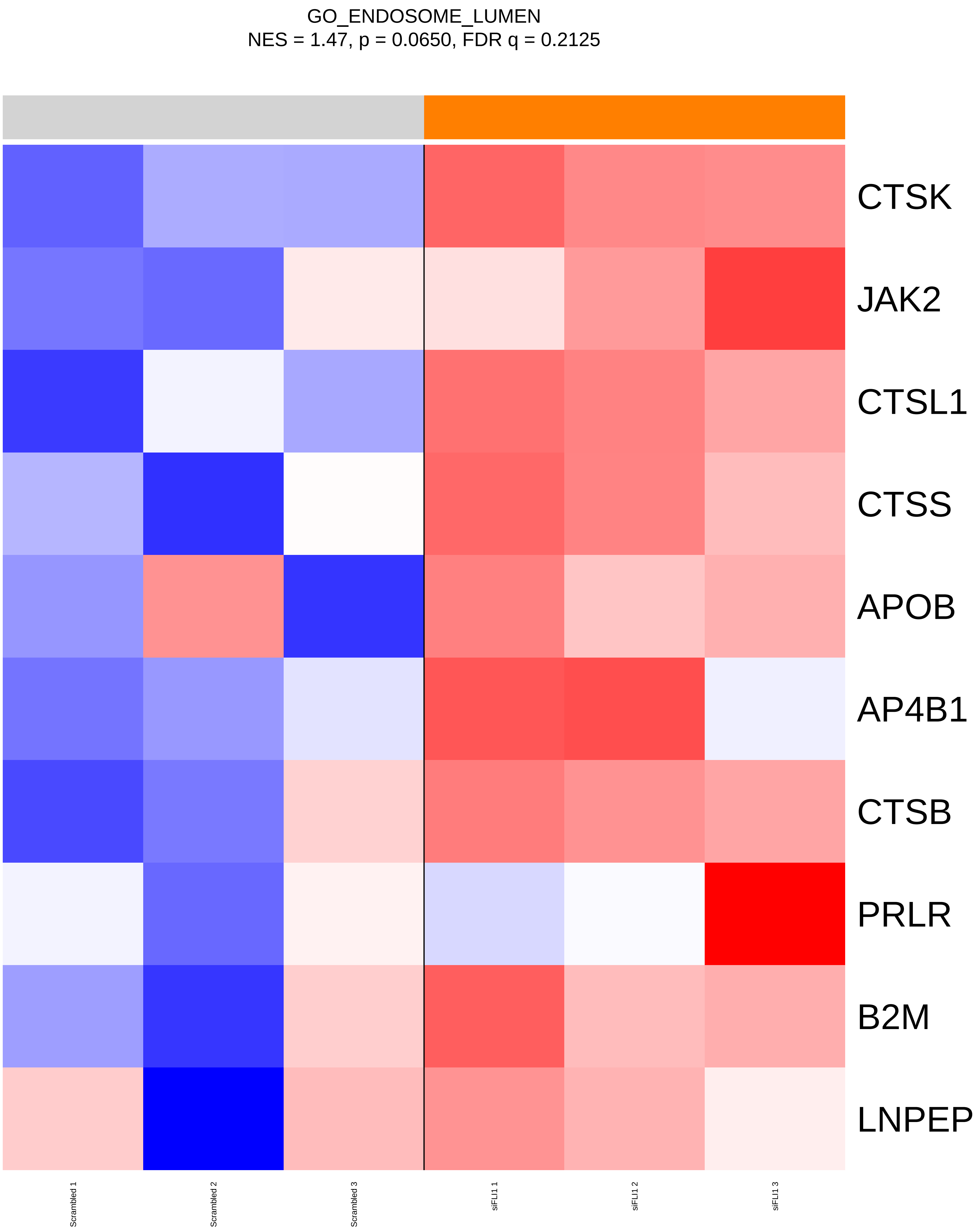
GO_ENDOSOME_LUMEN
NES = 1.47, p = 0.0650, FDR q = 0.2125
CTSK
JAK2
CTSL1
CTSS
APOB
AP4B1
CTSB
PRLR
B2M
LNPEP
Scrambled 1
Scrambled 2
Scrambled 3
siFLI1 1
siFLI1 2
siFLI1 3

NRF2_01
NES = 1.47, p = 0.0043, FDR q = 0.2131

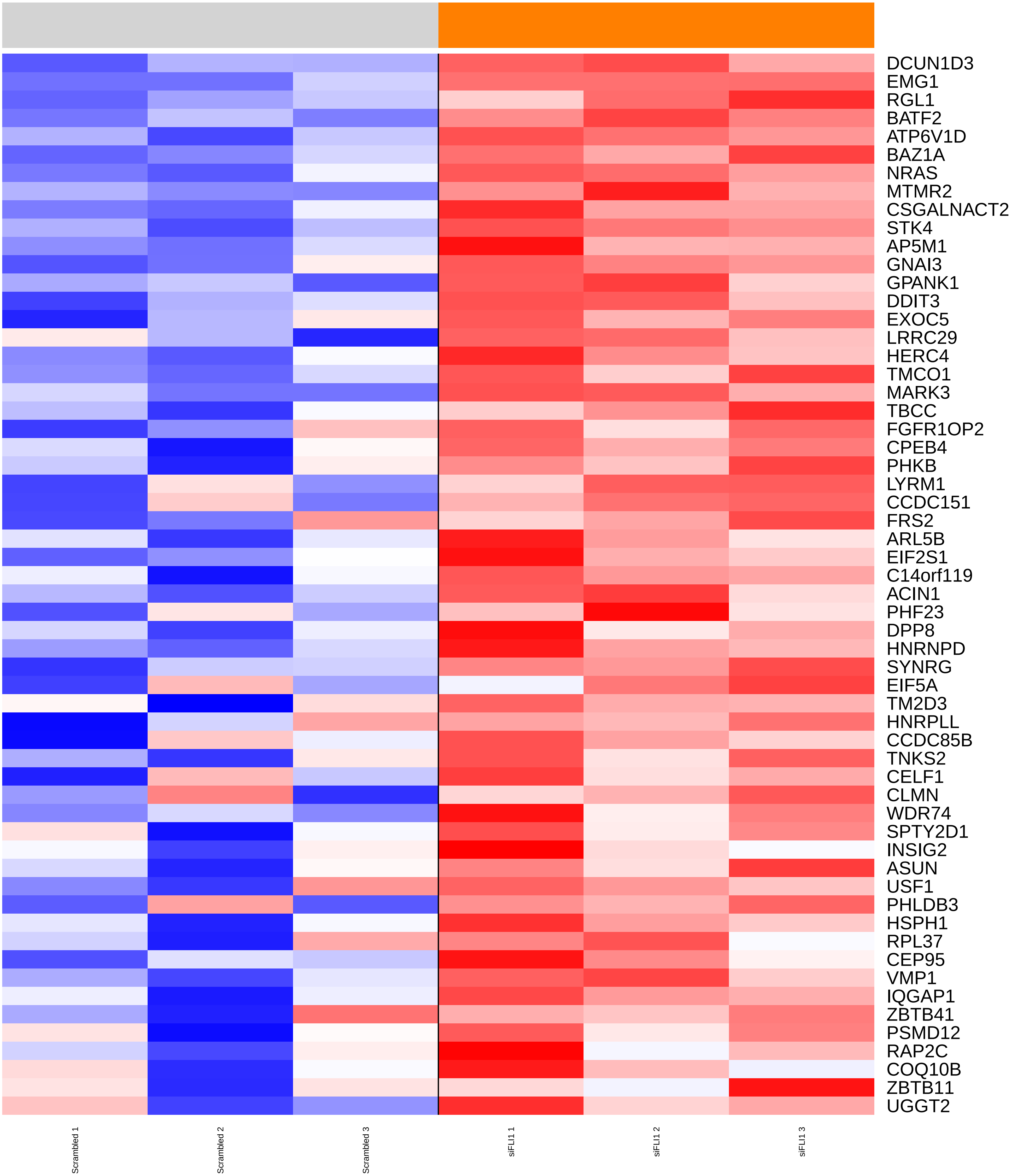

TACTTGA_MIR26A_MIR26B
NES = 1.46, p = 0.0000, FDR q = 0.2145

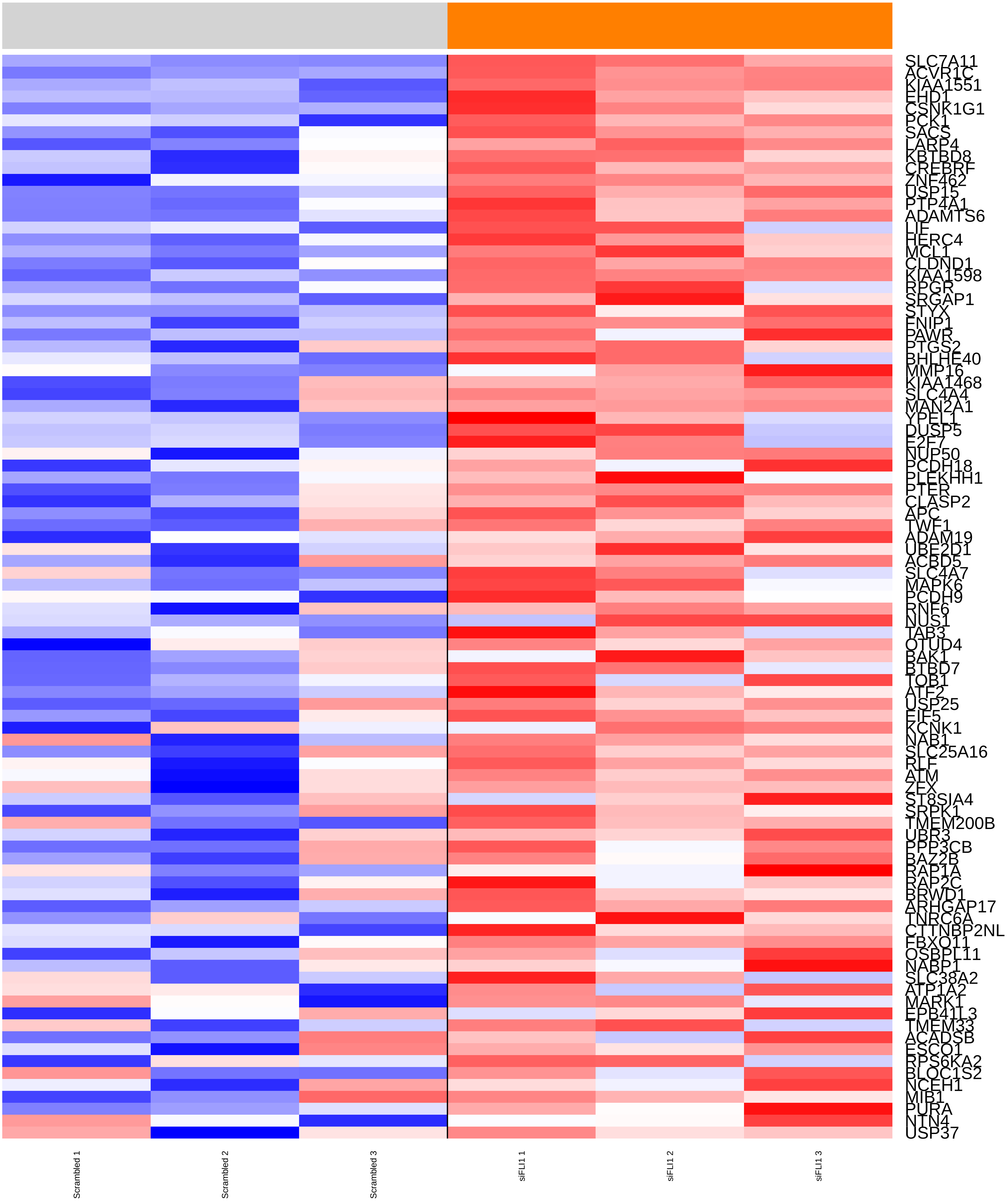

GO_REGULATION_OF_STRESS_ACTIVATED_PROTEIN_KINASE_SIGNALING_CASCADE
NES = 1.46, p = 0.0043, FDR q = 0.2160

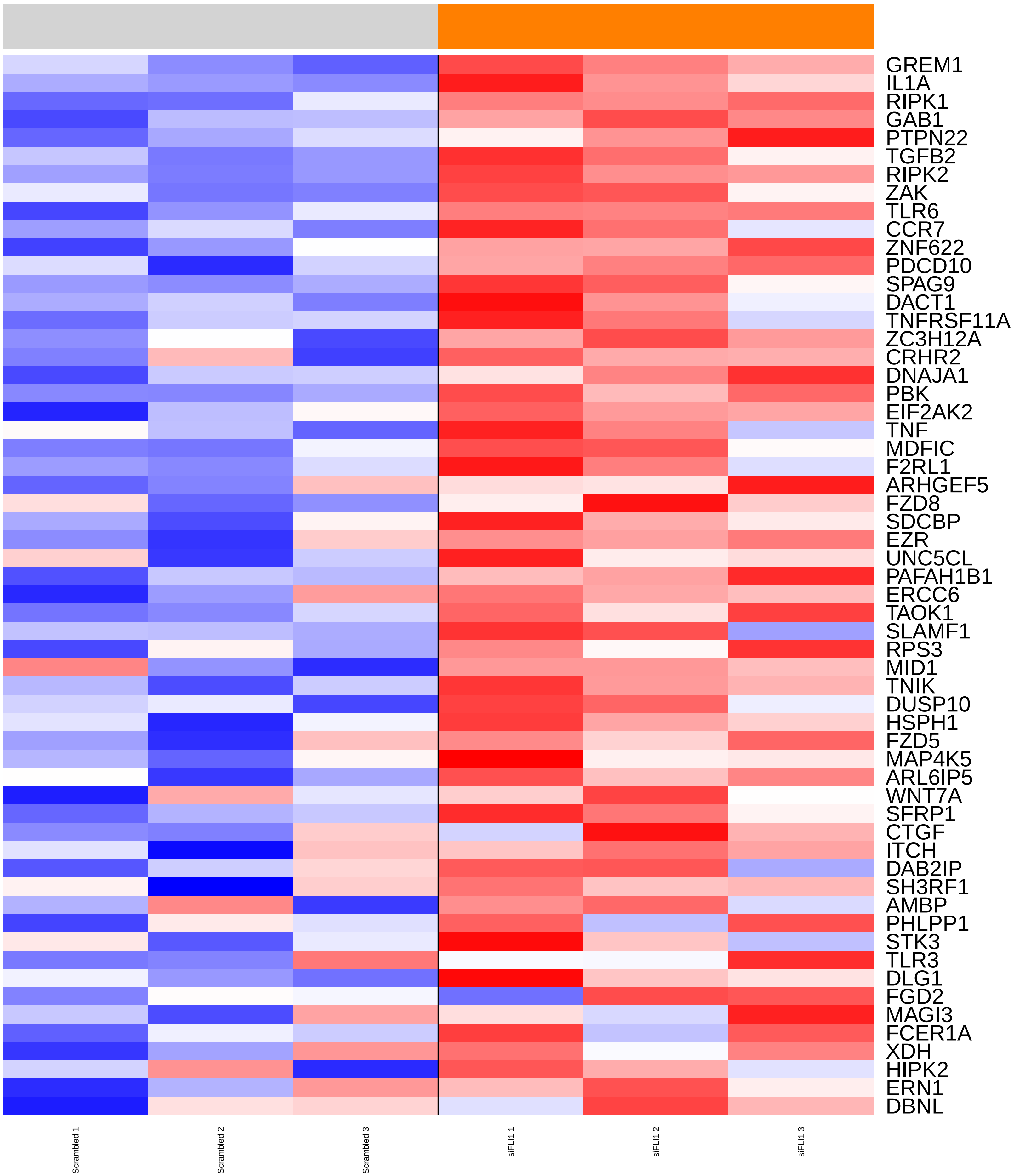

GO_ORGANELLE_MEMBRANE_FUSION
NES = 1.46, p = 0.0185, FDR q = 0.2164

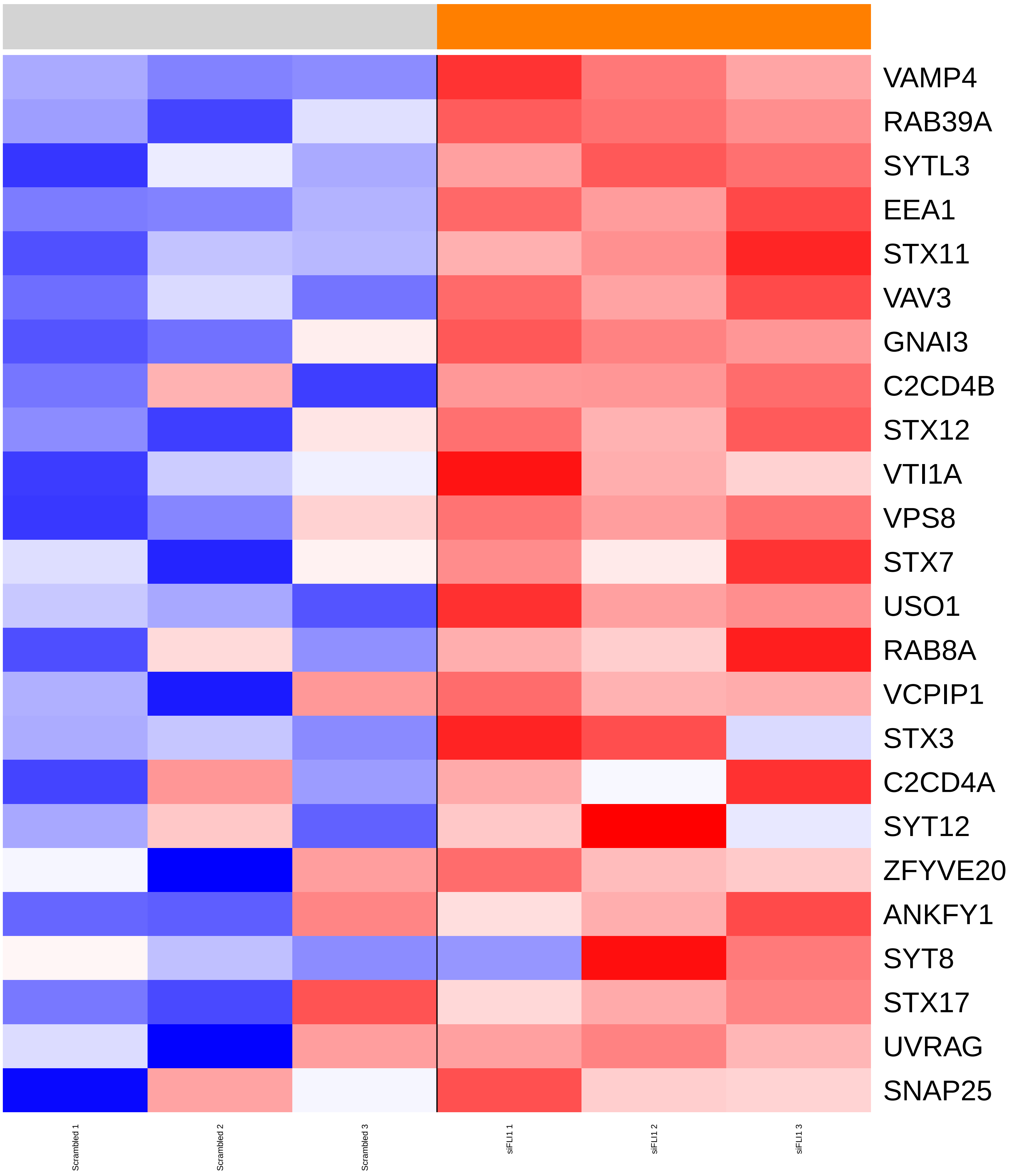

GO_REGULATION_OF_SMOOTH_MUSCLE_CELL_MIGRATION
NES = 1.46, p = 0.0309, FDR q = 0.2161

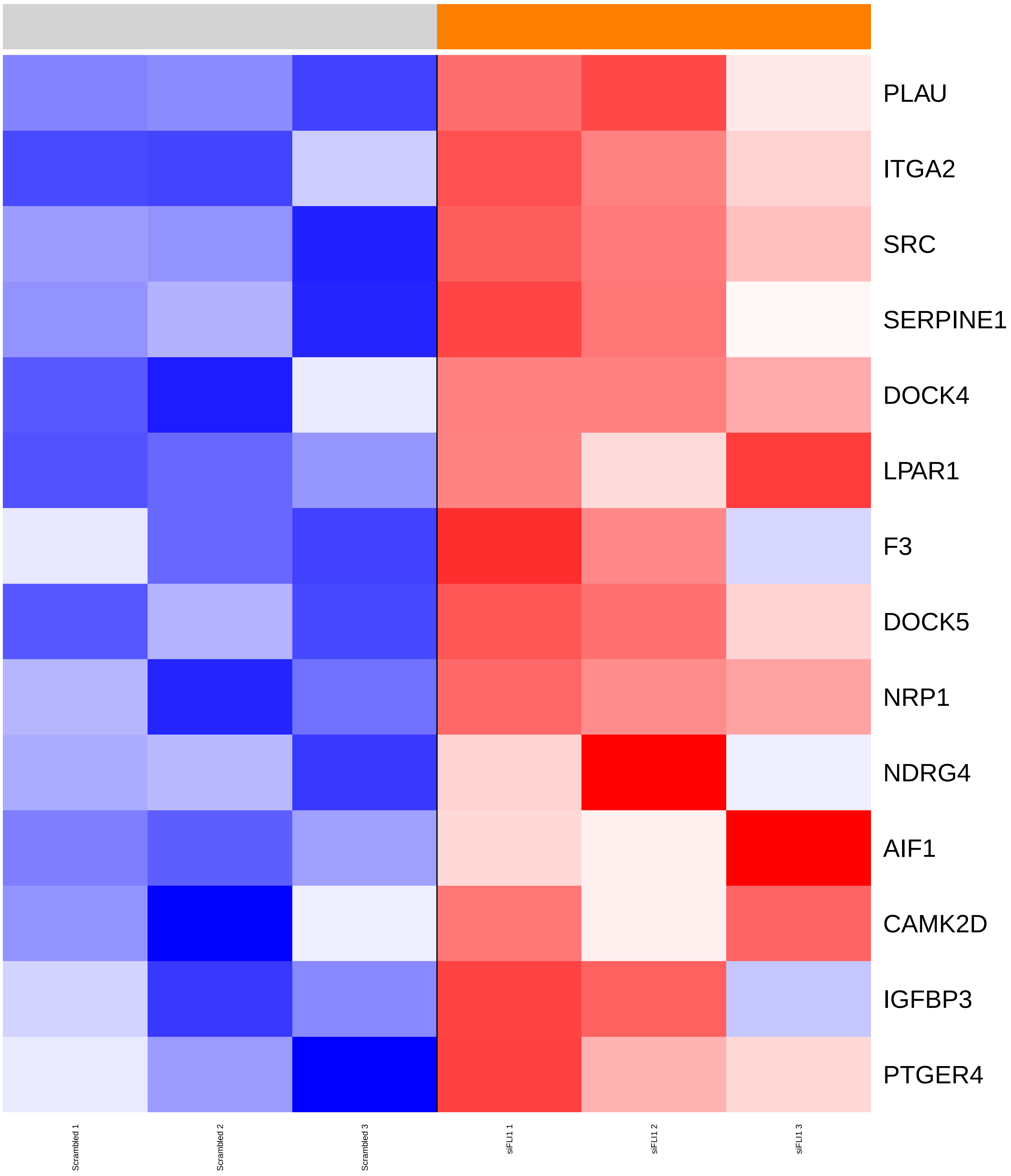

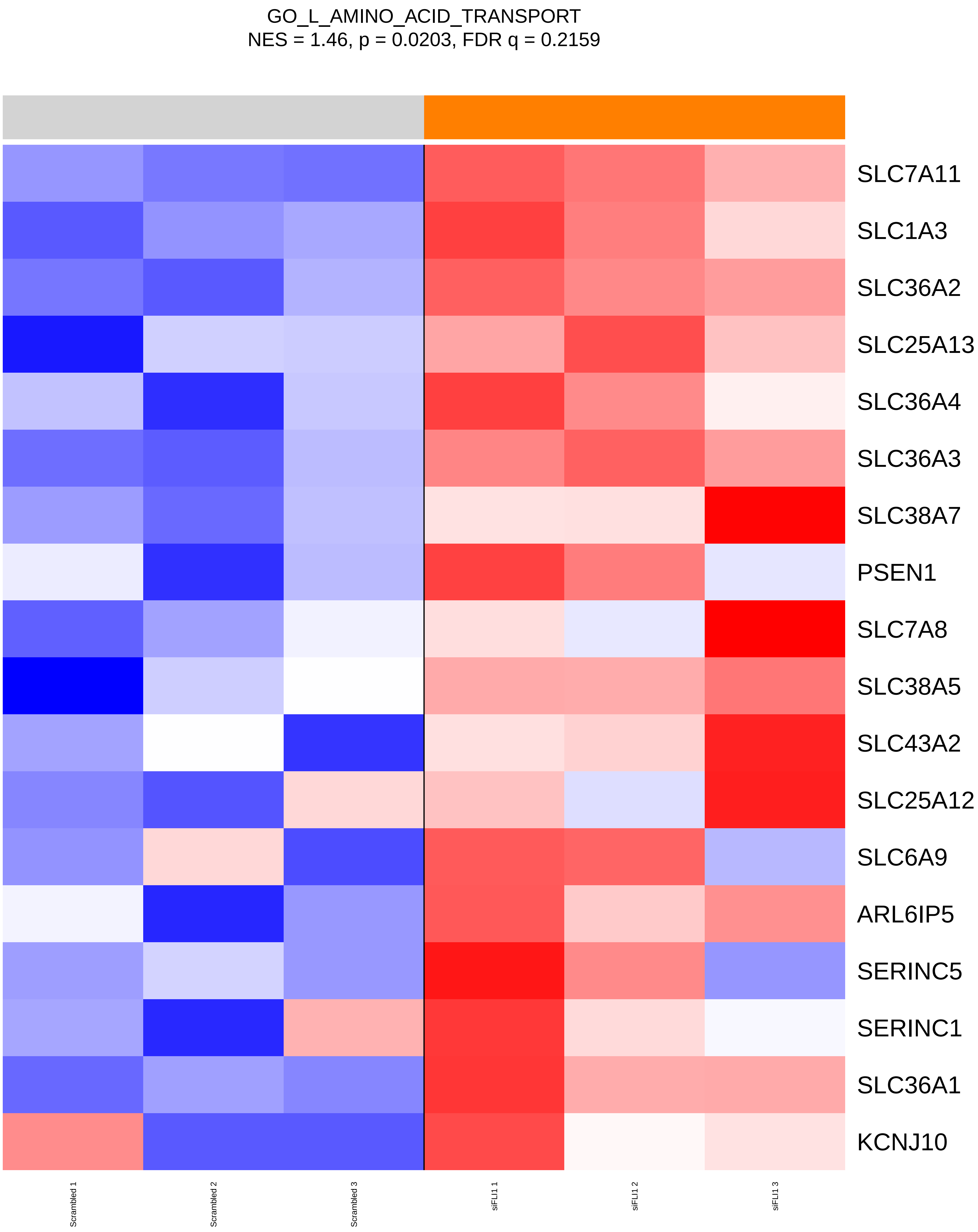
GO_L_AMINO_ACID_TRANSPORT
NES = 1.46, p = 0.0203, FDR q = 0.2159
SLC7A11
SLC1A3
SLC36A2
SLC25A13
SLC36A4
SLC36A3
SLC38A7
PSEN1
SLC7A8
SLC38A5
SLC43A2
SLC25A12
SLC6A9
ARL6IP5
SERINC5
SERINC1
SLC36A1
KCNJ10
Scrambled 1
Scrambled 2
Scrambled 3
siFLI1 1
siFLI1 2
siFLI1 3

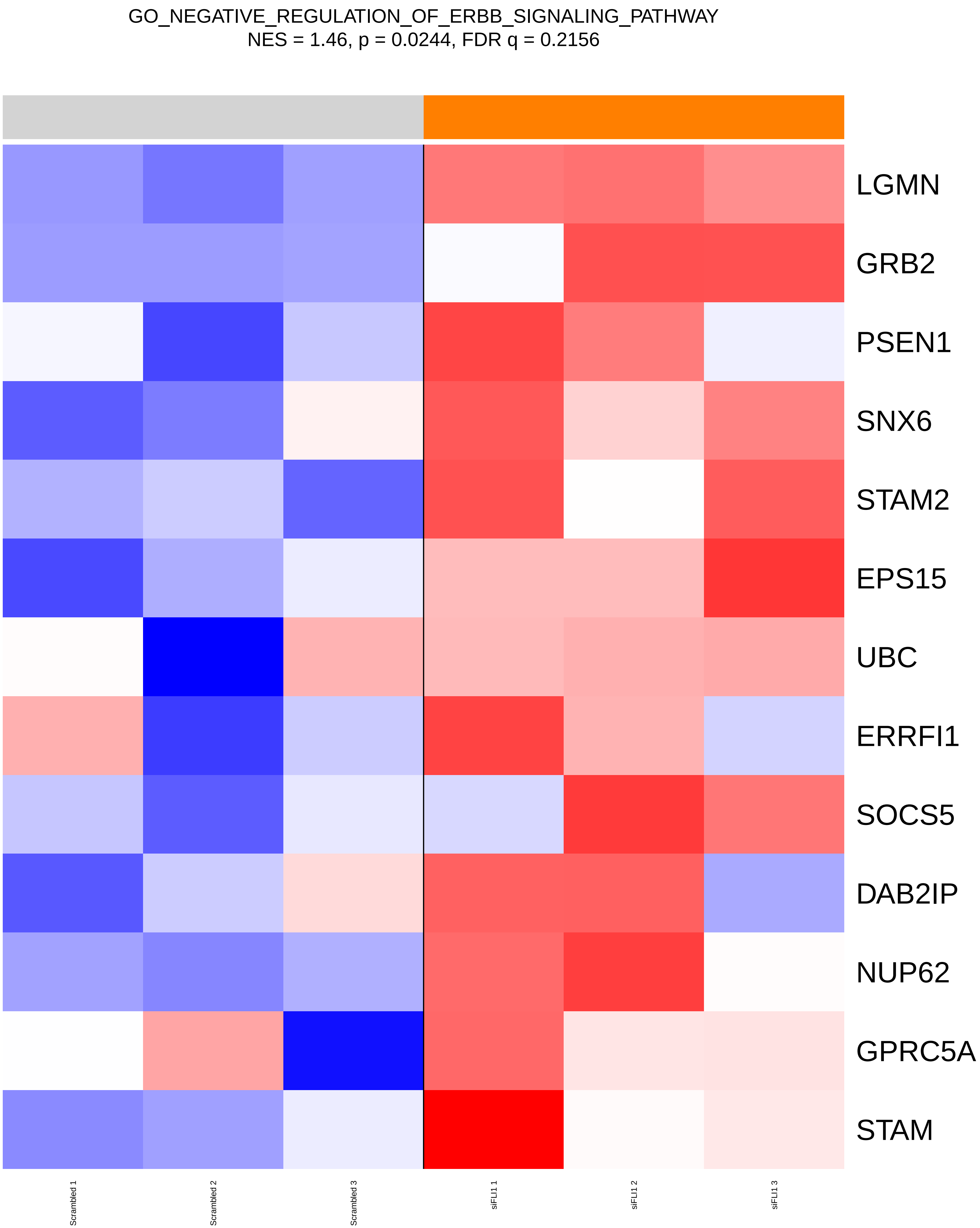
GO_NEGATIVE_REGULATION_OF_ERBB_SIGNALING_PATHWAY
NES = 1.46, p = 0.0244, FDR q = 0.2156
LGMN
GRB2
PSEN1
SNX6
STAM2
EPS15
UBC
ERRFI1
SOCS5
DAB2IP
NUP62
GPRC5A
STAM
Scrambled 1
Scrambled 2
Scrambled 3
siFLI1 1
siFLI1 2
siFLI1 3

AATGTGA_MIR23A_MIR23B
NES = 1.46, p = 0.0000, FDR q = 0.2155

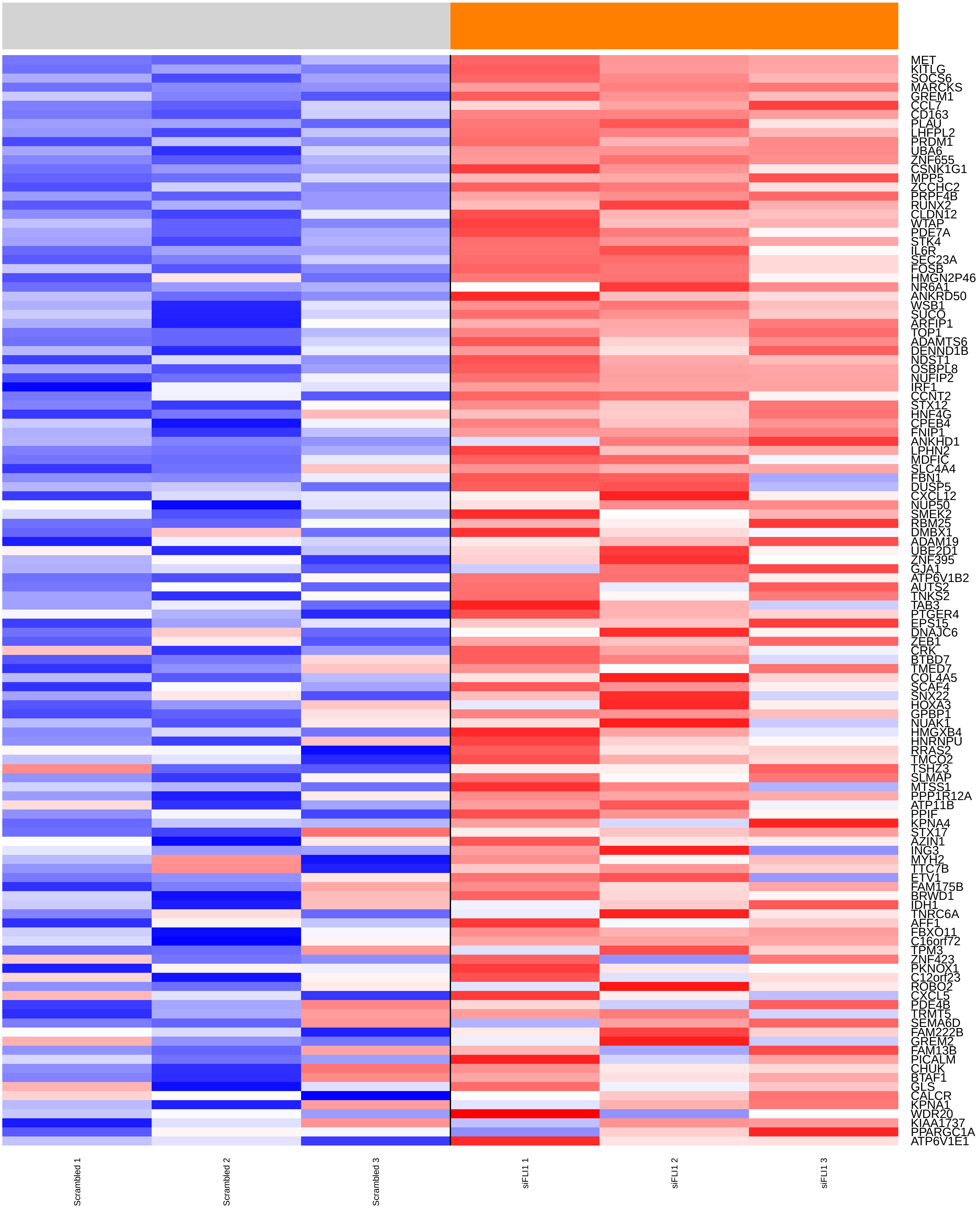

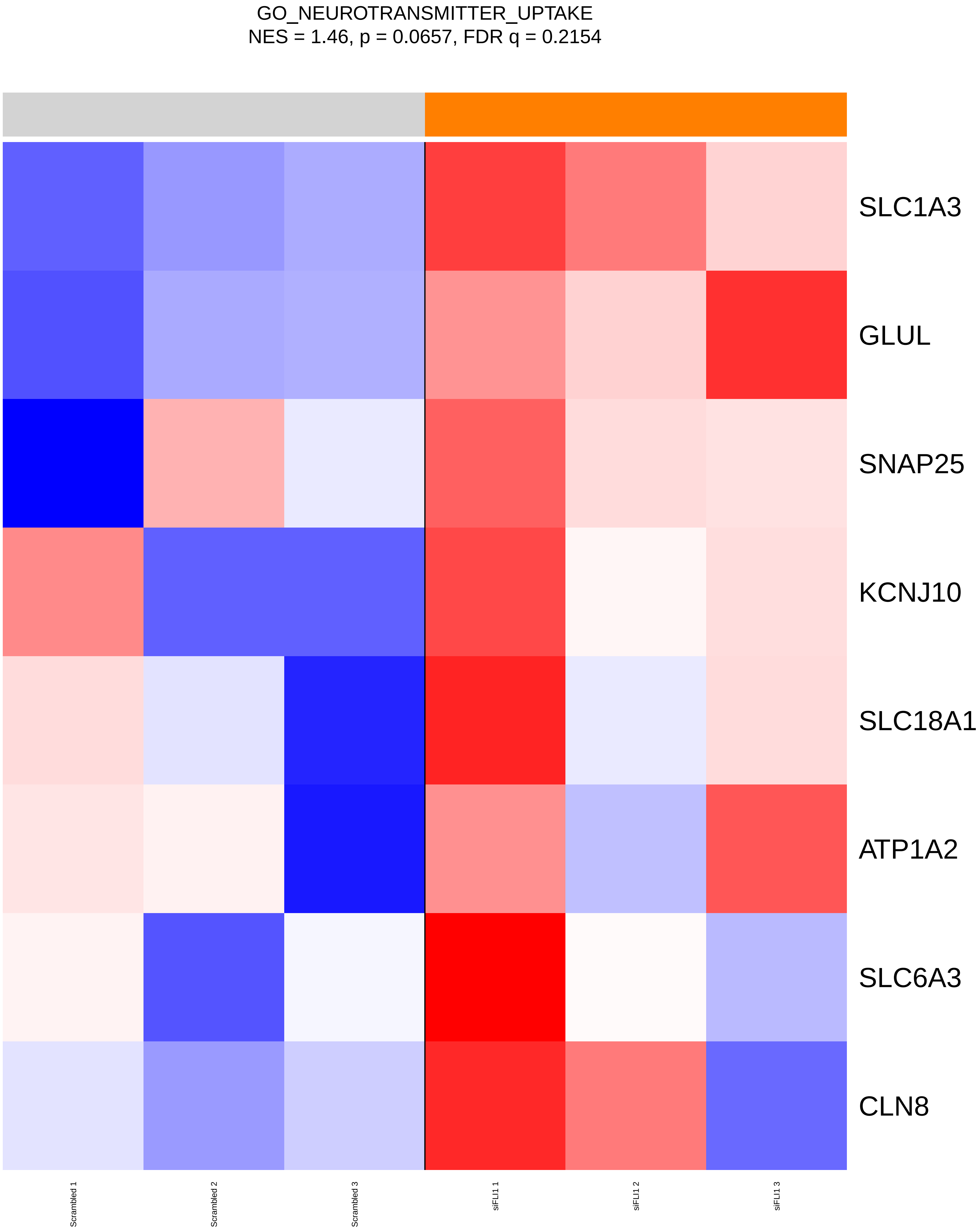
GO_NEUROTRANSMITTER_UPTAKE
NES = 1.46, p = 0.0657, FDR q = 0.2154
SLC1A3
GLUL
SNAP25
KCNJ10
SLC18A1
ATP1A2
SLC6A3
CLN8
Scrambled 1
Scrambled 2
Scrambled 3
siFLI1 1
siFLI1 2
siFLI1 3

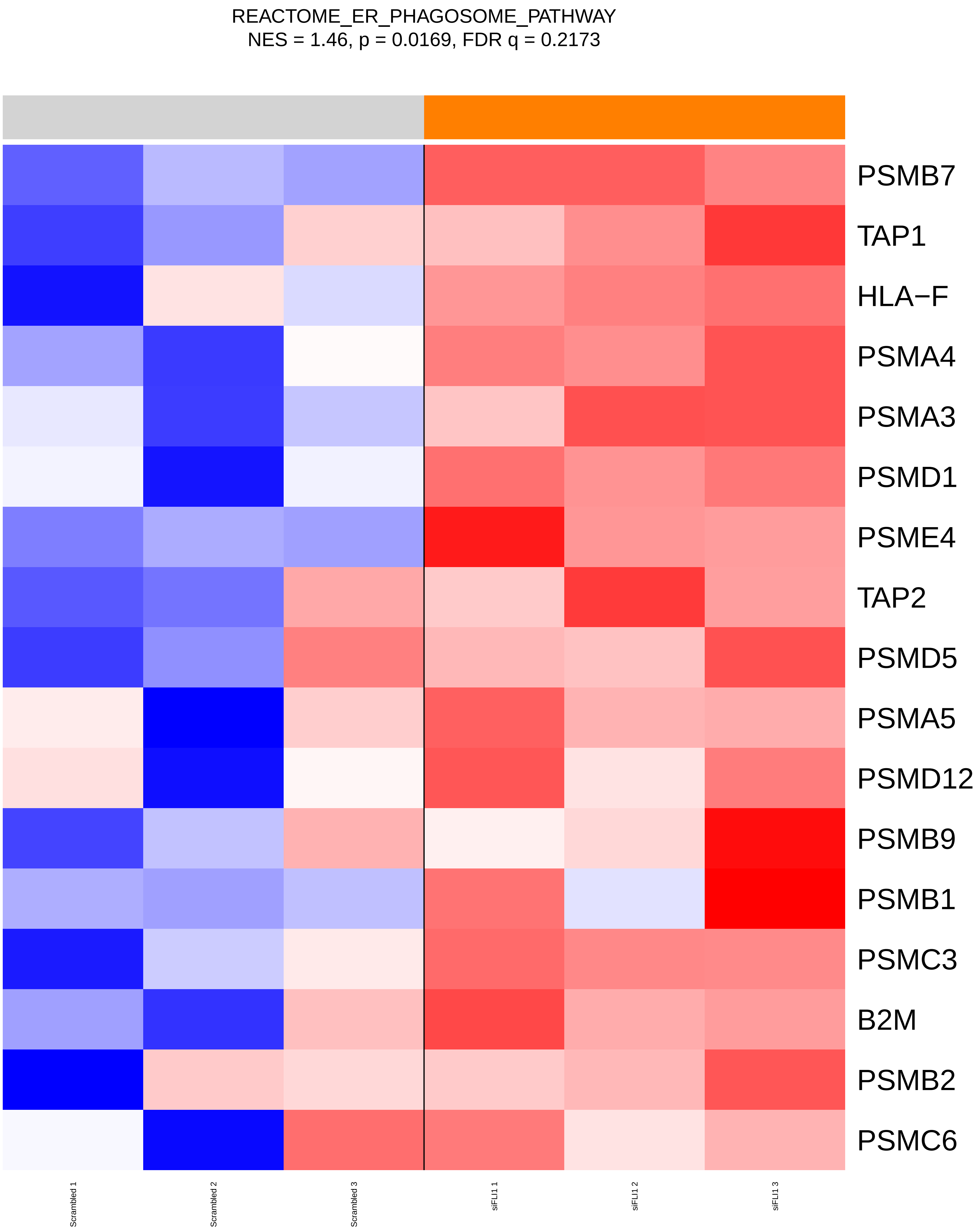

REACTOME_ER_PHAGOSOME_PATHWAY
NES = 1.46, p = 0.0169, FDR q = 0.2173
PSMB7
TAP1
HLA−F
PSMA4
PSMA3
PSMD1
PSME4
TAP2
PSMD5
PSMA5
PSMD12
PSMB9
PSMB1
PSMC3
B2M
PSMB2
PSMC6
Scrambled 1
Scrambled 2
Scrambled 3
siFLI1 1
siFLI1 2
siFLI1 3

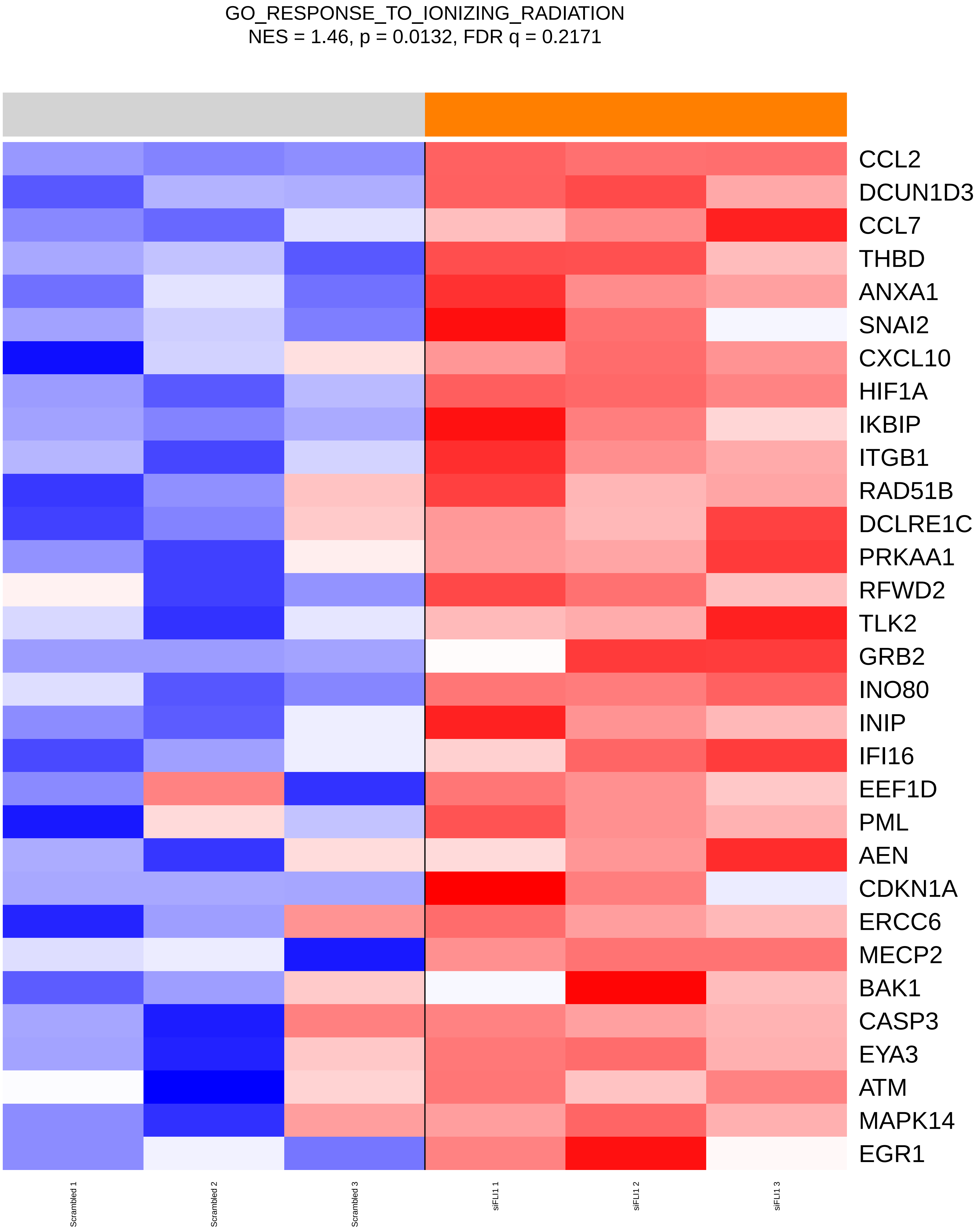
GO_RESPONSE_TO_IONIZING_RADIATION
NES = 1.46, p = 0.0132, FDR q = 0.2171
CCL2
DCUN1D3
CCL7
THBD
ANXA1
SNAI2
CXCL10
HIF1A
IKBIP
ITGB1
RAD51B
DCLRE1C
PRKAA1
RFWD2
TLK2
GRB2
INO80
INIP
IFI16
EEF1D
PML
AEN
CDKN1A
ERCC6
MECP2
BAK1
CASP3
EYA3
ATM
MAPK14
EGR1
Scrambled 1
Scrambled 2
Scrambled 3
siFLI1 1
siFLI1 2
siFLI1 3

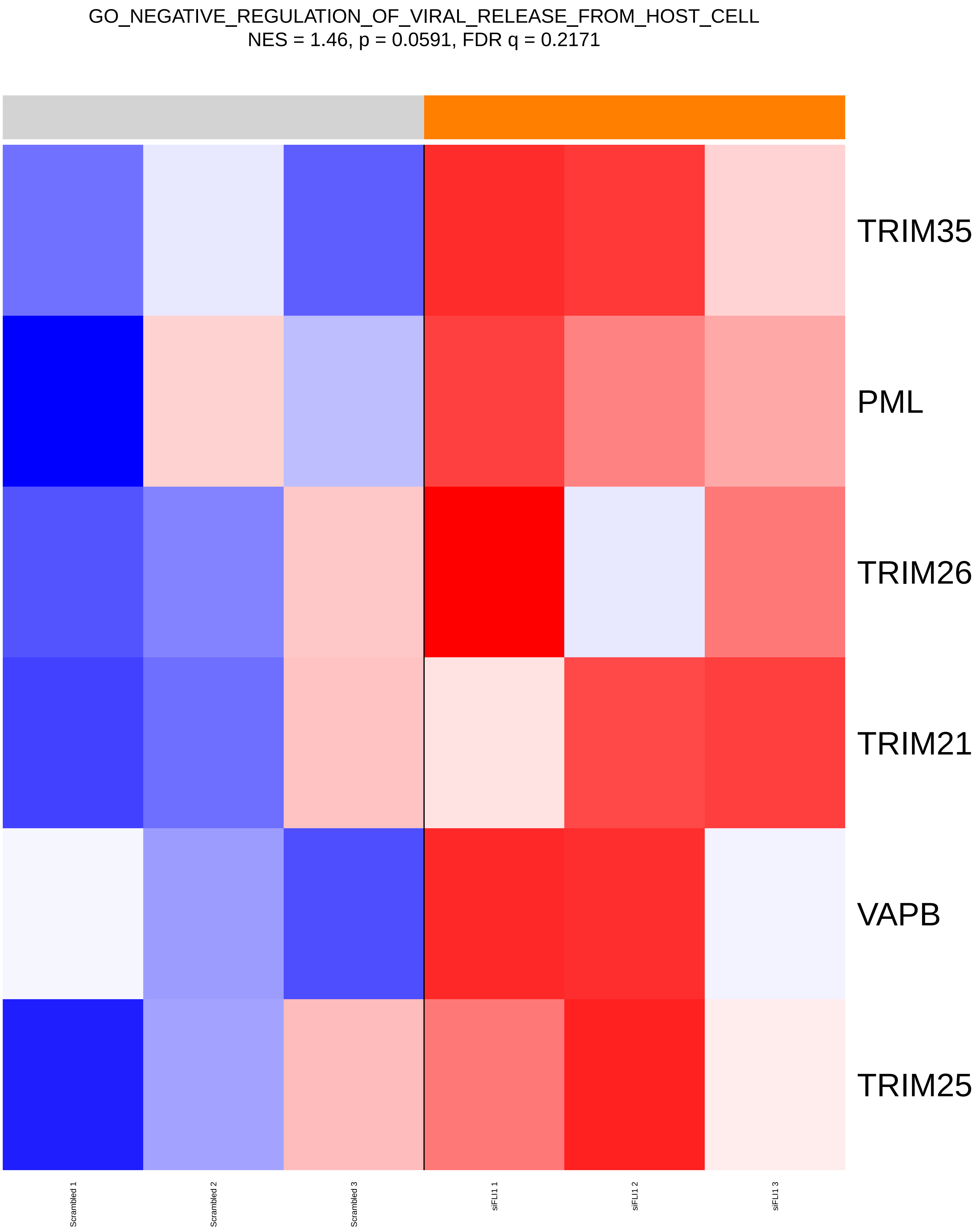
GO_NEGATIVE_REGULATION_OF_VIRAL_RELEASE_FROM_HOST_CELL
NES = 1.46, p = 0.0591, FDR q = 0.2171
TRIM35
PML
TRIM26
TRIM21
VAPB
TRIM25
Scrambled 1
Scrambled 2
Scrambled 3
siFLI1 1
siFLI1 2
siFLI1 3

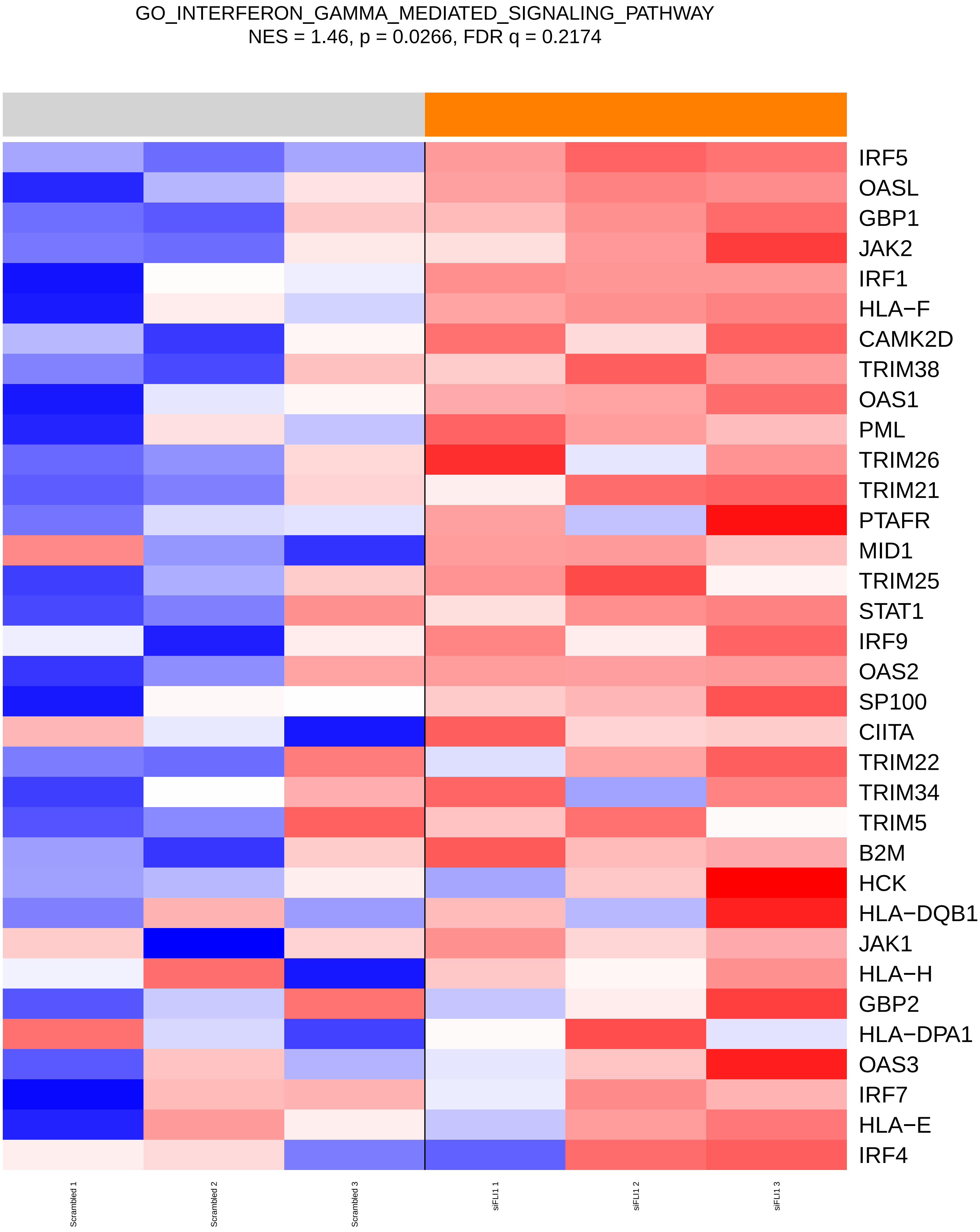

GO_INTERFERON_GAMMA_MEDIATED_SIGNALING_PATHWAY
NES = 1.46, p = 0.0266, FDR q = 0.2174
IRF5
OASL
GBP1
JAK2
IRF1
HLA−F
CAMK2D
TRIM38
OAS1
PML
TRIM26
TRIM21
PTAFR
MID1
TRIM25
STAT1
IRF9
OAS2
SP100
CIITA
TRIM22
TRIM34
TRIM5
B2M
HCK
HLA−DQB1
JAK1
HLA−H
GBP2
HLA−DPA1
OAS3
IRF7
HLA−E
IRF4
Scrambled 1
Scrambled 2
Scrambled 3
siFLI1 1
siFLI1 2
siFLI1 3

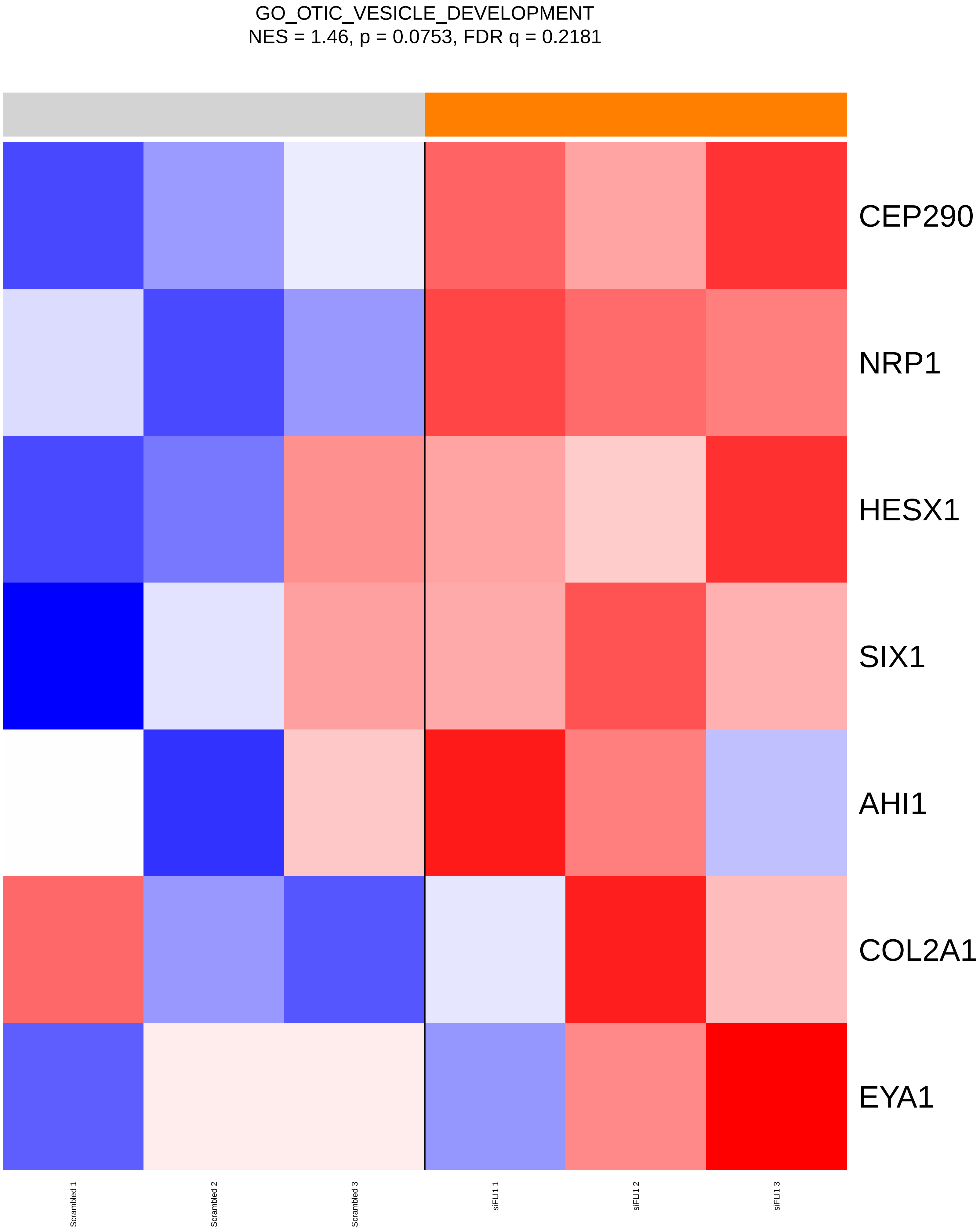
GO_OTIC_VESICLE_DEVELOPMENT
NES = 1.46, p = 0.0753, FDR q = 0.2181
CEP290
NRP1
HESX1
SIX1
AHI1
COL2A1
EYA1
Scrambled 1
Scrambled 2
Scrambled 3
siFLI1 1
siFLI1 2
siFLI1 3

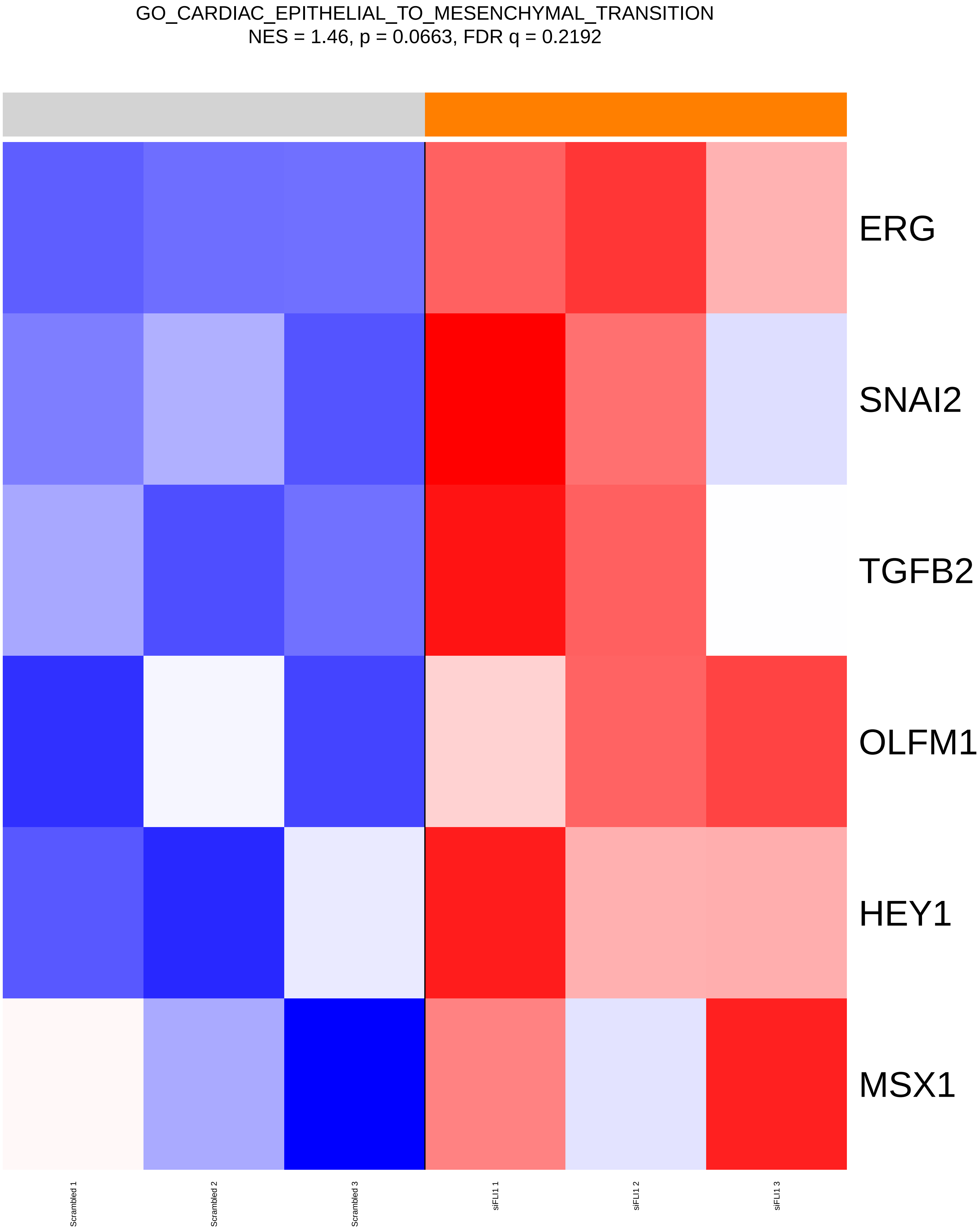
GO_CARDIAC_EPITHELIAL_TO_MESENCHYMAL_TRANSITION
NES = 1.46, p = 0.0663, FDR q = 0.2192
ERG
SNAI2
TGFB2
OLFM1
HEY1
MSX1
Scrambled 1
Scrambled 2
Scrambled 3
siFLI1 1
siFLI1 2
siFLI1 3

KEGG_EPITHELIAL_CELL_SIGNALING_IN_HELICOBACTER_PYLORI_INFECTION
NES = 1.46, p = 0.0370, FDR q = 0.2191

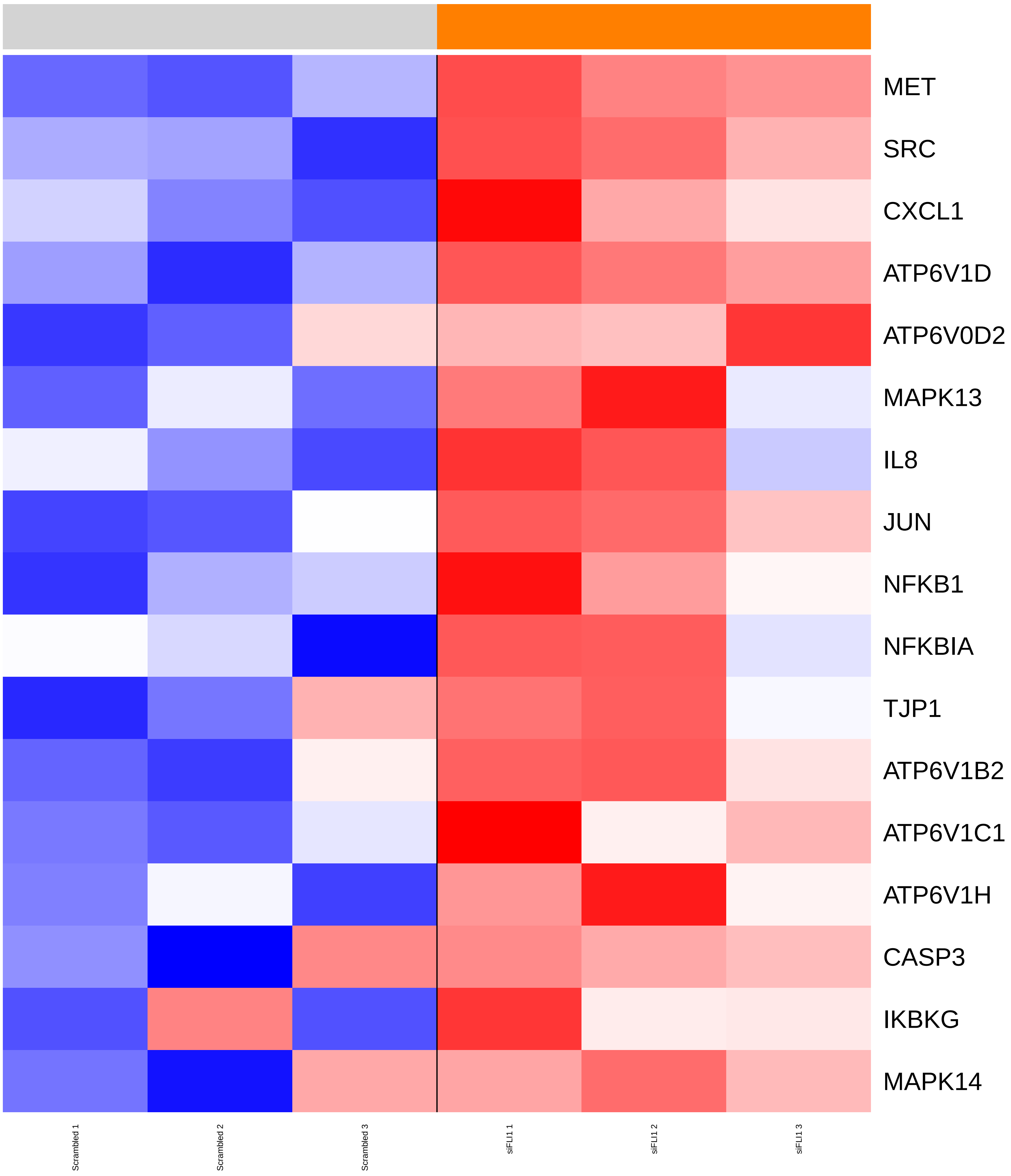

GO_ALPHA_TUBULIN_BINDING
NES = 1.46, p = 0.0631, FDR q = 0.2192

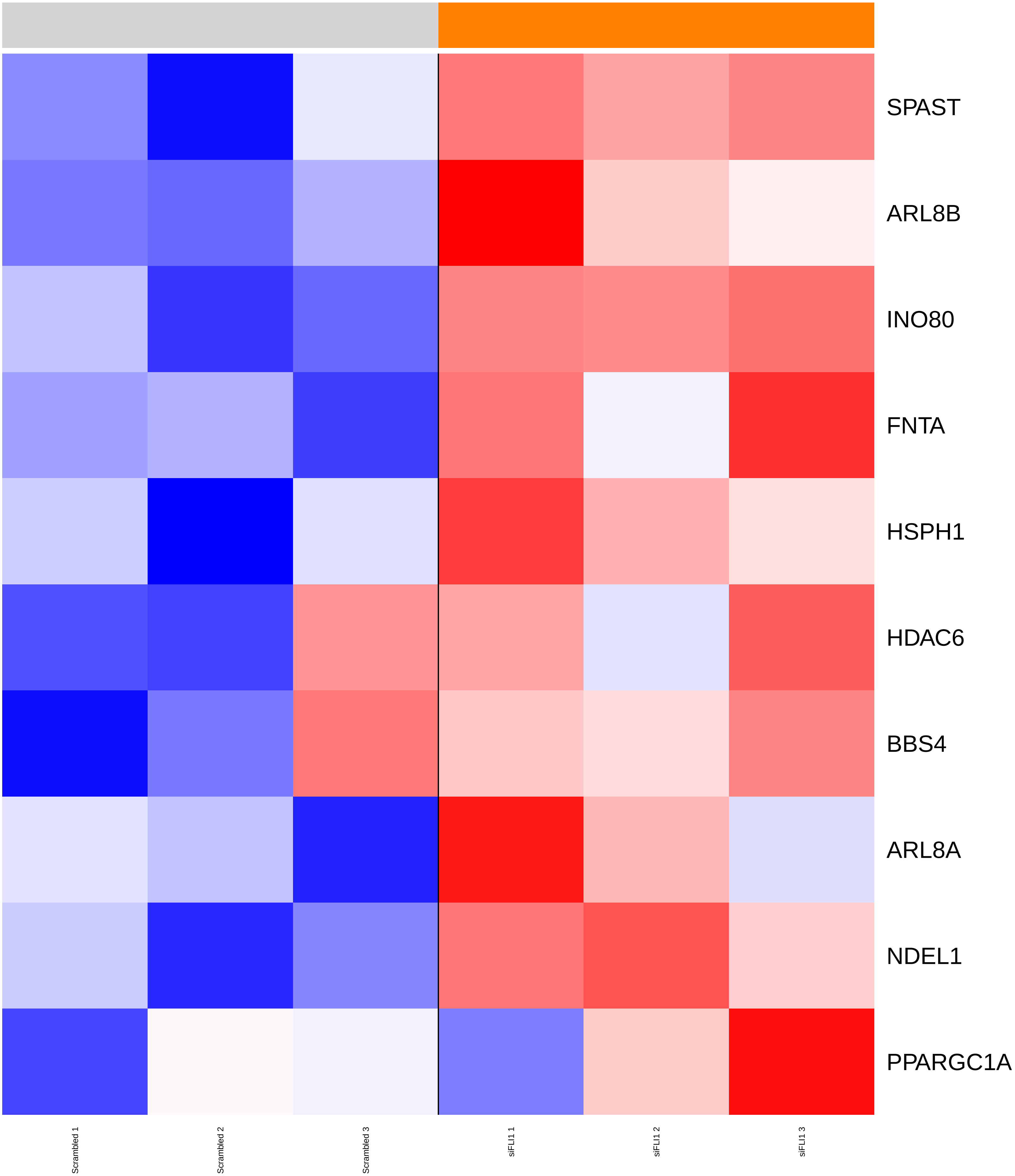

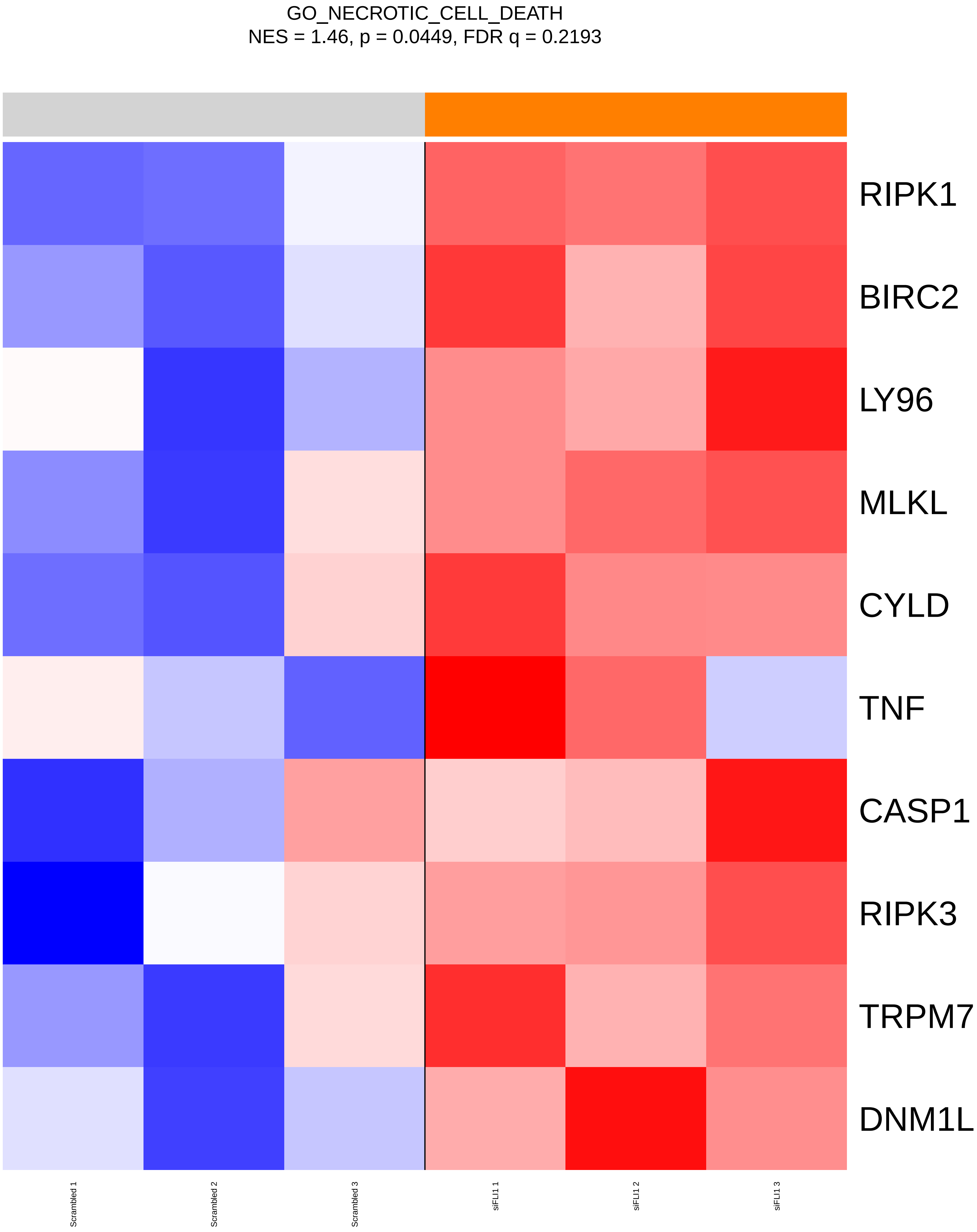
GO_NECROTIC_CELL_DEATH
NES = 1.46, p = 0.0449, FDR q = 0.2193
RIPK1
BIRC2
LY96
MLKL
CYLD
TNF
CASP1
RIPK3
TRPM7
DNM1L
Scrambled 1
Scrambled 2
Scrambled 3
siFLI1 1
siFLI1 2
siFLI1 3

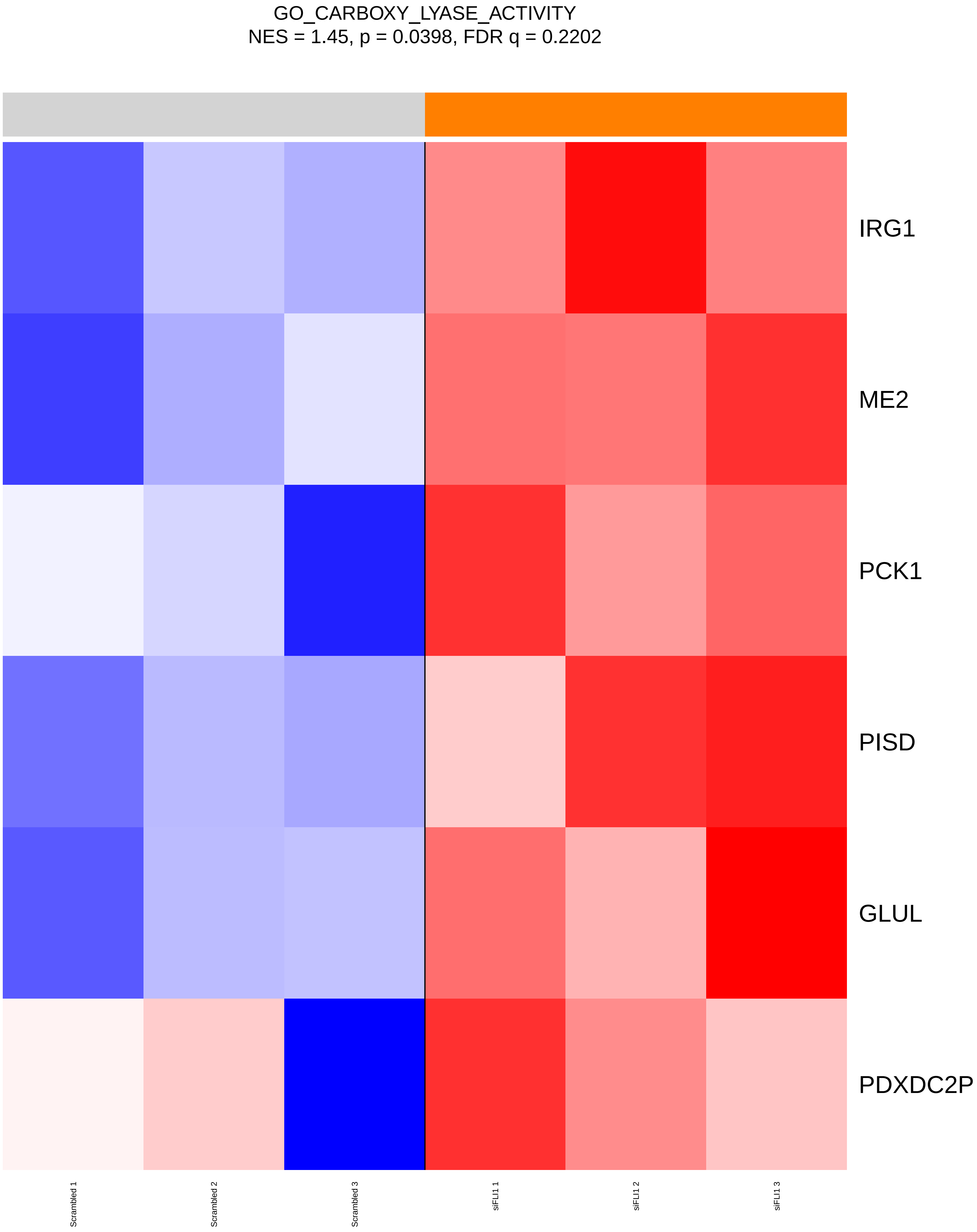
GO_CARBOXY_LYASE_ACTIVITY
NES = 1.45, p = 0.0398, FDR q = 0.2202
IRG1
ME2
PCK1
PISD
GLUL
PDXDC2P
Scrambled 1
Scrambled 2
Scrambled 3
siFLI1 1
siFLI1 2
siFLI1 3

GO_INTRACELLULAR_RECEPTOR_SIGNALING_PATHWAY
NES = 1.45, p = 0.0068, FDR q = 0.2213

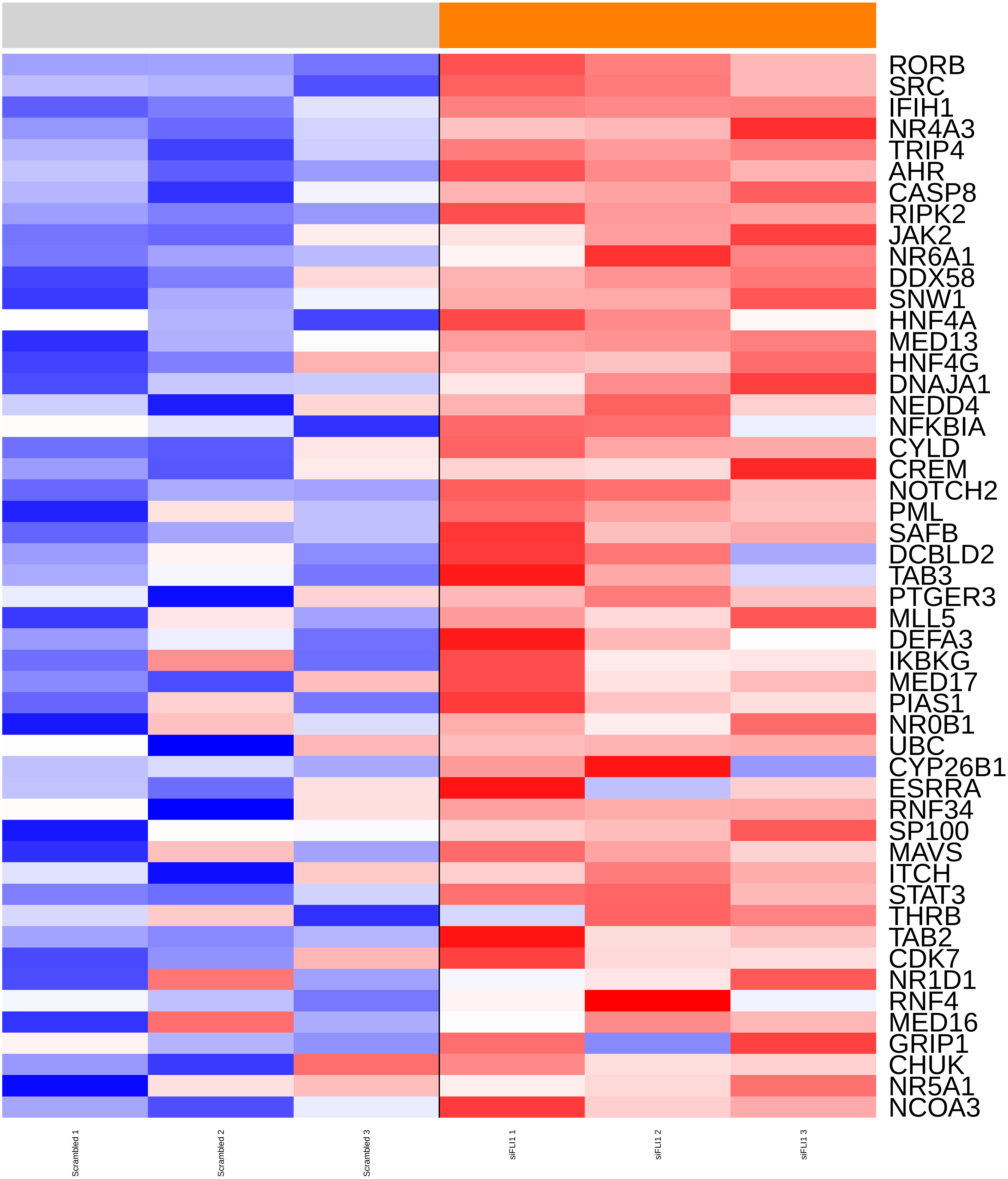

GO_REGULATION_OF_OSTEOBLAST_PROLIFERATION
NES = 1.45, p = 0.0602, FDR q = 0.2209

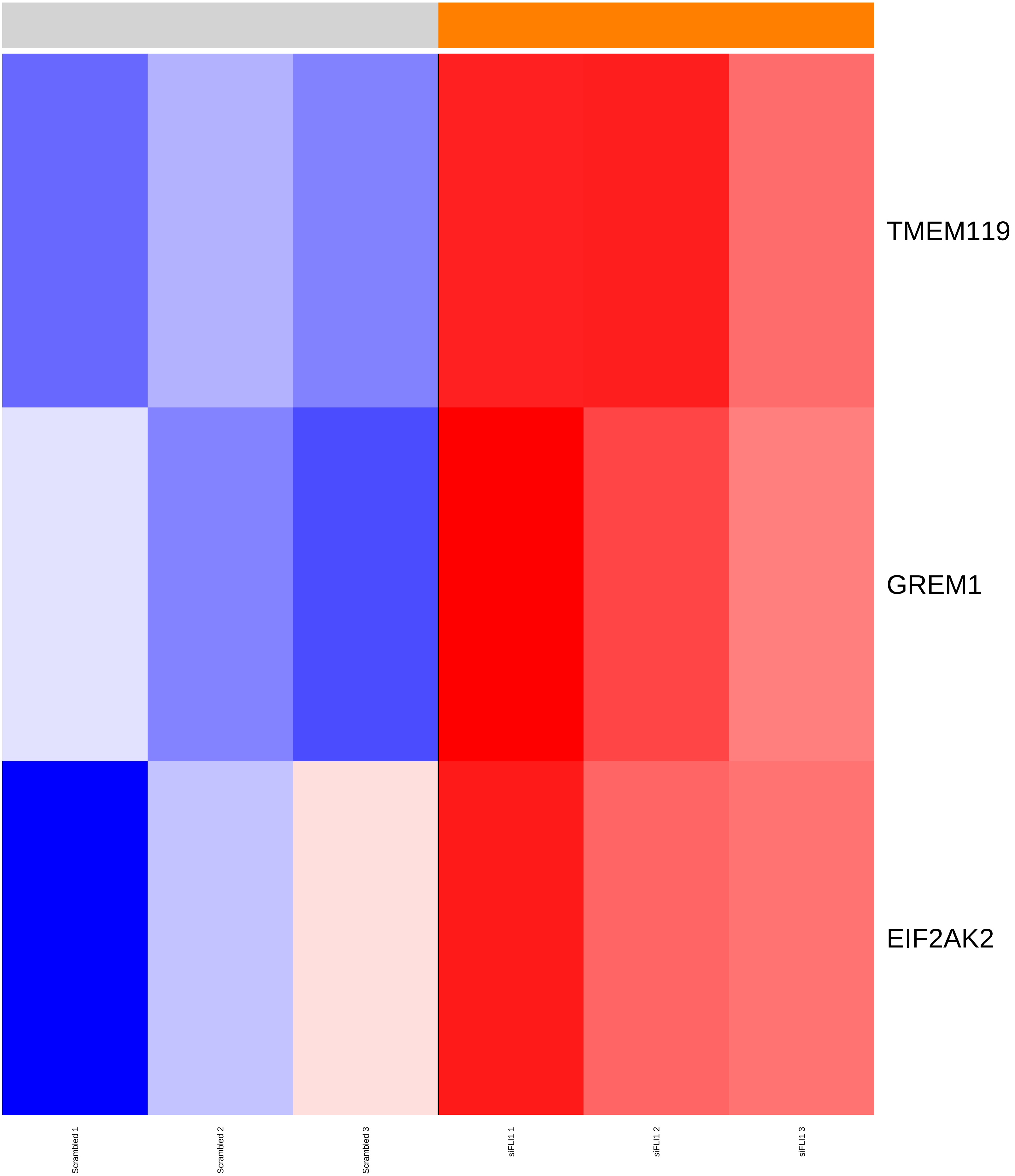

HALLMARK_ALLOGRAFT_REJECTION
NES = 1.45, p = 0.0040, FDR q = 0.2215

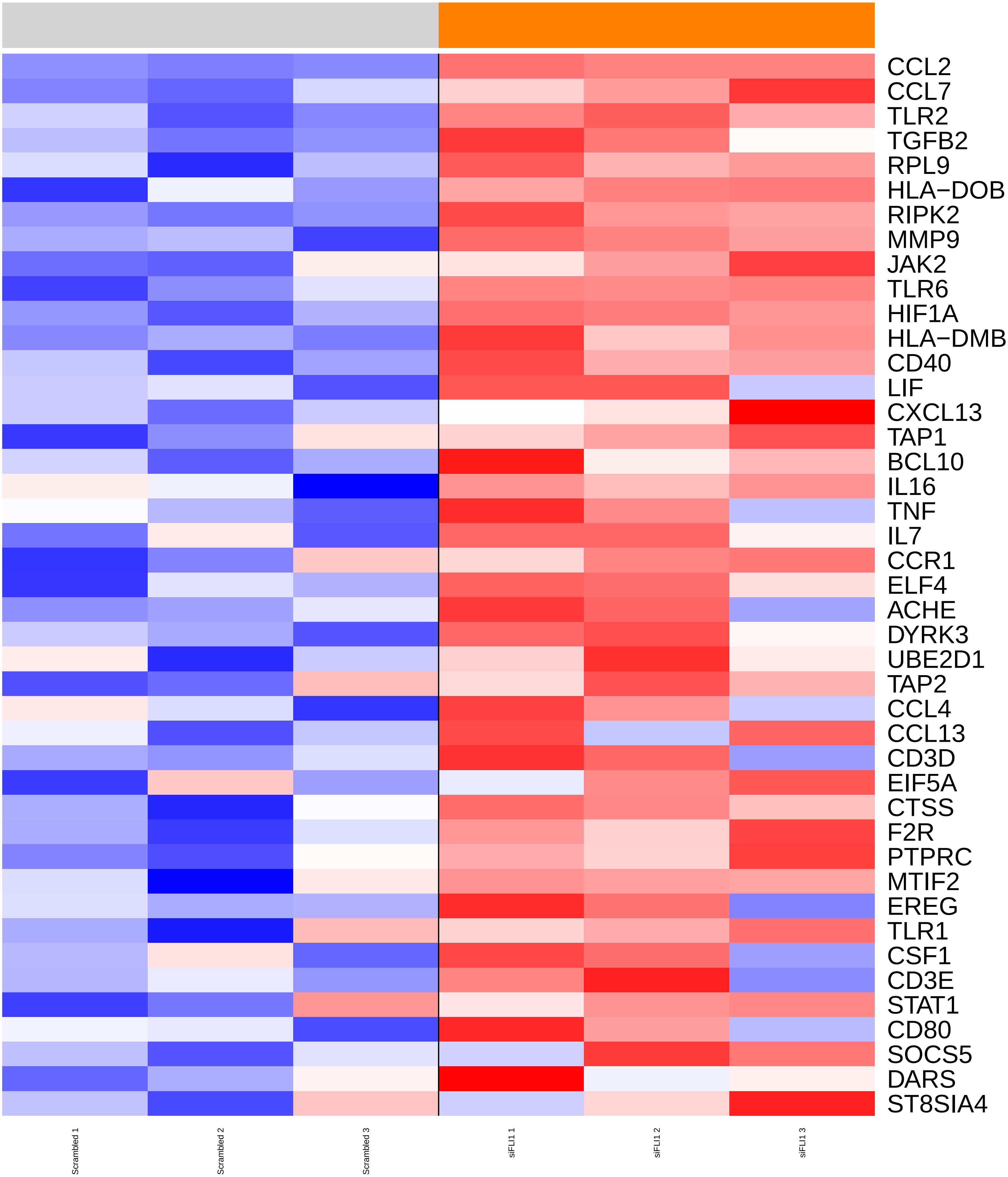

REACTOME_NUCLEAR_SIGNALING_BY_ERBB4
NES = 1.45, p = 0.0574, FDR q = 0.2211

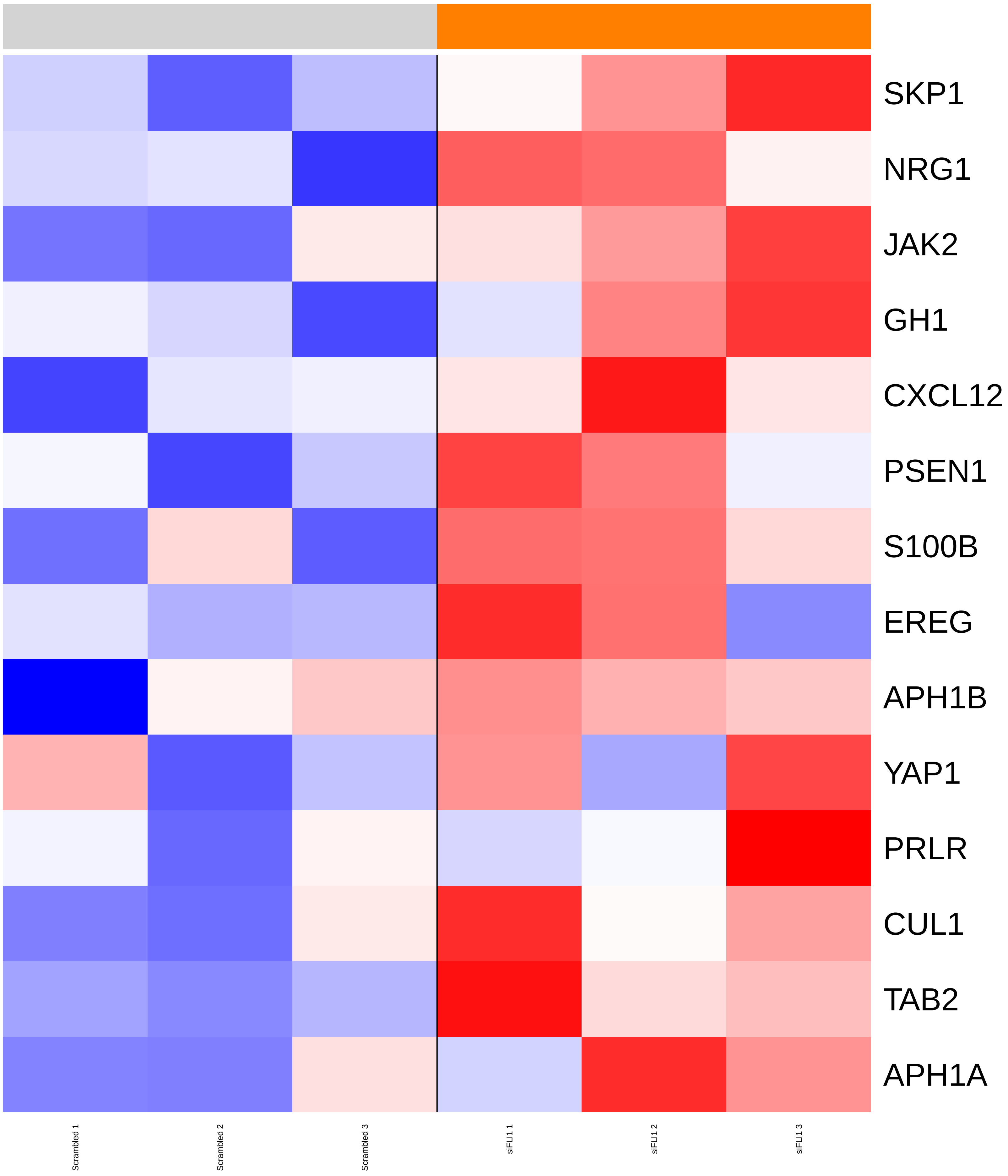

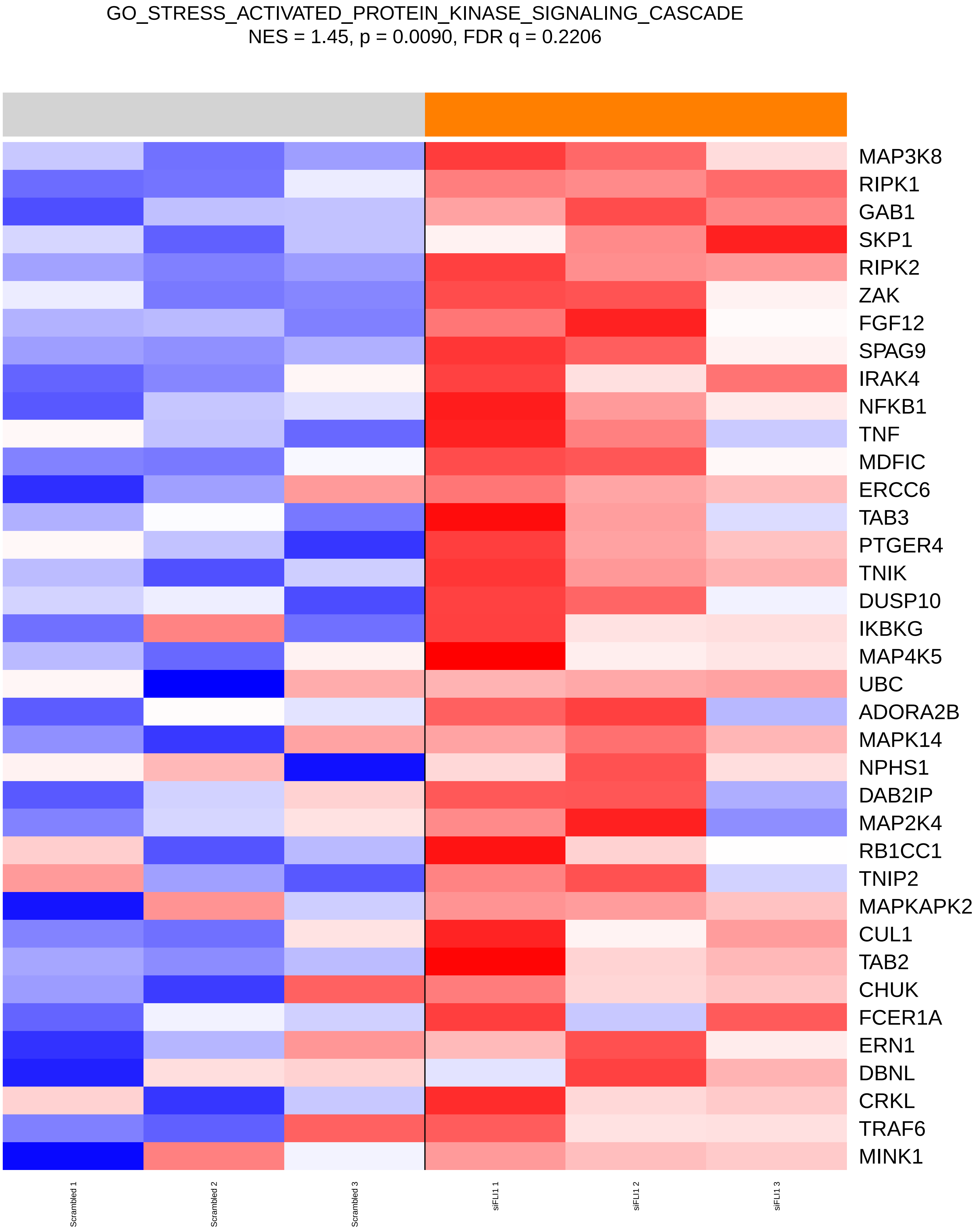
GO_STRESS_ACTIVATED_PROTEIN_KINASE_SIGNALING_CASCADE
NES = 1.45, p = 0.0090, FDR q = 0.2206
MAP3K8
RIPK1
GAB1
SKP1
RIPK2
ZAK
FGF12
SPAG9
IRAK4
NFKB1
TNF
MDFIC
ERCC6
TAB3
PTGER4
TNIK
DUSP10
IKBKG
MAP4K5
UBC
ADORA2B
MAPK14
NPHS1
DAB2IP
MAP2K4
RB1CC1
TNIP2
MAPKAPK2
CUL1
TAB2
CHUK
FCER1A
ERN1
DBNL
CRKL
TRAF6
MINK1
Scrambled 1
Scrambled 2
Scrambled 3
siFLI1 1
siFLI1 2
siFLI1 3

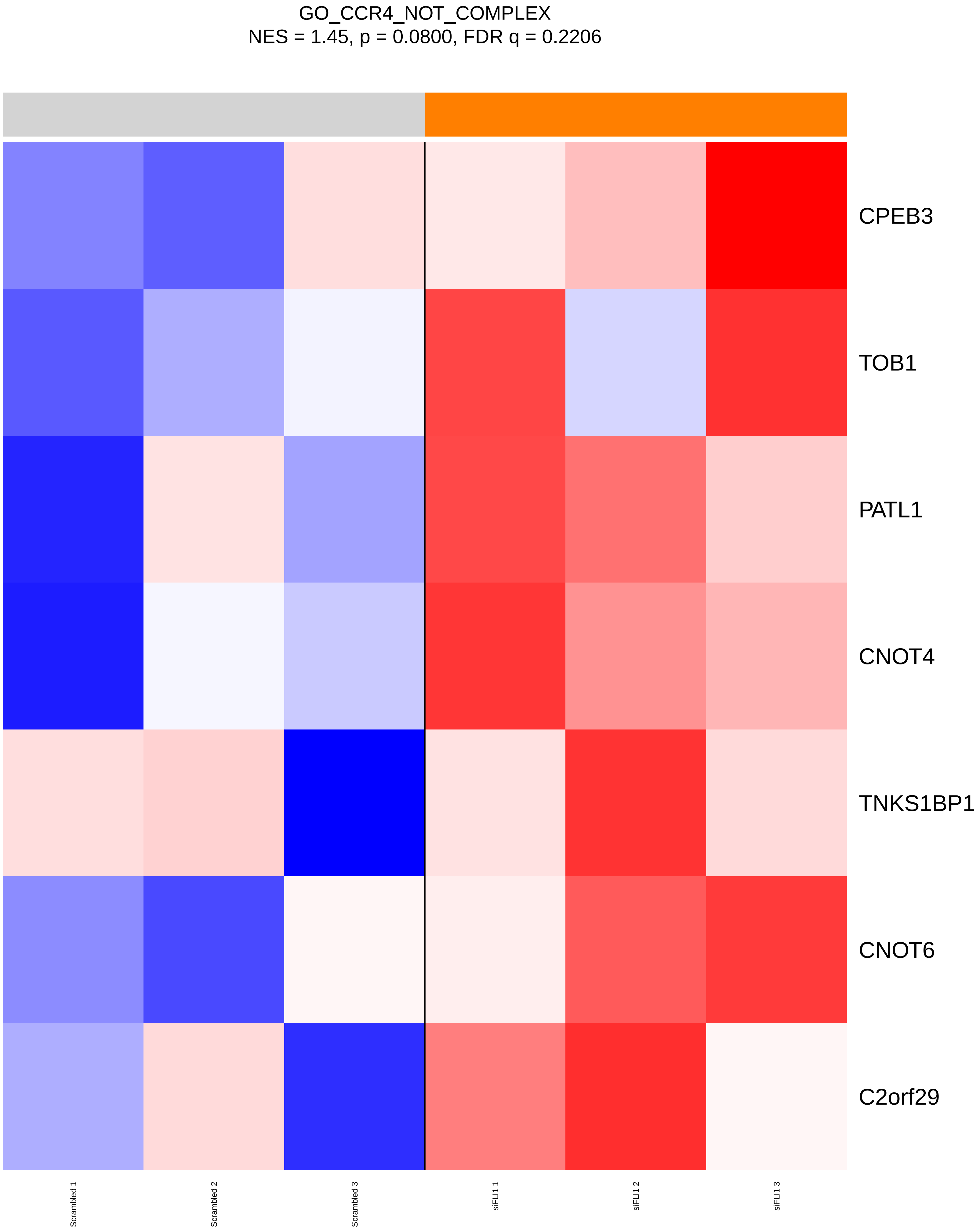
GO_CCR4_NOT_COMPLEX
NES = 1.45, p = 0.0800, FDR q = 0.2206
CPEB3
TOB1
PATL1
CNOT4
TNKS1BP1
CNOT6
C2orf29
Scrambled 1
Scrambled 2
Scrambled 3
siFLI1 1
siFLI1 2
siFLI1 3

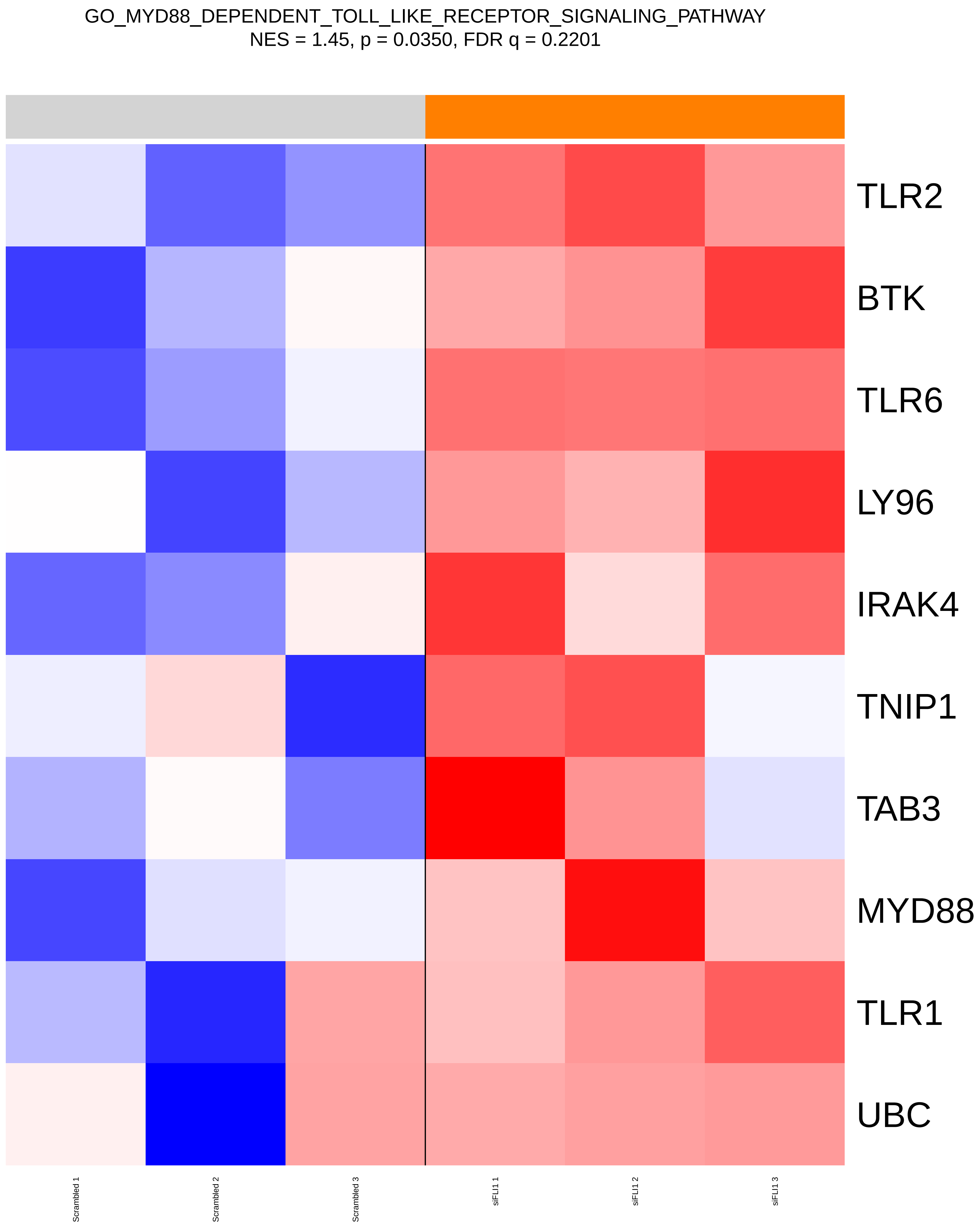
GO_MYD88_DEPENDENT_TOLL_LIKE_RECEPTOR_SIGNALING_PATHWAY
NES = 1.45, p = 0.0350, FDR q = 0.2201
TLR2
BTK
TLR6
LY96
IRAK4
TNIP1
TAB3
MYD88
TLR1
UBC
Scrambled 1
Scrambled 2
Scrambled 3
siFLI1 1
siFLI1 2
siFLI1 3

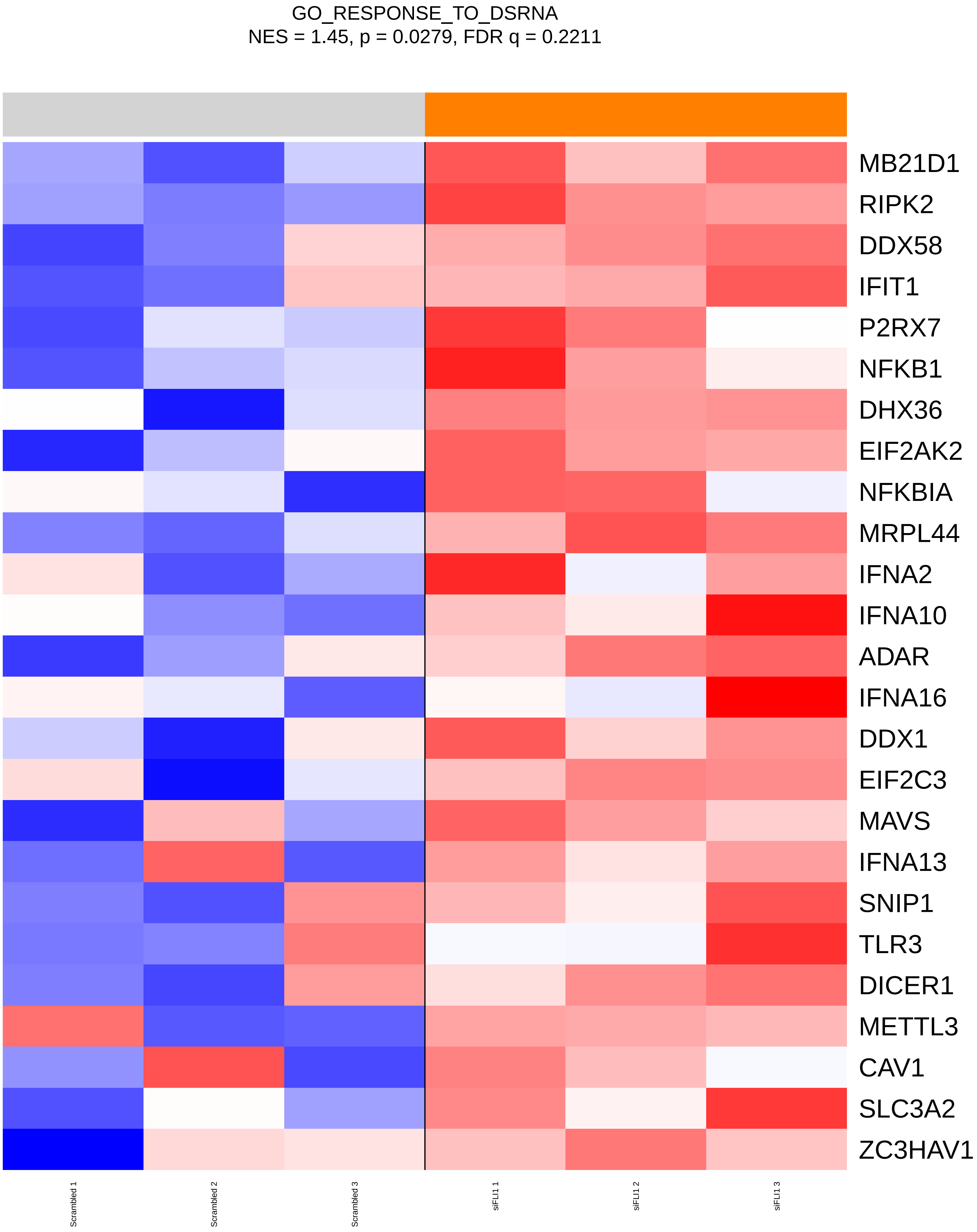
GO_RESPONSE_TO_DSRNA
NES = 1.45, p = 0.0279, FDR q = 0.2211
MB21D1
RIPK2
DDX58
IFIT1
P2RX7
NFKB1
DHX36
EIF2AK2
NFKBIA
MRPL44
IFNA2
IFNA10
ADAR
IFNA16
DDX1
EIF2C3
MAVS
IFNA13
SNIP1
TLR3
DICER1
METTL3
CAV1
SLC3A2
ZC3HAV1
Scrambled 1
Scrambled 2
Scrambled 3
siFLI1 1
siFLI1 2
siFLI1 3

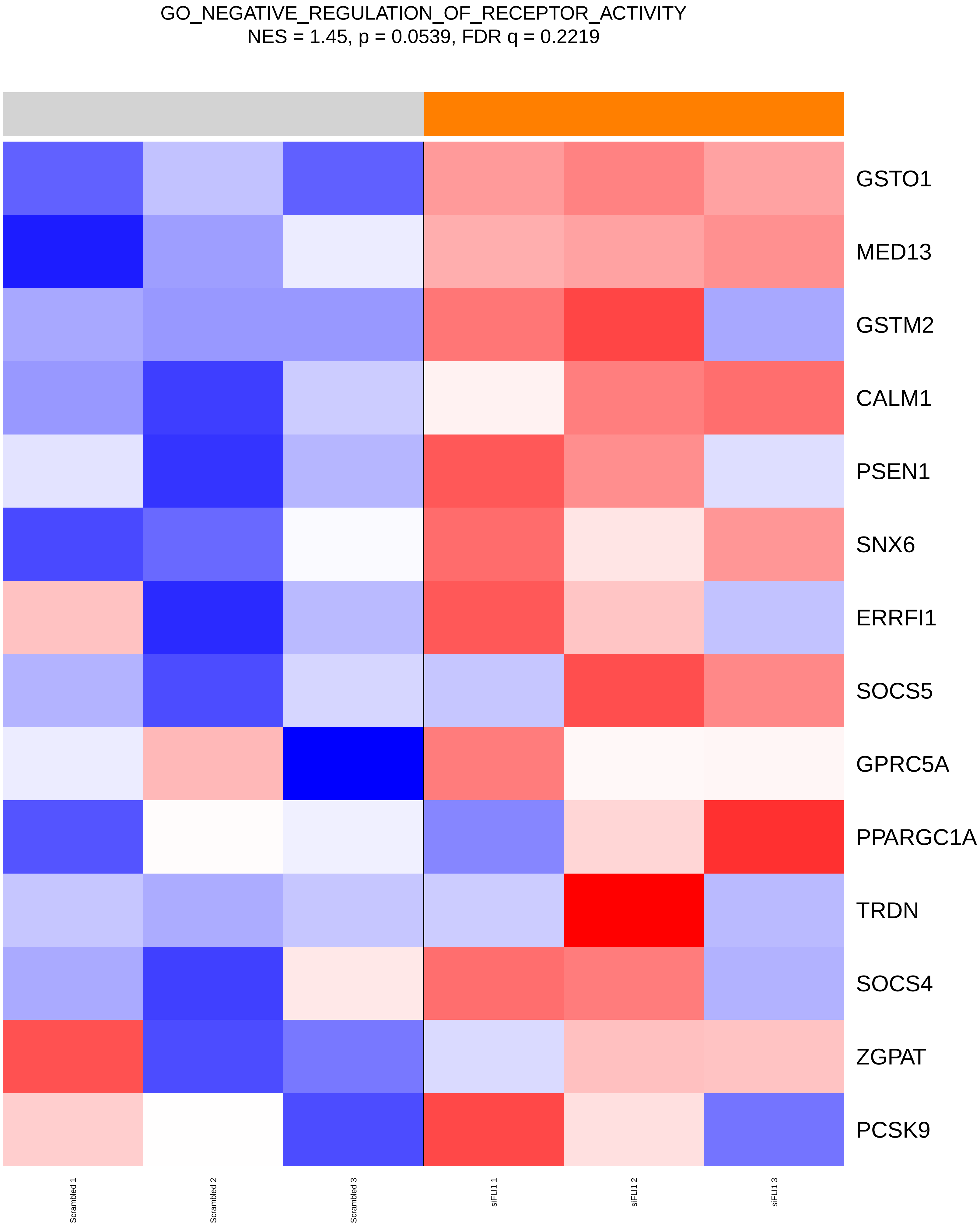

GO_NEGATIVE_REGULATION_OF_RECEPTOR_ACTIVITY
NES = 1.45, p = 0.0539, FDR q = 0.2219
GSTO1
MED13
GSTM2
CALM1
PSEN1
SNX6
ERRFI1
SOCS5
GPRC5A
PPARGC1A
TRDN
SOCS4
ZGPAT
PCSK9
Scrambled 1
Scrambled 2
Scrambled 3
siFLI1 1
siFLI1 2
siFLI1 3

GO_HISTONE_MRNA_METABOLIC_PROCESS
NES = 1.45, p = 0.0447, FDR q = 0.2225

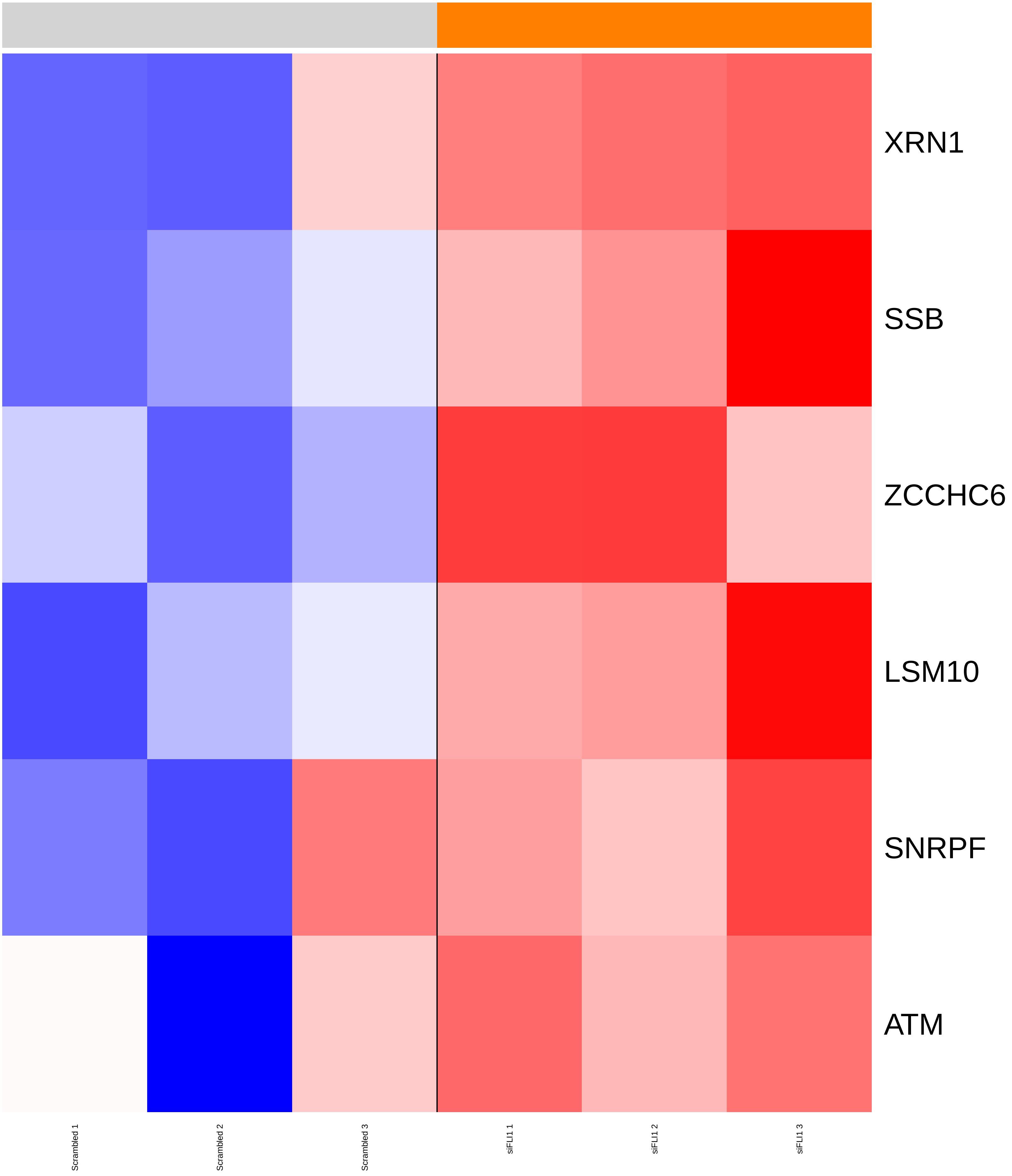

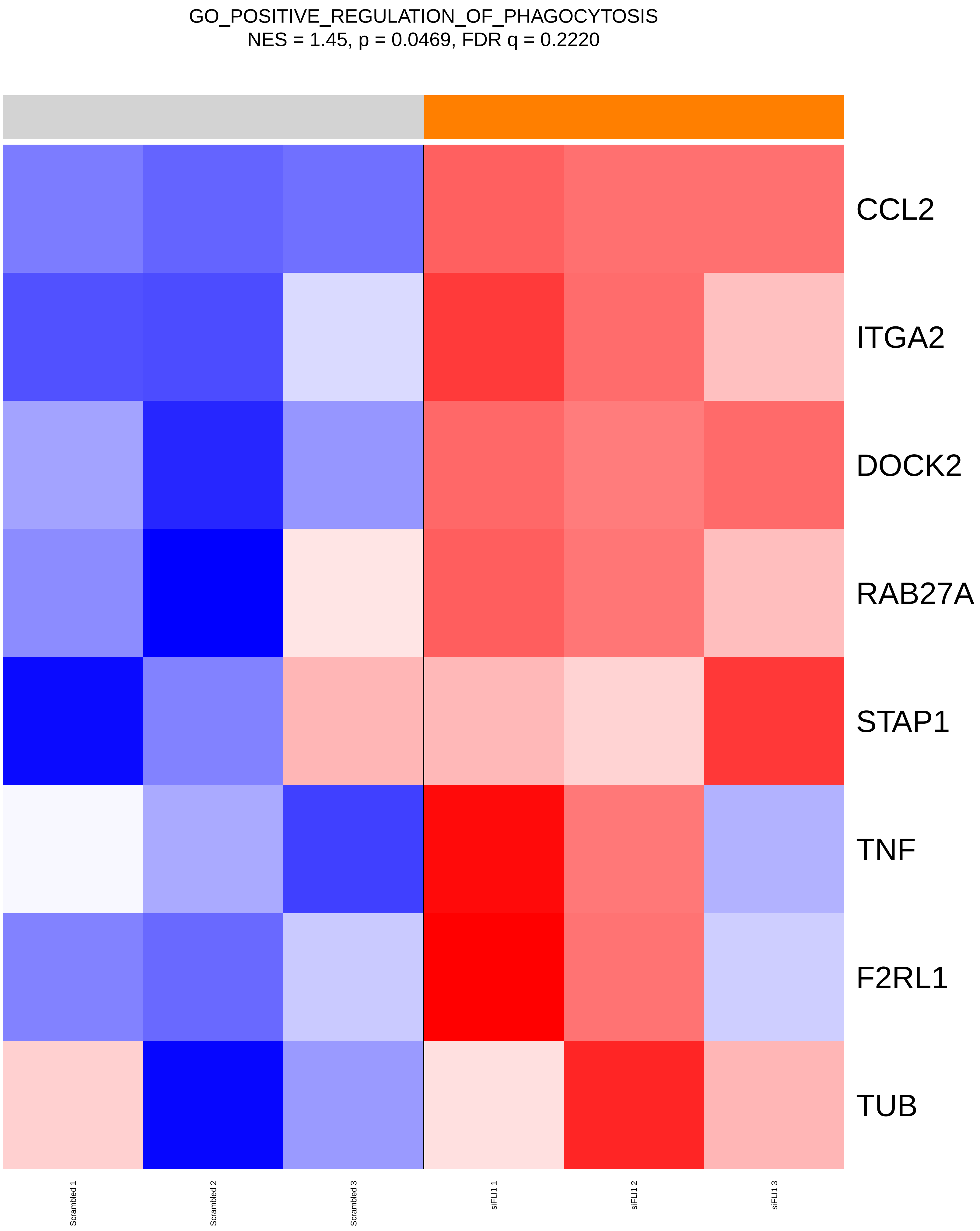
GO_POSITIVE_REGULATION_OF_PHAGOCYTOSIS
NES = 1.45, p = 0.0469, FDR q = 0.2220
CCL2
ITGA2
DOCK2
RAB27A
STAP1
TNF
F2RL1
TUB
Scrambled 1
Scrambled 2
Scrambled 3
siFLI1 1
siFLI1 2
siFLI1 3

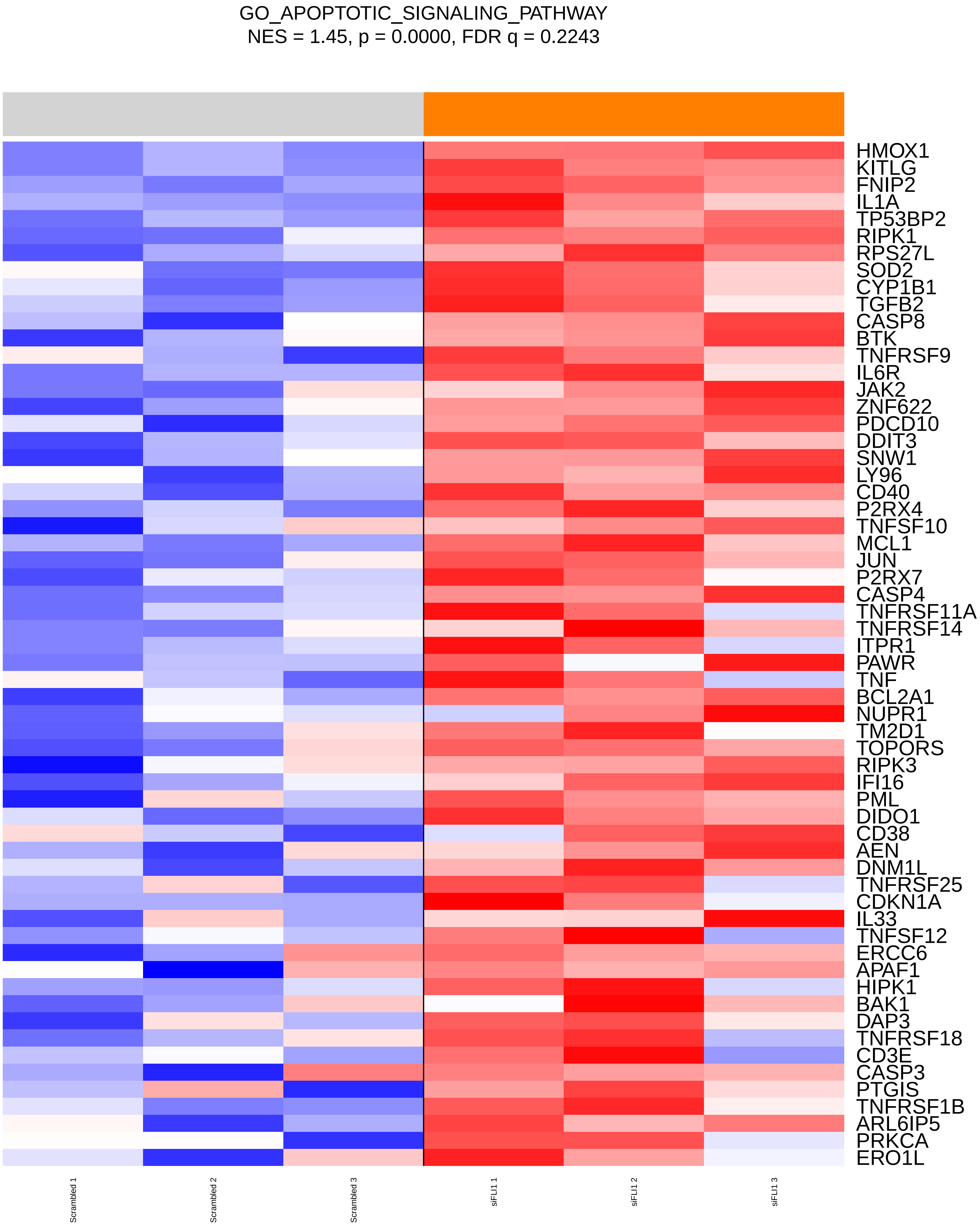

GO_APOPTOTIC_SIGNALING_PATHWAY
NES = 1.45, p = 0.0000, FDR q = 0.2243
HMOX1
KITLG
FNIP2
IL1A
TP53BP2
RIPK1
RPS27L
SOD2
CYP1B1
TGFB2
CASP8
BTK
TNFRSF9
IL6R
JAK2
ZNF622
PDCD10
DDIT3
SNW1
LY96
CD40
P2RX4
TNFSF10
MCL1
JUN
P2RX7
CASP4
TNFRSF11A
TNFRSF14
ITPR1
PAWR
TNF
BCL2A1
NUPR1
TM2D1
TOPORS
RIPK3
IFI16
PML
DIDO1
CD38
AEN
DNM1L
TNFRSF25
CDKN1A
IL33
TNFSF12
ERCC6
APAF1
HIPK1
BAK1
DAP3
TNFRSF18
CD3E
CASP3
PTGIS
TNFRSF1B
ARL6IP5
PRKCA
ERO1L
Scrambled 1
Scrambled 2
Scrambled 3
siFLI1 1
siFLI1 2
siFLI1 3

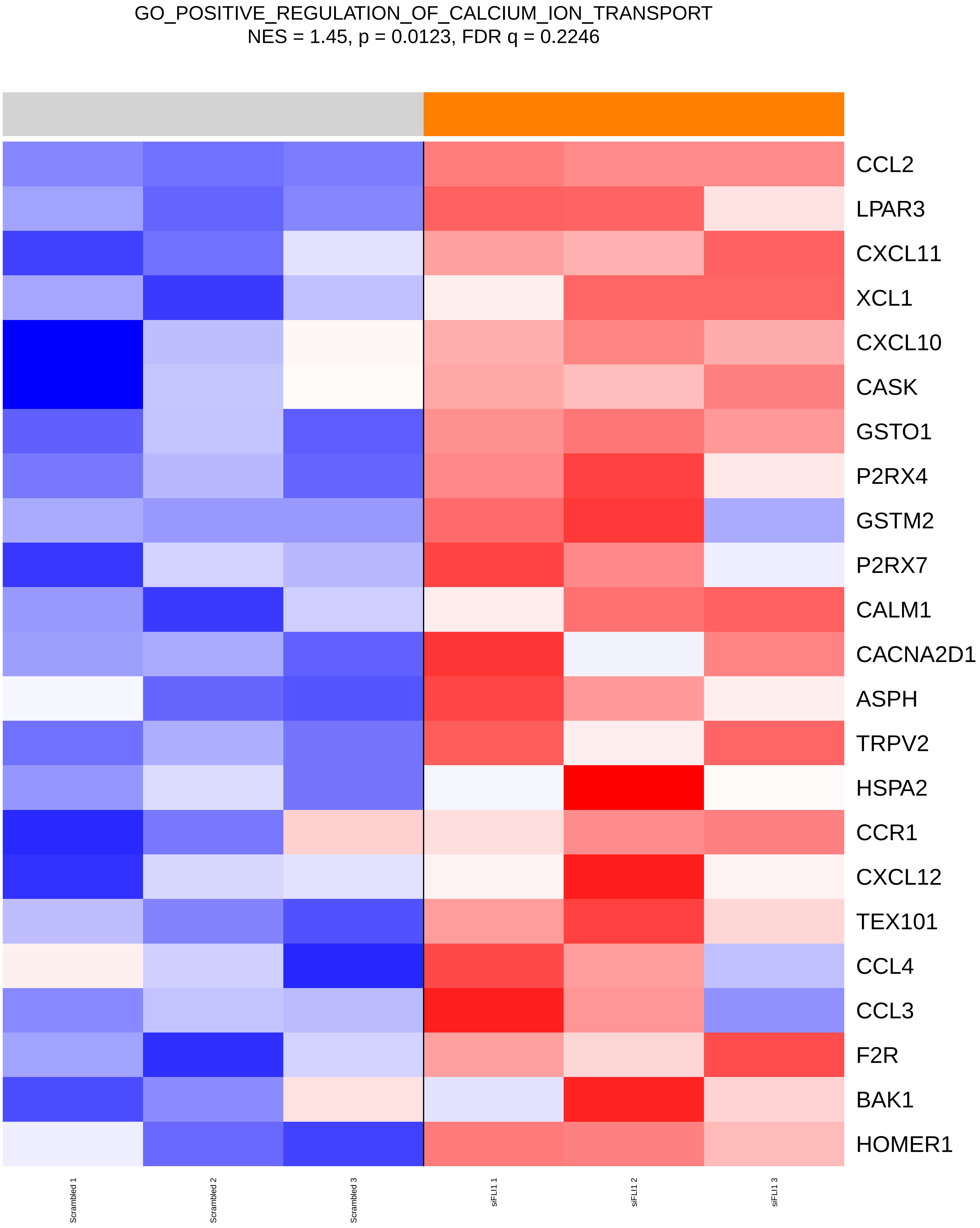
GO_POSITIVE_REGULATION_OF_CALCIUM_ION_TRANSPORT
NES = 1.45, p = 0.0123, FDR q = 0.2246
CCL2
LPAR3
CXCL11
XCL1
CXCL10
CASK
GSTO1
P2RX4
GSTM2
P2RX7
CALM1
CACNA2D1
ASPH
TRPV2
HSPA2
CCR1
CXCL12
TEX101
CCL4
CCL3
F2R
BAK1
HOMER1
Scrambled 1
Scrambled 2
Scrambled 3
siFLI1 1
siFLI1 2
siFLI1 3

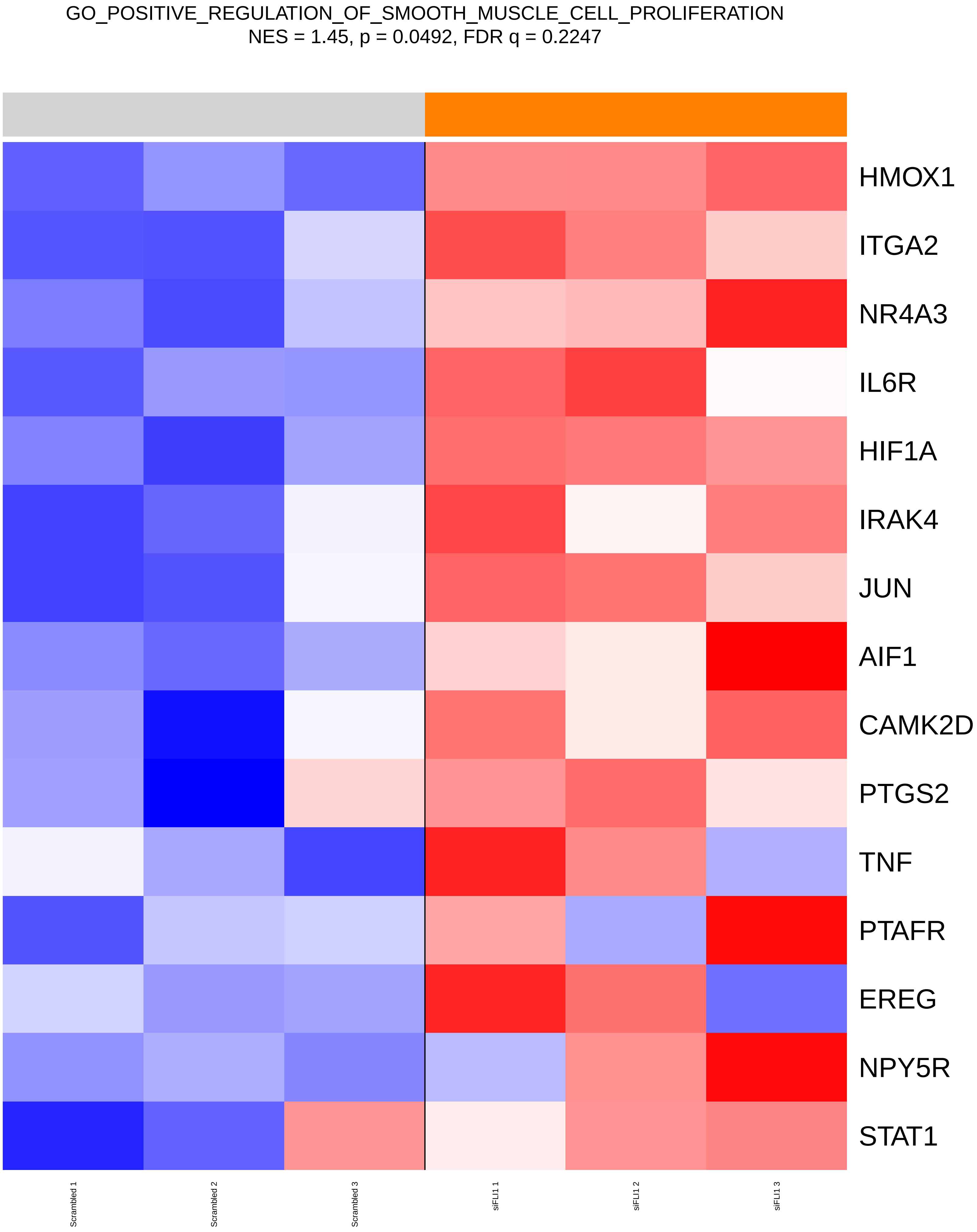
GO_POSITIVE_REGULATION_OF_SMOOTH_MUSCLE_CELL_PROLIFERATION
NES = 1.45, p = 0.0492, FDR q = 0.2247
HMOX1
ITGA2
NR4A3
IL6R
HIF1A
IRAK4
JUN
AIF1
CAMK2D
PTGS2
TNF
PTAFR
EREG
NPY5R
STAT1
Scrambled 1
Scrambled 2
Scrambled 3
siFLI1 1
siFLI1 2
siFLI1 3

GO_CELLULAR_RESPONSE_TO_RETINOIC_ACID
NES = 1.45, p = 0.0418, FDR q = 0.2248

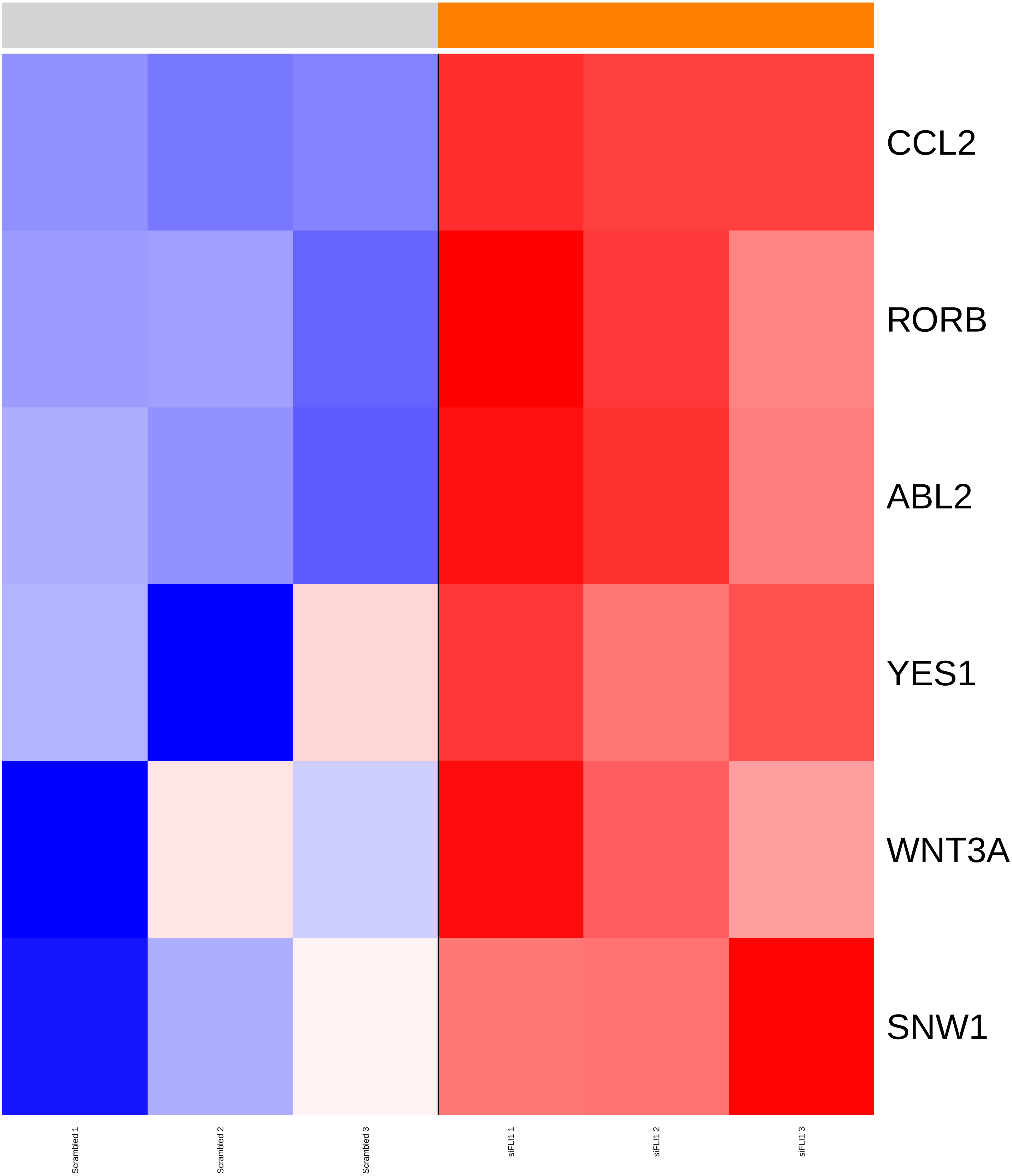

AAGCAAT_MIR137
NES = 1.44, p = 0.0000, FDR q = 0.2257

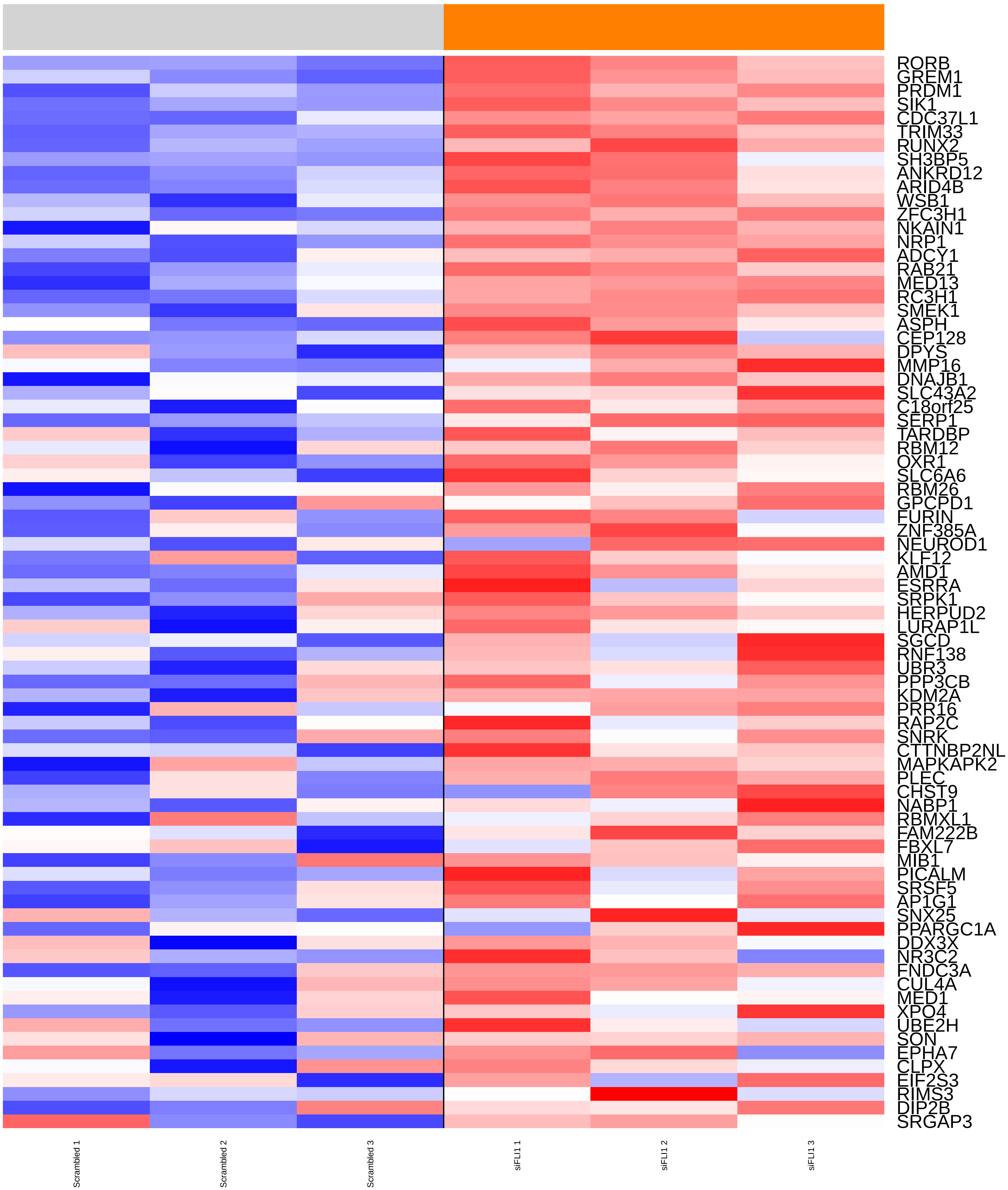

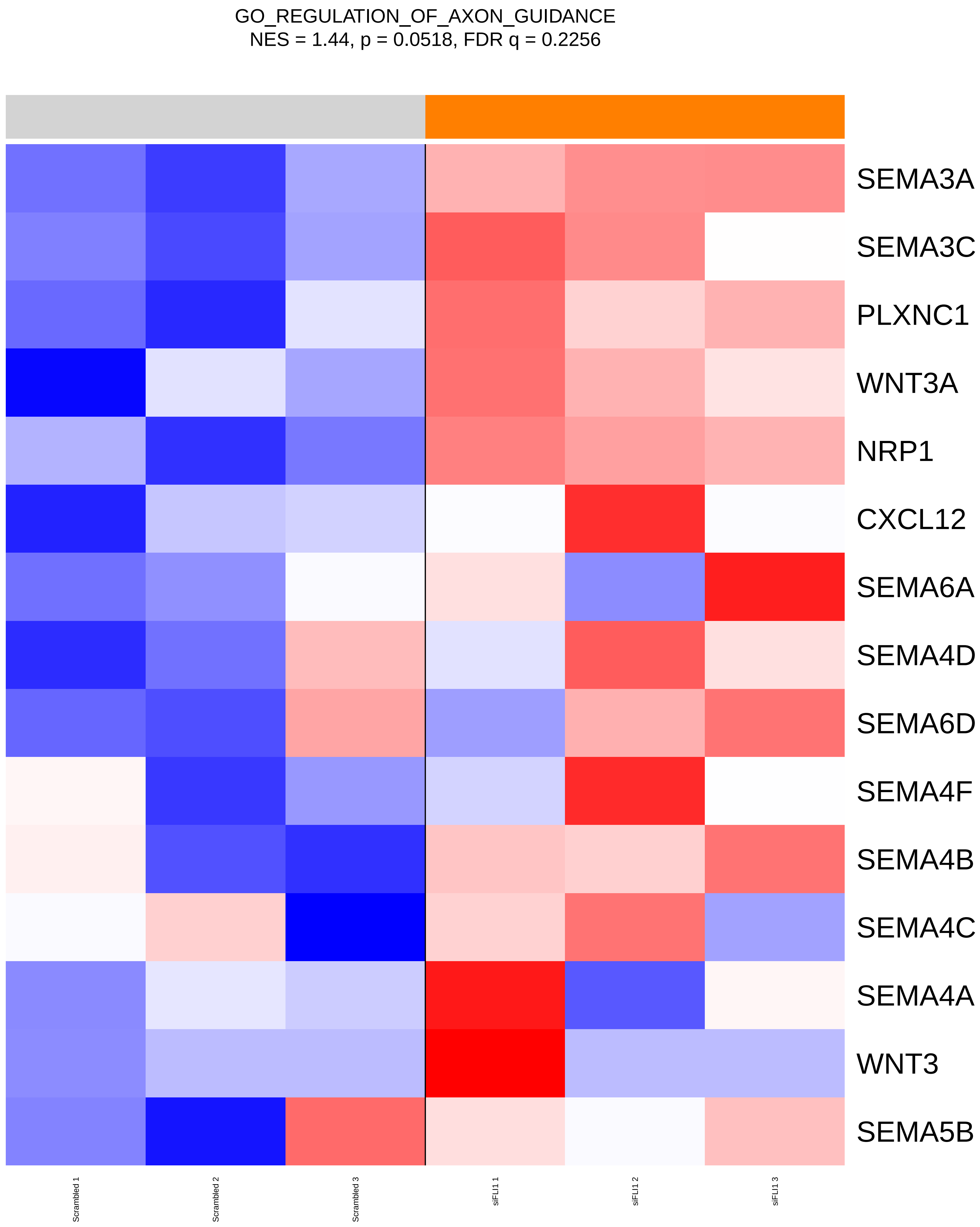
GO_REGULATION_OF_AXON_GUIDANCE
NES = 1.44, p = 0.0518, FDR q = 0.2256
SEMA3A
SEMA3C
PLXNC1
WNT3A
NRP1
CXCL12
SEMA6A
SEMA4D
SEMA6D
SEMA4F
SEMA4B
SEMA4C
SEMA4A
WNT3
SEMA5B
Scrambled 1
Scrambled 2
Scrambled 3
siFLI1 1
siFLI1 2
siFLI1 3

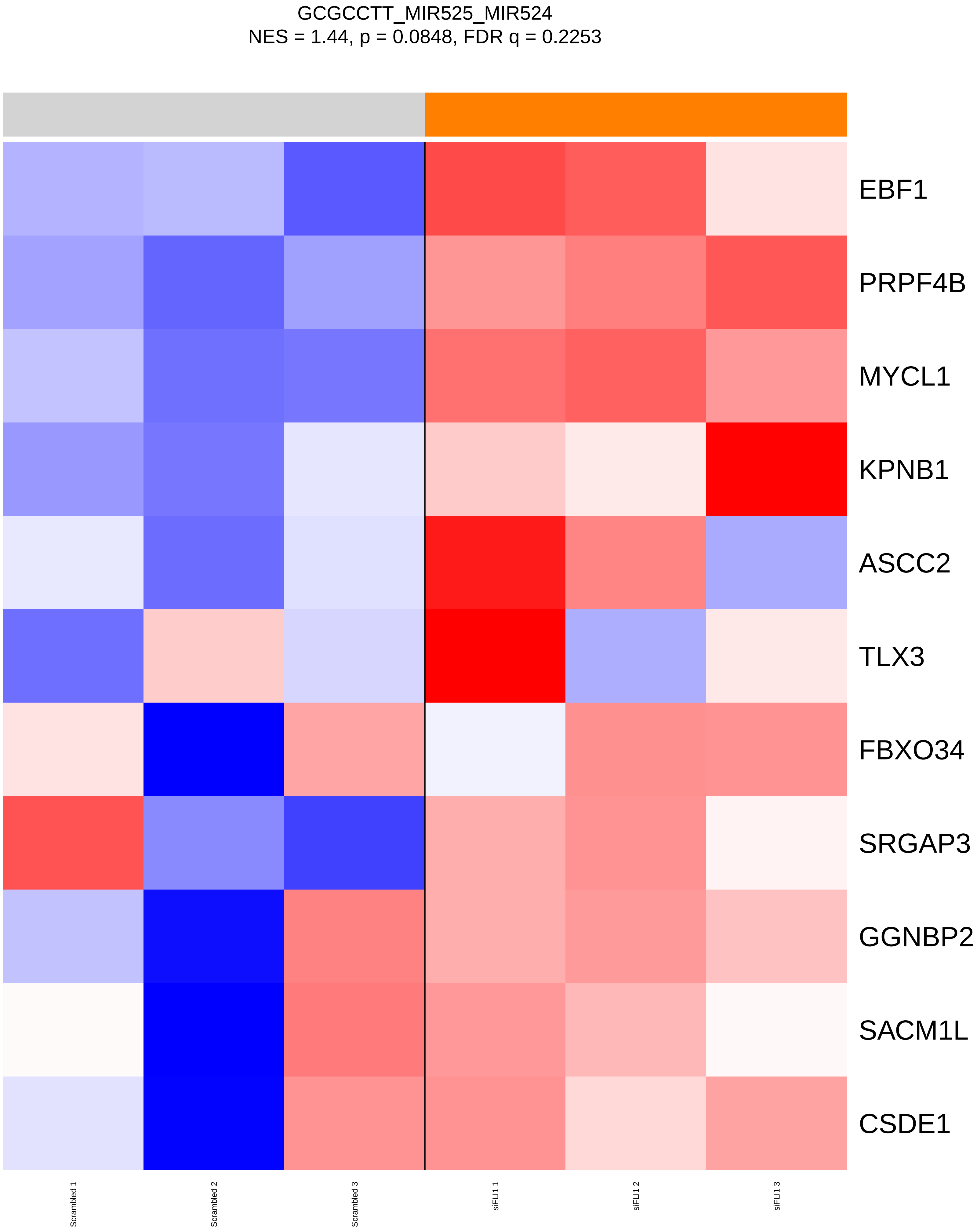
GCGCCTT_MIR525_MIR524
NES = 1.44, p = 0.0848, FDR q = 0.2253
EBF1
PRPF4B
MYCL1
KPNB1
ASCC2
TLX3
FBXO34
SRGAP3
GGNBP2
SACM1L
CSDE1
Scrambled 1
Scrambled 2
Scrambled 3
siFLI1 1
siFLI1 2
siFLI1 3

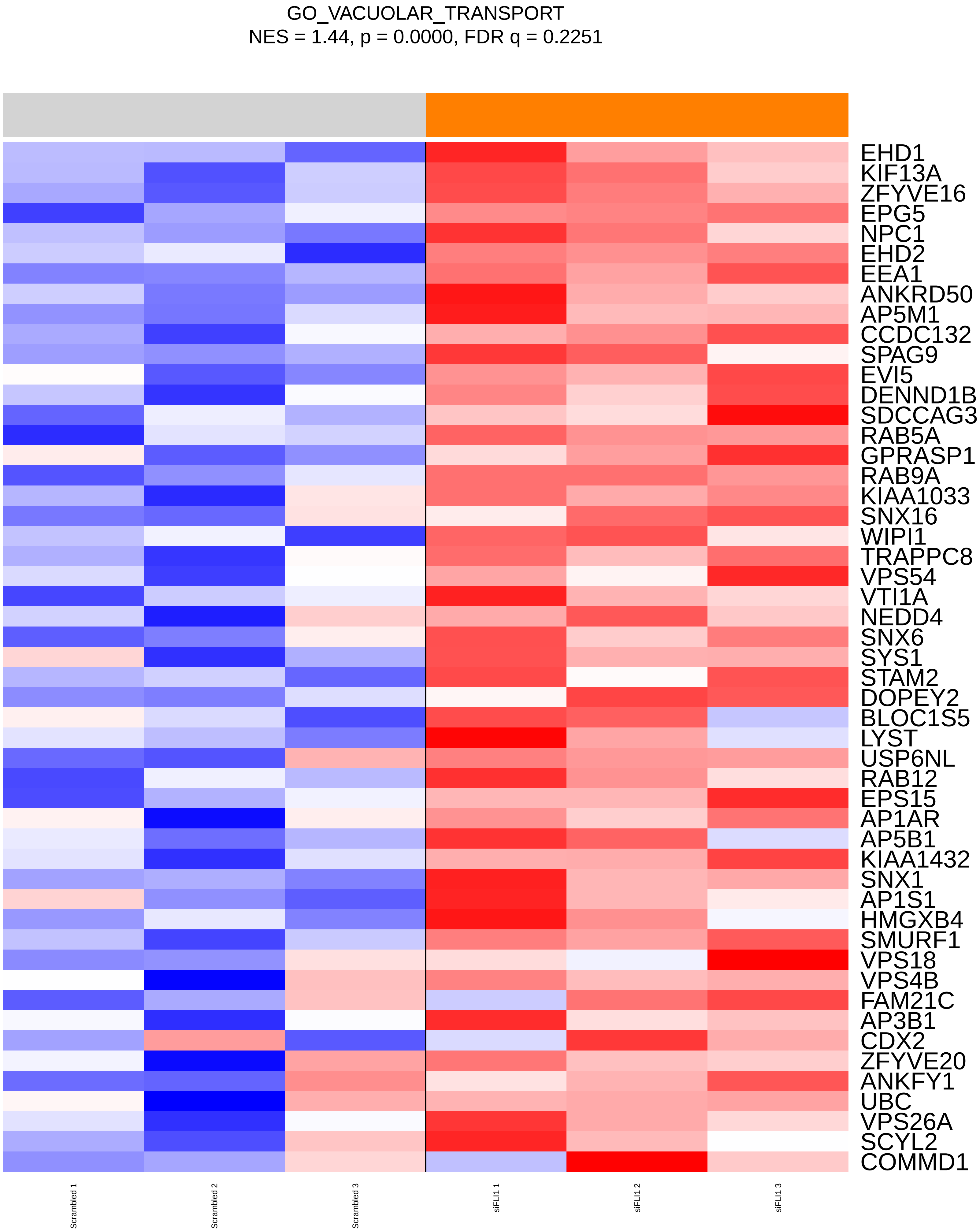
GO_VACUOLAR_TRANSPORT
NES = 1.44, p = 0.0000, FDR q = 0.2251
EHD1
KIF13A
ZFYVE16
EPG5
NPC1
EHD2
EEA1
ANKRD50
AP5M1
CCDC132
SPAG9
EVI5
DENND1B
SDCCAG3
RAB5A
GPRASP1
RAB9A
KIAA1033
SNX16
WIPI1
TRAPPC8
VPS54
VTI1A
NEDD4
SNX6
SYS1
STAM2
DOPEY2
BLOC1S5
LYST
USP6NL
RAB12
EPS15
AP1AR
AP5B1
KIAA1432
SNX1
AP1S1
HMGXB4
SMURF1
VPS18
VPS4B
FAM21C
AP3B1
CDX2
ZFYVE20
ANKFY1
UBC
VPS26A
SCYL2
COMMD1
Scrambled 1
Scrambled 2
Scrambled 3
siFLI1 1
siFLI1 2
siFLI1 3

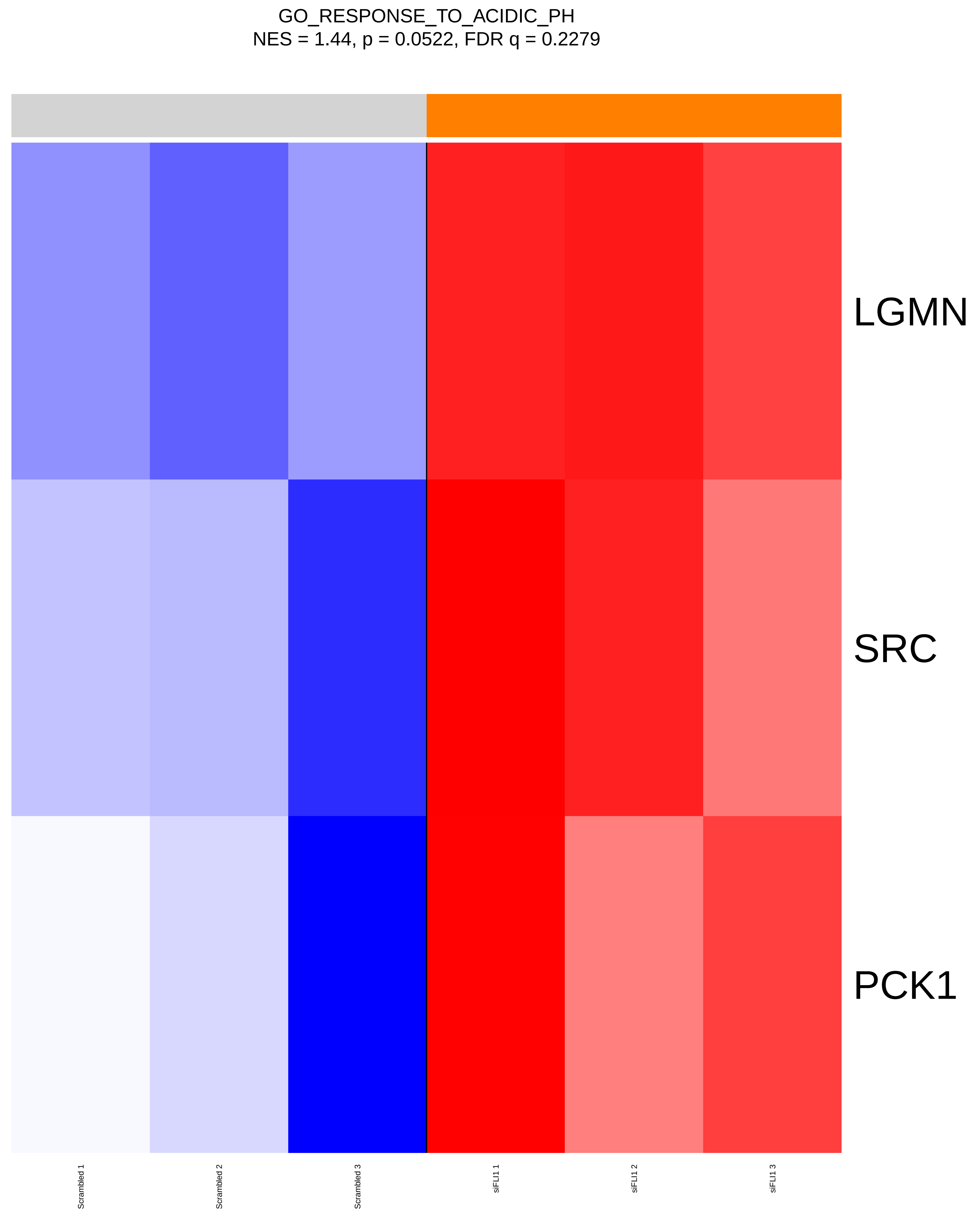
GO_RESPONSE_TO_ACIDIC_PH
NES = 1.44, p = 0.0522, FDR q = 0.2279
LGMN
SRC
PCK1
Scrambled 1
Scrambled 2
Scrambled 3
siFLI1 1
siFLI1 2
siFLI1 3

GO_POSITIVE_REGULATION_OF_NUCLEASE_ACTIVITY
NES = 1.44, p = 0.0958, FDR q = 0.2289

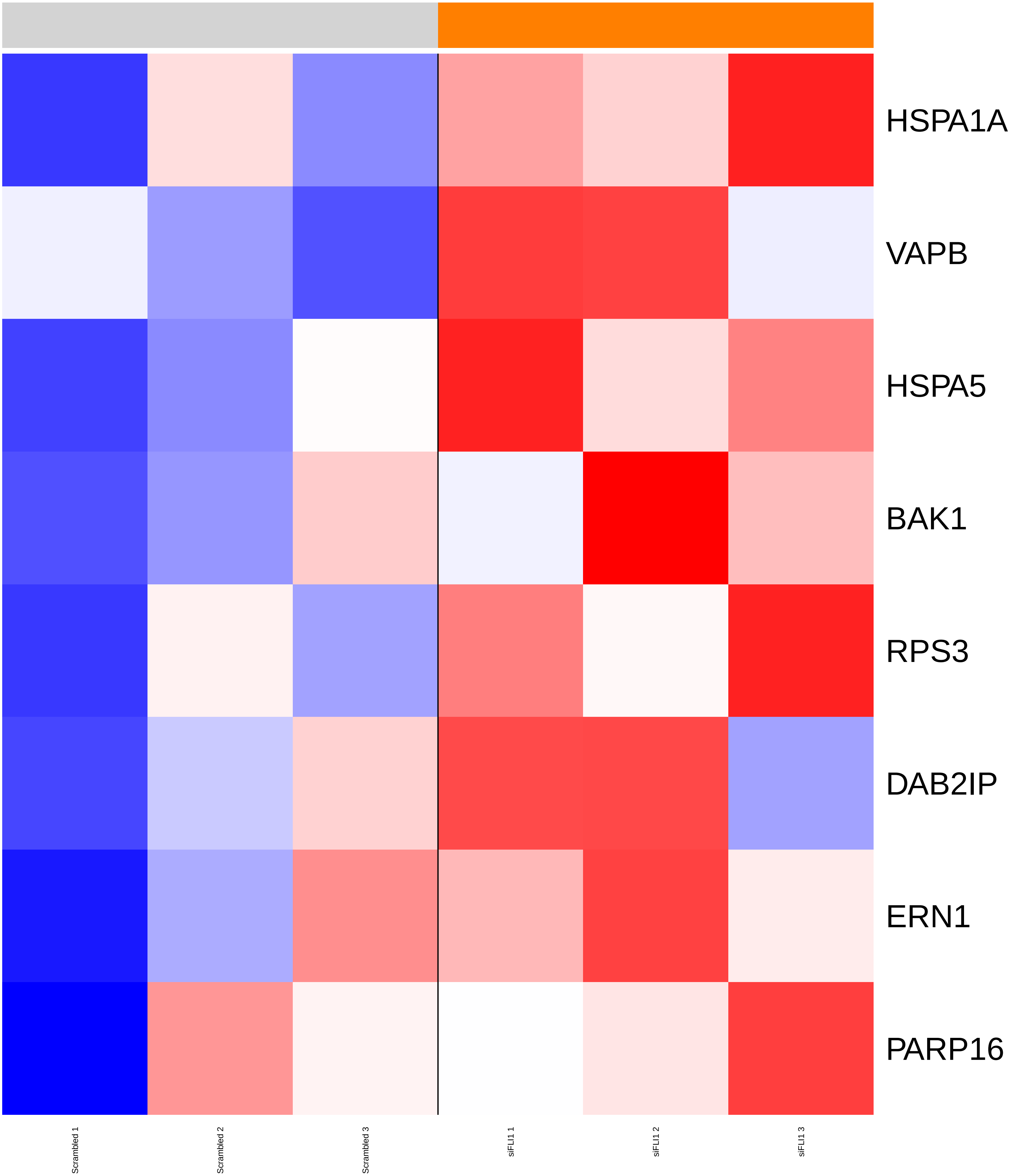

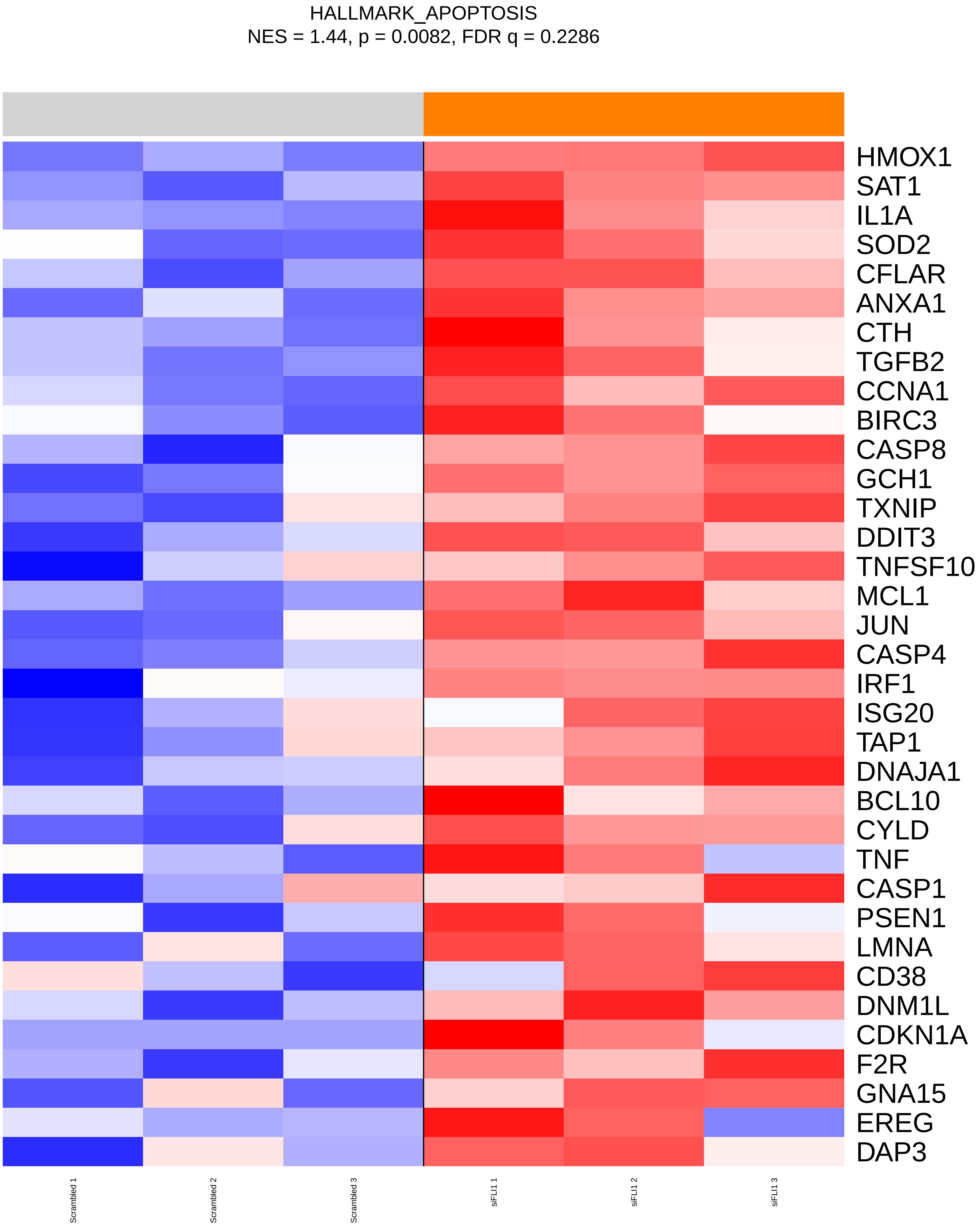
HALLMARK_APOPTOSIS
NES = 1.44, p = 0.0082, FDR q = 0.2286
HMOX1
SAT1
IL1A
SOD2
CFLAR
ANXA1
CTH
TGFB2
CCNA1
BIRC3
CASP8
GCH1
TXNIP
DDIT3
TNFSF10
MCL1
JUN
CASP4
IRF1
ISG20
TAP1
DNAJA1
BCL10
CYLD
TNF
CASP1
PSEN1
LMNA
CD38
DNM1L
CDKN1A
F2R
GNA15
EREG
DAP3
Scrambled 1
Scrambled 2
Scrambled 3
siFLI1 1
siFLI1 2
siFLI1 3

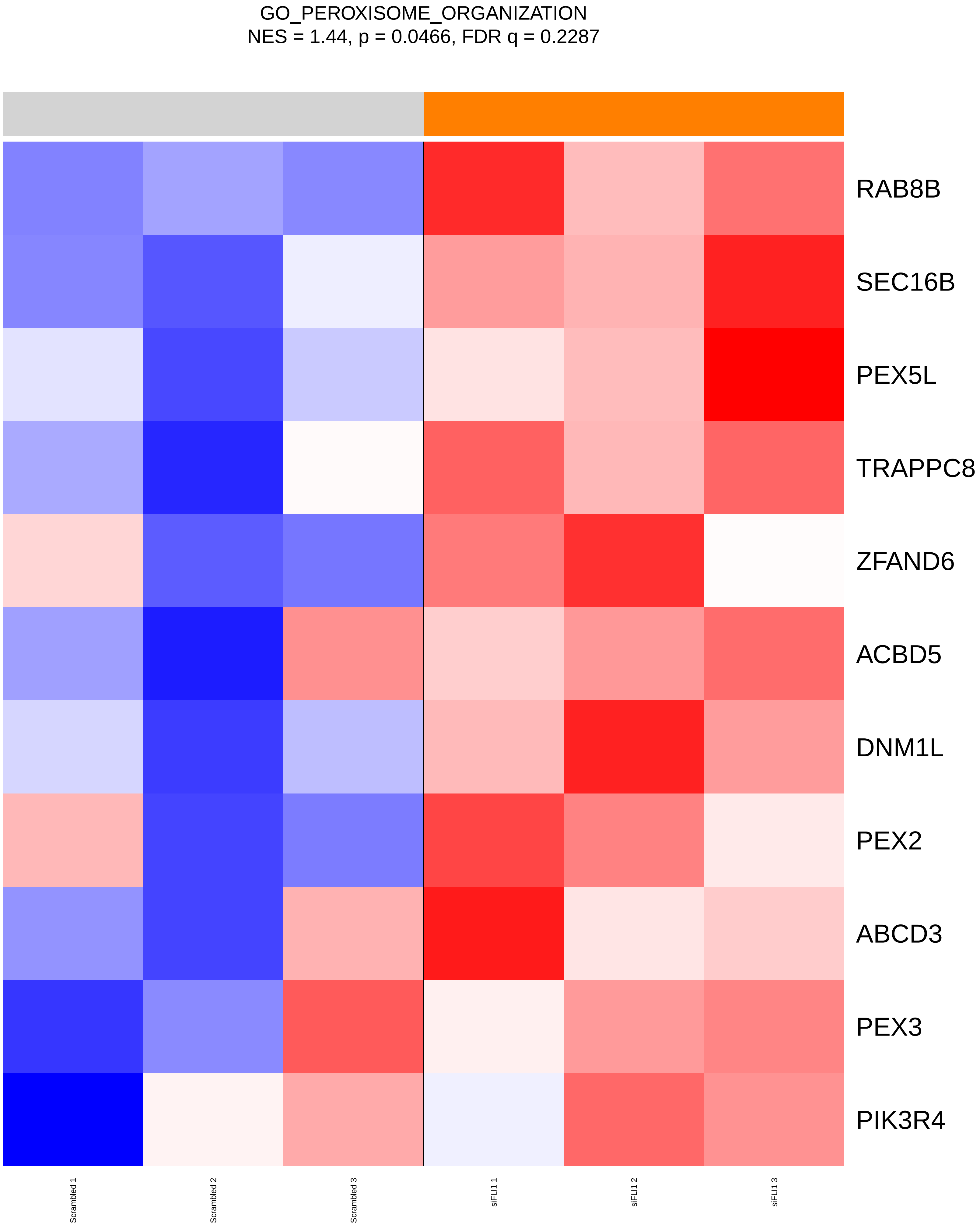
GO_PEROXISOME_ORGANIZATION
NES = 1.44, p = 0.0466, FDR q = 0.2287
RAB8B
SEC16B
PEX5L
TRAPPC8
ZFAND6
ACBD5
DNM1L
PEX2
ABCD3
PEX3
PIK3R4
Scrambled 1
Scrambled 2
Scrambled 3
siFLI1 1
siFLI1 2
siFLI1 3

GO_N_TERMINAL_PROTEIN_AMINO_ACID_MODIFICATION
NES = 1.44, p = 0.0550, FDR q = 0.2286

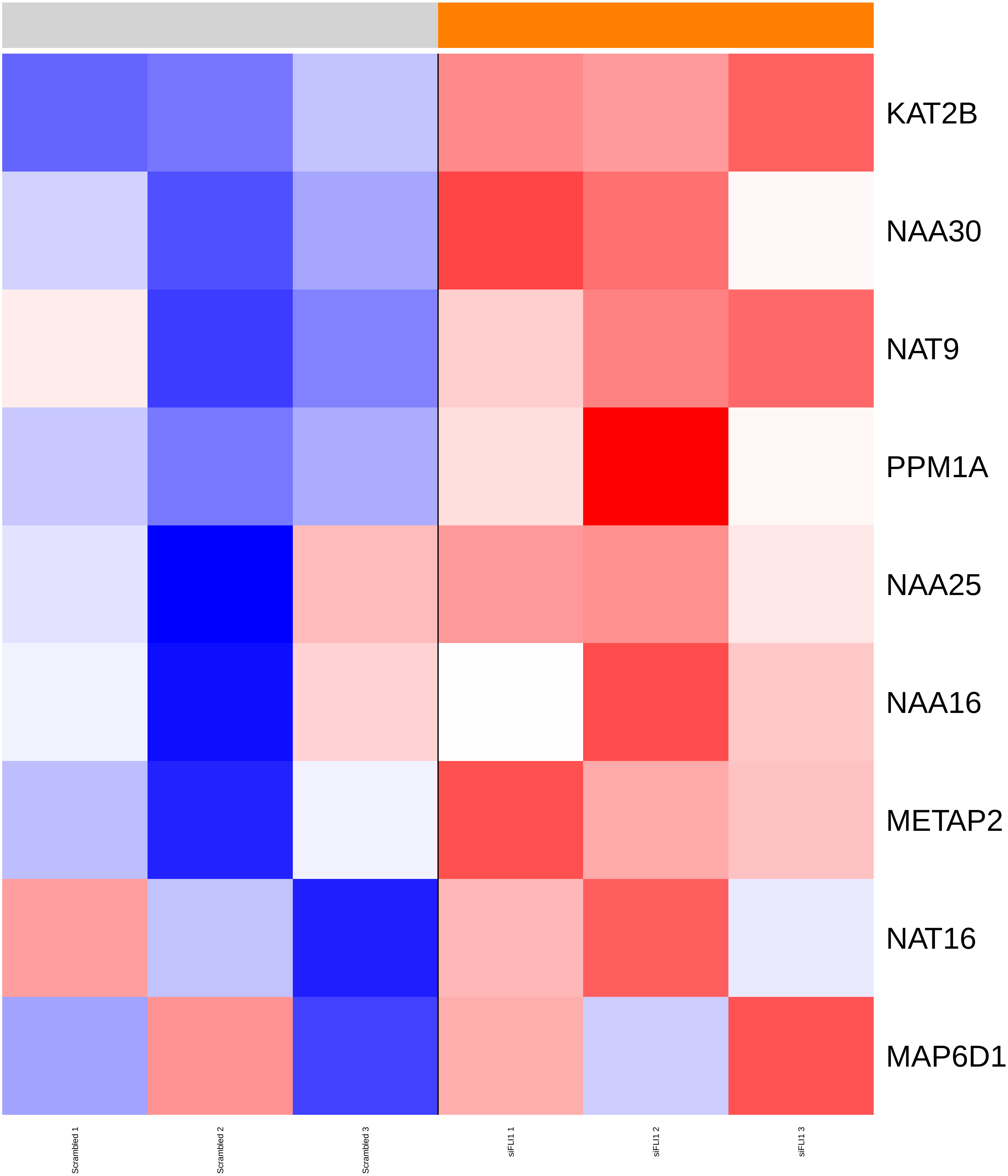

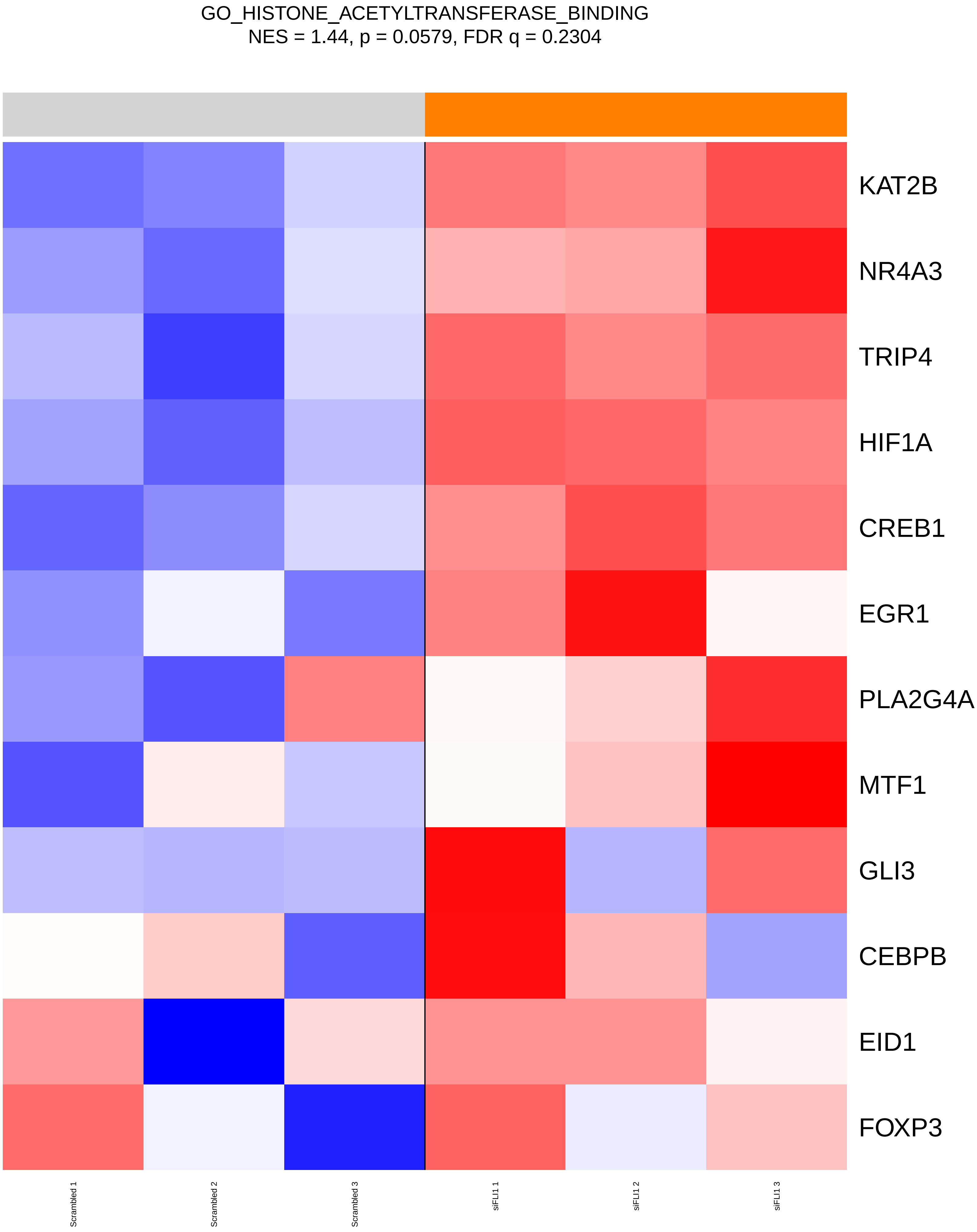
GO_HISTONE_ACETYLTRANSFERASE_BINDING
NES = 1.44, p = 0.0579, FDR q = 0.2304
KAT2B
NR4A3
TRIP4
HIF1A
CREB1
EGR1
PLA2G4A
MTF1
GLI3
CEBPB
EID1
FOXP3
Scrambled 1
Scrambled 2
Scrambled 3
siFLI1 1
siFLI1 2
siFLI1 3

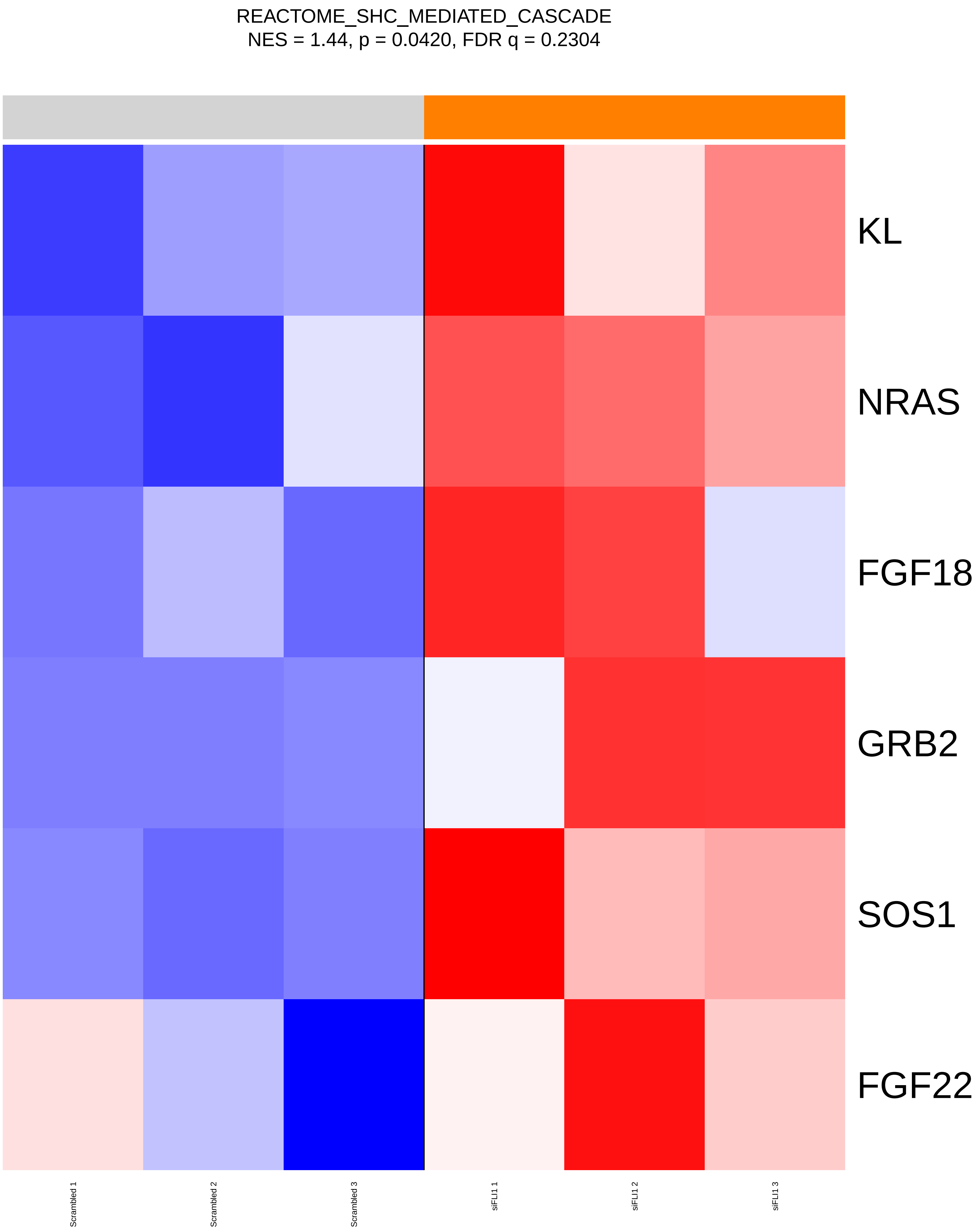
REACTOME_SHC_MEDIATED_CASCADE
NES = 1.44, p = 0.0420, FDR q = 0.2304
KL
NRAS
FGF18
GRB2
SOS1
FGF22
Scrambled 1
Scrambled 2
Scrambled 3
siFLI1 1
siFLI1 2
siFLI1 3

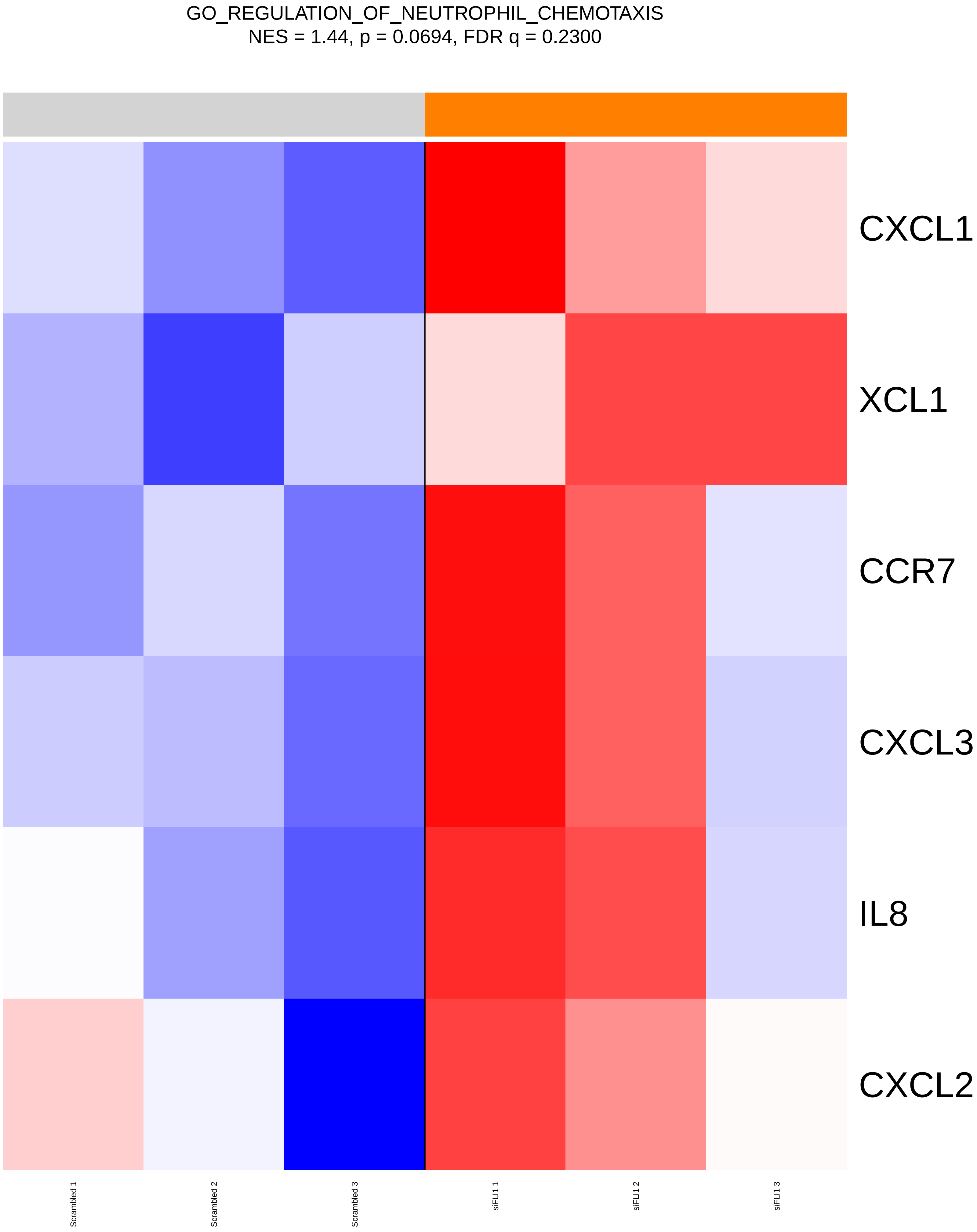
GO_REGULATION_OF_NEUTROPHIL_CHEMOTAXIS
NES = 1.44, p = 0.0694, FDR q = 0.2300
CXCL1
XCL1
CCR7
CXCL3
IL8
CXCL2
Scrambled 1
Scrambled 2
Scrambled 3
siFLI1 1
siFLI1 2
siFLI1 3

AAAGGGA_MIR204_MIR211
NES = 1.44, p = 0.0041, FDR q = 0.2303

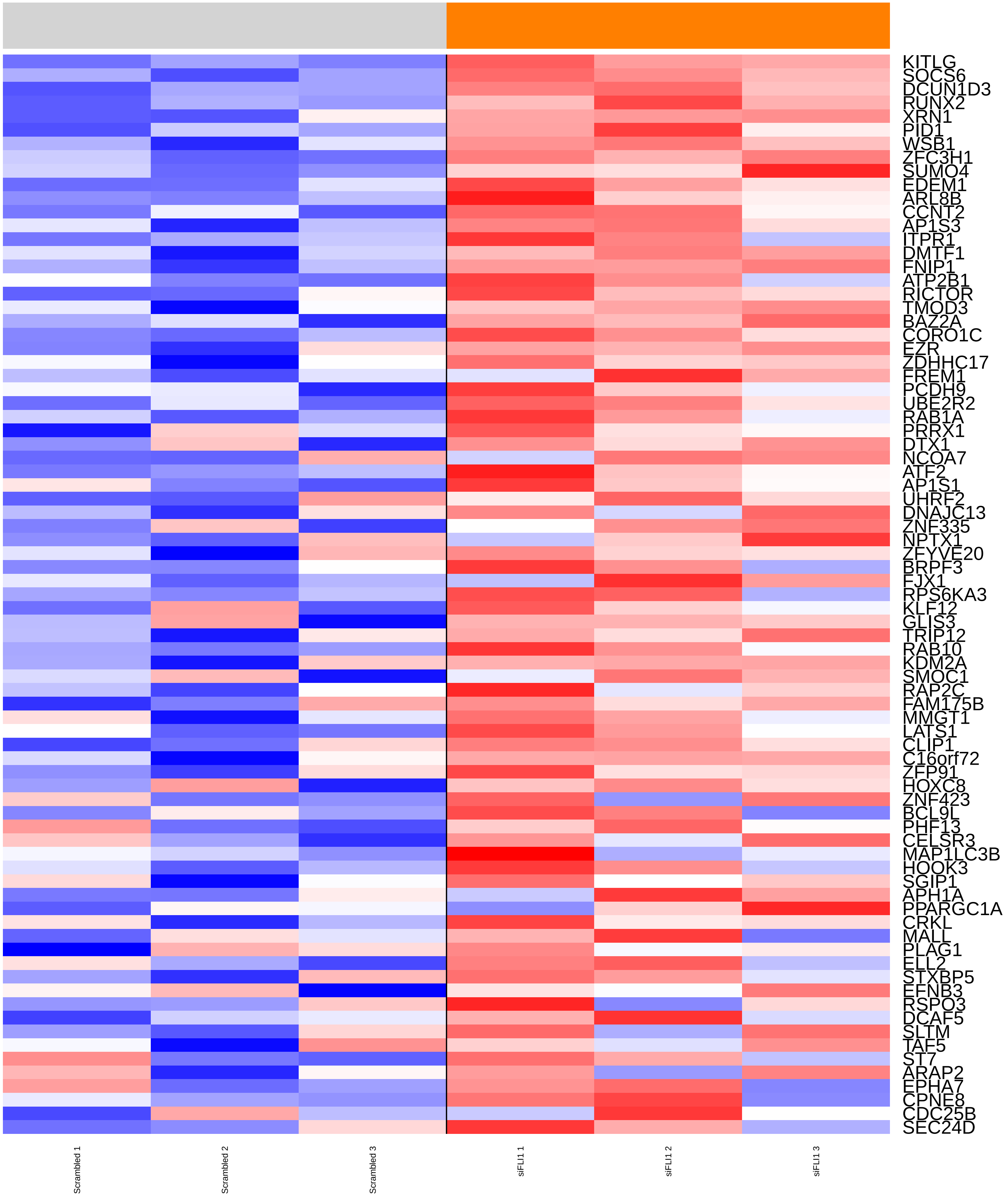

GO_REGULATION_OF_SYNAPTIC_VESICLE_EXOCYTOSIS
NES = 1.44, p = 0.0670, FDR q = 0.2298

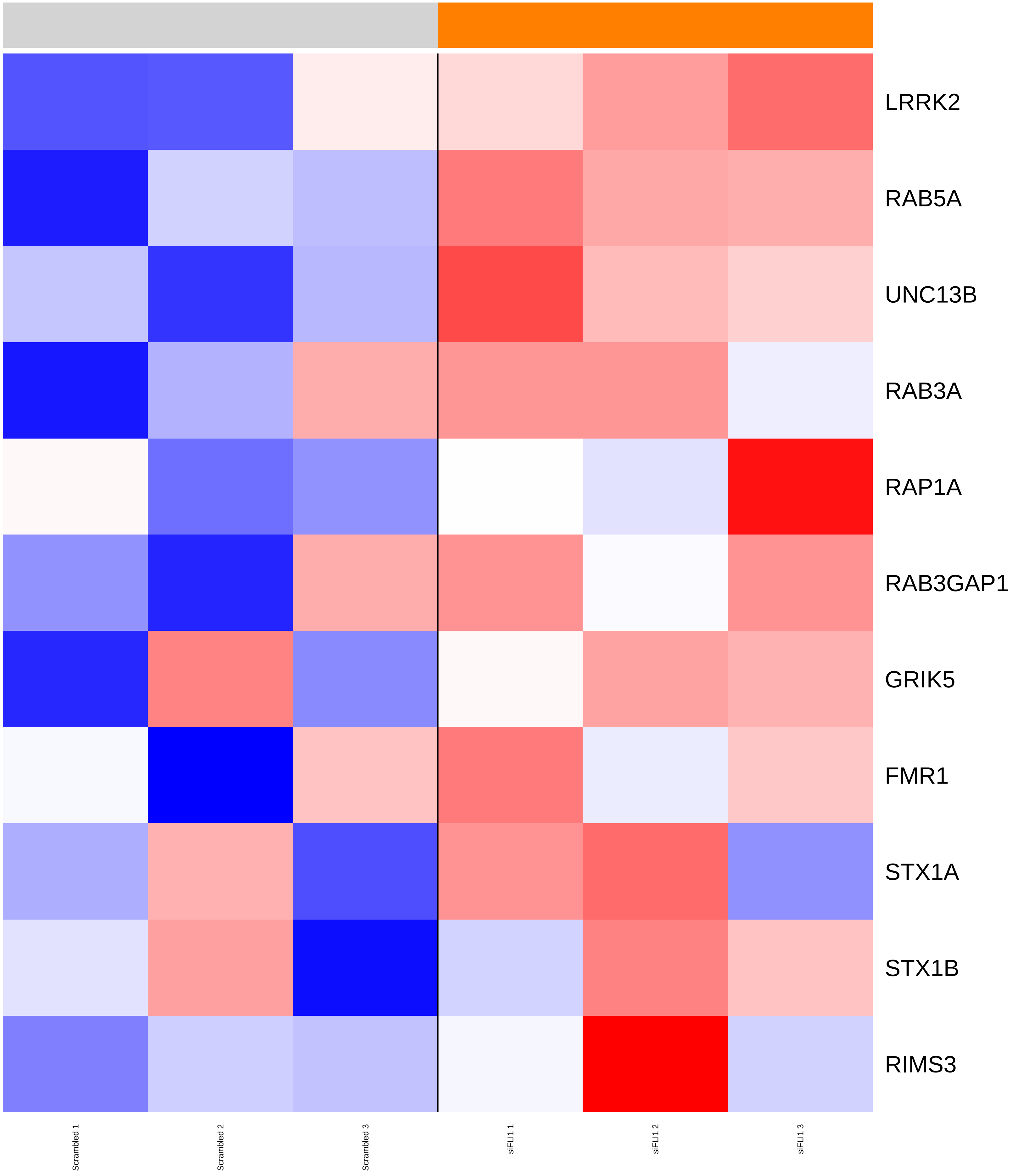

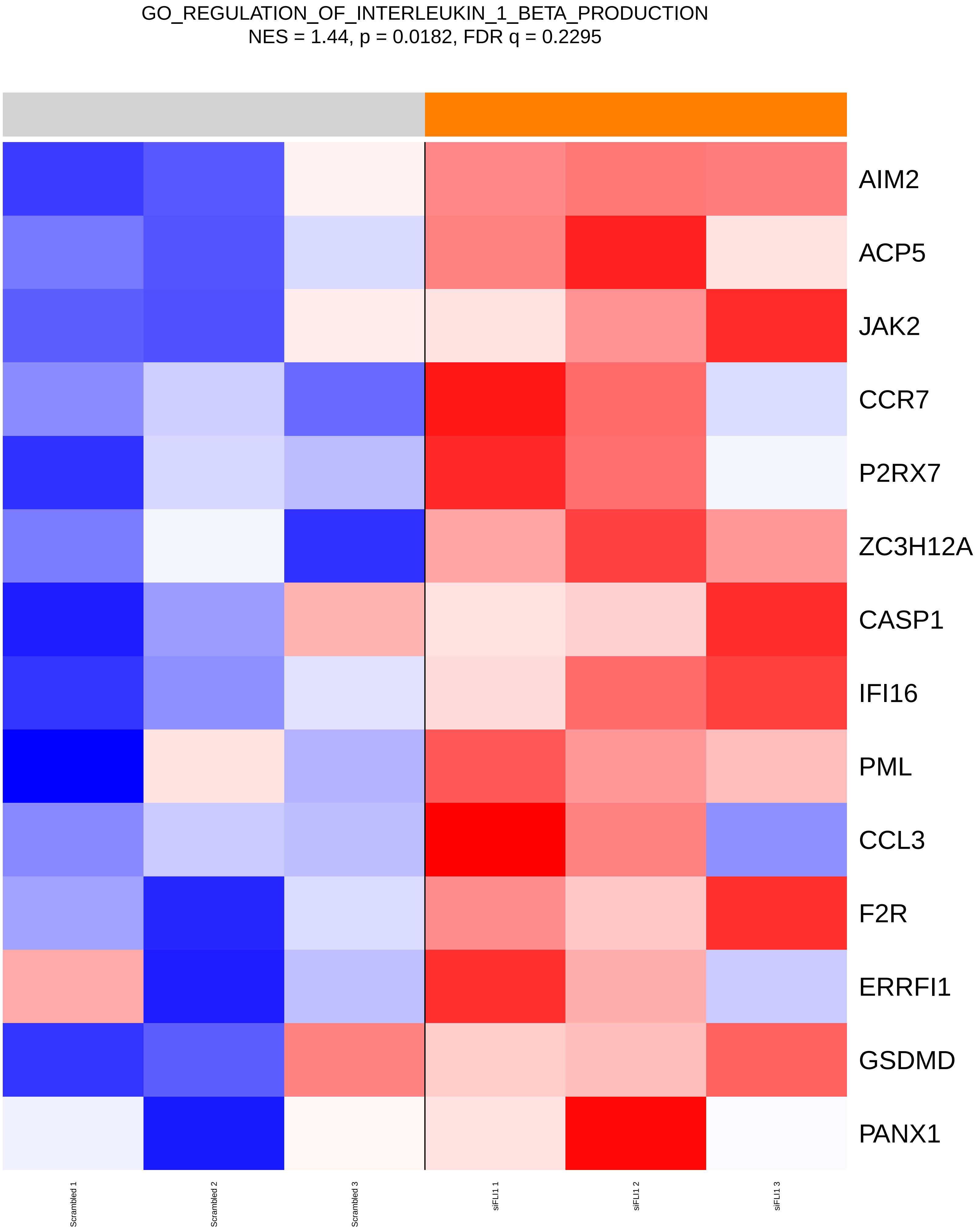
GO_REGULATION_OF_INTERLEUKIN_1_BETA_PRODUCTION
NES = 1.44, p = 0.0182, FDR q = 0.2295
AIM2
ACP5
JAK2
CCR7
P2RX7
ZC3H12A
CASP1
IFI16
PML
CCL3
F2R
ERRFI1
GSDMD
PANX1
Scrambled 1
Scrambled 2
Scrambled 3
siFLI1 1
siFLI1 2
siFLI1 3

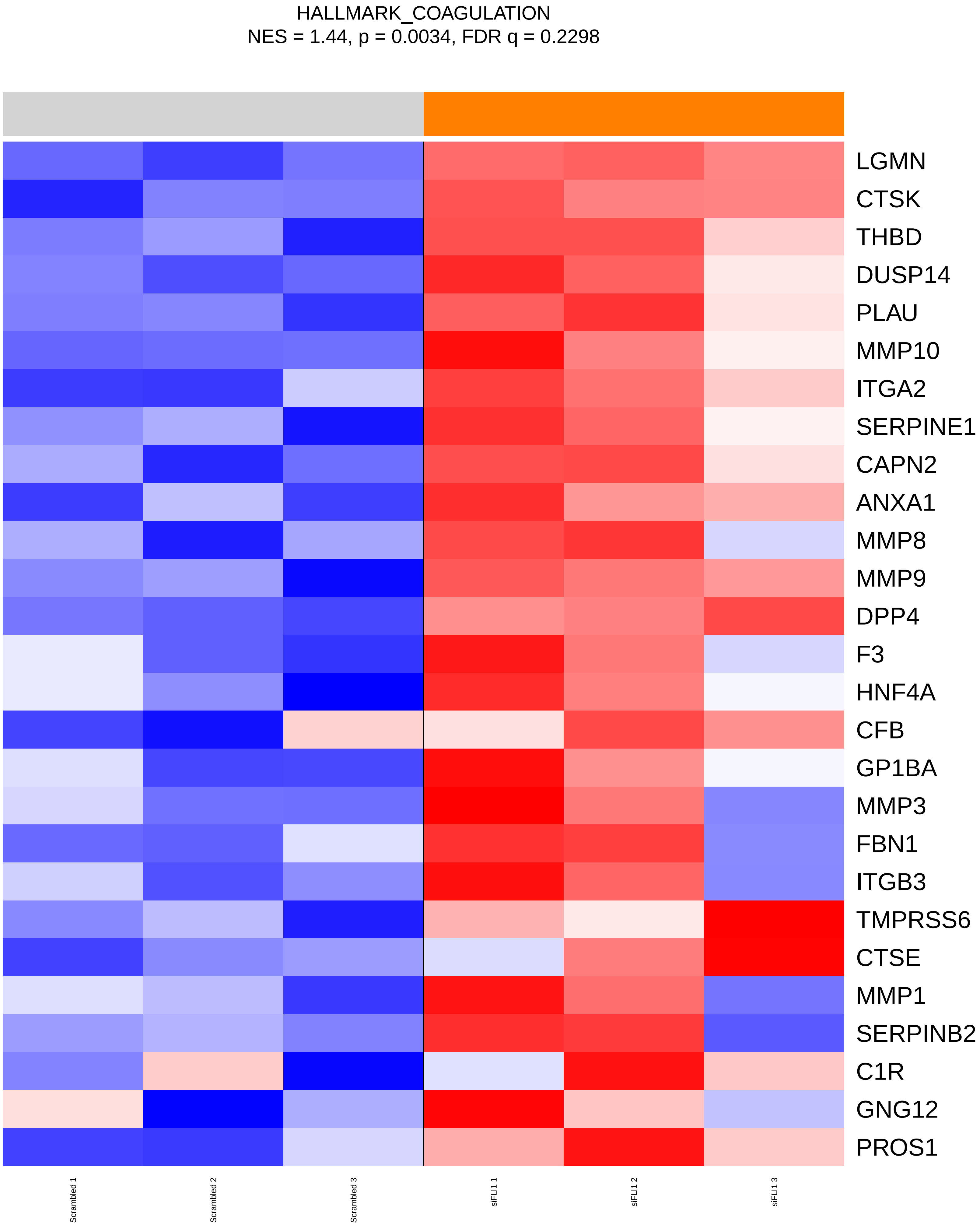
HALLMARK_COAGULATION
NES = 1.44, p = 0.0034, FDR q = 0.2298
LGMN
CTSK
THBD
DUSP14
PLAU
MMP10
ITGA2
SERPINE1
CAPN2
ANXA1
MMP8
MMP9
DPP4
F3
HNF4A
CFB
GP1BA
MMP3
FBN1
ITGB3
TMPRSS6
CTSE
MMP1
SERPINB2
C1R
GNG12
PROS1
Scrambled 1
Scrambled 2
Scrambled 3
siFLI1 1
siFLI1 2
siFLI1 3

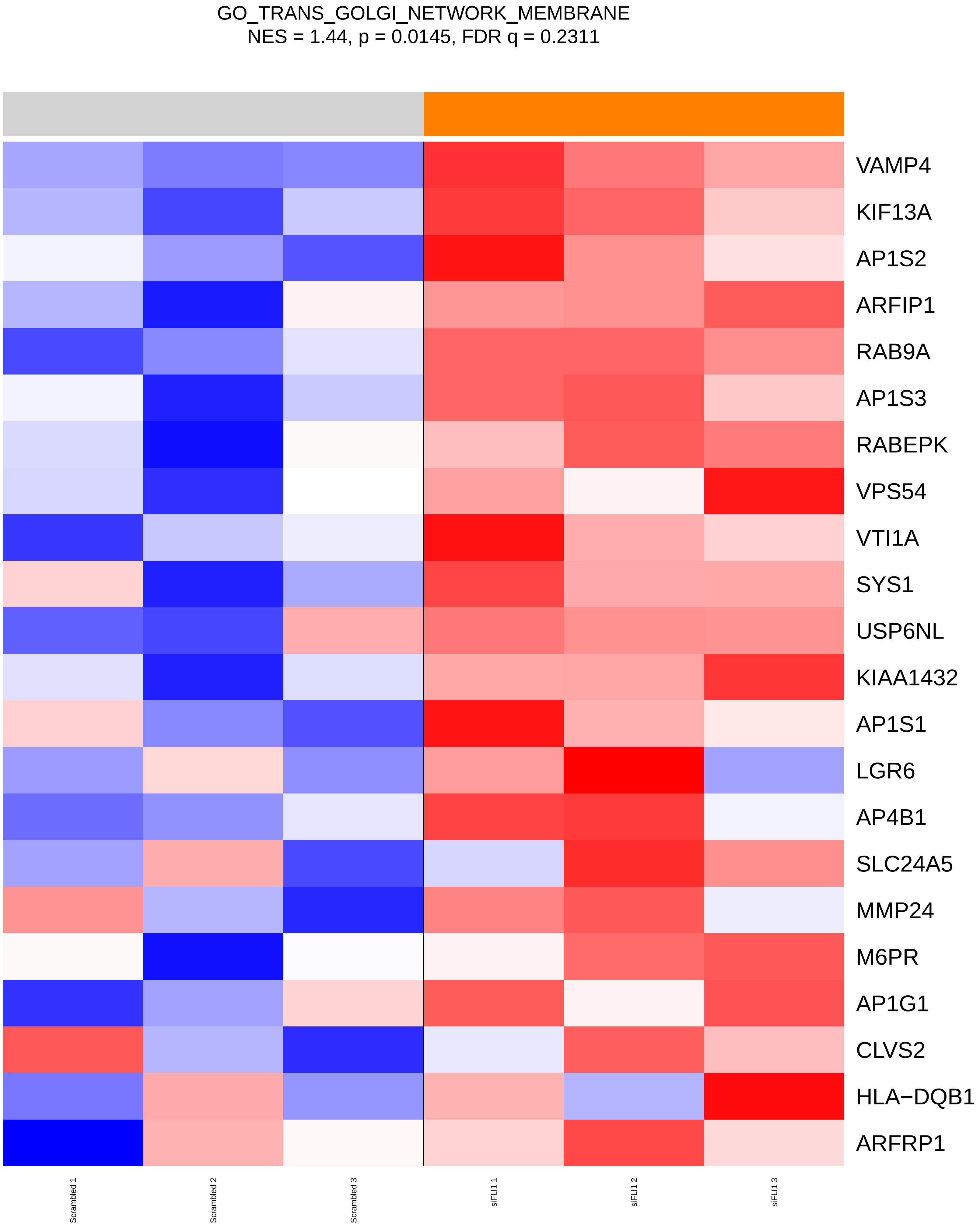
GO_TRANS_GOLGI_NETWORK_MEMBRANE
NES = 1.44, p = 0.0145, FDR q = 0.2311
VAMP4
KIF13A
AP1S2
ARFIP1
RAB9A
AP1S3
RABEPK
VPS54
VTI1A
SYS1
USP6NL
KIAA1432
AP1S1
LGR6
AP4B1
SLC24A5
MMP24
M6PR
AP1G1
CLVS2
HLA−DQB1
ARFRP1
Scrambled 1
Scrambled 2
Scrambled 3
siFLI1 1
siFLI1 2
siFLI1 3

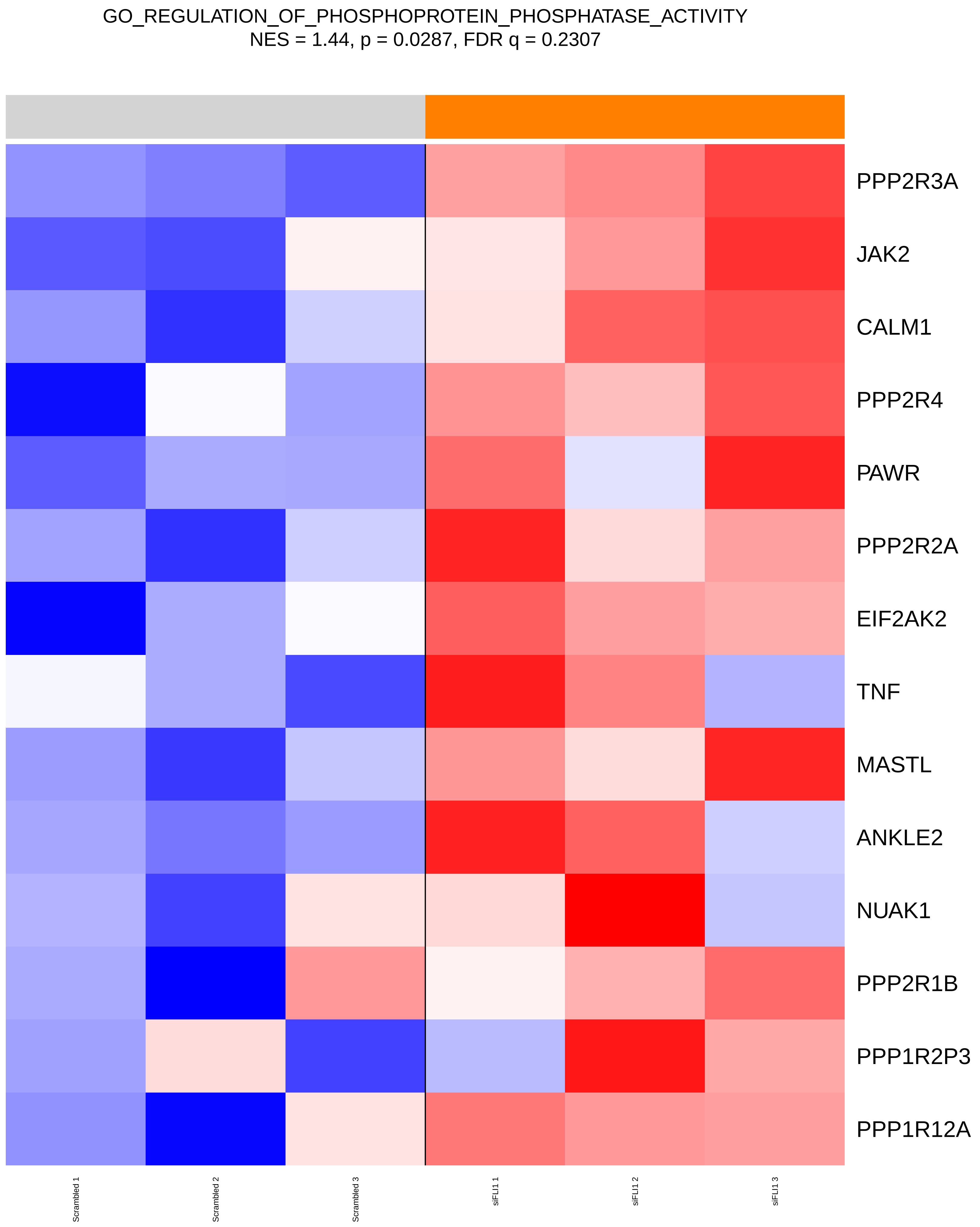
GO_REGULATION_OF_PHOSPHOPROTEIN_PHOSPHATASE_ACTIVITY
NES = 1.44, p = 0.0287, FDR q = 0.2307
PPP2R3A
JAK2
CALM1
PPP2R4
PAWR
PPP2R2A
EIF2AK2
TNF
MASTL
ANKLE2
NUAK1
PPP2R1B
PPP1R2P3
PPP1R12A
Scrambled 1
Scrambled 2
Scrambled 3
siFLI1 1
siFLI1 2
siFLI1 3

GO_AXON_CYTOPLASM
NES = 1.43, p = 0.0633, FDR q = 0.2315

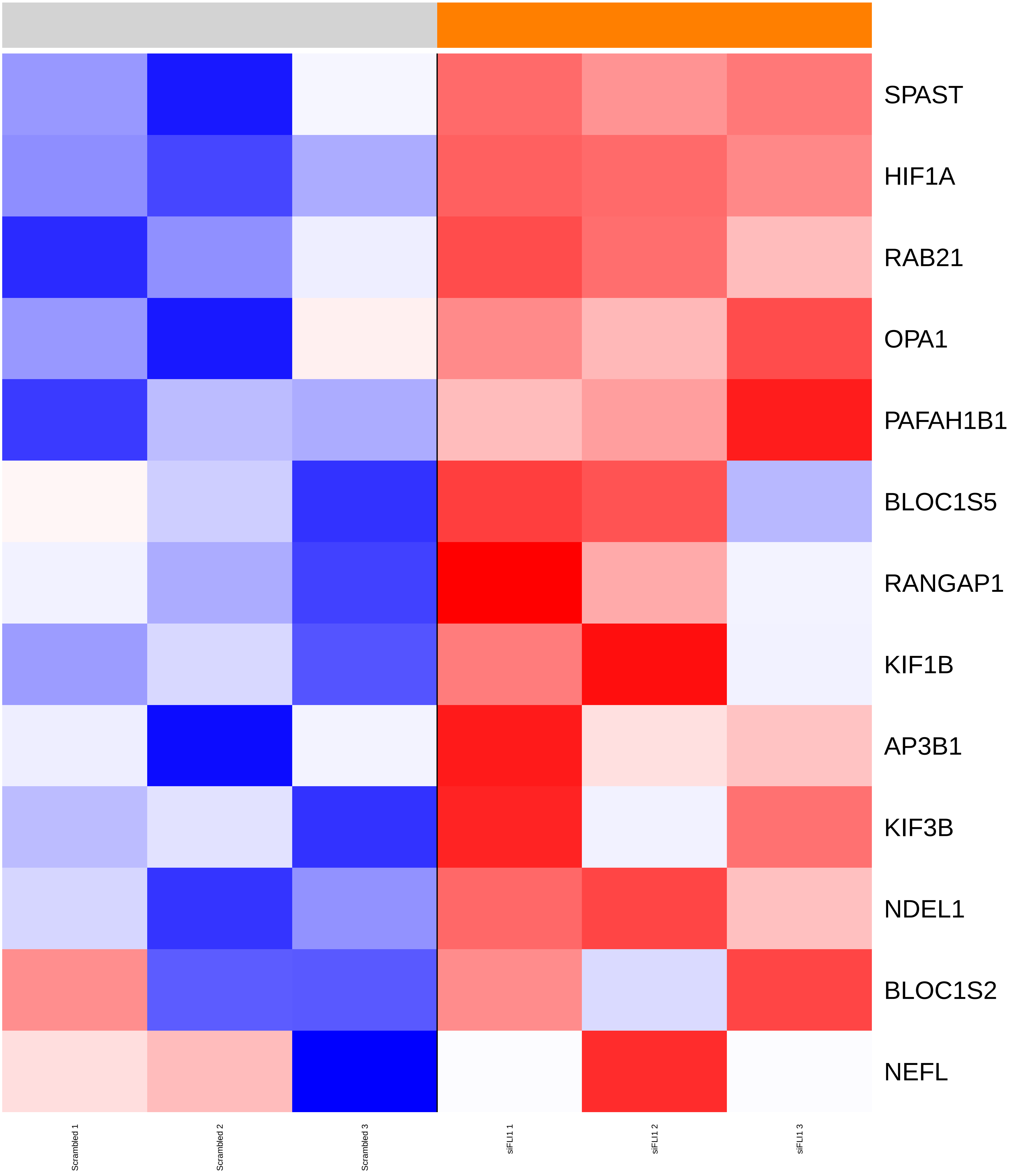

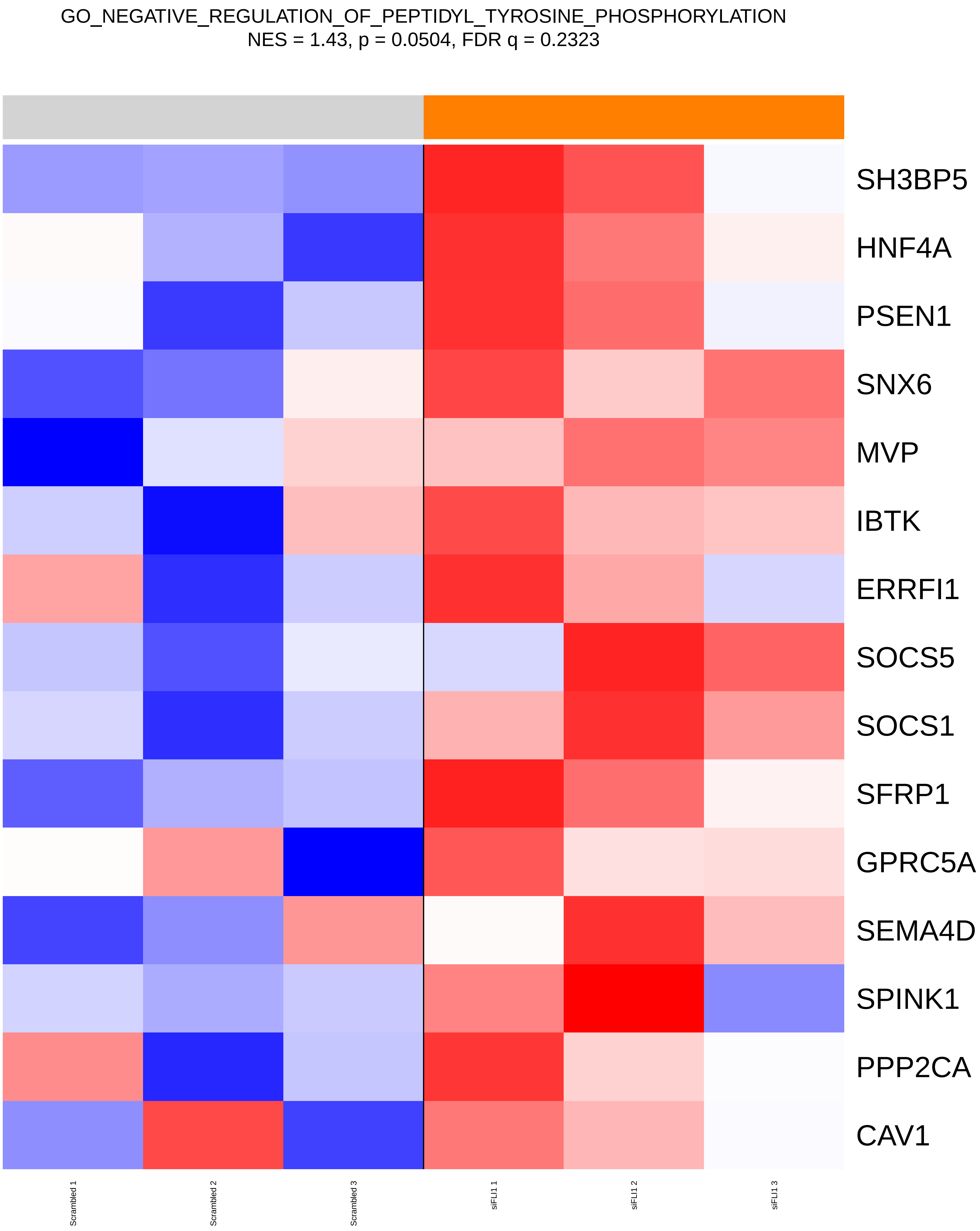
GO_NEGATIVE_REGULATION_OF_PEPTIDYL_TYROSINE_PHOSPHORYLATION
NES = 1.43, p = 0.0504, FDR q = 0.2323
SH3BP5
HNF4A
PSEN1
SNX6
MVP
IBTK
ERRFI1
SOCS5
SOCS1
SFRP1
GPRC5A
SEMA4D
SPINK1
PPP2CA
CAV1
Scrambled 1
Scrambled 2
Scrambled 3
siFLI1 1
siFLI1 2
siFLI1 3

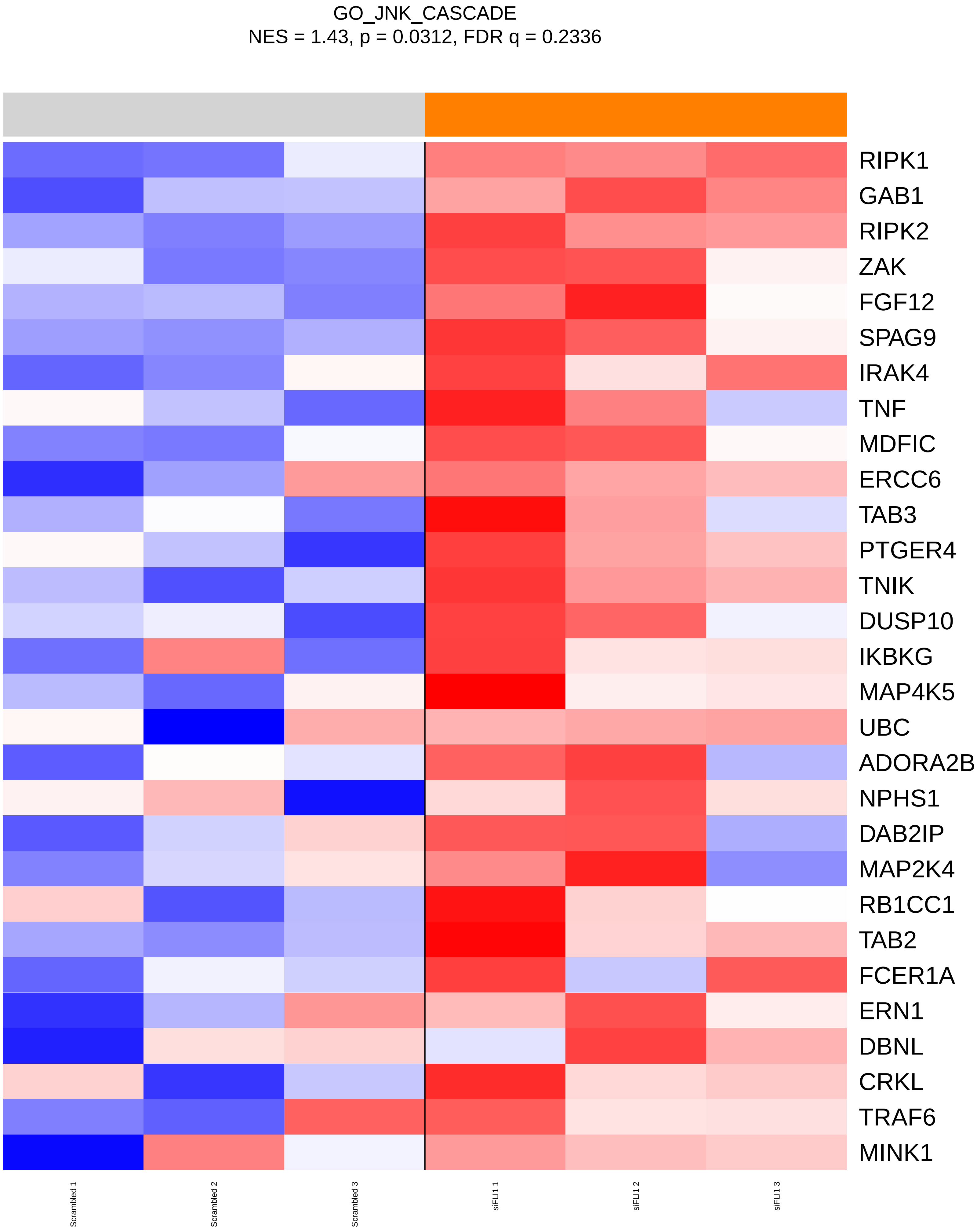
GO_JNK_CASCADE
NES = 1.43, p = 0.0312, FDR q = 0.2336
RIPK1
GAB1
RIPK2
ZAK
FGF12
SPAG9
IRAK4
TNF
MDFIC
ERCC6
TAB3
PTGER4
TNIK
DUSP10
IKBKG
MAP4K5
UBC
ADORA2B
NPHS1
DAB2IP
MAP2K4
RB1CC1
TAB2
FCER1A
ERN1
DBNL
CRKL
TRAF6
MINK1
Scrambled 1
Scrambled 2
Scrambled 3
siFLI1 1
siFLI1 2
siFLI1 3

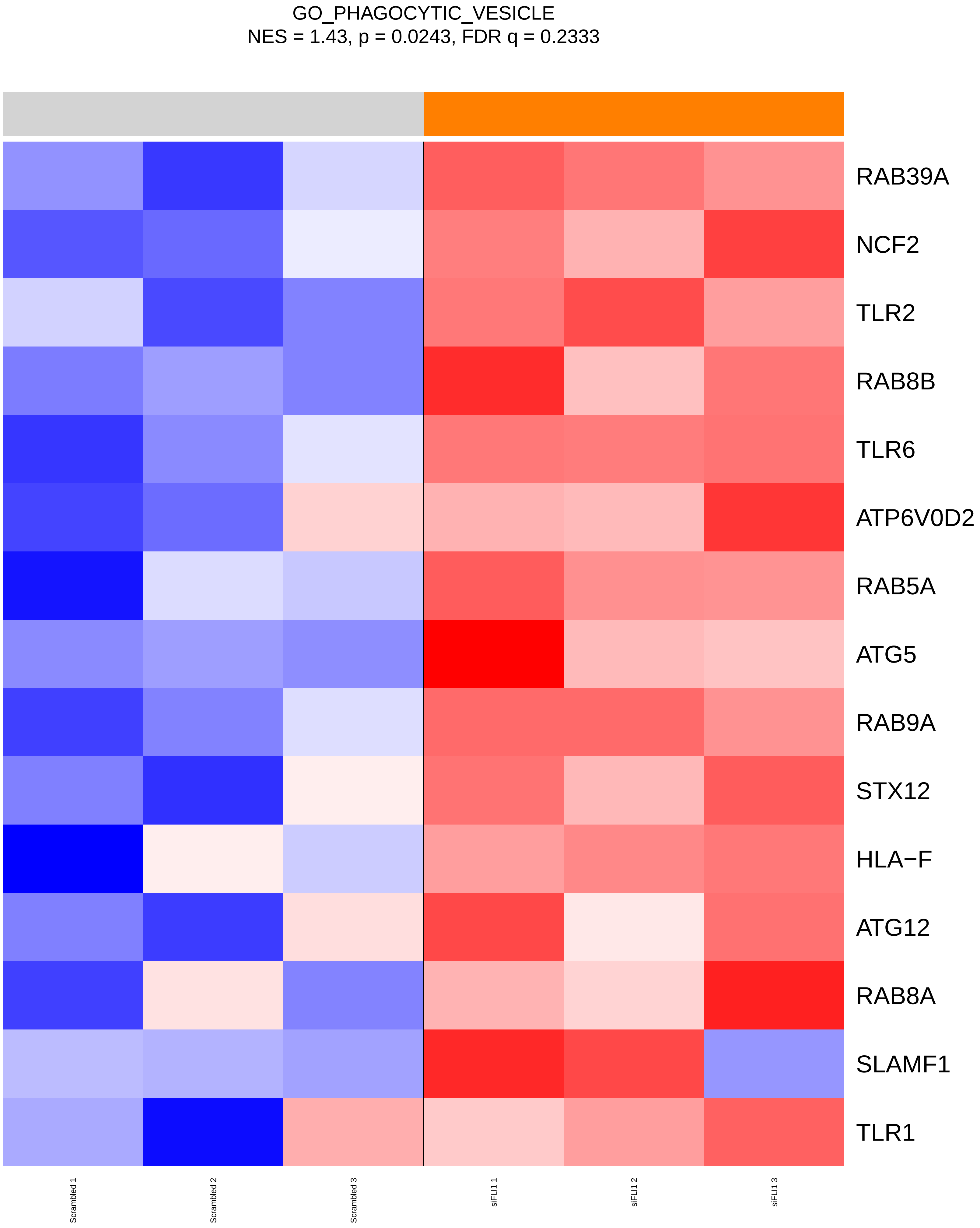
GO_PHAGOCYTIC_VESICLE
NES = 1.43, p = 0.0243, FDR q = 0.2333
RAB39A
NCF2
TLR2
RAB8B
TLR6
ATP6V0D2
RAB5A
ATG5
RAB9A
STX12
HLA−F
ATG12
RAB8A
SLAMF1
TLR1
Scrambled 1
Scrambled 2
Scrambled 3
siFLI1 1
siFLI1 2
siFLI1 3

GO_RESPONSE_TO_ARSENIC_CONTAINING_SUBSTANCE
NES = 1.43, p = 0.0474, FDR q = 0.2331

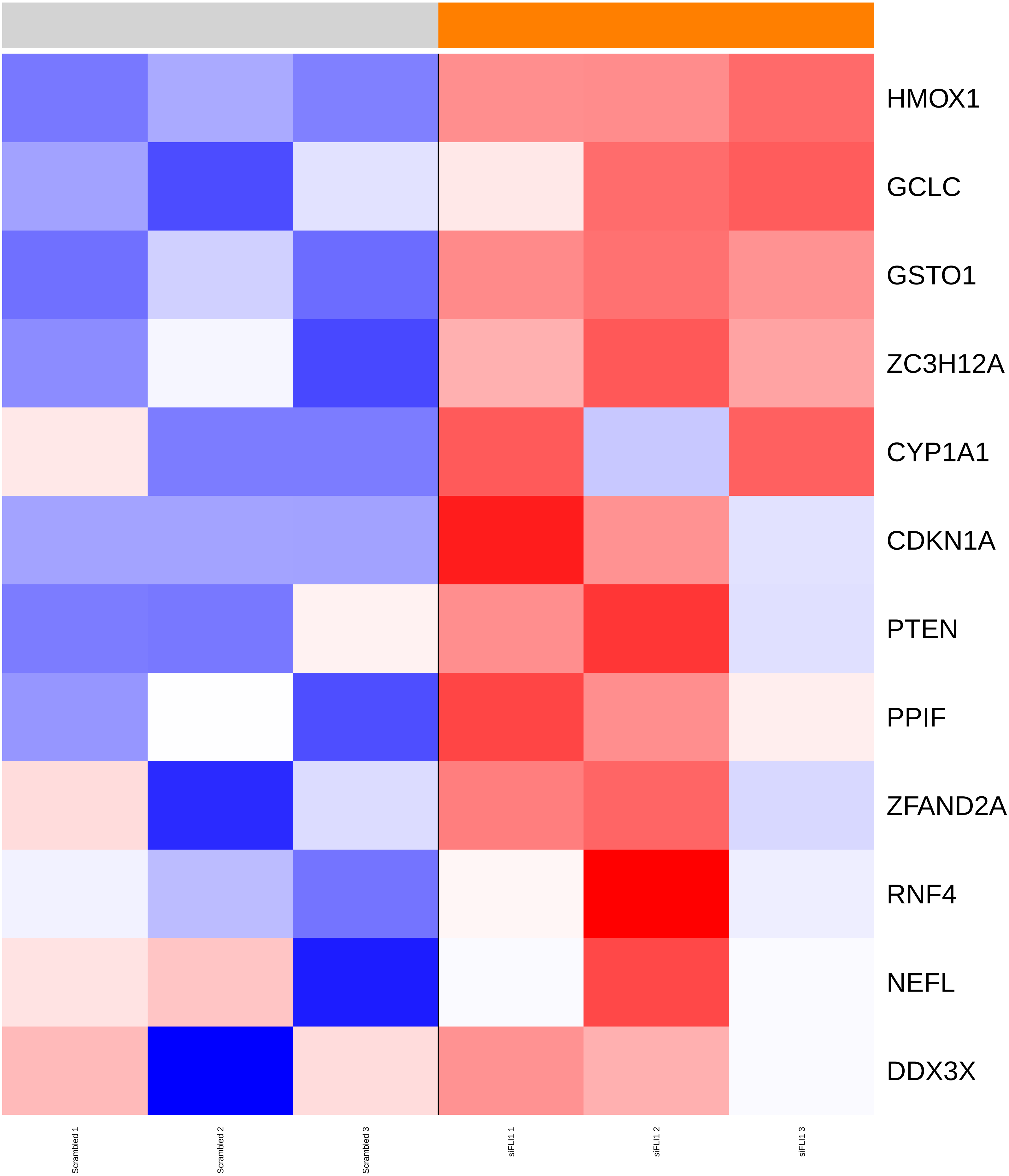

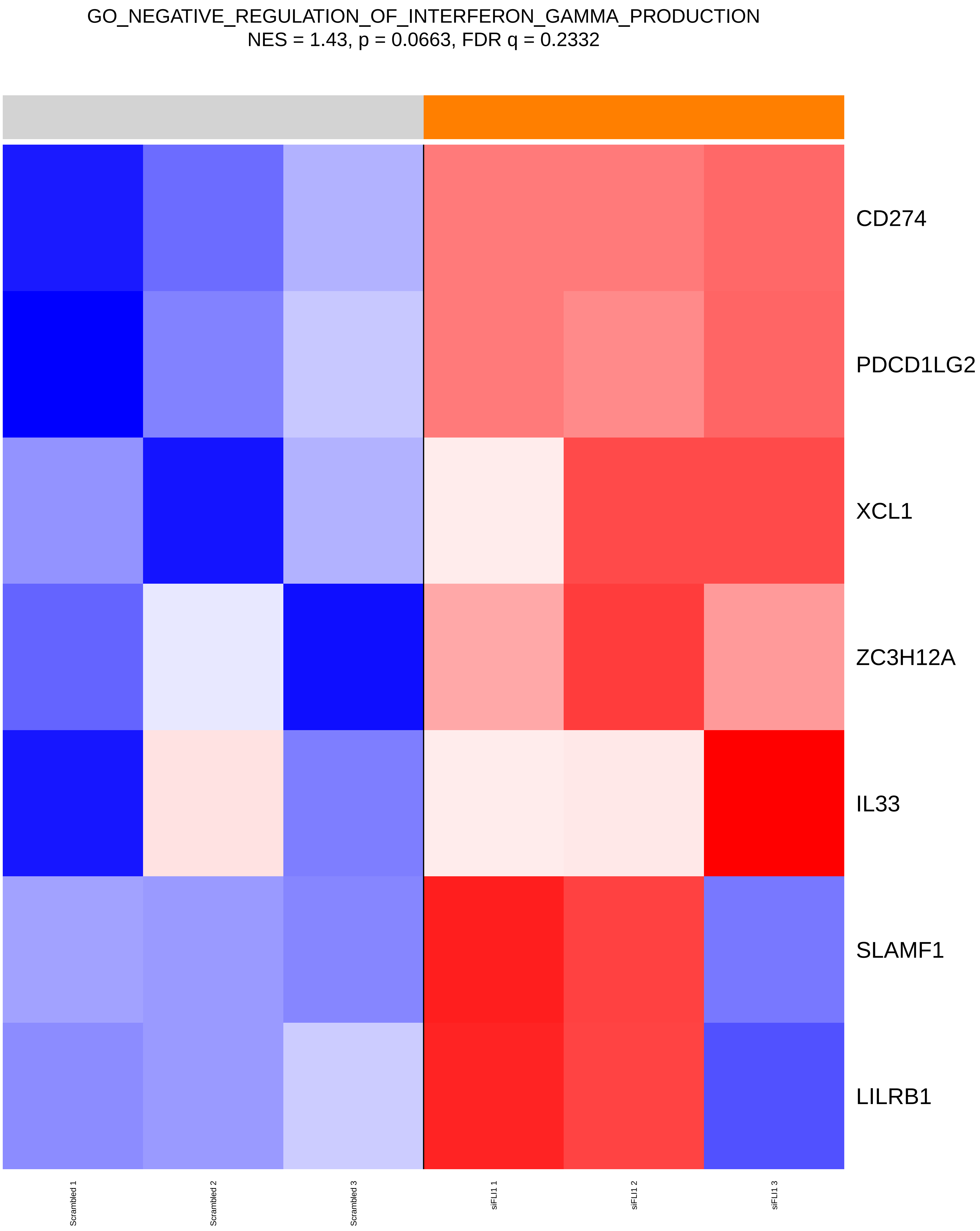
GO_NEGATIVE_REGULATION_OF_INTERFERON_GAMMA_PRODUCTION
NES = 1.43, p = 0.0663, FDR q = 0.2332
CD274
PDCD1LG2
XCL1
ZC3H12A
IL33
SLAMF1
LILRB1
Scrambled 1
Scrambled 2
Scrambled 3
siFLI1 1
siFLI1 2
siFLI1 3

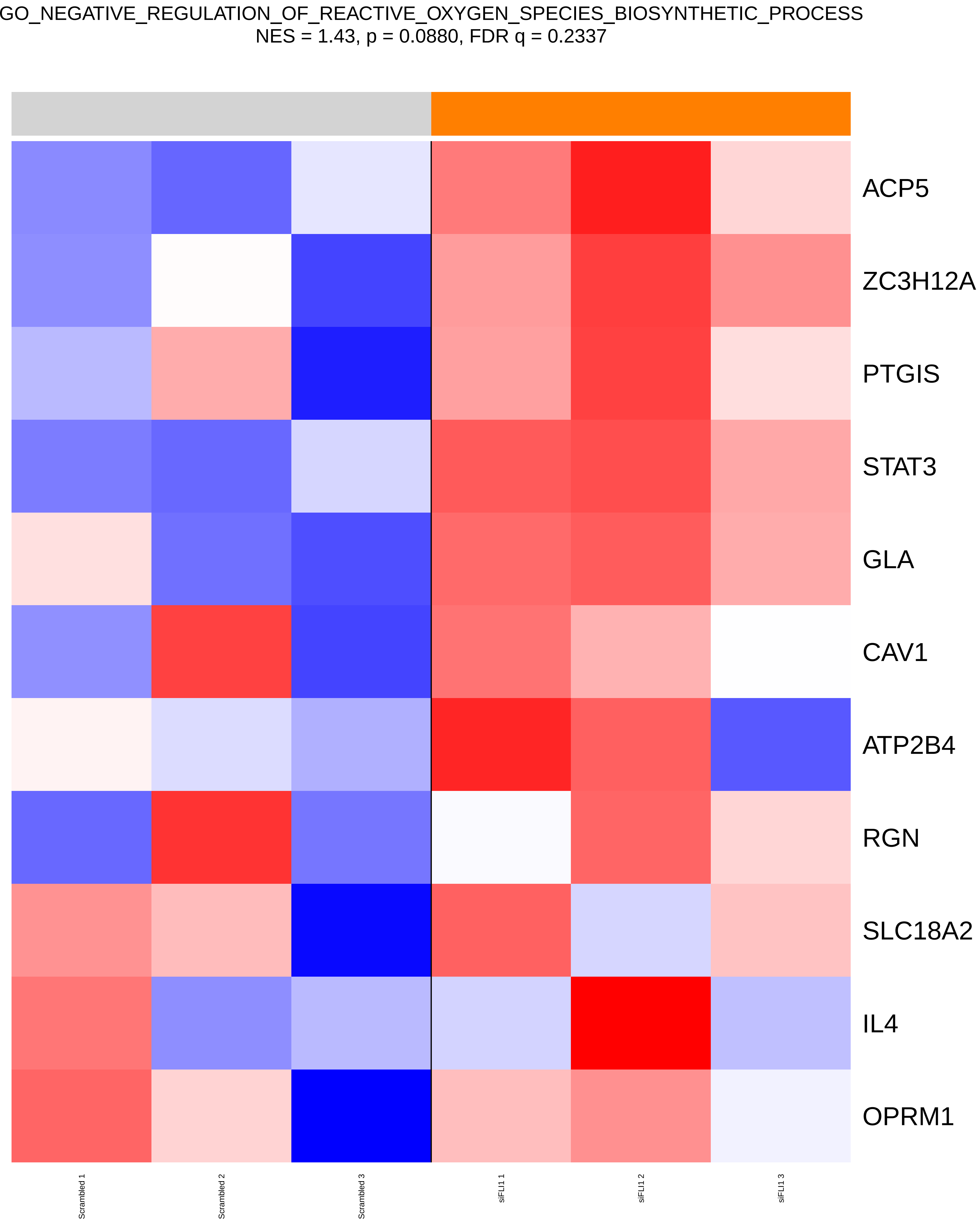
GO_NEGATIVE_REGULATION_OF_REACTIVE_OXYGEN_SPECIES_BIOSYNTHETIC_PROCESS
NES = 1.43, p = 0.0880, FDR q = 0.2337
ACP5
ZC3H12A
PTGIS
STAT3
GLA
CAV1
ATP2B4
RGN
SLC18A2
IL4
OPRM1
Scrambled 1
Scrambled 2
Scrambled 3
siFLI1 1
siFLI1 2
siFLI1 3

GO_REGULATION_OF_NEURON_MIGRATION
NES = 1.43, p = 0.0497, FDR q = 0.2333

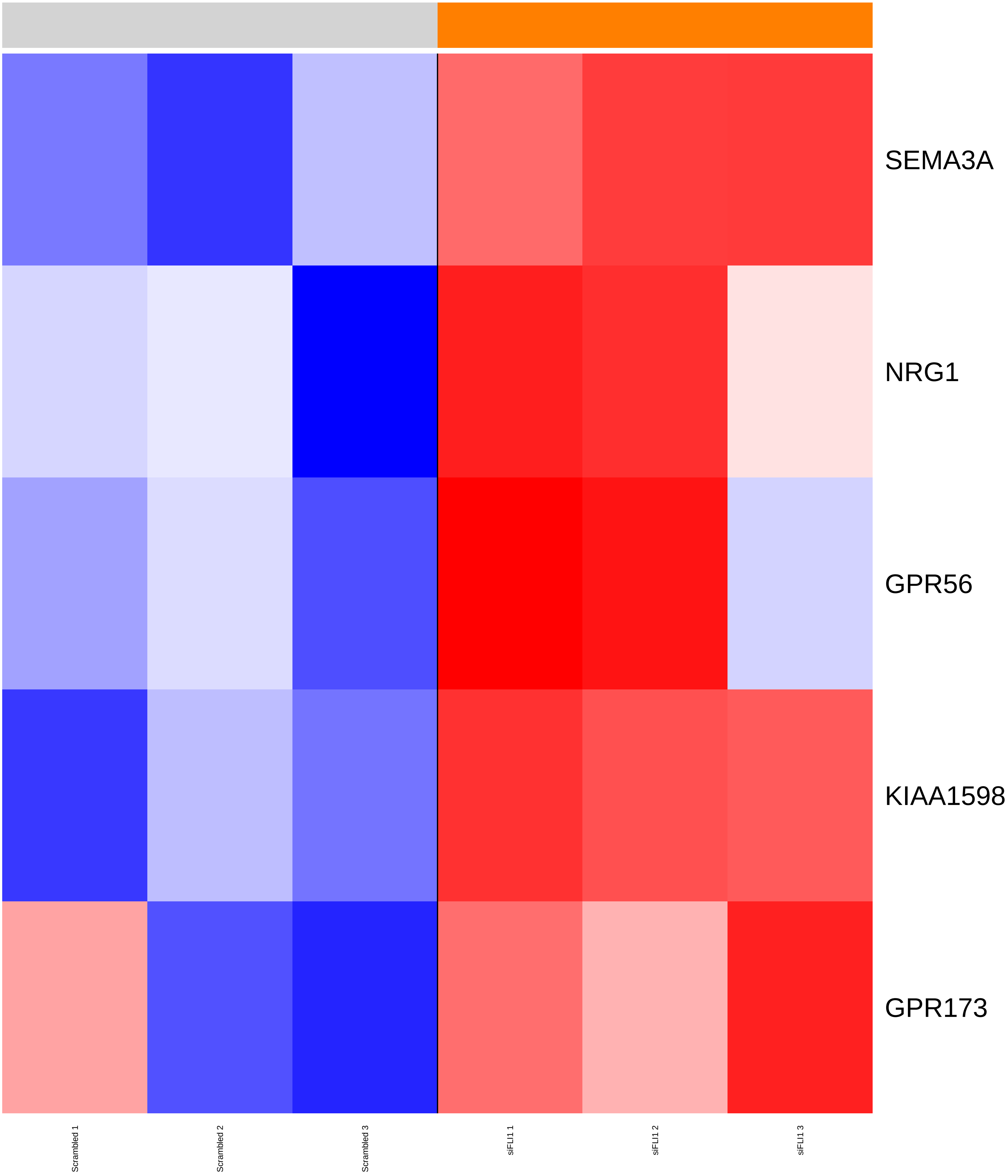

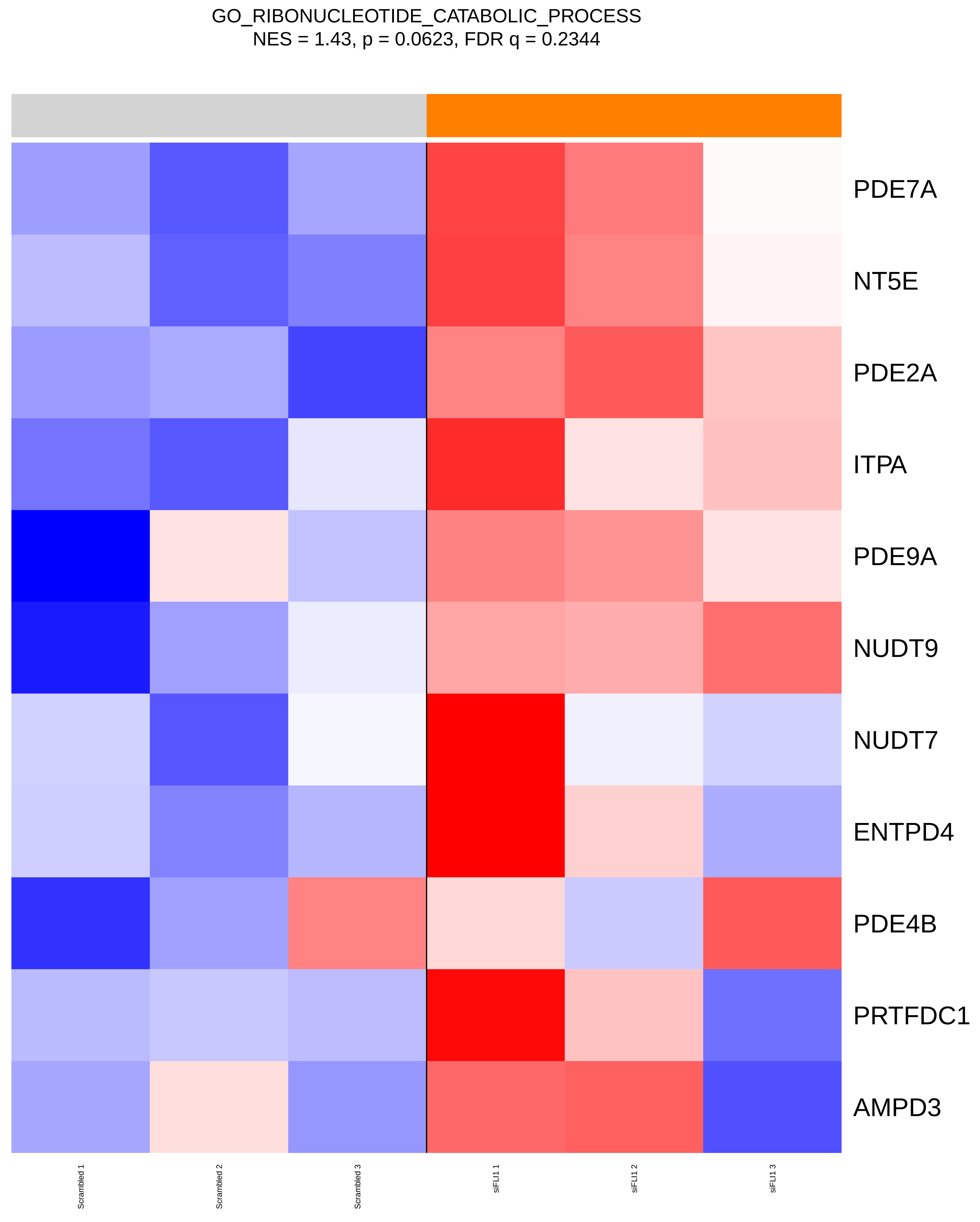

GO_RIBONUCLEOTIDE_CATABOLIC_PROCESS
NES = 1.43, p = 0.0623, FDR q = 0.2344
PDE7A
NT5E
PDE2A
ITPA
PDE9A
NUDT9
NUDT7
ENTPD4
PDE4B
PRTFDC1
AMPD3
Scrambled 1
Scrambled 2
Scrambled 3
siFLI1 1
siFLI1 2
siFLI1 3

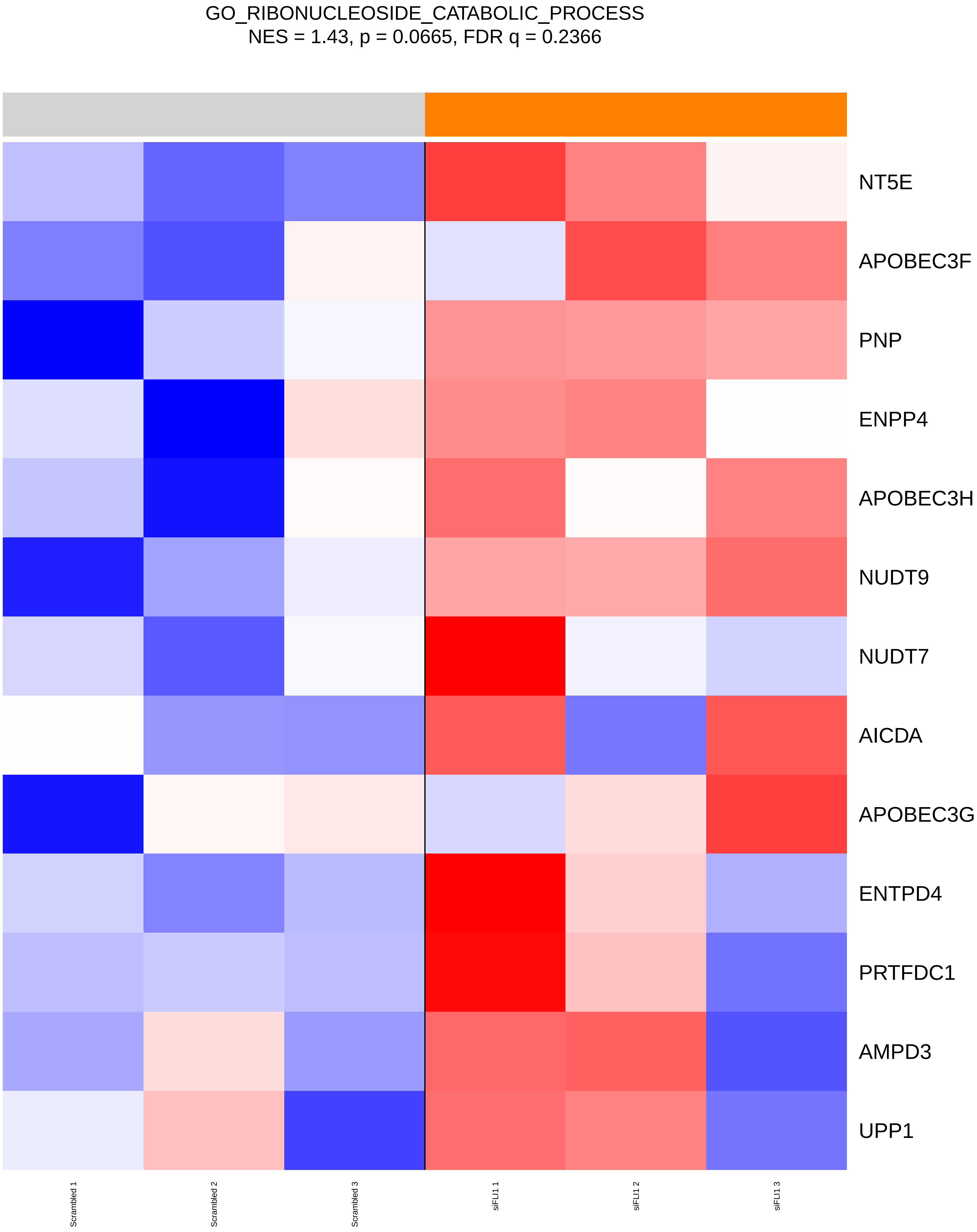
GO_RIBONUCLEOSIDE_CATABOLIC_PROCESS
NES = 1.43, p = 0.0665, FDR q = 0.2366
NT5E
APOBEC3F
PNP
ENPP4
APOBEC3H
NUDT9
NUDT7
AICDA
APOBEC3G
ENTPD4
PRTFDC1
AMPD3
UPP1
Scrambled 1
Scrambled 2
Scrambled 3
siFLI1 1
siFLI1 2
siFLI1 3

GO_VESICLE_ORGANIZATION
NES = 1.43, p = 0.0000, FDR q = 0.2378

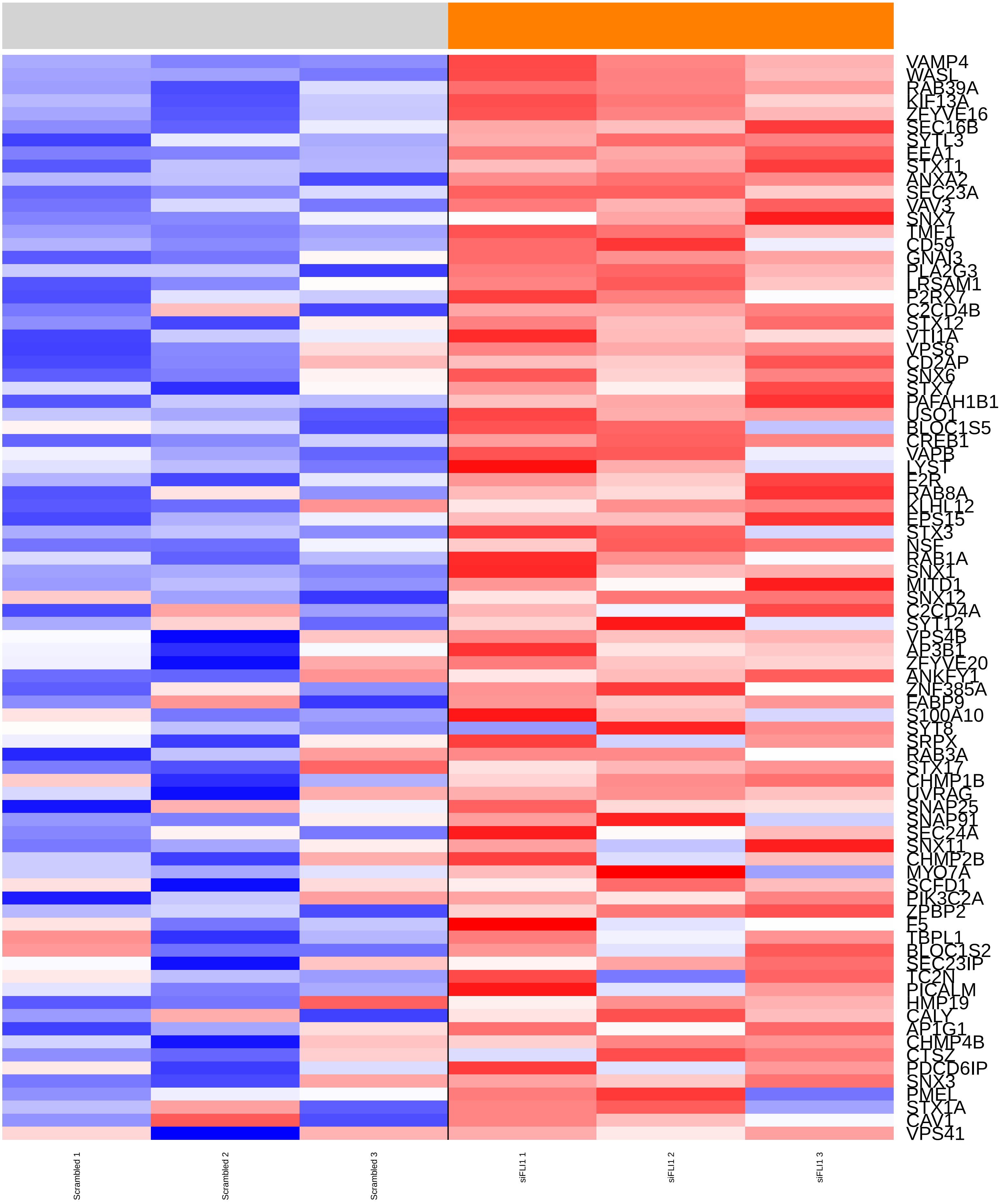

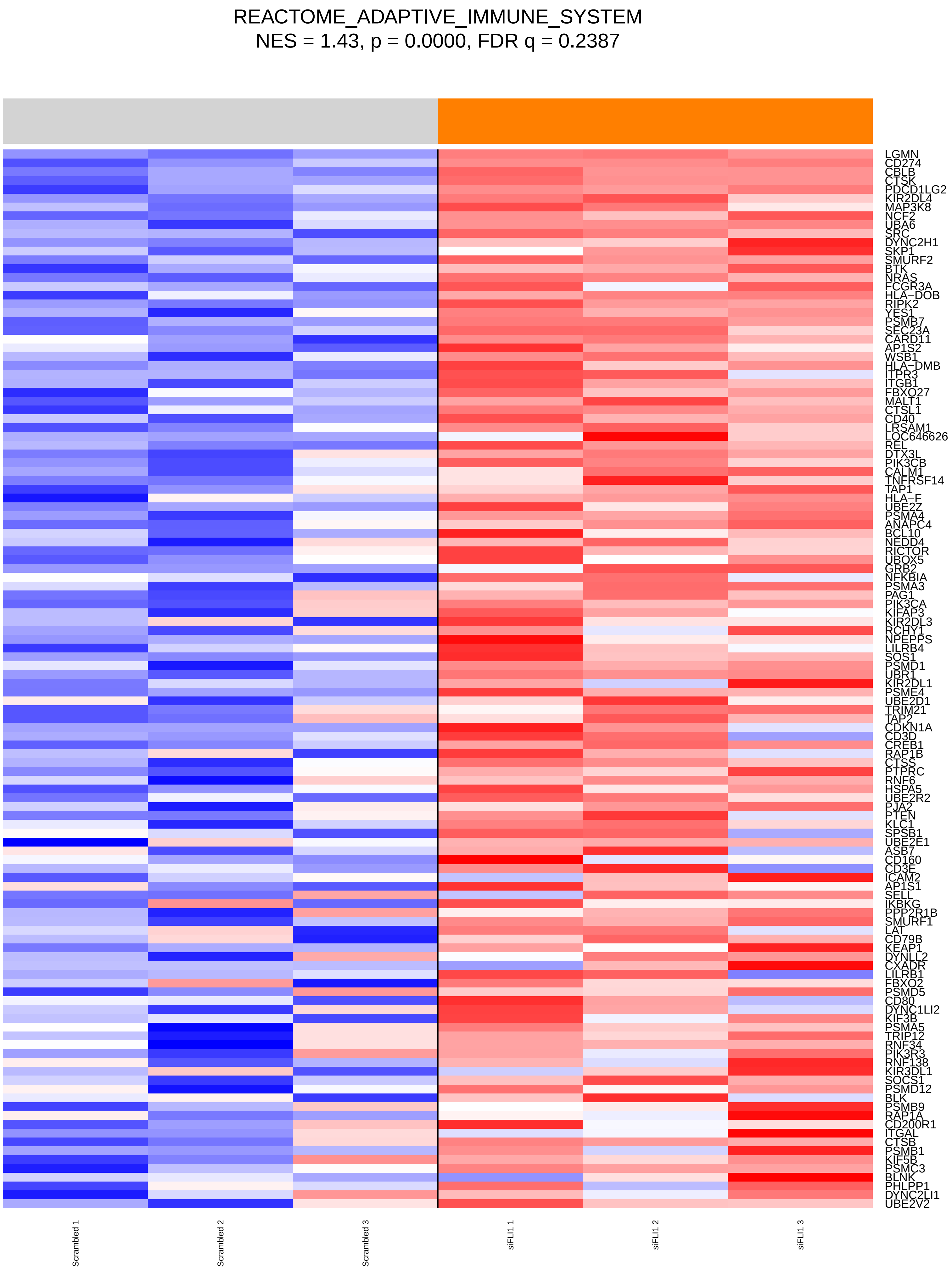
REACTOME_ADAPTIVE_IMMUNE_SYSTEM
NES = 1.43, p = 0.0000, FDR q = 0.2387
LGMN
CD274
CBLB
CTSK
PDCD1LG2
KIR2DL4
MAP3K8
NCF2
UBA6
SRC
DYNC2H1
SKP1
SMURF2
BTK
NRAS
FCGR3A
HLA−DOB
RIPK2
YES1
PSMB7
SEC23A
CARD11
AP1S2
WSB1
HLA−DMB
ITPR3
ITGB1
FBXO27
MALT1
CTSL1
CD40
LRSAM1
LOC646626
REL
DTX3L
PIK3CB
CALM1
TNFRSF14
TAP1
HLA−F
UBE2Z
PSMA4
ANAPC4
BCL10
NEDD4
RICTOR
UBOX5
GRB2
NFKBIA
PSMA3
PAG1
PIK3CA
KIFAP3
KIR2DL3
RCHY1
NPEPPS
LILRB4
SOS1
PSMD1
UBR1
KIR2DL1
PSME4
UBE2D1
TRIM21
TAP2
CDKN1A
CD3D
CREB1
RAP1B
CTSS
PTPRC
RNF6
HSPA5
UBE2R2
PJA2
PTEN
KLC1
SPSB1
UBE2E1
ASB7
CD160
CD3E
ICAM2
AP1S1
SELL
IKBKG
PPP2R1B
SMURF1
LAT
CD79B
KEAP1
DYNLL2
CXADR
LILRB1
FBXO2
PSMD5
CD80
DYNC1LI2
KIF3B
PSMA5
TRIP12
RNF34
PIK3R3
RNF138
KIR3DL1
SOCS1
PSMD12
BLK
PSMB9
RAP1A
CD200R1
ITGAL
CTSB
PSMB1
KIF5B
PSMC3
BLNK
PHLPP1
DYNC2LI1
UBE2V2
Scrambled 1
Scrambled 2
Scrambled 3
siFLI1 1
siFLI1 2
siFLI1 3

ACACTAC_MIR1423P
NES = 1.43, p = 0.0129, FDR q = 0.2385

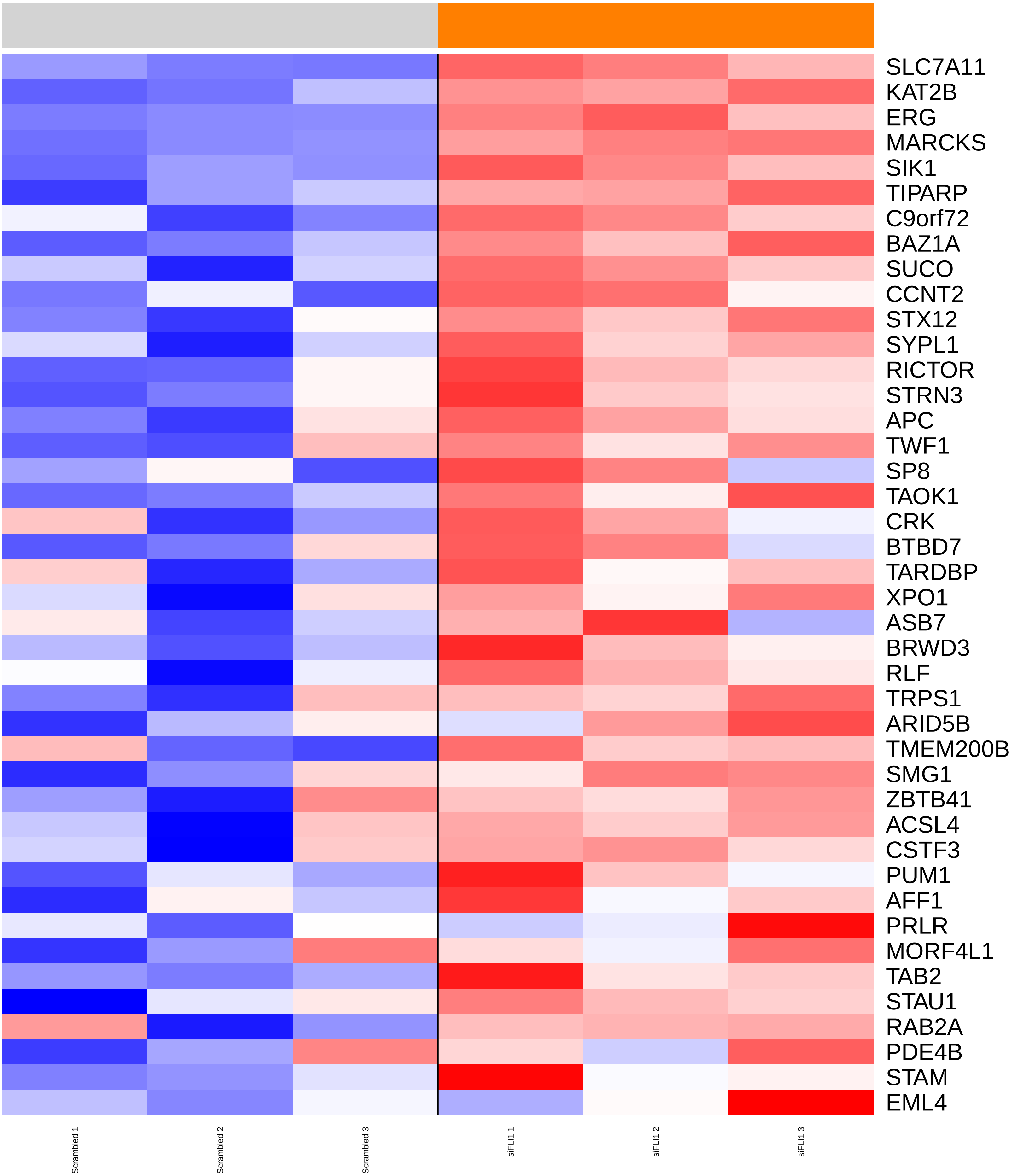

GO_UBIQUITIN_LIKE_PROTEIN_BINDING
NES = 1.43, p = 0.0169, FDR q = 0.2382

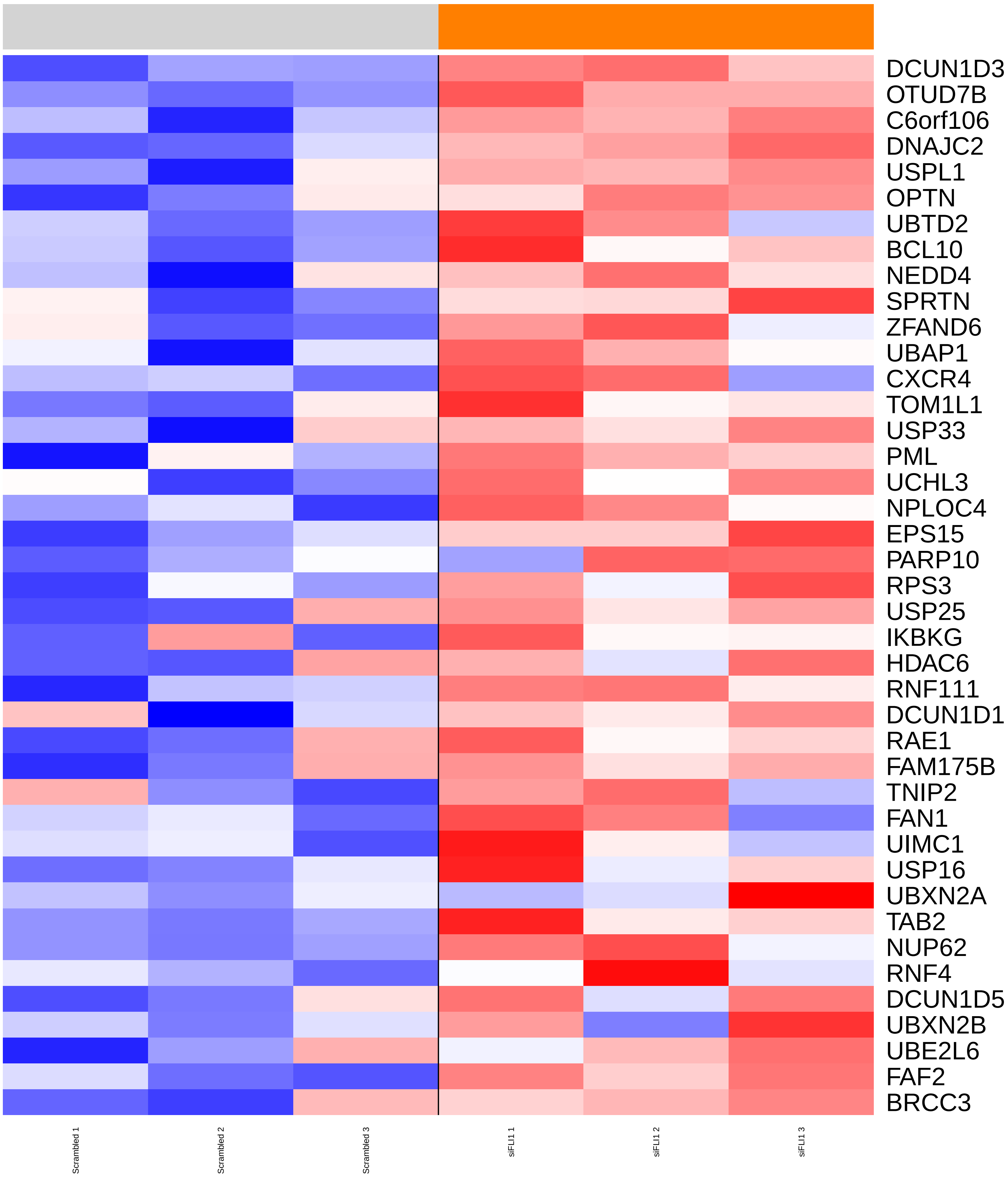

YAATNANRNNNCAG_UNKNOWN
NES = 1.43, p = 0.0204, FDR q = 0.2381

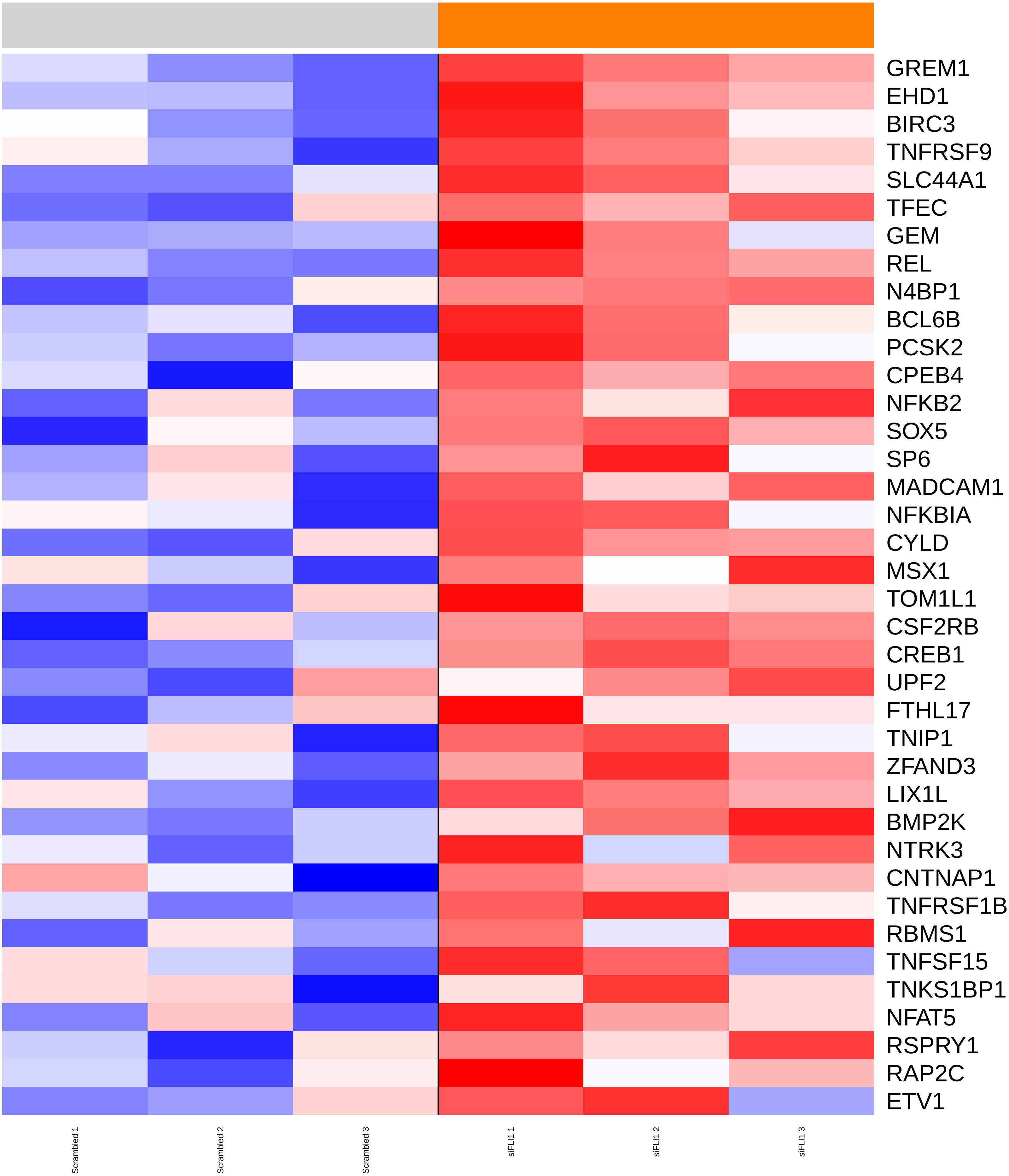

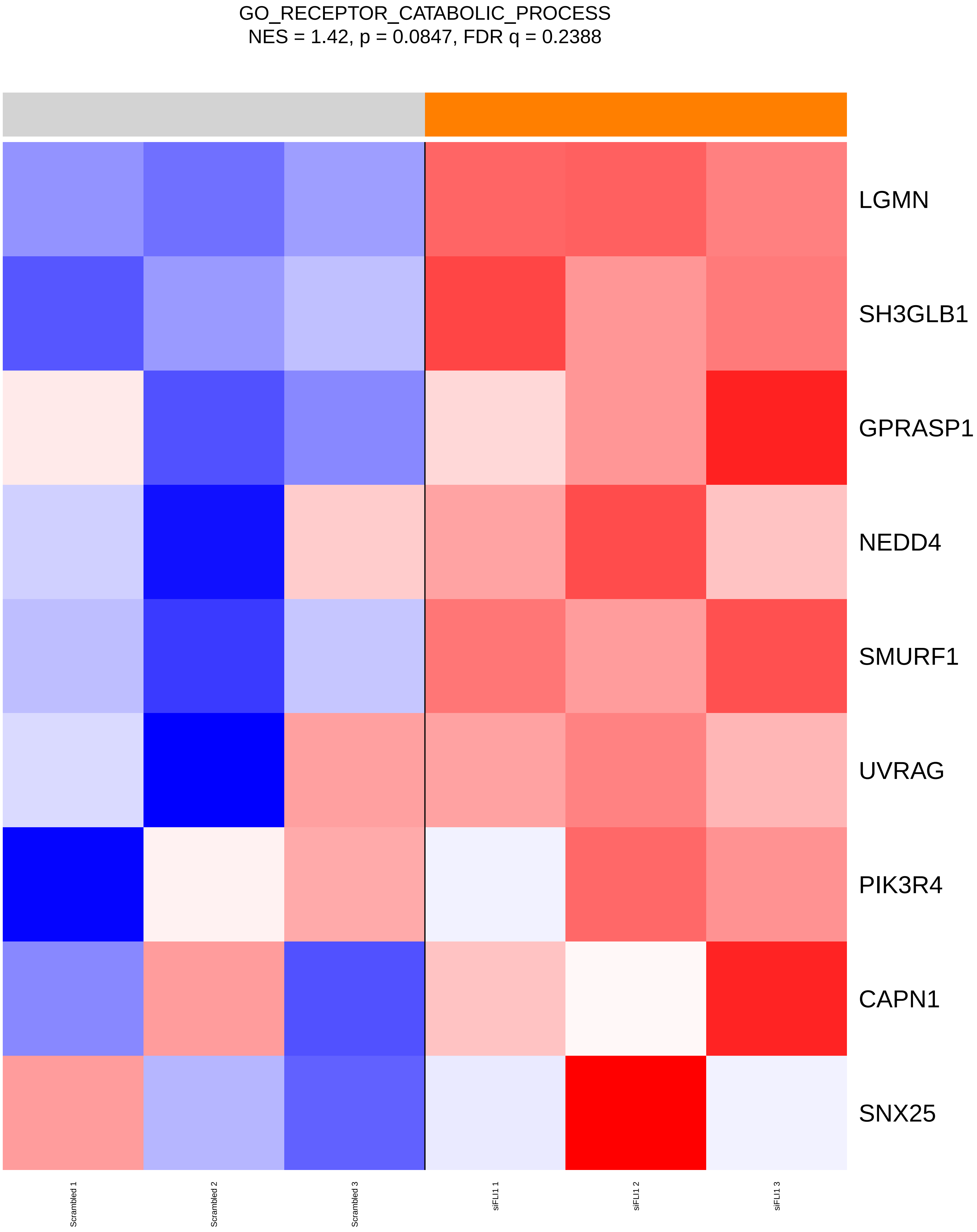
GO_RECEPTOR_CATABOLIC_PROCESS
NES = 1.42, p = 0.0847, FDR q = 0.2388
LGMN
SH3GLB1
GPRASP1
NEDD4
SMURF1
UVRAG
PIK3R4
CAPN1
SNX25
Scrambled 1
Scrambled 2
Scrambled 3
siFLI1 1
siFLI1 2
siFLI1 3

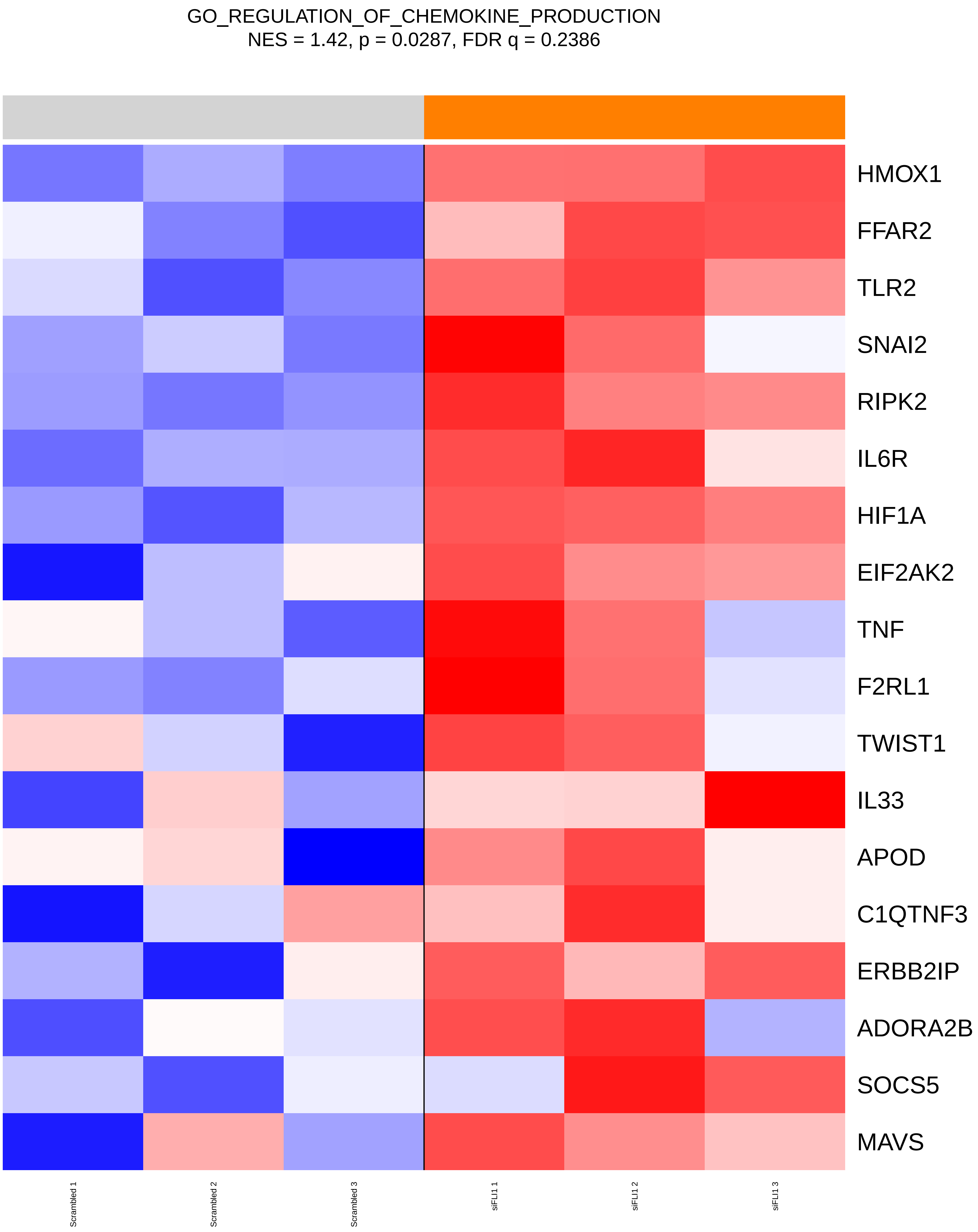
GO_REGULATION_OF_CHEMOKINE_PRODUCTION
NES = 1.42, p = 0.0287, FDR q = 0.2386
HMOX1
FFAR2
TLR2
SNAI2
RIPK2
IL6R
HIF1A
EIF2AK2
TNF
F2RL1
TWIST1
IL33
APOD
C1QTNF3
ERBB2IP
ADORA2B
SOCS5
MAVS
Scrambled 1
Scrambled 2
Scrambled 3
siFLI1 1
siFLI1 2
siFLI1 3

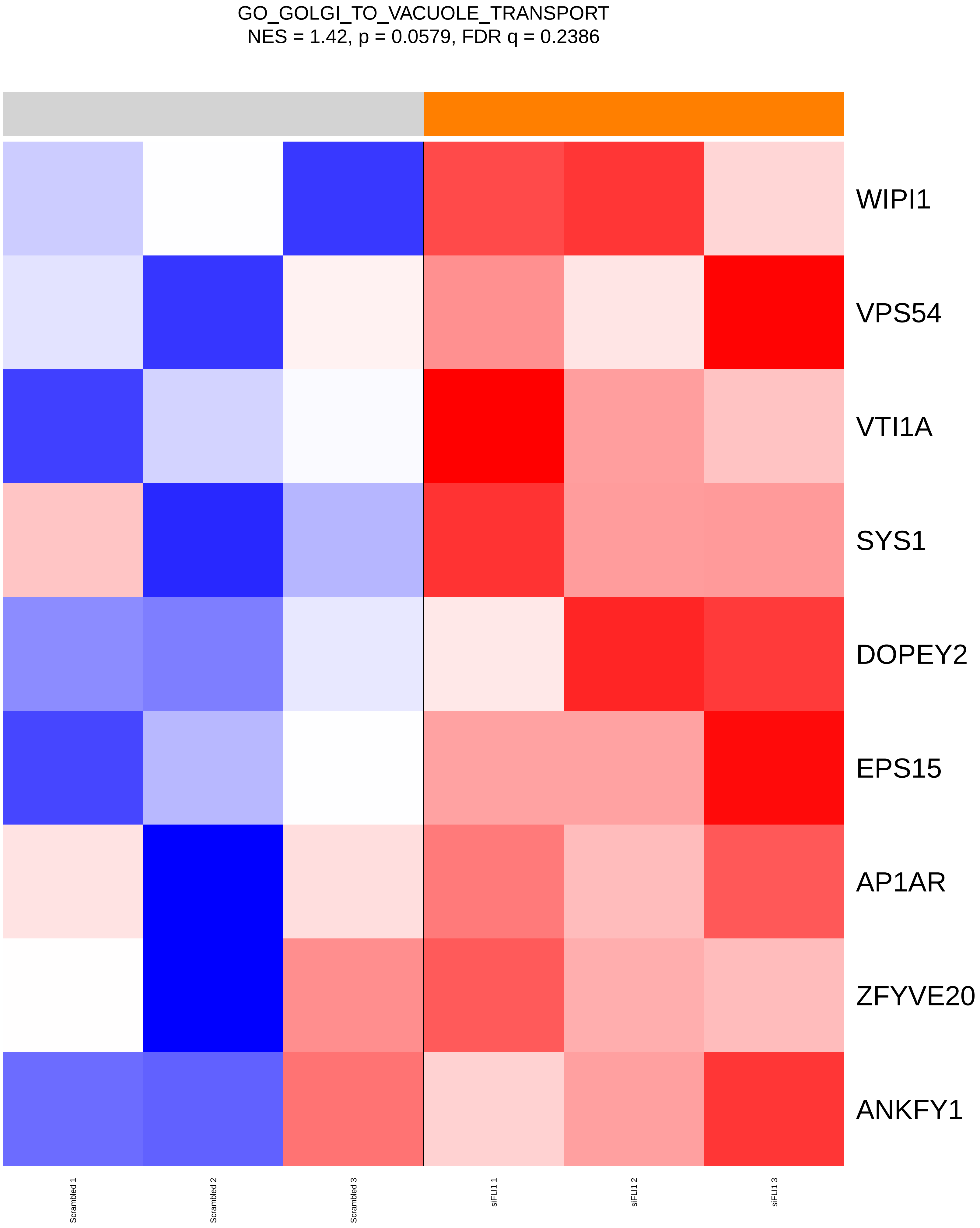

GO_GOLGI_TO_VACUOLE_TRANSPORT
NES = 1.42, p = 0.0579, FDR q = 0.2386
WIPI1
VPS54
VTI1A
SYS1
DOPEY2
EPS15
AP1AR
ZFYVE20
ANKFY1
Scrambled 1
Scrambled 2
Scrambled 3
siFLI1 1
siFLI1 2
siFLI1 3

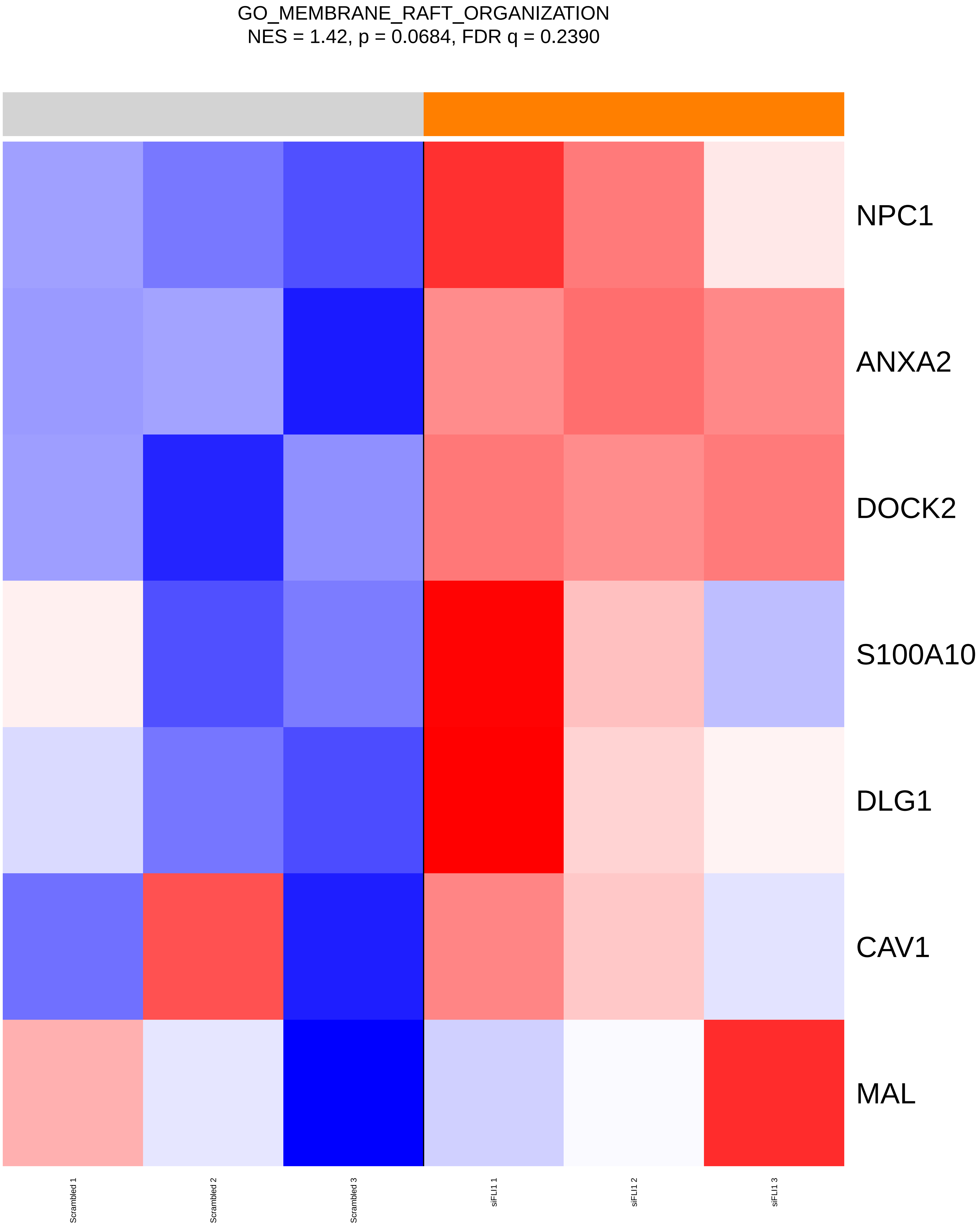
GO_MEMBRANE_RAFT_ORGANIZATION
NES = 1.42, p = 0.0684, FDR q = 0.2390
NPC1
ANXA2
DOCK2
S100A10
DLG1
CAV1
MAL
Scrambled 1
Scrambled 2
Scrambled 3
siFLI1 1
siFLI1 2
siFLI1 3

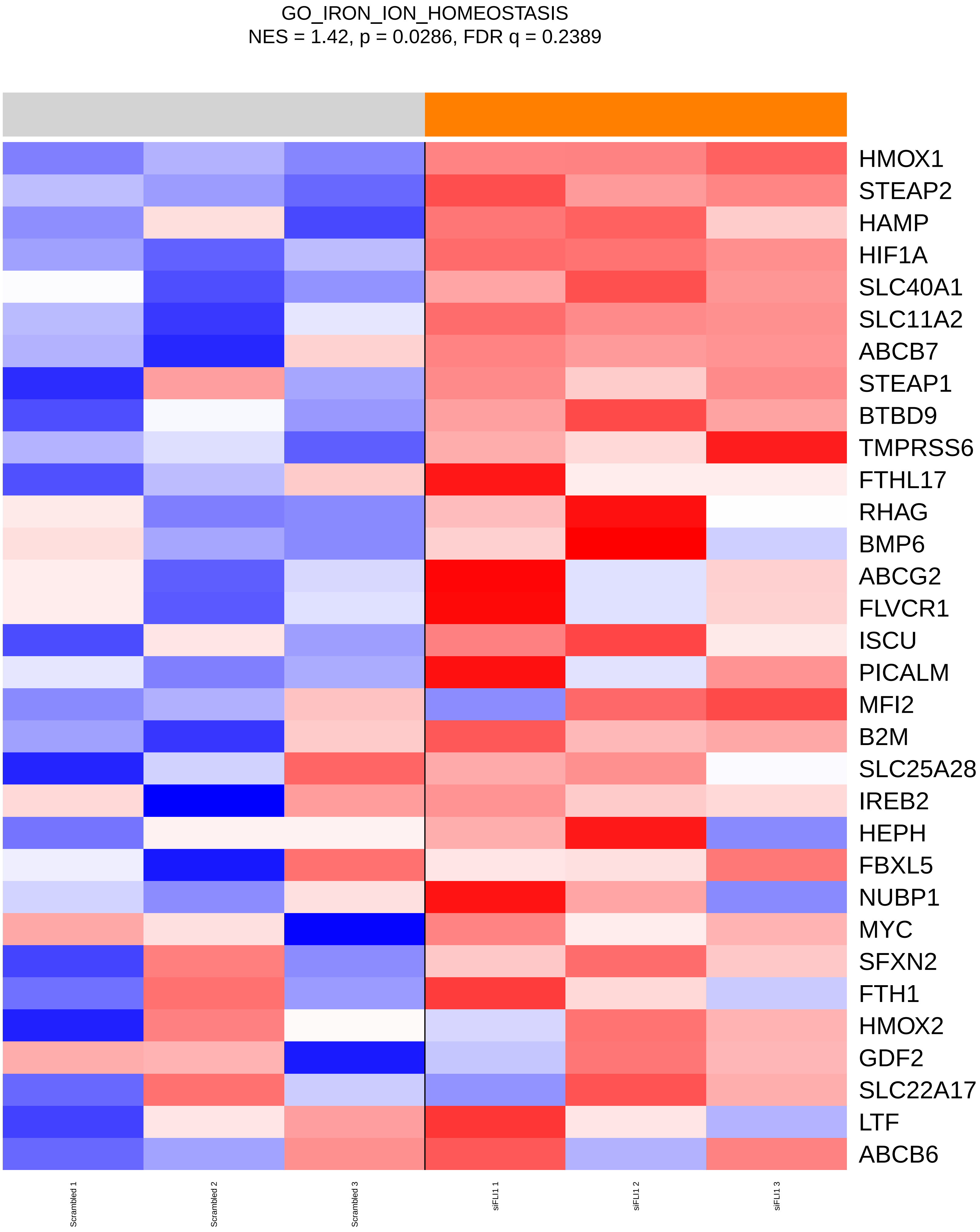
GO_IRON_ION_HOMEOSTASIS
NES = 1.42, p = 0.0286, FDR q = 0.2389
HMOX1
STEAP2
HAMP
HIF1A
SLC40A1
SLC11A2
ABCB7
STEAP1
BTBD9
TMPRSS6
FTHL17
RHAG
BMP6
ABCG2
FLVCR1
ISCU
PICALM
MFI2
B2M
SLC25A28
IREB2
HEPH
FBXL5
NUBP1
MYC
SFXN2
FTH1
HMOX2
GDF2
SLC22A17
LTF
ABCB6
Scrambled 1
Scrambled 2
Scrambled 3
siFLI1 1
siFLI1 2
siFLI1 3

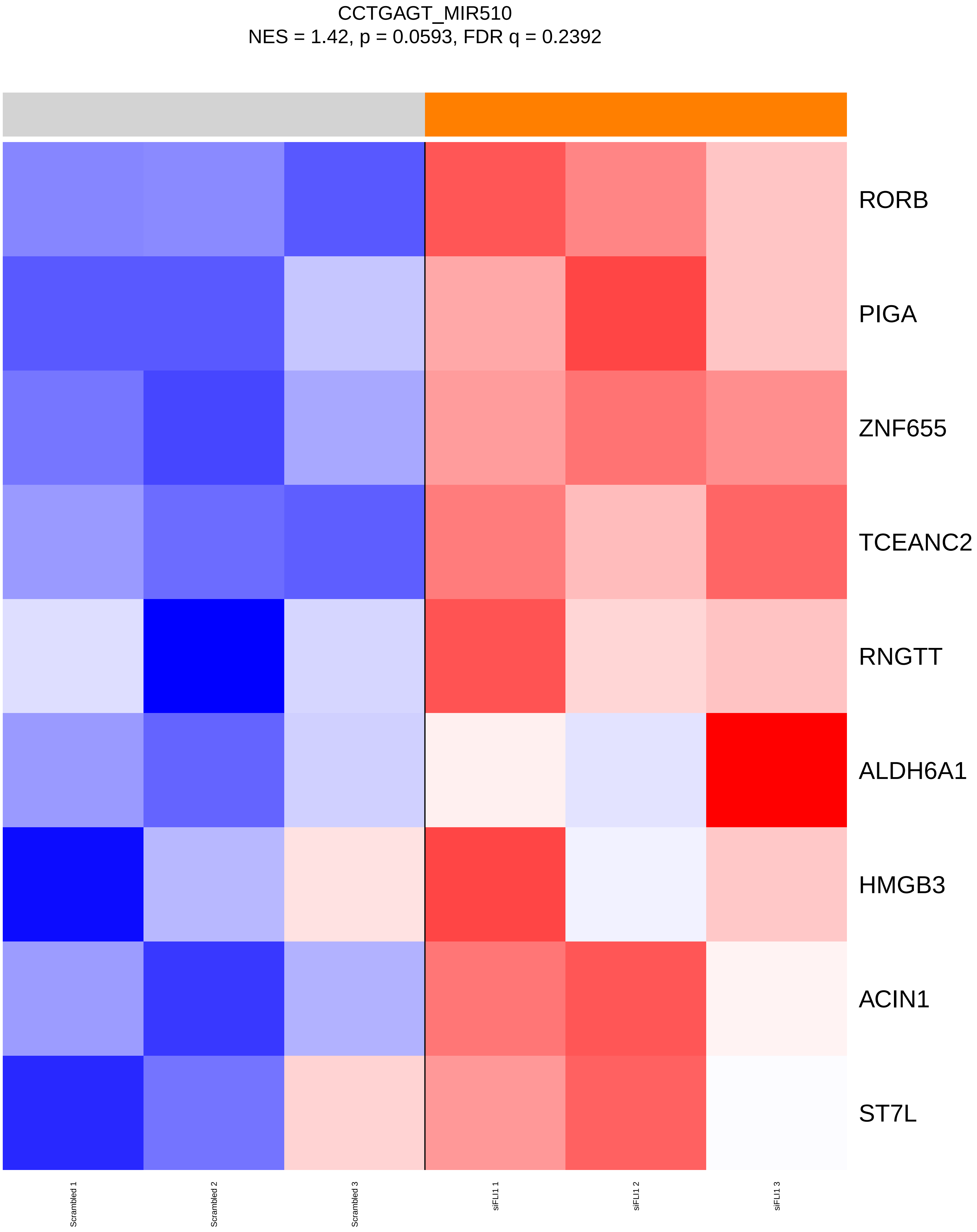
CCTGAGT_MIR510
NES = 1.42, p = 0.0593, FDR q = 0.2392
RORB
PIGA
ZNF655
TCEANC2
RNGTT
ALDH6A1
HMGB3
ACIN1
ST7L
Scrambled 1
Scrambled 2
Scrambled 3
siFLI1 1
siFLI1 2
siFLI1 3

GO_PHAGOCYTIC_VESICLE_MEMBRANE
NES = 1.42, p = 0.0526, FDR q = 0.2407

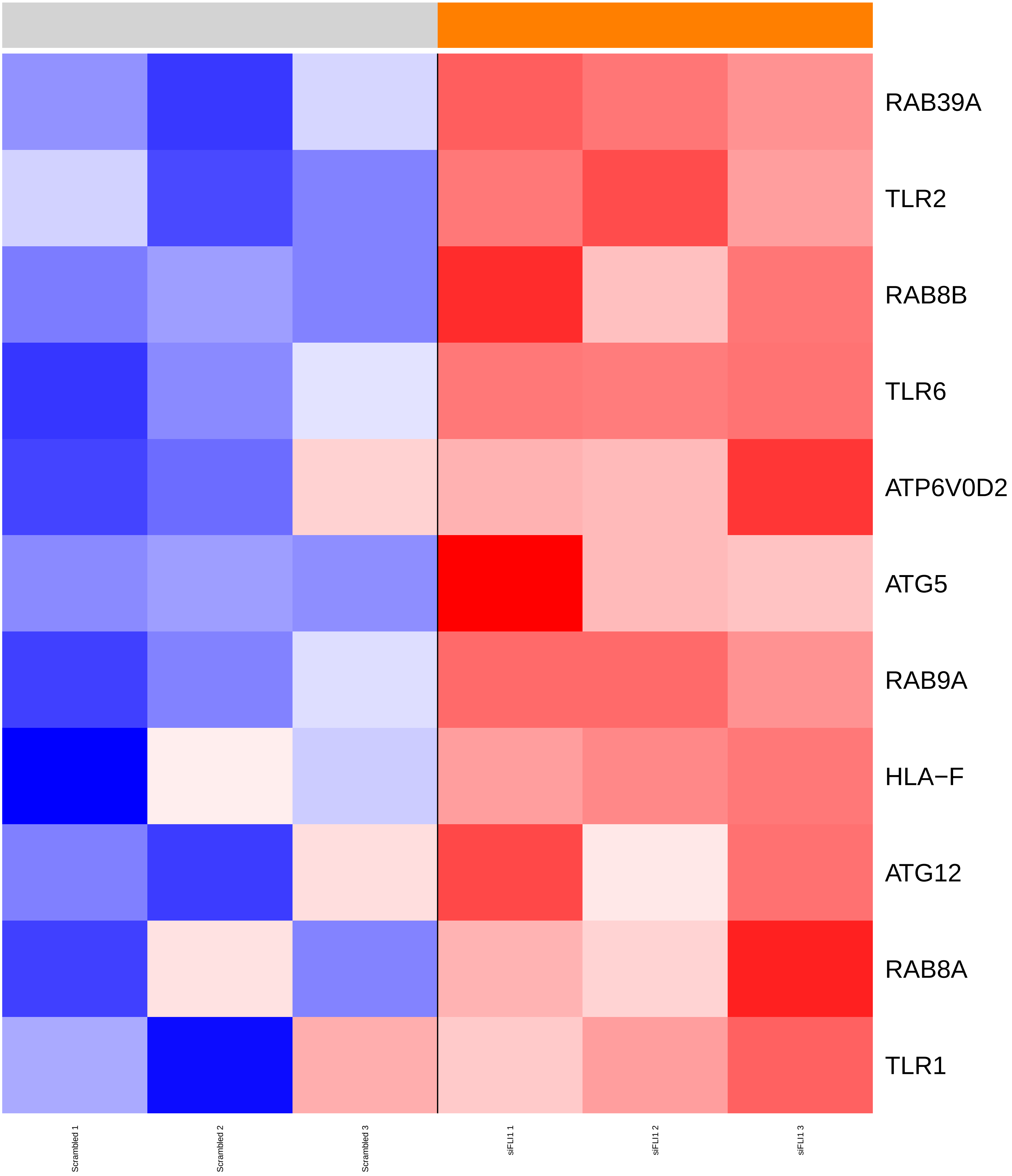

GO_GTPASE_ACTIVITY
NES = 1.42, p = 0.0041, FDR q = 0.2404

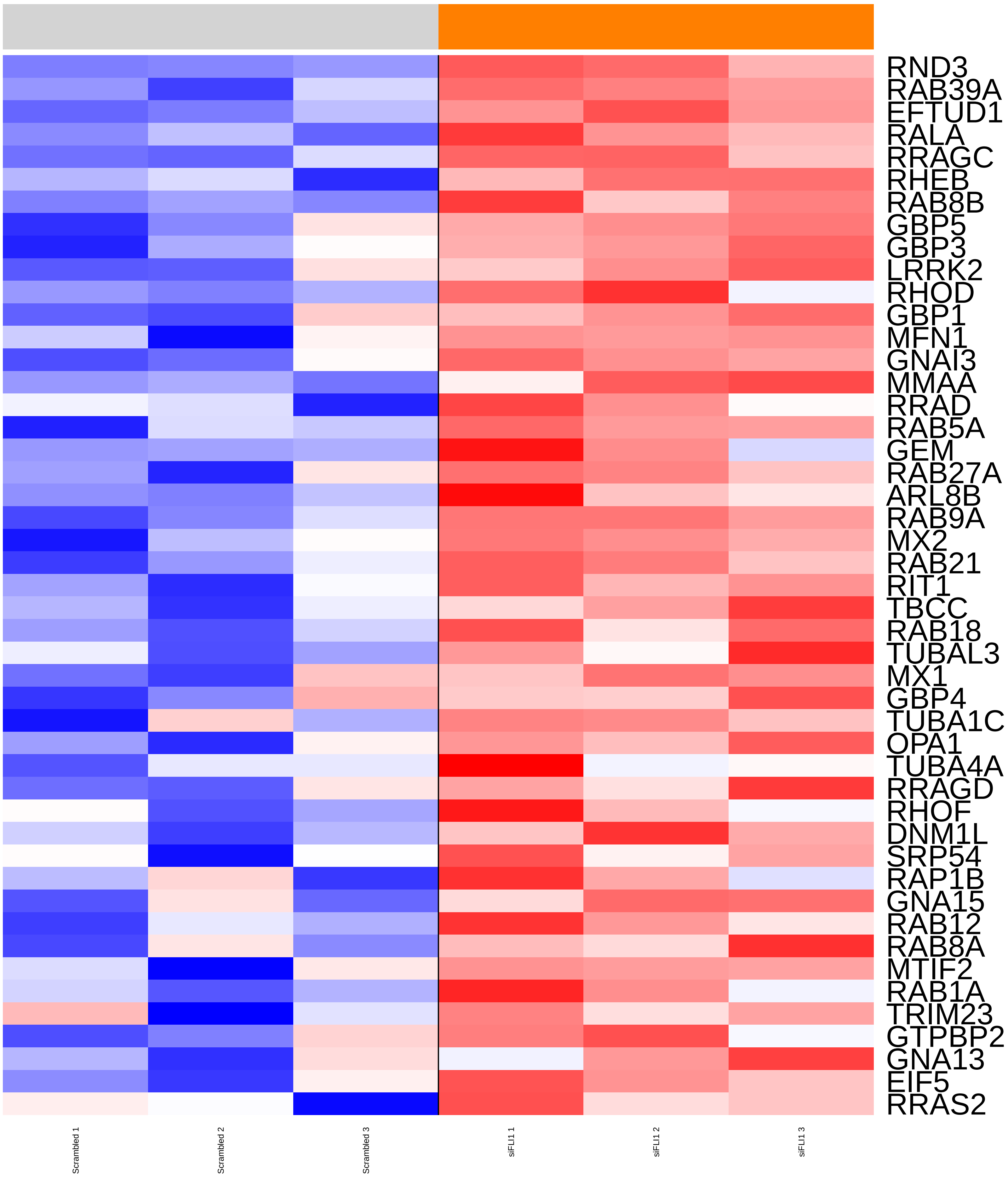

GO_TRANSFORMING_GROWTH_FACTOR_BETA_RECEPTOR_SIGNALING_PATHWAY
NES = 1.42, p = 0.0280, FDR q = 0.2400

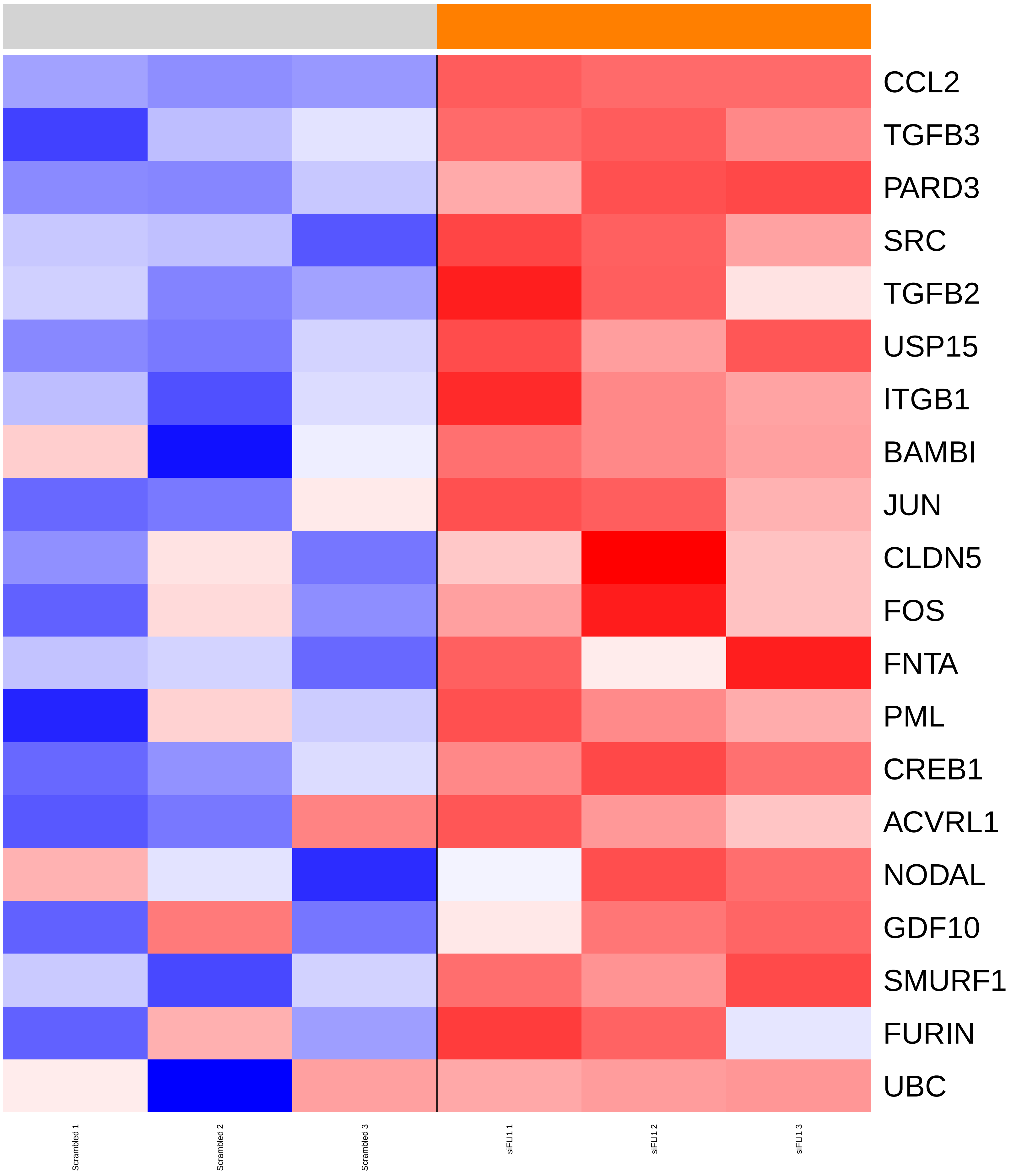

GO_POLYUBIQUITIN_BINDING
NES = 1.42, p = 0.0383, FDR q = 0.2406

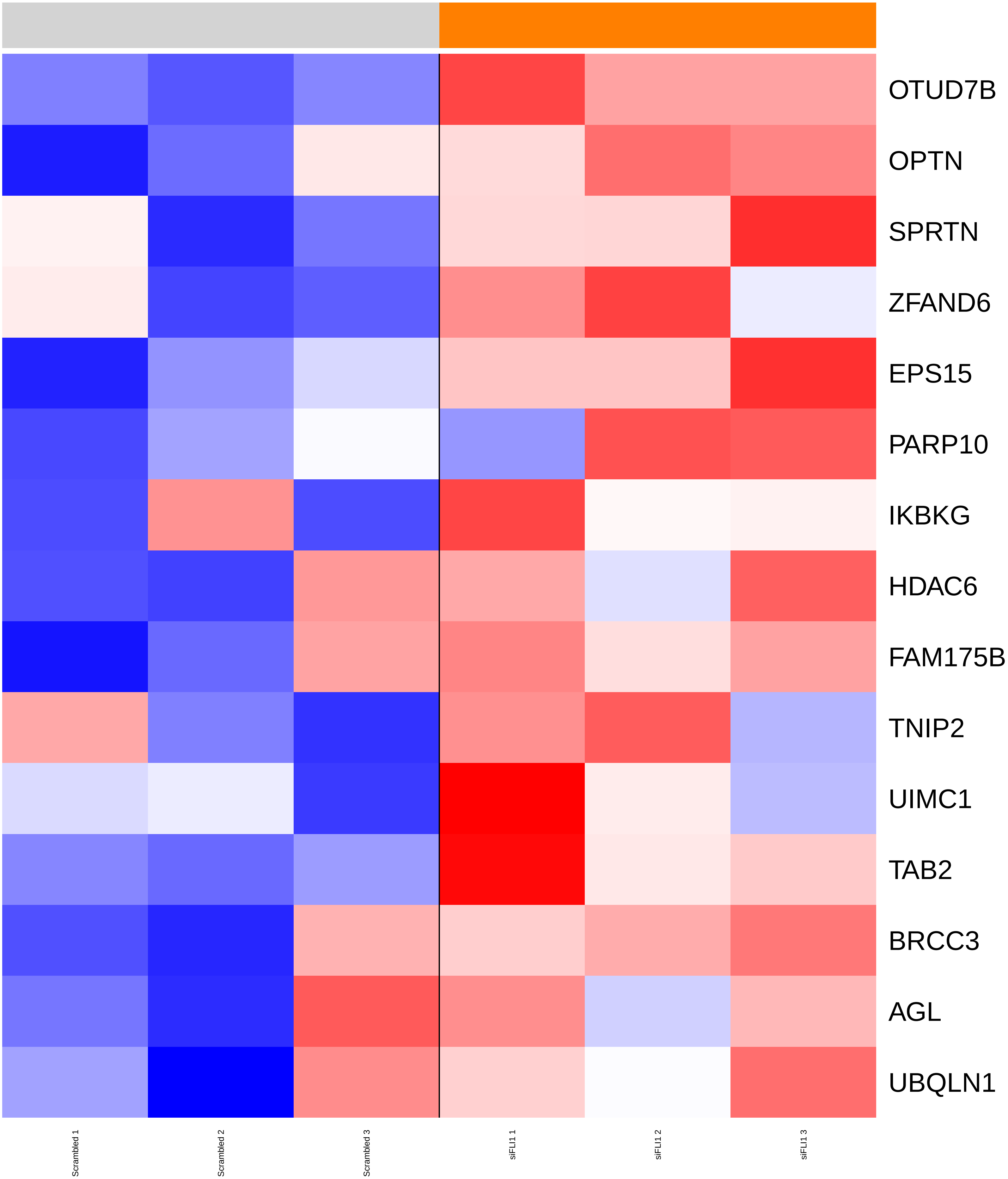

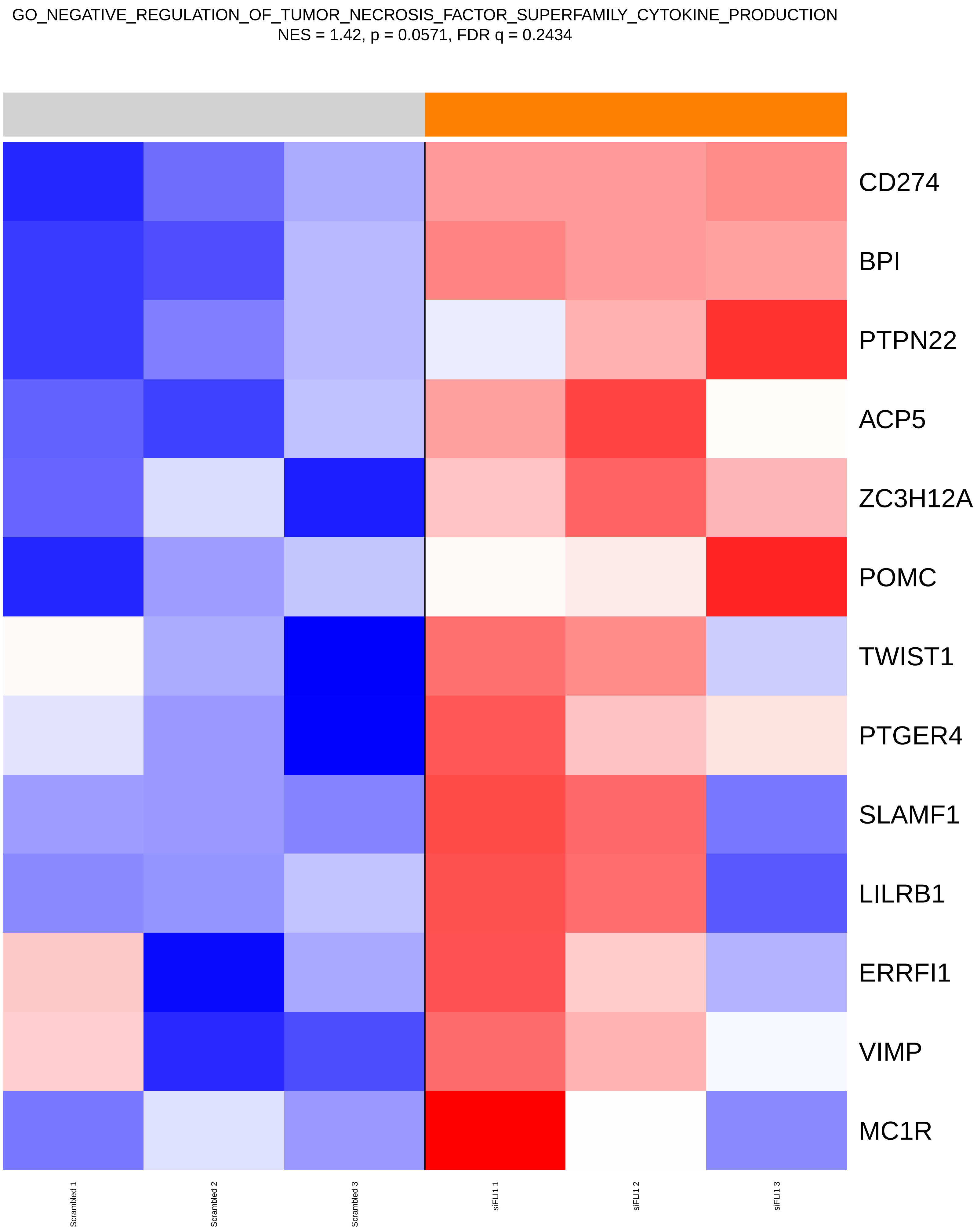
GO_NEGATIVE_REGULATION_OF_TUMOR_NECROSIS_FACTOR_SUPERFAMILY_CYTOKINE_PRODUCTION
NES = 1.42, p = 0.0571, FDR q = 0.2434
CD274
BPI
PTPN22
ACP5
ZC3H12A
POMC
TWIST1
PTGER4
SLAMF1
LILRB1
ERRFI1
VIMP
MC1R
Scrambled 1
Scrambled 2
Scrambled 3
siFLI1 1
siFLI1 2
siFLI1 3

GO_POSITIVE_REGULATION_OF_TUMOR_NECROSIS_FACTOR_SUPERFAMILY_CYTOKINE_PRODUCTION
NES = 1.42, p = 0.0378, FDR q = 0.2452

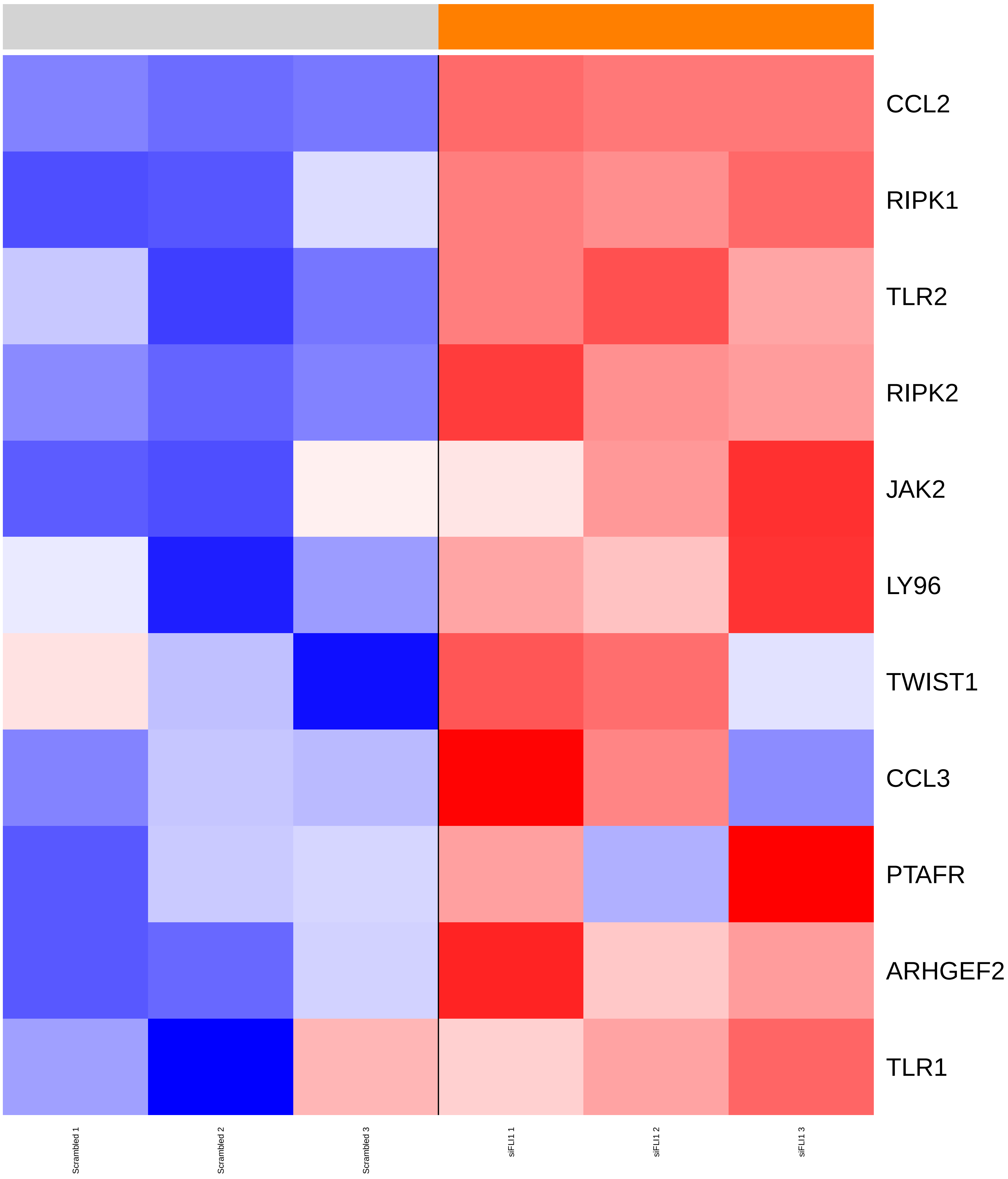

REACTOME_POST_CHAPERONIN_TUBULIN_FOLDING_PATHWAY
NES = 1.42, p = 0.0940, FDR q = 0.2459

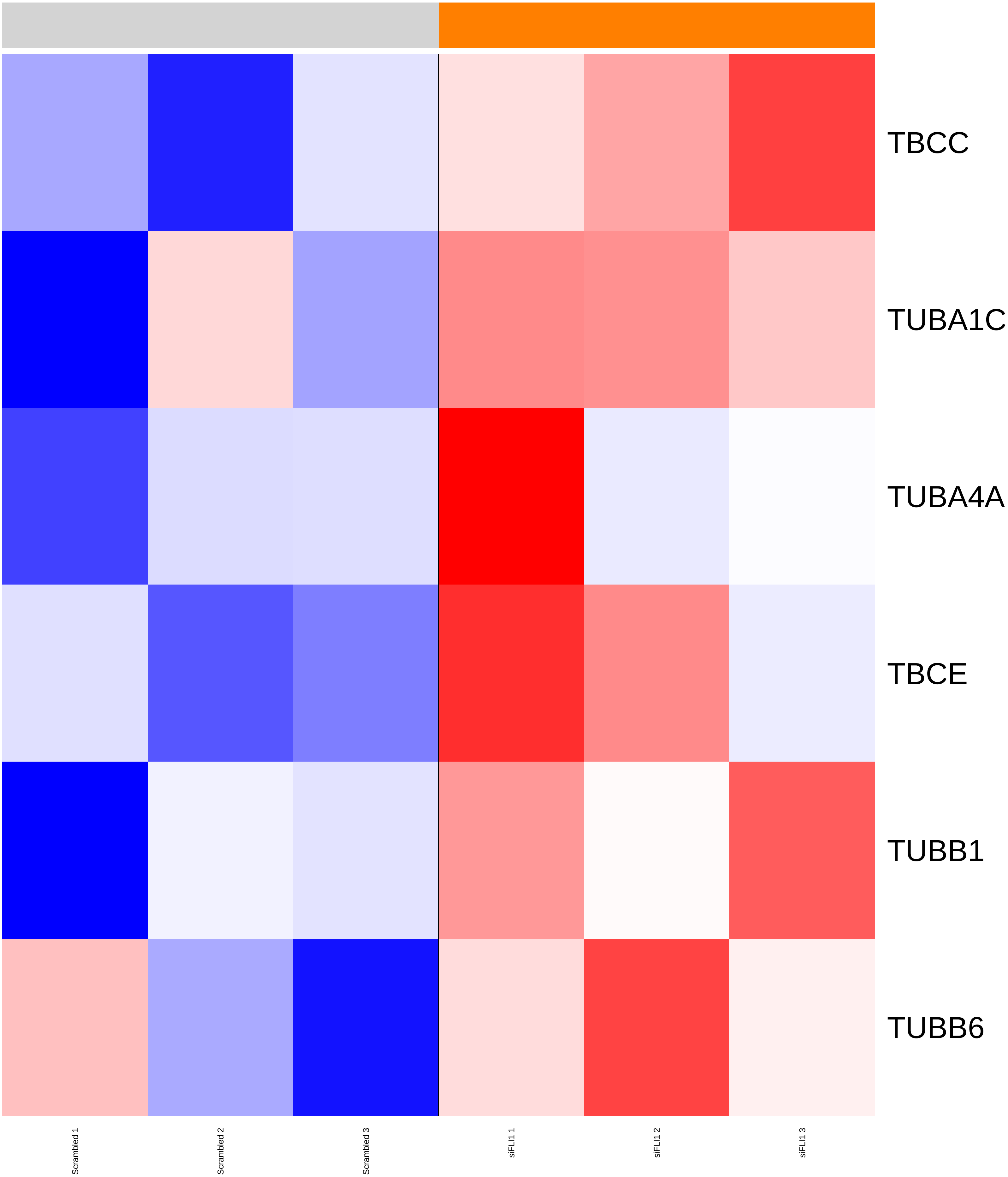

GO_REGULATION_OF_VESICLE_MEDIATED_TRANSPORT
NES = 1.42, p = 0.0000, FDR q = 0.2457

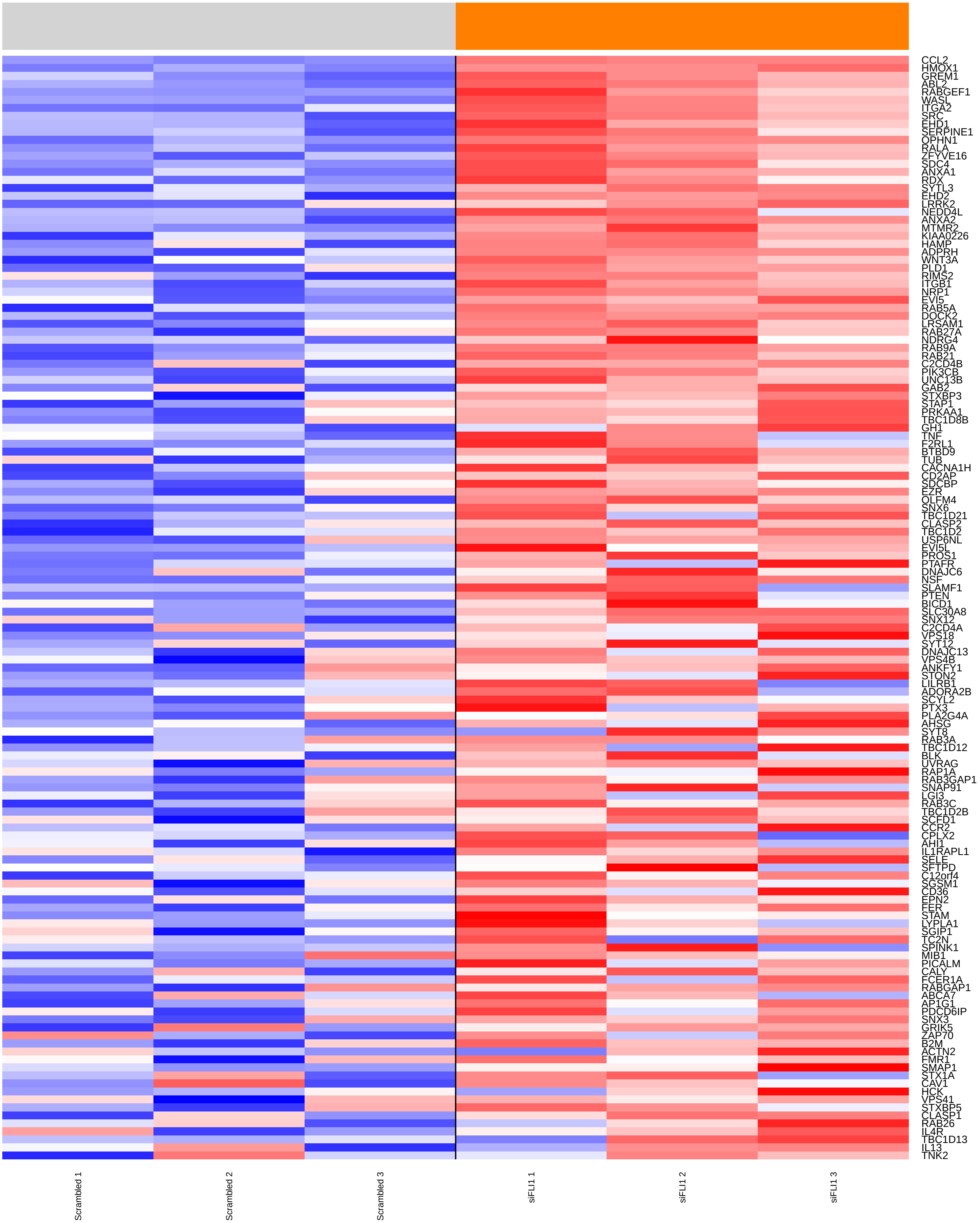

GO_POSITIVE_REGULATION_OF_ENDOCYTOSIS
NES = 1.41, p = 0.0224, FDR q = 0.2456

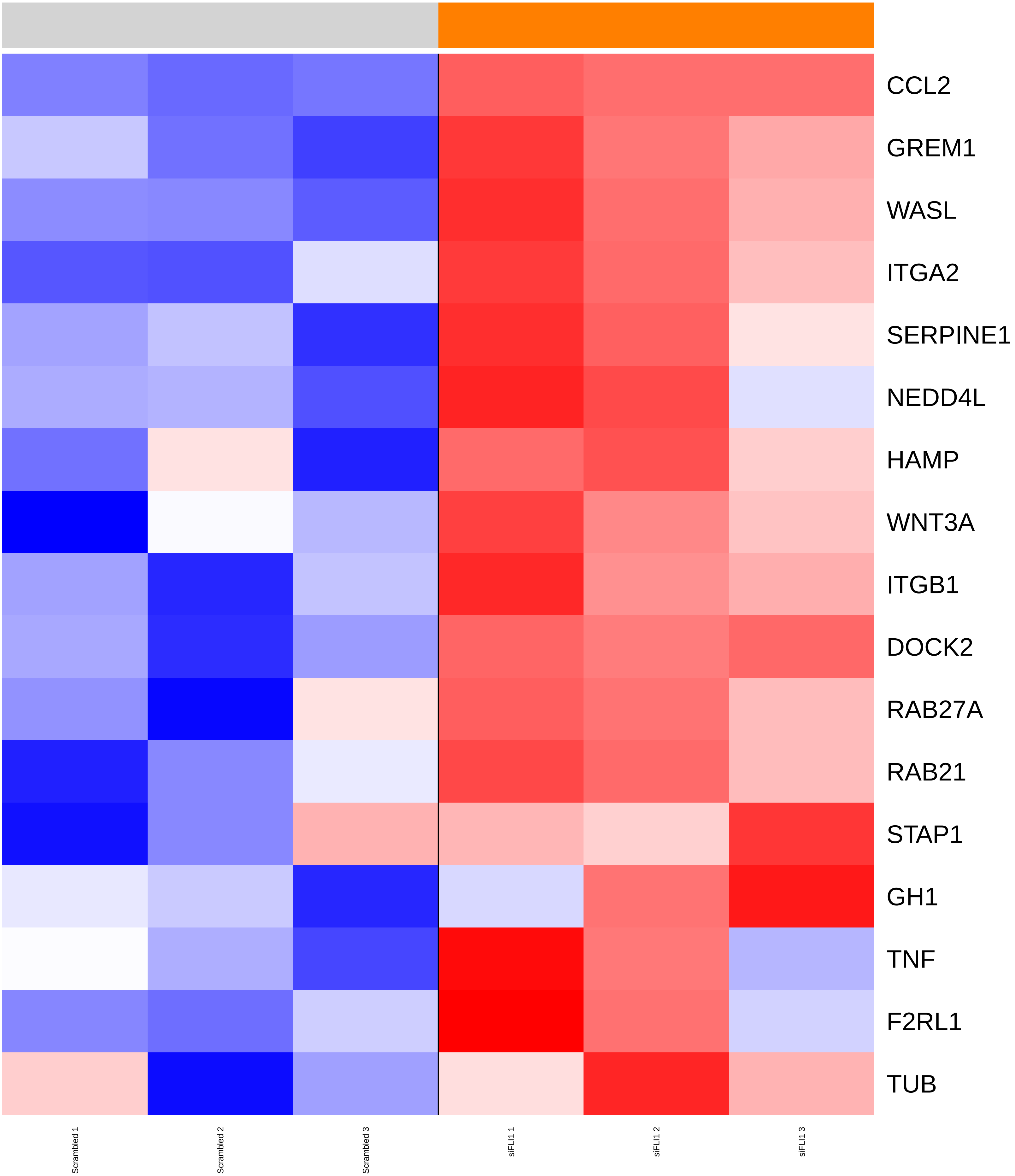

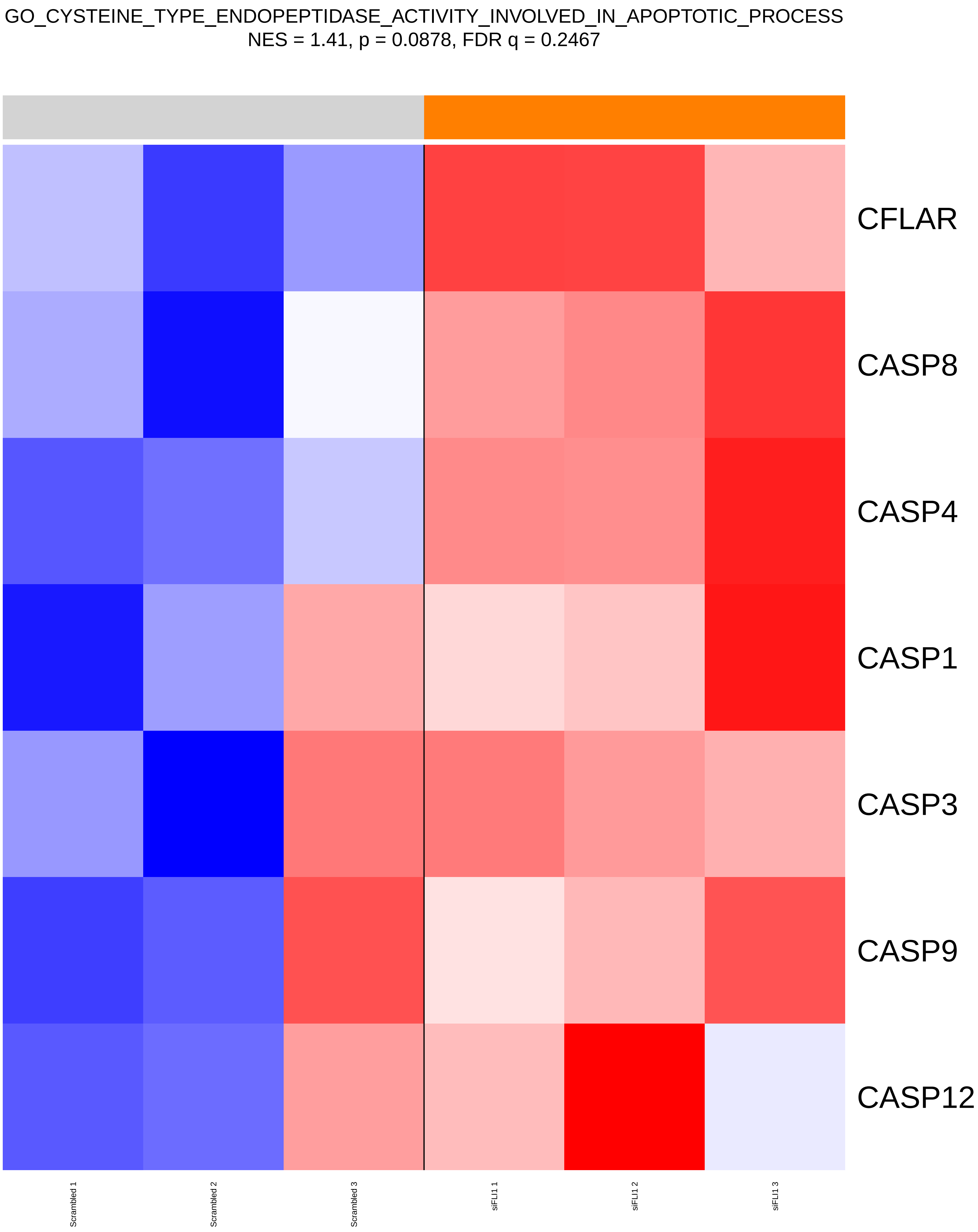
GO_CYSTEINE_TYPE_ENDOPEPTIDASE_ACTIVITY_INVOLVED_IN_APOPTOTIC_PROCESS
NES = 1.41, p = 0.0878, FDR q = 0.2467
CFLAR
CASP8
CASP4
CASP1
CASP3
CASP9
CASP12
Scrambled 1
Scrambled 2
Scrambled 3
siFLI1 1
siFLI1 2
siFLI1 3

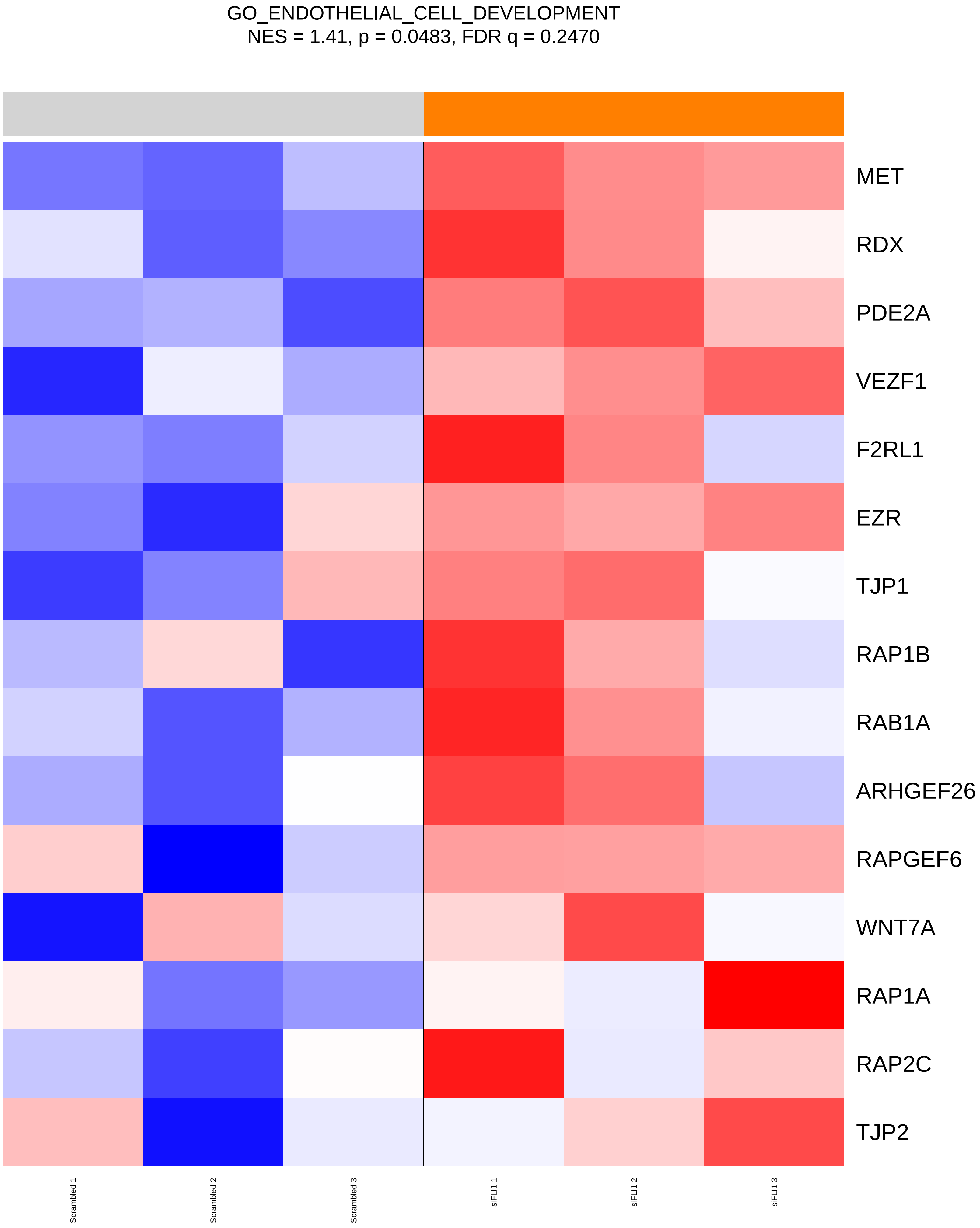
GO_ENDOTHELIAL_CELL_DEVELOPMENT
NES = 1.41, p = 0.0483, FDR q = 0.2470
MET
RDX
PDE2A
VEZF1
F2RL1
EZR
TJP1
RAP1B
RAB1A
ARHGEF26
RAPGEF6
WNT7A
RAP1A
RAP2C
TJP2
Scrambled 1
Scrambled 2
Scrambled 3
siFLI1 1
siFLI1 2
siFLI1 3

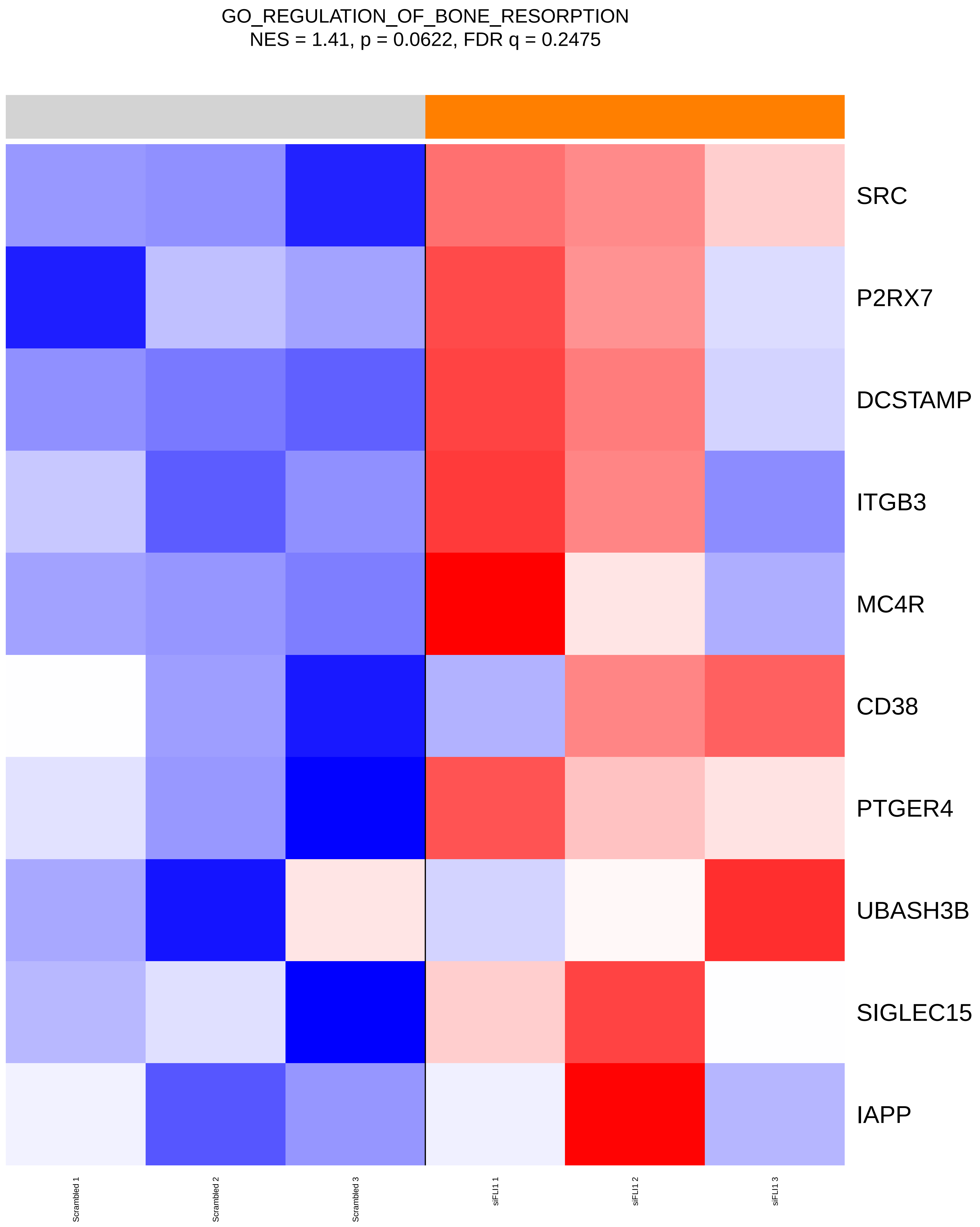
GO_REGULATION_OF_BONE_RESORPTION
NES = 1.41, p = 0.0622, FDR q = 0.2475
SRC
P2RX7
DCSTAMP
ITGB3
MC4R
CD38
PTGER4
UBASH3B
SIGLEC15
IAPP
Scrambled 1
Scrambled 2
Scrambled 3
siFLI1 1
siFLI1 2
siFLI1 3

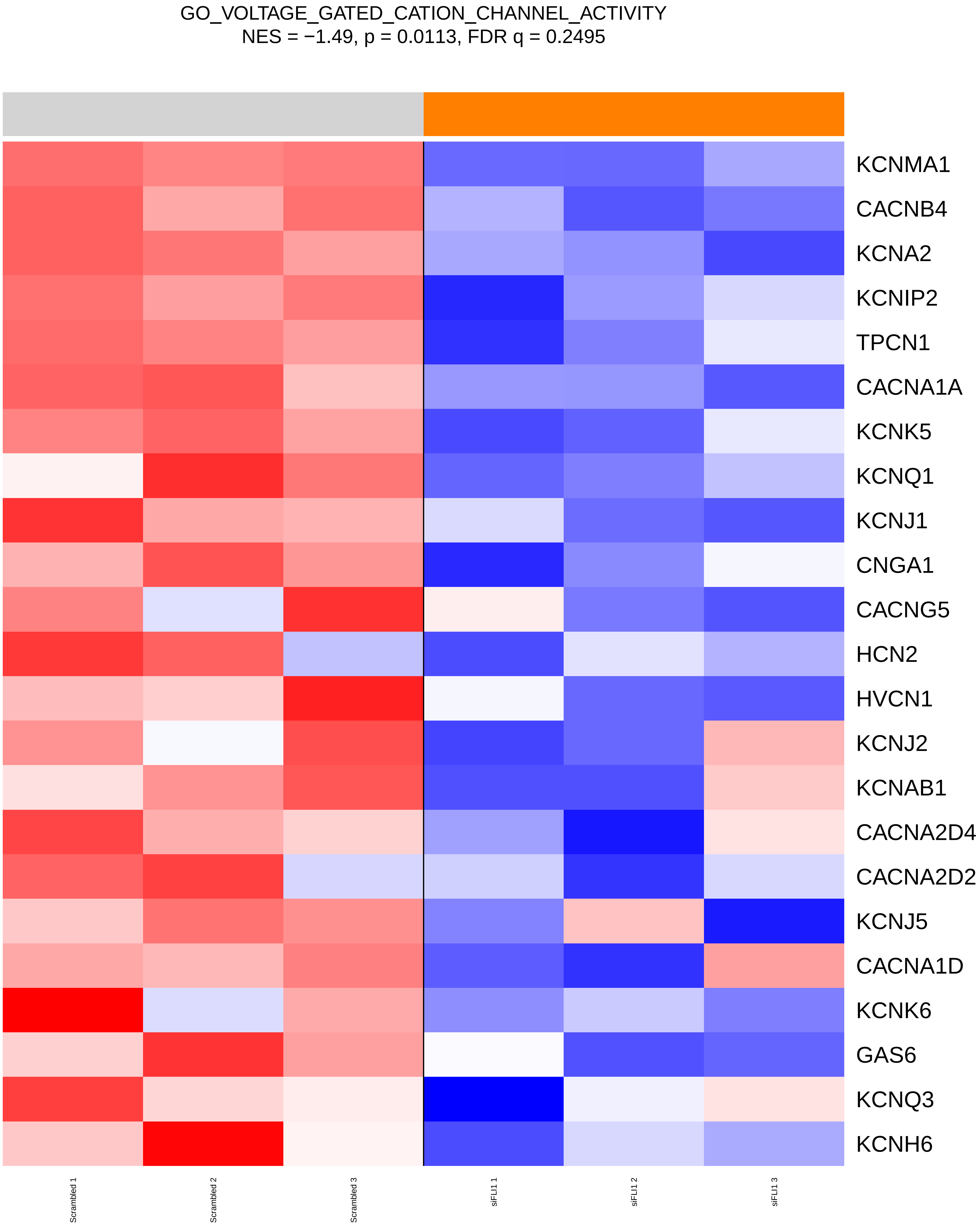
GO_VOLTAGE_GATED_CATION_CHANNEL_ACTIVITY
NES = −1.49, p = 0.0113, FDR q = 0.2495
KCNMA1
CACNB4
KCNA2
KCNIP2
TPCN1
CACNA1A
KCNK5
KCNQ1
KCNJ1
CNGA1
CACNG5
HCN2
HVCN1
KCNJ2
KCNAB1
CACNA2D4
CACNA2D2
KCNJ5
CACNA1D
KCNK6
GAS6
KCNQ3
KCNH6
Scrambled 1
Scrambled 2
Scrambled 3
siFLI1 1
siFLI1 2
siFLI1 3

GO_POSITIVE_REGULATION_OF_NATURAL_KILLER_CELL_MEDIATED_IMMUNITY
NES = −1.49, p = 0.0430, FDR q = 0.2494

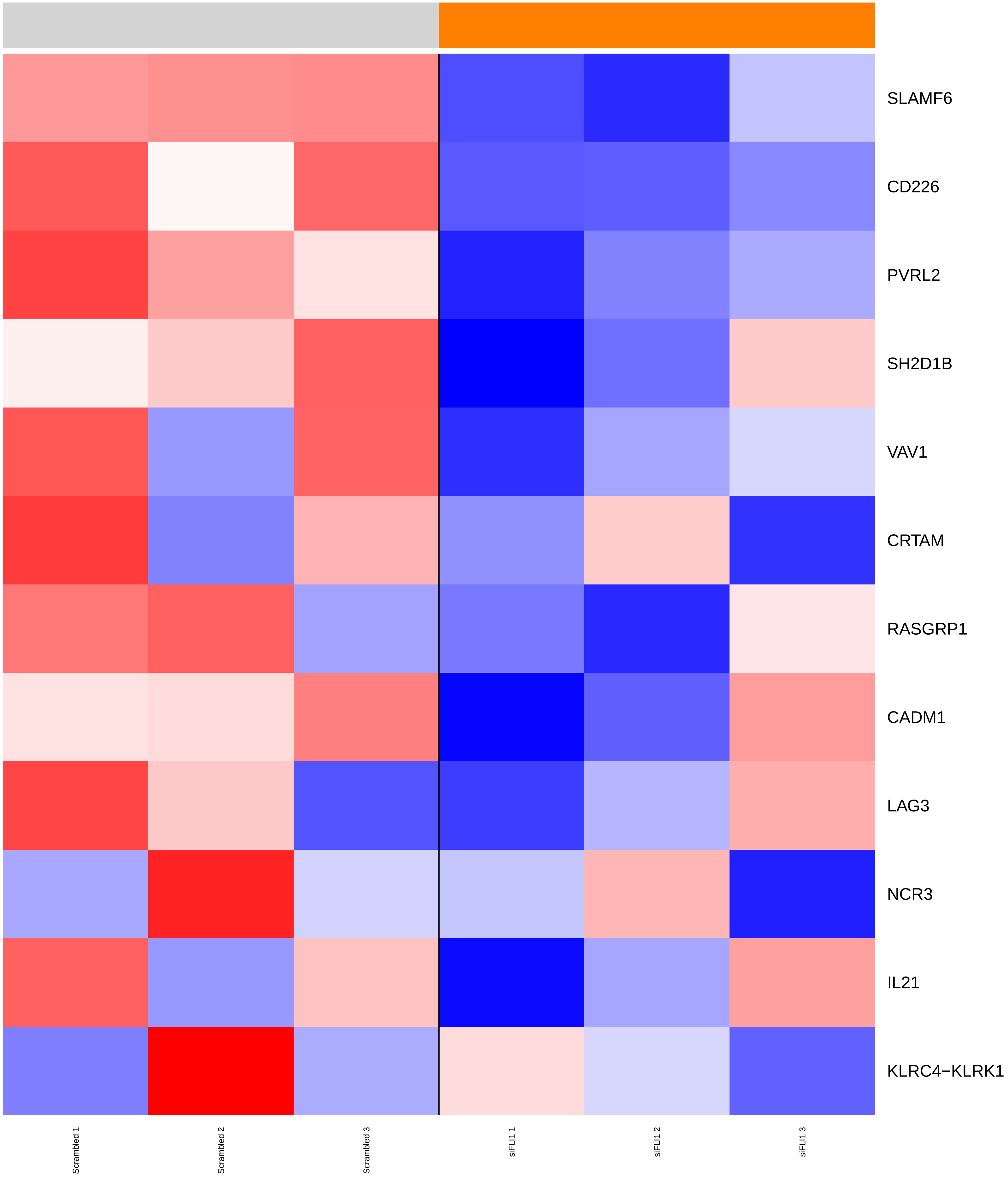

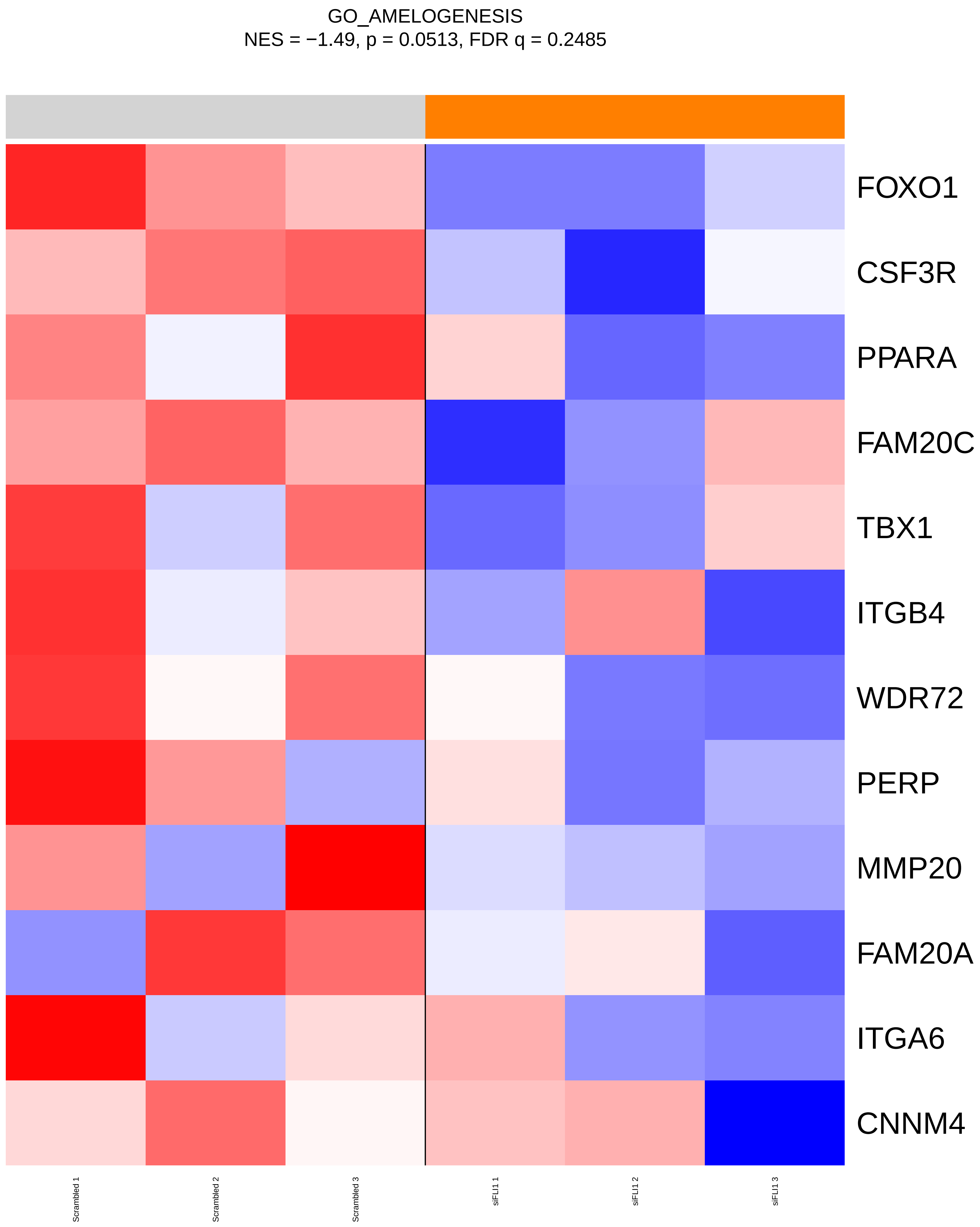
GO_AMELOGENESIS
NES = −1.49, p = 0.0513, FDR q = 0.2485
FOXO1
CSF3R
PPARA
FAM20C
TBX1
ITGB4
WDR72
PERP
MMP20
FAM20A
ITGA6
CNNM4
Scrambled 1
Scrambled 2
Scrambled 3
siFLI1 1
siFLI1 2
siFLI1 3

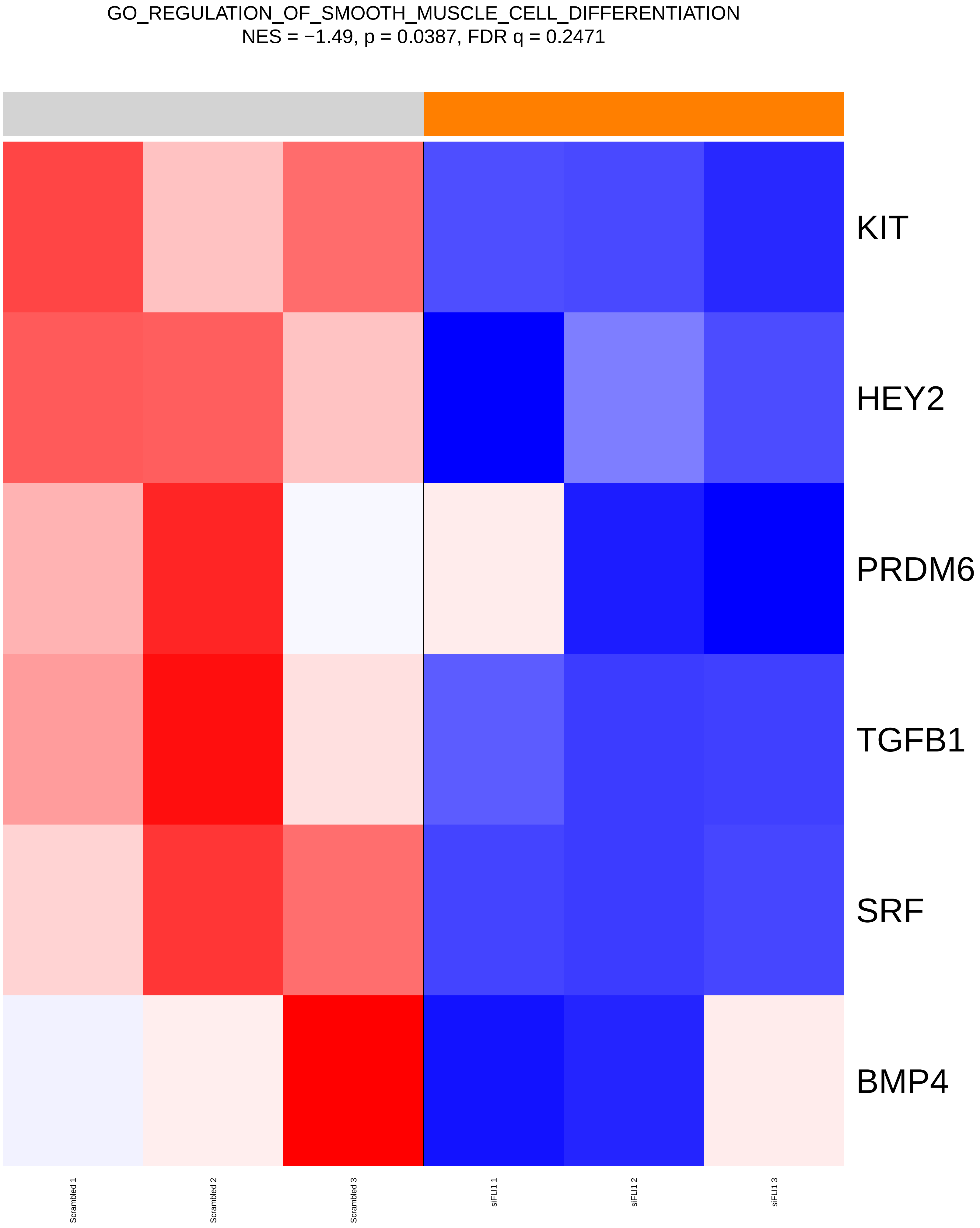
GO_REGULATION_OF_SMOOTH_MUSCLE_CELL_DIFFERENTIATION
NES = −1.49, p = 0.0387, FDR q = 0.2471
KIT
HEY2
PRDM6
TGFB1
SRF
BMP4
Scrambled 1
Scrambled 2
Scrambled 3
siFLI1 1
siFLI1 2
siFLI1 3

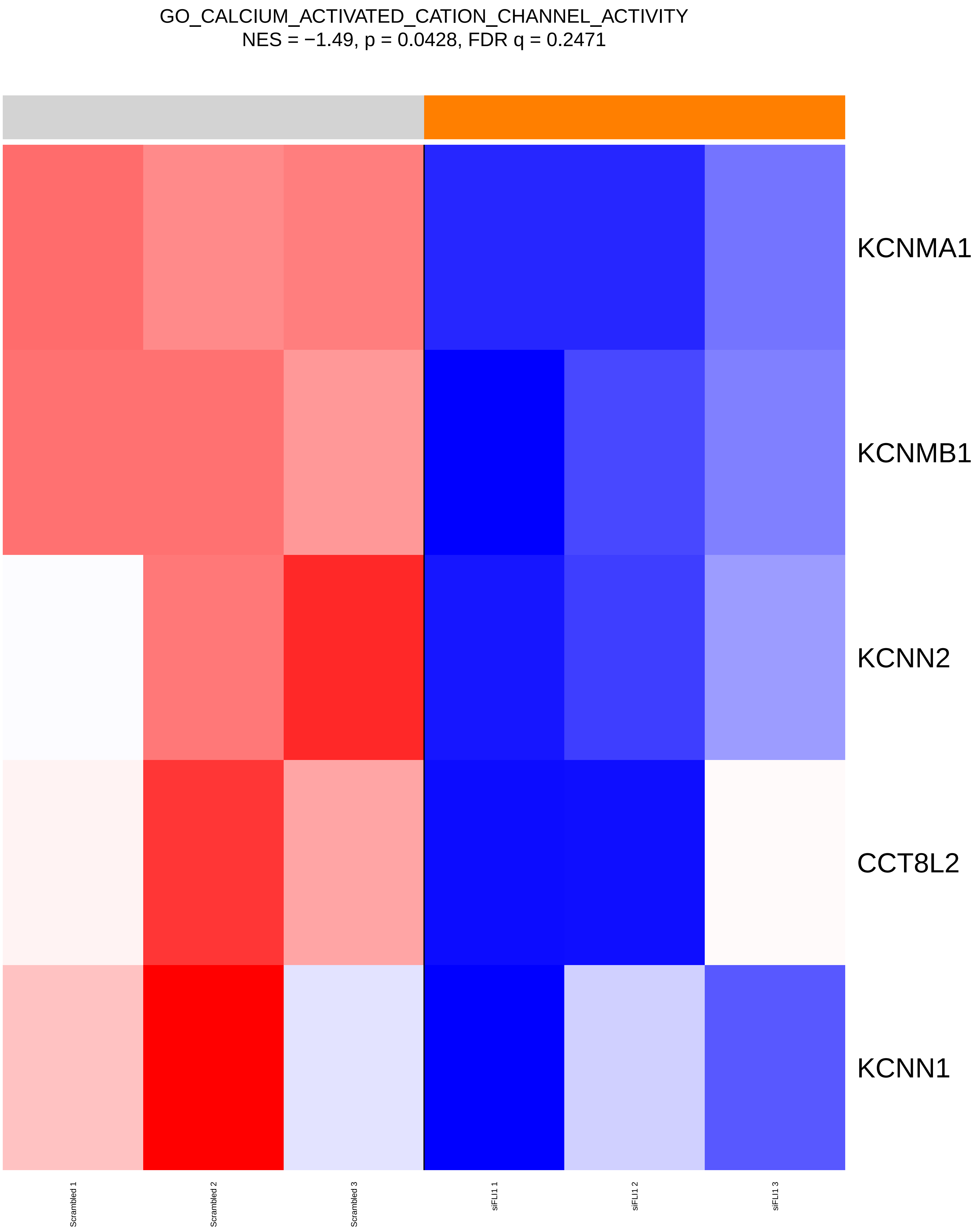
GO_CALCIUM_ACTIVATED_CATION_CHANNEL_ACTIVITY
NES = −1.49, p = 0.0428, FDR q = 0.2471
KCNMA1
KCNMB1
KCNN2
CCT8L2
KCNN1
Scrambled 1
Scrambled 2
Scrambled 3
siFLI1 1
siFLI1 2
siFLI1 3

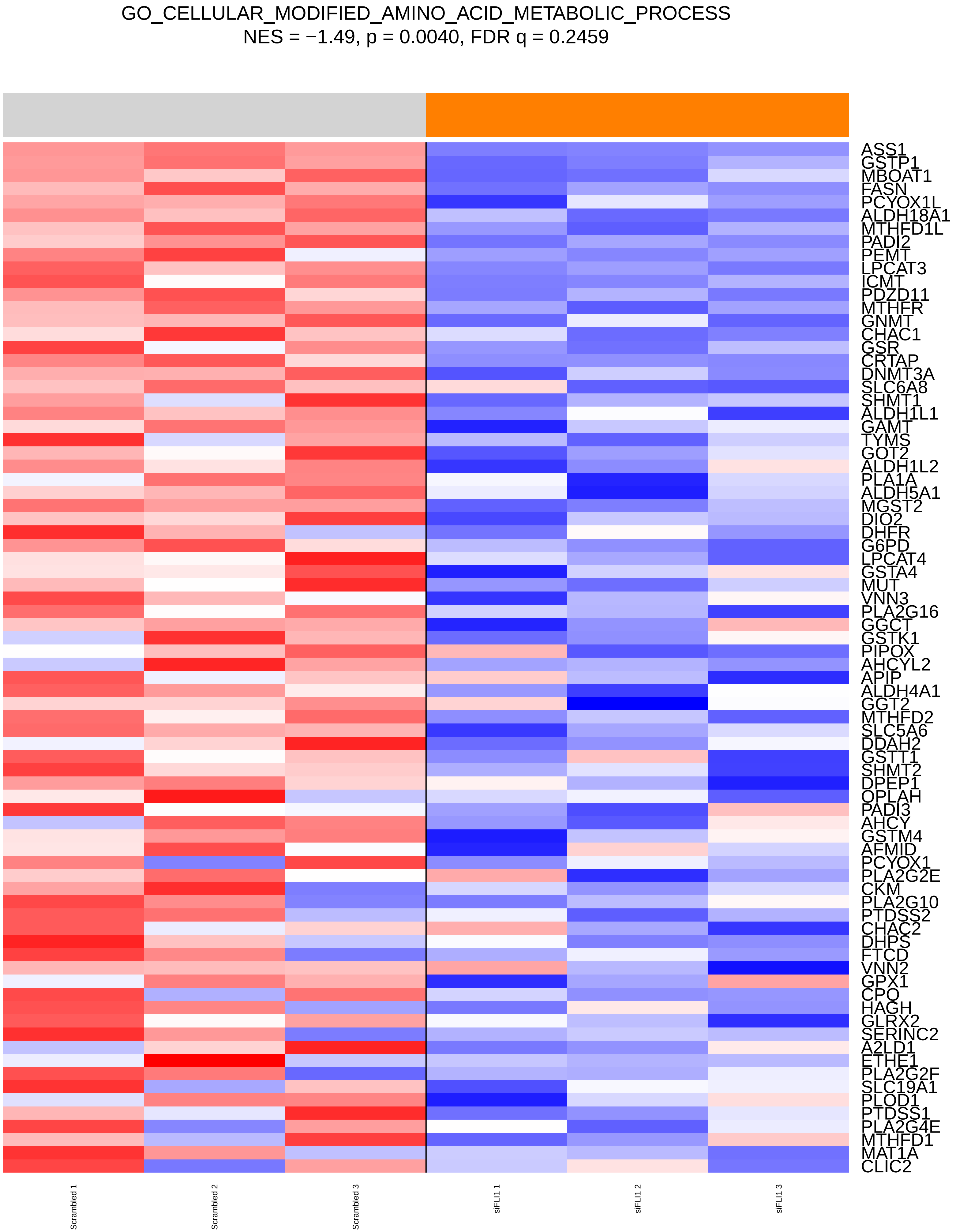

GO_CELLULAR_MODIFIED_AMINO_ACID_METABOLIC_PROCESS
NES = −1.49, p = 0.0040, FDR q = 0.2459
ASS1
GSTP1
MBOAT1
FASN
PCYOX1L
ALDH18A1
MTHFD1L
PADI2
PEMT
LPCAT3
ICMT
PDZD11
MTHFR
GNMT
CHAC1
GSR
CRTAP
DNMT3A
SLC6A8
SHMT1
ALDH1L1
GAMT
TYMS
GOT2
ALDH1L2
PLA1A
ALDH5A1
MGST2
DIO2
DHFR
G6PD
LPCAT4
GSTA4
MUT
VNN3
PLA2G16
GGCT
GSTK1
PIPOX
AHCYL2
APIP
ALDH4A1
GGT2
MTHFD2
SLC5A6
DDAH2
GSTT1
SHMT2
DPEP1
OPLAH
PADI3
AHCY
GSTM4
AFMID
PCYOX1
PLA2G2E
CKM
PLA2G10
PTDSS2
CHAC2
DHPS
FTCD
VNN2
GPX1
CPQ
HAGH
GLRX2
SERINC2
A2LD1
ETHE1
PLA2G2F
SLC19A1
PLOD1
PTDSS1
PLA2G4E
MTHFD1
MAT1A
CLIC2
Scrambled 1
Scrambled 2
Scrambled 3
siFLI1 1
siFLI1 2
siFLI1 3

GO_OXIDOREDUCTASE_COMPLEX
NES = −1.49, p = 0.0118, FDR q = 0.2450

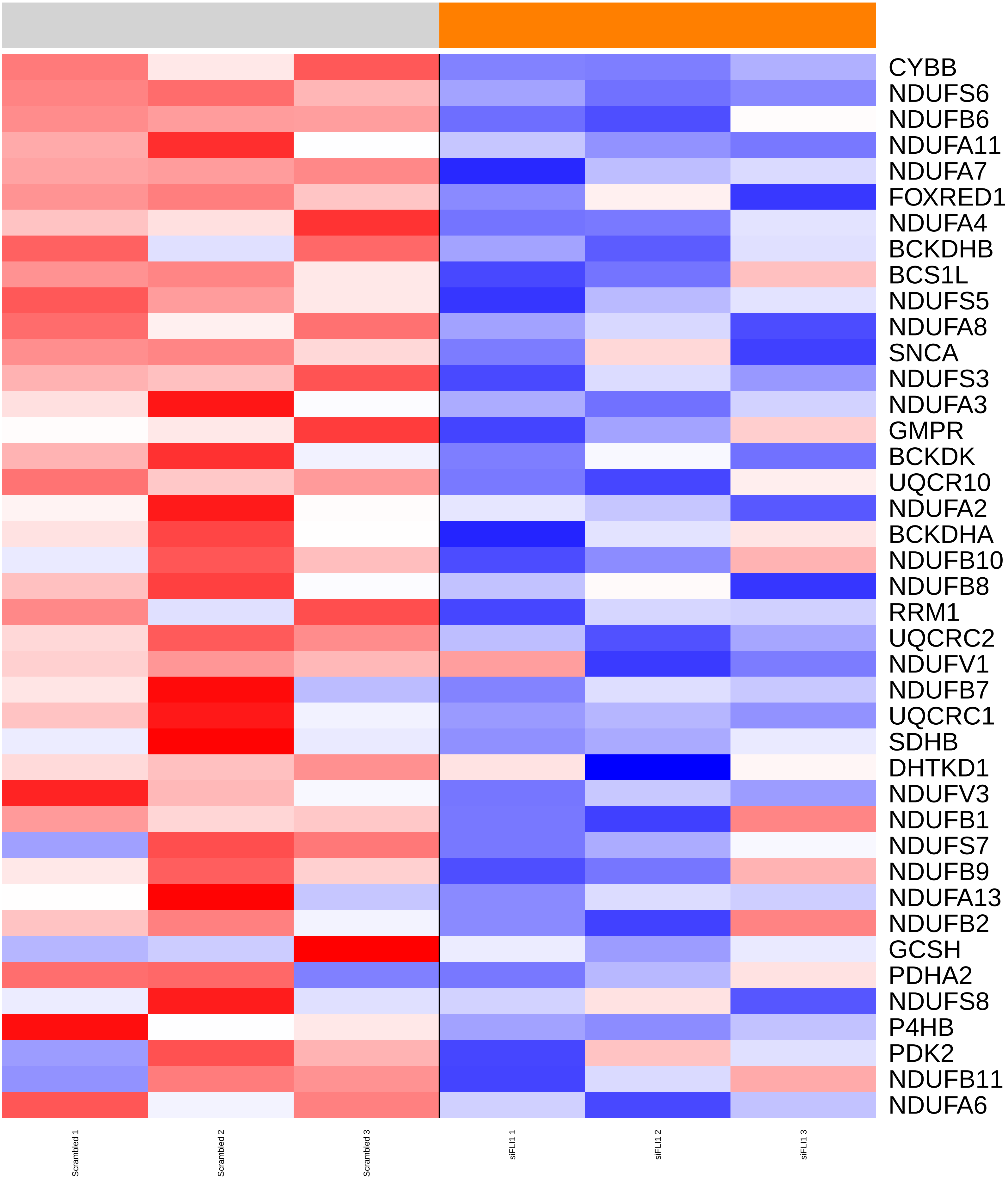

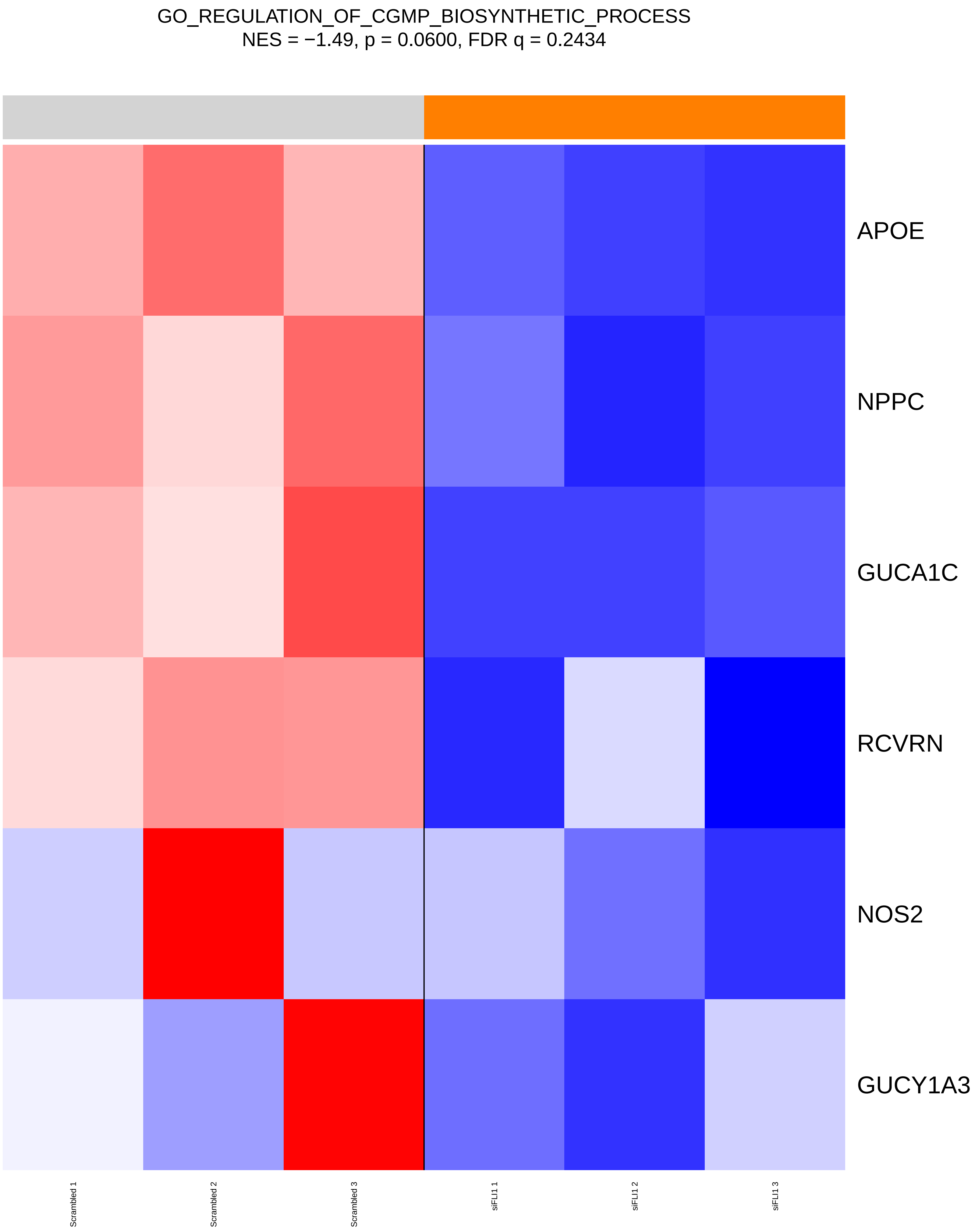
GO_REGULATION_OF_CGMP_BIOSYNTHETIC_PROCESS
NES = −1.49, p = 0.0600, FDR q = 0.2434
APOE
NPPC
GUCA1C
RCVRN
NOS2
GUCY1A3
Scrambled 1
Scrambled 2
Scrambled 3
siFLI1 1
siFLI1 2
siFLI1 3

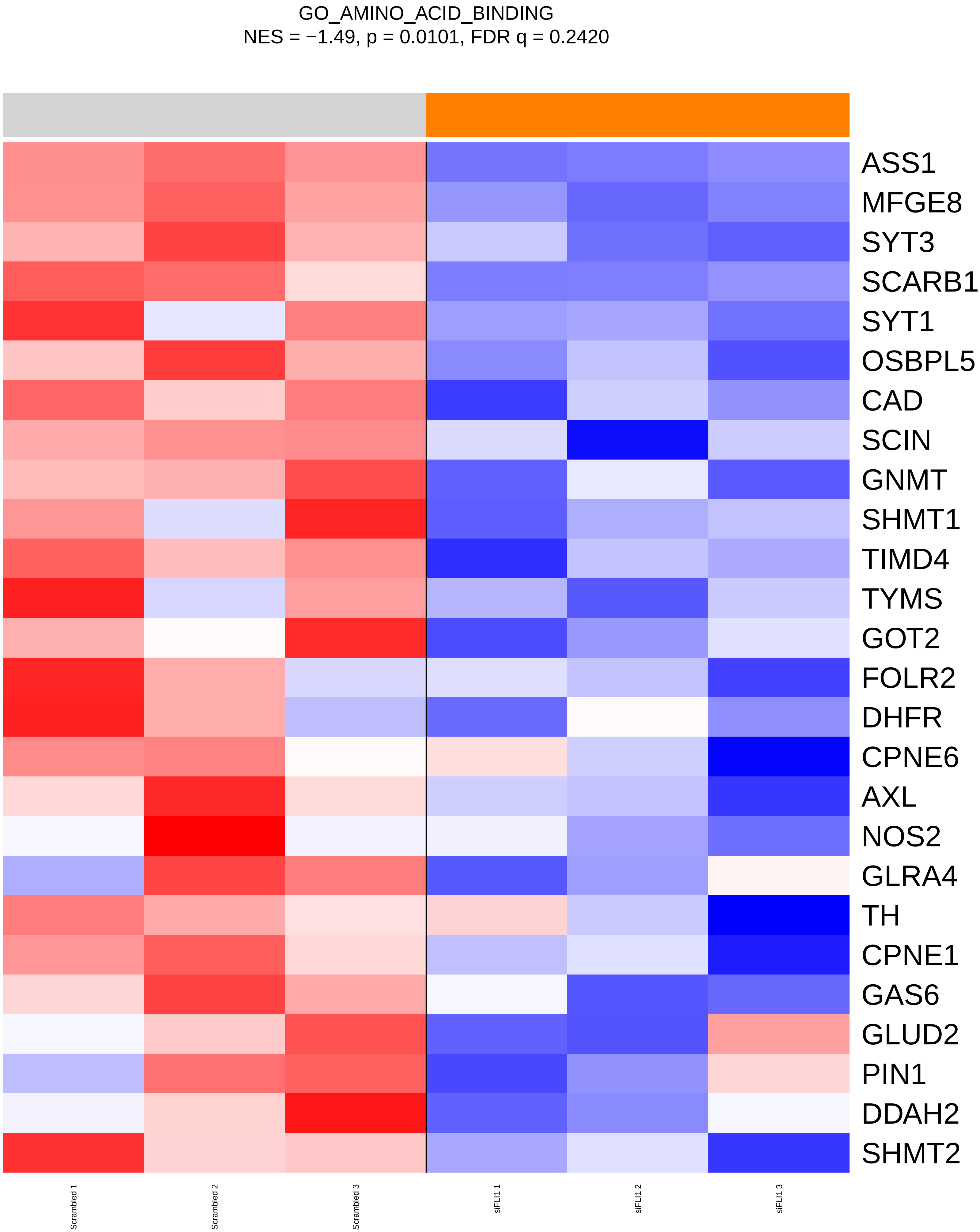
GO_AMINO_ACID_BINDING
NES = −1.49, p = 0.0101, FDR q = 0.2420
ASS1
MFGE8
SYT3
SCARB1
SYT1
OSBPL5
CAD
SCIN
GNMT
SHMT1
TIMD4
TYMS
GOT2
FOLR2
DHFR
CPNE6
AXL
NOS2
GLRA4
TH
CPNE1
GAS6
GLUD2
PIN1
DDAH2
SHMT2
Scrambled 1
Scrambled 2
Scrambled 3
siFLI1 1
siFLI1 2
siFLI1 3

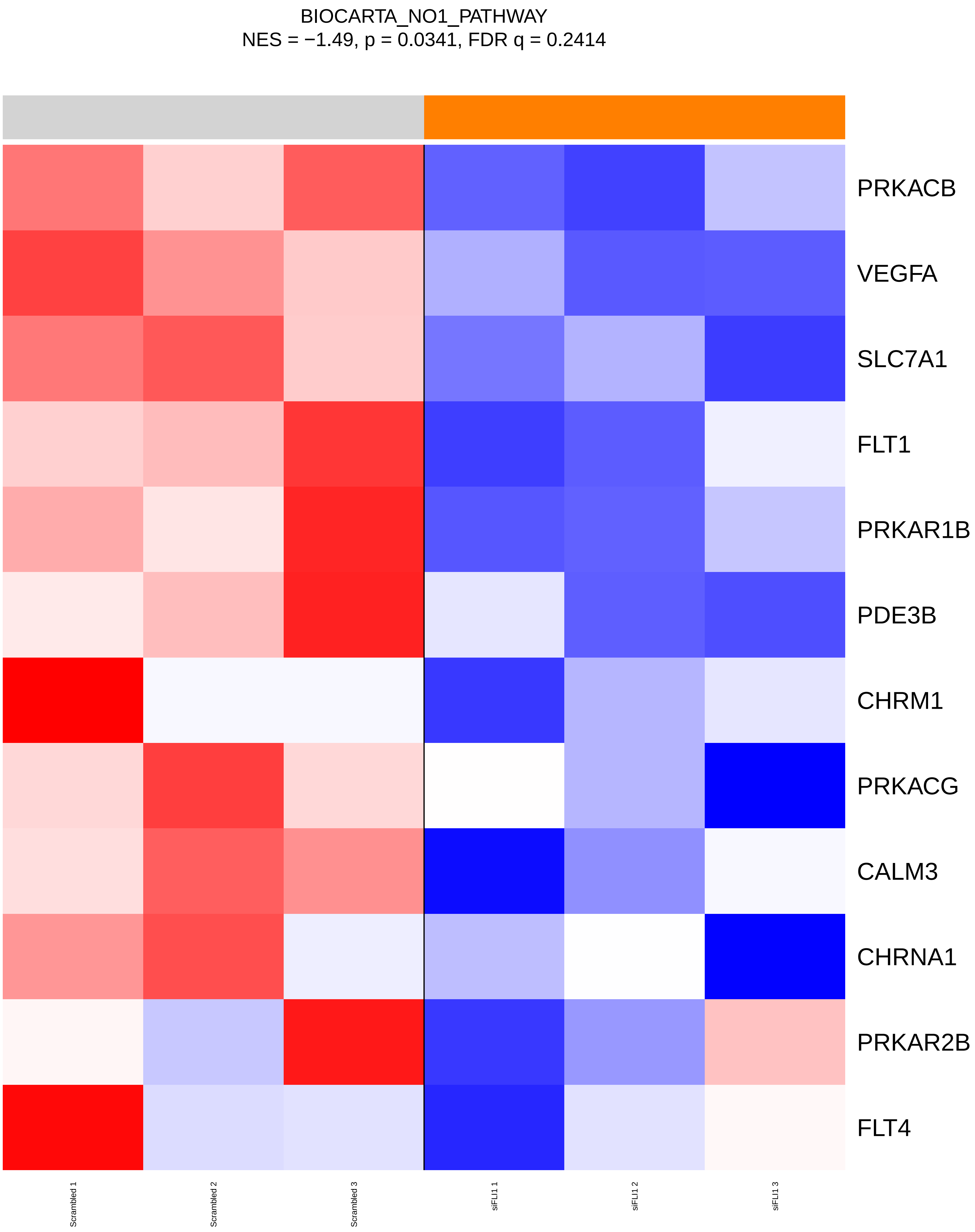

BIOCARTA_NO1_PATHWAY
NES = −1.49, p = 0.0341, FDR q = 0.2414
PRKACB
VEGFA
SLC7A1
FLT1
PRKAR1B
PDE3B
CHRM1
PRKACG
CALM3
CHRNA1
PRKAR2B
FLT4
Scrambled 1
Scrambled 2
Scrambled 3
siFLI1 1
siFLI1 2
siFLI1 3

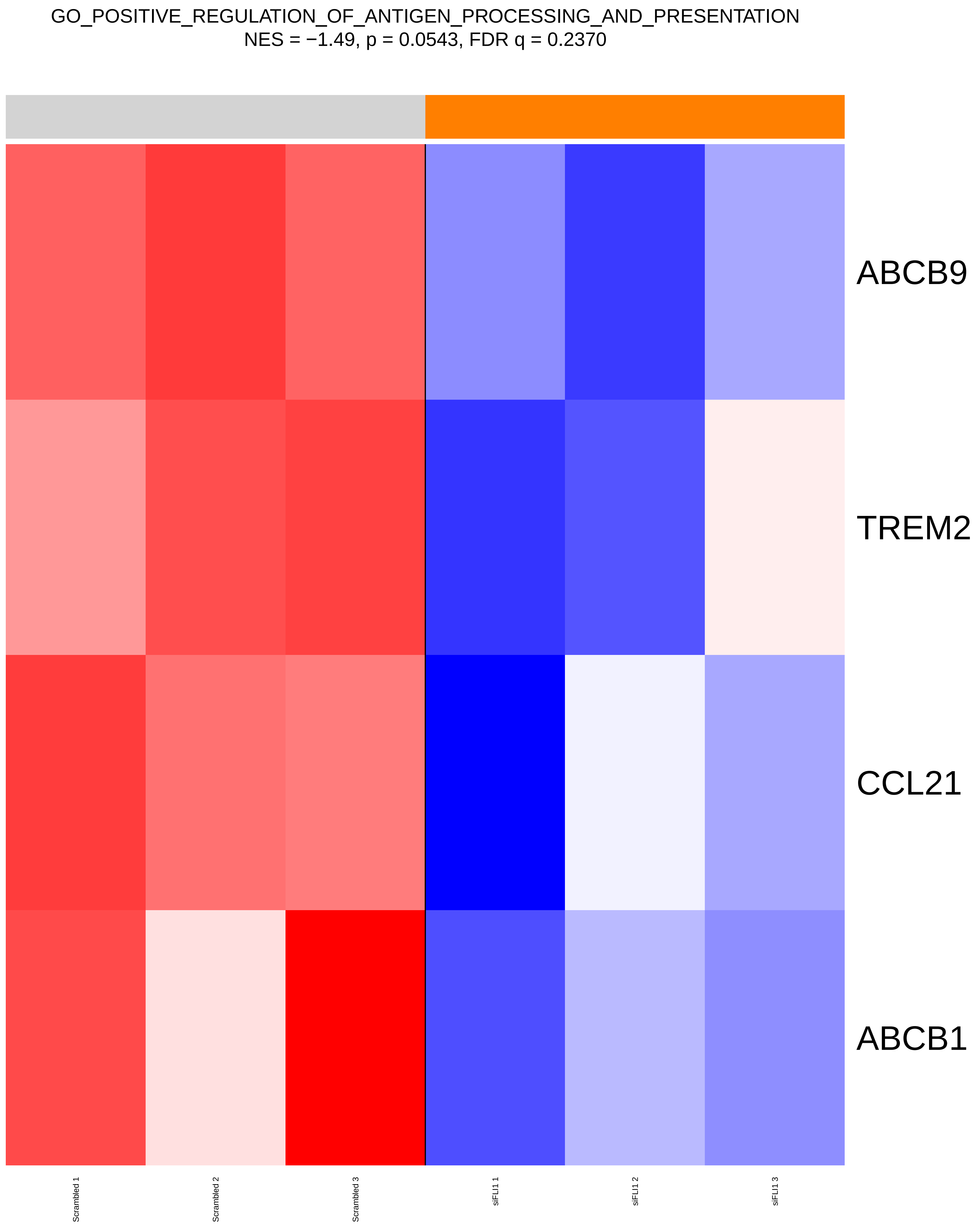
GO_POSITIVE_REGULATION_OF_ANTIGEN_PROCESSING_AND_PRESENTATION
NES = −1.49, p = 0.0543, FDR q = 0.2370
ABCB9
TREM2
CCL21
ABCB1
Scrambled 1
Scrambled 2
Scrambled 3
siFLI1 1
siFLI1 2
siFLI1 3

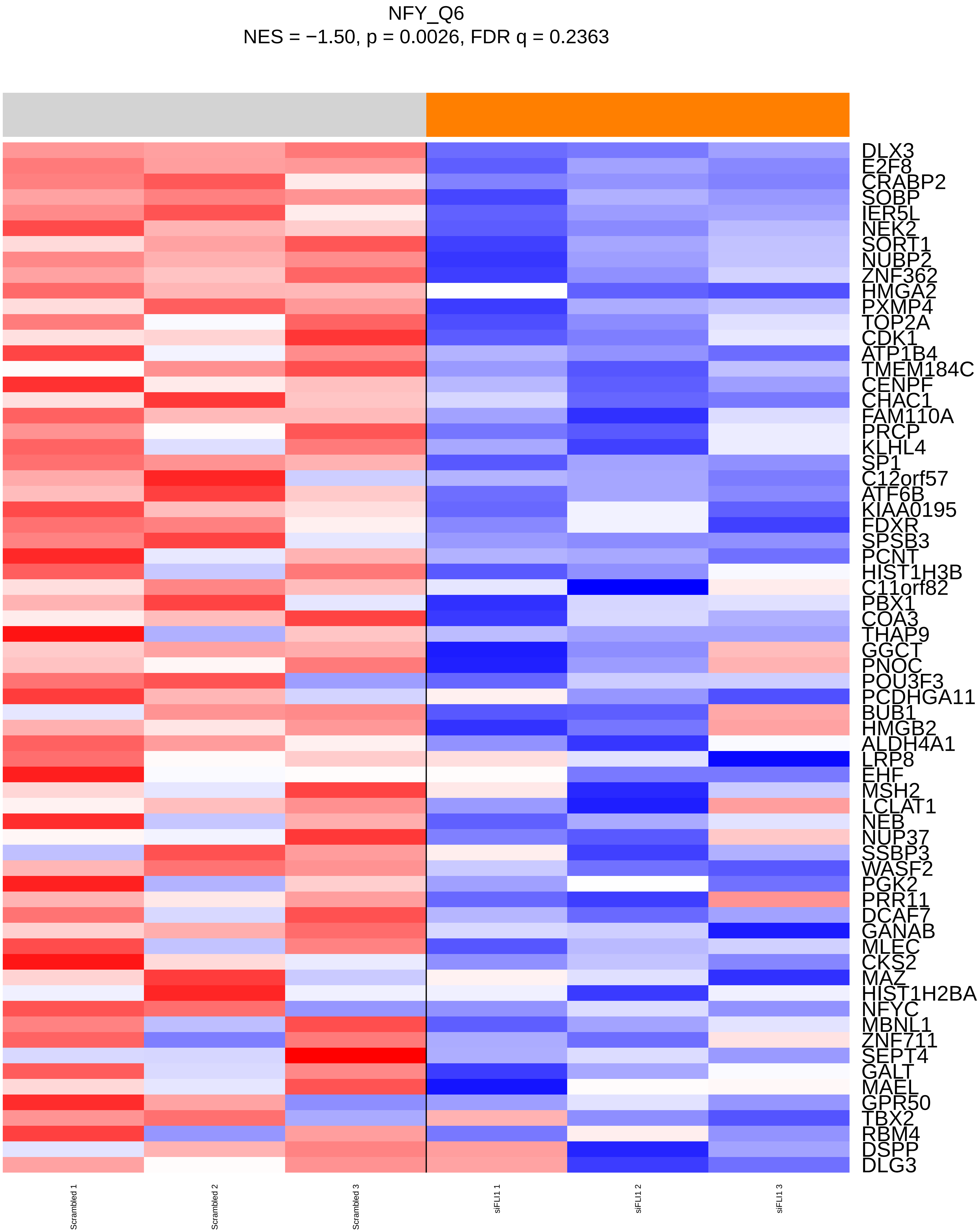
NFY_Q6
NES = −1.50, p = 0.0026, FDR q = 0.2363
DLX3
E2F8
CRABP2
SOBP
IER5L
NEK2
SORT1
NUBP2
ZNF362
HMGA2
PXMP4
TOP2A
CDK1
ATP1B4
TMEM184C
CENPF
CHAC1
FAM110A
PRCP
KLHL4
SP1
C12orf57
ATF6B
KIAA0195
FDXR
SPSB3
PCNT
HIST1H3B
C11orf82
PBX1
COA3
THAP9
GGCT
PNOC
POU3F3
PCDHGA11
BUB1
HMGB2
ALDH4A1
LRP8
EHF
MSH2
LCLAT1
NEB
NUP37
SSBP3
WASF2
PGK2
PRR11
DCAF7
GANAB
MLEC
CKS2
MAZ
HIST1H2BA
NFYC
MBNL1
ZNF711
SEPT4
GALT
MAEL
GPR50
TBX2
RBM4
DSPP
DLG3
Scrambled 1
Scrambled 2
Scrambled 3
siFLI1 1
siFLI1 2
siFLI1 3

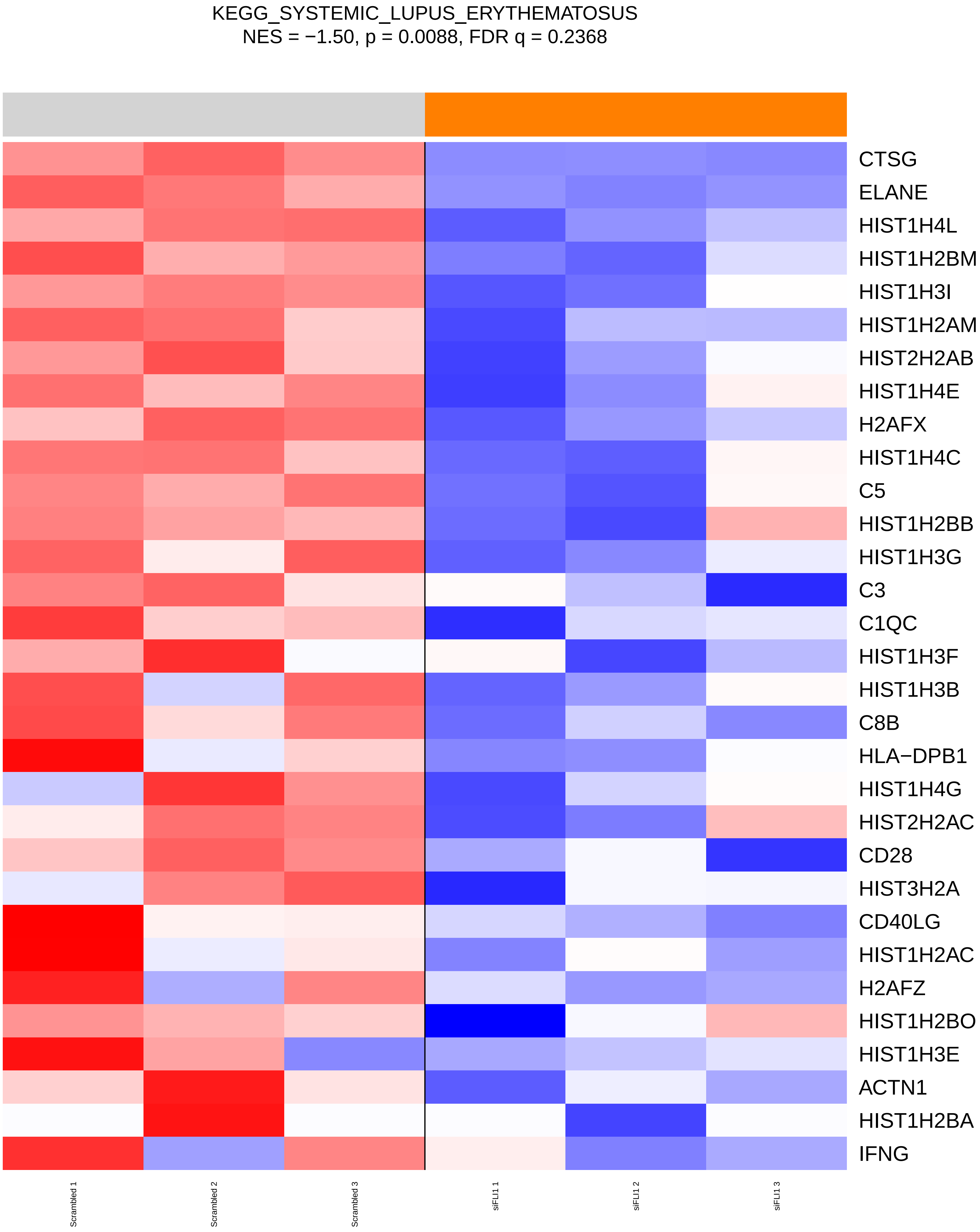
KEGG_SYSTEMIC_LUPUS_ERYTHEMATOSUS
NES = −1.50, p = 0.0088, FDR q = 0.2368
CTSG
ELANE
HIST1H4L
HIST1H2BM
HIST1H3I
HIST1H2AM
HIST2H2AB
HIST1H4E
H2AFX
HIST1H4C
C5
HIST1H2BB
HIST1H3G
C3
C1QC
HIST1H3F
HIST1H3B
C8B
HLA−DPB1
HIST1H4G
HIST2H2AC
CD28
HIST3H2A
CD40LG
HIST1H2AC
H2AFZ
HIST1H2BO
HIST1H3E
ACTN1
HIST1H2BA
IFNG
Scrambled 1
Scrambled 2
Scrambled 3
siFLI1 1
siFLI1 2
siFLI1 3

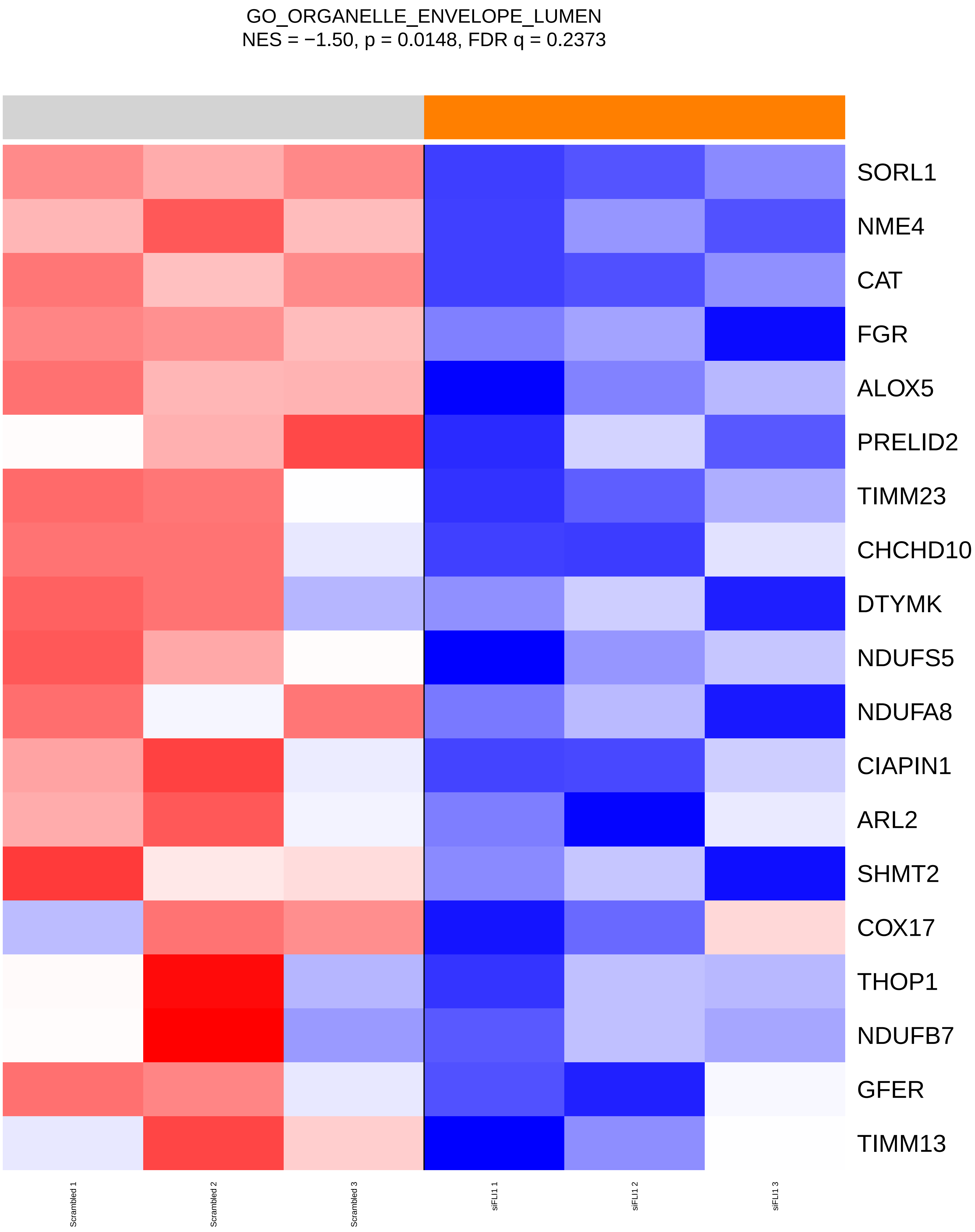
GO_ORGANELLE_ENVELOPE_LUMEN
NES = −1.50, p = 0.0148, FDR q = 0.2373
SORL1
NME4
CAT
FGR
ALOX5
PRELID2
TIMM23
CHCHD10
DTYMK
NDUFS5
NDUFA8
CIAPIN1
ARL2
SHMT2
COX17
THOP1
NDUFB7
GFER
TIMM13
Scrambled 1
Scrambled 2
Scrambled 3
siFLI1 1
siFLI1 2
siFLI1 3

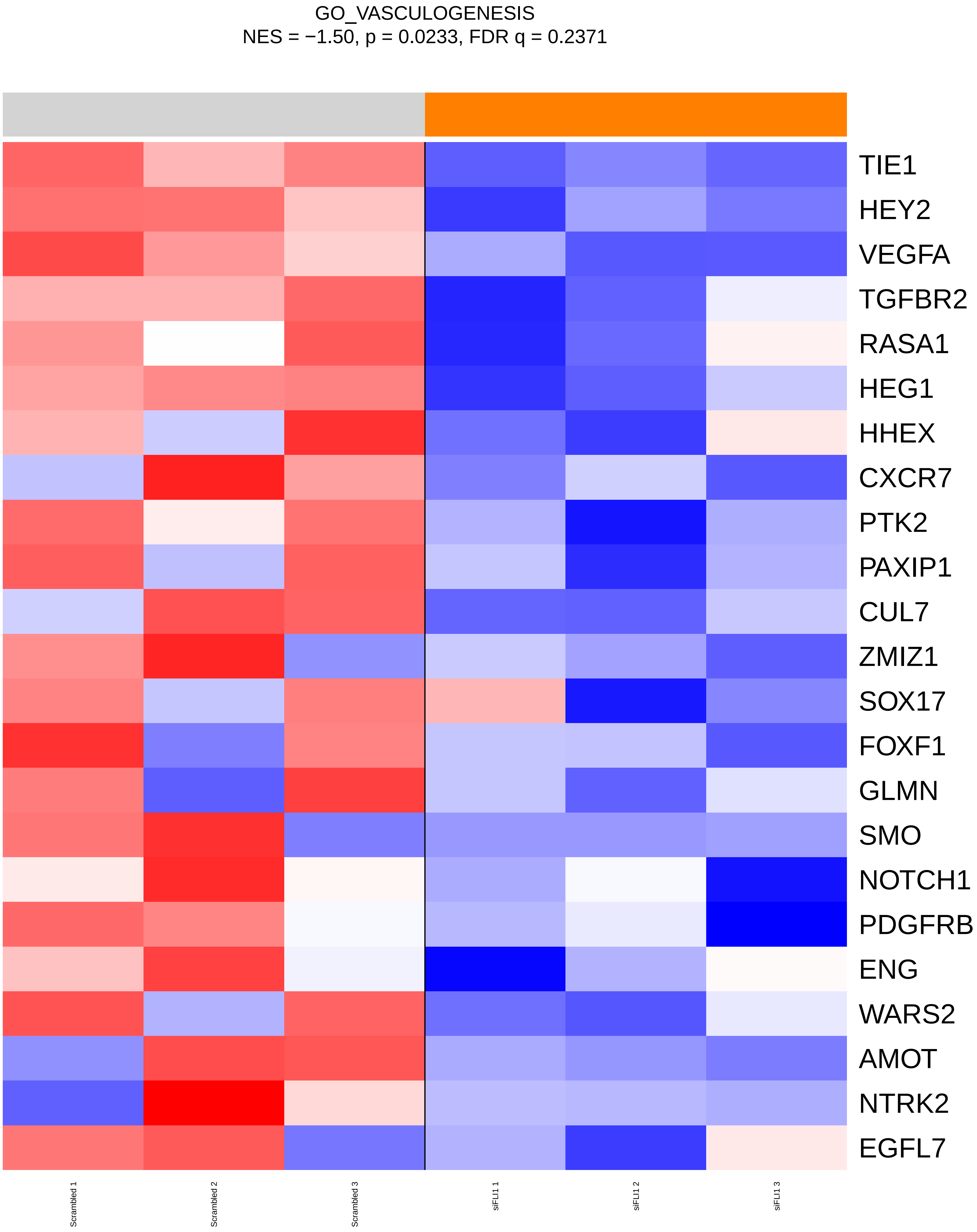
GO_VASCULOGENESIS
NES = −1.50, p = 0.0233, FDR q = 0.2371
TIE1
HEY2
VEGFA
TGFBR2
RASA1
HEG1
HHEX
CXCR7
PTK2
PAXIP1
CUL7
ZMIZ1
SOX17
FOXF1
GLMN
SMO
NOTCH1
PDGFRB
ENG
WARS2
AMOT
NTRK2
EGFL7
Scrambled 1
Scrambled 2
Scrambled 3
siFLI1 1
siFLI1 2
siFLI1 3

REACTOME_REGULATION_OF_WATER_BALANCE_BY_RENAL_AQUAPORINS
NES = −1.50, p = 0.0352, FDR q = 0.2363

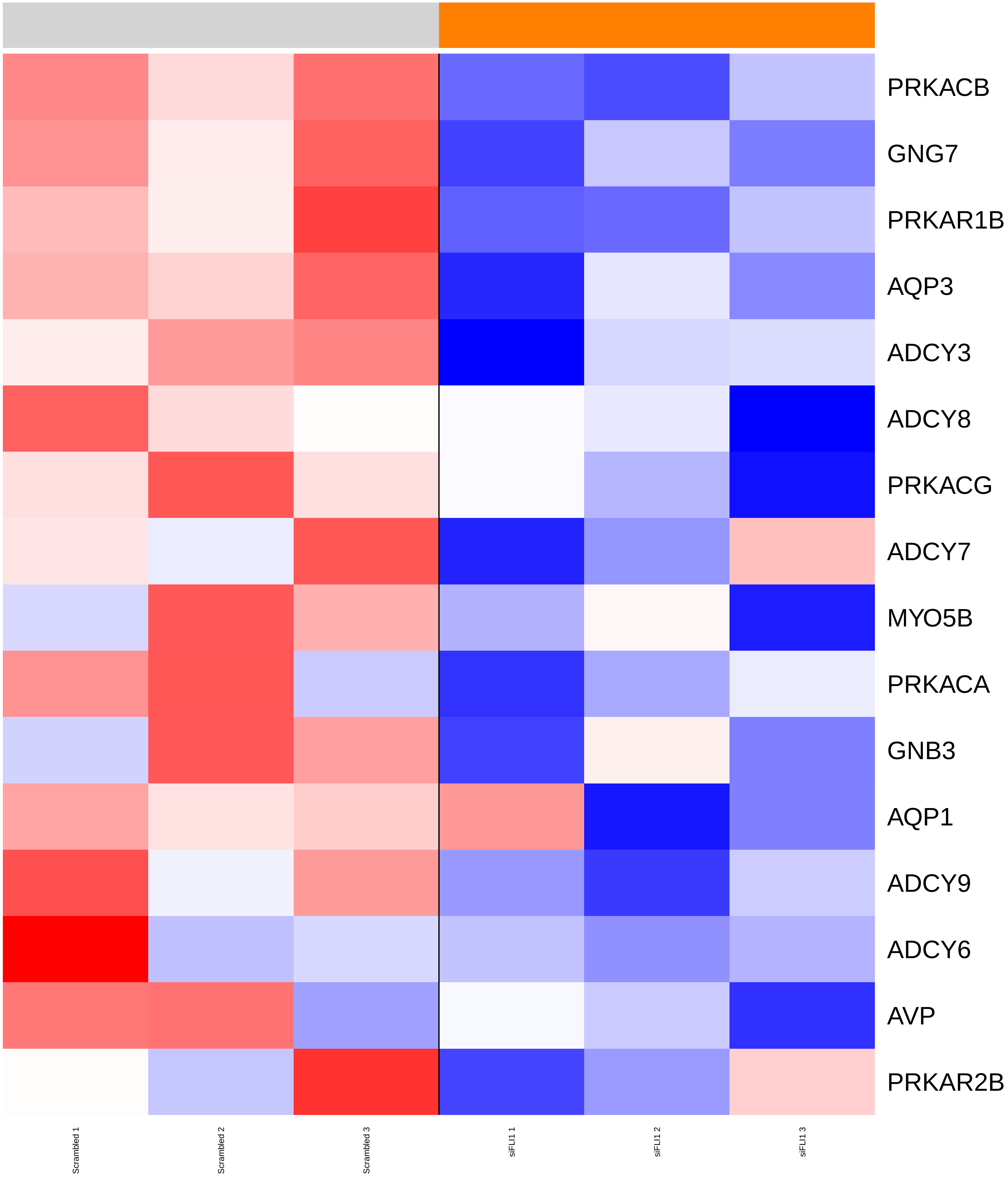

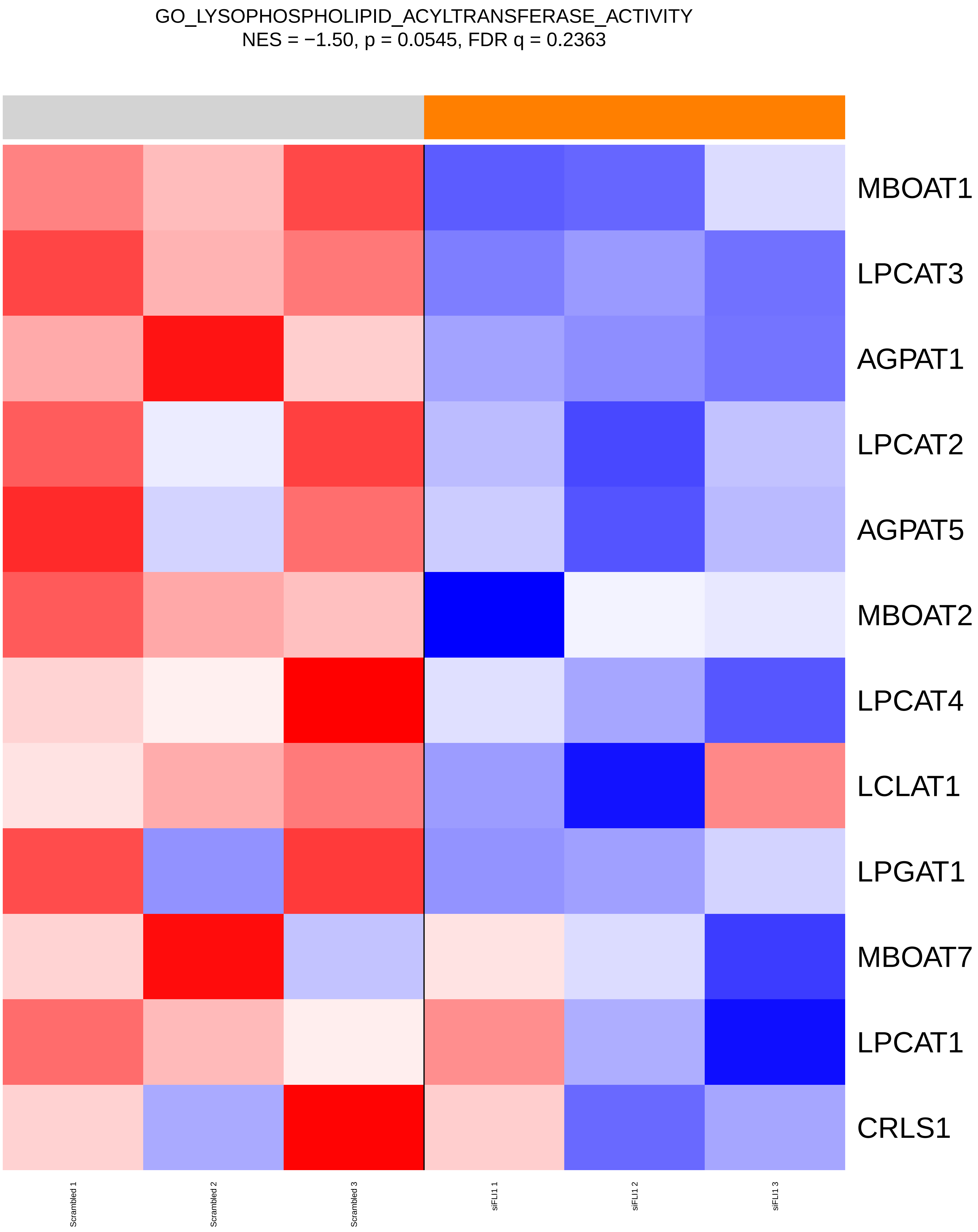
GO_LYSOPHOSPHOLIPID_ACYLTRANSFERASE_ACTIVITY
NES = −1.50, p = 0.0545, FDR q = 0.2363
MBOAT1
LPCAT3
AGPAT1
LPCAT2
AGPAT5
MBOAT2
LPCAT4
LCLAT1
LPGAT1
MBOAT7
LPCAT1
CRLS1
Scrambled 1
Scrambled 2
Scrambled 3
siFLI1 1
siFLI1 2
siFLI1 3

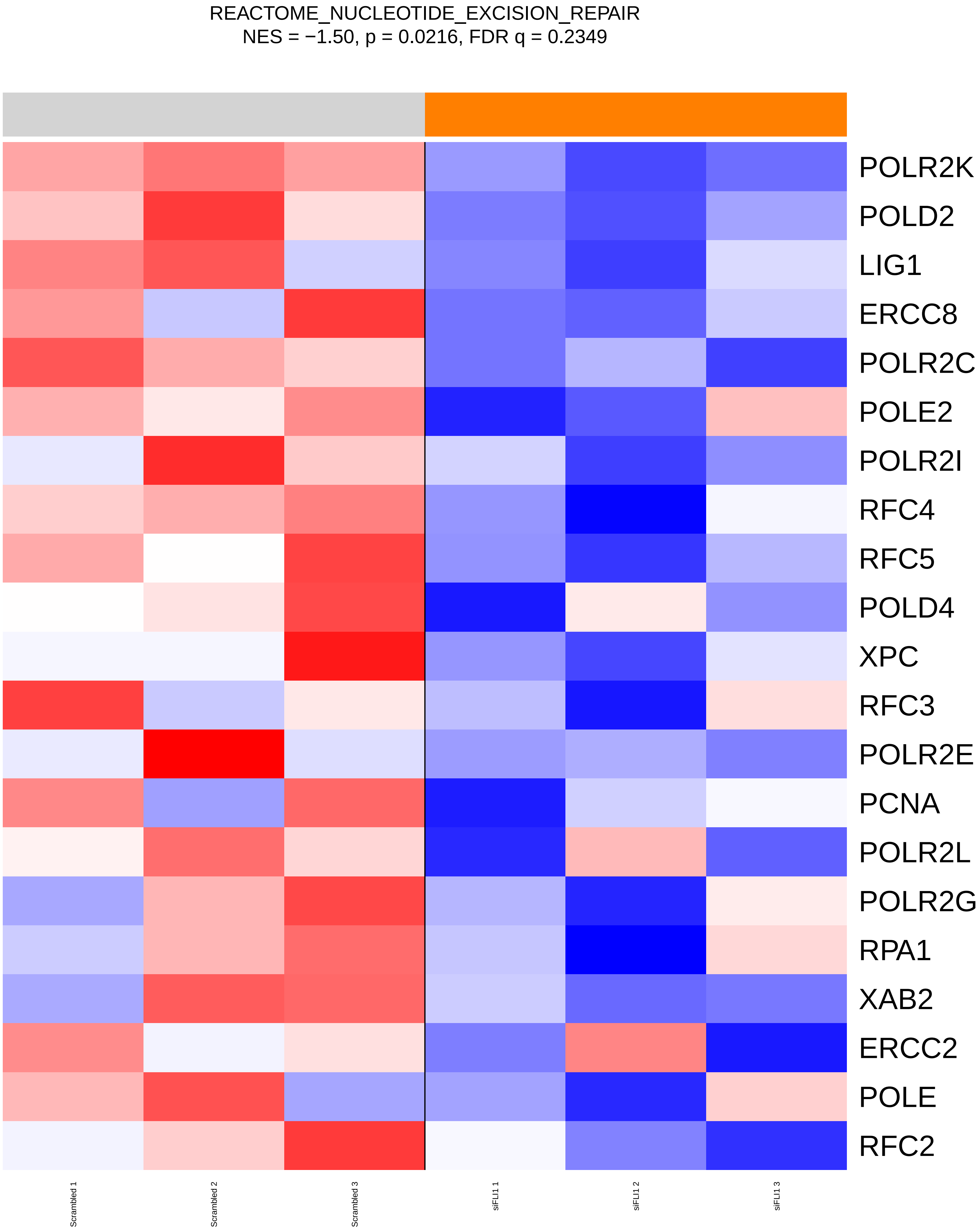
REACTOME_NUCLEOTIDE_EXCISION_REPAIR
NES = −1.50, p = 0.0216, FDR q = 0.2349
POLR2K
POLD2
LIG1
ERCC8
POLR2C
POLE2
POLR2I
RFC4
RFC5
POLD4
XPC
RFC3
POLR2E
PCNA
POLR2L
POLR2G
RPA1
XAB2
ERCC2
POLE
RFC2
Scrambled 1
Scrambled 2
Scrambled 3
siFLI1 1
siFLI1 2
siFLI1 3

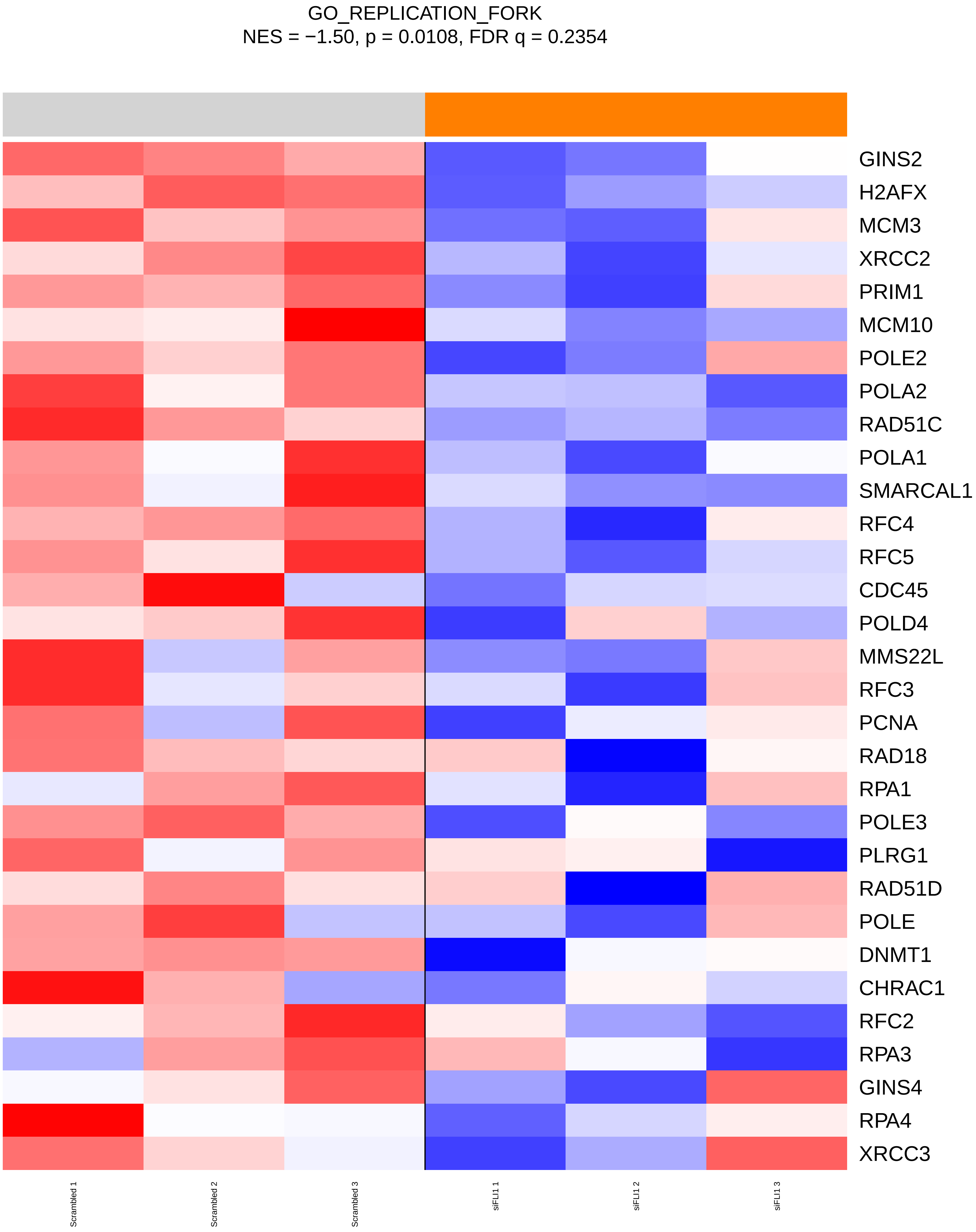
GO_REPLICATION_FORK
NES = −1.50, p = 0.0108, FDR q = 0.2354
GINS2
H2AFX
MCM3
XRCC2
PRIM1
MCM10
POLE2
POLA2
RAD51C
POLA1
SMARCAL1
RFC4
RFC5
CDC45
POLD4
MMS22L
RFC3
PCNA
RAD18
RPA1
POLE3
PLRG1
RAD51D
POLE
DNMT1
CHRAC1
RFC2
RPA3
GINS4
RPA4
XRCC3
Scrambled 1
Scrambled 2
Scrambled 3
siFLI1 1
siFLI1 2
siFLI1 3

GO_WATER_TRANSPORT
NES = −1.50, p = 0.0482, FDR q = 0.2351

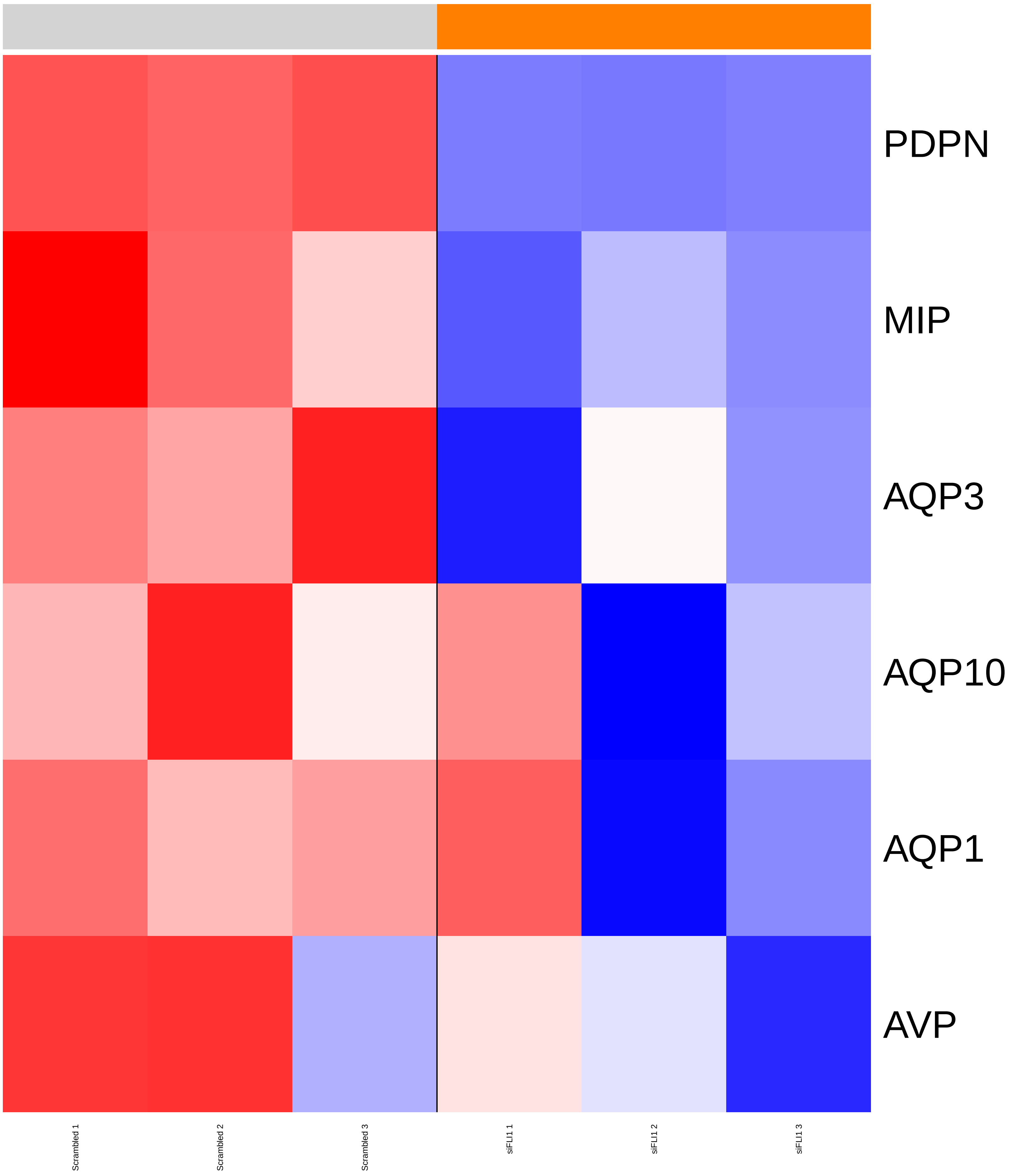

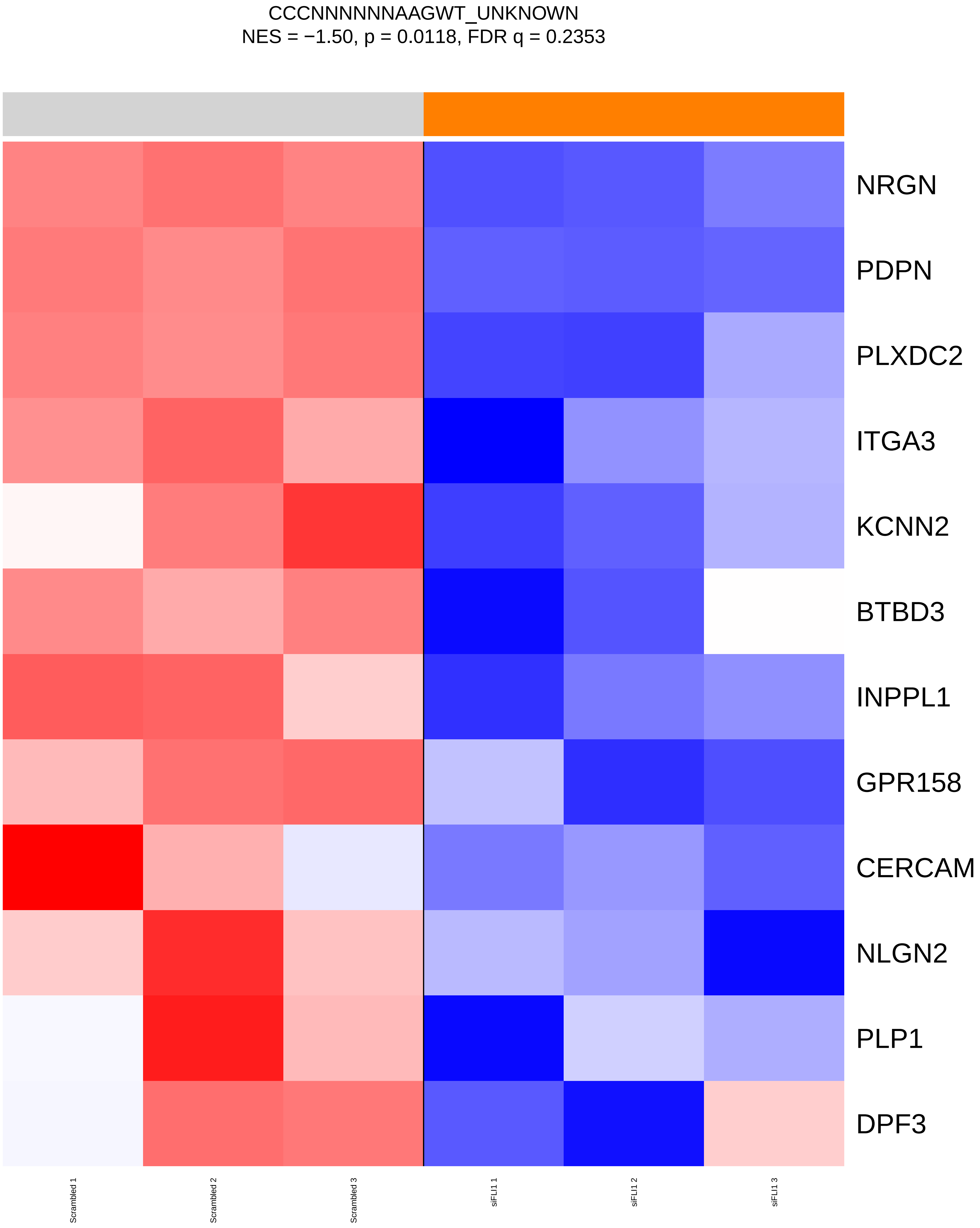
CCCNNNNNNAAGWT_UNKNOWN
NES = −1.50, p = 0.0118, FDR q = 0.2353
NRGN
PDPN
PLXDC2
ITGA3
KCNN2
BTBD3
INPPL1
GPR158
CERCAM
NLGN2
PLP1
DPF3
Scrambled 1
Scrambled 2
Scrambled 3
siFLI1 1
siFLI1 2
siFLI1 3

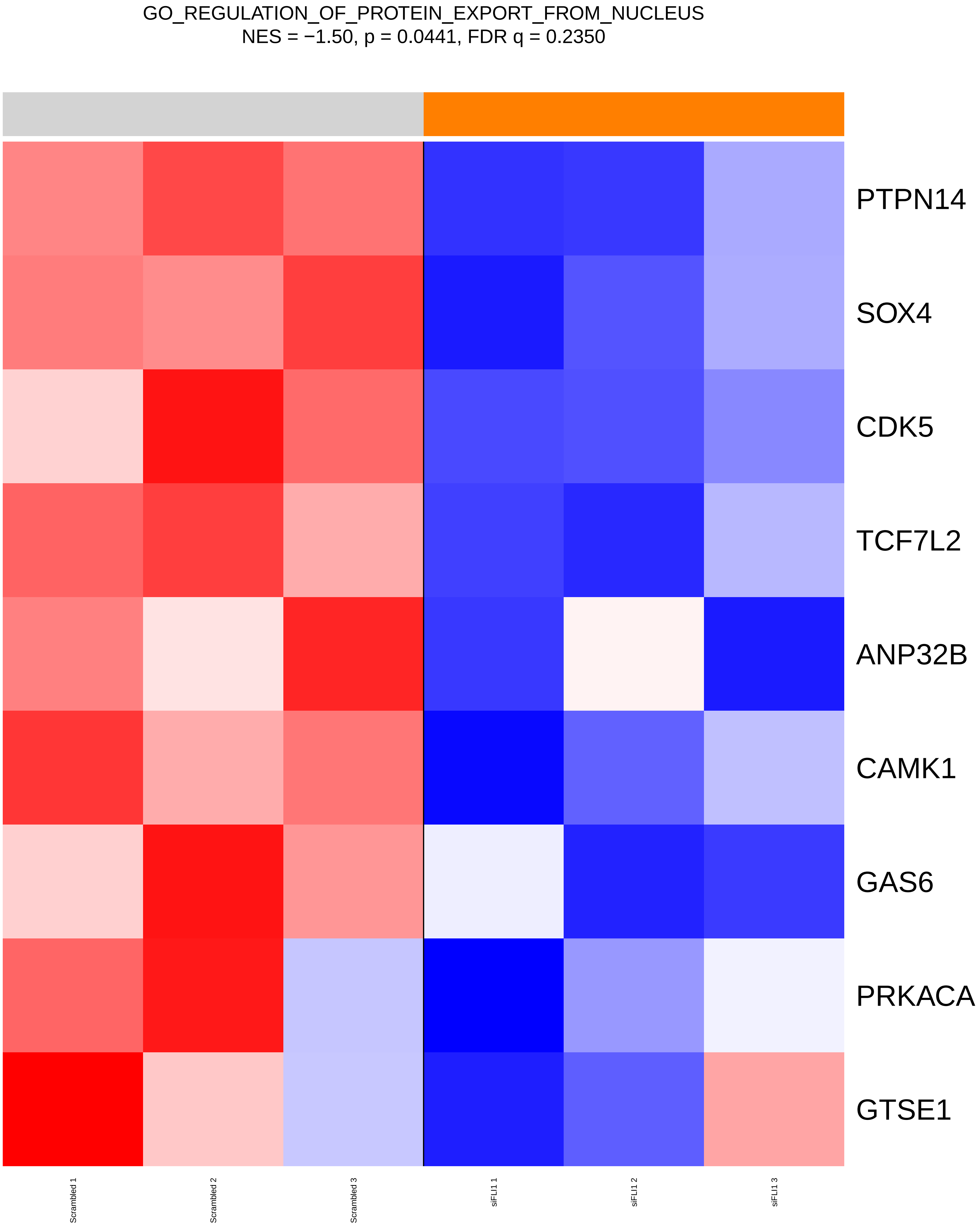

GO_REGULATION_OF_PROTEIN_EXPORT_FROM_NUCLEUS
NES = −1.50, p = 0.0441, FDR q = 0.2350
PTPN14
SOX4
CDK5
TCF7L2
ANP32B
CAMK1
GAS6
PRKACA
GTSE1
Scrambled 1
Scrambled 2
Scrambled 3
siFLI1 1
siFLI1 2
siFLI1 3

GO_STEROID_METABOLIC_PROCESS
NES = −1.50, p = 0.0013, FDR q = 0.2355

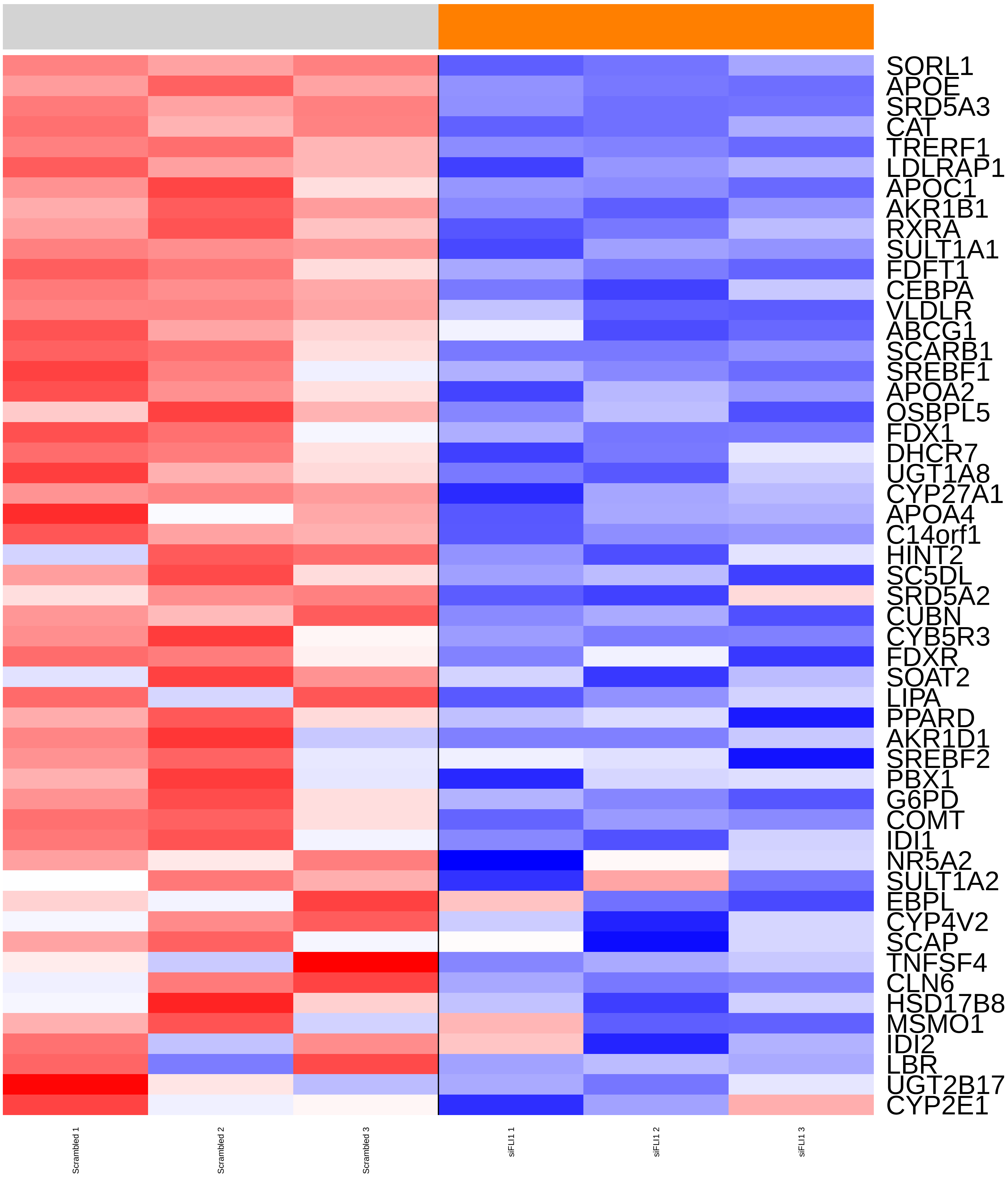

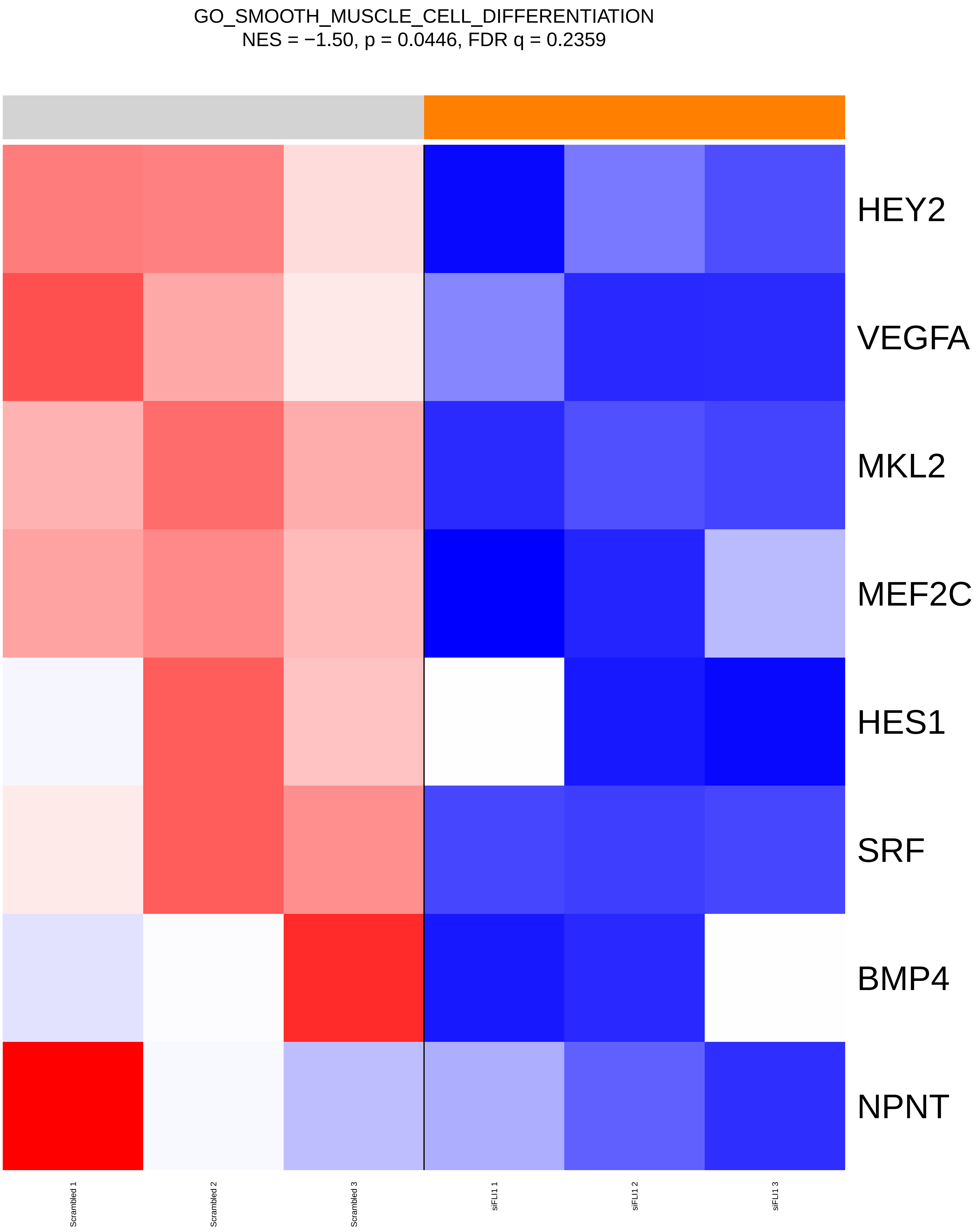
GO_SMOOTH_MUSCLE_CELL_DIFFERENTIATION
NES = −1.50, p = 0.0446, FDR q = 0.2359
HEY2
VEGFA
MKL2
MEF2C
HES1
SRF
BMP4
NPNT
Scrambled 1
Scrambled 2
Scrambled 3
siFLI1 1
siFLI1 2
siFLI1 3

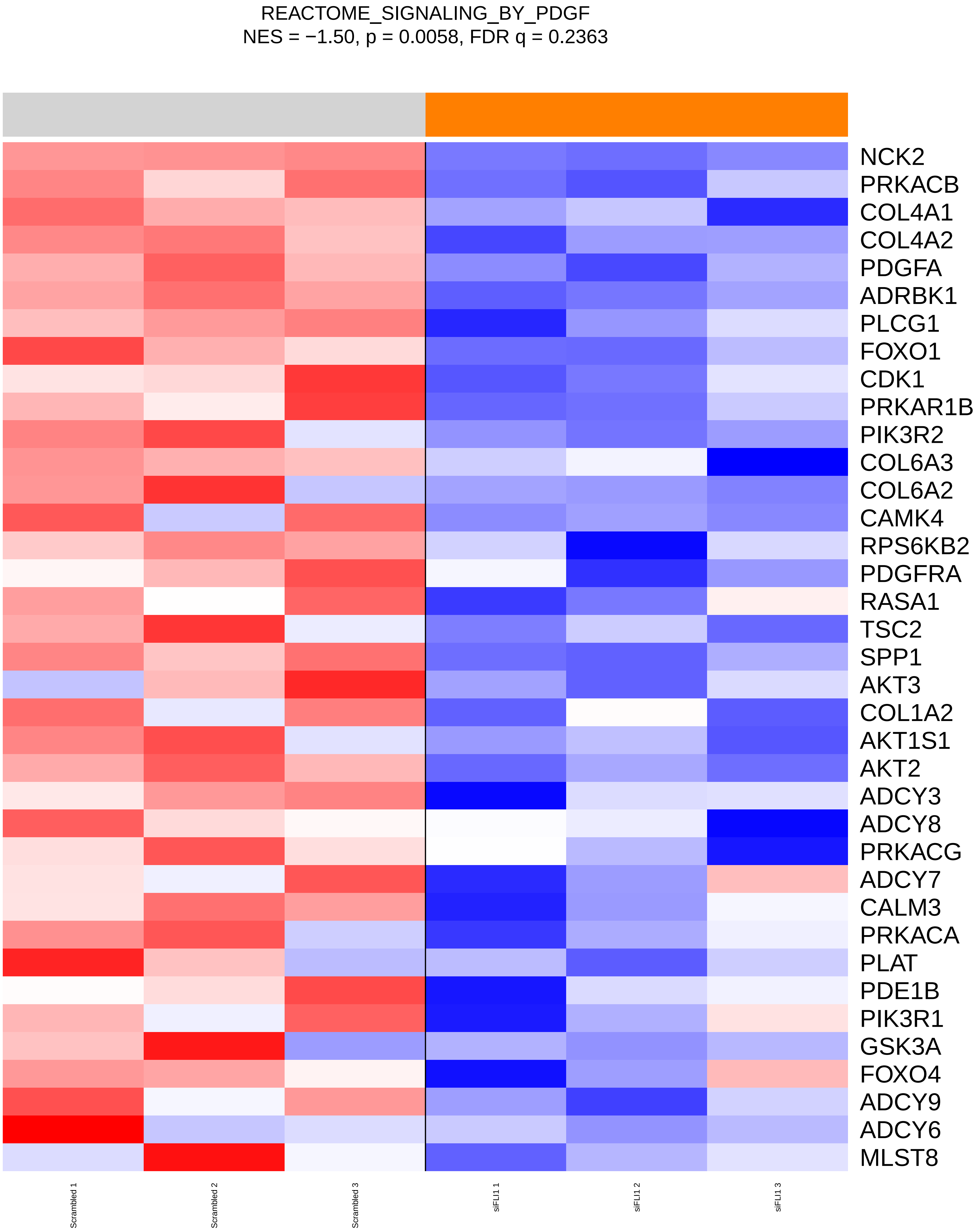
REACTOME_SIGNALING_BY_PDGF
NES = −1.50, p = 0.0058, FDR q = 0.2363
NCK2
PRKACB
COL4A1
COL4A2
PDGFA
ADRBK1
PLCG1
FOXO1
CDK1
PRKAR1B
PIK3R2
COL6A3
COL6A2
CAMK4
RPS6KB2
PDGFRA
RASA1
TSC2
SPP1
AKT3
COL1A2
AKT1S1
AKT2
ADCY3
ADCY8
PRKACG
ADCY7
CALM3
PRKACA
PLAT
PDE1B
PIK3R1
GSK3A
FOXO4
ADCY9
ADCY6
MLST8
Scrambled 1
Scrambled 2
Scrambled 3
siFLI1 1
siFLI1 2
siFLI1 3

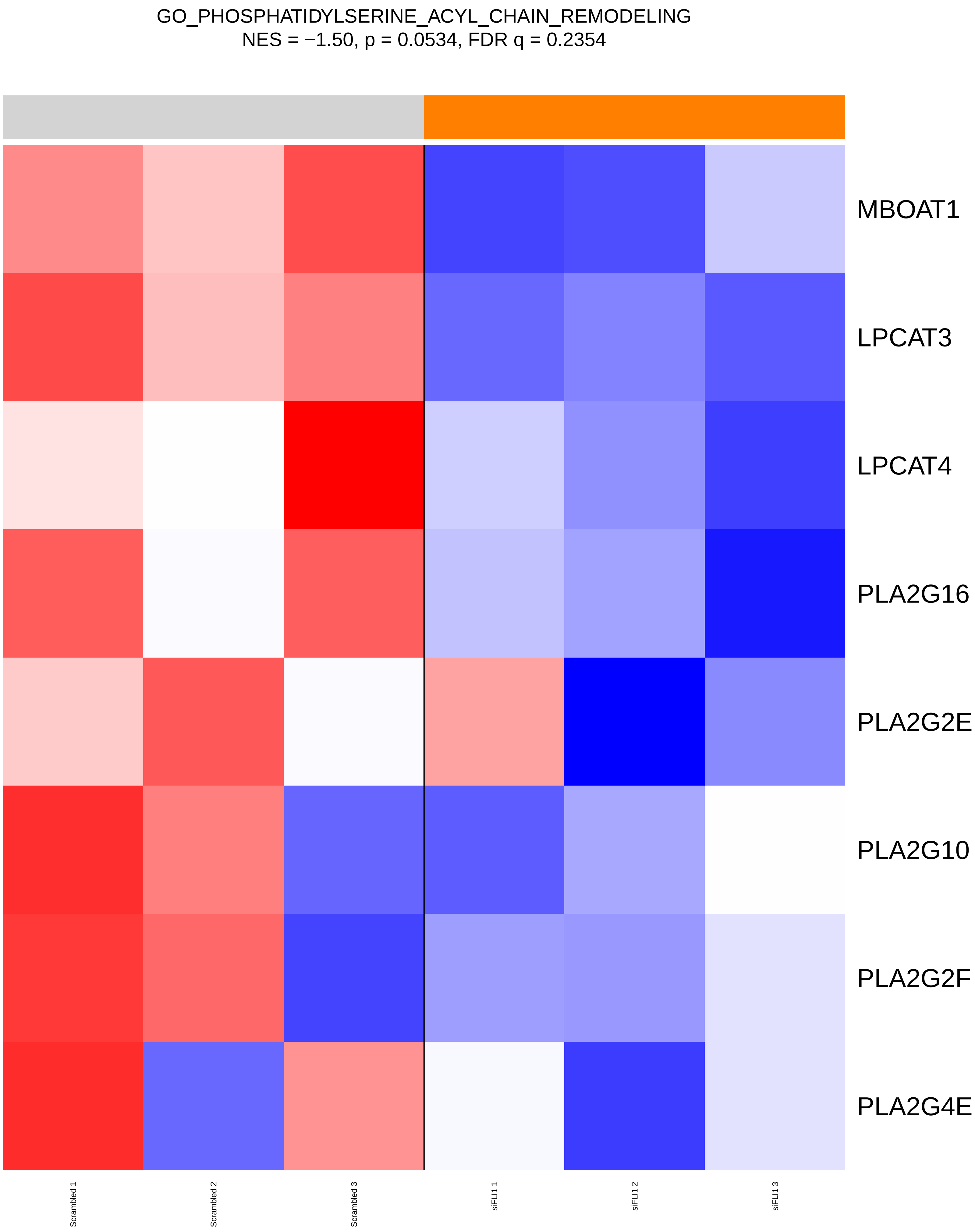
GO_PHOSPHATIDYLSERINE_ACYL_CHAIN_REMODELING
NES = −1.50, p = 0.0534, FDR q = 0.2354
MBOAT1
LPCAT3
LPCAT4
PLA2G16
PLA2G2E
PLA2G10
PLA2G2F
PLA2G4E
Scrambled 1
Scrambled 2
Scrambled 3
siFLI1 1
siFLI1 2
siFLI1 3

ALPHACP1_01
NES = −1.50, p = 0.0026, FDR q = 0.2359

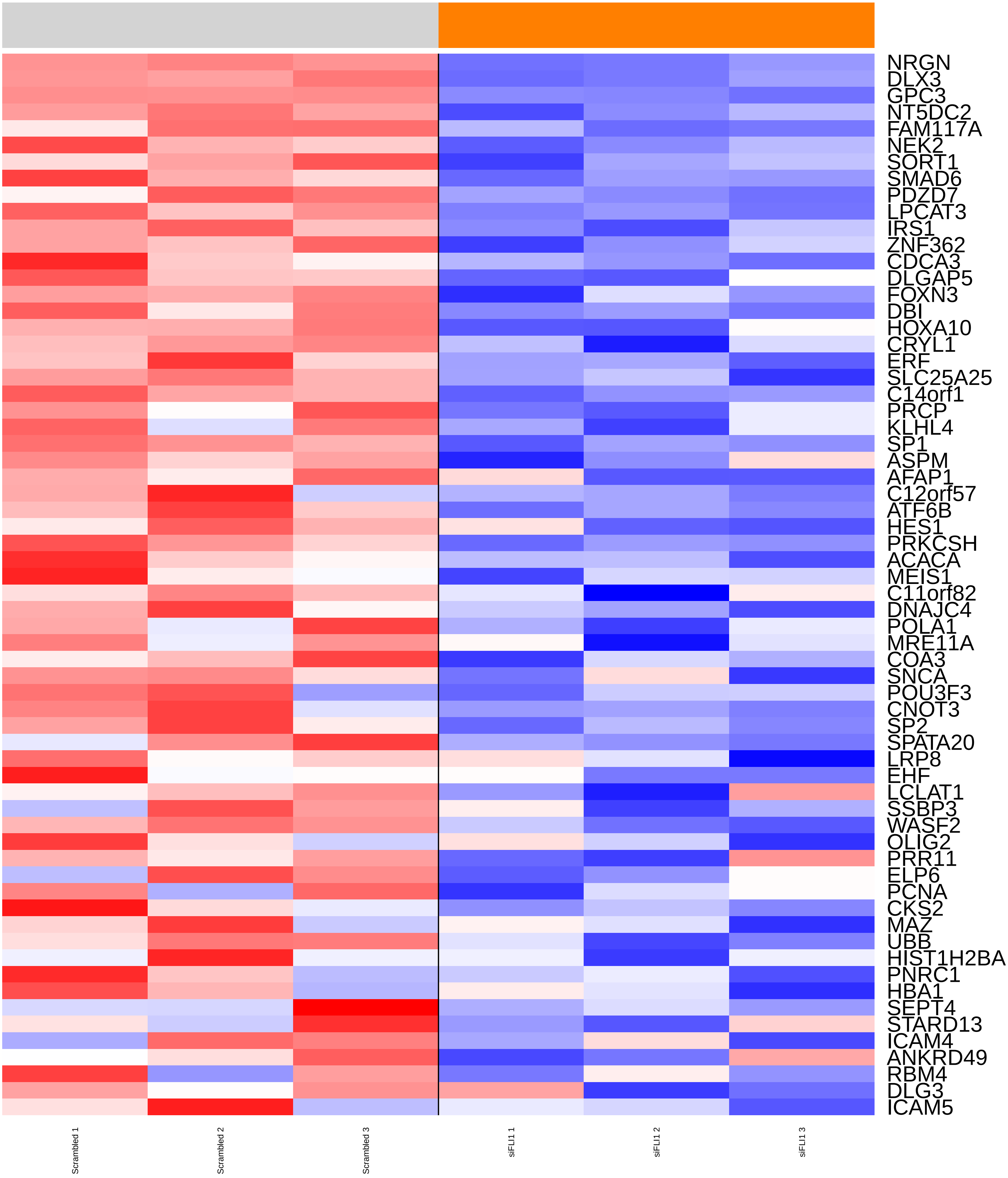

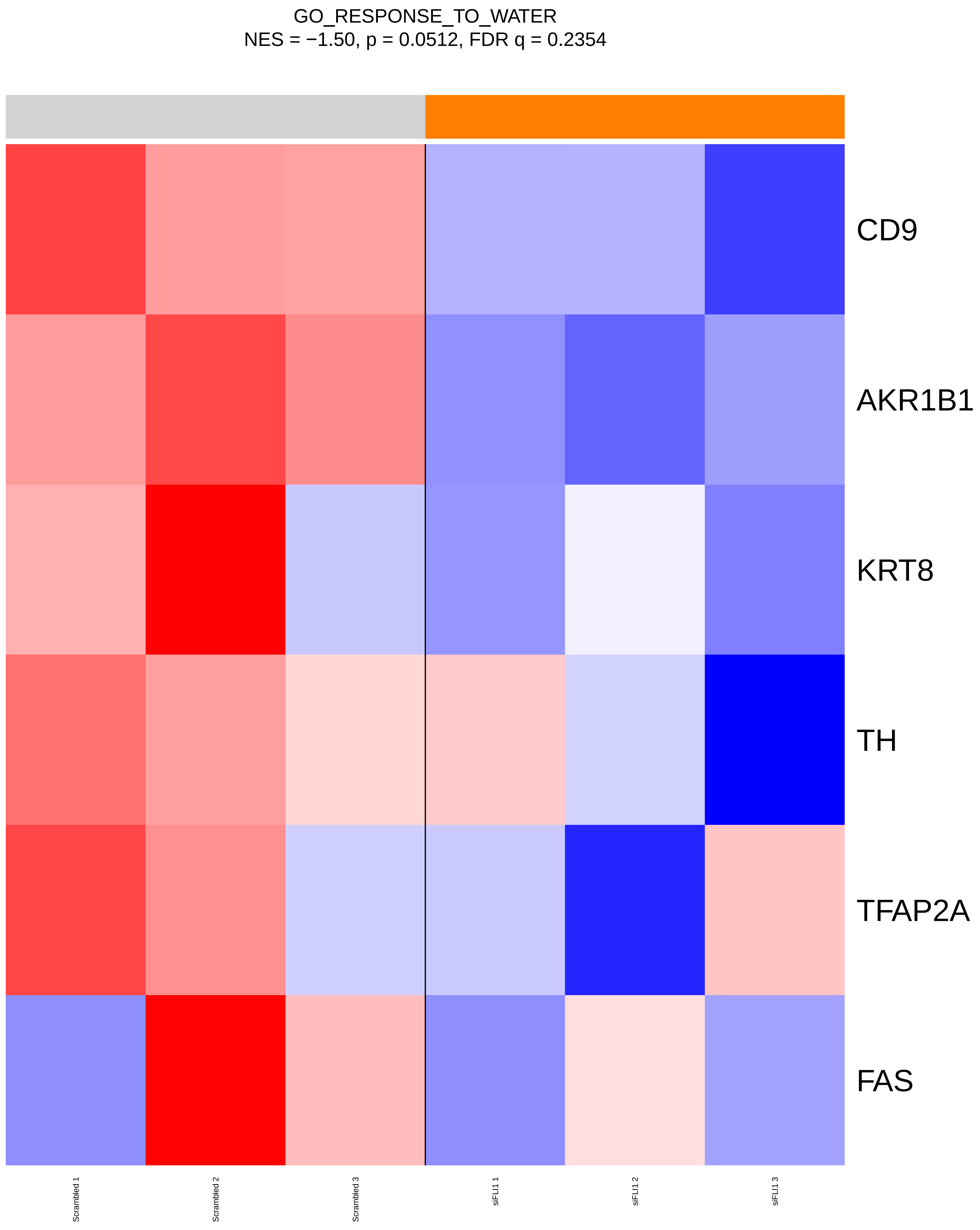
GO_RESPONSE_TO_WATER
NES = −1.50, p = 0.0512, FDR q = 0.2354
CD9
AKR1B1
KRT8
TH
TFAP2A
FAS
Scrambled 1
Scrambled 2
Scrambled 3
siFLI1 1
siFLI1 2
siFLI1 3

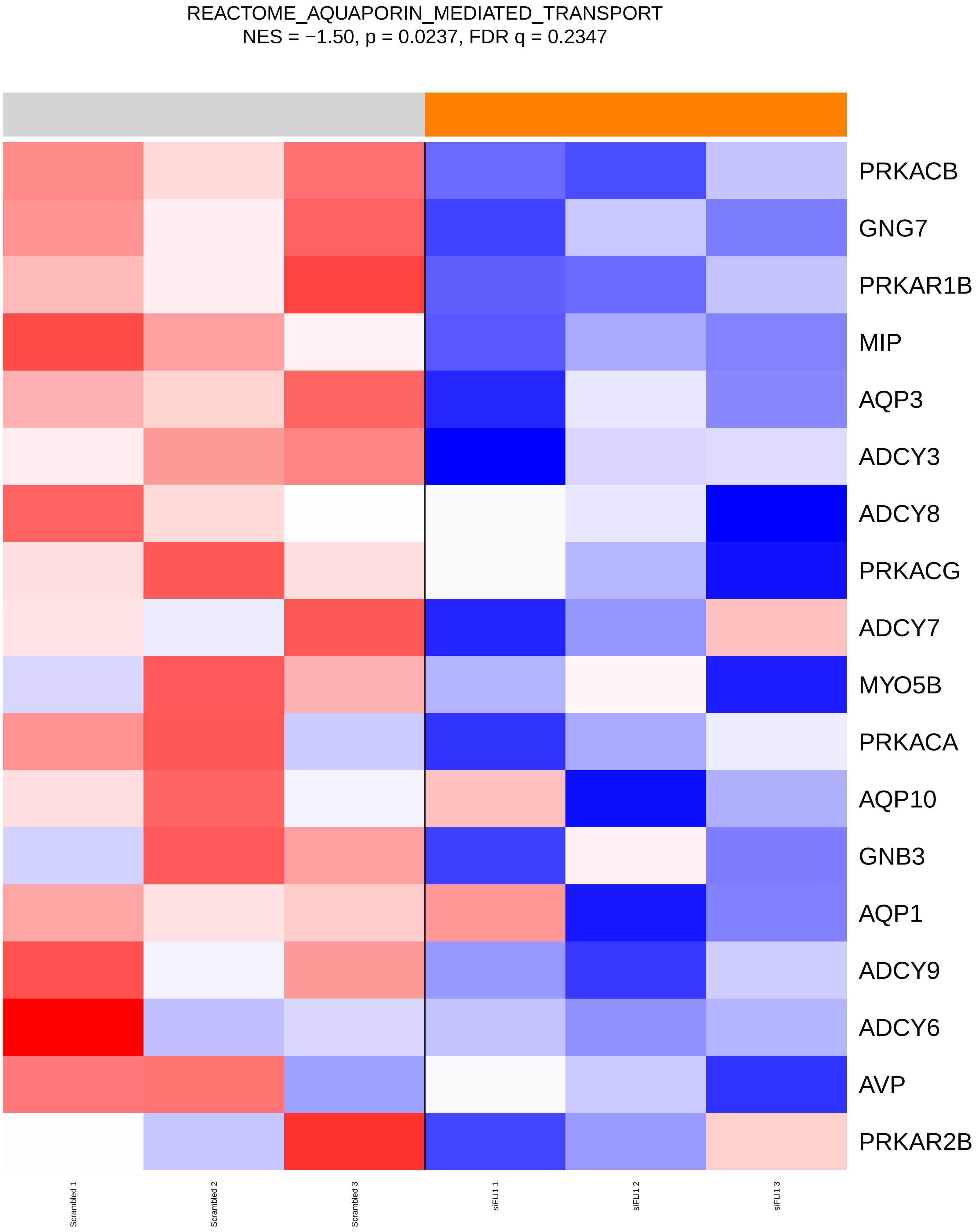
REACTOME_AQUAPORIN_MEDIATED_TRANSPORT
NES = −1.50, p = 0.0237, FDR q = 0.2347
PRKACB
GNG7
PRKAR1B
MIP
AQP3
ADCY3
ADCY8
PRKACG
ADCY7
MYO5B
PRKACA
AQP10
GNB3
AQP1
ADCY9
ADCY6
AVP
PRKAR2B
Scrambled 1
Scrambled 2
Scrambled 3
siFLI1 1
siFLI1 2
siFLI1 3

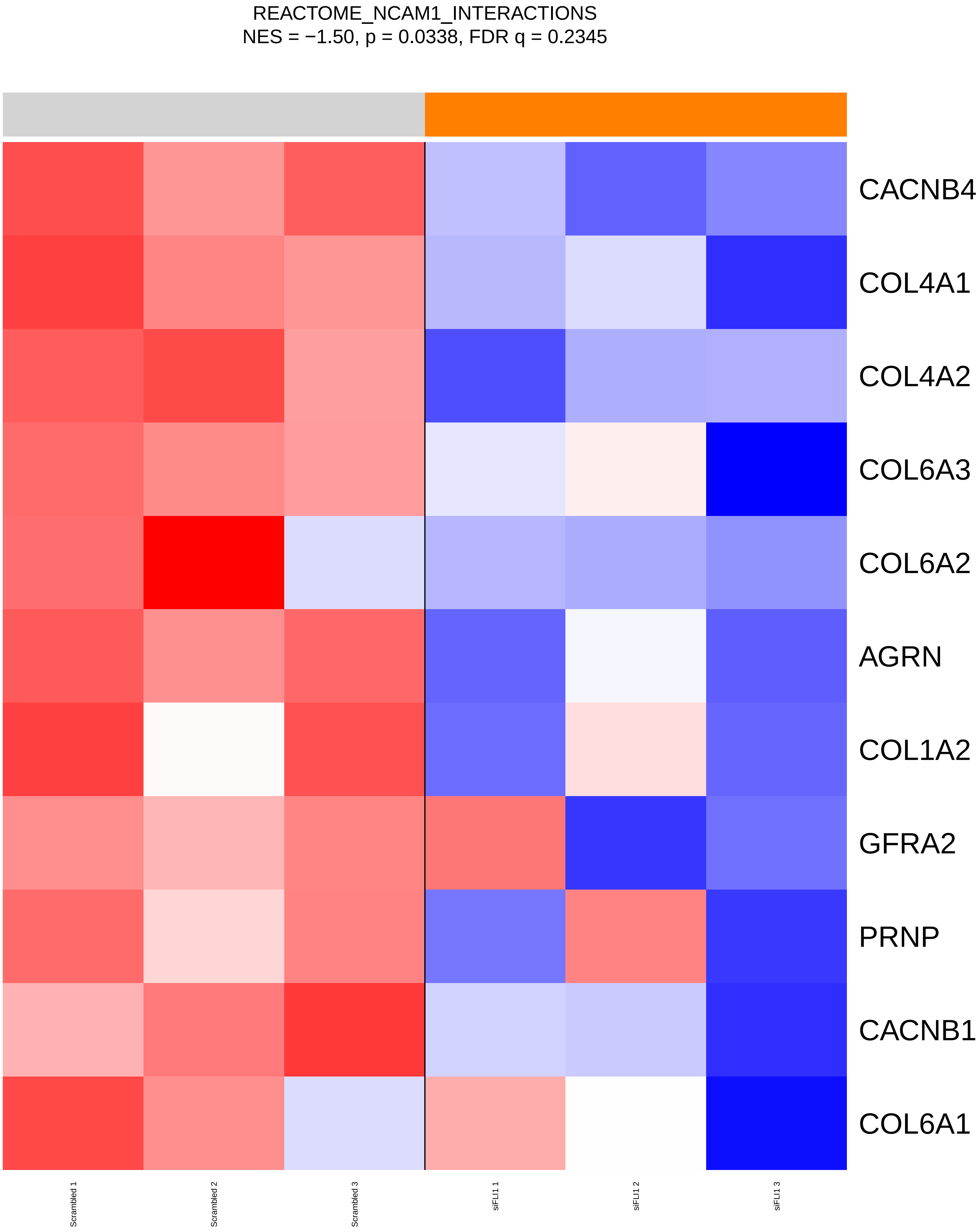
REACTOME_NCAM1_INTERACTIONS
NES = −1.50, p = 0.0338, FDR q = 0.2345
CACNB4
COL4A1
COL4A2
COL6A3
COL6A2
AGRN
COL1A2
GFRA2
PRNP
CACNB1
COL6A1
Scrambled 1
Scrambled 2
Scrambled 3
siFLI1 1
siFLI1 2
siFLI1 3

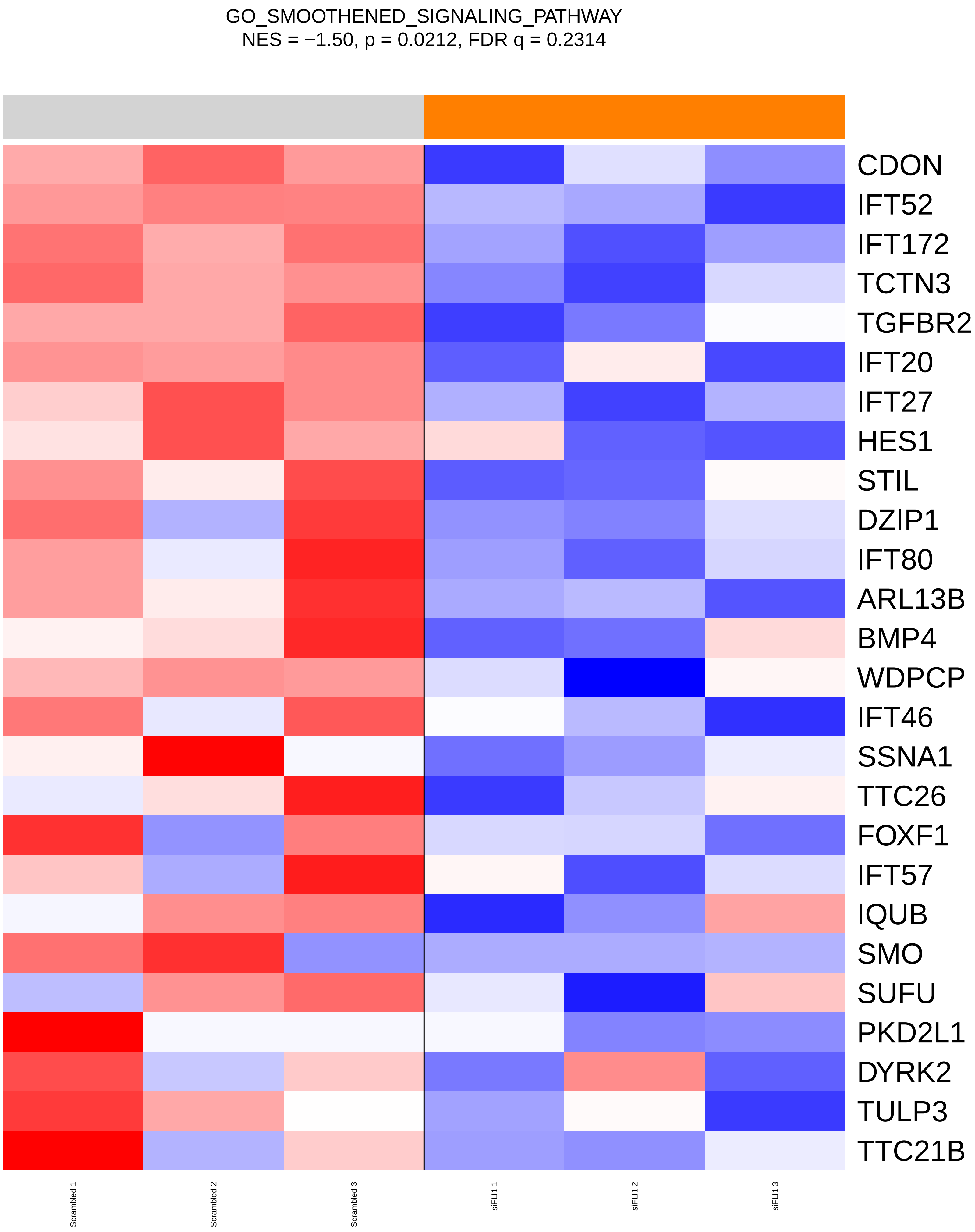
GO_SMOOTHENED_SIGNALING_PATHWAY
NES = −1.50, p = 0.0212, FDR q = 0.2314
CDON
IFT52
IFT172
TCTN3
TGFBR2
IFT20
IFT27
HES1
STIL
DZIP1
IFT80
ARL13B
BMP4
WDPCP
IFT46
SSNA1
TTC26
FOXF1
IFT57
IQUB
SMO
SUFU
PKD2L1
DYRK2
TULP3
TTC21B
Scrambled 1
Scrambled 2
Scrambled 3
siFLI1 1
siFLI1 2
siFLI1 3

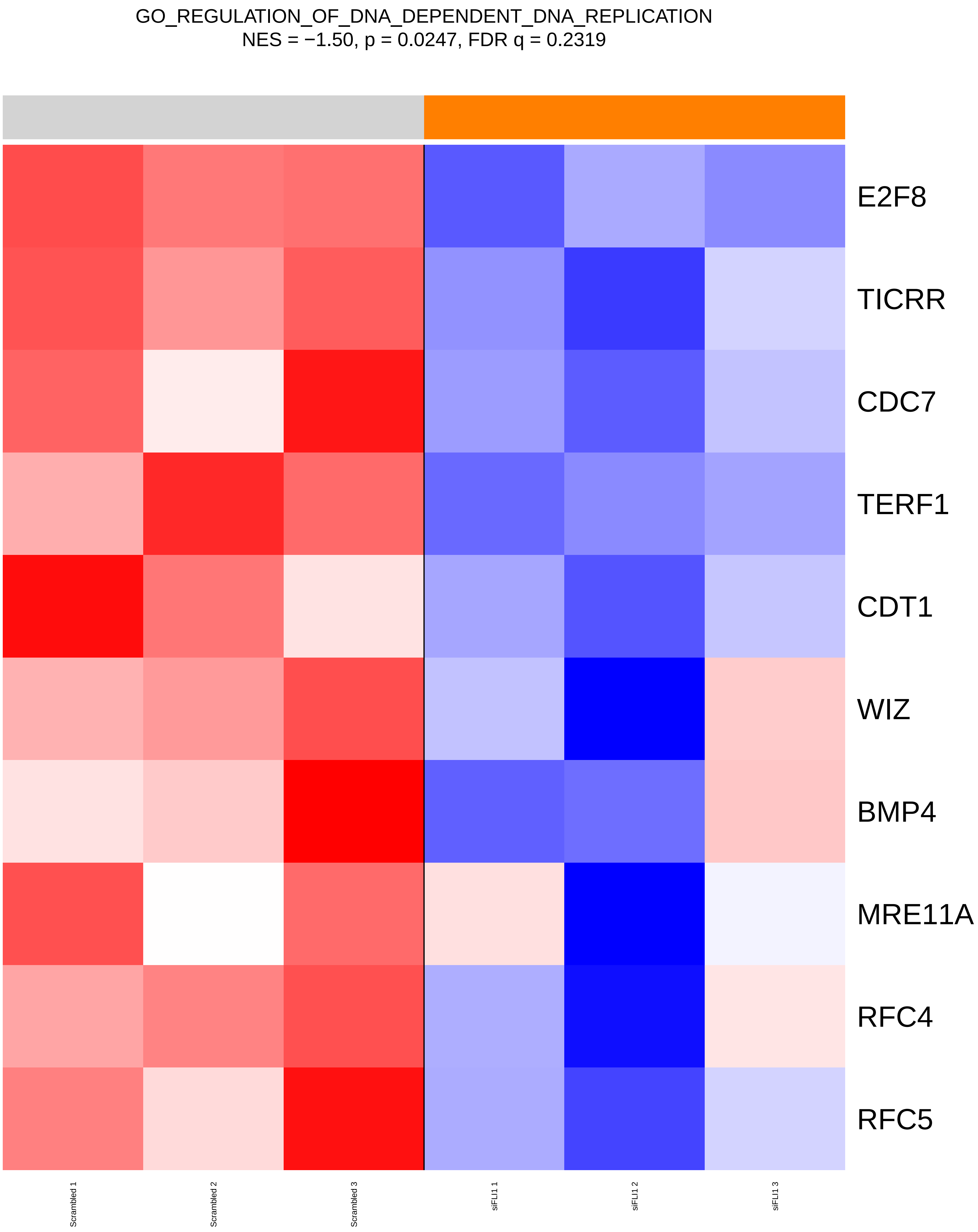
GO_REGULATION_OF_DNA_DEPENDENT_DNA_REPLICATION
NES = −1.50, p = 0.0247, FDR q = 0.2319
E2F8
TICRR
CDC7
TERF1
CDT1
WIZ
BMP4
MRE11A
RFC4
RFC5
Scrambled 1
Scrambled 2
Scrambled 3
siFLI1 1
siFLI1 2
siFLI1 3

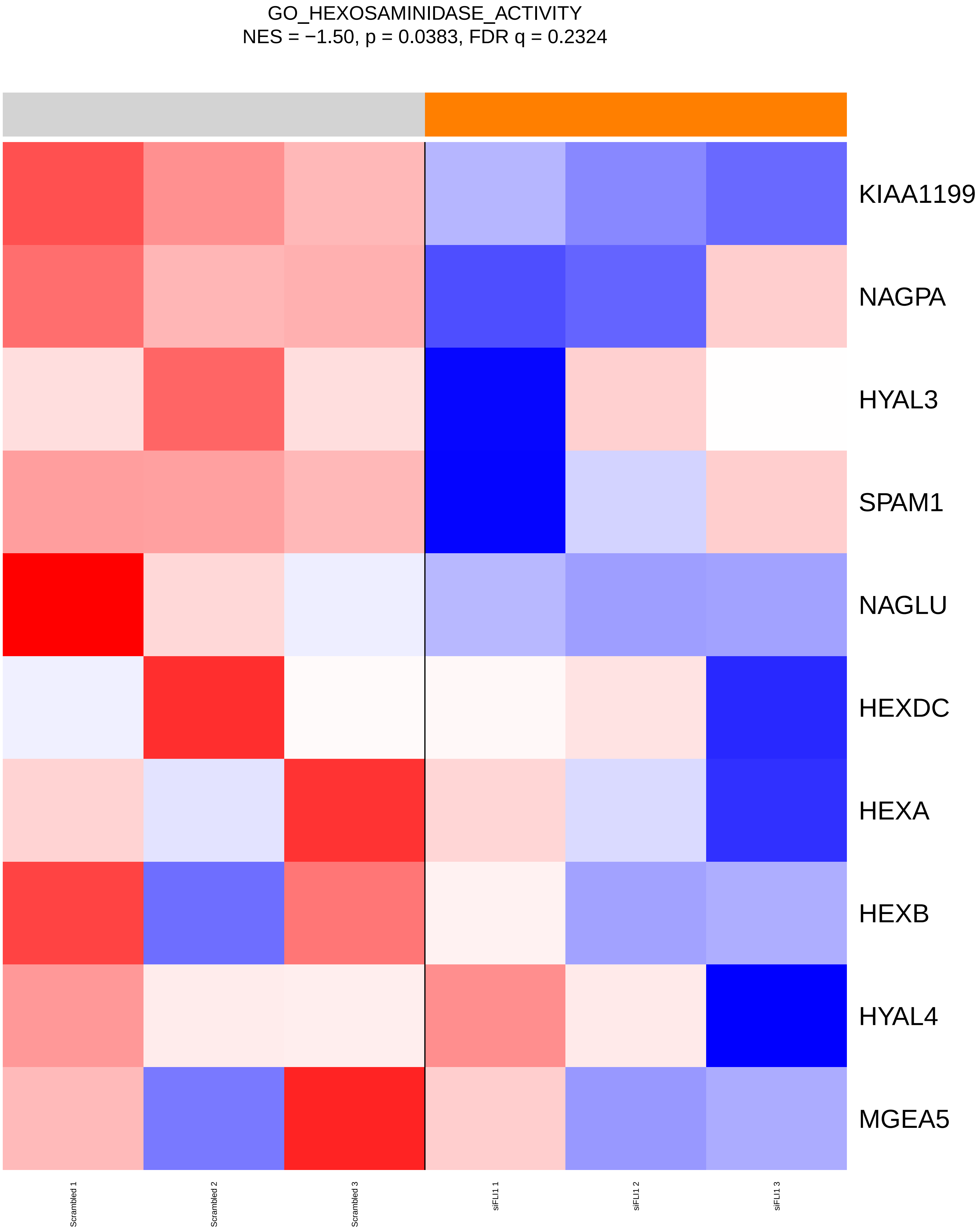
GO_HEXOSAMINIDASE_ACTIVITY
NES = −1.50, p = 0.0383, FDR q = 0.2324
KIAA1199
NAGPA
HYAL3
SPAM1
NAGLU
HEXDC
HEXA
HEXB
HYAL4
MGEA5
Scrambled 1
Scrambled 2
Scrambled 3
siFLI1 1
siFLI1 2
siFLI1 3

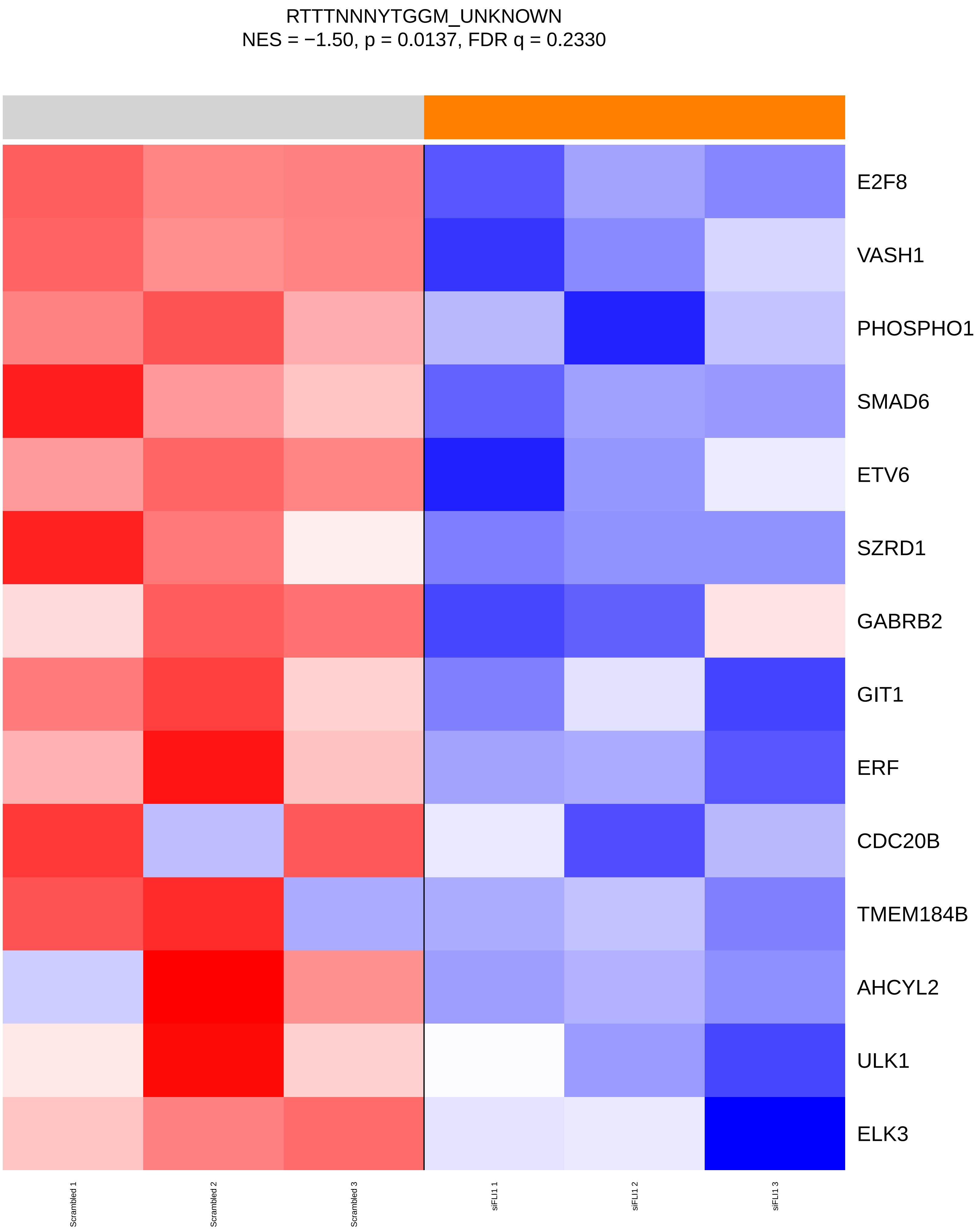
RTTTNNNYTGGM_UNKNOWN
NES = −1.50, p = 0.0137, FDR q = 0.2330
E2F8
VASH1
PHOSPHO1
SMAD6
ETV6
SZRD1
GABRB2
GIT1
ERF
CDC20B
TMEM184B
AHCYL2
ULK1
ELK3
Scrambled 1
Scrambled 2
Scrambled 3
siFLI1 1
siFLI1 2
siFLI1 3

GO_ODONTOGENESIS_OF_DENTIN_CONTAINING_TOOTH
NES = −1.50, p = 0.0108, FDR q = 0.2334

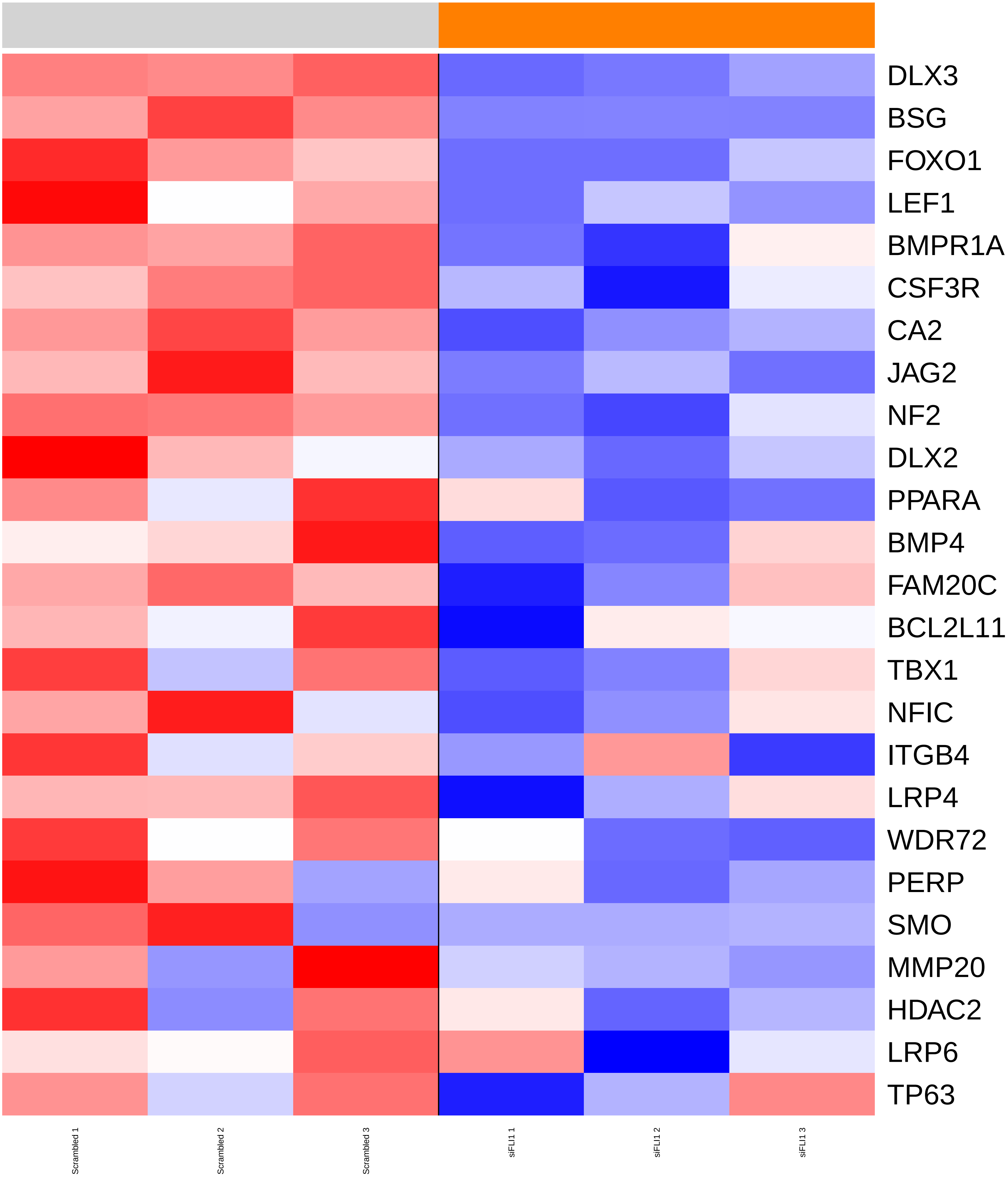

GO_ACTIN_FILAMENT_BUNDLE
NES = −1.51, p = 0.0158, FDR q = 0.2337

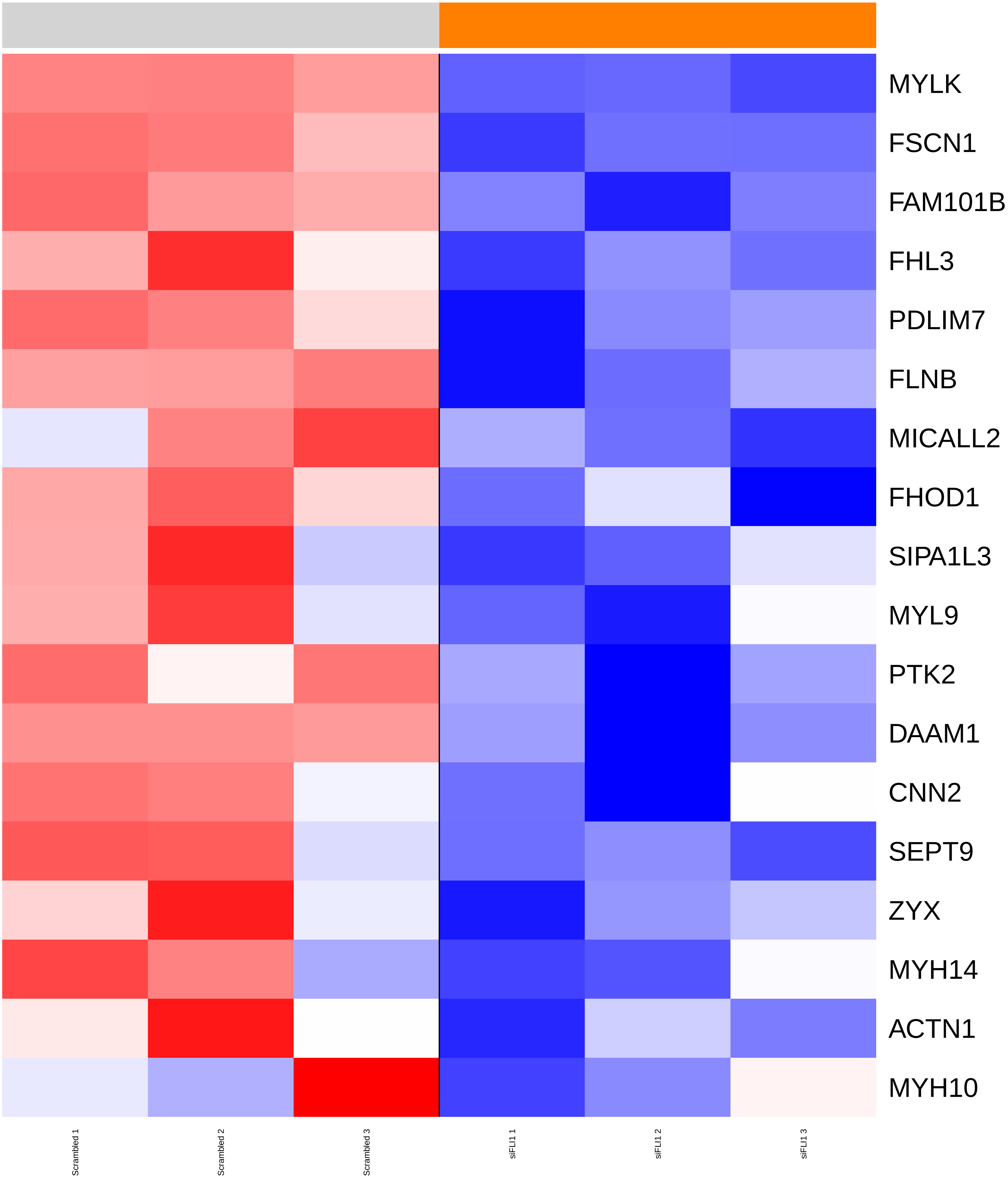

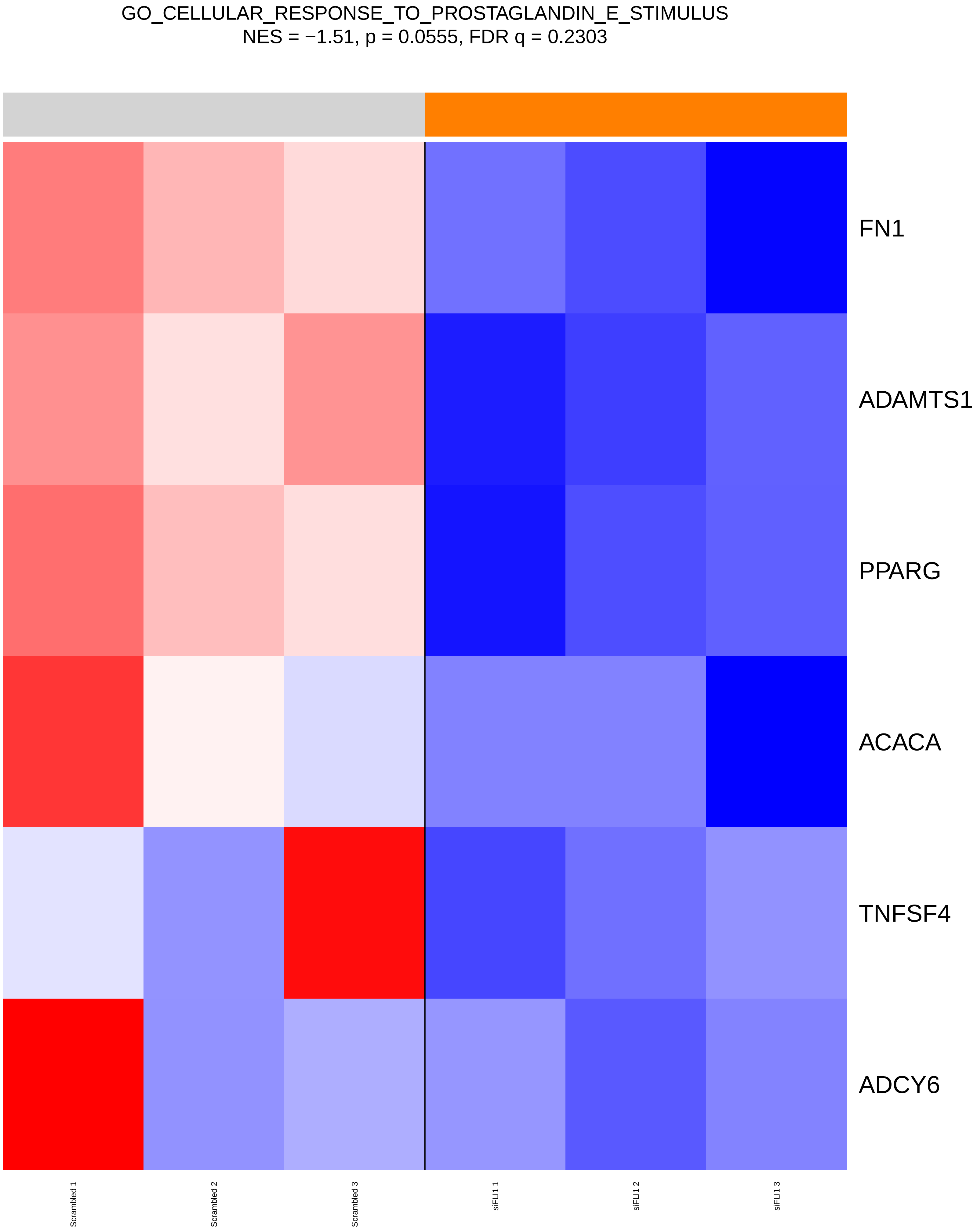
GO_CELLULAR_RESPONSE_TO_PROSTAGLANDIN_E_STIMULUS
NES = −1.51, p = 0.0555, FDR q = 0.2303
FN1
ADAMTS1
PPARG
ACACA
TNFSF4
ADCY6
Scrambled 1
Scrambled 2
Scrambled 3
siFLI1 1
siFLI1 2
siFLI1 3

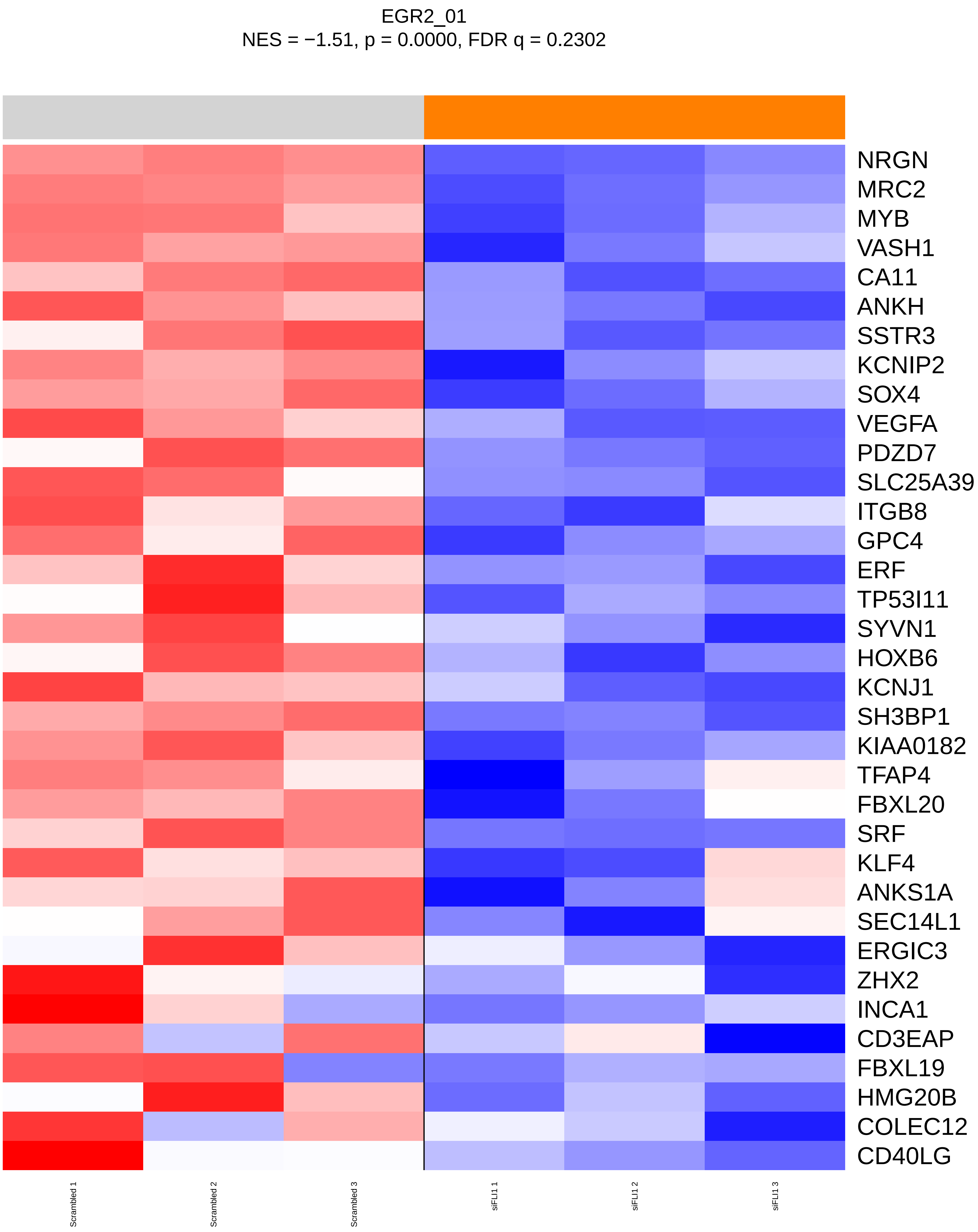
EGR2_01
NES = −1.51, p = 0.0000, FDR q = 0.2302
NRGN
MRC2
MYB
VASH1
CA11
ANKH
SSTR3
KCNIP2
SOX4
VEGFA
PDZD7
SLC25A39
ITGB8
GPC4
ERF
TP53I11
SYVN1
HOXB6
KCNJ1
SH3BP1
KIAA0182
TFAP4
FBXL20
SRF
KLF4
ANKS1A
SEC14L1
ERGIC3
ZHX2
INCA1
CD3EAP
FBXL19
HMG20B
COLEC12
CD40LG
Scrambled 1
Scrambled 2
Scrambled 3
siFLI1 1
siFLI1 2
siFLI1 3

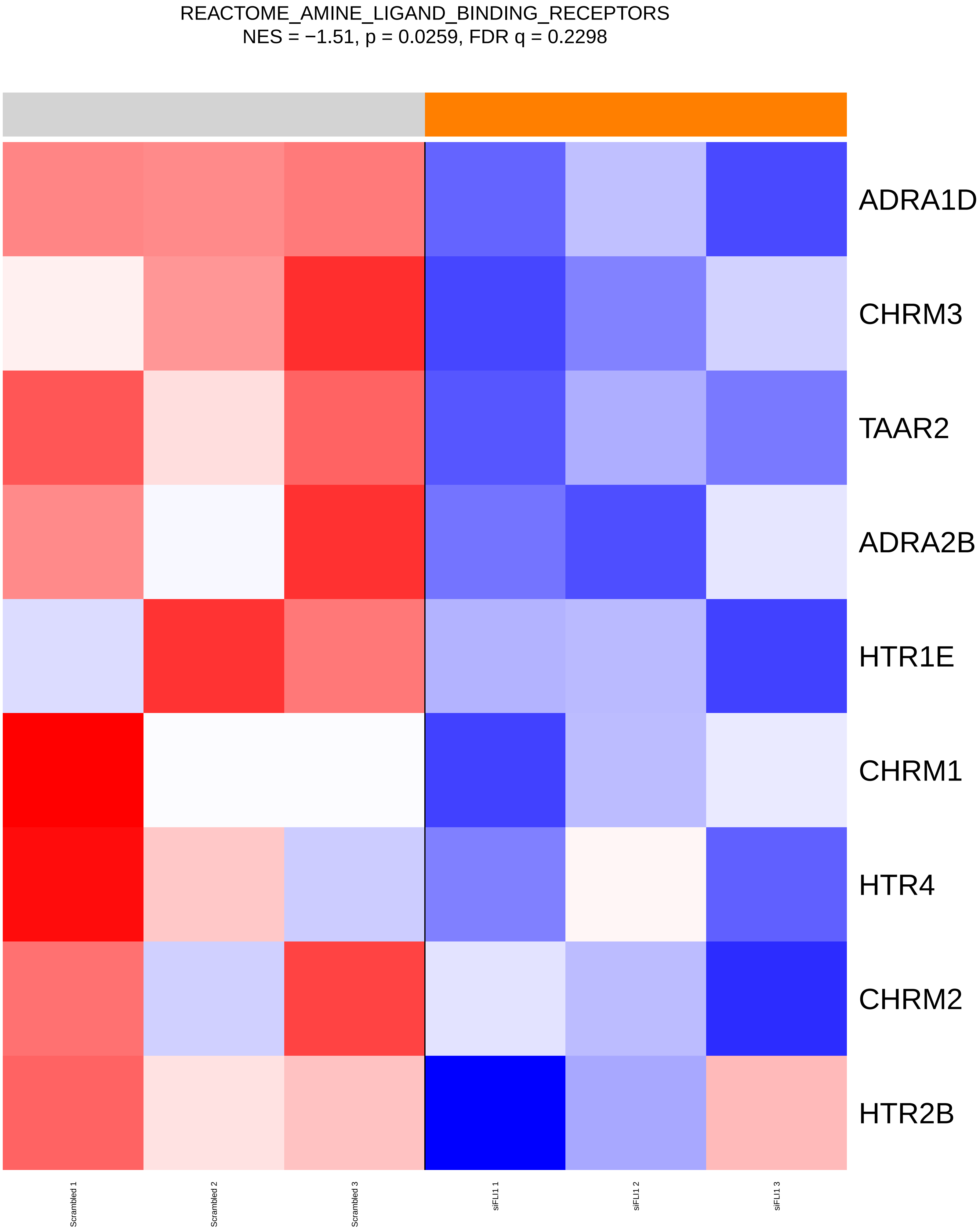
REACTOME_AMINE_LIGAND_BINDING_RECEPTORS
NES = −1.51, p = 0.0259, FDR q = 0.2298
ADRA1D
CHRM3
TAAR2
ADRA2B
HTR1E
CHRM1
HTR4
CHRM2
HTR2B
Scrambled 1
Scrambled 2
Scrambled 3
siFLI1 1
siFLI1 2
siFLI1 3

KEGG_PENTOSE_AND_GLUCURONATE_INTERCONVERSIONS
NES = −1.51, p = 0.0354, FDR q = 0.2292

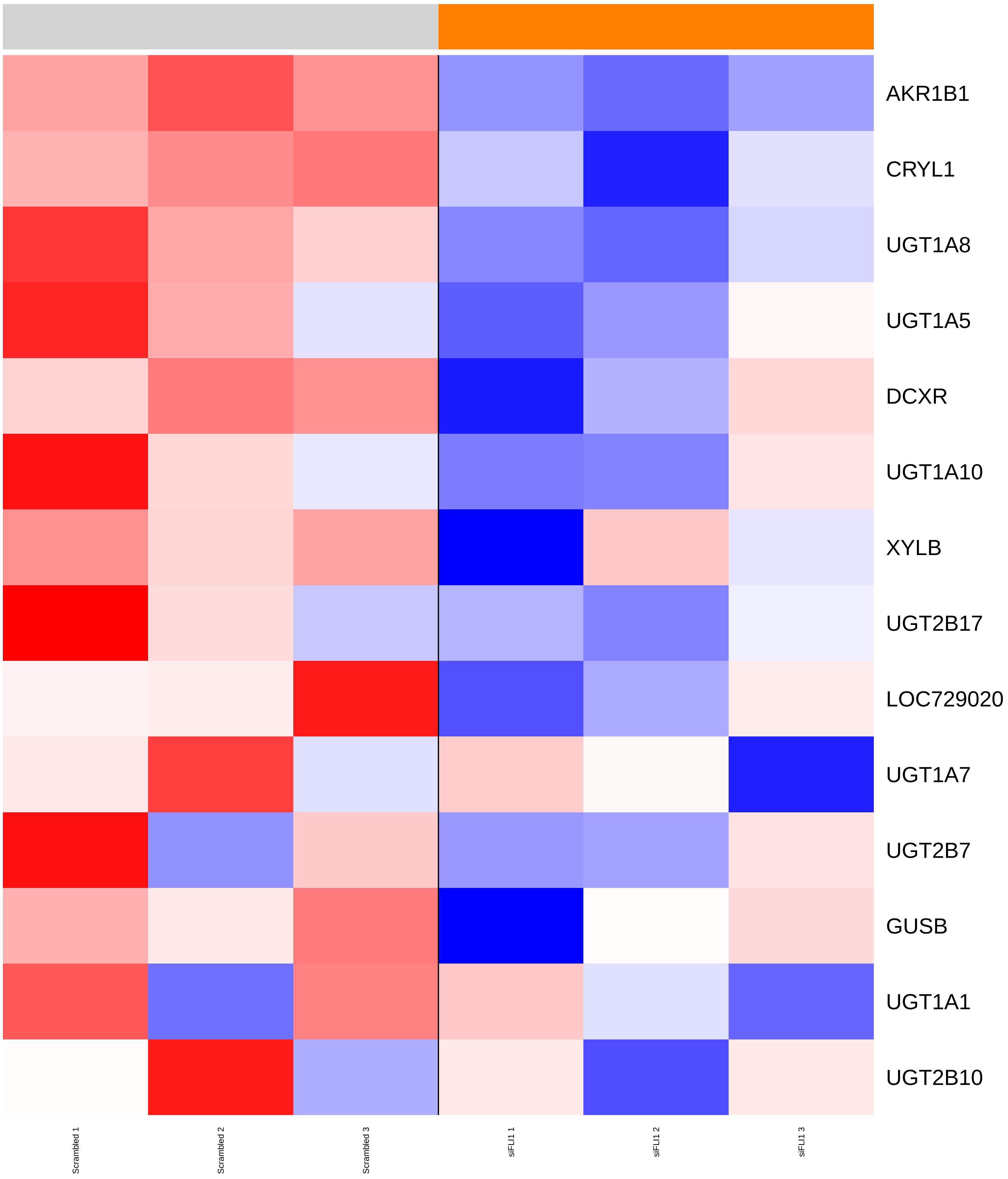

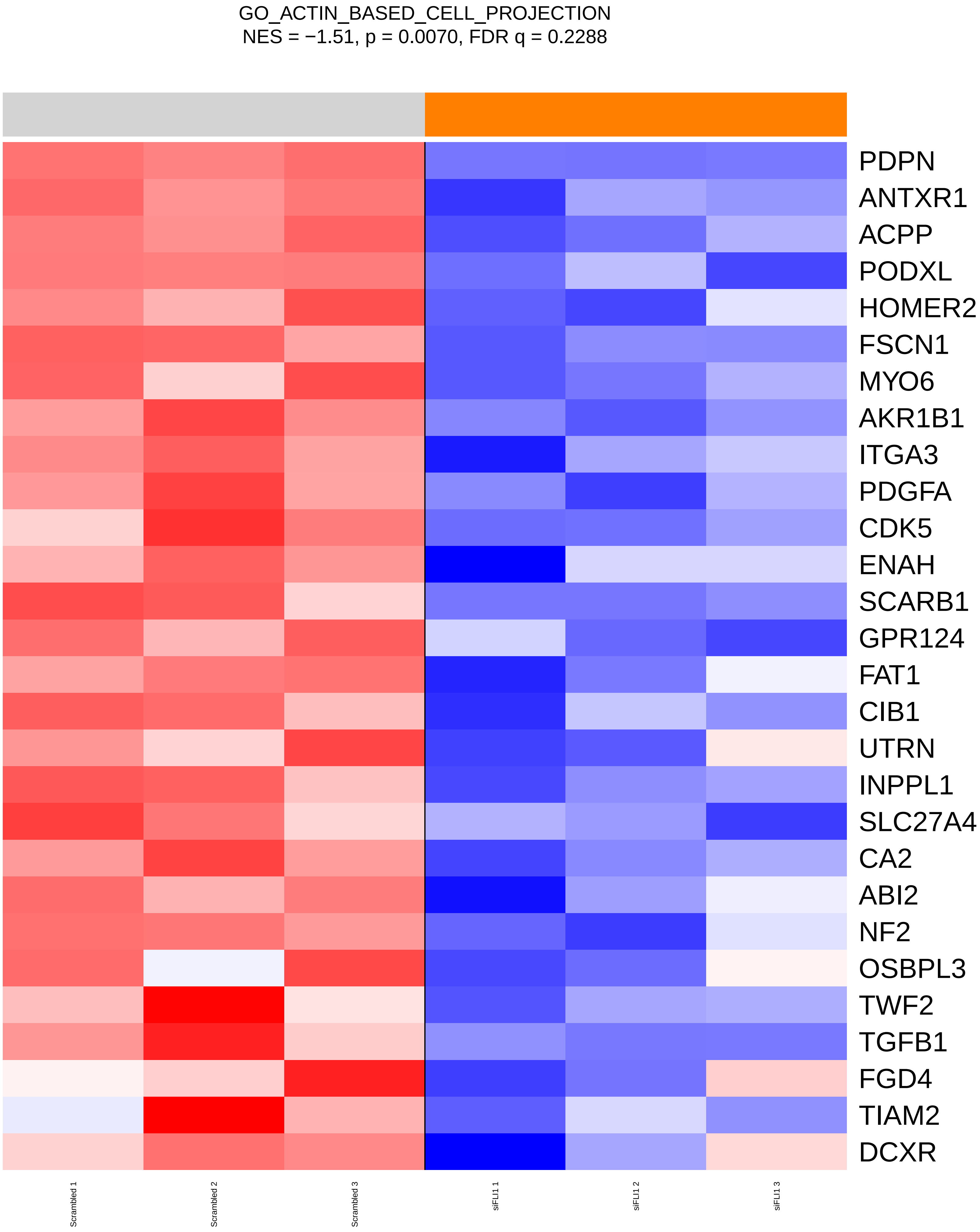
GO_ACTIN_BASED_CELL_PROJECTION
NES = −1.51, p = 0.0070, FDR q = 0.2288
PDPN
ANTXR1
ACPP
PODXL
HOMER2
FSCN1
MYO6
AKR1B1
ITGA3
PDGFA
CDK5
ENAH
SCARB1
GPR124
FAT1
CIB1
UTRN
INPPL1
SLC27A4
CA2
ABI2
NF2
OSBPL3
TWF2
TGFB1
FGD4
TIAM2
DCXR
Scrambled 1
Scrambled 2
Scrambled 3
siFLI1 1
siFLI1 2
siFLI1 3

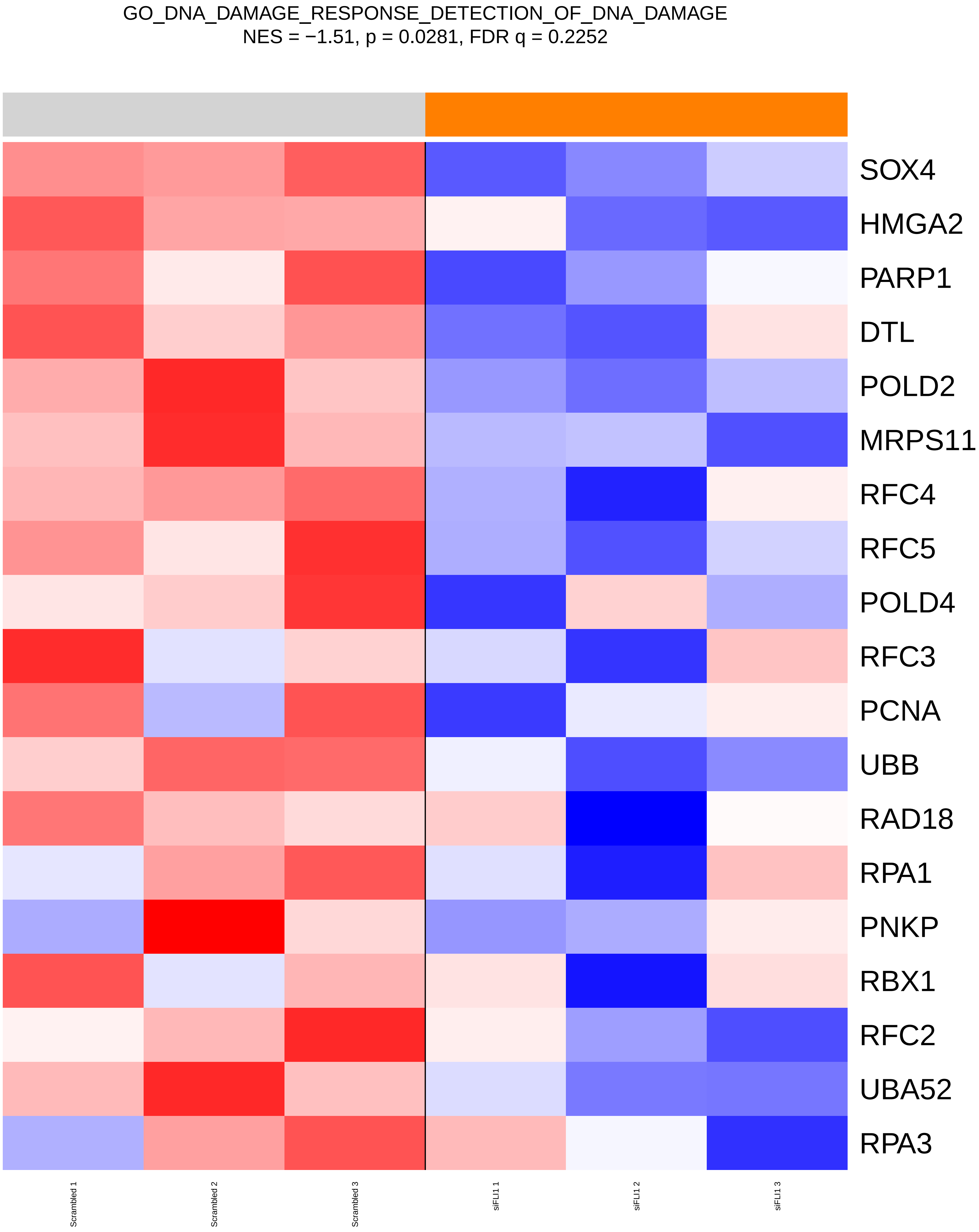
GO_DNA_DAMAGE_RESPONSE_DETECTION_OF_DNA_DAMAGE
NES = −1.51, p = 0.0281, FDR q = 0.2252
SOX4
HMGA2
PARP1
DTL
POLD2
MRPS11
RFC4
RFC5
POLD4
RFC3
PCNA
UBB
RAD18
RPA1
PNKP
RBX1
RFC2
UBA52
RPA3
Scrambled 1
Scrambled 2
Scrambled 3
siFLI1 1
siFLI1 2
siFLI1 3

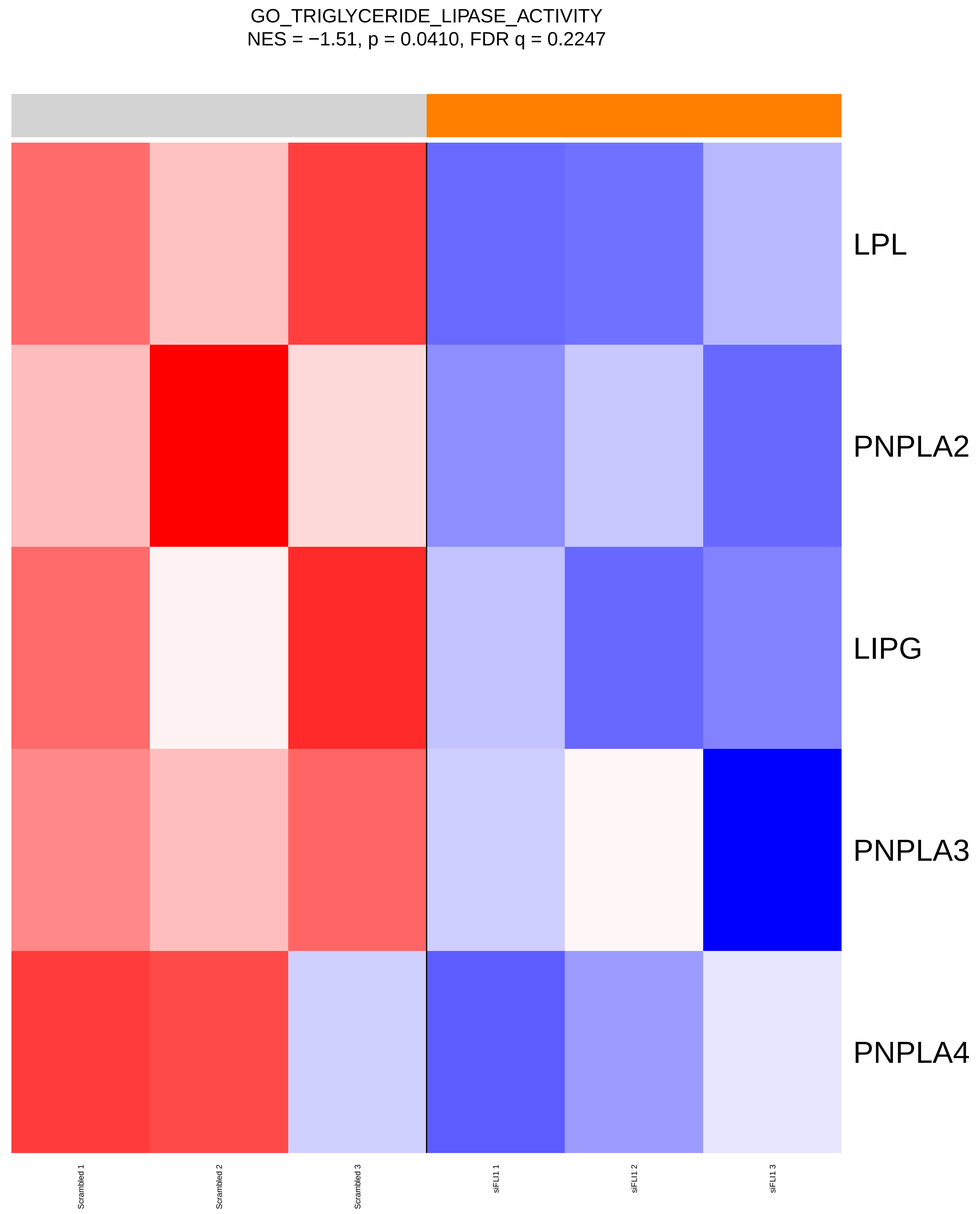
GO_TRIGLYCERIDE_LIPASE_ACTIVITY
NES = −1.51, p = 0.0410, FDR q = 0.2247
LPL
PNPLA2
LIPG
PNPLA3
PNPLA4
Scrambled 1
Scrambled 2
Scrambled 3
siFLI1 1
siFLI1 2
siFLI1 3

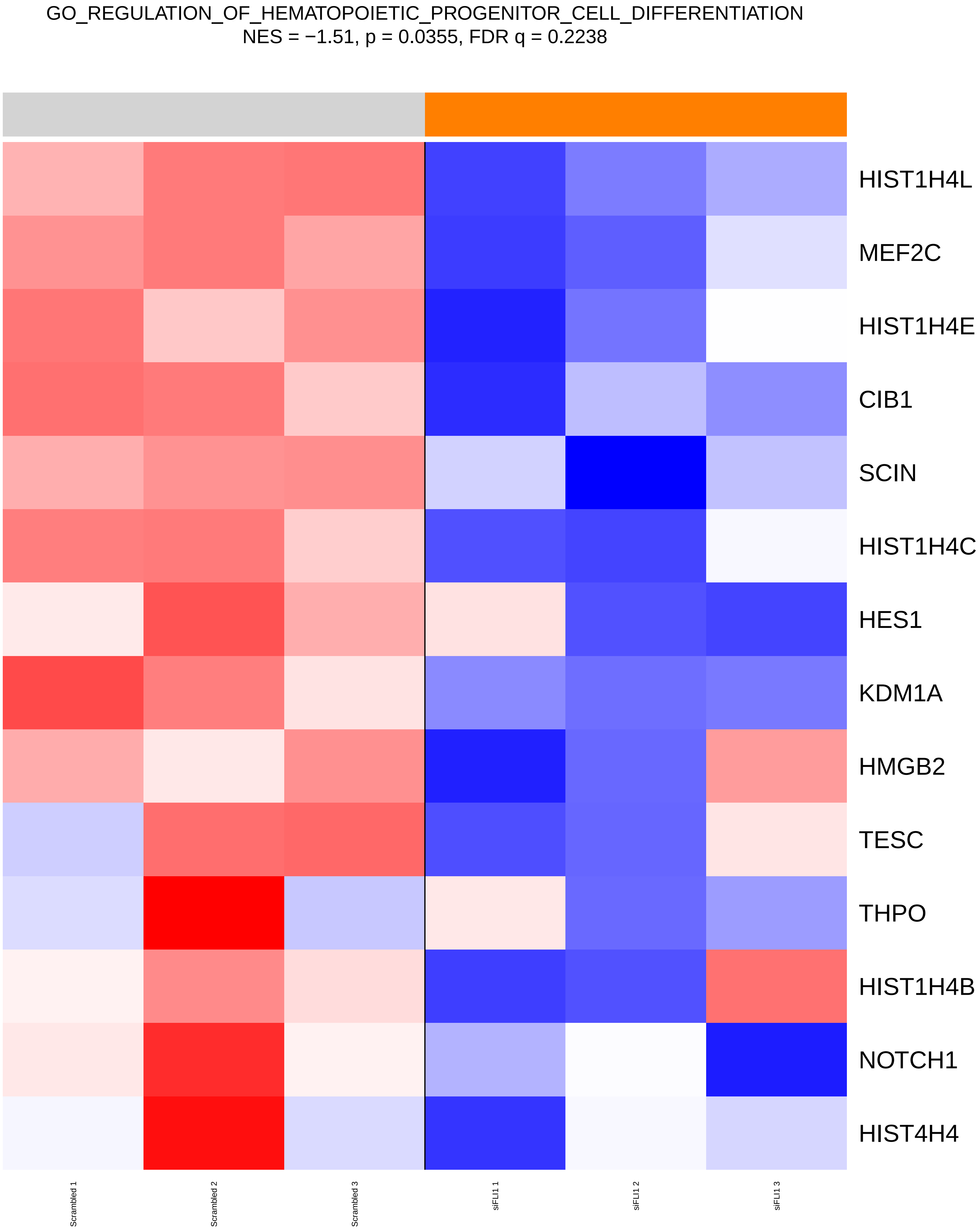
GO_REGULATION_OF_HEMATOPOIETIC_PROGENITOR_CELL_DIFFERENTIATION
NES = −1.51, p = 0.0355, FDR q = 0.2238
HIST1H4L
MEF2C
HIST1H4E
CIB1
SCIN
HIST1H4C
HES1
KDM1A
HMGB2
TESC
THPO
HIST1H4B
NOTCH1
HIST4H4
Scrambled 1
Scrambled 2
Scrambled 3
siFLI1 1
siFLI1 2
siFLI1 3

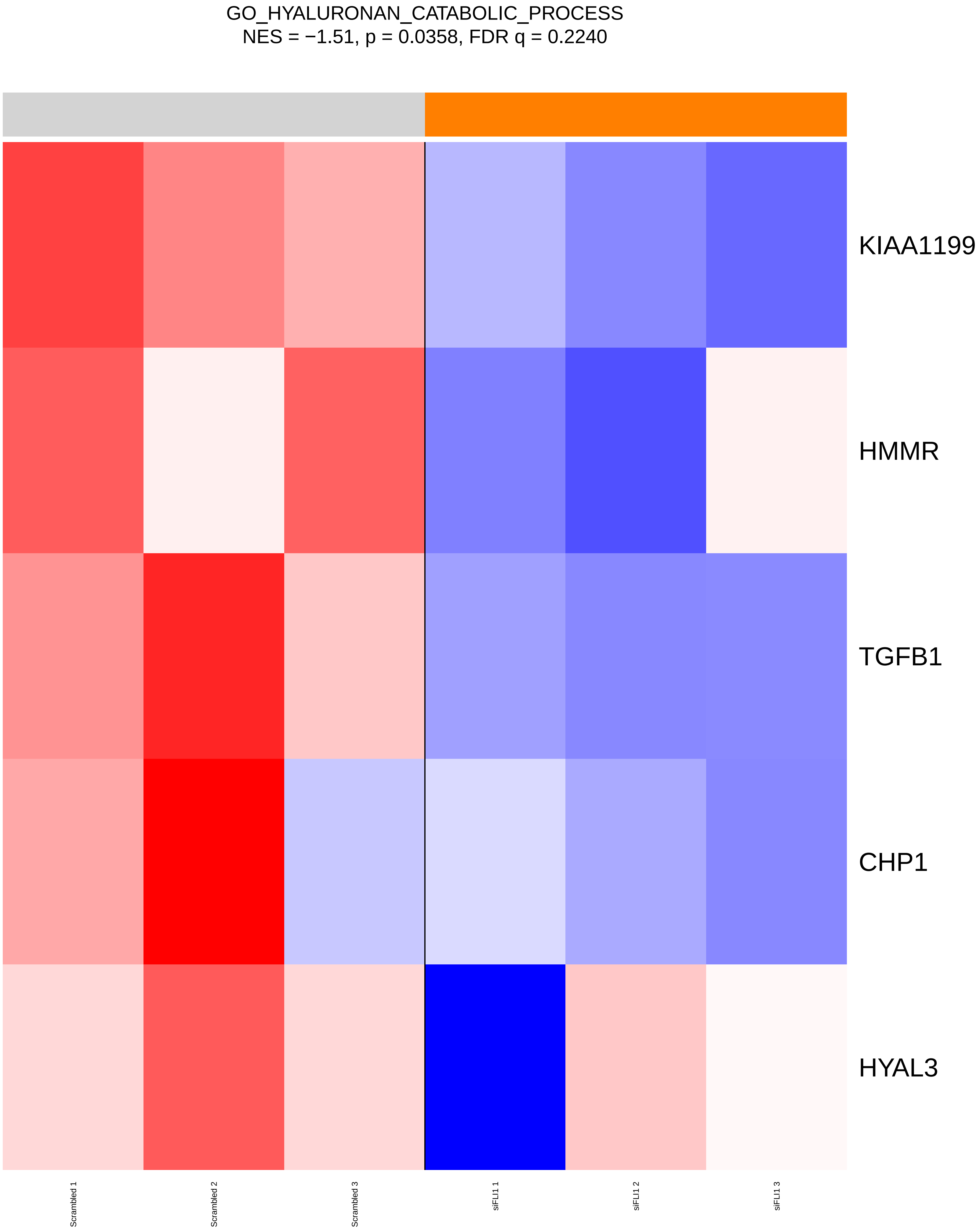
GO_HYALURONAN_CATABOLIC_PROCESS
NES = −1.51, p = 0.0358, FDR q = 0.2240
KIAA1199
HMMR
TGFB1
CHP1
HYAL3
Scrambled 1
Scrambled 2
Scrambled 3
siFLI1 1
siFLI1 2
siFLI1 3

GO_RESPONSE_TO_MERCURY_ION
NES = −1.51, p = 0.0538, FDR q = 0.2243

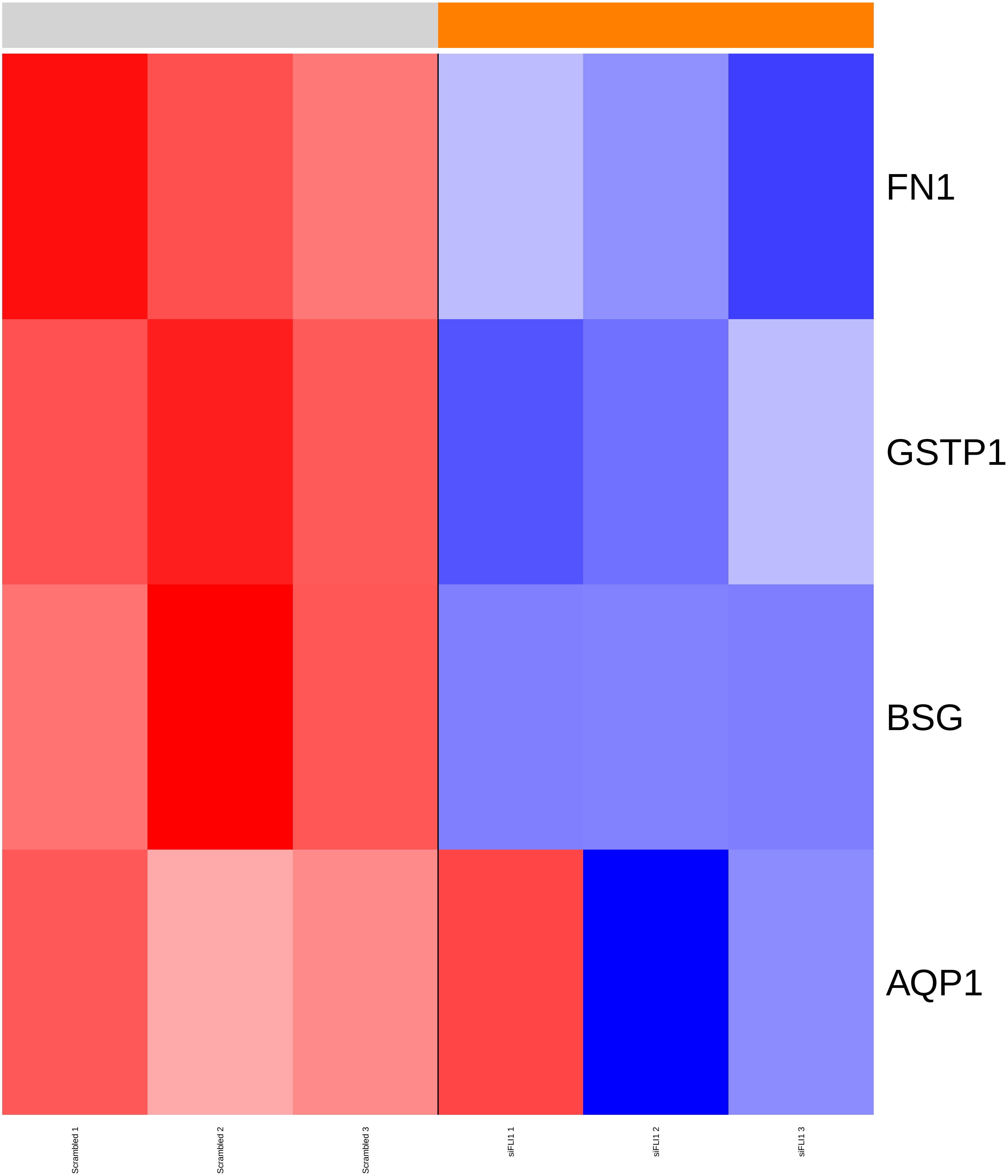

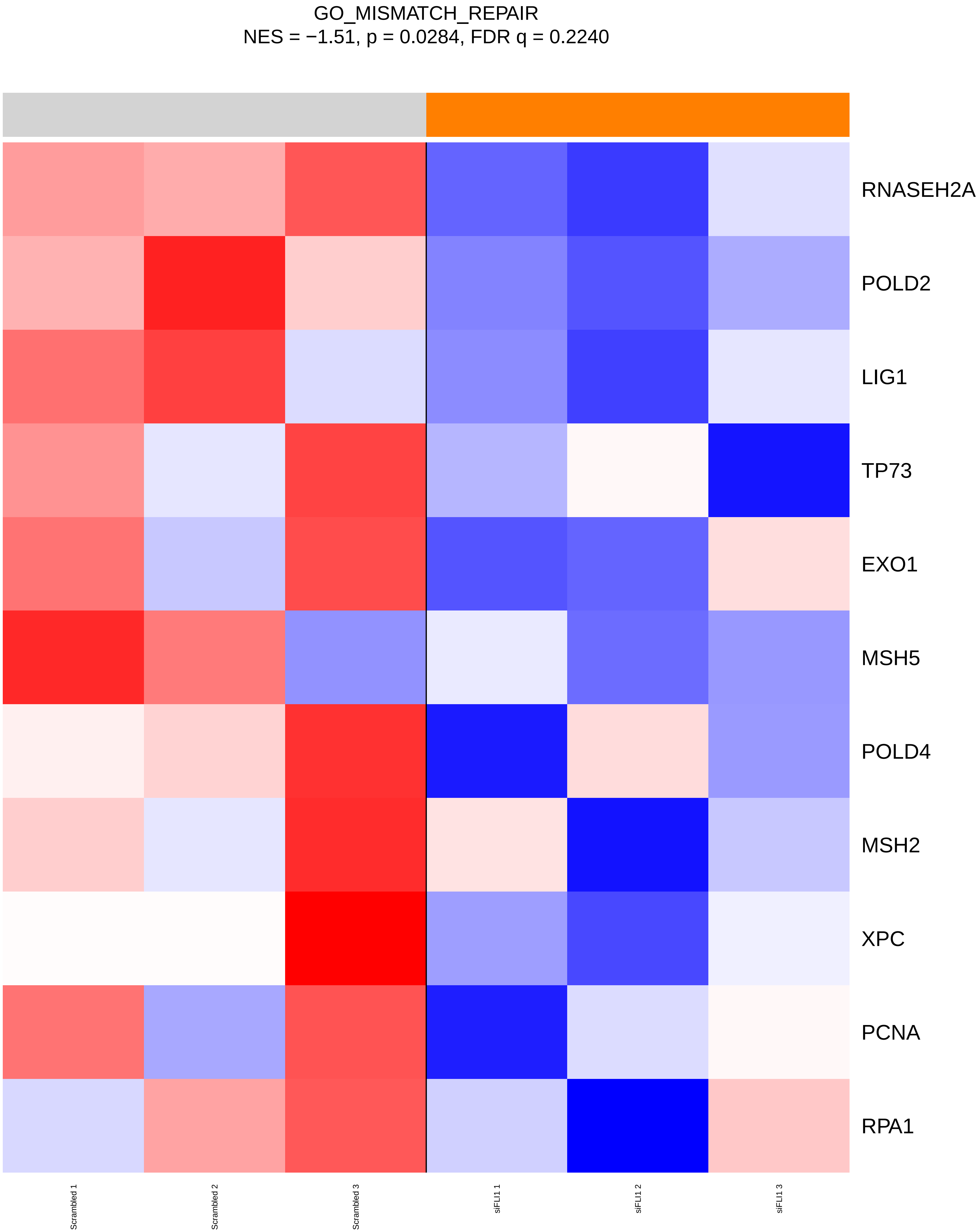
GO_MISMATCH_REPAIR
NES = −1.51, p = 0.0284, FDR q = 0.2240
RNASEH2A
POLD2
LIG1
TP73
EXO1
MSH5
POLD4
MSH2
XPC
PCNA
RPA1
Scrambled 1
Scrambled 2
Scrambled 3
siFLI1 1
siFLI1 2
siFLI1 3

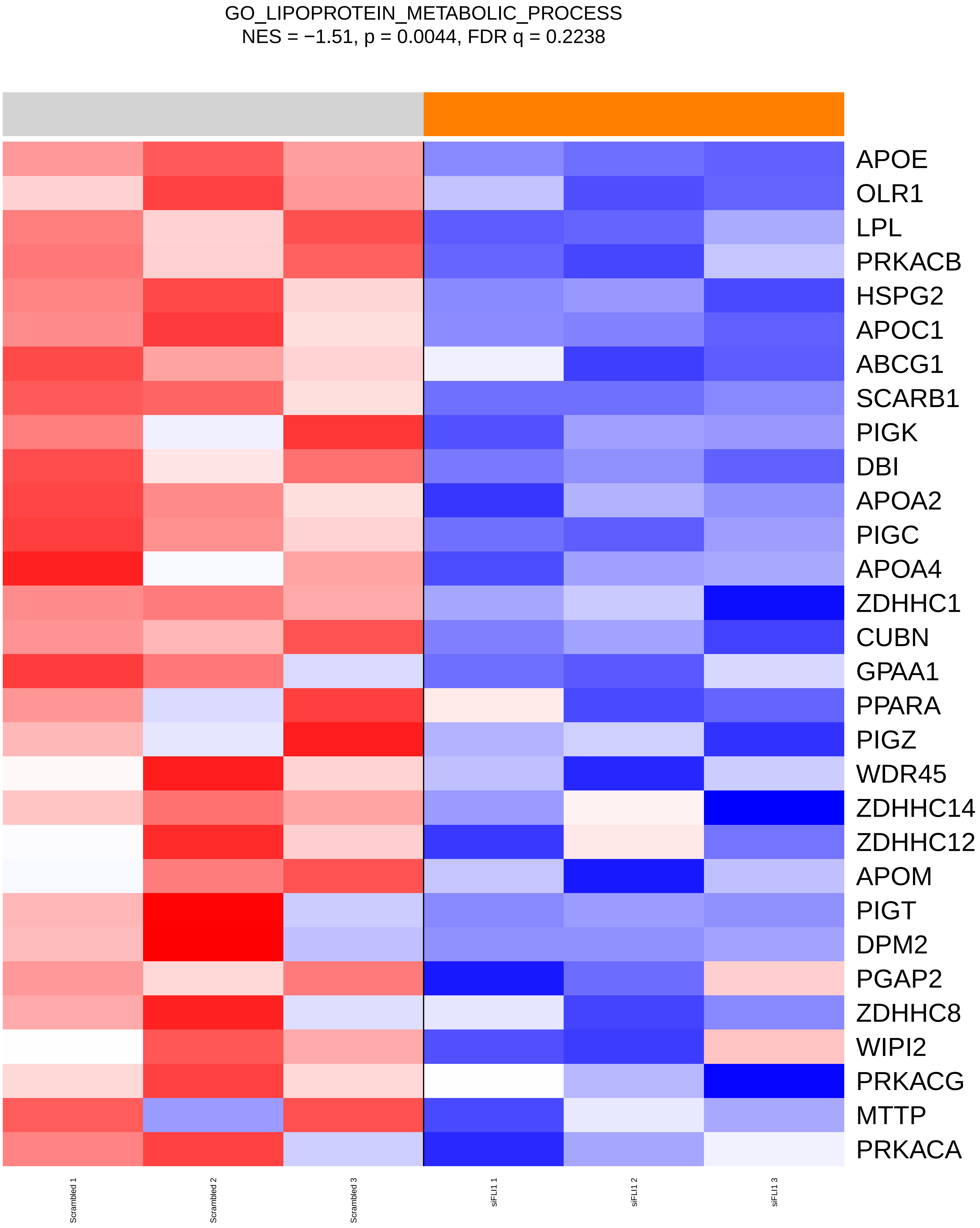
GO_LIPOPROTEIN_METABOLIC_PROCESS
NES = −1.51, p = 0.0044, FDR q = 0.2238
APOE
OLR1
LPL
PRKACB
HSPG2
APOC1
ABCG1
SCARB1
PIGK
DBI
APOA2
PIGC
APOA4
ZDHHC1
CUBN
GPAA1
PPARA
PIGZ
WDR45
ZDHHC14
ZDHHC12
APOM
PIGT
DPM2
PGAP2
ZDHHC8
WIPI2
PRKACG
MTTP
PRKACA
Scrambled 1
Scrambled 2
Scrambled 3
siFLI1 1
siFLI1 2
siFLI1 3

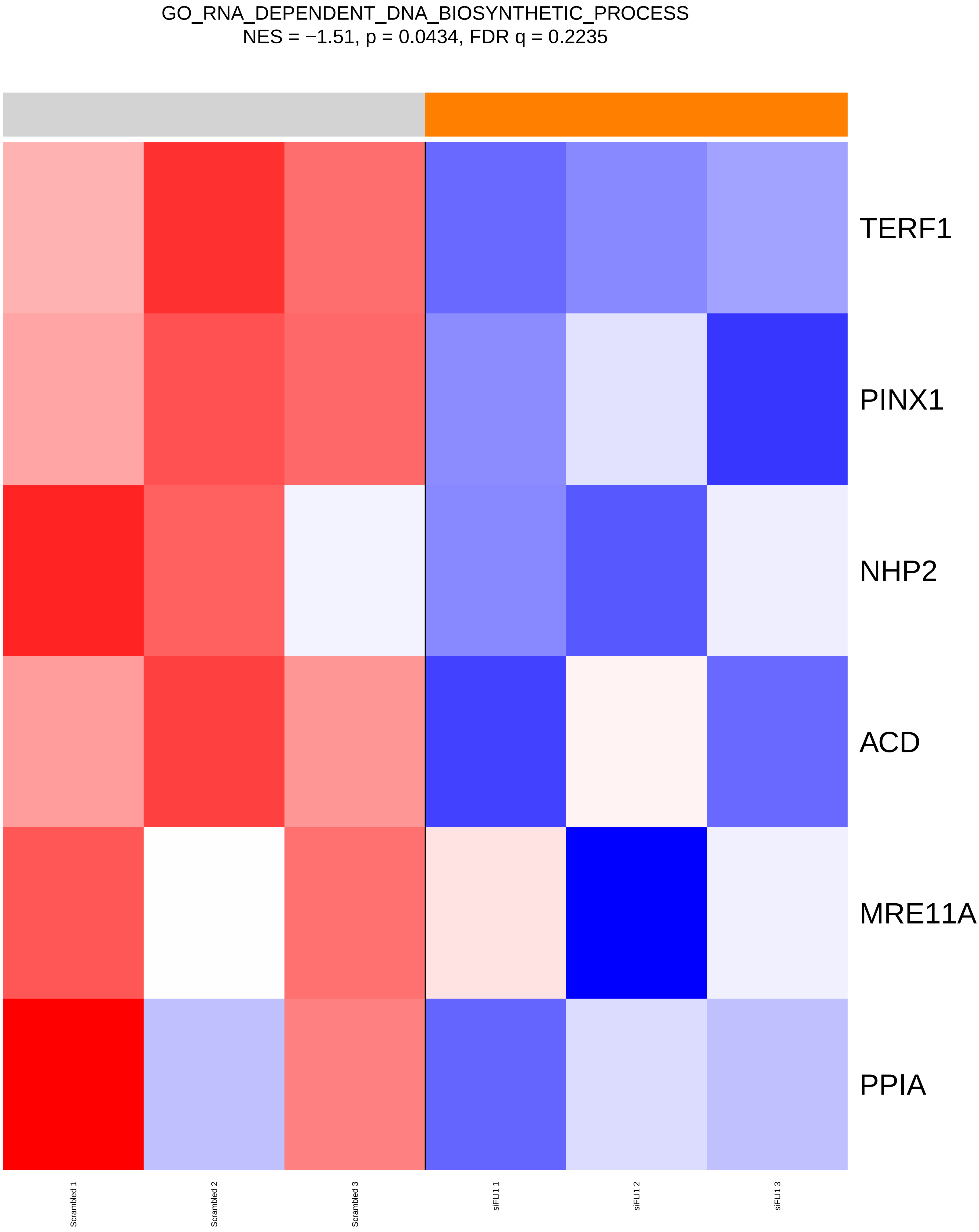

GO_RNA_DEPENDENT_DNA_BIOSYNTHETIC_PROCESS
NES = −1.51, p = 0.0434, FDR q = 0.2235
TERF1
PINX1
NHP2
ACD
MRE11A
PPIA
Scrambled 1
Scrambled 2
Scrambled 3
siFLI1 1
siFLI1 2
siFLI1 3

GO_REGULATION_OF_SYNAPSE_STRUCTURE_OR_ACTIVITY
NES = −1.52, p = 0.0026, FDR q = 0.2220

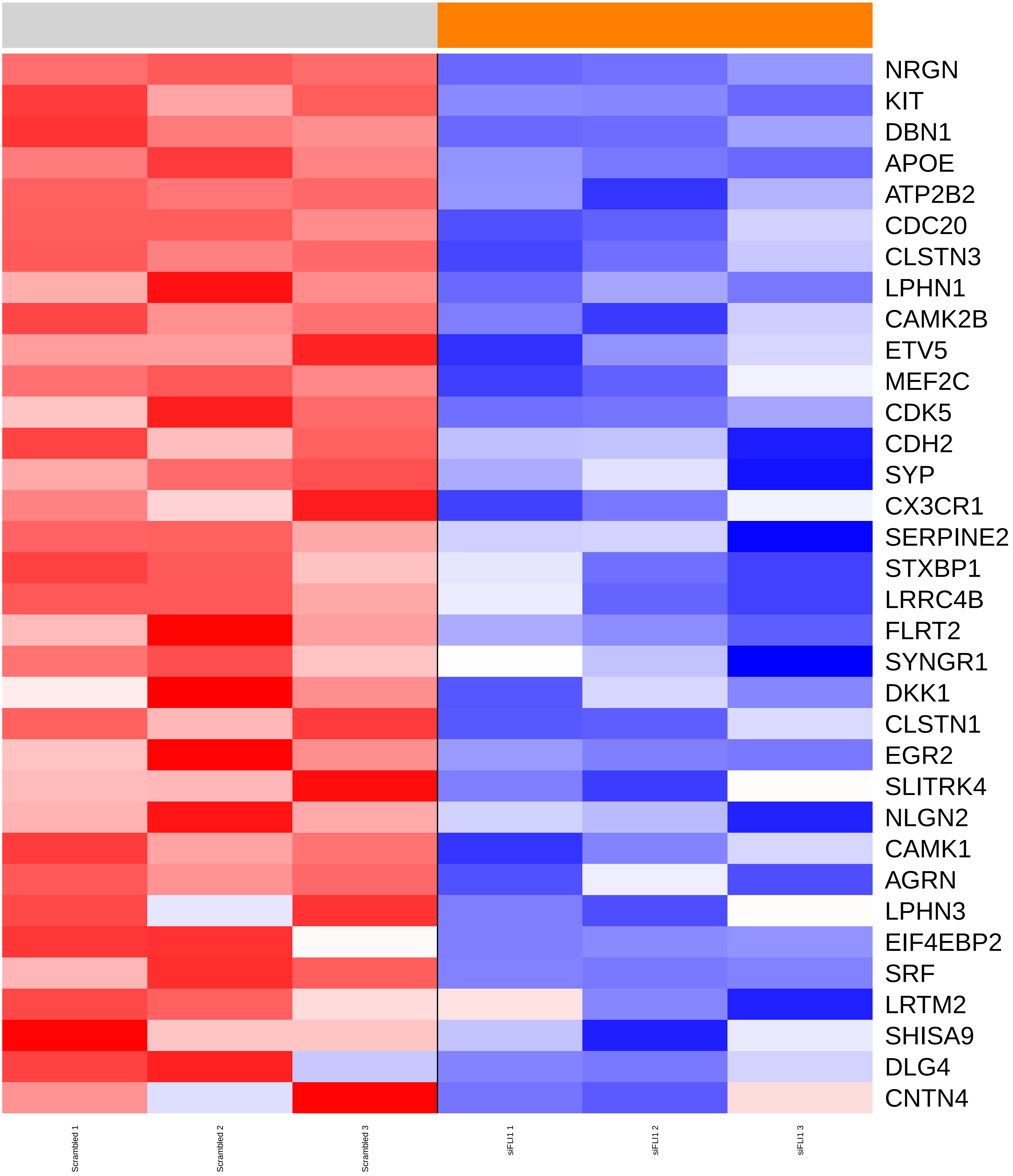

GO_NUCLEOSIDE_MONOPHOSPHATE_METABOLIC_PROCESS
NES = −1.52, p = 0.0027, FDR q = 0.2223

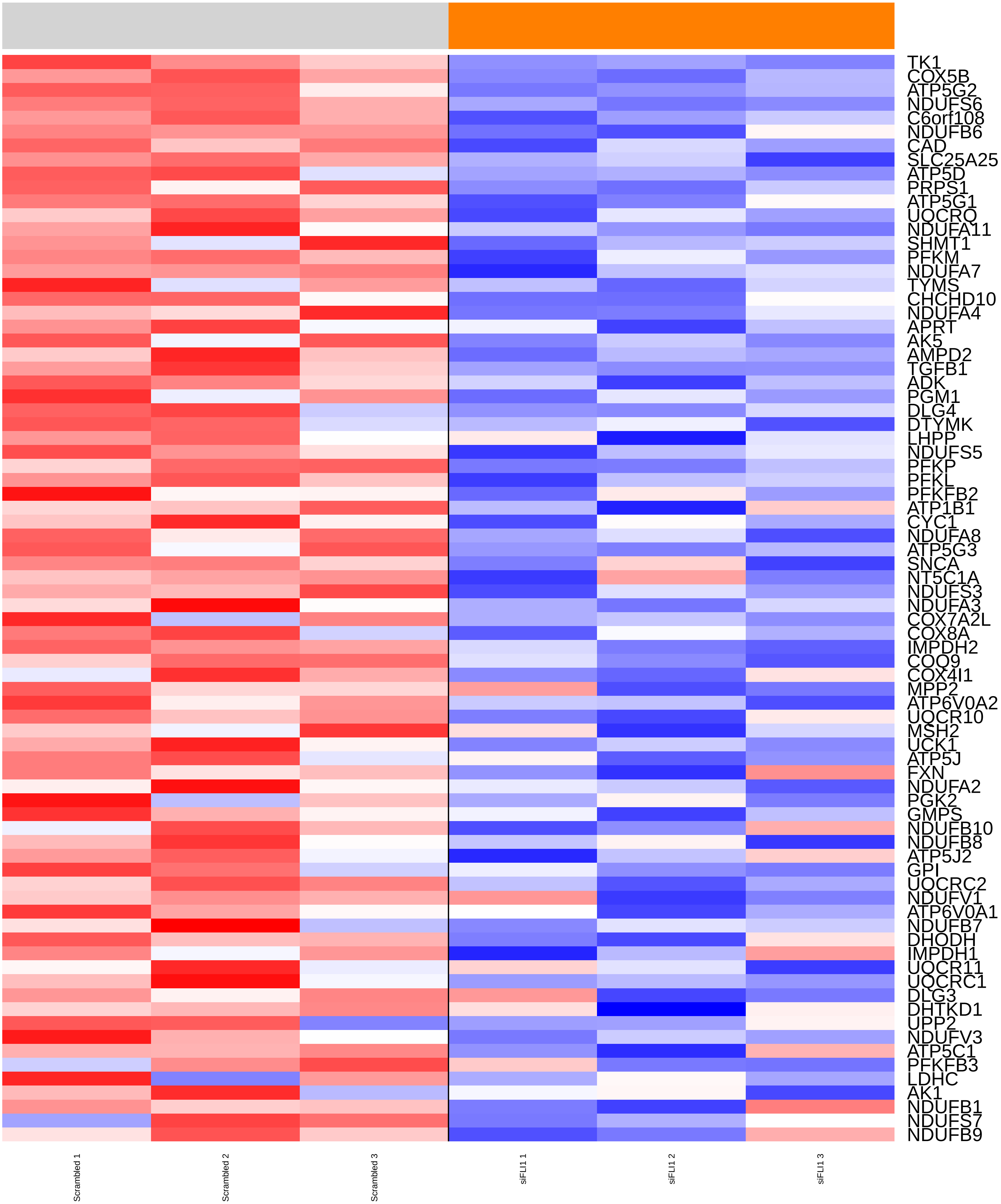

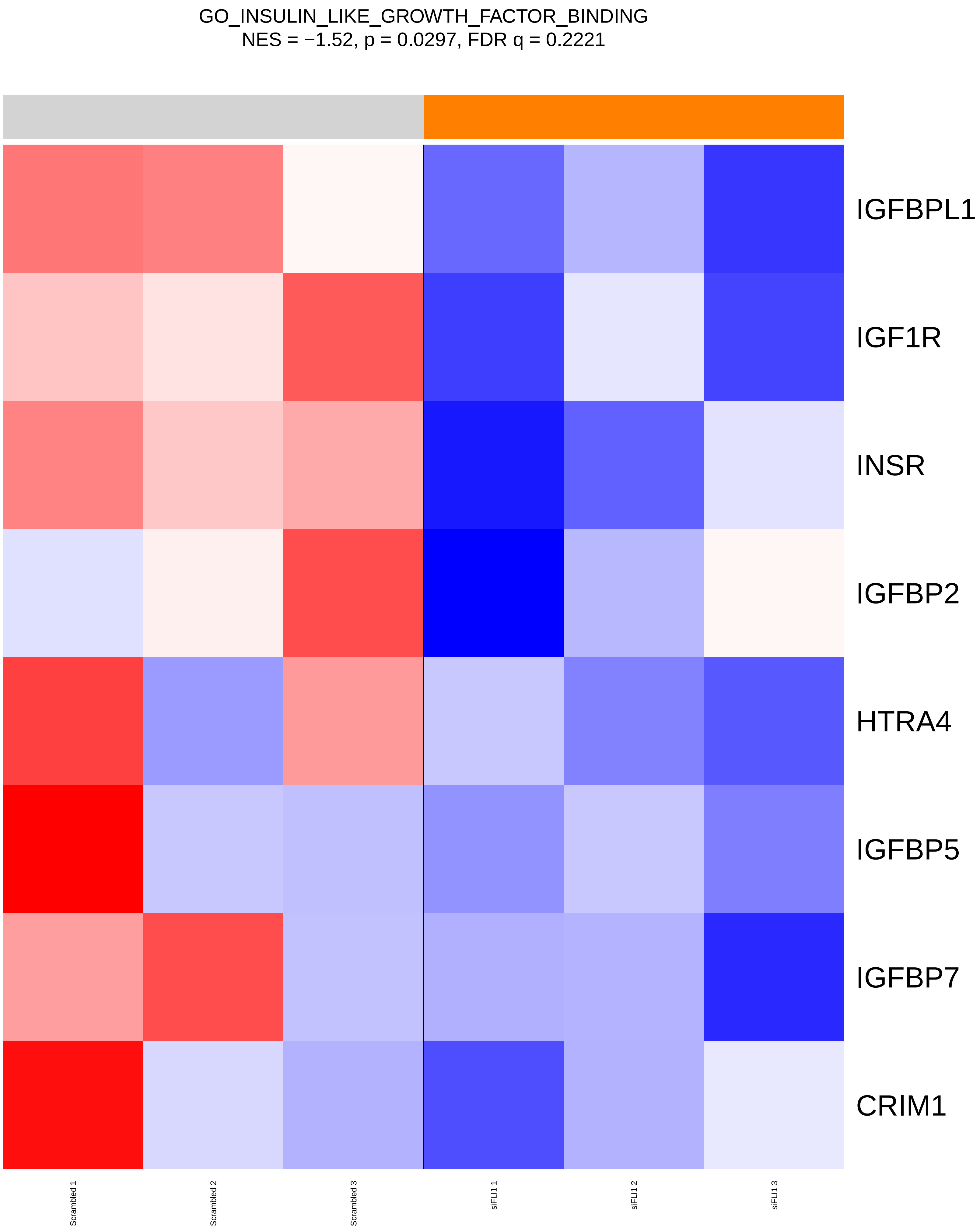

GO_INSULIN_LIKE_GROWTH_FACTOR_BINDING
NES = −1.52, p = 0.0297, FDR q = 0.2221
IGFBPL1
IGF1R
INSR
IGFBP2
HTRA4
IGFBP5
IGFBP7
CRIM1
Scrambled 1
Scrambled 2
Scrambled 3
siFLI1 1
siFLI1 2
siFLI1 3

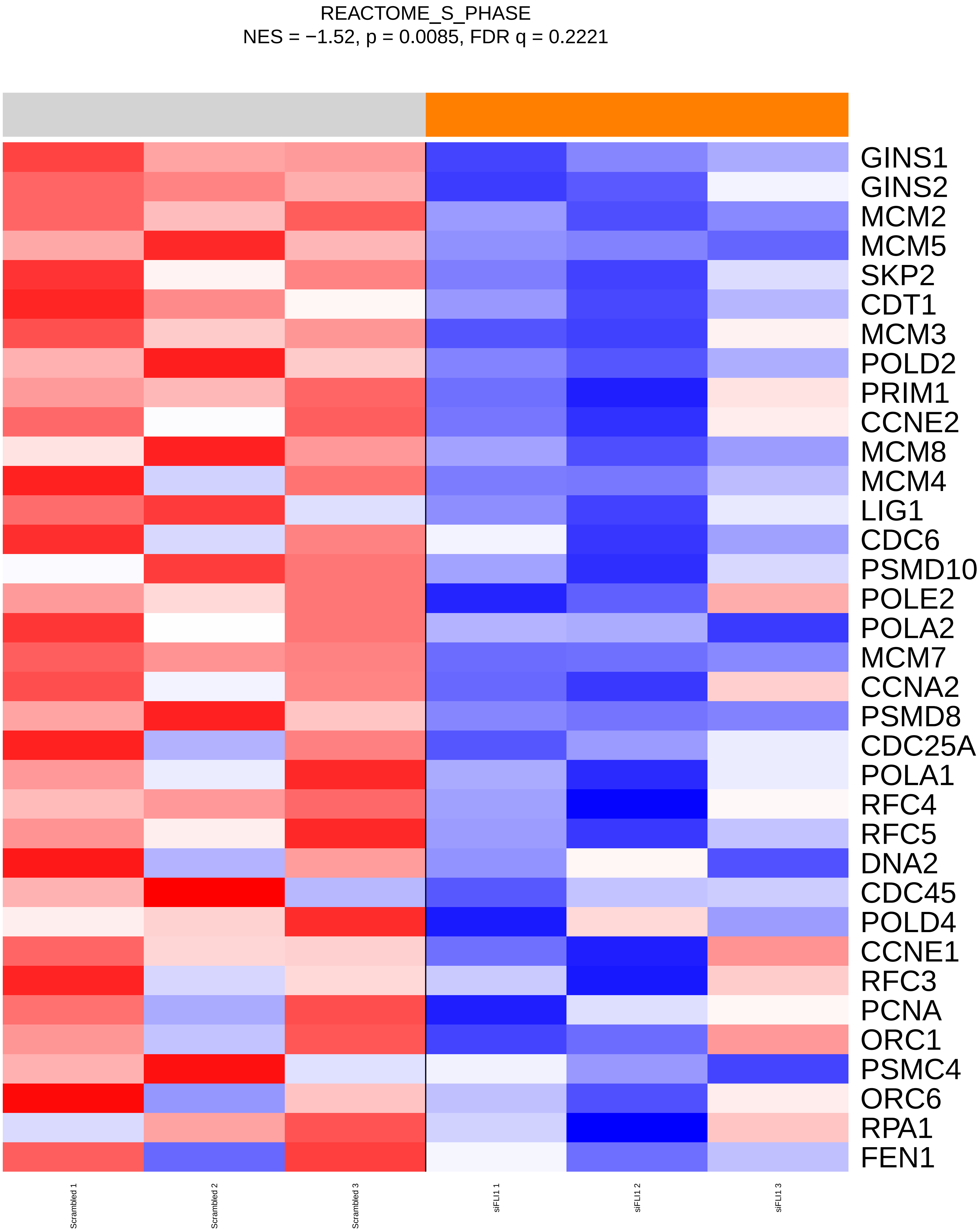
REACTOME_S_PHASE
NES = −1.52, p = 0.0085, FDR q = 0.2221
GINS1
GINS2
MCM2
MCM5
SKP2
CDT1
MCM3
POLD2
PRIM1
CCNE2
MCM8
MCM4
LIG1
CDC6
PSMD10
POLE2
POLA2
MCM7
CCNA2
PSMD8
CDC25A
POLA1
RFC4
RFC5
DNA2
CDC45
POLD4
CCNE1
RFC3
PCNA
ORC1
PSMC4
ORC6
RPA1
FEN1
Scrambled 1
Scrambled 2
Scrambled 3
siFLI1 1
siFLI1 2
siFLI1 3

GO_INSULIN_LIKE_GROWTH_FACTOR_RECEPTOR_BINDING
NES = −1.52, p = 0.0485, FDR q = 0.2226

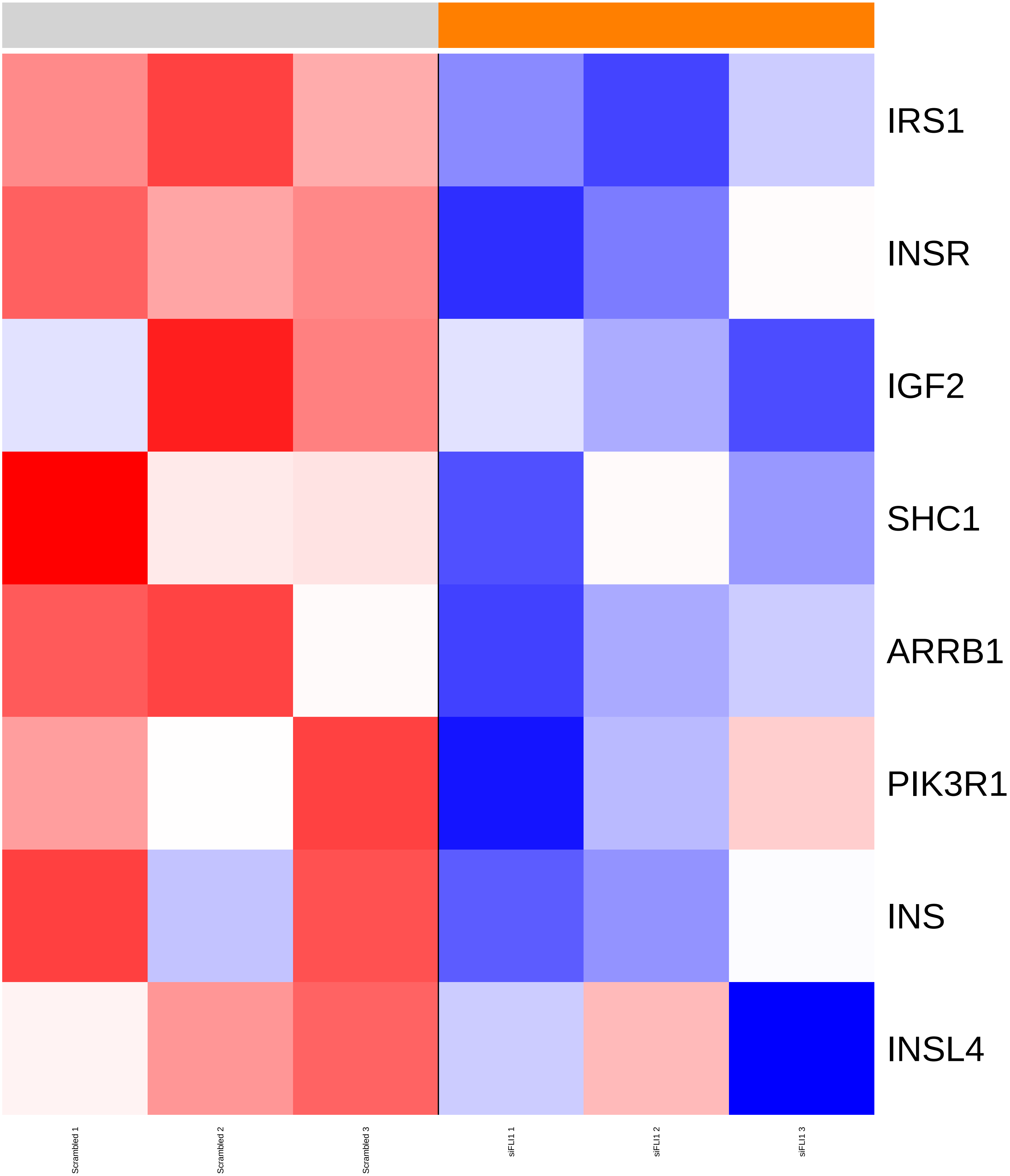

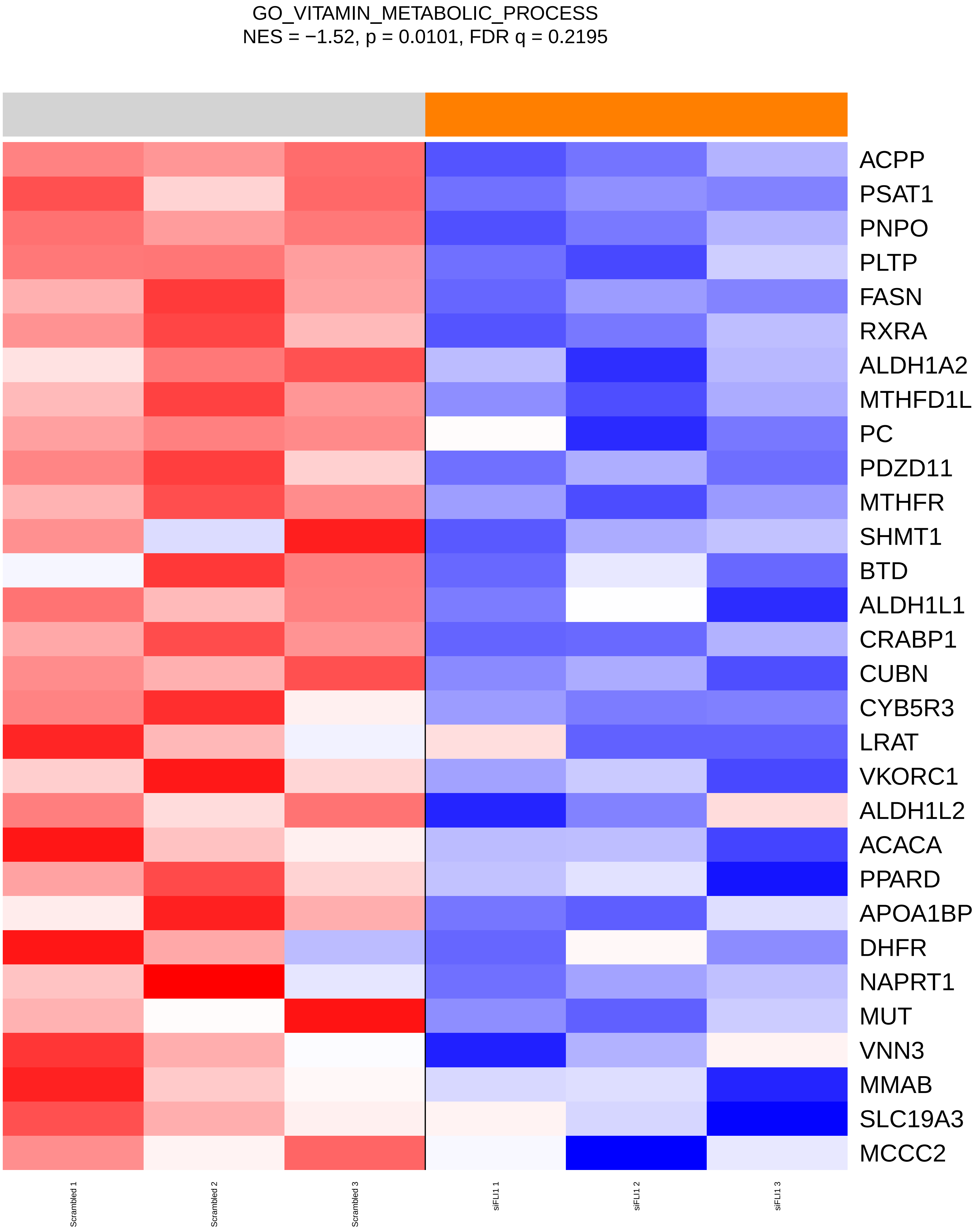
GO_VITAMIN_METABOLIC_PROCESS
NES = −1.52, p = 0.0101, FDR q = 0.2195
ACPP
PSAT1
PNPO
PLTP
FASN
RXRA
ALDH1A2
MTHFD1L
PC
PDZD11
MTHFR
SHMT1
BTD
ALDH1L1
CRABP1
CUBN
CYB5R3
LRAT
VKORC1
ALDH1L2
ACACA
PPARD
APOA1BP
DHFR
NAPRT1
MUT
VNN3
MMAB
SLC19A3
MCCC2
Scrambled 1
Scrambled 2
Scrambled 3
siFLI1 1
siFLI1 2
siFLI1 3

GO_RECIPROCAL_DNA_RECOMBINATION
NES = −1.52, p = 0.0235, FDR q = 0.2194

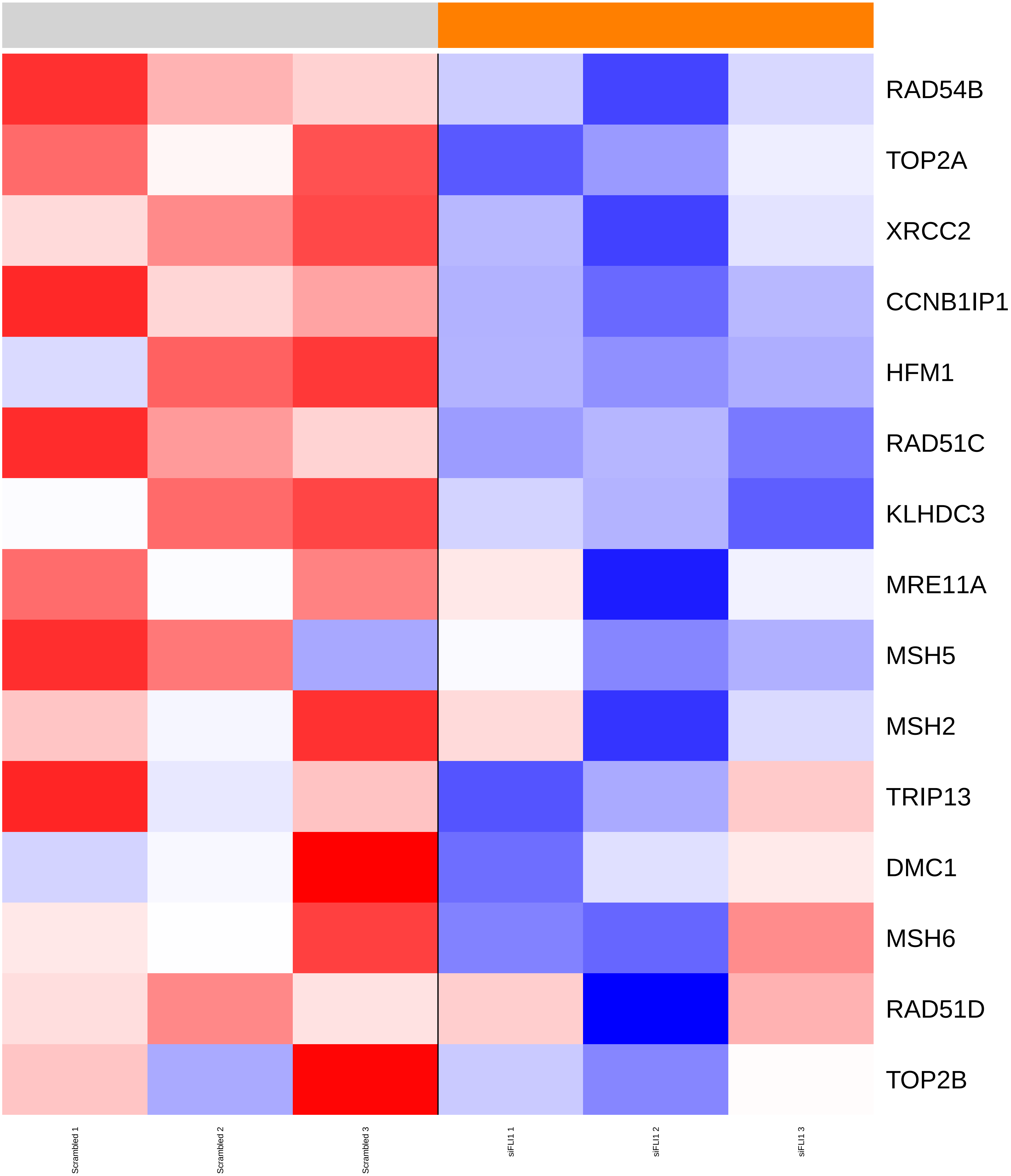

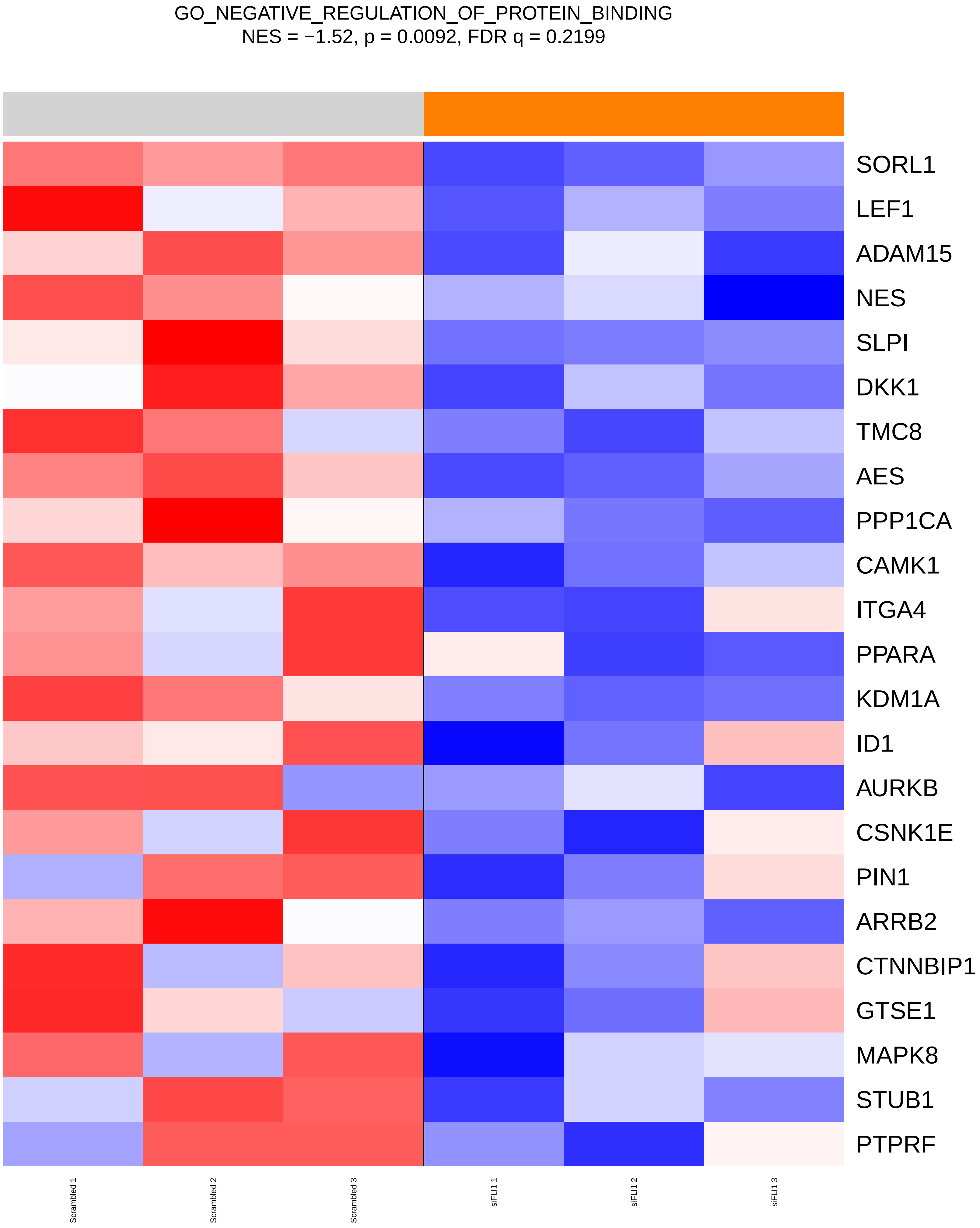
GO_NEGATIVE_REGULATION_OF_PROTEIN_BINDING
NES = −1.52, p = 0.0092, FDR q = 0.2199
SORL1
LEF1
ADAM15
NES
SLPI
DKK1
TMC8
AES
PPP1CA
CAMK1
ITGA4
PPARA
KDM1A
ID1
AURKB
CSNK1E
PIN1
ARRB2
CTNNBIP1
GTSE1
MAPK8
STUB1
PTPRF
Scrambled 1
Scrambled 2
Scrambled 3
siFLI1 1
siFLI1 2
siFLI1 3

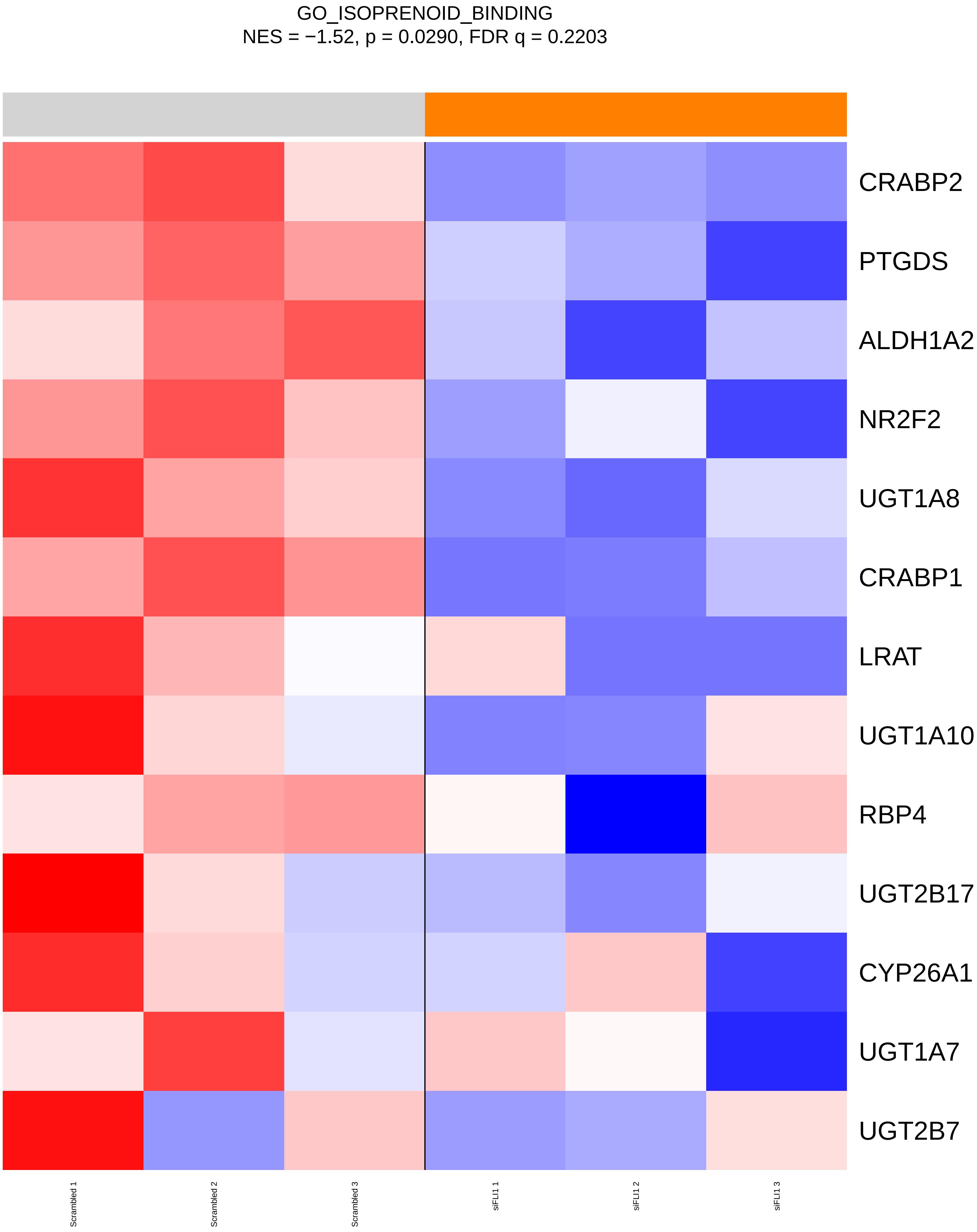
GO_ISOPRENOID_BINDING
NES = −1.52, p = 0.0290, FDR q = 0.2203
CRABP2
PTGDS
ALDH1A2
NR2F2
UGT1A8
CRABP1
LRAT
UGT1A10
RBP4
UGT2B17
CYP26A1
UGT1A7
UGT2B7
Scrambled 1
Scrambled 2
Scrambled 3
siFLI1 1
siFLI1 2
siFLI1 3

GO_DNA_REPLICATION_INDEPENDENT_NUCLEOSOME_ORGANIZATION
NES = −1.52, p = 0.0212, FDR q = 0.2201

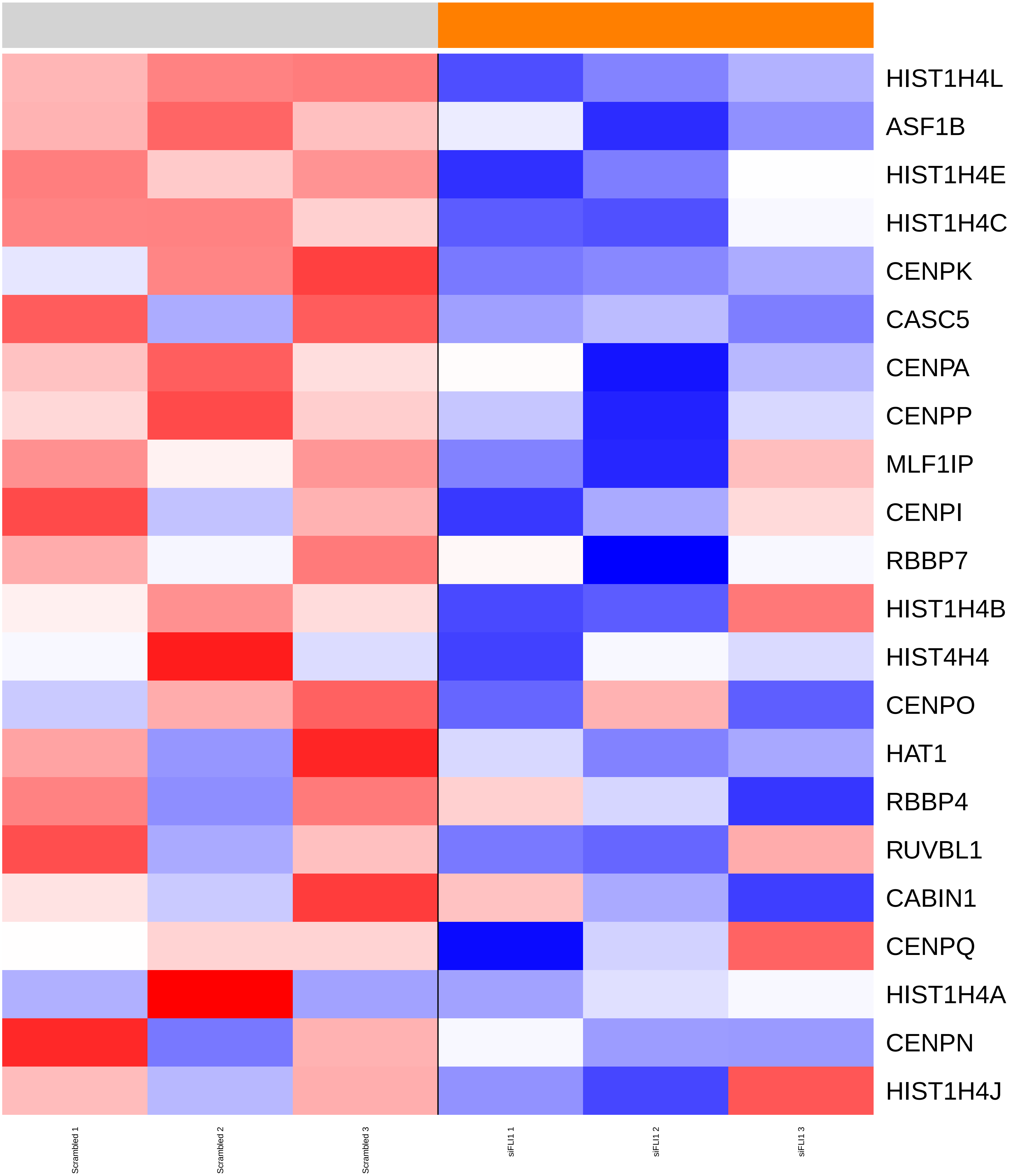

GO_NEGATIVE_REGULATION_OF_CELL_CYCLE_PROCESS
NES = −1.52, p = 0.0000, FDR q = 0.2205

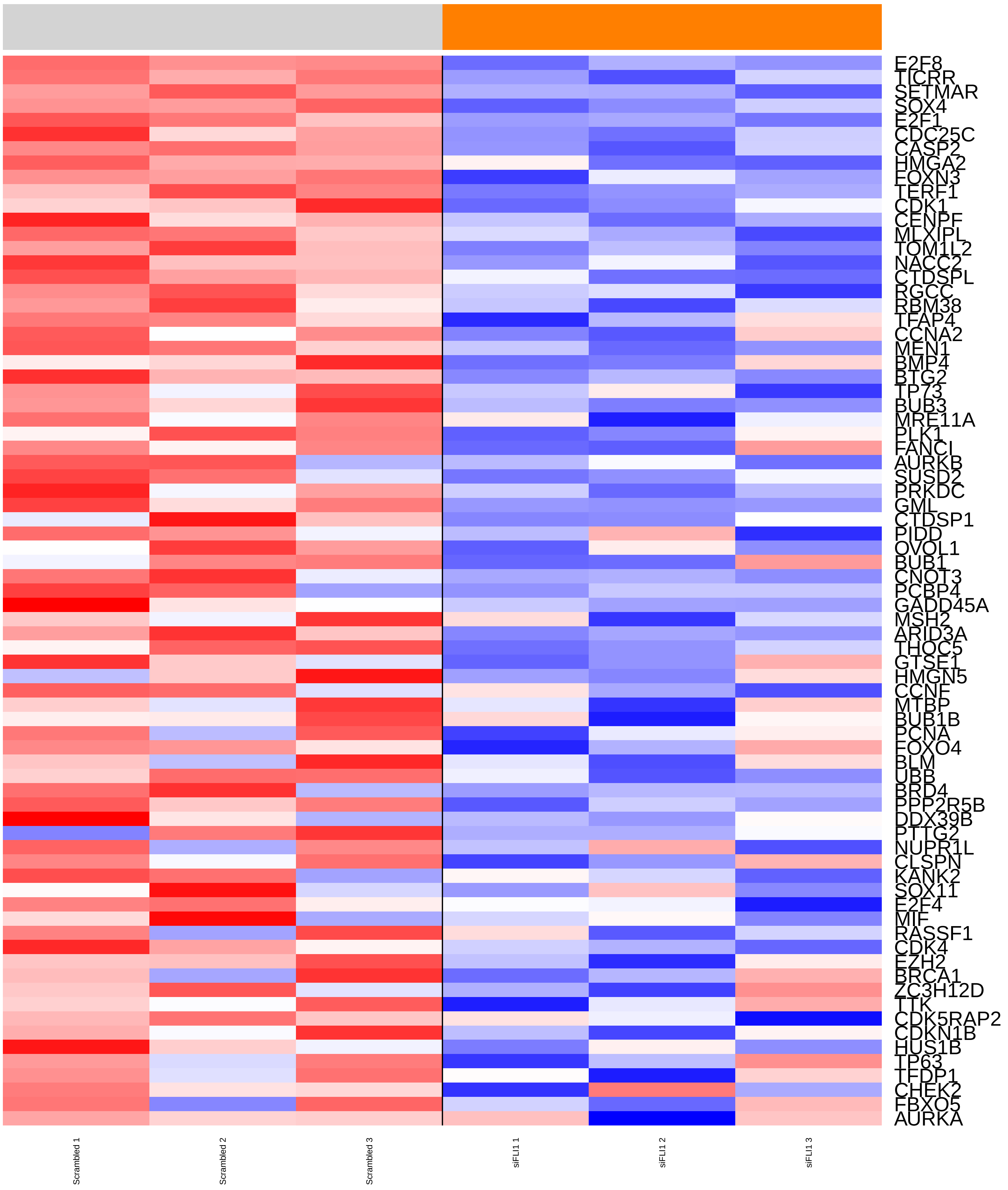

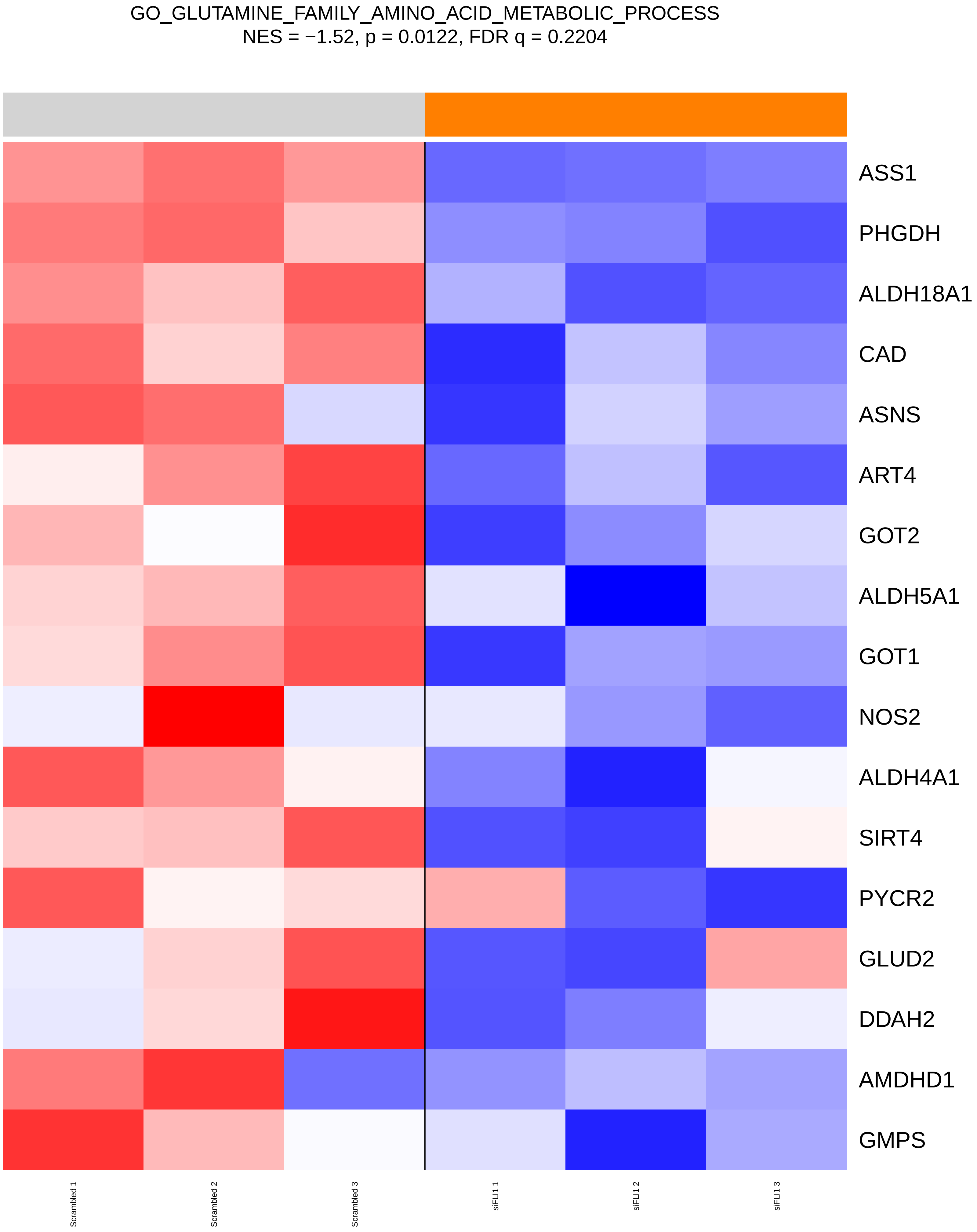
GO_GLUTAMINE_FAMILY_AMINO_ACID_METABOLIC_PROCESS
NES = −1.52, p = 0.0122, FDR q = 0.2204
ASS1
PHGDH
ALDH18A1
CAD
ASNS
ART4
GOT2
ALDH5A1
GOT1
NOS2
ALDH4A1
SIRT4
PYCR2
GLUD2
DDAH2
AMDHD1
GMPS
Scrambled 1
Scrambled 2
Scrambled 3
siFLI1 1
siFLI1 2
siFLI1 3

GO_ENDODERM_DEVELOPMENT
NES = −1.52, p = 0.0104, FDR q = 0.2202

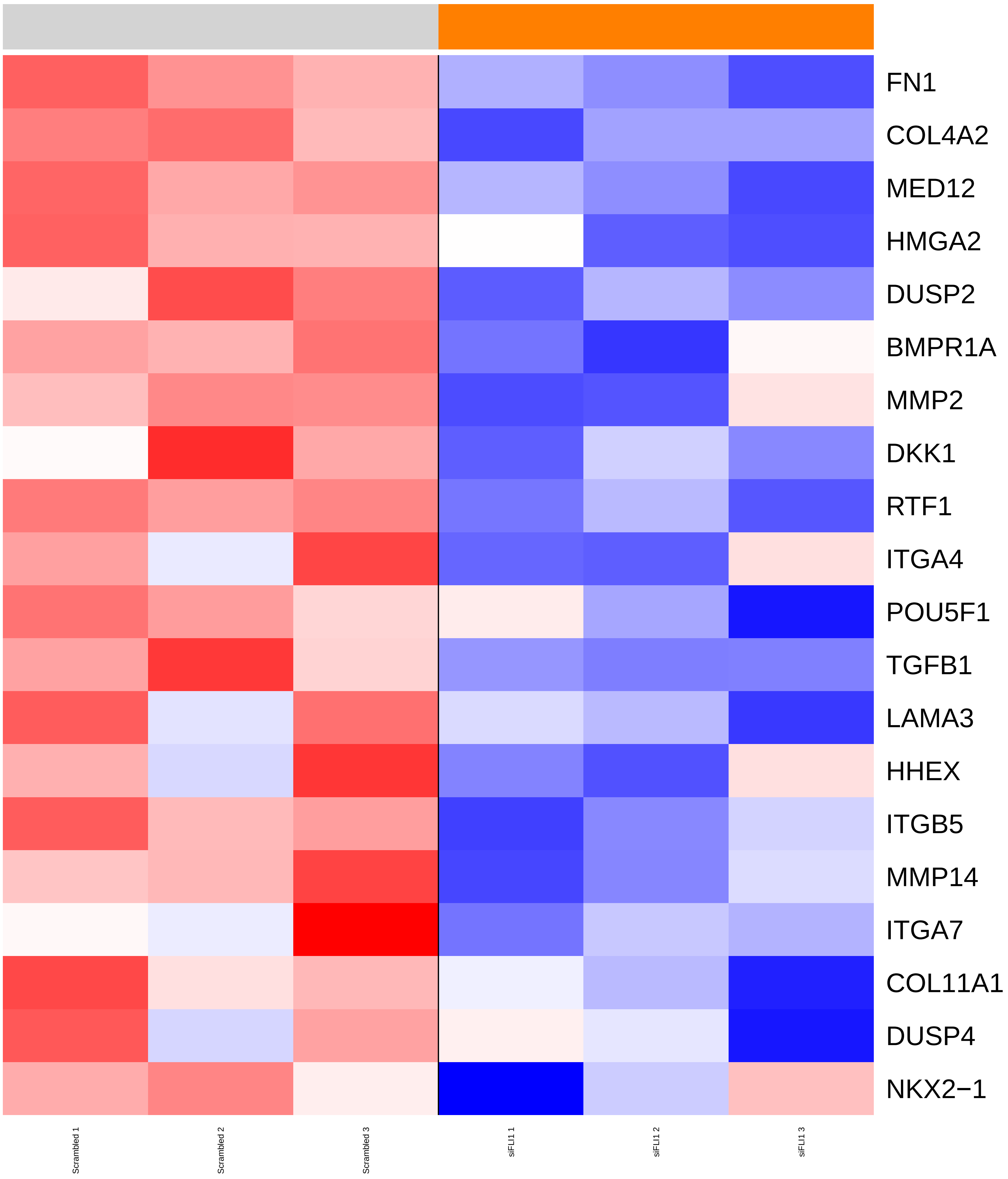

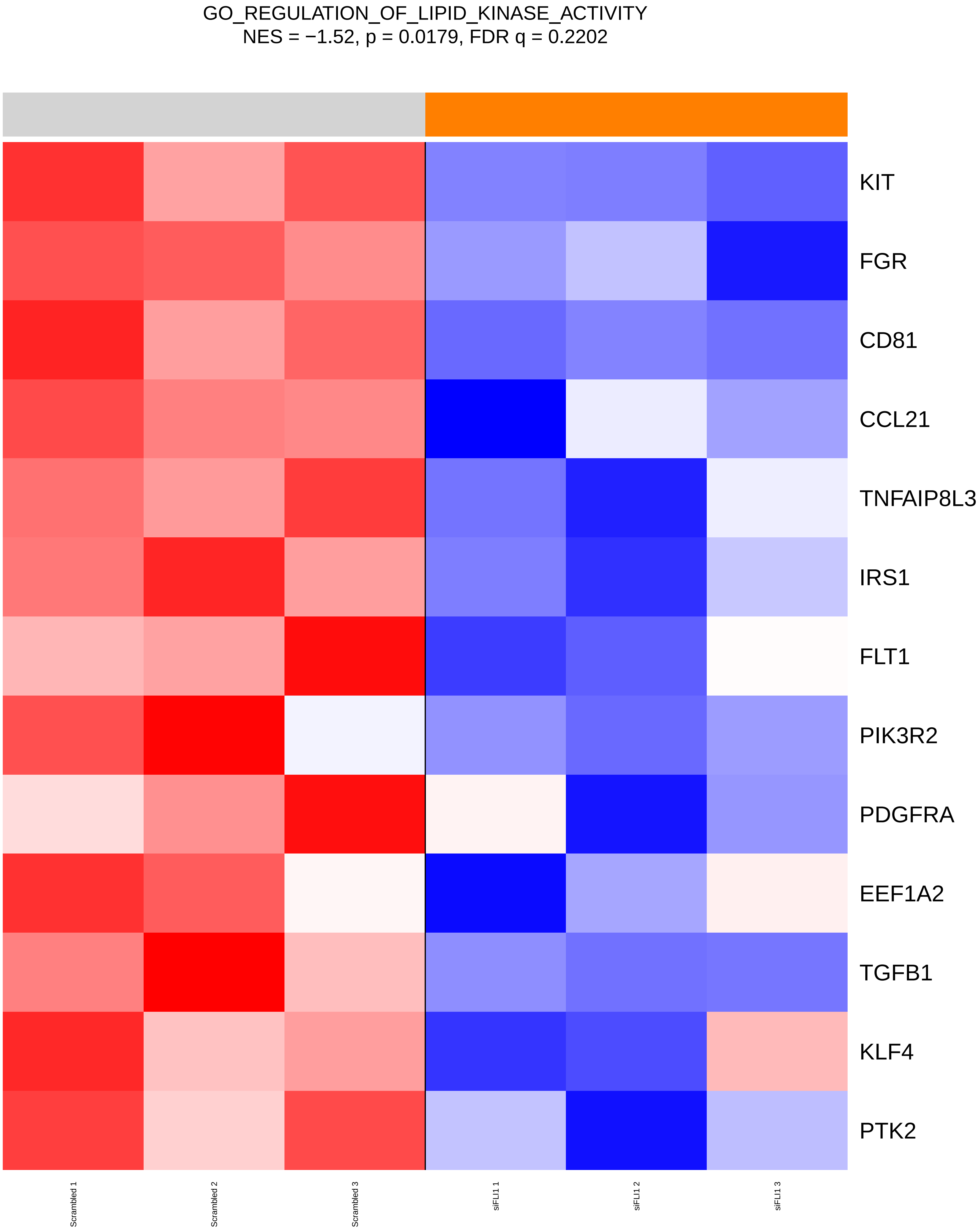
GO_REGULATION_OF_LIPID_KINASE_ACTIVITY
NES = −1.52, p = 0.0179, FDR q = 0.2202
KIT
FGR
CD81
CCL21
TNFAIP8L3
IRS1
FLT1
PIK3R2
PDGFRA
EEF1A2
TGFB1
KLF4
PTK2
Scrambled 1
Scrambled 2
Scrambled 3
siFLI1 1
siFLI1 2
siFLI1 3

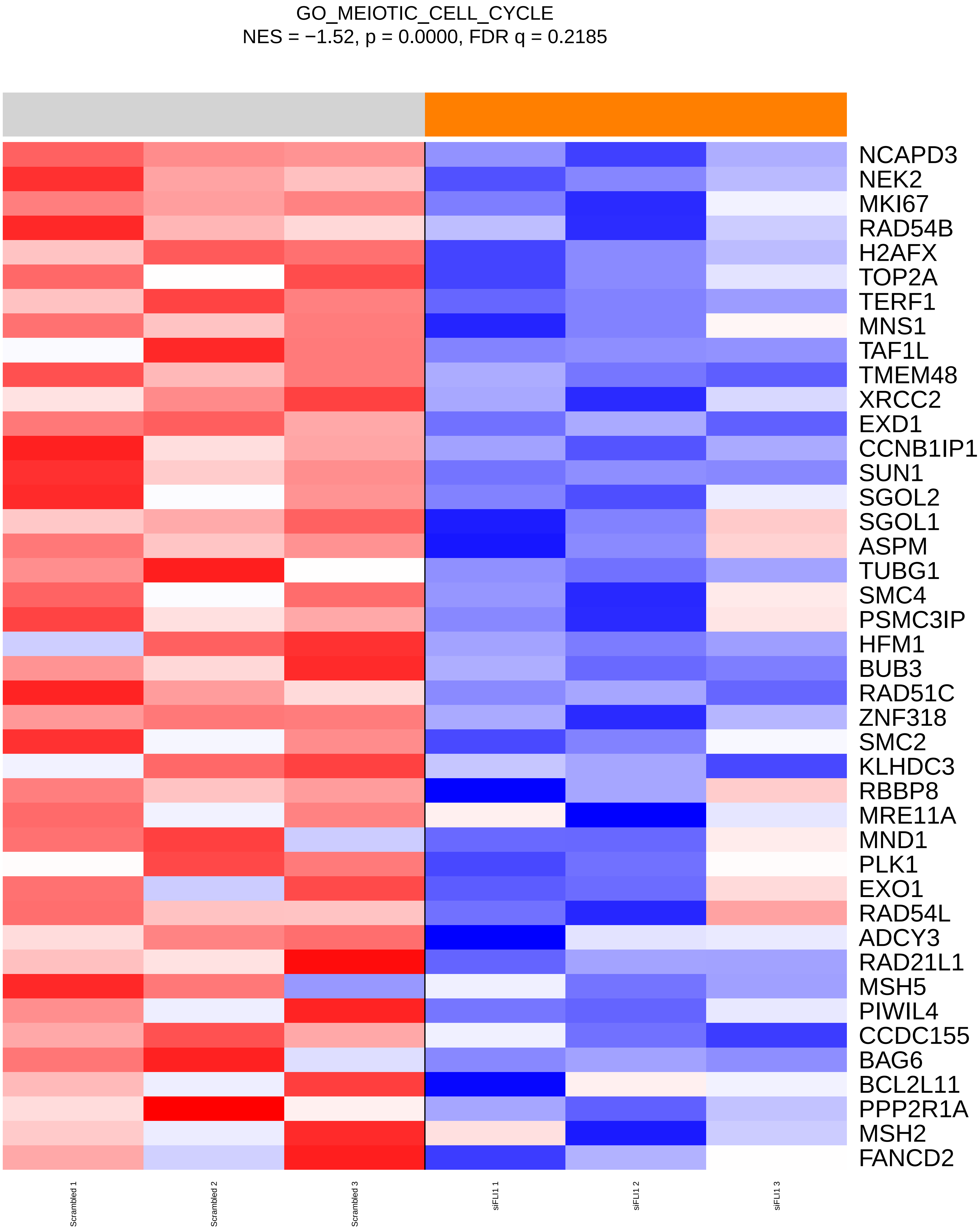
GO_MEIOTIC_CELL_CYCLE
NES = −1.52, p = 0.0000, FDR q = 0.2185
NCAPD3
NEK2
MKI67
RAD54B
H2AFX
TOP2A
TERF1
MNS1
TAF1L
TMEM48
XRCC2
EXD1
CCNB1IP1
SUN1
SGOL2
SGOL1
ASPM
TUBG1
SMC4
PSMC3IP
HFM1
BUB3
RAD51C
ZNF318
SMC2
KLHDC3
RBBP8
MRE11A
MND1
PLK1
EXO1
RAD54L
ADCY3
RAD21L1
MSH5
PIWIL4
CCDC155
BAG6
BCL2L11
PPP2R1A
MSH2
FANCD2
Scrambled 1
Scrambled 2
Scrambled 3
siFLI1 1
siFLI1 2
siFLI1 3

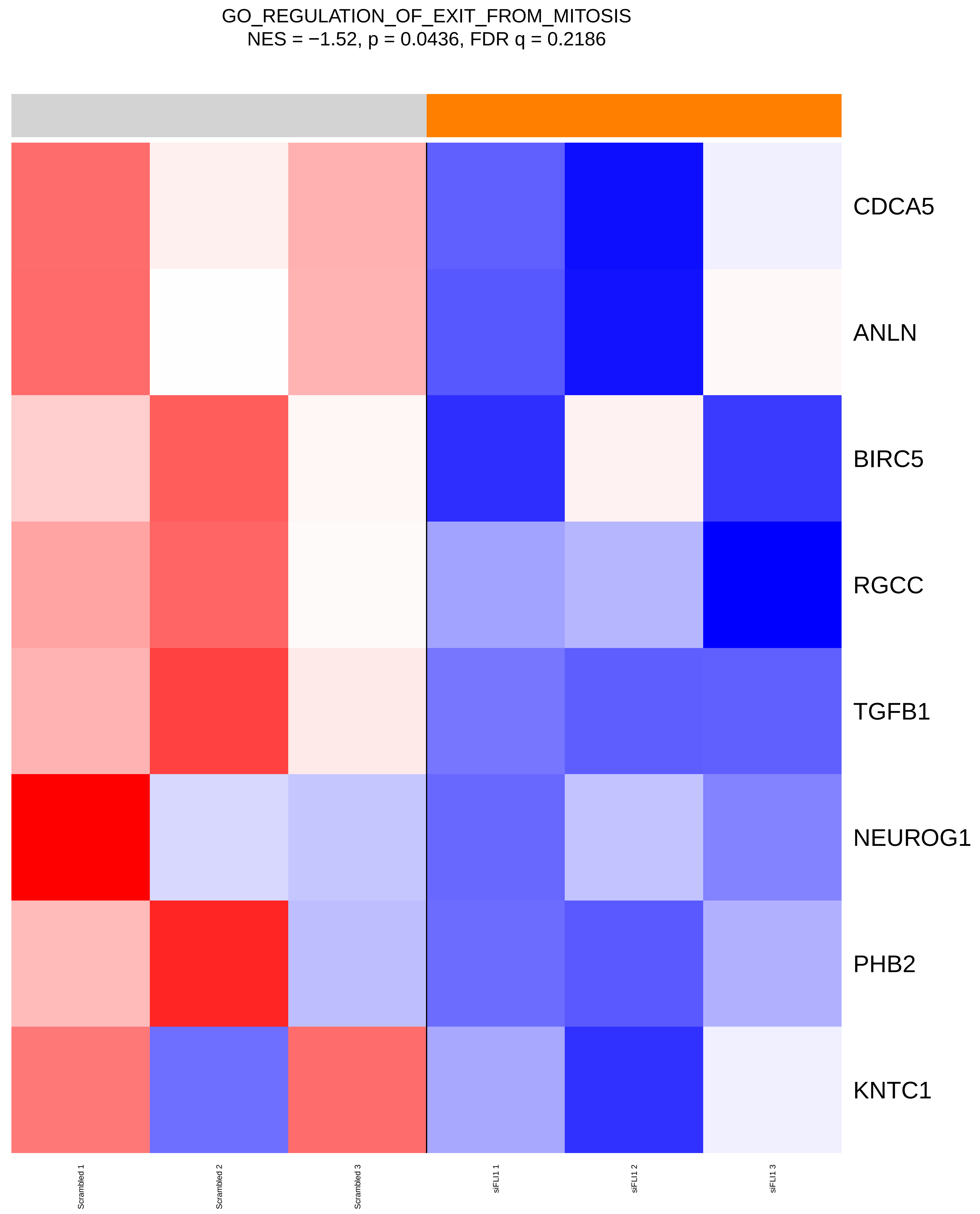
GO_REGULATION_OF_EXIT_FROM_MITOSIS
NES = −1.52, p = 0.0436, FDR q = 0.2186
CDCA5
ANLN
BIRC5
RGCC
TGFB1
NEUROG1
PHB2
KNTC1
Scrambled 1
Scrambled 2
Scrambled 3
siFLI1 1
siFLI1 2
siFLI1 3

GO_ORGANIC_CATION_TRANSMEMBRANE_TRANSPORTER_ACTIVITY
NES = −1.53, p = 0.0317, FDR q = 0.2132

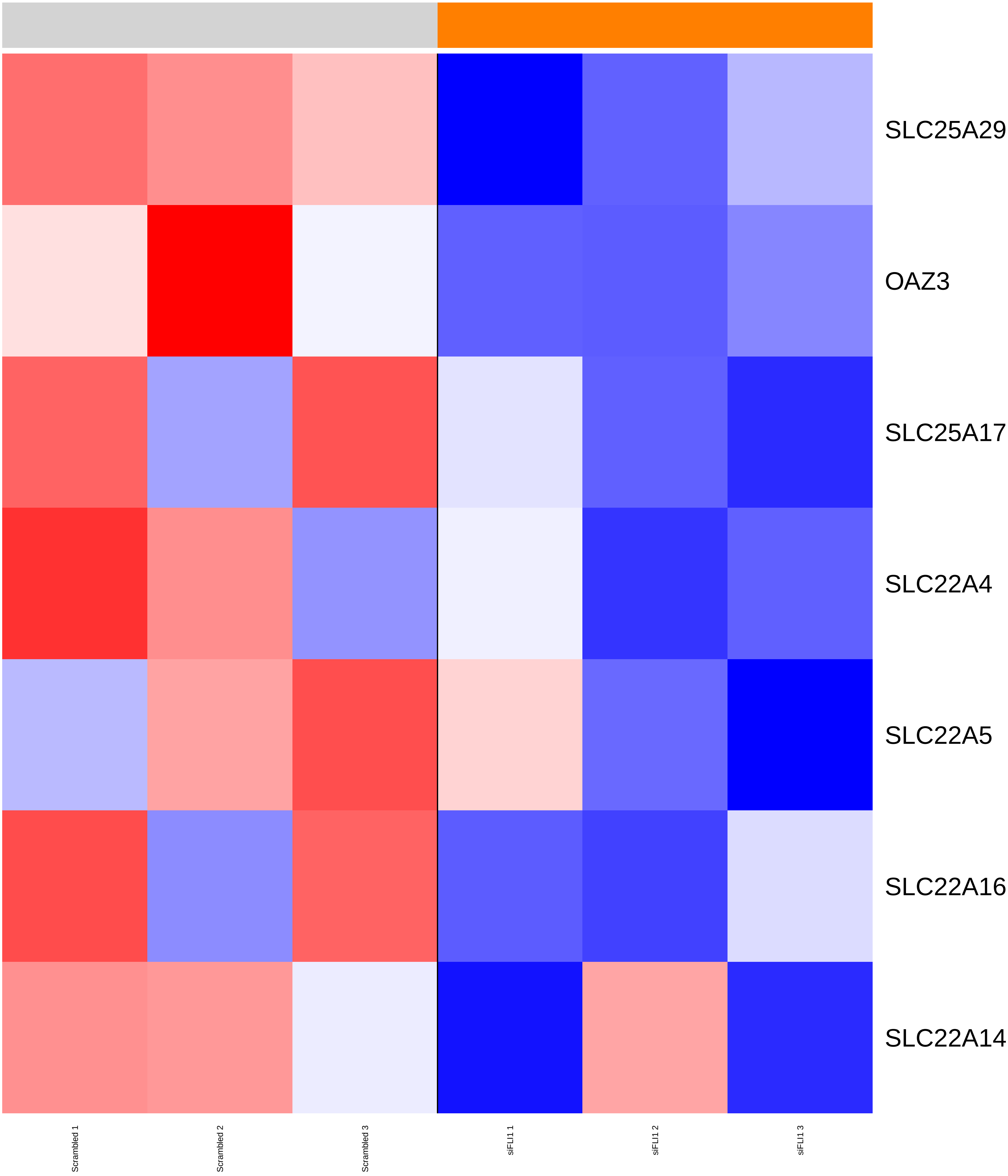

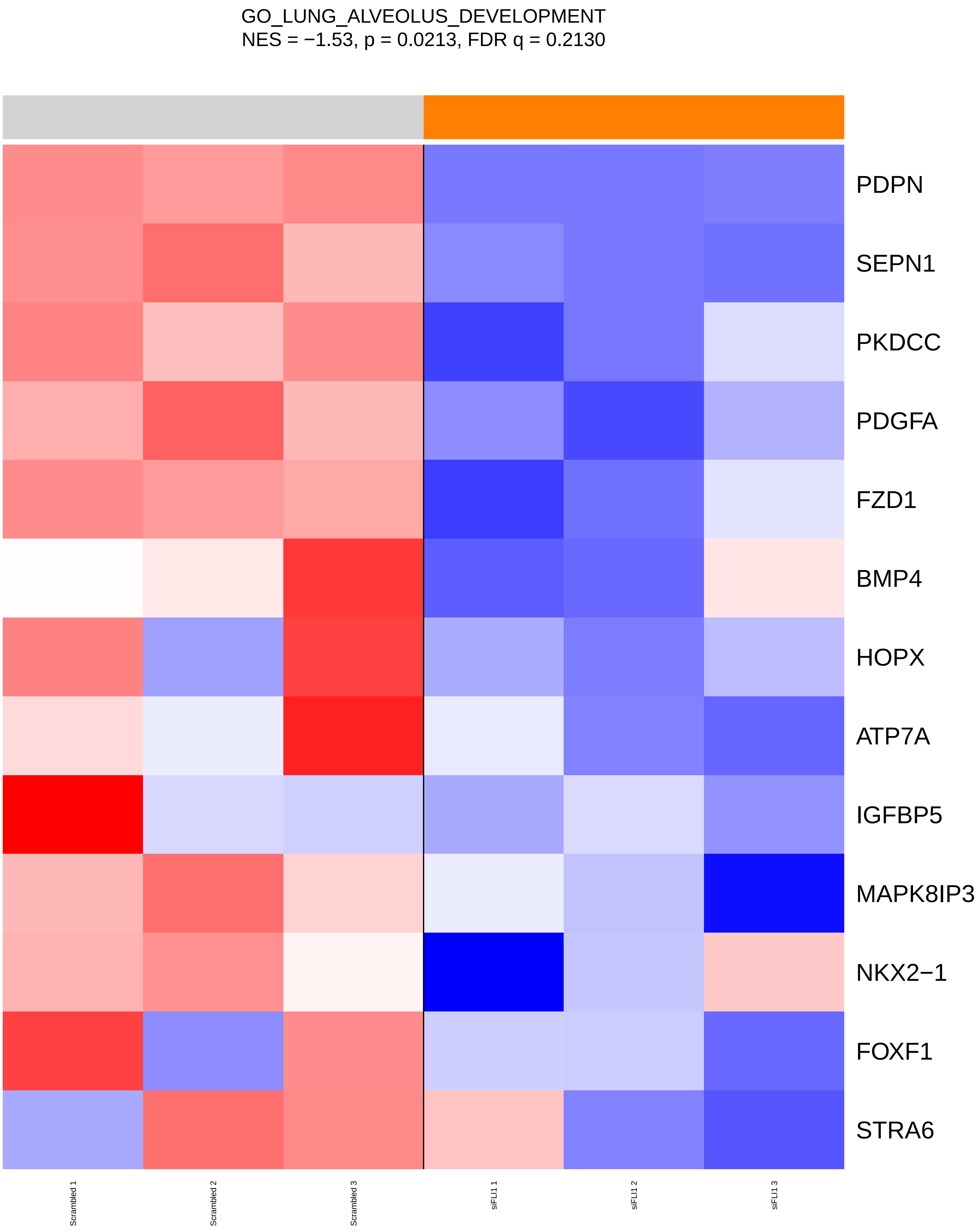
GO_LUNG_ALVEOLUS_DEVELOPMENT
NES = −1.53, p = 0.0213, FDR q = 0.2130
PDPN
SEPN1
PKDCC
PDGFA
FZD1
BMP4
HOPX
ATP7A
IGFBP5
MAPK8IP3
NKX2−1
FOXF1
STRA6
Scrambled 1
Scrambled 2
Scrambled 3
siFLI1 1
siFLI1 2
siFLI1 3

REACTOME_TRANSPORT_TO_THE_GOLGI_AND_SUBSEQUENT_MODIFICATION
NES = −1.53, p = 0.0413, FDR q = 0.2128

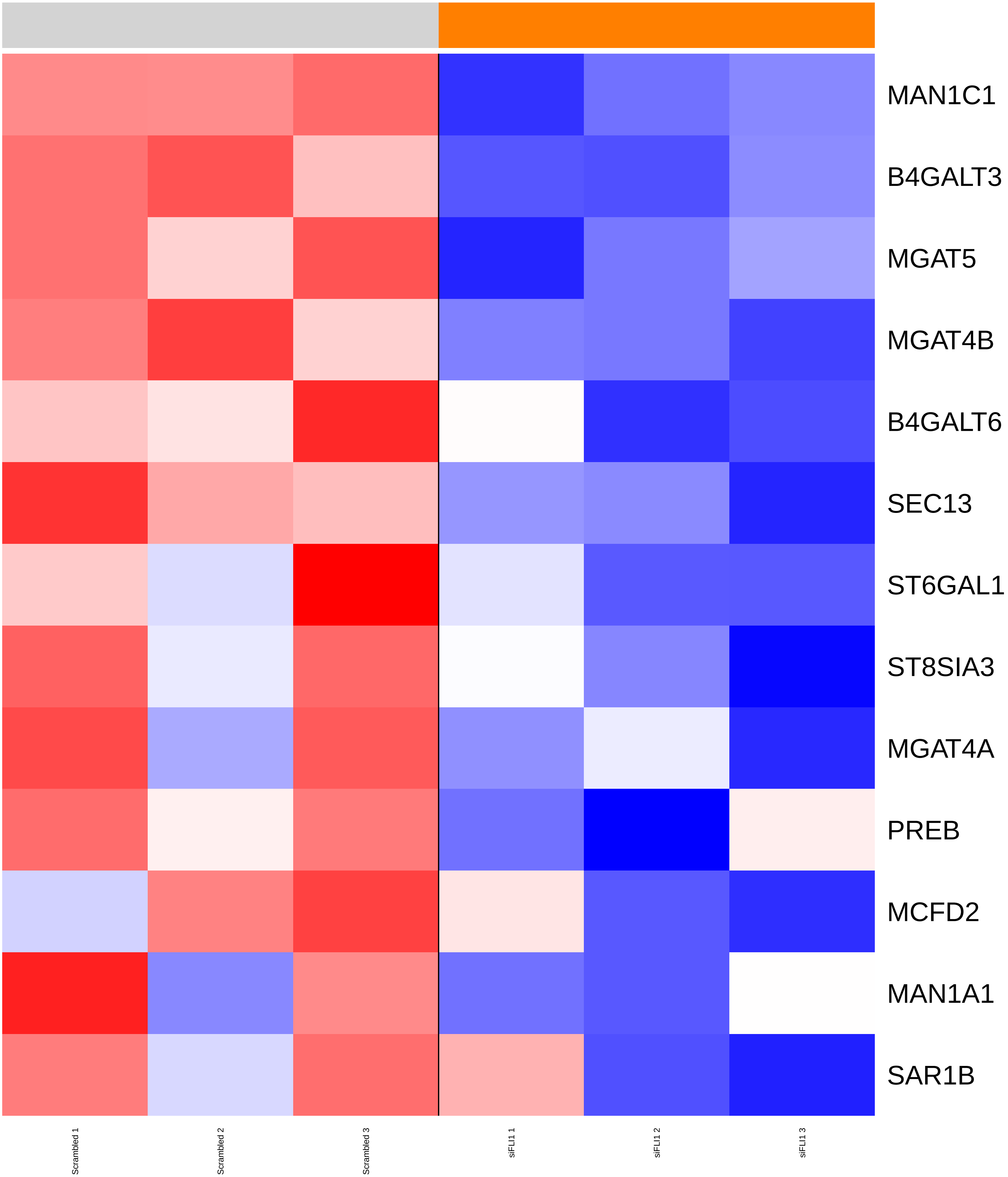

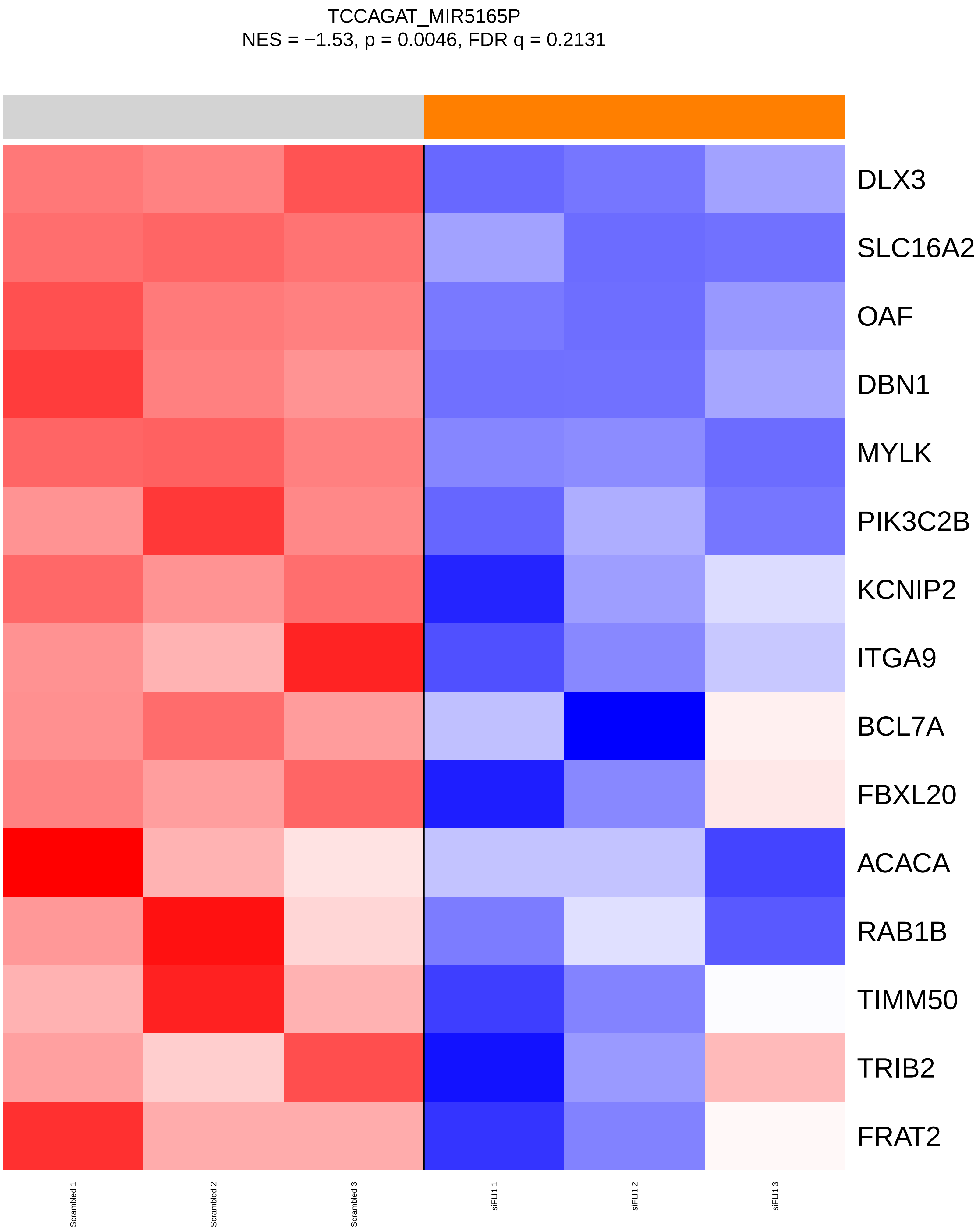

TCCAGAT_MIR5165P
NES = −1.53, p = 0.0046, FDR q = 0.2131
DLX3
SLC16A2
OAF
DBN1
MYLK
PIK3C2B
KCNIP2
ITGA9
BCL7A
FBXL20
ACACA
RAB1B
TIMM50
TRIB2
FRAT2
Scrambled 1
Scrambled 2
Scrambled 3
siFLI1 1
siFLI1 2
siFLI1 3

REACTOME_MEIOSIS
NES = −1.53, p = 0.0088, FDR q = 0.2136

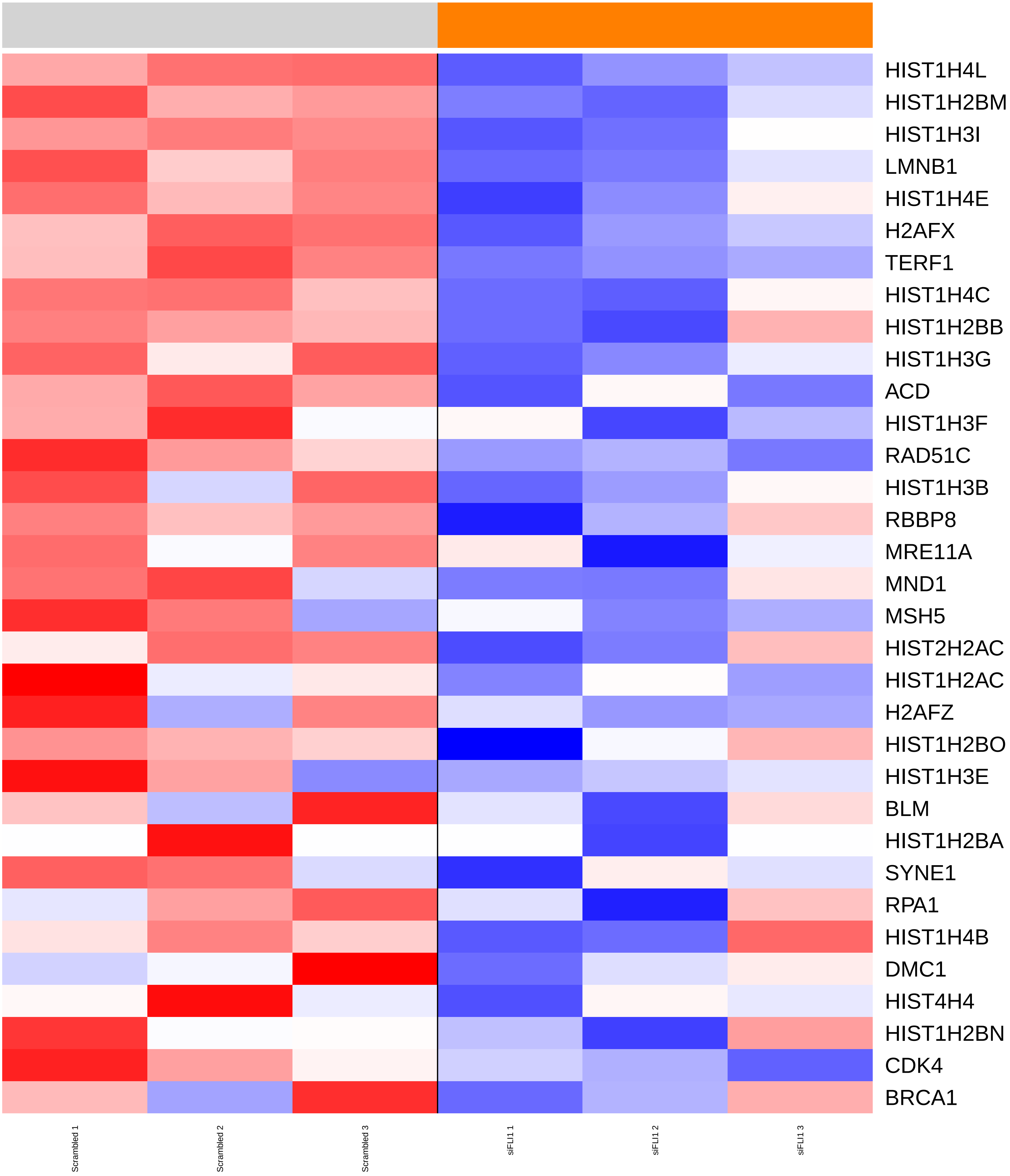

REACTOME_CA_DEPENDENT_EVENTS
NES = −1.53, p = 0.0272, FDR q = 0.2133

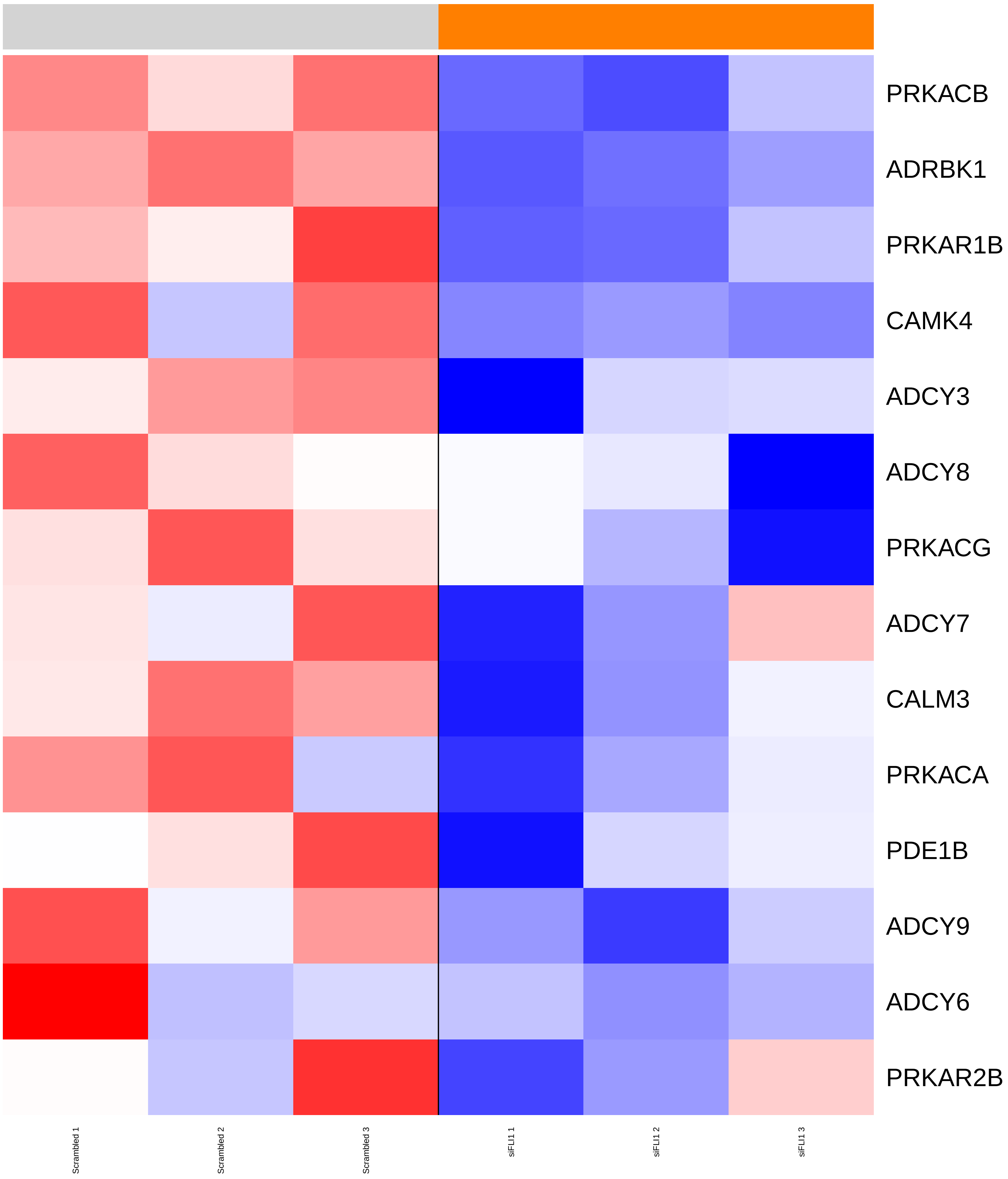

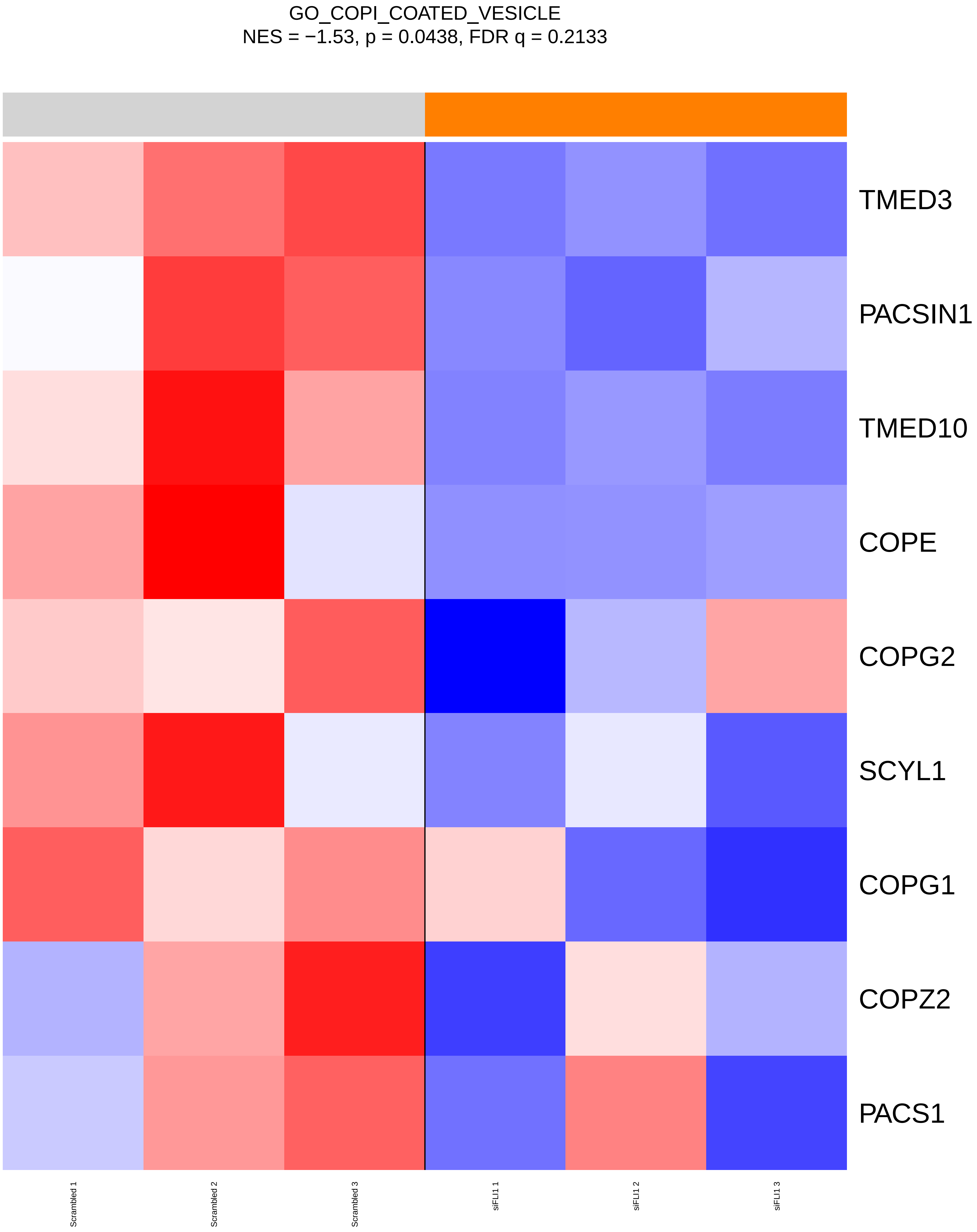

GO_COPI_COATED_VESICLE
NES = −1.53, p = 0.0438, FDR q = 0.2133
TMED3
PACSIN1
TMED10
COPE
COPG2
SCYL1
COPG1
COPZ2
PACS1
Scrambled 1
Scrambled 2
Scrambled 3
siFLI1 1
siFLI1 2
siFLI1 3

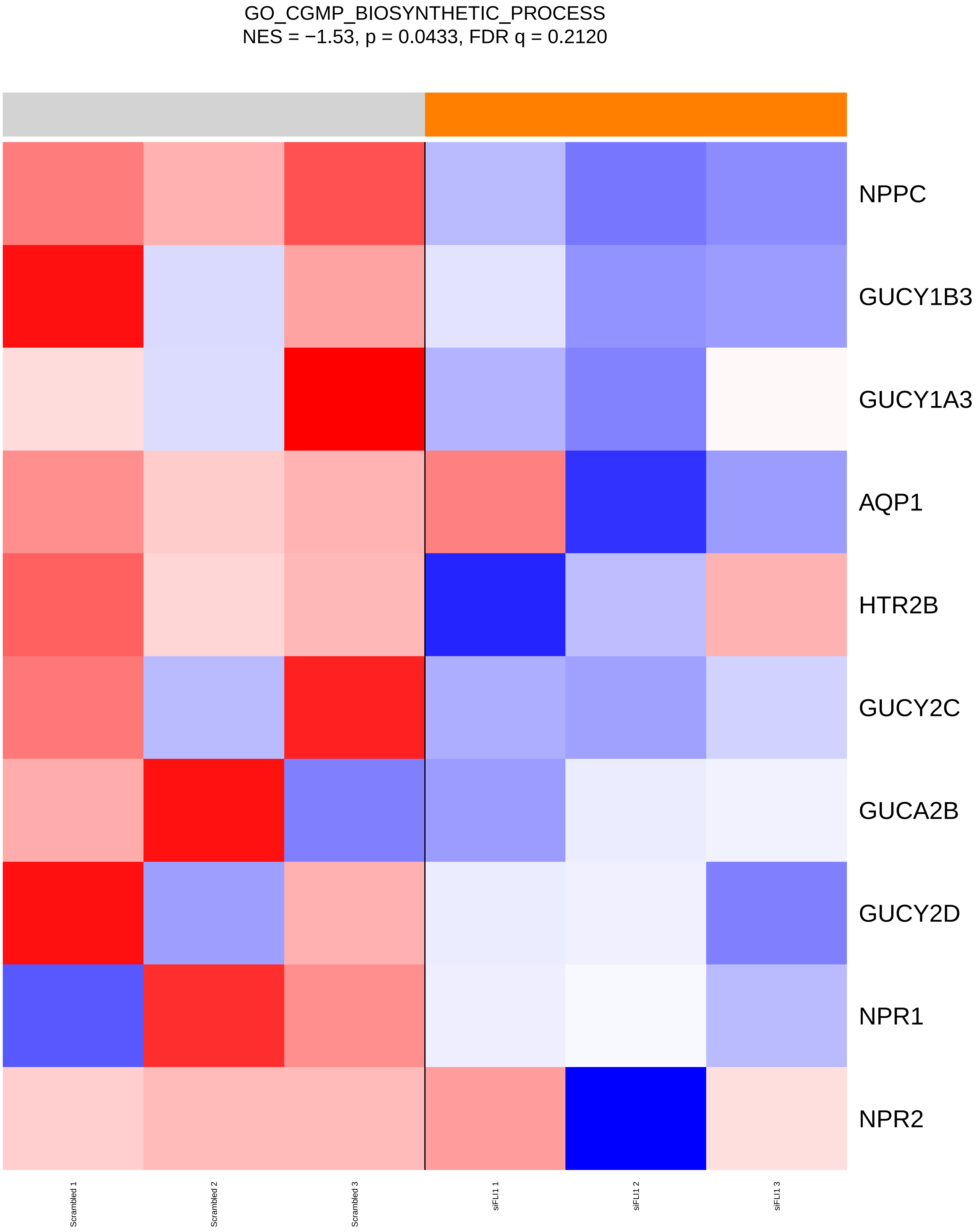
GO_CGMP_BIOSYNTHETIC_PROCESS
NES = −1.53, p = 0.0433, FDR q = 0.2120
NPPC
GUCY1B3
GUCY1A3
AQP1
HTR2B
GUCY2C
GUCA2B
GUCY2D
NPR1
NPR2
Scrambled 1
Scrambled 2
Scrambled 3
siFLI1 1
siFLI1 2
siFLI1 3

GO_POTASSIUM_CHANNEL_ACTIVITY
NES = −1.53, p = 0.0086, FDR q = 0.2125

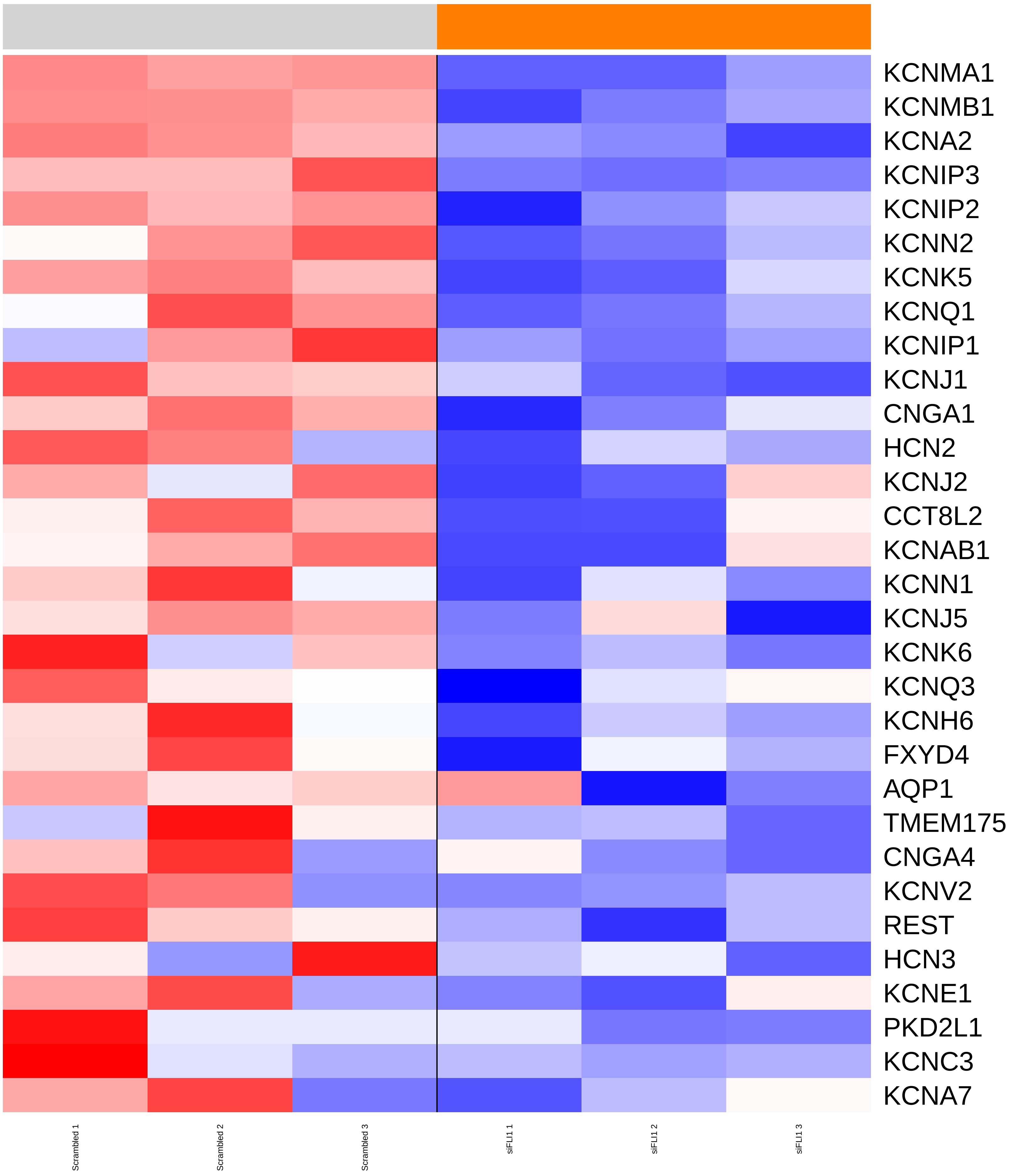

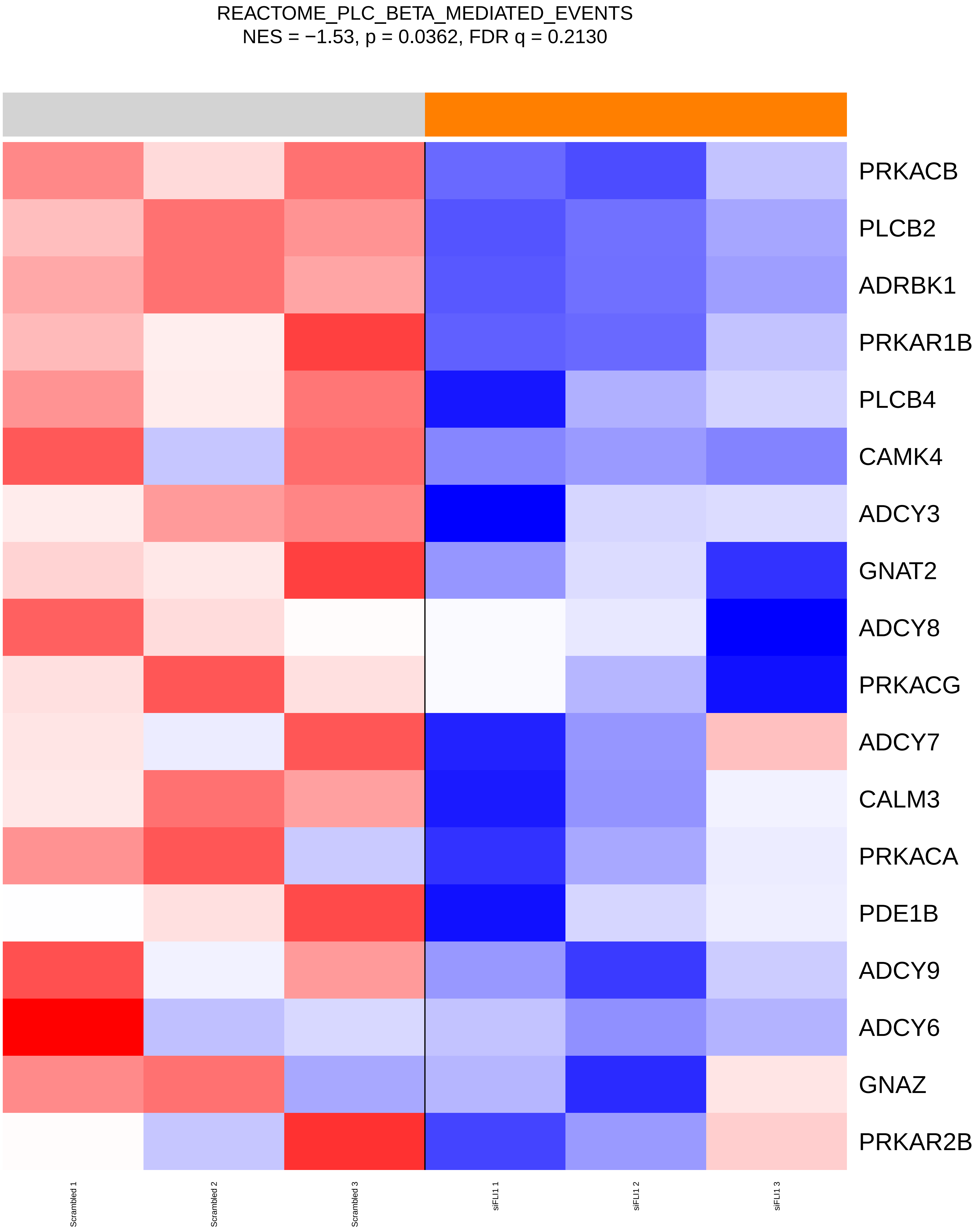
REACTOME_PLC_BETA_MEDIATED_EVENTS
NES = −1.53, p = 0.0362, FDR q = 0.2130
PRKACB
PLCB2
ADRBK1
PRKAR1B
PLCB4
CAMK4
ADCY3
GNAT2
ADCY8
PRKACG
ADCY7
CALM3
PRKACA
PDE1B
ADCY9
ADCY6
GNAZ
PRKAR2B
Scrambled 1
Scrambled 2
Scrambled 3
siFLI1 1
siFLI1 2
siFLI1 3

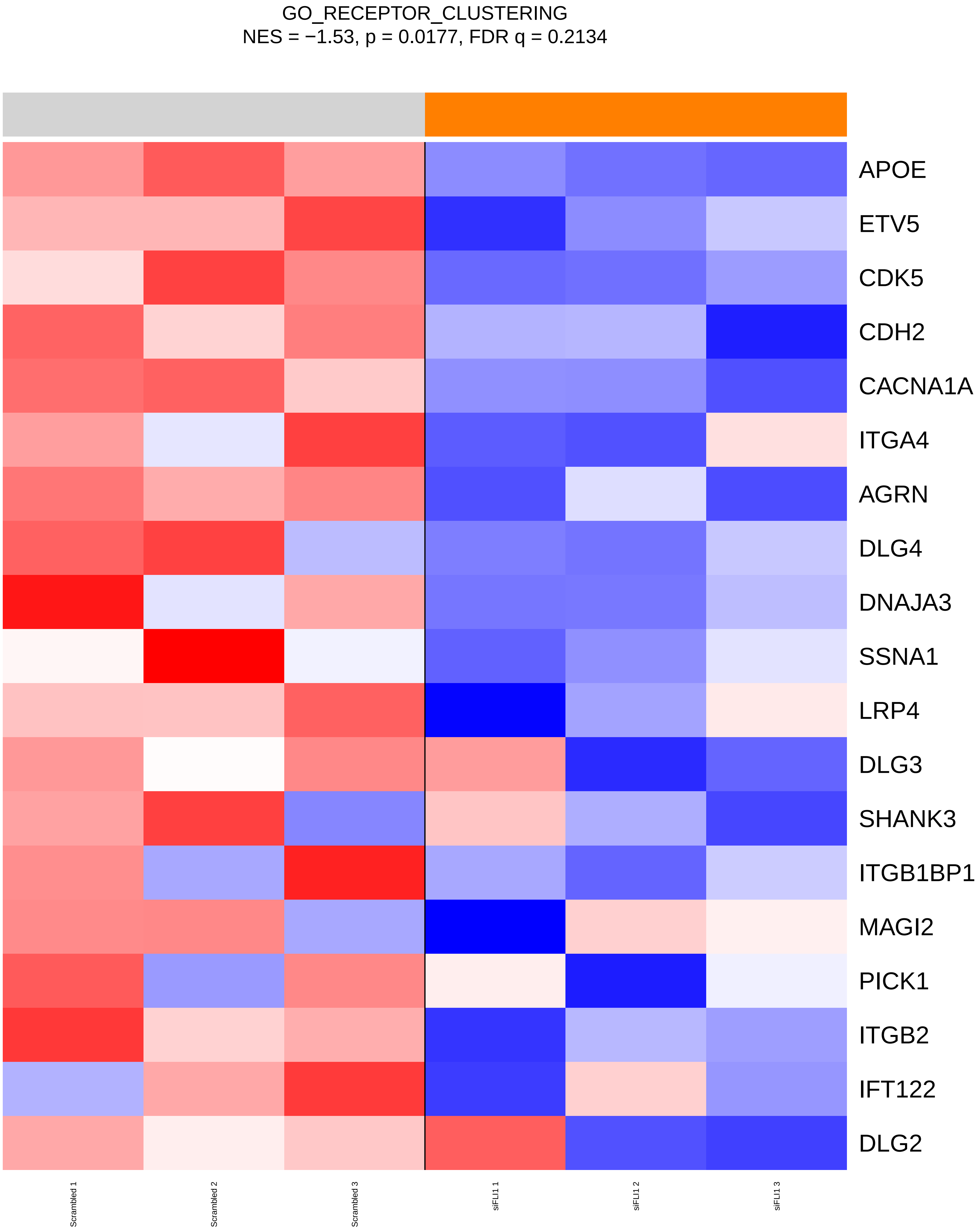
GO_RECEPTOR_CLUSTERING
NES = −1.53, p = 0.0177, FDR q = 0.2134
APOE
ETV5
CDK5
CDH2
CACNA1A
ITGA4
AGRN
DLG4
DNAJA3
SSNA1
LRP4
DLG3
SHANK3
ITGB1BP1
MAGI2
PICK1
ITGB2
IFT122
DLG2
Scrambled 1
Scrambled 2
Scrambled 3
siFLI1 1
siFLI1 2
siFLI1 3

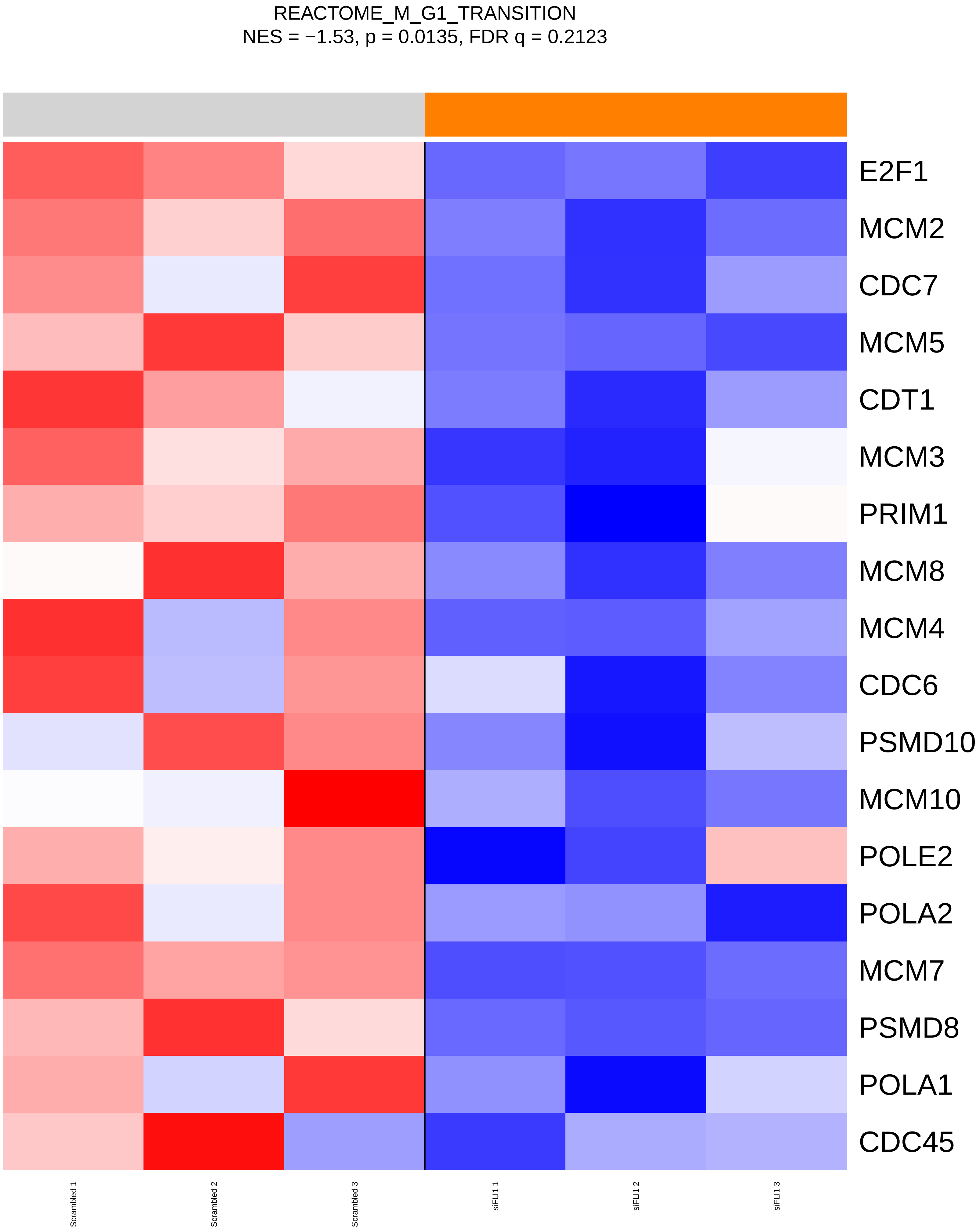
REACTOME_M_G1_TRANSITION
NES = −1.53, p = 0.0135, FDR q = 0.2123
E2F1
MCM2
CDC7
MCM5
CDT1
MCM3
PRIM1
MCM8
MCM4
CDC6
PSMD10
MCM10
POLE2
POLA2
MCM7
PSMD8
POLA1
CDC45
Scrambled 1
Scrambled 2
Scrambled 3
siFLI1 1
siFLI1 2
siFLI1 3

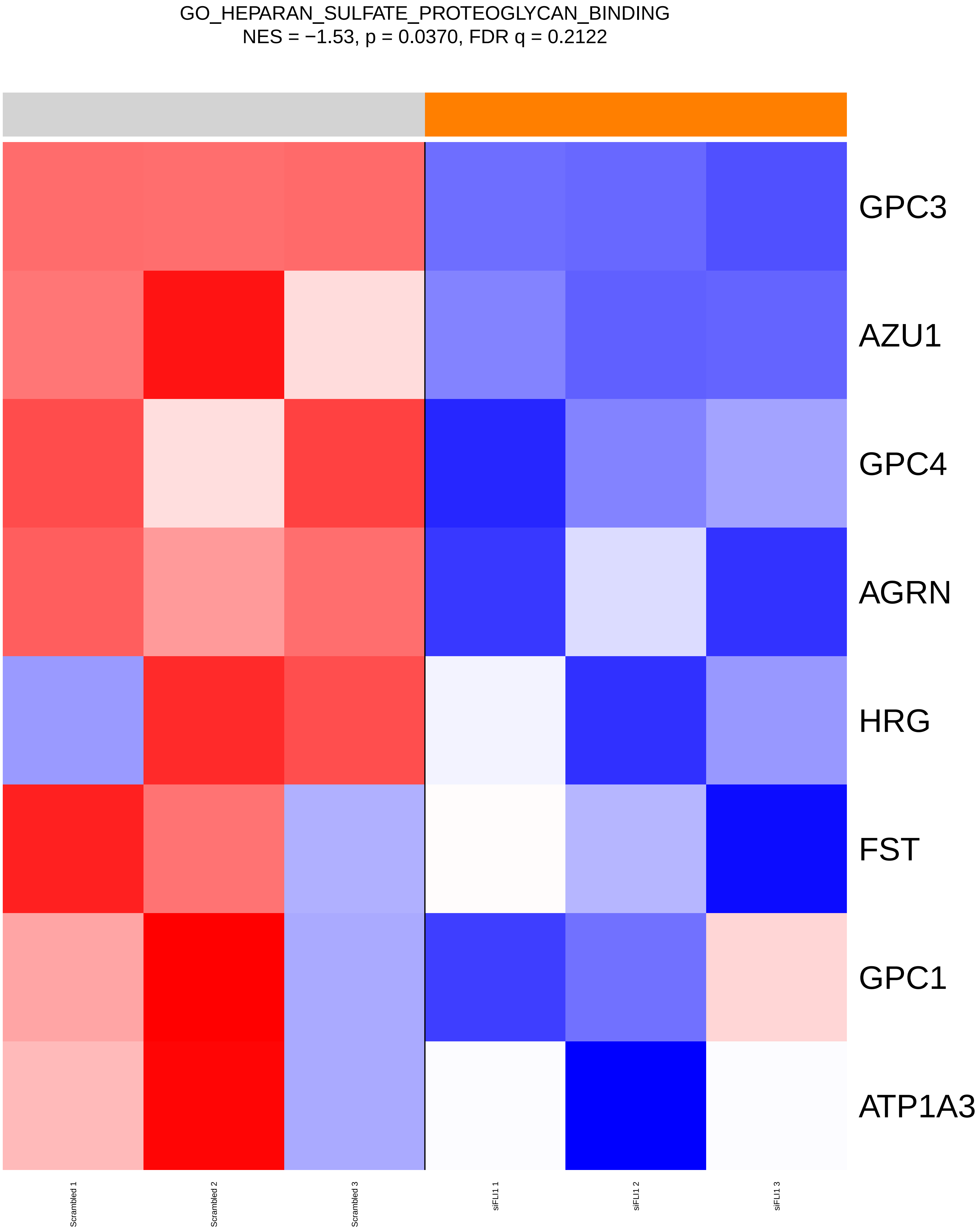
GO_HEPARAN_SULFATE_PROTEOGLYCAN_BINDING
NES = −1.53, p = 0.0370, FDR q = 0.2122
GPC3
AZU1
GPC4
AGRN
HRG
FST
GPC1
ATP1A3
Scrambled 1
Scrambled 2
Scrambled 3
siFLI1 1
siFLI1 2
siFLI1 3

GO_REGULATION_OF_CELL_CYCLE_ARREST
NES = −1.53, p = 0.0072, FDR q = 0.2128

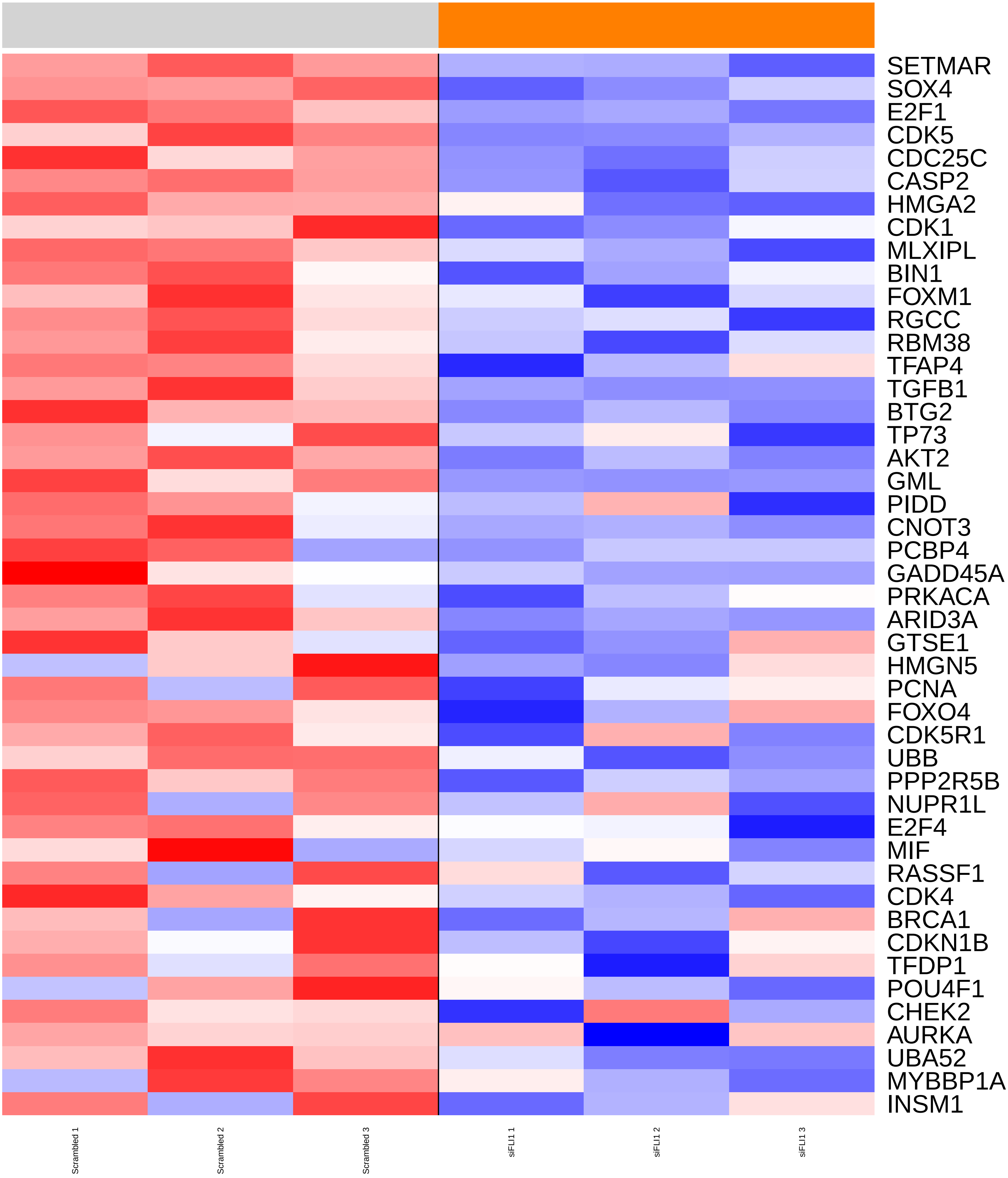

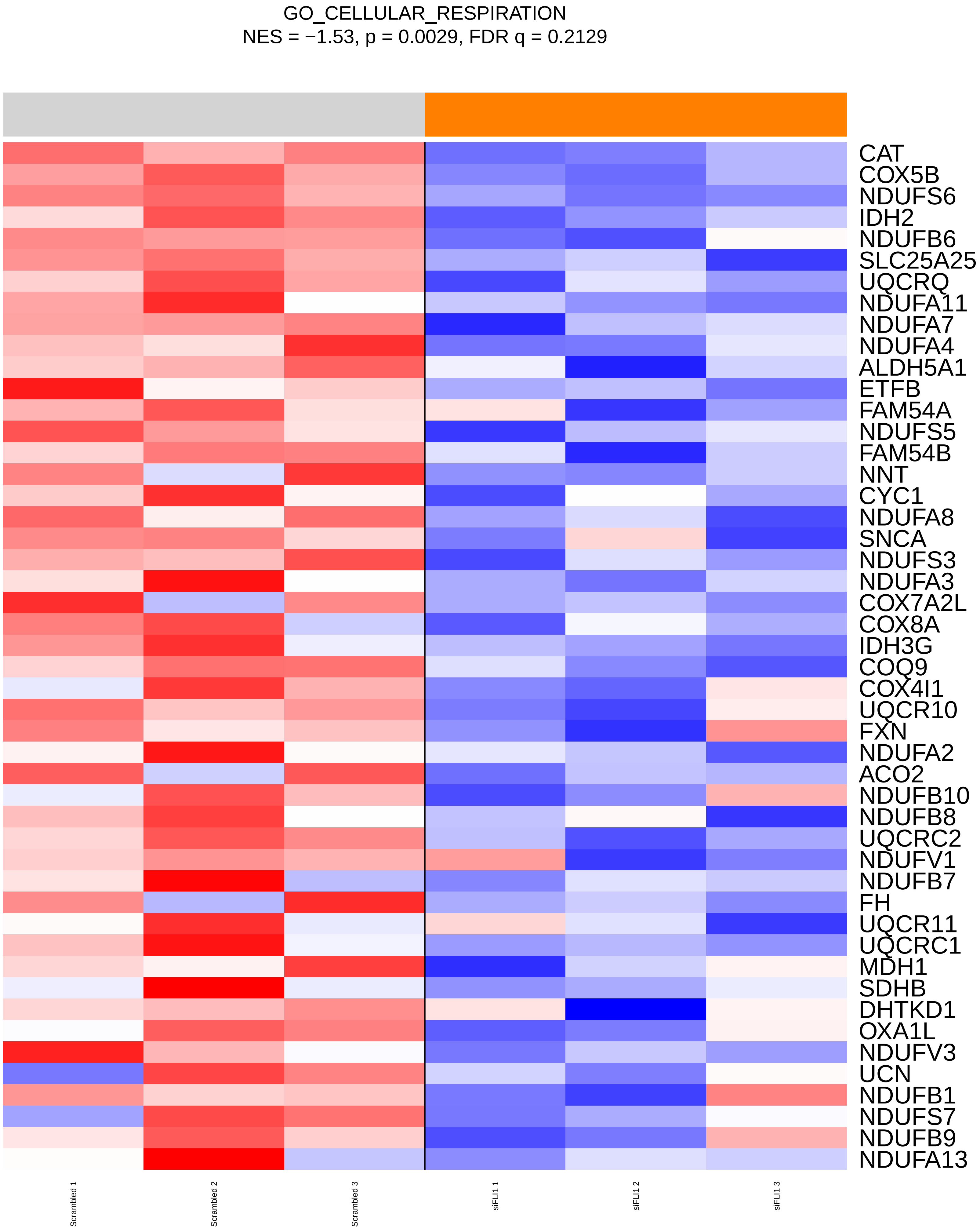
GO_CELLULAR_RESPIRATION
NES = −1.53, p = 0.0029, FDR q = 0.2129
CAT
COX5B
NDUFS6
IDH2
NDUFB6
SLC25A25
UQCRQ
NDUFA11
NDUFA7
NDUFA4
ALDH5A1
ETFB
FAM54A
NDUFS5
FAM54B
NNT
CYC1
NDUFA8
SNCA
NDUFS3
NDUFA3
COX7A2L
COX8A
IDH3G
COQ9
COX4I1
UQCR10
FXN
NDUFA2
ACO2
NDUFB10
NDUFB8
UQCRC2
NDUFV1
NDUFB7
FH
UQCR11
UQCRC1
MDH1
SDHB
DHTKD1
OXA1L
NDUFV3
UCN
NDUFB1
NDUFS7
NDUFB9
NDUFA13
Scrambled 1
Scrambled 2
Scrambled 3
siFLI1 1
siFLI1 2
siFLI1 3

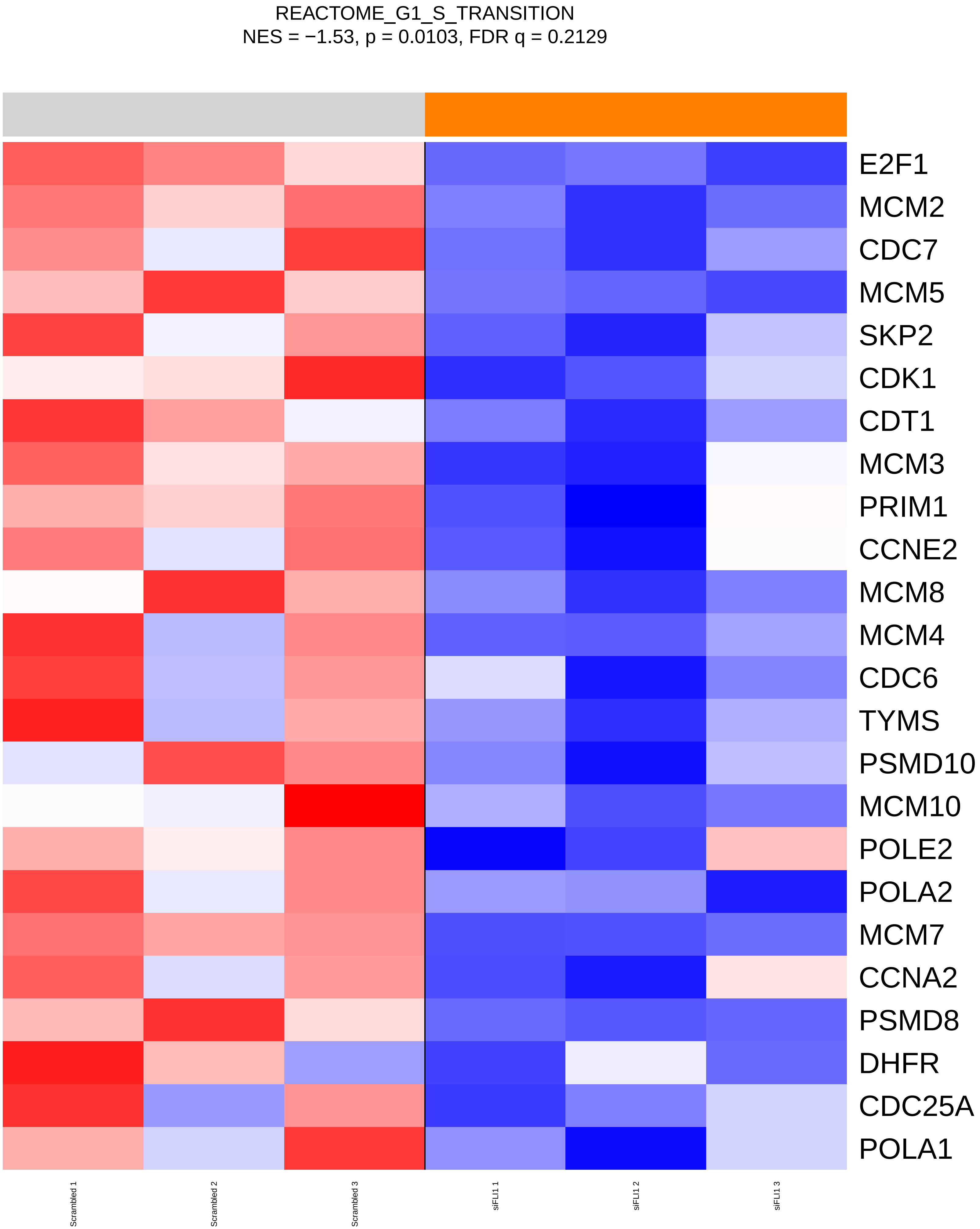

REACTOME_G1_S_TRANSITION
NES = −1.53, p = 0.0103, FDR q = 0.2129
E2F1
MCM2
CDC7
MCM5
SKP2
CDK1
CDT1
MCM3
PRIM1
CCNE2
MCM8
MCM4
CDC6
TYMS
PSMD10
MCM10
POLE2
POLA2
MCM7
CCNA2
PSMD8
DHFR
CDC25A
POLA1
Scrambled 1
Scrambled 2
Scrambled 3
siFLI1 1
siFLI1 2
siFLI1 3

GO_G_PROTEIN_COUPLED_AMINE_RECEPTOR_ACTIVITY
NES = −1.53, p = 0.0171, FDR q = 0.2134

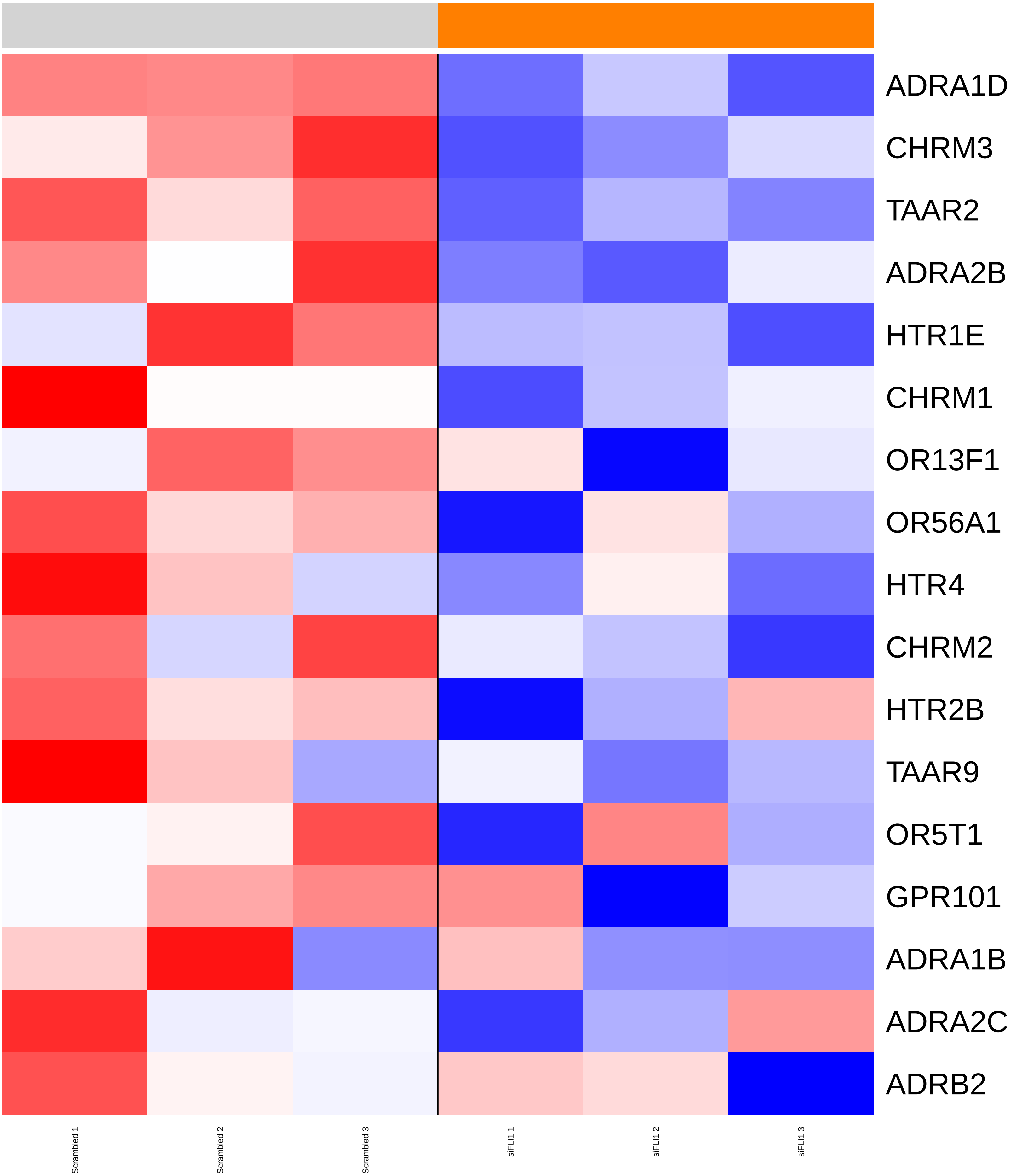

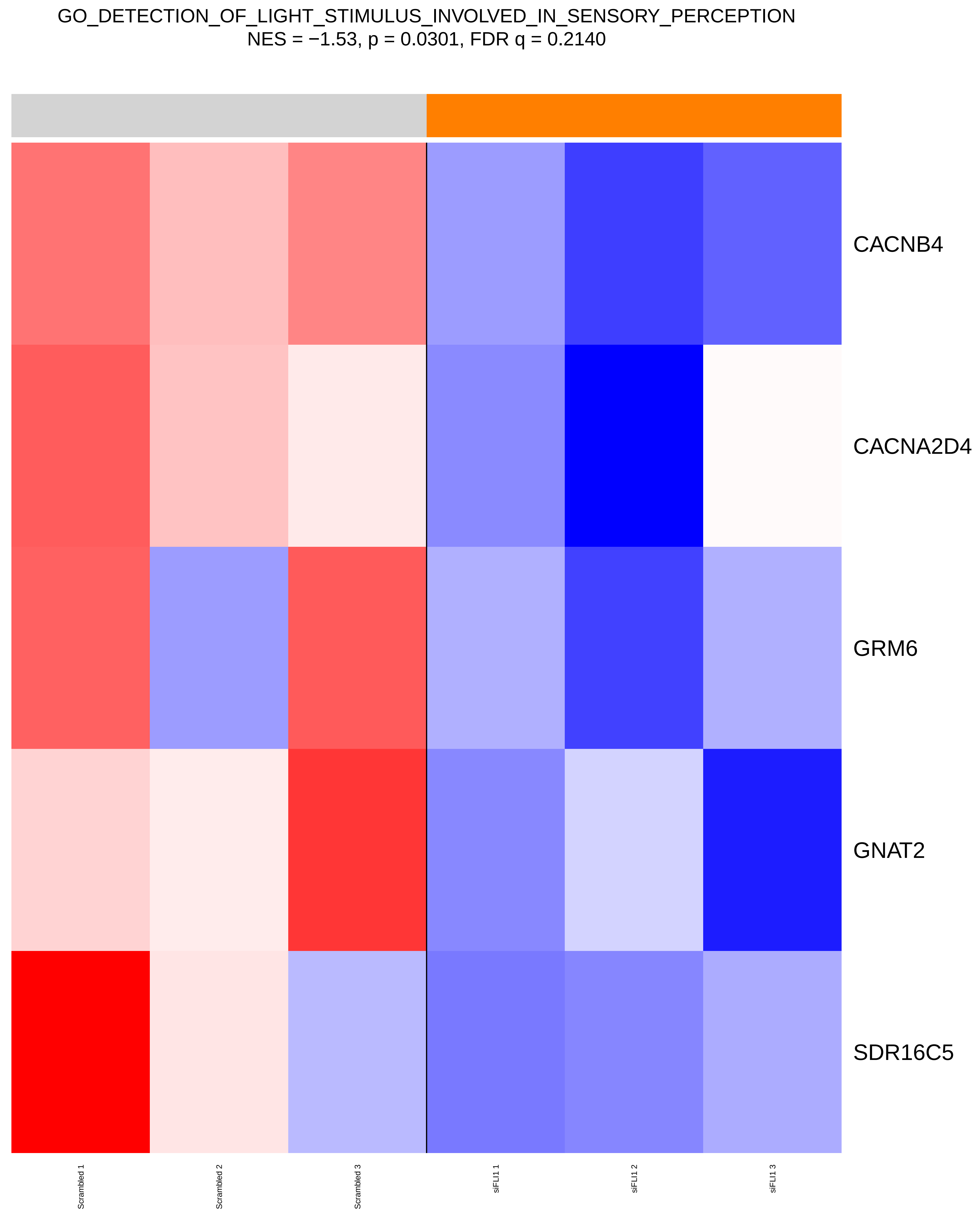
GO_DETECTION_OF_LIGHT_STIMULUS_INVOLVED_IN_SENSORY_PERCEPTION
NES = −1.53, p = 0.0301, FDR q = 0.2140
CACNB4
CACNA2D4
GRM6
GNAT2
SDR16C5
Scrambled 1
Scrambled 2
Scrambled 3
siFLI1 1
siFLI1 2
siFLI1 3

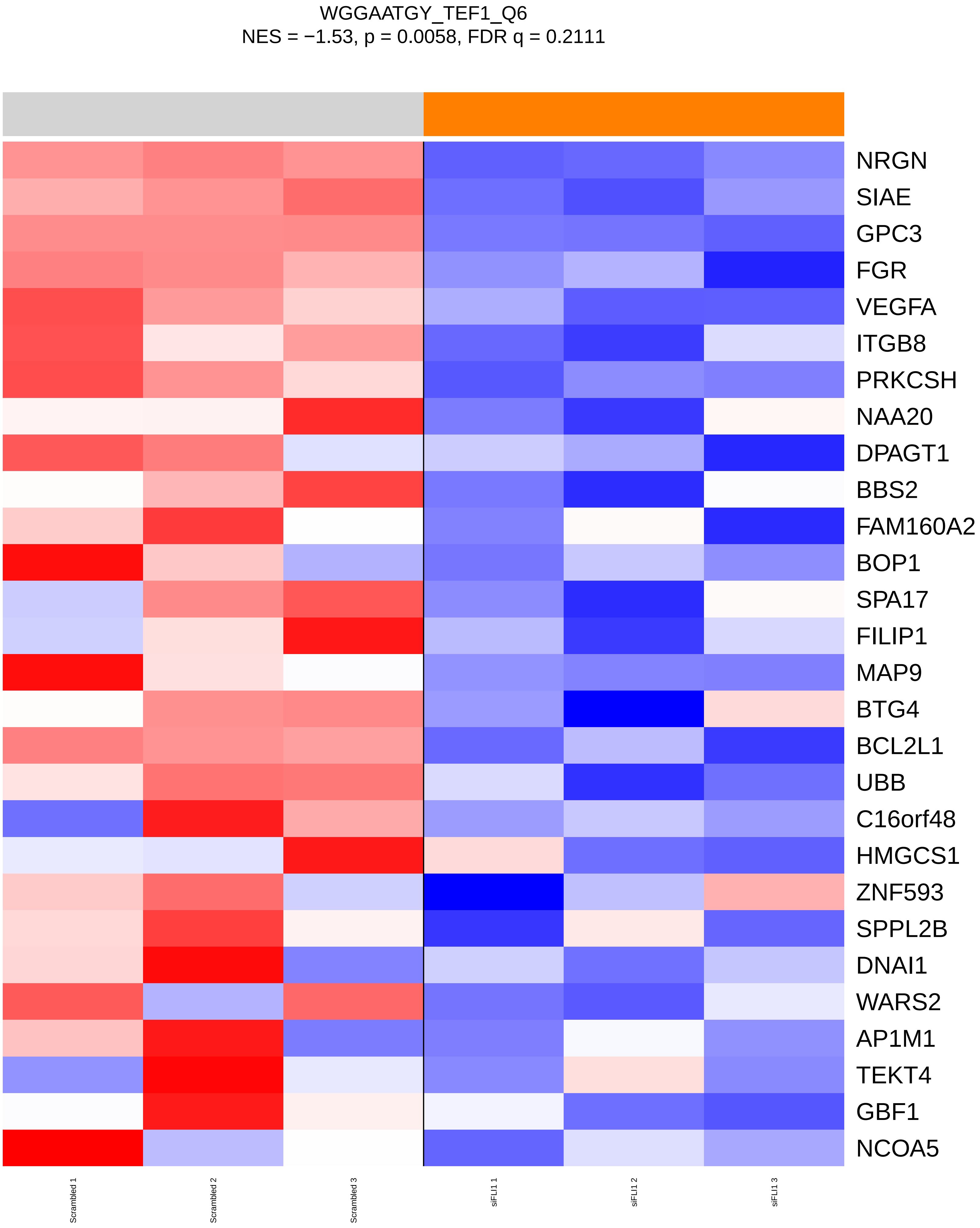
WGGAATGY_TEF1_Q6
NES = −1.53, p = 0.0058, FDR q = 0.2111
NRGN
SIAE
GPC3
FGR
VEGFA
ITGB8
PRKCSH
NAA20
DPAGT1
BBS2
FAM160A2
BOP1
SPA17
FILIP1
MAP9
BTG4
BCL2L1
UBB
C16orf48
HMGCS1
ZNF593
SPPL2B
DNAI1
WARS2
AP1M1
TEKT4
GBF1
NCOA5
Scrambled 1
Scrambled 2
Scrambled 3
siFLI1 1
siFLI1 2
siFLI1 3

GO_POSTSYNAPTIC_MEMBRANE_ORGANIZATION
NES = −1.54, p = 0.0285, FDR q = 0.2111

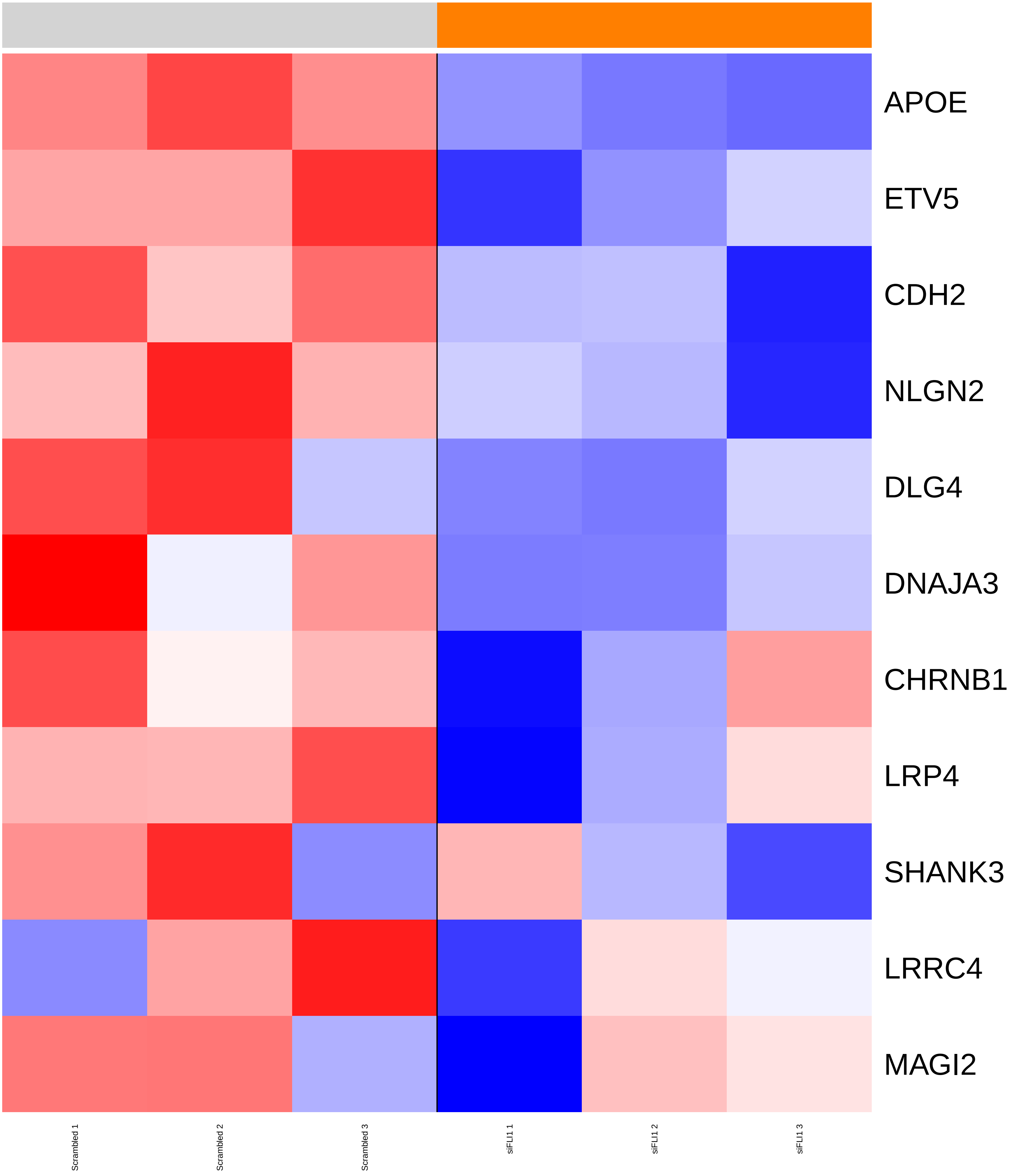

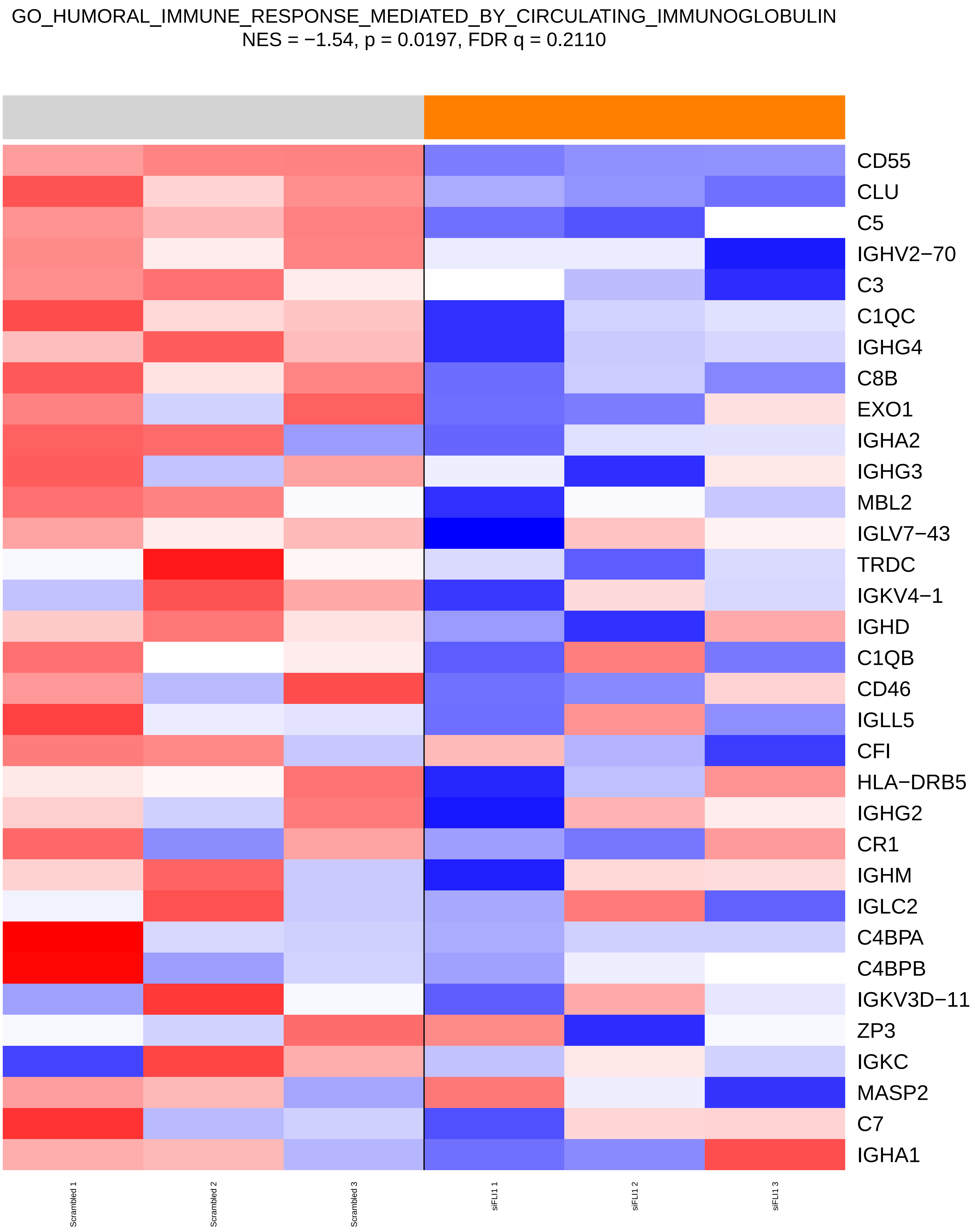

GO_HUMORAL_IMMUNE_RESPONSE_MEDIATED_BY_CIRCULATING_IMMUNOGLOBULIN
NES = −1.54, p = 0.0197, FDR q = 0.2110
CD55
CLU
C5
IGHV2−70
C3
C1QC
IGHG4
C8B
EXO1
IGHA2
IGHG3
MBL2
IGLV7−43
TRDC
IGKV4−1
IGHD
C1QB
CD46
IGLL5
CFI
HLA−DRB5
IGHG2
CR1
IGHM
IGLC2
C4BPA
C4BPB
IGKV3D−11
ZP3
IGKC
MASP2
C7
IGHA1
Scrambled 1
Scrambled 2
Scrambled 3
siFLI1 1
siFLI1 2
siFLI1 3

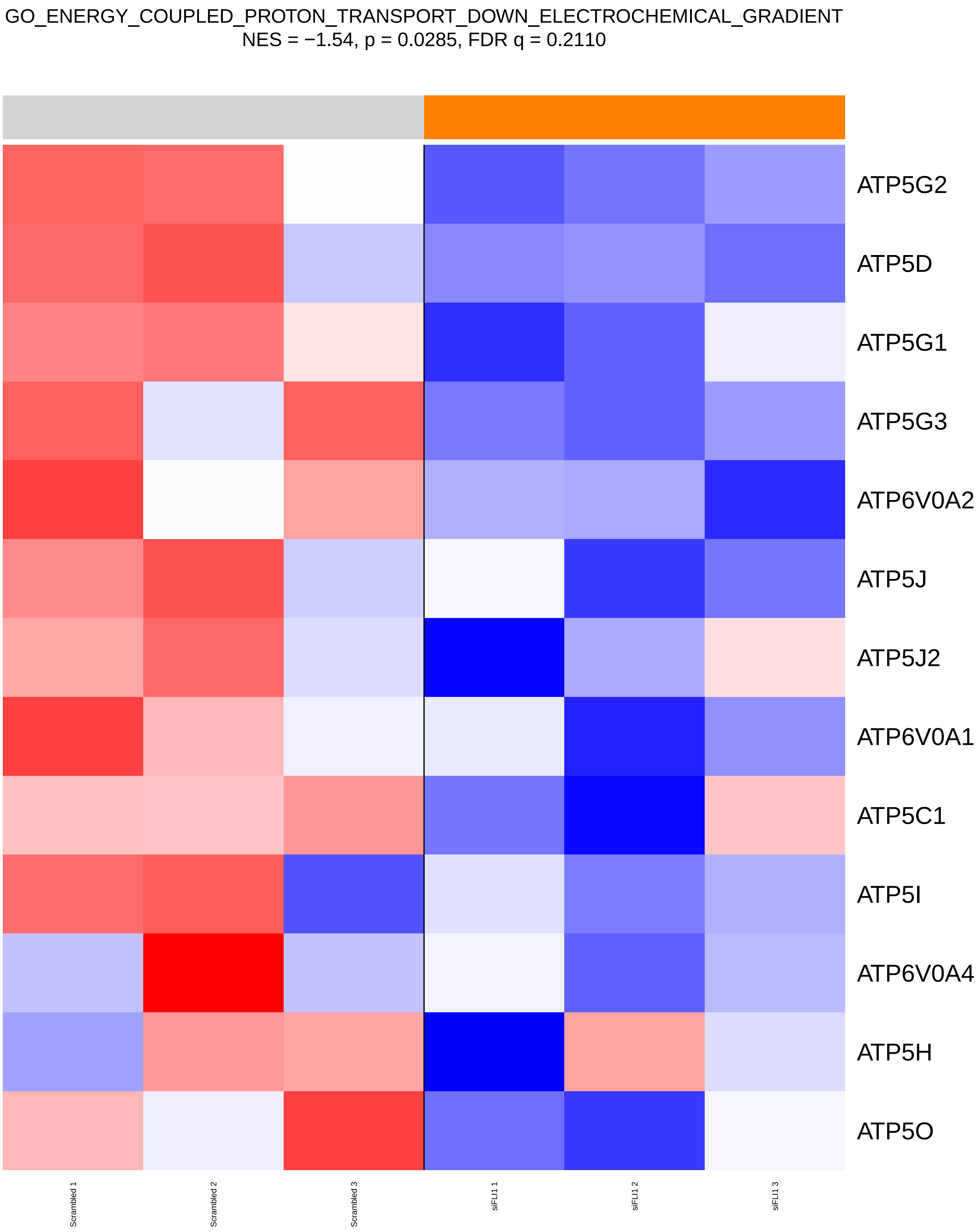
GO_ENERGY_COUPLED_PROTON_TRANSPORT_DOWN_ELECTROCHEMICAL_GRADIENT
NES = −1.54, p = 0.0285, FDR q = 0.2110
ATP5G2
ATP5D
ATP5G1
ATP5G3
ATP6V0A2
ATP5J
ATP5J2
ATP6V0A1
ATP5C1
ATP5I
ATP6V0A4
ATP5H
ATP5O
Scrambled 1
Scrambled 2
Scrambled 3
siFLI1 1
siFLI1 2
siFLI1 3

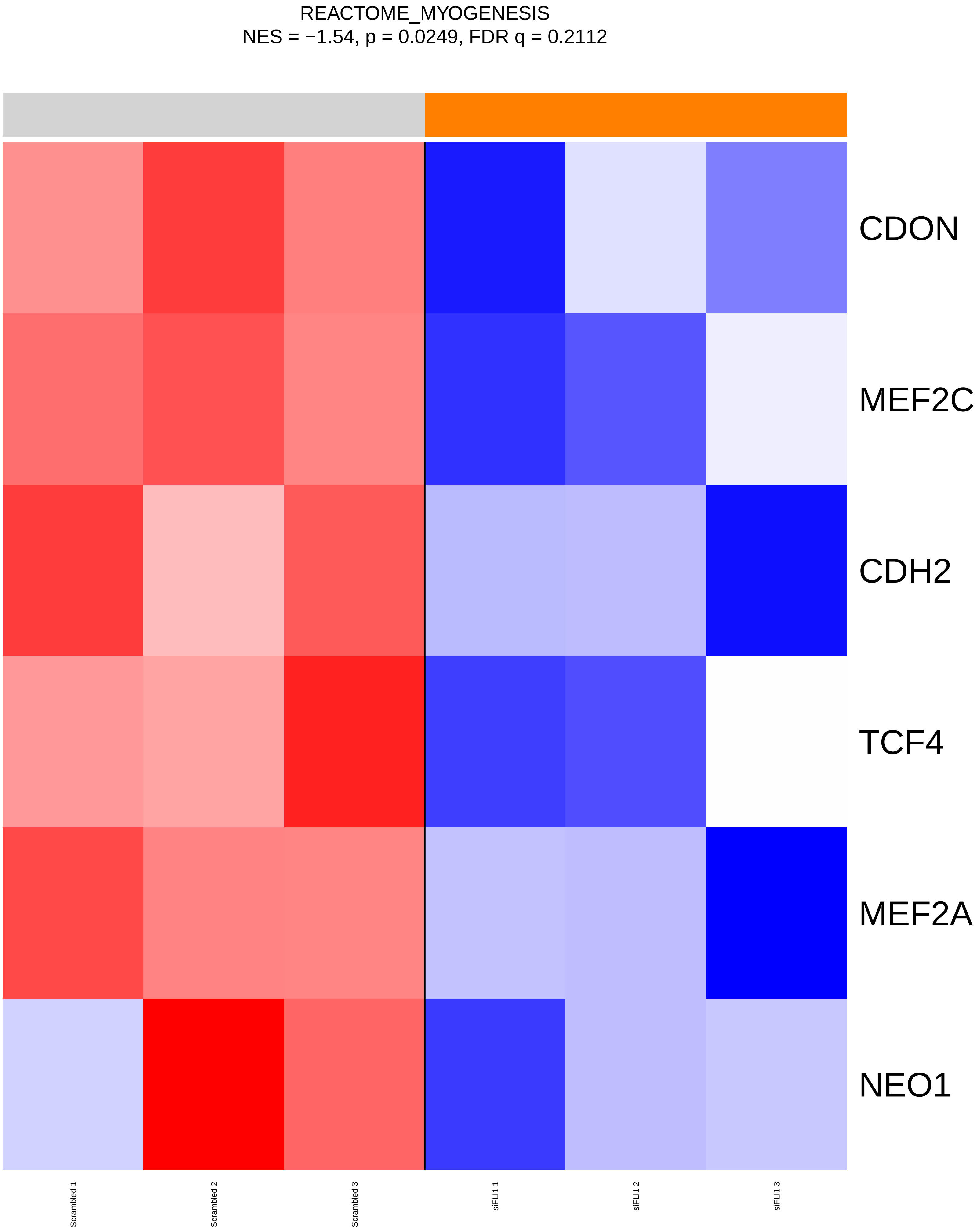
REACTOME_MYOGENESIS
NES = −1.54, p = 0.0249, FDR q = 0.2112
CDON
MEF2C
CDH2
TCF4
MEF2A
NEO1
Scrambled 1
Scrambled 2
Scrambled 3
siFLI1 1
siFLI1 2
siFLI1 3

GO_VENTRICULAR_CARDIAC_MUSCLE_CELL_DIFFERENTIATION
NES = −1.54, p = 0.0324, FDR q = 0.2088

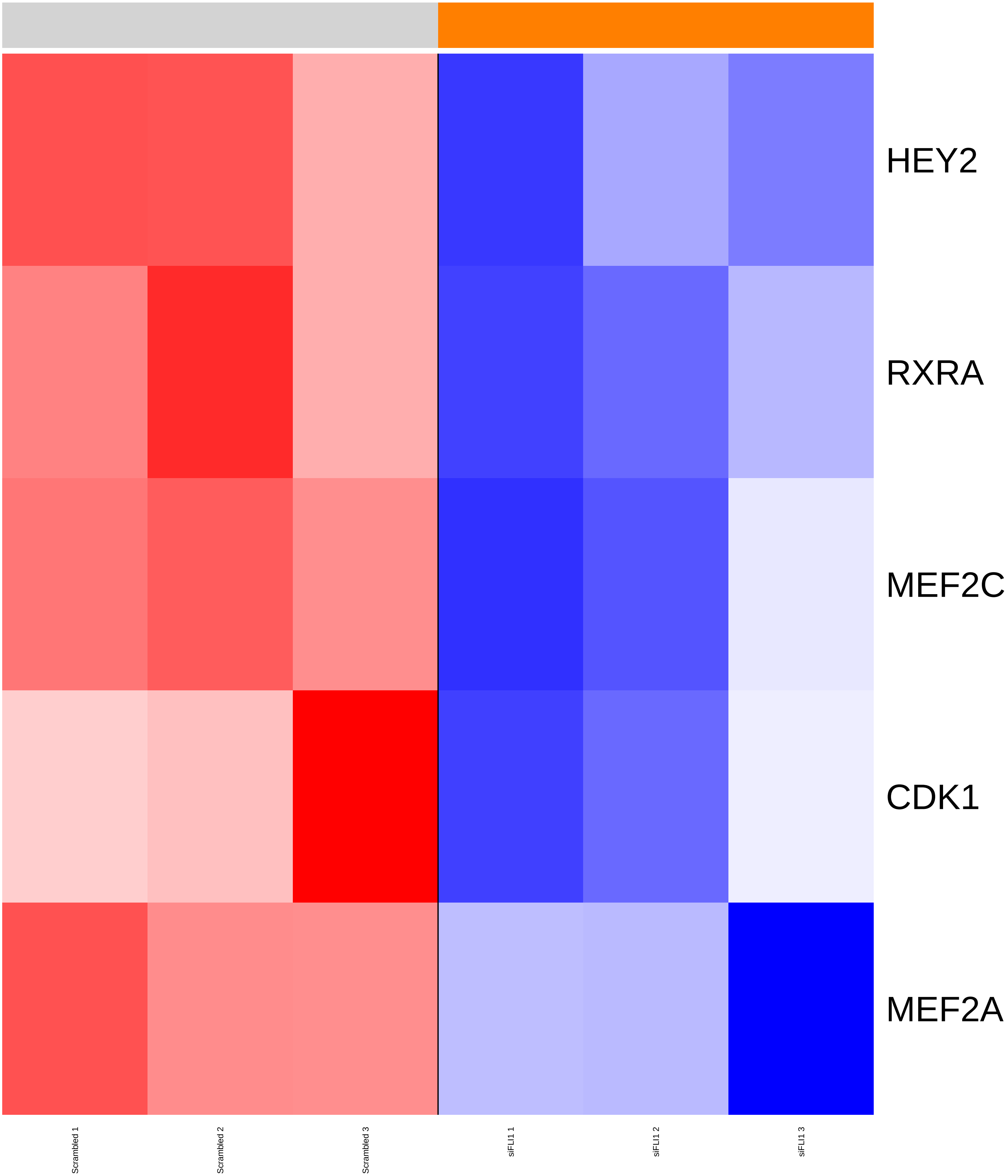

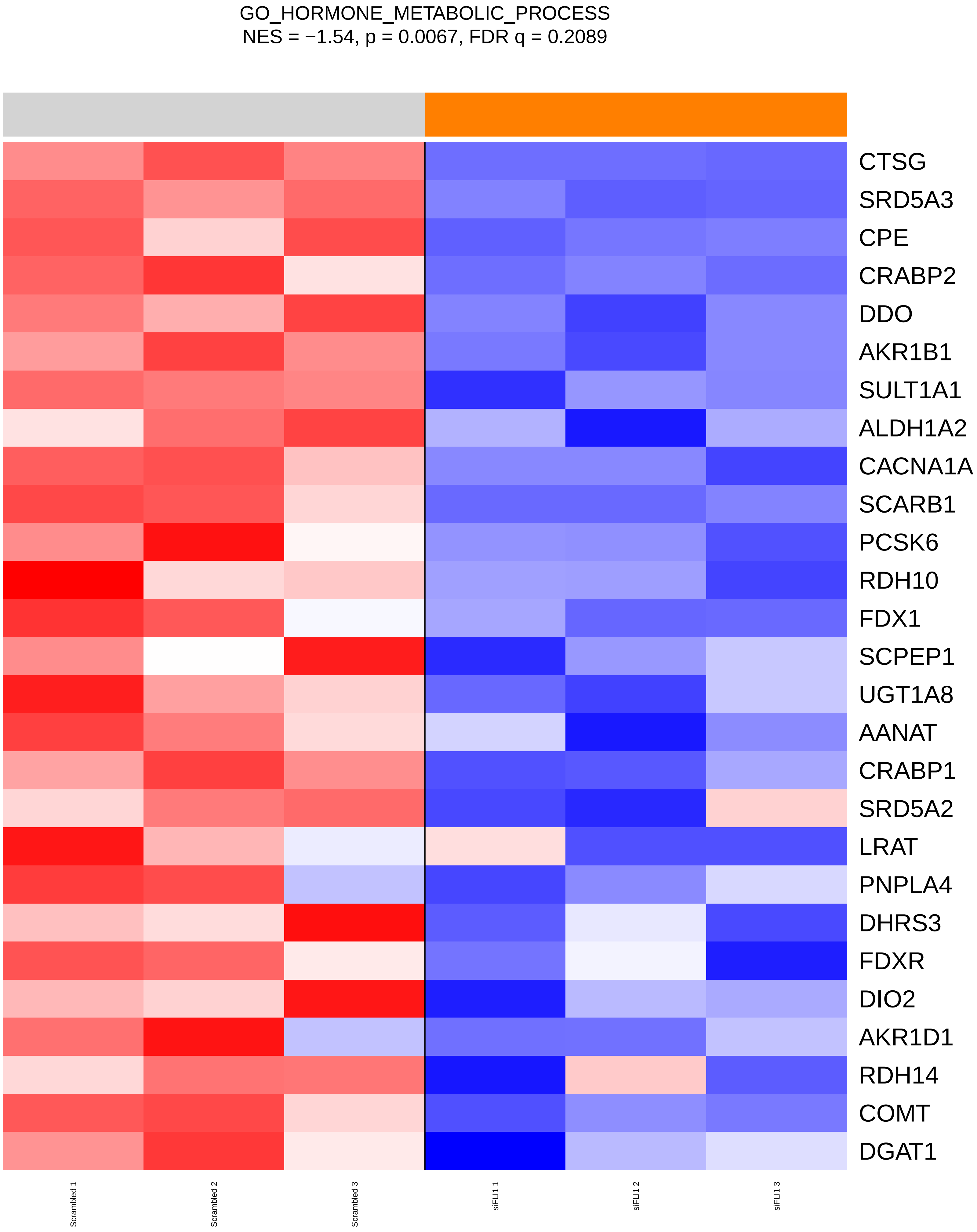
GO_HORMONE_METABOLIC_PROCESS
NES = −1.54, p = 0.0067, FDR q = 0.2089
CTSG
SRD5A3
CPE
CRABP2
DDO
AKR1B1
SULT1A1
ALDH1A2
CACNA1A
SCARB1
PCSK6
RDH10
FDX1
SCPEP1
UGT1A8
AANAT
CRABP1
SRD5A2
LRAT
PNPLA4
DHRS3
FDXR
DIO2
AKR1D1
RDH14
COMT
DGAT1
Scrambled 1
Scrambled 2
Scrambled 3
siFLI1 1
siFLI1 2
siFLI1 3

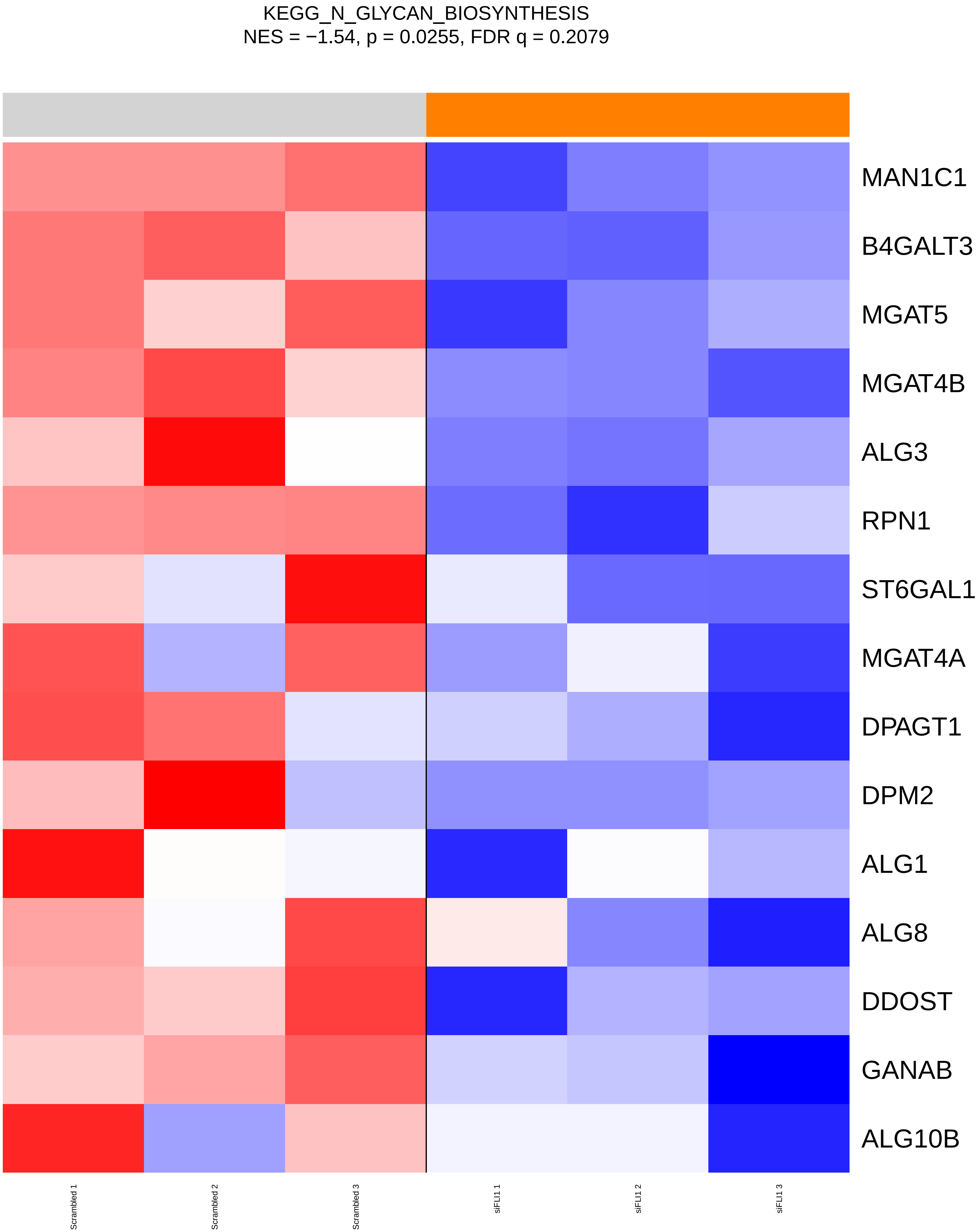
KEGG_N_GLYCAN_BIOSYNTHESIS
NES = −1.54, p = 0.0255, FDR q = 0.2079
MAN1C1
B4GALT3
MGAT5
MGAT4B
ALG3
RPN1
ST6GAL1
MGAT4A
DPAGT1
DPM2
ALG1
ALG8
DDOST
GANAB
ALG10B
Scrambled 1
Scrambled 2
Scrambled 3
siFLI1 1
siFLI1 2
siFLI1 3

GO_NITRIC_OXIDE_MEDIATED_SIGNAL_TRANSDUCTION
NES = −1.54, p = 0.0298, FDR q = 0.2069

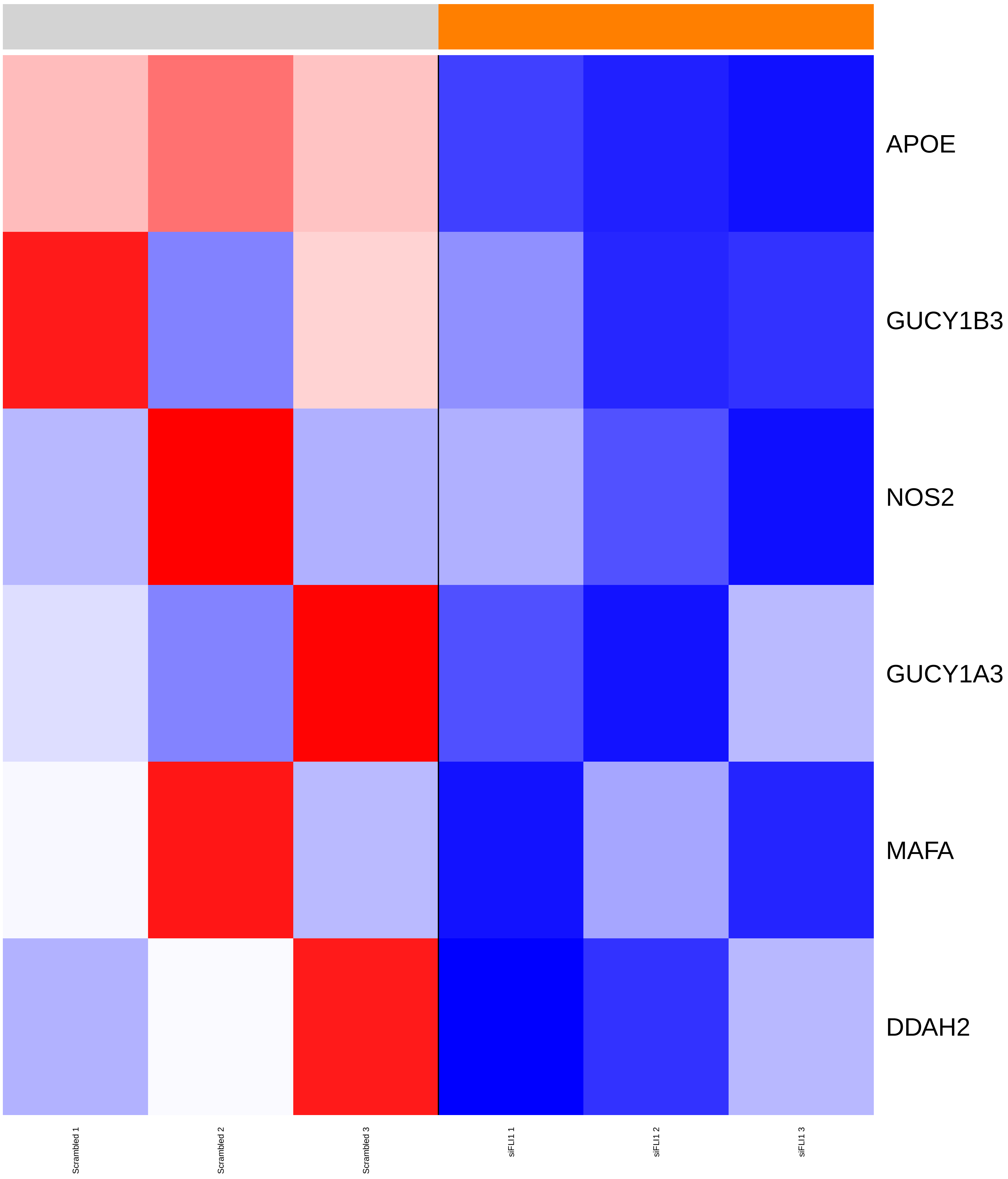

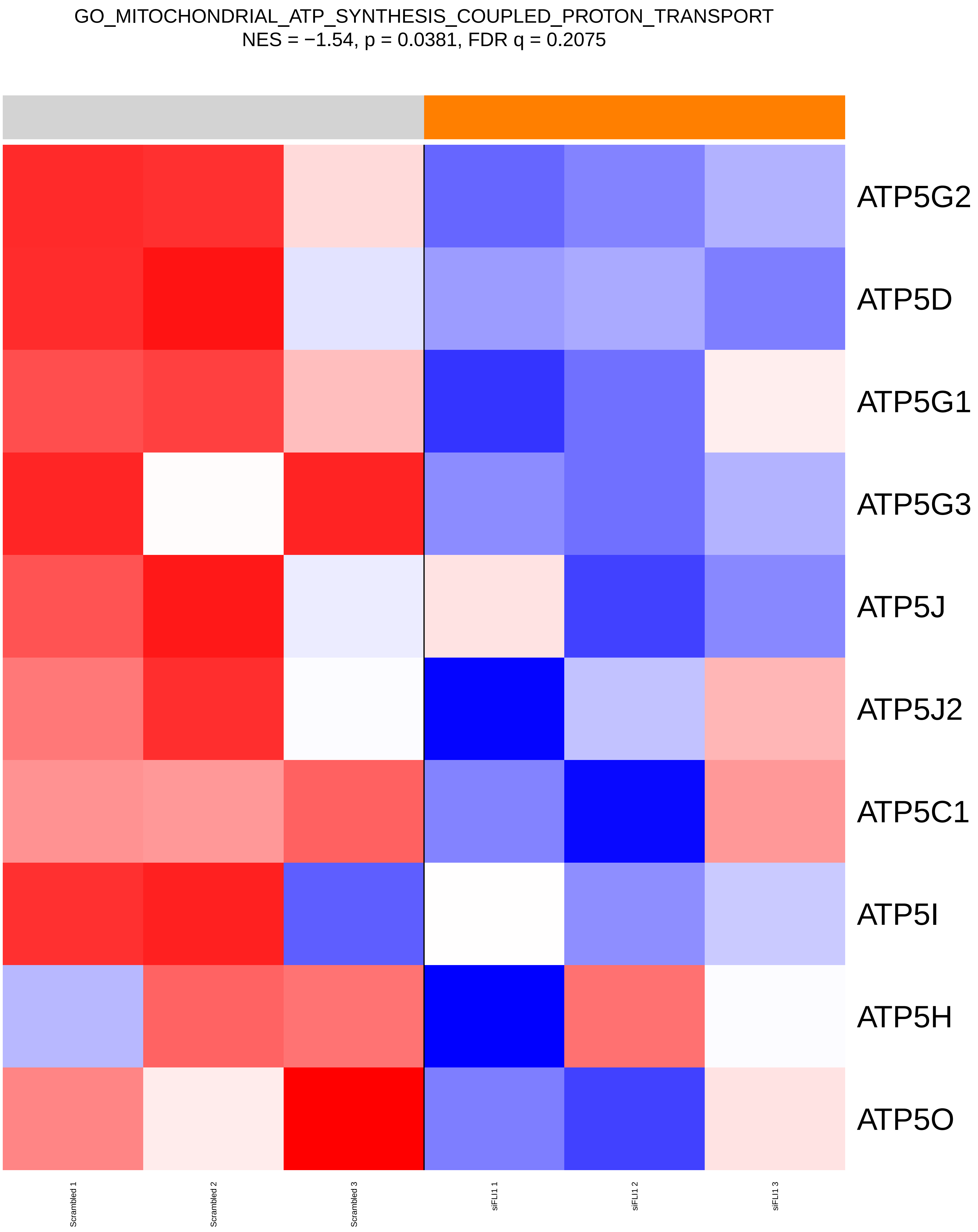
GO_MITOCHONDRIAL_ATP_SYNTHESIS_COUPLED_PROTON_TRANSPORT
NES = −1.54, p = 0.0381, FDR q = 0.2075
ATP5G2
ATP5D
ATP5G1
ATP5G3
ATP5J
ATP5J2
ATP5C1
ATP5I
ATP5H
ATP5O
Scrambled 1
Scrambled 2
Scrambled 3
siFLI1 1
siFLI1 2
siFLI1 3

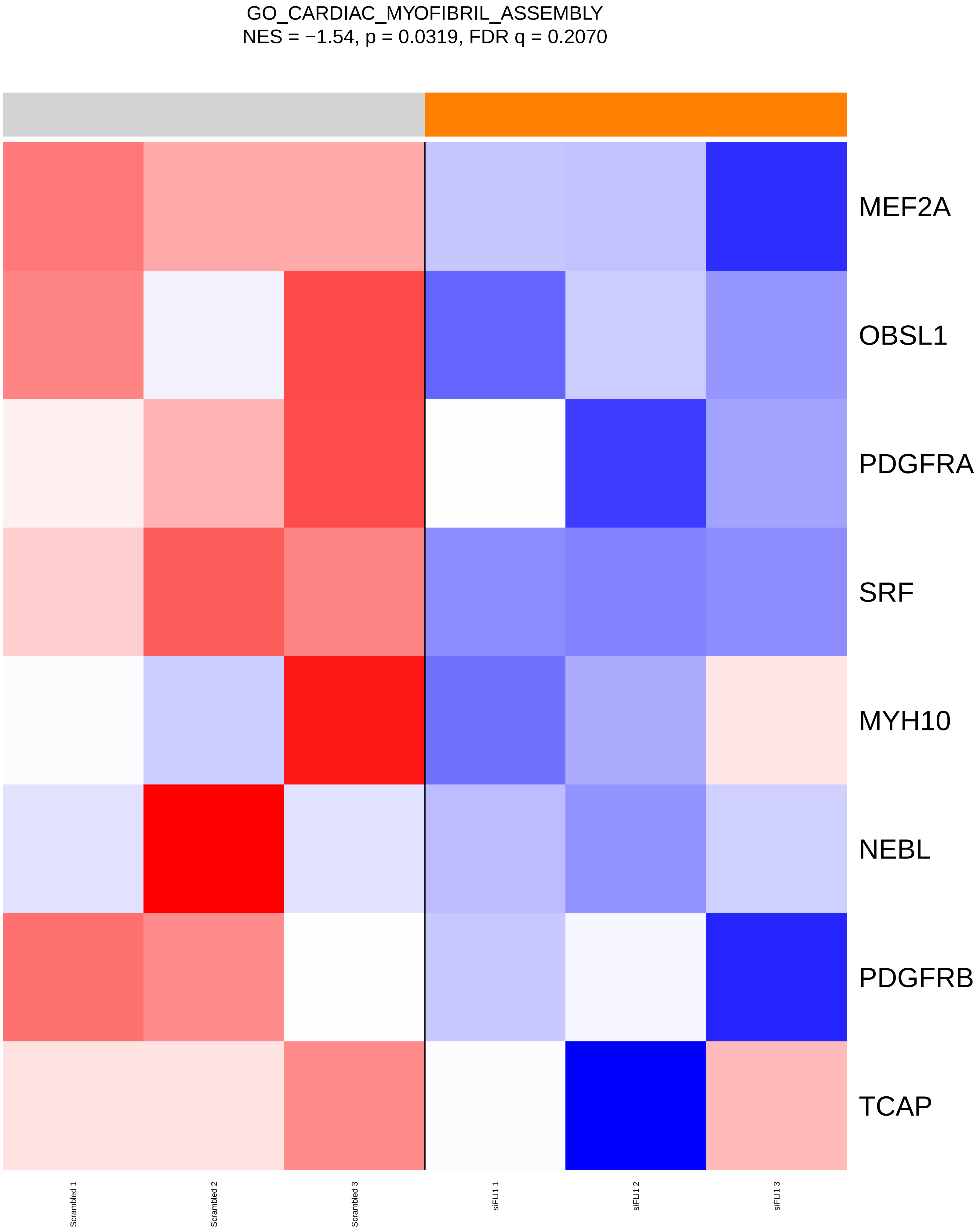
GO_CARDIAC_MYOFIBRIL_ASSEMBLY
NES = −1.54, p = 0.0319, FDR q = 0.2070
MEF2A
OBSL1
PDGFRA
SRF
MYH10
NEBL
PDGFRB
TCAP
Scrambled 1
Scrambled 2
Scrambled 3
siFLI1 1
siFLI1 2
siFLI1 3

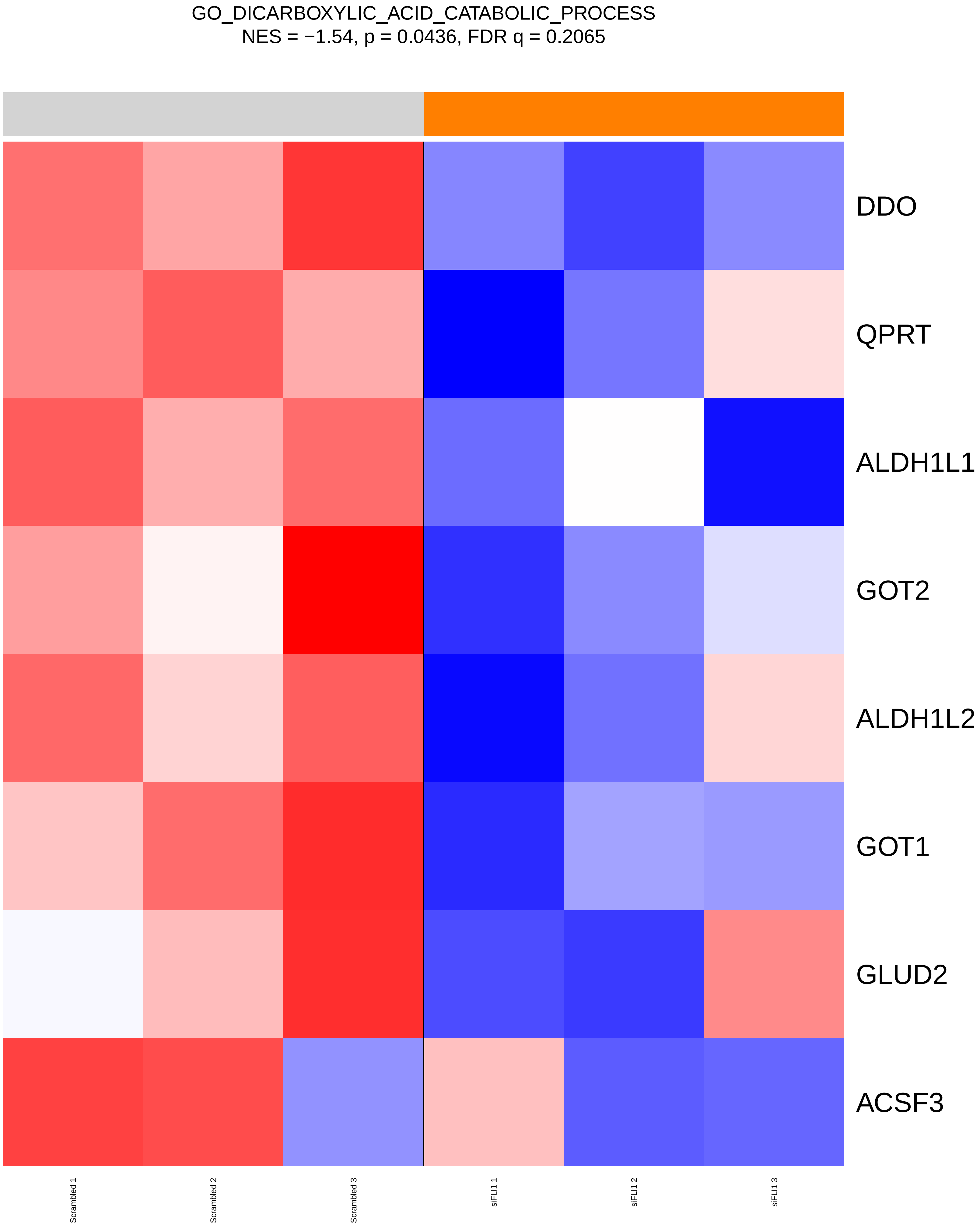
GO_DICARBOXYLIC_ACID_CATABOLIC_PROCESS
NES = −1.54, p = 0.0436, FDR q = 0.2065
DDO
QPRT
ALDH1L1
GOT2
ALDH1L2
GOT1
GLUD2
ACSF3
Scrambled 1
Scrambled 2
Scrambled 3
siFLI1 1
siFLI1 2
siFLI1 3

GO_REGULATION_OF_CELLULAR_SENESCENCE
NES = −1.54, p = 0.0310, FDR q = 0.2067

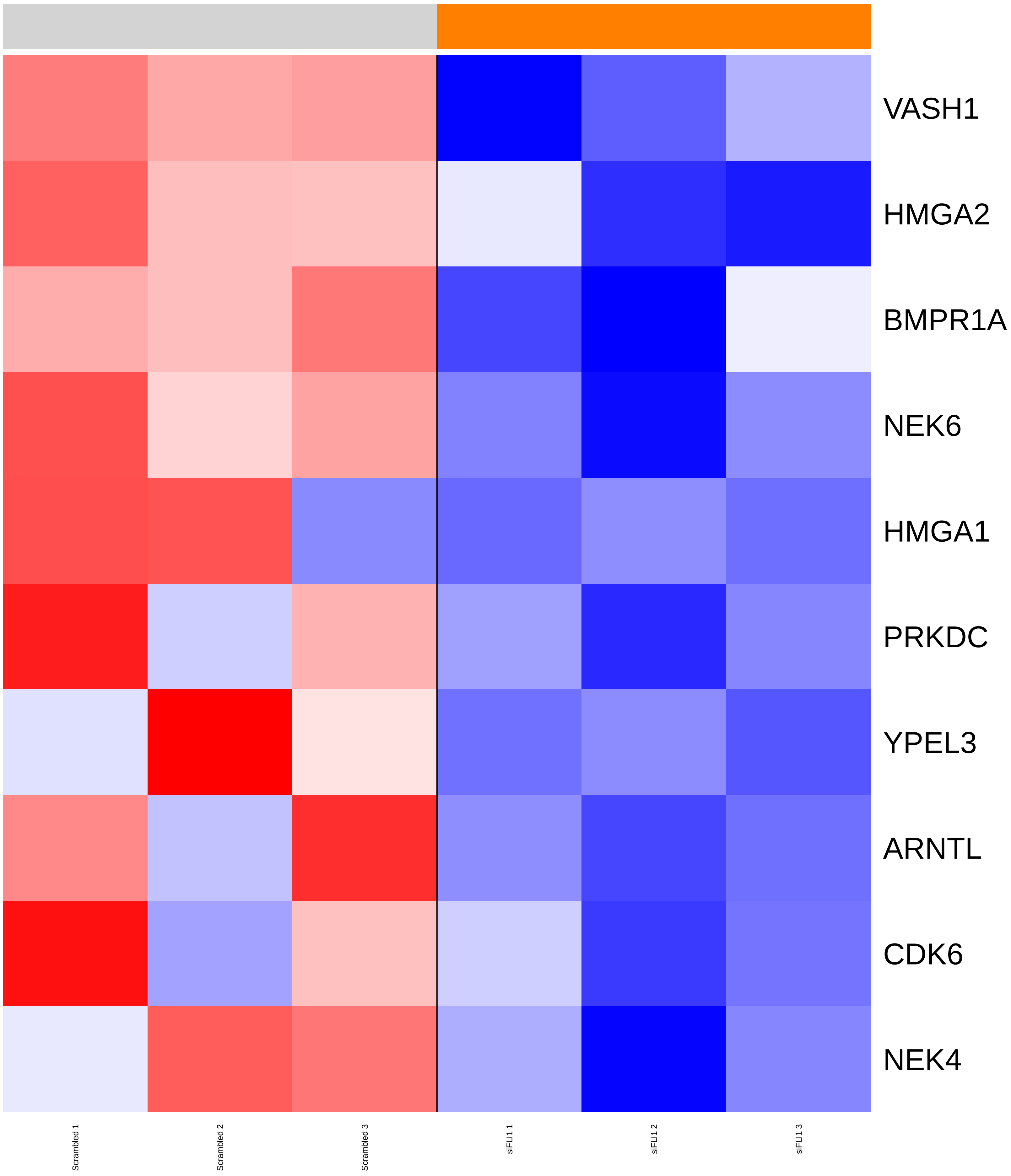

GO_PLATELET_ALPHA_GRANULE
NES = −1.54, p = 0.0106, FDR q = 0.2067

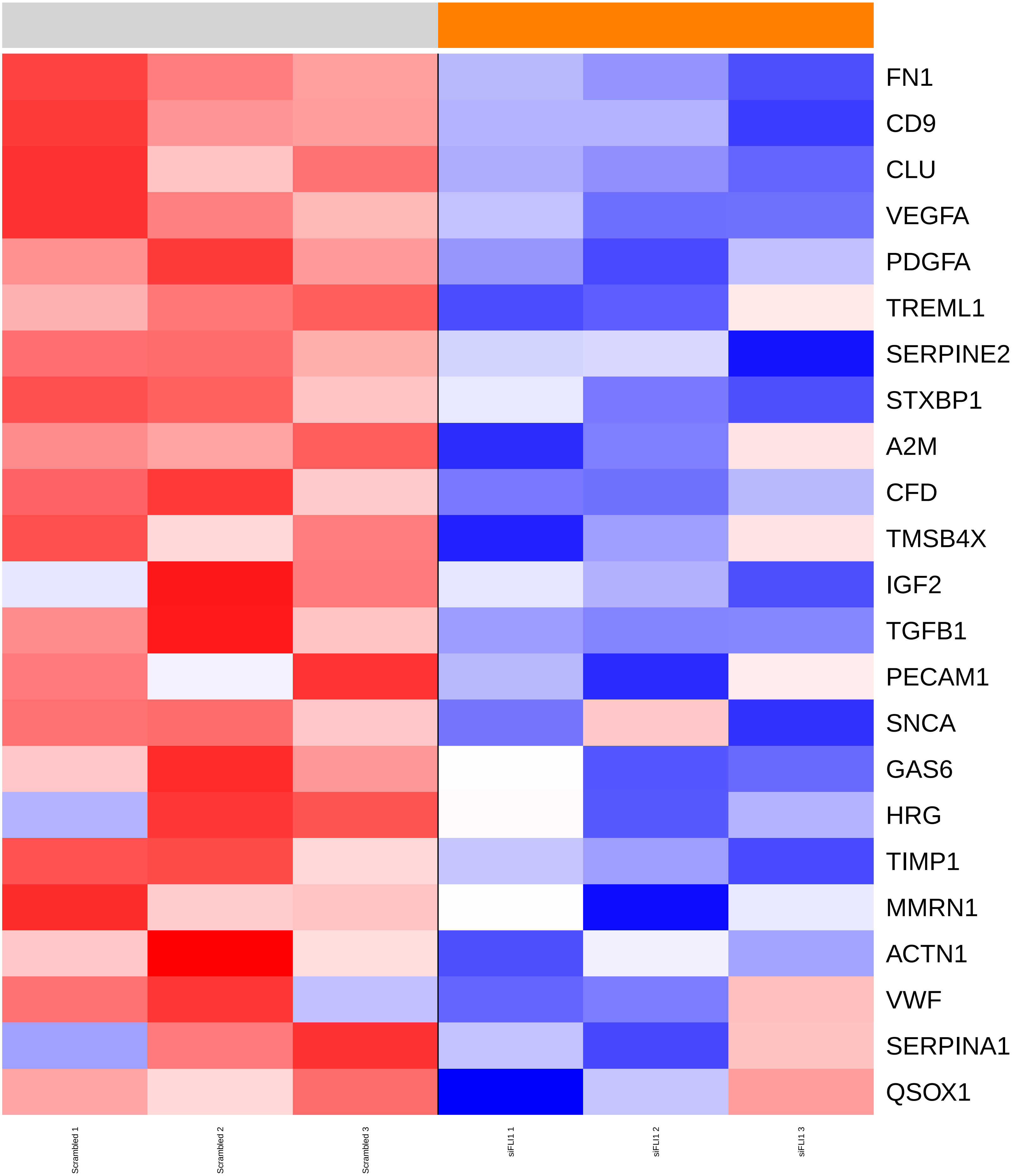

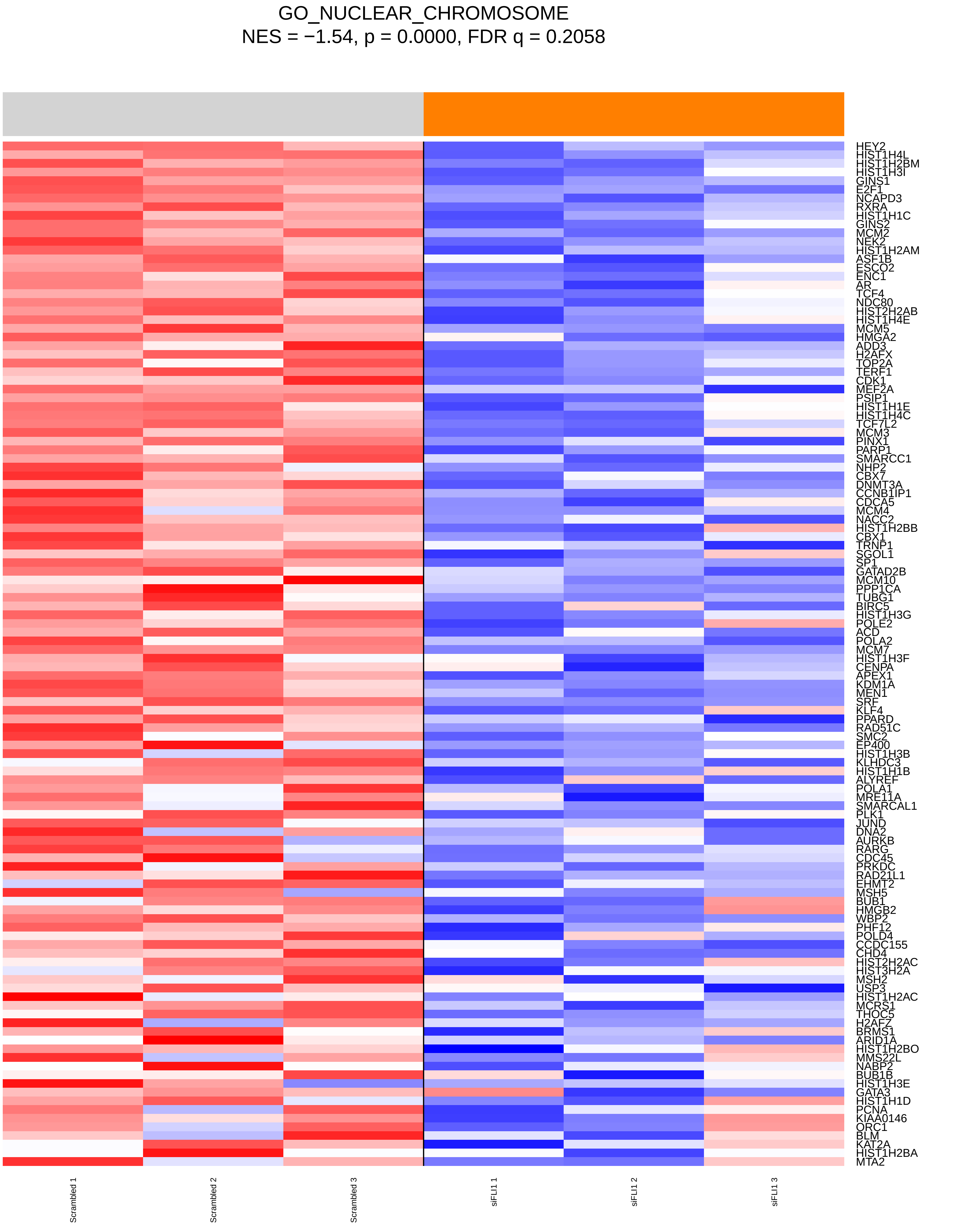
GO_NUCLEAR_CHROMOSOME
NES = −1.54, p = 0.0000, FDR q = 0.2058
HEY2
HIST1H4L
HIST1H2BM
HIST1H3I
GINS1
E2F1
NCAPD3
RXRA
HIST1H1C
GINS2
MCM2
NEK2
HIST1H2AM
ASF1B
ESCO2
ENC1
AR
TCF4
NDC80
HIST2H2AB
HIST1H4E
MCM5
HMGA2
ADD3
H2AFX
TOP2A
TERF1
CDK1
MEF2A
PSIP1
HIST1H1E
HIST1H4C
TCF7L2
MCM3
PINX1
PARP1
SMARCC1
NHP2
CBX7
DNMT3A
CCNB1IP1
CDCA5
MCM4
NACC2
HIST1H2BB
CBX1
TRNP1
SGOL1
SP1
GATAD2B
MCM10
PPP1CA
TUBG1
BIRC5
HIST1H3G
POLE2
ACD
POLA2
MCM7
HIST1H3F
CENPA
APEX1
KDM1A
MEN1
SRF
KLF4
PPARD
RAD51C
SMC2
EP400
HIST1H3B
KLHDC3
HIST1H1B
ALYREF
POLA1
MRE11A
SMARCAL1
PLK1
JUND
DNA2
AURKB
RARG
CDC45
PRKDC
RAD21L1
EHMT2
MSH5
BUB1
HMGB2
WBP2
PHF12
POLD4
CCDC155
CHD4
HIST2H2AC
HIST3H2A
MSH2
USP3
HIST1H2AC
MCRS1
THOC5
H2AFZ
BRMS1
ARID1A
HIST1H2BO
MMS22L
NABP2
BUB1B
HIST1H3E
GATA3
HIST1H1D
PCNA
KIAA0146
ORC1
BLM
KAT2A
HIST1H2BA
MTA2
Scrambled 1
Scrambled 2
Scrambled 3
siFLI1 1
siFLI1 2
siFLI1 3

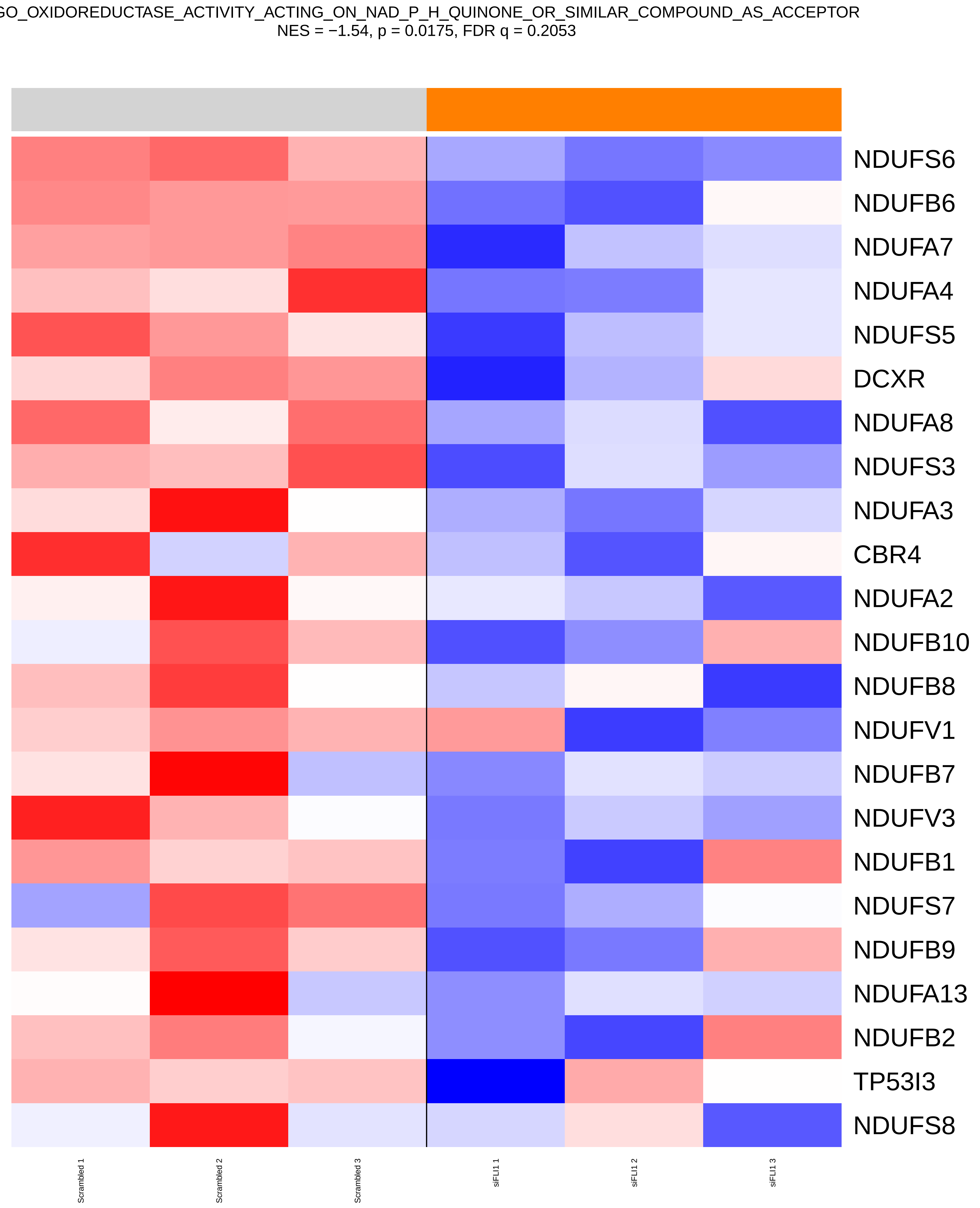
GO_OXIDOREDUCTASE_ACTIVITY_ACTING_ON_NAD_P_H_QUINONE_OR_SIMILAR_COMPOUND_AS_ACCEPTOR
NES = −1.54, p = 0.0175, FDR q = 0.2053
NDUFS6
NDUFB6
NDUFA7
NDUFA4
NDUFS5
DCXR
NDUFA8
NDUFS3
NDUFA3
CBR4
NDUFA2
NDUFB10
NDUFB8
NDUFV1
NDUFB7
NDUFV3
NDUFB1
NDUFS7
NDUFB9
NDUFA13
NDUFB2
TP53I3
NDUFS8
Scrambled 1
Scrambled 2
Scrambled 3
siFLI1 1
siFLI1 2
siFLI1 3

GO_CHROMATIN
NES = −1.54, p = 0.0000, FDR q = 0.2055

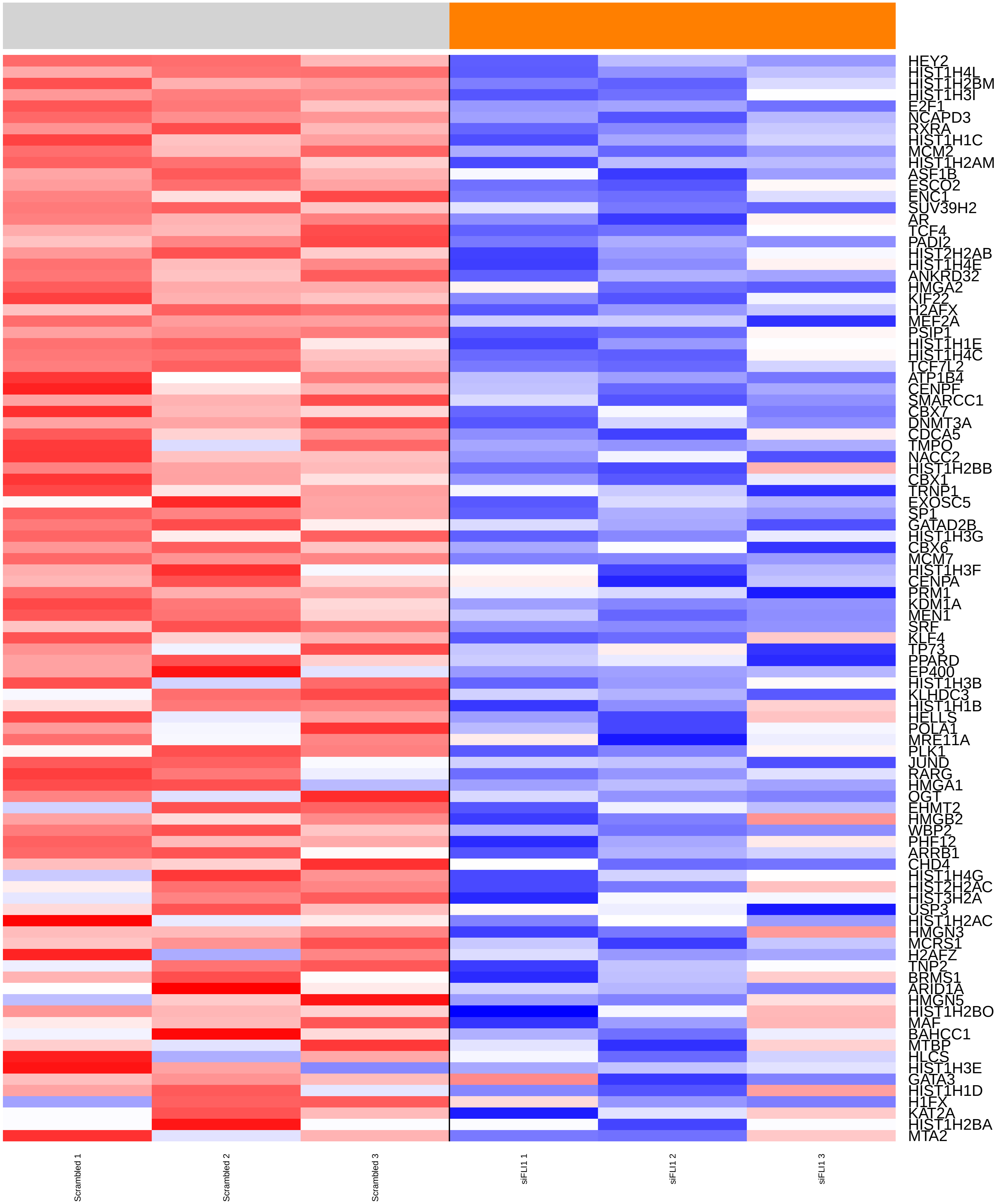

GO_MEMBRANE_INVAGINATION
NES = −1.54, p = 0.0251, FDR q = 0.2059

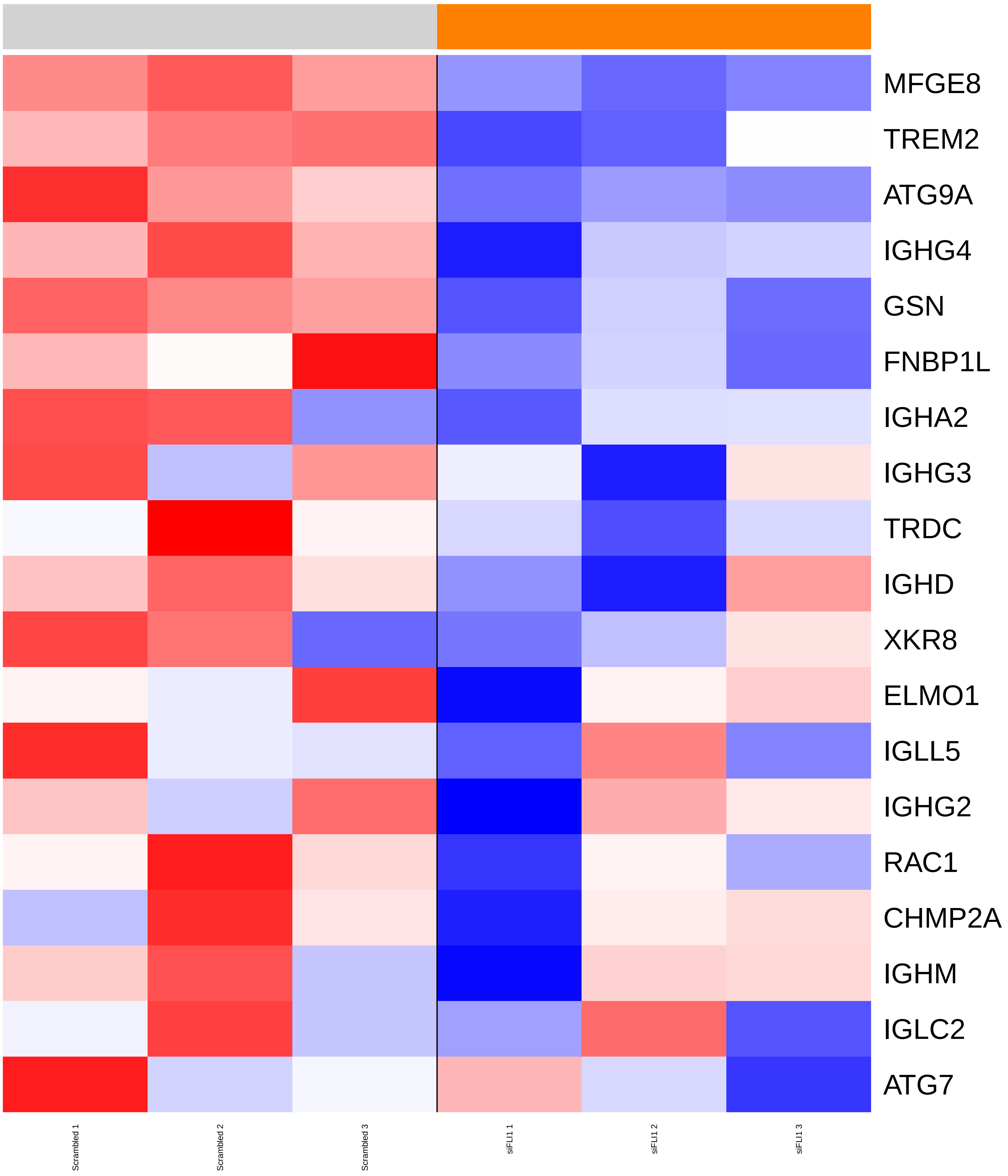

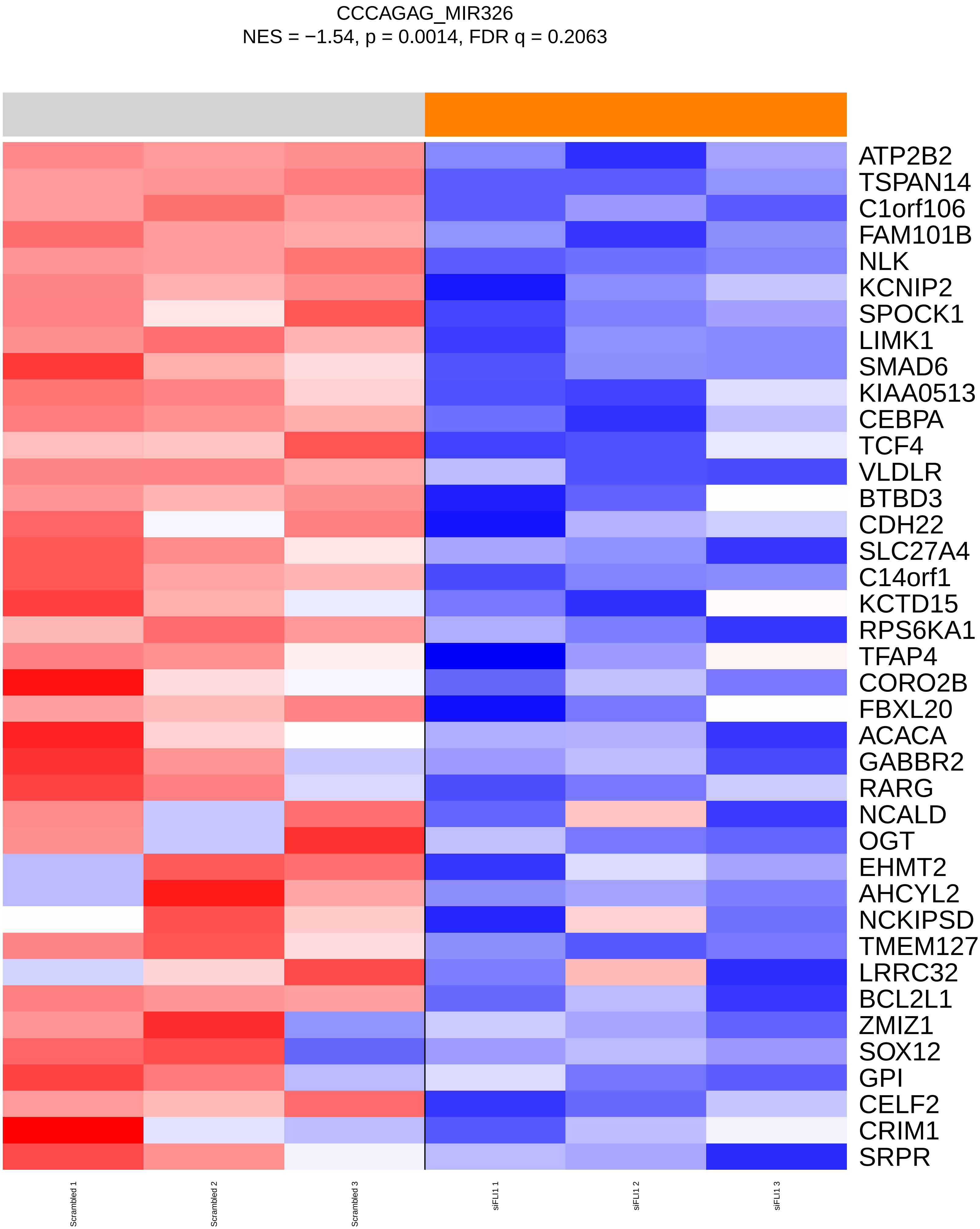
CCCAGAG_MIR326
NES = −1.54, p = 0.0014, FDR q = 0.2063
ATP2B2
TSPAN14
C1orf106
FAM101B
NLK
KCNIP2
SPOCK1
LIMK1
SMAD6
KIAA0513
CEBPA
TCF4
VLDLR
BTBD3
CDH22
SLC27A4
C14orf1
KCTD15
RPS6KA1
TFAP4
CORO2B
FBXL20
ACACA
GABBR2
RARG
NCALD
OGT
EHMT2
AHCYL2
NCKIPSD
TMEM127
LRRC32
BCL2L1
ZMIZ1
SOX12
GPI
CELF2
CRIM1
SRPR
Scrambled 1
Scrambled 2
Scrambled 3
siFLI1 1
siFLI1 2
siFLI1 3

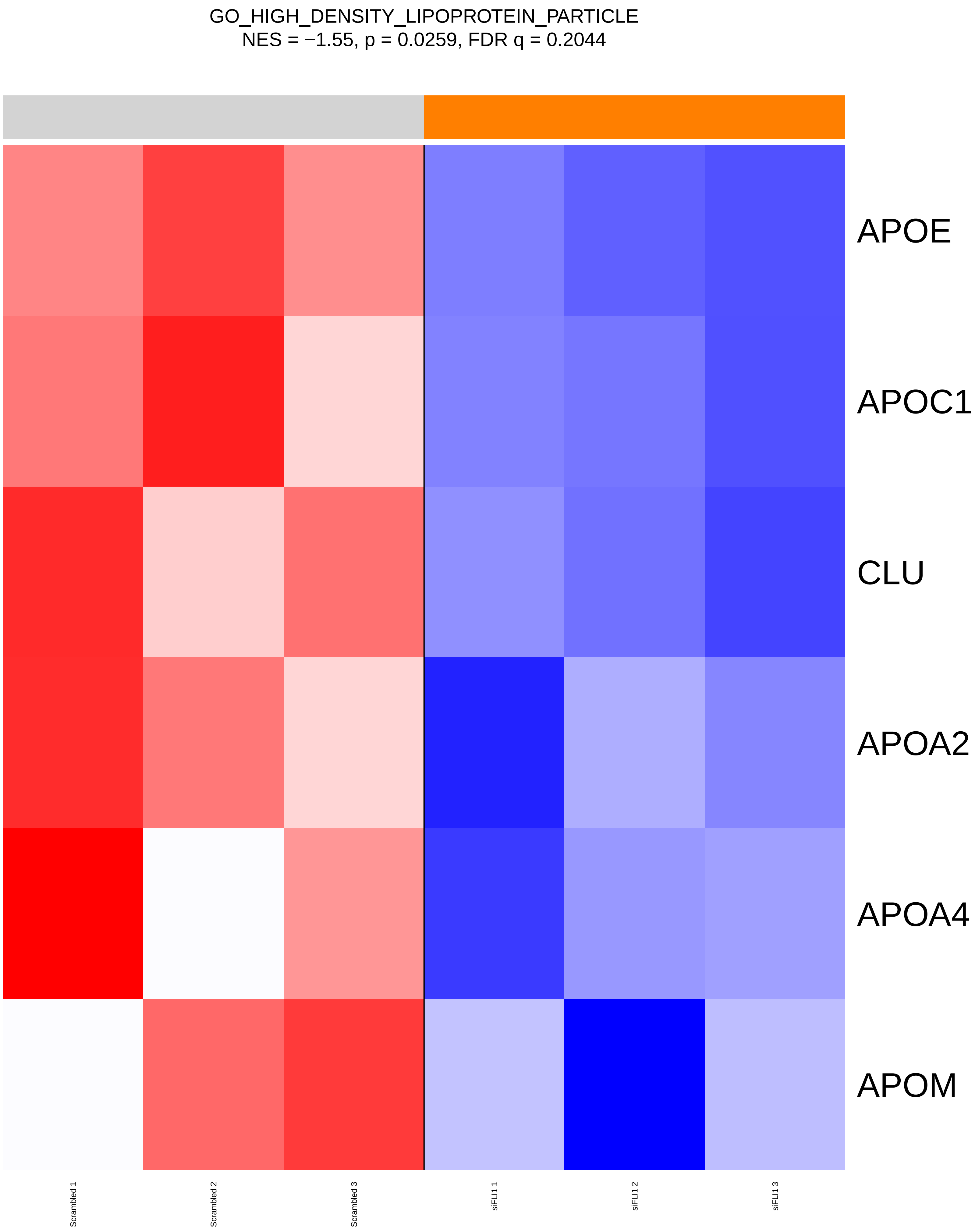

GO_HIGH_DENSITY_LIPOPROTEIN_PARTICLE
NES = −1.55, p = 0.0259, FDR q = 0.2044
APOE
APOC1
CLU
APOA2
APOA4
APOM
Scrambled 1
Scrambled 2
Scrambled 3
siFLI1 1
siFLI1 2
siFLI1 3

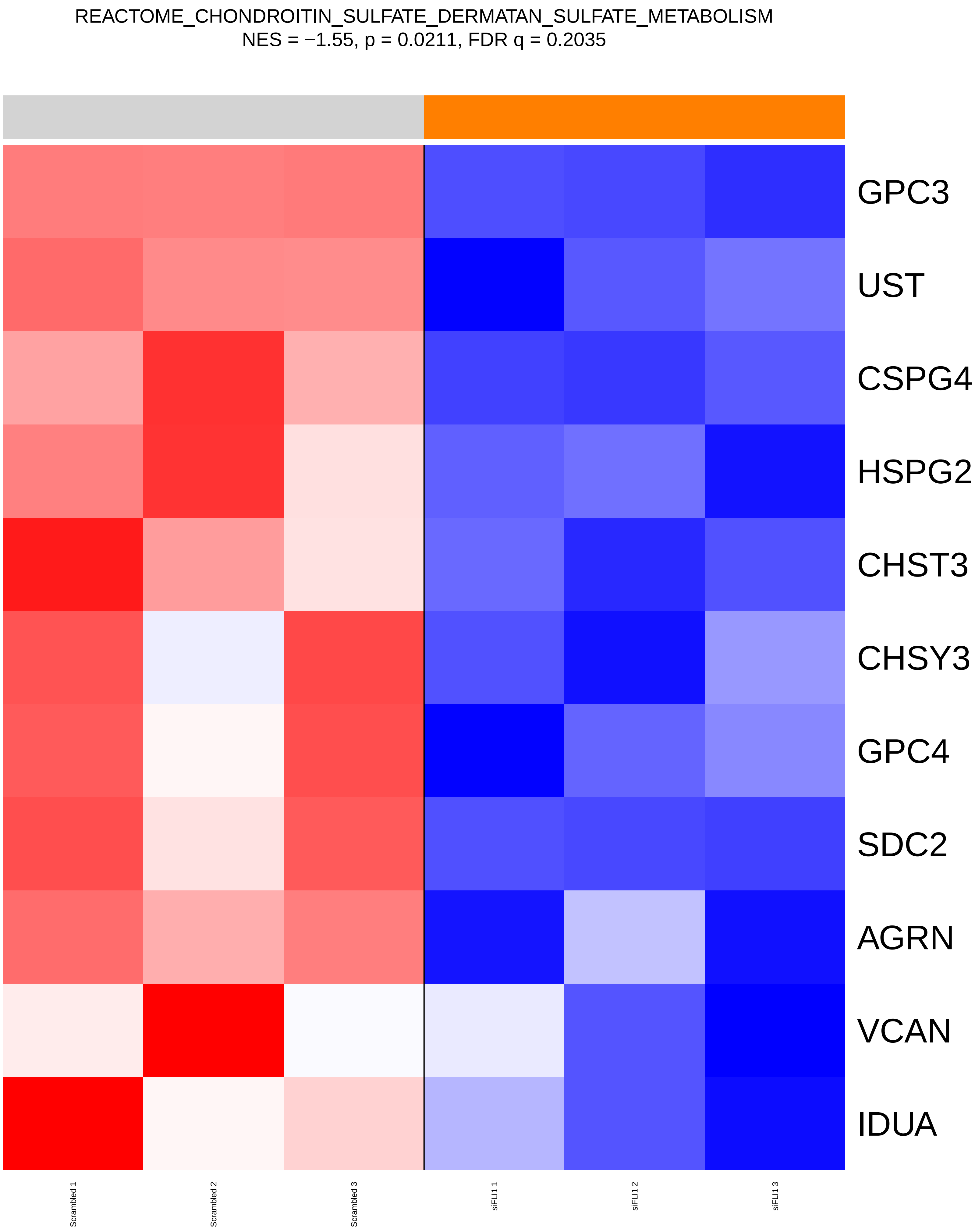
REACTOME_CHONDROITIN_SULFATE_DERMATAN_SULFATE_METABOLISM
NES = −1.55, p = 0.0211, FDR q = 0.2035
GPC3
UST
CSPG4
HSPG2
CHST3
CHSY3
GPC4
SDC2
AGRN
VCAN
IDUA
Scrambled 1
Scrambled 2
Scrambled 3
siFLI1 1
siFLI1 2
siFLI1 3

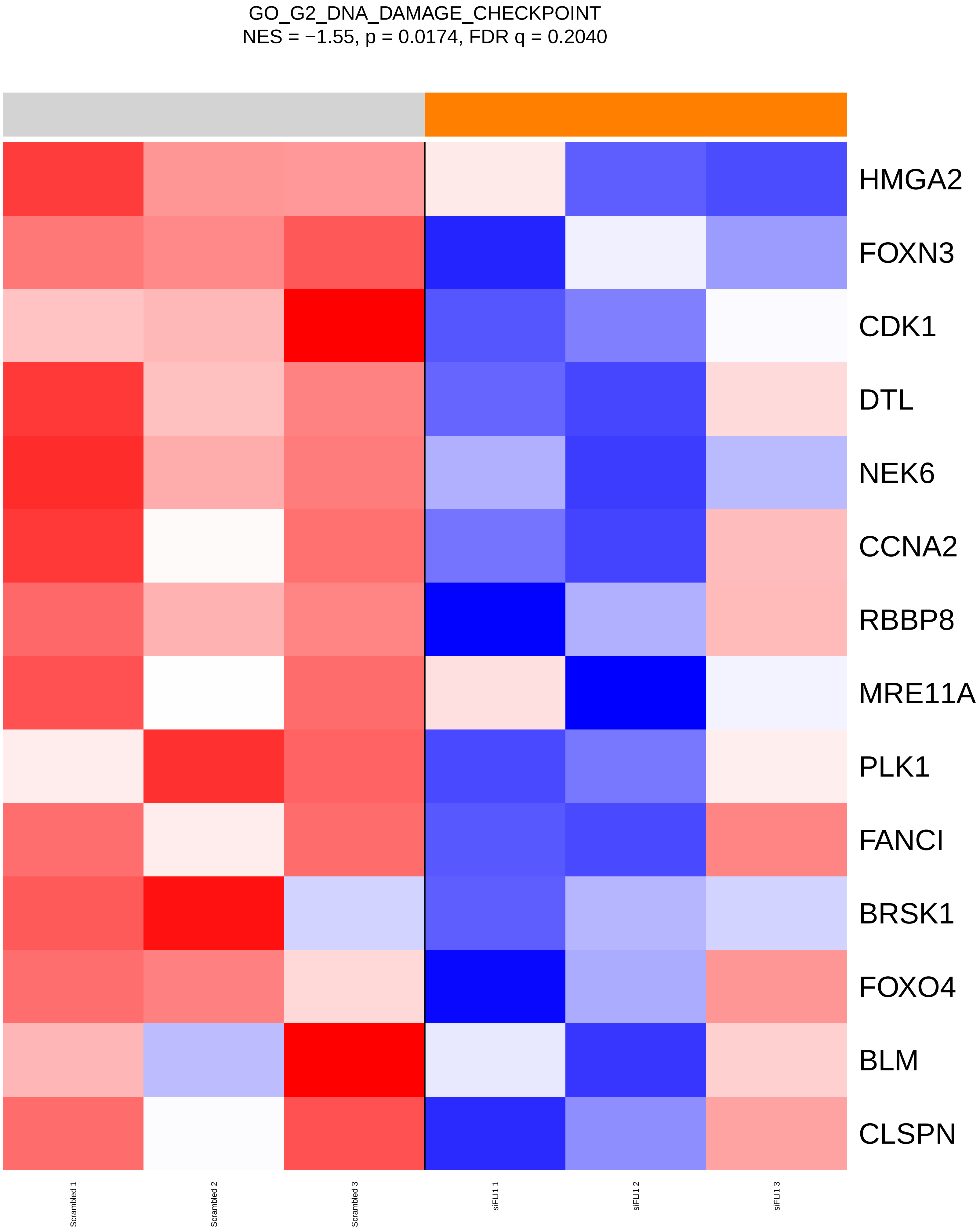
GO_G2_DNA_DAMAGE_CHECKPOINT
NES = −1.55, p = 0.0174, FDR q = 0.2040
HMGA2
FOXN3
CDK1
DTL
NEK6
CCNA2
RBBP8
MRE11A
PLK1
FANCI
BRSK1
FOXO4
BLM
CLSPN
Scrambled 1
Scrambled 2
Scrambled 3
siFLI1 1
siFLI1 2
siFLI1 3

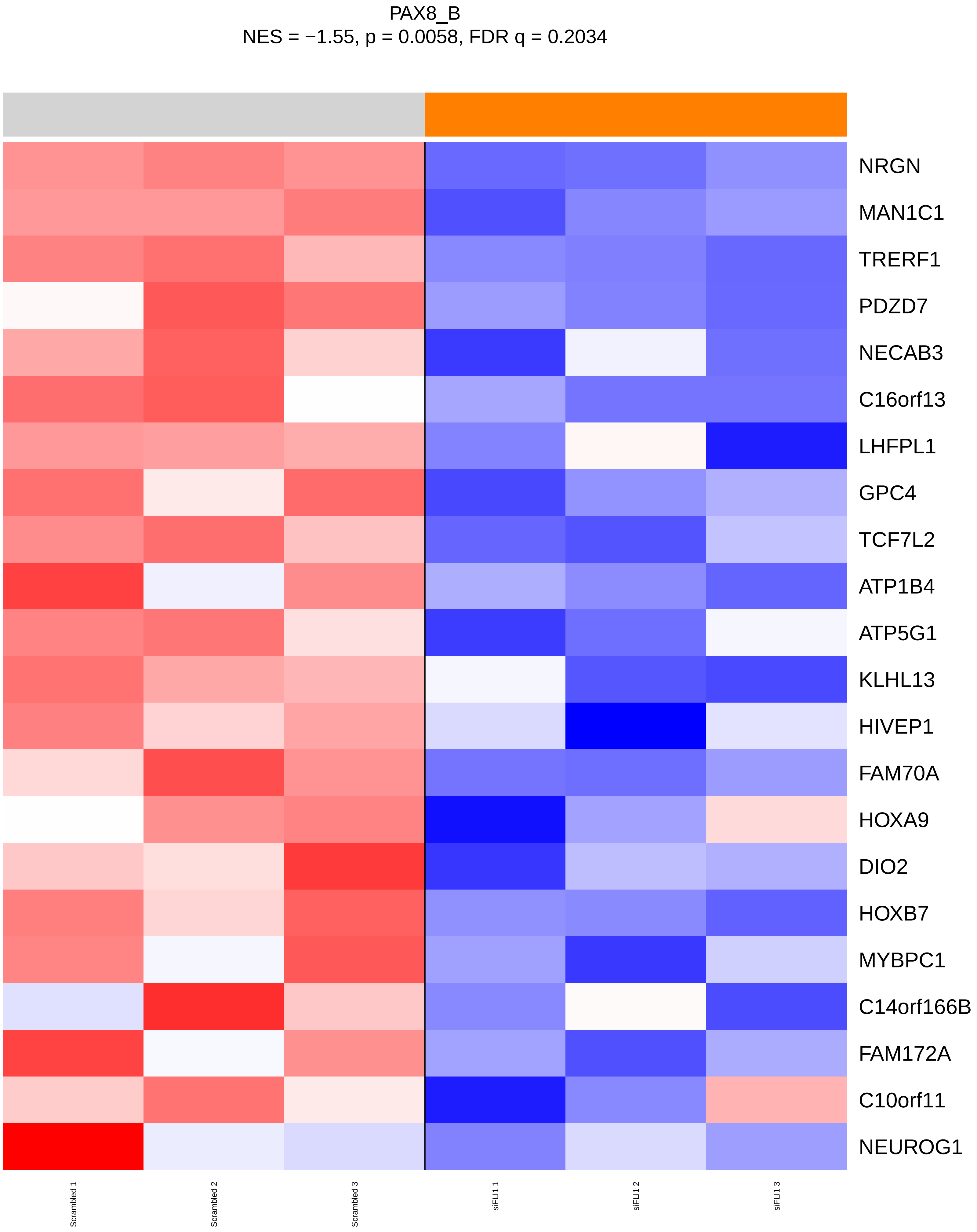
PAX8_B
NES = −1.55, p = 0.0058, FDR q = 0.2034
NRGN
MAN1C1
TRERF1
PDZD7
NECAB3
C16orf13
LHFPL1
GPC4
TCF7L2
ATP1B4
ATP5G1
KLHL13
HIVEP1
FAM70A
HOXA9
DIO2
HOXB7
MYBPC1
C14orf166B
FAM172A
C10orf11
NEUROG1
Scrambled 1
Scrambled 2
Scrambled 3
siFLI1 1
siFLI1 2
siFLI1 3

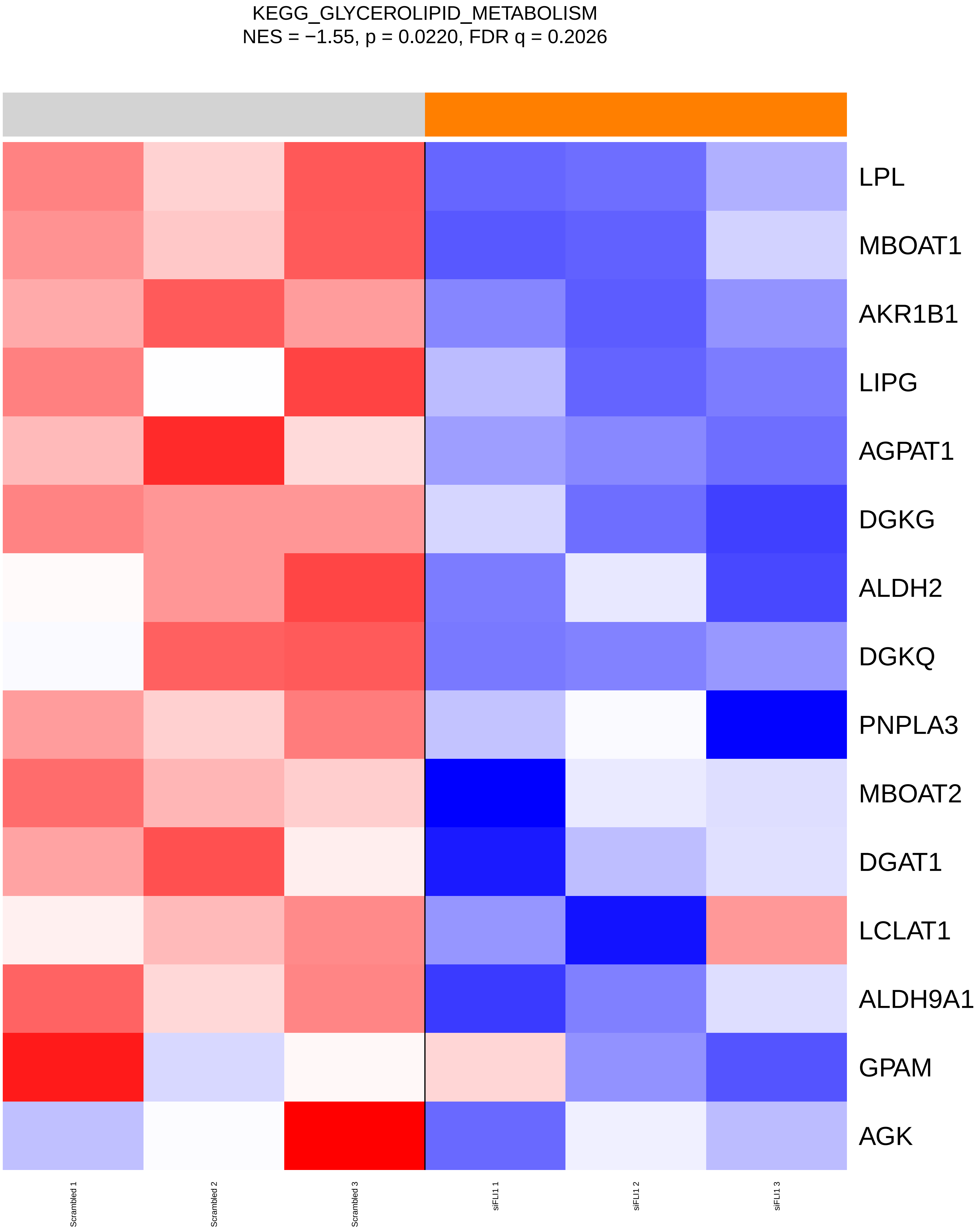
KEGG_GLYCEROLIPID_METABOLISM
NES = −1.55, p = 0.0220, FDR q = 0.2026
LPL
MBOAT1
AKR1B1
LIPG
AGPAT1
DGKG
ALDH2
DGKQ
PNPLA3
MBOAT2
DGAT1
LCLAT1
ALDH9A1
GPAM
AGK
Scrambled 1
Scrambled 2
Scrambled 3
siFLI1 1
siFLI1 2
siFLI1 3

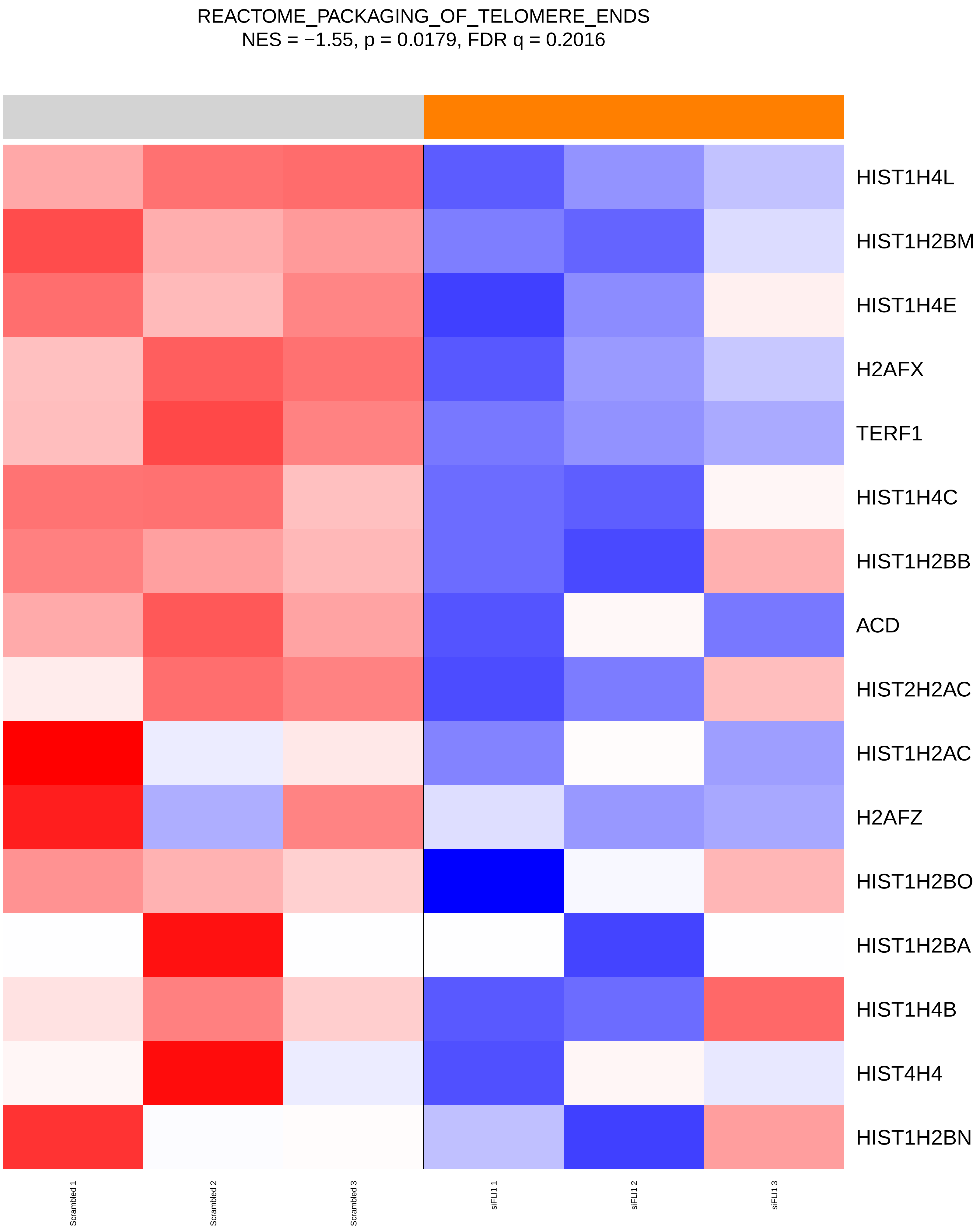

REACTOME_PACKAGING_OF_TELOMERE_ENDS
NES = −1.55, p = 0.0179, FDR q = 0.2016
HIST1H4L
HIST1H2BM
HIST1H4E
H2AFX
TERF1
HIST1H4C
HIST1H2BB
ACD
HIST2H2AC
HIST1H2AC
H2AFZ
HIST1H2BO
HIST1H2BA
HIST1H4B
HIST4H4
HIST1H2BN
Scrambled 1
Scrambled 2
Scrambled 3
siFLI1 1
siFLI1 2
siFLI1 3

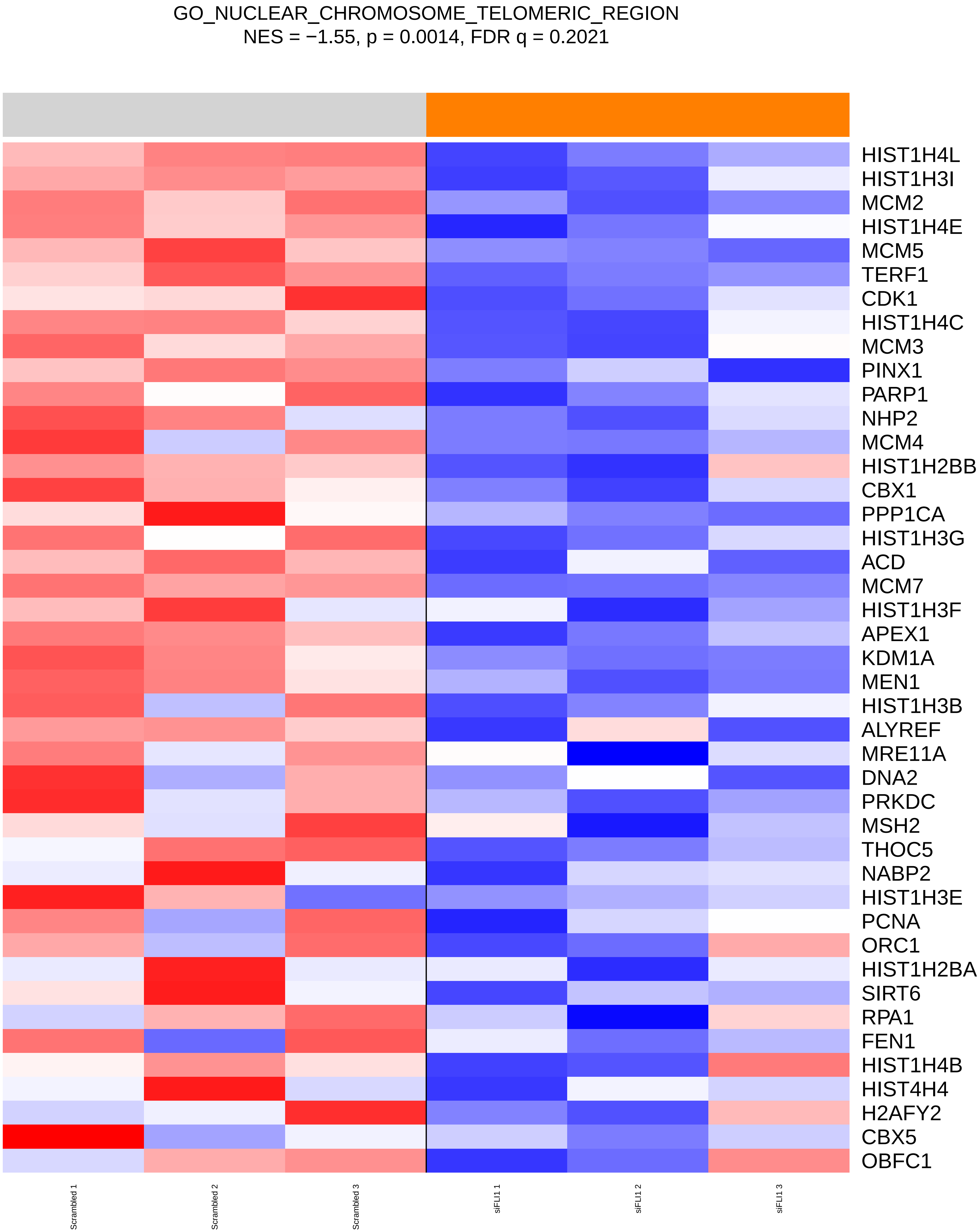
GO_NUCLEAR_CHROMOSOME_TELOMERIC_REGION
NES = −1.55, p = 0.0014, FDR q = 0.2021
HIST1H4L
HIST1H3I
MCM2
HIST1H4E
MCM5
TERF1
CDK1
HIST1H4C
MCM3
PINX1
PARP1
NHP2
MCM4
HIST1H2BB
CBX1
PPP1CA
HIST1H3G
ACD
MCM7
HIST1H3F
APEX1
KDM1A
MEN1
HIST1H3B
ALYREF
MRE11A
DNA2
PRKDC
MSH2
THOC5
NABP2
HIST1H3E
PCNA
ORC1
HIST1H2BA
SIRT6
RPA1
FEN1
HIST1H4B
HIST4H4
H2AFY2
CBX5
OBFC1
Scrambled 1
Scrambled 2
Scrambled 3
siFLI1 1
siFLI1 2
siFLI1 3

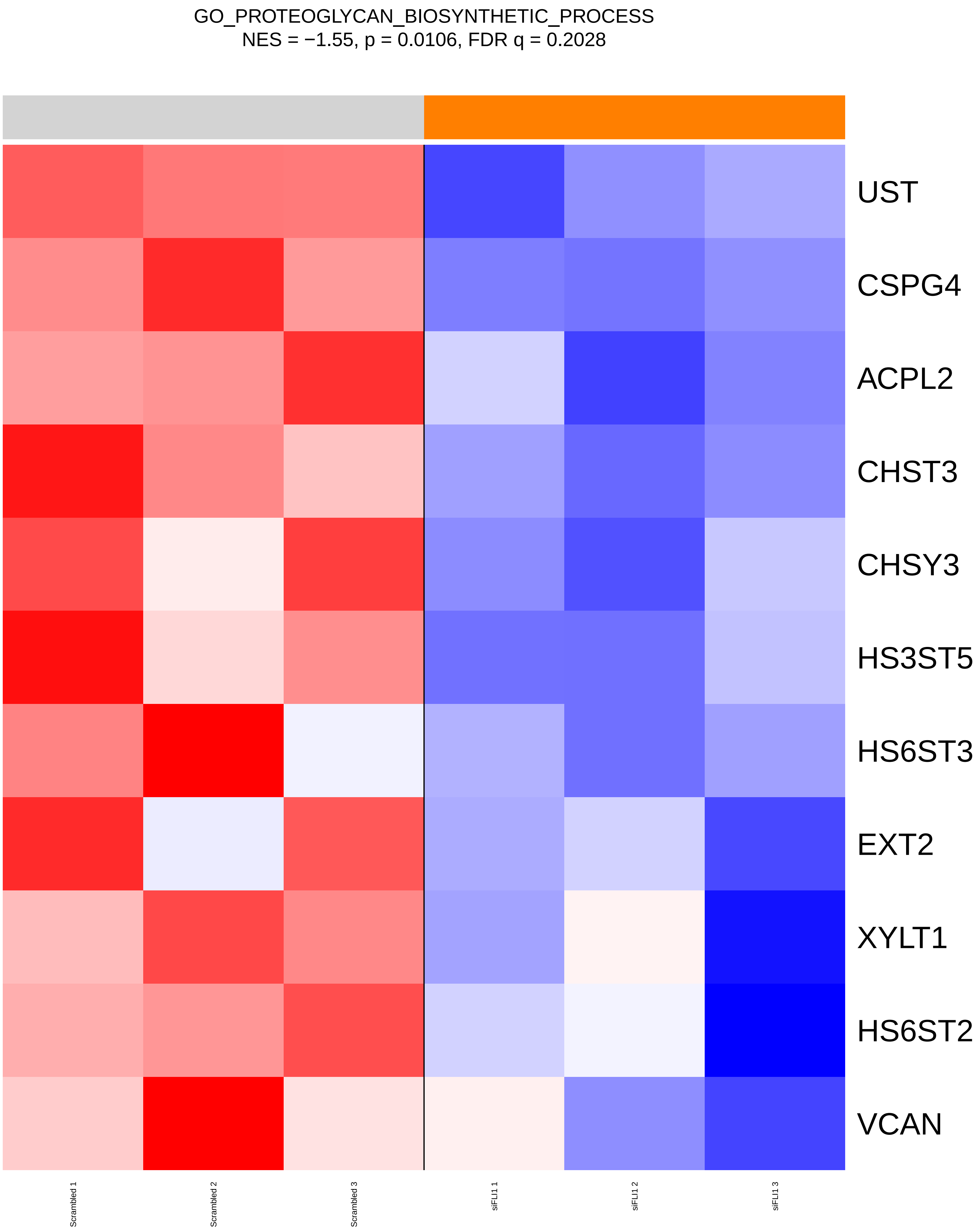
GO_PROTEOGLYCAN_BIOSYNTHETIC_PROCESS
NES = −1.55, p = 0.0106, FDR q = 0.2028
UST
CSPG4
ACPL2
CHST3
CHSY3
HS3ST5
HS6ST3
EXT2
XYLT1
HS6ST2
VCAN
Scrambled 1
Scrambled 2
Scrambled 3
siFLI1 1
siFLI1 2
siFLI1 3

GO_CILIARY_BASE
NES = −1.55, p = 0.0334, FDR q = 0.1996

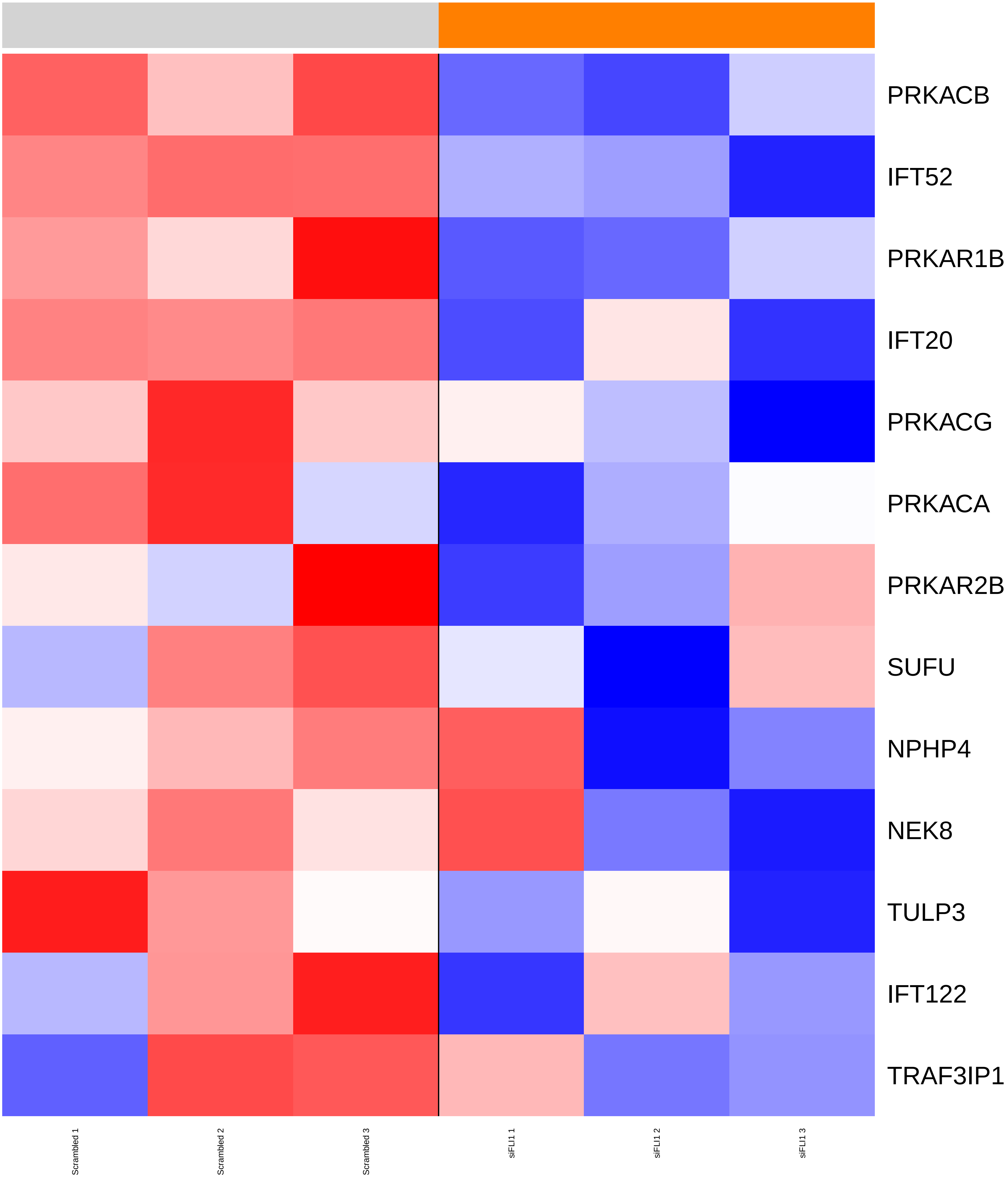

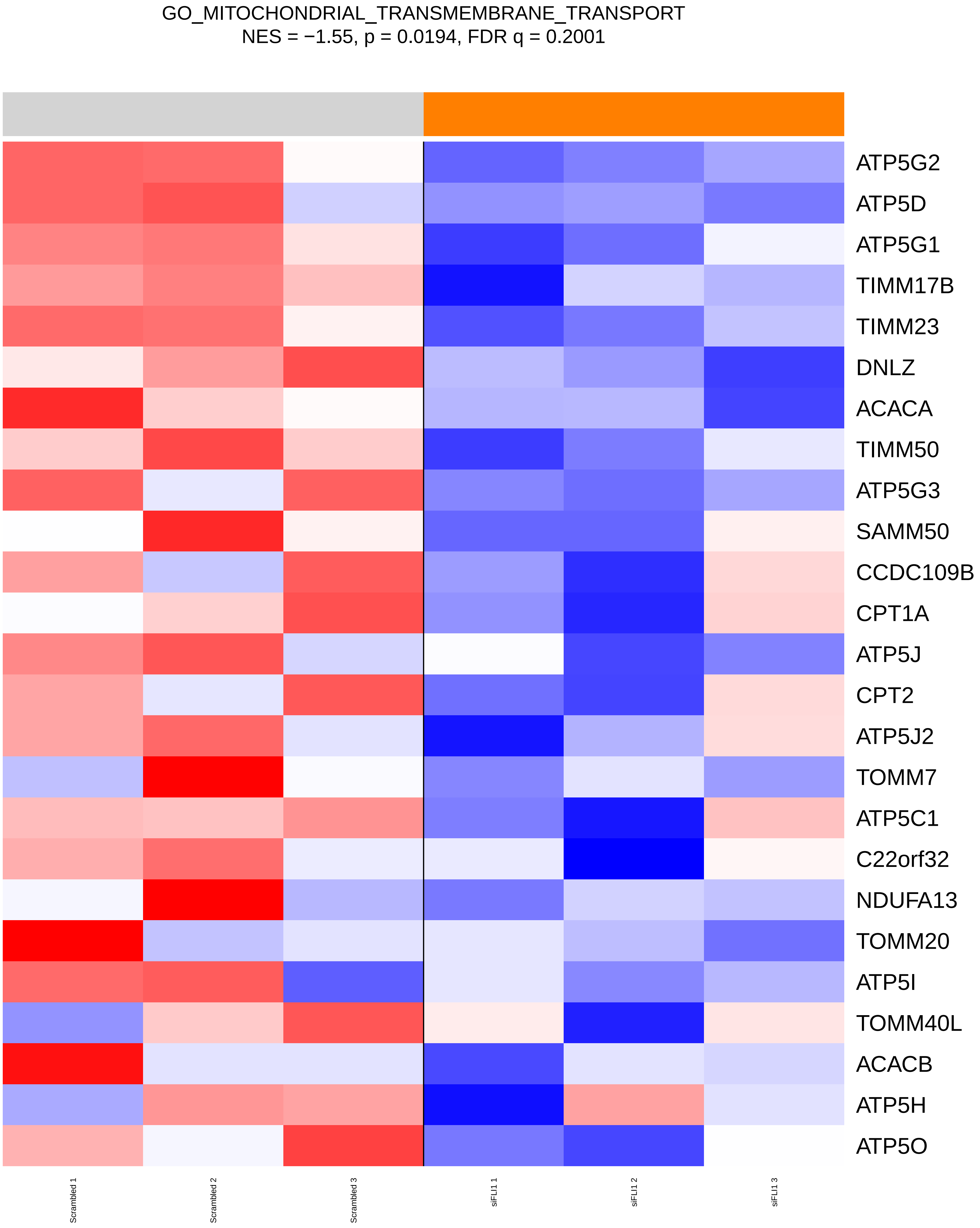
GO_MITOCHONDRIAL_TRANSMEMBRANE_TRANSPORT
NES = −1.55, p = 0.0194, FDR q = 0.2001
ATP5G2
ATP5D
ATP5G1
TIMM17B
TIMM23
DNLZ
ACACA
TIMM50
ATP5G3
SAMM50
CCDC109B
CPT1A
ATP5J
CPT2
ATP5J2
TOMM7
ATP5C1
C22orf32
NDUFA13
TOMM20
ATP5I
TOMM40L
ACACB
ATP5H
ATP5O
Scrambled 1
Scrambled 2
Scrambled 3
siFLI1 1
siFLI1 2
siFLI1 3

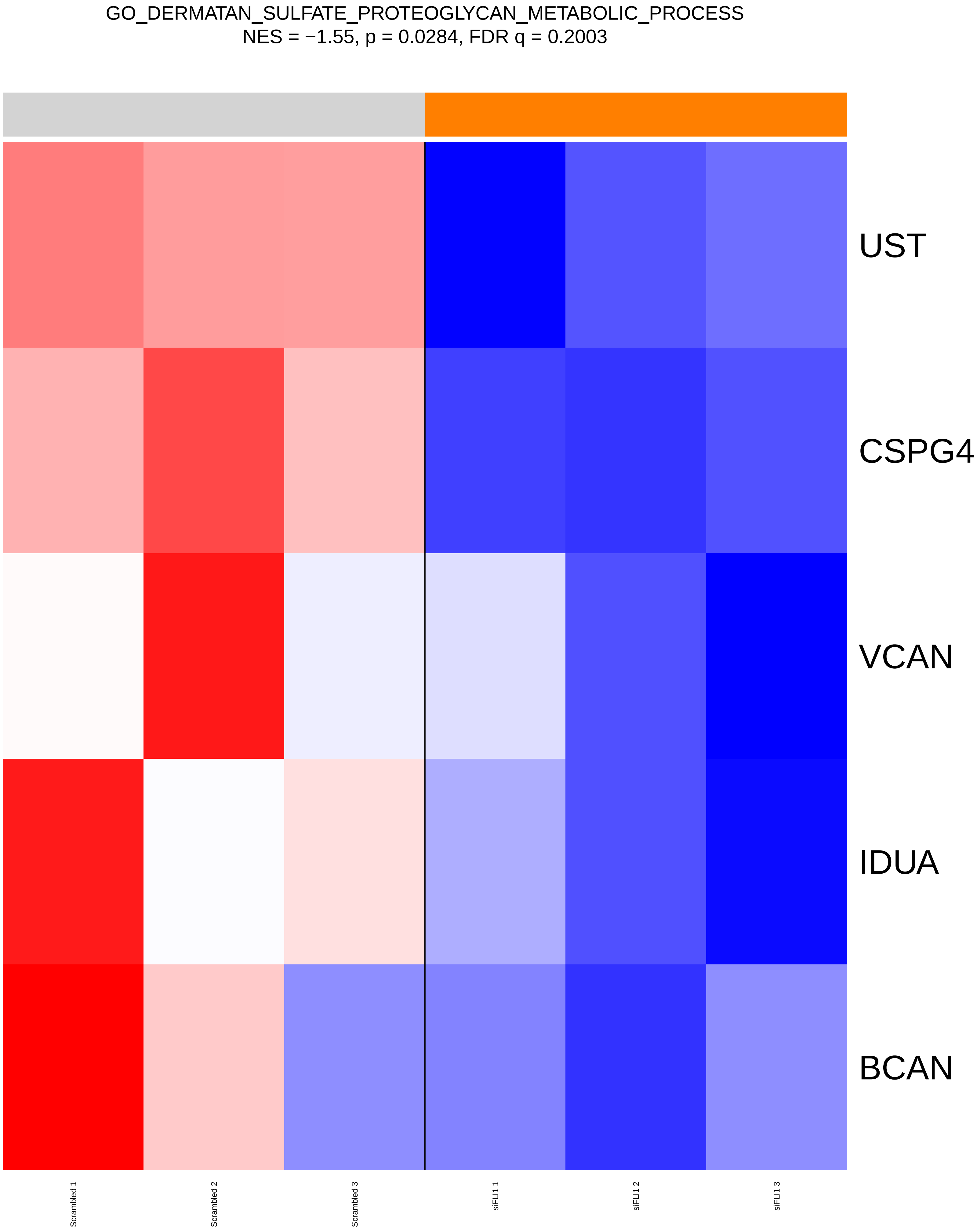

GO_DERMATAN_SULFATE_PROTEOGLYCAN_METABOLIC_PROCESS
NES = −1.55, p = 0.0284, FDR q = 0.2003
UST
CSPG4
VCAN
IDUA
BCAN
Scrambled 1
Scrambled 2
Scrambled 3
siFLI1 1
siFLI1 2
siFLI1 3

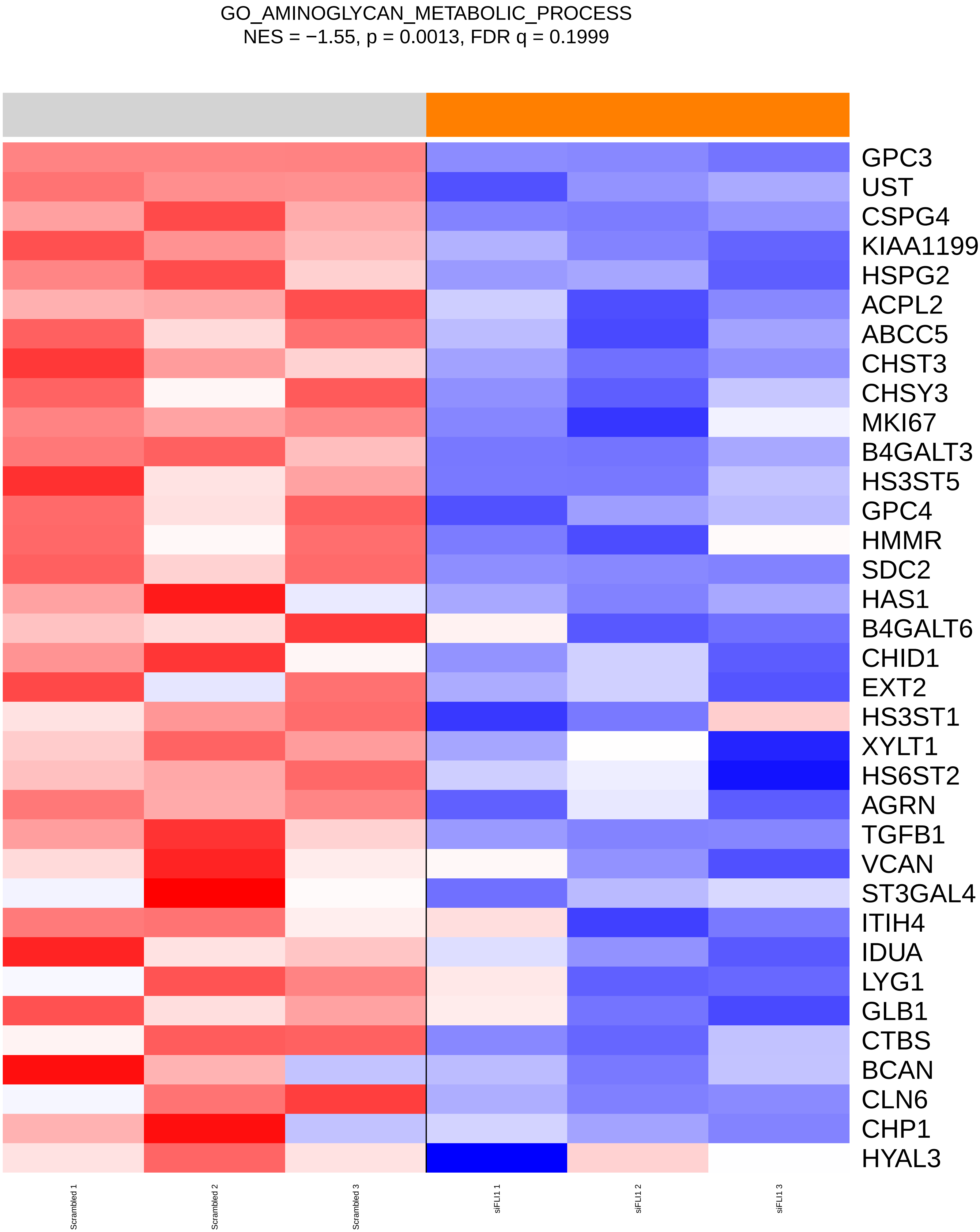
GO_AMINOGLYCAN_METABOLIC_PROCESS
NES = −1.55, p = 0.0013, FDR q = 0.1999
GPC3
UST
CSPG4
KIAA1199
HSPG2
ACPL2
ABCC5
CHST3
CHSY3
MKI67
B4GALT3
HS3ST5
GPC4
HMMR
SDC2
HAS1
B4GALT6
CHID1
EXT2
HS3ST1
XYLT1
HS6ST2
AGRN
TGFB1
VCAN
ST3GAL4
ITIH4
IDUA
LYG1
GLB1
CTBS
BCAN
CLN6
CHP1
HYAL3
Scrambled 1
Scrambled 2
Scrambled 3
siFLI1 1
siFLI1 2
siFLI1 3

REACTOME_DAG_AND_IP3_SIGNALING
NES = −1.55, p = 0.0279, FDR q = 0.2001

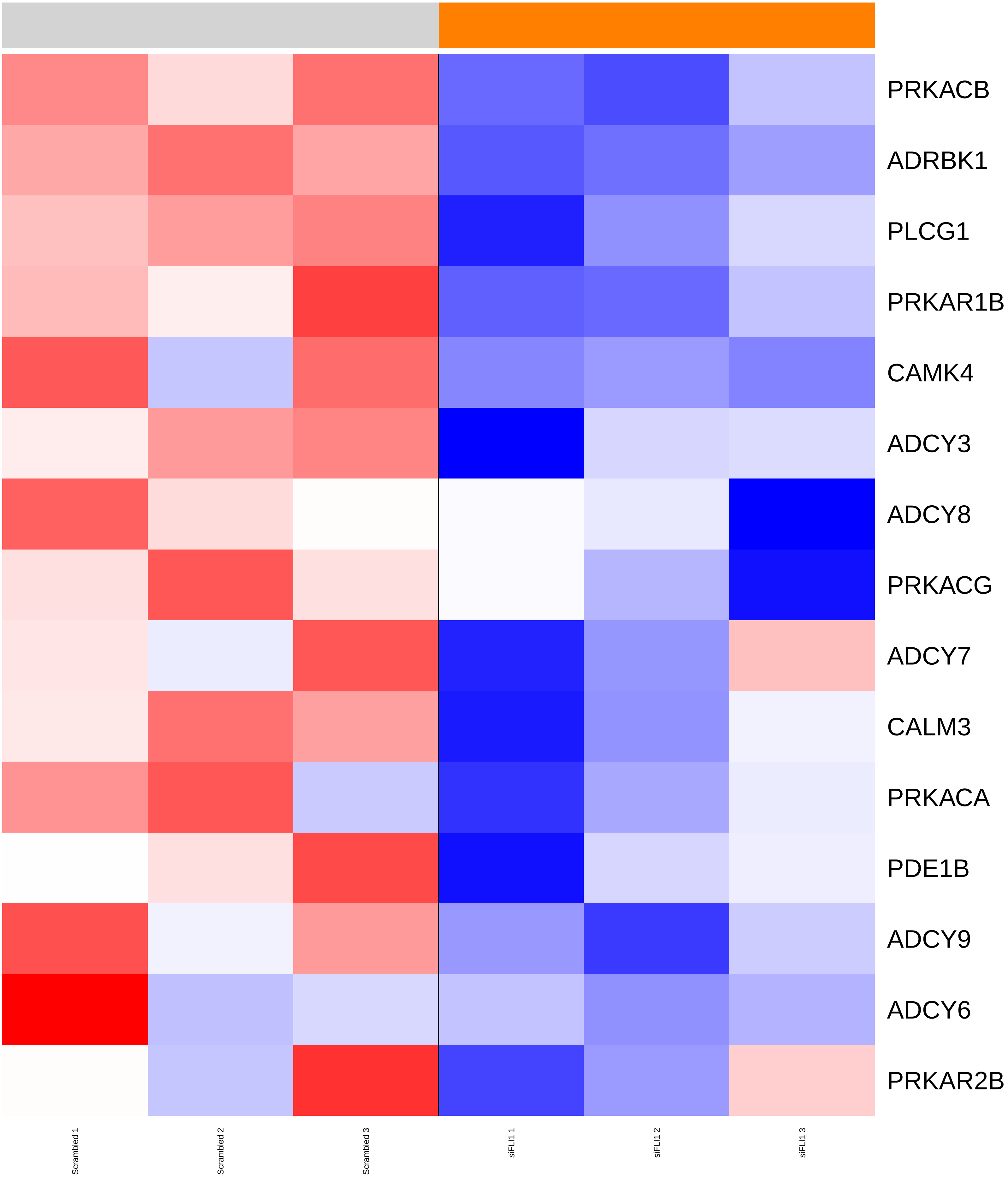

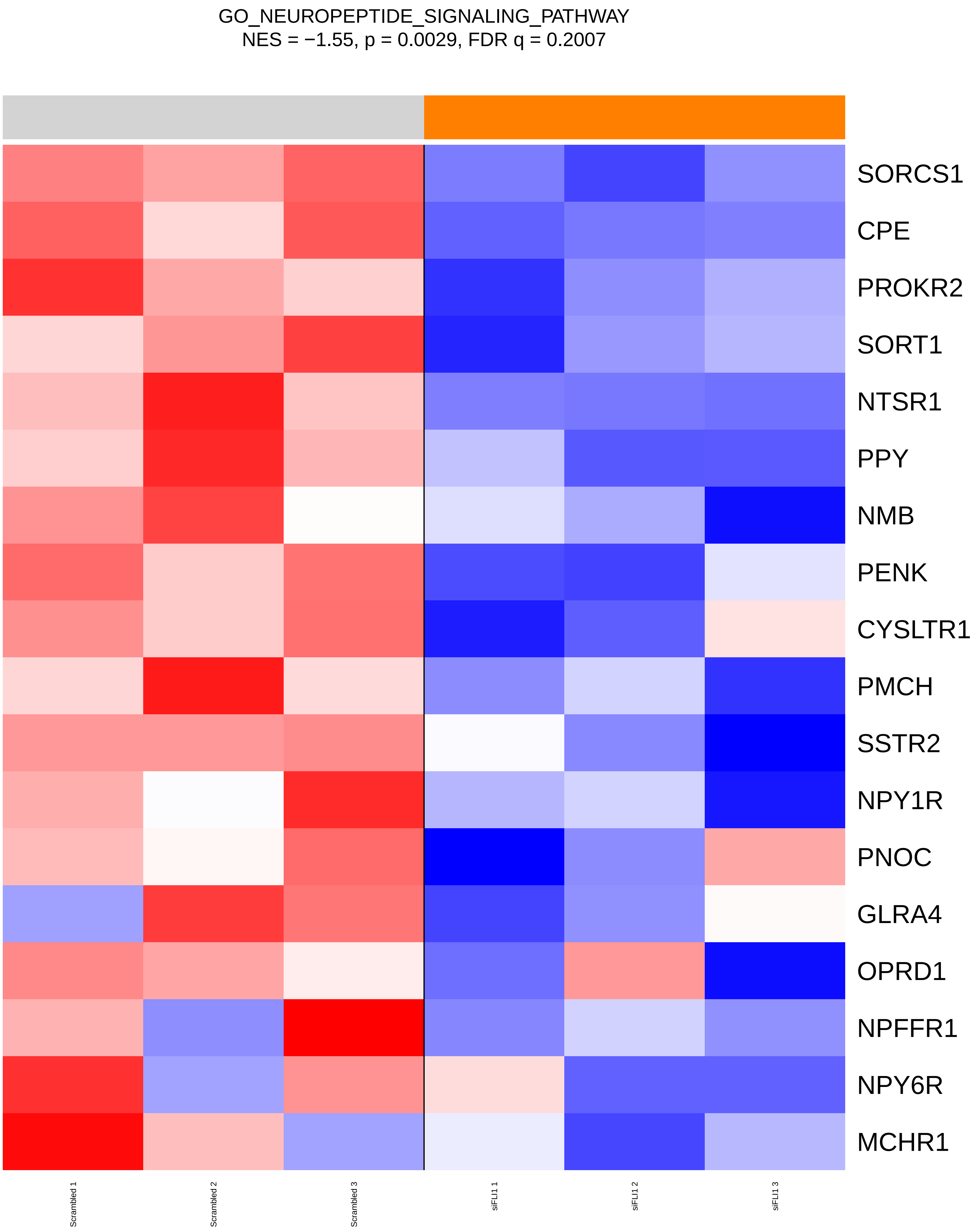
GO_NEUROPEPTIDE_SIGNALING_PATHWAY
NES = −1.55, p = 0.0029, FDR q = 0.2007
SORCS1
CPE
PROKR2
SORT1
NTSR1
PPY
NMB
PENK
CYSLTR1
PMCH
SSTR2
NPY1R
PNOC
GLRA4
OPRD1
NPFFR1
NPY6R
MCHR1
Scrambled 1
Scrambled 2
Scrambled 3
siFLI1 1
siFLI1 2
siFLI1 3

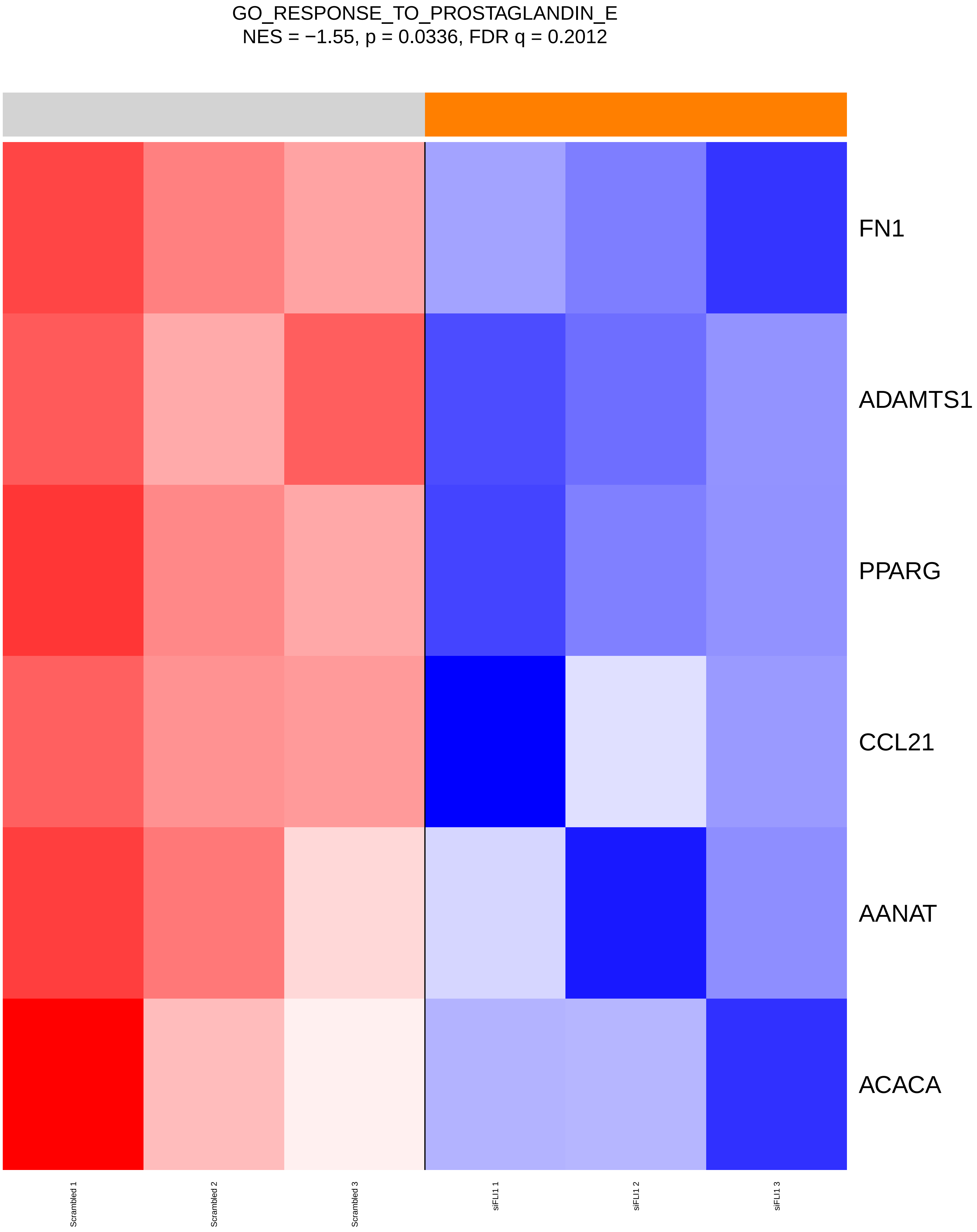
GO_RESPONSE_TO_PROSTAGLANDIN_E
NES = −1.55, p = 0.0336, FDR q = 0.2012
FN1
ADAMTS1
PPARG
CCL21
AANAT
ACACA
Scrambled 1
Scrambled 2
Scrambled 3
siFLI1 1
siFLI1 2
siFLI1 3

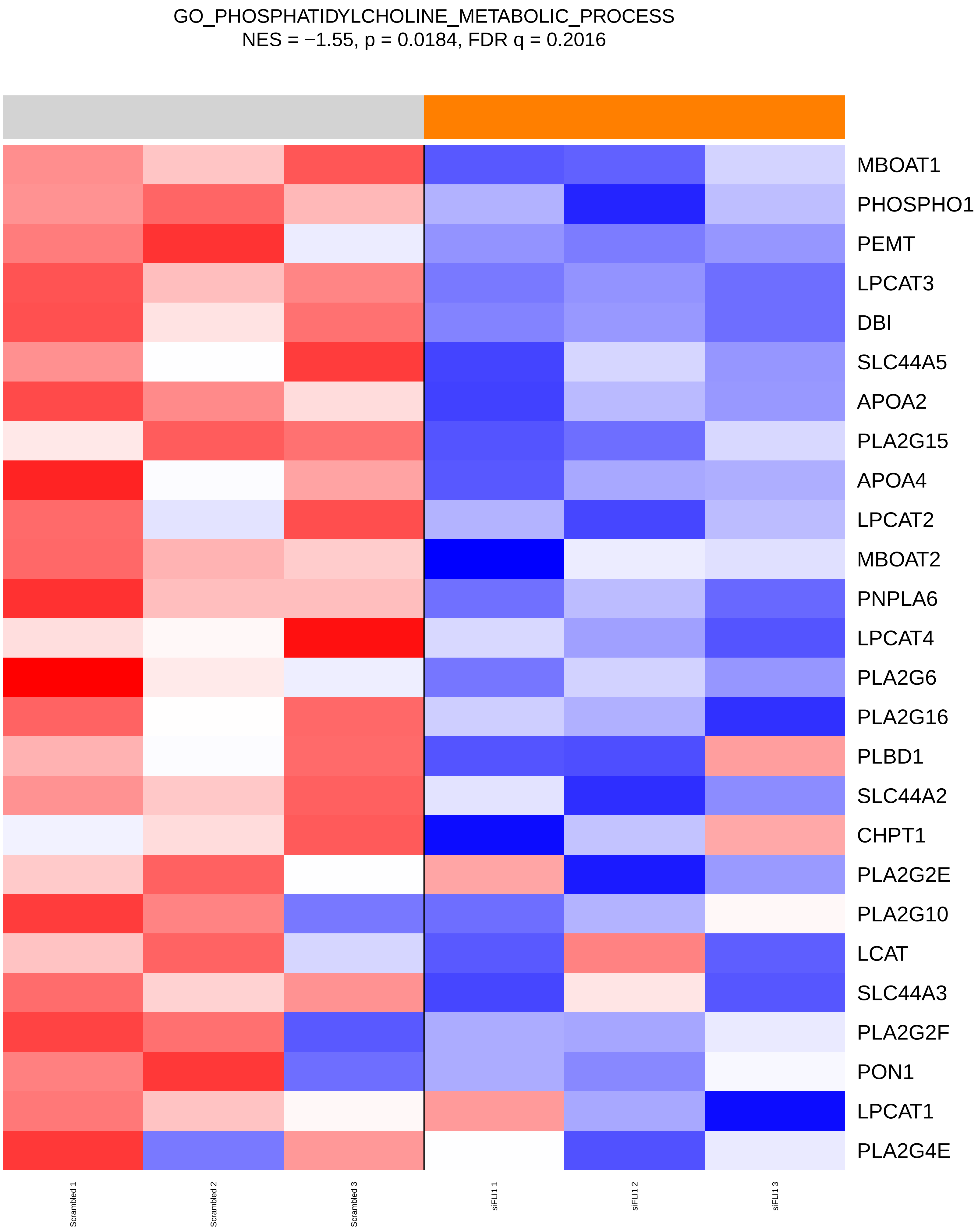
GO_PHOSPHATIDYLCHOLINE_METABOLIC_PROCESS
NES = −1.55, p = 0.0184, FDR q = 0.2016
MBOAT1
PHOSPHO1
PEMT
LPCAT3
DBI
SLC44A5
APOA2
PLA2G15
APOA4
LPCAT2
MBOAT2
PNPLA6
LPCAT4
PLA2G6
PLA2G16
PLBD1
SLC44A2
CHPT1
PLA2G2E
PLA2G10
LCAT
SLC44A3
PLA2G2F
PON1
LPCAT1
PLA2G4E
Scrambled 1
Scrambled 2
Scrambled 3
siFLI1 1
siFLI1 2
siFLI1 3

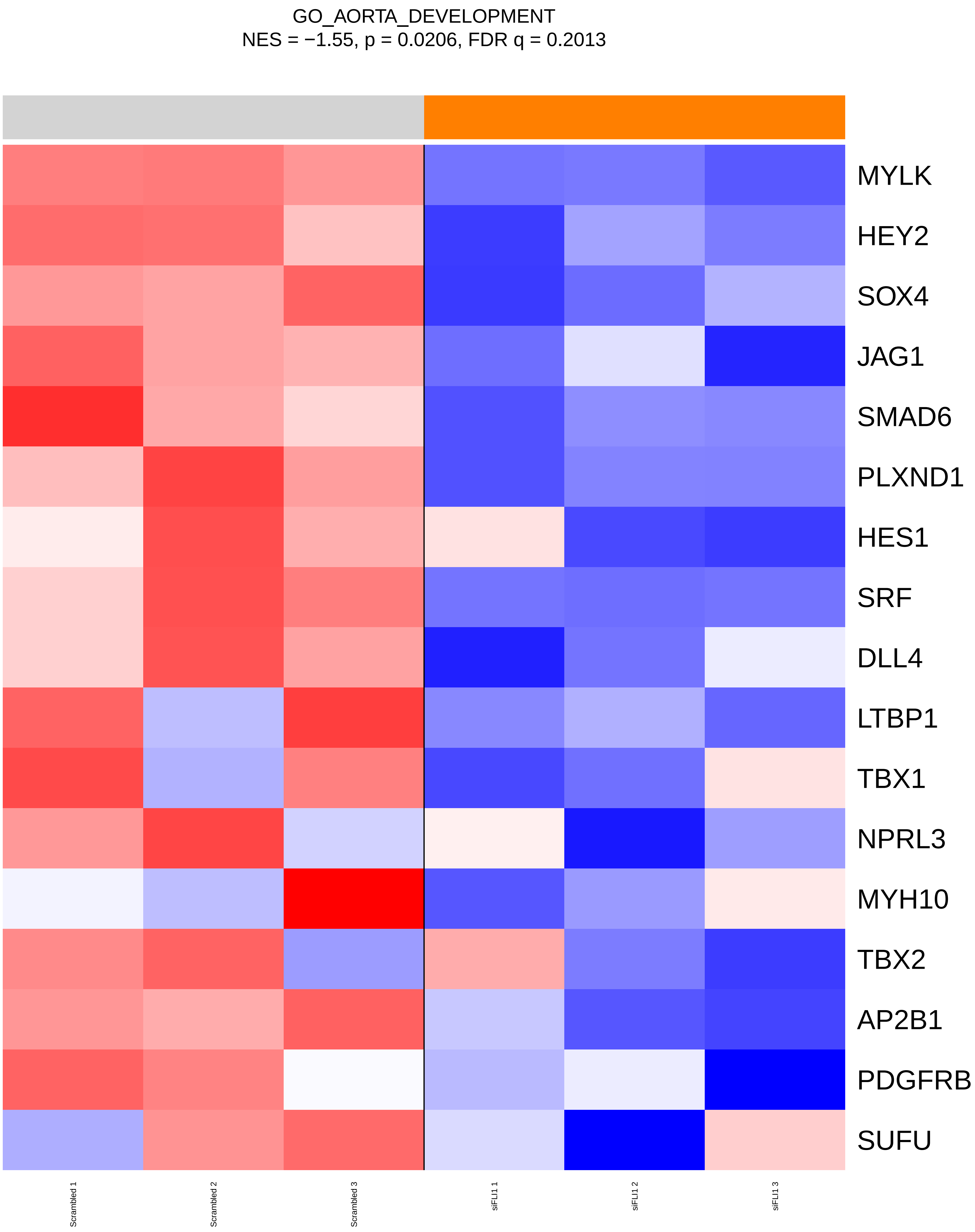
GO_AORTA_DEVELOPMENT
NES = −1.55, p = 0.0206, FDR q = 0.2013
MYLK
HEY2
SOX4
JAG1
SMAD6
PLXND1
HES1
SRF
DLL4
LTBP1
TBX1
NPRL3
MYH10
TBX2
AP2B1
PDGFRB
SUFU
Scrambled 1
Scrambled 2
Scrambled 3
siFLI1 1
siFLI1 2
siFLI1 3

E2F_Q4_01
NES = −1.56, p = 0.0000, FDR q = 0.2002

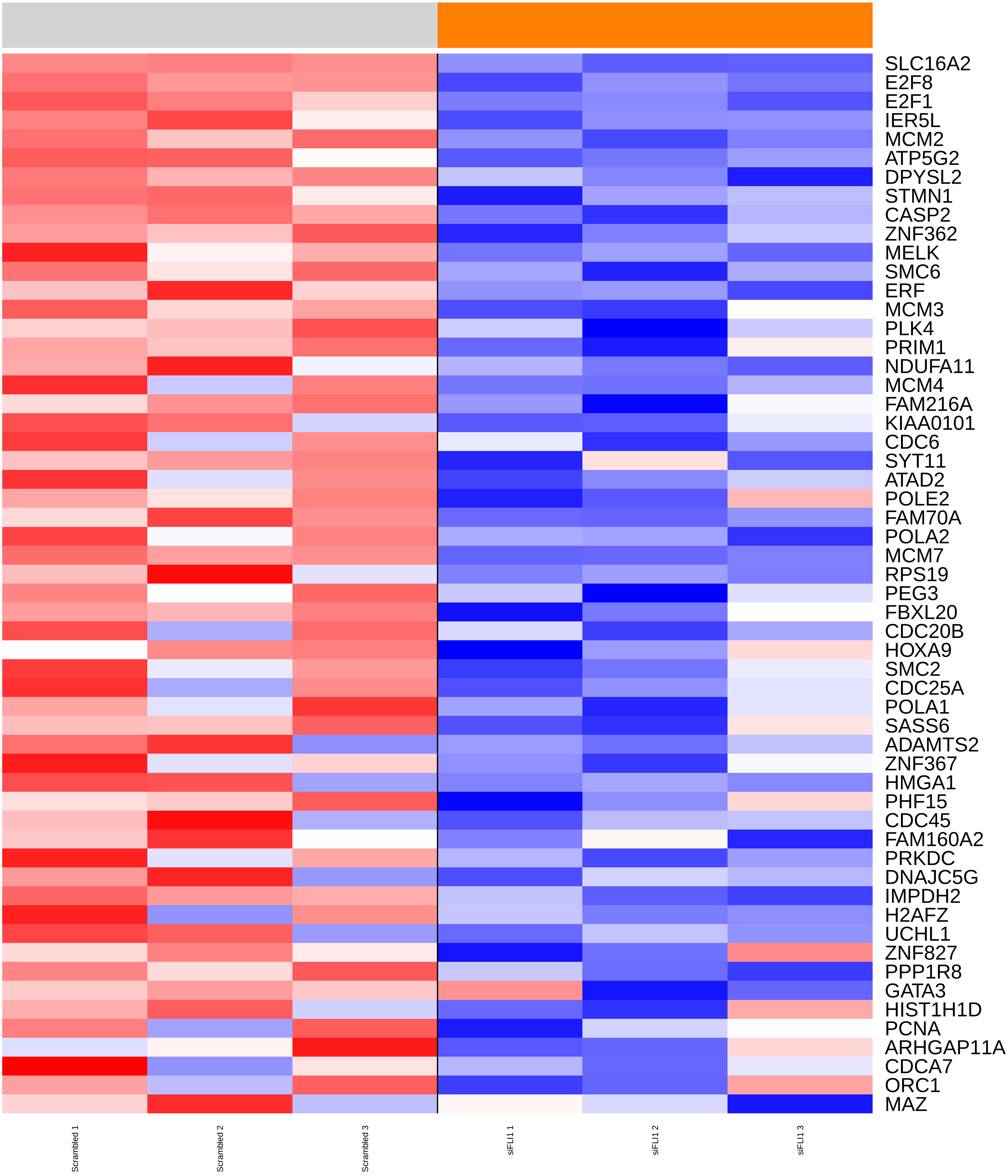

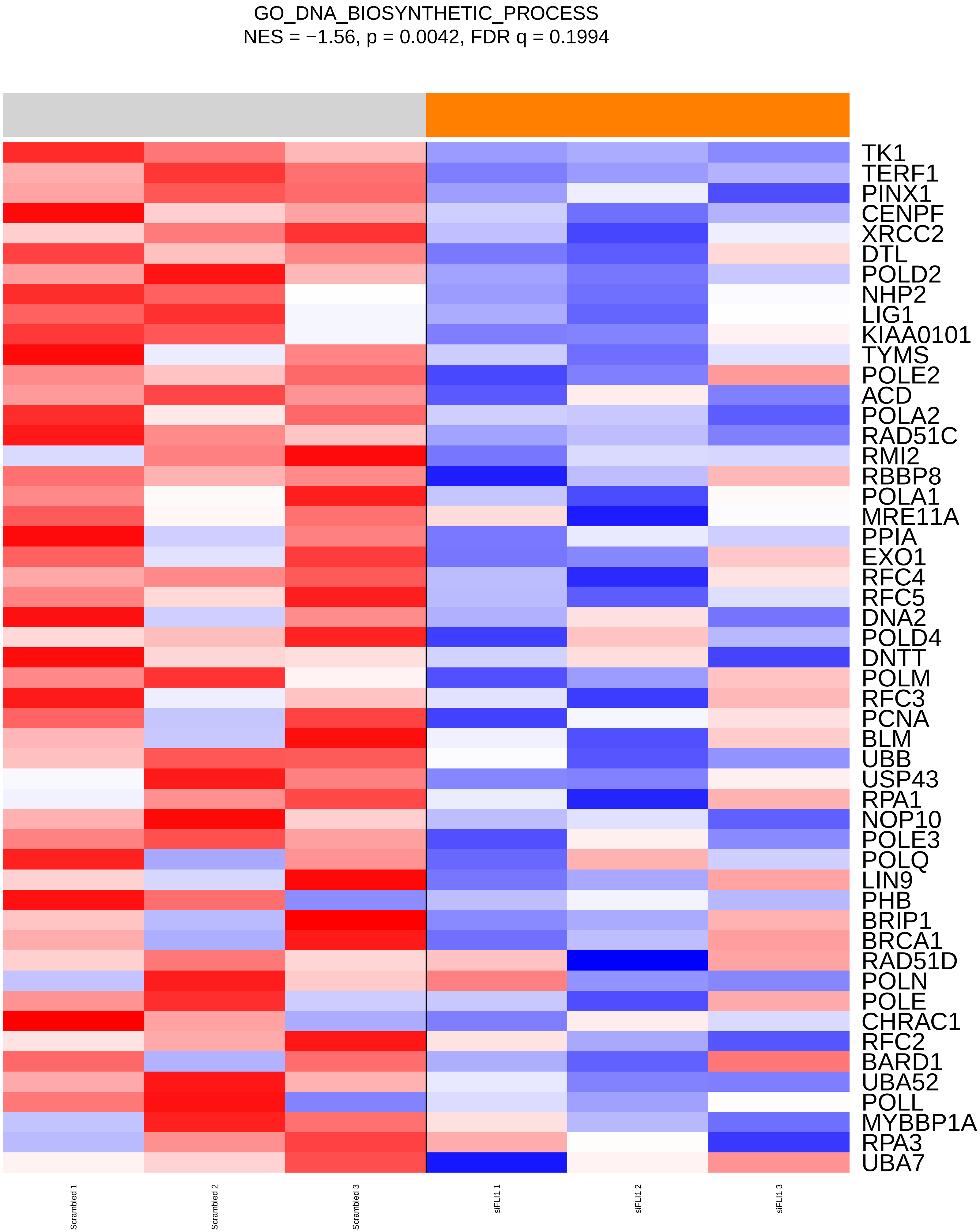
GO_DNA_BIOSYNTHETIC_PROCESS
NES = −1.56, p = 0.0042, FDR q = 0.1994
TK1
TERF1
PINX1
CENPF
XRCC2
DTL
POLD2
NHP2
LIG1
KIAA0101
TYMS
POLE2
ACD
POLA2
RAD51C
RMI2
RBBP8
POLA1
MRE11A
PPIA
EXO1
RFC4
RFC5
DNA2
POLD4
DNTT
POLM
RFC3
PCNA
BLM
UBB
USP43
RPA1
NOP10
POLE3
POLQ
LIN9
PHB
BRIP1
BRCA1
RAD51D
POLN
POLE
CHRAC1
RFC2
BARD1
UBA52
POLL
MYBBP1A
RPA3
UBA7
Scrambled 1
Scrambled 2
Scrambled 3
siFLI1 1
siFLI1 2
siFLI1 3

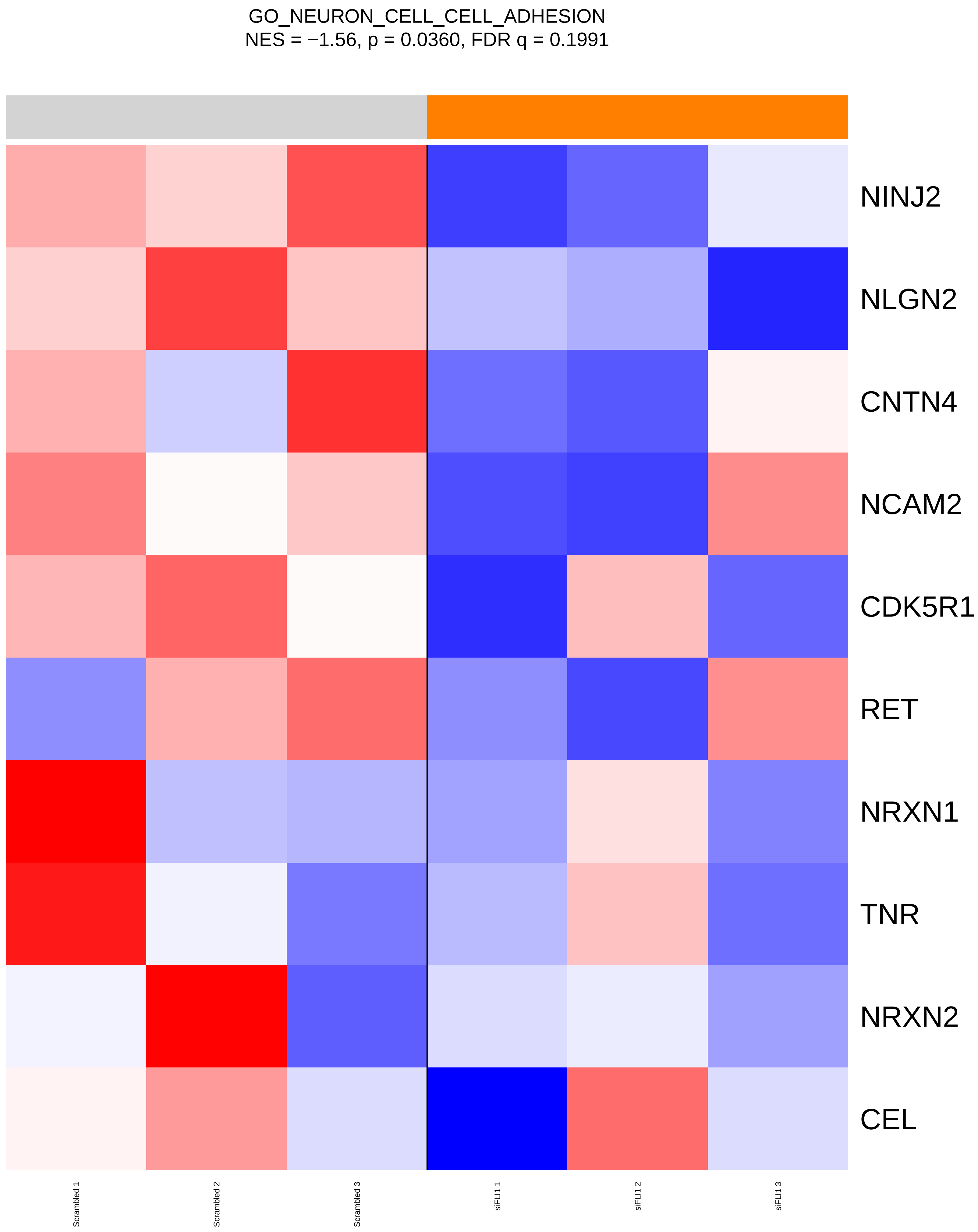
GO_NEURON_CELL_CELL_ADHESION
NES = −1.56, p = 0.0360, FDR q = 0.1991
NINJ2
NLGN2
CNTN4
NCAM2
CDK5R1
RET
NRXN1
TNR
NRXN2
CEL
Scrambled 1
Scrambled 2
Scrambled 3
siFLI1 1
siFLI1 2
siFLI1 3

GGGGCCC_MIR296
NES = −1.56, p = 0.0123, FDR q = 0.1998

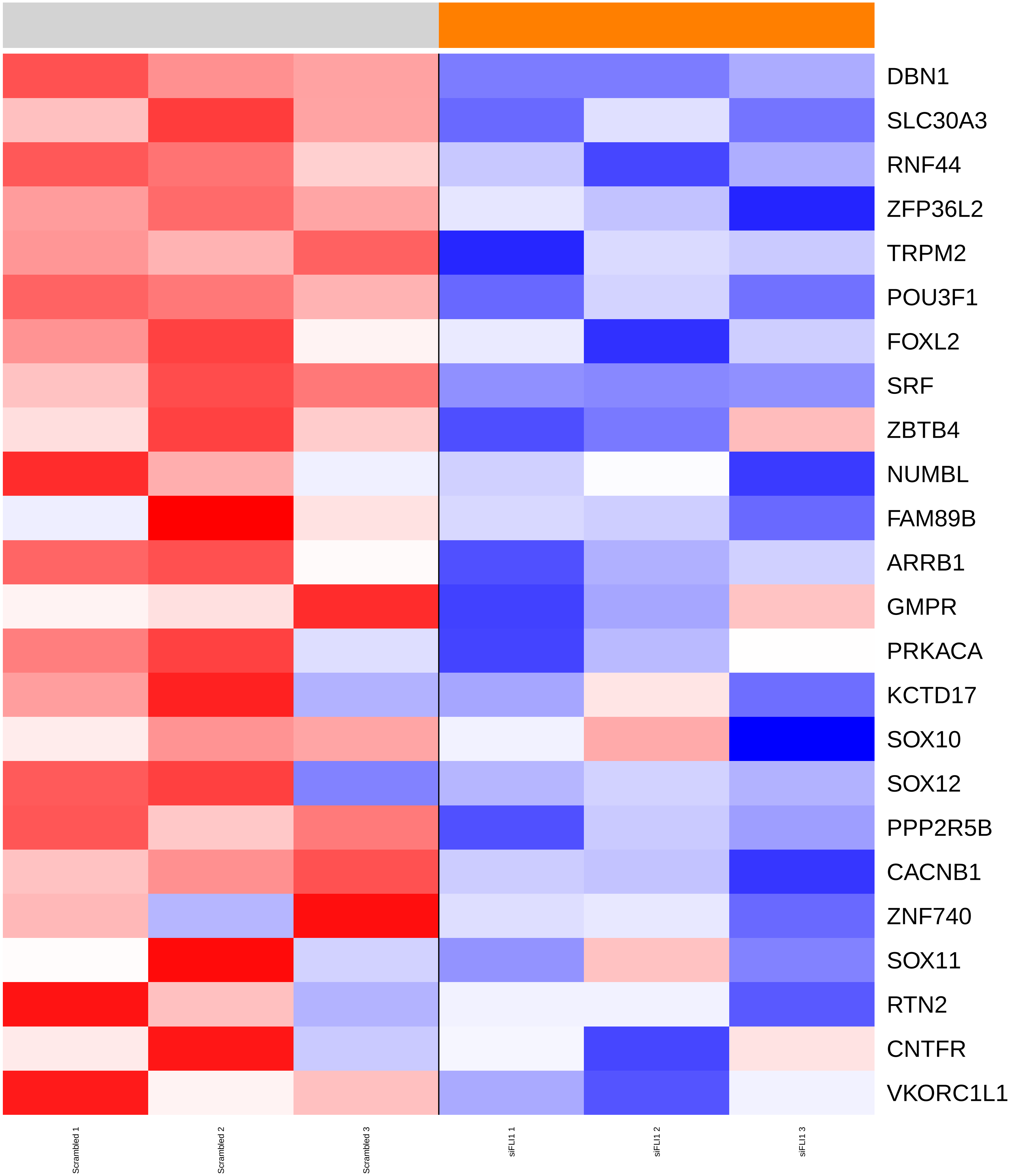

GO_REGULATION_OF_SYNAPTIC_PLASTICITY
NES = −1.56, p = 0.0028, FDR q = 0.1990

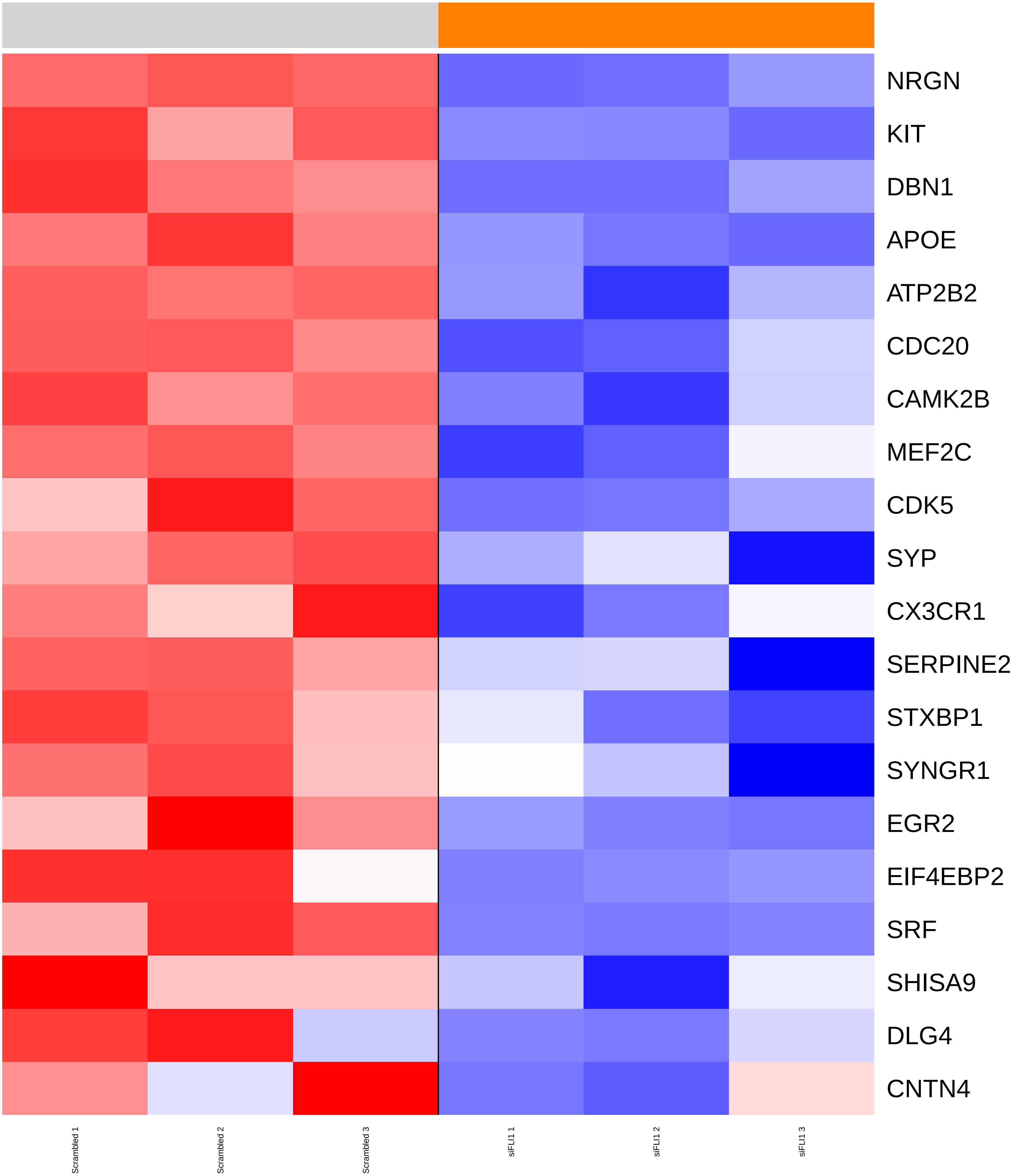

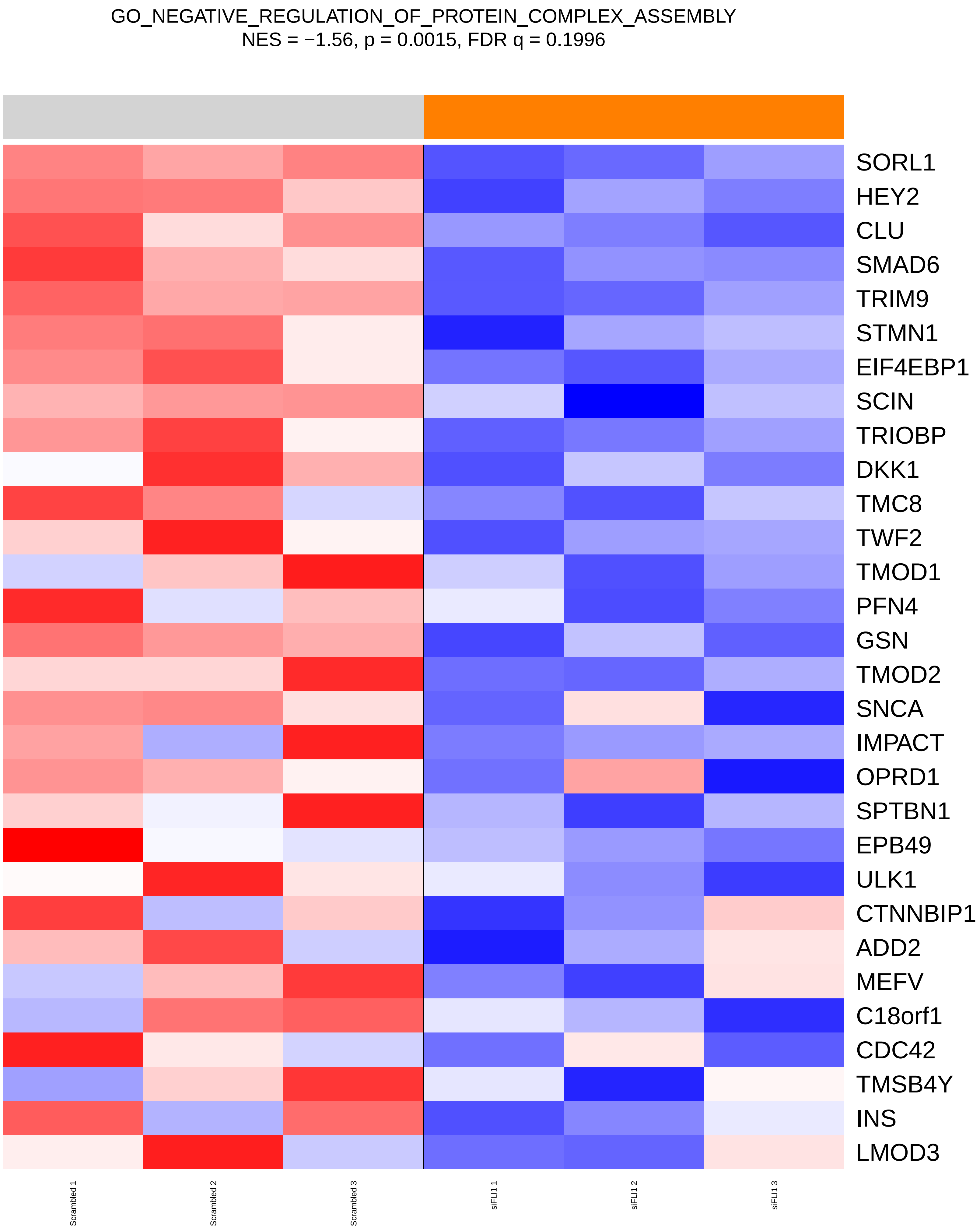
GO_NEGATIVE_REGULATION_OF_PROTEIN_COMPLEX_ASSEMBLY
NES = −1.56, p = 0.0015, FDR q = 0.1996
SORL1
HEY2
CLU
SMAD6
TRIM9
STMN1
EIF4EBP1
SCIN
TRIOBP
DKK1
TMC8
TWF2
TMOD1
PFN4
GSN
TMOD2
SNCA
IMPACT
OPRD1
SPTBN1
EPB49
ULK1
CTNNBIP1
ADD2
MEFV
C18orf1
CDC42
TMSB4Y
INS
LMOD3
Scrambled 1
Scrambled 2
Scrambled 3
siFLI1 1
siFLI1 2
siFLI1 3

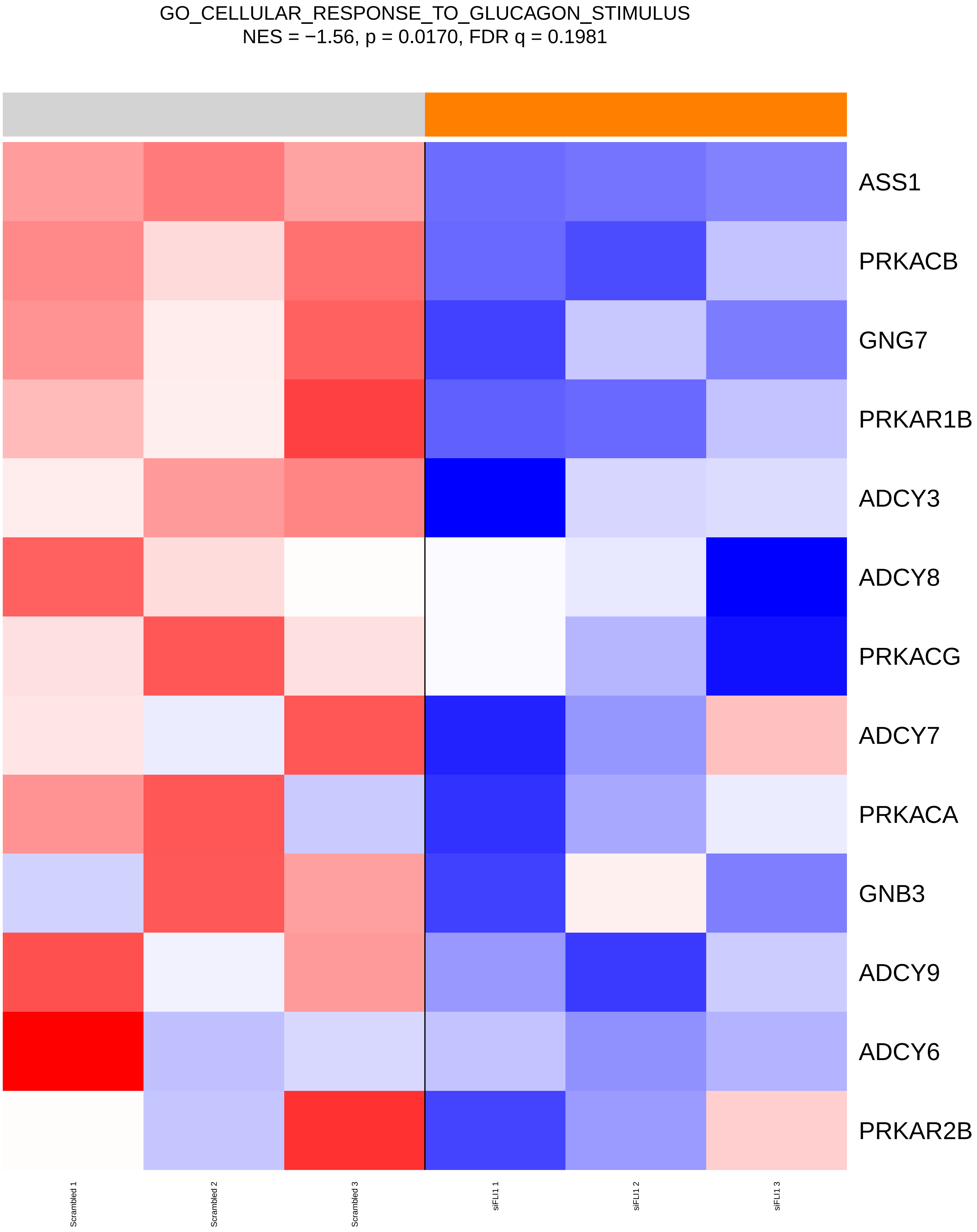
GO_CELLULAR_RESPONSE_TO_GLUCAGON_STIMULUS
NES = −1.56, p = 0.0170, FDR q = 0.1981
ASS1
PRKACB
GNG7
PRKAR1B
ADCY3
ADCY8
PRKACG
ADCY7
PRKACA
GNB3
ADCY9
ADCY6
PRKAR2B
Scrambled 1
Scrambled 2
Scrambled 3
siFLI1 1
siFLI1 2
siFLI1 3

GO_LIPID_LOCALIZATION
NES = −1.56, p = 0.0000, FDR q = 0.1979

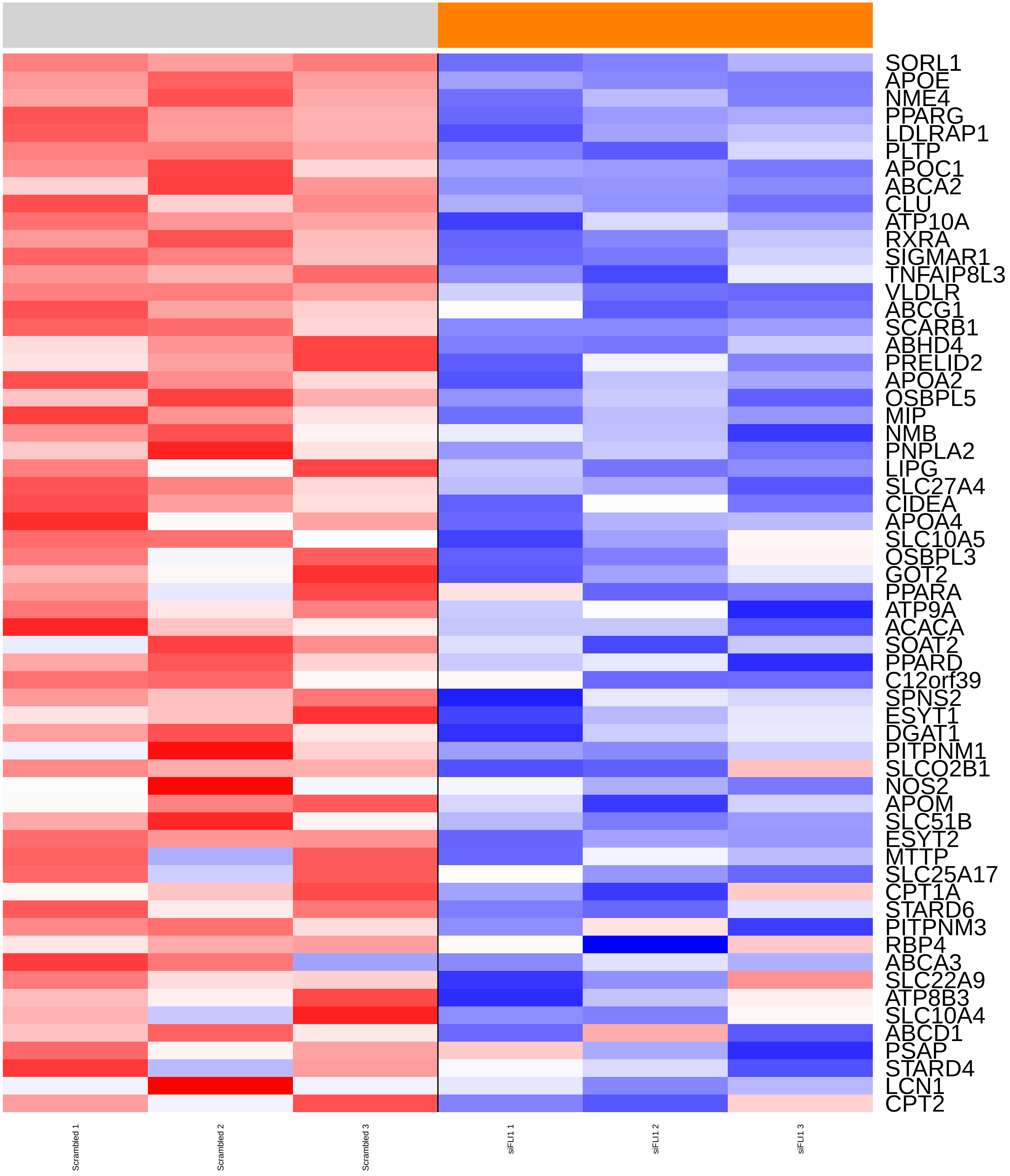

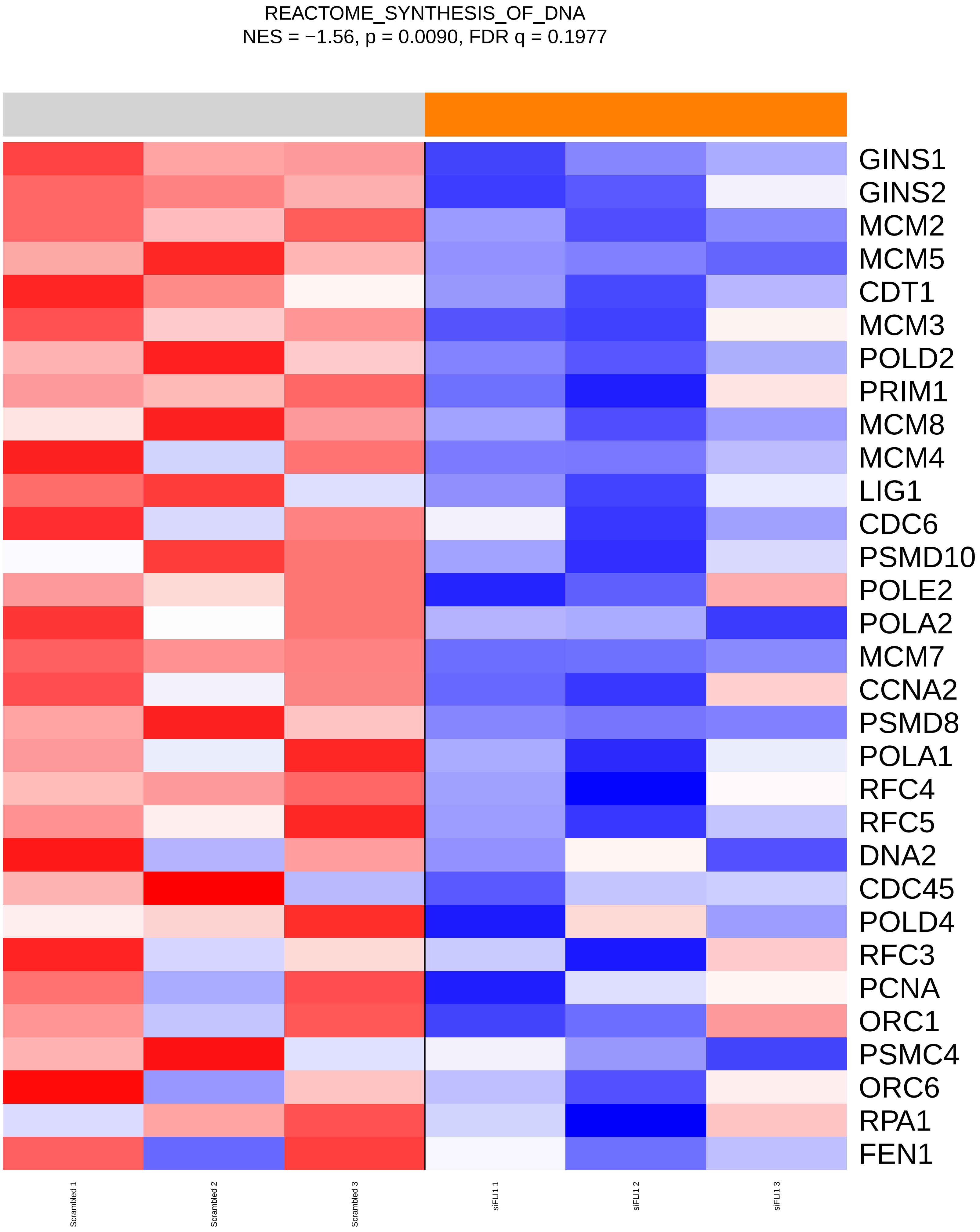
REACTOME_SYNTHESIS_OF_DNA
NES = −1.56, p = 0.0090, FDR q = 0.1977
GINS1
GINS2
MCM2
MCM5
CDT1
MCM3
POLD2
PRIM1
MCM8
MCM4
LIG1
CDC6
PSMD10
POLE2
POLA2
MCM7
CCNA2
PSMD8
POLA1
RFC4
RFC5
DNA2
CDC45
POLD4
RFC3
PCNA
ORC1
PSMC4
ORC6
RPA1
FEN1
Scrambled 1
Scrambled 2
Scrambled 3
siFLI1 1
siFLI1 2
siFLI1 3

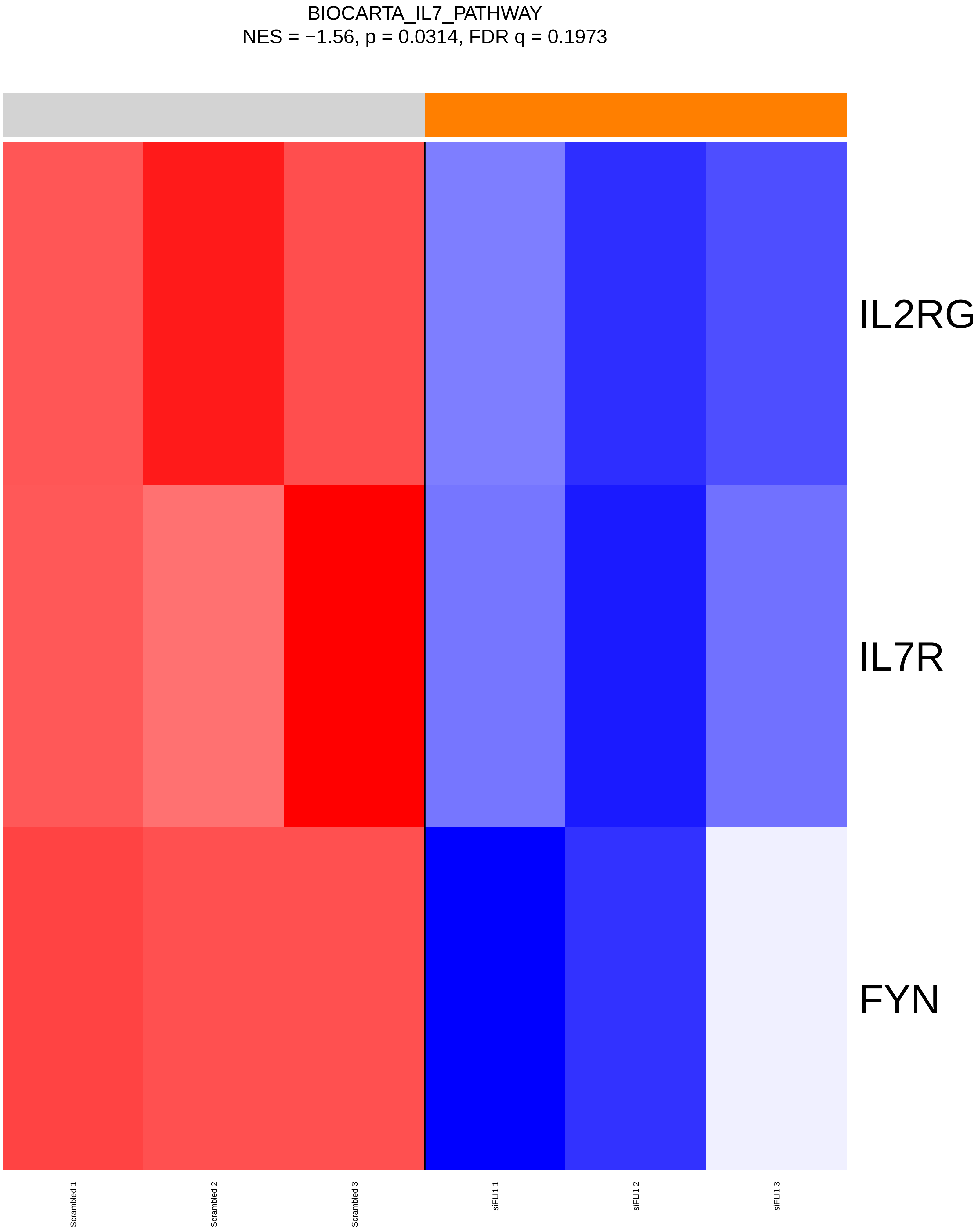
BIOCARTA_IL7_PATHWAY
NES = −1.56, p = 0.0314, FDR q = 0.1973
IL2RG
IL7R
FYN
Scrambled 1
Scrambled 2
Scrambled 3
siFLI1 1
siFLI1 2
siFLI1 3

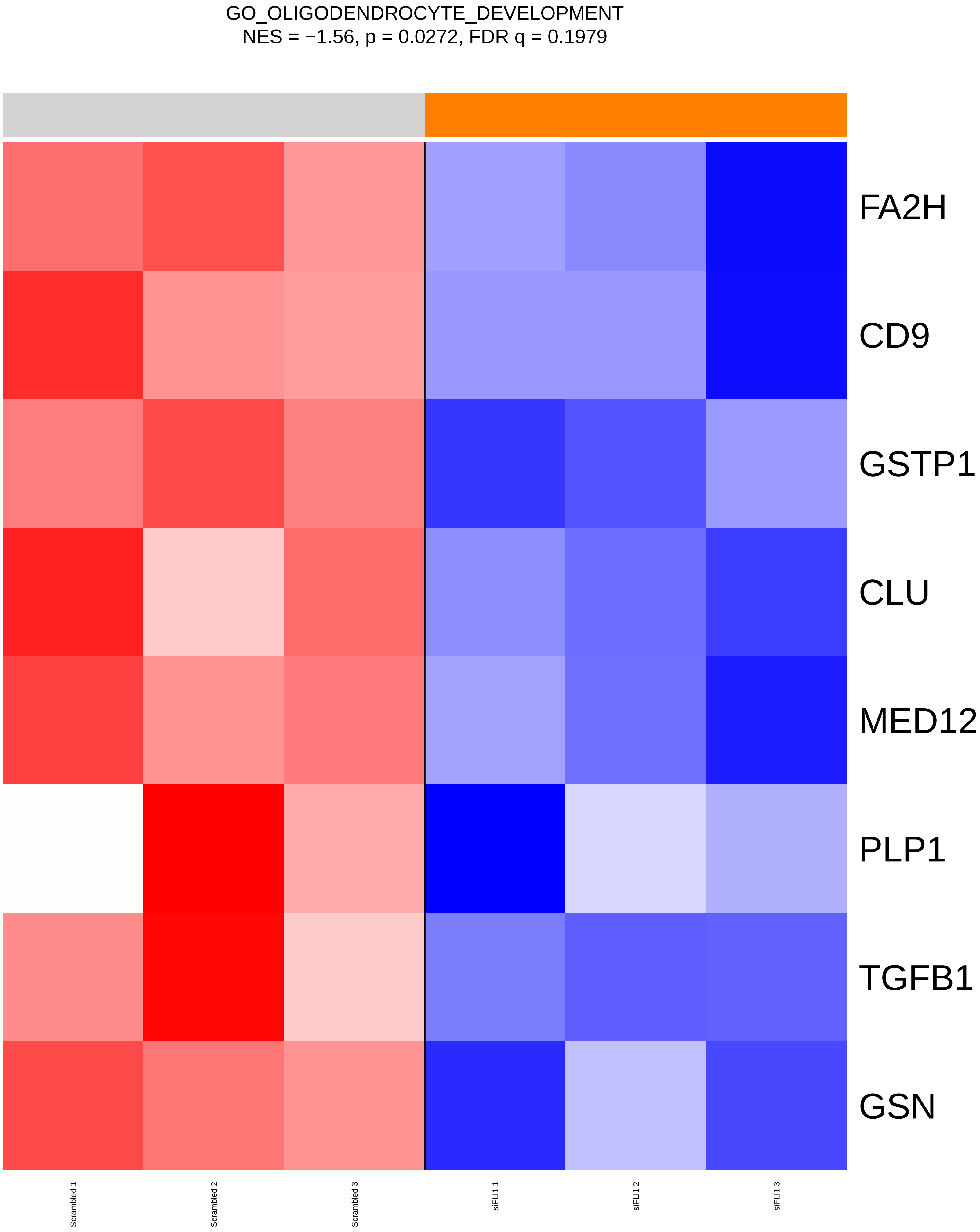
GO_OLIGODENDROCYTE_DEVELOPMENT
NES = −1.56, p = 0.0272, FDR q = 0.1979
FA2H
CD9
GSTP1
CLU
MED12
PLP1
TGFB1
GSN
Scrambled 1
Scrambled 2
Scrambled 3
siFLI1 1
siFLI1 2
siFLI1 3

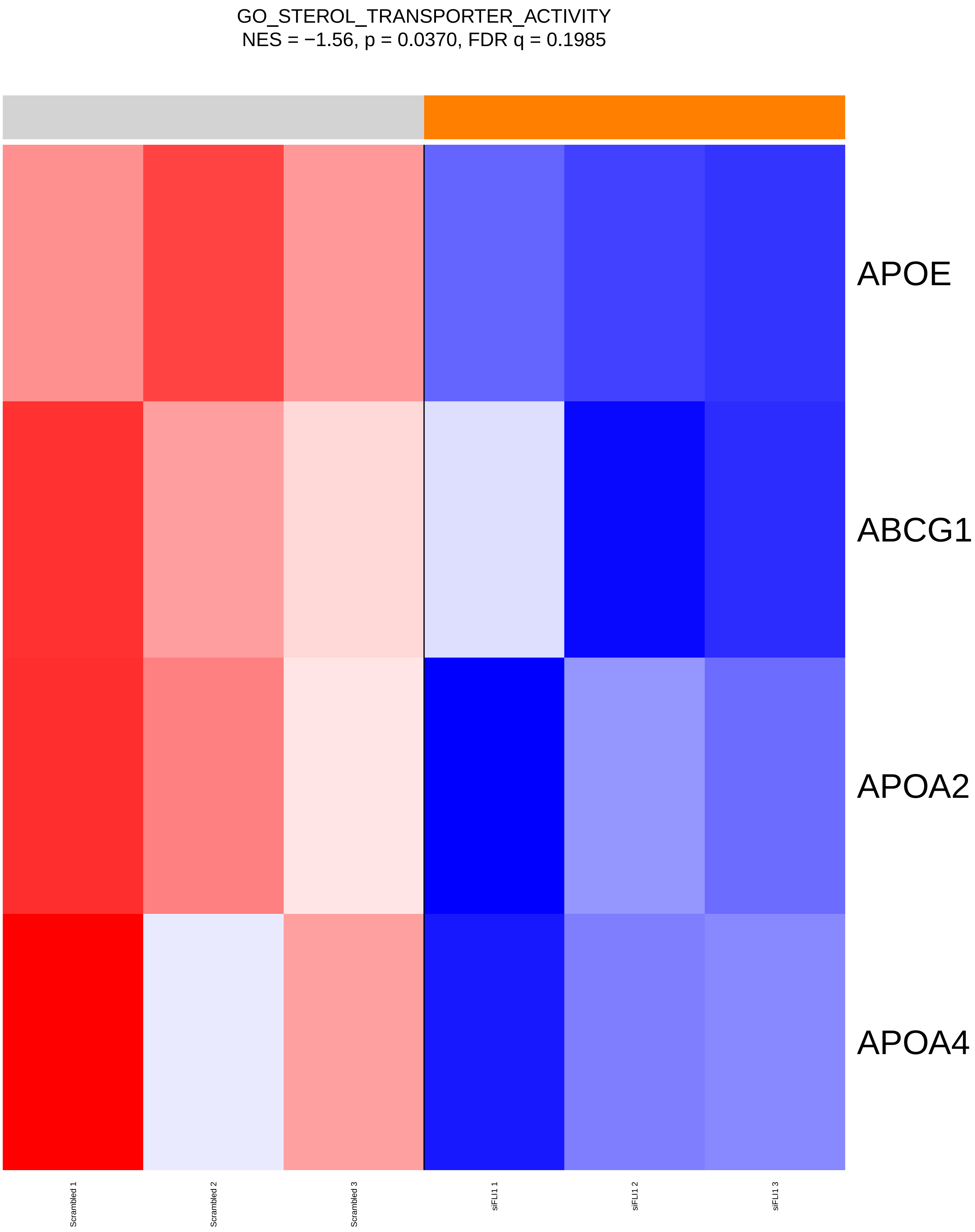
GO_STEROL_TRANSPORTER_ACTIVITY
NES = −1.56, p = 0.0370, FDR q = 0.1985
APOE
ABCG1
APOA2
APOA4
Scrambled 1
Scrambled 2
Scrambled 3
siFLI1 1
siFLI1 2
siFLI1 3

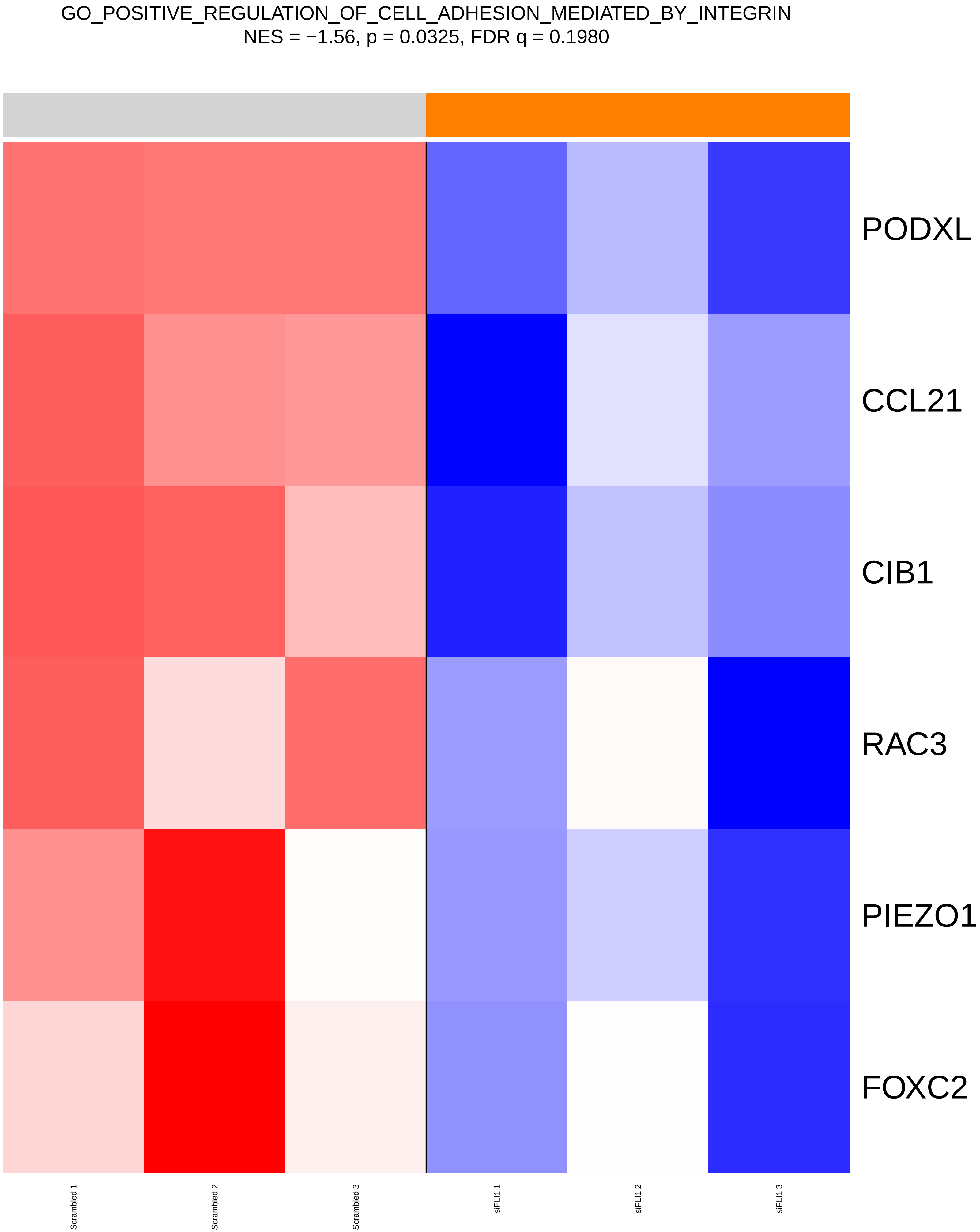
GO_POSITIVE_REGULATION_OF_CELL_ADHESION_MEDIATED_BY_INTEGRIN
NES = −1.56, p = 0.0325, FDR q = 0.1980
PODXL
CCL21
CIB1
RAC3
PIEZO1
FOXC2
Scrambled 1
Scrambled 2
Scrambled 3
siFLI1 1
siFLI1 2
siFLI1 3

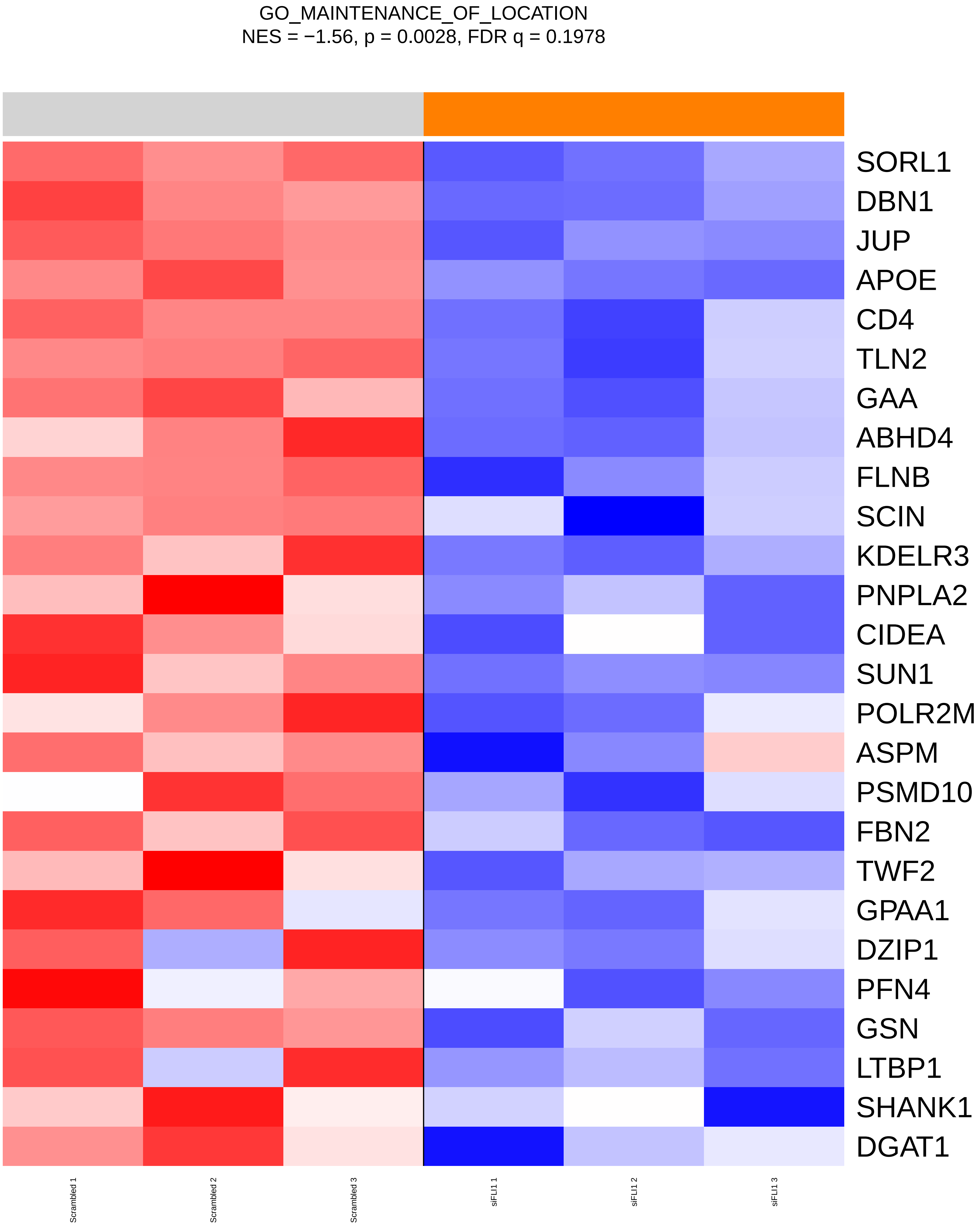
GO_MAINTENANCE_OF_LOCATION
NES = −1.56, p = 0.0028, FDR q = 0.1978
SORL1
DBN1
JUP
APOE
CD4
TLN2
GAA
ABHD4
FLNB
SCIN
KDELR3
PNPLA2
CIDEA
SUN1
POLR2M
ASPM
PSMD10
FBN2
TWF2
GPAA1
DZIP1
PFN4
GSN
LTBP1
SHANK1
DGAT1
Scrambled 1
Scrambled 2
Scrambled 3
siFLI1 1
siFLI1 2
siFLI1 3

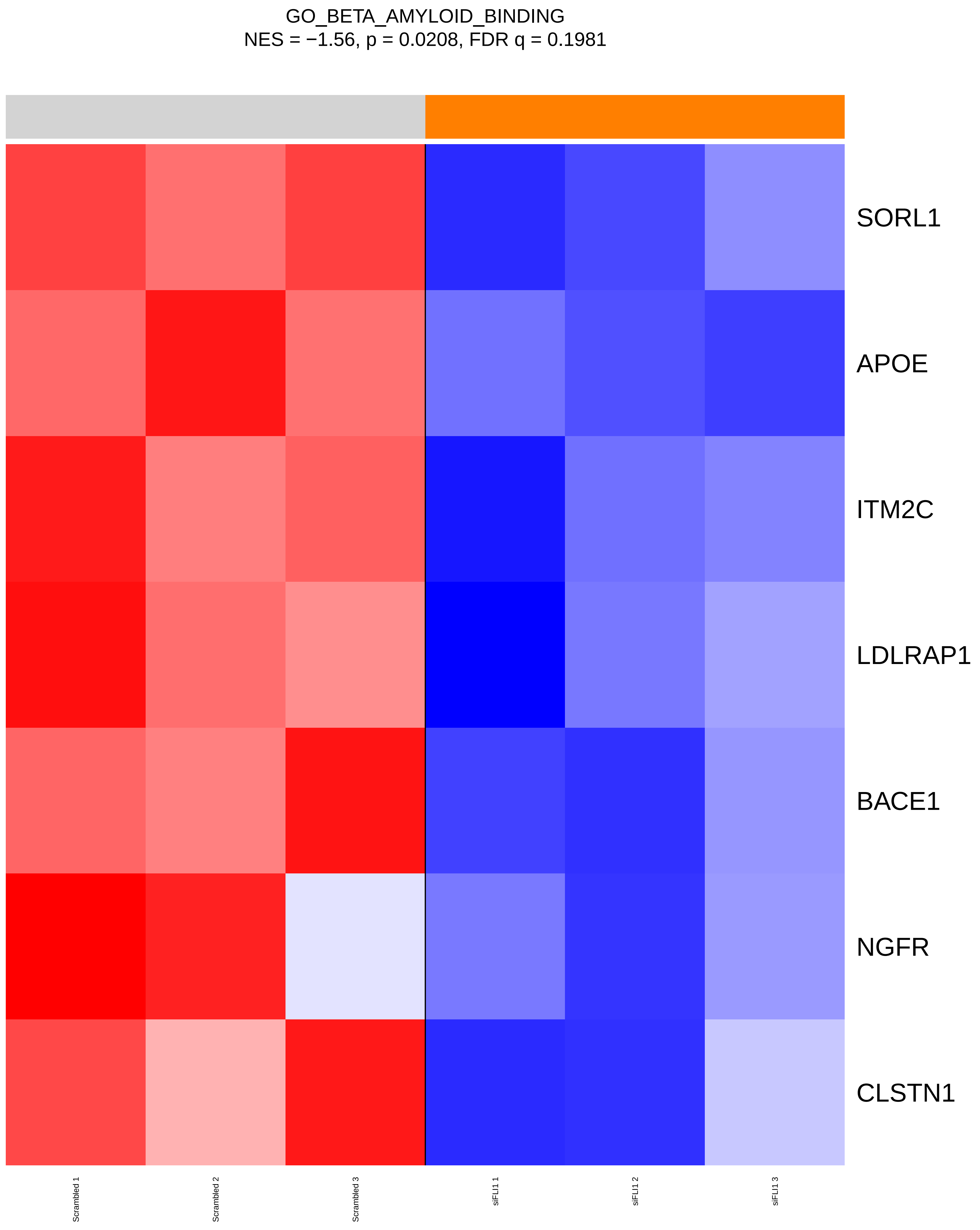
GO_BETA_AMYLOID_BINDING
NES = −1.56, p = 0.0208, FDR q = 0.1981
SORL1
APOE
ITM2C
LDLRAP1
BACE1
NGFR
CLSTN1
Scrambled 1
Scrambled 2
Scrambled 3
siFLI1 1
siFLI1 2
siFLI1 3

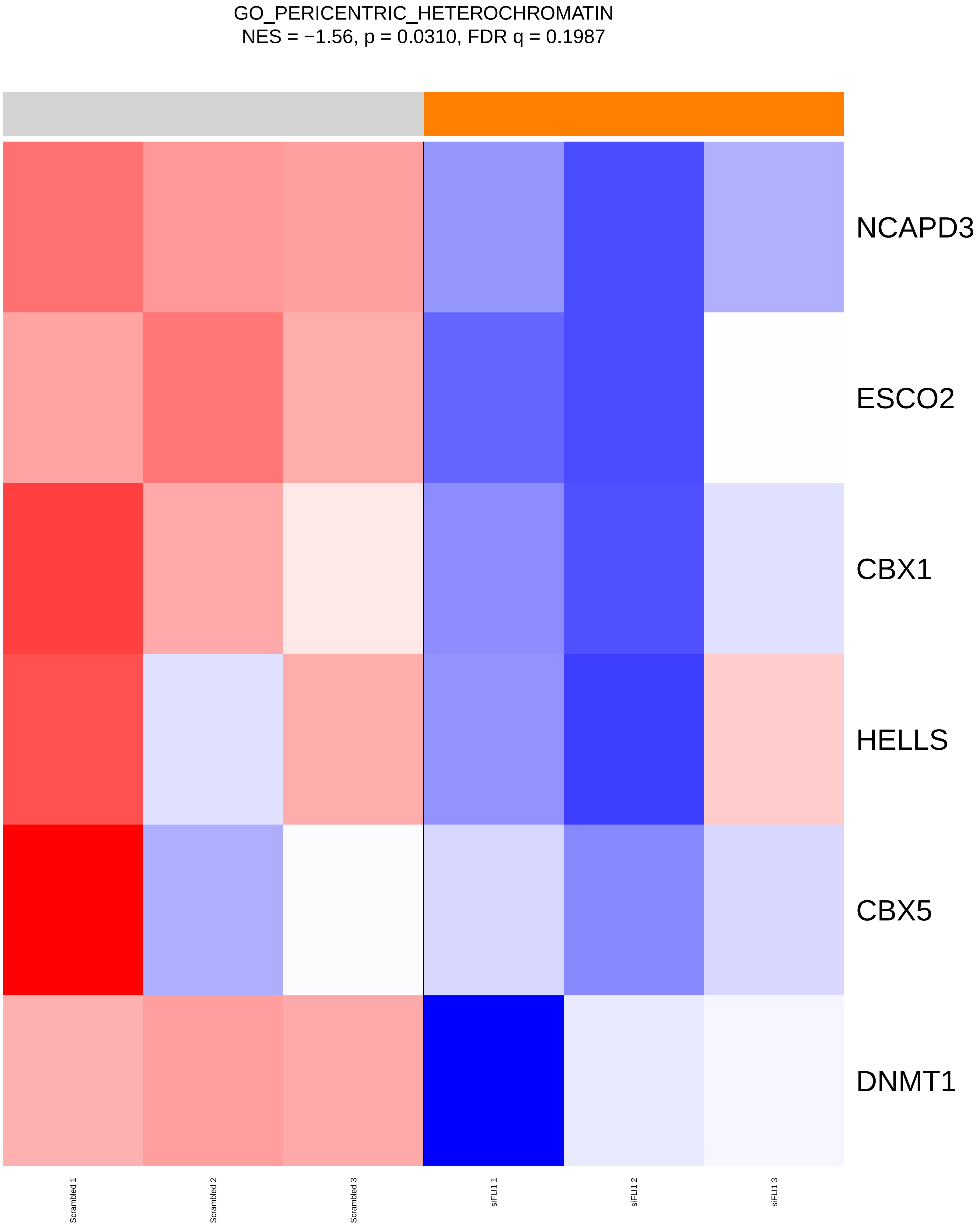
GO_PERICENTRIC_HETEROCHROMATIN
NES = −1.56, p = 0.0310, FDR q = 0.1987
NCAPD3
ESCO2
CBX1
HELLS
CBX5
DNMT1
Scrambled 1
Scrambled 2
Scrambled 3
siFLI1 1
siFLI1 2
siFLI1 3

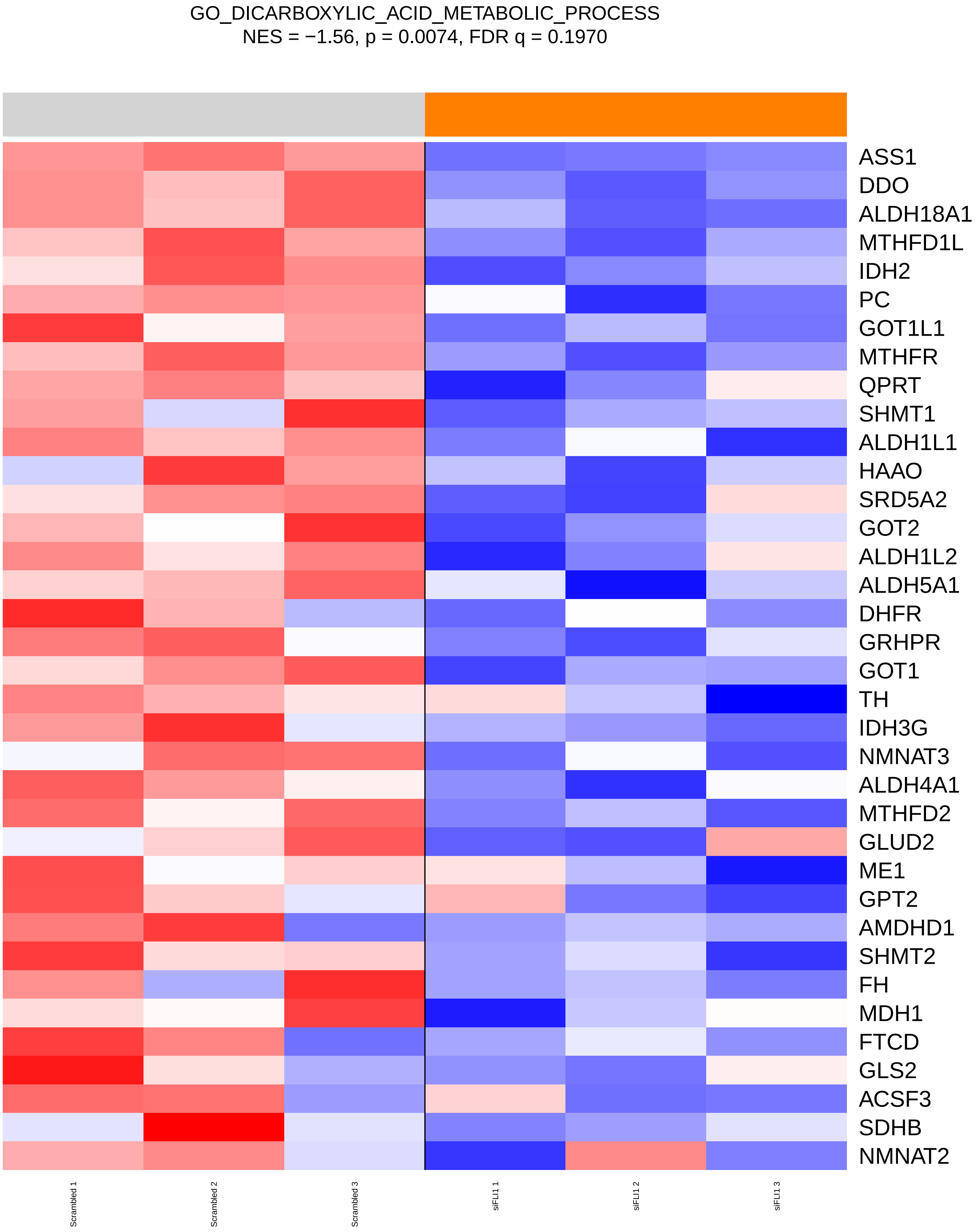
GO_DICARBOXYLIC_ACID_METABOLIC_PROCESS
NES = −1.56, p = 0.0074, FDR q = 0.1970
ASS1
DDO
ALDH18A1
MTHFD1L
IDH2
PC
GOT1L1
MTHFR
QPRT
SHMT1
ALDH1L1
HAAO
SRD5A2
GOT2
ALDH1L2
ALDH5A1
DHFR
GRHPR
GOT1
TH
IDH3G
NMNAT3
ALDH4A1
MTHFD2
GLUD2
ME1
GPT2
AMDHD1
SHMT2
FH
MDH1
FTCD
GLS2
ACSF3
SDHB
NMNAT2
Scrambled 1
Scrambled 2
Scrambled 3
siFLI1 1
siFLI1 2
siFLI1 3

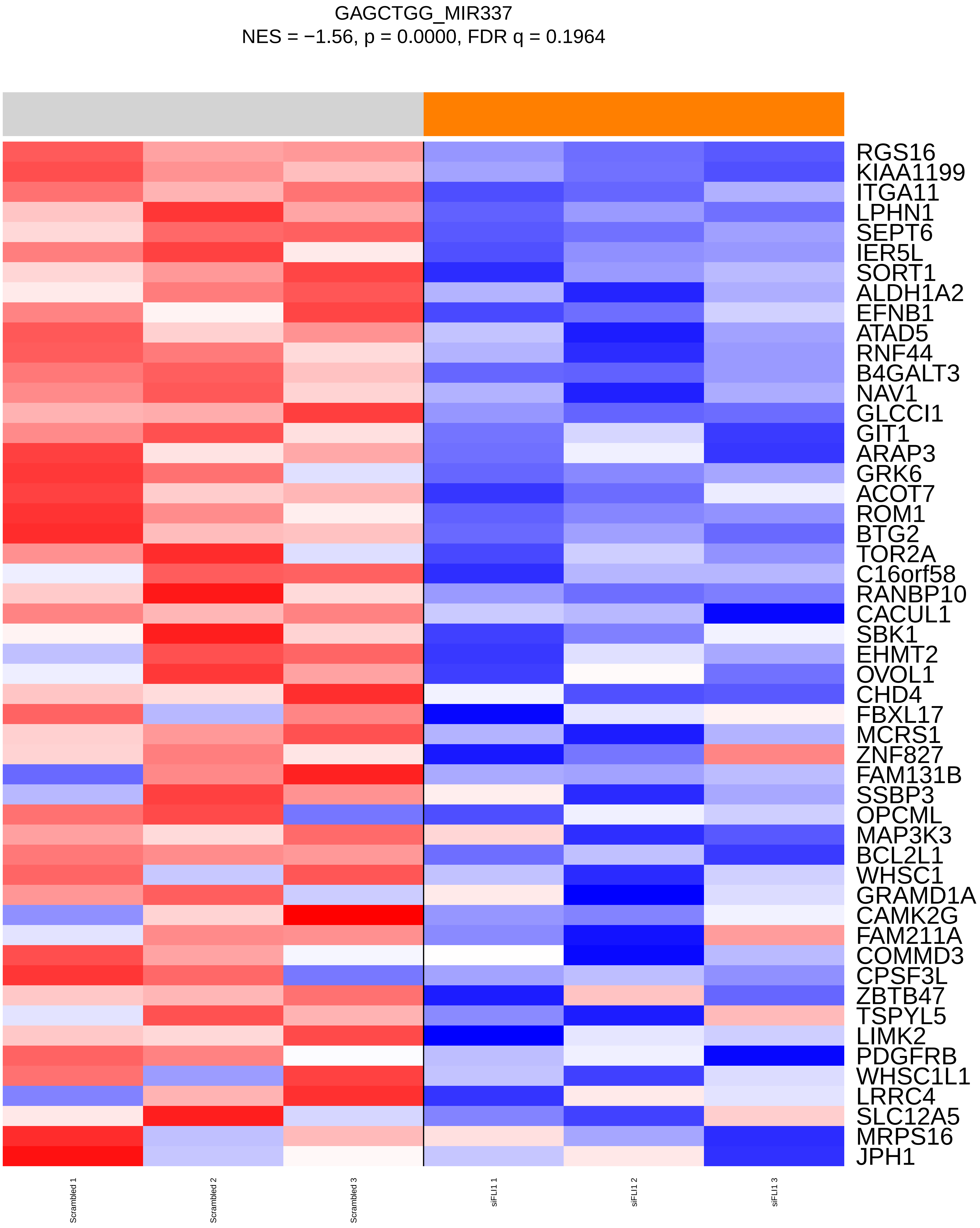
GAGCTGG_MIR337
NES = −1.56, p = 0.0000, FDR q = 0.1964
RGS16
KIAA1199
ITGA11
LPHN1
SEPT6
IER5L
SORT1
ALDH1A2
EFNB1
ATAD5
RNF44
B4GALT3
NAV1
GLCCI1
GIT1
ARAP3
GRK6
ACOT7
ROM1
BTG2
TOR2A
C16orf58
RANBP10
CACUL1
SBK1
EHMT2
OVOL1
CHD4
FBXL17
MCRS1
ZNF827
FAM131B
SSBP3
OPCML
MAP3K3
BCL2L1
WHSC1
GRAMD1A
CAMK2G
FAM211A
COMMD3
CPSF3L
ZBTB47
TSPYL5
LIMK2
PDGFRB
WHSC1L1
LRRC4
SLC12A5
MRPS16
JPH1
Scrambled 1
Scrambled 2
Scrambled 3
siFLI1 1
siFLI1 2
siFLI1 3

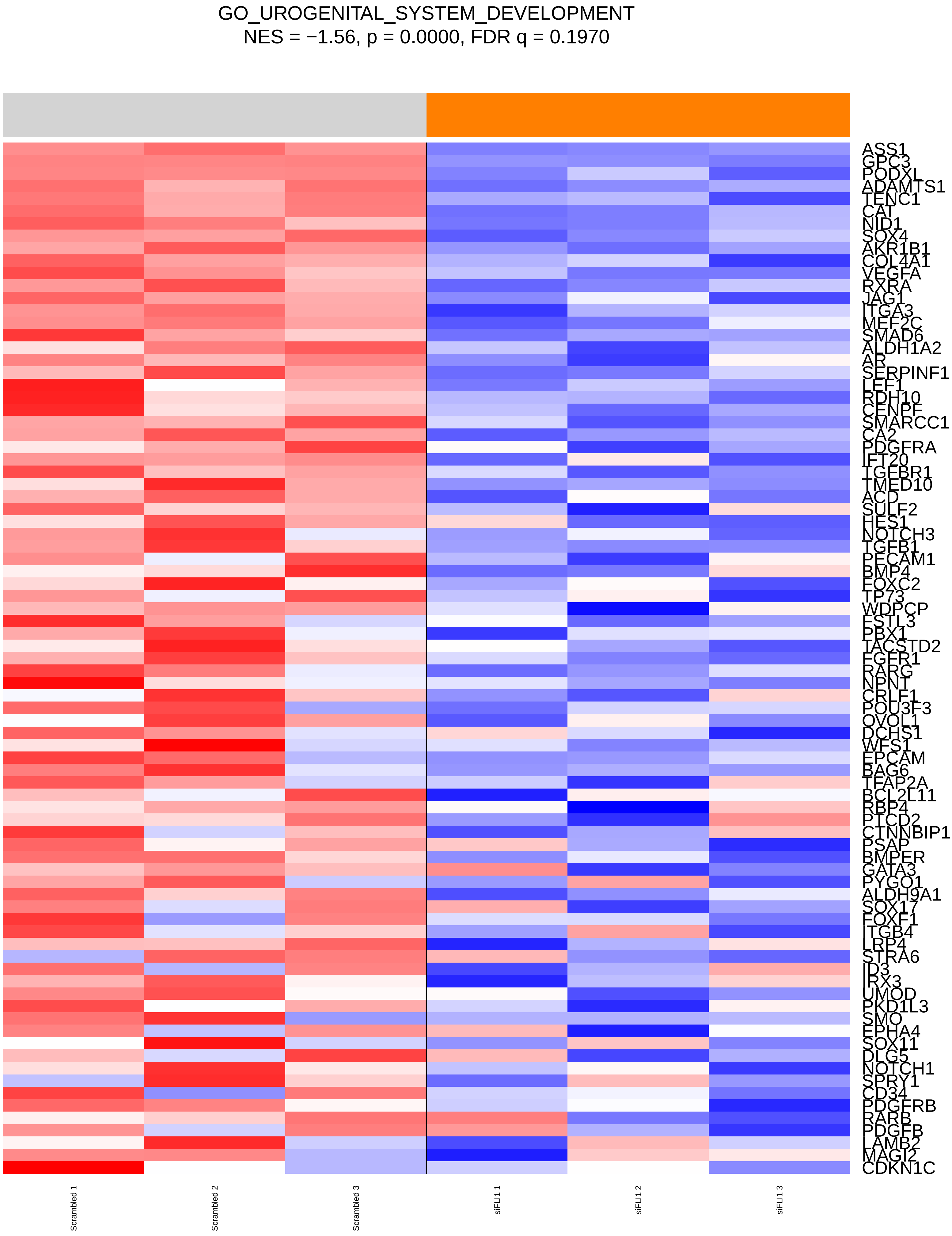
GO_UROGENITAL_SYSTEM_DEVELOPMENT
NES = −1.56, p = 0.0000, FDR q = 0.1970
ASS1
GPC3
PODXL
ADAMTS1
TENC1
CAT
NID1
SOX4
AKR1B1
COL4A1
VEGFA
RXRA
JAG1
ITGA3
MEF2C
SMAD6
ALDH1A2
AR
SERPINF1
LEF1
RDH10
CENPF
SMARCC1
CA2
PDGFRA
IFT20
TGFBR1
TMED10
ACD
SULF2
HES1
NOTCH3
TGFB1
PECAM1
BMP4
FOXC2
TP73
WDPCP
FSTL3
PBX1
TACSTD2
FGFR1
RARG
NPNT
CRLF1
POU3F3
OVOL1
DCHS1
WFS1
EPCAM
BAG6
TFAP2A
BCL2L11
RBP4
PTCD2
CTNNBIP1
PSAP
BMPER
GATA3
PYGO1
ALDH9A1
SOX17
FOXF1
ITGB4
LRP4
STRA6
ID3
IRX3
UMOD
PKD1L3
SMO
EPHA4
SOX11
DLG5
NOTCH1
SPRY1
CD34
PDGFRB
RARB
PDGFB
LAMB2
MAGI2
CDKN1C
Scrambled 1
Scrambled 2
Scrambled 3
siFLI1 1
siFLI1 2
siFLI1 3

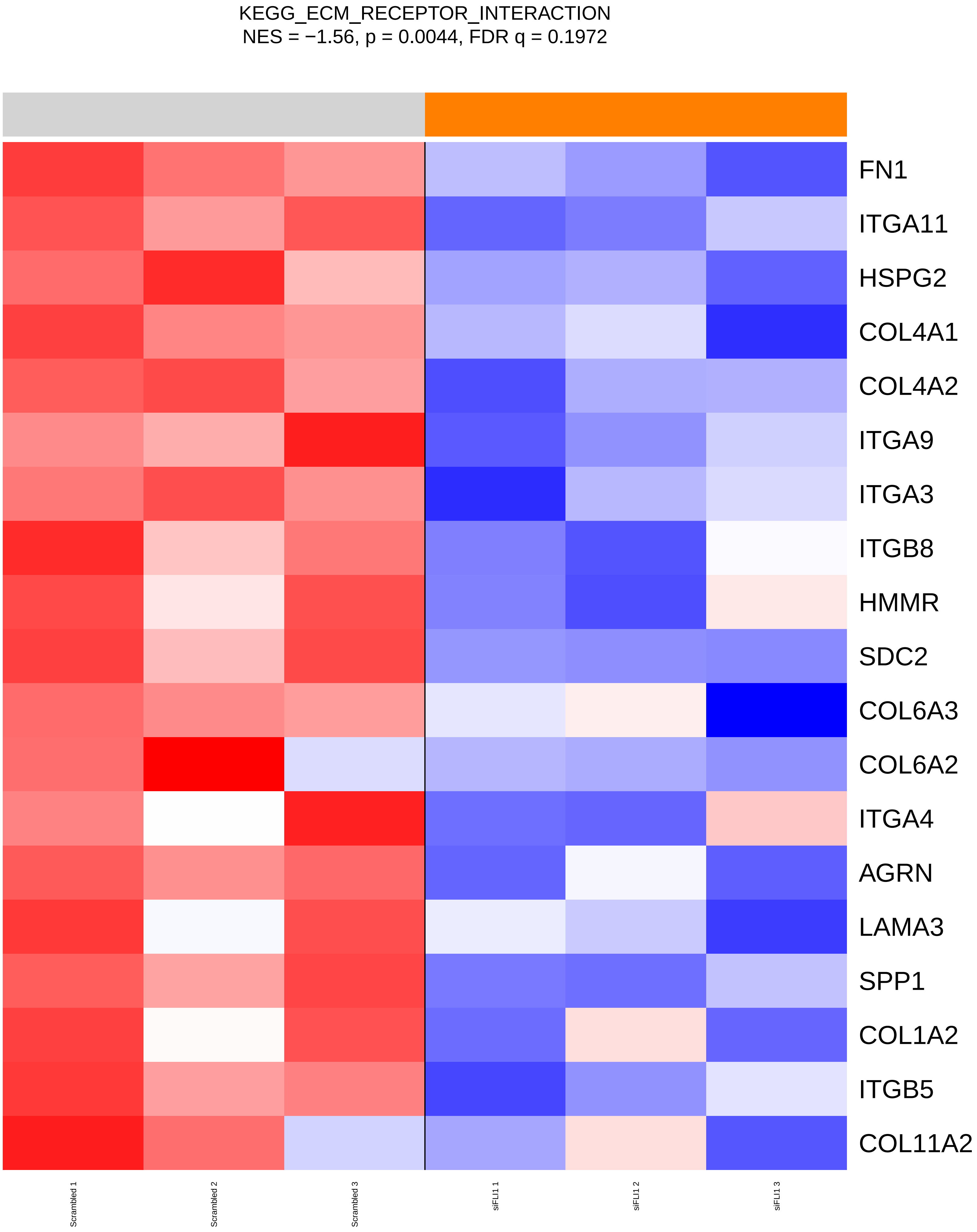

KEGG_ECM_RECEPTOR_INTERACTION
NES = −1.56, p = 0.0044, FDR q = 0.1972
FN1
ITGA11
HSPG2
COL4A1
COL4A2
ITGA9
ITGA3
ITGB8
HMMR
SDC2
COL6A3
COL6A2
ITGA4
AGRN
LAMA3
SPP1
COL1A2
ITGB5
COL11A2
Scrambled 1
Scrambled 2
Scrambled 3
siFLI1 1
siFLI1 2
siFLI1 3

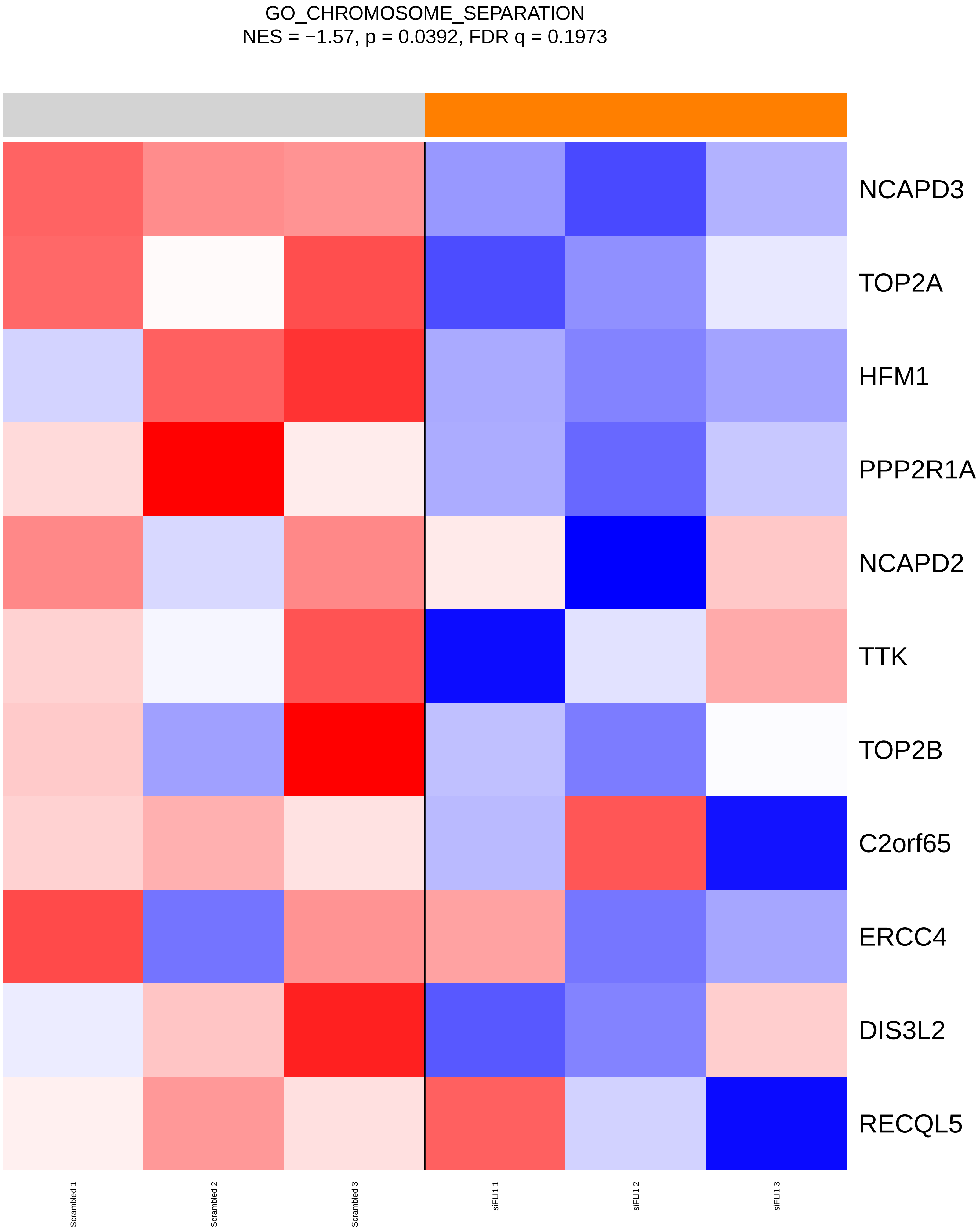
GO_CHROMOSOME_SEPARATION
NES = −1.57, p = 0.0392, FDR q = 0.1973
NCAPD3
TOP2A
HFM1
PPP2R1A
NCAPD2
TTK
TOP2B
C2orf65
ERCC4
DIS3L2
RECQL5
Scrambled 1
Scrambled 2
Scrambled 3
siFLI1 1
siFLI1 2
siFLI1 3

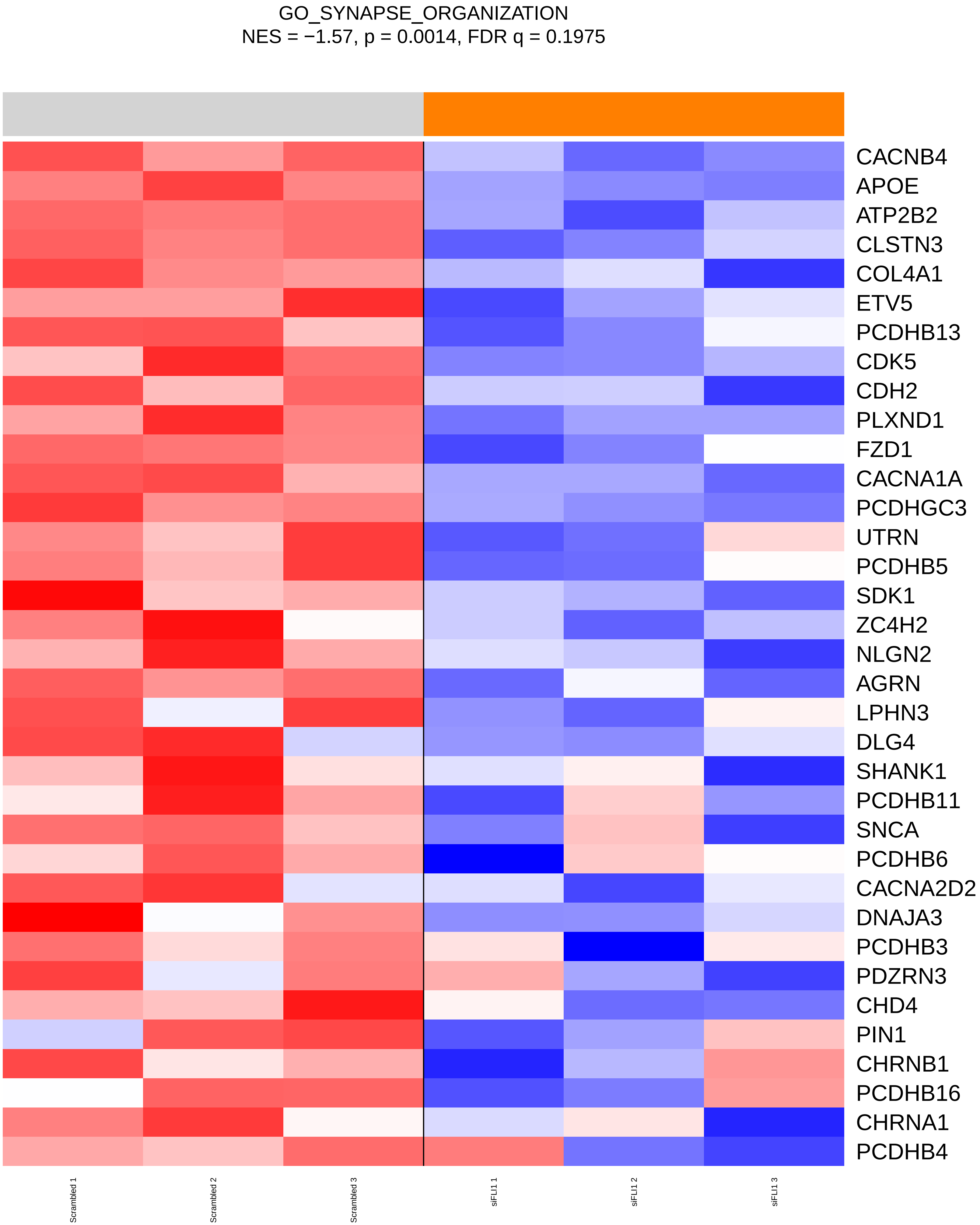
GO_SYNAPSE_ORGANIZATION
NES = −1.57, p = 0.0014, FDR q = 0.1975
CACNB4
APOE
ATP2B2
CLSTN3
COL4A1
ETV5
PCDHB13
CDK5
CDH2
PLXND1
FZD1
CACNA1A
PCDHGC3
UTRN
PCDHB5
SDK1
ZC4H2
NLGN2
AGRN
LPHN3
DLG4
SHANK1
PCDHB11
SNCA
PCDHB6
CACNA2D2
DNAJA3
PCDHB3
PDZRN3
CHD4
PIN1
CHRNB1
PCDHB16
CHRNA1
PCDHB4
Scrambled 1
Scrambled 2
Scrambled 3
siFLI1 1
siFLI1 2
siFLI1 3

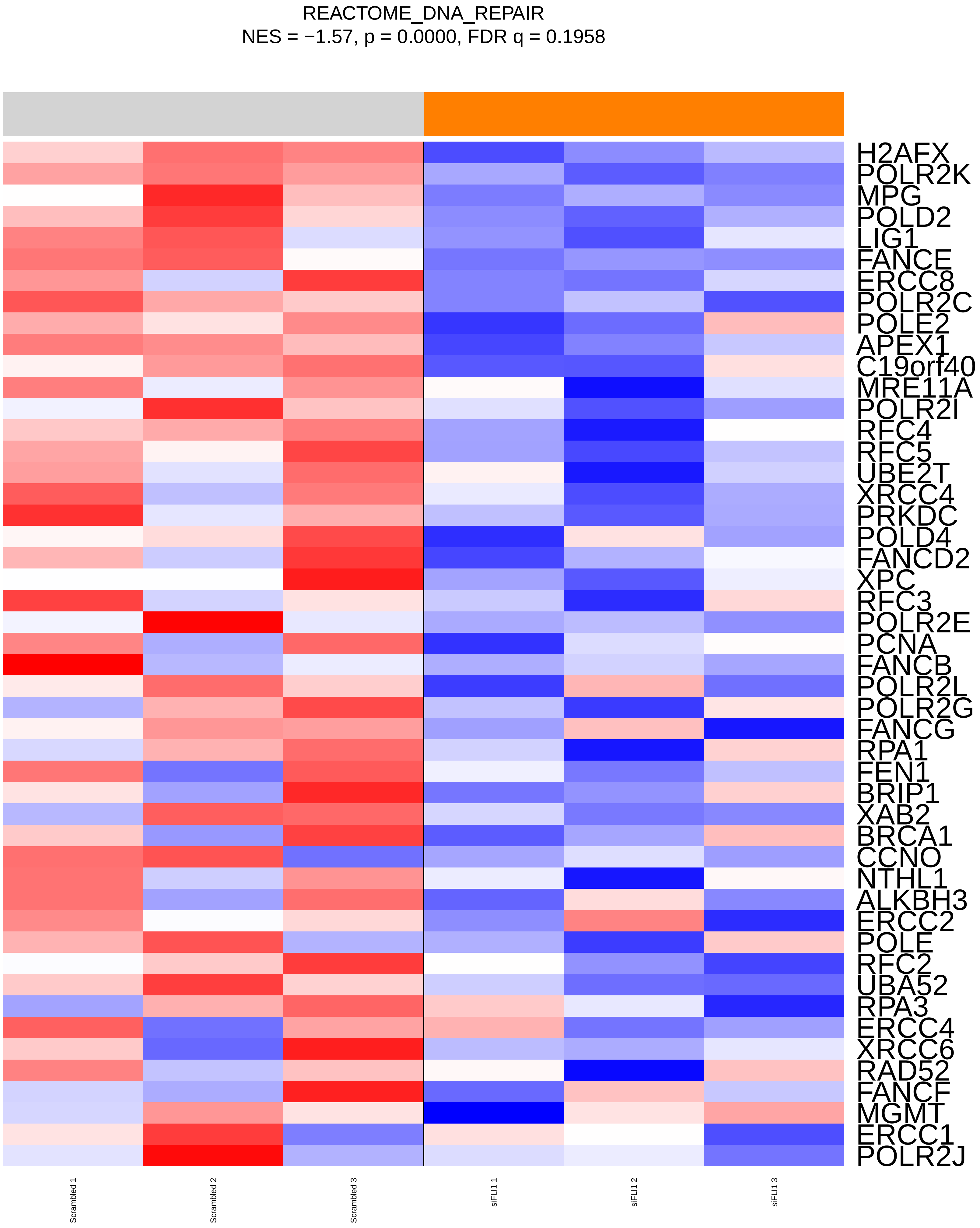
REACTOME_DNA_REPAIR
NES = −1.57, p = 0.0000, FDR q = 0.1958
H2AFX
POLR2K
MPG
POLD2
LIG1
FANCE
ERCC8
POLR2C
POLE2
APEX1
C19orf40
MRE11A
POLR2I
RFC4
RFC5
UBE2T
XRCC4
PRKDC
POLD4
FANCD2
XPC
RFC3
POLR2E
PCNA
FANCB
POLR2L
POLR2G
FANCG
RPA1
FEN1
BRIP1
XAB2
BRCA1
CCNO
NTHL1
ALKBH3
ERCC2
POLE
RFC2
UBA52
RPA3
ERCC4
XRCC6
RAD52
FANCF
MGMT
ERCC1
POLR2J
Scrambled 1
Scrambled 2
Scrambled 3
siFLI1 1
siFLI1 2
siFLI1 3

GO_PROSTANOID_METABOLIC_PROCESS
NES = −1.57, p = 0.0221, FDR q = 0.1955

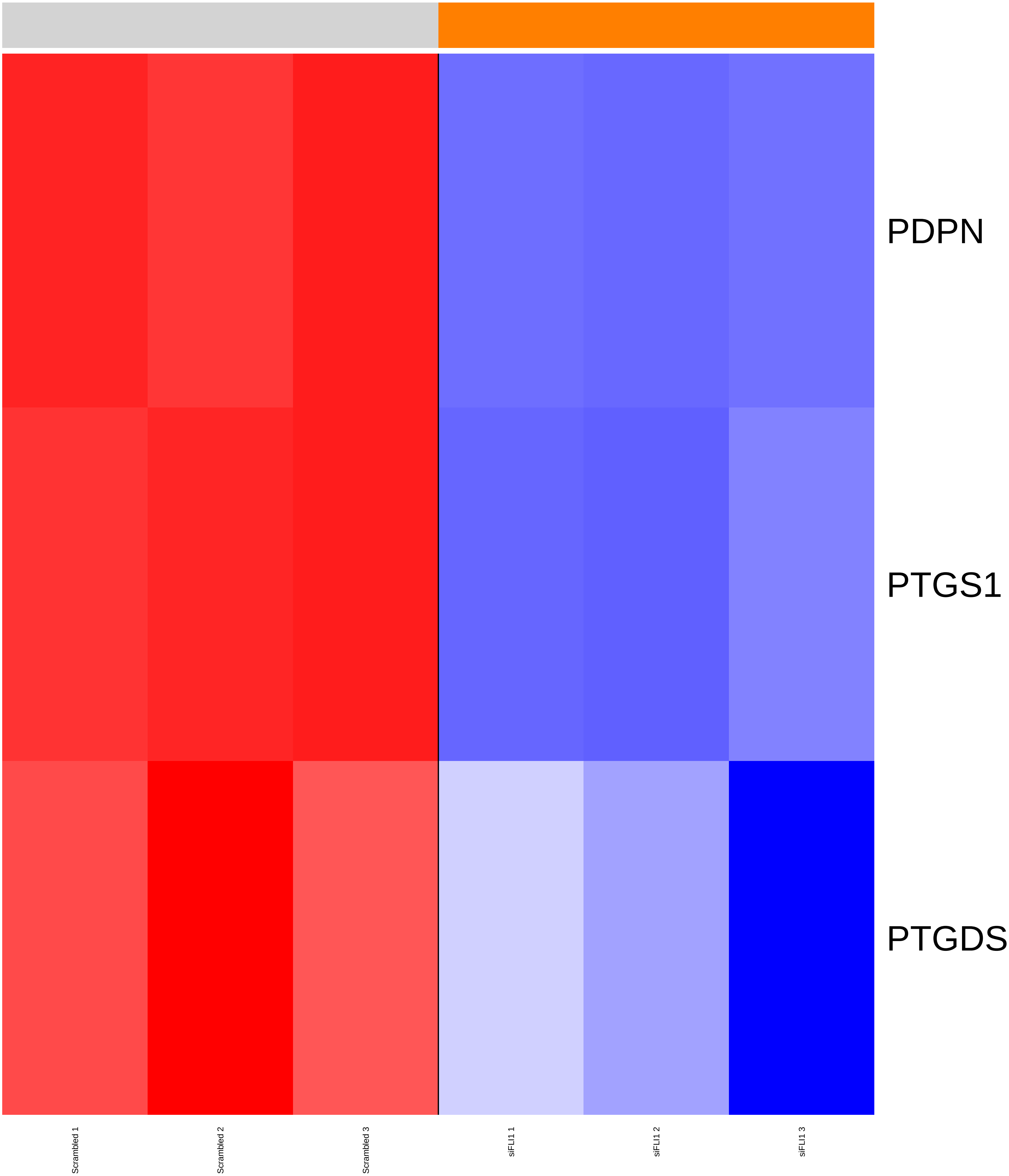

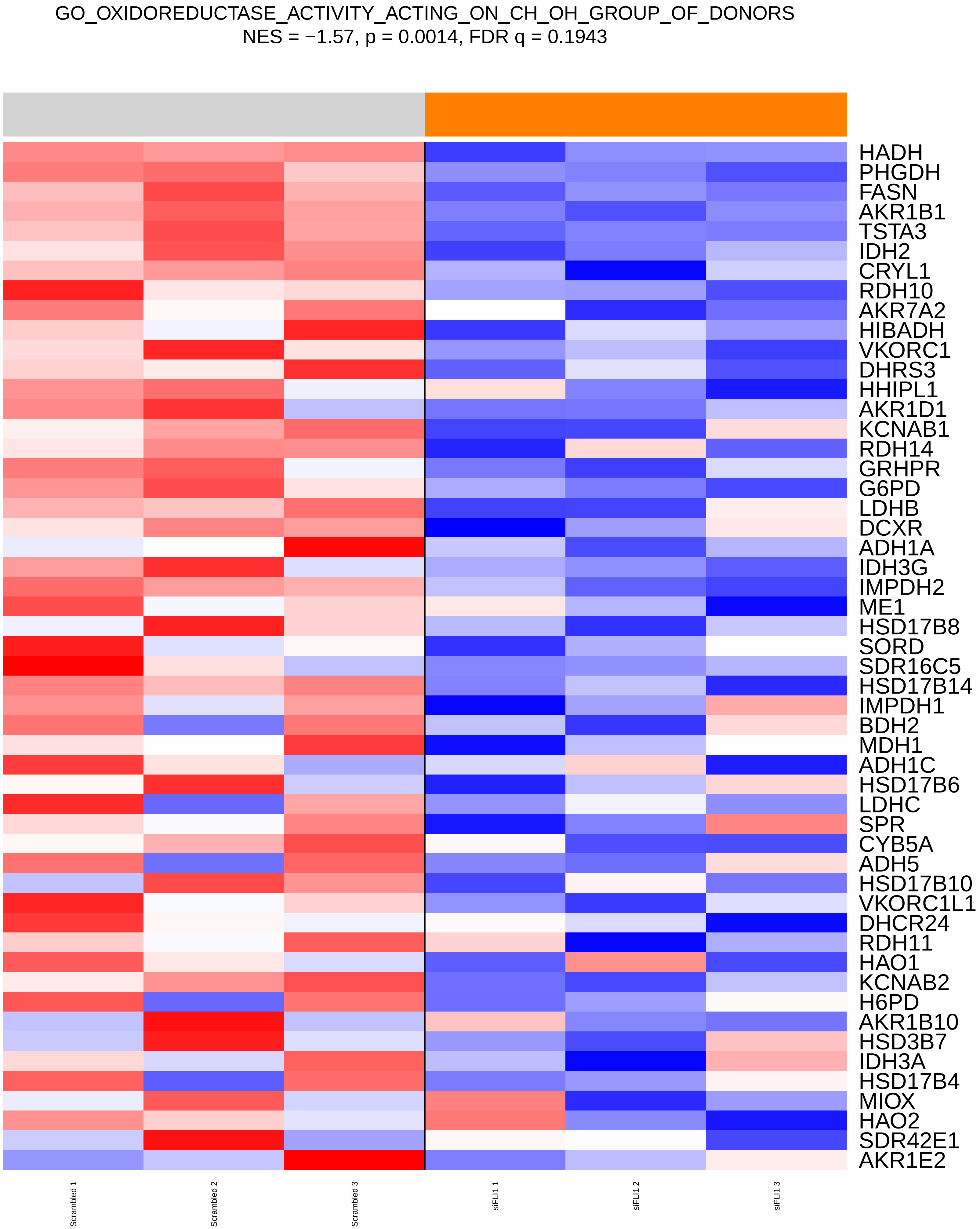
GO_OXIDOREDUCTASE_ACTIVITY_ACTING_ON_CH_OH_GROUP_OF_DONORS
NES = −1.57, p = 0.0014, FDR q = 0.1943
HADH
PHGDH
FASN
AKR1B1
TSTA3
IDH2
CRYL1
RDH10
AKR7A2
HIBADH
VKORC1
DHRS3
HHIPL1
AKR1D1
KCNAB1
RDH14
GRHPR
G6PD
LDHB
DCXR
ADH1A
IDH3G
IMPDH2
ME1
HSD17B8
SORD
SDR16C5
HSD17B14
IMPDH1
BDH2
MDH1
ADH1C
HSD17B6
LDHC
SPR
CYB5A
ADH5
HSD17B10
VKORC1L1
DHCR24
RDH11
HAO1
KCNAB2
H6PD
AKR1B10
HSD3B7
IDH3A
HSD17B4
MIOX
HAO2
SDR42E1
AKR1E2
Scrambled 1
Scrambled 2
Scrambled 3
siFLI1 1
siFLI1 2
siFLI1 3

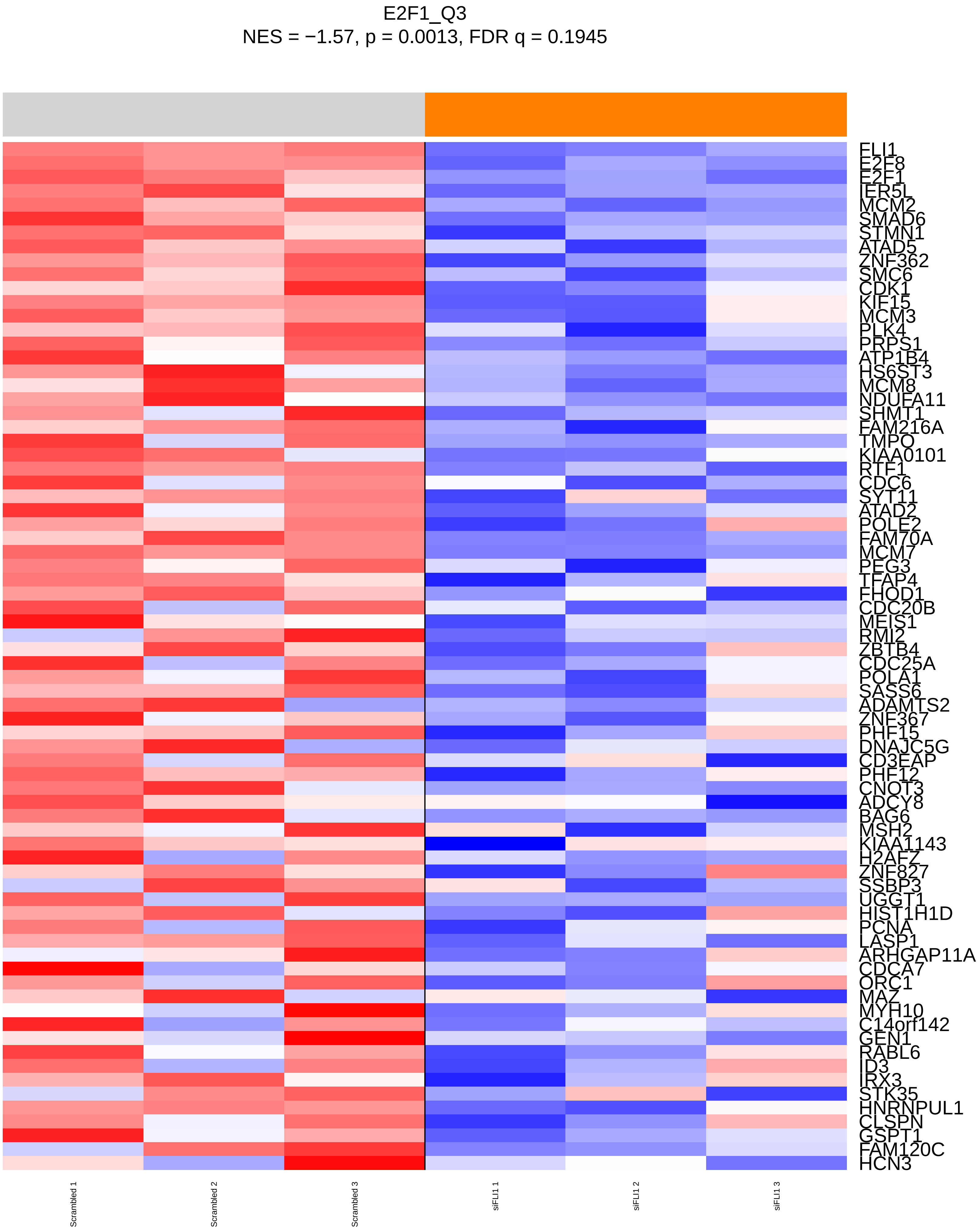
E2F1_Q3
NES = −1.57, p = 0.0013, FDR q = 0.1945
FLI1
E2F8
E2F1
IER5L
MCM2
SMAD6
STMN1
ATAD5
ZNF362
SMC6
CDK1
KIF15
MCM3
PLK4
PRPS1
ATP1B4
HS6ST3
MCM8
NDUFA11
SHMT1
FAM216A
TMPO
KIAA0101
RTF1
CDC6
SYT11
ATAD2
POLE2
FAM70A
MCM7
PEG3
TFAP4
FHOD1
CDC20B
MEIS1
RMI2
ZBTB4
CDC25A
POLA1
SASS6
ADAMTS2
ZNF367
PHF15
DNAJC5G
CD3EAP
PHF12
CNOT3
ADCY8
BAG6
MSH2
KIAA1143
H2AFZ
ZNF827
SSBP3
UGGT1
HIST1H1D
PCNA
LASP1
ARHGAP11A
CDCA7
ORC1
MAZ
MYH10
C14orf142
GEN1
RABL6
ID3
IRX3
STK35
HNRNPUL1
CLSPN
GSPT1
FAM120C
HCN3
Scrambled 1
Scrambled 2
Scrambled 3
siFLI1 1
siFLI1 2
siFLI1 3

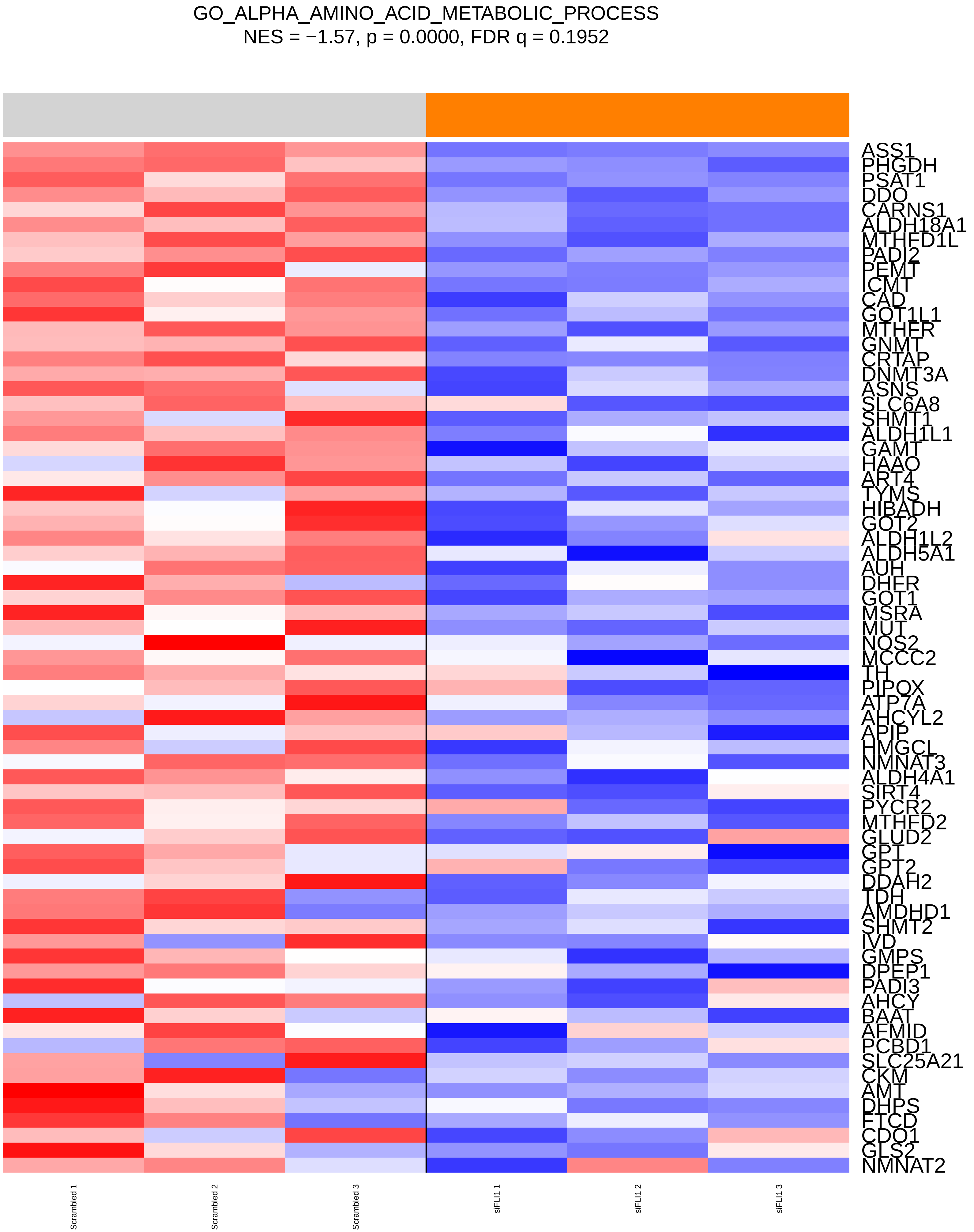
GO_ALPHA_AMINO_ACID_METABOLIC_PROCESS
NES = −1.57, p = 0.0000, FDR q = 0.1952
ASS1
PHGDH
PSAT1
DDO
CARNS1
ALDH18A1
MTHFD1L
PADI2
PEMT
ICMT
CAD
GOT1L1
MTHFR
GNMT
CRTAP
DNMT3A
ASNS
SLC6A8
SHMT1
ALDH1L1
GAMT
HAAO
ART4
TYMS
HIBADH
GOT2
ALDH1L2
ALDH5A1
AUH
DHFR
GOT1
MSRA
MUT
NOS2
MCCC2
TH
PIPOX
ATP7A
AHCYL2
APIP
HMGCL
NMNAT3
ALDH4A1
SIRT4
PYCR2
MTHFD2
GLUD2
GPT
GPT2
DDAH2
TDH
AMDHD1
SHMT2
IVD
GMPS
DPEP1
PADI3
AHCY
BAAT
AFMID
PCBD1
SLC25A21
CKM
AMT
DHPS
FTCD
CDO1
GLS2
NMNAT2
Scrambled 1
Scrambled 2
Scrambled 3
siFLI1 1
siFLI1 2
siFLI1 3

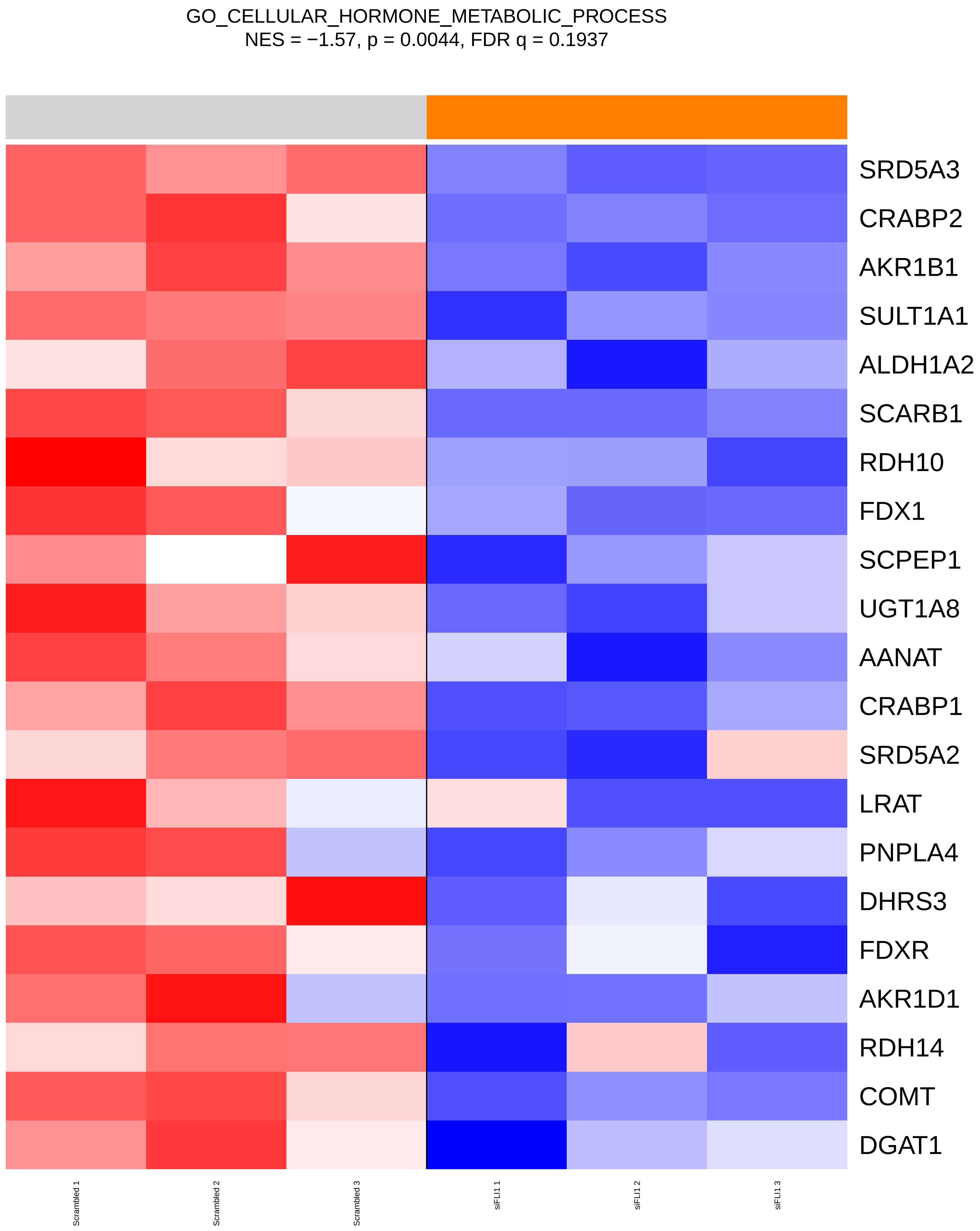
GO_CELLULAR_HORMONE_METABOLIC_PROCESS
NES = −1.57, p = 0.0044, FDR q = 0.1937
SRD5A3
CRABP2
AKR1B1
SULT1A1
ALDH1A2
SCARB1
RDH10
FDX1
SCPEP1
UGT1A8
AANAT
CRABP1
SRD5A2
LRAT
PNPLA4
DHRS3
FDXR
AKR1D1
RDH14
COMT
DGAT1
Scrambled 1
Scrambled 2
Scrambled 3
siFLI1 1
siFLI1 2
siFLI1 3

GO_RNA_PHOSPHODIESTER_BOND_HYDROLYSIS_ENDONUCLEOLYTIC
NES = −1.57, p = 0.0146, FDR q = 0.1927

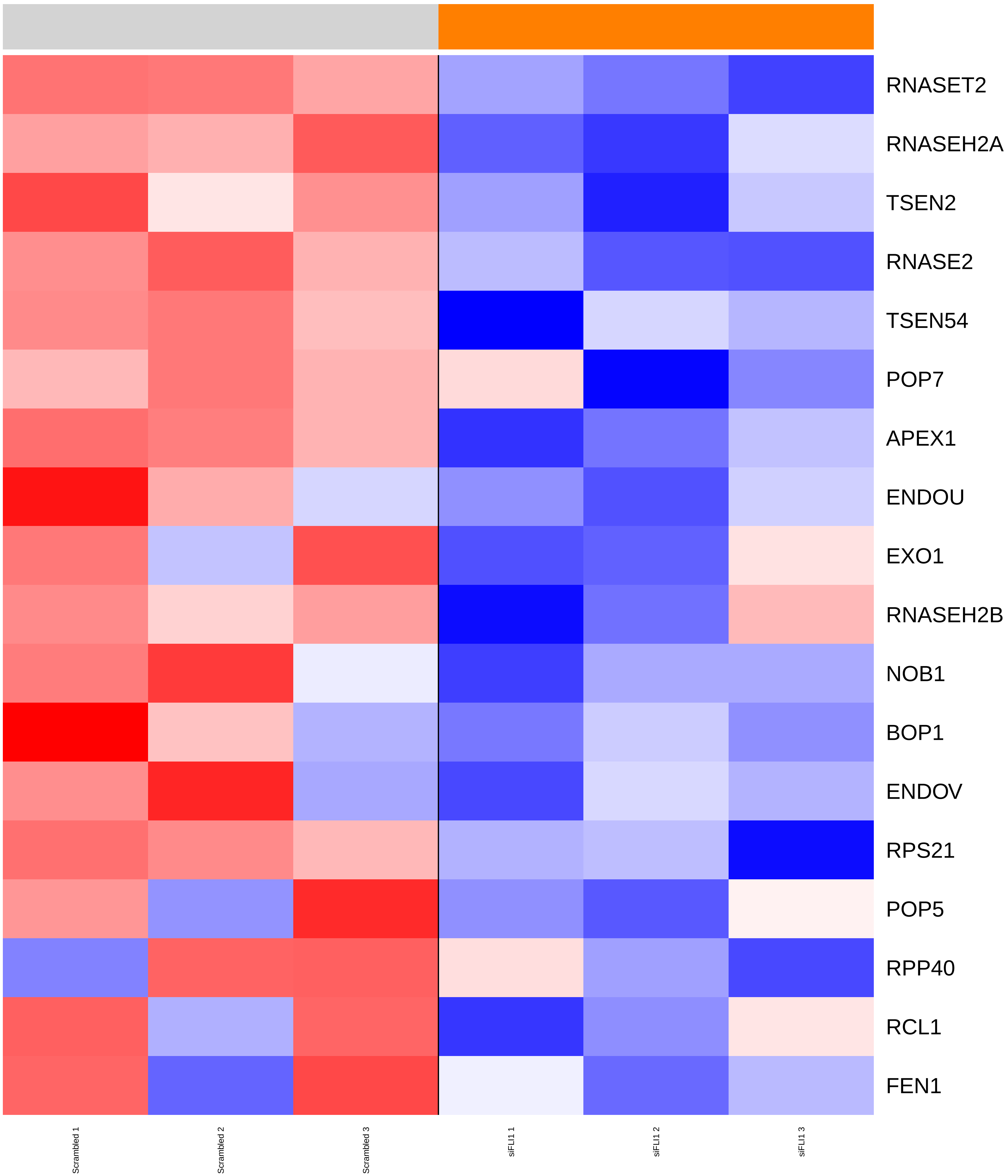

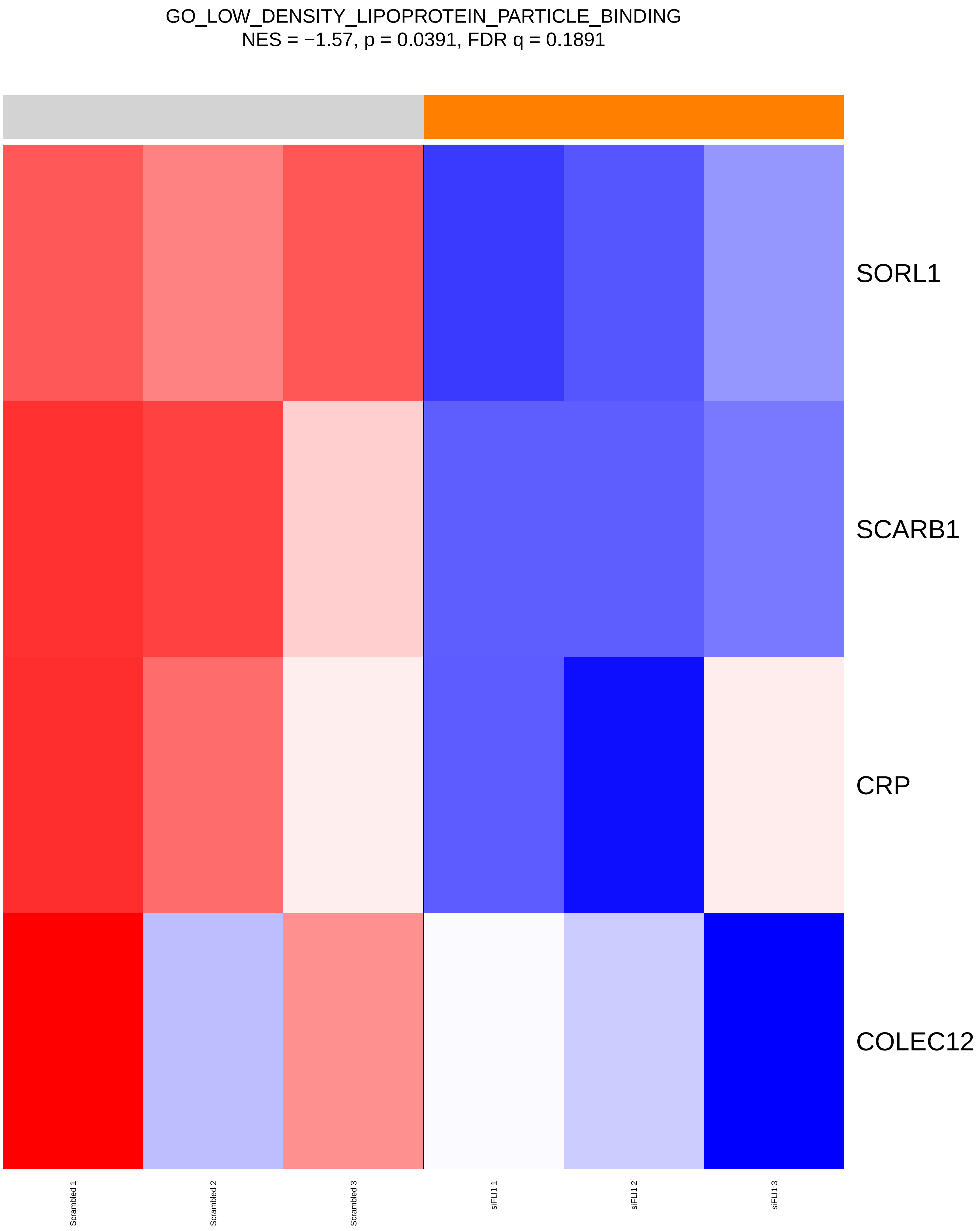
GO_LOW_DENSITY_LIPOPROTEIN_PARTICLE_BINDING
NES = −1.57, p = 0.0391, FDR q = 0.1891
SORL1
SCARB1
CRP
COLEC12
Scrambled 1
Scrambled 2
Scrambled 3
siFLI1 1
siFLI1 2
siFLI1 3

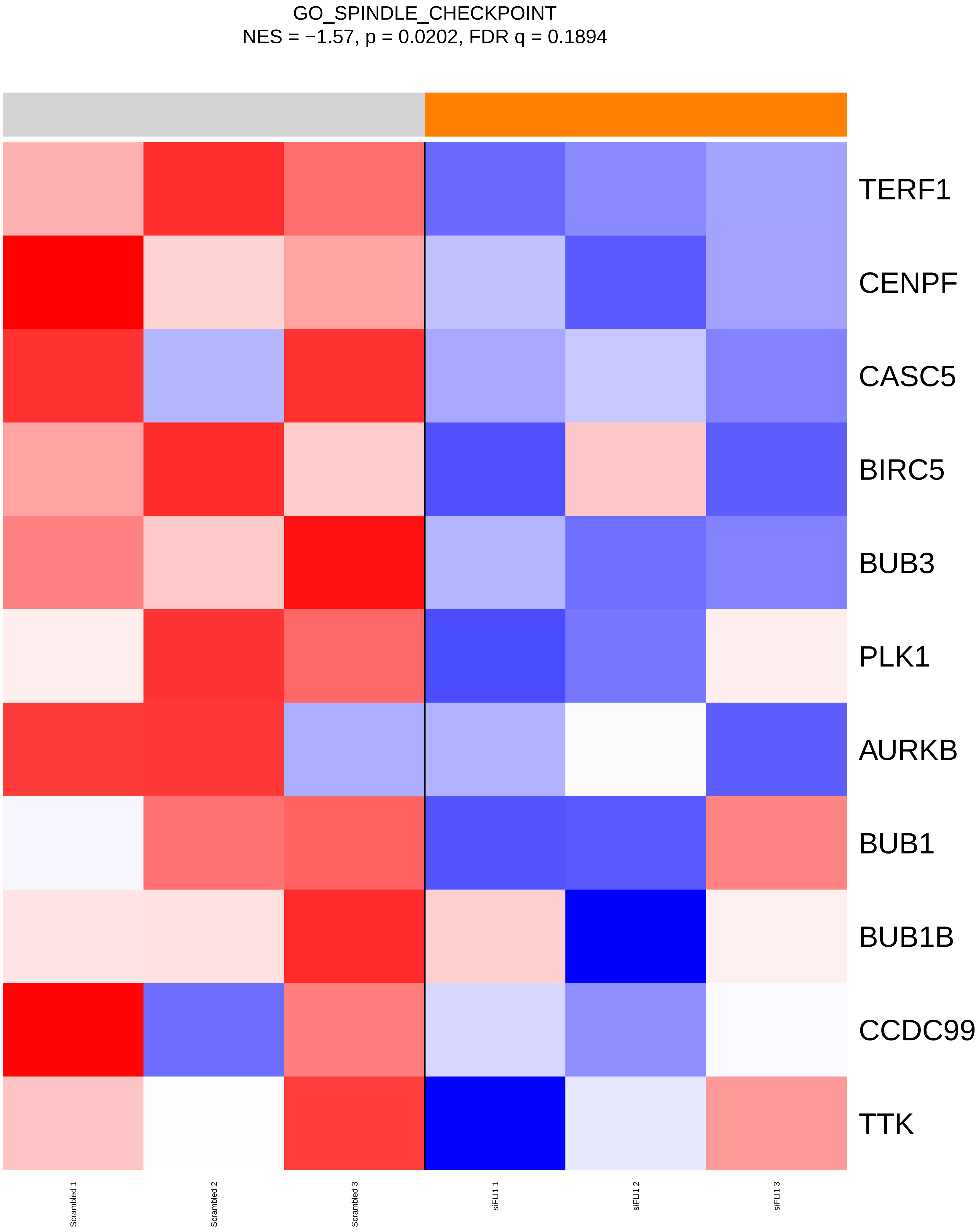
GO_SPINDLE_CHECKPOINT
NES = −1.57, p = 0.0202, FDR q = 0.1894
TERF1
CENPF
CASC5
BIRC5
BUB3
PLK1
AURKB
BUB1
BUB1B
CCDC99
TTK
Scrambled 1
Scrambled 2
Scrambled 3
siFLI1 1
siFLI1 2
siFLI1 3

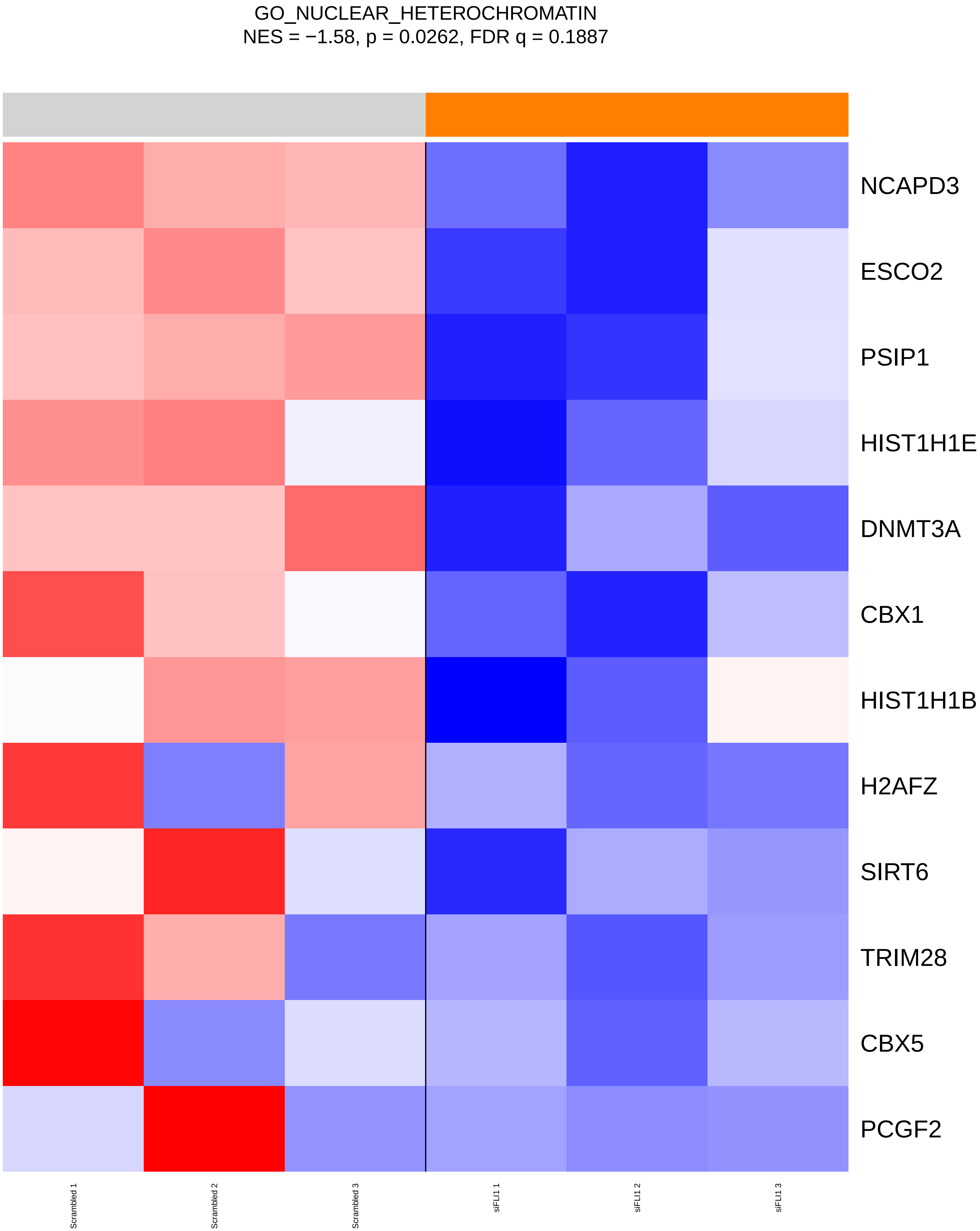

GO_NUCLEAR_HETEROCHROMATIN
NES = −1.58, p = 0.0262, FDR q = 0.1887
NCAPD3
ESCO2
PSIP1
HIST1H1E
DNMT3A
CBX1
HIST1H1B
H2AFZ
SIRT6
TRIM28
CBX5
PCGF2
Scrambled 1
Scrambled 2
Scrambled 3
siFLI1 1
siFLI1 2
siFLI1 3

GO_PHAGOCYTOSIS_ENGULFMENT
NES = −1.58, p = 0.0199, FDR q = 0.1884

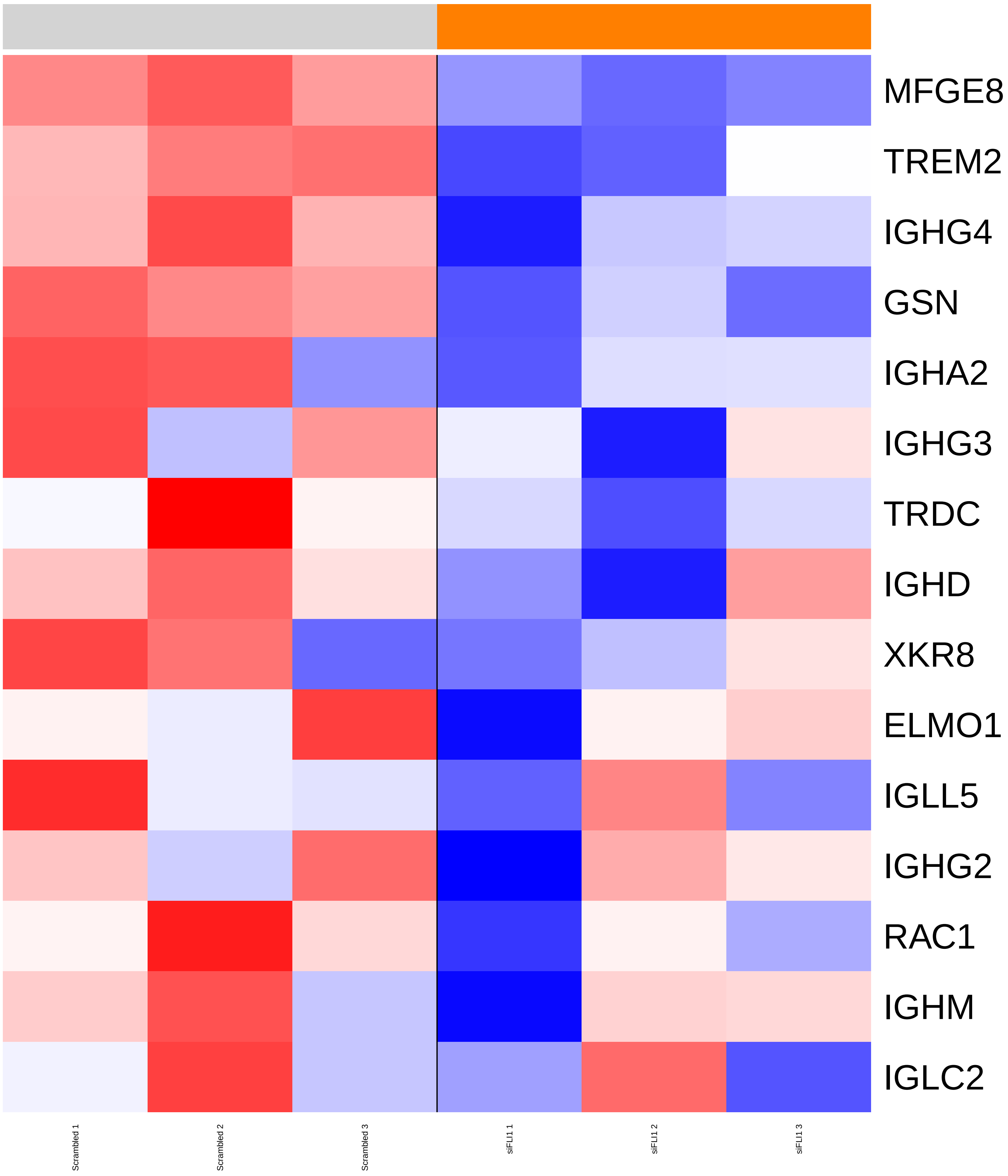

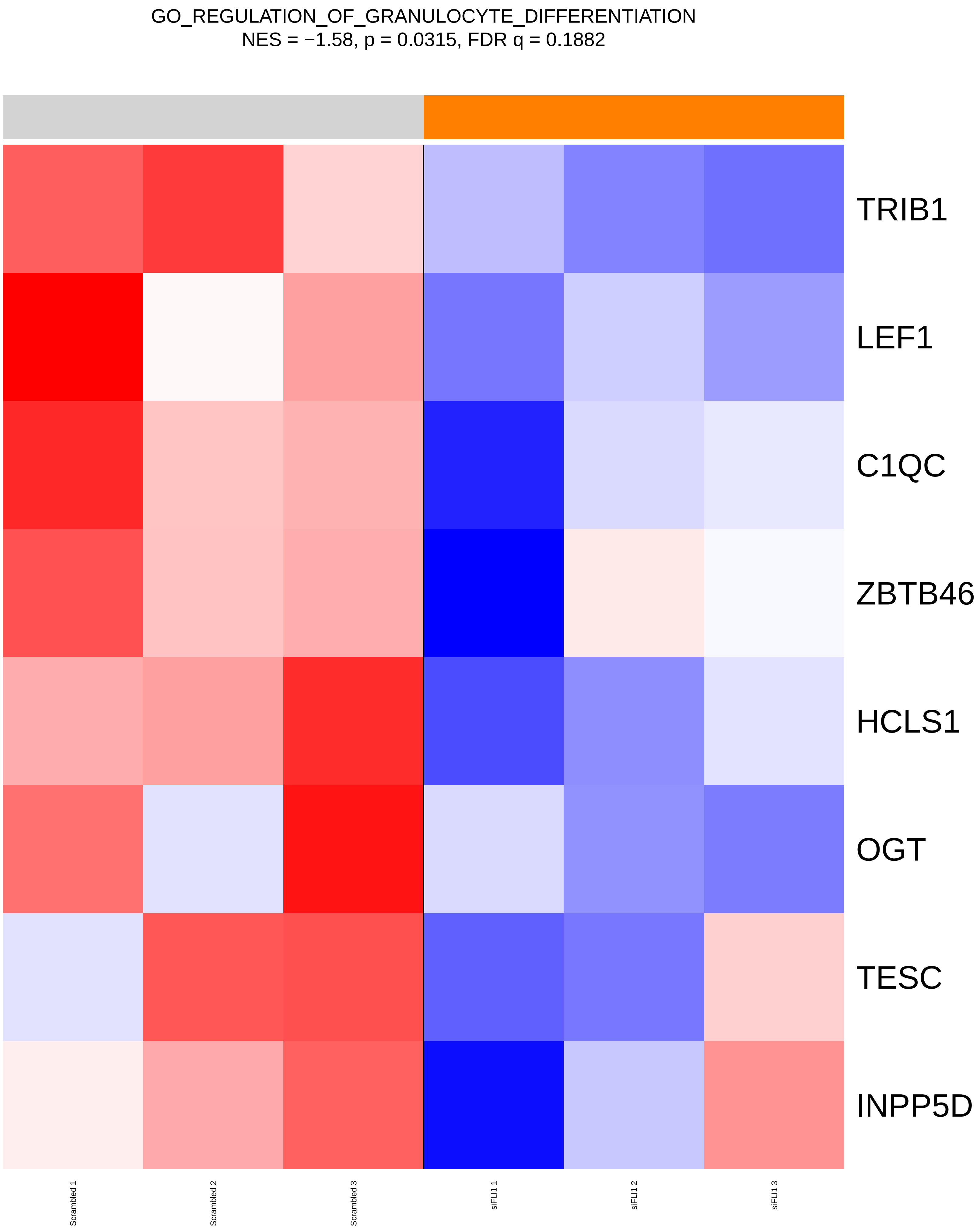
GO_REGULATION_OF_GRANULOCYTE_DIFFERENTIATION
NES = −1.58, p = 0.0315, FDR q = 0.1882
TRIB1
LEF1
C1QC
ZBTB46
HCLS1
OGT
TESC
INPP5D
Scrambled 1
Scrambled 2
Scrambled 3
siFLI1 1
siFLI1 2
siFLI1 3

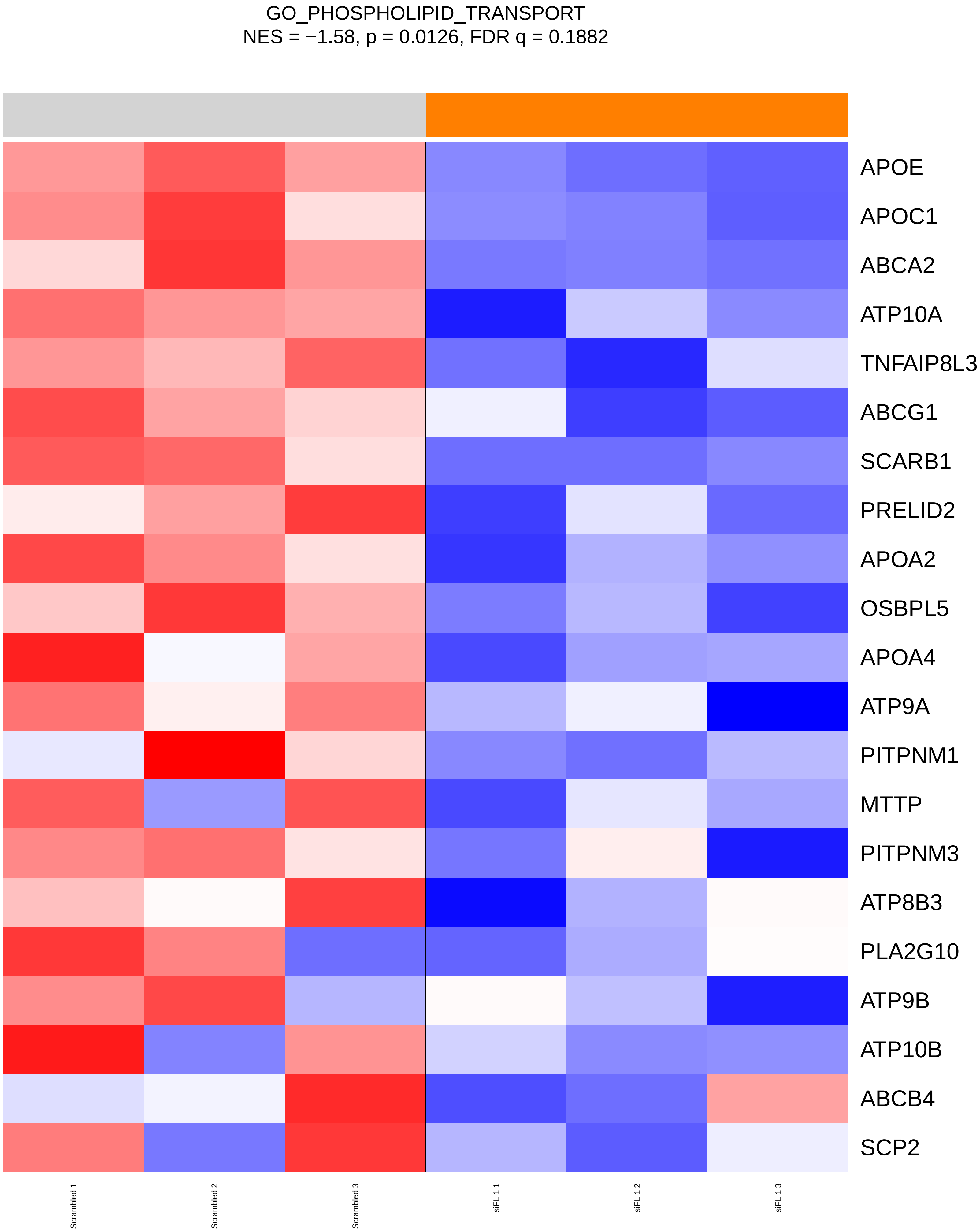
GO_PHOSPHOLIPID_TRANSPORT
NES = −1.58, p = 0.0126, FDR q = 0.1882
APOE
APOC1
ABCA2
ATP10A
TNFAIP8L3
ABCG1
SCARB1
PRELID2
APOA2
OSBPL5
APOA4
ATP9A
PITPNM1
MTTP
PITPNM3
ATP8B3
PLA2G10
ATP9B
ATP10B
ABCB4
SCP2
Scrambled 1
Scrambled 2
Scrambled 3
siFLI1 1
siFLI1 2
siFLI1 3

GO_POSITIVE_REGULATION_OF_LIPID_CATABOLIC_PROCESS
NES = −1.58, p = 0.0246, FDR q = 0.1883

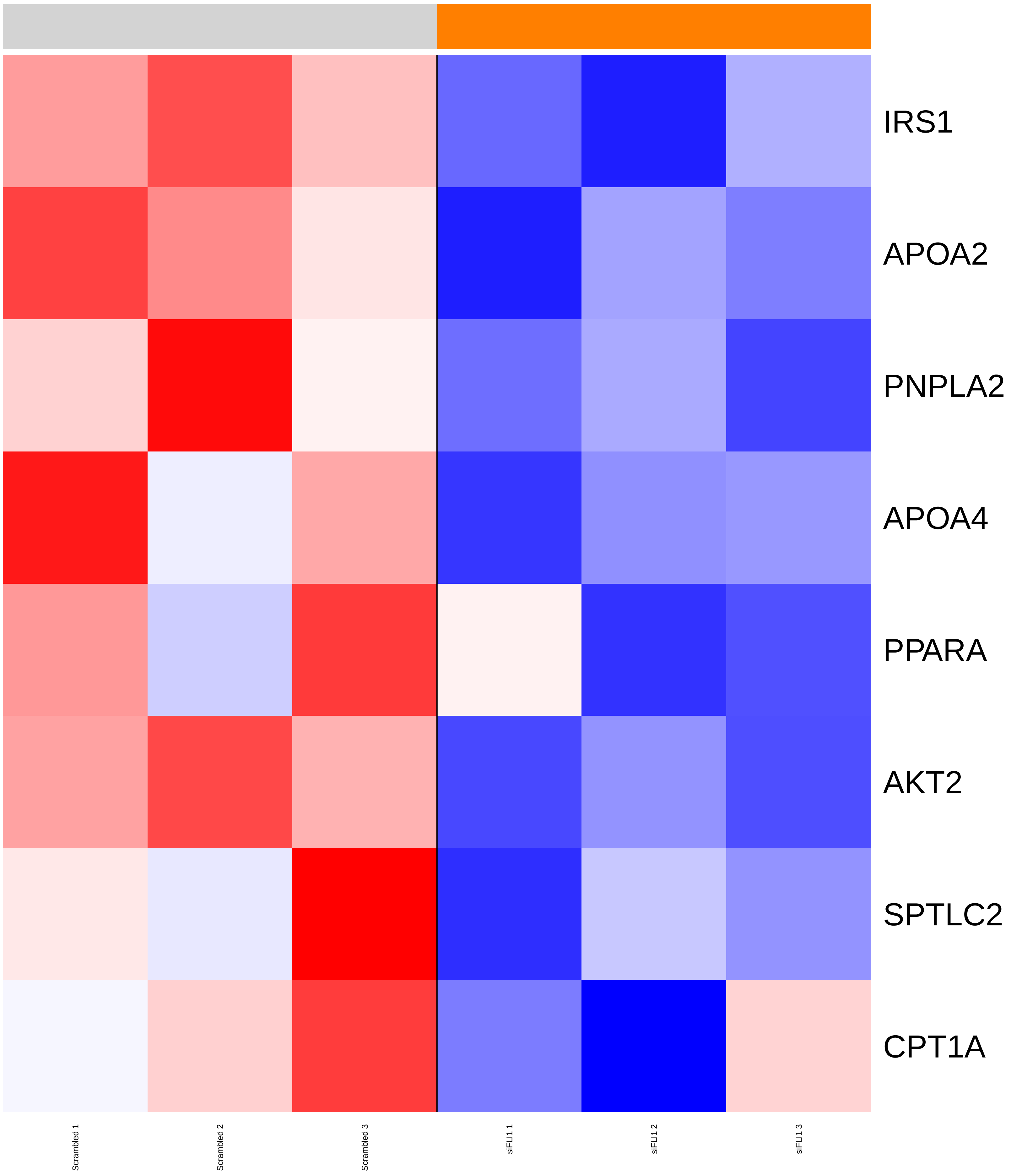

GO_REGULATION_OF_LONG_TERM_NEURONAL_SYNAPTIC_PLASTICITY
NES = −1.58, p = 0.0249, FDR q = 0.1871

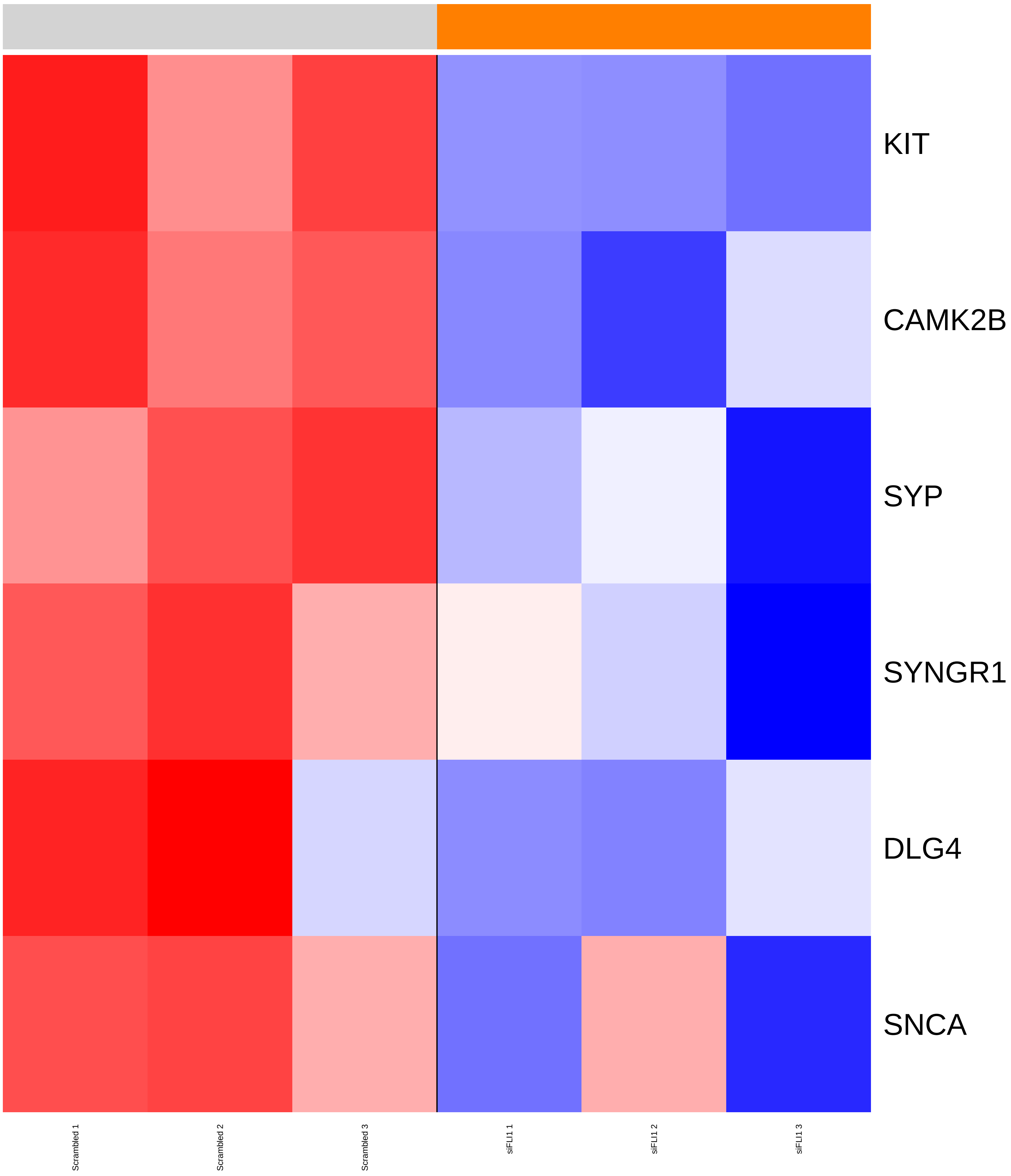

GO_TETRAHYDROFOLATE_METABOLIC_PROCESS
NES = −1.58, p = 0.0285, FDR q = 0.1874

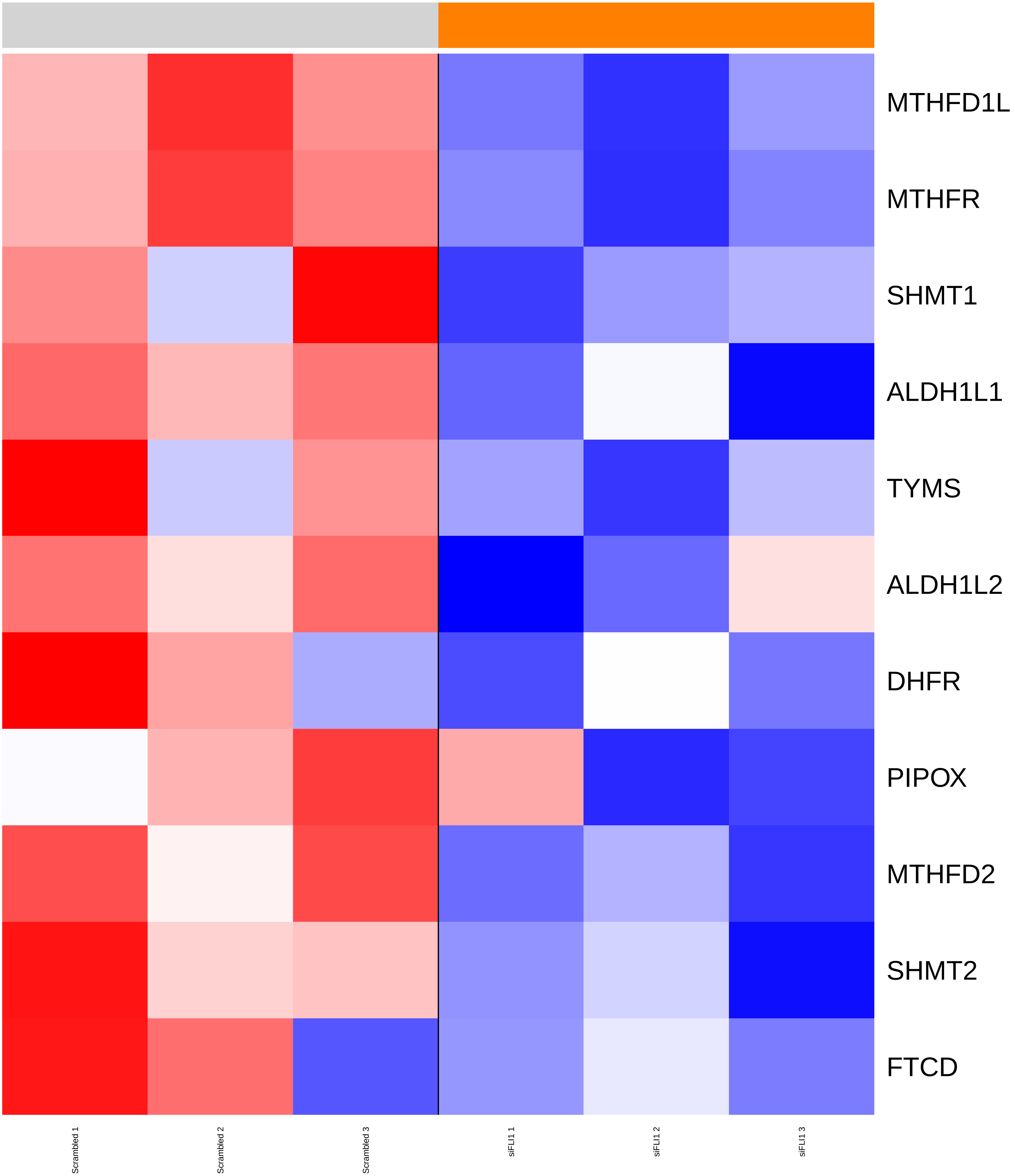

GO_COFACTOR_BINDING
NES = −1.58, p = 0.0000, FDR q = 0.1849

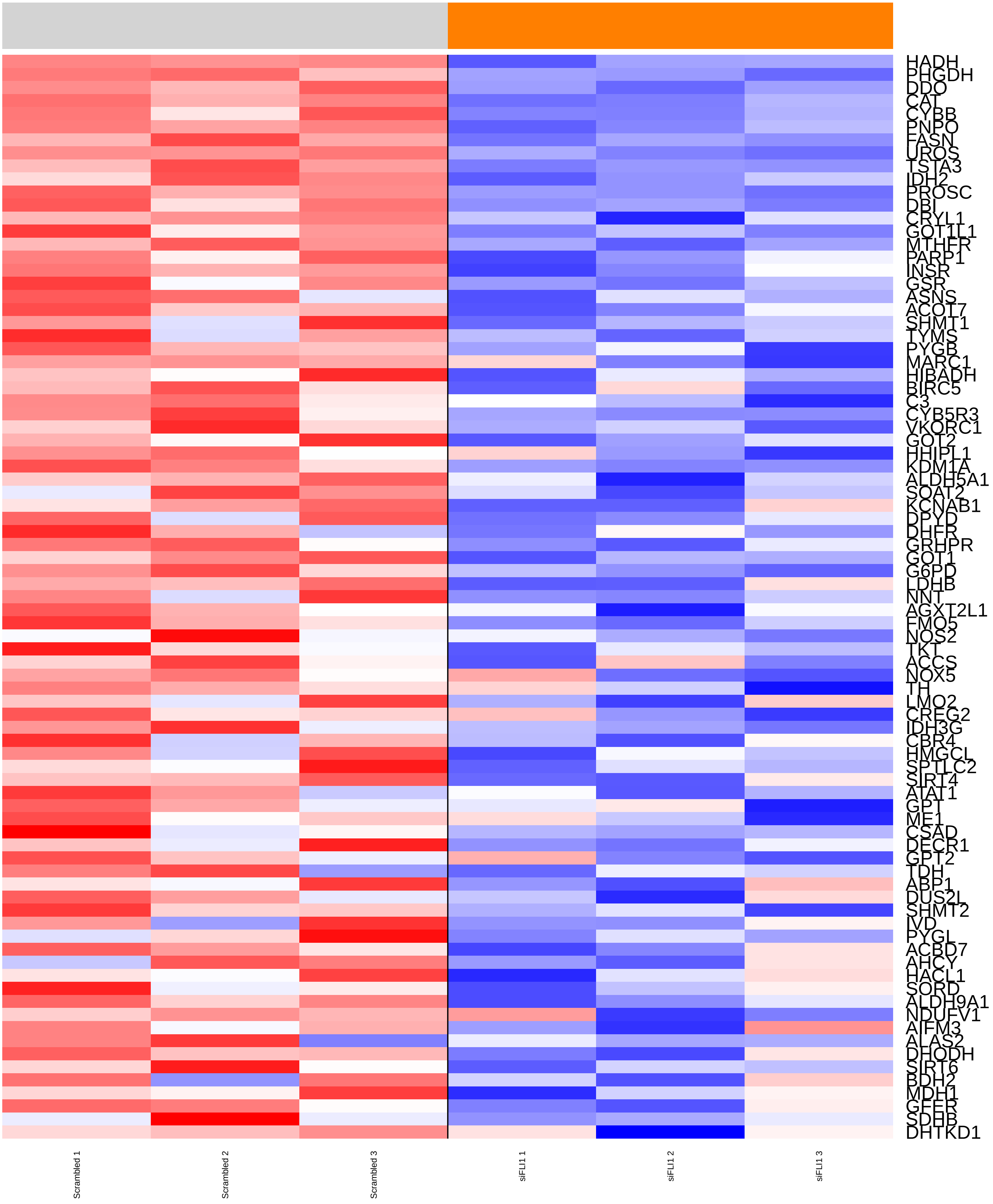

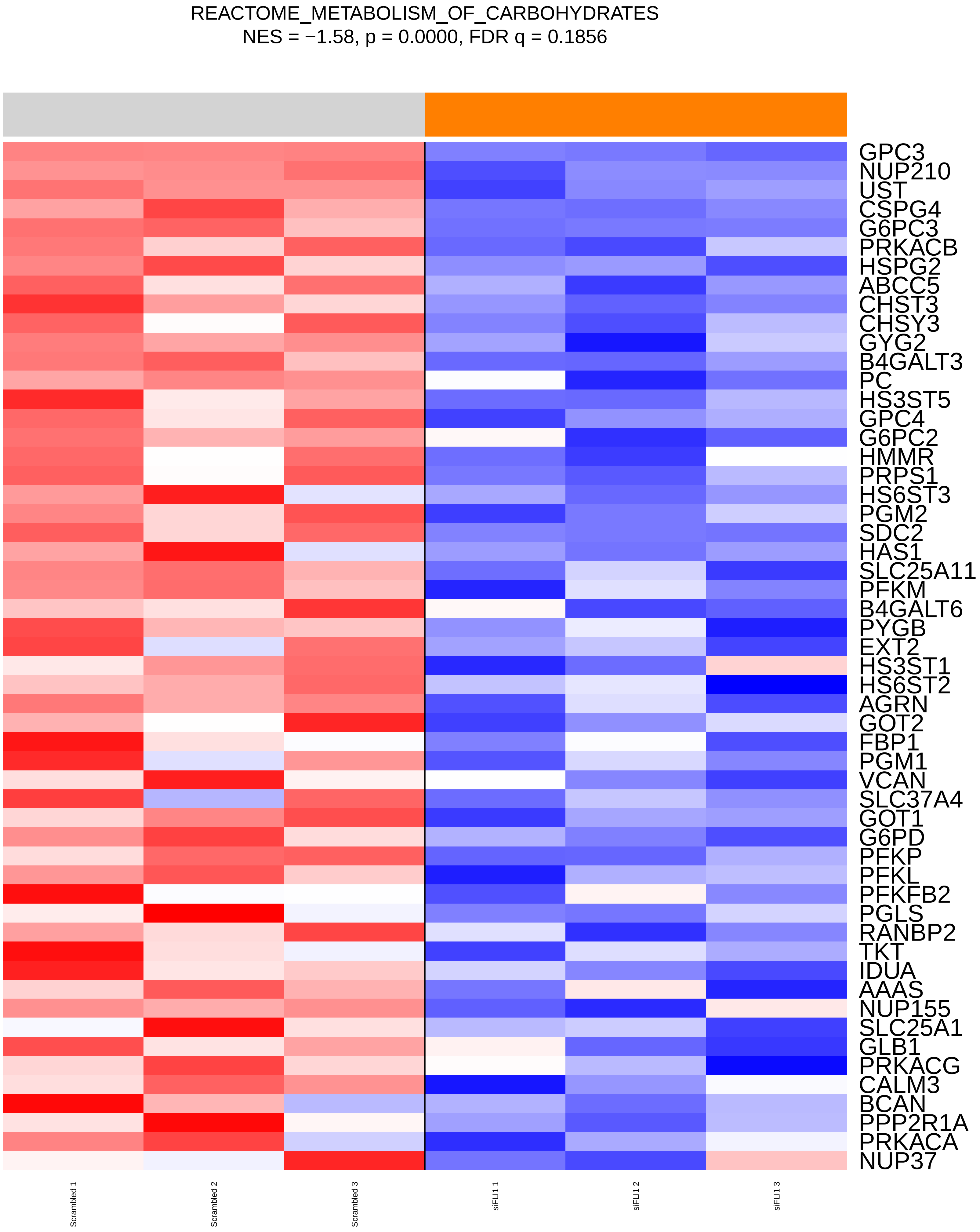
REACTOME_METABOLISM_OF_CARBOHYDRATES
NES = −1.58, p = 0.0000, FDR q = 0.1856
GPC3
NUP210
UST
CSPG4
G6PC3
PRKACB
HSPG2
ABCC5
CHST3
CHSY3
GYG2
B4GALT3
PC
HS3ST5
GPC4
G6PC2
HMMR
PRPS1
HS6ST3
PGM2
SDC2
HAS1
SLC25A11
PFKM
B4GALT6
PYGB
EXT2
HS3ST1
HS6ST2
AGRN
GOT2
FBP1
PGM1
VCAN
SLC37A4
GOT1
G6PD
PFKP
PFKL
PFKFB2
PGLS
RANBP2
TKT
IDUA
AAAS
NUP155
SLC25A1
GLB1
PRKACG
CALM3
BCAN
PPP2R1A
PRKACA
NUP37
Scrambled 1
Scrambled 2
Scrambled 3
siFLI1 1
siFLI1 2
siFLI1 3

E2F1_Q6_01
NES = −1.58, p = 0.0000, FDR q = 0.1853

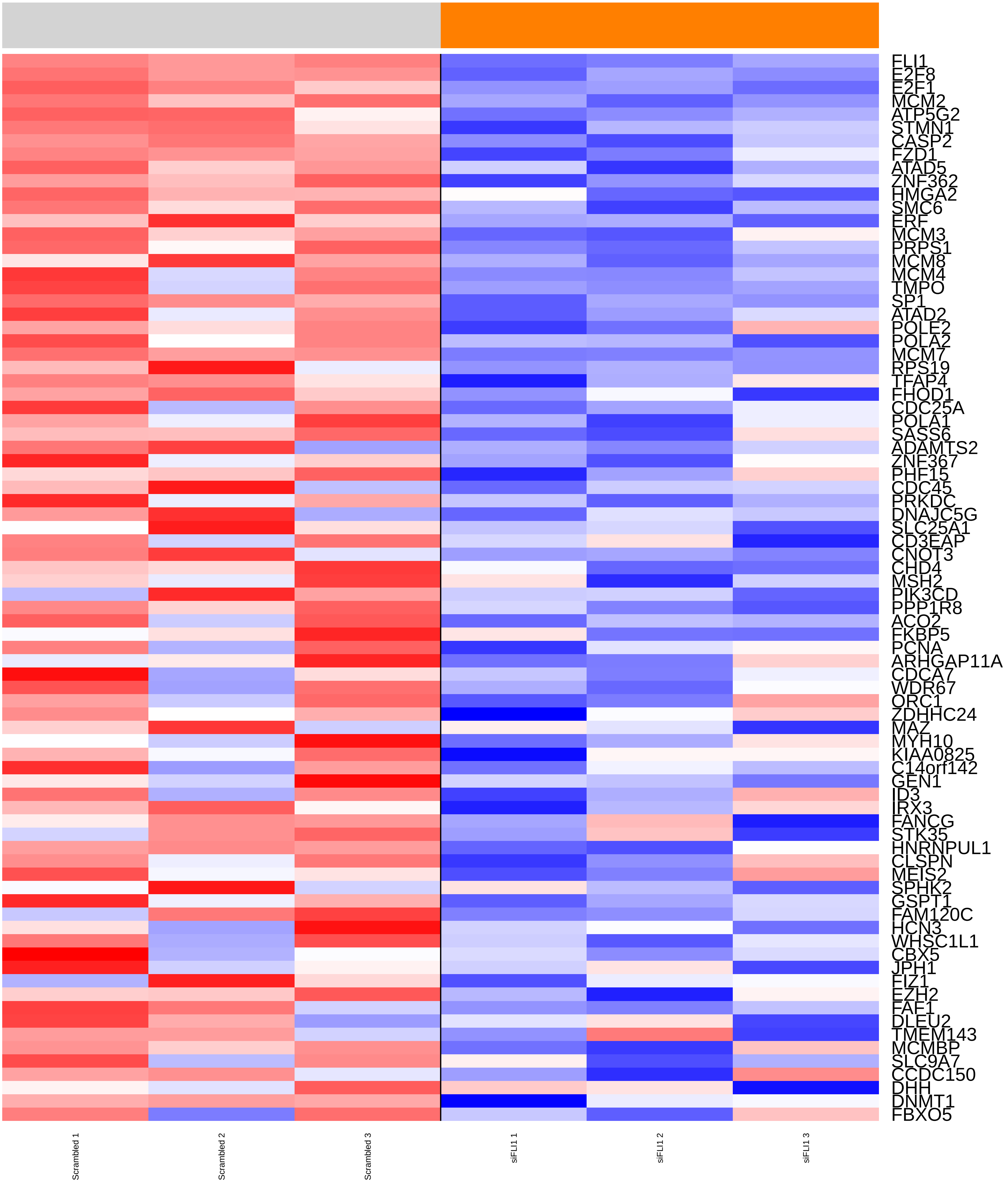

HALLMARK_CHOLESTEROL_HOMEOSTASIS
NES = −1.58, p = 0.0031, FDR q = 0.1840

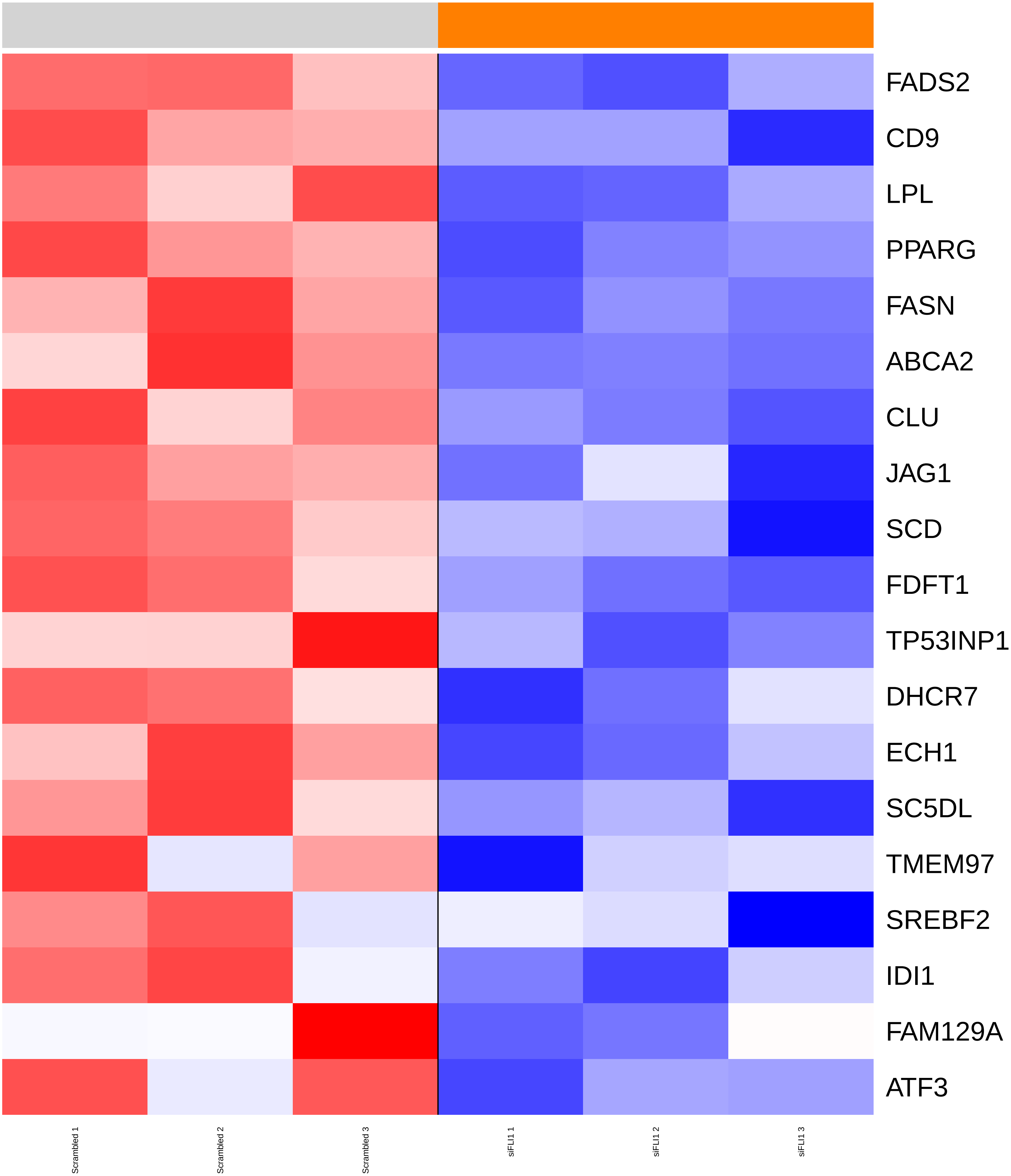

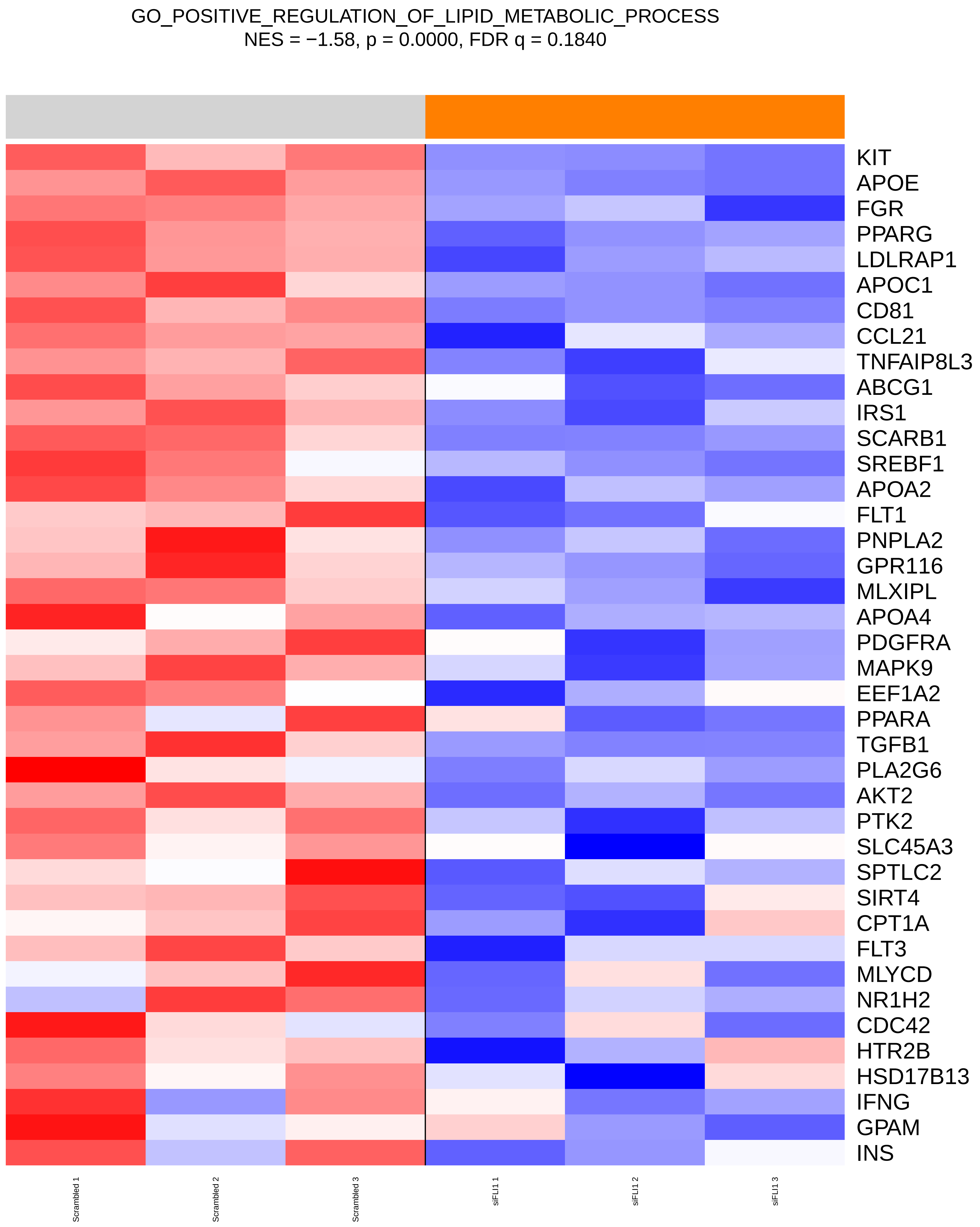
GO_POSITIVE_REGULATION_OF_LIPID_METABOLIC_PROCESS
NES = −1.58, p = 0.0000, FDR q = 0.1840
KIT
APOE
FGR
PPARG
LDLRAP1
APOC1
CD81
CCL21
TNFAIP8L3
ABCG1
IRS1
SCARB1
SREBF1
APOA2
FLT1
PNPLA2
GPR116
MLXIPL
APOA4
PDGFRA
MAPK9
EEF1A2
PPARA
TGFB1
PLA2G6
AKT2
PTK2
SLC45A3
SPTLC2
SIRT4
CPT1A
FLT3
MLYCD
NR1H2
CDC42
HTR2B
HSD17B13
IFNG
GPAM
INS
Scrambled 1
Scrambled 2
Scrambled 3
siFLI1 1
siFLI1 2
siFLI1 3

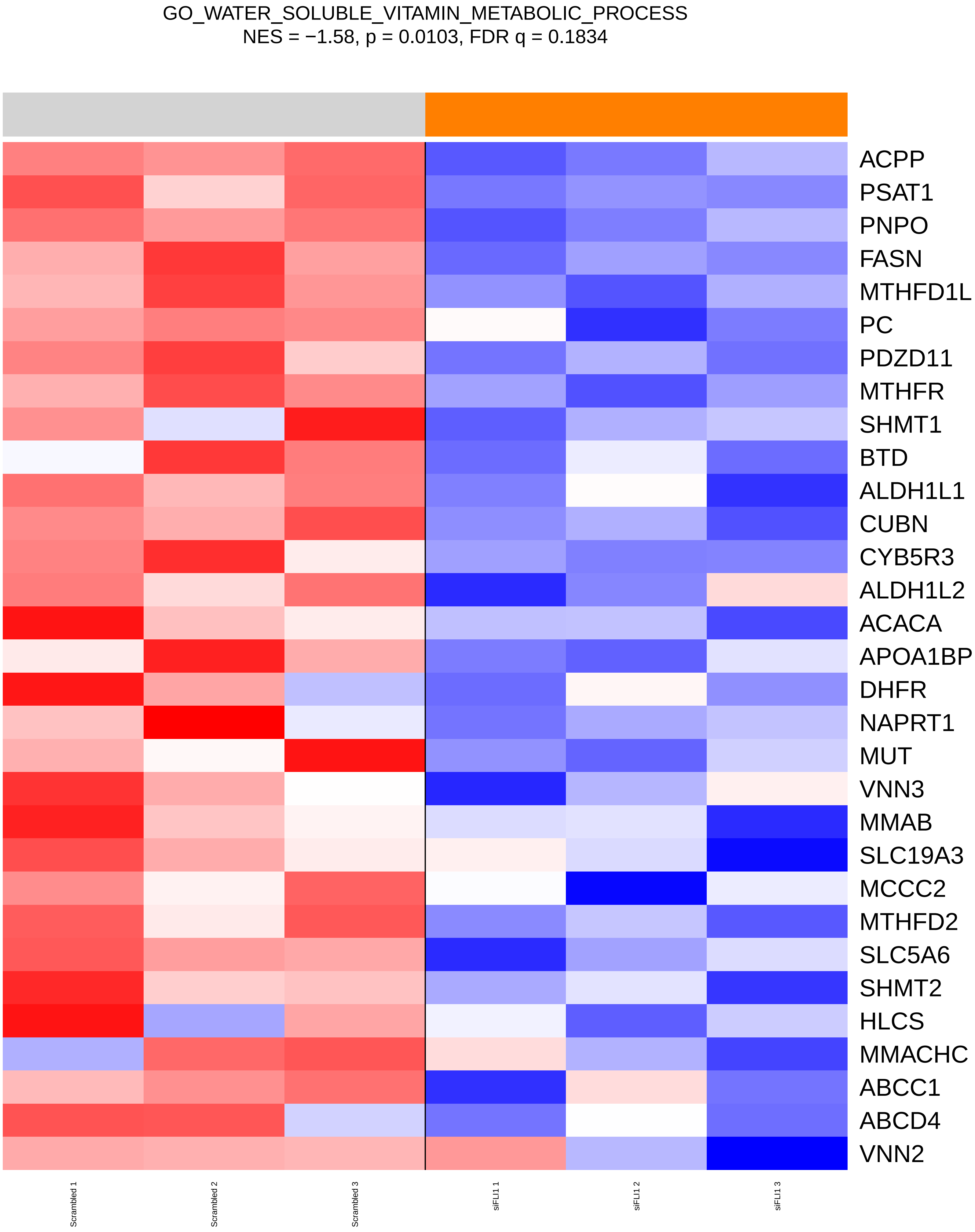
GO_WATER_SOLUBLE_VITAMIN_METABOLIC_PROCESS
NES = −1.58, p = 0.0103, FDR q = 0.1834
ACPP
PSAT1
PNPO
FASN
MTHFD1L
PC
PDZD11
MTHFR
SHMT1
BTD
ALDH1L1
CUBN
CYB5R3
ALDH1L2
ACACA
APOA1BP
DHFR
NAPRT1
MUT
VNN3
MMAB
SLC19A3
MCCC2
MTHFD2
SLC5A6
SHMT2
HLCS
MMACHC
ABCC1
ABCD4
VNN2
Scrambled 1
Scrambled 2
Scrambled 3
siFLI1 1
siFLI1 2
siFLI1 3

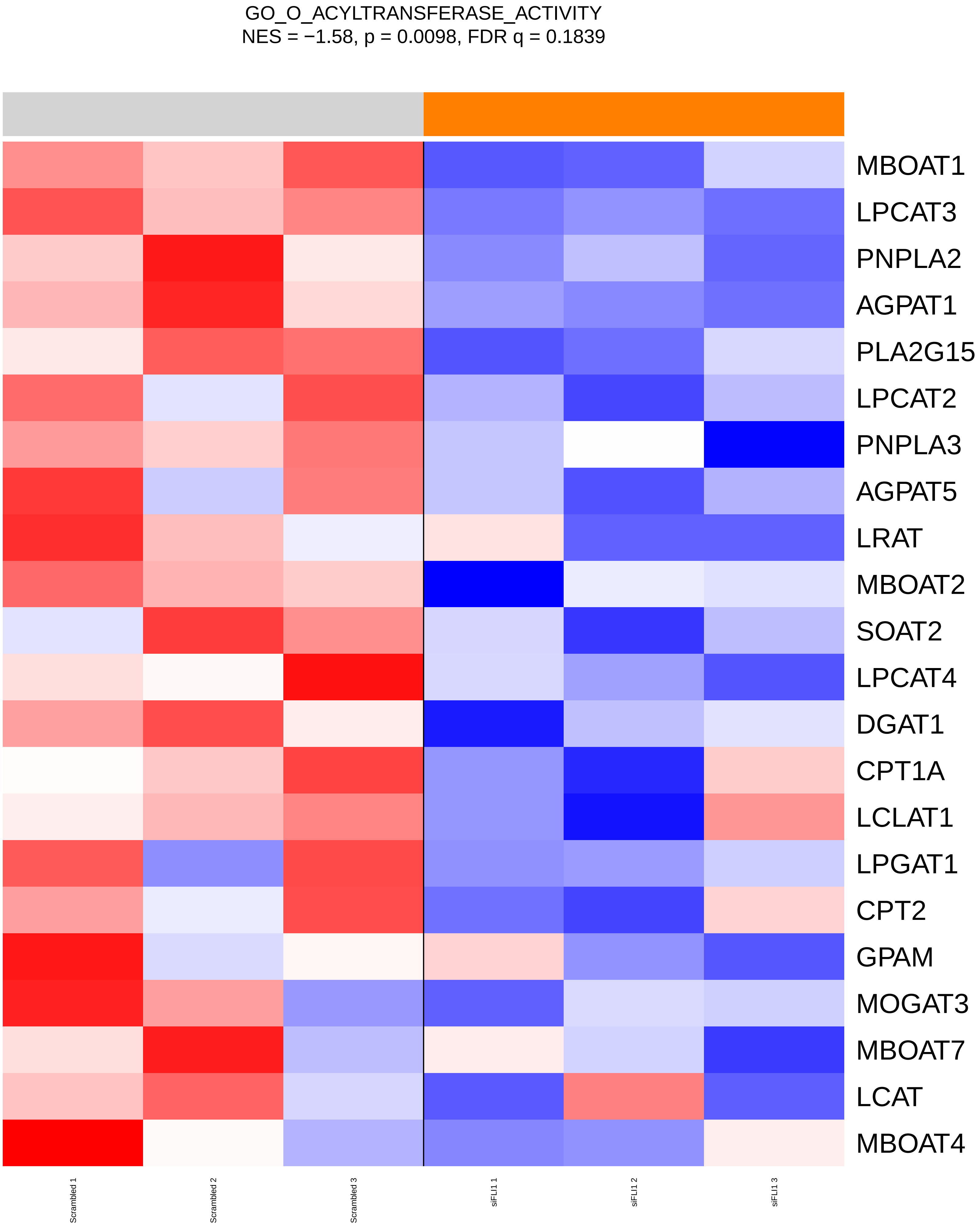
GO_O_ACYLTRANSFERASE_ACTIVITY
NES = −1.58, p = 0.0098, FDR q = 0.1839
MBOAT1
LPCAT3
PNPLA2
AGPAT1
PLA2G15
LPCAT2
PNPLA3
AGPAT5
LRAT
MBOAT2
SOAT2
LPCAT4
DGAT1
CPT1A
LCLAT1
LPGAT1
CPT2
GPAM
MOGAT3
MBOAT7
LCAT
MBOAT4
Scrambled 1
Scrambled 2
Scrambled 3
siFLI1 1
siFLI1 2
siFLI1 3

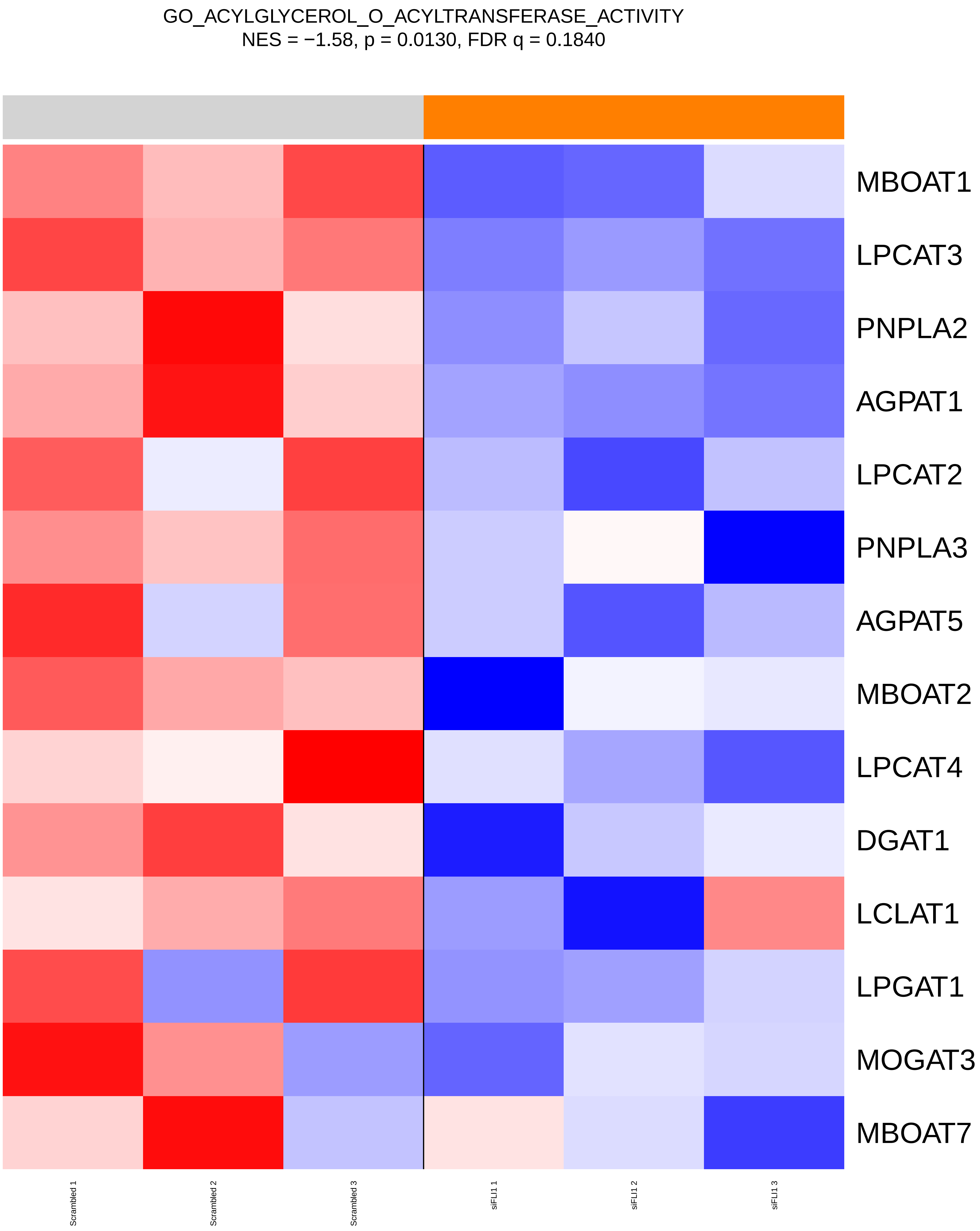
GO_ACYLGLYCEROL_O_ACYLTRANSFERASE_ACTIVITY
NES = −1.58, p = 0.0130, FDR q = 0.1840
MBOAT1
LPCAT3
PNPLA2
AGPAT1
LPCAT2
PNPLA3
AGPAT5
MBOAT2
LPCAT4
DGAT1
LCLAT1
LPGAT1
MOGAT3
MBOAT7
Scrambled 1
Scrambled 2
Scrambled 3
siFLI1 1
siFLI1 2
siFLI1 3

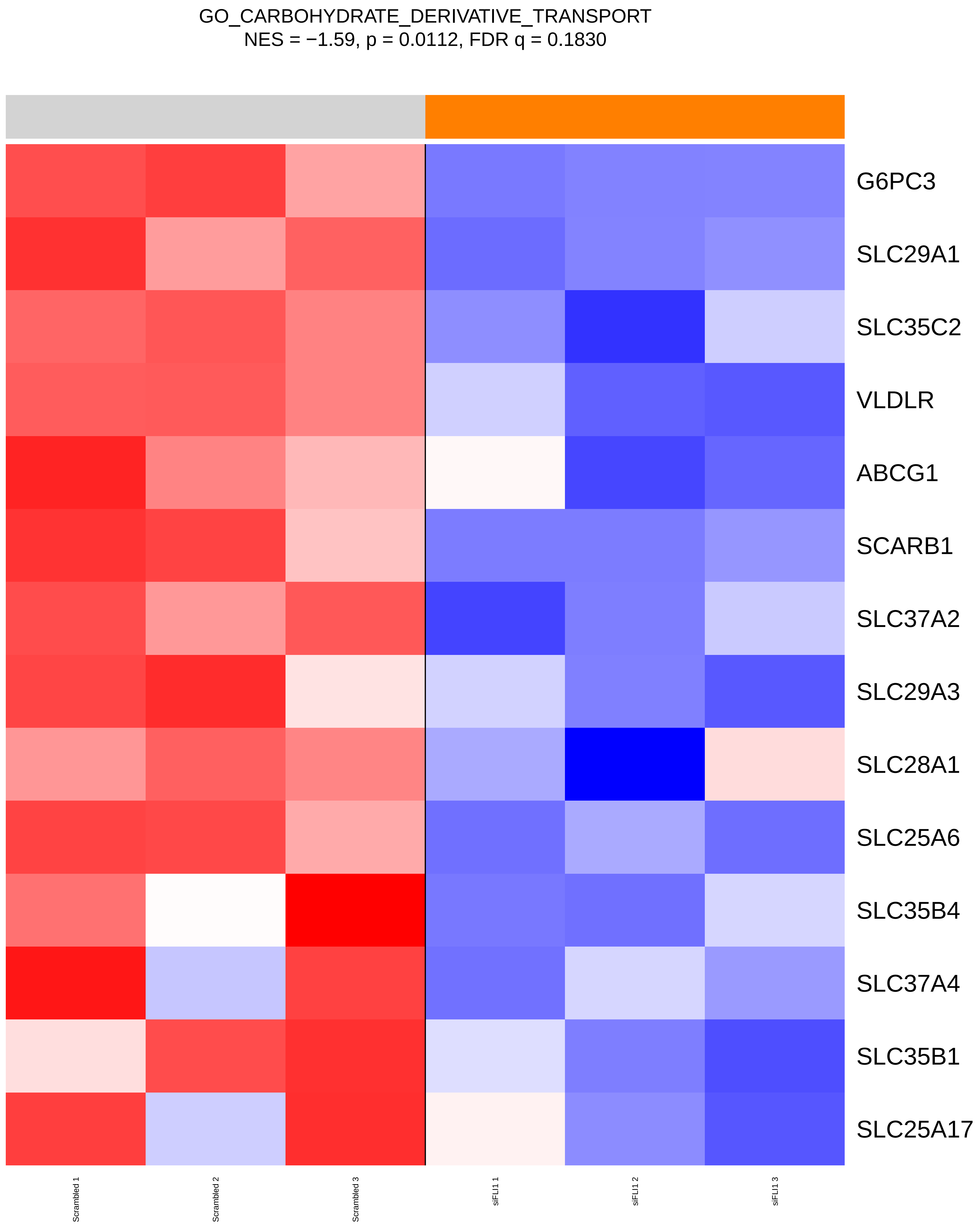
GO_CARBOHYDRATE_DERIVATIVE_TRANSPORT
NES = −1.59, p = 0.0112, FDR q = 0.1830
G6PC3
SLC29A1
SLC35C2
VLDLR
ABCG1
SCARB1
SLC37A2
SLC29A3
SLC28A1
SLC25A6
SLC35B4
SLC37A4
SLC35B1
SLC25A17
Scrambled 1
Scrambled 2
Scrambled 3
siFLI1 1
siFLI1 2
siFLI1 3

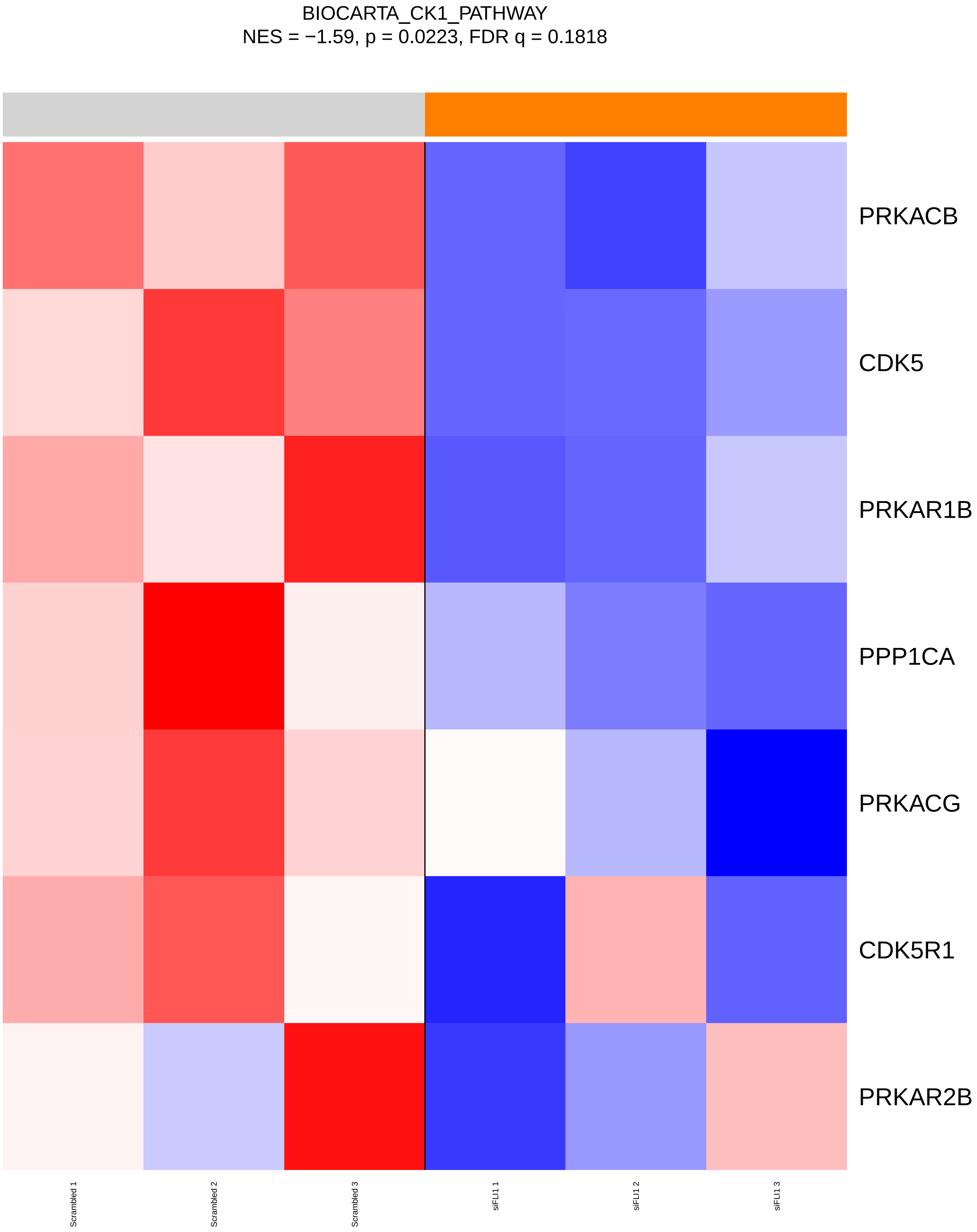

BIOCARTA_CK1_PATHWAY
NES = −1.59, p = 0.0223, FDR q = 0.1818
PRKACB
CDK5
PRKAR1B
PPP1CA
PRKACG
CDK5R1
PRKAR2B
Scrambled 1
Scrambled 2
Scrambled 3
siFLI1 1
siFLI1 2
siFLI1 3

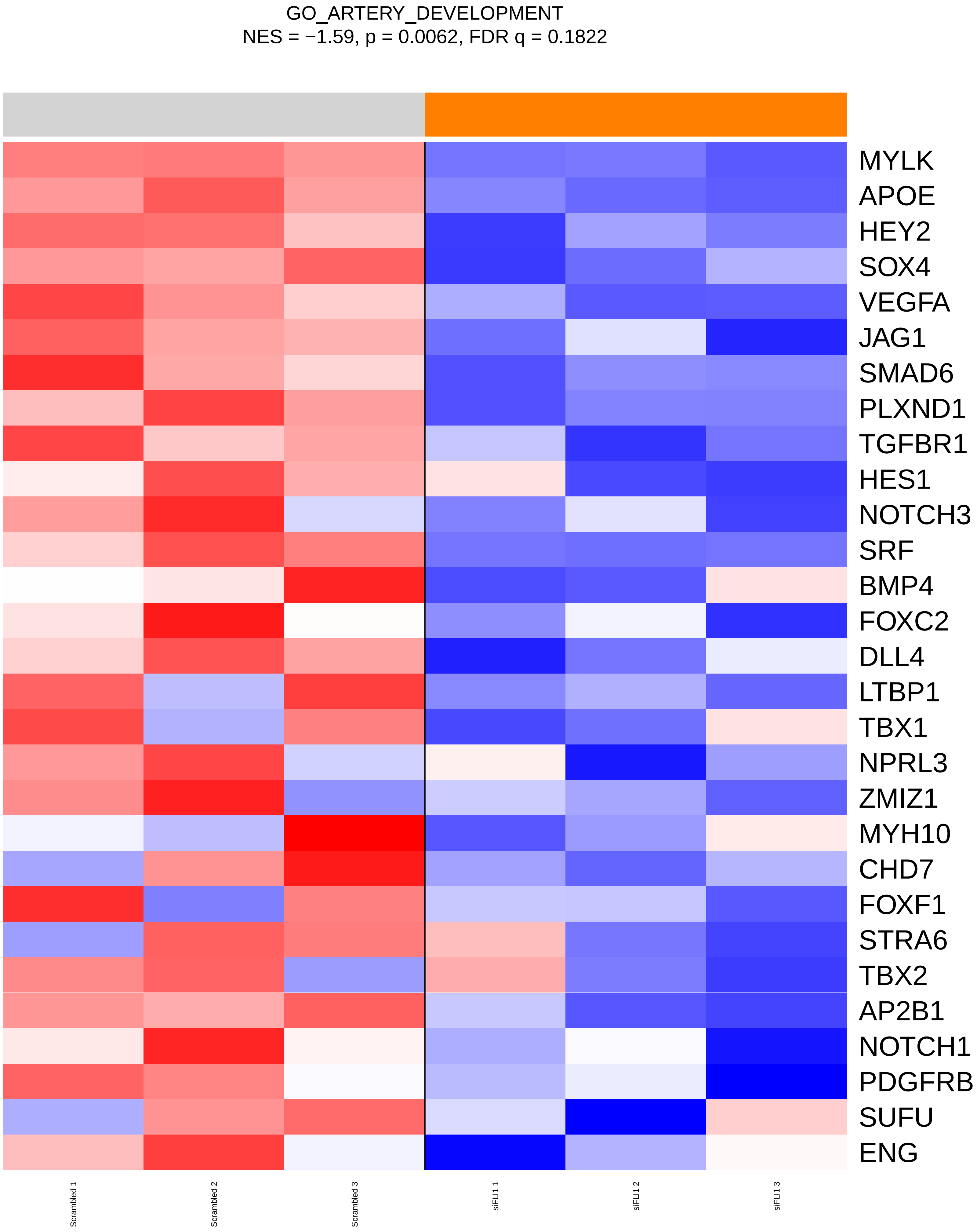
GO_ARTERY_DEVELOPMENT
NES = −1.59, p = 0.0062, FDR q = 0.1822
MYLK
APOE
HEY2
SOX4
VEGFA
JAG1
SMAD6
PLXND1
TGFBR1
HES1
NOTCH3
SRF
BMP4
FOXC2
DLL4
LTBP1
TBX1
NPRL3
ZMIZ1
MYH10
CHD7
FOXF1
STRA6
TBX2
AP2B1
NOTCH1
PDGFRB
SUFU
ENG
Scrambled 1
Scrambled 2
Scrambled 3
siFLI1 1
siFLI1 2
siFLI1 3

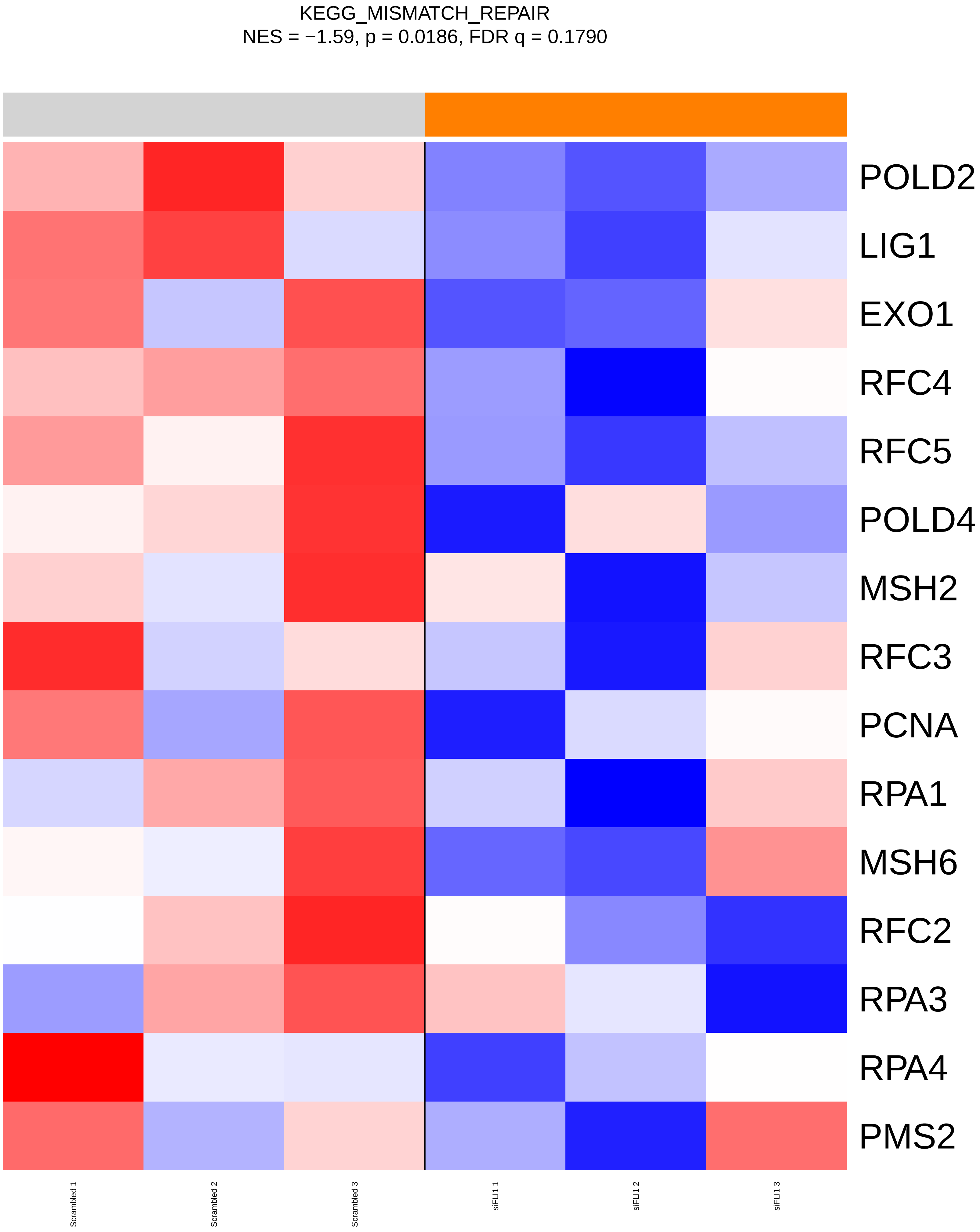
KEGG_MISMATCH_REPAIR
NES = −1.59, p = 0.0186, FDR q = 0.1790
POLD2
LIG1
EXO1
RFC4
RFC5
POLD4
MSH2
RFC3
PCNA
RPA1
MSH6
RFC2
RPA3
RPA4
PMS2
Scrambled 1
Scrambled 2
Scrambled 3
siFLI1 1
siFLI1 2
siFLI1 3

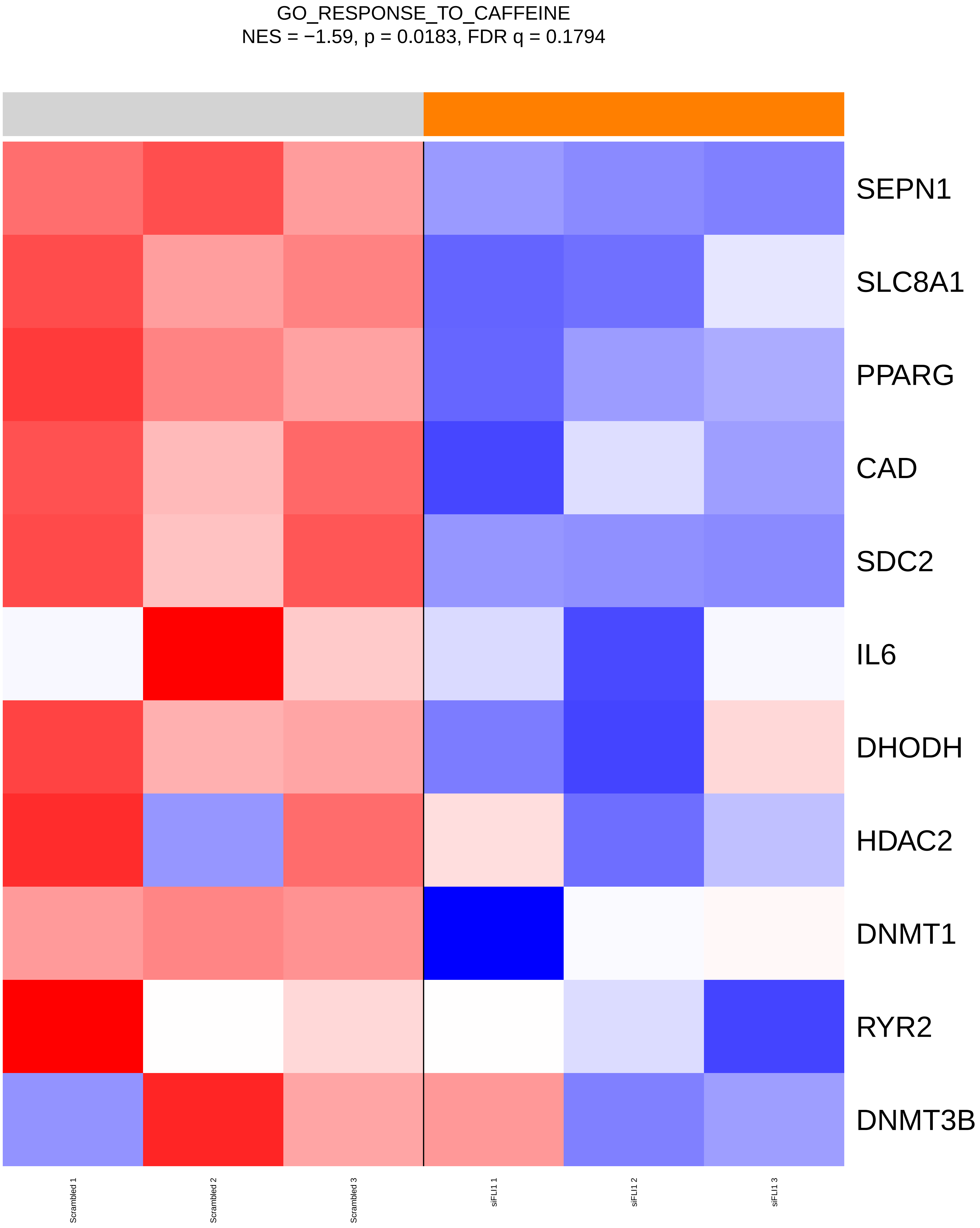

GO_RESPONSE_TO_CAFFEINE
NES = −1.59, p = 0.0183, FDR q = 0.1794
SEPN1
SLC8A1
PPARG
CAD
SDC2
IL6
DHODH
HDAC2
DNMT1
RYR2
DNMT3B
Scrambled 1
Scrambled 2
Scrambled 3
siFLI1 1
siFLI1 2
siFLI1 3

GO_CONDENSED_NUCLEAR_CHROMOSOME_CENTROMERIC_REGION
NES = −1.59, p = 0.0321, FDR q = 0.1800

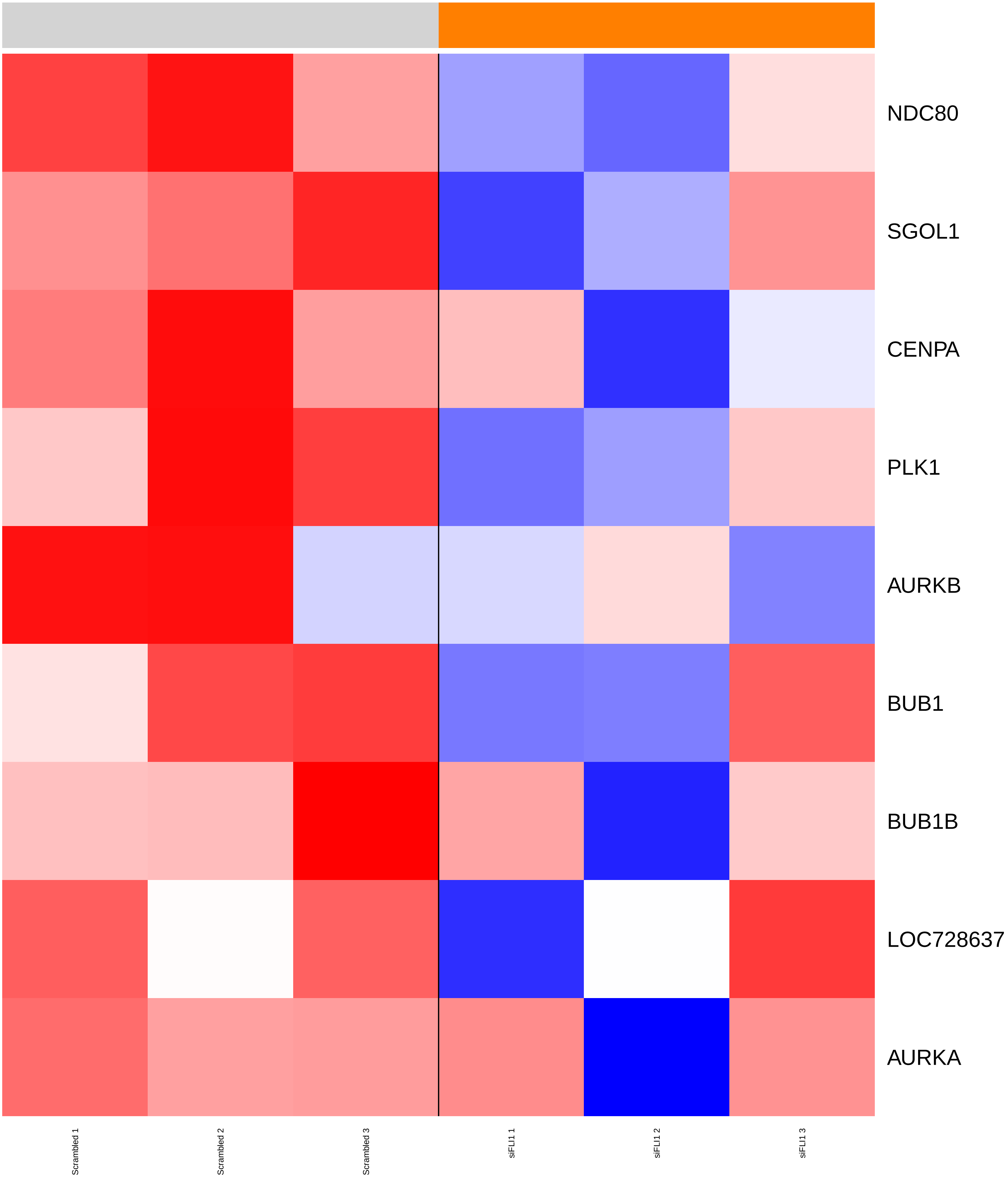

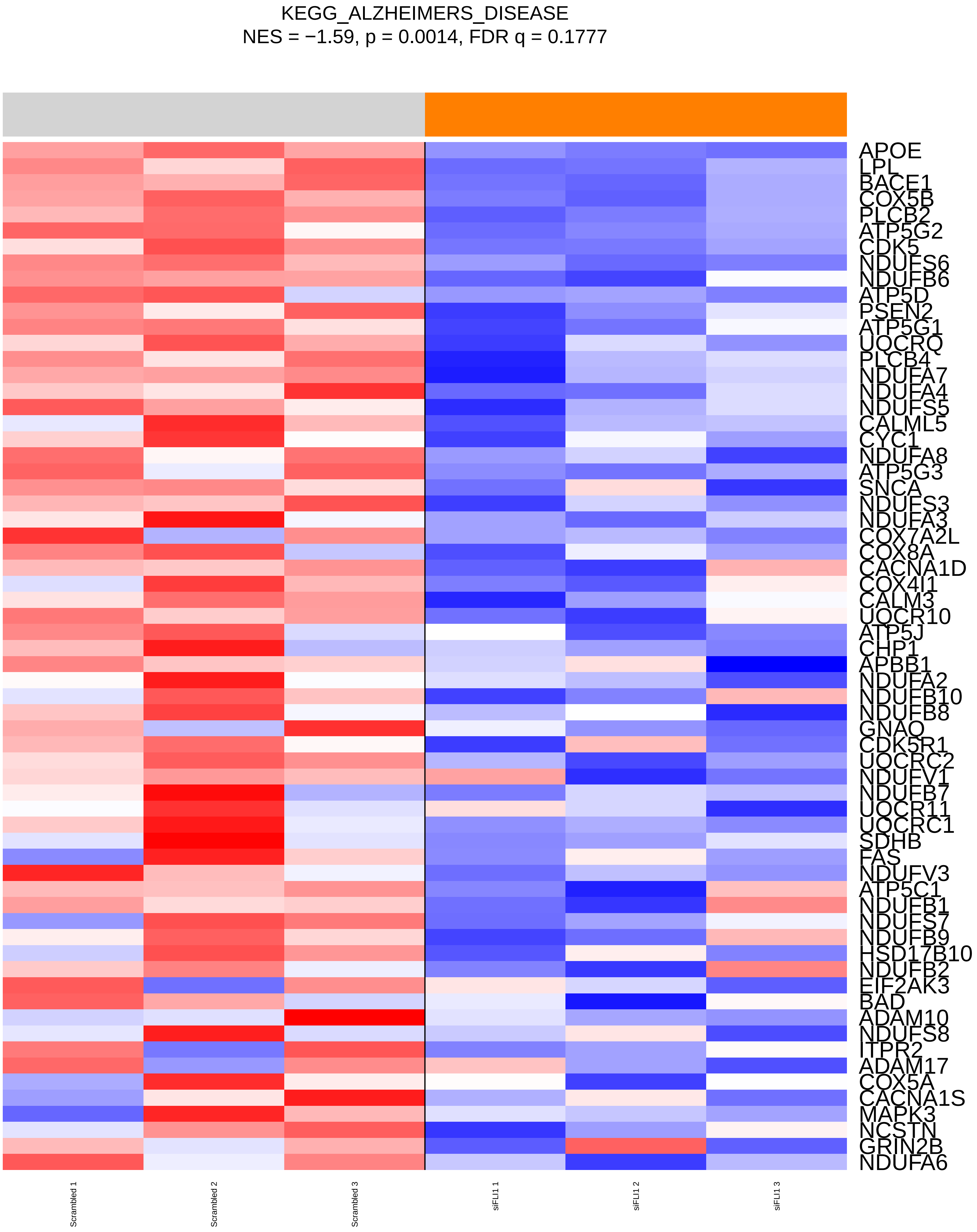
KEGG_ALZHEIMERS_DISEASE
NES = −1.59, p = 0.0014, FDR q = 0.1777
APOE
LPL
BACE1
COX5B
PLCB2
ATP5G2
CDK5
NDUFS6
NDUFB6
ATP5D
PSEN2
ATP5G1
UQCRQ
PLCB4
NDUFA7
NDUFA4
NDUFS5
CALML5
CYC1
NDUFA8
ATP5G3
SNCA
NDUFS3
NDUFA3
COX7A2L
COX8A
CACNA1D
COX4I1
CALM3
UQCR10
ATP5J
CHP1
APBB1
NDUFA2
NDUFB10
NDUFB8
GNAQ
CDK5R1
UQCRC2
NDUFV1
NDUFB7
UQCR11
UQCRC1
SDHB
FAS
NDUFV3
ATP5C1
NDUFB1
NDUFS7
NDUFB9
HSD17B10
NDUFB2
EIF2AK3
BAD
ADAM10
NDUFS8
ITPR2
ADAM17
COX5A
CACNA1S
MAPK3
NCSTN
GRIN2B
NDUFA6
Scrambled 1
Scrambled 2
Scrambled 3
siFLI1 1
siFLI1 2
siFLI1 3

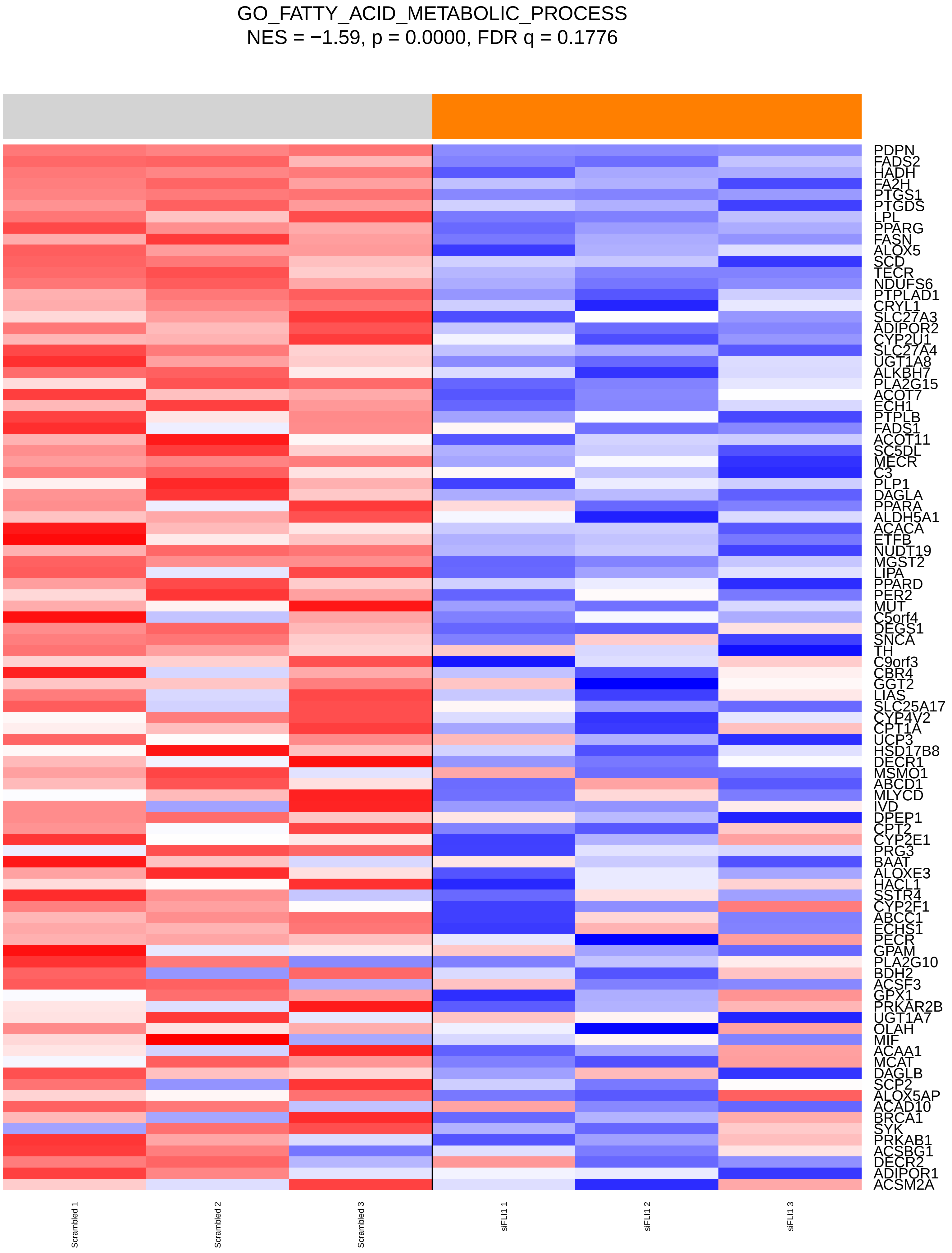
GO_FATTY_ACID_METABOLIC_PROCESS
NES = −1.59, p = 0.0000, FDR q = 0.1776
PDPN
FADS2
HADH
FA2H
PTGS1
PTGDS
LPL
PPARG
FASN
ALOX5
SCD
TECR
NDUFS6
PTPLAD1
CRYL1
SLC27A3
ADIPOR2
CYP2U1
SLC27A4
UGT1A8
ALKBH7
PLA2G15
ACOT7
ECH1
PTPLB
FADS1
ACOT11
SC5DL
MECR
C3
PLP1
DAGLA
PPARA
ALDH5A1
ACACA
ETFB
NUDT19
MGST2
LIPA
PPARD
PER2
MUT
C5orf4
DEGS1
SNCA
TH
C9orf3
CBR4
GGT2
LIAS
SLC25A17
CYP4V2
CPT1A
UCP3
HSD17B8
DECR1
MSMO1
ABCD1
MLYCD
IVD
DPEP1
CPT2
CYP2E1
PRG3
BAAT
ALOXE3
HACL1
SSTR4
CYP2F1
ABCC1
ECHS1
PECR
GPAM
PLA2G10
BDH2
ACSF3
GPX1
PRKAR2B
UGT1A7
OLAH
MIF
ACAA1
MCAT
DAGLB
SCP2
ALOX5AP
ACAD10
BRCA1
SYK
PRKAB1
ACSBG1
DECR2
ADIPOR1
ACSM2A
Scrambled 1
Scrambled 2
Scrambled 3
siFLI1 1
siFLI1 2
siFLI1 3

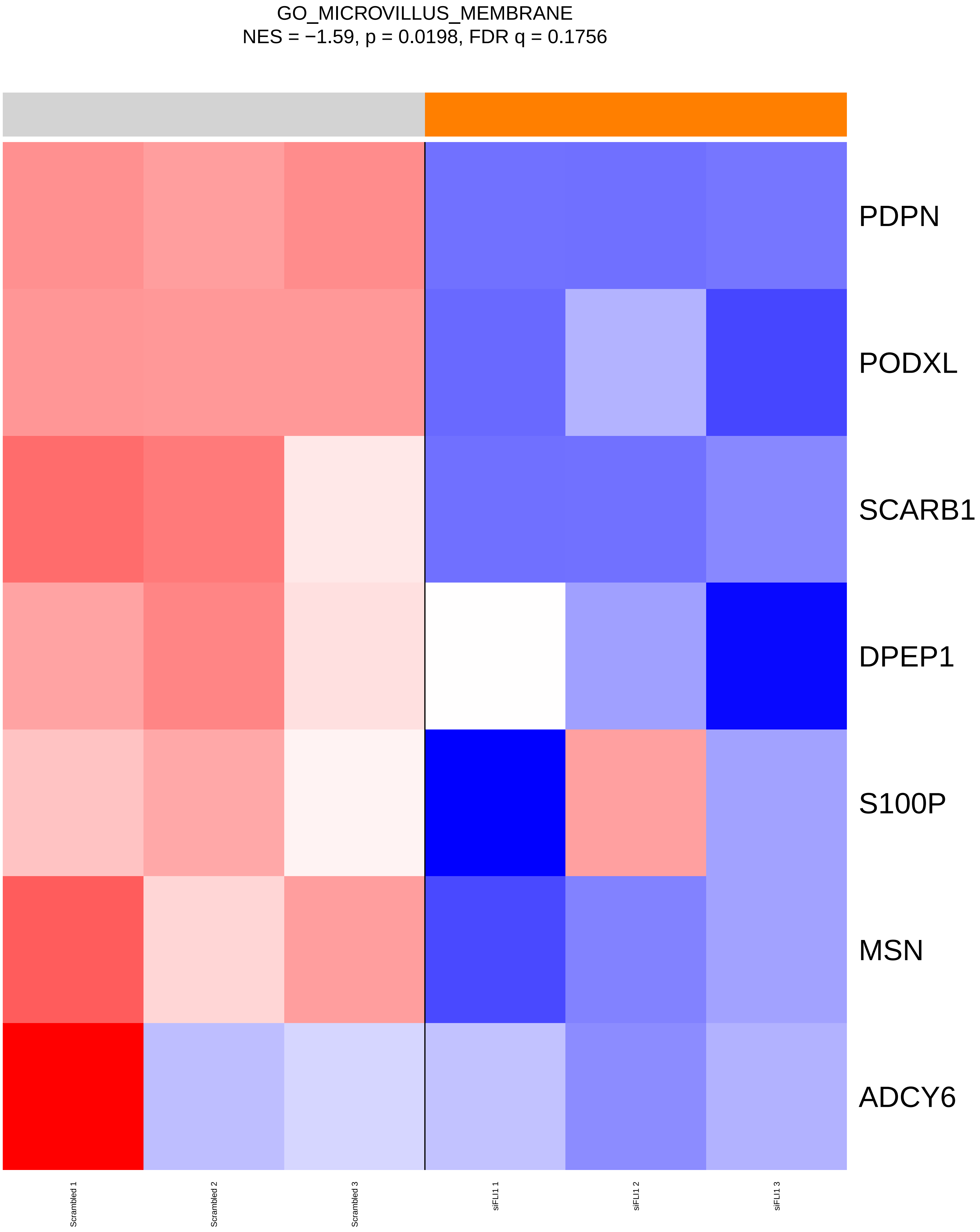
GO_MICROVILLUS_MEMBRANE
NES = −1.59, p = 0.0198, FDR q = 0.1756
PDPN
PODXL
SCARB1
DPEP1
S100P
MSN
ADCY6
Scrambled 1
Scrambled 2
Scrambled 3
siFLI1 1
siFLI1 2
siFLI1 3

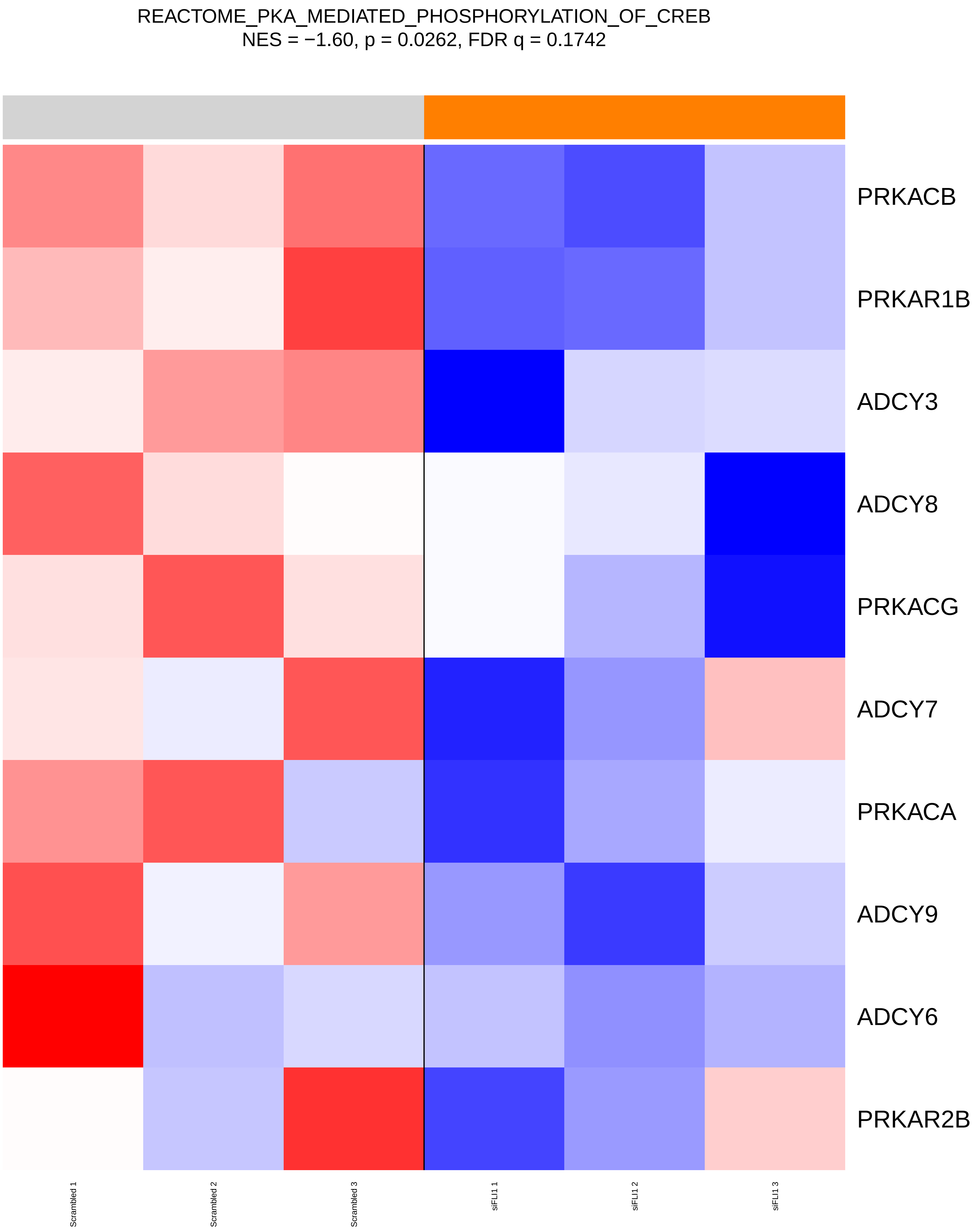
REACTOME_PKA_MEDIATED_PHOSPHORYLATION_OF_CREB
NES = −1.60, p = 0.0262, FDR q = 0.1742
PRKACB
PRKAR1B
ADCY3
ADCY8
PRKACG
ADCY7
PRKACA
ADCY9
ADCY6
PRKAR2B
Scrambled 1
Scrambled 2
Scrambled 3
siFLI1 1
siFLI1 2
siFLI1 3

GO_GOLGI_ASSOCIATED_VESICLE_MEMBRANE
NES = −1.60, p = 0.0123, FDR q = 0.1745

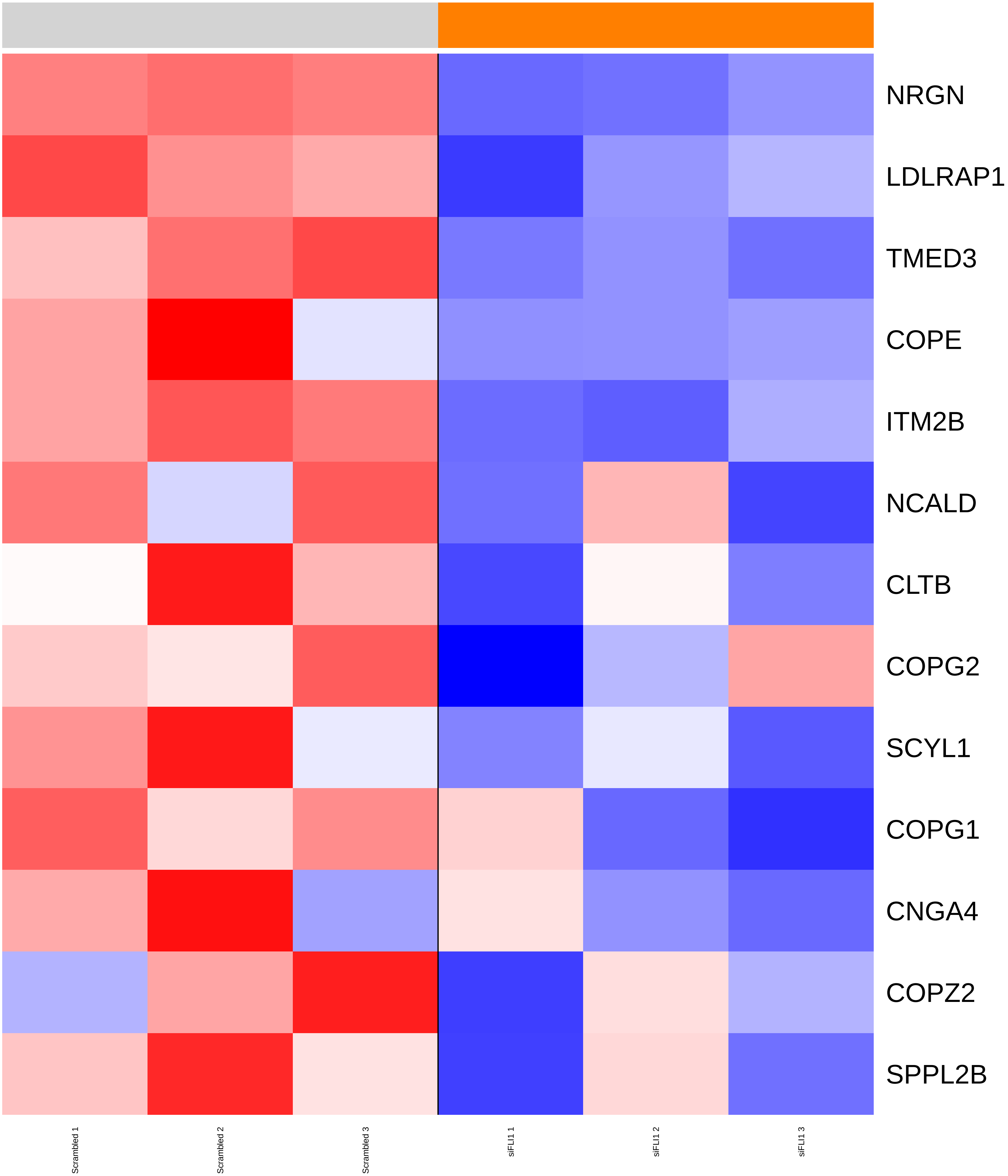

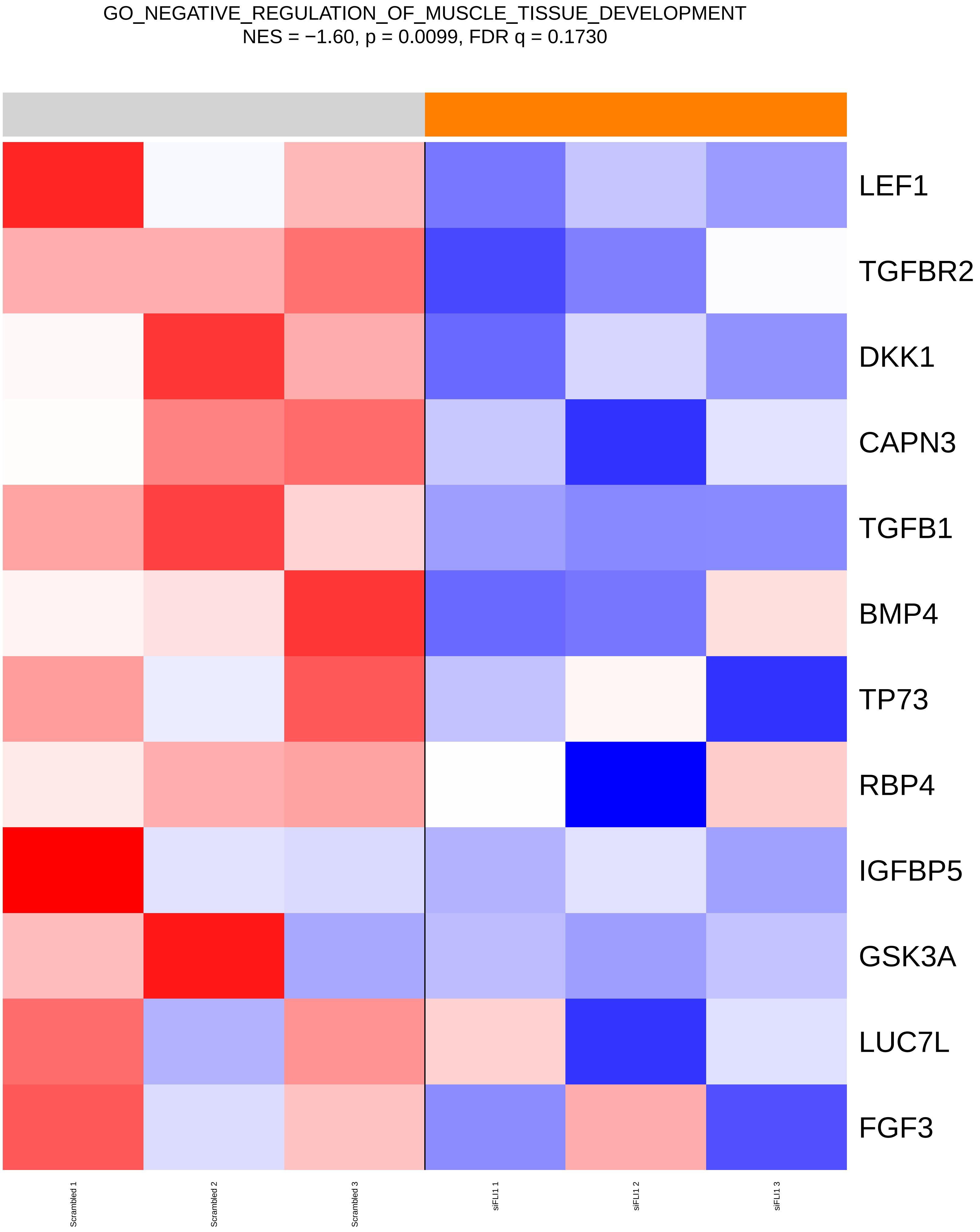
GO_NEGATIVE_REGULATION_OF_MUSCLE_TISSUE_DEVELOPMENT
NES = −1.60, p = 0.0099, FDR q = 0.1730
LEF1
TGFBR2
DKK1
CAPN3
TGFB1
BMP4
TP73
RBP4
IGFBP5
GSK3A
LUC7L
FGF3
Scrambled 1
Scrambled 2
Scrambled 3
siFLI1 1
siFLI1 2
siFLI1 3

GO_PURINE_NUCLEOSIDE_MONOPHOSPHATE_BIOSYNTHETIC_PROCESS
NES = −1.60, p = 0.0046, FDR q = 0.1728

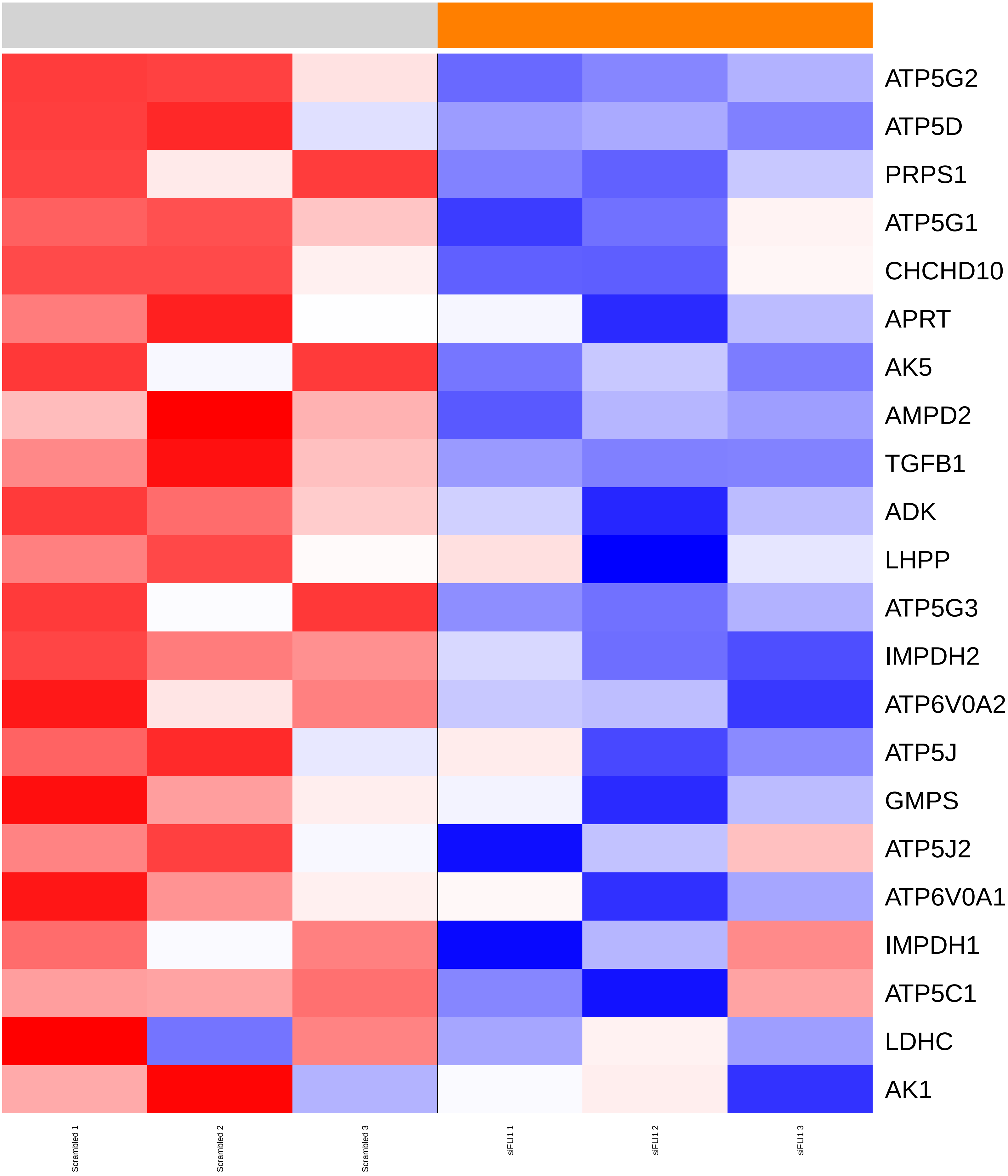

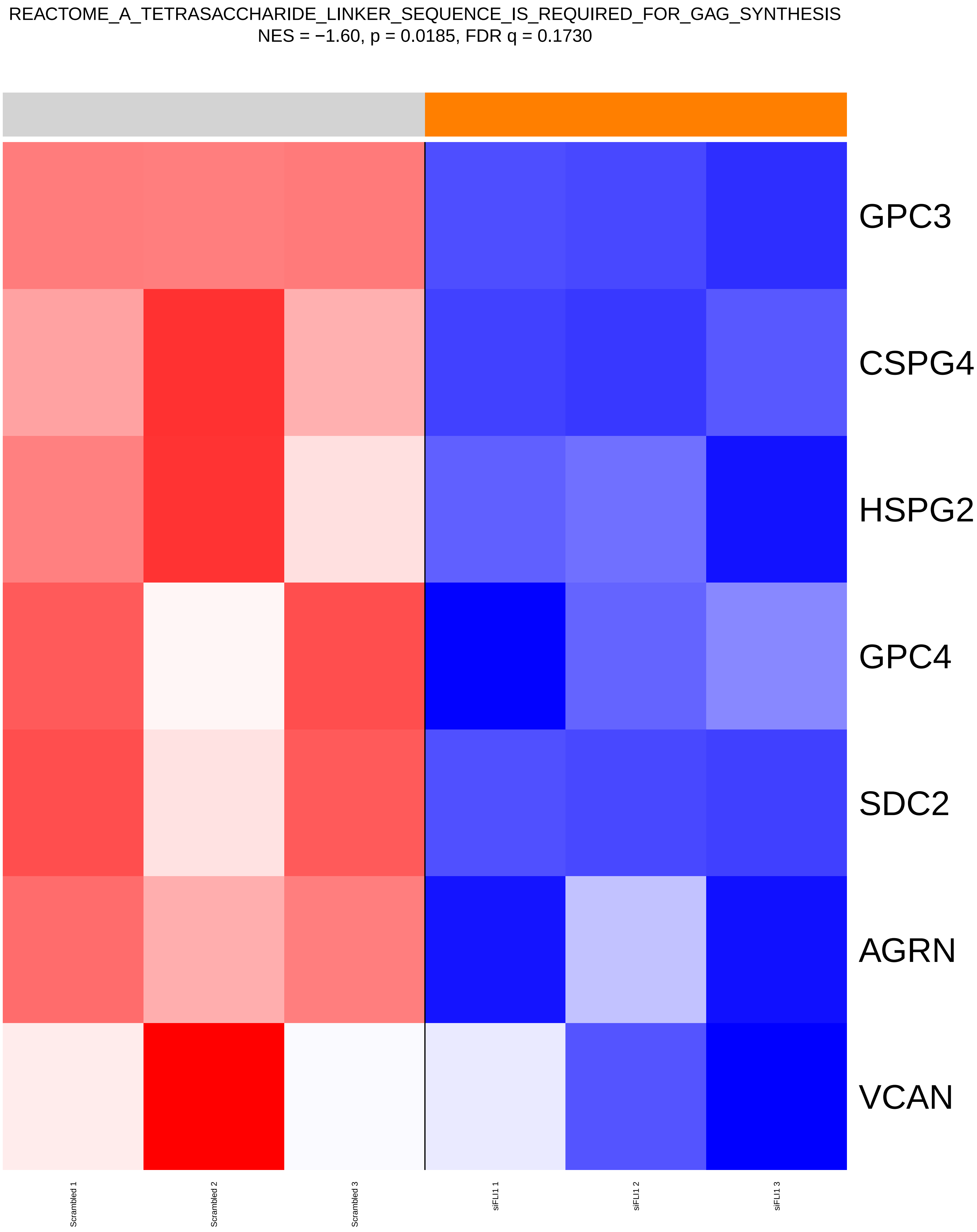
REACTOME_A_TETRASACCHARIDE_LINKER_SEQUENCE_IS_REQUIRED_FOR_GAG_SYNTHESIS
NES = −1.60, p = 0.0185, FDR q = 0.1730
GPC3
CSPG4
HSPG2
GPC4
SDC2
AGRN
VCAN
Scrambled 1
Scrambled 2
Scrambled 3
siFLI1 1
siFLI1 2
siFLI1 3

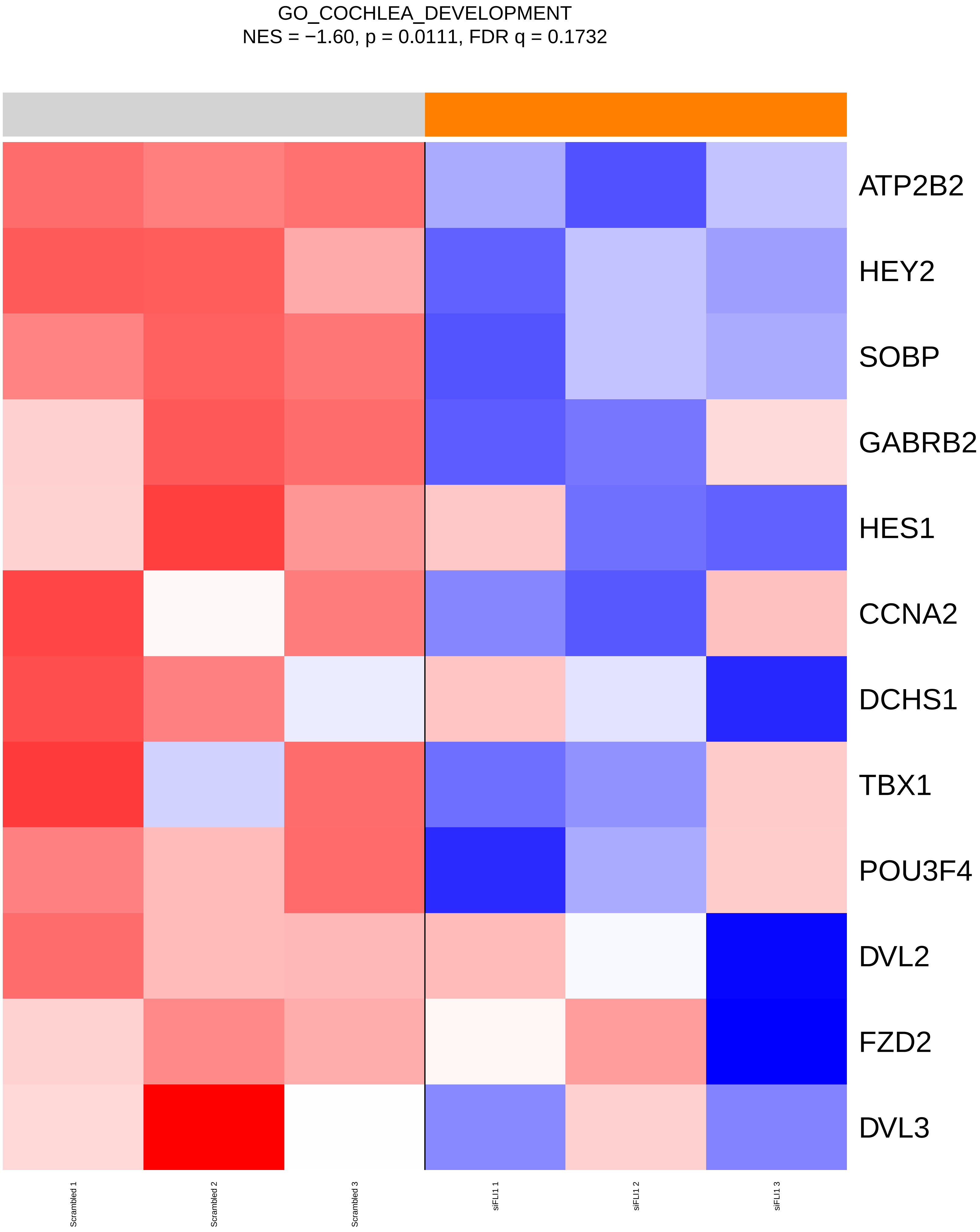
GO_COCHLEA_DEVELOPMENT
NES = −1.60, p = 0.0111, FDR q = 0.1732
ATP2B2
HEY2
SOBP
GABRB2
HES1
CCNA2
DCHS1
TBX1
POU3F4
DVL2
FZD2
DVL3
Scrambled 1
Scrambled 2
Scrambled 3
siFLI1 1
siFLI1 2
siFLI1 3

GO_REGULATION_OF_NEURONAL_SYNAPTIC_PLASTICITY
NES = −1.60, p = 0.0045, FDR q = 0.1707

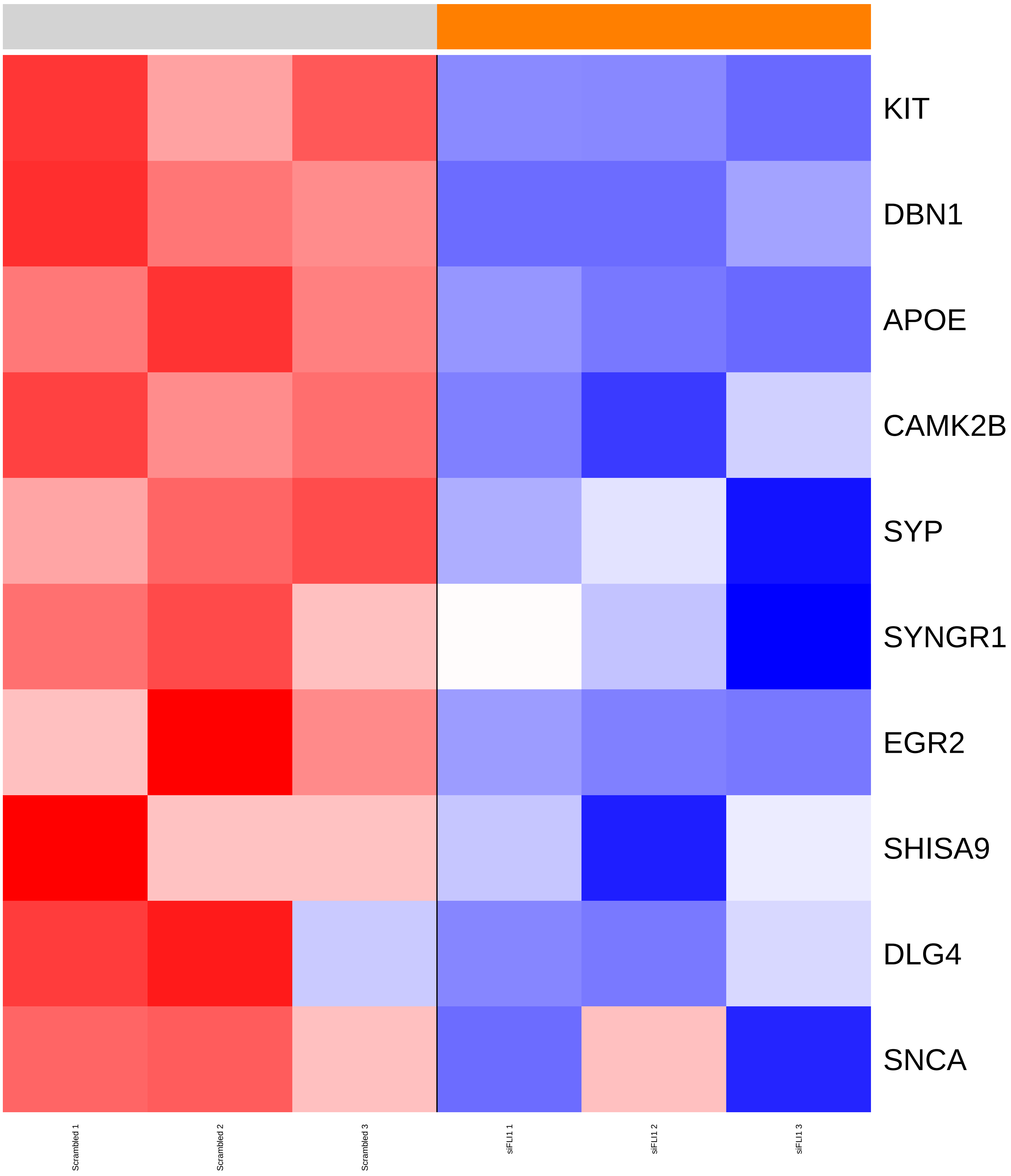

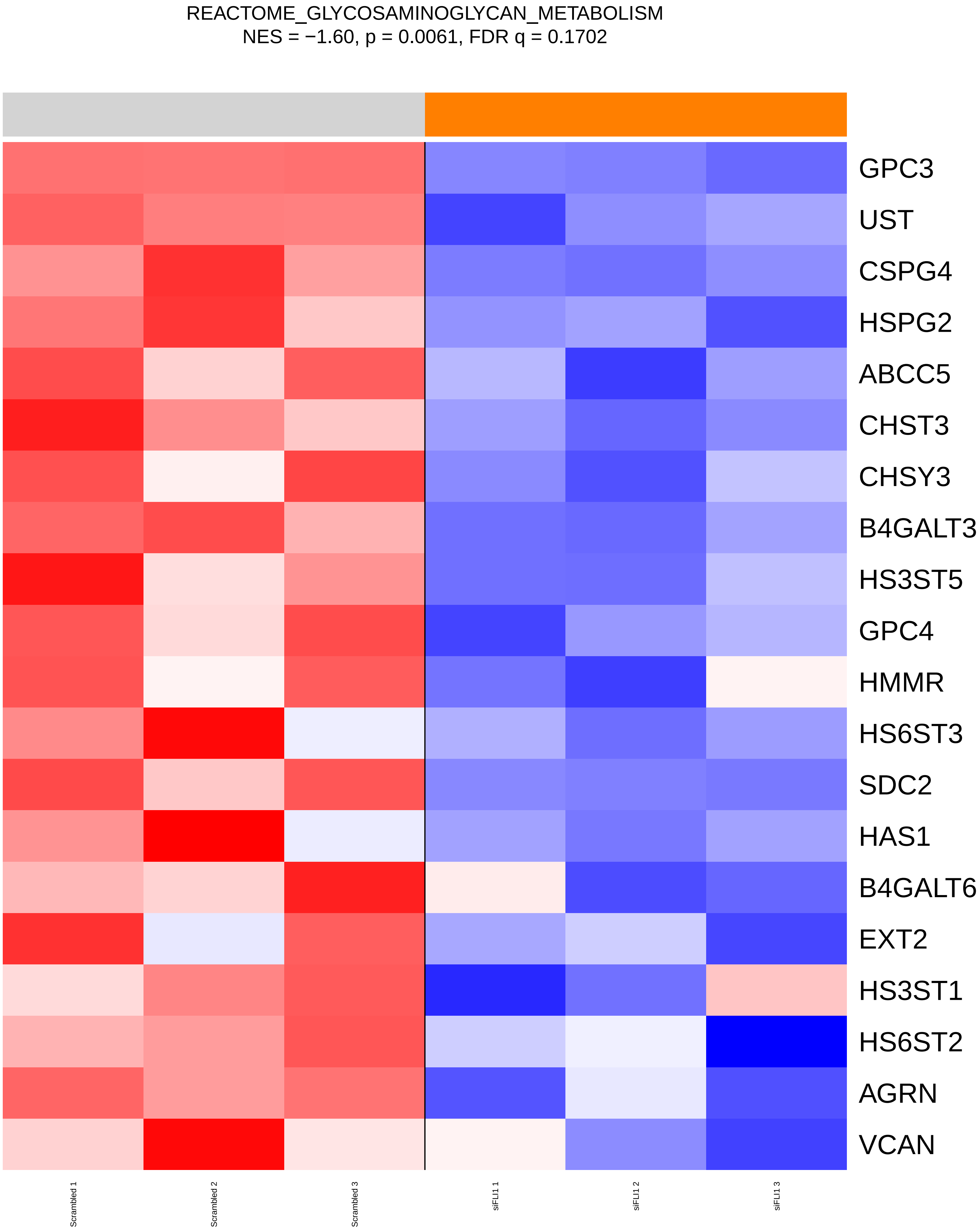
REACTOME_GLYCOSAMINOGLYCAN_METABOLISM
NES = −1.60, p = 0.0061, FDR q = 0.1702
GPC3
UST
CSPG4
HSPG2
ABCC5
CHST3
CHSY3
B4GALT3
HS3ST5
GPC4
HMMR
HS6ST3
SDC2
HAS1
B4GALT6
EXT2
HS3ST1
HS6ST2
AGRN
VCAN
Scrambled 1
Scrambled 2
Scrambled 3
siFLI1 1
siFLI1 2
siFLI1 3

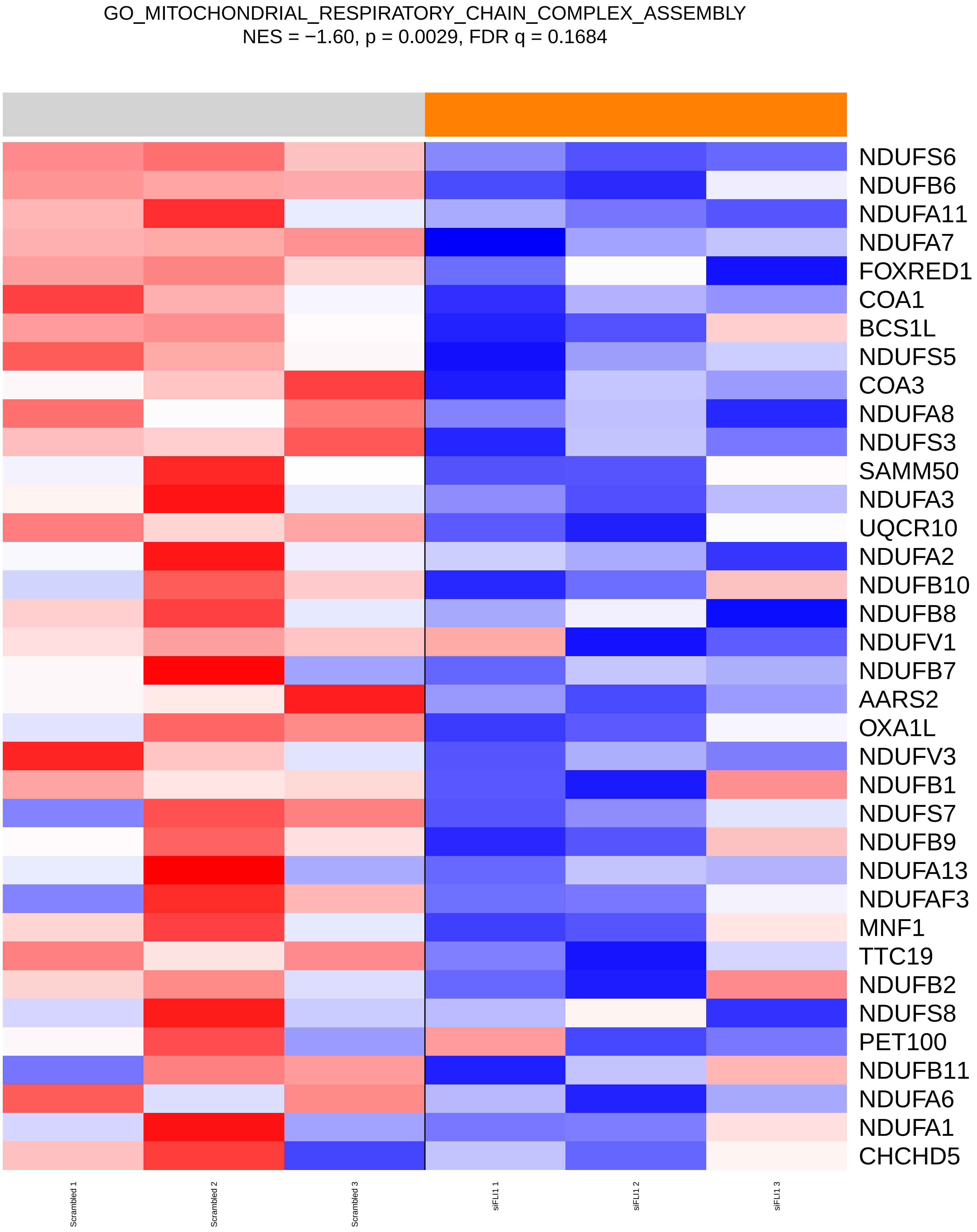
GO_MITOCHONDRIAL_RESPIRATORY_CHAIN_COMPLEX_ASSEMBLY
NES = −1.60, p = 0.0029, FDR q = 0.1684
NDUFS6
NDUFB6
NDUFA11
NDUFA7
FOXRED1
COA1
BCS1L
NDUFS5
COA3
NDUFA8
NDUFS3
SAMM50
NDUFA3
UQCR10
NDUFA2
NDUFB10
NDUFB8
NDUFV1
NDUFB7
AARS2
OXA1L
NDUFV3
NDUFB1
NDUFS7
NDUFB9
NDUFA13
NDUFAF3
MNF1
TTC19
NDUFB2
NDUFS8
PET100
NDUFB11
NDUFA6
NDUFA1
CHCHD5
Scrambled 1
Scrambled 2
Scrambled 3
siFLI1 1
siFLI1 2
siFLI1 3

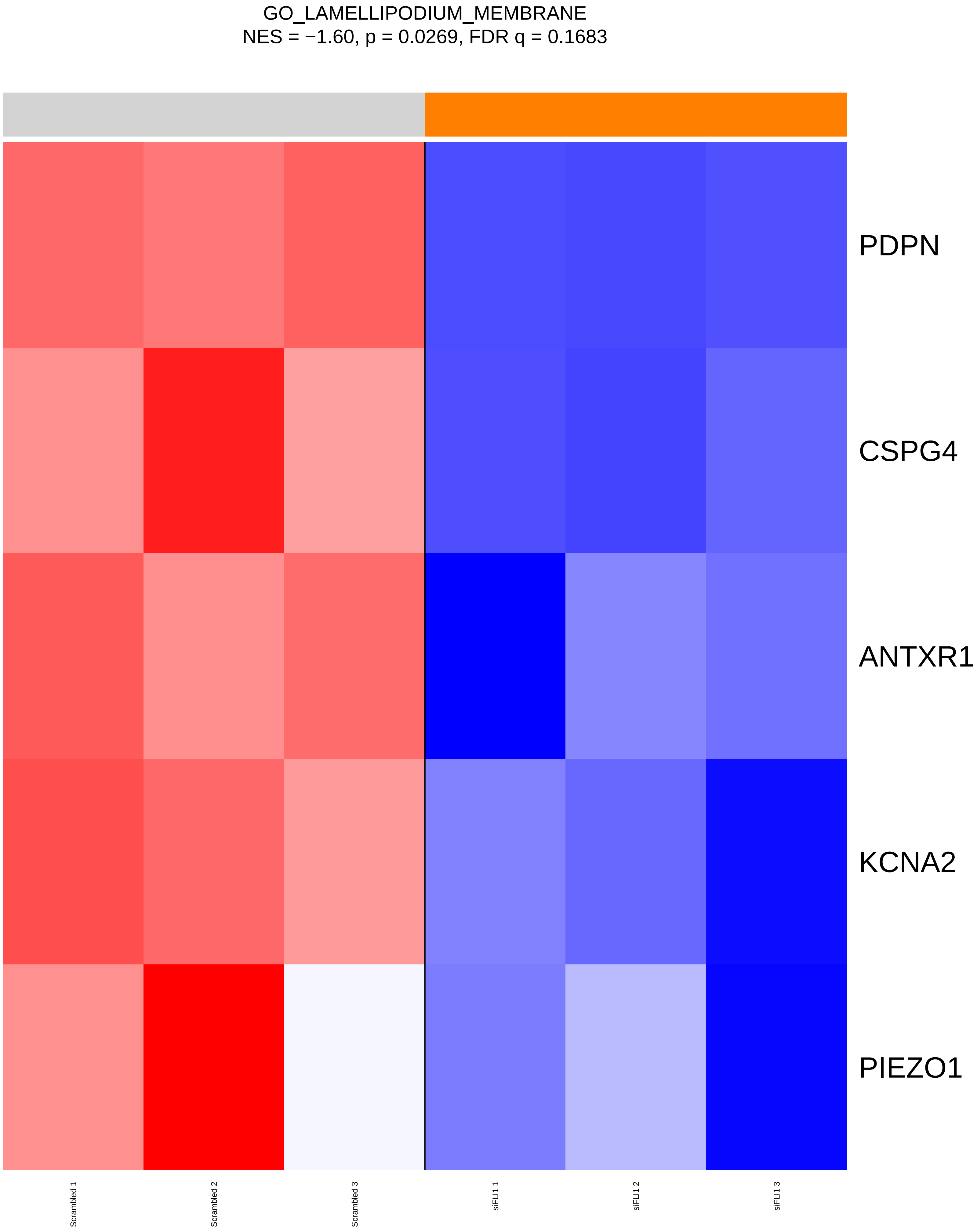
GO_LAMELLIPODIUM_MEMBRANE
NES = −1.60, p = 0.0269, FDR q = 0.1683
PDPN
CSPG4
ANTXR1
KCNA2
PIEZO1
Scrambled 1
Scrambled 2
Scrambled 3
siFLI1 1
siFLI1 2
siFLI1 3

GO_REGULATION_OF_CGMP_METABOLIC_PROCESS
NES = −1.60, p = 0.0137, FDR q = 0.1678

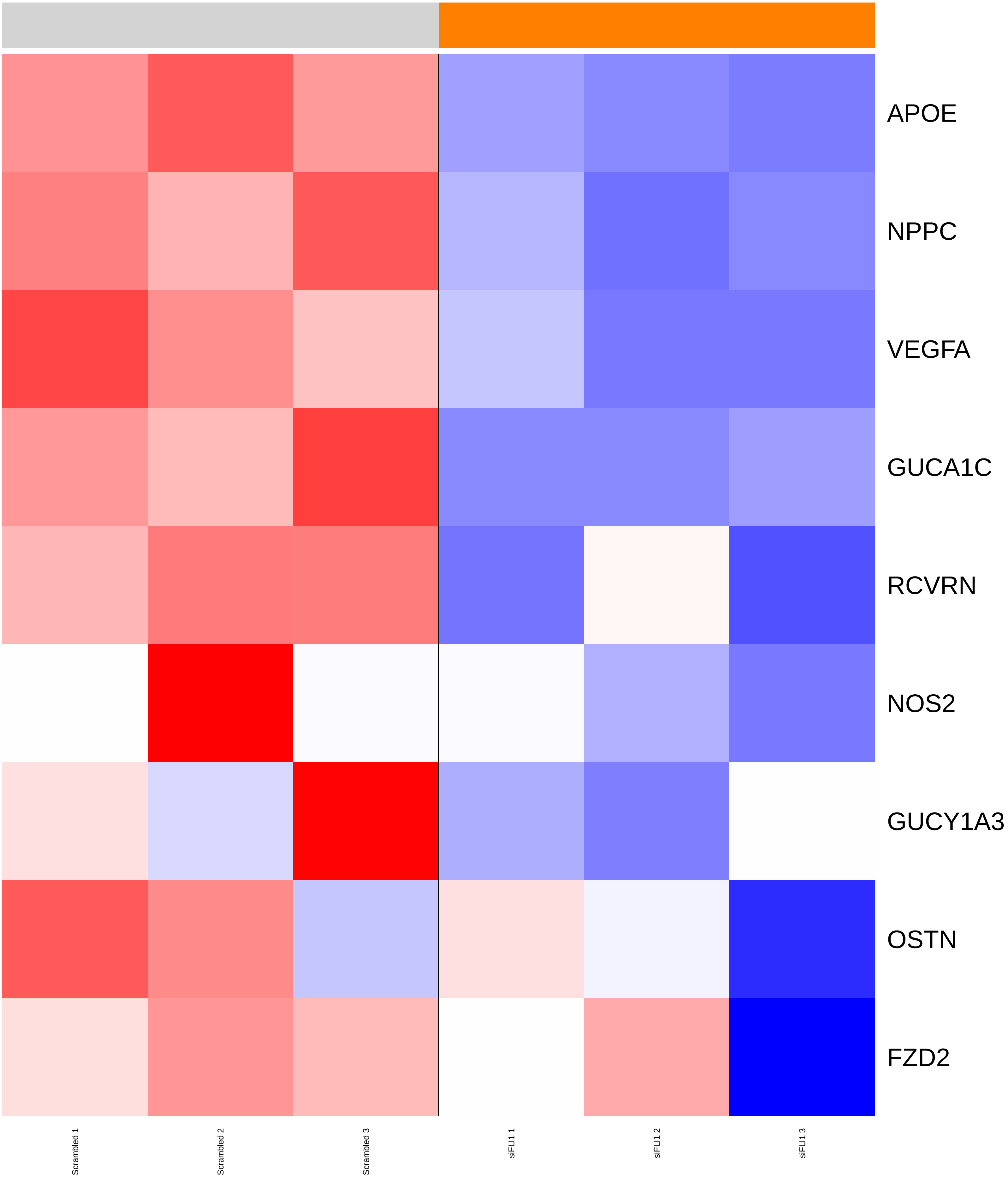

GO_ORGANIC_HYDROXY_COMPOUND_METABOLIC_PROCESS
NES = −1.61, p = 0.0000, FDR q = 0.1668

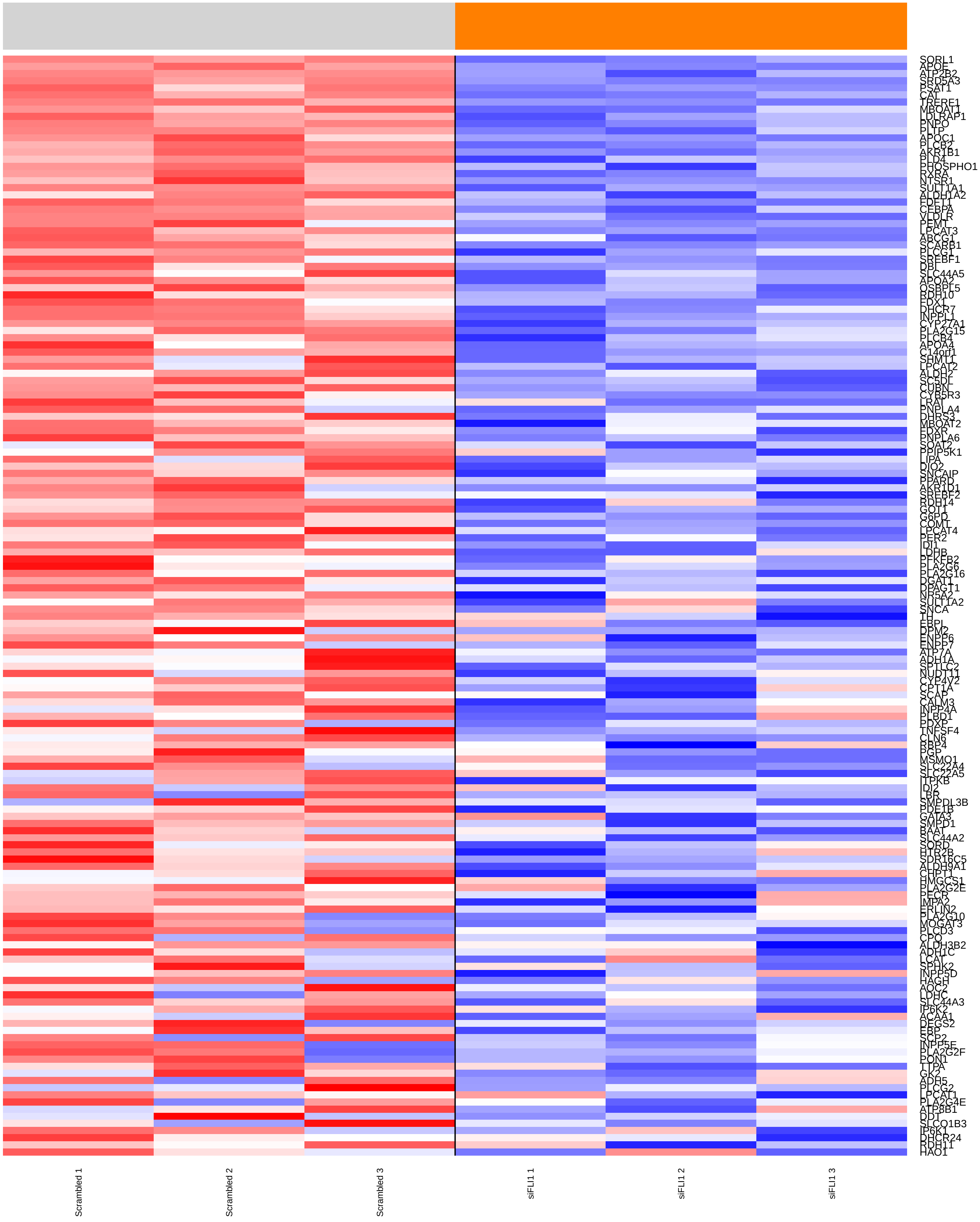

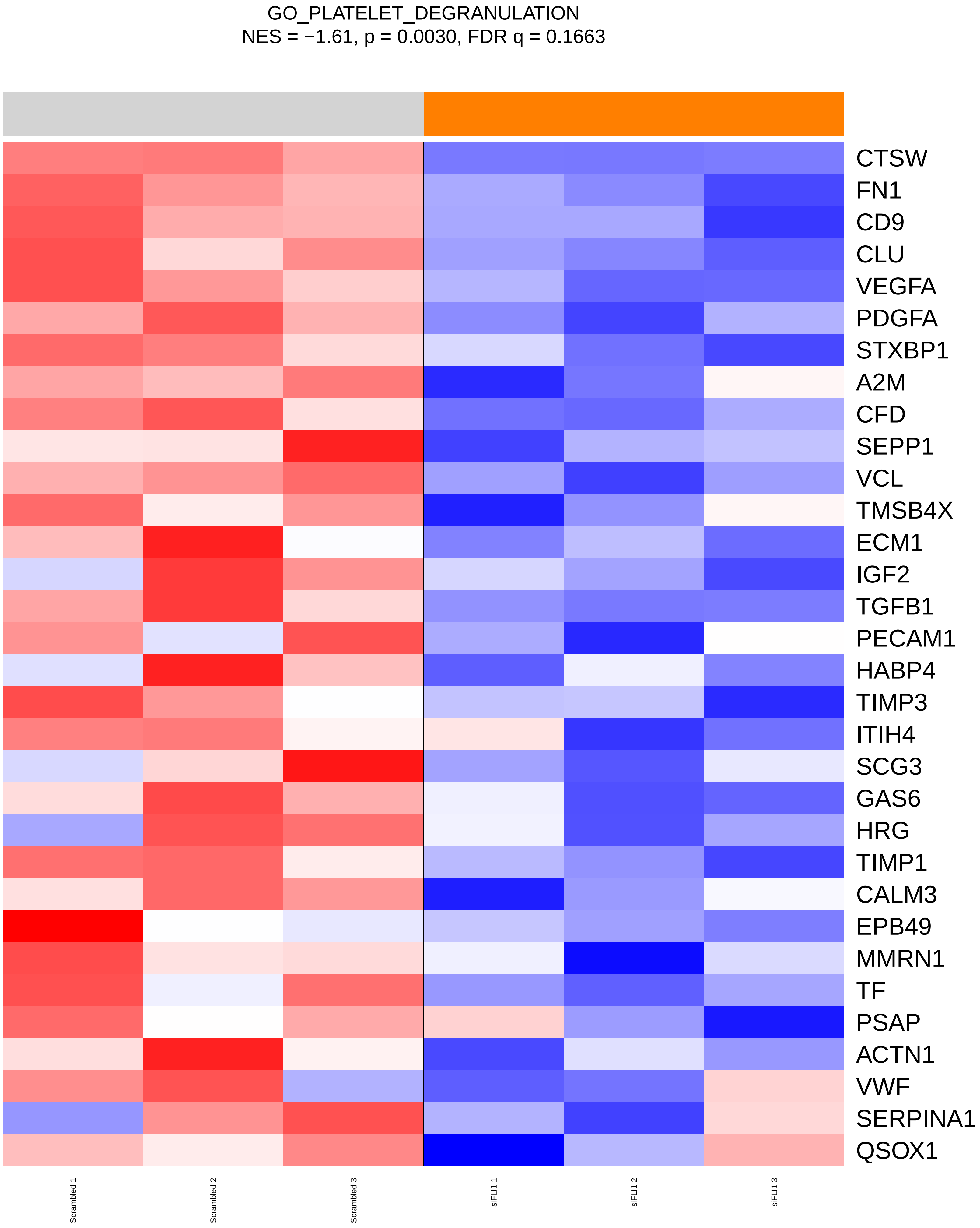
GO_PLATELET_DEGRANULATION
NES = −1.61, p = 0.0030, FDR q = 0.1663
CTSW
FN1
CD9
CLU
VEGFA
PDGFA
STXBP1
A2M
CFD
SEPP1
VCL
TMSB4X
ECM1
IGF2
TGFB1
PECAM1
HABP4
TIMP3
ITIH4
SCG3
GAS6
HRG
TIMP1
CALM3
EPB49
MMRN1
TF
PSAP
ACTN1
VWF
SERPINA1
QSOX1
Scrambled 1
Scrambled 2
Scrambled 3
siFLI1 1
siFLI1 2
siFLI1 3

GO_POSITIVE_REGULATION_OF_STEROL_TRANSPORT
NES = −1.61, p = 0.0151, FDR q = 0.1624

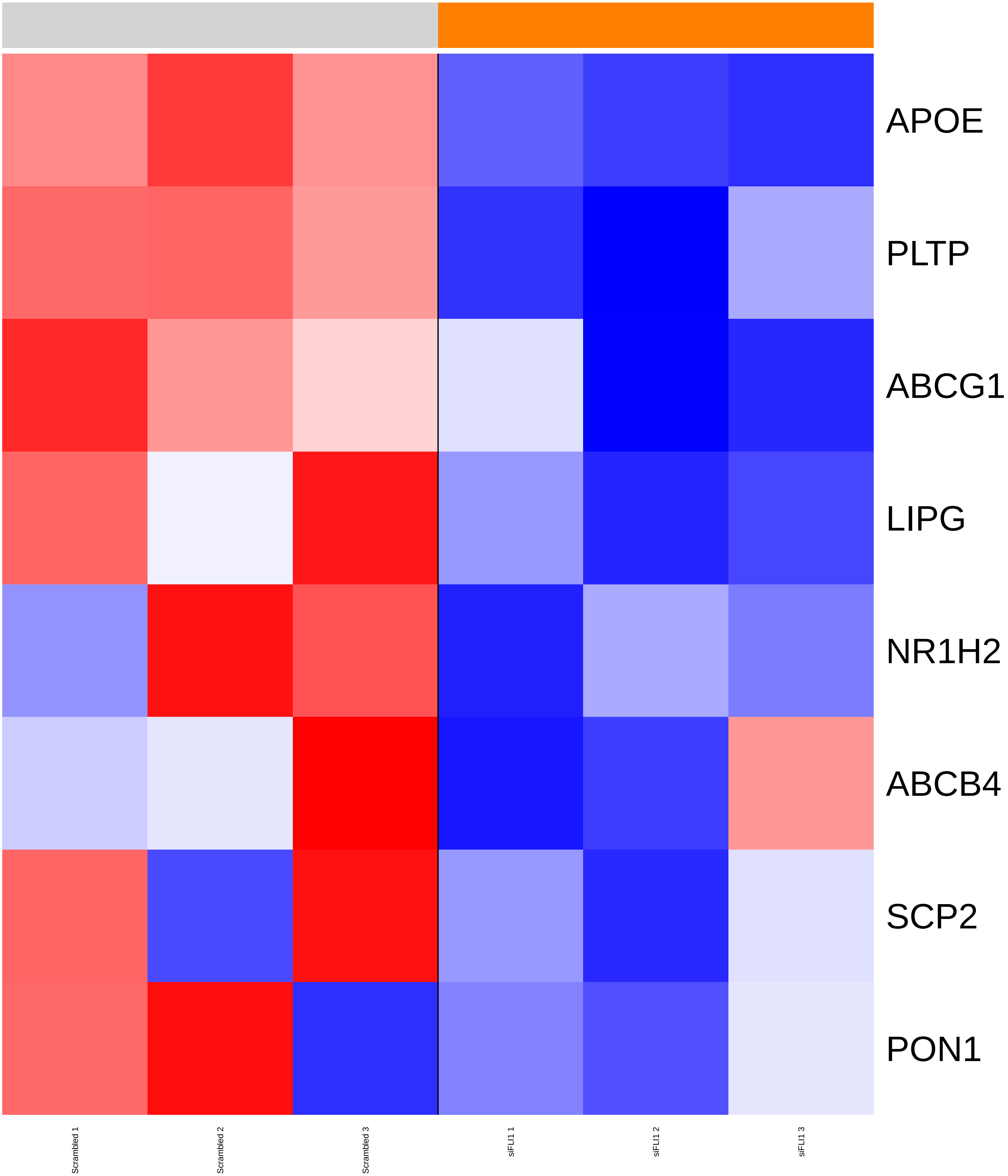

RGTTAMWNATT_HNF1_01
NES = −1.61, p = 0.0029, FDR q = 0.1616

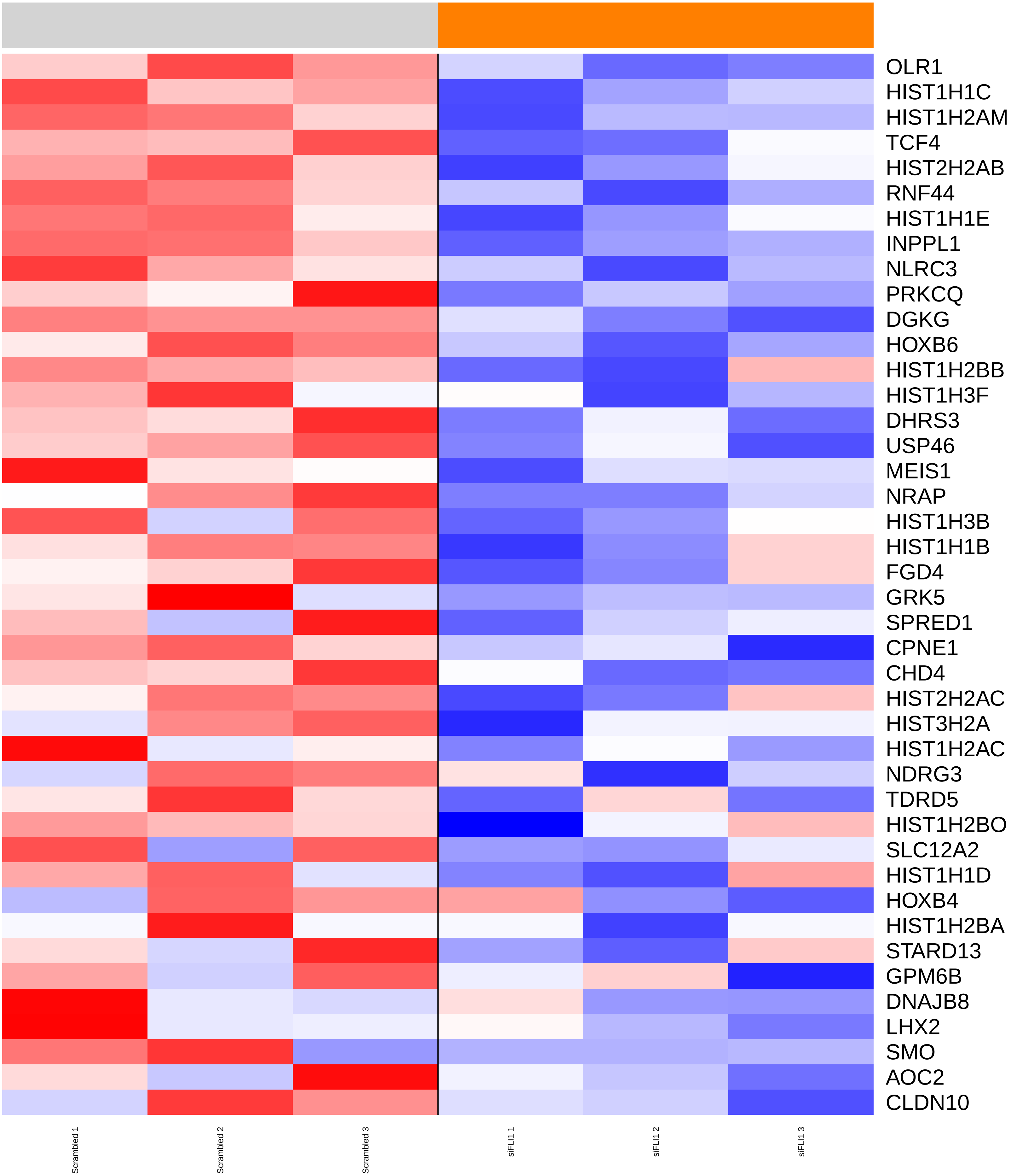

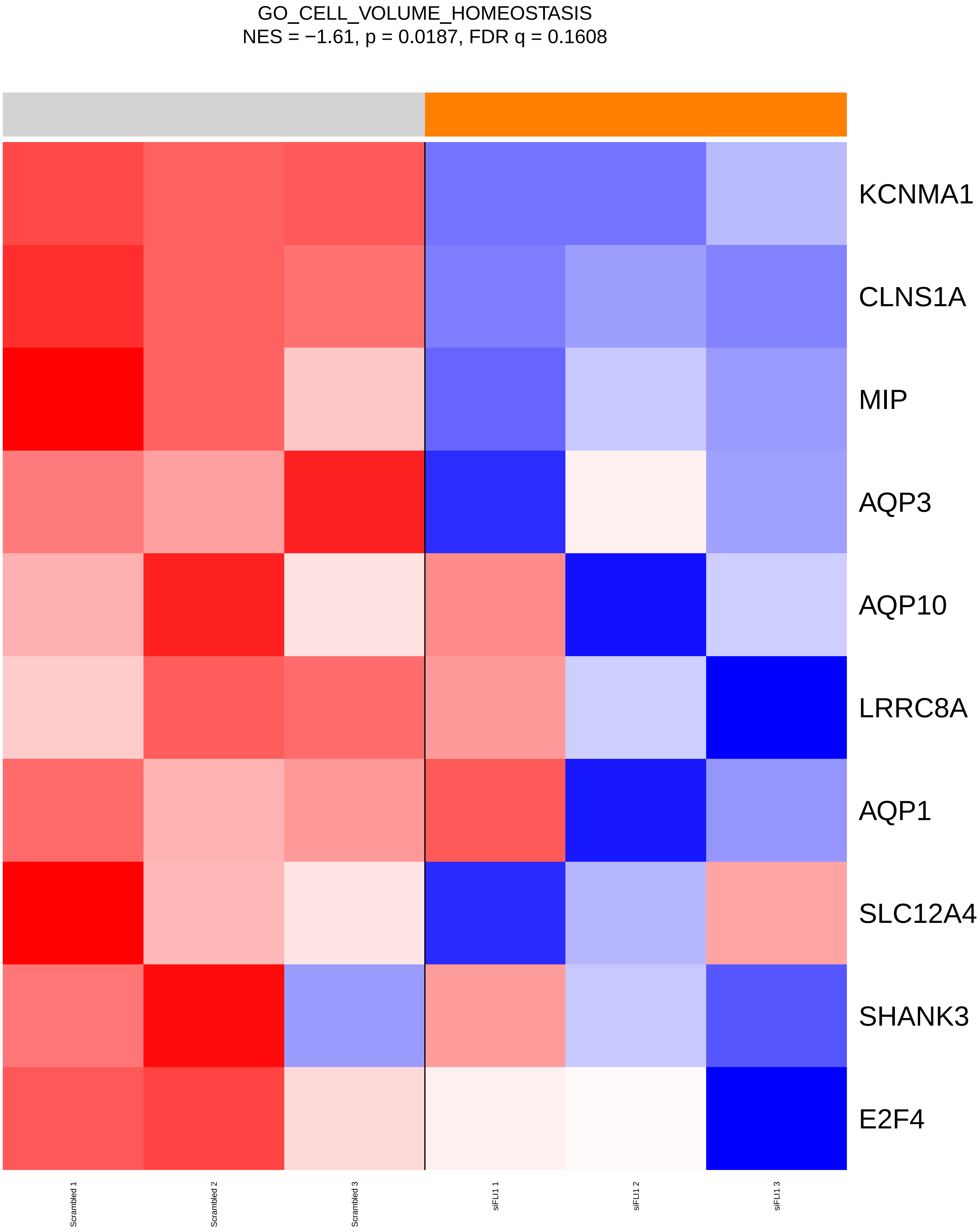
GO_CELL_VOLUME_HOMEOSTASIS
NES = −1.61, p = 0.0187, FDR q = 0.1608
KCNMA1
CLNS1A
MIP
AQP3
AQP10
LRRC8A
AQP1
SLC12A4
SHANK3
E2F4
Scrambled 1
Scrambled 2
Scrambled 3
siFLI1 1
siFLI1 2
siFLI1 3

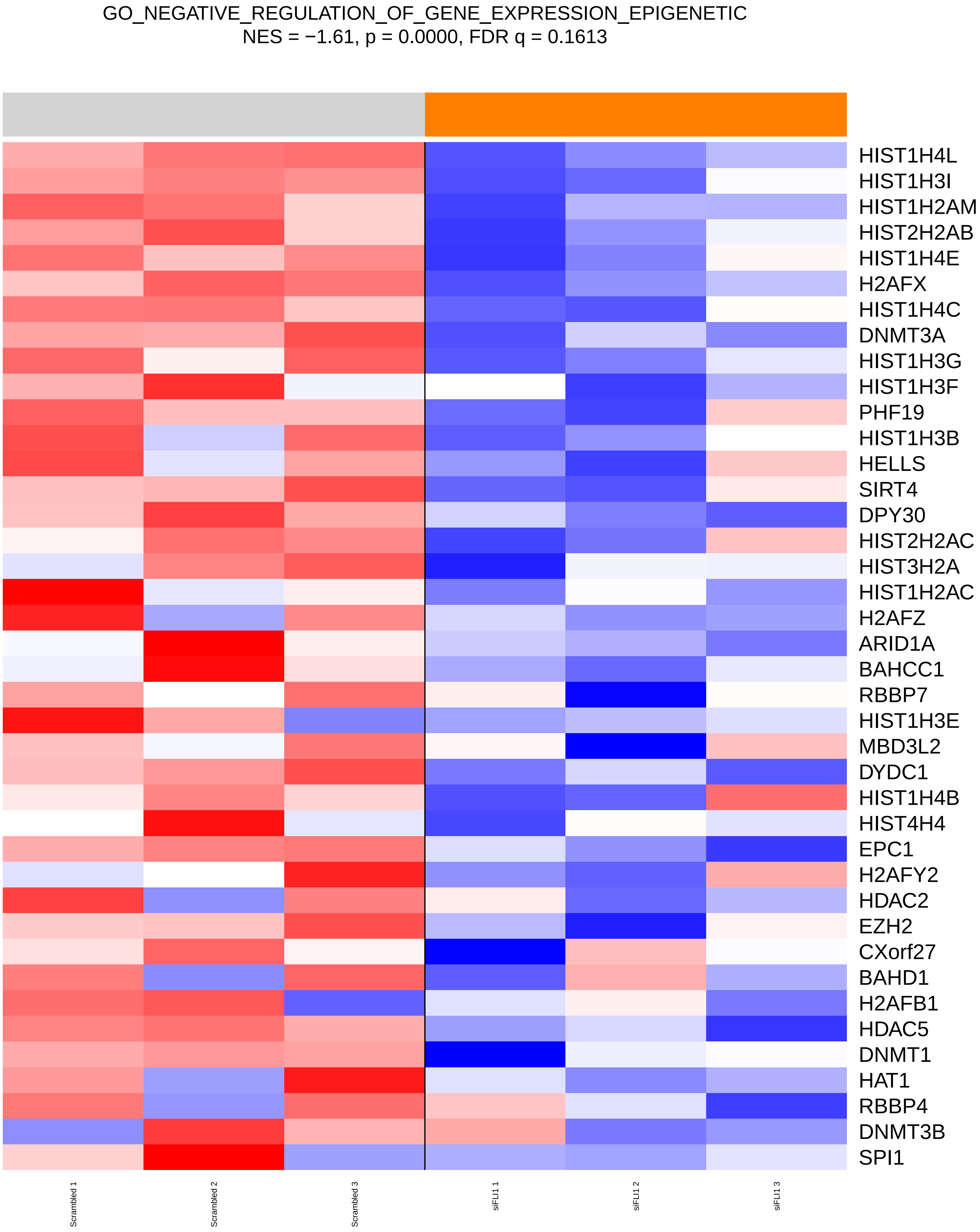

GO_NEGATIVE_REGULATION_OF_GENE_EXPRESSION_EPIGENETIC
NES = −1.61, p = 0.0000, FDR q = 0.1613
HIST1H4L
HIST1H3I
HIST1H2AM
HIST2H2AB
HIST1H4E
H2AFX
HIST1H4C
DNMT3A
HIST1H3G
HIST1H3F
PHF19
HIST1H3B
HELLS
SIRT4
DPY30
HIST2H2AC
HIST3H2A
HIST1H2AC
H2AFZ
ARID1A
BAHCC1
RBBP7
HIST1H3E
MBD3L2
DYDC1
HIST1H4B
HIST4H4
EPC1
H2AFY2
HDAC2
EZH2
CXorf27
BAHD1
H2AFB1
HDAC5
DNMT1
HAT1
RBBP4
DNMT3B
SPI1
Scrambled 1
Scrambled 2
Scrambled 3
siFLI1 1
siFLI1 2
siFLI1 3

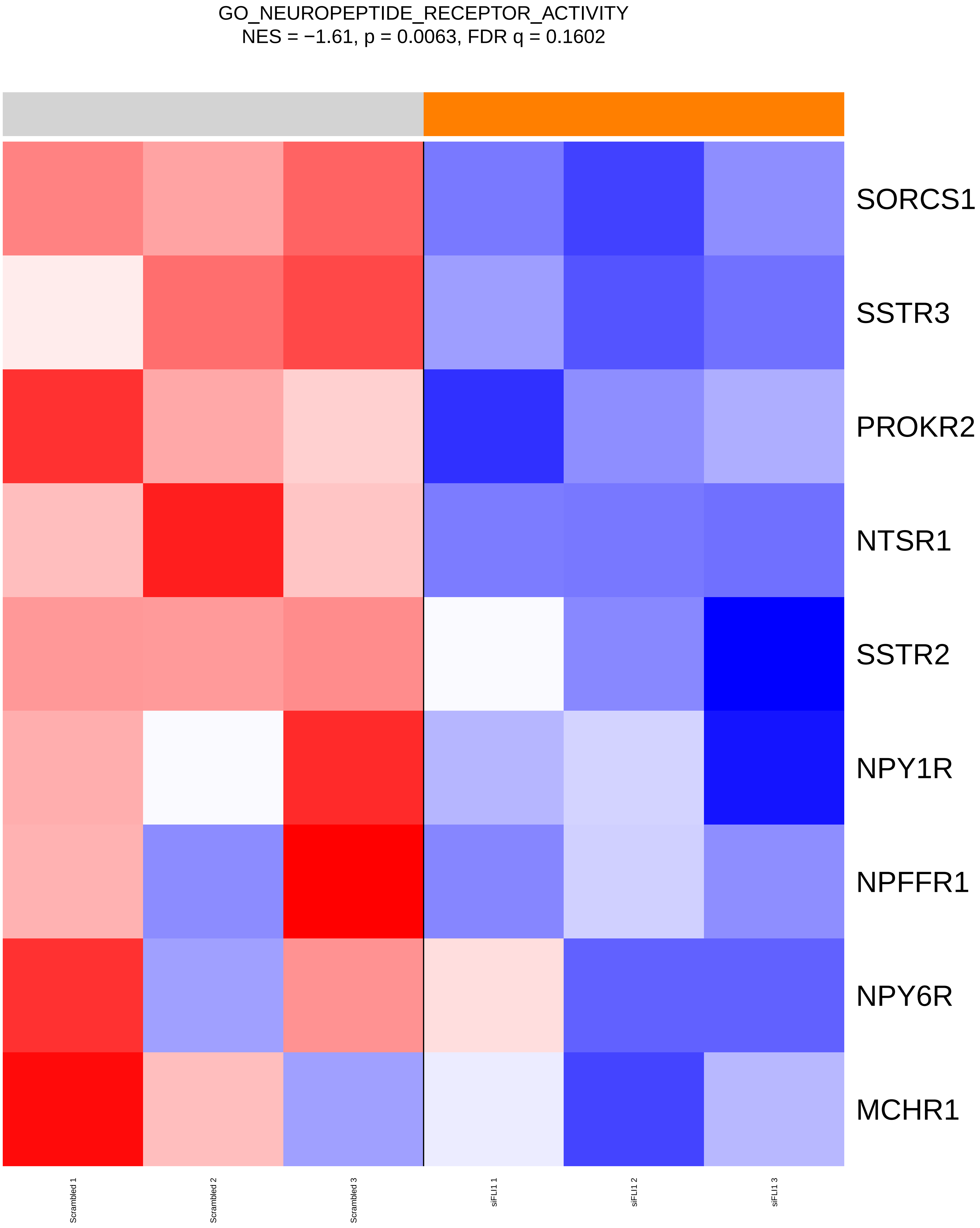
GO_NEUROPEPTIDE_RECEPTOR_ACTIVITY
NES = −1.61, p = 0.0063, FDR q = 0.1602
SORCS1
SSTR3
PROKR2
NTSR1
SSTR2
NPY1R
NPFFR1
NPY6R
MCHR1
Scrambled 1
Scrambled 2
Scrambled 3
siFLI1 1
siFLI1 2
siFLI1 3

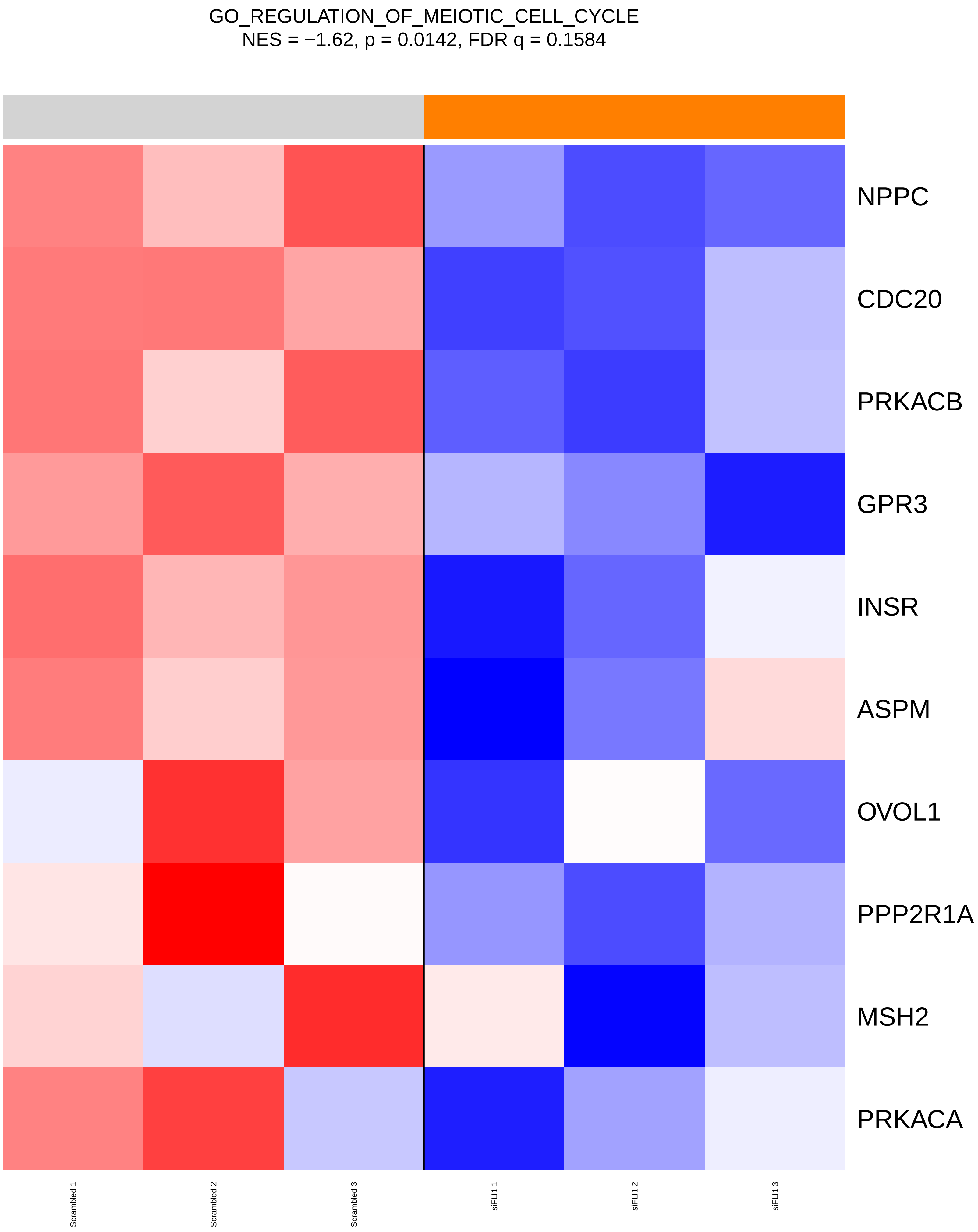
GO_REGULATION_OF_MEIOTIC_CELL_CYCLE
NES = −1.62, p = 0.0142, FDR q = 0.1584
NPPC
CDC20
PRKACB
GPR3
INSR
ASPM
OVOL1
PPP2R1A
MSH2
PRKACA
Scrambled 1
Scrambled 2
Scrambled 3
siFLI1 1
siFLI1 2
siFLI1 3

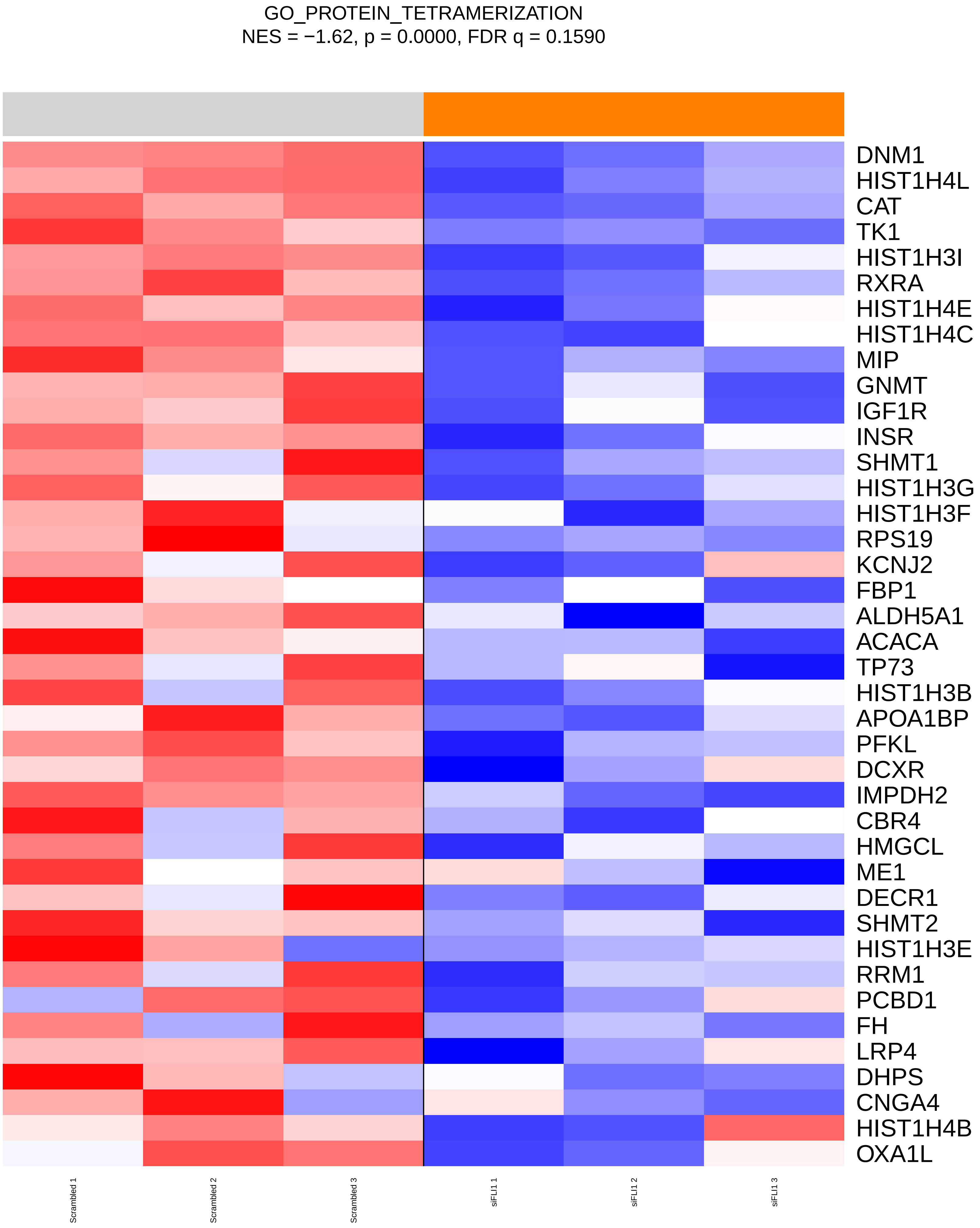
GO_PROTEIN_TETRAMERIZATION
NES = −1.62, p = 0.0000, FDR q = 0.1590
DNM1
HIST1H4L
CAT
TK1
HIST1H3I
RXRA
HIST1H4E
HIST1H4C
MIP
GNMT
IGF1R
INSR
SHMT1
HIST1H3G
HIST1H3F
RPS19
KCNJ2
FBP1
ALDH5A1
ACACA
TP73
HIST1H3B
APOA1BP
PFKL
DCXR
IMPDH2
CBR4
HMGCL
ME1
DECR1
SHMT2
HIST1H3E
RRM1
PCBD1
FH
LRP4
DHPS
CNGA4
HIST1H4B
OXA1L
Scrambled 1
Scrambled 2
Scrambled 3
siFLI1 1
siFLI1 2
siFLI1 3

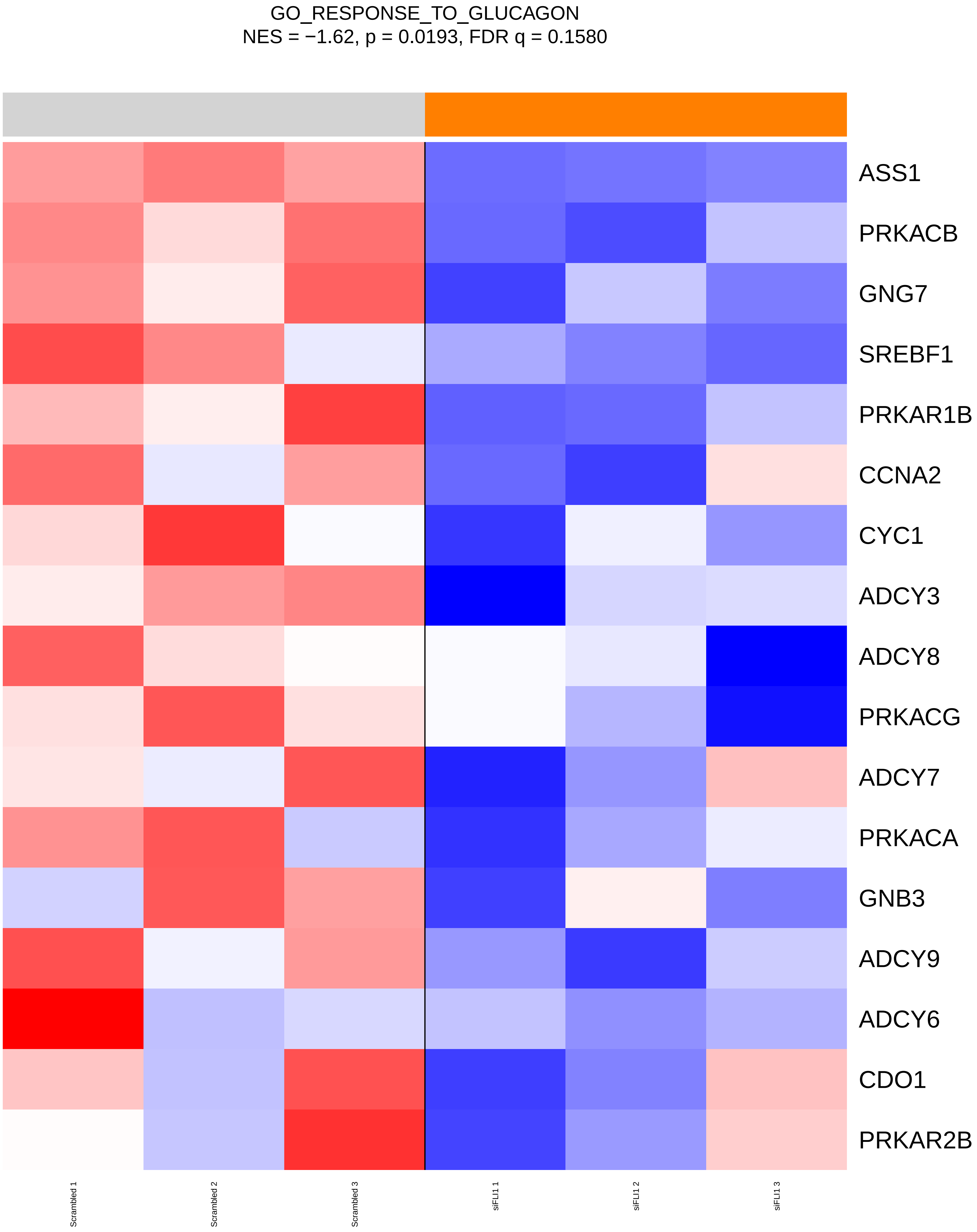
GO_RESPONSE_TO_GLUCAGON
NES = −1.62, p = 0.0193, FDR q = 0.1580
ASS1
PRKACB
GNG7
SREBF1
PRKAR1B
CCNA2
CYC1
ADCY3
ADCY8
PRKACG
ADCY7
PRKACA
GNB3
ADCY9
ADCY6
CDO1
PRKAR2B
Scrambled 1
Scrambled 2
Scrambled 3
siFLI1 1
siFLI1 2
siFLI1 3

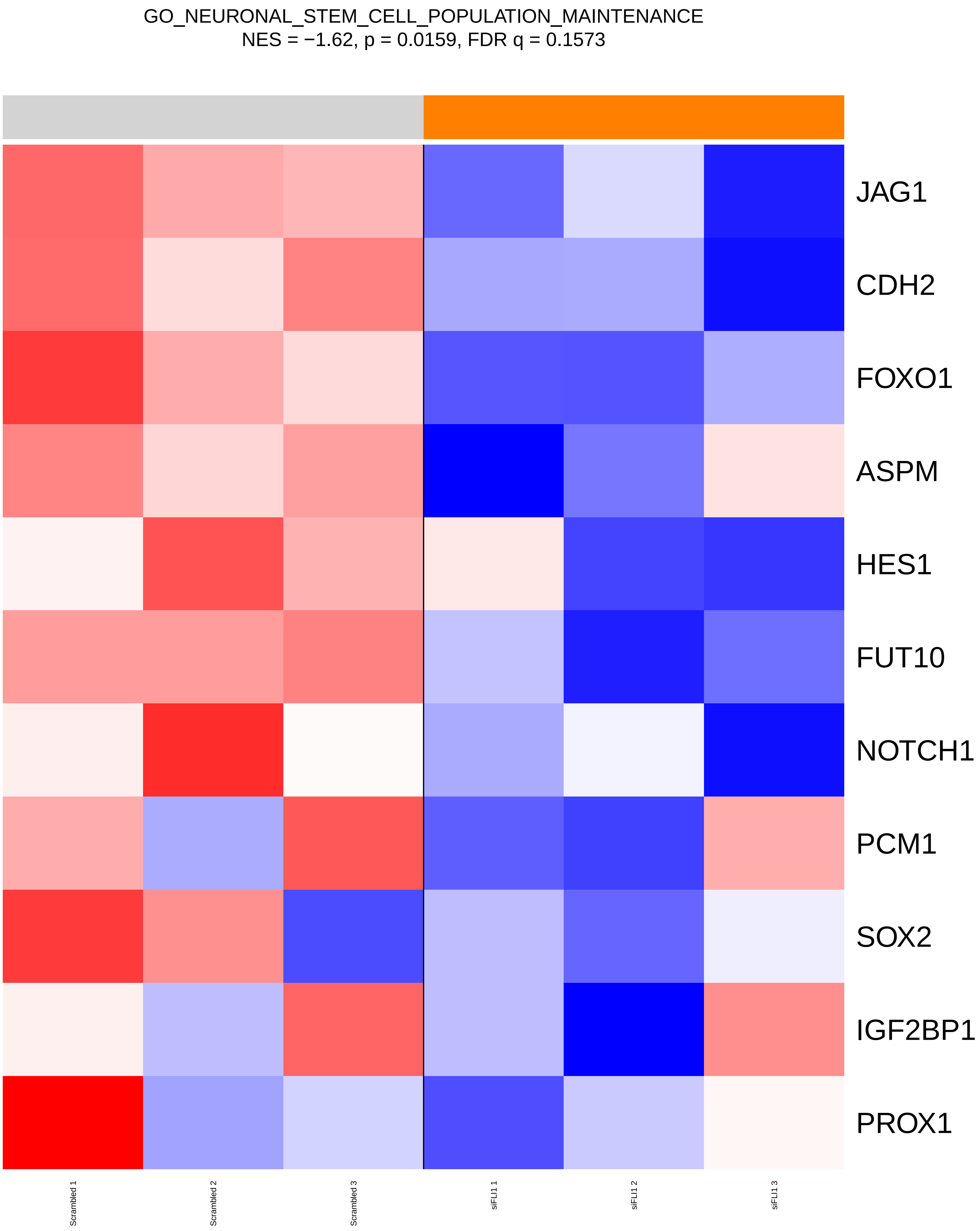
GO_NEURONAL_STEM_CELL_POPULATION_MAINTENANCE
NES = −1.62, p = 0.0159, FDR q = 0.1573
JAG1
CDH2
FOXO1
ASPM
HES1
FUT10
NOTCH1
PCM1
SOX2
IGF2BP1
PROX1
Scrambled 1
Scrambled 2
Scrambled 3
siFLI1 1
siFLI1 2
siFLI1 3

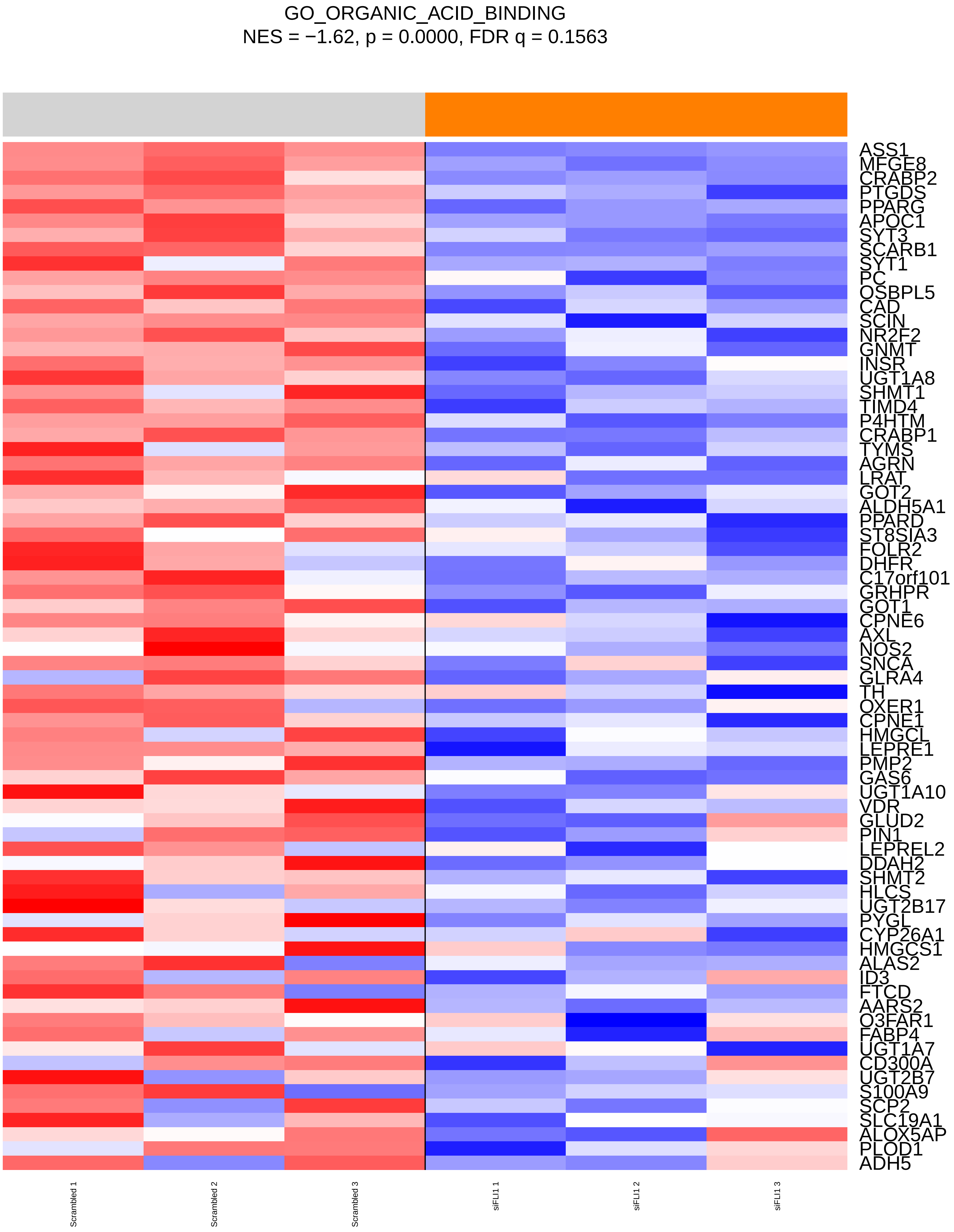
GO_ORGANIC_ACID_BINDING
NES = −1.62, p = 0.0000, FDR q = 0.1563
ASS1
MFGE8
CRABP2
PTGDS
PPARG
APOC1
SYT3
SCARB1
SYT1
PC
OSBPL5
CAD
SCIN
NR2F2
GNMT
INSR
UGT1A8
SHMT1
TIMD4
P4HTM
CRABP1
TYMS
AGRN
LRAT
GOT2
ALDH5A1
PPARD
ST8SIA3
FOLR2
DHFR
C17orf101
GRHPR
GOT1
CPNE6
AXL
NOS2
SNCA
GLRA4
TH
OXER1
CPNE1
HMGCL
LEPRE1
PMP2
GAS6
UGT1A10
VDR
GLUD2
PIN1
LEPREL2
DDAH2
SHMT2
HLCS
UGT2B17
PYGL
CYP26A1
HMGCS1
ALAS2
ID3
FTCD
AARS2
O3FAR1
FABP4
UGT1A7
CD300A
UGT2B7
S100A9
SCP2
SLC19A1
ALOX5AP
PLOD1
ADH5
Scrambled 1
Scrambled 2
Scrambled 3
siFLI1 1
siFLI1 2
siFLI1 3

GO_DNA_RECOMBINATION
NES = −1.62, p = 0.0013, FDR q = 0.1559

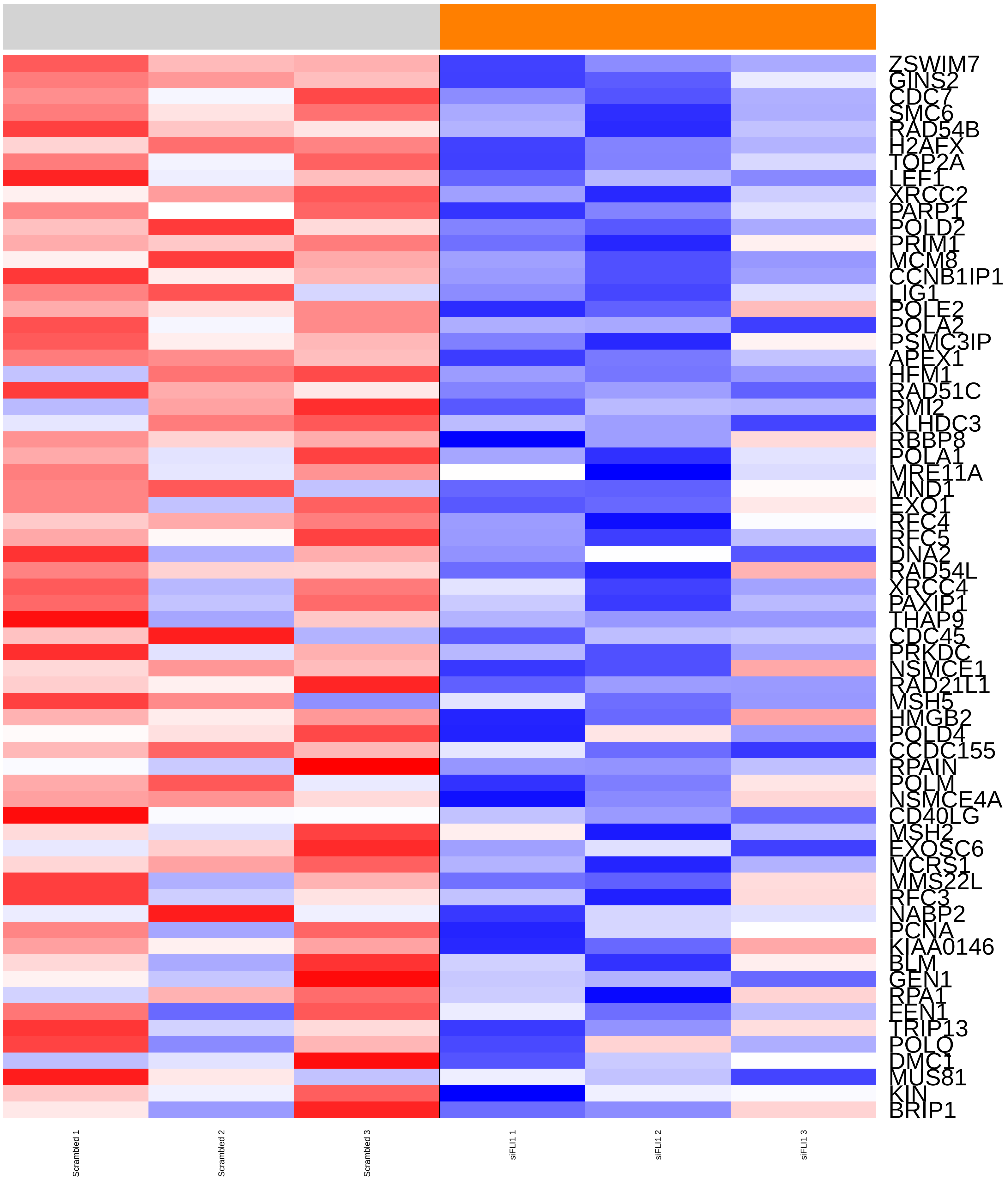

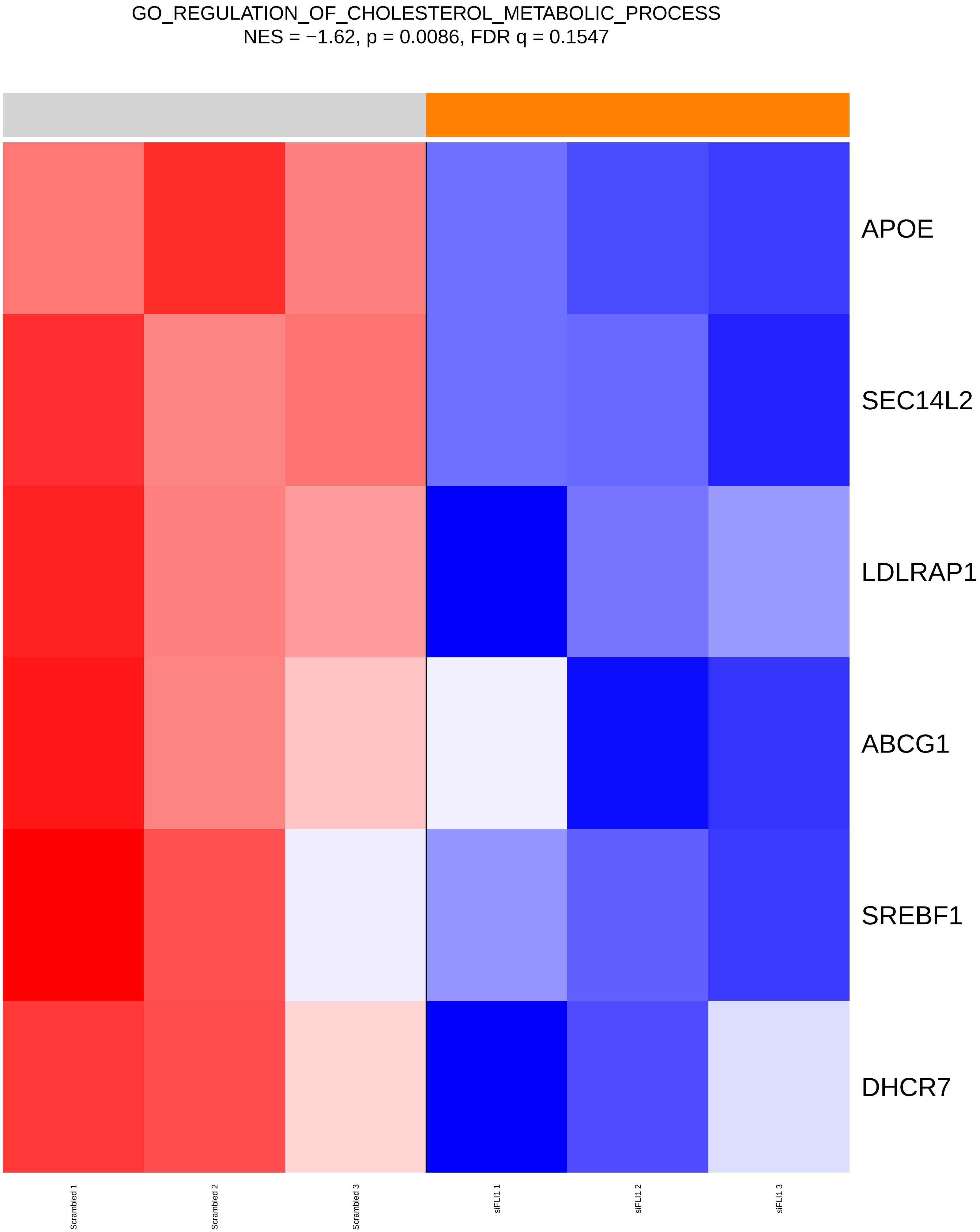

GO_REGULATION_OF_CHOLESTEROL_METABOLIC_PROCESS
NES = −1.62, p = 0.0086, FDR q = 0.1547
APOE
SEC14L2
LDLRAP1
ABCG1
SREBF1
DHCR7
Scrambled 1
Scrambled 2
Scrambled 3
siFLI1 1
siFLI1 2
siFLI1 3

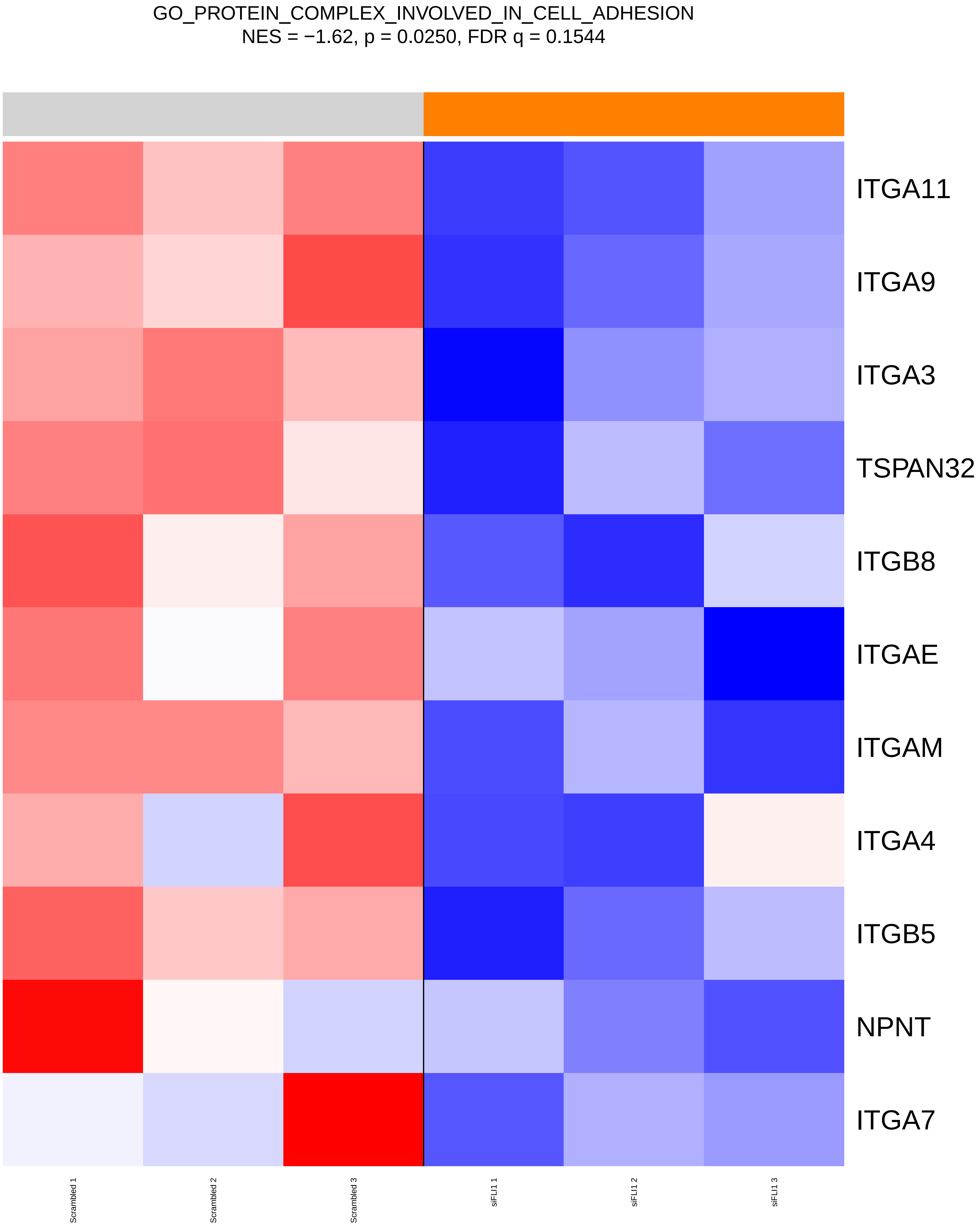
GO_PROTEIN_COMPLEX_INVOLVED_IN_CELL_ADHESION
NES = −1.62, p = 0.0250, FDR q = 0.1544
ITGA11
ITGA9
ITGA3
TSPAN32
ITGB8
ITGAE
ITGAM
ITGA4
ITGB5
NPNT
ITGA7
Scrambled 1
Scrambled 2
Scrambled 3
siFLI1 1
siFLI1 2
siFLI1 3

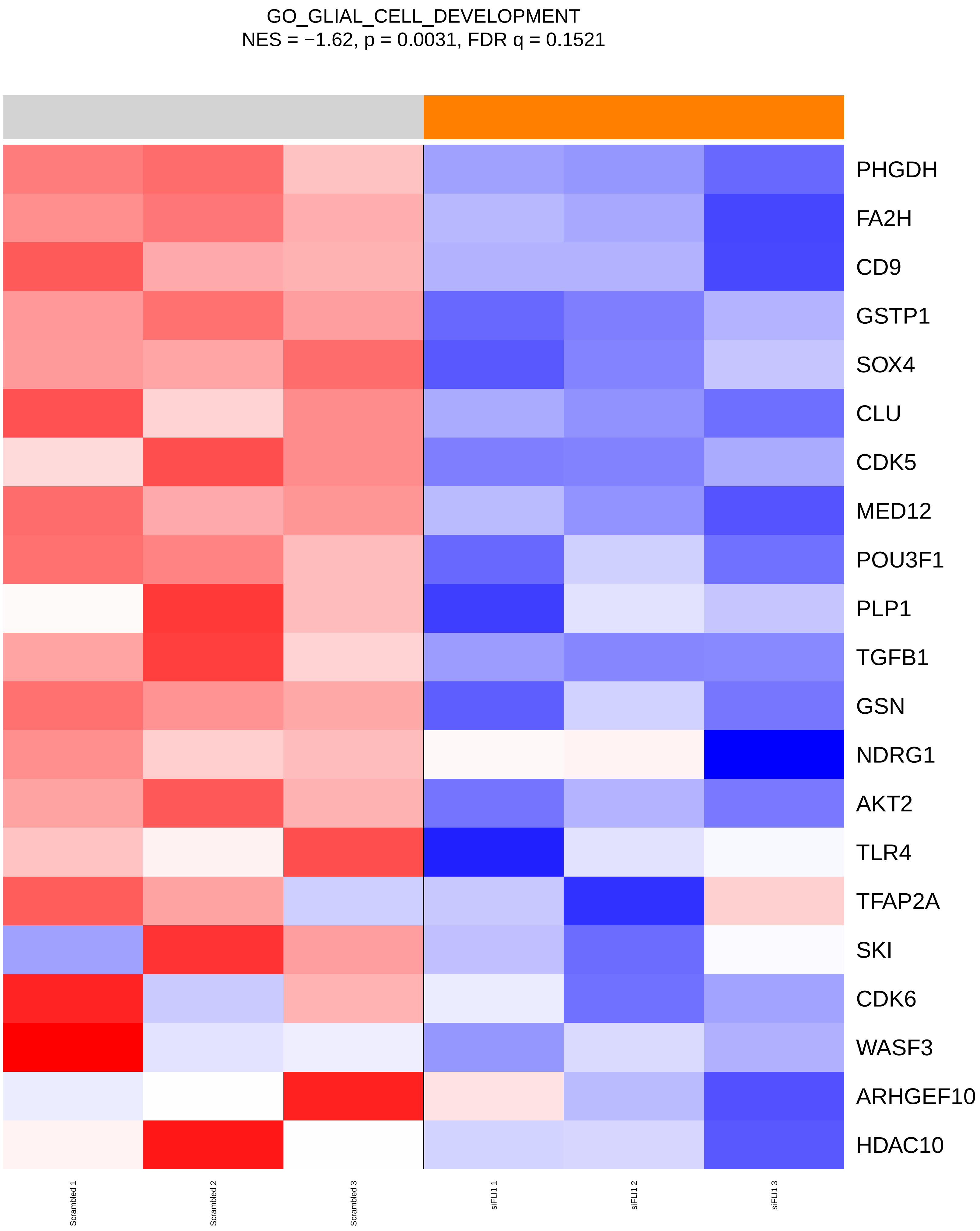
GO_GLIAL_CELL_DEVELOPMENT
NES = −1.62, p = 0.0031, FDR q = 0.1521
PHGDH
FA2H
CD9
GSTP1
SOX4
CLU
CDK5
MED12
POU3F1
PLP1
TGFB1
GSN
NDRG1
AKT2
TLR4
TFAP2A
SKI
CDK6
WASF3
ARHGEF10
HDAC10
Scrambled 1
Scrambled 2
Scrambled 3
siFLI1 1
siFLI1 2
siFLI1 3

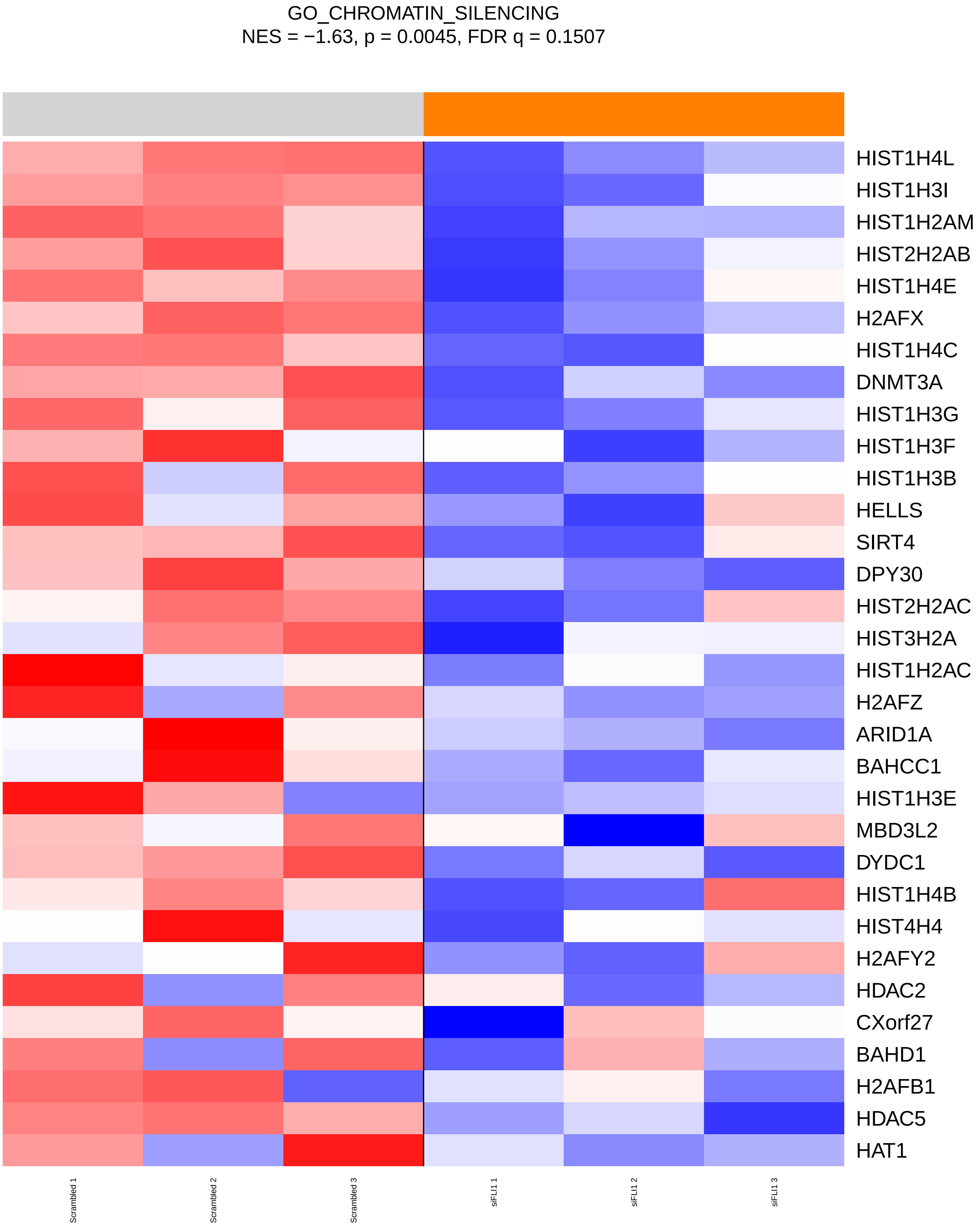

GO_CHROMATIN_SILENCING
NES = −1.63, p = 0.0045, FDR q = 0.1507
HIST1H4L
HIST1H3I
HIST1H2AM
HIST2H2AB
HIST1H4E
H2AFX
HIST1H4C
DNMT3A
HIST1H3G
HIST1H3F
HIST1H3B
HELLS
SIRT4
DPY30
HIST2H2AC
HIST3H2A
HIST1H2AC
H2AFZ
ARID1A
BAHCC1
HIST1H3E
MBD3L2
DYDC1
HIST1H4B
HIST4H4
H2AFY2
HDAC2
CXorf27
BAHD1
H2AFB1
HDAC5
HAT1
Scrambled 1
Scrambled 2
Scrambled 3
siFLI1 1
siFLI1 2
siFLI1 3

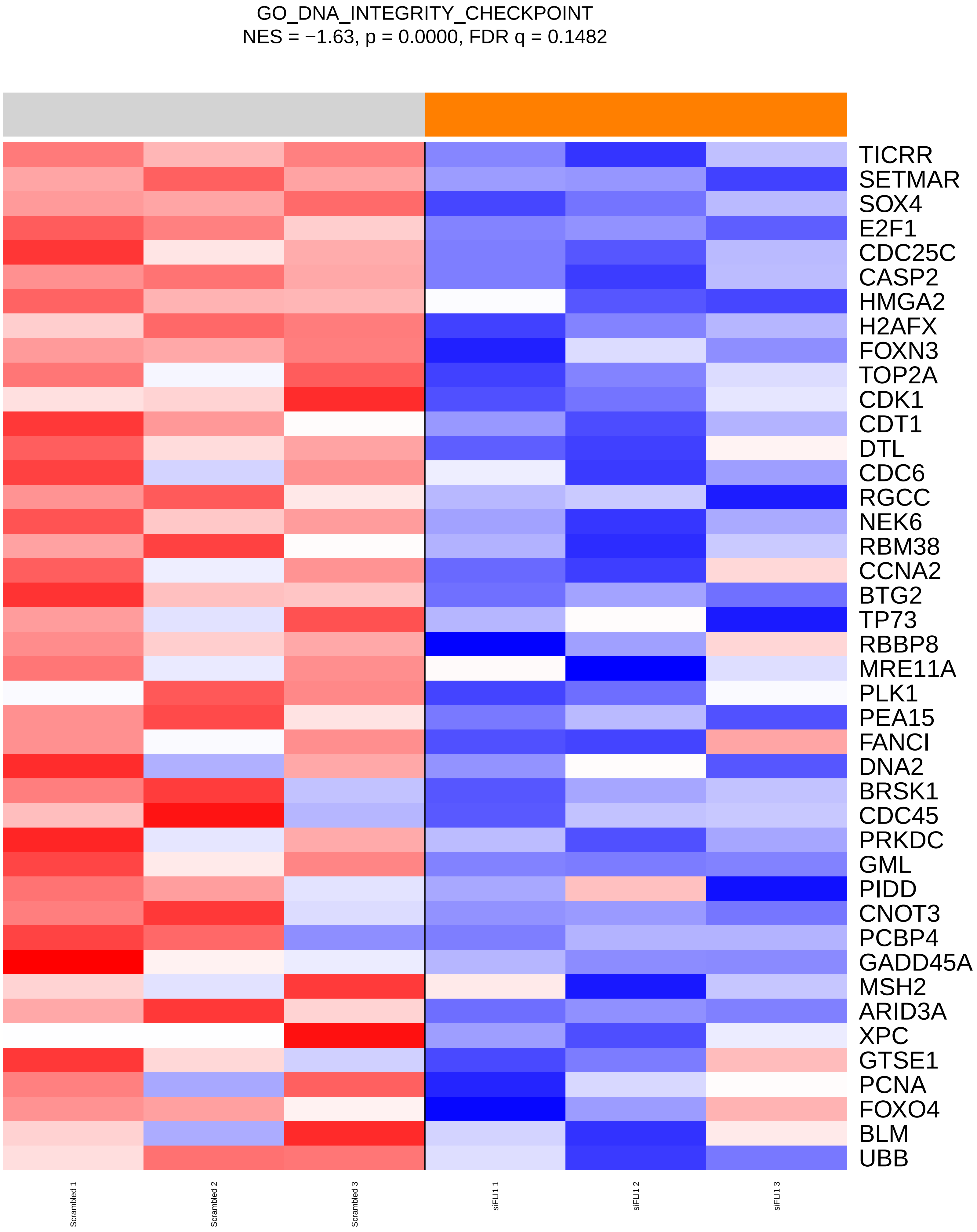
GO_DNA_INTEGRITY_CHECKPOINT
NES = −1.63, p = 0.0000, FDR q = 0.1482
TICRR
SETMAR
SOX4
E2F1
CDC25C
CASP2
HMGA2
H2AFX
FOXN3
TOP2A
CDK1
CDT1
DTL
CDC6
RGCC
NEK6
RBM38
CCNA2
BTG2
TP73
RBBP8
MRE11A
PLK1
PEA15
FANCI
DNA2
BRSK1
CDC45
PRKDC
GML
PIDD
CNOT3
PCBP4
GADD45A
MSH2
ARID3A
XPC
GTSE1
PCNA
FOXO4
BLM
UBB
Scrambled 1
Scrambled 2
Scrambled 3
siFLI1 1
siFLI1 2
siFLI1 3

REACTOME_CELL_CYCLE
NES = −1.63, p = 0.0000, FDR q = 0.1469

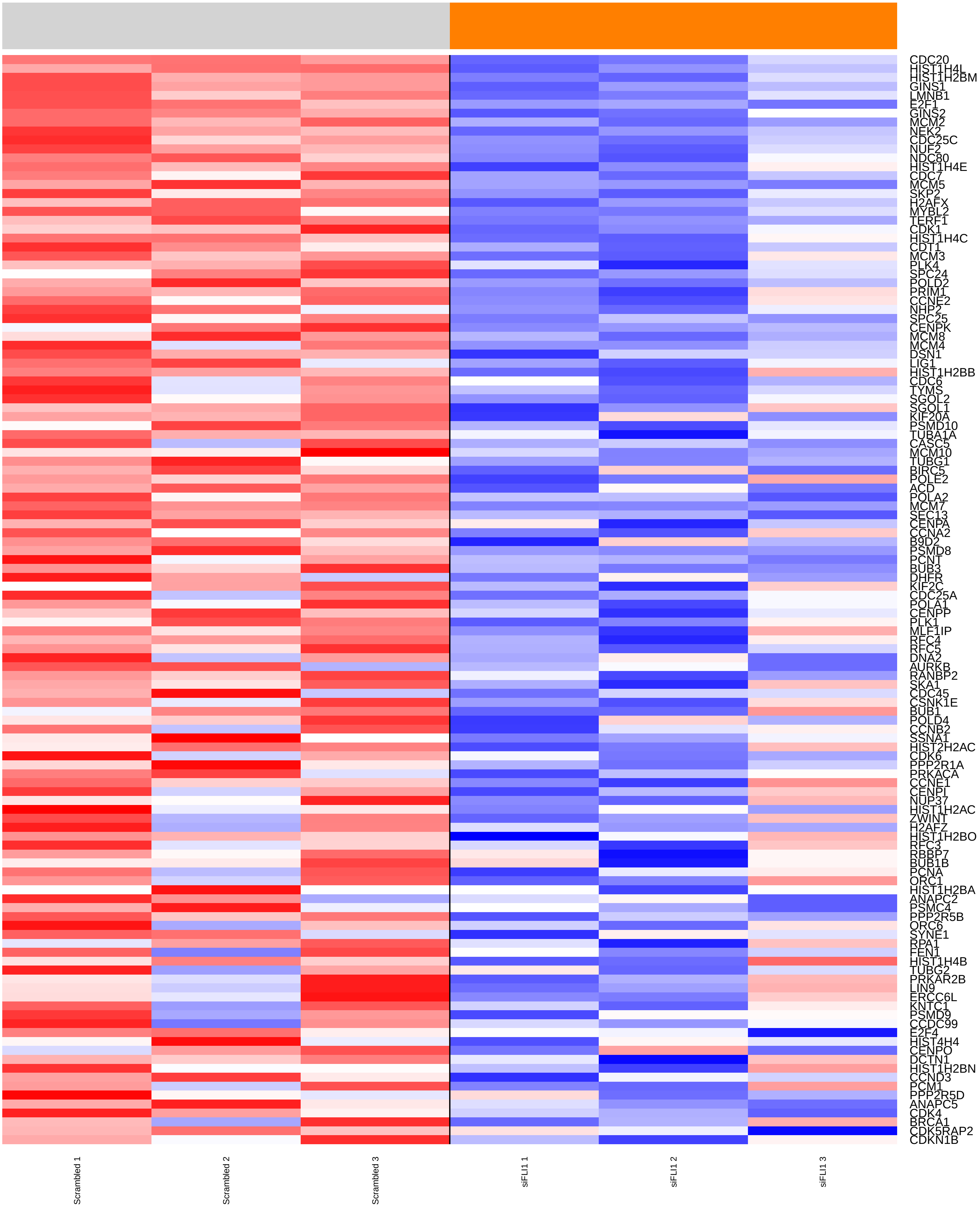

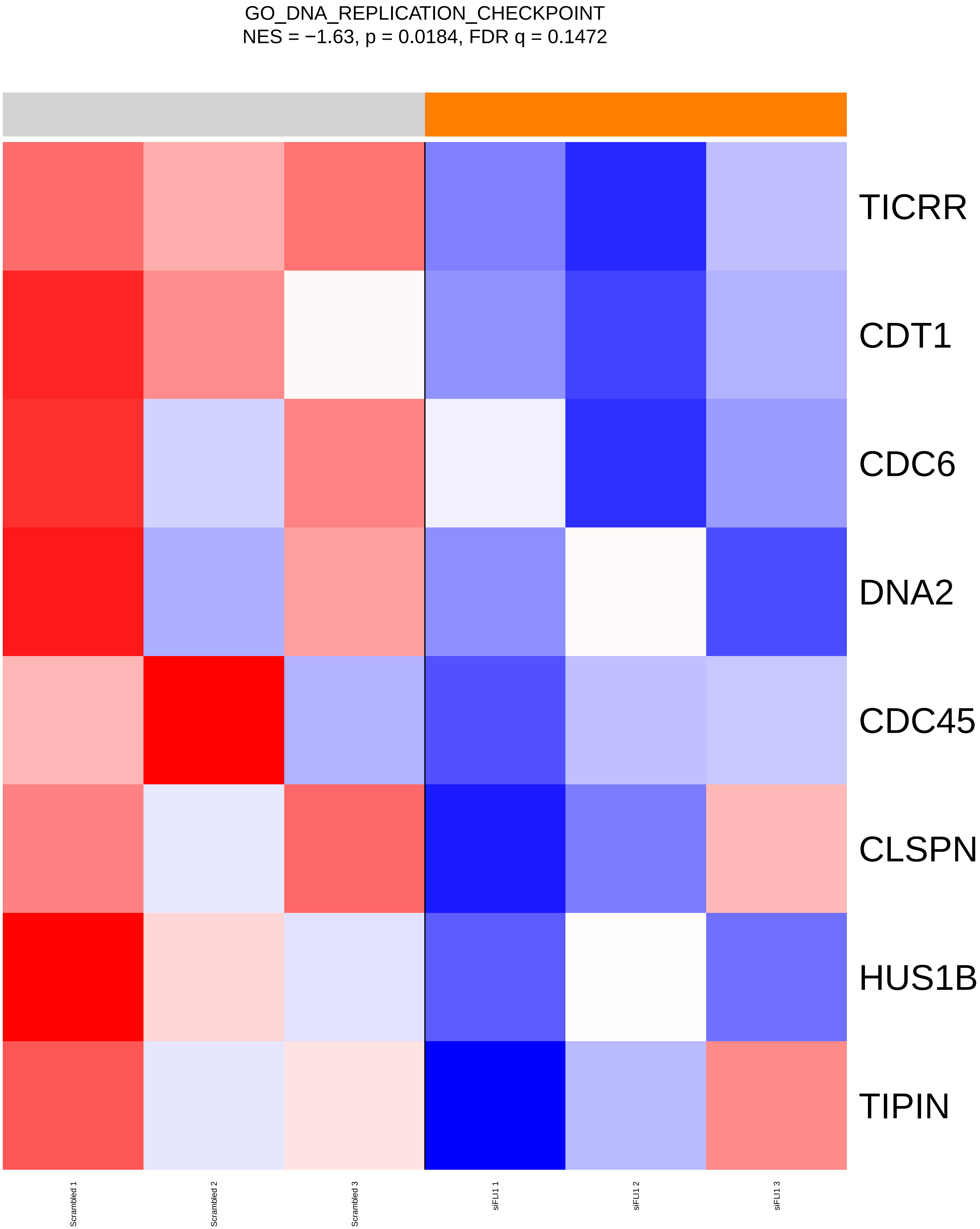
GO_DNA_REPLICATION_CHECKPOINT
NES = −1.63, p = 0.0184, FDR q = 0.1472
TICRR
CDT1
CDC6
DNA2
CDC45
CLSPN
HUS1B
TIPIN
Scrambled 1
Scrambled 2
Scrambled 3
siFLI1 1
siFLI1 2
siFLI1 3

GO_ALCOHOL_DEHYDROGENASE_NADP_ACTIVITY
NES = −1.63, p = 0.0208, FDR q = 0.1458

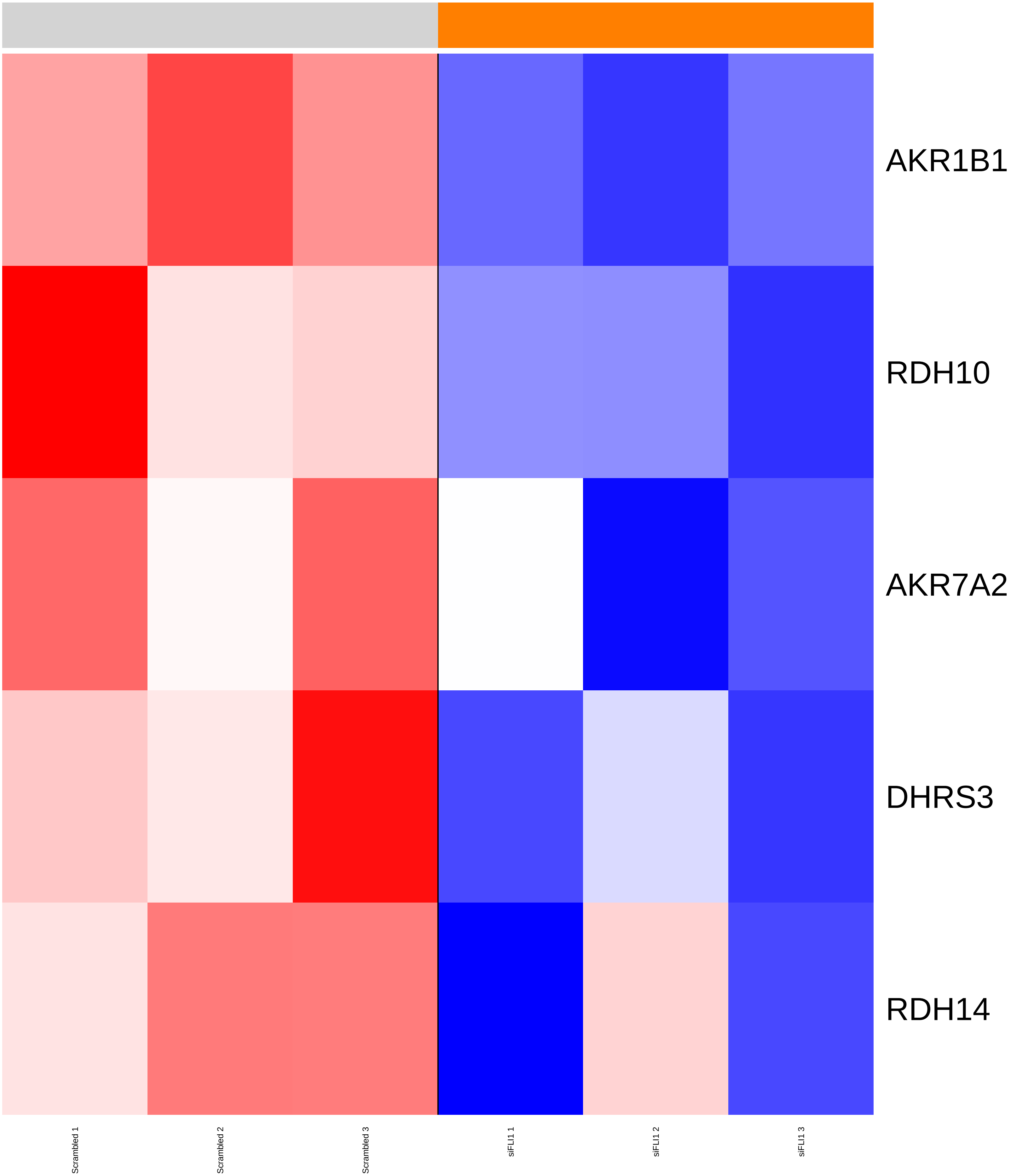

GO_MITOCHONDRIAL_RESPIRATORY_CHAIN_COMPLEX_I_BIOGENESIS
NES = −1.63, p = 0.0079, FDR q = 0.1464

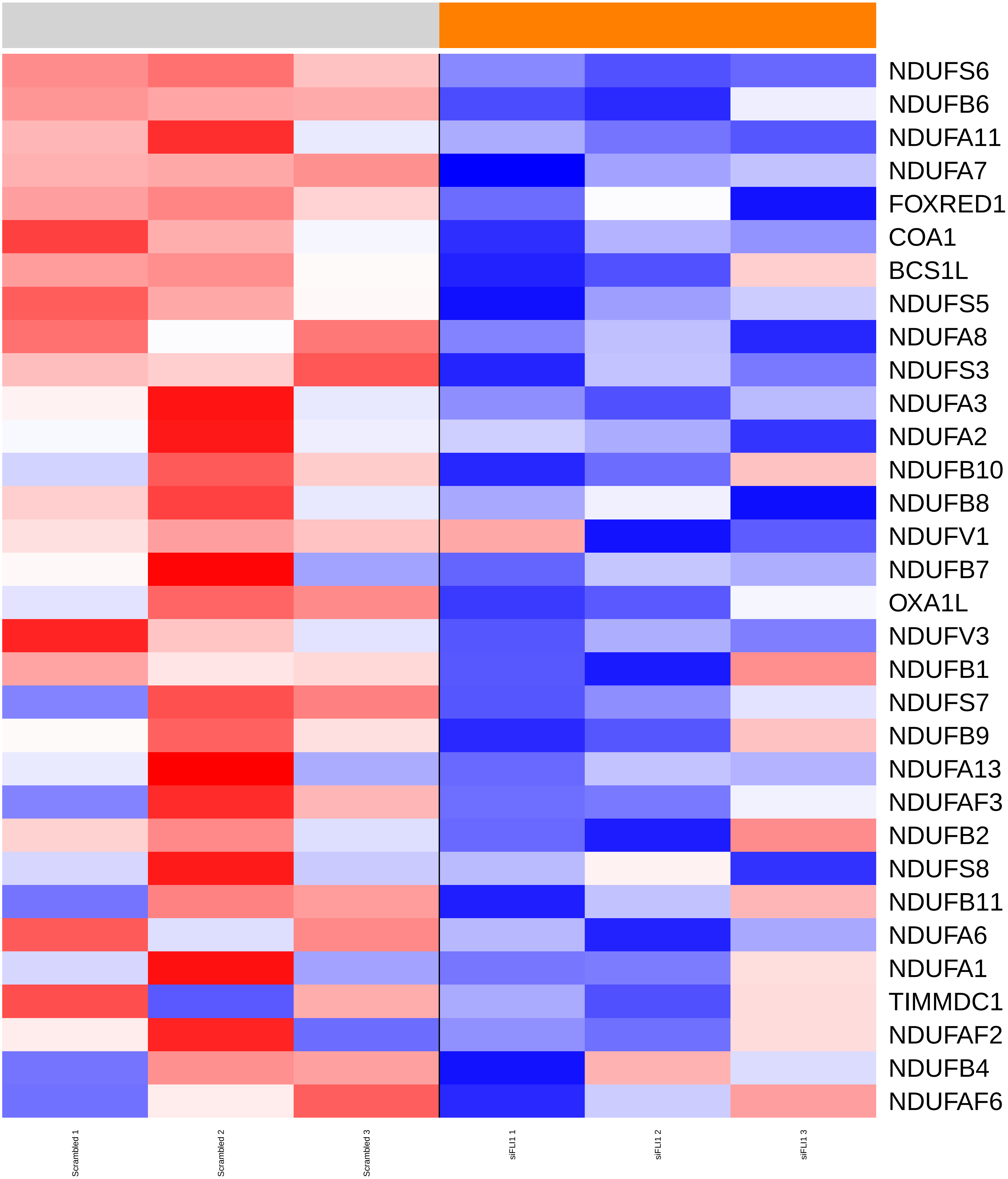

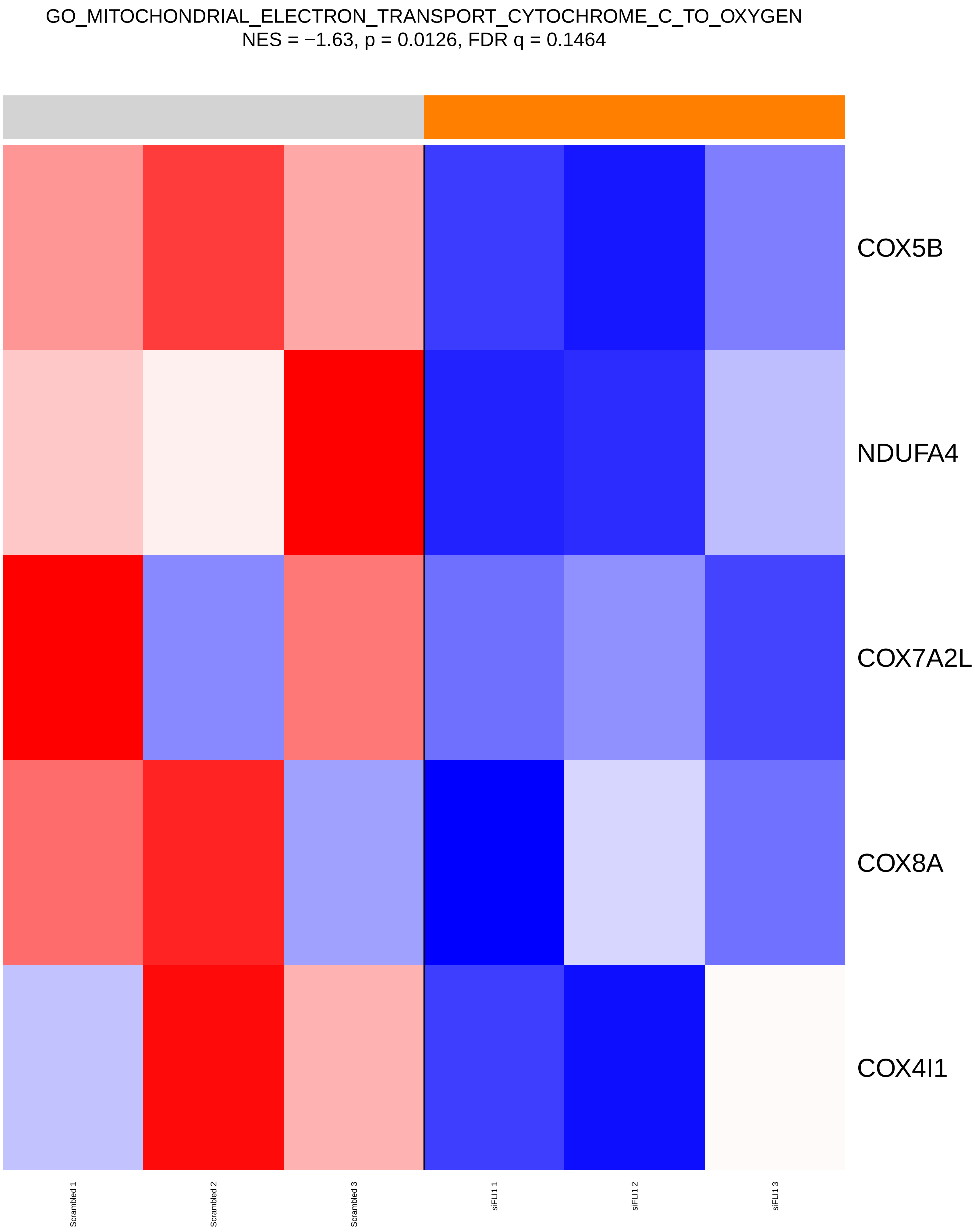
GO_MITOCHONDRIAL_ELECTRON_TRANSPORT_CYTOCHROME_C_TO_OXYGEN
NES = −1.63, p = 0.0126, FDR q = 0.1464
COX5B
NDUFA4
COX7A2L
COX8A
COX4I1
Scrambled 1
Scrambled 2
Scrambled 3
siFLI1 1
siFLI1 2
siFLI1 3

GO_PURINE_NUCLEOBASE_METABOLIC_PROCESS
NES = −1.63, p = 0.0197, FDR q = 0.1462

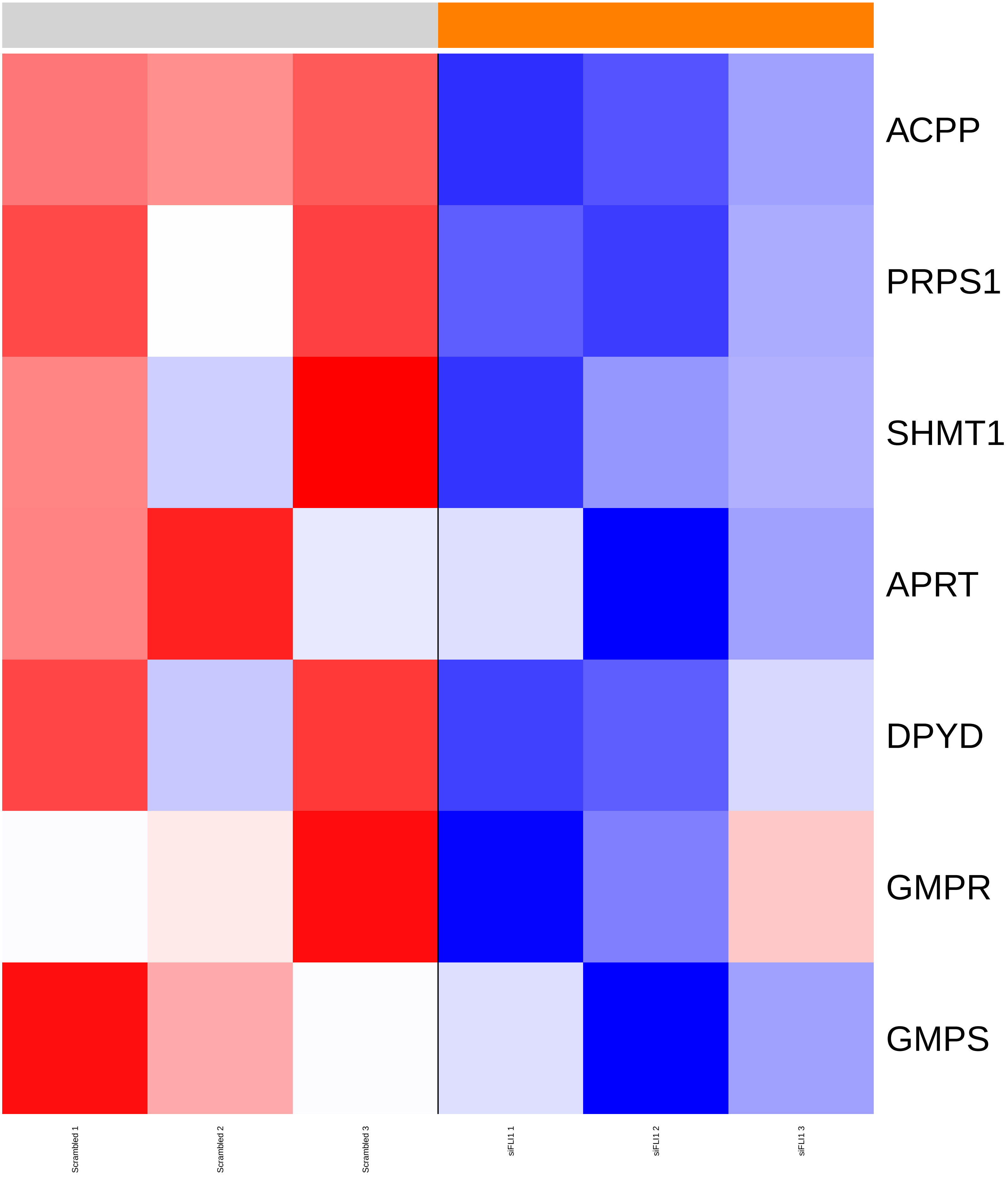

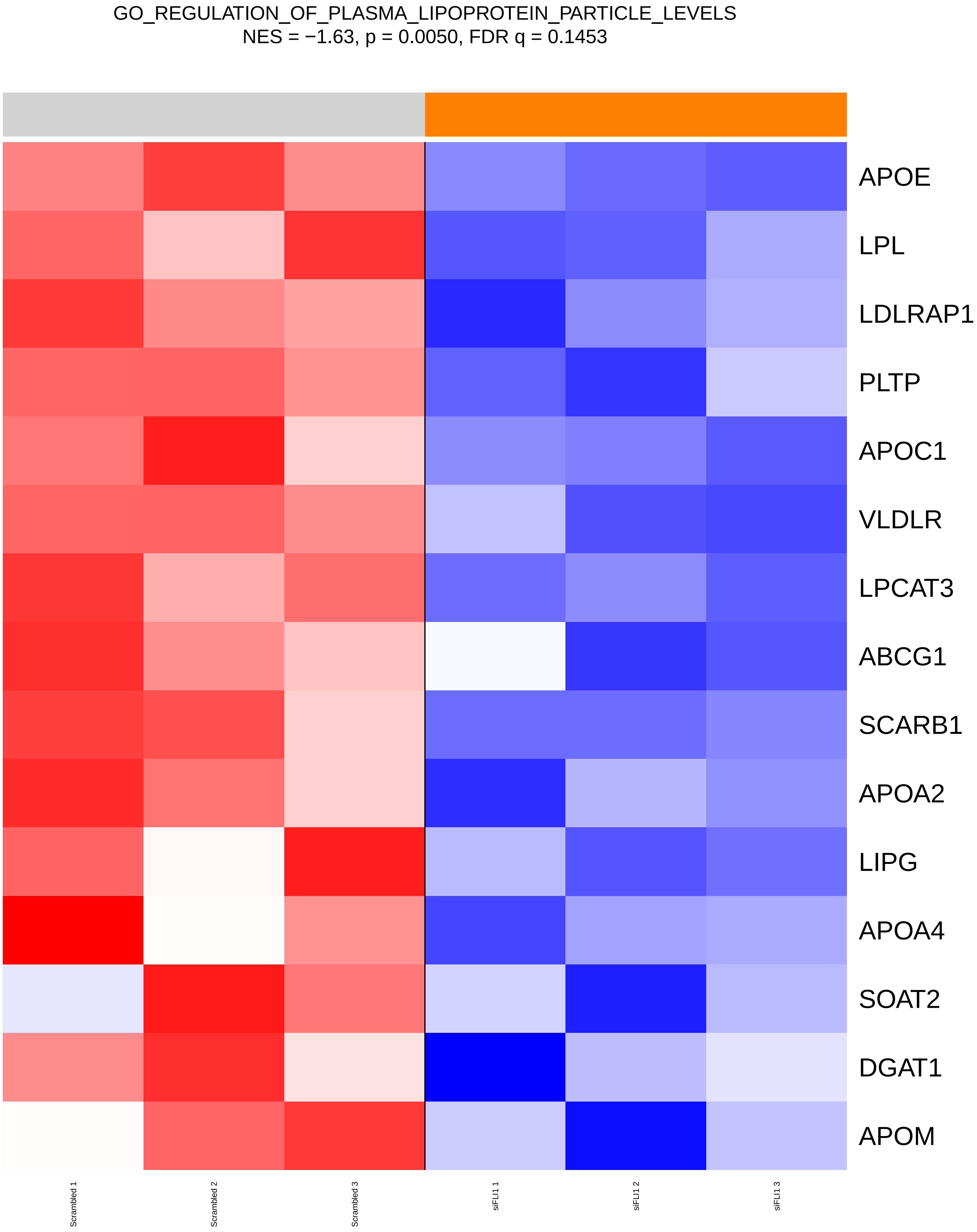
GO_REGULATION_OF_PLASMA_LIPOPROTEIN_PARTICLE_LEVELS
NES = −1.63, p = 0.0050, FDR q = 0.1453
APOE
LPL
LDLRAP1
PLTP
APOC1
VLDLR
LPCAT3
ABCG1
SCARB1
APOA2
LIPG
APOA4
SOAT2
DGAT1
APOM
Scrambled 1
Scrambled 2
Scrambled 3
siFLI1 1
siFLI1 2
siFLI1 3

GO_MONOCARBOXYLIC_ACID_BIOSYNTHETIC_PROCESS
NES = −1.63, p = 0.0013, FDR q = 0.1447

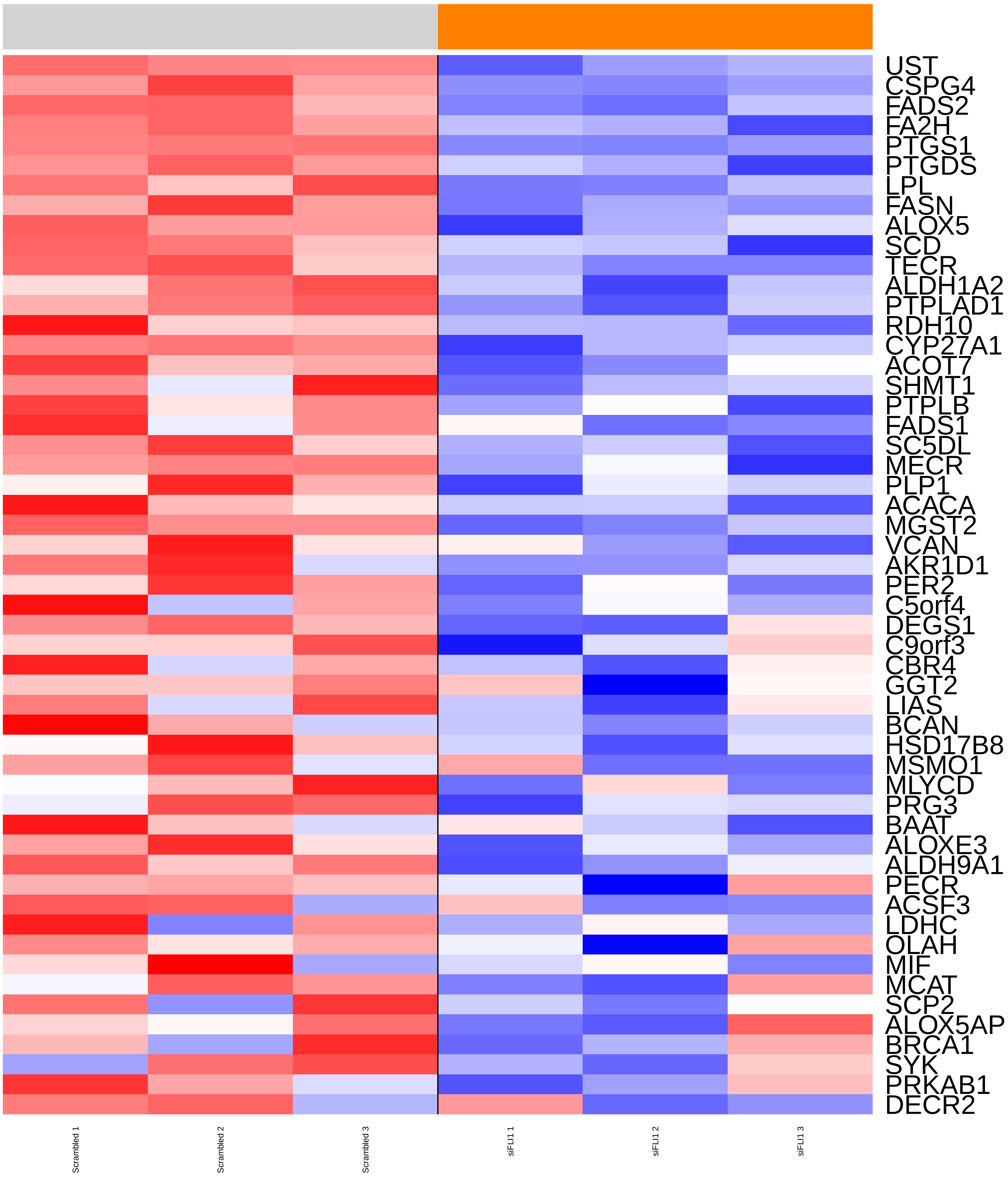

GO_RETINOIC_ACID_METABOLIC_PROCESS
NES = −1.64, p = 0.0166, FDR q = 0.1442

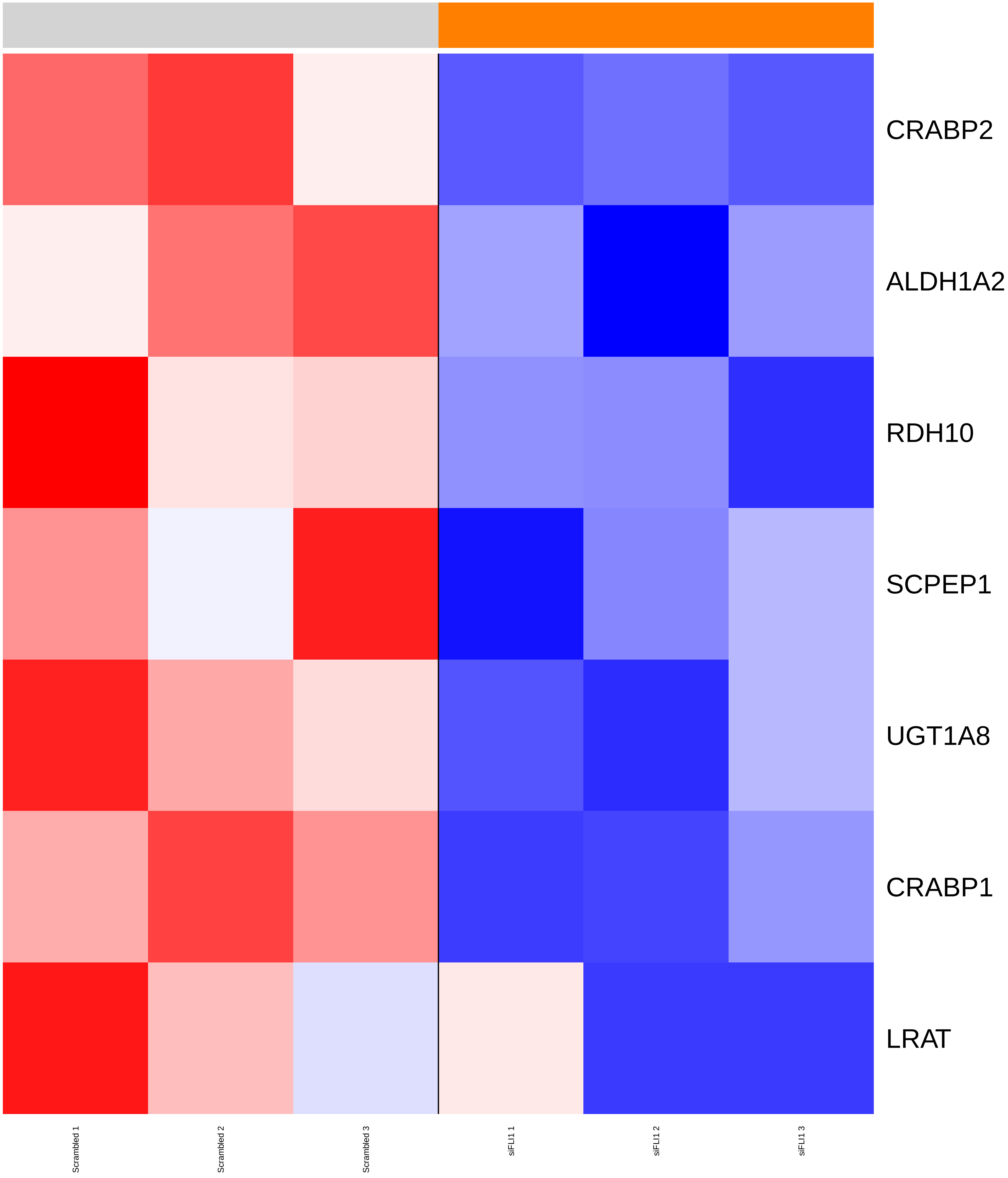

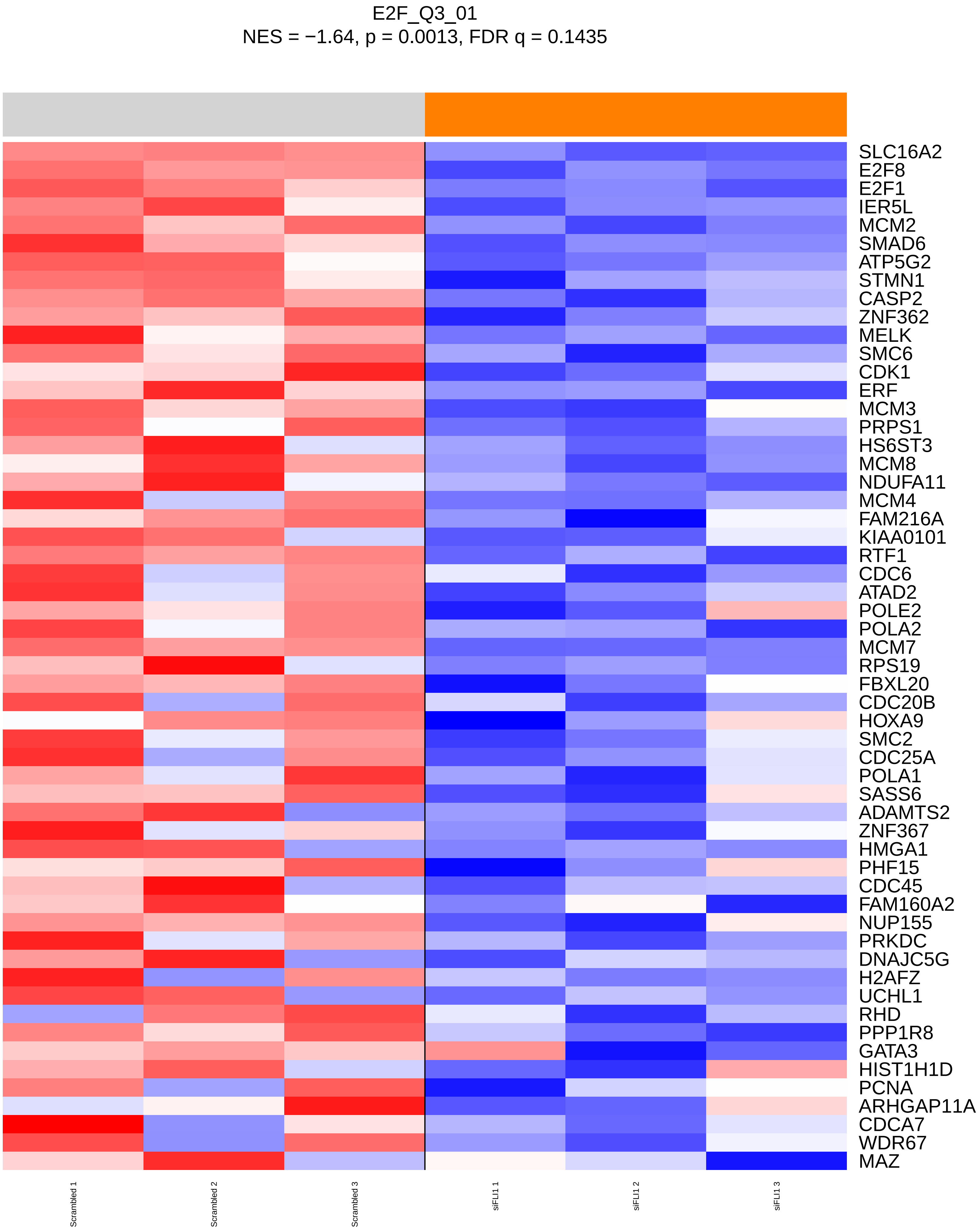
E2F_Q3_01
NES = −1.64, p = 0.0013, FDR q = 0.1435
SLC16A2
E2F8
E2F1
IER5L
MCM2
SMAD6
ATP5G2
STMN1
CASP2
ZNF362
MELK
SMC6
CDK1
ERF
MCM3
PRPS1
HS6ST3
MCM8
NDUFA11
MCM4
FAM216A
KIAA0101
RTF1
CDC6
ATAD2
POLE2
POLA2
MCM7
RPS19
FBXL20
CDC20B
HOXA9
SMC2
CDC25A
POLA1
SASS6
ADAMTS2
ZNF367
HMGA1
PHF15
CDC45
FAM160A2
NUP155
PRKDC
DNAJC5G
H2AFZ
UCHL1
RHD
PPP1R8
GATA3
HIST1H1D
PCNA
ARHGAP11A
CDCA7
WDR67
MAZ
Scrambled 1
Scrambled 2
Scrambled 3
siFLI1 1
siFLI1 2
siFLI1 3

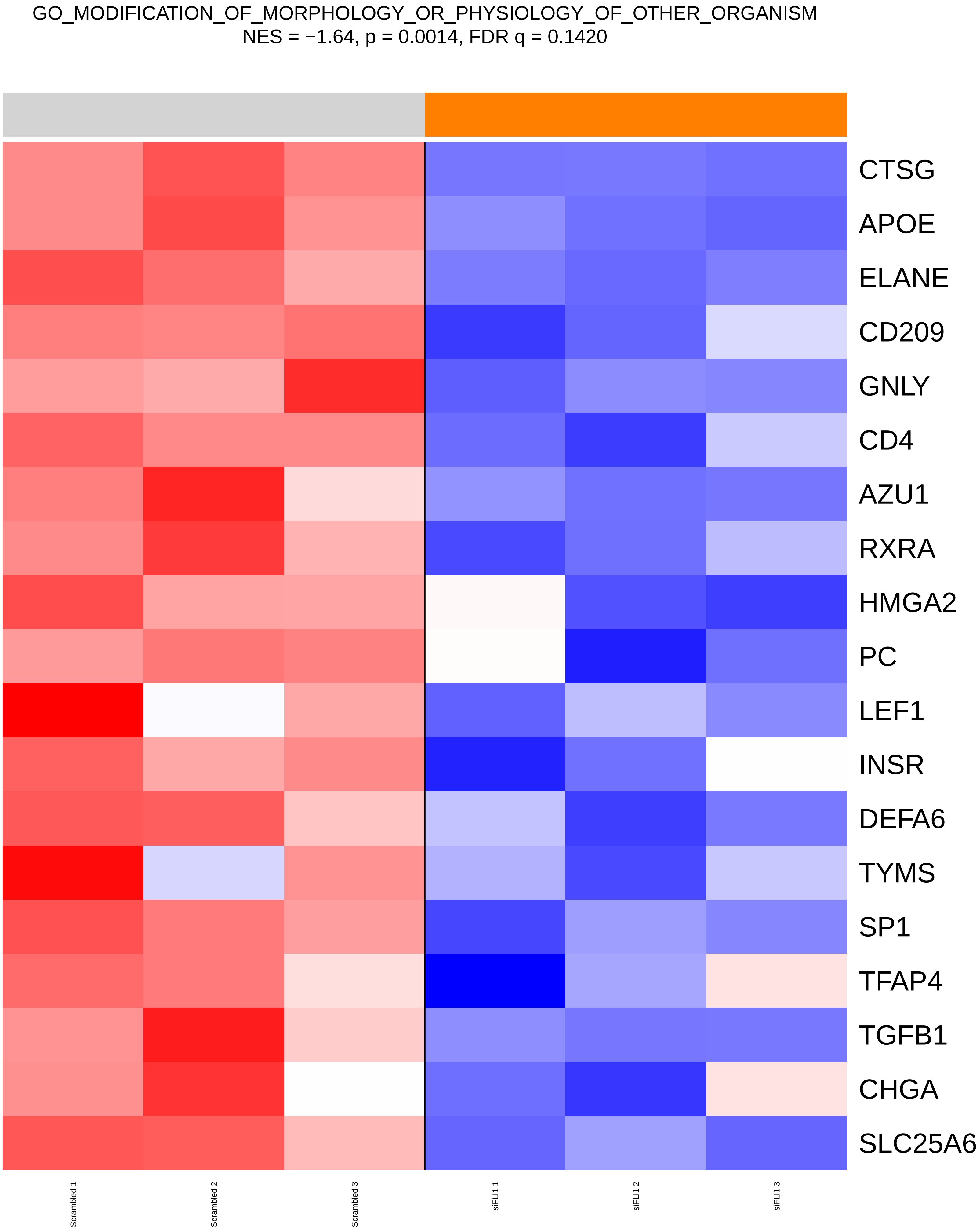
GO_MODIFICATION_OF_MORPHOLOGY_OR_PHYSIOLOGY_OF_OTHER_ORGANISM
NES = −1.64, p = 0.0014, FDR q = 0.1420
CTSG
APOE
ELANE
CD209
GNLY
CD4
AZU1
RXRA
HMGA2
PC
LEF1
INSR
DEFA6
TYMS
SP1
TFAP4
TGFB1
CHGA
SLC25A6
Scrambled 1
Scrambled 2
Scrambled 3
siFLI1 1
siFLI1 2
siFLI1 3

GO_SMOOTH_MUSCLE_CONTRACTION
NES = −1.64, p = 0.0064, FDR q = 0.1425

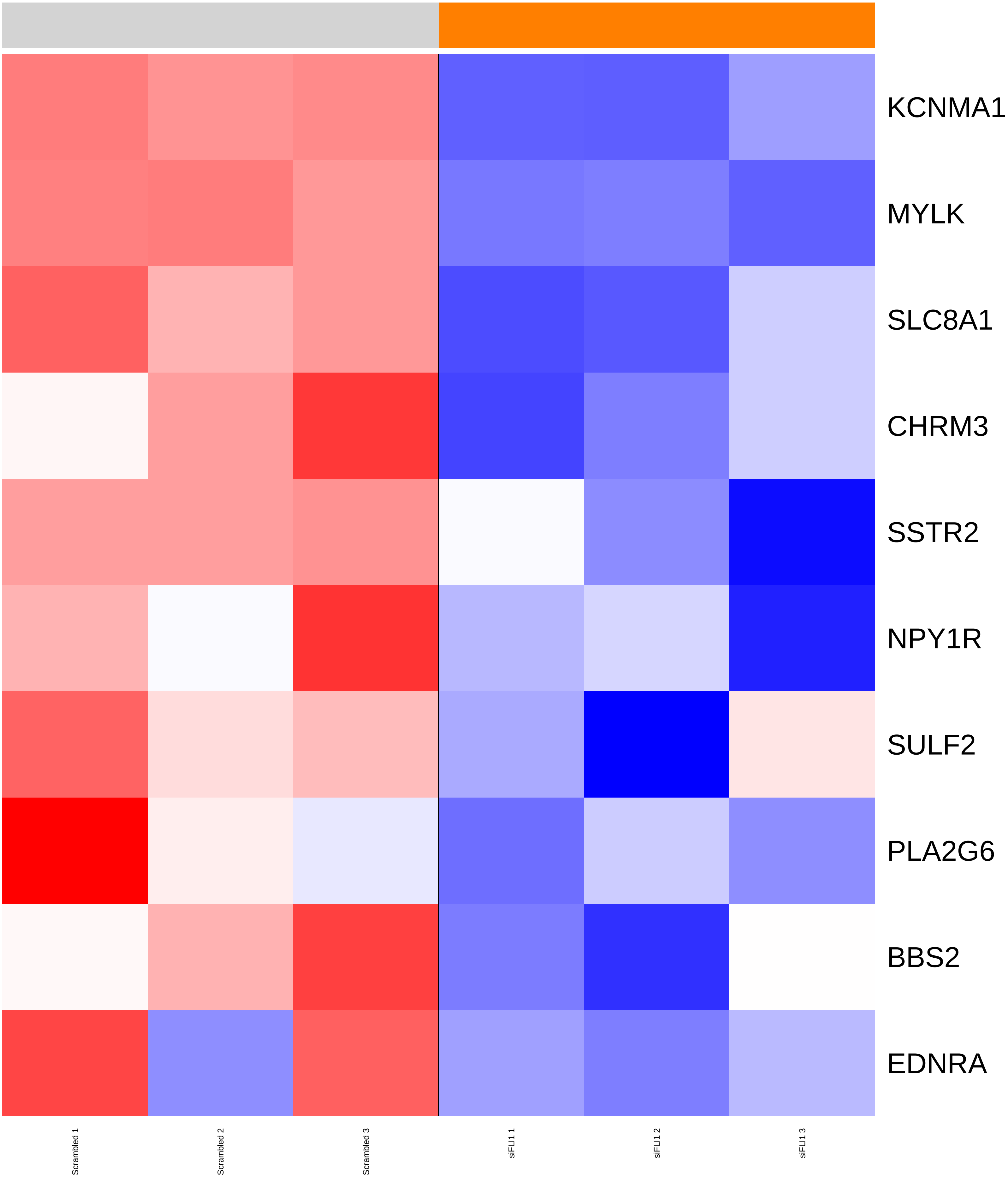

GO_URONIC_ACID_METABOLIC_PROCESS
NES = −1.64, p = 0.0147, FDR q = 0.1431

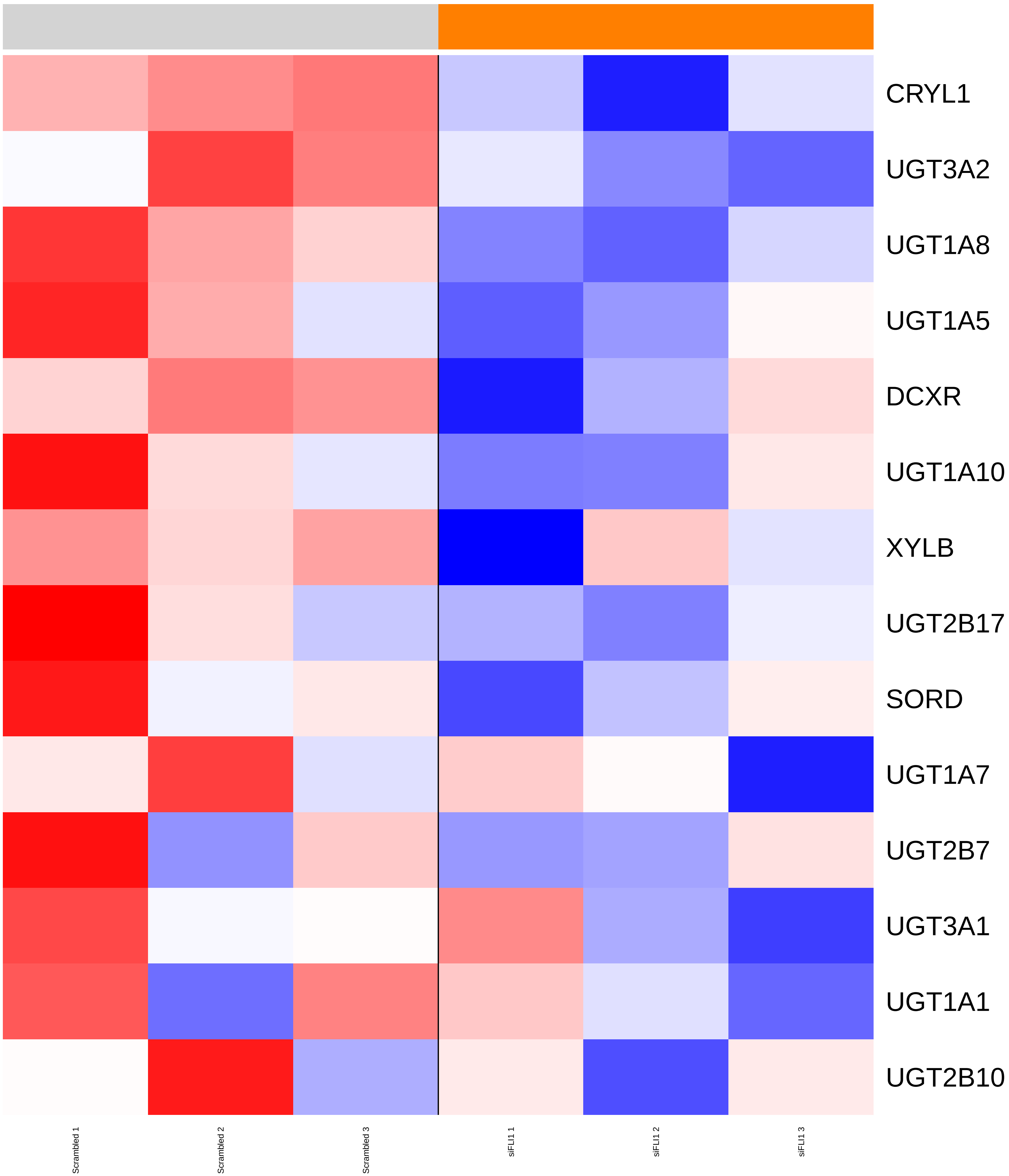

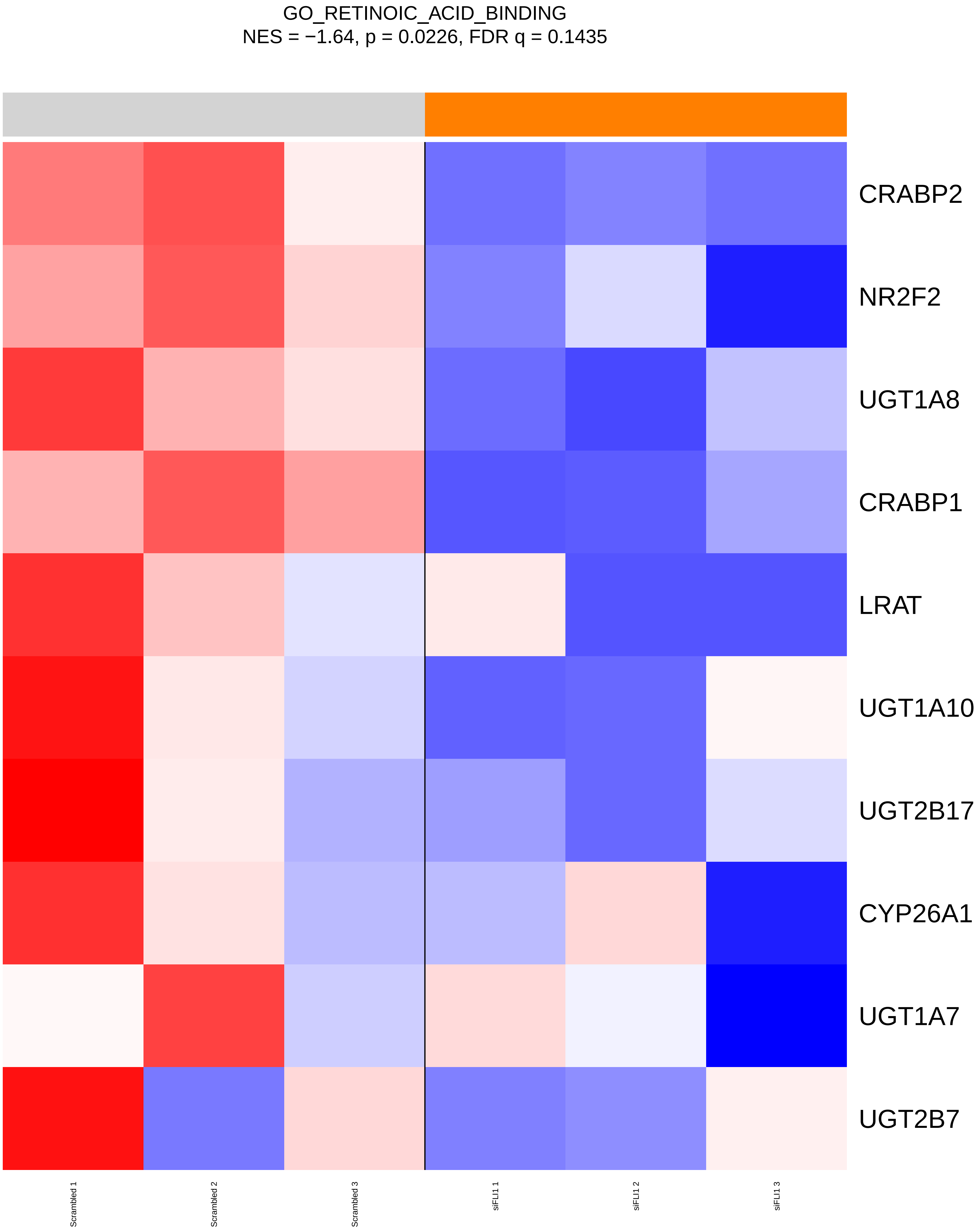
GO_RETINOIC_ACID_BINDING
NES = −1.64, p = 0.0226, FDR q = 0.1435
CRABP2
NR2F2
UGT1A8
CRABP1
LRAT
UGT1A10
UGT2B17
CYP26A1
UGT1A7
UGT2B7
Scrambled 1
Scrambled 2
Scrambled 3
siFLI1 1
siFLI1 2
siFLI1 3

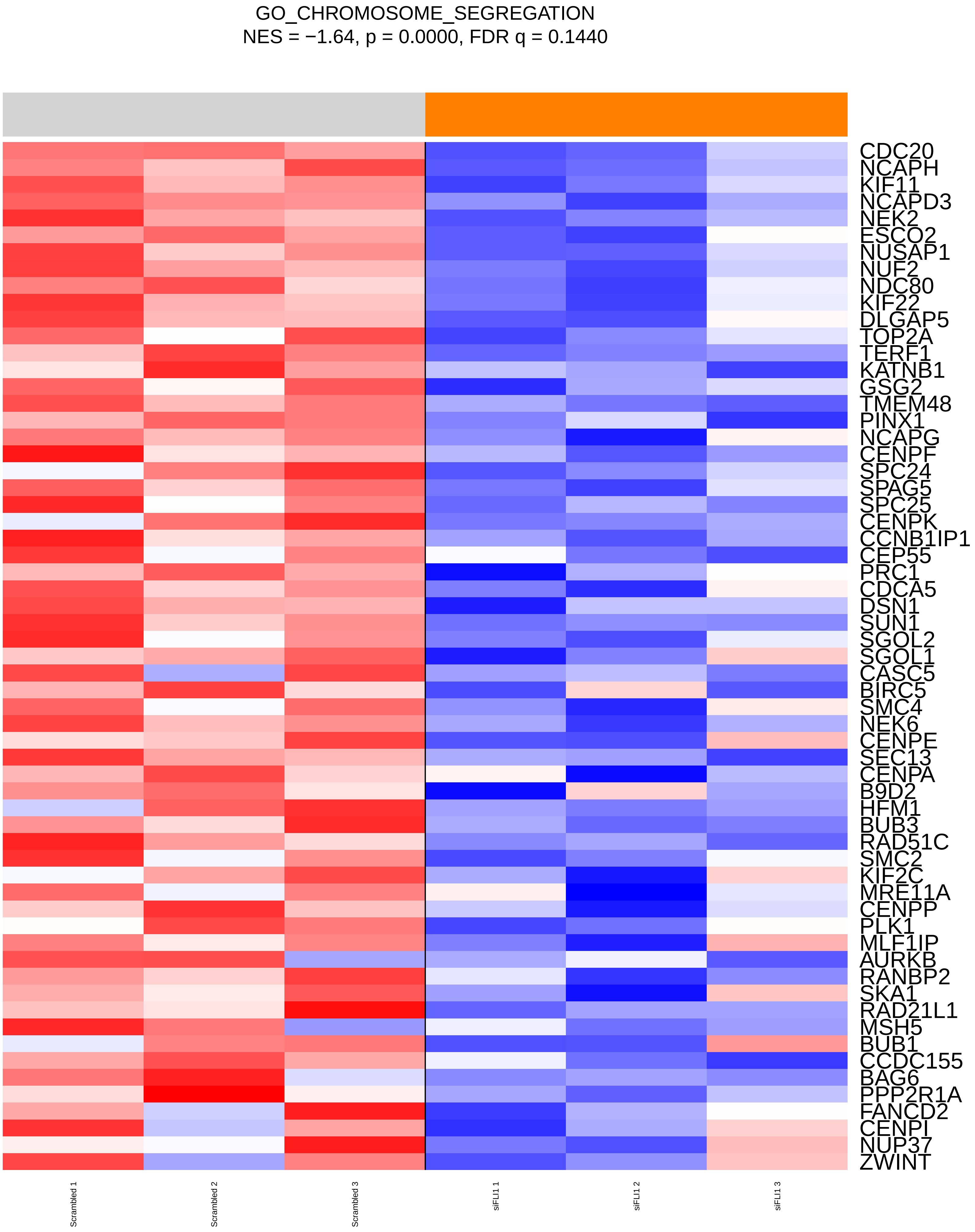
GO_CHROMOSOME_SEGREGATION
NES = −1.64, p = 0.0000, FDR q = 0.1440
CDC20
NCAPH
KIF11
NCAPD3
NEK2
ESCO2
NUSAP1
NUF2
NDC80
KIF22
DLGAP5
TOP2A
TERF1
KATNB1
GSG2
TMEM48
PINX1
NCAPG
CENPF
SPC24
SPAG5
SPC25
CENPK
CCNB1IP1
CEP55
PRC1
CDCA5
DSN1
SUN1
SGOL2
SGOL1
CASC5
BIRC5
SMC4
NEK6
CENPE
SEC13
CENPA
B9D2
HFM1
BUB3
RAD51C
SMC2
KIF2C
MRE11A
CENPP
PLK1
MLF1IP
AURKB
RANBP2
SKA1
RAD21L1
MSH5
BUB1
CCDC155
BAG6
PPP2R1A
FANCD2
CENPI
NUP37
ZWINT
Scrambled 1
Scrambled 2
Scrambled 3
siFLI1 1
siFLI1 2
siFLI1 3

GO_MONOCARBOXYLIC_ACID_METABOLIC_PROCESS
NES = −1.64, p = 0.0000, FDR q = 0.1436

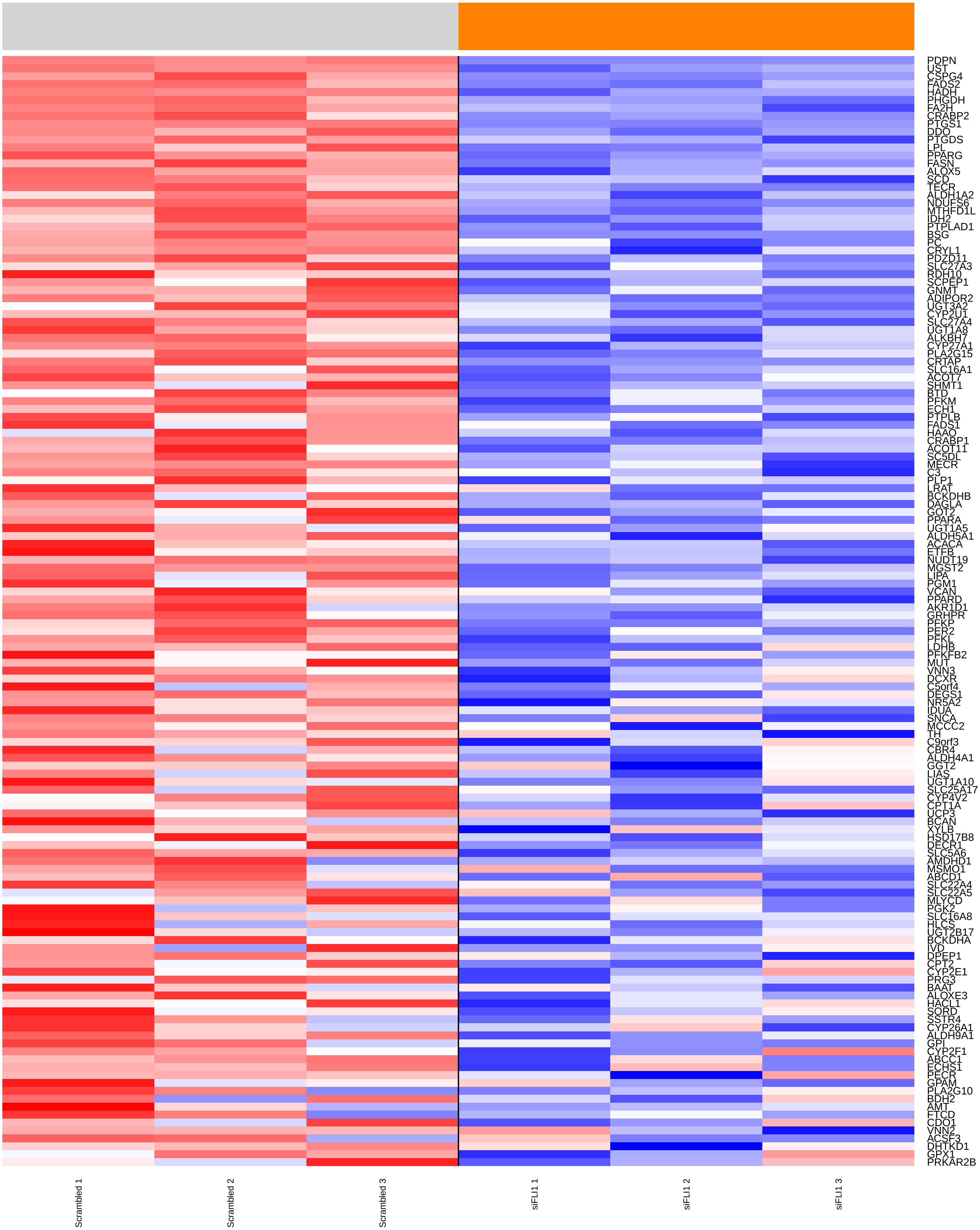

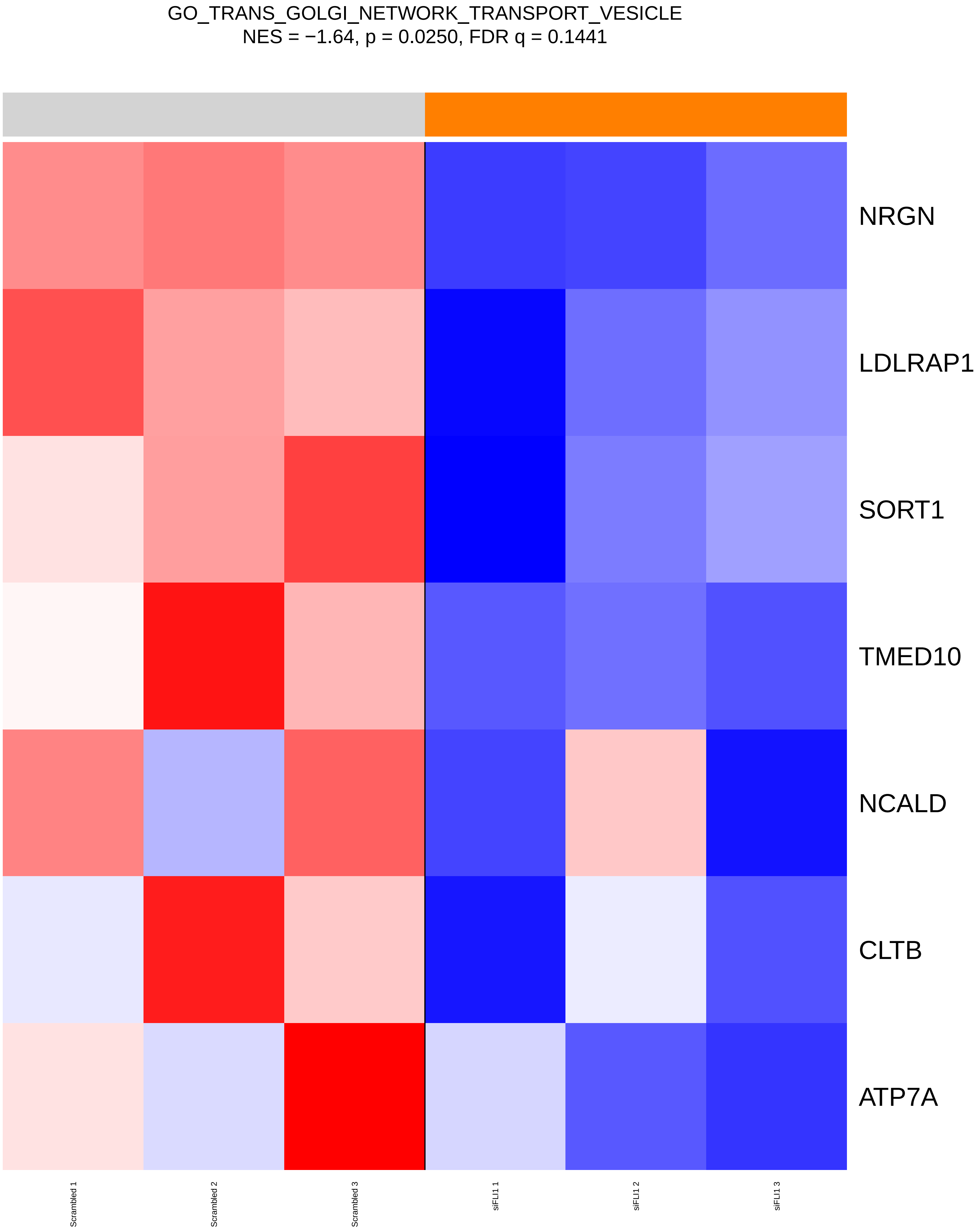
GO_TRANS_GOLGI_NETWORK_TRANSPORT_VESICLE
NES = −1.64, p = 0.0250, FDR q = 0.1441
NRGN
LDLRAP1
SORT1
TMED10
NCALD
CLTB
ATP7A
Scrambled 1
Scrambled 2
Scrambled 3
siFLI1 1
siFLI1 2
siFLI1 3

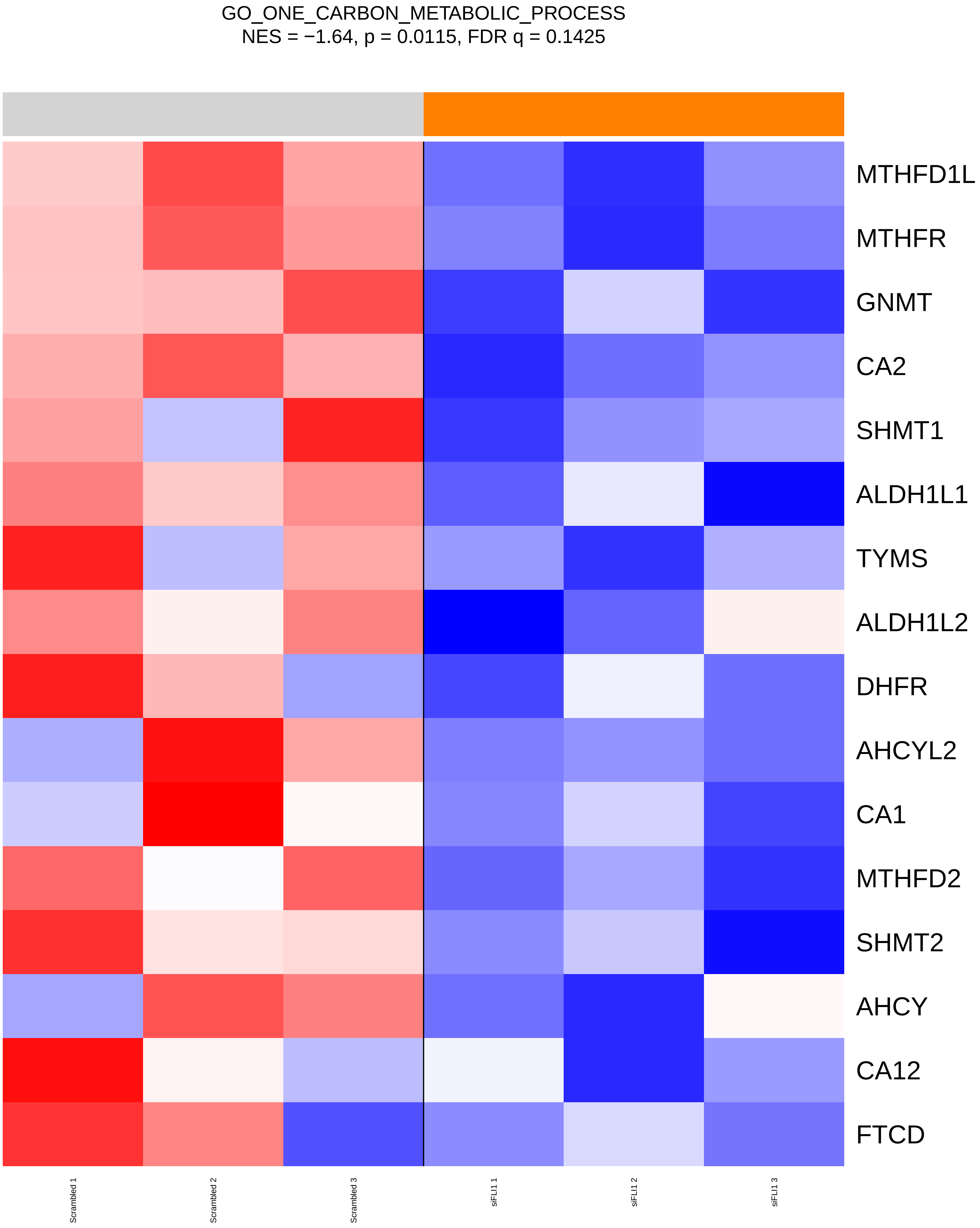
GO_ONE_CARBON_METABOLIC_PROCESS
NES = −1.64, p = 0.0115, FDR q = 0.1425
MTHFD1L
MTHFR
GNMT
CA2
SHMT1
ALDH1L1
TYMS
ALDH1L2
DHFR
AHCYL2
CA1
MTHFD2
SHMT2
AHCY
CA12
FTCD
Scrambled 1
Scrambled 2
Scrambled 3
siFLI1 1
siFLI1 2
siFLI1 3

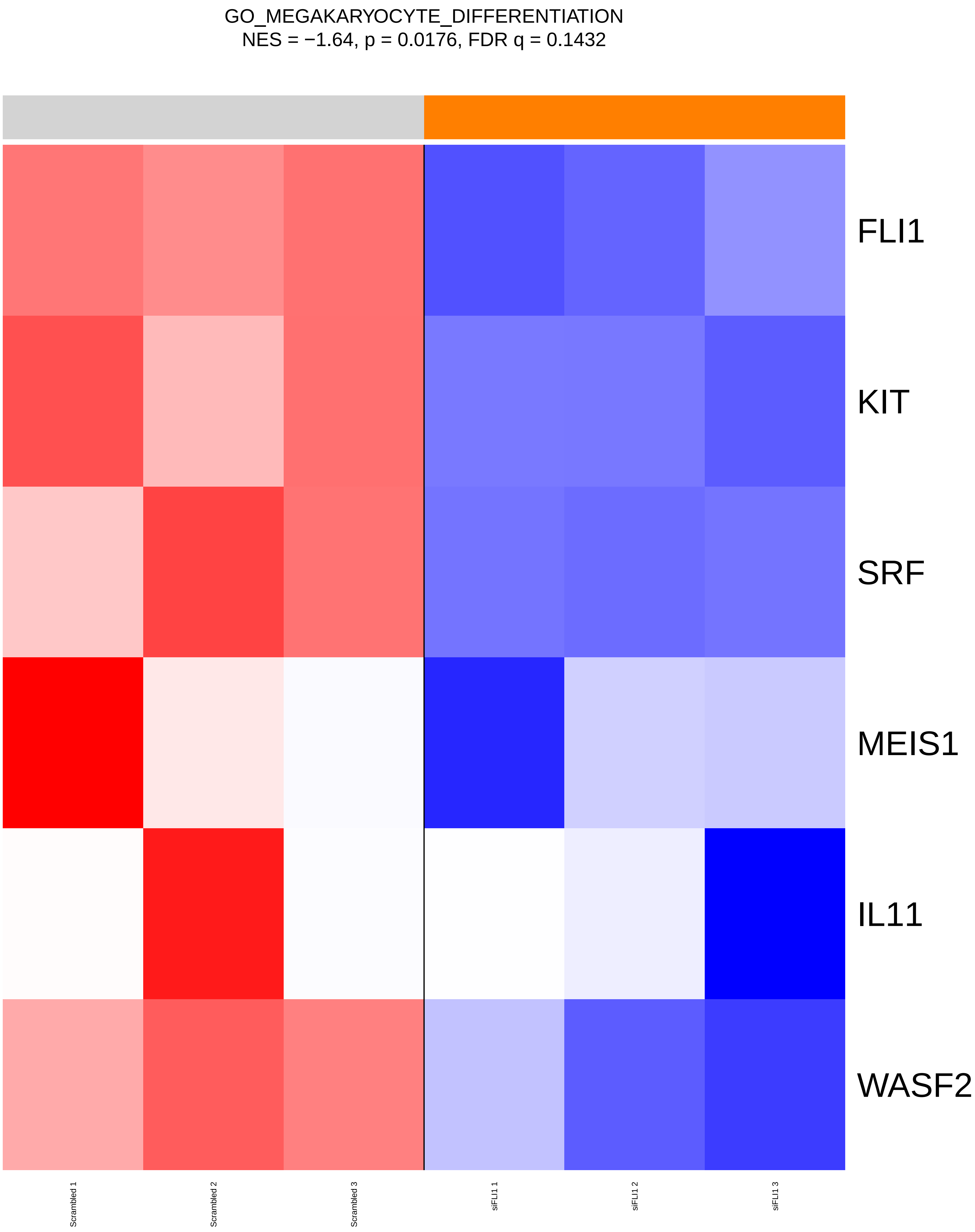
GO_MEGAKARYOCYTE_DIFFERENTIATION
NES = −1.64, p = 0.0176, FDR q = 0.1432
FLI1
KIT
SRF
MEIS1
IL11
WASF2
Scrambled 1
Scrambled 2
Scrambled 3
siFLI1 1
siFLI1 2
siFLI1 3

GO_REGULATION_OF_NOTCH_SIGNALING_PATHWAY
NES = −1.64, p = 0.0000, FDR q = 0.1431

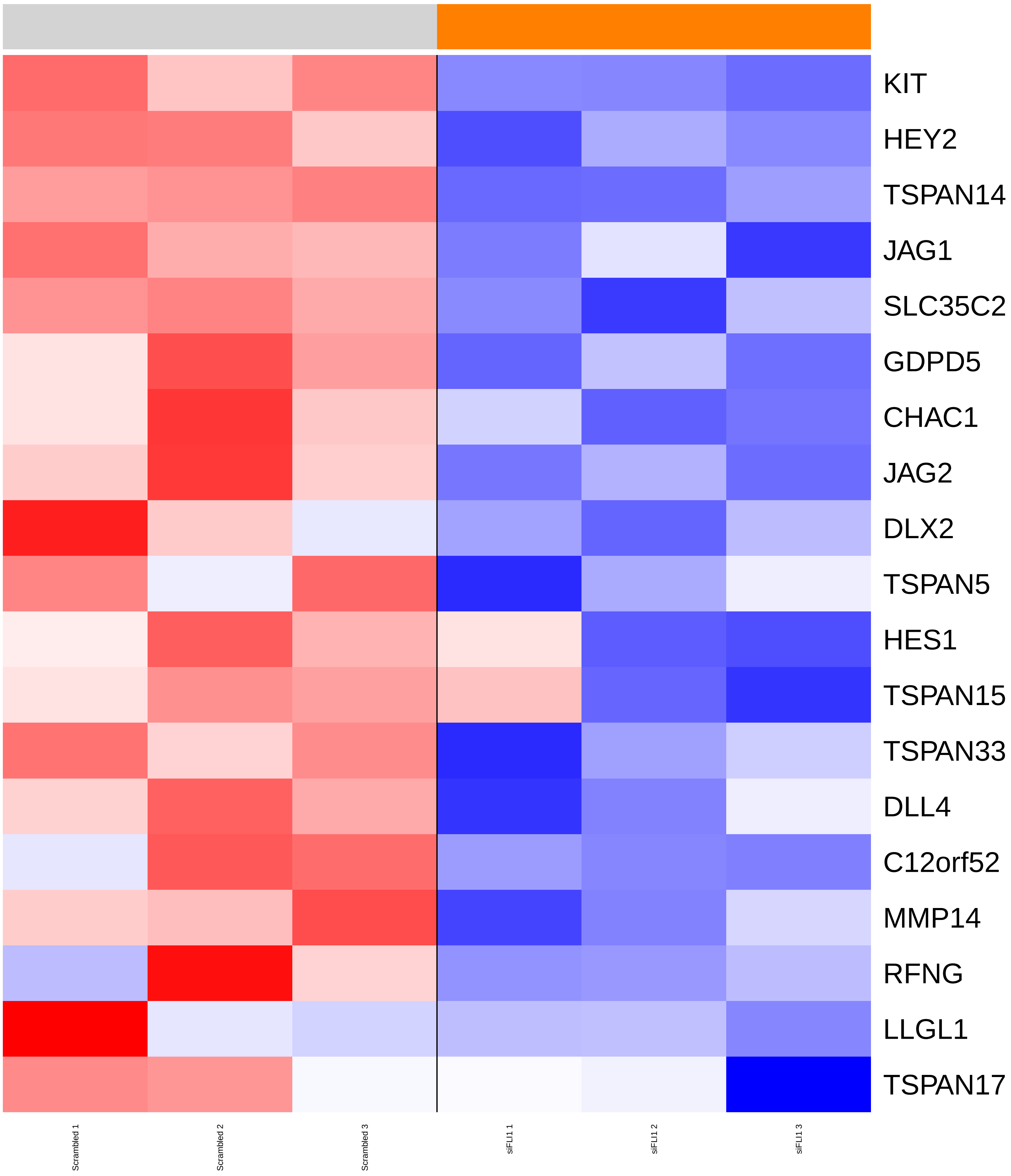

GO_REGULATION_OF_HISTONE_H3_K9_METHYLATION
NES = −1.64, p = 0.0187, FDR q = 0.1429

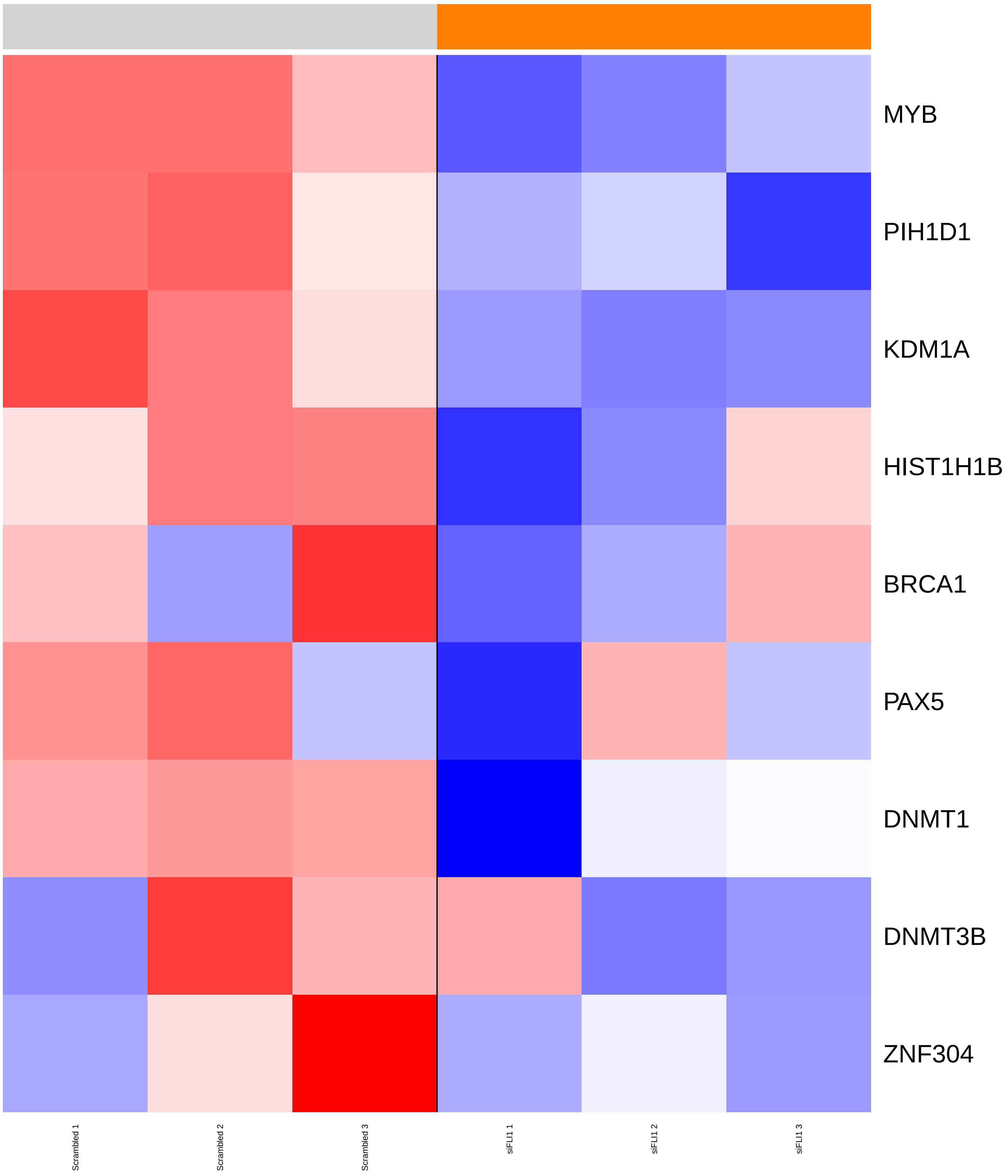

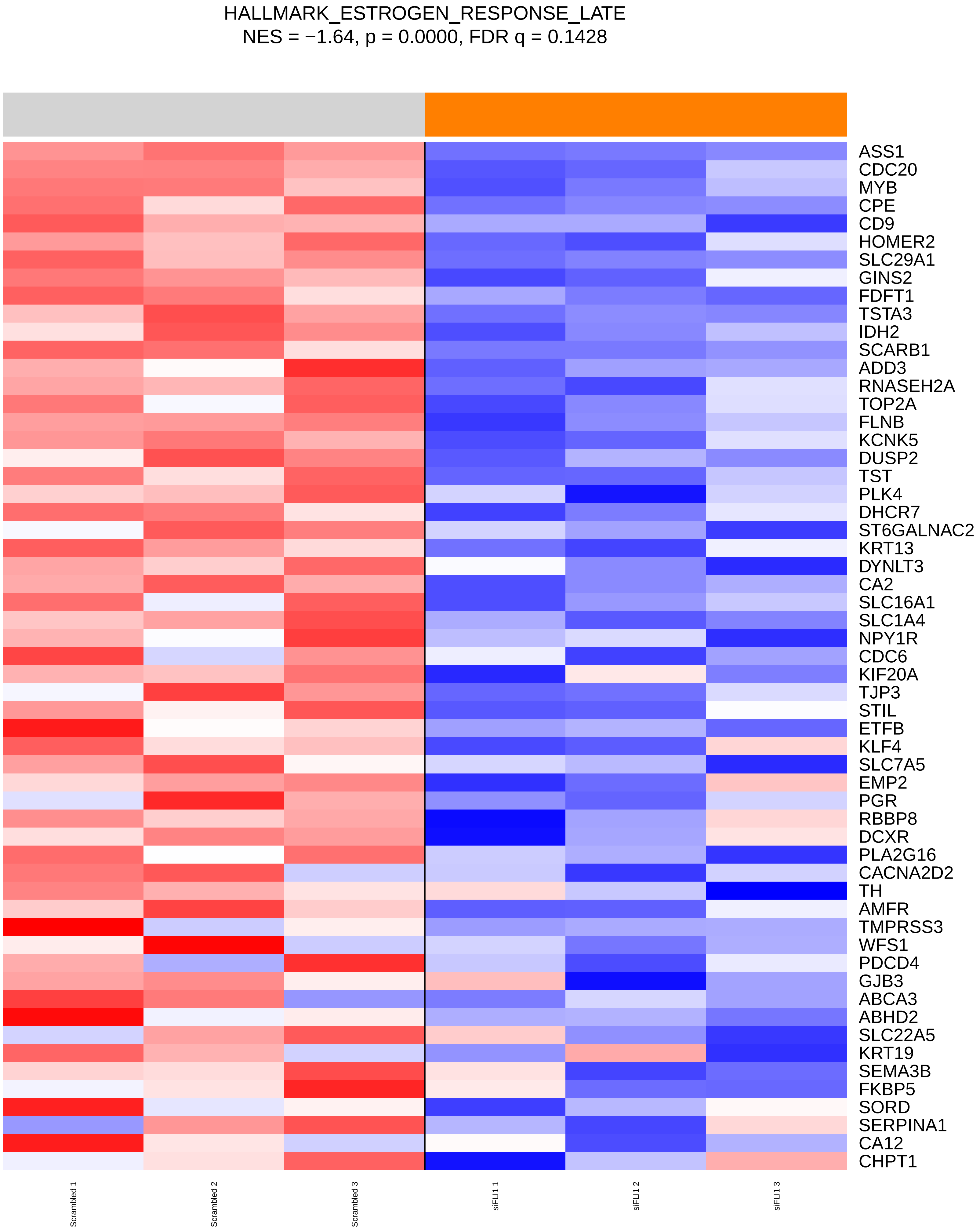
HALLMARK_ESTROGEN_RESPONSE_LATE
NES = −1.64, p = 0.0000, FDR q = 0.1428
ASS1
CDC20
MYB
CPE
CD9
HOMER2
SLC29A1
GINS2
FDFT1
TSTA3
IDH2
SCARB1
ADD3
RNASEH2A
TOP2A
FLNB
KCNK5
DUSP2
TST
PLK4
DHCR7
ST6GALNAC2
KRT13
DYNLT3
CA2
SLC16A1
SLC1A4
NPY1R
CDC6
KIF20A
TJP3
STIL
ETFB
KLF4
SLC7A5
EMP2
PGR
RBBP8
DCXR
PLA2G16
CACNA2D2
TH
AMFR
TMPRSS3
WFS1
PDCD4
GJB3
ABCA3
ABHD2
SLC22A5
KRT19
SEMA3B
FKBP5
SORD
SERPINA1
CA12
CHPT1
Scrambled 1
Scrambled 2
Scrambled 3
siFLI1 1
siFLI1 2
siFLI1 3

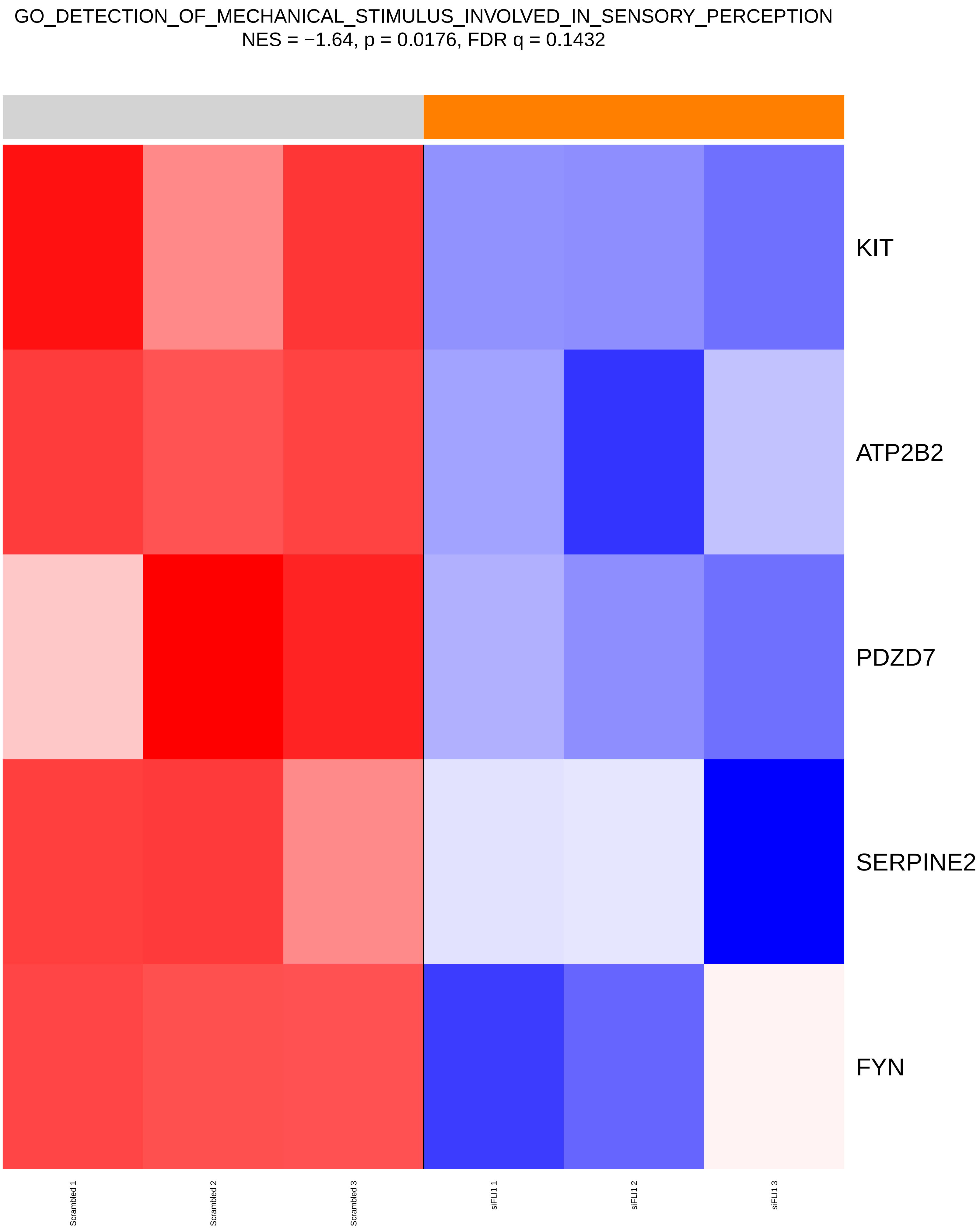
GO_DETECTION_OF_MECHANICAL_STIMULUS_INVOLVED_IN_SENSORY_PERCEPTION
NES = −1.64, p = 0.0176, FDR q = 0.1432
KIT
ATP2B2
PDZD7
SERPINE2
FYN
Scrambled 1
Scrambled 2
Scrambled 3
siFLI1 1
siFLI1 2
siFLI1 3

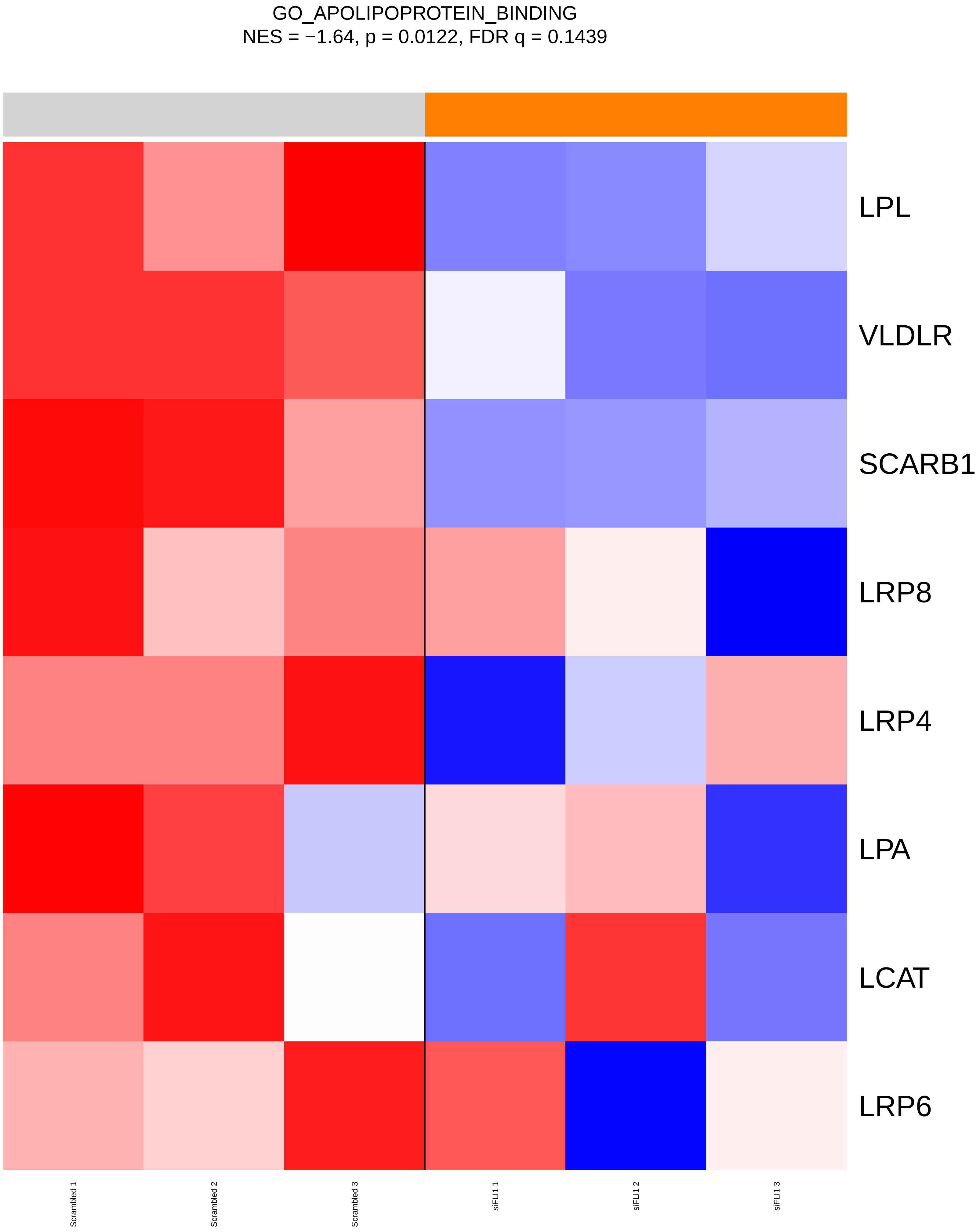
GO_APOLIPOPROTEIN_BINDING
NES = −1.64, p = 0.0122, FDR q = 0.1439
LPL
VLDLR
SCARB1
LRP8
LRP4
LPA
LCAT
LRP6
Scrambled 1
Scrambled 2
Scrambled 3
siFLI1 1
siFLI1 2
siFLI1 3

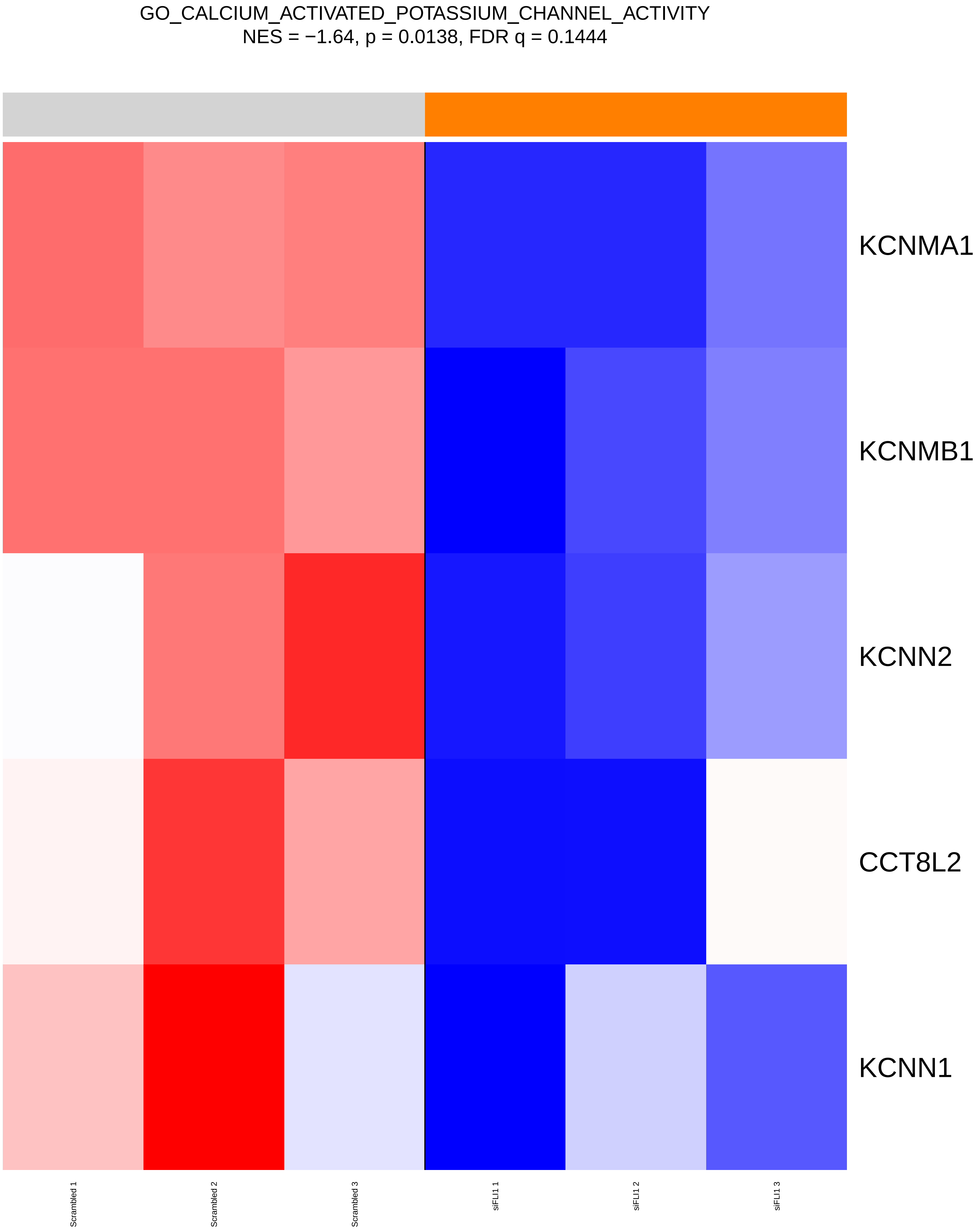
GO_CALCIUM_ACTIVATED_POTASSIUM_CHANNEL_ACTIVITY
NES = −1.64, p = 0.0138, FDR q = 0.1444
KCNMA1
KCNMB1
KCNN2
CCT8L2
KCNN1
Scrambled 1
Scrambled 2
Scrambled 3
siFLI1 1
siFLI1 2
siFLI1 3

GO_PROTEIN_HETEROTETRAMERIZATION
NES = −1.65, p = 0.0098, FDR q = 0.1406

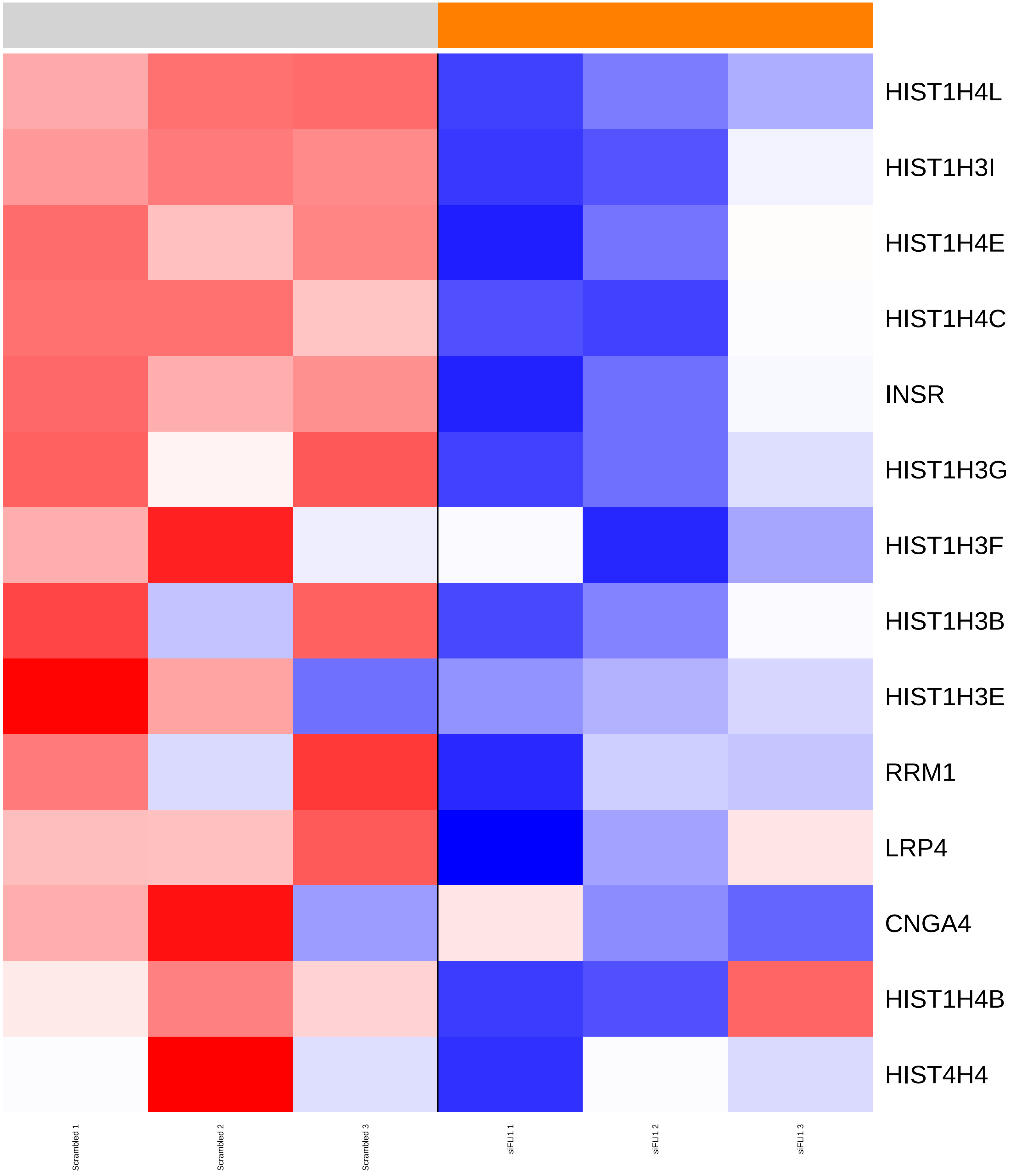

GO_LOW_DENSITY_LIPOPROTEIN_PARTICLE_RECEPTOR_BINDING
NES = −1.65, p = 0.0244, FDR q = 0.1413

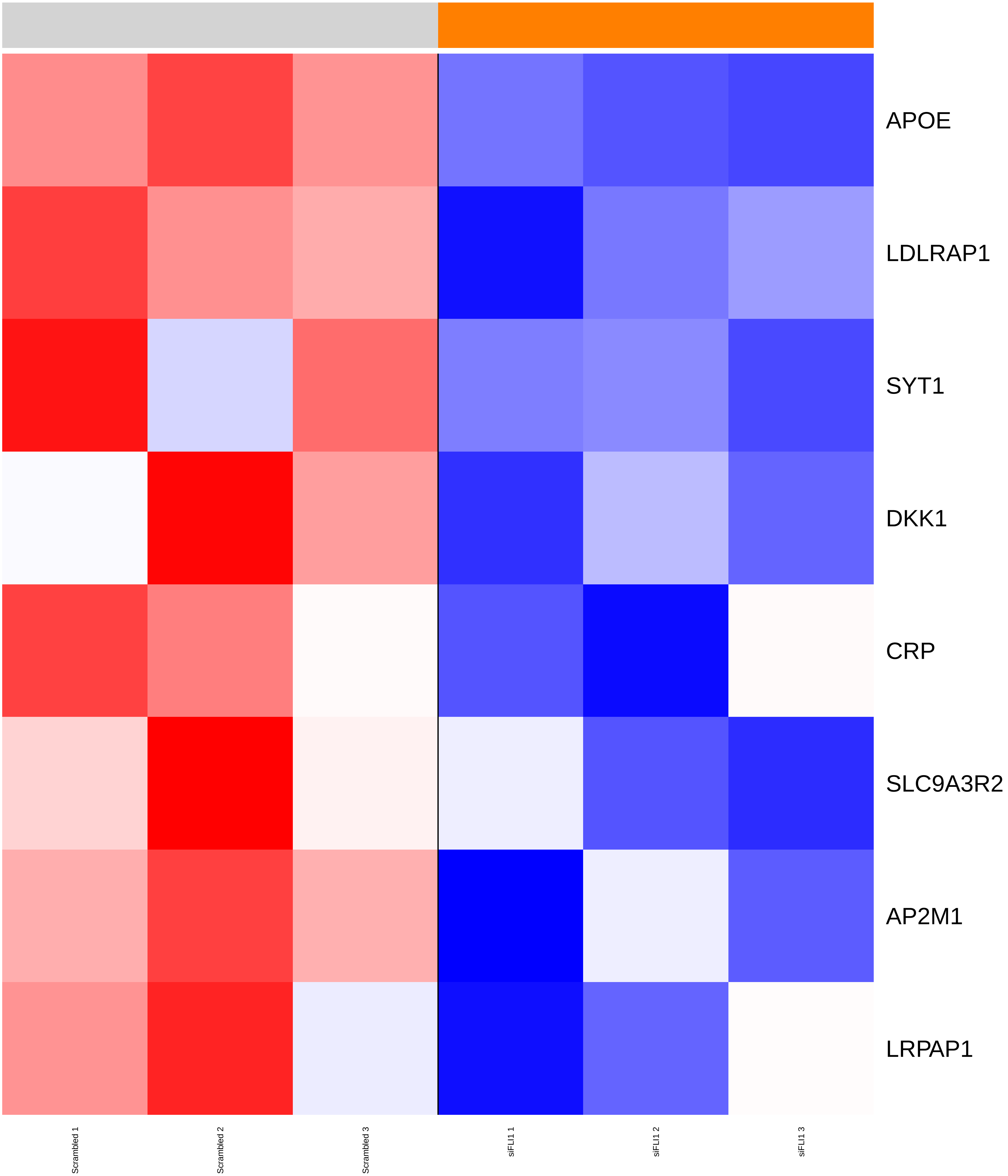

REACTOME_G0_AND_EARLY_G1
NES = −1.65, p = 0.0086, FDR q = 0.1418

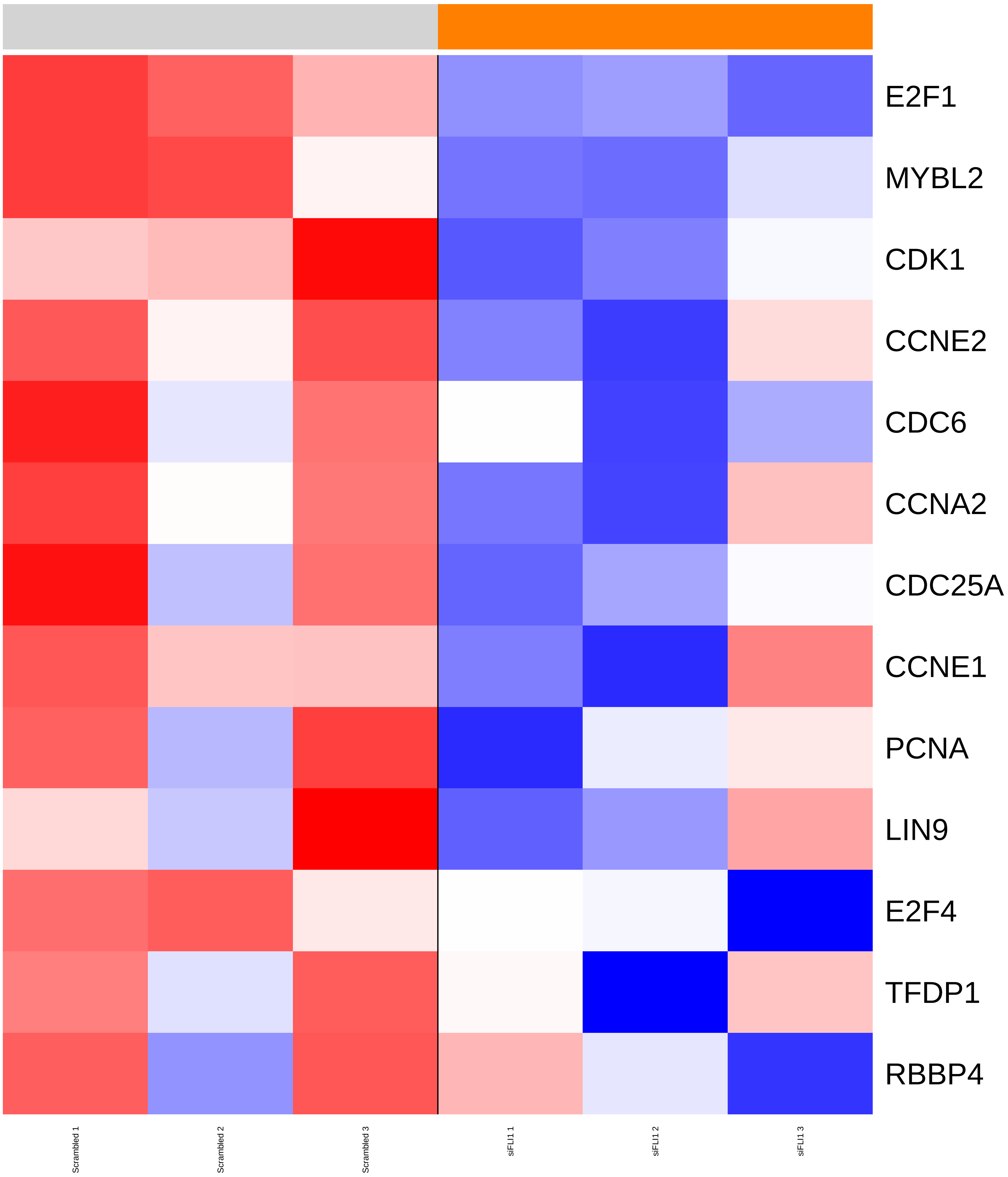

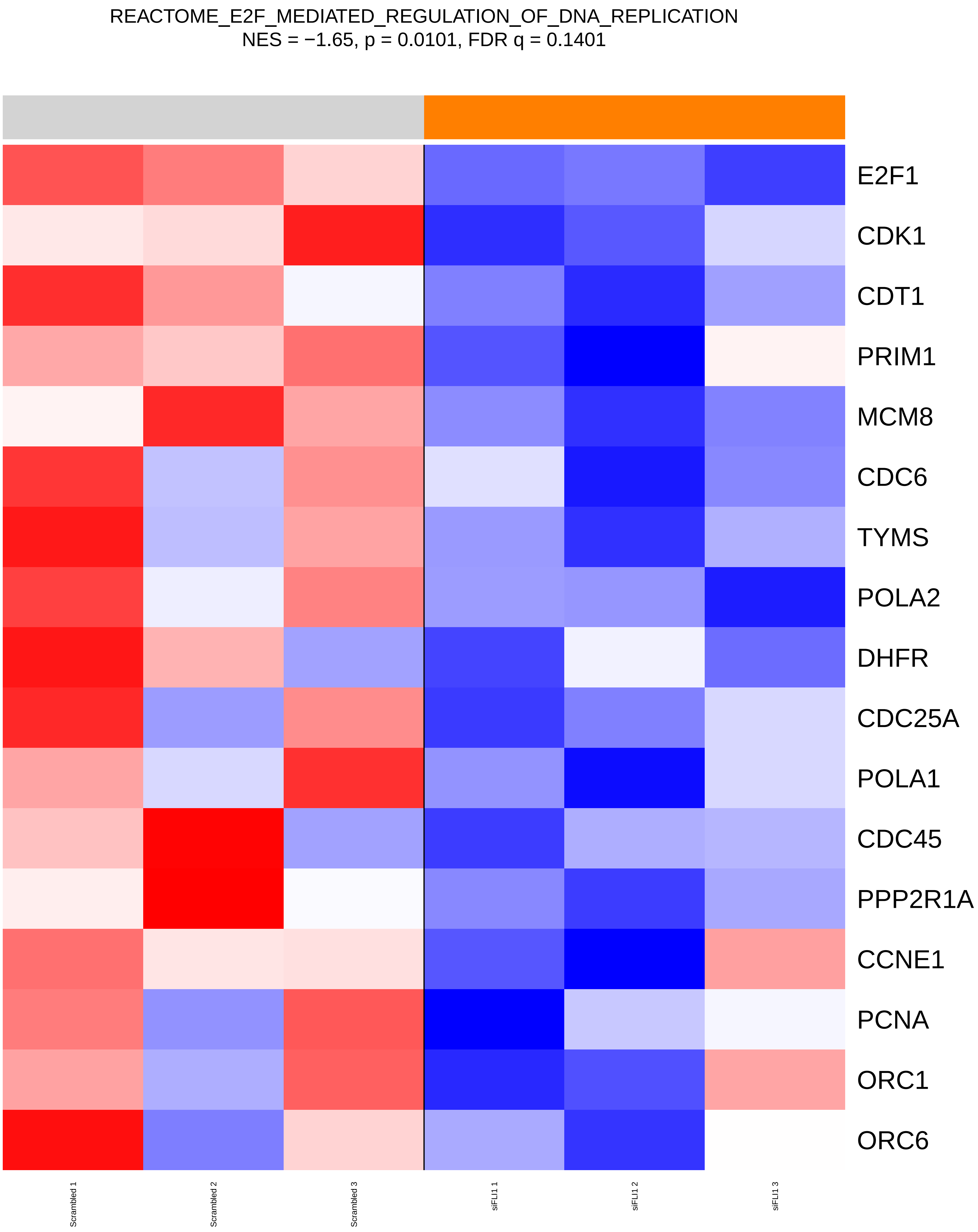

REACTOME_E2F_MEDIATED_REGULATION_OF_DNA_REPLICATION
NES = −1.65, p = 0.0101, FDR q = 0.1401
E2F1
CDK1
CDT1
PRIM1
MCM8
CDC6
TYMS
POLA2
DHFR
CDC25A
POLA1
CDC45
PPP2R1A
CCNE1
PCNA
ORC1
ORC6
Scrambled 1
Scrambled 2
Scrambled 3
siFLI1 1
siFLI1 2
siFLI1 3

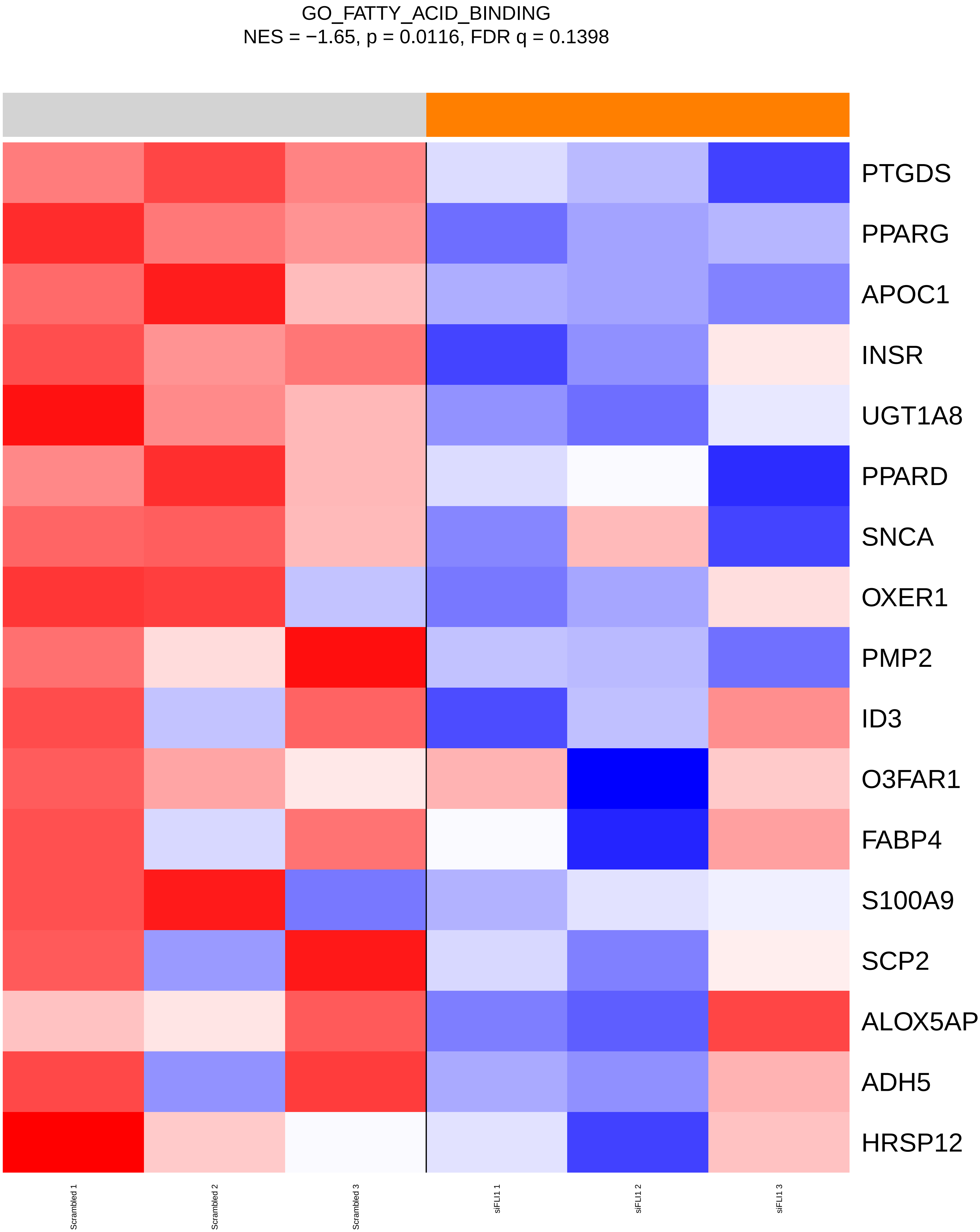
GO_FATTY_ACID_BINDING
NES = −1.65, p = 0.0116, FDR q = 0.1398
PTGDS
PPARG
APOC1
INSR
UGT1A8
PPARD
SNCA
OXER1
PMP2
ID3
O3FAR1
FABP4
S100A9
SCP2
ALOX5AP
ADH5
HRSP12
Scrambled 1
Scrambled 2
Scrambled 3
siFLI1 1
siFLI1 2
siFLI1 3

GO_MITOTIC_DNA_INTEGRITY_CHECKPOINT
NES = −1.65, p = 0.0000, FDR q = 0.1397

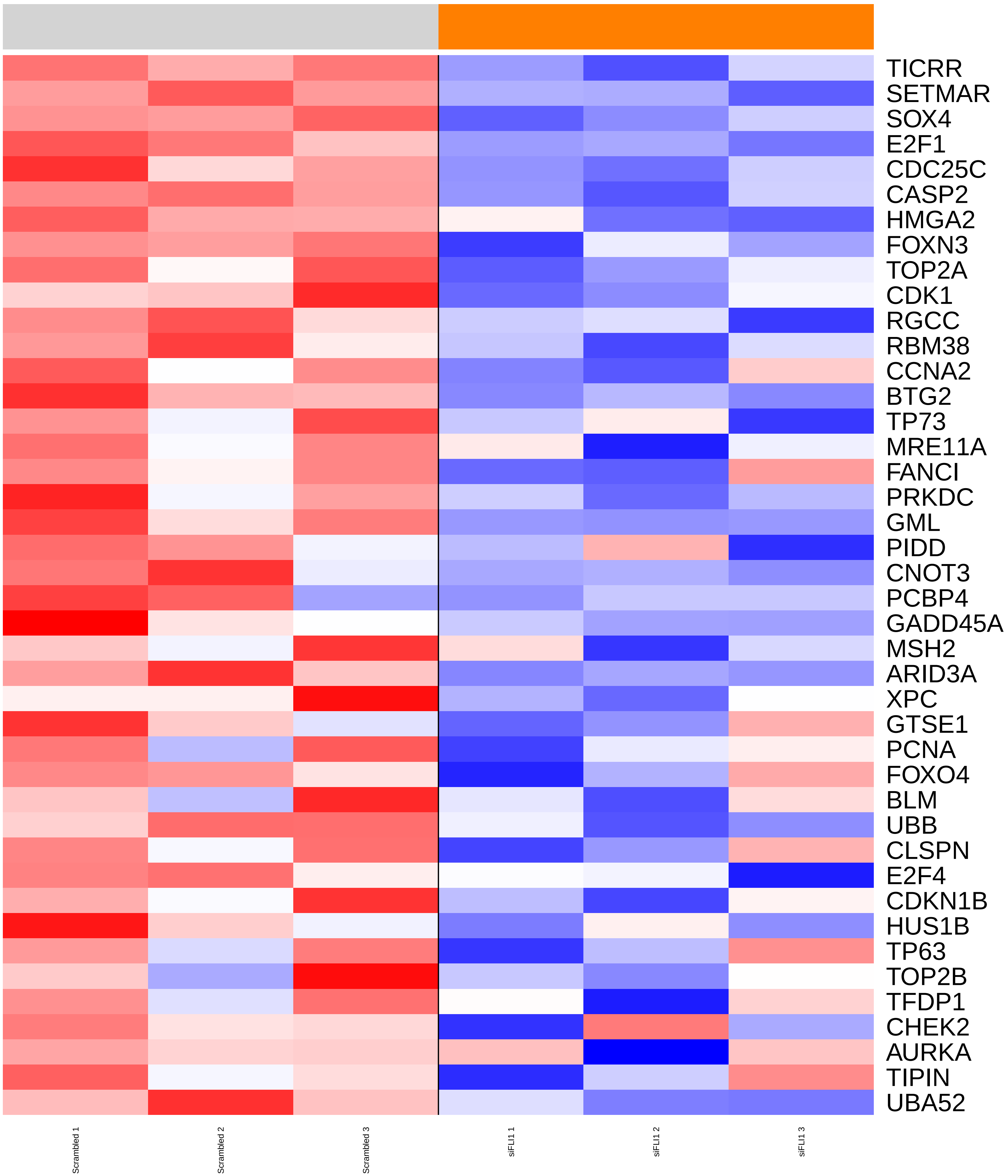

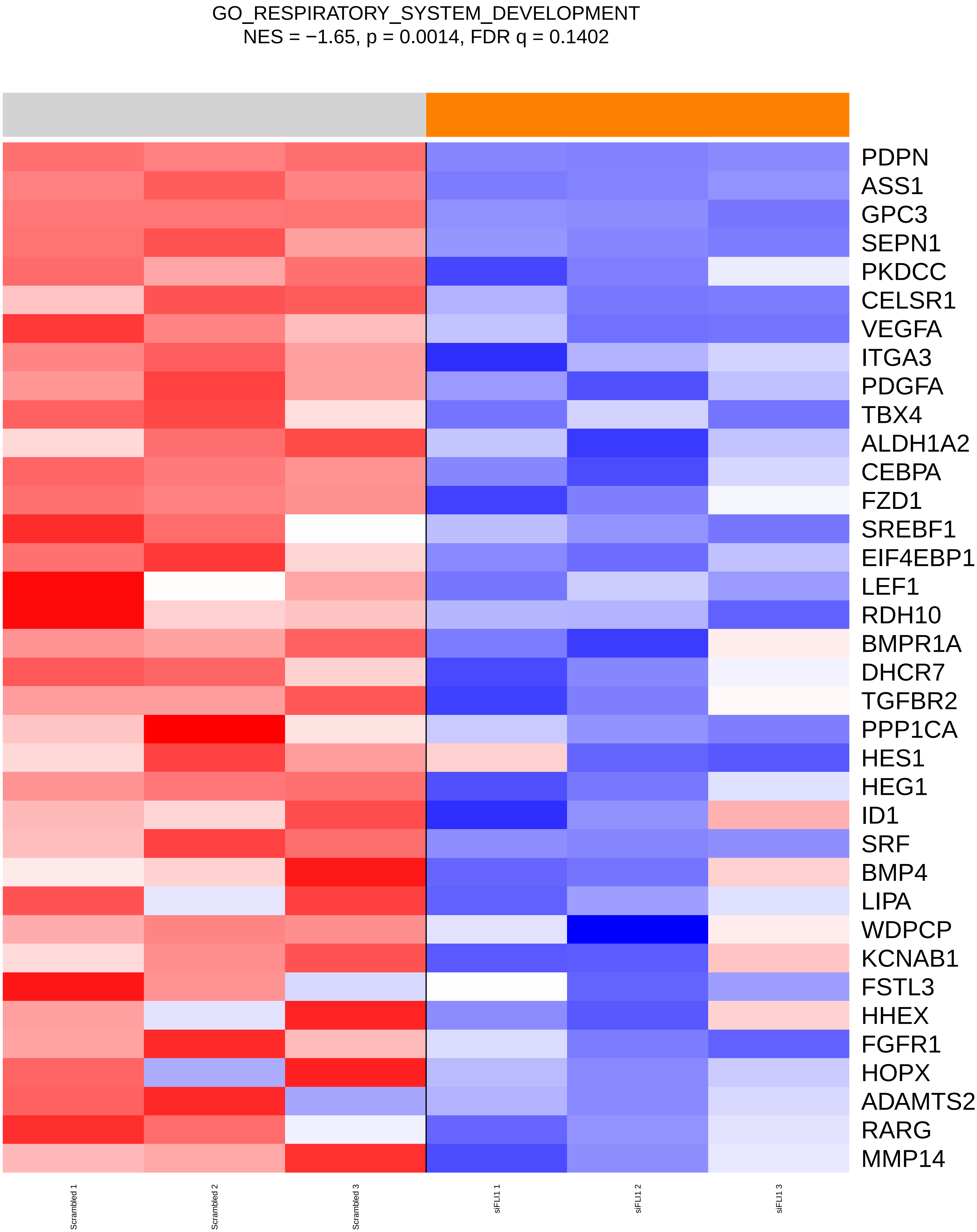
GO_RESPIRATORY_SYSTEM_DEVELOPMENT
NES = −1.65, p = 0.0014, FDR q = 0.1402
PDPN
ASS1
GPC3
SEPN1
PKDCC
CELSR1
VEGFA
ITGA3
PDGFA
TBX4
ALDH1A2
CEBPA
FZD1
SREBF1
EIF4EBP1
LEF1
RDH10
BMPR1A
DHCR7
TGFBR2
PPP1CA
HES1
HEG1
ID1
SRF
BMP4
LIPA
WDPCP
KCNAB1
FSTL3
HHEX
FGFR1
HOPX
ADAMTS2
RARG
MMP14
Scrambled 1
Scrambled 2
Scrambled 3
siFLI1 1
siFLI1 2
siFLI1 3

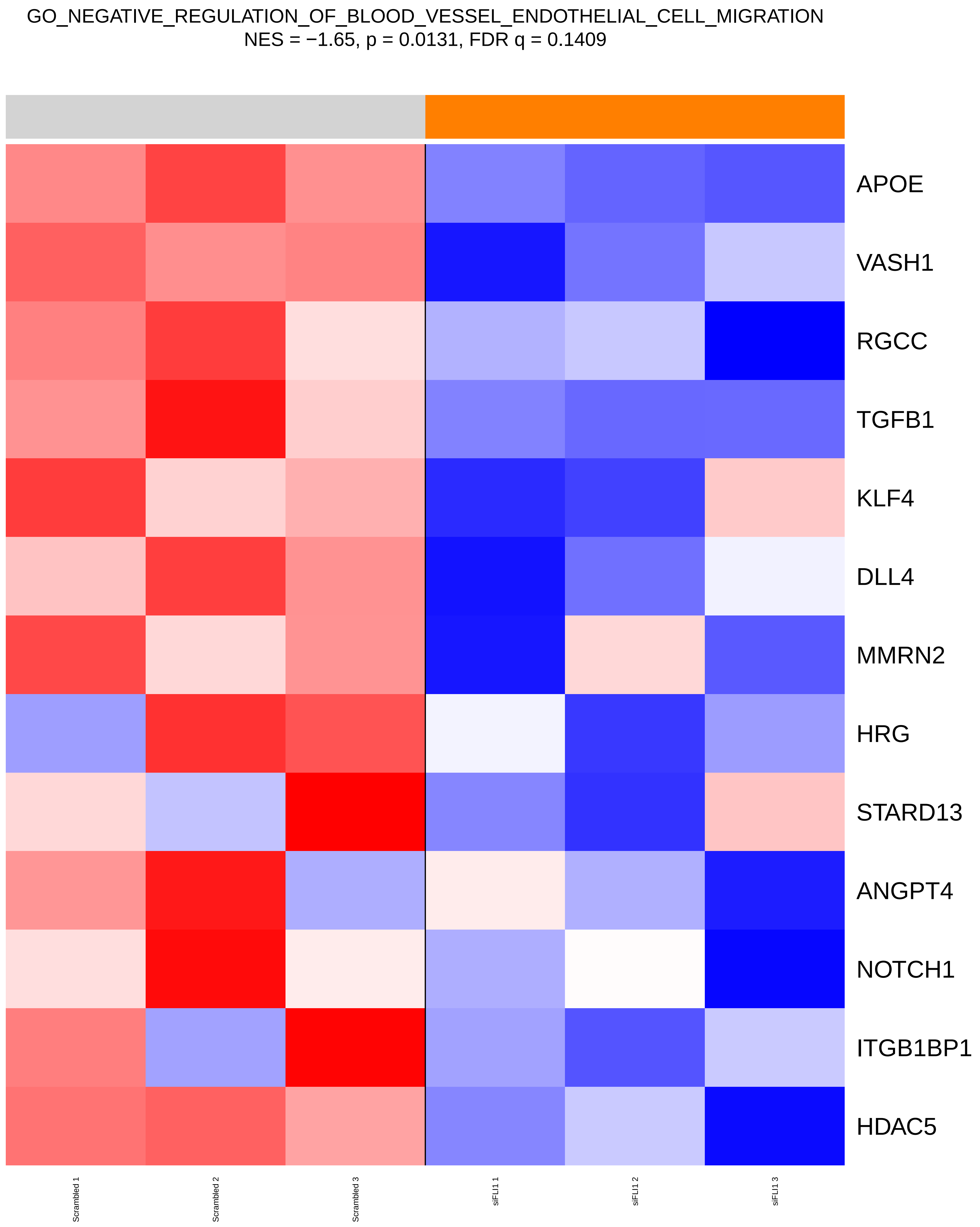
GO_NEGATIVE_REGULATION_OF_BLOOD_VESSEL_ENDOTHELIAL_CELL_MIGRATION
NES = −1.65, p = 0.0131, FDR q = 0.1409
APOE
VASH1
RGCC
TGFB1
KLF4
DLL4
MMRN2
HRG
STARD13
ANGPT4
NOTCH1
ITGB1BP1
HDAC5
Scrambled 1
Scrambled 2
Scrambled 3
siFLI1 1
siFLI1 2
siFLI1 3

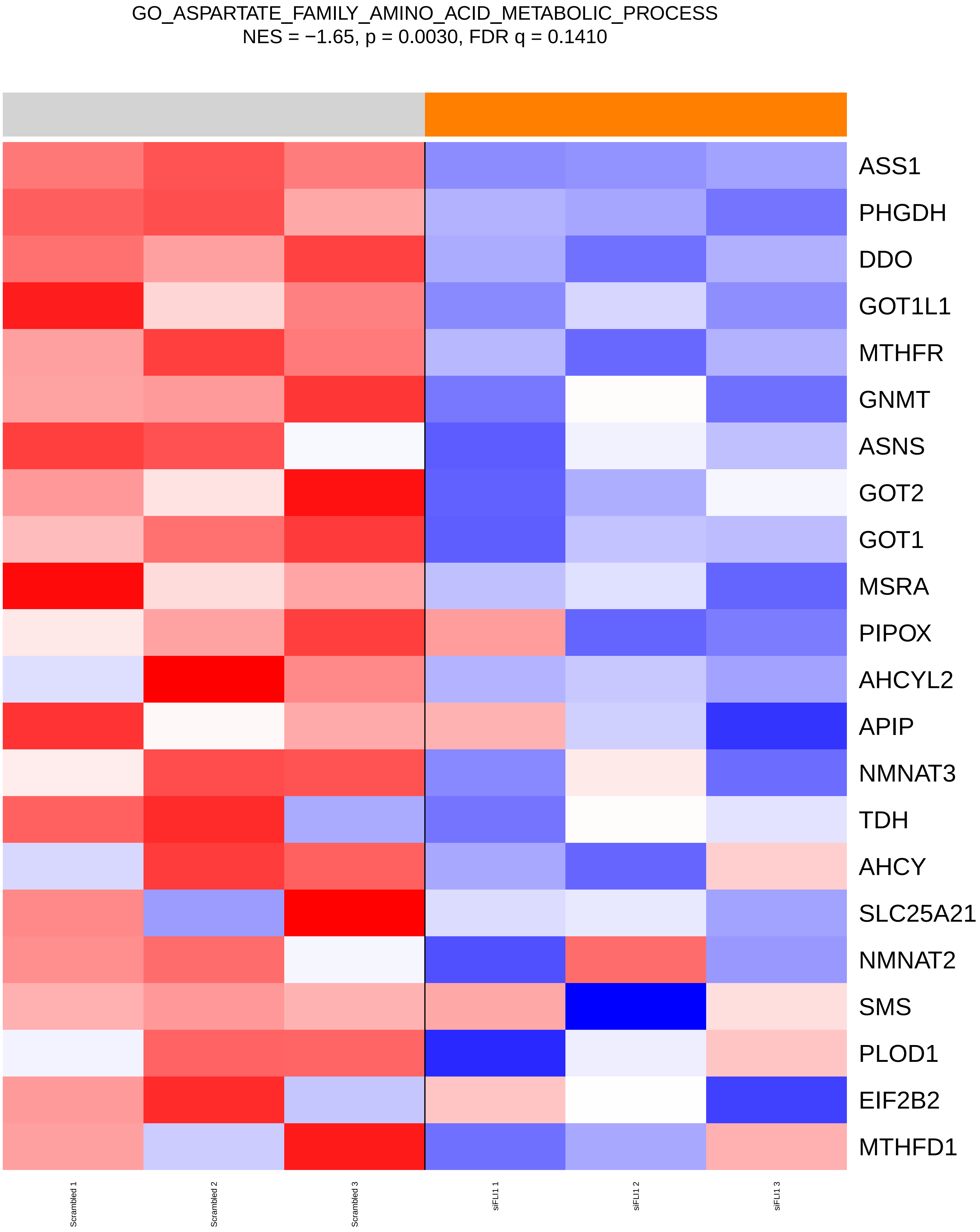
GO_ASPARTATE_FAMILY_AMINO_ACID_METABOLIC_PROCESS
NES = −1.65, p = 0.0030, FDR q = 0.1410
ASS1
PHGDH
DDO
GOT1L1
MTHFR
GNMT
ASNS
GOT2
GOT1
MSRA
PIPOX
AHCYL2
APIP
NMNAT3
TDH
AHCY
SLC25A21
NMNAT2
SMS
PLOD1
EIF2B2
MTHFD1
Scrambled 1
Scrambled 2
Scrambled 3
siFLI1 1
siFLI1 2
siFLI1 3

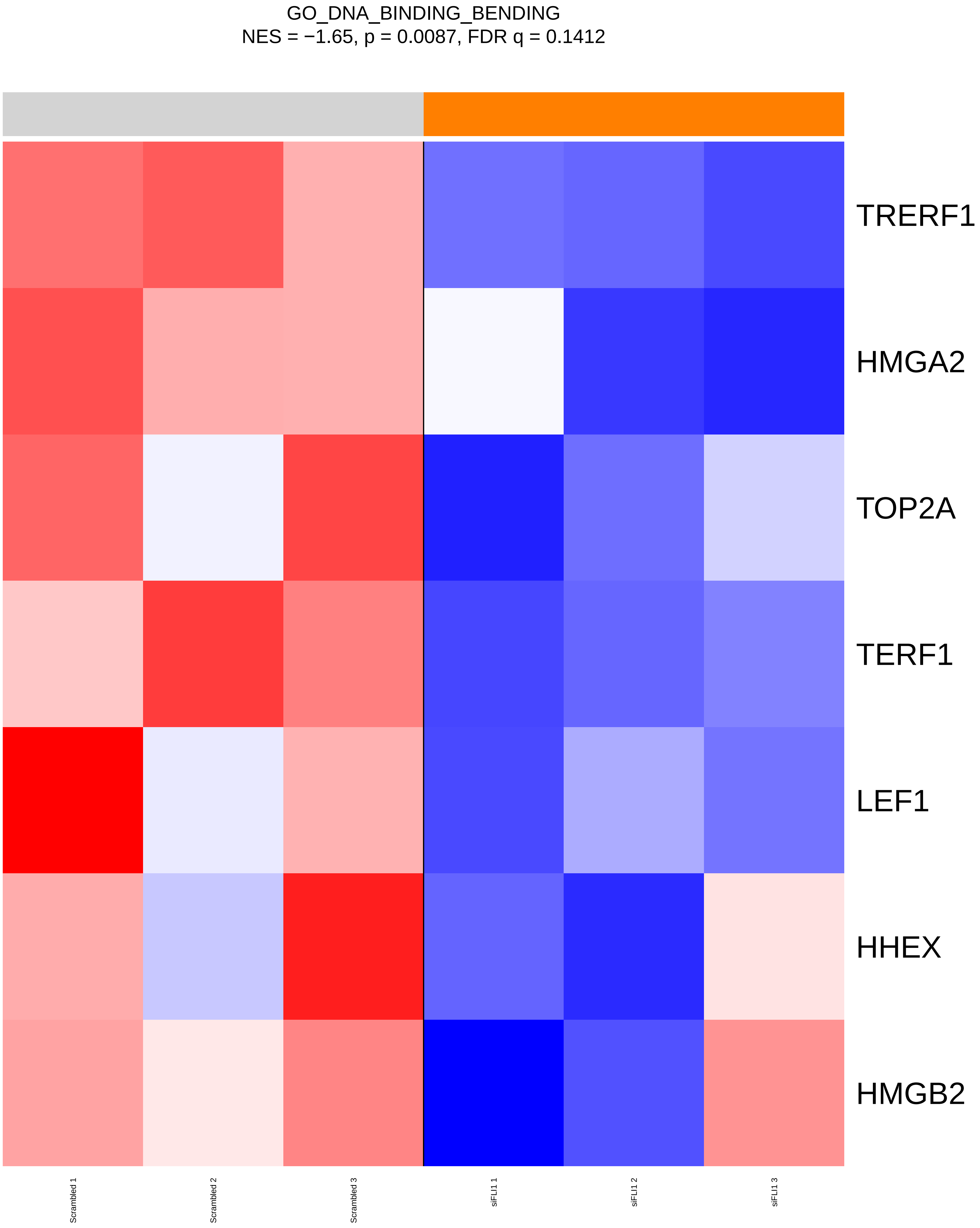
GO_DNA_BINDING_BENDING
NES = −1.65, p = 0.0087, FDR q = 0.1412
TRERF1
HMGA2
TOP2A
TERF1
LEF1
HHEX
HMGB2
Scrambled 1
Scrambled 2
Scrambled 3
siFLI1 1
siFLI1 2
siFLI1 3

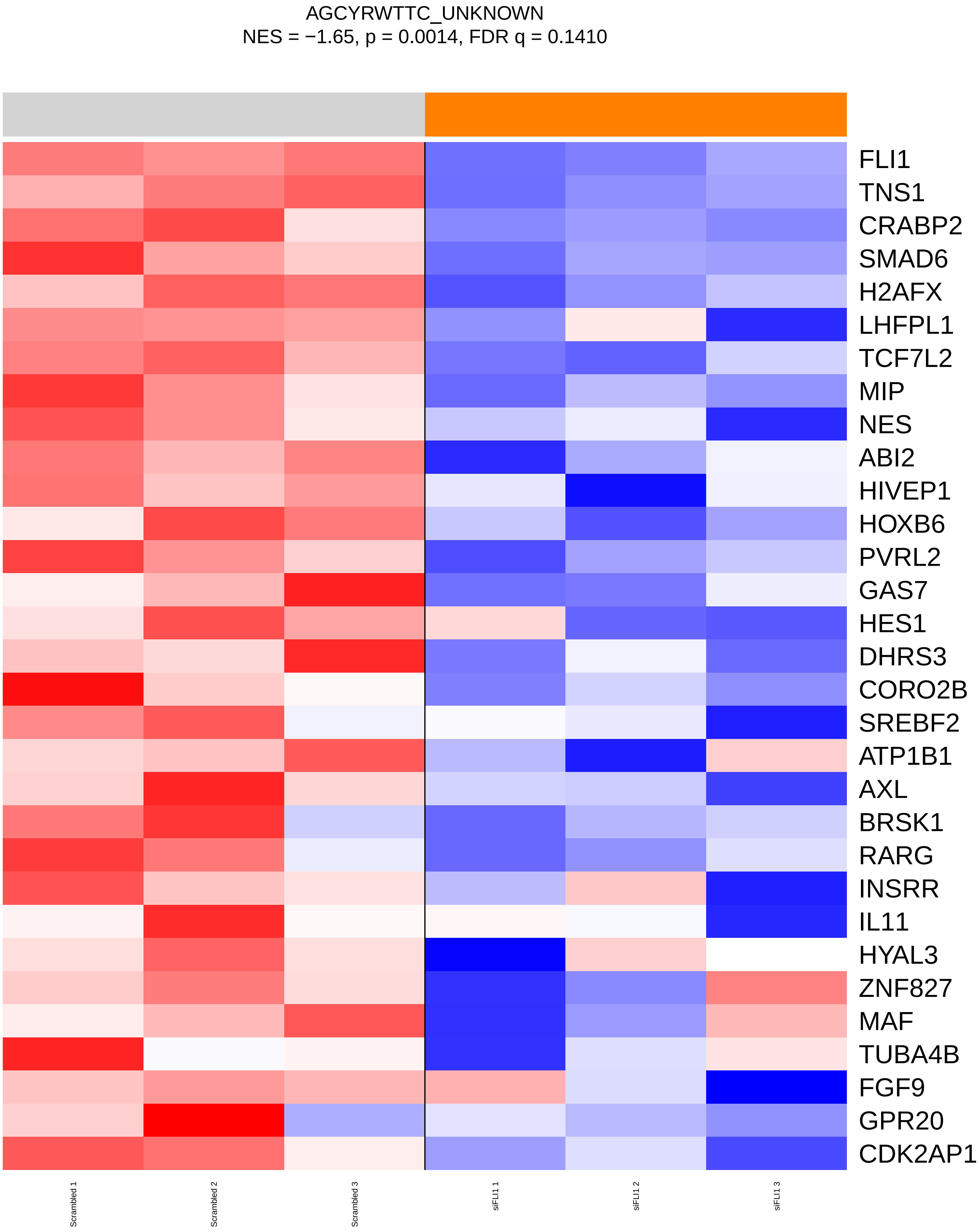
AGCYRWTTC_UNKNOWN
NES = −1.65, p = 0.0014, FDR q = 0.1410
FLI1
TNS1
CRABP2
SMAD6
H2AFX
LHFPL1
TCF7L2
MIP
NES
ABI2
HIVEP1
HOXB6
PVRL2
GAS7
HES1
DHRS3
CORO2B
SREBF2
ATP1B1
AXL
BRSK1
RARG
INSRR
IL11
HYAL3
ZNF827
MAF
TUBA4B
FGF9
GPR20
CDK2AP1
Scrambled 1
Scrambled 2
Scrambled 3
siFLI1 1
siFLI1 2
siFLI1 3

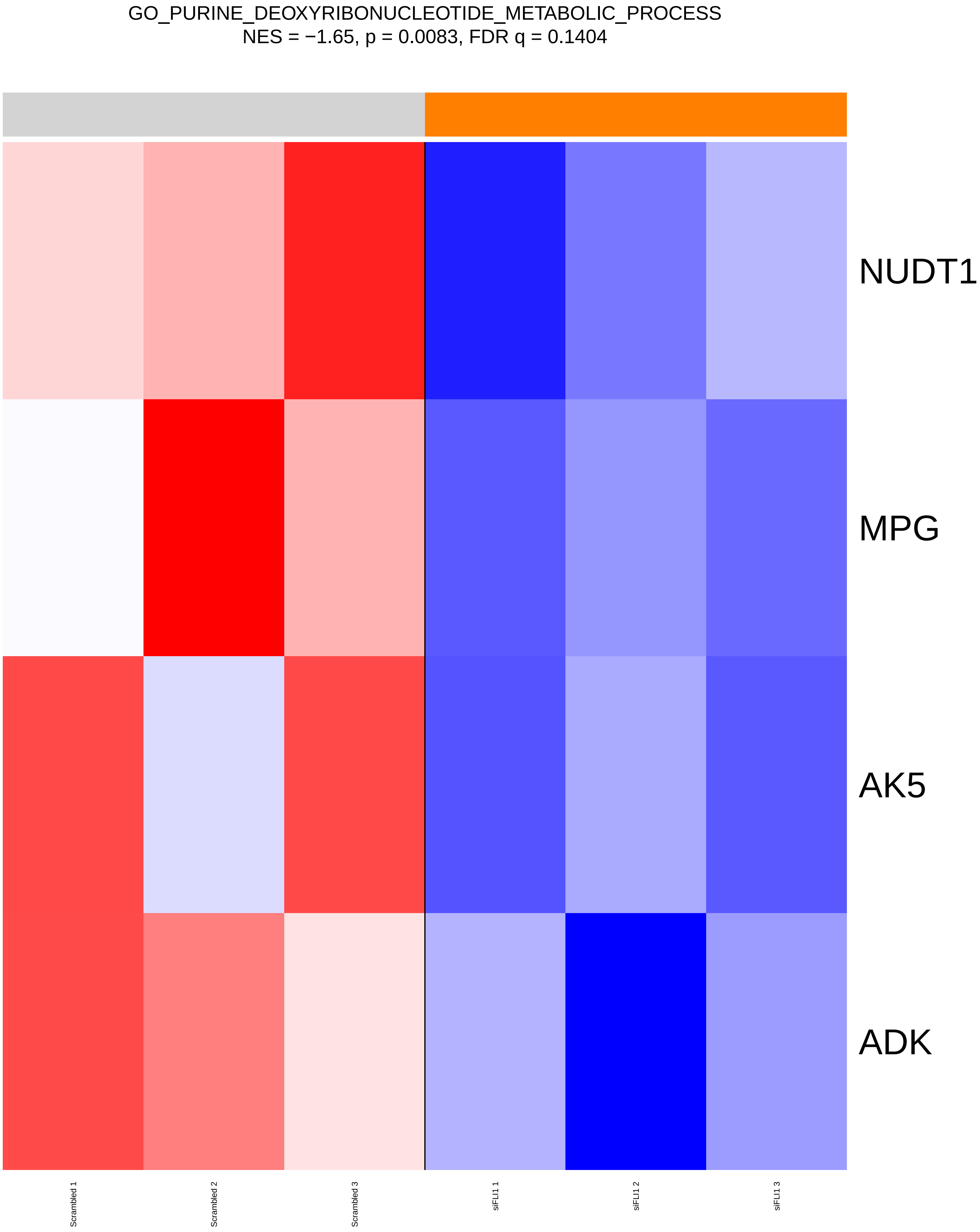
GO_PURINE_DEOXYRIBONUCLEOTIDE_METABOLIC_PROCESS
NES = −1.65, p = 0.0083, FDR q = 0.1404
NUDT1
MPG
AK5
ADK
Scrambled 1
Scrambled 2
Scrambled 3
siFLI1 1
siFLI1 2
siFLI1 3

GO_RETINOID_X_RECEPTOR_BINDING
NES = −1.65, p = 0.0070, FDR q = 0.1410

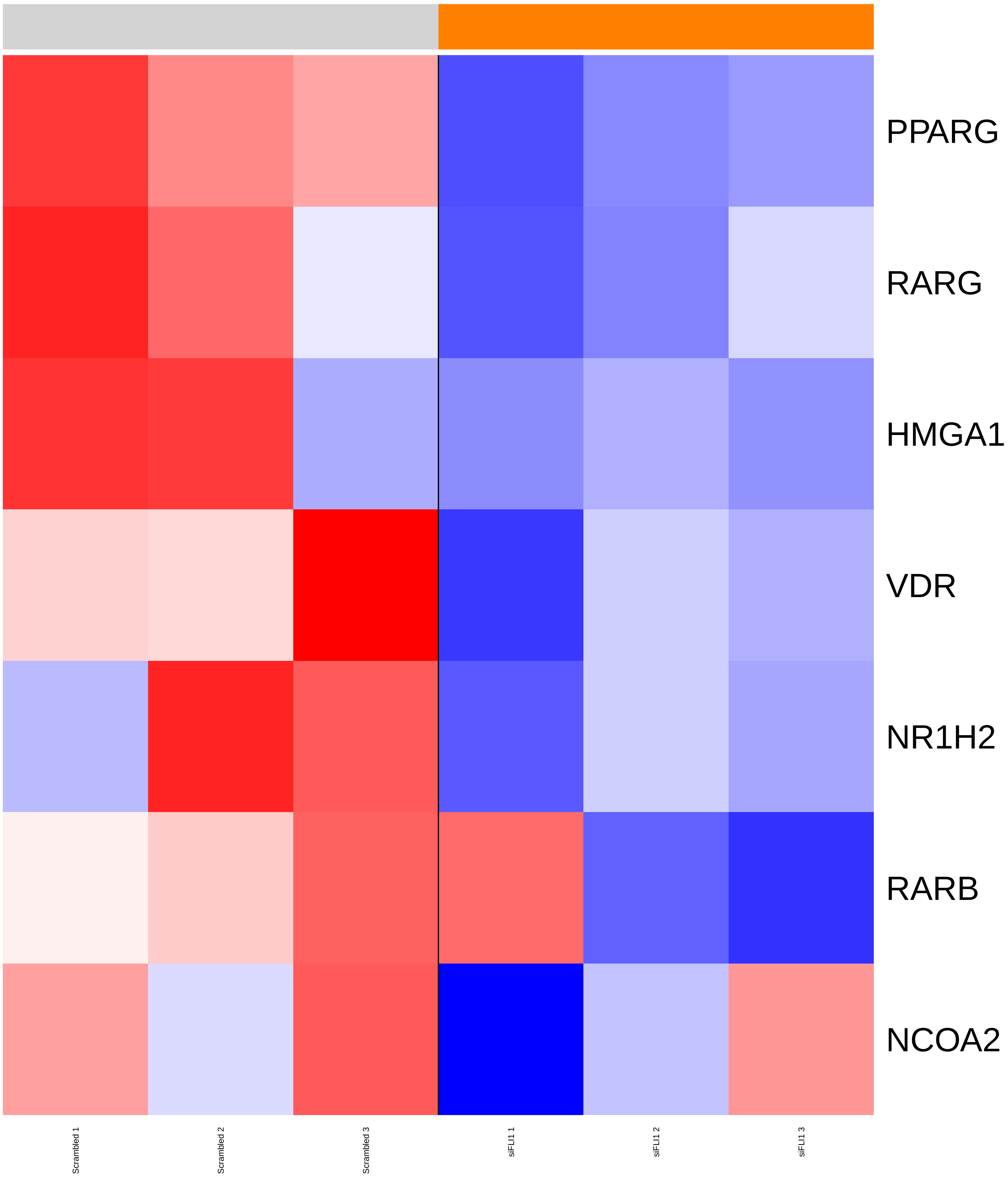

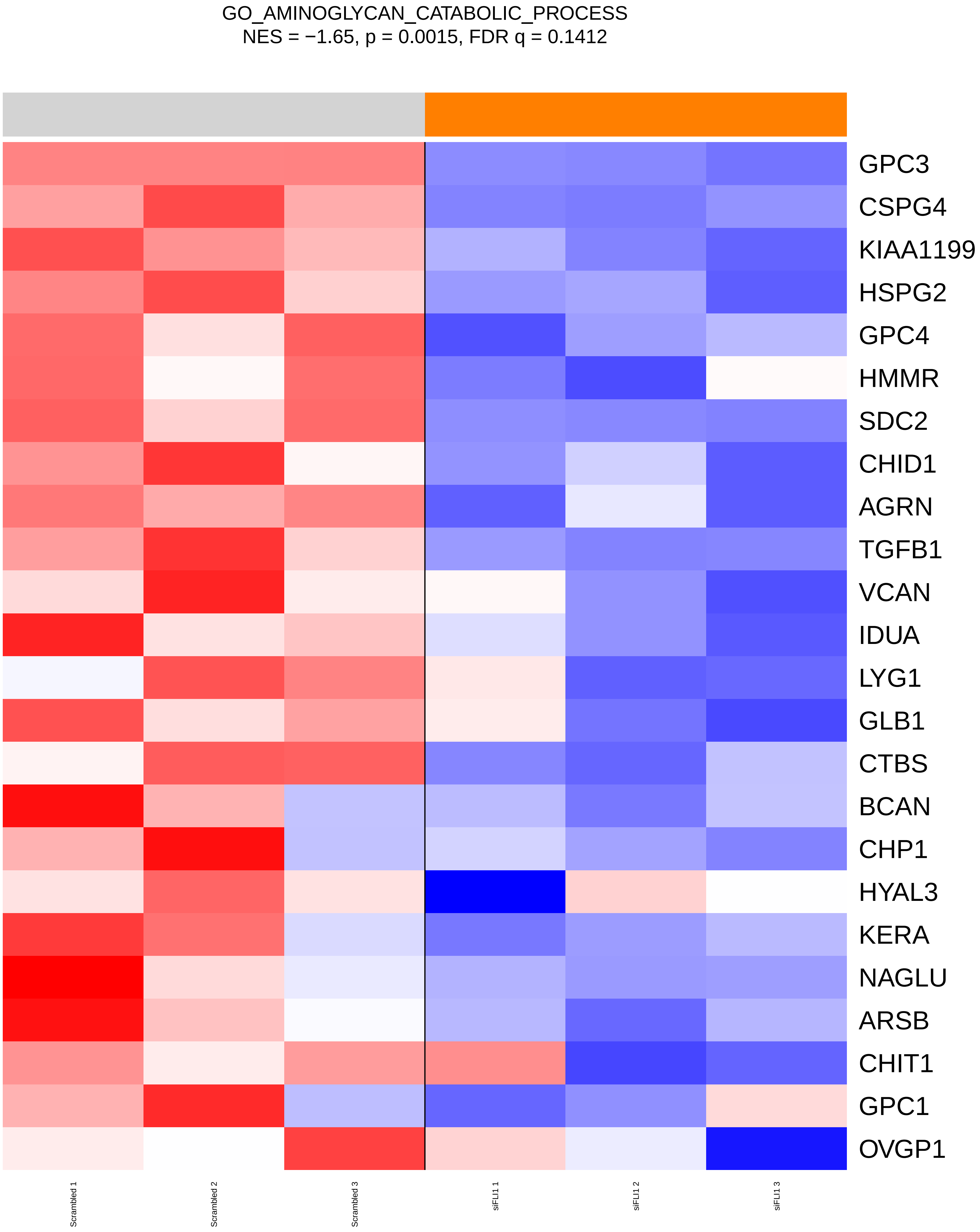
GO_AMINOGLYCAN_CATABOLIC_PROCESS
NES = −1.65, p = 0.0015, FDR q = 0.1412
GPC3
CSPG4
KIAA1199
HSPG2
GPC4
HMMR
SDC2
CHID1
AGRN
TGFB1
VCAN
IDUA
LYG1
GLB1
CTBS
BCAN
CHP1
HYAL3
KERA
NAGLU
ARSB
CHIT1
GPC1
OVGP1
Scrambled 1
Scrambled 2
Scrambled 3
siFLI1 1
siFLI1 2
siFLI1 3

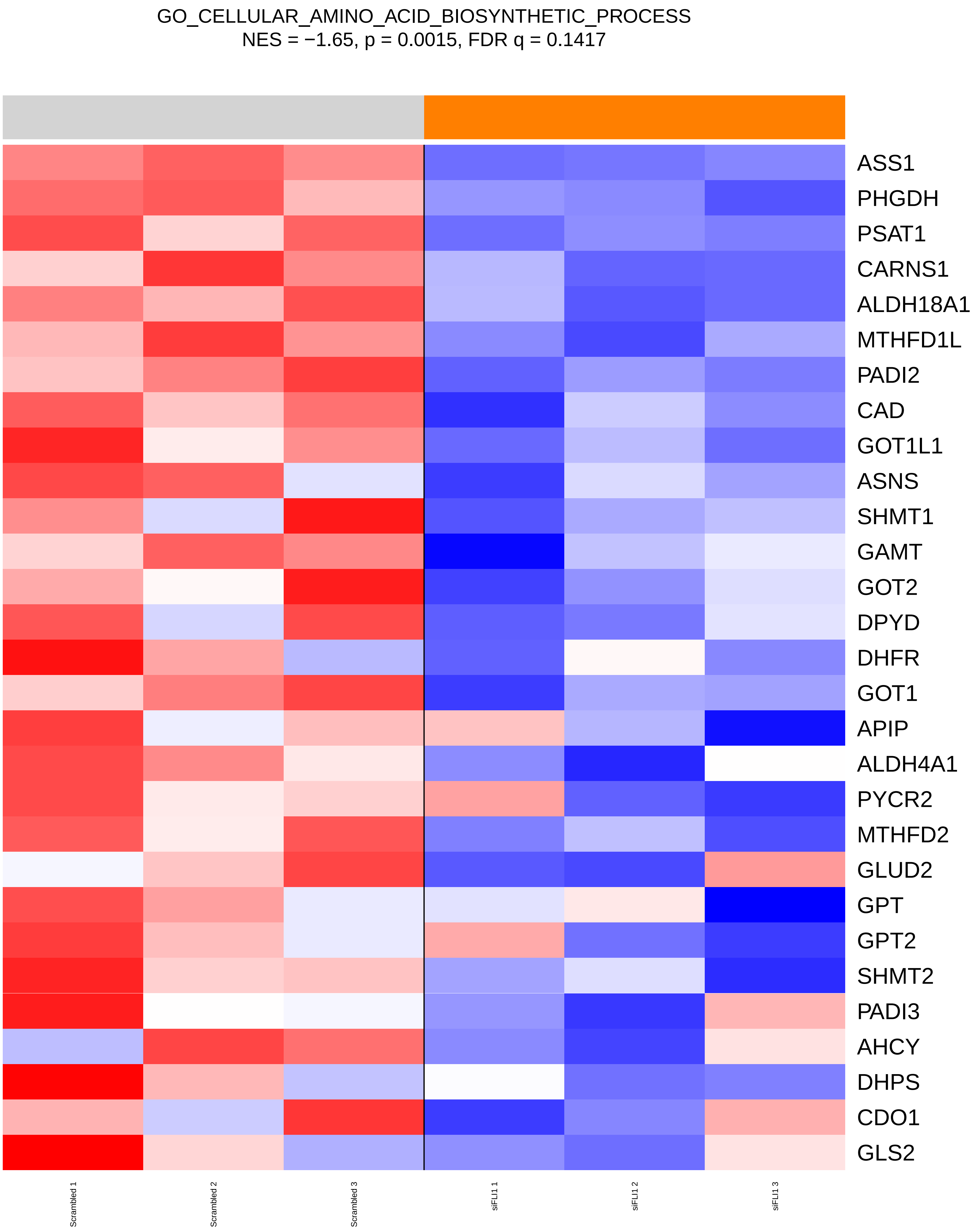
GO_CELLULAR_AMINO_ACID_BIOSYNTHETIC_PROCESS
NES = −1.65, p = 0.0015, FDR q = 0.1417
ASS1
PHGDH
PSAT1
CARNS1
ALDH18A1
MTHFD1L
PADI2
CAD
GOT1L1
ASNS
SHMT1
GAMT
GOT2
DPYD
DHFR
GOT1
APIP
ALDH4A1
PYCR2
MTHFD2
GLUD2
GPT
GPT2
SHMT2
PADI3
AHCY
DHPS
CDO1
GLS2
Scrambled 1
Scrambled 2
Scrambled 3
siFLI1 1
siFLI1 2
siFLI1 3

REACTOME_CELL_CYCLE_MITOTIC
NES = −1.65, p = 0.0000, FDR q = 0.1424

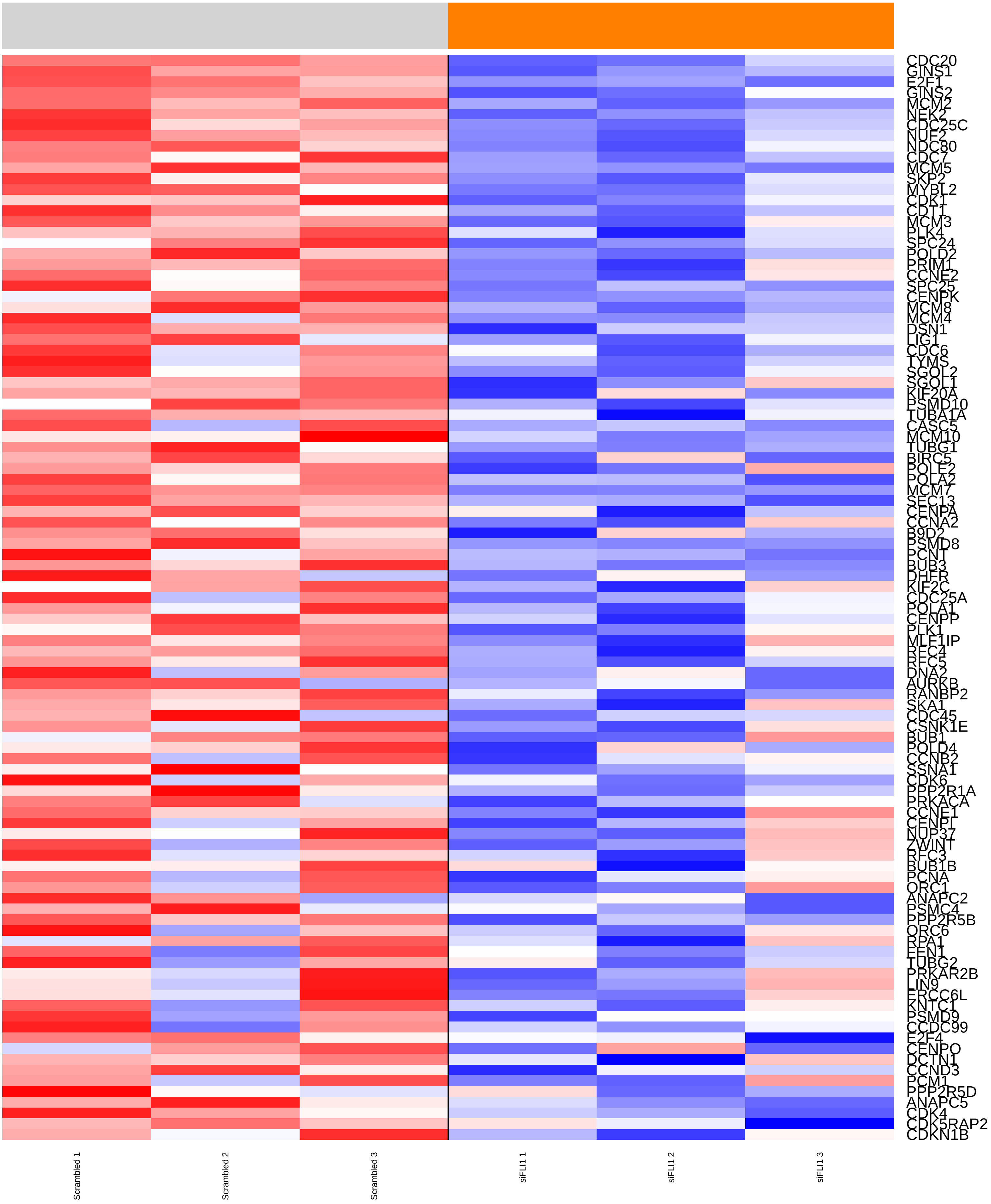

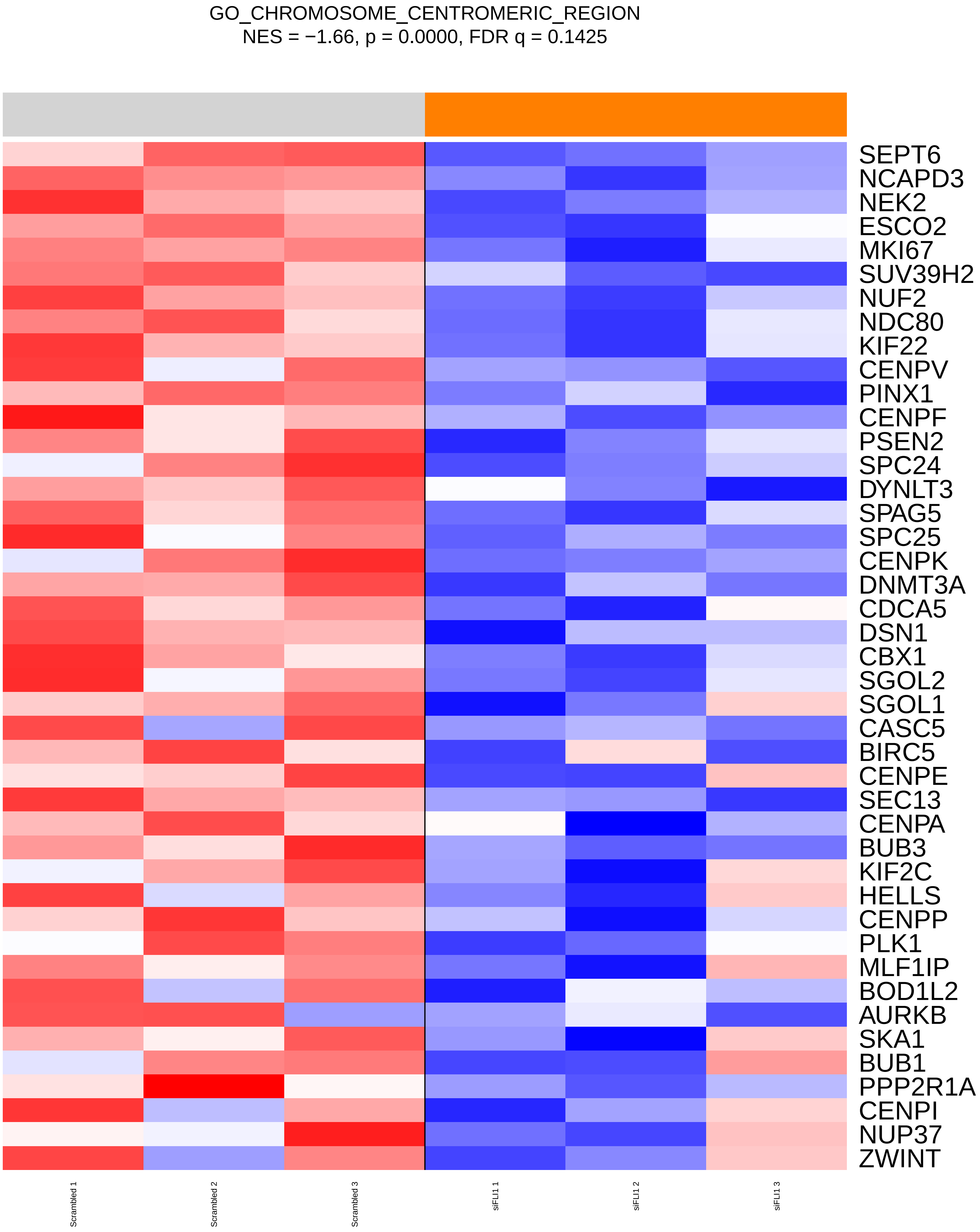
GO_CHROMOSOME_CENTROMERIC_REGION
NES = −1.66, p = 0.0000, FDR q = 0.1425
SEPT6
NCAPD3
NEK2
ESCO2
MKI67
SUV39H2
NUF2
NDC80
KIF22
CENPV
PINX1
CENPF
PSEN2
SPC24
DYNLT3
SPAG5
SPC25
CENPK
DNMT3A
CDCA5
DSN1
CBX1
SGOL2
SGOL1
CASC5
BIRC5
CENPE
SEC13
CENPA
BUB3
KIF2C
HELLS
CENPP
PLK1
MLF1IP
BOD1L2
AURKB
SKA1
BUB1
PPP2R1A
CENPI
NUP37
ZWINT
Scrambled 1
Scrambled 2
Scrambled 3
siFLI1 1
siFLI1 2
siFLI1 3

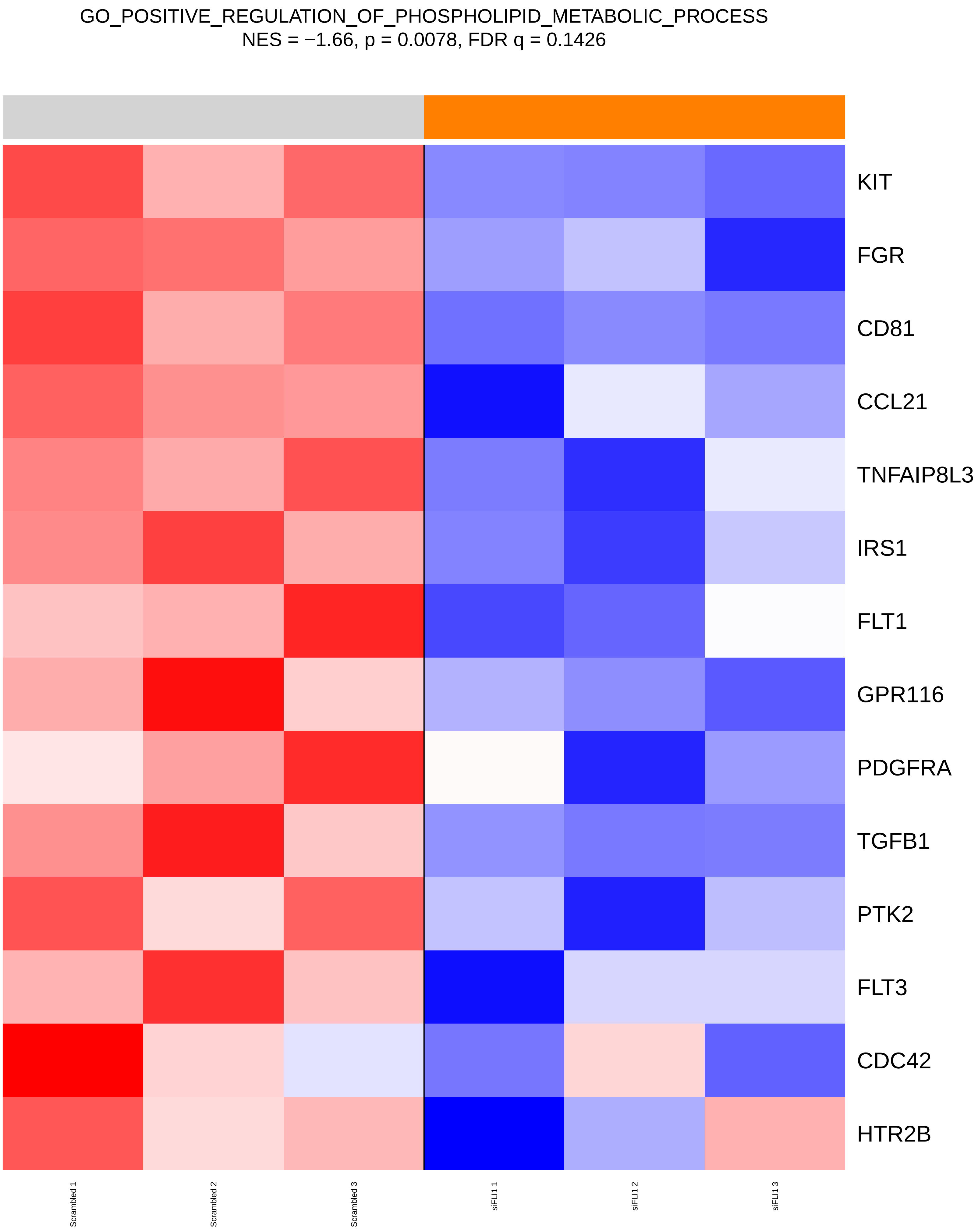
GO_POSITIVE_REGULATION_OF_PHOSPHOLIPID_METABOLIC_PROCESS
NES = −1.66, p = 0.0078, FDR q = 0.1426
KIT
FGR
CD81
CCL21
TNFAIP8L3
IRS1
FLT1
GPR116
PDGFRA
TGFB1
PTK2
FLT3
CDC42
HTR2B
Scrambled 1
Scrambled 2
Scrambled 3
siFLI1 1
siFLI1 2
siFLI1 3

GO_DETECTION_OF_MECHANICAL_STIMULUS
NES = −1.66, p = 0.0064, FDR q = 0.1433

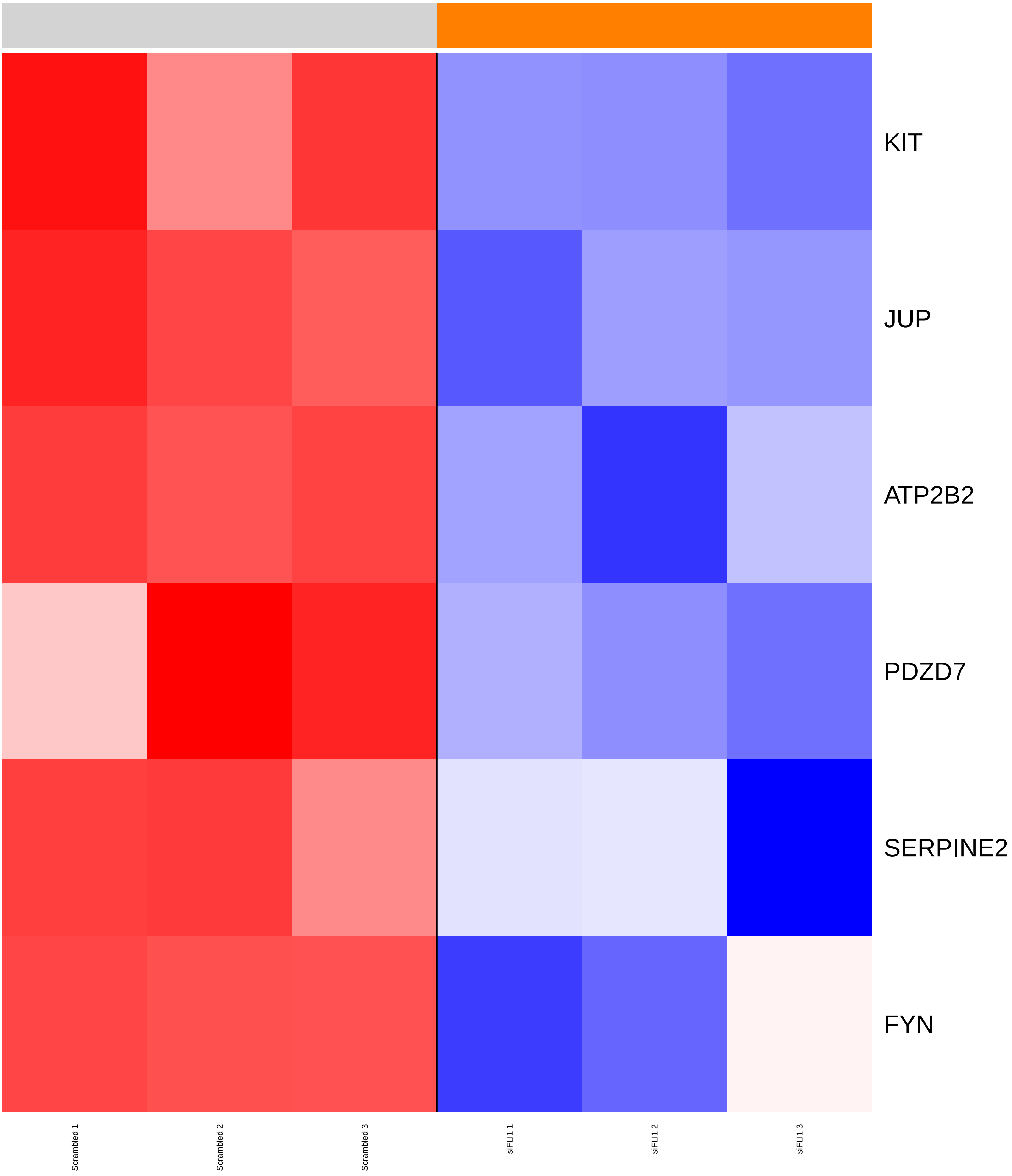

GO_CHROMOSOMAL_REGION
NES = −1.66, p = 0.0000, FDR q = 0.1435

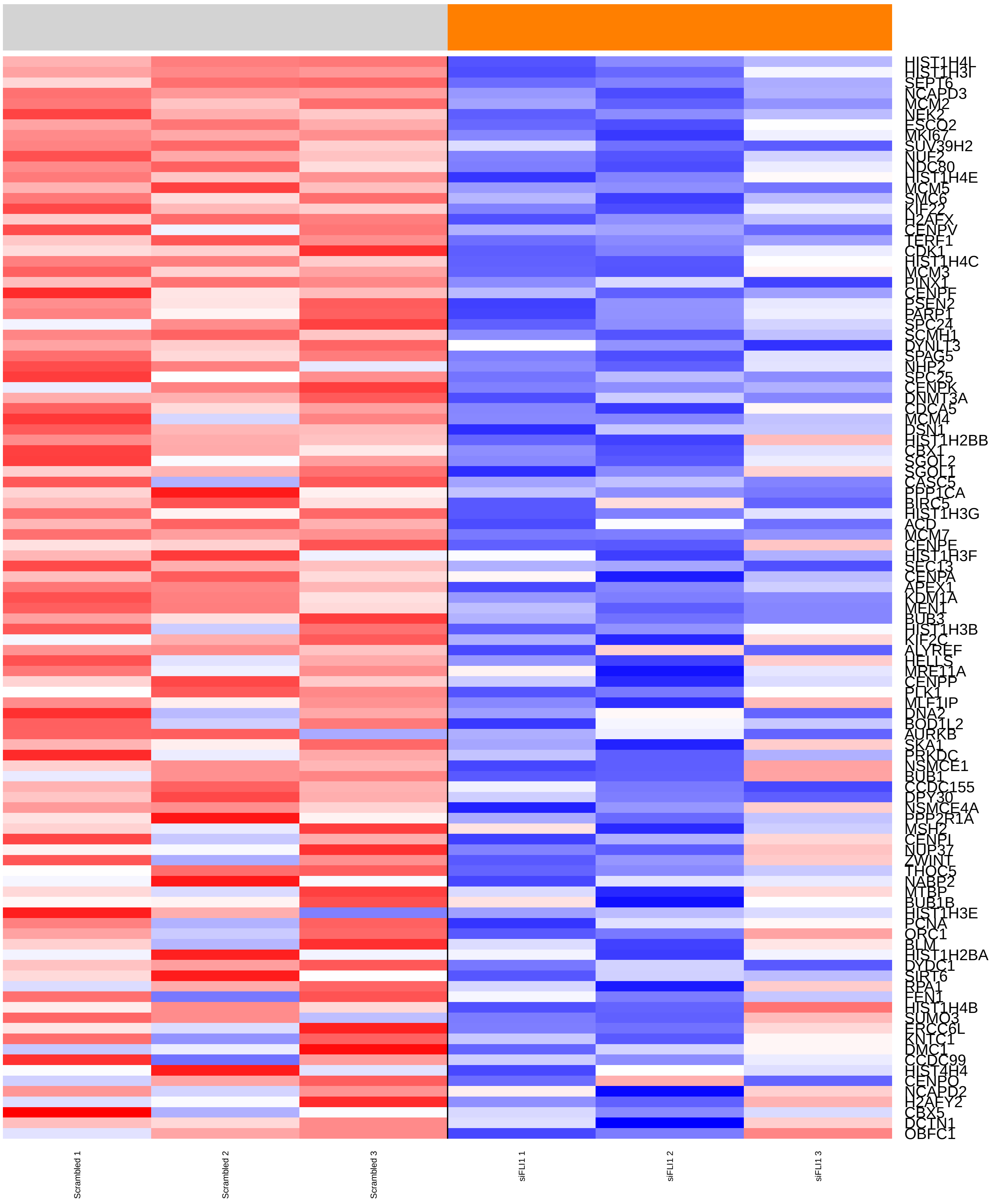

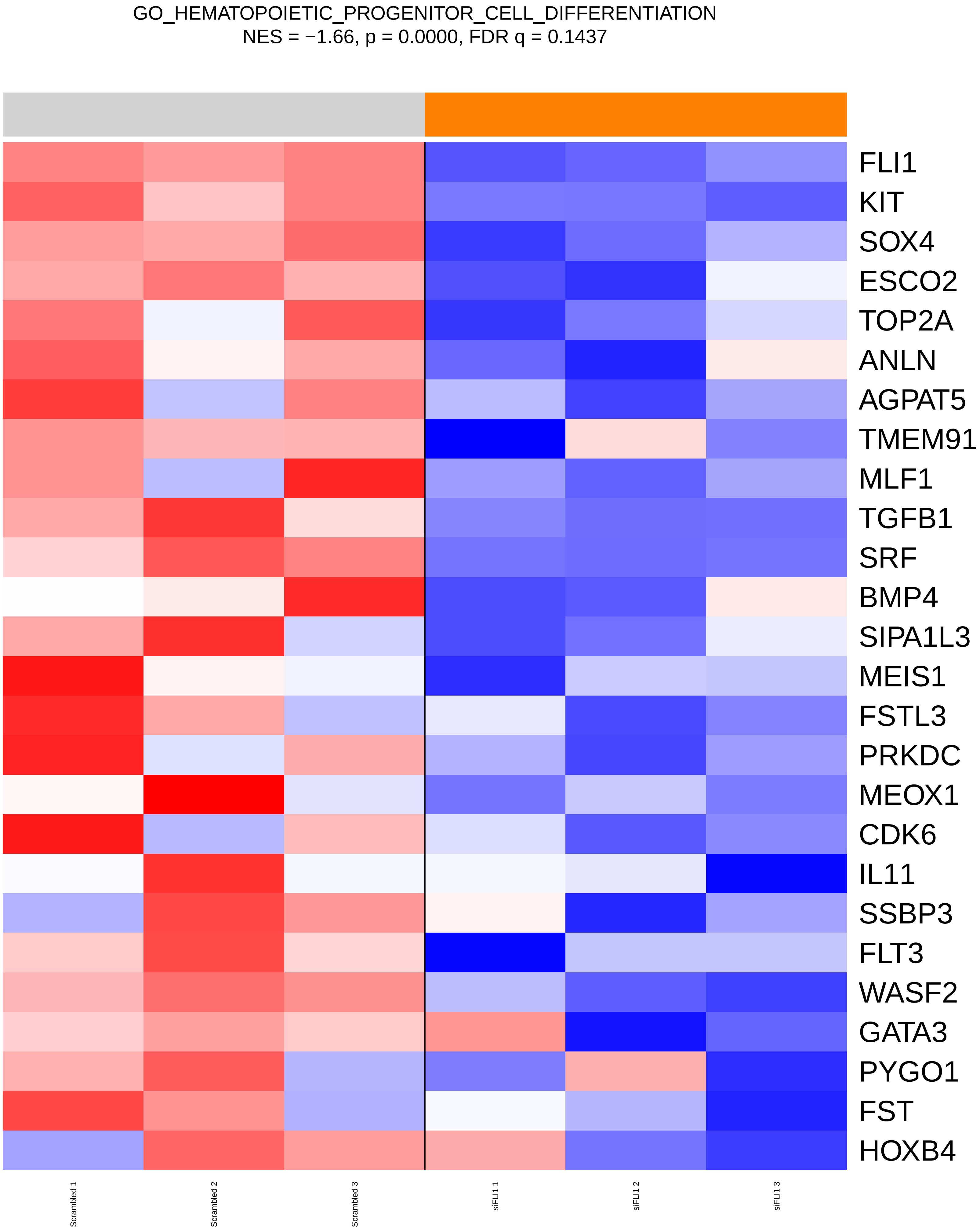
GO_HEMATOPOIETIC_PROGENITOR_CELL_DIFFERENTIATION
NES = −1.66, p = 0.0000, FDR q = 0.1437
FLI1
KIT
SOX4
ESCO2
TOP2A
ANLN
AGPAT5
TMEM91
MLF1
TGFB1
SRF
BMP4
SIPA1L3
MEIS1
FSTL3
PRKDC
MEOX1
CDK6
IL11
SSBP3
FLT3
WASF2
GATA3
PYGO1
FST
HOXB4
Scrambled 1
Scrambled 2
Scrambled 3
siFLI1 1
siFLI1 2
siFLI1 3

GO_REGULATION_OF_SULFUR_METABOLIC_PROCESS
NES = −1.66, p = 0.0174, FDR q = 0.1442

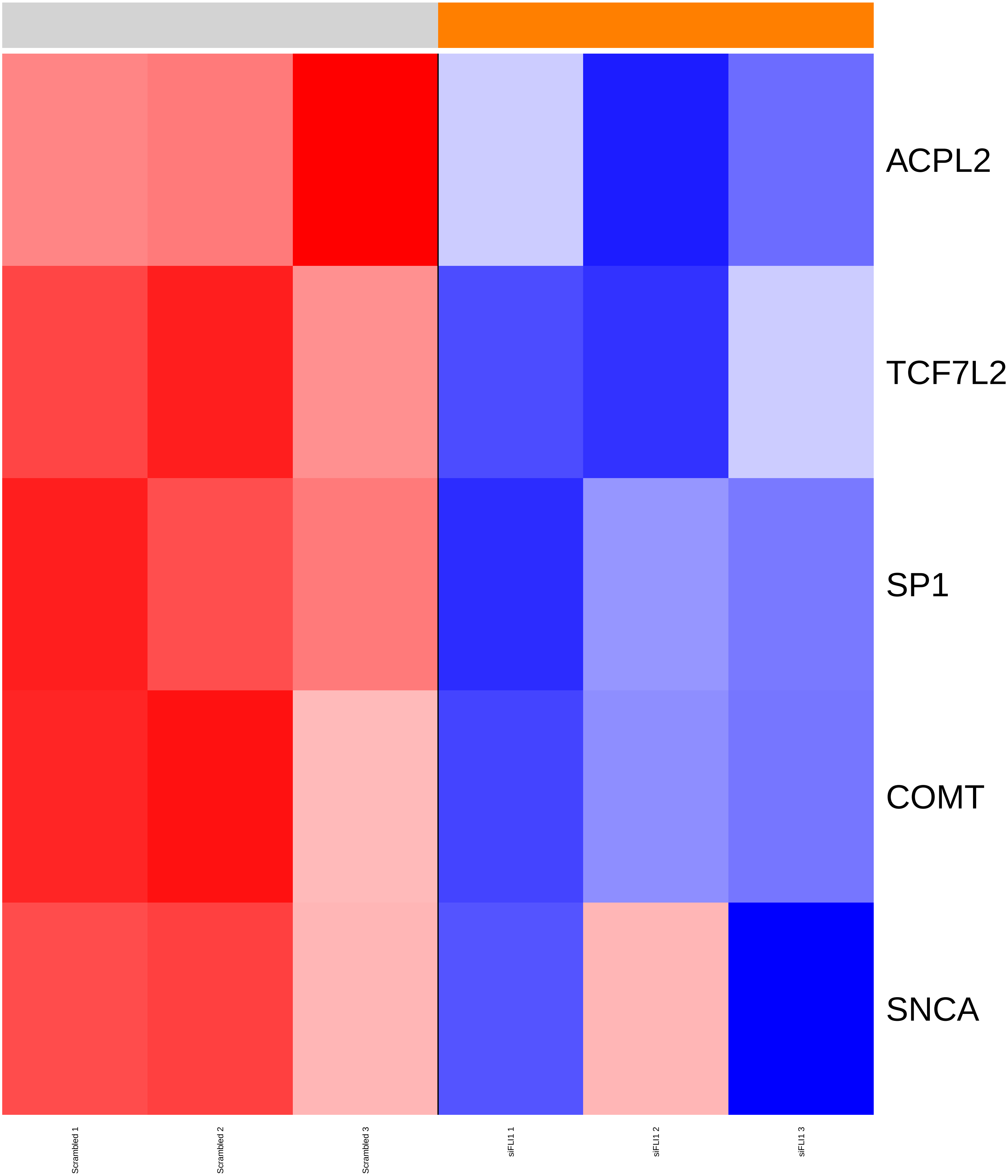

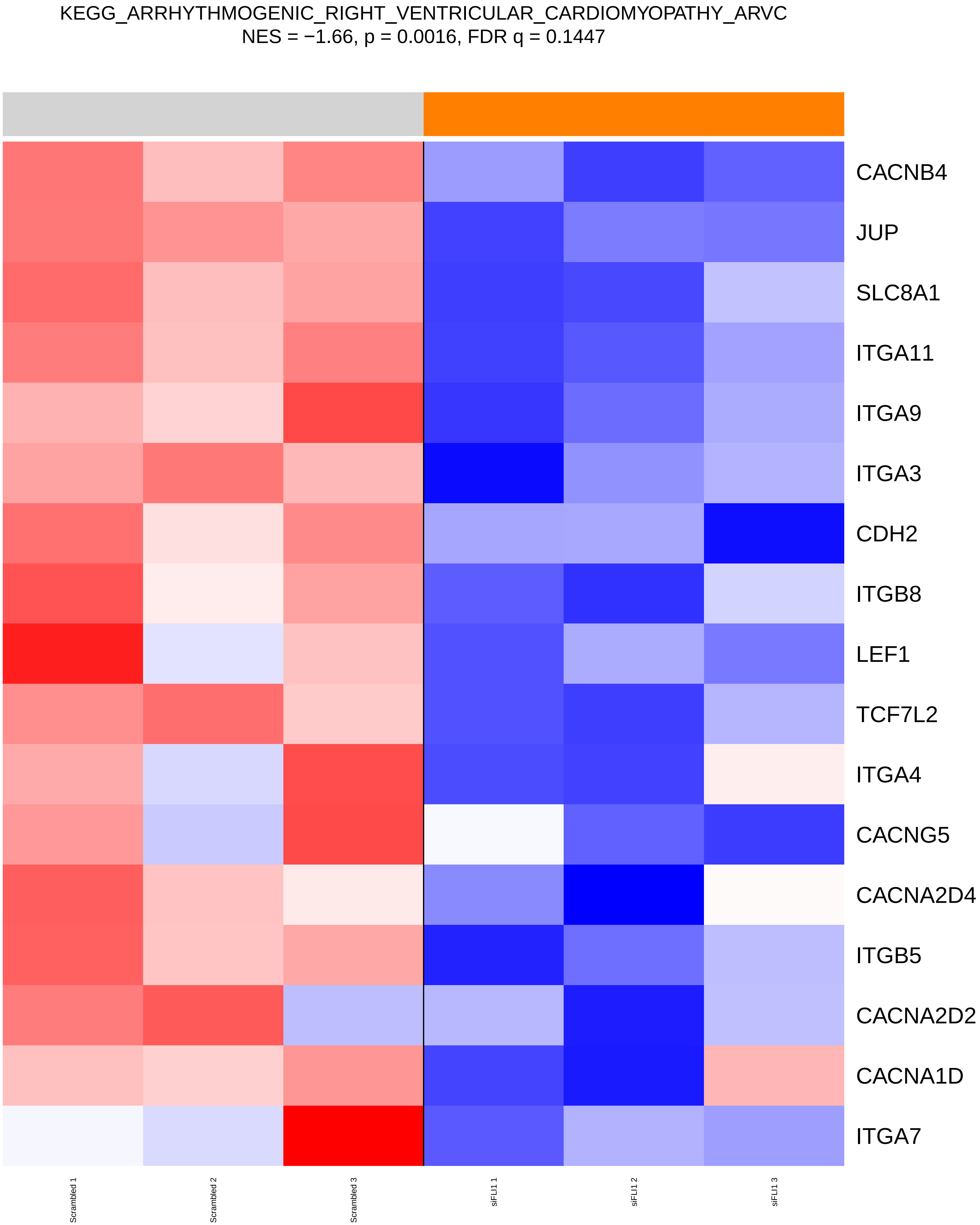
KEGG_ARRHYTHMOGENIC_RIGHT_VENTRICULAR_CARDIOMYOPATHY_ARVC
NES = −1.66, p = 0.0016, FDR q = 0.1447
CACNB4
JUP
SLC8A1
ITGA11
ITGA9
ITGA3
CDH2
ITGB8
LEF1
TCF7L2
ITGA4
CACNG5
CACNA2D4
ITGB5
CACNA2D2
CACNA1D
ITGA7
Scrambled 1
Scrambled 2
Scrambled 3
siFLI1 1
siFLI1 2
siFLI1 3

GO_POSITIVE_REGULATION_OF_PHOSPHATIDYLINOSITOL_3_KINASE_SIGNALING
NES = −1.66, p = 0.0016, FDR q = 0.1455

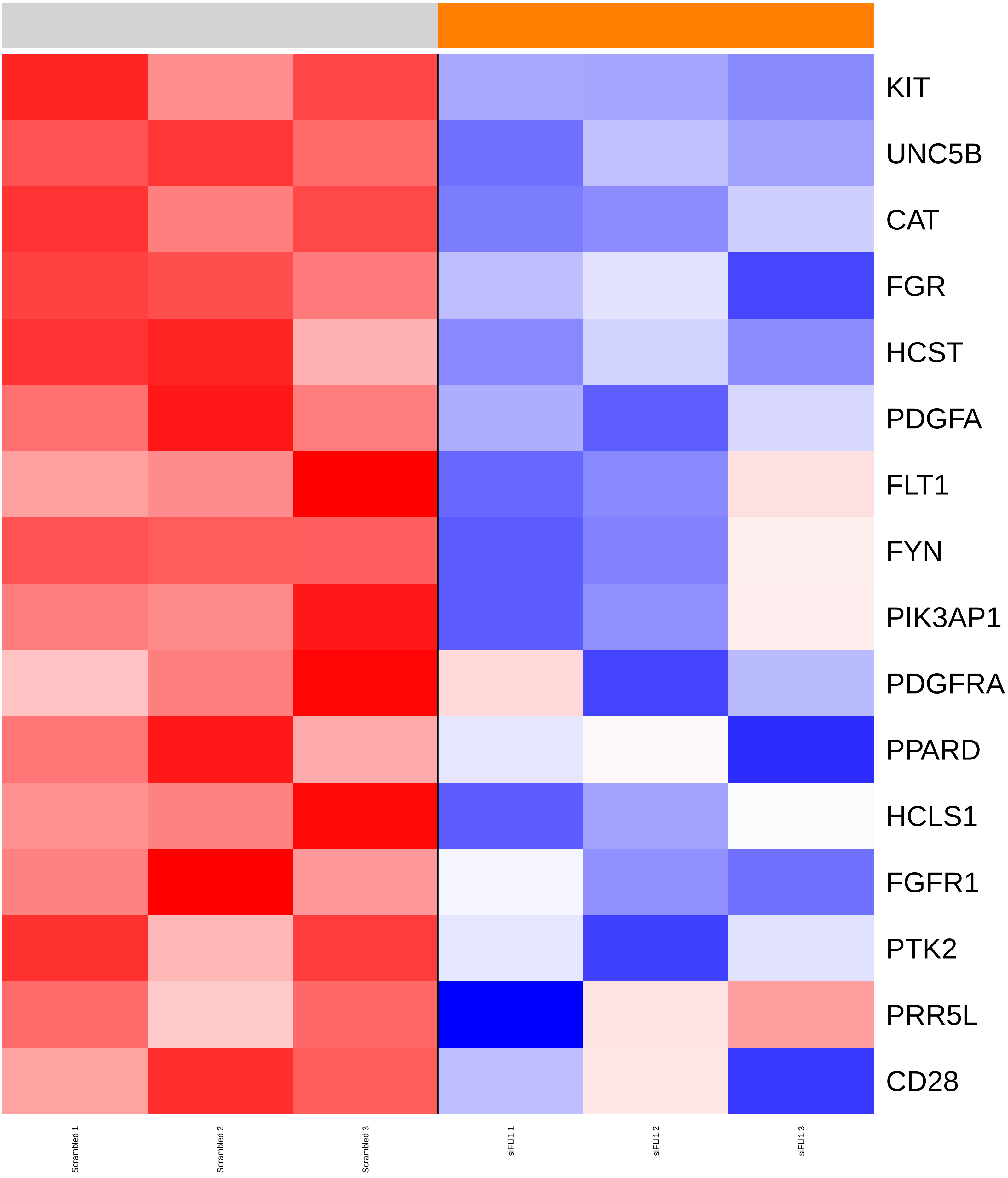

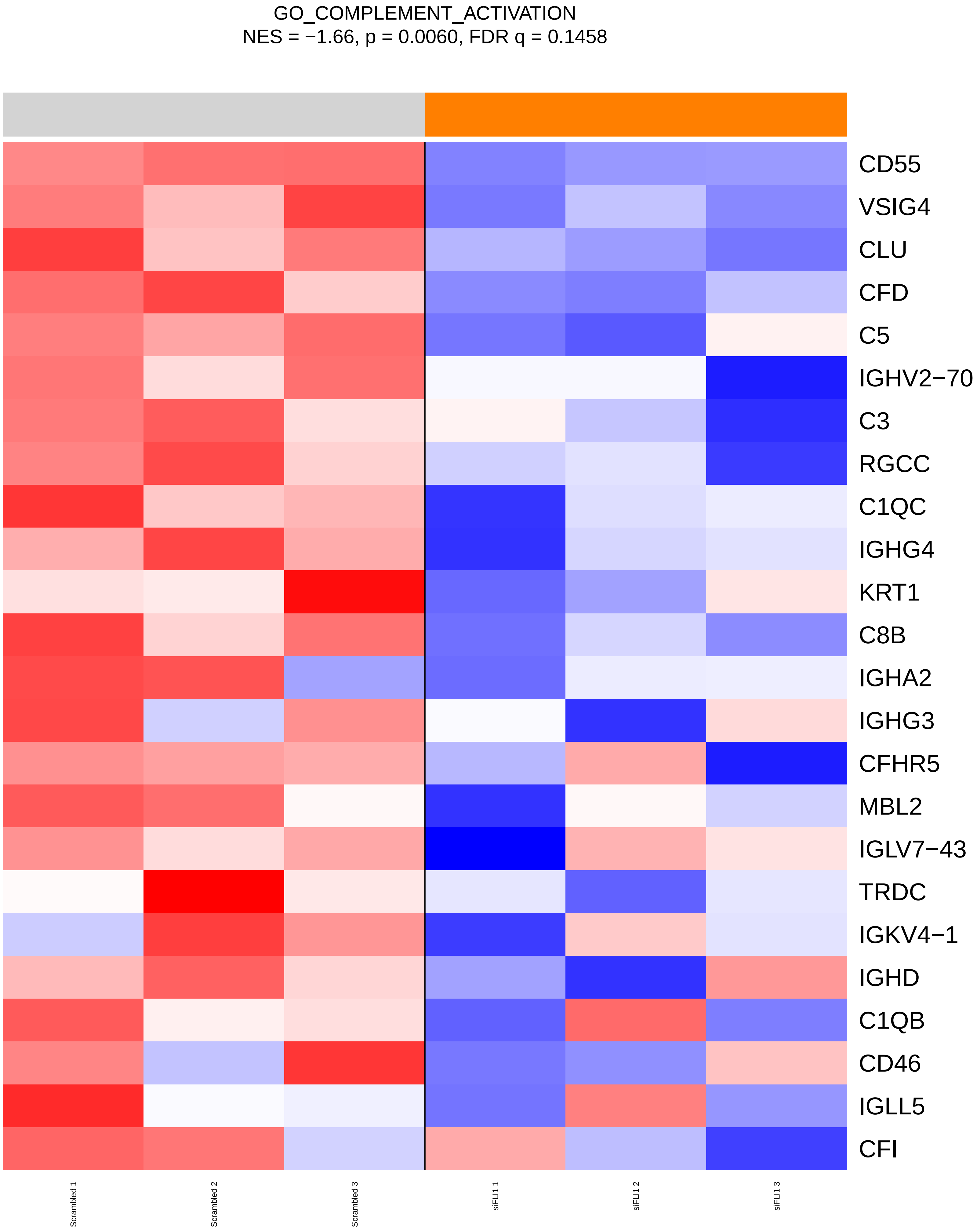
GO_COMPLEMENT_ACTIVATION
NES = −1.66, p = 0.0060, FDR q = 0.1458
CD55
VSIG4
CLU
CFD
C5
IGHV2−70
C3
RGCC
C1QC
IGHG4
KRT1
C8B
IGHA2
IGHG3
CFHR5
MBL2
IGLV7−43
TRDC
IGKV4−1
IGHD
C1QB
CD46
IGLL5
CFI
Scrambled 1
Scrambled 2
Scrambled 3
siFLI1 1
siFLI1 2
siFLI1 3

REACTOME_HEPARAN_SULFATE_HEPARIN_HS_GAG_METABOLISM
NES = −1.66, p = 0.0016, FDR q = 0.1457

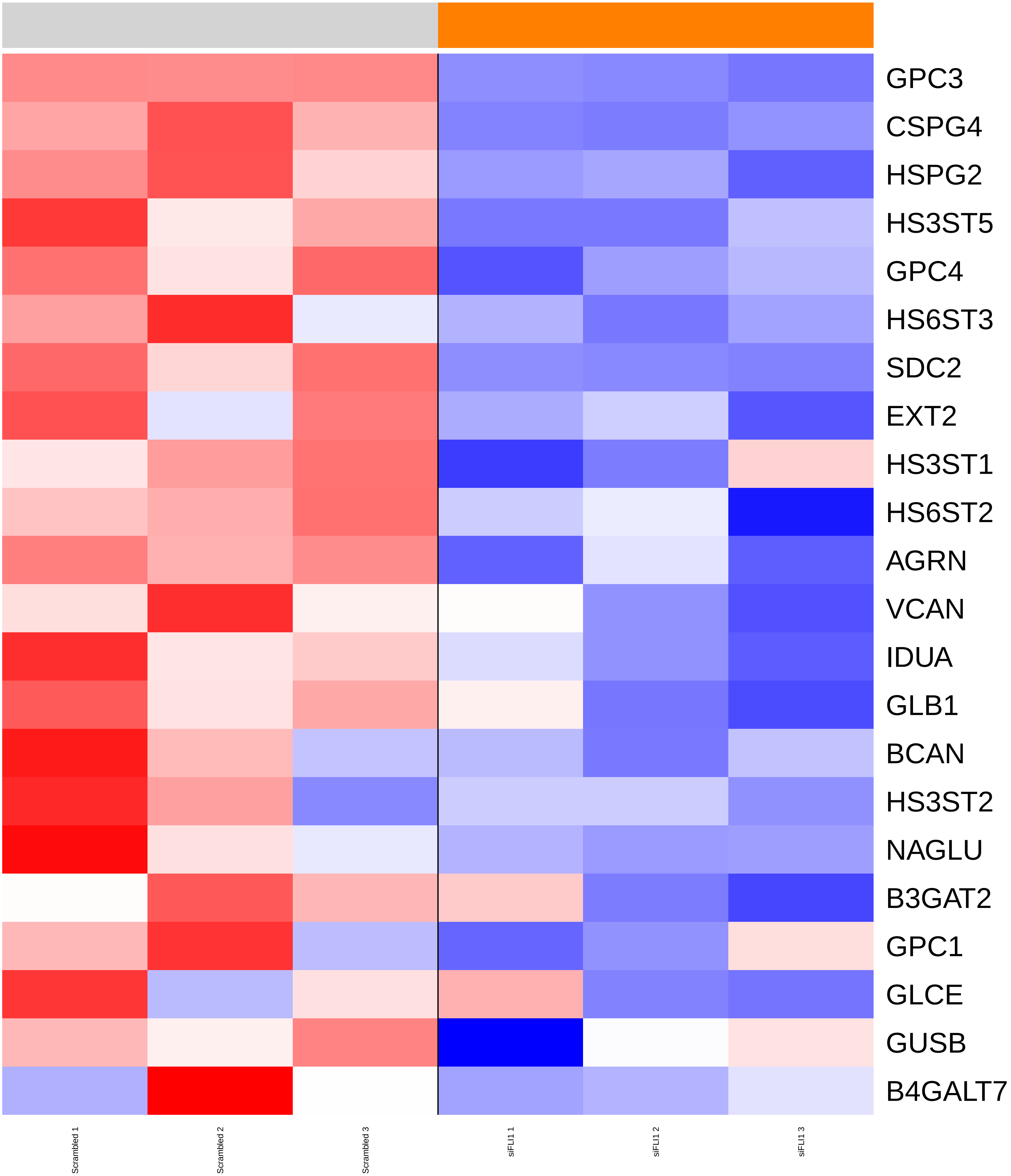

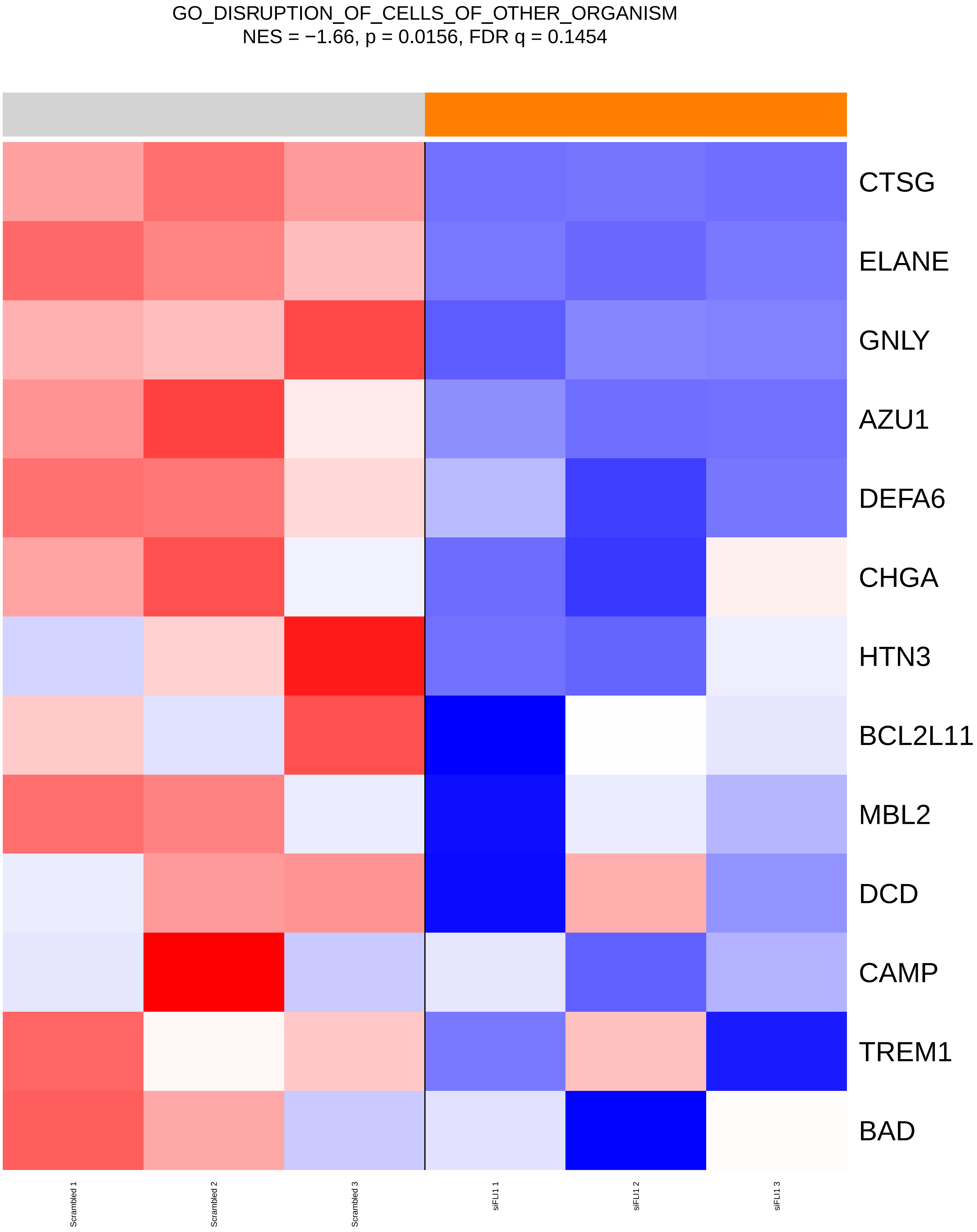
GO_DISRUPTION_OF_CELLS_OF_OTHER_ORGANISM
NES = −1.66, p = 0.0156, FDR q = 0.1454
CTSG
ELANE
GNLY
AZU1
DEFA6
CHGA
HTN3
BCL2L11
MBL2
DCD
CAMP
TREM1
BAD
Scrambled 1
Scrambled 2
Scrambled 3
siFLI1 1
siFLI1 2
siFLI1 3

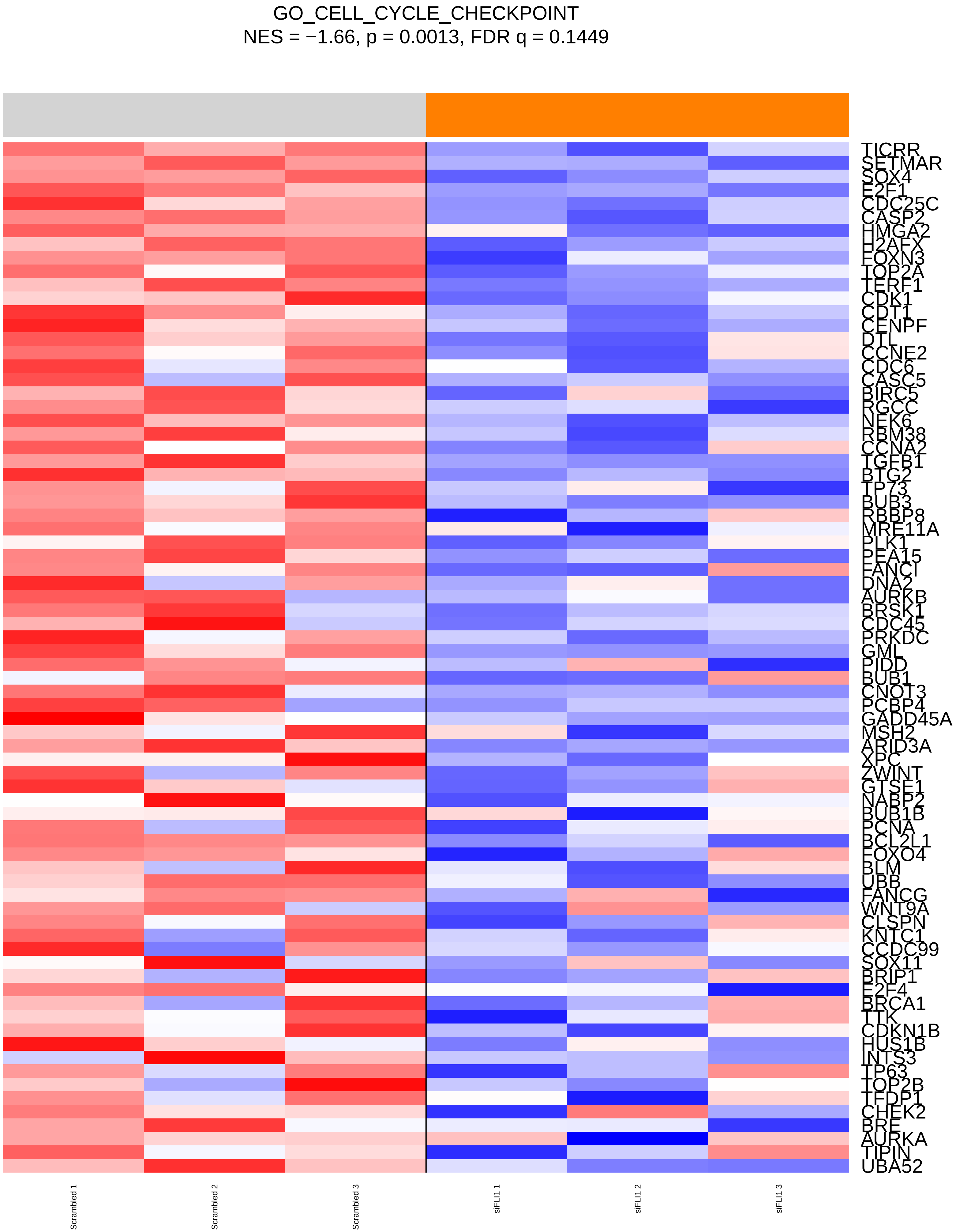
GO_CELL_CYCLE_CHECKPOINT
NES = −1.66, p = 0.0013, FDR q = 0.1449
TICRR
SETMAR
SOX4
E2F1
CDC25C
CASP2
HMGA2
H2AFX
FOXN3
TOP2A
TERF1
CDK1
CDT1
CENPF
DTL
CCNE2
CDC6
CASC5
BIRC5
RGCC
NEK6
RBM38
CCNA2
TGFB1
BTG2
TP73
BUB3
RBBP8
MRE11A
PLK1
PEA15
FANCI
DNA2
AURKB
BRSK1
CDC45
PRKDC
GML
PIDD
BUB1
CNOT3
PCBP4
GADD45A
MSH2
ARID3A
XPC
ZWINT
GTSE1
NABP2
BUB1B
PCNA
BCL2L1
FOXO4
BLM
UBB
FANCG
WNT9A
CLSPN
KNTC1
CCDC99
SOX11
BRIP1
E2F4
BRCA1
TTK
CDKN1B
HUS1B
INTS3
TP63
TOP2B
TFDP1
CHEK2
BRE
AURKA
TIPIN
UBA52
Scrambled 1
Scrambled 2
Scrambled 3
siFLI1 1
siFLI1 2
siFLI1 3

GO_ENDORIBONUCLEASE_ACTIVITY
NES = −1.66, p = 0.0081, FDR q = 0.1442

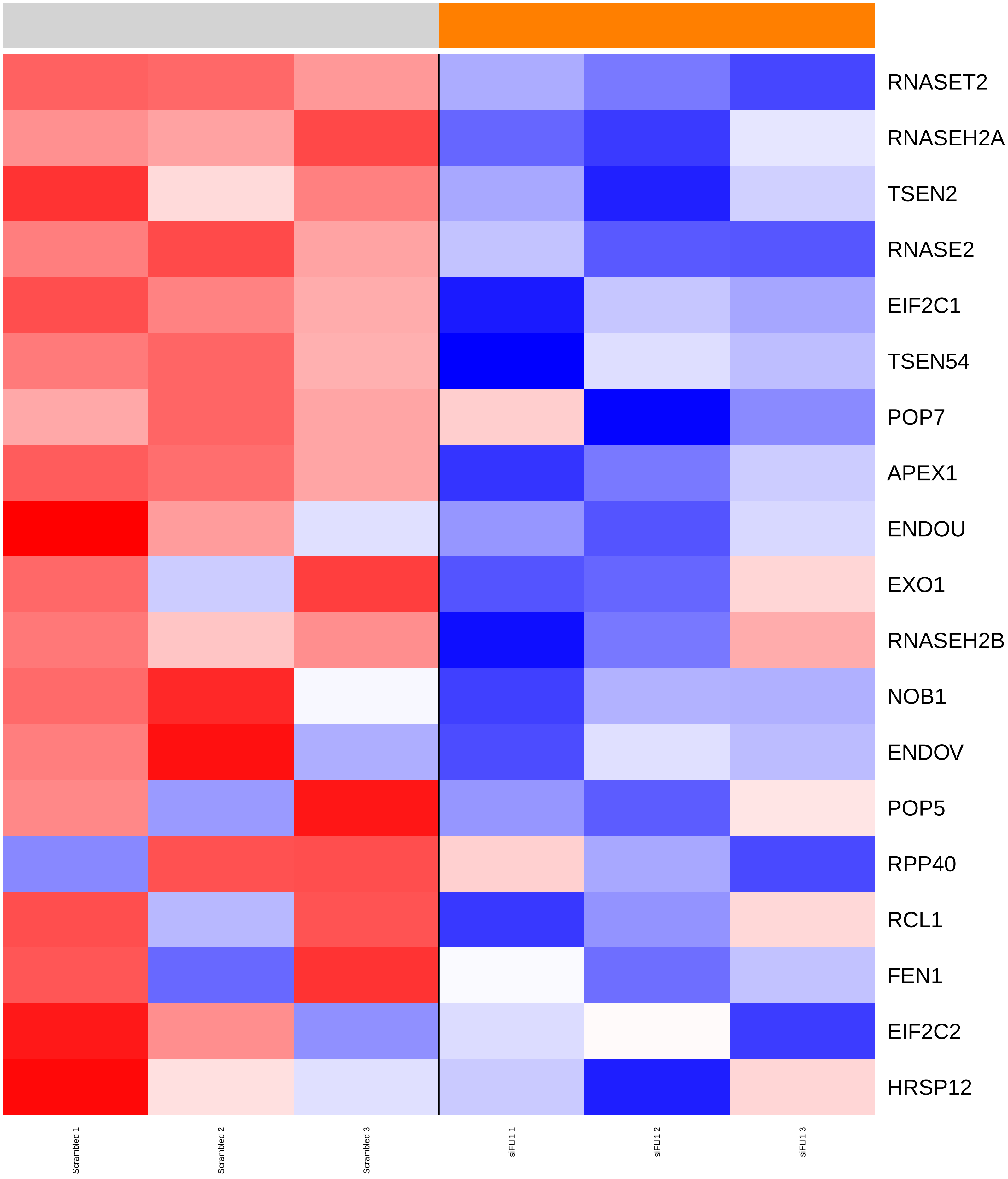

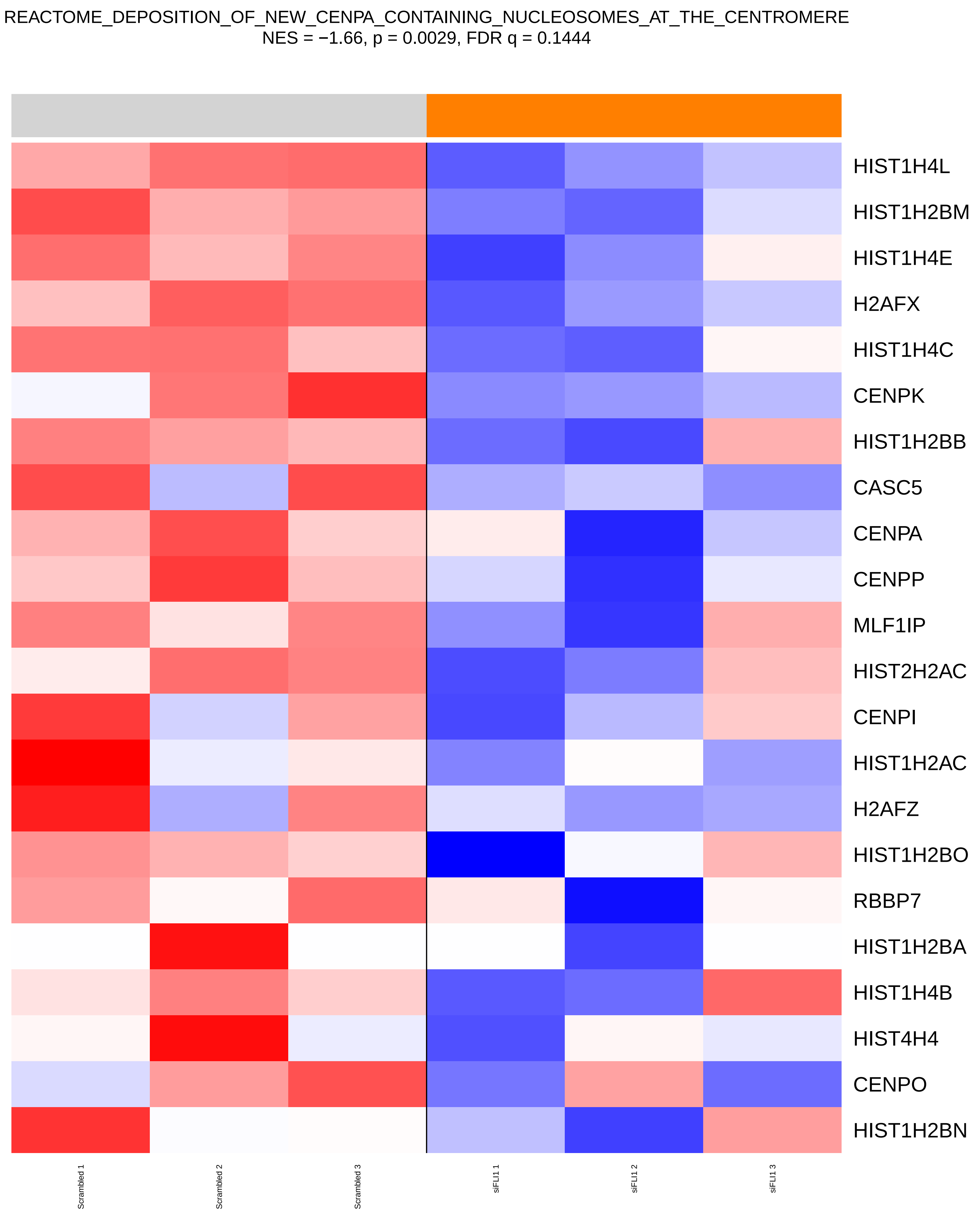
REACTOME_DEPOSITION_OF_NEW_CENPA_CONTAINING_NUCLEOSOMES_AT_THE_CENTROMERE
NES = −1.66, p = 0.0029, FDR q = 0.1444
HIST1H4L
HIST1H2BM
HIST1H4E
H2AFX
HIST1H4C
CENPK
HIST1H2BB
CASC5
CENPA
CENPP
MLF1IP
HIST2H2AC
CENPI
HIST1H2AC
H2AFZ
HIST1H2BO
RBBP7
HIST1H2BA
HIST1H4B
HIST4H4
CENPO
HIST1H2BN
Scrambled 1
Scrambled 2
Scrambled 3
siFLI1 1
siFLI1 2
siFLI1 3

GO_MODIFIED_AMINO_ACID_BINDING
NES = −1.66, p = 0.0015, FDR q = 0.1449

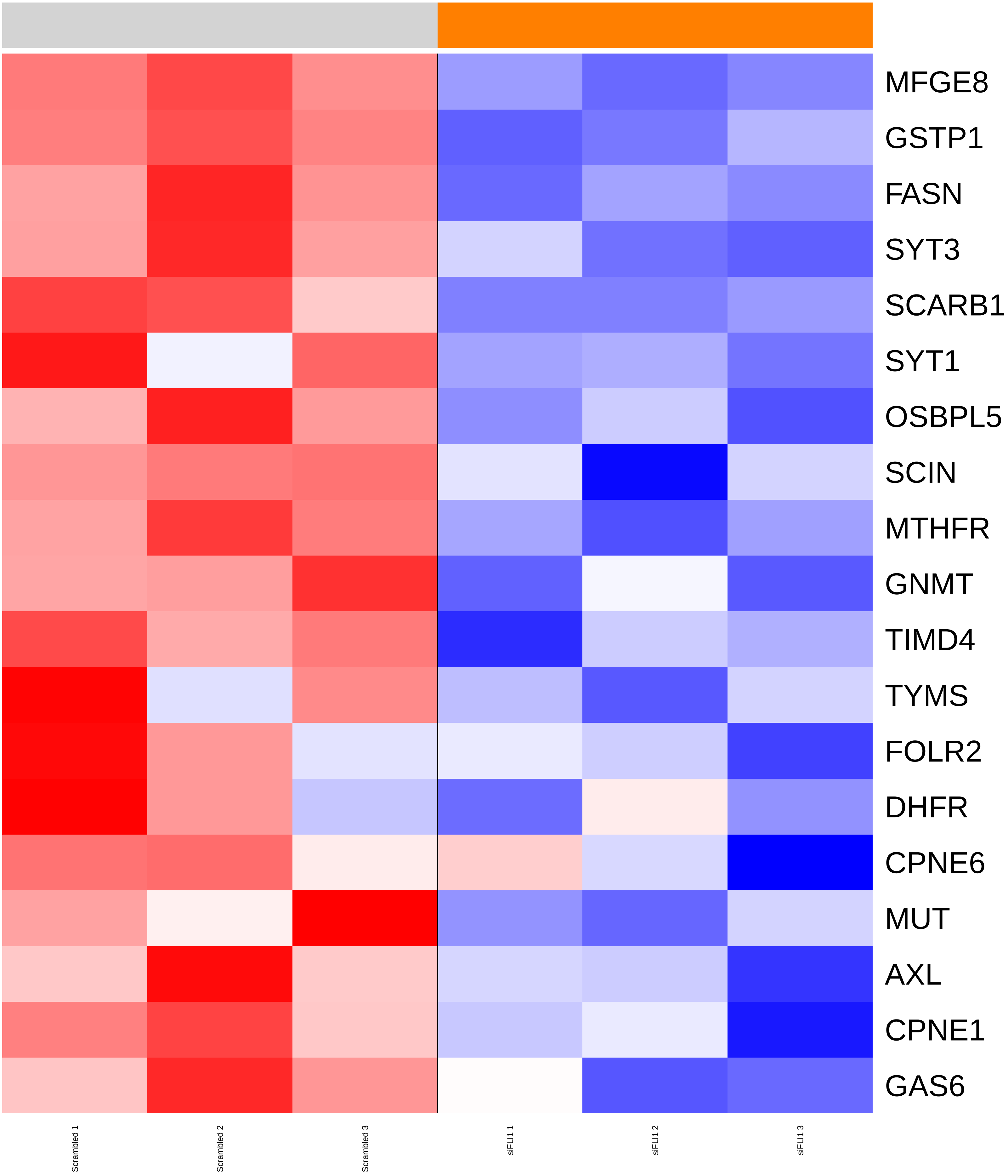

GO_OXIDOREDUCTASE_ACTIVITY_ACTING_ON_THE_CH_OH_GROUP_OF_DONORS_NAD_OR_NADP_AS_ACCEPTOR
NES = −1.67, p = 0.0014, FDR q = 0.1409

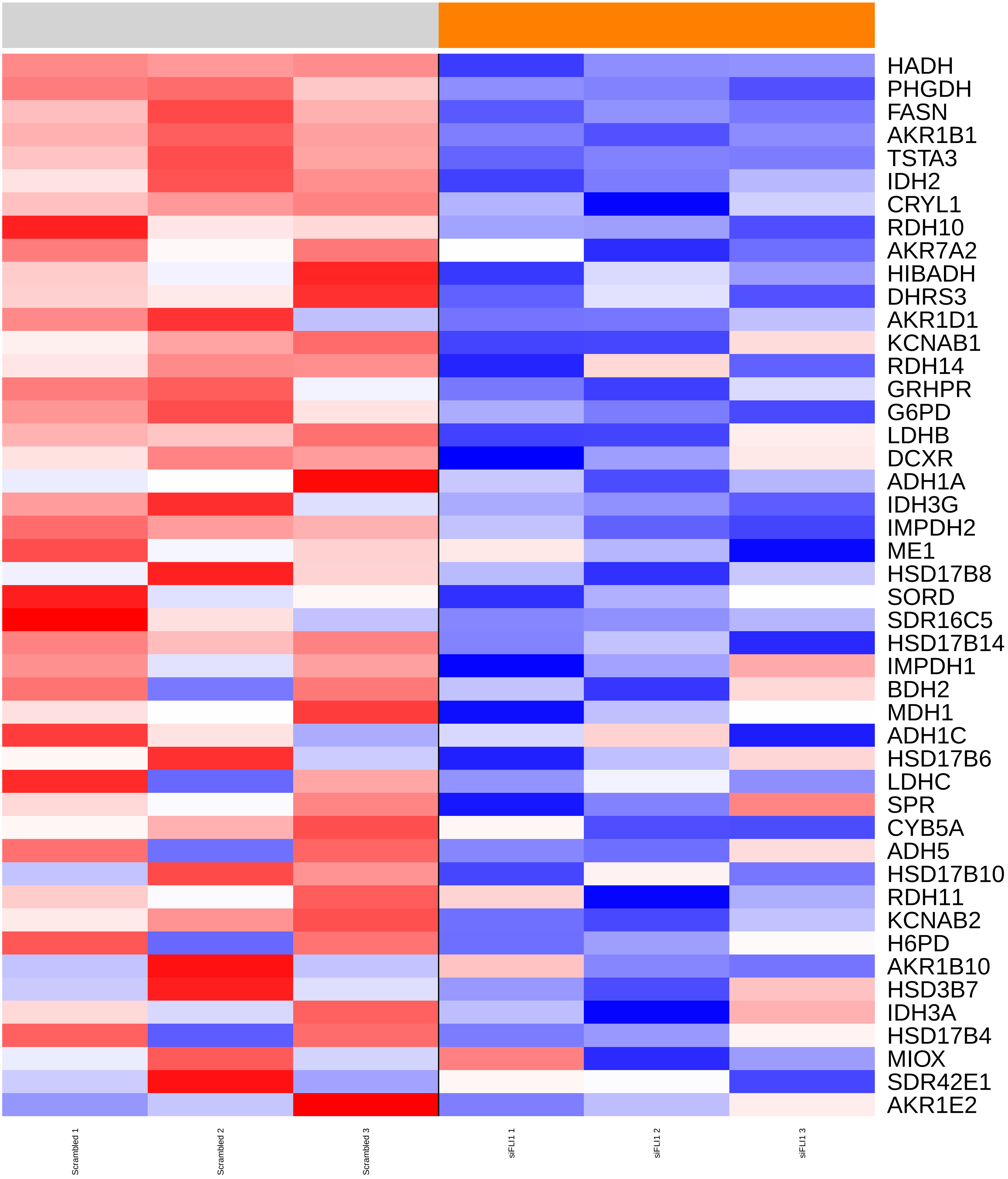

HALLMARK_OXIDATIVE_PHOSPHORYLATION
NES = −1.67, p = 0.0000, FDR q = 0.1413

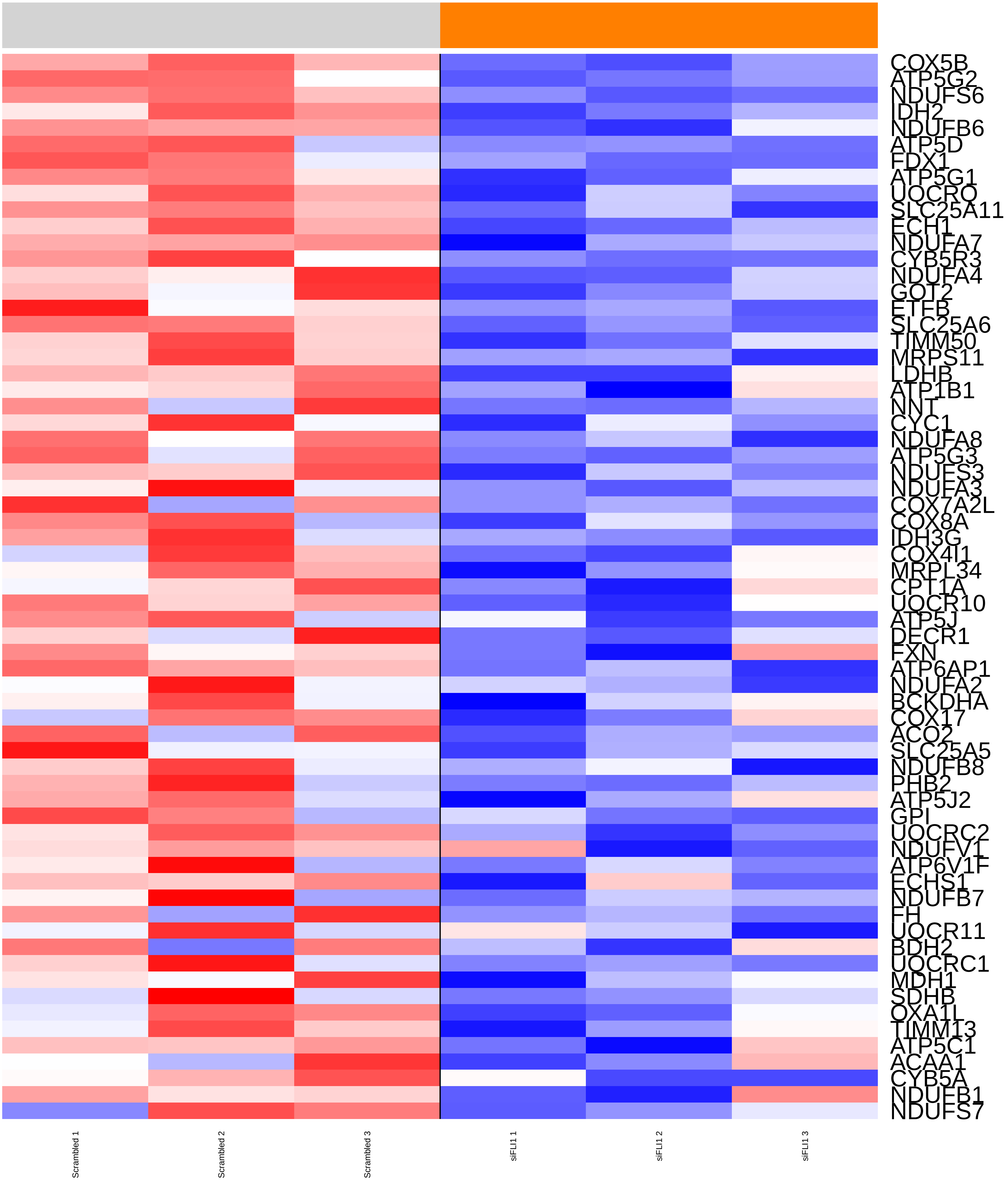

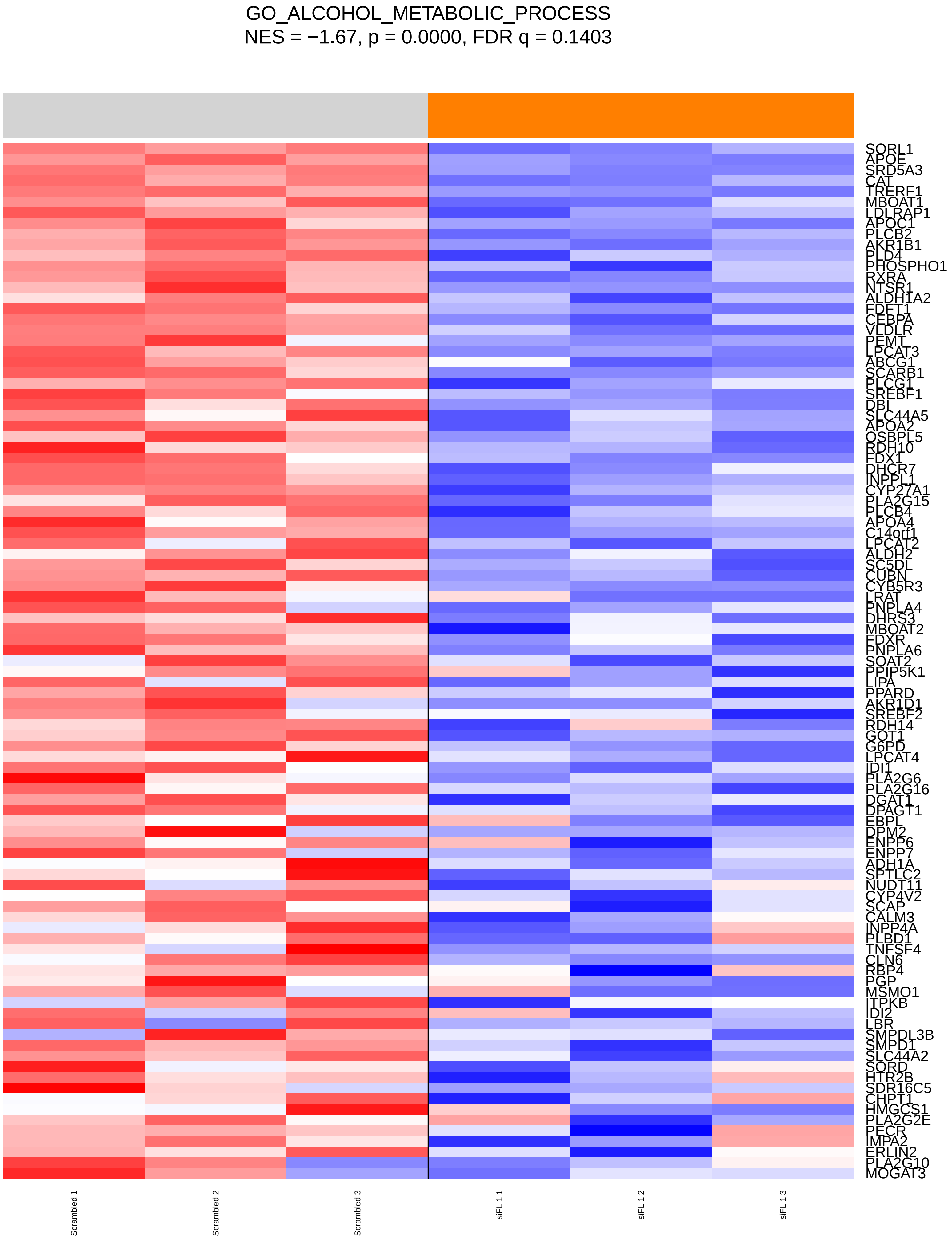
GO_ALCOHOL_METABOLIC_PROCESS
NES = −1.67, p = 0.0000, FDR q = 0.1403
SORL1
APOE
SRD5A3
CAT
TRERF1
MBOAT1
LDLRAP1
APOC1
PLCB2
AKR1B1
PLD4
PHOSPHO1
RXRA
NTSR1
ALDH1A2
FDFT1
CEBPA
VLDLR
PEMT
LPCAT3
ABCG1
SCARB1
PLCG1
SREBF1
DBI
SLC44A5
APOA2
OSBPL5
RDH10
FDX1
DHCR7
INPPL1
CYP27A1
PLA2G15
PLCB4
APOA4
C14orf1
LPCAT2
ALDH2
SC5DL
CUBN
CYB5R3
LRAT
PNPLA4
DHRS3
MBOAT2
FDXR
PNPLA6
SOAT2
PPIP5K1
LIPA
PPARD
AKR1D1
SREBF2
RDH14
GOT1
G6PD
LPCAT4
IDI1
PLA2G6
PLA2G16
DGAT1
DPAGT1
EBPL
DPM2
ENPP6
ENPP7
ADH1A
SPTLC2
NUDT11
CYP4V2
SCAP
CALM3
INPP4A
PLBD1
TNFSF4
CLN6
RBP4
PGP
MSMO1
ITPKB
IDI2
LBR
SMPDL3B
SMPD1
SLC44A2
SORD
HTR2B
SDR16C5
CHPT1
HMGCS1
PLA2G2E
PECR
IMPA2
ERLIN2
PLA2G10
MOGAT3
Scrambled 1
Scrambled 2
Scrambled 3
siFLI1 1
siFLI1 2
siFLI1 3

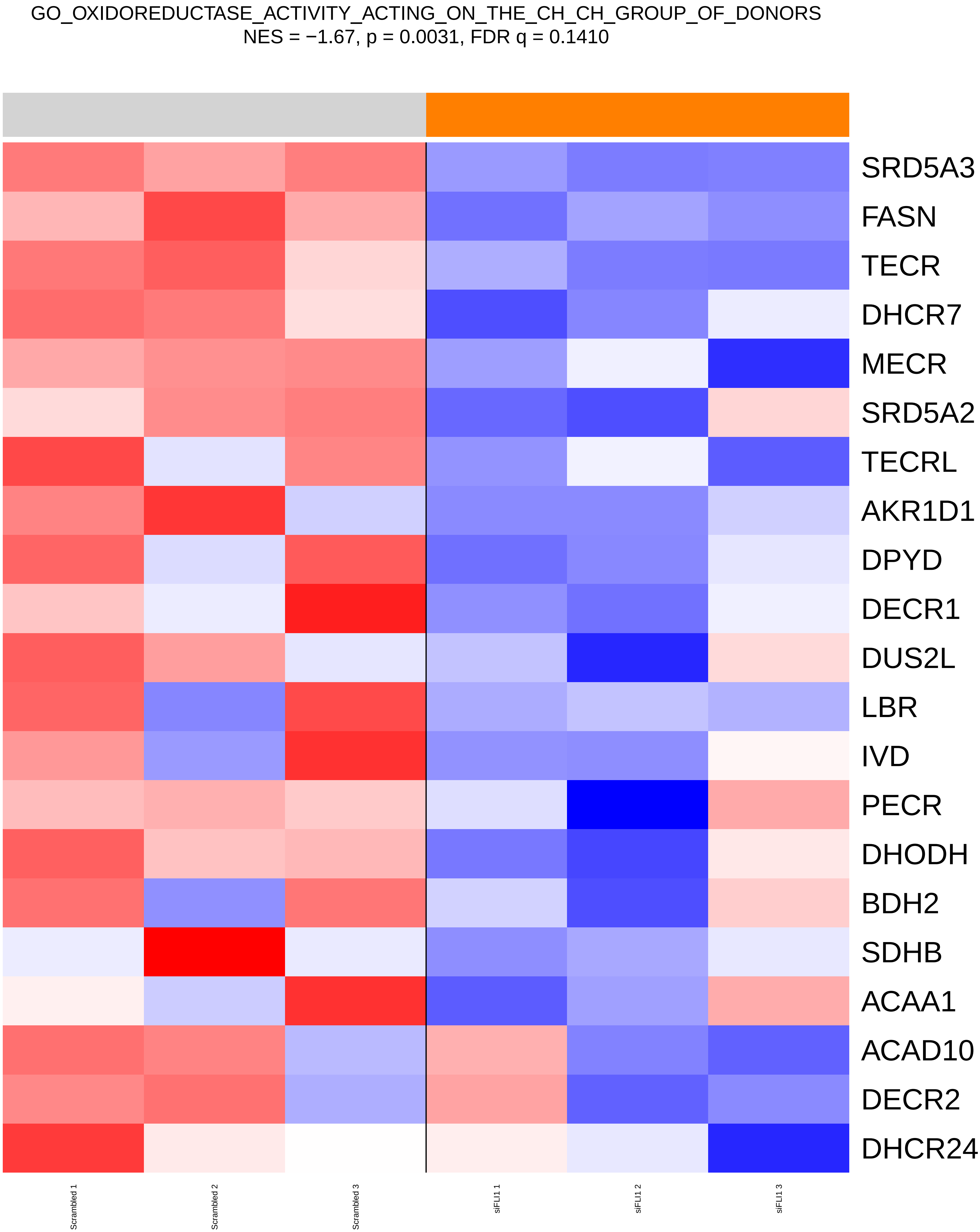
GO_OXIDOREDUCTASE_ACTIVITY_ACTING_ON_THE_CH_CH_GROUP_OF_DONORS
NES = −1.67, p = 0.0031, FDR q = 0.1410
SRD5A3
FASN
TECR
DHCR7
MECR
SRD5A2
TECRL
AKR1D1
DPYD
DECR1
DUS2L
LBR
IVD
PECR
DHODH
BDH2
SDHB
ACAA1
ACAD10
DECR2
DHCR24
Scrambled 1
Scrambled 2
Scrambled 3
siFLI1 1
siFLI1 2
siFLI1 3

GO_ARTERY_MORPHOGENESIS
NES = −1.67, p = 0.0031, FDR q = 0.1414

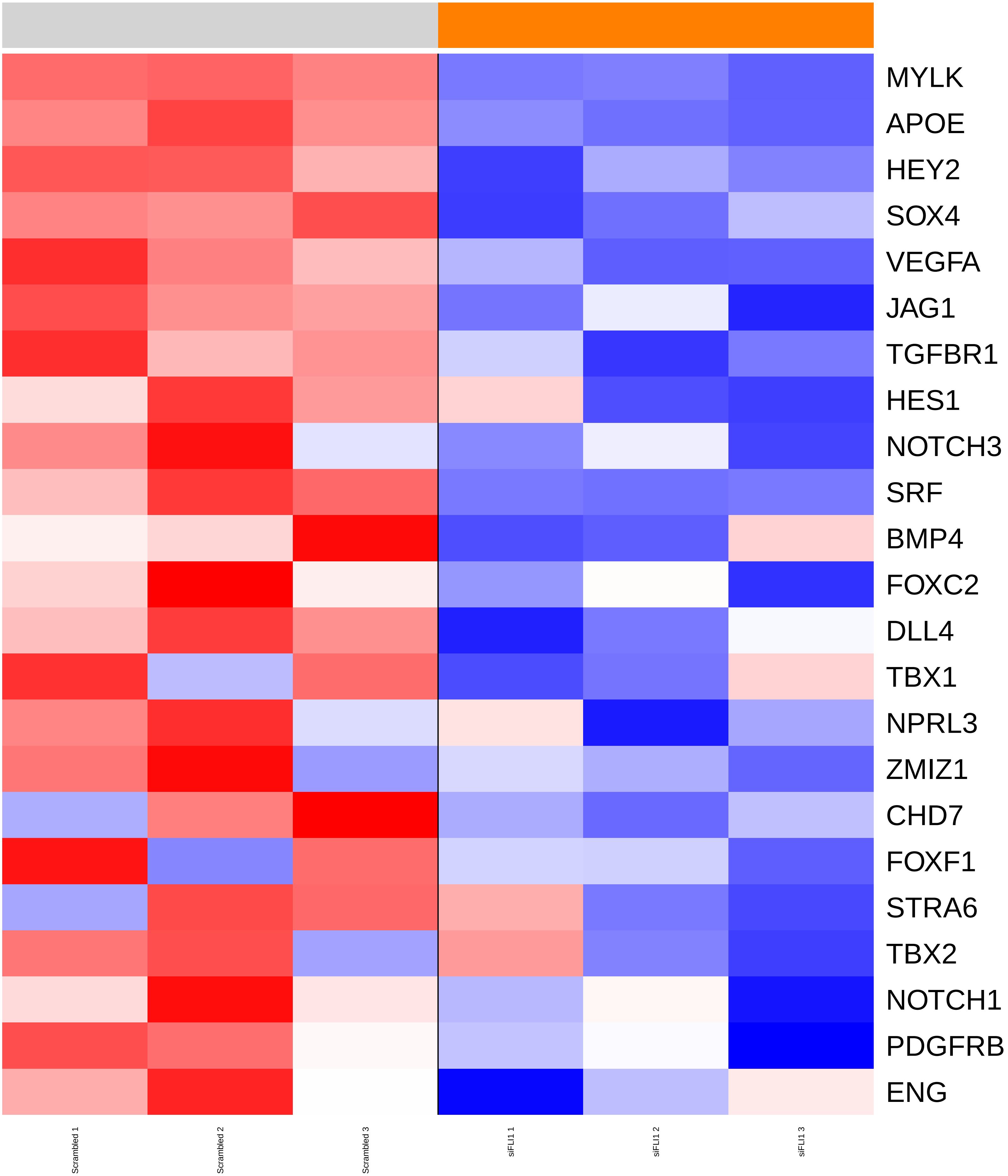

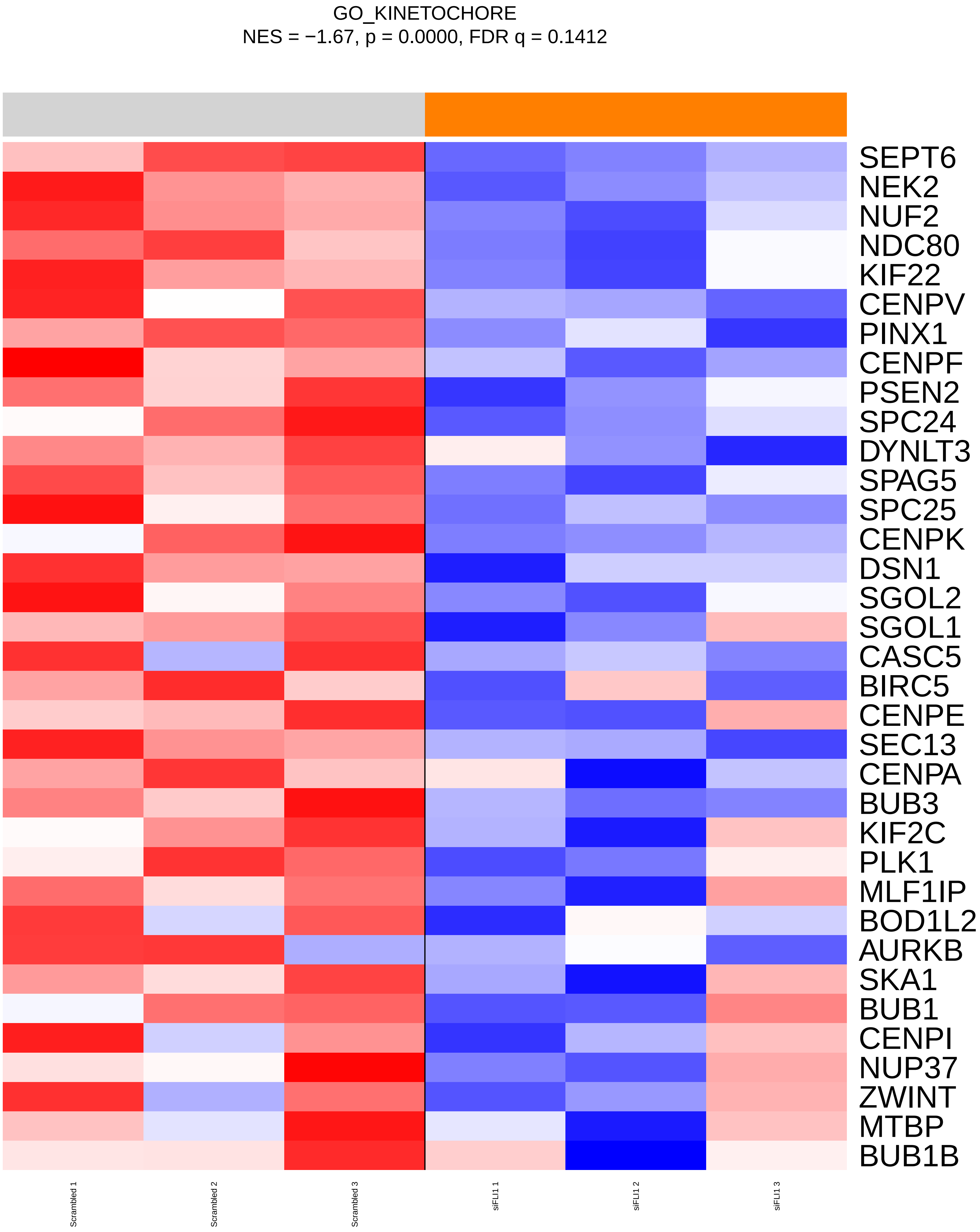
GO_KINETOCHORE
NES = −1.67, p = 0.0000, FDR q = 0.1412
SEPT6
NEK2
NUF2
NDC80
KIF22
CENPV
PINX1
CENPF
PSEN2
SPC24
DYNLT3
SPAG5
SPC25
CENPK
DSN1
SGOL2
SGOL1
CASC5
BIRC5
CENPE
SEC13
CENPA
BUB3
KIF2C
PLK1
MLF1IP
BOD1L2
AURKB
SKA1
BUB1
CENPI
NUP37
ZWINT
MTBP
BUB1B
Scrambled 1
Scrambled 2
Scrambled 3
siFLI1 1
siFLI1 2
siFLI1 3

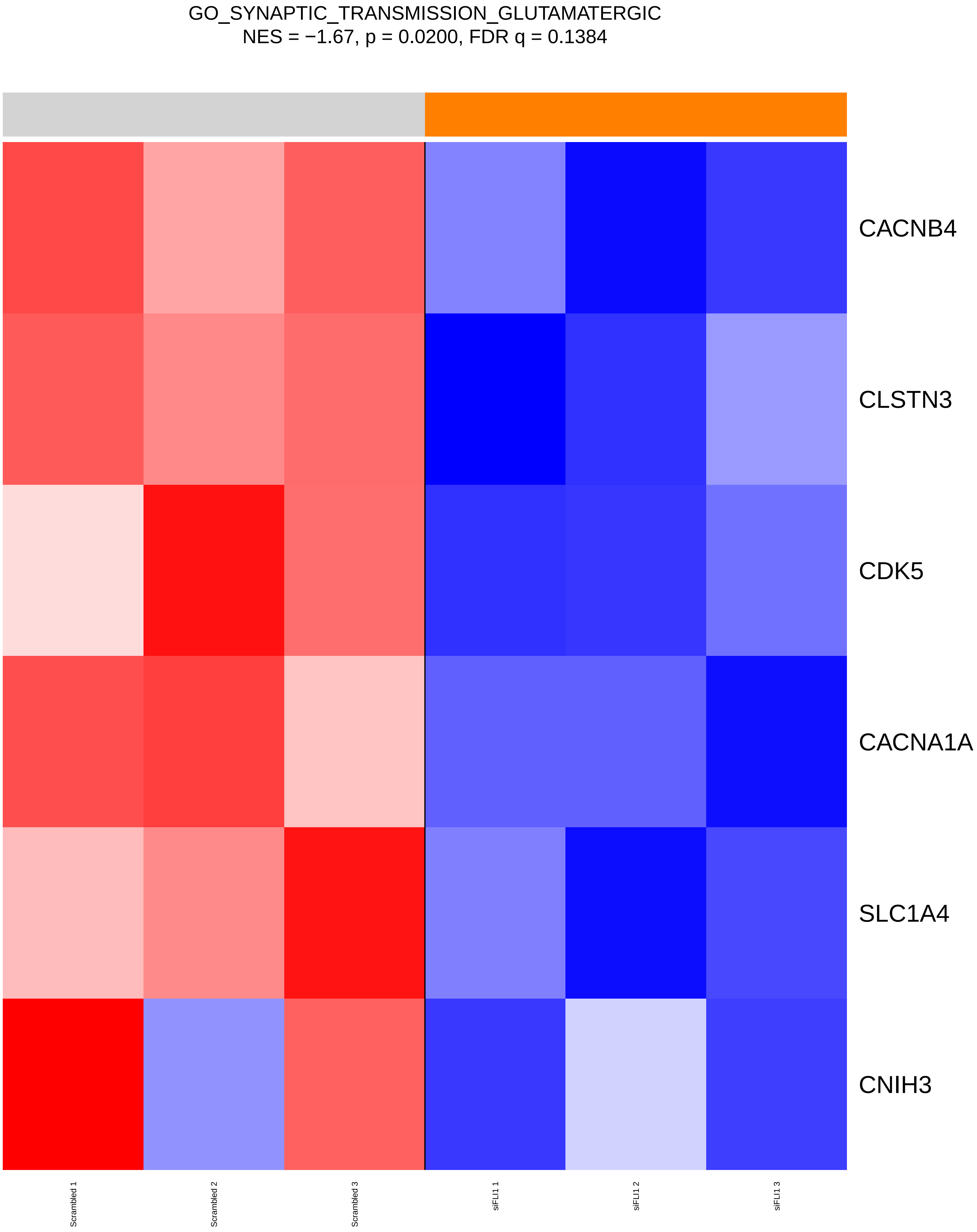
GO_SYNAPTIC_TRANSMISSION_GLUTAMATERGIC
NES = −1.67, p = 0.0200, FDR q = 0.1384
CACNB4
CLSTN3
CDK5
CACNA1A
SLC1A4
CNIH3
Scrambled 1
Scrambled 2
Scrambled 3
siFLI1 1
siFLI1 2
siFLI1 3

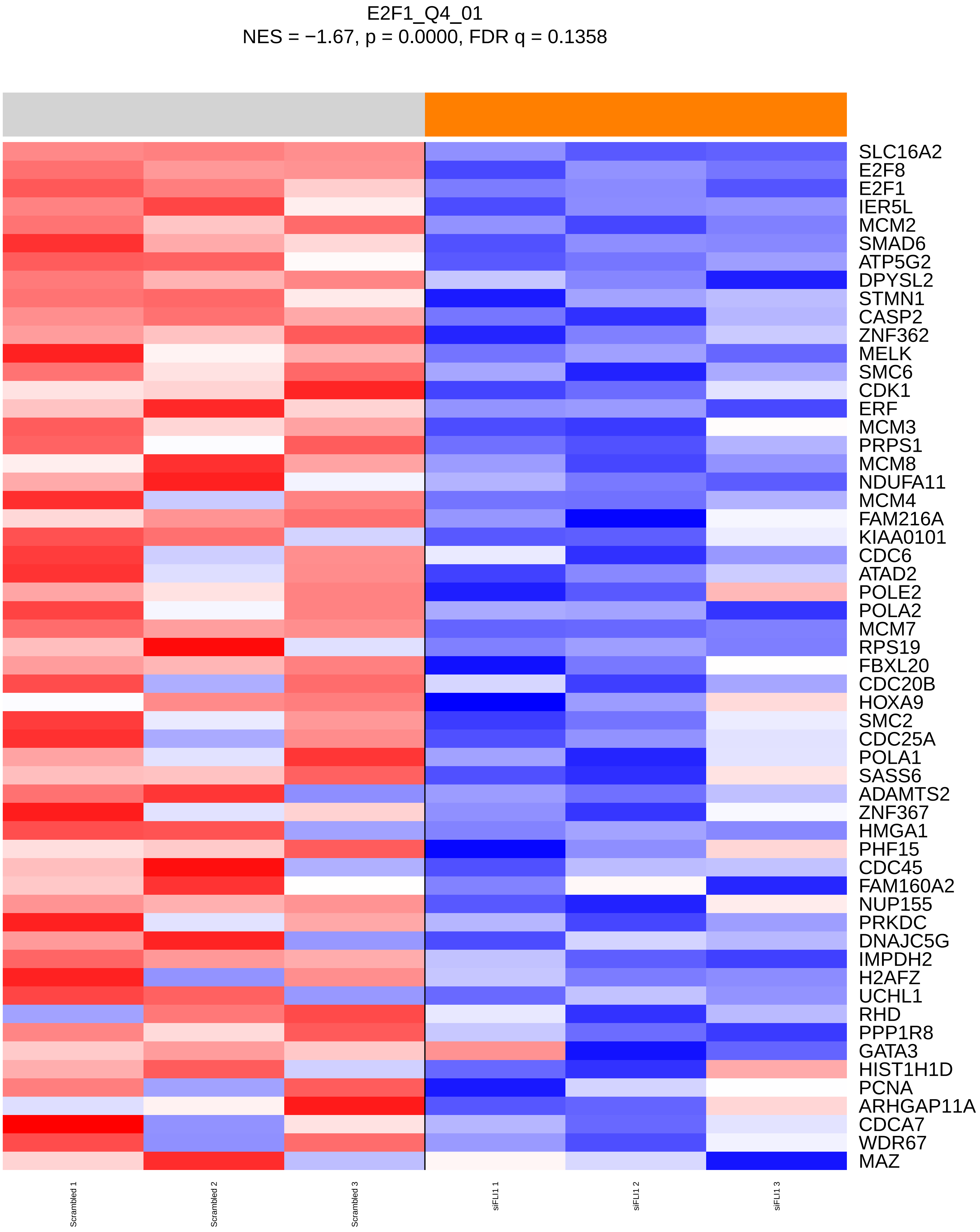
E2F1_Q4_01
NES = −1.67, p = 0.0000, FDR q = 0.1358
SLC16A2
E2F8
E2F1
IER5L
MCM2
SMAD6
ATP5G2
DPYSL2
STMN1
CASP2
ZNF362
MELK
SMC6
CDK1
ERF
MCM3
PRPS1
MCM8
NDUFA11
MCM4
FAM216A
KIAA0101
CDC6
ATAD2
POLE2
POLA2
MCM7
RPS19
FBXL20
CDC20B
HOXA9
SMC2
CDC25A
POLA1
SASS6
ADAMTS2
ZNF367
HMGA1
PHF15
CDC45
FAM160A2
NUP155
PRKDC
DNAJC5G
IMPDH2
H2AFZ
UCHL1
RHD
PPP1R8
GATA3
HIST1H1D
PCNA
ARHGAP11A
CDCA7
WDR67
MAZ
Scrambled 1
Scrambled 2
Scrambled 3
siFLI1 1
siFLI1 2
siFLI1 3

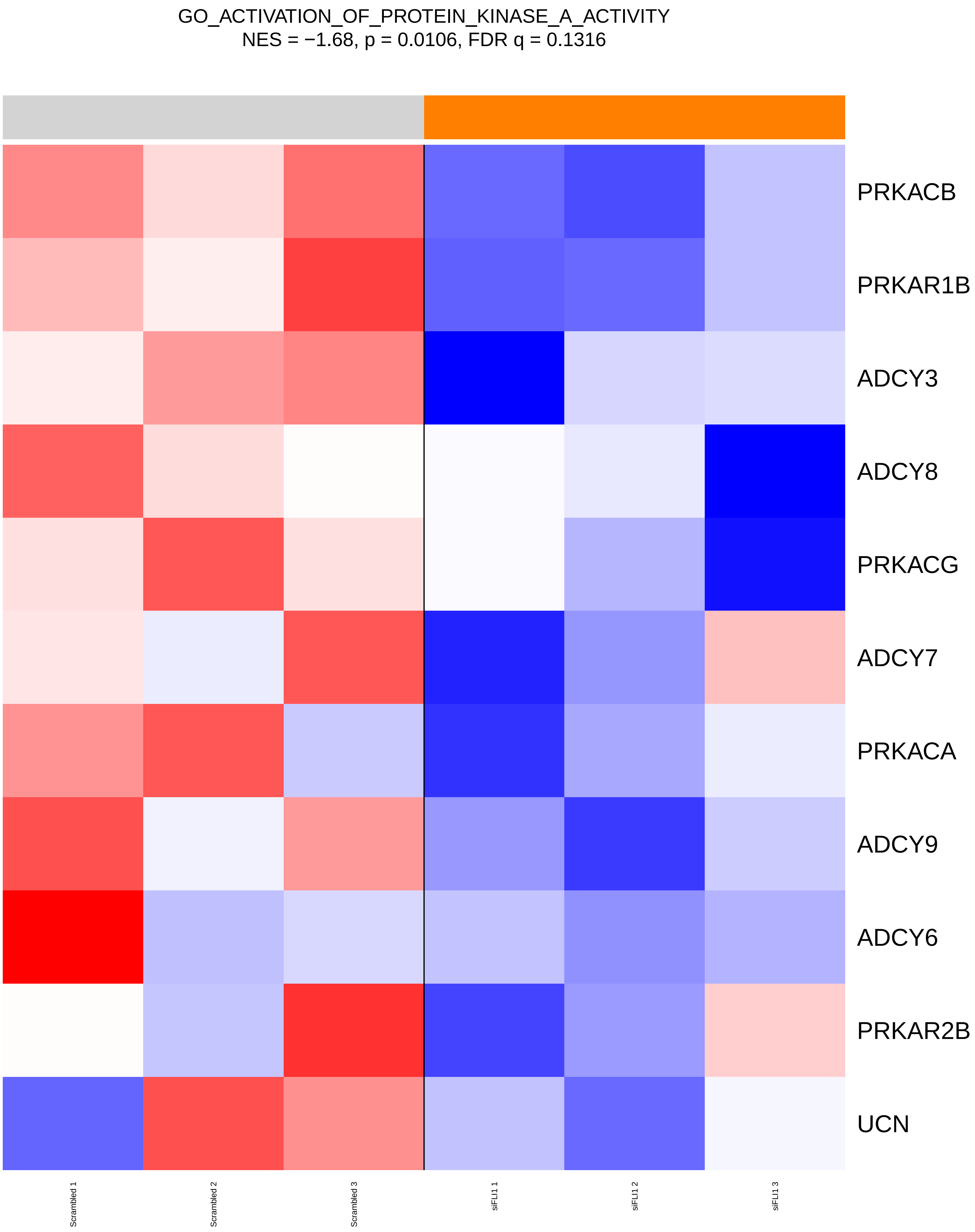
GO_ACTIVATION_OF_PROTEIN_KINASE_A_ACTIVITY
NES = −1.68, p = 0.0106, FDR q = 0.1316
PRKACB
PRKAR1B
ADCY3
ADCY8
PRKACG
ADCY7
PRKACA
ADCY9
ADCY6
PRKAR2B
UCN
Scrambled 1
Scrambled 2
Scrambled 3
siFLI1 1
siFLI1 2
siFLI1 3

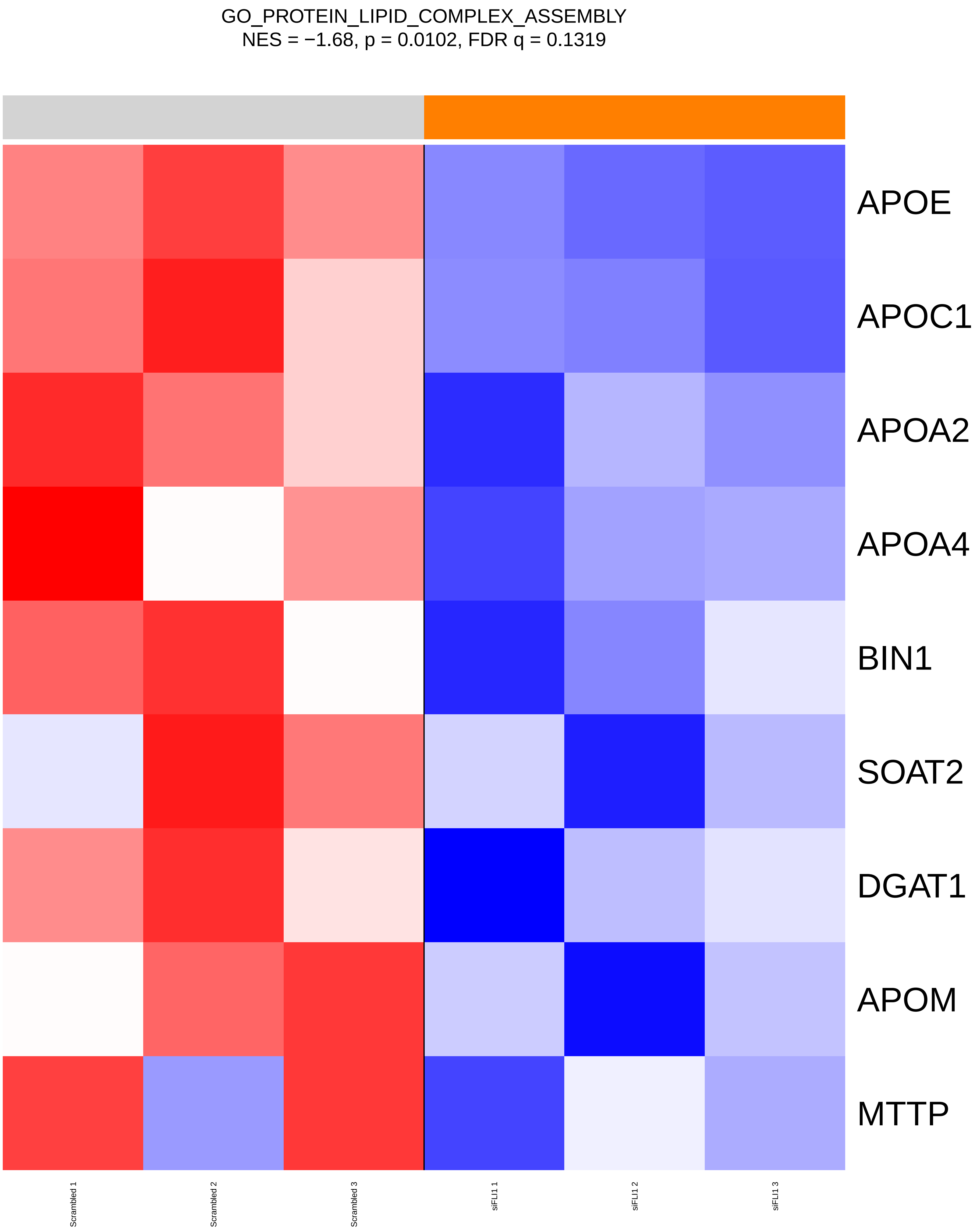
GO_PROTEIN_LIPID_COMPLEX_ASSEMBLY
NES = −1.68, p = 0.0102, FDR q = 0.1319
APOE
APOC1
APOA2
APOA4
BIN1
SOAT2
DGAT1
APOM
MTTP
Scrambled 1
Scrambled 2
Scrambled 3
siFLI1 1
siFLI1 2
siFLI1 3

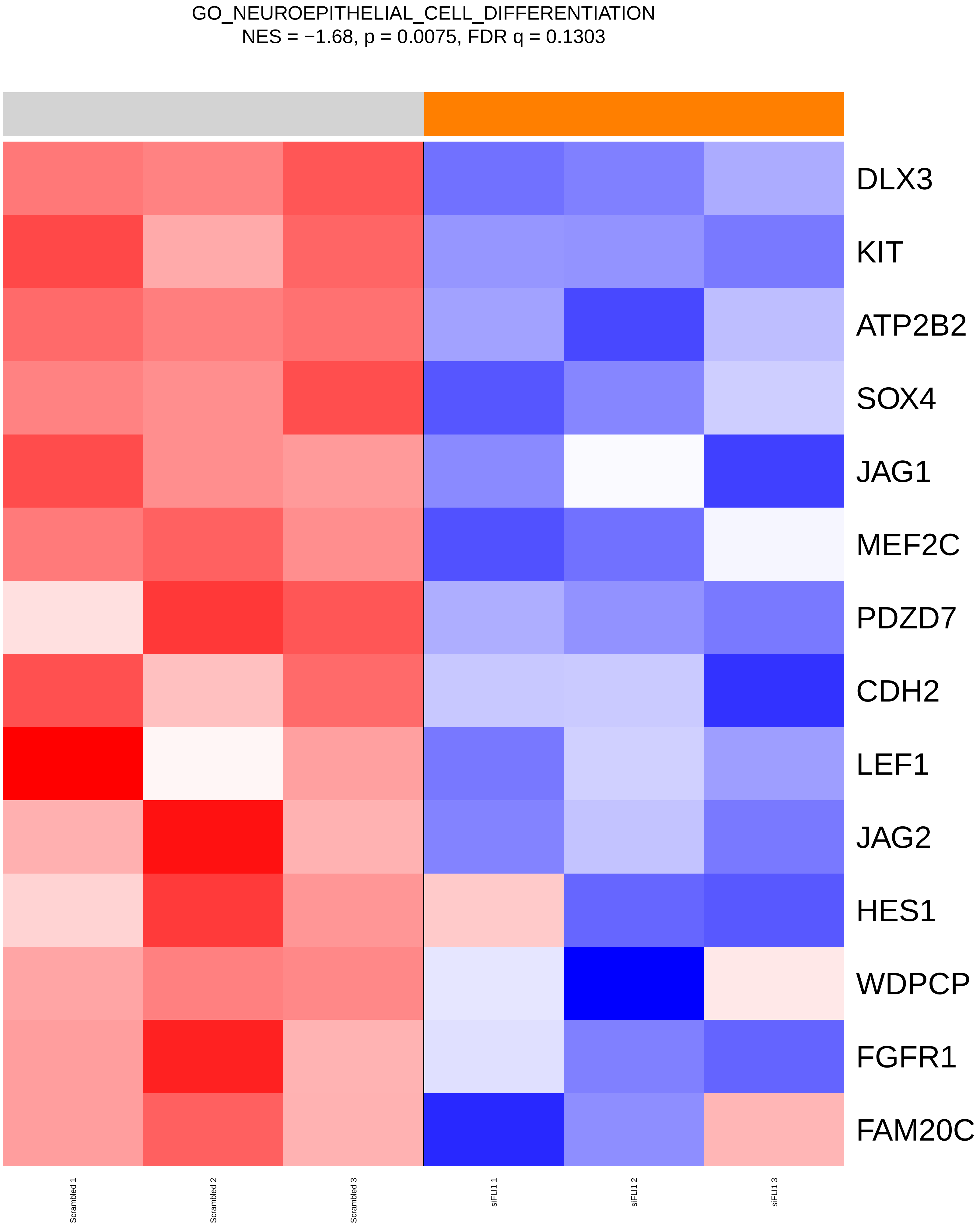
GO_NEUROEPITHELIAL_CELL_DIFFERENTIATION
NES = −1.68, p = 0.0075, FDR q = 0.1303
DLX3
KIT
ATP2B2
SOX4
JAG1
MEF2C
PDZD7
CDH2
LEF1
JAG2
HES1
WDPCP
FGFR1
FAM20C
Scrambled 1
Scrambled 2
Scrambled 3
siFLI1 1
siFLI1 2
siFLI1 3

GO_SITE_OF_DOUBLE_STRAND_BREAK
NES = −1.68, p = 0.0033, FDR q = 0.1300

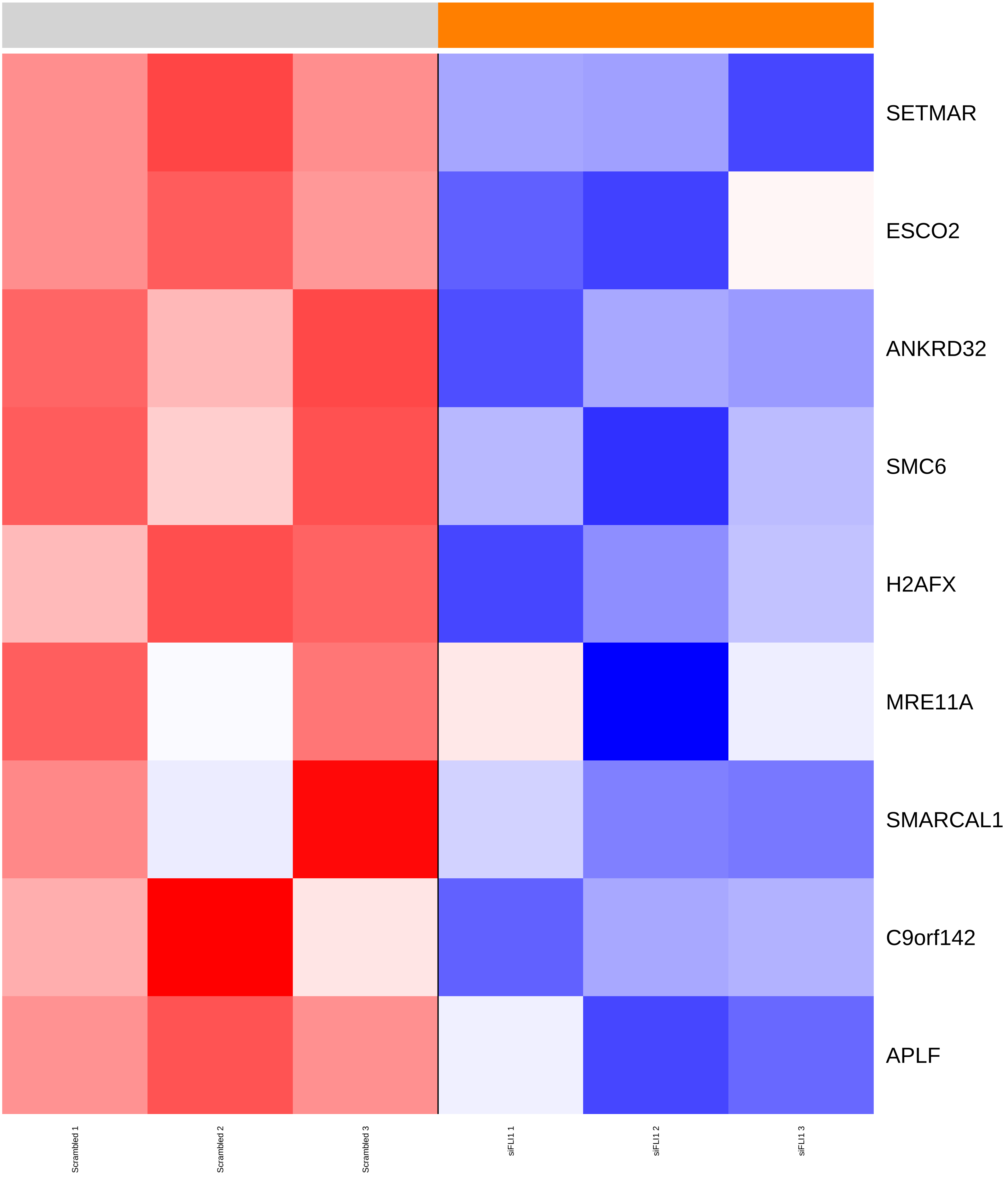

GO_CHROMATIN_SILENCING_AT_RDNA
NES = −1.68, p = 0.0050, FDR q = 0.1285

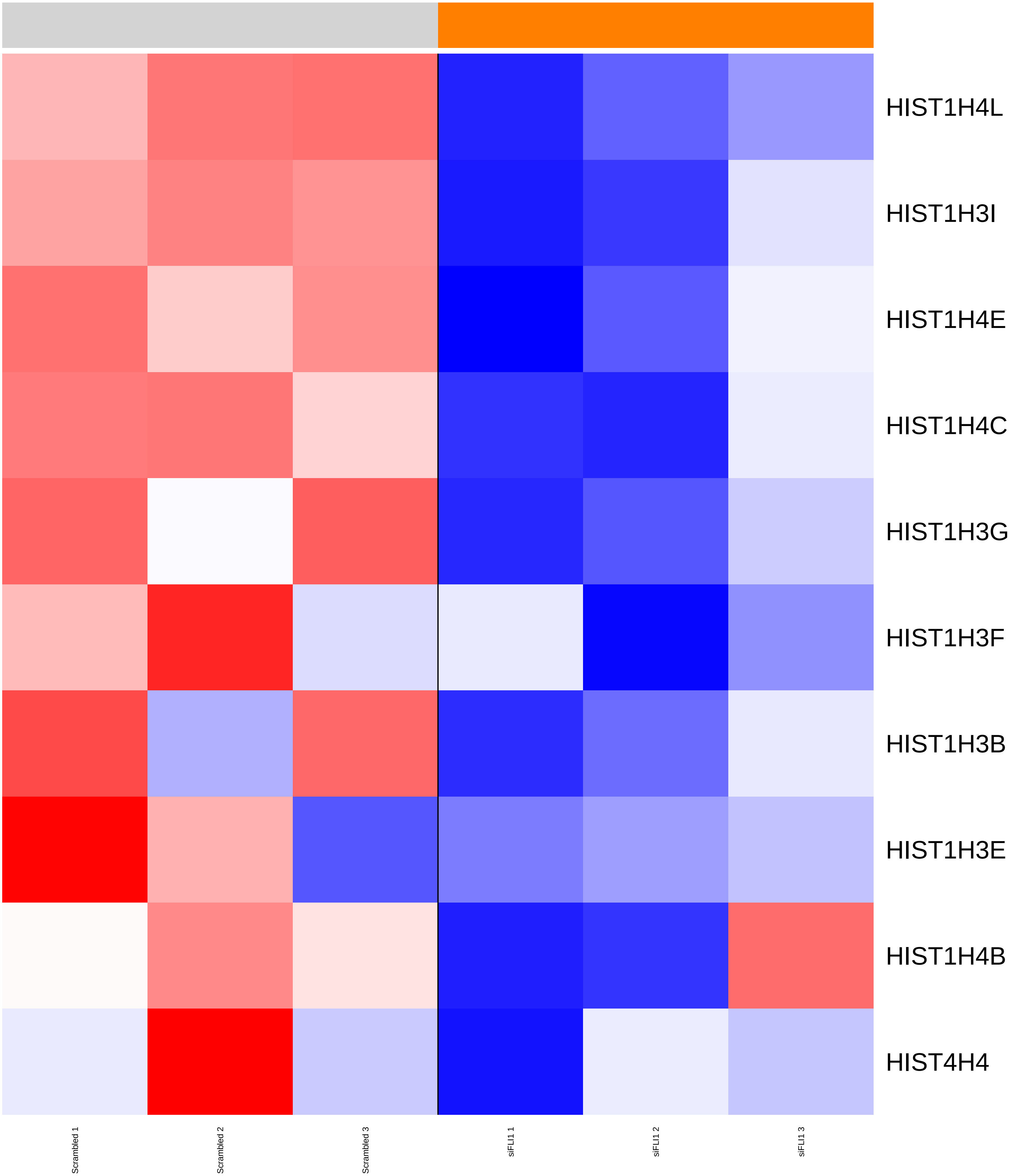

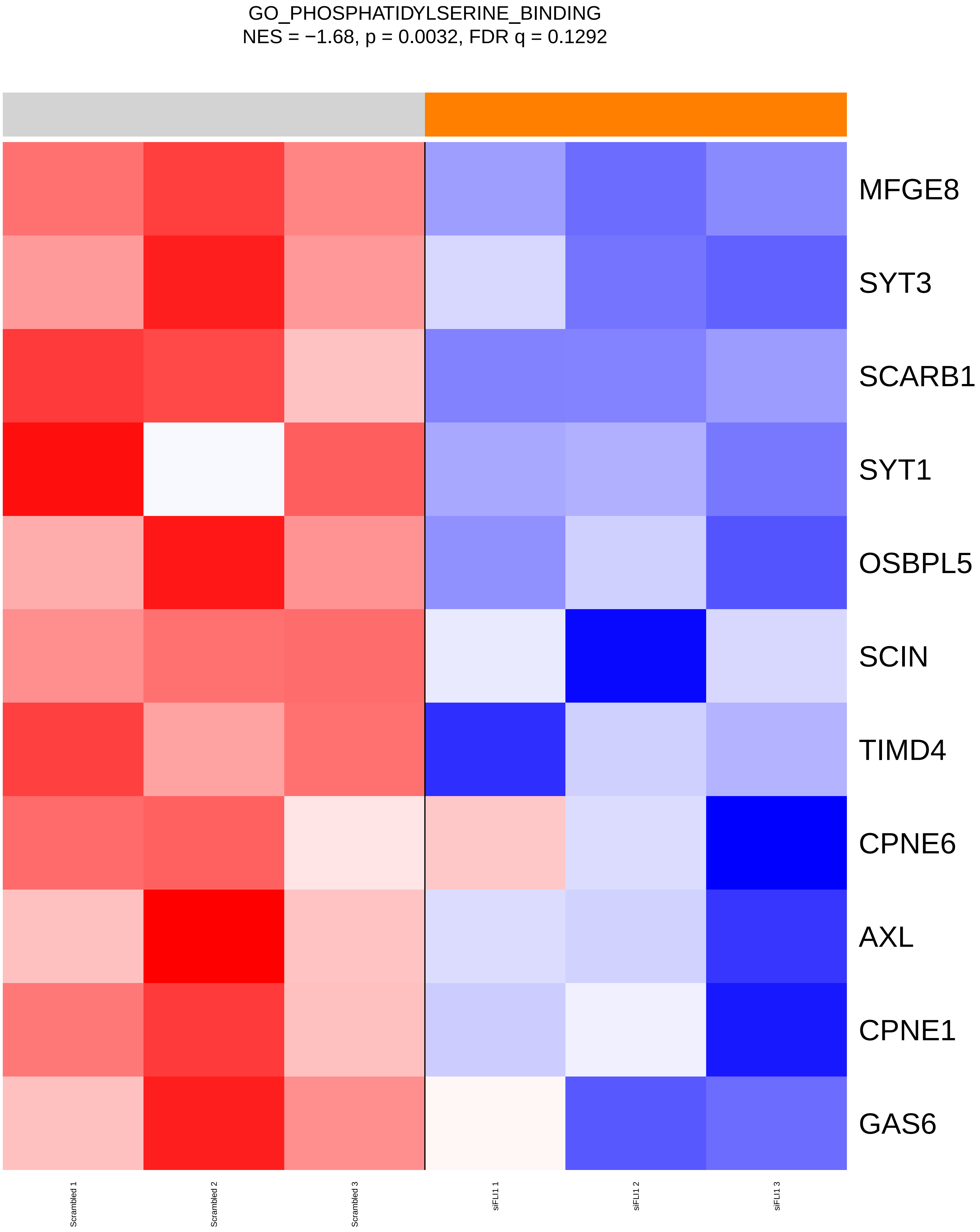
GO_PHOSPHATIDYLSERINE_BINDING
NES = −1.68, p = 0.0032, FDR q = 0.1292
MFGE8
SYT3
SCARB1
SYT1
OSBPL5
SCIN
TIMD4
CPNE6
AXL
CPNE1
GAS6
Scrambled 1
Scrambled 2
Scrambled 3
siFLI1 1
siFLI1 2
siFLI1 3

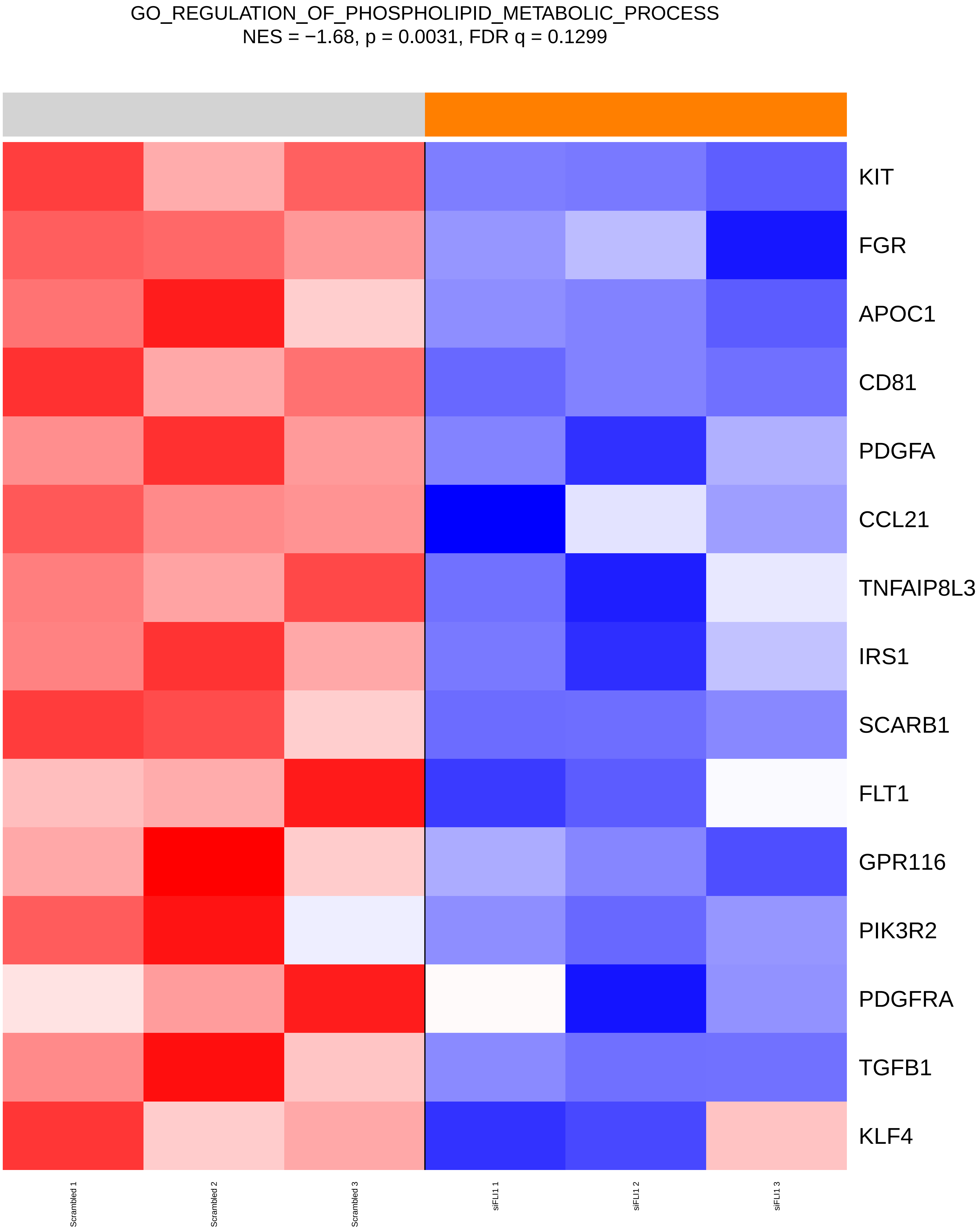
GO_REGULATION_OF_PHOSPHOLIPID_METABOLIC_PROCESS
NES = −1.68, p = 0.0031, FDR q = 0.1299
KIT
FGR
APOC1
CD81
PDGFA
CCL21
TNFAIP8L3
IRS1
SCARB1
FLT1
GPR116
PIK3R2
PDGFRA
TGFB1
KLF4
Scrambled 1
Scrambled 2
Scrambled 3
siFLI1 1
siFLI1 2
siFLI1 3

KEGG_PPAR_SIGNALING_PATHWAY
NES = −1.68, p = 0.0015, FDR q = 0.1307

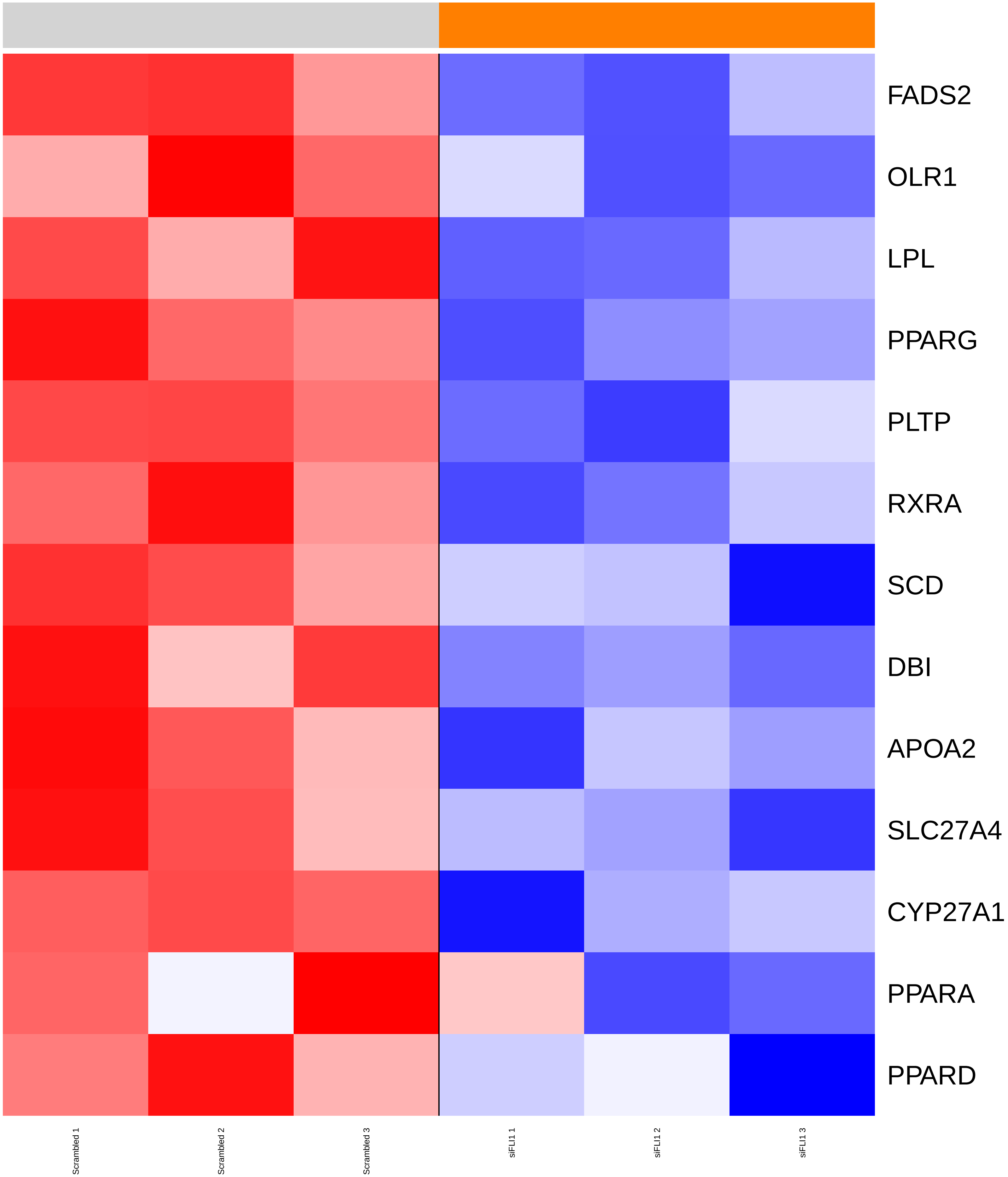

GO_SMALL_MOLECULE_BIOSYNTHETIC_PROCESS
NES = −1.69, p = 0.0000, FDR q = 0.1287

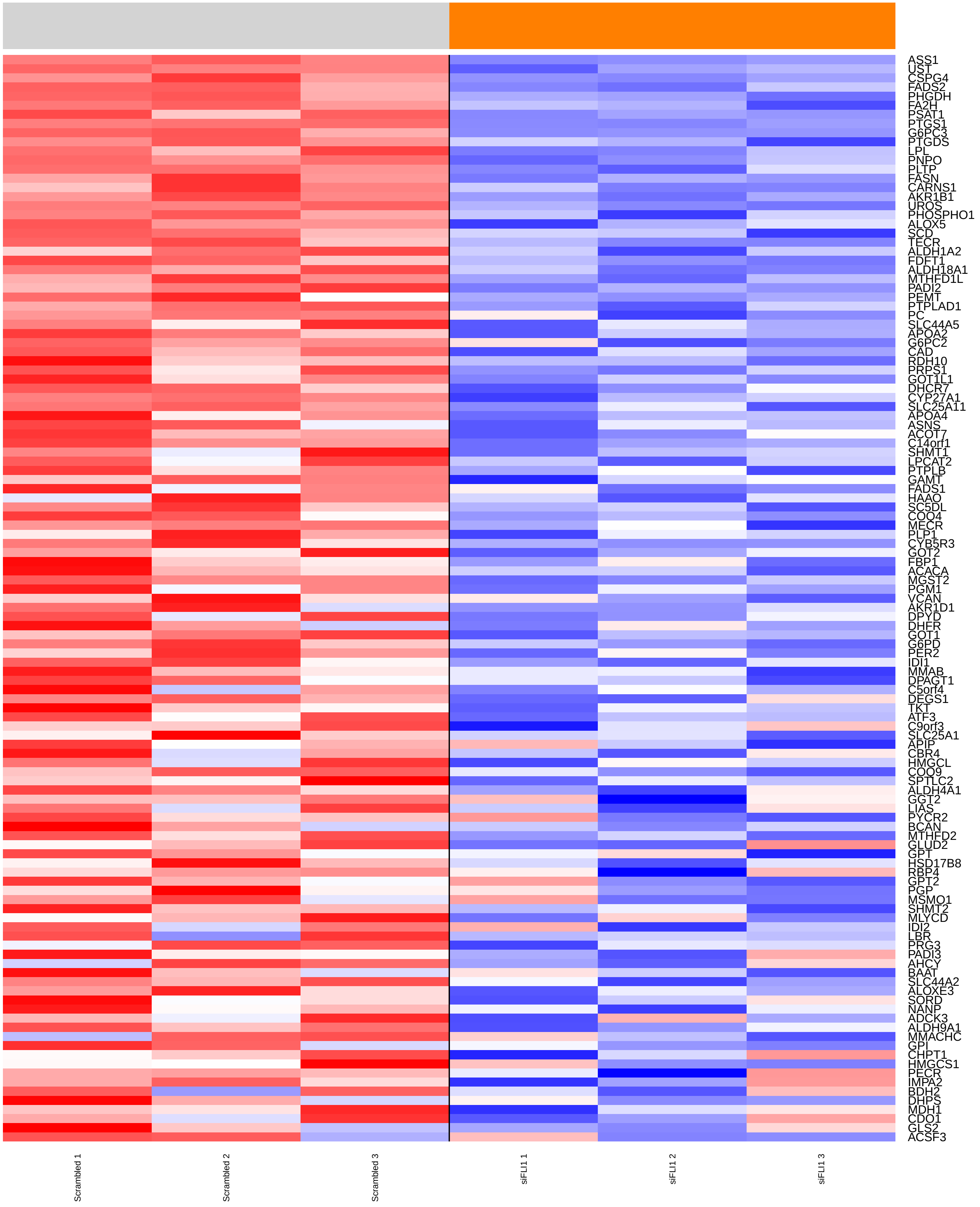

GO_REGULATION_OF_TRANSCRIPTION_INVOLVED_IN_CELL_FATE_COMMITMENT
NES = −1.69, p = 0.0152, FDR q = 0.1293

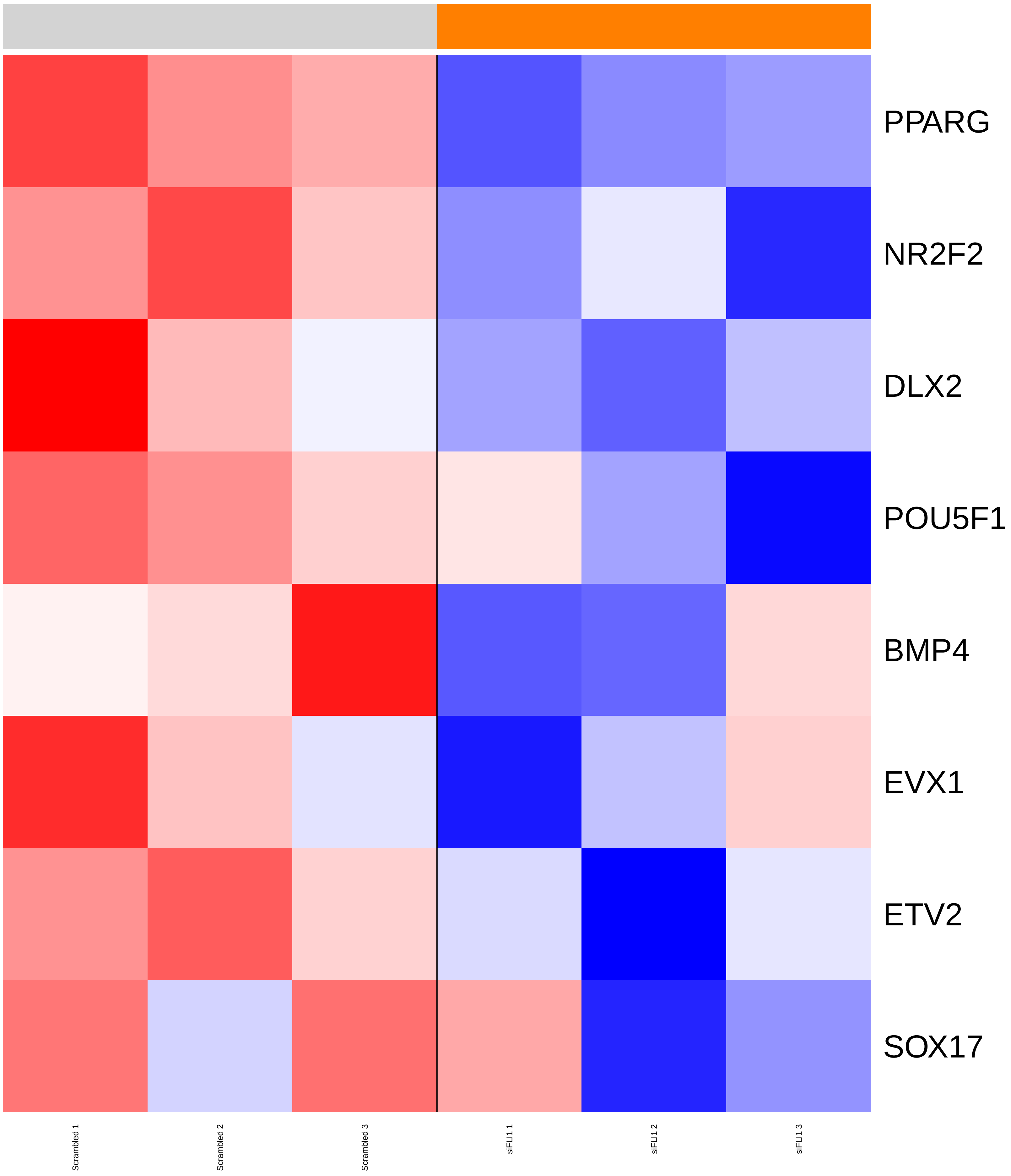

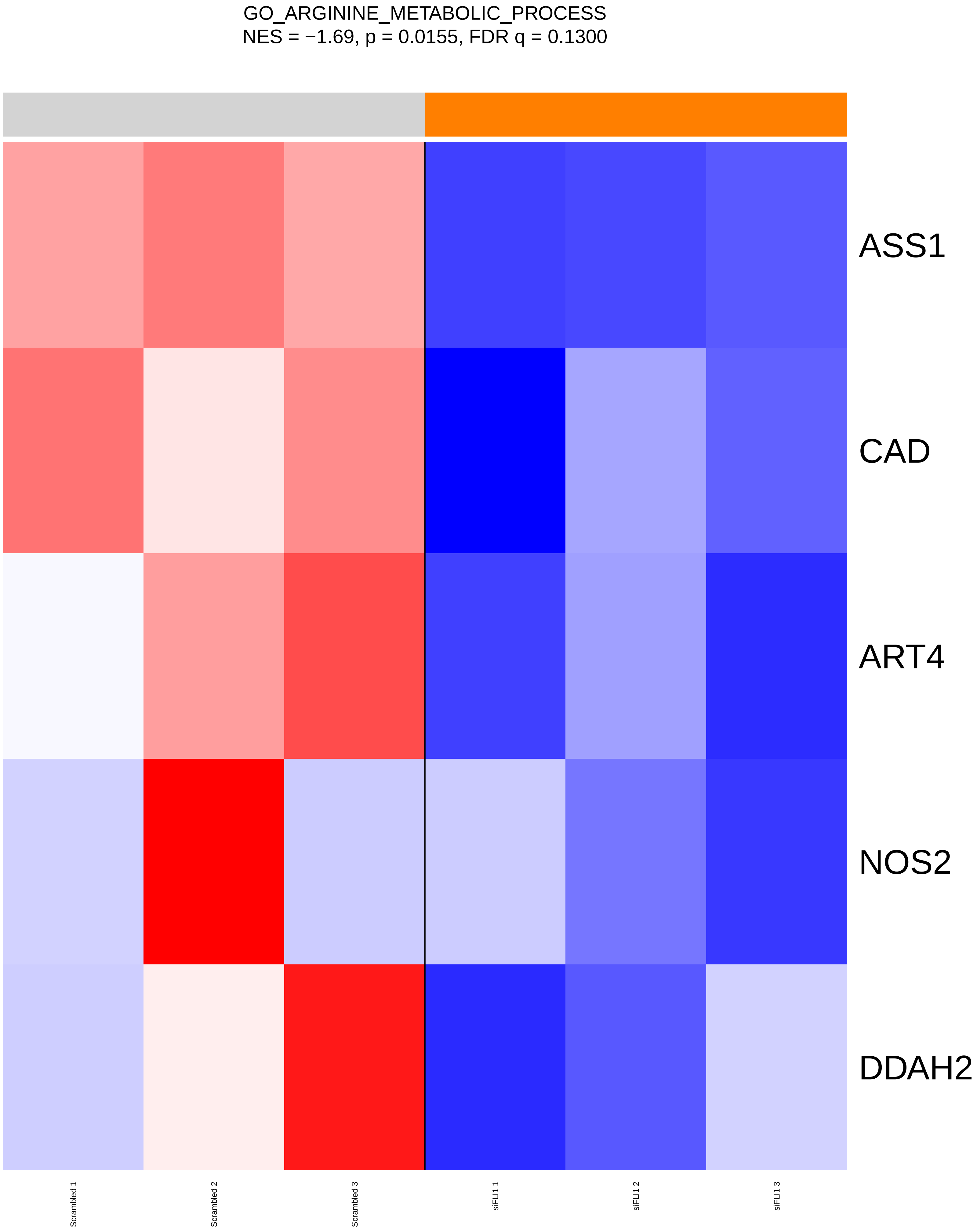
GO_ARGININE_METABOLIC_PROCESS
NES = −1.69, p = 0.0155, FDR q = 0.1300
ASS1
CAD
ART4
NOS2
DDAH2
Scrambled 1
Scrambled 2
Scrambled 3
siFLI1 1
siFLI1 2
siFLI1 3

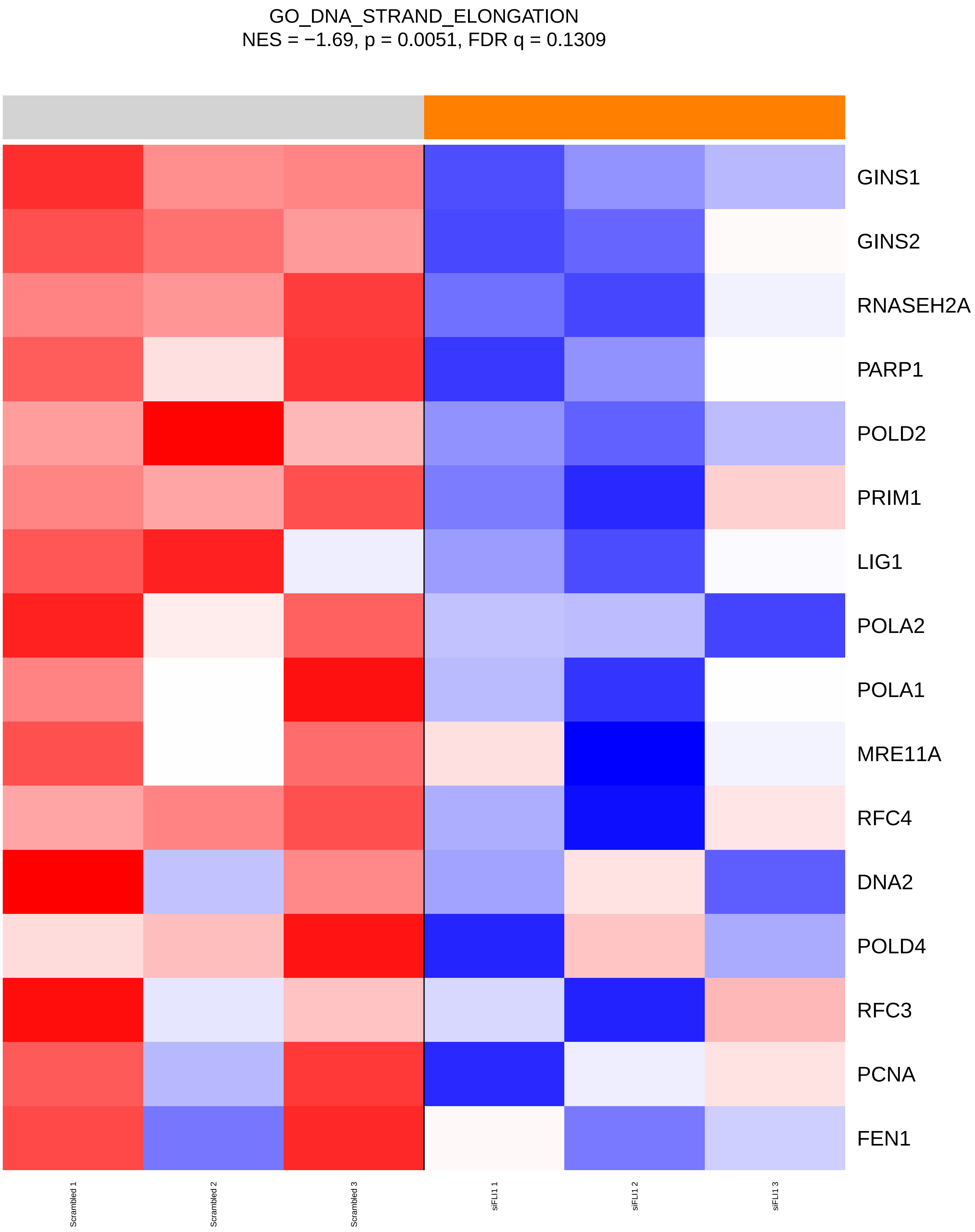
GO_DNA_STRAND_ELONGATION
NES = −1.69, p = 0.0051, FDR q = 0.1309
GINS1
GINS2
RNASEH2A
PARP1
POLD2
PRIM1
LIG1
POLA2
POLA1
MRE11A
RFC4
DNA2
POLD4
RFC3
PCNA
FEN1
Scrambled 1
Scrambled 2
Scrambled 3
siFLI1 1
siFLI1 2
siFLI1 3

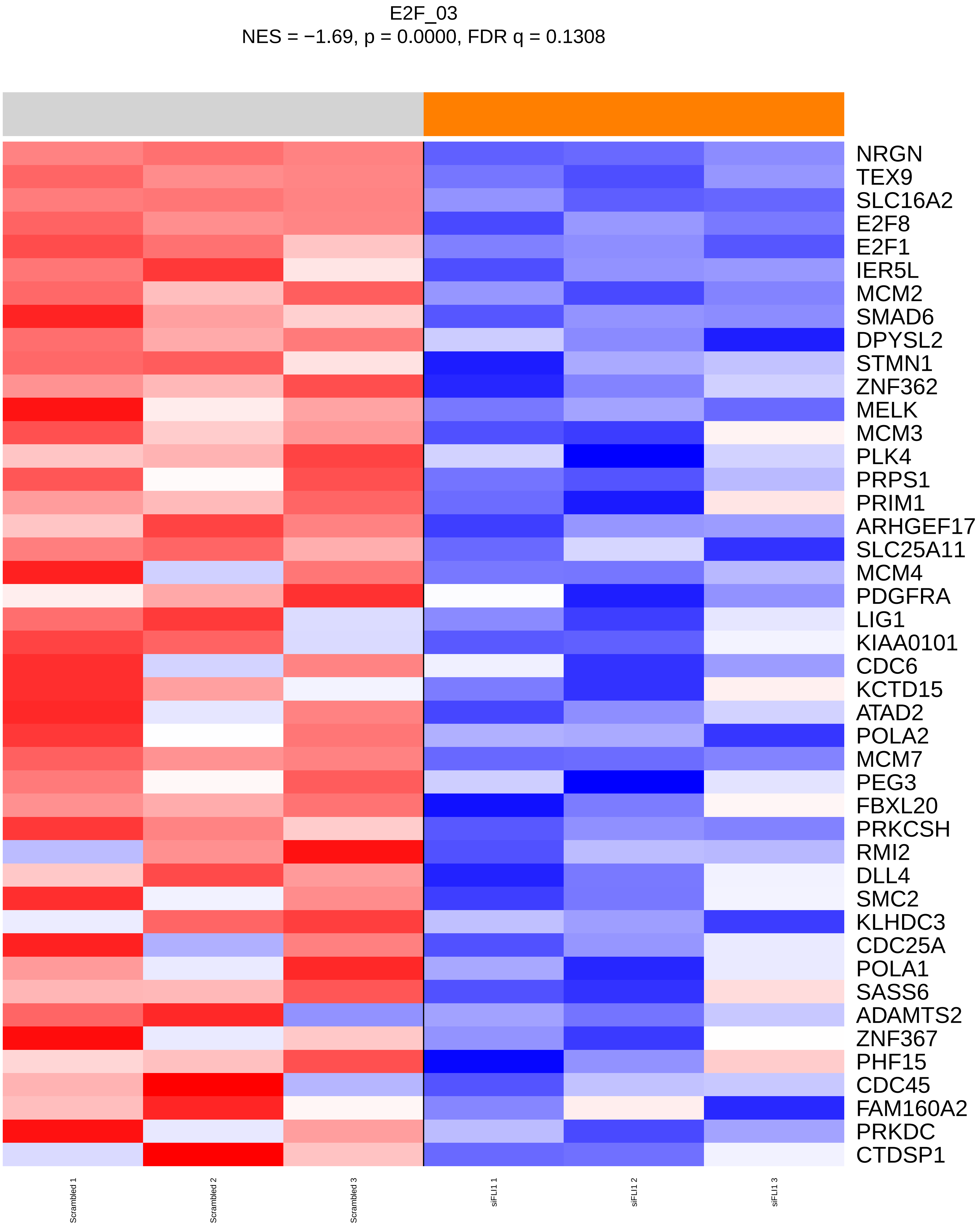
E2F_03
NES = −1.69, p = 0.0000, FDR q = 0.1308
NRGN
TEX9
SLC16A2
E2F8
E2F1
IER5L
MCM2
SMAD6
DPYSL2
STMN1
ZNF362
MELK
MCM3
PLK4
PRPS1
PRIM1
ARHGEF17
SLC25A11
MCM4
PDGFRA
LIG1
KIAA0101
CDC6
KCTD15
ATAD2
POLA2
MCM7
PEG3
FBXL20
PRKCSH
RMI2
DLL4
SMC2
KLHDC3
CDC25A
POLA1
SASS6
ADAMTS2
ZNF367
PHF15
CDC45
FAM160A2
PRKDC
CTDSP1
Scrambled 1
Scrambled 2
Scrambled 3
siFLI1 1
siFLI1 2
siFLI1 3

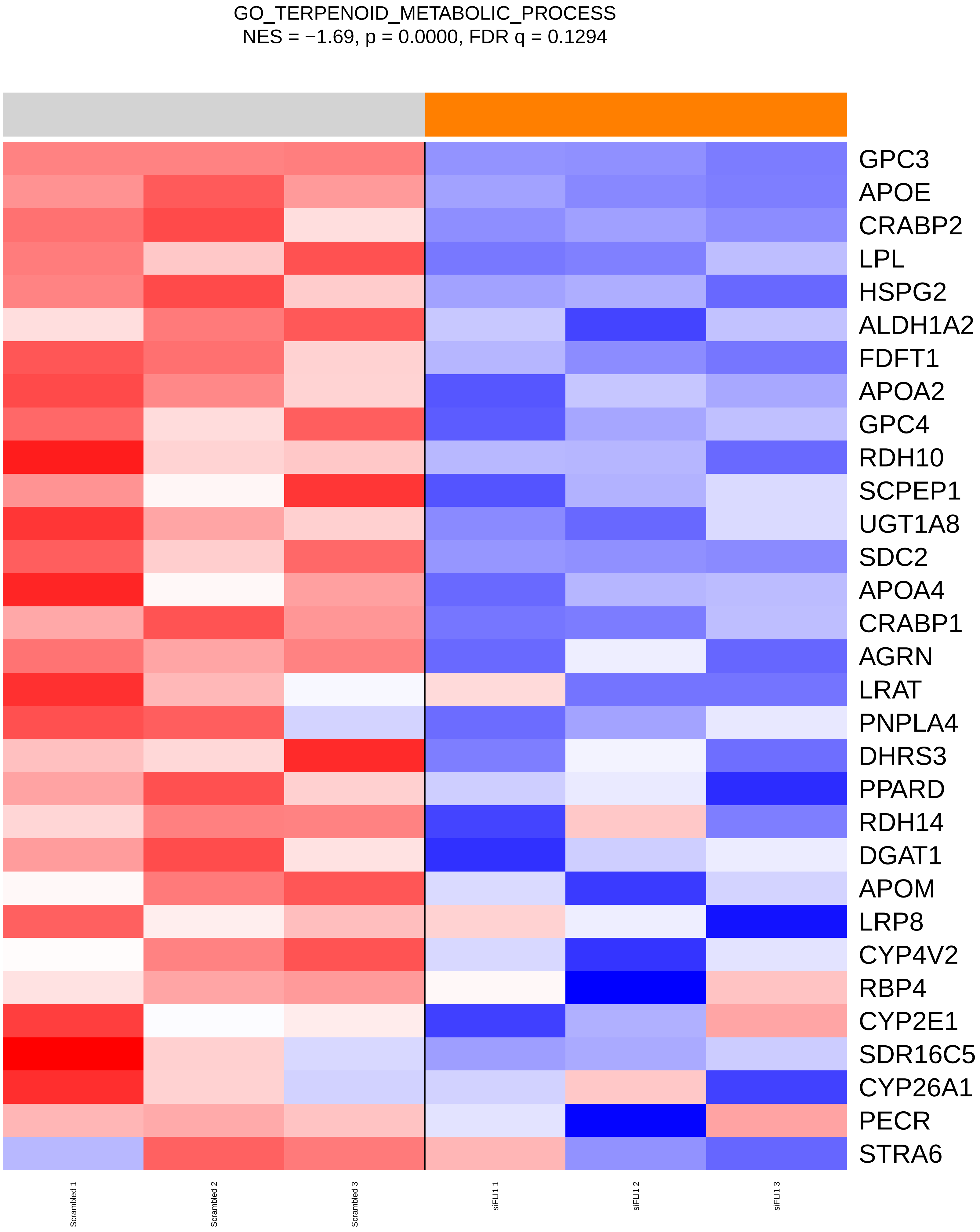
GO_TERPENOID_METABOLIC_PROCESS
NES = −1.69, p = 0.0000, FDR q = 0.1294
GPC3
APOE
CRABP2
LPL
HSPG2
ALDH1A2
FDFT1
APOA2
GPC4
RDH10
SCPEP1
UGT1A8
SDC2
APOA4
CRABP1
AGRN
LRAT
PNPLA4
DHRS3
PPARD
RDH14
DGAT1
APOM
LRP8
CYP4V2
RBP4
CYP2E1
SDR16C5
CYP26A1
PECR
STRA6
Scrambled 1
Scrambled 2
Scrambled 3
siFLI1 1
siFLI1 2
siFLI1 3

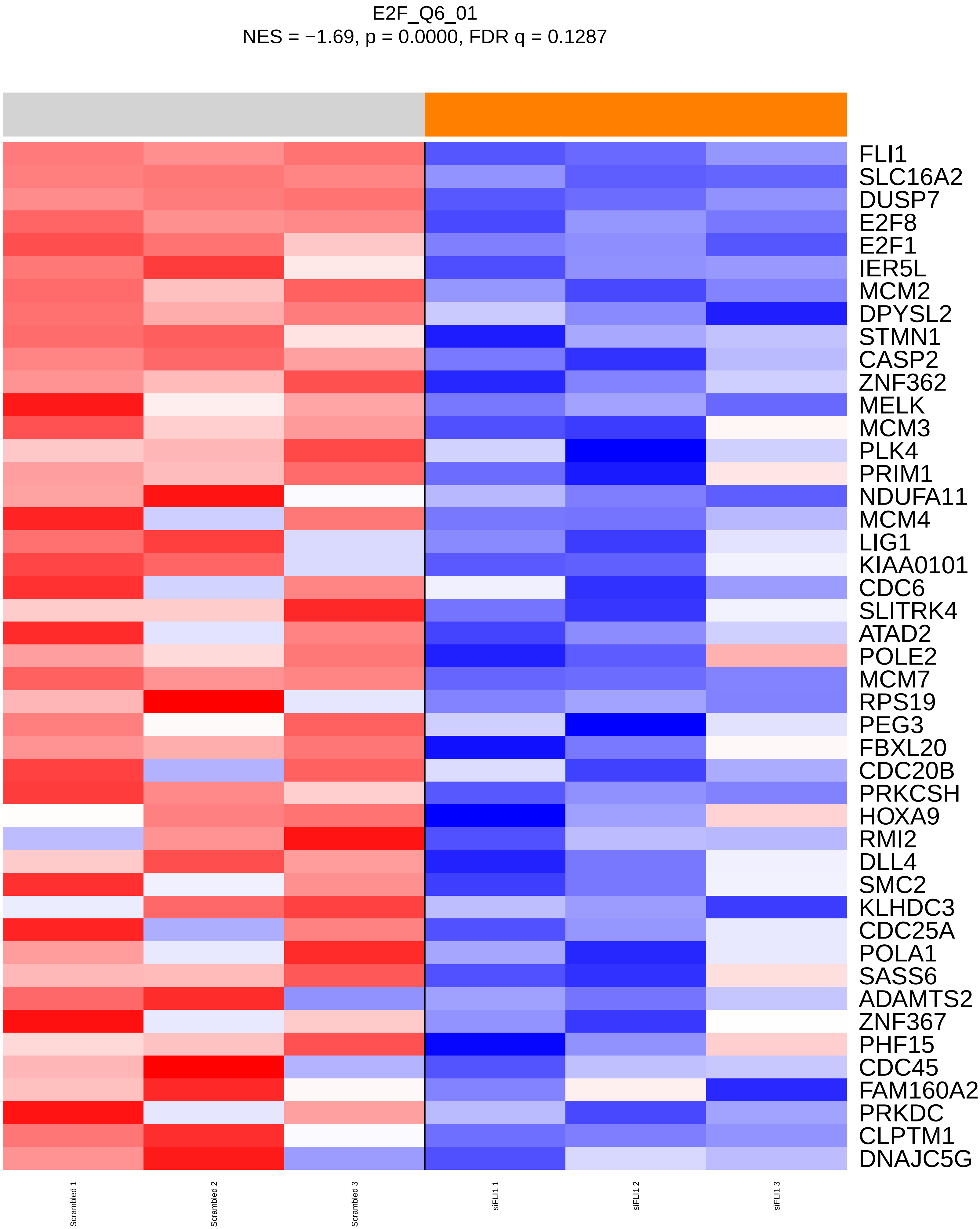
E2F_Q6_01
NES = −1.69, p = 0.0000, FDR q = 0.1287
FLI1
SLC16A2
DUSP7
E2F8
E2F1
IER5L
MCM2
DPYSL2
STMN1
CASP2
ZNF362
MELK
MCM3
PLK4
PRIM1
NDUFA11
MCM4
LIG1
KIAA0101
CDC6
SLITRK4
ATAD2
POLE2
MCM7
RPS19
PEG3
FBXL20
CDC20B
PRKCSH
HOXA9
RMI2
DLL4
SMC2
KLHDC3
CDC25A
POLA1
SASS6
ADAMTS2
ZNF367
PHF15
CDC45
FAM160A2
PRKDC
CLPTM1
DNAJC5G
Scrambled 1
Scrambled 2
Scrambled 3
siFLI1 1
siFLI1 2
siFLI1 3

GO_NEURON_NEURON_SYNAPTIC_TRANSMISSION
NES = −1.69, p = 0.0031, FDR q = 0.1291

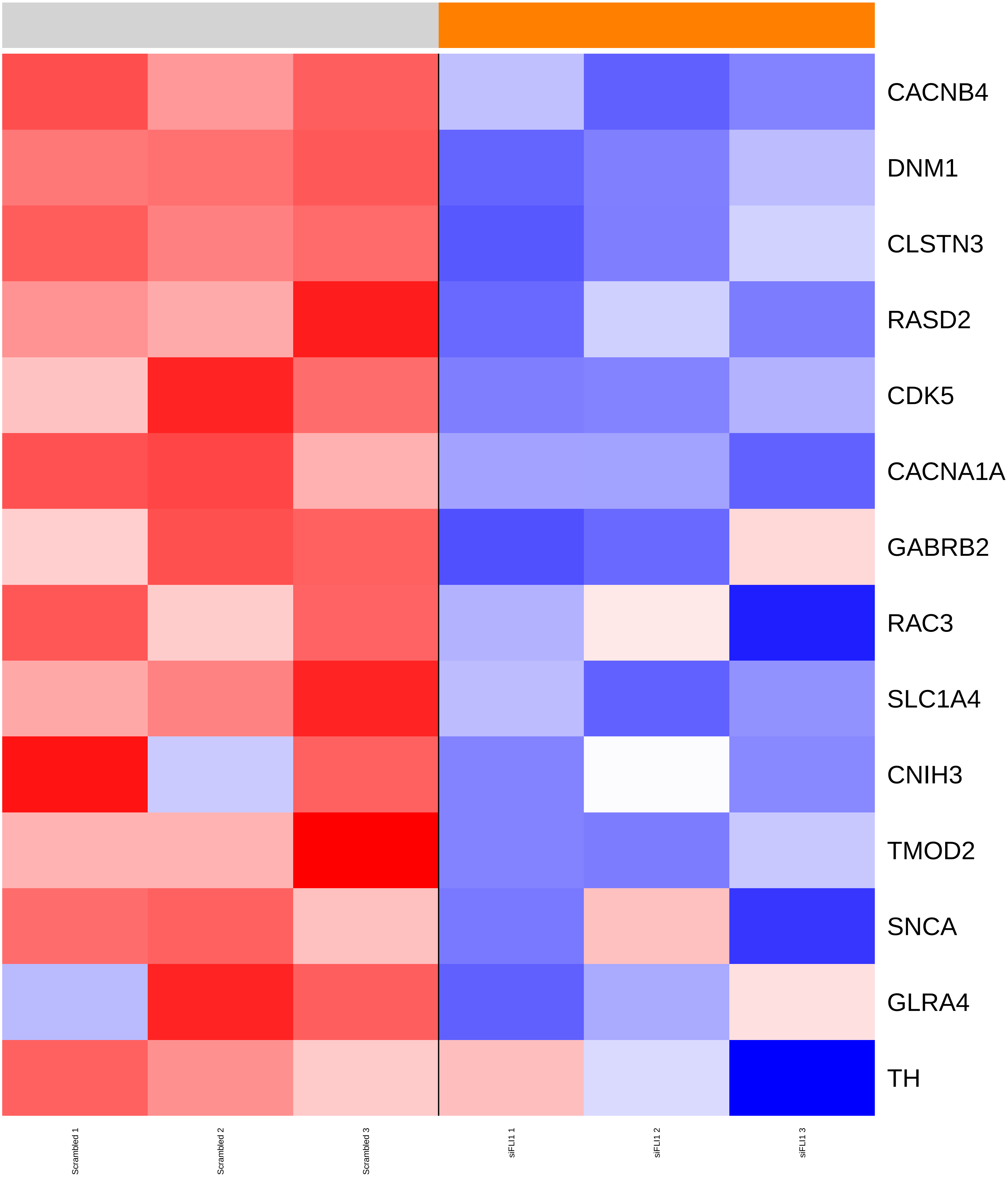

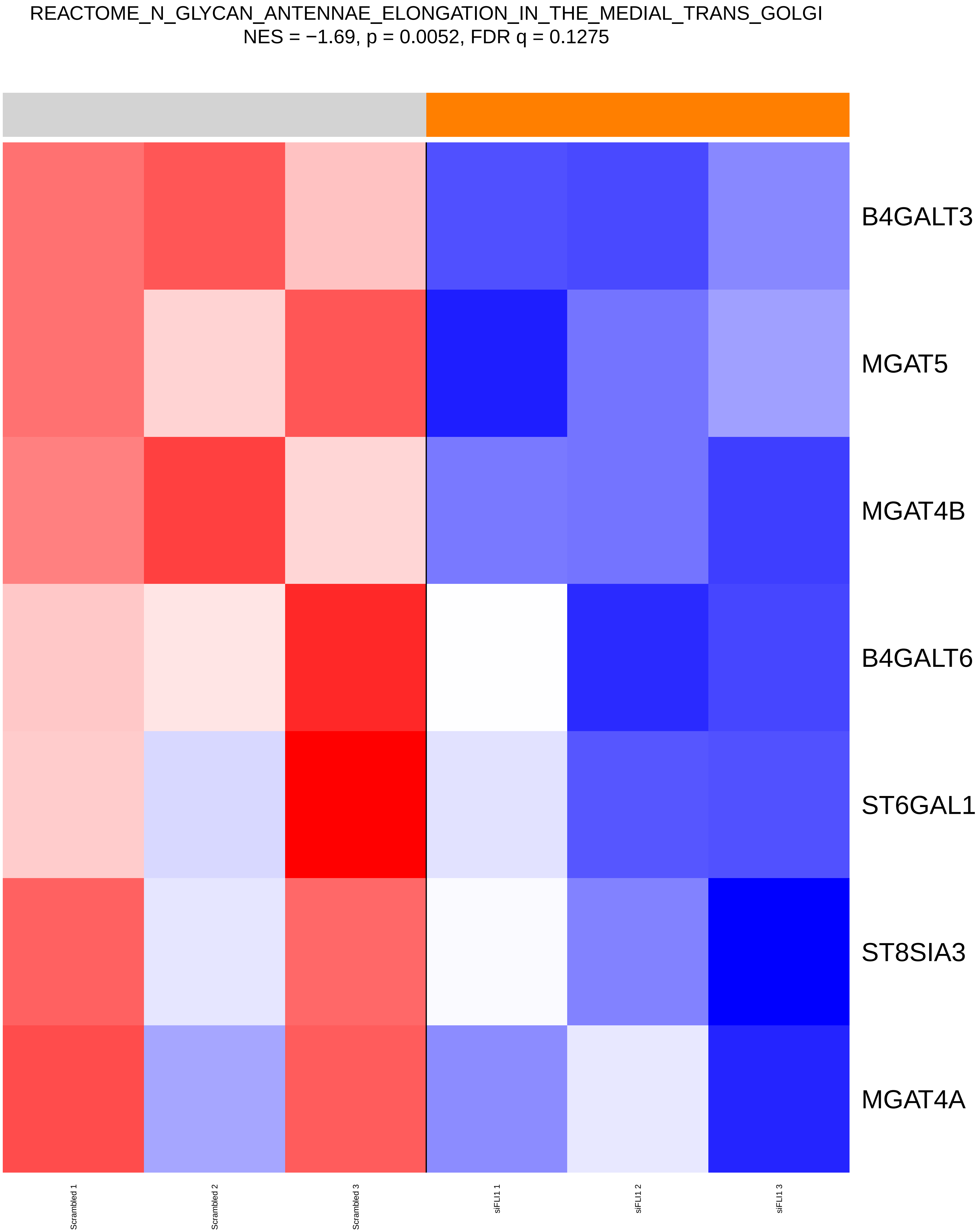

REACTOME_N_GLYCAN_ANTENNAE_ELONGATION_IN_THE_MEDIAL_TRANS_GOLGI
NES = −1.69, p = 0.0052, FDR q = 0.1275
B4GALT3
MGAT5
MGAT4B
B4GALT6
ST6GAL1
ST8SIA3
MGAT4A
Scrambled 1
Scrambled 2
Scrambled 3
siFLI1 1
siFLI1 2
siFLI1 3

GO_ORGANOPHOSPHATE_ESTER_TRANSPORT
NES = −1.69, p = 0.0015, FDR q = 0.1283

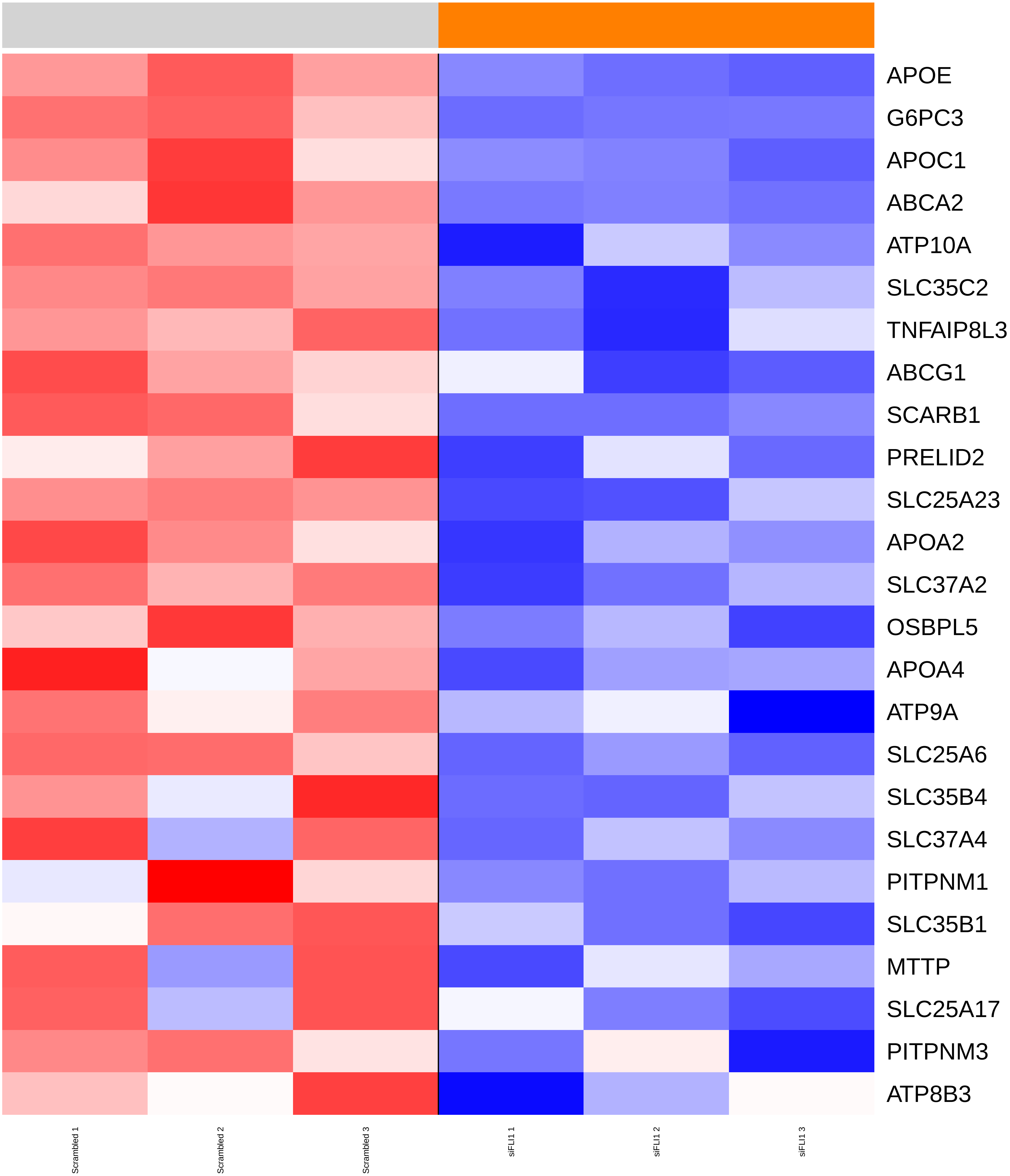

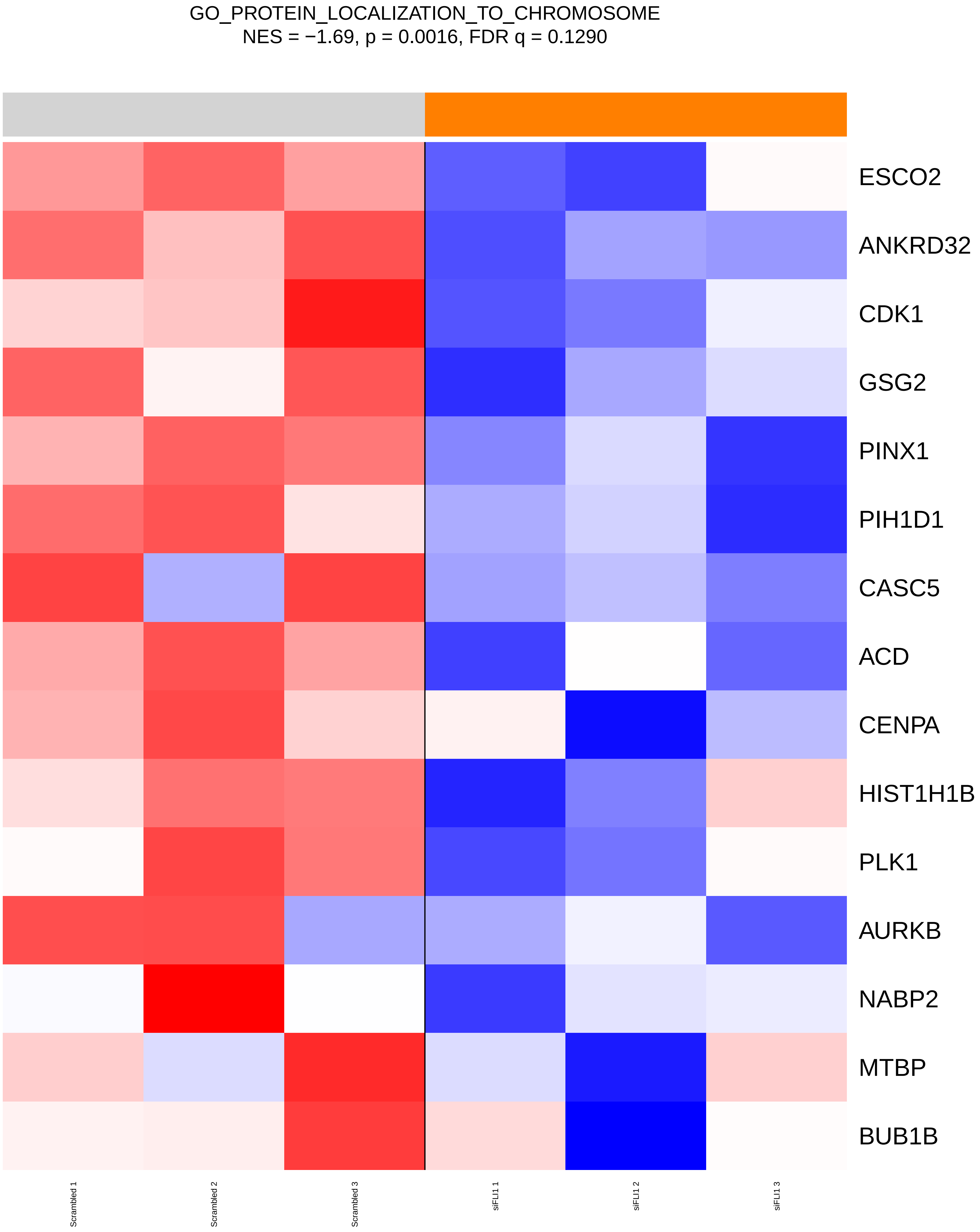
GO_PROTEIN_LOCALIZATION_TO_CHROMOSOME
NES = −1.69, p = 0.0016, FDR q = 0.1290
ESCO2
ANKRD32
CDK1
GSG2
PINX1
PIH1D1
CASC5
ACD
CENPA
HIST1H1B
PLK1
AURKB
NABP2
MTBP
BUB1B
Scrambled 1
Scrambled 2
Scrambled 3
siFLI1 1
siFLI1 2
siFLI1 3

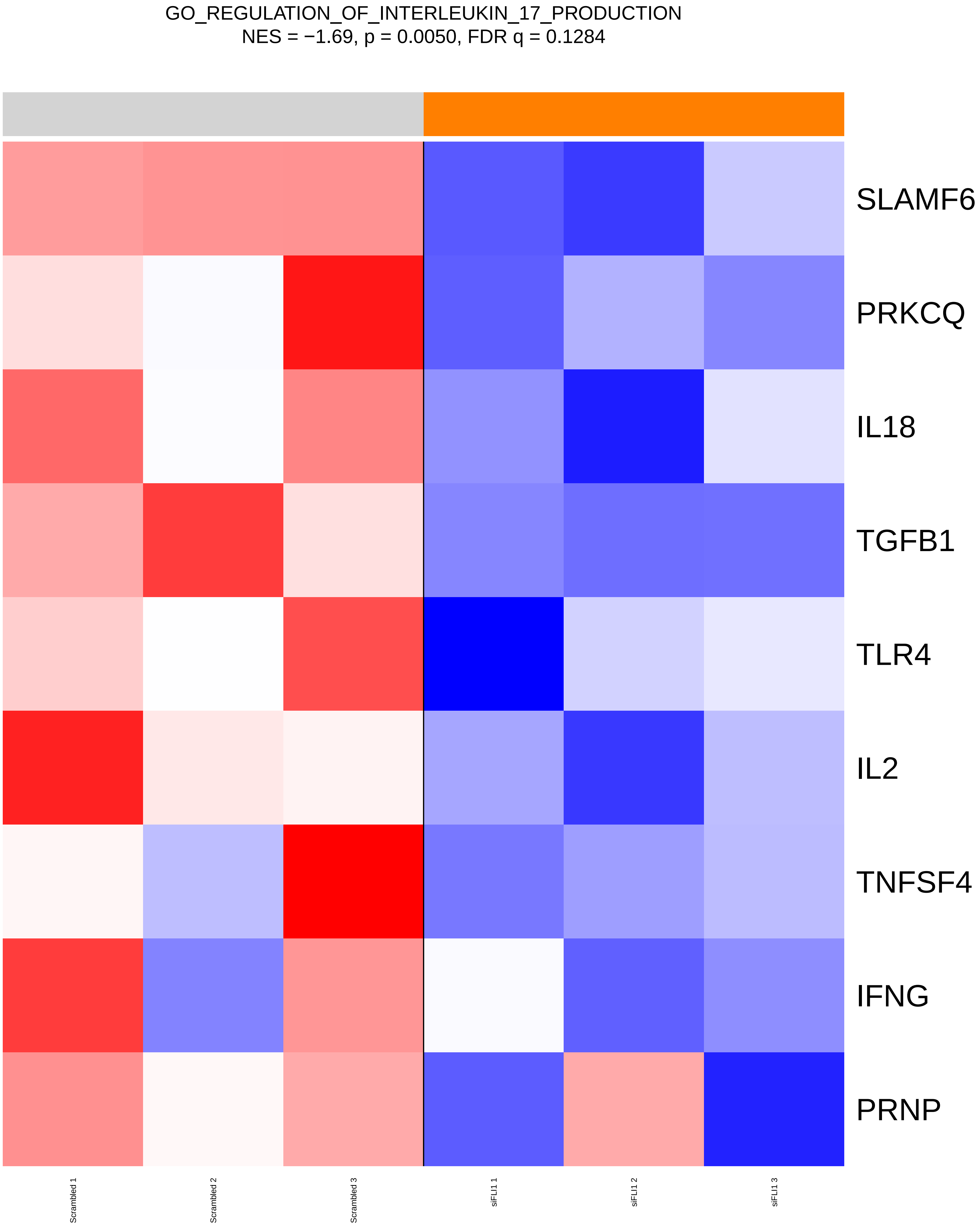
GO_REGULATION_OF_INTERLEUKIN_17_PRODUCTION
NES = −1.69, p = 0.0050, FDR q = 0.1284
SLAMF6
PRKCQ
IL18
TGFB1
TLR4
IL2
TNFSF4
IFNG
PRNP
Scrambled 1
Scrambled 2
Scrambled 3
siFLI1 1
siFLI1 2
siFLI1 3

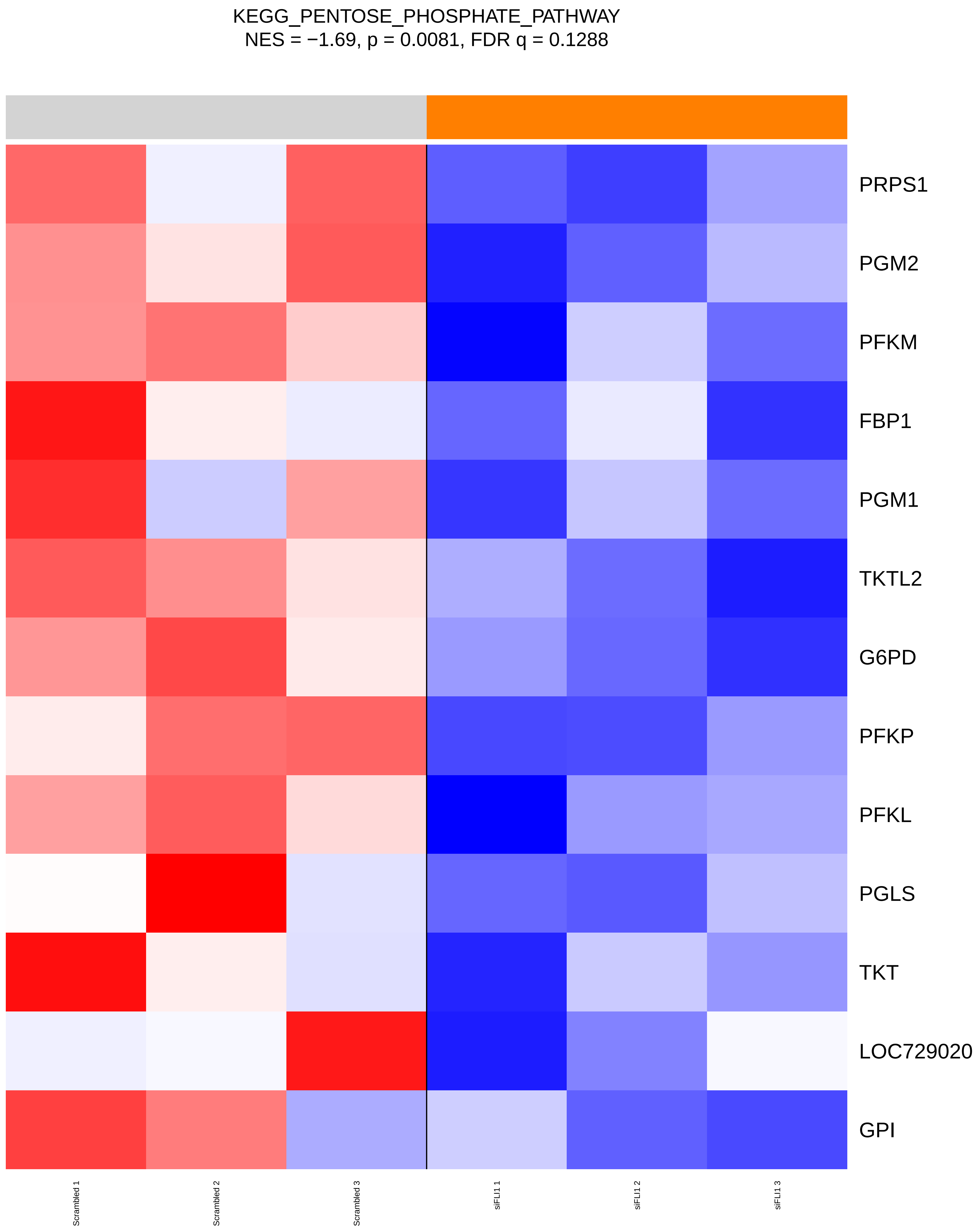

KEGG_PENTOSE_PHOSPHATE_PATHWAY
NES = −1.69, p = 0.0081, FDR q = 0.1288
PRPS1
PGM2
PFKM
FBP1
PGM1
TKTL2
G6PD
PFKP
PFKL
PGLS
TKT
LOC729020
GPI
Scrambled 1
Scrambled 2
Scrambled 3
siFLI1 1
siFLI1 2
siFLI1 3

GO_CELL_CYCLE_G1_S_PHASE_TRANSITION
NES = −1.70, p = 0.0000, FDR q = 0.1291

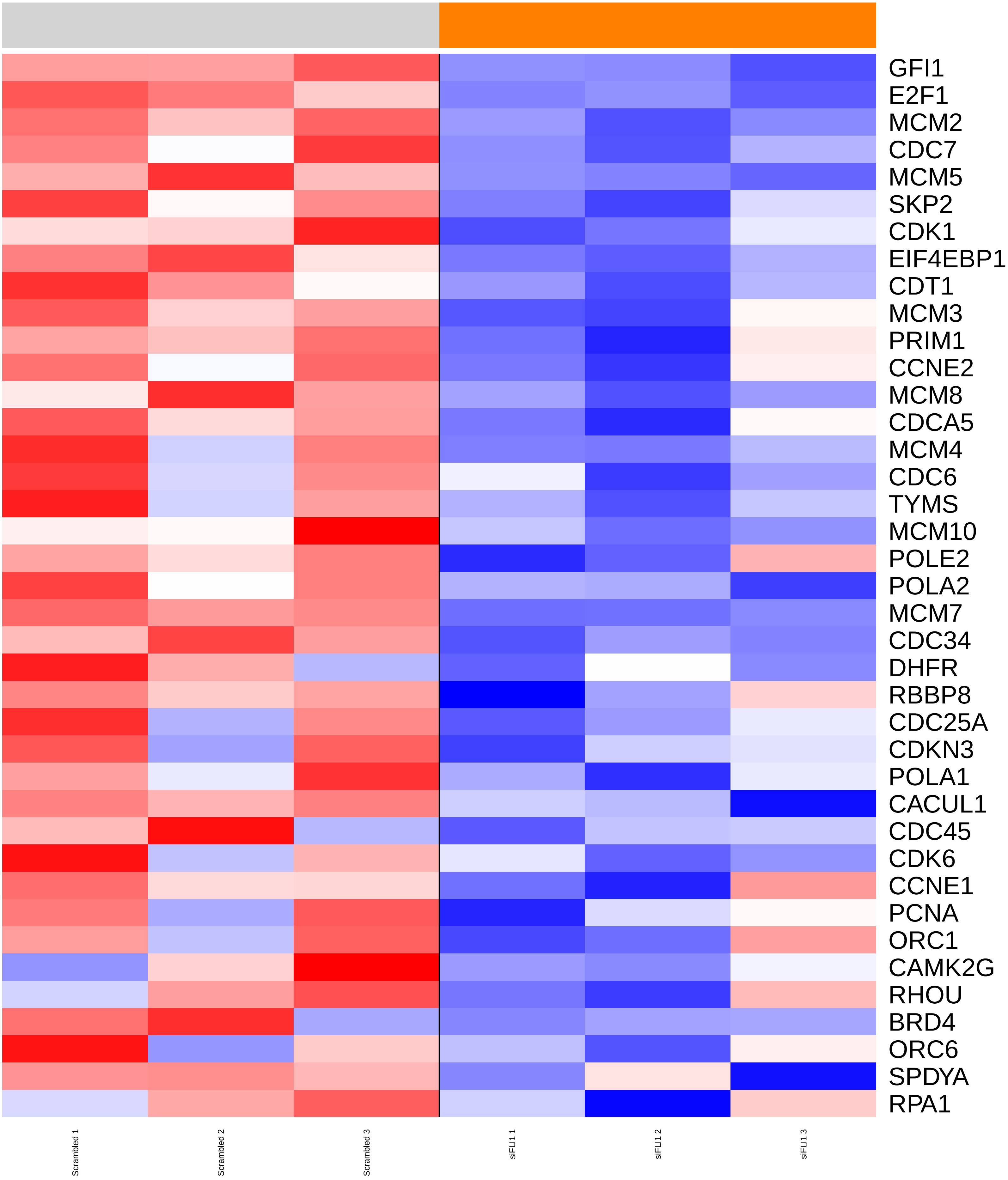

GO_NEGATIVE_REGULATION_OF_HEMATOPOIETIC_PROGENITOR_CELL_DIFFERENTIATION
NES = −1.70, p = 0.0084, FDR q = 0.1293

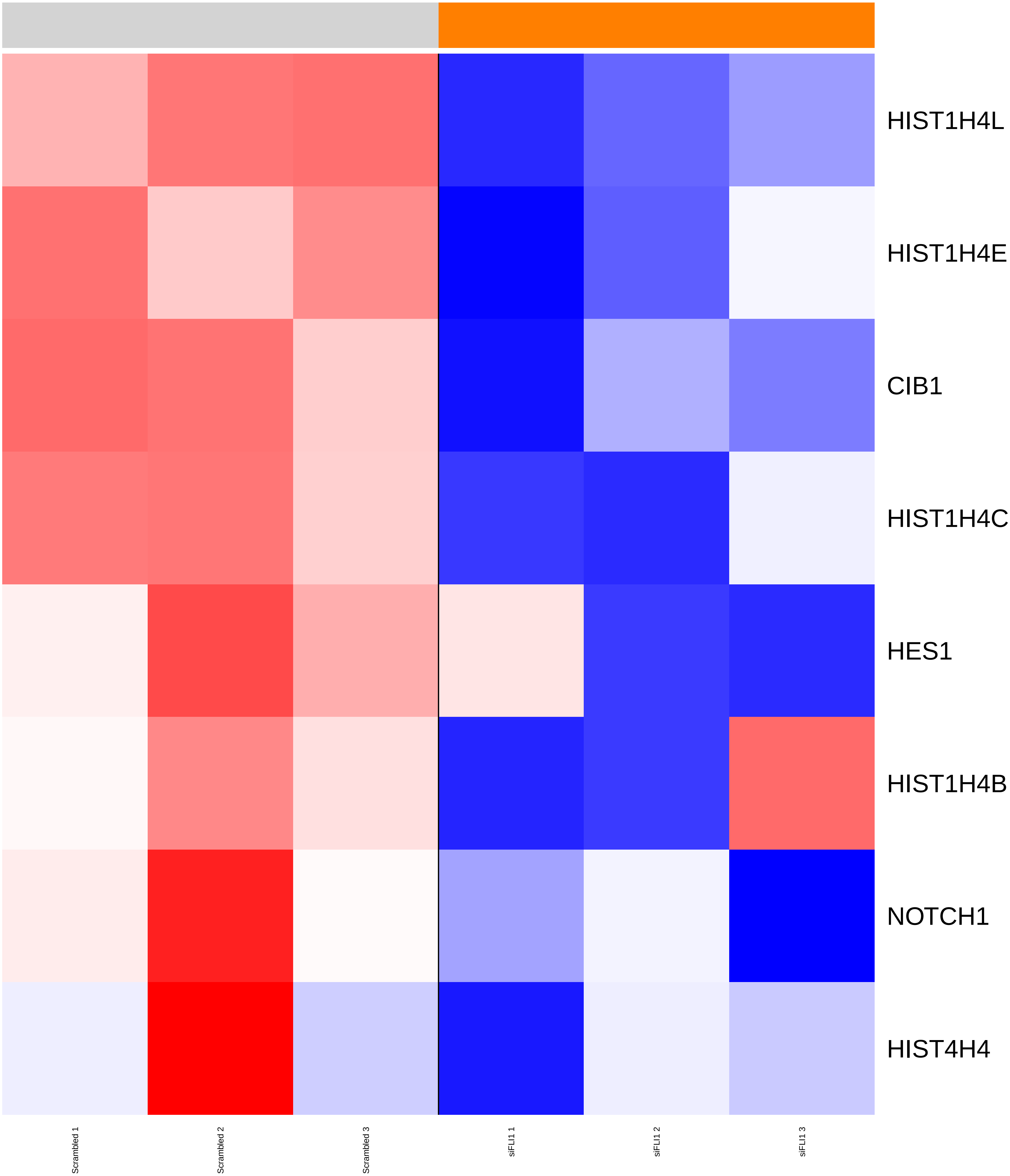

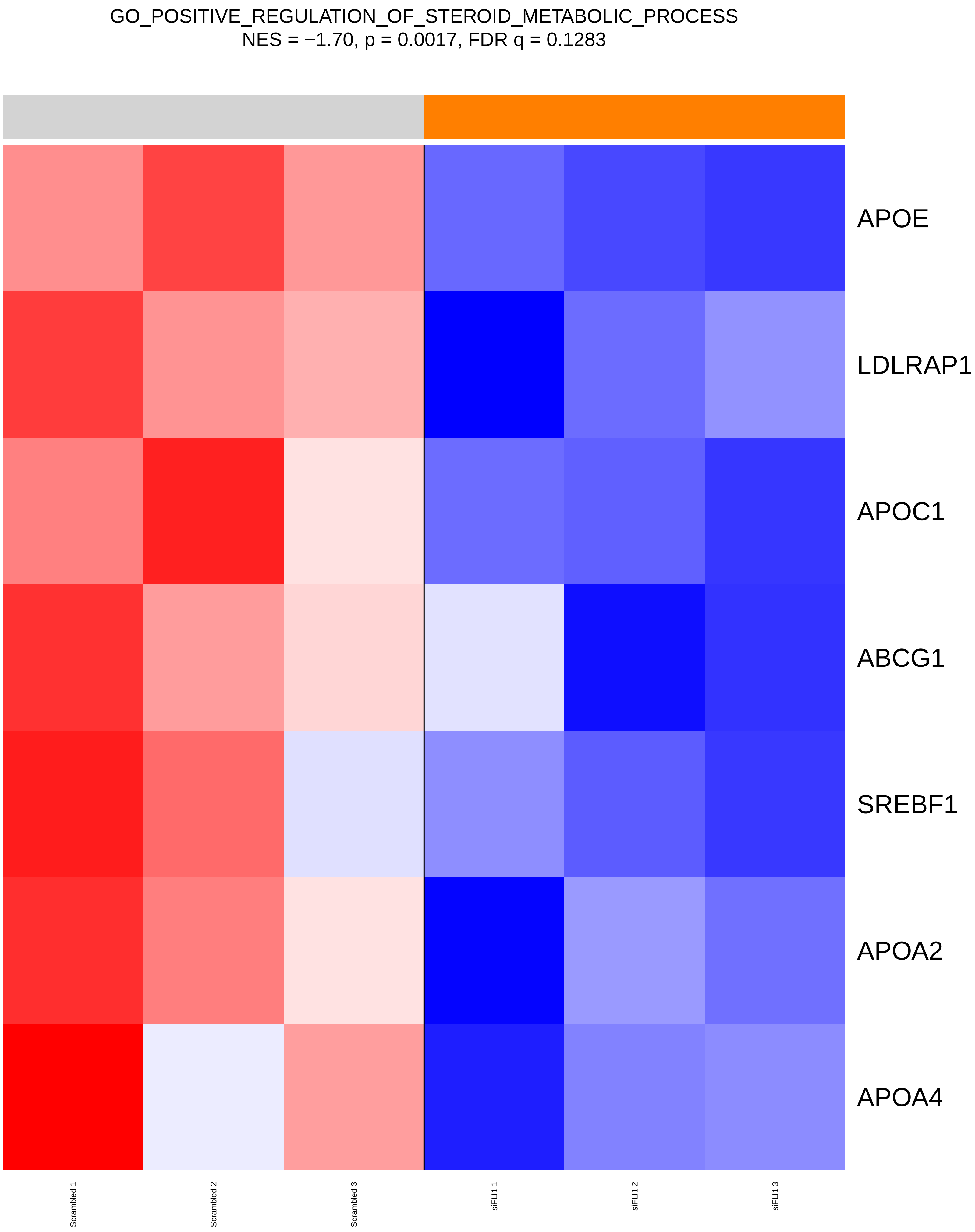
GO_POSITIVE_REGULATION_OF_STEROID_METABOLIC_PROCESS
NES = −1.70, p = 0.0017, FDR q = 0.1283
APOE
LDLRAP1
APOC1
ABCG1
SREBF1
APOA2
APOA4
Scrambled 1
Scrambled 2
Scrambled 3
siFLI1 1
siFLI1 2
siFLI1 3

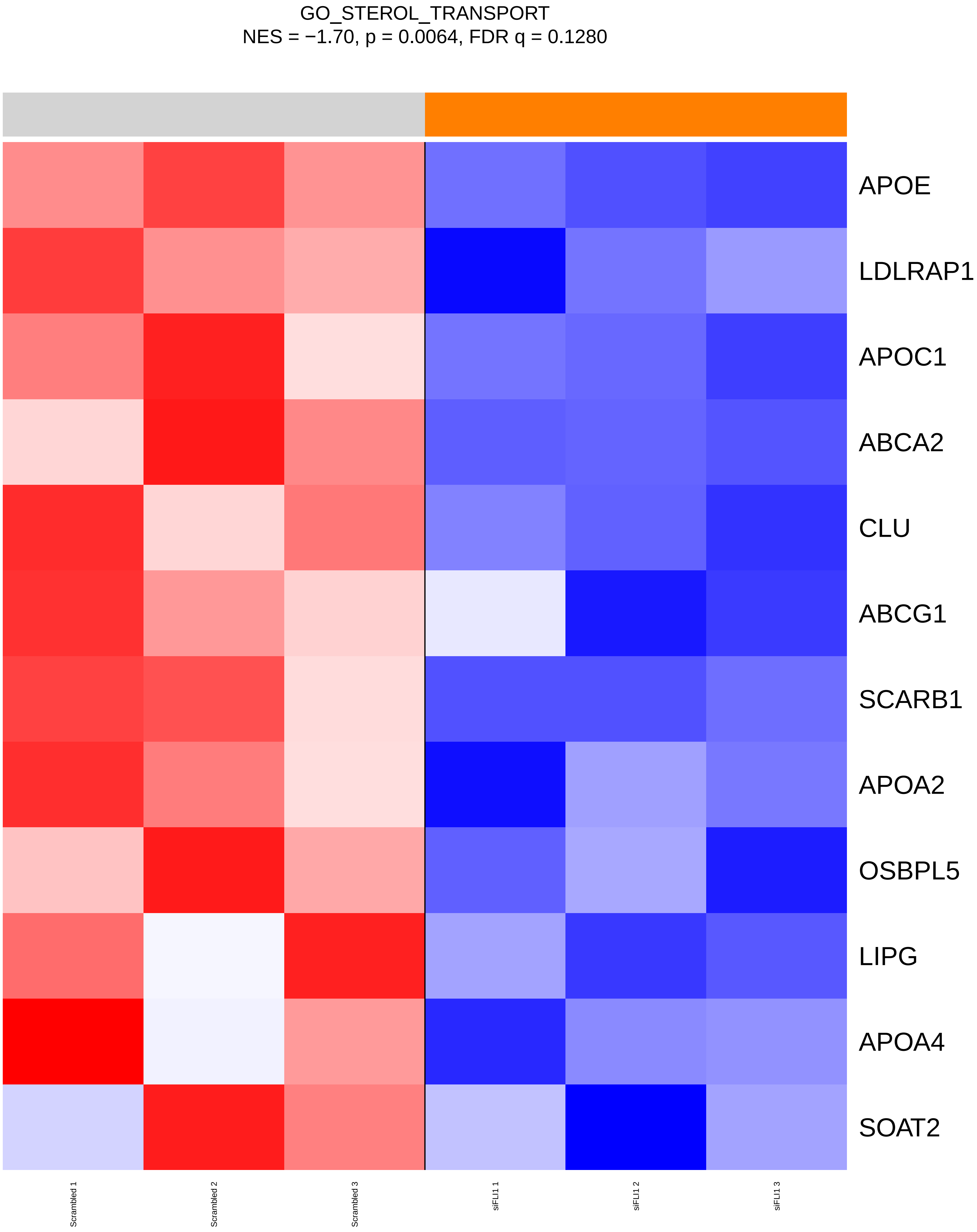
GO_STEROL_TRANSPORT
NES = −1.70, p = 0.0064, FDR q = 0.1280
APOE
LDLRAP1
APOC1
ABCA2
CLU
ABCG1
SCARB1
APOA2
OSBPL5
LIPG
APOA4
SOAT2
Scrambled 1
Scrambled 2
Scrambled 3
siFLI1 1
siFLI1 2
siFLI1 3

GO_TELOMERE_ORGANIZATION
NES = −1.70, p = 0.0015, FDR q = 0.1260

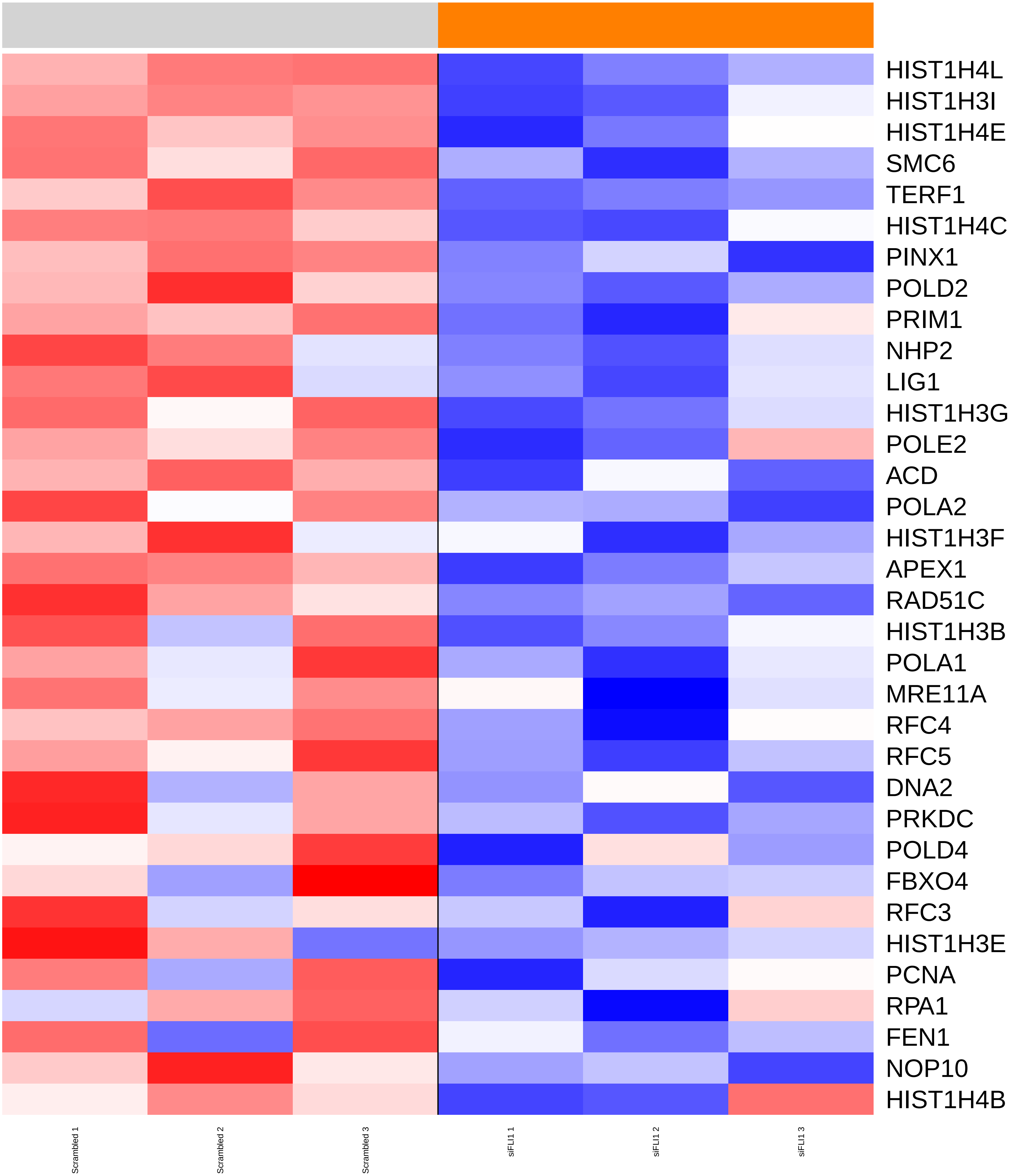

GO_LYMPH_VESSEL_DEVELOPMENT
NES = −1.70, p = 0.0000, FDR q = 0.1268

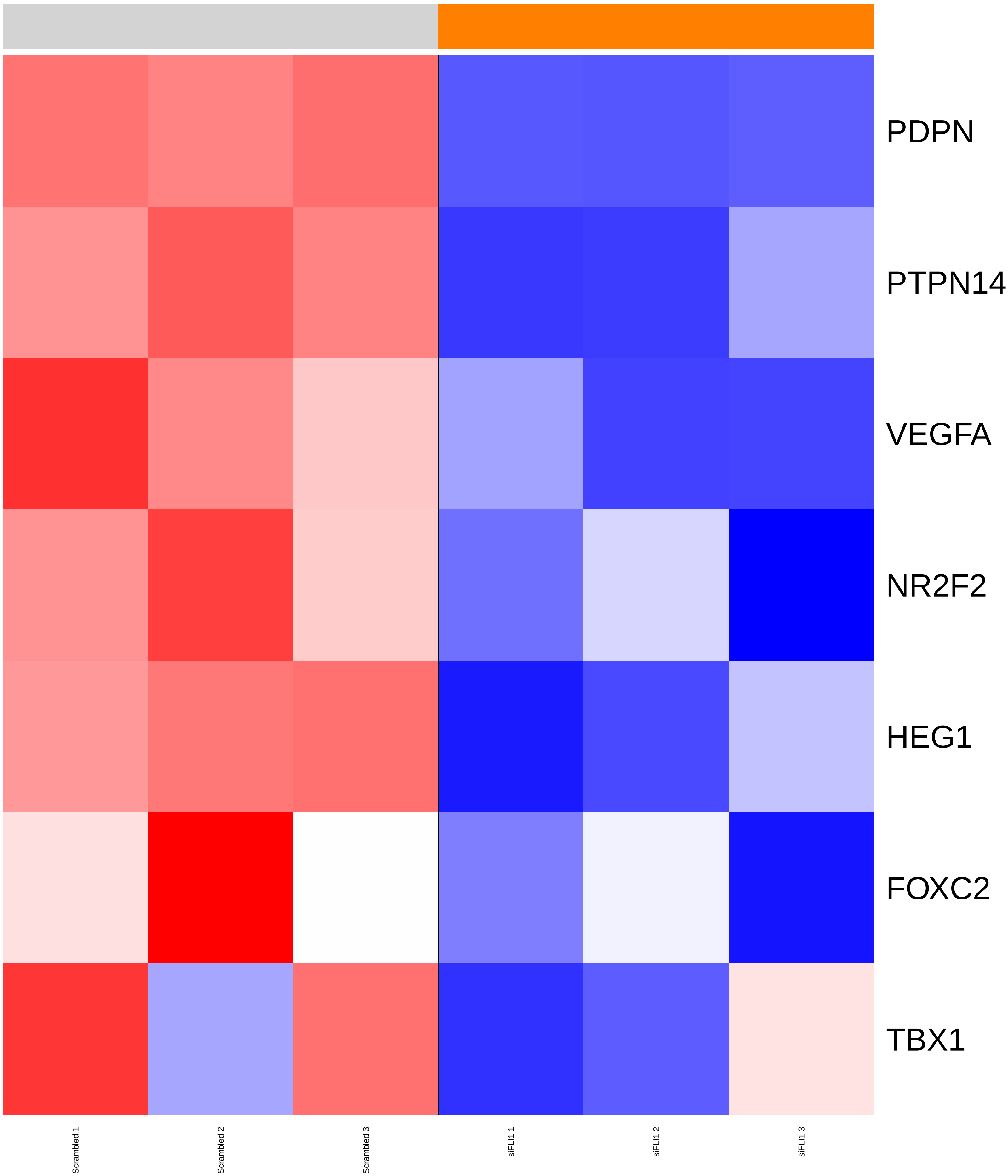

GO_MITOCHONDRIAL_MEMBRANE_PART
NES = −1.70, p = 0.0000, FDR q = 0.1251

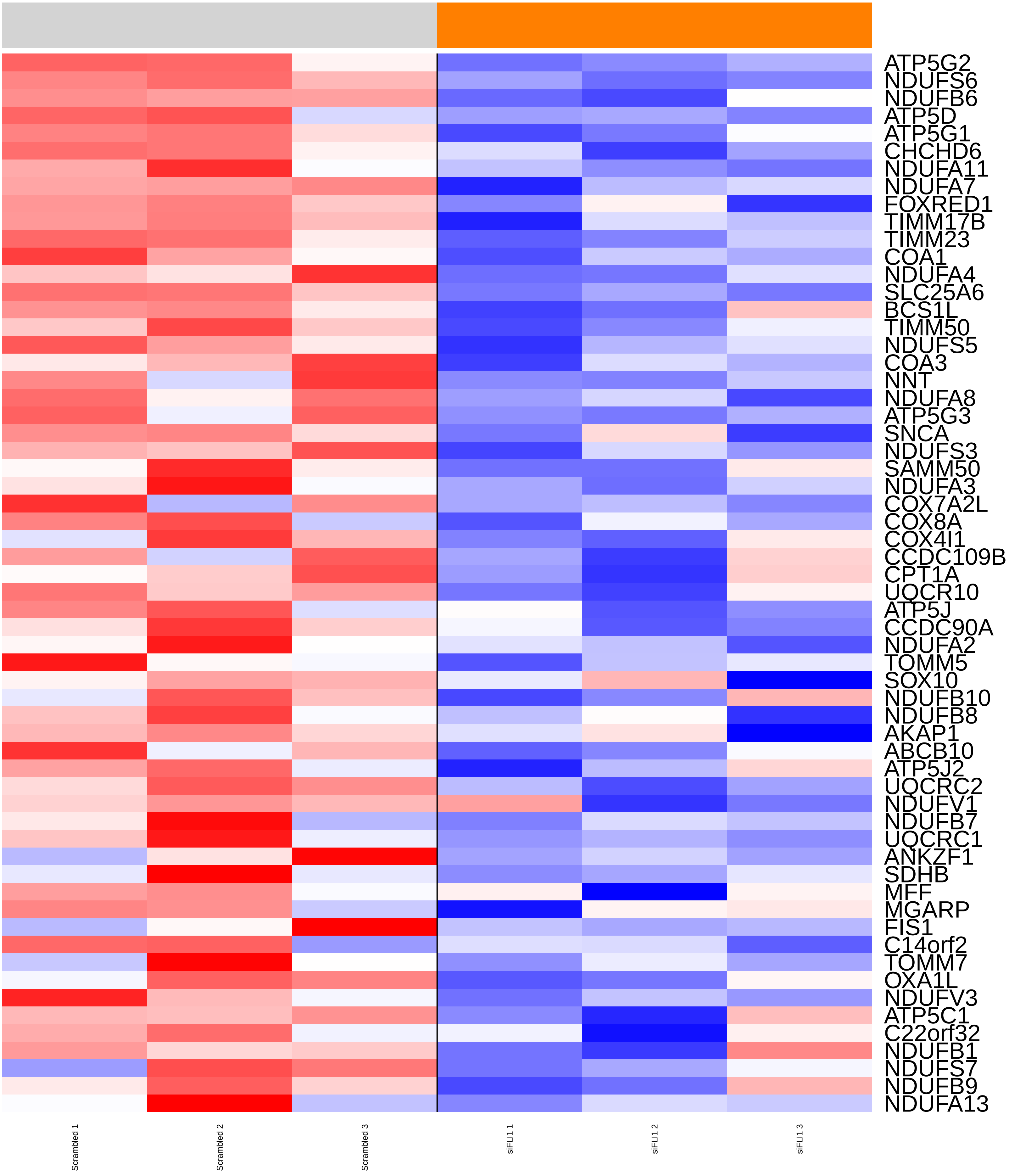

GO_TRIGLYCERIDE_RICH_LIPOPROTEIN_PARTICLE
NES = −1.71, p = 0.0144, FDR q = 0.1192

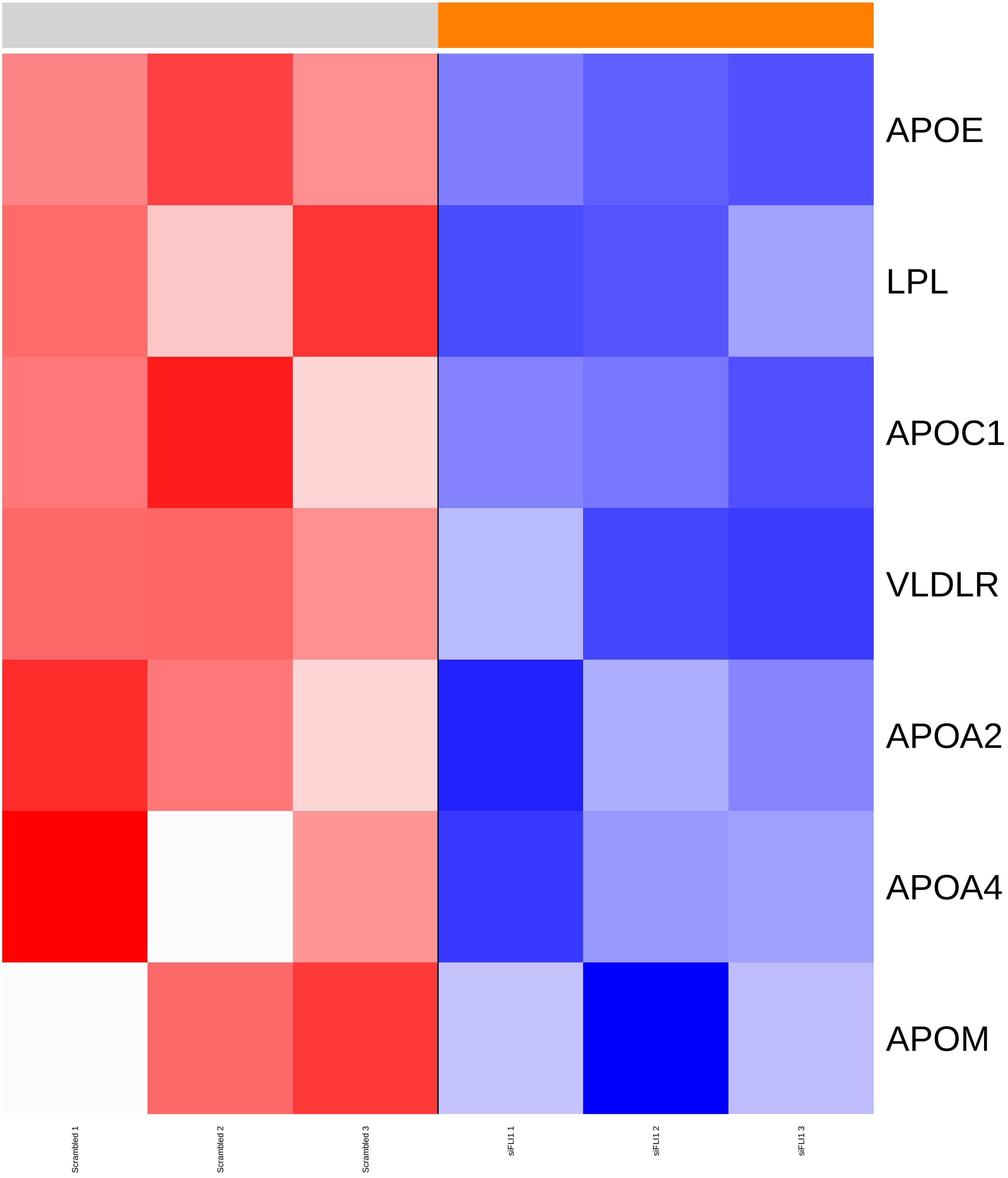

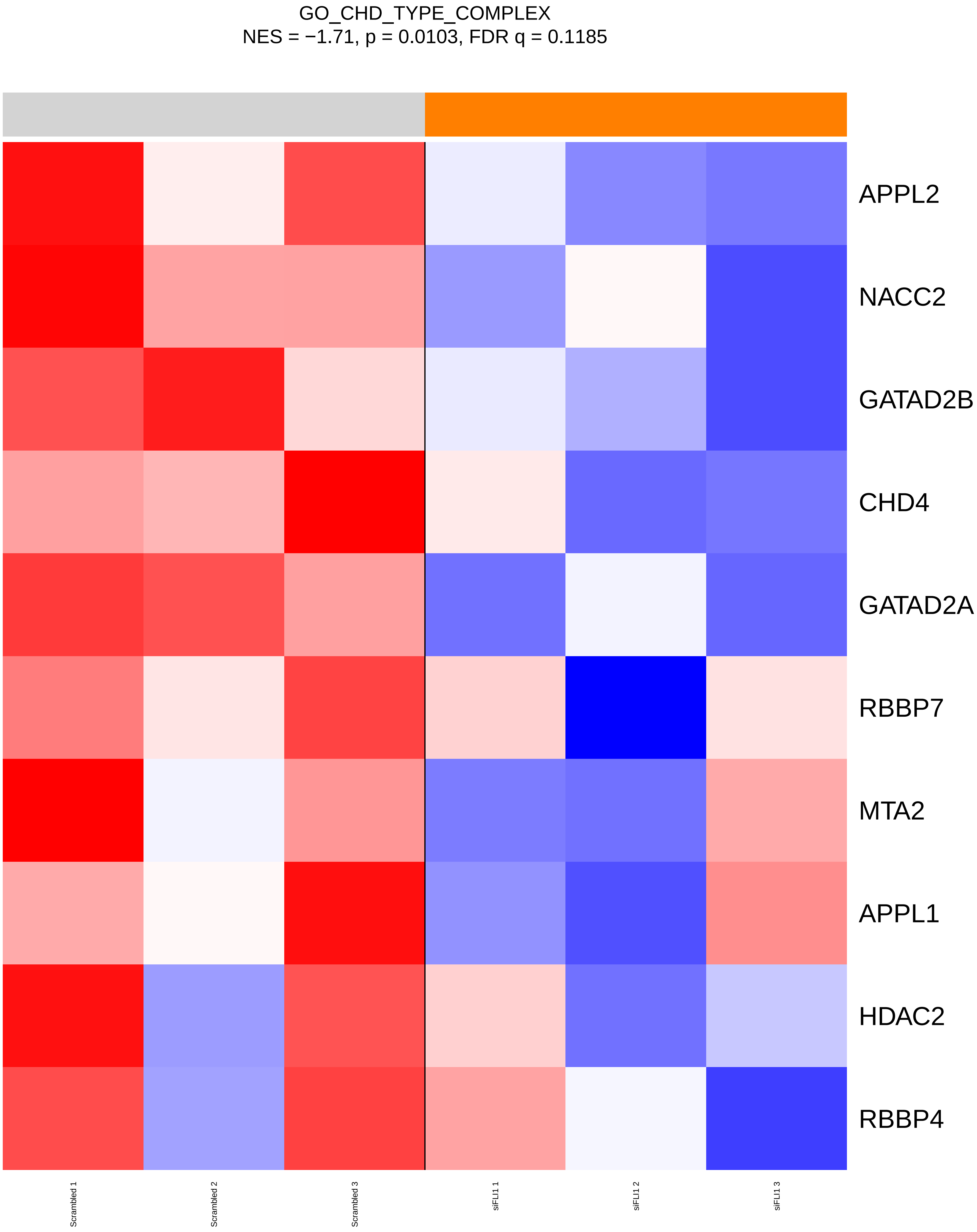
GO_CHD_TYPE_COMPLEX
NES = −1.71, p = 0.0103, FDR q = 0.1185
APPL2
NACC2
GATAD2B
CHD4
GATAD2A
RBBP7
MTA2
APPL1
HDAC2
RBBP4
Scrambled 1
Scrambled 2
Scrambled 3
siFLI1 1
siFLI1 2
siFLI1 3

GO_XY_BODY
NES = −1.71, p = 0.0104, FDR q = 0.1150

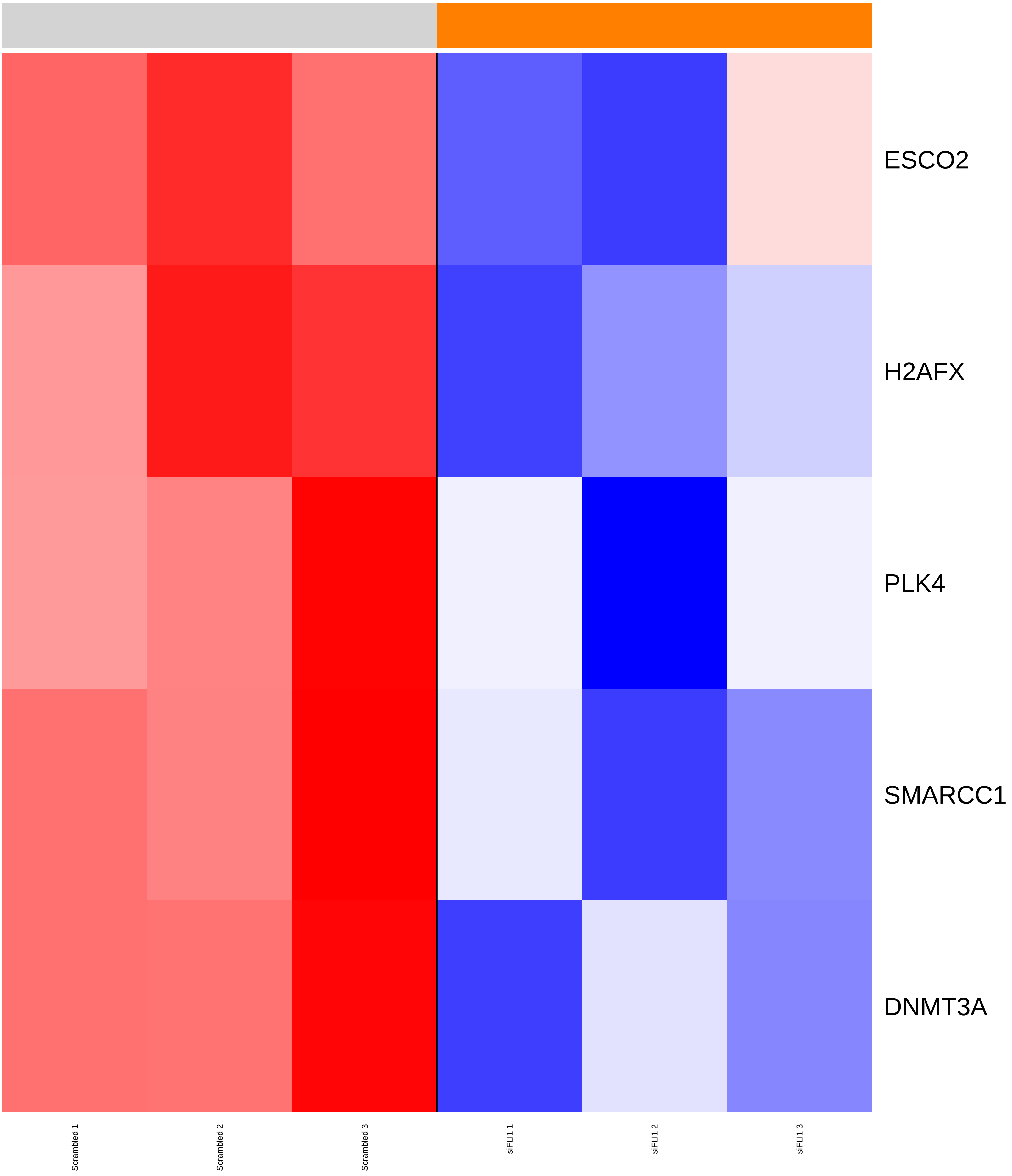

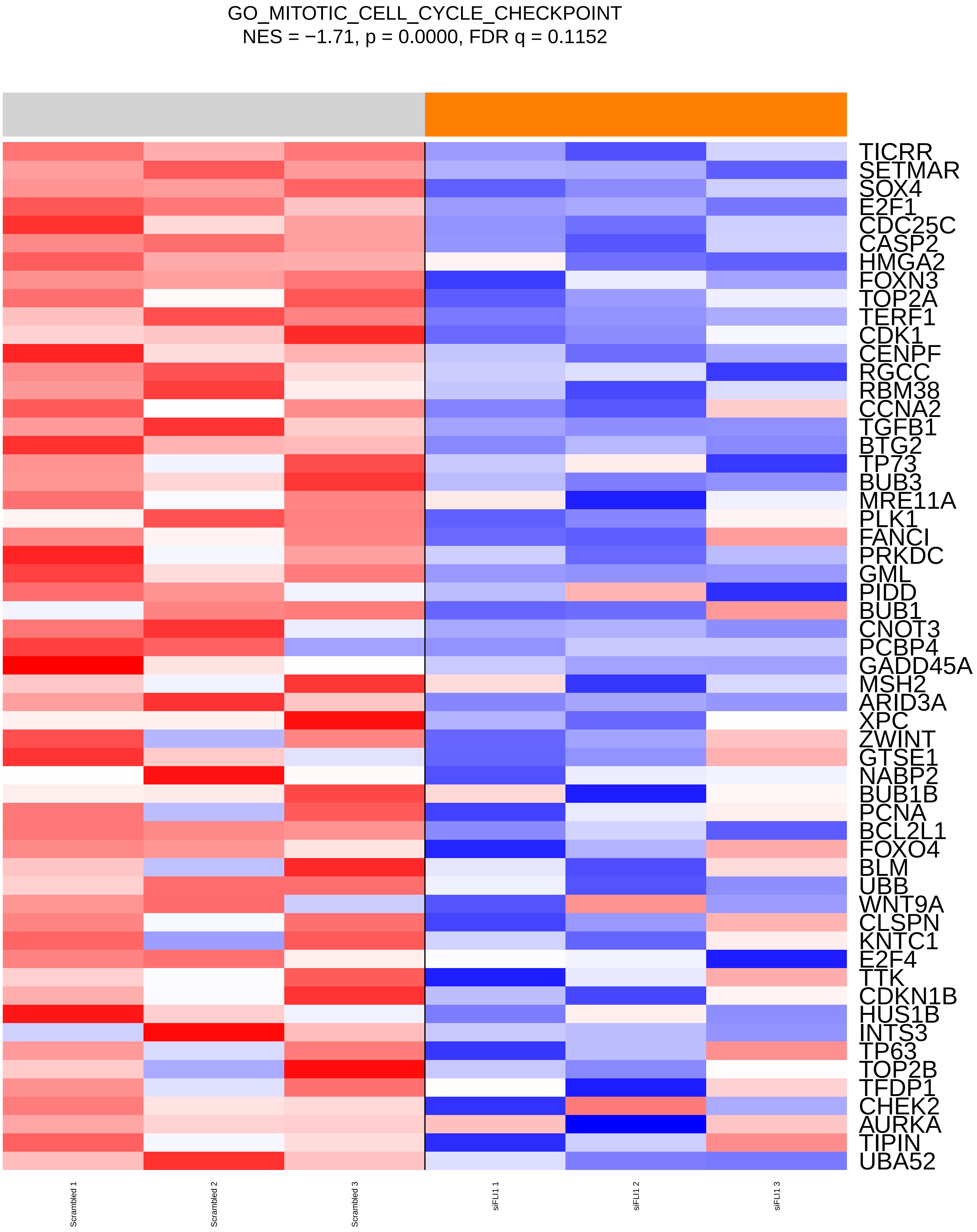
GO_MITOTIC_CELL_CYCLE_CHECKPOINT
NES = −1.71, p = 0.0000, FDR q = 0.1152
TICRR
SETMAR
SOX4
E2F1
CDC25C
CASP2
HMGA2
FOXN3
TOP2A
TERF1
CDK1
CENPF
RGCC
RBM38
CCNA2
TGFB1
BTG2
TP73
BUB3
MRE11A
PLK1
FANCI
PRKDC
GML
PIDD
BUB1
CNOT3
PCBP4
GADD45A
MSH2
ARID3A
XPC
ZWINT
GTSE1
NABP2
BUB1B
PCNA
BCL2L1
FOXO4
BLM
UBB
WNT9A
CLSPN
KNTC1
E2F4
TTK
CDKN1B
HUS1B
INTS3
TP63
TOP2B
TFDP1
CHEK2
AURKA
TIPIN
UBA52
Scrambled 1
Scrambled 2
Scrambled 3
siFLI1 1
siFLI1 2
siFLI1 3

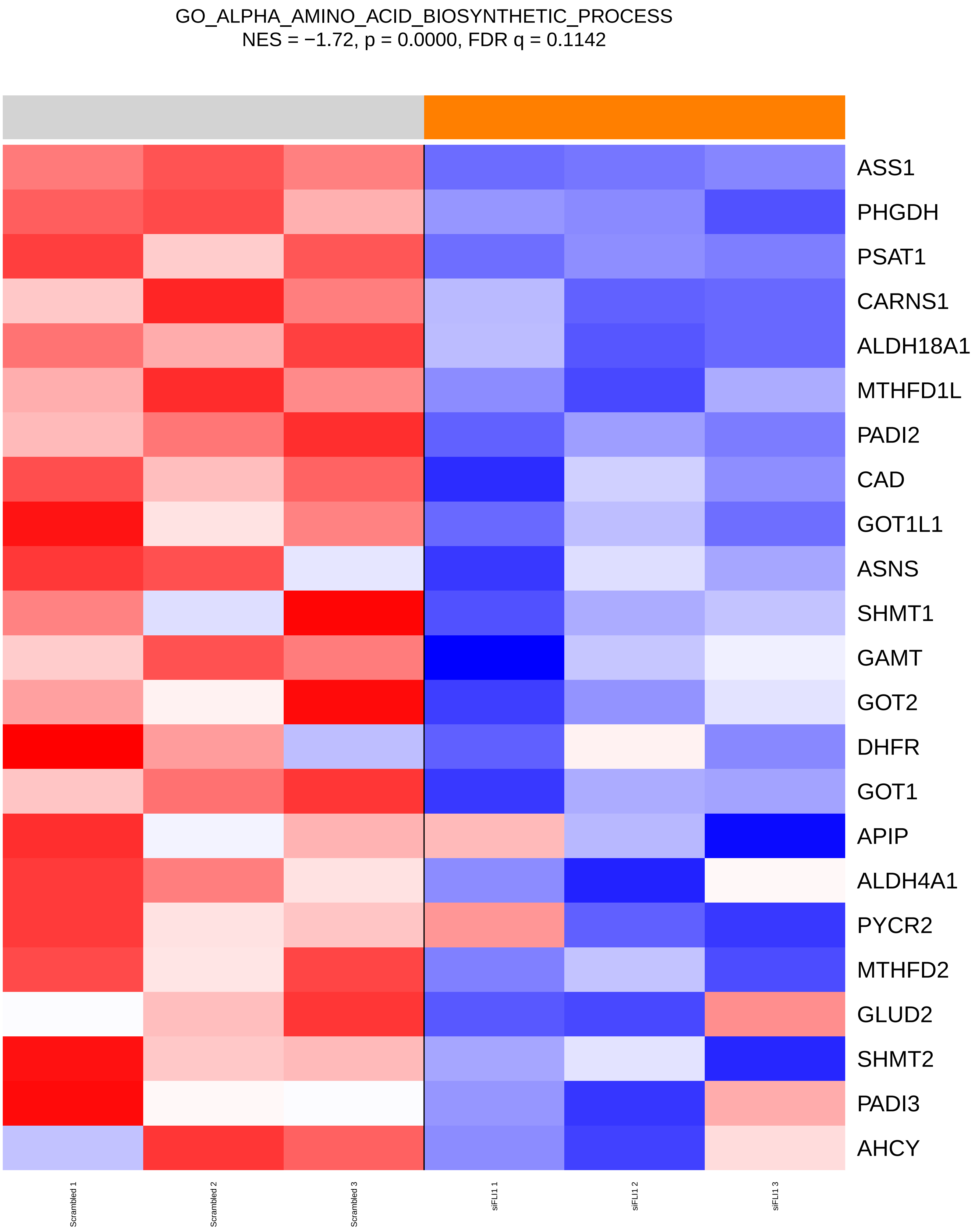
GO_ALPHA_AMINO_ACID_BIOSYNTHETIC_PROCESS
NES = −1.72, p = 0.0000, FDR q = 0.1142
ASS1
PHGDH
PSAT1
CARNS1
ALDH18A1
MTHFD1L
PADI2
CAD
GOT1L1
ASNS
SHMT1
GAMT
GOT2
DHFR
GOT1
APIP
ALDH4A1
PYCR2
MTHFD2
GLUD2
SHMT2
PADI3
AHCY
Scrambled 1
Scrambled 2
Scrambled 3
siFLI1 1
siFLI1 2
siFLI1 3

GO_NAD_BINDING
NES = −1.72, p = 0.0031, FDR q = 0.1150

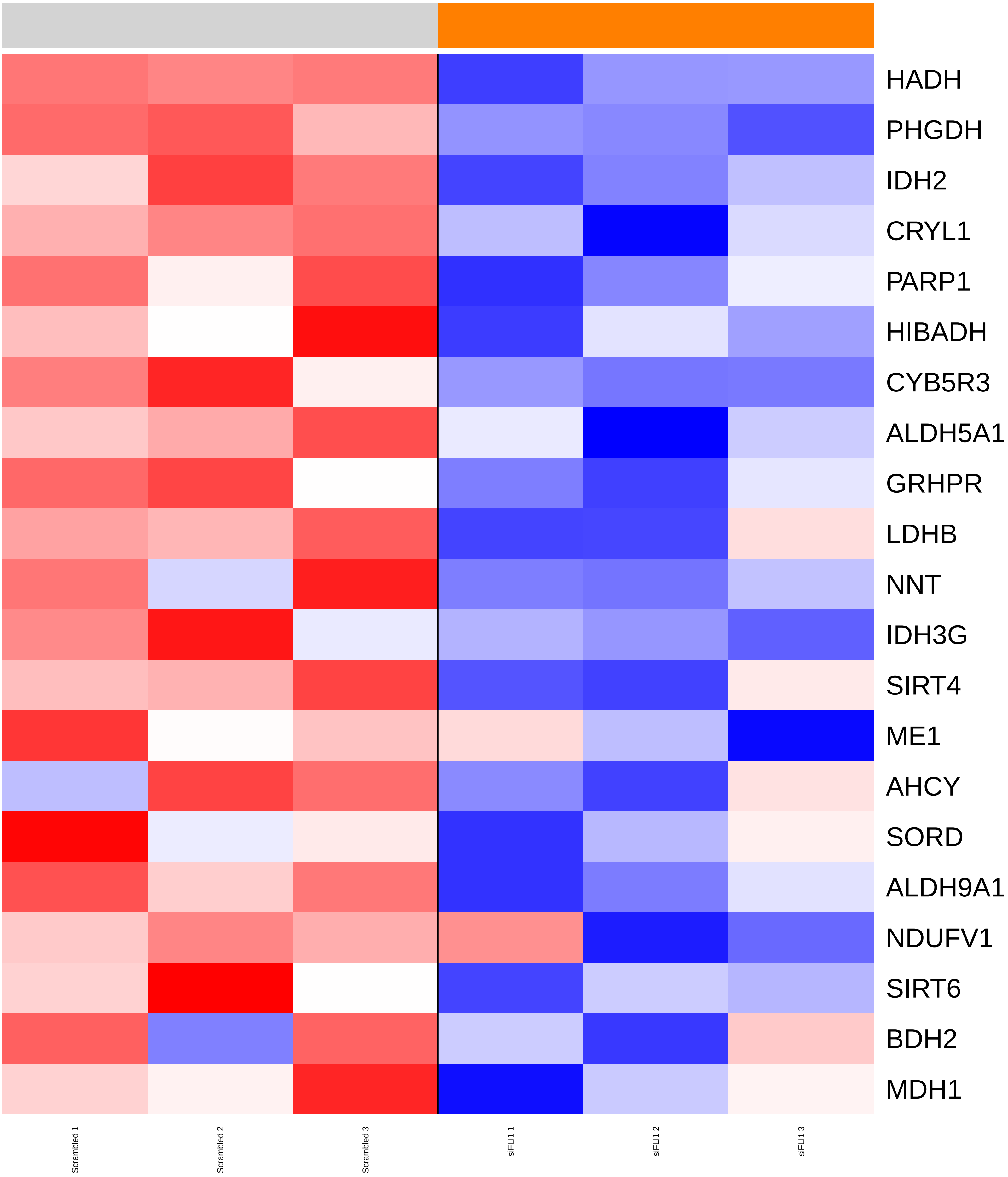

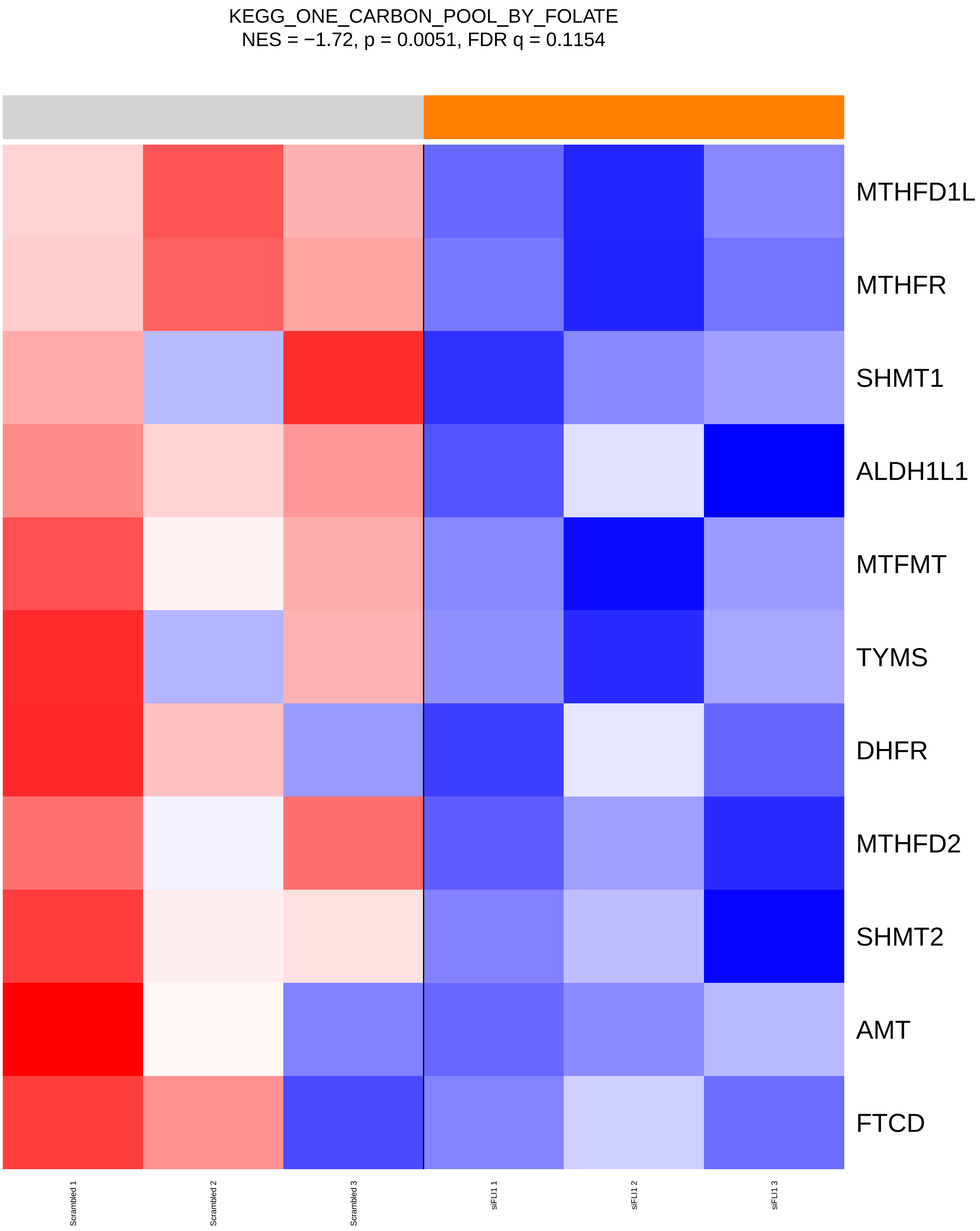

KEGG_ONE_CARBON_POOL_BY_FOLATE
NES = −1.72, p = 0.0051, FDR q = 0.1154
MTHFD1L
MTHFR
SHMT1
ALDH1L1
MTFMT
TYMS
DHFR
MTHFD2
SHMT2
AMT
FTCD
Scrambled 1
Scrambled 2
Scrambled 3
siFLI1 1
siFLI1 2
siFLI1 3

GO_REGULATION_OF_TRANSCRIPTION_INVOLVED_IN_G1_S_TRANSITION_OF_MITOTIC_CELL_CYCLE
NES = −1.72, p = 0.0016, FDR q = 0.1162

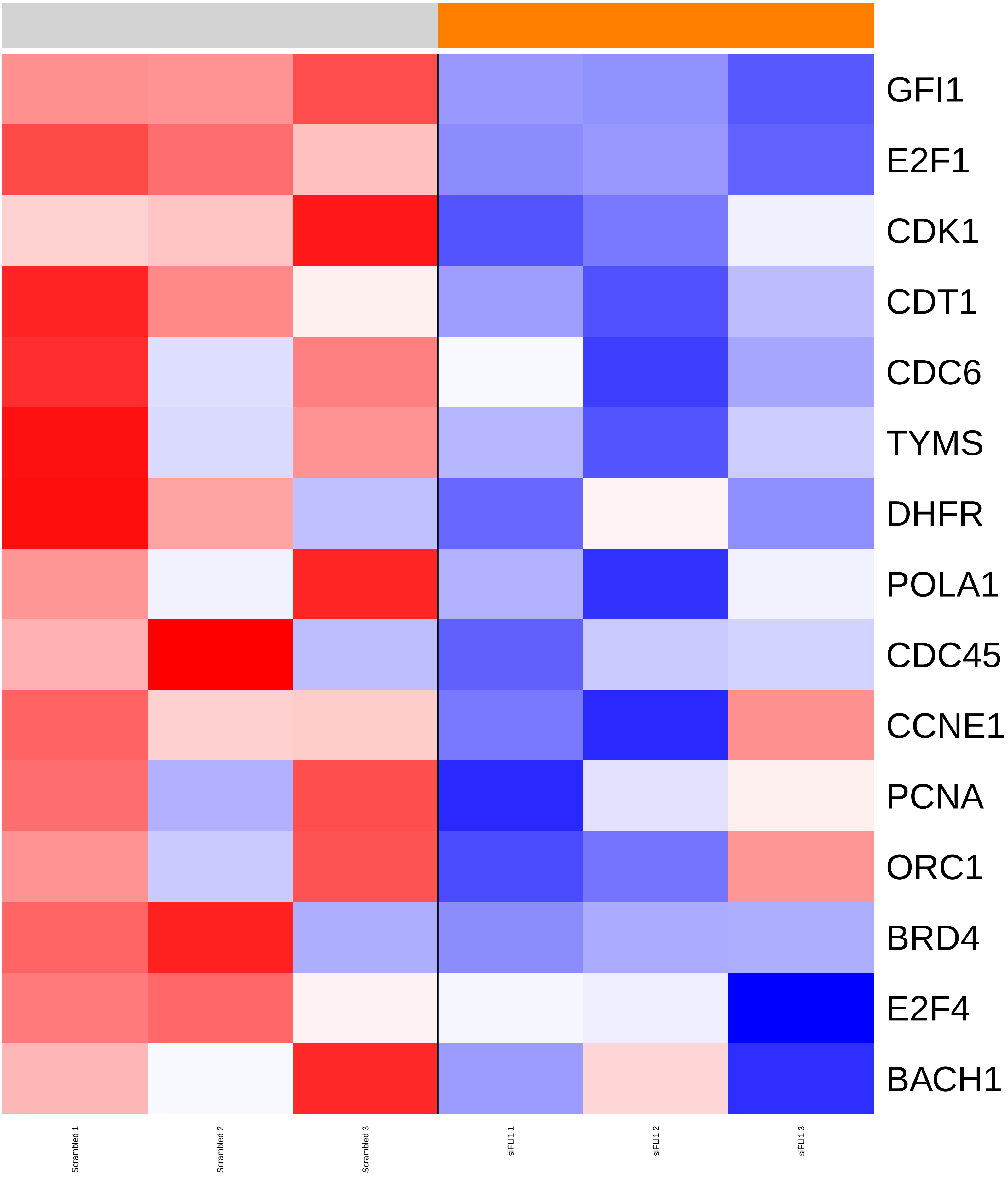

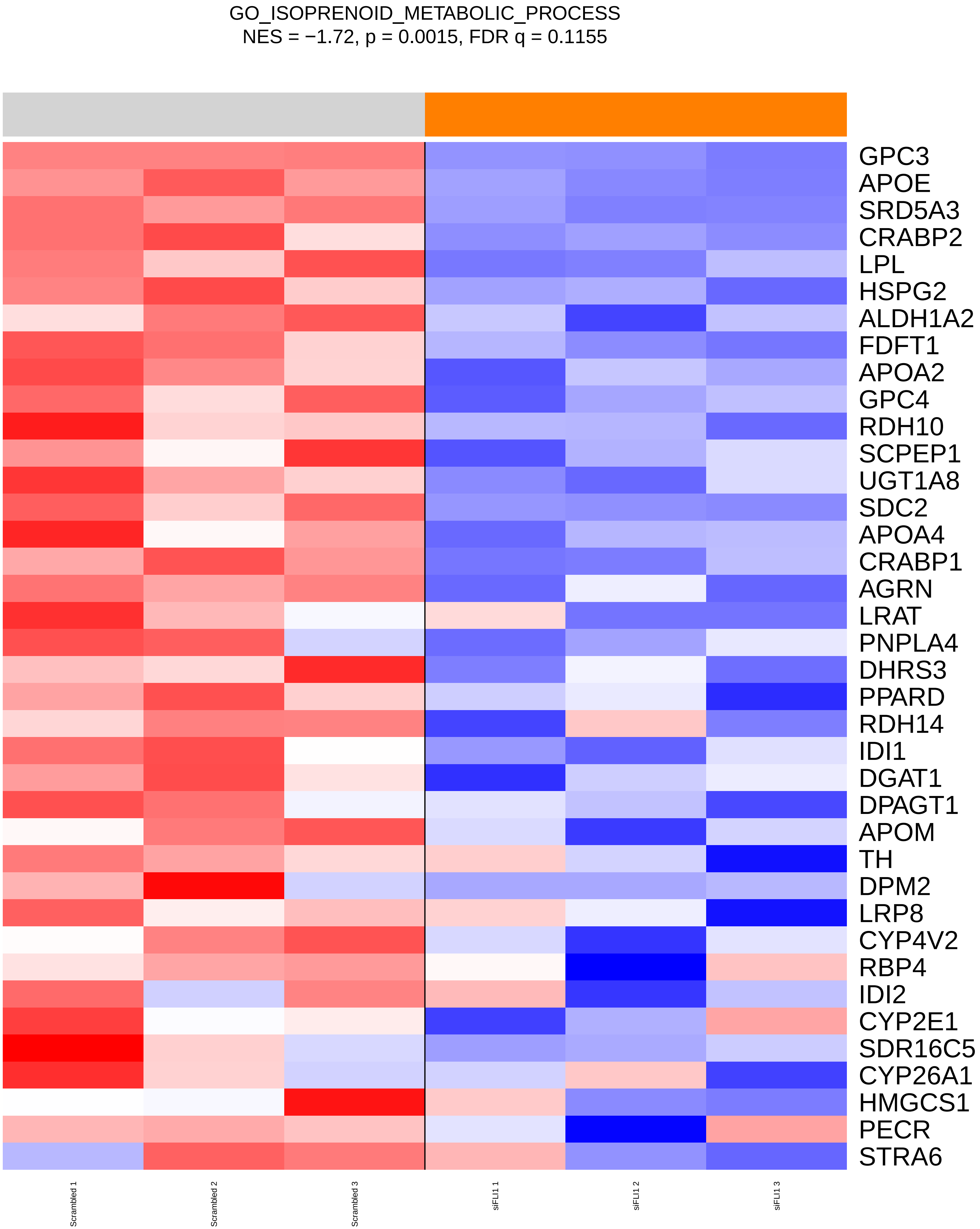
GO_ISOPRENOID_METABOLIC_PROCESS
NES = −1.72, p = 0.0015, FDR q = 0.1155
GPC3
APOE
SRD5A3
CRABP2
LPL
HSPG2
ALDH1A2
FDFT1
APOA2
GPC4
RDH10
SCPEP1
UGT1A8
SDC2
APOA4
CRABP1
AGRN
LRAT
PNPLA4
DHRS3
PPARD
RDH14
IDI1
DGAT1
DPAGT1
APOM
TH
DPM2
LRP8
CYP4V2
RBP4
IDI2
CYP2E1
SDR16C5
CYP26A1
HMGCS1
PECR
STRA6
Scrambled 1
Scrambled 2
Scrambled 3
siFLI1 1
siFLI1 2
siFLI1 3

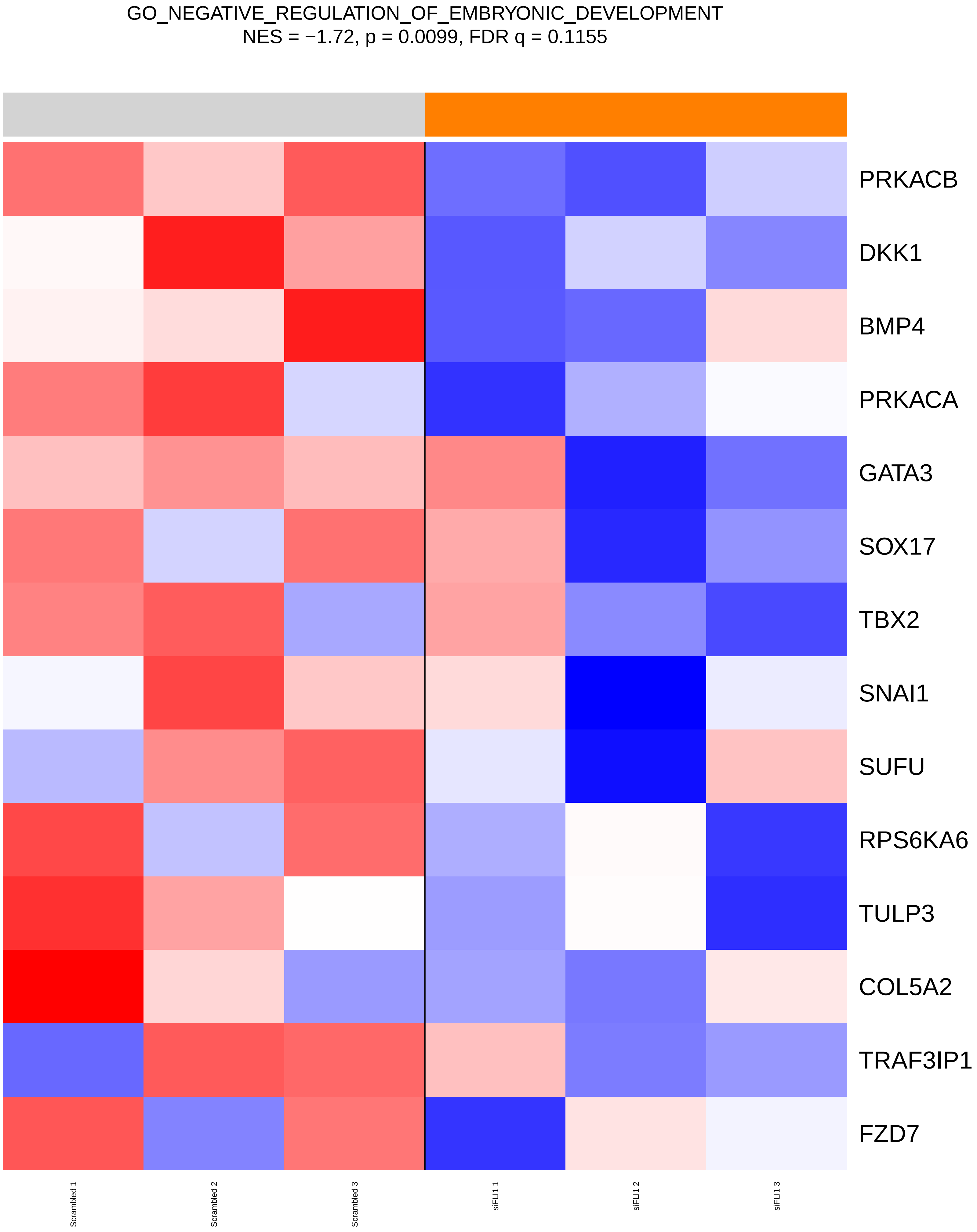

GO_NEGATIVE_REGULATION_OF_EMBRYONIC_DEVELOPMENT
NES = −1.72, p = 0.0099, FDR q = 0.1155
PRKACB
DKK1
BMP4
PRKACA
GATA3
SOX17
TBX2
SNAI1
SUFU
RPS6KA6
TULP3
COL5A2
TRAF3IP1
FZD7
Scrambled 1
Scrambled 2
Scrambled 3
siFLI1 1
siFLI1 2
siFLI1 3

REACTOME_MEIOTIC_RECOMBINATION
NES = −1.72, p = 0.0030, FDR q = 0.1103

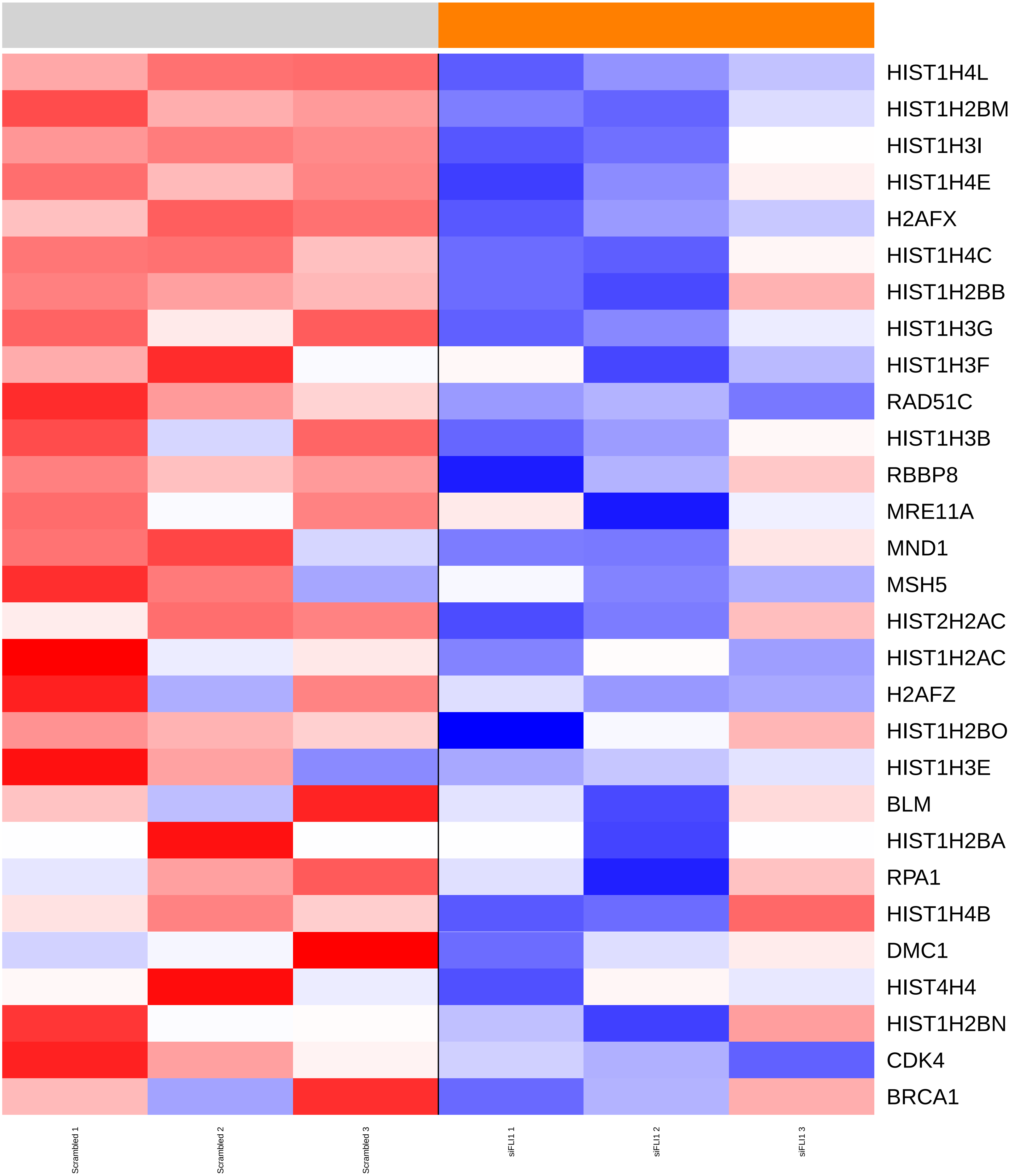

GO_MODIFIED_AMINO_ACID_TRANSPORT
NES = −1.72, p = 0.0032, FDR q = 0.1112

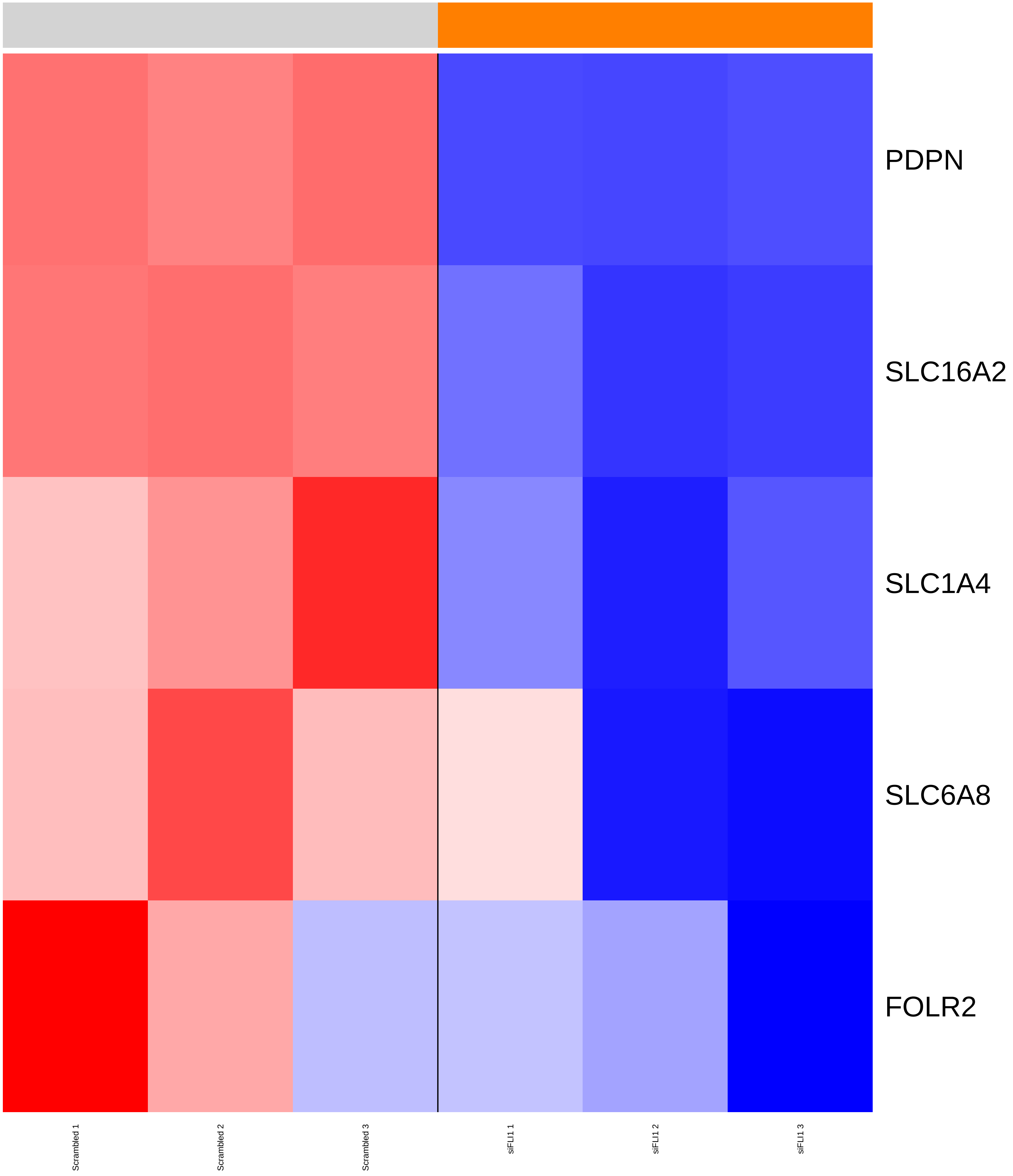

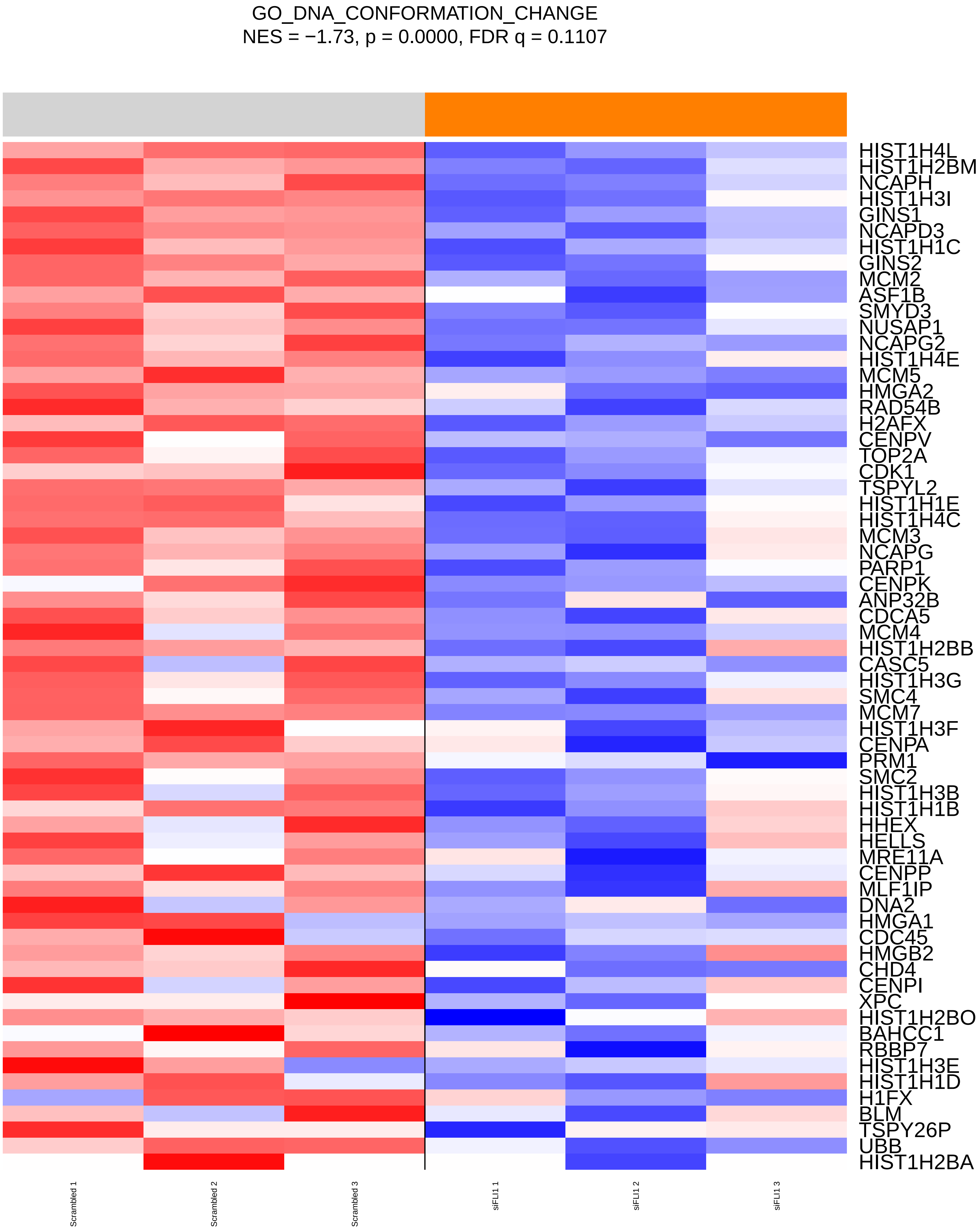
GO_DNA_CONFORMATION_CHANGE
NES = −1.73, p = 0.0000, FDR q = 0.1107
HIST1H4L
HIST1H2BM
NCAPH
HIST1H3I
GINS1
NCAPD3
HIST1H1C
GINS2
MCM2
ASF1B
SMYD3
NUSAP1
NCAPG2
HIST1H4E
MCM5
HMGA2
RAD54B
H2AFX
CENPV
TOP2A
CDK1
TSPYL2
HIST1H1E
HIST1H4C
MCM3
NCAPG
PARP1
CENPK
ANP32B
CDCA5
MCM4
HIST1H2BB
CASC5
HIST1H3G
SMC4
MCM7
HIST1H3F
CENPA
PRM1
SMC2
HIST1H3B
HIST1H1B
HHEX
HELLS
MRE11A
CENPP
MLF1IP
DNA2
HMGA1
CDC45
HMGB2
CHD4
CENPI
XPC
HIST1H2BO
BAHCC1
RBBP7
HIST1H3E
HIST1H1D
H1FX
BLM
TSPY26P
UBB
HIST1H2BA
Scrambled 1
Scrambled 2
Scrambled 3
siFLI1 1
siFLI1 2
siFLI1 3

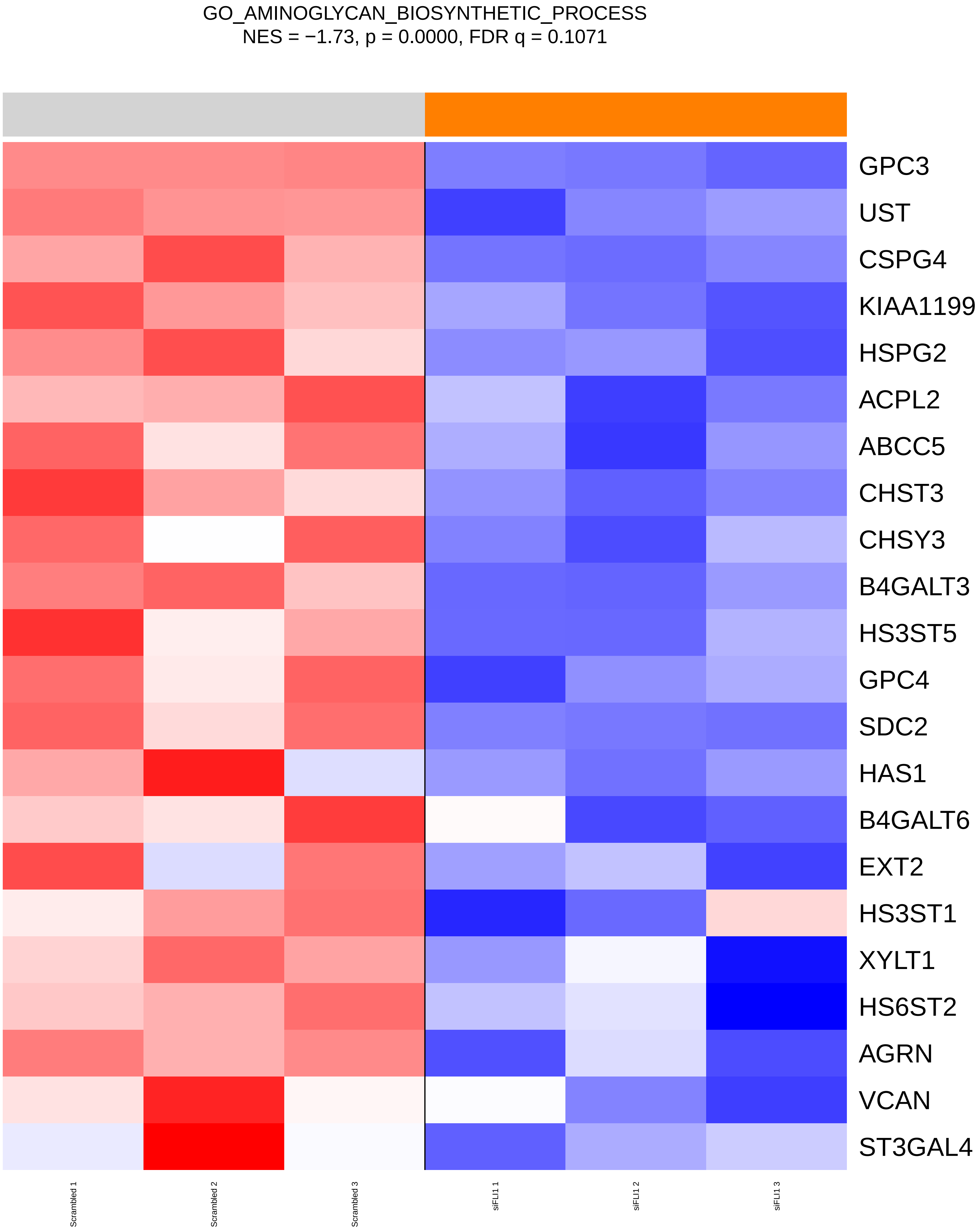
GO_AMINOGLYCAN_BIOSYNTHETIC_PROCESS
NES = −1.73, p = 0.0000, FDR q = 0.1071
GPC3
UST
CSPG4
KIAA1199
HSPG2
ACPL2
ABCC5
CHST3
CHSY3
B4GALT3
HS3ST5
GPC4
SDC2
HAS1
B4GALT6
EXT2
HS3ST1
XYLT1
HS6ST2
AGRN
VCAN
ST3GAL4
Scrambled 1
Scrambled 2
Scrambled 3
siFLI1 1
siFLI1 2
siFLI1 3

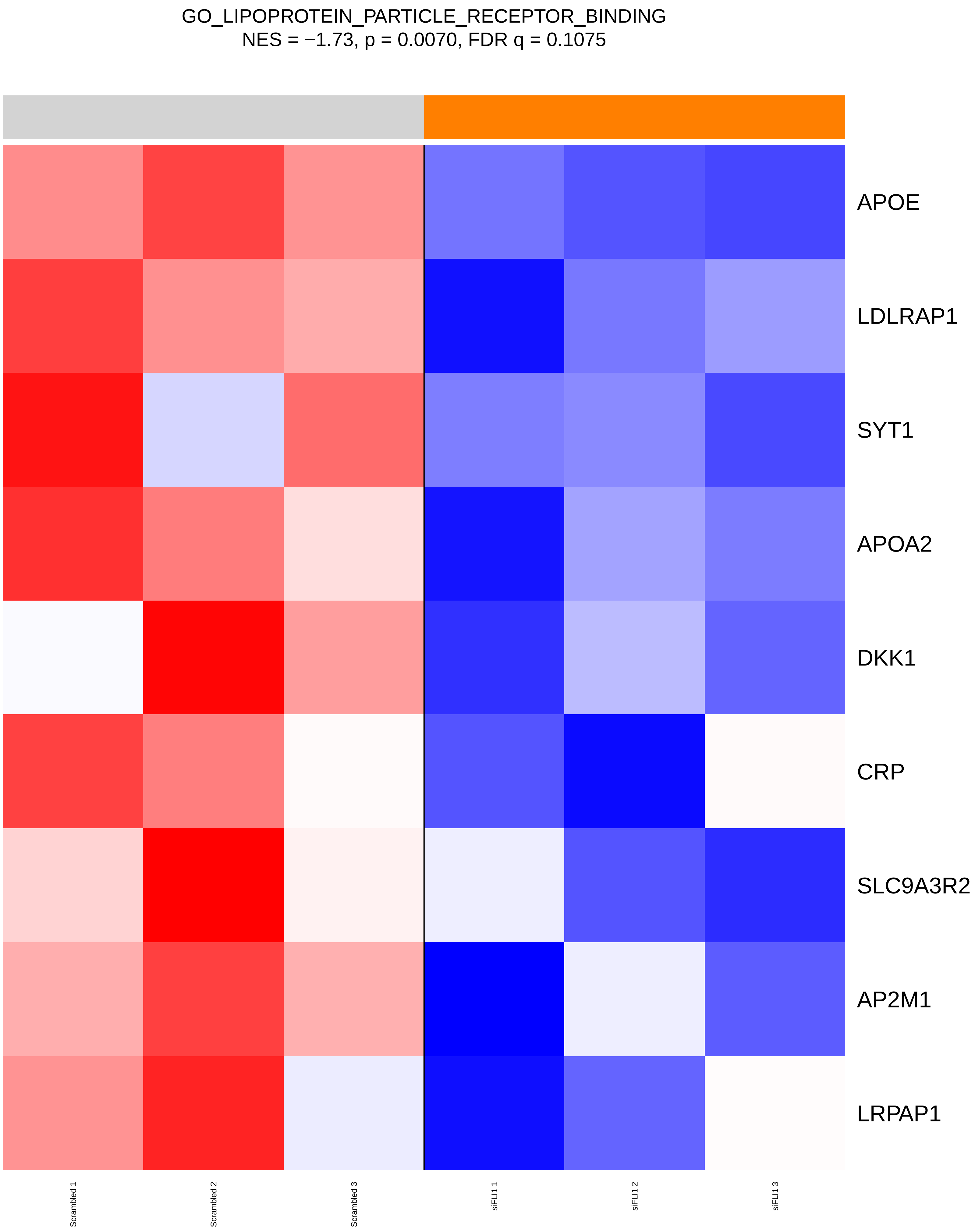
GO_LIPOPROTEIN_PARTICLE_RECEPTOR_BINDING
NES = −1.73, p = 0.0070, FDR q = 0.1075
APOE
LDLRAP1
SYT1
APOA2
DKK1
CRP
SLC9A3R2
AP2M1
LRPAP1
Scrambled 1
Scrambled 2
Scrambled 3
siFLI1 1
siFLI1 2
siFLI1 3

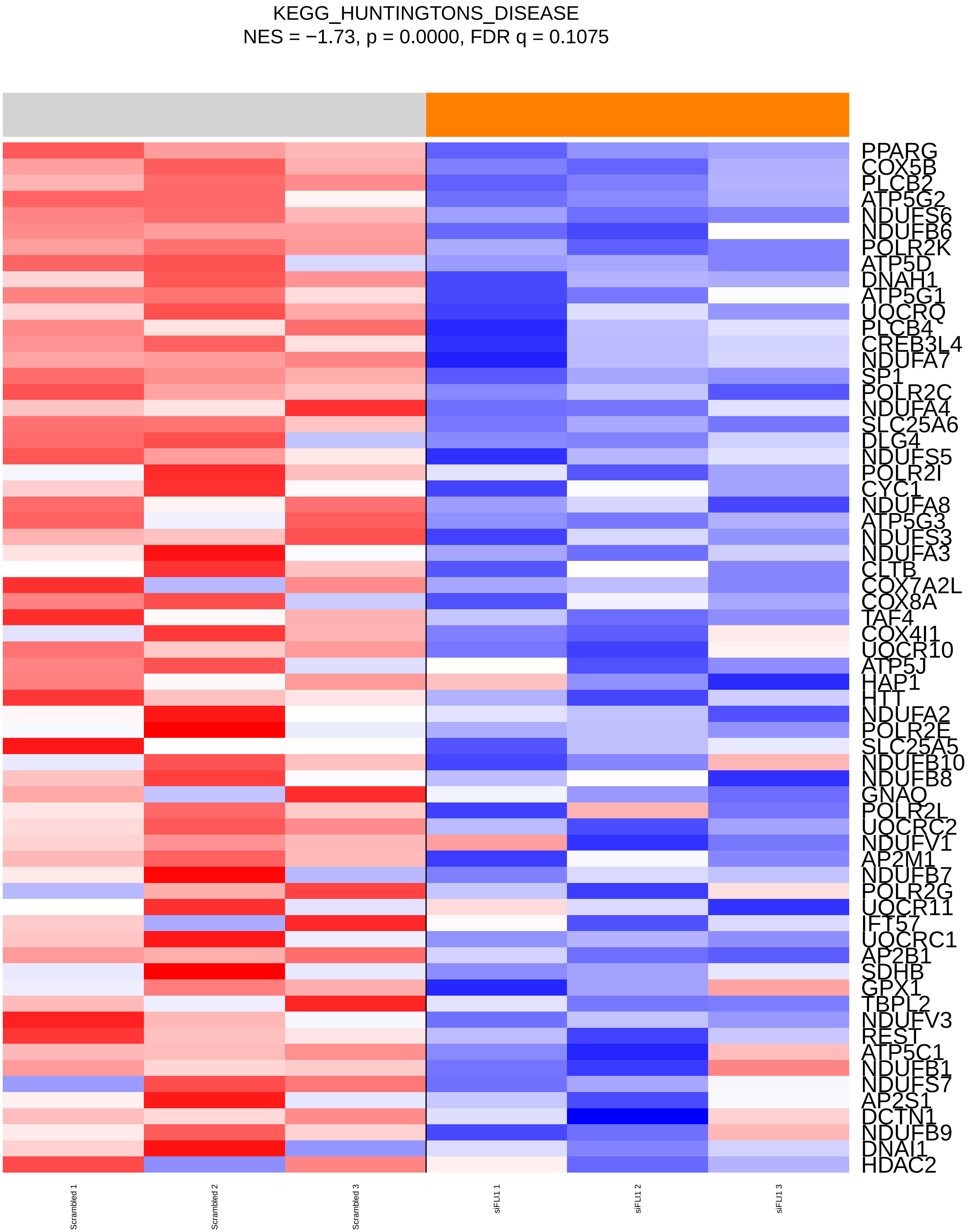
KEGG_HUNTINGTONS_DISEASE
NES = −1.73, p = 0.0000, FDR q = 0.1075
PPARG
COX5B
PLCB2
ATP5G2
NDUFS6
NDUFB6
POLR2K
ATP5D
DNAH1
ATP5G1
UQCRQ
PLCB4
CREB3L4
NDUFA7
SP1
POLR2C
NDUFA4
SLC25A6
DLG4
NDUFS5
POLR2I
CYC1
NDUFA8
ATP5G3
NDUFS3
NDUFA3
CLTB
COX7A2L
COX8A
TAF4
COX4I1
UQCR10
ATP5J
HAP1
HTT
NDUFA2
POLR2E
SLC25A5
NDUFB10
NDUFB8
GNAQ
POLR2L
UQCRC2
NDUFV1
AP2M1
NDUFB7
POLR2G
UQCR11
IFT57
UQCRC1
AP2B1
SDHB
GPX1
TBPL2
NDUFV3
REST
ATP5C1
NDUFB1
NDUFS7
AP2S1
DCTN1
NDUFB9
DNAI1
HDAC2
Scrambled 1
Scrambled 2
Scrambled 3
siFLI1 1
siFLI1 2
siFLI1 3

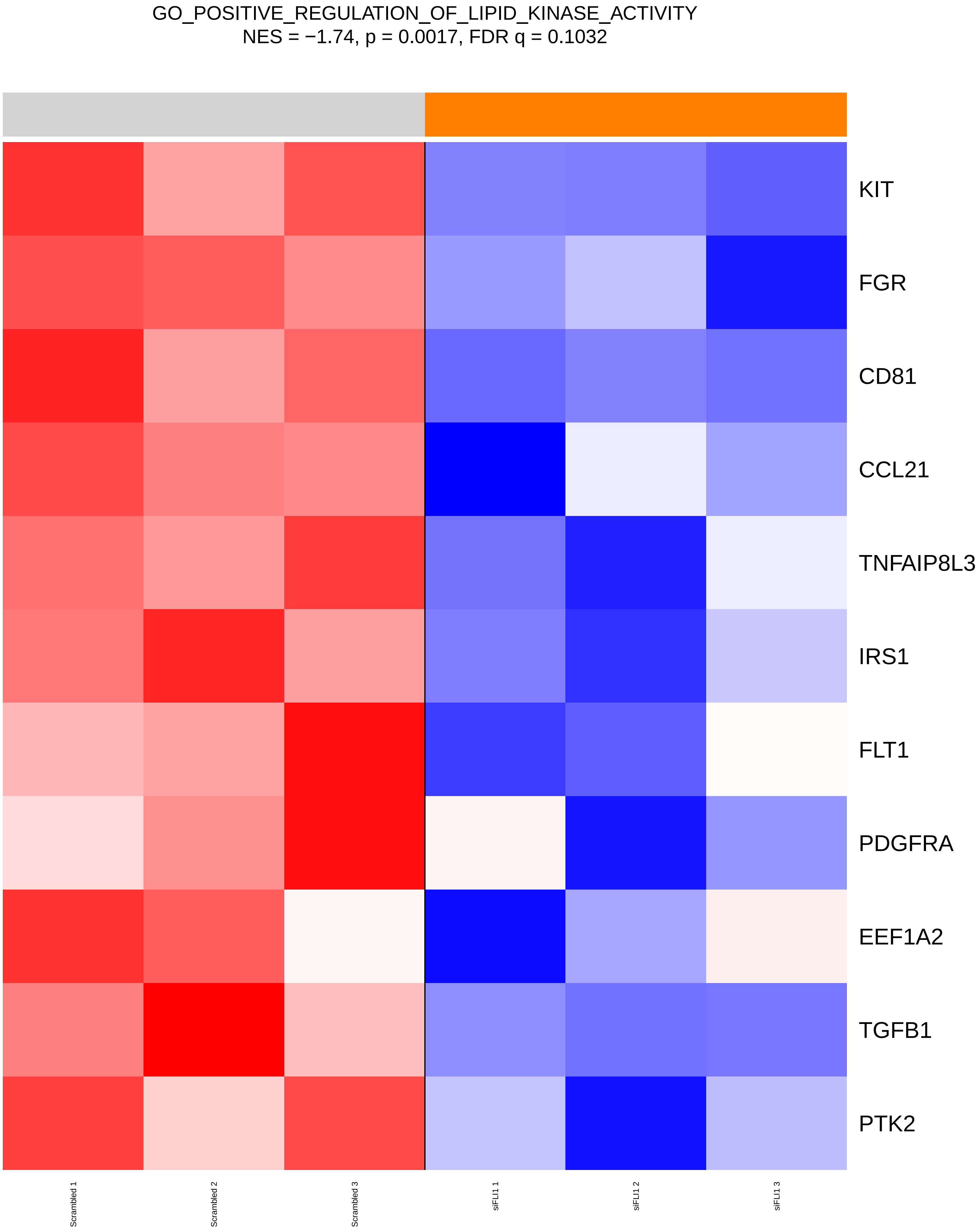
GO_POSITIVE_REGULATION_OF_LIPID_KINASE_ACTIVITY
NES = −1.74, p = 0.0017, FDR q = 0.1032
KIT
FGR
CD81
CCL21
TNFAIP8L3
IRS1
FLT1
PDGFRA
EEF1A2
TGFB1
PTK2
Scrambled 1
Scrambled 2
Scrambled 3
siFLI1 1
siFLI1 2
siFLI1 3

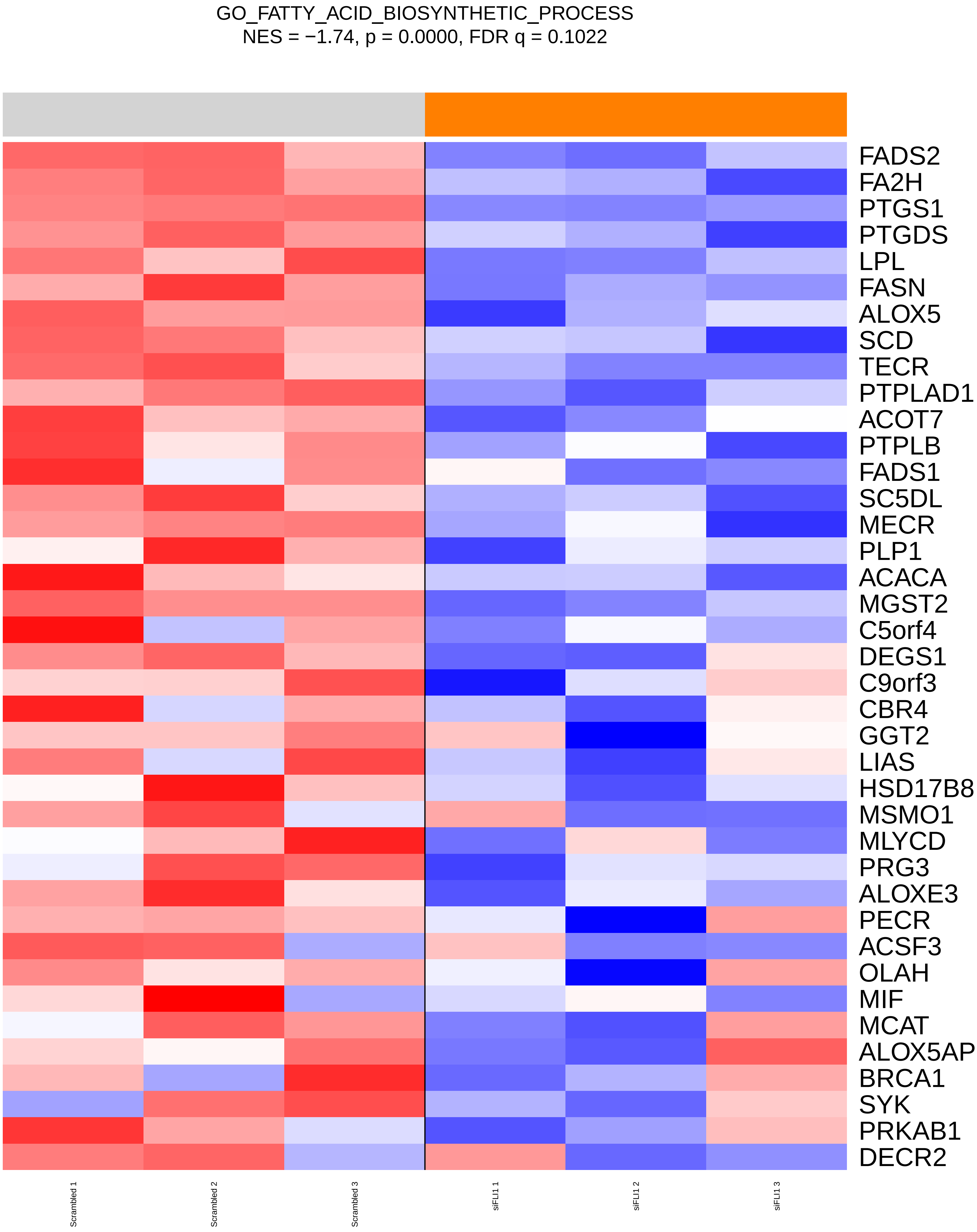
GO_FATTY_ACID_BIOSYNTHETIC_PROCESS
NES = −1.74, p = 0.0000, FDR q = 0.1022
FADS2
FA2H
PTGS1
PTGDS
LPL
FASN
ALOX5
SCD
TECR
PTPLAD1
ACOT7
PTPLB
FADS1
SC5DL
MECR
PLP1
ACACA
MGST2
C5orf4
DEGS1
C9orf3
CBR4
GGT2
LIAS
HSD17B8
MSMO1
MLYCD
PRG3
ALOXE3
PECR
ACSF3
OLAH
MIF
MCAT
ALOX5AP
BRCA1
SYK
PRKAB1
DECR2
Scrambled 1
Scrambled 2
Scrambled 3
siFLI1 1
siFLI1 2
siFLI1 3

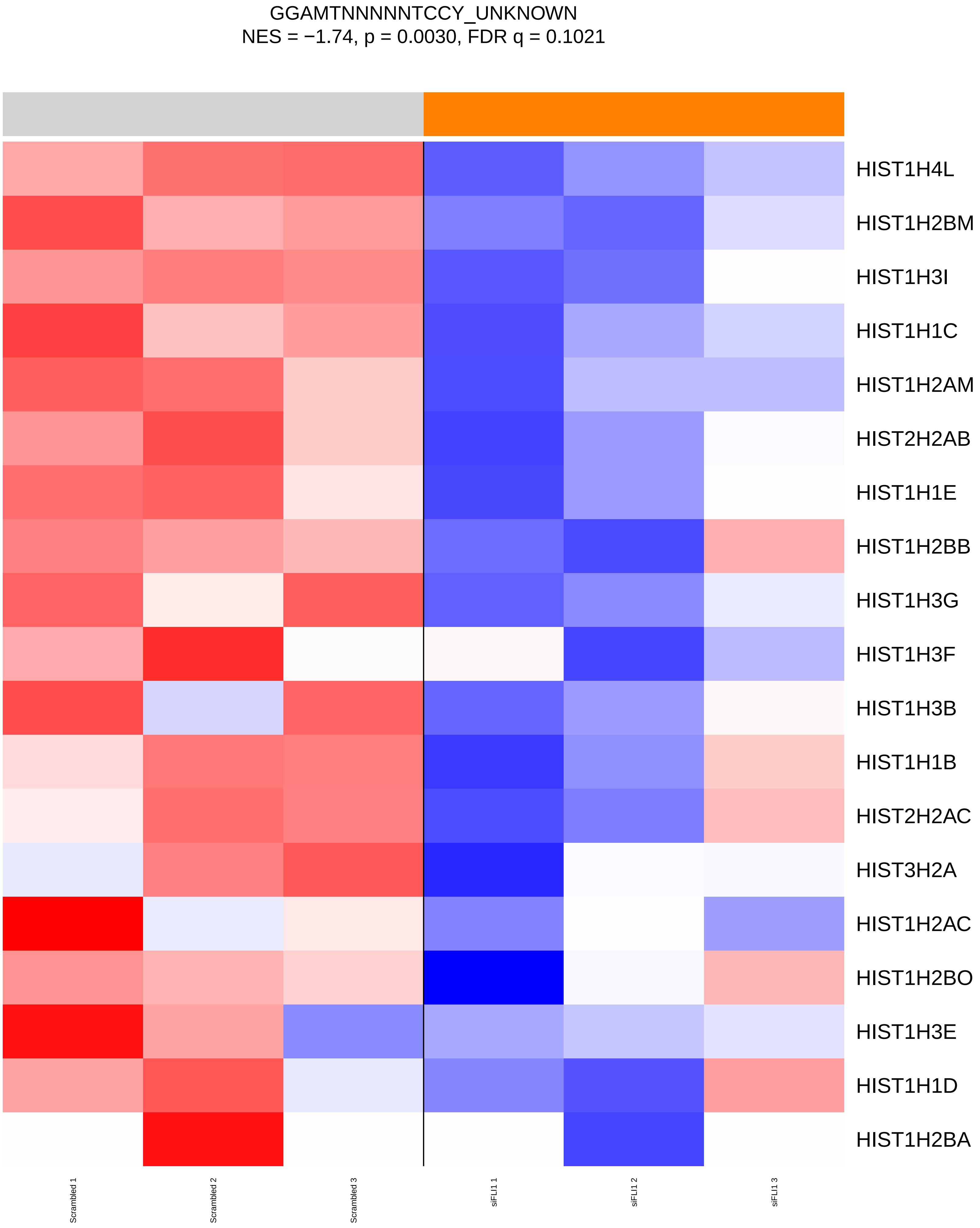
GGAMTNNNNNTCCY_UNKNOWN
NES = −1.74, p = 0.0030, FDR q = 0.1021
HIST1H4L
HIST1H2BM
HIST1H3I
HIST1H1C
HIST1H2AM
HIST2H2AB
HIST1H1E
HIST1H2BB
HIST1H3G
HIST1H3F
HIST1H3B
HIST1H1B
HIST2H2AC
HIST3H2A
HIST1H2AC
HIST1H2BO
HIST1H3E
HIST1H1D
HIST1H2BA
Scrambled 1
Scrambled 2
Scrambled 3
siFLI1 1
siFLI1 2
siFLI1 3

GO_DNA_REPLICATION
NES = −1.74, p = 0.0000, FDR q = 0.1022

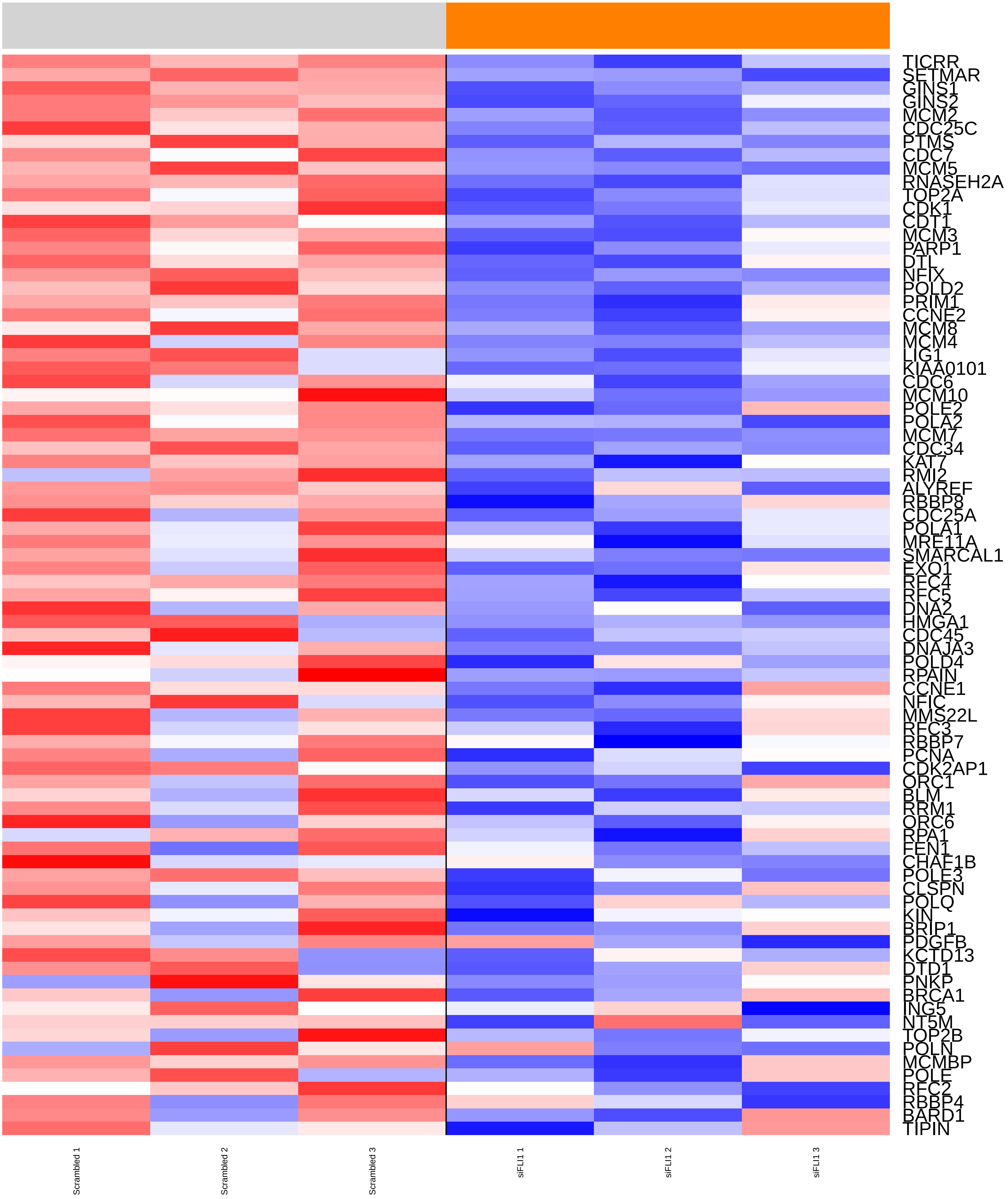

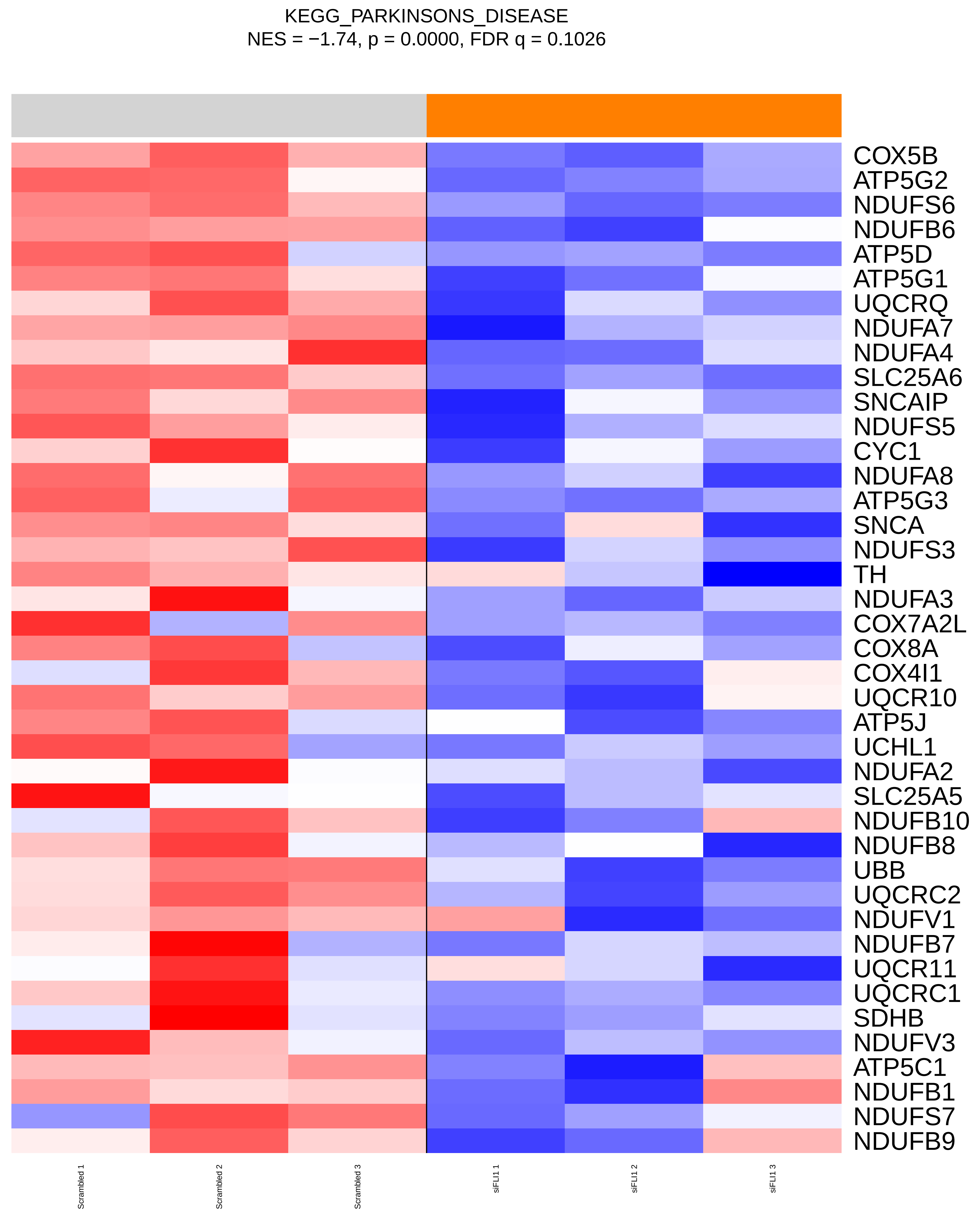

KEGG_PARKINSONS_DISEASE
NES = −1.74, p = 0.0000, FDR q = 0.1026
COX5B
ATP5G2
NDUFS6
NDUFB6
ATP5D
ATP5G1
UQCRQ
NDUFA7
NDUFA4
SLC25A6
SNCAIP
NDUFS5
CYC1
NDUFA8
ATP5G3
SNCA
NDUFS3
TH
NDUFA3
COX7A2L
COX8A
COX4I1
UQCR10
ATP5J
UCHL1
NDUFA2
SLC25A5
NDUFB10
NDUFB8
UBB
UQCRC2
NDUFV1
NDUFB7
UQCR11
UQCRC1
SDHB
NDUFV3
ATP5C1
NDUFB1
NDUFS7
NDUFB9
Scrambled 1
Scrambled 2
Scrambled 3
siFLI1 1
siFLI1 2
siFLI1 3

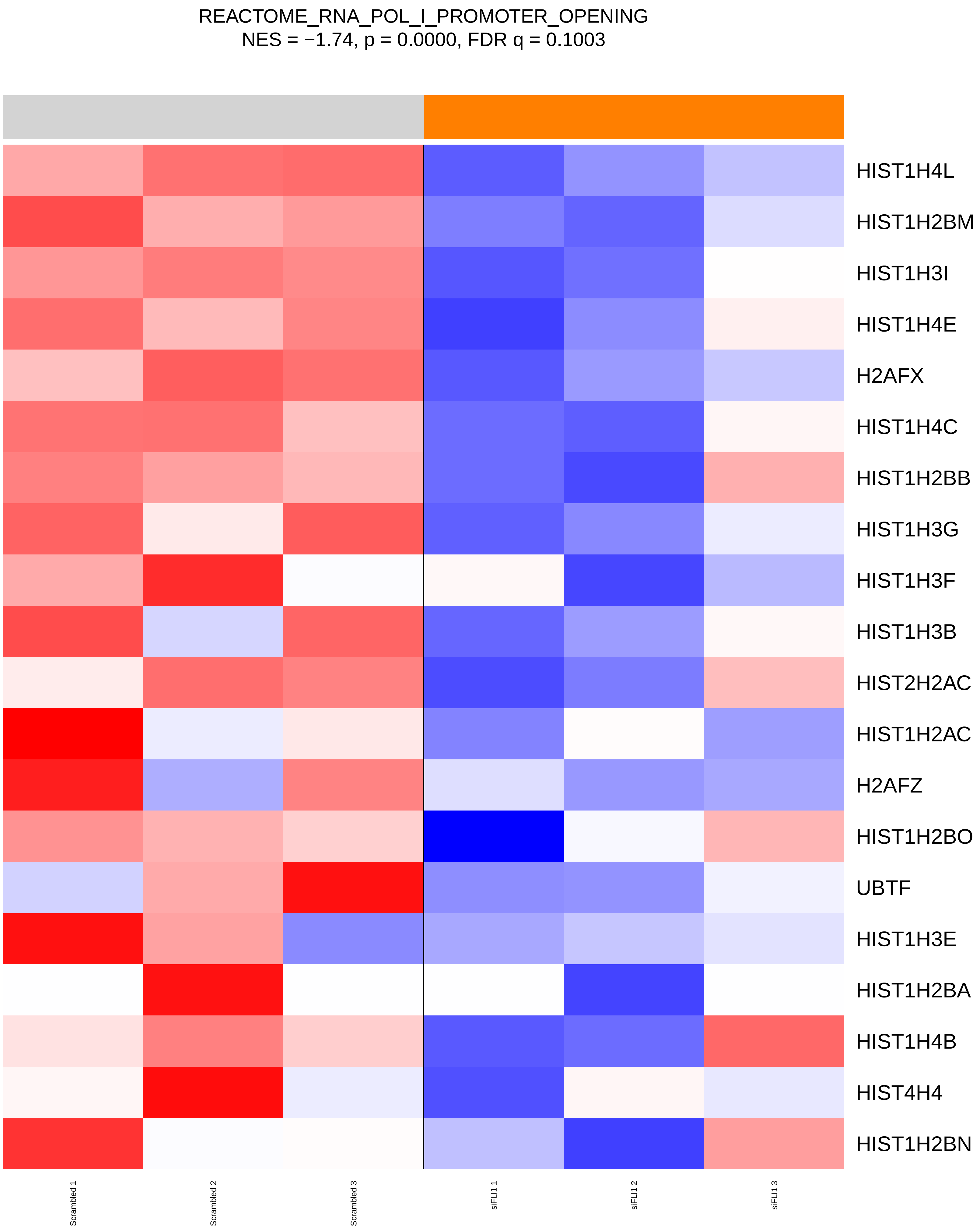
REACTOME_RNA_POL_I_PROMOTER_OPENING
NES = −1.74, p = 0.0000, FDR q = 0.1003
HIST1H4L
HIST1H2BM
HIST1H3I
HIST1H4E
H2AFX
HIST1H4C
HIST1H2BB
HIST1H3G
HIST1H3F
HIST1H3B
HIST2H2AC
HIST1H2AC
H2AFZ
HIST1H2BO
UBTF
HIST1H3E
HIST1H2BA
HIST1H4B
HIST4H4
HIST1H2BN
Scrambled 1
Scrambled 2
Scrambled 3
siFLI1 1
siFLI1 2
siFLI1 3

REACTOME_CHROMOSOME_MAINTENANCE
NES = −1.74, p = 0.0000, FDR q = 0.1011

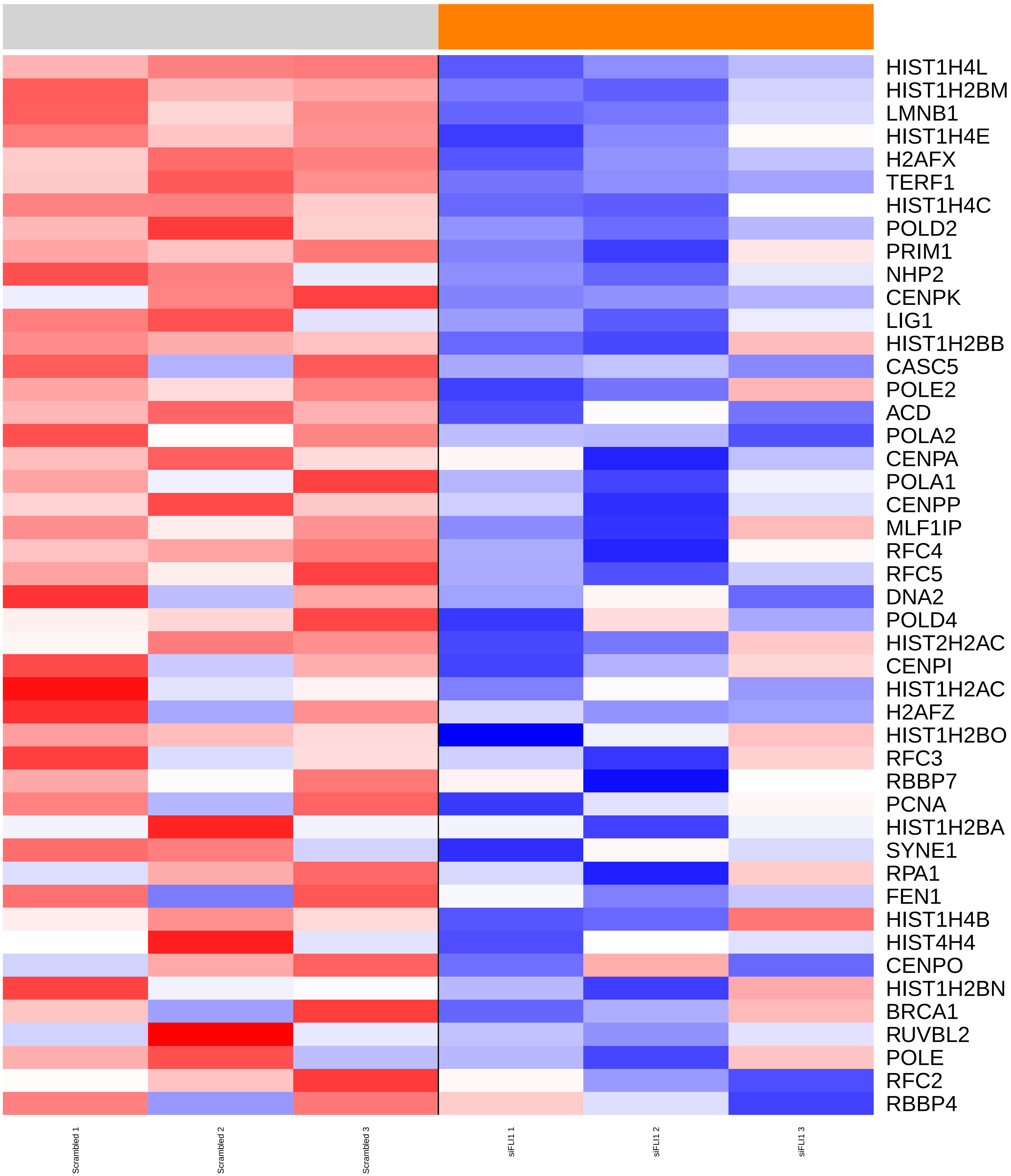

GO_PROTEIN_DNA_COMPLEX_SUBUNIT_ORGANIZATION
NES = −1.74, p = 0.0000, FDR q = 0.1018

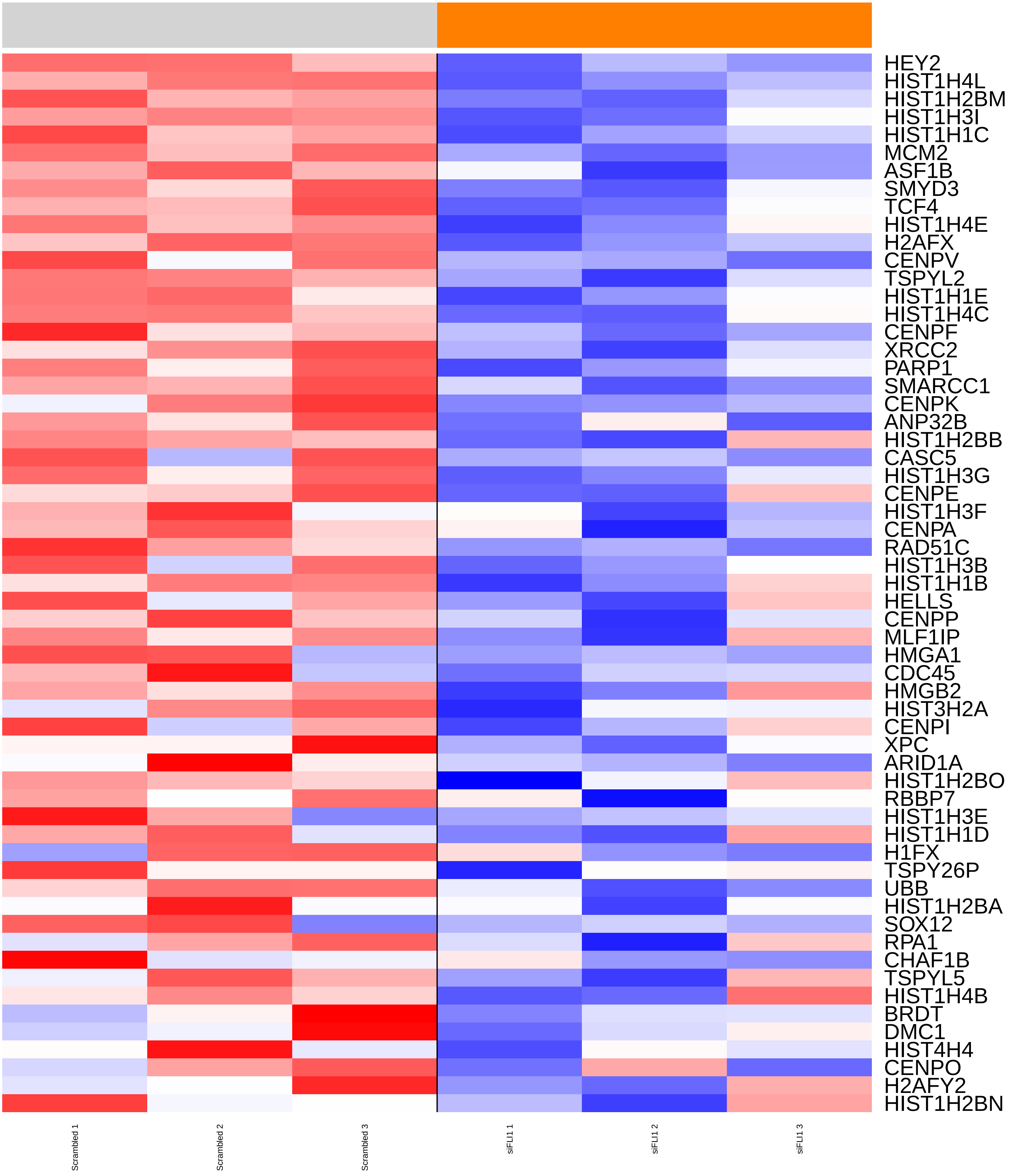

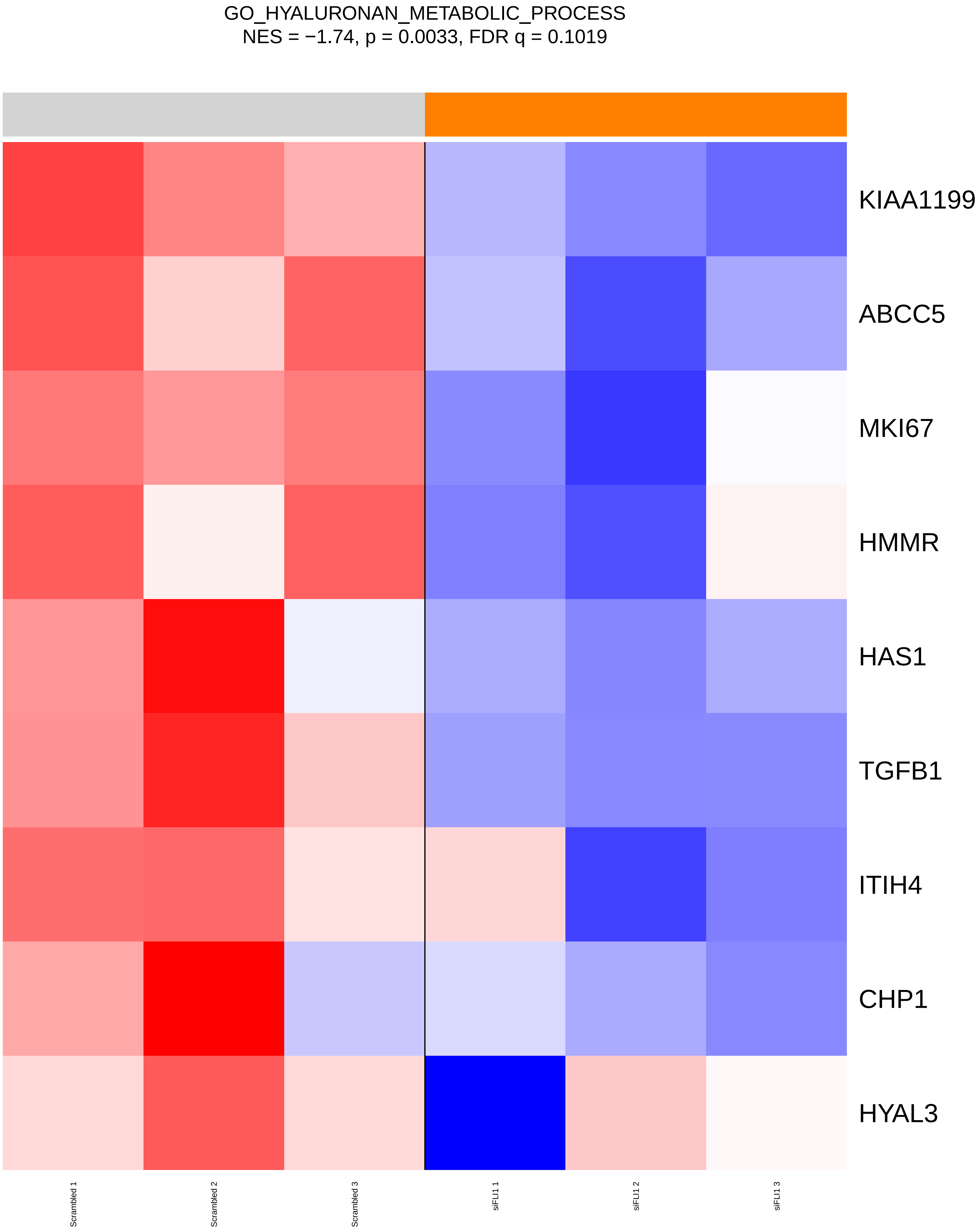
GO_HYALURONAN_METABOLIC_PROCESS
NES = −1.74, p = 0.0033, FDR q = 0.1019
KIAA1199
ABCC5
MKI67
HMMR
HAS1
TGFB1
ITIH4
CHP1
HYAL3
Scrambled 1
Scrambled 2
Scrambled 3
siFLI1 1
siFLI1 2
siFLI1 3

GO_NUCLEOSIDE_MONOPHOSPHATE_BIOSYNTHETIC_PROCESS
NES = −1.75, p = 0.0000, FDR q = 0.1007

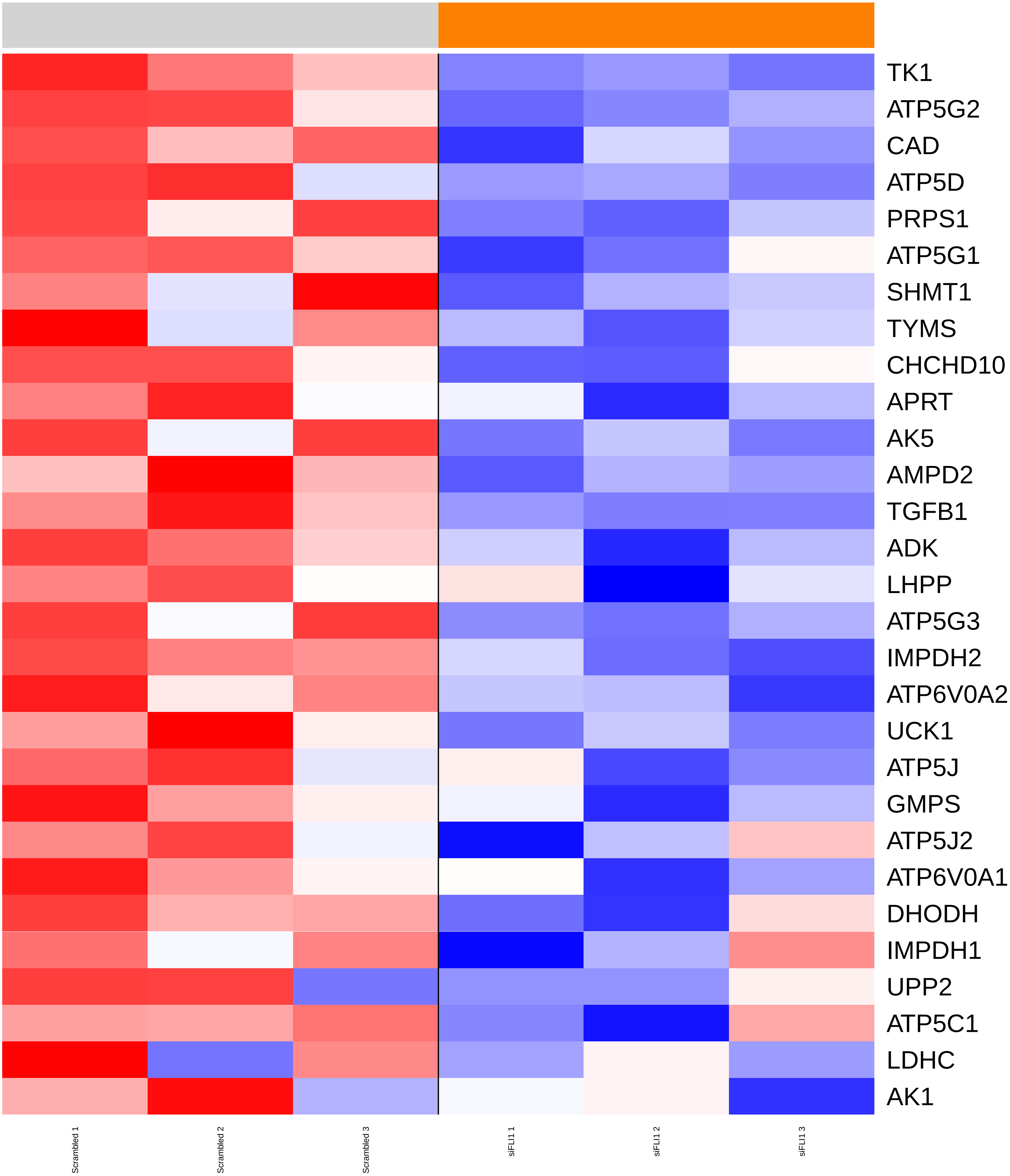

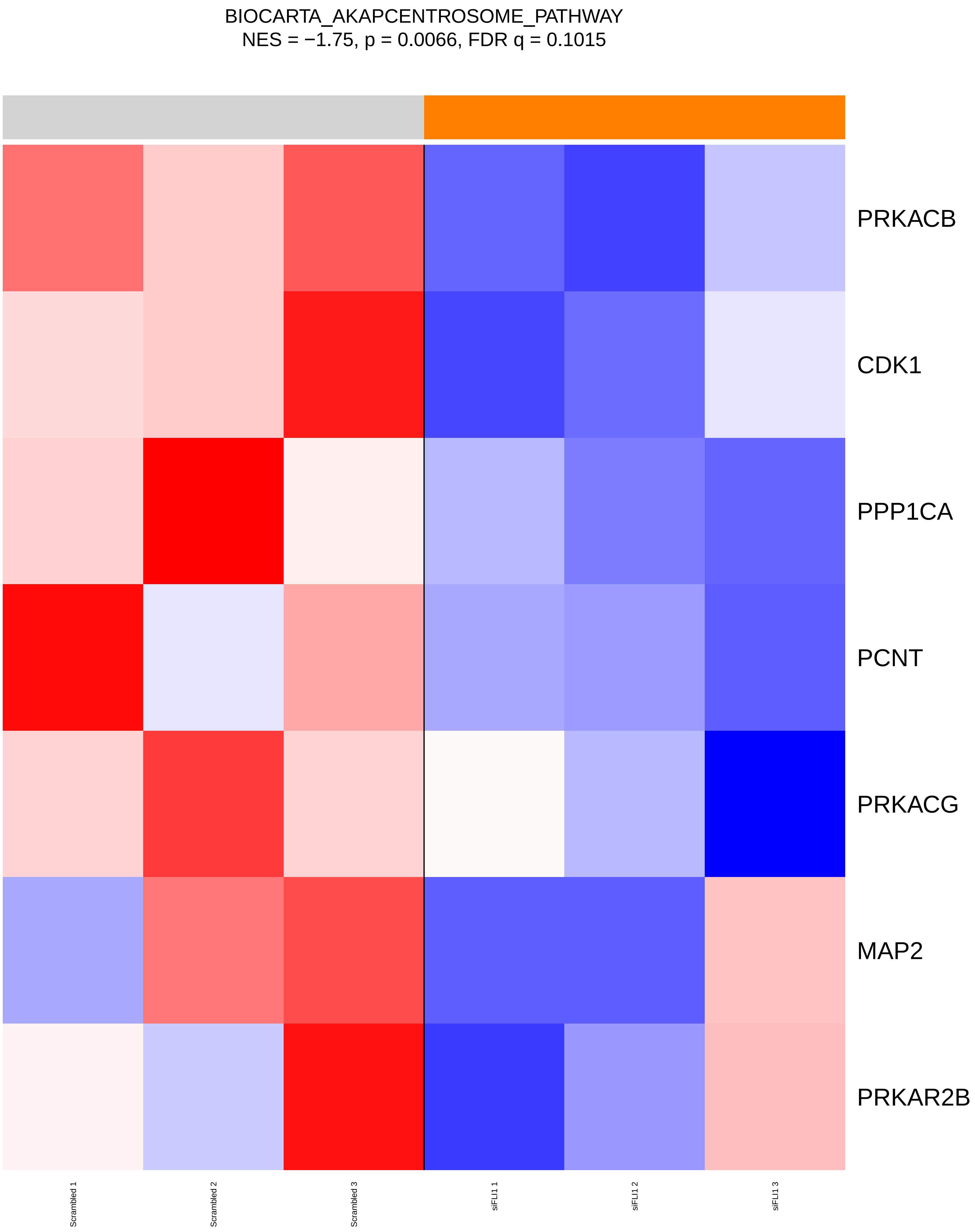
BIOCARTA_AKAPCENTROSOME_PATHWAY
NES = −1.75, p = 0.0066, FDR q = 0.1015
PRKACB
CDK1
PPP1CA
PCNT
PRKACG
MAP2
PRKAR2B
Scrambled 1
Scrambled 2
Scrambled 3
siFLI1 1
siFLI1 2
siFLI1 3

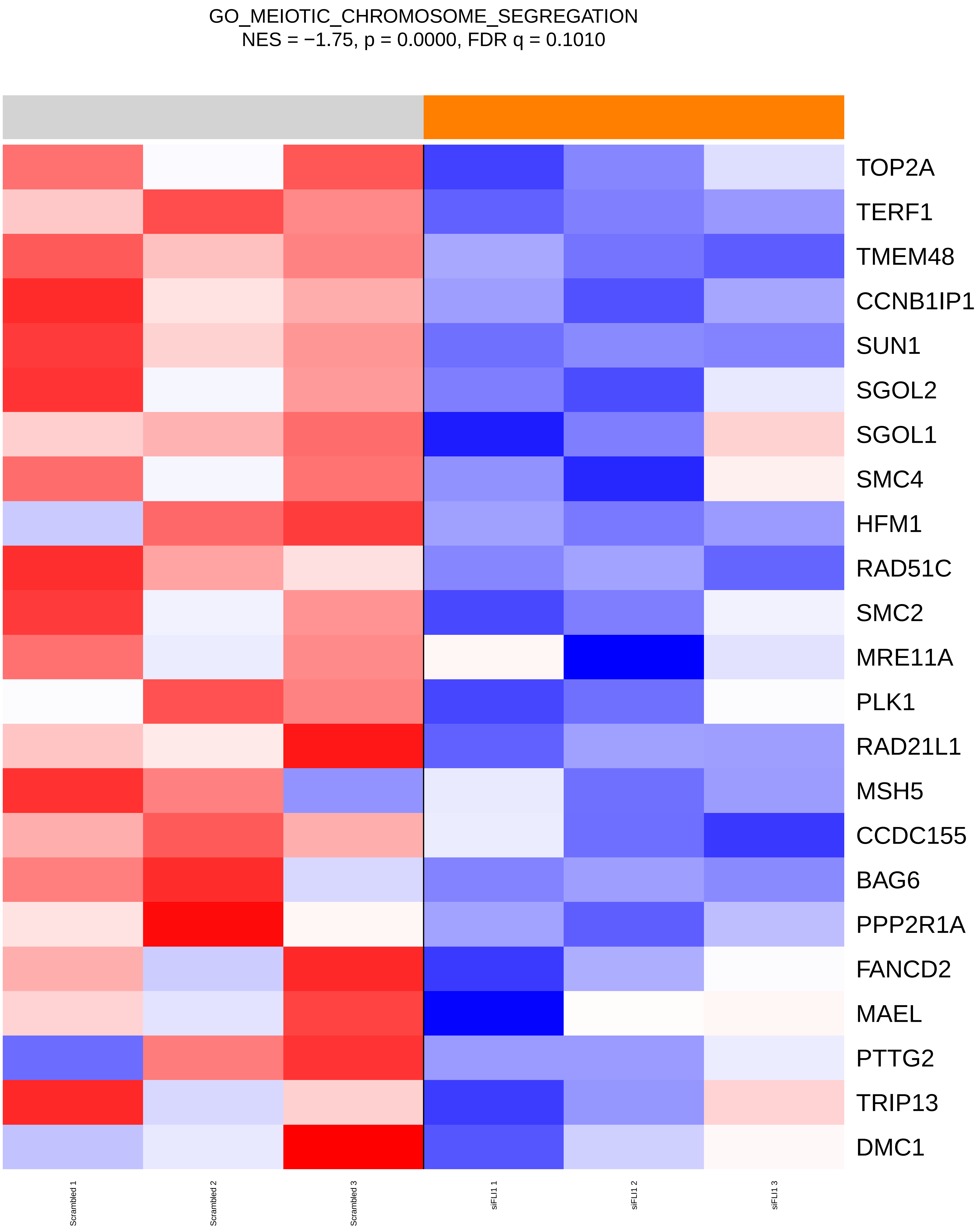

GO_MEIOTIC_CHROMOSOME_SEGREGATION
NES = −1.75, p = 0.0000, FDR q = 0.1010
TOP2A
TERF1
TMEM48
CCNB1IP1
SUN1
SGOL2
SGOL1
SMC4
HFM1
RAD51C
SMC2
MRE11A
PLK1
RAD21L1
MSH5
CCDC155
BAG6
PPP2R1A
FANCD2
MAEL
PTTG2
TRIP13
DMC1
Scrambled 1
Scrambled 2
Scrambled 3
siFLI1 1
siFLI1 2
siFLI1 3

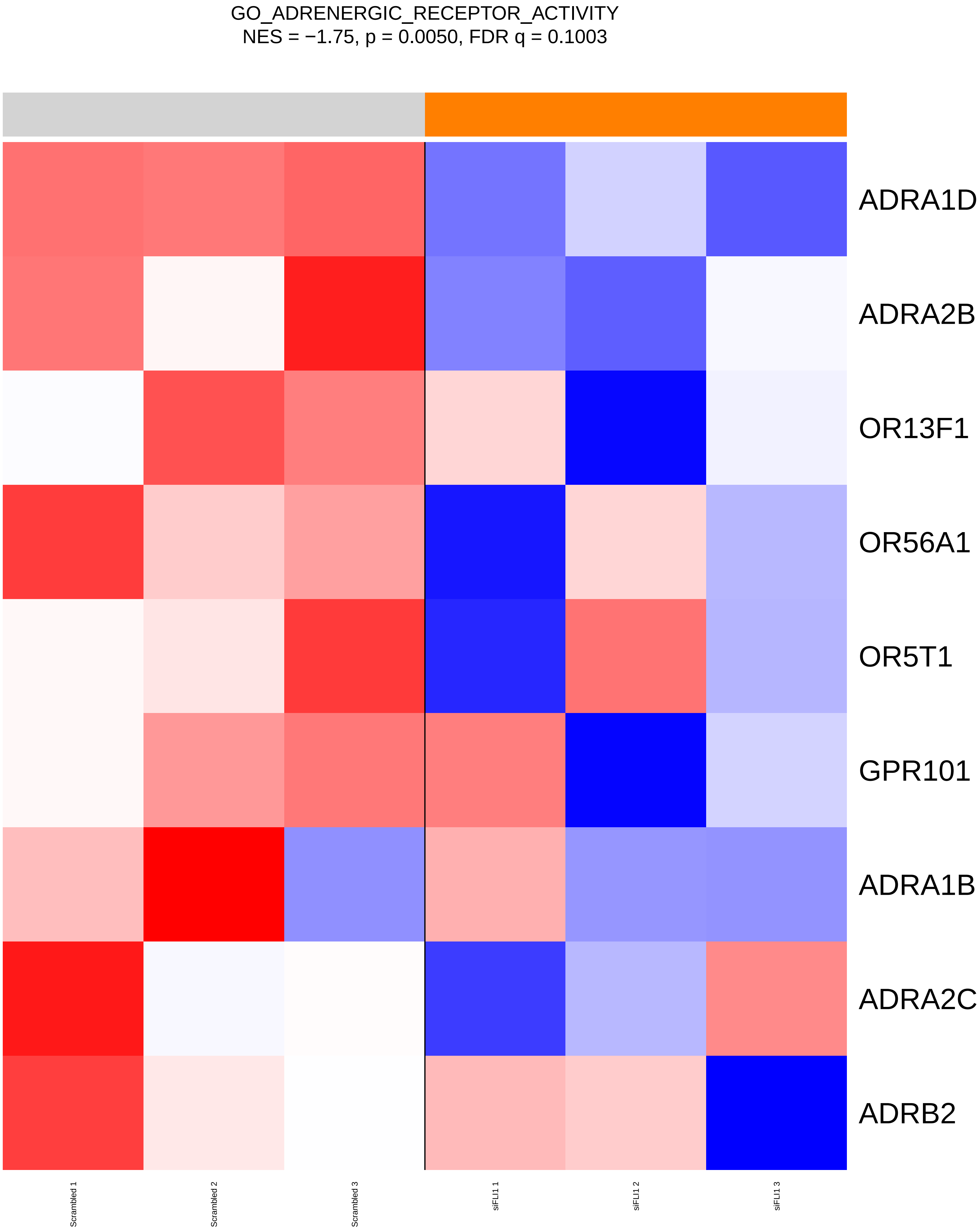
GO_ADRENERGIC_RECEPTOR_ACTIVITY
NES = −1.75, p = 0.0050, FDR q = 0.1003
ADRA1D
ADRA2B
OR13F1
OR56A1
OR5T1
GPR101
ADRA1B
ADRA2C
ADRB2
Scrambled 1
Scrambled 2
Scrambled 3
siFLI1 1
siFLI1 2
siFLI1 3

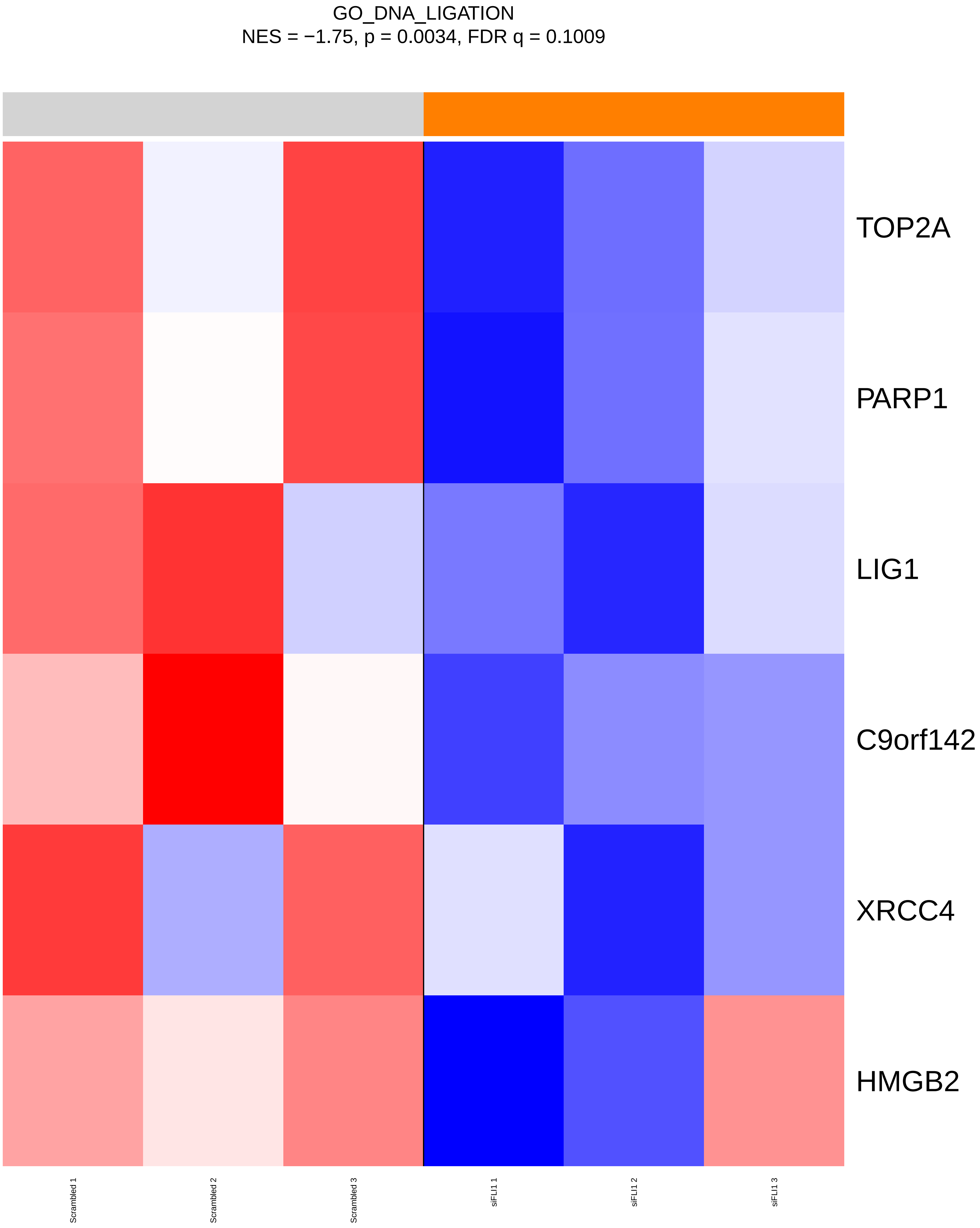
GO_DNA_LIGATION
NES = −1.75, p = 0.0034, FDR q = 0.1009
TOP2A
PARP1
LIG1
C9orf142
XRCC4
HMGB2
Scrambled 1
Scrambled 2
Scrambled 3
siFLI1 1
siFLI1 2
siFLI1 3

GO_REGULATION_OF_BLOOD_VESSEL_ENDOTHELIAL_CELL_MIGRATION
NES = −1.75, p = 0.0000, FDR q = 0.1006

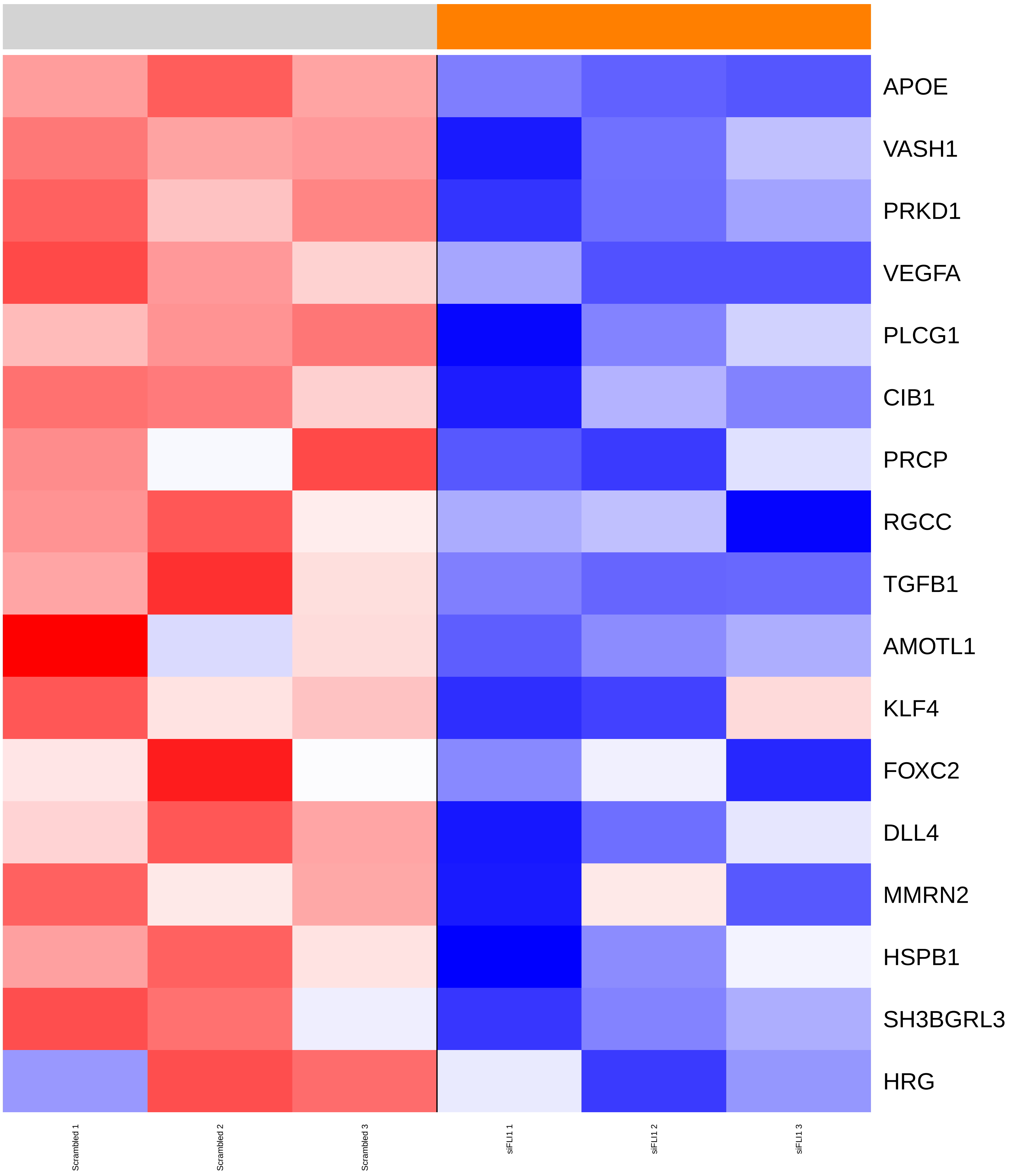

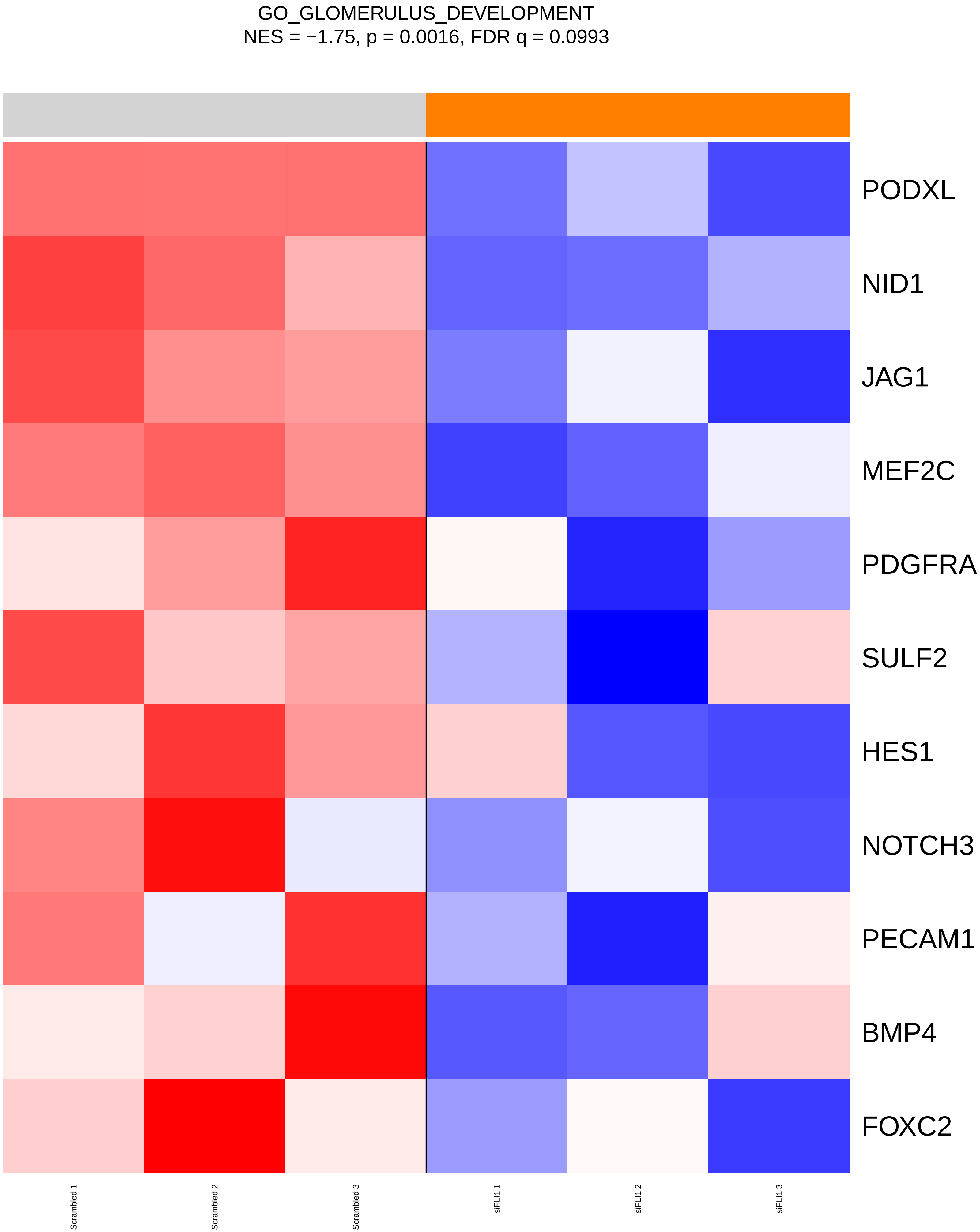
GO_GLOMERULUS_DEVELOPMENT
NES = −1.75, p = 0.0016, FDR q = 0.0993
PODXL
NID1
JAG1
MEF2C
PDGFRA
SULF2
HES1
NOTCH3
PECAM1
BMP4
FOXC2
Scrambled 1
Scrambled 2
Scrambled 3
siFLI1 1
siFLI1 2
siFLI1 3

KEGG_ALANINE_ASPARTATE_AND_GLUTAMATE_METABOLISM
NES = −1.75, p = 0.0000, FDR q = 0.1001

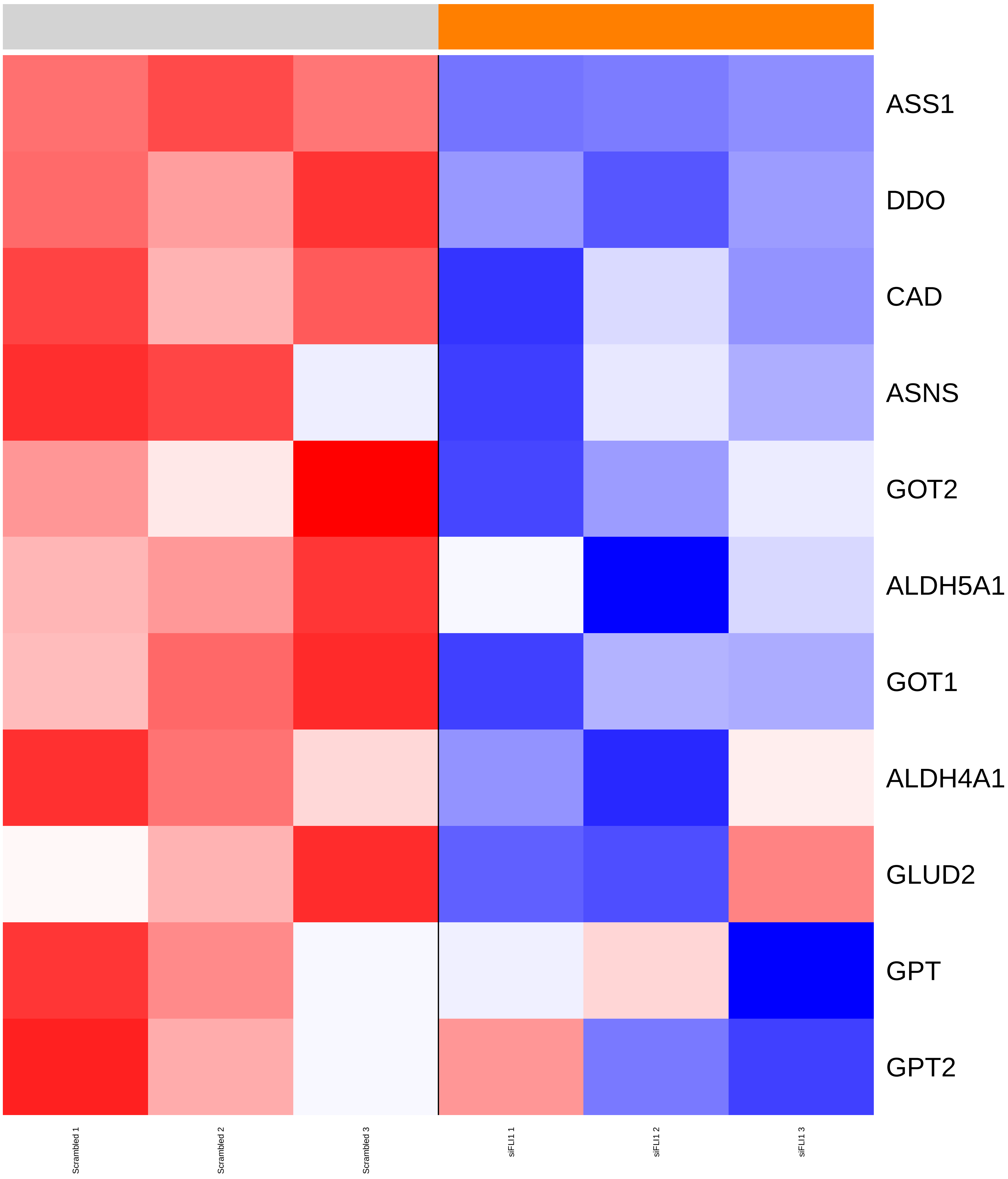

GO_DEFENSE_RESPONSE_TO_FUNGUS
NES = −1.75, p = 0.0000, FDR q = 0.1010

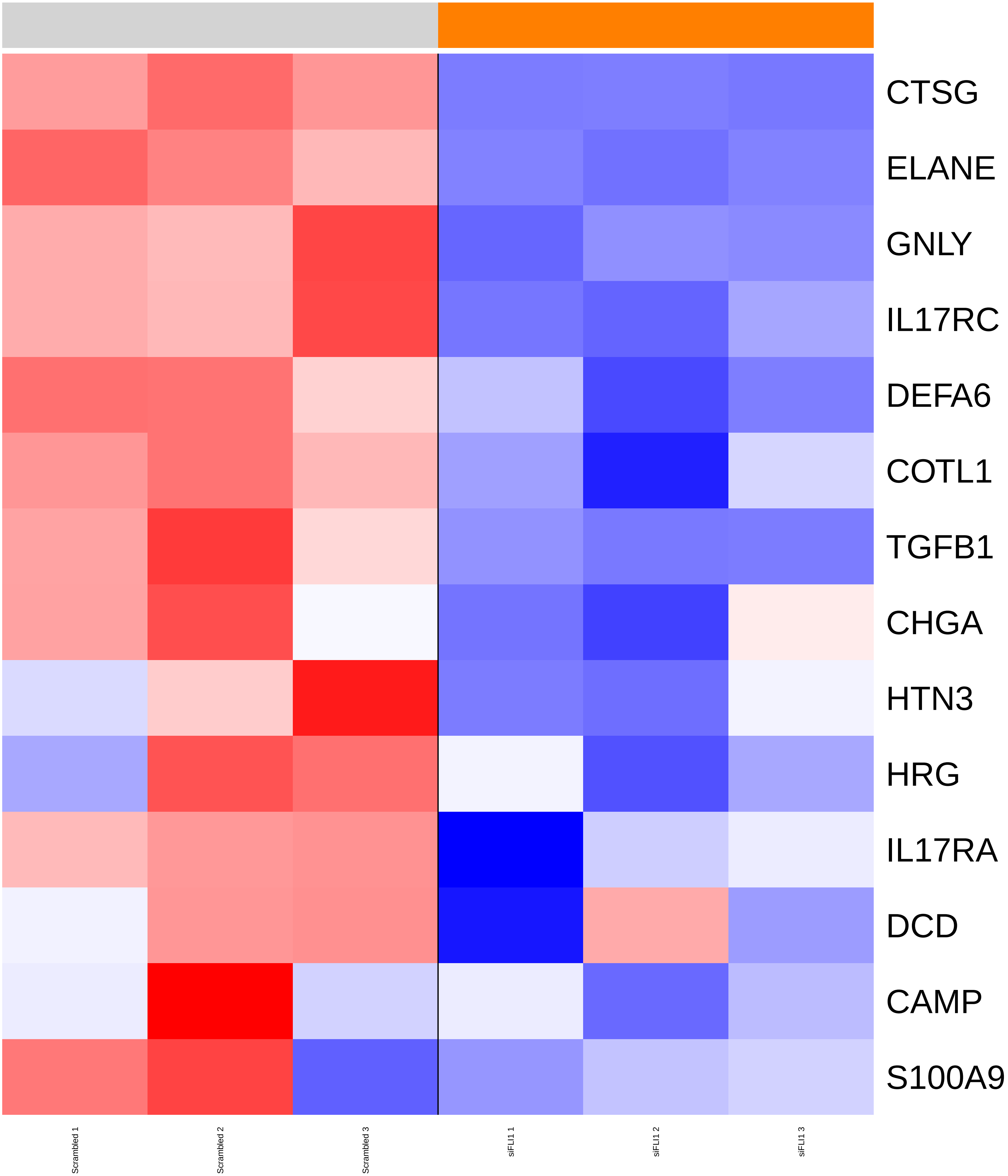

HALLMARK_ANGIOGENESIS
NES = −1.76, p = 0.0000, FDR q = 0.1014

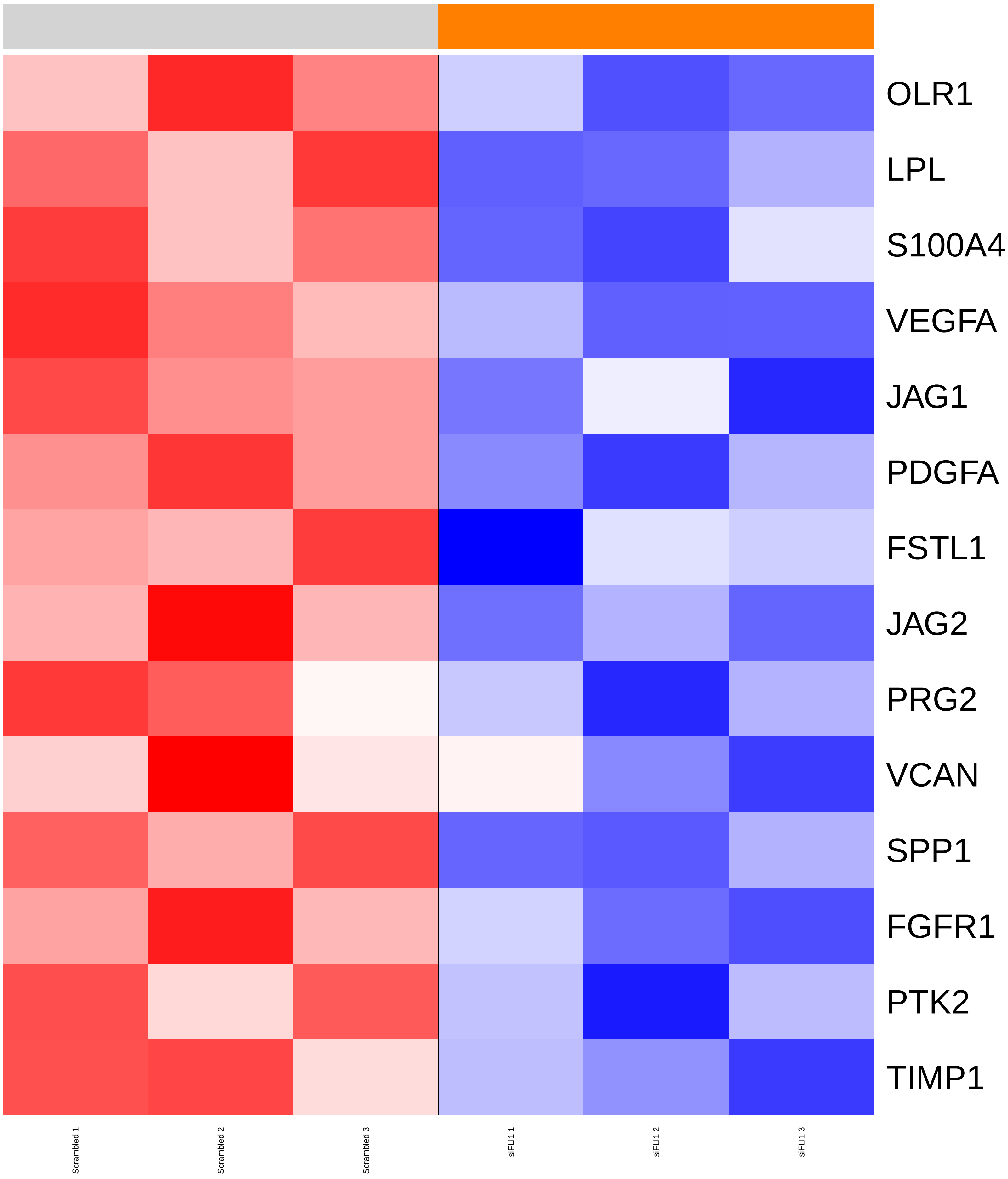

GO_MODULATION_OF_GROWTH_OF_SYMBIONT_INVOLVED_IN_INTERACTION_WITH_HOST
NES = −1.76, p = 0.0051, FDR q = 0.1003

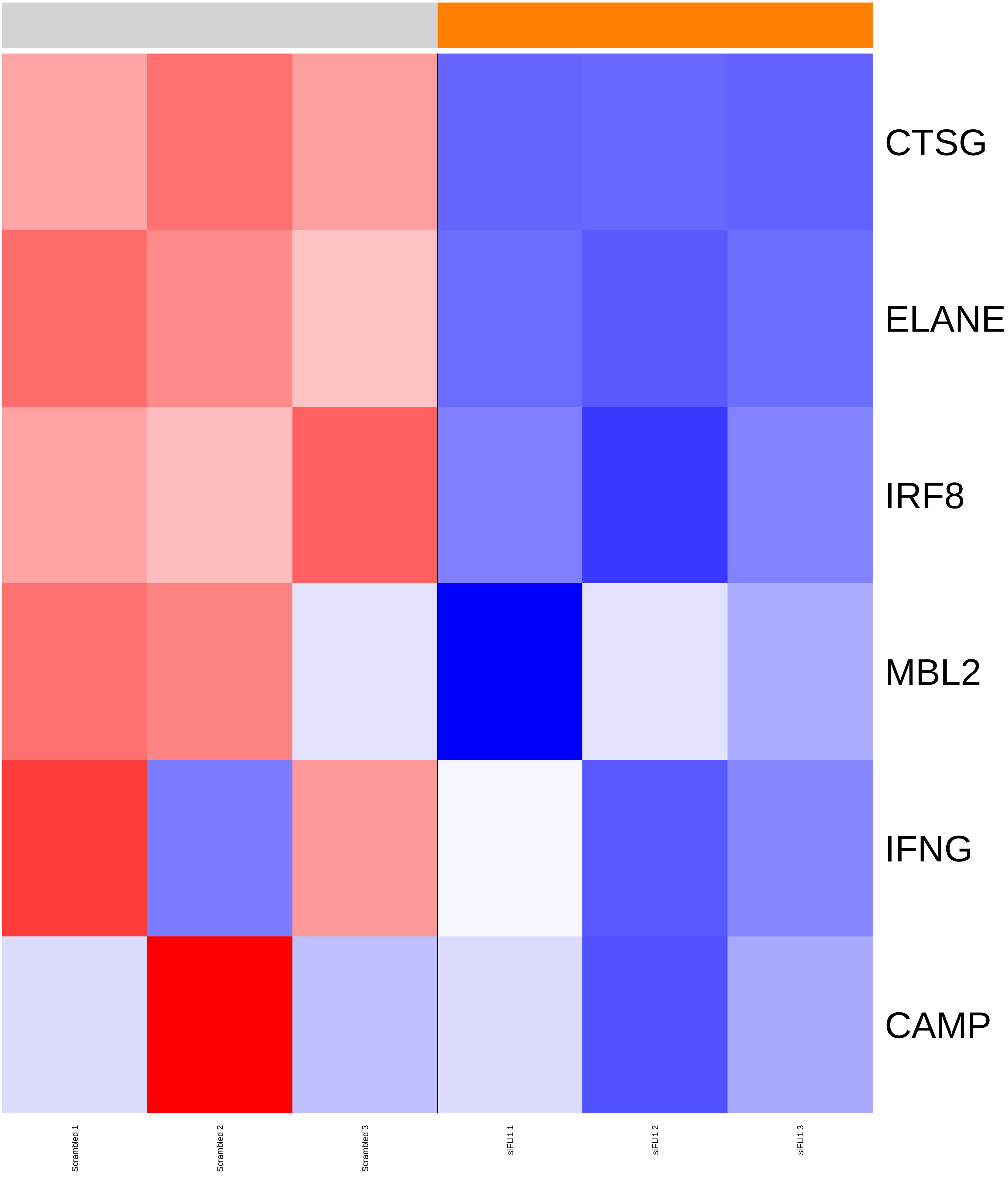

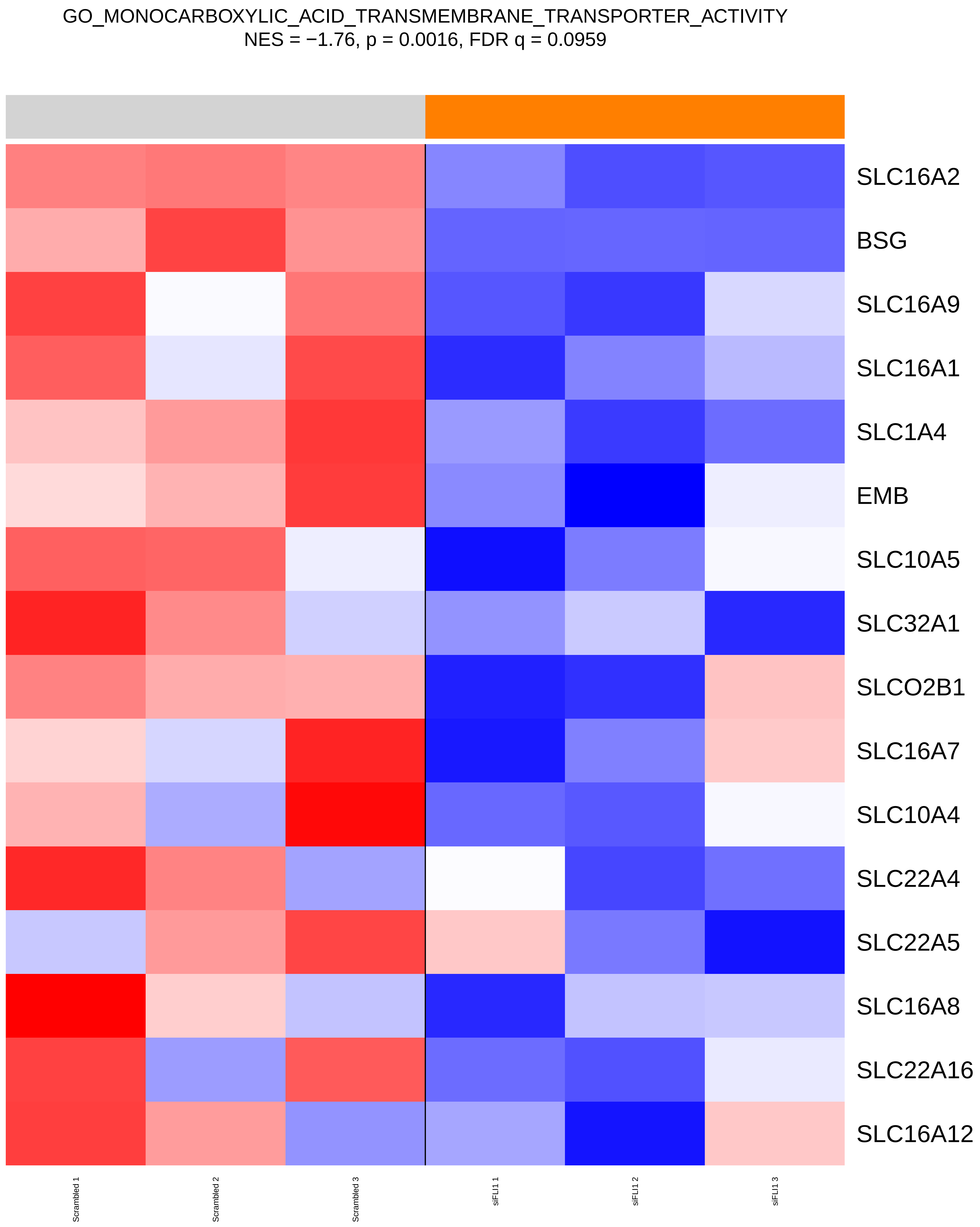
GO_MONOCARBOXYLIC_ACID_TRANSMEMBRANE_TRANSPORTER_ACTIVITY
NES = −1.76, p = 0.0016, FDR q = 0.0959
SLC16A2
BSG
SLC16A9
SLC16A1
SLC1A4
EMB
SLC10A5
SLC32A1
SLCO2B1
SLC16A7
SLC10A4
SLC22A4
SLC22A5
SLC16A8
SLC22A16
SLC16A12
Scrambled 1
Scrambled 2
Scrambled 3
siFLI1 1
siFLI1 2
siFLI1 3

GO_ORGANIC_ACID_BIOSYNTHETIC_PROCESS
NES = −1.76, p = 0.0000, FDR q = 0.0955

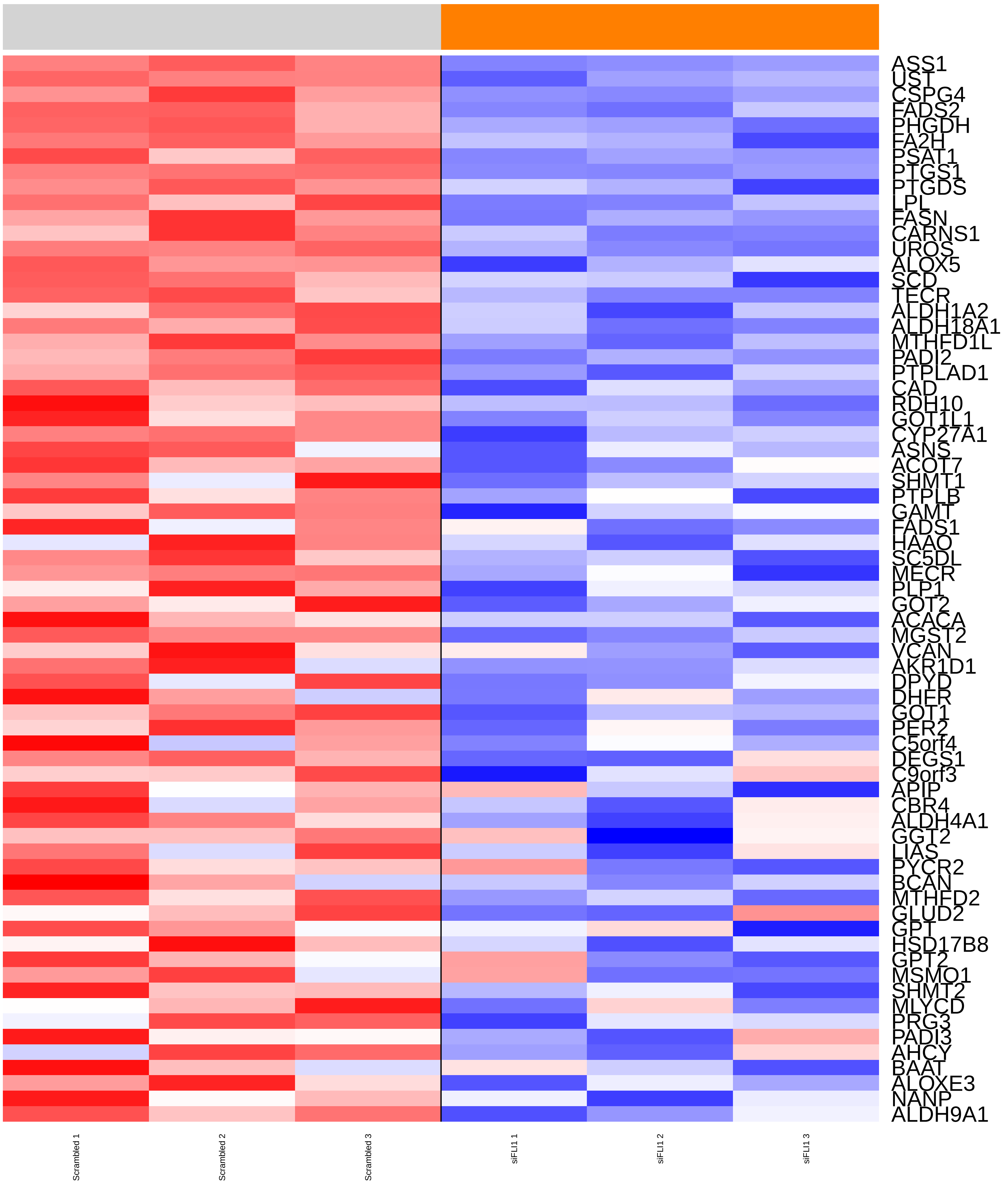

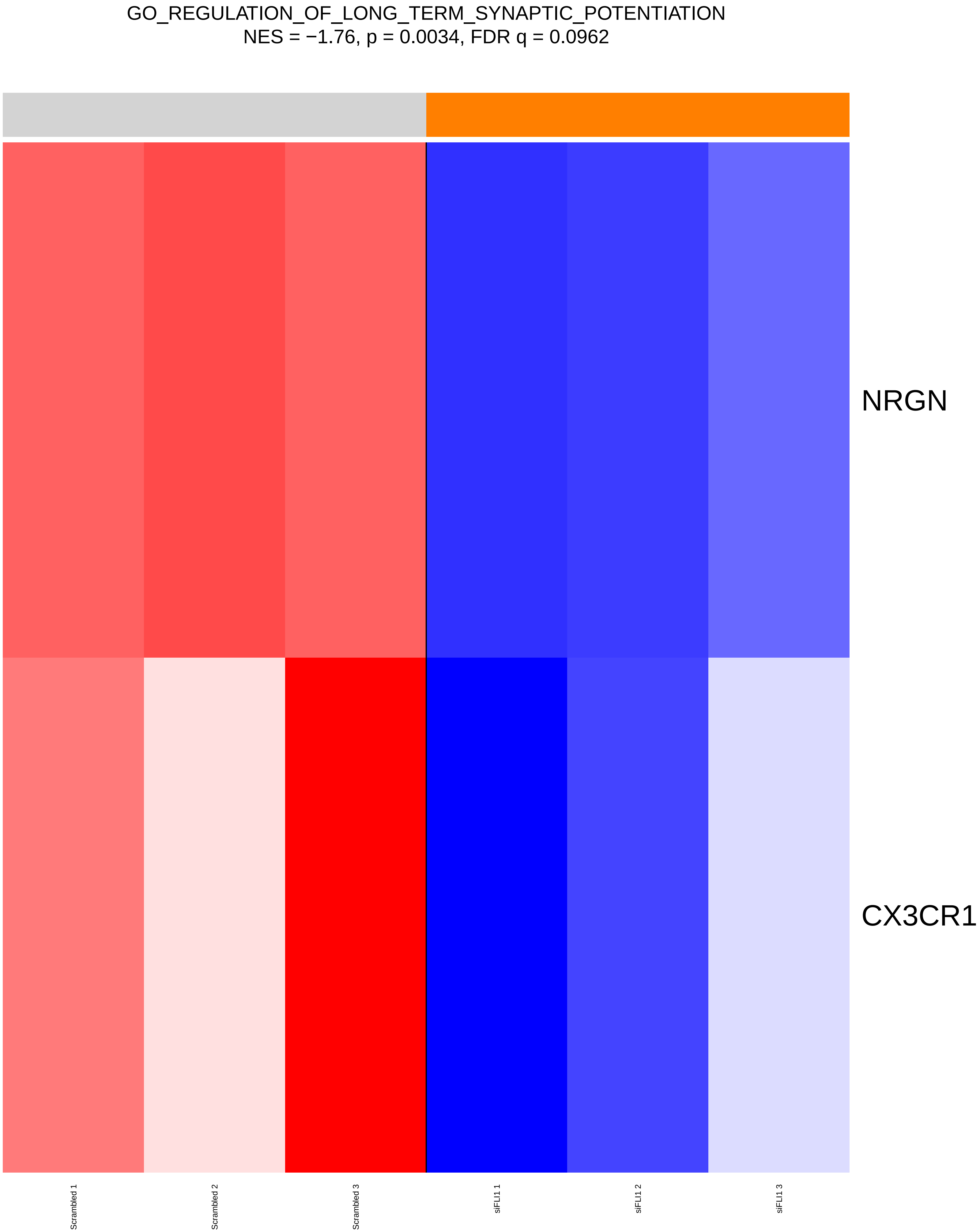
GO_REGULATION_OF_LONG_TERM_SYNAPTIC_POTENTIATION
NES = −1.76, p = 0.0034, FDR q = 0.0962
NRGN
CX3CR1
Scrambled 1
Scrambled 2
Scrambled 3
siFLI1 1
siFLI1 2
siFLI1 3

REACTOME_MITOTIC_PROMETAPHASE
NES = −1.76, p = 0.0000, FDR q = 0.0972

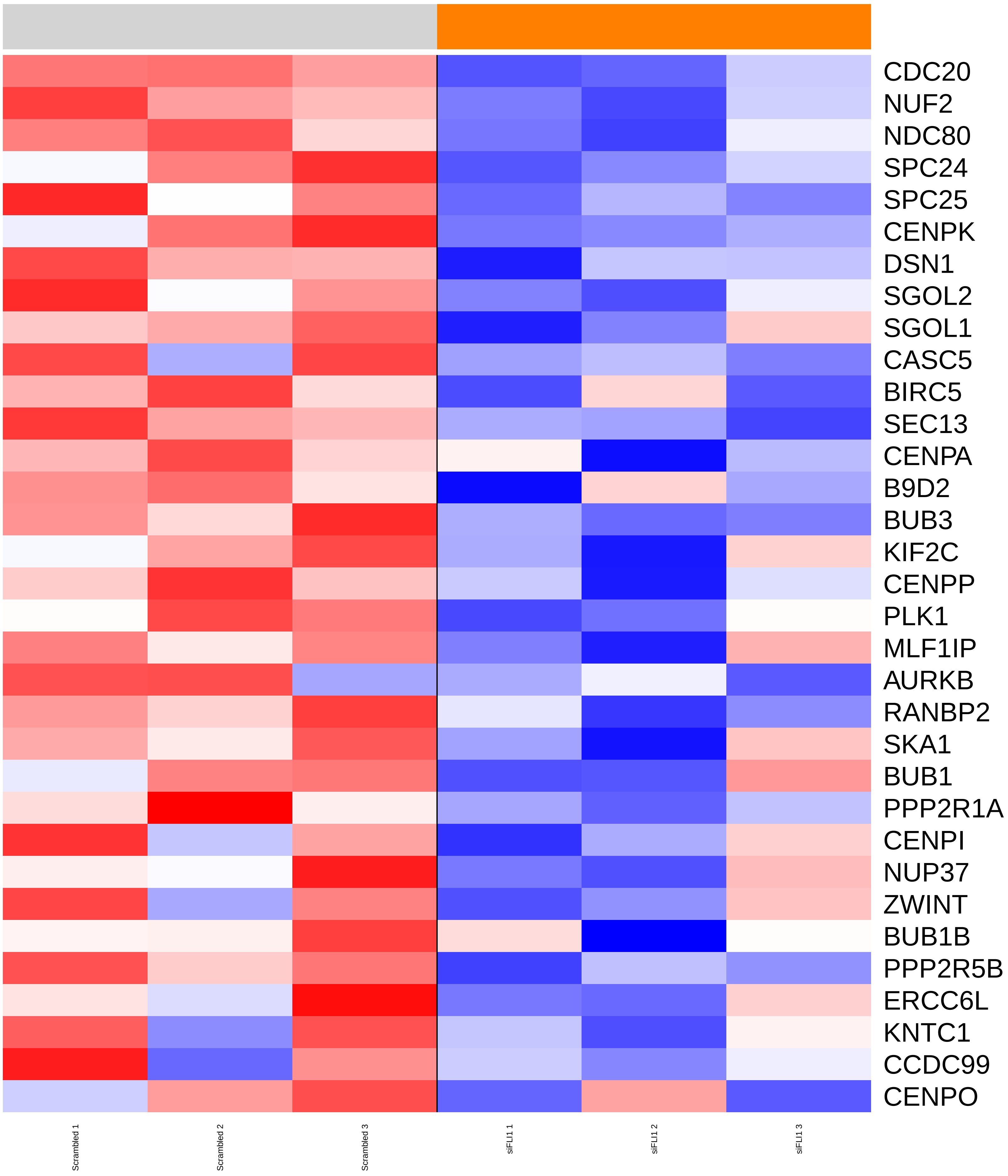

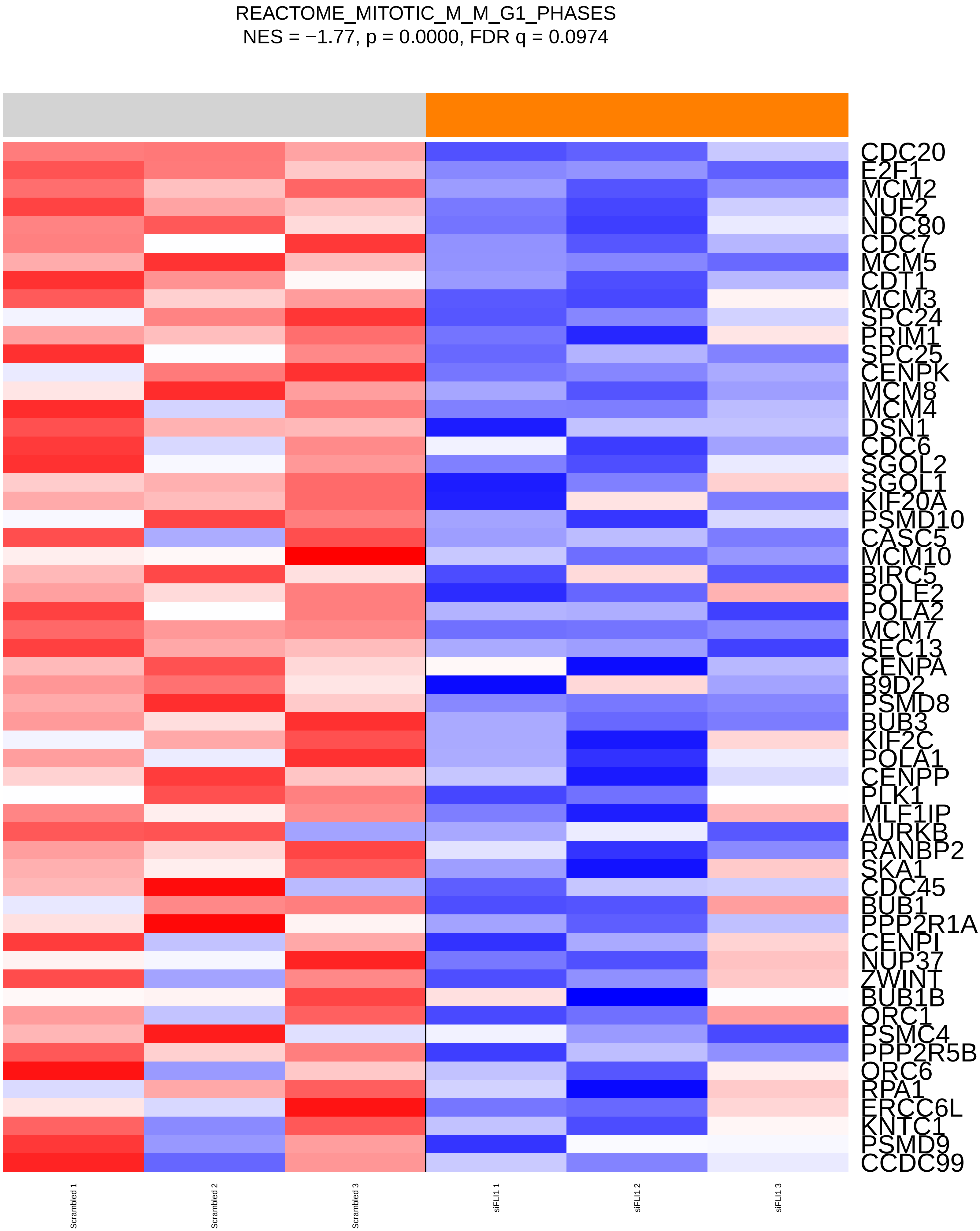
REACTOME_MITOTIC_M_M_G1_PHASES
NES = −1.77, p = 0.0000, FDR q = 0.0974
CDC20
E2F1
MCM2
NUF2
NDC80
CDC7
MCM5
CDT1
MCM3
SPC24
PRIM1
SPC25
CENPK
MCM8
MCM4
DSN1
CDC6
SGOL2
SGOL1
KIF20A
PSMD10
CASC5
MCM10
BIRC5
POLE2
POLA2
MCM7
SEC13
CENPA
B9D2
PSMD8
BUB3
KIF2C
POLA1
CENPP
PLK1
MLF1IP
AURKB
RANBP2
SKA1
CDC45
BUB1
PPP2R1A
CENPI
NUP37
ZWINT
BUB1B
ORC1
PSMC4
PPP2R5B
ORC6
RPA1
ERCC6L
KNTC1
PSMD9
CCDC99
Scrambled 1
Scrambled 2
Scrambled 3
siFLI1 1
siFLI1 2
siFLI1 3

KEGG_VASCULAR_SMOOTH_MUSCLE_CONTRACTION
NES = −1.77, p = 0.0000, FDR q = 0.0945

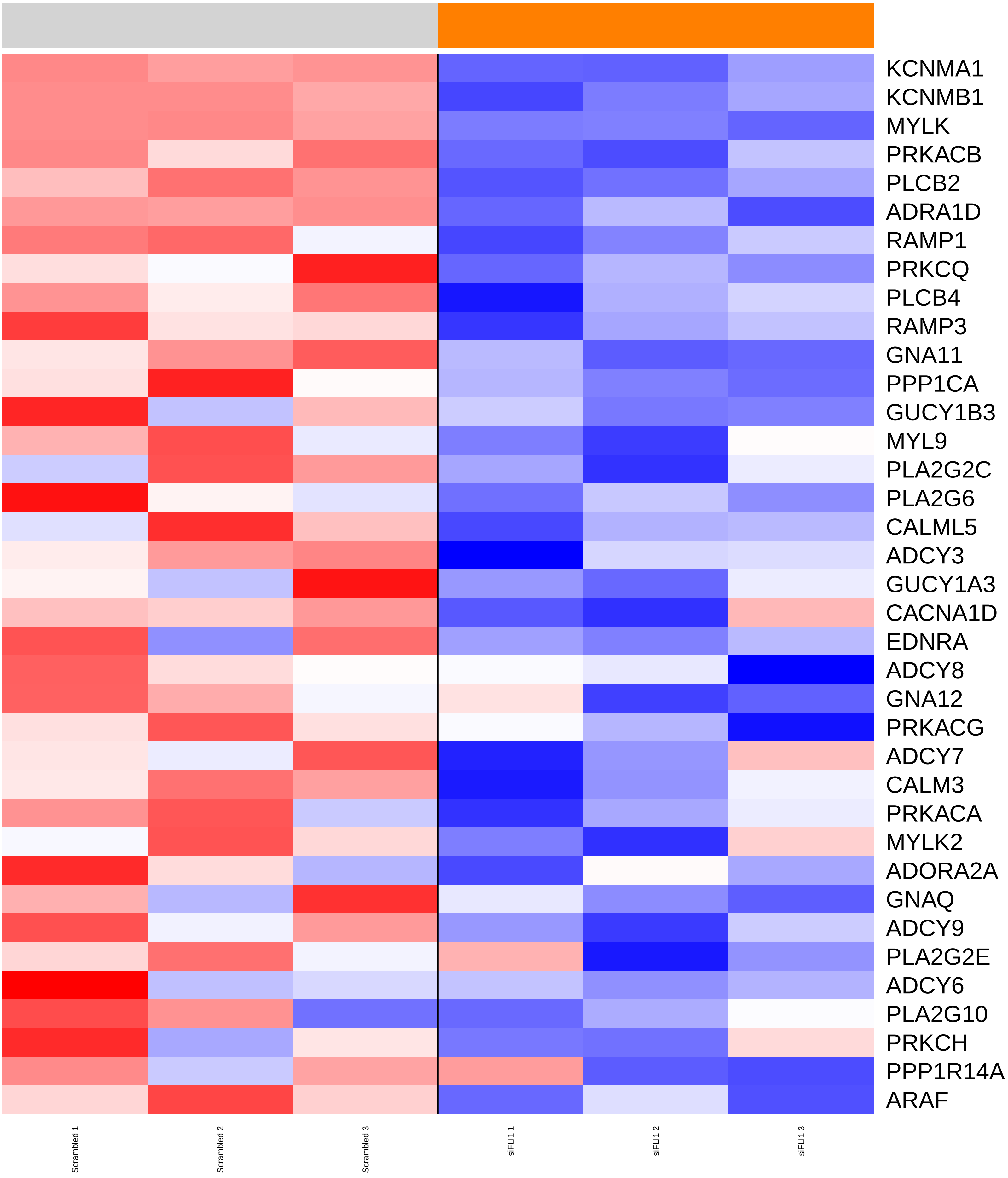

GO_RESPONSE_TO_VITAMIN_A
NES = −1.78, p = 0.0085, FDR q = 0.0862

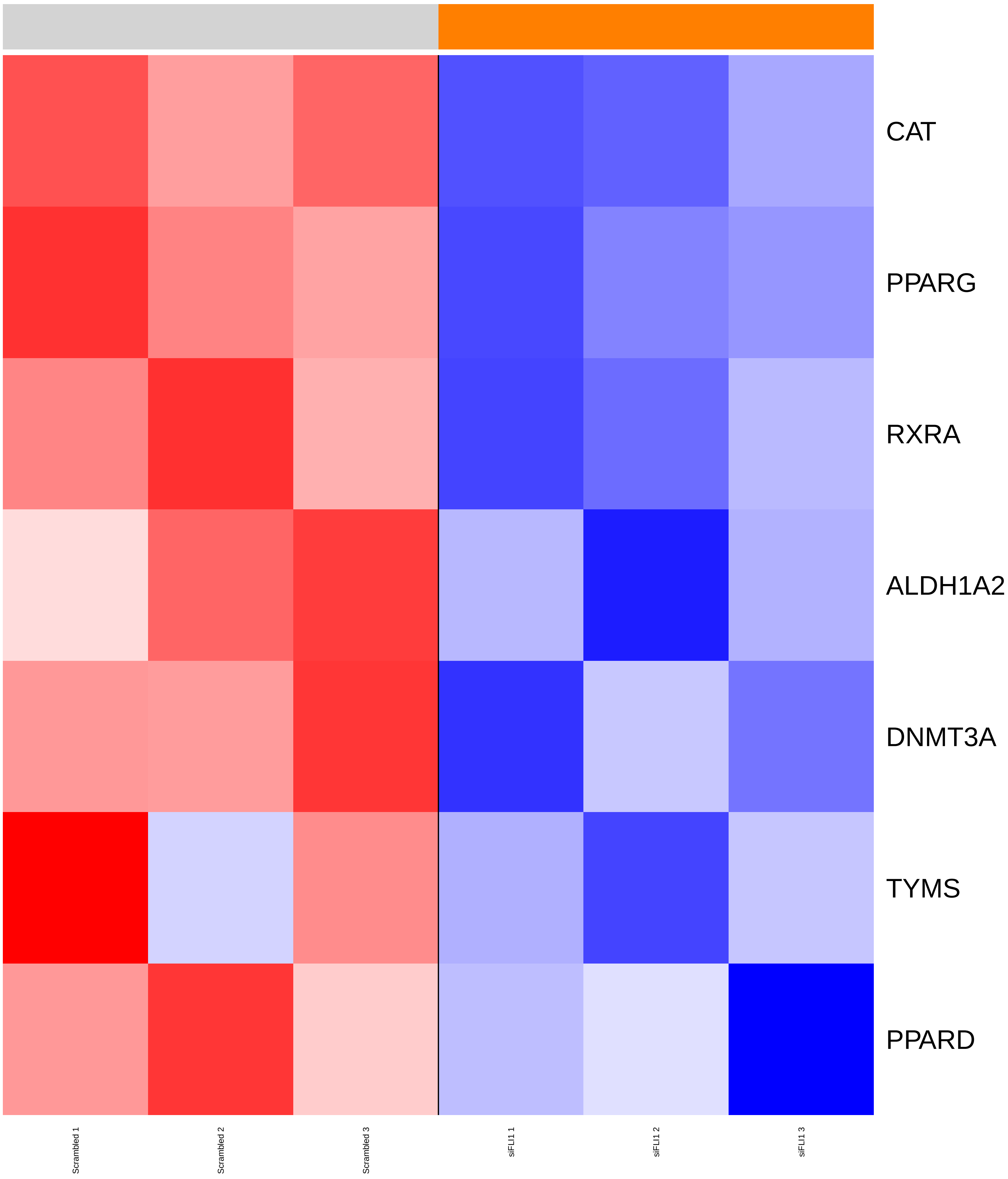

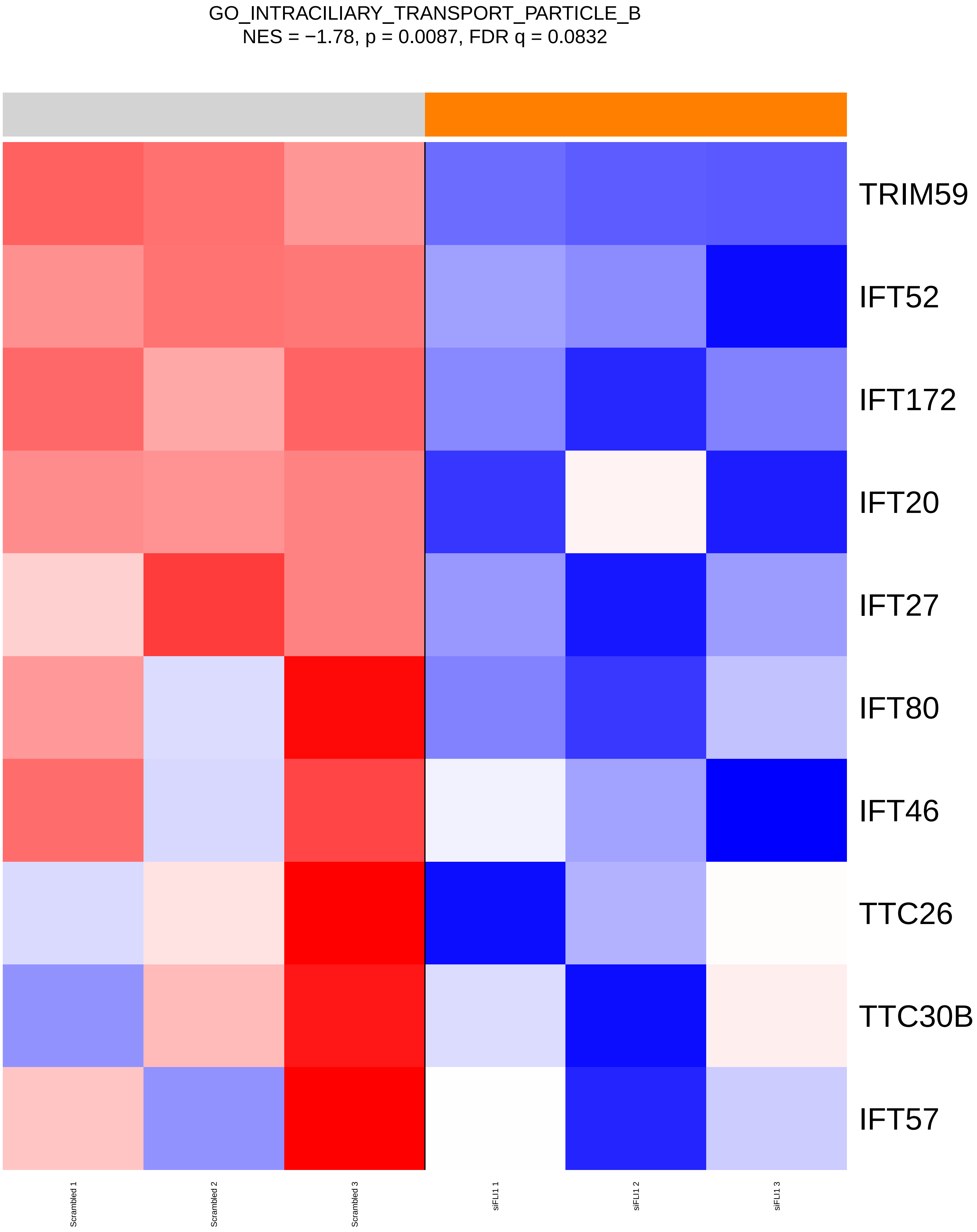
GO_INTRACILIARY_TRANSPORT_PARTICLE_B
NES = −1.78, p = 0.0087, FDR q = 0.0832
TRIM59
IFT52
IFT172
IFT20
IFT27
IFT80
IFT46
TTC26
TTC30B
IFT57
Scrambled 1
Scrambled 2
Scrambled 3
siFLI1 1
siFLI1 2
siFLI1 3

GO_AORTA_MORPHOGENESIS
NES = −1.78, p = 0.0033, FDR q = 0.0827

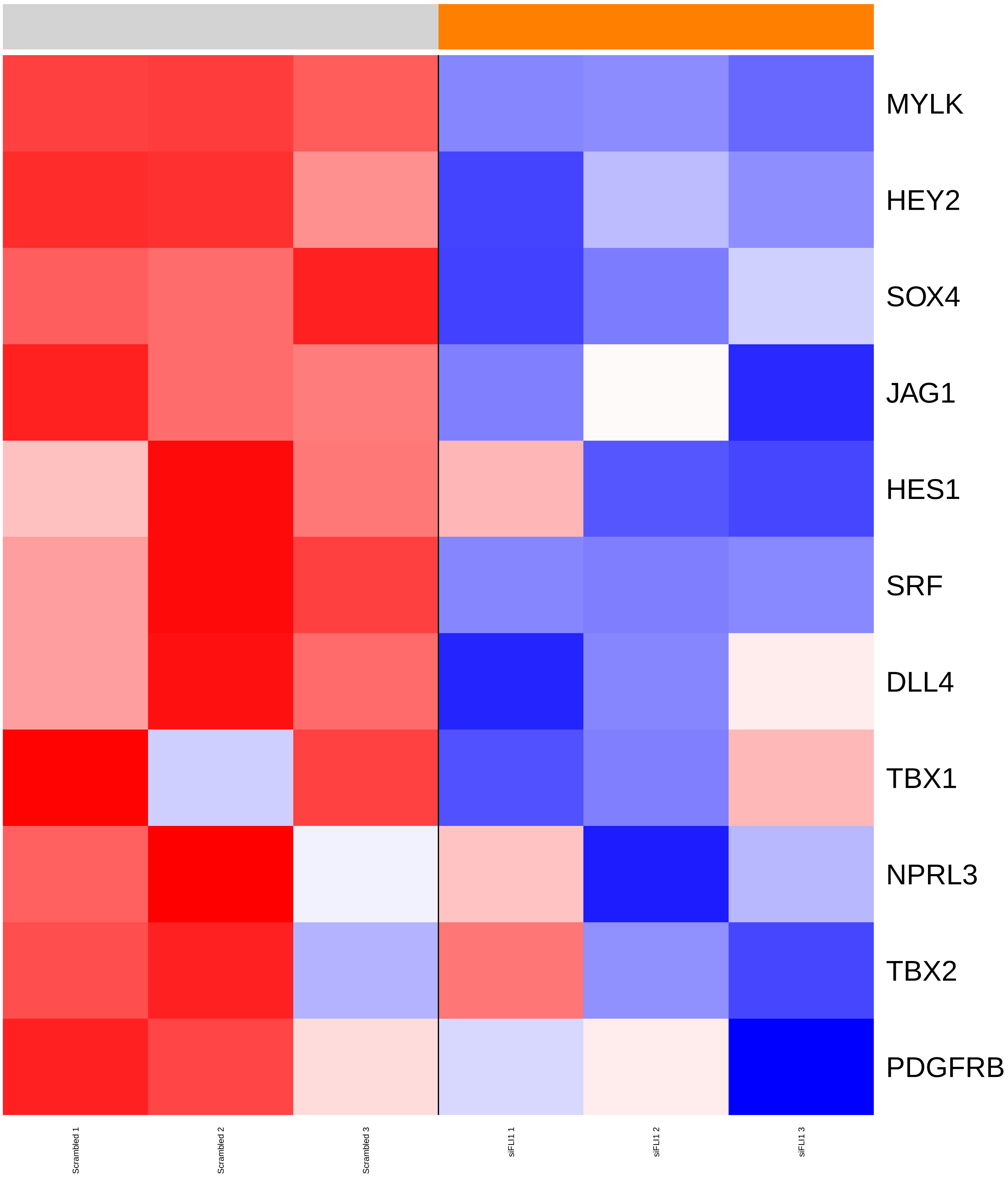

GO_MEGAKARYOCYTE_DEVELOPMENT
NES = −1.79, p = 0.0000, FDR q = 0.0828

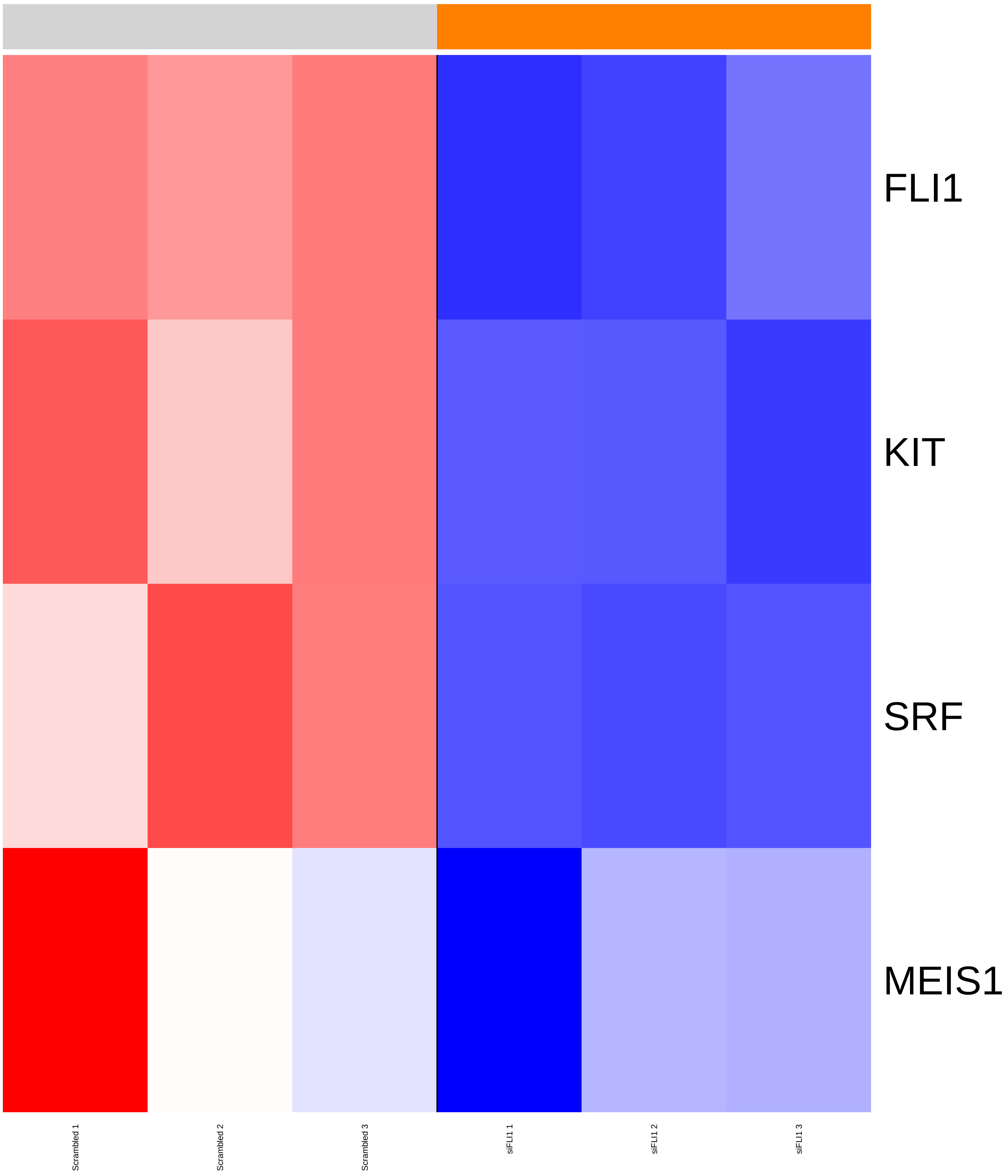

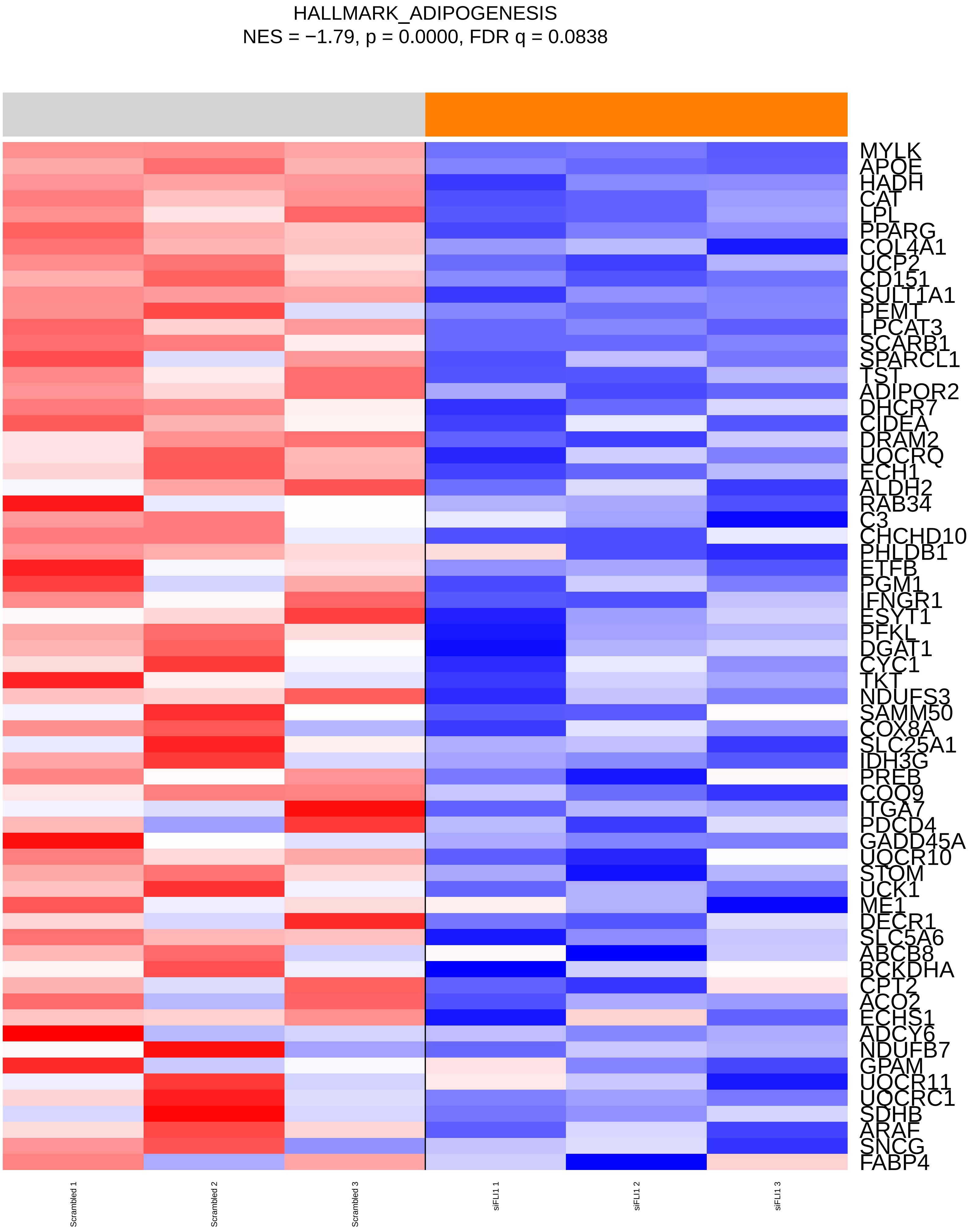
HALLMARK_ADIPOGENESIS
NES = −1.79, p = 0.0000, FDR q = 0.0838
MYLK
APOE
HADH
CAT
LPL
PPARG
COL4A1
UCP2
CD151
SULT1A1
PEMT
LPCAT3
SCARB1
SPARCL1
TST
ADIPOR2
DHCR7
CIDEA
DRAM2
UQCRQ
ECH1
ALDH2
RAB34
C3
CHCHD10
PHLDB1
ETFB
PGM1
IFNGR1
ESYT1
PFKL
DGAT1
CYC1
TKT
NDUFS3
SAMM50
COX8A
SLC25A1
IDH3G
PREB
COQ9
ITGA7
PDCD4
GADD45A
UQCR10
STOM
UCK1
ME1
DECR1
SLC5A6
ABCB8
BCKDHA
CPT2
ACO2
ECHS1
ADCY6
NDUFB7
GPAM
UQCR11
UQCRC1
SDHB
ARAF
SNCG
FABP4
Scrambled 1
Scrambled 2
Scrambled 3
siFLI1 1
siFLI1 2
siFLI1 3

GO_RETROGRADE_VESICLE_MEDIATED_TRANSPORT_GOLGI_TO_ER
NES = −1.79, p = 0.0000, FDR q = 0.0844

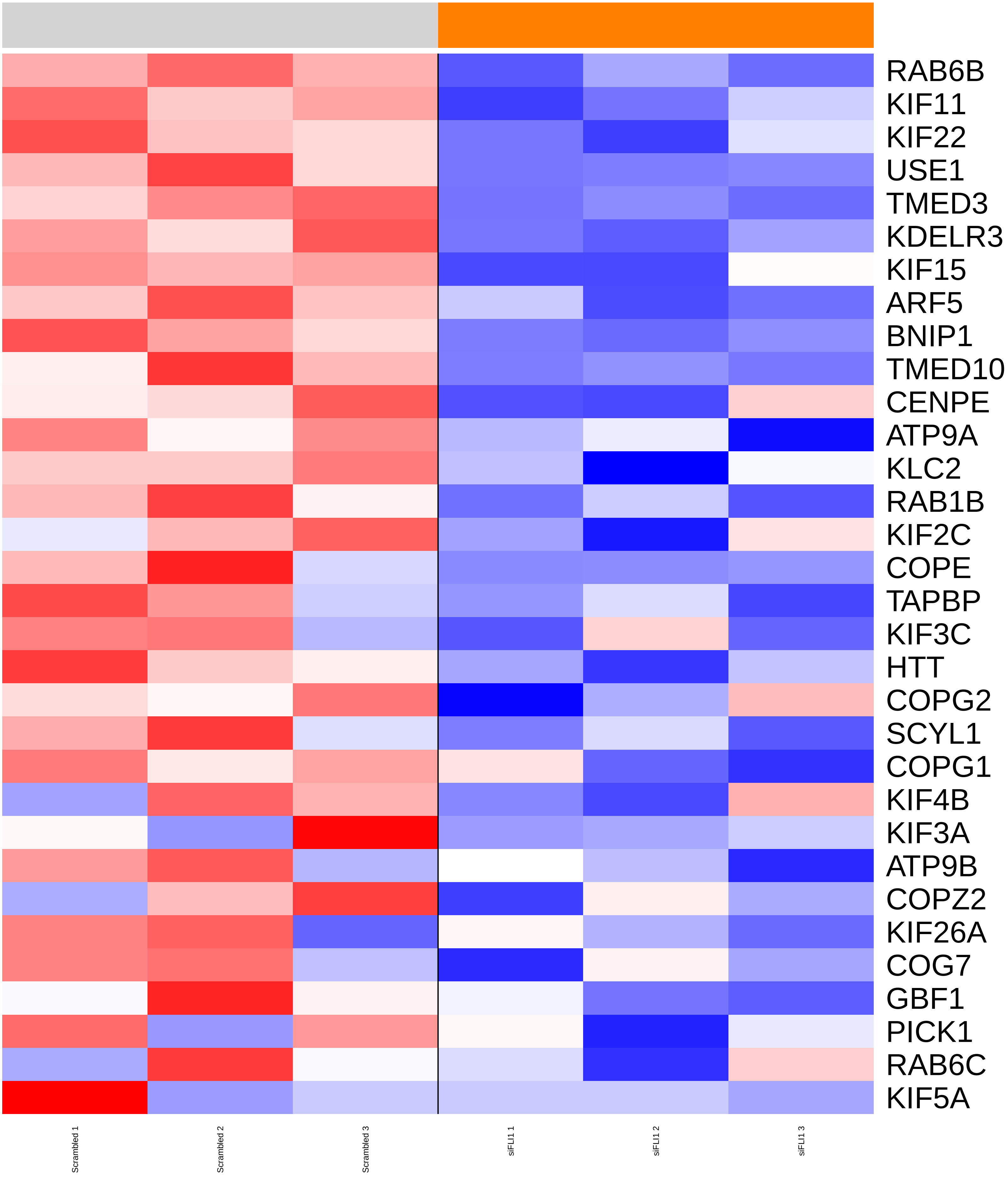

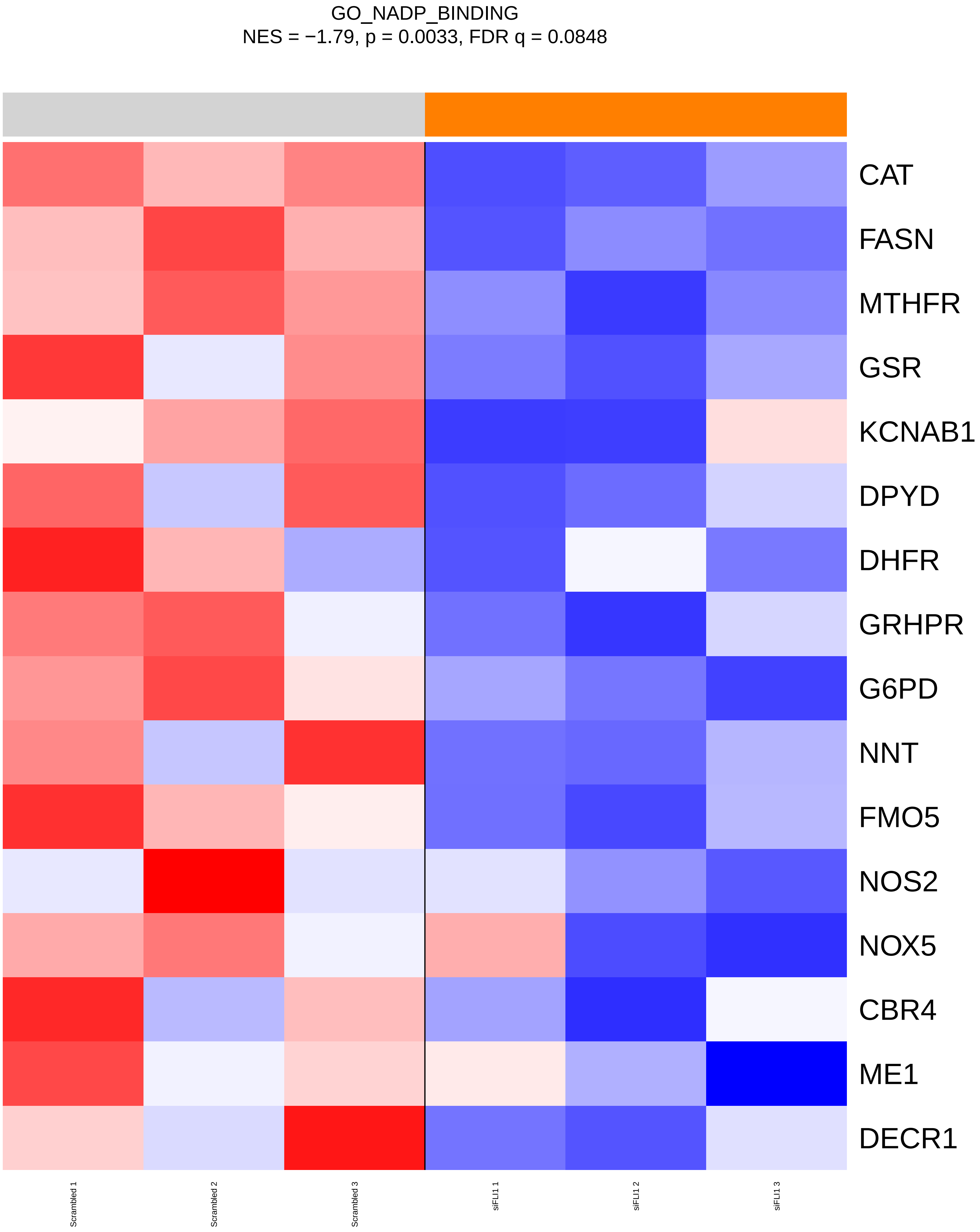
GO_NADP_BINDING
NES = −1.79, p = 0.0033, FDR q = 0.0848
CAT
FASN
MTHFR
GSR
KCNAB1
DPYD
DHFR
GRHPR
G6PD
NNT
FMO5
NOS2
NOX5
CBR4
ME1
DECR1
Scrambled 1
Scrambled 2
Scrambled 3
siFLI1 1
siFLI1 2
siFLI1 3

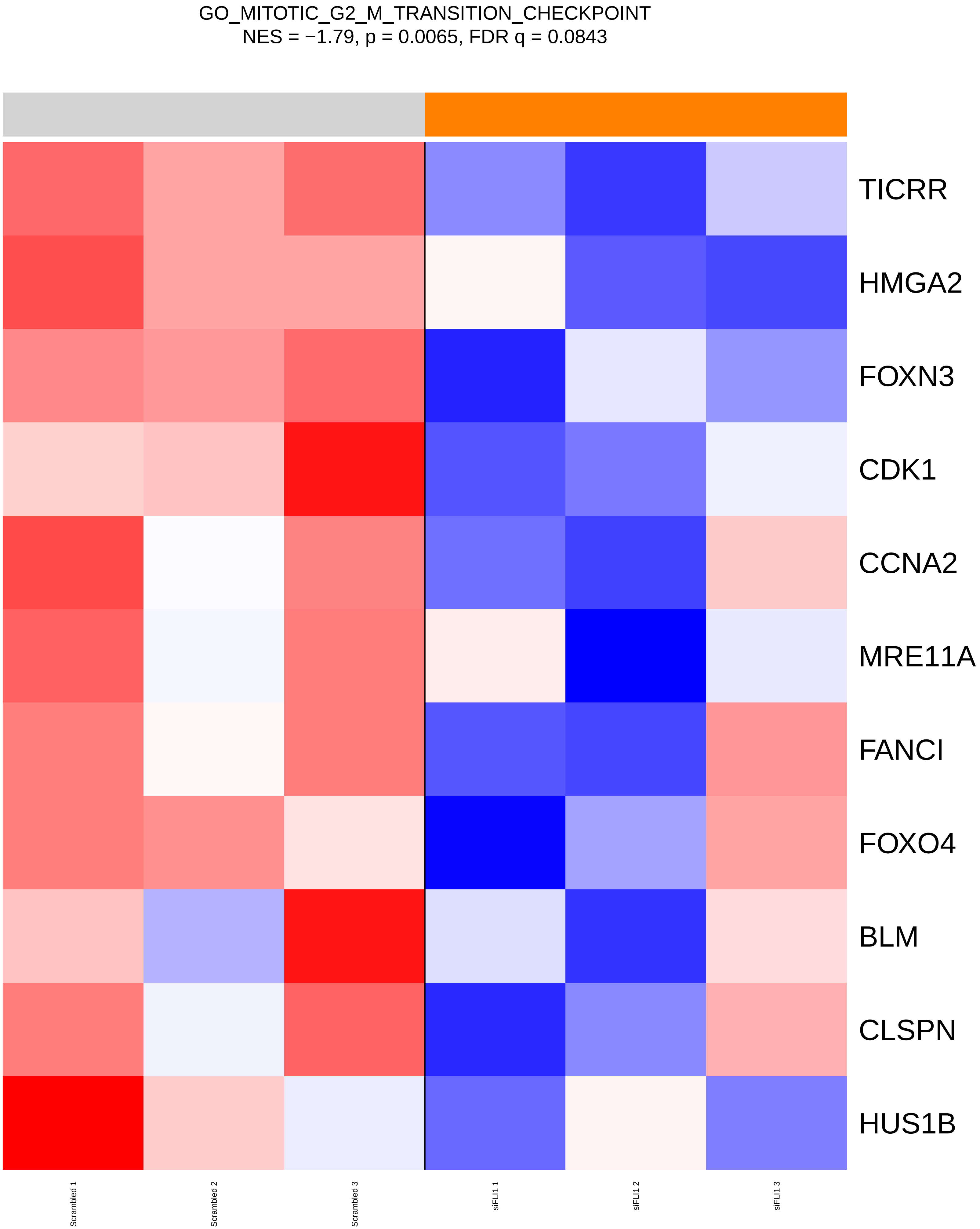
GO_MITOTIC_G2_M_TRANSITION_CHECKPOINT
NES = −1.79, p = 0.0065, FDR q = 0.0843
TICRR
HMGA2
FOXN3
CDK1
CCNA2
MRE11A
FANCI
FOXO4
BLM
CLSPN
HUS1B
Scrambled 1
Scrambled 2
Scrambled 3
siFLI1 1
siFLI1 2
siFLI1 3

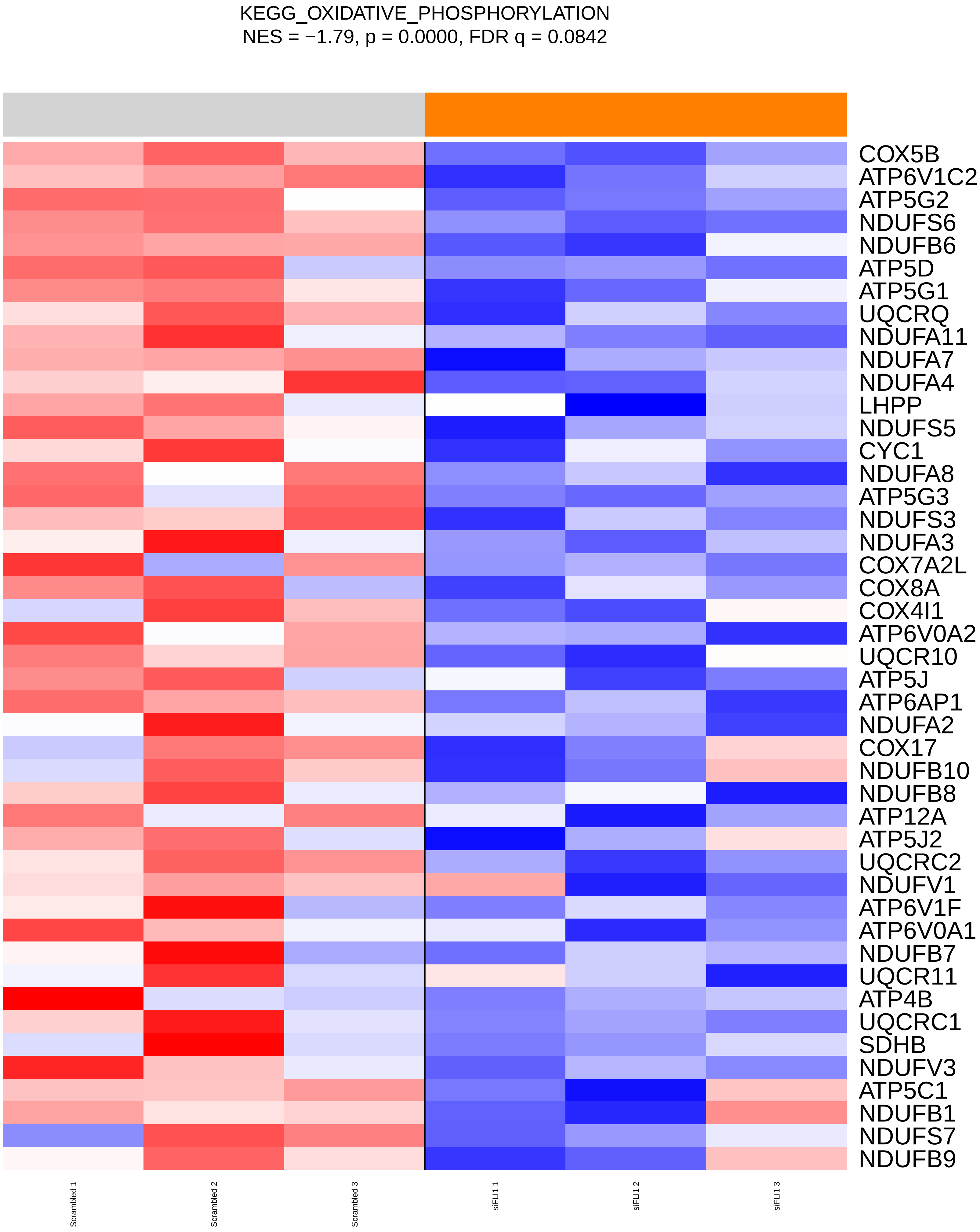
KEGG_OXIDATIVE_PHOSPHORYLATION
NES = −1.79, p = 0.0000, FDR q = 0.0842
COX5B
ATP6V1C2
ATP5G2
NDUFS6
NDUFB6
ATP5D
ATP5G1
UQCRQ
NDUFA11
NDUFA7
NDUFA4
LHPP
NDUFS5
CYC1
NDUFA8
ATP5G3
NDUFS3
NDUFA3
COX7A2L
COX8A
COX4I1
ATP6V0A2
UQCR10
ATP5J
ATP6AP1
NDUFA2
COX17
NDUFB10
NDUFB8
ATP12A
ATP5J2
UQCRC2
NDUFV1
ATP6V1F
ATP6V0A1
NDUFB7
UQCR11
ATP4B
UQCRC1
SDHB
NDUFV3
ATP5C1
NDUFB1
NDUFS7
NDUFB9
Scrambled 1
Scrambled 2
Scrambled 3
siFLI1 1
siFLI1 2
siFLI1 3

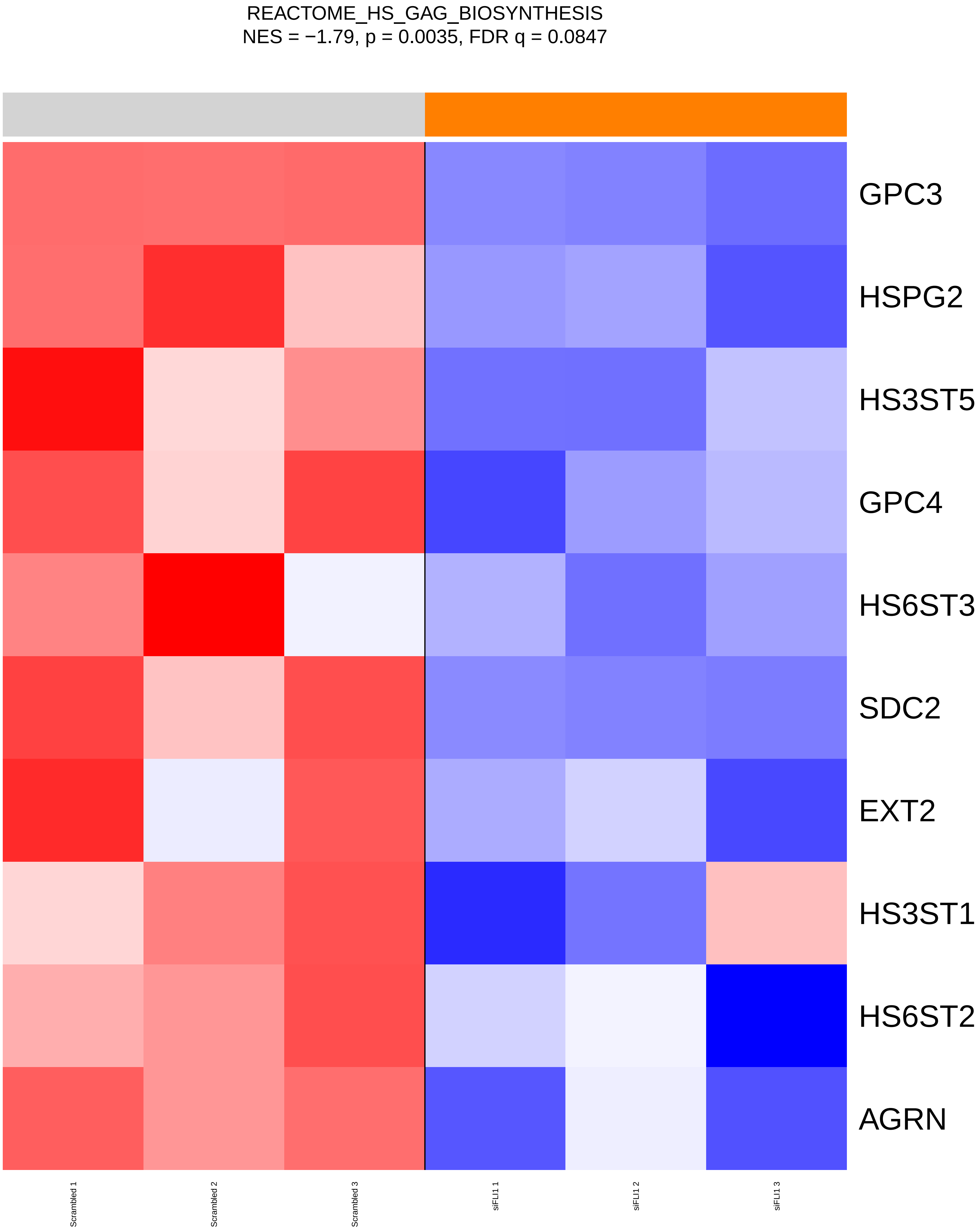
REACTOME_HS_GAG_BIOSYNTHESIS
NES = −1.79, p = 0.0035, FDR q = 0.0847
GPC3
HSPG2
HS3ST5
GPC4
HS6ST3
SDC2
EXT2
HS3ST1
HS6ST2
AGRN
Scrambled 1
Scrambled 2
Scrambled 3
siFLI1 1
siFLI1 2
siFLI1 3

GO_NUCLEAR_CHROMOSOME_SEGREGATION
NES = −1.81, p = 0.0000, FDR q = 0.0722

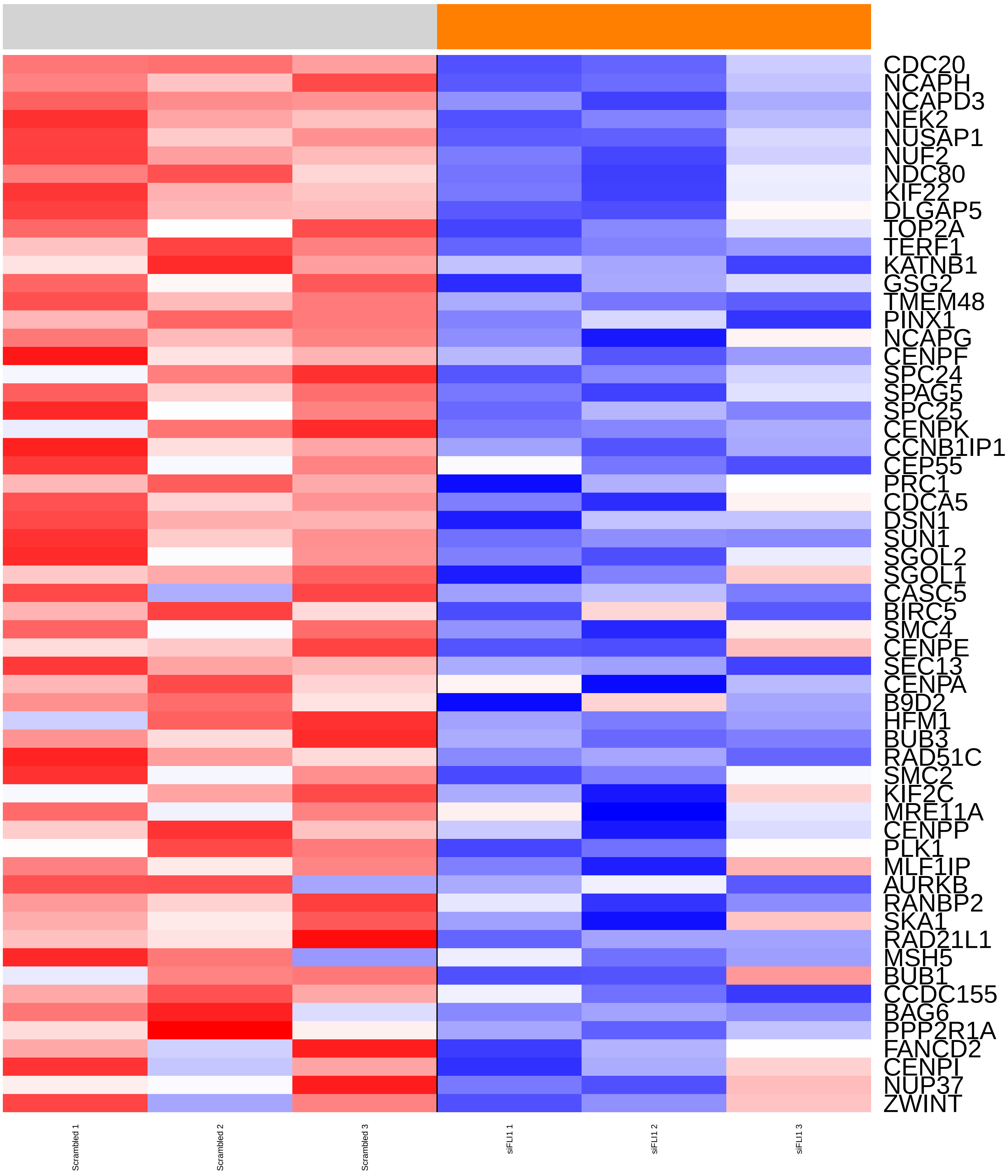

GO_OXIDOREDUCTASE_ACTIVITY_ACTING_ON_THE_CH_CH_GROUP_OF_DONORS_NAD_OR_NADP_AS_ACCEPTOR
NES = −1.81, p = 0.0017, FDR q = 0.0732

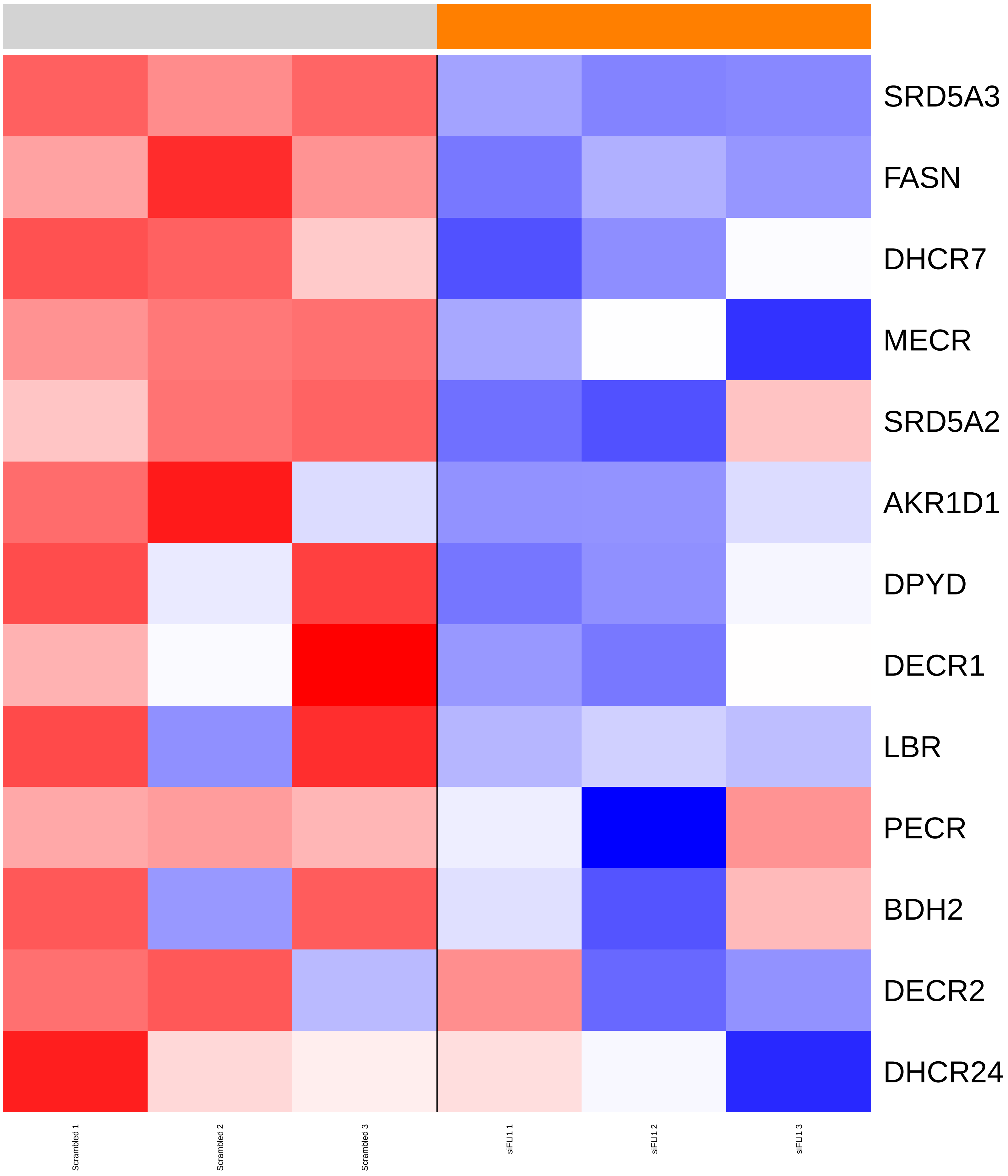

GO_MITOTIC_SISTER_CHROMATID_SEGREGATION
NES = −1.81, p = 0.0000, FDR q = 0.0732

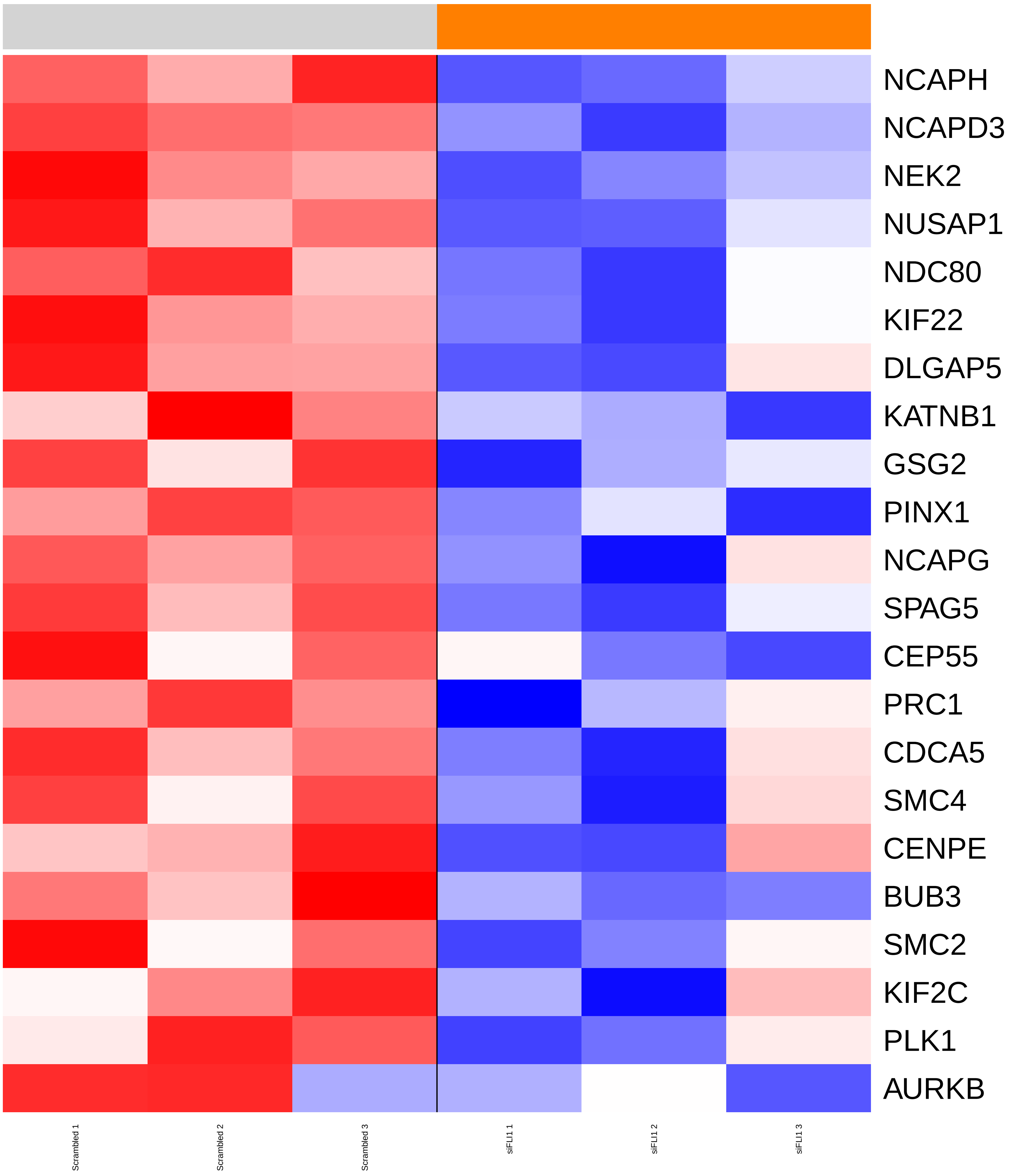

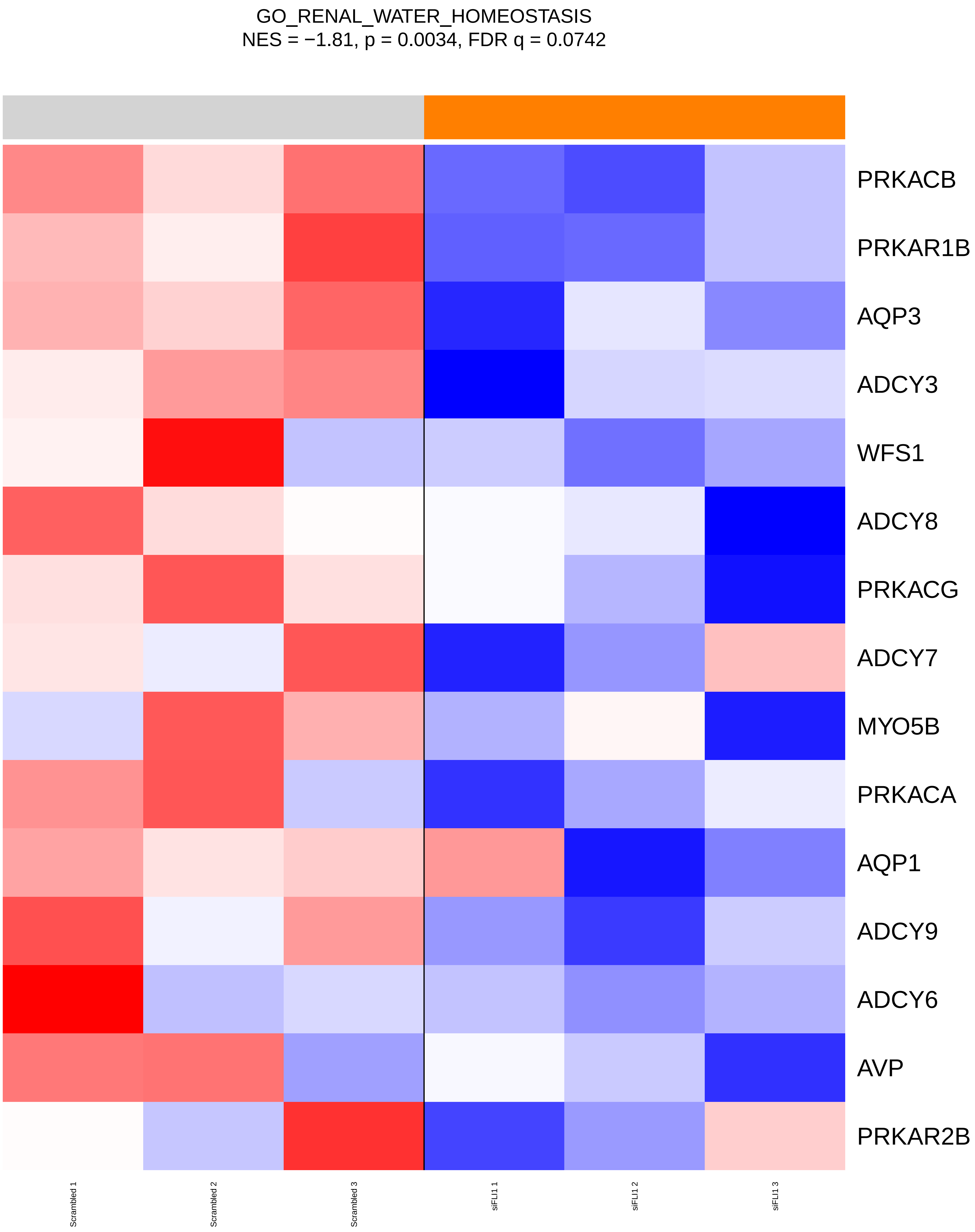
GO_RENAL_WATER_HOMEOSTASIS
NES = −1.81, p = 0.0034, FDR q = 0.0742
PRKACB
PRKAR1B
AQP3
ADCY3
WFS1
ADCY8
PRKACG
ADCY7
MYO5B
PRKACA
AQP1
ADCY9
ADCY6
AVP
PRKAR2B
Scrambled 1
Scrambled 2
Scrambled 3
siFLI1 1
siFLI1 2
siFLI1 3

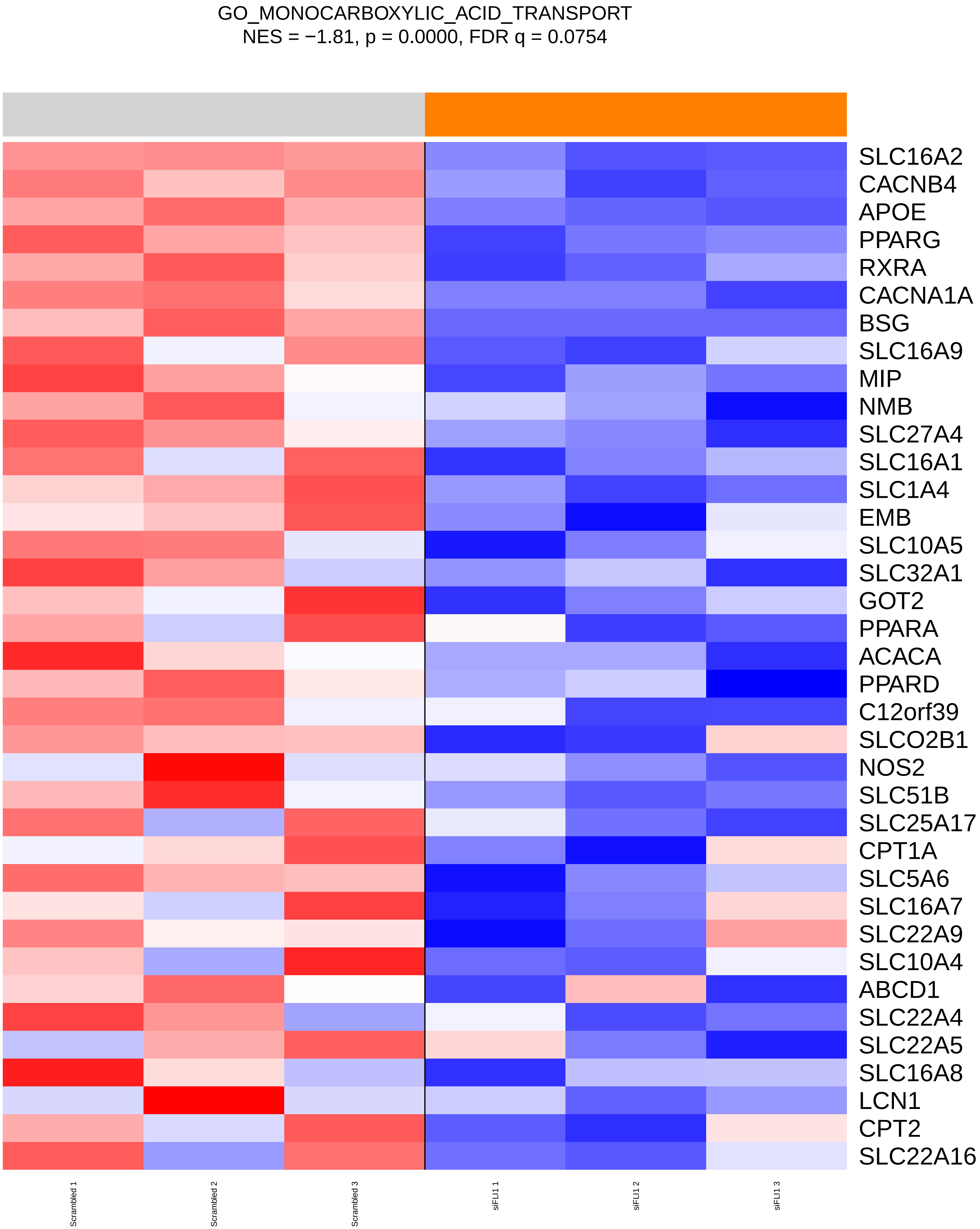
GO_MONOCARBOXYLIC_ACID_TRANSPORT
NES = −1.81, p = 0.0000, FDR q = 0.0754
SLC16A2
CACNB4
APOE
PPARG
RXRA
CACNA1A
BSG
SLC16A9
MIP
NMB
SLC27A4
SLC16A1
SLC1A4
EMB
SLC10A5
SLC32A1
GOT2
PPARA
ACACA
PPARD
C12orf39
SLCO2B1
NOS2
SLC51B
SLC25A17
CPT1A
SLC5A6
SLC16A7
SLC22A9
SLC10A4
ABCD1
SLC22A4
SLC22A5
SLC16A8
LCN1
CPT2
SLC22A16
Scrambled 1
Scrambled 2
Scrambled 3
siFLI1 1
siFLI1 2
siFLI1 3

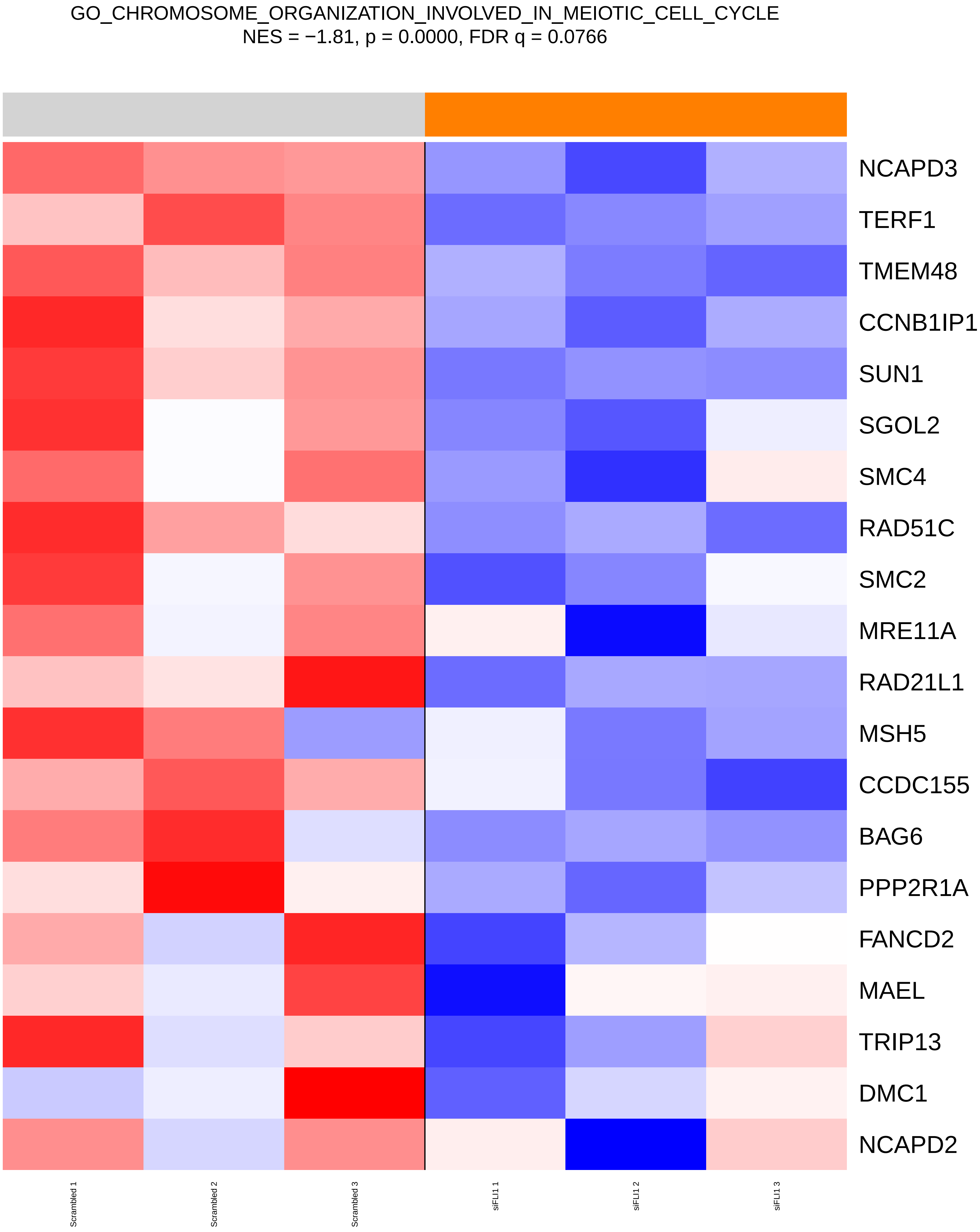
GO_CHROMOSOME_ORGANIZATION_INVOLVED_IN_MEIOTIC_CELL_CYCLE
NES = −1.81, p = 0.0000, FDR q = 0.0766
NCAPD3
TERF1
TMEM48
CCNB1IP1
SUN1
SGOL2
SMC4
RAD51C
SMC2
MRE11A
RAD21L1
MSH5
CCDC155
BAG6
PPP2R1A
FANCD2
MAEL
TRIP13
DMC1
NCAPD2
Scrambled 1
Scrambled 2
Scrambled 3
siFLI1 1
siFLI1 2
siFLI1 3

GO_POSITIVE_REGULATION_OF_NOTCH_SIGNALING_PATHWAY
NES = −1.81, p = 0.0000, FDR q = 0.0754

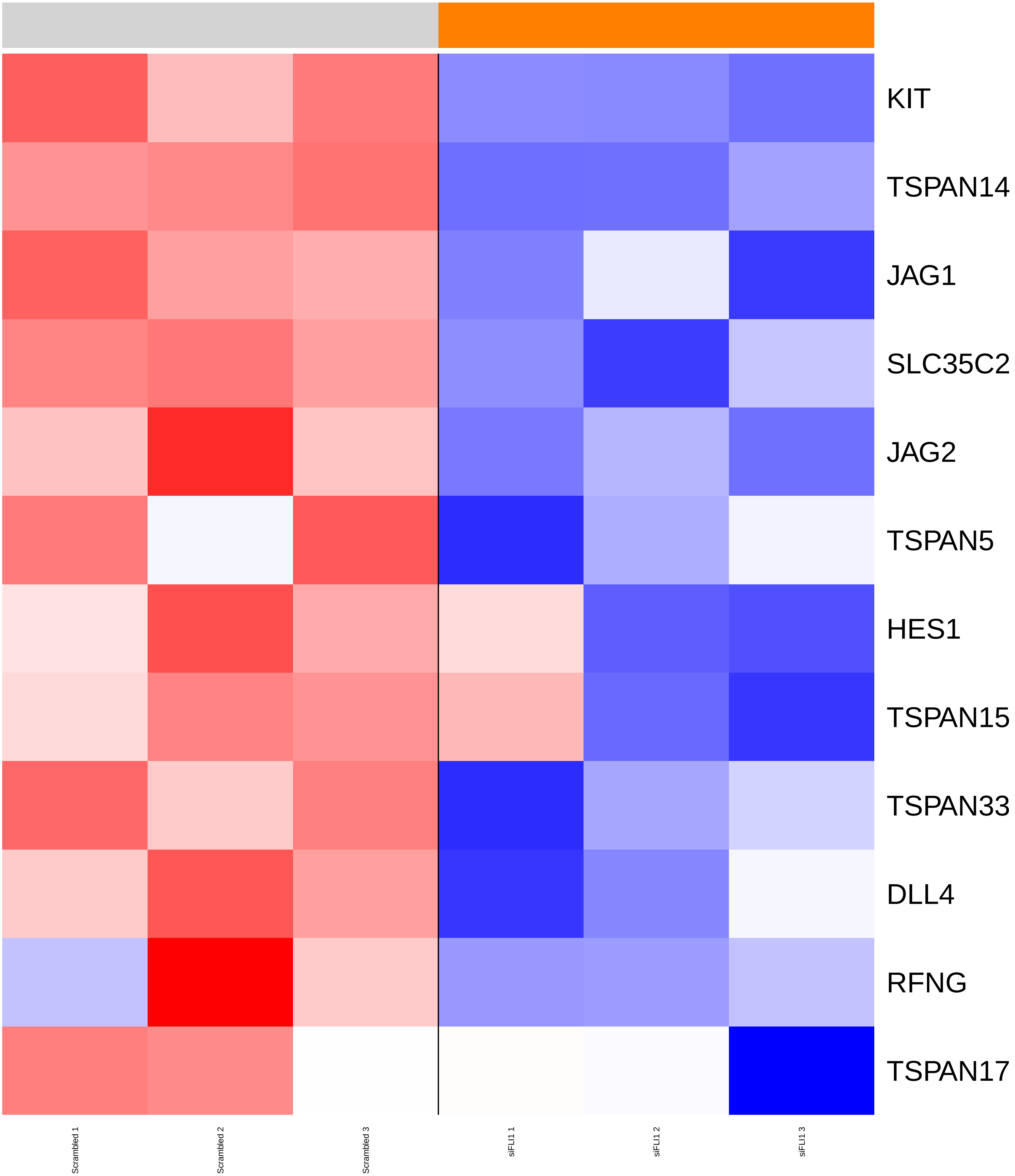

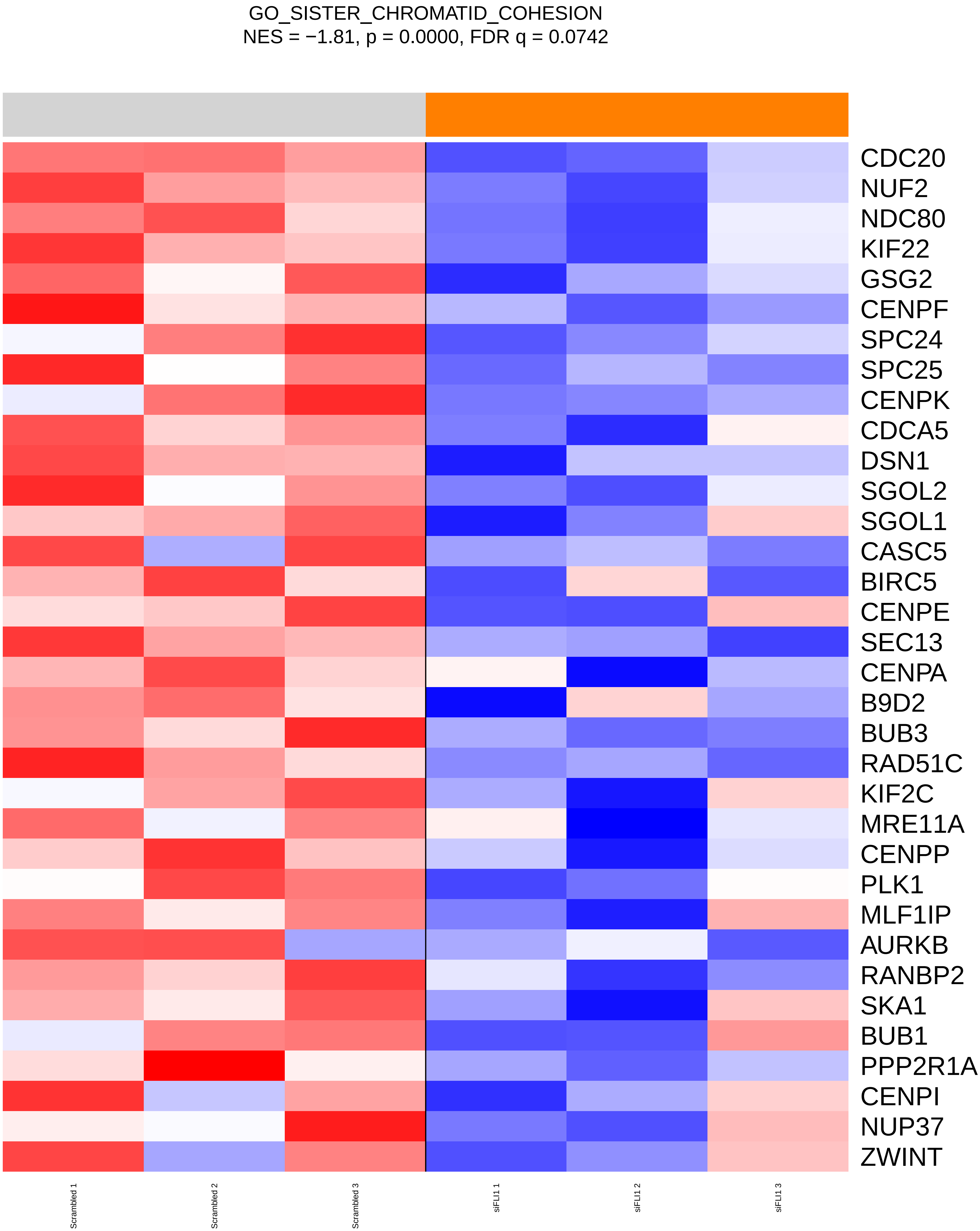
GO_SISTER_CHROMATID_COHESION
NES = −1.81, p = 0.0000, FDR q = 0.0742
CDC20
NUF2
NDC80
KIF22
GSG2
CENPF
SPC24
SPC25
CENPK
CDCA5
DSN1
SGOL2
SGOL1
CASC5
BIRC5
CENPE
SEC13
CENPA
B9D2
BUB3
RAD51C
KIF2C
MRE11A
CENPP
PLK1
MLF1IP
AURKB
RANBP2
SKA1
BUB1
PPP2R1A
CENPI
NUP37
ZWINT
Scrambled 1
Scrambled 2
Scrambled 3
siFLI1 1
siFLI1 2
siFLI1 3

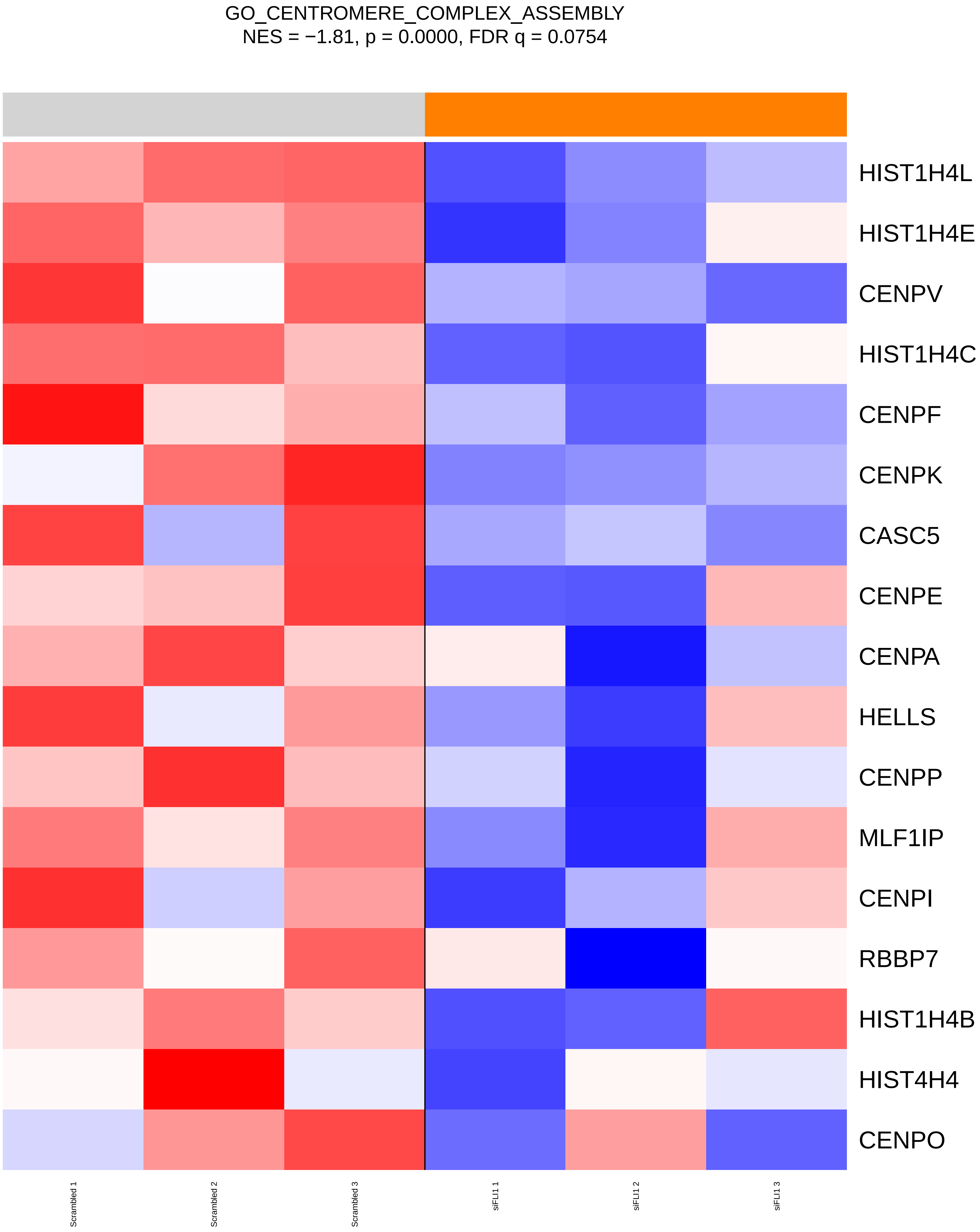

GO_CENTROMERE_COMPLEX_ASSEMBLY
NES = −1.81, p = 0.0000, FDR q = 0.0754
HIST1H4L
HIST1H4E
CENPV
HIST1H4C
CENPF
CENPK
CASC5
CENPE
CENPA
HELLS
CENPP
MLF1IP
CENPI
RBBP7
HIST1H4B
HIST4H4
CENPO
Scrambled 1
Scrambled 2
Scrambled 3
siFLI1 1
siFLI1 2
siFLI1 3

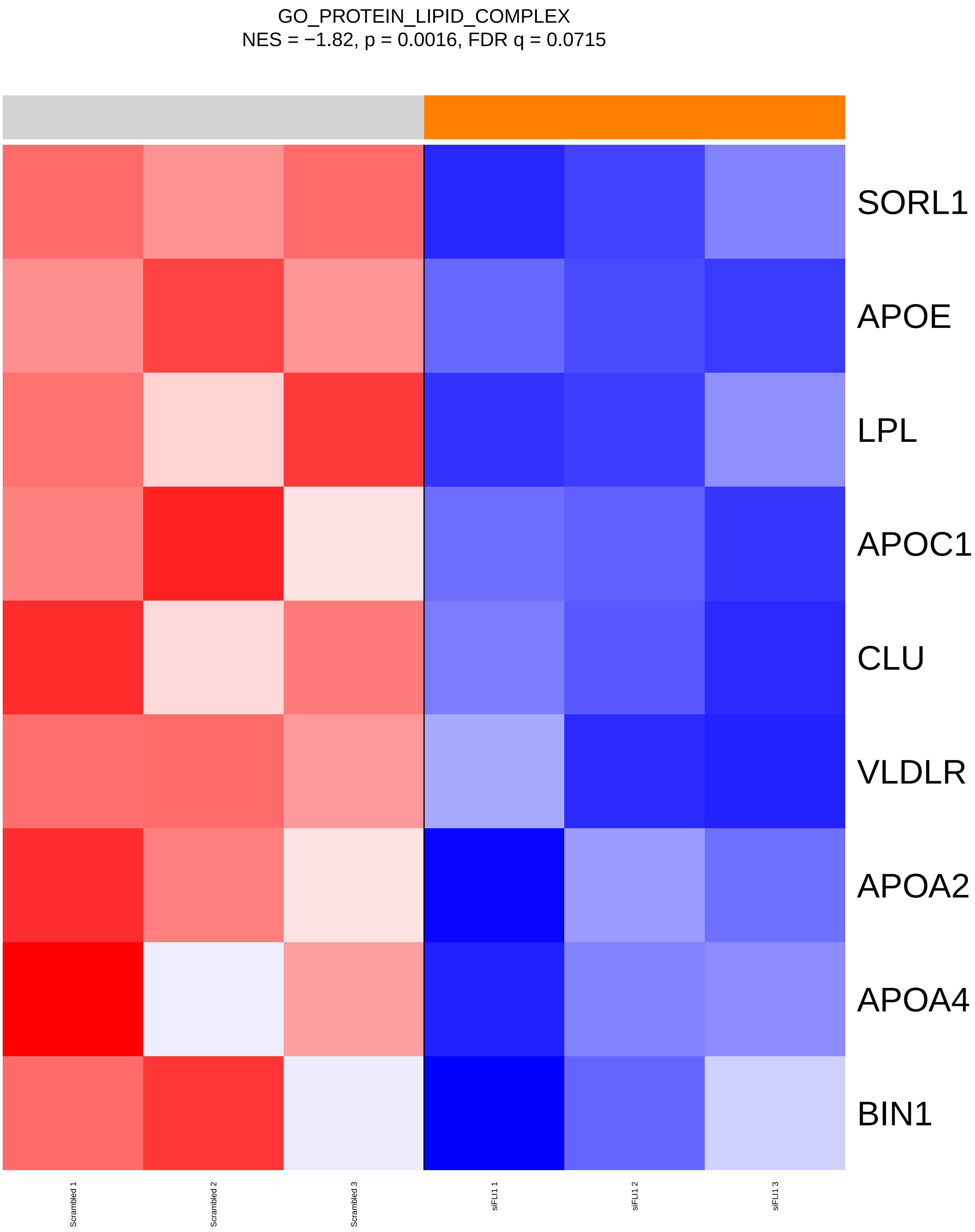
GO_PROTEIN_LIPID_COMPLEX
NES = −1.82, p = 0.0016, FDR q = 0.0715
SORL1
APOE
LPL
APOC1
CLU
VLDLR
APOA2
APOA4
BIN1
Scrambled 1
Scrambled 2
Scrambled 3
siFLI1 1
siFLI1 2
siFLI1 3

REACTOME_AMINO_ACID_SYNTHESIS_AND_INTERCONVERSION_TRANSAMINATION
NES = −1.83, p = 0.0000, FDR q = 0.0657

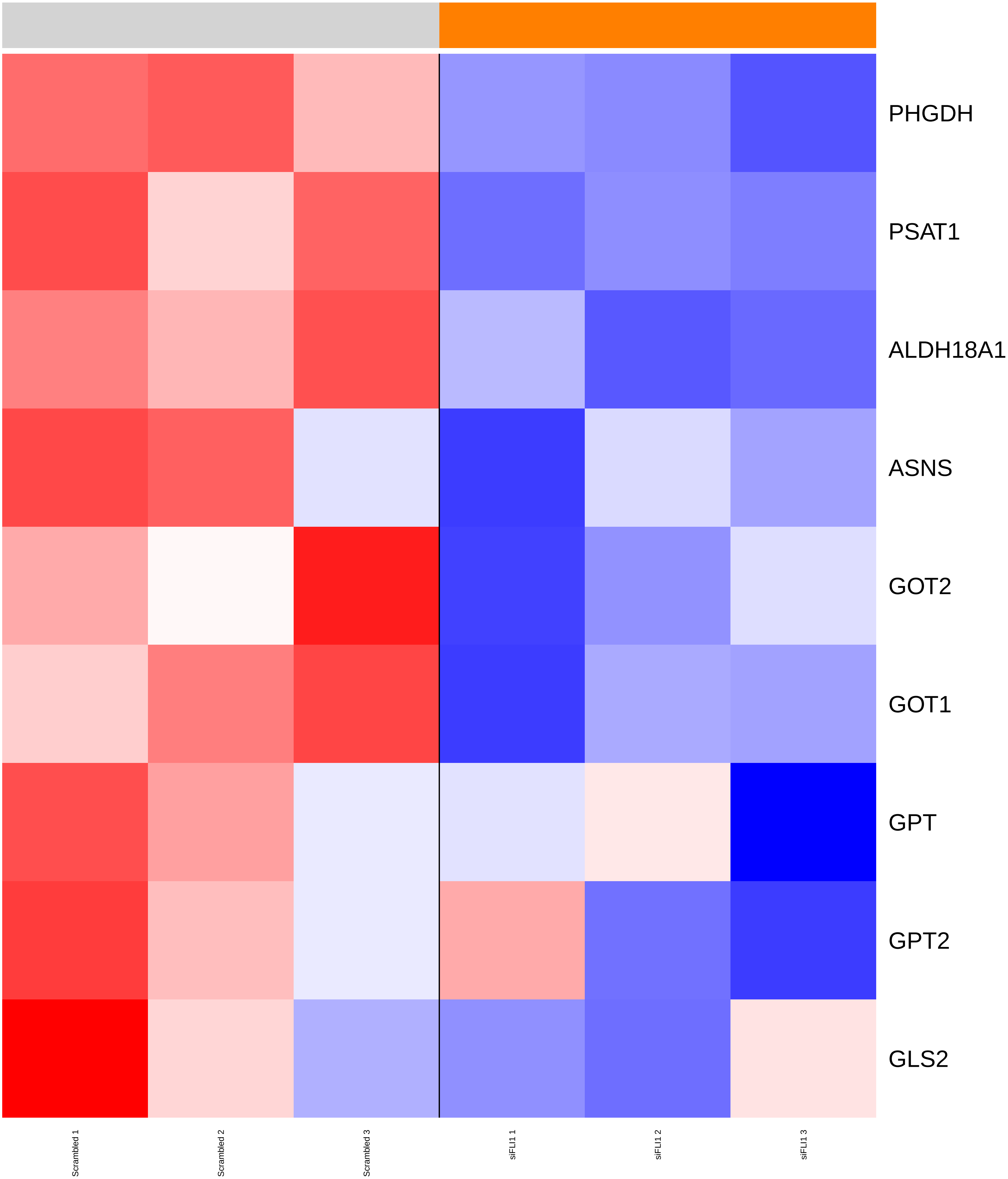

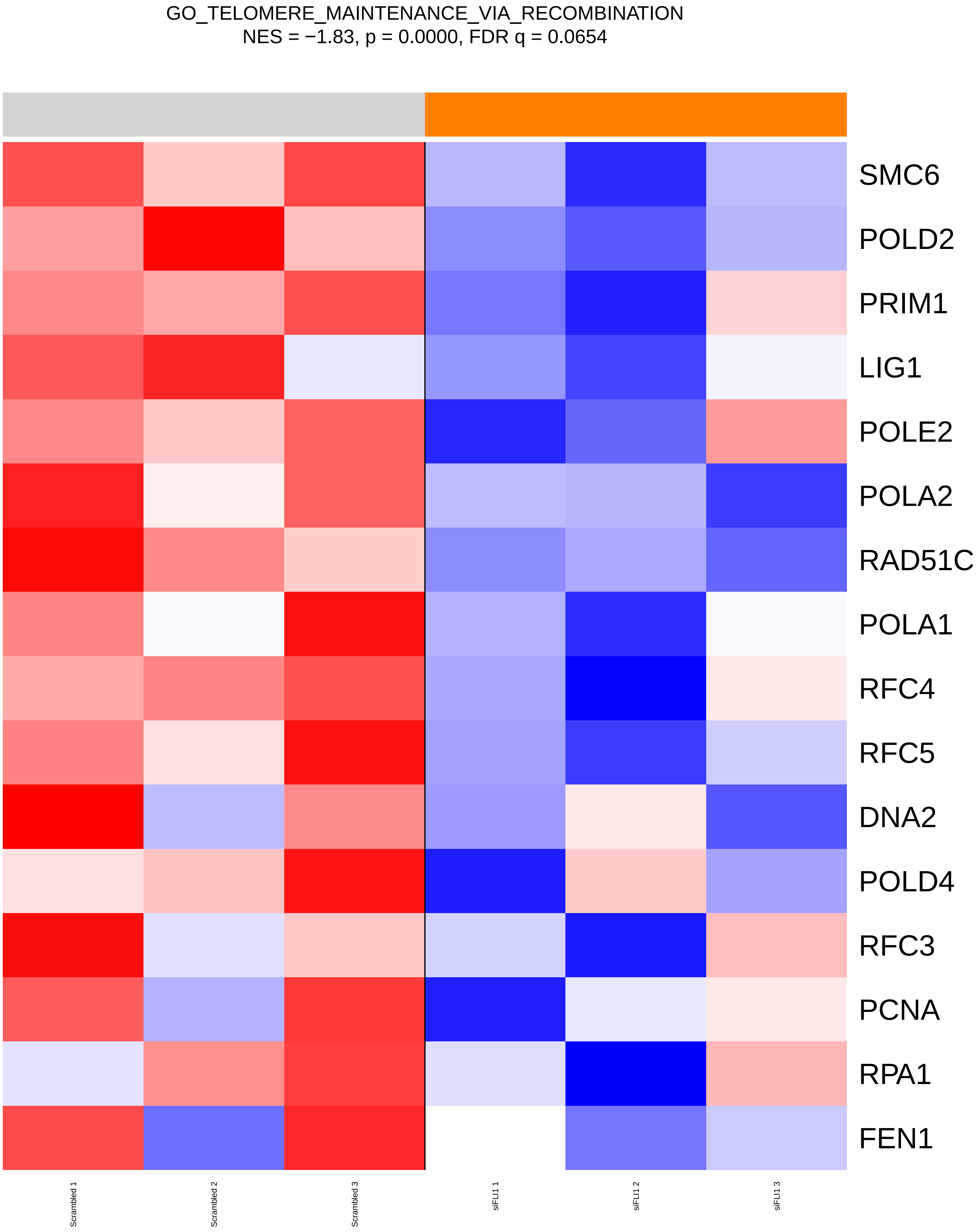
GO_TELOMERE_MAINTENANCE_VIA_RECOMBINATION
NES = −1.83, p = 0.0000, FDR q = 0.0654
SMC6
POLD2
PRIM1
LIG1
POLE2
POLA2
RAD51C
POLA1
RFC4
RFC5
DNA2
POLD4
RFC3
PCNA
RPA1
FEN1
Scrambled 1
Scrambled 2
Scrambled 3
siFLI1 1
siFLI1 2
siFLI1 3

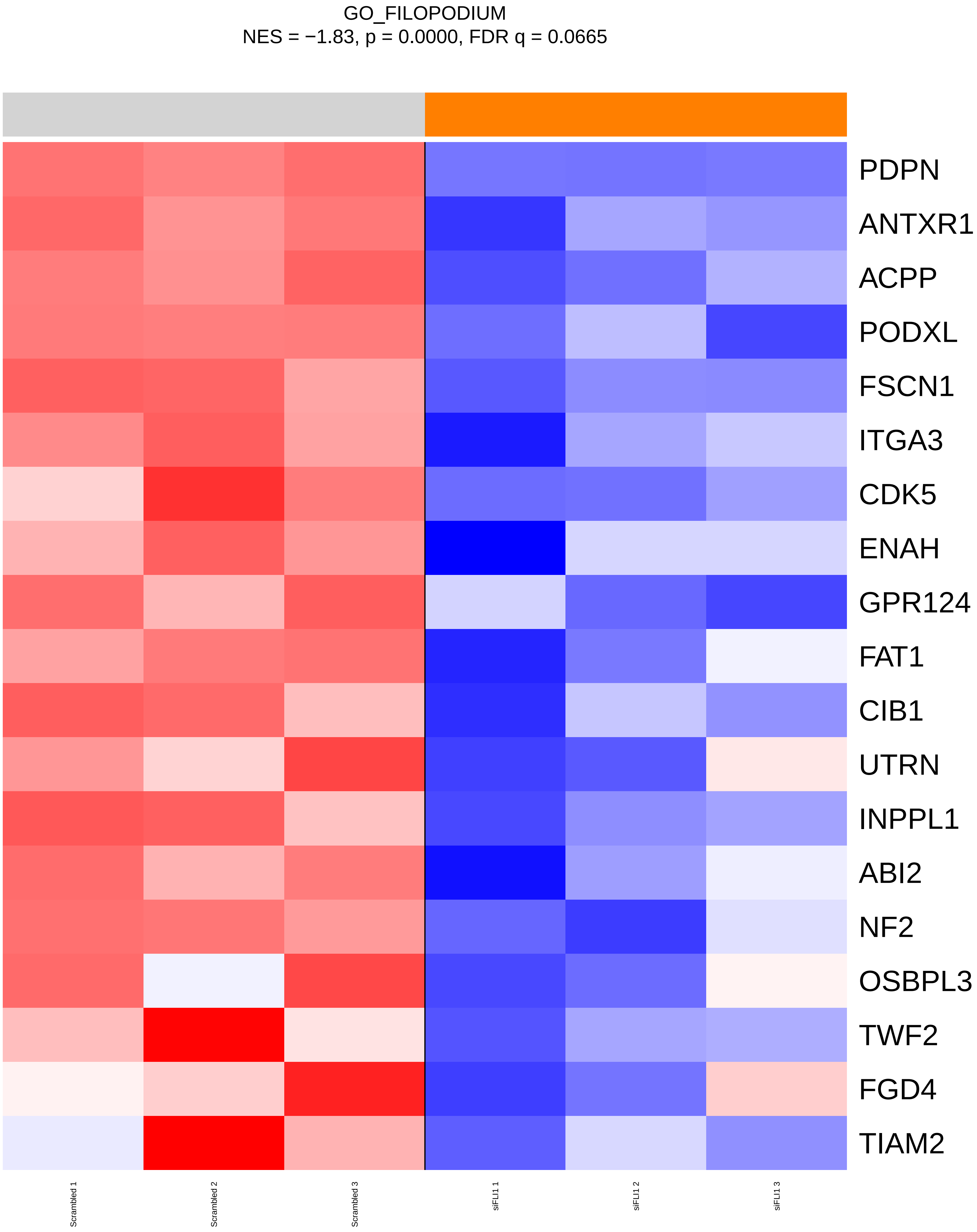
GO_FILOPODIUM
NES = −1.83, p = 0.0000, FDR q = 0.0665
PDPN
ANTXR1
ACPP
PODXL
FSCN1
ITGA3
CDK5
ENAH
GPR124
FAT1
CIB1
UTRN
INPPL1
ABI2
NF2
OSBPL3
TWF2
FGD4
TIAM2
Scrambled 1
Scrambled 2
Scrambled 3
siFLI1 1
siFLI1 2
siFLI1 3

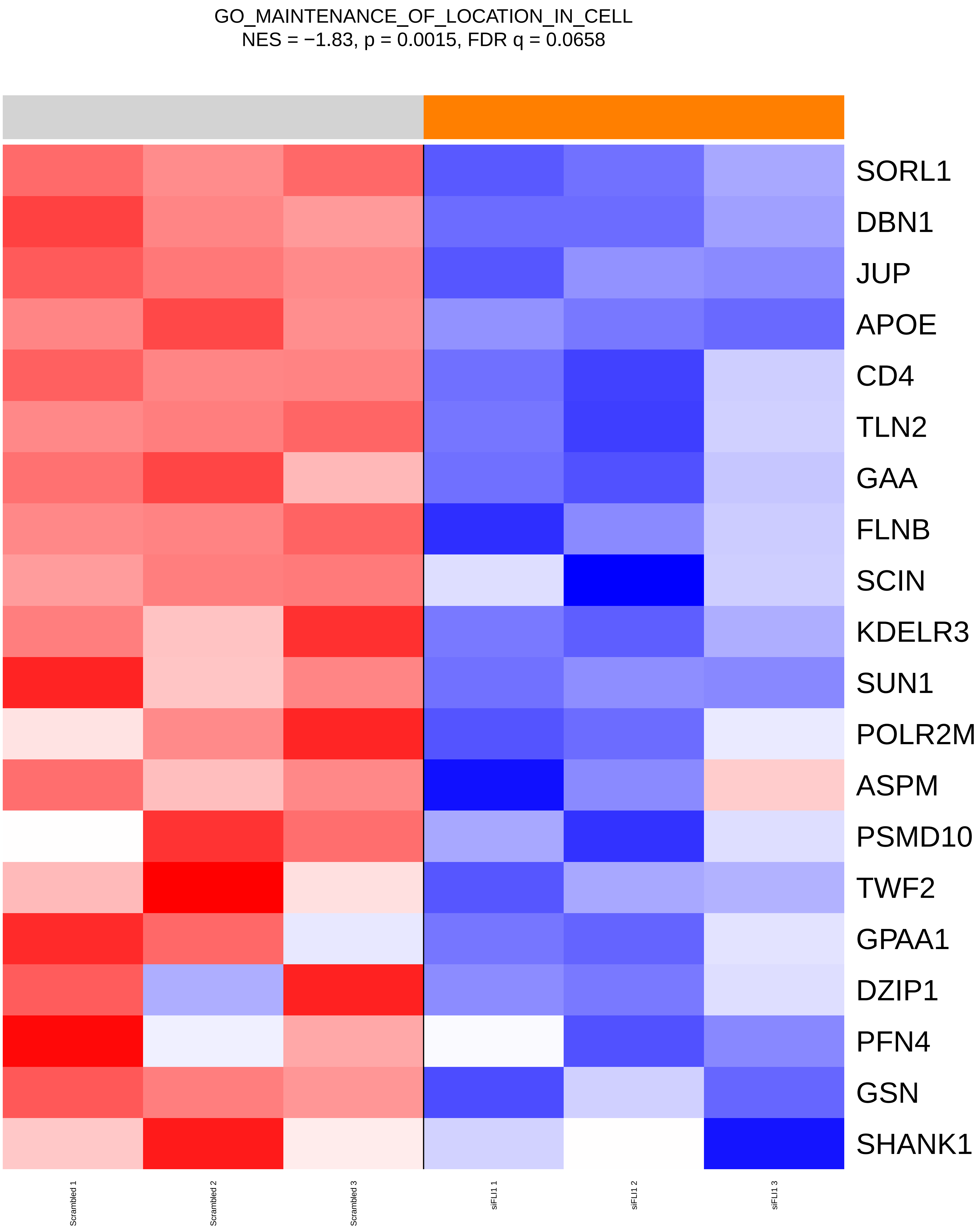
GO_MAINTENANCE_OF_LOCATION_IN_CELL
NES = −1.83, p = 0.0015, FDR q = 0.0658
SORL1
DBN1
JUP
APOE
CD4
TLN2
GAA
FLNB
SCIN
KDELR3
SUN1
POLR2M
ASPM
PSMD10
TWF2
GPAA1
DZIP1
PFN4
GSN
SHANK1
Scrambled 1
Scrambled 2
Scrambled 3
siFLI1 1
siFLI1 2
siFLI1 3

HALLMARK_WNT_BETA_CATENIN_SIGNALING
NES = −1.83, p = 0.0016, FDR q = 0.0669

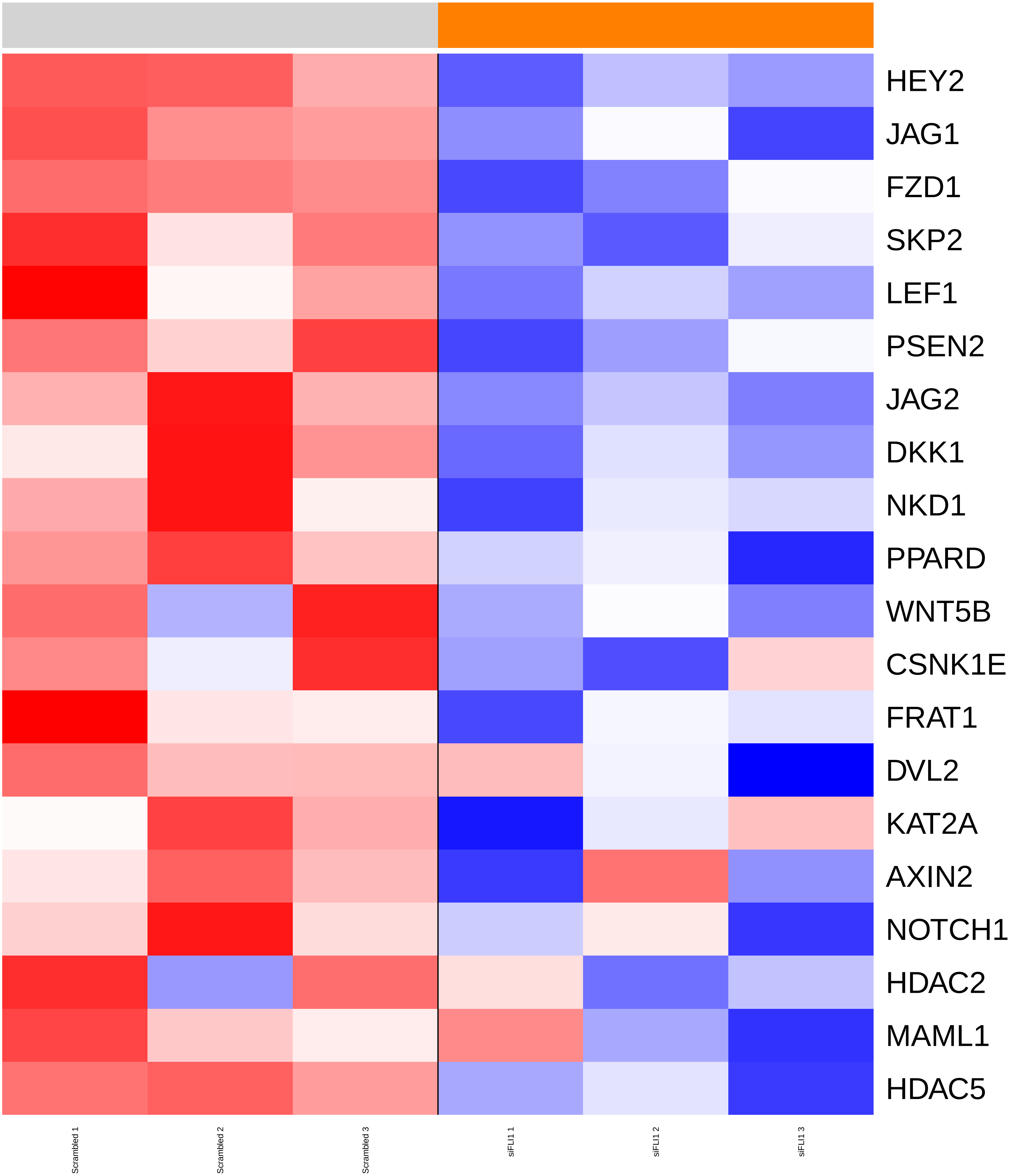

GO_DNA_STRAND_ELONGATION_INVOLVED_IN_DNA_REPLICATION
NES = −1.83, p = 0.0000, FDR q = 0.0677

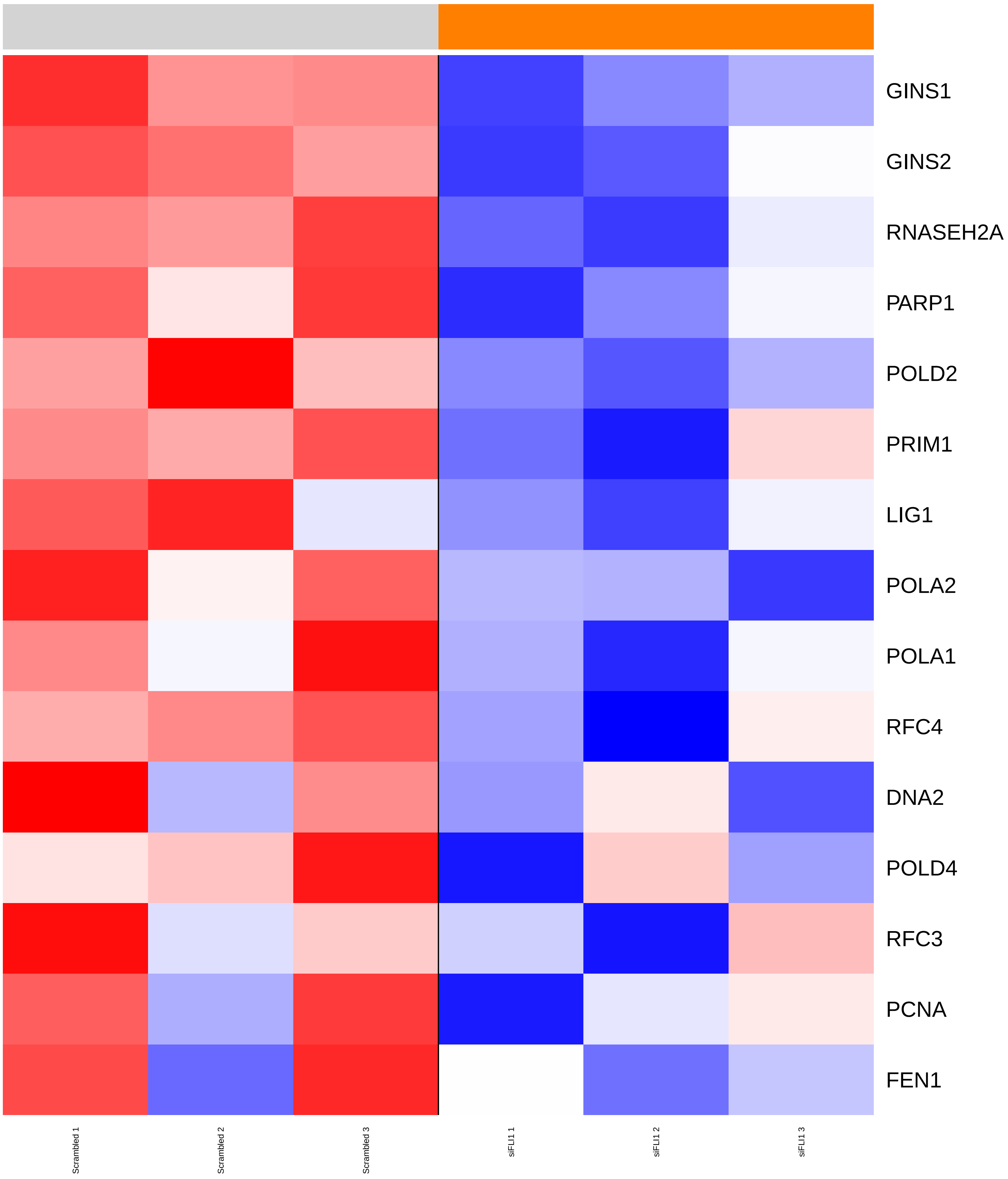

GO_OXIDATIVE_PHOSPHORYLATION
NES = −1.84, p = 0.0015, FDR q = 0.0647

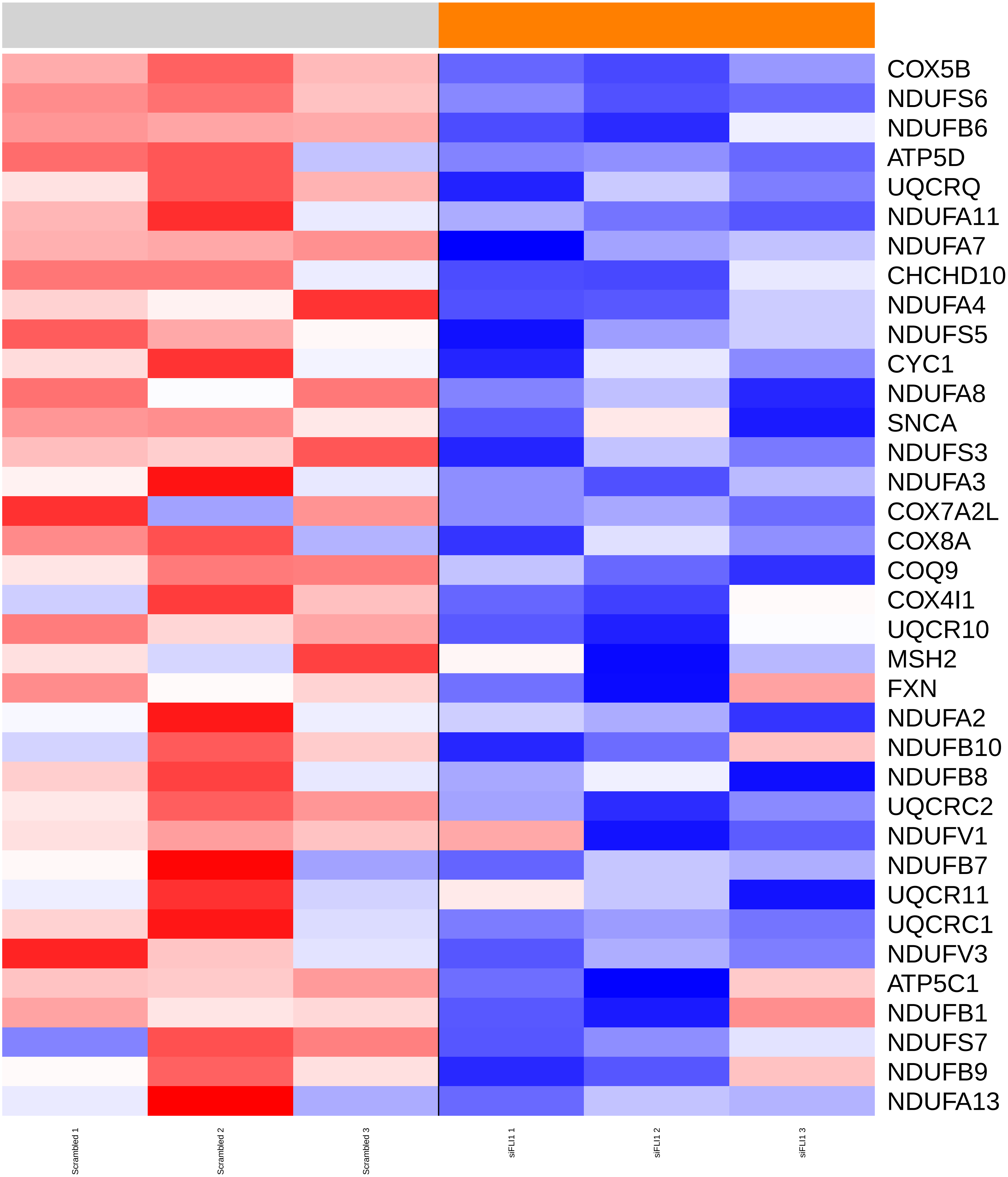

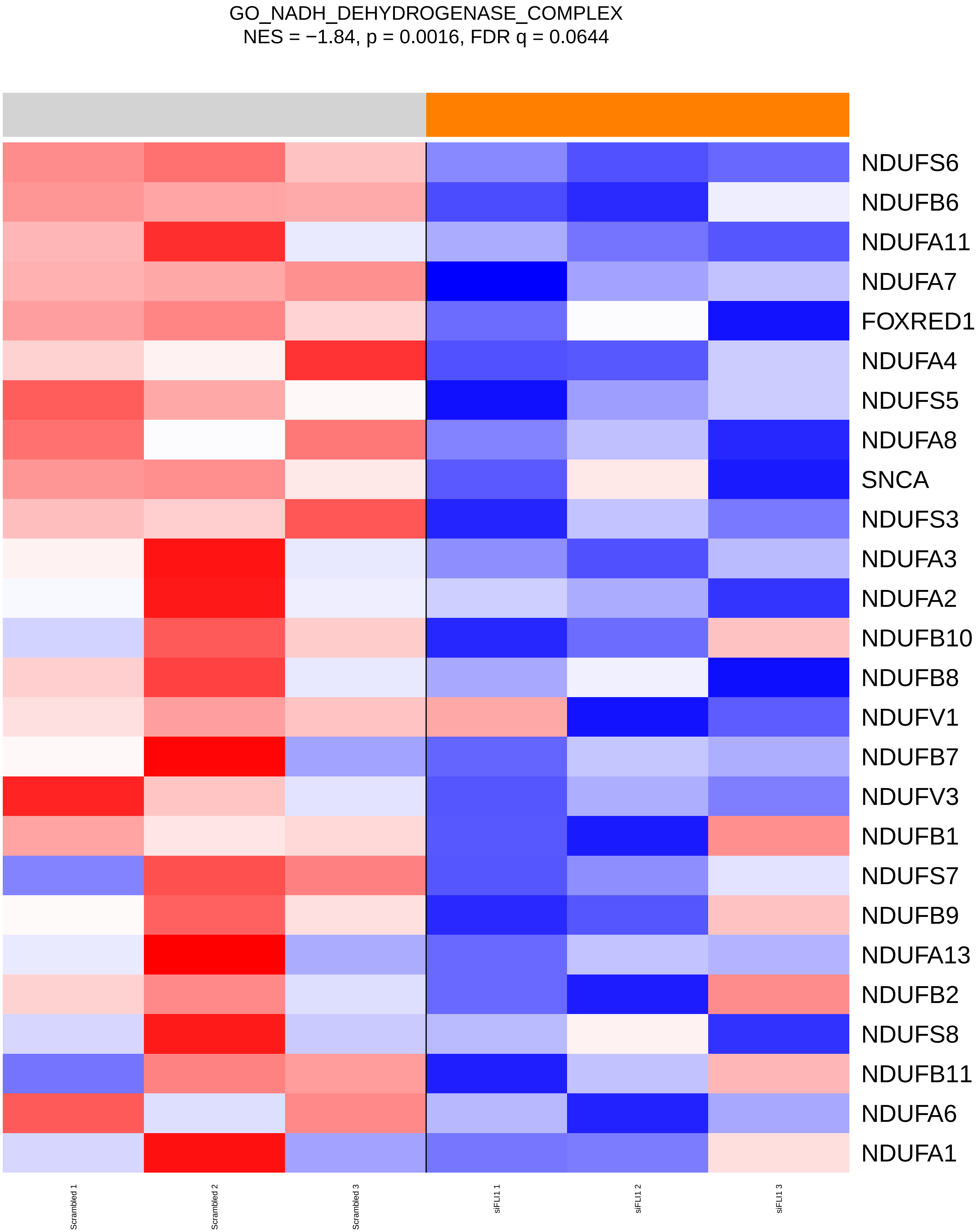
GO_NADH_DEHYDROGENASE_COMPLEX
NES = −1.84, p = 0.0016, FDR q = 0.0644
NDUFS6
NDUFB6
NDUFA11
NDUFA7
FOXRED1
NDUFA4
NDUFS5
NDUFA8
SNCA
NDUFS3
NDUFA3
NDUFA2
NDUFB10
NDUFB8
NDUFV1
NDUFB7
NDUFV3
NDUFB1
NDUFS7
NDUFB9
NDUFA13
NDUFB2
NDUFS8
NDUFB11
NDUFA6
NDUFA1
Scrambled 1
Scrambled 2
Scrambled 3
siFLI1 1
siFLI1 2
siFLI1 3

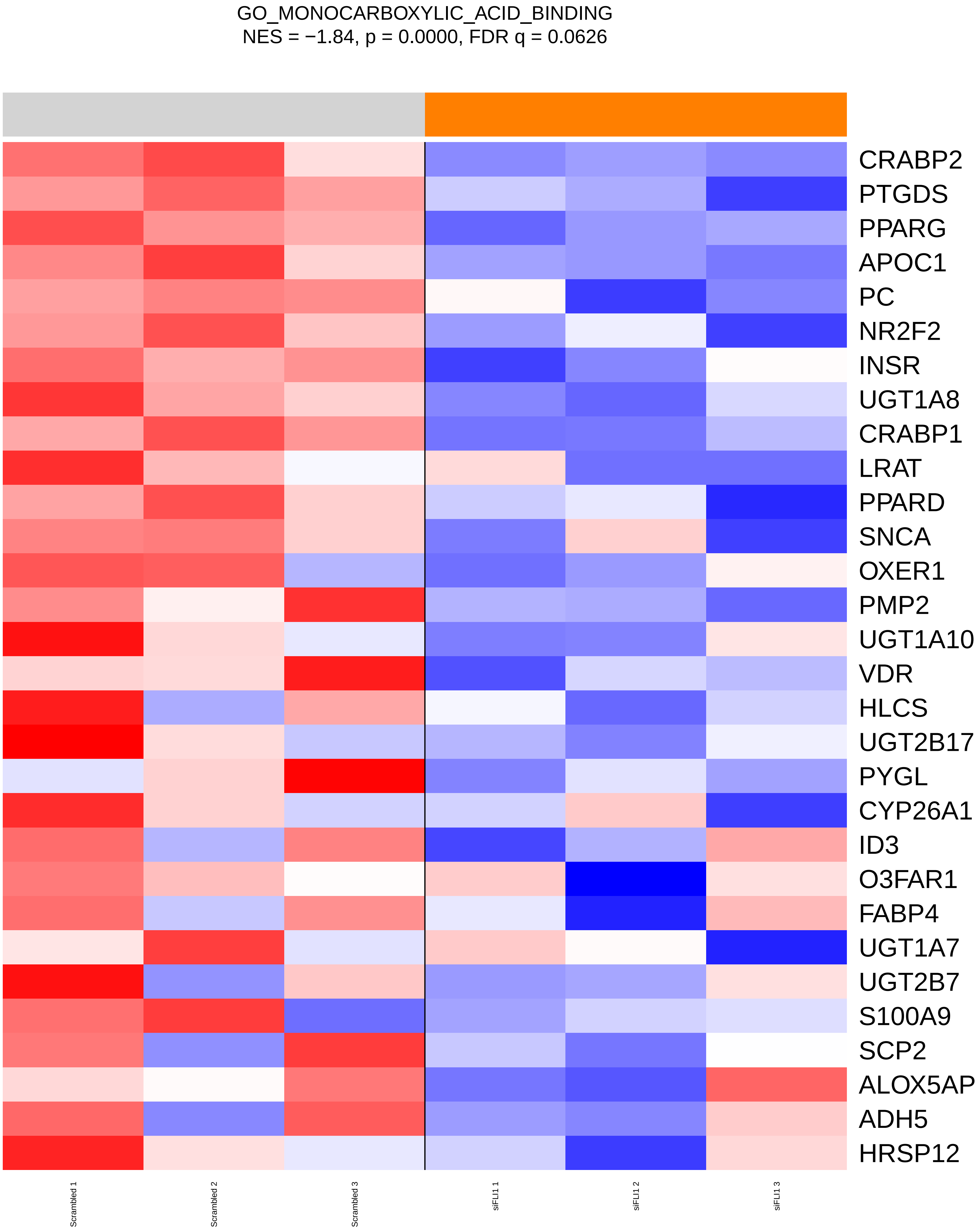
GO_MONOCARBOXYLIC_ACID_BINDING
NES = −1.84, p = 0.0000, FDR q = 0.0626
CRABP2
PTGDS
PPARG
APOC1
PC
NR2F2
INSR
UGT1A8
CRABP1
LRAT
PPARD
SNCA
OXER1
PMP2
UGT1A10
VDR
HLCS
UGT2B17
PYGL
CYP26A1
ID3
O3FAR1
FABP4
UGT1A7
UGT2B7
S100A9
SCP2
ALOX5AP
ADH5
HRSP12
Scrambled 1
Scrambled 2
Scrambled 3
siFLI1 1
siFLI1 2
siFLI1 3

KEGG_BIOSYNTHESIS_OF_UNSATURATED_FATTY_ACIDS
NES = −1.84, p = 0.0034, FDR q = 0.0639

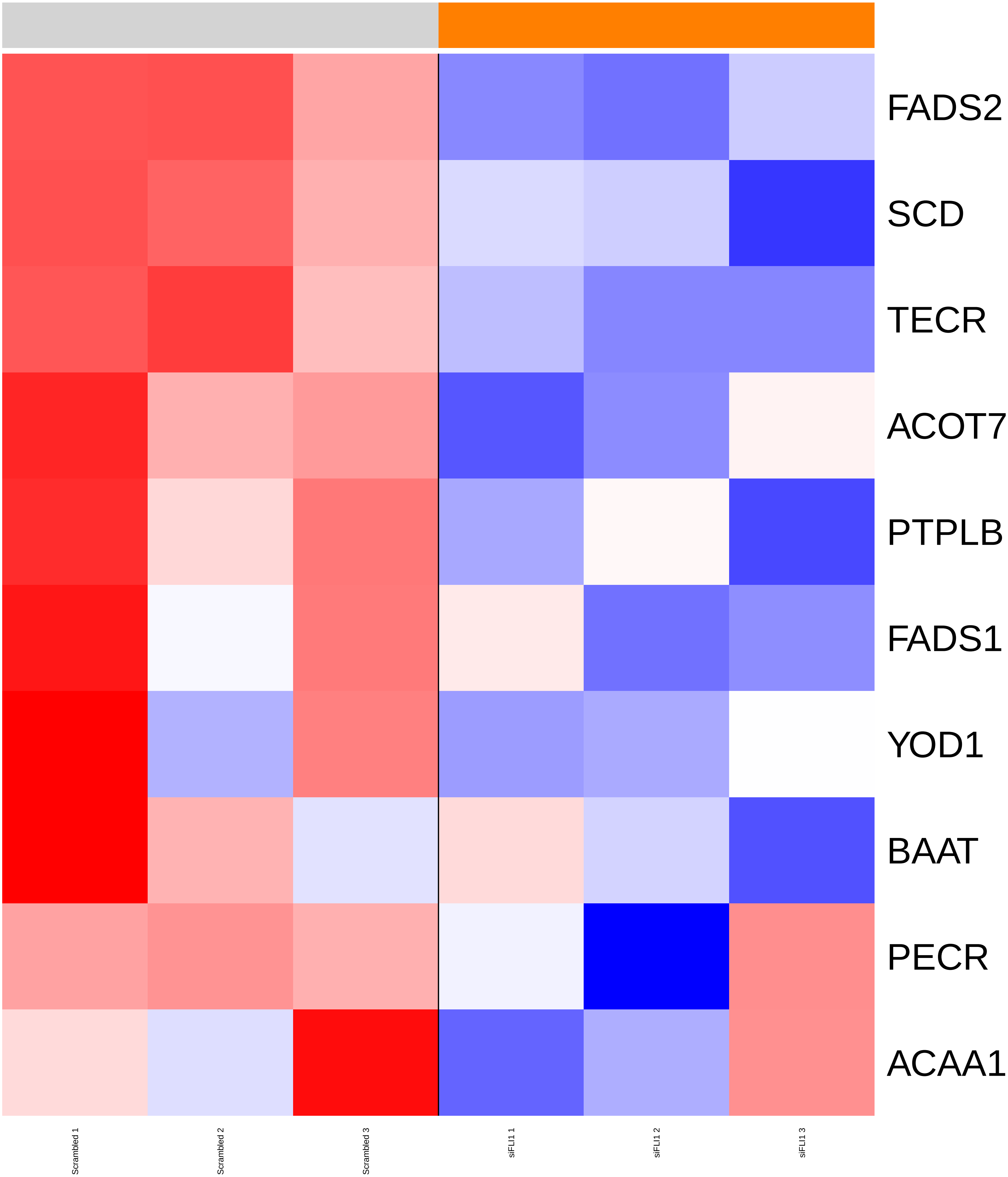

REACTOME_G1_S_SPECIFIC_TRANSCRIPTION
NES = −1.84, p = 0.0000, FDR q = 0.0650

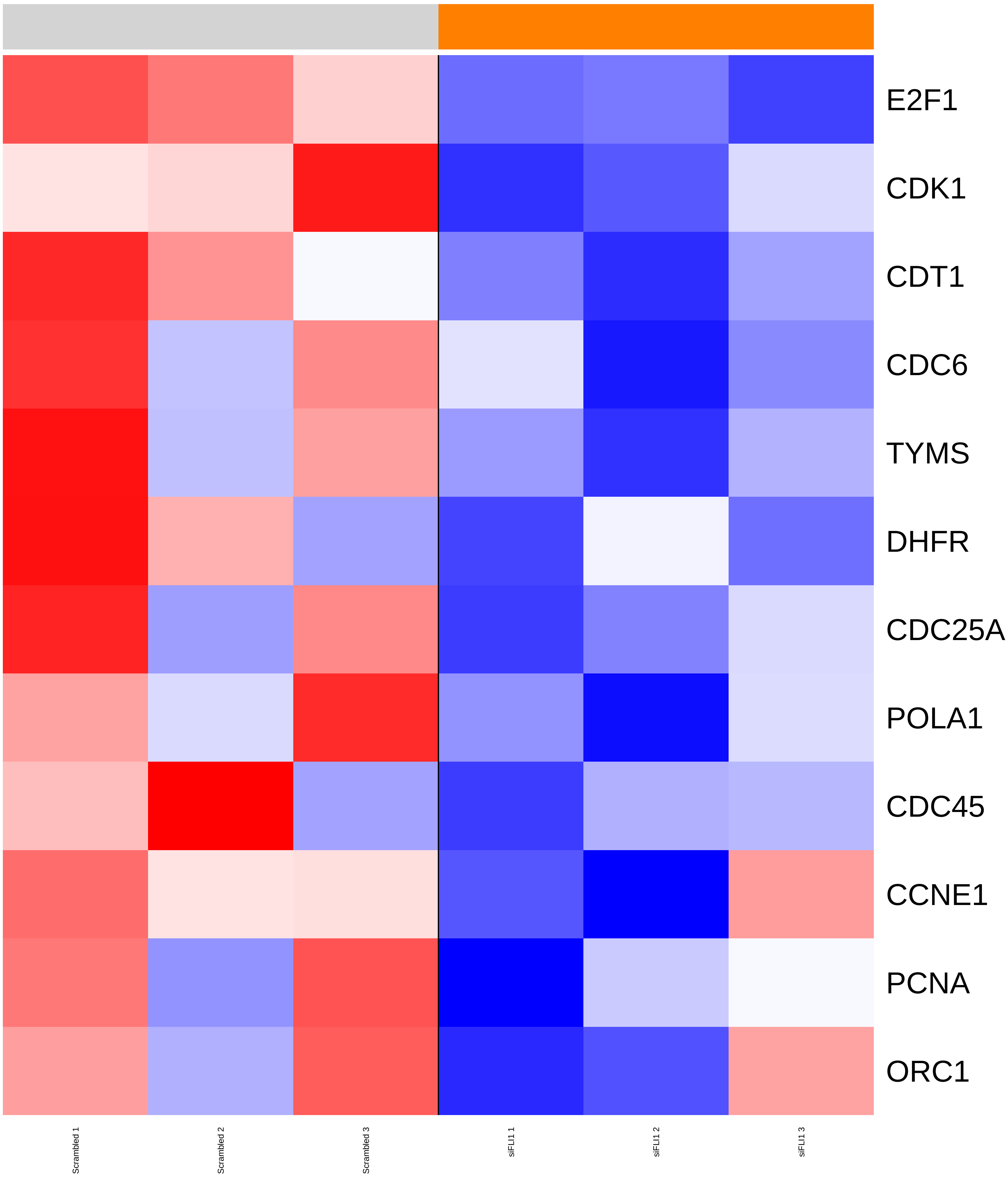

E2F_Q3
NES = −1.85, p = 0.0000, FDR q = 0.0578

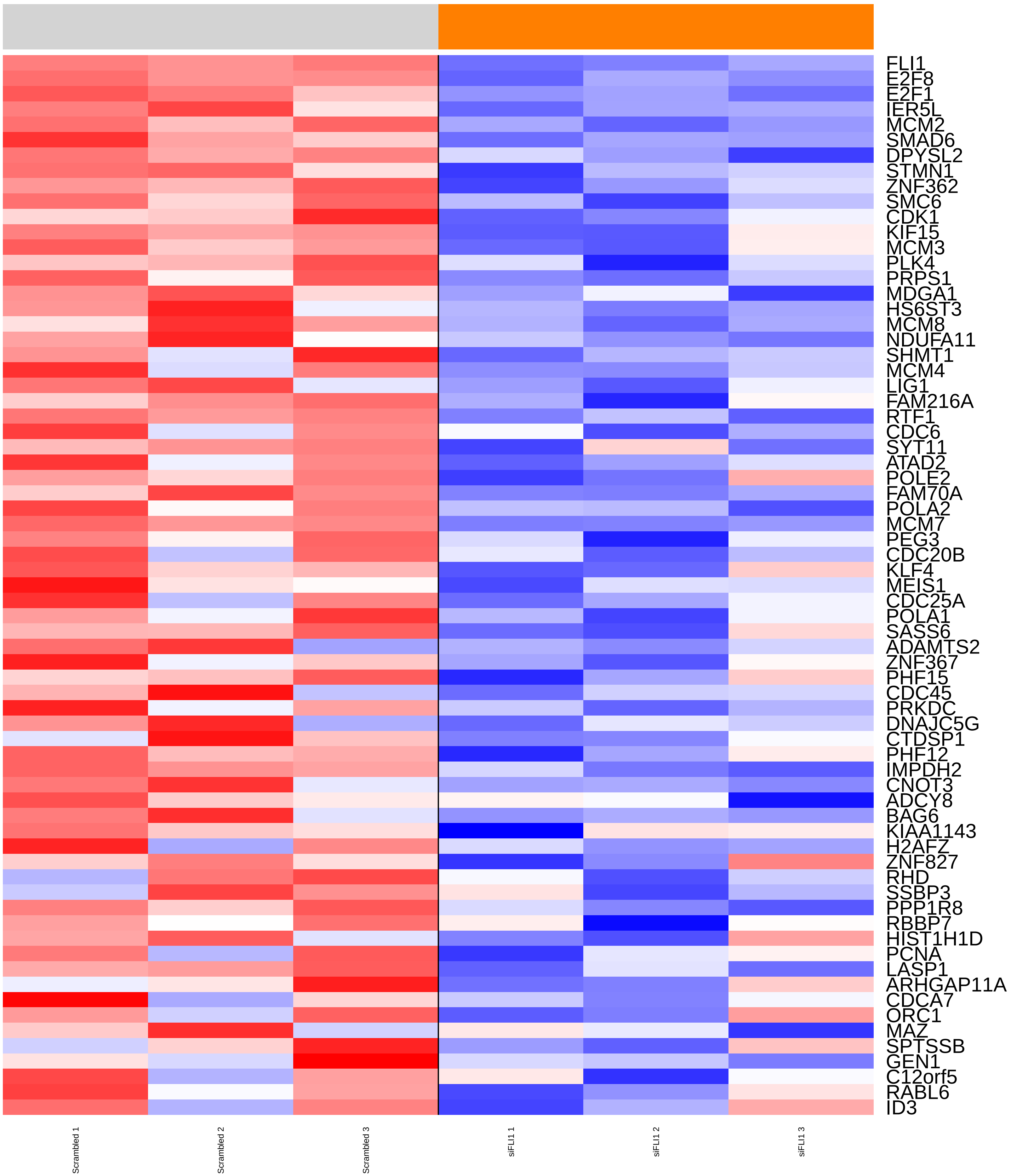

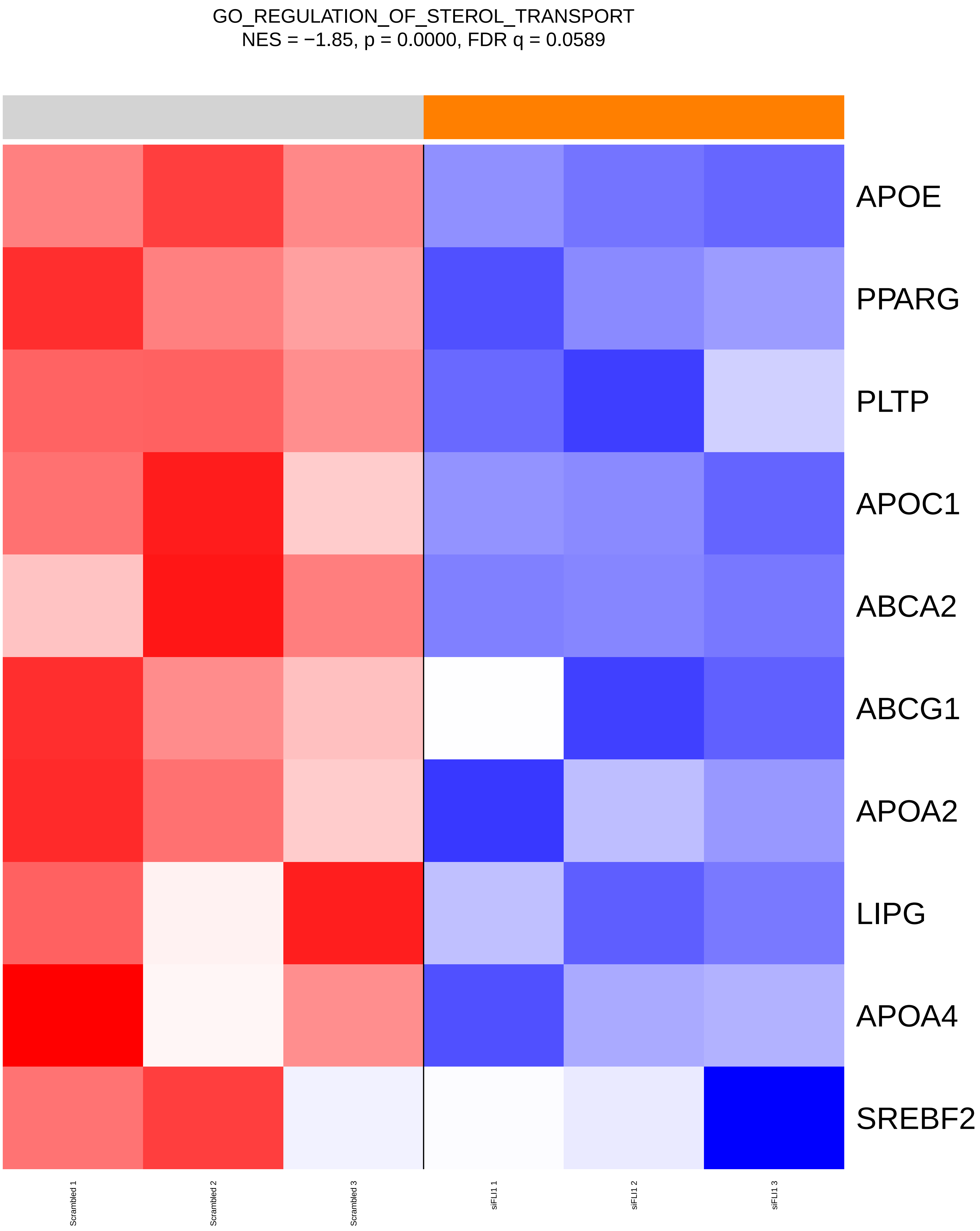
GO_REGULATION_OF_STEROL_TRANSPORT
NES = −1.85, p = 0.0000, FDR q = 0.0589
APOE
PPARG
PLTP
APOC1
ABCA2
ABCG1
APOA2
LIPG
APOA4
SREBF2
Scrambled 1
Scrambled 2
Scrambled 3
siFLI1 1
siFLI1 2
siFLI1 3

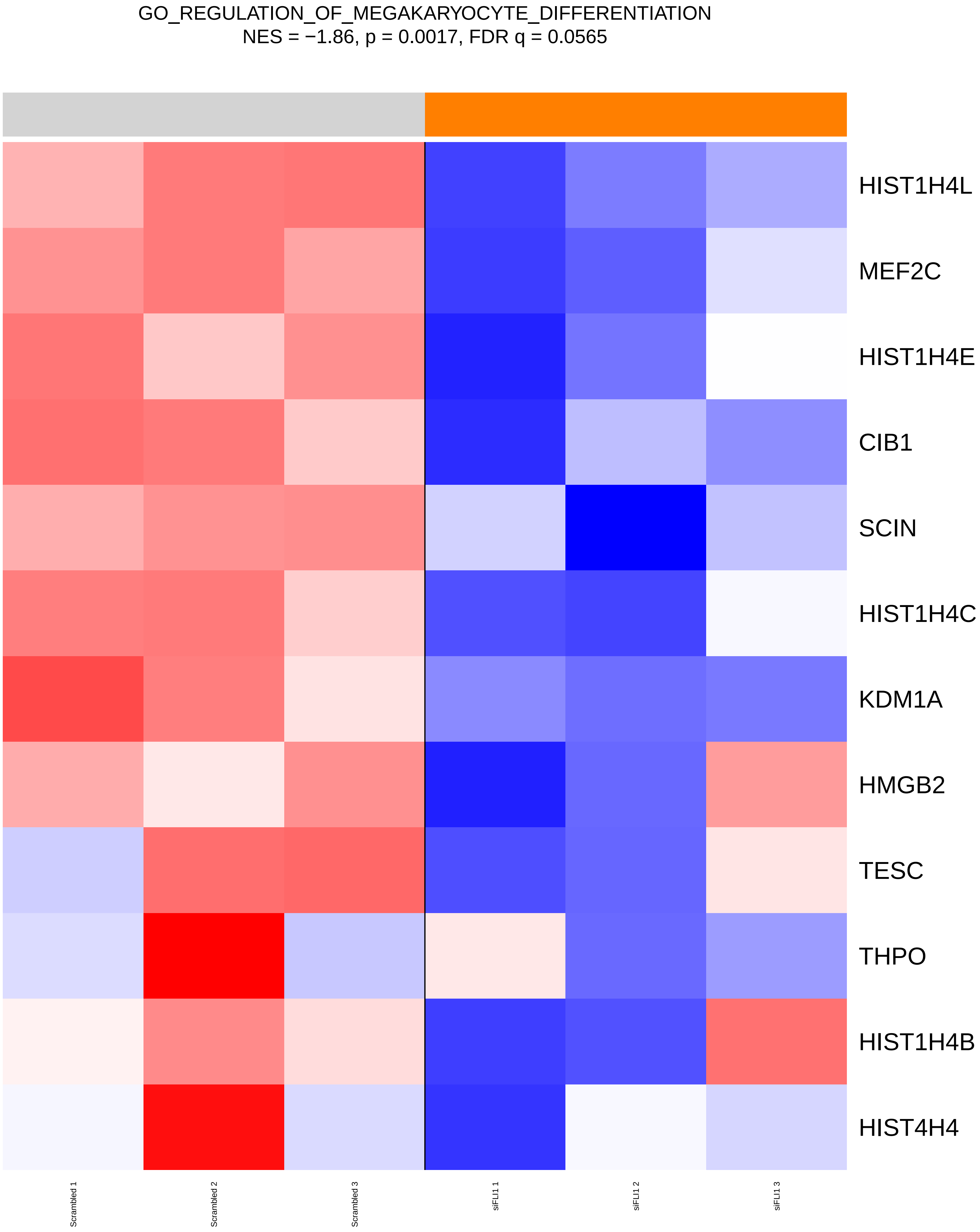
GO_REGULATION_OF_MEGAKARYOCYTE_DIFFERENTIATION
NES = −1.86, p = 0.0017, FDR q = 0.0565
HIST1H4L
MEF2C
HIST1H4E
CIB1
SCIN
HIST1H4C
KDM1A
HMGB2
TESC
THPO
HIST1H4B
HIST4H4
Scrambled 1
Scrambled 2
Scrambled 3
siFLI1 1
siFLI1 2
siFLI1 3

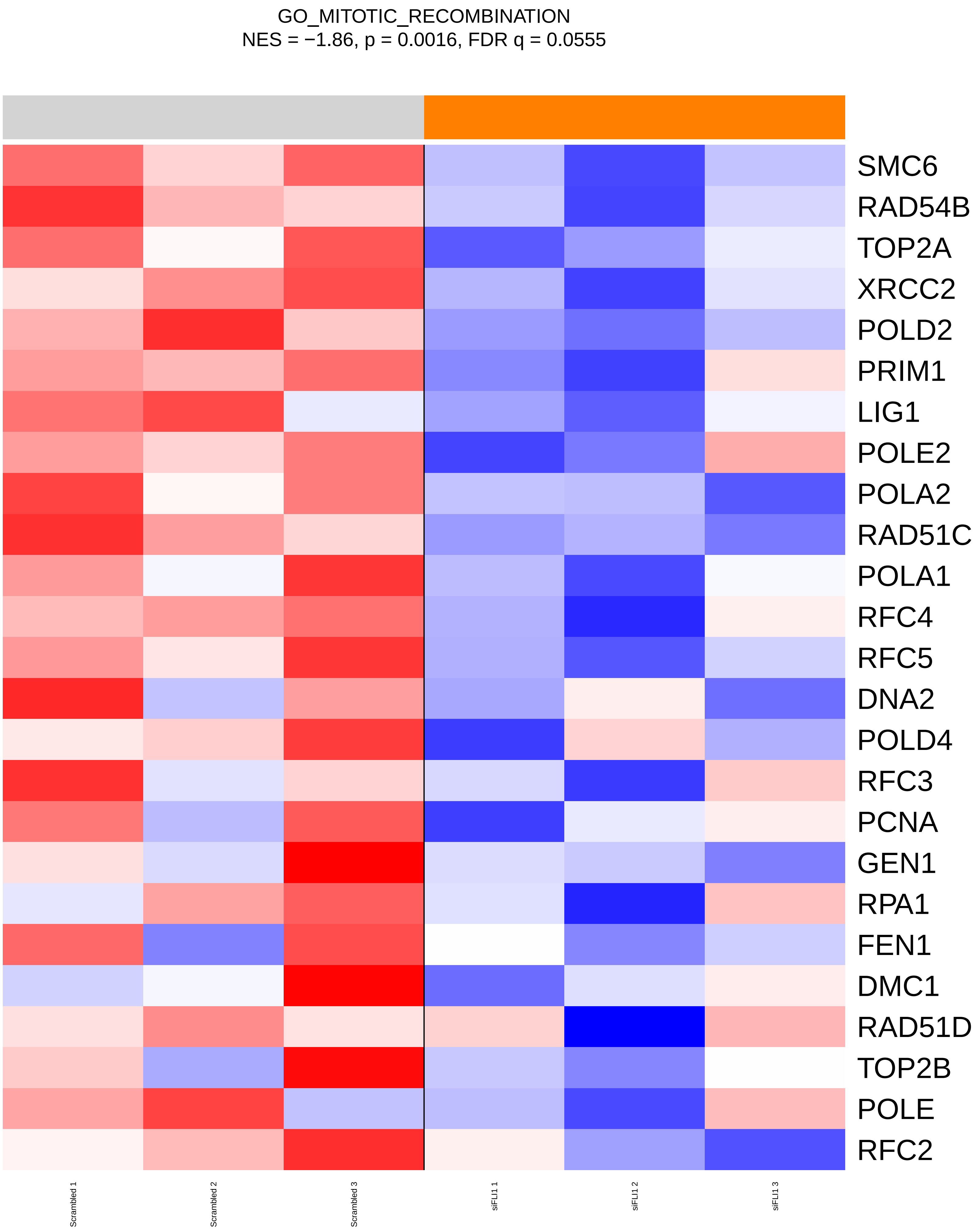
GO_MITOTIC_RECOMBINATION
NES = −1.86, p = 0.0016, FDR q = 0.0555
SMC6
RAD54B
TOP2A
XRCC2
POLD2
PRIM1
LIG1
POLE2
POLA2
RAD51C
POLA1
RFC4
RFC5
DNA2
POLD4
RFC3
PCNA
GEN1
RPA1
FEN1
DMC1
RAD51D
TOP2B
POLE
RFC2
Scrambled 1
Scrambled 2
Scrambled 3
siFLI1 1
siFLI1 2
siFLI1 3

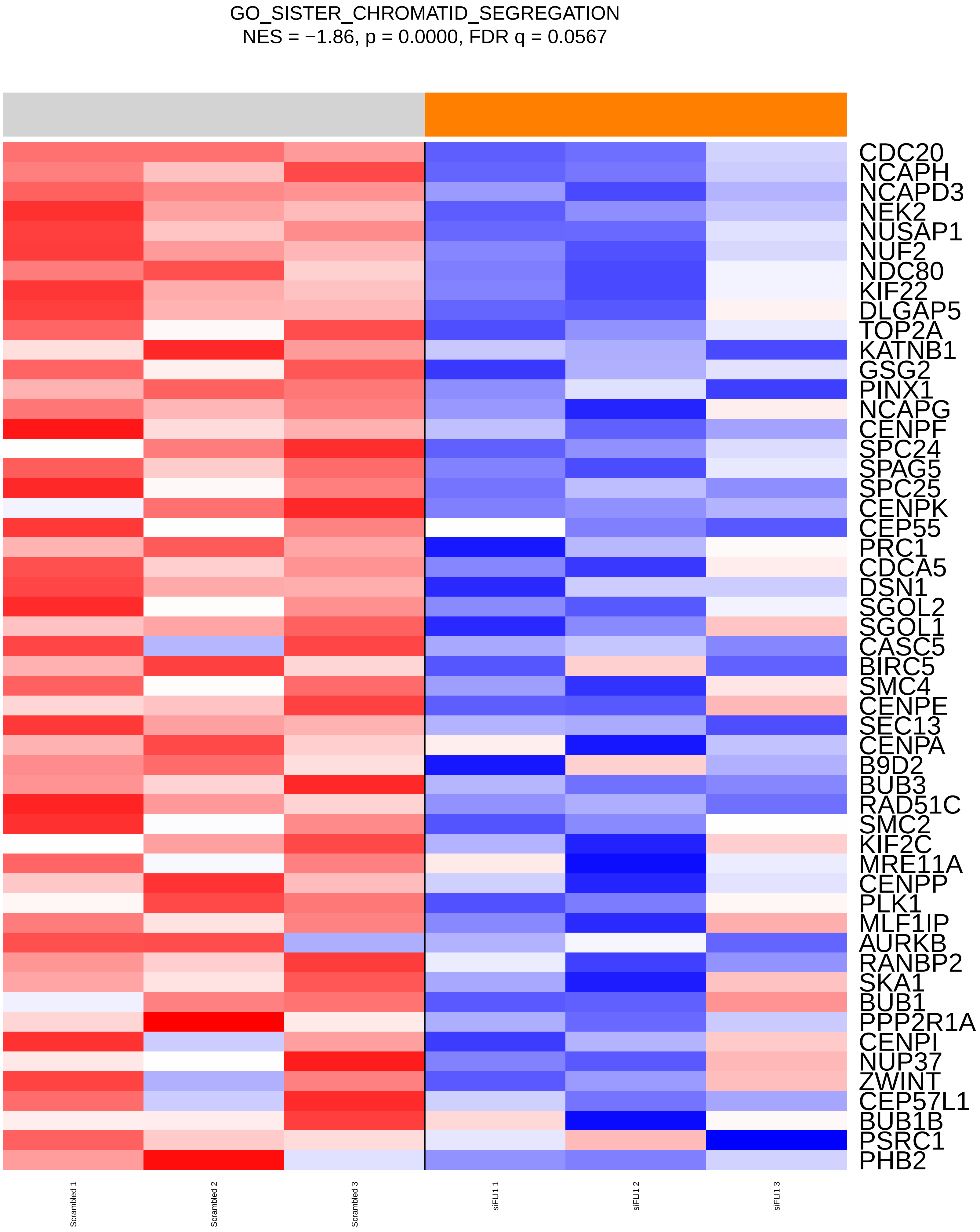
GO_SISTER_CHROMATID_SEGREGATION
NES = −1.86, p = 0.0000, FDR q = 0.0567
CDC20
NCAPH
NCAPD3
NEK2
NUSAP1
NUF2
NDC80
KIF22
DLGAP5
TOP2A
KATNB1
GSG2
PINX1
NCAPG
CENPF
SPC24
SPAG5
SPC25
CENPK
CEP55
PRC1
CDCA5
DSN1
SGOL2
SGOL1
CASC5
BIRC5
SMC4
CENPE
SEC13
CENPA
B9D2
BUB3
RAD51C
SMC2
KIF2C
MRE11A
CENPP
PLK1
MLF1IP
AURKB
RANBP2
SKA1
BUB1
PPP2R1A
CENPI
NUP37
ZWINT
CEP57L1
BUB1B
PSRC1
PHB2
Scrambled 1
Scrambled 2
Scrambled 3
siFLI1 1
siFLI1 2
siFLI1 3

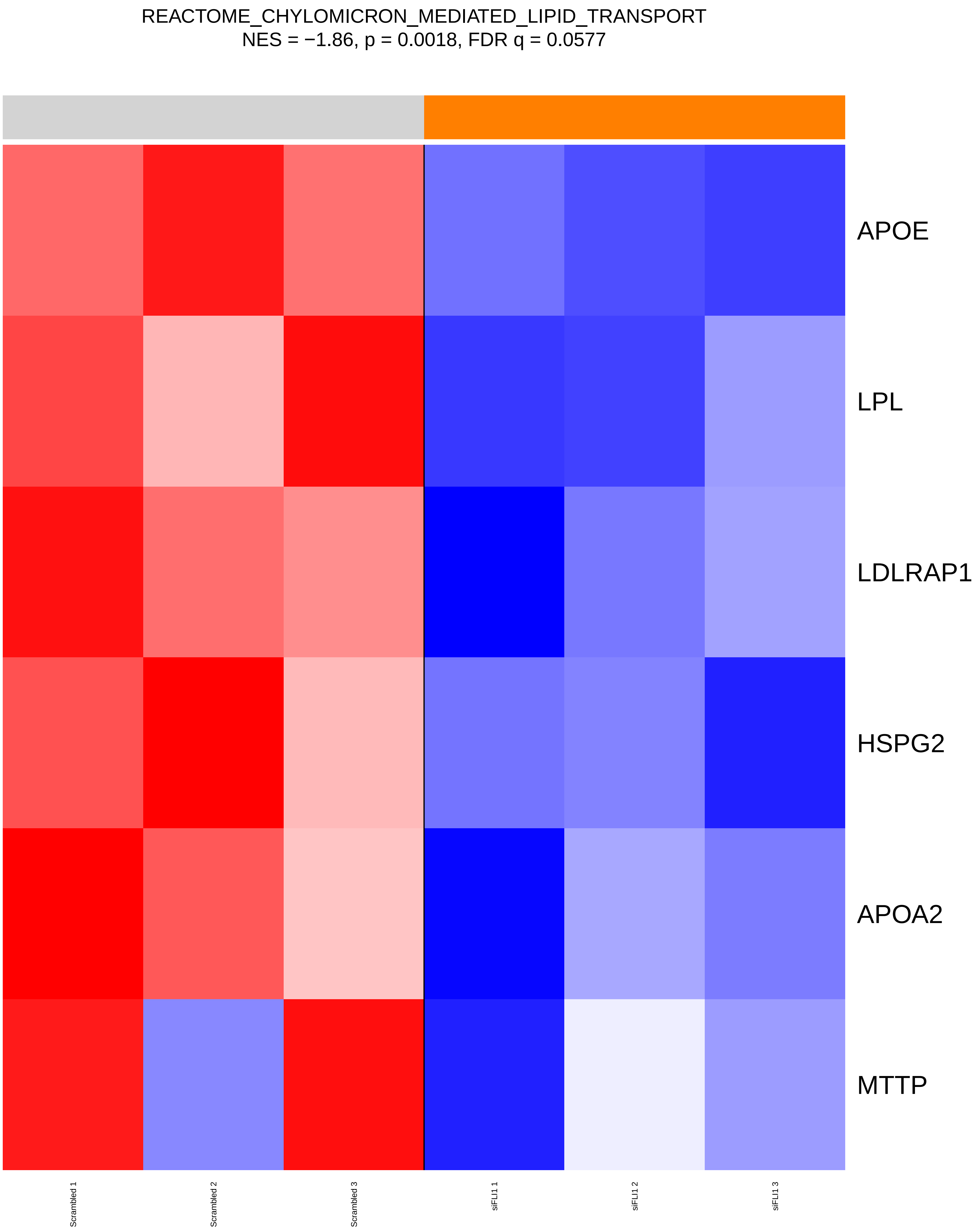

REACTOME_CHYLOMICRON_MEDIATED_LIPID_TRANSPORT
NES = −1.86, p = 0.0018, FDR q = 0.0577
APOE
LPL
LDLRAP1
HSPG2
APOA2
MTTP
Scrambled 1
Scrambled 2
Scrambled 3
siFLI1 1
siFLI1 2
siFLI1 3

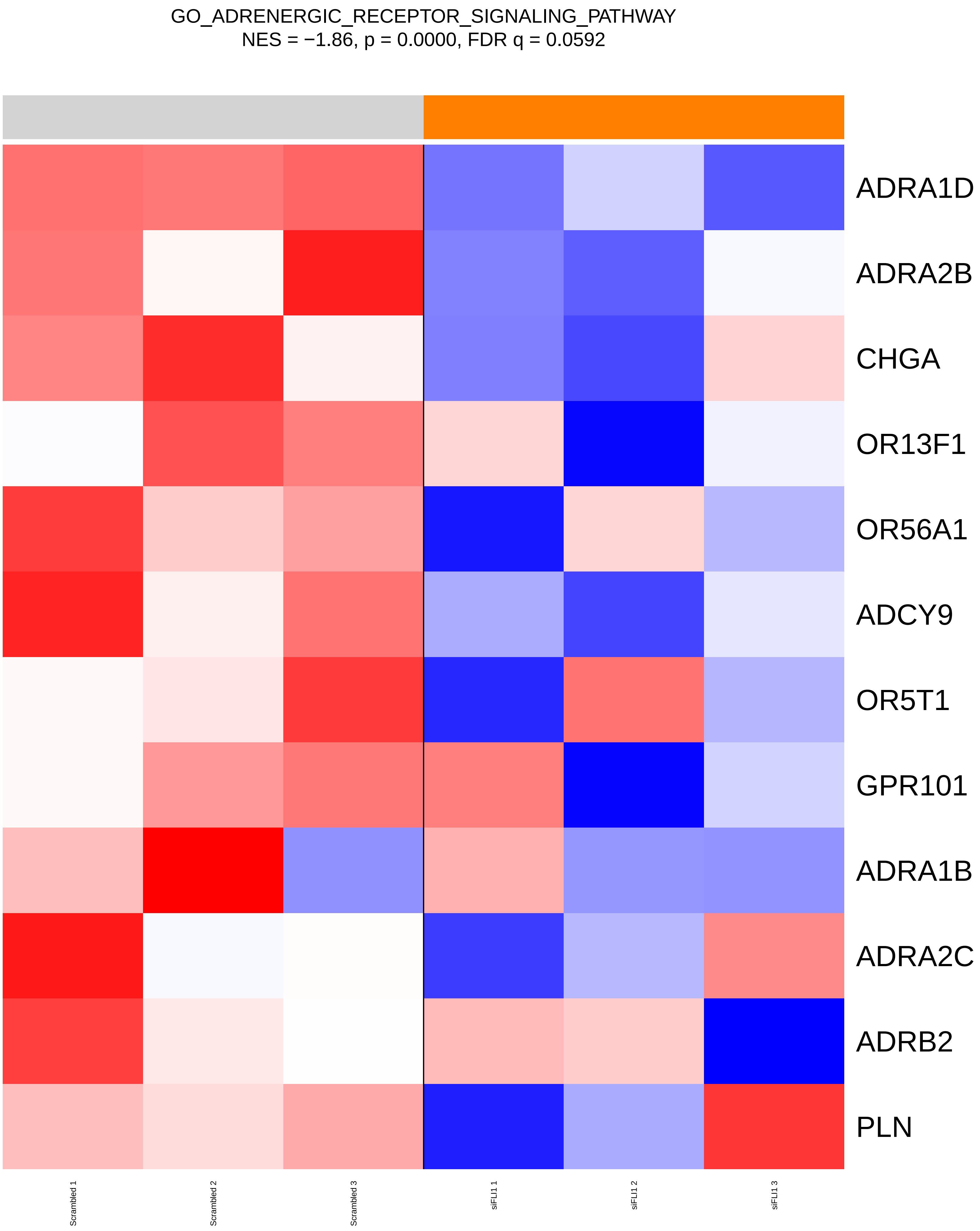
GO_ADRENERGIC_RECEPTOR_SIGNALING_PATHWAY
NES = −1.86, p = 0.0000, FDR q = 0.0592
ADRA1D
ADRA2B
CHGA
OR13F1
OR56A1
ADCY9
OR5T1
GPR101
ADRA1B
ADRA2C
ADRB2
PLN
Scrambled 1
Scrambled 2
Scrambled 3
siFLI1 1
siFLI1 2
siFLI1 3

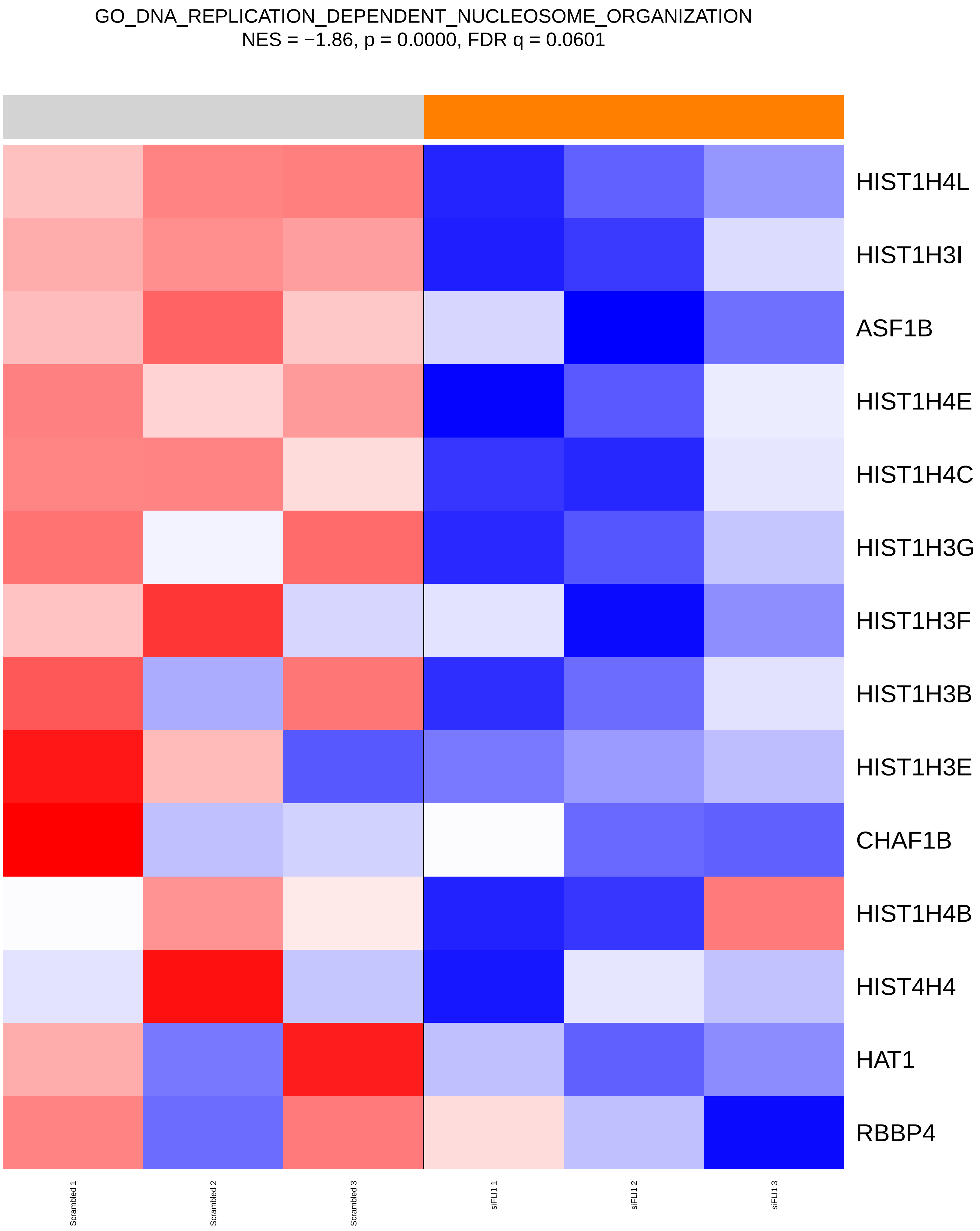
GO_DNA_REPLICATION_DEPENDENT_NUCLEOSOME_ORGANIZATION
NES = −1.86, p = 0.0000, FDR q = 0.0601
HIST1H4L
HIST1H3I
ASF1B
HIST1H4E
HIST1H4C
HIST1H3G
HIST1H3F
HIST1H3B
HIST1H3E
CHAF1B
HIST1H4B
HIST4H4
HAT1
RBBP4
Scrambled 1
Scrambled 2
Scrambled 3
siFLI1 1
siFLI1 2
siFLI1 3

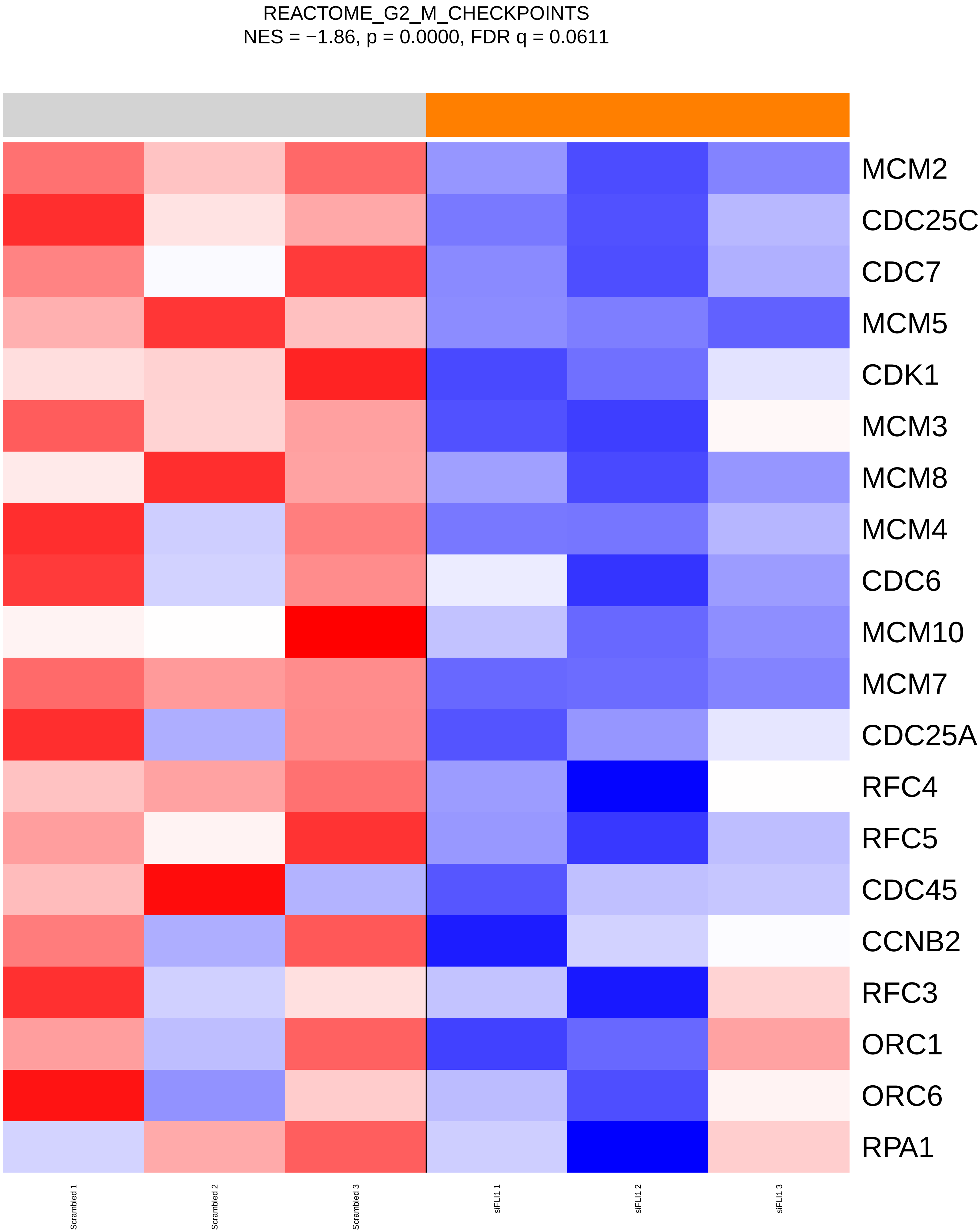

REACTOME_G2_M_CHECKPOINTS
NES = −1.86, p = 0.0000, FDR q = 0.0611
MCM2
CDC25C
CDC7
MCM5
CDK1
MCM3
MCM8
MCM4
CDC6
MCM10
MCM7
CDC25A
RFC4
RFC5
CDC45
CCNB2
RFC3
ORC1
ORC6
RPA1
Scrambled 1
Scrambled 2
Scrambled 3
siFLI1 1
siFLI1 2
siFLI1 3

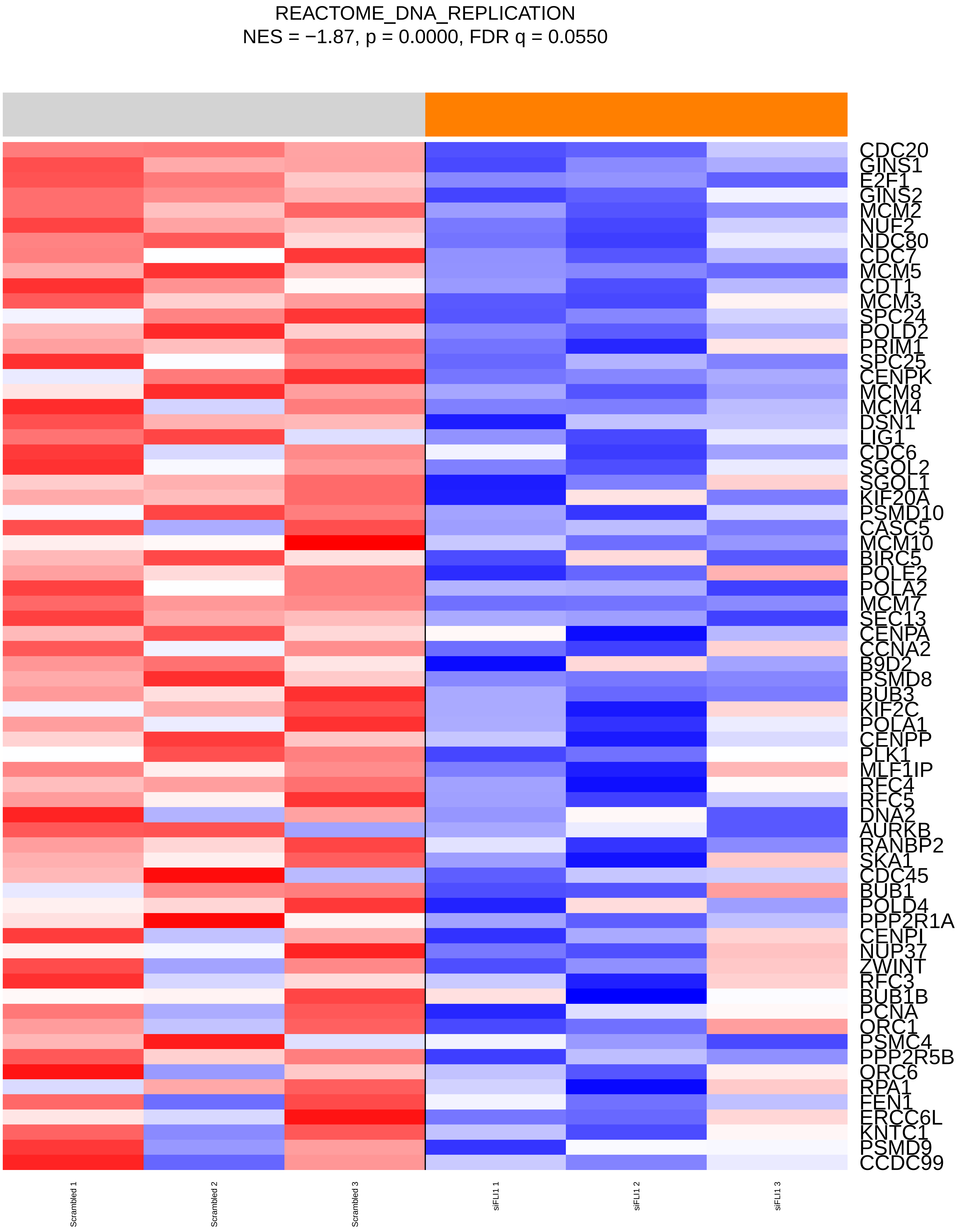
REACTOME_DNA_REPLICATION
NES = −1.87, p = 0.0000, FDR q = 0.0550
CDC20
GINS1
E2F1
GINS2
MCM2
NUF2
NDC80
CDC7
MCM5
CDT1
MCM3
SPC24
POLD2
PRIM1
SPC25
CENPK
MCM8
MCM4
DSN1
LIG1
CDC6
SGOL2
SGOL1
KIF20A
PSMD10
CASC5
MCM10
BIRC5
POLE2
POLA2
MCM7
SEC13
CENPA
CCNA2
B9D2
PSMD8
BUB3
KIF2C
POLA1
CENPP
PLK1
MLF1IP
RFC4
RFC5
DNA2
AURKB
RANBP2
SKA1
CDC45
BUB1
POLD4
PPP2R1A
CENPI
NUP37
ZWINT
RFC3
BUB1B
PCNA
ORC1
PSMC4
PPP2R5B
ORC6
RPA1
FEN1
ERCC6L
KNTC1
PSMD9
CCDC99
Scrambled 1
Scrambled 2
Scrambled 3
siFLI1 1
siFLI1 2
siFLI1 3

GO_CHROMATIN_ASSEMBLY_OR_DISASSEMBLY
NES = −1.88, p = 0.0000, FDR q = 0.0548

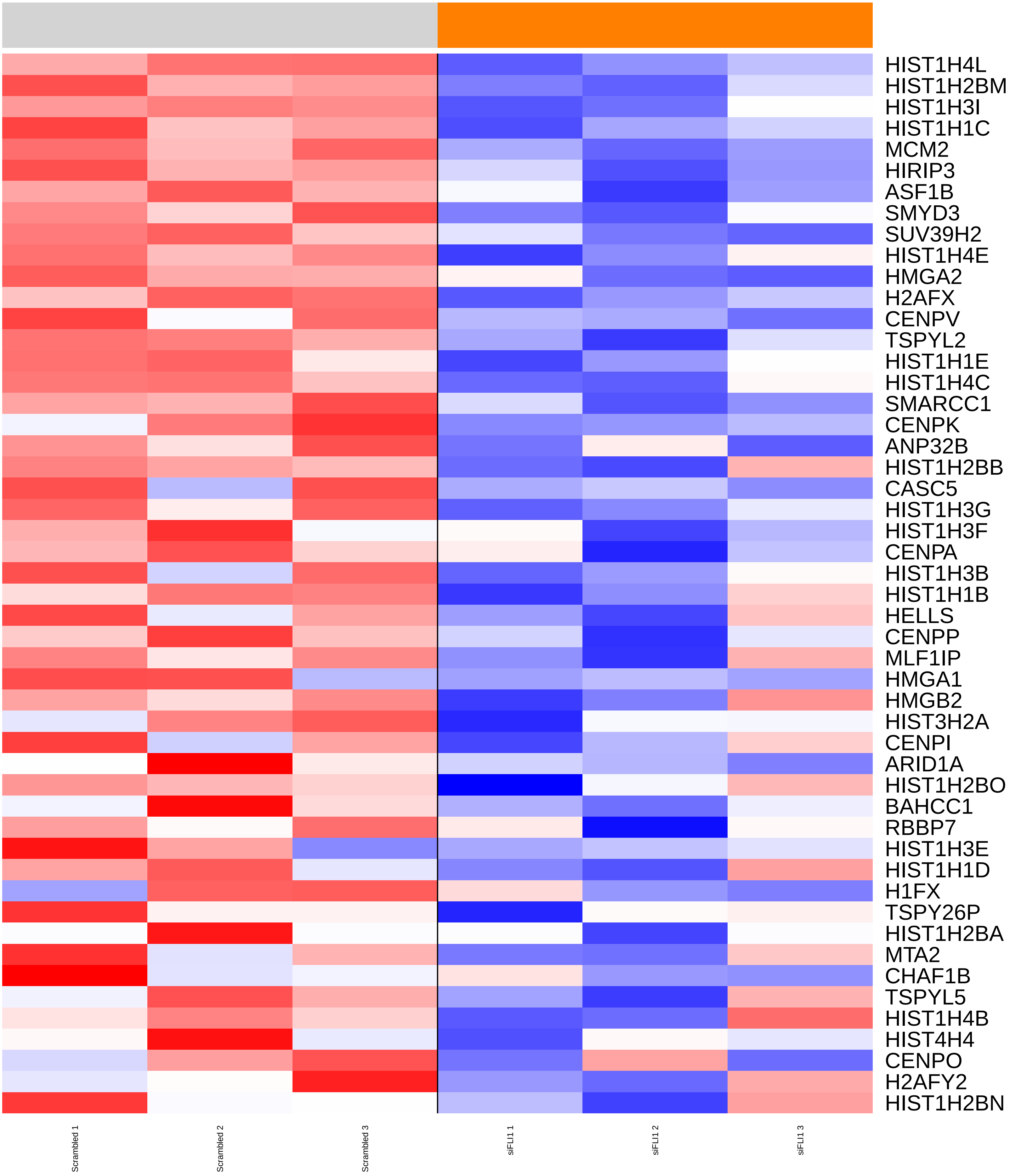

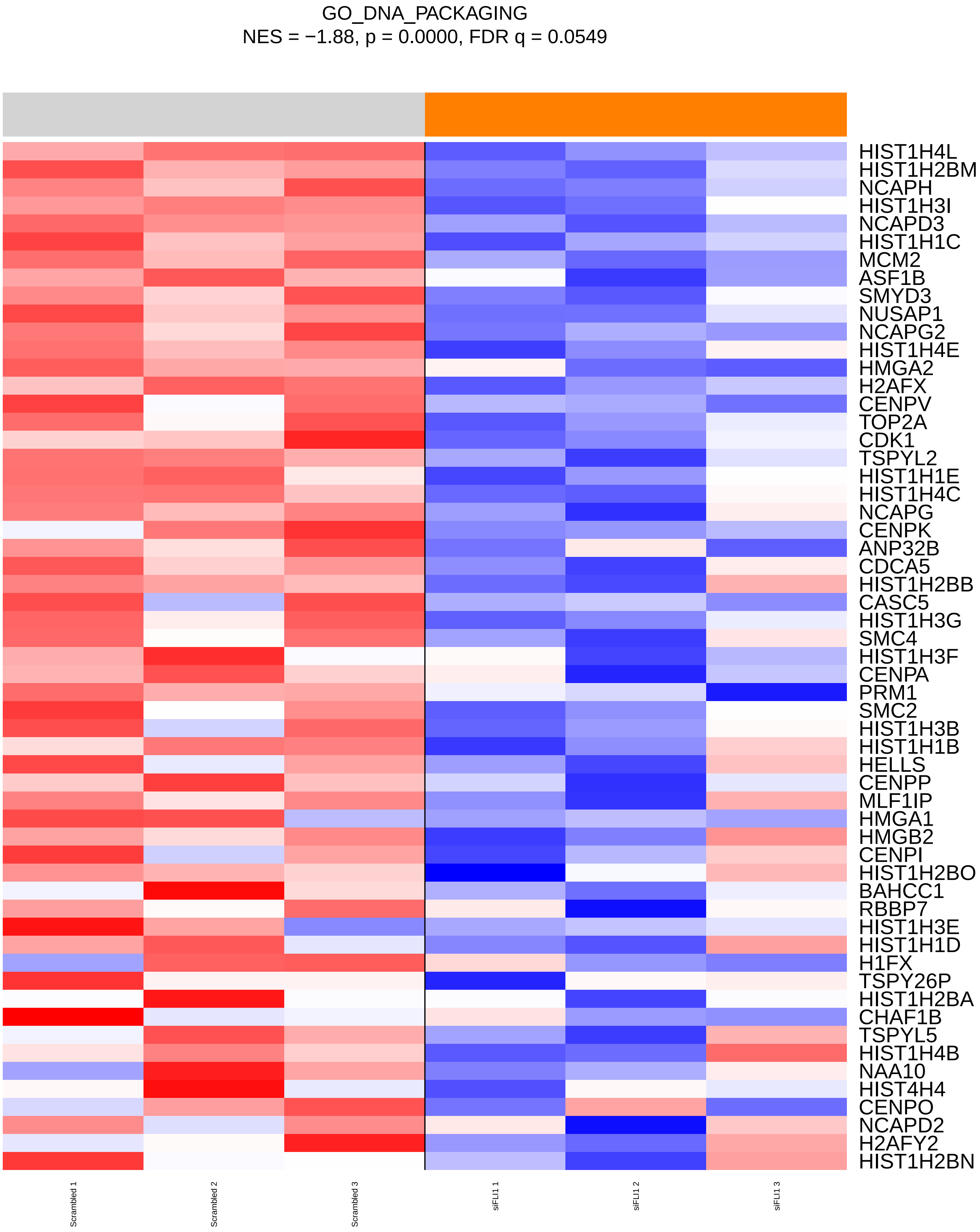
GO_DNA_PACKAGING
NES = −1.88, p = 0.0000, FDR q = 0.0549
HIST1H4L
HIST1H2BM
NCAPH
HIST1H3I
NCAPD3
HIST1H1C
MCM2
ASF1B
SMYD3
NUSAP1
NCAPG2
HIST1H4E
HMGA2
H2AFX
CENPV
TOP2A
CDK1
TSPYL2
HIST1H1E
HIST1H4C
NCAPG
CENPK
ANP32B
CDCA5
HIST1H2BB
CASC5
HIST1H3G
SMC4
HIST1H3F
CENPA
PRM1
SMC2
HIST1H3B
HIST1H1B
HELLS
CENPP
MLF1IP
HMGA1
HMGB2
CENPI
HIST1H2BO
BAHCC1
RBBP7
HIST1H3E
HIST1H1D
H1FX
TSPY26P
HIST1H2BA
CHAF1B
TSPYL5
HIST1H4B
NAA10
HIST4H4
CENPO
NCAPD2
H2AFY2
HIST1H2BN
Scrambled 1
Scrambled 2
Scrambled 3
siFLI1 1
siFLI1 2
siFLI1 3

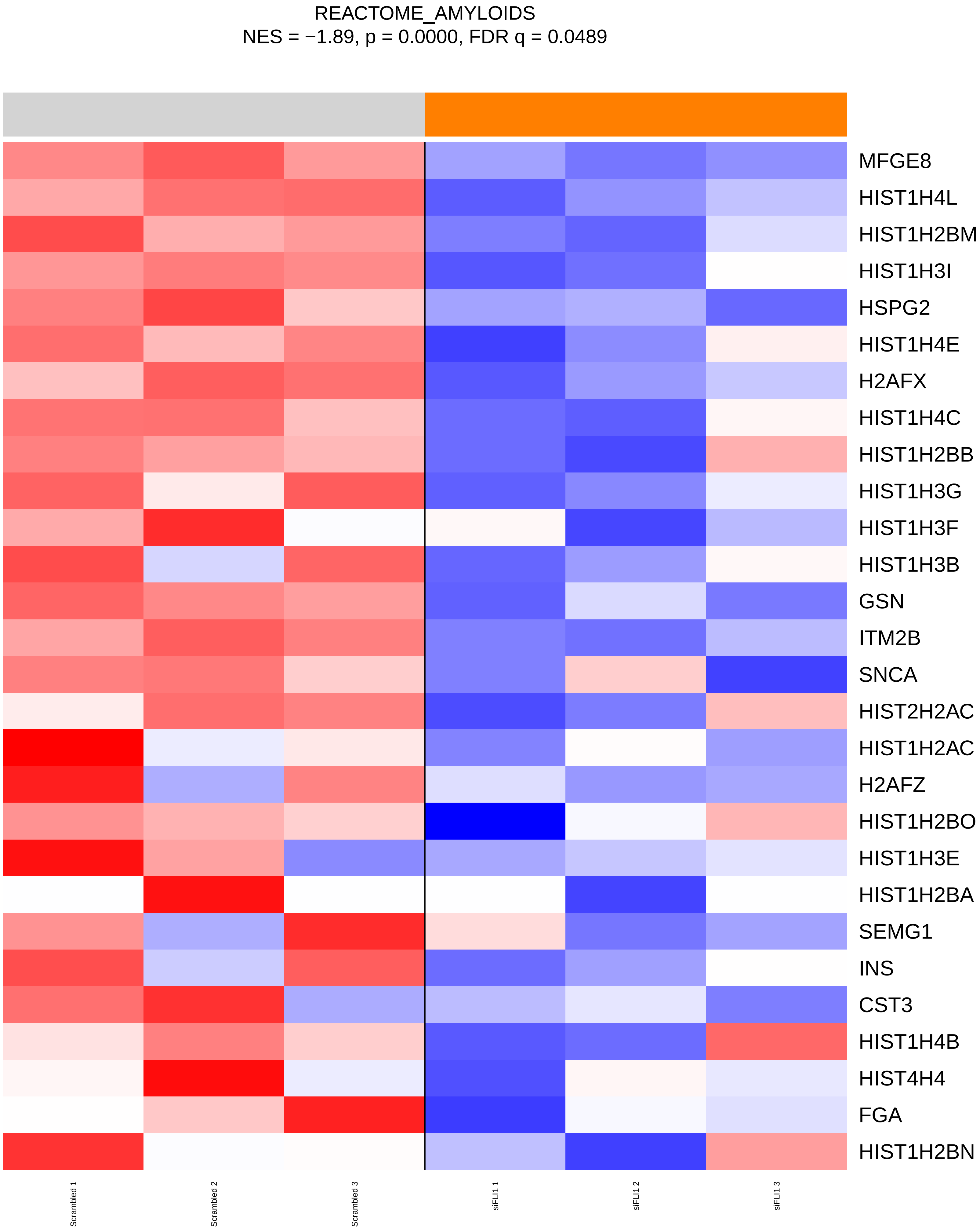

REACTOME_AMYLOIDS
NES = −1.89, p = 0.0000, FDR q = 0.0489
MFGE8
HIST1H4L
HIST1H2BM
HIST1H3I
HSPG2
HIST1H4E
H2AFX
HIST1H4C
HIST1H2BB
HIST1H3G
HIST1H3F
HIST1H3B
GSN
ITM2B
SNCA
HIST2H2AC
HIST1H2AC
H2AFZ
HIST1H2BO
HIST1H3E
HIST1H2BA
SEMG1
INS
CST3
HIST1H4B
HIST4H4
FGA
HIST1H2BN
Scrambled 1
Scrambled 2
Scrambled 3
siFLI1 1
siFLI1 2
siFLI1 3

GO_PROTEIN_LIPID_COMPLEX_BINDING
NES = −1.89, p = 0.0017, FDR q = 0.0497

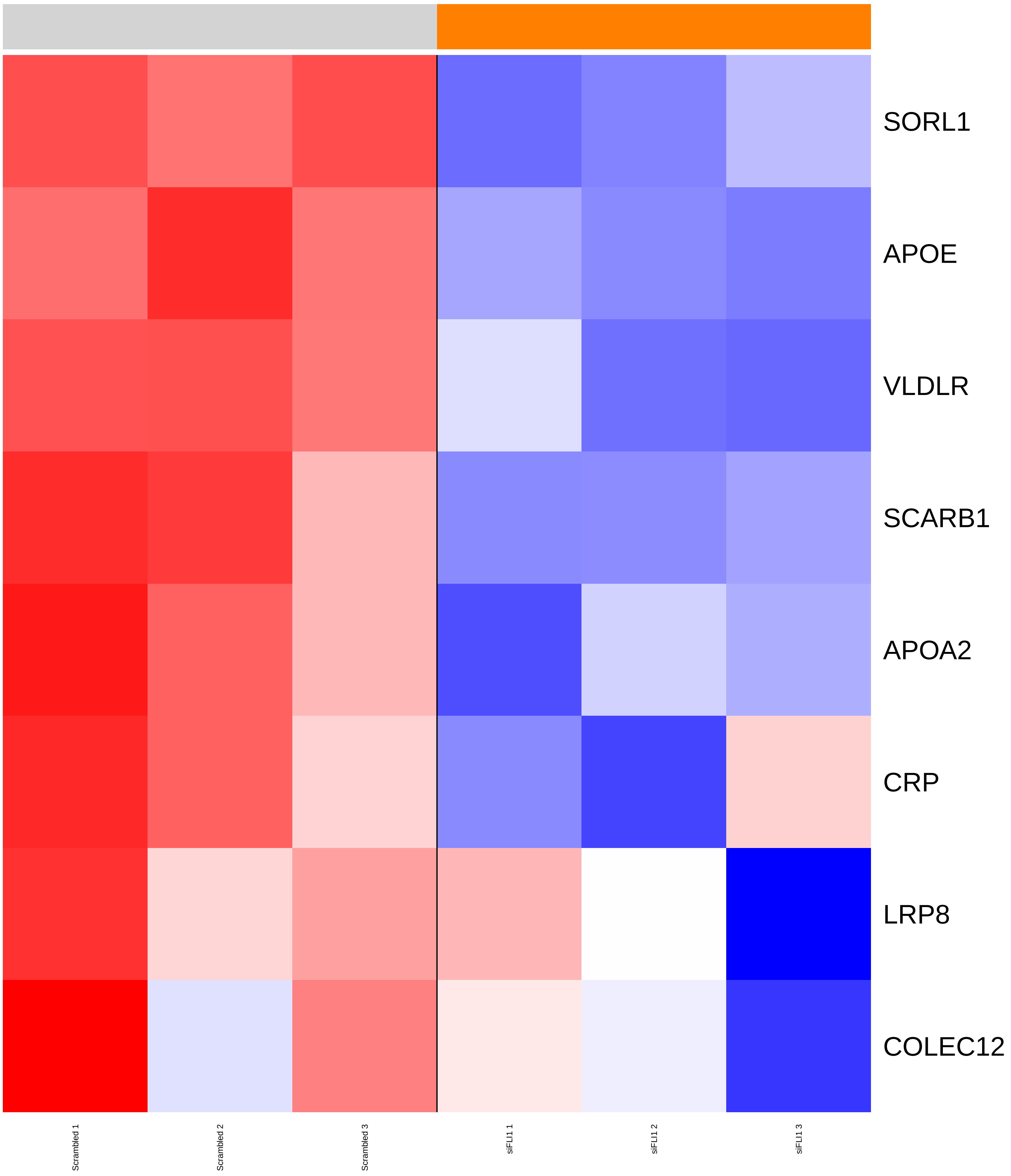

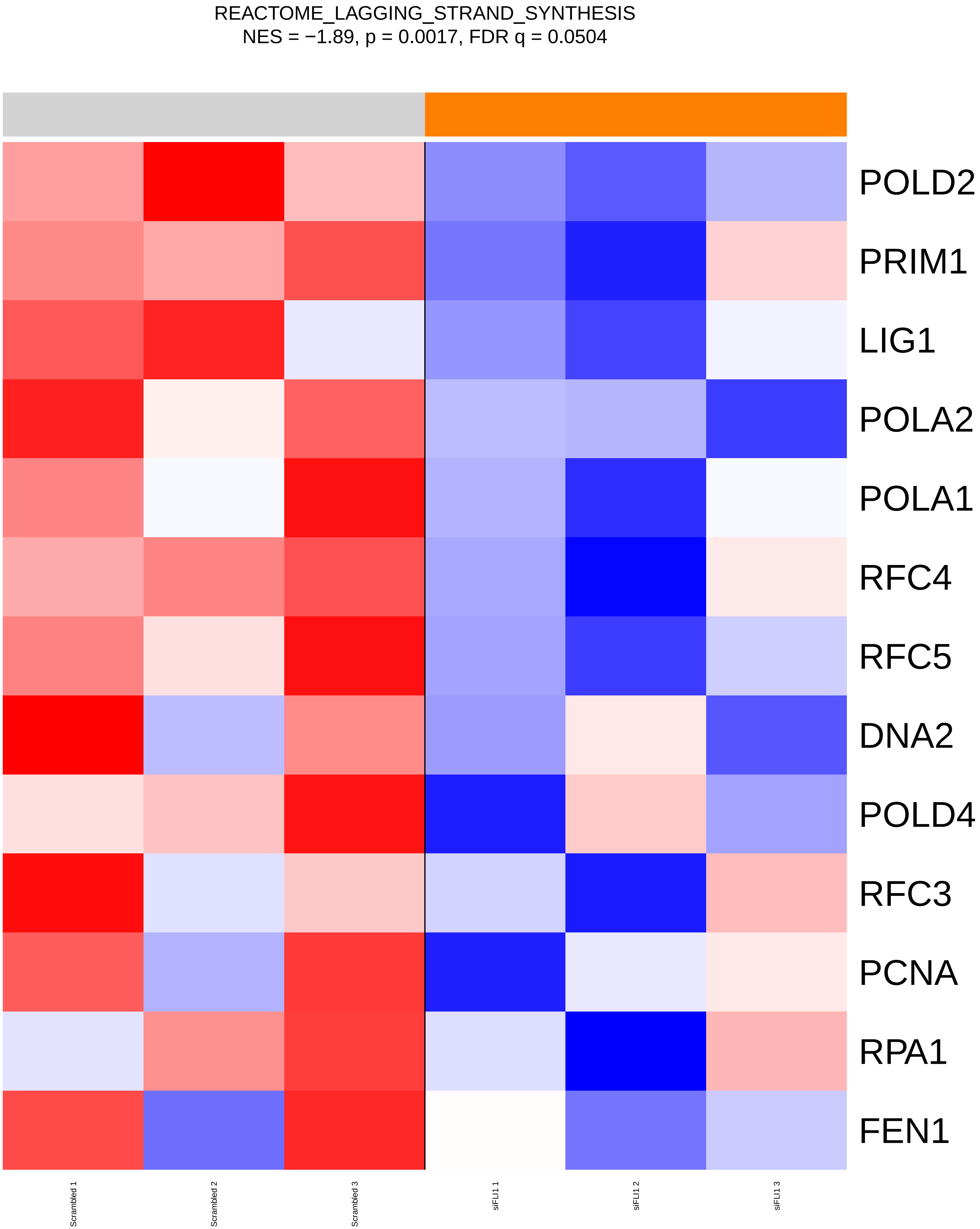
REACTOME_LAGGING_STRAND_SYNTHESIS
NES = −1.89, p = 0.0017, FDR q = 0.0504
POLD2
PRIM1
LIG1
POLA2
POLA1
RFC4
RFC5
DNA2
POLD4
RFC3
PCNA
RPA1
FEN1
Scrambled 1
Scrambled 2
Scrambled 3
siFLI1 1
siFLI1 2
siFLI1 3

GO_MITOCHONDRIAL_PROTEIN_COMPLEX
NES = −1.89, p = 0.0000, FDR q = 0.0504

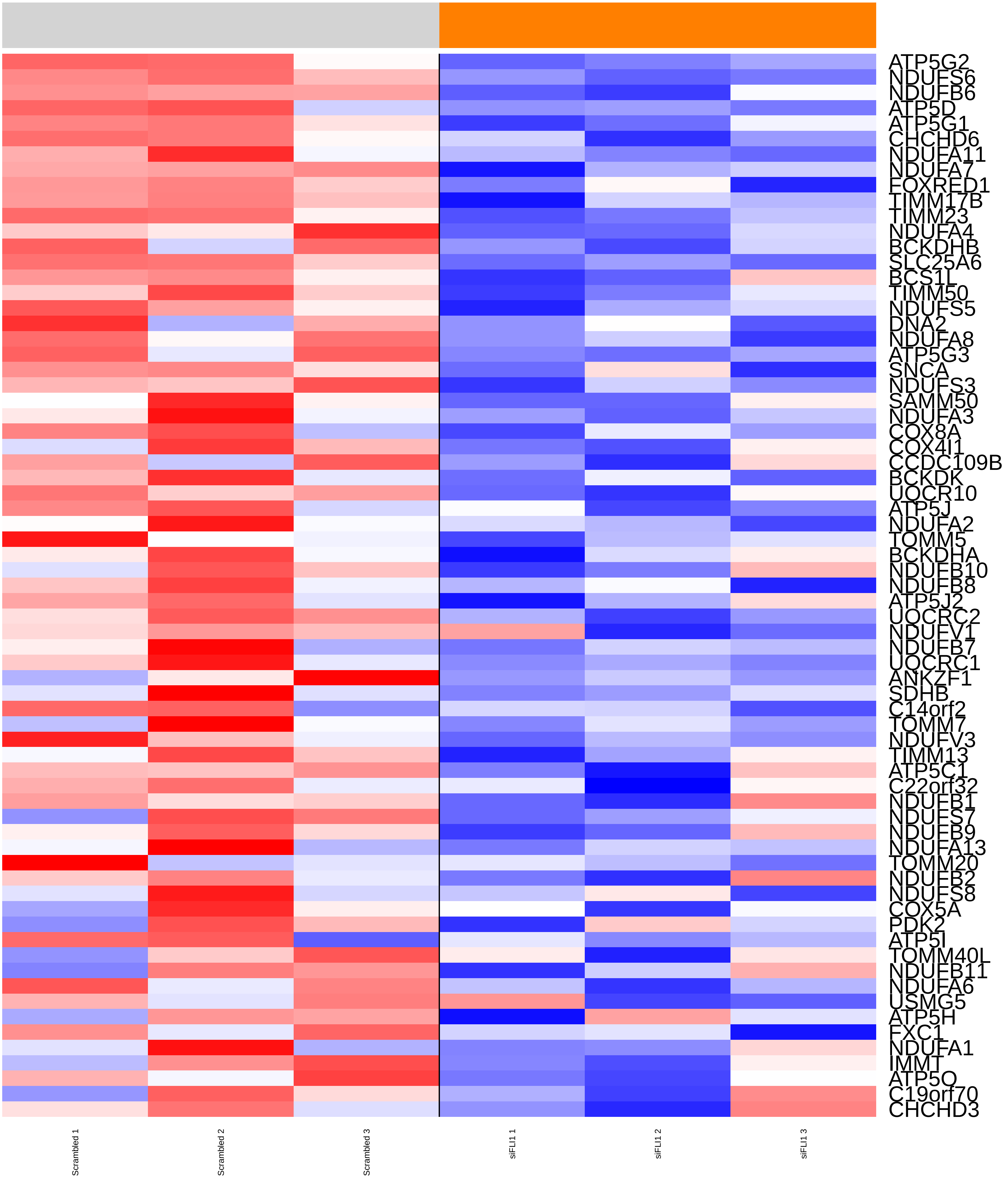

GO_INNER_MITOCHONDRIAL_MEMBRANE_PROTEIN_COMPLEX
NES = −1.90, p = 0.0000, FDR q = 0.0502

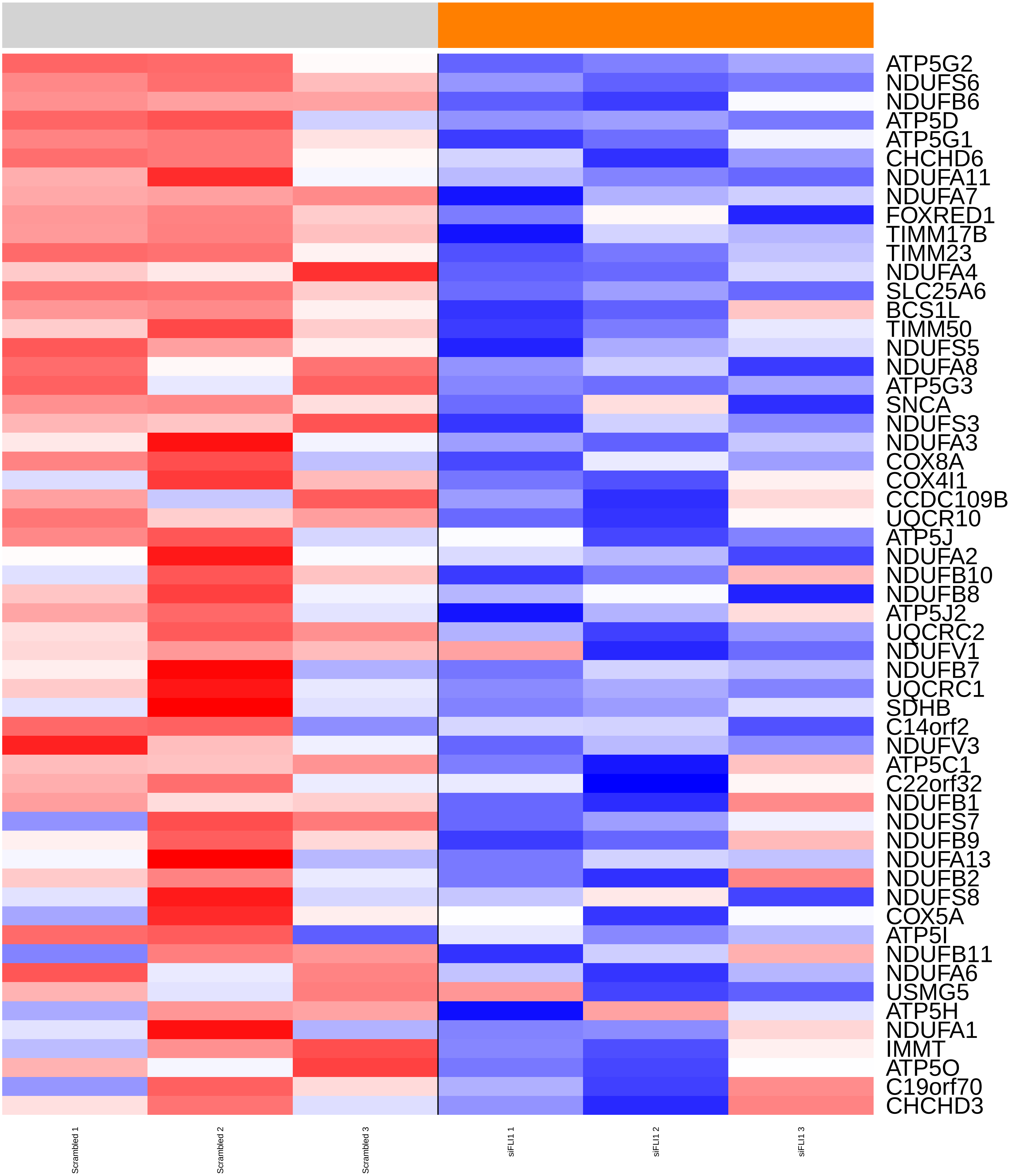

REACTOME_ACTIVATION_OF_ATR_IN_RESPONSE_TO_REPLICATION_STRESS
NES = −1.90, p = 0.0016, FDR q = 0.0511

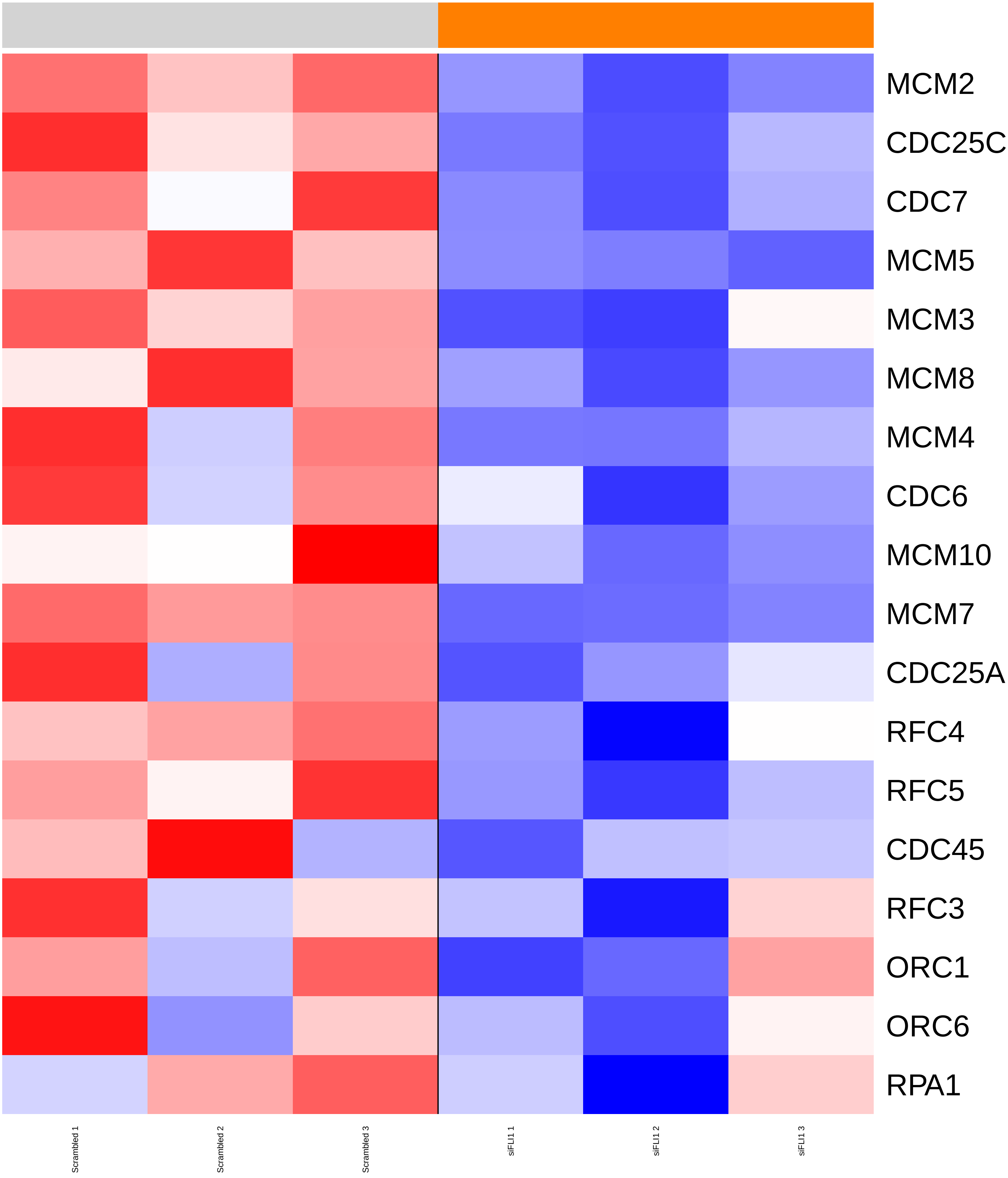

REACTOME_RESPIRATORY_ELECTRON_TRANSPORT
NES = −1.91, p = 0.0000, FDR q = 0.0485

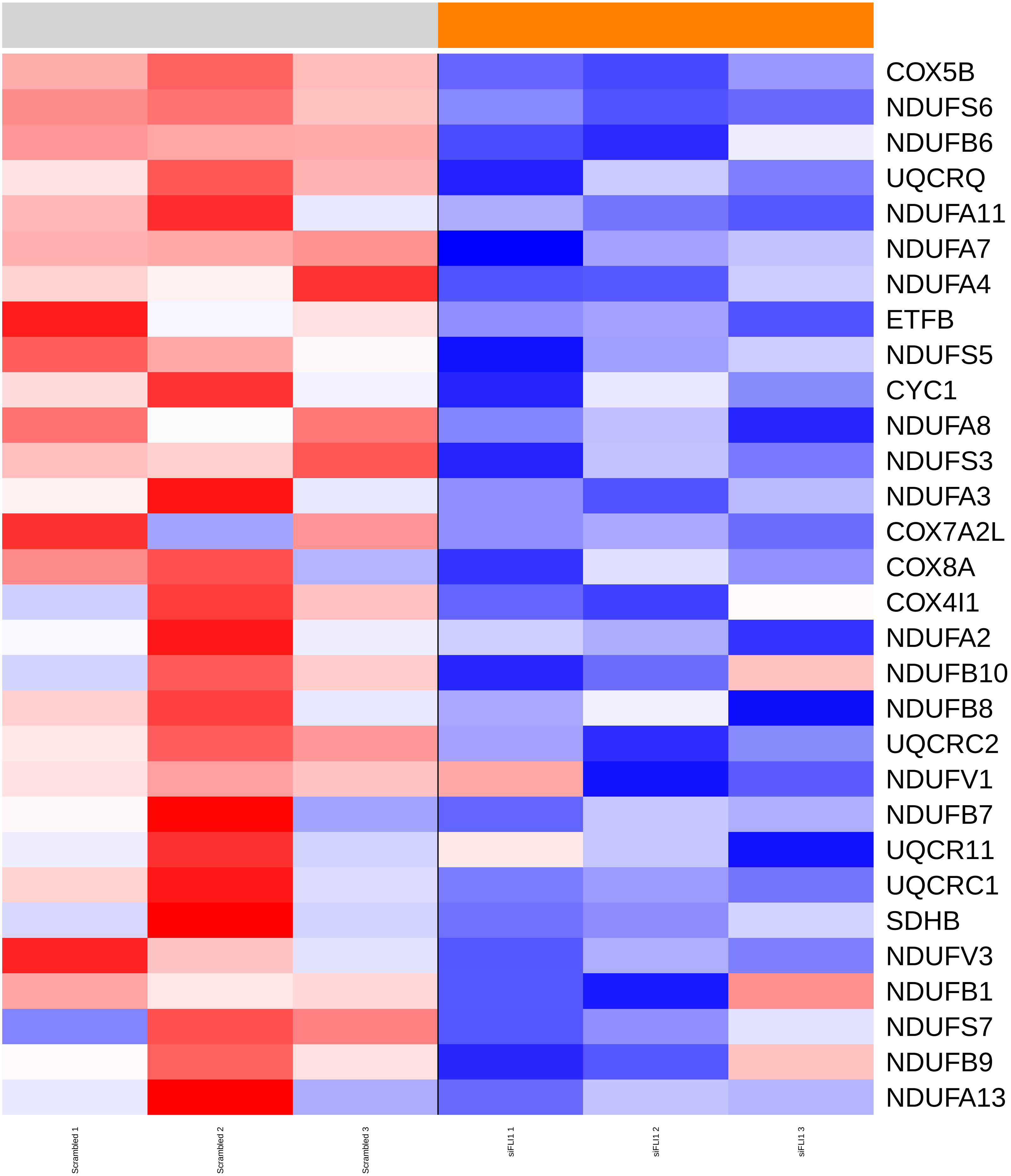

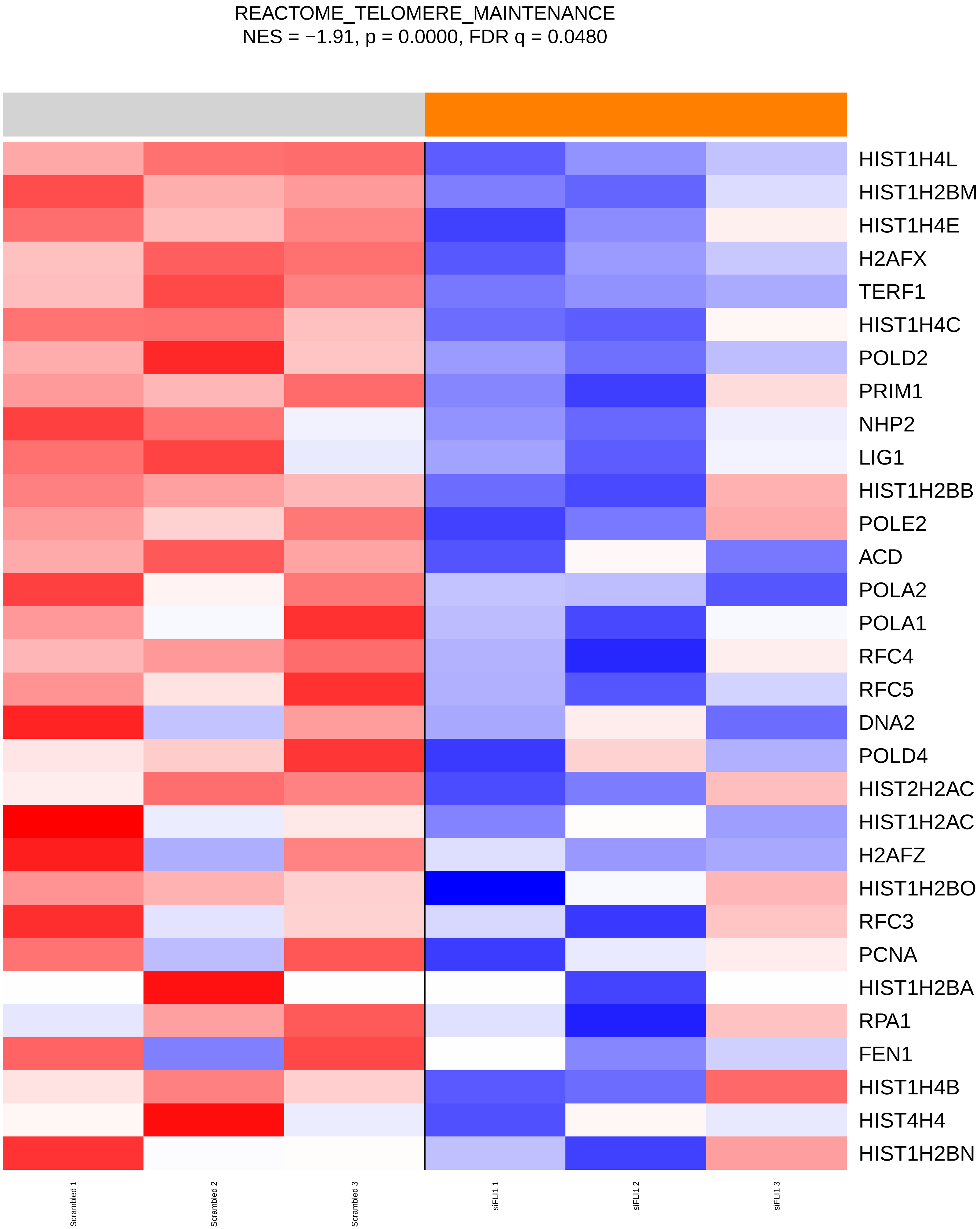
REACTOME_TELOMERE_MAINTENANCE
NES = −1.91, p = 0.0000, FDR q = 0.0480
HIST1H4L
HIST1H2BM
HIST1H4E
H2AFX
TERF1
HIST1H4C
POLD2
PRIM1
NHP2
LIG1
HIST1H2BB
POLE2
ACD
POLA2
POLA1
RFC4
RFC5
DNA2
POLD4
HIST2H2AC
HIST1H2AC
H2AFZ
HIST1H2BO
RFC3
PCNA
HIST1H2BA
RPA1
FEN1
HIST1H4B
HIST4H4
HIST1H2BN
Scrambled 1
Scrambled 2
Scrambled 3
siFLI1 1
siFLI1 2
siFLI1 3

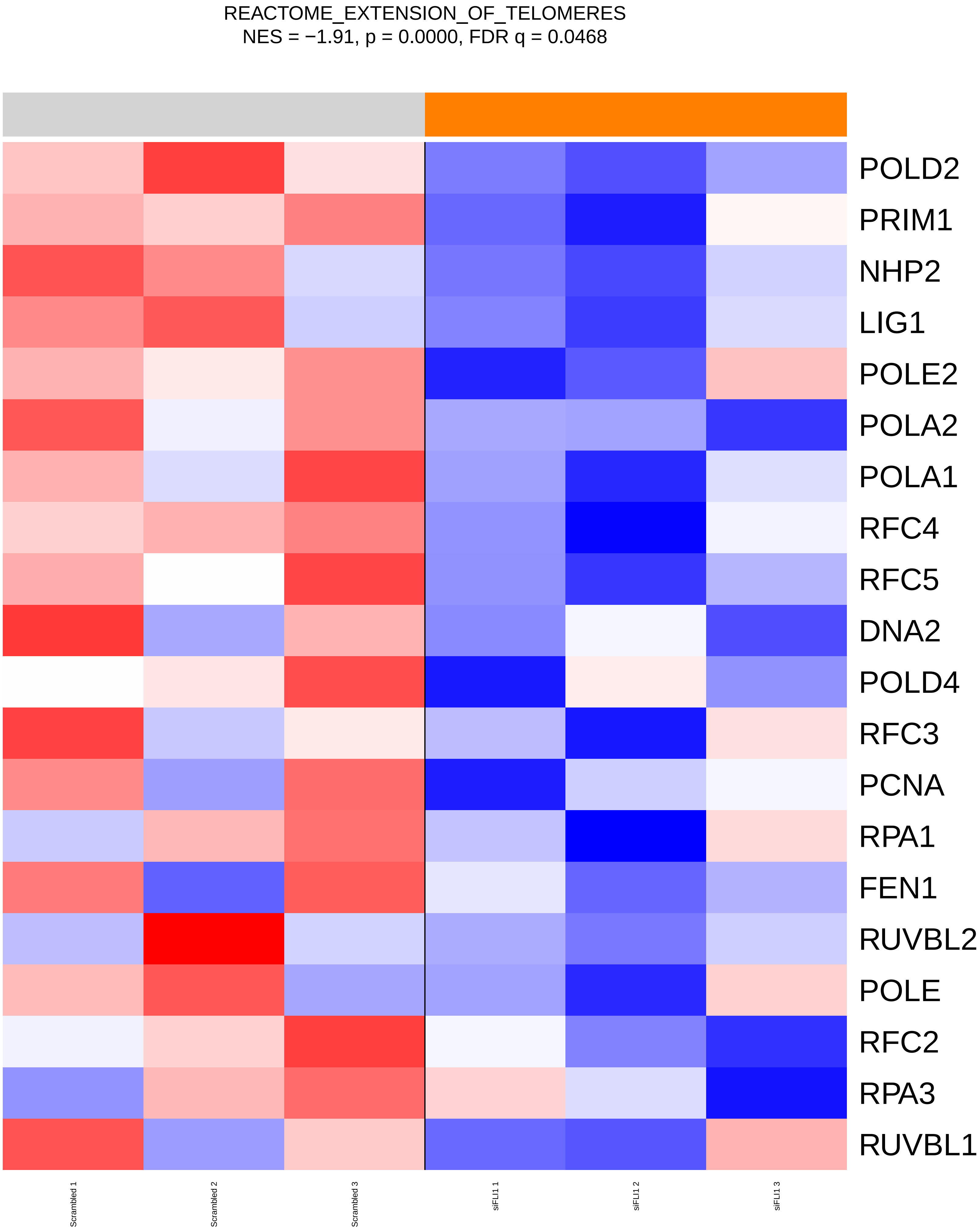
REACTOME_EXTENSION_OF_TELOMERES
NES = −1.91, p = 0.0000, FDR q = 0.0468
POLD2
PRIM1
NHP2
LIG1
POLE2
POLA2
POLA1
RFC4
RFC5
DNA2
POLD4
RFC3
PCNA
RPA1
FEN1
RUVBL2
POLE
RFC2
RPA3
RUVBL1
Scrambled 1
Scrambled 2
Scrambled 3
siFLI1 1
siFLI1 2
siFLI1 3

GO_CONDENSED_CHROMOSOME_CENTROMERIC_REGION
NES = −1.92, p = 0.0000, FDR q = 0.0433

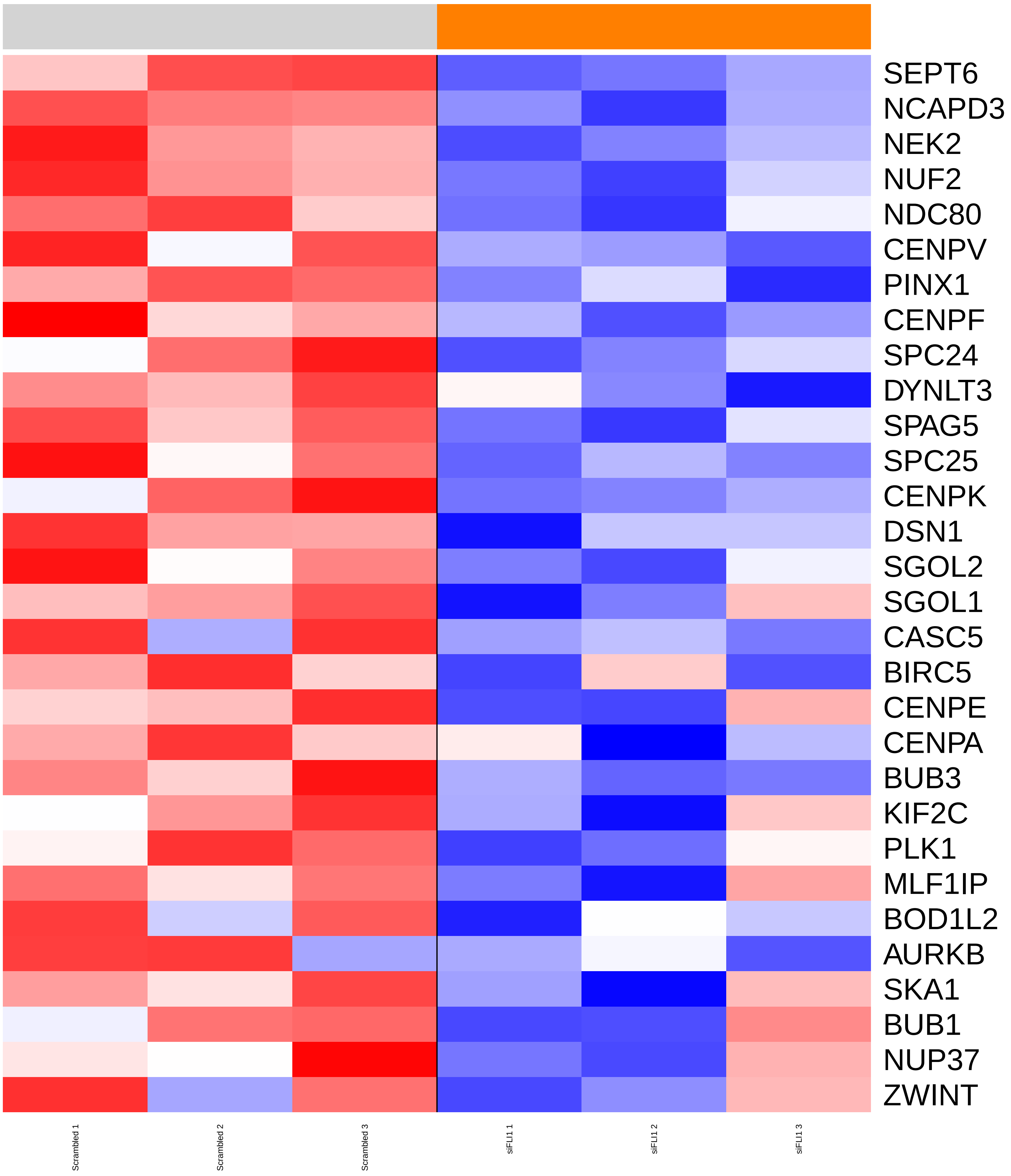

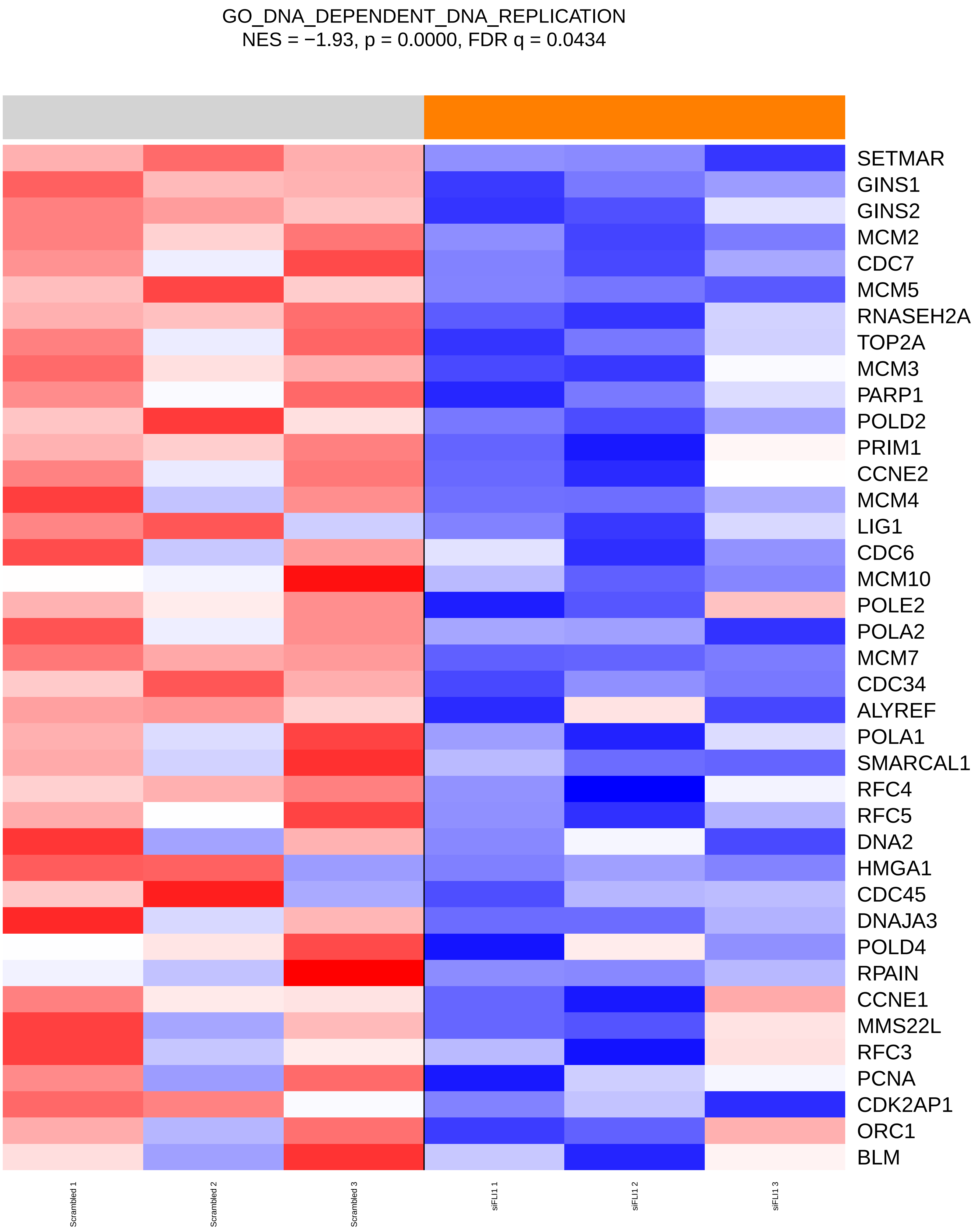
GO_DNA_DEPENDENT_DNA_REPLICATION
NES = −1.93, p = 0.0000, FDR q = 0.0434
SETMAR
GINS1
GINS2
MCM2
CDC7
MCM5
RNASEH2A
TOP2A
MCM3
PARP1
POLD2
PRIM1
CCNE2
MCM4
LIG1
CDC6
MCM10
POLE2
POLA2
MCM7
CDC34
ALYREF
POLA1
SMARCAL1
RFC4
RFC5
DNA2
HMGA1
CDC45
DNAJA3
POLD4
RPAIN
CCNE1
MMS22L
RFC3
PCNA
CDK2AP1
ORC1
BLM
Scrambled 1
Scrambled 2
Scrambled 3
siFLI1 1
siFLI1 2
siFLI1 3

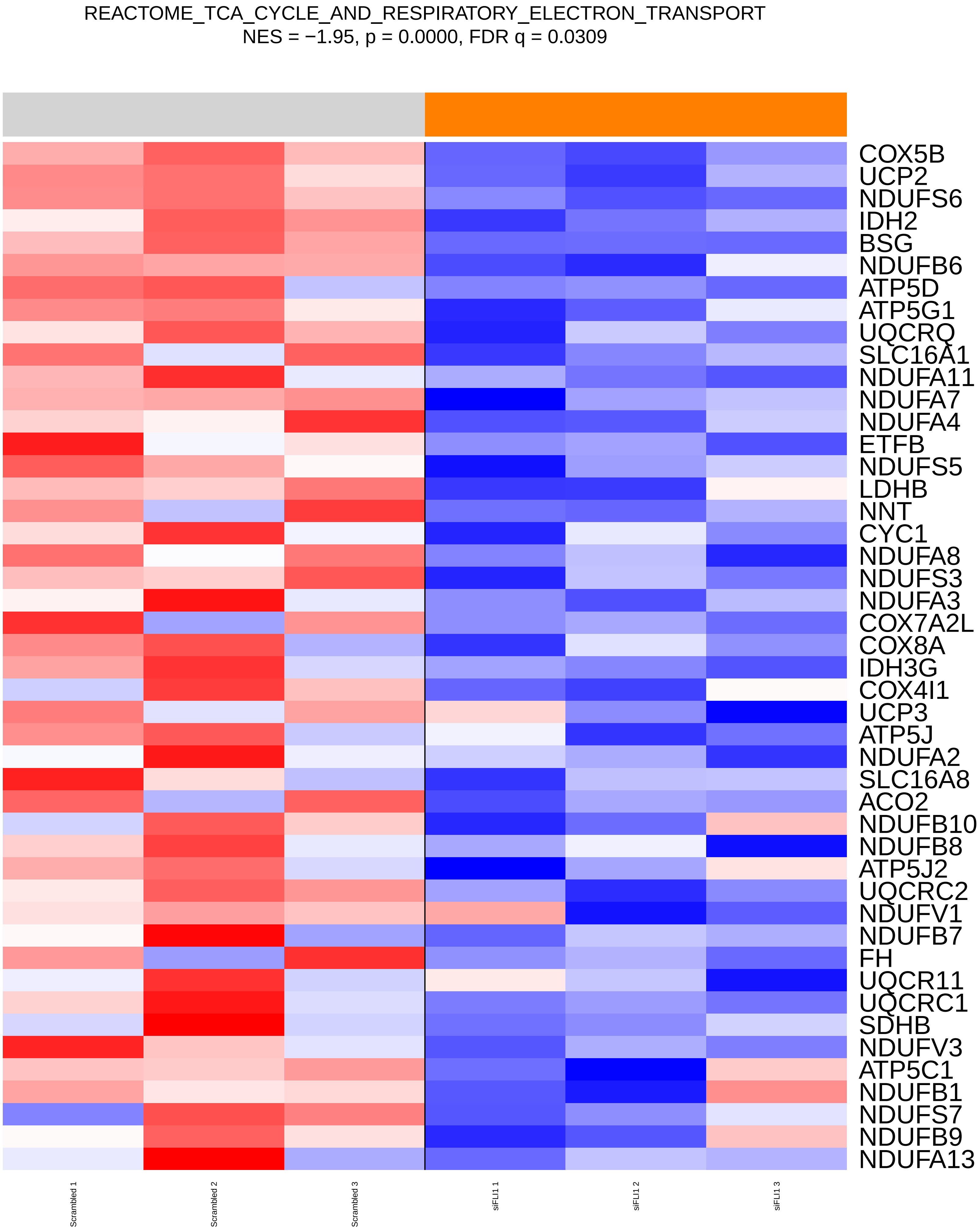
REACTOME_TCA_CYCLE_AND_RESPIRATORY_ELECTRON_TRANSPORT
NES = −1.95, p = 0.0000, FDR q = 0.0309
COX5B
UCP2
NDUFS6
IDH2
BSG
NDUFB6
ATP5D
ATP5G1
UQCRQ
SLC16A1
NDUFA11
NDUFA7
NDUFA4
ETFB
NDUFS5
LDHB
NNT
CYC1
NDUFA8
NDUFS3
NDUFA3
COX7A2L
COX8A
IDH3G
COX4I1
UCP3
ATP5J
NDUFA2
SLC16A8
ACO2
NDUFB10
NDUFB8
ATP5J2
UQCRC2
NDUFV1
NDUFB7
FH
UQCR11
UQCRC1
SDHB
NDUFV3
ATP5C1
NDUFB1
NDUFS7
NDUFB9
NDUFA13
Scrambled 1
Scrambled 2
Scrambled 3
siFLI1 1
siFLI1 2
siFLI1 3

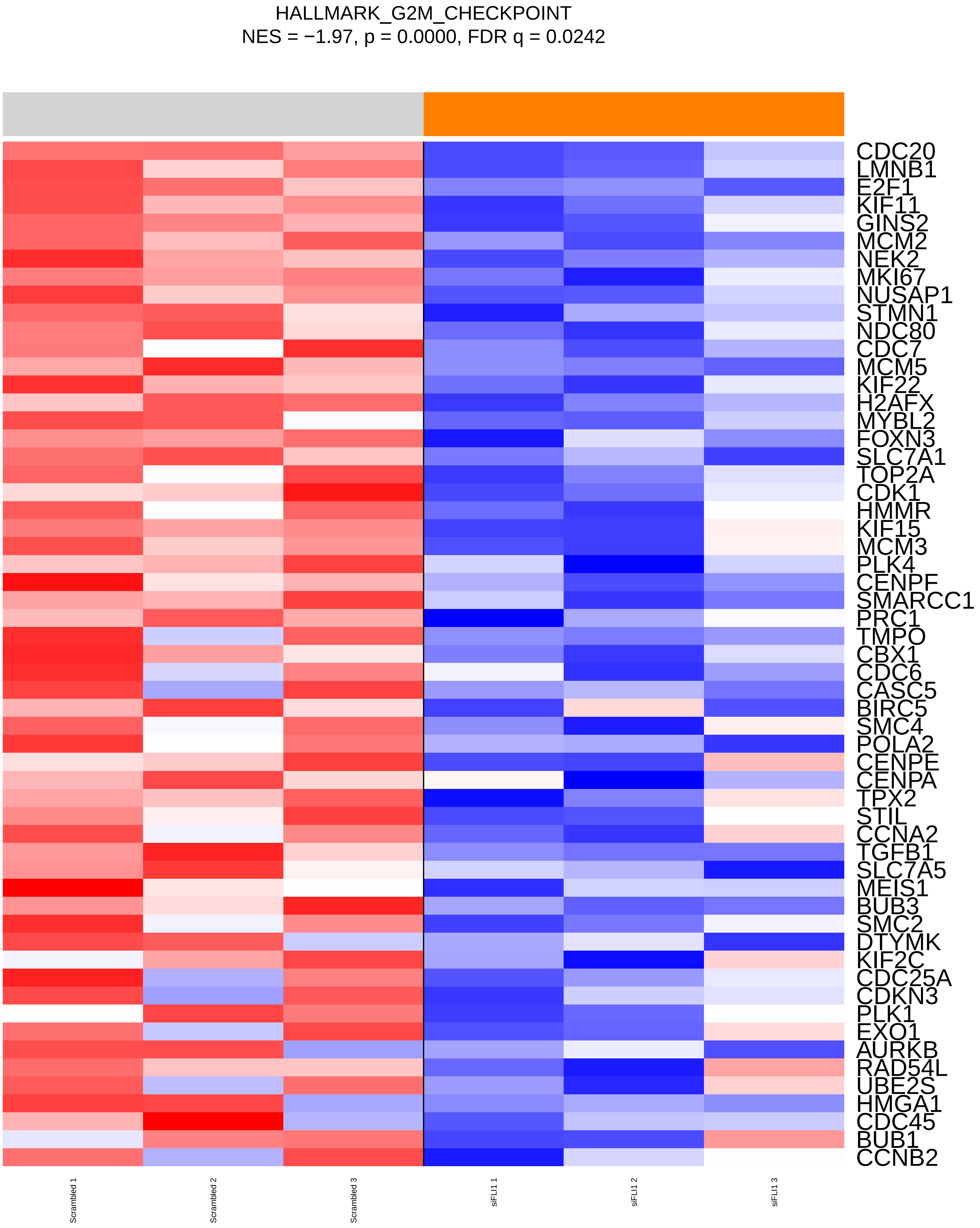
HALLMARK_G2M_CHECKPOINT
NES = −1.97, p = 0.0000, FDR q = 0.0242
CDC20
LMNB1
E2F1
KIF11
GINS2
MCM2
NEK2
MKI67
NUSAP1
STMN1
NDC80
CDC7
MCM5
KIF22
H2AFX
MYBL2
FOXN3
SLC7A1
TOP2A
CDK1
HMMR
KIF15
MCM3
PLK4
CENPF
SMARCC1
PRC1
TMPO
CBX1
CDC6
CASC5
BIRC5
SMC4
POLA2
CENPE
CENPA
TPX2
STIL
CCNA2
TGFB1
SLC7A5
MEIS1
BUB3
SMC2
DTYMK
KIF2C
CDC25A
CDKN3
PLK1
EXO1
AURKB
RAD54L
UBE2S
HMGA1
CDC45
BUB1
CCNB2
Scrambled 1
Scrambled 2
Scrambled 3
siFLI1 1
siFLI1 2
siFLI1 3

REACTOME_ACTIVATION_OF_THE_PRE_REPLICATIVE_COMPLEX
NES = −1.97, p = 0.0017, FDR q = 0.0236

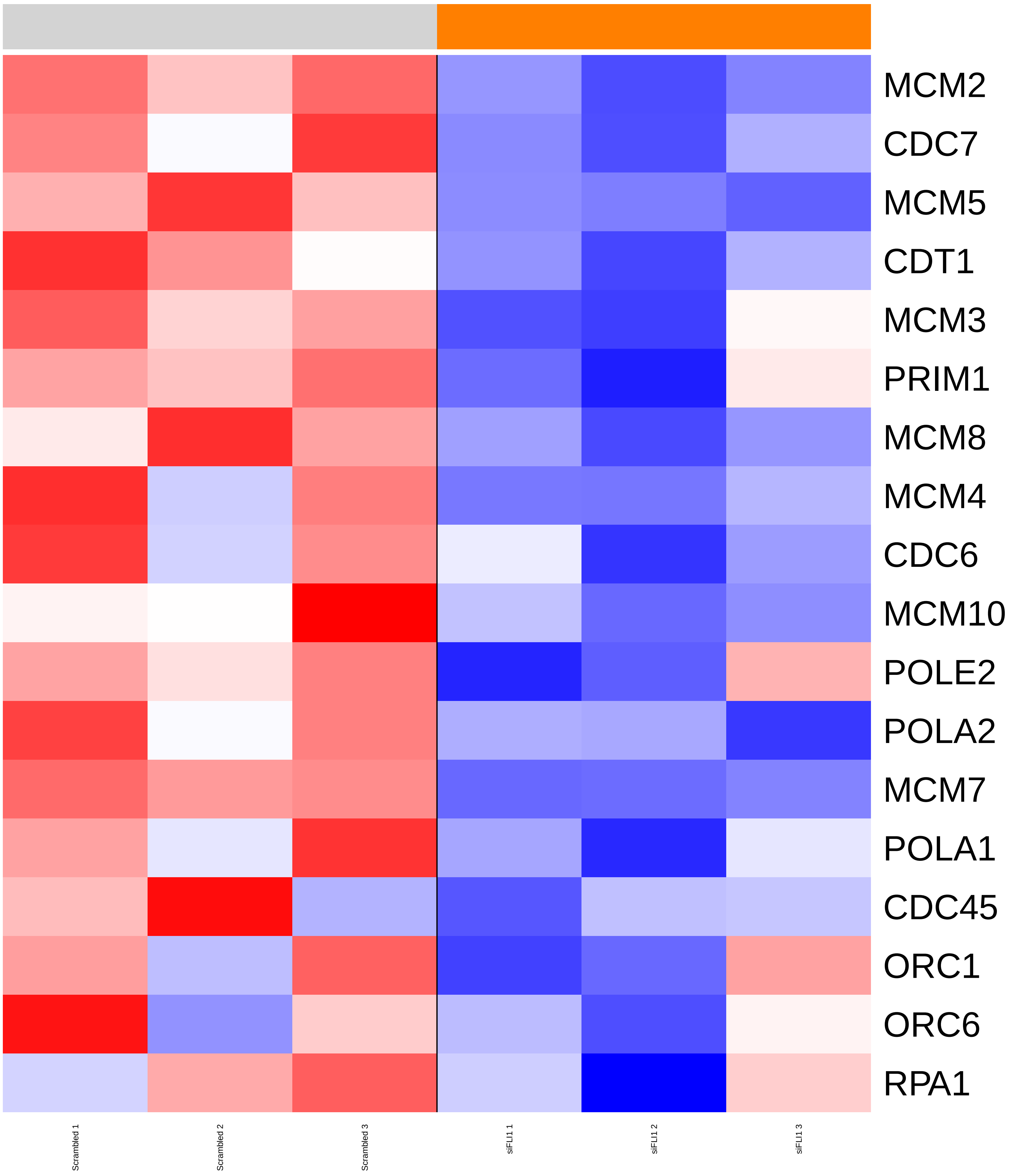

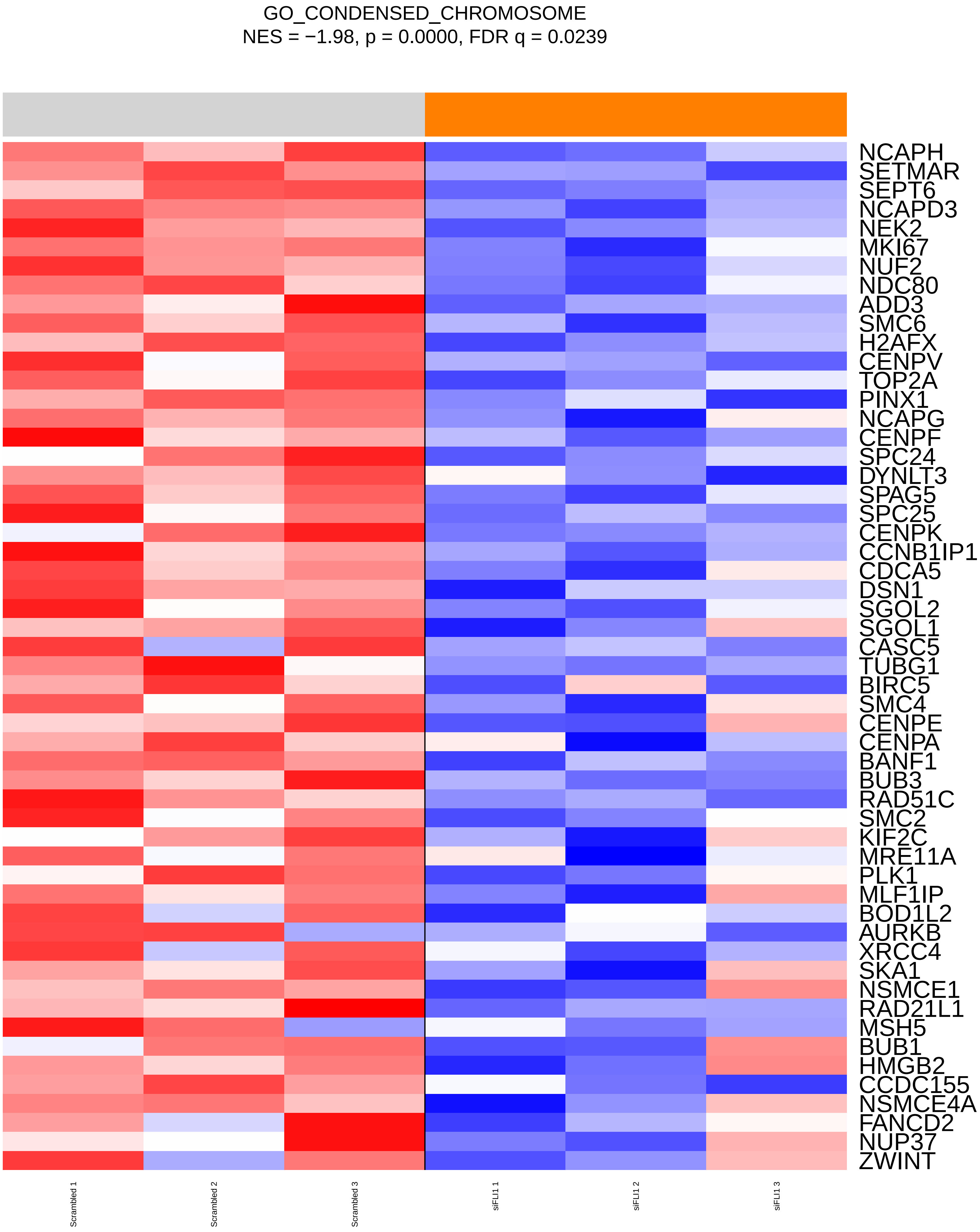

GO_CONDENSED_CHROMOSOME
NES = −1.98, p = 0.0000, FDR q = 0.0239
NCAPH
SETMAR
SEPT6
NCAPD3
NEK2
MKI67
NUF2
NDC80
ADD3
SMC6
H2AFX
CENPV
TOP2A
PINX1
NCAPG
CENPF
SPC24
DYNLT3
SPAG5
SPC25
CENPK
CCNB1IP1
CDCA5
DSN1
SGOL2
SGOL1
CASC5
TUBG1
BIRC5
SMC4
CENPE
CENPA
BANF1
BUB3
RAD51C
SMC2
KIF2C
MRE11A
PLK1
MLF1IP
BOD1L2
AURKB
XRCC4
SKA1
NSMCE1
RAD21L1
MSH5
BUB1
HMGB2
CCDC155
NSMCE4A
FANCD2
NUP37
ZWINT
Scrambled 1
Scrambled 2
Scrambled 3
siFLI1 1
siFLI1 2
siFLI1 3

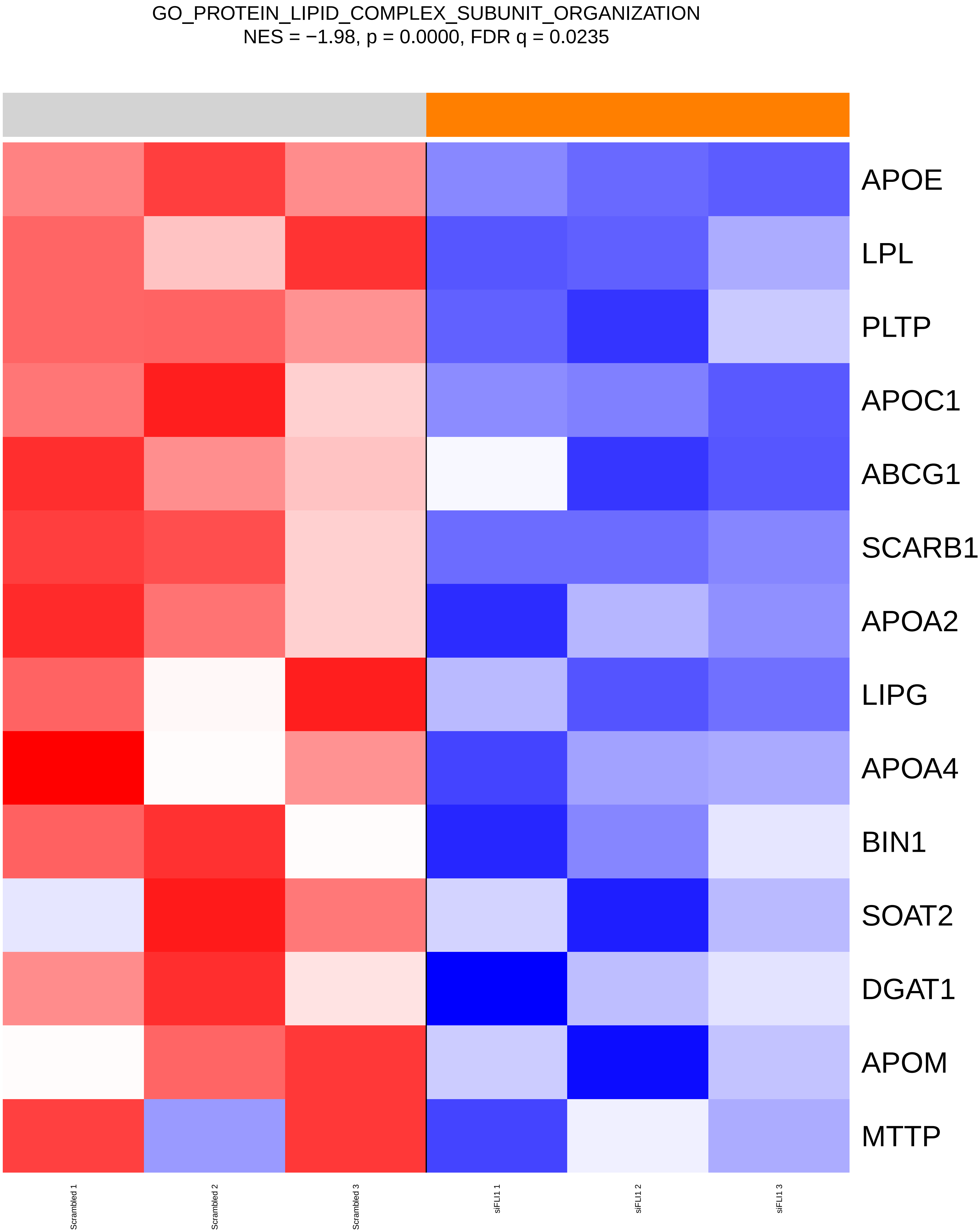
GO_PROTEIN_LIPID_COMPLEX_SUBUNIT_ORGANIZATION
NES = −1.98, p = 0.0000, FDR q = 0.0235
APOE
LPL
PLTP
APOC1
ABCG1
SCARB1
APOA2
LIPG
APOA4
BIN1
SOAT2
DGAT1
APOM
MTTP
Scrambled 1
Scrambled 2
Scrambled 3
siFLI1 1
siFLI1 2
siFLI1 3

REACTOME_LIPID_DIGESTION_MOBILIZATION_AND_TRANSPORT
NES = −2.00, p = 0.0000, FDR q = 0.0188

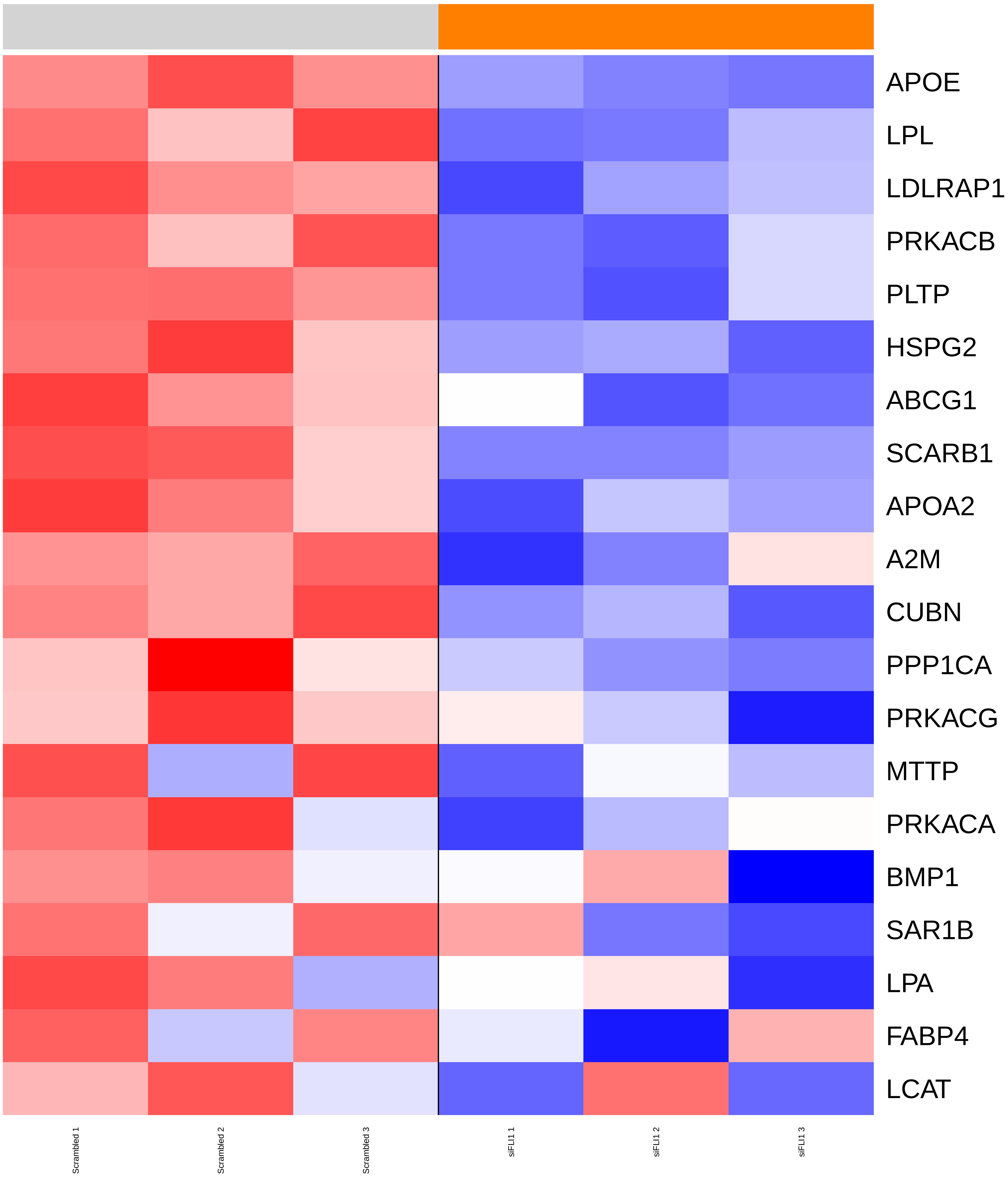

GO_DNA_REPLICATION_INITIATION
NES = −2.00, p = 0.0000, FDR q = 0.0192

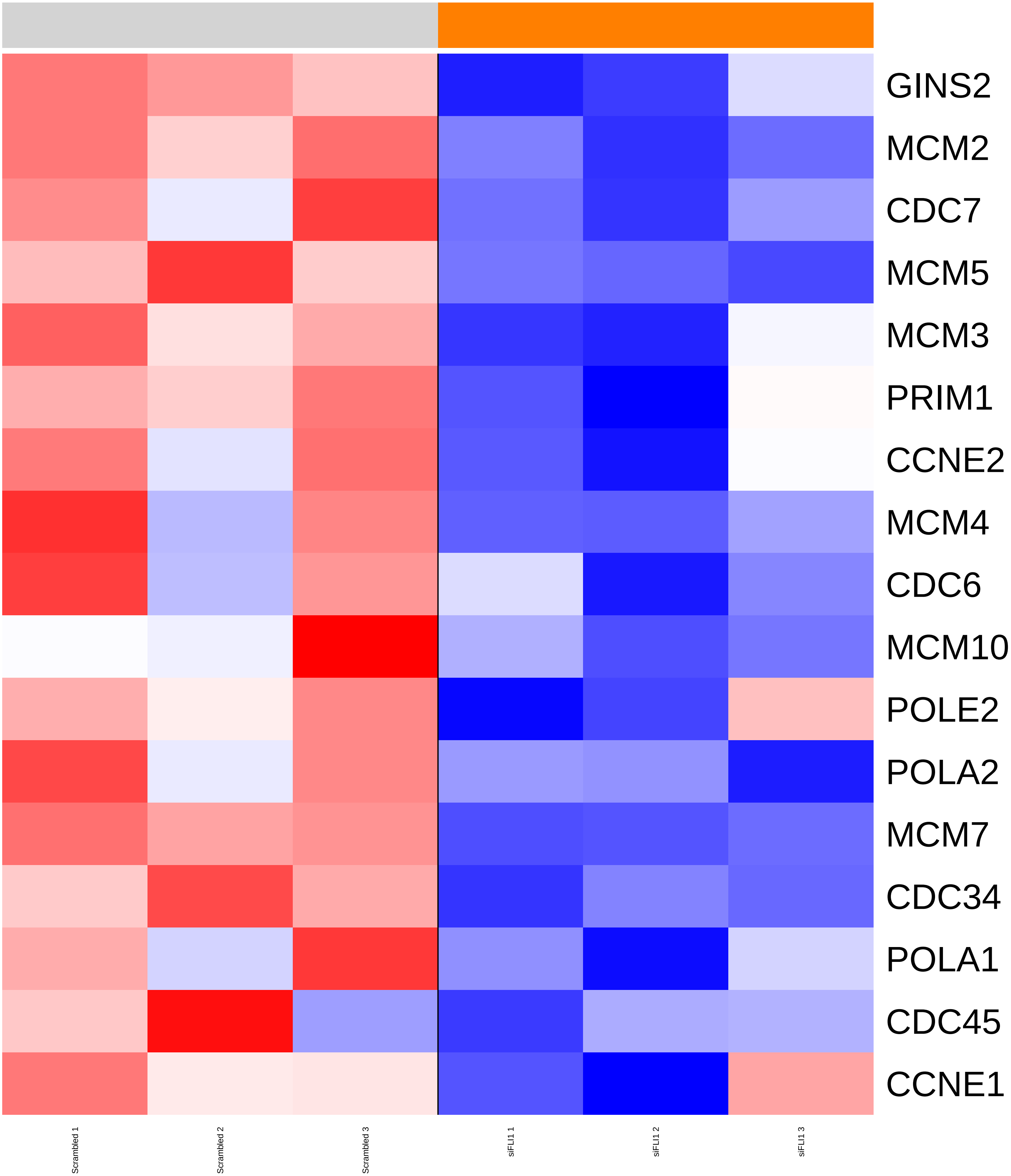

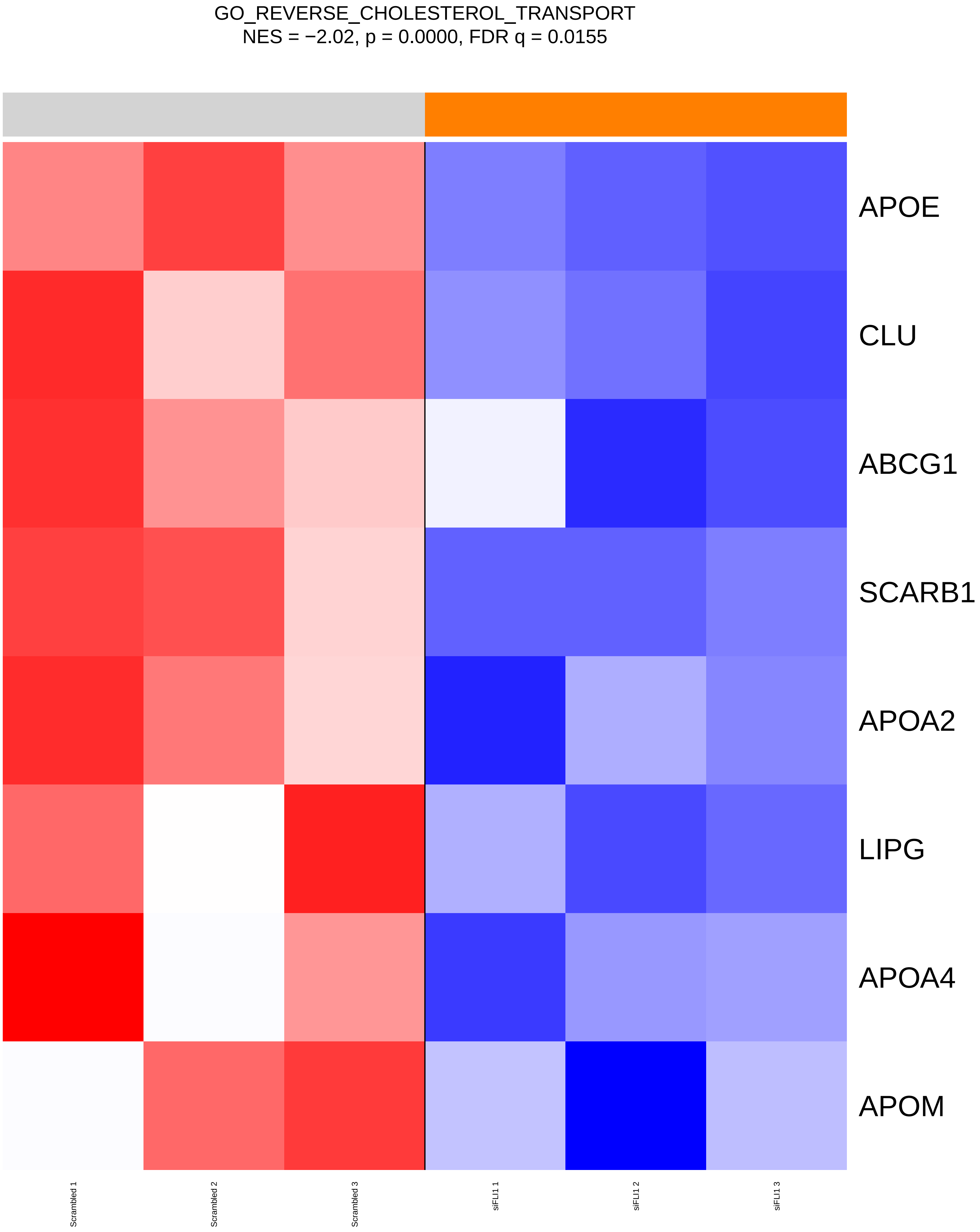

GO_REVERSE_CHOLESTEROL_TRANSPORT
NES = −2.02, p = 0.0000, FDR q = 0.0155
APOE
CLU
ABCG1
SCARB1
APOA2
LIPG
APOA4
APOM
Scrambled 1
Scrambled 2
Scrambled 3
siFLI1 1
siFLI1 2
siFLI1 3

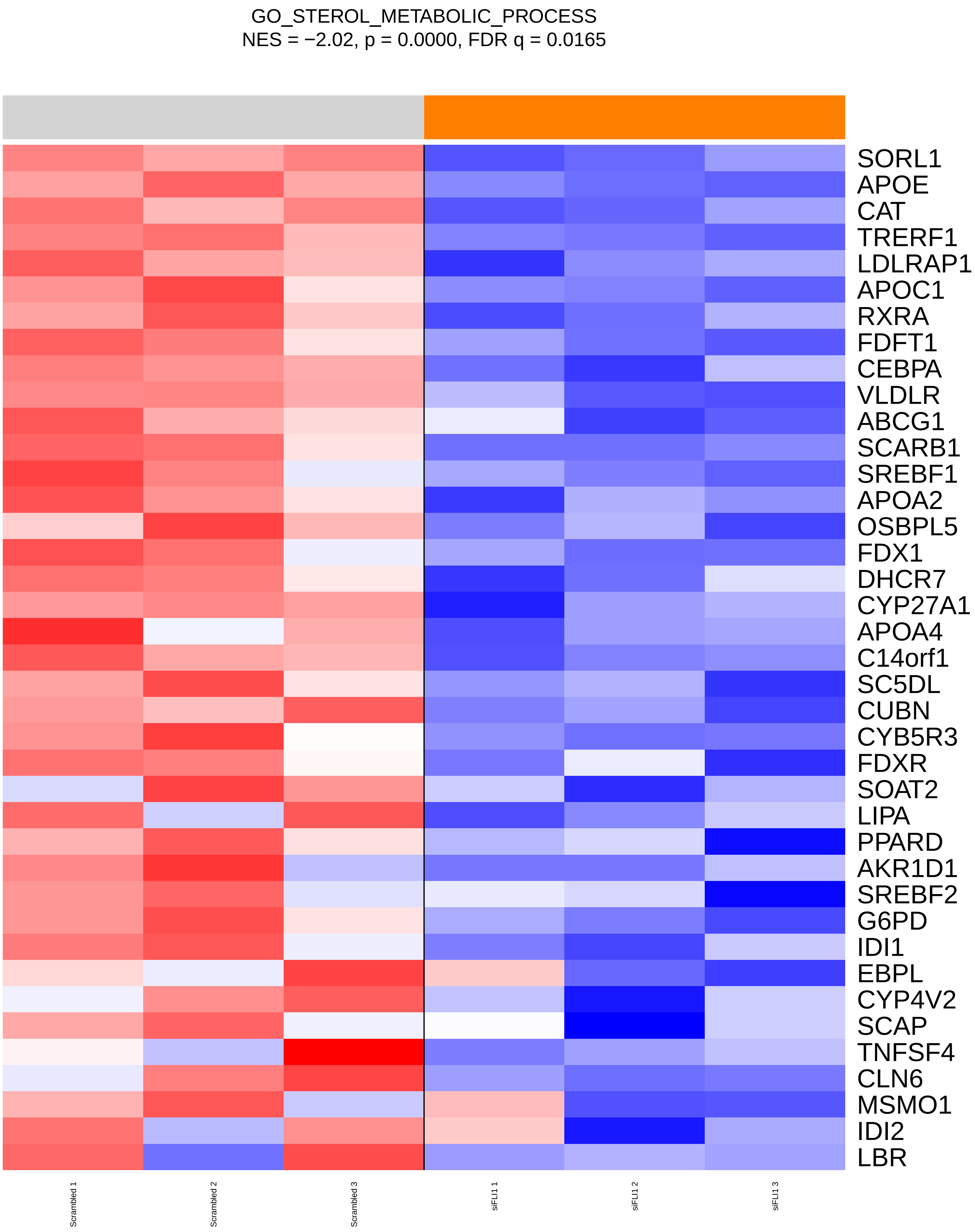
GO_STEROL_METABOLIC_PROCESS
NES = −2.02, p = 0.0000, FDR q = 0.0165
SORL1
APOE
CAT
TRERF1
LDLRAP1
APOC1
RXRA
FDFT1
CEBPA
VLDLR
ABCG1
SCARB1
SREBF1
APOA2
OSBPL5
FDX1
DHCR7
CYP27A1
APOA4
C14orf1
SC5DL
CUBN
CYB5R3
FDXR
SOAT2
LIPA
PPARD
AKR1D1
SREBF2
G6PD
IDI1
EBPL
CYP4V2
SCAP
TNFSF4
CLN6
MSMO1
IDI2
LBR
Scrambled 1
Scrambled 2
Scrambled 3
siFLI1 1
siFLI1 2
siFLI1 3

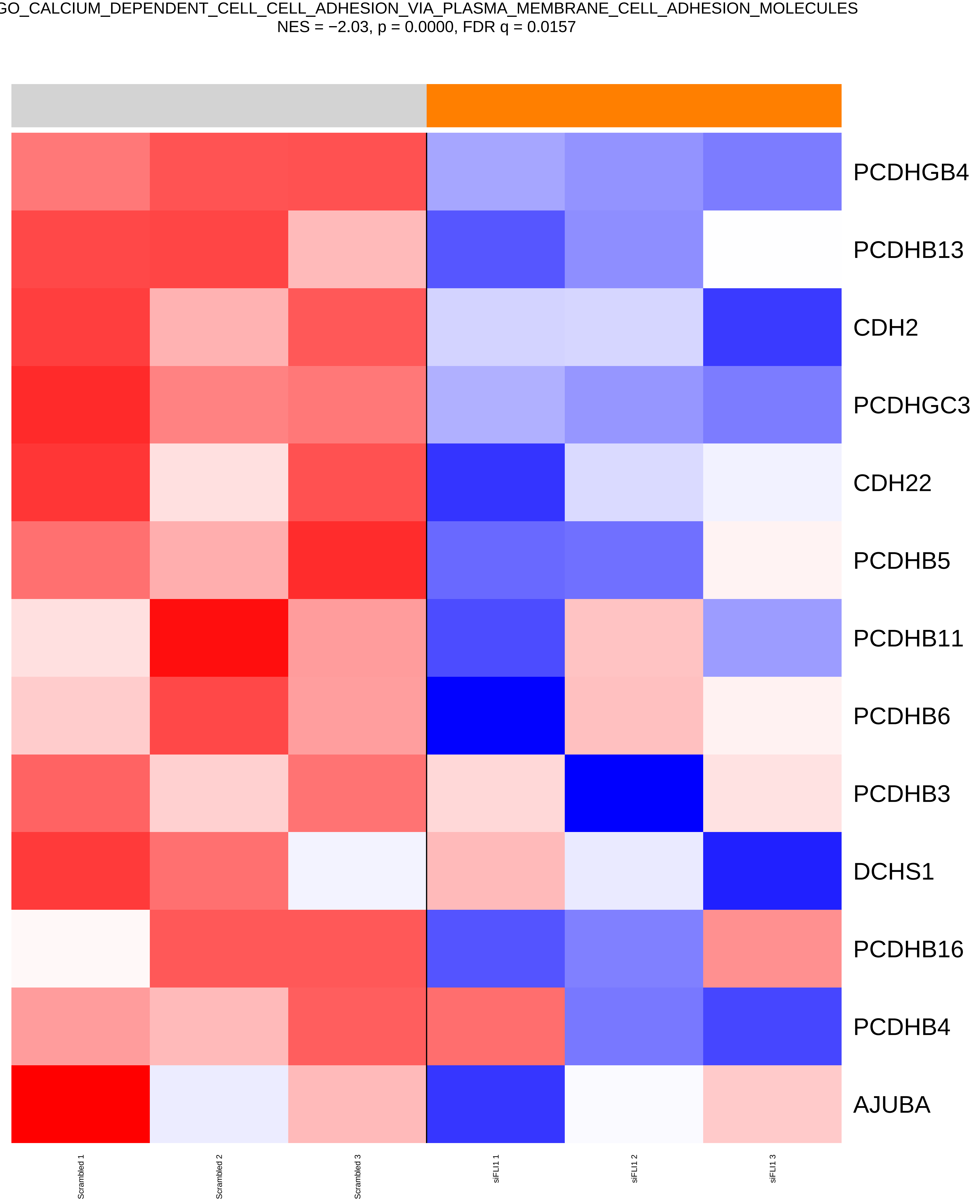
GO_CALCIUM_DEPENDENT_CELL_CELL_ADHESION_VIA_PLASMA_MEMBRANE_CELL_ADHESION_MOLECULES
NES = −2.03, p = 0.0000, FDR q = 0.0157
PCDHGB4
PCDHB13
CDH2
PCDHGC3
CDH22
PCDHB5
PCDHB11
PCDHB6
PCDHB3
DCHS1
PCDHB16
PCDHB4
AJUBA
Scrambled 1
Scrambled 2
Scrambled 3
siFLI1 1
siFLI1 2
siFLI1 3

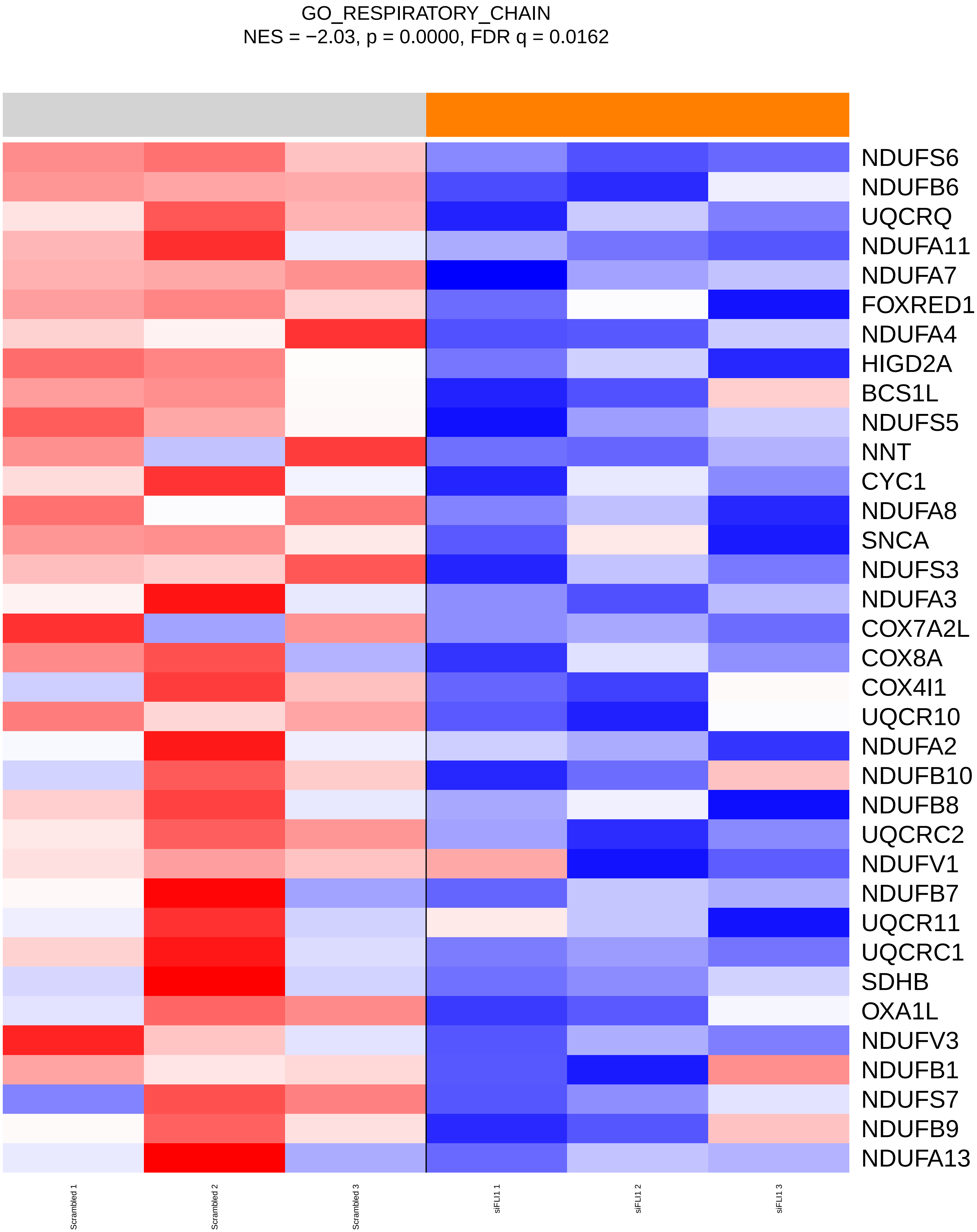
GO_RESPIRATORY_CHAIN
NES = −2.03, p = 0.0000, FDR q = 0.0162
NDUFS6
NDUFB6
UQCRQ
NDUFA11
NDUFA7
FOXRED1
NDUFA4
HIGD2A
BCS1L
NDUFS5
NNT
CYC1
NDUFA8
SNCA
NDUFS3
NDUFA3
COX7A2L
COX8A
COX4I1
UQCR10
NDUFA2
NDUFB10
NDUFB8
UQCRC2
NDUFV1
NDUFB7
UQCR11
UQCRC1
SDHB
OXA1L
NDUFV3
NDUFB1
NDUFS7
NDUFB9
NDUFA13
Scrambled 1
Scrambled 2
Scrambled 3
siFLI1 1
siFLI1 2
siFLI1 3

GO_DNA_PACKAGING_COMPLEX
NES = −2.03, p = 0.0000, FDR q = 0.0177

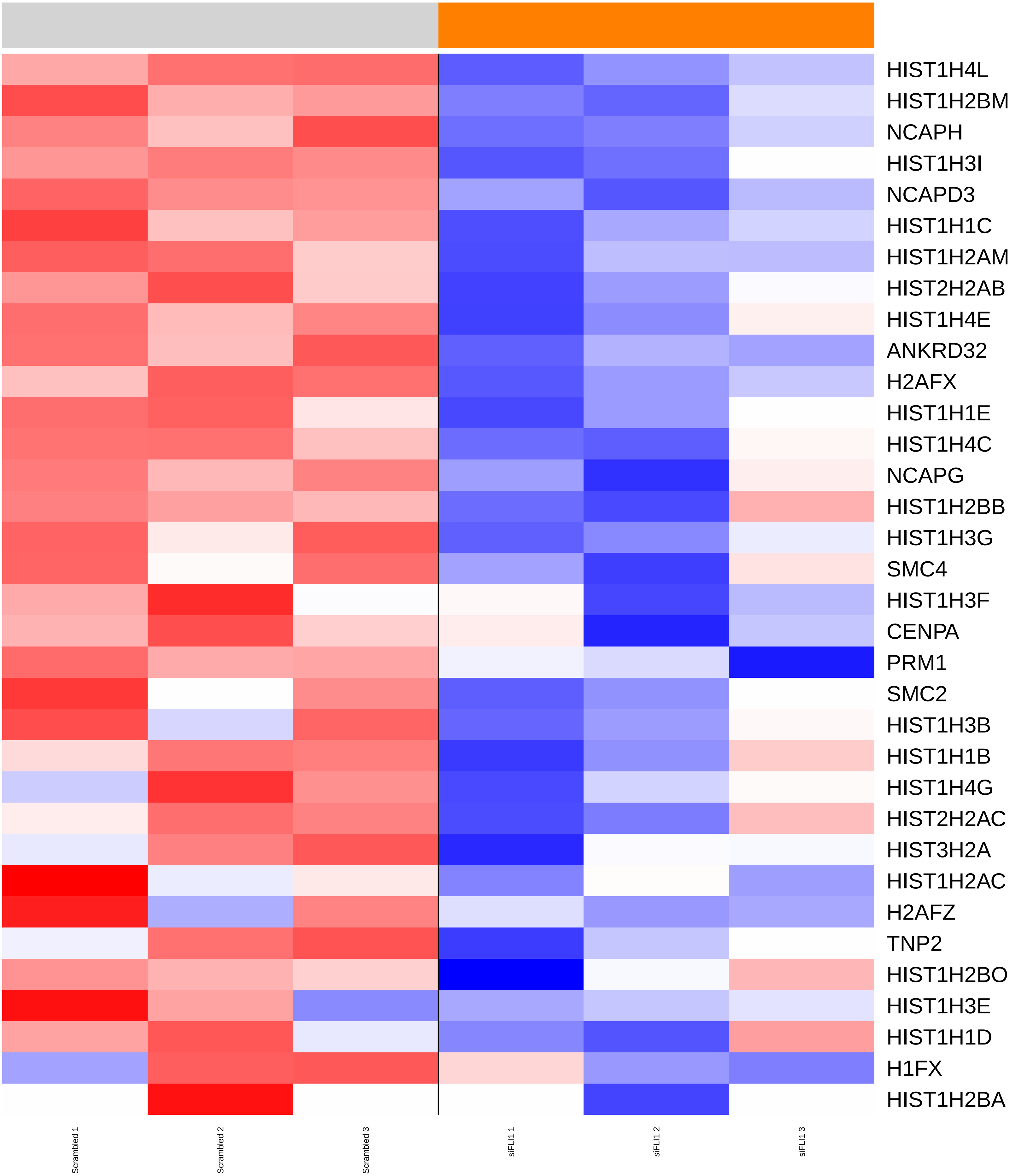

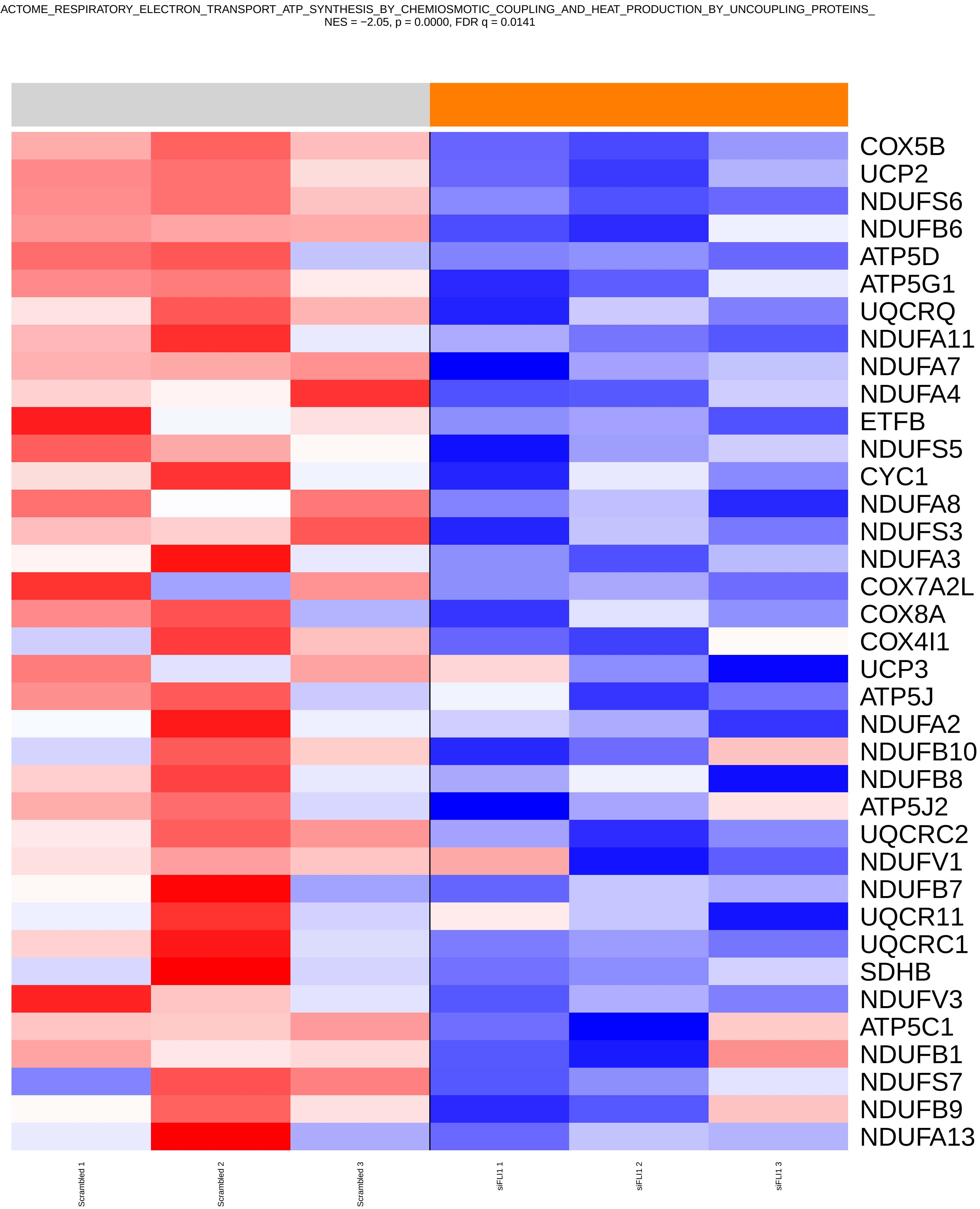

ACTOME_RESPIRATORY_ELECTRON_TRANSPORT_ATP_SYNTHESIS_BY_CHEMIOSMOTIC_COUPLING_AND_HEAT_PRODUCTION_BY_UNCOUPLING_PROTEINS_
NES = −2.05, p = 0.0000, FDR q = 0.0141
COX5B
UCP2
NDUFS6
NDUFB6
ATP5D
ATP5G1
UQCRQ
NDUFA11
NDUFA7
NDUFA4
ETFB
NDUFS5
CYC1
NDUFA8
NDUFS3
NDUFA3
COX7A2L
COX8A
COX4I1
UCP3
ATP5J
NDUFA2
NDUFB10
NDUFB8
ATP5J2
UQCRC2
NDUFV1
NDUFB7
UQCR11
UQCRC1
SDHB
NDUFV3
ATP5C1
NDUFB1
NDUFS7
NDUFB9
NDUFA13
Scrambled 1
Scrambled 2
Scrambled 3
siFLI1 1
siFLI1 2
siFLI1 3

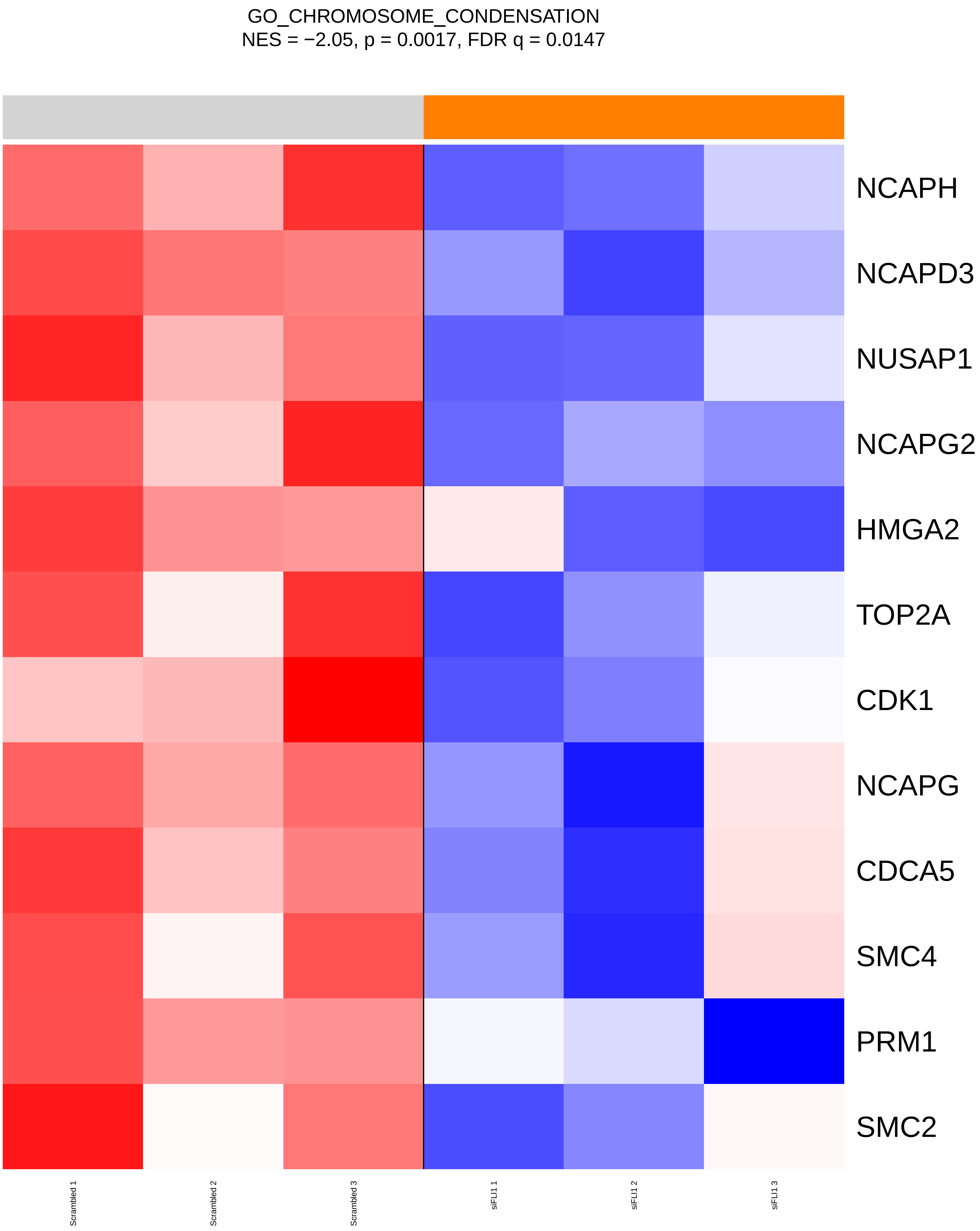
GO_CHROMOSOME_CONDENSATION
NES = −2.05, p = 0.0017, FDR q = 0.0147
NCAPH
NCAPD3
NUSAP1
NCAPG2
HMGA2
TOP2A
CDK1
NCAPG
CDCA5
SMC4
PRM1
SMC2
Scrambled 1
Scrambled 2
Scrambled 3
siFLI1 1
siFLI1 2
siFLI1 3

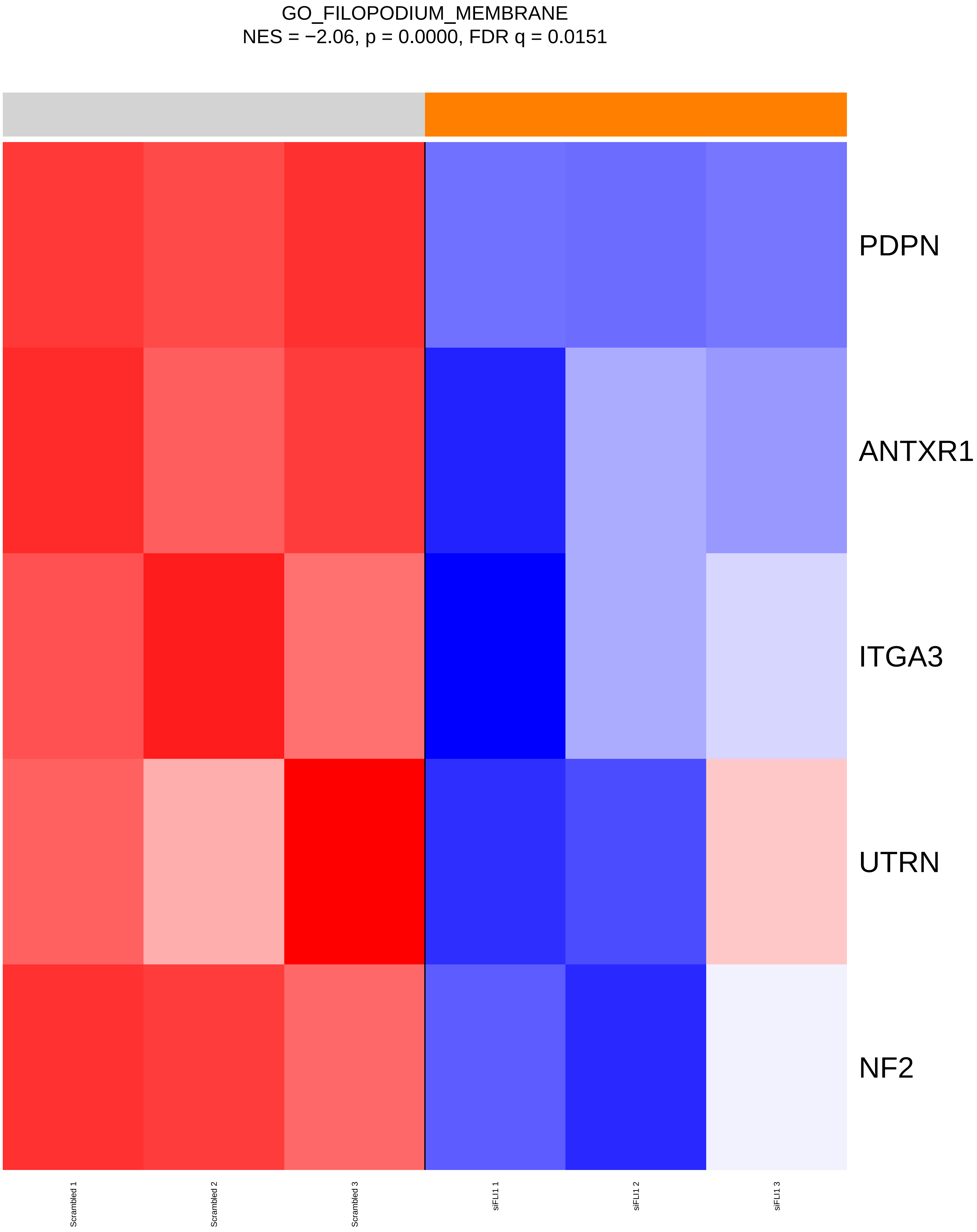
GO_FILOPODIUM_MEMBRANE
NES = −2.06, p = 0.0000, FDR q = 0.0151
PDPN
ANTXR1
ITGA3
UTRN
NF2
Scrambled 1
Scrambled 2
Scrambled 3
siFLI1 1
siFLI1 2
siFLI1 3

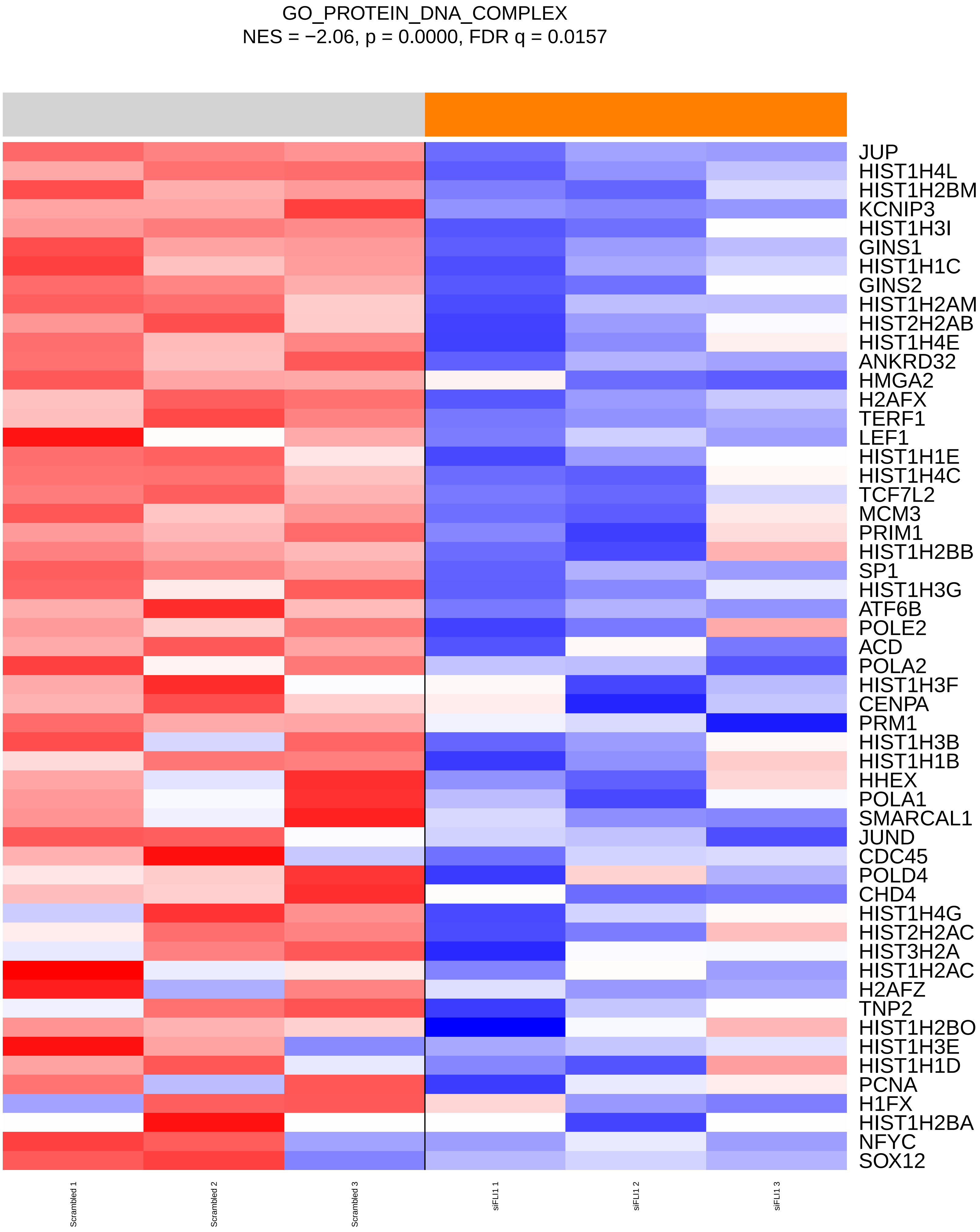
GO_PROTEIN_DNA_COMPLEX
NES = −2.06, p = 0.0000, FDR q = 0.0157
JUP
HIST1H4L
HIST1H2BM
KCNIP3
HIST1H3I
GINS1
HIST1H1C
GINS2
HIST1H2AM
HIST2H2AB
HIST1H4E
ANKRD32
HMGA2
H2AFX
TERF1
LEF1
HIST1H1E
HIST1H4C
TCF7L2
MCM3
PRIM1
HIST1H2BB
SP1
HIST1H3G
ATF6B
POLE2
ACD
POLA2
HIST1H3F
CENPA
PRM1
HIST1H3B
HIST1H1B
HHEX
POLA1
SMARCAL1
JUND
CDC45
POLD4
CHD4
HIST1H4G
HIST2H2AC
HIST3H2A
HIST1H2AC
H2AFZ
TNP2
HIST1H2BO
HIST1H3E
HIST1H1D
PCNA
H1FX
HIST1H2BA
NFYC
SOX12
Scrambled 1
Scrambled 2
Scrambled 3
siFLI1 1
siFLI1 2
siFLI1 3

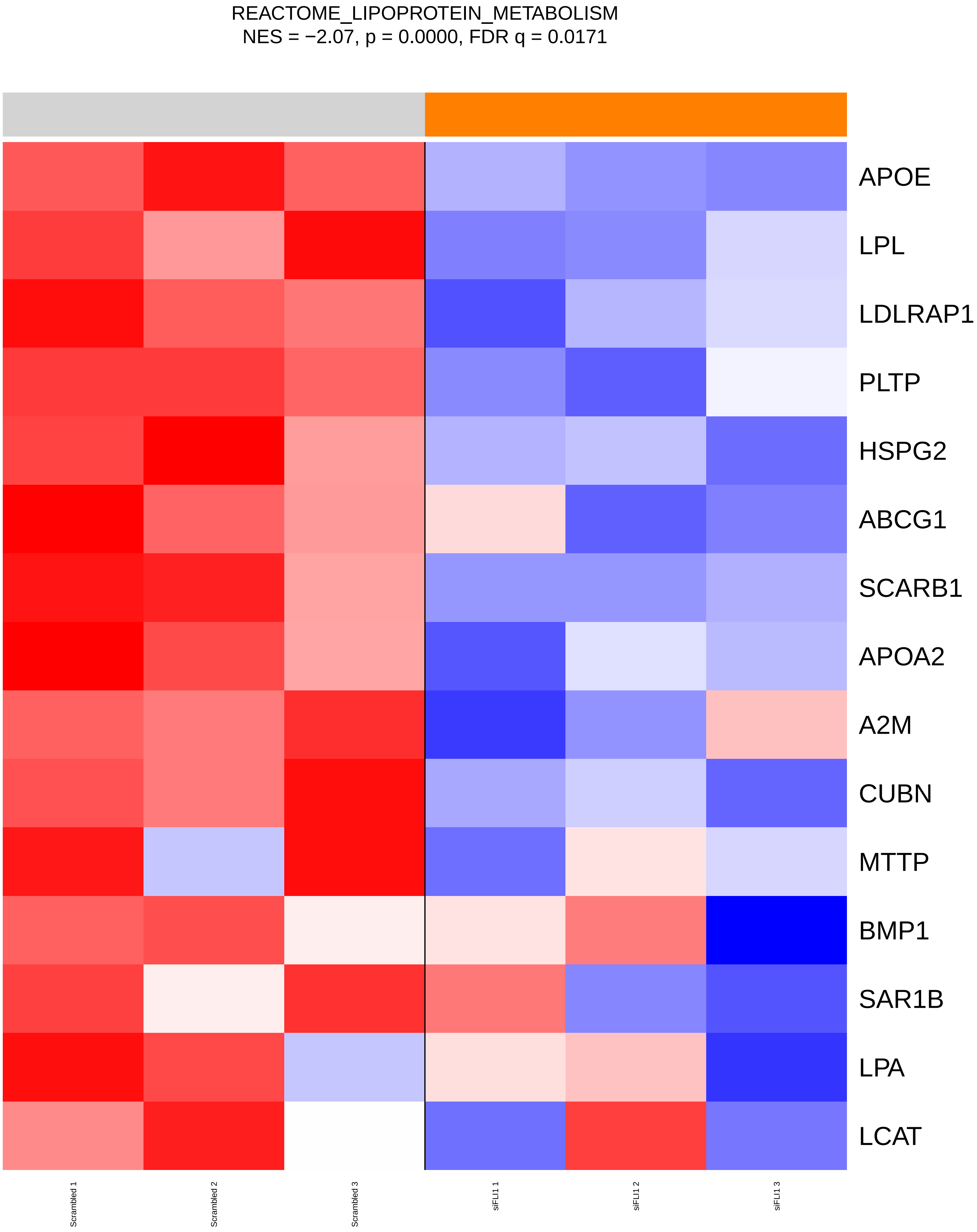
REACTOME_LIPOPROTEIN_METABOLISM
NES = −2.07, p = 0.0000, FDR q = 0.0171
APOE
LPL
LDLRAP1
PLTP
HSPG2
ABCG1
SCARB1
APOA2
A2M
CUBN
MTTP
BMP1
SAR1B
LPA
LCAT
Scrambled 1
Scrambled 2
Scrambled 3
siFLI1 1
siFLI1 2
siFLI1 3

HALLMARK_E2F_TARGETS
NES = −2.13, p = 0.0000, FDR q = 0.0075

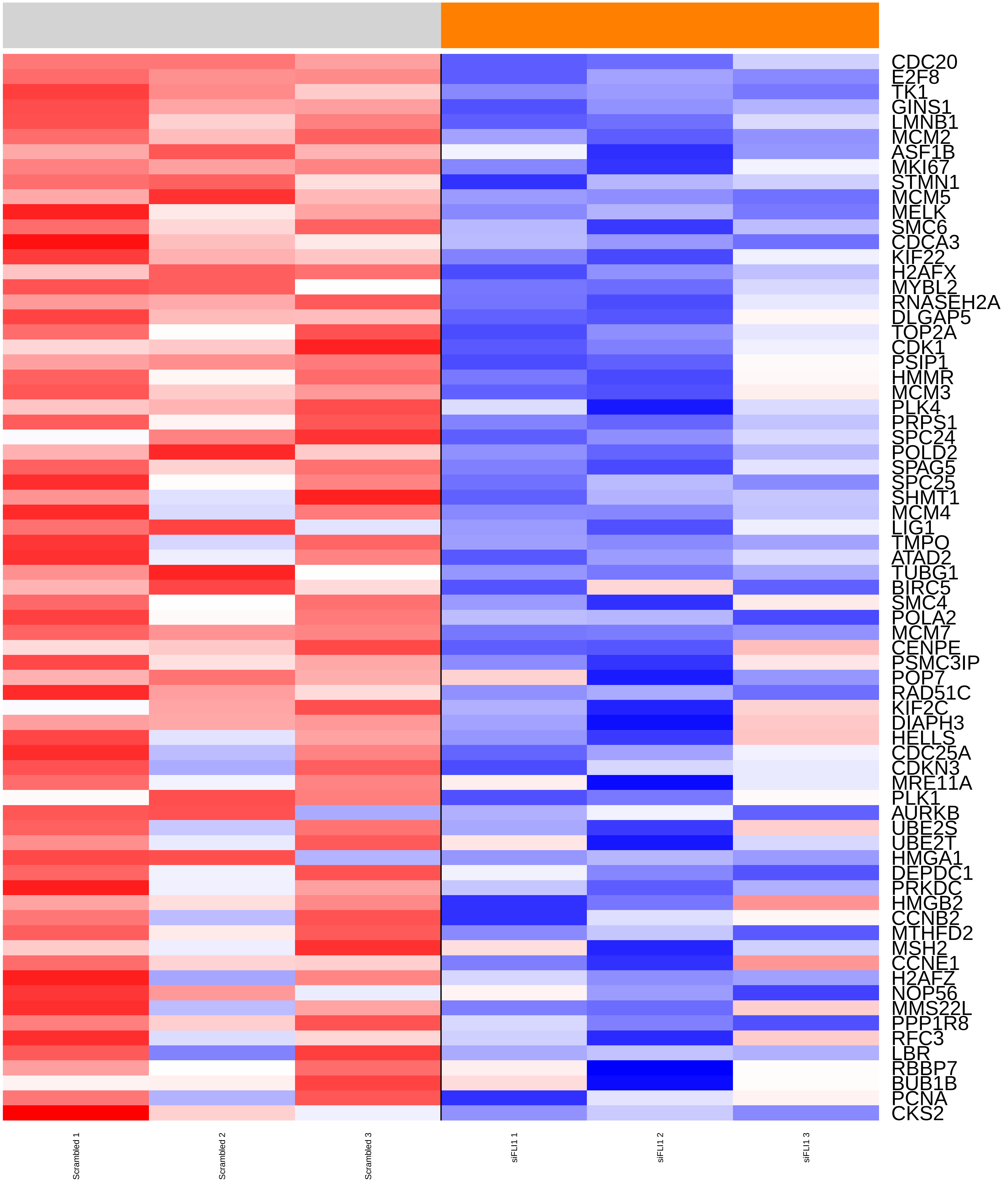

KEGG_DNA_REPLICATION
NES = −2.13, p = 0.0000, FDR q = 0.0086

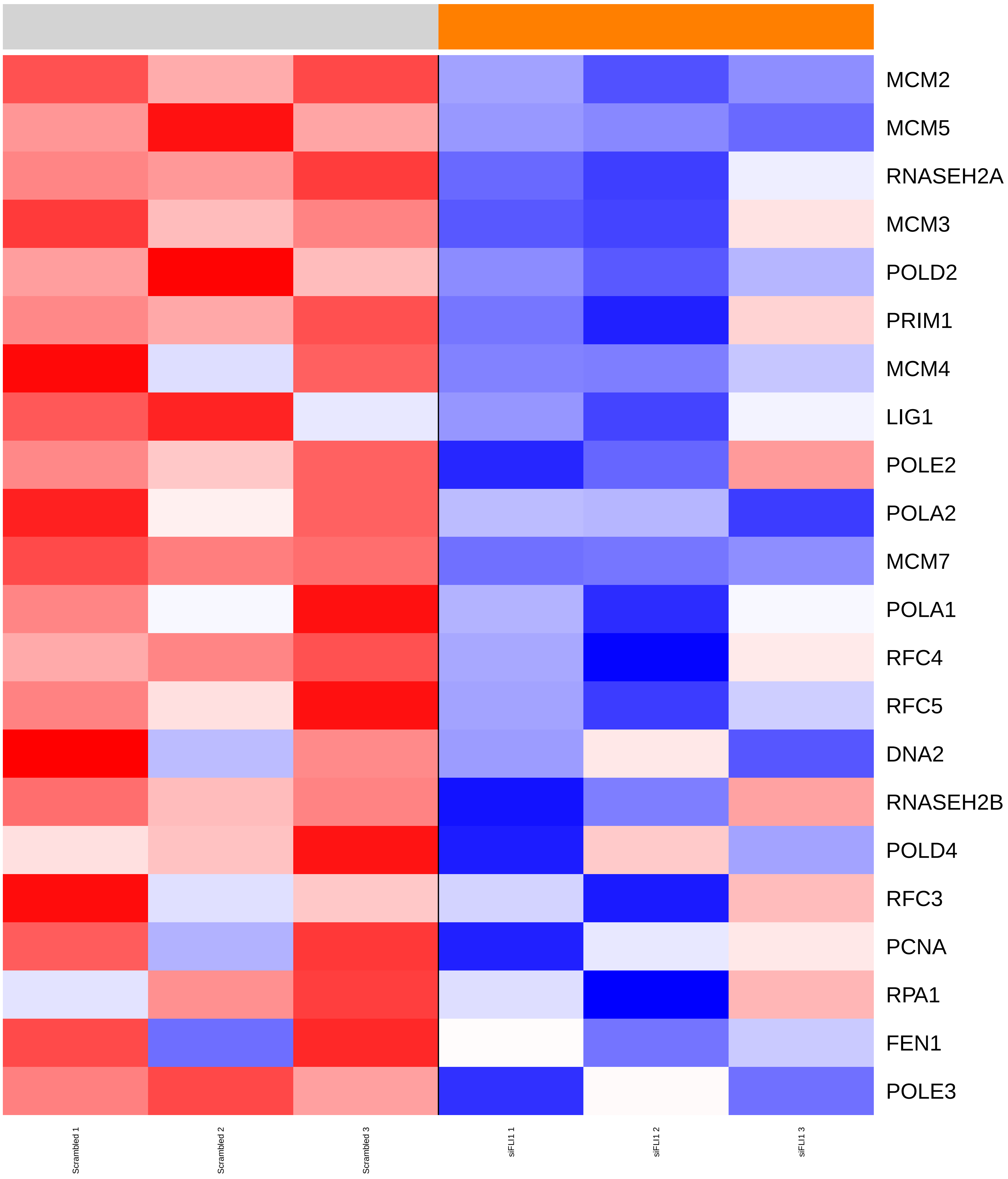

GO_MACROMOLECULAR_COMPLEX_REMODELING
NES = −2.14, p = 0.0000, FDR q = 0.0093

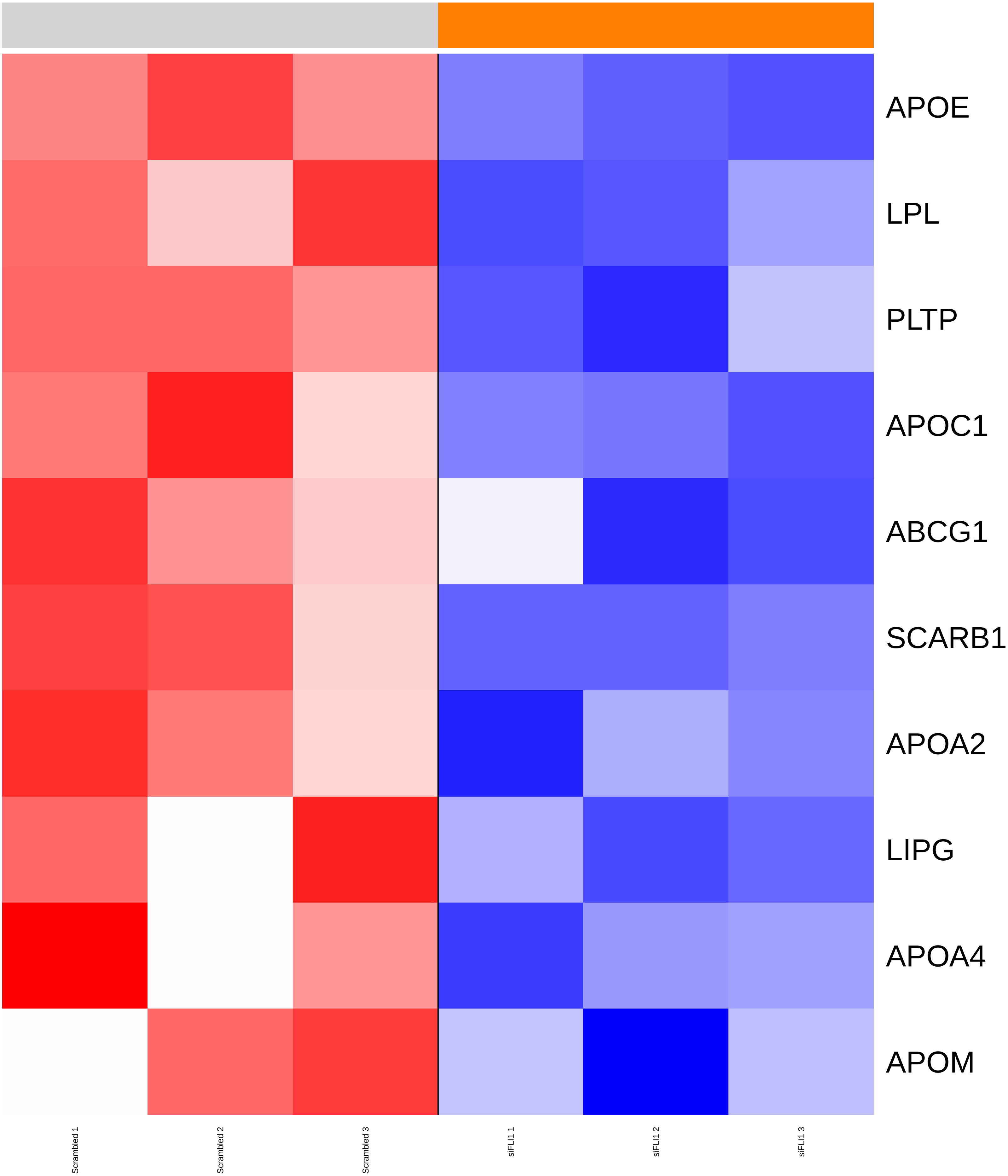

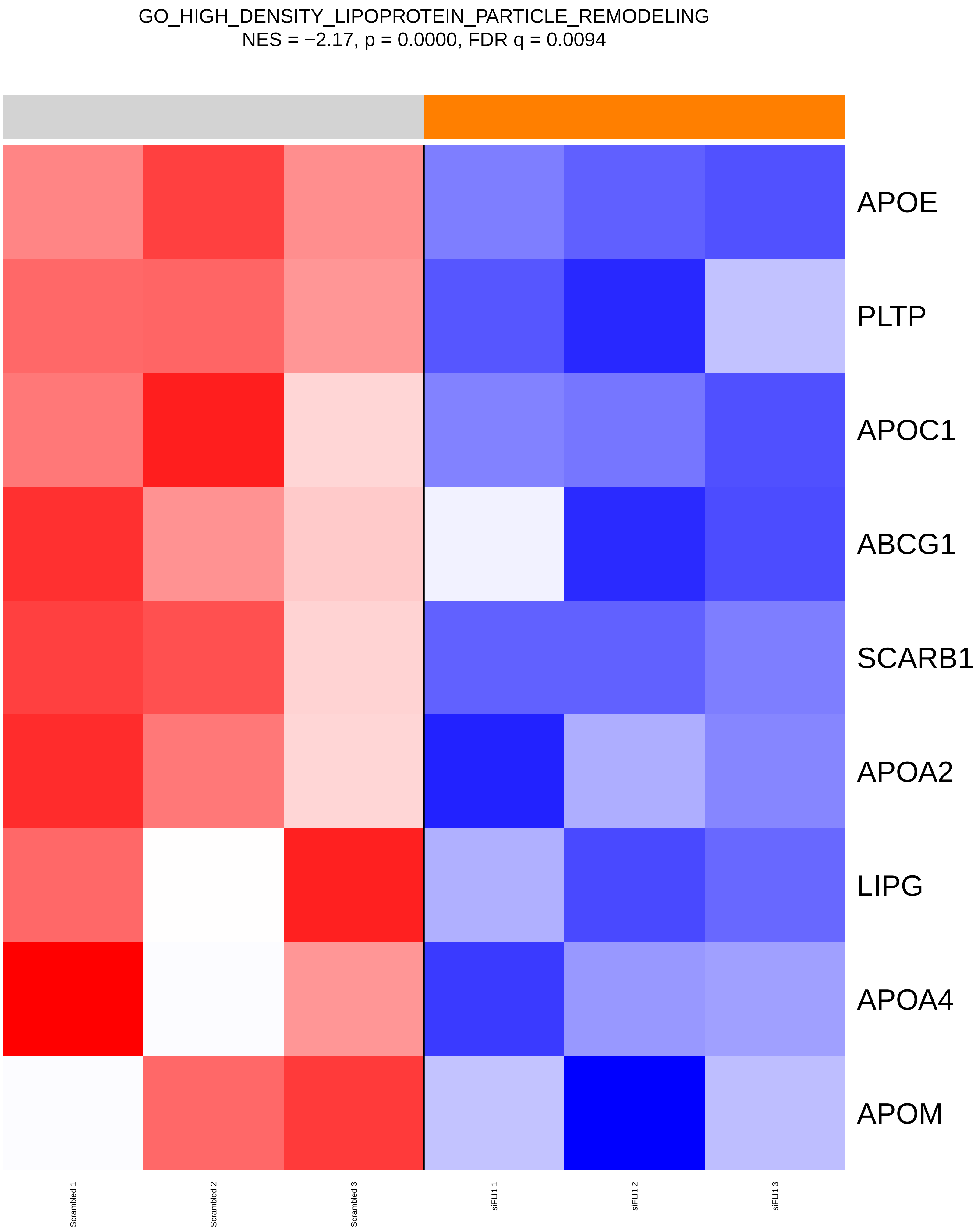
GO_HIGH_DENSITY_LIPOPROTEIN_PARTICLE_REMODELING
NES = −2.17, p = 0.0000, FDR q = 0.0094
APOE
PLTP
APOC1
ABCG1
SCARB1
APOA2
LIPG
APOA4
APOM
Scrambled 1
Scrambled 2
Scrambled 3
siFLI1 1
siFLI1 2
siFLI1 3

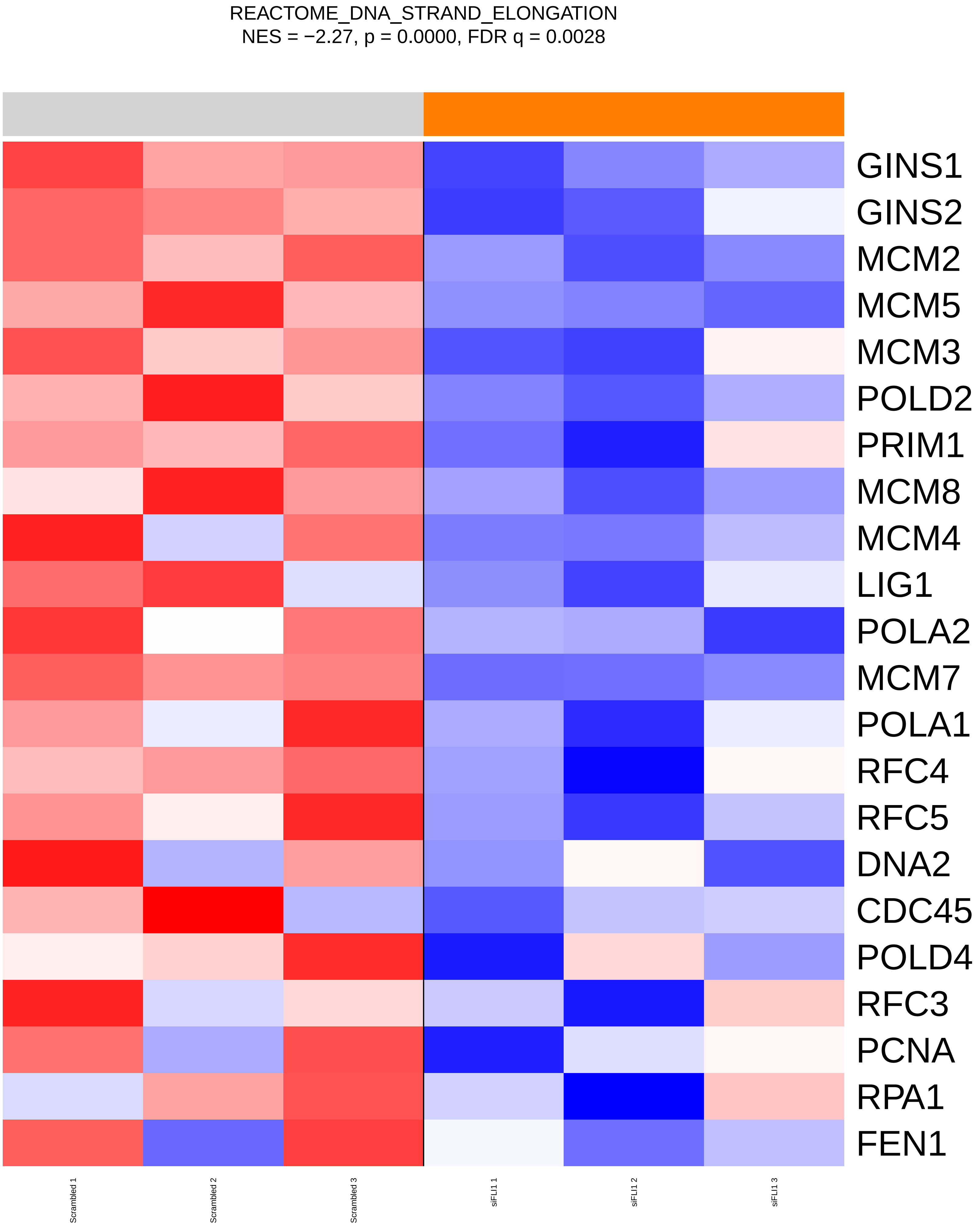
REACTOME_DNA_STRAND_ELONGATION
NES = −2.27, p = 0.0000, FDR q = 0.0028
GINS1
GINS2
MCM2
MCM5
MCM3
POLD2
PRIM1
MCM8
MCM4
LIG1
POLA2
MCM7
POLA1
RFC4
RFC5
DNA2
CDC45
POLD4
RFC3
PCNA
RPA1
FEN1
Scrambled 1
Scrambled 2
Scrambled 3
siFLI1 1
siFLI1 2
siFLI1 3
